# Novel lncRNA Panel as for Prognosis in Esophageal Squamous Cell Carcinoma Based on ceRNA Network Mechanism

**DOI:** 10.1155/2021/8020879

**Published:** 2021-09-24

**Authors:** Jin Zhang, Fei Xiao, Guangliang Qiang, Zhenrong Zhang, Qianli Ma, Yang Hao, Huajie Xing, Chaoyang Liang

**Affiliations:** Department of Thoracic Surgery, China-Japan Friendship Hospital, Beijing 100029, China

## Abstract

**Background:**

The competitive endogenous RNA (ceRNA) mechanism has been discovered recently and regulating cancer-related gene expressions. The ceRNA network participates in multiple processes, such as cell proliferation and metastasis, and potentially drives the progression of cancer. In this study, we focus on the ceRNA networks of esophageal squamous cell carcinoma and discovered a novel biomarker panel for cancer prognosis.

**Methods:**

RNA expression data of esophageal carcinoma from the TCGA database were achieved and constructed ceRNA network in esophageal carcinoma using R packages.

**Results:**

Four miRNAs were discovered as the core of the ceRNA model, including miR-93, miR-191, miR-99b, and miR-3615. Moreover, we constructed a ceRNA network in esophageal carcinoma, which included 4 miRNAs and 6 lncRNAs. After ceRNA network modeling, we investigated six lncRNAs which could be taken together as a panel for prognosis prediction of esophageal cancer, including LINC02575, LINC01087, LINC01816, AL136162.1, AC012073.1, and AC117402.1. Finally, we tested the predictive power of the panel in all TCGA samples.

**Conclusions:**

Our study discovered a new biomarker panel which may have potential values in the prediction of prognosis of esophageal carcinoma.

## 1. Introduction

Esophageal squamous cell carcinoma is one of the most frequently diagnosed malignancies among women worldwide. Despite the substantial research findings in investigating the intracellular mechanisms of esophageal cancer, it remains to be a significant cause of cancer death to women. One reason for this urgent situation is that we have not found effective methods for the early diagnosis and prognosis of esophageal cancer. Previous work has proved that some noncoding RNAs (NcRNAs), including circular RNA (circRNA), long noncoding RNAs (lncRNAs), and micro RNAs (miRNAs) [1], could interact with each other [2].

Based on the functions of NcRNAs, most researches focus on the functional NcRNAs, which also play essential roles in the ceRNA network [3], in brief, miRNAs bind with mRNAs via MREs, and the functions of mRNAs are thus inhibited by miRNAs, at the same if functional lncRNAs bind with the corresponding miRNAs, the functions of miRNAs are thus inhibited which indirectly release the mRNAs which were inhibited by miRNAs, and the interaction network are called ceRNETs [4]. Recently, more and more evidence have proved that the ceRNA network has key roles in the progression of cancer, and some ceRNAs could be further used to construct biomarkers for the prediction of patient prognosis [5, 6].

Esophageal cancer is one of the most aggressive cancer types and sixth most common cause of cancer death worldwide [7]. Esophageal cancer has two major subtypes: esophageal squamous cell carcinoma (ESCC) and esophageal adenocarcinoma (EAC). The incidence rate of ESCC is high in the Asian regions including Japan, China, and India [8, 9]. ESCC is one of the most deadly forms of human malignancy characterized by late metastasis, stage diagnosis, therapy resistance, and frequent recurrence [10]. Earlier diagnosing ESCC could improve treatment outcomes of patients as well as prolong patients' OS. ESCC has a close relation with aberrant expressions of genes, which makes clinical diagnosis more complicated to patients [11]. The ceRNA network could improve our insight into the progression of esophageal carcinoma, and it may help us to build a better biomarker panel for the diagnosis and prognosis of patients [12].

Numerous experimental studies have demonstrated ceRNA network had a crucial role in modulating the pathogenesis of human diseases. For example, lncRNA PART1 suppresses ESCC cell proliferation by regulating the miR-18a-5p/SOX6 signaling axis [13]. Linc00941 regulates ESCC via functioning as a competing endogenous RNA for miR-877-3p to modulate PMEPA1 expression [14]. Moreover, analysis of the ceRNA network has been utilized in biomarker construction, drug target discovery, and even drug resistance studies [15]. Based on the ceRNA network theory, Cantile et al. discovered that a single lncRNA, HOTAIR, was highly expressed in ESCC, which was the first breast cancer hallmark and a biomarker to predict the patient's OS [16]. Since a single lncRNA was not enough considering poor sensitivity and specificity in a statistic perspective [17], taking multiple dysregulated RNAs into evaluation of the prognosis of patients were gradually accepted by researchers.

Taken together, it has been generally approved that lncRNAs have great predict powers in the prediction of cancer stages and patients' prognosis. In this study, we discovered a ceRNA network by analyzing RNA expressions in esophageal squamous cell carcinoma. Through bioinformatic procedures, we further explored novel ESCC progression-related lncRNAs, then construct a prediction model, and evaluated the predictive power in TCGA samples.

## 2. Material and Methods

### 2.1. RNA-Seq Data Procession

The study was done in R environment, and RTCGAToolBox was used to download mRNA and miRNA database. GDC Data Portal was used to download the lncRNA database. The following R packages were selected for data filtration and analysis in this paper, including Hmisc, plyr, hash, ggplot2, R.utils, ggbiplot, pROC, gplots, survival, ggbiplot, edgeR, survminer, RColorBrewer, magrittr, GenomicDataCommons, rtracklayer, grid, corrplot, scales, and limma.

### 2.2. RNA-Seq Data Procession

ESCC RNA-seq data containing 161 tumor samples and 11 normal samples from the TCGA database were retrieved with the software R. All databases were opensource and free to use.

### 2.3. RNA Expression Analysis

All RNA expression profiles were divided into high and low expression groups with Edge R and limma as previously described by Anders and Huber [18]. GENCODE was used to analyze lncRNA expressions. In this study, log2 FC ≥ 1.5 and the FDR < 0.01 were set as threshold.

### 2.4. ceRNA Network Construction

MiRWalk (http://mirwalk.umm.uni-heidelberg.de/) was selected to screen miRNAs and corresponding target genes. The miRNA-lncRNA correlation > 0.45 and *P* < 0.05 was set to filter out powerful correlations [19]. Cytoscape 3.6.3 was used to construct the network model. Cluster Profiler was used to do GO and KEGG analysis followed by methods previously described [20].

A schematic view of this study is shown in Supplementary Figure 1 (Figure S1). In brief, the key procedures of this study could be listed as follows:

1. Download mRNA, miRNA, and lncRNA data from the TCGA portal
2. Data filtering and gather DERNAs
3. Analyze all DERNAs and gather information of all possible ceRNA network connections. In this study, all published or validated proof of ceRNA network is taken into account for the following analysis. MicroRNA survival analysis followed by correlation analysis generate all possible core miRNAs
4. Adopt unsupervised clustering strategy to further filter DElncRNAs to a proper amount to build ceRNA network
5. Training the lncRNA panel

## 3. Survival Analysis

We referred to a previously described method for the construction of CESC prognosis prediction model [21]. Survival package was selected to generate survival plots, which were then used to evaluate the prediction power of the biomarker panel. Cox regression statistic procedures were processed to test the predictive value of the panel. ROC and AUC curves were plotted with the Daim package to evaluate model specificity and sensitivity [22].

## 4. Results

### 4.1. Database Download and Clinical Features

**Figure 1:**
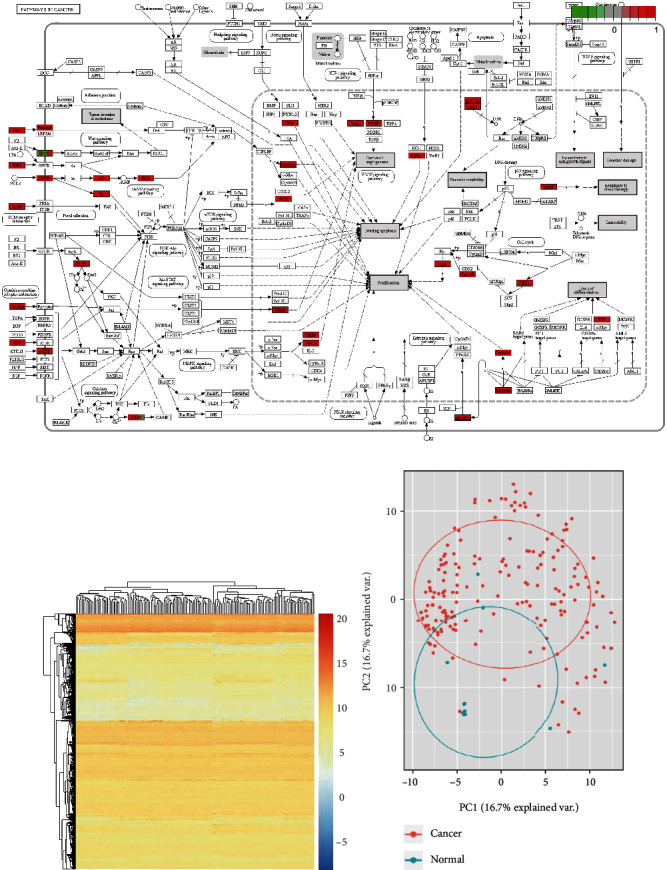
Transcriptome profiles in esophageal carcinoma. (a) Pathways of cancer. up or downregulated DEmRNAs were marked red or green, respectively. (b) The DEmRNAs were shown in heatmap. (c) PCA plot analysis of ESCA and normal samples based on DEmRNAs' expression.

All ESCC TCGA samples with level 2 RNA expression info were downloaded. The sample information was shown in Table S1. The transcriptome data of 161 ESCC samples and 11 normal samples were achieved. Log2 FC > 1.5 and *P* < 0.01 set as threshold. To help our understanding of the intracellular mechanisms which were involved in the development of tumor progression, KEGG analysis was also performed in the R-based ProfilerCluster package. The pathways in cancer (*P* < 0.01) were shown in Figure 1(a). Heatmap package was used to plot the heatmap with the top 1000 differentially expressed genes (Figure 1(b)), followed by PCA plots in Figure 1(c). Expression data were gathered in Table S2.

### 4.2. lncRNA and miRNA Profiles

**Figure 2:**
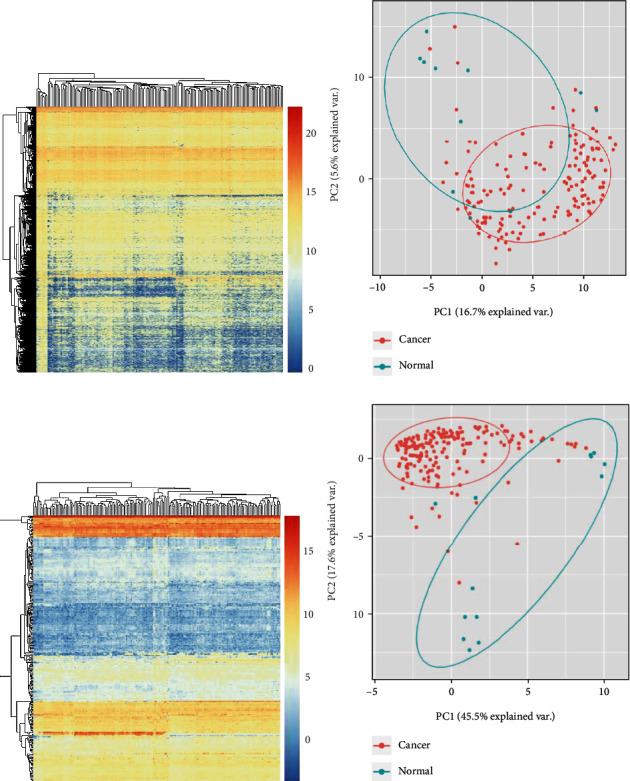
lncRNA and miRNA profiles in esophageal carcinoma. (a) The DElncRNAs were depicted in heatmap. (b) PCA plots of ESCA and normal samples based on DElncRNAs' expression. (c) The DEmiRNAs were shown in heatmap. (d) PCA plots of ESCA and normal samples based on DEmiRNAs' expression.

Log2 FC > 1.5 and *P* < 0.01 were set as threshold, as shown in Figure 2(a) (DEGs were shown in Table S3). Next, PCA plots were shown in Figure 2(b). Expression data were grouped into multiple categories based on clinical features to evaluate prognostic significance.

To generate ceRNA network, we further processed the miRNA expression data. Dysexpressed miRNAs (DEmiRNAs) were shown in Table S4. Heatmap was shown in Figure 2(c). The PCA plots were shown in Figure 2(d).

### 4.3. miRNA Survival Analysis

**Figure 3:**
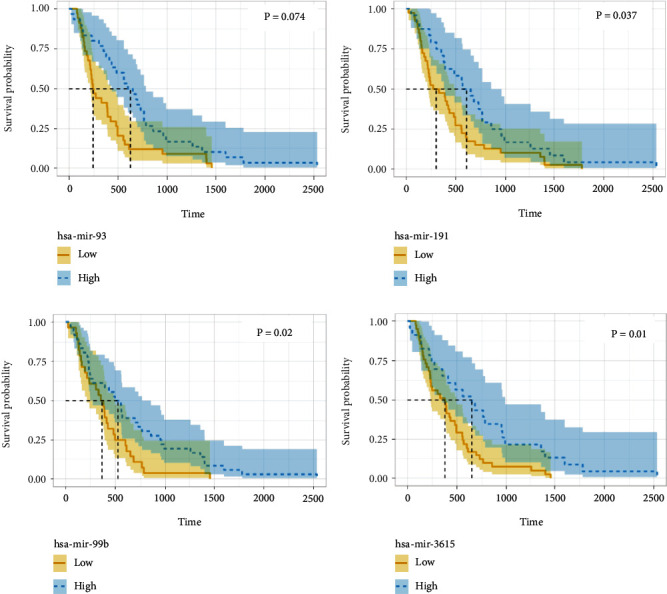
miRNA survival analysis in esophageal carcinoma. (a)–(d) The dysregulation of miR-93 (a), miR-191 (b), miR-99b (c), and miR-3615 (d) was correlated to overall survival in esophageal carcinoma.

As miRNAs form the core of ceRNA network, survival plots were generated to filter out the miRNAs with clinical significance. Significant correlation among four miRNAs, namely, miR-93, miR-191, miR-99b, and miR-3615 and the patients' OS were found (*P* < 0.05). To be more specific, all the four miRNAs were positively correlated with patients' OS (Figures 3(a)–3(d)).

### 4.4. Construction of ceRNA Network in Esophageal Carcinoma

**Figure 4:**
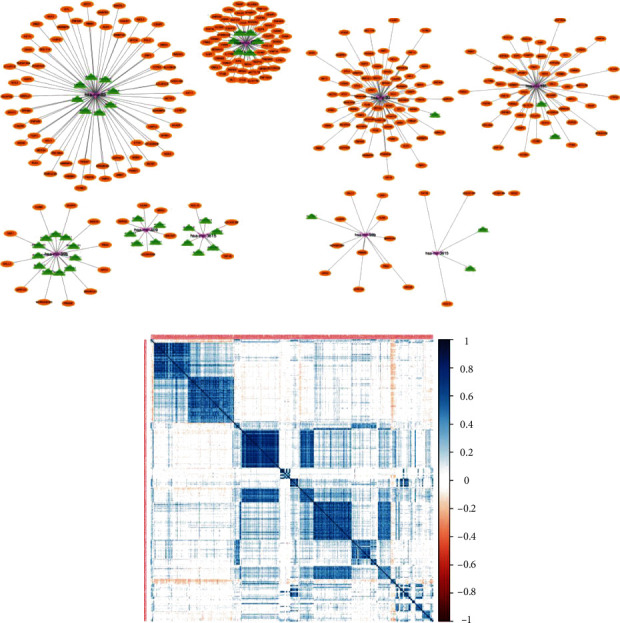
Construction of ceRNA network in esophageal squamous carcinoma. (a) the ceRNA network model. lncRNAs, miRNAs, and mRNAs are marked as green, brown, and purple, respectively. (b) After unsupervised clustering, we constructed an optimized lncRNA-miRNA-mRNA network in esophageal carcinoma. lncRNAs, miRNAs, and mRNAs were marked as green, purple, and brown, respectively. (c) miRNA-lncRNA network linear regression plots.

In the first step, we tried to find a hubLncRNA panel without training the lncRNA data. But the result showed that the smallest panel we could find contained as many as 50 hubLncRNAs, which were not appropriate for future application. The initial ceRNA network was shown in Figure 4(a). Then, we trained the lncRNAs data by unsupervised hierarchical clustering strategy. After unsupervised clustering, the number of lncRNAs was limited to a proper amount, and we constructed an initial ceRNA network that directly yielded four key miRNAs (Figure 4(b)). For instance, two lncRNAs, namely, LINC02575 and LINC01087 could competitively bind with miR-191. Two lncRNAs, namely, LINC01816 and AL136162.1, could competitively bind with miR-3615. One lncRNA, AC012073.1, could competitively bind with miR-93. One lncRNA, AC117402.1, could competitively bind with miR-99b. Next, DElncRNAs and DERNAs were used to build a linear regression model. Results proved a strong correlation between miRNAs, lncRNAs, and mRNAs (Figure 4(c)).

### 4.5. Generation of Prediction Model

**Figure 5:**
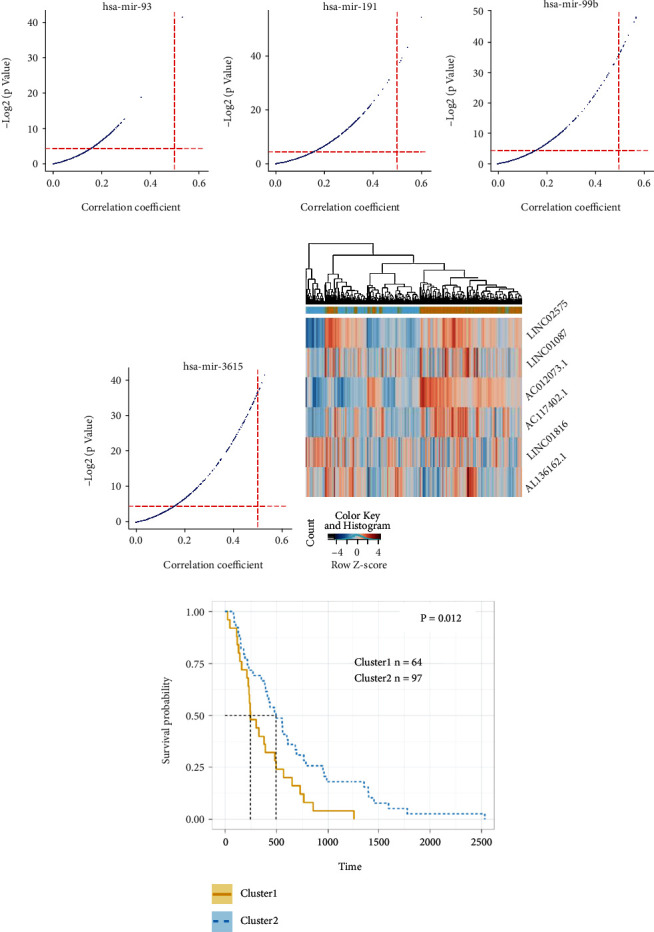
Statistical features of hublncRNAs in esophageal squamous carcinoma. (a)–(d) miRNA and lncRNA coefficiency plot analysis of miR-93 (a), miR-191 (b), miR-99b (c), and miR-3615 (d). (e) Heatmap plot analysis of six hub lncRNAs in ESCA and normal samples. (f) Survival plots of cluster 1 and cluster 2.

Topological analysis was selected to discover the potential hub lncRNAs in the construction of the prognosis prediction model (Figures 5(a)–5(d)). Finally, six hub lncRNAs were filtered out: *LINC02575*, *LINC01087*, *LINC01816*, *AL136162.1*, *AC012073.1*, and *AC117402.1*, and the interaction network of the hublncRNAs is shown in Table S5.

To clearly investigate the predictive power of the six hub genes, by unsupervised clustering strategy, all 161 patients were grouped into two clusters (Figure 5(e)). Survival analysis showed statistical significance (median OS, 494 vs. 247 days) (log-rank test *P* = 0.012; Figure 5(f)).

The survival data of the six HublncRNAs were taken to computing the patients' risk score which comes as below:

(1) $$\begin{array}{c} \text{Riskscore}=\left(-0.05084833\times \exp LINC02575\right)\pm \left(-0.07918478\times \exp LINC01087\right)\pm \left(0.08471654\times \exp AC012073.1\right)\pm \left(-0.00634161\times \exp AC117402.1\right)\pm \left(0.00279609\times \exp LINC01816\right)\pm \left(0.01202439\times \exp AL136162.1\right). \end{array}$$

Then, we analyzed the prediction model with all sample data via Cox regression analysis (Table 1).

### 4.6. Clinical Prognostic Value of Biomarker Panel

**Figure 6:**
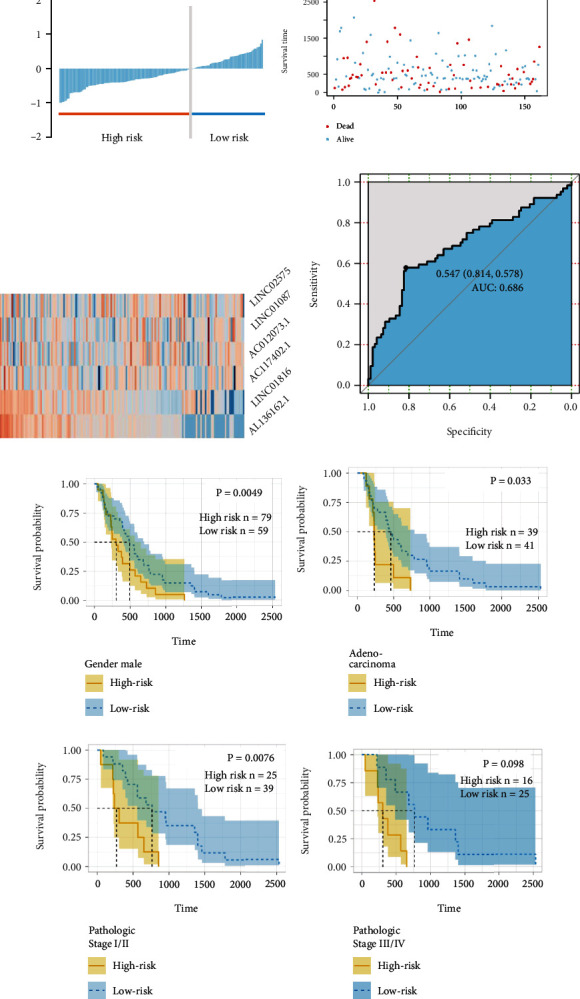
Clinical prognostic significance of biomarker panel. (a)–(c) Risk distribution plots of biomarker panel. Grey line indicated the cutoff value based on risk scores. (d) ROC plot analysis of biomarker panel in ESCC. (e) Overall survival plot analysis of high-risk and low-risk group in male esophageal cancer patients. (f) Overall survival plots analysis of high-risk and low-risk group in esophageal adenocarcinoma patients. (g) Overall survival plots analysis of high-risk and low-risk group in stage I/II esophageal cancer patients. (h) Overall survival plot analysis of high-risk and low-risk group in stage III/IV esophageal cancer patients.

Clinical relevances of all 161 samples were shown in Figures 6(a)–6(d). Six HublncRNAs were considered as key risk factors, including *LINC02575*, *LINC01087*, *LINC01816*, *AL136162.1*, *AC012073.1*, and *AC117402.1*. For example, the AUC value reached 0.686, which means a strong predictive power. When combined all together, the six lncRNAs constructed a powerful biomarker panel. For example, in male patients, the OS curves showed statistical significance (log-rank test *P* < 0.05, Figure 6(e)). AUC curve was shown in Figure 6(d). Furthermore, similar results could be found in stages I-II/III-IV as well (log-rank test *P* < 0.05, Figures 6(g)–6(h)).

## 5. Discussion

The ceRNA model is found on the basis of miRNA competitive binding. More and more evidence shows that these miRNAs and their competitive endogenous targets can form complex ceRNA networks [23].

Thanks to the free access to the comprehensive TCGA database, this study managed to develop an ESCC prognostic prediction biomarker panel. Our ceRNA network-based prediction model provided a deeper perspective into the intracellular mechanisms of ESCC.

As discussed in many studies, lncRNAs have great predict power in prognosis and diagnosis, which are sometimes even stronger than miRNAs and mRNAs [6, 17, 24, 25], some lncRNAs such as Fender have already been applied in practice. In esophageal cancer, several lncRNAs had been revealed to be related to the cancer prognosis and progression [26]. For example, overexpression of LEF1-AS1 predicts a poor prognosis in ESCC [26]. Li et al. reported an eight-lncRNA signature could predict the overall survival of ESCC [27]. Moreover, upregulation of HOTAIR was related to the poor prognosis of ESCC [28]. However, few studies on ceRNA have focused on predicting ESCC prognosis. Moreover, there are rare lncRNAs, mRNAs, or miRNAs related to ESCC could be treated as reliable biomarkers to detect ESCC and stratify the ESCC risk. Under this background and ceRNA network hypothesis, our study identified 95 dysexpressed lncRNAs, such as DIAPH3-AS1, NCBP2-AS1, ALG9-IT1, PRR7-AS1, SLC25A5-AS1, and TBL1XR1-AS1, which were strongly correlated with the progression of esophageal carcinoma. One lncRNA worth noting is LINC01087, it is the only lncRNA that has been reported to promote the cell division and metastasis of cancer [29]. As far as we know, the rest 5 lncRNAs are not reported to have prognostic value or molecular insights. One thing worth noting, at the beginning of this study, first, we tried to find a hub lncRNA panel without training the lncRNA data. But the result showed that the smallest panel we could find contained as many as 50 hub lncRNAs, which were not appropriate for future application. So we trained the lncRNA data by unsupervised hierarchical clustering strategy. Unsupervised clustering strategy has been adopted in many researches, for example, a multi-institutional study of the International Mesothelioma Panel from MESOPATH Reference Center proved that unsupervised clustering strategy is a powerful tool in achieving the predict accuracy of biomarker panel [30].

Given that any transcripts harbouring MREs could theoretically function as ceRNAs, as previously reported, they may represent in a widespread multiple forms of regulation in both pathology and physiology [31]. In this study, as shown in Figure 4(b), we found two lncRNAs, namely, LINC02575 and LINC01087, could competitively bind with miR-191. Two lncRNAs, namely, LINC01816 and AL136162.1, could competitively bind with miR-3615. One lncRNA, AC012073.1, could competitively bind with miR-93. One lncRNA, AC117402.1, could competitively bind with miR-99b. Among the core miRNAs found in this study, miR-191 has been previously reported in esophageal carcinoma which predicted poor prognosis and promoted cell proliferation and invasion [32]. Furthermore, miR-191 has been proved to be emerged as key participants of p53 signaling pathways in breast cancer [33]. miR-93 has been proved to promote cervical cancer progression by targeting THBS2/MMPS signal pathway [34]. miR-99b was previously reported to be a tumor suppressor by targeting IGF-1R in gastric cancer [35], while in this study, miR-99b is highly expressed in cancer patients and is supposed to be a tumor promoter. Moreover, miR-99b and miR-375 as a combination are found to be potential predictive response biomarker panel for preoperative chemoradiotherapy in rectal cancer [36]. miR-3615 was previously reported to be a potential oncogene in human cancer. For example, miR-3615 was overexpressed in liver cancer and related to the high TNM stage [37]. Although the ceRNA network we found in this study identifies many ESCC-related miRNAs, lncRNAs, and mRNAs, the correlation and the extent of ceRNA effects remain to be investigated by in vivo experiments. Recent studies have proved that by constitutive posttranscriptional regulations, binding free energy and repression feedbacks are pivotal factors for crosstalk between ceRNAs [38, 39], which means that there are many criteria for validating ceRNA networks, such as miRNAs and RBPs, kinetic parameters, the target ratio of miRNAs, the quantitative measurements of miRNAs, and the size and affinities of the competing targets [40, 41]. Moreover, we reported six lncRNAs which could be taken together as a panel for prognosis prediction of esophageal cancer, including LINC02575, LINC01087, LINC01816, AL136162.1, AC012073.1, and AC117402.1. Of note, several previous reports had implied the functional importance of these lncRNAs in cancers. For instance, LINC02575 was reported to promote the proliferation of laryngeal squamous cell carcinoma cells [42]. LINC01087 is highly expressed in breast cancer [43]. LINC01816 was found to promote the migration of thyroid carcinoma cells [44]. Therefore, our findings still need to be verified through in the future in vivo and in vitro experiments as well as clinical practice.

This study showed that the six lncRNAs together can be taken as a biomarker panel for the prognosis of esophageal cancer.

## 6. Conclusion

This study provides a novel biomarker for the prediction of prognosis to patients diagnosed with esophageal squamous carcinoma.

## Figures and Tables

**Table 1:** Univariate/multivariate Cox regression analysis in all patients.

Variables	Univariate statistics			Multivariate statistics		
	HR	95% CI of HR	*P* value	HR	95% CI of HR	*P* value
Patient age	1	0.98-1	0.96	0.99	0.97-1	0.616708
Stage						
Stage *T*1	0.63	0.32-1.8	0.048	0.2	0.023-1.8	0.153204
Stage *T*2	0.73	0.39-1.4	0.89	0.23	0.027-2	0.181557
Stage *T*3	1.6	0.94-2.8	0.083	0.39	0.048-3.2	0.382688
Stage *T*4	1.7	0.39-7	0.49	0.46	0.039-5.4	0.533969
Risk_score	0.55	0.32-0.95	0.031	0.52	0.28-0.97	0.0397278

Abbreviations: HR: hazard ratio; CI: confidence interval.

## Data Availability

The data used to support the findings of this study are included within the article. The data and materials in the current study are available from the corresponding author on reasonable request.

## Supplementary Materials

Supplementary Materials Supplementary Table 1: the sample information was shown in Table S1. Supplementary Table 2: expression data of genes were gathered in Table S2. Supplementary Table 3: differently expressed lncRNAs were shown in Table S3. Supplementary Table 4: differently expressed miRNAs were shown in Table S4. Supplementary Table 5: the interaction network of between lncRNAs and miRNAs is shown in Table S5.
