# Supplementary material for: Novel lncRNA Panel as for Prognosis in Esophageal Squamous Cell Carcinoma Based on ceRNA Network Mechanism
Source: Comput Math Methods Med. 2021 Sep 24;2021:8020879. doi: 10.1155/2021/8020879 (PMC8486540; doi:10.1155/2021/8020879)
Supplement: Supplementary Materials — Supplementary Table 1: the sample information was shown in Table S1. Supplementary Table 2: expression data of genes were gathered in Table S2. Supplementary Table 3: differently expressed lncRNAs were shown in Table S3. Supplementary Table 4: differently expressed miRNAs were shown in Table S4. Supplementary Table 5: the interaction network of between lncRNAs and miRNAs is shown in Table S5. [file 8020879.f1.zip › Table-S1.pdf]

Hybridization REF	Composite Element	years	vital_	days_to_death	days_to_last_followup	stage	T_stage	N_stage	M_stage	gender	date_of_initial_pathologic_diagnosis	radiation_therapy	karnofsky_performance_score	histological_type
tcga-ic-a6rf	value	69	0	NA	477	ia	t1	n0	m0	female	2013	no	NA	esophagus squamous cell carcinoma
tcga-jy-a6fb	value	77	0	NA	1837	i	t1	n0	m0	male	2007	no	NA	esophagus adenocarcinoma, nos
tcga-jy-a938	value	75	0	NA	1060	iib	t3	n0	m0	male	2012	no	NA	esophagus adenocarcinoma, nos
tcga-l5-a43i	value	77	1	556	NA	iiia	t3	n1	mx	male	2011	yes	NA	esophagus adenocarcinoma, nos
tcga-l5-a43j	value	90	1	131	NA	iib	t3	n0	mx	male	2011	NA	NA	esophagus squamous cell carcinoma
tcga-l5-a43m	value	84	0	NA	272	NA	t3	n2	mx	female	2009	no	NA	esophagus adenocarcinoma, nos
tcga-l5-a4oe	value	81	1	730	NA	iiib	t3	n2	NA	male	2011	no	NA	esophagus adenocarcinoma, nos

tcga-l5-a4og	value	79	0	NA	143	i	t1	n0	m0	female	2012	no	NA	esophagus adenocarcinoma, nos
tcga-l5-a4oi	value	79	0	NA	608	iiic	t3	n3	mx	male	2012	no	NA	esophagus adenocarcinoma, nos
tcga-l5-a88t	value	86	0	NA	694	iib	t2	n1	m0	male	2012	no	NA	esophagus adenocarcinoma, nos
tcga-l5-a88y	value	76	0	NA	11	NA	t1	n0	mx	male	2006	NA	NA	esophagus adenocarcinoma, nos
tcga-l5-a8ng	value	77	0	NA	1094	iii	t3	n1	m0	male	2001	no	NA	esophagus adenocarcinoma, nos
tcga-l5-a8nt	value	69	0	NA	825	iib	t3	n0	m0	male	2001	yes	NA	esophagus adenocarcinoma, nos
tcga-q9-a6fu	value	57	1	157	NA	iiib	t3	n2	m0	female	2012	no	NA	esophagus squamous cell carcinoma
tcga-s8-a6bv	value	76	0	NA	609	iiia	t3	n1	mx	male	2012	no	NA	esophagus adenocarcinoma, nos
tcga-2h-a9gf	value	67	1	784	NA	iii	t3	n1	m0	male	2001	no	NA	esophagus adenocarcinoma, nos

tcga-2h-a9gg	value	66	1	610	NA	iii	t3	n1	m0	male	1999	no	NA	esophagus adenocarcinoma, nos
tcga-2h-a9gh	value	44	1	951	NA	iib	t1	n1	m0	male	1998	no	NA	esophagus adenocarcinoma, nos
tcga-2h-a9gi	value	68	1	435	NA	iii	t3	n1	m0	male	1999	no	NA	esophagus adenocarcinoma, nos
tcga-2h-a9gj	value	57	1	###	NA	i	t1	n0	m0	male	2000	no	NA	esophagus adenocarcinoma, nos
tcga-2h-a9gk	value	43	1	232	NA	iii	t3	n1	m0	male	2000	no	NA	esophagus adenocarcinoma, nos
tcga-2h-a9gl	value	74	1	180	NA	iii	t3	n1	m0	male	2000	no	NA	esophagus adenocarcinoma, nos
tcga-2h-a9gm	value	53	1	424	NA	iib	t1	n1	m0	male	2000	no	NA	esophagus adenocarcinoma, nos
tcga-2h-a9gn	value	70	1	272	NA	iii	t3	n1	m0	male	2000	no	NA	esophagus adenocarcinoma, nos
tcga-2h-a9go	value	58	1	494	NA	iva	t3	n1	m1a	male	2000	no	NA	esophagus adenocarcinoma, nos

tcga-2h-a9gq	value	80	1	128	NA	iii	t3	n1	m0	male	2001	no	NA	esophagus adenocarcinoma, nos
tcga-2h-a9gr	value	80	1	987	NA	iia	t3	n0	m0	male	1999	no	NA	esophagus adenocarcinoma, nos
tcga-ic-a6re	value	59	0	NA	234	iib	t1	n1	m0	male	2013	no	NA	esophagus adenocarcinoma, nos
tcga-ig-a3i8	value	51	0	NA	1012	iia	t3	n0	m0	female	2011	no	80	esophagus squamous cell carcinoma
tcga-ig-a3ql	value	54	0	NA	1071	iia	t2	n0	m0	male	2011	no	80	esophagus squamous cell carcinoma
tcga-ig-a3y9	value	72	1	26	NA	iiia	t4	n0	m0	male	2012	no	80	esophagus squamous cell carcinoma
tcga-ig-a3ya	value	53	0	NA	632	iiia	t4	n0	m0	male	2012	no	80	esophagus squamous cell carcinoma
tcga-ig-a3yb	value	61	0	NA	80	iiia	t3	n1	m0	male	2012	yes	80	esophagus squamous cell carcinoma
tcga-ig-a3yc	value	62	0	NA	612	iiia	t3	n1	m0	male	2012	no	80	esophagus squamous cell carcinoma

tcga-ig-a4p3	value	48	1	567	NA	iib	t2	n1	m0	male	2012	yes	NA	esophagus squamous cell carcinoma
tcga-ig-a4qs	value	71	1	118	NA	iiib	t3	n2	m0	male	2012	no	NA	esophagus adenocarcinoma, nos
tcga-ig-a4qt	value	56	1	283	NA	iia	t3	n0	m0	male	2012	yes	NA	esophagus squamous cell carcinoma
tcga-ig-a50l	value	58	0	NA	16	iiia	t3	n1	m0	male	2012	NA	NA	esophagus squamous cell carcinoma
tcga-ig-a51d	value	63	0	NA	518	iib	t1	n1	m0	male	2012	no	80	esophagus squamous cell carcinoma
tcga-ig-a5b8	value	72	1	24	NA	ib	t3	n0	m0	male	2012	no	NA	esophagus squamous cell carcinoma
tcga-ig-a5s3	value	69	0	NA	712	iib	t3	n0	m0	female	2012	no	80	esophagus squamous cell carcinoma
tcga-ig-a625	value	60	1	390	NA	iiib	t3	n2	m0	male	2012	NA	NA	esophagus squamous cell carcinoma
tcga-ig-a6qs	value	54	1	303	NA	iib	t2	n1	m0	male	2013	no	NA	esophagus squamous cell carcinoma

tcga-ig-a7dp	value	50	0	NA	452	iiia	t4a	nx	mx	female	2013	no	90	esophagus adenocarcinoma, nos
tcga-ig-a8o2	value	62	1	142	NA	iiib	t3	n2	m0	male	2013	NA	NA	esophagus squamous cell carcinoma
tcga-ig-a97h	value	36	0	NA	441	iia	t3	nx	m0	male	2013	NA	NA	esophagus squamous cell carcinoma
tcga-ig-a97i	value	58	0	NA	370	iia	t2	n0	m0	male	2013	yes	NA	esophagus squamous cell carcinoma
tcga-jy-a6f8	value	56	0	NA	3714	i	t1	n0	m0	male	2003	no	NA	esophagus adenocarcinoma, nos
tcga-jy-a6fa	value	51	1	###	NA	iib	t2	n1	m0	male	2006	no	NA	esophagus squamous cell carcinoma
tcga-jy-a6fd	value	51	0	NA	2069	iia	t3	n0	m0	female	2008	no	NA	esophagus squamous cell carcinoma
tcga-jy-a6fe	value	49	1	112	NA	iii	t3	n1	m0	male	2009	no	NA	esophagus squamous cell carcinoma
tcga-jy-a6fg	value	50	1	###	NA	iii	t3	n1	m0	male	2009	no	NA	esophagus squamous cell carcinoma

tcga-jy-a6fh	value	53	0	NA	1441	iib	t2	n1	m0	male	2010	no	NA	esophagus adenocarcinoma, nos
tcga-jy-a939	value	77	0	NA	660	iib	t1	n1	m0	male	2012	no	NA	esophagus adenocarcinoma, nos
tcga-jy-a93c	value	47	0	NA	705	iiib	t3	n2	m0	male	2011	no	NA	esophagus adenocarcinoma, nos
tcga-jy-a93d	value	51	1	960	NA	iiic	t2	n3	m0	male	2012	yes	NA	esophagus adenocarcinoma, nos
tcga-jy-a93e	value	61	0	NA	767	iiia	t3	n1	m0	male	2012	no	NA	esophagus adenocarcinoma, nos
tcga-jy-a93f	value	58	0	NA	731	ib	t2	n0	m0	female	2012	no	NA	esophagus squamous cell carcinoma
tcga-kh-a6wc	value	82	0	NA	191	ia	t1	n0	m0	male	2013	yes	70	esophagus squamous cell carcinoma
tcga-l5-a43c	value	81	0	NA	96	NA	NA	NA	NA	male	2009	no	NA	esophagus adenocarcinoma, nos
tcga-l5-a43e	value	74	0	NA	920	i	t1	n0	m0	male	2011	no	NA	esophagus adenocarcinoma, nos

tcga-l5-a43h	value	75	1	9	NA	iii	t3	n1	m0	male	2011	no	NA	esophagus squamous cell carcinoma
tcga-l5-a4of	value	63	1	801	NA	iib	t1	n1	NA	male	2012	no	NA	esophagus adenocarcinoma, nos
tcga-l5-a4oh	value	71	0	NA	992	i	t1	n0	m0	male	2012	no	NA	esophagus adenocarcinoma, nos
tcga-l5-a4oj	value	70	0	NA	639	i	t1	n0	m0	female	2012	no	NA	esophagus adenocarcinoma, nos
tcga-l5-a4om	value	54	1	###	NA	ia	t1	n0	NA	female	2010	no	NA	esophagus squamous cell carcinoma
tcga-l5-a4on	value	65	1	558	NA	iib	t1	n1	NA	male	2010	NA	NA	esophagus adenocarcinoma, nos
tcga-l5-a4oo	value	75	0	NA	101	iiic	t3	n3	m0	male	2010	NA	NA	esophagus adenocarcinoma, nos
tcga-l5-a4op	value	67	0	NA	218	ia	t1	n0	mx	female	2010	NA	NA	esophagus adenocarcinoma, nos
tcga-l5-a4oq	value	75	1	42	NA	iiia	t2	n2	mx	male	2010	no	NA	esophagus adenocarcinoma, nos

tcga-l5-a4or	value	83	1	96	NA	ia	t1	n0	mx	male	2010	no	NA	esophagus adenocarcinoma, nos
tcga-l5-a4os	value	86	0	NA	1782	iib	t2	n1	m0	female	2010	no	NA	esophagus adenocarcinoma, nos
tcga-l5-a4ot	value	77	1	149	NA	iv	t3	n1	m1a	male	2006	no	NA	esophagus adenocarcinoma, nos
tcga-l5-a4ou	value	81	0	NA	882	iia	t3	n0	m0	male	2009	no	NA	esophagus adenocarcinoma, nos
tcga-l5-a4ow	value	56	1	217	NA	iib	t2	n1	m0	female	2006	no	NA	esophagus adenocarcinoma, nos
tcga-l5-a4ox	value	60	1	226	NA	iib	t2	n1	m0	male	2006	no	NA	esophagus adenocarcinoma, nos
tcga-l5-a88s	value	84	0	NA	471	ib	t3	n0	mx	male	2013	no	NA	esophagus squamous cell carcinoma
tcga-l5-a88v	value	60	0	NA	79	iii	t3	n1	mx	male	2007	no	NA	esophagus adenocarcinoma, nos
tcga-l5-a88w	value	67	1	764	NA	iia	t3	n0	mx	male	2007	yes	NA	esophagus squamous cell carcinoma

tcga-l5-a88z	value	70	0	NA	225	iia	t1	n1	m0	female	2005	no	NA	esophagus squamous cell carcinoma
tcga-l5-a891	value	51	0	NA	114	NA	t3	n1	m0	male	2005	NA	NA	esophagus adenocarcinoma, nos
tcga-l5-a893	value	71	0	NA	92	i	t1	n0	m0	female	2005	no	NA	esophagus adenocarcinoma, nos
tcga-l5-a8ne	value	77	0	NA	1688	iib	t2	n1	m0	male	2000	no	NA	esophagus adenocarcinoma, nos
tcga-l5-a8nf	value	57	1	81	NA	iva	t1	n0	m1a	male	2001	no	NA	esophagus adenocarcinoma, nos
tcga-l5-a8nh	value	54	1	393	NA	iv	t1	n0	m1	male	2001	NA	NA	esophagus adenocarcinoma, nos
tcga-l5-a8ni	value	79	1	410	NA	iii	t3	n1	m0	male	2004	no	NA	esophagus adenocarcinoma, nos
tcga-l5-a8nj	value	77	0	NA	501	iii	t3	n1	m0	male	2000	no	NA	esophagus adenocarcinoma, nos
tcga-l5-a8nk	value	84	0	NA	412	iia	t3	n0	m0	female	2004	NA	NA	esophagus squamous cell carcinoma

tcga-l5-a8nl	value	56	0	NA	402	iii	t3	n1	m0	male	2004	NA	NA	esophagus adenocarcinoma, nos
tcga-l5-a8nm	value	84	1	236	NA	iib	t2	n1	m0	female	2004	no	NA	esophagus adenocarcinoma, nos
tcga-l5-a8nn	value	81	0	NA	167	iii	t3	n1	m0	male	2004	no	NA	esophagus adenocarcinoma, nos
tcga-l5-a8nq	value	71	1	650	NA	iia	t2	n0	m0	male	2001	no	NA	esophagus squamous cell carcinoma
tcga-l5-a8nr	value	81	0	NA	265	iii	t3	n1	m0	female	2001	no	NA	esophagus adenocarcinoma, nos
tcga-l5-a8ns	value	76	0	NA	408	iib	t3	n0	m0	male	2001	yes	NA	esophagus adenocarcinoma, nos
tcga-l5-a8nu	value	84	1	###	NA	iia	t2	n0	m0	male	2001	NA	NA	esophagus adenocarcinoma, nos
tcga-l5-a8nv	value	75	1	###	NA	iia	t3	n0	m0	male	2001	no	NA	esophagus adenocarcinoma, nos
tcga-l5-a8nw	value	55	1	###	NA	ib	t2	n1	m0	male	2001	no	NA	esophagus adenocarcinoma, nos

tcga-l7-a56g	value	66	1	330	NA	NA	NA	NA	NA	male	2012	yes	NA	esophagus squamous cell carcinoma
tcga-l7-a6vz	value	62	0	NA	315	iiic	t3	n3	mx	male	2013	yes	NA	esophagus adenocarcinoma, nos
tcga-ln-a49k	value	66	1	180	NA	iia	t3	n0	m0	male	2010	no	60	esophagus squamous cell carcinoma
tcga-ln-a49l	value	44	1	318	NA	iia	t2	n0	m0	male	2010	yes	60	esophagus squamous cell carcinoma
tcga-ln-a49m	value	62	0	NA	385	iia	t2	n0	m0	male	2010	yes	60	esophagus squamous cell carcinoma
tcga-ln-a49n	value	50	0	NA	378	iib	t2	n1	m0	male	2010	no	80	esophagus squamous cell carcinoma
tcga-ln-a49o	value	47	0	NA	408	iia	t3	n0	m0	male	2011	yes	60	esophagus squamous cell carcinoma
tcga-ln-a49p	value	71	0	NA	375	iia	t3	n0	m0	male	2011	yes	40	esophagus squamous cell carcinoma
tcga-ln-a49r	value	46	0	NA	407	iii	t3	n1	m0	male	2011	yes	60	esophagus squamous cell carcinoma

tcga-ln-a49s	value	59	0	NA	400	iia	t3	n0	m0	male	2011	yes	60	esophagus squamous cell carcinoma
tcga-ln-a49u	value	62	0	NA	467	iia	t3	n0	m0	male	2011	yes	60	esophagus squamous cell carcinoma
tcga-ln-a49v	value	49	0	NA	383	iia	t3	n0	m0	male	2011	yes	60	esophagus squamous cell carcinoma
tcga-ln-a49w	value	73	0	NA	403	iii	t3	n1	m0	male	2011	yes	40	esophagus squamous cell carcinoma
tcga-ln-a49x	value	44	0	NA	384	iia	t3	n0	m0	male	2011	yes	60	esophagus squamous cell carcinoma
tcga-ln-a49y	value	77	0	NA	379	iia	t3	n0	m0	male	2011	no	20	esophagus squamous cell carcinoma
tcga-ln-a4a1	value	60	0	NA	383	iia	t3	n0	m0	male	2011	no	60	esophagus squamous cell carcinoma
tcga-ln-a4a2	value	57	0	NA	380	iia	t3	n0	m0	male	2011	yes	60	esophagus squamous cell carcinoma
tcga-ln-a4a3	value	61	0	NA	388	iii	t3	n1	m0	male	2011	yes	60	esophagus squamous cell carcinoma

tcga-ln-a4a4	value	36	0	NA	383	iii	t3	n1	m0	male	2011	yes	60	esophagus squamous cell carcinoma
tcga-ln-a4a5	value	49	1	681	NA	iia	t2	n0	m0	male	2011	no	60	esophagus squamous cell carcinoma
tcga-ln-a4a6	value	65	0	NA	391	ii	t2	n0	m0	male	2012	no	40	esophagus squamous cell carcinoma
tcga-ln-a4a8	value	52	0	NA	472	iia	t2	n0	m0	male	2012	no	60	esophagus squamous cell carcinoma
tcga-ln-a4a9	value	58	1	351	NA	iia	t2	n0	m0	male	2012	no	60	esophagus squamous cell carcinoma
tcga-ln-a4mq	value	46	0	NA	375	iii	t3	n1	m0	male	2010	no	60	esophagus squamous cell carcinoma
tcga-ln-a4mr	value	57	0	NA	402	iia	t2	n0	m0	male	2012	no	60	esophagus squamous cell carcinoma
tcga-ln-a5u5	value	57	1	136	NA	iv	t3	n1	m1	male	2012	no	70	esophagus squamous cell carcinoma
tcga-ln-a5u6	value	54	0	NA	375	iib	t2	n1	m0	male	2012	yes	70	esophagus squamous cell carcinoma

tcga-ln-a5u7	value	46	0	NA	768	iia	t2	n0	m0	male	2012	no	70	esophagus squamous cell carcinoma
tcga-ln-a7hv	value	58	0	NA	320	iia	t2	n0	m0	male	2012	yes	70	esophagus squamous cell carcinoma
tcga-ln-a7hw	value	59	0	NA	365	iia	t2	n0	m0	male	2013	no	70	esophagus squamous cell carcinoma
tcga-ln-a7hx	value	72	0	NA	372	iia	t2	n0	m0	male	2013	yes	50	esophagus squamous cell carcinoma
tcga-ln-a7hy	value	50	0	NA	366	iii	t3	n1	m0	male	2013	no	70	esophagus squamous cell carcinoma
tcga-ln-a7hz	value	49	0	NA	401	iia	t2	n0	m0	male	2013	no	90	esophagus squamous cell carcinoma
tcga-ln-a8hz	value	56	0	NA	375	iia	t2	n0	m0	male	2013	yes	NA	esophagus squamous cell carcinoma
tcga-ln-a8i0	value	52	0	NA	407	iia	t2	n0	m0	male	2013	no	NA	esophagus squamous cell carcinoma
tcga-ln-a8i1	value	67	0	NA	401	iia	t2	n0	m0	female	2013	yes	NA	esophagus squamous cell carcinoma

tcga-ln-a9fo	value	42	0	NA	4	iia	t2	n0	m0	male	2013	no	NA	esophagus squamous cell carcinoma
tcga-ln-a9fp	value	60	0	NA	366	iia	t2	n0	m0	female	2013	yes	NA	esophagus squamous cell carcinoma
tcga-ln-a9fq	value	62	0	NA	391	iia	t3	n0	m0	male	2013	no	NA	esophagus squamous cell carcinoma
tcga-ln-a9fr	value	70	0	NA	373	iib	t2	n1	m0	male	2013	yes	NA	esophagus squamous cell carcinoma
tcga-m9-a5m8	value	58	0	NA	1007	iia	t3	n0	m0	male	NA	no	NA	esophagus adenocarcinoma, nos
tcga-q9-a6fw	value	61	0	NA	238	iiib	t3	n2	m0	male	2013	no	NA	esophagus adenocarcinoma, nos
tcga-r6-a6dn	value	58	1	244	NA	NA	NA	NA	NA	male	2011	NA	NA	esophagus adenocarcinoma, nos
tcga-r6-a6dq	value	74	1	231	NA	NA	NA	NA	NA	female	2010	NA	NA	esophagus adenocarcinoma, nos
tcga-r6-a6kz	value	42	1	154	NA	NA	NA	NA	NA	male	2010	no	NA	esophagus adenocarcinoma, nos

tcga-r6-a6l4	value	27	1	496	NA	NA	NA	NA	NA	male	2012	no	NA	esophagus adenocarcinoma, nos
tcga-r6-a6l6	value	68	1	214	NA	NA	NA	NA	NA	male	2010	no	NA	esophagus adenocarcinoma, nos
tcga-r6-a6xg	value	64	0	NA	1168	NA	NA	NA	NA	male	2011	no	NA	esophagus adenocarcinoma, nos
tcga-r6-a6xq	value	58	1	193	NA	NA	NA	NA	NA	male	2008	no	NA	esophagus adenocarcinoma, nos
tcga-r6-a6y0	value	54	0	NA	1641	NA	NA	NA	NA	male	2005	no	NA	esophagus adenocarcinoma, nos
tcga-r6-a6y2	value	71	1	283	NA	NA	NA	NA	NA	male	2005	no	NA	esophagus adenocarcinoma, nos
tcga-r6-a8w5	value	60	1	480	NA	iva	t3	n1	m1a	male	2004	no	NA	esophagus adenocarcinoma, nos
tcga-r6-a8w8	value	72	1	88	NA	NA	NA	NA	NA	male	2012	no	NA	esophagus adenocarcinoma, nos
tcga-r6-a8wc	value	56	0	NA	70	NA	NA	NA	NA	male	2013	NA	NA	esophagus adenocarcinoma, nos

tcga-r6-a8wg	value	60	1	386	NA	NA	NA	NA	NA	male	2013	no	NA	esophagus adenocarcinoma, nos
tcga-re-a7bo	value	72	1	213	NA	iib	t1	n1	m0	female	2006	no	NA	esophagus adenocarcinoma, nos
tcga-s8-a6bw	value	51	0	NA	620	ib	t2	n0	mx	male	2012	no	NA	esophagus squamous cell carcinoma
tcga-v5-a7rb	value	59	1	161	NA	NA	t0	n1	mx	male	NA	no	90	esophagus adenocarcinoma, nos
tcga-v5-a7rc	value	55	1	104	NA	NA	NA	NA	NA	male	NA	yes	80	esophagus squamous cell carcinoma
tcga-v5-a7re	value	45	0	NA	500	ib	t1	n0	m0	male	NA	yes	90	esophagus adenocarcinoma, nos
tcga-v5-aasv	value	67	0	NA	467	iib	t3	n0	mx	male	NA	yes	80	esophagus squamous cell carcinoma
tcga-v5-aasw	value	72	0	NA	282	NA	NA	NA	NA	male	NA	yes	60	esophagus adenocarcinoma, nos
tcga-v5-aasx	value	74	0	NA	273	NA	NA	NA	NA	male	NA	NA	90	esophagus adenocarcinoma, nos

tcga-vr-a8eo	value	49	0	NA	785	iia	t3	n0	m0	male	2013	yes	90	esophagus squamous cell carcinoma
tcga-vr-a8ep	value	51	0	NA	824	iiib	t3	n2	m0	male	2013	no	90	esophagus squamous cell carcinoma
tcga-vr-a8eq	value	73	1	694	NA	iii	t3	n1	m0	male	2009	yes	90	esophagus adenocarcinoma, nos
tcga-vr-a8er	value	54	1	378	NA	iii	t4	n1	m0	male	2009	yes	80	esophagus squamous cell carcinoma
tcga-vr-a8et	value	64	1	47	NA	iia	t2	n0	m0	male	2010	no	90	esophagus squamous cell carcinoma
tcga-vr-a8eu	value	51	1	557	NA	iv	t1	n1	m1	male	2011	yes	80	esophagus squamous cell carcinoma
tcga-vr-a8ew	value	57	1	247	NA	iiib	t3	n2	m0	male	2012	no	100	esophagus squamous cell carcinoma
tcga-vr-a8ex	value	63	1	855	NA	iva	t1	n1	m1a	male	2009	no	90	esophagus squamous cell carcinoma
tcga-vr-a8ey	value	44	0	NA	1025	iia	t3	n0	m0	female	2012	no	90	esophagus squamous cell carcinoma

tcga-vr-a8ez	value	47	1	553	NA	iiic	t3	n3	m0	male	2013	no	90		esophagus squamous cell carcinoma
tcga-vr-a8q7	value	60	0	NA	1590	iiia	t3	n1	m0	male	2010	no	90		esophagus squamous cell carcinoma
tcga-vr-aa4d	value	53	1	###	NA	iib	t1	n1	m0	male	2010	yes	90		esophagus adenocarcinoma, nos
tcga-vr-aa4g	value	51	0	NA	549	iiia	t2	n2	m0	female	2013	no	90		esophagus squamous cell carcinoma
tcga-vr-aa7b	value	65	0	NA	342	iv	t3	n3	m1	female	2011	no	90		esophagus squamous cell carcinoma
tcga-vr-aa7d	value	58	1	279	NA	iiic	t3	n3	m0	male	2012	yes	90		esophagus squamous cell carcinoma
tcga-vr-aa7i	value	70	1	484	NA	iii	t4	n0	m0	male	2010	no	90		esophagus squamous cell carcinoma
tcga-x8-aaar	value	69	0	NA	554	NA	t1	n1	NA	male	2013	no	NA		esophagus adenocarcinoma, nos
tcga-xp-a8t6	value	54	1	763	NA	iib	t2	n1	m0	male	2009	no	90		esophagus squamous cell carcinoma

tcga-xp-a8t7	value	63	0	NA	1254	iia	t2	n0	m0	female	2010	no	90	esophagus squamous cell carcinoma
tcga-xp-a8t8	value	49	0	NA	437	iib	t1	n1	m0	male	2011	yes	90	esophagus squamous cell carcinoma
tcga-z6-a8jd	value	53	0	NA	104	iib	t3	n0	m0	male	2013	no	90	esophagus squamous cell carcinoma
tcga-z6-a8je	value	57	0	NA	64	iiia	t3	n1	m0	male	2013	no	80	esophagus squamous cell carcinoma
tcga-z6-a9vb	value	53	0	NA	40	iiia	t3	n1	m0	male	2013	no	NA	esophagus squamous cell carcinoma
tcga-z6-aapn	value	57	0	NA	81	iia	t3	n0	m0	male	2013	no	NA	esophagus squamous cell carcinoma
tcga-zr-a9cj	value	65	1	600	NA	iiic	t3	n3	mx	male	2012	no	NA	esophagus adenocarcinoma, nos
