# Supplementary material for: Novel lncRNA Panel as for Prognosis in Esophageal Squamous Cell Carcinoma Based on ceRNA Network Mechanism
Source: Comput Math Methods Med. 2021 Sep 24;2021:8020879. doi: 10.1155/2021/8020879 (PMC8486540; doi:10.1155/2021/8020879)
Supplement: Supplementary Materials — Supplementary Table 1: the sample information was shown in Table S1. Supplementary Table 2: expression data of genes were gathered in Table S2. Supplementary Table 3: differently expressed lncRNAs were shown in Table S3. Supplementary Table 4: differently expressed miRNAs were shown in Table S4. Supplementary Table 5: the interaction network of between lncRNAs and miRNAs is shown in Table S5. [file 8020879.f1.zip › Table-S2.pdf]

	logFC	AveExpr	t	PValue	FDR	B
ZBTB16	-5.708	0.478328	-16.2072	4.00E-38	5.59E-34	75.82938
GREM2	-5.19514	0.404614	-15.5538	3.88E-36	2.71E-32	71.30432
DPT	-4.87027	1.378178	-15.1062	9.00E-35	4.19E-31	68.29138
GPER	-3.87544	1.474592	-14.7611	1.02E-33	3.57E-30	65.8634
AFF3	-4.31782	0.635851	-14.4673	8.13E-33	2.27E-29	63.76381
NCAM1	-4.77437	0.999751	-14.3925	1.38E-32	3.21E-29	63.31673
FAM107A	-4.40799	1.547215	-14.0927	1.14E-31	2.28E-28	61.25766
KLF15	-4.52699	0.455686	-13.9093	4.17E-31	7.29E-28	59.89729
P2RY14	-3.51307	0.823192	-13.8114	8.33E-31	1.29E-27	59.16148
GPR133	-4.65729	1.080201	-13.4221	1.30E-29	1.82E-26	56.58294
CLEC3B	-3.61456	1.433502	-13.2327	4.95E-29	6.28E-26	55.24911
GNG7	-3.49441	1.479336	-13.0738	1.52E-28	1.77E-25	54.14625
KL	-3.54113	0.396847	-12.7412	1.58E-27	1.70E-24	51.69477
RBPMS2	-3.79491	2.018109	-12.6697	2.61E-27	2.56E-24	51.38372
PTGER3	-4.4979	1.581038	-12.6622	2.75E-27	2.56E-24	51.33001
SLC2A4	-3.99188	0.948888	-12.5618	5.57E-27	4.86E-24	50.59542
CAB39L	-2.82554	3.290735	-12.4631	1.11E-26	9.15E-24	49.96318
ABCA8	-5.09215	0.327674	-12.3578	2.33E-26	1.81E-23	49.1943
C7	-6.0889	1.055167	-12.0999	1.42E-25	1.04E-22	47.44555
ADAMTSL1	-3.834	1.558904	-11.98	3.28E-25	2.29E-22	46.61452
SCUBE2	-3.83871	1.113053	-11.7518	1.61E-24	1.07E-21	45.03468
MAMDC2	-4.17594	1.241133	-11.7185	2.03E-24	1.29E-21	44.81886
PLIN4	-3.9591	2.045846	-11.6823	2.61E-24	1.59E-21	44.57969
ZNF471	-3.3718	0.194441	-11.6086	4.36E-24	2.54E-21	43.90245
GFRA1	-4.31582	1.635311	-11.5412	6.96E-24	3.89E-21	43.6133
GPR155	-3.91064	4.068626	-11.52	8.07E-24	4.34E-21	43.46693
SLC16A7	-3.53822	1.346629	-11.4204	1.61E-23	8.03E-21	42.7754
CARNS1	-3.06796	0.719505	-11.4255	1.55E-23	8.03E-21	42.71662
PNPLA7	-3.38267	1.82562	-11.2768	4.35E-23	2.03E-20	41.80761
STX12	-1.82105	5.763368	-11.2768	4.35E-23	2.03E-20	41.80178
C3orf18	-2.64284	0.801599	-11.2479	5.31E-23	2.39E-20	41.47024
HSPB7	-4.25221	1.659471	-11.2411	5.57E-23	2.43E-20	41.56719
PHYHD1	-3.55307	1.147454	-11.1283	1.21E-22	5.13E-20	40.78756
MYOCD	-4.23868	1.072564	-11.1139	1.34E-22	5.50E-20	40.69996
GPR146	-2.12322	1.722042	-11.0883	1.60E-22	6.37E-20	40.47523
LYVE1	-3.33822	1.084444	-10.9395	4.44E-22	1.71E-19	39.50407
RNF180	-2.91672	0.93131	-10.9371	4.52E-22	1.71E-19	39.44744
OGN	-4.24617	1.397064	-10.8644	7.44E-22	2.73E-19	39.01666
MLYCD	-1.42351	3.137478	-10.85	8.21E-22	2.94E-19	38.91843
LONRF2	-4.62299	1.310689	-10.7745	1.38E-21	4.81E-19	38.41042
MKI67	3.344343	7.859228	10.76926	1.43E-21	4.86E-19	38.37502
CRY2	-1.79908	4.838847	-10.7612	1.51E-21	5.01E-19	38.3024
PARK2	-3.10299	0.034613	-10.7275	1.90E-21	6.17E-19	37.91832
ATOH8	-3.13148	0.813063	-10.7162	2.05E-21	6.51E-19	37.97458
RGMB	-2.48761	5.195186	-10.671	2.79E-21	8.67E-19	37.69679
CGNL1	-3.65256	3.107821	-10.6104	4.22E-21	1.28E-18	37.29552
NEGR1	-3.38341	1.849295	-10.5972	4.62E-21	1.37E-18	37.22092
TPX2	3.843326	6.80741	10.56339	5.81E-21	1.69E-18	36.92729
SLC25A4	-2.43131	4.299226	-10.5459	6.55E-21	1.87E-18	36.85461
HIF3A	-4.23415	1.38313	-10.4624	1.16E-20	3.23E-18	36.31908
CCL14	-3.91193	0.464062	-10.4331	1.41E-20	3.86E-18	36.11123
KIF4B	3.411734	0.008927	10.38465	1.96E-20	5.26E-18	34.26781
C7orf41	-2.5795	4.633811	-10.3242	2.95E-20	7.77E-18	35.37135
CHRDL1	-5.06559	0.765084	-10.2738	4.15E-20	1.07E-17	35.06459
RYR2	-3.93202	1.154009	-10.2377	5.29E-20	1.34E-17	34.82663
PGM5	-4.2535	1.969816	-10.2258	5.73E-20	1.43E-17	34.73675
FAM165B	-1.88439	2.57935	-10.2127	6.26E-20	1.53E-17	34.66146

DUSP19	-2.25061	2.200643	-10.2059	6.55E-20	1.58E-17	34.61628
RAB11FIP2	-1.84422	4.754311	-10.169	8.40E-20	1.99E-17	34.33703
HSPB6	-4.33774	3.218414	-10.1576	9.07E-20	2.11E-17	34.27331
ITGA8	-4.04988	1.151921	-10.1384	1.03E-19	2.37E-17	34.16976
ZNF626	-3.15625	0.868221	-10.0996	1.34E-19	3.02E-17	33.89849
FAM189A2	-3.77585	0.55737	-10.0339	2.09E-19	4.62E-17	33.4747
KIT	-3.35998	2.825792	-10.0127	2.40E-19	5.19E-17	33.31817
RGS11	-3.29521	0.023985	-10.0119	2.42E-19	5.19E-17	33.24847
TMOD1	-2.9306	2.535242	-10.005	2.53E-19	5.36E-17	33.27921
ADAM33	-3.30506	1.015645	-9.99418	2.72E-19	5.67E-17	33.21731
ALDH6A1	-2.21305	3.980441	-9.90122	5.07E-19	1.04E-16	32.57295
C5orf4	-2.56131	2.949548	-9.80491	9.64E-19	1.95E-16	31.95985
RCAN2	-2.87752	3.297744	-9.75582	1.34E-18	2.67E-16	31.62457
MMRN1	-3.56598	1.487707	-9.74318	1.45E-18	2.86E-16	31.57434
GCNT4	-2.9082	1.119176	-9.71308	1.77E-18	3.44E-16	31.37685
TMEM161B	-1.52366	3.493576	-9.57626	4.39E-18	8.41E-16	30.47536
KIAA0427	-2.00613	4.955107	-9.55342	5.11E-18	9.64E-16	30.28834
LEPR	-2.90363	3.750013	-9.48036	8.27E-18	1.54E-15	29.82132
FAM54B	-1.32594	4.727834	-9.4783	8.38E-18	1.54E-15	29.80679
PRX	-1.74965	1.967031	-9.47045	8.83E-18	1.60E-15	29.80708
CFD	-3.26268	3.446961	-9.44976	1.01E-17	1.81E-15	29.62606
MITF	-2.40894	3.237418	-9.39753	1.43E-17	2.52E-15	29.304
C22orf23	-2.44708	1.632654	-9.38384	1.56E-17	2.72E-15	29.25593
ZNF662	-2.84536	0.703001	-9.36573	1.76E-17	3.03E-15	29.11927
TOP2A	3.755411	7.853448	9.34596	2.00E-17	3.41E-15	29.00085
KPNA2	2.09841	7.330151	9.336926	2.12E-17	3.57E-15	28.90589
MFSD4	-4.58633	2.70429	-9.32748	2.26E-17	3.75E-15	28.8466
SIK2	-1.89319	5.576587	-9.31763	2.41E-17	3.96E-15	28.7633
EFHA2	-2.87346	0.51855	-9.29357	2.82E-17	4.58E-15	28.64785
CENPF	3.366858	7.024015	9.230643	4.25E-17	6.82E-15	28.27491
ALAD	-1.64357	5.467839	-9.20984	4.86E-17	7.72E-15	28.06781
GNAZ	-2.86273	2.032417	-9.1817	5.84E-17	9.07E-15	27.94842
RCC2	1.67512	7.919562	9.181764	5.84E-17	9.07E-15	27.88774
MT1G	-4.18057	2.912331	-9.16304	6.60E-17	1.01E-14	27.78737
LOC400043	-3.09045	2.340741	-9.13744	7.79E-17	1.18E-14	27.6504
PDE2A	-2.48383	2.40191	-9.13536	7.90E-17	1.19E-14	27.65022
CDH2	-3.91962	0.771565	-9.12148	8.64E-17	1.28E-14	27.57796
HOXC9	5.137225	1.258094	9.108306	9.41E-17	1.38E-14	26.52632
CKB	-4.14648	5.425591	-9.05323	1.35E-16	1.96E-14	27.09498
SPHAR	-1.40338	2.365058	-8.97091	2.29E-16	3.30E-14	26.63147
RAI2	-2.57505	2.765327	-8.87917	4.13E-16	5.89E-14	26.00861
C8orf84	-2.79034	2.172218	-8.76426	8.63E-16	1.22E-13	25.30543
ACAT1	-1.73065	4.783882	-8.75926	8.91E-16	1.24E-13	25.20842
TNXB	-4.51168	3.842447	-8.72912	1.08E-15	1.49E-13	25.03855
KIF11	2.875648	5.912534	8.680534	1.47E-15	2.02E-13	24.81584
DPCR1	-6.71826	0.823198	-8.67391	1.53E-15	2.08E-13	24.72983
CACNA2D2	-3.05855	0.361667	-8.66995	1.57E-15	2.09E-13	24.74132
ABI3BP	-3.80805	2.782478	-8.67023	1.57E-15	2.09E-13	24.67251
DCLK2	-2.4145	0.794131	-8.654	1.74E-15	2.30E-13	24.63881
IPW	-2.9127	2.059187	-8.58466	2.70E-15	3.53E-13	24.18603
ACACB	-2.90747	4.005346	-8.55049	3.36E-15	4.34E-13	23.90834
ADAMTS8	-3.41582	0.028743	-8.54553	3.46E-15	4.44E-13	23.97597
ANK2	-3.24901	2.22528	-8.53988	3.59E-15	4.56E-13	23.88988
LIFR	-3.66688	4.048743	-8.53733	3.65E-15	4.59E-13	23.83247
KIAA1191	-1.24975	6.591657	-8.52261	4.00E-15	4.99E-13	23.73439
C14orf159	-1.58194	4.769113	-8.51152	4.29E-15	5.31E-13	23.66338
BVES	-2.84744	2.250428	-8.50504	4.47E-15	5.48E-13	23.68619
ROR1	-2.78533	1.943277	-8.49417	4.79E-15	5.82E-13	23.63624

TCF21	-2.38646	1.778079	-8.48953	4.93E-15	5.94E-13	23.62825
SORBS2	-3.46652	3.416506	-8.47913	5.27E-15	6.29E-13	23.4732
ECT2	2.627933	6.809479	8.476772	5.35E-15	6.33E-13	23.52
C5orf53	-1.55314	3.334366	-8.46703	5.68E-15	6.67E-13	23.44616
ACADSB	-1.9351	4.527399	-8.45292	6.21E-15	7.23E-13	23.30073
LGI4	-2.34724	0.738536	-8.44923	6.36E-15	7.34E-13	23.37828
C7orf58	-2.81513	2.689192	-8.43593	6.91E-15	7.85E-13	23.23957
SDPR	-2.7897	3.507744	-8.43686	6.87E-15	7.85E-13	23.21417
CBX7	-2.18737	4.462753	-8.42852	7.24E-15	8.16E-13	23.14905
GALNTL2	-2.75024	1.677673	-8.4105	8.11E-15	9.06E-13	23.1342
AOX1	-3.52728	0.604513	-8.38425	9.56E-15	1.06E-12	22.99086
FAM149A	-2.9068	1.528458	-8.3796	9.84E-15	1.08E-12	22.94604
FOXM1	3.299487	6.611659	8.375716	1.01E-14	1.09E-12	22.94028
IL33	-3.42704	2.735755	-8.37686	1.00E-14	1.09E-12	22.85914
CNTD1	-1.85711	1.245963	-8.34913	1.19E-14	1.28E-12	22.77285
ARHGAP24	-2.04807	3.355605	-8.33771	1.28E-14	1.36E-12	22.6294
CPEB3	-1.61376	3.21501	-8.3187	1.44E-14	1.52E-12	22.53862
PRIMA1	-3.6549	1.726234	-8.30918	1.53E-14	1.61E-12	22.47992
EPM2A	-1.64585	2.177349	-8.29293	1.69E-14	1.76E-12	22.43349
RRM2	3.45912	6.368686	8.283559	1.79E-14	1.86E-12	22.38398
GBGT1	-2.45033	1.636914	-8.27356	1.91E-14	1.96E-12	22.31183
SPC24	3.210898	0.975219	8.264478	2.02E-14	2.06E-12	21.58262
NECAB1	-2.76675	0.713295	-8.24998	2.21E-14	2.24E-12	22.1806
PAIP2B	-2.90384	2.490535	-8.24905	2.22E-14	2.24E-12	22.10058
DIRAS1	-3.22273	0.738628	-8.23638	2.41E-14	2.40E-12	22.09404
SYNJ2BP	-1.45471	5.867023	-8.22361	2.61E-14	2.58E-12	21.88862
RIMS3	-2.59784	2.463321	-8.21884	2.68E-14	2.64E-12	21.92961
GCOM1	-3.35777	2.978862	-8.17659	3.49E-14	3.38E-12	21.6277
HOXC8	4.926706	1.239794	8.17667	3.49E-14	3.38E-12	21.08539
DAAM2	-2.92517	3.775507	-8.14753	4.18E-14	4.02E-12	21.43478
CDHR3	-3.0326	0.176812	-8.14102	4.35E-14	4.16E-12	21.51673
MAL	-5.06225	1.503178	-8.1286	4.69E-14	4.46E-12	21.36179
CDK1	3.080632	5.742513	8.12525	4.79E-14	4.52E-12	21.42693
TMEM8B	-1.87651	3.361984	-8.10401	5.46E-14	5.12E-12	21.2121
GHR	-2.89525	1.669575	-8.09562	5.75E-14	5.36E-12	21.2137
ZSCAN18	-2.71763	3.195826	-8.09162	5.90E-14	5.46E-12	21.11584
TTLL7	-2.77871	1.681252	-8.08426	6.17E-14	5.61E-12	21.14916
PXMP2	-1.705	3.105122	-8.08301	6.22E-14	5.61E-12	21.10915
VSIG2	-5.1457	1.108343	-8.08281	6.23E-14	5.61E-12	21.09701
CDC25C	3.652463	2.433562	8.083875	6.19E-14	5.61E-12	20.5691
PTGIS	-3.95654	2.446102	-8.07216	6.65E-14	5.95E-12	21.003
SIDT2	-1.74365	5.617597	-8.07062	6.71E-14	5.97E-12	20.9599
ZHX3	-1.49737	5.391478	-8.06868	6.79E-14	6.01E-12	20.94513
MYBL2	4.103713	6.452017	8.053307	7.47E-14	6.56E-12	20.98914
DBT	-1.46791	5.224703	-8.05233	7.51E-14	6.56E-12	20.84681
GTF2IRD2P1	-1.65795	1.853313	-8.04711	7.76E-14	6.73E-12	20.9606
SECISBP2L	-1.46712	5.629577	-8.03576	8.32E-14	7.17E-12	20.74691
TMEM220	-2.01634	1.584641	-8.03246	8.49E-14	7.28E-12	20.87222
BTD	-1.57031	3.437587	-8.02837	8.70E-14	7.41E-12	20.76556
ITIH5	-2.8779	3.704023	-8.01836	9.25E-14	7.84E-12	20.65506
ADAMTSL3	-3.12783	1.193229	-8.0016	1.03E-13	8.63E-12	20.66521
SEMA3E	-3.88351	0.244037	-7.98159	1.16E-13	9.70E-12	20.56179
KCNK3	-2.81448	0.424888	-7.96896	1.25E-13	1.04E-11	20.49599
GPRASP1	-2.08984	1.682899	-7.96543	1.28E-13	1.04E-11	20.4666
JAM2	-2.39745	1.575475	-7.96653	1.27E-13	1.04E-11	20.46654
PLCL1	-2.19127	1.993781	-7.96754	1.26E-13	1.04E-11	20.45845
WASF3	-2.69311	2.230995	-7.9655	1.28E-13	1.04E-11	20.40845
PLK1	3.273588	5.822849	7.960233	1.32E-13	1.07E-11	20.44265

ITM2A	-2.33889	2.481558	-7.9454	1.45E-13	1.16E-11	20.28949
PCNA	1.81982	6.985568	7.929687	1.59E-13	1.27E-11	20.14216
ERO1LB	-2.54704	3.680992	-7.92928	1.60E-13	1.27E-11	20.12438
PBXIP1	-1.77766	6.190789	-7.91998	1.69E-13	1.33E-11	20.06004
NICN1	-1.25834	2.728694	-7.91663	1.72E-13	1.35E-11	20.15931
PLN	-3.34407	1.677661	-7.91302	1.76E-13	1.38E-11	20.0992
UBE2C	4.01809	5.55308	7.910969	1.79E-13	1.39E-11	20.09213
SCNN1B	-4.17483	1.456954	-7.90605	1.84E-13	1.42E-11	20.04143
RAB27A	-1.69493	4.823795	-7.90368	1.87E-13	1.43E-11	19.95614
REEP1	-2.75984	1.881815	-7.89175	2.01E-13	1.53E-11	19.98564
BUB1	3.912821	5.174111	7.882028	2.13E-13	1.62E-11	19.89219
GNAO1	-2.58686	1.990198	-7.87566	2.21E-13	1.67E-11	19.8913
ITGA9	-2.71881	2.242199	-7.86031	2.43E-13	1.83E-11	19.77895
GTF2IRD2	-1.43072	3.398486	-7.85867	2.46E-13	1.83E-11	19.75894
PBLD	-2.43984	2.866105	-7.85819	2.46E-13	1.83E-11	19.7429
PCBD2	-1.0863	3.673213	-7.85525	2.51E-13	1.85E-11	19.73886
PRC1	2.515768	6.046988	7.846465	2.64E-13	1.94E-11	19.74851
NUSAP1	3.325848	5.713013	7.83956	2.76E-13	2.02E-11	19.72758
ADCY5	-3.49423	1.577009	-7.82259	3.06E-13	2.22E-11	19.56085
MCM4	2.19665	6.818446	7.814867	3.20E-13	2.32E-11	19.48649
CILP	-3.60336	0.872856	-7.81022	3.29E-13	2.36E-11	19.52314
NPR1	-2.27488	1.903606	-7.80953	3.31E-13	2.36E-11	19.52157
ZBTB47	-1.56213	3.852707	-7.81093	3.28E-13	2.36E-11	19.44074
KIFC1	3.439532	4.573026	7.8035	3.43E-13	2.43E-11	19.41713
ARHGEF37	-2.27124	4.372336	-7.80032	3.50E-13	2.47E-11	19.34207
BUB1B	3.825576	4.982033	7.78376	3.87E-13	2.72E-11	19.30988
PRKAA2	-3.66452	1.649519	-7.77624	4.05E-13	2.81E-11	19.27556
DARC	-3.5127	2.017891	-7.77671	4.04E-13	2.81E-11	19.2642
HDGF	1.201418	8.644763	7.773396	4.12E-13	2.85E-11	19.18297
PTPN21	-1.48201	4.653985	-7.7684	4.24E-13	2.92E-11	19.15697
KIF18B	3.773454	5.013211	7.764692	4.34E-13	2.97E-11	19.20964
PPP1R3C	-3.06777	2.1966	-7.76184	4.41E-13	3.01E-11	19.18336
ZNF415	-2.59607	0.33421	-7.74477	4.89E-13	3.32E-11	19.17217
ASPM	3.745434	5.843482	7.724637	5.53E-13	3.73E-11	19.04707
FHL1	-3.47076	4.869128	-7.71436	5.88E-13	3.95E-11	18.8459
ANLN	3.15468	6.561632	7.692329	6.71E-13	4.49E-11	18.84561
TSPYL4	-1.47026	4.89382	-7.68445	7.04E-13	4.68E-11	18.65523
CKAP2	2.4184	5.840592	7.682007	7.14E-13	4.73E-11	18.78736
PDGFD	-2.49201	2.614505	-7.67743	7.34E-13	4.84E-11	18.68651
FAM72A	3.414793	1.043048	7.663011	8.01E-13	5.25E-11	18.19686
KIF2C	3.541987	5.071388	7.65837	8.23E-13	5.37E-11	18.62724
FANCA	2.929204	4.920474	7.655712	8.36E-13	5.44E-11	18.64087
F10	-2.70603	0.200748	-7.65169	8.57E-13	5.54E-11	18.63058
ETFDH	-1.56647	4.302801	-7.64893	8.71E-13	5.61E-11	18.46153
NCAPG	3.256558	4.976568	7.647121	8.81E-13	5.64E-11	18.57616
CDC20	3.829255	5.458725	7.59848	1.18E-12	7.50E-11	18.29327
CLSPN	3.241896	4.357834	7.598195	1.18E-12	7.50E-11	18.23817
AMT	-1.93026	1.761039	-7.58869	1.25E-12	7.86E-11	18.25472
DMD	-2.83957	3.777658	-7.58886	1.25E-12	7.86E-11	18.10193
ZNF132	-1.58534	1.23196	-7.58725	1.26E-12	7.90E-11	18.2606
PCCA	-1.62378	3.541602	-7.58483	1.28E-12	7.97E-11	18.12515
DIXDC1	-1.94911	3.784121	-7.58005	1.32E-12	8.17E-11	18.06823
SNX1	-0.91944	6.500217	-7.57683	1.34E-12	8.29E-11	18.02142
KANK3	-1.67164	1.963045	-7.56988	1.40E-12	8.60E-11	18.14605
SGOL1	3.34939	2.922465	7.562385	1.46E-12	8.96E-11	17.75162
APLP1	-3.24317	1.74914	-7.54887	1.58E-12	9.67E-11	17.955
CIRBP	-1.27336	6.275448	-7.54787	1.59E-12	9.68E-11	17.85369
ARHGAP11A	2.592447	5.539703	7.542213	1.65E-12	9.97E-11	17.99767

PEG3	-2.53113	0.389102	-7.53938	1.68E-12	1.01E-10	17.98491
C1QTNF3	-2.29719	2.138301	-7.53265	1.74E-12	1.05E-10	17.8816
CCNA2	2.828868	5.446181	7.528803	1.79E-12	1.06E-10	17.92606
CEP55	3.666184	5.380406	7.528172	1.79E-12	1.06E-10	17.89709
ACO2	-1.22159	7.208849	-7.52832	1.79E-12	1.06E-10	17.75426
PSCA	-5.61439	2.939733	-7.52672	1.81E-12	1.07E-10	17.77099
CDC25B	2.393874	6.86042	7.52186	1.86E-12	1.09E-10	17.77425
CBX3	1.274507	7.114626	7.51836	1.90E-12	1.11E-10	17.68611
ITPR1	-2.21481	4.311872	-7.5025	2.09E-12	1.22E-10	17.59195
KLHDC1	-1.89081	0.932952	-7.49965	2.12E-12	1.23E-10	17.75701
FAM63A	-1.73785	4.314596	-7.49584	2.17E-12	1.25E-10	17.5613
SPC25	2.906325	2.779772	7.492409	2.22E-12	1.27E-10	17.42684
TTYH3	2.020872	7.817271	7.486101	2.30E-12	1.32E-10	17.49919
C6orf204	-1.80054	1.21457	-7.47169	2.51E-12	1.43E-10	17.59872
BCL2L2	-1.24128	5.468194	-7.47099	2.52E-12	1.43E-10	17.40086
MAP6	-2.62402	0.585598	-7.46807	2.56E-12	1.45E-10	17.57733
TRIM3	-1.0118	3.2763	-7.46557	2.60E-12	1.46E-10	17.48703
PDK4	-4.68167	4.167025	-7.46314	2.64E-12	1.48E-10	17.38978
MELK	3.639829	4.70613	7.45385	2.79E-12	1.56E-10	17.41892
KAT2B	-1.98964	4.516489	-7.44178	2.99E-12	1.67E-10	17.23795
INPP5A	-1.22739	4.72909	-7.43388	3.13E-12	1.74E-10	17.20088
IQGAP3	3.327666	5.877701	7.424866	3.31E-12	1.83E-10	17.33116
C4orf34	-1.47141	4.376117	-7.41056	3.60E-12	1.98E-10	17.07226
NUP62	0.949453	6.403827	7.395203	3.94E-12	2.16E-10	16.98726
FANCI	2.112018	5.93768	7.386997	4.13E-12	2.26E-10	17.0483
FAM54A	3.407651	2.487915	7.378858	4.34E-12	2.36E-10	16.65371
LMNB2	1.6888	7.144195	7.373006	4.49E-12	2.43E-10	16.85746
NEK2	4.134901	4.246856	7.367856	4.63E-12	2.50E-10	16.80448
MND1	3.025693	1.555837	7.363283	4.75E-12	2.55E-10	16.56201
HOXC11	5.595484	1.072661	7.363096	4.76E-12	2.55E-10	16.55794
KIF22	1.55926	5.70851	7.350456	5.12E-12	2.72E-10	16.8127
DDX39	1.551763	6.026134	7.351107	5.10E-12	2.72E-10	16.79086
FAM49B	1.501773	6.062502	7.350179	5.13E-12	2.72E-10	16.7798
C16orf45	-1.79071	2.779471	-7.34868	5.18E-12	2.73E-10	16.80182
BDH2	-1.36708	3.504205	-7.34422	5.31E-12	2.79E-10	16.74771
NCAPH	3.737163	4.470659	7.341748	5.39E-12	2.82E-10	16.75358
MAPT	-2.80726	0.358545	-7.34066	5.43E-12	2.83E-10	16.85234
C1orf175	-2.45924	1.166555	-7.33679	5.55E-12	2.88E-10	16.81447
FABP3	-2.35344	1.228757	-7.33221	5.70E-12	2.94E-10	16.78961
GTSE1	3.272175	4.273412	7.331979	5.71E-12	2.94E-10	16.72731
KIF23	3.048508	5.150806	7.320323	6.11E-12	3.14E-10	16.73427
MYOM1	-2.914	1.163445	-7.3198	6.13E-12	3.14E-10	16.69173
RAD54L	3.482722	3.454496	7.312631	6.40E-12	3.26E-10	16.46747
RAF1	-0.93363	6.585658	-7.30879	6.54E-12	3.32E-10	16.46989
PIK3C2G	-4.357	0.258888	-7.30552	6.67E-12	3.38E-10	16.59964
TENC1	-2.05812	5.707047	-7.29925	6.92E-12	3.49E-10	16.41899
CENPA	4.015438	3.201527	7.295765	7.06E-12	3.55E-10	16.21691
ZMAT1	-2.69959	1.215693	-7.2796	7.76E-12	3.89E-10	16.47354
HPGD	-3.86873	4.007188	-7.27338	8.05E-12	4.02E-10	16.28218
RECK	-1.98223	2.535646	-7.27144	8.14E-12	4.05E-10	16.3679
MAD2L1	2.785568	4.734406	7.267395	8.33E-12	4.13E-10	16.43211
WDR62	3.408384	4.336663	7.261279	8.64E-12	4.26E-10	16.32932
TADA2B	-1.08173	4.851692	-7.26026	8.69E-12	4.27E-10	16.20245
TIMELESS	1.920479	5.655014	7.257839	8.81E-12	4.32E-10	16.31993
LMNB1	2.341727	5.659295	7.246271	9.43E-12	4.60E-10	16.28683
EEF1A2	-3.60786	0.731214	-7.24183	9.67E-12	4.71E-10	16.24131
ZNF778	-1.06663	2.296107	-7.23553	1.00E-11	4.85E-10	16.24372
PPP1R12C	-1.20815	5.689583	-7.23607	1.00E-11	4.85E-10	16.04863

SPAG5	3.012595	5.559124	7.2329	1.02E-11	4.91E-10	16.2446
DLGAP5	3.873238	4.739529	7.222731	1.08E-11	5.19E-10	16.11158
ABCA6	-2.90201	0.53556	-7.22174	1.09E-11	5.20E-10	16.17458
ASF1B	3.192327	4.857218	7.219953	1.10E-11	5.24E-10	16.15665
CDCA8	2.904365	4.862462	7.206392	1.19E-11	5.65E-10	16.09157
ANO5	-3.29302	1.044127	-7.20103	1.23E-11	5.81E-10	16.00478
TROAP	3.598868	4.490695	7.182667	1.36E-11	6.44E-10	15.89131
RNF14	-0.89054	5.104995	-7.17032	1.47E-11	6.89E-10	15.68719
KCNMB1	-2.52326	2.463334	-7.15151	1.63E-11	7.66E-10	15.66141
ME3	-2.39673	1.929221	-7.14964	1.65E-11	7.72E-10	15.70268
NCRNA00087	-2.13723	-0.09024	-7.12851	1.87E-11	8.69E-10	15.64129
CCNB1	2.789015	5.586161	7.115918	2.01E-11	9.32E-10	15.58293
PPP1R9A	-3.5482	1.504001	-7.10946	2.08E-11	9.64E-10	15.44115
C11orf67	-1.29056	1.956649	-7.10885	2.09E-11	9.64E-10	15.5405
UBL3	-1.95783	5.893392	-7.10591	2.13E-11	9.77E-10	15.32076
LMOD1	-3.6311	4.047446	-7.1005	2.19E-11	1.00E-09	15.29812
TBC1D14	-1.39563	6.318895	-7.10089	2.19E-11	1.00E-09	15.29022
CDCA5	3.436484	4.620919	7.095514	2.26E-11	1.03E-09	15.44011
SLIT2	-3.19597	1.249889	-7.0945	2.27E-11	1.03E-09	15.39327
SLC2A12	-2.58555	3.94594	-7.0938	2.28E-11	1.03E-09	15.25553
ZNF470	-2.3629	0.808143	-7.09301	2.29E-11	1.03E-09	15.46068
METTL7A	-2.8872	4.916651	-7.08788	2.36E-11	1.06E-09	15.2215
SUCLG2	-1.67892	5.195961	-7.0877	2.36E-11	1.06E-09	15.20931
TACC3	2.278384	5.755047	7.084489	2.41E-11	1.07E-09	15.3643
TAPT1	-1.03735	4.278418	-7.07571	2.53E-11	1.13E-09	15.18771
MYEF2	-2.86651	1.39365	-7.07499	2.54E-11	1.13E-09	15.29412
XPO1	0.943948	8.037493	7.074341	2.55E-11	1.13E-09	15.13521
CDC45	4.226611	3.679566	7.071364	2.59E-11	1.14E-09	15.07546
STMN1	2.070761	6.996222	7.068418	2.64E-11	1.16E-09	15.15306
CENPM	2.991915	2.931629	7.067662	2.65E-11	1.16E-09	15.12627
IL11RA	-1.64845	1.678385	-7.06219	2.73E-11	1.19E-09	15.27898
EXO1	3.770993	3.782244	7.060494	2.76E-11	1.20E-09	15.11146
TK1	2.600194	6.188269	7.058693	2.79E-11	1.21E-09	15.20687
AUH	-1.05424	3.30126	-7.05813	2.80E-11	1.21E-09	15.16406
NACAD	-2.23107	1.831874	-7.05584	2.84E-11	1.22E-09	15.19462
RAB4A	-1.01716	5.053484	-7.05277	2.89E-11	1.24E-09	15.02227
GTF2IRD2B	-1.59069	2.064649	-7.05092	2.92E-11	1.25E-09	15.19407
CDCA2	3.588002	3.902676	7.050244	2.93E-11	1.25E-09	15.09917
POU6F1	-1.53266	2.323965	-7.04787	2.97E-11	1.26E-09	15.15912
HMGA1	1.900507	8.475713	7.045972	3.00E-11	1.27E-09	14.97337
PRKAR2B	-2.33803	2.919162	-7.04353	3.04E-11	1.29E-09	15.03084
FYCO1	-1.59627	5.937112	-7.04247	3.06E-11	1.29E-09	14.95935
STIL	2.973604	4.86692	7.038983	3.12E-11	1.31E-09	15.16361
ZFP28	-2.36275	0.720925	-7.03857	3.13E-11	1.31E-09	15.1613
FLJ33630	-1.15658	4.053593	-7.03648	3.17E-11	1.33E-09	14.97631
ZNF542	-2.08636	1.784568	-7.0315	3.26E-11	1.36E-09	15.07361
SYNE1	-2.70804	4.628095	-7.02036	3.48E-11	1.45E-09	14.83809
ZNF844	-2.40967	1.439436	-7.01779	3.53E-11	1.46E-09	15.00338
TMEM47	-2.26143	4.082318	-7.00659	3.76E-11	1.56E-09	14.76619
HCFC2	-1.11998	4.098038	-6.99988	3.91E-11	1.61E-09	14.77023
ANKDD1A	-1.56382	1.681038	-6.99701	3.97E-11	1.63E-09	14.92113
FOXN3	-1.40573	6.804289	-6.99498	4.02E-11	1.65E-09	14.70426
AOC3	-2.64595	3.928292	-6.99204	4.09E-11	1.67E-09	14.68426
DKC1	1.259113	6.50976	6.984802	4.26E-11	1.74E-09	14.66766
UBE2T	3.196338	4.132956	6.981039	4.35E-11	1.77E-09	14.79502
LDHD	-2.49824	2.117042	-6.97689	4.46E-11	1.81E-09	14.71364
PINK1	-1.3064	5.717773	-6.97121	4.60E-11	1.86E-09	14.55543
CNN1	-3.71851	4.107543	-6.96364	4.81E-11	1.94E-09	14.53232

PRELP	-3.2132	3.654371	-6.95335	5.10E-11	2.05E-09	14.47195
PRICKLE2	-2.22255	4.073782	-6.9462	5.31E-11	2.13E-09	14.43032
ATPAF1	-0.87666	5.431573	-6.94238	5.43E-11	2.17E-09	14.39829
CKS2	2.685121	5.057604	6.939247	5.52E-11	2.20E-09	14.61859
KCNAB1	-1.5455	1.070119	-6.934	5.69E-11	2.25E-09	14.59153
TPCN2	-1.98187	4.630079	-6.9342	5.68E-11	2.25E-09	14.35407
PTPRN2	-3.28995	2.678551	-6.92764	5.90E-11	2.33E-09	14.3629
CKS1B	2.078253	5.467041	6.925924	5.96E-11	2.35E-09	14.49245
TCEB3	-0.95545	6.424295	-6.91961	6.18E-11	2.42E-09	14.27141
KIAA0141	-0.95688	5.136717	-6.90805	6.60E-11	2.58E-09	14.21346
KLF9	-2.37651	5.614106	-6.90357	6.76E-11	2.64E-09	14.19194
FAM114A2	-0.88637	3.962798	-6.90161	6.84E-11	2.66E-09	14.24836
KIAA0494	-1.10247	7.565856	-6.90153	6.84E-11	2.66E-09	14.19509
TUBB	1.206298	9.877496	6.899244	6.93E-11	2.68E-09	14.18839
MCM2	2.650072	6.298074	6.898035	6.98E-11	2.69E-09	14.30977
CRBN	-0.94724	4.185969	-6.89466	7.12E-11	2.74E-09	14.1892
KIAA0101	3.365246	4.805594	6.889007	7.35E-11	2.81E-09	14.33204
RGAG4	-2.06308	1.530836	-6.88947	7.33E-11	2.81E-09	14.3108
SORBS1	-3.506	5.395496	-6.88597	7.47E-11	2.85E-09	14.11312
BRP44L	-1.50282	4.41832	-6.87066	8.15E-11	3.10E-09	14.01707
KIF4A	3.692852	4.709366	6.86793	8.28E-11	3.14E-09	14.19508
PDZD4	-2.61577	0.555043	-6.86698	8.32E-11	3.15E-09	14.21666
KIAA0430	-0.94078	6.67804	-6.86525	8.40E-11	3.17E-09	13.97357
NBEA	-2.75983	1.711062	-6.86135	8.59E-11	3.23E-09	14.08849
BCKDHB	-1.48636	4.015193	-6.86108	8.60E-11	3.23E-09	13.98615
TCEAL3	-1.61587	3.08368	-6.85871	8.72E-11	3.27E-09	14.03429
ORC1L	3.715461	3.511776	6.855301	8.89E-11	3.32E-09	13.98592
LAMA2	-2.63353	3.844479	-6.85102	9.11E-11	3.39E-09	13.90365
UPRT	-1.31541	2.872937	-6.83936	9.73E-11	3.61E-09	13.97191
PHF17	-1.84202	4.947135	-6.83245	1.01E-10	3.75E-09	13.78818
SNRPB	1.235008	7.72654	6.829858	1.03E-10	3.79E-09	13.77071
KNTC1	1.76097	5.70482	6.828207	1.04E-10	3.82E-09	13.90135
SCUBE1	-3.13875	0.493093	-6.82737	1.04E-10	3.83E-09	13.98004
MYST4	-1.06424	5.758233	-6.81995	1.09E-10	3.98E-09	13.71604
NEXN	-2.29828	3.753443	-6.81771	1.10E-10	4.02E-09	13.72959
TOR1AIP1	-0.96447	6.165674	-6.81512	1.12E-10	4.07E-09	13.69108
EIF4E3	-1.64896	4.533388	-6.81078	1.14E-10	4.16E-09	13.67864
ACER2	-2.16808	2.658422	-6.80811	1.16E-10	4.21E-09	13.75914
ASAP3	-1.80443	4.22269	-6.803	1.19E-10	4.32E-09	13.64289
UHRF1	3.181726	4.308303	6.800424	1.21E-10	4.37E-09	13.83918
SOBP	-2.08324	2.676914	-6.7988	1.22E-10	4.40E-09	13.70902
MOAP1	-1.10013	4.457246	-6.7965	1.24E-10	4.45E-09	13.62246
XYLT2	-1.51831	5.386482	-6.79576	1.24E-10	4.45E-09	13.58451
CCNF	2.337838	4.909363	6.794296	1.25E-10	4.48E-09	13.82499
GIN1	-0.78533	2.212451	-6.79269	1.27E-10	4.51E-09	13.81098
HADHB	-0.85945	6.357314	-6.79113	1.28E-10	4.54E-09	13.56013
PDE1A	-2.23351	0.713777	-6.77883	1.37E-10	4.85E-09	13.74519
HN1	1.665504	6.685825	6.770049	1.44E-10	5.08E-09	13.49368
ZNF467	-1.68843	2.614499	-6.76553	1.47E-10	5.20E-09	13.56269
LMF1	-1.43515	3.066296	-6.76412	1.49E-10	5.23E-09	13.531
ID4	-2.10046	3.109464	-6.75777	1.54E-10	5.40E-09	13.4491
NFIC	-1.59213	6.50286	-6.75682	1.55E-10	5.42E-09	13.38491
SPRYD3	-1.01536	5.813396	-6.75449	1.57E-10	5.48E-09	13.3567
CENPI	3.090932	2.381406	6.747384	1.63E-10	5.68E-09	13.32495
MOSC2	-1.8777	2.513092	-6.74226	1.68E-10	5.83E-09	13.43092
RACGAP1	1.682012	5.922938	6.741878	1.68E-10	5.83E-09	13.39988
KCNIP4	-1.37	-0.01685	-6.72772	1.82E-10	6.27E-09	13.43688
YPEL1	-1.66255	1.883401	-6.72843	1.81E-10	6.27E-09	13.43337

PTBP1	0.722642	8.701353	6.727759	1.82E-10	6.27E-09	13.22846
PRKACB	-2.07102	5.208868	-6.7212	1.89E-10	6.48E-09	13.18051
BZRAP1	-2.2261	2.352936	-6.71748	1.93E-10	6.60E-09	13.28393
CCNB2	3.878556	5.114535	6.714306	1.96E-10	6.69E-09	13.38903
GPD1L	-1.86559	5.080288	-6.71417	1.96E-10	6.69E-09	13.13967
TOX	-2.41167	2.452769	-6.70921	2.02E-10	6.86E-09	13.2195
CLN8	-1.24038	4.199136	-6.69826	2.15E-10	7.28E-09	13.09485
NUF2	3.916921	3.94976	6.697713	2.15E-10	7.28E-09	13.19636
CYFIP2	-2.57237	4.087019	-6.69474	2.19E-10	7.39E-09	13.04226
NR3C2	-2.76706	2.275638	-6.68426	2.32E-10	7.79E-09	13.07779
SFRP1	-3.70393	2.29494	-6.68468	2.31E-10	7.79E-09	13.03581
PLK4	2.536927	4.146588	6.683783	2.33E-10	7.79E-09	13.23731
FAM172A	-1.14299	4.546845	-6.68205	2.35E-10	7.85E-09	12.99072
DGKD	-2.14564	5.429524	-6.67825	2.40E-10	8.00E-09	12.94966
FANCB	2.37861	1.350041	6.676344	2.42E-10	8.07E-09	12.94073
KIF18A	3.252482	3.91151	6.670941	2.50E-10	8.29E-09	13.1193
DEPDC1	3.39244	4.623198	6.669259	2.52E-10	8.35E-09	13.14918
GADD45B	-1.97955	4.442689	-6.66742	2.55E-10	8.42E-09	12.89208
PTTG1	3.27229	4.860945	6.664063	2.60E-10	8.51E-09	13.13354
DEAF1	-1.02069	4.636438	-6.66409	2.60E-10	8.51E-09	12.89421
FBXL5	-0.97809	6.27484	-6.66452	2.59E-10	8.51E-09	12.86959
STK32B	-2.37136	0.432443	-6.66073	2.64E-10	8.63E-09	13.11586
THRB	-2.49117	3.664539	-6.66096	2.64E-10	8.63E-09	12.87315
TCP11L2	-1.50794	3.614181	-6.64629	2.87E-10	9.33E-09	12.83981
GABRB3	-3.61158	0.69989	-6.64501	2.89E-10	9.38E-09	12.94626
PECI	-1.58032	4.370622	-6.64119	2.95E-10	9.55E-09	12.76161
SKA3	3.457612	4.00142	6.63849	2.99E-10	9.68E-09	12.94009
KIF14	3.849683	4.392965	6.637893	3.00E-10	9.69E-09	12.93958
DCLK1	-2.58837	1.161363	-6.63515	3.05E-10	9.81E-09	12.92502
PMM1	-1.27303	3.871144	-6.63267	3.09E-10	9.92E-09	12.76085
PLA2G12A	-0.96935	4.893961	-6.62836	3.16E-10	1.01E-08	12.69032
KCNMA1	-3.26518	3.314068	-6.6255	3.22E-10	1.03E-08	12.68112
GARNL3	-1.64043	1.238836	-6.61858	3.34E-10	1.07E-08	12.88554
HJURP	3.552149	4.299318	6.615212	3.40E-10	1.08E-08	12.83629
SKA1	3.245746	3.192212	6.613103	3.44E-10	1.09E-08	12.74005
RAD51	2.975336	3.404591	6.612272	3.46E-10	1.09E-08	12.78907
SGSM2	-1.16745	5.546744	-6.61233	3.46E-10	1.09E-08	12.5848
BLM	2.855188	3.904351	6.606672	3.57E-10	1.13E-08	12.80823
ESPL1	3.16781	5.313924	6.60188	3.66E-10	1.15E-08	12.80435
PDCD4	-1.64198	6.225555	-6.60022	3.70E-10	1.16E-08	12.5304
CSRNP1	-1.79595	5.254562	-6.59557	3.79E-10	1.19E-08	12.49679
SHCBP1	2.877958	3.91588	6.590204	3.91E-10	1.22E-08	12.7223
ARHGAP10	-1.58035	4.002889	-6.58334	4.06E-10	1.27E-08	12.46846
CAPN6	-3.67685	0.785853	-6.58029	4.13E-10	1.28E-08	12.58686
FHL5	-2.29005	0.143419	-6.57805	4.18E-10	1.30E-08	12.68135
NME1	1.71377	6.003288	6.567059	4.44E-10	1.38E-08	12.45049
CCNI	-1.09904	7.611112	-6.56654	4.45E-10	1.38E-08	12.3681
CKAP2L	3.37244	4.032537	6.565302	4.48E-10	1.38E-08	12.56923
DNMT1	1.272777	6.842358	6.565513	4.48E-10	1.38E-08	12.3545
CALCOCO1	-1.33046	5.65452	-6.56072	4.60E-10	1.41E-08	12.3074
TSPYL2	-1.46148	3.787485	-6.55899	4.64E-10	1.42E-08	12.35857
PAICS	1.327965	7.174829	6.557568	4.68E-10	1.43E-08	12.30102
SOCS2	-1.7724	3.415735	-6.55343	4.79E-10	1.46E-08	12.34008
CCDC150	2.829959	1.285976	6.550063	4.88E-10	1.48E-08	12.29811
CD36	-2.4268	3.149486	-6.54669	4.97E-10	1.51E-08	12.28851
ZNF568	-2.33489	0.256965	-6.54446	5.03E-10	1.52E-08	12.50317
C17orf103	-1.38284	3.698926	-6.54447	5.03E-10	1.52E-08	12.29284
USP53	-1.48089	5.712629	-6.54315	5.06E-10	1.53E-08	12.21492

BIRC5	3.732804	4.333232	6.539512	5.17E-10	1.56E-08	12.43275
RCC1	2.929169	4.918396	6.537082	5.24E-10	1.57E-08	12.46488
LIG1	1.521091	5.062743	6.535501	5.28E-10	1.58E-08	12.36574
ZFYVE20	-0.93139	5.122391	-6.53391	5.33E-10	1.59E-08	12.17466
ACADS	-1.4075	4.141731	-6.52729	5.53E-10	1.65E-08	12.16848
OIP5	3.131777	2.607881	6.517323	5.84E-10	1.74E-08	12.18037
DTL	3.192302	4.785664	6.515224	5.90E-10	1.75E-08	12.35009
EME1	2.446039	2.262314	6.514497	5.93E-10	1.76E-08	12.20797
RECQL4	2.758252	4.908816	6.513494	5.96E-10	1.76E-08	12.33867
AURKA	2.621306	5.005835	6.508217	6.13E-10	1.81E-08	12.30449
DEPDC6	-2.61237	3.492108	-6.5033	6.30E-10	1.86E-08	12.03023
WIPF3	-2.48862	0.538933	-6.50105	6.38E-10	1.88E-08	12.26307
LY6E	2.06983	8.062714	6.497142	6.52E-10	1.91E-08	11.97321
INMT	-2.13396	1.465969	-6.49673	6.53E-10	1.91E-08	12.19428
CENPK	2.640864	3.06698	6.495139	6.59E-10	1.93E-08	12.18109
SCNN1G	-3.5937	0.190309	-6.49095	6.74E-10	1.97E-08	12.17253
AURKB	4.003625	4.198897	6.488472	6.83E-10	1.99E-08	12.13512
GSG2	2.509203	3.185749	6.484425	6.99E-10	2.03E-08	12.14652
CCDC149	-1.61685	3.311077	-6.48375	7.01E-10	2.03E-08	11.98627
CDT1	3.191902	4.8647	6.482575	7.06E-10	2.04E-08	12.18039
NTN4	-2.11942	4.192474	-6.48211	7.08E-10	2.04E-08	11.90017
AK3	-1.10843	6.488214	-6.47859	7.21E-10	2.08E-08	11.87442
RGNEF	-1.68084	4.746946	-6.47337	7.42E-10	2.13E-08	11.84643
CBFB	1.194328	6.023648	6.472376	7.46E-10	2.14E-08	11.90326
MOCS1	-1.45929	3.257829	-6.4718	7.49E-10	2.14E-08	11.93767
SLC25A26	-0.76818	3.499911	-6.45676	8.13E-10	2.32E-08	11.89042
MCM10	3.948524	3.672342	6.456074	8.16E-10	2.33E-08	11.91552
KIF20A	3.731985	4.968872	6.453887	8.25E-10	2.35E-08	12.02421
NRTN	-1.80139	0.792519	-6.45211	8.34E-10	2.36E-08	12.01798
CCDC107	-1.30255	3.614794	-6.45231	8.33E-10	2.36E-08	11.81477
ACADM	-1.20522	5.224469	-6.44558	8.64E-10	2.44E-08	11.69543
PSMB2	0.940827	7.208367	6.44506	8.66E-10	2.44E-08	11.69069
PRDM16	-3.02984	2.024438	-6.42976	9.41E-10	2.65E-08	11.72109
SGOL2	2.302471	4.068932	6.428923	9.46E-10	2.66E-08	11.90149
HBB	-2.73215	2.237015	-6.42595	9.61E-10	2.70E-08	11.70081
MFAP5	-3.31456	2.385945	-6.42399	9.71E-10	2.71E-08	11.64692
TSC22D3	-2.26717	6.107057	-6.42403	9.71E-10	2.71E-08	11.59859
CA5B	-1.19597	3.131465	-6.42291	9.77E-10	2.72E-08	11.71195
CHEK2	1.875606	3.284988	6.419658	9.95E-10	2.77E-08	11.84853
PPM1K	-1.4716	3.286265	-6.41725	1.01E-09	2.80E-08	11.64474
CENPE	2.643913	5.128702	6.413367	1.03E-09	2.85E-08	11.80296
SYNPO2	-4.27676	5.228081	-6.413	1.03E-09	2.85E-08	11.56734
POLQ	3.286738	4.413241	6.393005	1.15E-09	3.17E-08	11.70591
KIF15	3.265608	3.836629	6.392707	1.15E-09	3.17E-08	11.67394
CDCA3	2.851495	3.902062	6.387322	1.19E-09	3.26E-08	11.67233
TGFBR3	-2.61591	4.446645	-6.37575	1.26E-09	3.46E-08	11.33
C1orf112	1.632386	3.618499	6.373979	1.27E-09	3.49E-08	11.61602
C14orf37	-1.82218	1.444735	-6.36006	1.37E-09	3.76E-08	11.50251
SLC26A9	-3.70888	1.892828	-6.35632	1.40E-09	3.82E-08	11.30949
TAP2	1.452235	6.669932	6.355643	1.41E-09	3.83E-08	11.25781
CACNB2	-2.11591	1.152394	-6.3463	1.48E-09	4.02E-08	11.43292
GINS1	2.806403	4.369379	6.342486	1.51E-09	4.10E-08	11.45495
ALG3	1.38023	6.567583	6.340922	1.52E-09	4.12E-08	11.18275
TMEM191A	2.349173	-0.02364	6.339833	1.53E-09	4.14E-08	11.2367
SLC4A4	-3.55548	1.818704	-6.3364	1.56E-09	4.21E-08	11.21661
FEN1	1.782474	5.319932	6.334777	1.57E-09	4.24E-08	11.30523
SRPX	-2.1411	2.692468	-6.33443	1.58E-09	4.24E-08	11.21688
PXK	-0.95567	3.205478	-6.31582	1.74E-09	4.68E-08	11.16506

APBB1	-1.84102	2.975854	-6.31386	1.76E-09	4.72E-08	11.10293
TRAIP	1.998977	2.100225	6.31181	1.78E-09	4.76E-08	11.21396
VAMP2	-1.25829	5.611041	-6.30835	1.81E-09	4.84E-08	10.9681
KIAA0495	-1.7386	3.054032	-6.30704	1.83E-09	4.86E-08	11.0678
FZD4	-1.8467	4.395712	-6.30587	1.84E-09	4.89E-08	10.96848
FAM110B	-2.03368	1.092955	-6.3044	1.85E-09	4.91E-08	11.22705
CAMTA2	-0.86988	5.996261	-6.30045	1.89E-09	5.01E-08	10.92583
LOC728264	-2.33043	3.094305	-6.29998	1.90E-09	5.01E-08	10.99065
GLUL	-2.29368	8.777403	-6.29604	1.94E-09	5.11E-08	10.98992
XPA	-1.06643	3.382934	-6.29405	1.96E-09	5.16E-08	11.02149
ORC6L	2.601734	3.35712	6.292485	1.98E-09	5.18E-08	11.17432
ERH	0.983176	5.828299	6.292544	1.98E-09	5.18E-08	10.95586
PSMA7	0.92835	7.1122	6.288915	2.01E-09	5.27E-08	10.86952
SMAD9	-2.31518	1.235278	-6.2872	2.03E-09	5.31E-08	11.10553
PBK	3.281711	4.264294	6.286058	2.05E-09	5.32E-08	11.15487
ARHGAP11B	2.225013	1.632679	6.286133	2.04E-09	5.32E-08	11.00116
CASC5	3.116586	4.759681	6.28344	2.07E-09	5.39E-08	11.15281
C16orf59	2.98365	2.301533	6.280721	2.11E-09	5.46E-08	10.97208
P2RX1	-2.46637	0.244627	-6.26902	2.24E-09	5.79E-08	11.06996
DTNA	-2.53738	1.855547	-6.26935	2.24E-09	5.79E-08	10.92455
CDKN3	3.055541	3.629822	6.267194	2.26E-09	5.83E-08	11.03811
C17orf53	2.729287	3.080118	6.266811	2.27E-09	5.83E-08	11.01836
RAE1	1.036278	5.514293	6.266492	2.27E-09	5.83E-08	10.85061
FAM72D	3.09989	2.666181	6.264492	2.30E-09	5.89E-08	10.92737
ADRM1	0.948918	6.914297	6.257961	2.38E-09	6.08E-08	10.71319
HERC1	-1.00959	6.290616	-6.25708	2.39E-09	6.10E-08	10.70348
FAM72B	3.067953	3.554194	6.253204	2.44E-09	6.22E-08	10.96187
ABL1	-1.02967	7.183162	-6.25244	2.45E-09	6.23E-08	10.69528
EZH2	2.027895	4.506772	6.245441	2.54E-09	6.46E-08	10.9371
ZNF737	-2.31481	1.681133	-6.24386	2.56E-09	6.50E-08	10.8352
KIAA1908	-1.53627	1.693325	-6.24253	2.58E-09	6.54E-08	10.89596
NEIL3	3.481028	1.949365	6.241886	2.59E-09	6.55E-08	10.76633
SNRPN	-2.14123	3.857848	-6.23941	2.63E-09	6.62E-08	10.63547
MCM7	1.690849	7.06848	6.237968	2.65E-09	6.66E-08	10.63281
NUCB2	-1.4216	5.290639	-6.23626	2.67E-09	6.71E-08	10.59316
ACTL6A	1.441924	6.142215	6.230829	2.75E-09	6.89E-08	10.6441
BBS1	-0.95795	4.809187	-6.22427	2.85E-09	7.13E-08	10.55408
FBXW11	-0.90692	5.572133	-6.22098	2.90E-09	7.24E-08	10.51414
KIF1C	-1.24905	7.452336	-6.21847	2.94E-09	7.32E-08	10.53042
POLD1	1.453568	5.141877	6.205942	3.14E-09	7.82E-08	10.62266
ZNF229	-2.65493	0.186057	-6.20487	3.16E-09	7.83E-08	10.73805
GMPR	-2.18028	1.483173	-6.20514	3.15E-09	7.83E-08	10.67223
KLHDC2	-0.8752	5.217715	-6.20199	3.20E-09	7.94E-08	10.42471
C12orf48	2.39119	3.684297	6.196703	3.30E-09	8.15E-08	10.71017
CYP1B1	-2.65623	3.018652	-6.19609	3.31E-09	8.16E-08	10.44042
HNRNPL	0.5724	7.41696	6.188597	3.44E-09	8.48E-08	10.34441
ZWILCH	1.532903	5.058733	6.187144	3.47E-09	8.52E-08	10.54577
SMC4	1.366663	7.456772	6.187024	3.47E-09	8.52E-08	10.3418
HMMR	3.214927	4.570091	6.184453	3.52E-09	8.62E-08	10.64995
CTSF	-2.04192	3.463788	-6.17802	3.64E-09	8.91E-08	10.34555
ZNF528	-2.06467	1.809445	-6.17504	3.70E-09	9.03E-08	10.48999
SLMAP	-1.1692	6.349204	-6.1724	3.75E-09	9.14E-08	10.26742
SGSM3	-1.14366	5.670063	-6.17164	3.77E-09	9.17E-08	10.25705
FBXO9	-0.93779	5.389684	-6.16976	3.80E-09	9.24E-08	10.25214
C17orf91	-1.42825	3.0467	-6.16858	3.83E-09	9.28E-08	10.37596
PGCP	-1.69784	3.63197	-6.16732	3.85E-09	9.33E-08	10.29749
PKMYT1	3.858548	4.784764	6.162971	3.94E-09	9.53E-08	10.53442
TTK	2.736253	4.542318	6.159466	4.02E-09	9.69E-08	10.52218

GABARAPL1	-1.56335	5.296484	-6.15913	4.02E-09	9.69E-08	10.1941
SF3B4	0.898091	7.107214	6.156054	4.09E-09	9.83E-08	10.17975
PARD3B	-2.06683	2.07262	-6.15299	4.16E-09	9.98E-08	10.347
FKBP5	-2.42104	6.460187	-6.1505	4.21E-09	1.01E-07	10.18196
TMC6	1.535854	5.715402	6.149469	4.23E-09	1.01E-07	10.27242
EIF2C4	-1.02623	4.289567	-6.14165	4.41E-09	1.05E-07	10.156
AKD1	-1.41375	2.057766	-6.13251	4.63E-09	1.10E-07	10.30863
ZWINT	2.214235	5.03063	6.127922	4.75E-09	1.13E-07	10.31303
TDO2	4.276072	1.331983	6.125294	4.81E-09	1.14E-07	10.19526
CXCL12	-2.30459	3.894649	-6.12498	4.82E-09	1.14E-07	10.03867
TUBA1B	1.05904	9.762493	6.120528	4.93E-09	1.17E-07	10.032
WDR67	1.775688	3.746117	6.111452	5.18E-09	1.22E-07	10.27803
GSN	-1.82105	8.57123	-6.10723	5.29E-09	1.25E-07	9.998521
C22orf39	-0.97174	4.147471	-6.09365	5.68E-09	1.34E-07	9.924723
PDZRN3	-2.28538	3.803227	-6.09079	5.77E-09	1.36E-07	9.867729
RUVBL1	1.486317	5.526919	6.089744	5.80E-09	1.36E-07	9.982642
REC8	-1.65654	2.559406	-6.08897	5.83E-09	1.37E-07	10.004
ZBTB3	-0.84127	2.936172	-6.08576	5.92E-09	1.39E-07	10.0266
HOXC6	3.952386	2.06073	6.077592	6.18E-09	1.45E-07	9.967725
BUB3	0.810856	6.802685	6.076113	6.23E-09	1.45E-07	9.774857
ZNF790	-1.97912	0.81577	-6.07411	6.30E-09	1.47E-07	10.07344
ASB16	-1.26051	0.127546	-6.06872	6.48E-09	1.51E-07	10.06472
RAD51AP1	2.873902	4.003824	6.06658	6.55E-09	1.52E-07	10.05768
ZNF425	-1.51223	0.995629	-6.06456	6.62E-09	1.53E-07	10.03702
NISCH	-1.03228	6.232031	-6.06173	6.72E-09	1.55E-07	9.696387
HELLS	2.252228	4.199836	6.058008	6.85E-09	1.58E-07	10.00986
ZNF25	-1.1522	3.137411	-6.05743	6.87E-09	1.59E-07	9.822595
NAT15	-0.75099	6.297039	-6.05462	6.98E-09	1.61E-07	9.657657
EDA	-2.06378	1.799662	-6.05363	7.01E-09	1.61E-07	9.873042
PDHB	-0.8338	5.014686	-6.04388	7.38E-09	1.69E-07	9.622618
ERCC6L	2.508939	3.167828	6.038493	7.59E-09	1.74E-07	9.903203
ARPC1B	1.563641	7.431955	6.036985	7.65E-09	1.75E-07	9.57858
C8orf79	-2.60204	0.35808	-6.03557	7.71E-09	1.76E-07	9.87566
FAHD2B	-1.65163	1.879847	-6.03421	7.76E-09	1.77E-07	9.808179
LRIG1	-2.14689	6.22086	-6.02365	8.20E-09	1.87E-07	9.521866
LOC646471	-1.25701	1.809315	-6.02014	8.35E-09	1.90E-07	9.778046
LOC729234	-1.46041	0.369907	-6.01982	8.37E-09	1.90E-07	9.828079
POLE2	2.294845	2.738394	6.019173	8.40E-09	1.90E-07	9.796278
RAPGEF3	-1.6596	3.502486	-6.01688	8.50E-09	1.92E-07	9.543314
CITED2	-1.53227	5.171899	-6.01603	8.54E-09	1.93E-07	9.463119
ZNF483	-2.12572	0.177339	-6.01446	8.61E-09	1.94E-07	9.796564
UBE2S	2.240916	4.837456	6.007507	8.92E-09	2.01E-07	9.723169
COL14A1	-2.48707	4.549039	-6.00376	9.10E-09	2.04E-07	9.406092
ZNF570	-1.98608	0.224054	-6.00021	9.27E-09	2.07E-07	9.727351
ARHGAP6	-2.07347	1.749475	-6.00009	9.28E-09	2.07E-07	9.608208
SMARCD3	-1.71996	2.752046	-6.00061	9.25E-09	2.07E-07	9.525854
APOD	-2.86286	3.129091	-5.99771	9.39E-09	2.10E-07	9.411768
CHRFAM7A	-2.27823	0.611975	-5.99535	9.51E-09	2.12E-07	9.678723
FLNC	-3.46927	5.011146	-5.99565	9.49E-09	2.12E-07	9.385201
NECAB3	-1.14138	4.167426	-5.99295	9.63E-09	2.14E-07	9.400997
FOXF1	-1.84986	3.314585	-5.99139	9.70E-09	2.15E-07	9.416293
LOC541471	2.35912	3.505133	5.984189	1.01E-08	2.23E-07	9.650892
PSMA4	0.841967	6.846757	5.984016	1.01E-08	2.23E-07	9.306718
WDR37	-0.96027	4.447749	-5.97337	1.07E-08	2.35E-07	9.292642
PRUNE2	-3.33689	3.675428	-5.97368	1.06E-08	2.35E-07	9.266061
KCNJ11	-2.40658	0.67521	-5.96934	1.09E-08	2.39E-07	9.537282
NCAPD2	1.387286	7.403086	5.961629	1.13E-08	2.49E-07	9.193144
GRK4	-1.07633	1.653197	-5.96075	1.14E-08	2.50E-07	9.505126

ITGA7	-1.94513	3.366869	-5.95906	1.15E-08	2.51E-07	9.243542
CDC37L1	-0.89072	3.451853	-5.95688	1.16E-08	2.53E-07	9.30498
CCT5	1.171484	8.114935	5.956984	1.16E-08	2.53E-07	9.164429
WNK4	-2.90938	1.287819	-5.95408	1.18E-08	2.57E-07	9.350534
C14orf132	-2.11335	3.529455	-5.95326	1.18E-08	2.57E-07	9.192705
NACC2	-1.18692	2.747408	-5.95273	1.19E-08	2.58E-07	9.340937
C11orf82	3.088873	3.796733	5.952232	1.19E-08	2.58E-07	9.485517
HNRNPC	0.632027	8.172372	5.951486	1.19E-08	2.59E-07	9.14473
TTC28	-1.74214	4.507884	-5.94902	1.21E-08	2.61E-07	9.135108
PPIA	1.141617	8.149028	5.948785	1.21E-08	2.61E-07	9.123916
ISCU	-0.85862	5.729078	-5.94793	1.22E-08	2.62E-07	9.117226
CORO2B	-2.06098	0.46899	-5.94287	1.25E-08	2.69E-07	9.434163
ILF2	0.930542	7.369448	5.941219	1.26E-08	2.71E-07	9.083218
FAM174B	-1.94441	3.162245	-5.94004	1.27E-08	2.71E-07	9.166802
TCF3	1.12371	6.701679	5.940023	1.27E-08	2.71E-07	9.101034
C6orf167	1.535276	4.44649	5.938104	1.28E-08	2.74E-07	9.355122
FBXL17	-0.99858	4.702792	-5.93756	1.28E-08	2.74E-07	9.09415
CHEK1	2.086616	3.891018	5.931694	1.32E-08	2.82E-07	9.388104
PRKAG2	-1.17051	4.307124	-5.93177	1.32E-08	2.82E-07	9.080932
BRCA1	1.934626	5.180406	5.924645	1.37E-08	2.92E-07	9.248339
PDE7B	-1.71612	1.467547	-5.92217	1.39E-08	2.95E-07	9.284197
NDC80	3.193106	3.943484	5.919619	1.41E-08	2.98E-07	9.328523
CELF2	-2.11696	3.986774	-5.90939	1.48E-08	3.14E-07	8.94639
PDE4D	-1.56389	4.511815	-5.90808	1.49E-08	3.16E-07	8.934215
STARD8	-1.4732	2.568434	-5.90498	1.52E-08	3.20E-07	9.094791
PRR11	3.180242	3.738904	5.902834	1.54E-08	3.23E-07	9.240402
PATL1	0.834318	6.802346	5.901339	1.55E-08	3.26E-07	8.892166
FMO5	-2.99415	2.19129	-5.8954	1.59E-08	3.35E-07	8.96395
TFRC	1.864232	8.341625	5.895114	1.60E-08	3.35E-07	8.852735
MCM5	1.578192	6.433382	5.894803	1.60E-08	3.35E-07	8.920587
DMXL1	-1.11529	5.626181	-5.88967	1.64E-08	3.44E-07	8.824867
DSCR3	-0.77679	5.160179	-5.88067	1.72E-08	3.59E-07	8.796225
SACM1L	-0.80838	5.415587	-5.87972	1.73E-08	3.61E-07	8.781887
MAOB	-2.70824	3.223702	-5.87928	1.73E-08	3.61E-07	8.816897
HNRNPA2B1	0.639666	9.376301	5.876056	1.76E-08	3.66E-07	8.795427
RAN	0.975839	7.675147	5.874874	1.77E-08	3.68E-07	8.750373
C10orf26	-0.91478	5.601438	-5.87205	1.80E-08	3.73E-07	8.738192
PGRMC2	-0.80825	5.905682	-5.87131	1.81E-08	3.74E-07	8.732454
MAGI1	-1.84089	5.172097	-5.8708	1.81E-08	3.74E-07	8.733712
IL1RL1	-2.5724	0.266242	-5.86949	1.82E-08	3.76E-07	9.062015
TYMS	2.354767	5.304812	5.868635	1.83E-08	3.77E-07	9.000537
ZNF57	-1.12954	2.97339	-5.86888	1.83E-08	3.77E-07	8.902125
TAP1	1.702839	7.526762	5.868147	1.83E-08	3.77E-07	8.73018
MOCS2	-0.86838	4.411246	-5.86683	1.85E-08	3.79E-07	8.767134
LAD1	2.332785	8.16735	5.863103	1.88E-08	3.86E-07	8.704402
RFWD3	1.139092	5.980866	5.858781	1.93E-08	3.94E-07	8.744646
MASP1	-2.53321	0.080445	-5.85842	1.93E-08	3.94E-07	9.017538
EPB41L4A	-1.76531	3.931665	-5.85801	1.93E-08	3.94E-07	8.706389
C20orf20	1.331644	4.377741	5.853615	1.98E-08	4.03E-07	8.925071
MCM6	1.313701	5.952886	5.851432	2.00E-08	4.07E-07	8.724229
CDC6	3.468938	4.876927	5.850605	2.01E-08	4.08E-07	8.998122
CDCA7	3.264374	5.298555	5.849991	2.01E-08	4.08E-07	8.976131
FEM1C	-1.05405	5.671905	-5.84827	2.03E-08	4.11E-07	8.618381
RNASEH2A	1.997218	4.355874	5.847623	2.04E-08	4.12E-07	8.950764
C6orf174	-1.95504	0.94228	-5.84622	2.05E-08	4.15E-07	8.935353
EPR1	3.66135	3.710648	5.843929	2.08E-08	4.19E-07	8.932874
FAM82B	-0.72589	5.140597	-5.83684	2.15E-08	4.34E-07	8.580742
KIAA1524	2.619399	3.964648	5.833857	2.19E-08	4.40E-07	8.918338

TMEM25	-1.37675	3.226647	-5.82989	2.23E-08	4.48E-07	8.652185
BRIP1	2.614579	3.262696	5.825864	2.28E-08	4.57E-07	8.871887
GPT2	-1.98076	5.45445	-5.81419	2.42E-08	4.84E-07	8.456271
PIF1	2.240569	2.499997	5.80908	2.48E-08	4.96E-07	8.773068
FILIP1	-1.79458	2.42864	-5.80819	2.49E-08	4.98E-07	8.596232
ZCCHC24	-1.72363	4.32468	-5.80132	2.58E-08	5.14E-07	8.405673
FGD4	-1.95679	5.30871	-5.80155	2.58E-08	5.14E-07	8.391959
C15orf17	-0.87675	4.939842	-5.79943	2.61E-08	5.18E-07	8.398903
H2AFX	1.514718	6.249464	5.790215	2.73E-08	5.41E-07	8.411869
SLIT3	-2.33583	4.461	-5.79017	2.73E-08	5.41E-07	8.337807
SFRS2	0.640261	7.71554	5.790207	2.73E-08	5.41E-07	8.333252
DCAF13	1.131668	5.653907	5.789035	2.75E-08	5.43E-07	8.42775
COL4A3BP	-0.8991	5.299004	-5.78622	2.79E-08	5.50E-07	8.319146
TBC1D9	-1.49129	5.06597	-5.78622	2.79E-08	5.50E-07	8.314779
SESN1	-1.39497	4.440343	-5.78205	2.85E-08	5.61E-07	8.317201
DDR2	-2.05777	2.948846	-5.78094	2.86E-08	5.63E-07	8.38618
ACADVL	-1.02766	7.237126	-5.78037	2.87E-08	5.64E-07	8.303863
MAGI3	-1.267	4.908385	-5.78005	2.88E-08	5.64E-07	8.29203
SH3BP1	2.366222	6.106225	5.779346	2.89E-08	5.65E-07	8.46159
SLC25A42	-1.21905	2.674195	-5.77414	2.97E-08	5.79E-07	8.46195
DLX4	3.324748	0.484208	5.771762	3.00E-08	5.86E-07	8.512083
MTHFD1L	1.680973	5.039272	5.769943	3.03E-08	5.90E-07	8.473647
KIAA0232	-1.1321	5.883909	-5.76211	3.15E-08	6.13E-07	8.192954
PSMD14	0.896132	6.053768	5.760102	3.18E-08	6.19E-07	8.23365
TOM1L2	-1.53629	6.527538	-5.75854	3.21E-08	6.22E-07	8.190906
PRKAB1	-1.03028	5.262678	-5.75879	3.21E-08	6.22E-07	8.18225
UCK2	1.185777	4.742175	5.757248	3.23E-08	6.25E-07	8.390406
ZNF347	-2.1339	1.082101	-5.75618	3.25E-08	6.28E-07	8.471055
LIMS2	-1.89031	3.808421	-5.75386	3.29E-08	6.34E-07	8.193224
WDHD1	2.278836	4.342979	5.752578	3.31E-08	6.38E-07	8.504479
EFTUD2	0.867982	6.920059	5.748945	3.37E-08	6.48E-07	8.134177
PDE4C	-2.71693	0.11662	-5.74772	3.39E-08	6.52E-07	8.471301
FZD8	-1.75964	2.824669	-5.74598	3.42E-08	6.56E-07	8.250874
THOC4	1.166859	5.3477	5.744564	3.45E-08	6.60E-07	8.244841
C6orf225	-1.61086	1.188201	-5.74224	3.49E-08	6.67E-07	8.433851
NFKBIL2	1.999862	5.2809	5.739215	3.54E-08	6.77E-07	8.331712
ASB1	-0.80839	4.391286	-5.73878	3.55E-08	6.77E-07	8.140017
C15orf42	3.015623	4.634637	5.732231	3.67E-08	6.98E-07	8.422595
PSKH1	-0.96982	5.301045	-5.73249	3.66E-08	6.98E-07	8.053256
GLT25D1	1.093354	7.018785	5.724294	3.82E-08	7.26E-07	8.016778
ATP1B2	-1.91657	0.622087	-5.72274	3.85E-08	7.30E-07	8.35936
ADAMTS1	-2.26832	5.315498	-5.72065	3.89E-08	7.37E-07	7.998513
CENPN	1.291501	5.085199	5.719493	3.91E-08	7.40E-07	8.171972
DNAH3	3.223862	0.531467	5.717834	3.94E-08	7.44E-07	8.262293
MRVI1	-2.21006	4.609283	-5.71772	3.95E-08	7.44E-07	7.980357
PTGES3	0.771489	7.940536	5.717996	3.94E-08	7.44E-07	7.979952
FGF13	-2.35326	0.85322	-5.7126	4.05E-08	7.62E-07	8.263414
KIAA0513	-1.63448	4.429217	-5.71204	4.06E-08	7.64E-07	7.965721
ZNF493	-1.95541	2.256513	-5.70897	4.12E-08	7.74E-07	8.118143
TNFSF12	-1.29344	3.143399	-5.70881	4.13E-08	7.74E-07	8.074126
TTC37	-0.90546	6.164502	-5.70529	4.20E-08	7.87E-07	7.914487
PRIM2	1.390942	3.589087	5.704431	4.22E-08	7.89E-07	8.269562
FAM60A	1.291731	6.608408	5.701668	4.28E-08	7.99E-07	7.935095
ELL2	-1.88668	6.143355	-5.70114	4.29E-08	8.00E-07	7.908906
ZNF85	-1.96353	0.768132	-5.69853	4.35E-08	8.10E-07	8.232155
FAM13A	-1.4664	4.413011	-5.69609	4.40E-08	8.19E-07	7.89415
CMAH	-1.67071	1.71285	-5.69463	4.43E-08	8.24E-07	8.147069
C21orf2	-0.99427	3.461632	-5.69299	4.47E-08	8.29E-07	7.990113

HMGB3	2.161468	5.472048	5.68987	4.54E-08	8.41E-07	8.085703
FANCD2	2.271445	4.38605	5.680504	4.76E-08	8.81E-07	8.155919
HBA1	-2.3093	2.066255	-5.6791	4.79E-08	8.85E-07	7.962814
ROGDI	-1.03657	4.336221	-5.67891	4.80E-08	8.85E-07	7.837158
CCDC69	-1.9793	4.21893	-5.67523	4.89E-08	9.01E-07	7.784595
SIK3	-0.79286	5.798477	-5.67172	4.97E-08	9.16E-07	7.749989
GPM6B	-1.6766	2.470914	-5.66921	5.04E-08	9.26E-07	7.92548
CYB5D2	-1.03875	3.271362	-5.669	5.04E-08	9.26E-07	7.892177
NEK1	-0.9839	3.982383	-5.66694	5.09E-08	9.34E-07	7.8117
RBM5	-0.90945	6.01897	-5.66461	5.15E-08	9.44E-07	7.715109
PPAP2B	-1.54901	5.520592	-5.66415	5.17E-08	9.45E-07	7.715371
OAS3	1.661824	7.674966	5.661163	5.24E-08	9.58E-07	7.705936
SNTB1	-2.16268	4.250038	-5.65955	5.29E-08	9.64E-07	7.703881
STAT1	1.457052	8.378896	5.658805	5.31E-08	9.67E-07	7.689477
HLF	-2.43847	2.346468	-5.65374	5.44E-08	9.90E-07	7.794825
RTKN	1.335654	5.660091	5.652461	5.48E-08	9.95E-07	7.779518
F11R	1.130933	8.444201	5.649176	5.57E-08	1.01E-06	7.64788
FAM64A	3.25831	2.763244	5.64392	5.72E-08	1.04E-06	7.954696
CARKD	-0.93913	5.276846	-5.63774	5.90E-08	1.07E-06	7.592934
ATAD5	2.014474	4.008302	5.636215	5.94E-08	1.07E-06	7.952202
NDUFA5	-0.96419	5.597286	-5.63544	5.96E-08	1.08E-06	7.574805
RANBP1	1.277877	6.279556	5.634457	5.99E-08	1.08E-06	7.630773
CFL2	-1.5192	4.57001	-5.63253	6.05E-08	1.09E-06	7.576258
FAM83H	2.140412	8.338162	5.629515	6.14E-08	1.10E-06	7.548879
LAMB2	-1.5401	7.093787	-5.62776	6.20E-08	1.11E-06	7.567154
ST5	-1.21526	6.49907	-5.62505	6.28E-08	1.13E-06	7.533476
SYTL4	-1.31265	3.229927	-5.62294	6.35E-08	1.14E-06	7.647733
S1PR1	-1.81375	4.005091	-5.62205	6.38E-08	1.14E-06	7.542616
MTBP	2.03759	3.251263	5.621494	6.39E-08	1.14E-06	7.90688
WLS	-1.43351	6.039977	-5.62149	6.39E-08	1.14E-06	7.512566
SEC14L1	-1.05577	6.372369	-5.61957	6.46E-08	1.15E-06	7.502485
E2F1	1.87832	4.399934	5.615373	6.59E-08	1.17E-06	7.817634
WFDC1	-1.8024	2.118381	-5.61325	6.66E-08	1.18E-06	7.690478
CRYZL1	-0.79125	3.304326	-5.61337	6.66E-08	1.18E-06	7.646283
JUP	2.206696	10.78904	5.613102	6.67E-08	1.18E-06	7.506236
E2F2	2.69599	3.900032	5.610047	6.77E-08	1.20E-06	7.852472
PURA	-0.71976	4.738714	-5.60946	6.79E-08	1.20E-06	7.491072
DACT3	-1.86527	2.018354	-5.60555	6.92E-08	1.22E-06	7.656226
SNRPG	1.243221	4.861133	5.601908	7.05E-08	1.24E-06	7.628757
SLAIN1	-2.45398	1.270253	-5.60075	7.09E-08	1.25E-06	7.669009
FGF2	-1.91903	2.388549	-5.59751	7.21E-08	1.27E-06	7.564403
FAM111B	2.753282	4.236845	5.593681	7.34E-08	1.29E-06	7.769482
FILIP1L	-2.02712	5.48287	-5.59386	7.34E-08	1.29E-06	7.381081
ATP6V0A2	-0.77909	4.812059	-5.59226	7.40E-08	1.30E-06	7.400313
EIF4A3	0.929475	6.536066	5.590611	7.46E-08	1.31E-06	7.380936
DUSP3	-0.95807	6.461182	-5.58806	7.55E-08	1.32E-06	7.350418
SAP30L	-0.75567	5.062033	-5.58741	7.58E-08	1.32E-06	7.363868
TH1L	0.958807	6.546607	5.585337	7.66E-08	1.34E-06	7.356555
VPS13D	-1.06212	6.850429	-5.58506	7.67E-08	1.34E-06	7.344504
PPP1R12B	-2.42352	6.200121	-5.57895	7.90E-08	1.37E-06	7.33054
PITPNM2	-1.31627	4.96982	-5.57788	7.94E-08	1.38E-06	7.304608
KIF20B	1.647869	5.30656	5.576853	7.99E-08	1.39E-06	7.500124
PIP5K1C	-0.94302	6.6749	-5.57086	8.23E-08	1.43E-06	7.270881
ESCO2	2.838813	3.708928	5.566139	8.42E-08	1.46E-06	7.649076
HEMK1	-0.95555	3.341191	-5.56563	8.44E-08	1.46E-06	7.395401
MCM3	1.169899	6.983976	5.565167	8.46E-08	1.46E-06	7.24914
ZNF793	-2.25216	0.787655	-5.56416	8.50E-08	1.47E-06	7.56765
ZNF429	-1.96614	1.475101	-5.56103	8.64E-08	1.49E-06	7.506525

JPH2	-2.30842	2.424832	-5.5608	8.65E-08	1.49E-06	7.349527
APOC2	4.175197	1.029781	5.55924	8.71E-08	1.50E-06	7.538719
C18orf32	-0.95097	5.060472	-5.55882	8.73E-08	1.50E-06	7.219657
SNRPA	0.797447	6.008512	5.558237	8.76E-08	1.50E-06	7.250763
KLF2	-2.01046	4.898843	-5.55803	8.77E-08	1.50E-06	7.204881
KIAA1737	-0.87928	5.057351	-5.55747	8.79E-08	1.50E-06	7.215702
NFIA	-1.48982	6.294602	-5.55766	8.78E-08	1.50E-06	7.210147
SATL1	-0.99796	0.41447	-5.55687	8.82E-08	1.50E-06	7.605535
FRY	-1.89279	3.820744	-5.55358	8.96E-08	1.53E-06	7.221425
OSR1	-2.00852	1.081794	-5.54879	9.18E-08	1.56E-06	7.487819
FAM190B	-0.87702	6.018262	-5.54857	9.19E-08	1.56E-06	7.154951
FUS	0.750241	7.405443	5.546397	9.29E-08	1.58E-06	7.144061
CENPW	2.578306	3.4213	5.54551	9.33E-08	1.58E-06	7.553146
ATAD2	1.432504	6.457253	5.54352	9.42E-08	1.60E-06	7.191662
C13orf33	-2.00666	2.120205	-5.53992	9.59E-08	1.62E-06	7.315219
DMRTA1	-2.70775	0.022002	-5.5393	9.62E-08	1.62E-06	7.48352
C1orf135	2.15525	1.957918	5.537304	9.71E-08	1.64E-06	7.480443
PID1	-1.89683	2.352431	-5.53193	9.97E-08	1.67E-06	7.26013
DNAJC9	1.066715	4.966451	5.532251	9.96E-08	1.67E-06	7.258507
CLIC6	-3.20647	3.155158	-5.53206	9.97E-08	1.67E-06	7.11272
MTMR10	-0.91451	5.570578	-5.53248	9.95E-08	1.67E-06	7.080355
PGR	-2.17034	0.349436	-5.52748	1.02E-07	1.71E-06	7.434941
U2AF2	0.644538	7.014601	5.527404	1.02E-07	1.71E-06	7.054921
C19orf40	1.718009	1.231213	5.525184	1.03E-07	1.73E-06	7.402123
SLC4A2	-1.35506	6.670411	-5.52448	1.03E-07	1.73E-06	7.056298
CLDN4	3.929714	7.437253	5.52396	1.04E-07	1.73E-06	7.248643
ALG9	-0.75854	4.614154	-5.52353	1.04E-07	1.73E-06	7.085805
ASTN2	-2.00922	1.691901	-5.52231	1.05E-07	1.74E-06	7.292177
TUBA1C	1.263284	8.86968	5.518931	1.06E-07	1.77E-06	7.027075
ZKSCAN3	-1.09352	1.6433	-5.51859	1.07E-07	1.77E-06	7.368071
ST6GALNAC6	-1.16665	4.415697	-5.51734	1.07E-07	1.78E-06	7.045634
ARMCX1	-1.62879	2.085944	-5.51615	1.08E-07	1.79E-06	7.250295
VPS39	-0.56641	6.374517	-5.51572	1.08E-07	1.79E-06	6.998023
C6orf72	-0.74611	4.599868	-5.5122	1.10E-07	1.82E-06	7.033357
OMD	-2.62465	0.219568	-5.51051	1.11E-07	1.83E-06	7.338361
RFC3	1.699292	4.828821	5.510034	1.11E-07	1.83E-06	7.254225
ACOX2	-1.93378	0.577892	-5.50955	1.11E-07	1.83E-06	7.349687
ECHDC2	-1.49067	4.287711	-5.50737	1.13E-07	1.85E-06	6.99036
CHAF1A	1.305659	4.887684	5.505961	1.13E-07	1.86E-06	7.177238
FGD6	1.649503	5.84387	5.505804	1.13E-07	1.86E-06	7.08811
PHYHIP	-2.03133	0.542561	-5.50315	1.15E-07	1.88E-06	7.315136
DEPDC1B	2.958081	3.827805	5.496192	1.19E-07	1.95E-06	7.325048
HOXA11	4.673049	0.688551	5.494757	1.20E-07	1.96E-06	7.246672
SMOC2	-2.3681	3.770208	-5.49482	1.20E-07	1.96E-06	6.925768
C9orf130	-1.2981	2.229003	-5.49439	1.20E-07	1.96E-06	7.169451
CACNA1H	-2.28759	4.208659	-5.49405	1.20E-07	1.96E-06	6.907799
GINS4	2.681547	3.499439	5.49331	1.21E-07	1.96E-06	7.311801
NBAS	-0.73081	5.791538	-5.49291	1.21E-07	1.97E-06	6.890517
DISP1	-1.4111	2.986231	-5.49236	1.21E-07	1.97E-06	7.042704
ULBP1	3.521439	0.582924	5.489223	1.23E-07	2.00E-06	7.221205
POC1A	1.873026	3.111848	5.488795	1.23E-07	2.00E-06	7.288017
KCNE4	-1.76198	1.958431	-5.48854	1.23E-07	2.00E-06	7.122935
SATB1	-1.42328	4.381754	-5.48629	1.25E-07	2.02E-06	6.887623
NCRNA00152	2.144989	3.379256	5.484612	1.26E-07	2.03E-06	7.268042
RFX2	-1.36477	3.953493	-5.48264	1.27E-07	2.05E-06	6.903975
C14orf28	-0.88655	2.217786	-5.48044	1.28E-07	2.07E-06	7.148945
TUBE1	-0.86733	3.371781	-5.47941	1.29E-07	2.08E-06	6.99085
ANP32E	1.139165	6.267791	5.474698	1.32E-07	2.12E-06	6.857252

OBFC1	-1.0735	4.678293	-5.47301	1.33E-07	2.14E-06	6.824141
PSMD11	0.949363	6.867377	5.472666	1.33E-07	2.14E-06	6.804557
MTMR12	-0.99607	5.902621	-5.4721	1.34E-07	2.14E-06	6.790814
SYTL3	-1.39604	2.000912	-5.47053	1.35E-07	2.16E-06	7.075747
CENPO	1.572133	3.231754	5.468954	1.36E-07	2.17E-06	7.184453
TRIP13	3.01097	4.763968	5.464776	1.39E-07	2.22E-06	7.155546
SSC5D	-2.03404	3.224456	-5.46394	1.39E-07	2.22E-06	6.832762
XRCC2	2.533806	2.654993	5.463001	1.40E-07	2.23E-06	7.159533
BID	1.437709	4.912216	5.463094	1.40E-07	2.23E-06	6.988607
C19orf48	1.344775	5.624997	5.455601	1.45E-07	2.31E-06	6.843372
PTPRM	-1.63962	4.671459	-5.45492	1.46E-07	2.31E-06	6.720219
CCDC159	-0.99178	2.173983	-5.45373	1.46E-07	2.32E-06	7.0198
DNALI1	-2.35779	1.099796	-5.45178	1.48E-07	2.34E-06	6.998283
AZI2	-0.67871	5.028699	-5.45199	1.48E-07	2.34E-06	6.723354
CHADL	-1.50724	1.103987	-5.45121	1.48E-07	2.34E-06	7.067052
TM7SF2	-1.58689	3.62324	-5.45059	1.49E-07	2.35E-06	6.765937
TRIM59	2.063219	4.205601	5.449759	1.49E-07	2.35E-06	7.066583
RPS6KA5	-1.21468	3.408421	-5.44944	1.50E-07	2.36E-06	6.809342
CRISPLD2	-2.08898	5.85867	-5.44763	1.51E-07	2.37E-06	6.689953
UFSP2	-0.80597	4.060229	-5.4456	1.52E-07	2.39E-06	6.759551
GCNT2	-1.98463	3.395164	-5.44541	1.53E-07	2.39E-06	6.734634
BAX	1.158925	4.43728	5.444065	1.54E-07	2.40E-06	6.929764
STARD13	-1.60724	4.166725	-5.4442	1.53E-07	2.40E-06	6.692937
HERC3	-1.14558	5.001465	-5.4442	1.53E-07	2.40E-06	6.670822
SENP8	-0.93673	0.753743	-5.44242	1.55E-07	2.42E-06	7.070236
FAM13B	-0.98921	4.812614	-5.44226	1.55E-07	2.42E-06	6.674894
ITGA10	-1.61695	0.34971	-5.44154	1.55E-07	2.42E-06	7.059578
PER1	-1.91879	6.15711	-5.4417	1.55E-07	2.42E-06	6.664227
TLE2	-1.78442	3.23087	-5.44076	1.56E-07	2.43E-06	6.740939
C20orf194	-1.6392	4.507151	-5.43783	1.58E-07	2.46E-06	6.644936
FOXO3	-0.92157	6.788305	-5.43712	1.59E-07	2.47E-06	6.635944
RNLS	-2.27983	0.768028	-5.43323	1.62E-07	2.51E-06	6.953603
DNAJC27	-0.76176	2.92092	-5.43305	1.62E-07	2.51E-06	6.848525
BRCA2	2.379529	4.394222	5.431142	1.64E-07	2.53E-06	6.98765
CENPL	1.368837	3.678715	5.430282	1.64E-07	2.54E-06	6.968444
ARL6IP1	1.030679	8.135618	5.42753	1.66E-07	2.57E-06	6.584383
SERPINH1	1.6121	7.661057	5.426185	1.68E-07	2.58E-06	6.580757
GEN1	1.27487	4.960484	5.423599	1.70E-07	2.61E-06	6.775578
PTGDS	-2.18378	2.934223	-5.4238	1.69E-07	2.61E-06	6.660807
NUDT1	2.157108	3.480257	5.422274	1.71E-07	2.62E-06	6.97876
HMGB2	1.428394	6.461132	5.421461	1.71E-07	2.63E-06	6.612017
C9orf45	-1.29196	2.659841	-5.42009	1.73E-07	2.65E-06	6.760035
USP47	-0.66936	6.517367	-5.41468	1.77E-07	2.71E-06	6.522188
ACTG2	-3.67456	5.56239	-5.41382	1.78E-07	2.72E-06	6.562108
CDKN1C	-1.70008	3.848503	-5.41238	1.79E-07	2.74E-06	6.559422
C22orf32	-0.83405	4.593593	-5.41172	1.80E-07	2.74E-06	6.55323
DMPK	-1.29387	5.005868	-5.41066	1.81E-07	2.76E-06	6.509059
CACNB4	-2.1437	0.454393	-5.40668	1.84E-07	2.80E-06	6.868562
APOLD1	-1.58205	4.405305	-5.40671	1.84E-07	2.80E-06	6.504492
C9orf5	-0.98414	6.659733	-5.40569	1.85E-07	2.81E-06	6.486773
FAM188A	-0.78547	4.00524	-5.40445	1.86E-07	2.82E-06	6.573225
CCT3	0.832108	7.939625	5.404466	1.86E-07	2.82E-06	6.475444
ZBTB4	-1.0823	6.70562	-5.40289	1.88E-07	2.84E-06	6.476117
LOC728743	-1.3338	2.597073	-5.40082	1.90E-07	2.87E-06	6.67567
TACR2	-2.31569	0.542781	-5.39986	1.90E-07	2.88E-06	6.815325
GINS2	2.789248	3.584693	5.39737	1.93E-07	2.91E-06	6.872773
CRTC1	-0.93349	4.191027	-5.39646	1.94E-07	2.92E-06	6.506694
WBSCR17	-2.17256	0.259235	-5.39588	1.94E-07	2.93E-06	6.828428

CCNG1	-1.2057	6.084642	-5.39551	1.94E-07	2.93E-06	6.433622
PSMA3	0.896675	6.105581	5.39494	1.95E-07	2.93E-06	6.476574
LRRN4CL	-1.68497	0.717167	-5.39388	1.96E-07	2.94E-06	6.817745
NR2F2	-1.8184	5.617206	-5.39269	1.97E-07	2.96E-06	6.423355
OXCT1	-1.86204	4.987887	-5.3907	1.99E-07	2.98E-06	6.41068
CLIP3	-1.74013	3.638779	-5.38172	2.08E-07	3.11E-06	6.428968
C9orf140	2.62064	5.538625	5.378064	2.12E-07	3.17E-06	6.657422
TEAD4	1.681134	4.386359	5.377224	2.12E-07	3.18E-06	6.682904
DNA2	1.916358	3.468923	5.374071	2.16E-07	3.22E-06	6.751001
NOP56	0.938142	6.959753	5.3723	2.18E-07	3.24E-06	6.328828
ARHGEF9	-1.4218	3.989764	-5.3701	2.20E-07	3.28E-06	6.367093
NEURL3	3.620253	0.069159	5.368428	2.22E-07	3.30E-06	6.681516
SNRPD1	1.182478	5.622849	5.363651	2.27E-07	3.37E-06	6.396036
MAGI2	-1.57086	1.964546	-5.36248	2.28E-07	3.39E-06	6.553996
ARPC5	0.699333	7.46822	5.361334	2.29E-07	3.40E-06	6.269307
ATXN7	-0.81809	5.174675	-5.35992	2.31E-07	3.42E-06	6.278362
SLC22A17	-1.69403	2.497937	-5.35311	2.39E-07	3.53E-06	6.421361
MMP14	1.474818	8.162586	5.353106	2.39E-07	3.53E-06	6.231749
GRINA	0.977836	7.616544	5.350316	2.42E-07	3.57E-06	6.217777
KCNJ12	-2.25221	0.162232	-5.34806	2.45E-07	3.61E-06	6.612007
NOSTRIN	-2.38992	1.96062	-5.3451	2.48E-07	3.66E-06	6.384699
S100A11	1.589617	9.597227	5.343614	2.50E-07	3.68E-06	6.210859
DNAJC6	-2.05284	1.444359	-5.34338	2.50E-07	3.68E-06	6.481208
RELL1	-1.16009	4.887036	-5.34171	2.52E-07	3.71E-06	6.194128
CDCA4	1.943115	5.055961	5.340366	2.54E-07	3.73E-06	6.460574
DSN1	1.297413	4.488257	5.339975	2.54E-07	3.73E-06	6.456599
INPP5J	-1.66915	2.306175	-5.33739	2.58E-07	3.77E-06	6.384095
ZFYVE28	-1.43534	2.907202	-5.33662	2.59E-07	3.78E-06	6.322017
SYNGR1	-2.08945	3.183082	-5.33609	2.59E-07	3.79E-06	6.233824
SLC19A2	-1.17947	4.500864	-5.33462	2.61E-07	3.81E-06	6.17994
SNHG1	1.143334	5.364785	5.333871	2.62E-07	3.82E-06	6.283062
LTBP4	-1.66114	7.50132	-5.33352	2.62E-07	3.82E-06	6.186068
UTRN	-1.40363	6.332617	-5.33009	2.67E-07	3.88E-06	6.134913
AKAP6	-1.76235	2.371909	-5.32975	2.67E-07	3.88E-06	6.320727
LRFN4	1.998124	5.208228	5.325928	2.72E-07	3.95E-06	6.380932
ADAMTS15	-2.25201	1.322733	-5.32469	2.74E-07	3.97E-06	6.392079
ATP9B	-0.8703	4.381818	-5.3245	2.74E-07	3.97E-06	6.15979
NDUFS1	-0.82398	6.342867	-5.32355	2.75E-07	3.99E-06	6.095919
RNF150	-2.3774	0.881436	-5.32179	2.78E-07	4.02E-06	6.414454
ARRB1	-1.83065	3.625835	-5.32111	2.79E-07	4.02E-06	6.144373
CSE1L	0.931736	7.542022	5.320044	2.80E-07	4.04E-06	6.076895
DTYMK	1.335123	4.498495	5.319299	2.81E-07	4.05E-06	6.364438
FAM13C	-1.76466	1.498411	-5.318	2.83E-07	4.07E-06	6.387423
TMEM206	1.522385	3.16089	5.3175	2.83E-07	4.08E-06	6.490749
MFAP4	-2.61784	5.230281	-5.31629	2.85E-07	4.10E-06	6.075291
JAM3	-1.51804	3.930749	-5.31527	2.86E-07	4.11E-06	6.110256
SYP	-1.87916	0.272046	-5.31218	2.91E-07	4.17E-06	6.461438
KANK2	-1.78052	5.82905	-5.31189	2.91E-07	4.17E-06	6.048748
SNPH	-1.67525	2.513705	-5.31067	2.93E-07	4.19E-06	6.22637
TCEA3	-1.66792	4.144923	-5.30691	2.98E-07	4.26E-06	6.049571
PIGU	0.954075	4.994744	5.301984	3.05E-07	4.36E-06	6.160921
TOB2	-0.89163	6.564032	-5.29572	3.15E-07	4.49E-06	5.971045
E2F7	3.826774	4.033841	5.290063	3.23E-07	4.60E-06	6.387055
TMEM26	2.478601	0.632782	5.29011	3.23E-07	4.60E-06	6.339143
C17orf108	-1.32388	1.606841	-5.28996	3.23E-07	4.60E-06	6.292999
ANAPC16	-0.7524	6.1127	-5.28916	3.25E-07	4.61E-06	5.933834
MFSD7	-1.61877	1.496542	-5.28577	3.30E-07	4.69E-06	6.258552
COBLL1	-1.56968	6.182519	-5.28372	3.33E-07	4.73E-06	5.920347

C5	-1.80619	2.371346	-5.28269	3.35E-07	4.75E-06	6.104263
CDH23	-2.118	0.53378	-5.28215	3.36E-07	4.75E-06	6.293425
AGAP11	-1.78465	0.656465	-5.28089	3.38E-07	4.77E-06	6.300354
FBXO38	-0.54092	5.157224	-5.28131	3.37E-07	4.77E-06	5.92558
PRMT1	0.927488	6.480809	5.280974	3.38E-07	4.77E-06	5.924041
CSRP1	-1.40118	7.707965	-5.27994	3.39E-07	4.78E-06	5.93713
SNX18	-0.92323	5.009804	-5.27808	3.42E-07	4.82E-06	5.902004
LRP2BP	-1.15293	0.949892	-5.27674	3.45E-07	4.85E-06	6.2976
PSMB3	1.125724	6.923816	5.275327	3.47E-07	4.88E-06	5.885902
MLF1IP	2.007268	4.514031	5.274256	3.49E-07	4.89E-06	6.229301
APOOL	-0.899	3.654574	-5.2743	3.49E-07	4.89E-06	5.995801
CDADC1	-0.79506	2.55	-5.26897	3.58E-07	5.01E-06	6.13874
NMI	1.319314	5.005851	5.263376	3.67E-07	5.14E-06	6.032074
GPX3	-2.64881	5.5265	-5.26203	3.70E-07	5.16E-06	5.829762
TSC2	-0.7132	6.766354	-5.26202	3.70E-07	5.16E-06	5.816067
AG2	-1.91723	4.619351	-5.26158	3.70E-07	5.17E-06	5.815028
RDH12	-2.78025	0.232865	-5.25784	3.77E-07	5.26E-06	6.161614
SMPD1	-1.01374	4.617899	-5.25611	3.80E-07	5.30E-06	5.817904
SEMA3G	-1.59147	2.361822	-5.25372	3.85E-07	5.35E-06	5.996529
ATP1B3	1.667963	7.801787	5.252925	3.86E-07	5.37E-06	5.772294
ZNF167	-1.67849	-0.01749	-5.25236	3.87E-07	5.38E-06	6.210869
TAL1	-1.59725	0.624641	-5.25092	3.90E-07	5.41E-06	6.178236
PARP14	1.246961	7.376339	5.247966	3.95E-07	5.48E-06	5.74964
MYO1B	1.483157	7.244494	5.247729	3.96E-07	5.48E-06	5.759891
ERGIC1	-0.96132	7.186467	-5.2473	3.96E-07	5.48E-06	5.761661
RAVER1	0.690911	7.054921	5.247482	3.96E-07	5.48E-06	5.743325
MPRIP	-0.82629	6.804458	-5.24389	4.03E-07	5.56E-06	5.735198
HEATR1	0.888118	6.601522	5.240422	4.10E-07	5.65E-06	5.729434
TSPYL1	-0.8611	6.181676	-5.23981	4.11E-07	5.66E-06	5.707903
FOXS1	2.267984	0.503978	5.238081	4.14E-07	5.70E-06	6.112199
HSPE1	1.163362	6.34227	5.238118	4.14E-07	5.70E-06	5.750498
C16orf75	2.455352	3.863017	5.236854	4.17E-07	5.72E-06	6.13431
SEC22C	-0.75993	3.858739	-5.2363	4.18E-07	5.73E-06	5.812239
PVT1	2.454068	3.171708	5.233034	4.24E-07	5.82E-06	6.135182
SGEF	-2.03154	3.217952	-5.23109	4.28E-07	5.87E-06	5.74972
SPINT2	1.415057	8.38958	5.230536	4.29E-07	5.88E-06	5.667837
TEX2	-0.79005	5.983074	-5.22484	4.41E-07	6.03E-06	5.637717
CCL21	-2.95339	2.572526	-5.21647	4.59E-07	6.27E-06	5.683215
RBL1	1.672164	4.292876	5.216119	4.60E-07	6.27E-06	5.95712
SGSM1	-2.46157	0.749166	-5.21464	4.63E-07	6.31E-06	5.938533
EBNA1BP2	0.865897	5.906982	5.21478	4.63E-07	6.31E-06	5.656024
SNTA1	-1.23864	3.510129	-5.21105	4.71E-07	6.41E-06	5.691373
C18orf56	1.944616	0.15251	5.208811	4.76E-07	6.47E-06	5.984409
SLC7A2	-2.45022	3.578221	-5.20799	4.78E-07	6.49E-06	5.596338
CFL1	0.804211	9.67942	5.205992	4.82E-07	6.54E-06	5.598098
TSN	0.779527	6.510593	5.204145	4.87E-07	6.59E-06	5.562885
SLC41A1	-0.90314	5.607767	-5.20409	4.87E-07	6.59E-06	5.545228
PDE1B	-1.83726	0.705475	-5.20288	4.90E-07	6.61E-06	5.93962
TNS1	-2.47046	7.314746	-5.20272	4.90E-07	6.61E-06	5.598895
ZMYND11	-0.83878	6.41653	-5.20302	4.89E-07	6.61E-06	5.542045
NOP2	1.065361	5.790152	5.20111	4.94E-07	6.66E-06	5.620202
DYNC1LI2	-0.68515	7.034602	-5.20085	4.94E-07	6.66E-06	5.540044
PTPLA	-1.82716	0.919999	-5.20022	4.96E-07	6.67E-06	5.9111
LOC200030	-0.8955	4.500366	-5.19953	4.97E-07	6.69E-06	5.5741
SELENBP1	-2.77909	4.161191	-5.19762	5.02E-07	6.74E-06	5.525899
HIST1H2AE	2.741399	0.928578	5.19724	5.03E-07	6.75E-06	5.936752
PLXNA4	-2.16399	1.367615	-5.19275	5.14E-07	6.89E-06	5.789572
TCF19	1.380872	4.949825	5.190506	5.19E-07	6.95E-06	5.716385

ZNF181	-0.87757	2.985711	-5.18969	5.21E-07	6.97E-06	5.703311
SLC13A3	-2.27263	0.922446	-5.18389	5.36E-07	7.16E-06	5.800177
CAD	1.0695	6.90994	5.18329	5.37E-07	7.17E-06	5.462645
APC2	-1.56646	1.106171	-5.18263	5.39E-07	7.19E-06	5.83618
STX6	0.933916	6.04174	5.18131	5.42E-07	7.23E-06	5.497702
ZNF43	-1.83629	2.046346	-5.18109	5.43E-07	7.23E-06	5.680656
ZFP64	0.965677	5.168138	5.180051	5.45E-07	7.26E-06	5.581316
ZNF331	-1.84518	3.094462	-5.17508	5.58E-07	7.41E-06	5.519382
TMEM167B	-0.63214	5.567669	-5.1751	5.58E-07	7.41E-06	5.418603
RSPO3	-2.5387	1.254208	-5.17102	5.69E-07	7.55E-06	5.662605
CCDC99	1.324587	4.141933	5.170816	5.70E-07	7.55E-06	5.733479
NFIX	-1.34655	7.036386	-5.16868	5.75E-07	7.62E-06	5.407805
CLDN7	4.493644	6.182772	5.158371	6.04E-07	7.99E-06	5.761295
CDK2	1.059637	5.014555	5.157842	6.05E-07	8.00E-06	5.513574
SVEP1	-1.77499	3.717881	-5.15735	6.07E-07	8.01E-06	5.388431
PODN	-2.18645	3.876995	-5.15144	6.24E-07	8.23E-06	5.331945
CDK4	1.089186	6.415901	5.150616	6.26E-07	8.26E-06	5.341667
XPR1	1.287194	6.563958	5.148226	6.33E-07	8.34E-06	5.333353
SLC25A16	-0.67645	3.550255	-5.1476	6.35E-07	8.36E-06	5.455468
PRKCE	-0.87999	3.569795	-5.14584	6.41E-07	8.42E-06	5.421926
ACOX3	-1.08221	4.091895	-5.14482	6.44E-07	8.45E-06	5.346584
NCALD	-1.52323	3.089789	-5.14356	6.47E-07	8.49E-06	5.405627
RAP1A	-0.84971	5.81017	-5.14355	6.48E-07	8.49E-06	5.268179
C1orf106	2.120214	7.005414	5.143292	6.48E-07	8.49E-06	5.338722
GNAQ	-0.92242	6.582013	-5.13989	6.59E-07	8.62E-06	5.258919
DUSP22	-0.97581	4.674328	-5.13633	6.70E-07	8.75E-06	5.270401
RERE	-0.99366	7.312256	-5.13477	6.75E-07	8.81E-06	5.252982
INCENP	1.158347	5.434354	5.134342	6.76E-07	8.82E-06	5.363576
HDHD2	-0.88224	4.391297	-5.13255	6.82E-07	8.89E-06	5.279746
ZBED3	-1.61866	1.612118	-5.13219	6.83E-07	8.89E-06	5.551851
ERN1	-1.26333	3.886289	-5.13099	6.87E-07	8.93E-06	5.290698
KIAA1529	-2.17038	0.007516	-5.12973	6.91E-07	8.97E-06	5.643859
FAM110A	1.714631	4.59524	5.129712	6.91E-07	8.97E-06	5.537548
USP2	-1.73653	1.747848	-5.12911	6.93E-07	8.99E-06	5.50473
C14orf139	-1.48975	1.062452	-5.12649	7.01E-07	9.09E-06	5.596197
TMEM108	-1.99352	0.827031	-5.12536	7.05E-07	9.13E-06	5.568995
SIGLEC7	2.401796	-0.15179	5.121141	7.19E-07	9.30E-06	5.607396
LIMCH1	-1.86688	4.131726	-5.12093	7.20E-07	9.31E-06	5.192025
TACSTD2	2.666888	8.155893	5.118101	7.30E-07	9.41E-06	5.180837
MDH1	-0.66054	6.509287	-5.11797	7.30E-07	9.41E-06	5.155063
CPEB4	-1.23207	5.71349	-5.11784	7.30E-07	9.41E-06	5.152909
HOXA10	5.164091	3.322482	5.116445	7.35E-07	9.47E-06	5.594278
F8	-1.32779	2.901726	-5.11254	7.49E-07	9.63E-06	5.309642
TYMP	2.385684	6.965704	5.112396	7.49E-07	9.63E-06	5.224478
PFDN2	1.233429	4.943984	5.110192	7.57E-07	9.71E-06	5.33458
TPM3	0.729478	8.509451	5.110156	7.57E-07	9.71E-06	5.133711
GIPC3	-1.30064	2.356807	-5.10986	7.58E-07	9.72E-06	5.382728
C7orf11	1.151276	4.550174	5.1092	7.61E-07	9.74E-06	5.379319
CKAP5	0.847322	7.557171	5.103961	7.79E-07	9.97E-06	5.089154
BCL2L12	1.326302	3.958766	5.103415	7.81E-07	9.99E-06	5.454719
ATP5A1	-1.04153	8.188115	-5.10214	7.86E-07	1.00E-05	5.132035
ATL1	-1.20228	2.373173	-5.10189	7.87E-07	1.00E-05	5.357115
ABCC9	-1.89503	2.707213	-5.10086	7.91E-07	1.01E-05	5.22257
CHTF18	1.621262	4.270541	5.099754	7.95E-07	1.01E-05	5.434007
ENO1	1.11784	9.869741	5.095715	8.10E-07	1.03E-05	5.09631
MYO9A	-0.95125	4.915691	-5.09473	8.14E-07	1.03E-05	5.070533
CRELD1	-0.97311	3.742519	-5.09389	8.17E-07	1.04E-05	5.160621
NR4A3	-2.04571	3.025207	-5.09377	8.17E-07	1.04E-05	5.14623

HNRNPR	0.557512	7.134291	5.092707	8.21E-07	1.04E-05	5.038198
EIF4EBP3	-1.34254	2.368836	-5.09182	8.25E-07	1.04E-05	5.298689
C17orf48	-0.76726	2.408749	-5.09131	8.27E-07	1.05E-05	5.360772
FHIT	-1.84213	0.420203	-5.0907	8.29E-07	1.05E-05	5.464423
DTWD1	-0.66259	3.650132	-5.08978	8.33E-07	1.05E-05	5.183557
CCPG1	-1.29112	6.062019	-5.08862	8.37E-07	1.06E-05	5.025746
SHE	-1.49167	1.948109	-5.08832	8.38E-07	1.06E-05	5.322953
GPRASP2	-1.1591	2.46992	-5.08782	8.40E-07	1.06E-05	5.285278
DLEU2	1.797988	2.271923	5.08717	8.43E-07	1.06E-05	5.497529
ZSWIM6	-0.92573	4.777369	-5.08671	8.45E-07	1.06E-05	5.04335
C12orf51	-0.8828	6.500391	-5.08631	8.46E-07	1.06E-05	5.015653
PRIM1	1.618916	3.129146	5.085105	8.51E-07	1.07E-05	5.460738
ZC3H6	-1.16597	4.083913	-5.08325	8.58E-07	1.08E-05	5.063887
LAMC2	2.560204	8.282533	5.083316	8.58E-07	1.08E-05	5.01408
ZNF569	-1.7252	0.828375	-5.08273	8.60E-07	1.08E-05	5.404025
ZNF10	-1.26111	2.892026	-5.08282	8.60E-07	1.08E-05	5.187131
CSDC2	-1.87266	0.118874	-5.08171	8.65E-07	1.08E-05	5.440376
C4orf31	-2.21345	0.762334	-5.08058	8.69E-07	1.09E-05	5.360281
KAT2A	0.884135	6.026071	5.080276	8.70E-07	1.09E-05	5.039707
YIF1B	1.069483	4.91386	5.079757	8.72E-07	1.09E-05	5.180821
VSIG1	-3.42665	2.153867	-5.07937	8.74E-07	1.09E-05	5.076926
ZER1	-0.80273	6.374917	-5.07454	8.94E-07	1.11E-05	4.95996
CENPH	1.837427	3.153716	5.074307	8.95E-07	1.11E-05	5.421864
FERMT1	2.55187	6.472215	5.073446	8.98E-07	1.11E-05	5.126865
LTF	-3.29579	1.57255	-5.07352	8.98E-07	1.11E-05	5.11106
GALNT12	-1.84596	4.51774	-5.07144	9.07E-07	1.12E-05	4.955569
PACSIN2	-0.89394	5.620596	-5.07072	9.10E-07	1.13E-05	4.942107
PSMC4	0.809084	6.415447	5.070111	9.13E-07	1.13E-05	4.963143
SLC25A34	-1.48301	0.401763	-5.06929	9.16E-07	1.13E-05	5.390574
RASGRP2	-1.85704	0.846488	-5.0689	9.18E-07	1.13E-05	5.330675
LOC202181	-1.67524	1.871465	-5.06902	9.17E-07	1.13E-05	5.226453
TCEAL1	-1.02532	3.023075	-5.06568	9.31E-07	1.15E-05	5.119547
CLDN5	-1.63996	3.208153	-5.05854	9.63E-07	1.19E-05	5.001558
TMTC2	-1.21291	4.24032	-5.0577	9.67E-07	1.19E-05	4.934398
IL6ST	-1.506	5.586589	-5.05662	9.72E-07	1.19E-05	4.879991
CTDSPL	-0.9665	6.072374	-5.05673	9.71E-07	1.19E-05	4.878838
KIAA1109	-1.01601	6.427314	-5.05291	9.88E-07	1.21E-05	4.867074
GGCT	1.114389	5.743225	5.050134	1.00E-06	1.23E-05	4.94856
SLC16A13	1.90836	2.655182	5.048475	1.01E-06	1.24E-05	5.327489
CAND2	-1.88161	1.366571	-5.04805	1.01E-06	1.24E-05	5.175235
THSD4	-2.03378	4.941059	-5.04356	1.03E-06	1.26E-05	4.823743
DDX27	0.779797	6.213689	5.042278	1.04E-06	1.27E-05	4.849457
MON2	-0.68965	6.001699	-5.03783	1.06E-06	1.29E-05	4.792904
HAGH	-0.78005	3.984317	-5.0348	1.07E-06	1.31E-05	4.888113
HOXB7	4.181261	3.392663	5.034395	1.08E-06	1.31E-05	5.248086
HOMER2	-2.48292	3.076709	-5.03384	1.08E-06	1.32E-05	4.846272
TCOF1	0.969311	6.587668	5.029887	1.10E-06	1.34E-05	4.782602
MXI1	-1.09146	5.198829	-5.02875	1.11E-06	1.34E-05	4.76058
CHAF1B	1.800557	3.565796	5.027927	1.11E-06	1.35E-05	5.194227
ADD1	-0.76087	7.64547	-5.02786	1.11E-06	1.35E-05	4.777138
FGF14	-2.26218	0.136931	-5.02733	1.11E-06	1.35E-05	5.179871
CYYR1	-1.30576	3.244639	-5.025	1.12E-06	1.36E-05	4.877794
IKZF4	-0.80314	3.729583	-5.0238	1.13E-06	1.37E-05	4.863843
N6AMT1	-0.83924	2.592121	-5.0205	1.15E-06	1.39E-05	5.012656
LARGE	-1.52783	4.26929	-5.02009	1.15E-06	1.39E-05	4.747503
ACYP1	1.317075	2.563293	5.019208	1.16E-06	1.39E-05	5.187777
MT1X	-2.05915	4.667207	-5.01774	1.16E-06	1.40E-05	4.710439
NTN1	-2.30204	4.677068	-5.01729	1.17E-06	1.40E-05	4.708893

ANKRD29	-1.94709	1.842672	-5.017	1.17E-06	1.41E-05	4.96925
CAPN7	-0.68551	5.055429	-5.01658	1.17E-06	1.41E-05	4.726012
PJA2	-0.91967	6.945942	-5.01665	1.17E-06	1.41E-05	4.713437
ZNF397	-0.88207	3.556457	-5.01389	1.18E-06	1.42E-05	4.83384
DHX34	0.790894	5.626781	5.012748	1.19E-06	1.43E-05	4.765548
TTLL11	-0.93158	1.978004	-5.01202	1.19E-06	1.43E-05	5.046224
ITGA2	1.750021	6.817379	5.010244	1.20E-06	1.44E-05	4.729103
ADCY1	-1.70662	1.880935	-5.00642	1.23E-06	1.47E-05	4.943338
SETBP1	-2.00168	3.928831	-5.0064	1.23E-06	1.47E-05	4.685941
PARVA	-0.94647	5.989551	-5.0041	1.24E-06	1.48E-05	4.643848
PTTG1IP	-0.80425	7.66828	-4.99827	1.27E-06	1.52E-05	4.647678
KIAA0895	-1.35187	3.771889	-4.99782	1.27E-06	1.52E-05	4.69982
GPT	-2.38201	0.439208	-4.99653	1.28E-06	1.53E-05	5.015577
XKR8	-0.75295	3.069802	-4.98945	1.32E-06	1.58E-05	4.815346
ADAR	0.6521	8.835778	4.98899	1.33E-06	1.58E-05	4.60305
ZFP106	-0.80944	6.555879	-4.98645	1.34E-06	1.60E-05	4.570722
RHOU	-2.02835	4.115177	-4.98088	1.38E-06	1.64E-05	4.563657
DCAF8	-0.58891	6.164368	-4.97644	1.41E-06	1.67E-05	4.520387
MSRB3	-1.88189	4.300313	-4.97545	1.41E-06	1.68E-05	4.535093
TRIM23	-0.76038	3.207141	-4.97484	1.42E-06	1.68E-05	4.72579
TMSB10	1.194917	9.164278	4.974956	1.42E-06	1.68E-05	4.537215
HOXC10	6.096472	2.863037	4.973102	1.43E-06	1.69E-05	4.988453
MTERFD3	-0.87024	2.947085	-4.97224	1.43E-06	1.69E-05	4.74216
RALY	0.644004	7.022965	4.97088	1.44E-06	1.70E-05	4.497759
SYNM	-3.00367	5.174014	-4.97029	1.45E-06	1.71E-05	4.516909
ULBP2	4.556057	2.414963	4.964411	1.49E-06	1.75E-05	4.951587
HIGD1A	-0.97639	6.578526	-4.96338	1.49E-06	1.76E-05	4.471969
NCAPG2	1.657641	5.204523	4.961406	1.51E-06	1.77E-05	4.69742
C6orf115	1.386952	4.927385	4.958848	1.52E-06	1.79E-05	4.690797
RNASE4	-2.12533	4.330031	-4.9578	1.53E-06	1.80E-05	4.451824
DSEL	-1.55413	2.141375	-4.95641	1.54E-06	1.81E-05	4.70393
MSH2	1.073073	5.201722	4.955264	1.55E-06	1.82E-05	4.588036
HMGN4	0.782814	5.844831	4.953703	1.56E-06	1.83E-05	4.485095
ALPK3	-1.63905	3.644987	-4.95293	1.57E-06	1.83E-05	4.491622
EIF4B	-0.6959	7.943457	-4.9524	1.57E-06	1.84E-05	4.450535
REEP5	-0.70482	6.876122	-4.94992	1.59E-06	1.86E-05	4.413539
PRPF19	0.839927	6.708576	4.948911	1.60E-06	1.86E-05	4.413708
CD80	2.465212	0.188495	4.94849	1.60E-06	1.87E-05	4.881569
SALL4	4.087402	1.550713	4.946716	1.61E-06	1.88E-05	4.876762
ATP5J	-0.8947	6.08137	-4.94577	1.62E-06	1.89E-05	4.386737
NNT	-1.27129	6.076322	-4.94543	1.62E-06	1.89E-05	4.389918
EIF3B	0.793901	7.849954	4.945366	1.62E-06	1.89E-05	4.387679
G3BP1	0.667492	7.040641	4.944858	1.62E-06	1.89E-05	4.383357
CNRIP1	-1.25801	2.09562	-4.94433	1.63E-06	1.89E-05	4.696382
NOP58	0.867671	6.436491	4.94349	1.64E-06	1.90E-05	4.404137
DBF4	1.601918	4.402608	4.941873	1.65E-06	1.91E-05	4.723017
MAN1C1	-1.55536	2.628253	-4.94193	1.65E-06	1.91E-05	4.565991
TOMM40	1.058651	6.013717	4.941797	1.65E-06	1.91E-05	4.440139
FERMT2	-1.73153	4.900398	-4.94208	1.65E-06	1.91E-05	4.375033
CMTM4	-1.55933	5.994953	-4.94013	1.66E-06	1.92E-05	4.369943
NOX4	2.986463	0.961626	4.938977	1.67E-06	1.93E-05	4.843602
KIAA1683	-1.73091	1.751778	-4.93856	1.67E-06	1.93E-05	4.663503
SNED1	-1.56956	3.806495	-4.93826	1.67E-06	1.93E-05	4.417982
FOXP1	-1.20733	6.124081	-4.93814	1.68E-06	1.93E-05	4.357625
ARRDC4	-1.8184	5.111915	-4.93842	1.67E-06	1.93E-05	4.357499
FAT4	-1.69495	3.20021	-4.93754	1.68E-06	1.93E-05	4.463199
FOXO3B	-0.754	5.186294	-4.93596	1.69E-06	1.94E-05	4.361045
DES	-4.98575	4.892981	-4.93442	1.70E-06	1.96E-05	4.404479

EIF2AK1	0.659915	7.826965	4.932189	1.72E-06	1.97E-05	4.331312
SF3B3	0.737252	8.001125	4.93018	1.74E-06	1.99E-05	4.324286
CDC7	1.699771	3.499285	4.928854	1.75E-06	2.00E-05	4.76381
COL1A1	2.37941	11.19358	4.927775	1.76E-06	2.01E-05	4.358259
SASH1	-1.51316	5.402475	-4.92678	1.76E-06	2.02E-05	4.304897
ARSK	-0.93004	2.692037	-4.92489	1.78E-06	2.03E-05	4.567846
HMGN5	-1.69505	2.162271	-4.92499	1.78E-06	2.03E-05	4.546809
TXNIP	-2.14756	8.148753	-4.92274	1.80E-06	2.05E-05	4.363735
DLC1	-1.50036	4.476313	-4.92085	1.81E-06	2.07E-05	4.299989
TPSB2	-1.93879	2.953436	-4.92034	1.82E-06	2.07E-05	4.395638
F12	3.444934	3.05141	4.919845	1.82E-06	2.07E-05	4.779215
CLK4	-0.96435	4.094701	-4.92002	1.82E-06	2.07E-05	4.35612
ZNF439	-1.56	0.714352	-4.91952	1.82E-06	2.07E-05	4.715345
MCEE	-0.85979	2.084176	-4.91935	1.83E-06	2.07E-05	4.640028
GAMT	-1.97984	2.335924	-4.91857	1.83E-06	2.08E-05	4.455911
DHRS7	-1.11606	5.421913	-4.91633	1.85E-06	2.10E-05	4.259832
AGRN	1.276314	9.062365	4.915746	1.86E-06	2.10E-05	4.273112
INTS7	0.937792	5.254473	4.914423	1.87E-06	2.11E-05	4.386256
DDX17	-0.55982	8.896821	-4.91299	1.88E-06	2.12E-05	4.302252
NUP85	0.820755	5.231614	4.909375	1.91E-06	2.16E-05	4.354451
FAM129A	-2.04731	5.960146	-4.90823	1.92E-06	2.17E-05	4.238652
SSB	0.779419	6.120968	4.906114	1.94E-06	2.19E-05	4.255501
YDJC	1.261367	4.734008	4.902674	1.97E-06	2.22E-05	4.458
PUS1	1.007055	4.398921	4.90205	1.97E-06	2.22E-05	4.470749
PNPLA4	-1.38065	3.296625	-4.90049	1.99E-06	2.24E-05	4.319457
ARMCX2	-1.63368	2.892175	-4.8993	2.00E-06	2.25E-05	4.336074
FMOD	-2.05962	5.425889	-4.8991	2.00E-06	2.25E-05	4.190953
MASP2	-1.46521	0.319902	-4.89748	2.02E-06	2.26E-05	4.654641
NOL11	0.734941	5.679141	4.897455	2.02E-06	2.26E-05	4.249848
SNRPC	0.947609	5.40526	4.896921	2.02E-06	2.26E-05	4.294074
SPATA6	-1.50924	1.35356	-4.89466	2.04E-06	2.29E-05	4.551842
TMEM189	1.128895	6.62063	4.894537	2.04E-06	2.29E-05	4.193591
ILDR1	4.035589	2.90769	4.893884	2.05E-06	2.29E-05	4.659473
CNST	-0.83761	5.349522	-4.89329	2.05E-06	2.29E-05	4.165773
RPS6KA2	-1.40064	5.030383	-4.89295	2.06E-06	2.30E-05	4.161598
TECPR2	-0.76264	5.174779	-4.89223	2.06E-06	2.30E-05	4.170137
SCN4B	-1.68321	1.60367	-4.89204	2.07E-06	2.30E-05	4.488177
TEK	-1.51261	2.560512	-4.88937	2.09E-06	2.33E-05	4.355083
ARL6IP6	1.145466	4.928184	4.88799	2.10E-06	2.34E-05	4.345904
PKP3	2.173784	7.443937	4.888114	2.10E-06	2.34E-05	4.17698
PDK2	-0.89669	5.293355	-4.88644	2.12E-06	2.35E-05	4.136349
HAUS8	1.183567	2.865388	4.885826	2.13E-06	2.36E-05	4.592359
TMEM41A	0.872461	5.458954	4.885706	2.13E-06	2.36E-05	4.232253
SUOX	-0.88098	3.780734	-4.8848	2.14E-06	2.37E-05	4.242111
ATP5O	-0.82484	6.327927	-4.88422	2.14E-06	2.37E-05	4.119583
TXNDC12	0.655083	6.360515	4.883673	2.15E-06	2.37E-05	4.136434
LYRM7	-0.76908	4.357135	-4.87661	2.22E-06	2.45E-05	4.15801
ATP8A1	-2.37752	3.832739	-4.87661	2.22E-06	2.45E-05	4.110153
POLI	-0.98696	3.547848	-4.87569	2.22E-06	2.46E-05	4.218997
GYPC	-1.39475	3.015869	-4.87493	2.23E-06	2.46E-05	4.238598
MRGPRF	-1.67502	2.697349	-4.87476	2.23E-06	2.46E-05	4.248252
PSMB4	0.70935	7.128553	4.871219	2.27E-06	2.50E-05	4.061347
PLCE1	-1.98141	3.850618	-4.87088	2.27E-06	2.50E-05	4.098441
PDPK1	-0.66574	6.272123	-4.86994	2.28E-06	2.51E-05	4.055217
VDR	1.668863	5.731105	4.869498	2.29E-06	2.51E-05	4.216992
RFC4	1.865335	4.40127	4.867821	2.31E-06	2.53E-05	4.433468
ZNF853	-1.76169	2.060065	-4.86684	2.32E-06	2.54E-05	4.298753
SLC30A9	-0.70999	5.862932	-4.86677	2.32E-06	2.54E-05	4.041932

FBXO5	1.286627	3.765742	4.865839	2.33E-06	2.55E-05	4.436208
WDR75	0.626686	5.773211	4.865756	2.33E-06	2.55E-05	4.095944
DCLRE1B	1.143212	3.742981	4.865404	2.33E-06	2.55E-05	4.421637
FIGNL1	1.389626	4.539363	4.865431	2.33E-06	2.55E-05	4.344806
MDK	1.882226	6.956092	4.864445	2.34E-06	2.55E-05	4.090299
RNF185	-0.65015	5.644899	-4.86435	2.34E-06	2.55E-05	4.03613
ECHDC3	-2.42171	1.878771	-4.86312	2.35E-06	2.56E-05	4.231662
BRIX1	1.141814	5.264591	4.863357	2.35E-06	2.56E-05	4.187645
NUP107	1.139837	5.45513	4.863217	2.35E-06	2.56E-05	4.162473
KLHL3	-1.37807	2.218639	-4.86263	2.36E-06	2.57E-05	4.310819
RHBDF2	1.313028	6.066459	4.861638	2.37E-06	2.58E-05	4.108239
CHRD	-1.67421	1.604012	-4.86101	2.38E-06	2.58E-05	4.356001
SHC2	-1.46588	2.667239	-4.8586	2.40E-06	2.61E-05	4.212497
LDB2	-1.21934	2.707933	-4.85701	2.42E-06	2.62E-05	4.231632
E2F3	1.217017	4.793959	4.855981	2.43E-06	2.63E-05	4.2392
LRRC70	-1.14969	0.57486	-4.85522	2.44E-06	2.64E-05	4.476752
LAMB3	2.233198	8.395982	4.855093	2.44E-06	2.64E-05	3.99609
NRIP2	-1.15039	1.087173	-4.85433	2.45E-06	2.65E-05	4.43715
DDX11	1.183686	5.284475	4.851671	2.48E-06	2.67E-05	4.140626
SYBU	-2.27975	3.003358	-4.85171	2.48E-06	2.67E-05	4.068779
SF3B14	0.801043	5.961744	4.851747	2.48E-06	2.67E-05	4.032898
RUVBL2	0.813482	6.400806	4.851686	2.48E-06	2.67E-05	4.003268
AKAP12	-2.14637	4.873466	-4.85139	2.48E-06	2.67E-05	3.980698
EFNB1	1.70995	6.851139	4.850925	2.49E-06	2.68E-05	4.026232
SMTN	-1.33863	6.620029	-4.85047	2.49E-06	2.68E-05	3.986704
MEG3	-1.71606	1.132771	-4.8483	2.52E-06	2.70E-05	4.359205
GPR172A	1.167699	6.094128	4.847867	2.52E-06	2.71E-05	4.033748
MKI67IP	0.917272	5.612261	4.845232	2.55E-06	2.74E-05	4.045624
CDC25A	1.986373	3.218155	4.843264	2.58E-06	2.76E-05	4.435318
AKAP2	-1.83312	4.430207	-4.84293	2.58E-06	2.76E-05	3.953254
TOPBP1	1.041259	6.407192	4.840536	2.61E-06	2.79E-05	3.968234
CRADD	-0.74883	2.263291	-4.83676	2.65E-06	2.83E-05	4.27426
TMEM48	1.234541	5.068112	4.836244	2.66E-06	2.84E-05	4.114331
CKLF	1.559107	3.777056	4.834175	2.68E-06	2.86E-05	4.326485
RAB33B	-0.69396	4.00724	-4.83315	2.70E-06	2.87E-05	4.01123
KIAA1370	-0.93111	5.074234	-4.83066	2.73E-06	2.90E-05	3.901836
RAD54B	1.761876	3.263868	4.829243	2.74E-06	2.92E-05	4.36181
CAPZA1	0.713748	7.319002	4.829379	2.74E-06	2.92E-05	3.879237
LRRC17	-2.11566	1.301001	-4.82761	2.76E-06	2.94E-05	4.203272
OTX1	3.235562	2.583501	4.825938	2.78E-06	2.96E-05	4.38431
PRKDC	0.988096	8.694648	4.825548	2.79E-06	2.96E-05	3.879151
ACOT7	1.355355	5.659964	4.824447	2.80E-06	2.97E-05	3.993998
MGC12982	2.410914	2.355609	4.823789	2.81E-06	2.98E-05	4.383853
ANXA2	1.270874	9.152313	4.823754	2.81E-06	2.98E-05	3.876405
KALRN	-1.87399	4.350361	-4.82346	2.81E-06	2.98E-05	3.871774
H2AFZ	0.947422	6.891978	4.823308	2.82E-06	2.98E-05	3.864614
UAP1	-0.81579	5.427318	-4.82137	2.84E-06	3.00E-05	3.852713
ZNF204P	-1.40855	2.554141	-4.82055	2.85E-06	3.01E-05	4.073537
HMGCL	-0.79821	4.566396	-4.81933	2.87E-06	3.03E-05	3.891495
MPHOSPH8	-0.82121	5.693047	-4.81603	2.91E-06	3.07E-05	3.82391
NRM	1.587824	4.669	4.815534	2.92E-06	3.07E-05	4.138663
PRR7	2.305338	3.584125	4.813634	2.94E-06	3.10E-05	4.306175
MAGOH	0.934726	4.193069	4.812754	2.95E-06	3.11E-05	4.108679
C12orf72	-1.02275	0.540938	-4.81243	2.96E-06	3.11E-05	4.305381
DDX12	1.613509	3.282757	4.811905	2.96E-06	3.11E-05	4.278198
DSCC1	1.748008	3.504836	4.8114	2.97E-06	3.12E-05	4.268297
NUP37	1.141203	4.250039	4.810769	2.98E-06	3.13E-05	4.120321
WDR12	0.982791	4.485211	4.809826	2.99E-06	3.14E-05	4.056986

TTF2	1.196748	5.086438	4.808743	3.01E-06	3.15E-05	3.98767
HIPK3	-1.0946	6.514084	-4.8078	3.02E-06	3.16E-05	3.79622
ACSL3	-0.89675	6.857689	-4.80687	3.03E-06	3.17E-05	3.79524
SLC44A2	-0.99941	7.462034	-4.80575	3.05E-06	3.18E-05	3.80776
FXC1	-0.74762	5.044901	-4.80514	3.06E-06	3.19E-05	3.800854
PALB2	0.924579	4.540913	4.804595	3.06E-06	3.20E-05	4.016951
WNT3	2.157235	-0.06503	4.803734	3.07E-06	3.21E-05	4.286483
SGPL1	0.784342	6.727127	4.803022	3.08E-06	3.21E-05	3.777702
C13orf34	1.383424	3.893347	4.802561	3.09E-06	3.22E-05	4.164283
LDHA	1.191244	9.826649	4.800587	3.12E-06	3.24E-05	3.797738
FOXP3	2.937179	1.634663	4.799136	3.14E-06	3.26E-05	4.270252
ZNF345	-1.76956	0.493932	-4.7989	3.14E-06	3.26E-05	4.206516
STS	-1.67582	4.33337	-4.79894	3.14E-06	3.26E-05	3.773115
PART1	-2.29757	0.418056	-4.79872	3.14E-06	3.26E-05	4.172005
CDCP1	1.42091	7.349621	4.794699	3.20E-06	3.32E-05	3.741817
KLHDC10	-0.70773	5.093009	-4.79427	3.21E-06	3.32E-05	3.753584
NIPAL3	-0.79949	5.404114	-4.79138	3.25E-06	3.36E-05	3.725005
PTGS1	-1.87307	4.797419	-4.78904	3.28E-06	3.40E-05	3.712208
FICD	-0.98162	1.387359	-4.78783	3.30E-06	3.41E-05	4.143625
EZH1	-0.79592	4.711093	-4.78485	3.35E-06	3.45E-05	3.732233
WDTC1	-0.76734	6.746879	-4.78499	3.34E-06	3.45E-05	3.697105
PIGP	-0.78342	3.40438	-4.7839	3.36E-06	3.46E-05	3.867256
C1orf69	-0.71745	3.624641	-4.7839	3.36E-06	3.46E-05	3.843868
ZNF71	-1.56501	1.606865	-4.77821	3.45E-06	3.55E-05	4.015212
SLC9A3	-3.31024	2.893278	-4.7773	3.46E-06	3.56E-05	3.707746
CLDN1	3.653584	7.145088	4.773467	3.52E-06	3.62E-05	3.875946
SMC2	1.079512	6.020188	4.7734	3.52E-06	3.62E-05	3.713605
SLC25A23	-1.28615	6.388195	-4.77298	3.53E-06	3.62E-05	3.648208
IRAK1	0.925504	7.524887	4.770859	3.56E-06	3.65E-05	3.62844
PRKAB2	-0.91643	5.34765	-4.76883	3.59E-06	3.68E-05	3.627521
ZNF287	-1.679	1.009581	-4.76797	3.61E-06	3.69E-05	4.034541
KIAA1958	-1.21069	2.751951	-4.7673	3.62E-06	3.70E-05	3.845314
SRPK1	0.842869	6.818338	4.766144	3.64E-06	3.72E-05	3.618741
CLN6	1.040361	5.545882	4.764704	3.66E-06	3.74E-05	3.719709
MCM8	1.357966	4.676814	4.764371	3.66E-06	3.74E-05	3.889306
CPSF3	0.810878	5.448352	4.762984	3.69E-06	3.76E-05	3.700084
BANF1	1.020128	6.577938	4.762856	3.69E-06	3.76E-05	3.622966
ESM1	3.859618	2.009398	4.760564	3.73E-06	3.80E-05	4.114461
DCAF15	0.845546	4.968155	4.760647	3.73E-06	3.80E-05	3.751221
ARL1	-0.58616	6.364164	-4.75883	3.76E-06	3.82E-05	3.577559
BGN	1.961774	8.242536	4.758465	3.76E-06	3.83E-05	3.579106
C9orf103	-1.0608	1.256715	-4.75425	3.83E-06	3.90E-05	4.007894
SERHL2	-1.00429	0.369989	-4.75378	3.84E-06	3.90E-05	4.070725
HBA2	-2.18977	3.054198	-4.75389	3.84E-06	3.90E-05	3.651045
AIMP2	1.175138	4.43224	4.749698	3.91E-06	3.97E-05	3.837726
SNRPE	1.013523	4.538991	4.745446	3.99E-06	4.04E-05	3.779253
PPM1L	-1.42584	2.857624	-4.74477	4.00E-06	4.04E-05	3.701531
EVI5	-0.77081	4.9494	-4.74491	4.00E-06	4.04E-05	3.548033
COX6B2	3.261135	0.185372	4.742088	4.05E-06	4.09E-05	4.037944
TXNDC15	-0.70789	4.74831	-4.74105	4.06E-06	4.11E-05	3.548498
PTGER2	-2.03335	1.587043	-4.74073	4.07E-06	4.11E-05	3.805774
IGFBP5	-2.2969	7.941503	-4.74013	4.08E-06	4.12E-05	3.575559
PAQR4	1.462395	5.276145	4.739454	4.09E-06	4.13E-05	3.704027
PRMT10	-0.7117	3.069524	-4.73926	4.10E-06	4.13E-05	3.741641
C21orf45	1.338781	3.256903	4.738786	4.11E-06	4.13E-05	3.953872
SGCD	-1.84131	2.900475	-4.73884	4.10E-06	4.13E-05	3.626958
AASS	-1.33946	3.48361	-4.73766	4.13E-06	4.15E-05	3.602109
VLDLR	-1.97145	3.762586	-4.7371	4.14E-06	4.15E-05	3.530918

ATIC	0.914292	6.190905	4.736513	4.15E-06	4.16E-05	3.530067
USP54	-1.29782	5.961187	-4.73623	4.15E-06	4.16E-05	3.485465
BCHE	-2.63339	0.445458	-4.73541	4.17E-06	4.18E-05	3.865642
C5orf34	1.890759	1.673851	4.733296	4.21E-06	4.21E-05	4.012837
FBXL7	-1.37397	3.123645	-4.73327	4.21E-06	4.21E-05	3.621219
TTC3	-0.90904	7.329667	-4.73311	4.21E-06	4.21E-05	3.491934
R3HCC1	-0.73085	4.209633	-4.73268	4.22E-06	4.22E-05	3.558485
ISOC1	-0.73408	4.62103	-4.73034	4.26E-06	4.26E-05	3.511155
LEPROT	-0.78687	4.886673	-4.72904	4.29E-06	4.28E-05	3.483557
NDFIP1	-0.57126	6.81032	-4.72901	4.29E-06	4.28E-05	3.456709
AFF1	-0.92906	6.594567	-4.72796	4.31E-06	4.29E-05	3.454251
DCAF11	-0.65855	6.181547	-4.72741	4.32E-06	4.30E-05	3.443418
AFF4	-0.89863	7.339054	-4.72719	4.32E-06	4.30E-05	3.466802
LOC286367	-1.325	0.318718	-4.72544	4.36E-06	4.33E-05	3.940433
C2orf7	-0.79221	2.874216	-4.72451	4.37E-06	4.35E-05	3.700375
RELT	1.550355	3.519859	4.722432	4.41E-06	4.38E-05	3.879109
PSME2	1.214446	5.707324	4.722158	4.42E-06	4.38E-05	3.53904
SLC2A1	2.45629	8.354999	4.721991	4.42E-06	4.39E-05	3.432573
ZNF404	-1.35557	0.452703	-4.72181	4.43E-06	4.39E-05	3.915636
CSTF2	1.040624	4.444234	4.721282	4.44E-06	4.39E-05	3.696077
IGF1	-2.10866	2.764956	-4.72104	4.44E-06	4.39E-05	3.543459
XK	-2.27639	3.262232	-4.72105	4.44E-06	4.39E-05	3.486689
NHSL2	-1.69581	0.654702	-4.71853	4.49E-06	4.43E-05	3.86228
UTP6	0.618141	5.240927	4.71849	4.49E-06	4.43E-05	3.514792
RBPMS	-1.48088	5.548518	-4.71786	4.50E-06	4.44E-05	3.405839
C8orf76	0.97545	4.234657	4.715479	4.55E-06	4.49E-05	3.697723
SPARCL1	-2.08471	6.857064	-4.71543	4.55E-06	4.49E-05	3.434472
PYCARD	1.837597	5.125839	4.714453	4.57E-06	4.50E-05	3.677419
NDRG2	-1.64601	5.062307	-4.71231	4.62E-06	4.54E-05	3.38364
PPM1G	0.687002	7.156502	4.710304	4.66E-06	4.58E-05	3.371684
C11orf54	-1.05526	4.159586	-4.70865	4.69E-06	4.61E-05	3.436249
DCUN1D4	-0.75272	4.848695	-4.70713	4.72E-06	4.64E-05	3.395207
ZNF880	-1.89332	0.604386	-4.70622	4.74E-06	4.65E-05	3.800825
PAFAH2	-1.03762	4.425207	-4.70502	4.77E-06	4.67E-05	3.400181
FBXL22	-1.2506	0.153008	-4.70307	4.81E-06	4.71E-05	3.8613
AKR7A3	-2.98656	1.815635	-4.70275	4.81E-06	4.71E-05	3.507208
C10orf32	-0.80218	4.105341	-4.70047	4.86E-06	4.75E-05	3.426626
PHKA1	-0.952	4.329761	-4.70045	4.86E-06	4.75E-05	3.393829
LIMK1	1.104545	6.098418	4.699736	4.88E-06	4.77E-05	3.396305
SDC1	2.025857	8.888217	4.699273	4.89E-06	4.77E-05	3.328758
IL11	3.254018	0.886448	4.699114	4.89E-06	4.77E-05	3.866626
ZNF649	-1.3722	2.386213	-4.69797	4.92E-06	4.79E-05	3.584306
FNIP2	-1.31675	5.193762	-4.69704	4.94E-06	4.81E-05	3.319894
ENPP5	-2.36795	1.476791	-4.69652	4.95E-06	4.82E-05	3.591091
AKAP10	-0.60014	4.440654	-4.69604	4.96E-06	4.82E-05	3.392199
SPATA18	-1.35766	2.406414	-4.69436	4.99E-06	4.86E-05	3.571297
DNAJB11	0.847372	6.091972	4.688674	5.12E-06	4.98E-05	3.33098
C17orf51	-1.26648	3.142318	-4.68846	5.13E-06	4.98E-05	3.442977
HYAL1	-1.84103	2.918036	-4.68744	5.15E-06	5.00E-05	3.413212
SH2D2A	2.042156	2.52367	4.685449	5.19E-06	5.04E-05	3.812226
MARCKSL1	1.545657	7.216252	4.684621	5.21E-06	5.05E-05	3.284982
PEX19	-0.59492	6.090013	-4.68408	5.23E-06	5.06E-05	3.260378
SNRPD2	0.859807	6.534264	4.683271	5.24E-06	5.07E-05	3.280255
ACAD8	-0.87088	3.804295	-4.6815	5.29E-06	5.11E-05	3.373602
FAM20A	-1.73565	2.247903	-4.67968	5.33E-06	5.15E-05	3.484519
CALM1	-0.77811	8.703574	-4.67933	5.34E-06	5.15E-05	3.30178
TSC1	-0.6365	5.579353	-4.67771	5.37E-06	5.19E-05	3.241474
CTDSP1	-0.59385	6.713839	-4.67739	5.38E-06	5.19E-05	3.237599

ZNF862	-1.23362	4.025784	-4.67647	5.40E-06	5.21E-05	3.299013
PEX7	-0.92431	2.756591	-4.67463	5.45E-06	5.25E-05	3.494591
ANKAR	-0.99229	0.989993	-4.67211	5.51E-06	5.30E-05	3.694802
SULF2	1.582667	7.544931	4.665184	5.68E-06	5.46E-05	3.19154
LOC100125556	1.751675	1.863116	4.663622	5.72E-06	5.49E-05	3.731128
SOX17	-1.38579	0.940518	-4.66385	5.71E-06	5.49E-05	3.634703
BEX4	-1.64874	3.03718	-4.66366	5.72E-06	5.49E-05	3.311866
ACCS	-0.99762	2.84596	-4.66141	5.77E-06	5.54E-05	3.411961
PLAU	2.621027	6.468177	4.661119	5.78E-06	5.54E-05	3.35482
TMEM70	-0.69251	4.565023	-4.66054	5.79E-06	5.55E-05	3.225281
MDFI	2.882789	4.804583	4.659654	5.82E-06	5.57E-05	3.614933
TUBA8	-1.08361	1.011993	-4.65939	5.82E-06	5.57E-05	3.633908
RNF146	-0.6586	4.229321	-4.65798	5.86E-06	5.60E-05	3.247986
IFI30	1.440345	6.773778	4.656809	5.89E-06	5.62E-05	3.188398
PPAP2A	-1.0924	4.736358	-4.65694	5.89E-06	5.62E-05	3.173748
FAM13AOS	-1.24713	1.093416	-4.65641	5.90E-06	5.63E-05	3.603218
DSTN	-0.94548	8.432383	-4.6547	5.94E-06	5.66E-05	3.194933
RNF10	-0.60735	7.620242	-4.65306	5.99E-06	5.70E-05	3.155237
NPM3	1.56554	2.981382	4.652576	6.00E-06	5.71E-05	3.635594
DHRS12	-0.90509	2.29992	-4.65266	6.00E-06	5.71E-05	3.47605
EMCN	-1.42062	2.657029	-4.64992	6.07E-06	5.77E-05	3.334868
ZBTB40	-0.64066	5.415826	-4.64893	6.10E-06	5.79E-05	3.126205
GAB2	-1.32054	4.457717	-4.6487	6.10E-06	5.79E-05	3.145082
PPP1R16B	-1.53622	3.127368	-4.64718	6.14E-06	5.83E-05	3.245013
SEMA3B	-2.08529	4.061969	-4.64646	6.16E-06	5.84E-05	3.127576
TFAP4	1.240597	4.227307	4.645822	6.18E-06	5.85E-05	3.445712
EML1	-1.4992	3.934057	-4.64592	6.18E-06	5.85E-05	3.160306
TXNRD2	-0.74587	3.815484	-4.64419	6.22E-06	5.88E-05	3.228754
HIPK2	-1.25592	7.126216	-4.64411	6.23E-06	5.88E-05	3.120488
PIK3R1	-1.17437	5.835773	-4.64275	6.26E-06	5.92E-05	3.089081
C6orf89	-0.6624	6.643181	-4.64146	6.30E-06	5.95E-05	3.086972
SIP1	1.078883	2.443856	4.640282	6.33E-06	5.97E-05	3.591945
C1orf183	-1.09196	1.08533	-4.63978	6.34E-06	5.98E-05	3.545827
NUDT5	1.094955	5.231046	4.638684	6.37E-06	6.01E-05	3.233913
RAB5B	-0.62922	7.484207	-4.63678	6.43E-06	6.05E-05	3.084136
EIF2AK3	-0.84938	5.483928	-4.6346	6.49E-06	6.11E-05	3.059037
AAK1	-0.79269	6.595123	-4.63251	6.55E-06	6.16E-05	3.050869
TFF1	-4.37217	1.214109	-4.63047	6.61E-06	6.21E-05	3.170878
RUNDC2C	-1.30205	0.585783	-4.62968	6.63E-06	6.22E-05	3.531891
PARP9	1.124577	6.703184	4.629066	6.65E-06	6.24E-05	3.057793
ZNF367	1.681484	4.114281	4.627108	6.70E-06	6.29E-05	3.437361
BTG2	-1.62423	6.685563	-4.62554	6.75E-06	6.33E-05	3.040297
PUS7	1.321352	5.068738	4.62368	6.80E-06	6.37E-05	3.229201
APEX2	0.85597	5.3786	4.619366	6.93E-06	6.49E-05	3.108165
LRP8	2.908692	4.32888	4.618428	6.96E-06	6.51E-05	3.487425
ZNF207	0.431703	7.667659	4.615917	7.04E-06	6.58E-05	2.981312
CCDC106	-1.26344	2.788191	-4.61484	7.07E-06	6.60E-05	3.187523
C14orf106	0.958157	5.067012	4.614717	7.08E-06	6.60E-05	3.140717
LMBRD1	-0.7996	5.452402	-4.61077	7.20E-06	6.71E-05	2.961693
BTBD3	-1.19181	5.733114	-4.6063	7.34E-06	6.84E-05	2.936803
CLCC1	-0.66796	5.184571	-4.60583	7.35E-06	6.85E-05	2.955862
DEXI	-0.70037	4.455767	-4.60132	7.50E-06	6.98E-05	2.987232
MLL3	-0.84637	7.051717	-4.60087	7.51E-06	6.99E-05	2.929053
CDH3	4.219092	6.890395	4.599266	7.57E-06	7.03E-05	3.272388
AMMECR1	1.199843	5.490029	4.599276	7.57E-06	7.03E-05	3.048581
STBD1	-1.261	3.466256	-4.59907	7.57E-06	7.03E-05	3.031602
BTC	-1.81474	0.173774	-4.59655	7.66E-06	7.10E-05	3.393218
NKIRAS1	-0.68321	2.806619	-4.59565	7.69E-06	7.12E-05	3.189131

RASSF3	-1.2037	3.547359	-4.59553	7.69E-06	7.12E-05	3.013956
NUTF2	0.853576	6.288508	4.595724	7.68E-06	7.12E-05	2.929017
PKIB	-2.02503	1.49153	-4.59338	7.76E-06	7.18E-05	3.210391
RNPS1	0.658927	7.178273	4.591477	7.83E-06	7.24E-05	2.874224
WBP1	-0.64314	4.862425	-4.59127	7.83E-06	7.24E-05	2.916728
DAZAP1	0.567991	6.537844	4.589302	7.90E-06	7.29E-05	2.8764
NLGN3	-1.43873	0.372458	-4.58829	7.93E-06	7.32E-05	3.369754
PPP3CB	-0.61248	5.543286	-4.58753	7.96E-06	7.34E-05	2.86719
POLL	-0.61193	4.131039	-4.5872	7.97E-06	7.35E-05	2.96915
BOLA2	1.063614	5.709141	4.583551	8.10E-06	7.46E-05	2.945375
PSMA6	0.828869	6.129837	4.581955	8.15E-06	7.51E-05	2.881739
UTP18	0.770734	4.983979	4.581412	8.17E-06	7.52E-05	2.989882
BOC	-1.87109	3.405954	-4.5812	8.18E-06	7.52E-05	2.91263
LRRFIP2	-0.76122	5.308421	-4.58115	8.18E-06	7.52E-05	2.844802
HOXA13	5.319782	0.933404	4.580055	8.22E-06	7.55E-05	3.396057
TXNDC9	0.690483	5.02001	4.578761	8.27E-06	7.58E-05	2.964499
C10orf55	2.536468	0.122593	4.577566	8.31E-06	7.62E-05	3.385538
CBX8	1.18197	2.184095	4.577007	8.33E-06	7.63E-05	3.3573
CLEC5A	2.711742	1.030823	4.574728	8.41E-06	7.70E-05	3.375186
RPA3	1.236016	4.17679	4.573667	8.45E-06	7.73E-05	3.155962
SURF1	-0.73138	4.480083	-4.57166	8.53E-06	7.80E-05	2.860708
MASTL	1.103568	4.604623	4.570029	8.59E-06	7.85E-05	3.053465
PPIL5	1.013073	3.408469	4.569795	8.59E-06	7.85E-05	3.214924
TRMT6	0.948629	4.455771	4.568903	8.63E-06	7.87E-05	3.04881
GNPDA1	0.806505	5.226808	4.568235	8.65E-06	7.89E-05	2.908842
WDR34	1.128069	5.693788	4.568061	8.66E-06	7.89E-05	2.889374
EIF2AK2	1.088956	5.620377	4.567329	8.69E-06	7.91E-05	2.890403
DPH3	-0.73388	4.740876	-4.56687	8.70E-06	7.92E-05	2.819088
SPOP	-0.59167	5.403373	-4.56644	8.72E-06	7.93E-05	2.785859
ADARB1	-1.25768	4.173979	-4.56613	8.73E-06	7.94E-05	2.825555
TBL1XR1	0.863411	7.95717	4.56517	8.77E-06	7.96E-05	2.769937
BCKDHA	-0.89072	5.387002	-4.56375	8.82E-06	8.01E-05	2.766987
TLN1	-0.97932	9.010698	-4.56249	8.87E-06	8.05E-05	2.830256
NEDD4L	-1.37932	6.360973	-4.56181	8.89E-06	8.06E-05	2.763759
LOC643008	-1.95169	0.935164	-4.56097	8.93E-06	8.08E-05	3.162999
CRLF3	0.845866	4.588044	4.560969	8.93E-06	8.08E-05	2.978102
ASB8	-0.67021	4.466447	-4.56107	8.92E-06	8.08E-05	2.82243
SLC5A12	4.03175	0.738578	4.560255	8.95E-06	8.10E-05	3.318524
TMEM201	0.927134	5.457047	4.559597	8.98E-06	8.12E-05	2.859607
C20orf24	1.039907	6.773584	4.558544	9.02E-06	8.15E-05	2.757985
E2F8	2.365725	3.980702	4.558249	9.03E-06	8.15E-05	3.234191
TFDP1	0.93266	6.90311	4.554874	9.16E-06	8.27E-05	2.733759
TSPAN2	-1.68303	2.08813	-4.5543	9.19E-06	8.28E-05	2.99371
WDR60	-0.88914	4.291286	-4.55386	9.20E-06	8.29E-05	2.791676
NCL	0.666896	8.924475	4.553108	9.23E-06	8.31E-05	2.745605
CACNA1C	-1.53031	3.361163	-4.55121	9.31E-06	8.38E-05	2.821398
CALR	0.582301	9.386096	4.550959	9.32E-06	8.38E-05	2.752974
POPDC2	-1.41483	0.060777	-4.54985	9.36E-06	8.41E-05	3.237219
ABCA3	-1.94768	3.225043	-4.54846	9.42E-06	8.46E-05	2.791097
ZNF577	-1.49831	1.646526	-4.5458	9.53E-06	8.55E-05	3.056074
WDR13	-0.76824	5.546686	-4.5457	9.53E-06	8.55E-05	2.691134
PPARGC1A	-2.22881	2.702365	-4.53721	9.89E-06	8.86E-05	2.778988
PSD	-1.7306	0.505255	-4.53683	9.90E-06	8.87E-05	3.129979
ARHGAP20	-1.65881	0.899761	-4.53655	9.92E-06	8.87E-05	3.095627
MRPL9	0.749305	5.250032	4.535985	9.94E-06	8.89E-05	2.768144
CHP	-0.91655	7.330029	-4.53557	9.96E-06	8.90E-05	2.668549
KLHDC7A	-2.96569	-0.08487	-4.5326	1.01E-05	9.01E-05	3.070389
CHRM3	-1.68468	2.677907	-4.52992	1.02E-05	9.10E-05	2.804398

SH2B1	-0.60981	5.106172	-4.52991	1.02E-05	9.10E-05	2.649951
NUDCD1	0.986947	4.731273	4.529672	1.02E-05	9.11E-05	2.848804
MEST	2.305589	5.463897	4.529402	1.02E-05	9.11E-05	2.930766
FNBP1	-1.46062	5.530539	-4.52932	1.02E-05	9.11E-05	2.620692
ZC4H2	-1.61846	2.040171	-4.52836	1.03E-05	9.14E-05	2.908282
HINT3	-0.80849	4.938648	-4.5283	1.03E-05	9.14E-05	2.64339
S100A1	-1.56577	0.343604	-4.52807	1.03E-05	9.14E-05	3.122742
MYH11	-4.3171	7.507395	-4.52731	1.03E-05	9.16E-05	2.731363
PFDN4	1.072034	4.144583	4.525674	1.04E-05	9.22E-05	2.941425
ZFP3	-1.65886	2.664303	-4.52554	1.04E-05	9.22E-05	2.789352
DLG4	-1.08146	2.478281	-4.52471	1.04E-05	9.25E-05	2.896597
ARIH2	-0.61688	5.677354	-4.52362	1.05E-05	9.29E-05	2.600383
PAFAH1B3	1.441822	4.505343	4.522068	1.06E-05	9.34E-05	2.925198
GPRIN1	2.393902	4.195148	4.520105	1.06E-05	9.41E-05	3.063177
SRRT	0.553601	6.36444	4.520041	1.06E-05	9.41E-05	2.597962
SPCS3	-0.69307	7.055784	-4.51939	1.07E-05	9.43E-05	2.590695
IDH2	-1.24411	6.801662	-4.51923	1.07E-05	9.43E-05	2.59575
VEGFB	-0.88452	5.485785	-4.519	1.07E-05	9.44E-05	2.580956
MSRA	-1.13931	2.743766	-4.51764	1.08E-05	9.48E-05	2.816421
ST14	1.540555	7.964501	4.513109	1.10E-05	9.66E-05	2.553461
CACYBP	0.843987	5.679331	4.512796	1.10E-05	9.67E-05	2.637699
DCTPP1	1.009589	5.250854	4.511953	1.10E-05	9.70E-05	2.699285
TRIM14	1.081928	6.236208	4.510058	1.11E-05	9.77E-05	2.596678
C15orf52	-1.54421	3.570021	-4.5097	1.11E-05	9.78E-05	2.629397
RIMBP2	-2.49867	0.554824	-4.50806	1.12E-05	9.84E-05	2.936756
PPIL1	0.889309	5.177421	4.507012	1.13E-05	9.88E-05	2.67397
MXD4	-0.91269	5.648089	-4.50693	1.13E-05	9.88E-05	2.528443
SNX10	2.58412	4.391551	4.504864	1.14E-05	9.95E-05	3.00006
SASS6	1.220184	3.704319	4.505002	1.13E-05	9.95E-05	2.940396
CSDE1	-0.70596	8.910761	-4.50335	1.14E-05	0.0001	2.5782
PSRC1	1.698823	2.771117	4.501039	1.15E-05	0.000101	3.047226
C3orf19	-0.62878	3.849576	-4.49935	1.16E-05	0.000102	2.640263
ODF2	0.761417	5.427437	4.49846	1.17E-05	0.000102	2.59736
WNT2	3.518731	1.170836	4.497915	1.17E-05	0.000102	3.076971
NAP1L5	-1.28022	1.679399	-4.49666	1.18E-05	0.000103	2.879907
C12orf32	1.048726	4.917819	4.493887	1.19E-05	0.000104	2.681402
SPINT1	1.436693	7.890444	4.49226	1.20E-05	0.000105	2.468161
ALDH1L1	-2.55939	1.026359	-4.49156	1.20E-05	0.000105	2.790996
DGUOK	0.800412	4.988737	4.491196	1.20E-05	0.000105	2.624434
HSPB8	-2.4844	4.459454	-4.4908	1.21E-05	0.000105	2.470358
TACC1	-1.39979	7.1274	-4.49011	1.21E-05	0.000105	2.489705
BAZ1A	0.842795	6.957896	4.488706	1.22E-05	0.000106	2.459068
MEGF8	-1.01976	5.838674	-4.48864	1.22E-05	0.000106	2.452762
ATG4A	-0.77147	3.779032	-4.48762	1.22E-05	0.000106	2.587079
FAM46C	-1.99619	3.678269	-4.48709	1.22E-05	0.000106	2.498038
MRPL47	0.927737	5.211239	4.486682	1.23E-05	0.000106	2.591457
SCAPER	-0.81047	4.423298	-4.4858	1.23E-05	0.000107	2.508408
UNC13B	-1.40577	5.613309	-4.48548	1.23E-05	0.000107	2.441805
DPP3	0.844888	6.098637	4.484433	1.24E-05	0.000107	2.486164
TNFSF10	1.982923	6.516247	4.480343	1.26E-05	0.000109	2.528487
C11orf46	-0.6461	3.848073	-4.47994	1.26E-05	0.000109	2.559212
COL4A4	-2.10988	1.553025	-4.47948	1.27E-05	0.000109	2.721232
DBF4B	1.248289	3.781438	4.47932	1.27E-05	0.000109	2.830891
TBRG1	-0.75083	5.179232	-4.47881	1.27E-05	0.000109	2.431632
POU2F1	0.740863	4.568276	4.474991	1.29E-05	0.000111	2.616784
KIF5C	-1.75934	0.429809	-4.47306	1.30E-05	0.000112	2.881153
S100A3	2.391607	0.85964	4.472257	1.30E-05	0.000112	2.978057
TMEM143	-0.74269	2.920819	-4.47157	1.31E-05	0.000113	2.65909

COQ7	-0.63997	3.826259	-4.47116	1.31E-05	0.000113	2.527417
TMEM50B	-0.81586	5.121527	-4.46981	1.32E-05	0.000113	2.39533
ADAMTS13	-1.40926	1.391245	-4.46924	1.32E-05	0.000114	2.795928
FBL	0.869115	6.774006	4.468463	1.33E-05	0.000114	2.383484
CD300LF	2.109433	0.170702	4.467979	1.33E-05	0.000114	2.961275
DONSON	1.033025	3.963305	4.466384	1.34E-05	0.000115	2.725484
KLHL15	-0.93702	4.105287	-4.46466	1.35E-05	0.000115	2.441768
FAM149B1	-0.56955	4.00035	-4.46323	1.36E-05	0.000116	2.481685
WDR7	-0.77328	4.6447	-4.46289	1.36E-05	0.000116	2.399477
CPA3	-1.85347	2.371702	-4.46173	1.36E-05	0.000117	2.552058
STIP1	0.784458	7.313564	4.461754	1.36E-05	0.000117	2.343301
COX7A1	-1.27442	1.140399	-4.46095	1.37E-05	0.000117	2.802591
F2RL3	-1.49179	2.264586	-4.45903	1.38E-05	0.000118	2.611994
CPEB2	-1.19903	4.943865	-4.45902	1.38E-05	0.000118	2.346263
CEP72	1.591048	2.855015	4.458431	1.38E-05	0.000118	2.867715
CRYL1	-1.29778	3.85728	-4.45782	1.39E-05	0.000118	2.411862
IL23A	2.422386	0.394039	4.456067	1.40E-05	0.000119	2.915781
ZFPM2	-1.61441	1.559818	-4.45561	1.40E-05	0.000119	2.687949
HIST1H1E	1.894433	0.607417	4.454704	1.41E-05	0.00012	2.910654
DDX18	0.692845	6.658593	4.454754	1.41E-05	0.00012	2.326202
MECP2	-0.6193	6.271513	-4.45359	1.41E-05	0.00012	2.310244
TRIP11	-0.79832	5.68375	-4.45124	1.43E-05	0.000121	2.303131
PSMG3	1.092675	4.266504	4.449573	1.44E-05	0.000122	2.619112
FAM3B	-2.94929	1.790071	-4.44872	1.44E-05	0.000122	2.464447
PPP3CC	-0.75501	3.470158	-4.44818	1.44E-05	0.000122	2.47045
TNFRSF25	1.722692	3.484818	4.447772	1.45E-05	0.000123	2.782771
CCDC125	-0.89675	2.992983	-4.44701	1.45E-05	0.000123	2.527394
SERINC1	-0.74509	7.369437	-4.4466	1.45E-05	0.000123	2.303706
HDAC1	0.709872	7.196459	4.446615	1.45E-05	0.000123	2.282423
MARVELD3	2.438842	3.886654	4.443707	1.47E-05	0.000124	2.791342
ZNF329	-1.38786	2.321138	-4.44121	1.49E-05	0.000126	2.540865
PTK7	1.730777	6.766261	4.438415	1.51E-05	0.000127	2.313475
C13orf37	1.037833	4.842301	4.437429	1.51E-05	0.000128	2.465833
CYB561D1	-0.88268	3.970635	-4.43604	1.52E-05	0.000128	2.345967
UCN2	3.298292	0.562747	4.435776	1.52E-05	0.000128	2.838374
POLR3GL	-0.80294	3.835192	-4.43328	1.54E-05	0.00013	2.357139
THOP1	0.852342	5.460491	4.433285	1.54E-05	0.00013	2.338753
ZNF423	-1.40443	2.460082	-4.43313	1.54E-05	0.00013	2.483123
LAMP3	3.760559	4.742708	4.43289	1.54E-05	0.00013	2.775052
SRR	-0.87506	2.699265	-4.43173	1.55E-05	0.00013	2.51611
CSTF3	0.689168	5.061453	4.429389	1.56E-05	0.000131	2.352205
MAPK8IP1	-1.31472	2.697429	-4.42851	1.57E-05	0.000132	2.440087
SNX21	-0.7519	3.84311	-4.42779	1.57E-05	0.000132	2.338573
PREX2	-2.00455	0.66378	-4.42654	1.58E-05	0.000133	2.651482
PLA2G7	3.138111	2.369055	4.425025	1.59E-05	0.000133	2.798089
EXOSC5	1.098994	4.228259	4.424398	1.60E-05	0.000134	2.52564
GKAP1	-1.47822	0.934614	-4.4228	1.61E-05	0.000134	2.655995
SCNM1	0.834248	4.399788	4.422789	1.61E-05	0.000134	2.449575
PARP12	1.080861	5.808294	4.422014	1.61E-05	0.000135	2.280247
NCOA1	-0.72772	6.594511	-4.42174	1.62E-05	0.000135	2.18721
HNRNPM	0.383918	7.557145	4.420167	1.63E-05	0.000136	2.180323
PHF1	-0.70089	4.946316	-4.4192	1.63E-05	0.000136	2.206358
MAPKAPK3	-0.82084	5.242483	-4.41801	1.64E-05	0.000137	2.181218
PKD1	-1.03599	6.776795	-4.41546	1.66E-05	0.000138	2.17066
USP25	-0.76197	5.34334	-4.41167	1.69E-05	0.00014	2.153655
STK33	-2.09169	0.036164	-4.41144	1.69E-05	0.00014	2.647723
ATF4	-0.64091	7.767854	-4.41001	1.70E-05	0.000141	2.165139
LSM12	0.615627	5.932108	4.409447	1.70E-05	0.000141	2.180335

PNPT1	0.825991	5.422678	4.40858	1.71E-05	0.000142	2.241741
LOC375190	-1.38267	0.018618	-4.40819	1.71E-05	0.000142	2.683052
CYB5R1	-1.10203	5.233143	-4.40728	1.72E-05	0.000142	2.131308
TMPO	0.940414	6.70779	4.407011	1.72E-05	0.000142	2.141703
MAN2C1	-0.55931	5.145802	-4.40645	1.72E-05	0.000143	2.150449
ANXA2P2	1.249952	8.437751	4.405632	1.73E-05	0.000143	2.124517
CCT6A	0.898368	7.696308	4.405243	1.73E-05	0.000143	2.11654
BCAP31	0.728497	7.378325	4.404898	1.73E-05	0.000143	2.114199
DNAJC4	-0.76494	4.622401	-4.40325	1.75E-05	0.000144	2.162298
MPP2	-1.71183	1.441252	-4.40211	1.75E-05	0.000145	2.480345
ZNF441	-0.97095	2.391968	-4.40137	1.76E-05	0.000145	2.433373
CCBL2	-0.74144	4.44955	-4.39972	1.77E-05	0.000146	2.164736
CDC123	0.866123	5.731413	4.397491	1.79E-05	0.000148	2.169385
RELL2	1.930772	2.116867	4.395195	1.81E-05	0.000149	2.673728
DENND2A	-1.35252	2.259324	-4.39521	1.81E-05	0.000149	2.374839
F13A1	-1.93665	2.688915	-4.39457	1.81E-05	0.000149	2.229176
CYBRD1	-1.83319	6.021465	-4.3942	1.81E-05	0.000149	2.086336
KIAA0319L	-0.76433	6.443425	-4.39237	1.83E-05	0.00015	2.067904
FAM161B	-0.99148	1.158139	-4.39073	1.84E-05	0.000151	2.550422
ANXA10	-3.53326	1.145281	-4.39063	1.84E-05	0.000151	2.264066
RAP1GAP2	-1.43646	5.561801	-4.38948	1.85E-05	0.000152	2.055199
PRCC	0.586929	6.008729	4.389056	1.85E-05	0.000152	2.091343
TNFRSF9	2.315788	0.183215	4.38867	1.86E-05	0.000152	2.659395
SET	0.636161	8.006334	4.38727	1.87E-05	0.000153	2.05275
DTX3L	0.927401	7.191382	4.386556	1.87E-05	0.000153	2.044435
POLK	-0.66104	4.484861	-4.38445	1.89E-05	0.000155	2.106794
POLR2H	0.845872	5.336931	4.383191	1.90E-05	0.000155	2.152287
LOC388796	1.105869	4.527548	4.381702	1.91E-05	0.000156	2.307556
PGK1	0.795826	8.493577	4.380868	1.92E-05	0.000157	2.034382
NIP7	0.873285	5.070257	4.380188	1.92E-05	0.000157	2.176303
VBP1	0.692849	5.355838	4.380045	1.92E-05	0.000157	2.121878
SSBP3	-0.89359	5.078297	-4.37996	1.92E-05	0.000157	2.033484
RAB37	-1.76324	0.56633	-4.37528	1.96E-05	0.00016	2.482944
CISH	-0.81068	3.346158	-4.37545	1.96E-05	0.00016	2.192577
ZNF254	-1.50492	3.08219	-4.37537	1.96E-05	0.00016	2.145956
CRY1	-0.70448	4.508804	-4.37391	1.97E-05	0.000161	2.059282
KCND3	-1.98577	1.141188	-4.37328	1.98E-05	0.000161	2.377671
RPP14	-0.52186	4.447164	-4.37332	1.98E-05	0.000161	2.077357
LRCH2	-1.57651	0.878523	-4.37056	2.00E-05	0.000163	2.446953
EPS15	-0.52474	6.354836	-4.37002	2.01E-05	0.000163	1.975152
C20orf72	1.027041	4.670751	4.369367	2.01E-05	0.000163	2.222247
FGL2	-2.04172	4.188986	-4.36886	2.02E-05	0.000163	1.990453
PER2	-1.15911	5.766117	-4.3684	2.02E-05	0.000164	1.969949
MAOA	-1.9731	6.005362	-4.36825	2.02E-05	0.000164	1.985387
DCAF6	-0.63389	6.009962	-4.36799	2.02E-05	0.000164	1.967192
RASIP1	-1.20924	3.28186	-4.36645	2.04E-05	0.000165	2.117709
RUFY3	-0.72419	4.856884	-4.36657	2.04E-05	0.000165	2.000838
SERPINB5	4.70122	7.102524	4.365876	2.04E-05	0.000165	2.366028
NR2C2AP	0.972281	3.878962	4.365836	2.04E-05	0.000165	2.330595
C17orf39	-0.76894	4.151641	-4.36466	2.05E-05	0.000166	2.050995
EIF6	0.928209	7.19783	4.364476	2.05E-05	0.000166	1.956411
C1orf89	-0.74762	1.378703	-4.36282	2.07E-05	0.000167	2.44112
ACO1	-1.18339	5.537093	-4.36238	2.07E-05	0.000167	1.946269
RSAD1	-0.71209	4.13674	-4.35928	2.10E-05	0.000169	2.037575
MUT	-0.70483	5.192345	-4.35918	2.10E-05	0.000169	1.952885
GOLGA4	-0.99949	7.400232	-4.35885	2.10E-05	0.000169	1.960035
ABCD3	-0.9052	5.876117	-4.35893	2.10E-05	0.000169	1.931353
C3orf26	1.31876	3.847432	4.358033	2.11E-05	0.000169	2.349778

REPS2	-1.87011	3.72054	-4.35634	2.12E-05	0.000171	1.97835
MRTO4	0.799432	5.370771	4.353273	2.15E-05	0.000173	2.024573
CNTN4	-1.93967	0.871351	-4.35218	2.16E-05	0.000173	2.340835
TBRG4	0.811065	6.088404	4.351676	2.17E-05	0.000174	1.952166
MPP7	-1.56092	5.0166	-4.35102	2.17E-05	0.000174	1.90512
MTMR3	-0.70906	6.044464	-4.34931	2.19E-05	0.000175	1.892805
SMPD4	0.552428	7.01775	4.348661	2.19E-05	0.000175	1.890958
TNFRSF4	1.970721	0.946215	4.348067	2.20E-05	0.000176	2.506664
SHFM1	1.075285	6.682747	4.346034	2.22E-05	0.000177	1.906617
GPR19	2.076796	0.318135	4.34419	2.23E-05	0.000178	2.492027
LSM7	1.05756	4.634553	4.343586	2.24E-05	0.000179	2.130211
ENPP4	-2.04673	3.893863	-4.34345	2.24E-05	0.000179	1.90509
ADAMTSL4	-1.7379	4.47254	-4.34046	2.27E-05	0.000181	1.875063
BMP1	1.294886	5.924745	4.338436	2.29E-05	0.000182	1.955192
HIST1H3D	2.133678	0.162343	4.336453	2.31E-05	0.000184	2.463023
ADPGK	0.594851	5.530441	4.335485	2.32E-05	0.000184	1.919423
MT1F	-1.58935	2.375495	-4.33487	2.32E-05	0.000185	2.090135
IFITM3	1.266342	8.388551	4.334715	2.32E-05	0.000185	1.841406
SCD	1.639848	8.248575	4.333836	2.33E-05	0.000185	1.832984
FLAD1	0.786532	5.507479	4.333545	2.34E-05	0.000185	1.931112
SLC2A11	-1.1881	1.546484	-4.33236	2.35E-05	0.000186	2.259213
CPNE1	1.043747	7.037062	4.332465	2.35E-05	0.000186	1.83643
SENP7	-1.0498	3.975193	-4.33063	2.36E-05	0.000187	1.910531
PCMTD1	-0.79596	5.752334	-4.33077	2.36E-05	0.000187	1.82117
HIST1H2BJ	2.735823	1.696962	4.329209	2.38E-05	0.000188	2.436071
NUP93	0.827557	5.68144	4.328921	2.38E-05	0.000188	1.898921
HADH	-0.88395	5.403107	-4.32893	2.38E-05	0.000188	1.819683
LSG1	0.854284	6.058863	4.328472	2.38E-05	0.000189	1.865915
PYROXD1	-0.77982	4.367944	-4.32807	2.39E-05	0.000189	1.885077
PPAP2C	1.783908	5.748092	4.327195	2.40E-05	0.000189	1.994374
TSC22D1	-0.94915	7.454413	-4.32723	2.40E-05	0.000189	1.835188
CPT2	-0.68287	5.081455	-4.32615	2.41E-05	0.00019	1.829251
SCMH1	-0.80636	5.259126	-4.32552	2.41E-05	0.00019	1.812988
PSMD3	1.092979	7.28057	4.323622	2.43E-05	0.000192	1.79636
POLE3	0.768591	6.259337	4.323348	2.44E-05	0.000192	1.825138
MRPS36	-0.67024	3.601549	-4.32232	2.45E-05	0.000193	1.962365
FXYD6	-1.45482	3.150089	-4.32095	2.46E-05	0.000194	1.927232
EEF2	-0.78518	11.18866	-4.32086	2.46E-05	0.000194	1.919886
ZNF433	-1.51345	0.966352	-4.31469	2.52E-05	0.000198	2.22617
ABHD6	-0.90119	3.302034	-4.31447	2.53E-05	0.000198	1.946625
PLCB4	-2.12337	3.68013	-4.31408	2.53E-05	0.000199	1.799656
PSMA5	0.673428	6.114105	4.314152	2.53E-05	0.000199	1.792924
GABPB1	0.634065	4.492676	4.313493	2.54E-05	0.000199	1.970839
TRAK2	-0.83885	5.490784	-4.31355	2.54E-05	0.000199	1.757683
ZNF175	-1.16461	2.533315	-4.31305	2.54E-05	0.000199	2.032881
PPIH	0.954096	4.337578	4.310858	2.57E-05	0.000201	2.038237
THOC6	1.14131	4.207122	4.310595	2.57E-05	0.000201	2.086922
TYW3	-0.5957	4.63148	-4.31049	2.57E-05	0.000201	1.805871
GMPS	0.997344	6.35699	4.308321	2.59E-05	0.000202	1.774026
LAPTM4B	1.049692	7.490333	4.308385	2.59E-05	0.000202	1.732533
GAB1	-1.0291	5.208269	-4.30709	2.61E-05	0.000203	1.736043
KCNH2	-1.92537	1.845535	-4.30648	2.61E-05	0.000204	2.012159
ALS2CR8	-0.89146	2.792094	-4.30645	2.61E-05	0.000204	2.00307
RCN1	1.034363	6.71926	4.305817	2.62E-05	0.000204	1.744212
MAP3K3	-0.70279	4.97699	-4.30491	2.63E-05	0.000205	1.750506
PPAT	1.082471	4.473734	4.304143	2.64E-05	0.000205	2.008783
IQSEC1	-0.86379	6.07071	-4.30402	2.64E-05	0.000205	1.715003
GIMAP6	-1.23087	3.103664	-4.30387	2.64E-05	0.000205	1.892572

PXMP4	-0.80173	4.204848	-4.30365	2.64E-05	0.000205	1.803033
ACAD10	-0.74984	4.639439	-4.30267	2.65E-05	0.000206	1.763662
C3orf37	0.891477	5.535504	4.302504	2.66E-05	0.000206	1.815889
KBTBD3	-0.89679	1.669699	-4.3012	2.67E-05	0.000207	2.155962
ACSM3	-2.50841	1.228545	-4.29907	2.69E-05	0.000209	2.013018
LOC100132707	-0.96934	1.49366	-4.29885	2.70E-05	0.000209	2.160512
TDRD3	-0.72299	3.865577	-4.29778	2.71E-05	0.00021	1.824732
ING3	-0.64907	3.198442	-4.29635	2.72E-05	0.000211	1.930277
TARSL2	-0.66813	3.298875	-4.29592	2.73E-05	0.000211	1.907803
ILK	-0.76978	6.240199	-4.29568	2.73E-05	0.000211	1.682813
DHX9	0.488727	7.750094	4.295448	2.73E-05	0.000211	1.68725
TPM2	-1.66308	6.270637	-4.29525	2.74E-05	0.000211	1.696134
NUDT12	-1.15959	3.711107	-4.29509	2.74E-05	0.000211	1.791191
GLYR1	-0.54431	6.73497	-4.29457	2.74E-05	0.000212	1.68213
THUMPD1	-0.62066	5.548495	-4.29259	2.77E-05	0.000213	1.678759
CTSB	1.179274	10.15264	4.291834	2.77E-05	0.000214	1.720735
PLLP	-1.93034	3.936618	-4.29157	2.78E-05	0.000214	1.704204
MKL2	-0.67215	6.331321	-4.29005	2.80E-05	0.000215	1.660657
CDON	-1.39039	2.877269	-4.28942	2.80E-05	0.000216	1.848614
UGP2	-0.53898	6.482845	-4.28875	2.81E-05	0.000216	1.655723
LRRK2	-1.7558	2.561851	-4.28811	2.82E-05	0.000217	1.844199
CCDC138	1.3077	2.159393	4.287217	2.83E-05	0.000217	2.234486
LOC100128191	1.503617	2.655179	4.287277	2.83E-05	0.000217	2.212988
SQLE	1.64615	6.381219	4.284237	2.86E-05	0.00022	1.729366
CXCL17	-3.16198	3.206794	-4.28409	2.86E-05	0.00022	1.675764
NFATC2	-1.69526	2.833942	-4.28336	2.87E-05	0.00022	1.799353
MRPL3	0.746695	6.613499	4.282788	2.88E-05	0.000221	1.646225
EIF5A	0.874044	7.946337	4.282593	2.88E-05	0.000221	1.634672
IL17RC	-0.76309	4.379483	-4.28116	2.90E-05	0.000222	1.701022
GNB1L	1.172959	2.530469	4.280406	2.91E-05	0.000222	2.172654
HMGN2	0.741123	7.642203	4.278235	2.93E-05	0.000224	1.614938
ETV4	3.663803	5.036966	4.27777	2.94E-05	0.000225	2.145479
FOXP2	-2.30308	1.378306	-4.27657	2.95E-05	0.000225	1.917857
MORC2	0.606327	5.707933	4.276655	2.95E-05	0.000225	1.672936
TBC1D13	-0.63884	5.238968	-4.27046	3.03E-05	0.000231	1.604279
SOSTDC1	-2.61701	1.694722	-4.26829	3.06E-05	0.000233	1.797715
GADD45G	-1.35476	1.744011	-4.26777	3.06E-05	0.000233	1.959898
PPP4C	0.769268	6.332618	4.266396	3.08E-05	0.000235	1.597536
CHRNA5	1.836498	2.454371	4.265064	3.10E-05	0.000236	2.160875
SMYD5	0.676124	5.134393	4.264245	3.11E-05	0.000236	1.68956
ADAM28	-2.11304	2.916789	-4.26427	3.11E-05	0.000236	1.678108
PIGN	-0.89598	4.679829	-4.26244	3.13E-05	0.000238	1.593948
GGTA1	-1.52102	0.827803	-4.2613	3.15E-05	0.000239	2.035003
ASB2	-1.67599	2.001156	-4.25965	3.17E-05	0.00024	1.835871
C14orf1	0.77974	5.481687	4.2587	3.18E-05	0.000241	1.639782
SF3A2	0.738182	6.938211	4.257925	3.19E-05	0.000242	1.538871
RPH3AL	-1.52195	2.807772	-4.25575	3.22E-05	0.000244	1.714972
TNRC6C	-0.90009	4.603179	-4.25455	3.23E-05	0.000245	1.568154
TSSC1	0.869858	4.535985	4.253802	3.24E-05	0.000245	1.768417
C10orf108	-2.40021	0.68594	-4.25219	3.26E-05	0.000247	1.927091
C20orf3	0.773552	6.532399	4.251787	3.27E-05	0.000247	1.530156
FXYD5	1.457839	6.519067	4.251487	3.27E-05	0.000247	1.573098
GIMAP5	-1.24499	2.470819	-4.25112	3.28E-05	0.000248	1.790343
C5orf33	-0.74446	4.761994	-4.25109	3.28E-05	0.000248	1.552955
EEPD1	-1.40047	3.993624	-4.25074	3.28E-05	0.000248	1.57078
C17orf107	-1.21958	1.407799	-4.25038	3.29E-05	0.000248	1.959017
REEP4	1.274951	5.532899	4.249201	3.31E-05	0.000249	1.649829
IFI6	2.283551	7.166984	4.247985	3.32E-05	0.00025	1.572113

LMCD1	-1.13301	3.783276	-4.24716	3.33E-05	0.000251	1.597099
ZZEF1	-0.81452	6.685184	-4.24337	3.38E-05	0.000255	1.48593
ANKRD12	-0.95384	6.45104	-4.24282	3.39E-05	0.000255	1.482073
TUBG2	-0.91835	3.431891	-4.24124	3.41E-05	0.000257	1.638244
PFN1	0.711646	9.11687	4.23569	3.49E-05	0.000262	1.48284
BOP1	2.030885	4.609718	4.235419	3.50E-05	0.000262	1.859684
CCL3	2.50842	0.547783	4.234688	3.51E-05	0.000263	2.085802
POLA2	1.086633	4.739927	4.233501	3.52E-05	0.000264	1.689051
TLCD1	1.636221	3.308146	4.23097	3.56E-05	0.000267	1.954554
ZNF271	-0.65878	4.594224	-4.22974	3.58E-05	0.000268	1.4897
SFN	2.887638	8.276585	4.229524	3.58E-05	0.000268	1.459309
CCDC77	1.187991	2.792672	4.228545	3.60E-05	0.000269	1.95432
SNRPF	1.048324	5.481004	4.228485	3.60E-05	0.000269	1.550087
AES	-0.73274	7.949051	-4.22604	3.63E-05	0.000272	1.449129
CRYBG3	-0.89177	4.745723	-4.22445	3.66E-05	0.000273	1.442019
DEF8	-0.668	5.018175	-4.22455	3.65E-05	0.000273	1.436884
GLOD4	-0.67049	5.489144	-4.22399	3.66E-05	0.000273	1.412421
GGA2	-0.62835	5.983371	-4.22325	3.67E-05	0.000274	1.399916
TMEM56	-1.86246	3.288167	-4.221	3.71E-05	0.000276	1.488385
DIP2C	-1.03397	4.647218	-4.21958	3.73E-05	0.000278	1.421608
FAM91A1	0.808394	6.75038	4.218195	3.75E-05	0.000279	1.392306
SLC25A20	-1.15035	3.09382	-4.21725	3.76E-05	0.00028	1.57078
SBF2	-0.66203	5.828849	-4.21705	3.77E-05	0.00028	1.377493
KDM4C	-0.71064	4.903013	-4.21684	3.77E-05	0.00028	1.412587
FOXO4	-0.88959	3.663429	-4.21662	3.77E-05	0.000281	1.516891
TSPAN12	-1.67901	2.716599	-4.21558	3.79E-05	0.000282	1.556288
PHF6	0.730473	5.35095	4.210858	3.86E-05	0.000287	1.464228
ZNF717	-1.68053	0.799804	-4.20914	3.89E-05	0.000289	1.825792
TRAF2	0.906464	4.409204	4.207993	3.91E-05	0.00029	1.620713
CD302	-1.29874	4.778512	-4.20401	3.97E-05	0.000294	1.34328
CRAT	-1.51268	5.4572	-4.20247	4.00E-05	0.000296	1.322052
SHKBP1	0.617625	6.4813	4.201086	4.02E-05	0.000298	1.329783
MTHFR	-1.05057	5.354268	-4.20006	4.04E-05	0.000299	1.31555
SUMO2	0.626829	6.906603	4.199051	4.05E-05	0.0003	1.309452
ZRANB1	-0.49163	5.614332	-4.19733	4.08E-05	0.000302	1.310314
TRPM2	1.964025	3.09628	4.194983	4.12E-05	0.000304	1.863135
BCL11B	2.834906	4.558411	4.194863	4.12E-05	0.000304	1.802529
C2orf88	-1.51212	2.207169	-4.19502	4.12E-05	0.000304	1.584167
CSGALNACT1	-1.25448	3.894436	-4.19519	4.12E-05	0.000304	1.375829
ZNF652	-0.73258	6.090988	-4.19495	4.12E-05	0.000304	1.290449
EXT1	0.940784	6.533147	4.193953	4.14E-05	0.000305	1.314833
CTSC	1.38127	7.569331	4.194007	4.14E-05	0.000305	1.291817
KIAA0753	-0.64024	4.362487	-4.19045	4.20E-05	0.000309	1.361187
ZNF295	-0.66034	4.996082	-4.18948	4.21E-05	0.00031	1.303606
SFRS1	0.422474	7.726838	4.187601	4.25E-05	0.000312	1.269483
LARP1B	-0.70043	4.589067	-4.18578	4.28E-05	0.000314	1.318227
TAGLN	-2.08027	7.012444	-4.18493	4.29E-05	0.000315	1.299712
NUP62CL	2.50298	1.491666	4.184174	4.30E-05	0.000316	1.901012
MIA3	-0.67951	6.675942	-4.1842	4.30E-05	0.000316	1.255268
UGCG	-0.98703	4.476629	-4.18355	4.32E-05	0.000316	1.298758
HSDL2	-0.78099	5.727164	-4.18361	4.31E-05	0.000316	1.24901
CDH24	1.64985	3.855691	4.180627	4.37E-05	0.00032	1.704868
CASP8	0.992177	5.375756	4.179494	4.39E-05	0.000321	1.369095
BNC2	-1.61998	2.547527	-4.17837	4.41E-05	0.000323	1.440687
ME2	-0.79679	5.421423	-4.17739	4.42E-05	0.000324	1.231882
TEX10	0.787151	4.837307	4.176021	4.45E-05	0.000325	1.404717
TNFAIP8L3	-1.44675	1.752845	-4.17588	4.45E-05	0.000325	1.591856
WDR43	0.703326	6.055615	4.175532	4.46E-05	0.000326	1.261226

INPP5B	-0.66378	4.392984	-4.17509	4.47E-05	0.000326	1.297763
N4BP3	-1.14075	3.526826	-4.1747	4.47E-05	0.000326	1.347823
SNRPA1	0.895965	4.017043	4.170622	4.55E-05	0.000332	1.544305
CCDC58	1.082813	3.051297	4.169399	4.57E-05	0.000333	1.696495
SKP2	1.578275	4.259307	4.169168	4.57E-05	0.000333	1.599005
TMEM129	-0.8111	5.045591	-4.16911	4.57E-05	0.000333	1.215353
RGS2	-1.59107	4.928513	-4.16838	4.59E-05	0.000334	1.195274
NME2	0.809839	8.045187	4.167833	4.60E-05	0.000334	1.193494
CYP4X1	-1.96683	2.177908	-4.16698	4.61E-05	0.000335	1.414851
PSMB8	1.154568	6.278623	4.167107	4.61E-05	0.000335	1.244811
TMEM88	-1.14696	0.596012	-4.16562	4.64E-05	0.000337	1.729408
SMC6	0.875669	5.614665	4.165393	4.64E-05	0.000337	1.276106
C2orf55	-1.55764	3.312838	-4.1637	4.68E-05	0.000339	1.292853
C5orf41	-0.87003	4.413839	-4.16361	4.68E-05	0.000339	1.235467
XPC	-0.60544	4.930395	-4.16349	4.68E-05	0.000339	1.212023
HOXD9	2.462707	1.476849	4.162912	4.69E-05	0.00034	1.823843
WDR48	-0.59759	5.14215	-4.16277	4.69E-05	0.00034	1.195757
KIAA1161	-1.54985	4.930416	-4.16248	4.70E-05	0.00034	1.173508
HDLBP	-0.66226	9.319143	-4.16178	4.71E-05	0.000341	1.242348
PPP2R3A	-1.7469	4.751115	-4.16019	4.74E-05	0.000343	1.165797
MICB	2.6422	2.909437	4.159607	4.75E-05	0.000343	1.782595
C10orf118	-0.9887	5.439529	-4.15869	4.77E-05	0.000344	1.156157
SH3BGRL	-1.13701	6.355873	-4.1565	4.81E-05	0.000347	1.151382
PLAUR	1.712463	5.587835	4.156353	4.82E-05	0.000347	1.348072
GAD1	3.330441	0.420656	4.154612	4.85E-05	0.00035	1.794152
GULP1	-1.67067	4.170185	-4.15431	4.86E-05	0.00035	1.169892
MEF2D	-0.6819	6.459154	-4.15262	4.89E-05	0.000352	1.131233
ITGB3	-1.4758	3.102217	-4.15125	4.92E-05	0.000354	1.276356
TRAF3	0.822885	4.377626	4.150379	4.93E-05	0.000355	1.391417
TMEM170B	-1.35114	2.235554	-4.1486	4.97E-05	0.000357	1.423789
SDS	3.208967	2.080258	4.147943	4.98E-05	0.000358	1.769499
ST3GAL6	-1.29321	1.372943	-4.14561	5.03E-05	0.000361	1.557088
PPP1R14A	-1.2951	1.721112	-4.14391	5.06E-05	0.000363	1.496076
SH3GLB2	-0.7265	5.752393	-4.14391	5.06E-05	0.000363	1.097603
UBE2I	0.629815	6.294052	4.14341	5.07E-05	0.000364	1.118672
ELAC1	-0.73546	1.763284	-4.14146	5.11E-05	0.000367	1.553979
ZFAND5	-0.69203	7.576012	-4.14073	5.13E-05	0.000367	1.109777
GSTP1	1.278107	9.066398	4.140786	5.13E-05	0.000367	1.103358
ARID4A	-0.75452	5.279609	-4.14097	5.12E-05	0.000367	1.099265
YWHAZ	0.839395	10.21829	4.140332	5.14E-05	0.000367	1.147626
TEF	-1.07087	4.487351	-4.14035	5.14E-05	0.000367	1.127466
RMND5B	-0.81156	4.713454	-4.14022	5.14E-05	0.000367	1.126361
IFNAR1	-0.61078	6.207641	-4.14025	5.14E-05	0.000367	1.081122
ZIC5	4.358173	0.646797	4.139632	5.15E-05	0.000368	1.739991
KCNIP3	-1.45499	1.617275	-4.1385	5.17E-05	0.000369	1.472531
TIPIN	1.051742	2.81114	4.138276	5.18E-05	0.000369	1.602218
CYB5A	-1.07386	4.732917	-4.13776	5.19E-05	0.00037	1.101443
KCTD5	0.723692	5.665239	4.137471	5.20E-05	0.00037	1.151134
CYP27B1	2.090826	1.747927	4.137163	5.20E-05	0.00037	1.724474
MAP9	-1.69827	3.04834	-4.13632	5.22E-05	0.000371	1.204496
NDUFS4	-0.71205	4.356189	-4.13632	5.22E-05	0.000371	1.149902
SLC16A3	1.284794	5.975017	4.135377	5.24E-05	0.000373	1.163178
POLD2	0.842168	6.417421	4.134403	5.26E-05	0.000374	1.088724
SMARCD1	0.586471	6.420548	4.134202	5.26E-05	0.000374	1.075481
C5orf44	-0.48944	4.051792	-4.13381	5.27E-05	0.000374	1.194602
TGFBRAP1	-0.70979	4.783218	-4.13188	5.31E-05	0.000377	1.096292
ATXN2L	0.530389	8.065374	4.131625	5.32E-05	0.000377	1.060571
REV3L	-0.75183	5.513278	-4.13171	5.32E-05	0.000377	1.056061

SMAD4		-0.88926	5.948324	-4.12944	5.36E-05	0.00038	1.040588
BMPR1B		-2.11397	1.114898	-4.12835	5.39E-05	0.000382	1.42707
MAPRE2		-0.93724	5.528586	-4.12812	5.39E-05	0.000382	1.038788
HSPBP1		0.683213	5.53946	4.127176	5.41E-05	0.000383	1.120544
C4orf46		1.14262	3.925824	4.12673	5.42E-05	0.000383	1.42991
AGTRAP		1.185699	5.232132	4.124822	5.46E-05	0.000386	1.206507
GAS6		-1.2528	6.294728	-4.12396	5.48E-05	0.000387	1.028595
DNMT3B		2.65874	3.330837	4.121877	5.53E-05	0.00039	1.620964
IPP		-0.58255	3.811184	-4.12198	5.53E-05	0.00039	1.170859
SETDB2		-0.67694	3.809	-4.12186	5.53E-05	0.00039	1.160047
C3orf70		-1.45155	2.717413	-4.12165	5.53E-05	0.00039	1.221628
ZNF546		-1.02322	1.407618	-4.12099	5.55E-05	0.000391	1.489657
DARS2		0.986157	5.652151	4.120761	5.55E-05	0.000391	1.11408
LYAR		1.046129	4.498236	4.120241	5.57E-05	0.000392	1.293025
STAM2		-0.56133	5.566733	-4.11897	5.59E-05	0.000394	1.011044
ARHGEF6		-1.18592	3.434624	-4.11656	5.65E-05	0.000397	1.132391
CRIPAK		-0.83063	3.613244	-4.11613	5.66E-05	0.000398	1.147983
C5orf32		-2.31313	6.06213	-4.11588	5.66E-05	0.000398	1.016089
FZD2		2.419077	2.754401	4.115611	5.67E-05	0.000398	1.618449
TCF25		-0.52874	6.194217	-4.11503	5.68E-05	0.000399	0.985267
IL24		3.659702	0.131183	4.114678	5.69E-05	0.000399	1.650553
BBS2		-0.70268	4.553188	-4.11364	5.71E-05	0.000401	1.046016
UTP14A		0.708722	4.703816	4.11159	5.76E-05	0.000404	1.170586
MAN2B2		-0.74297	6.101475	-4.11168	5.76E-05	0.000404	0.97308
ABHD5		-0.78743	4.906397	-4.10881	5.83E-05	0.000408	0.995296
H2AFY		0.599431	7.424786	4.108297	5.84E-05	0.000408	0.960809
VPS53		-0.56139	6.041065	-4.1081	5.84E-05	0.000409	0.95973
SNX8		1.027458	4.201709	4.107103	5.87E-05	0.00041	1.293928
TBC1D17		-0.6016	5.236509	-4.10706	5.87E-05	0.00041	0.978693
MYL9		-2.05738	6.834815	-4.10687	5.87E-05	0.00041	0.996734
FAM63B		-0.80203	4.33481	-4.10585	5.89E-05	0.000411	1.028932
OXR1		-0.6981	5.625522	-4.10542	5.90E-05	0.000412	0.954799
ABHD14A		-0.83665	2.843083	-4.10359	5.95E-05	0.000415	1.228042
ECE2		1.397971	3.687633	4.103375	5.95E-05	0.000415	1.409381
UQCRQ		-0.78716	5.906057	-4.103	5.96E-05	0.000415	0.940452
PCDHGB6		-1.47852	0.980122	-4.10093	6.01E-05	0.000419	1.418016
FAHD2A		-0.69905	3.297147	-4.10066	6.02E-05	0.000419	1.157119
AKAP13		-0.87442	7.780264	-4.10015	6.03E-05	0.000419	0.967305
FABP4		-2.65992	0.408895	-4.09991	6.04E-05	0.000419	1.355363
C1orf107		0.683139	5.382886	4.099851	6.04E-05	0.000419	1.03362
BBS4		-0.58549	3.412028	-4.09958	6.04E-05	0.00042	1.149891
SLC9A3R2		-0.88977	5.267222	-4.09953	6.05E-05	0.00042	0.938734
DOCK3		-1.73042	1.112534	-4.09907	6.06E-05	0.00042	1.362673
ABCA2		-1.4054	5.919957	-4.0982	6.08E-05	0.000421	0.928032
NRARP		1.483698	6.313549	4.097636	6.09E-05	0.000422	1.00556
SLC38A7		0.689108	4.515347	4.097348	6.10E-05	0.000422	1.146543
CTPS		1.016801	5.320927	4.09732	6.10E-05	0.000422	1.065846
PTDSS1		0.890692	6.568918	4.096708	6.11E-05	0.000423	0.940262
	2-Mar	-0.76471	4.072666	-4.09389	6.18E-05	0.000428	1.01431
GALNT10		-1.16156	5.612489	-4.09255	6.22E-05	0.00043	0.901943
HSD17B4		-0.60049	6.263937	-4.09212	6.23E-05	0.00043	0.899162
GPN1		0.529926	5.416449	4.091722	6.24E-05	0.000431	0.985282
LDLRAD2		-0.84508	3.136266	-4.09062	6.26E-05	0.000432	1.125347
VAV2		1.125342	6.006872	4.088829	6.31E-05	0.000435	0.970347
COQ6		-0.50291	3.471636	-4.08787	6.33E-05	0.000436	1.107504
ZNF33A		-0.81194	5.6938	-4.08788	6.33E-05	0.000436	0.885486
RFC5		1.038122	4.367088	4.08682	6.36E-05	0.000438	1.190463
HSPD1		0.799008	8.458054	4.086753	6.36E-05	0.000438	0.894511

MAPK1	-0.56863	7.696554	-4.08531	6.40E-05	0.00044	0.900572
PPP1CA	0.75204	7.011983	4.084663	6.41E-05	0.000441	0.874734
ACTA2	-1.89801	7.186865	-4.08239	6.47E-05	0.000445	0.910857
LGALS8	0.613663	5.734812	4.081506	6.49E-05	0.000446	0.9241
CREBL2	-0.64821	5.57886	-4.07813	6.58E-05	0.000452	0.854361
UBXN6	-0.62964	5.602606	-4.07774	6.59E-05	0.000453	0.85262
ARID5B	-1.04648	6.32992	-4.07731	6.60E-05	0.000453	0.849839
FANCG	1.009097	3.956512	4.076892	6.61E-05	0.000454	1.220101
RHOBTB2	-1.05291	4.53386	-4.07675	6.62E-05	0.000454	0.885063
PDLIM3	-1.8154	4.836134	-4.07644	6.63E-05	0.000454	0.846444
MAZ	0.705086	7.879719	4.075944	6.64E-05	0.000455	0.844098
RANGAP1	0.677715	7.170771	4.075136	6.66E-05	0.000456	0.835795
COLEC12	-1.90074	2.204701	-4.07344	6.70E-05	0.000459	1.061769
HERPUD1	-1.03808	6.64237	-4.07303	6.72E-05	0.000459	0.83913
ZNF33B	-0.76432	5.016877	-4.0721	6.74E-05	0.000461	0.85196
SSRP1	0.61338	7.520272	4.071263	6.76E-05	0.000462	0.822346
IP6K1	-0.56474	5.834188	-4.07085	6.77E-05	0.000463	0.822504
BSN	-1.45605	0.548788	-4.06486	6.94E-05	0.000473	1.335395
CENPQ	1.132149	2.803529	4.064219	6.95E-05	0.000474	1.336718
TIGD1	1.059955	2.855728	4.062844	6.99E-05	0.000477	1.319575
SLBP	0.71659	5.801747	4.061392	7.03E-05	0.000479	0.851271
COL7A1	3.504112	6.571977	4.059775	7.08E-05	0.000482	1.106863
NDUFA10	-0.57898	6.364591	-4.0588	7.10E-05	0.000484	0.774738
COBL	-2.60064	4.408253	-4.05861	7.11E-05	0.000484	0.784107
EIF3L	-0.67788	7.79951	-4.0578	7.13E-05	0.000485	0.803455
TRIOBP	-0.70125	6.421179	-4.05763	7.14E-05	0.000485	0.772376
POLR2D	0.628547	4.956609	4.05558	7.20E-05	0.000489	0.91183
NFASC	-1.68661	3.877741	-4.05379	7.25E-05	0.000492	0.810073
ZNF763	-1.2478	0.159223	-4.05278	7.28E-05	0.000494	1.344447
VILL	-2.1429	3.613992	-4.05251	7.28E-05	0.000494	0.803333
SAPS2	-0.54713	5.754842	-4.05081	7.33E-05	0.000497	0.749607
CYP20A1	-0.69789	5.198211	-4.05014	7.35E-05	0.000498	0.762988
ZNF671	-1.13313	1.311275	-4.04845	7.40E-05	0.000501	1.221452
ZNF710	-0.73282	4.989937	-4.04833	7.40E-05	0.000501	0.766281
CYS1	-1.59115	1.055287	-4.04808	7.41E-05	0.000502	1.200136
FBXW4	-0.6624	5.28666	-4.04715	7.44E-05	0.000503	0.749002
FAM122A	-0.59635	4.607883	-4.04656	7.46E-05	0.000504	0.798116
CD59	-0.99703	8.416671	-4.04641	7.46E-05	0.000504	0.789071
EFNA4	1.275621	3.527431	4.045963	7.47E-05	0.000505	1.203881
ANKRD43	-2.26055	0.921168	-4.04521	7.50E-05	0.000506	1.135059
MT1E	-1.94433	4.345241	-4.04506	7.50E-05	0.000506	0.740241
GPRC5C	-1.85679	4.917557	-4.04477	7.51E-05	0.000507	0.726965
FTSJ2	0.699853	4.805123	4.044515	7.52E-05	0.000507	0.902192
MMRN2	-1.09578	4.509306	-4.04357	7.54E-05	0.000508	0.760081
ACOX1	-0.9641	6.548505	-4.04338	7.55E-05	0.000509	0.725221
PHKB	-0.71834	6.408612	-4.04096	7.62E-05	0.000513	0.710253
CHPT1	-1.36229	4.887638	-4.04022	7.64E-05	0.000515	0.716958
QSOX1	-1.25986	7.565775	-4.03373	7.84E-05	0.000528	0.722925
PTRH2	0.868553	4.200554	4.033244	7.86E-05	0.000528	0.993835
PA2G4	0.682196	7.171025	4.032922	7.87E-05	0.000529	0.678141
MKNK2	-1.04208	7.560854	-4.0328	7.87E-05	0.000529	0.713053
EGFL8	-1.08367	1.437753	-4.03187	7.90E-05	0.00053	1.150416
SNRK	-0.75009	5.632158	-4.03182	7.90E-05	0.00053	0.677929
MYO1C	-0.66345	8.018498	-4.03076	7.93E-05	0.000532	0.709122
SNRPD3	0.635361	6.159897	4.030499	7.94E-05	0.000533	0.702586
GLA	1.078442	4.530444	4.029261	7.98E-05	0.000535	0.952984
RAP1GAP	-1.83789	4.561209	-4.02881	8.00E-05	0.000536	0.674747
ATP6V1H	-0.56384	5.60074	-4.02831	8.01E-05	0.000536	0.669825

EPT1	0.844298	6.742595	4.026755	8.06E-05	0.000539	0.668704
C11orf92	-2.14231	2.608094	-4.02645	8.07E-05	0.00054	0.808507
ZNF248	-0.65772	3.651691	-4.02478	8.12E-05	0.000543	0.820645
ST6GALNAC1	-2.65718	3.343361	-4.02455	8.13E-05	0.000543	0.694819
CAPG	1.135256	6.367331	4.023949	8.15E-05	0.000544	0.697021
KHDRBS1	0.382059	7.314851	4.023392	8.17E-05	0.000545	0.643658
NR2F1	-1.20542	3.026449	-4.02021	8.27E-05	0.000552	0.828569
NXT1	0.810813	4.321278	4.018232	8.33E-05	0.000556	0.905971
CRYAB	-2.00889	3.678902	-4.01546	8.43E-05	0.000562	0.665099
ZNF420	-1.40496	1.448758	-4.01479	8.45E-05	0.000563	1.045498
TUB	-1.93506	1.350708	-4.01436	8.46E-05	0.000564	0.986196
SLC5A6	1.12808	5.581265	4.014173	8.47E-05	0.000564	0.737937
GMPPB	-0.78253	3.740393	-4.01382	8.48E-05	0.000564	0.755109
GOT1	-0.77406	5.854198	-4.01386	8.48E-05	0.000564	0.607554
PDIA4	0.848579	7.723811	4.013551	8.49E-05	0.000564	0.607385
HOXA1	2.308175	2.155965	4.01273	8.52E-05	0.000566	1.271083
FBXO3	-0.64623	5.015029	-4.01262	8.52E-05	0.000566	0.636572
C20orf27	1.013386	4.909892	4.01184	8.55E-05	0.000567	0.81081
YEATS2	1.002784	6.448838	4.011626	8.55E-05	0.000567	0.636167
ARHGEF15	-1.17394	2.534111	-4.01026	8.60E-05	0.00057	0.883043
COQ9	-0.624	5.615288	-4.00837	8.66E-05	0.000574	0.593893
USP4	-0.49472	5.546863	-4.00734	8.70E-05	0.000576	0.596067
SPAST	0.732575	5.352706	4.004315	8.80E-05	0.000583	0.685862
LOC92659	1.521015	1.561853	4.004169	8.81E-05	0.000583	1.236066
ST6GALNAC3	-1.4101	0.821827	-4.00366	8.82E-05	0.000584	1.087506
MRPL14	0.993948	5.096619	4.003216	8.84E-05	0.000584	0.74631
TOMM34	0.838082	5.654626	4.003275	8.84E-05	0.000584	0.660119
C14orf143	0.822492	1.874122	4.002253	8.87E-05	0.000586	1.17099
CCDC21	0.962051	4.539659	4.001893	8.88E-05	0.000587	0.832438
UQCRC1	-0.65793	7.300848	-4.00152	8.90E-05	0.000587	0.579904
MTL5	2.639856	2.544389	4.001353	8.90E-05	0.000587	1.227923
CCDC48	-1.48065	0.759226	-4.00079	8.92E-05	0.000588	1.07756
FKBP1A	0.581351	7.921623	4.000766	8.92E-05	0.000588	0.566887
DNAJB5	-1.38586	3.350285	-4.00026	8.94E-05	0.000589	0.689388
RTN4R	1.859994	3.263579	4.000028	8.95E-05	0.000589	1.123426
FLJ35390	-1.09084	1.201497	-3.9998	8.96E-05	0.00059	1.06101
MAPRE1	0.488662	7.21292	3.999154	8.98E-05	0.000591	0.552313
RORC	-2.51412	1.338415	-3.99882	8.99E-05	0.000591	0.860664
PRR19	1.626316	0.5833	3.998324	9.01E-05	0.000592	1.238224
C9orf7	-1.0878	4.270653	-3.99721	9.05E-05	0.000594	0.608971
INSIG1	-1.15951	5.747372	-3.9963	9.08E-05	0.000596	0.543184
B3GALNT2	-0.65457	5.881234	-3.994	9.16E-05	0.000601	0.534794
LOC401093	-1.3977	2.133977	-3.99221	9.23E-05	0.000605	0.849078
FSCN1	2.476845	7.877044	3.992194	9.23E-05	0.000605	0.563044
IGFBP2	-2.3611	6.510827	-3.99206	9.23E-05	0.000605	0.567658
KIAA1143	-0.61915	5.129053	-3.98959	9.32E-05	0.000611	0.545858
UQCRC2	-0.64114	7.047505	-3.98648	9.44E-05	0.000618	0.518319
FBLN5	-1.32734	4.376002	-3.98563	9.47E-05	0.00062	0.541266
C16orf93	1.150733	0.598376	3.984775	9.50E-05	0.000621	1.188742
PRKCB	-1.46103	2.653441	-3.98459	9.51E-05	0.000621	0.720662
IFITM1	1.675217	6.991426	3.983285	9.55E-05	0.000624	0.538279
GGH	1.771456	5.139383	3.982488	9.58E-05	0.000626	0.787324
VPS37C	-0.58865	5.361559	-3.98239	9.59E-05	0.000626	0.508651
TPSAB1	-1.75124	3.85375	-3.97965	9.69E-05	0.000632	0.534303
TMEM138	0.660248	5.024644	3.978832	9.72E-05	0.000634	0.623736
CCNY	-0.55802	6.023436	-3.97803	9.75E-05	0.000636	0.475169
RPL3	-0.70086	9.622717	-3.97747	9.77E-05	0.000637	0.562922
RHOB	-1.2657	7.869266	-3.97743	9.77E-05	0.000637	0.524423

GNPDA2	-0.76867	3.917428	-3.97724	9.78E-05	0.000637	0.597948
TAF1A	0.889219	2.566042	3.975919	9.83E-05	0.00064	1.01829
VPS11	-0.55844	5.230573	-3.97583	9.84E-05	0.00064	0.49239
CABC1	-1.21547	5.291231	-3.97511	9.86E-05	0.000641	0.468345
TGFBR2	-1.41583	6.84954	-3.97455	9.88E-05	0.000642	0.487016
PITPNC1	-0.96484	3.755087	-3.97349	9.93E-05	0.000645	0.585478
ISG15	2.56396	5.629301	3.972341	9.97E-05	0.000647	0.795796
FIGN	-2.00008	0.045361	-3.97213	9.98E-05	0.000648	1.001677
RNF220	0.435156	5.685841	3.971418	0.0001	0.000649	0.506079
HDAC5	-0.69455	5.682931	-3.97065	0.0001	0.000651	0.451737
RAD51L1	1.071437	1.875633	3.969822	0.000101	0.000652	1.07236
IKZF5	-0.51259	4.742415	-3.96984	0.000101	0.000652	0.509061
MRPL13	0.767239	5.084576	3.969424	0.000101	0.000653	0.593856
GJB7	3.106397	-0.67713	3.968455	0.000101	0.000655	1.135078
PRPF4	0.787585	5.512034	3.966989	0.000102	0.000658	0.536616
PYCRL	1.146222	4.144753	3.9662	0.000102	0.00066	0.803674
HSP90AA1	0.744373	10.196	3.963456	0.000103	0.000667	0.489822
BICD1	1.316798	3.322662	3.96331	0.000103	0.000667	0.930824
CYP2U1	-1.1665	2.641638	-3.96259	0.000104	0.000669	0.688743
SETMAR	-0.73722	2.87714	-3.96111	0.000104	0.000672	0.708447
RGS19	1.222701	3.797433	3.960542	0.000104	0.000673	0.84799
TECPR1	-0.7869	4.177974	-3.9606	0.000104	0.000673	0.507398
PRPF40A	0.430732	7.405963	3.960617	0.000104	0.000673	0.412831
VSTM2L	-2.02803	0.414297	-3.95997	0.000105	0.000674	0.915524
PWWP2A	-0.53837	4.333442	-3.95957	0.000105	0.000675	0.509188
FAR1	-0.76147	7.084548	-3.95933	0.000105	0.000675	0.42181
B3GNT5	1.420688	6.786146	3.957223	0.000106	0.00068	0.439868
LOC387647	-0.6246	4.012621	-3.95534	0.000106	0.000685	0.521871
PHF5A	0.654305	4.14222	3.954822	0.000107	0.000686	0.681618
TPM1	-1.28287	7.134767	-3.95473	0.000107	0.000686	0.418953
LOC100130776	1.801128	3.460532	3.951679	0.000108	0.000694	0.92287
TUSC2	-0.66894	3.907934	-3.95152	0.000108	0.000694	0.516448
ICOS	2.23132	0.524485	3.951375	0.000108	0.000694	1.074749
GIMAP7	-1.24254	2.041298	-3.95025	0.000109	0.000696	0.740035
MBD5	-0.7872	3.920905	-3.95022	0.000109	0.000696	0.49738
ALG1L	3.400403	1.578258	3.950045	0.000109	0.000696	1.069237
CCDC18	1.256225	2.540478	3.94945	0.000109	0.000697	0.95823
AHCY	0.729396	7.658923	3.949474	0.000109	0.000697	0.372055
RNF149	0.645068	6.11128	3.949278	0.000109	0.000697	0.407388
FAM8A1	-0.66341	5.581085	-3.94851	0.000109	0.000699	0.373896
MYCT1	-1.12048	2.023989	-3.94748	0.00011	0.000702	0.751819
CYBA	1.329302	6.577246	3.947415	0.00011	0.000702	0.413711
ACBD4	-1.00112	2.346397	-3.94699	0.00011	0.000702	0.712967
PARP1	0.652819	7.5008	3.946688	0.00011	0.000703	0.360781
CTHRC1	3.52177	4.624883	3.944692	0.000111	0.000708	0.941819
CCDC34	1.503801	3.685856	3.943423	0.000112	0.000711	0.838015
CCT7	0.548828	7.64803	3.942629	0.000112	0.000713	0.34916
EIF4EBP2	-0.63535	7.724235	-3.94179	0.000112	0.000715	0.371815
ROD1	0.72335	8.237945	3.941137	0.000113	0.000716	0.351928
ZNF571	-1.29747	1.180295	-3.93848	0.000114	0.000724	0.820508
ST20	1.144167	1.211852	3.937258	0.000114	0.000727	1.001063
PPFIBP2	-1.14998	5.045047	-3.93705	0.000114	0.000727	0.337224
MTR	-0.71639	5.738959	-3.93667	0.000114	0.000728	0.326082
TREM1	2.874137	0.638637	3.935102	0.000115	0.000732	1.018322
UBE2A	0.576254	6.255305	3.934791	0.000115	0.000732	0.341542
TSHZ2	-1.39169	3.16201	-3.93442	0.000115	0.000733	0.470985
SH3BP5	-0.95873	4.246408	-3.93421	0.000116	0.000733	0.38857
C11orf2	-0.6529	6.480094	-3.9342	0.000116	0.000733	0.317092

MTERFD1	0.898013	4.032615	3.933032	0.000116	0.000736	0.662085
GSPT2	-1.49769	1.650644	-3.9329	0.000116	0.000736	0.702907
ATP6V0E2	-1.45346	3.210969	-3.93202	0.000117	0.000738	0.450973
DNAJC2	0.848538	5.506234	3.932135	0.000116	0.000738	0.416572
MPDZ	-1.39873	3.550707	-3.93122	0.000117	0.00074	0.411405
USP16	-0.54169	5.418037	-3.93087	0.000117	0.00074	0.319278
TMEM110	-0.604	2.76921	-3.93057	0.000117	0.000741	0.640051
HNRNPF	0.510153	7.821837	3.929641	0.000118	0.000743	0.305469
RUNX1T1	-1.56694	0.425602	-3.92913	0.000118	0.000744	0.846572
SLC12A8	1.603402	4.417243	3.92596	0.000119	0.000753	0.686237
PRRT1	-1.12412	1.041032	-3.92512	0.00012	0.000755	0.806993
C4orf21	1.140855	3.255633	3.924998	0.00012	0.000755	0.783732
HELQ	-0.49028	3.108927	-3.9248	0.00012	0.000756	0.575253
PHYH	-1.11735	4.089367	-3.92335	0.000121	0.000759	0.353009
CST3	-1.06262	8.254094	-3.92275	0.000121	0.000761	0.331129
KHNYN	-0.57316	6.687391	-3.92209	0.000121	0.000762	0.274999
RIOK2	-0.48119	4.173292	-3.92171	0.000121	0.000763	0.39501
CBR4	-0.59898	4.077262	-3.92159	0.000121	0.000763	0.393567
HOXC13	4.302632	1.319372	3.921027	0.000122	0.000764	0.969123
SARDH	-1.7067	0.282515	-3.92107	0.000122	0.000764	0.823368
TMEM194A	0.966799	5.421542	3.919748	0.000122	0.000768	0.393472
NCRNA00219	-0.77376	2.842193	-3.91935	0.000122	0.000768	0.558367
PAQR8	-1.9966	4.678101	-3.91891	0.000123	0.000769	0.266404
FGF7	-1.6016	2.278958	-3.9184	0.000123	0.00077	0.524113
FGFR1	-1.55123	5.035876	-3.91843	0.000123	0.00077	0.262191
SNRNP40	0.668819	4.762891	3.918027	0.000123	0.000771	0.440668
NLRC5	1.343035	6.191985	3.917257	0.000123	0.000773	0.338898
AP1S1	0.988051	4.833977	3.915699	0.000124	0.000777	0.471286
UBE2D1	0.624857	4.981346	3.914532	0.000125	0.00078	0.389991
CTSZ	0.964693	6.614396	3.914628	0.000125	0.00078	0.268692
ITGB4	1.327056	9.483853	3.914345	0.000125	0.00078	0.272737
SLC39A10	1.09647	4.98032	3.913253	0.000125	0.000783	0.454006
INTS8	0.682706	5.735564	3.913241	0.000125	0.000783	0.309588
RAD18	0.849637	3.733782	3.912683	0.000126	0.000784	0.632899
CYP2C9	-2.55522	-0.58708	-3.91099	0.000126	0.000789	0.803424
CCNE1	2.124909	3.385403	3.910388	0.000127	0.00079	0.81375
NR6A1	1.450861	0.075995	3.909926	0.000127	0.000791	0.932269
TDP1	0.700055	4.211733	3.909825	0.000127	0.000791	0.513948
DAXX	0.494903	5.935142	3.909686	0.000127	0.000791	0.266111
S1PR3	-1.59102	4.450949	-3.90954	0.000127	0.000791	0.2473
FBXO21	-0.49985	5.832954	-3.90952	0.000127	0.000791	0.229085
CAT	-0.926	5.947031	-3.90953	0.000127	0.000791	0.225241
HES1	0.895823	6.374096	3.908129	0.000128	0.000795	0.25576
MED30	1.034947	3.740355	3.907294	0.000128	0.000797	0.639421
SUV420H2	0.954657	3.74719	3.905701	0.000129	0.000802	0.62247
NOLC1	0.627767	7.252436	3.904529	0.00013	0.000805	0.206448
LPP	-0.93833	6.917244	-3.90435	0.00013	0.000805	0.221596
ZNF383	-0.74332	2.176823	-3.90314	0.00013	0.000808	0.620644
PAFAH1B1	-0.56132	7.173997	-3.90174	0.000131	0.000812	0.210266
ZNF695	2.814594	1.062162	3.901227	0.000131	0.000814	0.901368
C4orf32	-0.93749	4.091314	-3.90062	0.000131	0.000815	0.285048
OLR1	3.379905	1.570239	3.899983	0.000132	0.000817	0.896636
TRIM68	-0.76296	3.367905	-3.89976	0.000132	0.000817	0.396172
LSR	1.214355	7.673014	3.899758	0.000132	0.000817	0.19049
METTL1	0.875234	3.744558	3.899362	0.000132	0.000818	0.587127
BMPR1A	-0.62065	4.891868	-3.8986	0.000133	0.00082	0.229777
RAPGEF2	-0.82451	5.182229	-3.89675	0.000133	0.000825	0.195715
STON1	-1.53087	4.009905	-3.89593	0.000134	0.000827	0.229243

SGK223	1.003023	5.905535	3.895304	0.000134	0.000829	0.256952
ERMAP	-0.67557	3.800909	-3.89441	0.000135	0.000831	0.321357
FAM174A	-0.7925	3.451597	-3.89384	0.000135	0.000833	0.356096
TMEM69	0.71785	5.235364	3.893605	0.000135	0.000833	0.293625
LOC399959	-1.51813	2.40531	-3.89251	0.000136	0.000836	0.420514
ADAMDEC1	3.288756	1.517228	3.892055	0.000136	0.000837	0.86952
GINS3	1.287757	3.56163	3.892028	0.000136	0.000837	0.643426
GAS7	-1.53882	4.452621	-3.89182	0.000136	0.000837	0.18522
PTPN6	0.847711	5.157277	3.891369	0.000136	0.000838	0.311144
F8A1	0.829164	5.255131	3.891302	0.000136	0.000838	0.295176
SMNDC1	0.449951	5.079229	3.891274	0.000136	0.000838	0.275621
COG1	-0.48174	5.264157	-3.88722	0.000138	0.000851	0.171953
ADCY6	-1.33359	5.161052	-3.88705	0.000139	0.000851	0.149018
SLC25A27	-1.38776	1.754792	-3.88608	0.000139	0.000854	0.53652
FRMD8	0.76409	6.304788	3.885333	0.000139	0.000856	0.169857
FLVCR2	2.146627	2.606463	3.884686	0.00014	0.000857	0.789702
TTC26	0.980132	2.180362	3.884743	0.00014	0.000857	0.740356
IL4I1	1.701912	2.720379	3.884133	0.00014	0.000859	0.747437
WDFY3	-0.69135	6.127848	-3.88379	0.00014	0.000859	0.131696
ZC3H12A	1.468525	6.22541	3.882301	0.000141	0.000864	0.220783
C6orf129	0.945041	3.328603	3.881134	0.000142	0.000867	0.596136
SOS2	-0.62301	5.862056	-3.88112	0.000142	0.000867	0.124063
RPLP0P2	3.259238	1.414231	3.880499	0.000142	0.000868	0.8301
ROM1	-0.79229	0.694454	-3.88052	0.000142	0.000868	0.715874
TMBIM6	-0.62727	9.838962	-3.87998	0.000142	0.00087	0.21364
CLIC1	0.815899	8.042084	3.879505	0.000143	0.000871	0.123323
TNRC6A	-0.47174	6.170409	-3.87922	0.000143	0.000872	0.115127
NEDD1	0.786117	4.984232	3.87891	0.000143	0.000872	0.282328
KLHL36	-0.60295	4.395163	-3.87805	0.000143	0.000875	0.201612
MPPE1	-0.58256	4.475403	-3.87658	0.000144	0.000879	0.189805
PDSS1	1.279259	3.168346	3.875538	0.000145	0.000882	0.63323
CACHD1	-1.28499	4.011906	-3.87539	0.000145	0.000882	0.173144
ZNF354C	-1.64934	0.106064	-3.87491	0.000145	0.000883	0.680327
BYSL	1.022022	4.403267	3.874767	0.000145	0.000883	0.406364
TATDN3	-0.56708	3.112704	-3.87477	0.000145	0.000883	0.383111
AP2S1	0.751036	5.936604	3.875047	0.000145	0.000883	0.159474
PSMB9	1.939037	5.22406	3.872021	0.000147	0.000892	0.404052
ENPP2	-1.44838	3.637569	-3.87191	0.000147	0.000892	0.185199
MTMR6	-0.63505	5.357754	-3.87172	0.000147	0.000892	0.104758
KIF24	1.25127	3.629336	3.871227	0.000147	0.000893	0.557892
ADSSL1	-1.36276	2.092212	-3.87093	0.000147	0.000894	0.428111
C21orf63	-1.16429	3.705939	-3.87086	0.000147	0.000894	0.199277
DCTN2	-0.53843	6.482846	-3.87093	0.000147	0.000894	0.086852
TAF2	0.608841	6.101103	3.86852	0.000149	0.000901	0.113499
ABCA10	-1.68466	-0.01736	-3.86822	0.000149	0.000902	0.668709
ZNF267	0.860261	4.537062	3.868042	0.000149	0.000902	0.331639
MAGEH1	-1.11703	2.514291	-3.86733	0.000149	0.000904	0.377667
HAUS6	0.816543	4.777606	3.867362	0.000149	0.000904	0.279239
6-Sep	-1.0442	4.665174	-3.86589	0.00015	0.000908	0.102528
PSMA1	0.545432	6.912604	3.865936	0.00015	0.000908	0.069444
CENPJ	1.098461	3.765676	3.865723	0.00015	0.000908	0.496078
ZNF219	-0.86536	5.386482	-3.86509	0.000151	0.00091	0.072926
RNF11	-0.69357	6.756199	-3.86424	0.000151	0.000912	0.069323
NSUN5	0.788937	4.989312	3.864065	0.000151	0.000913	0.22908
STIM1	-0.83078	6.397129	-3.86362	0.000151	0.000914	0.063352
FAM95B1	-1.73186	-0.03603	-3.86304	0.000152	0.000915	0.646556
GIGYF2	-0.47547	6.468337	-3.86298	0.000152	0.000915	0.057535
LIN9	0.98006	3.629194	3.860171	0.000153	0.000924	0.481912

PAPSS1	-0.64146	5.732966	-3.86014	0.000153	0.000924	0.050852
CADPS2	-1.53714	4.310274	-3.86	0.000154	0.000924	0.079427
L3MBTL4	-2.06412	0.390191	-3.85906	0.000154	0.000927	0.553984
CEP135	1.01693	3.850799	3.855036	0.000156	0.000941	0.433546
PLSCR1	0.94696	6.529976	3.854955	0.000157	0.000941	0.057562
JUB	1.608789	5.385971	3.854551	0.000157	0.000942	0.252914
PVRL1	1.889777	7.815263	3.853672	0.000157	0.000945	0.035602
BLCAP	0.746323	6.427664	3.852984	0.000158	0.000947	0.045647
NAT10	0.656321	6.256874	3.852825	0.000158	0.000947	0.049989
TSTD2	-0.54057	4.517644	-3.85181	0.000158	0.00095	0.100592
KRBA2	-0.93841	1.578857	-3.85032	0.000159	0.000955	0.496192
ROR2	-1.67441	3.376697	-3.84979	0.00016	0.000957	0.1142
RUSC2	-0.92389	4.841719	-3.84932	0.00016	0.000958	0.038545
TIMM8A	0.76157	2.887567	3.84817	0.000161	0.000962	0.514632
RAMP1	-1.61212	2.575415	-3.84781	0.000161	0.000962	0.221353
HTT	-0.68612	7.044337	-3.8479	0.000161	0.000962	0.016419
YWHAB	0.487329	8.953344	3.847407	0.000161	0.000963	0.038353
PFKP	0.902224	7.025633	3.847312	0.000161	0.000963	0.007513
GNL2	0.611799	5.796412	3.845733	0.000162	0.000969	0.055609
ELMOD3	-0.4913	3.455321	-3.84503	0.000163	0.000971	0.227059
YWHAG	0.712193	7.794427	3.843954	0.000163	0.000975	-0.00685
SNAPC3	-0.62716	4.613043	-3.84345	0.000164	0.000976	0.054789
CXCL16	1.150512	6.622603	3.842287	0.000164	0.00098	0.018697
SNX2	-0.53659	5.647274	-3.84226	0.000164	0.00098	-0.00851
C10orf4	-0.54888	3.671871	-3.84156	0.000165	0.000982	0.167837
OAS2	1.552702	6.910374	3.83913	0.000166	0.00099	0.013921
CLOCK	-0.77455	5.023156	-3.83794	0.000167	0.000994	-0.00513
WRAP53	0.803294	3.326627	3.837657	0.000167	0.000995	0.424334
C21orf58	1.408919	2.4792	3.837462	0.000167	0.000995	0.583314
NDUFB8	-0.59313	5.517195	-3.83596	0.000168	0.001001	-0.02816
MKLN1	-0.53688	6.353993	-3.83527	0.000169	0.001003	-0.04195
RPS19	0.735095	8.483569	3.833796	0.00017	0.001008	-0.0297
PI4KA	-0.63751	6.873737	-3.83193	0.000171	0.001015	-0.04486
ZNF565	-0.67434	1.874979	-3.83096	0.000171	0.001018	0.420036
PDHA1	-0.71123	6.243936	-3.8304	0.000172	0.00102	-0.05864
BRMS1	0.796714	5.200359	3.830007	0.000172	0.001021	0.079942
CYTSB	-0.91668	4.802246	-3.82948	0.000172	0.001022	-0.02904
CCDC137	0.673898	4.79071	3.82875	0.000173	0.001025	0.117084
SREBF1	0.939401	7.358254	3.828401	0.000173	0.001026	-0.06526
RBL2	-0.6336	6.652879	-3.82643	0.000174	0.001033	-0.06839
RAD21	0.659048	8.153978	3.825408	0.000175	0.001037	-0.06537
KRI1	0.685515	5.111643	3.823094	0.000177	0.001045	0.053025
KLF12	-1.23332	3.908035	-3.82304	0.000177	0.001045	0.000496
HNRNPU	0.386106	8.868583	3.820861	0.000178	0.001053	-0.056
PSMC2	0.682632	6.275284	3.819527	0.000179	0.001058	-0.06824
C3orf23	-0.64793	4.554725	-3.81899	0.000179	0.00106	-0.02855
NOC2L	0.674213	6.596743	3.818718	0.00018	0.00106	-0.08745
ABLIM1	-1.37323	8.002599	-3.81833	0.00018	0.001061	-0.04264
RNF160	-0.55185	5.781137	-3.81735	0.00018	0.001065	-0.10116
NASP	0.673584	6.482266	3.816808	0.000181	0.001067	-0.08928
PLCXD1	-1.0932	4.488611	-3.81586	0.000181	0.00107	-0.06524
PML	0.85281	7.497473	3.815655	0.000182	0.00107	-0.11173
RPP40	1.104806	2.736922	3.815138	0.000182	0.001072	0.451575
PTPN12	0.785535	6.981059	3.815235	0.000182	0.001072	-0.10826
LAGE3	0.976901	3.990907	3.81221	0.000184	0.001083	0.252475
ZNF358	-0.96206	5.837003	-3.81206	0.000184	0.001083	-0.12409
DCTN4	-0.4717	6.295723	-3.81162	0.000184	0.001085	-0.12659
LOC100130093	-0.84246	1.581367	-3.81081	0.000185	0.001087	0.369271

NONO	0.543967	8.324835	3.810762	0.000185	0.001087	-0.11135
PMEPA1	1.637141	6.959813	3.809764	0.000186	0.001091	-0.0879
CCDC86	0.833311	5.469804	3.809538	0.000186	0.001091	-0.021
ZC3H3	0.658287	6.041005	3.807859	0.000187	0.001098	-0.095
MAGIX	-1.36784	1.460336	-3.80747	0.000187	0.001099	0.313401
ZNF280C	1.149242	3.594133	3.806165	0.000188	0.001104	0.319352
CBX4	0.735168	5.360066	3.804512	0.000189	0.00111	-0.03617
TMEM66	-0.63577	7.3555	-3.80422	0.00019	0.001111	-0.13255
PLCD4	-0.87702	1.776155	-3.80357	0.00019	0.001113	0.311325
ESRP1	1.564022	7.330349	3.803249	0.00019	0.001114	-0.13686
CTH	-1.40143	1.491208	-3.80297	0.000191	0.001115	0.288231
STAT5B	-0.5952	6.038775	-3.80274	0.000191	0.001115	-0.15743
TMEM38A	-1.46657	1.474171	-3.8026	0.000191	0.001115	0.279486
RADIL	-1.71597	0.27586	-3.80143	0.000192	0.001119	0.400077
RNF123	-0.55948	5.405438	-3.80144	0.000192	0.001119	-0.14495
POLR1B	0.703025	5.498949	3.80125	0.000192	0.00112	-0.06608
HIF1AN	-0.5287	5.658809	-3.80115	0.000192	0.00112	-0.15465
FLJ39653	-0.89317	1.959823	-3.79946	0.000193	0.001126	0.269605
QSOX2	0.823402	5.045762	3.799331	0.000193	0.001126	-0.00397
BCL2	-1.13839	3.092882	-3.79897	0.000193	0.001127	0.02687
SPG20	-1.58074	4.453383	-3.79851	0.000194	0.001129	-0.14957
OBFC2B	0.677556	5.029658	3.797753	0.000194	0.001131	-0.02624
NDUFV3	-0.56076	4.463582	-3.79789	0.000194	0.001131	-0.08749
TMX4	-0.82783	5.08484	-3.79774	0.000194	0.001131	-0.15349
PTMA	0.607603	9.419343	3.797525	0.000194	0.001131	-0.12723
RGS5	-1.8403	6.908428	-3.79718	0.000195	0.001132	-0.13835
CGRRF1	-0.59666	2.47974	-3.7959	0.000196	0.001137	0.213108
MAT2A	-0.69277	7.495666	-3.79538	0.000196	0.001139	-0.15862
SGMS2	-1.1903	5.563702	-3.79496	0.000196	0.00114	-0.18363
TP53INP1	-0.99624	5.194365	-3.79453	0.000197	0.001142	-0.17522
PRLR	-1.98849	2.432688	-3.7944	0.000197	0.001142	0.011647
APPL1	-0.60478	6.144897	-3.79417	0.000197	0.001142	-0.18815
EPN1	-0.69952	7.767226	-3.79272	0.000198	0.001148	-0.1597
HADHA	-0.49294	7.466939	-3.79264	0.000198	0.001148	-0.17393
USP39	0.520735	5.796737	3.792015	0.000199	0.00115	-0.14187
MYLK	-1.866	6.706762	-3.79187	0.000199	0.00115	-0.16258
RGL3	-2.39225	0.387082	-3.79165	0.000199	0.00115	0.289076
LYN	1.310673	5.813827	3.791507	0.000199	0.00115	-0.07293
GHITM	-0.5622	7.487556	-3.79162	0.000199	0.00115	-0.17536
TFG	0.622339	7.407398	3.791124	0.000199	0.001151	-0.19822
ZP3	1.738594	2.582689	3.789926	0.0002	0.001156	0.436917
CCDC25	-0.66508	5.210457	-3.78998	0.0002	0.001156	-0.18026
AVPR1A	-1.37366	0.135831	-3.78946	0.0002	0.001157	0.405364
TMEM185B	0.742901	5.392265	3.789315	0.000201	0.001157	-0.09285
WDR76	1.215205	3.470442	3.787146	0.000202	0.001166	0.27899
SP3	0.484326	6.867921	3.787255	0.000202	0.001166	-0.21057
PSMD1	0.468646	7.39725	3.786353	0.000203	0.001169	-0.21386
ZNF280D	-0.50588	4.918386	-3.7862	0.000203	0.001169	-0.16529
B3GNT1	-0.80092	3.874867	-3.78608	0.000203	0.001169	-0.08553
USP20	-0.51246	4.842278	-3.78515	0.000204	0.001173	-0.1633
AMZ2P1	-0.722	2.077348	-3.78465	0.000204	0.001174	0.222522
LSM4	0.784452	6.087537	3.783119	0.000205	0.001181	-0.17687
PSMB1	0.57397	6.959233	3.781786	0.000206	0.001186	-0.22987
DDX56	0.608887	6.069573	3.78087	0.000207	0.00119	-0.19567
PCGF5	-0.70778	6.367221	-3.78055	0.000207	0.001191	-0.2338
DENR	0.526963	6.204467	3.779682	0.000208	0.001194	-0.21325
LOXL2	1.656547	5.817225	3.77912	0.000208	0.001196	-0.07462
EXOSC2	0.643292	4.635376	3.778962	0.000208	0.001196	-0.03674

ZNF323	-1.08755	3.198179	-3.7778	0.000209	0.001201	-0.05601
SLC4A11	2.861535	4.253949	3.777338	0.00021	0.001202	0.33213
LCLAT1	0.638421	5.320727	3.777398	0.00021	0.001202	-0.1369
TRIM29	4.094878	7.762167	3.775802	0.000211	0.001209	-0.03288
ATP10A	-1.26812	2.420709	-3.7755	0.000211	0.001209	0.04584
SNX19	-0.82233	6.484458	-3.77501	0.000212	0.001211	-0.24997
FAM102A	-1.02906	7.304526	-3.77428	0.000212	0.001214	-0.22959
STAC3	1.467978	0.141768	3.772687	0.000213	0.001221	0.468094
TAPBP	0.664967	8.640064	3.771037	0.000215	0.001228	-0.24597
HMGXB4	0.54363	5.311133	3.769849	0.000216	0.001233	-0.17194
MAP4K3	-0.6331	5.571881	-3.76952	0.000216	0.001234	-0.26606
XPOT	0.79491	6.836536	3.768697	0.000217	0.001236	-0.26831
AHCYL2	-1.39909	5.559541	-3.76878	0.000217	0.001236	-0.27493
SCARB1	1.297124	5.615634	3.768501	0.000217	0.001237	-0.13048
DUSP11	0.627274	4.796588	3.768072	0.000217	0.001238	-0.10437
CPE	-1.71439	4.456915	-3.76733	0.000218	0.001241	-0.26377
KCTD18	-0.5056	4.118982	-3.76708	0.000218	0.001242	-0.15222
TRIM11	0.534892	5.137245	3.766265	0.000219	0.001245	-0.16572
ELK1	0.617016	5.336087	3.765534	0.000219	0.001248	-0.1826
C11orf84	1.011809	4.260724	3.762372	0.000222	0.001262	0.034837
XPO5	0.859152	6.685181	3.76244	0.000222	0.001262	-0.28225
FAM73B	-0.65116	4.750649	-3.76212	0.000222	0.001263	-0.24629
ZNF24	-0.65284	6.248413	-3.76151	0.000223	0.001265	-0.30243
GSS	0.656331	5.709769	3.760961	0.000223	0.001267	-0.23248
SGIP1	-1.22089	1.624164	-3.75985	0.000224	0.001272	0.138466
TTC18	-1.43732	0.352492	-3.7593	0.000224	0.001274	0.275646
G0S2	-1.50976	2.235852	-3.75921	0.000224	0.001274	-0.00975
TUBGCP6	-0.58825	5.651181	-3.75703	0.000226	0.001284	-0.31117
KRT17	5.958209	8.619494	3.756772	0.000226	0.001284	0.06443
LARP6	-1.28835	2.754027	-3.75622	0.000227	0.001286	-0.08515
KIAA0913	-0.60433	6.436178	-3.7554	0.000228	0.00129	-0.32248
PPP2R5A	-0.67003	6.476834	-3.75448	0.000228	0.001294	-0.32432
TIMP3	-1.78535	7.260133	-3.75324	0.000229	0.001299	-0.28327
PER3	-1.46019	5.127352	-3.75326	0.000229	0.001299	-0.3269
IRAK4	-0.54181	4.525918	-3.75206	0.000231	0.001304	-0.25235
ABCA5	-0.97961	4.444722	-3.75128	0.000231	0.001307	-0.28209
CTBS	-0.64051	5.04484	-3.75046	0.000232	0.001311	-0.30811
PRKD2	0.504898	6.24009	3.74977	0.000232	0.001313	-0.32105
CASKIN2	-0.67939	5.756317	-3.74817	0.000234	0.001321	-0.34629
RUNX1	0.892132	6.980981	3.747085	0.000235	0.001326	-0.34535
TOP1MT	1.20545	5.049069	3.746451	0.000235	0.001328	-0.13303
ATRX	-0.64262	7.476734	-3.74586	0.000236	0.001331	-0.33424
CCNDBP1	-0.61376	5.048645	-3.74563	0.000236	0.001331	-0.32365
LHPP	-0.76396	4.14046	-3.74478	0.000237	0.001335	-0.25765
BCAS1	-2.6689	2.673868	-3.74416	0.000237	0.001337	-0.2553
CCT2	0.866458	7.053826	3.743986	0.000238	0.001338	-0.35877
NSUN2	0.653059	6.437692	3.743459	0.000238	0.00134	-0.34618
ARHGAP39	1.186549	4.367183	3.742976	0.000238	0.001341	-0.0231
LOC25845	-1.52515	5.050019	-3.74298	0.000238	0.001341	-0.36204
CDC14C	-1.05017	0.114813	-3.74216	0.000239	0.001344	0.273104
CCT6B	-1.19892	0.076633	-3.74217	0.000239	0.001344	0.263738
SMG7	0.454956	7.361132	3.742066	0.000239	0.001344	-0.3694
USP19	-0.53042	5.851602	-3.74194	0.000239	0.001344	-0.36762
TBCE	0.699598	4.037409	3.740248	0.000241	0.001352	-0.05203
MID2	-1.22761	4.341694	-3.74008	0.000241	0.001352	-0.33025
SETD7	-0.70313	6.845359	-3.73945	0.000242	0.001355	-0.37001
PAQR5	-1.49708	3.448455	-3.73894	0.000242	0.001357	-0.26491
ZNF654	-0.62127	4.224526	-3.73877	0.000242	0.001357	-0.27452

MRPL35	-0.47234	5.221675	-3.73885	0.000242	0.001357	-0.35077
ZFP112	-0.86154	2.3207	-3.73854	0.000242	0.001357	0.001683
DET1	-0.82014	2.063185	-3.73742	0.000243	0.001362	0.048922
BTNL9	-1.65013	1.901746	-3.73741	0.000243	0.001362	-0.04851
STXBP6	-2.04482	0.790055	-3.73692	0.000244	0.001364	0.077842
TMEM80	-0.74413	3.345531	-3.73602	0.000245	0.001368	-0.17531
ZNF573	-1.16018	1.004386	-3.73524	0.000245	0.00137	0.14566
ICA1L	-1.05815	2.176376	-3.73525	0.000245	0.00137	-0.01697
GAPDH	0.945266	11.22166	3.735318	0.000245	0.00137	-0.29817
ACTR8	-0.5197	4.013761	-3.73464	0.000246	0.001372	-0.25349
PAM	-0.89708	6.788929	-3.73461	0.000246	0.001372	-0.38445
HIST1H2BK	1.620317	5.802843	3.732736	0.000248	0.001381	-0.24066
PCDHGB7	-1.25801	2.311838	-3.73262	0.000248	0.001381	-0.08227
HSP90AB1	0.693923	10.55484	3.731963	0.000248	0.001384	-0.32376
ARSG	-1.003	1.462394	-3.73153	0.000249	0.001386	0.0917
MCFD2	-0.48019	6.836857	-3.73019	0.00025	0.001392	-0.40616
CLCN2	1.332698	3.662474	3.729929	0.00025	0.001393	0.071787
ANKRD13D	0.778278	5.107464	3.729317	0.000251	0.001395	-0.26438
PLEKHA3	-0.51676	4.325343	-3.72939	0.000251	0.001395	-0.30824
GORASP1	-0.60289	5.176009	-3.7292	0.000251	0.001395	-0.38821
AHCYL1	-0.51417	7.189734	-3.72903	0.000251	0.001395	-0.40275
LRRC8D	0.816199	5.50503	3.728329	0.000252	0.001399	-0.31069
DERL1	0.562191	6.619596	3.72768	0.000252	0.001401	-0.41174
NCBP2	0.698775	6.21101	3.727532	0.000252	0.001402	-0.38615
FBLN1	-2.17051	6.363285	-3.72711	0.000253	0.001403	-0.39054
IFNGR2	0.687734	6.084123	3.726564	0.000253	0.001405	-0.38131
CCNK	0.502078	6.239853	3.726331	0.000254	0.001406	-0.40287
KLF16	0.664392	5.733057	3.726179	0.000254	0.001406	-0.35539
SLC12A9	0.820092	5.666601	3.724965	0.000255	0.001412	-0.33892
ANO7	-1.538	0.927489	-3.72414	0.000256	0.001416	0.082795
VWA5A	-1.21973	4.179149	-3.72336	0.000256	0.001419	-0.3732
ITSN2	-0.66647	6.448705	-3.72315	0.000257	0.00142	-0.43394
GBA2	-0.85102	5.495624	-3.72289	0.000257	0.001421	-0.43156
GNG11	-0.92721	3.242373	-3.72194	0.000258	0.001425	-0.23498
USP12	-0.74387	4.312105	-3.72127	0.000258	0.001428	-0.35594
TET3	0.840916	6.550798	3.721272	0.000258	0.001428	-0.42018
RBP7	-1.40684	0.551708	-3.72103	0.000259	0.001428	0.123409
DCHS2	-2.02448	0.420168	-3.72047	0.000259	0.001431	0.077229
DCAF10	-0.5387	5.830472	-3.71862	0.000261	0.00144	-0.44844
CHST3	-1.55848	5.333327	-3.7182	0.000261	0.001442	-0.45078
SNX9	-0.7805	6.410271	-3.71793	0.000262	0.001443	-0.45104
NRXN3	-1.88628	1.103323	-3.71488	0.000265	0.001458	-0.03196
WTIP	-1.02539	2.353256	-3.71414	0.000265	0.001462	-0.11504
C19orf51	2.06467	0.139198	3.712222	0.000267	0.001472	0.268081
SLC25A3	-0.50485	8.375334	-3.7117	0.000268	0.001474	-0.42903
PRMT2	-0.61602	5.518614	-3.71112	0.000268	0.001476	-0.46751
NAGS	1.943587	1.70925	3.710402	0.000269	0.00148	0.239604
FBXO22OS	0.989706	1.019393	3.710303	0.000269	0.00148	0.230829
TMPRSS4	2.611969	6.423805	3.709819	0.00027	0.001482	-0.26842
SUV39H2	0.812208	3.897955	3.708846	0.000271	0.001486	-0.11646
CCDC80	-1.88773	5.235467	-3.70849	0.000271	0.001488	-0.48163
CNKSR3	-0.86061	4.536945	-3.70824	0.000271	0.001489	-0.43125
HOXA9	3.364913	1.358591	3.707449	0.000272	0.001492	0.25058
DCHS1	-1.26309	5.029538	-3.70704	0.000272	0.001494	-0.48228
GEFT	-1.34631	2.520762	-3.70535	0.000274	0.001502	-0.23182
ITSN1	-0.6515	5.101961	-3.70531	0.000274	0.001502	-0.46932
TPPP	-1.5661	4.663939	-3.70502	0.000274	0.001503	-0.48401
WDR31	-1.03979	1.342486	-3.70428	0.000275	0.001507	0.011616

AZGP1	-2.69365	1.868212	-3.70361	0.000276	0.00151	-0.30085
ACVR2A	-0.68865	4.104279	-3.70336	0.000276	0.001511	-0.39029
NKRF	0.694881	4.513605	3.701661	0.000278	0.00152	-0.27522
FAM107B	-1.60099	5.737085	-3.70013	0.000279	0.001528	-0.50959
C9orf152	-2.59106	2.002066	-3.69968	0.00028	0.00153	-0.32288
KBTBD4	-0.5075	3.665512	-3.6994	0.00028	0.001531	-0.32287
ZC3H8	0.746106	2.995061	3.697597	0.000282	0.00154	-0.02091
LSM2	0.697359	4.682641	3.697156	0.000282	0.001542	-0.32008
SFXN1	0.768799	5.672837	3.69718	0.000282	0.001542	-0.44061
SAMD11	-1.60547	0.408587	-3.69687	0.000283	0.001543	0.03833
SOX21	-2.52243	1.59876	-3.6966	0.000283	0.001544	-0.26491
GORAB	0.703148	3.495344	3.696359	0.000283	0.001544	-0.10585
RALGPS1	-1.33415	4.152188	-3.69645	0.000283	0.001544	-0.47162
ALG6	0.615821	4.094372	3.694012	0.000286	0.001556	-0.23574
LIPA	0.710181	6.346462	3.692421	0.000287	0.001565	-0.51543
FBXL6	1.079538	4.852866	3.691836	0.000288	0.001568	-0.30479
R3HDM1	0.582143	5.656173	3.691416	0.000288	0.00157	-0.47524
FANCL	0.908955	3.703545	3.69095	0.000289	0.001572	-0.12765
TNNT1	4.556696	0.812917	3.690024	0.00029	0.001576	0.194125
CPOX	0.730676	4.903149	3.689812	0.00029	0.001577	-0.37732
PHF10	-0.62811	5.907306	-3.68922	0.000291	0.00158	-0.55263
FOXD2	2.225913	1.815105	3.688979	0.000291	0.001581	0.177664
CHD2	-0.45226	6.980506	-3.68777	0.000292	0.001587	-0.55083
CTSE	-3.86924	2.309609	-3.68589	0.000294	0.001597	-0.48974
SH3BGRL2	-2.33491	5.492092	-3.68541	0.000295	0.0016	-0.55101
HMOX2	-0.79919	5.341539	-3.68502	0.000295	0.001601	-0.5564
RIBC2	2.384565	-0.14114	3.684726	0.000296	0.001602	0.178508
CARM1	0.584918	6.737858	3.683078	0.000297	0.001612	-0.56882
SLC3A2	0.987069	7.471649	3.681368	0.000299	0.001621	-0.58015
TMEM86B	-0.93633	2.463753	-3.67779	0.000303	0.001642	-0.24108
LEPRE1	0.961725	5.210967	3.677396	0.000304	0.001644	-0.43364
IER5L	1.124096	4.827671	3.676626	0.000305	0.001648	-0.34762
NAALADL2	-1.51844	2.189427	-3.67511	0.000306	0.001656	-0.29268
RQCD1	0.632307	3.916485	3.674623	0.000307	0.001658	-0.26668
TMEM177	0.926489	3.216873	3.673892	0.000308	0.001662	-0.10805
DDX49	0.611018	5.151378	3.673974	0.000308	0.001662	-0.47962
HNRNPD	0.465787	7.59624	3.673284	0.000308	0.001665	-0.60415
DPY30	0.650873	4.874524	3.673134	0.000309	0.001665	-0.44066
C1orf123	-0.52562	4.20296	-3.67154	0.00031	0.001673	-0.49495
MTHFD2	0.721826	6.356581	3.671616	0.00031	0.001673	-0.58686
GTF2F2	0.715005	4.643888	3.671126	0.000311	0.001674	-0.40026
SUMO1	0.496762	5.892566	3.671061	0.000311	0.001674	-0.5716
VTA1	0.573316	6.030571	3.671096	0.000311	0.001674	-0.57651
RRP9	0.882515	3.915176	3.670813	0.000311	0.001675	-0.23799
HOXC5	-1.033	0.431781	-3.67063	0.000311	0.001676	0.003796
AATK	-1.48265	2.896456	-3.6694	0.000313	0.001683	-0.4323
DDX31	0.619833	3.990006	3.669125	0.000313	0.001684	-0.3009
RAPGEF4	-1.07032	2.633121	-3.66894	0.000313	0.001684	-0.32333
DPH2	0.666966	4.928967	3.667714	0.000315	0.00169	-0.46504
GTPBP4	0.704042	6.070781	3.667811	0.000315	0.00169	-0.5819
JMY	-0.93917	5.211094	-3.66759	0.000315	0.001691	-0.61586
TARDBP	0.314028	7.242127	3.666582	0.000316	0.001696	-0.63037
LOC284900	-0.6112	2.472603	-3.66635	0.000316	0.001697	-0.23404
PDE4DIP	-0.83327	5.933642	-3.66619	0.000316	0.001697	-0.63284
RGL2	-0.60302	5.707598	-3.66592	0.000317	0.001698	-0.62845
HOXB9	4.805486	1.762059	3.665381	0.000317	0.001701	0.112533
ESPN	3.291646	4.528635	3.66495	0.000318	0.001703	-0.03793
NFATC4	-1.02904	3.536502	-3.66457	0.000318	0.001704	-0.49024

SERTAD1	-0.84713	3.961451	-3.66458	0.000318	0.001704	-0.5222
GRASP	-0.91018	2.338383	-3.66382	0.000319	0.001707	-0.2667
GOLGA3	-0.51732	7.043911	-3.66387	0.000319	0.001707	-0.63054
WDR19	-0.66536	3.692331	-3.66324	0.00032	0.001708	-0.47279
LOC92249	-1.08104	4.667046	-3.66336	0.00032	0.001708	-0.60916
KCNQ1	-2.06925	4.20796	-3.66341	0.00032	0.001708	-0.62215
TUBB2C	0.776777	8.613025	3.66329	0.00032	0.001708	-0.62282
POP1	0.989013	3.647743	3.662583	0.000321	0.001712	-0.20373
ASMTL	-0.73726	3.571559	-3.66234	0.000321	0.001713	-0.46587
POC5	0.662945	3.183342	3.661546	0.000322	0.001717	-0.18003
MTCH2	0.582238	6.155543	3.661043	0.000322	0.00172	-0.61891
UBE2L6	1.191551	6.048649	3.660789	0.000323	0.001721	-0.56382
NFE2L3	1.775588	5.543809	3.659805	0.000324	0.001726	-0.41982
ARSD	-1.21433	5.531757	-3.65978	0.000324	0.001726	-0.6534
EIF5AL1	0.740551	6.181208	3.659332	0.000324	0.001728	-0.61671
C4orf52	-0.65627	3.562026	-3.6585	0.000325	0.001732	-0.46582
REEP2	-1.60151	0.537903	-3.65762	0.000327	0.001737	-0.10938
MAP3K5	-0.79465	5.384696	-3.65733	0.000327	0.001738	-0.65286
ZBTB8A	-0.58185	3.94064	-3.657	0.000327	0.00174	-0.51846
TGFB3	-1.17281	3.40246	-3.65517	0.000329	0.001751	-0.51886
ZNF709	-1.08694	1.336917	-3.65465	0.00033	0.001752	-0.16191
ADCY4	-1.04068	2.475666	-3.65472	0.00033	0.001752	-0.34129
BAK1	0.90284	5.144443	3.654639	0.00033	0.001752	-0.50896
EVPL	2.008606	7.315403	3.654353	0.00033	0.001753	-0.62908
CDC42BPA	-1.04115	6.667398	-3.65443	0.00033	0.001753	-0.66071
BCL2L11	0.702953	5.282296	3.653824	0.000331	0.001755	-0.55357
CAPS2	-1.19835	0.224151	-3.65326	0.000332	0.001758	-0.04825
TFR2	-1.71192	1.686102	-3.65333	0.000332	0.001758	-0.30189
CTNS	-0.57807	4.123361	-3.65225	0.000333	0.001764	-0.55598
S100A10	1.193251	8.788625	3.651229	0.000334	0.001769	-0.66844
COX7C	-0.68049	6.131492	-3.65086	0.000335	0.001771	-0.68553
PRKAR2A	-0.7438	4.601637	-3.65033	0.000335	0.001774	-0.62661
SAT2	-0.66432	3.930941	-3.64968	0.000336	0.001777	-0.55075
MLXIP	-0.69709	6.797586	-3.64952	0.000336	0.001778	-0.68121
SFRS3	0.364796	7.652893	3.648654	0.000337	0.001783	-0.6859
PBX3	-0.85633	4.257306	-3.64849	0.000338	0.001783	-0.61024
CTLA4	2.135829	1.018397	3.648148	0.000338	0.001785	0.057791
NUP160	0.537366	6.311217	3.647606	0.000339	0.001788	-0.67588
CCDC109B	1.206304	3.655283	3.645477	0.000341	0.001801	-0.23251
KLF5	1.299955	8.552125	3.645217	0.000342	0.001802	-0.69566
DENND4C	-0.72413	6.185293	-3.64457	0.000342	0.001805	-0.70651
GADD45A	-0.82364	4.757668	-3.64264	0.000345	0.001817	-0.67109
LSM5	0.668881	5.058871	3.64196	0.000346	0.001821	-0.57028
DCN	-1.67769	7.313772	-3.64154	0.000346	0.001823	-0.66995
SARM1	-1.14884	3.860574	-3.64086	0.000347	0.001827	-0.61996
CARD9	1.989563	0.650898	3.640297	0.000348	0.00183	0.032986
RDH5	-1.33638	0.830712	-3.63975	0.000348	0.001833	-0.17343
RG9MTD3	-0.59619	2.704291	-3.63734	0.000352	0.001849	-0.37078
RTKN2	2.167126	3.372288	3.636017	0.000353	0.001857	-0.12034
LONRF1	-0.86506	4.312316	-3.63556	0.000354	0.001859	-0.66046
TNS4	4.860172	7.1528	3.635167	0.000354	0.001861	-0.27955
TTC7B	-1.12029	3.987539	-3.63448	0.000355	0.001865	-0.65285
SHOX2	2.615811	0.264746	3.632916	0.000357	0.001874	0.009755
SCAMP3	0.577645	6.19217	3.632944	0.000357	0.001874	-0.71708
PLOD1	0.843447	7.081074	3.633051	0.000357	0.001874	-0.74192
LYRM5	-1.04838	3.664922	-3.63189	0.000359	0.00188	-0.61762
REXO4	0.622807	4.441305	3.630902	0.00036	0.001886	-0.51491
MEN1	0.598342	5.725493	3.630276	0.000361	0.00189	-0.6889

SEC63	-0.50566	6.479843	-3.63012	0.000361	0.00189	-0.75507
SP140L	1.134537	4.516598	3.62961	0.000362	0.001892	-0.44479
ARHGEF10	-1.15817	4.397709	-3.62955	0.000362	0.001892	-0.70943
COL5A2	1.587801	7.453084	3.62854	0.000363	0.001899	-0.74664
PLEKHM3	-0.66663	4.389152	-3.62643	0.000366	0.001913	-0.68214
PRICKLE4	-1.2931	1.45016	-3.62482	0.000368	0.001923	-0.30212
IKBIP	1.154695	3.890558	3.623318	0.00037	0.001933	-0.35193
PHC1	-0.88839	4.118823	-3.62292	0.00037	0.001935	-0.68651
SERGEF	-0.55867	3.06083	-3.6227	0.000371	0.001935	-0.48146
FAM53C	-0.51821	5.40891	-3.62264	0.000371	0.001935	-0.76272
C17orf49	0.728296	5.320702	3.621532	0.000372	0.001942	-0.66507
NPDC1	-1.33777	5.016974	-3.62142	0.000372	0.001943	-0.77656
PLEKHG5	1.177621	5.471599	3.621171	0.000373	0.001944	-0.635
LTBP3	-1.07628	6.778021	-3.62032	0.000374	0.001949	-0.77371
HPSE	1.717229	2.925375	3.619513	0.000375	0.001954	-0.17104
H1FX	0.896727	6.946708	3.61929	0.000375	0.001955	-0.78389
PCDH7	-1.80953	5.295873	-3.61895	0.000376	0.001956	-0.7889
PSME3	0.488191	7.588295	3.617825	0.000377	0.001964	-0.79368
AKTIP	-0.62314	4.152934	-3.61752	0.000378	0.001965	-0.68305
CBFA2T3	-1.30819	2.116268	-3.61729	0.000378	0.001966	-0.44668
ASCC3	0.621691	6.447423	3.61688	0.000379	0.001968	-0.78303
C12orf34	-1.11754	3.024056	-3.61544	0.000381	0.001978	-0.58545
ANGPT2	1.608002	3.85673	3.61516	0.000381	0.001979	-0.30833
ANKRD28	-0.59982	5.93318	-3.6134	0.000383	0.001991	-0.81154
SYT15	-1.27026	1.232848	-3.61231	0.000385	0.001998	-0.31269
ZYG11B	-0.49552	5.665841	-3.61182	0.000386	0.002001	-0.80884
PNO1	0.589691	4.823299	3.61159	0.000386	0.002001	-0.65075
CTGF	-1.54919	6.739233	-3.61164	0.000386	0.002001	-0.79268
CCT4	0.537034	7.690977	3.611493	0.000386	0.002001	-0.81409
DHX29	-0.50047	5.407167	-3.61124	0.000386	0.002002	-0.80043
ABAT	-1.23542	3.58088	-3.6105	0.000387	0.002007	-0.69873
SIGLEC12	2.363675	-0.20068	3.610119	0.000388	0.002009	-0.06265
CC2D2A	-1.02426	3.011213	-3.60957	0.000389	0.002012	-0.59458
UMPS	0.740222	5.01425	3.606687	0.000393	0.002032	-0.67496
TMEM192	-0.71697	3.9463	-3.60621	0.000394	0.002034	-0.7059
FAM114A1	-0.65417	5.000424	-3.6062	0.000394	0.002034	-0.80143
MITD1	0.697311	3.113521	3.605773	0.000394	0.002037	-0.35152
DCXR	-0.82388	5.245623	-3.60558	0.000394	0.002037	-0.82422
SAC3D1	1.021055	3.594689	3.605127	0.000395	0.00204	-0.38342
PPT2	-0.59989	4.266777	-3.60483	0.000395	0.00204	-0.7357
SPNS1	0.530384	5.63814	3.604884	0.000395	0.00204	-0.77275
CASP2	0.708817	5.819803	3.604767	0.000396	0.00204	-0.77472
APOE	2.323078	6.104627	3.604485	0.000396	0.002042	-0.61745
MALAT1	-1.16855	11.51751	-3.60361	0.000397	0.002047	-0.67704
APOC1	3.324828	3.142025	3.603485	0.000397	0.002047	-0.11998
RABEPK	0.695905	4.06188	3.603289	0.000398	0.002048	-0.52371
IL32	2.108642	5.764313	3.602981	0.000398	0.00205	-0.59923
GIMAP8	-1.07646	2.619531	-3.60231	0.000399	0.002054	-0.55269
GATA4	-2.80472	2.22113	-3.60211	0.000399	0.002055	-0.70346
PMS2	0.558476	4.811205	3.600906	0.000401	0.002063	-0.68883
USO1	-0.60209	6.719813	-3.60005	0.000402	0.002068	-0.85202
AHNAK	-1.16824	10.32738	-3.59962	0.000403	0.002071	-0.73035
ANXA6	-1.12316	5.385765	-3.59884	0.000404	0.002076	-0.8581
PHACTR2	-0.91297	5.771719	-3.59838	0.000405	0.002079	-0.86275
VAT1L	-1.53422	0.429183	-3.59791	0.000405	0.002081	-0.2859
SCD5	-1.2228	4.07516	-3.59704	0.000407	0.002087	-0.79606
RAD9A	0.766608	3.83164	3.596445	0.000408	0.002091	-0.49148
PGM1	-0.77767	5.784595	-3.59569	0.000409	0.002096	-0.8711

KIAA1147	-0.90942	5.879124	-3.59548	0.000409	0.002097	-0.87292
HOXB13	4.32387	0.642093	3.593996	0.000411	0.002104	-0.11597
LSM11	-0.57327	3.393834	-3.59426	0.000411	0.002104	-0.64309
SNX24	-0.724	3.316197	-3.59422	0.000411	0.002104	-0.6513
PRICKLE3	0.84919	4.712202	3.594062	0.000411	0.002104	-0.65175
KLHL26	-0.64899	3.577062	-3.59388	0.000411	0.002104	-0.68753
C2CD2	-0.74443	5.044344	-3.5939	0.000411	0.002104	-0.85063
FBXO45	0.926649	5.43397	3.593257	0.000412	0.002108	-0.75276
ELAVL1	0.386727	6.4679	3.593231	0.000412	0.002108	-0.87216
TRIM36	-1.5092	1.711607	-3.59255	0.000413	0.002112	-0.47996
EDNRB	-1.34532	3.133221	-3.59208	0.000414	0.002115	-0.71468
KIF13B	-1.48871	6.112716	-3.59178	0.000415	0.002116	-0.87525
ADRB1	-1.58919	0.390522	-3.5915	0.000415	0.002118	-0.309
ENPP1	-1.39623	2.766024	-3.59059	0.000416	0.002124	-0.66767
FIG4	-0.60167	3.797363	-3.59045	0.000417	0.002124	-0.72592
TRAK1	-0.8776	6.726988	-3.59039	0.000417	0.002124	-0.87968
TREM2	2.78022	1.811889	3.589892	0.000417	0.002127	-0.13069
DDR1	0.839631	8.663108	3.589485	0.000418	0.002129	-0.87332
CCDC121	-0.6422	1.611012	-3.58873	0.000419	0.002133	-0.36467
PAMR1	-1.20061	1.881369	-3.58872	0.000419	0.002133	-0.48054
SERAC1	-0.68953	3.848016	-3.58892	0.000419	0.002133	-0.74765
LOC644538	-1.29156	0.492503	-3.58847	0.00042	0.002134	-0.30245
PALM	-1.35308	3.157134	-3.58818	0.00042	0.002134	-0.73491
ZADH2	-0.63666	4.948461	-3.58821	0.00042	0.002134	-0.85743
CLK1	-0.69529	6.09145	-3.58831	0.00042	0.002134	-0.89753
TSPYL5	-1.59955	2.792857	-3.58732	0.000421	0.002139	-0.7126
NOTCH1	0.978064	7.467613	3.586548	0.000422	0.002145	-0.9026
NSA2	-0.55853	5.501187	-3.58638	0.000423	0.002145	-0.89048
TBXA2R	-0.95067	1.270205	-3.5858	0.000424	0.002149	-0.36671
SSBP2	-1.10955	3.094438	-3.58555	0.000424	0.002149	-0.70309
ABCE1	0.565279	6.521094	3.585563	0.000424	0.002149	-0.89396
MRPL4	0.674794	5.942965	3.584874	0.000425	0.002154	-0.8552
NT5C3	1.064397	4.962153	3.584323	0.000426	0.002157	-0.69098
USP18	1.511213	3.634447	3.584095	0.000426	0.002158	-0.3937
RPN1	0.545782	8.382011	3.583861	0.000427	0.002159	-0.89288
DUSP9	3.59747	0.251038	3.583682	0.000427	0.00216	-0.14811
WDR18	0.783778	5.162165	3.5831	0.000428	0.002164	-0.76896
CARD14	2.459691	3.320064	3.582595	0.000428	0.002165	-0.26429
IKBKE	1.181307	4.37476	3.582577	0.000429	0.002165	-0.56795
FUT1	-1.01974	3.36339	-3.5827	0.000428	0.002165	-0.73883
CCL4L2	2.499453	0.909548	3.581422	0.00043	0.002173	-0.15635
DTX2	0.93417	5.773977	3.581429	0.00043	0.002173	-0.82869
CENPP	1.37517	2.489912	3.580858	0.000431	0.002176	-0.28578
ACTR3	0.555532	8.308721	3.579071	0.000434	0.00219	-0.91102
SYNC	-1.44406	2.232562	-3.57852	0.000435	0.002193	-0.62273
MTHFD1	0.648377	6.407371	3.578358	0.000435	0.002194	-0.90975
PRMT5	0.633352	6.027061	3.577299	0.000437	0.002201	-0.89028
COMMD10	-0.52057	3.593532	-3.57663	0.000438	0.002206	-0.72833
PIGW	0.828677	3.718875	3.57478	0.000441	0.00222	-0.53444
TIRAP	-0.71776	3.316324	-3.5743	0.000441	0.002221	-0.7168
SLC41A2	-1.62092	3.201503	-3.57445	0.000441	0.002221	-0.80764
SPON1	-1.61732	4.288485	-3.57438	0.000441	0.002221	-0.91348
SYDE2	-1.2922	2.202141	-3.57387	0.000442	0.002224	-0.60407
MIF	0.965287	8.214069	3.573733	0.000442	0.002224	-0.93835
RAC1	0.552002	8.568653	3.573201	0.000443	0.002227	-0.9238
ARPC3	0.585964	6.911265	3.573255	0.000443	0.002227	-0.94535
C7orf40	0.899463	3.993086	3.571905	0.000445	0.002235	-0.5823
HCP5	1.516166	5.734831	3.571957	0.000445	0.002235	-0.79141

ZNF449	-0.58788	3.096752	-3.57166	0.000446	0.002236	-0.66264
PLAGL1	-1.18335	4.003515	-3.57089	0.000447	0.002242	-0.87419
TFAP2A	3.863987	5.208589	3.570494	0.000447	0.002244	-0.36568
ULK2	-0.95872	4.293733	-3.57047	0.000448	0.002244	-0.88591
PPA1	0.711983	6.957741	3.570343	0.000448	0.002244	-0.95364
TMEM59	-0.6336	7.745134	-3.57023	0.000448	0.002244	-0.92699
RGP1	-0.66151	3.371365	-3.56898	0.00045	0.002253	-0.73538
KIAA1328	-0.68193	2.351261	-3.56809	0.000451	0.00226	-0.55346
HABP4	-0.83777	2.914157	-3.56779	0.000452	0.00226	-0.68438
ACTR2	0.402495	8.209139	3.567872	0.000452	0.00226	-0.9477
FCGR1A	2.043206	0.774372	3.566155	0.000455	0.002272	-0.20462
NUDT16	-0.81964	4.368032	-3.56623	0.000454	0.002272	-0.89528
PFKFB4	1.822232	3.150775	3.565917	0.000455	0.002272	-0.3618
KLHL2	-0.69251	4.360635	-3.56593	0.000455	0.002272	-0.88513
EIF1B	-0.57459	4.724189	-3.56583	0.000455	0.002272	-0.91015
FAM120B	-0.41425	4.944411	-3.56319	0.000459	0.002293	-0.92569
CTDSP2	-0.57067	7.990224	-3.56249	0.000461	0.002297	-0.94697
EIF4A1	0.59507	8.548441	3.562576	0.00046	0.002297	-0.96096
HLA-B	1.246404	9.740244	3.561979	0.000461	0.002301	-0.9442
C6orf223	3.837918	0.954466	3.561406	0.000462	0.002304	-0.22016
OSCP1	-0.78738	1.546624	-3.56063	0.000464	0.002309	-0.4664
ADAM17	0.925124	5.48509	3.560723	0.000463	0.002309	-0.86774
ARL6IP5	-0.69592	6.344858	-3.56046	0.000464	0.00231	-0.98907
FAM83B	2.639953	5.010162	3.558919	0.000466	0.002322	-0.5227
OBFC2A	1.215305	4.205312	3.557801	0.000468	0.002329	-0.61571
RCHY1	-0.56319	4.254921	-3.55789	0.000468	0.002329	-0.88832
DNMBP	-0.92539	6.084861	-3.55776	0.000468	0.002329	-0.99824
C16orf5	-1.14833	4.10585	-3.55688	0.00047	0.002335	-0.92817
ATG2B	-0.57351	5.855983	-3.55634	0.000471	0.002339	-1.00167
MMP11	4.488257	5.947381	3.55582	0.000472	0.002343	-0.42928
KIAA0406	0.581889	5.493059	3.555664	0.000472	0.002343	-0.91906
MOV10	0.66479	6.052834	3.554591	0.000474	0.002351	-0.96567
NCAPD3	0.797835	5.664297	3.553347	0.000476	0.002361	-0.92416
DPM1	0.53676	5.343138	3.552167	0.000478	0.00237	-0.91956
TRIM39	-0.46147	3.810766	-3.55148	0.000479	0.002375	-0.84053
PDE5A	-1.39477	4.74501	-3.54981	0.000482	0.002388	-1.00956
MMP12	5.301835	4.083723	3.549539	0.000482	0.002389	-0.26133
HSP90B1	0.547177	9.633077	3.549515	0.000482	0.002389	-0.96915
C11orf80	1.309205	3.840649	3.549291	0.000483	0.00239	-0.56655
MICALL1	-1.36496	6.814955	-3.54846	0.000484	0.002396	-1.00735
RET	-1.81854	0.362816	-3.54768	0.000486	0.002402	-0.47111
SLC2A13	-1.07399	3.834684	-3.5471	0.000487	0.002406	-0.9247
PPM1A	-0.55172	6.122577	-3.54696	0.000487	0.002407	-1.0359
MED6	0.484025	4.186998	3.546653	0.000487	0.002408	-0.77299
BCAT2	-0.64726	4.890447	-3.54667	0.000487	0.002408	-0.99268
FAM176A	2.684685	0.845831	3.54496	0.00049	0.002419	-0.2719
TRMT1	0.699832	4.65625	3.545113	0.00049	0.002419	-0.82932
ALDH2	-1.25421	7.052397	-3.54491	0.00049	0.002419	-1.0153
ERRFI1	-1.24232	6.218611	-3.54491	0.00049	0.002419	-1.03459
NAE1	0.62134	5.455065	3.54323	0.000493	0.002433	-0.95338
USP22	-0.5713	7.602671	-3.54178	0.000496	0.002445	-1.02775
PPP1R13L	1.32543	6.640896	3.541359	0.000497	0.002447	-1.008
PSMC3IP	1.026614	2.159373	3.540591	0.000498	0.002453	-0.41446
RPS21	0.737767	8.237642	3.540305	0.000498	0.002455	-1.04554
ZNF445	-0.62076	4.849667	-3.54016	0.000499	0.002455	-1.00938
RBM6	-0.51146	5.387456	-3.53953	0.0005	0.002459	-1.04023
C4orf41	-0.4806	5.531776	-3.53955	0.0005	0.002459	-1.04559
SUSD4	-2.09203	3.174364	-3.53803	0.000503	0.002471	-0.97013

YTHDF1	0.468574	6.407097	3.536969	0.000504	0.00248	-1.05531
GLIPR2	-0.96897	3.021066	-3.53574	0.000507	0.002489	-0.83338
AGXT2L2	-0.63873	4.241623	-3.53573	0.000507	0.002489	-0.96722
CPXM2	-1.43742	3.018673	-3.5354	0.000507	0.00249	-0.90091
EPB41L1	-1.27084	6.60607	-3.53547	0.000507	0.00249	-1.05798
SCP2	-0.62126	6.903948	-3.53471	0.000508	0.002495	-1.06686
ADCK5	1.007455	3.362053	3.533833	0.00051	0.002502	-0.58377
PPARGC1B	-0.86253	2.741911	-3.53393	0.00051	0.002502	-0.76626
DNAJC18	-0.8538	2.150894	-3.53332	0.000511	0.002504	-0.65931
FAM168B	-0.56582	6.873344	-3.53342	0.000511	0.002504	-1.07272
SAMM50	-0.47586	5.281938	-3.53187	0.000514	0.002516	-1.05821
ZFYVE21	-0.58415	4.767616	-3.53164	0.000514	0.002518	-1.02839
STK3	0.734184	4.865758	3.529037	0.000519	0.00254	-0.9128
TTLL3	-0.87413	4.122664	-3.52845	0.00052	0.002545	-1.00094
TBC1D7	0.858786	3.713416	3.528134	0.000521	0.002546	-0.68195
NUPL2	0.657818	4.574137	3.527986	0.000521	0.002547	-0.87707
LPAR1	-1.20667	3.669252	-3.52768	0.000521	0.002549	-0.98361
ZNF615	-1.05217	2.98491	-3.52558	0.000525	0.002566	-0.87255
GTF3C5	0.698074	5.731668	3.525643	0.000525	0.002566	-1.03178
RAB8A	0.533005	6.499323	3.524231	0.000528	0.002577	-1.09878
CDC14A	-0.79833	3.266788	-3.52148	0.000533	0.002602	-0.8955
GLTSCR2	-0.71312	7.056499	-3.52073	0.000534	0.002608	-1.10827
C1orf26	-0.54128	2.885082	-3.52028	0.000535	0.002611	-0.78289
SAMD4A	-1.0999	4.053453	-3.51979	0.000536	0.002615	-1.04213
HSD17B6	-1.27551	0.337383	-3.5179	0.00054	0.002631	-0.51135
PDZD2	-1.25374	4.223974	-3.51747	0.000541	0.002634	-1.07767
SELP	-1.84648	1.53336	-3.5167	0.000542	0.002641	-0.75935
KIAA0556	-0.47709	5.144962	-3.51557	0.000544	0.00265	-1.10348
ZNF554	-0.69022	2.092576	-3.51509	0.000545	0.002653	-0.6838
PTEN	-0.51126	6.453177	-3.51515	0.000545	0.002653	-1.14041
TCEANC	-0.71995	0.659492	-3.5145	0.000546	0.002658	-0.50356
TNFAIP8L1	0.873108	3.731143	3.513876	0.000548	0.002663	-0.72975
PAK1IP1	0.843828	4.266655	3.511794	0.000552	0.002681	-0.84183
SLC1A5	0.783232	7.72024	3.511443	0.000552	0.002684	-1.15111
ZNF681	-1.62059	1.037224	-3.51125	0.000553	0.002684	-0.66082
SECISBP2	-0.50457	5.085865	-3.51121	0.000553	0.002684	-1.11521
MMP13	5.208106	1.254364	3.509024	0.000557	0.002704	-0.38579
TBCEL	-1.0023	3.180147	-3.50871	0.000558	0.002706	-0.95572
PTPN2	0.651325	4.943788	3.507872	0.000559	0.002713	-1.00525
PPP1R14B	1.002655	5.405895	3.507618	0.00056	0.002715	-1.02574
UBXN10	-1.90608	1.378377	-3.50676	0.000561	0.002722	-0.76942
ITGB8	1.407367	6.680135	3.504823	0.000565	0.00274	-1.12564
STK40	-0.71487	6.357449	-3.50395	0.000567	0.002747	-1.17609
RPAP3	0.540767	5.009647	3.503279	0.000568	0.002752	-1.04276
EFNA1	0.928913	6.264928	3.503329	0.000568	0.002752	-1.1329
NUDT15	0.76821	4.609743	3.502447	0.00057	0.002759	-0.94939
PTPRB	-1.29736	4.605621	-3.50188	0.000571	0.002764	-1.1575
TMEM150A	-0.58826	3.368426	-3.50045	0.000574	0.002777	-0.95012
FASTKD1	0.639269	4.54151	3.50034	0.000574	0.002777	-0.96489
IFI27	1.597653	7.426765	3.499192	0.000577	0.002787	-1.1776
IQGAP2	-2.01626	4.639807	-3.4973	0.000581	0.002805	-1.19165
C10orf116	-1.43061	4.212867	-3.49642	0.000582	0.002812	-1.15623
TRNP1	-1.77976	4.549868	-3.49648	0.000582	0.002812	-1.18832
SUPT16H	0.53843	7.098973	3.495894	0.000583	0.002816	-1.20472
RUNDC2A	-0.73527	1.808776	-3.49538	0.000584	0.002819	-0.7098
PBX1	-1.44234	5.623934	-3.49536	0.000585	0.002819	-1.20313
ZFP30	-1.28089	2.024502	-3.49508	0.000585	0.002821	-0.83043
PES1	0.544515	6.001041	3.494601	0.000586	0.002825	-1.1686

PGPEP1	-1.24039	4.440451	-3.49416	0.000587	0.002828	-1.16931
PAK2	0.591502	7.707645	3.493912	0.000588	0.00283	-1.20653
TK2	-0.6226	4.213752	-3.4938	0.000588	0.00283	-1.10113
SAA2	3.745345	1.051786	3.493081	0.000589	0.002835	-0.43512
CD44	1.240457	9.100231	3.493126	0.000589	0.002835	-1.19129
PSMB7	0.648847	6.380548	3.493029	0.000589	0.002835	-1.19152
HAPLN3	1.517908	3.493725	3.492395	0.000591	0.00284	-0.6755
ZBTB20	-1.05044	2.127954	-3.49194	0.000592	0.002843	-0.81968
RAB11B	-0.49617	6.977058	-3.49192	0.000592	0.002843	-1.20888
ZNF366	-1.27161	0.265075	-3.49171	0.000592	0.002843	-0.58569
E2F4	0.547059	6.449084	3.491704	0.000592	0.002843	-1.20351
C20orf160	-1.06398	0.681136	-3.49132	0.000593	0.002846	-0.61409
ANKHD1-EIF4EBP3	-0.8981	5.358382	-3.48856	0.000599	0.002873	-1.21999
RIPK2	0.913016	4.562796	3.488135	0.0006	0.002876	-0.96214
RASL12	-1.20077	2.818219	-3.48584	0.000604	0.002898	-0.99975
GRK5	-1.06658	4.128036	-3.48576	0.000605	0.002898	-1.15706
OLFML2B	2.337333	4.109047	3.485395	0.000605	0.002901	-0.67938
GJB3	2.965597	5.366609	3.484419	0.000608	0.002908	-0.77629
LGALS9C	-1.96159	1.088853	-3.48452	0.000607	0.002908	-0.79167
THY1	1.42008	6.157959	3.484364	0.000608	0.002908	-1.1432
C16orf52	-0.58315	3.908266	-3.48336	0.00061	0.002918	-1.09424
PRKD1	-1.3489	1.523775	-3.48307	0.00061	0.00292	-0.79202
PON2	1.120785	5.761272	3.482419	0.000612	0.002925	-1.13718
GOLGA6L10	-0.71708	2.511504	-3.47936	0.000618	0.002956	-0.87612
C12orf56	3.350613	-0.13676	3.479021	0.000619	0.002958	-0.47643
SH2D3A	1.218863	4.511372	3.479103	0.000619	0.002958	-0.92449
TRMT112	0.83835	5.574772	3.478625	0.00062	0.002961	-1.15754
MMP1	4.784129	6.11084	3.477219	0.000623	0.002974	-0.66653
BRPF3	-0.65058	6.067745	-3.47669	0.000624	0.002979	-1.26772
KPTN	0.837	2.571861	3.476407	0.000625	0.002981	-0.68631
OPHN1	-0.72005	3.848603	-3.47593	0.000626	0.002985	-1.12545
POLDIP3	-0.45337	6.402179	-3.47484	0.000628	0.002995	-1.27377
ZFP82	-1.36034	0.258359	-3.47423	0.00063	0.003	-0.65155
C18orf45	1.036411	3.614439	3.473768	0.000631	0.003004	-0.81484
TMEM120A	-0.6458	4.298605	-3.47281	0.000633	0.003014	-1.18099
PKM2	0.818165	10.43042	3.472382	0.000634	0.003017	-1.20576
PRDM5	-1.43743	0.668831	-3.47209	0.000634	0.003018	-0.71417
EDEM2	0.641275	4.846061	3.472111	0.000634	0.003018	-1.10815
WWC2	-0.91288	4.505658	-3.47154	0.000636	0.003023	-1.22753
SLMO2	0.629923	6.724797	3.471331	0.000636	0.003024	-1.27719
TMEM102	0.871538	3.801422	3.470049	0.000639	0.003037	-0.88277
EPHX2	-1.25184	3.579703	-3.46954	0.00064	0.003041	-1.16656
LOC400027	-0.60343	2.885838	-3.46821	0.000643	0.003054	-0.96698
LOH3CR2A	-0.92566	1.175452	-3.46712	0.000646	0.003065	-0.73851
STAP2	1.335494	5.012117	3.467051	0.000646	0.003065	-1.04021
PLEK2	2.48625	4.544172	3.466788	0.000646	0.003066	-0.775
PFDN6	0.931865	3.493099	3.466416	0.000647	0.003069	-0.83424
PALM2-AKAP2	-1.0419	5.361021	-3.46619	0.000648	0.003071	-1.2963
PSMD2	0.724842	7.872051	3.465996	0.000648	0.003072	-1.29704
RAB6B	-1.31314	2.866235	-3.46556	0.000649	0.003075	-1.08857
C1orf59	2.193239	2.894098	3.46446	0.000652	0.003086	-0.62504
TLE1	-0.81407	5.423132	-3.46432	0.000652	0.003087	-1.29917
CSAD	-0.7102	3.621922	-3.46419	0.000652	0.003087	-1.13079
IGFBP6	-1.7121	4.09146	-3.46396	0.000653	0.003088	-1.26964
C3orf39	-0.75585	4.063982	-3.46354	0.000654	0.003092	-1.19592
FAM70A	-1.56684	0.679945	-3.46335	0.000654	0.003092	-0.75747
SFRS12IP1	-0.6725	5.724851	-3.46333	0.000654	0.003092	-1.30741
SOD3	-1.52427	4.969314	-3.45928	0.000664	0.003135	-1.31742

TWISTNB	0.570795	5.3311	3.456502	0.00067	0.003164	-1.22964
LPCAT1	1.055137	6.004939	3.456473	0.00067	0.003164	-1.25336
SVIP	-1.40454	3.206556	-3.45629	0.000671	0.003164	-1.17851
SYNPO	-1.1187	6.921838	-3.45625	0.000671	0.003164	-1.31397
SLC25A12	-0.76526	4.69159	-3.45601	0.000671	0.003166	-1.2835
PGAM5	0.702011	5.091235	3.455015	0.000673	0.003176	-1.19169
NBPF1	-0.69939	4.673415	-3.45283	0.000679	0.003199	-1.2874
ATP13A3	0.615025	7.891286	3.452314	0.00068	0.003204	-1.3395
PMAIP1	1.608632	4.072085	3.448896	0.000688	0.003241	-0.8883
C7orf28B	0.664226	5.165111	3.448644	0.000689	0.003243	-1.22641
ARHGEF12	-0.51362	7.80893	-3.44819	0.00069	0.003247	-1.33058
CCBP2	-1.32376	0.666108	-3.44794	0.00069	0.003249	-0.77747
KLHL22	-0.59014	3.980909	-3.44779	0.000691	0.003249	-1.21985
RNF2	0.623323	4.666224	3.444697	0.000698	0.003284	-1.17119
NUAK2	1.782308	4.986317	3.444501	0.000699	0.003285	-1.03327
YWHAE	0.520125	8.661299	3.443766	0.0007	0.003292	-1.34689
ZNF501	-0.91728	1.320848	-3.443	0.000702	0.0033	-0.83186
POR	0.734987	6.718399	3.442668	0.000703	0.003302	-1.36704
AMPH	-1.33583	0.272126	-3.4425	0.000704	0.003303	-0.75178
CLU	-1.9937	7.209721	-3.44239	0.000704	0.003303	-1.32568
POP7	0.849596	4.288795	3.441961	0.000705	0.003307	-1.07057
DHFR	1.033094	3.992011	3.441548	0.000706	0.003311	-0.98438
NAA25	0.44267	5.557248	3.440662	0.000708	0.00332	-1.31554
FAM40B	2.172364	2.766484	3.439864	0.00071	0.003328	-0.69238
UBA6	0.723521	6.424404	3.439621	0.000711	0.003329	-1.36424
KDM1A	0.610623	6.489836	3.439623	0.000711	0.003329	-1.37255
CXorf36	-0.97019	3.213167	-3.43873	0.000713	0.003338	-1.18243
C1QTNF6	1.952672	4.231898	3.438519	0.000713	0.003339	-0.89328
TRIM8	-0.56332	6.79898	-3.43809	0.000714	0.003343	-1.38658
ATP13A4	-2.31199	1.373172	-3.43638	0.000719	0.003361	-1.05428
FOSL2	-0.80997	7.899605	-3.43637	0.000719	0.003361	-1.35756
EPCAM	2.127189	7.611339	3.434683	0.000723	0.00338	-1.37036
MAML3	-0.86621	4.078802	-3.43416	0.000724	0.003385	-1.30364
OSCAR	1.719493	1.145228	3.43389	0.000725	0.003387	-0.62492
HSD17B12	-0.61811	6.737557	-3.43293	0.000727	0.003397	-1.40344
C3orf57	-2.06911	1.914804	-3.43157	0.000731	0.003412	-1.13508
PELI2	-1.34222	4.143591	-3.43117	0.000732	0.003415	-1.35821
ARHGAP8	1.53297	3.965917	3.429354	0.000737	0.003435	-0.93767
CALD1	-1.41024	8.31456	-3.4294	0.000736	0.003435	-1.34969
SLC22A5	-0.58316	3.692753	-3.42796	0.00074	0.00345	-1.24053
FUNDC2	-0.53868	5.034137	-3.4272	0.000742	0.003458	-1.388
CAMK2D	-0.75139	6.022608	-3.4269	0.000743	0.003461	-1.42943
TEAD1	-0.69612	6.633707	-3.4264	0.000744	0.003466	-1.4251
ZNF221	-1.17321	0.178737	-3.42565	0.000746	0.003474	-0.77778
PDCL3	0.575741	3.805425	3.425075	0.000748	0.003479	-1.08202
KCNJ8	-1.05474	2.236655	-3.42428	0.00075	0.003488	-1.06057
MIIP	0.779064	4.009751	3.423215	0.000752	0.003499	-1.08921
CPM	-1.40774	4.640094	-3.42315	0.000753	0.003499	-1.42005
CLDN18	-3.69606	3.258446	-3.42246	0.000754	0.003506	-1.40922
KIAA0415	0.533544	5.088332	3.421807	0.000756	0.003513	-1.31737
FOXD1	3.232535	2.317775	3.421206	0.000758	0.003519	-0.65915
NAA10	0.582372	5.436795	3.420689	0.000759	0.003524	-1.3556
PPARA	-0.72534	5.913566	-3.4204	0.00076	0.003527	-1.44992
SEC16A	-0.56858	7.954102	-3.41858	0.000765	0.003547	-1.42012
TMEM57	-0.51112	5.235006	-3.41863	0.000764	0.003547	-1.42731
C1orf21	-0.91595	5.355976	-3.41785	0.000767	0.003555	-1.45021
PSMD10	0.524789	5.436971	3.417262	0.000768	0.003561	-1.37204
ILF3	0.400056	8.145924	3.415123	0.000774	0.003586	-1.4505

CTSA	0.676125	7.591548	3.413354	0.000779	0.003607	-1.47124
RNF214	-0.4633	4.012821	-3.41318	0.000779	0.003608	-1.32094
ADCY3	0.805268	6.092223	3.412715	0.00078	0.003612	-1.42345
SYTL2	-1.62747	4.271773	-3.41118	0.000784	0.00363	-1.44747
OAS1	1.465543	5.845453	3.410973	0.000785	0.003632	-1.34027
QDPR	-0.61114	4.049374	-3.41069	0.000786	0.003634	-1.35009
TAF6	0.667589	6.296017	3.41018	0.000787	0.003639	-1.45488
ETV7	1.738263	3.079491	3.408861	0.000791	0.003655	-0.86346
GPRC5B	-1.57587	4.204067	-3.40861	0.000791	0.003656	-1.44921
SPATA20	-0.75439	5.375442	-3.4087	0.000791	0.003656	-1.47599
SLC25A22	0.804352	5.389444	3.407116	0.000796	0.003673	-1.37101
C1orf96	0.811747	4.361878	3.406376	0.000798	0.003682	-1.2052
FAM115A	-0.77939	3.475006	-3.40596	0.000799	0.003686	-1.30368
GLE1	0.584559	5.651014	3.405189	0.000801	0.003694	-1.42637
NT5DC2	1.018675	5.845142	3.404268	0.000803	0.003705	-1.41005
ADAMTS14	2.138422	3.048428	3.403928	0.000804	0.003708	-0.83465
HSPBAP1	0.731462	3.218644	3.401965	0.00081	0.003732	-1.02558
TBCB	0.713952	5.426448	3.401514	0.000811	0.003736	-1.40362
SFRS2B	-0.56023	5.54726	-3.39957	0.000817	0.00376	-1.50573
LNX1	-1.03882	3.760662	-3.39913	0.000818	0.003765	-1.39512
RCE1	0.709736	4.418368	3.398017	0.000821	0.003778	-1.25988
DNTTIP1	0.679423	5.092543	3.397429	0.000823	0.003783	-1.37993
BRP44	-0.71975	5.164263	-3.39744	0.000823	0.003783	-1.50119
IVD	-1.00327	5.571526	-3.39712	0.000824	0.003786	-1.52296
AIM1L	3.770881	3.762193	3.396994	0.000824	0.003787	-0.78645
QTRTD1	0.627812	4.957887	3.396258	0.000826	0.003795	-1.37192
SUMF1	-0.68993	4.640273	-3.39617	0.000826	0.003795	-1.46619
SLC9A2	-2.08977	2.792781	-3.39607	0.000826	0.003795	-1.38494
CNTNAP1	-1.05416	3.65324	-3.39461	0.000831	0.003813	-1.39954
EHMT2	0.571247	6.407867	3.393537	0.000834	0.003826	-1.51883
PRR5	0.814324	4.41889	3.392752	0.000836	0.003835	-1.25824
GAS2L3	1.614121	2.366479	3.392483	0.000837	0.003837	-0.85075
MMD	1.215907	4.015597	3.392313	0.000837	0.003838	-1.11743
PGF	2.204633	3.320626	3.390995	0.000841	0.003854	-0.89953
IRF9	0.820067	5.847445	3.389601	0.000845	0.003872	-1.47439
WDR4	0.688222	3.786471	3.38813	0.000849	0.003889	-1.17469
DPY19L1	0.93018	5.811281	3.388028	0.00085	0.003889	-1.4656
HNRNPAB	0.514888	7.747237	3.388016	0.00085	0.003889	-1.54783
WBSCR27	2.045351	0.786931	3.387831	0.00085	0.003889	-0.75812
PPT1	0.809176	7.116421	3.387798	0.00085	0.003889	-1.55211
MAGOHB	0.761654	3.752686	3.386497	0.000854	0.003904	-1.16044
FBXL19	0.672386	5.050274	3.386499	0.000854	0.003904	-1.41035
C1orf85	0.603599	5.336332	3.386233	0.000855	0.003906	-1.45275
C18orf54	1.30489	2.725936	3.384677	0.00086	0.003926	-0.94582
TPRG1L	-0.60557	6.044106	-3.38444	0.00086	0.003928	-1.56569
SLC22A4	1.352059	0.489263	3.383762	0.000862	0.003936	-0.76972
PPP1R9B	0.50462	6.519608	3.382493	0.000866	0.003952	-1.56125
USP43	1.395553	3.71291	3.382312	0.000867	0.003953	-1.0675
STOX2	-1.414	1.838256	-3.38217	0.000867	0.003954	-1.17909
SLC2A10	-1.23783	4.214494	-3.38195	0.000868	0.003955	-1.51478
NLN	0.788179	4.486095	3.381793	0.000868	0.003956	-1.31151
FLJ45340	-0.63299	6.13108	-3.38112	0.00087	0.003964	-1.57662
YARS2	0.626478	4.110002	3.380984	0.000871	0.003965	-1.27022
RASSF2	-1.12855	3.768387	-3.38085	0.000871	0.003965	-1.46481
RAC2	1.527208	5.216249	3.38006	0.000873	0.003975	-1.32483
JUND	-0.77896	7.620484	-3.37946	0.000875	0.003982	-1.5501
NUP155	0.822707	5.685155	3.379015	0.000876	0.003986	-1.49191
MAPK9	-0.52538	5.658098	-3.3785	0.000878	0.003992	-1.57589

PRR5L	2.069962	3.043204	3.378182	0.000879	0.003995	-0.92263
KDM1B	0.782035	5.435799	3.377518	0.000881	0.004002	-1.47381
UBAP2L	0.391763	7.816962	3.37751	0.000881	0.004002	-1.57797
PUF60	0.618275	6.898174	3.377286	0.000882	0.004004	-1.58518
SHROOM3	-2.01581	6.423407	-3.37717	0.000882	0.004004	-1.55888
OSM	1.852445	0.036583	3.37663	0.000884	0.004009	-0.7903
BEND7	-1.53089	2.876039	-3.37665	0.000884	0.004009	-1.40214
FHOD1	0.703142	4.817163	3.376468	0.000884	0.00401	-1.40208
KCTD7	-0.72553	3.338562	-3.37608	0.000885	0.004014	-1.36778
DCBLD1	1.182847	4.871372	3.375772	0.000886	0.004017	-1.33335
NUP205	0.611442	6.801804	3.375264	0.000888	0.004022	-1.58966
GART	0.537096	6.383789	3.374763	0.000889	0.004028	-1.57913
LNPEP	-0.7233	4.353271	-3.37417	0.000891	0.004033	-1.51183
DEK	0.605018	7.632307	3.374089	0.000891	0.004033	-1.59551
SFRS18	-0.70254	6.386048	-3.3741	0.000891	0.004033	-1.59645
TPD52L2	0.523703	6.970073	3.373387	0.000894	0.004042	-1.60016
UBE2G2	-0.48716	5.404413	-3.3716	0.000899	0.004065	-1.58617
ACSS1	-1.2291	5.587215	-3.37136	0.0009	0.004067	-1.60606
ADRB2	-1.62975	1.687373	-3.3712	0.0009	0.004068	-1.21977
IRAK3	-1.36832	4.036122	-3.37065	0.000902	0.004075	-1.54422
CNOT6L	-0.60775	5.791672	-3.37032	0.000903	0.004078	-1.60727
RAB3D	-0.88019	6.296109	-3.37005	0.000904	0.00408	-1.60815
BECN1	-0.39637	6.030525	-3.36909	0.000907	0.004093	-1.6124
ZEB2	-1.03677	4.369665	-3.36887	0.000908	0.004094	-1.55613
MAPK6	0.815386	7.247497	3.368768	0.000908	0.004094	-1.6146
HIST2H4A	1.680096	3.448396	3.367654	0.000911	0.004109	-1.04559
TM4SF19	3.736467	1.070457	3.367215	0.000913	0.004114	-0.82128
TBC1D4	-0.9918	5.53637	-3.36686	0.000914	0.004117	-1.61888
SFRS9	0.478198	6.291891	3.366035	0.000916	0.004128	-1.60515
TRIB3	1.815324	4.782186	3.365891	0.000917	0.004128	-1.23708
IPO4	0.754144	6.004807	3.365783	0.000917	0.004129	-1.57004
SIX1	3.06922	1.776365	3.365326	0.000919	0.004134	-0.82819
DPY19L3	-0.72842	4.140497	-3.36432	0.000922	0.004147	-1.52068
MFI2	3.270648	4.292505	3.364176	0.000922	0.004147	-0.98098
CCNE2	1.297714	3.624	3.363925	0.000923	0.00415	-1.12731
GLRX3	0.717807	5.661761	3.361497	0.000931	0.004183	-1.55533
COMMD4	0.578522	5.174556	3.36123	0.000932	0.004185	-1.51628
FBXO8	-0.49951	4.002173	-3.36065	0.000933	0.004192	-1.49172
BMS1	0.426874	6.165086	3.36061	0.000934	0.004192	-1.61838
PLCL2	-1.22938	2.35073	-3.36048	0.000934	0.004192	-1.31015
LILRB5	-1.50466	0.285491	-3.35823	0.000941	0.004222	-1.03222
ZFYVE9	-0.60726	4.820489	-3.35825	0.000941	0.004222	-1.59665
RHOT1	-0.4539	5.29297	-3.35668	0.000946	0.004243	-1.62588
SAR1B	-0.5441	4.66201	-3.35584	0.000949	0.004254	-1.58486
RNF38	-0.56703	5.689598	-3.35543	0.00095	0.004258	-1.65128
LOC113230	-1.28986	2.707621	-3.3553	0.000951	0.004259	-1.40124
SELK	-0.49591	4.340426	-3.3548	0.000952	0.004264	-1.55089
SMARCA2	-0.85656	6.212809	-3.35475	0.000952	0.004264	-1.65809
PSAP	-0.55365	9.972127	-3.35381	0.000956	0.004276	-1.56248
NCLN	0.555317	7.005235	3.352679	0.000959	0.004292	-1.66594
CCDC96	-0.71118	0.359881	-3.35181	0.000962	0.004303	-0.98343
PDRG1	0.62204	3.904064	3.351182	0.000964	0.004311	-1.32551
PDCD10	0.602264	5.611984	3.350541	0.000966	0.004318	-1.59518
PLOD3	1.069253	6.544194	3.350533	0.000966	0.004318	-1.63664
FAM100B	0.621248	6.087873	3.350114	0.000968	0.004322	-1.63529
NUDT13	-0.8789	0.734974	-3.34909	0.000971	0.004336	-1.04821
C9orf95	-0.78887	3.745092	-3.34767	0.000976	0.004356	-1.53046
PASK	0.836453	3.934291	3.34744	0.000977	0.004358	-1.30329

TACC2	-0.95108	6.876426	-3.34695	0.000978	0.004364	-1.66947
CREB3L2	-0.75114	6.621841	-3.34634	0.00098	0.004371	-1.68016
MRS2	-0.51173	4.939459	-3.34601	0.000981	0.004375	-1.63809
ZIC2	3.851418	1.50582	3.345721	0.000982	0.004378	-0.88681
KRT80	3.687365	5.299309	3.344625	0.000986	0.004393	-1.11762
SGCB	-0.87381	5.243811	-3.34403	0.000988	0.004401	-1.68084
ESYT2	-0.70557	7.260305	-3.34372	0.000989	0.004404	-1.67575
EFHC2	-1.49976	-0.06035	-3.34283	0.000992	0.004416	-1.04168
MAPK10	-1.52845	1.689687	-3.34223	0.000994	0.004422	-1.29654
RNPEP	0.621066	6.789722	3.34227	0.000994	0.004422	-1.69378
PPIB	0.584867	7.947034	3.341936	0.000995	0.004425	-1.69154
USP42	0.504331	5.109144	3.341357	0.000997	0.004432	-1.57962
ATR	0.656928	5.905747	3.341161	0.000998	0.004434	-1.64681
SAAL1	0.708381	3.301045	3.340725	0.000999	0.004439	-1.23336
NMB	1.660489	3.04796	3.339601	0.001003	0.004455	-1.08494
SLC30A4	-0.75631	2.730655	-3.33912	0.001005	0.00446	-1.37294
XBP1	-0.98794	7.420699	-3.33907	0.001005	0.00446	-1.67837
TOR3A	0.635392	5.189495	3.338508	0.001007	0.004467	-1.58398
OLA1	0.538021	6.446235	3.338429	0.001007	0.004467	-1.69706
LOC283174	-1.71589	1.248134	-3.33701	0.001012	0.004487	-1.26308
LRP10	-0.80824	8.255167	-3.33691	0.001012	0.004487	-1.66354
MSMP	-0.64235	1.230391	-3.33643	0.001014	0.004493	-1.12293
C1orf130	-2.11395	0.117556	-3.33548	0.001017	0.004506	-1.1413
SFMBT2	-1.20371	1.149925	-3.33518	0.001018	0.004509	-1.18253
NT5DC1	-0.61351	5.039809	-3.3349	0.001019	0.004512	-1.68702
TIGD6	-0.41075	2.263694	-3.33385	0.001023	0.004525	-1.24833
ASAM	-1.36653	2.789957	-3.33393	0.001023	0.004525	-1.50406
C7orf70	0.631831	4.31037	3.333532	0.001024	0.004529	-1.45833
C1orf131	0.644591	4.142951	3.332741	0.001027	0.00454	-1.42417
AGPAT9	-1.2991	2.798785	-3.33233	0.001028	0.004545	-1.49387
PIK3C2A	-0.58888	6.635849	-3.33143	0.001031	0.004557	-1.72956
C14orf80	0.834466	3.398841	3.33129	0.001032	0.004558	-1.25907
RPGRIP1L	0.917485	2.944593	3.3303	0.001035	0.004572	-1.18551
BTRC	-0.46258	4.816671	-3.32976	0.001037	0.004577	-1.67512
ADI1	-0.55999	5.975274	-3.32985	0.001037	0.004577	-1.73775
GTF2H3	0.676213	4.014302	3.329266	0.001039	0.004583	-1.40611
RIPK1	-0.47826	5.577108	-3.32853	0.001042	0.004593	-1.72979
ATP11A	1.123234	6.859188	3.327683	0.001045	0.004605	-1.72444
G6PD	1.357633	7.009135	3.327079	0.001047	0.004613	-1.72263
BAZ2B	-0.6227	5.869469	-3.32606	0.00105	0.004628	-1.74889
ATXN1L	-0.46435	6.192108	-3.32422	0.001057	0.004655	-1.7568
ALDH4A1	-0.86496	4.993101	-3.32256	0.001063	0.00468	-1.73623
SLC28A3	3.426238	1.76105	3.322433	0.001063	0.004681	-0.95691
GNAL	-1.28407	2.379884	-3.32198	0.001065	0.004686	-1.45312
WDR46	0.515332	5.226997	3.321827	0.001065	0.004687	-1.65345
CD3EAP	0.639677	4.443935	3.320702	0.00107	0.004704	-1.52227
AHSA1	0.56146	6.315968	3.319492	0.001074	0.004722	-1.74959
PRKG1	-1.19367	1.66546	-3.3188	0.001076	0.004731	-1.31271
EPAS1	-0.862	8.002702	-3.31716	0.001082	0.004756	-1.7323
TGFA	1.520152	5.472596	3.316751	0.001084	0.00476	-1.57158
CSK	0.626598	6.559847	3.316705	0.001084	0.00476	-1.76659
TAF4	0.526269	5.404398	3.31623	0.001086	0.004767	-1.68898
USP46	-0.63222	4.649591	-3.31577	0.001088	0.004773	-1.7174
AATF	0.494472	5.936857	3.315545	0.001088	0.004775	-1.74176
ZC3H12B	-1.31939	1.295132	-3.31525	0.00109	0.004778	-1.27864
DHX32	-0.52221	5.583734	-3.31292	0.001098	0.004814	-1.78046
PPP2R1B	-0.66112	5.501207	-3.31124	0.001104	0.00484	-1.78682
ACAA1	-0.71046	5.07284	-3.31091	0.001106	0.004844	-1.76961

SKA2	0.702597	5.35939	3.310747	0.001106	0.004845	-1.6839
ZNF846	-0.73069	1.855371	-3.30978	0.00111	0.00486	-1.30355
TIMP1	1.160854	7.734263	3.309605	0.001111	0.004861	-1.80184
ZBTB33	0.534572	5.947166	3.309075	0.001113	0.004868	-1.76003
TP53INP2	-1.17459	6.508121	-3.30668	0.001122	0.004906	-1.79945
CUEDC1	-0.76466	4.137554	-3.30586	0.001125	0.004918	-1.70792
C20orf199	0.785979	5.969769	3.304676	0.001129	0.004936	-1.75674
SNX33	-0.62583	5.609325	-3.30134	0.001142	0.004991	-1.82044
LETMD1	-0.56113	4.951646	-3.30095	0.001144	0.004996	-1.78383
IL8	4.346318	5.111869	3.300724	0.001144	0.004998	-1.15613
MEIS2	-0.81118	3.547317	-3.30043	0.001145	0.005001	-1.65332
FOSB	-2.03269	5.588401	-3.29983	0.001148	0.00501	-1.82085
MFSD2A	1.867408	3.961767	3.29942	0.001149	0.005015	-1.30327
C5orf30	-0.80432	3.748922	-3.29709	0.001158	0.005053	-1.69001
C10orf72	-1.11992	3.012902	-3.29696	0.001159	0.005054	-1.62073
SLC39A4	1.412549	5.154002	3.296187	0.001162	0.005066	-1.59584
ADORA2B	2.519421	3.140299	3.295816	0.001163	0.005067	-1.14312
PSME1	0.613863	6.970181	3.295966	0.001163	0.005067	-1.84288
ANKS1A	-0.56923	6.258421	-3.29585	0.001163	0.005067	-1.84553
CYB5R3	-0.50486	8.062616	-3.2951	0.001166	0.005078	-1.80981
GTF2H4	0.560463	4.16656	3.294872	0.001167	0.00508	-1.56236
HYAL2	-0.48844	5.39879	-3.29471	0.001168	0.00508	-1.82804
VMA21	0.579564	6.49075	3.294698	0.001168	0.00508	-1.83437
CTSL2	2.787142	4.258115	3.293276	0.001173	0.005101	-1.24747
SMCR7	-0.52082	3.246258	-3.29334	0.001173	0.005101	-1.57626
MT2A	-1.56391	6.290573	-3.29319	0.001174	0.005101	-1.83814
IMMP2L	-0.78839	1.370224	-3.29292	0.001175	0.005104	-1.29188
MAD1L1	0.708059	4.865298	3.292623	0.001176	0.005108	-1.67339
TGS1	0.557999	5.286508	3.290081	0.001186	0.00515	-1.75473
EBF1	-0.97934	2.639839	-3.28994	0.001187	0.005151	-1.54572
KHSRP	0.471554	7.434559	3.289713	0.001188	0.005153	-1.86244
RILP	-0.76643	2.84667	-3.28952	0.001188	0.005155	-1.54882
LRWD1	0.744121	4.187322	3.288942	0.001191	0.005164	-1.55239
BDP1	-0.51672	6.012113	-3.28871	0.001192	0.005166	-1.86657
XRCC4	0.870648	2.992045	3.288382	0.001193	0.00517	-1.32515
MAPK13	0.891587	6.226537	3.28781	0.001195	0.005178	-1.8223
SNHG3	1.001633	2.822256	3.287636	0.001196	0.00518	-1.29003
HLA-C	0.995545	9.39036	3.287092	0.001198	0.005186	-1.83481
HEATR2	0.628642	6.200086	3.287152	0.001198	0.005186	-1.84008
PTGER4	-1.13662	3.905182	-3.28584	0.001203	0.005206	-1.77796
SH2D5	2.827927	0.319527	3.285247	0.001206	0.005214	-1.06411
SPATS2L	0.626656	6.947954	3.285262	0.001206	0.005214	-1.87566
RNF114	0.570641	6.316632	3.284875	0.001207	0.005218	-1.85711
LSM6	0.703355	3.503137	3.284565	0.001208	0.005222	-1.44381
C9orf172	-1.05346	1.878808	-3.28334	0.001213	0.005242	-1.43831
C14orf145	0.88042	2.709193	3.282653	0.001216	0.005253	-1.30501
ZNF397OS	-0.6621	3.436076	-3.28205	0.001219	0.005262	-1.66943
CCDC66	-0.50706	3.18068	-3.28133	0.001222	0.005273	-1.59707
ANG	-1.33078	2.589162	-3.27986	0.001228	0.005297	-1.62038
PRAME	5.446364	1.482447	3.27966	0.001228	0.005299	-1.08349
SLC2A5	2.122108	1.384435	3.278623	0.001233	0.005316	-1.08766
ZC3H13	-0.60302	7.302532	-3.2767	0.001241	0.005349	-1.88678
FTSJ1	0.579299	5.39184	3.276497	0.001242	0.005351	-1.80647
PLEKHH3	-0.72486	5.019459	-3.27621	0.001243	0.005354	-1.87545
KRCC1	-0.78673	4.256667	-3.27611	0.001243	0.005354	-1.81517
THOC3	0.972495	5.696946	3.275925	0.001244	0.005356	-1.803
MAPKSP1	-0.46531	5.42138	-3.27419	0.001251	0.005386	-1.89195
CMTM1	1.080343	2.452016	3.273922	0.001252	0.005389	-1.27561

FBXW8	-0.48227	4.538109	-3.27378	0.001253	0.00539	-1.82464
TKT	0.890781	8.064645	3.273182	0.001255	0.005399	-1.90937
ARMC6	0.548251	4.992451	3.272762	0.001257	0.005405	-1.77454
CYB5B	0.644014	6.403131	3.272479	0.001258	0.005409	-1.89658
OSBPL3	0.78041	6.020999	3.272165	0.00126	0.005413	-1.86252
MRPS17	0.915255	3.984977	3.270801	0.001266	0.005436	-1.53907
PARM1	-1.53666	4.426614	-3.26882	0.001274	0.00547	-1.90231
SNRPB2	0.68805	5.428206	3.268384	0.001276	0.005477	-1.82464
SLC22A20	2.203902	0.368611	3.26789	0.001278	0.005484	-1.11525
PRDM2	-0.54531	5.969292	-3.26702	0.001282	0.005498	-1.93362
CBX2	2.797239	3.876347	3.266421	0.001284	0.005508	-1.28349
GLO1	0.556639	7.386639	3.266298	0.001285	0.005508	-1.93652
STX17	-0.48384	5.568917	-3.26605	0.001286	0.005511	-1.92457
CBWD3	1.232046	4.144712	3.264817	0.001291	0.005532	-1.53272
RBM42	0.535345	5.762208	3.264625	0.001292	0.005534	-1.88327
GRN	0.632229	9.085357	3.26426	0.001294	0.005539	-1.90509
APOBEC3B	2.620334	3.14848	3.263204	0.001298	0.005557	-1.23193
MTMR9L	-0.916	1.086784	-3.26269	0.0013	0.005565	-1.36064
IL6R	-0.98261	3.930934	-3.26219	0.001303	0.005572	-1.84069
SCARB2	-0.60626	7.89888	-3.26157	0.001305	0.005582	-1.91613
AZI1	0.636742	4.122923	3.261266	0.001307	0.005586	-1.64368
WBP2	-0.48622	6.819143	-3.26074	0.001309	0.005594	-1.94852
HIP1R	-0.77078	6.178208	-3.26056	0.00131	0.005596	-1.95408
ITPA	0.668913	4.934128	3.258678	0.001318	0.005628	-1.79435
GSTT1	-2.64379	2.653374	-3.25873	0.001318	0.005628	-1.8483
ISG20L2	0.468291	5.432798	3.257941	0.001321	0.00564	-1.8786
TNFRSF18	2.770197	1.88305	3.25687	0.001326	0.005659	-1.15095
ISY1	0.527263	4.494514	3.255713	0.001331	0.005679	-1.75249
KCTD2	-0.39978	5.335524	-3.25541	0.001332	0.005683	-1.94223
B3GNTL1	0.887383	1.561265	3.255076	0.001334	0.005686	-1.2541
FUBP1	0.427293	6.246002	3.255165	0.001334	0.005686	-1.95223
ZSWIM4	0.834973	5.200696	3.254933	0.001335	0.005687	-1.82313
LOC729991-MEF2B	1.062052	0.441511	3.254077	0.001338	0.0057	-1.15854
TIPRL	0.419103	5.429385	3.254045	0.001339	0.0057	-1.89476
MSL3L2	1.815165	3.333835	3.252906	0.001344	0.005717	-1.36612
TBC1D8	-0.70599	5.451376	-3.25306	0.001343	0.005717	-1.9672
PPP1R3B	-1.05398	5.338006	-3.25293	0.001344	0.005717	-1.97237
PERP	1.542155	9.63078	3.252217	0.001347	0.005728	-1.95035
ZNF14	-0.8692	2.683192	-3.25184	0.001348	0.005733	-1.65348
S100B	-1.30265	0.934413	-3.25127	0.001351	0.005743	-1.42163
C7orf27	0.521568	6.205555	3.250477	0.001355	0.005756	-1.95988
ACSF2	-0.93229	3.369575	-3.24976	0.001358	0.005768	-1.79676
C5orf24	-0.49965	6.108815	-3.24902	0.001361	0.005781	-1.99037
NFKBIE	1.250433	4.056487	3.248852	0.001362	0.005782	-1.56178
FAM155B	-2.15451	-0.14719	-3.24864	0.001363	0.005784	-1.38277
WDR74	0.647579	4.876793	3.2483	0.001364	0.005789	-1.8207
CBLC	2.157522	5.040854	3.248136	0.001365	0.005789	-1.58573
TMEM175	-0.62104	4.180022	-3.24805	0.001366	0.005789	-1.87731
MTUS1	-0.97722	6.954732	-3.24802	0.001366	0.005789	-1.97554
CYP7B1	-1.37188	1.269608	-3.24774	0.001367	0.005792	-1.49022
C2orf86	-0.67063	2.164246	-3.24771	0.001367	0.005792	-1.53505
B3GALNT1	-0.90552	3.761743	-3.24759	0.001368	0.005792	-1.85791
PPIEL	-1.00743	0.843038	-3.24619	0.001374	0.005814	-1.38985
GLS	0.842232	6.809704	3.246194	0.001374	0.005814	-1.98723
DOPEY2	-0.97363	5.728219	-3.24632	0.001373	0.005814	-1.99793
FAP	3.273371	3.362783	3.245724	0.001376	0.005818	-1.25045
TBKBP1	-0.74409	3.528596	-3.24578	0.001376	0.005818	-1.81152
FAM160B1	-0.43613	5.277546	-3.24583	0.001376	0.005818	-1.9701

AKIRIN2	0.526538	5.535617	3.245246	0.001378	0.005825	-1.92293
ZBTB9	0.609516	3.767061	3.24511	0.001379	0.005826	-1.62902
HIST2H2AA3	1.755697	3.979538	3.244323	0.001383	0.005839	-1.48822
TCF20	0.557957	6.812888	3.244291	0.001383	0.005839	-2.00104
PHC2	-0.53831	6.825627	-3.24396	0.001384	0.005843	-1.99928
CROCCL2	-0.76348	1.968349	-3.2438	0.001385	0.005845	-1.52721
CYP39A1	-1.41435	1.200047	-3.24344	0.001387	0.00585	-1.49789
MMP10	4.482564	2.244555	3.242253	0.001392	0.005867	-1.1945
ADAM12	3.698986	4.062473	3.242457	0.001391	0.005867	-1.28534
RBMS3	-1.21116	0.76886	-3.24221	0.001392	0.005867	-1.41621
PRDX1	0.679015	8.420058	3.242277	0.001392	0.005867	-1.99344
DDX21	0.593323	7.480819	3.242097	0.001393	0.005867	-2.01044
MREG	1.35458	4.146321	3.241979	0.001393	0.005868	-1.58214
FNDC3A	-0.78215	6.544321	-3.24145	0.001396	0.005876	-2.0079
WRB	-0.60311	3.913516	-3.24047	0.0014	0.005894	-1.86622
RNF152	-1.15582	1.959937	-3.2403	0.001401	0.005895	-1.59519
SUV39H1	0.932524	4.062141	3.239966	0.001403	0.0059	-1.64537
C9orf156	-0.42629	3.162036	-3.23907	0.001407	0.005916	-1.70867
POLR2G	0.554927	5.24002	3.237259	0.001416	0.005948	-1.91283
RHOBTB3	-1.21763	5.390738	-3.23732	0.001415	0.005948	-2.02356
TGFB1I1	-1.01483	4.889766	-3.23714	0.001416	0.005949	-2.00275
GRHL2	1.468881	5.784137	3.236769	0.001418	0.005954	-1.87526
PSMB5	0.557477	6.24129	3.235469	0.001424	0.005978	-2.0063
ANKLE2	0.505124	6.536382	3.234266	0.00143	0.006	-2.0256
CCDC104	-0.54064	4.354511	-3.23281	0.001437	0.006028	-1.93655
C22orf25	-0.44357	4.432049	-3.23263	0.001437	0.006029	-1.93576
NOTCH3	1.249617	8.675004	3.231545	0.001443	0.00605	-2.03167
KIAA1199	3.116563	5.120252	3.231278	0.001444	0.006053	-1.50981
TRPC1	-1.20689	1.70281	-3.23098	0.001445	0.006057	-1.59159
VPS26B	-0.57329	5.846677	-3.22809	0.001459	0.006114	-2.05173
PLP2	0.923697	7.722018	3.227787	0.001461	0.006118	-2.05465
SAMD9	1.629071	6.936344	3.226916	0.001465	0.006134	-2.01164
IPO9	0.502708	7.083811	3.226532	0.001467	0.00614	-2.0597
MFAP2	2.694495	4.453315	3.226258	0.001468	0.006144	-1.48828
RPP25	1.086069	4.799203	3.225795	0.00147	0.006151	-1.80253
TPST2	0.784574	4.855762	3.225519	0.001472	0.006153	-1.86732
PSMD4	0.476546	6.63851	3.225425	0.001472	0.006153	-2.05664
NUP188	0.542782	7.279254	3.225501	0.001472	0.006153	-2.06271
SART3	0.341899	5.992382	3.224668	0.001476	0.006167	-2.03634
HNRNPK	0.280478	8.501237	3.224225	0.001478	0.006174	-2.03679
SYNJ2	-0.71337	5.544738	-3.22323	0.001483	0.006193	-2.062
ZNF177	-1.26657	0.003215	-3.22265	0.001486	0.006203	-1.38801
ZNF549	-1.13174	2.074737	-3.22133	0.001492	0.006228	-1.67326
SEC31B	-0.93188	2.00046	-3.22055	0.001496	0.006242	-1.6287
KIAA0776	-0.51214	5.800922	-3.22004	0.001499	0.006251	-2.07423
C6orf150	1.800674	3.433083	3.218839	0.001505	0.006274	-1.48332
CSRP2BP	-0.52991	4.843264	-3.21862	0.001506	0.006277	-2.02699
SR140	0.545391	7.226646	3.21854	0.001506	0.006277	-2.08419
TRA2B	0.401981	7.112944	3.218088	0.001509	0.006284	-2.08567
RSRC1	0.74115	4.476796	3.217897	0.001509	0.006286	-1.82761
ACSL1	-0.90476	6.277797	-3.21565	0.001521	0.006331	-2.08913
PEX12	-0.49505	2.785898	-3.21555	0.001521	0.006332	-1.71907
DPF1	2.509893	0.068495	3.215299	0.001523	0.006335	-1.26784
GATA6	-2.062	4.224198	-3.21491	0.001524	0.006341	-2.07474
POFUT1	0.649772	6.913417	3.214781	0.001525	0.006342	-2.09164
ZNF19	-0.64147	1.068701	-3.21342	0.001532	0.006369	-1.4756
ZNF629	-0.52761	5.251136	-3.21298	0.001534	0.006376	-2.07369
ZNF708	-0.95159	2.546686	-3.2128	0.001535	0.006378	-1.75854

RAD50	-0.49372	6.070958	-3.21264	0.001536	0.006379	-2.10137
ZNF764	-0.52686	3.19488	-3.21228	0.001538	0.006385	-1.81387
CA8	-2.18043	0.786678	-3.21066	0.001546	0.006417	-1.63038
TRAF4	0.754128	6.042091	3.210286	0.001548	0.006423	-2.05583
RBPJ	-0.46602	5.543979	-3.20974	0.001551	0.006433	-2.09574
FARSA	0.534252	5.929513	3.209493	0.001552	0.006437	-2.06603
AMIGO1	-1.03845	1.633229	-3.20885	0.001555	0.006448	-1.61709
C7orf28A	0.633275	5.207457	3.207575	0.001562	0.006474	-1.99101
KLF11	-0.62082	4.667514	-3.20738	0.001563	0.006476	-2.05273
RUSC1	0.484035	5.843569	3.207194	0.001564	0.006478	-2.07
SLC38A6	0.813969	2.531654	3.206397	0.001568	0.006493	-1.51852
ZNF641	-0.52305	3.108001	-3.2062	0.001569	0.006495	-1.81619
RABIF	0.541204	4.20883	3.205305	0.001574	0.006513	-1.84994
ADIPOR2	-0.57801	6.59175	-3.20462	0.001577	0.006525	-2.12288
GRIN2D	4.067568	4.35467	3.204382	0.001578	0.006525	-1.39318
HSFX2	-0.80313	0.582925	-3.20439	0.001578	0.006525	-1.46232
ZNF704	-1.20769	2.827434	-3.20435	0.001579	0.006525	-1.8832
GAR1	0.519134	4.000221	3.203701	0.001582	0.006536	-1.81676
ZNF37A	-0.61907	4.595405	-3.20373	0.001582	0.006536	-2.05671
SPINK1	-3.09649	1.601714	-3.20283	0.001587	0.006548	-1.92694
PDHX	-0.53587	5.037295	-3.20289	0.001586	0.006548	-2.09067
ROCK1	-0.61363	6.904995	-3.20295	0.001586	0.006548	-2.12182
HIF1A	0.762617	8.404104	3.202651	0.001587	0.006551	-2.11661
SMAD7	-0.66027	4.03588	-3.20253	0.001588	0.006551	-2.0035
CEP152	0.824518	3.411512	3.201597	0.001593	0.006569	-1.65877
RPL15	-0.52123	9.014686	-3.20147	0.001594	0.00657	-2.06626
WISP1	2.427121	2.174793	3.201242	0.001595	0.006573	-1.34706
CYP3A5	-2.15157	2.715434	-3.19989	0.001602	0.006601	-1.9902
C7orf47	0.879496	3.636851	3.199363	0.001605	0.00661	-1.69681
C15orf23	0.856674	4.417631	3.199167	0.001606	0.006613	-1.8505
WBSCR22	0.622506	5.668797	3.198169	0.001611	0.00663	-2.07052
ATP8B1	-1.1127	6.841972	-3.19824	0.001611	0.00663	-2.12735
HLA-A	0.968869	9.370443	3.197978	0.001612	0.006633	-2.10844
GABARAP	-0.52402	7.372851	-3.19764	0.001614	0.006638	-2.12935
RAP2C	0.525817	5.526471	3.19683	0.001618	0.006654	-2.06951
NIT2	0.64913	4.838811	3.196561	0.00162	0.006658	-1.97252
KDELC1	1.016201	2.376855	3.195558	0.001625	0.006678	-1.50909
GJB2	2.758786	7.133969	3.1942	0.001632	0.006706	-2.02643
PLEKHN1	2.319821	2.575529	3.193806	0.001634	0.006711	-1.40913
ADAM10	0.601529	6.715681	3.193888	0.001634	0.006711	-2.15144
LEO1	0.548651	4.938613	3.190603	0.001652	0.00678	-2.01834
SULT1A1	-0.99505	2.237952	-3.19016	0.001654	0.006788	-1.77305
CDRT4	-0.64742	2.924499	-3.19006	0.001655	0.006788	-1.84881
ZC3HAV1L	0.946715	2.731098	3.189458	0.001658	0.006799	-1.58054
OTUD1	-0.69916	4.669997	-3.18871	0.001662	0.006814	-2.11514
ANAPC7	0.518725	5.715866	3.18846	0.001663	0.006817	-2.11352
CIRH1A	0.594961	5.989944	3.188377	0.001664	0.006817	-2.1306
SIL1	-0.556	4.690244	-3.18796	0.001666	0.006825	-2.10786
SUN2	-0.65331	7.028451	-3.18725	0.00167	0.006839	-2.16617
CACNA2D1	-1.64759	1.923305	-3.18702	0.001671	0.006842	-1.83475
ABCB1	-1.40082	1.348045	-3.18634	0.001675	0.006855	-1.69368
NGLY1	-0.51221	4.25348	-3.18549	0.00168	0.006872	-2.06555
CTF1	-1.17548	2.307262	-3.1845	0.001685	0.006893	-1.83825
PRMT3	0.653715	4.59992	3.183511	0.001691	0.006913	-1.96978
CDC14B	-0.88667	4.512428	-3.18293	0.001694	0.006924	-2.13254
SLC25A46	-0.4813	4.941101	-3.18223	0.001698	0.006938	-2.14197
RPL36A	0.932374	2.89734	3.181313	0.001703	0.006957	-1.62852
STRA6	3.946002	2.656533	3.1802	0.001709	0.006981	-1.37481

DCUN1D5	0.822427	4.34114	3.179941	0.001711	0.006984	-1.90157
RBM12B	0.590618	4.706476	3.179773	0.001712	0.006984	-2.00952
TOMM5	0.700118	5.508756	3.179769	0.001712	0.006984	-2.10283
MRPS25	-0.55651	4.992406	-3.17952	0.001713	0.006988	-2.15937
RBM34	0.546598	4.83824	3.179352	0.001714	0.00699	-2.03867
RHOJ	-0.92342	2.469358	-3.1777	0.001723	0.007026	-1.846
C3orf21	0.999716	4.736677	3.177558	0.001724	0.007027	-1.95564
UQCR11	-0.53376	5.763938	-3.17676	0.001729	0.007043	-2.20508
PCSK9	4.24606	4.051391	3.174987	0.001739	0.00708	-1.43726
ZNF527	-0.70954	1.909485	-3.17498	0.001739	0.00708	-1.71781
AP1G1	-0.44845	7.096937	-3.17452	0.001741	0.007089	-2.20701
FCGR3A	2.631767	4.109095	3.174294	0.001743	0.007092	-1.60546
AMPD2	0.578734	5.497472	3.173711	0.001746	0.007099	-2.13143
SEPP1	-1.68512	6.874449	-3.17389	0.001745	0.007099	-2.18493
DIP2A	-0.51654	5.488021	-3.17375	0.001746	0.007099	-2.20417
OSBPL1A	-0.85704	5.196408	-3.17362	0.001746	0.007099	-2.20423
ESAM	-0.87897	4.458803	-3.17346	0.001747	0.007101	-2.15639
SLMO1	1.269984	0.754877	3.173034	0.00175	0.007109	-1.40135
SLC35A2	0.728835	5.288103	3.172867	0.001751	0.007111	-2.09484
ZNF552	-0.7357	3.047669	-3.17257	0.001752	0.007115	-1.93898
HPS3	0.729558	5.619398	3.172496	0.001753	0.007115	-2.13309
MFSD8	-0.45654	3.907049	-3.17169	0.001757	0.007132	-2.05531
LOC401052	-0.71657	0.150589	-3.17084	0.001762	0.007147	-1.5044
MAGEE1	-1.37431	0.83657	-3.17077	0.001763	0.007147	-1.65877
PSMD12	0.492195	5.817849	3.170787	0.001763	0.007147	-2.17741
ZNF879	-1.32028	0.483983	-3.17006	0.001767	0.007162	-1.60763
LRRC4B	-1.05743	0.924251	-3.16974	0.001769	0.007167	-1.63533
S100A16	1.223099	8.076541	3.169556	0.00177	0.00717	-2.23012
C22orf9	0.824209	6.771535	3.168975	0.001773	0.007181	-2.22151
FEZ2	-0.43193	4.842405	-3.16887	0.001774	0.007181	-2.17009
VPS24	-0.3851	7.131199	-3.16879	0.001774	0.007181	-2.22476
CST1	6.44899	3.727719	3.168095	0.001778	0.007196	-1.41104
LSM14B	0.508455	5.021696	3.167488	0.001782	0.007208	-2.10345
KCNJ14	0.929841	1.835102	3.166832	0.001786	0.007221	-1.53999
DCAF5	-0.50528	6.296279	-3.16673	0.001786	0.007222	-2.2413
NOC4L	0.616718	4.848505	3.164682	0.001798	0.007268	-2.0739
CPXM1	2.426308	3.039821	3.164586	0.001799	0.007268	-1.52889
ZNF668	0.458753	3.709294	3.163153	0.001807	0.0073	-1.89208
P4HTM	-0.97035	3.784816	-3.16146	0.001817	0.007338	-2.12763
CA11	-1.01094	2.125009	-3.16128	0.001818	0.007339	-1.84569
DIABLO	0.550105	4.628381	3.161319	0.001818	0.007339	-2.05858
LOC650623	-0.93367	1.902682	-3.16113	0.001819	0.00734	-1.79131
EPHA3	-1.28575	1.844461	-3.16051	0.001823	0.007353	-1.83748
SDCCAG3	0.625115	4.835996	3.159351	0.00183	0.007379	-2.08704
CRTAP	-0.59053	7.225064	-3.15883	0.001833	0.007389	-2.24865
P2RY6	1.872315	2.387194	3.158369	0.001836	0.007398	-1.53484
SNAP29	-0.42515	5.590576	-3.15762	0.00184	0.007414	-2.25374
OVOL1	2.590897	4.445509	3.155495	0.001853	0.007463	-1.7076
INTS6	-0.48226	5.575041	-3.15504	0.001856	0.007472	-2.26287
THAP10	-0.65384	2.00443	-3.1549	0.001857	0.007474	-1.7825
PMS2L1	0.867122	2.398799	3.154574	0.001859	0.007479	-1.64901
C1orf174	0.460677	4.035083	3.154245	0.001861	0.007485	-1.98147
CD276	0.91775	6.81685	3.152762	0.00187	0.007519	-2.26839
MOBKL1B	0.403917	6.161346	3.152567	0.001871	0.007522	-2.26098
CYP2R1	-0.63182	3.786421	-3.15234	0.001872	0.007523	-2.1175
IGF2BP2	1.363994	5.979462	3.152363	0.001872	0.007523	-2.16822
BOD1L	-0.53516	6.517301	-3.15171	0.001876	0.007536	-2.28422
GRB7	2.101299	5.608381	3.14911	0.001892	0.007598	-2.00436

DNAJB4	-0.95845	4.43314	-3.14873	0.001894	0.007606	-2.23512
L1CAM	-1.73549	2.979961	-3.14727	0.001903	0.007638	-2.14822
ABLIM3	-1.23772	3.899124	-3.14726	0.001903	0.007638	-2.20903
ATP5F1	-0.47263	6.916688	-3.14698	0.001905	0.007642	-2.29282
WHSC1	0.640099	6.959542	3.145453	0.001915	0.007678	-2.30205
MRAS	-0.97893	3.429823	-3.14529	0.001916	0.00768	-2.13244
MLLT3	-0.82321	3.361755	-3.14436	0.001921	0.007701	-2.09928
SPRN	-0.71952	2.252641	-3.14184	0.001937	0.007762	-1.87256
TESC	-2.18022	1.968154	-3.14159	0.001939	0.007767	-2.05298
ATP2C1	0.550406	7.353833	3.14145	0.00194	0.007768	-2.31635
ZNF134	-1.05218	2.91679	-3.13957	0.001952	0.007813	-2.07119
PSIMCT-1	1.05932	0.060698	3.139268	0.001954	0.007819	-1.4853
TTC33	-0.62208	4.240901	-3.1388	0.001956	0.007828	-2.21541
SLC25A43	0.844406	5.231802	3.138327	0.00196	0.007838	-2.17897
PDGFRA	-1.23328	5.471355	-3.13766	0.001964	0.007853	-2.32609
CTNNAL1	-0.82564	4.601857	-3.13718	0.001967	0.007863	-2.27393
SPARC	1.129523	9.178072	3.137089	0.001967	0.007863	-2.30199
RPN2	0.54171	8.591804	3.136933	0.001968	0.007865	-2.30448
BAT2L2	0.378831	8.801451	3.135989	0.001974	0.007886	-2.29637
SFRS5	-0.48375	6.775445	-3.13585	0.001975	0.007888	-2.32844
SCN1B	-1.02022	1.230221	-3.13475	0.001982	0.007914	-1.77506
RABEP1	-0.46281	5.729351	-3.13438	0.001985	0.007919	-2.32958
EFEMP1	-1.50776	4.983117	-3.13436	0.001985	0.007919	-2.33089
MED7	-0.39857	2.837809	-3.13401	0.001987	0.007926	-1.95635
LRRC42	0.519158	4.75699	3.133167	0.001993	0.007943	-2.16851
EEF2K	-0.53122	5.901712	-3.1332	0.001992	0.007943	-2.33873
TNFRSF10B	0.815814	6.216926	3.132985	0.001994	0.007946	-2.29817
C1QBP	0.667602	6.579092	3.132077	0.002	0.007967	-2.3303
LILRB4	2.106316	2.132163	3.131698	0.002002	0.00797	-1.56756
ZNF141	-1.17463	1.314355	-3.13175	0.002002	0.00797	-1.81567
UBXN8	-0.70305	2.398636	-3.13167	0.002002	0.00797	-1.92814
ALDH1A1	-2.6787	6.124889	-3.1316	0.002003	0.00797	-2.30489
MANBA	-0.53011	4.759066	-3.13105	0.002006	0.007982	-2.28325
TRAPPC2	-0.5012	3.414749	-3.13067	0.002009	0.00799	-2.09697
KCTD6	-0.57727	2.306404	-3.12931	0.002018	0.008023	-1.90088
C1orf126	-1.33638	2.235582	-3.12886	0.002021	0.008032	-2.00806
SNX29	-0.66865	4.858512	-3.12855	0.002023	0.008038	-2.30947
SAE1	0.469948	6.198359	3.128091	0.002026	0.008047	-2.33279
AGL	-0.64424	5.743089	-3.12781	0.002028	0.008052	-2.35347
SRA1	-0.47432	4.536086	-3.12772	0.002028	0.008053	-2.26638
C17orf68	-0.53775	4.707147	-3.12726	0.002031	0.008062	-2.29039
TDG	0.574267	5.17744	3.127034	0.002033	0.008066	-2.23601
PHLDA2	1.467024	5.422887	3.126393	0.002037	0.00808	-2.1534
MRPL17	0.599877	5.168886	3.126333	0.002037	0.00808	-2.23404
RRM1	0.647858	6.54595	3.125245	0.002044	0.008106	-2.35002
TMEM49	0.587476	7.130454	3.125134	0.002045	0.008106	-2.36532
RNF219	0.595918	4.193632	3.124176	0.002052	0.008129	-2.07832
LIMD2	1.317493	3.674927	3.123662	0.002055	0.00814	-1.86178
ITGB3BP	0.58714	3.399689	3.12313	0.002058	0.008152	-1.92866
CAPN2	-0.66231	8.261375	-3.12296	0.00206	0.008154	-2.32232
FER1L6	-2.4698	1.293597	-3.12233	0.002064	0.008168	-2.02131
HEATR5B	-0.38034	5.586148	-3.12226	0.002064	0.008168	-2.35741
BCL2A1	1.830783	1.45999	3.121298	0.002071	0.008191	-1.56292
CORO2A	1.081194	6.002459	3.120701	0.002075	0.008205	-2.29395
APIP	-0.58734	3.741279	-3.11999	0.002079	0.008221	-2.20097
CCDC55	-0.57541	5.490202	-3.11991	0.00208	0.008221	-2.36746
TRIM66	-0.67128	3.981677	-3.11942	0.002083	0.008232	-2.24602
EIF2S2	0.488434	6.253519	3.119045	0.002086	0.00824	-2.36162

SLC25A32	0.549234	5.183542	3.118904	0.002087	0.008241	-2.26341
EIF4ENIF1	-0.36608	4.766145	-3.11877	0.002088	0.008242	-2.30725
ENOPH1	0.449768	5.595013	3.117773	0.002094	0.008266	-2.31953
DYM	-0.51755	5.13366	-3.11757	0.002096	0.00827	-2.35264
RWDD1	-0.46765	4.561255	-3.11697	0.0021	0.008283	-2.30072
ZNF621	-0.55373	4.88921	-3.11636	0.002104	0.008297	-2.34007
C14orf49	-1.10393	1.011853	-3.11594	0.002107	0.008306	-1.80836
PIGC	0.516038	4.41867	3.114232	0.002119	0.00835	-2.16511
BAIAP3	-1.12945	1.423434	-3.11367	0.002122	0.008363	-1.87611
NFIB	-1.0364	6.556026	-3.11341	0.002124	0.008367	-2.38962
FBXW7	-0.55262	4.442711	-3.11287	0.002128	0.008375	-2.30793
UHRF1BP1L	-0.4883	4.844479	-3.11288	0.002128	0.008375	-2.34185
TMEM219	-0.56608	5.588942	-3.11277	0.002129	0.008375	-2.39188
RAD23B	0.463799	7.629933	3.112776	0.002129	0.008375	-2.39677
SPG7	-0.39752	5.714759	-3.11171	0.002136	0.008401	-2.39454
COX4I1	-0.57076	7.836881	-3.11108	0.00214	0.008416	-2.37421
GPI	0.640803	8.551787	3.110713	0.002143	0.008421	-2.38599
MAP4K4	0.634511	7.552369	3.11074	0.002143	0.008421	-2.40628
GPSM2	0.833696	5.715168	3.10979	0.002149	0.008444	-2.32116
HECTD1	-0.54464	7.696691	-3.10946	0.002151	0.008451	-2.38407
CXorf56	0.558926	2.623236	3.109241	0.002153	0.008454	-1.8489
ATPAF2	-0.46173	3.553313	-3.1089	0.002155	0.008461	-2.18232
CHI3L1	3.228061	4.510665	3.10841	0.002159	0.008472	-1.77813
ATP7A	-0.5657	4.90084	-3.10783	0.002163	0.008486	-2.36714
RNASE1	-1.41053	5.900312	-3.10758	0.002164	0.00849	-2.41136
PABPC1L	1.236271	4.047569	3.107233	0.002167	0.008497	-1.98303
GREB1	-1.23692	1.585698	-3.10625	0.002174	0.008522	-1.94149
THBS1	-1.32791	8.310178	-3.10586	0.002177	0.00853	-2.35219
C17orf42	0.523105	3.268824	3.105394	0.00218	0.008538	-1.96701
RAPGEF5	0.72216	5.508073	3.105417	0.00218	0.008538	-2.32254
SRPR	-0.61928	7.681604	-3.1044	0.002187	0.008563	-2.39739
C19orf47	0.466583	4.19594	3.103914	0.00219	0.008574	-2.16246
PCDHB4	-1.15849	1.035514	-3.10346	0.002193	0.008584	-1.85655
TNFSF9	2.416053	1.935313	3.102912	0.002197	0.008592	-1.61148
DNAJC7	0.452535	5.901554	3.102956	0.002197	0.008592	-2.38892
STAT2	0.591684	6.605958	3.102996	0.002197	0.008592	-2.42022
PREPL	-0.49629	6.161503	-3.10251	0.0022	0.008601	-2.43262
C5orf39	1.277899	1.034473	3.101656	0.002206	0.008622	-1.62386
WSB2	-0.47076	6.477457	-3.10062	0.002214	0.008649	-2.43734
PRPF3	0.443332	5.098494	3.100048	0.002218	0.008662	-2.32026
MAP6D1	1.00985	1.6695	3.098845	0.002226	0.008693	-1.71148
FBXW5	-0.51528	6.837241	-3.09854	0.002228	0.008699	-2.43747
PCF11	-0.48066	5.49629	-3.09842	0.002229	0.0087	-2.42786
NSDHL	0.711872	4.751345	3.098279	0.00223	0.008702	-2.2415
TSEN54	0.657991	4.498898	3.098182	0.002231	0.008702	-2.20212
RSBN1	-0.44745	4.86174	-3.09792	0.002233	0.008707	-2.38428
ICAM1	1.339945	6.033127	3.097241	0.002238	0.008724	-2.34194
GNPTG	-0.49114	4.88217	-3.09604	0.002246	0.008755	-2.39475
ACTR5	0.593335	3.644977	3.094864	0.002255	0.008786	-2.05665
GABRA3	3.343314	-0.1281	3.094559	0.002257	0.008788	-1.61094
LAS1L	0.507032	5.2788	3.094679	0.002256	0.008788	-2.35038
SPG21	0.381712	6.236624	3.094513	0.002257	0.008788	-2.4381
C4orf43	0.584028	3.840602	3.093749	0.002263	0.008807	-2.10035
TGIF1	0.652251	6.183805	3.093432	0.002265	0.008814	-2.42399
XRCC3	0.666259	4.697708	3.092621	0.002271	0.008834	-2.2545
PRR5-ARHGAP8	1.439079	1.11975	3.092239	0.002274	0.00884	-1.64459
SLC43A1	-1.43316	3.235306	-3.09229	0.002274	0.00884	-2.30806
RALGAPA1	-0.56186	4.962562	-3.09162	0.002279	0.008855	-2.41948

NCF2	1.849334	3.145345	3.091434	0.00228	0.008858	-1.81613
LOC653501	-0.84755	2.061263	-3.09127	0.002281	0.008858	-2.01146
ING5	-0.50192	4.372518	-3.09126	0.002281	0.008858	-2.35928
FAM73A	-0.57081	4.480025	-3.09073	0.002285	0.008871	-2.3787
HSPA14	0.533569	4.907179	3.090086	0.00229	0.008887	-2.31505
UCKL1	0.45159	5.243299	3.089634	0.002293	0.008895	-2.36666
PDCD6	0.652115	6.116519	3.089616	0.002293	0.008895	-2.43059
NMU	2.675719	2.051852	3.088706	0.0023	0.008919	-1.64089
FKBP1B	-0.9714	2.217858	-3.08732	0.00231	0.008956	-2.06779
CDH1	1.386597	8.71523	3.087068	0.002312	0.008961	-2.46678
DBI	0.719795	6.408291	3.085962	0.00232	0.00899	-2.45611
NCRNA00176	1.504275	0.310107	3.085482	0.002324	0.009002	-1.63732
YTHDC2	-0.44445	5.376951	-3.08495	0.002328	0.009014	-2.4599
ADAMTS12	3.058739	2.743846	3.084819	0.002329	0.009016	-1.67882
CCDC123	0.555893	4.158853	3.084548	0.002331	0.009021	-2.19596
PPP1R15B	-0.47892	6.967814	-3.08417	0.002334	0.009029	-2.47793
GBP1	1.400803	5.669245	3.083629	0.002338	0.009043	-2.32944
RAB26	-1.32621	0.193444	-3.08317	0.002341	0.009053	-1.819
ZNF595	-1.20961	2.190196	-3.08273	0.002344	0.009064	-2.12113
RAMP3	-0.92996	2.437642	-3.08243	0.002347	0.00907	-2.12122
SRD5A1	1.313445	4.664674	3.082237	0.002348	0.009073	-2.16503
NDUFAF1	-0.46315	3.026494	-3.08117	0.002356	0.009102	-2.1592
AMIGO3	-0.70021	2.577197	-3.08098	0.002358	0.009103	-2.11054
KIAA0467	-0.48984	6.253702	-3.08095	0.002358	0.009103	-2.49635
IGF2BP3	5.139622	4.052599	3.080862	0.002358	0.009103	-1.65778
GPR115	3.8214	2.192309	3.079741	0.002367	0.009128	-1.6582
ZNF274	-0.69904	3.98548	-3.07988	0.002366	0.009128	-2.36719
ITPKB	-0.86656	5.642415	-3.07975	0.002367	0.009128	-2.49681
YY1	0.352035	6.523634	3.07925	0.002371	0.00914	-2.49464
8-Mar	-0.63732	3.879379	-3.07914	0.002372	0.009141	-2.34878
EVI5L	-0.64251	4.535345	-3.07906	0.002372	0.009141	-2.425
C9orf93	-0.80112	1.094262	-3.07867	0.002375	0.00915	-1.89226
LIAS	-0.51834	2.548747	-3.07821	0.002379	0.00916	-2.08427
GJB4	3.476305	2.182166	3.078019	0.00238	0.009163	-1.66294
TNFRSF10A	1.050054	3.299334	3.077767	0.002382	0.009168	-1.97319
WDR11	-0.41235	5.765345	-3.07743	0.002385	0.009176	-2.49766
RELB	0.783218	4.838788	3.077215	0.002386	0.009179	-2.30599
MECOM	-1.65796	5.4684	-3.07668	0.00239	0.009193	-2.50377
DDX55	0.509481	4.475219	3.076552	0.002391	0.009193	-2.28728
MLLT6	-0.63737	7.581691	-3.07649	0.002392	0.009193	-2.48207
CEP78	0.631147	4.132369	3.076021	0.002395	0.009204	-2.20155
MEF2A	-0.58687	6.396799	-3.07516	0.002402	0.009227	-2.51183
RICH2	-1.25582	2.688404	-3.07471	0.002405	0.009238	-2.24638
CLYBL	-0.93923	1.51997	-3.07463	0.002406	0.009238	-1.98088
FAM108C1	1.031257	6.222254	3.074299	0.002409	0.009245	-2.45537
KIF26B	1.954308	4.094025	3.07372	0.002413	0.009257	-1.97951
RAD51L3	0.674292	2.970583	3.073726	0.002413	0.009257	-1.98715
RAD17	-0.34053	4.549167	-3.07346	0.002415	0.009262	-2.41528
SRRM2	-0.40233	10.13107	-3.07331	0.002416	0.009264	-2.41538
TUBG1	0.621235	5.756008	3.073049	0.002418	0.009269	-2.45187
HPDL	2.288058	2.542404	3.072653	0.002421	0.009279	-1.75601
METTL14	-0.38245	3.460521	-3.07176	0.002428	0.009303	-2.26053
CD99L2	-0.69625	4.963306	-3.07157	0.00243	0.009306	-2.4871
THTPA	-0.51525	3.018344	-3.07002	0.002442	0.009349	-2.19958
C16orf80	0.552281	4.470627	3.069349	0.002447	0.009362	-2.3008
AIF1L	-1.27824	3.693902	-3.06943	0.002446	0.009362	-2.42088
IL1R1	-0.98267	6.181244	-3.0694	0.002447	0.009362	-2.52628
GDE1	-0.47572	6.56942	-3.0691	0.002449	0.009367	-2.52885

UCK1	-0.45925	4.141482	-3.06895	0.00245	0.009369	-2.39276
PRRG4	1.411199	4.666206	3.068769	0.002451	0.009371	-2.18719
FCHO1	2.388295	2.605727	3.068628	0.002453	0.009373	-1.7649
KYNU	3.16881	3.416083	3.068122	0.002457	0.009375	-1.77607
IFI35	1.10437	4.258792	3.068171	0.002456	0.009375	-2.16171
MYBBP1A	0.621814	6.328395	3.068352	0.002455	0.009375	-2.5083
KLHL9	-0.75499	5.278938	-3.06817	0.002456	0.009375	-2.51714
APBB2	-0.69673	5.625618	-3.06817	0.002456	0.009375	-2.52753
CDK5RAP1	0.465997	4.394775	3.068027	0.002457	0.009376	-2.30501
C17orf96	1.422245	3.375656	3.066911	0.002466	0.009404	-1.96899
TUG1	0.39592	8.094876	3.066939	0.002466	0.009404	-2.52091
CXCL1	4.286729	4.931121	3.066424	0.00247	0.009415	-1.82894
PPP1R12A	-0.54097	6.549478	-3.06638	0.00247	0.009415	-2.53622
PLEKHG4	1.554725	4.715414	3.066133	0.002472	0.00942	-2.17579
PVRL4	2.422834	5.155634	3.065519	0.002477	0.009433	-2.10759
CNOT2	0.458836	6.076794	3.065539	0.002477	0.009433	-2.5105
P4HA3	1.648083	0.714247	3.06534	0.002478	0.009434	-1.69455
RAB3A	-0.7913	1.193372	-3.06533	0.002479	0.009434	-1.94331
CCDC103	1.104341	0.709256	3.063881	0.00249	0.009475	-1.71654
MET	1.009264	7.134048	3.063767	0.002491	0.009475	-2.53927
TMEM223	0.679616	3.642159	3.063201	0.002495	0.00949	-2.13162
ITFG2	-0.49903	4.014066	-3.06244	0.002501	0.00951	-2.3988
KCTD3	-0.56723	6.285236	-3.06236	0.002502	0.00951	-2.55037
ZNF488	2.204433	2.139	3.061868	0.002506	0.009522	-1.75716
UNC93B1	0.856332	5.866018	3.061792	0.002507	0.009522	-2.47424
PPM1M	-0.59111	3.272112	-3.06057	0.002516	0.009557	-2.29353
PITPNM3	-1.30393	4.674675	-3.05979	0.002523	0.009578	-2.53372
ZNF2	-0.39789	2.378591	-3.05927	0.002527	0.009589	-2.08817
SFPQ	0.331352	7.876436	3.059313	0.002527	0.009589	-2.54682
POMP	0.578238	6.48602	3.05898	0.002529	0.009595	-2.54531
AP3M2	0.784823	4.443606	3.058536	0.002533	0.009603	-2.2852
NVL	0.413754	4.480331	3.058455	0.002533	0.009603	-2.35763
KIAA0182	-0.6123	6.510224	-3.0585	0.002533	0.009603	-2.55876
TGFBI	1.565703	7.490007	3.05826	0.002535	0.009607	-2.54943
ORMDL2	0.645322	4.706857	3.057569	0.002541	0.009625	-2.36283
PDE12	-0.51236	5.244115	-3.05691	0.002546	0.009643	-2.53754
XPO6	0.407988	7.111229	3.056547	0.002549	0.009651	-2.56761
CNOT3	0.392734	6.20838	3.056425	0.00255	0.009652	-2.54786
VPS72	0.45512	5.336198	3.055512	0.002557	0.009678	-2.47624
CD83	1.276279	3.201955	3.055217	0.00256	0.009684	-1.99687
PALLD	-1.12242	7.900352	-3.05486	0.002563	0.009693	-2.52094
PKD2	-0.69538	4.837131	-3.05451	0.002565	0.009698	-2.5278
SMG6	-0.42024	5.807496	-3.05454	0.002565	0.009698	-2.5661
VAMP4	-0.47786	4.348399	-3.05432	0.002567	0.009701	-2.46213
ODF2L	1.123562	4.054826	3.052344	0.002583	0.00976	-2.16625
SLC8A1	-0.95489	3.005705	-3.05217	0.002585	0.009763	-2.32846
ZNF788	-1.14356	0.659598	-3.052	0.002586	0.009764	-1.95273
DSG2	1.004839	8.257773	3.051959	0.002586	0.009764	-2.57218
GPR137B	0.784418	4.434569	3.051873	0.002587	0.009764	-2.30406
ABHD11	1.156684	4.838311	3.051555	0.00259	0.009771	-2.3159
LPGAT1	0.62522	6.539569	3.05115	0.002593	0.009781	-2.56844
EPC1	-0.46815	4.152839	-3.05089	0.002595	0.009787	-2.44764
C1orf31	0.995135	4.055582	3.049634	0.002606	0.009821	-2.19614
ALKBH7	-0.5929	4.535894	-3.04965	0.002605	0.009821	-2.5063
GIT1	0.576061	6.775056	3.048691	0.002613	0.009847	-2.58485
RUFY2	-0.42509	3.853555	-3.04817	0.002618	0.00986	-2.40765
C5orf25	-0.84227	4.159288	-3.04811	0.002618	0.00986	-2.49375
ANO8	-0.70406	3.821489	-3.04709	0.002627	0.009889	-2.44156

LOC729375	0.998054	0.808614	3.046818	0.002629	0.009895	-1.78016
ARFIP2	-0.55284	5.477768	-3.04585	0.002637	0.009923	-2.58333
AMN1	-0.6943	2.693804	-3.04512	0.002643	0.009944	-2.23688
RCOR2	1.875093	1.750125	3.044743	0.002646	0.009949	-1.79701
TRIM65	0.544967	4.536597	3.044754	0.002646	0.009949	-2.38567
DLD	-0.53191	6.386048	-3.04469	0.002647	0.009949	-2.60137
FBLN2	-1.52366	5.330714	-3.0446	0.002648	0.009949	-2.59914
DOCK8	-1.01512	4.392147	-3.04426	0.00265	0.009957	-2.54312
TMEM8A	-0.65663	6.383035	-3.04209	0.002669	0.010023	-2.60762
ITIH4	-1.26981	0.590042	-3.04136	0.002675	0.010044	-1.98556
FAM125B	-0.87757	3.712581	-3.04076	0.00268	0.010061	-2.46545
ZC3H10	-0.43919	2.18745	-3.0406	0.002681	0.010063	-2.11557
NDN	-1.14213	3.420659	-3.04044	0.002683	0.010065	-2.45874
PVRL3	-1.39868	2.558961	-3.04009	0.002686	0.010074	-2.34306
POLD3	0.806837	4.926865	3.039774	0.002688	0.010081	-2.42609
MAL2	1.223943	8.288796	3.038765	0.002697	0.010111	-2.61333
EPHB2	2.839059	4.994109	3.038233	0.002702	0.010122	-2.09388
NBEAL1	-0.85692	4.17034	-3.0383	0.002701	0.010122	-2.52383
PLCB3	0.772861	6.481656	3.037702	0.002706	0.010137	-2.59821
NGDN	0.539371	4.140404	3.037409	0.002709	0.010143	-2.3319
FIBCD1	2.89712	0.330836	3.036601	0.002716	0.010166	-1.77301
DNASE1	-0.96221	2.527456	-3.03618	0.002719	0.010174	-2.27401
TCEAL4	-0.73671	5.157489	-3.03619	0.002719	0.010174	-2.60395
SCO2	0.928039	4.404691	3.036039	0.00272	0.010176	-2.31729
EIF4EBP1	1.02552	5.995803	3.035768	0.002723	0.010181	-2.54712
YIPF5	-0.41159	5.402887	-3.03573	0.002723	0.010181	-2.60329
KDM4B	-0.50265	5.619065	-3.03562	0.002724	0.010182	-2.61715
HKR1	-0.62202	4.380778	-3.0348	0.002731	0.010205	-2.53652
ZBTB2	0.40492	4.66817	3.034509	0.002734	0.010212	-2.45933
SNHG3-RCC1	0.890449	3.732666	3.03428	0.002736	0.010214	-2.19763
TTC17	-0.42292	5.948103	-3.03433	0.002735	0.010214	-2.62831
AS3MT	-1.32483	1.353462	-3.03406	0.002737	0.010218	-2.12658
RHBDD3	0.59622	3.810774	3.033662	0.002741	0.010229	-2.26493
TMEM204	-0.77459	2.874197	-3.0326	0.00275	0.010257	-2.32599
TMEM120B	-0.47521	4.537849	-3.03255	0.002751	0.010257	-2.54532
MAP1A	-1.07229	4.176648	-3.03253	0.002751	0.010257	-2.56203
TMEM150C	-0.91727	1.543253	-3.03123	0.002762	0.010297	-2.1063
MAP1B	-1.36668	5.996015	-3.03002	0.002773	0.010333	-2.63749
FOXN2	0.66883	5.395187	3.029766	0.002775	0.010339	-2.53576
NACC1	0.427201	7.085788	3.028928	0.002782	0.010364	-2.64777
GDAP2	-0.45569	4.809887	-3.02875	0.002784	0.010367	-2.58184
CTTNBP2	-1.5026	1.683047	-3.02857	0.002785	0.010368	-2.23232
RBBP8	0.853454	5.608787	3.028539	0.002786	0.010368	-2.54473
ZNF75D	-0.55203	3.899061	-3.02839	0.002787	0.01037	-2.48774
ATAD3A	0.731894	4.77122	3.027735	0.002793	0.010383	-2.44694
DDX3X	-0.43652	8.34757	-3.02776	0.002793	0.010383	-2.60494
MTA2	0.433181	6.760461	3.027829	0.002792	0.010383	-2.64793
HPCAL1	-0.70123	5.370217	-3.02658	0.002803	0.010419	-2.63982
SMS	0.702695	5.553695	3.026433	0.002804	0.010421	-2.55977
FADD	1.276213	5.465941	3.025785	0.00281	0.010436	-2.48385
TMEM131	-0.38756	7.072574	-3.02578	0.00281	0.010436	-2.64752
MPHOSPH9	0.70462	4.170439	3.025647	0.002811	0.010438	-2.34034
SLC2A6	1.40054	2.399207	3.025108	0.002816	0.010453	-1.96144
LRPAP1	-0.53952	6.051231	-3.02475	0.002819	0.010462	-2.65907
C10orf125	1.253102	1.989705	3.024603	0.002821	0.010463	-1.92998
TM9SF3	-0.62074	8.254312	-3.02456	0.002821	0.010463	-2.61171
ANK3	-0.75151	5.369558	-3.02336	0.002832	0.0105	-2.65097
S100A6	1.005622	8.75861	3.023182	0.002833	0.010503	-2.64456

NR2C2	-0.46891	6.03804	-3.02279	0.002837	0.010513	-2.66392
CADM1	-1.25797	3.037521	-3.02066	0.002856	0.010581	-2.47182
TXN	0.882212	7.636484	3.019925	0.002862	0.010603	-2.67275
SHMT2	0.680845	6.578161	3.019247	0.002869	0.010623	-2.66018
MGC21881	-0.70294	2.116964	-3.01862	0.002874	0.010639	-2.2078
HAUS5	0.707371	4.230566	3.018604	0.002874	0.010639	-2.37316
POLA1	0.658636	4.908836	3.018226	0.002878	0.010649	-2.50777
MUL1	-0.39694	4.963316	-3.01735	0.002886	0.010675	-2.62384
ZNF79	-0.38687	2.39782	-3.0169	0.00289	0.010688	-2.2112
HDAC2	0.476046	6.773535	3.016203	0.002896	0.010708	-2.6809
AMACR	-0.92049	4.539193	-3.01484	0.002909	0.010751	-2.63304
SLC25A38	-0.48798	4.50163	-3.01459	0.002911	0.010757	-2.59467
CYP2E1	-1.72132	0.820677	-3.0136	0.00292	0.010788	-2.15196
CISD2	0.446975	4.714724	3.013505	0.002921	0.010788	-2.52034
LOC220930	1.145613	2.132574	3.013272	0.002923	0.010791	-1.99012
IDUA	-0.78578	3.553301	-3.01334	0.002922	0.010791	-2.50846
FAM184A	-1.58267	0.245297	-3.013	0.002926	0.010797	-2.05938
FBXO25	-0.55613	4.129747	-3.01253	0.00293	0.01081	-2.56427
C1orf172	1.151332	4.382119	3.012145	0.002934	0.01082	-2.33306
RAB11FIP3	-0.60159	4.995887	-3.01191	0.002936	0.010826	-2.65665
COL4A1	0.988635	8.775216	3.010923	0.002945	0.010856	-2.6791
CNTN1	-2.28315	1.443078	-3.01067	0.002947	0.010862	-2.36674
APH1A	0.393726	6.823747	3.010495	0.002949	0.010865	-2.69946
TDRD5	3.138586	0.835447	3.009776	0.002955	0.010887	-1.84799
MRPL12	0.653928	5.496335	3.00947	0.002958	0.010894	-2.607
CRTC2	0.372884	5.515619	3.008455	0.002968	0.010926	-2.63735
BRWD1	-0.49638	6.13228	-3.00839	0.002968	0.010926	-2.70659
UBE3B	-0.37432	5.913482	-3.00766	0.002975	0.010948	-2.70339
GMNN	0.889323	4.125011	3.007579	0.002976	0.010948	-2.34754
ENTPD5	-0.65727	3.511464	-3.00695	0.002982	0.010967	-2.50038
SERPINI1	-0.92584	1.718214	-3.00666	0.002985	0.010974	-2.20243
ARL11	1.669069	0.791028	3.005638	0.002994	0.011006	-1.85892
CHKB	-0.54674	2.63884	-3.00544	0.002996	0.01101	-2.31553
GOLGA2	-0.4236	6.311257	-3.00516	0.002999	0.011017	-2.71636
RAD54L2	-0.47632	4.240555	-3.00469	0.003003	0.011028	-2.59228
LGTN	-0.46146	4.893427	-3.00467	0.003003	0.011028	-2.65907
CILP2	2.207209	0.840222	3.004443	0.003005	0.01103	-1.8624
PDCD2L	0.804755	2.971487	3.004496	0.003005	0.01103	-2.16691
RAP2B	0.829974	6.191345	3.004352	0.003006	0.01103	-2.67154
C2CD4B	-1.66837	1.645823	-3.00397	0.00301	0.011038	-2.31432
PCYOX1	-0.5142	6.851287	-3.00398	0.00301	0.011038	-2.71231
EID1	-0.54464	6.362924	-3.00318	0.003017	0.011062	-2.72124
MBNL2	-0.69872	6.833791	-3.00219	0.003027	0.011094	-2.71454
EMG1	0.676648	4.556419	3.001178	0.003036	0.011126	-2.49212
CSNK1E	0.464093	6.665259	3.000092	0.003047	0.011161	-2.72502
C1orf115	-1.02565	3.918312	-2.99955	0.003052	0.011177	-2.6226
IFI27L2	1.220148	3.755365	2.998886	0.003058	0.011198	-2.24937
MST4	0.722751	5.410498	2.998467	0.003062	0.01121	-2.62235
STK31	2.734694	1.310997	2.998214	0.003065	0.011216	-1.8807
ZNF154	-0.89224	1.345837	-2.99735	0.003073	0.011243	-2.17053
TBX19	0.761505	1.816595	2.997047	0.003076	0.011251	-2.03871
AK3L1	-1.06141	4.335851	-2.99693	0.003077	0.011252	-2.6772
HAUS1	0.820395	3.084068	2.996811	0.003078	0.011253	-2.20436
TLL1	-1.50913	0.707131	-2.99524	0.003093	0.011306	-2.15999
M6PR	0.425446	6.782282	2.995051	0.003095	0.01131	-2.74262
PA2G4P4	0.68782	3.554315	2.994614	0.0031	0.011322	-2.31196
RRP12	0.535359	5.800351	2.994278	0.003103	0.011331	-2.69038
ZNF585A	-0.91336	1.992063	-2.99317	0.003114	0.011368	-2.29001

HAP1	3.16299	0.9013	2.992436	0.003121	0.011391	-1.89537
C1orf113	1.638384	2.971307	2.99232	0.003122	0.011392	-2.09769
CLN5	-0.51139	4.509721	-2.99213	0.003124	0.011396	-2.66176
CPNE2	0.904388	5.099179	2.991987	0.003125	0.011398	-2.5787
HAT1	0.482581	5.647068	2.990569	0.003139	0.011446	-2.69168
EXD2	-0.42811	4.839035	-2.99049	0.00314	0.011446	-2.69221
EPHA2	1.088305	7.301131	2.990229	0.003142	0.011452	-2.75383
C10orf119	0.318181	6.608919	2.989847	0.003146	0.011463	-2.75621
ATP2A1	1.355957	0.169393	2.989469	0.00315	0.01147	-1.9009
ZMYM6	-0.37295	4.698813	-2.9895	0.00315	0.01147	-2.67667
UBE2J1	-0.53757	6.544735	-2.9893	0.003152	0.011473	-2.75897
RAB3GAP1	-0.4065	6.168633	-2.9889	0.003156	0.011485	-2.76212
SUCLA2	-0.46982	5.238781	-2.98812	0.003163	0.01151	-2.73332
LMBRD2	-0.63803	3.501148	-2.98774	0.003167	0.01152	-2.55047
ATP8B2	-1.04218	4.427154	-2.98745	0.00317	0.011528	-2.7119
C11orf52	-1.0977	1.745117	-2.98661	0.003178	0.011555	-2.28772
MYH3	-1.05466	0.453264	-2.9861	0.003183	0.01157	-2.10027
WDR53	0.842855	2.74615	2.985526	0.003189	0.011588	-2.18354
C2orf74	-1.26918	1.635602	-2.98357	0.003209	0.011656	-2.31001
RTN1	-1.21906	1.038459	-2.98309	0.003213	0.011666	-2.20758
PRDM8	-1.09139	1.459335	-2.9832	0.003212	0.011666	-2.25404
CHURC1	-0.48508	5.731185	-2.98306	0.003214	0.011666	-2.77134
ALOX12P2	3.22491	0.558799	2.982282	0.003222	0.011691	-1.92191
POLG2	0.555158	2.475046	2.981238	0.003232	0.011716	-2.19245
RCSD1	-0.95284	2.567956	-2.9812	0.003232	0.011716	-2.44336
MAP2K5	-0.42203	3.61437	-2.98146	0.00323	0.011716	-2.5559
RNPEPL1	-0.57221	7.173006	-2.98141	0.00323	0.011716	-2.76912
SLC29A1	-0.87859	5.778352	-2.98136	0.003231	0.011716	-2.78307
DIP2B	0.691504	7.116109	2.980494	0.00324	0.011738	-2.78517
CDX2	4.658245	0.873335	2.980375	0.003241	0.01174	-1.92821
ASCL2	3.31557	2.45082	2.980167	0.003243	0.011744	-1.93242
TRABD	0.532437	5.928128	2.980074	0.003244	0.011745	-2.74143
SEL1L	-0.57408	7.118787	-2.97999	0.003245	0.011745	-2.77432
ANKRD32	0.65018	2.980153	2.979855	0.003246	0.011747	-2.25857
C10orf2	0.719175	4.099848	2.979675	0.003248	0.011749	-2.45563
POGK	0.401256	6.530088	2.979633	0.003248	0.011749	-2.78136
LPHN1	-0.83731	6.00238	-2.9795	0.00325	0.01175	-2.78889
CHRAC1	0.560339	5.982335	2.977795	0.003267	0.01181	-2.7503
RWDD2A	-0.61572	1.694555	-2.97763	0.003269	0.011813	-2.23877
ZNF70	-0.53856	3.412356	-2.97704	0.003275	0.011832	-2.5493
CPAMD8	-1.11473	1.548938	-2.9768	0.003277	0.011838	-2.28699
HAUS2	0.456986	5.012037	2.976274	0.003282	0.011854	-2.66651
MIOS	-0.45413	5.011767	-2.97571	0.003288	0.011872	-2.75098
F2R	1.113448	5.75319	2.974876	0.003297	0.0119	-2.68665
PDAP1	0.605931	6.256486	2.974195	0.003304	0.011922	-2.7764
ZMIZ1	-0.52029	7.625072	-2.97408	0.003305	0.011923	-2.77941
YPEL2	-0.77793	4.472394	-2.97337	0.003312	0.011946	-2.73479
MTIF3	-0.53202	4.86743	-2.9733	0.003313	0.011946	-2.75138
ZBTB42	-0.62606	3.490929	-2.97259	0.00332	0.011966	-2.58967
UBR4	-0.42067	8.324159	-2.9726	0.00332	0.011966	-2.7641
MGRN1	-0.46611	6.695362	-2.97203	0.003326	0.011984	-2.80693
SORT1	-0.97591	6.94588	-2.97183	0.003328	0.011985	-2.79305
SLC35E2	-0.67485	6.376279	-2.97188	0.003328	0.011985	-2.80901
NFE2L2	-0.80013	7.97026	-2.97022	0.003345	0.012042	-2.77137
TM9SF4	0.365656	7.220751	2.9699	0.003348	0.012051	-2.81589
SIRT1	-0.48646	4.782442	-2.96964	0.003351	0.012058	-2.75107
TOLLIP	-0.59481	6.242463	-2.96915	0.003356	0.012073	-2.81869
CERK	-0.6452	5.75548	-2.96842	0.003364	0.012098	-2.81694

KCNK15	-1.59407	0.595913	-2.96791	0.003369	0.012114	-2.23009
IGSF10	-1.65578	1.496208	-2.96705	0.003378	0.012143	-2.39112
HIPK1	-0.57849	6.852985	-2.96661	0.003383	0.012157	-2.81774
ATP6V1B1	2.234382	0.28315	2.96604	0.003389	0.012172	-1.9647
TNFAIP2	1.14471	6.846054	2.966088	0.003388	0.012172	-2.80412
CD68	0.837122	6.931035	2.96547	0.003395	0.01219	-2.82114
RCN3	1.557469	4.228942	2.965334	0.003396	0.012192	-2.36968
RNF7	0.590522	5.945364	2.96494	0.0034	0.012204	-2.7818
SERPINB9	1.264882	4.881178	2.96444	0.003406	0.012214	-2.5551
CNIH4	0.487254	5.293623	2.964466	0.003405	0.012214	-2.73013
ZNF83	-1.10021	4.11485	-2.96443	0.003406	0.012214	-2.75227
SALL2	-1.23718	1.821377	-2.96406	0.00341	0.012225	-2.39668
SLC39A1	0.454883	6.767437	2.963868	0.003412	0.012229	-2.83065
RASA4P	-0.80096	2.263064	-2.96378	0.003413	0.01223	-2.40513
GRAMD1C	-0.93673	2.975957	-2.96337	0.003417	0.012242	-2.56661
TPM4	0.531579	9.346746	2.963085	0.00342	0.01225	-2.78518
EGFL6	1.724703	3.077359	2.962708	0.003424	0.012261	-2.18332
PCM1	-0.58578	6.824798	-2.96224	0.003429	0.012276	-2.83052
PPP1R3E	-0.6521	1.779286	-2.95997	0.003453	0.012359	-2.30713
WDR54	1.152688	2.865028	2.959861	0.003455	0.01236	-2.23202
SLC10A3	0.623562	5.566762	2.959672	0.003457	0.012364	-2.75952
LOC728554	1.616865	3.932743	2.959488	0.003459	0.012368	-2.32994
ZNF197	-0.48155	3.924596	-2.95934	0.00346	0.012371	-2.6799
FAM38A	0.600593	7.821735	2.959047	0.003463	0.012379	-2.84057
LCN2	2.826565	7.787361	2.958455	0.00347	0.012398	-2.77612
BRI3BP	0.891754	3.958572	2.957672	0.003478	0.012425	-2.45493
C16orf55	0.716782	3.091996	2.957405	0.003481	0.012433	-2.32845
STX7	-0.53382	5.829597	-2.95715	0.003484	0.012439	-2.8485
SLC44A1	0.576597	7.768861	2.956234	0.003494	0.012471	-2.8491
DCK	0.509536	5.262363	2.956069	0.003495	0.012474	-2.74764
CTSO	-0.85689	4.600248	-2.95592	0.003497	0.012474	-2.8021
AK2	0.415762	6.246287	2.955943	0.003497	0.012474	-2.83695
DNAH1	-1.03577	3.307438	-2.95567	0.0035	0.012481	-2.66644
ZNF333	-0.51856	2.94871	-2.95504	0.003507	0.012502	-2.51568
KIAA0564	-0.56068	5.443437	-2.9541	0.003517	0.012535	-2.84503
TBC1D10B	0.390338	6.236478	2.953957	0.003518	0.012537	-2.84322
PTPRS	-1.61705	5.925899	-2.9536	0.003522	0.012548	-2.85255
TAZ	0.529633	4.414384	2.953092	0.003528	0.012565	-2.62605
MPND	-0.49077	3.437016	-2.95227	0.003537	0.012594	-2.61444
ANKRD13C	-0.35326	4.855285	-2.95195	0.00354	0.012603	-2.79689
TBX15	1.786378	0.132904	2.951574	0.003545	0.012611	-2.00298
JUN	-0.84802	7.750637	-2.95159	0.003544	0.012611	-2.82993
IFT52	0.545243	4.329353	2.951363	0.003547	0.012616	-2.61276
RERG	-1.07923	1.483513	-2.95011	0.003561	0.012659	-2.3499
APBA1	-0.80158	2.94171	-2.95015	0.00356	0.012659	-2.5779
CSTF1	0.412783	5.141016	2.950023	0.003562	0.012659	-2.76066
COMMD5	0.488304	4.150995	2.949938	0.003563	0.012659	-2.59074
THRA	-0.90577	5.17209	-2.94926	0.00357	0.012683	-2.85917
COL24A1	2.069218	0.50389	2.94856	0.003578	0.012706	-2.0123
ZNF767	-0.52661	3.180313	-2.9485	0.003578	0.012706	-2.58155
UBA5	-0.45742	5.153834	-2.94664	0.003599	0.012776	-2.84432
HDAC11	-0.59551	3.818628	-2.94428	0.003626	0.012867	-2.72128
GEMIN6	0.602047	2.638982	2.944123	0.003627	0.012869	-2.31286
AKT1	-0.42721	7.423966	-2.94391	0.00363	0.012875	-2.87242
KIAA2026	-0.48252	5.591059	-2.94323	0.003637	0.012898	-2.87919
RPL26L1	0.681407	2.636791	2.943044	0.003639	0.012902	-2.3062
BMP8A	1.368349	3.773667	2.942977	0.00364	0.012902	-2.38397
PODNL1	1.994138	2.364862	2.942728	0.003643	0.012908	-2.12724

TMED7	-0.42102	6.944657	-2.94265	0.003644	0.012908	-2.88637
FAM160B2	-0.44683	5.955794	-2.94227	0.003648	0.01292	-2.89162
CNPY3	0.736671	5.731619	2.94154	0.003656	0.012946	-2.81613
PANK4	-0.36673	4.140841	-2.94128	0.003659	0.012954	-2.74779
H3F3B	0.501403	8.802767	2.941163	0.003661	0.012955	-2.86418
NDST1	-0.78129	7.120912	-2.94066	0.003666	0.012972	-2.88118
AGAP5	-0.6289	1.673851	-2.94051	0.003668	0.012974	-2.34035
SEMA5B	1.790723	0.875854	2.939593	0.003678	0.013007	-2.03737
RNMTL1	-0.48287	3.343469	-2.93953	0.003679	0.013007	-2.6317
MMP9	3.261006	4.480123	2.939447	0.00368	0.013007	-2.24617
MRPL54	-0.50956	3.708388	-2.93876	0.003688	0.013031	-2.70621
MRPL11	0.585018	4.762431	2.93865	0.003689	0.013031	-2.7209
NUCB1	-0.43649	7.99682	-2.9386	0.00369	0.013031	-2.87051
TUBB3	1.567206	5.024029	2.937417	0.003703	0.013076	-2.60653
AGPS	0.518688	6.18684	2.936646	0.003712	0.013104	-2.88308
FAM76A	-0.41575	3.192353	-2.9365	0.003714	0.013106	-2.59735
PPAPDC2	-0.52489	3.611248	-2.93643	0.003714	0.013106	-2.69723
HSPA5	0.439497	9.57895	2.935987	0.00372	0.01312	-2.85107
ZNF461	-0.88588	1.47683	-2.93546	0.003726	0.013138	-2.3614
GIMAP1	-0.93014	0.709314	-2.93465	0.003735	0.013168	-2.26089
TSPAN14	0.580254	7.360805	2.93449	0.003737	0.013171	-2.91612
FAM86C	1.033559	3.423229	2.934354	0.003738	0.013173	-2.40219
MGP	-1.38278	5.199911	-2.93347	0.003749	0.013206	-2.91494
HEXIM2	-0.6074	1.256273	-2.93326	0.003751	0.013208	-2.29907
BMS1P5	-0.78399	2.378105	-2.93331	0.00375	0.013208	-2.50474
ZNF217	0.512356	6.487822	2.932809	0.003756	0.013223	-2.90866
DUS4L	0.657098	3.088652	2.932668	0.003758	0.013225	-2.40606
IGSF6	1.381927	1.539203	2.932046	0.003765	0.013247	-2.12844
EEF1A1	-0.50466	11.26637	-2.93143	0.003772	0.013269	-2.77931
DUT	0.576067	5.336254	2.931366	0.003773	0.013269	-2.81862
WDFY2	-0.74259	4.224177	-2.9312	0.003775	0.013272	-2.82489
COL28A1	-1.4858	0.962115	-2.93088	0.003779	0.013281	-2.37242
PDCD5	0.636423	4.828252	2.930826	0.003779	0.013281	-2.74625
MOBKL1A	-0.51668	5.257301	-2.92932	0.003797	0.013339	-2.90333
ANKRD39	0.697703	2.815531	2.929066	0.0038	0.013346	-2.36817
ZMYM1	0.504283	4.561565	2.928024	0.003812	0.013386	-2.72819
AP2M1	0.605305	8.209878	2.92724	0.003821	0.013413	-2.92218
MAN1A2	-0.50974	5.921354	-2.92714	0.003822	0.013413	-2.93443
HN1L	0.546617	6.894414	2.927122	0.003823	0.013413	-2.93479
CA2	-2.02578	5.401348	-2.92701	0.003824	0.013414	-2.92792
CSF1R	-1.09304	4.44714	-2.92648	0.00383	0.013433	-2.88859
CLSTN1	0.698182	8.661156	2.926181	0.003834	0.013442	-2.91569
PTCD1	0.639556	4.768143	2.925823	0.003838	0.013453	-2.74979
ELF4	0.509901	6.589986	2.925668	0.00384	0.013456	-2.93237
PCGF1	0.550756	3.428351	2.924505	0.003854	0.013501	-2.50678
HPRT1	0.618899	5.468895	2.924379	0.003855	0.013503	-2.84818
COL11A1	5.30829	2.050643	2.92417	0.003858	0.013505	-2.08188
FAM175A	-0.48557	2.920215	-2.92417	0.003858	0.013505	-2.59069
NEIL1	-0.87102	1.556679	-2.92356	0.003865	0.013523	-2.40186
ZNF107	0.890805	4.110426	2.923486	0.003866	0.013523	-2.58312
RNF43	1.494309	5.462004	2.923515	0.003865	0.013523	-2.73457
ZNF516	-0.74761	4.472081	-2.92229	0.00388	0.013569	-2.8765
DHCR7	1.149566	6.70974	2.922013	0.003883	0.013578	-2.91978
GJA3	3.107439	1.452735	2.921415	0.00389	0.013599	-2.08757
ERBB2IP	-0.45064	6.805261	-2.92119	0.003893	0.013605	-2.94859
SERINC2	1.103257	7.971995	2.920749	0.003898	0.01362	-2.95263
CRIPT	0.402832	4.260958	2.920507	0.003901	0.013627	-2.71112
PLXNA3	0.727744	6.226353	2.920235	0.003905	0.013635	-2.91891

NKIRAS2	0.447471	5.90564	2.919846	0.003909	0.013648	-2.91574
COX4NB	0.467846	4.686359	2.919234	0.003917	0.01367	-2.7801
CASZ1	-0.83102	6.346256	-2.91792	0.003932	0.013722	-2.95926
MSH6	0.639529	6.125144	2.916635	0.003948	0.013773	-2.92782
SLC27A1	-0.70264	4.698121	-2.91602	0.003955	0.013796	-2.91073
STUB1	-0.49143	5.678579	-2.91588	0.003957	0.013798	-2.95922
PRIC285	0.806896	7.514226	2.915374	0.003963	0.013816	-2.96972
RPS6KA4	0.668469	6.252956	2.914859	0.00397	0.013835	-2.9396
CD58	0.673304	4.140207	2.914759	0.003971	0.013835	-2.65403
POLR3G	1.314264	2.483648	2.914129	0.003978	0.013855	-2.28771
TYSND1	0.793686	4.535228	2.914158	0.003978	0.013855	-2.71241
VASN	-0.85269	4.210386	-2.9127	0.003996	0.013912	-2.88637
CGGBP1	-0.41477	6.426495	-2.91263	0.003997	0.013912	-2.97777
NPTX2	-1.30422	0.782979	-2.9119	0.004006	0.013939	-2.37871
SOX4	0.844174	7.225744	2.911848	0.004006	0.013939	-2.97793
NFKB2	0.698395	6.2593	2.911728	0.004008	0.01394	-2.94675
KANK1	-0.87318	5.6858	-2.91112	0.004015	0.013963	-2.97984
CD86	1.573529	2.594952	2.910543	0.004022	0.013984	-2.28408
INHBA	3.037773	4.333653	2.909975	0.004029	0.014005	-2.33654
XRN2	0.426868	6.923013	2.909831	0.004031	0.014008	-2.98501
HOXD11	3.711636	-0.28239	2.909288	0.004038	0.014027	-2.11443
UBE4A	-0.50034	6.606172	-2.90897	0.004042	0.014038	-2.98514
C3orf34	1.457935	1.249015	2.908642	0.004046	0.014048	-2.15827
GTF2E1	0.547123	3.944088	2.908502	0.004048	0.014051	-2.65492
CHMP4B	0.434712	6.611289	2.908092	0.004053	0.014065	-2.98421
TPRKB	0.552535	4.09859	2.907013	0.004066	0.014105	-2.68864
MPP5	-0.47439	5.471779	-2.90703	0.004066	0.014105	-2.97493
ITGAX	1.739169	3.490096	2.906442	0.004073	0.014126	-2.39341
PSMD13	0.432741	6.445467	2.906245	0.004076	0.014131	-2.98416
TP73	3.021122	2.693425	2.90575	0.004082	0.014149	-2.16795
CXCL6	3.409526	2.364297	2.904783	0.004094	0.014179	-2.13374
EAF2	0.894312	1.277628	2.90473	0.004095	0.014179	-2.21857
OSBP	-0.39476	6.635965	-2.90491	0.004093	0.014179	-2.99744
SIPA1L1	-0.46374	6.534963	-2.90483	0.004093	0.014179	-2.99808
C9orf102	-0.53719	3.365157	-2.9045	0.004098	0.014185	-2.7421
ANKRA2	-0.39774	3.422531	-2.90439	0.004099	0.014187	-2.72974
BBC3	1.089863	2.901545	2.904227	0.004101	0.014187	-2.3973
SLC35E1	-0.37007	6.722194	-2.90422	0.004101	0.014187	-2.99846
CDH13	1.721916	4.872608	2.903694	0.004108	0.014206	-2.6385
SRM	0.611015	6.87197	2.90339	0.004112	0.014216	-2.99943
ATXN7L3	0.378268	6.32027	2.903191	0.004114	0.014221	-2.98972
C17orf100	0.809427	1.556803	2.902133	0.004127	0.014264	-2.26406
CADM4	-0.90922	3.227161	-2.902	0.004129	0.014266	-2.78832
PABPC1	0.588525	11.07191	2.901886	0.004131	0.014268	-2.9017
MTMR7	-0.77036	1.186236	-2.90156	0.004135	0.014278	-2.39656
FLJ35220	-0.64667	2.291131	-2.90143	0.004136	0.014281	-2.55618
EFNA3	1.956599	3.842488	2.901191	0.004139	0.014287	-2.42941
NPTXR	-1.39242	3.191796	-2.90104	0.004141	0.014291	-2.84976
FABP6	2.36858	-0.43813	2.900564	0.004147	0.014308	-2.1366
IER5	1.038194	5.56206	2.898937	0.004168	0.014375	-2.88703
TCEB1	0.508677	4.563387	2.898668	0.004171	0.014384	-2.81047
PACS2	-0.44627	6.103973	-2.89852	0.004173	0.014387	-3.01621
UCA1	3.702512	1.1542	2.897886	0.004181	0.014411	-2.14915
KPNB1	0.332948	8.168985	2.897429	0.004187	0.014428	-3.00053
IRF3	0.495188	5.282395	2.897288	0.004189	0.01443	-2.91523
XRCC6	0.403641	8.003454	2.89707	0.004192	0.014436	-3.00698
COL5A3	1.693199	5.004396	2.896564	0.004198	0.014455	-2.69118
KIAA1841	0.685906	3.739076	2.896304	0.004202	0.014463	-2.62062

HECTD2	-0.68608	3.456958	-2.89608	0.004205	0.01447	-2.81046
ZNF596	-0.78291	1.097255	-2.89577	0.004208	0.014473	-2.40133
TAF6L	0.515296	3.810527	2.89575	0.004209	0.014473	-2.66756
MRPL18	0.547076	5.23079	2.895805	0.004208	0.014473	-2.90863
XDH	3.613223	3.817382	2.894661	0.004223	0.014518	-2.25042
SETD2	-0.42402	6.768991	-2.89346	0.004238	0.014568	-3.02681
CYTSA	-0.51577	6.140207	-2.89336	0.00424	0.014568	-3.03128
TTC23	-0.53685	3.896151	-2.89319	0.004242	0.014572	-2.86788
LEF1	1.55518	3.86795	2.892253	0.004254	0.014611	-2.51276
OTUD6B	0.566226	4.144655	2.891961	0.004258	0.014616	-2.73725
LOC339290	-0.85404	4.36372	-2.892	0.004257	0.014616	-2.95931
RNF115	-0.38726	4.710924	-2.89166	0.004262	0.014626	-2.95452
BCKDK	-0.45881	5.669387	-2.89041	0.004278	0.014679	-3.02873
ZNF502	-1.18886	1.250162	-2.88998	0.004284	0.014689	-2.49128
TBC1D12	-0.5332	3.968544	-2.88986	0.004285	0.014689	-2.88595
CASP3	0.48755	5.510047	2.889935	0.004284	0.014689	-2.96079
ZEB1	-0.96379	4.920839	-2.88999	0.004283	0.014689	-3.01535
PDP1	0.745395	5.80704	2.889109	0.004295	0.014719	-2.96911
PMFBP1	1.573749	0.010666	2.88885	0.004298	0.014727	-2.16892
FANCE	0.978729	3.739974	2.88871	0.0043	0.01473	-2.59319
CCDC56	-0.61691	5.499408	-2.88856	0.004302	0.014733	-3.03245
SMC3	0.426663	6.604309	2.887633	0.004314	0.014771	-3.04098
FLVCR1	0.957326	4.02503	2.88668	0.004327	0.01481	-2.65454
POLB	0.805369	3.768388	2.886222	0.004333	0.014824	-2.63369
COL3A1	1.372174	10.29109	2.886274	0.004332	0.014824	-2.99328
ELF2	-0.35674	5.200273	-2.885	0.004349	0.014875	-3.01354
DUSP1	-1.26782	7.492394	-2.88477	0.004352	0.014882	-3.01248
MFSD6	-0.69048	6.59298	-2.88427	0.004359	0.014901	-3.05102
HYI	-0.59698	2.89572	-2.88368	0.004367	0.014924	-2.7154
FAM161A	-0.7472	2.289608	-2.88324	0.004372	0.014934	-2.62143
ACBD6	0.500563	4.467307	2.883362	0.004371	0.014934	-2.83666
HNRNPA1	0.398895	7.775501	2.883228	0.004373	0.014934	-3.05003
DDTL	-0.64544	3.691951	-2.88077	0.004405	0.015042	-2.88349
C7orf46	-1.29332	2.727075	-2.87927	0.004426	0.015107	-2.8123
UBA2	0.459068	6.852066	2.879148	0.004427	0.015109	-3.06883
THAP2	-0.62852	2.28864	-2.87764	0.004448	0.015172	-2.61897
LRCH1	-0.58377	5.100311	-2.87763	0.004448	0.015172	-3.04041
ZNF506	-0.88083	4.024996	-2.87732	0.004452	0.015182	-2.96574
SLTM	-0.40745	6.920833	-2.87718	0.004454	0.015185	-3.0694
PSMG1	0.752788	4.585412	2.876573	0.004462	0.015209	-2.83385
UBE2Q1	0.332521	6.198715	2.876356	0.004465	0.015216	-3.06039
AGPAT1	-0.47207	6.153023	-2.87563	0.004475	0.015246	-3.08021
ATP2A2	0.409901	9.27187	2.875472	0.004477	0.015249	-3.02867
CAPS	-0.99803	2.647613	-2.87526	0.00448	0.015252	-2.75672
PPP1R8	0.340274	5.094376	2.875306	0.004479	0.015252	-2.97095
NPM1	0.539913	7.445373	2.874515	0.00449	0.015282	-3.08173
ARID5A	-0.68313	4.696567	-2.87411	0.004496	0.015298	-3.02615
RAB17	-1.71057	1.824404	-2.87368	0.004501	0.015314	-2.71381
RECQL	0.609807	5.481387	2.873421	0.004505	0.015322	-2.99217
CDK2AP1	0.599212	6.942238	2.873134	0.004509	0.015332	-3.08464
PRRX2	1.867768	2.757676	2.872822	0.004513	0.015341	-2.37165
CHL1	-1.59507	2.453862	-2.87278	0.004514	0.015341	-2.83146
FAM193A	-0.40682	5.238425	-2.87253	0.004517	0.015348	-3.05359
CASP1	1.500099	4.228255	2.871499	0.004531	0.015393	-2.64077
ANKRD13B	1.171022	3.814939	2.870858	0.00454	0.015419	-2.62392
KREMEN2	2.987909	1.424708	2.870375	0.004547	0.015431	-2.22199
ZNF606	-1.10243	2.582128	-2.87037	0.004547	0.015431	-2.78082
TRIP6	0.946766	5.616319	2.870496	0.004545	0.015431	-2.98116

GSTA4	-1.13742	4.419237	-2.87015	0.00455	0.015436	-3.04539
DYRK1A	-0.39368	6.211362	-2.87009	0.004551	0.015436	-3.09545
C19orf22	0.531233	6.368902	2.869924	0.004553	0.01544	-3.07755
AQP1	-1.29923	6.121402	-2.86969	0.004556	0.015448	-3.08889
NUDT3	0.443313	4.548078	2.868394	0.004574	0.015505	-2.90156
FASTKD3	0.520556	3.292778	2.868064	0.004579	0.015513	-2.64085
GATAD2A	0.408607	6.82469	2.868101	0.004578	0.015513	-3.09961
AP1M2	1.402649	5.829182	2.866976	0.004594	0.015555	-2.96166
CAPRIN1	0.408021	8.370346	2.867064	0.004593	0.015555	-3.08066
TES	-0.58312	6.337672	-2.86692	0.004595	0.015555	-3.1037
COL12A1	1.174974	7.894601	2.866776	0.004597	0.015558	-3.10356
FBXW2	-0.39284	6.440949	-2.86653	0.0046	0.015566	-3.10543
MMP19	-0.68869	3.910788	-2.86624	0.004604	0.015576	-2.96164
SMARCA1	-1.01575	4.765692	-2.8654	0.004616	0.015612	-3.07645
DOLPP1	0.652526	4.313263	2.864708	0.004626	0.015641	-2.82836
OGFR	0.473531	6.12265	2.864355	0.004631	0.015654	-3.08233
TMEM199	0.402133	4.101655	2.863874	0.004637	0.015673	-2.83477
BIK	1.705753	2.953728	2.863357	0.004645	0.015694	-2.43575
MLLT1	-0.37571	6.705638	-2.86295	0.00465	0.015709	-3.11262
ISL2	1.254203	0.657287	2.862078	0.004663	0.015747	-2.24689
HAGHL	1.518201	1.324448	2.861284	0.004674	0.015781	-2.28491
NXT2	0.755382	4.537106	2.861201	0.004675	0.015781	-2.86425
ITPR3	0.690269	8.73131	2.860835	0.00468	0.015795	-3.09442
GPAM	-0.61629	4.184206	-2.8597	0.004696	0.015846	-3.00567
C8orf73	1.861302	4.666886	2.859507	0.004699	0.01585	-2.6912
SLCO2A1	-1.17542	5.147827	-2.85945	0.0047	0.01585	-3.11566
TMEM194B	0.709621	2.990782	2.858475	0.004714	0.015893	-2.58521
LOC651250	-0.55481	5.937529	-2.85836	0.004715	0.015894	-3.12591
ZBED4	0.460838	5.820151	2.858137	0.004719	0.015901	-3.07856
EIF2B4	-0.33521	4.921282	-2.85798	0.004721	0.015905	-3.06276
MEA1	0.639999	5.465759	2.857126	0.004733	0.015942	-3.032
UPP1	1.500022	4.676079	2.856566	0.004741	0.015964	-2.77069
MCM3AP	-0.39942	6.629693	-2.85652	0.004742	0.015964	-3.13103
RGS20	2.568077	-0.02566	2.856122	0.004747	0.015975	-2.25452
OSBPL9	-0.42421	6.174369	-2.85619	0.004746	0.015975	-3.13348
COQ2	0.473418	3.438371	2.855845	0.004751	0.015985	-2.71036
AGMAT	2.618221	2.296698	2.855298	0.004759	0.016004	-2.29651
TTC9C	0.461874	3.330502	2.855284	0.004759	0.016004	-2.69186
COL4A6	-1.8096	3.420505	-2.85507	0.004762	0.016011	-3.04309
CD34	-0.89694	4.992976	-2.85491	0.004765	0.016012	-3.11254
TPI1	0.625451	8.266554	2.854879	0.004765	0.016012	-3.1216
MEX3A	1.937091	3.218058	2.853939	0.004779	0.016054	-2.47244
NKTR	-0.61921	6.360502	-2.85377	0.004781	0.016059	-3.13915
FCER1G	1.538655	4.5158	2.853521	0.004785	0.016067	-2.73805
MOGS	0.41504	6.049656	2.853047	0.004792	0.016086	-3.11195
ODF3B	1.026273	2.935518	2.852785	0.004795	0.016093	-2.55063
ZNF330	-0.35592	4.466694	-2.85274	0.004796	0.016093	-3.03191
RORA	-1.03337	4.510789	-2.85262	0.004798	0.016095	-3.09443
ATP5J2	0.716419	6.84979	2.852283	0.004803	0.016107	-3.13734
RGMA	-1.76639	4.25847	-2.85102	0.004821	0.016165	-3.12062
SDC2	-0.96681	4.961435	-2.85073	0.004825	0.016175	-3.12549
HTATIP2	0.807211	5.668678	2.850559	0.004828	0.01618	-3.05477
FAM117A	-0.78468	3.774201	-2.85035	0.004831	0.016185	-2.99869
CEP250	0.558118	5.889344	2.850297	0.004832	0.016185	-3.09852
GFM2	-0.38845	4.741509	-2.84977	0.004839	0.016207	-3.07207
TBC1D19	-0.62477	1.794744	-2.84901	0.00485	0.01624	-2.60943
TNFAIP3	1.102058	6.419679	2.848767	0.004854	0.016248	-3.10443
BAT1	0.327055	7.893905	2.848608	0.004856	0.016252	-3.14131

ECSIT	-0.50149	4.871852	-2.84796	0.004866	0.01628	-3.09849
DHX37	0.45599	5.188899	2.847764	0.004869	0.016286	-3.04519
COIL	0.414938	4.367327	2.847398	0.004874	0.0163	-2.92968
SLC1A1	-1.2907	2.902247	-2.84667	0.004885	0.016328	-2.93469
ZNF236	-0.42721	4.450255	-2.84668	0.004885	0.016328	-3.05346
BTN2A3	0.852902	0.441294	2.845461	0.004903	0.016383	-2.30151
NR1D1	0.884008	5.40371	2.84527	0.004905	0.016389	-3.03062
SIRT6	0.561116	3.902237	2.845123	0.004908	0.016392	-2.81537
VPS37B	-0.6809	6.245843	-2.84337	0.004934	0.016475	-3.16806
MED19	0.527812	3.835445	2.843242	0.004935	0.016478	-2.81301
PPAN	0.539214	4.727105	2.842295	0.00495	0.016521	-2.98837
VARS	0.511177	7.064932	2.84206	0.004953	0.016528	-3.17221
REG1A	-3.11688	1.429484	-2.84195	0.004955	0.016528	-2.93869
MRPS12	0.589719	4.721375	2.8419	0.004955	0.016528	-2.9795
ZSCAN2	-0.59275	2.550334	-2.84137	0.004963	0.016551	-2.75863
EPSTI1	1.818491	3.956605	2.840756	0.004973	0.016562	-2.62666
ANO10	-0.50338	4.535315	-2.84076	0.004972	0.016562	-3.08608
PHF14	0.557241	6.146128	2.840858	0.004971	0.016562	-3.14316
CLASP2	-0.43312	5.51654	-2.84097	0.004969	0.016562	-3.15732
FXYD3	1.394479	7.760365	2.84075	0.004973	0.016562	-3.1734
NBPF10	-0.62043	5.187225	-2.84045	0.004977	0.016573	-3.15
PSMA2	0.421307	6.725531	2.83985	0.004986	0.016599	-3.17505
LIPG	2.444327	3.425102	2.839547	0.004991	0.01661	-2.47585
SKAP2	0.618915	5.53735	2.839454	0.004992	0.016611	-3.09013
NGFR	-1.77141	2.632735	-2.83933	0.004994	0.016613	-2.98147
GNA14	-1.22375	0.903169	-2.83912	0.004997	0.01662	-2.58116
CXCL11	2.987388	1.885982	2.838491	0.005007	0.016647	-2.30606
FLJ23867	-0.92062	5.48235	-2.83719	0.005026	0.016709	-3.18026
ELN	-1.49218	4.51054	-2.83698	0.005029	0.016715	-3.16153
SNCG	-1.27982	1.352494	-2.83598	0.005045	0.016761	-2.66982
TTC15	-0.37541	5.154866	-2.83554	0.005051	0.01678	-3.14669
PDCD11	0.467765	6.254966	2.835285	0.005055	0.016789	-3.16958
MSH5	1.012955	4.225847	2.835164	0.005057	0.016791	-2.82317
HOXD10	3.46894	0.71409	2.834642	0.005065	0.016813	-2.31287
ERAL1	0.447758	5.911266	2.833916	0.005076	0.016846	-3.15265
RBM7	-0.39142	4.639105	-2.83365	0.00508	0.016855	-3.10617
NCOA4	-0.4044	8.085926	-2.83341	0.005084	0.016863	-3.15922
COX7A2	-0.52618	6.538139	-2.83163	0.005111	0.01695	-3.19851
OTUB1	0.475749	7.033899	2.830764	0.005124	0.01699	-3.20299
RETSAT	-0.70504	5.89574	-2.8303	0.005131	0.01701	-3.20336
SPG11	-0.41174	6.531278	-2.82973	0.00514	0.017035	-3.20508
NOXO1	2.000041	0.239722	2.829534	0.005143	0.017041	-2.32419
C1orf53	0.913932	0.436419	2.829405	0.005145	0.017043	-2.33893
C12orf47	0.444134	3.752845	2.828795	0.005155	0.01707	-2.85115
HAVCR2	1.561423	2.664541	2.828519	0.005159	0.01708	-2.51334
RFC2	0.70277	4.966718	2.828429	0.00516	0.017081	-3.03994
IRAK1BP1	-0.63222	0.978902	-2.82777	0.005171	0.017111	-2.54956
ACTR1B	-0.45872	5.541086	-2.82755	0.005174	0.017118	-3.19589
CCDC74A	1.457365	1.577763	2.826931	0.005183	0.017146	-2.40437
CYTIP	-0.96921	2.703388	-2.82659	0.005189	0.017155	-2.89618
SUSD2	-1.06221	3.143436	-2.82652	0.00519	0.017155	-2.99794
SOS1	-0.38348	6.511792	-2.82658	0.005189	0.017155	-3.21409
TAF5	0.486656	2.981357	2.826156	0.005196	0.017169	-2.70381
MKNK1	-0.37032	4.969135	-2.82572	0.005202	0.01718	-3.15767
EPB49	-0.93972	4.871778	-2.8258	0.005201	0.01718	-3.18664
PDIA3	0.475668	8.59178	2.825725	0.005202	0.01718	-3.18872
EFHD1	-1.07752	2.000829	-2.82548	0.005206	0.017188	-2.78005
FBXO31	-0.38986	5.076675	-2.8248	0.005217	0.017219	-3.17083

LPHN2	-1.06687	5.57263	-2.82409	0.005228	0.017251	-3.21922
ZNF211	-0.65121	3.117573	-2.82342	0.005238	0.017282	-2.93423
ARHGAP31	-0.78431	4.498456	-2.82316	0.005242	0.017291	-3.15485
TRIM28	0.551495	8.475749	2.822058	0.00526	0.017345	-3.204
TUBBP5	2.60504	0.844778	2.821967	0.005261	0.017345	-2.34581
MTDH	0.384771	8.134371	2.82094	0.005277	0.017395	-3.21214
FANCC	0.568518	4.058142	2.819758	0.005296	0.017449	-2.91335
C7orf30	0.483477	3.996619	2.81974	0.005296	0.017449	-2.91878
FOXO1	-0.73996	5.525725	-2.8192	0.005305	0.017473	-3.22658
C2orf29	0.456116	6.049683	2.816253	0.005352	0.01762	-3.20967
PLEKHA6	-2.03048	4.775327	-2.8163	0.005351	0.01762	-3.23534
BUD31	0.615603	5.819696	2.816026	0.005356	0.017627	-3.18102
C16orf57	0.539748	6.128301	2.815596	0.005362	0.017646	-3.21165
MRM1	0.714074	2.376063	2.815372	0.005366	0.017654	-2.60992
SLC15A2	1.45792	2.660481	2.813526	0.005396	0.017747	-2.56493
CCDC74B	1.544114	0.170758	2.813327	0.005399	0.017753	-2.36552
STK4	0.47447	6.268198	2.812187	0.005417	0.01781	-3.23248
WDR5	0.483839	5.788497	2.81187	0.005423	0.017822	-3.19998
GPR172B	2.382333	0.94908	2.811452	0.005429	0.017841	-2.3731
KCNJ15	-1.61902	2.901896	-2.81105	0.005436	0.017858	-3.07625
CNN3	-0.77706	6.073297	-2.81083	0.005439	0.017865	-3.25664
ALKBH6	0.612259	3.314111	2.810433	0.005446	0.017882	-2.78558
NIPSNAP3B	-0.84807	0.424607	-2.81031	0.005448	0.017885	-2.54947
FAM117B	0.701383	5.117765	2.809331	0.005464	0.017933	-3.11318
AIM1	1.048235	6.939418	2.808489	0.005478	0.017974	-3.24845
SPOCD1	1.748651	1.695477	2.807777	0.005489	0.018003	-2.43936
LMO4	-0.76513	5.938227	-2.80783	0.005488	0.018003	-3.26474
HIST1H4H	1.650163	2.189441	2.806724	0.005506	0.018052	-2.50275
RUNX3	1.781174	4.653726	2.806785	0.005505	0.018052	-2.84429
MBTPS1	-0.37239	6.986129	-2.80618	0.005515	0.018077	-3.26229
EDN1	-0.86482	3.471284	-2.80591	0.00552	0.018087	-3.08188
MAP4	-0.50945	8.08449	-2.8058	0.005522	0.018089	-3.23083
COL10A1	4.752827	2.926796	2.805643	0.005524	0.018093	-2.39309
WWP1	-0.46075	5.959373	-2.80557	0.005525	0.018093	-3.26835
HLA-H	0.9972	5.751398	2.80292	0.005569	0.018232	-3.17378
ACAN	2.383818	2.200734	2.801667	0.00559	0.018293	-2.44564
SPAG7	-0.4874	4.280034	-2.80165	0.00559	0.018293	-3.16178
PPFIA3	1.220355	3.835341	2.801371	0.005595	0.018303	-2.80506
RFX5	0.52014	5.674819	2.801102	0.0056	0.018314	-3.216
CCNJL	-1.17966	1.6309	-2.80031	0.005613	0.018353	-2.78838
EPB41L5	-0.5786	4.294518	-2.7999	0.00562	0.018371	-3.17662
LRRN1	-1.65499	0.787488	-2.79938	0.005628	0.018395	-2.72531
THAP6	-0.38312	3.893765	-2.79922	0.005631	0.0184	-3.10251
GTPBP3	0.573038	3.94982	2.799093	0.005633	0.018402	-2.94773
COG8	-0.37711	4.415438	-2.79871	0.00564	0.018419	-3.1741
ESR1	-1.24107	0.264826	-2.79751	0.00566	0.01848	-2.60717
CREB3	-0.51925	4.931193	-2.7965	0.005677	0.018532	-3.24407
APBB3	-0.53347	3.158151	-2.79588	0.005687	0.018561	-2.99436
KARS	0.418556	7.020443	2.795815	0.005688	0.018561	-3.29758
TIFA	-0.55684	3.490623	-2.79464	0.005708	0.018622	-3.07092
C16orf88	0.617378	4.516304	2.794443	0.005712	0.018628	-3.06505
ADAMTS9	-1.14876	4.224473	-2.7943	0.005714	0.018631	-3.23519
EXOSC4	0.717971	4.081406	2.794117	0.005717	0.018637	-2.96001
IL1A	3.784879	2.010141	2.793286	0.005731	0.018679	-2.4226
CASK	0.636071	6.713084	2.792084	0.005752	0.018742	-3.29856
OCRL	-0.47857	5.880563	-2.79197	0.005754	0.018744	-3.30365
ZNF836	-0.50774	2.33501	-2.79172	0.005758	0.018753	-2.83824
FAM58A	0.551245	3.658034	2.790758	0.005774	0.018798	-2.91491

SFRS12	-0.39886	6.277289	-2.79082	0.005773	0.018798	-3.3111
C19orf24	0.583441	4.830473	2.790647	0.005776	0.0188	-3.13644
B3GALT6	0.544895	4.626128	2.790125	0.005785	0.018823	-3.11004
SLC25A39	0.500583	7.284704	2.790075	0.005786	0.018823	-3.31246
SCRIB	0.561463	7.460042	2.789335	0.005799	0.01886	-3.3132
TMOD2	-0.80515	4.108154	-2.78799	0.005822	0.018931	-3.21036
C19orf28	0.563642	6.146612	2.787774	0.005826	0.018939	-3.28621
PPIP5K2	-0.42122	5.518509	-2.78711	0.005837	0.018971	-3.30232
CHST1	1.992499	2.638429	2.78697	0.00584	0.018975	-2.57056
WWC3	-0.78526	5.660329	-2.78686	0.005842	0.018977	-3.31783
LGALS9B	-1.85535	-0.31378	-2.78662	0.005846	0.018986	-2.62338
ATXN7L3B	-0.51017	6.986087	-2.78647	0.005848	0.01899	-3.31279
EGLN1	-0.41427	6.229862	-2.78631	0.005851	0.018994	-3.32301
SPRY1	-0.80924	4.697433	-2.78565	0.005863	0.019028	-3.2748
ATP5B	-0.43252	9.049338	-2.78551	0.005865	0.019031	-3.25534
HIST1H2BD	1.481035	3.377747	2.785126	0.005872	0.019048	-2.73308
SHISA5	0.412639	7.371121	2.784858	0.005876	0.019059	-3.32472
HIST3H2A	1.539789	1.872757	2.784739	0.005879	0.019061	-2.53609
TMC7	1.792963	3.311423	2.783965	0.005892	0.019101	-2.68498
ANKRD16	-0.51202	2.898141	-2.78284	0.005912	0.01916	-2.97332
RASD1	-1.14979	2.620409	-2.78251	0.005918	0.019174	-3.03197
FAM46B	-1.44402	3.220706	-2.78215	0.005924	0.01919	-3.18134
ZNF407	-0.41356	4.066561	-2.78169	0.005932	0.019212	-3.17975
PSD3	-0.85963	5.608977	-2.78131	0.005939	0.019229	-3.33275
IFIT3	1.59852	5.065608	2.781131	0.005942	0.019235	-3.0294
ANKRD37	-0.92344	1.927406	-2.78096	0.005945	0.01924	-2.86007
SNW1	0.355658	5.661207	2.779625	0.005968	0.019312	-3.28553
HIST2H2BF	-0.96033	1.872435	-2.77904	0.005979	0.019341	-2.8573
ADRBK1	0.472757	7.341514	2.778346	0.005991	0.019376	-3.34311
RYBP	-0.43083	5.586405	-2.77782	0.006	0.019402	-3.33068
G2E3	0.541767	5.027137	2.777193	0.006011	0.019433	-3.20618
CBX6	-0.99465	4.823833	-2.77613	0.00603	0.01949	-3.3206
APEX1	0.462559	6.909292	2.775929	0.006034	0.019497	-3.34963
C8orf47	-1.70062	-0.27277	-2.77454	0.006059	0.019573	-2.64517
IFRD2	-0.56055	5.475558	-2.77444	0.00606	0.019574	-3.33943
ZNF444	-0.59673	4.574375	-2.77408	0.006067	0.019591	-3.27744
PPP1R13B	-0.5115	5.762719	-2.77322	0.006083	0.019636	-3.35147
LDOC1	-1.28093	3.132735	-2.77294	0.006087	0.019648	-3.17699
BACE1	-0.58394	5.554735	-2.77261	0.006093	0.019662	-3.34849
DPP9	0.375107	6.828426	2.772225	0.0061	0.01968	-3.35959
ELK4	0.57041	4.429758	2.771484	0.006114	0.019719	-3.11864
NANP	0.493264	3.586135	2.771364	0.006116	0.019721	-2.96216
ULBP3	1.112867	2.035572	2.770303	0.006135	0.019778	-2.63617
CCDC28B	0.855582	1.538365	2.770132	0.006138	0.01978	-2.60493
TSEN34	0.439508	5.151614	2.770124	0.006138	0.01978	-3.25174
ABCA13	5.040732	3.017071	2.769835	0.006144	0.019792	-2.48449
C6orf120	-0.39891	5.499385	-2.76905	0.006158	0.019834	-3.34864
TMEM181	-0.5775	6.406891	-2.76892	0.00616	0.019836	-3.3681
PCDHGB5	-1.35678	0.905928	-2.76855	0.006167	0.019853	-2.78746
ZNF23	-0.43605	3.02324	-2.76794	0.006178	0.019885	-3.02554
GSDMD	0.690101	5.20849	2.767771	0.006181	0.01989	-3.23618
UNC119B	-0.56804	5.123468	-2.76704	0.006195	0.019929	-3.34067
IL21R	1.738399	0.961329	2.764142	0.006248	0.020095	-2.49384
FBXO10	-0.57712	3.060081	-2.76383	0.006253	0.020109	-3.07045
PCDHGA12	-0.97709	1.221098	-2.76293	0.00627	0.020157	-2.79847
C2orf63	-0.71465	1.253466	-2.76285	0.006271	0.020157	-2.76811
GAS5	0.645214	5.720135	2.762453	0.006279	0.020177	-3.31267
MUC16	3.912027	1.05201	2.761752	0.006292	0.020214	-2.50008

CDS2	-0.37726	4.969467	-2.76055	0.006314	0.020281	-3.33308
MAD2L2	0.759684	4.514694	2.760317	0.006319	0.020282	-3.13105
SUZ12	0.443563	6.007102	2.760399	0.006317	0.020282	-3.35717
HNRNPA1L2	0.381919	6.105486	2.760288	0.006319	0.020282	-3.36723
MACROD1	-0.75282	3.576553	-2.75995	0.006325	0.020293	-3.20599
TMX1	0.483672	6.212295	2.759963	0.006325	0.020293	-3.36877
C21orf33	-0.50551	4.88093	-2.75978	0.006329	0.020299	-3.33708
LOC100132288	-1.00372	3.155581	-2.75935	0.006336	0.02032	-3.1741
NOTCH4	-0.84423	4.400697	-2.75815	0.006359	0.020386	-3.32415
OGDH	-0.53459	7.750695	-2.7581	0.00636	0.020386	-3.36832
POMT1	-0.5323	4.23505	-2.75759	0.006369	0.020411	-3.2782
RCBTB2	-0.6596	3.52382	-2.75732	0.006375	0.020423	-3.19286
SARS	-0.45514	6.638835	-2.75646	0.006391	0.02047	-3.39976
NAA15	0.388153	6.339695	2.756261	0.006394	0.020477	-3.389
HEXIM1	-0.44125	5.309479	-2.75456	0.006427	0.020575	-3.37869
AMY2B	-1.03012	0.854639	-2.75356	0.006445	0.020631	-2.77486
SLC9A9	-1.18695	2.595974	-2.75234	0.006469	0.020701	-3.1173
CSNK1G2	0.376658	6.223706	2.751695	0.006481	0.020735	-3.39637
C10orf10	-1.11215	5.400184	-2.75158	0.006483	0.020738	-3.41131
CD9	0.980292	8.347121	2.751395	0.006487	0.020744	-3.405
SIRPG	1.636496	0.426626	2.750998	0.006494	0.020763	-2.52467
ABCC6	-1.52543	0.942072	-2.75051	0.006503	0.020788	-2.85449
MAN1B1	0.440437	5.983294	2.750159	0.00651	0.020805	-3.38278
POLR1C	0.743074	4.583678	2.749886	0.006515	0.020817	-3.1753
N4BP2L2	-0.52103	5.868096	-2.74981	0.006517	0.020817	-3.41636
POLR3K	0.682489	3.193275	2.749474	0.006523	0.020833	-2.91219
SUMO1P3	0.428912	4.021153	2.749035	0.006532	0.020855	-3.12168
PPFIA1	0.956686	7.108213	2.748279	0.006546	0.020896	-3.41758
CDKL3	-0.48272	0.042664	-2.74784	0.006555	0.020919	-2.6281
ZNF426	-0.85565	3.051552	-2.74773	0.006557	0.020921	-3.16122
IFNGR1	0.57008	6.861898	2.747585	0.00656	0.020925	-3.42244
BARD1	0.749169	3.070871	2.747035	0.00657	0.020954	-2.88949
EZR	-0.7305	8.750494	-2.74666	0.006577	0.020972	-3.35956
PPHLN1	0.313731	5.345192	2.746303	0.006584	0.020989	-3.34813
KIAA1530	-0.55869	3.575848	-2.74585	0.006593	0.021008	-3.21406
CBX1	0.718964	5.552804	2.745916	0.006592	0.021008	-3.33272
ZNF131	0.545143	5.124543	2.745414	0.006601	0.021029	-3.30266
FA2H	-1.76654	3.75468	-2.74465	0.006616	0.021072	-3.36515
PIGL	-0.5394	2.400743	-2.74397	0.006629	0.021104	-2.97873
FAM105B	0.55443	5.341665	2.743979	0.006629	0.021104	-3.33076
COL27A1	1.291927	4.87555	2.74343	0.00664	0.021133	-3.14652
CHCHD10	-0.6976	5.824682	-2.74259	0.006656	0.02118	-3.43719
CEP170	0.70701	5.491898	2.742426	0.00666	0.021186	-3.336
ARFGAP3	-0.45349	5.776175	-2.74209	0.006666	0.021201	-3.433
USP8	-0.34001	6.101188	-2.74203	0.006667	0.021201	-3.43848
CELF6	-0.88809	0.612524	-2.74143	0.006679	0.021233	-2.75639
NCBP1	0.404854	5.922346	2.741055	0.006686	0.021252	-3.40489
TMEM39B	0.524743	4.11614	2.740942	0.006689	0.021254	-3.14512
BAHD1	-0.40851	5.43744	-2.74084	0.00669	0.021255	-3.42057
SNAPC1	1.070249	3.734805	2.740333	0.006701	0.021282	-2.97262
CREG2	2.126614	-0.10737	2.740118	0.006705	0.021291	-2.55005
NOP10	0.585235	5.532345	2.73985	0.00671	0.021302	-3.35899
PAPOLG	0.374846	4.68787	2.738908	0.006729	0.021356	-3.28154
CCL22	1.729509	2.072713	2.737982	0.006747	0.021394	-2.65938
EMILIN3	-1.22644	0.380482	-2.73787	0.006749	0.021394	-2.77415
MACROD2	-1.45591	0.626359	-2.73781	0.00675	0.021394	-2.83632
ZNF225	-0.56205	1.574524	-2.73776	0.006751	0.021394	-2.86057
YEATS4	0.766563	4.227224	2.737786	0.006751	0.021394	-3.13012

ATG16L2	-0.63779	3.74496	-2.73792	0.006748	0.021394	-3.27659
SEC61G	0.86602	6.039079	2.738131	0.006744	0.021394	-3.38743
TSPAN11	-0.94564	2.886163	-2.73742	0.006758	0.021411	-3.16921
CH25H	-1.34401	1.213176	-2.73644	0.006777	0.021456	-2.92055
RUNX2	1.388538	3.776623	2.736394	0.006778	0.021456	-2.9354
HAUS7	0.675989	3.611635	2.736413	0.006778	0.021456	-3.02574
AGPAT3	-0.4876	6.624874	-2.73652	0.006776	0.021456	-3.45219
ZNF800	-0.43571	4.965745	-2.73603	0.006786	0.021474	-3.40176
RHOV	3.430481	4.205589	2.734984	0.006806	0.021535	-2.73465
ZNF561	-0.46472	4.747535	-2.73486	0.006809	0.021538	-3.38748
ATAD3C	1.524759	0.37892	2.734683	0.006812	0.021539	-2.56535
WFS1	-0.76587	5.628472	-2.73468	0.006812	0.021539	-3.45532
HMGB1	0.458765	7.918606	2.734273	0.00682	0.021557	-3.44975
LDOC1L	-0.57122	5.561561	-2.73424	0.006821	0.021557	-3.44985
ZNF160	-0.99468	4.097192	-2.73221	0.006862	0.02168	-3.37524
SPEG	-1.39116	3.615169	-2.73179	0.00687	0.021702	-3.36028
C11orf9	-2.36696	4.806178	-2.73164	0.006873	0.021707	-3.45849
ZGPAT	0.382298	4.853447	2.731324	0.006879	0.021722	-3.32343
SPP1	4.011863	5.805661	2.730002	0.006906	0.021801	-2.89023
SP6	1.787394	3.085142	2.7293	0.00692	0.021841	-2.79829
SBNO2	0.498236	7.149782	2.727426	0.006958	0.021956	-3.47912
CCL20	4.58753	3.820392	2.726764	0.006972	0.021993	-2.60139
RCCD1	0.66315	3.716097	2.726598	0.006975	0.021999	-3.07344
TMEM161A	0.564626	4.64777	2.726508	0.006977	0.022	-3.27871
C9orf16	0.659069	6.195774	2.725897	0.006989	0.022034	-3.44761
ARHGEF18	-0.47179	6.408504	-2.7255	0.006997	0.022055	-3.48384
ITFG1	-0.38812	5.537084	-2.72541	0.006999	0.022055	-3.46534
FOXF2	-0.88375	3.556937	-2.7253	0.007001	0.022057	-3.31489
NCK1	0.653786	5.245537	2.723952	0.007029	0.022135	-3.36193
FBXO30	-0.50025	4.976024	-2.72399	0.007028	0.022135	-3.43937
POLR2K	0.48839	5.689151	2.723298	0.007043	0.02217	-3.42632
RPL28	0.597712	9.754263	2.723252	0.007043	0.02217	-3.42695
EVC	-1.14668	4.291936	-2.72119	0.007086	0.022298	-3.43602
RSF1	-0.42015	5.941934	-2.72099	0.00709	0.022306	-3.49201
FN3K	-0.96992	3.730735	-2.71972	0.007116	0.022384	-3.35961
RPS2	0.589551	8.712028	2.71916	0.007128	0.022415	-3.47197
PADI3	3.721977	0.814487	2.718709	0.007137	0.02244	-2.6071
ZBTB12	0.871063	1.761211	2.718578	0.00714	0.02244	-2.76232
SLC12A2	-1.38473	6.494403	-2.71855	0.007141	0.02244	-3.48553
SLAIN2	-0.4533	6.407319	-2.71827	0.007147	0.022454	-3.50293
DIAPH3	0.90582	3.962584	2.716996	0.007173	0.022532	-3.10052
CNTNAP3	-1.35272	1.960458	-2.71523	0.00721	0.022634	-3.11532
TMEM65	0.817235	4.708561	2.715351	0.007207	0.022634	-3.27775
AKIRIN1	-0.43401	5.788653	-2.71521	0.00721	0.022634	-3.50349
KIF2A	0.466609	5.039454	2.715093	0.007213	0.022637	-3.38026
BCDIN3D	-0.45246	2.485574	-2.71443	0.007227	0.022675	-3.05788
ARHGAP17	-0.41743	6.014934	-2.71392	0.007238	0.022704	-3.51187
EIF3C	1.289108	4.274874	2.71375	0.007241	0.02271	-3.1113
RNF130	-0.7894	5.272753	-2.71357	0.007245	0.022716	-3.49988
MRPL51	0.513677	5.565408	2.713434	0.007248	0.022717	-3.43819
MRPL34	-0.45524	4.76602	-2.71341	0.007248	0.022717	-3.44445
C1orf35	0.540439	3.751453	2.712424	0.007269	0.022777	-3.14074
KLHDC9	-1.06538	0.151802	-2.71184	0.007281	0.022811	-2.79234
C16orf70	0.458256	4.858841	2.71161	0.007286	0.022821	-3.36714
FANCM	0.672052	3.474877	2.711224	0.007294	0.022841	-3.06541
APPBP2	-0.37986	5.57045	-2.71097	0.0073	0.022851	-3.50444
GPR124	-0.8736	5.552131	-2.71092	0.007301	0.022851	-3.51773
HCN2	-1.13988	0.650584	-2.71068	0.007306	0.022857	-2.87235

RPL23AP53	-0.54812	2.927226	-2.71072	0.007305	0.022857	-3.17598
PPP1CB	-0.49847	8.471771	-2.7105	0.00731	0.022859	-3.47065
NIPA2	0.413224	6.133017	2.710521	0.007309	0.022859	-3.49797
ZNF346	-0.38913	3.604375	-2.71022	0.007316	0.022872	-3.28701
TADA1	0.374993	4.109835	2.709999	0.00732	0.022877	-3.25143
ZNF589	-0.53089	3.623471	-2.71004	0.00732	0.022877	-3.31386
NCRNA00081	-0.438	3.2158	-2.70898	0.007342	0.022939	-3.2182
CNKSR1	1.00601	4.611133	2.708696	0.007348	0.022953	-3.23486
ZFX	-0.47034	5.3975	-2.70859	0.00735	0.022955	-3.50584
FCHO2	-0.62234	5.465272	-2.70844	0.007354	0.02296	-3.51532
COL5A1	1.270836	7.90139	2.708308	0.007356	0.022964	-3.5281
ATF7	-0.36923	6.532202	-2.70774	0.007369	0.022996	-3.5301
C7orf63	-0.89052	1.309729	-2.70647	0.007396	0.023076	-2.94429
ST3GAL3	-0.80946	2.654154	-2.70586	0.007409	0.023112	-3.1809
ULK1	-0.66198	6.451706	-2.7056	0.007415	0.023124	-3.5332
SMPD3	-1.53413	3.422669	-2.70532	0.007421	0.023138	-3.4129
OCEL1	-0.58812	2.500997	-2.70464	0.007435	0.023178	-3.10778
DCUN1D3	-0.55864	3.209525	-2.7044	0.00744	0.023189	-3.25227
METRN	-0.83963	2.99641	-2.7041	0.007447	0.023204	-3.25746
MCTP2	0.817371	4.809861	2.703524	0.007459	0.023238	-3.32355
IBTK	-0.56978	6.462884	-2.70336	0.007463	0.023238	-3.54011
UBE4B	-0.46497	6.522833	-2.7034	0.007462	0.023238	-3.54052
LPPR2	-0.64602	4.826485	-2.70246	0.007482	0.023294	-3.49342
MADD	-0.43314	5.944984	-2.70195	0.007493	0.023323	-3.54198
SLC16A10	-1.11992	0.849673	-2.70105	0.007513	0.023377	-2.91751
HMBOX1	-0.5175	3.218315	-2.70092	0.007516	0.023377	-3.2536
TPT1	-0.62383	10.57527	-2.70095	0.007515	0.023377	-3.42136
CYB5R2	1.506039	3.264594	2.700833	0.007518	0.023378	-2.93319
SH3D19	-0.55433	6.488344	-2.70024	0.007531	0.023413	-3.54812
FAM120AOS	-0.40422	5.321585	-2.6994	0.007549	0.023465	-3.52198
GATA2	-0.89786	2.086017	-2.69854	0.007568	0.023518	-3.0989
EPOR	-0.81687	1.530698	-2.69798	0.00758	0.023551	-2.98752
ZNF296	0.843483	2.957562	2.697616	0.007588	0.023568	-2.9837
ACVR1	0.498872	5.512462	2.697577	0.007589	0.023568	-3.47546
FIBIN	-1.16604	1.793583	-2.6972	0.007597	0.023589	-3.09479
ZNF559	-0.93358	2.519601	-2.69702	0.007601	0.023596	-3.19677
ZNF136	-0.51365	3.052533	-2.6966	0.00761	0.023619	-3.23118
FJX1	1.427518	3.372641	2.695366	0.007638	0.023698	-2.97619
KCNC4	-0.64259	3.694286	-2.69498	0.007646	0.023709	-3.38096
ROBO4	-0.7968	4.049348	-2.6951	0.007644	0.023709	-3.44625
USP1	0.487052	5.93725	2.695028	0.007645	0.023709	-3.52075
DUSP12	0.404531	3.767745	2.694699	0.007652	0.023723	-3.21542
KDM3B	-0.33375	6.848949	-2.69447	0.007658	0.023734	-3.56079
MNDA	1.427159	1.761323	2.69438	0.007659	0.023734	-2.76569
CDKN2D	0.808996	3.278656	2.69426	0.007662	0.023737	-3.05254
CXCL10	3.228467	3.369101	2.693409	0.007681	0.023775	-2.76331
TMEM68	0.477832	4.577606	2.693449	0.00768	0.023775	-3.36558
ARL3	-0.46226	3.895199	-2.69351	0.007679	0.023775	-3.39236
ZFP36	-0.95751	8.31489	-2.69349	0.007679	0.023775	-3.50649
ZNF703	1.096857	6.209839	2.693246	0.007685	0.023781	-3.50093
MTX3	-0.68035	4.823821	-2.69238	0.007704	0.023835	-3.52162
PDE9A	-1.26272	1.439531	-2.69219	0.007708	0.023843	-3.0563
RNASEL	-0.51948	4.00387	-2.69204	0.007711	0.023848	-3.41883
SAMD1	0.50887	5.118666	2.691468	0.007724	0.02388	-3.44661
ABHD14B	-0.64653	5.620111	-2.69136	0.007727	0.02388	-3.56568
ACSS2	-0.69068	5.842259	-2.69135	0.007727	0.02388	-3.57133
MDM4	-0.49579	5.791499	-2.69105	0.007734	0.023895	-3.56787
LOC642846	0.760226	3.376254	2.690456	0.007747	0.023931	-3.08418

ZDHHC12	0.615456	5.676611	2.689382	0.007771	0.024	-3.50199
KAZALD1	-1.16785	1.355975	-2.68765	0.00781	0.024115	-3.0341
EPB41	-0.58292	5.862681	-2.68724	0.007819	0.024138	-3.5809
TGIF2	0.809492	4.922985	2.686982	0.007825	0.024151	-3.39055
SLC26A1	-0.84563	1.607502	-2.68662	0.007833	0.024171	-3.03448
SEC31A	-0.33734	7.941271	-2.68538	0.007861	0.024252	-3.55828
TPRXL	3.416507	2.050382	2.684637	0.007878	0.024293	-2.69479
MOB2	-0.42994	4.331436	-2.68466	0.007877	0.024293	-3.46994
BTBD9	-0.55872	4.54199	-2.68442	0.007883	0.024303	-3.50629
H3F3A	0.446091	7.718295	2.677612	0.008039	0.024779	-3.60115
RHOBTB1	-0.88599	3.665767	-2.67681	0.008058	0.024831	-3.45168
PRPF31	0.345048	5.533699	2.67656	0.008064	0.024843	-3.54551
ARHGEF3	-0.50804	5.017285	-2.675	0.0081	0.024949	-3.56994
C16orf87	0.603975	2.753512	2.674735	0.008106	0.024963	-3.04565
SNAPC4	0.455861	4.619599	2.674655	0.008108	0.024963	-3.42496
LOC100131193	-0.69947	1.246794	-2.67435	0.008115	0.02498	-2.99133
PPIL6	-0.78337	0.532305	-2.67404	0.008122	0.024996	-2.90432
GOLGA1	-0.34233	5.177907	-2.67391	0.008125	0.025	-3.57401
UACA	-0.65966	5.983641	-2.6736	0.008132	0.025017	-3.61854
FAM45A	0.45157	3.945492	2.671584	0.00818	0.025157	-3.30247
C3orf64	-0.48421	5.029274	-2.67146	0.008183	0.02516	-3.57877
SLC7A7	1.634418	3.848671	2.671196	0.008189	0.025174	-3.07773
ARMCX3	-0.79553	5.197799	-2.67093	0.008195	0.025188	-3.60716
ZC3H18	0.363843	5.682767	2.670442	0.008207	0.025217	-3.5728
C4orf29	-0.44554	3.064698	-2.66977	0.008222	0.02526	-3.28816
MED13L	-0.48222	7.048838	-2.66954	0.008228	0.025271	-3.61891
C15orf44	0.371433	5.353543	2.668765	0.008246	0.025322	-3.54523
CD300A	1.253723	1.544479	2.66763	0.008273	0.025399	-2.82538
HIATL1	0.404794	6.543236	2.66753	0.008276	0.025401	-3.6272
ARAF	-0.46003	6.0394	-2.66733	0.00828	0.02541	-3.63362
SCG2	-1.36645	0.271959	-2.6668	0.008293	0.025443	-2.9559
CHERP	0.348038	6.682431	2.666008	0.008312	0.025496	-3.63569
DOK7	-1.33272	0.351098	-2.66573	0.008318	0.025505	-2.964
DNAL1	-0.49211	3.509182	-2.66578	0.008317	0.025505	-3.39953
PDE6D	0.444333	3.98848	2.665441	0.008325	0.02552	-3.32813
SOCS6	-0.61699	5.344978	-2.66525	0.00833	0.025528	-3.62155
CYP2C19	-1.9638	-0.68162	-2.66408	0.008358	0.025604	-2.90397
KIAA2018	-0.56022	5.919467	-2.66407	0.008358	0.025604	-3.64138
ZNF91	-1.14791	3.914627	-2.66397	0.008361	0.025606	-3.54573
TMEM44	0.811247	3.765346	2.663425	0.008374	0.02564	-3.21892
DAPK1	-1.23348	5.152575	-2.66319	0.008379	0.025652	-3.6379
DHRSX	-0.56345	3.669112	-2.6623	0.008401	0.025711	-3.44785
SH3D20	1.097776	2.387754	2.662203	0.008403	0.025713	-2.95879
TNKS1BP1	-0.63052	7.766219	-2.6616	0.008418	0.025751	-3.61674
DLST	-0.35928	6.255431	-2.66153	0.008419	0.025751	-3.64999
FARSB	0.414928	5.778069	2.661345	0.008424	0.025759	-3.60045
TMCO4	-0.51606	4.682746	-2.66075	0.008438	0.025798	-3.5773
ZNF155	-0.70349	2.152823	-2.65979	0.008461	0.025863	-3.17832
ANKMY2	-0.56324	4.517159	-2.65887	0.008484	0.025923	-3.56939
PBRM1	-0.46059	6.247859	-2.65883	0.008484	0.025923	-3.65716
PIK3AP1	1.712736	4.355895	2.658518	0.008492	0.02594	-3.18567
RAB25	1.319496	6.542847	2.658005	0.008505	0.025973	-3.60184
DUSP10	1.071999	3.71049	2.656686	0.008537	0.026064	-3.18192
STK16	-0.37445	4.11485	-2.65664	0.008538	0.026064	-3.50737
SENP1	0.429037	5.167608	2.656415	0.008543	0.026074	-3.5512
EDIL3	-1.15041	3.819123	-2.65619	0.008549	0.026085	-3.55496
TOMM6	0.596506	6.338786	2.655778	0.008559	0.026111	-3.64154
ERI3	0.439975	5.394222	2.655142	0.008575	0.026152	-3.57835

TCN2	-0.8173	3.66077	-2.65485	0.008582	0.026169	-3.50188
SNX5	0.431724	6.321682	2.654773	0.008584	0.026169	-3.65072
FCGR2A	1.378008	4.769123	2.654007	0.008602	0.02622	-3.34283
RCN2	0.50828	6.246243	2.653174	0.008623	0.026277	-3.64762
PRSS22	2.4968	4.175208	2.652713	0.008634	0.026306	-3.05222
PIK3R3	-0.72393	4.753981	-2.65254	0.008639	0.026313	-3.62159
SPAG16	-0.84623	3.131353	-2.65208	0.00865	0.026338	-3.42332
AP1G2	0.542483	6.29364	2.652062	0.00865	0.026338	-3.65111
CHN1	1.061128	3.542567	2.649862	0.008705	0.026498	-3.17147
THUMPD2	0.409747	3.563112	2.649594	0.008712	0.026512	-3.28702
LOC144571	-0.78099	0.527453	-2.64887	0.00873	0.026561	-2.96743
RPS6KA1	0.6464	6.051038	2.648782	0.008732	0.026562	-3.63677
DAGLB	0.439433	4.693954	2.648601	0.008736	0.02657	-3.5067
CIB2	1.227932	2.380291	2.647957	0.008752	0.026613	-2.97673
FAM96A	0.448182	4.919544	2.647805	0.008756	0.026619	-3.53908
MARK2	0.430008	6.513866	2.646871	0.00878	0.026679	-3.67847
KIF21A	-0.57696	5.686271	-2.6469	0.008779	0.026679	-3.68037
TIGIT	1.629242	0.742398	2.646496	0.008789	0.026696	-2.78376
SC65	1.143269	4.863459	2.646493	0.008789	0.026696	-3.42389
MTF2	0.403736	4.629779	2.645983	0.008802	0.026723	-3.50793
PSMD7	0.377877	6.625524	2.646033	0.008801	0.026723	-3.68505
GYLTL1B	1.936819	4.768961	2.645378	0.008817	0.026763	-3.2535
H6PD	-0.54654	7.142504	-2.64521	0.008821	0.02677	-3.67797
MED27	0.533746	3.612947	2.644711	0.008834	0.026803	-3.28772
FAM113B	1.123614	2.931104	2.644542	0.008838	0.02681	-3.07802
ZNF608	-0.98898	3.361061	-2.64352	0.008864	0.026882	-3.50631
TOR2A	0.602456	4.218832	2.642312	0.008894	0.026965	-3.40221
ORC3L	0.462152	4.755605	2.64229	0.008895	0.026965	-3.52908
ZNF585B	-1.04405	1.120392	-2.64221	0.008897	0.026965	-3.09998
COL1A2	1.229746	10.23449	2.641847	0.008906	0.026987	-3.63974
FAM26F	1.647562	1.058799	2.640791	0.008933	0.027063	-2.80854
TARBP2	0.460763	3.70653	2.640526	0.00894	0.027077	-3.32973
TOMM22	0.442233	5.271243	2.639498	0.008966	0.027151	-3.60458
SAA1	3.754457	2.606814	2.638865	0.008982	0.027194	-2.80783
TRIM15	3.839361	0.274484	2.638689	0.008987	0.027202	-2.8014
SHPRH	-0.41601	4.626706	-2.63857	0.00899	0.027205	-3.61963
DDX6	-0.34674	7.111888	-2.638	0.009005	0.027244	-3.7008
ZNF628	0.38777	4.044701	2.637343	0.009021	0.027288	-3.42305
MMP3	4.815102	3.677048	2.637152	0.009026	0.027297	-2.81404
FGFR1OP	0.610103	3.324224	2.636468	0.009044	0.027345	-3.2361
NEB	2.876785	2.568998	2.635325	0.009073	0.027428	-2.85773
EBP	0.712911	5.009777	2.634906	0.009084	0.027454	-3.55158
PHB	0.442682	6.83642	2.63437	0.009098	0.02749	-3.71756
LOC100288778	-0.59652	3.853129	-2.63395	0.009109	0.027518	-3.55601
RANBP6	-0.44322	4.656409	-2.63329	0.009126	0.027563	-3.63884
CCDC142	0.468908	3.319623	2.633081	0.009131	0.027573	-3.26949
FAM132A	1.905244	0.302758	2.631307	0.009177	0.027707	-2.81891
METTL13	0.370234	5.14366	2.631106	0.009183	0.027713	-3.61844
RDX	-0.89219	5.858834	-2.63108	0.009183	0.027713	-3.72715
C12orf52	0.433283	4.364641	2.630884	0.009188	0.027722	-3.49476
ADAM8	1.610271	4.381699	2.630788	0.009191	0.027724	-3.27737
EBPL	0.697452	4.527469	2.630579	0.009196	0.027734	-3.48081
ATP2A3	-1.43272	5.820311	-2.63033	0.009203	0.027747	-3.724
SH3YL1	-0.76126	5.181506	-2.62958	0.009223	0.027801	-3.71033
MON1B	-0.32962	5.790166	-2.62811	0.009261	0.027912	-3.72476
SNHG9	-0.78745	0.119888	-2.62786	0.009268	0.027925	-2.96764
CLEC7A	1.841771	2.773766	2.627736	0.009271	0.027929	-3.00879
FKBP3	0.47982	5.453579	2.627618	0.009274	0.027932	-3.65071

POU6F2	2.586612	-0.64486	2.627182	0.009286	0.027955	-2.82501
PCLO	-1.76933	2.736978	-2.62718	0.009286	0.027955	-3.55379
ALOX15B	-1.62866	1.452028	-2.62702	0.00929	0.027957	-3.28041
VTI1B	-0.38345	5.346255	-2.627	0.00929	0.027957	-3.70812
TCP1	0.429042	7.6293	2.62645	0.009305	0.027995	-3.73334
ETV6	0.480879	6.215559	2.626175	0.009312	0.02801	-3.71596
SLC35C2	0.364673	5.582947	2.625443	0.009331	0.028063	-3.67871
DUSP2	1.180561	2.854929	2.624742	0.00935	0.028112	-3.11017
CLIC4	-0.74728	7.661727	-2.62467	0.009352	0.028112	-3.71083
PDE4A	-0.74926	4.449426	-2.62436	0.00936	0.028131	-3.66662
C9orf30	0.70623	4.143424	2.623363	0.009387	0.028204	-3.41934
EIF5A2	-0.77333	4.180441	-2.62305	0.009395	0.028223	-3.64316
CFH	-0.9981	5.599818	-2.62285	0.0094	0.028233	-3.74642
NAA38	0.500749	4.464094	2.622597	0.009407	0.028247	-3.52257
MED20	0.563954	4.621895	2.622432	0.009411	0.028255	-3.54159
AP1B1	-0.42846	7.485925	-2.62168	0.009432	0.028309	-3.732
ZNF256	-1.16226	1.061184	-2.62072	0.009457	0.02838	-3.16231
SLC39A8	1.145873	4.454079	2.619718	0.009484	0.028454	-3.40663
ZNF808	-0.88991	3.460266	-2.61951	0.00949	0.028465	-3.56942
WDR66	3.109853	2.64941	2.618687	0.009512	0.028503	-2.8859
HACL1	-0.48318	3.803132	-2.61888	0.009506	0.028503	-3.56999
BPNT1	0.537189	4.799283	2.618684	0.009512	0.028503	-3.58451
C16orf53	0.46603	4.935135	2.618659	0.009513	0.028503	-3.61352
TXNDC11	-0.43025	5.686218	-2.61867	0.009512	0.028503	-3.74797
ZFYVE1	-0.45848	4.708462	-2.6185	0.009517	0.02851	-3.68287
TLE4	-0.85396	4.835851	-2.61829	0.009522	0.02852	-3.72316
PHF2	-0.38563	5.886125	-2.6179	0.009533	0.028546	-3.7551
SNHG12	0.709799	3.546614	2.617714	0.009538	0.028555	-3.31051
PDCD6IP	-0.46443	7.645087	-2.61744	0.009546	0.028571	-3.7374
PICK1	-0.37678	4.078281	-2.61726	0.00955	0.028579	-3.60208
NOL10	0.376688	5.489095	2.617006	0.009557	0.028594	-3.69022
GORASP2	0.329808	7.203093	2.616752	0.009564	0.028608	-3.76306
SEMA3D	-1.73821	1.018798	-2.61638	0.009574	0.028632	-3.24482
QPCT	1.828748	2.270696	2.616164	0.00958	0.028637	-2.97462
RHBDD2	0.467844	6.866698	2.616204	0.009579	0.028637	-3.76365
IFIT2	1.508141	3.472417	2.615322	0.009603	0.028693	-3.17946
CAMLG	-0.35654	4.414753	-2.61538	0.009601	0.028693	-3.64911
LOC80154	-0.68724	5.312315	-2.6152	0.009606	0.028697	-3.75011
MAN2A2	-0.67695	5.928913	-2.61448	0.009626	0.028749	-3.76889
SF3A3	0.395074	6.144732	2.613932	0.009641	0.028788	-3.74747
ARFGAP2	-0.3126	6.111294	-2.61369	0.009647	0.028801	-3.76933
HACE1	-0.48122	3.971123	-2.61348	0.009653	0.028813	-3.61099
SRGAP2	0.463217	5.572483	2.613266	0.009659	0.028824	-3.70043
ATF7IP	0.489633	6.476616	2.61255	0.009678	0.028876	-3.76218
ASAP1	0.586307	6.062341	2.612119	0.00969	0.028905	-3.73545
C11orf74	-0.49637	2.195309	-2.61185	0.009697	0.028921	-3.27206
TNK2	0.644051	6.379079	2.611379	0.00971	0.028953	-3.75408
MEIS1	-0.81447	3.884919	-2.61114	0.009717	0.028967	-3.64421
ALDH9A1	-0.52689	6.206336	-2.61043	0.009737	0.029019	-3.78015
FBXL3	-0.37798	6.020616	-2.61014	0.009745	0.029036	-3.77765
S100A14	1.86681	6.964752	2.609881	0.009752	0.029051	-3.71667
ZNF451	-0.37468	5.248986	-2.60866	0.009785	0.029145	-3.74741
PNRC1	-0.63344	6.971427	-2.60846	0.009791	0.029156	-3.77314
ZNF187	-0.47841	3.009372	-2.60827	0.009796	0.029158	-3.44057
TRAPPC9	-0.4507	5.743405	-2.60831	0.009795	0.029158	-3.77672
ANAPC1	0.499685	5.935626	2.607238	0.009825	0.029237	-3.74406
TAGLN2	0.480393	9.430578	2.606409	0.009848	0.0293	-3.73408
PXDN	1.104301	6.457617	2.605906	0.009862	0.029335	-3.7442

ANKRD44	-0.8502	1.970428	-2.60572	0.009867	0.029344	-3.30436
TMED10P1	-0.50189	1.458032	-2.60507	0.009885	0.029392	-3.17022
JOSD1	0.417774	6.49439	2.604869	0.00989	0.029402	-3.78458
PTER	-0.65161	4.423117	-2.6033	0.009934	0.029526	-3.70805
USP30	-0.39465	3.848531	-2.60312	0.009939	0.029534	-3.60487
AP2A2	-0.35504	6.379542	-2.60277	0.009949	0.029557	-3.7996
CTU1	0.519419	3.378055	2.60235	0.009961	0.02958	-3.34911
C2orf67	-0.56496	4.353366	-2.60236	0.009961	0.02958	-3.69539
KLRA1	-0.72996	0.596677	-2.60207	0.009969	0.029597	-3.08431
DDX47	0.412467	5.641385	2.60089	0.010002	0.029689	-3.74206
CLEC4A	1.053233	0.304799	2.600755	0.010006	0.029691	-2.8922
POLE	0.47157	6.29593	2.600716	0.010007	0.029691	-3.78476
C18orf1	-0.97409	3.938554	-2.5996	0.010038	0.029778	-3.69535
C8orf30A	0.569621	5.302214	2.599492	0.010041	0.029781	-3.69558
IL2RA	1.525877	1.473865	2.597729	0.010091	0.029923	-2.96298
ENTPD4	-0.47431	4.655298	-2.59751	0.010098	0.029935	-3.73133
SYTL5	-1.72107	0.934029	-2.59726	0.010105	0.02995	-3.26847
C1orf163	0.483711	3.538967	2.596928	0.010114	0.029971	-3.40062
EPHA7	-1.83921	0.667011	-2.5966	0.010123	0.029986	-3.25224
FLJ10038	-0.45242	3.035941	-2.59663	0.010123	0.029986	-3.47113
KLF4	-1.03362	6.825371	-2.5965	0.010126	0.029989	-3.79824
ZNF146	0.435349	6.712902	2.595979	0.010141	0.030026	-3.81235
KIAA1279	-0.34241	5.031401	-2.59555	0.010153	0.030056	-3.76099
ANKRD9	-0.56335	4.584776	-2.59538	0.010158	0.030064	-3.73722
SCAMP1	-0.42962	6.175327	-2.59507	0.010167	0.030084	-3.81831
ZKSCAN2	-0.49059	2.847204	-2.59393	0.0102	0.030174	-3.44484
ZNF524	-0.48717	3.925304	-2.59328	0.010218	0.030222	-3.65305
TRAF6	-0.36966	3.670018	-2.59219	0.01025	0.030305	-3.59561
ZDBF2	-1.10902	3.353839	-2.59209	0.010253	0.030305	-3.65332
PTPN1	0.39105	6.49868	2.592093	0.010253	0.030305	-3.81757
CDK12	0.52401	6.718096	2.591898	0.010258	0.030315	-3.82072
ODC1	1.058771	7.651029	2.591716	0.010263	0.030324	-3.82632
WDR6	-0.46543	6.559767	-2.59129	0.010276	0.030353	-3.82623
GPSM1	-0.85774	3.600561	-2.59067	0.010294	0.0304	-3.66062
C12orf23	-0.57251	5.478884	-2.58985	0.010317	0.030464	-3.81698
SLAMF8	1.394706	3.403322	2.588514	0.010356	0.030559	-3.25195
KLHDC8B	-0.69999	3.203211	-2.58861	0.010353	0.030559	-3.57262
C9orf91	-0.51891	4.476655	-2.58852	0.010356	0.030559	-3.73939
MKRN2	-0.34622	4.63052	-2.58756	0.010384	0.030634	-3.74224
SULF1	1.383656	6.603721	2.58742	0.010388	0.03064	-3.7809
MUTYH	0.577879	3.373849	2.587172	0.010395	0.030648	-3.37437
WASL	-0.40118	6.691543	-2.58719	0.010395	0.030648	-3.8355
ARC	-1.33976	0.24985	-2.58697	0.010401	0.030654	-3.15049
RBM45	0.25439	2.792164	2.586952	0.010402	0.030654	-3.32152
CXXC1	-0.40116	4.977711	-2.58667	0.01041	0.030672	-3.78301
RASA4	-0.67917	3.594945	-2.58654	0.010414	0.030676	-3.64642
RCOR3	-0.40131	5.29619	-2.58632	0.01042	0.030689	-3.80847
AUP1	0.371482	6.875725	2.585986	0.01043	0.030711	-3.84076
PTGFR	-1.13909	0.383454	-2.58558	0.010442	0.03074	-3.14671
PLK2	0.959383	5.26235	2.585452	0.010445	0.030744	-3.68118
TARBP1	0.554602	5.55042	2.58477	0.010465	0.030797	-3.76094
KIAA0562	-0.31359	5.381623	-2.58453	0.010472	0.030811	-3.81342
NHLRC2	-0.45358	3.946496	-2.58338	0.010506	0.030904	-3.67731
CHML	0.795979	4.938173	2.583213	0.010511	0.030912	-3.65811
FZD6	0.950604	6.512223	2.583094	0.010515	0.030916	-3.81576
WRNIP1	0.430582	5.882638	2.582527	0.010531	0.030958	-3.80675
CBY1	-0.41734	3.636677	-2.58245	0.010534	0.030958	-3.62193
GRB2	0.331515	6.769422	2.581899	0.01055	0.031	-3.85027

MYO18A	-0.59464	7.06576	-2.58166	0.010557	0.031014	-3.83896
HIBCH	-0.49685	4.320442	-2.58077	0.010583	0.031085	-3.73825
ZNF684	-0.52967	1.400402	-2.58049	0.010592	0.031103	-3.22568
TECR	0.615608	6.696414	2.580021	0.010606	0.031137	-3.8473
A2M	-1.0947	8.508871	-2.57961	0.010618	0.031167	-3.78537
LOC728643	1.961601	0.633868	2.578197	0.01066	0.031277	-2.94704
BAD	-0.44953	4.679875	-2.57825	0.010658	0.031277	-3.77972
PDZD11	0.502209	5.163963	2.577879	0.010669	0.031298	-3.74084
CTDSPL2	0.424652	5.530197	2.577455	0.010682	0.031328	-3.78905
GSTM3	-1.58192	4.888525	-2.57735	0.010685	0.031331	-3.8542
C1orf159	0.529937	3.769417	2.577025	0.010695	0.031353	-3.48807
C9orf110	1.293288	1.202705	2.576321	0.010716	0.031408	-3.006
FLNA	-1.1418	10.4082	-2.57575	0.010733	0.031452	-3.72906
PAPD4	-0.30142	5.342494	-2.57482	0.010761	0.031527	-3.83419
C11orf31	0.5213	4.831582	2.574321	0.010776	0.031559	-3.70333
SDHA	-0.51697	7.03115	-2.57431	0.010776	0.031559	-3.8595
HK3	1.578415	0.706912	2.573774	0.010792	0.031599	-2.95768
SPSB2	0.606505	2.830893	2.573236	0.010809	0.03163	-3.3086
MBD4	0.429609	5.084857	2.573202	0.01081	0.03163	-3.75062
GNPTAB	-0.39684	6.056911	-2.57324	0.010809	0.03163	-3.87096
AMOTL2	-0.60804	6.095784	-2.57292	0.010818	0.031648	-3.87376
GOLGA6L9	-0.58623	3.522671	-2.57281	0.010821	0.031651	-3.64983
RRP8	-0.36167	3.211072	-2.57236	0.010835	0.031685	-3.54896
ADCY7	0.764715	5.35451	2.572043	0.010845	0.031706	-3.74969
FBXO42	-0.3341	4.9531	-2.57182	0.010851	0.031719	-3.81263
ACVRL1	-0.77026	3.681484	-2.57147	0.010862	0.031744	-3.70829
CPSF6	0.418903	6.583387	2.57123	0.010869	0.031758	-3.87134
SPON2	0.986592	5.346687	2.569996	0.010907	0.031854	-3.72663
ABCB7	-0.33497	4.44889	-2.57004	0.010906	0.031854	-3.76398
KCTD10	-0.43043	6.088759	-2.56993	0.010909	0.031854	-3.88007
ATP6AP2	0.382145	6.981431	2.56815	0.010963	0.032006	-3.88579
DGKG	-1.35999	1.908982	-2.56774	0.010976	0.032035	-3.47835
MAVS	-0.45912	7.729411	-2.5676	0.01098	0.032041	-3.85975
ZNF192	-0.55771	4.658187	-2.56745	0.010985	0.032048	-3.81415
DDHD2	-0.50021	5.3459	-2.56705	0.010997	0.032077	-3.86448
UBR3	-0.35593	6.165698	-2.56658	0.011011	0.032112	-3.88858
HSPH1	0.65502	7.572473	2.566507	0.011014	0.032112	-3.88765
MYL5	-0.54134	2.179318	-2.56584	0.011034	0.032166	-3.39009
C6orf27	-1.04112	1.971358	-2.56472	0.011069	0.032259	-3.43119
SLC20A1	0.569902	6.048343	2.564518	0.011075	0.032271	-3.8542
BTBD7	-0.38467	5.818573	-2.56368	0.011101	0.03234	-3.88878
FOXL1	1.67279	1.866048	2.563066	0.01112	0.032388	-3.07051
MMP24	-0.62055	3.085875	-2.56279	0.011128	0.032406	-3.59352
ITGA3	0.880958	8.298974	2.562433	0.011139	0.032432	-3.88807
ERF	0.509829	5.973551	2.562173	0.011147	0.032448	-3.85875
HMBS	0.684877	4.025359	2.562056	0.011151	0.032452	-3.54865
NAGLU	-0.43841	5.054846	-2.56171	0.011162	0.032477	-3.85391
PPME1	0.569059	6.237861	2.561487	0.011169	0.03249	-3.87465
LOC441089	0.557028	2.632599	2.560552	0.011198	0.032561	-3.31635
INPP5K	-0.38431	4.963584	-2.5606	0.011196	0.032561	-3.84517
PCDHB15	-0.88304	0.70824	-2.56003	0.011214	0.032602	-3.22285
SNHG10	0.544955	1.634634	2.559117	0.011243	0.032678	-3.17663
TAF1B	0.525625	4.009059	2.558929	0.011249	0.032688	-3.58574
SLC25A30	-0.43433	3.447778	-2.55811	0.011274	0.032756	-3.64708
TOPORS	-0.42907	5.085035	-2.55797	0.011279	0.032762	-3.86532
NDRG3	0.441774	5.679636	2.557576	0.011291	0.032791	-3.8508
CYP4F12	-1.63247	1.291776	-2.55745	0.011295	0.032796	-3.42095
DDX42	-0.27317	6.859471	-2.55735	0.011298	0.032798	-3.90887

MRRF	0.478205	5.123577	2.55702	0.011308	0.032821	-3.78983
UQCRB	-0.44684	6.556544	-2.55665	0.01132	0.032848	-3.91265
PIAS2	-0.51865	3.531141	-2.55648	0.011325	0.032851	-3.68325
FER1L4	-1.4772	2.880214	-2.55647	0.011326	0.032851	-3.70472
GOLGA6L5	-0.6516	0.712337	-2.55619	0.011335	0.032866	-3.20232
MALL	1.413178	6.838292	2.556161	0.011335	0.032866	-3.87565
LOC100270710	1.359275	1.702352	2.555942	0.011342	0.032879	-3.1049
DOPEY1	-0.49067	5.297962	-2.55412	0.0114	0.033039	-3.89309
WNT5A	1.352761	5.815627	2.553766	0.011411	0.033065	-3.78619
EMR2	1.281235	2.843506	2.553686	0.011414	0.033065	-3.26792
TRIAP1	0.408784	4.094548	2.553529	0.011419	0.033073	-3.63712
ZXDA	-0.6239	1.672592	-2.55342	0.011422	0.033076	-3.34661
PHLDB1	-0.62905	5.406806	-2.55323	0.011428	0.033086	-3.90699
SEMA4B	0.780085	8.102137	2.55294	0.011437	0.033106	-3.91385
TOX4	-0.32672	6.214706	-2.55255	0.01145	0.033135	-3.92356
SLC25A6	-0.63563	8.408268	-2.55185	0.011472	0.033184	-3.87094
C15orf39	0.464161	6.219234	2.551796	0.011474	0.033184	-3.90298
TBL1X	-0.76101	5.395388	-2.55183	0.011473	0.033184	-3.91438
SOX9	1.194768	7.26073	2.551117	0.011495	0.03324	-3.91886
RABGAP1	-0.42117	6.101706	-2.55104	0.011498	0.03324	-3.92687
LATS2	-0.56337	5.274919	-2.5509	0.011502	0.033246	-3.90346
PLEKHG2	0.632777	5.628492	2.550668	0.01151	0.03326	-3.84608
C14orf45	-0.6023	1.935989	-2.54983	0.011537	0.033331	-3.3964
C9orf40	0.689818	2.810478	2.549339	0.011552	0.03337	-3.35225
KBTBD2	0.33433	6.042018	2.54924	0.011555	0.033372	-3.90596
ACTC1	-1.66621	0.184983	-2.54901	0.011563	0.033386	-3.27442
MAP2K4	-0.44761	5.435988	-2.54875	0.011571	0.033404	-3.91206
NME2P1	0.705677	1.557743	2.548356	0.011584	0.033433	-3.17453
MID1IP1	-0.54584	5.610307	-2.54792	0.011598	0.033467	-3.92526
NYNRIN	-0.94934	5.269456	-2.54687	0.011632	0.033557	-3.92745
ST3GAL1	-0.88022	6.075693	-2.54592	0.011662	0.033639	-3.93936
EBF4	-0.88931	3.088429	-2.5452	0.011686	0.033699	-3.68993
HLA-F	1.203963	5.90178	2.545125	0.011688	0.033699	-3.83211
TUBA4A	0.92866	6.787982	2.544852	0.011697	0.033718	-3.92677
MAPKAP1	-0.32833	6.27918	-2.54457	0.011706	0.033737	-3.94389
SAMHD1	-0.78163	5.665543	-2.54285	0.011762	0.033887	-3.94453
THOC2	0.430623	6.754113	2.542817	0.011763	0.033887	-3.94523
OGFOD1	0.348207	5.285363	2.542584	0.011771	0.033902	-3.8578
RAET1L	2.640978	0.715798	2.542336	0.011779	0.033918	-3.03179
C13orf1	-0.46759	4.068376	-2.54214	0.011785	0.03393	-3.79885
TP53I13	0.505143	4.722122	2.541893	0.011793	0.033946	-3.76829
HDAC4	-0.58172	5.330519	-2.5417	0.011799	0.033957	-3.92987
PPP3R1	0.29229	6.278932	2.541222	0.011815	0.033995	-3.93912
NAP1L2	-1.48353	0.139922	-2.54099	0.011823	0.034003	-3.26464
OSBPL7	-1.0701	3.855534	-2.54103	0.011821	0.034003	-3.83695
HES4	1.180781	3.632067	2.540107	0.011852	0.034072	-3.43995
VRK2	0.430169	4.693703	2.540132	0.011851	0.034072	-3.77952
TTLL5	0.359539	5.318171	2.539734	0.011864	0.0341	-3.86697
SPDEF	-2.11327	0.745893	-2.53953	0.011871	0.034105	-3.42943
DUSP14	0.87032	4.609983	2.539548	0.01187	0.034105	-3.69561
ANXA2P1	1.106378	3.154659	2.538735	0.011897	0.034174	-3.37259
TIMM50	0.44231	5.267063	2.538331	0.01191	0.034205	-3.85667
DBR1	0.526373	4.543643	2.53777	0.011928	0.034251	-3.74315
TMEM216	0.557256	3.419972	2.537572	0.011935	0.034261	-3.50885
LAPTM5	0.985033	6.367777	2.537517	0.011937	0.034261	-3.91583
CYLD	-0.51313	5.679096	-2.53721	0.011947	0.034283	-3.95309
ATF6B	0.417827	6.635486	2.535957	0.011988	0.034395	-3.95975
MFN2	-0.47433	7.135338	-2.53549	0.012004	0.034432	-3.95397

SETD3	-0.33361	5.731987	-2.53503	0.012019	0.034468	-3.95479
PCBP4	-0.5365	4.47922	-2.53403	0.012052	0.03455	-3.87533
TBC1D2B	-0.42717	5.833666	-2.53407	0.012051	0.03455	-3.96321
TMTC1	-1.21708	4.269843	-2.53371	0.012063	0.034573	-3.91325
TMF1	-0.52823	5.843187	-2.53341	0.012073	0.034595	-3.96717
IL2RB	1.313711	3.921658	2.532868	0.012091	0.034639	-3.48656
OPA3	-0.32299	4.90378	-2.5324	0.012107	0.034677	-3.90415
STC2	1.978955	3.489354	2.53197	0.012121	0.034711	-3.32202
UBR1	-0.42099	5.473179	-2.53142	0.012139	0.034753	-3.9552
SDHD	-0.44734	6.360693	-2.53139	0.012141	0.034753	-3.97641
EFCAB2	-0.73298	1.895154	-2.53087	0.012158	0.034795	-3.45258
SNORD1C	0.69848	1.310342	2.530622	0.012166	0.03481	-3.18666
ISCA1	-0.38824	4.662305	-2.53057	0.012168	0.03481	-3.89023
VPS4A	-0.34802	6.110095	-2.53048	0.012171	0.034811	-3.97663
DSP	1.23671	10.34628	2.529536	0.012203	0.034895	-3.91635
ARL5B	0.622994	5.203199	2.529442	0.012206	0.034897	-3.85156
SEC22B	-0.44865	5.095036	-2.52889	0.012225	0.034943	-3.93866
HRH1	1.001385	3.896066	2.528457	0.012239	0.03497	-3.54955
FARP2	-0.58318	4.671062	-2.52847	0.012239	0.03497	-3.91265
EML5	-0.87079	1.466121	-2.52776	0.012263	0.03503	-3.41145
GAL3ST1	-1.80709	0.161767	-2.52767	0.012266	0.035032	-3.33076
CARD16	1.014426	1.880767	2.527596	0.012268	0.035032	-3.2294
RTN4RL2	1.161991	1.302907	2.527179	0.012282	0.035065	-3.14672
XRCC6BP1	0.520072	2.198028	2.527069	0.012286	0.035069	-3.33552
GNA11	-0.46325	5.63752	-2.52684	0.012294	0.035083	-3.97542
RPP21	0.564674	4.113772	2.526284	0.012313	0.03513	-3.67915
EPHB6	-1.28069	3.112541	-2.52547	0.01234	0.035202	-3.80335
ADSL	0.455961	5.191374	2.525129	0.012352	0.035228	-3.87906
SLCO1B3	3.409615	0.445273	2.524337	0.012379	0.035278	-3.07282
ZNF658	-0.75292	1.387886	-2.52442	0.012376	0.035278	-3.39105
NSUN7	-1.20982	1.717309	-2.52432	0.012379	0.035278	-3.5131
IFI16	1.162936	7.202929	2.524488	0.012374	0.035278	-3.98349
ARHGAP18	-0.82779	4.701917	-2.52385	0.012396	0.035316	-3.94469
PCOLCE2	-1.47264	0.444769	-2.52306	0.012423	0.035386	-3.34694
JAG2	1.034554	5.601102	2.522727	0.012434	0.035411	-3.87089
ENOX2	0.435692	4.56819	2.522471	0.012443	0.035429	-3.80112
VRK1	0.589037	3.876314	2.521671	0.01247	0.035493	-3.63437
ERCC4	-0.39244	3.680161	-2.52167	0.01247	0.035493	-3.7752
RBMX2	0.434853	4.399401	2.521526	0.012475	0.035499	-3.77208
RIOK1	0.493662	4.591485	2.520985	0.012494	0.035545	-3.79975
AXL	-0.84014	5.28152	-2.52063	0.012506	0.035572	-3.98913
FRZB	-1.0203	2.202793	-2.52056	0.012508	0.035572	-3.58682
GPR176	1.493438	2.121798	2.52041	0.012513	0.035579	-3.22235
C13orf27	0.723915	2.620227	2.519237	0.012554	0.035687	-3.391
GPR108	-0.37268	5.525222	-2.51876	0.01257	0.035727	-3.98668
C3orf1	0.429327	5.951596	2.518337	0.012585	0.035761	-3.97012
FBXL4	-0.35636	4.084338	-2.51706	0.012629	0.035879	-3.8485
NPRL2	-0.47098	3.586831	-2.51684	0.012637	0.035893	-3.78053
IL22RA1	1.69955	4.156755	2.515887	0.01267	0.035973	-3.50385
DNAJC3	-0.49421	6.518577	-2.51592	0.012669	0.035973	-4.01243
TMPRSS2	-2.29709	4.303905	-2.51578	0.012674	0.035976	-4.00068
LYSMD3	-0.42181	4.813699	-2.51511	0.012697	0.036035	-3.94597
FAM168A	-0.5357	3.275891	-2.51444	0.01272	0.036091	-3.73726
CCDC50	-0.53701	6.323876	-2.5144	0.012722	0.036091	-4.01755
FPGS	0.485592	5.783643	2.514277	0.012726	0.036096	-3.96257
RBM14	0.301319	6.373518	2.513889	0.01274	0.036126	-4.00916
DDOST	0.344745	7.679181	2.513826	0.012742	0.036126	-4.01061
COL18A1	0.671076	7.73272	2.513745	0.012745	0.036127	-4.01503

RBM43	-0.50191	2.698822	-2.51248	0.012789	0.036245	-3.61526
NPHP4	0.589853	4.208138	2.511939	0.012808	0.036292	-3.72746
LOC388152	-0.63162	4.5976	-2.51135	0.012829	0.036342	-3.95134
ANKRD13A	-0.39052	5.997123	-2.51129	0.012831	0.036342	-4.02201
FAM188B	0.767754	3.022999	2.511194	0.012834	0.036344	-3.46741
STX1A	1.00308	3.082675	2.511045	0.012839	0.036351	-3.44153
DHX8	0.352266	6.211254	2.510304	0.012866	0.036418	-4.00929
SEC62	-0.54422	7.818707	-2.50987	0.012881	0.036454	-3.99622
PTGES	1.782605	3.697973	2.509521	0.012893	0.036481	-3.43308
ANP32B	0.388311	7.1423	2.509374	0.012898	0.036489	-4.02988
PLXNA2	-0.74672	6.684975	-2.50909	0.012908	0.036509	-4.02246
CDKL5	-0.77384	1.718178	-2.50808	0.012944	0.0366	-3.48455
CYP27A1	-0.98773	3.295622	-2.50792	0.01295	0.0366	-3.83213
NR4A2	-0.97632	4.193325	-2.50795	0.012949	0.0366	-3.94898
AGPAT2	0.857589	6.199544	2.507889	0.012951	0.0366	-3.98411
EXOSC9	0.394576	4.187492	2.507391	0.012969	0.036643	-3.77138
GATA3	1.703483	1.643362	2.506992	0.012983	0.03667	-3.17895
TAOK2	-0.3279	6.172646	-2.50697	0.012984	0.03667	-4.0346
RPL22L1	0.919105	4.859141	2.506285	0.013008	0.036732	-3.81245
STEAP1	1.213713	4.116087	2.505926	0.013021	0.036761	-3.6046
DHDDS	-0.36892	5.001324	-2.50574	0.013028	0.036772	-3.98147
TTC12	-0.52031	2.674231	-2.50554	0.013035	0.036774	-3.62865
FOXRED2	0.675331	5.757112	2.505554	0.013034	0.036774	-3.9654
HBP1	-0.40937	6.17954	-2.5055	0.013036	0.036774	-4.03902
TMEM60	0.459172	3.574869	2.503673	0.013101	0.036946	-3.63999
EPM2AIP1	-0.55704	5.270251	-2.50364	0.013102	0.036946	-4.01855
GPHN	-0.56168	4.38949	-2.50306	0.013123	0.036998	-3.94345
PLEKHB2	-0.44532	7.083623	-2.50223	0.013153	0.037075	-4.03661
TMEM209	0.467289	4.946702	2.501324	0.013186	0.037151	-3.90457
ROMO1	0.548697	5.23321	2.501364	0.013184	0.037151	-3.93147
NXPH4	3.392057	2.603193	2.500994	0.013198	0.037177	-3.13661
C7orf55	-0.55319	2.797043	-2.50075	0.013207	0.037195	-3.67219
MXD3	0.637649	3.577768	2.50032	0.013222	0.037231	-3.61517
KSR2	-1.22661	0.097003	-2.50008	0.013231	0.037241	-3.32132
LYST	-0.59982	5.581458	-2.50011	0.01323	0.037241	-4.04254
GK3P	0.91247	-0.04342	2.499613	0.013248	0.037281	-3.1273
ZNF350	-0.93877	2.052857	-2.49912	0.013266	0.037323	-3.59507
MPHOSPH6	0.579062	4.187558	2.498382	0.013292	0.037391	-3.75924
ARHGEF17	-0.70422	5.660805	-2.4971	0.013339	0.037515	-4.05468
PPAPDC1B	-0.67623	4.672691	-2.49586	0.013384	0.037627	-3.99932
TRIM13	-0.4672	5.215255	-2.49592	0.013382	0.037627	-4.02889
TBC1D20	-0.32069	5.838713	-2.49545	0.013399	0.037661	-4.05454
ZNF707	0.473638	3.235392	2.494958	0.013417	0.037689	-3.59062
TNFRSF12A	0.972895	5.4948	2.49509	0.013413	0.037689	-3.93202
RALGAPA2	-1.00554	5.303631	-2.49497	0.013417	0.037689	-4.05628
RFX1	-0.27263	5.052459	-2.49326	0.01348	0.037857	-4.0086
ADAMTS5	-0.90653	2.884164	-2.49305	0.013487	0.037865	-3.77368
PGM2	0.629293	5.639637	2.493031	0.013488	0.037865	-3.98882
RAI14	0.73147	6.396813	2.49296	0.013491	0.037865	-4.0425
UNG	0.522771	5.405834	2.492681	0.013501	0.037886	-3.97458
MOCOS	1.214412	3.605446	2.492504	0.013507	0.037897	-3.54169
DAAM1	-0.6399	5.098612	-2.49195	0.013528	0.037947	-4.04054
CCDC43	0.372026	4.616771	2.491789	0.013534	0.037948	-3.89211
ALDH1L2	-1.09279	3.899161	-2.49181	0.013533	0.037948	-3.96504
GRAMD2	1.403126	1.429506	2.490913	0.013566	0.038023	-3.22049
DNAJB9	-0.44254	4.639367	-2.49084	0.013569	0.038023	-3.98892
NEAT1	-0.76294	8.800241	-2.49092	0.013566	0.038023	-4.00254
TMPRSS13	3.563635	2.618538	2.489648	0.013613	0.038132	-3.15951

C6orf35	-0.39325	3.750593	-2.48967	0.013612	0.038132	-3.86561
EPHA1	1.476645	4.771832	2.489455	0.01362	0.038144	-3.72723
KLC2	0.507204	5.756601	2.488803	0.013644	0.038205	-4.0203
CD177	2.891344	0.964569	2.488553	0.013654	0.038223	-3.15713
NRD1	0.280086	7.155854	2.487797	0.013682	0.038294	-4.08124
CLDN11	-1.05897	0.326855	-2.48743	0.013696	0.038325	-3.36357
GRK6	0.452051	5.554432	2.486298	0.013738	0.038435	-4.01209
ZNF598	0.423442	6.381224	2.486091	0.013746	0.038449	-4.07243
RNF213	0.598196	8.355203	2.485878	0.013754	0.038464	-4.06803
RBMX	0.261489	7.19683	2.485235	0.013778	0.038524	-4.08672
PPA2	-0.36301	5.339761	-2.48451	0.013805	0.038591	-4.05844
PMS2L5	0.676315	0.413322	2.484331	0.013812	0.038603	-3.19872
PDLIM5	-0.45155	7.480386	-2.4842	0.013816	0.038609	-4.07074
C19orf42	-0.33166	5.267019	-2.48408	0.013821	0.038614	-4.05256
C1orf51	-0.96976	1.925399	-2.48253	0.013879	0.038746	-3.61545
BST2	1.357314	5.885181	2.482751	0.013871	0.038746	-3.96538
GMEB2	0.332243	5.115534	2.482641	0.013875	0.038746	-3.98569
SPSB3	-0.39072	5.048097	-2.48255	0.013879	0.038746	-4.04331
HSD17B7	0.55372	3.245851	2.482268	0.013889	0.038766	-3.60882
ORC5L	0.52584	4.282418	2.481555	0.013916	0.038833	-3.82832
JAG1	0.883447	7.95215	2.481401	0.013922	0.038842	-4.09254
C6orf134	0.582429	3.648121	2.480923	0.01394	0.038885	-3.68611
FAM89A	1.043386	3.508995	2.480722	0.013948	0.03889	-3.58032
RRS1	0.542907	4.905848	2.480728	0.013948	0.03889	-3.93956
FGFR3	1.583236	6.040122	2.480277	0.013965	0.03893	-3.96474
GPC6	-1.093	3.606021	-2.47969	0.013987	0.038984	-3.96022
SEC23IP	-0.41402	5.979653	-2.47944	0.013997	0.039003	-4.09894
PCDHAC2	-1.57781	0.348102	-2.47921	0.014005	0.039019	-3.44929
CCDC28A	-0.43562	3.719626	-2.47894	0.014016	0.039041	-3.89008
GLTPD1	-0.55006	5.595498	-2.47787	0.014057	0.039146	-4.09511
C19orf53	0.495029	5.519826	2.477262	0.01408	0.039203	-4.02656
TELO2	0.414627	5.06941	2.47666	0.014103	0.039259	-3.98607
PRPS1	0.490235	4.935884	2.476202	0.01412	0.0393	-3.96128
BLOC1S3	0.427893	4.102754	2.47577	0.014137	0.039339	-3.82238
LSS	-0.54575	5.746896	-2.47541	0.014151	0.039369	-4.10569
SEMA7A	1.129691	3.126854	2.474746	0.014176	0.039417	-3.51813
FAM176B	0.69801	3.205206	2.474839	0.014173	0.039417	-3.59744
SPIRE1	-0.77235	4.942373	-2.47476	0.014175	0.039417	-4.07917
DHRS7B	-0.48421	3.773341	-2.47467	0.014179	0.039417	-3.91775
HK1	-0.68132	7.335302	-2.47412	0.0142	0.039468	-4.09329
C16orf62	-0.76788	4.704616	-2.47371	0.014216	0.039503	-4.06343
NAAA	-0.68122	3.60941	-2.47294	0.014246	0.039579	-3.92391
CST6	2.474991	0.35477	2.47179	0.01429	0.039694	-3.19296
SLC25A25	-0.65237	5.154694	-2.47061	0.014336	0.039814	-4.09578
SCUBE3	-1.19633	0.95352	-2.47019	0.014352	0.039835	-3.51273
TMCO6	0.46905	3.363324	2.470205	0.014352	0.039835	-3.67493
IGFBP3	1.072796	7.137076	2.470236	0.014351	0.039835	-4.11515
NBPF3	-0.7372	2.465609	-2.47009	0.014356	0.039838	-3.71062
SEPSECS	-0.53296	4.014772	-2.46987	0.014365	0.039854	-3.97331
PRKAR1A	-0.33645	7.817745	-2.46969	0.014372	0.039865	-4.09889
SRP9	0.337132	7.685926	2.469264	0.014388	0.039904	-4.11783
ITGB6	1.666215	6.037705	2.469009	0.014398	0.039923	-3.97782
CLEC1A	-0.78755	0.879148	-2.4689	0.014402	0.039927	-3.45043
TYRP1	-1.34865	0.029499	-2.4684	0.014422	0.039973	-3.40141
PQLC1	-0.39805	4.950793	-2.46833	0.014425	0.039973	-4.06953
PLCD1	-0.74683	3.944762	-2.46806	0.014435	0.039986	-3.99379
COL4A5	-1.42402	5.245484	-2.46813	0.014433	0.039986	-4.12566
JMJD5	-0.43899	1.513383	-2.46722	0.014468	0.040061	-3.50275

TSEN15	0.478624	5.012268	2.467234	0.014468	0.040061	-3.99415
SEC24B	-0.34822	6.102143	-2.46708	0.014474	0.040068	-4.12982
C2	1.580202	4.869661	2.46694	0.014479	0.040072	-3.78275
CDK16	0.419984	6.91823	2.466901	0.014481	0.040072	-4.13169
OBSL1	-1.03302	4.952251	-2.46627	0.014505	0.040133	-4.11278
ALKBH2	0.609019	2.798169	2.465815	0.014523	0.040174	-3.56095
TOR1A	0.335632	5.238391	2.465276	0.014545	0.040225	-4.04069
ANO1	1.453084	7.335648	2.464808	0.014563	0.04026	-4.12211
GARS	0.451108	7.363503	2.464829	0.014562	0.04026	-4.13556
RRP1	0.53503	4.76137	2.464342	0.014581	0.040303	-3.95806
PKIG	-0.6224	4.137101	-2.46339	0.014619	0.040399	-4.01751
C17orf69	-0.60902	1.617149	-2.4626	0.01465	0.040477	-3.5532
CAB39	-0.40567	7.158658	-2.46252	0.014653	0.040478	-4.13124
PGBD1	-0.60146	1.798896	-2.46157	0.014691	0.040566	-3.58444
PSMB10	0.811685	4.914215	2.461626	0.014689	0.040566	-3.94615
CCDC41	0.497345	3.25326	2.461496	0.014694	0.040566	-3.6683
KIAA1432	-0.46038	5.284592	-2.46103	0.014712	0.040609	-4.11661
FAM119B	-0.38871	3.47105	-2.45988	0.014758	0.040727	-3.88023
RIMKLA	-1.34788	1.589615	-2.45966	0.014767	0.040743	-3.66707
ZBTB1	-0.32078	5.494058	-2.45936	0.014779	0.040769	-4.12588
BAMBI	2.096902	3.259555	2.459081	0.01479	0.040791	-3.44267
ITFG3	-0.48384	6.823182	-2.45813	0.014828	0.040888	-4.14693
DCLRE1C	0.470527	4.606952	2.457459	0.014855	0.040954	-3.95776
LRRC49	-0.61158	1.928009	-2.45734	0.01486	0.040959	-3.61747
SNAI1	1.074481	2.324729	2.456869	0.014879	0.041003	-3.44974
SIAE	-0.85435	4.654619	-2.45658	0.01489	0.041027	-4.10504
USP10	0.339442	6.729285	2.456325	0.014901	0.041047	-4.15554
ZDHHC3	-0.43472	6.613409	-2.45592	0.014917	0.041084	-4.15636
ZNF268	-0.68704	3.968664	-2.45552	0.014933	0.04112	-4.02071
GPC3	-1.59401	2.920598	-2.45537	0.014939	0.041129	-3.97882
NOC3L	0.395871	4.673187	2.455233	0.014945	0.041136	-3.98536
NOV	-0.9204	2.670168	-2.45468	0.014967	0.041189	-3.82647
NTHL1	0.541165	3.069446	2.454172	0.014987	0.041238	-3.6443
POP5	0.535994	3.439668	2.453137	0.015029	0.041345	-3.71787
FBXO7	-0.26308	6.291143	-2.45303	0.015034	0.041348	-4.16487
GEM	-0.92302	4.371945	-2.4527	0.015047	0.041378	-4.09606
TCTEX1D2	1.163274	2.150602	2.451721	0.015087	0.041478	-3.42592
UCHL3	0.62	4.499138	2.451412	0.015099	0.041505	-3.9266
ACLY	0.580094	7.611242	2.450258	0.015146	0.041626	-4.16828
CHD9	-0.46064	6.39248	-2.44946	0.015179	0.041707	-4.17371
BMS1P4	-0.58717	0.91479	-2.44899	0.015198	0.041751	-3.47482
NETO2	1.69294	4.382297	2.448765	0.015207	0.041763	-3.70837
MAMLD1	-0.80038	2.913064	-2.44874	0.015208	0.041763	-3.86794
ZNF642	0.632259	2.12649	2.448427	0.015221	0.041774	-3.4964
PIP5K1B	-1.70782	2.56602	-2.4485	0.015218	0.041774	-3.94241
WWP2	-0.38763	6.307422	-2.44847	0.015219	0.041774	-4.17642
GPR180	0.504527	4.41344	2.448157	0.015232	0.041796	-3.93671
PYCR2	0.580003	6.063357	2.447906	0.015243	0.041816	-4.13738
PTPRE	0.634613	5.053564	2.447803	0.015247	0.041819	-4.02693
DAP3	0.338494	6.198586	2.447322	0.015267	0.041865	-4.1604
SP110	0.642758	4.624949	2.447084	0.015276	0.041875	-3.95616
NDUFB2	-0.44301	5.026949	-2.44702	0.015279	0.041875	-4.12989
MGEA5	-0.31903	7.052678	-2.44716	0.015273	0.041875	-4.1715
SPHK1	1.419315	4.529639	2.446526	0.015299	0.041922	-3.79699
PTBP2	-0.54074	3.82261	-2.44637	0.015306	0.041932	-4.00456
ALKBH5	-0.35992	6.663923	-2.44621	0.015312	0.041941	-4.17969
BNIP3	-1.24313	4.245742	-2.44568	0.015334	0.041993	-4.12473
IQCB1	0.526167	4.31711	2.444402	0.015387	0.042129	-3.92405

ITGA6	0.806457	8.915469	2.44408	0.0154	0.042158	-4.15705
SPATA5L1	0.506004	3.682105	2.443807	0.015412	0.04218	-3.79656
AP3S2	-0.39354	5.698387	-2.44205	0.015485	0.042372	-4.17942
C11orf61	-0.49155	3.441686	-2.44169	0.0155	0.042404	-3.93552
CBLN3	-0.61491	0.880133	-2.44157	0.015504	0.04241	-3.4923
QPCTL	0.506258	2.762553	2.440897	0.015533	0.04247	-3.62711
CCDC90A	0.533736	4.327775	2.440955	0.01553	0.04247	-3.9338
FAM20C	-0.80978	5.417585	-2.43988	0.015575	0.042577	-4.18704
ZNF395	-0.53314	6.146257	-2.43962	0.015586	0.042599	-4.19711
ZNF747	-0.41088	3.27501	-2.43935	0.015597	0.042614	-3.89364
VPS25	0.378994	5.670784	2.439374	0.015596	0.042614	-4.14072
C9orf142	0.552582	3.869611	2.438913	0.015615	0.042655	-3.83647
VWF	-0.99317	7.226225	-2.43883	0.015619	0.042657	-4.1722
METTL8	0.564351	4.860479	2.438247	0.015643	0.042715	-4.03218
PDIA3P	0.399553	7.369971	2.438	0.015654	0.042734	-4.19863
PPCS	-0.34161	5.076316	-2.43727	0.015684	0.042809	-4.14966
LOC647121	1.29516	1.078534	2.436587	0.015713	0.04288	-3.31832
HIST1H2AC	1.013688	5.021783	2.436379	0.015722	0.042896	-3.99203
RNF126	0.455408	5.832803	2.436279	0.015726	0.042899	-4.15591
HPS6	-0.38436	4.249092	-2.43616	0.015731	0.042904	-4.06796
BMP6	-0.68446	2.044425	-2.43596	0.01574	0.042919	-3.69812
MDC1	0.467397	5.811488	2.43586	0.015744	0.042922	-4.15408
LOC162632	-1.29685	1.627817	-2.43577	0.015748	0.042924	-3.7244
ZNF76	-0.37638	4.283427	-2.43562	0.015754	0.04293	-4.07292
ALDH1B1	-0.80346	5.212205	-2.43557	0.015756	0.04293	-4.18885
PGLYRP4	2.831866	0.037903	2.435483	0.01576	0.042932	-3.27371
LOC374443	0.94535	2.17954	2.435091	0.015776	0.042968	-3.49547
C6orf145	-0.55033	4.635308	-2.43463	0.015796	0.043013	-4.13251
PRKCSH	0.320565	8.155404	2.433555	0.015841	0.043129	-4.1916
PRDX5	-0.49664	6.409441	-2.43345	0.015846	0.043132	-4.2112
KIAA1614	-0.79785	1.383974	-2.4333	0.015852	0.043142	-3.61198
CALCOCO2	-0.40003	6.720825	-2.43247	0.015888	0.04323	-4.2108
MRPL2	0.612367	4.596144	2.432133	0.015902	0.04326	-3.99166
C4orf3	-0.56395	6.314131	-2.43195	0.01591	0.043273	-4.21494
RNPC3	-0.41977	2.890253	-2.43164	0.015923	0.043284	-3.83059
ARFGEF2	-0.44074	7.098396	-2.43175	0.015918	0.043284	-4.20478
MLF2	0.414666	7.481865	2.431698	0.015921	0.043284	-4.21199
FAM108A1	-0.37472	5.094577	-2.43134	0.015936	0.043305	-4.16771
UQCRFS1	-0.51118	6.424077	-2.43131	0.015937	0.043305	-4.21597
CENPC1	-0.43203	3.864048	-2.43112	0.015945	0.043319	-4.03059
ATAD3B	0.643003	4.136874	2.430874	0.015956	0.043338	-3.89538
RAB7A	0.406887	8.477114	2.430799	0.015959	0.043339	-4.19117
SYT11	-0.78176	3.376702	-2.43056	0.015969	0.043358	-4.0003
SCAND2	-0.30402	3.575967	-2.4304	0.015976	0.04336	-3.95651
ATP1B1	-1.01737	8.26262	-2.43039	0.015976	0.04336	-4.15693
MRPL36	0.623431	4.367215	2.429799	0.016002	0.043421	-3.9504
GATSL3	-0.79667	2.64301	-2.42957	0.016012	0.043424	-3.85736
REXO2	-0.43034	4.845584	-2.42956	0.016012	0.043424	-4.1544
PTCH1	-0.85239	4.557704	-2.42968	0.016007	0.043424	-4.16104
MSR1	1.601281	3.11813	2.429233	0.016026	0.043448	-3.55988
SOCS7	-0.48858	2.50371	-2.42919	0.016028	0.043448	-3.76919
DNAJC24	-0.37339	3.613149	-2.42913	0.01603	0.043448	-3.97955
FAM134B	-1.15675	3.66132	-2.42872	0.016048	0.043469	-4.0931
MYL6	-0.4313	8.640949	-2.42867	0.01605	0.043469	-4.166
TM4SF1	0.782805	8.143663	2.428787	0.016045	0.043469	-4.21237
AGFG1	0.37095	7.013379	2.428672	0.01605	0.043469	-4.22314
VMAC	-0.43224	2.218744	-2.42822	0.016069	0.043512	-3.70704
ATHL1	1.237995	4.466862	2.428083	0.016075	0.04352	-3.85476

AP3B1	-0.3261	6.148879	-2.42796	0.016081	0.043526	-4.22307
PLEKHO2	-0.54623	4.895836	-2.42751	0.0161	0.043569	-4.17306
XRCC5	0.293599	8.203559	2.427268	0.01611	0.043589	-4.2045
NAPEPLD	-0.51849	4.815554	-2.42715	0.016115	0.043594	-4.16383
ABCD1	0.696765	5.166629	2.427067	0.016119	0.043596	-4.08333
ERG	-0.65338	3.379693	-2.42698	0.016123	0.043597	-3.98592
EEF1A1P9	-0.54997	2.995603	-2.42684	0.016129	0.043605	-3.88936
SAFB2	-0.22016	5.939949	-2.42671	0.016134	0.043612	-4.21888
VOPP1	0.658849	5.423352	2.426507	0.016143	0.043627	-4.11982
MYO5C	-1.30006	5.01235	-2.42638	0.016149	0.043633	-4.21753
NOL8	0.339614	5.330139	2.425354	0.016193	0.043745	-4.1445
APEH	-0.47913	6.085562	-2.42514	0.016202	0.043762	-4.23047
ATP13A1	0.372442	6.891231	2.425037	0.016207	0.043765	-4.23112
RSC1A1	0.488878	4.341125	2.424656	0.016223	0.043801	-3.98281
DGKH	1.039459	3.117055	2.424389	0.016235	0.043824	-3.6476
STX19	-1.07944	0.781521	-2.42386	0.016258	0.043875	-3.57426
USP34	-0.33505	7.540561	-2.42381	0.01626	0.043875	-4.21545
HOXB5	1.987787	1.672749	2.423641	0.016267	0.043886	-3.34155
SIGMAR1	0.554354	6.655602	2.423212	0.016286	0.043928	-4.22742
RHOG	0.429876	6.199963	2.422977	0.016296	0.043947	-4.2138
AMD1	-0.35506	6.951997	-2.42267	0.01631	0.043975	-4.2306
SMAP1	-0.43938	5.740002	-2.42252	0.016316	0.043984	-4.22831
CSNK2B	0.410481	7.215314	2.422176	0.016331	0.044016	-4.23778
TRAPPC10	-0.32388	6.20188	-2.42166	0.016354	0.044068	-4.23857
EXOC8	-0.2925	4.869832	-2.42125	0.016372	0.044108	-4.16459
SNX25	-0.39385	3.685789	-2.42087	0.016388	0.044145	-4.01762
TLR9	-0.8939	0.300794	-2.42071	0.016395	0.044155	-3.49516
ARNT	-0.26985	5.855575	-2.42063	0.016399	0.044155	-4.23176
RPF2	0.507839	4.181099	2.42034	0.016411	0.044174	-3.9561
ZNF780B	-0.53294	3.947001	-2.42033	0.016412	0.044174	-4.08225
CSPG4	-1.22236	6.133168	-2.42023	0.016416	0.044177	-4.23598
NFX1	-0.36173	5.704726	-2.41975	0.016437	0.044226	-4.23122
WARS	0.788073	7.735293	2.418691	0.016484	0.044342	-4.2436
TAB2	-0.34621	6.825643	-2.41845	0.016494	0.044362	-4.24287
VGLL3	-0.97132	3.037621	-2.41827	0.016502	0.044374	-3.99641
C20orf135	0.651498	3.203969	2.417937	0.016517	0.044405	-3.73627
C1orf170	1.373701	1.022482	2.41759	0.016532	0.044438	-3.34874
AADAC	-1.72148	0.678797	-2.41729	0.016546	0.044465	-3.65591
ARL6IP4	-0.33374	6.184461	-2.41675	0.016569	0.044521	-4.24997
TWIST1	1.544769	1.946186	2.41653	0.016579	0.044538	-3.43781
LTB	1.539006	2.090015	2.415818	0.016611	0.044592	-3.45597
MORN4	-0.75582	1.554848	-2.41581	0.016611	0.044592	-3.67174
FBXW9	0.505226	2.959774	2.415789	0.016612	0.044592	-3.72011
NCOA7	0.773836	6.754387	2.41585	0.016609	0.044592	-4.24085
EXOSC8	0.530086	4.160681	2.415001	0.016647	0.044673	-3.95896
C19orf61	0.415325	4.652048	2.414961	0.016649	0.044673	-4.07358
MAGED2	-0.4532	6.267866	-2.41446	0.016671	0.044724	-4.25657
TMEM158	1.48624	4.03204	2.414062	0.016688	0.044754	-3.76263
PIGH	-0.3622	3.703152	-2.41408	0.016688	0.044754	-4.02908
SCARNA7	-0.75112	1.263309	-2.41384	0.016698	0.044772	-3.63022
CTCF	0.244511	6.199886	2.413272	0.016724	0.044831	-4.24458
OLFML1	-0.80423	2.458426	-2.41305	0.016734	0.04485	-3.8547
PARP10	0.542768	5.731334	2.412519	0.016757	0.044904	-4.19556
MED9	-0.36065	3.537059	-2.41089	0.01683	0.045091	-4.00459
EIF3A	-0.2897	8.515079	-2.41072	0.016838	0.045103	-4.21663
GNAI3	0.37525	6.934835	2.409975	0.016871	0.045183	-4.26676
WASH2P	-0.41031	3.353934	-2.4098	0.016879	0.045195	-3.97894
FCF1	0.367305	4.856061	2.409443	0.016895	0.04523	-4.12319

RHOC	0.501407	6.848888	2.40921	0.016905	0.045249	-4.26603
GSTO1	0.470632	5.841296	2.409107	0.01691	0.045253	-4.21937
OXTR	1.206605	0.774723	2.408756	0.016926	0.045287	-3.35942
RAP1B	0.4712	6.785935	2.408484	0.016938	0.045311	-4.26714
CLK2	0.372199	5.032157	2.408354	0.016944	0.045313	-4.14707
DDX52	0.348711	5.365562	2.408318	0.016946	0.045313	-4.1873
OSBPL5	-0.53024	4.624312	-2.40818	0.016952	0.045322	-4.19146
OLFML3	-0.86626	3.585131	-2.40805	0.016958	0.045325	-4.10068
STT3B	-0.43029	6.696987	-2.408	0.01696	0.045325	-4.26814
ENPEP	1.067677	3.025637	2.407541	0.016981	0.045372	-3.66709
CMTM6	0.418737	6.709962	2.406867	0.017011	0.045445	-4.27041
NHLRC1	1.287086	1.793321	2.406357	0.017034	0.045471	-3.46894
PYROXD2	-0.97978	1.090884	-2.40655	0.017025	0.045471	-3.65028
LOC643837	-0.39714	2.271319	-2.40635	0.017034	0.045471	-3.76327
GAK	-0.51123	6.897654	-2.40629	0.017037	0.045471	-4.26728
NCOA6	0.323181	7.223444	2.406449	0.01703	0.045471	-4.27378
GSTT2	-0.98219	1.080959	-2.40616	0.017043	0.045474	-3.65289
DDB2	0.609203	4.245481	2.406056	0.017048	0.045474	-3.98509
C11orf48	0.562599	4.459067	2.406106	0.017045	0.045474	-4.03391
LOC728323	-0.58254	0.258008	-2.40548	0.017074	0.045534	-3.48673
CA9	-1.91474	3.106015	-2.40537	0.017079	0.045539	-4.14605
BZW1	0.351958	7.651384	2.404315	0.017127	0.045658	-4.27215
JDP2	-0.52756	3.793872	-2.40375	0.017153	0.045718	-4.09592
EFR3B	-0.85208	1.152023	-2.40239	0.017215	0.045875	-3.65486
NEK9	-0.39018	6.107321	-2.40208	0.017228	0.045903	-4.28386
CCDC24	0.785337	2.89428	2.401611	0.01725	0.045952	-3.69917
C16orf58	-0.34824	6.590967	-2.40119	0.017269	0.045994	-4.28647
RPL39L	1.916004	2.609421	2.400526	0.0173	0.046067	-3.51388
HCN3	0.696392	2.838608	2.400261	0.017312	0.046091	-3.70667
CCDC144A	-1.20685	0.013519	-2.40008	0.01732	0.046099	-3.53977
ZNF137	-0.98197	0.55559	-2.39991	0.017328	0.046099	-3.58622
ANO9	1.277855	4.175395	2.400011	0.017323	0.046099	-3.84825
TBL2	0.443639	5.345242	2.39995	0.017326	0.046099	-4.19509
KATNB1	0.459164	4.658466	2.39976	0.017335	0.046108	-4.10374
ZNF630	-0.85728	1.168848	-2.39931	0.017356	0.046147	-3.6633
B2M	0.61108	10.96664	2.399293	0.017357	0.046147	-4.18782
NAA50	0.441903	7.691144	2.39771	0.01743	0.046333	-4.2883
RPL7L1	0.524682	5.188892	2.397445	0.017442	0.046357	-4.17505
TAF1D	0.565573	4.753174	2.396943	0.017465	0.046402	-4.10962
TMCO3	-0.56358	5.199215	-2.39693	0.017466	0.046402	-4.26731
ZNF252	-0.42705	4.894331	-2.39597	0.01751	0.046511	-4.23693
ZNF564	-0.41136	3.244008	-2.3956	0.017527	0.046549	-3.98729
EIF2B2	0.400012	4.590676	2.395522	0.017531	0.046549	-4.11158
ACD	0.539415	4.437658	2.395315	0.01754	0.046566	-4.06061
PDIA6	0.357828	8.015924	2.395181	0.017547	0.046573	-4.28571
PTPRD	-1.3768	1.485457	-2.39482	0.017564	0.04661	-3.80557
HMGA2	3.155099	3.307239	2.394089	0.017597	0.04669	-3.4725
AHR	0.618019	7.869428	2.393938	0.017605	0.0467	-4.29654
HDGFRP2	0.326256	5.254577	2.393511	0.017624	0.046744	-4.21217
ELMO3	0.874841	5.08031	2.393385	0.01763	0.046751	-4.12196
C14orf33	0.878672	1.442027	2.393001	0.017648	0.046765	-3.50156
SGK2	-1.85227	1.014535	-2.39291	0.017652	0.046765	-3.78327
GPN3	0.456068	3.789767	2.392934	0.017651	0.046765	-3.9472
STARD10	-0.90133	6.383761	-2.39308	0.017644	0.046765	-4.3006
BAG1	-0.60641	5.813728	-2.39307	0.017645	0.046765	-4.30271
AHI1	-0.57369	4.228035	-2.39251	0.017671	0.046806	-4.1877
NUDT7	-0.8573	1.139352	-2.39051	0.017765	0.047045	-3.67968
ASB3	0.360965	3.719521	2.389881	0.017795	0.047115	-3.95738

CEP68	-0.52578	5.003225	-2.38952	0.017811	0.04715	-4.26899
SEC16B	-0.98997	3.508759	-2.38922	0.017826	0.047179	-4.14429
ARSB	-0.5848	4.079687	-2.38906	0.017833	0.047191	-4.17982
MED15	0.407426	7.186396	2.388415	0.017864	0.047262	-4.31674
TPPP3	-1.0316	4.388481	-2.38814	0.017877	0.047288	-4.25637
ZNF200	0.389202	3.438404	2.387389	0.017912	0.047372	-3.89912
PLEKHH1	-0.97588	4.551818	-2.38709	0.017926	0.047401	-4.26806
GIT2	-0.30434	5.607163	-2.38677	0.017941	0.047432	-4.30155
RAB27B	-0.99015	2.900795	-2.38649	0.017955	0.047453	-4.0381
CLK3	-0.2876	5.174808	-2.38646	0.017956	0.047453	-4.27268
ARHGEF1	0.383005	6.678481	2.386299	0.017964	0.047464	-4.31829
MYADM	-0.94628	7.148854	-2.38491	0.01803	0.04763	-4.3018
ZNF189	-0.47659	4.463228	-2.3844	0.018054	0.047685	-4.22398
C3orf42	-0.58853	0.091513	-2.38316	0.018113	0.047823	-3.51667
ZNF44	-0.48853	3.253816	-2.38318	0.018112	0.047823	-4.03265
RAD23A	0.370471	7.115568	2.382494	0.018145	0.047898	-4.33071
PI3	4.065651	6.510658	2.382073	0.018165	0.047942	-3.85018
DNLZ	0.625869	2.391005	2.381267	0.018204	0.048035	-3.69131
CDK5	0.574648	3.139753	2.380844	0.018224	0.048079	-3.82157
EPHB4	0.657482	7.052775	2.379358	0.018296	0.048259	-4.33615
PREX1	-0.75034	4.780413	-2.37902	0.018312	0.048287	-4.29083
C22orf13	-0.35921	6.84149	-2.37899	0.018313	0.048287	-4.33422
APOBEC3F	0.947362	2.88204	2.378889	0.018318	0.048291	-3.72841
RPUSD1	0.477374	4.610228	2.378387	0.018343	0.048328	-4.14161
AIFM1	-0.40912	5.355724	-2.37842	0.018341	0.048328	-4.31178
SCAP	-0.42274	6.556574	-2.37839	0.018342	0.048328	-4.33894
PAWR	0.647954	4.992744	2.378207	0.018351	0.048342	-4.17981
PAQR7	-0.53228	3.817898	-2.37813	0.018355	0.048342	-4.16111
CHORDC1	0.558427	5.0036	2.378063	0.018358	0.048342	-4.19285
CCL4	1.368513	1.788832	2.377967	0.018363	0.048345	-3.52306
LIMD1	-0.44698	5.558111	-2.37758	0.018382	0.048385	-4.32615
THBS3	-0.50384	4.200731	-2.37712	0.018404	0.048434	-4.21355
TSSK4	-0.53964	0.415417	-2.37653	0.018432	0.048491	-3.56735
HLCS	-0.40027	4.775529	-2.37655	0.018432	0.048491	-4.26864
LOC152217	0.648648	4.163599	2.376117	0.018453	0.048534	-4.02739
SLU7	-0.28629	5.401876	-2.37606	0.018456	0.048534	-4.31365
USP14	0.444849	6.547419	2.375541	0.018481	0.048582	-4.33823
LPIN2	-1.07536	5.789561	-2.37559	0.018478	0.048582	-4.34546
PTGES2	0.475308	5.638161	2.37544	0.018485	0.048585	-4.27879
NIPAL2	-0.66566	4.270687	-2.3739	0.018561	0.048773	-4.24504
PEBP1	-0.4674	6.886385	-2.37337	0.018587	0.048833	-4.34461
SELO	-0.40144	4.78673	-2.37305	0.018602	0.048864	-4.27767
GRWD1	0.346574	5.240691	2.372912	0.018609	0.048873	-4.25627
TCHH	2.280953	0.517428	2.372747	0.018617	0.048881	-3.41463
THSD7A	-1.1152	0.253066	-2.37265	0.018622	0.048881	-3.62363
MRPL37	0.401405	6.214372	2.37263	0.018623	0.048881	-4.33292
KDR	-0.78796	4.375544	-2.37136	0.018685	0.049036	-4.27387
ZNF579	-0.55686	4.315374	-2.37102	0.018702	0.04907	-4.24588
RGS1	1.471368	4.736213	2.370519	0.018726	0.049126	-4.0038
8-Sep	-0.33342	5.896713	-2.37045	0.01873	0.049126	-4.3515
PLA2G4E	3.055386	0.216848	2.370176	0.018743	0.049152	-3.41917
KCTD13	0.531687	3.729865	2.369957	0.018754	0.049171	-3.97239
PECAM1	-0.72219	4.850075	-2.36987	0.018758	0.049173	-4.31554
GPR109B	2.107048	0.602976	2.369295	0.018787	0.049227	-3.42249
CDKN2C	0.814737	3.765022	2.369363	0.018783	0.049227	-3.93139
MEMO1	0.475956	4.381137	2.369236	0.01879	0.049227	-4.1212
PTPN4	-0.3932	4.01164	-2.36891	0.018806	0.049261	-4.19188
PPM1J	0.89827	0.773896	2.368815	0.01881	0.049263	-3.47615

GFI1	1.198339	1.051768	2.368537	0.018824	0.04929	-3.47625
RPE	0.390766	5.015612	2.368379	0.018832	0.049301	-4.23563
GPX1	0.482085	6.624586	2.366759	0.018912	0.049502	-4.35976
15-Sep	0.32932	6.765686	2.366546	0.018923	0.04952	-4.36622
CCDC23	0.480274	2.891221	2.366436	0.018928	0.049523	-3.82586
TANC2	0.756249	6.14114	2.36638	0.018931	0.049523	-4.32103
IGSF9	1.353235	5.338484	2.366233	0.018938	0.049533	-4.15082
ARVCF	-0.54093	3.59512	-2.36532	0.018984	0.049643	-4.15272
ARNTL2	1.096696	4.703678	2.364783	0.019011	0.049703	-4.08128
LONP2	-0.38274	6.082665	-2.36387	0.019056	0.049813	-4.37176
NECAP1	-0.33144	4.817723	-2.36334	0.019082	0.049873	-4.29676
AKAP1	-0.62053	6.546821	-2.36305	0.019097	0.049902	-4.37176
CPT1B	-0.58678	3.719213	-2.3625	0.019125	0.049964	-4.18818
ZNF837	-0.6316	1.158303	-2.36234	0.019133	0.049976	-3.71516
SLC38A10	-0.34616	6.831676	-2.36219	0.01914	0.049987	-4.37325
FDPS	0.423548	6.206714	2.36198	0.019151	0.049995	-4.35597
LBR	0.541628	6.684212	2.36203	0.019148	0.049995	-4.37069
ZRSR2	-0.47123	2.773796	-2.36178	0.019161	0.050007	-3.9767
FKBP8	-0.36992	7.581909	-2.36175	0.019162	0.050007	-4.35759
SMPDL3A	-0.69224	3.84599	-2.36104	0.019198	0.05009	-4.22277
LOC440905	2.744173	0.422929	2.360787	0.019211	0.050114	-3.44045
PDSS2	-0.38229	4.299426	-2.36059	0.019221	0.050131	-4.24939
C5orf15	0.425536	5.9561	2.360334	0.019234	0.050155	-4.34383
NDUFS6	0.543911	5.379032	2.360215	0.01924	0.050161	-4.28082
PDXDC1	-0.52148	7.09796	-2.35988	0.019256	0.050196	-4.37028
CNP	0.470375	6.281436	2.359696	0.019266	0.050211	-4.36287
NOB1	0.435801	5.82124	2.359125	0.019295	0.050277	-4.33581
MYLIP	-0.68846	4.453057	-2.35876	0.019313	0.050316	-4.30312
EIF3I	0.335632	7.139897	2.357532	0.019375	0.050468	-4.38782
UBXN2A	0.408269	4.283402	2.357411	0.019381	0.050475	-4.14128
PRKX	0.872529	5.363824	2.356979	0.019403	0.050522	-4.25113
SEC11C	-0.60912	4.225982	-2.35644	0.019431	0.050584	-4.27357
FLCN	-0.35311	4.863985	-2.35556	0.019476	0.050688	-4.3211
PLXNA1	0.71759	7.719612	2.355516	0.019478	0.050688	-4.38929
NAA40	0.379169	4.811199	2.354443	0.019532	0.050821	-4.24186
LINGO1	1.25107	2.150457	2.354279	0.019541	0.050833	-3.63262
GPATCH2	0.395623	4.191723	2.35421	0.019544	0.050833	-4.13101
FAM36A	-0.41011	4.932461	-2.35411	0.019549	0.050836	-4.33569
STXBP4	-0.47969	3.030212	-2.35375	0.019568	0.050875	-4.05142
CXorf23	-0.43622	3.224522	-2.35341	0.019585	0.050901	-4.08463
TRIM37	0.391984	5.242486	2.353448	0.019583	0.050901	-4.29669
CCDC9	-0.31298	4.260419	-2.35314	0.019599	0.050928	-4.25383
MSX2	1.454618	2.592707	2.352569	0.019628	0.050994	-3.67201
RNF208	-0.79439	2.63988	-2.35234	0.01964	0.051016	-4.03045
CD63	-0.48544	7.937885	-2.35183	0.019667	0.051074	-4.36633
ADK	0.528093	5.223887	2.351644	0.019676	0.051078	-4.28475
DNAJC14	0.286226	5.484171	2.351639	0.019676	0.051078	-4.335
TTC19	-0.38925	5.332281	-2.35159	0.019679	0.051078	-4.371
NBPF14	-0.5385	4.083831	-2.35097	0.01971	0.05115	-4.26196
IL1RL2	1.159495	1.224029	2.350745	0.019722	0.051171	-3.53875
FIP1L1	0.344633	4.931886	2.350032	0.019759	0.051257	-4.27234
COMMD2	0.450501	5.412008	2.349855	0.019768	0.051271	-4.31744
TSPAN18	-1.00417	4.617999	-2.34924	0.0198	0.051345	-4.36372
ZNF519	0.806093	1.380792	2.349165	0.019804	0.051345	-3.5996
WWTR1	-0.77846	5.650463	-2.349	0.019812	0.051358	-4.40364
C8orf55	0.708584	5.679751	2.348638	0.019831	0.051396	-4.32324
TBC1D5	-0.3857	6.203723	-2.34698	0.019917	0.05161	-4.41196
LOC100170939	-0.52997	3.077354	-2.34667	0.019933	0.051642	-4.087

SIRT2	-0.39465	5.492869	-2.34623	0.019956	0.051691	-4.39274
TCN1	-1.91433	1.62691	-2.34608	0.019964	0.051703	-4.02709
NFKBIB	0.416461	4.803636	2.345952	0.01997	0.05171	-4.25555
RPL12	0.58344	6.725074	2.345672	0.019985	0.051738	-4.40813
ORC2L	0.358425	4.801827	2.345472	0.019995	0.051755	-4.26356
C9orf89	0.571512	3.958931	2.345044	0.020018	0.051794	-4.06949
WDR47	-0.59358	4.666132	-2.34507	0.020016	0.051794	-4.34672
GTF3C3	0.33008	5.312789	2.344957	0.020022	0.051796	-4.32945
ZNF605	-0.78889	3.848393	-2.34431	0.020056	0.051874	-4.27475
KDM6A	-0.53734	5.385232	-2.34411	0.020066	0.051891	-4.39827
IGJ	-1.86638	4.890652	-2.34386	0.020079	0.051915	-4.41326
ISCA1P1	-0.38387	1.234968	-2.34359	0.020094	0.051942	-3.73566
CIT	0.589105	5.570218	2.343168	0.020116	0.05199	-4.33536
ZRANB3	0.64212	2.181165	2.34304	0.020123	0.051998	-3.74209
ZMYND19	0.518064	4.042561	2.342482	0.020152	0.052064	-4.10273
NDUFA8	-0.41117	4.730569	-2.34239	0.020157	0.052067	-4.343
SNHG11	0.461133	2.789742	2.341921	0.020181	0.052121	-3.86535
C14orf79	0.534972	2.855885	2.34146	0.020206	0.052164	-3.86663
HOXB3	1.058537	4.067286	2.341466	0.020205	0.052164	-4.00648
SLC39A14	-0.89938	7.127413	-2.34013	0.020276	0.052336	-4.40619
TUBD1	0.43851	3.230893	2.339858	0.02029	0.052354	-3.95513
NAA20	0.581561	6.009367	2.339848	0.020291	0.052354	-4.38419
GAS2L1	-0.6824	5.441716	-2.33953	0.020308	0.052389	-4.41669
RIF1	0.44422	6.193996	2.339071	0.020332	0.052441	-4.40651
TFB2M	0.46826	4.193584	2.338989	0.020336	0.052443	-4.1525
CD14	0.963301	5.015581	2.338419	0.020366	0.052511	-4.22786
KDM2B	0.373958	5.666693	2.337067	0.020438	0.052686	-4.37789
TRAP1	0.416729	6.64809	2.336984	0.020442	0.052688	-4.42997
PCSK1N	-1.77724	0.601895	-2.33681	0.020451	0.052701	-3.84008
STAG2	0.353028	7.437976	2.336315	0.020478	0.05276	-4.43253
SRBD1	-0.38009	4.01224	-2.33621	0.020483	0.052765	-4.26516
NRN1	-1.09265	1.417289	-2.33598	0.020496	0.052786	-3.88015
NANOS1	-0.48928	2.461888	-2.33472	0.020563	0.05295	-3.97849
RNF19B	0.540945	5.826473	2.334583	0.02057	0.052959	-4.38423
PLA2G15	0.422806	4.426331	2.334306	0.020585	0.052984	-4.21774
VPS13C	-0.61019	6.357625	-2.33426	0.020588	0.052984	-4.44004
TAF12	0.470615	3.69921	2.334103	0.020596	0.052996	-4.05873
C17orf28	-1.55882	5.125815	-2.33386	0.020609	0.053019	-4.43756
SIGLEC10	1.233674	2.161863	2.333717	0.020617	0.053029	-3.68554
CXorf40A	-0.30834	3.242784	-2.33322	0.020643	0.053088	-4.11025
NUDT21	0.36104	6.856126	2.332754	0.020668	0.053143	-4.444
FDX1	-0.47553	4.677412	-2.33247	0.020683	0.053153	-4.3657
FGFRL1	-0.73166	5.364367	-2.33255	0.020679	0.053153	-4.43096
PSIP1	-0.52647	5.588358	-2.33247	0.020684	0.053153	-4.43349
SLC16A9	-1.28333	2.542931	-2.33238	0.020688	0.053155	-4.14647
LYSMD2	0.566988	3.628735	2.332071	0.020705	0.053188	-4.02993
TNFRSF6B	1.967247	4.041892	2.331673	0.020726	0.053225	-3.86889
TMBIM1	-0.59295	8.234397	-2.33166	0.020727	0.053225	-4.39881
NAT14	0.745694	2.280002	2.331315	0.020746	0.053263	-3.76979
GFPT2	-1.13463	2.754561	-2.33098	0.020764	0.053292	-4.16921
GTF3C2	0.31824	6.529572	2.330969	0.020764	0.053292	-4.44299
ARPC5L	0.408868	5.56942	2.330836	0.020771	0.0533	-4.38
USP40	-0.38479	5.529438	-2.32957	0.02084	0.053466	-4.43196
SCLT1	0.489609	3.247468	2.329338	0.020852	0.053486	-3.97348
ARHGAP27	0.703021	5.944378	2.329283	0.020855	0.053486	-4.3933
CSRNP2	-0.36425	5.324825	-2.32912	0.020864	0.053499	-4.42041
MFSD10	0.525191	5.823468	2.328343	0.020906	0.053597	-4.39879
CLEC14A	-0.66315	3.389571	-2.32819	0.020915	0.053609	-4.21777

FNDC4	-0.77856	1.821116	-2.32778	0.020937	0.053646	-3.92323
NIN	-0.65371	5.893861	-2.32778	0.020937	0.053646	-4.45438
PEX11A	-0.48891	1.921273	-2.32732	0.020962	0.053692	-3.89205
PMVK	-0.47125	4.916237	-2.3273	0.020963	0.053692	-4.39963
C19orf73	0.68485	0.153295	2.327145	0.020971	0.053704	-3.52151
C5orf45	-0.55773	1.440972	-2.3264	0.021012	0.053792	-3.82825
CLCN6	-0.41896	4.417557	-2.32632	0.021016	0.053792	-4.34624
HERC2	-0.37226	6.849698	-2.3263	0.021017	0.053792	-4.45426
NDST2	-0.29239	4.612518	-2.32615	0.021025	0.053803	-4.35619
GAL3ST4	1.094659	3.028304	2.326078	0.021029	0.053804	-3.85116
FAM96B	0.469508	4.828799	2.32443	0.021119	0.054024	-4.3017
RRAGB	-0.44519	2.971794	-2.32356	0.021167	0.054136	-4.10202
DDX41	0.371739	5.723528	2.323285	0.021182	0.054155	-4.41397
CCNYL1	-0.49018	5.111753	-2.32329	0.021182	0.054155	-4.42602
TLR2	1.006351	3.932264	2.322959	0.0212	0.054191	-4.03325
PSENEN	0.494904	5.099368	2.322549	0.021223	0.054238	-4.3386
KLRAQ1	-0.30888	4.701073	-2.32212	0.021246	0.054289	-4.37683
CAMKK2	-0.31245	5.969614	-2.32159	0.021275	0.054353	-4.46429
NAPB	-0.46732	3.730718	-2.32151	0.02128	0.054355	-4.26554
MAGEF1	0.634961	5.103935	2.320852	0.021316	0.054427	-4.32663
CCDC3	-0.75184	4.675247	-2.32086	0.021315	0.054427	-4.415
CCDC146	-0.71058	2.813489	-2.32066	0.021327	0.054445	-4.12397
ZNF791	-0.35233	4.046422	-2.32048	0.021336	0.05446	-4.30328
CDC27	0.310438	6.295638	2.320109	0.021357	0.054502	-4.46005
KLK8	3.805569	1.979988	2.319263	0.021404	0.054612	-3.53588
TSPAN7	-1.25327	4.029254	-2.31917	0.021409	0.054615	-4.39792
CSF2RA	1.195658	1.515627	2.318564	0.021442	0.054671	-3.63976
CCDC136	-0.91521	0.690691	-2.31856	0.021443	0.054671	-3.7821
PLEKHJ1	-0.48448	4.489854	-2.31865	0.021438	0.054671	-4.37696
PSAT1	0.847955	5.759925	2.318169	0.021464	0.054716	-4.38633
LOC100128288	-0.69664	0.365617	-2.31753	0.0215	0.054797	-3.71076
HGSNAT	-0.41081	6.934192	-2.31744	0.021505	0.054799	-4.47208
PSMC1	0.432879	4.590205	2.316191	0.021574	0.054967	-4.28718
DTD1	0.541742	4.139485	2.316086	0.02158	0.054967	-4.17909
USF1	0.340977	4.853096	2.316047	0.021582	0.054967	-4.3393
HOXD8	1.042008	1.375545	2.315731	0.0216	0.055002	-3.6459
C6orf108	0.684993	4.545147	2.315474	0.021614	0.055028	-4.2356
ICT1	0.429327	4.132759	2.315365	0.02162	0.055034	-4.20035
NME7	0.505468	3.199386	2.315278	0.021625	0.055036	-3.99316
ABCA12	4.472926	3.316288	2.315143	0.021633	0.055045	-3.54813
RABL3	-0.3864	4.856551	-2.31456	0.021666	0.055115	-4.41619
MRFAP1	-0.32281	7.122656	-2.31451	0.021668	0.055115	-4.47655
FAM185A	-0.37178	2.321508	-2.31438	0.021675	0.055123	-3.97605
SPTLC3	-1.04223	2.607894	-2.31336	0.021733	0.055259	-4.15815
FGD3	0.901954	2.799912	2.313157	0.021744	0.055278	-3.8675
C15orf63	0.528923	5.680563	2.312299	0.021792	0.055391	-4.42199
MAP1LC3A	-0.66342	3.453676	-2.31179	0.021821	0.055453	-4.26757
ASAH1	-0.48651	6.686589	-2.31152	0.021836	0.055482	-4.48858
ABI1	0.36397	6.547624	2.31141	0.021842	0.055488	-4.48648
ALDH3B1	-1.09838	3.91716	-2.31014	0.021914	0.05566	-4.38995
PILRA	1.088833	1.128067	2.309968	0.021924	0.055674	-3.62475
DDX10	0.478485	4.923909	2.309049	0.021976	0.055796	-4.34845
SLC35B1	0.398444	5.232174	2.308755	0.021992	0.055828	-4.39517
CELSR3	1.735075	4.170564	2.306995	0.022092	0.056072	-3.98196
SHOC2	-0.28085	5.890492	-2.30672	0.022108	0.056101	-4.49466
WDR44	-0.41555	4.967357	-2.30646	0.022123	0.056129	-4.447
RBM15B	-0.33168	6.628638	-2.3062	0.022138	0.056156	-4.50338
ZNF510	-0.38791	3.929344	-2.3057	0.022166	0.056218	-4.32221

DEDD	0.323684	5.429312	2.305323	0.022188	0.056263	-4.43155
UFSP1	0.68182	0.463741	2.304518	0.022234	0.056369	-3.60255
POLR1A	0.425973	6.0255	2.304208	0.022251	0.056404	-4.47598
RPS27L	-0.49338	4.775956	-2.30318	0.02231	0.056532	-4.44252
ELF3	1.394903	7.527274	2.303206	0.022309	0.056532	-4.50413
TMBIM4	-0.51551	5.237512	-2.30233	0.022359	0.056646	-4.48316
ADH7	-2.3947	1.458097	-2.30095	0.022439	0.056837	-4.18624
LTV1	0.365383	4.616596	2.300788	0.022448	0.056848	-4.33628
DENND5A	-0.61859	5.433491	-2.30067	0.022455	0.056848	-4.50199
INSR	-0.68086	6.386887	-2.30067	0.022455	0.056848	-4.51453
NFATC1	-0.71427	4.490161	-2.30046	0.022467	0.056868	-4.44021
AP1AR	0.375256	4.983007	2.299881	0.022501	0.056942	-4.3886
ABHD2	-0.86866	8.166627	-2.29976	0.022508	0.05695	-4.4644
NIF3L1	0.323825	4.410073	2.298857	0.02256	0.057071	-4.31052
ENY2	0.41344	4.497115	2.29869	0.022569	0.057086	-4.31244
S100A7A	4.113415	0.77803	2.298512	0.02258	0.057101	-3.57621
IRF7	0.935943	4.829078	2.298389	0.022587	0.057109	-4.28464
SERPINB7	2.79779	1.047187	2.297606	0.022632	0.057193	-3.57889
TTLL1	-0.53143	1.680567	-2.29762	0.022632	0.057193	-3.92686
PEX3	-0.35655	4.066681	-2.29768	0.022628	0.057193	-4.35728
SCN3B	-1.06973	0.418008	-2.29749	0.022639	0.0572	-3.80704
WDR72	3.507308	4.746794	2.297417	0.022643	0.0572	-3.83294
RPL30	0.398507	8.97048	2.297292	0.022651	0.057208	-4.48115
STX8	-0.38114	3.546394	-2.29714	0.02266	0.05722	-4.26923
LYRM1	-0.48979	4.141928	-2.29707	0.022664	0.05722	-4.38497
CAPN8	-2.30521	0.278475	-2.29642	0.022701	0.057305	-3.93528
VWA2	-1.26805	2.568896	-2.29594	0.02273	0.057366	-4.22211
LIF	1.652715	3.568819	2.295178	0.022774	0.057467	-3.92176
FLRT3	-1.36092	3.225314	-2.29352	0.022871	0.057702	-4.36331
PHF21A	-0.41233	5.226959	-2.29342	0.022877	0.057706	-4.49679
PCGF6	0.387274	3.011492	2.292968	0.022903	0.057763	-4.02695
PCDHB8	1.626945	0.74186	2.292542	0.022928	0.057806	-3.58818
LTB4R	1.589344	4.759709	2.292503	0.022931	0.057806	-4.16251
LLPH	0.49884	4.692629	2.292468	0.022933	0.057806	-4.34763
ADAP2	0.861635	3.155646	2.291831	0.02297	0.057858	-3.97893
DOCK2	-0.95503	3.221013	-2.29184	0.02297	0.057858	-4.31377
SSPN	-0.85005	4.162134	-2.29193	0.022964	0.057858	-4.43786
PNPLA2	-0.5913	6.787838	-2.29184	0.02297	0.057858	-4.52909
SURF2	0.548386	3.517245	2.29135	0.022999	0.057915	-4.10071
TMEM165	0.354495	6.259869	2.29124	0.023005	0.057915	-4.52159
SLC9A1	-0.58676	7.093109	-2.2913	0.023002	0.057915	-4.52406
RASSF6	-1.1474	3.866696	-2.29037	0.023056	0.058031	-4.4302
VEZF1	-0.39458	6.043995	-2.29031	0.02306	0.058031	-4.5376
THBS4	-1.7908	0.946107	-2.28982	0.023089	0.058094	-4.01185
TCEA2	-0.72453	2.575194	-2.2895	0.023108	0.058132	-4.14798
DTNBP1	-0.39773	3.295673	-2.28925	0.023123	0.058159	-4.23683
ARHGDIB	0.713947	5.729235	2.288983	0.023139	0.058188	-4.46248
BLOC1S1	-0.38387	4.946284	-2.28821	0.023184	0.058292	-4.48327
OSTC	0.34337	5.721359	2.287595	0.023221	0.058374	-4.49576
UNC119	0.537333	4.289622	2.287378	0.023234	0.058396	-4.27572
IGSF9B	-1.24202	1.310327	-2.28666	0.023277	0.058493	-3.99433
EFCAB4B	1.311031	2.547701	2.286366	0.023294	0.058513	-3.82974
METTL11A	0.466331	3.758734	2.286313	0.023297	0.058513	-4.1772
SFRS17A	-0.42488	5.265726	-2.28635	0.023295	0.058513	-4.51559
KIAA1310	-0.25336	6.2976	-2.28622	0.023303	0.058517	-4.54862
KBTBD8	-0.66028	1.623324	-2.28592	0.023321	0.058552	-3.96167
RPS3A	-0.42779	6.941181	-2.28572	0.023333	0.058569	-4.54265
YPEL5	-0.36054	6.062674	-2.28566	0.023336	0.058569	-4.54778

WDR90	0.520992	5.251448	2.285511	0.023345	0.058581	-4.43663
TRPT1	-0.37311	3.936658	-2.2847	0.023394	0.058693	-4.3668
TRIM44	-0.40419	6.218963	-2.28448	0.023407	0.058714	-4.55289
HCK	1.140371	3.20894	2.283897	0.023442	0.058792	-3.96636
PUSL1	0.52781	3.590143	2.283502	0.023465	0.058834	-4.1371
CCDC14	0.485781	5.450681	2.283475	0.023467	0.058834	-4.46697
GK	1.012614	3.214208	2.282682	0.023515	0.058932	-3.98382
LEPREL1	-1.2244	4.523276	-2.28272	0.023513	0.058932	-4.51887
SUZ12P	0.501304	2.667416	2.282261	0.02354	0.058985	-3.97177
BCL2L13	-0.42066	6.004868	-2.28189	0.023563	0.059029	-4.55601
ABCF1	0.288533	6.748584	2.281826	0.023566	0.059029	-4.5579
LOC285830	-0.72163	0.196309	-2.28173	0.023572	0.059034	-3.76776
CNPY2	0.43415	5.297574	2.2815	0.023586	0.059048	-4.45979
TSC22D2	-0.55529	5.703774	-2.28149	0.023586	0.059048	-4.55239
MPV17L	-1.30984	1.112198	-2.28094	0.02362	0.059121	-3.98173
RGPD3	-0.40689	3.816692	-2.28068	0.023635	0.05915	-4.36159
KIAA1217	0.557676	7.393606	2.28044	0.02365	0.059175	-4.56096
FAM136A	0.396353	5.65292	2.280085	0.023671	0.059218	-4.50227
NKD2	1.795378	2.423406	2.279988	0.023677	0.059222	-3.76983
FRMD4A	-0.58951	4.500001	-2.27933	0.023717	0.059312	-4.47713
NMNAT1	-0.49024	3.061313	-2.2791	0.023731	0.059336	-4.22594
CBS	-1.29466	2.738639	-2.27877	0.023751	0.059375	-4.30494
APOA1BP	0.441906	6.044748	2.276946	0.023862	0.059641	-4.53687
C9orf9	-0.64556	2.185086	-2.27676	0.023873	0.059658	-4.0806
ZNF511	0.504364	4.201261	2.27607	0.023915	0.059753	-4.28721
NRAS	0.530742	6.611532	2.275595	0.023944	0.059815	-4.56352
RWDD4A	-0.35289	4.275757	-2.27482	0.023991	0.059922	-4.43672
CASC2	-0.62272	0.856866	-2.27427	0.024025	0.059996	-3.8629
RFXANK	0.429902	4.496361	2.274079	0.024037	0.060014	-4.36415
CCDC30	-0.76314	0.188272	-2.27363	0.024064	0.060072	-3.7888
C12orf29	-0.40273	4.472254	-2.2735	0.024072	0.060081	-4.46901
PMS2CL	0.391732	2.942212	2.273295	0.024085	0.060102	-4.05637
C7orf25	0.374176	3.749567	2.272394	0.02414	0.060219	-4.22546
SEC24A	-0.47344	6.070097	-2.27243	0.024138	0.060219	-4.57864
ZSCAN5A	0.581504	1.918708	2.27212	0.024157	0.06025	-3.86698
GSTM2	-1.30738	3.439869	-2.27194	0.024168	0.060256	-4.43946
PLBD1	1.02938	5.854209	2.272005	0.024164	0.060256	-4.48255
MRPS5	0.328029	5.408659	2.271386	0.024202	0.060331	-4.50415
DCPS	0.601977	3.704928	2.271234	0.024212	0.060343	-4.17446
CHST6	1.279971	1.654213	2.270843	0.024236	0.060393	-3.75231
HES2	4.006173	3.549564	2.270153	0.024278	0.060488	-3.68462
EVL	-0.72674	4.972493	-2.27005	0.024285	0.060493	-4.5498
GIYD2	0.598008	5.026398	2.269904	0.024294	0.060505	-4.43334
MED10	0.485557	4.303843	2.269806	0.0243	0.060509	-4.32639
GBP5	2.200636	3.754224	2.269721	0.024305	0.060511	-3.92947
SUPT3H	0.584036	2.449723	2.268893	0.024356	0.060628	-3.95465
LOC100286793	0.571518	2.616163	2.268562	0.024377	0.060669	-3.98328
FAM116A	-0.33317	5.029246	-2.26833	0.024391	0.060693	-4.53096
DKK2	1.346805	0.430183	2.268166	0.024401	0.060694	-3.63818
SRGAP3	-0.83114	3.070812	-2.26819	0.0244	0.060694	-4.31756
TBK1	0.361947	5.151949	2.26812	0.024404	0.060694	-4.4807
YRDC	0.387019	4.249504	2.267961	0.024414	0.060707	-4.33742
UBE2V1	0.335506	7.225407	2.26782	0.024423	0.060718	-4.58867
IMP4	0.362998	5.021951	2.267268	0.024457	0.060793	-4.46691
DUSP7	0.915314	6.072982	2.266957	0.024477	0.06083	-4.5269
CDC42BPB	-0.38963	7.977051	-2.26627	0.024519	0.060925	-4.55984
TCTA	-0.45451	4.531588	-2.26598	0.024538	0.06096	-4.49667
CAPN5	-1.52504	5.594028	-2.2658	0.024549	0.060976	-4.58999

KLF6	-0.59588	7.512537	-2.26533	0.024578	0.061038	-4.5706
BAIAP2L2	2.342936	3.968425	2.264653	0.02462	0.061133	-3.94667
GABBR1	-0.72132	4.120685	-2.26445	0.024633	0.061154	-4.48084
GNA15	1.813018	4.834202	2.263956	0.024664	0.061216	-4.19899
KDELR1	0.346086	7.732481	2.263909	0.024667	0.061216	-4.58882
LTBR	0.441052	7.194942	2.263646	0.024683	0.061246	-4.5989
MAST4	-0.90517	6.008685	-2.26345	0.024696	0.061266	-4.59852
PTPRZ1	-1.9204	3.990244	-2.26305	0.024721	0.061317	-4.56146
ATXN1	-0.47664	5.626905	-2.26293	0.024728	0.061325	-4.58866
RBM33	-0.32546	6.096888	-2.26201	0.024786	0.061457	-4.60016
PWP1	0.305721	5.454022	2.261583	0.024813	0.061513	-4.53252
CARD8	-0.50853	4.092642	-2.26131	0.02483	0.061539	-4.45973
KIAA0247	-0.47122	6.579011	-2.26128	0.024832	0.061539	-4.60214
PPP2CB	-0.37023	6.333468	-2.26106	0.024846	0.061562	-4.60505
C12orf49	-0.47143	5.063124	-2.26094	0.024854	0.06157	-4.55969
RDH16	1.420424	0.011487	2.260333	0.024892	0.061632	-3.65269
ST3GAL2	0.695044	4.704451	2.260341	0.024892	0.061632	-4.388
HUS1	0.396354	4.607574	2.260349	0.024891	0.061632	-4.41937
LMAN1	-0.45109	7.438444	-2.2599	0.024919	0.061688	-4.58818
LYPD1	1.386115	1.007522	2.259662	0.024934	0.061715	-3.68934
KHDRBS3	-0.9388	1.728094	-2.25943	0.024949	0.06173	-4.08098
SOX12	0.704753	5.170844	2.259457	0.024947	0.06173	-4.46409
TMEM115	-0.31098	4.995079	-2.25923	0.024962	0.061739	-4.546
SGK1	-0.9954	7.290408	-2.25927	0.024959	0.061739	-4.57966
ZNF234	-0.47676	2.81524	-2.25884	0.024987	0.06179	-4.21629
MZF1	-0.5066	4.386208	-2.25874	0.024993	0.061794	-4.50135
S1PR5	2.793914	1.8384	2.258319	0.025019	0.061827	-3.66436
MNAT1	0.468429	3.663222	2.258329	0.025019	0.061827	-4.21958
RPL37	0.555288	8.926857	2.258442	0.025012	0.061827	-4.57341
HMGN1	0.399657	6.550069	2.258139	0.025031	0.061845	-4.60397
KIF1B	-0.41641	6.773638	-2.25788	0.025048	0.061875	-4.60743
OXNAD1	-0.39947	3.215716	-2.25731	0.025084	0.061942	-4.28908
DRAP1	0.559037	5.579965	2.257313	0.025083	0.061942	-4.53209
SMARCB1	0.381354	6.090864	2.256406	0.025141	0.062073	-4.58856
ECSCR	-0.70528	0.975636	-2.2559	0.025173	0.062142	-3.93215
ZNF226	-0.45805	3.225978	-2.25558	0.025194	0.062181	-4.30671
VEZT	0.331896	6.211393	2.255168	0.02522	0.062235	-4.60007
JAZF1	-0.5961	3.734844	-2.25414	0.025286	0.062387	-4.43585
CHIC2	0.460196	3.379761	2.25358	0.025322	0.062464	-4.17262
MRPL48	0.598411	4.280857	2.252757	0.025375	0.062583	-4.33714
CKMT1B	1.230571	5.827651	2.252332	0.025402	0.06264	-4.49968
DDA1	0.308381	5.691041	2.251824	0.025435	0.062698	-4.57486
DDX54	0.318177	6.481544	2.251849	0.025433	0.062698	-4.61802
PYGO2	0.290284	5.959462	2.25072	0.025506	0.062852	-4.59812
ARRDC3	-0.53574	6.060517	-2.25071	0.025506	0.062852	-4.62691
MBD1	-0.38075	5.70086	-2.25054	0.025517	0.062868	-4.61554
MYD88	0.473903	6.5531	2.250475	0.025522	0.062868	-4.61873
TBC1D2	0.914228	5.46635	2.249039	0.025614	0.063086	-4.50044
RNF141	-0.54065	5.929926	-2.24858	0.025644	0.063148	-4.62987
PRKCD	-0.51143	5.983836	-2.24759	0.025708	0.063294	-4.63251
ZBTB43	-0.42865	4.831802	-2.24718	0.025735	0.06335	-4.56676
SIPA1L3	0.488236	7.300377	2.24689	0.025754	0.063385	-4.63522
HSPG2	-0.68606	9.525621	-2.24602	0.02581	0.063512	-4.54319
CCT8	0.358719	6.797183	2.245813	0.025824	0.063535	-4.63698
PGM2L1	1.11538	4.697324	2.24574	0.025829	0.063535	-4.3393
GNRHR2	-0.46443	3.967836	-2.24552	0.025843	0.06356	-4.47207
CDK17	-0.39624	5.233794	-2.24514	0.025868	0.06361	-4.60283
B3GAT3	0.451065	4.200696	2.243792	0.025956	0.063804	-4.36683

ATP5G3	-0.42077	7.232477	-2.24381	0.025955	0.063804	-4.6291
C6orf153	0.443057	5.312711	2.243167	0.025997	0.063893	-4.54515
FAM40A	-0.29185	4.872418	-2.24272	0.026026	0.063954	-4.56886
NOL6	0.443827	6.445372	2.242383	0.026049	0.063997	-4.63358
KIAA0368	-0.30636	7.316956	-2.2421	0.026067	0.064031	-4.63322
IFIH1	0.698246	5.224472	2.241635	0.026098	0.064084	-4.50976
MATN2	-0.97642	4.987075	-2.2417	0.026094	0.064084	-4.62517
ZFHX3	-0.49551	6.588995	-2.24141	0.026113	0.064109	-4.6452
ZNF580	-0.44301	4.738173	-2.24103	0.026138	0.064159	-4.57257
TTC32	0.639306	1.505518	2.240496	0.026173	0.064235	-3.86996
KLHDC7B	2.603416	2.471932	2.239775	0.026221	0.06434	-3.7718
KAT5	-0.26957	5.264681	-2.23923	0.026257	0.064406	-4.6096
COG3	-0.43282	5.364015	-2.23927	0.026254	0.064406	-4.62576
C1QC	1.098093	5.782261	2.238439	0.026309	0.064523	-4.54059
THBS2	1.303391	6.417573	2.237936	0.026342	0.064594	-4.5883
HOXB6	2.252057	2.308138	2.237068	0.0264	0.064724	-3.79013
MEX3D	0.512419	5.392236	2.236789	0.026419	0.064758	-4.56117
MC1R	0.811503	2.53709	2.23636	0.026447	0.064817	-4.00584
KCTD17	0.717368	3.050994	2.236192	0.026458	0.064833	-4.10502
NBPF9	-0.41711	3.378827	-2.23601	0.02647	0.064851	-4.37528
TRIM2	-0.85455	6.895771	-2.2358	0.026484	0.064874	-4.64526
RARG	0.885256	6.003933	2.235495	0.026505	0.064913	-4.59204
RNF103	-0.53593	5.64436	-2.23456	0.026567	0.065054	-4.65307
IL1RAP	1.121801	5.283991	2.234081	0.026599	0.065121	-4.47998
ACOT11	-0.68544	4.399598	-2.23364	0.026629	0.065183	-4.57449
C1orf124	0.399631	3.879496	2.233008	0.026671	0.065274	-4.33433
SYNGR2	0.487171	7.583505	2.232396	0.026712	0.065363	-4.66293
CHCHD8	0.573623	5.059652	2.232144	0.026729	0.065393	-4.52421
WDR89	0.342287	3.790512	2.231788	0.026753	0.065441	-4.32804
ZNF230	-0.43745	1.782868	-2.23125	0.026789	0.065505	-4.07296
SMN2	0.419041	5.389236	2.231214	0.026792	0.065505	-4.58208
U2AF1	0.327062	6.049401	2.231185	0.026794	0.065505	-4.64415
B4GALT2	0.456122	5.766867	2.231044	0.026803	0.065517	-4.61553
FAIM	0.496766	3.330469	2.230942	0.02681	0.065522	-4.20492
UFD1L	0.339999	5.685644	2.230476	0.026842	0.065588	-4.61859
MAP7	0.694463	6.114492	2.230304	0.026853	0.065605	-4.62699
RPA1	0.417482	6.580937	2.229831	0.026885	0.065671	-4.66626
SEH1L	0.415372	5.868214	2.22935	0.026918	0.065739	-4.63043
TYROBP	1.180268	4.313332	2.229257	0.026924	0.065743	-4.28437
ANKRD26	-0.4811	4.151267	-2.22894	0.026945	0.065784	-4.53524
PDE8A	-0.48232	5.49687	-2.22878	0.026956	0.065799	-4.65785
LIX1L	-0.57208	4.428313	-2.22858	0.026969	0.065808	-4.57939
RBM9	-0.41831	6.534468	-2.2286	0.026968	0.065808	-4.67473
AGAP1	-0.54364	4.928215	-2.22699	0.027078	0.066061	-4.62813
FBXL15	-0.39758	3.621732	-2.22531	0.027192	0.066328	-4.44293
FAM92A1	0.870915	2.30075	2.22497	0.027215	0.066373	-3.98717
PCK2	0.599471	4.850814	2.224595	0.027241	0.066424	-4.50578
MYB	2.42137	3.020737	2.224359	0.027257	0.066452	-3.88804
TSPAN31	-0.33372	4.834239	-2.22374	0.027299	0.066543	-4.60964
LOC619207	-0.74658	0.680565	-2.2235	0.027316	0.066572	-3.9615
DAPP1	1.41307	3.749397	2.223404	0.027322	0.066576	-4.14158
FAM124A	-0.90601	0.775022	-2.2232	0.027337	0.066599	-3.99999
FAM162A	-0.54433	5.50635	-2.22268	0.027372	0.066674	-4.67387
ZNF639	0.474302	5.288612	2.221948	0.027422	0.066754	-4.58564
MRPS10	0.436773	5.852577	2.221957	0.027422	0.066754	-4.64364
HM13	0.368173	7.129684	2.221924	0.027424	0.066754	-4.69007
CDC37	0.304294	6.932285	2.221926	0.027424	0.066754	-4.69039
FOLH1	1.525225	1.124882	2.221641	0.027443	0.066766	-3.76694

CAPRIN2	-0.55836	4.181656	-2.22165	0.027442	0.066766	-4.5635
SELM	-0.72456	4.856893	-2.2217	0.027439	0.066766	-4.64706
PARP16	-0.32767	3.53663	-2.22148	0.027455	0.066782	-4.42269
RAG1AP1	0.516155	4.784464	2.220931	0.027492	0.066862	-4.51527
DPY19L4	-0.39117	5.722866	-2.21905	0.027622	0.067162	-4.6852
CNN2	0.576134	7.209752	2.219004	0.027625	0.067162	-4.69646
OSR2	0.961457	3.092953	2.218856	0.027636	0.067175	-4.11
CRKL	0.425355	7.111399	2.218575	0.027655	0.067211	-4.69757
NID2	1.049111	4.867173	2.218411	0.027666	0.067227	-4.4462
WNT7B	4.556775	4.217251	2.217399	0.027737	0.067374	-3.81218
JMJD1C	-0.4346	6.624527	-2.21744	0.027734	0.067374	-4.69758
DNAH2	2.23109	0.900287	2.216861	0.027774	0.067453	-3.74685
IRF1	0.719203	6.00804	2.216691	0.027786	0.06747	-4.64635
TTC5	0.401781	3.240077	2.216089	0.027828	0.06756	-4.23547
SART1	0.307753	6.159955	2.215733	0.027852	0.067608	-4.68444
DLX5	2.743835	1.093939	2.215159	0.027892	0.067694	-3.75122
TIMM10	0.472524	3.926127	2.214981	0.027905	0.0677	-4.36863
RFWD2	0.296436	5.653186	2.215035	0.027901	0.0677	-4.6527
TGFB1	0.847827	6.620701	2.21464	0.027929	0.067746	-4.68495
IRF6	1.001824	6.475893	2.214192	0.02796	0.06781	-4.66751
TMEM151B	-0.71997	0.355777	-2.21386	0.027983	0.067854	-3.93306
ARID3A	1.045107	3.473327	2.213786	0.027988	0.067856	-4.17316
HARS	-0.2927	5.493729	-2.21244	0.028083	0.068073	-4.68461
MTPAP	0.347739	4.800187	2.211999	0.028114	0.068136	-4.56041
EIF1AX	-0.3885	6.26117	-2.2115	0.028148	0.068208	-4.71278
SLC23A2	-0.45193	5.560165	-2.2114	0.028156	0.068215	-4.69727
ZNF41	-0.37451	3.753737	-2.21097	0.028186	0.068275	-4.49644
TRPV4	1.947187	3.114351	2.210663	0.028207	0.068316	-3.99576
IRAK2	1.277348	3.86236	2.210448	0.028223	0.068329	-4.21424
FAM65A	-0.48043	6.045743	-2.21047	0.028221	0.068329	-4.71366
TBCCD1	0.407846	4.093776	2.209564	0.028285	0.068444	-4.42785
DYNLL2	-0.38834	4.780102	-2.2097	0.028275	0.068444	-4.63985
NAPG	-0.36738	5.30415	-2.20963	0.028281	0.068444	-4.6828
RC3H1	-0.32397	6.196993	-2.20865	0.02835	0.068589	-4.71788
ZC3H7A	-0.27551	5.522629	-2.20852	0.028359	0.068591	-4.69388
ZC3H7B	-0.3376	7.019334	-2.2085	0.02836	0.068591	-4.71163
CABYR	1.713989	1.647552	2.208285	0.028375	0.068616	-3.83478
LOC729020	0.405986	0.49231	2.207804	0.02841	0.068662	-3.83681
UBXN11	0.535521	3.559761	2.207853	0.028406	0.068662	-4.29265
ZDHHC18	0.463053	5.19178	2.207922	0.028401	0.068662	-4.60597
ITPRIPL1	1.104541	0.506977	2.207386	0.028439	0.068717	-3.77107
RCOR1	0.467493	6.542774	2.207346	0.028442	0.068717	-4.71221
DLG3	-0.51956	5.413243	-2.20687	0.028476	0.068788	-4.70271
DMAP1	-0.29411	4.378322	-2.20623	0.028521	0.068885	-4.59228
DCAF12	0.394498	6.412279	2.206097	0.028531	0.068896	-4.71269
THNSL2	-1.34628	2.426162	-2.20548	0.028575	0.068986	-4.41029
PFDN5	-0.35367	6.721928	-2.20543	0.028578	0.068986	-4.72313
NBR1	-0.3161	7.22046	-2.20526	0.02859	0.069003	-4.71491
RGPD4	-0.37392	2.791954	-2.20466	0.028633	0.069095	-4.30797
FZR1	0.359228	6.0262	2.203843	0.028691	0.069224	-4.7001
CEACAM6	3.122439	6.642281	2.203492	0.028717	0.069273	-4.4454
RPL38	0.460952	8.518847	2.202733	0.028771	0.069392	-4.70474
MRPS30	0.419435	4.905301	2.202594	0.028781	0.069404	-4.58606
ZNF701	-0.85515	1.807687	-2.20202	0.028822	0.069479	-4.20301
CMTM7	1.052277	3.713487	2.202065	0.028819	0.069479	-4.24052
MSTO1	0.405416	4.674868	2.201906	0.02883	0.069487	-4.55528
AMFR	-0.35399	6.622852	-2.20178	0.028839	0.069497	-4.73232
RG9MTD2	-0.41071	2.564925	-2.20163	0.02885	0.06951	-4.27275

PLEKHA7	-0.83309	4.96276	-2.20117	0.028883	0.069579	-4.70408
BCO2	-0.89768	0.319791	-2.2008	0.02891	0.069619	-3.97903
MTMR15	-0.32792	4.746058	-2.2008	0.02891	0.069619	-4.64967
LOC338799	-0.54859	3.209411	-2.20056	0.028927	0.069647	-4.43843
PBX4	1.072924	0.341269	2.200002	0.028967	0.069733	-3.77905
PIAS1	-0.30349	5.273552	-2.19993	0.028973	0.069734	-4.69736
GPBP1L1	-0.24995	6.933358	-2.19979	0.028982	0.069745	-4.73335
IARS2	0.316501	7.291906	2.199337	0.029015	0.069812	-4.73659
C12orf76	0.443729	1.80983	2.199216	0.029024	0.069821	-4.02388
TTLL4	0.505172	5.326581	2.199097	0.029032	0.06983	-4.63501
GRAMD4	-0.44911	5.656042	-2.19894	0.029044	0.069845	-4.72782
CMPK2	1.137505	4.098712	2.198796	0.029054	0.069846	-4.30874
LRBA	-0.46033	6.734167	-2.1988	0.029054	0.069846	-4.73561
WSB1	-0.4656	6.362511	-2.19853	0.029073	0.06988	-4.74056
CCDC152	-0.968	1.540285	-2.19825	0.029094	0.069917	-4.18024
RBM17	0.326619	6.146487	2.19814	0.029102	0.069924	-4.72072
AMOTL1	-0.79009	6.489622	-2.19797	0.029114	0.069941	-4.73626
BEGAIN	-0.84745	0.597007	-2.1975	0.029148	0.069989	-4.01774
RGS10	0.602133	3.991005	2.197396	0.029156	0.069989	-4.39629
EFCAB4A	-1.1833	3.017383	-2.19741	0.029154	0.069989	-4.50993
POFUT2	-0.37508	4.519359	-2.19757	0.029143	0.069989	-4.63612
FOXK2	0.314756	6.633887	2.197354	0.029159	0.069989	-4.73986
CYP24A1	2.972296	0.920822	2.19638	0.029229	0.070134	-3.78897
ZMYND8	-0.38127	6.575483	-2.19642	0.029226	0.070134	-4.74403
ANAPC10	0.380477	2.417302	2.196253	0.029239	0.070144	-4.13319
LOC400931	-0.5852	2.473743	-2.19531	0.029307	0.070296	-4.29973
AXIN1	0.39715	5.669479	2.194914	0.029336	0.070354	-4.68925
FBXO41	0.936771	4.323213	2.194676	0.029353	0.070383	-4.40734
TTC22	1.125361	4.564963	2.194082	0.029397	0.070475	-4.41839
TEX264	-0.38498	4.74737	-2.194	0.029403	0.070478	-4.66924
SMAP2	-0.46594	5.472148	-2.19382	0.029416	0.070497	-4.7314
FLJ42627	-0.61886	1.443991	-2.19341	0.029446	0.070545	-4.12431
ACTN4	0.369113	10.08862	2.193468	0.029441	0.070545	-4.66921
GOLGA2B	-0.67256	2.55729	-2.19258	0.029507	0.070679	-4.3393
TUFT1	0.691429	5.283836	2.191954	0.029552	0.070776	-4.6261
FGD5	-0.7745	3.914533	-2.19149	0.029587	0.070846	-4.6172
BIVM	-0.42315	4.195472	-2.19074	0.029642	0.070953	-4.61619
LAPTM4A	-0.24995	8.078024	-2.19075	0.029641	0.070953	-4.7245
ARHGEF10L	-0.70454	5.589902	-2.19062	0.02965	0.070961	-4.75041
TRIB1	-0.61095	6.838325	-2.19004	0.029693	0.071051	-4.74988
TMCO1	0.390506	6.579117	2.189924	0.029702	0.07106	-4.7528
PDXDC2	-0.61562	3.925681	-2.18916	0.029758	0.071183	-4.60405
ZNF253	-0.81787	2.520693	-2.18826	0.029824	0.071328	-4.3692
XAF1	0.964084	5.906204	2.187426	0.029886	0.071465	-4.67743
HSD17B10	0.394818	5.439748	2.187275	0.029897	0.071479	-4.68419
ZNF273	0.630018	3.014146	2.186914	0.029924	0.071531	-4.21669
PWWP2B	-0.43535	4.354711	-2.18663	0.029945	0.071569	-4.64573
KIAA1377	-0.85048	2.364848	-2.18623	0.029975	0.071628	-4.34922
FAM101A	-1.68818	2.3527	-2.18561	0.030021	0.071725	-4.4823
PPP3CA	-0.39363	5.99668	-2.18485	0.030077	0.071835	-4.76645
OCIAD1	-0.35724	6.438814	-2.18491	0.030073	0.071835	-4.76989
C12orf11	0.529061	5.123073	2.184522	0.030102	0.071882	-4.63991
IPO8	-0.36696	6.243235	-2.1844	0.030111	0.071891	-4.77064
ABT1	0.338002	4.774434	2.183826	0.030154	0.071981	-4.61837
UBA7	-0.68043	4.798949	-2.1837	0.030163	0.071992	-4.72021
FAM46A	-0.79627	5.546937	-2.18347	0.03018	0.07202	-4.76646
VPS33B	-0.29465	3.699936	-2.18331	0.030192	0.072036	-4.53049
GOLT1B	0.387726	5.817345	2.182913	0.030222	0.072095	-4.72791

GALK1	0.504418	3.423767	2.182622	0.030244	0.072122	-4.32329
TNKS2	-0.30004	6.577518	-2.18263	0.030243	0.072122	-4.77427
GALNS	0.541166	5.012996	2.182549	0.030249	0.072123	-4.62844
NPHP3	-0.42167	4.302724	-2.18227	0.03027	0.072161	-4.64819
C6orf170	0.633318	2.791299	2.181813	0.030304	0.072205	-4.18594
GABPA	-0.30246	5.21403	-2.18183	0.030303	0.072205	-4.73151
LDHB	-0.90511	7.325647	-2.1819	0.030298	0.072205	-4.74846
THADA	0.238619	5.817397	2.181575	0.030322	0.072235	-4.74094
ZNF529	-0.61037	3.861233	-2.18066	0.03039	0.072386	-4.61563
KIF26A	-0.80769	2.9594	-2.18054	0.030399	0.072395	-4.47522
BTAF1	-0.33753	5.985187	-2.18009	0.030433	0.072463	-4.7753
FAM86A	0.442621	3.834111	2.17977	0.030458	0.072509	-4.42943
TIGD5	0.580554	4.047921	2.179412	0.030485	0.072561	-4.44952
GBE1	-0.38903	4.458453	-2.1783	0.030569	0.072737	-4.67206
KCTD12	-0.80604	6.463409	-2.17831	0.030568	0.072737	-4.77833
ZFP161	-0.28036	3.712645	-2.178	0.030592	0.072778	-4.54348
RALA	0.356985	6.159342	2.17729	0.030645	0.072893	-4.7645
NRBF2	0.391418	4.454622	2.176449	0.030709	0.073032	-4.57369
KIAA0020	0.480886	4.881377	2.176376	0.030714	0.073032	-4.63123
HEPHL1	3.439317	1.861981	2.175654	0.030769	0.073138	-3.83438
SNX14	-0.41072	5.725998	-2.1757	0.030766	0.073138	-4.77871
BBS5	-0.51332	2.81152	-2.17544	0.030785	0.073164	-4.40283
ARHGAP29	-0.81629	4.831283	-2.17519	0.030804	0.073197	-4.74998
SLCO3A1	0.834287	5.497773	2.175058	0.030814	0.073197	-4.67268
TOP2B	-0.39966	7.036165	-2.17505	0.030815	0.073197	-4.78167
LMX1B	2.111258	0.481461	2.173792	0.030911	0.0734	-3.83266
NUDT8	0.947156	3.058734	2.173848	0.030906	0.0734	-4.20011
GDPD5	1.136453	3.180342	2.173509	0.030932	0.073437	-4.19329
GIPC2	-1.30768	2.127794	-2.17345	0.030937	0.073437	-4.40619
MEX3B	0.930256	2.055731	2.172794	0.030987	0.073505	-4.05266
ACYP2	-0.50965	2.182369	-2.17284	0.030983	0.073505	-4.27914
SLC35D1	-0.48463	5.3998	-2.17288	0.03098	0.073505	-4.77298
RBCK1	0.371176	6.495873	2.17299	0.030972	0.073505	-4.78689
HSPA2	-1.02983	4.473346	-2.17253	0.031007	0.073528	-4.7426
METAP1	0.382161	6.179148	2.172547	0.031006	0.073528	-4.77439
MYOF	0.669666	7.762383	2.172285	0.031026	0.07356	-4.79159
C20orf177	0.504869	3.93472	2.171961	0.031051	0.073607	-4.4568
NOP16	0.48401	3.708827	2.171247	0.031105	0.073708	-4.41364
IFI44	1.119831	4.860398	2.171243	0.031106	0.073708	-4.53227
EIF2C2	0.527952	5.191649	2.171194	0.031109	0.073708	-4.67665
EPB41L3	-0.85467	3.492965	-2.17019	0.031186	0.073878	-4.61363
IL1F9	3.225775	0.522684	2.169506	0.031239	0.073991	-3.84175
C13orf15	-0.55152	4.127599	-2.1689	0.031286	0.074089	-4.66796
MCAM	-0.70314	6.532385	-2.16836	0.031327	0.074175	-4.79995
B4GALNT3	-1.00407	5.399075	-2.16818	0.031341	0.074195	-4.79903
CALM2	0.301938	8.303721	2.168013	0.031354	0.074213	-4.78093
SLC7A5	0.926456	8.457729	2.167793	0.031371	0.07424	-4.79142
MYOM3	2.113033	1.407381	2.166296	0.031487	0.074502	-3.8549
SLC16A1	0.715977	6.687884	2.165584	0.031542	0.07462	-4.79797
ZNF469	1.429442	3.819539	2.164783	0.031604	0.074754	-4.27448
ZNF643	0.707736	1.250578	2.164596	0.031619	0.074776	-3.98491
GPATCH4	0.380797	5.470768	2.163907	0.031672	0.07489	-4.73774
IGDCC4	-0.77435	1.988416	-2.16344	0.031709	0.074939	-4.30449
ACTR1A	-0.30274	6.710355	-2.16348	0.031706	0.074939	-4.81334
PSMD8	0.338942	6.926969	2.163549	0.0317	0.074939	-4.81505
SLC25A17	0.312298	4.500261	2.163098	0.031735	0.074976	-4.6226
RAPGEFL1	1.121428	6.431448	2.163121	0.031734	0.074976	-4.76475
PLCXD2	0.942957	2.568208	2.162908	0.03175	0.074998	-4.14582

LHFP	-0.63388	3.776323	-2.16256	0.031778	0.07505	-4.64364
MAPK12	1.054078	2.925261	2.162304	0.031797	0.075072	-4.1899
ZNF784	-0.35629	2.708578	-2.16237	0.031792	0.075072	-4.37608
GNLY	1.844188	1.463312	2.161842	0.031834	0.075125	-3.89565
IKBKG	0.40842	4.541259	2.16176	0.03184	0.075125	-4.61787
CEBPD	-0.62171	6.97189	-2.16174	0.031841	0.075125	-4.80702
LYPLA1	0.359807	6.730167	2.161746	0.031841	0.075125	-4.81647
RARRES2	-0.76256	4.011627	-2.16152	0.031859	0.075153	-4.69264
CDK7	0.431664	3.952144	2.161253	0.03188	0.075189	-4.4965
IGSF1	-1.0983	-0.01244	-2.16094	0.031904	0.075234	-4.03979
TMEM37	-0.84885	2.759436	-2.16051	0.031938	0.075301	-4.47933
MIER1	-0.29914	6.059426	-2.16023	0.03196	0.075341	-4.81829
MPHOSPH10	0.304908	5.243695	2.159544	0.032014	0.075455	-4.73065
MAFF	-0.57476	5.12595	-2.15871	0.03208	0.075585	-4.79134
AKAP9	-0.56874	7.404157	-2.15871	0.032079	0.075585	-4.80405
C19orf6	-0.26522	6.930432	-2.15862	0.032087	0.075588	-4.82065
TAB3	-0.4238	5.769927	-2.15846	0.032099	0.075604	-4.81709
EMILIN1	-0.85954	5.55771	-2.1577	0.032159	0.075733	-4.82261
RGPD6	-0.50061	5.455062	-2.15753	0.032172	0.075752	-4.80905
SNORA8	0.503361	4.75991	2.157177	0.0322	0.075805	-4.64926
STOX1	-0.91709	1.494622	-2.15692	0.03222	0.075839	-4.25309
ATP2C2	1.941493	3.880281	2.156446	0.032258	0.075915	-4.22144
FAM65B	-0.92111	1.413259	-2.15631	0.032269	0.075928	-4.24011
TLR6	1.160222	0.771902	2.155747	0.032313	0.07602	-3.89942
NDUFS7	-0.37896	5.38011	-2.15562	0.032323	0.07603	-4.80309
LONRF3	-1.28078	1.362834	-2.15525	0.032353	0.076087	-4.28548
KLC3	2.510575	2.260804	2.155048	0.032369	0.0761	-3.93108
TNNI1	1.014873	1.980101	2.155043	0.032369	0.0761	-4.06246
LOC653653	-0.54386	1.103986	-2.15471	0.032396	0.076137	-4.14376
GUSBL2	0.530518	2.236917	2.154726	0.032394	0.076137	-4.16982
UBXN7	0.526085	5.144267	2.154573	0.032406	0.076149	-4.70619
GNL3L	0.34871	6.365979	2.15371	0.032475	0.076298	-4.82387
EML4	0.385419	7.058137	2.153228	0.032513	0.076375	-4.83687
SHANK3	-0.46685	5.564203	-2.1526	0.032564	0.07648	-4.82307
ARFGAP1	0.343642	6.004952	2.152357	0.032583	0.076512	-4.80877
RPS7	0.486585	6.415939	2.151671	0.032638	0.076628	-4.82504
CUL5	-0.29689	5.414993	-2.15123	0.032673	0.076698	-4.81016
CPNE7	1.689469	1.047044	2.150088	0.032764	0.0769	-3.88739
FXR2	-0.30619	5.15289	-2.14997	0.032774	0.076909	-4.79418
THSD1	1.046913	2.676109	2.149171	0.032838	0.077047	-4.17763
CEP350	-0.31053	6.612105	-2.14887	0.032862	0.07709	-4.84513
ATXN7L2	0.469611	2.563629	2.147996	0.032932	0.077231	-4.24464
ADAMTS17	-0.84865	1.806225	-2.14799	0.032933	0.077231	-4.31489
SH3BGRL3	0.497054	8.328416	2.147109	0.033004	0.077384	-4.82899
ZNF266	-0.39183	4.70637	-2.147	0.033013	0.077392	-4.76546
BZW2	0.44187	6.702315	2.146762	0.033032	0.077424	-4.84588
NPLOC4	0.27934	7.410429	2.146579	0.033047	0.077445	-4.84598
C15orf37	-0.36469	1.475059	-2.14644	0.033058	0.07746	-4.1917
PGBD4	-0.42529	1.114481	-2.14617	0.03308	0.077497	-4.14535
QARS	-0.39222	6.867312	-2.1454	0.033142	0.077631	-4.8475
LOC100133161	0.863088	3.066954	2.145	0.033175	0.077693	-4.27436
STAM	-0.33987	5.162422	-2.14426	0.033234	0.07782	-4.80893
ZNF813	-1.06505	0.841843	-2.14418	0.033241	0.077822	-4.1962
NCKAP1	-0.33408	8.301362	-2.14401	0.033255	0.077843	-4.81321
TTPAL	0.473342	5.311208	2.143763	0.033275	0.077876	-4.75447
SENP6	-0.26673	6.265631	-2.14341	0.033304	0.07793	-4.85655
SULT1A3	0.643674	5.008328	2.143161	0.033324	0.077943	-4.69637
TIAF1	-0.52207	4.584264	-2.14314	0.033326	0.077943	-4.77213

MUC1	-1.57988	7.02412	-2.14322	0.033319	0.077943	-4.81966
PCDHB9	1.003891	1.491499	2.142747	0.033358	0.078004	-4.0274
FAM24B	1.031003	0.201806	2.142138	0.033407	0.078107	-3.89442
NOS2	2.906	1.390322	2.141368	0.03347	0.078227	-3.9014
PTGR1	-0.7947	5.35166	-2.14141	0.033466	0.078227	-4.84892
APOM	-0.63437	0.271801	-2.14084	0.033513	0.078316	-4.06163
FNDC1	2.11544	3.643165	2.140361	0.033552	0.078394	-4.20003
CHFR	-0.70247	4.119249	-2.13995	0.033586	0.07846	-4.74509
HIST1H2BC	1.157836	2.017975	2.139368	0.033634	0.078557	-4.08535
LOC399744	-0.67139	2.783116	-2.13926	0.033642	0.078565	-4.49916
KIF3C	1.0121	3.993104	2.13919	0.033648	0.078565	-4.44209
ZNF839	-0.33199	3.118802	-2.13909	0.033657	0.078571	-4.50776
ALDH16A1	0.382215	5.180449	2.138951	0.033668	0.078585	-4.75863
METTL4	0.456913	3.853797	2.13811	0.033737	0.078721	-4.51787
STYXL1	0.494244	4.304875	2.138105	0.033737	0.078721	-4.60492
KIAA1012	-0.37714	5.924219	-2.1379	0.033754	0.078746	-4.86344
ZNF302	-0.50156	4.101178	-2.13774	0.033768	0.078765	-4.72521
BLOC1S2	0.387938	4.932933	2.137212	0.033811	0.078853	-4.73167
COCH	2.201818	2.358152	2.136892	0.033837	0.078901	-4.01167
GSDMC	3.137365	1.425511	2.136662	0.033856	0.078919	-3.91091
C17orf67	0.545847	0.864388	2.13668	0.033855	0.078919	-4.01156
FAM189B	0.401702	6.083036	2.136535	0.033867	0.07893	-4.84359
NOS1AP	-0.75377	2.811493	-2.13606	0.033906	0.079008	-4.52476
ZNF503	-0.69642	5.14156	-2.13584	0.033924	0.079024	-4.84679
EPPK1	0.91868	6.679346	2.135842	0.033924	0.079024	-4.85171
KIAA1797	-0.54828	5.012513	-2.13572	0.033934	0.079034	-4.82926
PGAM2	-0.71478	0.645481	-2.13555	0.033948	0.079041	-4.13638
C11orf17	0.409896	3.504596	2.135585	0.033945	0.079041	-4.45707
WDR1	-0.33209	8.171227	-2.13483	0.034008	0.079167	-4.83689
TWF2	0.371304	4.749492	2.134382	0.034045	0.079239	-4.71457
ACBD7	1.323071	1.077861	2.134193	0.03406	0.079263	-3.95897
GRAP	0.86806	1.377858	2.134004	0.034076	0.079286	-4.04487
SCARA3	-0.94122	4.720606	-2.13393	0.034082	0.079287	-4.83736
MKRN3	2.246283	0.220179	2.13363	0.034107	0.079319	-3.91169
TOM1L1	-0.47227	5.382398	-2.13366	0.034105	0.079319	-4.85368
LRPPRC	0.333661	7.841539	2.133439	0.034123	0.079342	-4.86583
MYO19	0.411233	6.209019	2.13317	0.034145	0.079381	-4.85708
ASH1L	-0.34781	7.056844	-2.1325	0.034201	0.079497	-4.87158
RNF122	-0.56428	2.651053	-2.132	0.034243	0.079581	-4.46933
SLC11A1	0.99461	2.776761	2.131632	0.034273	0.079626	-4.23663
FURIN	0.47716	7.58035	2.131682	0.034269	0.079626	-4.8771
ING4	-0.40176	3.434999	-2.13033	0.034382	0.079865	-4.6082
PABPN1	0.317439	6.881682	2.12917	0.034479	0.080077	-4.88693
MAP3K11	0.304876	6.985466	2.128946	0.034498	0.080107	-4.88769
COL6A3	0.945776	8.911311	2.128049	0.034573	0.080269	-4.86341
GLUD2	-0.41617	4.802928	-2.12788	0.034587	0.080287	-4.81709
CSNK2A1	0.297436	6.371036	2.127603	0.034611	0.080317	-4.88026
RFC1	-0.33222	5.824519	-2.1276	0.034611	0.080317	-4.88081
POTEF	1.114898	0.966856	2.127358	0.034631	0.08035	-3.98323
C1QB	1.093581	5.953483	2.126935	0.034667	0.080419	-4.79714
NR4A1	-1.04684	6.401159	-2.12677	0.034681	0.080439	-4.88354
NHSL1	0.584362	6.065923	2.126563	0.034698	0.080465	-4.85171
C8orf51	0.890378	0.113183	2.126274	0.034723	0.080508	-3.92535
HSPA1L	-0.46008	0.555061	-2.12606	0.034741	0.080537	-4.10973
DVL1	-0.44868	6.366091	-2.12536	0.0348	0.080661	-4.89495
ZNF776	-0.3872	4.183956	-2.12522	0.034812	0.080675	-4.7484
C14orf169	0.372943	3.762994	2.12487	0.034841	0.08073	-4.54248
LAMA3	1.055667	7.41378	2.124751	0.034851	0.08074	-4.89362

PUS3	-0.35438	3.086438	-2.12467	0.034858	0.080741	-4.5352
CETN2	0.409702	5.276098	2.124569	0.034867	0.080748	-4.79715
ANKRD11	-0.29359	8.098781	-2.1235	0.034957	0.080944	-4.86397
NHLRC3	-0.45573	4.457449	-2.12142	0.035134	0.081341	-4.797
LOC401010	0.539112	0.593697	2.121333	0.035141	0.081344	-4.00999
ESCO1	0.457027	4.958581	2.120675	0.035197	0.081449	-4.76078
MLPH	-1.56173	4.01981	-2.12052	0.03521	0.081449	-4.84592
CSNK2A2	-0.31952	5.092984	-2.12046	0.035216	0.081449	-4.85152
ATN1	-0.38107	8.009446	-2.12065	0.0352	0.081449	-4.87027
LNX2	-0.41366	5.468282	-2.1205	0.035212	0.081449	-4.88309
ACOT8	-0.32221	4.037851	-2.11988	0.035265	0.08155	-4.72938
FAM157A	1.418844	1.230328	2.119639	0.035286	0.081557	-3.99465
ASPHD2	-0.69478	2.743007	-2.1197	0.03528	0.081557	-4.53141
KRT18	1.017475	8.119309	2.11975	0.035276	0.081557	-4.90042
PLEKHF2	-0.33163	5.228028	-2.11948	0.035299	0.081575	-4.86518
CEP120	-0.33513	4.467789	-2.11883	0.035355	0.081689	-4.79175
METAP2	0.317055	6.713667	2.118644	0.035371	0.081713	-4.9069
COPS5	0.309904	5.549146	2.118458	0.035387	0.081737	-4.84629
LAMC3	1.506192	2.8341	2.117742	0.035448	0.081851	-4.20026
IL15RA	0.6581	4.084496	2.117786	0.035444	0.081851	-4.56875
PRDM11	-0.5161	0.898274	-2.11744	0.035474	0.081898	-4.18477
HOMEZ	-0.44352	4.243495	-2.11682	0.035527	0.081993	-4.7798
ATP5D	-0.45446	6.291545	-2.11686	0.035524	0.081993	-4.91273
NMT1	0.249099	7.111107	2.116323	0.03557	0.082079	-4.91286
LMBR1	-0.36872	5.745488	-2.1152	0.035667	0.082288	-4.9048
C20orf11	0.304918	6.524782	2.114562	0.035722	0.082402	-4.9117
CLNS1A	0.403599	6.184651	2.114487	0.035728	0.082403	-4.89502
TMEM182	0.497039	2.51473	2.114294	0.035745	0.082406	-4.2996
MMP23B	-0.7386	1.584202	-2.11427	0.035747	0.082406	-4.32809
ANKRD6	-0.87203	2.329351	-2.1143	0.035745	0.082406	-4.49586
IGSF8	0.436025	5.557233	2.114031	0.035768	0.08244	-4.84516
SGK196	0.790319	3.1966	2.113699	0.035797	0.082492	-4.37274
IFIT1	1.587194	4.1739	2.113601	0.035805	0.082498	-4.42163
CHSY3	0.914945	1.192358	2.113221	0.035838	0.082547	-4.05821
SLC2A9	1.14309	2.36874	2.113222	0.035838	0.082547	-4.19198
LILRB2	1.066761	1.687475	2.112748	0.035879	0.082627	-4.10564
SLC10A7	-0.37629	3.931878	-2.11264	0.035889	0.082636	-4.73447
IRS2	-0.98552	6.052511	-2.11161	0.035978	0.082828	-4.92107
GPR135	-0.64022	0.101544	-2.11145	0.035992	0.082847	-4.10013
CASP9	-0.39139	3.628349	-2.11091	0.036038	0.08294	-4.68502
KBTBD11	-1.07771	2.472884	-2.11025	0.036097	0.08306	-4.56516
C6orf1	0.567596	3.568411	2.109256	0.036183	0.083237	-4.49707
STRA13	0.490604	4.749249	2.109211	0.036187	0.083237	-4.74912
RHEB	0.411071	5.418791	2.10916	0.036191	0.083237	-4.84404
SVIL	-0.67489	7.24014	-2.10897	0.036208	0.083262	-4.90955
RPL35	0.510716	8.065654	2.108549	0.036245	0.083332	-4.9158
RPIA	0.503193	4.141802	2.108236	0.036272	0.083381	-4.63061
MRPL39	-0.33834	3.607686	-2.10754	0.036333	0.083508	-4.67918
ZNF616	-0.32087	2.749328	-2.10589	0.036478	0.083826	-4.49538
TOX2	-0.83972	3.190082	-2.10516	0.036542	0.083961	-4.69252
ATRN	-0.50696	6.889976	-2.10404	0.036641	0.084173	-4.93087
ITGBL1	-1.20513	2.001179	-2.10345	0.036693	0.084279	-4.50982
RRP7A	0.412097	5.041958	2.102802	0.03675	0.08438	-4.81321
NEURL4	-0.27693	4.587834	-2.10275	0.036755	0.08438	-4.83308
KIAA0090	0.359154	6.267343	2.102852	0.036746	0.08438	-4.925
FAAH	-0.73772	3.412008	-2.10195	0.036826	0.084529	-4.71633
LOC728392	-0.71963	1.685159	-2.10165	0.036853	0.084577	-4.36893
DVL3	0.440089	7.80565	2.101363	0.036878	0.084621	-4.93487

FNIP1	-0.40509	5.190763	-2.10113	0.036898	0.084654	-4.9049
NUP133	0.291033	5.825323	2.100964	0.036913	0.084675	-4.90663
ADAMTS7	0.819462	3.616203	2.100638	0.036942	0.084713	-4.474
AGFG2	-0.59859	4.568609	-2.10069	0.036938	0.084713	-4.86609
HGS	0.295924	7.205686	2.100432	0.036961	0.084741	-4.94493
C6orf141	1.466691	1.777999	2.098995	0.037089	0.085011	-4.09526
TMEM154	1.561631	4.646278	2.098973	0.037091	0.085011	-4.55432
LPHN3	-0.95112	2.298598	-2.09849	0.037134	0.085096	-4.53122
CYP4V2	-0.65567	4.519493	-2.09842	0.03714	0.085096	-4.87052
ANPEP	2.498782	4.845044	2.09812	0.037167	0.085142	-4.41644
GTF2IRD1	0.456362	5.85787	2.098063	0.037172	0.085142	-4.90355
POU2AF1	-1.17297	3.117694	-2.09768	0.037206	0.085207	-4.73926
UBE2B	-0.31193	5.280613	-2.09751	0.037221	0.085227	-4.91312
ERI1	0.524356	4.562383	2.096781	0.037287	0.085363	-4.73632
CD101	0.709878	1.577884	2.096346	0.037326	0.085438	-4.1672
LANCL1	-0.3902	6.280189	-2.09586	0.037369	0.085524	-4.95585
CCDC52	0.411898	4.547224	2.095545	0.037397	0.085574	-4.75453
H3F3C	0.548193	0.799906	2.095346	0.037415	0.08559	-4.08626
DEDD2	0.418222	5.044866	2.095333	0.037416	0.08559	-4.8284
PPP2R5D	0.400133	5.843995	2.094744	0.037469	0.085697	-4.91313
CACNB1	0.705271	2.8801	2.093931	0.037543	0.085851	-4.37302
MBIP	-0.42117	4.168919	-2.09353	0.037579	0.085919	-4.81543
NHP2	0.427978	5.290781	2.093251	0.037604	0.085963	-4.86118
RCAN1	-0.53609	5.305015	-2.09262	0.037661	0.086079	-4.93735
TXNDC16	-0.50022	3.848646	-2.0924	0.037681	0.086111	-4.78113
PHF20L1	0.32325	6.220662	2.092293	0.037691	0.086118	-4.94601
ZNF566	-0.54051	2.933075	-2.09209	0.037709	0.086146	-4.60706
COX18	-0.33435	3.565603	-2.09186	0.037729	0.086179	-4.70049
PSMC3	0.311024	6.336803	2.091415	0.03777	0.086258	-4.95316
FER	-0.57675	2.22254	-2.09107	0.037802	0.086315	-4.46622
ADAMTSL2	0.817175	2.770471	2.090754	0.03783	0.086366	-4.34379
RTP4	1.231659	2.812037	2.09068	0.037837	0.086367	-4.29018
LOC645676	0.436297	1.263161	2.090362	0.037866	0.086419	-4.16826
C17orf101	-0.31999	3.518058	-2.09016	0.037884	0.086447	-4.69143
CXorf38	0.536019	3.449817	2.08993	0.037905	0.086481	-4.51378
ZNRD1	0.425885	3.911596	2.089861	0.037911	0.086481	-4.63406
BCL7A	0.585139	4.618583	2.089561	0.037938	0.086515	-4.75237
NID1	-0.79158	7.053193	-2.0896	0.037935	0.086515	-4.95126
GGT7	-0.74746	3.582161	-2.08935	0.037958	0.086545	-4.77812
IL27RA	1.00877	3.836079	2.089265	0.037965	0.086548	-4.50733
KITLG	-0.68845	5.809997	-2.08887	0.038001	0.086615	-4.96758
SYVN1	-0.27992	6.848066	-2.08858	0.038027	0.086661	-4.967
C1orf213	-0.49864	0.323107	-2.08742	0.038134	0.08689	-4.16018
BEX2	-1.47865	0.458424	-2.08696	0.038176	0.086914	-4.30942
LOC285074	0.37874	3.807767	2.087096	0.038163	0.086914	-4.62961
WDR52	-0.66901	3.062914	-2.08701	0.038171	0.086914	-4.66376
CDC23	0.283108	5.233209	2.087019	0.03817	0.086914	-4.88175
ZNF238	-0.67168	4.583759	-2.08719	0.038155	0.086914	-4.90082
WNT10A	1.995347	2.601509	2.086694	0.0382	0.086955	-4.16593
ZFAND3	-0.28574	6.138596	-2.08604	0.03826	0.087078	-4.97336
ZNF860	0.889097	1.528927	2.085114	0.038345	0.087256	-4.15913
TMEM133	-0.73501	2.326659	-2.08424	0.038425	0.087425	-4.52232
RNFT2	1.241618	2.167111	2.083707	0.038474	0.087522	-4.20672
C9orf68	-0.81463	1.155385	-2.08359	0.038485	0.087532	-4.32827
C20orf29	0.371458	3.465346	2.083093	0.038531	0.087622	-4.56267
ST13	-0.29316	7.04884	-2.08295	0.038544	0.087638	-4.97467
CLMN	-0.98712	5.027222	-2.08257	0.038579	0.087702	-4.96249
WHAMML1	-0.76234	0.745168	-2.08227	0.038607	0.087752	-4.26477

LAMA1	2.54524	1.259911	2.082043	0.038628	0.087785	-4.01717
NCOR1	-0.39652	7.565959	-2.08177	0.038652	0.087827	-4.96308
TBC1D9B	-0.3174	7.406609	-2.08031	0.038788	0.088121	-4.97196
NCKAP5	-0.89043	2.298359	-2.07973	0.038841	0.088228	-4.56012
TIMM16	0.493376	3.351352	2.079138	0.038897	0.08834	-4.52391
C13orf29	1.054232	2.043176	2.078585	0.038948	0.088427	-4.22439
ISPD	-0.65088	1.718975	-2.0786	0.038947	0.088427	-4.40912
KIAA1324	-1.74278	4.646175	-2.07852	0.038954	0.088427	-4.97809
TFAP2E	1.072302	0.160998	2.078148	0.038989	0.088477	-4.01953
VPS37A	-0.32274	5.20284	-2.0782	0.038984	0.088477	-4.94737
LGALS1	0.865546	6.681122	2.077981	0.039004	0.088498	-4.97297
GOLGB1	-0.40849	7.776108	-2.07749	0.03905	0.088587	-4.96534
EGR1	-0.87193	7.662922	-2.07678	0.039116	0.088723	-4.95709
NOTUM	2.23138	0.801222	2.076577	0.039135	0.088737	-4.02581
FMO4	-0.61227	1.909523	-2.07661	0.039133	0.088737	-4.43825
NR2F6	-0.43344	6.214949	-2.07583	0.039205	0.088881	-4.9965
PAAF1	-0.46643	3.663865	-2.07571	0.039216	0.088892	-4.77734
WWC1	-0.67507	5.53279	-2.07564	0.039223	0.088892	-4.98718
CACNB3	0.524575	4.283632	2.074555	0.039325	0.089109	-4.7287
ARPM1	0.715137	1.194619	2.074312	0.039347	0.089146	-4.15853
MLH1	-0.38076	4.653407	-2.0742	0.039358	0.089155	-4.90886
MOCS3	0.418525	3.648818	2.073701	0.039405	0.089232	-4.61322
FUCA1	-0.64468	5.199298	-2.07373	0.039402	0.089232	-4.97482
DLK2	1.890042	0.795657	2.072644	0.039504	0.089419	-4.03328
CEP97	0.629612	3.620541	2.07264	0.039505	0.089419	-4.56993
DIS3L	-0.35288	4.80021	-2.07262	0.039507	0.089419	-4.92492
ACOT1	-0.58774	1.792558	-2.07251	0.039516	0.089427	-4.4254
IL12RB1	1.215868	0.842664	2.072233	0.039543	0.089458	-4.06542
CA13	-0.91097	2.881142	-2.07227	0.039539	0.089458	-4.70107
ZNF417	-0.47851	3.369835	-2.07176	0.039588	0.089531	-4.72728
F2RL1	0.916261	5.896136	2.0718	0.039584	0.089531	-4.91995
C16orf63	0.443305	5.339556	2.071465	0.039615	0.089578	-4.90917
ZMYND15	0.987292	1.378319	2.071133	0.039647	0.089628	-4.15535
ABCG1	-0.57611	4.380955	-2.0711	0.03965	0.089628	-4.90307
AP3M1	-0.28512	6.290369	-2.07076	0.039682	0.089686	-5.00649
MST1P9	-1.20384	0.798952	-2.0702	0.039735	0.089791	-4.35065
ZNF655	-0.84728	5.59666	-2.06984	0.039769	0.089853	-5.00449
TNFSF13	-0.72962	4.079188	-2.06967	0.039785	0.089874	-4.88442
ZMAT5	-0.43182	3.372975	-2.06923	0.039827	0.089955	-4.72542
FOLR2	-0.93458	1.348094	-2.06883	0.039864	0.089988	-4.40902
FLJ10357	-0.59075	4.76708	-2.06897	0.039852	0.089988	-4.94872
C10orf54	-0.59713	5.615863	-2.0688	0.039867	0.089988	-5.00183
CTTN	0.65804	7.885024	2.068904	0.039858	0.089988	-5.00314
PRDX6	-0.37297	7.342307	-2.06819	0.039925	0.090104	-4.99687
SLC35A4	-0.31063	7.044465	-2.06742	0.039998	0.090255	-5.00593
PUM1	0.204865	7.37114	2.067266	0.040013	0.090274	-5.00869
PARN	0.299168	5.548635	2.06697	0.040042	0.090323	-4.95235
MTX1	0.551786	4.2762	2.06637	0.040099	0.090437	-4.73622
MIER2	0.303479	4.952637	2.066262	0.040109	0.090446	-4.88908
TXNRD3IT1	-0.65665	2.573881	-2.06617	0.040118	0.090451	-4.60383
MRPS28	0.414834	3.74376	2.065739	0.040159	0.090529	-4.64976
TLR4	-0.75743	3.005654	-2.06538	0.040193	0.090592	-4.71611
ATM	-0.46846	5.728673	-2.06487	0.040242	0.090688	-5.00997
DVL2	0.481658	5.219942	2.064018	0.040324	0.090848	-4.90706
ABCF2	0.379837	6.048516	2.063988	0.040327	0.090848	-4.99151
ZMYND17	-0.40669	1.898945	-2.06356	0.040368	0.090885	-4.43301
C7orf44	0.280754	4.872512	2.063712	0.040353	0.090885	-4.88659
FBP1	-1.14604	3.878099	-2.06365	0.040359	0.090885	-4.91279

AKT3	-0.79325	4.468367	-2.06355	0.040369	0.090885	-4.94922
HLX	0.649487	2.036295	2.06324	0.040398	0.090937	-4.30758
C19orf55	0.445058	3.554806	2.062331	0.040486	0.091119	-4.61081
SH2B3	0.594757	5.377776	2.061719	0.040544	0.091236	-4.91712
TRPA1	-1.33332	1.829589	-2.06113	0.040601	0.091335	-4.57251
UTP20	0.408126	5.538668	2.061134	0.040601	0.091335	-4.9539
CYB5RL	-0.48864	2.072654	-2.06097	0.040616	0.091354	-4.48012
STARD5	0.89581	3.269284	2.060681	0.040645	0.091403	-4.47611
CMTM3	0.835535	4.20761	2.06047	0.040665	0.091434	-4.67821
PRDM1	0.775049	5.68986	2.060227	0.040688	0.091472	-4.93719
ZNF607	-0.76679	2.530581	-2.05997	0.040714	0.091514	-4.62778
GPR109A	2.35948	1.448746	2.059731	0.040736	0.09155	-4.06079
MTERFD2	-0.30375	3.403917	-2.05938	0.04077	0.091609	-4.7273
ITGB2	1.028466	4.771795	2.059325	0.040775	0.091609	-4.76274
C15orf57	-0.36273	3.498161	-2.05857	0.040849	0.091712	-4.75846
FRAT2	0.584295	4.355	2.058708	0.040835	0.091712	-4.76024
TTC4	0.329889	4.582407	2.058565	0.040849	0.091712	-4.8492
PRR15L	-1.65934	3.145169	-2.05852	0.040854	0.091712	-4.87845
TOB1	-0.59112	6.184444	-2.05851	0.040854	0.091712	-5.03155
PKN1	-0.60674	6.06164	-2.0581	0.040895	0.091788	-5.03209
LOC100289341	0.797509	1.073541	2.058017	0.040902	0.09179	-4.16529
URB2	0.405414	4.863944	2.057544	0.040948	0.091878	-4.88321
CRISPLD1	1.280979	2.660185	2.05712	0.040989	0.091927	-4.3295
NUDCD2	0.377124	3.890725	2.057129	0.040988	0.091927	-4.70698
LUM	0.874556	7.920338	2.057149	0.040987	0.091927	-5.02959
C2CD4D	0.877358	0.602607	2.056907	0.04101	0.091958	-4.10045
FIS1	-0.35113	5.320276	-2.05672	0.041028	0.091984	-5.00102
C8orf59	0.39965	4.606066	2.056466	0.041053	0.092025	-4.84667
HORMAD1	2.255879	-0.17235	2.056326	0.041067	0.092041	-4.06007
TOR1B	0.376291	5.034663	2.05602	0.041096	0.092093	-4.91121
ZBTB49	-0.30419	2.08113	-2.05586	0.041112	0.092113	-4.46469
CDH5	-0.67974	4.828448	-2.05566	0.041132	0.092143	-4.98757
PALM2	1.208181	0.925661	2.055424	0.041154	0.092178	-4.10956
MBP	-0.38167	5.616791	-2.05511	0.041186	0.092233	-5.02236
NAT9	0.413255	4.027414	2.054673	0.041228	0.092262	-4.7324
SNHG6	0.529056	5.439662	2.054638	0.041231	0.092262	-4.9457
GPC4	-0.83748	4.699373	-2.05468	0.041227	0.092262	-4.99039
DENND4A	-0.38359	5.315255	-2.05476	0.041219	0.092262	-5.00652
PCDH1	-1.02906	6.605745	-2.05464	0.041231	0.092262	-5.02721
TMEM111	-0.35407	4.974539	-2.05456	0.041239	0.092264	-4.97782
PIM2	0.884581	4.329236	2.054108	0.041283	0.092348	-4.70627
PITPNA	-0.30941	6.603992	-2.05343	0.04135	0.092482	-5.0411
TMEM126A	-0.3886	3.47564	-2.05317	0.041375	0.092524	-4.76925
DQX1	1.408825	2.12356	2.053084	0.041383	0.092528	-4.23697
KRBA1	-0.85657	2.820409	-2.05262	0.041429	0.0926	-4.72185
LBH	0.685214	5.496608	2.052654	0.041425	0.0926	-4.94048
C11orf41	2.214893	3.098884	2.052402	0.04145	0.092622	-4.2756
ARNT2	-0.96596	3.328002	-2.05238	0.041452	0.092622	-4.84274
VCL	-0.44661	8.108441	-2.05197	0.041493	0.092698	-5.00492
LST1	0.966109	1.55136	2.051676	0.041521	0.092719	-4.22069
C10orf76	-0.29978	5.21858	-2.05177	0.041512	0.092719	-5.00025
RANBP9	-0.40292	6.035582	-2.05167	0.041522	0.092719	-5.0428
VANGL1	0.532258	5.92808	2.050788	0.041609	0.092883	-4.9993
SLC7A8	-1.16802	6.463338	-2.05084	0.041603	0.092883	-5.03498
GCAT	-0.54256	2.97893	-2.05071	0.041616	0.092885	-4.69824
OCIAD2	0.56475	5.325288	2.050232	0.041663	0.092976	-4.93674
TMEM98	-0.75225	4.586545	-2.04967	0.041719	0.093084	-4.98319
RP9	0.483536	3.439343	2.049443	0.041741	0.09312	-4.60372

TATDN1	0.486321	3.468753	2.048415	0.041843	0.093331	-4.61074
RABGAP1L	-0.32489	4.43564	-2.0481	0.041874	0.093386	-4.93072
MEGF9	-0.60989	5.443869	-2.04771	0.041912	0.093456	-5.03818
WDR70	0.358225	4.178628	2.047643	0.041919	0.093457	-4.79069
CCDC51	0.456856	3.612818	2.047258	0.041957	0.093527	-4.65089
GBA	0.435518	5.282636	2.045955	0.042087	0.093801	-4.95471
PTENP1	-0.32938	3.220738	-2.04581	0.042101	0.093813	-4.71935
PTN	-0.97251	2.757566	-2.04576	0.042106	0.093813	-4.74754
HSPA1B	0.737477	6.290696	2.045151	0.042167	0.093932	-5.0238
PITPNM1	0.474471	6.262923	2.045092	0.042173	0.093932	-5.03669
ARHGAP19	0.429962	4.161353	2.044742	0.042207	0.093995	-4.77723
ANTXR2	-0.74274	5.54288	-2.04459	0.042223	0.094014	-5.05187
KIAA1731	0.386257	4.762229	2.044284	0.042253	0.094066	-4.89721
RPL8	0.465004	9.891547	2.043992	0.042282	0.094116	-4.98746
KLHL18	-0.34858	4.664975	-2.04372	0.04231	0.094162	-4.9681
ZNF286A	0.521657	3.78186	2.043162	0.042365	0.094271	-4.68492
RHBDD1	-0.38339	4.247383	-2.043	0.042381	0.094281	-4.92353
C17orf85	-0.25511	5.096632	-2.0429	0.042391	0.094281	-5.00431
NARG2	-0.25358	5.525666	-2.04284	0.042397	0.094281	-5.03649
PCSK7	-0.46705	5.622006	-2.04287	0.042394	0.094281	-5.05009
ZNF17	-0.30898	2.780336	-2.04138	0.042544	0.094593	-4.63059
LOC283314	0.630216	2.809852	2.041081	0.042574	0.094644	-4.47609
FMR1	0.342174	5.974816	2.040632	0.042619	0.094729	-5.03578
MME	1.535501	3.067273	2.040445	0.042637	0.094756	-4.38799
NAA16	-0.35154	4.384366	-2.04038	0.042644	0.094756	-4.94219
FAM171A2	1.239492	1.213043	2.039699	0.042712	0.094892	-4.17055
SOD2	0.576626	8.264878	2.039086	0.042774	0.095014	-5.05325
RGS16	0.977749	3.446515	2.038719	0.042811	0.095082	-4.54139
SLC27A2	2.022424	2.410661	2.038605	0.042823	0.095092	-4.23036
KCNC3	1.065677	2.179844	2.03841	0.042842	0.095106	-4.32219
COX16	-0.32195	4.97806	-2.03844	0.04284	0.095106	-5.00743
KLK9	2.529519	-0.14425	2.038133	0.04287	0.095152	-4.0949
LIG4	-0.41189	4.332676	-2.03712	0.042973	0.095365	-4.94855
PNRC2	-0.35223	6.484808	-2.03699	0.042986	0.095378	-5.07457
SPCS1	-0.35825	5.990189	-2.03633	0.043053	0.095512	-5.07176
ZFYVE16	-0.29721	5.554617	-2.03566	0.043121	0.095647	-5.05467
D2HGDH	-0.38962	4.315175	-2.03558	0.043128	0.095649	-4.94691
SFXN4	0.448736	3.913424	2.03506	0.043182	0.095752	-4.74007
CXCL9	2.203308	4.48937	2.03487	0.043201	0.09578	-4.53098
PDGFRB	0.767286	6.908545	2.034557	0.043233	0.095835	-5.07229
PALMD	-0.79793	3.714471	-2.03432	0.043257	0.095873	-4.91747
SIAH1	0.379861	4.812496	2.033989	0.043291	0.095933	-4.92606
ADO	0.280037	5.169676	2.033756	0.043314	0.095955	-4.98206
PRPF38A	0.251331	5.164946	2.033799	0.04331	0.095955	-4.98432
PRRT3	-0.43108	1.828762	-2.0324	0.043452	0.096245	-4.48779
ZNF699	-0.64256	1.072034	-2.03194	0.043499	0.096334	-4.3965
NT5DC3	-0.5894	4.349608	-2.03185	0.043509	0.096341	-4.98062
TRIM9	-1.05567	0.169308	-2.03175	0.043519	0.096342	-4.31628
KIAA0831	-0.33716	4.561374	-2.03171	0.043523	0.096342	-4.97953
ZNF619	-0.43456	1.790092	-2.03155	0.04354	0.096363	-4.48129
ALDH3B2	4.026307	4.022138	2.031362	0.043559	0.096389	-4.2188
KIAA0922	-0.57499	4.610369	-2.03122	0.043573	0.096406	-5.00852
ATP6V0D1	-0.33297	6.293658	-2.0311	0.043585	0.096418	-5.08635
DCTN3	-0.34916	4.75141	-2.03081	0.043615	0.096453	-5.00307
AHDC1	-0.5598	6.38917	-2.03082	0.043614	0.096453	-5.08591
AQP9	1.408553	1.09005	2.030525	0.043644	0.096502	-4.15413
ALG8	0.450786	4.862058	2.030108	0.043687	0.096582	-4.93115
CCM2	0.392773	5.141306	2.030022	0.043696	0.096586	-4.97441

PRRT2	-0.71611	1.162175	-2.02991	0.043708	0.096597	-4.42307
HLA-DRA	0.881558	8.028739	2.029452	0.043755	0.096685	-5.08309
NRBP2	-0.49715	4.995702	-2.02936	0.043763	0.096689	-5.04054
WDR77	0.349493	5.454694	2.029088	0.043792	0.096737	-5.01475
C3orf67	1.4082	2.138787	2.02853	0.043849	0.096831	-4.2906
C17orf95	0.347421	4.125813	2.028477	0.043855	0.096831	-4.81775
ARL4C	0.926138	5.506863	2.028474	0.043855	0.096831	-4.96403
NDUFS2	-0.30535	6.267524	-2.02806	0.043898	0.09691	-5.09199
SUMO3	0.330089	6.377471	2.027919	0.043912	0.096926	-5.08181
P2RY8	-0.80107	1.647938	-2.02748	0.043958	0.097012	-4.52206
RPRD1A	-0.41331	6.046309	-2.02721	0.043985	0.097056	-5.09193
SLC35B3	-0.36088	4.484929	-2.02703	0.044004	0.097079	-4.98139
GNG12	-0.37729	7.646271	-2.02698	0.04401	0.097079	-5.07137
SPDYE3	0.569367	1.074901	2.026676	0.044041	0.097126	-4.25168
PARP2	0.381209	4.202516	2.026607	0.044048	0.097126	-4.83262
PANX1	0.466626	4.849764	2.026538	0.044055	0.097126	-4.9353
SURF6	0.352194	5.049134	2.026505	0.044058	0.097126	-4.9748
MRPL21	0.726201	4.787187	2.025959	0.044115	0.097235	-4.88671
TCEA1	0.33477	6.057907	2.024894	0.044225	0.097447	-5.07254
PLAGL2	0.48656	6.515256	2.024897	0.044225	0.097447	-5.08754
SHB	0.443125	5.906415	2.024632	0.044252	0.097492	-5.05614
IGBP1	-0.32245	5.713171	-2.02452	0.044264	0.0975	-5.08537
SMAD2	-0.37144	6.314827	-2.02446	0.04427	0.0975	-5.0997
UBE2Z	0.259443	6.897702	2.0241	0.044307	0.097567	-5.10043
LYPD5	1.325981	2.973081	2.023964	0.044321	0.097583	-4.43747
MORF4L2	0.274555	7.614151	2.023886	0.04433	0.097585	-5.09201
MMP7	3.9646	3.869894	2.023322	0.044388	0.097699	-4.22174
RASSF8	-0.79803	3.835383	-2.02259	0.044464	0.09785	-4.95839
UBD	2.77709	4.238493	2.022388	0.044485	0.097882	-4.42642
FAM83A	5.06628	4.524886	2.021432	0.044585	0.09807	-4.18034
RNF44	0.274852	6.464113	2.021489	0.044579	0.09807	-5.09895
WHSC2	0.340915	5.141883	2.020842	0.044647	0.098191	-4.99807
ZNF700	-0.36888	3.857262	-2.02075	0.044656	0.098196	-4.90557
NLE1	0.428574	4.11508	2.020639	0.044668	0.098206	-4.81647
FBXO36	-0.51674	1.809573	-2.02039	0.044694	0.098247	-4.51899
TNRC18	0.331648	8.53212	2.020005	0.044734	0.098321	-5.07736
CLDN12	0.618078	6.175682	2.019299	0.044808	0.098453	-5.07431
G3BP2	-0.34555	7.211628	-2.01936	0.044802	0.098453	-5.09792
C10orf84	-0.22612	4.220606	-2.01909	0.04483	0.098472	-4.94822
ADSS	0.305234	6.034047	2.019081	0.044831	0.098472	-5.08427
OXSM	-0.3324	2.815109	-2.01861	0.044881	0.09855	-4.68495
PIK3C3	-0.33003	4.437835	-2.01867	0.044874	0.09855	-4.98998
SKP1	-0.27283	6.975819	-2.01841	0.044901	0.098579	-5.10578
DAPK2	-0.69899	2.686777	-2.01755	0.044992	0.098762	-4.72351
LOC606724	0.691931	0.454831	2.016829	0.045068	0.098854	-4.18074
FRAT1	-0.45681	1.976291	-2.01702	0.045048	0.098854	-4.54361
BTN2A2	0.601174	3.066735	2.016822	0.045069	0.098854	-4.57511
LRRC37A4	-0.69707	2.992542	-2.01688	0.045063	0.098854	-4.79989
MTMR1	0.389655	5.737512	2.017018	0.045048	0.098854	-5.06153
ITPR2	-0.63753	5.393272	-2.01577	0.045179	0.099081	-5.10036
WDR20	-0.23831	4.273641	-2.0155	0.045209	0.09913	-4.96411
SYNJ1	-0.35494	4.286238	-2.01504	0.045257	0.099212	-4.98077
KCNQ1OT1	-0.80461	4.856347	-2.015	0.045261	0.099212	-5.07839
GZMA	1.43079	1.097631	2.014114	0.045355	0.099403	-4.18284
RBM28	0.3396	4.37989	2.013825	0.045385	0.099409	-4.89977
TIE1	-0.60188	3.763008	-2.01402	0.045365	0.099409	-4.93826
SGSH	-0.38274	5.151225	-2.01382	0.045386	0.099409	-5.07603
GRHPR	-0.36084	5.345278	-2.01382	0.045386	0.099409	-5.08864

PTGFRN	0.621809	7.495584	2.013306	0.04544	0.099513	-5.11996
ERP27	-1.00249	0.278614	-2.01245	0.045532	0.099697	-4.3574
ABCC10	0.429958	5.366671	2.012156	0.045563	0.099749	-5.03128
GJB5	3.400932	2.724248	2.011587	0.045623	0.099834	-4.1697
FMO2	-1.35663	2.873914	-2.01152	0.04563	0.099834	-4.9044
TAF11	0.290229	4.454171	2.011572	0.045625	0.099834	-4.92647
HDHD1A	-0.62977	4.007267	-2.01158	0.045624	0.099834	-4.97978
PRSS23	-0.68585	5.902047	-2.01141	0.045642	0.099846	-5.1241
HNF1B	-2.12641	0.320354	-2.01083	0.045704	0.099935	-4.51156
MINPP1	0.397825	4.475938	2.010824	0.045704	0.099935	-4.9128
WDR24	-0.31266	4.30235	-2.01087	0.045699	0.099935	-4.98594
ZNF34	-0.34252	2.068539	-2.00977	0.045817	0.10015	-4.55891
SREBF2	-0.44114	8.24057	-2.00977	0.045816	0.10015	-5.08423
WNT2B	-0.926	1.873238	-2.00969	0.045826	0.100153	-4.6186
ZNF318	0.465381	5.969618	2.009426	0.045854	0.100198	-5.08942
LINS1	0.345159	4.164018	2.009057	0.045893	0.100269	-4.86505
C1orf83	-0.28066	3.356384	-2.00884	0.045916	0.100288	-4.81304
PHTF2	0.488544	4.942497	2.008884	0.045912	0.100288	-4.98115
CSTF2T	-0.32398	4.690466	-2.00823	0.045982	0.100415	-5.03896
CAPN13	-1.68968	0.44957	-2.00803	0.046003	0.100446	-4.47918
SETDB1	0.304915	5.580348	2.007686	0.04604	0.100511	-5.07264
GATM	-1.15348	4.987559	-2.00721	0.046091	0.100591	-5.11767
ITPRIP	-0.67641	6.02082	-2.00725	0.046087	0.100591	-5.13314
IRF5	0.770547	3.649632	2.006892	0.046125	0.10065	-4.67902
FUNDC1	-0.40293	3.35189	-2.00661	0.046156	0.100701	-4.83744
SH2D3C	-0.51719	3.203281	-2.00631	0.046188	0.100755	-4.8307
TBC1D1	-0.51239	5.569514	-2.00613	0.046207	0.100782	-5.12248
LOC100129034	-0.57235	5.996704	-2.00571	0.046253	0.100865	-5.13541
KRT23	3.010629	2.665433	2.005535	0.046271	0.10089	-4.2153
ATP6V1C2	0.843402	3.053322	2.005177	0.04631	0.100958	-4.55563
EFHD2	0.495539	7.542222	2.004809	0.04635	0.101029	-5.13443
MLL2	-0.33328	7.647799	-2.00469	0.046362	0.101041	-5.11636
RANBP17	-1.27078	0.151708	-2.00381	0.046457	0.101232	-4.39132
C12orf35	0.528964	6.557338	2.003277	0.046515	0.101342	-5.13015
COX10	-0.30473	4.289296	-2.00276	0.046571	0.101448	-4.99929
C4orf48	0.85585	2.311361	2.002363	0.046614	0.101526	-4.43419
IDO1	2.464447	2.774682	2.00208	0.046645	0.101577	-4.29772
FXR1	0.416315	6.461118	2.001644	0.046692	0.101665	-5.13374
DFNB31	-0.63356	3.314668	-2.00091	0.046772	0.101822	-4.88803
RAG1	1.14277	1.906406	2.000236	0.046845	0.10195	-4.3437
NDUFA4	-0.348	6.618413	-2.00029	0.046839	0.10195	-5.14588
1-Mar	0.673896	1.166047	2.000057	0.046865	0.101961	-4.30212
SYNRG	-0.31242	5.797418	-2.00006	0.046865	0.101961	-5.1365
ADAMTS2	1.358891	4.220282	1.999517	0.046924	0.102073	-4.69724
FBXO6	0.682901	3.718009	1.999411	0.046935	0.102082	-4.72337
HK2	0.717699	6.945685	1.999051	0.046974	0.102152	-5.1445
MEF2C	-0.61318	3.888322	-1.99855	0.047029	0.102255	-4.99075
LYNX1	-1.35838	4.027853	-1.99816	0.047072	0.102332	-5.081
ZNF362	-0.44916	4.787269	-1.99794	0.047096	0.102369	-5.08082
TMEM180	0.618994	2.942458	1.996054	0.047302	0.102791	-4.58641
LRRK1	0.438902	5.379721	1.996029	0.047305	0.102791	-5.06378
KIAA0196	0.378371	6.454094	1.995378	0.047377	0.10293	-5.14696
EXOSC10	0.276527	6.069326	1.995293	0.047386	0.102935	-5.1344
ZBTB7A	-0.34765	7.169469	-1.99464	0.047458	0.103076	-5.14732
HSPA8	-0.35801	10.11366	-1.99455	0.047468	0.103081	-5.05283
PMP22	-0.65262	5.324861	-1.99148	0.047807	0.103802	-5.14561
CHIC1	-0.68244	3.55516	-1.9912	0.047838	0.103852	-4.96085
ProSAPiP1	-0.81533	4.668642	-1.99105	0.047855	0.103872	-5.11068

CNIH	0.28502	6.298483	1.990962	0.047864	0.103877	-5.15307
ANKS3	-0.30897	3.243688	-1.99087	0.047874	0.103881	-4.82873
ALOXE3	1.979398	0.689241	1.990397	0.047927	0.103964	-4.18822
F2RL2	1.429705	3.341797	1.990422	0.047924	0.103964	-4.54457
WBP11	0.310849	6.720314	1.990114	0.047958	0.104016	-5.16525
FYB	1.130949	4.422285	1.990003	0.047971	0.104027	-4.80478
DMRTA2	2.20865	-0.25204	1.989068	0.048075	0.104223	-4.18608
ARID1B	-0.24325	7.010777	-1.98905	0.048076	0.104223	-5.16319
ALG13	-0.29176	4.653573	-1.98898	0.048084	0.104224	-5.06901
FOSL1	1.173511	5.408513	1.988668	0.048119	0.104284	-4.99828
CHST10	-0.8097	1.95584	-1.98852	0.048135	0.104287	-4.65726
STK39	-0.61753	5.432206	-1.98856	0.048131	0.104287	-5.15464
RPL36AL	-0.3487	6.663785	-1.98809	0.048184	0.104375	-5.16913
LACTB2	0.656495	4.546128	1.987644	0.048233	0.104467	-4.92593
CLEC2D	0.455705	4.007555	1.986516	0.048359	0.104723	-4.85566
MRE11A	0.364286	4.679975	1.984418	0.048594	0.105216	-5.00576
FAM128A	0.456456	4.727307	1.983785	0.048666	0.105354	-5.00171
FBLIM1	0.474427	6.644285	1.983274	0.048723	0.105462	-5.17364
NBN	0.338639	6.229732	1.981813	0.048888	0.105802	-5.16589
EIF3F	-0.34337	6.325289	-1.98083	0.048998	0.106025	-5.18519
NKAP	0.283007	3.795228	1.980509	0.049035	0.106072	-4.85481
DGKA	0.751965	5.833353	1.980517	0.049034	0.106072	-5.11171
ANKRD46	-0.38191	4.115245	-1.98007	0.049085	0.106163	-5.02887
TWF1	0.340579	7.318965	1.979355	0.049166	0.106322	-5.18548
ACTB	0.316992	12.68945	1.97902	0.049204	0.106388	-5.01192
WDR25	-0.30567	2.483247	-1.97853	0.049259	0.106491	-4.68959
ST7	-0.33035	4.935262	-1.97841	0.049273	0.106505	-5.12204
FARS2	-0.30411	3.297091	-1.97822	0.049295	0.106524	-4.86337
CMIP	0.367196	7.746414	1.978197	0.049297	0.106524	-5.18043
MTMR9	-0.44676	4.550764	-1.97812	0.049306	0.106526	-5.0941
QRICH1	-0.25033	6.062645	-1.97798	0.049322	0.106544	-5.1859
RABEP2	-0.28724	4.235591	-1.97722	0.049409	0.106715	-5.03948
PTP4A3	-0.62775	4.225314	-1.97692	0.049443	0.106772	-5.0775
ST8SIA1	-0.83965	0.993756	-1.97657	0.049483	0.106841	-4.51998
PPP1R14C	3.082579	3.408756	1.976033	0.049544	0.106957	-4.35549
PAK4	0.309349	6.468786	1.974581	0.04971	0.107299	-5.1899
ATP11B	-0.4484	7.487097	-1.97434	0.049738	0.107342	-5.17703
XYLT1	-0.68059	4.600753	-1.97412	0.049763	0.10738	-5.12816
TMEM184B	0.343297	7.084795	1.973793	0.0498	0.107444	-5.19864
TMEM104	0.318839	5.212667	1.971611	0.050051	0.107968	-5.1045
SLC22A3	-1.01025	3.057821	-1.9709	0.050132	0.108127	-4.95563
CRLF1	-1.33131	0.633873	-1.97077	0.050147	0.108143	-4.54255
C5orf22	0.363069	5.176112	1.970272	0.050205	0.108234	-5.09832
GPD2	0.390426	6.568997	1.97031	0.050201	0.108234	-5.19883
UPF3B	0.481998	4.398722	1.969876	0.050251	0.108316	-4.96459
LTB4R2	1.24196	3.732896	1.969216	0.050327	0.108464	-4.68414
ARF1	0.22892	8.958942	1.968799	0.050375	0.108551	-5.1597
RFX7	0.369963	5.440289	1.968576	0.050401	0.10859	-5.12981
MRPL15	0.358581	5.268589	1.968235	0.050441	0.108659	-5.11325
ANXA8L2	4.117596	3.538733	1.967578	0.050517	0.108806	-4.27195
LTK	-1.28678	0.090783	-1.9675	0.050526	0.108809	-4.44992
TRAFD1	0.320263	5.15713	1.965577	0.05075	0.109268	-5.11026
PDPR	-0.44427	5.872483	-1.96553	0.050755	0.109268	-5.20935
TMEM184C	-0.32774	4.858986	-1.96452	0.050874	0.109506	-5.14133
WAC	-0.2268	7.225272	-1.96426	0.050903	0.109552	-5.20725
MIPOL1	-0.4888	2.498101	-1.96374	0.050964	0.109667	-4.75535
TFCP2	0.349631	4.807479	1.96289	0.051064	0.109848	-5.06797
UTP14C	-0.39431	5.813204	-1.9629	0.051062	0.109848	-5.2115

PREP	0.383437	5.804812	1.962712	0.051084	0.109876	-5.17303
DUSP16	-0.48216	5.837534	-1.96211	0.051155	0.110011	-5.2158
NDUFV1	-0.39127	6.424095	-1.96196	0.051172	0.11003	-5.2215
LRRC27	-0.4851	1.753778	-1.96129	0.051251	0.110179	-4.61798
BTG3	0.469555	5.041954	1.96124	0.051257	0.110179	-5.08814
CMBL	-0.95635	4.667022	-1.96031	0.051366	0.110397	-5.17973
SLFN5	0.642202	5.331647	1.959854	0.05142	0.110496	-5.10749
GOSR1	-0.25689	5.998155	-1.95944	0.051469	0.110584	-5.22013
KDM5B	0.411051	6.992373	1.959316	0.051484	0.110598	-5.2265
SUSD5	-0.85386	0.504332	-1.95898	0.051523	0.110666	-4.47592
SYK	0.655009	5.813709	1.958711	0.051555	0.110717	-5.15965
DNAJB2	-0.30884	5.432252	-1.95857	0.051571	0.110735	-5.19871
PAX9	2.405487	3.752562	1.958443	0.051587	0.110751	-4.52894
ACOT4	0.736769	1.379385	1.958261	0.051608	0.11078	-4.40107
CD82	0.524643	6.04338	1.957952	0.051645	0.110842	-5.19066
LOC645332	0.495044	2.034211	1.957796	0.051663	0.110864	-4.52977
SLC46A1	-0.65295	3.977276	-1.95715	0.05174	0.111011	-5.08663
C8orf42	-0.91141	1.924887	-1.95679	0.051783	0.111087	-4.72857
WDR85	0.302573	3.918648	1.956256	0.051846	0.111187	-4.92254
LPIN3	0.502834	4.661988	1.956284	0.051842	0.111187	-5.03621
NUDT4	-0.43566	5.284014	-1.95563	0.05192	0.111331	-5.20206
LCK	1.213581	2.684006	1.955074	0.051986	0.111454	-4.5357
SPEF2	-0.85579	1.133958	-1.95375	0.052143	0.111774	-4.58405
ONECUT2	3.174501	3.470916	1.953595	0.052162	0.111797	-4.39023
CCDC87	0.848594	0.029858	1.953506	0.052173	0.111803	-4.25241
MRPS22	0.356886	4.808465	1.953176	0.052212	0.11187	-5.08603
ZNF497	0.64639	1.718573	1.952455	0.052298	0.112038	-4.47232
COX11	-0.28914	5.23548	-1.95229	0.052317	0.112062	-5.19549
OTUD7B	-0.27274	5.308338	-1.95196	0.052358	0.112131	-5.20085
CYP26B1	1.262607	2.796086	1.951649	0.052394	0.112175	-4.55349
ALS2CR4	0.630767	4.530262	1.951701	0.052388	0.112175	-4.99964
CTNNA1	-0.30741	8.745153	-1.95136	0.052429	0.112231	-5.18405
HNMT	-0.7694	3.767657	-1.95103	0.052468	0.112299	-5.08138
HPS1	-0.31735	6.022903	-1.9508	0.052496	0.11234	-5.23859
MCF2L	-0.81451	5.040045	-1.95061	0.052518	0.112372	-5.2163
CCL18	1.855841	3.018633	1.950519	0.05253	0.112379	-4.50785
FAM158A	0.432287	2.36847	1.95045	0.052538	0.112379	-4.60641
FMN1	-0.88225	1.900082	-1.95018	0.05257	0.112426	-4.72487
CUEDC2	-0.33243	4.61486	-1.95007	0.052584	0.112426	-5.1439
MLLT4	-0.46925	7.37738	-1.95007	0.052584	0.112426	-5.22608
KCNQ5	-1.34768	0.205058	-1.94992	0.052601	0.112446	-4.51874
MGLL	-0.87291	6.383276	-1.94985	0.05261	0.112446	-5.23946
LGALS3BP	0.456305	9.716713	1.949718	0.052626	0.112464	-5.17711
S100A13	0.433627	4.528408	1.948535	0.052768	0.112751	-5.039
TTN	-0.59917	3.127115	-1.94819	0.052809	0.112821	-4.9395
PSPH	0.679492	3.955285	1.947953	0.052838	0.112866	-4.87245
NR3C1	-0.60719	5.932765	-1.94754	0.052888	0.112956	-5.24757
ZNF675	-0.81171	2.217822	-1.94713	0.052938	0.11301	-4.78953
FAM3D	-1.68822	2.312027	-1.94709	0.052943	0.11301	-4.95984
BRAF	-0.33388	3.635116	-1.94707	0.052945	0.11301	-5.00098
USP37	0.397725	5.093574	1.94706	0.052946	0.11301	-5.12929
PABPC3	0.457608	6.104205	1.946916	0.052963	0.11303	-5.22012
NUPR1	-0.82181	4.81052	-1.94663	0.052998	0.113069	-5.20964
SLC38A1	0.442042	7.889612	1.946683	0.052992	0.113069	-5.23937
PPP1CC	0.224525	7.422937	1.946015	0.053072	0.11321	-5.24614
ITGA1	-0.69466	5.440376	-1.94593	0.053083	0.113215	-5.2399
HBEGF	-0.72811	4.884368	-1.94505	0.053189	0.113424	-5.21152
TBX5	1.324488	0.771581	1.944461	0.053261	0.113543	-4.29015

EDARADD	-0.83507	2.857674	-1.9445	0.053256	0.113543	-4.93338
PRSS21	2.548575	0.673459	1.944252	0.053286	0.113579	-4.27283
CAND1	0.371871	7.471825	1.944034	0.053312	0.113618	-5.25135
FLII	-0.30167	7.466495	-1.94339	0.053391	0.113767	-5.24035
IFFO2	0.873166	6.270779	1.943322	0.053399	0.113768	-5.2119
ILKAP	0.23946	3.854355	1.943193	0.053415	0.113784	-4.94645
TAF13	-0.34639	2.446898	-1.94312	0.053424	0.113786	-4.758
PIGF	0.343293	3.598536	1.941799	0.053584	0.114111	-4.87098
LOC150381	-0.49333	1.170353	-1.94157	0.053613	0.114137	-4.56016
CNGA1	-0.87904	1.080362	-1.94156	0.053613	0.114137	-4.60032
EVC2	-0.95028	1.186396	-1.94137	0.053637	0.114146	-4.63352
PLXND1	0.582083	6.574458	1.941401	0.053633	0.114146	-5.24836
CCDC85C	-0.43764	5.913456	-1.94133	0.053642	0.114146	-5.25648
PLB1	0.862955	1.514165	1.940743	0.053713	0.114264	-4.43708
BOK	-0.49258	5.457506	-1.94081	0.053705	0.114264	-5.24282
FOXP4	-0.56863	7.413771	-1.9405	0.053744	0.114311	-5.24089
DBN1	-0.60306	5.947417	-1.94026	0.053772	0.114354	-5.26156
ZNF84	-0.44542	4.618883	-1.93915	0.053908	0.114625	-5.17616
PLTP	-0.89936	6.569582	-1.93898	0.05393	0.114654	-5.25638
VPS13A	-0.48476	6.014061	-1.9384	0.054	0.114787	-5.26475
MAPRE3	-0.58454	3.805441	-1.93793	0.054058	0.114874	-5.09015
LZTS2	-0.35302	6.025216	-1.93795	0.054056	0.114874	-5.26383
C20orf111	0.291284	5.179838	1.937724	0.054083	0.114911	-5.16879
CDK5R1	1.096954	3.168468	1.937249	0.054142	0.115017	-4.66703
CDA	1.857491	3.223278	1.93713	0.054157	0.115031	-4.56276
MKL1	-0.30132	5.864148	-1.93639	0.054248	0.115208	-5.26122
SON	-0.24907	8.662798	-1.93625	0.054265	0.115226	-5.21746
EHF	1.094961	6.573253	1.936064	0.054288	0.115257	-5.23173
ADAMTS6	0.915983	1.352949	1.935761	0.054325	0.115284	-4.41643
ZSCAN22	-0.33102	2.812237	-1.93589	0.054309	0.115284	-4.84415
EIF2S1	0.308603	6.383675	1.935806	0.05432	0.115284	-5.26147
GLDN	-0.74828	1.221098	-1.93534	0.054377	0.115378	-4.62109
STEAP4	-1.12329	3.947891	-1.9348	0.054443	0.1155	-5.17398
PRR3	0.427775	3.413298	1.934407	0.054492	0.115586	-4.83279
KIAA0040	-0.48877	5.818894	-1.93431	0.054505	0.115595	-5.26853
IFT20	-0.31214	3.594446	-1.93391	0.054553	0.115681	-5.01077
UBE2D4	-0.3389	3.236979	-1.9336	0.054592	0.115728	-4.94269
ABCB6	0.7124	4.992114	1.933608	0.054591	0.115728	-5.10325
S100A2	4.162581	6.313707	1.933403	0.054617	0.115745	-4.72808
TMEM179B	0.337722	5.514374	1.933453	0.054611	0.115745	-5.20657
LOC284441	0.350857	1.823267	1.932501	0.054729	0.115947	-4.56443
TAF15	0.269775	7.049211	1.932518	0.054727	0.115947	-5.27748
KLK6	4.261232	3.851124	1.93159	0.054842	0.116132	-4.35519
POLR3C	0.233976	4.639481	1.931577	0.054843	0.116132	-5.11877
HLA-E	0.438362	8.726371	1.931627	0.054837	0.116132	-5.24503
SEPW1	-0.39257	6.441754	-1.93153	0.054849	0.116132	-5.27945
NDUFB10	-0.3612	5.565952	-1.93109	0.054904	0.116225	-5.26107
THRAP3	-0.17857	7.179867	-1.93104	0.05491	0.116225	-5.27242
STX2	-0.47487	3.561325	-1.93076	0.054945	0.116282	-5.04181
ZNF706	0.384152	5.588633	1.930617	0.054963	0.116302	-5.21527
TRIM4	-0.36237	5.088093	-1.92997	0.055043	0.116454	-5.23125
FOXK1	0.371327	6.831728	1.92873	0.055198	0.116765	-5.28377
SPATA5	0.393248	4.033362	1.928485	0.055229	0.116794	-4.98253
TMEM106C	0.414927	5.882469	1.928491	0.055228	0.116794	-5.24208
GAS8	-0.34299	4.113838	-1.92835	0.055246	0.116812	-5.12293
TYRO3	-0.48289	4.407924	-1.92826	0.055258	0.116819	-5.17649
FMO1	1.523633	0.50854	1.927743	0.055322	0.116938	-4.30156
SLC25A36	-0.44869	6.651914	-1.92714	0.055398	0.117081	-5.28481

FAM200B	-0.31228	3.724986	-1.92692	0.055426	0.117122	-5.05379
TNRC6B	-0.3227	6.380553	-1.9268	0.05544	0.117134	-5.28873
SH2B2	0.649957	2.083662	1.92672	0.05545	0.117138	-4.57618
CCDC46	-0.65627	1.931819	-1.92656	0.05547	0.117162	-4.74323
ZNF7	0.30512	4.523676	1.925552	0.055597	0.117413	-5.10137
MGC72080	0.692409	2.44312	1.924673	0.055708	0.117559	-4.62831
DLEU1	0.498318	2.5331	1.924468	0.055734	0.117559	-4.67298
PPP4R1L	0.567946	2.631368	1.924732	0.0557	0.117559	-4.67651
FPR3	1.025475	3.145775	1.924805	0.055691	0.117559	-4.69767
DUS3L	0.36716	4.329787	1.92447	0.055733	0.117559	-5.0571
RSPRY1	-0.2844	4.513128	-1.92459	0.055718	0.117559	-5.17531
TTL	0.370624	5.720023	1.924634	0.055713	0.117559	-5.23983
CTNNB1	0.35578	8.354309	1.924878	0.055682	0.117559	-5.26715
PMM2	0.369303	5.837525	1.924304	0.055754	0.117585	-5.24963
ACAP2	0.43072	6.9019	1.923598	0.055843	0.117755	-5.29356
EDEM3	-0.46691	6.609155	-1.92346	0.055861	0.117774	-5.29214
SLC6A14	3.240315	3.232185	1.922192	0.056021	0.118094	-4.40828
PDE3A	-0.95568	2.03487	-1.92208	0.056035	0.118106	-4.8264
DAGLA	-0.77309	3.767427	-1.92192	0.056056	0.118132	-5.13793
CLEC16A	-0.271	5.569737	-1.92176	0.056075	0.118155	-5.27483
CYB5D1	-0.29441	4.203819	-1.92146	0.056113	0.118217	-5.14241
MCF2L2	0.775411	0.380186	1.919727	0.056334	0.118664	-4.34265
CIC	-0.31348	7.103668	-1.9194	0.056376	0.118735	-5.29353
C13orf23	0.400402	6.559452	1.919225	0.056398	0.118763	-5.29562
EED	0.33536	4.174698	1.919092	0.056415	0.11878	-5.04117
C9orf69	0.366018	5.908235	1.919015	0.056425	0.118783	-5.26536
ING2	-0.3333	3.11736	-1.91832	0.056513	0.118933	-4.94536
MRPS35	0.461201	6.022248	1.918323	0.056513	0.118933	-5.2684
CXorf42	0.449954	-0.0528	1.917863	0.056572	0.119039	-4.32941
SPATA2	0.367307	5.253923	1.917751	0.056586	0.119051	-5.20651
SNHG5	-0.68756	5.477176	-1.9172	0.056656	0.11918	-5.29525
GTPBP2	0.401518	5.989492	1.916995	0.056682	0.119218	-5.27243
ZNF263	-0.23316	4.752102	-1.91674	0.056714	0.119268	-5.21228
RAB28	-0.28783	3.499091	-1.91619	0.056786	0.1194	-5.02181
MRPS21	0.422712	5.26202	1.9155	0.056874	0.119567	-5.20645
LRRTM2	-0.60499	0.115987	-1.91515	0.056919	0.119643	-4.47001
CNTROB	0.319868	4.839429	1.915026	0.056935	0.119658	-5.16665
IFNAR2	0.399038	4.232539	1.914796	0.056964	0.119703	-5.05056
RIN1	0.693346	4.615199	1.914713	0.056975	0.119707	-5.0733
HEATR6	0.325824	4.513457	1.914199	0.057041	0.119828	-5.11821
NCDN	-0.32881	5.302933	-1.91359	0.05712	0.119975	-5.2766
CLCN3	-0.46272	6.201449	-1.91343	0.05714	0.120001	-5.31359
COPS4	-0.24527	5.089051	-1.91298	0.057197	0.120102	-5.25447
TRRAP	0.375113	7.33509	1.912847	0.057215	0.120121	-5.31248
ZBTB7C	-1.17306	4.708873	-1.91242	0.05727	0.12022	-5.28643
TAF4B	0.663831	3.763789	1.912163	0.057303	0.12027	-4.90297
IPPK	0.594996	4.774392	1.912073	0.057315	0.120276	-5.12563
MOBKL2C	-0.37172	4.551969	-1.91155	0.057383	0.120401	-5.21379
ALDH7A1	-0.64163	4.632675	-1.91112	0.057438	0.120481	-5.24728
GNA13	0.309185	7.337086	1.911132	0.057436	0.120481	-5.31481
NPTN	-0.29736	6.832431	-1.91094	0.057461	0.12051	-5.31467
TRMT61B	0.304073	3.737111	1.910797	0.057479	0.120514	-4.96944
CDKN1A	-0.60546	7.061499	-1.9108	0.057479	0.120514	-5.30499
ADAMTSL5	0.98549	3.079625	1.910687	0.057494	0.120525	-4.71716
CRLS1	-0.388	5.602305	-1.91046	0.057523	0.120568	-5.30261
CDK20	-0.74622	1.372465	-1.90912	0.057696	0.120914	-4.69231
LYPD3	2.72837	5.740412	1.908431	0.057786	0.121048	-4.92894
TSFM	0.297374	4.351958	1.908479	0.05778	0.121048	-5.10358

SLC35A3	-0.50573	4.552035	-1.90854	0.057772	0.121048	-5.23105
MED24	0.431965	6.635986	1.908266	0.057808	0.121075	-5.31746
BAG4	0.475318	3.656462	1.907756	0.057874	0.121196	-4.92375
GABARAPL2	-0.2777	5.479579	-1.90755	0.057901	0.121235	-5.29663
RAD1	0.329055	4.672007	1.907279	0.057936	0.121289	-5.15656
GZMB	1.170926	1.703901	1.906874	0.057989	0.121327	-4.4871
PROCR	0.756358	4.186888	1.906939	0.05798	0.121327	-4.98552
MEIS3P1	-0.72113	3.11225	-1.90691	0.057984	0.121327	-5.04187
SSBP1	0.345047	5.433507	1.90701	0.057971	0.121327	-5.24812
C5orf13	0.66629	5.318191	1.905867	0.05812	0.121583	-5.2045
GUCY1A2	0.90764	0.114775	1.905733	0.058138	0.121584	-4.33873
TMEM149	0.553781	2.366935	1.905731	0.058138	0.121584	-4.67154
UBFD1	0.289208	5.980015	1.905554	0.058161	0.121614	-5.30004
EXOSC3	0.37324	3.791277	1.905193	0.058208	0.121676	-4.97837
ANKFY1	-0.32051	6.669503	-1.90523	0.058204	0.121676	-5.32743
ORAI2	0.624103	4.607777	1.905091	0.058222	0.121686	-5.1026
TBC1D22A	-0.29745	4.906698	-1.90492	0.058245	0.121687	-5.25636
HS1BP3	-0.30315	4.945516	-1.90497	0.058238	0.121687	-5.26044
PTRF	-0.67279	7.499115	-1.90489	0.058248	0.121687	-5.3029
RWDD3	-0.28847	2.969905	-1.90474	0.058268	0.12171	-4.92735
DPP8	-0.25651	4.982639	-1.90452	0.058297	0.121752	-5.26107
GATC	0.36666	1.30408	1.904369	0.058316	0.121774	-4.53585
EIF3H	0.32928	7.994682	1.903967	0.058369	0.121866	-5.31536
FAM179B	-0.31201	4.785928	-1.90373	0.0584	0.121914	-5.24793
TPCN1	-0.47661	5.853951	-1.90283	0.058518	0.122142	-5.32839
PANK2	0.296975	4.357229	1.902587	0.05855	0.122171	-5.11693
SMARCA4	0.333503	7.586535	1.902632	0.058544	0.122171	-5.3267
DECR1	-0.33645	5.283766	-1.90251	0.05856	0.122174	-5.29621
RABGEF1	-0.30653	5.64147	-1.90233	0.058584	0.122206	-5.31654
DHX30	-0.24695	6.400158	-1.90155	0.058686	0.1224	-5.33602
MMP25	0.769275	1.529474	1.90141	0.058705	0.122421	-4.5223
SEMA4F	0.56595	3.305822	1.901221	0.05873	0.122443	-4.84408
C14orf126	0.498726	4.153295	1.901196	0.058733	0.122443	-5.04015
ZNF193	-0.40531	2.059839	-1.90069	0.0588	0.122565	-4.7727
TBX3	0.802267	4.515395	1.900138	0.058872	0.122697	-5.0581
ZDHHC11	-0.88	2.038742	-1.89962	0.058941	0.122821	-4.85045
PSTPIP1	0.8189	1.0444	1.899211	0.058995	0.122916	-4.45592
GYG2	-0.98934	1.723136	-1.8991	0.05901	0.122929	-4.80474
LRRC32	-0.62049	4.749839	-1.89866	0.059068	0.123031	-5.28041
JPH1	1.307862	3.513748	1.898348	0.059109	0.123099	-4.76456
ZNF165	0.710129	2.342016	1.897846	0.059176	0.123219	-4.65828
BMP8B	0.838663	2.859713	1.897239	0.059256	0.123368	-4.72533
PTAFR	0.794089	2.990499	1.896509	0.059353	0.123552	-4.75733
DCP1B	-0.5019	3.624581	-1.89632	0.059378	0.123585	-5.12409
LOC100130932	0.435491	2.314409	1.8956	0.059474	0.123767	-4.69848
ZNF486	-1.0622	0.215716	-1.8949	0.059568	0.123925	-4.57883
APITD1	0.445484	3.210754	1.894901	0.059567	0.123925	-4.85885
PLA2G6	-0.42096	4.010019	-1.89426	0.059653	0.124084	-5.1801
ZNF555	-0.31228	1.746692	-1.89386	0.059706	0.124175	-4.71778
PIM3	-0.40714	6.744735	-1.89365	0.059735	0.124216	-5.34671
CLDN23	-0.9316	3.229393	-1.89348	0.059757	0.124245	-5.11684
HLA-DQA1	1.317212	4.718152	1.89326	0.059786	0.124287	-5.01853
GANAB	0.272802	8.932341	1.893076	0.059811	0.124319	-5.30517
ANAPC11	0.344842	5.52738	1.892946	0.059828	0.124337	-5.28335
PLA1A	-1.14611	0.048969	-1.89214	0.059936	0.124524	-4.56917
DHRS3	-0.56364	5.883256	-1.89216	0.059933	0.124524	-5.3504
NSMCE2	0.402436	3.712063	1.891589	0.06001	0.124659	-4.98002
GRSF1	-0.23942	6.582208	-1.89114	0.060069	0.124764	-5.35521

AMH	1.434225	0.413125	1.891022	0.060086	0.12478	-4.36643
GPX2	1.52696	7.35155	1.890822	0.060113	0.124817	-5.33784
C9orf123	-0.39422	4.439313	-1.89062	0.060139	0.124817	-5.24126
RAP2A	0.359108	5.597133	1.890664	0.060134	0.124817	-5.29343
RBM12	0.208931	6.601354	1.890661	0.060134	0.124817	-5.35398
YIF1A	0.346396	5.805185	1.889689	0.060265	0.125059	-5.31345
C9orf72	-0.47672	3.917609	-1.88911	0.060344	0.125204	-5.18398
QTRT1	0.314475	4.293489	1.888463	0.06043	0.125365	-5.12671
RNASEN	0.308386	6.11633	1.888227	0.060462	0.125412	-5.33898
DDX19B	-0.38063	3.928959	-1.88786	0.060512	0.125496	-5.1738
RDH14	-0.22908	3.77599	-1.88771	0.060532	0.125501	-5.12144
SBDS	-0.3913	6.36263	-1.88771	0.060532	0.125501	-5.36181
TARS	0.343251	6.799073	1.887349	0.060581	0.125583	-5.36113
RAB5A	-0.25724	5.893161	-1.8872	0.060601	0.125607	-5.35309
WNT11	1.847103	1.189621	1.886879	0.060644	0.125659	-4.38528
TMEM208	0.359454	4.557201	1.886909	0.06064	0.125659	-5.17016
ZNF140	-0.39213	3.783765	-1.88633	0.060719	0.125786	-5.154
SLC25A13	0.46025	5.311632	1.886296	0.060723	0.125786	-5.26206
POU2F3	1.217109	1.236392	1.88619	0.060737	0.125797	-4.45647
C12orf62	-0.30009	4.151535	-1.88529	0.06086	0.126032	-5.2027
CHD3	-0.39465	7.443292	-1.88517	0.060876	0.126046	-5.3481
METTL9	-0.25191	6.441114	-1.88481	0.060925	0.126129	-5.36724
C11orf83	0.440273	3.338894	1.883144	0.061151	0.126579	-4.9066
MPV17	0.337105	5.276002	1.88299	0.061172	0.126603	-5.27712
ZNF286B	0.611631	0.821099	1.882805	0.061198	0.126635	-4.4801
SORCS2	-0.93882	1.883776	-1.88273	0.061208	0.126635	-4.86727
KLF3	-0.34357	7.371653	-1.88268	0.061215	0.126635	-5.35554
PDF	0.372003	2.991421	1.882597	0.061226	0.126639	-4.85076
SAMSN1	0.866043	2.569589	1.882136	0.061289	0.126751	-4.7037
RXRB	-0.24171	5.051462	-1.88187	0.061325	0.126807	-5.30848
DDX20	0.296877	4.366395	1.881725	0.061345	0.126829	-5.15639
SPI1	0.883486	3.756391	1.881165	0.061421	0.126968	-4.91881
LPAR3	2.353015	0.673568	1.880864	0.061463	0.127035	-4.38579
CD55	0.866094	7.094842	1.880776	0.061475	0.127041	-5.36943
GALNT11	-0.3953	4.901898	-1.88009	0.061568	0.127215	-5.31016
CCDC61	-0.30544	2.571348	-1.87995	0.061588	0.127238	-4.89056
ARHGEF7	-0.30546	6.20818	-1.8798	0.061609	0.127261	-5.37509
AVL9	0.40797	4.583798	1.879596	0.061636	0.127281	-5.18064
CSNK1D	-0.21251	7.161123	-1.87962	0.061634	0.127281	-5.36826
KRT15	3.084191	5.693289	1.879028	0.061714	0.127423	-4.8981
ATF1	0.278303	4.737863	1.878323	0.061811	0.127604	-5.22618
RPL13A	-0.4407	7.500326	-1.87756	0.061916	0.127803	-5.35954
NUDT2	-0.36438	3.026668	-1.87704	0.061987	0.127911	-5.00545
CDK14	-0.72855	3.94343	-1.87708	0.061982	0.127911	-5.24098
DCUN1D1	0.396872	5.099485	1.876604	0.062048	0.128017	-5.26221
KIAA1468	-0.42919	5.566794	-1.87619	0.062105	0.128116	-5.36633
UBA1	0.258457	8.892719	1.876063	0.062122	0.128133	-5.33758
SLC22A23	-0.6056	6.385303	-1.87579	0.06216	0.128192	-5.38208
ZBED2	2.019103	1.170816	1.874981	0.062272	0.128387	-4.39821
ZNF620	-0.70335	1.126704	-1.87497	0.062273	0.128387	-4.70673
PDPN	1.093233	4.977171	1.873703	0.062449	0.12873	-5.14751
CD4	0.773543	4.710674	1.873436	0.062486	0.128788	-5.15384
TUSC1	-0.70285	3.008668	-1.87307	0.062536	0.128873	-5.0755
NAGA	0.257891	5.653905	1.87214	0.062666	0.129121	-5.34029
PLSCR4	-0.61011	4.287367	-1.87152	0.062752	0.12924	-5.28187
CARHSP1	0.478814	5.840123	1.871523	0.062752	0.12924	-5.3403
EXOC7	-0.26011	7.256585	-1.87154	0.06275	0.12924	-5.3803
RNGTT	0.281884	4.782756	1.871392	0.06277	0.129259	-5.24461

ATP8B3		1.096352	0.985264	1.87125	0.06279	0.129281	-4.46694
PIP4K2A		-0.35307	4.686233	-1.87095	0.062831	0.129347	-5.30178
PAK6		0.811464	4.702899	1.870857	0.062845	0.129355	-5.15007
TEX9		-0.68648	1.100986	-1.86966	0.063012	0.129681	-4.70993
WDR8		0.273609	4.291069	1.869156	0.063082	0.129787	-5.1686
GTF2A2		0.31347	4.975318	1.869221	0.063073	0.129787	-5.26958
IFT80		0.439005	5.041268	1.868356	0.063194	0.129998	-5.26508
NDUFA6		-0.30087	5.52445	-1.86812	0.063228	0.130048	-5.37348
FAM59A		-0.5744	4.295739	-1.86768	0.063289	0.130156	-5.2847
SLC7A11		1.220076	5.614602	1.867405	0.063327	0.130186	-5.24579
RNF34		0.236613	4.920788	1.867481	0.063316	0.130186	-5.27445
DHX16		0.237132	5.543456	1.86737	0.063332	0.130186	-5.34099
C14orf176		1.045353	0.396952	1.867293	0.063343	0.130189	-4.4109
PCDHGA1		0.965503	0.181988	1.866816	0.06341	0.13028	-4.40754
APOBEC3G		0.955545	3.006933	1.866778	0.063415	0.13028	-4.79031
CWF19L2		-0.36705	4.456235	-1.86682	0.063409	0.13028	-5.28461
TMEM218		-0.30172	2.802154	-1.86638	0.063471	0.130376	-4.96335
CHD7		0.474437	5.747632	1.866202	0.063496	0.130408	-5.34204
PANK1		-0.55355	3.100507	-1.866	0.063524	0.130446	-5.0767
WNK2		-1.03267	5.515473	-1.86581	0.063552	0.130484	-5.39868
PAXIP1		0.356703	4.670282	1.865323	0.06362	0.130605	-5.22927
TTC21A		-0.58363	1.019253	-1.86489	0.06368	0.13071	-4.69063
KLK10		2.492867	5.618521	1.864741	0.063702	0.13072	-5.02816
ARID4B		-0.31922	6.325866	-1.86472	0.063704	0.13072	-5.40397
ZNF32		-0.36681	4.400367	-1.86446	0.063741	0.130758	-5.28211
CRIM1		-0.43311	6.403724	-1.86452	0.063733	0.130758	-5.40415
CXCL5		2.791927	2.076304	1.864346	0.063757	0.130772	-4.42486
TOP1		0.315222	7.437529	1.863885	0.063822	0.130886	-5.40071
NECAP2		0.254931	5.546864	1.863459	0.063883	0.13099	-5.34699
VPS18		-0.30262	5.111293	-1.86305	0.063941	0.13109	-5.3532
KIAA1632		-0.36357	5.463367	-1.8626	0.064005	0.131202	-5.3832
EEFSEC		0.347608	4.801844	1.862351	0.064039	0.131254	-5.25564
NLGN4Y		2.01488	0.920222	1.862105	0.064074	0.131287	-4.41958
AGAP4		-0.38593	3.001895	-1.86213	0.06407	0.131287	-5.03041
NDUFB7		-0.32902	5.413006	-1.86196	0.064094	0.131308	-5.37945
C1orf109		0.317236	4.177755	1.861772	0.064121	0.131326	-5.15197
SAFB		0.2046	6.102852	1.861781	0.06412	0.131326	-5.3917
NPR2		-0.67468	2.645354	-1.86167	0.064136	0.131336	-5.01146
GLMN		0.385574	2.845369	1.860452	0.064309	0.13166	-4.86239
BFAR		0.274004	5.701898	1.860421	0.064313	0.13166	-5.36423
PCBP1		0.212334	8.468731	1.860112	0.064357	0.131731	-5.37984
NSFL1C		0.288727	6.238832	1.859246	0.06448	0.131964	-5.39898
ZNF586		-0.36831	3.286314	-1.85886	0.064535	0.132057	-5.09538
HRAS		0.531099	4.686555	1.858609	0.064571	0.132111	-5.21931
LUC7L		-0.30433	4.451369	-1.85748	0.064733	0.132418	-5.29397
PCGF3		-0.30499	5.920955	-1.85743	0.06474	0.132418	-5.41
MRFAP1L1		-0.27501	5.178654	-1.85733	0.064754	0.132428	-5.3674
NRBP1		0.296173	6.720004	1.857197	0.064773	0.132429	-5.41609
DDX46		-0.19881	6.612153	-1.85719	0.064774	0.132429	-5.4177
FGGY		-0.45954	1.948415	-1.85638	0.06489	0.132647	-4.84117
SUSD3		0.837008	1.119747	1.856228	0.064911	0.132671	-4.53981
PKIA		-0.80798	1.160557	-1.8558	0.064973	0.132777	-4.76527
ALAS1		-0.38331	5.78654	-1.85524	0.065052	0.13292	-5.41174
GBAP1		0.564905	2.388595	1.855063	0.065078	0.132954	-4.76454
RPGR		-0.38539	3.503199	-1.85441	0.065173	0.133127	-5.1529
	7-Sep	0.277579	7.051687	1.854029	0.065227	0.133218	-5.42324
UBE2N		0.247781	6.337965	1.853242	0.06534	0.13343	-5.41519
RMI1		0.390333	4.50716	1.853099	0.06536	0.133453	-5.21769

CHCHD2	0.363619	7.134844	1.851467	0.065596	0.133914	-5.4278
FKBP10	1.075657	5.615289	1.850539	0.06573	0.134168	-5.29667
TPBG	0.71831	5.717532	1.849593	0.065867	0.134428	-5.34916
MRPS2	0.359669	5.476811	1.849373	0.065899	0.134474	-5.3571
C6orf168	-1.03467	1.629972	-1.84918	0.065927	0.134503	-4.8972
SAP130	0.247047	5.768976	1.848971	0.065957	0.134503	-5.3924
PDXK	-0.43452	7.078138	-1.84894	0.065962	0.134503	-5.42199
NR1H2	-0.30244	6.008794	-1.84905	0.065946	0.134503	-5.42753
ARGLU1	-0.4344	6.375752	-1.84913	0.065934	0.134503	-5.43232
CRIP2	-0.61574	5.633371	-1.8485	0.066026	0.134615	-5.42548
PROM1	-1.72007	2.73666	-1.84667	0.066292	0.135138	-5.23348
ETF1	-0.2599	7.005252	-1.84655	0.066309	0.135152	-5.4309
YAP1	-0.40555	7.308238	-1.84621	0.066359	0.135234	-5.42246
LCP2	0.785561	3.669116	1.845214	0.066504	0.135511	-4.98152
C19orf66	0.411087	4.764658	1.845013	0.066534	0.135551	-5.27294
ZNF557	-0.3197	3.145673	-1.84483	0.06656	0.135575	-5.0815
S100A9	2.011192	7.670741	1.844798	0.066565	0.135575	-5.41557
GCLM	0.856496	5.166737	1.844404	0.066623	0.135653	-5.27528
ZDHHC17	-0.31193	4.564905	-1.84446	0.066615	0.135653	-5.33261
NDUFAF2	-0.33197	2.64229	-1.84424	0.066647	0.135682	-4.9773
TSHZ3	-0.6839	3.375981	-1.84345	0.066763	0.135899	-5.20517
WASF1	0.908831	3.569218	1.843341	0.066778	0.135911	-4.94651
MSC	1.250645	3.155634	1.843019	0.066825	0.135987	-4.81365
TNFAIP6	1.147387	2.161525	1.842941	0.066837	0.13599	-4.6714
TMEM67	0.522039	3.443709	1.842645	0.06688	0.136059	-4.9873
CD164	-0.38605	8.570124	-1.84249	0.066903	0.136085	-5.39
BAZ1B	0.256154	7.16884	1.841756	0.067011	0.136285	-5.44423
ALDH3A2	-0.53725	6.713791	-1.84162	0.067031	0.136306	-5.44053
PIGX	0.525982	5.312689	1.841188	0.067095	0.136415	-5.33828
ATP5S	-0.27794	2.482755	-1.84026	0.067232	0.136652	-4.94126
TOX3	-1.81685	1.30434	-1.8402	0.067241	0.136652	-4.96056
MTA1	0.30967	6.392312	1.840242	0.067234	0.136652	-5.43865
FMNL2	0.489584	6.063425	1.840069	0.067259	0.136671	-5.41357
ZNF526	0.260493	4.32349	1.839922	0.067281	0.136695	-5.22998
ZNF599	-0.52485	1.572019	-1.83945	0.067351	0.136818	-4.81806
ZNF223	-0.58259	1.356351	-1.83898	0.06742	0.136937	-4.79117
ZNF593	0.44536	3.998092	1.838359	0.067512	0.137104	-5.12993
EIF4A2	-0.32643	8.387015	-1.83821	0.067534	0.137129	-5.40604
SLC47A1	1.451403	0.789211	1.837806	0.067594	0.137231	-4.46567
TMCC1	0.36749	5.262522	1.837701	0.067609	0.137242	-5.35538
LRP3	-0.7514	4.889624	-1.83748	0.067642	0.137288	-5.41346
RASL11A	-0.80349	1.932169	-1.83718	0.067687	0.137359	-4.93368
AIF1	0.829761	2.783867	1.836887	0.06773	0.137427	-4.82557
ZBP1	1.358235	0.782085	1.836532	0.067783	0.137494	-4.47336
HTRA2	0.273059	4.398058	1.836563	0.067778	0.137494	-5.24843
RAB38	1.780502	3.448542	1.836028	0.067857	0.137606	-4.79579
SUCLG1	-0.30992	5.859636	-1.83609	0.067849	0.137606	-5.44683
COL21A1	-0.98664	0.263118	-1.83595	0.067869	0.137609	-4.68213
RFNG	-0.27886	5.175441	-1.83583	0.067886	0.137624	-5.40609
INPPL1	-0.40363	6.842391	-1.83575	0.067899	0.13763	-5.45101
KEAP1	0.346787	6.234008	1.835072	0.068	0.137814	-5.44027
FBXO22	0.269002	4.202806	1.834577	0.068073	0.137944	-5.21408
RPUSD4	-0.36292	4.217503	-1.83398	0.068162	0.138104	-5.31247
RNF24	0.506961	4.620677	1.833743	0.068198	0.138155	-5.25705
CTAGE4	0.899979	2.473796	1.833496	0.068234	0.13819	-4.76688
DYNLRB1	0.315913	6.786974	1.833551	0.068226	0.13819	-5.45952
MDGA1	-0.94544	2.800016	-1.83328	0.068267	0.138235	-5.15412
DOHH	0.328317	4.01475	1.833174	0.068283	0.138247	-5.16708

CBWD2	0.283184	4.62927	1.832671	0.068358	0.138379	-5.29258
KIAA0754	-0.65636	3.901153	-1.83208	0.068446	0.138538	-5.30709
IPO13	-0.30337	5.728179	-1.83155	0.068526	0.13868	-5.44989
MTA3	-0.26666	5.148676	-1.83024	0.068723	0.139058	-5.41322
LRRC1	0.401103	5.4938	1.82998	0.068761	0.139115	-5.38982
TSPAN8	-2.21971	3.552367	-1.82988	0.068776	0.139126	-5.40673
LAT2	0.721553	2.141728	1.828919	0.06892	0.139346	-4.75106
LHX6	-0.68553	1.532095	-1.82889	0.068925	0.139346	-4.85654
ILVBL	-0.36433	5.515862	-1.82905	0.068901	0.139346	-5.44666
PDLIM1	0.426319	7.9015	1.828968	0.068913	0.139346	-5.45671
SYT12	1.384145	1.352683	1.828581	0.068971	0.139419	-4.55371
CAMK2G	-0.3308	5.562759	-1.82847	0.068987	0.139432	-5.44891
CHPF	0.373215	7.747273	1.828358	0.069005	0.139447	-5.46011
MGMT	-0.66372	3.022424	-1.82726	0.069171	0.139763	-5.153
FKRP	-0.27265	3.885885	-1.82615	0.069338	0.140079	-5.26291
ADPRHL1	-0.61502	1.792328	-1.82593	0.069372	0.140107	-4.88875
DPH1	-0.26934	4.724525	-1.82595	0.069368	0.140107	-5.37938
ERO1L	0.55855	7.241462	1.82581	0.06939	0.140123	-5.47427
TMEM30A	-0.26962	7.598395	-1.82559	0.069422	0.140169	-5.45541
ZNF815	0.509297	0.518291	1.825474	0.06944	0.140185	-4.55207
PDE10A	-0.71887	2.256902	-1.82515	0.069489	0.140263	-4.99799
GRTP1	0.604673	3.591571	1.824766	0.069548	0.140361	-5.03279
GJC2	-0.58401	0.387535	-1.82457	0.069578	0.140382	-4.66755
ZSCAN12	-0.54006	2.691708	-1.82461	0.069571	0.140382	-5.06222
RNFT1	0.427448	3.864757	1.824068	0.069654	0.140513	-5.12907
SHPK	-0.27925	4.622263	-1.824	0.069663	0.140513	-5.37184
CHAC2	0.490828	2.245815	1.823842	0.069688	0.140543	-4.80754
PUS10	0.393469	3.271947	1.823612	0.069723	0.140593	-5.01042
GRHL3	2.721652	3.471293	1.823441	0.069749	0.14062	-4.68733
PEAR1	-0.57085	3.041076	-1.82339	0.069756	0.14062	-5.14304
AGPHD1	-0.43783	1.232133	-1.82283	0.069842	0.140772	-4.77839
PABPC4	-0.39462	7.515014	-1.82247	0.069896	0.14086	-5.46023
FAS	0.586277	4.00299	1.822016	0.069966	0.140981	-5.13435
CAMK1	-0.42674	2.739306	-1.82188	0.069986	0.141001	-5.05547
RCAN3	-0.33878	3.857316	-1.82176	0.070005	0.141005	-5.27459
RANBP2	-0.28501	7.65123	-1.82174	0.070008	0.141005	-5.46052
VAMP8	0.401652	6.193437	1.820716	0.070164	0.141299	-5.46128
PLXDC1	0.653155	3.899221	1.820105	0.070257	0.141426	-5.10124
ZBTB34	-0.29225	4.359059	-1.82018	0.070246	0.141426	-5.34821
CRK	-0.2025	5.976124	-1.82014	0.070252	0.141426	-5.47627
FZD10	2.113645	3.187521	1.819923	0.070285	0.141461	-4.73472
SLC48A1	-0.41985	4.778545	-1.81915	0.070404	0.14168	-5.41131
EPB41L4B	-0.74382	4.990605	-1.81824	0.070543	0.14194	-5.45323
ZNF782	-0.34385	1.896928	-1.81784	0.070604	0.142041	-4.88242
CMYA5	-0.7692	1.802691	-1.81722	0.0707	0.142213	-4.93855
ACN9	0.669616	3.67019	1.817156	0.070709	0.142213	-5.05455
KIDINS220	-0.26639	6.757024	-1.81707	0.070722	0.142218	-5.48794
HDAC10	0.346001	4.640609	1.816986	0.070736	0.142224	-5.31215
SLC15A1	1.952987	1.158453	1.815524	0.070961	0.142657	-4.50077
BLVRB	-0.47285	5.706056	-1.81523	0.071007	0.142728	-5.48371
PFN2	0.951176	6.356495	1.814862	0.071063	0.142821	-5.44924
TCERG1	0.220669	6.119104	1.814508	0.071117	0.14291	-5.47702
IFT27	-0.44597	3.679373	-1.81346	0.071279	0.143215	-5.27331
MGAT5	-0.53447	4.235084	-1.81322	0.071316	0.143248	-5.37074
LRSAM1	-0.27692	4.654045	-1.81324	0.071313	0.143248	-5.39492
PCDH19	-1.22121	1.251116	-1.81279	0.071383	0.143353	-4.91674
UBN2	-0.29005	5.544932	-1.81275	0.071389	0.143353	-5.47403
RHBDF1	0.409046	5.198556	1.812457	0.071435	0.143424	-5.38878

JMJD4	0.343326	4.123034	1.812068	0.071495	0.143524	-5.22443
DPP7	-0.40417	5.756242	-1.812	0.071506	0.143525	-5.48922
SCAND3	-1.31295	-0.09164	-1.81124	0.071624	0.143741	-4.71478
SEC61A2	0.409007	2.7788	1.810542	0.071732	0.143938	-4.93392
C14orf156	0.361267	4.684889	1.80958	0.071882	0.144218	-5.33128
PPP2R5C	-0.24752	6.757101	-1.80929	0.071927	0.144288	-5.50204
CYP2W1	2.112853	0.654846	1.808879	0.071991	0.144385	-4.50961
ARHGAP1	-0.2803	7.562413	-1.80885	0.071996	0.144385	-5.486
FAM38B	-0.81891	1.406847	-1.80868	0.072021	0.144415	-4.88781
PNPO	0.344068	5.585953	1.808499	0.07205	0.14444	-5.4421
PFKM	-0.43811	5.385908	-1.80844	0.07206	0.14444	-5.4802
PELI1	0.451936	6.363129	1.808405	0.072065	0.14444	-5.48942
LOC654342	-0.58212	3.279621	-1.8082	0.072097	0.144484	-5.22644
HSF4	-0.52436	2.446146	-1.80755	0.072198	0.144666	-5.03509
FAM127C	-0.66727	3.359487	-1.80705	0.072277	0.144804	-5.26613
ARL15	-0.40079	3.457647	-1.80665	0.07234	0.144908	-5.23258
SRD5A3	0.515583	5.110932	1.806311	0.072393	0.144993	-5.3763
DYX1C1	0.884247	0.76962	1.805425	0.072532	0.145233	-4.5761
C10orf58	0.605708	5.970832	1.805413	0.072534	0.145233	-5.46106
PSPC1	0.319793	4.921737	1.804883	0.072617	0.145379	-5.37829
KIAA0240	-0.37656	5.392076	-1.80437	0.072697	0.145518	-5.48435
SCIN	-0.97836	2.578601	-1.8043	0.072708	0.14552	-5.1517
DYNLT1	0.314864	5.685077	1.803722	0.072799	0.145682	-5.46201
PLEKHA9	0.325955	1.955976	1.803535	0.072829	0.14572	-4.82054
INTU	-0.49041	2.163789	-1.8033	0.072866	0.145774	-4.98193
CXCL13	1.946383	2.339445	1.803129	0.072893	0.145806	-4.66096
LIN54	0.282617	4.841238	1.80297	0.072918	0.145836	-5.37554
KLRG2	1.983529	-0.18748	1.802791	0.072946	0.145871	-4.51546
C20orf43	0.223818	6.198988	1.802667	0.072966	0.145889	-5.50147
SERHL	-0.32405	1.680564	-1.80226	0.07303	0.145997	-4.87193
GTF3C1	-0.29551	7.220322	-1.80132	0.073179	0.146252	-5.507
COPZ1	0.245508	7.10341	1.801327	0.073177	0.146252	-5.51722
LAG3	1.033159	1.801846	1.800836	0.073255	0.146384	-4.70617
TMEM39A	0.317098	4.918917	1.800653	0.073284	0.146421	-5.38505
CCDC7	-0.32531	1.209814	-1.80055	0.073299	0.146431	-4.79748
HSPA12B	-0.53023	2.003509	-1.80032	0.073337	0.146484	-4.96266
UBE2V2	0.313418	5.299774	1.800255	0.073347	0.146484	-5.43135
DNAJB14	-0.28467	4.253908	-1.79972	0.073432	0.146633	-5.36945
ZNF264	-0.41008	5.685834	-1.79957	0.073456	0.14666	-5.50895
SMAD1	0.359446	5.066016	1.799362	0.073489	0.146704	-5.40141
B3GALT4	-0.43048	3.087329	-1.79925	0.073506	0.146718	-5.1707
CBWD6	0.506843	3.292008	1.799059	0.073537	0.146758	-5.03566
MTX2	0.257425	4.685358	1.798707	0.073592	0.146849	-5.36464
AGPAT5	0.475161	5.564224	1.79815	0.073681	0.147004	-5.44698
JMJD8	-0.29415	5.879001	-1.79783	0.073731	0.147084	-5.51532
UBE2Q2P1	-0.472	0.606188	-1.79706	0.073854	0.147296	-4.73145
C14orf4	-0.45761	6.300458	-1.79703	0.073859	0.147296	-5.52574
LRRC29	-0.48378	0.55854	-1.79632	0.073973	0.147502	-4.72825
CDC42EP1	-0.51902	7.602793	-1.79588	0.074043	0.147622	-5.50171
MATN3	0.974215	1.100184	1.795808	0.074054	0.147622	-4.62521
ATP6V1E2	0.544972	1.31268	1.795668	0.074077	0.147625	-4.70587
KIAA1244	-1.15034	4.290162	-1.79567	0.074076	0.147625	-5.46404
WDR45	-0.30119	4.936691	-1.79525	0.074144	0.147738	-5.45876
CXCL3	1.955213	2.330186	1.795037	0.074177	0.147783	-4.66807
KLF10	-0.45507	6.770587	-1.79473	0.074227	0.147862	-5.52459
SETD1B	-0.23941	6.406548	-1.79431	0.074294	0.147975	-5.53056
APPL2	-0.32759	5.734486	-1.79372	0.074389	0.148142	-5.51832
GDAP1	0.743512	3.43461	1.793565	0.074413	0.148169	-5.03445

LMLN	0.48106	4.276154	1.793342	0.074449	0.148198	-5.26276
WDR91	-0.5147	4.222113	-1.79336	0.074445	0.148198	-5.40327
CCNH	-0.25622	4.408573	-1.7919	0.074681	0.148638	-5.40058
AKNA	-0.49234	5.327364	-1.79173	0.074707	0.14867	-5.50875
SC4MOL	0.532497	6.058586	1.790775	0.074861	0.148955	-5.49857
SPTBN1	-0.56077	9.231607	-1.79057	0.074894	0.148999	-5.45472
CCNB1IP1	0.390825	4.886515	1.790399	0.074922	0.149034	-5.39099
REEP6	1.06078	3.698417	1.790081	0.074973	0.149114	-5.03112
TOP1P1	0.323309	2.78822	1.789903	0.075002	0.14915	-4.98455
ZNF35	-0.27817	3.127489	-1.78964	0.075045	0.149214	-5.16844
CD93	-0.63949	5.800782	-1.78908	0.075134	0.14937	-5.53622
NHP2L1	0.258609	6.346397	1.788847	0.075173	0.149425	-5.53027
CS	-0.24381	7.881714	-1.78816	0.075283	0.14961	-5.51443
DCTN1	-0.23588	7.794895	-1.78814	0.075287	0.14961	-5.51726
OLFM2	1.29651	3.402848	1.787816	0.075339	0.149693	-4.94653
ZFP14	-0.46457	2.632226	-1.7877	0.075358	0.149709	-5.10034
L3MBTL3	0.419618	3.274911	1.7874	0.075407	0.149785	-5.07036
LACE1	-0.35592	1.15508	-1.78722	0.075435	0.149804	-4.81644
ATP6V1F	0.368374	5.914743	1.787206	0.075438	0.149804	-5.50532
TMEM40	2.884496	1.17346	1.787029	0.075467	0.14984	-4.54883
BTN3A1	0.567331	4.78314	1.786758	0.075511	0.149906	-5.35548
PHAX	-0.22367	4.135018	-1.7865	0.075553	0.14995	-5.36808
SLC25A10	0.518182	5.213307	1.78649	0.075555	0.14995	-5.4235
TUBGCP2	-0.25451	6.144043	-1.78633	0.07558	0.14998	-5.54154
GPR137	-0.28655	4.387513	-1.786	0.075635	0.150067	-5.41196
C10orf78	0.473869	3.09806	1.785907	0.075649	0.150074	-5.02489
FAT1	0.586493	8.700445	1.78527	0.075753	0.150258	-5.51682
CARD11	1.689958	3.759178	1.78498	0.0758	0.150297	-4.94744
MAPKAPK5	0.23256	4.724817	1.784952	0.075804	0.150297	-5.3971
CIAPIN1	0.26688	5.243686	1.784998	0.075797	0.150297	-5.45657
ZDHHC2	-0.85595	4.56999	-1.78483	0.075825	0.150315	-5.48789
PI4KAP2	-0.47982	3.339935	-1.78452	0.075874	0.150374	-5.26009
SERPINF1	-0.71244	5.191398	-1.78451	0.075876	0.150374	-5.52477
RBMXL1	-0.2353	5.299609	-1.78411	0.075942	0.150483	-5.50534
SMCR8	-0.2917	5.939737	-1.78396	0.075966	0.15051	-5.54156
PHACTR1	-0.66788	0.117983	-1.78268	0.076175	0.150857	-4.7107
GMCL1	0.361496	4.541387	1.782748	0.076164	0.150857	-5.35483
TOR1AIP2	-0.30699	5.433644	-1.78262	0.076184	0.150857	-5.52147
DDAH1	-1.01942	5.435656	-1.78273	0.076166	0.150857	-5.54564
MRPL24	0.377792	5.244116	1.78187	0.076308	0.15108	-5.4507
GPR3	0.79768	1.052963	1.781545	0.076361	0.151101	-4.66381
E2F6	0.287426	3.975667	1.781634	0.076346	0.151101	-5.25758
DRG1	0.278762	5.381901	1.781541	0.076361	0.151101	-5.47588
KIAA1949	0.537447	6.041783	1.781557	0.076359	0.151101	-5.51348
C1orf84	0.409153	1.098555	1.781406	0.076383	0.151123	-4.71678
SSX2IP	0.39739	5.038493	1.781188	0.076419	0.151172	-5.42581
C3	-1.17286	7.502833	-1.78111	0.076432	0.151177	-5.5118
ATF3	-0.73411	5.291496	-1.78076	0.076489	0.151268	-5.53694
C5orf42	-0.65661	4.1656	-1.78067	0.076503	0.151274	-5.43447
C19orf60	0.334072	4.219955	1.780509	0.076531	0.151295	-5.30141
KDELR2	0.344241	8.337032	1.78048	0.076535	0.151295	-5.52978
RGS3	-0.42069	5.173408	-1.78025	0.076573	0.151348	-5.51438
ZNF462	0.646442	4.942906	1.780079	0.076601	0.15136	-5.38479
GNAI1	-0.75341	4.842277	-1.78014	0.07659	0.15136	-5.5117
BCL2L15	-1.49587	1.578055	-1.77968	0.076667	0.15147	-5.06954
PCSK6	-0.61171	4.494992	-1.77897	0.076783	0.151676	-5.46978
SLC39A3	0.299197	4.563146	1.778554	0.076852	0.151791	-5.37525
ZNF100	-0.59072	2.951934	-1.77825	0.076901	0.151868	-5.20915

SSSCA1	0.430568	3.920507	1.77782	0.076973	0.151987	-5.22396
PPM1F	-0.31728	5.168248	-1.77773	0.076988	0.151996	-5.5117
LOC400759	1.198525	0.38248	1.777229	0.07707	0.152123	-4.56102
GPBP1	-0.21692	6.253685	-1.7772	0.077074	0.152123	-5.55889
ZNF833	0.532157	0.30016	1.777043	0.077101	0.152154	-4.60528
NOL7	0.309362	4.927601	1.776883	0.077127	0.152185	-5.42908
ZNF222	-0.4498	0.333293	-1.77643	0.077203	0.152297	-4.725
ZNF688	-0.32478	3.051991	-1.77636	0.077214	0.152297	-5.18227
DPYSL3	-0.89122	6.540606	-1.77634	0.077217	0.152297	-5.55357
SESN2	-0.42223	4.398534	-1.77559	0.077341	0.15252	-5.44564
FTSJ3	0.274007	5.775701	1.775432	0.077367	0.152551	-5.52196
B4GALNT1	1.747178	2.163889	1.77506	0.077429	0.15265	-4.70975
KLHL7	0.334038	4.96502	1.774591	0.077506	0.152782	-5.43547
C1orf55	0.266811	5.260148	1.774501	0.077521	0.15279	-5.47677
RSAD2	1.168031	3.881079	1.774146	0.07758	0.152885	-5.07657
PCDHB5	-0.9407	0.469318	-1.77403	0.0776	0.152902	-4.8158
C16orf68	-0.24741	3.217464	-1.77381	0.077637	0.152952	-5.20949
RAB22A	0.269339	6.442422	1.773689	0.077656	0.152969	-5.55956
KIAA1755	-0.79001	1.81989	-1.77359	0.077673	0.152982	-5.01891
RHPN1	0.896057	3.190891	1.773081	0.077757	0.153121	-4.99367
TSHZ1	-0.35275	5.05561	-1.77303	0.077766	0.153121	-5.51318
CCL11	-0.86269	1.349059	-1.77294	0.077781	0.153129	-4.94501
HSPB1	0.900314	9.712229	1.772817	0.077801	0.153147	-5.51384
PRPSAP2	0.338881	4.584349	1.772751	0.077812	0.153147	-5.3832
ZNF250	-0.33053	3.305588	-1.77134	0.078047	0.153559	-5.24974
SENP3	0.294396	5.401862	1.771408	0.078036	0.153559	-5.49421
PSMD5	0.254758	5.984719	1.771294	0.078055	0.153559	-5.54436
LAIR1	0.837673	3.190492	1.770421	0.0782	0.153824	-5.01046
TMC8	0.758018	3.854377	1.770295	0.078221	0.153844	-5.15774
C1orf74	0.74155	2.591557	1.769805	0.078303	0.153984	-4.91999
CPSF4	0.420975	4.317066	1.769632	0.078332	0.154019	-5.3237
TADA3	-0.26277	6.01654	-1.76951	0.078353	0.154037	-5.56818
HUNK	-0.80749	1.451504	-1.7691	0.078421	0.15415	-4.96254
TRPM4	-0.50345	5.853166	-1.76878	0.078475	0.154235	-5.57043
PILRB	0.444712	5.357151	1.768642	0.078498	0.154258	-5.47939
GRAMD1B	-1.16222	1.258527	-1.76825	0.078563	0.154365	-4.97604
SLC25A15	0.469139	3.923078	1.76813	0.078584	0.154383	-5.23038
ACTR6	0.315637	4.236293	1.768048	0.078597	0.154385	-5.33091
C1orf77	0.203561	6.253782	1.76799	0.078607	0.154385	-5.5652
C3orf62	-0.35174	2.540588	-1.76752	0.078686	0.154518	-5.09382
DZIP3	-0.45091	3.744208	-1.76733	0.078718	0.15456	-5.36811
GCH1	0.508778	4.118654	1.767061	0.078763	0.154626	-5.27066
CCHCR1	0.358172	4.238868	1.766739	0.078817	0.154711	-5.32358
CHKA	-0.67398	4.650714	-1.76631	0.078889	0.154809	-5.51185
MAP2K1	0.274957	5.920352	1.766375	0.078878	0.154809	-5.54794
PMS2L2	0.411878	2.008768	1.766224	0.078904	0.154816	-4.88096
RASSF1	-0.35524	4.101848	-1.76608	0.078928	0.154842	-5.41518
UBE2D3	-0.22367	7.404051	-1.76531	0.079057	0.155073	-5.56746
DNM2	-0.31688	7.90524	-1.76503	0.079105	0.155146	-5.552
LOC201651	1.498874	0.005046	1.764847	0.079136	0.155184	-4.5796
HIST2H2BE	0.746965	4.661772	1.763533	0.079358	0.155528	-5.34547
PCGF2	0.467444	4.984035	1.763345	0.079389	0.155528	-5.44126
B4GALT3	0.281935	5.305966	1.763383	0.079383	0.155528	-5.49888
CSNK1G1	0.308977	5.453948	1.763468	0.079369	0.155528	-5.51171
GLUD1	-0.28668	7.219497	-1.76342	0.079377	0.155528	-5.57355
NADK	-0.28222	5.951826	-1.76363	0.07934	0.155528	-5.57709
SFRS13A	0.210487	6.436795	1.763369	0.079385	0.155528	-5.57879
IL12RB2	1.514332	1.490519	1.761005	0.079786	0.156283	-4.67269

UROS	-0.29392	4.116943	-1.76068	0.079841	0.15637	-5.41883
ASNA1	0.274049	5.496606	1.7605	0.079871	0.156407	-5.52411
KIAA0355	-0.31469	5.330451	-1.76029	0.079907	0.156432	-5.55405
RBM47	-0.6938	7.211486	-1.76031	0.079904	0.156432	-5.57043
COQ10A	-0.35565	1.804337	-1.75901	0.080126	0.156839	-4.97111
PANX2	2.126964	2.217059	1.758733	0.080172	0.156875	-4.70043
C5orf28	0.396506	4.235263	1.758811	0.080159	0.156875	-5.32981
FAM57A	0.439772	5.427284	1.758701	0.080178	0.156875	-5.50513
CWF19L1	0.261054	4.314339	1.758354	0.080237	0.156968	-5.37157
CTAGE9	0.886267	3.77199	1.757851	0.080323	0.157114	-5.13361
SGCE	-0.70225	2.991435	-1.75715	0.080442	0.157326	-5.27806
UTP23	0.282914	4.678454	1.756519	0.08055	0.157515	-5.43379
ZCRB1	-0.2903	5.523322	-1.75645	0.080563	0.157518	-5.57158
DENND5B	-0.73219	3.630403	-1.75598	0.080643	0.157653	-5.40939
SLC20A2	0.588981	6.407214	1.755772	0.080678	0.157677	-5.57766
GPC1	0.964082	7.50996	1.755791	0.080675	0.157677	-5.59686
ATP2B4	-0.55338	7.548091	-1.7556	0.080708	0.157713	-5.57287
SS18L2	0.331783	3.635108	1.755513	0.080723	0.15772	-5.21739
CRAMP1L	-0.27027	5.657267	-1.75538	0.080745	0.157742	-5.57958
NCOA2	-0.41384	5.55955	-1.75523	0.080771	0.15777	-5.58111
GMFB	0.284079	6.280264	1.755092	0.080795	0.157795	-5.58568
CDKN2AIP	-0.30505	4.547796	-1.75501	0.080809	0.1578	-5.48761
C14orf43	-0.3301	6.495991	-1.7547	0.080861	0.15788	-5.59944
EXOSC1	0.2882	4.085281	1.753702	0.081033	0.158195	-5.32752
SNRNP200	0.244198	8.543885	1.75363	0.081046	0.158197	-5.56721
SCARF1	-0.46752	2.645709	-1.75346	0.081075	0.158209	-5.16113
DTWD2	-0.32303	3.650196	-1.75344	0.081079	0.158209	-5.35204
CARD6	-0.60057	4.231543	-1.75333	0.081097	0.158209	-5.48232
COMMD6	-0.33677	5.48244	-1.75335	0.081095	0.158209	-5.57685
MGAT3	-0.95186	2.800657	-1.75302	0.08115	0.158289	-5.2857
GADD45GIP1	0.35667	5.170373	1.752635	0.081217	0.158376	-5.49554
LYPLA2	0.322358	5.825326	1.752638	0.081217	0.158376	-5.5617
PTPN11	-0.22485	7.34736	-1.7525	0.08124	0.158399	-5.59085
GALNT3	0.696769	6.502313	1.752267	0.08128	0.158455	-5.58296
C17orf65	0.352956	3.095978	1.75205	0.081318	0.158506	-5.10497
UGT1A1	1.690253	-0.32771	1.751297	0.081448	0.158715	-4.60003
APLN	1.210896	2.308174	1.751325	0.081443	0.158715	-4.83647
GNB2	0.328945	7.98012	1.751168	0.08147	0.158736	-5.58985
PROS1	-0.65028	4.513198	-1.75068	0.081554	0.158872	-5.52576
MYO1E	0.441122	6.730263	1.750634	0.081562	0.158872	-5.60263
SH3GLB1	-0.27018	7.000167	-1.75044	0.081595	0.158914	-5.60063
EMP1	-0.86215	7.682208	-1.75018	0.081641	0.158981	-5.56901
SPATA13	-0.76067	4.366067	-1.74978	0.081709	0.159092	-5.51922
NEK11	0.584539	2.33221	1.74971	0.081722	0.159095	-4.93598
LOC646851	0.767624	0.815214	1.749309	0.081792	0.159207	-4.68993
RBKS	0.470788	1.418646	1.748967	0.081851	0.159278	-4.8088
DEPDC7	0.885899	1.945295	1.748971	0.08185	0.159278	-4.83161
TMEM171	-0.86253	2.432033	-1.74889	0.081864	0.159282	-5.18319
SDCCAG1	-0.23102	5.82265	-1.74815	0.081993	0.15951	-5.59788
UBL4A	0.264856	5.733166	1.748011	0.082017	0.159534	-5.56672
SCOC	-0.45319	5.714893	-1.74709	0.082177	0.159825	-5.60277
DDX24	-0.22425	6.985957	-1.7466	0.082262	0.159968	-5.60825
AP1S3	0.692507	4.25138	1.746029	0.082361	0.160116	-5.29463
UBR5	0.289358	7.476035	1.746028	0.082362	0.160116	-5.60856
SCLY	0.399681	4.117918	1.745746	0.082411	0.16019	-5.32702
PKP1	3.290218	6.755859	1.745567	0.082442	0.160192	-5.3266
C9orf114	0.291704	4.710841	1.745574	0.082441	0.160192	-5.45548
ACAP3	-0.39948	5.851548	-1.74554	0.082446	0.160192	-5.60833

MED11	-0.2955	3.064685	-1.74546	0.08246	0.160197	-5.2324
FASTKD2	-0.22278	5.721001	-1.74533	0.082484	0.160221	-5.59795
VAPA	-0.2578	7.259455	-1.74526	0.082496	0.160222	-5.60457
RNF167	-0.27472	6.247124	-1.74507	0.082528	0.160262	-5.61509
EHD4	0.364589	6.166663	1.744753	0.082584	0.160337	-5.59482
BCOR	-0.32825	5.958746	-1.74472	0.08259	0.160337	-5.61099
C16orf91	0.364649	2.901821	1.744486	0.082631	0.160394	-5.07848
NT5C	0.373828	4.41961	1.743621	0.082782	0.160665	-5.39789
STARD7	0.282183	7.258194	1.743474	0.082807	0.160693	-5.61646
CEP164	0.253555	4.871225	1.743329	0.082833	0.160719	-5.4861
DGKZ	0.258505	5.323686	1.743138	0.082866	0.160723	-5.53805
RPL5	-0.28456	8.730918	-1.7432	0.082855	0.160723	-5.5624
GPR125	-0.4094	5.139092	-1.74312	0.082869	0.160723	-5.57537
NUP35	0.304059	3.985502	1.742517	0.082975	0.160896	-5.32351
FIZ1	-0.24962	4.374389	-1.74242	0.082992	0.160896	-5.48129
PTMS	0.472746	7.400938	1.742414	0.082993	0.160896	-5.61857
PNPLA8	-0.28441	5.447965	-1.74168	0.083121	0.161123	-5.59211
SOCS1	0.729347	3.600965	1.741607	0.083135	0.161126	-5.15681
TNFRSF11B	1.596421	1.631072	1.740432	0.083341	0.161498	-4.71143
STON2	1.063204	4.4472	1.740385	0.08335	0.161498	-5.27779
SLC19A1	0.543999	3.979633	1.739757	0.08346	0.161689	-5.27456
ALG10	0.408826	2.794938	1.739537	0.083499	0.161732	-5.05721
STAMBP	0.224543	5.467466	1.739502	0.083505	0.161732	-5.56156
TNFSF13B	0.860182	1.171974	1.739118	0.083573	0.16184	-4.74255
LRRC58	0.342791	6.928098	1.739021	0.08359	0.161851	-5.62639
SH3GL1	0.360457	7.07691	1.738047	0.083762	0.162161	-5.62812
TIMP2	-0.60212	7.884715	-1.73791	0.083787	0.162187	-5.59107
MTCH1	-0.23452	6.993237	-1.73775	0.083814	0.162217	-5.62309
ZNF430	-0.39343	2.937031	-1.73737	0.083882	0.162326	-5.23962
LAMA5	0.43509	8.535571	1.736286	0.084073	0.162674	-5.60267
LOC100131691	-0.43061	0.147408	-1.7361	0.084106	0.162698	-4.76344
CEBPG	0.3448	6.257355	1.736086	0.084109	0.162698	-5.61498
WIPI1	-0.42718	4.69077	-1.73559	0.084198	0.162847	-5.54827
SNX13	-0.29017	6.074976	-1.73501	0.0843	0.163023	-5.62962
CTU2	0.331885	3.25789	1.734806	0.084336	0.163047	-5.1726
NF2	-0.27748	5.548416	-1.73483	0.084331	0.163047	-5.6095
PRODH	1.662961	2.776257	1.734627	0.084368	0.163086	-4.88144
KMO	0.83634	0.244269	1.7345	0.08439	0.163093	-4.63768
UBASH3B	0.730053	3.764058	1.734476	0.084394	0.163093	-5.20657
SULT2B1	2.276378	3.724691	1.734165	0.08445	0.163177	-4.93972
MYSM1	-0.37617	3.511896	-1.73409	0.084464	0.163181	-5.36457
ANGPTL4	-0.75147	3.46026	-1.73394	0.08449	0.16321	-5.42205
C2orf89	1.702417	1.35173	1.733849	0.084506	0.163218	-4.67476
LOC441294	0.801001	2.882712	1.733776	0.084519	0.16322	-5.02017
CDSN	1.9417	2.208082	1.733341	0.084596	0.163315	-4.76039
DOK3	0.62187	2.793141	1.733303	0.084603	0.163315	-5.03682
NOSIP	0.293576	4.685812	1.733334	0.084598	0.163315	-5.47271
DSC2	0.969841	7.613847	1.733201	0.084621	0.163328	-5.63574
MRPL33	-0.2185	4.206468	-1.73309	0.084642	0.163345	-5.47005
ZNF432	-0.46612	3.008395	-1.73301	0.084655	0.163348	-5.27464
ARIH1	-0.22163	6.558909	-1.73289	0.084676	0.163365	-5.63708
DUSP18	0.374388	3.872019	1.732787	0.084695	0.16338	-5.30075
RANBP3	-0.19415	5.835119	-1.73258	0.084732	0.163429	-5.62368
RECQL5	-0.26729	4.78364	-1.73245	0.084756	0.163452	-5.54858
FBN1	-0.84165	6.437234	-1.73224	0.084793	0.163501	-5.63228
TGDS	0.35314	3.730402	1.731784	0.084874	0.163635	-5.27432
CAPN1	0.389272	8.449867	1.731634	0.084901	0.163664	-5.61195
C1orf203	-0.37085	1.008093	-1.73145	0.084933	0.163703	-4.88932

IL16	-0.54465	3.512663	-1.73137	0.084948	0.163709	-5.40313
BRD1	-0.24256	5.949689	-1.73121	0.084977	0.163742	-5.63175
ECH1	-0.43807	6.620247	-1.73093	0.085027	0.163816	-5.63781
RRN3P3	-0.31929	2.335009	-1.73083	0.085045	0.163829	-5.10837
SLC44A5	1.905066	2.941783	1.730746	0.08506	0.163834	-4.88034
PPP2R3C	-0.25414	4.040733	-1.73047	0.085109	0.163907	-5.45268
HGD	-1.7201	0.187409	-1.73024	0.085149	0.163928	-4.93803
TRMT61A	0.323762	4.400082	1.73021	0.085155	0.163928	-5.42615
HR	0.868034	6.561071	1.73021	0.085155	0.163928	-5.6157
CAP1	0.374735	8.427597	1.729791	0.085231	0.164048	-5.6154
UBP1	-0.24217	6.313317	-1.72973	0.085241	0.164048	-5.64173
SLC5A3	0.39961	5.460337	1.729491	0.085284	0.164108	-5.56256
ARHGEF11	-0.23984	6.747443	-1.72935	0.08531	0.164135	-5.64129
CORO1A	0.68695	4.492445	1.728982	0.085376	0.164239	-5.38111
MYH9	0.344302	10.89399	1.728725	0.085422	0.164305	-5.53214
TFAM	0.313981	4.726809	1.728412	0.085478	0.164346	-5.48446
DIAPH2	-0.44595	4.646496	-1.72828	0.085502	0.164346	-5.55826
FCHSD2	-0.3787	5.10723	-1.72831	0.085496	0.164346	-5.59621
LRRFIP1	-0.35096	7.565018	-1.7283	0.085498	0.164346	-5.62425
ADH5	-0.3059	6.440276	-1.72853	0.085456	0.164346	-5.6446
NRGN	0.639918	1.818724	1.72756	0.085631	0.164554	-4.88238
ANXA4	0.609032	7.053863	1.727546	0.085633	0.164554	-5.64467
TMEM9B	-0.27083	5.339344	-1.72722	0.085691	0.164642	-5.60826
NDUFA13	-0.29623	6.098889	-1.72706	0.085721	0.164676	-5.64373
PIKFYVE	-0.28585	5.576157	-1.72653	0.085816	0.164836	-5.62547
CYP2C18	-1.22529	3.079797	-1.7263	0.085858	0.164875	-5.42892
SHROOM4	-0.59418	3.760987	-1.72629	0.085859	0.164875	-5.46356
GEMIN7	0.300711	3.467482	1.726221	0.085872	0.164876	-5.23643
MYO1G	0.814654	2.700733	1.725743	0.085958	0.164997	-5.00356
ASB7	0.254882	4.62189	1.725767	0.085954	0.164997	-5.48141
LDLRAD3	0.668046	3.76488	1.725258	0.086046	0.165142	-5.23557
PRKCZ	0.482087	4.933462	1.724392	0.086202	0.165419	-5.49859
ZDHHC7	-0.31177	5.918096	-1.72425	0.086227	0.165445	-5.64439
OSBPL11	0.329733	4.823512	1.724049	0.086264	0.165493	-5.50431
PDZK1IP1	1.65139	5.162819	1.723806	0.086308	0.165555	-5.33499
PDIA5	-0.40313	4.401173	-1.7237	0.086327	0.165569	-5.53237
CCDC59	0.308359	4.274823	1.723432	0.086376	0.165639	-5.41553
TMSL3	-0.45837	9.740513	-1.72315	0.086426	0.165713	-5.55685
TRIM21	0.401196	4.25838	1.722657	0.086517	0.165857	-5.39576
MED14	0.306333	6.892467	1.722609	0.086525	0.165857	-5.65436
VAV1	0.890466	2.149576	1.721996	0.086636	0.166047	-4.9116
APOBEC3A	1.836835	1.333216	1.721556	0.086716	0.166155	-4.68224
CTSL1	0.604496	5.534458	1.721561	0.086715	0.166155	-5.56458
TRIM41	-0.26726	4.834315	-1.72053	0.086903	0.166489	-5.57432
MCOLN2	1.010851	0.764764	1.720435	0.08692	0.1665	-4.70162
ZNHIT6	0.251401	4.328324	1.720151	0.086972	0.166576	-5.44246
GGA1	-0.21024	5.816038	-1.72005	0.08699	0.166587	-5.64468
ARV1	-0.3052	3.968015	-1.71971	0.087052	0.16666	-5.46664
MOBKL2B	-0.6478	4.584066	-1.71976	0.087043	0.16666	-5.58301
SNN	-0.4437	4.715019	-1.7196	0.087072	0.166676	-5.58042
SLC4A1AP	0.201861	4.854773	1.718568	0.087261	0.167014	-5.53192
TTC27	0.268748	4.272889	1.718457	0.087281	0.16703	-5.43091
FLJ45445	0.961802	4.605302	1.718055	0.087354	0.167131	-5.36747
ATP5L	-0.35315	6.525967	-1.71804	0.087358	0.167131	-5.66173
HOXA5	0.598875	1.800207	1.71721	0.087509	0.167392	-4.90449
NAV1	0.698958	6.71589	1.717161	0.087518	0.167392	-5.6523
C1orf151	-0.26795	5.683732	-1.71663	0.087614	0.167554	-5.64687
DLGAP4	0.257352	6.86569	1.716013	0.087728	0.167749	-5.66562

MYO7A		0.863616	3.015727	1.715443	0.087833	0.167926	-5.06514
TAB1		-0.21385	4.81065	-1.71535	0.087851	0.167936	-5.57576
C6orf105		-0.96079	1.968983	-1.71503	0.087908	0.168024	-5.1649
DUSP4		-0.96496	4.200146	-1.71463	0.087983	0.168143	-5.57818
COPS6		0.329344	6.353881	1.714473	0.088011	0.168175	-5.65632
GSTCD		0.37022	3.980801	1.714217	0.088058	0.168241	-5.35662
ZBTB17		-0.23493	4.657031	-1.71369	0.088155	0.168403	-5.56317
DHRS13		0.500474	3.355477	1.71316	0.088253	0.168457	-5.19755
MACC1		1.29706	5.15659	1.713146	0.088256	0.168457	-5.42342
RAB34		0.698772	5.244234	1.713188	0.088248	0.168457	-5.53458
	5-Sep	-0.82954	4.073937	-1.71326	0.088234	0.168457	-5.55747
CD109		1.137011	5.906257	1.713357	0.088217	0.168457	-5.56619
TTC38		-0.46562	4.612986	-1.71339	0.08821	0.168457	-5.5803
MLKL		0.420764	4.224382	1.712669	0.088343	0.168602	-5.4018
ZNF321		-0.77131	1.542111	-1.71163	0.088536	0.168923	-5.0653
GTF2H5		-0.32111	4.305397	-1.71164	0.088533	0.168923	-5.53321
NKPD1		1.070022	0.567521	1.710572	0.088731	0.169212	-4.69049
NME6		-0.24858	3.148003	-1.71054	0.088737	0.169212	-5.30044
ALPK1		-0.37646	4.463694	-1.71069	0.088709	0.169212	-5.56023
ATP6V1C1		0.247256	6.227632	1.71051	0.088742	0.169212	-5.66086
APOL6		0.498883	7.087253	1.710482	0.088748	0.169212	-5.67471
TMED9		0.254429	6.430594	1.710386	0.088765	0.169222	-5.66802
REL		0.49265	3.761898	1.71032	0.088778	0.169223	-5.29143
RAB11A		-0.31785	7.549306	-1.71016	0.088807	0.169255	-5.65611
IL2RG		1.106097	4.737585	1.709954	0.088845	0.169305	-5.38016
SYT13		-1.50943	1.950614	-1.70895	0.089031	0.169636	-5.27108
MCCC1		-0.38888	4.938959	-1.70848	0.08912	0.169759	-5.61553
RRAGA		-0.27324	5.80164	-1.70852	0.089112	0.169759	-5.6659
C11orf73		0.342339	4.179316	1.708167	0.089177	0.169845	-5.41644
TMLHE		-0.26737	3.404084	-1.70745	0.089311	0.170077	-5.36658
PGGT1B		-0.23653	3.834429	-1.70694	0.089406	0.170234	-5.45165
ANXA11		-0.43816	7.97124	-1.7061	0.089561	0.170507	-5.64688
FAM111A		0.384448	5.468873	1.705882	0.089603	0.170562	-5.60388
GTF2E2		0.349569	4.848036	1.705321	0.089707	0.170739	-5.53565
MERTK		-0.57108	3.463111	-1.70523	0.089725	0.170749	-5.43951
MTO1		-0.23819	4.358368	-1.70514	0.08974	0.170755	-5.54093
DDX5		-0.21097	8.915294	-1.70506	0.089756	0.170761	-5.62291
KPNA5		-0.44499	1.317059	-1.70471	0.089822	0.170863	-4.99451
APC		-0.3101	5.7235	-1.70422	0.089913	0.171014	-5.6712
ORC4L		0.239528	4.751633	1.703716	0.090008	0.17117	-5.53904
STAG3L4		-0.31785	2.75282	-1.70312	0.090119	0.171359	-5.24033
NOD1		0.347627	4.097581	1.703032	0.090136	0.171367	-5.40751
MAP3K7		0.227445	5.147415	1.702787	0.090182	0.171431	-5.59059
HLA-DRB1		0.937757	5.922298	1.702683	0.090201	0.171433	-5.60497
DLAT		-0.27389	5.684858	-1.70265	0.090207	0.171433	-5.67069
NFU1		-0.26738	3.905794	-1.70226	0.090281	0.171551	-5.47837
ICAM2		-0.59193	2.555098	-1.7016	0.090404	0.17176	-5.25202
CYBASC3		0.287964	5.593607	1.701354	0.090451	0.171824	-5.63222
PGD		0.652573	7.24985	1.701295	0.090462	0.171824	-5.69
ABTB1		-0.39579	4.369505	-1.70111	0.090496	0.171865	-5.56632
EAF1		-0.30376	5.313122	-1.70059	0.090595	0.172029	-5.65345
HMHA1		0.466594	4.619639	1.70011	0.090685	0.172177	-5.49318
TFAP2C		2.267911	3.625895	1.699926	0.09072	0.172219	-4.9828
AP1S2		-0.43381	3.256039	-1.69973	0.090758	0.172239	-5.38259
UROD		-0.25373	4.679948	-1.69971	0.09076	0.172239	-5.5915
CCDC124		0.294938	5.189037	1.699675	0.090767	0.172239	-5.5937
LPAR2		0.424503	4.36409	1.698984	0.090897	0.172455	-5.4486
DOCK1		-0.28707	6.431573	-1.69894	0.090906	0.172455	-5.69452

ELFN1	0.779793	2.193385	1.698778	0.090936	0.172467	-4.96954
SPTLC1	0.249388	6.560108	1.69883	0.090926	0.172467	-5.69076
RPF1	0.231156	4.806643	1.698271	0.091032	0.17258	-5.55668
RAPGEF1	-0.3054	6.953347	-1.69838	0.091011	0.17258	-5.68921
HOOK3	-0.3713	6.822166	-1.69826	0.091033	0.17258	-5.6907
SIRT3	-0.25059	4.404973	-1.69757	0.091165	0.172807	-5.56087
CNOT8	-0.20042	5.490346	-1.69583	0.091494	0.173407	-5.66758
TAF5L	0.225301	4.934233	1.695445	0.091567	0.173522	-5.57837
MYBL1	0.603519	2.277745	1.695375	0.091581	0.173524	-5.01494
C20orf103	1.306606	0.209869	1.695295	0.091596	0.173529	-4.69317
IK	-0.19977	5.703441	-1.69506	0.091641	0.173592	-5.68117
CTAGE6	0.663461	0.024838	1.694164	0.091811	0.173889	-4.69662
PMS2L11	0.5467	2.592997	1.693962	0.091849	0.173939	-5.0772
ZC3H15	0.279831	6.097832	1.69365	0.091909	0.174028	-5.68171
NEDD8	0.271304	5.775592	1.693283	0.091979	0.174127	-5.66218
MORF4L1	0.218419	6.204249	1.693244	0.091986	0.174127	-5.68993
RNF187	0.341729	6.803313	1.691984	0.092227	0.174559	-5.70474
RTTN	0.379745	3.798342	1.691641	0.092292	0.174659	-5.3522
KIAA1704	-0.27067	3.869068	-1.69121	0.092374	0.174791	-5.49042
KLC1	-0.26647	6.031783	-1.69066	0.09248	0.174967	-5.70286
RPRD1B	0.216786	6.039115	1.690121	0.092583	0.175128	-5.68751
LIMA1	0.421327	7.562695	1.690083	0.09259	0.175128	-5.70362
TNFAIP8L2	0.667215	0.623925	1.689859	0.092633	0.175162	-4.77432
PDCL	0.247304	4.42035	1.689828	0.092639	0.175162	-5.50986
MTIF2	0.249385	5.31207	1.689795	0.092646	0.175162	-5.62753
TPRG1	1.561966	1.982703	1.689671	0.092669	0.175183	-4.84913
SLC29A2	0.76469	4.077121	1.68944	0.092714	0.175219	-5.33815
JRKL	0.33095	4.003321	1.689451	0.092712	0.175219	-5.40972
BSDC1	-0.19868	6.49231	-1.6893	0.09274	0.175245	-5.71058
PIGK	-0.28439	5.093764	-1.68913	0.092774	0.175286	-5.65347
SLC22A18AS	0.917518	0.316154	1.688962	0.092805	0.175298	-4.70875
HLA-DQB2	1.303843	1.083107	1.689001	0.092798	0.175298	-4.76175
CREM	-0.31364	3.947113	-1.68785	0.093019	0.175677	-5.51829
FAM83C	2.945712	1.520213	1.687543	0.093078	0.175728	-4.71198
SPATA7	-0.36578	2.245806	-1.68763	0.093061	0.175728	-5.16983
ST7L	-0.34598	3.769678	-1.68752	0.093083	0.175728	-5.49096
VCAN	0.843205	6.913361	1.687216	0.093141	0.175813	-5.70425
ACAT2	0.580448	4.545145	1.686885	0.093205	0.175909	-5.48189
ANKRD57	0.549893	6.334695	1.68666	0.093248	0.175967	-5.69294
NOXA1	-0.63975	2.015742	-1.68575	0.093423	0.17625	-5.173
COQ10B	-0.24597	4.663565	-1.68581	0.093412	0.17625	-5.61189
FAR2	1.065417	2.625736	1.685062	0.093557	0.176478	-5.01957
LOC493754	-0.21679	4.676246	-1.6848	0.093606	0.176524	-5.61209
HNRNPA3	0.246329	7.670001	1.684832	0.093601	0.176524	-5.70727
FZD3	-0.50832	3.188554	-1.68409	0.093745	0.176753	-5.4062
CORO7	0.349787	5.271535	1.684046	0.093753	0.176753	-5.62282
PRKCA	-0.6118	5.614629	-1.68386	0.093789	0.176797	-5.71052
NOS3	-0.57951	3.811433	-1.68359	0.093841	0.176872	-5.54131
CDK2AP2	0.381841	6.187823	1.683265	0.093904	0.176967	-5.69873
FAM35B2	-0.27255	1.534635	-1.68301	0.093953	0.177035	-5.03987
ZNF624	0.365162	2.364928	1.682932	0.093969	0.177041	-5.08507
NAPRT1	0.595663	5.994187	1.682836	0.093988	0.177052	-5.67313
SNHG8	-0.37075	4.236883	-1.68228	0.094096	0.177232	-5.57806
FAM81A	1.043085	1.410198	1.681638	0.09422	0.177395	-4.84464
CHCHD4	-0.2597	3.336333	-1.68167	0.094213	0.177395	-5.3929
LENG8	-0.35271	7.204687	-1.68167	0.094215	0.177395	-5.7111
APOB48R	0.693294	2.669462	1.680531	0.094436	0.177752	-5.09265
PTPRJ	-0.67972	5.177469	-1.68059	0.094423	0.177752	-5.69854

FAM122B	0.316725	5.53161	1.680464	0.094449	0.177753	-5.65897
C18orf55	-0.30875	3.675186	-1.68011	0.094518	0.177859	-5.47895
C16orf61	0.295586	4.014206	1.679631	0.094611	0.17801	-5.4359
TCTE3	0.430747	1.141797	1.679261	0.094683	0.178104	-4.88841
HLA-DQB1	1.118172	5.111021	1.679244	0.094686	0.178104	-5.50877
LIPH	-1.129	3.976748	-1.67827	0.094876	0.178437	-5.62775
SLC9A5	0.566194	0.276549	1.678001	0.094929	0.178496	-4.75924
ASB9	-0.74354	0.742558	-1.67798	0.094933	0.178496	-4.9859
FCGR2C	-0.71939	0.293587	-1.67781	0.094967	0.178535	-4.9172
FIBP	0.291947	5.391092	1.677574	0.095013	0.178598	-5.65221
RNF145	0.32015	6.679959	1.677373	0.095052	0.178648	-5.72743
BAP1	-0.23427	6.216604	-1.67713	0.095099	0.178713	-5.72856
KRTCAP3	0.972684	3.86249	1.677059	0.095113	0.178715	-5.26536
LIN7C	-0.27436	6.143431	-1.67677	0.095171	0.178798	-5.72845
MSL1	0.337417	6.323602	1.676665	0.095191	0.178812	-5.71783
MRPS34	0.333842	5.547769	1.676437	0.095235	0.178872	-5.66555
LOC729799	-0.49578	0.062429	-1.67619	0.095284	0.178939	-4.85738
ZXDC	-0.26599	6.079741	-1.6759	0.09534	0.17902	-5.72847
TCFL5	0.354675	4.490173	1.675191	0.09548	0.179259	-5.53012
C5orf56	-0.52025	1.711924	-1.67468	0.095581	0.179424	-5.11889
TXN2	-0.25205	5.585736	-1.67417	0.095681	0.179589	-5.71194
COMMD7	0.326303	5.290938	1.67375	0.095763	0.179718	-5.64471
GPR158	1.598279	1.290233	1.673184	0.095875	0.179904	-4.77725
NMT2	-0.34559	3.660859	-1.6731	0.095891	0.179909	-5.4929
TRIP12	-0.23413	7.58003	-1.67304	0.095904	0.179909	-5.71907
EWSR1	0.149247	7.636822	1.672748	0.095961	0.179992	-5.72613
LOC222699	0.800696	0.487909	1.67264	0.095982	0.180008	-4.76961
COX19	0.319286	4.356922	1.672552	0.095999	0.180015	-5.51279
YIPF1	-0.24489	4.509206	-1.67249	0.096011	0.180015	-5.61475
SDHAP3	-0.50842	1.462512	-1.67238	0.096033	0.180032	-5.0806
CAST	-0.40762	8.075259	-1.67231	0.096048	0.180035	-5.70053
ZNF828	-0.28575	5.540037	-1.67158	0.096192	0.180281	-5.71537
KCNMB3	0.538435	2.099236	1.671356	0.096236	0.180339	-5.03327
SLFN11	0.698632	4.897446	1.67124	0.096259	0.180357	-5.55135
MTF1	-0.29823	5.123017	-1.67053	0.0964	0.180584	-5.68815
PLCD3	-0.59488	6.156694	-1.6705	0.096405	0.180584	-5.74134
FAF2	0.204646	6.473307	1.670053	0.096494	0.180725	-5.73744
DUS2L	0.355791	3.424704	1.66982	0.09654	0.180787	-5.31148
MED1	0.332752	6.29904	1.669618	0.09658	0.180838	-5.72863
IDH1	0.439369	6.925052	1.669288	0.096645	0.180936	-5.74253
BTN3A2	0.529032	5.374301	1.668757	0.096751	0.181103	-5.64155
SH3RF1	-0.49209	5.467128	-1.66871	0.09676	0.181103	-5.72536
SMC1A	0.264359	7.357622	1.668358	0.09683	0.181209	-5.74076
FUT8	-0.54928	5.158086	-1.66828	0.096845	0.181213	-5.71071
ATP6V0A1	-0.3033	6.201061	-1.66745	0.09701	0.181498	-5.74515
EMILIN2	0.56195	3.824863	1.666721	0.097155	0.181745	-5.36366
OLFM1	-0.96676	3.032654	-1.6664	0.097219	0.18184	-5.49089
KRR1	0.232141	5.300844	1.666266	0.097246	0.181866	-5.66714
ZDHHC21	-0.43876	3.90673	-1.66607	0.097286	0.181905	-5.56571
ENOSF1	-0.3636	4.371301	-1.66603	0.097293	0.181905	-5.62134
DNM1	0.837088	4.41714	1.665783	0.097343	0.181973	-5.43914
BTN3A3	0.60988	4.13872	1.665628	0.097373	0.182007	-5.42384
MXRA7	-0.57209	6.173868	-1.66506	0.097487	0.182194	-5.7503
LSM14A	0.229449	6.890684	1.664907	0.097518	0.182227	-5.75076
KIFC2	0.569612	4.605938	1.664403	0.097618	0.182387	-5.53184
DENND3	-0.47765	4.443076	-1.66435	0.097629	0.182387	-5.64473
C1orf58	0.328711	4.54361	1.664202	0.097658	0.182393	-5.56049
FNBP1L	-0.43891	5.951447	-1.66423	0.097652	0.182393	-5.74801

EXOC3	-0.29765	5.978525	-1.66411	0.097678	0.182404	-5.74601
DHX57	0.249137	4.755128	1.663941	0.097711	0.182442	-5.60462
FLJ45244	-0.45095	0.011537	-1.66368	0.097763	0.182503	-4.86462
LRRC28	-0.28265	3.841362	-1.66365	0.09777	0.182503	-5.53198
EIF3M	0.302552	6.825663	1.662913	0.097917	0.182753	-5.75337
PGS1	0.246868	4.640042	1.662842	0.097931	0.182755	-5.58981
HIST1H1C	0.866925	4.407227	1.662412	0.098017	0.182892	-5.43496
MICAL2	-0.57074	6.940419	-1.66226	0.098047	0.182923	-5.74448
COMMD9	0.307092	4.898616	1.662141	0.098071	0.182944	-5.61935
LPAR6	0.679689	4.447068	1.661872	0.098125	0.183021	-5.48626
ISOC2	0.352791	4.518198	1.661795	0.098141	0.183025	-5.55667
SLC44A3	-0.82726	3.164861	-1.66153	0.098194	0.1831	-5.48756
PRDM4	-0.20223	5.491399	-1.66145	0.098211	0.183107	-5.72456
TSC22D4	0.319741	5.736423	1.661273	0.098246	0.183148	-5.70837
NUMA1	-0.34932	8.735976	-1.66058	0.098386	0.183384	-5.69852
A2LD1	-0.4388	2.227593	-1.66027	0.098447	0.183474	-5.22125
ZDHHC9	0.353795	6.500072	1.660117	0.098478	0.183508	-5.75073
TMEM116	-0.59296	2.841085	-1.65991	0.09852	0.183536	-5.38261
ECHDC1	-0.24756	5.536669	-1.65994	0.098513	0.183536	-5.73225
ALX3	-0.48199	0.466472	-1.65935	0.098633	0.183698	-4.94068
PI4KB	-0.2071	6.389033	-1.65935	0.098633	0.183698	-5.75957
NUPL1	0.273934	6.146052	1.659162	0.098671	0.183744	-5.74127
CYTH1	-0.26654	5.813627	-1.65902	0.0987	0.183773	-5.74811
CSMD2	1.064364	0.312955	1.658929	0.098718	0.183782	-4.75109
SORD	0.517551	5.538948	1.658566	0.098791	0.183894	-5.67712
FECH	-0.41069	4.378389	-1.65836	0.098833	0.183947	-5.64085
NPL	0.73721	3.742544	1.657844	0.098937	0.184117	-5.32877
SMAD3	0.410766	7.023874	1.65753	0.099001	0.18421	-5.76262
GTPBP5	0.270525	4.478277	1.657412	0.099024	0.18423	-5.5692
ZNF613	-0.57882	1.632039	-1.6569	0.099129	0.184399	-5.14446
NIPSNAP3A	-0.30634	4.131949	-1.65672	0.099165	0.184441	-5.59746
TRAF7	0.303107	7.342069	1.656657	0.099177	0.184441	-5.76071
PEX11G	-0.39147	0.720303	-1.65625	0.099259	0.18452	-4.97012
REST	-0.26788	5.078663	-1.65626	0.099258	0.18452	-5.70515
DARS	0.276912	6.721117	1.656265	0.099257	0.18452	-5.7634
AAAS	0.27393	5.071996	1.6561	0.09929	0.184553	-5.65415
EIF2B1	0.216338	5.565463	1.655579	0.099396	0.184724	-5.71122
KRT16	4.39486	6.134614	1.655254	0.099462	0.184822	-5.1433
SIRPB1	0.707204	1.443816	1.655062	0.099501	0.184845	-4.93627
SFRS8	0.194347	5.489699	1.655102	0.099493	0.184845	-5.7069
CAPN12	0.697505	2.836457	1.654734	0.099567	0.184908	-5.15684
SHROOM1	-0.44896	4.07408	-1.65469	0.099577	0.184908	-5.6095
KIAA1598	0.321214	5.922778	1.654555	0.099604	0.184908	-5.73302
SQSTM1	0.362126	8.464739	1.65457	0.099601	0.184908	-5.73912
FAM160A2	-0.30864	5.502551	-1.65444	0.099626	0.184908	-5.74222
CPSF2	0.229913	6.382576	1.654442	0.099627	0.184908	-5.75994
QSER1	0.417448	6.568721	1.654594	0.099596	0.184908	-5.76004
MARK3	-0.28257	6.080613	-1.65319	0.099881	0.185355	-5.766
C11orf24	0.377164	5.693142	1.651665	0.100193	0.185908	-5.71568
UBE2CBP	0.370565	2.076421	1.651434	0.10024	0.185971	-5.08629
IFFO1	-0.45793	1.987382	-1.6512	0.100288	0.186016	-5.19857
PRDX2	-0.39772	6.797727	-1.65118	0.100291	0.186016	-5.76833
OS9	-0.21	7.724026	-1.65094	0.100341	0.186085	-5.75195
SBF1	-0.32742	7.370852	-1.65084	0.100361	0.186098	-5.75848
C5orf36	-0.40481	0.651887	-1.64996	0.100541	0.186406	-4.97333
KPNA6	-0.21271	7.078796	-1.64972	0.10059	0.186472	-5.76862
HSD11B1	-0.74911	0.044616	-1.64919	0.1007	0.186651	-4.92891
C4orf19	-1.21329	2.648621	-1.64894	0.10075	0.186719	-5.46783

ZNF185	1.079415	6.34731	1.648679	0.100804	0.186794	-5.72186
MGAT4A	-0.78337	4.832213	-1.6482	0.100903	0.186953	-5.73475
COX5B	-0.33316	6.801682	-1.64733	0.10108	0.187257	-5.77551
TRPV1	-0.43977	2.790581	-1.64719	0.10111	0.187287	-5.36283
IQSEC2	-0.34409	4.234147	-1.64693	0.101164	0.187363	-5.63292
BCL3	0.393886	6.84278	1.646857	0.101178	0.187364	-5.77879
SYN1	-0.69658	0.766382	-1.64648	0.101255	0.187432	-5.0373
INTS4	0.238172	4.645824	1.646545	0.101243	0.187432	-5.61807
SERP1	-0.27366	7.818218	-1.64661	0.10123	0.187432	-5.75458
DISP2	-0.79596	1.758635	-1.64598	0.10136	0.187551	-5.21245
IL18BP	0.571033	3.968672	1.646006	0.101354	0.187551	-5.42834
UBQLN4	0.265137	6.135835	1.646075	0.101339	0.187551	-5.76245
VCAM1	0.869841	4.061336	1.645629	0.101431	0.187658	-5.38977
MX1	0.698743	6.650643	1.645337	0.101491	0.187745	-5.76866
PHF7	-0.35426	1.089203	-1.64519	0.101522	0.187777	-5.04097
C3orf54	0.932233	0.750556	1.644752	0.101612	0.187919	-4.83209
KATNA1	0.241441	3.688682	1.644628	0.101638	0.187932	-5.43327
TNFAIP8	0.502251	4.426456	1.644587	0.101646	0.187932	-5.54199
DPH3B	0.386201	0.404313	1.643946	0.101779	0.188152	-4.8504
CHST14	0.451576	4.441052	1.643495	0.101872	0.1883	-5.55534
UAP1L1	0.629532	4.474103	1.643064	0.101961	0.18844	-5.52748
CCDC45	0.266645	4.55731	1.642451	0.102088	0.188594	-5.60731
CLSTN3	0.386718	5.583988	1.642483	0.102082	0.188594	-5.72021
SKI	-0.31608	7.146925	-1.6424	0.102099	0.188594	-5.7773
ALCAM	-0.67726	6.390801	-1.64258	0.102062	0.188594	-5.78442
PPDPF	-0.38179	8.605715	-1.64233	0.102114	0.188597	-5.73164
ACVR2B	-0.36805	3.913531	-1.64221	0.102139	0.188608	-5.59416
NDOR1	-0.3156	5.715205	-1.64217	0.102147	0.188608	-5.77312
PTGIR	0.639319	0.626752	1.642053	0.102171	0.188628	-4.85235
ZDHHC13	0.40946	4.931125	1.640424	0.102509	0.189227	-5.64632
TMEM107	0.450447	2.676726	1.640143	0.102568	0.18931	-5.19418
C17orf90	0.335866	3.681818	1.639844	0.10263	0.189375	-5.41929
C1orf57	0.339108	4.075525	1.639901	0.102618	0.189375	-5.50582
NFKBID	0.449341	1.824424	1.639513	0.102699	0.189477	-5.0539
C19orf54	0.27695	4.234861	1.638974	0.102811	0.189659	-5.55156
LOC441869	-0.82687	2.220785	-1.63869	0.102871	0.189744	-5.33345
DKK1	2.012847	2.909785	1.638383	0.102934	0.189836	-5.0067
COL17A1	1.657784	6.696312	1.63821	0.102971	0.189878	-5.72027
NLGN4X	-0.88414	1.575969	-1.63807	0.102999	0.189905	-5.21434
LOC91316	-0.3554	3.806525	-1.63801	0.103012	0.189905	-5.57898
INPP5E	0.319752	4.187871	1.637809	0.103054	0.189957	-5.5375
PELI3	-0.39064	3.377313	-1.6377	0.103077	0.189974	-5.5012
C9orf6	0.271965	4.555778	1.637539	0.103111	0.190011	-5.61419
NEK8	0.403345	1.963676	1.637079	0.103207	0.190163	-5.085
TRIM16	0.578711	6.403326	1.636978	0.103228	0.190177	-5.77624
ZMAT3	-0.33007	3.689949	-1.63684	0.103257	0.190197	-5.55561
NEK7	-0.30664	6.624869	-1.6368	0.103266	0.190197	-5.79535
PCNX	-0.29292	6.361512	-1.63657	0.103313	0.190258	-5.79676
DDX58	0.527742	5.045761	1.636424	0.103344	0.19029	-5.65418
FDXR	0.543155	3.853729	1.636187	0.103394	0.190331	-5.4231
C2orf79	0.352396	3.77258	1.636201	0.103391	0.190331	-5.44219
C19orf21	1.942805	5.338975	1.635973	0.103438	0.190389	-5.45535
SATB2	0.680723	3.119062	1.635454	0.103547	0.190564	-5.24455
RNF168	0.47546	4.322536	1.6352	0.103601	0.190628	-5.54008
ICA1	-1.03319	3.431135	-1.63514	0.103614	0.190628	-5.61694
WBP4	-0.31837	4.19916	-1.63509	0.103623	0.190628	-5.64411
FAM45B	0.337716	2.940299	1.635001	0.103642	0.190638	-5.26963
ZNF785	-0.30318	3.455418	-1.63481	0.103683	0.190689	-5.50339

SFT2D3	0.287318	4.301429	1.634125	0.103826	0.190927	-5.57114
RFK	-0.49326	5.270143	-1.63401	0.10385	0.190945	-5.77077
CSGALNACT2	0.39882	4.99533	1.633753	0.103904	0.19102	-5.66764
MTFMT	0.237112	3.039625	1.633335	0.103992	0.191157	-5.31101
FBXO33	-0.2396	4.534844	-1.6325	0.104168	0.191435	-5.68255
C1RL	-0.34233	5.133216	-1.63249	0.104171	0.191435	-5.75407
DNAJA2	-0.2012	6.060972	-1.63145	0.104389	0.191809	-5.799
SSBP4	0.303634	4.647832	1.631051	0.104474	0.191941	-5.63523
TPR	0.228602	7.587348	1.630893	0.104507	0.191977	-5.79672
C6orf154	-0.65524	0.272572	-1.63065	0.104559	0.192046	-4.979
ASCC2	-0.26035	6.072075	-1.63054	0.104581	0.192061	-5.80202
MTMR11	0.804997	4.343973	1.630309	0.10463	0.192127	-5.48625
RNF113A	0.281256	3.620408	1.629968	0.104703	0.192235	-5.43318
HHEX	0.761519	2.706936	1.629848	0.104728	0.192256	-5.16833
DHX36	0.312498	5.912631	1.629583	0.104784	0.192334	-5.77351
ANKRD35	-0.90516	1.53098	-1.62931	0.104843	0.192416	-5.22574
DSG3	3.971737	6.204265	1.629015	0.104904	0.192504	-5.26776
ELP2	-0.30386	4.92636	-1.62846	0.105023	0.19267	-5.73872
POGZ	-0.25647	6.684422	-1.62848	0.105018	0.19267	-5.80858
ZNF718	-0.53909	1.612077	-1.62762	0.105201	0.192971	-5.18194
RP9P	0.396487	3.315061	1.627515	0.105223	0.192986	-5.34803
ZNF702P	-0.88942	0.837201	-1.62728	0.105272	0.193052	-5.1024
CD274	0.835121	0.998121	1.627198	0.10529	0.193059	-4.90107
TLK2	0.198073	4.660369	1.62673	0.105389	0.193216	-5.65791
ADCK4	-0.2399	4.628671	-1.62653	0.105433	0.19327	-5.70356
PRR16	0.652843	1.221472	1.626344	0.105472	0.193316	-4.95536
SUPT7L	0.15393	5.681367	1.626278	0.105486	0.193317	-5.77241
MEOX1	-0.72572	0.620897	-1.62509	0.105738	0.193754	-5.05196
NMD3	-0.24836	6.080345	-1.62451	0.105862	0.193955	-5.81164
TPD52L1	-0.90225	4.562074	-1.62442	0.105883	0.193968	-5.75961
GUF1	-0.31677	5.606306	-1.62427	0.105914	0.194	-5.79707
ESR2	0.555843	0.323327	1.624047	0.105961	0.194061	-4.85035
SHISA4	-0.54021	2.351512	-1.62384	0.106006	0.194096	-5.32748
GBF1	-0.22907	7.383517	-1.62383	0.106008	0.194096	-5.80374
FAM22A	-0.53013	0.529304	-1.62355	0.106067	0.194167	-5.01261
PEMT	0.41572	4.131107	1.623452	0.106088	0.194167	-5.53021
NDUFA2	-0.27992	4.675192	-1.62347	0.106084	0.194167	-5.71761
PYGL	1.103916	5.411508	1.622764	0.106236	0.194385	-5.65589
BIN1	-0.57535	4.77401	-1.62278	0.106232	0.194385	-5.75488
DHRS4	-0.44199	3.18603	-1.62234	0.106327	0.194527	-5.49176
SIK1	-0.55338	6.506379	-1.62196	0.106407	0.194648	-5.8177
C18orf19	0.365669	4.166651	1.621359	0.106537	0.194859	-5.55119
COL13A1	0.718821	1.105658	1.621173	0.106577	0.194907	-4.93725
PPIP5K1	-0.29633	5.231517	-1.62044	0.106733	0.195168	-5.77764
SLC35B2	0.412426	5.462359	1.620147	0.106797	0.195258	-5.74109
UBE2K	-0.22064	6.756854	-1.61994	0.106842	0.195316	-5.82179
PLVAP	-0.51711	6.702909	-1.61967	0.106899	0.195394	-5.81881
MMP28	-0.83919	3.076015	-1.61955	0.106925	0.195416	-5.54675
SMUG1	0.257696	4.4346	1.619473	0.106942	0.195421	-5.6242
LRRC8E	0.766337	3.923197	1.619272	0.106985	0.195475	-5.41919
HEY1	0.999718	3.067543	1.619059	0.107031	0.195533	-5.20999
GCNT1	-0.87659	4.192497	-1.61899	0.107046	0.195535	-5.72608
C5AR1	0.754898	3.402992	1.618222	0.107211	0.195811	-5.31537
CCNT1	-0.32205	3.643641	-1.61767	0.107331	0.195979	-5.57501
OAT	-0.35039	6.496493	-1.61767	0.107331	0.195979	-5.82679
FAM134A	-0.20145	6.546662	-1.6176	0.107345	0.195979	-5.82738
KRT5	3.506291	7.719107	1.617537	0.107359	0.195979	-5.67065
TTBK2	-0.41024	3.155171	-1.61731	0.107408	0.196043	-5.48838

LIG3	0.318336	5.049035	1.617233	0.107425	0.196047	-5.70919
MUC12	1.814874	0.578637	1.617074	0.107459	0.196084	-4.81657
DKFZP586I1420	-0.35077	3.828415	-1.61689	0.107499	0.196131	-5.61728
SUGT1	0.294104	5.1453	1.616722	0.107535	0.196172	-5.7239
LASS4	-0.92331	3.940963	-1.61649	0.107584	0.196236	-5.70955
KIAA1644	-0.81049	1.605448	-1.61559	0.107779	0.196566	-5.24218
DPY19L2P2	-0.74523	0.206481	-1.61445	0.108026	0.196991	-5.00749
RB1	0.37145	6.795756	1.614388	0.10804	0.196991	-5.83062
RPS6KC1	-0.28975	4.904307	-1.61389	0.108149	0.197164	-5.75854
RHOA	-0.22674	8.633052	-1.61373	0.108184	0.1972	-5.7814
CRABP2	1.552132	4.720568	1.613513	0.10823	0.19726	-5.45218
CABLES2	0.372613	4.336957	1.613332	0.10827	0.19728	-5.59659
POLR2J	0.370637	5.344475	1.613372	0.108261	0.19728	-5.7438
RPP38	0.282227	3.962079	1.612713	0.108404	0.197474	-5.53527
NCAPH2	0.291936	5.16164	1.612761	0.108394	0.197474	-5.73245
HOXA3	0.659871	3.283791	1.611659	0.108634	0.19784	-5.32206
RAB8B	-0.34672	4.781006	-1.61168	0.108629	0.19784	-5.75439
CHRNB1	0.553338	2.273025	1.611527	0.108663	0.197855	-5.15577
PLK1S1	-0.38842	3.494293	-1.61147	0.108674	0.197855	-5.56451
ZFAND2B	-0.30309	3.889453	-1.61143	0.108684	0.197855	-5.62817
MCART1	-0.23728	3.391451	-1.61114	0.108747	0.197943	-5.51506
KRT222	-0.45555	0.395141	-1.61056	0.108873	0.198092	-5.00288
FGF11	1.266065	2.972143	1.61044	0.1089	0.198092	-5.16664
RNF217	-0.88733	2.605094	-1.61053	0.108881	0.198092	-5.47287
ANKRD5	0.45437	3.94062	1.61047	0.108893	0.198092	-5.49748
ZKSCAN1	-0.39244	6.406716	-1.61068	0.108846	0.198092	-5.83813
NR2C1	0.339922	4.19474	1.610038	0.108987	0.198226	-5.57813
MUC4	2.758975	5.268002	1.608985	0.109217	0.198619	-5.33269
WDR35	-0.39994	4.231296	-1.60866	0.109289	0.198723	-5.70068
SDR39U1	-0.25267	3.989368	-1.60833	0.109361	0.198803	-5.64443
ARL5A	-0.22524	6.02551	-1.60835	0.109357	0.198803	-5.8355
KIAA0195	-0.29241	6.646894	-1.60809	0.109413	0.198871	-5.84114
GALNT7	-0.50221	6.210237	-1.60731	0.109585	0.199158	-5.84344
TLR8	0.960239	0.356589	1.606504	0.109761	0.1994	-4.8383
DERA	0.346258	5.138031	1.606549	0.109751	0.1994	-5.73391
SPTBN2	0.7418	6.516327	1.606586	0.109743	0.1994	-5.82285
EFEMP2	-0.54807	4.154845	-1.60633	0.1098	0.199445	-5.71152
TBC1D8B	-0.47017	4.240379	-1.60609	0.109851	0.199477	-5.71154
RAB31	0.625558	6.139856	1.606052	0.109861	0.199477	-5.80724
ITGA5	-0.57441	7.157204	-1.60609	0.109853	0.199477	-5.8301
ANXA3	-0.71468	5.195796	-1.6058	0.109916	0.199551	-5.82233
GCC2	-0.48193	6.180305	-1.60567	0.109945	0.199578	-5.84589
ABR	-0.32036	7.214738	-1.60555	0.109972	0.1996	-5.83476
PAPOLA	0.193903	7.99584	1.605203	0.110048	0.199712	-5.82762
ZNF514	-0.31868	3.562099	-1.60513	0.110064	0.199716	-5.57542
ZNF563	-0.43286	0.541707	-1.60495	0.110104	0.199753	-5.03065
GRHL1	0.936061	5.031364	1.604906	0.110113	0.199753	-5.64563
TTC14	-0.3893	4.864523	-1.60482	0.110133	0.199763	-5.77757
PPP1R10	-0.24575	6.590296	-1.60456	0.110188	0.199838	-5.84766
LILRB1	0.806696	1.329102	1.604426	0.110219	0.199867	-4.98549
USP32	0.245363	6.119254	1.604356	0.110234	0.199869	-5.82937
S100P	-1.40571	4.766293	-1.60412	0.110287	0.199939	-5.83048
DCP1A	-0.23757	5.619879	-1.60341	0.110442	0.200194	-5.82736
CHMP7	-0.2378	5.095909	-1.60329	0.110469	0.200218	-5.78913
ARHGDIA	0.265578	8.726434	1.602918	0.110551	0.200315	-5.81053
PRELID1	0.371256	6.407409	1.602919	0.110551	0.200315	-5.83906
HIC1	-0.482	3.461952	-1.60277	0.110584	0.200348	-5.59199
MRPS26	0.314154	4.635068	1.602435	0.110658	0.200456	-5.67724

C9orf78	0.231951	5.571423	1.602315	0.110685	0.200478	-5.79582
CLIP2	-0.41219	5.987658	-1.60179	0.110801	0.200662	-5.84878
VAV3	-0.87239	4.255313	-1.60117	0.110938	0.200885	-5.76366
IL18	0.787995	4.60884	1.60107	0.11096	0.200899	-5.59558
CLDN3	3.557942	3.528572	1.600958	0.110985	0.2009	-4.94525
ADCY9	-0.3628	5.385988	-1.60094	0.11099	0.2009	-5.82416
TUBGCP3	0.288355	4.880524	1.600581	0.111069	0.201017	-5.71791
UBOX5	-0.25921	4.060842	-1.60026	0.111139	0.201118	-5.67
RTEL1	0.392739	5.203558	1.600045	0.111188	0.201128	-5.74653
MRPL30	0.231689	5.560338	1.60006	0.111184	0.201128	-5.79839
MYCBP2	-0.36524	6.921728	-1.60012	0.111171	0.201128	-5.84843
GNL3	0.283692	6.055233	1.599744	0.111254	0.201211	-5.83151
TALDO1	0.403944	7.555294	1.59971	0.111262	0.201211	-5.84993
C14orf167	-0.3191	4.508395	-1.59846	0.11154	0.201665	-5.74209
MED23	-0.21511	5.540223	-1.59845	0.111542	0.201665	-5.82941
LETM1	-0.30569	6.322057	-1.59676	0.111919	0.202319	-5.86006
SNAPIN	0.267541	4.256	1.596655	0.111942	0.202336	-5.62625
ERCC5	-0.28655	5.698884	-1.59572	0.11215	0.202685	-5.84542
CEP63	-0.26563	4.309552	-1.59554	0.112191	0.202733	-5.71571
CNPY4	0.428658	2.411966	1.595372	0.112229	0.202775	-5.22227
VTCN1	1.863527	1.056091	1.595144	0.112279	0.202801	-4.85204
ARL14	-1.345	1.443102	-1.59505	0.112301	0.202801	-5.31959
PTPN14	-0.45536	4.734742	-1.59505	0.112301	0.202801	-5.78616
RPL19	0.339497	9.158832	1.595055	0.112299	0.202801	-5.8101
SYT17	-0.66118	2.185714	-1.59471	0.112377	0.202911	-5.36141
SRI	0.408744	5.994255	1.594223	0.112486	0.203082	-5.82912
SETD1A	0.192779	6.28043	1.593821	0.112576	0.203218	-5.85471
C6orf147	0.581324	-0.03651	1.593348	0.112682	0.203383	-4.85288
IL17RE	-0.55993	4.031528	-1.5931	0.112737	0.203456	-5.71288
ANKRD40	-0.23587	6.114862	-1.59261	0.112848	0.20363	-5.86279
DNASE2	0.279712	5.68114	1.592351	0.112906	0.203682	-5.81712
GFM1	0.25743	6.228551	1.592353	0.112905	0.203682	-5.85267
METTL5	0.248541	4.760859	1.591927	0.113001	0.203828	-5.72044
DPP4	1.570765	3.793904	1.591537	0.113089	0.20396	-5.28286
KRT6A	3.297961	7.51126	1.591468	0.113104	0.203961	-5.71225
PIGA	0.33738	4.216374	1.590825	0.113249	0.204196	-5.61404
METTL7B	1.886942	1.93083	1.590227	0.113384	0.204386	-4.95196
CWC27	-0.23276	4.079393	-1.59018	0.113394	0.204386	-5.68563
PCNXL3	0.238489	7.166487	1.590163	0.113398	0.204386	-5.8688
TMEM123	0.291183	8.31164	1.589897	0.113458	0.204468	-5.84524
C2CD4A	1.569619	2.131931	1.589685	0.113506	0.204527	-5.01679
LOC100190939	-0.3997	2.253404	-1.58952	0.113543	0.204568	-5.33364
ACVR1C	0.884949	2.514211	1.58938	0.113575	0.204579	-5.17567
MGAT2	0.231459	5.356441	1.589364	0.113578	0.204579	-5.79588
DYNLL1	0.301854	7.504429	1.589271	0.113599	0.20459	-5.86565
RASD2	0.870456	2.692492	1.589	0.11366	0.204674	-5.21605
ZNF277	-0.29527	4.401352	-1.58818	0.113845	0.20498	-5.7421
CD81	-0.41685	7.832823	-1.5879	0.11391	0.20507	-5.84379
NFKBIL1	-0.24563	3.472384	-1.58769	0.113956	0.2051	-5.56973
PPWD1	-0.19353	4.016288	-1.58771	0.113952	0.2051	-5.6721
LGR4	-0.62886	6.191663	-1.58713	0.114082	0.205301	-5.87494
STIM2	0.314535	4.910672	1.586713	0.114178	0.20542	-5.73996
PELP1	0.244044	6.365819	1.586742	0.114171	0.20542	-5.86708
PLXNB2	-0.28461	9.20714	-1.58641	0.114248	0.205519	-5.80284
TMED8	-0.24176	4.368333	-1.58587	0.114369	0.20571	-5.73588
KIAA0649	-0.37679	5.125358	-1.58511	0.114542	0.205996	-5.83041
CDC5L	0.277826	5.905062	1.584807	0.114611	0.206093	-5.84606
ANKRD22	-0.77578	4.50957	-1.58454	0.114672	0.206177	-5.805

METT11D1	0.224201	4.214117	1.584161	0.114758	0.206277	-5.64418
TMEM222	-0.24029	4.723672	-1.58417	0.114755	0.206277	-5.78118
ACPL2	0.482097	3.889708	1.58369	0.114865	0.206443	-5.5264
HDAC8	0.224268	3.690005	1.58342	0.114926	0.206528	-5.53371
VIM	-0.51827	8.747103	-1.58315	0.114987	0.20661	-5.8166
DSTYK	-0.25425	4.848679	-1.58304	0.115014	0.206632	-5.79861
TMEM38B	0.446812	3.991339	1.582631	0.115106	0.206771	-5.55616
BAG3	-0.47472	7.046831	-1.58248	0.115141	0.206806	-5.87166
PHKA2	-0.31092	4.806309	-1.5824	0.11516	0.206813	-5.79995
ZNF518A	-0.37063	5.327915	-1.58213	0.11522	0.206895	-5.85002
PRRX1	1.028463	4.285632	1.582021	0.115246	0.206915	-5.51006
PLEKHA8	0.344932	1.99502	1.581911	0.115271	0.206933	-5.18569
LGI2	-0.81726	1.828388	-1.58156	0.115351	0.207051	-5.34309
FLT4	-0.4801	3.42792	-1.58123	0.115426	0.20716	-5.61478
IFT140	-0.30884	4.254046	-1.58107	0.115463	0.207199	-5.73593
NEURL2	-0.40446	0.813469	-1.58092	0.115497	0.207233	-5.10487
CCNL1	0.315503	6.483442	1.580536	0.115585	0.207364	-5.87838
TMEFF1	0.616168	2.029165	1.580202	0.115661	0.207475	-5.15439
NSMCE4A	-0.23056	4.662162	-1.58011	0.115683	0.207487	-5.77969
IGFL1	1.942256	-0.28283	1.579847	0.115743	0.207567	-4.86761
PGP	0.314924	4.452897	1.579495	0.115823	0.207685	-5.6816
PYCR1	0.525654	5.981727	1.579126	0.115908	0.207811	-5.843
THOC1	0.31901	4.380407	1.578945	0.11595	0.207832	-5.66884
JAK2	-0.48662	4.631103	-1.57896	0.115946	0.207832	-5.80284
P4HA1	0.440024	5.426751	1.578662	0.116014	0.207921	-5.80009
C16orf54	-0.77058	0.555391	-1.57801	0.116165	0.208117	-5.1191
ZNF773	-0.50668	1.492309	-1.57799	0.116168	0.208117	-5.23444
ZCCHC14	-0.23756	6.177751	-1.57802	0.116163	0.208117	-5.88686
C18orf25	-0.34235	5.234002	-1.5777	0.116235	0.20821	-5.84875
KRT6B	4.209898	6.04266	1.577459	0.116291	0.208283	-5.2798
PPP1R2P3	0.290404	1.764055	1.577116	0.11637	0.208318	-5.16365
FANK1	-0.53023	1.208042	-1.57719	0.116354	0.208318	-5.18838
C8orf38	0.268088	3.029691	1.577171	0.116357	0.208318	-5.39022
PFKL	-0.2672	7.481224	-1.57715	0.116361	0.208318	-5.87429
DHODH	0.32687	2.413321	1.576979	0.116401	0.208347	-5.26512
ZNFX1	0.344558	7.215443	1.576656	0.116476	0.208454	-5.89041
PLIN3	0.377634	6.53213	1.576577	0.116494	0.20846	-5.88433
FGFBP1	2.930376	4.471229	1.576408	0.116533	0.208476	-5.21606
ZNF317	-0.18112	5.476198	-1.57647	0.116518	0.208476	-5.85784
FLJ44606	-0.60665	0.992937	-1.57624	0.116571	0.208491	-5.16672
NOL12	0.27038	3.403573	1.576248	0.11657	0.208491	-5.47077
LOC387646	1.031598	0.909238	1.575917	0.116646	0.208588	-4.944
SCN8A	1.019759	1.388618	1.575877	0.116655	0.208588	-5.0092
MN1	-0.81951	4.25724	-1.5756	0.11672	0.208677	-5.79988
PTGR2	-0.3363	3.431542	-1.57544	0.116756	0.208694	-5.59699
GSTK1	-0.38685	6.219473	-1.57543	0.116759	0.208694	-5.89314
ASPH	-0.37022	7.903303	-1.57466	0.116937	0.208985	-5.86344
NEURL	-0.73006	1.294711	-1.57449	0.116976	0.209027	-5.23768
FOXJ2	-0.28411	5.532016	-1.57421	0.117039	0.209114	-5.87053
KLF13	-0.33391	7.212927	-1.57407	0.117073	0.209148	-5.88386
C11orf45	0.715872	1.477933	1.573491	0.117206	0.209332	-5.06545
PKN3	0.452978	4.047865	1.573512	0.117201	0.209332	-5.58015
ZFP41	0.305923	4.732719	1.573363	0.117236	0.209359	-5.73807
ZBTB7B	-0.35591	6.925857	-1.57328	0.117254	0.209364	-5.89048
CLP1	0.227326	3.408726	1.5729	0.117343	0.209469	-5.48641
TBPL1	0.262749	3.759586	1.572918	0.117339	0.209469	-5.5563
CES1	-1.36635	2.671411	-1.57227	0.117488	0.209702	-5.64354
TTLL12	0.429333	7.04031	1.57198	0.117556	0.209797	-5.89875

ID3	-0.42405	5.149973	-1.57175	0.11761	0.209848	-5.85687
LEMD2	0.197899	5.984303	1.571727	0.117615	0.209848	-5.87568
OMA1	-0.28214	3.702766	-1.57155	0.117657	0.209895	-5.6502
NOMO3	-0.32686	6.196038	-1.57144	0.117681	0.209911	-5.89855
IL20RB	1.862846	3.249924	1.570724	0.117848	0.210123	-5.18591
TSGA14	0.494677	3.473103	1.570675	0.117859	0.210123	-5.45126
MACF1	-0.4061	8.379389	-1.57071	0.117852	0.210123	-5.85221
CYR61	-0.61725	5.944864	-1.57079	0.117832	0.210123	-5.8993
GOLGA7B	1.198331	2.538066	1.570275	0.117952	0.210227	-5.16512
ZNF324	-0.31856	3.279121	-1.57023	0.117963	0.210227	-5.57042
FOS	-0.72837	7.959602	-1.57032	0.117943	0.210227	-5.85754
C12orf66	0.342439	3.027688	1.569844	0.118053	0.210333	-5.38815
DOK4	-0.48301	5.192805	-1.56988	0.118045	0.210333	-5.86633
CIB1	0.4014	6.31855	1.569641	0.1181	0.21039	-5.88626
SERPINB4	2.702092	1.258859	1.569037	0.118241	0.210614	-4.89177
RNASE7	2.031371	0.15008	1.568757	0.118306	0.210623	-4.88652
GPR87	3.017592	1.987672	1.568765	0.118304	0.210623	-4.89504
PPM1N	0.685746	0.457421	1.568862	0.118281	0.210623	-4.93632
KLHL20	-0.20101	4.298936	-1.56894	0.118264	0.210623	-5.74802
RIMKLB	-0.65049	3.418006	-1.56854	0.118357	0.21066	-5.66799
GPATCH8	0.19868	6.509366	1.568603	0.118342	0.21066	-5.9003
FAM109B	-0.35083	3.744025	-1.56838	0.118393	0.210671	-5.67605
KIAA0652	-0.21315	6.597572	-1.56842	0.118384	0.210671	-5.90433
CERKL	0.63761	1.200885	1.568211	0.118433	0.210716	-5.04265
GRAMD3	-0.37924	3.776082	-1.56784	0.11852	0.210842	-5.68806
DGCR8	-0.19232	5.257453	-1.56766	0.118563	0.210892	-5.85554
PHLDB2	-0.83441	5.59467	-1.56693	0.118732	0.211166	-5.90212
GAS1	-0.85098	3.145496	-1.56684	0.118754	0.211179	-5.64927
DCUN1D2	-0.29196	3.755435	-1.5666	0.118811	0.211253	-5.6712
ZNF385A	0.823359	6.298082	1.566293	0.118881	0.211352	-5.86747
ZNF30	-0.42684	1.742139	-1.56601	0.118948	0.211443	-5.28155
C12orf75	0.746252	5.402137	1.565751	0.119008	0.211523	-5.78328
TYW1	0.268363	4.790216	1.565572	0.11905	0.211544	-5.7627
UPF3A	-0.29119	4.559281	-1.56559	0.119046	0.211544	-5.79634
STK38	0.271567	6.394364	1.564968	0.119192	0.211769	-5.90103
MUTED	-0.2391	3.466646	-1.56444	0.119316	0.211963	-5.60341
SLC7A5P2	0.515937	0.212101	1.564086	0.119399	0.212082	-4.9311
ZNF512B	-0.55278	4.723415	-1.5638	0.119466	0.212175	-5.84226
HSCB	-0.24096	2.616663	-1.56372	0.119484	0.21218	-5.41982
ALG12	-0.2319	5.062888	-1.56338	0.119564	0.212295	-5.84775
INO80D	-0.28496	5.639752	-1.56303	0.119648	0.212416	-5.89353
PTPDC1	-0.30247	3.624928	-1.56288	0.119683	0.212452	-5.65148
BCORL1	0.359072	4.867919	1.562731	0.119717	0.212486	-5.76694
STK32C	0.631837	2.386096	1.561531	0.12	0.212961	-5.23803
MICA	-0.29739	3.497392	-1.56113	0.120094	0.213078	-5.62702
LCP1	0.676336	6.020814	1.561122	0.120097	0.213078	-5.86385
INTS2	0.261468	4.785669	1.560897	0.12015	0.213145	-5.77026
CCDC15	0.43113	1.687585	1.560552	0.120231	0.213229	-5.15499
CKMT1A	1.072698	4.593702	1.560528	0.120237	0.213229	-5.59231
ZNF692	0.324112	3.936968	1.560503	0.120243	0.213229	-5.6025
FOXN1	2.295384	1.264911	1.560133	0.12033	0.213357	-4.90477
CCDC68	-0.98362	2.457717	-1.55999	0.120364	0.213386	-5.52246
ITGB1BP1	0.238527	5.070142	1.559933	0.120377	0.213386	-5.81015
CCRL1	-0.69725	1.648862	-1.55975	0.120421	0.213437	-5.31097
B3GNT2	0.29815	5.41589	1.559266	0.120535	0.213612	-5.84249
C1QA	0.792755	5.251789	1.559108	0.120572	0.213651	-5.77109
EFNA5	-0.63812	3.496177	-1.55883	0.120638	0.213659	-5.69292
RPS16	0.329794	8.585928	1.558867	0.120629	0.213659	-5.88586

IGF1R	-0.6351	6.736068	-1.55897	0.120606	0.213659	-5.91134
SAPS1	0.232278	7.202543	1.558842	0.120635	0.213659	-5.9169
HSPA1A	0.597128	8.594618	1.558584	0.120696	0.213735	-5.89348
MSX1	0.703422	1.849055	1.558392	0.120742	0.213788	-5.1463
GCDH	-0.28085	3.933249	-1.55813	0.120803	0.213843	-5.71696
AKR7A2	-0.28247	4.872768	-1.55817	0.120796	0.213843	-5.84151
SFRS7	0.181744	6.537797	1.55803	0.120828	0.213859	-5.91748
MFAP3	-0.20898	5.559486	-1.55787	0.120865	0.213899	-5.89349
MANBAL	0.235425	5.462437	1.557318	0.120997	0.214104	-5.8559
TRUB2	0.246626	4.868866	1.557101	0.121048	0.214168	-5.78864
ALKBH4	0.324299	3.110246	1.556736	0.121135	0.214279	-5.42782
RNF8	0.219193	4.047034	1.556708	0.121141	0.214279	-5.65394
GGT6	-1.05529	3.277883	-1.55662	0.121163	0.21429	-5.71447
POLR3H	-0.23458	5.321595	-1.55622	0.121258	0.21443	-5.8813
C19orf10	0.279497	6.071338	1.555937	0.121325	0.214499	-5.9006
SDC3	-0.43824	6.117335	-1.55592	0.121328	0.214499	-5.92262
AKAP5	0.589366	1.537167	1.555403	0.121452	0.214691	-5.11969
MRPL42	0.243347	5.397967	1.555138	0.121515	0.214776	-5.85194
FAM98C	-0.28995	3.455018	-1.55477	0.121602	0.214903	-5.62429
NLRX1	-0.46324	4.952535	-1.55469	0.121621	0.214909	-5.86999
NAPA	-0.2523	7.076676	-1.55387	0.121818	0.215229	-5.91932
AMIGO2	0.851654	4.082436	1.553374	0.121935	0.215411	-5.54206
SNX20	0.69617	0.668205	1.553282	0.121957	0.215422	-4.9857
KDELR3	0.641127	4.95947	1.553146	0.12199	0.215452	-5.75349
SYMPK	0.188468	6.81691	1.552786	0.122076	0.215577	-5.92854
ARSA	-0.32542	5.102407	-1.55244	0.122158	0.215695	-5.87547
OSTF1	0.357165	5.079864	1.552159	0.122226	0.215787	-5.81048
C9orf100	0.541644	3.650301	1.55206	0.12225	0.215802	-5.50486
CCDC40	-0.54394	1.059365	-1.55183	0.122305	0.215873	-5.20769
AKAP11	-0.28816	6.502542	-1.55156	0.122369	0.215958	-5.93039
LRRC3	-0.47138	1.5748	-1.55146	0.122392	0.215971	-5.28098
ZCCHC2	-0.2959	4.68651	-1.55131	0.122428	0.216008	-5.83381
IRGQ	-0.27353	5.685237	-1.55093	0.122521	0.216144	-5.91389
SERPINF2	0.847933	1.338839	1.55082	0.122546	0.216162	-5.06436
COMMD8	0.317502	4.311274	1.550627	0.122593	0.216216	-5.69966
AFTPH	-0.21227	6.311352	-1.55026	0.122681	0.216344	-5.93139
HCG18	-0.30887	5.021477	-1.5499	0.122766	0.216467	-5.87106
ABCG2	-0.68513	1.63129	-1.54948	0.122868	0.21662	-5.32602
SLC25A19	0.371092	3.452326	1.549406	0.122886	0.216624	-5.50233
TRIM47	0.447118	5.780267	1.549059	0.122969	0.216743	-5.88009
ZNF500	-0.21568	3.547114	-1.54894	0.122997	0.216765	-5.6407
ARL6	-0.3416	1.38509	-1.54857	0.123087	0.216896	-5.23595
ARMC5	-0.23396	4.08282	-1.54844	0.123118	0.216924	-5.75146
BEND3	0.369763	3.992319	1.5481	0.1232	0.21704	-5.62297
TBC1D15	-0.23178	5.530179	-1.54782	0.123268	0.217133	-5.90827
ECE1	0.329074	7.618473	1.547386	0.123372	0.217288	-5.92869
CREB3L4	-0.40694	3.017994	-1.54722	0.123411	0.217303	-5.55926
COPB2	0.23268	7.695587	1.547259	0.123402	0.217303	-5.92554
C18orf10	0.366685	5.863931	1.547095	0.123442	0.21733	-5.89577
GPR98	1.725626	1.200394	1.546351	0.123621	0.217618	-4.94493
PNMA1	-0.35798	5.316004	-1.54622	0.123652	0.217644	-5.90428
ZMIZ2	0.275199	7.338532	1.5461	0.123682	0.21767	-5.93489
RARRES1	1.280607	3.438154	1.545885	0.123734	0.217734	-5.34414
HIBADH	-0.33575	5.156759	-1.54575	0.123766	0.217762	-5.89095
PSMC6	0.233925	5.522561	1.544766	0.124004	0.218155	-5.8806
PLEKHG6	0.871589	4.782243	1.544504	0.124068	0.218239	-5.69793
CRNKL1	0.235695	5.263722	1.544417	0.124089	0.218249	-5.85548
TFB1M	0.298656	3.054744	1.54313	0.124401	0.21877	-5.44155

PEX10	0.270593	4.290545	1.543015	0.124428	0.218791	-5.71476
DUOX1	-0.92209	5.891771	-1.54265	0.124516	0.218918	-5.94305
ST6GALNAC4	0.414672	4.329396	1.541969	0.124682	0.219182	-5.69827
TMEM50A	0.193438	6.887819	1.5419	0.124699	0.219185	-5.94523
NDUFB1	-0.25387	4.483384	-1.54171	0.124746	0.219239	-5.81975
SH3TC2	0.977698	1.734934	1.541343	0.124835	0.219367	-5.11747
C9orf119	0.304963	3.437251	1.541157	0.12488	0.219368	-5.52609
SH3KBP1	0.607005	5.375996	1.541158	0.12488	0.219368	-5.83443
EMD	0.252711	5.494694	1.541147	0.124882	0.219368	-5.88213
KIAA1539	-0.36014	4.623206	-1.54081	0.124964	0.219484	-5.84881
COL22A1	1.514209	0.899086	1.540344	0.125078	0.219617	-4.93987
PLCH2	1.551158	3.391539	1.540363	0.125073	0.219617	-5.30046
LOX	0.729377	5.065873	1.540307	0.125087	0.219617	-5.78248
MYL12B	0.365559	7.94933	1.539787	0.125213	0.219784	-5.93386
ARID2	-0.24483	5.907204	-1.53984	0.125202	0.219784	-5.93872
ARHGEF16	0.579822	5.080805	1.539459	0.125294	0.219897	-5.8018
CUTC	-0.23644	3.111785	-1.53933	0.125325	0.219924	-5.56332
SLK	-0.35491	7.184491	-1.53904	0.125396	0.220022	-5.93786
CA5BP	0.298682	3.79066	1.538503	0.125527	0.220143	-5.6092
HARS2	-0.19485	4.54409	-1.5386	0.125502	0.220143	-5.8255
RAVER2	-0.36683	5.138701	-1.5385	0.125528	0.220143	-5.90297
FZD5	-0.65491	5.461489	-1.53866	0.125488	0.220143	-5.93668
TMEM200A	-0.69723	2.345375	-1.53809	0.125627	0.220289	-5.4815
CUL3	-0.21541	6.343777	-1.53776	0.125709	0.220405	-5.95082
FAM83D	0.512461	5.86874	1.537684	0.125727	0.220409	-5.90057
PPP1R3D	0.303608	4.375679	1.537224	0.12584	0.220578	-5.73523
C1orf27	-0.28448	5.015642	-1.53713	0.125861	0.220589	-5.88743
MIER3	-0.27353	5.023726	-1.53704	0.125885	0.220603	-5.88782
NCSTN	0.194272	7.396691	1.536787	0.125946	0.220683	-5.94679
CDIPT	-0.23087	6.232991	-1.53667	0.125975	0.220706	-5.95125
C16orf72	-0.22667	6.046966	-1.53597	0.126147	0.220979	-5.94831
LIPT2	0.536438	1.230204	1.535832	0.126181	0.22101	-5.10722
SKAP1	1.121438	1.323748	1.535675	0.126219	0.221022	-5.04659
GBP3	0.762529	4.756189	1.535735	0.126204	0.221022	-5.72636
SUV420H1	-0.26151	5.609693	-1.53528	0.126316	0.221164	-5.93331
FBXO46	0.252611	5.211406	1.534135	0.126597	0.221629	-5.86362
CBFA2T2	0.288263	5.774094	1.533735	0.126696	0.221773	-5.91436
B4GALT4	0.46945	5.465475	1.533101	0.126852	0.222019	-5.87164
GVIN1	-0.66028	1.657596	-1.53213	0.127092	0.222382	-5.35319
AAMP	-0.19757	6.29452	-1.53213	0.12709	0.222382	-5.9586
FLJ10213	-0.37909	0.819142	-1.53191	0.127147	0.222403	-5.17414
APOL1	0.66803	6.563444	1.531887	0.127151	0.222403	-5.9438
CREG1	0.33706	7.372205	1.53201	0.127121	0.222403	-5.95662
RASGRP4	-0.56041	0.088956	-1.53108	0.127349	0.222722	-5.09081
SLC39A6	0.490326	6.407191	1.530713	0.127441	0.222854	-5.94616
FGFR4	1.438731	4.042531	1.530602	0.127469	0.222875	-5.44484
XRN1	0.300654	6.308345	1.53029	0.127546	0.222982	-5.9499
SORBS3	-0.33206	6.014499	-1.52962	0.127712	0.223244	-5.9594
RPS3	0.365615	9.462481	1.529057	0.127851	0.223459	-5.90192
OAZ2	-0.19238	5.426136	-1.52867	0.127948	0.223601	-5.9282
C20orf151	0.987618	1.724619	1.528275	0.128045	0.223742	-5.13113
ASPN	0.921709	3.963235	1.527304	0.128286	0.224135	-5.54281
ARSI	1.283276	2.12615	1.527192	0.128314	0.224156	-5.15739
C19orf57	0.591681	1.348309	1.526911	0.128384	0.2242	-5.13283
PLEKHB1	-1.06161	2.81557	-1.52685	0.128399	0.2242	-5.67284
MRPL52	0.30685	4.43692	1.526963	0.128371	0.2242	-5.7595
ABCC5	-0.70077	6.662958	-1.52683	0.128403	0.2242	-5.96047
LOC151534	0.664454	3.360296	1.526451	0.128498	0.224332	-5.45664

CNOT4	-0.17157	4.780724	-1.5264	0.128511	0.224332	-5.86956
TRMT2A	0.244031	4.310398	1.525957	0.128621	0.224496	-5.74834
DNAJC5	-0.25212	7.138404	-1.52501	0.128857	0.22488	-5.96194
RG9MTD1	0.278069	4.627454	1.524534	0.128976	0.225059	-5.80116
TLR3	-0.55226	2.946542	-1.52444	0.129	0.225074	-5.6104
LCORL	-0.2997	2.652202	-1.52433	0.129027	0.225092	-5.49645
C10orf41	-0.57102	1.435046	-1.52409	0.129086	0.225167	-5.31545
HS6ST1	0.477389	5.92485	1.52331	0.129281	0.225456	-5.92862
ARHGAP21	-0.32281	7.050485	-1.5233	0.129284	0.225456	-5.96497
RPS24	0.315436	8.622751	1.523178	0.129314	0.225481	-5.93848
CHM	-0.27218	5.093477	-1.52258	0.129463	0.225713	-5.91597
HOXB2	0.760192	3.262859	1.522145	0.129573	0.225876	-5.43414
MYC	0.451217	7.39101	1.522017	0.129605	0.225903	-5.97288
LZTS1	0.70736	3.392063	1.52175	0.129672	0.225992	-5.47004
SH3RF2	-0.64891	5.561036	-1.52147	0.129742	0.226086	-5.96614
HSD17B1	0.852843	1.634989	1.521249	0.129797	0.226126	-5.14887
FTL	0.439552	10.52125	1.521279	0.12979	0.226126	-5.8797
MAST2	0.285395	6.397202	1.521184	0.129814	0.226126	-5.96736
IFRD1	-0.34155	5.409158	-1.52109	0.129837	0.226139	-5.9471
ARHGAP42	-0.63294	3.452696	-1.5209	0.129885	0.226174	-5.73376
ERC1	-0.34755	6.542421	-1.52088	0.12989	0.226174	-5.97628
KCNS3	0.969728	3.504768	1.520488	0.129988	0.226287	-5.44788
PHKG2	0.235184	4.598249	1.520506	0.129984	0.226287	-5.80761
RANBP10	-0.1964	5.177411	-1.52043	0.130003	0.226287	-5.92086
UBE2E3	0.20895	5.836311	1.519872	0.130143	0.226502	-5.94491
PPP2R2A	0.283506	6.03478	1.518464	0.130497	0.22709	-5.9554
CNO	0.263529	3.547643	1.517778	0.13067	0.227363	-5.59361
TRAF5	-0.48639	4.364028	-1.51729	0.130794	0.22755	-5.86701
PTGS2	1.090647	4.181351	1.517202	0.130815	0.227559	-5.57284
PCBP2	-0.19345	8.369238	-1.51624	0.131058	0.227953	-5.94208
TTC39A	-0.76654	3.920708	-1.51599	0.131121	0.228034	-5.83993
CPPED1	-0.45968	4.371369	-1.51579	0.131173	0.228095	-5.868
LOC90834	-0.37764	1.044435	-1.51549	0.131248	0.22816	-5.23289
NKD1	-0.81607	1.555634	-1.51553	0.131237	0.22816	-5.38642
KLHL21	-0.34791	6.57615	-1.51545	0.131259	0.22816	-5.98408
LASS5	0.2062	4.892207	1.515118	0.131342	0.228276	-5.86026
MRPS16	0.230843	5.967249	1.513857	0.131661	0.228802	-5.961
ASPRV1	-0.60548	1.24725	-1.51366	0.131712	0.228862	-5.30433
SNX15	-0.25329	4.371948	-1.51358	0.131731	0.228867	-5.84774
MMAA	-0.28992	3.484972	-1.5135	0.131753	0.228876	-5.69392
ATXN7L1	-0.2736	3.860493	-1.51341	0.131775	0.228885	-5.7686
AQP11	-0.56535	0.172378	-1.51313	0.131846	0.228981	-5.12743
IL1B	1.153899	3.482231	1.512906	0.131903	0.229043	-5.41611
DYNC2H1	-0.5886	3.379743	-1.51286	0.131915	0.229043	-5.73187
TMEM173	0.455411	4.553296	1.512711	0.131952	0.22908	-5.78017
MFHAS1	0.430839	5.794214	1.51157	0.132242	0.229555	-5.93875
DSC3	3.32907	5.99401	1.511365	0.132294	0.229596	-5.52617
SLC15A3	0.576342	4.339521	1.511314	0.132307	0.229596	-5.71698
UBE2O	0.234172	5.98336	1.511257	0.132322	0.229596	-5.96573
SUDS3	-0.212	5.805422	-1.51122	0.132332	0.229596	-5.97677
NFXL1	0.295021	3.893925	1.51104	0.132377	0.229647	-5.6736
FH	-0.22298	5.971201	-1.51074	0.132455	0.229752	-5.98402
RNF40	0.192095	6.882818	1.5104	0.13254	0.229873	-5.99268
CAMKK1	0.478856	3.39084	1.509901	0.132668	0.230065	-5.53113
TRIT1	0.50958	3.920321	1.50927	0.132829	0.230258	-5.64178
C2CD2L	-0.31018	3.939877	-1.50933	0.132813	0.230258	-5.79554
SCAMP4	0.231518	6.450874	1.509379	0.132801	0.230258	-5.98807
GTPBP10	0.341827	4.381132	1.509129	0.132865	0.230292	-5.76997

MRC1	-0.74587	2.921433	-1.50767	0.133236	0.230908	-5.66692
CCDC53	-0.25885	4.111567	-1.50747	0.133289	0.23097	-5.82002
RAET1G	1.230007	0.287795	1.507308	0.13333	0.231013	-4.97571
KLHL25	-0.36949	4.154885	-1.50649	0.13354	0.231349	-5.84207
SLC36A1	0.343444	4.921997	1.506386	0.133566	0.231365	-5.86143
NCEH1	0.559738	5.69267	1.505624	0.133762	0.231675	-5.92683
CAPZB	0.193593	7.840327	1.505426	0.133813	0.231734	-5.98455
MAMSTR	0.49531	0.435156	1.505137	0.133887	0.231788	-5.04838
TRIM52	-0.32586	2.874125	-1.50507	0.133904	0.231788	-5.57849
MYPOP	0.241046	3.645281	1.505029	0.133915	0.231788	-5.6387
PFAS	0.306405	4.969122	1.504983	0.133927	0.231788	-5.8731
ASS1	0.801763	6.13585	1.505083	0.133901	0.231788	-5.94852
STRADA	-0.22492	4.450839	-1.50453	0.134043	0.231961	-5.86812
C7orf36	0.326207	3.228281	1.503861	0.134215	0.232229	-5.5322
CNNM4	0.407535	5.654119	1.503503	0.134307	0.23236	-5.9398
GNS	-0.29075	7.538181	-1.50336	0.134345	0.232397	-5.98448
TMEM136	-0.4325	1.315898	-1.50314	0.134401	0.232465	-5.30495
SARNP	0.199974	4.887476	1.503059	0.134422	0.232472	-5.87815
HCG26	0.645075	1.278389	1.502397	0.134592	0.232628	-5.14923
ADAL	-0.29562	2.595682	-1.50242	0.134587	0.232628	-5.51679
KCTD14	-0.87852	2.588493	-1.50256	0.13455	0.232628	-5.61718
KDM3A	0.25206	6.152209	1.502583	0.134545	0.232628	-5.98663
SKIV2L2	-0.19183	5.889405	-1.50239	0.134595	0.232628	-5.99265
PET112L	-0.26044	3.736281	-1.50208	0.134674	0.232735	-5.7599
WDR45L	0.209988	6.700509	1.501807	0.134745	0.2328	-6.00442
FAM20B	-0.28053	6.432276	-1.50186	0.134732	0.2328	-6.00543
PGLS	0.310381	5.503423	1.501664	0.134782	0.232835	-5.93714
ENDOG	-0.34691	3.349742	-1.50159	0.1348	0.232837	-5.69207
ZAK	-0.4538	6.561711	-1.50135	0.134863	0.232917	-6.00406
RBM8A	0.211511	6.273543	1.500845	0.134993	0.233114	-5.99582
TNFSF15	0.924654	1.06309	1.500702	0.13503	0.23312	-5.08498
DEF6	0.433846	4.412538	1.500727	0.135024	0.23312	-5.77054
SIAH2	0.397961	6.079309	1.500577	0.135063	0.233147	-5.97844
LRTOMT	-0.36945	2.858232	-1.50041	0.135105	0.233188	-5.58984
PAIP1	0.28186	4.977399	1.500356	0.13512	0.233188	-5.88404
BIRC6	-0.22632	7.395318	-1.50002	0.135206	0.233308	-5.99424
VPS33A	0.186061	4.856736	1.499433	0.135359	0.233543	-5.88098
OTUB2	0.629457	2.762391	1.499314	0.13539	0.233568	-5.39431
KIAA2013	0.252701	6.596002	1.499217	0.135415	0.233582	-6.00605
PNMAL2	-0.64011	0.155905	-1.49875	0.135536	0.233763	-5.1586
CARS	-0.21969	5.530786	-1.49867	0.135557	0.23377	-5.9814
CCDC78	0.735945	-0.0192	1.498199	0.135679	0.233951	-4.98664
S1PR2	-0.34373	2.888055	-1.49799	0.135732	0.234015	-5.59732
SF3B2	0.173127	7.543232	1.497753	0.135795	0.234093	-6.00203
INO80B	0.291519	3.814487	1.49738	0.135892	0.234231	-5.67692
YLPM1	-0.21116	6.415345	-1.4972	0.135937	0.234281	-6.01212
PLEK	0.768383	3.15633	1.497057	0.135975	0.234289	-5.44896
TNIK	-0.77661	3.804345	-1.49711	0.135961	0.234289	-5.85508
SARS2	0.298025	4.478068	1.496883	0.136021	0.234338	-5.81359
TTC39B	-0.39157	3.556592	-1.49677	0.136051	0.234361	-5.75295
ZNF284	-0.48514	0.838387	-1.4967	0.136069	0.234363	-5.24316
C10orf35	0.62919	1.720673	1.496523	0.136114	0.234411	-5.22745
TADA2A	0.241076	3.044696	1.496462	0.13613	0.234411	-5.52117
PCDHB14	0.625721	2.775406	1.496254	0.136184	0.234475	-5.40567
FLI1	-0.43701	3.11276	-1.49589	0.136279	0.234609	-5.66919
LY6K	1.741082	2.242474	1.495565	0.136364	0.234726	-5.15869
MBD3	-0.23999	6.017757	-1.49548	0.136385	0.234734	-6.00841
CAPNS2	2.166841	0.417504	1.495342	0.136422	0.234769	-4.99356

TXNL1	-0.23535	5.483536	-1.49497	0.136519	0.234907	-5.98499
SKIL	0.419779	5.768344	1.49458	0.136621	0.235053	-5.96253
RBMS2	-0.35594	5.722344	-1.49444	0.136657	0.235087	-6.00344
USP38	-0.24429	5.75499	-1.49428	0.136698	0.235128	-6.00094
RAB20	-0.54204	3.567745	-1.49404	0.136763	0.235211	-5.78237
ACOT2	-0.39193	2.732539	-1.49387	0.136807	0.235257	-5.57885
IGFBP4	-0.49004	8.134401	-1.49374	0.13684	0.235285	-5.97451
BAHCC1	-0.50279	4.860114	-1.49358	0.136882	0.235328	-5.95658
KCNK1	0.662876	5.292511	1.493182	0.136986	0.235478	-5.88902
INSIG2	-0.27371	4.56367	-1.49294	0.137049	0.235557	-5.90387
MFNG	-0.50892	2.296853	-1.49234	0.137205	0.235798	-5.51143
ROBO2	1.147737	1.534674	1.491681	0.137379	0.236029	-5.13904
CARD10	0.539385	5.377372	1.491635	0.137391	0.236029	-5.91603
ICK	-0.38283	5.863558	-1.49165	0.137388	0.236029	-6.01322
COG7	-0.20279	4.990321	-1.49077	0.137618	0.23639	-5.94754
WASF2	-0.29751	8.150459	-1.49018	0.137773	0.236627	-5.98515
WNT5B	-0.59428	2.564334	-1.48982	0.137867	0.23676	-5.58977
APLNR	0.848746	3.77645	1.489653	0.137911	0.236761	-5.56959
MMGT1	-0.21408	5.826923	-1.4897	0.1379	0.236761	-6.00967
CAPZA2	-0.24999	6.670332	-1.48963	0.137918	0.236761	-6.02236
NEDD4	0.373374	4.972348	1.489481	0.137956	0.236797	-5.88897
SRGAP1	0.400319	4.958932	1.489415	0.137974	0.236798	-5.88415
RAB23	-0.36033	4.580696	-1.48929	0.138007	0.236826	-5.92183
FAM164A	-0.43406	3.201265	-1.48893	0.138102	0.23696	-5.70162
FUT6	2.0053	2.051135	1.488606	0.138187	0.237076	-5.10295
TRNT1	-0.25444	3.558886	-1.48836	0.138252	0.237153	-5.73952
TPP1	0.249534	8.037634	1.488307	0.138266	0.237153	-6.00616
LUZP6	0.259985	7.545061	1.488185	0.138298	0.237179	-6.01764
ZFP36L2	-0.42119	8.464047	-1.48808	0.138325	0.237196	-5.97334
PCDH17	0.619721	3.614977	1.487978	0.138352	0.237214	-5.58007
PPP1R3F	-0.44936	1.4031	-1.4875	0.138479	0.23738	-5.34695
GMPR2	-0.19665	4.987797	-1.48748	0.138483	0.23738	-5.9517
COQ3	0.311896	2.781574	1.486552	0.138728	0.237772	-5.46882
SPRY2	-0.47948	4.410422	-1.48633	0.138788	0.237816	-5.91715
GCC1	-0.20523	4.854729	-1.48637	0.138776	0.237816	-5.9402
DNM3	-0.61738	1.527185	-1.48623	0.138815	0.237832	-5.39038
HLTF	0.513323	5.634215	1.486157	0.138833	0.237834	-5.95456
SELE	1.291275	2.052602	1.485153	0.139098	0.238259	-5.20725
AMDHD2	0.300727	2.920299	1.48494	0.139155	0.238313	-5.50048
NARS	-0.26302	7.294415	-1.48491	0.139164	0.238313	-6.01803
GUSBP3	-0.37073	1.111811	-1.48479	0.139194	0.238336	-5.28816
COX7B	-0.32129	6.531396	-1.48439	0.139301	0.23849	-6.03071
TGFBR1	-0.32553	6.433365	-1.48418	0.139357	0.238556	-6.03146
ARMC10	0.260972	4.992301	1.483977	0.13941	0.238617	-5.91227
KIAA1033	0.223074	6.167658	1.483903	0.139429	0.238622	-6.01601
GXYLT2	-0.79153	1.610382	-1.48365	0.139495	0.238705	-5.43791
TMED7-TICAM2	1.133576	2.109682	1.483474	0.139543	0.238723	-5.23779
RPL7	0.693804	3.761878	1.483448	0.13955	0.238723	-5.60718
DNAJB12	-0.19158	5.602308	-1.48342	0.139557	0.238723	-6.00678
EPHB1	1.10614	1.026077	1.483296	0.13959	0.23875	-5.08843
LRRC45	0.281029	4.214855	1.48317	0.139624	0.23875	-5.78478
OSMR	0.509159	6.336247	1.483205	0.139614	0.23875	-6.01267
NOD2	1.019517	2.930006	1.482785	0.139726	0.238895	-5.3893
C1QL1	-0.72799	0.851923	-1.48263	0.139766	0.238906	-5.30204
PPP2R5B	-0.2907	4.093667	-1.48267	0.139756	0.238906	-5.85799
ZBTB22	-0.24354	4.030301	-1.48242	0.139824	0.238916	-5.84144
ARRDC2	-0.3306	4.605831	-1.48233	0.139847	0.238916	-5.93064
RPL17	-0.40593	4.880662	-1.48229	0.139858	0.238916	-5.96698

RBM38	0.352202	5.665817	1.482476	0.139808	0.238916	-5.97718
CDV3	-0.2099	7.584749	-1.48251	0.139798	0.238916	-6.01587
FAM82A1	-0.37674	1.460806	-1.4821	0.139907	0.238971	-5.35144
COX15	-0.19442	5.767734	-1.48197	0.139942	0.239002	-6.0176
AXIN2	-0.7378	3.527415	-1.48181	0.139986	0.239022	-5.82884
CNOT6	0.239324	5.934571	1.481797	0.139989	0.239022	-6.00601
CCDC144B	-0.84565	0.703251	-1.48117	0.140156	0.239278	-5.29438
LTBP1	-0.61963	6.466863	-1.48111	0.140173	0.239278	-6.03293
IGSF11	-1.09083	0.0171	-1.47978	0.140525	0.239851	-5.22987
ANXA8	3.519056	4.098692	1.479585	0.140578	0.239911	-5.2175
RPL13P5	0.375549	0.630215	1.479071	0.140715	0.240116	-5.12478
TRPV3	1.055183	0.977598	1.478841	0.140777	0.240162	-5.09004
MPZL1	0.201074	7.547158	1.478861	0.140771	0.240162	-6.03021
LOC90110	-0.27562	3.114313	-1.47818	0.140954	0.240382	-5.66197
SNUPN	0.232418	4.051836	1.478237	0.140938	0.240382	-5.76776
C9orf125	-0.60506	4.537872	-1.47817	0.140957	0.240382	-5.95574
UEVLD	-0.22904	4.082104	-1.47783	0.141046	0.240504	-5.85473
TBP	0.186889	3.700537	1.477259	0.1412	0.240737	-5.70141
ARTN	1.358907	1.458793	1.476967	0.141278	0.240812	-5.1228
GHDC	-0.35508	4.536514	-1.47701	0.141265	0.240812	-5.93232
KLK7	2.601392	3.423311	1.476482	0.141408	0.241004	-5.24318
TMTC3	0.437311	6.050862	1.473946	0.142089	0.242135	-6.01351
SFXN5	-0.29808	3.706546	-1.47319	0.142293	0.242453	-5.80209
PARD6A	-0.575	1.02645	-1.47227	0.14254	0.242844	-5.32103
SEMA3C	-0.65799	6.263334	-1.47206	0.142598	0.242914	-6.04803
CXXC5	-0.46325	4.107651	-1.47143	0.142766	0.243141	-5.89834
BLMH	0.427467	5.209132	1.471472	0.142756	0.243141	-5.93899
ZNF689	-0.27593	3.103921	-1.47125	0.142816	0.243167	-5.67012
UCHL5	0.239668	4.768781	1.471253	0.142815	0.243167	-5.90474
STX5	-0.1908	5.29546	-1.47115	0.142842	0.243182	-6.00293
WHAMM	-0.20187	4.102697	-1.47069	0.142967	0.243335	-5.86452
CCAR1	0.193908	6.347089	1.470733	0.142955	0.243335	-6.04296
TRERF1	0.478472	5.035488	1.469731	0.143226	0.243717	-5.91394
NUP88	0.208364	5.514951	1.469792	0.14321	0.243717	-5.99349
TIPARP	-0.39681	5.807827	-1.46963	0.143253	0.243733	-6.04406
FOXA3	-1.40567	1.446835	-1.46952	0.143284	0.243756	-5.51166
XPNPEP1	0.249888	6.160837	1.469008	0.143422	0.243961	-6.03631
UQCC	-0.23418	5.128437	-1.46831	0.143612	0.244195	-5.99624
STK11	-0.27399	5.814439	-1.46837	0.143596	0.244195	-6.04238
RXRA	-0.36134	6.879483	-1.46843	0.14358	0.244195	-6.04864
VSNL1	1.998537	3.930713	1.468193	0.143643	0.244218	-5.43103
HSF1	0.289021	6.83758	1.468057	0.14368	0.244251	-6.05447
MED25	-0.21989	6.446213	-1.46793	0.143715	0.24428	-6.05512
NINJ1	0.390815	5.292919	1.467432	0.14385	0.24448	-5.9577
ARHGAP23	0.598805	6.957482	1.467113	0.143937	0.244598	-6.05351
TMEM117	0.389298	3.803846	1.466977	0.143973	0.244631	-5.70214
MR1	0.38727	3.19457	1.466748	0.144036	0.244707	-5.56972
ZNF438	-0.29714	3.220096	-1.4665	0.144103	0.244791	-5.70725
FAM83F	0.845091	3.926919	1.466314	0.144154	0.244821	-5.63074
GPR116	-0.48509	4.690438	-1.46631	0.144155	0.244821	-5.97792
VHL	0.27951	5.517442	1.466234	0.144175	0.244825	-5.99285
PDK1	-0.33797	5.069062	-1.46575	0.144307	0.245018	-6.00289
STXBP2	0.31518	6.268107	1.46568	0.144326	0.245021	-6.04308
RBM10	0.175789	5.953338	1.464458	0.144659	0.245557	-6.03577
DCI	-0.26205	4.836192	-1.46386	0.144824	0.245806	-5.97636
JARID2	0.407669	5.437169	1.463676	0.144873	0.24586	-5.97753
DOCK11	-0.52236	4.191652	-1.46315	0.145016	0.246072	-5.93035
SLC36A4	-0.37378	3.321763	-1.46262	0.145162	0.24629	-5.75093

USMG5	-0.28267	5.463846	-1.4623	0.145249	0.246409	-6.03388
SIDT1	-0.75531	1.997169	-1.4622	0.145275	0.246409	-5.53306
CDCA7L	0.448846	4.953062	1.462159	0.145288	0.246409	-5.91691
ARHGEF5	0.513965	5.396182	1.462105	0.145302	0.246409	-5.96427
MUC15	-1.26269	0.683	-1.46169	0.145415	0.24654	-5.38506
SCAND1	0.307485	5.339651	1.461711	0.14541	0.24654	-5.9791
PISD	0.200253	4.785441	1.461476	0.145475	0.246612	-5.92544
TMEM71	-0.63529	0.10412	-1.46119	0.145554	0.246716	-5.20383
RIMBP3	0.685799	0.238831	1.461086	0.145581	0.246733	-5.06326
TM9SF1	0.211331	6.218935	1.46101	0.145602	0.246738	-6.05188
PDCD2	0.247903	5.069205	1.460828	0.145652	0.246793	-5.95627
CPSF1	0.259715	6.9748	1.460438	0.145759	0.246944	-6.06587
LOC100128292	0.391069	0.029151	1.460274	0.145804	0.246991	-5.07113
SLC35A1	-0.35362	3.984543	-1.46018	0.145831	0.247006	-5.88078
PEX2	-0.2094	5.168542	-1.46001	0.145877	0.247053	-6.00968
SPNS2	-0.7947	5.158993	-1.45977	0.145943	0.247136	-6.0448
ZNF275	-0.31241	5.124617	-1.45941	0.146041	0.247272	-6.01485
KPNA4	0.242071	7.162946	1.458694	0.146238	0.247576	-6.0667
AVEN	-0.2675	3.18951	-1.45798	0.146436	0.24788	-5.70561
GDI2	0.219951	7.875299	1.457865	0.146467	0.247902	-6.05384
RPTOR	-0.21028	6.002473	-1.45775	0.146499	0.247915	-6.0623
UFM1	-0.25632	6.173475	-1.45771	0.14651	0.247915	-6.06731
ITPKA	-0.67874	2.171282	-1.45745	0.146582	0.248008	-5.55417
PADI2	1.329448	2.800231	1.456851	0.146746	0.248255	-5.35369
ORAI1	0.284367	4.958967	1.45669	0.14679	0.2483	-5.94488
YIPF6	-0.23477	4.129879	-1.45651	0.146841	0.248355	-5.89356
FAM78A	0.509447	2.319251	1.456105	0.146952	0.248513	-5.3995
CPNE5	-0.54786	2.269751	-1.45565	0.147077	0.248663	-5.56297
KBTBD7	-0.28907	3.360878	-1.45571	0.147061	0.248663	-5.7491
BACH2	-0.63323	2.177142	-1.45556	0.147103	0.248679	-5.56394
ACAD11	-0.3326	3.891198	-1.45525	0.147189	0.248792	-5.87155
TERF2IP	-0.22204	5.770945	-1.45487	0.147294	0.24894	-6.05826
WDR78	-0.54858	0.028091	-1.45464	0.147357	0.248987	-5.18846
BAT2	0.192507	8.963576	1.454692	0.147343	0.248987	-6.02348
RBM19	0.282508	5.292968	1.454276	0.147458	0.249127	-5.98755
ARHGEF35	0.528582	5.113548	1.454067	0.147516	0.249185	-5.93979
ERCC3	0.165346	5.350656	1.454023	0.147528	0.249185	-6.00442
CORO1B	0.264765	6.590767	1.453532	0.147664	0.249385	-6.07234
BNIPL	1.669514	0.993693	1.453402	0.1477	0.249405	-5.05893
IQCH	0.483063	0.714512	1.453359	0.147712	0.249405	-5.15748
COX5A	-0.29387	6.453632	-1.45295	0.147824	0.249564	-6.07678
PLS3	0.434946	7.891027	1.452269	0.148014	0.249856	-6.06579
YPEL3	-0.35576	5.097287	-1.45183	0.148136	0.25003	-6.0267
GYG1	-0.26916	5.384021	-1.45152	0.148223	0.250147	-6.0437
OPTN	-0.27967	6.33283	-1.45138	0.148262	0.250183	-6.07869
DHX33	0.293934	4.376424	1.451045	0.148354	0.250309	-5.86308
FADS2	0.826813	4.978224	1.450976	0.148374	0.250311	-5.88574
TJP2	-0.40073	7.006545	-1.45091	0.148392	0.250313	-6.07077
TESK1	-0.25164	5.799292	-1.45006	0.148629	0.250681	-6.06744
IL7	0.700031	1.444109	1.449924	0.148667	0.250714	-5.24045
NEO1	-0.50582	6.390515	-1.44978	0.148705	0.25075	-6.08048
ANKIB1	0.317202	6.535572	1.449588	0.14876	0.250812	-6.07557
JHDM1D	-0.34743	5.838938	-1.449	0.148925	0.251059	-6.07337
C3orf63	-0.21354	6.401853	-1.44836	0.149103	0.251328	-6.08312
LOC84740	2.174272	2.347763	1.448032	0.149194	0.251453	-5.1788
IL1F5	2.413571	0.765075	1.447118	0.14945	0.251853	-5.06209
PPP1R7	-0.18927	5.37954	-1.44639	0.149653	0.252166	-6.04528
CCDC85B	0.372022	4.80179	1.446258	0.149691	0.252167	-5.92916

PQBP1	0.256307	5.054523	1.446275	0.149686	0.252167	-5.97477
ATP1A1	0.330247	9.877657	1.446179	0.149713	0.252174	-6.00828
PCDHB13	0.668046	1.786772	1.445703	0.149846	0.252359	-5.30546
SLC43A2	0.395599	5.028752	1.445659	0.149858	0.252359	-5.95616
SND1	-0.26294	7.613351	-1.44547	0.149912	0.252419	-6.06741
FABP5	1.506668	4.004563	1.444998	0.150044	0.25261	-5.56044
PGLYRP3	2.151523	0.696562	1.444009	0.150321	0.253003	-5.06589
FAM122C	-0.36246	1.545255	-1.44407	0.150305	0.253003	-5.41656
RPUSD2	0.265052	2.905669	1.443974	0.150331	0.253003	-5.56276
RHBDL1	-0.62222	0.360538	-1.4437	0.150409	0.253103	-5.26214
AQP5	-1.38291	0.433136	-1.44336	0.150503	0.253212	-5.37704
SLC5A1	1.952086	3.443125	1.443338	0.15051	0.253212	-5.3813
ATP6V1D	-0.25251	5.51338	-1.4431	0.150578	0.253295	-6.06293
LOC613037	-0.57774	2.260643	-1.44231	0.150798	0.253605	-5.58512
ZNF460	-0.34975	4.306836	-1.44238	0.15078	0.253605	-5.95422
C3orf10	-0.22737	6.143422	-1.44183	0.150934	0.253803	-6.08901
FAM22G	-0.37562	0.181785	-1.44155	0.151015	0.253878	-5.20718
ELOVL4	-0.87371	1.495627	-1.44159	0.151003	0.253878	-5.49434
THYN1	-0.30717	3.973255	-1.44057	0.151289	0.254308	-5.90144
HOMER3	0.808801	4.013411	1.440394	0.15134	0.254363	-5.70338
ABCF3	0.236633	5.601574	1.440198	0.151395	0.254426	-6.04121
MAP3K14	-0.33615	4.702803	-1.44006	0.151434	0.254459	-6.00393
ANKS6	-0.35103	5.13063	-1.43985	0.151493	0.254528	-6.04578
ZNF614	-0.50026	3.194576	-1.4396	0.151565	0.254618	-5.78152
SERPINE1	0.78489	7.109331	1.439182	0.151682	0.254786	-6.09301
EDAR	1.448952	1.93822	1.438896	0.151763	0.254891	-5.22642
C20orf114	-1.75197	0.596343	-1.43845	0.151889	0.255071	-5.46128
LOC153684	0.505669	0.783565	1.437046	0.152288	0.25571	-5.18831
KGFLP2	-0.45557	1.562772	-1.43668	0.152393	0.255856	-5.44514
IFT81	0.349185	3.409649	1.436478	0.152449	0.25592	-5.66413
ZNF304	-0.30091	3.750028	-1.43632	0.152494	0.255964	-5.86621
GSDMA	1.364154	0.263677	1.435905	0.152612	0.256115	-5.07431
SIRPA	0.567166	5.804615	1.435874	0.152621	0.256115	-6.03909
PINX1	0.426612	2.733995	1.435742	0.152658	0.256148	-5.51243
TIMM13	0.308856	5.194468	1.435192	0.152815	0.25638	-6.00085
PCP4L1	-1.06048	0.25025	-1.43473	0.152945	0.256567	-5.32517
LOXL3	0.467513	1.917378	1.434444	0.153028	0.256644	-5.36968
LRRC37A	-0.32233	3.819476	-1.43449	0.153014	0.256644	-5.88349
ADAT3	0.336431	2.996496	1.434289	0.153072	0.256687	-5.58056
VDAC3	0.355734	6.194643	1.433345	0.153341	0.257108	-6.08445
KIAA1462	-0.4974	4.586097	-1.43302	0.153434	0.257233	-6.01662
STAB1	-0.50309	5.161738	-1.43213	0.153688	0.257628	-6.06982
SEPT7P2	-0.26599	2.586108	-1.43205	0.15371	0.257634	-5.61069
TM6SF1	-0.47714	1.350752	-1.43188	0.153759	0.257685	-5.42057
CROCCL1	-0.27932	4.101843	-1.43124	0.153943	0.257963	-5.9319
FNTA	0.270916	6.352027	1.430949	0.154026	0.25807	-6.09785
LMF2	0.259331	6.316078	1.430811	0.154065	0.258105	-6.09715
ZNF786	0.33033	3.293398	1.430665	0.154107	0.258145	-5.65099
CSTB	0.75738	8.045386	1.430421	0.154177	0.258231	-6.0995
KTELC1	0.239496	4.345657	1.430267	0.154221	0.258243	-5.89617
OPLAH	-0.43593	4.979335	-1.43029	0.154213	0.258243	-6.05254
EPHX3	1.747484	2.373103	1.430176	0.154247	0.258251	-5.26937
PHF19	0.315752	5.183662	1.430121	0.154263	0.258251	-6.00673
MTRF1L	-0.22029	3.273179	-1.42979	0.154357	0.258377	-5.75586
ATP6V1E1	-0.21529	6.15449	-1.42953	0.154433	0.258473	-6.10649
AIM2	1.441873	1.117069	1.429124	0.154549	0.258637	-5.13276
ORAOV1	0.617887	4.311398	1.42891	0.15461	0.258709	-5.81939
KCNN3	-0.56553	1.132551	-1.42868	0.154678	0.258772	-5.3982

HNRNPH1	0.183659	7.24337	1.428649	0.154685	0.258772	-6.10765
ZNF184	0.248061	3.374453	1.428345	0.154773	0.258877	-5.68757
CEPT1	-0.19188	4.631115	-1.4283	0.154785	0.258877	-5.99681
KLRK1	-0.69251	0.047139	-1.42817	0.154823	0.258879	-5.24716
VNN1	1.970994	2.812613	1.428214	0.15481	0.258879	-5.30145
EHBP1L1	-0.29376	6.774519	-1.4281	0.154844	0.258883	-6.10915
MBLAC2	-0.28251	3.197598	-1.42801	0.154869	0.258892	-5.75564
LOC728190	0.435504	3.654096	1.427334	0.155063	0.259187	-5.71476
ATXN2	-0.20464	5.981708	-1.42716	0.155113	0.259239	-6.1051
VPS26A	0.188019	6.332263	1.427039	0.155148	0.259267	-6.10522
RAB3IP	0.452583	5.047566	1.426805	0.155216	0.25932	-5.97873
CMAS	0.283604	5.447222	1.426801	0.155217	0.25932	-6.04242
TPRN	-0.42804	4.809876	-1.4262	0.155389	0.259576	-6.04152
BCAR3	-0.42203	4.505122	-1.42613	0.15541	0.259579	-6.00906
AMZ2	0.238251	5.544795	1.425728	0.155526	0.259743	-6.05669
EYA2	-1.01518	2.91679	-1.42526	0.155661	0.259937	-5.83895
BTBD11	1.491746	3.729068	1.424722	0.155816	0.260166	-5.53738
MUC5B	-1.61755	3.058085	-1.42447	0.155888	0.260255	-5.94575
FLJ42709	-0.53504	0.946739	-1.4243	0.155937	0.260305	-5.37159
TUBA1A	-0.46267	6.445978	-1.42407	0.156004	0.260385	-6.11691
SLC30A5	-0.18039	5.56542	-1.42393	0.156044	0.260421	-6.08929
GJD3	0.449849	2.89966	1.423588	0.156144	0.260502	-5.55914
TTC1	-0.20663	5.00189	-1.4236	0.156142	0.260502	-6.04586
SHC1	0.265997	7.430072	1.423574	0.156148	0.260502	-6.11283
LIMS3-LOC440895	-0.72663	0.552362	-1.42315	0.15627	0.260673	-5.33471
KCNAB2	0.54578	4.040303	1.42278	0.156378	0.260823	-5.78571
MAPK8IP3	-0.28737	6.11354	-1.42251	0.156458	0.260925	-6.11679
FAM19A5	0.817719	1.23558	1.42222	0.15654	0.261031	-5.23399
EREG	1.730331	3.041496	1.422122	0.156569	0.261047	-5.38763
HIRA	-0.22013	5.627873	-1.42145	0.156763	0.26134	-6.09853
ATP7B	-0.73631	3.611848	-1.4213	0.156808	0.261384	-5.92588
C6orf203	-0.27475	2.737972	-1.42106	0.156877	0.261467	-5.65866
LAP3	0.299176	6.248085	1.420681	0.156987	0.26162	-6.1072
EIF2B5	0.222756	5.847361	1.420441	0.157057	0.261705	-6.08926
DOCK7	-0.26046	5.611386	-1.42026	0.157109	0.261761	-6.10118
DNPEP	0.194717	5.947939	1.42009	0.157159	0.261812	-6.09766
NUP153	0.239834	6.812666	1.419832	0.157234	0.261906	-6.12346
REEP3	-0.30057	5.433671	-1.41975	0.157258	0.261916	-6.09355
CNOT10	0.207358	4.750976	1.419574	0.157309	0.261969	-5.97961
CD52	0.718704	2.435026	1.418468	0.157631	0.262459	-5.44223
TMEM45A	1.121285	4.065382	1.418369	0.157659	0.262459	-5.68073
TBCD	-0.21359	6.139631	-1.41841	0.157646	0.262459	-6.12178
C17orf61	-0.32823	3.535701	-1.41782	0.15782	0.262632	-5.84984
NIPSNAP1	0.295133	5.310525	1.417858	0.157809	0.262632	-6.03948
SPTLC2	-0.31596	6.420856	-1.41784	0.157813	0.262632	-6.12659
CTNNBIP1	-0.40686	5.454974	-1.4177	0.157854	0.262658	-6.10322
IPCEF1	-0.53522	1.212323	-1.4176	0.157883	0.262675	-5.42282
SBDSP1	-0.23951	3.997023	-1.41727	0.15798	0.262805	-5.92831
PIWIL4	-0.76016	1.637279	-1.41703	0.158049	0.262857	-5.52675
MXRA5	0.656966	7.356796	1.417082	0.158035	0.262857	-6.12707
LSP1	-0.5173	4.901049	-1.41674	0.158135	0.262969	-6.07216
BAIAP2L1	0.438614	6.921451	1.416575	0.158183	0.262986	-6.1272
CELSR1	0.622285	7.156977	1.416574	0.158183	0.262986	-6.12757
SERBP1	0.190752	8.384337	1.416222	0.158286	0.263126	-6.09789
CAMK2N2	0.782854	0.560241	1.416025	0.158344	0.263191	-5.15355
PARL	0.25713	5.272544	1.415884	0.158385	0.263228	-6.04207
RWDD2B	-0.29408	3.632495	-1.41574	0.158428	0.263267	-5.86887
AP3D1	-0.18004	7.878412	-1.41462	0.158756	0.263782	-6.10562

PAPD7	0.246557	5.689634	1.414497	0.158791	0.263808	-6.08421
FAM76B	0.229577	4.210891	1.414344	0.158836	0.263852	-5.89252
ELTD1	-0.45783	3.903675	-1.41428	0.158856	0.263853	-5.94919
PITX2	1.561554	1.503962	1.414032	0.158927	0.263941	-5.19332
WEE1	-0.31451	6.144481	-1.41339	0.159117	0.264224	-6.1306
DEPDC5	-0.17868	4.348476	-1.41306	0.159212	0.26435	-5.98068
KDM4A	0.223223	5.772832	1.411753	0.159597	0.264958	-6.09609
ERLEC1	-0.21366	5.877031	-1.4115	0.159672	0.265052	-6.12367
HOPX	-0.86004	4.459821	-1.41118	0.159765	0.265174	-6.06591
ITGA11	0.928345	3.960572	1.410677	0.159914	0.265389	-5.70386
MFSD9	-0.2944	3.736986	-1.41043	0.159986	0.265475	-5.89425
LRRC8B	0.296863	5.591056	1.410373	0.160003	0.265475	-6.07745
MBOAT1	-0.39807	4.131944	-1.41009	0.160087	0.265582	-5.97961
C8orf37	0.377153	1.851187	1.409543	0.160248	0.265818	-5.4067
OVOL2	0.700979	2.971832	1.409169	0.160358	0.265938	-5.54184
ALDOA	0.311349	10.10766	1.409179	0.160355	0.265938	-6.05206
GPR75	-0.36826	1.358408	-1.40898	0.160416	0.265991	-5.43549
MAP3K1	0.322933	5.57471	1.408811	0.160464	0.265991	-6.07596
DYRK2	0.351599	5.797493	1.408838	0.160456	0.265991	-6.09298
STOML2	0.276419	5.815422	1.408802	0.160467	0.265991	-6.0998
C11orf58	-0.17691	6.763627	-1.40872	0.160491	0.266	-6.1379
GLRX2	0.24598	2.62648	1.408036	0.160693	0.266303	-5.56073
CYBB	0.606262	4.531268	1.407953	0.160718	0.266313	-5.89774
CG030	-0.38702	1.830375	-1.40781	0.160759	0.266318	-5.51823
USP33	-0.17456	5.80752	-1.40787	0.160741	0.266318	-6.12444
C14orf109	0.227563	3.477769	1.407704	0.160791	0.26634	-5.74187
NCRNA00182	-0.32398	1.929168	-1.40692	0.161024	0.266693	-5.52827
PTOV1	-0.20949	6.409215	-1.40675	0.161074	0.266745	-6.14186
MRPL28	0.296965	5.116002	1.406518	0.161143	0.266827	-6.03373
ZFAND2A	0.434103	3.67302	1.406201	0.161236	0.266951	-5.7486
HOXA7	1.338938	2.293538	1.405765	0.161366	0.267006	-5.34483
SPRY3	-0.4096	1.581919	-1.406	0.161297	0.267006	-5.48259
C2orf68	0.210934	4.798042	1.405771	0.161364	0.267006	-6.00445
NDUFC1	-0.26825	4.564347	-1.40578	0.161362	0.267006	-6.02846
ARPC1A	0.311417	7.085649	1.405877	0.161332	0.267006	-6.14277
FAM3C	-0.30605	6.372997	-1.40564	0.161403	0.267036	-6.14352
ADA	-0.46569	3.738504	-1.40411	0.161857	0.267755	-5.93126
ARFRP1	0.248236	4.512644	1.403737	0.161968	0.267908	-5.9607
RPL23	0.291641	8.857961	1.40335	0.162083	0.268067	-6.10278
EFTUD1	0.235212	4.704884	1.402973	0.162196	0.268178	-5.99294
COG6	-0.2583	4.831482	-1.40299	0.16219	0.268178	-6.06127
LPCAT2	0.414485	5.512894	1.402932	0.162208	0.268178	-6.07007
GNG2	-0.43009	3.450118	-1.40274	0.162264	0.268239	-5.87408
KCNG1	1.313492	1.084215	1.402613	0.162303	0.268271	-5.18186
FAM43A	0.553062	4.817657	1.402233	0.162416	0.268395	-5.96878
EEA1	-0.31455	6.022396	-1.40223	0.162416	0.268395	-6.14368
C17orf80	-0.21204	4.455464	-1.40214	0.162444	0.26841	-6.01403
C19orf33	0.97384	4.988078	1.400975	0.162791	0.268951	-5.92919
NCOA3	0.274958	7.007562	1.400005	0.163081	0.269398	-6.15122
C17orf97	-0.49745	1.766036	-1.39992	0.163106	0.269408	-5.53736
SIX4	0.88348	3.361114	1.399626	0.163195	0.269492	-5.60293
ZNF653	-0.19754	2.600239	-1.39962	0.163196	0.269492	-5.64533
C2orf77	-0.50609	1.01104	-1.39944	0.16325	0.269519	-5.40951
BTBD6	-0.22849	5.456256	-1.39944	0.163251	0.269519	-6.1192
ZBTB37	-0.30912	0.81305	-1.39921	0.163318	0.269587	-5.35087
CD6	0.675838	2.155375	1.399171	0.163331	0.269587	-5.42609
TMED1	0.210983	3.85322	1.398912	0.163408	0.269652	-5.84114
TMEM184A	0.759263	5.298964	1.398963	0.163393	0.269652	-6.01314

SLC44A4	-1.56614	3.608182	-1.39881	0.163439	0.26967	-6.04588
NDUFA3	-0.33923	4.80759	-1.39866	0.163483	0.269711	-6.07247
DDX23	0.14917	6.758446	1.398245	0.163608	0.269886	-6.15373
MGC2752	-0.23243	4.595551	-1.39768	0.163777	0.270101	-6.04023
SLC12A4	-0.32998	5.365134	-1.3977	0.163772	0.270101	-6.12175
HELB	0.449189	2.366777	1.397617	0.163797	0.270101	-5.49745
SERPING1	-0.58932	7.105322	-1.39728	0.163897	0.270235	-6.14052
RDBP	0.241142	5.74917	1.397173	0.16393	0.270257	-6.11333
SGK494	0.416526	2.409382	1.396766	0.164052	0.270365	-5.51081
MECR	-0.21091	3.579119	-1.39687	0.16402	0.270365	-5.86647
RPA2	0.241134	4.855437	1.396761	0.164054	0.270365	-6.02109
YWHAQ	0.259952	8.767506	1.396641	0.164089	0.270393	-6.11425
PDGFC	-0.49942	3.821269	-1.3964	0.164162	0.270481	-5.96596
ARPC2	0.209733	8.010783	1.396104	0.164251	0.270595	-6.13733
UBTD1	0.350072	3.40268	1.394813	0.164639	0.271203	-5.72137
GPR157	0.49955	2.969884	1.394476	0.164741	0.271243	-5.60306
PIR	0.637077	3.72512	1.394499	0.164734	0.271243	-5.73698
SIRT5	-0.24886	3.705115	-1.39455	0.164718	0.271243	-5.90372
ECHS1	-0.27623	6.308688	-1.39466	0.164687	0.271243	-6.15826
TP53	0.547042	5.899604	1.394359	0.164776	0.271268	-6.1069
GFOD1	-0.46046	3.18255	-1.3942	0.164825	0.271317	-5.83308
KCNE3	1.417513	4.398303	1.392674	0.165285	0.271977	-5.72044
RGS14	0.414314	3.876965	1.392769	0.165256	0.271977	-5.81606
BSPRY	-0.61578	3.376487	-1.39273	0.165267	0.271977	-5.89687
SCN9A	-0.99874	1.458773	-1.39243	0.165359	0.272067	-5.57634
TLN2	-0.51524	4.887365	-1.39217	0.165438	0.272164	-6.10345
ERN2	-1.53433	1.786701	-1.39202	0.165481	0.272204	-5.71365
FMNL3	0.334106	5.326323	1.39164	0.165597	0.272363	-6.07389
SFMBT1	-0.33679	4.121264	-1.39141	0.165667	0.272445	-5.99761
TIGD2	0.351757	2.82102	1.389991	0.166097	0.273088	-5.60349
ATF5	0.384302	4.929775	1.390021	0.166088	0.273088	-6.02316
AKAP7	-0.46294	2.591884	-1.38922	0.16633	0.27344	-5.70886
MSN	0.52707	8.707419	1.388593	0.166521	0.273709	-6.1354
ZNF687	0.212003	6.06701	1.388554	0.166533	0.273709	-6.14698
H19	1.098314	6.569586	1.387965	0.166712	0.273971	-6.12895
CDH11	0.702059	5.146917	1.387727	0.166785	0.274026	-6.01818
PCCB	-0.24712	5.483247	-1.38776	0.166776	0.274026	-6.13819
FITM2	-0.34024	1.488456	-1.38766	0.166806	0.274029	-5.48066
ATP9A	-0.43164	6.997014	-1.38714	0.166963	0.274255	-6.15985
CHCHD1	0.235874	4.523492	1.386798	0.167067	0.274393	-5.98721
ANKRD23	0.361729	1.364288	1.386375	0.167196	0.27453	-5.36158
TLR5	-0.46857	2.807135	-1.38623	0.16724	0.27453	-5.76148
SDHAP1	0.350139	3.683166	1.386314	0.167215	0.27453	-5.79199
CREB5	-0.61372	3.489976	-1.3862	0.167248	0.27453	-5.93539
CASC4	-0.26355	6.758882	-1.3863	0.167219	0.27453	-6.16793
A1BG	0.568409	1.053971	1.386006	0.167309	0.27458	-5.28768
FLJ35776	0.459306	2.210948	1.385972	0.167319	0.27458	-5.48576
HLA-DMB	0.66262	4.27905	1.384983	0.167621	0.274913	-5.8656
ARHGEF19	0.604424	4.362373	1.385107	0.167583	0.274913	-5.89414
SLC24A3	-0.6321	3.323831	-1.38506	0.167597	0.274913	-5.91221
MRPL10	-0.19152	5.413124	-1.38506	0.167596	0.274913	-6.1337
DICER1	-0.24409	6.793549	-1.38492	0.167639	0.274913	-6.16958
FOXJ3	-0.18219	6.279045	-1.38493	0.167638	0.274913	-6.1703
GUK1	-0.2452	6.892009	-1.38472	0.1677	0.274979	-6.16828
PTPN20B	-1.08043	-0.19864	-1.38422	0.167853	0.27519	-5.32198
EPRS	0.225085	7.212136	1.384174	0.167868	0.27519	-6.17054
DNAJC8	0.177182	5.845291	1.383646	0.168029	0.275423	-6.14293
CUL9	-0.32242	5.600025	-1.3829	0.168257	0.275765	-6.15525

STX16	0.204038	6.436853	1.382516	0.168375	0.275925	-6.16962
LY86	0.708594	0.757307	1.382171	0.168481	0.276066	-5.23551
EDNRA	0.589321	3.72482	1.381979	0.16854	0.27613	-5.76112
SIM2	-0.76078	3.9308	-1.38171	0.168621	0.276221	-6.03353
IRF2BP1	-0.23483	5.346851	-1.38167	0.168635	0.276221	-6.13629
CHMP4C	0.570257	3.899701	1.381357	0.168731	0.276346	-5.80073
KLK11	-1.15592	4.207303	-1.38043	0.169016	0.276781	-6.10573
GGT1	-0.69498	3.114393	-1.38007	0.169127	0.27693	-5.87202
NUDT16L1	-0.20202	3.707622	-1.37976	0.16922	0.277031	-5.91563
LRP1	-0.38785	8.952726	-1.37974	0.169228	0.277031	-6.11086
SLC9A7	-0.46535	2.865135	-1.37958	0.169275	0.277046	-5.77995
PPP6C	-0.15477	6.234276	-1.37958	0.169277	0.277046	-6.17637
ATP5I	-0.30462	5.326504	-1.37943	0.169323	0.277088	-6.14231
C10orf81	2.345778	1.623543	1.379268	0.169373	0.277136	-5.15934
XKRX	0.834734	0.466899	1.379141	0.169412	0.277136	-5.18512
APLP2	-0.2484	9.034118	-1.37914	0.16941	0.277136	-6.11307
SPA17	0.42754	2.355407	1.379065	0.169435	0.277142	-5.52381
CNOT7	0.220874	6.369055	1.378816	0.169512	0.277235	-6.17232
SGMS1	-0.25553	5.218639	-1.37874	0.169534	0.27724	-6.13146
FAM10A4	-0.20365	3.469474	-1.37796	0.169776	0.277602	-5.86797
PCDHGB1	1.12582	0.752057	1.376232	0.170309	0.278441	-5.19336
C6orf130	-0.29291	3.775168	-1.3755	0.170534	0.278777	-5.95084
ZSCAN16	-0.49246	1.697938	-1.37527	0.170607	0.278864	-5.55591
IFIT5	0.336603	4.513171	1.375029	0.170681	0.278951	-5.98733
POLR3B	-0.24518	4.038648	-1.37479	0.170756	0.279041	-5.99499
P2RY2	0.855005	3.274875	1.374375	0.170883	0.279217	-5.62838
SLC17A5	-0.41091	4.326479	-1.37377	0.171072	0.279459	-6.0576
SDHAF2	0.188699	4.996272	1.373822	0.171055	0.279459	-6.07564
BATF2	0.993354	2.872395	1.373483	0.17116	0.279571	-5.53089
C17orf75	0.265087	3.481113	1.373342	0.171204	0.279609	-5.78293
KIAA0907	0.255948	5.699668	1.373111	0.171275	0.279694	-6.14108
LILRA6	0.686456	0.922734	1.371938	0.17164	0.28021	-5.27342
ADAMTS4	-0.54182	4.796647	-1.37177	0.171692	0.28021	-6.12693
FTO	-0.23325	5.738036	-1.37188	0.171657	0.28021	-6.17317
DCTD	-0.19662	5.89412	-1.37187	0.171662	0.28021	-6.17832
RPS6KA3	0.269313	6.649408	1.371776	0.17169	0.28021	-6.1875
ATL3	-0.31075	5.458301	-1.37168	0.171718	0.280221	-6.16208
ELP4	0.228175	3.770933	1.371389	0.17181	0.280338	-5.85771
NDUFA4L2	1.049456	5.077934	1.371212	0.171865	0.280395	-5.98085
TOMM7	-0.28455	5.836973	-1.37113	0.171891	0.280404	-6.18001
LRP4	-0.54759	4.317695	-1.37102	0.171927	0.28043	-6.07599
HAS3	1.361713	4.947183	1.370624	0.172049	0.280596	-5.89074
IFT46	-0.25906	3.568118	-1.37034	0.172138	0.280708	-5.91019
TRAM2	0.311366	6.069434	1.369946	0.17226	0.280875	-6.16771
HSPC159	-0.38776	5.090078	-1.36937	0.17244	0.281135	-6.14322
ZNF548	-0.29363	3.396389	-1.36913	0.172516	0.281226	-5.88187
SLC39A9	-0.18718	6.844464	-1.36883	0.172609	0.281346	-6.19149
ZNF814	-0.38476	3.930642	-1.36841	0.172738	0.281523	-6.00483
FAM35B	-0.22804	2.485273	-1.36816	0.172817	0.281585	-5.66955
DNHD1	-0.37971	3.472118	-1.36817	0.172815	0.281585	-5.9162
TMEM93	0.266225	4.07979	1.367581	0.172998	0.281848	-5.92279
GOSR2	-0.18034	5.545443	-1.36751	0.17302	0.281852	-6.16596
FKBP2	-0.27149	5.416485	-1.36723	0.173109	0.281963	-6.16303
AUTS2	-0.57032	5.234277	-1.36643	0.173357	0.282334	-6.16945
DGAT1	0.423211	5.515527	1.366135	0.173451	0.282454	-6.11955
ZNF180	-0.2367	3.13624	-1.3659	0.173525	0.282543	-5.81561
UST	-0.61681	3.445919	-1.36539	0.173685	0.282769	-5.96012
ZCWPW1	-0.51313	0.96696	-1.36413	0.17408	0.28338	-5.45211

FAM193B	-0.25455	4.808181	-1.36385	0.174168	0.28349	-6.11224
GAB3	-0.42963	1.39137	-1.36338	0.174315	0.283696	-5.51166
ESYT3	-0.77613	0.771621	-1.36328	0.174348	0.283716	-5.45928
LOC150786	0.485344	1.151566	1.363123	0.174396	0.283762	-5.34308
NOTCH2	-0.33276	7.548259	-1.36164	0.174863	0.284488	-6.18385
UBAP1	-0.26335	6.230795	-1.36143	0.17493	0.284564	-6.20253
PLAC4	-0.63201	1.006177	-1.36135	0.174955	0.284572	-5.4773
SEC11A	0.209311	5.98092	1.361254	0.174985	0.284587	-6.17967
TDRKH	0.520616	3.167727	1.360893	0.175099	0.284706	-5.68162
THAP5	-0.22495	4.309288	-1.36096	0.175079	0.284706	-6.05221
DNAH17	1.25151	2.537822	1.360152	0.175333	0.285024	-5.45767
CEBPA	0.651811	5.098891	1.360145	0.175335	0.285024	-6.05408
PTPN7	0.668594	2.672712	1.359984	0.175386	0.285073	-5.5692
C1orf91	0.267351	2.873029	1.359627	0.175499	0.285224	-5.66949
DHX35	0.222798	4.329971	1.359514	0.175534	0.285248	-5.99323
ZNF780A	-0.27384	3.632009	-1.35911	0.175663	0.285357	-5.94067
KCTD1	0.541472	5.189008	1.359125	0.175658	0.285357	-6.08092
CDK18	-0.43302	5.159568	-1.35911	0.175662	0.285357	-6.16511
MUM1	-0.18802	4.901138	-1.35894	0.175714	0.285408	-6.12287
C14orf181	0.355304	0.988999	1.358678	0.175799	0.285512	-5.34088
C14orf73	-0.84785	0.312975	-1.35854	0.175841	0.285548	-5.39686
RAD51C	0.270637	3.254332	1.357568	0.17615	0.286017	-5.75296
C19orf29	0.193176	4.906588	1.356907	0.17636	0.286291	-6.08733
STYX	-0.23342	5.283315	-1.35695	0.176347	0.286291	-6.16476
DEM1	0.446186	2.413969	1.356649	0.176442	0.28639	-5.56282
CCNT2	-0.21708	5.714537	-1.35634	0.17654	0.286516	-6.19227
SIVA1	0.235623	5.434528	1.356157	0.176598	0.286577	-6.14207
ZFPL1	0.216787	4.905285	1.355676	0.176751	0.2867	-6.08576
C17orf62	0.204699	5.74655	1.355732	0.176733	0.2867	-6.17192
RPL35A	0.288031	8.272106	1.355661	0.176756	0.2867	-6.18696
TMEM87B	0.251869	6.509222	1.355706	0.176741	0.2867	-6.2066
RTN2	0.451988	2.765776	1.355254	0.176885	0.286876	-5.62481
WDR92	0.200265	3.091691	1.354671	0.177071	0.287131	-5.73546
FAM109A	-0.46314	4.11729	-1.35463	0.177084	0.287131	-6.0605
NME1-NME2	0.480284	1.432341	1.354461	0.177137	0.287185	-5.39777
BET1L	-0.21815	6.180788	-1.35438	0.177163	0.287194	-6.21046
LOC100130015	-0.3818	0.586356	-1.35428	0.177193	0.28721	-5.38652
LGALS7	2.712623	2.471654	1.354009	0.177281	0.287319	-5.25755
RBAK	0.206015	5.253731	1.353845	0.177334	0.28737	-6.12985
KLHL17	0.366836	2.825002	1.353094	0.177573	0.287725	-5.65177
WNT9A	-0.81285	0.510769	-1.35301	0.177599	0.287733	-5.44023
PCSK4	-0.52409	0.641404	-1.35271	0.177695	0.287811	-5.41474
CCND1	0.542398	8.418361	1.352669	0.177708	0.287811	-6.19319
CHTF8	-0.15921	6.478807	-1.3527	0.177698	0.287811	-6.21574
ZNF214	-0.59005	-0.07406	-1.35249	0.177766	0.28787	-5.31774
DR1	0.183376	5.879729	1.35195	0.177938	0.288116	-6.18759
KIAA1407	-0.3664	2.990534	-1.35146	0.178093	0.288314	-5.8279
TRIM33	-0.2034	6.478987	-1.35144	0.178102	0.288314	-6.21752
LOC642587	2.780111	1.542084	1.350566	0.178381	0.288665	-5.1928
LOC285033	-0.21077	1.443893	-1.35057	0.17838	0.288665	-5.50401
ARL2	0.288448	5.309303	1.350656	0.178352	0.288665	-6.13232
NEU3	-0.33676	2.360307	-1.35031	0.178461	0.288762	-5.68675
MCM3APAS	0.347948	1.099722	1.349901	0.178594	0.288935	-5.36993
KRT19	1.148937	8.798859	1.349851	0.178609	0.288935	-6.20071
IQCK	-0.3083	3.554555	-1.34972	0.178652	0.288971	-5.94239
TMEM79	0.858249	4.364624	1.349568	0.1787	0.289015	-5.89532
DOK6	-0.56063	0.518234	-1.34922	0.178811	0.28916	-5.40521
C9orf86	-0.21394	6.871702	-1.34904	0.178869	0.28922	-6.2173

B3GNT9	0.375645	4.386919	1.348652	0.178994	0.28931	-5.99259
ARG2	-0.3063	3.862715	-1.34861	0.179007	0.28931	-6.00898
POP4	0.328296	4.701378	1.348637	0.178999	0.28931	-6.05379
PSME4	0.200826	7.166123	1.348761	0.178959	0.28931	-6.21877
ZNF750	2.366926	3.610629	1.34814	0.179158	0.289521	-5.48542
NDUFV2	-0.24878	5.409094	-1.34806	0.179185	0.289531	-6.18715
SFI1	0.240533	4.248679	1.347826	0.179259	0.289617	-5.98876
AEBP2	0.317366	5.722531	1.347661	0.179312	0.289669	-6.17303
RENBP	0.649092	1.615639	1.347406	0.179394	0.289768	-5.41251
NAALADL1	-0.45853	0.337653	-1.34725	0.179444	0.289774	-5.36831
CSRP2	-0.57559	3.715996	-1.3472	0.17946	0.289774	-6.02868
ZBTB44	-0.23063	6.084032	-1.3473	0.179427	0.289774	-6.21812
C11orf57	-0.1961	4.827409	-1.34684	0.179576	0.289928	-6.13187
PLCB1	-0.47609	4.654451	-1.34676	0.179602	0.289937	-6.13985
ATG7	-0.2172	4.788104	-1.34653	0.179674	0.29002	-6.12979
GPR56	0.359296	8.053872	1.346254	0.179764	0.29011	-6.20712
AKR1C1	-1.19836	5.481962	-1.34623	0.179772	0.29011	-6.22172
NAGPA	-0.20676	3.789351	-1.34608	0.179821	0.290156	-5.97852
RABL2B	0.276285	3.661694	1.345824	0.179903	0.290254	-5.85862
NUDT14	0.329955	3.867884	1.34518	0.18011	0.290555	-5.89199
GSPT1	-0.17421	7.610675	-1.34507	0.180146	0.290579	-6.20806
GIMAP2	0.554168	2.086149	1.344944	0.180186	0.290611	-5.50397
DNAJC21	-0.21165	5.979047	-1.34473	0.180257	0.290664	-6.21821
SPPL3	0.270107	6.434671	1.344713	0.180261	0.290664	-6.21893
EGR3	-0.60269	3.783317	-1.34421	0.180423	0.290875	-6.04715
CD2AP	0.378697	6.932539	1.344178	0.180433	0.290875	-6.22669
SLC25A14	0.231872	2.567456	1.344036	0.180479	0.290916	-5.63817
STK36	-0.2853	4.443962	-1.34392	0.180515	0.29094	-6.09964
LOC147804	0.469032	2.73506	1.343414	0.18068	0.291172	-5.6309
ARHGAP12	-0.39313	5.604611	-1.34316	0.180761	0.291269	-6.21167
SLC35F2	0.461132	4.606997	1.343062	0.180794	0.291288	-6.02495
KRT7	1.91746	4.783144	1.342736	0.180899	0.291424	-5.77818
C10orf18	0.212135	6.872238	1.342411	0.181004	0.29156	-6.22948
MAP3K12	-0.36189	2.413817	-1.34185	0.181186	0.291617	-5.7169
MAP2	-0.67609	3.587905	-1.34193	0.181161	0.291617	-6.02764
MAPK7	-0.23886	4.694486	-1.34197	0.181146	0.291617	-6.12809
AIG1	-0.26549	4.892441	-1.34196	0.181151	0.291617	-6.15207
RAB11FIP5	-0.22095	5.554527	-1.34188	0.181176	0.291617	-6.20313
SUPT6H	-0.19182	7.546776	-1.3419	0.18117	0.291617	-6.21347
FBRSL1	-0.22185	6.320752	-1.34201	0.181134	0.291617	-6.22915
SEMA6A	-0.63508	4.295382	-1.34156	0.181281	0.291736	-6.12239
BCL6B	-0.4224	2.738035	-1.34145	0.181317	0.291761	-5.79633
YTHDF3	-0.18749	6.87054	-1.33936	0.181995	0.292818	-6.23058
SYAP1	-0.23034	5.520959	-1.33914	0.182064	0.292895	-6.20508
CCDC64	0.460848	4.574333	1.338903	0.182142	0.292987	-6.0253
MGAT1	-0.21287	6.934713	-1.33855	0.182256	0.293137	-6.23025
CD72	0.584392	0.55161	1.338404	0.182304	0.293181	-5.27802
C2orf18	0.198946	6.356769	1.33833	0.182328	0.293186	-6.227
NUP98	0.172075	7.349233	1.33751	0.182595	0.293581	-6.23031
GBP4	0.677182	5.157846	1.337172	0.182705	0.293724	-6.08937
H1F0	0.358738	7.443336	1.336649	0.182875	0.293964	-6.23267
RNF26	0.22333	5.798744	1.336517	0.182919	0.294	-6.20016
CHST12	0.248567	4.311662	1.336372	0.182966	0.294042	-6.01555
DAG1	-0.3233	7.908415	-1.336	0.183088	0.294204	-6.20764
COMMD1	-0.22847	3.578303	-1.33592	0.183114	0.294213	-5.95258
RCBTB1	-0.30128	4.720671	-1.3354	0.183284	0.294452	-6.14524
ZNF354B	-0.27721	2.454066	-1.33474	0.183499	0.294764	-5.7169
CDC16	-0.21164	5.868448	-1.33439	0.183613	0.294911	-6.22798

EXOG	-0.2258	2.624407	-1.33394	0.183762	0.295083	-5.7443
ACAA2	-0.58872	4.843464	-1.334	0.183741	0.295083	-6.18452
YY2	0.361184	0.267123	1.332964	0.18408	0.29556	-5.27274
DZIP1	-0.5801	3.214534	-1.33228	0.184303	0.295885	-5.95041
GIMAP4	-0.42285	3.531367	-1.33205	0.18438	0.295975	-5.98583
ANAPC4	-0.17596	4.331226	-1.3319	0.184427	0.296016	-6.08767
FBF1	0.364684	3.531056	1.331585	0.184532	0.296116	-5.83091
SIPA1L2	0.486886	5.909067	1.331594	0.184529	0.296116	-6.19742
TNFSF12-TNFSF13	-0.48344	2.924612	-1.33141	0.184589	0.296139	-5.86021
ACP5	0.475955	5.307787	1.331422	0.184586	0.296139	-6.13815
IFI44L	1.004933	4.446394	1.331215	0.184654	0.296209	-5.90467
WDR41	-0.22917	4.744903	-1.33089	0.18476	0.296346	-6.14732
SERPINB2	2.245796	2.793465	1.330811	0.184786	0.296354	-5.39443
ZNF235	-0.30505	1.874789	-1.33065	0.184838	0.296402	-5.61697
NFATC3	0.212172	5.574193	1.330368	0.184932	0.296519	-6.19147
SLC29A4	-0.69042	2.806397	-1.32971	0.185148	0.296832	-5.87791
ZBTB10	-0.56322	3.733184	-1.32962	0.185179	0.296847	-6.05161
SPRYD4	0.251949	3.73533	1.328969	0.185392	0.297098	-5.90054
CHUK	-0.18424	5.129112	-1.32903	0.185371	0.297098	-6.18449
YTHDC1	-0.12652	6.105891	-1.32895	0.185399	0.297098	-6.24061
FUZ	-0.54555	2.026611	-1.32877	0.185458	0.297158	-5.68765
TINF2	-0.21573	5.39282	-1.32844	0.185568	0.2973	-6.20987
MRPL43	-0.18728	5.124338	-1.32835	0.185595	0.29731	-6.18532
ATXN10	0.218133	6.221501	1.328197	0.185646	0.297358	-6.23494
SAMD9L	0.644851	5.450438	1.327783	0.185783	0.297542	-6.1416
TMEM128	-0.22079	4.058574	-1.32724	0.185964	0.297798	-6.05737
PACS1	-0.27834	7.520055	-1.32709	0.186013	0.297843	-6.2318
RCL1	0.256852	3.811474	1.326707	0.186138	0.297929	-5.91988
CHDH	-0.83235	2.89556	-1.32663	0.186165	0.297929	-5.92544
SLC43A3	0.527594	5.282305	1.326695	0.186142	0.297929	-6.13676
SERF2	-0.23118	7.835339	-1.32677	0.186116	0.297929	-6.2246
KREMEN1	-0.54694	6.321558	-1.3266	0.186173	0.297929	-6.24979
INADL	-0.24466	6.59451	-1.32635	0.186257	0.298029	-6.25037
ADD3	-0.3721	6.910525	-1.32605	0.186354	0.29815	-6.24457
PTRH1	0.336243	2.694451	1.325795	0.18644	0.298218	-5.66671
OSGIN1	-0.62094	3.048736	-1.32586	0.186418	0.298218	-5.92513
RASGEF1A	1.158898	1.375577	1.325677	0.186478	0.298242	-5.33929
B3GALT5	-1.09295	0.530687	-1.32556	0.186518	0.298242	-5.51014
HCFC1	0.19152	8.008591	1.325578	0.186511	0.298242	-6.23188
ATP5EP2	-0.29851	0.831447	-1.32523	0.186628	0.29828	-5.44883
FAM190A	-0.7521	1.138417	-1.32529	0.186606	0.29828	-5.56521
CHST2	0.857133	4.188977	1.325258	0.186617	0.29828	-5.8868
GOLGA8B	-0.42283	4.418403	-1.32537	0.186579	0.29828	-6.13585
AMN	1.072274	2.837349	1.324996	0.186704	0.298368	-5.57368
STXBP5	-0.30544	5.154534	-1.32493	0.186727	0.29837	-6.2019
RPS15A	0.269335	8.102852	1.324613	0.186831	0.298502	-6.23246
KPNA1	0.210448	6.747614	1.32453	0.186858	0.298512	-6.25253
WIBG	0.242762	4.124165	1.324459	0.186882	0.298515	-5.99542
ZNF816A	-0.4955	2.532405	-1.32394	0.187053	0.298721	-5.78636
MUDENG	-0.18945	5.452891	-1.324	0.187034	0.298721	-6.21832
PGAP2	0.271246	4.403298	1.32379	0.187103	0.298735	-6.04444
UQCR10	-0.20957	5.670239	-1.32379	0.187105	0.298735	-6.23277
RPS23	-0.25528	8.261994	-1.32372	0.187126	0.298735	-6.21401
PHPT1	-0.2491	5.119812	-1.32334	0.187253	0.298903	-6.19638
GPRC5D	0.713306	0.269066	1.322707	0.187463	0.299204	-5.24469
INO80E	0.219829	4.756707	1.322496	0.187533	0.299282	-6.11002
ST6GALNAC5	-0.58201	0.974631	-1.32211	0.18766	0.29945	-5.51896
BARX2	1.162117	3.170043	1.321893	0.187733	0.299501	-5.62377

IAH1	0.227219	4.264661	1.321889	0.187735	0.299501	-6.02948
FLG	-1.11087	0.97482	-1.3217	0.187798	0.299568	-5.60072
FAM126B	-0.27032	5.560209	-1.32161	0.187827	0.299579	-6.23255
TIGD7	-0.37946	2.343224	-1.3215	0.187865	0.299606	-5.73131
HMGXB3	-0.1753	5.972373	-1.32131	0.187926	0.299669	-6.24774
BNIP2	0.205314	5.586755	1.320808	0.188094	0.299903	-6.20561
NPRL3	0.219988	5.69521	1.320529	0.188187	0.300017	-6.21355
JAK1	-0.21541	7.701987	-1.32035	0.188248	0.30008	-6.23728
EXT2	0.203252	6.240406	1.320222	0.188289	0.300112	-6.24667
COX8A	-0.24595	7.089709	-1.3194	0.188563	0.300514	-6.252
NUP210	0.697706	6.200489	1.318895	0.188732	0.300749	-6.22102
C2orf24	-0.22712	5.669399	-1.31836	0.18891	0.300964	-6.24067
CLDND1	0.330361	6.503135	1.318401	0.188897	0.300964	-6.25398
ZNF37B	-0.33585	3.964091	-1.31823	0.188956	0.301002	-6.07075
C16orf74	1.068539	1.865165	1.31808	0.189004	0.30101	-5.43141
MKS1	0.250662	3.342642	1.318125	0.188989	0.30101	-5.82776
C4orf23	-0.23738	3.169371	-1.31788	0.18907	0.301081	-5.88694
MAMDC4	-0.46827	1.914407	-1.31719	0.189302	0.301417	-5.66454
KIF21B	0.670921	3.744306	1.317018	0.189359	0.301473	-5.83145
CEACAM1	1.097733	5.25849	1.316745	0.189451	0.301584	-6.07199
ING1	0.226572	4.267821	1.316378	0.189573	0.301691	-6.03721
GPR153	0.598374	5.419018	1.316306	0.189598	0.301691	-6.15903
NUP54	0.191694	5.366448	1.316286	0.189604	0.301691	-6.1922
TXNDC5	-0.27034	8.01877	-1.31633	0.189588	0.301691	-6.23136
IL28RA	0.496173	3.684376	1.315869	0.189744	0.301741	-5.85824
ZNF584	-0.24072	3.189321	-1.3159	0.189734	0.301741	-5.89518
ZNF434	-0.15304	4.067921	-1.31601	0.189698	0.301741	-6.06366
MYST1	-0.18787	4.548912	-1.31599	0.189702	0.301741	-6.13884
TIMM17A	0.179336	5.815766	1.31599	0.189703	0.301741	-6.23113
TP53BP1	0.191644	5.715056	1.315408	0.189899	0.301953	-6.22374
RAMP2	-0.38895	3.661386	-1.31524	0.189954	0.301972	-6.02672
DUSP8	-0.45526	4.163552	-1.31528	0.189942	0.301972	-6.11877
NSUN5P2	-0.29944	3.63714	-1.31494	0.190054	0.302097	-6.00296
C13orf18	0.664669	0.98752	1.314571	0.190179	0.302262	-5.35996
ZNF789	0.429056	2.31631	1.313338	0.190593	0.302885	-5.60209
SOCS5	-0.17827	5.133128	-1.31282	0.190769	0.30313	-6.20616
TPMT	0.313652	5.178359	1.312547	0.190859	0.303239	-6.16477
ZNF771	0.321984	2.558161	1.312348	0.190926	0.303311	-5.66449
PLEC	0.355321	10.88259	1.311956	0.191058	0.303476	-6.15713
RPS29	0.256665	7.72615	1.311912	0.191073	0.303476	-6.25781
CCDC92	-0.27076	5.225231	-1.31128	0.191286	0.30378	-6.22317
RARB	-0.53397	2.623547	-1.31113	0.191337	0.303826	-5.83731
MYCBP	0.205626	5.174288	1.310479	0.191556	0.304138	-6.17831
TAOK3	-0.2604	5.871185	-1.31042	0.191577	0.304138	-6.26098
ARHGAP15	-0.52137	1.018495	-1.31031	0.191613	0.30415	-5.53163
ROCK2	-0.30117	6.10326	-1.31027	0.191628	0.30415	-6.26852
EEF1E1	0.293554	3.975691	1.309447	0.191905	0.30455	-5.97164
AGGF1	-0.15806	5.322468	-1.30939	0.191924	0.30455	-6.22514
STAG3L1	0.384905	2.464623	1.30902	0.192049	0.304714	-5.64082
APH1B	-0.30708	3.19482	-1.30891	0.192085	0.304736	-5.91703
ZNF45	-0.23611	3.376738	-1.30865	0.192176	0.304811	-5.94469
TANC1	-0.22902	6.350453	-1.30869	0.19216	0.304811	-6.27325
YTHDF2	0.158491	6.431489	1.307296	0.192633	0.305501	-6.27063
CDK8	-0.23868	3.750616	-1.30616	0.19302	0.306069	-6.02938
TMEM54	0.360921	6.04983	1.30611	0.193035	0.306069	-6.24823
ZNF697	0.403376	3.316554	1.305873	0.193115	0.306142	-5.8064
C14orf118	-0.20647	3.785626	-1.30574	0.19316	0.306142	-6.03147
MLL	-0.2117	7.109391	-1.30572	0.193168	0.306142	-6.27001

PHC3	-0.26135	6.805061	-1.30578	0.193146	0.306142	-6.27451
CEL	1.418543	1.633554	1.305095	0.19338	0.306442	-5.36617
LPIN1	-0.37621	5.552644	-1.30501	0.193409	0.306454	-6.25881
MRPL44	0.174592	4.517418	1.30394	0.193772	0.306981	-6.10535
SNX11	-0.1509	4.390578	-1.3039	0.193785	0.306981	-6.12939
COBRA1	0.197053	6.219397	1.303612	0.193884	0.307102	-6.26761
LOC652276	0.273543	1.639038	1.303172	0.194034	0.307304	-5.5246
PTAR1	-0.23804	5.988757	-1.30309	0.19406	0.307312	-6.27373
PTCD3	0.160775	5.88719	1.30299	0.194096	0.307333	-6.25349
PGRMC1	0.21274	6.842832	1.302819	0.194154	0.30739	-6.28127
FCGR2B	-0.63798	1.269822	-1.30264	0.194217	0.307455	-5.60143
GTF3C4	0.257307	4.983027	1.302481	0.194269	0.307503	-6.16106
SLC25A24	0.272448	6.117243	1.300483	0.194951	0.308548	-6.26384
FAM125A	0.22327	4.471431	1.300305	0.195012	0.308609	-6.09552
ENTPD3	-0.74203	2.749294	-1.30011	0.195077	0.308678	-5.91621
C11orf1	-0.30625	3.565722	-1.29996	0.195129	0.308724	-6.0101
3-Sep	0.896543	1.951342	1.299309	0.195353	0.309044	-5.4917
IL15	0.500828	1.912867	1.299157	0.195405	0.309091	-5.54299
PRUNE	0.200186	5.046958	1.299043	0.195444	0.309118	-6.17935
MST1P2	-0.52322	1.392292	-1.29888	0.1955	0.30917	-5.6055
SYNCRIP	0.171695	7.780005	1.298817	0.195522	0.30917	-6.27187
DUSP23	0.505245	4.271408	1.298665	0.195574	0.309218	-6.00886
MRPS11	0.204635	4.620464	1.298444	0.19565	0.309286	-6.12395
PROSC	-0.28862	5.778153	-1.29841	0.195661	0.309286	-6.27391
TTC21B	-0.24149	4.00456	-1.29822	0.195726	0.309353	-6.0895
HSD3B7	0.394238	4.796803	1.297985	0.195807	0.309446	-6.1251
ZNF627	-0.18998	3.638646	-1.29792	0.19583	0.309447	-6.00655
YIPF2	0.21671	5.119704	1.297679	0.195912	0.309542	-6.18734
LEPREL2	-0.45868	3.409845	-1.29731	0.196038	0.309707	-6.00951
AP2A1	0.171762	7.125419	1.2972	0.196076	0.309732	-6.2865
SCAF1	0.174314	7.193917	1.297116	0.196105	0.309742	-6.28565
CYGB	-0.40392	4.059805	-1.29621	0.196415	0.310197	-6.12319
S100A8	1.727356	6.546595	1.296101	0.196454	0.310223	-6.19515
RDH13	-0.339	3.99719	-1.29594	0.196509	0.310274	-6.10423
MTCP1NB	-0.25376	2.988157	-1.29529	0.196733	0.310593	-5.87936
ZNF597	-0.47439	1.203487	-1.29502	0.196827	0.310707	-5.57373
PIK3C2B	-0.39812	5.763357	-1.29484	0.196887	0.310767	-6.28147
C7orf68	0.536571	3.459319	1.294074	0.197152	0.311149	-5.83069
C17orf59	-0.21031	3.094722	-1.29338	0.197391	0.311492	-5.89605
ABCC2	1.088004	0.552689	1.292522	0.197687	0.311924	-5.27421
ADPRHL2	0.205147	4.339635	1.292171	0.197808	0.31208	-6.0864
MRPL38	0.208445	5.710776	1.291903	0.197901	0.31219	-6.25262
LOC728024	0.223192	0.039323	1.291544	0.198025	0.312351	-5.3107
TYK2	0.178076	6.229329	1.291478	0.198048	0.312352	-6.28422
SPRY4	0.437743	5.261789	1.290661	0.198331	0.312762	-6.18954
PPARG	-0.81967	3.116832	-1.28991	0.198591	0.313138	-6.0189
SYT7	0.939957	3.858522	1.289801	0.198629	0.313162	-5.84084
IER2	0.290513	6.942996	1.289624	0.19869	0.313223	-6.29818
DUSP6	0.430671	6.178699	1.289429	0.198757	0.313294	-6.2737
MED29	-0.16023	5.822148	-1.28933	0.198791	0.313312	-6.28268
ACTR3C	-0.49373	0.861126	-1.28821	0.199181	0.313891	-5.52695
COL15A1	-0.49038	6.303362	-1.2881	0.199219	0.313915	-6.30001
MYO9B	0.181417	7.259159	1.288025	0.199245	0.313921	-6.29634
GEMIN8	-0.22531	3.572087	-1.28789	0.199291	0.313959	-6.01063
LOC283267	-0.26329	3.828458	-1.2876	0.199394	0.314085	-6.07389
BHLHB9	-0.34114	2.2452	-1.28738	0.199468	0.314166	-5.7489
NCRNA00085	-0.5129	0.182725	-1.28642	0.199803	0.314658	-5.43017
C10orf28	0.175925	4.372275	1.286305	0.199843	0.314686	-6.10351

TCEB2	0.231819	6.398954	1.285959	0.199964	0.31484	-6.29538
C12orf26	-0.22593	1.482208	-1.28581	0.200014	0.314884	-5.59549
SERPINE2	0.759338	5.279423	1.285393	0.200161	0.315079	-6.16375
RHOQ	-0.27438	5.633798	-1.28527	0.200203	0.31511	-6.28384
FN3KRP	0.241859	4.499653	1.285068	0.200274	0.315187	-6.11664
MRPL50	0.230507	4.675449	1.284999	0.200298	0.315189	-6.1456
TBC1D25	-0.20914	4.647108	-1.28489	0.200335	0.315212	-6.1935
ZSWIM7	-0.20211	2.958499	-1.28467	0.200412	0.315262	-5.87472
CREBBP	-0.18199	7.59745	-1.28472	0.200394	0.315262	-6.28677
SPIN1	-0.17809	6.751424	-1.28373	0.200743	0.315747	-6.30454
ZFHX4	-0.71273	2.230232	-1.28346	0.200836	0.315786	-5.82125
BANP	0.169188	3.497037	1.283534	0.20081	0.315786	-5.92374
TTC31	0.159015	4.733445	1.283485	0.200827	0.315786	-6.16444
MRPL55	0.221136	4.29036	1.283229	0.200916	0.315841	-6.08438
VPS41	-0.19245	5.882946	-1.28325	0.200909	0.315841	-6.29418
ZC3H12C	-0.44667	4.488968	-1.28315	0.200944	0.315849	-6.20185
ESRRA	-0.25743	5.71216	-1.28233	0.20123	0.316264	-6.29037
PPIG	-0.23174	6.487714	-1.28216	0.20129	0.316322	-6.30789
PARP8	-0.34467	3.981823	-1.28181	0.201412	0.316478	-6.1202
SEC24C	-0.19666	7.130493	-1.28166	0.201466	0.316528	-6.30068
HOXC4	-0.41139	2.125919	-1.28149	0.201523	0.316582	-5.74241
FYN	-0.34886	4.461907	-1.28101	0.201694	0.316815	-6.19052
LDB1	-0.23335	5.899671	-1.28086	0.201746	0.316861	-6.29917
LRRN2	-0.67906	1.315125	-1.2806	0.201838	0.31697	-5.6467
SEPX1	0.357535	4.875494	1.280529	0.201861	0.316971	-6.16437
TBC1D24	-0.23535	4.083085	-1.27998	0.202056	0.317241	-6.12356
COASY	0.186976	6.237536	1.279726	0.202143	0.317342	-6.29919
HSPA4	0.203908	6.978834	1.279385	0.202263	0.317494	-6.31097
ADAMTS10	-0.44664	1.763066	-1.27909	0.202368	0.317624	-5.68613
ASB13	0.329457	4.32264	1.278405	0.202607	0.317928	-6.07751
MAEA	-0.22079	6.075112	-1.27846	0.202587	0.317928	-6.3071
IQGAP1	0.241761	9.015124	1.27815	0.202697	0.317998	-6.26167
ZNF609	-0.20259	6.324185	-1.2782	0.20268	0.317998	-6.31177
FDPSL2A	0.466248	1.127098	1.277913	0.202781	0.318093	-5.44826
ARHGAP26	-0.48565	5.27457	-1.27753	0.202917	0.318234	-6.28309
RALB	0.278061	6.291383	1.277584	0.202897	0.318234	-6.30082
CEBPB	0.353961	7.247771	1.277097	0.203068	0.318436	-6.31255
CASP8AP2	0.221849	5.045808	1.276939	0.203124	0.318488	-6.20492
YBX1	0.196332	8.486528	1.276517	0.203272	0.318685	-6.28075
EID2	0.206186	3.396566	1.275938	0.203477	0.318948	-5.90426
PTK2	0.210061	7.005545	1.275912	0.203486	0.318948	-6.31522
RPS13	-0.26845	7.734	-1.275	0.203807	0.319416	-6.29327
ATPBD4	0.242386	3.141939	1.274898	0.203844	0.319437	-5.84105
GDF11	0.415511	2.993987	1.274492	0.203987	0.319626	-5.78047
GPSM3	0.446806	3.00092	1.274265	0.204068	0.319716	-5.77498
CNTLN	-0.57024	2.613413	-1.27399	0.204166	0.31981	-5.88832
EIF4G1	0.2349	9.308001	1.273921	0.204189	0.31981	-6.2565
DNM1L	0.191881	6.671857	1.273902	0.204196	0.31981	-6.31709
CRTC3	-0.206	6.054578	-1.27364	0.204289	0.31992	-6.31237
C10orf99	2.331864	1.125126	1.273542	0.204323	0.319938	-5.28606
GJA5	-0.48432	2.606312	-1.27338	0.20438	0.319991	-5.86733
BICD2	0.522622	6.937106	1.273287	0.204413	0.320007	-6.3171
TCTN2	0.319872	3.82238	1.272257	0.204778	0.320542	-5.97967
RNF6	0.208689	6.003336	1.271651	0.204993	0.320843	-6.29775
TYW1B	0.354262	1.888283	1.271428	0.205072	0.320931	-5.59515
ACSF3	-0.19915	4.077304	-1.27128	0.205126	0.320979	-6.12873
GJB6	2.948224	3.546531	1.2712	0.205153	0.320986	-5.48621
PNMA2	-0.78893	1.441747	-1.27091	0.205255	0.321109	-5.69008

NADSYN1	0.349516	5.774148	1.269706	0.205684	0.321744	-6.27554
ZBTB8OS	0.230966	3.421556	1.269377	0.205801	0.321891	-5.91099
MCM9	0.22959	2.157542	1.269233	0.205852	0.321935	-5.66118
A4GALT	-0.55644	4.277107	-1.269	0.205934	0.322026	-6.20656
DOCK9	-0.3035	6.695982	-1.26885	0.205988	0.322075	-6.32263
BCAN	0.889751	0.495682	1.268638	0.206064	0.322158	-5.31772
NPHP1	-0.45059	1.263004	-1.26838	0.206157	0.322268	-5.61365
UBR7	-0.18731	5.517278	-1.26757	0.206445	0.322681	-6.29493
LY75	0.779777	5.238466	1.267177	0.206585	0.322864	-6.17672
C14orf104	0.212409	3.84371	1.267068	0.206624	0.322889	-6.01212
OSGIN2	0.249234	4.768485	1.26644	0.206848	0.323202	-6.18048
WDR36	0.188491	5.938399	1.266204	0.206932	0.323298	-6.30186
PRKACA	-0.16416	6.044729	-1.26612	0.206962	0.323309	-6.32054
NPW	1.080251	1.471912	1.265768	0.207088	0.323469	-5.43444
ARL10	-0.50311	1.310091	-1.26539	0.207221	0.323642	-5.63507
DCAF7	0.190115	7.297752	1.265308	0.207252	0.323654	-6.32454
HMGCS1	0.379358	6.754406	1.264693	0.207472	0.323961	-6.32722
TMEM63B	0.316097	6.622987	1.263602	0.207863	0.324535	-6.32728
MTP18	0.306687	3.801893	1.263306	0.207969	0.324664	-5.98696
ZDHHC5	0.192064	7.662569	1.263018	0.208072	0.324789	-6.32029
IMPA2	-0.42171	5.754111	-1.26282	0.208144	0.324865	-6.32244
CD7	0.654516	2.683057	1.262642	0.208207	0.324927	-5.69609
GPRC5A	0.904905	6.882137	1.262205	0.208364	0.325135	-6.32025
TMEM42	-0.21906	2.145667	-1.26211	0.208397	0.32515	-5.73737
C8orf4	-0.66723	3.864062	-1.26185	0.208492	0.325263	-6.17259
SLC6A8	0.495094	7.008693	1.261604	0.20858	0.325363	-6.33283
DHX15	0.159531	7.265193	1.261526	0.208608	0.325371	-6.32937
RNF181	0.223205	5.199107	1.261098	0.208762	0.325574	-6.24201
XG	1.233078	1.101366	1.260855	0.208849	0.325638	-5.37015
GABRE	1.375751	3.391937	1.260858	0.208848	0.325638	-5.71424
STK19	-0.21035	2.819013	-1.26066	0.208918	0.325709	-5.87582
GMIP	0.302961	4.966461	1.260221	0.209077	0.325884	-6.20631
TMEM64	-0.3083	4.93755	-1.26022	0.209076	0.325884	-6.26538
PADI1	1.64111	0.569989	1.259497	0.209338	0.326254	-5.29981
ANGPT1	-0.57394	1.917903	-1.25923	0.209436	0.32637	-5.75946
TNC	0.872583	7.991853	1.258634	0.209649	0.326666	-6.33103
SFRP2	-1.13863	4.830661	-1.25799	0.209881	0.326955	-6.3146
GALNT2	0.252943	7.196224	1.258019	0.209871	0.326955	-6.33606
C3orf38	-0.19087	4.231881	-1.25764	0.210009	0.327099	-6.17036
CFB	0.870977	6.261325	1.257605	0.21002	0.327099	-6.2909
ZKSCAN5	0.256005	4.23907	1.25718	0.210174	0.327301	-6.10098
FNDC3B	0.304753	7.312322	1.2568	0.210311	0.327479	-6.33655
CHI3L2	1.058987	0.089879	1.256625	0.210374	0.327541	-5.30029
C9orf85	0.207607	2.73735	1.256529	0.210409	0.327558	-5.78593
BBS9	-0.23034	3.561195	-1.25646	0.210435	0.327563	-6.05159
TJP1	-0.25203	7.489968	-1.25638	0.210461	0.327567	-6.32352
PARP3	-0.29939	3.926119	-1.25626	0.210506	0.3276	-6.13749
C1orf25	-0.16868	4.523808	-1.25512	0.21092	0.328208	-6.21094
USPL1	-0.2436	4.821215	-1.25442	0.211172	0.328563	-6.25444
ABHD8	-0.30209	3.404544	-1.25434	0.2112	0.32857	-6.03327
GOLPH3L	-0.26175	5.278222	-1.25428	0.211224	0.328571	-6.2988
ARPP19	-0.19534	7.921158	-1.25393	0.211351	0.328732	-6.31603
TCF7	0.505438	4.29039	1.253725	0.211425	0.328804	-6.06569
POLR2C	0.175922	5.804351	1.25367	0.211444	0.328804	-6.30982
CAPN3	-0.41709	2.011365	-1.25339	0.211546	0.328925	-5.7563
ZCCHC8	0.162744	4.761717	1.253291	0.211582	0.328944	-6.20567
STAU1	0.150105	7.550156	1.252741	0.211782	0.329218	-6.3347
RPL14	-0.27817	6.986325	-1.25252	0.21186	0.329304	-6.33862

CTAGE5	-0.26766	5.744171	-1.25245	0.211888	0.32931	-6.32969
PPP2R3B	0.361089	3.043577	1.252327	0.211932	0.329342	-5.82666
ZNF669	0.252978	2.559621	1.251984	0.212057	0.3295	-5.75035
FHOD3	-0.67617	2.594251	-1.25191	0.212083	0.329504	-5.93528
SRXN1	0.481146	6.405349	1.251756	0.21214	0.329555	-6.33017
SLC35A5	-0.19614	4.798836	-1.25149	0.212236	0.329668	-6.25101
PPFIA4	0.897125	0.280454	1.251194	0.212344	0.329799	-5.31417
C12orf10	0.194628	4.444083	1.251017	0.212409	0.329863	-6.15766
PHLDA1	-0.45176	7.109847	-1.25075	0.212507	0.329905	-6.33515
DPYSL2	-0.35654	6.202139	-1.25085	0.212469	0.329905	-6.34611
PPFIBP1	-0.31001	6.356766	-1.2508	0.212489	0.329905	-6.34704
FAM53B	-0.24596	5.980565	-1.25065	0.212544	0.329926	-6.34001
USP27X	-0.28936	1.822217	-1.25051	0.212592	0.329964	-5.70693
IMPAD1	-0.16401	7.488167	-1.25021	0.212703	0.330099	-6.33311
PRPF4B	-0.19303	6.614675	-1.25006	0.212759	0.33015	-6.34786
TOP3A	0.19913	4.868531	1.249865	0.212829	0.330221	-6.21961
NCS1	-0.54267	5.269159	-1.24977	0.212864	0.330239	-6.32133
FAM100A	-0.1795	4.914396	-1.24961	0.21292	0.33029	-6.26438
ALG5	-0.25906	4.468064	-1.24948	0.212967	0.330326	-6.21997
PLA2G4A	0.759501	3.768504	1.249352	0.213016	0.330328	-5.90614
LRP6	-0.2977	5.795039	-1.24939	0.213003	0.330328	-6.33678
SACS	-0.39272	5.027461	-1.24923	0.213059	0.330359	-6.29441
C20orf108	-0.3166	6.502081	-1.24903	0.213131	0.330434	-6.34907
EXTL2	-0.2918	3.673165	-1.24863	0.21328	0.330628	-6.09589
CYB5R4	0.234346	4.293326	1.248547	0.213309	0.330637	-6.12632
CTTNBP2NL	0.220449	5.863755	1.248205	0.213434	0.330794	-6.31806
GJB1	-1.39962	0.549483	-1.24794	0.213531	0.330907	-5.64765
IWS1	0.144914	5.738788	1.247863	0.213559	0.330914	-6.31431
SELPLG	0.526699	3.404103	1.247543	0.213676	0.331021	-5.87721
SIPA1	0.272506	5.364712	1.247553	0.213672	0.331021	-6.27198
C1orf97	0.420031	2.131324	1.247172	0.213812	0.331158	-5.65259
ERGIC3	0.225701	7.643314	1.247232	0.21379	0.331158	-6.34095
DSE	0.451215	5.018849	1.247039	0.213861	0.331197	-6.21413
GIPC1	0.287341	7.148316	1.246672	0.213995	0.331361	-6.35109
MKRN1	-0.18009	6.463124	-1.24662	0.214014	0.331361	-6.35225
ZC3H12D	0.746016	1.108214	1.246433	0.214083	0.331401	-5.44692
GNAS	-0.20155	9.969769	-1.24636	0.214111	0.331401	-6.25484
SLPI	0.805552	6.481952	1.246359	0.214109	0.331401	-6.32522
MAP2K6	0.656002	2.116424	1.245535	0.214411	0.331829	-5.61766
UBE2M	0.233291	5.338828	1.245285	0.214503	0.331935	-6.27575
RER1	-0.19551	6.45977	-1.24492	0.214637	0.332106	-6.35438
RBM23	-0.1463	5.746422	-1.24337	0.215207	0.33295	-6.33614
SMYD3	0.351816	3.078306	1.243249	0.21525	0.33298	-5.84647
ADORA3	0.565852	0.86358	1.242891	0.215382	0.333147	-5.4411
IMMP1L	0.238027	1.626896	1.242481	0.215533	0.333344	-5.60242
FLJ40330	-0.7818	0.012885	-1.24217	0.215646	0.333445	-5.49012
OSBPL2	-0.2209	6.131202	-1.24219	0.21564	0.333445	-6.35375
CCR6	0.585683	1.565569	1.242009	0.215707	0.333502	-5.54257
KIAA1257	0.709799	0.23107	1.241816	0.215778	0.333575	-5.33804
CASP6	0.307397	3.56775	1.241504	0.215892	0.333712	-5.96067
SDF2	0.194171	4.952176	1.241445	0.215914	0.333712	-6.24074
TRIM17	0.529486	0.188802	1.241057	0.216057	0.333799	-5.3555
TMEM81	0.258725	0.992105	1.241107	0.216039	0.333799	-5.50113
PARVG	0.569662	2.163403	1.240966	0.216091	0.333799	-5.64605
RNF139	0.213195	5.487522	1.240903	0.216114	0.333799	-6.29731
FLT1	-0.3986	5.361603	-1.24121	0.216	0.333799	-6.32935
RAB10	0.257859	8.072972	1.24094	0.2161	0.333799	-6.33926
PPP1R2	0.187951	6.122869	1.240418	0.216293	0.334038	-6.34355

MSL3	0.182682	4.824279	1.239695	0.21656	0.334414	-6.2284
C1orf116	-0.73081	6.421258	-1.23944	0.216652	0.334519	-6.35718
TRIM7	0.897936	3.299706	1.239357	0.216685	0.334532	-5.7983
C14orf142	0.207703	3.226862	1.239176	0.216751	0.334599	-5.90931
ANGEL1	-0.20851	5.122913	-1.23889	0.216859	0.334727	-6.30043
RBM26	-0.18679	5.911157	-1.23857	0.216976	0.334871	-6.35084
GDA	1.986803	3.053679	1.237971	0.217197	0.335175	-5.57905
CLEC2B	0.75129	3.431685	1.237774	0.21727	0.33525	-5.85657
IRS1	-0.38663	6.002127	-1.23729	0.217448	0.335451	-6.36004
LGMN	-0.25423	6.60165	-1.23731	0.217443	0.335451	-6.3633
ARL16	-0.206	3.703507	-1.23716	0.217499	0.335493	-6.10097
LSAMP	-0.59513	1.347066	-1.23639	0.217782	0.335892	-5.68772
DCP2	0.211132	5.443608	1.236315	0.21781	0.335899	-6.29882
AOAH	0.76457	1.259767	1.236067	0.217902	0.33593	-5.4794
ZNF92	0.297146	3.820187	1.236144	0.217874	0.33593	-6.02875
ZHX1	-0.25412	5.852996	-1.23612	0.217883	0.33593	-6.35388
BCL9L	0.225084	7.516201	1.235743	0.218023	0.336066	-6.35769
MRC2	-0.46548	6.435285	-1.2357	0.218039	0.336066	-6.36492
PRKD3	-0.30053	5.442828	-1.2345	0.218485	0.336717	-6.33752
KRT14	3.797867	6.2088	1.23419	0.218599	0.336856	-5.85395
C12orf45	0.281087	2.779265	1.233822	0.218736	0.336992	-5.80778
BHLHE41	0.658381	4.594926	1.233868	0.218719	0.336992	-6.12854
LRRC16A	0.329592	5.589869	1.233276	0.218939	0.337268	-6.30578
NDUFB6	-0.21133	4.68019	-1.23306	0.219021	0.337356	-6.26161
MAN2A1	-0.29391	5.408722	-1.23278	0.219126	0.33748	-6.33675
LOC100130557	-0.31799	0.903048	-1.23257	0.219203	0.337491	-5.57784
ANXA9	-0.70032	1.575823	-1.23256	0.219205	0.337491	-5.74878
CCND2	-0.58358	6.00679	-1.23258	0.219196	0.337491	-6.36838
RASGRP1	0.646596	3.031778	1.232309	0.219299	0.337599	-5.79877
C2orf44	0.206746	3.505045	1.232095	0.219379	0.337685	-5.98086
ZBTB48	-0.20096	3.769226	-1.23158	0.219569	0.337866	-6.12015
PTPRH	-0.97688	3.505753	-1.23163	0.219552	0.337866	-6.18553
ACER3	0.251819	5.428903	1.231668	0.219538	0.337866	-6.2994
ELK3	0.314825	5.236669	1.230644	0.21992	0.338369	-6.27518
LOC440173	1.296422	0.454422	1.230189	0.22009	0.338578	-5.33345
MRPL46	-0.18274	3.445706	-1.23015	0.220105	0.338578	-6.04699
UGDH	-0.3309	6.042194	-1.22998	0.22017	0.338641	-6.36883
C19orf43	0.221088	6.452294	1.229865	0.220211	0.338667	-6.36701
SEMA6C	-0.3541	2.91138	-1.22933	0.220412	0.338859	-5.9668
TMEM5	0.205988	3.629569	1.229268	0.220435	0.338859	-6.01284
KIAA0391	0.198378	5.081883	1.229291	0.220426	0.338859	-6.27069
TMTC4	-0.31691	4.643829	-1.22921	0.220457	0.338859	-6.27231
RASAL2	0.278744	6.16346	1.229293	0.220425	0.338859	-6.35523
SCEL	2.247525	2.728609	1.229074	0.220507	0.338891	-5.51012
PPM1B	-0.14334	5.726	-1.229	0.220535	0.338891	-6.35252
MMP15	-0.52671	6.037585	-1.22896	0.220551	0.338891	-6.37263
MDP1	-0.22358	2.217977	-1.22862	0.220676	0.339046	-5.79381
TIMM17B	0.26729	4.899929	1.22852	0.220714	0.339068	-6.24188
OXA1L	-0.18244	6.639413	-1.22827	0.220807	0.339174	-6.37452
ATXN3	-0.18538	4.640859	-1.22811	0.220866	0.339226	-6.26061
LOC147727	0.28873	3.535126	1.227621	0.221051	0.339473	-5.97641
BAZ2A	0.150504	7.484439	1.226773	0.221369	0.339924	-6.36795
RING1	0.189057	4.738459	1.226516	0.221465	0.340034	-6.23247
P2RY1	1.058063	2.442626	1.225904	0.221695	0.34035	-5.63962
PIK3R4	0.221983	5.651102	1.225195	0.221962	0.340722	-6.33002
C20orf54	0.724209	3.97887	1.225079	0.222005	0.340751	-5.98857
BRD9	0.245786	5.176614	1.224878	0.222081	0.340829	-6.28183
PSMB6	0.224507	5.947833	1.224024	0.222402	0.341285	-6.35258

MAPK3	-0.27876	6.391775	-1.22394	0.222435	0.341298	-6.38002
TFEC	0.635597	1.618638	1.22378	0.222494	0.341313	-5.56808
SEPHS1	0.197417	5.411064	1.223839	0.222471	0.341313	-6.31209
EPHX1	-0.47453	6.597436	-1.2236	0.222563	0.341382	-6.37762
ZNF525	-0.57098	2.566859	-1.22336	0.222652	0.34148	-5.93926
C19orf20	-0.25358	2.710066	-1.22304	0.222771	0.341626	-5.90716
LOC151162	-0.30953	6.30626	-1.22282	0.222857	0.341721	-6.3811
SHISA2	1.12766	1.841579	1.222593	0.222941	0.341812	-5.53144
XRRA1	-0.2869	4.140984	-1.2219	0.223201	0.342136	-6.21284
TARS2	0.195325	4.629654	1.221912	0.223198	0.342136	-6.22196
PLD1	0.354758	5.643572	1.221702	0.223277	0.342215	-6.32347
PCDHB10	0.469759	1.858165	1.221103	0.223503	0.342486	-5.63492
SUMF2	0.294705	6.600038	1.221156	0.223483	0.342486	-6.37945
TMEM17	0.393376	1.41115	1.220862	0.223594	0.342588	-5.57279
RBM4	0.133509	6.17707	1.220743	0.223639	0.342619	-6.37184
DAP	-0.30355	7.458296	-1.2202	0.223845	0.342897	-6.36752
MAP1S	0.224807	6.06703	1.220106	0.22388	0.342913	-6.36385
MBOAT2	0.537574	5.346993	1.219945	0.223941	0.342969	-6.27784
TRMT12	0.331406	3.543674	1.219447	0.224129	0.34322	-5.97978
WDYHV1	0.259792	3.379871	1.219126	0.224251	0.343354	-5.95677
CBWD1	-0.33598	4.426792	-1.21909	0.224266	0.343354	-6.26173
LOC284232	-0.3208	0.755416	-1.21844	0.22451	0.34369	-5.57288
PCDHB7	-0.47349	0.156051	-1.21807	0.224653	0.34387	-5.50236
S100A7	4.273656	3.958872	1.217982	0.224684	0.343881	-5.4311
HOMER1	0.405364	3.699071	1.217416	0.224899	0.344135	-6.00567
SOX7	0.554293	4.743445	1.217415	0.224899	0.344135	-6.19678
CYP2J2	0.838979	2.023702	1.216759	0.225148	0.344478	-5.60955
CTR9	-0.15714	5.855422	-1.21665	0.22519	0.344505	-6.37419
BCR	-0.23446	7.006308	-1.21651	0.225242	0.344546	-6.38301
CBX5	-0.29183	7.35575	-1.21596	0.225451	0.344827	-6.37538
XKR6	-0.55066	0.142194	-1.21588	0.225482	0.344837	-5.51284
BASP1	0.751736	4.355277	1.215741	0.225535	0.344843	-6.08073
SLC39A13	-0.21412	5.164127	-1.21575	0.22553	0.344843	-6.333
CDH17	2.708824	2.513502	1.215597	0.22559	0.344889	-5.42875
PCIF1	0.154537	6.112965	1.215316	0.225697	0.345015	-6.37494
MTHFS	0.290178	3.437321	1.21514	0.225764	0.34508	-5.96647
TRIP10	0.305622	5.715894	1.214818	0.225886	0.345229	-6.34203
ATP5C1	-0.22691	7.318842	-1.21418	0.22613	0.345563	-6.37963
CBR3	0.69464	2.658879	1.213214	0.226497	0.346087	-5.74864
WFIKKN1	-0.51454	0.424465	-1.2127	0.226693	0.346349	-5.55189
FAM115C	0.52707	3.589065	1.212635	0.226719	0.346349	-5.96261
STRBP	0.271831	5.247499	1.211687	0.227081	0.346864	-6.30295
MED16	-0.17644	6.181808	-1.21102	0.227334	0.347213	-6.39188
PRMT7	0.209664	4.401617	1.210815	0.227414	0.347259	-6.19692
LOC339047	-0.30036	5.069963	-1.21077	0.22743	0.347259	-6.33762
PSMG2	0.200927	5.61155	1.210751	0.227438	0.347259	-6.34576
LRRC16B	0.803643	0.62177	1.210034	0.227713	0.34764	-5.41825
MYEOV	1.310056	3.199923	1.209964	0.22774	0.347643	-5.74428
SLC16A4	-0.53285	1.60311	-1.20982	0.227796	0.347692	-5.75444
PRKY	1.43024	1.315373	1.20916	0.228047	0.347999	-5.439
POMGNT1	-0.18319	5.714071	-1.20918	0.228039	0.347999	-6.37758
RMND1	0.194255	3.729286	1.208998	0.22811	0.348056	-6.06088
MAD2L1BP	0.309318	4.212206	1.208904	0.228145	0.348073	-6.14312
RBX1	0.215937	5.148548	1.208533	0.228288	0.348252	-6.30164
RNF216L	0.220635	3.752486	1.207676	0.228617	0.348715	-6.06226
FAM86D	-0.39374	2.490056	-1.20742	0.228714	0.348826	-5.90476
RYK	0.238356	5.769469	1.207069	0.22885	0.348995	-6.36022
LOC728875	0.577051	0.214834	1.206886	0.22892	0.349058	-5.39217

HSBP1L1	-0.3002	2.393146	-1.20676	0.228969	0.349058	-5.86669
UBAC1	0.229277	5.24786	1.206701	0.228991	0.349058	-6.31356
HDHD3	-0.3828	5.124827	-1.20681	0.228948	0.349058	-6.35215
ADNP	0.169921	7.30503	1.206305	0.229143	0.349252	-6.39636
SEC23A	0.241166	6.472928	1.205851	0.229318	0.34948	-6.39607
C14orf119	0.183994	5.505188	1.205742	0.22936	0.349506	-6.34372
ARID1A	-0.17566	7.67219	-1.20568	0.229385	0.349506	-6.38248
SLC1A4	0.449312	6.206335	1.205278	0.229539	0.349678	-6.37816
PRKCI	0.289558	6.814954	1.205253	0.229548	0.349678	-6.40187
PSMF1	0.179808	7.21009	1.205036	0.229632	0.349767	-6.3996
KIF5B	-0.23938	8.226676	-1.20477	0.229736	0.349888	-6.36458
PURB	0.20224	7.027773	1.204625	0.22979	0.349933	-6.40264
MICAL1	-0.31792	4.824347	-1.20432	0.229906	0.350071	-6.32222
HLA-DQA2	1.102723	1.733642	1.204104	0.229991	0.350162	-5.5431
ZBTB46	-0.35131	2.537396	-1.2038	0.230109	0.350281	-5.9102
CSF1	-0.37676	4.886577	-1.20371	0.230144	0.350281	-6.33518
GPX4	0.249787	6.866811	1.20373	0.230135	0.350281	-6.40426
IFT172	-0.21772	4.27648	-1.20354	0.230207	0.350338	-6.24557
MSI2	0.354854	3.979541	1.203088	0.230383	0.350567	-6.09144
CSDA	0.314425	7.969532	1.202556	0.230588	0.350842	-6.38964
ACY1	0.404163	4.791383	1.202434	0.230636	0.350876	-6.24
CDC42SE1	0.206824	6.853008	1.202292	0.23069	0.350921	-6.40605
RPS19BP1	-0.18564	4.838635	-1.20204	0.230788	0.351031	-6.31443
ABI2	-0.25931	5.517396	-1.20194	0.230826	0.351051	-6.37929
DNAJC1	-0.26681	4.608856	-1.20161	0.230956	0.35121	-6.29652
MMP17	0.809066	1.103345	1.201127	0.231141	0.351377	-5.49387
TTC30B	0.291299	3.041158	1.201193	0.231115	0.351377	-5.8991
SLC9A8	-0.24662	5.72194	-1.20115	0.231134	0.351377	-6.39034
UGGT2	-0.25884	4.173032	-1.20091	0.231225	0.351465	-6.2388
ANKMY1	-0.23288	3.18822	-1.19992	0.231609	0.352012	-6.03462
C9orf23	0.285116	3.554556	1.199832	0.231642	0.352024	-6.01315
TMEM132A	0.461711	6.213991	1.199657	0.23171	0.352088	-6.38456
DDX60L	0.406206	5.238179	1.199372	0.231821	0.352218	-6.30178
SDHB	-0.17121	5.916675	-1.19869	0.232086	0.352583	-6.39871
S100A4	0.476088	4.565831	1.197813	0.232426	0.353061	-6.19899
SRRM3	0.850351	2.392222	1.197651	0.232489	0.353088	-5.69643
ZNHIT3	0.154257	4.529974	1.197636	0.232495	0.353088	-6.24185
C17orf86	0.265866	2.110487	1.197118	0.232696	0.353356	-5.73436
CPA4	1.529111	0.460025	1.196951	0.232761	0.3534	-5.37159
ZMPSTE24	0.197192	6.312084	1.196914	0.232775	0.3534	-6.40313
DAB2IP	-0.27098	7.005416	-1.19666	0.232873	0.353509	-6.40616
TBC1D16	-0.25766	4.48071	-1.19622	0.233044	0.353731	-6.28655
PAX8	0.697503	2.491172	1.196098	0.233093	0.353752	-5.73205
TAF10	0.262014	5.564122	1.196058	0.233108	0.353752	-6.35397
KTI12	0.153008	3.129553	1.195726	0.233238	0.35391	-5.95098
C11orf10	0.219138	5.542687	1.195636	0.233273	0.353924	-6.35608
ZNF259	0.248872	4.852333	1.195406	0.233362	0.353983	-6.27757
IDH3A	0.215666	6.125461	1.195464	0.23334	0.353983	-6.39674
LY6G5C	-0.36227	0.26883	-1.19477	0.23361	0.35432	-5.52911
CORO1C	0.262846	7.267661	1.194615	0.233671	0.354374	-6.41224
ZSCAN20	-0.23502	1.941117	-1.19409	0.233875	0.354645	-5.78552
CNTNAP2	1.196607	2.660155	1.193799	0.233989	0.35478	-5.6917
FAT2	2.293569	5.009104	1.193252	0.234203	0.355065	-5.94215
C12orf57	-0.25406	5.178955	-1.19272	0.234411	0.355284	-6.36492
PGAM1	0.272106	7.781834	1.192688	0.234423	0.355284	-6.4048
VPS13B	-0.21498	6.102581	-1.19272	0.234409	0.355284	-6.41268
C22orf27	0.243712	2.048389	1.192412	0.234531	0.355408	-5.73278
DGCR2	-0.19175	7.132128	-1.19234	0.234558	0.35541	-6.41021

LAMA4	-0.40997	6.126784	-1.19211	0.23465	0.355512	-6.41687
CNFN	1.914358	3.857536	1.191885	0.234737	0.355528	-5.78931
HSPB11	0.232046	3.694805	1.191956	0.234709	0.355528	-6.06673
WDR26	-0.19191	7.234448	-1.19191	0.234728	0.355528	-6.40867
LARS	-0.15358	6.82439	-1.19174	0.234795	0.355578	-6.41675
SERPINB3	2.688011	3.015452	1.191403	0.234926	0.355656	-5.53697
SNX12	0.23513	3.111382	1.191457	0.234904	0.355656	-5.93714
DCBLD2	-0.62052	4.912666	-1.19134	0.234949	0.355656	-6.37172
GNA12	0.222217	6.609728	1.191524	0.234878	0.355656	-6.41637
EXOC4	-0.17489	6.108675	-1.19124	0.234988	0.355677	-6.41366
ITGAM	0.584345	2.470728	1.190615	0.235234	0.356011	-5.75537
GEMIN5	0.187479	5.075994	1.190343	0.235341	0.356134	-6.31802
PDCD1	0.651623	0.38353	1.190257	0.235375	0.356135	-5.42519
SLC27A4	0.318336	5.967055	1.190211	0.235393	0.356135	-6.38897
YKT6	0.187986	6.548651	1.189581	0.23564	0.35647	-6.41816
SASH3	0.548234	2.780898	1.189164	0.235803	0.356602	-5.81897
MTHFD2L	-0.24249	3.361223	-1.1892	0.235788	0.356602	-6.08889
TBCK	-0.20922	4.506531	-1.18924	0.235773	0.356602	-6.29281
C2orf81	0.434894	1.823989	1.189045	0.23585	0.356634	-5.66999
TMEM183A	0.164923	5.512659	1.188862	0.235922	0.356678	-6.36604
PRPF6	0.155154	6.792661	1.188841	0.23593	0.356678	-6.42201
SRFBP1	0.186839	3.642002	1.188208	0.236179	0.356992	-6.06838
MSL2	0.209426	5.60902	1.188182	0.236189	0.356992	-6.37181
NBPF15	0.326893	3.504762	1.187822	0.236331	0.357167	-6.01156
AZIN1	0.202276	7.578155	1.187685	0.236385	0.357201	-6.41373
SSU72	0.18444	6.643007	1.187635	0.236404	0.357201	-6.42205
ATAD2B	0.188192	4.744143	1.187532	0.236445	0.357224	-6.27985
TNFRSF11A	0.827868	2.755636	1.187028	0.236643	0.357485	-5.76805
IL1RN	0.901256	6.465405	1.186572	0.236822	0.357717	-6.39071
NRIP3	-0.5256	1.523299	-1.1865	0.23685	0.357721	-5.76634
HNRPLL	-0.12409	5.21584	-1.18619	0.236975	0.35787	-6.36564
PFKFB2	-0.40596	5.436096	-1.18579	0.237129	0.358047	-6.40057
LHFPL2	-0.28514	5.580642	-1.18576	0.237143	0.358047	-6.40304
CP	-0.92897	1.849521	-1.18534	0.237307	0.358217	-5.89811
PNKP	0.185203	4.6962	1.185355	0.237302	0.358217	-6.27577
VCP	0.195632	8.535222	1.185101	0.237402	0.358322	-6.39053
LOC285359	-0.22835	0.477606	-1.18467	0.237572	0.35854	-5.5551
SPAG4	0.545214	1.058657	1.184361	0.237694	0.358685	-5.5366
TP63	2.809293	5.215019	1.183669	0.237968	0.359043	-5.89539
ZNF558	-0.34623	4.045354	-1.18363	0.237983	0.359043	-6.25281
C18orf18	-0.42583	0.518517	-1.18338	0.23808	0.359151	-5.58992
HSD17B8	-0.28875	2.979425	-1.18304	0.238215	0.359277	-6.01996
SMG1	0.180848	7.779001	1.183084	0.238198	0.359277	-6.41436
FGF12	-0.61856	0.560993	-1.18277	0.238322	0.359397	-5.626
LZTFL1	-0.21269	4.214402	-1.18271	0.238346	0.359397	-6.26106
FUT3	1.28909	4.002666	1.182392	0.238472	0.359548	-5.92835
GPR114	0.78746	1.833605	1.182169	0.238561	0.359612	-5.62565
MMS19	0.17878	5.948996	1.182155	0.238566	0.359612	-6.40497
HSBP1	0.186453	6.702067	1.181925	0.238657	0.35971	-6.42938
CD74	0.475631	9.914034	1.18165	0.238766	0.359836	-6.35515
TLK1	-0.15939	6.08391	-1.18145	0.238846	0.359917	-6.42418
ABLIM2	0.602136	3.104812	1.181146	0.238966	0.359981	-5.87955
FRG1	-0.17735	4.380401	-1.18126	0.238922	0.359981	-6.28253
SPOPL	-0.23776	5.258342	-1.18118	0.238953	0.359981	-6.38371
ZNF574	0.17767	5.475042	1.18086	0.239079	0.360113	-6.37115
POLR2I	0.245372	4.440143	1.18034	0.239285	0.360346	-6.23429
RBM22	0.136617	5.681993	1.180382	0.239268	0.360346	-6.39186
RMND5A	0.19251	6.736093	1.180266	0.239314	0.360351	-6.4316

KDM4D	0.364991	0.02839	1.179963	0.239435	0.360487	-5.42327
UBTD2	0.175794	4.968085	1.17991	0.239456	0.360487	-6.3186
CHRDL2	-0.8833	1.29668	-1.17929	0.239701	0.360816	-5.79658
POLRMT	0.197055	5.60257	1.17908	0.239785	0.360866	-6.38279
ATP10D	-0.39346	5.226228	-1.17911	0.239774	0.360866	-6.39459
PLA2G2A	-1.18683	2.559937	-1.17838	0.240064	0.361246	-6.10845
MLST8	0.204971	5.047879	1.177811	0.24029	0.361547	-6.32728
UBAP2	0.202206	6.461873	1.177696	0.240335	0.361577	-6.42993
RNF125	-0.4609	2.92656	-1.17737	0.240466	0.361734	-6.04677
TMEM14A	0.327451	4.463625	1.177206	0.24053	0.361793	-6.22948
C16orf13	0.265309	5.078777	1.176951	0.240632	0.361867	-6.32549
NUP50	0.160306	6.516217	1.176984	0.240619	0.361867	-6.43281
DPH5	-0.18835	3.800994	-1.17673	0.24072	0.361959	-6.19
MED8	0.181042	4.938323	1.176668	0.240745	0.361959	-6.31806
SPAG1	0.386637	4.412027	1.176258	0.240908	0.362088	-6.20904
COMMD3	0.181219	4.47759	1.176339	0.240876	0.362088	-6.25458
CHD1	-0.20314	5.759367	-1.17632	0.240884	0.362088	-6.4196
SPIN4	0.533898	4.124901	1.176031	0.240999	0.362185	-6.12144
FHAD1	0.670834	0.331226	1.175881	0.241059	0.362197	-5.42847
CHD6	-0.22868	6.3772	-1.17594	0.241035	0.362197	-6.43689
ENTPD7	0.285279	5.413073	1.17573	0.241119	0.362248	-6.36108
COL4A2	0.389298	8.865	1.175429	0.241239	0.362351	-6.39642
DNAJA1	0.22098	6.679534	1.175462	0.241225	0.362351	-6.43634
RBP1	0.955784	4.288846	1.17527	0.241302	0.362407	-6.0753
TMEM11	0.184683	4.380461	1.174702	0.241529	0.362708	-6.23948
TMEM9	0.219286	5.45902	1.174635	0.241556	0.36271	-6.37311
RPL39	0.335108	5.171133	1.174334	0.241676	0.362851	-6.33219
RSL1D1	0.186594	7.233428	1.174145	0.241751	0.362926	-6.43576
USH1G	1.35361	-0.00089	1.173897	0.24185	0.362996	-5.3946
MTMR2	-0.17329	5.831844	-1.17394	0.241834	0.362996	-6.4244
ZXDB	-0.25001	4.687134	-1.17369	0.241931	0.363079	-6.33732
C14orf128	0.376202	1.595719	1.173404	0.242047	0.363214	-5.6603
STAT6	-0.18513	7.784646	-1.17286	0.242264	0.3635	-6.41782
BEST1	-0.32992	0.629313	-1.17265	0.242349	0.363588	-5.60576
MEI1	-0.56972	0.188248	-1.1723	0.242489	0.36376	-5.56937
RBM18	-0.16135	4.977454	-1.17215	0.242548	0.363769	-6.36194
SUB1	0.200445	6.926727	1.172163	0.242544	0.363769	-6.4415
TMEM121	-0.41708	0.289096	-1.17178	0.242698	0.363956	-5.5678
ALG14	-0.2137	2.706783	-1.1712	0.242931	0.364267	-5.95604
RPS6KL1	0.627208	1.505286	1.170676	0.24314	0.364293	-5.61246
PCDHGA6	-0.40562	0.618951	-1.17107	0.242981	0.364293	-5.61774
GLYCTK	-0.49931	2.34885	-1.17099	0.243016	0.364293	-5.93107
LMBR1L	-0.18686	3.964723	-1.17059	0.243175	0.364293	-6.22888
CCDC82	-0.21837	4.513048	-1.17072	0.243122	0.364293	-6.31568
C2CD3	0.230382	5.421637	1.170826	0.24308	0.364293	-6.37312
ARF4	-0.20414	7.4962	-1.17064	0.243156	0.364293	-6.42751
GBAS	-0.23012	6.191183	-1.17094	0.243035	0.364293	-6.44031
USP5	0.213973	6.622141	1.170567	0.243184	0.364293	-6.44127
CLEC11A	0.470694	2.849701	1.170447	0.243232	0.364326	-5.86719
C2orf64	-0.18044	3.700247	-1.1701	0.243371	0.364456	-6.17368
NUDT9	-0.18503	4.134536	-1.17015	0.243352	0.364456	-6.25924
TRMU	0.172408	3.531427	1.168447	0.244035	0.365412	-6.06884
CCDC88C	0.28296	6.223232	1.168372	0.244066	0.365419	-6.43004
UBE2Q2	-0.27315	5.814811	-1.16784	0.24428	0.3657	-6.43448
LOC595101	0.373059	2.925826	1.167584	0.244383	0.365815	-5.90052
U2AF1L4	-0.23408	2.332378	-1.16724	0.24452	0.365981	-5.89032
ZNF121	0.258083	3.657634	1.166499	0.24482	0.366391	-6.08329
BBS12	-0.24788	1.901069	-1.16613	0.244968	0.366456	-5.81301

C8orf40	-0.28955	4.252354	-1.16618	0.244948	0.366456	-6.29497
RIN3	0.351936	4.939732	1.166319	0.244893	0.366456	-6.31118
AGBL5	-0.23104	4.717618	-1.16626	0.244917	0.366456	-6.34761
ZNF16	-0.2015	3.319633	-1.16552	0.245215	0.366746	-6.1004
AIP	0.223033	4.920035	1.165539	0.245207	0.366746	-6.32438
ERP29	-0.20736	6.496434	-1.16525	0.245323	0.366868	-6.44966
NIPA1	-0.24433	5.410643	-1.16516	0.245359	0.366883	-6.41425
DDC	1.761957	0.659057	1.163727	0.245939	0.367711	-5.40975
EXD3	-0.25154	3.172988	-1.16317	0.246165	0.367852	-6.07583
PTDSS2	-0.22644	4.244705	-1.1633	0.246112	0.367852	-6.28927
NAB2	0.30809	4.766572	1.163322	0.246103	0.367852	-6.29773
SOX13	0.343766	5.482141	1.163229	0.246141	0.367852	-6.37788
C17orf63	0.218398	6.106367	1.163173	0.246163	0.367852	-6.43344
NINJ2	0.429507	1.689369	1.162588	0.2464	0.368164	-5.67818
BHLHE40	0.333798	7.738884	1.161971	0.24665	0.368498	-6.44277
LITAF	0.225843	7.150574	1.161797	0.246721	0.368564	-6.45175
LRRC25	0.485651	1.817815	1.161452	0.246861	0.368734	-5.6932
DDAH2	0.354853	4.863085	1.161107	0.247	0.368894	-6.30656
CHMP2A	-0.23146	5.908627	-1.16106	0.247021	0.368894	-6.44431
MTRR	0.203142	5.115439	1.160798	0.247126	0.369012	-6.35517
PTPLAD2	-0.43879	3.112239	-1.16059	0.247208	0.369091	-6.10998
CEBPZ	0.164887	6.027112	1.160536	0.247232	0.369091	-6.43494
EMP2	0.351649	7.228773	1.160067	0.247422	0.369336	-6.45412
FASTKD5	0.199537	4.544438	1.15962	0.247604	0.369568	-6.28249
ZNF324B	-0.24486	2.790595	-1.15936	0.247709	0.369634	-5.99672
TTC9	-0.56983	4.280198	-1.15935	0.247713	0.369634	-6.3392
C17orf37	0.491617	5.640423	1.159315	0.247728	0.369634	-6.3843
CDR2	-0.28401	4.53476	-1.15895	0.247877	0.369818	-6.33974
RDH11	0.179274	6.279135	1.158674	0.247989	0.369945	-6.44722
BOLA3	0.265152	3.489913	1.15789	0.248308	0.370381	-6.05124
GALE	-0.41768	5.156959	-1.15752	0.248457	0.370564	-6.41502
PLBD2	0.211358	6.057501	1.157236	0.248574	0.370699	-6.43808
ZNF398	-0.19491	4.898836	-1.15594	0.249104	0.371449	-6.37571
TMPPE	-0.28183	2.579291	-1.1557	0.2492	0.371488	-5.96065
TOP3B	0.154752	4.360608	1.155717	0.249194	0.371488	-6.26225
PUM2	-0.13807	7.623064	-1.15568	0.24921	0.371488	-6.44312
CCDC76	-0.18589	3.939707	-1.15546	0.249298	0.371581	-6.24045
BATF	0.626313	1.793429	1.154802	0.249567	0.371942	-5.67513
VASH2	-0.50167	1.4835	-1.15471	0.249603	0.371956	-5.79465
PPP1R1C	0.638698	-0.01315	1.15443	0.249719	0.372089	-5.41823
PLA2G16	-0.67526	3.769563	-1.15435	0.249752	0.372098	-6.28521
ACTR10	-0.16384	5.079986	-1.15395	0.249917	0.372264	-6.39373
RFFL	0.242958	5.723016	1.153996	0.249897	0.372264	-6.41818
PLEKHM1	-0.27569	6.19756	-1.15349	0.250103	0.372501	-6.46121
C7orf31	-0.35216	2.659814	-1.15277	0.250401	0.372905	-5.9942
CTSK	0.502955	5.471616	1.152548	0.25049	0.372998	-6.37405
MAK16	0.257918	4.713818	1.152321	0.250582	0.373057	-6.30776
NAV2	-0.36053	6.08741	-1.15236	0.250565	0.373057	-6.46187
CCRN4L	0.333932	3.215803	1.152043	0.250697	0.373187	-5.98513
C19orf23	0.327119	0.453321	1.15187	0.250767	0.373252	-5.51549
LARP4	-0.18401	6.243781	-1.15174	0.250822	0.373294	-6.46265
FLOT2	0.210738	6.837349	1.151081	0.251091	0.373655	-6.46578
SLC34A2	1.690353	1.37779	1.150794	0.251209	0.373729	-5.47646
JAK3	0.460722	3.982479	1.150764	0.251221	0.373729	-6.13402
CDYL	0.198713	5.134638	1.150876	0.251175	0.373729	-6.36931
FDXACB1	0.246124	1.566134	1.150616	0.251282	0.37374	-5.6985
TSKU	0.364561	6.543952	1.150649	0.251268	0.37374	-6.45939
C5orf35	-0.32364	1.935242	-1.14998	0.251544	0.37409	-5.84777

LMO2	-0.33621	2.834919	-1.14944	0.251766	0.374335	-6.03633
C17orf89	0.289527	3.426421	1.149418	0.251774	0.374335	-6.043
USP48	-0.15676	5.86879	-1.14938	0.251789	0.374335	-6.45365
OPN3	0.366328	4.337085	1.14903	0.251934	0.37451	-6.22975
C7orf26	0.152798	5.064981	1.148029	0.252346	0.375083	-6.36903
EIF1AD	0.131159	4.825869	1.147864	0.252414	0.375143	-6.34298
CDC42EP3	-0.38725	4.750938	-1.14779	0.252445	0.37515	-6.38778
H2AFV	-0.17933	7.457944	-1.14682	0.252845	0.375704	-6.45631
NCRNA00120	0.291208	0.486338	1.146671	0.252905	0.375742	-5.53064
MED26	0.151097	4.178074	1.146567	0.252948	0.375742	-6.23952
GSR	-0.38276	5.755105	-1.14656	0.252951	0.375742	-6.46007
C9orf82	-0.26978	4.216606	-1.14639	0.25302	0.375805	-6.3102
HSPA12A	-0.43546	2.958774	-1.14624	0.253083	0.375859	-6.09138
ZNF721	-0.23479	4.559803	-1.14602	0.253174	0.375953	-6.35224
SMARCE1	0.26965	6.613001	1.145849	0.253244	0.376018	-6.46858
C5orf23	-0.77168	1.620785	-1.14533	0.253457	0.376257	-5.87186
GLCE	0.239143	5.321815	1.14533	0.253459	0.376257	-6.39126
PHB2	0.207325	6.991439	1.144773	0.253689	0.376558	-6.47264
FAM195A	-0.2922	4.040868	-1.14429	0.253889	0.376816	-6.28659
C8orf33	0.196636	5.719512	1.143123	0.254372	0.377491	-6.43361
HLA-DMA	0.53871	4.755129	1.143034	0.254409	0.377506	-6.28433
ISYNA1	0.502682	4.512313	1.142437	0.254656	0.377815	-6.24942
GPS1	0.168346	6.284027	1.142402	0.254671	0.377815	-6.46627
OVGP1	-0.40619	0.83473	-1.14212	0.254787	0.377907	-5.68284
C1orf38	0.433181	3.830388	1.142173	0.254765	0.377907	-6.11292
LRMP	-0.59242	0.967832	-1.142	0.254836	0.377939	-5.73561
NEK4	-0.19931	4.66992	-1.14124	0.255153	0.378369	-6.36701
MAP2K7	-0.15278	5.324009	-1.14103	0.255242	0.378461	-6.42925
MYL6B	0.335098	3.480912	1.140952	0.255272	0.378466	-6.05775
MYO1D	0.280479	7.49158	1.140775	0.255345	0.378534	-6.47097
DSG1	2.044718	1.775839	1.14056	0.255435	0.378627	-5.49945
IRF2BP2	0.151511	8.143792	1.140345	0.255524	0.378719	-6.45335
RHCG	2.872876	4.505547	1.139928	0.255697	0.378936	-5.81004
FAM108B1	0.193184	4.554645	1.139625	0.255823	0.379082	-6.30755
RPS17	0.243062	8.870798	1.13933	0.255946	0.379223	-6.43304
SEMA6B	-0.34255	3.561736	-1.13918	0.25601	0.379278	-6.21207
TPRA1	0.178674	4.846413	1.138722	0.256199	0.379518	-6.35026
MORC3	-0.1942	5.566621	-1.13789	0.256545	0.37999	-6.45302
ZNF720	-0.20654	3.913736	-1.1378	0.256584	0.380007	-6.25924
CCDC71	-0.17431	3.607953	-1.13753	0.256697	0.380134	-6.18997
C22orf36	-0.30831	2.903976	-1.13726	0.256807	0.38024	-6.0574
SAV1	-0.22693	5.31957	-1.13722	0.256823	0.38024	-6.43848
GMEB1	0.176153	3.343459	1.136867	0.256972	0.380421	-6.06122
STK11IP	0.162439	4.419548	1.136708	0.257038	0.380459	-6.29292
SNTB2	-0.21789	6.042723	-1.13664	0.257065	0.380459	-6.47598
BCAR1	-0.23002	6.802362	-1.13661	0.25708	0.380459	-6.47973
LOC146880	0.300517	4.563891	1.136393	0.25717	0.380552	-6.29673
GGNBP2	-0.11458	5.971461	-1.13614	0.257275	0.380667	-6.47134
MAPKBP1	-0.32382	5.783576	-1.13576	0.257435	0.380864	-6.47158
TRAPPC3	0.151678	5.133785	1.135453	0.257562	0.381012	-6.39117
GLG1	-0.15921	8.024135	-1.13534	0.257609	0.381041	-6.45423
FADS3	0.396765	3.722017	1.135192	0.257671	0.381093	-6.10657
STRN3	-0.21771	5.248616	-1.13489	0.257796	0.381237	-6.43491
TFCP2L1	-0.57308	5.585258	-1.13479	0.257838	0.381258	-6.47283
ARCN1	-0.1773	7.78982	-1.13469	0.25788	0.38128	-6.46153
MRPS27	-0.16079	5.630138	-1.1342	0.258085	0.381543	-6.45917
TASP1	0.17981	3.45153	1.133516	0.258372	0.381927	-6.08851
CABLES1	-0.49491	4.105727	-1.13324	0.258489	0.38202	-6.33485

CYP2S1	0.627018	6.757855	1.133284	0.258469	0.38202	-6.47793
KLC4	-0.37128	4.32478	-1.13235	0.258863	0.382531	-6.35252
PWP2	0.204132	4.894483	1.132084	0.258972	0.382644	-6.36141
UCP2	0.4471	5.829411	1.132032	0.258994	0.382644	-6.43631
HLA-DOB	0.560459	0.855392	1.131946	0.25903	0.382657	-5.566
COMP	1.284628	0.362204	1.131789	0.259096	0.382714	-5.44253
HSD17B7P2	-0.45999	0.044384	-1.13094	0.259451	0.383197	-5.58063
LACTB	0.204089	3.773474	1.130838	0.259495	0.383222	-6.15799
TJP3	-1.00527	3.835918	-1.13015	0.259786	0.383611	-6.3572
PTPRF	0.266747	9.393022	1.129859	0.259907	0.383732	-6.42601
DDX60	0.43635	5.905548	1.129821	0.259923	0.383732	-6.44534
LRG1	-0.71133	3.607424	-1.12947	0.260069	0.383908	-6.28979
METTL3	0.168188	4.501941	1.129044	0.260249	0.384133	-6.31469
ZNF766	-0.17426	4.064139	-1.12886	0.260326	0.384178	-6.29248
CCDC6	-0.21148	7.1395	-1.12884	0.260335	0.384178	-6.48281
CD3D	0.593436	1.335673	1.127608	0.260854	0.384904	-5.63777
LOC338758	-0.31109	0.527588	-1.12741	0.260938	0.384947	-5.6389
NF1	0.224108	7.131257	1.127452	0.26092	0.384947	-6.49097
LY6G6C	1.307876	0.621698	1.126993	0.261113	0.385107	-5.45169
LMTK3	0.547379	3.159772	1.126954	0.26113	0.385107	-5.96036
CDC40	-0.15225	5.12119	-1.12697	0.261124	0.385107	-6.427
YIPF4	-0.13291	5.448371	-1.12683	0.261184	0.385146	-6.45357
SNAI2	0.803965	4.493979	1.126747	0.261217	0.385155	-6.20437
DPYD	-0.47279	4.414603	-1.12655	0.261299	0.385223	-6.38252
MRP63	0.208568	5.059197	1.126507	0.261318	0.385223	-6.38675
FLJ42393	-0.3641	0.539103	-1.12573	0.261646	0.385624	-5.64766
PIGT	0.227816	7.520624	1.125796	0.261619	0.385624	-6.48635
SCPEP1	-0.3962	6.026102	-1.12557	0.261716	0.385686	-6.49157
NOP14	0.193151	5.779418	1.125192	0.261874	0.385879	-6.45834
EYA4	-0.76838	0.098696	-1.12464	0.262109	0.386185	-5.63705
PDCD7	-0.14077	4.471827	-1.1243	0.262251	0.386354	-6.35551
SH3TC1	-0.38683	4.748261	-1.12421	0.262288	0.386367	-6.41324
DCAF4	0.235824	3.767889	1.123985	0.262384	0.38645	-6.15914
STK17B	0.265116	5.57196	1.123948	0.2624	0.38645	-6.43722
CLDN15	0.618912	3.221774	1.123453	0.262609	0.386718	-5.96156
CDK5RAP3	-0.19623	5.974141	-1.12322	0.262707	0.38682	-6.48835
MFRP	0.408375	3.608682	1.12311	0.262755	0.38685	-6.09037
MPI	-0.19632	4.638169	-1.12301	0.262796	0.38687	-6.38305
LGALS7B	2.173613	2.04663	1.12284	0.262869	0.386937	-5.53826
LOC730101	-0.50998	3.029559	-1.12278	0.262897	0.386937	-6.14403
CNNM3	-0.25054	5.628938	-1.1224	0.263056	0.387131	-6.47651
ARID3B	-0.26365	3.751089	-1.12232	0.263091	0.387142	-6.25189
4-Sep	-0.31638	1.831666	-1.12198	0.263235	0.387312	-5.86141
ZNF498	0.222228	4.257235	1.121525	0.263427	0.387554	-6.2706
ZNRF3	-0.32462	4.696224	-1.12144	0.263465	0.387569	-6.40522
EIF2C3	-0.18054	4.022177	-1.12047	0.263873	0.388088	-6.29566
FAM83G	0.387976	5.433381	1.120495	0.263864	0.388088	-6.41718
HIP1	0.338444	5.529925	1.119881	0.264125	0.388417	-6.43222
KIAA1609	0.346683	4.650578	1.119607	0.264242	0.388548	-6.32356
LRRC15	0.907273	2.766301	1.119124	0.264447	0.388668	-5.83683
NUDT18	-0.24849	2.412429	-1.11909	0.264462	0.388668	-5.95919
SIGIRR	-0.3684	4.373701	-1.11929	0.264376	0.388668	-6.37235
C7orf50	0.232578	5.203886	1.119178	0.264424	0.388668	-6.4087
CYP51A1	0.314563	6.605546	1.11922	0.264406	0.388668	-6.49748
SRP54	0.160119	5.614345	1.119014	0.264494	0.388674	-6.4548
C11orf71	-0.26629	2.429572	-1.11882	0.264578	0.388757	-5.96828
FAM84A	0.739497	5.34033	1.118404	0.264753	0.388973	-6.37283
ERCC8	-0.15542	2.860427	-1.11826	0.264814	0.389019	-6.03979

IPO5	-0.18736	7.655388	-1.1182	0.26484	0.389019	-6.48343
NUBP1	-0.17417	4.092954	-1.11785	0.264989	0.389155	-6.30941
OTUD4	0.179311	6.507657	1.117859	0.264985	0.389155	-6.49962
POLR2F	0.207113	4.678542	1.117786	0.265017	0.389156	-6.34816
ALG11	0.33189	0.054939	1.117173	0.265278	0.389439	-5.49712
WWOX	-0.27394	2.982176	-1.11701	0.265349	0.389439	-6.09485
MRPS24	0.212551	5.467339	1.117075	0.26532	0.389439	-6.43996
CPSF7	0.129356	6.665815	1.117027	0.265341	0.389439	-6.50357
MCCC2	-0.19012	6.595484	-1.11712	0.265302	0.389439	-6.504
TSPAN13	0.421598	6.256375	1.116788	0.265442	0.389535	-6.48374
NRF1	0.098476	3.822538	1.116432	0.265594	0.389717	-6.20767
KIAA1826	-0.23012	3.768672	-1.11625	0.265673	0.389758	-6.25964
SMAGP	0.416698	4.942208	1.116235	0.265678	0.389758	-6.35964
DDT	-0.2218	4.858548	-1.11604	0.26576	0.389837	-6.41849
TMEM87A	0.16467	6.057194	1.115924	0.265811	0.389871	-6.48681
PIPSL	0.161644	3.033903	1.115121	0.266154	0.39031	-6.02059
BRD4	0.164255	7.560045	1.115093	0.266166	0.39031	-6.49633
NHEDC2	-0.33833	3.25718	-1.11492	0.266238	0.390334	-6.17191
WDR3	0.219017	5.380443	1.114966	0.266221	0.390334	-6.43351
ZNF282	0.192756	5.460441	1.114753	0.266311	0.3904	-6.44356
MLXIPL	1.091318	1.676154	1.114415	0.266456	0.390571	-5.63352
HKDC1	1.891407	1.783735	1.114337	0.26649	0.390579	-5.54163
PQLC2	0.18047	3.908212	1.114144	0.266572	0.390659	-6.21173
PLEKHH2	-0.42601	3.606109	-1.11387	0.266689	0.390707	-6.26428
ANKRD17	-0.16612	7.427422	-1.11392	0.266668	0.390707	-6.49416
ACACA	0.230927	6.924929	1.113921	0.266667	0.390707	-6.50759
PLCB2	0.455481	2.730111	1.113283	0.266941	0.391035	-5.91053
C15orf48	0.792982	4.363111	1.112808	0.267144	0.391292	-6.18605
GREM1	-0.76785	6.33816	-1.11259	0.267237	0.391387	-6.50573
SENP5	0.210818	5.699586	1.11244	0.267302	0.391441	-6.46536
CD3E	0.575024	3.047244	1.112249	0.267384	0.391499	-5.95112
RNF138	0.214931	5.052982	1.112217	0.267397	0.391499	-6.40136
DTX3	-0.4323	3.563412	-1.1119	0.267535	0.39166	-6.26227
CCNC	-0.17147	5.906399	-1.11137	0.267761	0.39195	-6.49834
DLX3	1.184217	0.316805	1.110815	0.267999	0.392257	-5.46443
KLHL31	0.402539	0.560323	1.110606	0.268089	0.392347	-5.56598
SLC31A2	-0.30203	3.490558	-1.11027	0.268235	0.39252	-6.21779
TMED4	-0.17082	5.548482	-1.10969	0.268483	0.392842	-6.48187
C2orf47	0.159208	3.816834	1.10895	0.268801	0.393228	-6.20099
ERI2	-0.19546	4.607493	-1.10894	0.268804	0.393228	-6.3945
ZSWIM5	-0.51845	3.008078	-1.10814	0.26915	0.393694	-6.15596
VAC14	0.158106	6.403689	1.108061	0.269184	0.393702	-6.50845
PSMG4	0.282799	2.696103	1.107613	0.269377	0.393943	-5.93673
HEXDC	-0.22009	3.69512	-1.10711	0.269593	0.394177	-6.24955
ARHGEF4	0.970509	4.959992	1.10715	0.269576	0.394177	-6.29626
IDI2	-0.24707	1.240841	-1.10692	0.269677	0.394258	-5.76469
ALKBH3	-0.32434	3.545972	-1.10671	0.269768	0.39435	-6.23908
ZNF517	-0.23661	3.269449	-1.1065	0.269856	0.394437	-6.16262
MDH2	0.200069	7.764894	1.106436	0.269884	0.394438	-6.50209
PLEKHA5	-0.21835	5.50219	-1.10588	0.270123	0.394745	-6.48588
GNPAT	-0.16023	5.849013	-1.10572	0.270192	0.394804	-6.50175
FAM127B	0.349413	5.511198	1.105364	0.270347	0.39499	-6.44585
CD200	-0.40419	2.400662	-1.105	0.270506	0.39518	-6.00563
EXOC3L	0.281961	1.418837	1.10459	0.270682	0.39533	-5.71887
FAM177A1	-0.19336	5.365637	-1.10462	0.270669	0.39533	-6.47606
ATP5E	0.211242	6.651796	1.104564	0.270693	0.39533	-6.51627
RNF39	0.594269	2.980782	1.104144	0.270875	0.395513	-5.94012
KIF27	-0.25427	2.240633	-1.10417	0.270862	0.395513	-5.94449

FREM2	-1.00988	1.96567	-1.10389	0.270984	0.395606	-6.0167
MYL12A	0.252169	7.732862	1.103866	0.270995	0.395606	-6.50664
PITX1	0.577905	6.690469	1.103767	0.271038	0.395627	-6.51024
ATG4C	-0.18583	2.851302	-1.1034	0.271198	0.395819	-6.05948
RABL5	0.277888	4.090692	1.103331	0.271227	0.39582	-6.24533
FAM104A	0.133603	5.449628	1.103201	0.271283	0.395861	-6.46001
ERCC2	0.200412	4.190626	1.10265	0.271522	0.396126	-6.28087
SLC27A3	-0.24209	4.218163	-1.10271	0.271494	0.396126	-6.35614
LRRC47	-0.14233	5.912086	-1.10236	0.271646	0.396266	-6.5075
LOC100129550	-0.30313	4.005284	-1.1021	0.27176	0.396391	-6.33225
SESTD1	-0.30948	5.330189	-1.10161	0.271971	0.396657	-6.48428
ETV1	0.535538	3.324536	1.101505	0.272018	0.396685	-6.02686
CCDC22	-0.16847	4.202224	-1.10125	0.272129	0.396792	-6.34478
CASP4	0.304971	5.576467	1.101205	0.272148	0.396792	-6.45976
ZNF233	-0.36713	0.115921	-1.10094	0.272263	0.396826	-5.61112
TEAD2	-0.34025	4.309468	-1.10089	0.272285	0.396826	-6.38329
MAX	0.146702	5.380554	1.100928	0.272268	0.396826	-6.45542
7-Mar	0.17454	6.760656	1.100943	0.272262	0.396826	-6.52167
UQCRHL	0.244359	4.074008	1.100776	0.272334	0.396856	-6.25086
ITM2B	-0.24405	9.455108	-1.09946	0.272908	0.397651	-6.44189
CLTCL1	-0.46124	2.379862	-1.09908	0.273072	0.397848	-6.01952
USP49	0.317307	1.150539	1.098784	0.273201	0.397952	-5.67789
CSF3R	0.586883	2.148233	1.098805	0.273191	0.397952	-5.80275
IRF4	-0.57862	1.693588	-1.09823	0.273442	0.398262	-5.9028
HEATR7A	-0.21465	6.077184	-1.09814	0.27348	0.398276	-6.51944
TSPAN17	0.199804	5.117064	1.097957	0.273561	0.398352	-6.42577
PCMTD2	0.19789	5.579287	1.09736	0.273821	0.398648	-6.47291
PHGDH	-0.53917	5.568543	-1.09737	0.273818	0.398648	-6.51305
NRIP1	0.296317	6.461502	1.097216	0.273883	0.398698	-6.51846
CPLX1	-0.49473	1.892726	-1.09678	0.274076	0.398811	-5.92566
C6orf211	0.182176	4.567986	1.096866	0.274036	0.398811	-6.35778
GET4	0.210347	5.559134	1.096839	0.274048	0.398811	-6.47057
ZNF335	0.121103	5.570022	1.096881	0.27403	0.398811	-6.47843
BST1	0.465563	0.553494	1.096464	0.274211	0.39893	-5.57207
JKAMP	0.152828	5.355708	1.096458	0.274214	0.39893	-6.45686
HAAO	-0.53261	1.605189	-1.09577	0.274515	0.399293	-5.88173
SSNA1	0.208031	5.000898	1.095755	0.274521	0.399293	-6.4135
FAM164C	-0.29594	0.75245	-1.09565	0.274567	0.399317	-5.70417
MAN1A1	-0.42228	5.591585	-1.09533	0.274707	0.39948	-6.51175
INTS1	0.177052	7.979343	1.09482	0.274929	0.399762	-6.50906
ZNF224	-0.25104	3.03338	-1.0946	0.275024	0.399858	-6.12222
HMCN1	-0.58898	3.889111	-1.09399	0.275292	0.400206	-6.3649
SLC15A4	0.171212	4.575041	1.09356	0.275481	0.400397	-6.36458
MANSC1	-0.46566	5.605107	-1.09357	0.275475	0.400397	-6.51592
MCPH1	-0.19118	4.4328	-1.09327	0.275606	0.400537	-6.38955
RAB6A	0.204653	7.648788	1.092386	0.275995	0.401061	-6.52008
PVR	0.27631	6.268481	1.092082	0.276128	0.401212	-6.5178
MED28	-0.15889	4.098623	-1.09175	0.276275	0.401342	-6.33667
MCL1	-0.21029	8.980505	-1.09181	0.276249	0.401342	-6.46758
SYT8	1.003505	1.677012	1.091432	0.276413	0.401501	-5.67082
FAM134C	-0.12887	6.152029	-1.09103	0.276588	0.401672	-6.52718
IMMT	-0.12956	6.849456	-1.09107	0.276573	0.401672	-6.53069
CAMTA1	-0.1904	4.472038	-1.09094	0.276628	0.401689	-6.39763
BEND6	0.738819	0.633495	1.090789	0.276695	0.401744	-5.55752
HLA-DRB6	1.008369	0.729937	1.090562	0.276795	0.401847	-5.53411
BRF2	0.253215	3.624889	1.090463	0.276838	0.401869	-6.16001
MRPS15	0.197794	5.517543	1.090184	0.276961	0.402005	-6.47525
ZFAND1	-0.1706	5.148488	-1.08921	0.277389	0.402584	-6.47241

BDNF		0.661537	0.675889	1.088484	0.277708	0.402892	-5.57453
TRAPPC5		0.241611	4.778824	1.088544	0.277682	0.402892	-6.38959
ETS2		0.289991	7.75123	1.088467	0.277716	0.402892	-6.52371
LRP11		0.227415	6.289676	1.088507	0.277698	0.402892	-6.52428
LRRC37A2		-0.25472	2.808344	-1.08816	0.277853	0.403048	-6.07972
CLTA		0.206218	6.900853	1.087894	0.277968	0.403173	-6.53605
RIPK3		-0.47121	2.986567	-1.08713	0.278303	0.4036	-6.1616
VEGFA		0.327098	7.242303	1.087095	0.27832	0.4036	-6.53502
ANKRD36B		0.346903	2.278496	1.086935	0.278391	0.403661	-5.87239
PIM1		-0.36804	5.926063	-1.08685	0.278428	0.403674	-6.53127
APLF		0.334889	1.620322	1.086219	0.278707	0.404035	-5.76552
KIAA0100		-0.19939	8.002962	-1.08615	0.278736	0.404035	-6.50795
C17orf58		-0.21398	3.001758	-1.08592	0.278838	0.404141	-6.11716
NCCRP1		1.659992	2.512559	1.085768	0.278905	0.404197	-5.71921
CCRL2		0.627907	1.711896	1.08561	0.278976	0.404257	-5.73502
APRT		0.240923	5.980026	1.085171	0.279169	0.404496	-6.51253
	6-Mar	-0.19888	6.920168	-1.08401	0.279684	0.405199	-6.53629
PCDHGA11		-0.41356	0.228676	-1.08376	0.279792	0.405295	-5.65388
RPL11		-0.2009	8.967596	-1.08373	0.279808	0.405295	-6.47699
MPZL3		0.411458	1.824608	1.083644	0.279844	0.405305	-5.78735
RRAS		-0.27513	5.381858	-1.08349	0.279912	0.405361	-6.50538
OR2A7		0.508996	3.636378	1.083114	0.280079	0.405561	-6.11627
CELSR2		0.52493	6.841511	1.082596	0.280308	0.405851	-6.53821
MATR3		-0.09416	8.08307	-1.08247	0.280366	0.405893	-6.51237
MYO7B		1.794985	2.08992	1.082085	0.280535	0.406053	-5.63177
CPT1C		0.504826	1.347201	1.082106	0.280525	0.406053	-5.70191
HNRNPUL1		0.126387	8.471927	1.08199	0.280577	0.406072	-6.50652
PJA1		0.274035	4.325504	1.081647	0.280729	0.40625	-6.31928
CXCR7		0.631353	5.17203	1.081278	0.280893	0.406445	-6.40321
GDI1		0.15505	7.161788	1.08076	0.281122	0.406735	-6.54092
UNKL		-0.20603	3.937358	-1.08035	0.281306	0.406959	-6.32786
TMCO7		-0.20582	3.96255	-1.08005	0.281438	0.407108	-6.33236
HTRA3		0.525455	5.273642	1.079292	0.281775	0.407552	-6.43017
C19orf62		0.144041	5.38758	1.079069	0.281874	0.407653	-6.47967
CYTH4		0.446912	2.635273	1.078696	0.282039	0.407851	-5.93047
SLC25A37		0.265003	4.815245	1.078353	0.282192	0.407916	-6.40243
C1GALT1		0.385339	5.133369	1.078478	0.282136	0.407916	-6.42804
ASH2L		-0.20937	5.475007	-1.07833	0.282203	0.407916	-6.51342
CLIP1		-0.27375	7.160095	-1.07845	0.282151	0.407916	-6.53647
CDK5RAP2		-0.20839	6.088661	-1.07827	0.282231	0.407916	-6.5411
RPS20		0.220674	9.027255	1.078129	0.282292	0.407963	-6.49408
SPRR1B		2.897339	5.113465	1.077964	0.282365	0.407984	-5.97822
BET1		0.25053	4.580573	1.078017	0.282342	0.407984	-6.37018
LXN		0.439877	2.957355	1.077609	0.282523	0.408049	-5.9915
DPAGT1		0.243038	5.502147	1.077581	0.282536	0.408049	-6.4834
DENND1A		-0.17443	5.451504	-1.07757	0.28254	0.408049	-6.51037
RABAC1		-0.22379	5.433215	-1.07754	0.282556	0.408049	-6.51213
MAP1LC3B		-0.16948	6.474182	-1.07757	0.28254	0.408049	-6.54711
ZNF638		-0.13995	6.621591	-1.07746	0.282589	0.408054	-6.5473
PPP2R5E		0.168674	5.726372	1.077344	0.282641	0.408088	-6.50849
PLXNC1		0.610675	2.529262	1.076839	0.282866	0.40837	-5.88869
FOXC2		0.773557	0.45762	1.075871	0.283298	0.408923	-5.54225
DDX3Y		1.548447	3.047681	1.075849	0.283308	0.408923	-5.83813
YBX2		0.915814	1.357547	1.075526	0.283452	0.409047	-5.65259
C20orf196		0.292433	1.275597	1.075535	0.283448	0.409047	-5.72542
PDDC1		-0.19485	5.320623	-1.0747	0.283822	0.409538	-6.50479
MAPK1IP1L		-0.13963	7.238921	-1.07451	0.283905	0.409616	-6.5415
PAF1		-0.14696	5.613808	-1.07434	0.283982	0.409685	-6.52303

CAMSAP1	0.217138	6.04065	1.07403	0.28412	0.409842	-6.52923
CDK9	0.13472	5.451837	1.07379	0.284227	0.409912	-6.49206
BAIAP2	-0.39535	6.340822	-1.07385	0.284202	0.409912	-6.5511
FDFT1	0.337989	7.000364	1.073655	0.284288	0.409957	-6.5512
C14orf102	-0.13907	3.799131	-1.07343	0.284387	0.410057	-6.2945
VAMP1	-0.23677	2.715791	-1.07321	0.284485	0.410114	-6.07338
ASL	-0.38986	4.780954	-1.07326	0.284466	0.410114	-6.47191
ATG9B	0.744976	1.655062	1.071657	0.285182	0.41105	-5.72671
BIN2	0.437329	2.250974	1.071633	0.285193	0.41105	-5.87171
GPR137C	0.413209	0.369179	1.071466	0.285268	0.411115	-5.57959
MRPS18C	-0.1533	3.534916	-1.07101	0.285473	0.411369	-6.24231
ACPP	0.723648	3.234571	1.070942	0.285503	0.41137	-6.00468
AKR1B10	-1.08538	5.362006	-1.07072	0.285601	0.411469	-6.54835
ARPC4	0.170171	6.551971	1.070602	0.285656	0.411504	-6.55192
ZNF384	0.136061	6.156361	1.070519	0.285693	0.411515	-6.54159
KCTD15	-0.46558	5.009295	-1.07028	0.285801	0.411629	-6.50434
GCA	-0.29683	4.141998	-1.06953	0.286137	0.41207	-6.38641
FAM82A2	-0.1585	4.995694	-1.06941	0.286189	0.412103	-6.4777
KANK4	-0.76	0.286518	-1.06918	0.286292	0.412209	-5.71963
SOX15	1.340379	3.463918	1.069047	0.286354	0.412213	-5.94995
AARS2	0.245435	4.641324	1.06907	0.286343	0.412213	-6.39014
PDCD1LG2	0.51819	1.055549	1.068883	0.286427	0.412248	-5.66807
GPX7	0.505215	2.456936	1.068795	0.286467	0.412248	-5.9009
PAN2	-0.20426	5.15643	-1.06886	0.28644	0.412248	-6.49768
LRRC4	-0.65973	2.292325	-1.06851	0.286593	0.412387	-6.07212
MYO1F	0.449499	3.20824	1.068315	0.286683	0.412474	-6.05458
NARS2	0.212001	3.944561	1.068034	0.28681	0.412614	-6.26313
ZFP1	0.181554	3.446219	1.067811	0.28691	0.412715	-6.16123
CSNK1A1	-0.19937	8.092048	-1.0677	0.286961	0.412746	-6.52482
RSPH3	-0.19307	3.239894	-1.067	0.287273	0.413152	-6.1886
EYA3	0.214026	3.617401	1.066823	0.287354	0.413227	-6.19221
NUP43	0.163398	5.27664	1.065964	0.287742	0.413742	-6.48083
PI4K2B	-0.17219	5.5041	-1.06564	0.287887	0.413908	-6.52647
C4orf33	-0.27297	3.265792	-1.06528	0.288051	0.414101	-6.20883
AFAP1L2	0.440144	4.989966	1.065196	0.288088	0.414112	-6.41964
EIF2B3	0.165805	3.843099	1.06476	0.288285	0.41431	-6.25513
C9orf25	0.241141	4.652	1.064764	0.288283	0.41431	-6.39759
SLC25A5	-0.20483	8.396059	-1.06459	0.28836	0.414375	-6.51741
CDRT1	0.573224	-0.05913	1.064379	0.288457	0.414471	-5.5128
FAM192A	0.140238	5.603513	1.06339	0.288904	0.415059	-6.51563
RPS25	-0.2373	7.297318	-1.06334	0.288926	0.415059	-6.55029
REV1	-0.15	5.330589	-1.06319	0.288993	0.415114	-6.51477
BRD3	0.183545	5.729079	1.06294	0.289107	0.415235	-6.52296
HPS5	0.160723	4.753079	1.062623	0.289251	0.415399	-6.42374
TMEM14C	-0.20062	5.710903	-1.06255	0.289286	0.415407	-6.54351
TTC8	0.214575	3.827125	1.062048	0.289511	0.415681	-6.24579
USP24	-0.14719	6.619547	-1.06199	0.289536	0.415681	-6.5637
PHLPP1	-0.25607	5.291542	-1.06137	0.289818	0.416043	-6.52123
ZNF845	-0.26651	3.326527	-1.06111	0.289938	0.416128	-6.22936
POMT2	0.201998	4.330886	1.061131	0.289927	0.416128	-6.35336
GPR65	0.432952	0.586773	1.060827	0.290065	0.416253	-5.61882
ZNF518B	-0.53464	3.668039	-1.06078	0.290084	0.416253	-6.35477
RIN2	0.238763	6.161647	1.059814	0.290524	0.416841	-6.54915
LY6D	2.711151	4.172061	1.059432	0.290698	0.417043	-5.86499
NT5E	0.544572	5.308475	1.059373	0.290724	0.417043	-6.45262
SMAD5	-0.1689	6.357045	-1.05917	0.290815	0.417131	-6.56567
NUDT6	-0.26261	0.663947	-1.05892	0.29093	0.417253	-5.72406
C20orf94	0.272589	1.251732	1.058626	0.291064	0.417401	-5.74077

MTMR4	-0.17612	5.827628	-1.05816	0.291274	0.417659	-6.55253
S1PR4	-0.43711	0.763084	-1.05752	0.291568	0.418039	-5.76692
ITGB5	0.234268	7.041112	1.057282	0.291675	0.418149	-6.56806
SLC9A3R1	0.358698	7.608207	1.056879	0.291858	0.418369	-6.56152
TBC1D10A	-0.2451	4.530318	-1.05659	0.29199	0.418515	-6.44777
LYSMD4	-0.202	3.359908	-1.05649	0.292033	0.418534	-6.22775
BNC1	2.24513	2.68439	1.055774	0.292362	0.418962	-5.69605
TUBGCP4	0.165974	4.387614	1.055652	0.292417	0.418999	-6.37493
ARMC1	0.157594	5.253956	1.055451	0.292509	0.419087	-6.49031
NAV3	-0.59082	1.06158	-1.05516	0.292642	0.419234	-5.84059
DGCR6L	-0.22955	4.530402	-1.05502	0.292708	0.419286	-6.44764
ATMIN	-0.14514	6.2721	-1.05483	0.292794	0.419366	-6.56867
DDIT3	0.283177	4.051935	1.054665	0.292868	0.419429	-6.28724
NME4	-0.31545	4.911092	-1.05453	0.292927	0.419471	-6.49914
LYRM4	0.232047	3.812568	1.054221	0.29307	0.419633	-6.24662
MAP3K10	-0.17427	4.330882	-1.05397	0.293183	0.419752	-6.41572
FAM133B	0.251326	2.363432	1.053847	0.293241	0.419791	-5.93817
FOXC1	0.451371	4.523345	1.053769	0.293276	0.419799	-6.35652
EXOC3L2	0.336088	1.761376	1.053394	0.293448	0.419984	-5.82053
SHANK2	-0.54242	4.908576	-1.05336	0.293466	0.419984	-6.51819
MMACHC	0.271629	2.614099	1.053053	0.293604	0.420139	-5.97852
RPL18	0.201755	8.973674	1.052723	0.293755	0.420312	-6.52224
MTRF1	-0.25965	2.910121	-1.0518	0.294179	0.420876	-6.13925
HES6	0.537943	2.499369	1.051411	0.294355	0.421041	-5.91671
ANKRD42	-0.24609	3.195872	-1.05144	0.294343	0.421041	-6.20407
LGR5	1.165044	1.561867	1.051142	0.294478	0.421131	-5.67101
RTN3	0.160672	7.025071	1.051183	0.294459	0.421131	-6.57406
CELF1	0.129772	6.64661	1.050939	0.294571	0.421221	-6.5745
TXNRD1	0.375174	7.260392	1.050079	0.294966	0.421742	-6.5746
C8orf41	0.215323	3.611856	1.04985	0.29507	0.421848	-6.20726
FAM21A	-0.25215	4.752524	-1.04963	0.295171	0.421948	-6.48176
TOMM70A	0.156332	6.754347	1.049549	0.295209	0.42196	-6.5766
FRS3	0.25063	2.149947	1.048911	0.295501	0.422335	-5.90478
PLD2	0.284587	5.425347	1.048562	0.295662	0.422521	-6.50339
SOCS4	-0.16876	5.450868	-1.04812	0.295863	0.422766	-6.54106
CBR1	-0.42138	5.971477	-1.04785	0.295987	0.422856	-6.57472
EDEM1	-0.19366	6.207899	-1.04787	0.295981	0.422856	-6.57551
RNF41	-0.13094	5.153548	-1.04775	0.296035	0.422882	-6.51387
SMO	-0.57509	3.805517	-1.04721	0.296285	0.423195	-6.39984
RLTPR	0.648976	0.750195	1.046816	0.296464	0.423408	-5.62819
KLF8	-0.55341	2.969841	-1.04607	0.296809	0.423857	-6.22944
DNTTIP2	0.142711	6.019829	1.045796	0.296934	0.423992	-6.56122
SALL1	0.759126	0.808301	1.045413	0.297111	0.424128	-5.61946
CD247	0.515715	0.675316	1.045477	0.297081	0.424128	-5.63614
ZNF20	-0.2252	2.150004	-1.04539	0.29712	0.424128	-5.98405
ARHGAP25	0.371938	2.858886	1.045303	0.297161	0.424138	-6.02015
UBQLN1	0.148219	7.168296	1.045245	0.297188	0.424138	-6.57821
ZNF124	0.296858	1.999733	1.044997	0.297302	0.424228	-5.87598
CYP4F11	1.458971	3.366352	1.044886	0.297353	0.424228	-5.9325
DGAT2	0.561346	3.910296	1.044844	0.297373	0.424228	-6.20934
FAM120A	0.162981	8.69833	1.044943	0.297327	0.424228	-6.53883
KDM5D	1.45584	2.389835	1.044388	0.297583	0.424478	-5.7758
TRPC6	-0.35953	1.981485	-1.04433	0.297609	0.424478	-5.97949
LPXN	0.356704	3.221829	1.044125	0.297704	0.42457	-6.09824
ITGA4	0.451379	3.939727	1.044061	0.297734	0.42457	-6.24149
TMEM62	0.313737	4.238715	1.043733	0.297885	0.424743	-6.33093
CEP110	0.230096	4.799435	1.043661	0.297919	0.424747	-6.4407
NDRG1	0.508322	9.288198	1.04342	0.29803	0.424862	-6.5304

LMAN2	0.177611	6.536874	1.043045	0.298203	0.425066	-6.58044
SLC16A6	0.516068	0.75855	1.042929	0.298256	0.425098	-5.65184
N4BP2L1	-0.35927	2.70612	-1.0428	0.298318	0.425143	-6.12802
CHMP2B	-0.2438	5.56946	-1.04241	0.298497	0.425355	-6.55903
BTBD10	0.164626	5.067166	1.042101	0.298639	0.425513	-6.48309
CHST11	0.500027	3.363245	1.04178	0.298788	0.425682	-6.10306
TNPO3	-0.15279	5.926832	-1.04132	0.299	0.425941	-6.57336
C4orf10	-0.22475	4.007864	-1.04062	0.299325	0.42636	-6.38438
BCL11A	0.726999	4.002047	1.040105	0.299563	0.426563	-6.20546
POLR3F	0.167044	3.64362	1.040052	0.299588	0.426563	-6.23504
SLC16A2	-0.41475	3.76276	-1.03999	0.299614	0.426563	-6.37018
SCYL3	-0.13829	4.107558	-1.0399	0.299657	0.426563	-6.38978
ANKRD50	-0.25445	5.75971	-1.03979	0.299711	0.426563	-6.57177
ALDH3A1	-0.99692	5.389157	-1.04021	0.299516	0.426563	-6.57959
VAT1	-0.23619	6.882395	-1.03985	0.299682	0.426563	-6.58294
SEC24D	-0.25122	6.171437	-1.03997	0.299625	0.426563	-6.58395
LIN37	0.220593	2.603613	1.039171	0.299996	0.426918	-6.00219
EIF2C1	-0.17708	5.93432	-1.03912	0.300022	0.426918	-6.57671
NTM	0.588335	1.899797	1.038799	0.300169	0.427051	-5.82469
FBXO17	-0.53006	2.090895	-1.03878	0.300177	0.427051	-6.03573
TEAD3	-0.18899	6.017089	-1.03808	0.300503	0.427472	-6.58097
KIAA0892	-0.13334	6.440838	-1.03801	0.300534	0.427473	-6.58827
LUC7L2	-0.12781	6.505271	-1.03792	0.300576	0.427488	-6.58874
ATE1	-0.1938	4.078728	-1.0378	0.300631	0.427523	-6.39562
B3GNT8	0.515527	2.851493	1.037547	0.30075	0.427649	-6.00211
RYR1	1.065946	2.362271	1.037473	0.300785	0.427655	-5.83544
ZNF611	-0.30942	3.199915	-1.03734	0.300844	0.427696	-6.23299
APTX	0.189608	4.274874	1.03722	0.300902	0.427734	-6.369
PAPD5	-0.16295	4.622642	-1.03628	0.301341	0.428227	-6.47091
COPA	0.136776	8.239889	1.036325	0.301318	0.428227	-6.56235
SLC6A6	0.282246	6.125385	1.036359	0.301302	0.428227	-6.56991
ITGAE	0.215079	3.674208	1.036116	0.301416	0.42829	-6.23727
SH2D4A	-0.33647	4.403384	-1.03597	0.301485	0.428345	-6.46136
FAM199X	0.167632	5.145357	1.035893	0.301519	0.42835	-6.49779
KIAA1009	-0.18075	3.235736	-1.03576	0.301581	0.428395	-6.21675
PLAC8	-0.77192	3.417301	-1.03541	0.301746	0.428542	-6.36488
SLC31A1	-0.19758	6.150639	-1.03546	0.301723	0.428542	-6.58726
FAM50A	0.202234	5.369714	1.035304	0.301794	0.428566	-6.51866
FAH	0.264022	3.622634	1.035219	0.301833	0.428578	-6.21542
PIP5K1A	0.146122	6.130985	1.035096	0.30189	0.428616	-6.57722
ACHE	0.945771	2.100884	1.035006	0.301932	0.428632	-5.80534
BSG	-0.20815	9.12618	-1.03466	0.302093	0.428817	-6.52277
ALOX12B	1.161159	0.935907	1.034401	0.302214	0.428901	-5.60283
ANAPC2	-0.14072	5.308083	-1.03445	0.302191	0.428901	-6.54195
CCDC93	-0.16565	5.567367	-1.03429	0.302265	0.42893	-6.56297
MRPS6	0.200739	4.474242	1.034057	0.302374	0.429042	-6.40761
ZNF496	-0.20422	4.351831	-1.03378	0.302502	0.429136	-6.44352
ELL	-0.14281	4.898713	-1.03383	0.302483	0.429136	-6.50359
GUSB	0.240759	5.856833	1.033516	0.302627	0.429269	-6.55876
CDK13	0.136763	6.36167	1.033445	0.30266	0.429272	-6.58719
PTPN18	-0.24887	5.942727	-1.03337	0.302695	0.429278	-6.58501
WDFY1	0.16746	6.207736	1.033265	0.302744	0.429305	-6.58142
NDUFA11	-0.17272	5.440924	-1.03177	0.303444	0.430254	-6.55749
TMUB2	0.13482	5.262207	1.031684	0.303482	0.430264	-6.51769
C4orf27	-0.17831	3.262814	-1.0315	0.303568	0.430343	-6.22872
DDX28	0.205942	3.815227	1.031122	0.303745	0.43055	-6.27598
ZNF117	0.485022	5.941931	1.03083	0.303882	0.430695	-6.5517
ABHD4	-0.27143	5.871425	-1.03077	0.303909	0.430695	-6.58602

ATP6AP1	0.135444	7.217535	1.030691	0.303947	0.430704	-6.59224
CPNE8	0.46763	3.787572	1.030416	0.304076	0.430799	-6.21942
OASL	0.60652	4.043312	1.030446	0.304062	0.430799	-6.23821
C15orf38	-0.21745	4.262518	-1.03021	0.304172	0.430849	-6.43533
CYHR1	0.18474	4.781166	1.030213	0.304171	0.430849	-6.45763
ALOX5AP	0.40855	3.109874	1.029881	0.304326	0.430981	-6.08102
ZSWIM1	0.151655	4.39347	1.029752	0.304386	0.430981	-6.4051
TMX3	-0.19192	5.597796	-1.02978	0.304372	0.430981	-6.57109
ARHGAP5	-0.23669	7.432133	-1.02971	0.304406	0.430981	-6.58223
UBQLN2	0.176826	6.529807	1.02968	0.30442	0.430981	-6.5941
AIFM2	0.275287	4.328374	1.028962	0.304757	0.431327	-6.37469
PDGFB	0.32101	4.588219	1.029052	0.304714	0.431327	-6.41307
UTP11L	0.158516	5.015358	1.029008	0.304735	0.431327	-6.49154
LOC388955	0.27251	4.216127	1.028536	0.304956	0.431565	-6.35105
ZNF512	-0.21695	4.710118	-1.02696	0.305694	0.432563	-6.49678
TGOLN2	-0.21881	8.198313	-1.0269	0.305723	0.432563	-6.563
PRR4	-0.38706	1.136312	-1.02639	0.305965	0.432686	-5.85133
NAT6	-0.18895	2.825877	-1.02659	0.305869	0.432686	-6.13366
ZNF337	-0.19632	4.267341	-1.02647	0.305924	0.432686	-6.43771
LARS2	-0.16045	4.626602	-1.02645	0.305935	0.432686	-6.48105
PMPCB	-0.15389	6.070459	-1.02645	0.305937	0.432686	-6.5935
BFSP1	0.417412	1.046821	1.026289	0.306011	0.432708	-5.72375
C9orf116	0.384918	0.177811	1.025781	0.30625	0.433002	-5.60142
KLF7	-0.42822	3.164922	-1.02531	0.306472	0.433185	-6.26316
KRTCAP2	0.213138	6.127908	1.02533	0.306462	0.433185	-6.5843
RB1CC1	-0.19367	6.689175	-1.02533	0.306462	0.433185	-6.60097
GRB14	0.839081	0.239128	1.025153	0.306545	0.433244	-5.55765
11-Sep	0.196688	6.918189	1.025085	0.306577	0.433246	-6.6019
CRELD2	-0.2047	4.974669	-1.02404	0.307072	0.4339	-6.5264
LASS3	1.929922	1.939146	1.023231	0.307451	0.434392	-5.65788
SPRR2D	2.259371	2.546462	1.023095	0.307515	0.434438	-5.70062
TFPT	0.211731	3.015567	1.022569	0.307763	0.434704	-6.10288
CCDC113	-0.42418	2.626058	-1.02256	0.307765	0.434704	-6.14177
DNAJB6	0.227415	5.819303	1.022268	0.307905	0.434858	-6.5689
FAM50B	-0.42416	2.650928	-1.02199	0.308034	0.434996	-6.14721
RREB1	-0.20713	7.175126	-1.0214	0.308314	0.435348	-6.59683
C16orf7	-0.23901	4.149118	-1.0212	0.308408	0.435436	-6.43147
TMEM160	0.20797	3.23206	1.021123	0.308446	0.435446	-6.15262
PRSS53	0.316131	0.751425	1.020882	0.30856	0.435563	-5.69681
KIAA0586	0.178076	4.331237	1.020079	0.30894	0.436055	-6.4002
RNASEH2B	0.192824	4.183812	1.019859	0.309043	0.436157	-6.36902
ALKBH1	-0.12365	3.611722	-1.01964	0.309147	0.43626	-6.30613
NFYA	0.212961	5.483299	1.019463	0.309231	0.436333	-6.54488
TIMM9	0.1585	4.448466	1.019162	0.309373	0.43649	-6.42398
CHD1L	0.176325	5.243644	1.01891	0.309493	0.436579	-6.52475
SLC35F5	-0.17609	5.622743	-1.0189	0.309498	0.436579	-6.58261
ZNF473	0.197707	3.907928	1.018622	0.309629	0.436719	-6.31014
FOXE1	2.128308	2.028216	1.018324	0.30977	0.436874	-5.65086
UPK1B	-1.04725	0.80033	-1.018	0.309924	0.437002	-5.90161
TIMM8B	0.235376	4.100938	1.018013	0.309918	0.437002	-6.34603
KIAA0802	0.390913	4.900675	1.017868	0.309986	0.437002	-6.46263
MRPL41	-0.21948	4.663412	-1.0179	0.30997	0.437002	-6.49979
RAB7L1	0.247018	4.279523	1.017694	0.310069	0.437075	-6.38019
RPS6	-0.24297	9.314626	-1.01674	0.310522	0.437669	-6.53354
DDI2	0.239037	3.50594	1.01655	0.310612	0.437751	-6.214
RNF4	-0.14324	6.12787	-1.01626	0.310749	0.437901	-6.60511
CHMP1A	0.159907	6.549031	1.015906	0.310917	0.438094	-6.60871
COPS7B	0.149952	4.794898	1.015751	0.310991	0.438153	-6.47807

DOLK	0.170905	4.362202	1.015663	0.311033	0.438168	-6.41017
SIX5	-0.22231	4.762038	-1.01511	0.311297	0.438495	-6.5153
DNAJC12	0.690037	0.563013	1.014758	0.311463	0.438686	-5.62782
IRX4	1.661878	0.823106	1.014318	0.311673	0.438892	-5.56426
ATG4D	0.222434	4.564903	1.014367	0.311649	0.438892	-6.43859
ZNF544	0.23573	4.740156	1.014197	0.31173	0.438929	-6.46283
CES3	0.963895	1.632829	1.013729	0.311953	0.439066	-5.74732
IL18R1	-0.40408	2.189481	-1.01386	0.311891	0.439066	-6.05251
SEZ6L2	0.549925	5.493945	1.013733	0.311951	0.439066	-6.51817
ANXA7	0.139308	6.502653	1.013765	0.311936	0.439066	-6.61039
FBN2	0.880956	2.917596	1.013345	0.312136	0.439279	-5.9762
MFSD3	0.277515	3.524551	1.013272	0.31217	0.439283	-6.20943
ATG4B	-0.12808	5.471134	-1.01308	0.31226	0.439365	-6.57575
ZNF841	-0.33331	3.894971	-1.01293	0.312331	0.439377	-6.40921
C11orf49	-0.20468	4.447362	-1.01297	0.312313	0.439377	-6.4766
NDEL1	-0.20665	5.767844	-1.01285	0.312373	0.439392	-6.5977
C10orf95	0.37577	0.560801	1.012532	0.312523	0.439558	-5.66812
VKORC1L1	0.185476	6.071202	1.011802	0.312871	0.440004	-6.59652
SNX30	0.202388	5.03174	1.011171	0.313172	0.440338	-6.50636
UGGT1	0.151505	7.301755	1.011199	0.313159	0.440338	-6.61079
ITK	-0.48635	1.556555	-1.01073	0.313381	0.440523	-5.9551
USP21	0.169289	4.796109	1.010698	0.313398	0.440523	-6.48149
ALMS1	0.207622	5.604895	1.010765	0.313366	0.440523	-6.56492
FUCA2	0.256743	5.917759	1.010591	0.313449	0.440551	-6.58516
PRR12	0.149812	6.660946	1.010305	0.313586	0.440698	-6.61604
SYT1	0.767327	2.525635	1.010024	0.31372	0.440842	-5.93386
KIAA1211	0.686473	3.317179	1.009276	0.314078	0.441301	-6.0868
GGCX	0.166809	4.853364	1.009139	0.314143	0.441348	-6.49009
ENTPD1	-0.22419	5.830841	-1.00892	0.31425	0.441454	-6.60511
PMS2L3	0.20478	1.222569	1.008561	0.31442	0.441604	-5.79637
CLTB	-0.30104	6.728417	-1.00859	0.314405	0.441604	-6.61621
CDYL2	-0.38221	1.402646	-1.00834	0.314526	0.441708	-5.91354
ACAD9	0.167758	5.434244	1.008143	0.31462	0.441796	-6.55569
CD2	0.52683	2.508038	1.007364	0.314993	0.442231	-5.96741
TRA2A	0.147175	5.325054	1.007425	0.314964	0.442231	-6.54743
SNRNP48	0.165874	4.529481	1.007217	0.315063	0.442241	-6.44716
SLC25A11	-0.14944	5.171735	-1.00728	0.315032	0.442241	-6.55823
ZFY	1.230026	1.642348	1.006933	0.3152	0.442371	-5.72629
EPS8L3	-1.38897	0.968517	-1.00689	0.315219	0.442371	-5.97952
L3MBTL	-0.37906	2.604821	-1.0068	0.315262	0.442387	-6.14594
FBXO28	0.138709	6.201365	1.006479	0.315417	0.442559	-6.60934
CALU	0.206347	7.342519	1.006241	0.315532	0.442676	-6.61591
STX18	-0.17843	4.032122	-1.00601	0.315644	0.442789	-6.41612
STEAP3	-0.28172	5.989219	-1.00594	0.315677	0.44279	-6.61496
NPAT	0.159347	5.000459	1.005438	0.315917	0.443083	-6.51301
DPCD	0.236199	2.762844	1.004825	0.316211	0.443428	-6.06463
RFXAP	-0.21757	2.506198	-1.00479	0.316227	0.443428	-6.09466
TRAPPC6B	-0.14854	4.893732	-1.00464	0.316301	0.443488	-6.53313
CLPTM1	-0.13564	7.461642	-1.00443	0.316401	0.443584	-6.60913
L2HGDH	-0.1914	4.07683	-1.00341	0.316891	0.444226	-6.42936
RPL29	-0.24281	8.452351	-1.00308	0.317048	0.444401	-6.57738
CEACAM5	1.284002	7.148152	1.002737	0.317215	0.444591	-6.60759
SIN3B	-0.14856	6.184828	-1.00228	0.317434	0.444854	-6.62068
KLHL12	0.151333	5.259592	1.002023	0.317559	0.444984	-6.54585
C7orf43	0.192232	4.592009	1.001777	0.317678	0.445061	-6.45872
HLA-DPA1	0.472843	6.507494	1.001801	0.317666	0.445061	-6.61396
C17orf71	0.143235	4.360592	1.00147	0.317825	0.445159	-6.42933
UHMK1	0.250352	5.300715	1.001433	0.317843	0.445159	-6.54114

LARP7	-0.15243	5.179174	-1.00144	0.317841	0.445159	-6.56505
PI4KAP1	-0.27237	3.637979	-1.00123	0.317941	0.445251	-6.35904
BMP2	-0.44011	4.376323	-1.00114	0.317984	0.445268	-6.50501
C14orf149	0.285184	2.3163	1.000817	0.31814	0.445401	-5.97855
RFT1	-0.15387	4.235245	-1.00081	0.318143	0.445401	-6.45229
TRAF3IP3	0.418594	1.329976	1.000399	0.318342	0.445616	-5.79158
ECM2	-0.31941	2.736651	-1.00029	0.318393	0.445616	-6.16829
USP9X	-0.18204	7.873071	-1.00031	0.318385	0.445616	-6.60137
PLEKHM2	-0.17419	6.678522	-1.00022	0.318428	0.44562	-6.62648
PPP2R2D	-0.1257	4.621816	-0.99978	0.318641	0.445874	-6.50371
DCLRE1A	0.190421	4.179604	0.999633	0.318712	0.445929	-6.38699
BIN3	-0.18321	3.995943	-0.99954	0.318755	0.445945	-6.41585
ARHGAP22	0.399256	0.98387	0.999243	0.3189	0.446102	-5.74387
NRP2	-0.39624	5.530848	-0.9981	0.319451	0.446828	-6.60922
ZNF749	0.207268	2.516089	0.997918	0.31954	0.446864	-6.02918
ZNF74	0.237347	4.068515	0.997967	0.319517	0.446864	-6.35826
ZUFSP	0.15642	3.193559	0.99767	0.319661	0.446905	-6.17784
UBE2E1	0.152885	5.29688	0.99766	0.319666	0.446905	-6.55423
RNF5	-0.19259	5.506225	-0.99772	0.319635	0.446905	-6.5975
METT10D	-0.13803	5.212305	-0.99731	0.319834	0.447094	-6.57108
NEURL1B	0.330345	5.632511	0.997249	0.319865	0.447094	-6.57152
ZCCHC4	-0.15716	2.586545	-0.99694	0.320014	0.447213	-6.1078
ANP32A	0.157853	5.927288	0.996874	0.320046	0.447213	-6.60504
SGK269	-0.2396	6.016708	-0.997	0.319984	0.447213	-6.62367
CFI	0.537177	3.786143	0.996624	0.320167	0.447301	-6.23525
MARVELD1	0.353364	5.708677	0.996611	0.320173	0.447301	-6.5771
LILRB3	0.468025	0.744509	0.996338	0.320306	0.447307	-5.69969
RAD52	-0.20148	2.624715	-0.99641	0.320271	0.447307	-6.12294
GLRX	-0.32192	4.02046	-0.99651	0.320222	0.447307	-6.44557
NIT1	0.152424	4.495378	0.996401	0.320275	0.447307	-6.45476
C11orf75	-0.30074	3.0938	-0.99619	0.320377	0.447314	-6.24282
TP53I11	0.361258	6.548607	0.996129	0.320407	0.447314	-6.62425
PPRC1	0.151217	6.744217	0.996156	0.320394	0.447314	-6.63083
ATP10B	1.138468	4.417128	0.995141	0.320886	0.447938	-6.24963
SLC46A3	-0.39637	4.075209	-0.99501	0.320951	0.447984	-6.46592
BBX	-0.2084	6.297006	-0.9946	0.321148	0.448215	-6.63124
CCNG2	-0.26432	5.334219	-0.99445	0.32122	0.448271	-6.59337
RILPL2	0.210228	3.564773	0.994371	0.321259	0.448281	-6.25546
CTDP1	-0.14296	4.882995	-0.99364	0.321613	0.44873	-6.54207
GALNT1	0.227117	6.921664	0.992978	0.321936	0.449136	-6.63411
FAM102B	0.308005	5.256547	0.992703	0.32207	0.449277	-6.53943
NDUFB3	0.163969	5.033538	0.992582	0.322129	0.449314	-6.52911
HTR7P1	0.300601	1.534928	0.992284	0.322274	0.449427	-5.85111
CHCHD7	-0.20265	4.283664	-0.99231	0.322261	0.449427	-6.47485
TGM1	1.578797	3.676441	0.992213	0.322308	0.44943	-6.02264
ZNF775	-0.24889	2.116317	-0.99165	0.32258	0.449752	-6.03557
ARHGEF2	-0.19494	6.084457	-0.99161	0.322603	0.449752	-6.62976
LOC284023	0.253295	2.293169	0.991503	0.322654	0.449777	-5.98945
DIS3	0.156337	6.121431	0.991354	0.322726	0.449834	-6.62041
CUX1	-0.18708	7.337163	-0.99108	0.32286	0.449975	-6.62412
GJA4	0.36572	2.444887	0.990937	0.322929	0.450027	-5.99898
OSGEPL1	0.172973	2.520723	0.990701	0.323044	0.450097	-6.04183
ARF6	0.176566	7.421	0.990739	0.323026	0.450097	-6.62935
E2F5	0.47302	2.412696	0.990528	0.323129	0.450125	-5.97461
ARHGAP33	0.311753	3.440331	0.99055	0.323118	0.450125	-6.20949
SFT2D1	0.183957	4.421921	0.990396	0.323193	0.45017	-6.44272
ARFIP1	-0.14646	5.389271	-0.99026	0.323259	0.450216	-6.59339
KDM6B	-0.17801	6.516528	-0.9899	0.323435	0.450418	-6.63725

C9orf80	0.136559	3.580973	0.989753	0.323506	0.450471	-6.27586
PLA2R1	0.408138	3.770542	0.989656	0.323553	0.450492	-6.26952
RPS15	0.228242	7.598549	0.989411	0.323673	0.450614	-6.62801
STARD3	0.372757	5.335616	0.98889	0.323927	0.450878	-6.54535
AFG3L2	-0.18004	6.014689	-0.98893	0.323908	0.450878	-6.63007
CYP4F3	1.134328	2.90108	0.988383	0.324175	0.451125	-5.96062
ADM	0.486262	4.700678	0.988329	0.324201	0.451125	-6.44902
TMED5	-0.17253	6.301069	-0.98841	0.324162	0.451125	-6.63697
PIH1D1	0.153621	4.924682	0.988134	0.324296	0.451212	-6.52147
TCIRG1	0.248746	6.161645	0.987313	0.324698	0.451725	-6.62236
SPDYA	0.263715	0.13999	0.986436	0.325127	0.452277	-5.64906
KIAA0895L	-0.17533	3.642919	-0.98618	0.325251	0.452405	-6.35715
EMB	0.533497	4.919393	0.985515	0.325577	0.452814	-6.47824
TMEM33	0.160695	6.715938	0.984979	0.32584	0.453134	-6.64158
KLK1	0.801663	0.700659	0.984729	0.325962	0.453259	-5.65801
AMICA1	-0.42671	1.87388	-0.98445	0.326101	0.453407	-6.02806
HSPA6	0.698275	3.336139	0.983932	0.326353	0.453712	-6.12217
KIAA0528	-0.17867	5.764083	-0.98353	0.326548	0.453893	-6.62537
WDR82	-0.1189	6.571315	-0.98357	0.326528	0.453893	-6.64346
SRRM5	0.256564	0.443471	0.983301	0.326663	0.453986	-5.69591
C14orf101	0.177664	4.41746	0.983265	0.32668	0.453986	-6.45044
CXCR3	0.561705	0.734964	0.983146	0.326739	0.454022	-5.69909
RNF121	0.214573	3.850142	0.982613	0.327001	0.454341	-6.32878
PIN1	-0.14638	4.907992	-0.98251	0.327052	0.454368	-6.55601
MICALCL	0.472624	1.694169	0.982357	0.327126	0.454425	-5.85746
MAPKAPK2	-0.13396	7.087487	-0.98216	0.327224	0.454515	-6.63886
PTCD2	-0.15642	2.475577	-0.98201	0.327299	0.454529	-6.09986
CITED4	0.38853	4.7235	0.982062	0.327271	0.454529	-6.47181
SAMD4B	0.135349	7.433573	0.980787	0.327898	0.455316	-6.63814
MUC17	2.64343	1.850717	0.980611	0.327985	0.455391	-5.60141
NDUFB4	0.17793	6.402786	0.980455	0.328061	0.455452	-6.6403
KIAA1712	-0.18567	3.805651	-0.98037	0.328102	0.455464	-6.39937
RPL18A	0.260288	4.544195	0.980016	0.328277	0.455662	-6.46379
BACE2	-0.32329	6.511703	-0.9798	0.328383	0.455764	-6.64672
BIRC3	-0.53322	4.867242	-0.97918	0.328689	0.456142	-6.58812
CPT1A	-0.32433	7.076172	-0.97911	0.328723	0.456144	-6.63874
MAP3K6	-0.29343	5.680636	-0.97898	0.328788	0.456189	-6.63078
TDRD7	0.213998	4.776297	0.978472	0.329038	0.456491	-6.50526
NDUFS3	-0.15866	5.57284	-0.97828	0.329135	0.45658	-6.61891
LOC100128842	-0.27594	1.218104	-0.97787	0.329337	0.456679	-5.89441
KLHL8	-0.16593	4.154581	-0.97787	0.329333	0.456679	-6.46434
POLR3E	-0.12958	5.212042	-0.97792	0.329313	0.456679	-6.58915
ARF5	0.188477	6.109821	0.977929	0.329306	0.456679	-6.63176
HMOX1	0.359746	4.2889	0.977557	0.32949	0.456846	-6.40471
SEPHS2	0.240016	6.540887	0.977084	0.329723	0.457124	-6.64553
ZNF575	-0.19167	1.379021	-0.97661	0.329957	0.457357	-5.91161
SRF	-0.1891	6.587747	-0.97663	0.329948	0.457357	-6.65011
SLC4A8	-0.49899	0.894985	-0.97626	0.330131	0.457553	-5.87519
CDS1	0.304544	5.481882	0.976172	0.330174	0.457567	-6.57926
EMID1	-0.54658	1.645098	-0.97587	0.330324	0.457729	-6.00798
AVIL	-0.37769	0.971087	-0.9754	0.330556	0.457914	-5.87259
MED18	-0.16959	3.345988	-0.97546	0.330524	0.457914	-6.29798
TNFRSF10D	-0.41734	3.135967	-0.97546	0.330525	0.457914	-6.29972
CCS	-0.15063	3.977605	-0.97514	0.330682	0.458044	-6.43196
ARAP2	0.29905	5.288057	0.97503	0.330738	0.458076	-6.56142
HTRA1	0.321366	5.975512	0.974875	0.330815	0.458092	-6.62068
FLOT1	0.158846	6.744687	0.974896	0.330805	0.458092	-6.65161
ZNF623	0.165958	4.934836	0.97477	0.330867	0.458119	-6.5346

NUFIP1	0.186532	3.219071	0.973631	0.331431	0.458855	-6.20148
FAM84B	-0.33822	6.190311	-0.97341	0.33154	0.458959	-6.65195
ZNF416	-0.21662	2.246938	-0.97293	0.331777	0.459197	-6.07412
RHOD	0.444746	5.020699	0.972941	0.331773	0.459197	-6.51617
C7orf10	0.652299	0.347025	0.972292	0.332095	0.459591	-5.6461
C1orf104	0.270934	2.105848	0.972119	0.332181	0.459664	-5.96946
NAA30	-0.13635	5.032159	-0.97204	0.332221	0.459674	-6.57831
ZNF195	0.237917	4.273601	0.971736	0.332371	0.459836	-6.42439
PEX13	0.140584	5.600123	0.971118	0.332678	0.460215	-6.60856
LAT	0.358808	1.874211	0.970888	0.332792	0.460308	-5.91925
ZNF521	-0.36462	2.695335	-0.97085	0.332811	0.460308	-6.19594
ATP5G2	-0.17307	6.977101	-0.97036	0.333052	0.460596	-6.65166
GPX8	0.353277	4.123651	0.970098	0.333184	0.46068	-6.37445
SUPV3L1	0.143372	4.788054	0.970149	0.333159	0.46068	-6.52308
ZMYM4	-0.12114	6.13174	-0.97004	0.333211	0.46068	-6.65032
CSNK2A1P	0.168249	5.338085	0.969802	0.333332	0.4608	-6.58368
GOLM1	0.494661	7.05182	0.969328	0.333568	0.461081	-6.65667
REPIN1	-0.30151	6.869262	-0.96921	0.333627	0.461118	-6.65264
NELF	0.231568	6.311043	0.969096	0.333683	0.461149	-6.64691
AP4M1	0.194867	3.696574	0.968632	0.333914	0.461407	-6.31076
RBM25	-0.13813	7.375898	-0.96859	0.333935	0.461407	-6.64611
DCAF17	0.141964	4.988166	0.968225	0.334117	0.461611	-6.54995
PRKAR1B	0.265677	3.637613	0.96603	0.335211	0.463078	-6.28723
GAL	1.158894	0.326223	0.965652	0.3354	0.463279	-5.60686
TAX1BP1	-0.1934	7.199364	-0.96561	0.335423	0.463279	-6.65167
DUSP28	-0.15466	2.160462	-0.96519	0.335629	0.463487	-6.05362
LOC388692	-0.28301	4.179038	-0.96517	0.33564	0.463487	-6.49723
KLK5	1.49362	0.499191	0.964862	0.335794	0.463609	-5.6087
DPF2	0.169994	5.800909	0.964927	0.335762	0.463609	-6.62772
TSNAX	0.134001	5.545263	0.964715	0.335868	0.463664	-6.61077
CFDP1	0.173555	4.499267	0.964556	0.335947	0.463728	-6.48392
GPRIN2	-0.56016	2.946506	-0.96353	0.33646	0.464325	-6.29491
SEC22A	0.14827	3.999729	0.963539	0.336456	0.464325	-6.39359
RTCD1	-0.15647	4.921193	-0.96349	0.336479	0.464325	-6.57632
ITM2C	-0.32005	6.043541	-0.96317	0.336638	0.464498	-6.65904
C19orf70	-0.18989	4.303516	-0.96266	0.336895	0.464807	-6.50469
ETAA1	0.17367	4.266091	0.96156	0.337447	0.465522	-6.44522
GZF1	-0.17136	4.493176	-0.96128	0.337585	0.465621	-6.53003
IPO11	-0.13045	4.745919	-0.96131	0.33757	0.465621	-6.55666
LMAN2L	0.165881	4.474099	0.960765	0.337846	0.465934	-6.48428
FAM104B	-0.18994	3.048022	-0.96038	0.338041	0.466013	-6.25166
RBM15	0.149216	4.401596	0.960457	0.338	0.466013	-6.47515
TRIB2	-0.33065	5.327447	-0.96043	0.338012	0.466013	-6.62971
SERTAD2	0.265233	5.98204	0.960368	0.338045	0.466013	-6.63822
VPS4B	-0.20947	6.232059	-0.96032	0.338069	0.466013	-6.66357
TRPM7	-0.15133	6.137913	-0.96013	0.338162	0.466095	-6.66066
BCAS2	0.169418	4.714711	0.95989	0.338285	0.466218	-6.52034
LSM10	0.147799	3.990518	0.959459	0.338501	0.46647	-6.39605
PTK6	0.546255	4.82556	0.959321	0.338571	0.46652	-6.48393
APOBEC3C	0.359691	4.177754	0.959163	0.33865	0.466524	-6.39647
MED4	0.151905	5.204241	0.959114	0.338674	0.466524	-6.58186
USP6NL	0.214093	5.489672	0.959236	0.338613	0.466524	-6.60472
TGFB2	0.480232	3.114055	0.958813	0.338826	0.466687	-6.13924
PIGR	-1.46627	5.116978	-0.95861	0.33893	0.466784	-6.66138
DEFB1	0.798308	2.500762	0.9579	0.339285	0.467227	-5.96956
QKI	-0.29352	6.379631	-0.95773	0.339368	0.467296	-6.66824
ZW10	0.138414	4.486292	0.957486	0.339493	0.467422	-6.49333
NEIL2	0.328067	3.623538	0.957339	0.339567	0.467478	-6.27881

EIF3G	-0.15285	6.475723	-0.95723	0.339622	0.467507	-6.66869
TROVE2	-0.1316	5.806409	-0.95703	0.339723	0.4676	-6.6511
EPC2	-0.13186	4.72522	-0.9567	0.33989	0.467784	-6.55899
GTPBP1	-0.13919	6.537047	-0.95619	0.340147	0.468092	-6.66983
LGALS4	-1.24085	3.180331	-0.95592	0.340284	0.468234	-6.46501
TRAPPC2L	-0.18685	4.335105	-0.9558	0.340343	0.468269	-6.5152
5-Mar	-0.12971	5.432811	-0.9551	0.340696	0.468708	-6.62973
RFTN2	-0.2685	1.417478	-0.95465	0.340924	0.468927	-5.9514
HECW2	0.338635	3.304741	0.954584	0.340955	0.468927	-6.20914
POLR3A	0.157381	4.913809	0.954682	0.340906	0.468927	-6.55218
KHDC1	0.396435	0.484275	0.954353	0.341072	0.469041	-5.7111
TCF7L1	-0.36059	3.975208	-0.95428	0.341107	0.469043	-6.48738
YWHAH	0.15354	7.26912	0.954126	0.341187	0.469107	-6.66714
HEATR3	0.14086	3.993017	0.953823	0.341339	0.46927	-6.40281
IER3	0.331807	6.757828	0.953597	0.341454	0.469381	-6.67044
PSMC5	0.141951	6.625487	0.952942	0.341785	0.46979	-6.67171
TSPAN3	0.317904	7.71664	0.952752	0.341881	0.469876	-6.66252
USP45	-0.17005	3.801711	-0.95246	0.342027	0.47003	-6.42177
ZNF696	0.177807	3.396508	0.952317	0.342101	0.470086	-6.26255
C2orf16	-0.27168	1.213942	-0.95208	0.34222	0.470203	-5.91914
LRRCC1	0.243737	4.598127	0.951868	0.342328	0.470306	-6.50105
HTATSF1	0.128008	6.166594	0.951777	0.342374	0.470322	-6.6616
ELOVL6	-0.3278	4.575232	-0.95158	0.342476	0.470416	-6.56577
P2RX4	0.268734	3.902658	0.951303	0.342614	0.470559	-6.35686
RUFY1	-0.15308	5.197052	-0.95123	0.342649	0.470561	-6.61549
TERF2	-0.11368	4.993496	-0.95114	0.342694	0.470577	-6.59217
IDE	-0.17851	5.673392	-0.95104	0.342749	0.470607	-6.65204
ST8SIA4	0.357906	3.070919	0.950957	0.342789	0.470615	-6.16007
SCML1	0.338459	4.099473	0.950814	0.342861	0.470668	-6.38789
GNAI2	0.165928	8.003235	0.950643	0.342948	0.47074	-6.65462
FRYL	-0.18506	6.47324	-0.95056	0.342988	0.47075	-6.67509
C2orf76	-0.24057	1.337348	-0.95034	0.343102	0.47086	-5.9378
C17orf81	0.172589	4.671473	0.950159	0.343193	0.470938	-6.52333
PHF23	0.148503	4.779952	0.949859	0.343345	0.471101	-6.54136
CNNM1	0.740886	2.339703	0.948681	0.343943	0.471874	-5.96217
WNK1	-0.22293	8.572082	-0.94801	0.344283	0.472295	-6.62716
MALT1	0.23076	5.415464	0.947615	0.344484	0.472524	-6.60732
LOC729082	-0.15003	5.017761	-0.94719	0.3447	0.472774	-6.60175
PHOSPHO2	-0.21263	1.106846	-0.94676	0.344916	0.473024	-5.8963
RPS27	0.322029	3.549734	0.946557	0.345022	0.47307	-6.27395
ZNF805	-0.14328	4.533993	-0.94651	0.345045	0.47307	-6.5461
HERC4	0.140773	5.584236	0.946446	0.345078	0.47307	-6.63072
STRN4	0.115565	6.925683	0.946432	0.345085	0.47307	-6.67854
MANF	0.189022	5.27769	0.945866	0.345373	0.473372	-6.59831
ENTPD6	0.243535	6.399003	0.945872	0.34537	0.473372	-6.6715
PRDM15	0.124433	3.763991	0.94552	0.345549	0.473567	-6.36394
C17orf57	-0.33692	1.227715	-0.94535	0.345636	0.47364	-5.93667
MIAT	0.451053	2.739116	0.944455	0.346092	0.474217	-6.08169
ZNF320	-0.40918	3.627738	-0.94437	0.346133	0.474224	-6.43789
ASNSD1	-0.10899	5.191229	-0.94431	0.346165	0.474224	-6.61772
C1D	0.139753	3.899814	0.944241	0.3462	0.474227	-6.39103
FXN	-0.16513	3.538923	-0.94397	0.34634	0.474347	-6.37113
DIO2	0.45235	5.114995	0.94387	0.34639	0.474347	-6.5555
RPL27	0.178647	8.52779	0.943931	0.346359	0.474347	-6.64506
ABL2	0.187031	5.980843	0.943277	0.346692	0.474714	-6.65834
PRR15	0.908817	2.937189	0.943196	0.346733	0.474724	-6.03551
SERPINB13	2.02794	1.826166	0.942039	0.347324	0.475486	-5.70722
SPRR2A	2.196033	2.875568	0.941717	0.347488	0.475664	-5.83732

PPP5C	0.116529	5.752238	0.941525	0.347587	0.475716	-6.64982
UBA52	0.17914	8.251666	0.94151	0.347594	0.475716	-6.65631
SRPX2	0.46018	3.278759	0.94094	0.347886	0.476045	-6.1973
FAM98B	0.180586	3.321267	0.940907	0.347902	0.476045	-6.25738
RUNDC1	-0.16206	5.035978	-0.94078	0.347965	0.476084	-6.61039
ABI3	0.330775	2.855056	0.940518	0.348101	0.476224	-6.12925
BMPR2	-0.17934	7.197897	-0.94044	0.348141	0.476231	-6.6758
ACP6	-0.30915	3.478127	-0.93989	0.348423	0.47657	-6.3911
HYLS1	0.229039	2.393305	0.939676	0.348532	0.476673	-6.05722
RAB35	0.111201	5.837693	0.939229	0.348761	0.476939	-6.65777
CLPX	-0.1261	5.328712	-0.93915	0.3488	0.476941	-6.63609
BCLAF1	-0.102	7.667726	-0.93909	0.348831	0.476941	-6.66804
ZNF227	-0.16941	3.542215	-0.93882	0.34897	0.477039	-6.37845
MTSS1	-0.52071	5.957424	-0.93883	0.348965	0.477039	-6.68366
HSD17B11	0.395466	5.143554	0.938316	0.349229	0.477345	-6.5693
EIF1AY	1.084773	1.149543	0.938098	0.34934	0.477405	-5.73725
MARK4	-0.17103	5.636032	-0.93813	0.349323	0.477405	-6.6618
RPS4Y1	1.567875	3.577502	0.937901	0.349441	0.477449	-6.06574
ANO6	-0.20263	6.687703	-0.93794	0.349419	0.477449	-6.68615
FPGT	-0.13636	4.156071	-0.9373	0.34975	0.477795	-6.49794
AKAP8	0.113153	5.179174	0.937274	0.349763	0.477795	-6.6034
NEK3	0.373551	2.982152	0.937167	0.349817	0.477823	-6.14539
INTS3	-0.12694	6.628853	-0.93693	0.349941	0.477945	-6.68795
PTHLH	1.301856	3.50539	0.936636	0.35009	0.478102	-6.09109
STOML1	-0.19326	3.096195	-0.93573	0.350558	0.478694	-6.28346
MOXD1	-0.48186	3.36959	-0.93566	0.350593	0.478695	-6.40931
TMEM189-UBE2V1	1.022836	1.69134	0.935275	0.350789	0.478916	-5.82456
SNHG7	0.219884	4.336767	0.93511	0.350874	0.478985	-6.47589
HIC2	-0.22371	3.56427	-0.9347	0.351087	0.479228	-6.39772
THAP9	-0.14923	2.541185	-0.93456	0.351159	0.479281	-6.15582
EAPP	0.142925	4.754906	0.93411	0.351388	0.479536	-6.55295
MAT2B	0.125444	5.91618	0.934059	0.351415	0.479536	-6.6664
CYP27C1	0.858374	0.658121	0.933634	0.351633	0.479787	-5.69678
GMFG	0.324784	2.161864	0.933379	0.351764	0.479825	-6.00846
SNX7	-0.1927	4.308402	-0.93346	0.351724	0.479825	-6.5335
UBE2J2	0.142535	5.145718	0.933431	0.351737	0.479825	-6.6006
GTF2A1	-0.17838	3.326038	-0.93272	0.352105	0.480196	-6.33681
SAR1A	-0.11787	6.081559	-0.93278	0.352074	0.480196	-6.68406
MBD2	0.220207	6.229685	0.932648	0.352141	0.480198	-6.67872
RARS2	-0.11912	4.64889	-0.93219	0.352376	0.480425	-6.57089
PIK3CB	0.173372	5.981184	0.932248	0.352347	0.480425	-6.66928
NFAM1	0.379922	2.262225	0.931959	0.352496	0.480495	-6.02045
VPS8	-0.15729	5.477872	-0.93202	0.352466	0.480495	-6.65642
FAM118A	-0.17622	4.114946	-0.93139	0.35279	0.480849	-6.50372
SF1	-0.08865	7.953839	-0.93126	0.352855	0.48089	-6.66771
RAB30	-0.25602	1.957067	-0.93109	0.352946	0.480967	-6.0651
SDF2L1	0.214618	4.330508	0.930752	0.353119	0.481156	-6.47792
NBEAL2	-0.29818	7.461374	-0.93059	0.3532	0.48122	-6.67669
NUMB	-0.13364	6.625618	-0.93036	0.35332	0.481337	-6.69403
LMO7	-0.37802	7.133392	-0.92997	0.35352	0.481562	-6.68314
NRXN2	-0.55766	0.559908	-0.92971	0.353654	0.481651	-5.87554
LRFN1	0.53423	2.039504	0.929727	0.353648	0.481651	-5.95976
PIAS4	0.133996	4.062712	0.929246	0.353896	0.481865	-6.44175
NQO1	-0.42111	7.388683	-0.92921	0.353915	0.481865	-6.677
FUBP3	0.108468	6.194531	0.929275	0.353881	0.481865	-6.68432
HNRNPH3	0.104573	6.62174	0.928868	0.354092	0.482059	-6.69456
C7orf60	0.210223	3.935147	0.928796	0.354129	0.482063	-6.40276
STT3A	0.16806	6.857779	0.928496	0.354284	0.482227	-6.69574

SLC7A6		0.181205	5.794435	0.928344	0.354363	0.482287	-6.66101
COL8A2		-0.46071	3.45697	-0.92781	0.354641	0.482545	-6.43286
NCOR2		-0.15302	8.666644	-0.92778	0.354656	0.482545	-6.6448
NAP1L1		0.183659	7.721514	0.927783	0.354653	0.482545	-6.68326
OXER1		-0.42789	0.360974	-0.92767	0.35471	0.482571	-5.8245
PEX5		0.146412	5.214941	0.927586	0.354755	0.482585	-6.61304
C1orf93		0.304636	5.606124	0.927435	0.354833	0.482645	-6.63713
TMEM63C		-0.73813	1.722364	-0.92708	0.355018	0.4828	-6.11119
GPRIN3		-0.46195	2.883463	-0.92709	0.35501	0.4828	-6.29338
CTSS		0.424983	6.260268	0.927015	0.355051	0.4828	-6.67657
C14orf135		0.147707	5.068262	0.926909	0.355106	0.482828	-6.5976
HMGN3		-0.2022	5.179005	-0.92664	0.355243	0.482967	-6.64062
ATP13A2		0.166403	6.613276	0.92646	0.355338	0.48305	-6.696
CLCA2		2.605858	4.334778	0.926137	0.355506	0.48323	-6.03788
THAP7		-0.15648	3.708089	-0.92594	0.355609	0.483324	-6.42421
PELO		-0.134	4.378497	-0.92545	0.355863	0.483622	-6.54389
RABL2A		0.204532	2.822823	0.925091	0.356048	0.483779	-6.15866
NDUFA1		-0.17845	5.719479	-0.92512	0.356035	0.483779	-6.67859
SMG5		0.126179	7.149814	0.924836	0.35618	0.483912	-6.69606
GPNMB		0.655909	7.468248	0.924626	0.356289	0.484013	-6.69799
XKR9		0.691838	0.641343	0.924289	0.356464	0.484132	-5.71958
DHRS9		-0.67342	1.811904	-0.92419	0.356515	0.484132	-6.10884
ARHGAP4		0.394741	4.019943	0.924221	0.3565	0.484132	-6.38816
STAU2		-0.16181	5.329945	-0.92426	0.356481	0.484132	-6.65269
SNRNP70		0.093594	6.921571	0.924043	0.356592	0.484189	-6.69926
PLXNB1		-0.18452	7.115078	-0.92378	0.356726	0.484324	-6.69279
UBXN2B		-0.16884	5.377625	-0.92368	0.356782	0.484353	-6.65748
POLR1D		-0.15436	6.51433	-0.923	0.357134	0.484783	-6.70081
PCDHGC3		-0.33879	6.209175	-0.92284	0.357218	0.48485	-6.69988
FSTL4		1.013737	0.832723	0.92255	0.357368	0.485006	-5.71444
FES		-0.28433	2.705211	-0.92222	0.35754	0.485193	-6.22882
CEACAM7		1.482019	1.627712	0.922026	0.35764	0.485282	-5.76081
BMP4		-0.4572	3.50152	-0.92173	0.357796	0.485447	-6.44
EGFLAM		0.37959	2.025303	0.921168	0.358087	0.485794	-5.98769
CROCC		-0.26343	4.689701	-0.9202	0.358592	0.486432	-6.60259
APBA2		0.511117	2.531477	0.919932	0.358731	0.486573	-6.06112
PROC		0.612887	0.44497	0.919667	0.358869	0.486688	-5.70431
ATP5G1		-0.19593	5.080829	-0.91961	0.358898	0.486688	-6.63715
PRPF38B		-0.159	6.213911	-0.91957	0.35892	0.486688	-6.70048
BLNK		-0.44885	3.318282	-0.91946	0.358979	0.48672	-6.39625
MAPK8IP2		0.590965	2.720833	0.919268	0.359077	0.486806	-6.07803
ICAM3		-0.27778	3.024306	-0.91916	0.359135	0.486834	-6.30016
ATAD1		-0.15541	5.741785	-0.9191	0.359167	0.486834	-6.68423
SH3RF3		-0.30298	3.460638	-0.91889	0.359274	0.486932	-6.40745
LCMT2		-0.19894	3.622723	-0.91842	0.359519	0.487185	-6.42176
RAB18		-0.1798	6.628357	-0.9184	0.359531	0.487185	-6.70478
MLH3		0.193167	4.374388	0.918139	0.359666	0.487322	-6.50282
RRP1B		0.152212	6.110745	0.918064	0.359706	0.487327	-6.68967
WTAP		0.117655	6.178568	0.917724	0.359883	0.48752	-6.69405
SCRN2		-0.18264	3.575842	-0.91741	0.360047	0.487648	-6.40758
FKTN		-0.15718	5.095801	-0.91742	0.360041	0.487648	-6.63724
MPST		-0.22297	5.860923	-0.91717	0.360172	0.48777	-6.69392
SLC6A20		1.301588	1.451397	0.916677	0.360431	0.488026	-5.7606
B4GALT6		-0.3235	3.01583	-0.91668	0.360428	0.488026	-6.313
WAS		0.37324	2.553198	0.916504	0.360521	0.488053	-6.08664
	10-Sep	-0.18072	6.630029	-0.91652	0.360511	0.488053	-6.70648
RPL34		-0.20894	7.411143	-0.91633	0.360609	0.488126	-6.69288
FAM131A		-0.22149	4.070595	-0.91615	0.360708	0.488212	-6.51679

NCF1	0.419602	1.809999	0.915769	0.360905	0.488431	-5.94923
PLDN	-0.1153	5.899503	-0.91556	0.361016	0.488534	-6.69314
GLT8D1	-0.13205	4.770031	-0.91481	0.361405	0.488973	-6.60262
ELOVL5	0.420279	5.572857	0.914804	0.36141	0.488973	-6.63689
CALML3	2.572003	4.041462	0.913931	0.361868	0.489536	-6.00183
KIF3B	0.179824	6.636101	0.913877	0.361896	0.489536	-6.70764
SNF8	0.132801	5.072919	0.913633	0.362024	0.489662	-6.61149
TMEM167A	-0.13261	6.438294	-0.91356	0.362061	0.489664	-6.70893
C1orf156	0.152426	2.544502	0.913174	0.362265	0.489892	-6.12241
SLC26A11	-0.2219	3.36848	-0.9124	0.362671	0.490346	-6.37519
PARG	0.133452	4.603796	0.912449	0.362646	0.490346	-6.55344
ZNF431	-0.24819	3.549896	-0.91154	0.36312	0.490906	-6.42201
KCP	0.484308	1.238724	0.911094	0.363357	0.491132	-5.85168
NUDT16P1	-0.48404	2.359666	-0.91113	0.36334	0.491132	-6.19821
BAG2	-0.32999	3.926028	-0.91095	0.363434	0.49114	-6.51387
UBTF	0.107819	6.694653	0.911011	0.3634	0.49114	-6.71151
LOC401588	0.220966	1.436598	0.910415	0.363714	0.491471	-5.92131
S100A12	1.062145	1.068703	0.909661	0.364111	0.491938	-5.75044
MUC20	0.76386	4.853594	0.909624	0.36413	0.491938	-6.50275
ZDHHC14	-0.2454	3.333061	-0.90921	0.36435	0.492188	-6.37207
THSD1P1	0.219712	2.56951	0.908827	0.36455	0.49241	-6.11881
GALK2	0.176137	4.242726	0.908525	0.364709	0.492529	-6.48769
SNX6	0.140813	6.211647	0.908573	0.364683	0.492529	-6.70281
NLRP2	-0.84729	2.553799	-0.90811	0.364928	0.492682	-6.31827
SRPRB	0.155456	5.502953	0.908233	0.364862	0.492682	-6.65767
UNC45A	-0.1383	6.355152	-0.90817	0.364893	0.492682	-6.71294
SERPINA1	0.806112	6.119527	0.907698	0.365145	0.492905	-6.65911
C14orf166	0.136112	6.447173	0.907662	0.365163	0.492905	-6.71065
FADS1	0.489435	4.534227	0.907444	0.365278	0.493013	-6.49503
PIGZ	-0.28737	2.954363	-0.90736	0.365322	0.493024	-6.29752
ABCC1	0.330745	7.601996	0.907021	0.365501	0.493219	-6.70743
TTF1	0.127869	3.963903	0.906814	0.365611	0.493318	-6.44193
HS6ST2	1.057828	1.687481	0.906696	0.365673	0.493355	-5.84847
PHLDB3	0.30657	3.53645	0.906249	0.365909	0.493626	-6.31332
NLRP3	-0.36564	0.541843	-0.90592	0.36608	0.493761	-5.86465
CLINT1	-0.19241	7.062041	-0.90597	0.366054	0.493761	-6.70986
ATG16L1	-0.15842	5.041687	-0.90585	0.366118	0.493765	-6.64252
NFKBIZ	0.331723	5.712943	0.905683	0.366208	0.493838	-6.66476
LATS1	-0.15539	5.244765	-0.9054	0.366355	0.493989	-6.66206
CEP290	-0.25701	4.502774	-0.90528	0.366419	0.494028	-6.5924
SP1	0.106251	7.636156	0.904981	0.366578	0.494195	-6.7044
SLC25A29	-0.21903	4.645464	-0.90456	0.366803	0.494449	-6.60706
NSL1	0.140256	4.396353	0.904012	0.367091	0.49479	-6.528
SFXN2	-0.30986	3.407929	-0.90339	0.367421	0.495187	-6.40478
NKG7	0.55394	1.413399	0.903001	0.367626	0.495362	-5.8765
PIGB	0.133254	3.516708	0.902967	0.367644	0.495362	-6.34273
COPS2	-0.12282	6.298978	-0.90294	0.367657	0.495362	-6.71655
C6orf182	0.167657	2.471887	0.902863	0.367699	0.495371	-6.11443
ATP1A3	-0.5465	0.458963	-0.90247	0.367909	0.495558	-5.88126
RSL24D1	-0.13495	6.02262	-0.90252	0.367881	0.495558	-6.71024
SP140	0.515789	1.094198	0.902306	0.367994	0.495625	-5.83166
GANC	-0.16547	4.295634	-0.90209	0.36811	0.495733	-6.55651
PIK3CD	0.327794	4.299346	0.901104	0.368631	0.496387	-6.48298
COG4	0.130218	5.861249	0.901021	0.368675	0.496399	-6.69308
IGFBP7	0.278049	7.514342	0.900505	0.368949	0.496671	-6.7141
SIN3A	0.11898	6.618813	0.900517	0.368942	0.496671	-6.72018
ACVR1B	-0.18418	6.17367	-0.90013	0.369147	0.496891	-6.71769
PCOLCE	0.382957	5.202699	0.899978	0.369228	0.496952	-6.61275

CAV1	-0.44185	6.601504	-0.89963	0.369412	0.497152	-6.71948
ZNF428	-0.17344	3.54728	-0.89943	0.369521	0.497251	-6.41722
SGK3	-0.19451	4.296749	-0.89925	0.369614	0.497327	-6.56269
RNASE6	0.401077	2.43345	0.899017	0.369739	0.497447	-6.0765
TMEM147	0.178529	5.535755	0.89873	0.369891	0.497605	-6.66759
RPL32P3	0.215611	3.383755	0.898351	0.370093	0.497798	-6.30048
RGL1	-0.25681	4.064134	-0.89833	0.370107	0.497798	-6.53795
TRAPPC4	-0.16261	4.5633	-0.89813	0.370209	0.497888	-6.59579
C1orf198	0.159443	6.200263	0.897522	0.370534	0.498269	-6.7117
TINAGL1	0.32555	6.419726	0.897466	0.370564	0.498269	-6.71407
ZNF101	0.18277	2.748458	0.897257	0.370674	0.49837	-6.1713
VIL1	1.966268	1.382249	0.89593	0.371381	0.499273	-5.6856
SPCS2	0.202137	4.427052	0.895784	0.371459	0.499281	-6.53015
GRLF1	-0.15801	7.272316	-0.8958	0.371448	0.499281	-6.71541
ASRGL1	-0.49403	2.16571	-0.89562	0.371549	0.499354	-6.17281
HIVEP1	0.17883	5.859604	0.89552	0.3716	0.499374	-6.6953
RGS4	-0.44752	1.704848	-0.8952	0.37177	0.499417	-6.08545
ANAPC13	-0.1549	5.906921	-0.89519	0.371774	0.499417	-6.71323
CNDP2	-0.19921	6.784913	-0.89531	0.37171	0.499417	-6.72389
GSK3A	0.130023	6.706818	0.895375	0.371677	0.499417	-6.72549
USP3	-0.14951	5.000082	-0.89493	0.371917	0.49956	-6.64721
GSDMB	0.700569	3.711424	0.894821	0.371972	0.499586	-6.27204
CPVL	0.509858	3.635138	0.894266	0.372269	0.49984	-6.30558
PTPN13	-0.61967	5.198683	-0.89427	0.372268	0.49984	-6.69935
C19orf2	0.196608	6.300526	0.894301	0.37225	0.49984	-6.71693
PQLC3	-0.18655	4.415481	-0.8939	0.372463	0.500053	-6.5825
C4orf14	-0.14045	5.24438	-0.89349	0.372681	0.500298	-6.67157
MEGF6	-0.36703	4.63113	-0.8933	0.372782	0.500385	-6.63126
EHHADH	-0.22648	4.470289	-0.89302	0.372934	0.500542	-6.5944
LOC100129534	-0.32196	0.354199	-0.89247	0.373227	0.500887	-5.84088
PEX16	0.166384	4.059985	0.892239	0.373351	0.500997	-6.46707
ARMC8	-0.13456	5.322501	-0.89218	0.373381	0.500997	-6.67913
ZNF12	0.136469	5.543262	0.891928	0.373517	0.501132	-6.67754
KIF3A	-0.15123	4.103802	-0.89168	0.373649	0.501259	-6.53357
CD79A	-0.64723	1.915296	-0.89127	0.373868	0.50149	-6.15674
ADAT1	0.157228	4.104862	0.891227	0.373892	0.50149	-6.47975
C1orf54	0.277578	1.334866	0.891038	0.373994	0.501578	-5.91366
FPR1	0.428562	1.255498	0.890689	0.37418	0.50178	-5.8811
VPRBP	-0.12971	5.780459	-0.89045	0.374308	0.501903	-6.71082
ABCA1	0.315454	5.682408	0.890315	0.374381	0.501904	-6.67751
R3HDM2	-0.11868	6.381387	-0.89034	0.374368	0.501904	-6.729
PIGV	-0.16359	4.072143	-0.89024	0.374419	0.501907	-6.52967
PDE3B	-0.33733	3.76127	-0.89016	0.374463	0.501919	-6.50312
NDUFC2	0.182105	6.226677	0.889876	0.374615	0.502074	-6.71867
SLC37A2	0.55624	3.835188	0.889499	0.374817	0.502297	-6.34894
PLAA	-0.19488	5.276434	-0.8893	0.374923	0.50239	-6.68225
TMEM170A	0.164198	4.723738	0.889165	0.374997	0.502441	-6.58676
GLT25D2	-0.60221	1.046317	-0.8888	0.375195	0.502615	-5.99559
C1orf216	0.172792	3.475553	0.888788	0.375199	0.502615	-6.34233
PHF16	-0.1814	4.181011	-0.88854	0.375332	0.502745	-6.55325
NPEPPS	-0.16137	6.947749	-0.88803	0.375605	0.503062	-6.72841
PRKAA1	-0.19004	6.916842	-0.88795	0.375649	0.503073	-6.72855
CNIH2	0.524124	0.693338	0.887732	0.375765	0.503084	-5.78347
TMEM139	-0.71843	0.782347	-0.88778	0.375737	0.503084	-5.966
BCL10	0.190995	5.081575	0.887736	0.375763	0.503084	-6.62959
SRC	0.174905	7.15469	0.887615	0.375828	0.50312	-6.73017
URM1	0.164374	5.697009	0.88753	0.375874	0.503133	-6.69154
HS3ST3A1	0.61046	1.752578	0.887396	0.375946	0.503182	-5.94041

AHCTF1	0.141144	6.592354	0.887237	0.376031	0.503248	-6.73141
CDKN2A	1.020794	2.690813	0.887101	0.376104	0.503297	-6.03083
NOTCH2NL	-0.1909	4.537035	-0.88687	0.376225	0.503411	-6.60638
TOMM40L	-0.16909	3.671451	-0.88663	0.376354	0.503482	-6.45519
LRCH4	0.192006	5.510806	0.886575	0.376387	0.503482	-6.67487
EHBP1	0.191792	6.246328	0.88668	0.37633	0.503482	-6.72196
EPDR1	-0.44339	2.443152	-0.88623	0.376573	0.503655	-6.2386
ATF7IP2	-0.36867	2.6802	-0.8862	0.376587	0.503655	-6.26601
FAM123B	-0.26406	3.93255	-0.88592	0.376739	0.503808	-6.52696
ELAC2	-0.12553	6.13217	-0.88485	0.377312	0.504527	-6.729
PCDHGB2	0.557536	1.758532	0.884581	0.377459	0.504627	-5.94716
SUPT4H1	0.132861	5.653656	0.884614	0.377441	0.504627	-6.69313
RASSF9	0.574291	1.495375	0.884253	0.377635	0.504814	-5.90526
FLJ37453	-0.20106	1.676133	-0.88385	0.377854	0.50501	-6.04947
TRDMT1	-0.21552	2.647415	-0.88385	0.377853	0.50501	-6.23584
PCDHGA9	-0.37642	1.245965	-0.88377	0.377893	0.505011	-6.00155
MDFIC	-0.30037	4.944033	-0.88371	0.377927	0.505011	-6.66611
POF1B	0.78627	5.204898	0.883313	0.378142	0.505201	-6.57885
NAB1	0.17039	6.65294	0.883352	0.37812	0.505201	-6.73525
TMEM22	0.356747	1.808211	0.883212	0.378196	0.505225	-5.98775
BAT5	0.158358	5.458858	0.882875	0.378377	0.50542	-6.67613
ELL3	0.244797	3.199677	0.882636	0.378507	0.505544	-6.26713
GALT	-0.16336	3.668895	-0.88206	0.378817	0.50591	-6.45496
STC1	-0.36908	5.113485	-0.88192	0.378895	0.505966	-6.68769
ATOX1	0.188414	4.496761	0.881847	0.378932	0.505967	-6.55725
CHST15	0.323335	5.307108	0.88169	0.379016	0.506031	-6.64739
ITGB1	0.188567	9.188244	0.881328	0.379212	0.506244	-6.67886
BUD13	0.134475	4.102646	0.881197	0.379283	0.50628	-6.49279
NBL1	-0.22041	6.8335	-0.88114	0.379312	0.50628	-6.73544
RNASEH2C	-0.15361	4.428445	-0.88106	0.379358	0.506294	-6.59245
KIAA1383	0.481373	1.829756	0.8809	0.379443	0.506334	-5.97368
C19orf56	-0.13561	5.840735	-0.88087	0.379461	0.506334	-6.72237
STARD4	-0.25749	3.363472	-0.88061	0.379601	0.506472	-6.40584
HINT2	-0.2437	3.449898	-0.88036	0.379733	0.506552	-6.4229
SRCAP	-0.1119	8.165315	-0.88038	0.379723	0.506552	-6.70618
FEM1A	-0.14761	5.532188	-0.88019	0.379828	0.50663	-6.70638
CCL5	0.536292	3.778132	0.879987	0.379936	0.506727	-6.34258
GBP6	1.746121	3.486976	0.879813	0.38003	0.506804	-6.06714
COL6A2	-0.37458	8.722739	-0.87956	0.380165	0.506936	-6.67924
UBL5	0.143668	6.002663	0.879277	0.38032	0.507093	-6.71953
BCCIP	0.127638	5.608485	0.8789	0.380524	0.507317	-6.69481
CDC34	0.152036	4.893784	0.878575	0.3807	0.507503	-6.61953
LOC400657	-0.19239	2.286282	-0.87822	0.380895	0.507659	-6.16262
CEP76	0.189246	3.447035	0.87809	0.380962	0.507659	-6.34013
ZNHIT1	0.182963	5.656664	0.87813	0.38094	0.507659	-6.69523
PTPLAD1	0.179526	6.920094	0.878223	0.38089	0.507659	-6.74077
SFRP4	1.044832	2.868266	0.877752	0.381145	0.507806	-6.07433
NUDT19	-0.17889	4.68711	-0.87781	0.381111	0.507806	-6.63092
TMEM125	-0.55566	2.674708	-0.87757	0.381242	0.507887	-6.30137
CDH6	0.395915	2.384174	0.876378	0.38189	0.508701	-6.08657
ERLIN2	-0.1975	6.300085	-0.8761	0.38204	0.508852	-6.74135
PAIP2	-0.12754	5.699058	-0.87601	0.382091	0.508873	-6.71926
STAG3L3	-0.21056	3.224112	-0.87561	0.382305	0.509108	-6.36894
ESYT1	0.15781	6.909101	0.875394	0.382423	0.509218	-6.74321
IQCC	0.162843	1.881295	0.875168	0.382546	0.509283	-6.03427
STX10	0.142133	3.31404	0.875196	0.382531	0.509283	-6.32035
MANEAL	0.507052	2.962983	0.874705	0.382797	0.50957	-6.17603
YES1	0.178332	6.72382	0.874581	0.382865	0.509611	-6.74355

TMEM106A	0.296108	2.46027	0.874467	0.382926	0.509644	-6.11556
HHAT	-0.31111	2.443957	-0.87426	0.383039	0.509745	-6.22117
FAM22D	-0.2369	1.921984	-0.8741	0.383128	0.509816	-6.10693
GATAD2B	0.120283	6.081092	0.87384	0.383267	0.509952	-6.72904
TLR1	0.347701	1.852059	0.873293	0.383564	0.510299	-6.00511
FAM48A	0.129104	5.494939	0.872891	0.383783	0.510541	-6.69053
LASP1	0.191214	8.274018	0.87273	0.38387	0.510609	-6.7179
SLC25A35	-0.21521	2.711822	-0.87231	0.384101	0.510867	-6.25792
GRPEL2	0.210567	4.900065	0.872155	0.384183	0.510928	-6.6197
BTBD19	-0.28659	0.328986	-0.87202	0.384255	0.510974	-5.84986
AK1	-0.22833	4.554781	-0.8719	0.384325	0.511019	-6.62444
C11orf63	-0.37504	1.292218	-0.87174	0.38441	0.511084	-6.02094
MCC	-0.42848	5.234422	-0.87153	0.384525	0.511188	-6.7108
USP31	0.225508	5.65385	0.871275	0.384662	0.511322	-6.6984
ZNF587	-0.22949	5.876745	-0.87111	0.38475	0.51139	-6.73569
LYPLA2P1	0.195639	1.866117	0.870897	0.384868	0.511498	-6.03044
CLSTN2	-0.42069	1.330001	-0.87064	0.385008	0.511586	-6.03402
FSTL1	-0.30507	7.595307	-0.87067	0.384991	0.511586	-6.72677
PMS1	0.135842	4.029835	0.870514	0.385077	0.511629	-6.48661
EXOC2	-0.12787	5.136643	-0.87017	0.385266	0.511832	-6.6811
PHF20	-0.13164	5.711078	-0.87001	0.385352	0.511898	-6.72522
EML6	-0.38317	1.53672	-0.8697	0.38552	0.512072	-6.0666
ATRIP	0.135963	3.199273	0.869523	0.385617	0.512104	-6.30184
MGC70857	0.181229	5.190198	0.869522	0.385617	0.512104	-6.65873
RBBP5	0.131488	4.606749	0.869161	0.385814	0.512229	-6.59225
DDX19A	0.116717	5.019122	0.869247	0.385768	0.512229	-6.6468
CABIN1	-0.13924	6.84898	-0.86915	0.385821	0.512229	-6.74672
EID3	-0.33857	0.171821	-0.869	0.385901	0.512237	-5.83618
COPG	0.144051	7.983191	0.869005	0.3859	0.512237	-6.72842
RASA2	0.198251	3.502563	0.868881	0.385968	0.51224	-6.35856
APAF1	0.222121	4.940632	0.868798	0.386013	0.51224	-6.62532
DAB2	0.29977	5.122529	0.868839	0.38599	0.51224	-6.63981
GLB1L	0.311852	2.145915	0.868641	0.386098	0.512305	-6.06518
USP15	-0.12192	5.957836	-0.86852	0.386166	0.512345	-6.73752
FRG1B	-0.2296	3.46313	-0.86816	0.386362	0.51254	-6.4353
BAG5	-0.11713	6.081731	-0.86811	0.386386	0.51254	-6.74197
EIF4H	-0.11868	7.631342	-0.868	0.386446	0.512571	-6.73244
DNAJC15	-0.31431	4.955415	-0.86765	0.386639	0.512779	-6.68117
TESK2	-0.19933	3.210008	-0.86747	0.386739	0.512862	-6.37345
IVL	1.836162	1.93459	0.867309	0.386826	0.512928	-5.81079
TRMT11	0.165738	3.742253	0.867163	0.386905	0.512986	-6.41948
PCID2	0.160685	5.010695	0.866369	0.38734	0.513513	-6.64337
GMDS	-0.39019	5.054632	-0.86606	0.38751	0.513672	-6.697
RPS11	0.174864	9.0138	0.866015	0.387533	0.513672	-6.69788
C1GALT1C1	0.173472	4.771382	0.865755	0.387675	0.513811	-6.61239
ACY3	0.725191	0.981895	0.864979	0.3881	0.514326	-5.81061
KCMF1	0.119143	6.207823	0.86457	0.388324	0.514574	-6.7422
WDR55	-0.14151	4.61205	-0.86406	0.388606	0.514849	-6.62932
HUWE1	-0.13568	9.000982	-0.86406	0.388602	0.514849	-6.69027
PLEKHO1	-0.2743	4.339838	-0.86361	0.388853	0.515079	-6.61254
HIGD2A	-0.16354	5.636894	-0.86363	0.388842	0.515079	-6.72812
CD2BP2	0.114714	5.374228	0.863465	0.38893	0.515132	-6.6889
NFKBIA	0.244313	6.98455	0.862992	0.389189	0.515426	-6.75389
CD96	0.407151	2.292382	0.86267	0.389366	0.515444	-6.08085
VSIG10L	0.743379	3.566905	0.862632	0.389386	0.515444	-6.27338
LOC729678	0.219567	3.275005	0.862875	0.389253	0.515444	-6.30788
ALG10B	-0.25405	3.814606	-0.86274	0.389325	0.515444	-6.52039
ABHD3	0.238123	5.256851	0.862765	0.389314	0.515444	-6.66552

HECA	-0.14174	6.257514	-0.86234	0.389549	0.515609	-6.75175
USP9Y	1.098354	2.068424	0.86197	0.38975	0.515729	-5.94411
FUT2	0.46825	4.665462	0.862037	0.389713	0.515729	-6.55527
DST	-0.32739	8.628154	-0.86202	0.38972	0.515729	-6.69922
DALRD3	-0.14722	4.268654	-0.86141	0.390055	0.516018	-6.58509
LOC100132287	0.199709	5.210382	0.86137	0.390079	0.516018	-6.66617
RPS6KB1	-0.12261	5.468106	-0.86144	0.390039	0.516018	-6.71637
SEPN1	-0.15473	7.683007	-0.86067	0.390466	0.516482	-6.7366
SERTAD3	-0.14791	3.892734	-0.8606	0.390505	0.516484	-6.52026
EIF4E2	-0.101	5.826851	-0.86038	0.390623	0.51659	-6.73804
MBNL3	-0.30576	3.279846	-0.86029	0.39067	0.516605	-6.41412
AMOT	-0.51382	3.450413	-0.8601	0.390777	0.516697	-6.49365
C22orf40	-0.13895	2.418888	-0.86003	0.390817	0.516701	-6.19481
LRRC37A3	0.28381	1.813253	0.8599	0.390887	0.51671	-6.01838
C3AR1	0.36779	2.545554	0.859814	0.390935	0.51671	-6.13484
WDR61	0.112131	4.776659	0.859847	0.390916	0.51671	-6.62557
POLH	0.18875	4.761883	0.859153	0.391298	0.517141	-6.61499
PTPRO	0.491521	0.79351	0.858652	0.391574	0.517408	-5.82784
MED31	0.181565	2.319998	0.858697	0.391549	0.517408	-6.12451
CALML5	1.435887	0.774081	0.858338	0.391747	0.517408	-5.7072
FKBP14	0.222064	3.547039	0.858288	0.391774	0.517408	-6.37265
RPAP2	0.116241	3.695489	0.858223	0.39181	0.517408	-6.42801
LSMD1	0.184764	4.250707	0.858442	0.391689	0.517408	-6.53395
TNFRSF14	-0.30196	5.103484	-0.85852	0.391648	0.517408	-6.70161
HEATR5A	-0.18187	5.365572	-0.8582	0.391825	0.517408	-6.71547
C14orf147	0.206851	6.209874	0.858181	0.391833	0.517408	-6.74466
RNF144B	-0.29702	4.426928	-0.85764	0.392132	0.517754	-6.62981
NAIP	-0.25526	1.22572	-0.85734	0.392295	0.517906	-6.00215
LRRC37B	0.171514	2.808419	0.857173	0.392388	0.517906	-6.22201
HNRNPA0	-0.09672	6.934748	-0.85716	0.392396	0.517906	-6.75633
PNN	0.133537	6.758938	0.857188	0.39238	0.517906	-6.75899
MNS1	0.391395	1.97537	0.856904	0.392537	0.518044	-6.03256
ZSCAN21	-0.16153	3.057791	-0.85684	0.392574	0.518044	-6.33922
ZNF300	-0.36488	1.885359	-0.85657	0.392723	0.518154	-6.1353
CHAC1	-0.37882	2.740578	-0.85655	0.392732	0.518154	-6.30161
ZNF215	0.57075	0.712422	0.856305	0.392867	0.518283	-5.80642
AHSA2	-0.18195	4.316883	-0.85606	0.393001	0.518394	-6.60114
METRNL	-0.25725	4.961612	-0.85602	0.393025	0.518394	-6.68705
ISLR2	-0.4426	0.540823	-0.85561	0.393253	0.518646	-5.91956
PSEN1	0.118283	6.653756	0.854064	0.394105	0.51972	-6.76108
SNAP47	0.133727	4.659399	0.853993	0.394144	0.519723	-6.61285
NKX3-1	0.440903	2.165393	0.853917	0.394186	0.519728	-6.06352
SPRR2E	1.792115	1.57191	0.853432	0.394454	0.519801	-5.77396
TBX18	0.728987	0.896703	0.853631	0.394344	0.519801	-5.81822
ZNF485	0.225058	1.629695	0.853476	0.39443	0.519801	-6.0004
MORN2	0.185822	2.964777	0.853473	0.394431	0.519801	-6.25302
TFEB	-0.20507	4.084192	-0.85341	0.394464	0.519801	-6.57158
CHCHD3	0.158299	4.889533	0.853419	0.394461	0.519801	-6.63988
KIAA1467	0.34328	2.962387	0.853228	0.394567	0.519888	-6.22761
TBCC	0.180199	4.313276	0.853067	0.394656	0.519956	-6.55081
FBXO18	-0.11672	6.229206	-0.85265	0.394884	0.520207	-6.75902
PAPLN	-0.31478	4.124555	-0.85192	0.395292	0.520647	-6.59589
TWSG1	-0.2307	6.217262	-0.85194	0.395278	0.520647	-6.76132
CD209	-0.39252	2.208206	-0.85167	0.395431	0.52078	-6.2058
HS3ST1	0.450697	3.782388	0.851259	0.395657	0.52092	-6.38255
EXOSC7	-0.12714	4.115884	-0.85121	0.395685	0.52092	-6.56639
UBXN1	-0.12718	5.647049	-0.85139	0.395585	0.52092	-6.73729
NT5C2	0.161648	6.531107	0.851207	0.395686	0.52092	-6.76124

BMP7	0.700392	4.894351	0.851056	0.395769	0.520981	-6.57329
C9orf84	0.518142	0.084621	0.850928	0.39584	0.521025	-5.72822
PIP4K2C	-0.13083	6.203451	-0.85081	0.395907	0.521064	-6.76026
LOC642826	-0.20214	2.405466	-0.85033	0.396173	0.521365	-6.21015
EXOC6B	-0.24156	4.221047	-0.84991	0.396404	0.521619	-6.60168
KIN	0.147474	3.122338	0.849656	0.396546	0.521757	-6.29772
ZNF646	-0.10983	5.247071	-0.84957	0.396595	0.521772	-6.70726
PKP2	0.436378	5.447046	0.849473	0.396647	0.521792	-6.67699
KLHDC5	-0.1884	5.624784	-0.84926	0.396763	0.521895	-6.74114
DDX50	0.105115	4.889974	0.848783	0.39703	0.522148	-6.65006
RPS10	0.174794	7.416679	0.848836	0.397001	0.522148	-6.75918
BACH1	0.161062	6.074686	0.848485	0.397196	0.522268	-6.74886
RBM16	-0.10121	5.95579	-0.8485	0.397187	0.522268	-6.75382
NPC2	0.156969	6.859192	0.847823	0.397563	0.522702	-6.76701
TRIM6	0.406712	1.283346	0.847431	0.397781	0.522856	-5.92461
CMC1	-0.15365	2.681914	-0.84741	0.397793	0.522856	-6.261
NOL9	-0.14374	4.565378	-0.8475	0.397742	0.522856	-6.6384
DRAM1	-0.22498	4.515985	-0.84648	0.398312	0.523489	-6.64145
RAB9A	-0.14951	4.375427	-0.84608	0.398531	0.523728	-6.61463
DERL2	-0.12537	4.491118	-0.84589	0.39864	0.523822	-6.62726
DOK1	-0.24608	2.837521	-0.84554	0.398831	0.524023	-6.31347
C12orf41	0.120341	4.419413	0.845145	0.399053	0.524232	-6.58614
ABHD12	-0.15345	5.916749	-0.84512	0.399065	0.524232	-6.75691
CD28	0.401437	0.607473	0.844656	0.399325	0.524476	-5.82308
TNKS	-0.17943	6.032697	-0.8447	0.399299	0.524476	-6.76209
SMARCAL1	0.10367	4.453164	0.84426	0.399546	0.524716	-6.59412
LRRC57	0.119225	4.017522	0.843382	0.400035	0.52531	-6.50983
PAN3	-0.13415	5.517893	-0.84317	0.400154	0.525415	-6.73624
HEYL	0.317364	4.027093	0.842532	0.40051	0.525785	-6.4735
EIF3K	-0.15407	6.710593	-0.84256	0.400492	0.525785	-6.77081
CCDC122	-0.2609	1.312613	-0.84218	0.400705	0.525893	-6.03015
METTL2A	0.134726	4.286018	0.84231	0.400634	0.525893	-6.56202
ULK3	-0.17698	5.193168	-0.84222	0.400686	0.525893	-6.71404
GM2A	0.28901	6.867153	0.842114	0.400743	0.525893	-6.7715
TM4SF18	-0.30862	0.980757	-0.84115	0.401284	0.526554	-5.97999
GNB1	0.114329	8.56233	0.840802	0.401476	0.526756	-6.73338
TGM3	-1.03992	1.832004	-0.84073	0.401515	0.526758	-6.25775
C6orf125	0.154838	4.960026	0.840645	0.401564	0.526772	-6.65924
LPPR4	0.421011	1.470722	0.839823	0.402023	0.527227	-5.95891
PRSS12	0.632842	2.863098	0.839845	0.402011	0.527227	-6.15816
RASEF	-0.53527	3.225954	-0.83984	0.402015	0.527227	-6.45558
RASSF7	0.282088	4.880064	0.83936	0.402283	0.527517	-6.63431
ANKRD19	0.271938	0.530301	0.839	0.402484	0.527682	-5.83307
CD163	-0.39707	3.673435	-0.83901	0.402477	0.527682	-6.53905
NAIF1	0.119241	3.400064	0.838762	0.402617	0.527807	-6.37603
OFD1	-0.1504	4.794942	-0.83864	0.402685	0.527841	-6.67354
HEXB	0.1505	6.283636	0.838581	0.402719	0.527841	-6.76574
RAB3GAP2	0.103409	6.203697	0.838203	0.40293	0.528069	-6.76484
CD248	-0.3023	4.976327	-0.83809	0.402993	0.528102	-6.70764
SDR42E1	-0.42814	2.945631	-0.83767	0.403228	0.528361	-6.38203
FAM98A	-0.10984	5.521633	-0.83755	0.403297	0.528401	-6.73978
SETD6	-0.19326	3.646789	-0.83711	0.403544	0.528675	-6.49683
RNF128	-0.60512	4.491285	-0.83693	0.403642	0.528754	-6.68305
FLYWCH2	0.184486	3.356491	0.836802	0.403716	0.528801	-6.35466
C14orf93	0.125192	3.16904	0.83664	0.403806	0.52882	-6.32384
L3MBTL2	-0.09605	5.18978	-0.83664	0.403804	0.52882	-6.71181
LRRC33	-0.27117	1.729479	-0.83601	0.404157	0.52918	-6.11065
NENF	0.169391	5.032924	0.836034	0.404147	0.52918	-6.67089

SNX4		-0.12277	5.356787	-0.83569	0.404342	0.529323	-6.72938
C11orf59		0.175225	6.319075	0.835688	0.404341	0.529323	-6.76863
PIGQ		-0.12848	5.244492	-0.83519	0.40462	0.529637	-6.72056
LOC93622		-0.1842	3.011051	-0.83445	0.405035	0.530132	-6.35329
IL13RA1		-0.17346	7.154403	-0.83421	0.405171	0.53026	-6.77052
TMX2		0.130917	6.28436	0.834115	0.405224	0.530279	-6.7701
PPP1R3G		-0.3644	0.222375	-0.834	0.40529	0.530316	-5.87803
UBE2W		-0.12183	5.291247	-0.83368	0.405469	0.5305	-6.72559
FCGRT		-0.29079	6.054913	-0.83351	0.405565	0.530576	-6.77453
UNC50		-0.10671	4.637174	-0.83339	0.405629	0.530611	-6.65448
CLDN2		1.143966	1.331632	0.833216	0.40573	0.530692	-5.83658
C12orf5		0.14749	4.415264	0.833063	0.405816	0.530755	-6.59114
ZNF283		-0.17869	1.769946	-0.8328	0.405964	0.530899	-6.10437
STXBP1		-0.29129	5.266229	-0.83268	0.406029	0.530935	-6.73676
CYorf15A		0.905323	0.75794	0.831556	0.406664	0.531694	-5.79402
RINL		0.319516	2.257738	0.831518	0.406685	0.531694	-6.11393
TJAP1		0.181763	5.239647	0.831158	0.406888	0.531909	-6.69651
STX1B		-0.30827	0.289095	-0.83083	0.407075	0.532103	-5.88082
TMSB15B		-0.38142	0.090619	-0.83034	0.407348	0.532312	-5.86224
KIAA1543		0.301586	5.670556	0.830368	0.407334	0.532312	-6.72747
NUB1		0.141742	5.967017	0.830341	0.407349	0.532312	-6.75931
RASGEF1B		0.264618	3.4167	0.830262	0.407393	0.53232	-6.35695
RBM41		0.158417	3.940458	0.829924	0.407584	0.53252	-6.49782
PHRF1		-0.11279	6.320924	-0.82979	0.407662	0.532571	-6.77984
HNRNPH2		0.093987	6.440005	0.82946	0.407846	0.532762	-6.77888
CYTH3		-0.16497	5.418045	-0.82882	0.408206	0.533183	-6.74307
RTN4RL1		-0.5797	0.640683	-0.82814	0.408593	0.533638	-5.97785
TFDP2		0.201149	4.217832	0.828053	0.40864	0.53365	-6.55019
KRT10		0.412824	5.981382	0.827986	0.408678	0.53365	-6.747
DMBT1		2.167501	3.158861	0.827783	0.408793	0.53375	-5.97738
ETNK1		-0.23029	6.383087	-0.82755	0.408926	0.533873	-6.78364
AAGAB		0.129481	5.662171	0.827304	0.409063	0.534003	-6.74267
C10orf12		0.189067	4.129351	0.827128	0.409163	0.534083	-6.53302
DYNC1H1		-0.15686	9.318443	-0.82691	0.409286	0.534193	-6.70986
SC5DL		-0.21076	6.357293	-0.8268	0.409346	0.534222	-6.7839
ZSWIM3		-0.1884	2.112052	-0.82649	0.409523	0.534279	-6.17173
GATS		-0.28676	2.859587	-0.82639	0.40958	0.534279	-6.34994
HLA-DRB5		0.588235	3.80046	0.826406	0.409571	0.534279	-6.38238
ATP5SL		-0.11346	5.376269	-0.82656	0.409484	0.534279	-6.73792
STAT3		0.13324	8.25772	0.826472	0.409534	0.534279	-6.75599
CROT		-0.2344	4.157442	-0.82584	0.409893	0.534637	-6.61207
PLSCR3		0.221235	5.154787	0.825673	0.409986	0.534708	-6.68835
SETD4		-0.13437	3.335682	-0.82506	0.410335	0.534913	-6.42407
GPATCH1		0.146873	3.753904	0.825252	0.410225	0.534913	-6.46153
PPIC		0.193189	4.775151	0.825062	0.410332	0.534913	-6.64537
RP2		0.179202	4.964058	0.825139	0.410289	0.534913	-6.67057
SBNO1		0.133689	5.454924	0.825241	0.410231	0.534913	-6.72688
KCTD21		-0.17405	3.71032	-0.82483	0.410464	0.535032	-6.51659
PIK3IP1		-0.21757	4.475321	-0.82472	0.410524	0.53506	-6.65399
	2-Sep	-0.10331	8.499601	-0.82436	0.41073	0.535278	-6.74242
DIMT1L		0.122451	4.446005	0.824097	0.410879	0.535423	-6.60796
MLLT11		0.495197	3.165643	0.824004	0.410932	0.535441	-6.26781
RAP1GDS1		-0.13708	5.108177	-0.82361	0.411152	0.535679	-6.71835
TRUB1		0.112733	4.936577	0.823292	0.411335	0.535867	-6.67582
EGLN3		-0.37196	4.277154	-0.82303	0.411482	0.536009	-6.64695
SSR4		-0.19685	6.682946	-0.82271	0.411664	0.536196	-6.78715
RPS18		0.216172	7.516507	0.822423	0.411828	0.536359	-6.77989
ITCH		-0.11621	6.456947	-0.82197	0.412087	0.536647	-6.78805

TMEM90B	0.559005	0.341753	0.821377	0.412422	0.537033	-5.78339
DYSF	-0.27773	4.498442	-0.82103	0.412621	0.537239	-6.66719
MRPL27	-0.13349	4.867785	-0.82096	0.412657	0.537239	-6.69456
EFHC1	-0.189	3.457404	-0.82073	0.412791	0.537362	-6.4651
HIST1H3E	0.334712	0.812762	0.820599	0.412864	0.537408	-5.88016
SAMD5	-0.55595	3.584322	-0.82044	0.412957	0.537478	-6.56155
UBR2	-0.17013	5.880618	-0.81995	0.413231	0.537786	-6.7769
SYF2	-0.11799	5.502342	-0.81954	0.413467	0.538042	-6.75368
ZNF714	0.321437	3.696381	0.81942	0.413535	0.53808	-6.41876
CD3G	0.47015	0.080487	0.81868	0.413956	0.538578	-5.75924
CD53	0.365289	3.946975	0.81847	0.414076	0.538684	-6.46919
TSPYL3	-0.30832	0.19189	-0.8183	0.414175	0.538763	-5.87652
OGFOD2	-0.11385	3.504647	-0.81802	0.41433	0.538914	-6.46179
B3GNT7	-0.50316	4.651634	-0.81793	0.414384	0.538934	-6.70736
ZMYM2	-0.14544	6.448006	-0.81682	0.415016	0.539706	-6.79238
MPPED2	-0.48251	0.211037	-0.81672	0.415076	0.539734	-5.90582
PLAC2	0.917032	1.983068	0.81662	0.41513	0.539754	-5.99251
C15orf40	-0.10917	3.14813	-0.81612	0.415413	0.539971	-6.38288
MSRB2	-0.18183	3.945003	-0.81614	0.415402	0.539971	-6.57244
ZNF281	0.153029	4.285781	0.816165	0.41539	0.539971	-6.58083
KCNJ2	0.349851	2.425	0.81557	0.415729	0.540332	-6.15145
SBSN	1.783687	2.713391	0.814653	0.416253	0.540836	-5.98371
SDHAP2	0.235632	4.021518	0.814796	0.416171	0.540836	-6.51231
TULP4	-0.14802	6.033557	-0.81462	0.416272	0.540836	-6.78607
CD99	-0.20356	6.443291	-0.81465	0.416254	0.540836	-6.79437
PEX6	-0.29612	4.372247	-0.81431	0.416451	0.541018	-6.65687
MORC4	0.20172	5.196541	0.814093	0.416572	0.541077	-6.70405
BCL6	-0.27868	6.049411	-0.81409	0.416574	0.541077	-6.79019
PRPS2	-0.1607	5.707703	-0.814	0.416626	0.541095	-6.77317
TTTY15	0.793021	0.11864	0.813824	0.416726	0.541124	-5.73693
TNFRSF21	0.27811	7.490924	0.813885	0.416691	0.541124	-6.78837
POT1	0.158394	4.562609	0.813697	0.416799	0.541131	-6.62866
ETFA	-0.12574	5.749479	-0.81368	0.416808	0.541131	-6.77406
TBXAS1	0.363942	2.498132	0.813564	0.416875	0.541167	-6.16282
CCR5	0.38461	1.317284	0.813446	0.416942	0.541204	-5.95936
IL7R	0.419817	2.650589	0.813207	0.417079	0.541281	-6.18121
AASDHPPT	0.128979	5.317801	0.813213	0.417076	0.541281	-6.72407
MCTP1	-0.33842	2.514703	-0.81302	0.417184	0.541316	-6.28971
EGLN2	-0.11627	5.452148	-0.81308	0.417153	0.541316	-6.75507
FAM71E1	0.313844	0.947454	0.81257	0.417444	0.541603	-5.91022
HOXB4	0.320781	2.317601	0.812266	0.417617	0.541778	-6.13899
GSTZ1	-0.19975	3.740232	-0.81197	0.417784	0.541824	-6.53652
SNX16	-0.16216	3.957442	-0.81211	0.417709	0.541824	-6.57483
ATG5	0.103731	5.393594	0.812	0.41777	0.541824	-6.73426
TRAPPC1	0.145306	5.996856	0.811933	0.417808	0.541824	-6.77583
CD8A	-0.42343	2.048249	-0.81164	0.417973	0.541988	-6.21244
FAM173A	-0.16997	2.605365	-0.81104	0.418322	0.54239	-6.27741
PLCG1	0.154857	6.284302	0.810534	0.418609	0.542712	-6.78872
ZFAT	0.12497	3.778377	0.809892	0.418977	0.543139	-6.4848
SDAD1	0.119404	5.496705	0.809516	0.419193	0.543368	-6.74454
TLR7	0.427145	0.319861	0.80937	0.419276	0.543426	-5.80608
HSD11B1L	-0.26617	1.413518	-0.80918	0.419384	0.543465	-6.07561
PRR24	-0.17156	3.086425	-0.80923	0.419357	0.543465	-6.38807
PSTK	-0.17508	0.806582	-0.80876	0.419625	0.543727	-5.95969
ADRA2A	0.726299	2.257682	0.807966	0.420083	0.544218	-6.06835
PRRG2	0.276643	2.751272	0.807966	0.420083	0.544218	-6.22507
USP35	0.198661	3.282736	0.806937	0.420674	0.544883	-6.36027
ELMOD2	-0.12136	3.093481	-0.80697	0.420652	0.544883	-6.3801

SDCCAG8	0.151184	4.386306	0.806575	0.420882	0.545102	-6.60693
KRT6C	2.099837	4.240411	0.806073	0.421171	0.545316	-6.20217
C14orf179	0.128572	3.352467	0.806016	0.421203	0.545316	-6.38925
ROBO1	0.314278	5.591334	0.806032	0.421194	0.545316	-6.74048
SRP14	-0.10848	7.278719	-0.80605	0.421183	0.545316	-6.79217
GDPD3	-0.42026	1.657019	-0.80551	0.421495	0.545644	-6.1429
PDIK1L	-0.14049	4.057909	-0.80476	0.421928	0.546153	-6.59561
SPOCK2	-0.36255	4.429873	-0.80449	0.422079	0.546299	-6.68146
TSSC4	-0.12284	4.380353	-0.80437	0.422151	0.546341	-6.64618
HIST4H4	-0.22056	0.136791	-0.80397	0.422382	0.546589	-5.86527
RNF144A	0.254068	4.512655	0.80371	0.422531	0.546731	-6.61623
APP	0.156895	9.443783	0.803081	0.422894	0.54715	-6.73444
AMBRA1	-0.10889	5.377873	-0.80294	0.422974	0.547202	-6.75676
EPN3	-0.40756	5.269594	-0.80246	0.423254	0.547514	-6.76778
PARP6	-0.25388	4.837247	-0.80211	0.423456	0.547704	-6.71883
NDUFB9	0.171946	6.750708	0.802067	0.423479	0.547704	-6.8043
EHD2	-0.29273	7.315937	-0.80171	0.423686	0.547922	-6.79133
FHL2	0.261429	6.306246	0.801304	0.423919	0.548172	-6.79343
IPMK	0.211928	2.53668	0.801191	0.423984	0.548205	-6.20452
GPR97	-0.35197	0.061637	-0.80094	0.424129	0.548342	-5.87511
SDR16C5	0.633282	3.595571	0.800709	0.424263	0.548464	-6.34203
PARD6G	0.513033	4.083455	0.800551	0.424354	0.548481	-6.4883
NPIP	-0.23914	3.577695	-0.80056	0.424349	0.548481	-6.52085
C2orf15	0.376731	0.982513	0.800346	0.424472	0.548528	-5.91561
MVD	0.19492	4.623193	0.800276	0.424513	0.548528	-6.64314
TSPAN1	-0.64926	4.871176	-0.80029	0.424504	0.548528	-6.75438
TBCA	-0.13592	5.513767	-0.80021	0.424548	0.548528	-6.77117
ZCCHC7	0.12826	4.311381	0.799924	0.424716	0.548695	-6.60239
CWC15	0.127276	4.690562	0.799551	0.424932	0.548797	-6.66203
SFXN3	0.203513	5.111658	0.799516	0.424952	0.548797	-6.70569
EXOC1	-0.11625	5.586319	-0.79956	0.424929	0.548797	-6.77546
TMEM127	-0.09455	7.293805	-0.79965	0.424872	0.548797	-6.79722
IFT57	-0.14634	4.715803	-0.79896	0.425276	0.549165	-6.69685
FAIM3	0.35199	2.279506	0.798743	0.425399	0.549272	-6.13834
FAM18B	0.146915	5.012495	0.798296	0.425658	0.549556	-6.70119
TACO1	0.137869	4.099497	0.798133	0.425752	0.549627	-6.55974
PDLIM2	0.270912	4.529237	0.798001	0.425829	0.549675	-6.62225
PKP4	0.13175	6.807391	0.797864	0.425908	0.549726	-6.80793
MESDC2	0.109301	5.849072	0.797411	0.42617	0.550014	-6.7809
CCDC120	0.208509	5.133544	0.797277	0.426248	0.550063	-6.70966
GOLGA7	0.142863	5.799173	0.797137	0.426329	0.550067	-6.7762
MAPK14	-0.09844	6.202038	-0.79714	0.426329	0.550067	-6.80358
PTPRR	-0.55192	0.338864	-0.79676	0.426546	0.550296	-5.94421
DOK2	0.346661	1.76433	0.796495	0.426701	0.550445	-6.05313
UGT8	0.696579	3.594295	0.796399	0.426757	0.550466	-6.32904
UHRF1BP1	-0.14452	5.629378	-0.79547	0.427294	0.551108	-6.78291
PEG10	-0.60278	3.07652	-0.79505	0.427536	0.55137	-6.48659
OAF	0.213	5.238688	0.794882	0.427637	0.551448	-6.72188
KHK	0.306634	2.11992	0.79471	0.427736	0.551526	-6.11998
AP1M1	0.147588	5.546886	0.794613	0.427793	0.551548	-6.7587
QRICH2	-0.28113	1.547527	-0.79421	0.428026	0.551797	-6.11273
SLFN13	0.444288	4.332009	0.793581	0.428392	0.552218	-6.55309
BTN2A1	0.15635	4.265655	0.793486	0.428448	0.552239	-6.59489
PRF1	0.41233	2.038058	0.793322	0.428543	0.552311	-6.09129
FLYWCH1	-0.18446	5.166183	-0.79316	0.428637	0.552381	-6.75223
LSM3	0.133016	5.034331	0.792392	0.429083	0.552854	-6.70992
LYRM2	-0.10374	4.939653	-0.79243	0.429061	0.552854	-6.7229
SYCP2	-0.47397	1.686906	-0.79224	0.429172	0.552867	-6.16683

C1QTNF1	-0.27225	5.242213	-0.79228	0.429147	0.552867	-6.76643
CCNJ	0.168173	3.445178	0.791878	0.429382	0.553086	-6.41388
C3orf59	0.242067	3.243865	0.791804	0.429425	0.553091	-6.35354
MAP4K5	0.13937	5.79626	0.791472	0.429618	0.553207	-6.78077
IDS	-0.18052	7.57689	-0.79145	0.429634	0.553207	-6.79564
MED13	0.127012	7.195763	0.791512	0.429595	0.553207	-6.80916
ATG10	-0.14286	2.0082	-0.79094	0.42993	0.553485	-6.17367
PHTF1	-0.18704	3.346921	-0.791	0.429895	0.553485	-6.46307
FOXA2	-0.82553	1.478184	-0.79083	0.42999	0.553512	-6.17245
BRI3	-0.1702	6.048118	-0.78978	0.430603	0.554251	-6.80693
SERPINB6	-0.2755	6.238717	-0.78965	0.43068	0.554299	-6.81305
TCP11L1	-0.16804	3.984804	-0.78954	0.430743	0.554329	-6.59983
LCAT	-0.2139	2.046766	-0.78941	0.430819	0.554375	-6.19383
TCHP	-0.12658	4.227213	-0.78918	0.430952	0.554494	-6.63624
FOXA1	-0.54941	4.839954	-0.78889	0.431121	0.554662	-6.75339
GALM	0.418714	3.934838	0.788413	0.431401	0.55497	-6.47374
MYCN	-0.51862	0.718493	-0.78806	0.431605	0.554995	-6.00879
C6orf64	0.150051	4.496248	0.788151	0.431554	0.554995	-6.63966
PMPCA	0.124222	5.389439	0.788241	0.431501	0.554995	-6.75122
ZNF148	0.137113	6.21044	0.788263	0.431489	0.554995	-6.80444
ZBTB38	-0.18201	6.751805	-0.78804	0.431618	0.554995	-6.81433
WDR33	0.076676	6.15747	0.787682	0.431828	0.555212	-6.80495
RNF20	-0.09542	5.933274	-0.78727	0.432067	0.555469	-6.80252
DNAJC16	-0.10619	5.077105	-0.7872	0.432107	0.55547	-6.74145
PTPLB	0.209255	4.475099	0.786995	0.432228	0.555574	-6.62907
ADRBK2	-0.25288	4.112192	-0.78657	0.432474	0.555839	-6.63832
LYPLAL1	-0.18111	3.692691	-0.78612	0.432742	0.556132	-6.54547
GTDC1	0.144353	3.852048	0.786025	0.432795	0.55615	-6.51554
RAB43	0.185138	3.976745	0.785834	0.432907	0.55624	-6.53367
SLC38A2	-0.23825	8.401885	-0.78577	0.432945	0.55624	-6.77257
KLHL35	0.442862	0.144517	0.78537	0.433178	0.556386	-5.79545
C12orf24	0.224536	1.867173	0.78539	0.433167	0.556386	-6.09771
LENG1	-0.11765	2.412385	-0.78546	0.433123	0.556386	-6.24957
SNRNP35	-0.1217	3.709615	-0.78503	0.433379	0.556387	-6.53698
PIBF1	0.144138	4.067314	0.785211	0.433271	0.556387	-6.56251
IFT122	-0.14905	4.600711	-0.78525	0.433249	0.556387	-6.69361
PRPF8	-0.13102	8.908054	-0.78514	0.43331	0.556387	-6.75841
VAMP7	0.104891	5.683659	0.785038	0.433372	0.556387	-6.77998
KIAA1671	-0.18698	7.051323	-0.78473	0.433554	0.556562	-6.81214
SMOC1	-0.61068	0.501848	-0.78432	0.433792	0.556816	-5.98677
RGPD1	0.317007	4.282608	0.784158	0.433887	0.556874	-6.57496
FYTTD1	0.172404	6.663211	0.784108	0.433917	0.556874	-6.81768
OTUD3	-0.19555	4.028803	-0.78345	0.434303	0.557318	-6.6169
AEN	0.162815	4.81844	0.783307	0.434385	0.557356	-6.68716
NXN	-0.35081	5.603922	-0.78326	0.434412	0.557356	-6.80107
AGAP2	0.343135	2.119872	0.783181	0.434459	0.557365	-6.12313
RPS28	-0.55321	2.358181	-0.7831	0.434506	0.557373	-6.32161
LRRC8A	0.237618	7.503491	0.783034	0.434546	0.557374	-6.812
ELOVL1	0.156751	6.699889	0.782204	0.435032	0.557946	-6.81968
C19orf76	0.291256	0.63678	0.781767	0.435288	0.558223	-5.8911
ABCB9	0.185792	3.179786	0.78134	0.435539	0.558493	-6.35831
MPP1	-0.25912	3.377466	-0.78113	0.435665	0.558604	-6.49677
PM20D2	0.191043	5.397905	0.780972	0.435754	0.558667	-6.75227
FAAH2	0.310636	2.212285	0.780642	0.435948	0.558865	-6.14395
UTY	0.934172	1.509947	0.780393	0.436094	0.558976	-5.94205
SRPK2	0.161249	5.61604	0.780357	0.436115	0.558976	-6.77437
EFCAB7	0.163596	2.2356	0.779882	0.436394	0.559283	-6.17572
FBXL20	-0.14456	3.743027	-0.77974	0.436475	0.559335	-6.55341

NANS	-0.17198	5.314578	-0.77964	0.436536	0.559362	-6.77404
EIF5	-0.11089	7.899643	-0.77944	0.436656	0.559465	-6.79788
DCAF16	-0.13815	5.157221	-0.7792	0.436798	0.559594	-6.75848
ST6GALNAC2	0.515699	4.023089	0.778406	0.437262	0.560105	-6.48717
CIR1	0.133579	5.002647	0.778381	0.437276	0.560105	-6.71726
ZNF532	0.223706	6.11348	0.777927	0.437544	0.560396	-6.80511
GPCPD1	-0.21858	5.794802	-0.77779	0.437625	0.560449	-6.80863
C3orf58	-0.25042	5.403195	-0.77737	0.437869	0.560709	-6.78881
LOC646762	-0.24608	3.947928	-0.77679	0.438215	0.561101	-6.61594
SLAMF7	0.477647	3.182898	0.776355	0.438468	0.56134	-6.30501
VIPAR	-0.10255	4.46158	-0.77633	0.438482	0.56134	-6.67716
DAPL1	1.073501	0.466965	0.77609	0.438625	0.561472	-5.7718
RSU1	-0.13756	5.95602	-0.77591	0.43873	0.561504	-6.81377
RAB21	-0.11806	6.026107	-0.77591	0.438728	0.561504	-6.81561
CCDC102A	0.231921	2.688111	0.775293	0.439094	0.561919	-6.25369
GDF15	0.720938	3.329115	0.774751	0.439413	0.562275	-6.2836
RINT1	0.126127	4.871827	0.774676	0.439458	0.562281	-6.70492
PRPF18	-0.09837	4.073619	-0.77419	0.439746	0.562598	-6.6155
C21orf57	-0.17052	2.255795	-0.77379	0.439978	0.562843	-6.238
ITGB7	0.323682	2.684411	0.773704	0.440032	0.56286	-6.23601
ASCC1	0.114153	4.725571	0.773051	0.440417	0.563301	-6.68925
GPN2	-0.09984	4.095689	-0.77282	0.440552	0.563423	-6.62154
MTERF	0.236874	3.377483	0.772382	0.440812	0.563704	-6.40001
FKBP4	0.177028	7.133842	0.771644	0.441248	0.564209	-6.82623
SCNN1D	-0.3736	1.454258	-0.77152	0.441321	0.564251	-6.12606
SBK1	0.50512	2.467622	0.770169	0.44212	0.565222	-6.17051
GPR132	0.320802	1.822466	0.770054	0.442188	0.565256	-6.08555
CD38	0.516165	1.53228	0.769696	0.4424	0.565424	-6.00917
UBC	-0.13776	10.8647	-0.76973	0.44238	0.565424	-6.70137
AFMID	-0.145	3.6566	-0.76856	0.443075	0.566081	-6.54082
ROBLD3	0.13683	4.507867	0.768635	0.443028	0.566081	-6.65794
TUBGCP5	-0.13048	4.268117	-0.7685	0.443108	0.566081	-6.65861
TULP3	0.139745	4.963258	0.768567	0.443069	0.566081	-6.71975
NFIL3	-0.19039	5.091267	-0.76849	0.443117	0.566081	-6.76534
NINL	-0.23013	4.177317	-0.7684	0.443167	0.566093	-6.65895
CDC42BPG	0.254583	5.508545	0.768185	0.443295	0.566205	-6.76571
RNASET2	-0.24929	5.405202	-0.76761	0.443639	0.566592	-6.79563
FAM167B	0.22759	1.168223	0.767505	0.443698	0.566617	-5.99124
MYEOV2	-0.1259	4.529965	-0.76727	0.44384	0.566746	-6.69578
ZCCHC6	-0.15175	5.730068	-0.76708	0.443953	0.566838	-6.81091
ADAM6	-0.69006	8.613716	-0.767	0.443996	0.566841	-6.76511
AK7	-0.33804	0.918119	-0.76634	0.444387	0.567289	-6.03302
KDM5C	-0.11048	7.107659	-0.76608	0.444544	0.567437	-6.82674
CERCAM	0.328123	5.156432	0.765734	0.444749	0.567647	-6.72423
TMEM43	-0.12935	6.917888	-0.76534	0.444982	0.567893	-6.83028
ANKZF1	0.125287	4.702068	0.764988	0.445192	0.568109	-6.69007
GMPPA	0.151861	5.236933	0.764913	0.445236	0.568114	-6.75078
TEP1	-0.16869	5.948679	-0.7644	0.445539	0.568448	-6.82322
RNF25	0.096972	3.84611	0.763887	0.445847	0.568788	-6.54152
FOXQ1	-0.39632	5.300653	-0.76361	0.446009	0.568944	-6.80025
ZNF436	-0.18332	4.949534	-0.76352	0.446064	0.568961	-6.75458
ANTXR1	0.312404	6.934513	0.762913	0.446426	0.569349	-6.83489
UBE2G1	-0.12857	6.494651	-0.76287	0.446449	0.569349	-6.83499
PGAM4	0.196456	2.665017	0.762651	0.446582	0.569466	-6.26323
CSF2RB	-0.28232	3.551083	-0.7623	0.44679	0.56968	-6.55169
FAM110C	0.432804	2.363851	0.762058	0.446935	0.569812	-6.16794
NARFL	-0.12187	4.276657	-0.76188	0.447041	0.569895	-6.66338
TAF3	-0.14164	4.306118	-0.76174	0.447123	0.569948	-6.67086

DAPK3	-0.12926	5.301656	-0.76167	0.447167	0.569953	-6.78427
FNBP4	0.114721	6.305207	0.761298	0.447387	0.570181	-6.82902
PPID	0.145254	4.81943	0.761143	0.44748	0.570247	-6.70656
MRAP2	-0.47824	1.677163	-0.76073	0.447728	0.570459	-6.18989
ATP5H	-0.14005	5.968067	-0.76074	0.447719	0.570459	-6.82584
HERPUD2	0.10036	5.121435	0.760472	0.44788	0.5706	-6.74769
DTX4	0.35358	5.21478	0.759879	0.448233	0.570999	-6.73203
PABPC4L	-0.36612	0.514853	-0.75968	0.448349	0.571094	-5.97964
FAM135A	0.227508	6.002522	0.759568	0.448419	0.571131	-6.81306
SPN	0.342794	2.628853	0.759021	0.448745	0.571494	-6.23365
GRAMD1A	-0.19895	5.727919	-0.7589	0.44882	0.571538	-6.81923
TMPRSS11D	1.40256	1.791752	0.758772	0.448894	0.571579	-5.9334
TREX1	0.170129	3.875928	0.758454	0.449084	0.57175	-6.53673
SPPL2B	-0.13956	5.731198	-0.75834	0.44915	0.57175	-6.81696
PSD4	-0.20397	6.158597	-0.75839	0.449122	0.57175	-6.83466
VNN2	0.604529	0.398755	0.757658	0.449559	0.572218	-5.83262
ABCA11P	-0.2055	2.130005	-0.75753	0.449635	0.572262	-6.23135
FUT10	-0.17661	2.944424	-0.75743	0.449695	0.572287	-6.39394
NRG1	0.636655	2.544247	0.756176	0.450445	0.573189	-6.17278
HINFP	-0.121	3.535733	-0.75591	0.450604	0.573263	-6.5197
C10orf137	-0.12107	4.2169	-0.75587	0.450626	0.573263	-6.65824
NFYB	-0.11505	4.780355	-0.75589	0.450617	0.573263	-6.73471
ZNF484	0.159188	2.896399	0.755752	0.450698	0.573303	-6.32124
CIDEB	0.205387	4.500383	0.755488	0.450856	0.573399	-6.65944
SF3A1	-0.08622	7.188717	-0.75551	0.450844	0.573399	-6.83364
RRN3P1	-0.29894	0.92467	-0.7552	0.45103	0.573568	-6.03729
KIAA0947	0.14259	6.199314	0.755025	0.451134	0.573648	-6.82938
LOC100130872	-0.31545	0.510154	-0.75461	0.451381	0.573774	-5.97072
VSIG4	-0.36637	2.519096	-0.75446	0.45147	0.573774	-6.34299
EPHA4	-0.37689	3.724685	-0.75446	0.451473	0.573774	-6.61427
RPUSD3	0.140541	4.38667	0.754624	0.451374	0.573774	-6.649
MFSD5	0.1309	4.822167	0.754547	0.45142	0.573774	-6.71384
ANKRD10	-0.1899	6.024338	-0.75445	0.45148	0.573774	-6.83392
C7orf64	0.186081	3.737596	0.75421	0.451622	0.573823	-6.50521
TSR2	-0.11766	4.497032	-0.75418	0.451641	0.573823	-6.70062
CAMK2N1	0.41821	5.7736	0.754186	0.451636	0.573823	-6.78847
IL20RA	-0.3537	3.415582	-0.75405	0.451715	0.573865	-6.53977
ADNP2	-0.11133	5.485599	-0.75387	0.451826	0.573954	-6.80344
IPO7	0.109381	7.784609	0.753787	0.451875	0.573964	-6.8258
IDH3G	0.128388	5.875369	0.753226	0.452212	0.574339	-6.81561
RPS5	0.172245	8.835613	0.752619	0.452575	0.574748	-6.79546
ERBB2	0.366417	7.885968	0.752466	0.452667	0.574812	-6.82964
TST	-0.25632	5.176863	-0.75229	0.452772	0.574894	-6.78955
LOC154761	0.311454	1.013804	0.75203	0.452928	0.57504	-5.96883
LCA5L	-0.201	0.391736	-0.75186	0.453031	0.575067	-5.94122
TMEM55B	-0.09795	4.374147	-0.75186	0.453032	0.575067	-6.68253
PLEKHF1	0.305947	3.339791	0.751256	0.453393	0.575473	-6.39634
TMEM52	0.349665	1.26121	0.75091	0.453601	0.575632	-5.99707
FBXO34	0.140903	5.52576	0.750951	0.453576	0.575632	-6.79079
GLS2	-0.33115	1.906347	-0.75063	0.453769	0.575686	-6.21773
SLC1A3	0.431402	3.349305	0.750651	0.453757	0.575686	-6.37612
NCRNA00094	-0.1465	4.300211	-0.75056	0.453808	0.575686	-6.67969
TMED3	-0.18365	6.285484	-0.75062	0.453775	0.575686	-6.84254
BPGM	0.209172	4.587369	0.750494	0.453851	0.575688	-6.67542
TMEM53	0.193502	3.215072	0.750336	0.453946	0.575756	-6.3861
ARL13B	0.172801	4.227897	0.750082	0.454098	0.575793	-6.61959
PMF1	0.124873	5.165798	0.750139	0.454064	0.575793	-6.75771
SPPL2A	-0.11382	5.394296	-0.75013	0.454067	0.575793	-6.79926

SDF4	0.118913	7.246751	0.749917	0.454198	0.575866	-6.8401
USH1C	-0.95384	1.350916	-0.74949	0.454454	0.576139	-6.2035
TSPAN15	-0.33492	5.14406	-0.74935	0.454538	0.576142	-6.79459
TRIM24	-0.17605	5.371504	-0.74935	0.454537	0.576142	-6.80245
ANXA1	0.494489	8.620475	0.748567	0.455009	0.576651	-6.81568
IMPDH2	0.155034	6.552062	0.748544	0.455023	0.576651	-6.84345
DOC2B	-0.41986	1.47864	-0.74804	0.455328	0.576986	-6.15773
SEMA3A	-0.35891	3.00992	-0.74783	0.455454	0.577093	-6.45887
SPIN3	-0.29705	2.907907	-0.74755	0.455623	0.577254	-6.42108
PRSS27	0.743172	1.915496	0.746608	0.456188	0.577917	-6.05719
FAM171A1	-0.35463	4.403286	-0.74654	0.456229	0.577917	-6.72077
ADM2	0.331027	3.281765	0.746456	0.45628	0.577929	-6.36792
TMEM159	0.205801	4.633422	0.746357	0.45634	0.577953	-6.6861
HSPA9	0.107503	7.799284	0.745998	0.456556	0.578175	-6.83121
CYB561	-0.15071	6.285393	-0.74527	0.456993	0.578675	-6.84604
CFTR	1.231871	1.51689	0.745096	0.4571	0.578741	-5.91854
ABCC4	0.239488	4.453893	0.74505	0.457128	0.578741	-6.65208
C7orf13	0.313542	2.180727	0.744731	0.45732	0.57888	-6.16714
KBTBD6	0.146587	4.41764	0.744795	0.457281	0.57888	-6.66059
RAB3B	0.576222	0.199394	0.744566	0.457419	0.578953	-5.81748
TMPRSS11A	1.218585	0.402224	0.743981	0.457772	0.579348	-5.78667
LOC148189	-0.27757	1.342911	-0.74364	0.45798	0.579558	-6.11473
SPDYE6	0.236973	2.237803	0.743199	0.458245	0.57984	-6.1907
EYS	-0.2168	0.038171	-0.74298	0.458377	0.579909	-5.89602
NPEPL1	-0.11559	5.134707	-0.74297	0.458382	0.579909	-6.78204
C21orf67	-0.20827	0.657728	-0.74242	0.458716	0.580279	-5.98996
C19orf44	-0.12252	2.621921	-0.74193	0.459013	0.580602	-6.32746
C5orf55	0.226095	1.136921	0.741478	0.459285	0.580893	-6.00691
SERPINA3	-0.61642	4.673272	-0.74112	0.459501	0.581114	-6.78088
CDC42	0.091421	7.527926	0.740996	0.459576	0.581157	-6.84075
THNSL1	0.25962	2.937234	0.740802	0.459694	0.581252	-6.32253
CRMP1	-0.34045	2.922986	-0.74029	0.460005	0.581593	-6.4424
SILV	0.250653	0.621129	0.740094	0.460122	0.581689	-5.92224
INE1	-0.19527	0.2207	-0.73999	0.460186	0.581717	-5.92221
TMEM163	-0.46558	1.069937	-0.73954	0.460456	0.582006	-6.09681
DOCK6	0.141801	6.542918	0.738975	0.4608	0.582388	-6.85059
USP13	0.22561	4.268083	0.738865	0.460867	0.58242	-6.62631
PMS2L4	-0.1623	0.074862	-0.73867	0.460982	0.582485	-5.89813
GALNT14	0.777249	1.933116	0.738612	0.46102	0.582485	-6.06212
RRBP1	-0.18106	8.471475	-0.73856	0.46105	0.582485	-6.80756
AEBP1	-0.33962	7.484185	-0.7385	0.461086	0.582485	-6.83474
NTAN1	-0.16154	4.085309	-0.73826	0.461236	0.582622	-6.65594
OGFRL1	-0.26705	3.70924	-0.738	0.461393	0.582716	-6.60383
NOMO2	0.173124	6.709132	0.738051	0.46136	0.582716	-6.85303
LIPT1	-0.13321	2.087581	-0.7379	0.461449	0.582733	-6.22561
XRCC1	0.122674	5.00382	0.736882	0.462069	0.583464	-6.7496
NSUN6	-0.12583	2.89645	-0.73669	0.462183	0.583555	-6.38908
JMJD6	-0.12249	4.592738	-0.73626	0.462447	0.583795	-6.72699
JRK	-0.1631	4.693654	-0.73624	0.462457	0.583795	-6.74322
SLC7A6OS	0.136649	2.971019	0.735656	0.462813	0.584192	-6.35655
DHRS1	-0.21409	4.077748	-0.73555	0.462879	0.584222	-6.66446
VPS45	-0.09793	5.125816	-0.73545	0.462936	0.584241	-6.7851
COPB1	0.093024	7.419291	0.735205	0.463087	0.58438	-6.84727
FMNL1	0.245275	4.406609	0.734677	0.463408	0.584732	-6.65122
RNF19A	-0.16274	6.320704	-0.73432	0.463625	0.584953	-6.85487
AP4B1	-0.14924	3.945547	-0.73407	0.463777	0.585092	-6.62919
LRRC8C	0.229211	5.019437	0.733938	0.463857	0.585137	-6.74249
FAM128B	0.150512	5.913151	0.733873	0.463897	0.585137	-6.83105

ARHGAP32	0.21187	6.933213	0.733208	0.464301	0.585594	-6.85712
KRTDAP	1.435237	0.702784	0.732943	0.464462	0.585692	-5.79703
HINT1	0.129956	6.841582	0.732979	0.464441	0.585692	-6.85738
CYorf15B	0.779463	0.555234	0.731856	0.465124	0.586474	-5.85556
IRX3	-0.52006	3.128717	-0.73172	0.46521	0.586528	-6.52399
ENO2	0.335021	4.459128	0.73136	0.465426	0.586749	-6.64976
LY96	0.304133	1.60746	0.731164	0.465546	0.586846	-6.08213
HSPA7	-0.43661	1.525966	-0.73065	0.465858	0.587187	-6.18327
HERC6	0.293857	5.077454	0.730408	0.466007	0.587322	-6.74547
FAM120C	-0.2078	3.701507	-0.73031	0.466064	0.587341	-6.59667
GRPEL1	-0.1239	4.7641	-0.73021	0.466128	0.587368	-6.75215
STK25	-0.09974	6.343946	-0.7299	0.466317	0.587553	-6.85759
LOC389333	-0.18905	2.091981	-0.72977	0.466398	0.587603	-6.24295
HS3ST3B1	-0.30876	0.334268	-0.72951	0.466557	0.58775	-5.96574
NDUFAF3	-0.1347	4.766117	-0.72926	0.466704	0.587883	-6.7542
ESF1	0.144586	5.184432	0.729137	0.466782	0.587927	-6.77342
GALNTL4	0.327571	3.825211	0.72876	0.467012	0.588164	-6.51955
STAT4	0.334176	1.272188	0.728043	0.46745	0.588621	-6.02311
TSR1	0.112807	6.050082	0.728028	0.467459	0.588621	-6.84425
KCNK10	-0.53098	0.671635	-0.72765	0.467693	0.588703	-6.04741
AFAP1L1	0.255428	3.438279	0.727837	0.467576	0.588703	-6.44518
HAUS3	-0.09982	4.106611	-0.72767	0.467676	0.588703	-6.65691
ZBTB11	0.115086	5.667127	0.727779	0.467611	0.588703	-6.82121
TRIO	0.182399	6.953205	0.727403	0.467841	0.588837	-6.86112
NDUFA12	0.115976	5.163389	0.727288	0.467911	0.588872	-6.77512
TBX2	-0.22705	3.986235	-0.72671	0.468265	0.589211	-6.65664
PPIE	0.150763	4.747324	0.726776	0.468224	0.589211	-6.72232
SRGN	-0.28042	5.507024	-0.72577	0.468839	0.58988	-6.83582
RNF216	0.099968	5.885572	0.725086	0.469258	0.590354	-6.83854
MANEA	-0.14076	5.03791	-0.72485	0.4694	0.590479	-6.78753
MLEC	-0.19312	7.999684	-0.72472	0.469481	0.590529	-6.83345
GLIS2	-0.23614	4.67573	-0.72439	0.469681	0.590727	-6.7586
ACE2	0.600235	1.551019	0.72378	0.470058	0.591147	-6.03058
ATG3	0.099364	5.608804	0.72295	0.470566	0.591734	-6.82126
MBOAT7	0.130688	6.545515	0.722685	0.470729	0.591884	-6.86267
UFC1	-0.14155	5.723201	-0.72205	0.471116	0.592319	-6.84346
RASL10B	-0.38393	0.464291	-0.72177	0.47129	0.592483	-6.00135
MAP1LC3B2	-0.10925	4.147147	-0.72156	0.471416	0.592589	-6.67047
TUBB1	-0.23062	0.072412	-0.72139	0.47152	0.592613	-5.91852
HBS1L	-0.13632	5.573655	-0.72143	0.4715	0.592613	-6.83489
CXCR6	0.34357	1.574441	0.72051	0.472063	0.593243	-6.07539
CCDC134	0.151195	1.910165	0.720141	0.47229	0.593474	-6.16124
MOSC1	-0.28947	3.187707	-0.7198	0.472496	0.593554	-6.50375
PLEKHM1P	0.183515	4.270776	0.719848	0.47247	0.593554	-6.64566
ZNF3	0.138778	5.101358	0.71976	0.472524	0.593554	-6.77157
TMOD3	0.147948	7.017145	0.719834	0.472478	0.593554	-6.86584
ZNF93	-0.35111	1.073761	-0.71853	0.473279	0.594449	-6.09796
DRAM2	-0.10827	4.979391	-0.71832	0.473409	0.594559	-6.78293
ZMYM5	-0.14119	3.804462	-0.71813	0.473527	0.594654	-6.6122
CD69	-0.33129	1.406529	-0.71744	0.473953	0.595136	-6.1535
DNM1P35	0.215167	0.120988	0.717057	0.474186	0.595369	-5.87122
RAB13	-0.12361	6.054994	-0.717	0.474224	0.595369	-6.86051
MAP4K1	0.385709	1.570444	0.71661	0.474462	0.595613	-6.07219
HSD11B2	0.329965	4.249505	0.71647	0.474548	0.595668	-6.61266
SLC22A15	-0.22738	2.870325	-0.71588	0.474912	0.596072	-6.41873
PPP2R2C	1.018065	2.969634	0.715662	0.475045	0.596078	-6.22215
D4S234E	-0.41399	2.684966	-0.71573	0.475003	0.596078	-6.41752
RIPK4	0.260858	5.702662	0.715737	0.474999	0.596078	-6.82199

RNF157	0.485416	2.223128	0.715314	0.47526	0.596146	-6.16704
RTN4IP1	-0.13206	2.833931	-0.71536	0.47523	0.596146	-6.39113
DMKN	0.618339	4.914279	0.715286	0.475277	0.596146	-6.69515
DRG2	0.111505	4.801403	0.71542	0.475195	0.596146	-6.74184
C12orf44	-0.0995	4.882444	-0.71523	0.475312	0.596146	-6.77386
CLTC	0.103041	9.335429	0.715009	0.475448	0.596262	-6.80303
FAHD1	0.133419	4.663592	0.71489	0.475521	0.596301	-6.72176
SIRPB2	0.299978	0.08708	0.714776	0.475592	0.596336	-5.85731
CALML4	0.279104	4.635325	0.714525	0.475747	0.596476	-6.69708
GAPVD1	-0.10308	6.371493	-0.71433	0.475864	0.59657	-6.86926
LOC729176	0.160885	0.101266	0.714053	0.476038	0.596681	-5.87759
KCNH8	-0.47266	0.565549	-0.71406	0.476032	0.596681	-6.03111
PDGFRL	-0.41645	0.456553	-0.71384	0.47617	0.596793	-6.00865
CCDC84	-0.14709	2.38979	-0.71292	0.47674	0.597454	-6.30396
NCRNA00105	-0.22534	1.255756	-0.71267	0.476894	0.597593	-6.11111
ZDHHC24	0.118186	3.751816	0.71257	0.476953	0.597614	-6.55151
TRIM31	1.080114	1.547341	0.711923	0.477353	0.598061	-5.96403
PTPN22	0.328531	2.15994	0.711805	0.477426	0.598098	-6.18439
RAB11FIP4	-0.2575	5.113248	-0.71145	0.477647	0.598323	-6.81329
CUL1	-0.10518	6.103688	-0.71133	0.477718	0.598358	-6.86557
SMOX	-0.20142	4.279237	-0.71098	0.477937	0.598578	-6.7107
BCAT1	0.399492	4.662354	0.710284	0.478366	0.599008	-6.68826
HS2ST1	0.126938	5.755753	0.710347	0.478327	0.599008	-6.83921
SULT1E1	0.769042	-0.03545	0.71	0.478542	0.599175	-5.80718
KLK13	-0.76166	2.222222	-0.70984	0.478643	0.599216	-6.39277
AQP3	0.529781	6.120032	0.709808	0.47866	0.599216	-6.83969
SCG5	-0.34476	0.523074	-0.70964	0.478766	0.599294	-6.00855
TMEM176B	0.335249	5.692337	0.709446	0.478885	0.599389	-6.81995
CP110	0.141751	4.755264	0.708797	0.479287	0.599838	-6.73715
ABO	-0.48061	2.227339	-0.70857	0.479425	0.599904	-6.33093
FLRT2	-0.43471	3.285979	-0.70862	0.479394	0.599904	-6.5598
SCO1	-0.09633	4.187388	-0.70836	0.479559	0.600018	-6.68446
SLC25A33	0.166529	3.261663	0.707895	0.479846	0.600323	-6.43546
UPK3B	0.563287	1.546571	0.706904	0.48046	0.600929	-6.04768
C10orf25	-0.21971	0.950539	-0.70696	0.480422	0.600929	-6.06439
RPLP2	0.165314	8.741089	0.706971	0.480418	0.600929	-6.83172
TM2D2	0.128616	5.058342	0.706525	0.480695	0.60117	-6.77694
TCF7L2	0.193295	5.482075	0.706222	0.480882	0.601351	-6.81453
TMCC3	-0.24585	4.585603	-0.70592	0.481067	0.601528	-6.76005
ENAH	-0.20114	7.502252	-0.70575	0.481178	0.601613	-6.86087
ZNF581	0.138303	3.924019	0.705176	0.481532	0.601994	-6.59292
ARMC7	0.147089	4.084222	0.705115	0.48157	0.601994	-6.62364
C20orf134	0.245886	1.448343	0.704645	0.481862	0.602305	-6.0788
ERICH1	-0.15678	4.029379	-0.70447	0.48197	0.602387	-6.66779
GNE	-0.24855	5.06873	-0.70433	0.482059	0.602444	-6.81465
RLIM	0.104533	6.643773	0.704132	0.48218	0.602542	-6.8774
KIFAP3	0.162609	5.037079	0.703888	0.482332	0.602624	-6.77366
POLR2A	0.119611	8.185898	0.703943	0.482298	0.602624	-6.85118
C12orf4	0.12309	4.202694	0.703699	0.482449	0.602716	-6.65519
DENND4B	0.100502	5.94289	0.703253	0.482726	0.603009	-6.85704
CSTA	0.843012	4.96586	0.702526	0.483179	0.60352	-6.67911
GPR37	-0.40045	0.701164	-0.70236	0.48328	0.603592	-6.05634
RAB2B	-0.11914	4.601317	-0.70212	0.483428	0.603723	-6.75221
ZNF674	-0.13429	1.616791	-0.70183	0.483611	0.603898	-6.16854
PTS	-0.14143	3.630026	-0.70129	0.48395	0.604267	-6.58478
EPB41L2	-0.23544	5.805944	-0.70097	0.484146	0.604458	-6.86625
COL8A1	0.442173	3.688016	0.700536	0.484417	0.604742	-6.48597
CMTM8	-0.24905	3.074963	-0.70043	0.484485	0.604773	-6.47683

CDHR1	0.888433	1.231052	0.700235	0.484605	0.604843	-5.96026
PDP2	-0.13201	3.323479	-0.7002	0.484628	0.604843	-6.51413
C19orf50	0.099569	5.999875	0.699871	0.484832	0.605043	-6.86237
TTC30A	0.208805	3.303003	0.699618	0.484989	0.605186	-6.4411
FLJ36031	-0.18	4.452662	-0.69951	0.485056	0.605215	-6.74191
MCTS1	0.096954	5.458472	0.699434	0.485104	0.605222	-6.8253
PGBD3	0.1455	2.295097	0.699057	0.485339	0.605435	-6.24664
CDKAL1	0.163103	4.021291	0.69902	0.485362	0.605435	-6.61236
DCTN6	-0.11694	4.55719	-0.69883	0.485483	0.605531	-6.74801
HGF	-0.40639	0.61271	-0.69845	0.485715	0.605713	-6.04377
MIB1	-0.134	6.598982	-0.6985	0.485686	0.605713	-6.88204
NGFRAP1	-0.35944	4.695007	-0.6977	0.486184	0.606244	-6.79235
LOC100134229	0.252033	3.064768	0.69745	0.486342	0.606332	-6.37826
MID1	0.179503	5.375018	0.697495	0.486314	0.606332	-6.8124
RANGRF	-0.14059	3.375403	-0.69732	0.48642	0.606376	-6.53035
ANGEL2	-0.11058	4.657427	-0.69711	0.486551	0.606485	-6.76143
EHD1	-0.15506	6.494352	-0.69699	0.486629	0.606529	-6.88296
CRB3	0.275101	3.908905	0.696895	0.486688	0.606548	-6.55995
MLL5	-0.1294	7.090735	-0.69665	0.486843	0.606687	-6.87732
ZNF26	0.141026	3.597689	0.695832	0.487352	0.607159	-6.52526
PLS1	0.455308	5.729916	0.695884	0.48732	0.607159	-6.82212
CXCL14	0.541502	6.604441	0.695906	0.487306	0.607159	-6.87335
HEG1	-0.23738	6.327628	-0.69573	0.487414	0.607182	-6.88319
SS18	-0.12274	6.239246	-0.69462	0.48811	0.607995	-6.88106
FBXO27	-0.46962	2.713783	-0.69451	0.488178	0.608025	-6.45522
SCYL2	-0.10121	6.23026	-0.69423	0.488353	0.608189	-6.88072
FBXL14	-0.1628	5.186937	-0.6936	0.488751	0.60863	-6.82562
TMEM176A	0.383289	4.96777	0.693483	0.488822	0.608664	-6.74623
FSD1L	0.247207	2.029895	0.693402	0.488872	0.608672	-6.18593
YOD1	-0.20399	5.357024	-0.69328	0.48895	0.608715	-6.84372
IL3RA	-0.20757	1.686794	-0.69294	0.48916	0.608814	-6.20007
UBIAD1	0.105764	4.669193	0.692999	0.489124	0.608814	-6.74074
SKIV2L	0.09763	5.631241	0.692972	0.489142	0.608814	-6.84424
PPPDE1	0.10708	6.354618	0.692868	0.489207	0.608818	-6.8801
EGR2	0.405365	2.997048	0.692331	0.489543	0.609182	-6.34764
NUAK1	0.251497	4.388329	0.691917	0.489802	0.60945	-6.67786
CPD	-0.17995	7.511056	-0.69184	0.489851	0.609456	-6.87076
HIVEP3	0.271046	4.122413	0.691564	0.490023	0.609617	-6.6226
TIAM2	-0.26111	2.445391	-0.69121	0.490247	0.609794	-6.34829
LOC654433	0.474366	3.348364	0.691198	0.490253	0.609794	-6.40116
IKZF2	-0.29308	3.989055	-0.69109	0.490324	0.609828	-6.69429
ERLIN1	0.121012	5.825581	0.690652	0.490596	0.610111	-6.85789
MNX1	0.580569	0.26539	0.690508	0.490686	0.610169	-5.85921
C16orf46	0.159924	0.862715	0.690354	0.490782	0.61018	-6.00713
TMCC2	0.372419	1.889393	0.690358	0.49078	0.61018	-6.14875
MSI1	-0.51175	1.074818	-0.69021	0.490874	0.61024	-6.13956
TSPAN33	0.258858	3.405656	0.689922	0.491053	0.610355	-6.46268
PL-5283	0.156892	5.958759	0.68998	0.491017	0.610355	-6.8645
SPATA24	0.133624	0.208048	0.689827	0.491113	0.610374	-5.91267
HDGFRP3	-0.33823	4.242712	-0.68973	0.491172	0.610394	-6.74172
BNIP1	0.112315	2.686267	0.68886	0.49172	0.611021	-6.33303
ACBD5	0.163802	5.546432	0.688514	0.491937	0.611236	-6.8351
CLPB	0.164194	4.950918	0.688359	0.492035	0.611271	-6.77328
TMEM214	-0.09785	7.220323	-0.68833	0.492053	0.611271	-6.88109
CXCL2	0.47304	3.135903	0.688202	0.492134	0.6113	-6.36064
CASD1	-0.14277	4.249922	-0.68815	0.492164	0.6113	-6.71633
CCDC112	-0.14913	3.092648	-0.68773	0.492429	0.611575	-6.47112
BRPF1	-0.10278	4.836337	-0.68717	0.492782	0.611959	-6.78883

MSH3	-0.09677	4.419368	-0.68682	0.493004	0.612181	-6.73532
SLC4A3	-0.39092	2.500774	-0.68664	0.493114	0.612263	-6.3973
ZNF576	0.112028	2.806841	0.686439	0.493242	0.612367	-6.36127
C1orf144	0.081373	7.208385	0.686144	0.493427	0.612542	-6.88581
APCDD1	-0.32312	4.639105	-0.68589	0.493584	0.612683	-6.78945
PARVB	-0.21143	3.970135	-0.6858	0.493642	0.612701	-6.68195
COQ4	-0.11242	4.992942	-0.68564	0.493747	0.612777	-6.80766
RAB15	0.253236	4.710656	0.685547	0.493803	0.612791	-6.73259
ZNF391	-0.28474	0.384281	-0.68528	0.493968	0.612942	-5.99816
GNB4	-0.27456	5.509145	-0.68522	0.494012	0.612942	-6.86426
PHF8	0.110066	5.947094	0.684839	0.494249	0.613181	-6.86957
C1orf204	-0.22052	0.236176	-0.68418	0.494662	0.613589	-5.96727
THAP8	0.148689	2.149627	0.684177	0.494665	0.613589	-6.2317
BTK	-0.30867	1.133788	-0.68312	0.495332	0.614361	-6.12654
HCFC1R1	0.170158	4.75398	0.682351	0.495817	0.614908	-6.75226
ZDHHC8	0.145219	5.802944	0.681714	0.496219	0.615352	-6.86125
GOLIM4	0.187857	5.870817	0.681527	0.496337	0.615427	-6.86319
STEAP2	-0.25958	5.55712	-0.68148	0.496368	0.615427	-6.86839
RPS27A	0.141333	7.354327	0.681409	0.496411	0.615427	-6.88733
ZBTB41	0.141707	5.274376	0.681331	0.49646	0.615433	-6.81646
EBAG9	-0.1157	4.540851	-0.68111	0.496599	0.615496	-6.75735
AQR	0.09547	6.051888	0.681112	0.496599	0.615496	-6.87785
UGT1A6	0.874476	3.7309	0.680384	0.497058	0.61601	-6.41515
OST4	0.128674	6.91244	0.680054	0.497267	0.616214	-6.89429
AKR1C2	-0.65719	5.241309	-0.67975	0.497462	0.616401	-6.87102
TSPAN4	-0.18352	3.570651	-0.67936	0.497704	0.616646	-6.59546
PCNT	-0.11321	5.909814	-0.67914	0.497842	0.616762	-6.88119
PCDHGA4	-0.31757	0.286049	-0.67892	0.497984	0.61683	-5.9936
GTF2H2C	-0.14561	3.84527	-0.67896	0.497956	0.61683	-6.64705
MGST3	-0.15541	5.596372	-0.67883	0.498041	0.616846	-6.867
SLC12A6	0.185744	6.007855	0.678603	0.498184	0.616968	-6.87346
SMARCC1	0.102158	7.160448	0.678342	0.498349	0.617117	-6.89221
RHOH	0.317164	1.422304	0.678201	0.498439	0.617173	-6.08322
FAM131B	-0.34795	0.208067	-0.67764	0.49879	0.617539	-5.98462
TICAM2	-0.29409	1.851232	-0.67734	0.498985	0.617539	-6.25018
IFT74	-0.20562	2.935611	-0.67749	0.498891	0.617539	-6.45875
DAK	0.139307	5.240789	0.677363	0.498969	0.617539	-6.81563
ZNF410	-0.08022	5.49332	-0.67728	0.499023	0.617539	-6.85661
USP36	-0.10109	6.026967	-0.67724	0.499043	0.617539	-6.88647
NUS1	-0.10124	6.297944	-0.67755	0.498848	0.617539	-6.89346
NSUN4	-0.07391	4.695043	-0.67714	0.499108	0.617564	-6.77554
PPPDE2	0.142402	4.478349	0.676882	0.499273	0.617714	-6.7192
SUSD1	-0.17863	3.78496	-0.67664	0.499428	0.617851	-6.63949
SDCBP	-0.12728	7.468212	-0.67647	0.499533	0.617926	-6.88334
EI24	0.118897	6.812128	0.676298	0.499643	0.618008	-6.8972
CREB3L1	-0.48728	6.089919	-0.67614	0.499746	0.61808	-6.89614
FBXO4	0.143054	3.038699	0.675869	0.499915	0.618234	-6.4119
NUDT17	0.167248	0.482183	0.675737	0.499998	0.618283	-5.95807
SMC5	-0.12727	5.877241	-0.67548	0.500162	0.618431	-6.88269
TRAF1	-0.20627	3.659344	-0.67528	0.500288	0.618522	-6.62296
PODXL	0.247547	6.087431	0.675223	0.500324	0.618522	-6.87675
ALS2CL	0.274987	4.769493	0.674893	0.500533	0.618725	-6.7463
NIPAL1	0.316743	2.98139	0.67462	0.500707	0.618885	-6.36261
ZC3H11A	0.082433	7.577963	0.674309	0.500904	0.619073	-6.8865
ADAM19	0.249773	4.803151	0.674026	0.501083	0.619241	-6.75589
MAST1	0.368176	0.176973	0.673791	0.501232	0.619301	-5.88974
GATSL2	-0.40795	0.234204	-0.67374	0.501265	0.619301	-6.0007
CORO6	-0.39735	0.732843	-0.67385	0.501193	0.619301	-6.08041

LRRC59		0.1323	7.67435	0.673515	0.501407	0.619422	-6.88584
C2orf72		-0.60968	1.75956	-0.67314	0.501646	0.619662	-6.27863
ANGPTL2		-0.24636	5.431285	-0.67291	0.501793	0.619734	-6.86599
SRP72		0.085172	7.072955	0.672957	0.501761	0.619734	-6.89696
NR5A2		0.47274	1.957925	0.672731	0.501905	0.619818	-6.1521
SUGT1L1		-0.2051	0.886814	-0.67245	0.502086	0.619877	-6.07355
ACOT9		0.151368	4.550762	0.672583	0.501999	0.619877	-6.73173
BCL7B		-0.09945	5.602826	-0.67249	0.502055	0.619877	-6.86868
GCNT3		0.939044	3.8109	0.671986	0.502378	0.620183	-6.41742
C16orf48		-0.16242	2.872729	-0.67152	0.502675	0.620419	-6.43855
LRRC61		0.2243	3.829348	0.671555	0.502652	0.620419	-6.57707
	9-Sep	0.108155	8.610541	0.671475	0.502703	0.620419	-6.8589
C6orf192		-0.19891	4.298641	-0.67126	0.502838	0.620532	-6.74081
TIAL1		0.059548	6.113934	0.670705	0.503192	0.620913	-6.88883
SERTAD4		0.403285	2.26653	0.670487	0.503331	0.62103	-6.22262
CTPS2		-0.15056	4.420451	-0.67028	0.503466	0.621141	-6.75293
CUL4B		-0.09198	6.62046	-0.67015	0.503544	0.621183	-6.90139
SPHK2		-0.13166	4.080952	-0.66985	0.503735	0.621338	-6.69674
TTC39C		0.182983	4.455726	0.669814	0.503759	0.621338	-6.71472
CLCA4		1.091802	1.347456	0.669614	0.503886	0.621404	-5.97097
SCAI		-0.12522	4.059855	-0.66938	0.504035	0.621404	-6.69336
KIAA1715		0.114885	5.420751	0.669505	0.503956	0.621404	-6.84103
SLC37A3		-0.10923	5.260497	-0.66942	0.504012	0.621404	-6.84443
LMTK2		0.17304	6.010874	0.669541	0.503933	0.621404	-6.88019
SELT		0.109019	6.500271	0.6692	0.50415	0.621491	-6.8993
FGD1		-0.25766	4.048726	-0.66892	0.504331	0.62166	-6.71517
C1orf50		-0.10759	2.70925	-0.66861	0.504523	0.621841	-6.39311
KIF9		-0.20989	1.992332	-0.66843	0.504637	0.621928	-6.2676
OLFM4		1.625432	3.391995	0.668127	0.504833	0.622113	-6.21855
PEX26		-0.11015	4.5829	-0.66782	0.505031	0.622303	-6.77166
TRIM5		-0.13573	4.31534	-0.66761	0.505163	0.62241	-6.73809
GXYLT1		-0.13939	4.361779	-0.66745	0.505262	0.622478	-6.74564
UGT1A10		-0.75547	0.981055	-0.667	0.505548	0.622776	-6.1753
N4BP2		-0.15263	3.750896	-0.66675	0.50571	0.62292	-6.63747
ZZZ3		-0.09018	5.471236	-0.66628	0.506011	0.623235	-6.86291
RPLP0		0.128199	9.55649	0.666097	0.506127	0.623323	-6.82977
ACRBP		0.341077	0.343729	0.665883	0.506263	0.623367	-5.91799
RND1		-0.40153	1.650118	-0.66587	0.506269	0.623367	-6.23856
WBP5		-0.22633	5.612451	-0.66583	0.506296	0.623367	-6.88061
VPS28		-0.12949	5.874287	-0.66567	0.506401	0.623441	-6.88915
MYNN		0.118384	4.662827	0.665396	0.506574	0.623545	-6.75723
SFRS11		-0.09529	7.181581	-0.6654	0.506569	0.623545	-6.89737
CDK19		-0.1145	5.298396	-0.66525	0.506669	0.623606	-6.85123
EXTL3		-0.15168	6.324184	-0.66473	0.507002	0.623961	-6.90327
C6orf57		-0.13964	1.314479	-0.66444	0.507182	0.624128	-6.14142
MEIS3		-0.26475	1.588695	-0.66434	0.507247	0.624153	-6.21176
STAG3L2		0.238025	2.334784	0.664262	0.507298	0.62416	-6.26106
PRKCDBP		-0.22254	4.614039	-0.664	0.507465	0.624311	-6.791
MDM1		0.169401	3.385374	0.663774	0.507609	0.624434	-6.49387
DHX58		0.182979	3.738239	0.663261	0.507937	0.624782	-6.56959
C10orf88		0.097034	3.171531	0.663154	0.508005	0.624811	-6.458
BCMO1		0.545003	0.000661	0.662776	0.508247	0.625053	-5.84685
ABP1		0.905209	3.500344	0.662505	0.50842	0.625211	-6.36326
ZBTB5		-0.12515	4.97521	-0.66205	0.508713	0.625516	-6.8234
FARP1		-0.21353	6.00045	-0.66186	0.508835	0.62561	-6.89915
ITPK1		-0.15381	6.662149	-0.66169	0.508939	0.625684	-6.90661
HARBI1		-0.10868	1.709567	-0.66146	0.509087	0.625775	-6.20667
TCEAL8		-0.17243	4.471222	-0.66144	0.509103	0.625775	-6.76937

MYH14	0.390958	8.231542	0.660852	0.509478	0.626181	-6.88604
FKBP9	0.136465	7.232877	0.660599	0.50964	0.626325	-6.90335
LOC145783	-0.1837	0.483163	-0.66007	0.509978	0.626685	-6.01518
DOCK4	-0.18972	4.00504	-0.65981	0.510146	0.626837	-6.69978
MBLAC1	-0.15073	0.197087	-0.65911	0.510591	0.627329	-5.96896
MBTD1	-0.12227	4.859935	-0.65884	0.510767	0.627489	-6.8122
NPR3	-0.50535	0.186367	-0.65863	0.510902	0.627524	-6.01663
SCAMP5	-0.29854	3.343698	-0.65858	0.510932	0.627524	-6.57689
EIF3CL	-0.11849	8.96071	-0.65846	0.511009	0.627524	-6.84797
MRPL20	0.114566	5.493537	0.658416	0.511038	0.627524	-6.85501
MGA	-0.10559	6.027306	-0.65851	0.51098	0.627524	-6.89911
AGR2	-0.78508	6.170565	-0.65837	0.511065	0.627524	-6.9075
CCBL1	-0.17345	2.339469	-0.65776	0.511456	0.627941	-6.33514
EPS15L1	-0.08946	5.563488	-0.6577	0.511494	0.627941	-6.87532
NDUFB11	-0.13858	5.763489	-0.65752	0.511614	0.628033	-6.88975
ZNF740	-0.10651	4.349191	-0.65745	0.51166	0.628034	-6.7467
TMEM18	-0.10343	4.274623	-0.65691	0.512007	0.628405	-6.73456
XIAP	0.111644	6.373797	0.656434	0.51231	0.628721	-6.90497
NIPAL4	0.782915	2.392801	0.656357	0.512359	0.628726	-6.19733
HNF1A	-0.50118	2.302644	-0.65614	0.512501	0.628845	-6.37757
TRIM26	0.100268	6.029563	0.655537	0.512885	0.629262	-6.89368
OSBPL6	-0.4381	1.539992	-0.65529	0.513045	0.629402	-6.23784
RPP30	0.106527	4.234049	0.654699	0.513423	0.629811	-6.69681
RASAL1	0.451602	3.427372	0.65446	0.513577	0.62989	-6.44384
FBXL18	0.145494	5.285271	0.654515	0.513542	0.62989	-6.83529
AKR1A1	-0.11307	6.161525	-0.65426	0.513708	0.629995	-6.90611
ZNF81	0.146836	3.23762	0.654161	0.513769	0.630015	-6.46977
BCS1L	0.10457	3.93168	0.653698	0.514067	0.630324	-6.63477
PEX14	0.11159	4.313248	0.653227	0.51437	0.630641	-6.71147
EP400	0.091969	6.934441	0.653102	0.51445	0.630684	-6.91183
C8orf83	-0.17121	4.852319	-0.65277	0.514666	0.630892	-6.8201
BDKRB2	-0.22936	4.818764	-0.65252	0.514824	0.630995	-6.82261
TNPO2	-0.0988	6.502078	-0.6525	0.51484	0.630995	-6.9127
ETHE1	0.219791	4.966699	0.652242	0.515004	0.63114	-6.79279
LOC727896	-0.20028	0.370791	-0.65188	0.515238	0.631372	-6.00366
CUL7	-0.14998	5.735677	-0.65178	0.515302	0.631395	-6.89261
PLEKHA1	0.119137	6.51701	0.651695	0.515356	0.631406	-6.91097
SLC30A6	0.109212	4.542734	0.651547	0.515451	0.631467	-6.75011
PRKCH	0.16449	5.110386	0.651343	0.515582	0.631573	-6.81717
SLC7A4	-0.4825	-0.13901	-0.65125	0.515643	0.631591	-5.96831
CLIC5	-0.44938	2.166512	-0.6511	0.515737	0.631611	-6.35357
SEMA3F	0.205892	6.405712	0.651083	0.51575	0.631611	-6.9071
ZNF770	0.146959	5.85469	0.650975	0.515819	0.631642	-6.88494
TRO	-0.28402	1.541554	-0.6508	0.515933	0.631725	-6.21363
RPL31	0.133286	8.299539	0.650459	0.516152	0.631938	-6.88406
GPR4	0.228389	2.938578	0.649208	0.516958	0.632759	-6.39093
PACRGL	-0.1161	2.764325	-0.64927	0.516916	0.632759	-6.41979
RNF207	0.252234	3.515987	0.649248	0.516933	0.632759	-6.51454
COX6A1	-0.11975	6.508513	-0.64885	0.517186	0.632982	-6.91519
A2ML1	1.36342	3.173631	0.648574	0.517368	0.633093	-6.24527
C2orf56	0.080691	3.405127	0.648584	0.517361	0.633093	-6.52467
TMEM119	-0.2736	2.815117	-0.64819	0.517613	0.633216	-6.46248
ZNF480	-0.17405	4.337641	-0.64828	0.517557	0.633216	-6.75936
SLC45A4	-0.19346	5.233183	-0.64822	0.517593	0.633216	-6.86203
TAF1C	-0.08909	5.418099	-0.64814	0.517649	0.633216	-6.87054
SHMT1	0.154709	5.107789	0.647836	0.517844	0.633399	-6.82008
DYRK1B	-0.18058	4.490173	-0.64658	0.518656	0.634281	-6.78196
C6orf48	0.149291	5.351955	0.646631	0.518622	0.634281	-6.84667

SORL1	0.252022	6.401418	0.646364	0.518795	0.634395	-6.90873
ZNHIT2	-0.57893	1.257513	-0.64618	0.518912	0.634484	-6.21089
VAMP5	-0.18831	3.112693	-0.64604	0.519002	0.634526	-6.5178
XPO4	0.122931	5.733995	0.645988	0.519038	0.634526	-6.88141
TPK1	-0.27056	1.690403	-0.64536	0.519446	0.634858	-6.23835
ISCA2	0.100516	3.379339	0.645487	0.519361	0.634858	-6.51426
RNF31	0.08642	5.770964	0.645411	0.51941	0.634858	-6.8866
C19orf26	0.342557	0.823846	0.644003	0.520321	0.635816	-6.00719
PFKFB3	-0.16067	6.471532	-0.64404	0.5203	0.635816	-6.91828
RABGGTB	0.098876	5.110147	0.643898	0.520389	0.635844	-6.82786
NFS1	0.105295	4.754094	0.643801	0.520451	0.635865	-6.78461
FHL3	0.180252	4.520608	0.643435	0.520689	0.636099	-6.74418
ZNF394	0.122245	4.559268	0.643083	0.520917	0.636322	-6.7567
TRPM6	0.434935	0.087488	0.642664	0.521187	0.636597	-5.88848
PAQR6	-0.24274	1.025316	-0.6424	0.521357	0.636638	-6.12318
DMWD	-0.09055	5.531356	-0.64248	0.521307	0.636638	-6.88296
RAB2A	-0.0858	5.940635	-0.64246	0.521318	0.636638	-6.90553
MCOLN3	-0.3951	1.88746	-0.64206	0.521582	0.636856	-6.29501
GTF2H2B	0.273456	1.383758	0.64189	0.521689	0.636931	-6.10469
RND3	-0.20294	5.89999	-0.6412	0.522136	0.637422	-6.90897
TSEN2	-0.12818	3.025943	-0.64073	0.522444	0.637742	-6.4854
C17orf56	0.097124	4.490239	0.640539	0.522565	0.637833	-6.75023
DDX51	-0.09489	4.436442	-0.64043	0.522636	0.637865	-6.76843
NXF1	-0.07194	6.275682	-0.64031	0.52271	0.637899	-6.917
TRIM32	0.121531	4.22262	0.640157	0.522812	0.637968	-6.70172
DGCR11	-0.13718	1.190119	-0.63953	0.523219	0.638408	-6.13626
XAB2	-0.09058	5.837628	-0.63904	0.523539	0.638743	-6.90334
PRNP	0.251426	7.412256	0.638941	0.523602	0.638764	-6.916
PCYT1A	0.169684	5.137927	0.638547	0.523858	0.63902	-6.82752
PION	-0.18033	4.030024	-0.63813	0.524126	0.639292	-6.71538
PSMD6	-0.0876	5.437817	-0.6379	0.524278	0.639422	-6.87866
C20orf46	0.329765	0.92936	0.636567	0.525144	0.640422	-6.02552
C1orf52	-0.08428	3.759809	-0.63641	0.525246	0.64049	-6.64572
LAMC1	-0.1685	8.149867	-0.63607	0.52547	0.640697	-6.88945
KRT8	0.444436	8.623975	0.636009	0.525507	0.640697	-6.89159
WDR83	0.106417	2.336357	0.635563	0.525797	0.640994	-6.30201
SMARCA5	0.094695	6.864793	0.635386	0.525912	0.641079	-6.92369
DHCR24	-0.20685	8.603041	-0.63523	0.526014	0.641147	-6.87275
NUDCD3	-0.09228	6.662711	-0.63498	0.526175	0.641288	-6.92417
NDFIP2	0.154752	6.437348	0.634592	0.526429	0.641541	-6.91962
AKT2	-0.08612	6.566486	-0.63448	0.526503	0.641576	-6.92447
FKBPL	0.140159	2.36702	0.63402	0.526802	0.641883	-6.303
WDR27	0.132748	3.27174	0.633635	0.527052	0.642132	-6.49139
PCMT1	-0.08994	5.798991	-0.63354	0.527117	0.642155	-6.90484
KDM2A	0.095213	7.726331	0.633335	0.527248	0.642259	-6.91011
TTC35	0.097939	5.001954	0.632495	0.527795	0.642869	-6.823
AFAP1	-0.13036	6.160021	-0.63218	0.528001	0.643064	-6.92067
C6orf162	0.147185	2.17499	0.631995	0.528121	0.643154	-6.26878
BCAP29	-0.12024	5.694485	-0.63156	0.528406	0.643445	-6.90203
SQRDL	0.181223	6.13761	0.63062	0.529018	0.644134	-6.91127
C8orf44	0.155816	0.854939	0.630201	0.529292	0.644355	-6.04436
SPRR1A	1.290838	3.445136	0.630259	0.529254	0.644355	-6.31498
SP2	-0.06852	5.717514	-0.62914	0.529982	0.645083	-6.90216
GTF2F1	-0.07299	6.18871	-0.62919	0.529955	0.645083	-6.92194
TMEM186	0.119761	2.758777	0.628575	0.530354	0.645479	-6.38687
LRIG3	-0.16561	5.653627	-0.6281	0.530666	0.645803	-6.90405
CD84	0.328916	1.385633	0.627619	0.530979	0.646018	-6.10779
RGPD5	-0.2246	2.480648	-0.62773	0.530907	0.646018	-6.39128

YME1L1	0.081002	7.280981	0.627615	0.530981	0.646018	-6.92278
OR2A4	0.343585	0.713383	0.6269	0.531449	0.646531	-5.99782
BBS10	-0.13371	3.839941	-0.62616	0.531931	0.64706	-6.67997
CCDC101	-0.12189	3.411067	-0.62565	0.532266	0.647412	-6.58294
RARS	0.083781	6.017668	0.62486	0.532784	0.647986	-6.91341
LRRC41	-0.08906	6.456095	-0.62419	0.533224	0.648464	-6.93022
CST7	0.286845	1.651897	0.624045	0.533319	0.648523	-6.16192
LANCL2	0.191321	4.954863	0.623954	0.533378	0.648539	-6.81294
FAM198B	-0.23907	5.398202	-0.6238	0.533477	0.648603	-6.89442
AGAP8	0.177821	1.691045	0.623618	0.533598	0.648637	-6.18318
PBX2	-0.09191	5.420017	-0.62364	0.533585	0.648637	-6.88657
EEF1G	0.1178	8.394222	0.623529	0.533657	0.648652	-6.8976
UTP3	-0.08721	4.571682	-0.62273	0.53418	0.649232	-6.79709
VRK3	-0.10233	4.574662	-0.62232	0.534452	0.649422	-6.79848
NCKAP5L	-0.14829	4.997387	-0.62228	0.534477	0.649422	-6.85344
C15orf24	-0.08686	5.379482	-0.62239	0.534401	0.649422	-6.88337
GOPC	0.093477	5.852677	0.6221	0.534594	0.649508	-6.90595
CASP7	0.134093	5.375393	0.622027	0.534642	0.64951	-6.86502
VASP	0.125499	6.810985	0.621895	0.534729	0.649559	-6.93237
TNIP2	0.100305	4.929591	0.621737	0.534832	0.649629	-6.82104
HVCN1	-0.20055	1.876929	-0.62165	0.534889	0.64964	-6.27892
LRRC40	0.093304	4.26526	0.621391	0.535059	0.649791	-6.72654
PDX1	0.837227	0.293448	0.620902	0.53538	0.650125	-5.87397
SFRS15	-0.08443	5.850369	-0.62083	0.535428	0.650126	-6.91512
MLLT10	0.097849	5.325696	0.620393	0.535714	0.650361	-6.86516
HLA-DPB1	0.291909	5.608886	0.620416	0.535699	0.650361	-6.87565
SNCAIP	0.399765	2.280842	0.620102	0.535906	0.650493	-6.26036
GABPB2	-0.12894	3.115912	-0.61994	0.53601	0.650493	-6.5181
SLC22A18	0.22994	4.494015	0.619988	0.535981	0.650493	-6.74357
AGPAT6	-0.12243	6.116733	-0.62006	0.535936	0.650493	-6.92687
CCDC91	-0.16182	4.69436	-0.61938	0.536381	0.650887	-6.82235
EIF4G2	-0.08022	10.11281	-0.61893	0.536679	0.651192	-6.83432
VPS35	0.078453	7.428773	0.618781	0.536774	0.651251	-6.92535
NUBP2	0.104653	4.843596	0.618501	0.536958	0.651418	-6.81227
C21orf59	0.105474	4.559732	0.617841	0.537392	0.651831	-6.77464
NFAT5	-0.12224	7.248619	-0.61785	0.537386	0.651831	-6.92598
OCLN	-0.34655	4.449803	-0.6175	0.537614	0.652043	-6.81057
EIF2A	0.102514	6.86156	0.617423	0.537667	0.652051	-6.93497
SHARPIN	0.115905	5.593491	0.617084	0.53789	0.652266	-6.88928
RAB36	0.239882	3.122516	0.616977	0.537961	0.652294	-6.45182
PPP1R16A	0.145934	5.318372	0.616604	0.538207	0.652536	-6.86184
BTF3	-0.1071	7.786102	-0.61622	0.53846	0.652787	-6.91468
GTF2H2	-0.22801	2.179768	-0.61611	0.538534	0.652819	-6.33959
SLITRK6	0.553377	2.113215	0.615952	0.538636	0.652884	-6.20703
AKT1S1	0.094892	6.203362	0.615884	0.538681	0.652884	-6.92607
CD200R1	0.274823	-0.07345	0.615759	0.538763	0.652927	-5.90312
ZNF232	0.148073	2.699145	0.615597	0.53887	0.652963	-6.37673
MDN1	-0.10645	6.284044	-0.61557	0.538886	0.652963	-6.93323
KIF12	0.666573	0.745543	0.615451	0.538965	0.653003	-5.96049
ADPRH	-0.1685	2.910246	-0.61458	0.53954	0.653585	-6.48594
CPSF3L	-0.09049	6.147912	-0.61459	0.539533	0.653585	-6.93033
CLIC2	-0.20913	3.005789	-0.61426	0.539748	0.653632	-6.51546
DLL4	0.233446	3.441809	0.614237	0.539766	0.653632	-6.52361
C9orf41	0.140601	3.531296	0.614305	0.539721	0.653632	-6.56147
TRIM35	0.132642	4.708068	0.614327	0.539706	0.653632	-6.79425
SENP2	0.113279	5.486216	0.613746	0.540089	0.653967	-6.88267
GPR89B	-0.26942	1.132966	-0.61348	0.540263	0.654064	-6.16108
KIRREL	0.257133	4.242725	0.613498	0.540253	0.654064	-6.69942

NSD1	0.098547	7.030487	0.613353	0.540349	0.654064	-6.93589
ICMT	0.093517	6.869933	0.613341	0.540357	0.654064	-6.93737
RRAGC	0.130766	3.98038	0.613236	0.540426	0.654092	-6.66774
CEACAM19	0.347522	3.369497	0.613098	0.540517	0.654145	-6.48675
PTPN9	0.075723	5.787701	0.61268	0.540793	0.654422	-6.90872
ZBTB39	-0.10605	4.239684	-0.61245	0.540948	0.654534	-6.75815
VDAC1	0.107989	7.658019	0.612399	0.540979	0.654534	-6.92489
STX4	0.092008	4.644841	0.612312	0.541036	0.654546	-6.79167
RNF32	0.172898	1.38297	0.612185	0.54112	0.654591	-6.13698
UBE2D2	0.076939	6.267999	0.611848	0.541342	0.654803	-6.93119
SCARF2	0.249974	4.063248	0.610762	0.54206	0.655615	-6.66554
KPNA7	0.434404	-0.06749	0.610541	0.542206	0.655668	-5.88183
NRCAM	0.441166	3.363719	0.610482	0.542245	0.655668	-6.46737
TAF9B	-0.11527	4.833282	-0.6106	0.542165	0.655668	-6.83987
SETD5	-0.08862	6.75961	-0.61017	0.542451	0.65586	-6.93901
MYO10	0.156666	7.645095	0.609965	0.542586	0.655967	-6.92757
SLC24A1	-0.11655	3.840074	-0.60951	0.542888	0.656275	-6.68424
ZAP70	-0.3029	1.252944	-0.60938	0.542971	0.656319	-6.19304
SLA2	0.298065	0.653422	0.60811	0.543813	0.65728	-6.00789
ETFB	-0.1654	5.815521	-0.60792	0.543937	0.657373	-6.92469
PCDHB12	0.264253	0.780575	0.607743	0.544057	0.657461	-6.03416
UHRF2	-0.1001	5.256096	-0.60744	0.544256	0.657644	-6.88279
SNRNP27	0.072706	4.629094	0.606859	0.544641	0.658053	-6.79487
ZNF792	0.21253	2.622618	0.606382	0.544957	0.658378	-6.35524
BMI1	0.120743	6.177981	0.606106	0.54514	0.658542	-6.93028
C17orf70	0.082708	5.593443	0.605965	0.545234	0.658599	-6.89867
TM7SF3	-0.15425	6.133502	-0.60588	0.54529	0.658609	-6.9367
CUL2	0.097416	5.303302	0.605787	0.545352	0.658627	-6.87184
C7orf42	-0.10119	7.343685	-0.60529	0.545679	0.658965	-6.93202
SGTA	0.088861	6.169323	0.605167	0.545763	0.65901	-6.93159
LYSMD1	-0.102	2.711416	-0.60434	0.546308	0.659497	-6.43289
LOC441208	-0.13608	3.296154	-0.60448	0.546218	0.659497	-6.5723
ZC3HAV1	-0.084	6.57604	-0.60436	0.546301	0.659497	-6.94309
STAG1	0.110927	5.851977	0.603707	0.546731	0.659894	-6.91626
PHF12	0.084743	6.189903	0.603724	0.54672	0.659894	-6.93329
PCSK5	0.318046	3.656542	0.603401	0.546934	0.660082	-6.56204
UPK3BL	0.451716	3.977517	0.603266	0.547024	0.660133	-6.60931
RPS14	-0.1212	8.769981	-0.60312	0.547122	0.660194	-6.88939
CACNA1A	-0.31952	0.700002	-0.60287	0.547286	0.660279	-6.10213
SFRS2IP	0.087974	7.257151	0.602925	0.547251	0.660279	-6.93848
C1orf56	-0.13352	2.685671	-0.60261	0.547462	0.660387	-6.43424
CREBZF	-0.11238	6.020909	-0.60259	0.547471	0.660387	-6.93419
RAB12	0.147618	5.166938	0.60166	0.54809	0.66102	-6.85622
CHMP5	0.106624	5.982244	0.601694	0.548068	0.66102	-6.92478
C20orf118	0.316156	1.76163	0.601521	0.548183	0.661075	-6.18722
HECTD3	-0.1158	6.395768	-0.60029	0.548998	0.662001	-6.9444
CENPT	-0.10939	4.578076	-0.59945	0.549563	0.662625	-6.81411
GFPT1	-0.17912	7.350255	-0.59904	0.549832	0.662891	-6.93412
CECR5	0.124699	4.457038	0.598912	0.549918	0.662901	-6.76764
ARAP1	0.121227	6.84051	0.598887	0.549935	0.662901	-6.94635
UGT1A9	0.687904	-0.21644	0.598349	0.550293	0.663263	-5.87514
OGT	-0.13346	7.44636	-0.59829	0.55033	0.663263	-6.93336
METTL10	-0.09179	3.467081	-0.5981	0.550456	0.663358	-6.60434
GLIPR1	0.216888	4.633145	0.597755	0.550689	0.663581	-6.78421
C10orf57	-0.15557	4.963939	-0.59755	0.550827	0.663691	-6.86538
RGS18	0.25555	-0.00426	0.597148	0.551093	0.663725	-5.92418
CYB561D2	-0.17382	3.264906	-0.59716	0.551088	0.663725	-6.57131
MRPL16	-0.10145	4.788435	-0.59716	0.551086	0.663725	-6.84027

ERGIC2	0.122184	5.187959	0.59735	0.550958	0.663725	-6.86288
PPTC7	-0.13642	5.387892	-0.59729	0.551001	0.663725	-6.90306
KAL1	0.356419	2.972652	0.595751	0.552024	0.664732	-6.41464
RNF135	0.122001	4.260447	0.595757	0.55202	0.664732	-6.73629
METTL12	0.127998	1.538571	0.595047	0.552494	0.665149	-6.1823
ARL17A	-0.13846	2.794037	-0.59498	0.552537	0.665149	-6.46172
HOOK2	0.111648	5.15884	0.594945	0.552561	0.665149	-6.86159
SLC25A44	0.077093	5.779482	0.595143	0.552429	0.665149	-6.91876
C20orf117	-0.20378	5.049692	-0.59481	0.552655	0.665205	-6.88058
ENO3	0.208612	0.583769	0.594597	0.552794	0.665315	-6.01707
LRRC14	0.096021	5.285124	0.59448	0.552872	0.665351	-6.87691
UXS1	0.105247	5.183121	0.594015	0.553182	0.665645	-6.8659
ZNF142	-0.08036	5.683902	-0.59397	0.553212	0.665645	-6.92219
TMPRSS3	0.72558	1.739471	0.593891	0.553265	0.665652	-6.11944
RPS26	-0.11893	5.218083	-0.59341	0.553584	0.665978	-6.8894
NCRNA00181	0.252096	0.601469	0.5931	0.553793	0.666173	-6.01485
MAGED1	-0.15877	7.048535	-0.59288	0.553943	0.666295	-6.94429
IFT88	0.13202	3.323124	0.592646	0.554096	0.666422	-6.52882
1-Sep	-0.20287	2.048384	-0.59188	0.554607	0.66698	-6.32726
RPAIN	-0.10273	4.412863	-0.59173	0.55471	0.66701	-6.79574
TRAF3IP2	-0.11722	5.605255	-0.5917	0.554728	0.66701	-6.92057
EDC3	0.093317	5.19425	0.590454	0.555561	0.667955	-6.87021
PPP2R1A	0.071047	7.975937	0.590327	0.555647	0.668	-6.92936
LAYN	0.282142	2.207148	0.590179	0.555746	0.668004	-6.28022
TERF1	-0.0888	4.68421	-0.5902	0.555732	0.668004	-6.83052
SAMD14	0.233249	0.31929	0.589981	0.555878	0.668105	-5.97619
MEF2B	0.229926	0.91784	0.589655	0.556096	0.668269	-6.06856
BTBD1	0.080499	6.285562	0.589635	0.55611	0.668269	-6.9449
ANKRD34A	0.178345	0.291767	0.589251	0.556366	0.66852	-5.98238
RPS10P7	-0.15635	1.441738	-0.58888	0.556613	0.668759	-6.21194
GPR160	-0.3266	4.097568	-0.58873	0.556719	0.668828	-6.77552
NEDD9	-0.21736	5.794211	-0.58819	0.557077	0.669201	-6.93747
STARD3NL	0.136283	4.366582	0.586666	0.558099	0.670371	-6.7576
ZNF75A	-0.16224	3.23184	-0.58622	0.558398	0.670519	-6.57448
NCRNA00201	-0.17139	5.356003	-0.58637	0.558295	0.670519	-6.90937
RPS9	-0.1056	8.38961	-0.5862	0.55841	0.670519	-6.91345
BTBD2	0.105812	6.90346	0.586195	0.558414	0.670519	-6.95345
SURF4	0.090581	8.196781	0.58578	0.558692	0.670796	-6.92588
PPL	0.350542	7.838045	0.58568	0.558759	0.670818	-6.94163
RAD9B	-0.11113	0.953974	-0.58492	0.559269	0.671315	-6.12686
OSBPL8	-0.10722	6.6087	-0.58497	0.559238	0.671315	-6.95467
GLIS3	0.289393	3.266928	0.58458	0.559498	0.671532	-6.49021
HAPLN1	-0.34426	1.064653	-0.58437	0.559641	0.671646	-6.17334
STK24	0.109086	8.02044	0.583316	0.560346	0.672435	-6.93312
C6orf70	-0.09729	3.575029	-0.58294	0.560597	0.672633	-6.63849
C4A	-0.32325	5.277392	-0.58293	0.560608	0.672633	-6.91634
MRPL45	0.088771	5.145899	0.582681	0.560773	0.672774	-6.87018
TNK1	0.127867	4.202925	0.582489	0.560902	0.67287	-6.72981
ZNF827	0.165226	4.34262	0.582352	0.560994	0.672923	-6.75191
RTF1	-0.07437	6.567251	-0.58209	0.561173	0.67308	-6.95622
FKBP9L	0.265247	0.98819	0.582009	0.561225	0.673084	-6.07804
SSH1	-0.09361	6.305424	-0.58191	0.561291	0.673106	-6.95353
NHEJ1	0.100707	3.96838	0.581666	0.561455	0.673245	-6.68847
STK10	0.126795	5.314734	0.581513	0.561558	0.67331	-6.88453
C7orf23	0.151881	3.741242	0.580986	0.561912	0.673678	-6.62683
CYCS	0.119334	7.634092	0.580757	0.562067	0.673805	-6.94448
BDKRB1	-0.27486	1.601525	-0.58045	0.562272	0.673993	-6.26633
DUOXA2	-0.43281	3.203857	-0.58016	0.562466	0.674061	-6.60963

POLR2L	0.12773	5.601652	0.580152	0.562473	0.674061	-6.91117
CDK6	0.207596	7.143649	0.580229	0.562422	0.674061	-6.95534
MMP16	-0.26585	0.423448	-0.57892	0.5633	0.674993	-6.06466
LRFN3	0.143678	4.611188	0.578775	0.563401	0.675057	-6.80033
NPIPL3	0.1733	6.053472	0.5786	0.563518	0.675082	-6.93881
SPEN	0.079692	7.668394	0.578621	0.563504	0.675082	-6.94417
LOC440957	-0.13974	2.38611	-0.57846	0.563611	0.675093	-6.38692
NCK2	0.096039	6.586774	0.578443	0.563624	0.675093	-6.95723
ZNF547	-0.17225	0.85277	-0.57825	0.563755	0.675183	-6.12221
FAM35A	-0.08802	4.98747	-0.57814	0.563826	0.675183	-6.87253
ESD	-0.09611	5.952048	-0.57812	0.563844	0.675183	-6.94557
RAB4B	0.120345	3.981429	0.577918	0.563978	0.675285	-6.6882
LOC678655	-0.13702	2.78047	-0.577	0.564596	0.675792	-6.47244
SLC25A40	0.125531	3.419003	0.576931	0.564643	0.675792	-6.56112
LOC641298	0.192825	3.952808	0.577067	0.564551	0.675792	-6.66996
C3orf52	-0.20794	3.824573	-0.57708	0.56454	0.675792	-6.71216
ALDH5A1	-0.19163	4.783185	-0.57715	0.564498	0.675792	-6.86136
RPS4X	-0.11466	9.741022	-0.57661	0.564856	0.675922	-6.87136
GTF2H1	0.073292	5.421409	0.576617	0.564855	0.675922	-6.9021
ACBD3	0.078589	6.685657	0.576554	0.564897	0.675922	-6.95932
TMEM101	0.089225	4.035576	0.575744	0.565444	0.676519	-6.70775
AP4E1	0.10629	4.534627	0.575446	0.565645	0.676701	-6.79615
RNF166	0.111879	3.332183	0.575252	0.565776	0.6768	-6.54681
SYPL1	0.125016	7.621567	0.575082	0.56589	0.676879	-6.94815
C20orf12	0.202408	1.057853	0.574727	0.56613	0.677003	-6.10229
DTNB	-0.15656	3.813477	-0.57471	0.566139	0.677003	-6.70739
SOLH	0.102208	6.779473	0.574788	0.566089	0.677003	-6.96047
MAFB	0.304636	5.656476	0.574572	0.566235	0.677059	-6.9064
EHMT1	0.071993	6.403882	0.574372	0.56637	0.677162	-6.95689
PXN	0.126206	7.397382	0.573939	0.566662	0.677454	-6.95347
TP53RK	0.077955	4.50237	0.573852	0.566721	0.677466	-6.796
MPP3	0.193562	2.112905	0.573228	0.567143	0.677912	-6.28635
HNRNPUL2	0.071184	7.063256	0.572971	0.567317	0.678062	-6.95901
PACSIN3	-0.21625	4.723597	-0.57269	0.567508	0.678233	-6.85945
BRD2	-0.06122	8.025442	-0.57233	0.567752	0.678466	-6.93485
VKORC1	-0.1068	5.071123	-0.5722	0.567838	0.678511	-6.88627
QRSL1	-0.09768	4.438909	-0.57169	0.568185	0.67883	-6.80976
PRSS8	0.319509	6.702058	0.571661	0.568202	0.67883	-6.95962
SEC14L2	0.207707	3.849566	0.571537	0.568286	0.678852	-6.64813
MTOR	-0.09046	6.779683	-0.57149	0.568317	0.678852	-6.9616
NSMCE1	-0.10864	5.055439	-0.57092	0.568705	0.679257	-6.88588
CIDECP	-0.10313	1.818322	-0.57073	0.568835	0.679296	-6.28138
ECD	0.065286	5.022289	0.570768	0.568806	0.679296	-6.86583
DYNLT3	-0.15074	5.643382	-0.57047	0.56901	0.679446	-6.93733
NUBPL	-0.10666	3.634063	-0.57014	0.569229	0.67965	-6.66267
FANCF	0.144502	4.093914	0.569889	0.569401	0.679789	-6.71136
DGKE	-0.13742	4.013151	-0.56983	0.569442	0.679789	-6.7482
TMEM200B	-0.28676	1.515619	-0.56962	0.569581	0.679838	-6.25879
RPS8	0.105153	9.064395	0.569681	0.569542	0.679838	-6.90561
MED17	0.083209	4.78002	0.569531	0.569644	0.679855	-6.83551
ZNF823	-0.12125	2.91876	-0.5691	0.569937	0.680147	-6.50209
TRIM16L	0.331086	3.584181	0.568868	0.570092	0.680274	-6.56387
CCDC90B	-0.09501	4.442959	-0.56853	0.570324	0.680492	-6.81221
SGPP1	-0.15838	4.444413	-0.56825	0.570514	0.680641	-6.8205
PRR13	-0.14177	6.34025	-0.5682	0.570546	0.680641	-6.96267
GLCCI1	-0.16717	3.396666	-0.56777	0.570835	0.680927	-6.62232
SYNGAP1	-0.11035	4.512887	-0.56759	0.570956	0.681014	-6.8251
FAM169A	0.250376	2.980237	0.567319	0.571142	0.681178	-6.44247

TBC1D22B	-0.08135	4.461996	-0.56719	0.571228	0.681222	-6.81386
CSF3	0.508716	1.121338	0.566934	0.571403	0.681358	-6.07731
PRPF39	-0.08393	4.735949	-0.56688	0.57144	0.681358	-6.84987
SNX27	-0.08225	5.929924	-0.56679	0.571502	0.681374	-6.95055
TMUB1	-0.11342	5.15865	-0.56651	0.57169	0.68154	-6.89853
RIOK3	0.144798	6.97321	0.566001	0.572036	0.681894	-6.96479
ALOX5	0.285494	3.499301	0.565909	0.572098	0.681911	-6.55654
CAP2	-0.21917	2.840491	-0.56576	0.572203	0.681977	-6.51276
C13orf31	-0.12224	3.924328	-0.56517	0.572602	0.682395	-6.73021
FKBP11	-0.15706	4.418072	-0.56502	0.572699	0.682452	-6.81678
SGTB	-0.12994	3.248513	-0.5647	0.57292	0.682657	-6.58488
SPTY2D1	-0.08179	5.731569	-0.56429	0.573198	0.68293	-6.9422
IRF2	-0.0749	5.516155	-0.56419	0.573264	0.68295	-6.9278
RPL23AP7	-0.11822	1.544447	-0.56379	0.573539	0.683196	-6.24
MARS2	0.125166	3.906721	0.563457	0.573763	0.683196	-6.67977
ATF2	-0.09322	4.870465	-0.56348	0.57375	0.683196	-6.86853
FTSJD2	0.075906	5.71108	0.563641	0.573638	0.683196	-6.93247
DUS1L	0.099891	6.241804	0.563512	0.573725	0.683196	-6.95794
PPP2R4	0.08678	6.70526	0.563654	0.573629	0.683196	-6.96669
PYGB	-0.19236	8.127039	-0.56318	0.573952	0.683363	-6.93301
MAP3K8	-0.169	3.823465	-0.56297	0.574093	0.683472	-6.72071
VPS54	-0.07806	5.51876	-0.56282	0.574194	0.683534	-6.92886
SMAD6	-0.22657	3.364047	-0.56244	0.574454	0.683786	-6.62478
ID2	-0.18886	4.994129	-0.56213	0.574663	0.683976	-6.89187
ZNF738	-0.23712	2.302836	-0.56094	0.575474	0.684829	-6.3986
HYOU1	-0.11246	7.827435	-0.56093	0.575478	0.684829	-6.94577
AREG	0.363716	4.168882	0.560309	0.575904	0.685277	-6.69343
CBLB	0.122518	5.162874	0.560188	0.575986	0.685317	-6.88117
WASH5P	0.115399	3.237243	0.560063	0.576071	0.68536	-6.53023
WIPI2	0.066678	6.146607	0.559301	0.57659	0.685919	-6.95811
APOBEC3D	0.234017	1.368419	0.559079	0.576741	0.68604	-6.15643
MAP3K4	0.098328	5.521031	0.55878	0.576945	0.686225	-6.91906
C3orf75	-0.07989	3.129156	-0.55839	0.57721	0.686481	-6.54738
C15orf58	0.14698	1.350835	0.558269	0.577293	0.686522	-6.16706
GSK3B	0.103363	6.645361	0.558137	0.577384	0.68657	-6.96926
ZSCAN12P1	0.258115	1.539208	0.557854	0.577576	0.686741	-6.18324
CLN3	0.100661	5.238835	0.557558	0.577778	0.686922	-6.89269
GJA1	0.318434	6.983201	0.557452	0.57785	0.68695	-6.97009
C8orf45	0.203642	0.517627	0.55718	0.578036	0.687112	-6.02839
KIAA0284	-0.13036	7.598123	-0.55708	0.578104	0.687135	-6.95359
ST7OT1	-0.16732	0.528862	-0.55668	0.578376	0.687399	-6.08119
SLC35D2	-0.14993	4.181828	-0.55625	0.578671	0.687633	-6.78532
LPCAT4	-0.14871	5.374697	-0.55629	0.578644	0.687633	-6.92598
C8orf58	0.11897	2.596471	0.556171	0.578724	0.687638	-6.39595
TAPBPL	0.152573	4.686637	0.556091	0.578778	0.687644	-6.82169
C16orf42	-0.10469	4.803338	-0.55595	0.578873	0.687698	-6.86624
DBNDD1	-0.20727	4.290465	-0.55559	0.579123	0.687936	-6.81231
PAFAH1B2	0.094891	4.907448	0.555428	0.579231	0.688006	-6.85756
LRP5	0.162101	7.438479	0.555085	0.579465	0.688225	-6.96389
CANT1	0.137944	6.789615	0.554952	0.579556	0.688275	-6.97159
SF3B5	0.098162	5.788629	0.553154	0.580784	0.689675	-6.94209
RAC3	0.245157	1.642987	0.55277	0.581046	0.689928	-6.20545
RAB1B	-0.08481	7.635126	-0.55266	0.581122	0.689958	-6.95613
NCRNA00171	0.135185	0.496244	0.552468	0.581253	0.690055	-6.03643
ZBTB24	0.084551	4.572706	0.552233	0.581413	0.690187	-6.81711
LTA4H	-0.10085	6.469458	-0.55215	0.581469	0.690195	-6.97271
POU2F2	0.205424	3.044521	0.551403	0.581981	0.690744	-6.48021
NARF	-0.08189	4.872812	-0.55083	0.582376	0.691154	-6.87472

LARP4B	-0.09655	6.605046	-0.55036	0.582697	0.691476	-6.97425
CLCN5	-0.15866	4.557241	-0.54999	0.582947	0.691715	-6.84424
BCL2L1	0.137013	6.898885	0.549767	0.583101	0.691838	-6.97427
LOC100128822	-0.13489	1.987963	-0.54955	0.583252	0.691938	-6.32718
C1orf43	0.07442	7.391465	0.549499	0.583284	0.691938	-6.96636
ACTG1	0.103677	12.0418	0.549134	0.583534	0.692176	-6.80994
ARL8B	-0.09416	6.863678	-0.54897	0.583648	0.692253	-6.97317
HOOK1	0.234684	5.496792	0.548475	0.583986	0.692594	-6.9106
GPR68	0.24904	3.291826	0.548181	0.584187	0.692715	-6.52488
GPR126	0.292536	5.271081	0.548244	0.584144	0.692715	-6.88284
AMPD3	0.179238	4.069723	0.548044	0.584281	0.692744	-6.71511
TMEM141	-0.13188	4.573514	-0.548	0.584311	0.692744	-6.84429
THAP1	0.07507	3.33413	0.547423	0.584707	0.693155	-6.5696
PIK3CA	0.11382	5.102476	0.547255	0.584821	0.693232	-6.88334
ZNF622	0.095573	4.469564	0.547033	0.584974	0.693354	-6.80307
HSPA4L	0.272552	4.243063	0.546757	0.585163	0.693519	-6.7351
C7orf29	-0.17902	2.914205	-0.5465	0.58534	0.69367	-6.52538
FBXL12	-0.06851	4.121384	-0.54623	0.585524	0.69383	-6.76978
C2orf42	-0.05982	3.416174	-0.54613	0.585596	0.693856	-6.61644
ELMO2	-0.07796	5.658026	-0.54603	0.585661	0.693873	-6.94787
LFNG	-0.24342	4.620432	-0.54569	0.585897	0.694094	-6.86301
TRPV2	0.203935	2.923681	0.544991	0.586375	0.694602	-6.45639
XYLB	0.191028	2.872774	0.544275	0.586866	0.695066	-6.44284
METT5D1	-0.08123	3.985921	-0.54435	0.586817	0.695066	-6.74569
SPDYE8P	0.176669	2.570293	0.543646	0.587298	0.695519	-6.38718
ATG12	-0.07268	4.488243	-0.54349	0.587403	0.695584	-6.82944
SLC6A1	0.26878	-0.02588	0.543211	0.587597	0.695696	-5.95018
MTG1	0.100808	4.179888	0.543265	0.58756	0.695696	-6.75229
EIF2AK4	0.079823	5.984098	0.542979	0.587757	0.695826	-6.95962
PIP4K2B	-0.09874	6.340703	-0.54209	0.588369	0.696492	-6.97657
PPOX	-0.10171	2.838861	-0.54131	0.588906	0.697068	-6.49712
ASNS	-0.17318	5.165465	-0.54121	0.58897	0.697085	-6.91795
C11orf95	0.113398	4.409415	0.540126	0.589719	0.697853	-6.79611
PLEKHA4	-0.1758	4.646549	-0.54018	0.589684	0.697853	-6.86431
GDPD1	-0.25576	0.422145	-0.54	0.589805	0.697896	-6.0831
SNAPC2	0.104518	3.788575	0.539553	0.590114	0.698051	-6.67337
DKFZp761E198	-0.09838	5.304183	-0.53958	0.590093	0.698051	-6.92583
NEBL	-0.29793	5.43671	-0.53952	0.590136	0.698051	-6.94948
SOCS3	-0.18745	6.73299	-0.53953	0.590127	0.698051	-6.97887
MED12	0.084765	6.62162	0.538801	0.590631	0.698577	-6.97978
ASPSCR1	0.110828	4.309635	0.538696	0.590704	0.698605	-6.77792
PGBD5	0.456259	1.88206	0.538603	0.590768	0.698621	-6.22373
GTF2IP1	-0.08244	8.15051	-0.53836	0.590937	0.698762	-6.94911
TNFAIP1	0.096374	6.602367	0.538256	0.591006	0.698785	-6.97977
ZFP36L1	-0.13566	8.637532	-0.53791	0.591246	0.699009	-6.9308
LOC349114	-0.12803	3.346405	-0.53771	0.591385	0.699114	-6.61818
DNASE1L3	-0.35951	0.539017	-0.53729	0.591675	0.699262	-6.12255
HIATL2	-0.1007	2.290479	-0.5373	0.591665	0.699262	-6.38366
ZNF138	0.119371	3.318141	0.537228	0.591715	0.699262	-6.5611
SOAT1	-0.14928	4.893565	-0.53738	0.591612	0.699262	-6.89081
TNIP1	-0.09255	7.084289	-0.53716	0.59176	0.699262	-6.97609
TOE1	0.105485	3.667014	0.536921	0.591927	0.699345	-6.64494
AP2B1	0.120656	8.199231	0.536915	0.591931	0.699345	-6.95398
HSD17B14	0.226432	0.95199	0.53571	0.592762	0.700209	-6.1046
ZNF408	0.077134	3.81405	0.535724	0.592753	0.700209	-6.68358
KIAA1549	0.291477	4.226148	0.535432	0.592954	0.700377	-6.72903
SCCPDH	-0.19083	4.554946	-0.53519	0.59312	0.700513	-6.85632
NOMO1	0.091766	7.511876	0.535033	0.593229	0.700583	-6.97203

FBLN7	-0.21849	0.663283	-0.53468	0.593475	0.700814	-6.12191
GOT2	-0.10101	7.303055	-0.53451	0.593592	0.700893	-6.97312
PAPPA	-0.23168	4.01834	-0.53425	0.593771	0.701045	-6.78328
HIAT1	-0.07397	5.980353	-0.53382	0.594066	0.701334	-6.97048
SAMD10	-0.1566	3.219674	-0.5334	0.594354	0.701484	-6.59723
CCL2	-0.26194	3.646482	-0.53355	0.594253	0.701484	-6.72317
MIB2	0.129753	5.588498	0.533347	0.594394	0.701484	-6.93616
NPAS2	-0.18415	5.856001	-0.53344	0.594332	0.701484	-6.96949
ZNF319	0.124857	4.328417	0.533159	0.594523	0.701577	-6.78358
LENG9	-0.13186	2.635383	-0.53223	0.595162	0.702272	-6.46097
SEC61B	0.093618	5.583572	0.531386	0.595749	0.702905	-6.93909
C5orf38	-0.40137	0.66404	-0.53095	0.596051	0.703143	-6.14621
ACIN1	-0.04901	7.201742	-0.53101	0.596007	0.703143	-6.97791
PI15	0.317196	3.047709	0.530787	0.596163	0.703216	-6.46753
ALOX12	0.454495	1.404041	0.530659	0.596252	0.703261	-6.14866
IFI27L1	-0.13976	1.66844	-0.53034	0.596474	0.703464	-6.28143
GTPBP8	0.084825	2.880047	0.529737	0.596889	0.703835	-6.47552
ANUBL1	-0.13632	2.778786	-0.52978	0.596858	0.703835	-6.49752
ZNF551	0.12272	3.117273	0.528857	0.597499	0.704471	-6.52107
EMP3	-0.18347	4.091627	-0.52881	0.59753	0.704471	-6.79351
SMCR7L	0.074758	5.838152	0.528669	0.597629	0.704529	-6.95968
ENSA	-0.07738	7.027045	-0.52825	0.597917	0.704809	-6.98207
LOC100190986	-0.15487	4.589468	-0.52808	0.598036	0.704889	-6.86008
CALCRL	-0.17232	4.58472	-0.52604	0.599452	0.706499	-6.86311
SH3PXD2A	-0.13805	7.257128	-0.52592	0.599537	0.70654	-6.97794
CCDC126	-0.11811	3.219091	-0.52564	0.59973	0.706708	-6.59336
TMEM85	-0.08017	5.79628	-0.52534	0.599937	0.706892	-6.9667
CGREF1	0.32121	2.391154	0.524404	0.600586	0.707596	-6.33806
POLR3D	0.08358	4.04174	0.524141	0.600769	0.707752	-6.73901
C1orf128	-0.08677	4.843088	-0.52397	0.600887	0.707832	-6.8865
MS4A14	0.233401	0.286367	0.523868	0.600958	0.707855	-6.00813
CCDC127	0.114867	3.577383	0.523701	0.601074	0.707932	-6.63164
RBBP4	0.079593	7.073302	0.522558	0.601868	0.708808	-6.98651
HERC5	0.234901	3.244807	0.522373	0.601996	0.7089	-6.53158
ZNRF2	-0.08537	5.361574	-0.52221	0.602108	0.708971	-6.93885
NFYC	0.083867	5.666946	0.521933	0.602302	0.70914	-6.9513
LOC221710	0.097097	4.358089	0.521859	0.602353	0.70914	-6.79909
CHMP6	-0.08679	4.086362	-0.52155	0.602569	0.709275	-6.77891
DMTF1	0.096022	5.619112	0.521575	0.602551	0.709275	-6.94709
SP100	0.110963	6.325815	0.521457	0.602633	0.70929	-6.98298
LOC646214	0.146979	2.436376	0.520847	0.603057	0.70973	-6.37809
SOX2	-0.50335	3.640683	-0.52059	0.603235	0.70988	-6.76506
DOCK5	-0.172	5.268828	-0.52044	0.603339	0.709943	-6.93805
ZNF761	0.166799	4.058272	0.520045	0.603615	0.710087	-6.72767
ATP6V0C	0.093137	8.00912	0.520092	0.603582	0.710087	-6.96783
RELA	0.067792	6.904522	0.520076	0.603593	0.710087	-6.9897
NACA	-0.08405	8.574445	-0.51973	0.603834	0.710285	-6.94423
POLR2J2	-0.29238	2.849919	-0.51961	0.60392	0.710327	-6.55115
PDS5B	-0.09463	5.695022	-0.51899	0.604349	0.710772	-6.9652
PRSS16	0.185839	3.619458	0.518591	0.604627	0.710919	-6.62048
VIPR1	-0.21477	3.298481	-0.51863	0.604596	0.710919	-6.63162
C1orf9	-0.09764	5.962633	-0.51866	0.604577	0.710919	-6.97851
AKR1E2	0.352526	0.095096	0.518353	0.604792	0.710993	-5.96761
B3GNT3	-0.41581	4.906839	-0.51838	0.604774	0.710993	-6.92397
DKFZP686I15217	-0.1416	0.701649	-0.51805	0.605003	0.711181	-6.1238
PHLDA3	0.242931	4.963936	0.517747	0.605215	0.71137	-6.87038
IVNS1ABP	0.098608	7.618674	0.517662	0.605274	0.71138	-6.97895
ODZ3	-0.40251	2.525621	-0.51754	0.605359	0.71142	-6.50091

GUSBP1	0.11965	2.248391	0.517222	0.60558	0.71162	-6.3518
SELL	0.284594	2.346713	0.516888	0.605813	0.711834	-6.34562
NCOA5	0.07163	5.400651	0.516673	0.605963	0.71195	-6.93317
ALDOC	0.216382	4.341013	0.516595	0.606017	0.711954	-6.77962
TMEM203	0.079005	4.953869	0.516368	0.606175	0.71208	-6.88581
DOCK10	0.210622	3.219083	0.516186	0.606302	0.712169	-6.53453
FAM131C	0.319131	0.03436	0.516074	0.60638	0.712201	-5.96369
MRPL1	-0.08602	3.851286	-0.51593	0.606479	0.712219	-6.73435
C19orf25	-0.0863	3.85871	-0.51591	0.606497	0.712219	-6.73529
OSTM1	-0.10267	5.302031	-0.51578	0.606584	0.712261	-6.93876
KIAA1429	-0.07149	6.32126	-0.51556	0.606736	0.71232	-6.98974
CYC1	0.111357	6.861842	0.515593	0.606716	0.71232	-6.99252
ENTPD2	0.303091	3.420419	0.515141	0.607031	0.712501	-6.55227
SP4	0.130866	3.787138	0.515232	0.606968	0.712501	-6.67823
APOL2	0.139745	4.999865	0.515123	0.607043	0.712501	-6.8854
C21orf91	0.182068	4.609071	0.514766	0.607292	0.712733	-6.8313
CXADR	0.171364	5.305604	0.514431	0.607526	0.712948	-6.9153
ABHD15	0.109206	4.292752	0.514166	0.607711	0.713105	-6.78826
TBC1D3C	-0.25108	1.286134	-0.51394	0.607866	0.713227	-6.24046
MYCL1	0.313411	4.010092	0.513607	0.608101	0.713442	-6.6879
BRE	0.06965	4.952446	0.513448	0.608212	0.713488	-6.8882
NNMT	-0.23843	4.721496	-0.51341	0.608242	0.713488	-6.89442
C9orf150	-0.17466	3.696328	-0.51327	0.608334	0.713537	-6.71961
LPL	-0.24711	2.135183	-0.51304	0.608494	0.713576	-6.39296
DFFB	0.137814	2.517086	0.513082	0.608468	0.713576	-6.39807
B3GALTL	-0.10757	3.557874	-0.513	0.608522	0.713576	-6.67508
CTSD	0.091743	9.552835	0.512819	0.608651	0.713669	-6.91873
DIDO1	0.079846	6.905308	0.512566	0.608828	0.713816	-6.99363
SOD1	0.097434	6.850288	0.512477	0.60889	0.713829	-6.99412
GPR110	0.569364	1.686629	0.512336	0.608988	0.713884	-6.18189
TRANK1	0.143035	5.010963	0.512263	0.609039	0.713884	-6.88758
CDKN1B	0.090373	5.293328	0.511513	0.609563	0.714438	-6.92394
RASL11B	-0.26941	0.764441	-0.51126	0.609741	0.714586	-6.15586
KIAA1586	0.140562	2.592173	0.511117	0.60984	0.714643	-6.4159
EXOC5	-0.08771	6.332712	-0.51072	0.61012	0.714911	-6.99271
ZNF830	-0.08304	3.535569	-0.5103	0.610412	0.715193	-6.66771
C15orf41	-0.0905	3.86066	-0.51	0.610619	0.715376	-6.74137
HSH2D	0.296214	2.755505	0.509635	0.610876	0.715557	-6.41505
OSGEP	0.08981	3.685939	0.509705	0.610827	0.715557	-6.667
UBAC2	0.079692	5.92533	0.509259	0.61114	0.715805	-6.97429
GAA	-0.18182	6.53227	-0.50909	0.61126	0.715887	-6.99605
GK5	0.114145	5.343649	0.508864	0.611416	0.715949	-6.92783
SLC40A1	-0.26781	6.553543	-0.50887	0.61141	0.715949	-6.99577
KIF13A	-0.13379	5.992123	-0.50814	0.61192	0.71648	-6.98622
TSGA10	-0.16091	1.524117	-0.50748	0.612386	0.716905	-6.2696
FAM105A	0.238017	3.550888	0.507503	0.612368	0.716905	-6.60206
TUBB2A	-0.18202	5.546564	-0.50736	0.612468	0.71694	-6.96683
NNAT	-0.21913	0.758313	-0.50721	0.612574	0.716944	-6.15125
HEBP2	-0.11523	5.876733	-0.50724	0.612553	0.716944	-6.9814
TSPAN5	-0.20343	2.153987	-0.50595	0.613455	0.717915	-6.39305
EFNB2	0.171301	6.592655	0.505803	0.613559	0.717977	-6.9956
SPATS2	0.086259	4.831436	0.505292	0.613917	0.718336	-6.87528
ALPL	-0.20266	2.884888	-0.50479	0.614267	0.718685	-6.54907
FUT11	-0.10057	3.786942	-0.50464	0.614377	0.718693	-6.73131
SNX3	-0.07481	6.921707	-0.50465	0.614369	0.718693	-6.99591
C17orf106	0.104412	3.427716	0.504422	0.614528	0.71881	-6.60612
CHN2	-0.29021	2.167658	-0.50408	0.614765	0.719027	-6.40839
IL17RA	-0.08961	4.694135	-0.50386	0.614919	0.719102	-6.87875

PDLIM4	-0.25495	4.599543	-0.50384	0.614932	0.719102	-6.88719
COG5	0.084752	5.788934	0.503737	0.615008	0.719131	-6.96885
SPR	0.125555	4.752315	0.503547	0.615141	0.719195	-6.86106
XPO7	0.074852	6.66524	0.503511	0.615166	0.719195	-6.99856
HCLS1	0.204728	3.864071	0.5026	0.615806	0.719883	-6.68938
SMYD4	-0.07968	3.963569	-0.50223	0.616064	0.720064	-6.76392
RGS12	-0.13716	5.207836	-0.50223	0.616063	0.720064	-6.93995
DNAJA3	0.07574	5.869303	0.50207	0.616178	0.720137	-6.97513
RPL23AP82	0.123754	1.906462	0.501918	0.616285	0.720191	-6.29877
ARHGAP30	0.158402	4.053981	0.501563	0.616534	0.720191	-6.7412
ZNF618	0.175556	4.837298	0.501575	0.616525	0.720191	-6.86767
RPS12	0.123003	7.940786	0.50167	0.616458	0.720191	-6.97982
PPARD	-0.12004	6.6908	-0.50177	0.616392	0.720191	-6.99937
ENG	0.138654	6.713034	0.501677	0.616454	0.720191	-6.99939
BICC1	-0.29753	1.416823	-0.50102	0.616918	0.72058	-6.27842
API5	0.06698	7.069704	0.500804	0.617067	0.720694	-6.9975
GEMIN8P4	0.140407	0.448757	0.500537	0.617255	0.720832	-6.05481
CIAO1	0.05371	6.247577	0.500489	0.617289	0.720832	-6.99293
P2RX5	0.211377	1.494564	0.500363	0.617377	0.720875	-6.21398
RASGRP3	-0.18416	2.995227	-0.50017	0.617509	0.720894	-6.56631
DNAJC25	-0.07795	3.302109	-0.50006	0.617587	0.720894	-6.61842
POLE4	0.089911	3.799098	0.500256	0.617452	0.720894	-6.69585
ARMCX5	-0.08994	3.94567	-0.50005	0.617599	0.720894	-6.76345
PLGLB2	0.187608	0.929137	0.499872	0.617722	0.720976	-6.12275
AIDA	0.067411	5.586479	0.499517	0.617972	0.721208	-6.95795
PARD3	-0.15291	6.126032	-0.49944	0.618027	0.721212	-6.99517
BRSK1	0.240345	0.810465	0.498914	0.618396	0.721582	-6.09894
ANKRD36	-0.13731	3.720272	-0.49857	0.618637	0.721804	-6.72361
P4HB	-0.08512	9.730262	-0.49849	0.618691	0.721806	-6.91439
ACAP1	0.216797	1.675089	0.497928	0.619089	0.722206	-6.2445
USP28	-0.08419	5.119386	-0.49786	0.619137	0.722206	-6.92916
MTFR1	0.092214	5.210844	0.497757	0.619209	0.72223	-6.92191
ULK4	0.142478	1.578582	0.49743	0.619439	0.722438	-6.23799
DUOXA1	-0.3587	3.350248	-0.49734	0.619502	0.722451	-6.68526
GLT8D2	-0.20239	2.580965	-0.49687	0.619834	0.722717	-6.48386
FAM69A	-0.12239	4.004411	-0.49693	0.619789	0.722717	-6.78344
C10orf47	0.207793	3.810887	0.496761	0.619911	0.722746	-6.67508
KIAA0317	-0.07972	5.759196	-0.49638	0.620177	0.722997	-6.97942
MAP2K3	-0.11117	6.108219	-0.49616	0.620336	0.723121	-6.9953
C20orf4	0.07515	5.452638	0.495494	0.620803	0.723605	-6.94812
FLNB	-0.13496	8.81198	-0.49539	0.620877	0.723632	-6.94631
PCDHGC5	0.354927	0.356818	0.495174	0.621028	0.72365	-6.0175
PCDHB2	0.282375	1.476042	0.495267	0.620962	0.72365	-6.20464
KCNMB4	-0.23659	1.034553	-0.49507	0.6211	0.72365	-6.20684
NPNT	0.231532	5.462861	0.495128	0.62106	0.72365	-6.93616
PCTP	-0.13557	3.722533	-0.49485	0.621253	0.72376	-6.72499
C2orf3	-0.07138	4.479125	-0.49479	0.621298	0.72376	-6.85357
JMJD7-PLA2G4B	-0.15031	4.938204	-0.49454	0.621473	0.723843	-6.91813
NDE1	0.117712	5.658555	0.494566	0.621457	0.723843	-6.96272
TTC7A	-0.08033	6.035044	-0.49439	0.621582	0.723909	-6.99291
SLCO4A1	-0.28496	4.205238	-0.49431	0.621638	0.723915	-6.83973
LIMK2	0.100632	6.266971	0.49378	0.62201	0.724288	-6.99555
THAP4	0.066161	5.759676	0.493412	0.62227	0.72453	-6.9733
LGALS9	-0.19298	6.033763	-0.49304	0.62253	0.724773	-6.99672
PTPMT1	0.07541	4.798823	0.492955	0.622592	0.724784	-6.879
ERBB3	-0.2281	7.36946	-0.49276	0.622733	0.724887	-6.99057
GALNT6	0.246597	5.275743	0.492337	0.623028	0.725171	-6.91494
NACA2	0.159162	1.013638	0.492141	0.623167	0.725211	-6.14509

ST6GAL1	-0.24846	5.460969	-0.49218	0.62314	0.725211	-6.97258
C14orf129	0.13395	5.403111	0.491933	0.623313	0.725322	-6.94021
3-Mar	-0.20429	2.522339	-0.49139	0.623699	0.725572	-6.47288
TUT1	-0.06865	4.293429	-0.4913	0.623757	0.725572	-6.8268
SEMA5A	-0.19441	4.293191	-0.49128	0.623777	0.725572	-6.84558
MYO15B	-0.28656	5.219244	-0.49126	0.623788	0.725572	-6.95703
PEA15	0.087393	7.246741	0.491505	0.623615	0.725572	-6.99948
ZNF22	0.101112	4.378182	0.491067	0.623925	0.72567	-6.81574
NUMBL	0.131087	5.006036	0.490856	0.624073	0.725752	-6.90014
PLIN2	-0.19704	4.730136	-0.4906	0.624255	0.725752	-6.90121
JAGN1	-0.08977	5.035688	-0.49071	0.624178	0.725752	-6.92413
LUZP1	0.08973	6.188001	0.490809	0.624107	0.725752	-6.99473
SH3PXD2B	-0.13828	6.464584	-0.49065	0.624222	0.725752	-7.00491
LEMD3	-0.08487	5.422235	-0.4904	0.624395	0.725855	-6.96011
PIGO	0.097582	5.571541	0.490211	0.624529	0.72595	-6.95868
BMP2K	-0.11899	4.361148	-0.49002	0.624666	0.726049	-6.846
DKK3	0.167236	5.992866	0.48981	0.624812	0.726158	-6.9834
CAMSAP1L1	0.113253	6.372982	0.489659	0.624919	0.726222	-7.00022
LOC100130581	0.132612	1.305489	0.489468	0.625054	0.726315	-6.19712
MFSD11	-0.0675	4.870961	-0.48921	0.625236	0.726315	-6.90486
CPNE3	-0.08772	7.284698	-0.48918	0.625259	0.726315	-6.99688
BPTF	-0.06904	7.120897	-0.48921	0.625236	0.726315	-7.00035
TNS3	-0.19291	6.680541	-0.48926	0.625198	0.726315	-7.00514
ALKBH8	-0.08727	3.122091	-0.489	0.625383	0.726398	-6.58402
RASGRF2	-0.19392	1.824975	-0.48845	0.625774	0.726617	-6.33814
SLC25A45	-0.13635	2.012259	-0.48853	0.625713	0.726617	-6.36399
SNX22	-0.0981	2.594129	-0.48844	0.625778	0.726617	-6.46752
TMEM109	-0.09637	6.769058	-0.48849	0.625748	0.726617	-7.00552
CHD4	-0.05003	8.213196	-0.48826	0.625906	0.726644	-6.97362
THBD	-0.25205	6.379818	-0.48831	0.625875	0.726644	-7.0061
KRIT1	0.106843	5.55337	0.488063	0.626047	0.726747	-6.95788
ATP11C	-0.1036	4.75612	-0.48764	0.626348	0.726976	-6.89629
FHDC1	-0.17464	4.877131	-0.48767	0.626327	0.726976	-6.91588
C17orf44	-0.12961	0.660736	-0.48752	0.626428	0.727008	-6.13235
PLEKHG3	0.133853	6.170257	0.487088	0.626736	0.727203	-6.9944
GOLPH3	-0.07441	7.194881	-0.48707	0.626752	0.727203	-6.99992
POM121C	0.081079	6.460367	0.487102	0.626727	0.727203	-7.00401
SHF	-0.1773	1.484228	-0.48694	0.626842	0.727247	-6.27827
ATG2A	-0.0867	6.070152	-0.48654	0.627126	0.727516	-6.99811
DGCR6	0.147728	3.149208	0.486242	0.627335	0.727698	-6.54491
BDH1	0.163932	5.150052	0.48599	0.627513	0.727845	-6.91457
DZIP1L	0.265325	2.426412	0.485693	0.627724	0.727968	-6.37588
LASS2	-0.09156	7.276236	-0.48569	0.627723	0.727968	-6.99865
SEMA4G	-0.35766	3.884455	-0.48554	0.627832	0.728033	-6.79689
FAM167A	0.267438	2.752818	0.485379	0.627946	0.728044	-6.43455
EP400NL	0.098321	2.878072	0.48542	0.627917	0.728044	-6.49368
PPM1D	-0.07519	3.90384	-0.4849	0.628284	0.728376	-6.75951
NGEF	0.236288	3.284297	0.484816	0.628344	0.728385	-6.55313
TMEM135	0.106572	4.223513	0.484594	0.628502	0.728507	-6.78926
HMG20A	-0.05662	5.040706	-0.48422	0.628767	0.728754	-6.92487
FZD7	0.229626	5.108488	0.483459	0.629306	0.729318	-6.90588
RPL19P12	-0.13749	0.358438	-0.48331	0.629409	0.729378	-6.08656
C22orf30	0.084647	6.31743	0.483212	0.62948	0.7294	-7.00256
DUSP5	-0.23388	4.570486	-0.4827	0.62984	0.729756	-6.89173
FAM119A	0.110579	3.394274	0.482531	0.629963	0.729838	-6.60797
C1orf162	0.19005	2.390505	0.482	0.63034	0.730068	-6.38415
KIFC3	0.147471	5.043467	0.482007	0.630335	0.730068	-6.9064
MNT	0.075554	5.814997	0.481957	0.63037	0.730068	-6.98185

MAFK	0.101287	6.02597	0.482082	0.630281	0.730068	-6.99186
LYZ	0.392589	7.62957	0.481781	0.630495	0.730151	-7.00193
KRT13	0.947635	4.876805	0.481365	0.63079	0.730384	-6.77625
LOC653566	0.094944	6.124805	0.481351	0.6308	0.730384	-6.99669
SYNGR3	-0.2086	0.434259	-0.48126	0.630867	0.7304	-6.11128
SYS1	-0.08476	4.945858	-0.48104	0.631022	0.73052	-6.919
TFE3	-0.07578	6.228541	-0.48096	0.631081	0.730527	-7.0051
PAX6	-0.19179	1.696262	-0.48071	0.631255	0.730668	-6.31865
LCMT1	0.077047	4.395258	0.480085	0.631699	0.731121	-6.82848
ECM1	0.269724	6.176967	0.479177	0.632343	0.731806	-6.99335
ZDHHC6	0.073426	5.135799	0.478614	0.632743	0.732148	-6.92502
INO80	-0.05855	5.804453	-0.47867	0.632705	0.732148	-6.98949
CD5	0.253885	1.688179	0.478427	0.632875	0.73224	-6.25019
ZNF550	-0.12884	2.951868	-0.47807	0.633127	0.732471	-6.55999
TRIM34	-0.11313	2.80657	-0.47773	0.633372	0.732693	-6.5218
BRCC3	0.087432	5.029358	0.477435	0.633581	0.732874	-6.91297
POLR2J4	-0.11304	3.712894	-0.47736	0.633635	0.732876	-6.7324
CASP10	0.216577	4.7851	0.477084	0.63383	0.733041	-6.8673
CIITA	0.257299	3.817547	0.476434	0.634292	0.733515	-6.67933
ABHD13	-0.08009	4.732195	-0.47635	0.634348	0.733519	-6.89602
DHRS4L2	-0.14858	2.544083	-0.4758	0.634743	0.733915	-6.4748
THAP3	-0.09268	2.996297	-0.47564	0.634854	0.733983	-6.56134
TRIM27	0.074296	6.083989	0.475558	0.634915	0.733993	-6.99852
LCOR	0.126351	3.965187	0.475462	0.634983	0.734011	-6.73583
NFKB1	0.076374	6.3391	0.475058	0.635271	0.734282	-7.00722
C6orf226	0.127743	1.386781	0.474912	0.635375	0.734307	-6.21919
THG1L	-0.08	2.845725	-0.47488	0.635397	0.734307	-6.52741
PIGS	0.08876	5.94018	0.47434	0.635782	0.734691	-6.99176
FMO3	0.219447	1.154891	0.474237	0.635855	0.734714	-6.16822
HOXA4	-0.18505	1.977212	-0.47407	0.635976	0.734793	-6.37634
BIRC2	0.076089	6.094012	0.473805	0.636162	0.734948	-6.9997
GFER	0.078347	3.741436	0.472847	0.636844	0.735675	-6.69894
TP53TG1	-0.14299	2.893878	-0.47231	0.637224	0.736053	-6.55341
GLI2	0.336995	3.869837	0.471806	0.637586	0.736367	-6.68084
PEPD	0.093029	5.674236	0.471784	0.637602	0.736367	-6.97621
CCNI2	0.397617	0.61144	0.471532	0.637782	0.736471	-6.05385
DKFZp686O24166	0.275876	3.47586	0.471511	0.637797	0.736471	-6.59892
PPP4R1	0.112999	7.139502	0.471225	0.638	0.736645	-7.01139
RAPGEF6	-0.09287	4.333773	-0.47109	0.638099	0.736698	-6.84698
PDZD8	-0.14135	5.753861	-0.47074	0.638347	0.736923	-6.99435
FAF1	0.060319	5.659039	0.470641	0.638416	0.736943	-6.97778
ZFC3H1	-0.07624	5.821406	-0.47029	0.63867	0.737174	-6.99517
DERL3	0.262875	2.887822	0.469859	0.638974	0.737427	-6.46659
IQCG	0.162716	2.97323	0.469797	0.639018	0.737427	-6.51157
C19orf63	0.093879	6.869906	0.469756	0.639047	0.737427	-7.01491
ZNF567	-0.11847	3.008734	-0.46957	0.639179	0.737518	-6.57591
SLAMF6	-0.24442	1.390247	-0.46892	0.639644	0.737994	-6.27944
CENPB	0.072244	6.687559	0.468716	0.63979	0.738101	-7.01555
GLI3	-0.27228	4.091575	-0.46856	0.639903	0.738171	-6.83779
C18orf22	-0.09085	3.270972	-0.46814	0.640198	0.738395	-6.63125
MKKS	0.076659	5.390229	0.468137	0.640203	0.738395	-6.95545
SLC30A1	-0.10863	4.428857	-0.468	0.640299	0.738445	-6.86454
C7orf54	-0.12636	1.453684	-0.46761	0.64058	0.738708	-6.27094
RASA3	-0.16886	4.323465	-0.4671	0.640946	0.739008	-6.85757
TGM2	-0.24125	7.197224	-0.46714	0.640916	0.739008	-7.00641
IQCE	0.130135	5.496917	0.466621	0.641286	0.739339	-6.96092
ALDH18A1	0.093939	6.627136	0.465842	0.641842	0.739919	-7.01629
ASB6	0.061329	5.292567	0.465653	0.641977	0.740013	-6.94857

RPL24	0.101941	7.585374	0.465245	0.642268	0.740288	-7.00547
DUOX2	-0.37936	6.002731	-0.46457	0.642748	0.74078	-7.01352
C1R	-0.18883	7.063666	-0.46407	0.643106	0.741071	-7.01141
PITRM1	-0.07788	6.564801	-0.46415	0.643055	0.741071	-7.01775
UBE2H	-0.08736	6.277739	-0.46392	0.64322	0.74114	-7.01442
MIF4GD	0.100346	4.219102	0.463686	0.643383	0.741268	-6.80235
CRYZ	-0.12027	4.685877	-0.46353	0.643492	0.741331	-6.9004
RPL36	0.106429	8.015381	0.463406	0.643584	0.741376	-6.99577
PRKG2	-0.2334	0.148107	-0.46315	0.643765	0.741524	-6.0811
GNRH1	0.13394	0.757604	0.463018	0.643861	0.741536	-6.12021
LOC729603	0.117708	1.51918	0.46299	0.643882	0.741536	-6.2486
RPL4	-0.08409	9.509768	-0.46237	0.644323	0.741983	-6.93939
UBE3C	-0.08006	7.045462	-0.46205	0.644553	0.742187	-7.01441
NAPSB	0.257684	0.052637	0.461618	0.644863	0.742483	-6.00199
C12orf36	-0.38351	1.617183	-0.46153	0.644924	0.742492	-6.33662
ANK1	-0.25684	1.626827	-0.46111	0.64523	0.742723	-6.32558
CC2D1B	-0.05579	5.203719	-0.4611	0.645231	0.742723	-6.95217
CCDC111	-0.08958	2.588569	-0.46058	0.64561	0.742925	-6.48065
PHF11	-0.08545	4.516464	-0.46057	0.645614	0.742925	-6.8764
NCKIPSD	-0.07988	4.909766	-0.46072	0.645507	0.742925	-6.92417
CTBP2	-0.08676	6.560397	-0.46056	0.645619	0.742925	-7.01941
ZNF713	-0.14329	1.522898	-0.46031	0.645802	0.743073	-6.29103
CDC42EP2	0.143596	4.127761	0.460227	0.64586	0.743079	-6.77573
PHF13	0.080891	4.622785	0.460021	0.646007	0.743187	-6.8721
HEXA	0.089039	5.889832	0.459851	0.646129	0.743267	-6.99578
BBS7	-0.07158	3.73273	-0.4596	0.646312	0.743415	-6.73403
ZSCAN29	-0.06073	4.904157	-0.45927	0.646543	0.743621	-6.92189
DHRS2	-0.37905	0.531452	-0.45907	0.646686	0.743663	-6.15386
SCAMP2	-0.095	7.051459	-0.45909	0.646678	0.743663	-7.01542
LOC100271836	0.103884	4.947806	0.458702	0.646953	0.743908	-6.91016
ORMDL1	-0.06166	4.897717	-0.45862	0.647013	0.743916	-6.92156
SLC35E4	0.145292	2.986102	0.458143	0.647353	0.744179	-6.51984
TMEM19	0.095122	5.30296	0.458082	0.647397	0.744179	-6.95043
CDK11B	-0.08308	5.350718	-0.458	0.647455	0.744179	-6.96936
GIGYF1	-0.09024	5.949103	-0.45801	0.64745	0.744179	-7.00726
SPIN2B	-0.15468	0.589853	-0.45781	0.647594	0.744216	-6.13802
LIN7B	-0.1251	0.901257	-0.45785	0.647562	0.744216	-6.18367
TXNDC17	0.126301	5.510424	0.457706	0.647667	0.744239	-6.9669
MEAF6	0.066165	5.510957	0.456765	0.648342	0.744953	-6.97226
LEPROTL1	0.079648	5.290841	0.45661	0.648453	0.744959	-6.95084
TIA1	0.079174	6.221996	0.456623	0.648444	0.744959	-7.0123
DHRS11	-0.15102	3.651156	-0.45651	0.648526	0.744981	-6.72971
CCDC102B	0.154048	1.420574	0.45597	0.648913	0.745284	-6.22893
HNF4A	-0.61445	2.140131	-0.45601	0.648881	0.745284	-6.46815
IL17RD	-0.18729	3.198785	-0.45592	0.64895	0.745284	-6.63819
ZNF664	-0.08338	6.978569	-0.45437	0.65006	0.746498	-7.01896
ZDHHC20	-0.10862	4.624694	-0.45398	0.650341	0.746698	-6.89589
TMEM106B	-0.08911	6.714252	-0.45402	0.650316	0.746698	-7.02216
TATDN2	-0.06526	5.921273	-0.45321	0.650898	0.747276	-7.0073
ACOT13	-0.10002	4.593427	-0.45292	0.651104	0.747451	-6.89167
FGFBP3	0.144811	0.489241	0.452693	0.651267	0.747576	-6.08282
TFPI2	0.354904	0.032595	0.452445	0.651445	0.74772	-5.99035
LOC100133331	0.099023	4.521878	0.452231	0.651599	0.747835	-6.8578
TMEM55A	-0.13336	2.757921	-0.452	0.651765	0.747964	-6.53185
DENND2D	-0.12617	4.755974	-0.45147	0.652145	0.748339	-6.91442
FST	-0.31768	2.756419	-0.45122	0.652328	0.748487	-6.56937
CCDC88B	0.170261	3.778379	0.451023	0.652468	0.748553	-6.69993
C9orf167	-0.26037	5.568935	-0.45099	0.652493	0.748553	-6.99873

SLC17A9	0.336737	3.564828	0.45069	0.652708	0.748663	-6.61053
ZNF213	-0.07278	5.068769	-0.45063	0.652749	0.748663	-6.94509
SLC7A1	0.107985	7.736003	0.450764	0.652655	0.748663	-7.00879
SLC39A7	0.093385	6.508721	0.45027	0.65301	0.748901	-7.02188
FAM21B	-0.09422	4.656478	-0.45015	0.653095	0.748937	-6.90028
TEC	-0.15512	1.299208	-0.44913	0.653834	0.749722	-6.25903
ZNF292	-0.09642	6.266649	-0.44901	0.653917	0.749757	-7.02114
PECR	-0.13944	2.360893	-0.44802	0.654631	0.750397	-6.44695
MRPS33	0.094539	4.034187	0.448074	0.654591	0.750397	-6.77154
SMEK2	-0.05761	7.052891	-0.44801	0.654637	0.750397	-7.02099
PRINS	0.178875	0.259023	0.447459	0.655034	0.750729	-6.04718
SLC30A7	0.080927	6.215761	0.447522	0.654989	0.750729	-7.01612
C21orf56	0.236997	2.627268	0.447222	0.655205	0.750801	-6.43557
MBTPS2	-0.08134	4.850713	-0.44724	0.65519	0.750801	-6.92374
FGF1	-0.20433	1.223253	-0.44703	0.655341	0.750896	-6.25711
TMEM2	-0.16989	6.395595	-0.44665	0.65562	0.751154	-7.02511
ZNF774	0.141912	1.052086	0.446274	0.655889	0.751366	-6.17316
FNTB	0.069526	5.032449	0.44624	0.655913	0.751366	-6.92986
LONP1	-0.06826	6.272452	-0.44589	0.656167	0.751595	-7.02214
PIK3CG	0.232456	1.607701	0.445059	0.656765	0.752219	-6.25392
N6AMT2	-0.10688	2.027157	-0.44491	0.656871	0.752254	-6.38193
PAQR3	0.104511	4.509769	0.444867	0.656903	0.752254	-6.86023
ATL2	0.082039	6.441047	0.444606	0.657092	0.752408	-7.0234
CMPK1	-0.09802	7.755805	-0.44445	0.6572	0.752471	-7.0065
ADAM9	-0.12572	7.606069	-0.44432	0.657296	0.752519	-7.00977
AGPAT4	0.160958	3.500419	0.443959	0.657558	0.752758	-6.64113
ZNF276	-0.07694	4.788428	-0.44283	0.658375	0.75363	-6.91814
ZNF182	-0.09554	3.02269	-0.44256	0.658568	0.753728	-6.58293
KCNN4	0.277435	3.60249	0.442618	0.658527	0.753728	-6.63345
ZKSCAN4	-0.07553	1.96304	-0.44226	0.658784	0.753914	-6.36608
MMAB	-0.08272	4.120247	-0.44209	0.658906	0.753991	-6.82304
GLB1L2	-0.29496	3.057888	-0.44146	0.659363	0.754453	-6.63217
MBNL1	0.079101	7.811231	0.44126	0.659507	0.754556	-7.01059
ATF6	-0.0555	6.176913	-0.44099	0.659703	0.754719	-7.02164
DEGS2	-0.30064	3.113149	-0.44087	0.659793	0.754759	-6.63713
RRP7B	0.079206	2.49593	0.440703	0.65991	0.754831	-6.43945
MLX	-0.06797	5.933462	-0.44049	0.660065	0.754947	-7.01351
DIRC2	0.090499	4.651459	0.440057	0.660377	0.755242	-6.88347
RBBP6	-0.06443	6.261237	-0.43988	0.660504	0.755326	-7.02448
MFAP3L	0.231753	2.382476	0.438883	0.661226	0.756089	-6.39388
IL34	-0.19786	1.162499	-0.43861	0.661424	0.7562	-6.24973
CAPNS1	0.077917	8.389353	0.438525	0.661486	0.7562	-6.99453
TPD52	0.123998	7.228463	0.43857	0.661453	0.7562	-7.02489
LRRC37B2	0.074034	4.286193	0.437865	0.661963	0.756684	-6.83032
CDC42EP4	-0.09382	6.740176	-0.43716	0.662474	0.757145	-7.02947
POLDIP2	0.060176	6.758776	0.437168	0.662467	0.757145	-7.02997
RSBN1L	-0.07243	4.695675	-0.43698	0.662603	0.75723	-6.90896
DNAJC19	-0.09275	3.93248	-0.4367	0.662803	0.757396	-6.79045
DHPS	-0.06605	4.931383	-0.43629	0.663101	0.75756	-6.93608
SH3BP4	-0.1099	7.162226	-0.43628	0.663109	0.75756	-7.02331
PTP4A2	-0.09297	6.577732	-0.43641	0.663014	0.75756	-7.03026
RNASEK	-0.07859	6.377542	-0.4362	0.663168	0.757565	-7.02853
FAM65C	0.188534	1.893652	0.436039	0.663285	0.757606	-6.31365
HAS2	-0.19708	2.120266	-0.43593	0.663366	0.757606	-6.41784
ZNF446	-0.08646	3.120709	-0.43595	0.663346	0.757606	-6.6085
C6orf136	0.091085	3.910717	0.435835	0.663432	0.75762	-6.74955
LOC220594	0.323734	0.552097	0.435729	0.663509	0.757646	-6.07357
TMEM134	-0.10934	4.591042	-0.43558	0.663621	0.757711	-6.90016

ARFGEF1	-0.07997	6.83968	-0.43548	0.663692	0.75773	-7.02933
MAPK11	-0.14276	2.201406	-0.43523	0.663868	0.757869	-6.42269
CES8	0.222621	1.020046	0.434873	0.664129	0.757982	-6.16244
VPS36	-0.07916	5.945325	-0.43497	0.664063	0.757982	-7.01689
NAP1L4	0.065529	6.389381	0.434906	0.664106	0.757982	-7.0269
PDE7A	0.121964	5.122669	0.434359	0.664502	0.758345	-6.93878
BRMS1L	0.108006	3.62363	0.434173	0.664637	0.758437	-6.68678
TBX1	0.386701	1.346074	0.433523	0.665108	0.758913	-6.19417
PHLPP2	-0.10786	3.912787	-0.43312	0.6654	0.75915	-6.79181
LRDD	0.085094	4.354283	0.433087	0.665425	0.75915	-6.84053
AHRR	-0.17314	1.725886	-0.43277	0.665654	0.75935	-6.34585
CCDC157	-0.09639	0.368232	-0.43256	0.665804	0.759397	-6.10451
ABCC3	0.284979	6.23319	0.43259	0.665785	0.759397	-7.0159
CDK11A	-0.0909	4.870698	-0.43191	0.666277	0.759874	-6.93358
ARL4D	0.332223	2.857066	0.431316	0.666709	0.760305	-6.47847
AKR1B1	0.169641	5.336324	0.43083	0.667062	0.760646	-6.95939
PFDN1	0.062536	5.320274	0.430631	0.667206	0.760748	-6.96709
CD97	-0.1829	5.997391	-0.43041	0.667365	0.760866	-7.02416
LOC100131434	-0.12856	0.767854	-0.43025	0.667486	0.760942	-6.17321
NOVA2	-0.13267	0.308635	-0.42995	0.667704	0.76103	-6.10162
TSNARE1	-0.09182	3.663337	-0.42999	0.66767	0.76103	-6.73362
ATG9A	0.064543	6.801705	0.429915	0.667726	0.76103	-7.03303
GOLT1A	-0.42366	-0.61451	-0.4296	0.667954	0.761228	-6.00004
DLL1	0.157163	4.079714	0.429503	0.668025	0.761247	-6.77876
SMARCC2	-0.04931	7.017563	-0.42916	0.668274	0.761468	-7.02993
GCN1L1	-0.06346	7.7153	-0.42886	0.66849	0.761652	-7.01519
ADCK1	-0.07437	3.352623	-0.42871	0.668602	0.761718	-6.65969
TM2D3	0.059059	4.567756	0.42863	0.66866	0.761721	-6.87997
LOC645166	-0.16237	1.79363	-0.42844	0.668798	0.761817	-6.35592
MST1	-0.15784	0.875828	-0.42819	0.668977	0.761959	-6.1949
PRCP	-0.08703	6.04665	-0.42795	0.669152	0.762096	-7.02411
PDGFA	-0.14843	4.499531	-0.42735	0.669594	0.762537	-6.89584
STMN3	0.210394	3.804639	0.42722	0.669685	0.762578	-6.70809
RAB14	-0.06054	7.056641	-0.42699	0.669852	0.762706	-7.03005
SRRD	-0.0614	3.541175	-0.42672	0.670045	0.762864	-6.70269
NCRNA00183	-0.07359	3.730373	-0.42651	0.670202	0.762981	-6.74813
DFFA	-0.06101	5.293655	-0.42609	0.670509	0.763269	-6.9766
CCDC8	-0.24913	1.441527	-0.42577	0.670737	0.763465	-6.3117
WHSC1L1	0.0846	6.070758	0.425696	0.670793	0.763467	-7.01999
CREB1	0.054652	5.882066	0.425349	0.671046	0.763673	-7.01234
UBXN4	0.05699	7.639236	0.425297	0.671083	0.763673	-7.02111
IGSF3	0.192846	6.515795	0.425192	0.67116	0.763698	-7.03127
TBL3	0.070528	5.422621	0.424993	0.671304	0.7638	-6.97825
CRIP1	-0.18339	4.99067	-0.4246	0.671589	0.764062	-6.95803
SLC45A3	-0.16481	3.813104	-0.42384	0.672147	0.764634	-6.77785
ZNF487	-0.09362	0.955784	-0.42338	0.672478	0.764948	-6.20192
SMEK1	-0.05538	6.478632	-0.42312	0.672666	0.7651	-7.03513
MARK1	-0.24517	3.858108	-0.42216	0.673371	0.765839	-6.81264
AGAP6	-0.09267	3.288042	-0.42166	0.673731	0.766186	-6.65232
PIK3R5	-0.17603	2.301659	-0.42107	0.674165	0.766618	-6.45518
KIAA1967	0.070134	6.440166	0.420854	0.67432	0.766731	-7.03382
EARS2	0.06902	4.64973	0.420515	0.674567	0.76695	-6.89343
RHPN2	0.311215	5.01448	0.420084	0.674881	0.767245	-6.90941
C6orf26	0.151221	0.829131	0.419922	0.674999	0.767263	-6.14598
TMEM164	-0.1017	5.369034	-0.41991	0.675007	0.767263	-6.9885
NT5C3L	0.138927	4.313509	0.419767	0.675112	0.76732	-6.83251
C17orf79	0.067126	4.4734	0.419584	0.675245	0.767409	-6.86924
LTBP2	0.156165	6.769626	0.419398	0.675381	0.767501	-7.03737

SDHAF1	0.073127	3.165145	0.418943	0.675713	0.767754	-6.59285
MRPS7	0.067192	5.34329	0.418946	0.675711	0.767754	-6.97361
CAPN10	-0.08078	3.821149	-0.41872	0.675874	0.767874	-6.77053
PNPLA6	-0.0623	6.037015	-0.41841	0.6761	0.768068	-7.02701
ZFYVE26	-0.07126	5.800076	-0.41833	0.67616	0.768074	-7.01685
DYNC1I2	0.058571	5.74637	0.4182	0.676256	0.76812	-7.00711
SPRR3	0.848669	4.037174	0.417376	0.676857	0.768676	-6.63734
NR1D2	-0.09254	5.793553	-0.41735	0.676878	0.768676	-7.01788
MICAL3	0.09616	6.334332	0.417303	0.67691	0.768676	-7.03237
FGFR2	-0.17387	5.330591	-0.41709	0.677066	0.76879	-6.99259
NAGK	0.097562	5.523771	0.417015	0.677121	0.76879	-6.98844
INTS9	0.075649	4.220747	0.416596	0.677426	0.769074	-6.82544
DNAJC10	-0.08107	6.990352	-0.41645	0.677531	0.769131	-7.03527
TAF7	0.062994	6.658339	0.416199	0.677716	0.769279	-7.03865
CEP70	0.094023	4.176629	0.415601	0.678153	0.769712	-6.81499
NEK6	0.12918	5.238278	0.415476	0.678245	0.769754	-6.95865
TRAPPC2P1	0.089396	2.332885	0.415055	0.678553	0.77004	-6.41811
FUT4	-0.24279	4.391289	-0.41439	0.679037	0.770528	-6.89613
TSSK6	-0.09146	0.89779	-0.41349	0.679696	0.77115	-6.19486
SPATA2L	0.083978	3.77749	0.413551	0.679652	0.77115	-6.73211
HCST	0.181548	1.212335	0.413064	0.680008	0.771316	-6.20988
RHBDL2	0.197251	2.798449	0.413134	0.679957	0.771316	-6.48639
ABCB8	-0.07178	5.002569	-0.41312	0.679965	0.771316	-6.95387
RPL13	-0.09219	9.548272	-0.4127	0.680272	0.771553	-6.95947
PSEN2	-0.10183	3.647626	-0.41219	0.68065	0.771611	-6.74124
ISG20	-0.16217	4.038017	-0.41218	0.680655	0.771611	-6.83122
NLRP1	0.192354	4.5828	0.412453	0.680455	0.771611	-6.873
SUFU	-0.06666	4.563798	-0.41222	0.680624	0.771611	-6.90223
MCRS1	0.053696	5.491555	0.412471	0.680441	0.771611	-6.9906
MARCKS	0.080145	7.427766	0.412383	0.680506	0.771611	-7.03151
SAPS3	0.070715	7.157973	0.412008	0.680781	0.771692	-7.03657
UNC5CL	-0.32809	2.316413	-0.4116	0.681078	0.771966	-6.47934
LZTR1	-0.06788	5.662648	-0.41135	0.681263	0.772113	-7.0118
VTI1A	-0.05663	4.553227	-0.41079	0.681673	0.772452	-6.89945
H2AFJ	-0.12125	5.273358	-0.41081	0.681657	0.772452	-6.98607
PSTPIP2	-0.16988	3.54148	-0.41064	0.68178	0.77251	-6.73308
SCFD2	0.074062	3.781449	0.410028	0.68223	0.772896	-6.73702
UBB	-0.09585	9.250753	-0.41003	0.68223	0.772896	-6.97069
CNNM2	-0.07179	3.8018	-0.40903	0.682961	0.773661	-6.76894
MAP7D3	-0.14602	3.52814	-0.4088	0.68313	0.773789	-6.72765
ODZ2	0.588908	3.601684	0.408713	0.683193	0.773798	-6.59561
MCAT	0.064409	3.699733	0.40837	0.683445	0.773911	-6.72013
TMEM41B	0.072378	5.333153	0.408373	0.683443	0.773911	-6.97626
MEX3C	-0.08094	5.527312	-0.40835	0.683459	0.773911	-7.00471
NSMAF	0.069638	5.285748	0.40826	0.683525	0.773924	-6.97256
THEM4	-0.10292	3.940857	-0.40773	0.683915	0.774302	-6.80469
LOC282997	-0.09694	0.604279	-0.40721	0.684294	0.774668	-6.15248
PRKRA	-0.05334	5.647423	-0.40684	0.684567	0.774914	-7.0119
KDELC2	-0.08849	5.759143	-0.40676	0.684625	0.774918	-7.02044
C1orf122	-0.07229	4.818997	-0.40638	0.684902	0.775107	-6.93637
MIDN	-0.07681	7.233319	-0.40638	0.684903	0.775107	-7.0351
ZNF354A	0.089106	3.553551	0.406237	0.685008	0.775163	-6.68459
TAF9	0.057791	5.652959	0.405954	0.685216	0.775335	-7.00562
ADAP1	0.218527	4.96701	0.405759	0.685359	0.775435	-6.92254
SPDYE1	0.11476	0.442462	0.405533	0.685525	0.775441	-6.09832
DACT1	-0.18249	2.685981	-0.40564	0.685444	0.775441	-6.54525
C22orf28	0.05338	6.048715	0.405523	0.685532	0.775441	-7.02863
ZNF205	0.067476	3.619581	0.40536	0.685652	0.775514	-6.70247

LOC388789	-0.07219	4.216661	-0.40523	0.685744	0.775556	-6.85346
SCRN3	-0.07156	4.279435	-0.40512	0.685825	0.775585	-6.86424
DENND1C	-0.1132	3.218975	-0.40496	0.685947	0.775644	-6.64546
CSPP1	0.083896	4.409692	0.404901	0.685988	0.775644	-6.86242
FBXL16	-0.19242	2.419081	-0.40465	0.68617	0.775786	-6.48875
PEX1	0.108352	4.873646	0.404511	0.686275	0.775842	-6.92427
FAM195B	-0.08627	4.295934	-0.40426	0.686456	0.775985	-6.86937
GALNT5	-0.26728	3.119527	-0.40393	0.6867	0.776197	-6.64944
KATNAL1	-0.12719	3.780821	-0.40377	0.686821	0.776271	-6.78213
C9orf37	0.074195	2.473589	0.403242	0.687207	0.776644	-6.44973
C20orf96	0.15086	2.57958	0.402871	0.687479	0.776884	-6.45841
COX17	-0.08402	4.032427	-0.4028	0.68753	0.776884	-6.82178
ZNF787	0.068081	4.980479	0.402369	0.687848	0.77718	-6.94205
UBE2L3	0.057201	6.40506	0.402128	0.688025	0.777317	-7.04107
C1orf226	-0.15546	4.808439	-0.40161	0.688406	0.777685	-6.94463
SSH2	-0.08306	5.221605	-0.40143	0.688536	0.777769	-6.98164
RAB24	-0.08422	3.815048	-0.40107	0.688801	0.778005	-6.7792
IREB2	-0.06009	6.195948	-0.40075	0.689038	0.77821	-7.03914
IL6	-0.2556	1.363389	-0.40037	0.689318	0.778401	-6.31162
MPV17L2	0.072101	3.821004	0.400371	0.689316	0.778401	-6.75057
SLC4A7	-0.0996	5.312371	-0.40023	0.689418	0.778451	-6.99217
AMMECR1L	-0.05096	5.185567	-0.39951	0.689948	0.778986	-6.97653
C19orf39	0.0814	1.050422	0.399294	0.690108	0.779055	-6.20097
ZNRF1	-0.0782	5.000807	-0.39922	0.69016	0.779055	-6.9606
STK35	0.063518	6.488591	0.399201	0.690177	0.779055	-7.04367
KILLIN	0.093212	0.229655	0.398746	0.690512	0.779371	-6.07217
ARSJ	-0.17513	3.836795	-0.39862	0.690606	0.779414	-6.80174
RAB3IL1	0.148451	2.80204	0.398482	0.690706	0.779464	-6.50937
P2RY11	0.105743	3.055553	0.398245	0.69088	0.779597	-6.57055
C2orf54	0.350543	2.9322	0.397586	0.691365	0.779922	-6.49613
PKNOX1	-0.04718	4.554084	-0.39755	0.691391	0.779922	-6.90442
PSMD9	0.061613	5.068528	0.397699	0.691281	0.779922	-6.9547
CHMP1B	-0.07281	6.520287	-0.39768	0.691294	0.779922	-7.04609
MGC23270	0.138696	0.189305	0.397372	0.691523	0.780008	-6.06214
CDC42EP5	0.251262	3.821335	0.39683	0.691921	0.780269	-6.70919
CSNK1G3	0.055263	5.130555	0.396914	0.69186	0.780269	-6.96229
CBARA1	-0.06493	5.611276	-0.3969	0.691868	0.780269	-7.01389
RAB32	0.129876	3.913552	0.396077	0.692476	0.78083	-6.7615
ARNTL	-0.08875	3.628211	-0.39594	0.692576	0.780881	-6.74301
IFITM2	0.143007	6.086498	0.395799	0.692681	0.780936	-7.03076
FBXO44	-0.0991	3.480302	-0.39569	0.692758	0.78096	-6.71006
PIK3R6	0.175625	-0.0143	0.395328	0.693028	0.781088	-6.02825
ARMCX6	0.142852	1.703711	0.395236	0.693095	0.781088	-6.30689
TRIP4	0.053227	4.416076	0.395457	0.692933	0.781088	-6.87159
TCTN3	-0.05534	5.147193	-0.39524	0.693091	0.781088	-6.97468
STOM	-0.11584	7.577404	-0.39508	0.693208	0.781089	-7.03138
OPA1	0.071032	7.085678	0.395129	0.693174	0.781089	-7.04454
B4GALT1	0.085189	7.772082	0.394622	0.693548	0.781409	-7.03111
CCNO	0.164789	1.913708	0.394177	0.693876	0.781713	-6.33359
UNC13D	0.185419	5.191024	0.394028	0.693986	0.781713	-6.95688
HNRPDL	-0.04712	7.247236	-0.39406	0.69396	0.781713	-7.04025
EIF3E	0.068291	7.641134	0.393465	0.694401	0.782055	-7.0343
MYH10	-0.18025	5.891986	-0.39347	0.694397	0.782055	-7.03572
TMEM229B	0.206627	2.736241	0.393201	0.694596	0.782148	-6.48118
SLC25A1	-0.07647	5.947831	-0.39326	0.694553	0.782148	-7.03412
SCML2	0.261764	0.879689	0.392288	0.695269	0.782754	-6.155
CCDC132	0.086924	4.809785	0.392221	0.695318	0.782754	-6.92364
FAM21C	-0.05863	5.94398	-0.39233	0.69524	0.782754	-7.03371

MGST1	-0.23419	6.330761	-0.39217	0.695358	0.782754	-7.04782
C4orf42	-0.06916	4.045361	-0.39188	0.695573	0.782933	-6.82713
C6orf97	0.200579	0.505757	0.391443	0.695892	0.783182	-6.10163
MTCP1	-0.08516	0.854988	-0.39142	0.695907	0.783182	-6.19701
CD48	-0.17441	2.205956	-0.39119	0.696078	0.783277	-6.44871
FAM126A	0.15754	5.189723	0.391158	0.696103	0.783277	-6.96181
MTAP	-0.17688	4.739638	-0.39051	0.696577	0.783748	-6.9442
OAZ1	0.061745	8.515089	0.390151	0.696845	0.783986	-7.00965
TIMM44	0.061292	4.616219	0.389799	0.697106	0.784216	-6.90256
PAOX	-0.10361	2.245248	-0.38956	0.697283	0.784353	-6.44493
LDLR	-0.11025	7.579163	-0.38937	0.697424	0.784447	-7.03367
LETM2	0.141709	1.368253	0.389218	0.697534	0.784508	-6.24933
JTB	-0.07197	6.559392	-0.38909	0.697631	0.784554	-7.04966
STRAP	0.062881	6.687345	0.3889	0.697769	0.784646	-7.04973
NCRNA00174	0.119581	4.172654	0.388546	0.698031	0.784677	-6.81869
PIGG	0.075525	4.922949	0.388407	0.698133	0.784677	-6.93994
FCGBP	-0.32603	5.18099	-0.38858	0.698005	0.784677	-7.00106
SIKE1	0.061503	5.658225	0.388538	0.698036	0.784677	-7.01274
TC2N	-0.16378	6.171434	-0.38846	0.698093	0.784677	-7.04562
MYO5B	-0.16651	6.270136	-0.38857	0.69801	0.784677	-7.0476
SLC9A6	0.055626	4.947218	0.388131	0.698337	0.784843	-6.94566
SLC12A7	0.149207	6.896999	0.387405	0.698873	0.785382	-7.05023
MFAP1	0.04589	4.879858	0.387305	0.698948	0.785403	-6.93879
CDKL1	0.118985	0.931814	0.387189	0.699034	0.785436	-6.18095
SUPT5H	0.043747	7.242799	0.386989	0.699181	0.785538	-7.04461
ANKHD1	-0.06031	7.338254	-0.38653	0.699518	0.785854	-7.0411
ITGAL	0.195725	3.298788	0.385672	0.700155	0.786506	-6.61301
C1orf66	-0.06569	4.1042	-0.38473	0.700854	0.787228	-6.83967
ETNK2	-0.23248	3.015807	-0.38455	0.700986	0.787313	-6.64554
GRIPAP1	-0.05225	5.236337	-0.38409	0.701328	0.787634	-6.9876
C12orf65	0.065981	3.419683	0.383986	0.701402	0.787654	-6.66594
WDFY4	-0.21788	2.156575	-0.38361	0.701679	0.787902	-6.45046
NCRNA00095	0.137473	1.656672	0.382899	0.702207	0.788431	-6.30121
MOBKL2A	-0.08858	6.263679	-0.38258	0.702439	0.788629	-7.04851
FAM171B	0.141327	2.946754	0.38231	0.702643	0.788645	-6.54625
C1orf210	-0.20855	2.981664	-0.38236	0.702609	0.788645	-6.61088
CCDC12	-0.06148	4.069421	-0.38229	0.702654	0.788645	-6.8335
COMTD1	0.106311	4.266772	0.38226	0.70268	0.788645	-6.8421
NDUFA7	-0.07296	4.499853	-0.38209	0.702806	0.788645	-6.90532
ZHX2	0.088256	5.164348	0.382054	0.702833	0.788645	-6.96872
MLL4	0.061717	6.85442	0.382031	0.702849	0.788645	-7.05212
FLJ43663	0.112192	2.006446	0.381742	0.703063	0.788725	-6.36875
LOC100216545	0.084129	2.618625	0.381696	0.703097	0.788725	-6.48668
HERC2P2	-0.09342	5.747334	-0.38163	0.703146	0.788725	-7.02975
LASS6	0.09151	6.699298	0.381689	0.703102	0.788725	-7.05245
GABRP	0.466022	3.321407	0.381349	0.703354	0.788895	-6.56259
NRL	-0.09057	0.124557	-0.38069	0.703845	0.789382	-6.08792
PARP11	0.103223	3.319355	0.379551	0.704686	0.790262	-6.63819
LAX1	-0.20901	0.566063	-0.3791	0.705019	0.790318	-6.17284
ZNF594	0.111515	2.45273	0.379178	0.704963	0.790318	-6.45151
SH3BP5L	0.058675	4.98491	0.379287	0.704883	0.790318	-6.95299
ERAP2	0.219676	5.247262	0.379102	0.705019	0.790318	-6.96668
PANK3	0.084337	5.235309	0.379379	0.704814	0.790318	-6.97721
ALS2	0.056416	5.144992	0.37898	0.70511	0.790357	-6.97049
DIS3L2	-0.04944	4.601277	-0.37858	0.705409	0.790628	-6.91782
PRICKLE1	-0.18855	2.809395	-0.37785	0.70595	0.790862	-6.58555
FAM18B2	0.075882	4.12381	0.377838	0.705957	0.790862	-6.82155
C1orf86	0.092041	4.604045	0.378144	0.70573	0.790862	-6.90154

FRMD6	0.225763	5.806817	0.378063	0.70579	0.790862	-7.01743
COPS7A	0.062458	5.931479	0.377975	0.705855	0.790862	-7.03352
NDUFS5	0.078281	6.192735	0.378032	0.705813	0.790862	-7.04411
PAPSS2	0.146398	4.651076	0.377626	0.706114	0.790975	-6.89994
N4BP1	-0.08306	6.683922	-0.3775	0.706205	0.791013	-7.05411
NMNAT3	0.142535	2.655065	0.376728	0.70678	0.791594	-6.48713
B4GALT5	0.091973	7.145218	0.376232	0.707148	0.791942	-7.05115
OSBPL10	0.11411	5.011376	0.376067	0.707271	0.792016	-6.95093
PPP4R2	-0.06244	4.197144	-0.37599	0.707329	0.792018	-6.85986
ERCC1	-0.07335	5.000134	-0.37578	0.707486	0.792067	-6.96865
MPDU1	-0.07377	5.051145	-0.3758	0.707466	0.792067	-6.97367
LOC90784	0.081079	4.189196	0.375565	0.707644	0.79218	-6.83594
SPAG17	0.363353	0.191026	0.375183	0.707927	0.792371	-6.04421
OLFML2A	-0.12961	5.888489	-0.37522	0.707903	0.792371	-7.04064
POLR2B	0.051456	6.835931	0.375048	0.708027	0.792419	-7.05482
MRPL19	0.047366	5.947176	0.374932	0.708114	0.792452	-7.03615
LOC407835	-0.06371	2.781257	-0.37467	0.708305	0.792539	-6.55132
ANKRD56	-0.19181	2.726327	-0.37472	0.708272	0.792539	-6.5584
GRINL1A	0.052993	5.766985	0.374424	0.70849	0.792683	-7.02601
C2orf60	-0.05299	3.744622	-0.37396	0.708833	0.793003	-6.76694
C11orf35	0.113732	1.351342	0.373116	0.709463	0.79358	-6.25416
BCAS4	-0.09261	3.406089	-0.37316	0.709432	0.79358	-6.69915
VSIG10	-0.12615	4.441945	-0.37234	0.710041	0.794164	-6.90756
STAMBPL1	0.140331	3.072396	0.372128	0.710196	0.794274	-6.57524
C20orf112	0.15646	5.442519	0.371626	0.71057	0.794628	-6.99335
MTHFSD	0.061993	3.88948	0.371448	0.710702	0.794713	-6.77861
NME3	-0.09298	3.767715	-0.37129	0.710821	0.794782	-6.78134
CLPP	0.062608	4.976836	0.371129	0.710939	0.79485	-6.95435
PIAS3	0.083179	5.838566	0.370914	0.711099	0.794966	-7.03015
TNPO1	-0.05659	7.126322	-0.36968	0.712016	0.795927	-7.05166
SLC24A6	-0.07089	5.294412	-0.3695	0.712152	0.795952	-6.9997
ADIPOR1	-0.05724	7.327917	-0.36956	0.712109	0.795952	-7.04778
NBR2	-0.08842	3.028753	-0.36937	0.712248	0.795958	-6.61275
SPTAN1	-0.06222	8.66648	-0.36934	0.712272	0.795958	-7.00826
IP6K2	-0.05766	5.604829	-0.36818	0.71313	0.796853	-7.02411
KCNK5	-0.23443	3.597844	-0.36778	0.713434	0.797129	-6.76791
DAD1	0.058797	6.279456	0.367146	0.713904	0.79759	-7.05139
GCFC1	-0.06573	5.114582	-0.36588	0.71485	0.798584	-6.98361
SHQ1	0.053533	4.059783	0.365332	0.715254	0.798919	-6.81791
VPS52	-0.05395	6.105365	-0.36532	0.715265	0.798919	-7.04985
UBE3A	-0.0484	6.899142	-0.36454	0.715846	0.799504	-7.05726
HP1BP3	-0.04796	7.7553	-0.36432	0.716006	0.799619	-7.04007
SEMA4A	0.154152	5.111894	0.364224	0.71608	0.799638	-6.96347
KIAA1919	-0.06252	3.420105	-0.36391	0.716315	0.799816	-6.69876
HDAC3	-0.04895	5.091584	-0.36386	0.716354	0.799816	-6.98066
ENDOD1	0.097739	6.031567	0.363511	0.716612	0.80004	-7.04233
HSF2	-0.06014	3.716667	-0.36327	0.716794	0.800116	-6.76758
COPE	0.058175	6.925109	0.363235	0.716818	0.800116	-7.05854
PHF3	-0.0562	6.591092	-0.36319	0.716852	0.800116	-7.05944
TAOK1	-0.08651	5.071486	-0.36284	0.717116	0.800347	-6.98228
KCTD20	-0.0689	6.184641	-0.36275	0.71718	0.800355	-7.05355
ZCCHC11	-0.10775	5.312048	-0.3625	0.717369	0.800502	-7.00716
FAM101B	-0.10567	3.830708	-0.36242	0.71743	0.800505	-6.8016
IMPACT	-0.08966	5.258906	-0.36225	0.717555	0.800582	-7.00088
ZNF18	-0.0677	3.018804	-0.36168	0.717975	0.800921	-6.61009
FAM118B	-0.08671	3.590971	-0.36175	0.717928	0.800921	-6.74226
ZDHHC1	0.108537	3.597275	0.361314	0.718251	0.801166	-6.70668
EIF1	-0.09312	9.088762	-0.36111	0.718407	0.801211	-6.99526

ASXL1	0.057661	6.630845	0.361167	0.718361	0.801211	-7.05985
HDAC9	0.20672	3.321606	0.360316	0.718996	0.801805	-6.62525
C5orf43	-0.05325	5.941638	-0.35998	0.71925	0.802024	-7.04546
PRSS3	0.42154	3.132739	0.359879	0.719322	0.802041	-6.53468
RMRP	0.106006	0.888531	0.359033	0.719954	0.802681	-6.1867
KCNK6	0.121982	4.864301	0.358827	0.720108	0.802724	-6.93827
SERPINB1	-0.15225	7.155156	-0.35884	0.720101	0.802724	-7.05344
ARMC2	-0.08526	0.919623	-0.35874	0.720171	0.802731	-6.21906
IL4R	-0.09122	6.437779	-0.35861	0.720271	0.802778	-7.06021
RPSAP58	-0.07003	8.331586	-0.35819	0.720584	0.803063	-7.02389
ABCD4	-0.05777	4.276828	-0.35778	0.720889	0.803339	-6.87902
PC	0.144245	5.143553	0.357684	0.720962	0.803356	-6.96937
FEM1B	-0.06888	6.805474	-0.35759	0.721031	0.803369	-7.06066
MRPS18B	0.060813	5.70122	0.357302	0.721247	0.803545	-7.02738
CACNG4	-0.29984	0.616749	-0.35708	0.721411	0.803664	-6.20168
TMEM51	0.106533	4.831701	0.356085	0.722157	0.804431	-6.9364
PHF15	0.111021	6.201355	0.355911	0.722287	0.804512	-7.05148
BCAM	-0.15023	6.417722	-0.35566	0.722474	0.804655	-7.0616
SLC37A1	-0.15799	4.805129	-0.35547	0.722614	0.804747	-6.9619
GTF2I	-0.06628	7.145615	-0.35524	0.722787	0.804876	-7.05637
EFHA1	-0.06511	5.095589	-0.35499	0.722973	0.805019	-6.98536
LOC284837	0.193875	1.216355	0.354471	0.723364	0.80539	-6.23105
PVRIG	0.108175	1.849987	0.354136	0.723615	0.805605	-6.3513
INPP4B	0.163636	3.64561	0.353569	0.724039	0.806013	-6.70843
SLC26A6	0.100628	4.137543	0.353346	0.724206	0.806135	-6.82523
DULLARD	0.047818	6.69895	0.353221	0.724299	0.806175	-7.063
MEPCE	0.064735	5.946033	0.35314	0.72436	0.806178	-7.04328
ZDHHC16	0.054245	4.7695	0.35287	0.724562	0.806256	-6.936
LOC647979	-0.05407	8.66937	-0.35284	0.724584	0.806256	-7.01436
POLR2J3	0.126579	5.623187	0.352815	0.724604	0.806256	-7.0195
DHX38	-0.04272	5.902472	-0.35255	0.724804	0.806415	-7.04605
ZNF672	-0.05824	5.100083	-0.35237	0.724938	0.8065	-6.98647
TRIM62	0.059027	3.88403	0.352082	0.725153	0.80661	-6.78542
GSTM4	-0.16664	4.590755	-0.35211	0.725133	0.80661	-6.94032
ANKRD54	0.049898	4.079759	0.351808	0.725358	0.80671	-6.82866
ELOVL7	-0.13614	4.152716	-0.35181	0.725356	0.80671	-6.87188
MYST2	-0.04428	5.756873	-0.35133	0.725714	0.807042	-7.03905
LOXL4	0.269911	2.166895	0.350639	0.726233	0.807555	-6.38649
HNF4G	0.38995	2.441498	0.350184	0.726574	0.807853	-6.40443
TMEM99	0.129303	3.14975	0.349987	0.726721	0.807853	-6.60383
CCDC94	0.063372	3.878409	0.350011	0.726704	0.807853	-6.7826
RPL10A	-0.06218	8.158157	-0.34997	0.726733	0.807853	-7.03289
CNBP	0.055396	8.305566	0.349676	0.726954	0.808035	-7.03156
HCG11	0.133469	3.721951	0.349478	0.727103	0.808132	-6.73783
CALM3	0.065433	7.816573	0.349406	0.727157	0.808132	-7.04635
PGM3	0.071505	4.703453	0.349291	0.727243	0.808163	-6.92641
C11orf93	0.217021	1.969578	0.348273	0.728006	0.808947	-6.35573
SPRED1	-0.07206	5.754932	-0.34776	0.728394	0.809313	-7.04151
CMKLR1	-0.14002	2.443276	-0.3476	0.728513	0.809382	-6.5054
ITGAV	0.09109	7.903957	0.347435	0.728634	0.809452	-7.04537
RASSF10	0.215014	1.769082	0.347242	0.728779	0.809542	-6.31983
LOC81691	-0.09373	2.245976	-0.34717	0.728831	0.809542	-6.45423
SIX2	0.332146	0.38028	0.346568	0.729285	0.809981	-6.08436
SLC35E3	0.083085	3.309009	0.346328	0.729465	0.810103	-6.65268
S100PBP	-0.05228	4.474675	-0.34619	0.729565	0.810103	-6.91288
ABCB10	0.056469	4.786949	0.346191	0.729568	0.810103	-6.94137
HSD17B2	0.347437	1.989917	0.34542	0.730146	0.810669	-6.33265
SEL1L3	-0.1521	6.680502	-0.34536	0.730194	0.810669	-7.06535

FAM32A	0.044621	5.83439	0.344972	0.730483	0.810925	-7.04106
PCDH12	0.122094	2.969586	0.344868	0.730561	0.810948	-6.56619
TUBB6	-0.15548	6.34334	-0.34471	0.730676	0.811011	-7.06466
C1orf201	0.084172	3.317343	0.344024	0.731194	0.811521	-6.65344
MRPL22	0.057588	4.271453	0.343904	0.731285	0.811558	-6.866
TMEM86A	-0.11208	2.206012	-0.34378	0.731381	0.811589	-6.45393
SNIP1	-0.04467	3.976065	-0.34368	0.731454	0.811589	-6.8272
RDH10	-0.1088	5.838714	-0.34363	0.731488	0.811589	-7.04887
YAF2	-0.05092	3.956662	-0.34323	0.731793	0.811863	-6.82508
CLCN4	-0.22642	2.455072	-0.3424	0.732416	0.812172	-6.52282
SLC16A14	0.165806	2.80018	0.342671	0.732211	0.812172	-6.52806
WNT4	0.190848	2.870023	0.342409	0.732408	0.812172	-6.53465
TSPAN9	0.101371	5.117736	0.342698	0.73219	0.812172	-6.97709
TFPI	-0.1487	4.940668	-0.34231	0.732478	0.812172	-6.98129
MXRA8	-0.13544	5.310125	-0.34246	0.732372	0.812172	-7.01648
KIAA0146	0.058205	5.566418	0.34234	0.732459	0.812172	-7.02288
LLGL1	0.073611	5.796311	0.342145	0.732606	0.812185	-7.03837
RIC8A	0.049405	6.171752	0.342214	0.732554	0.812185	-7.05713
SPOCK1	0.234817	3.257414	0.341743	0.732908	0.812455	-6.61579
KPNA3	0.050224	6.407794	0.34157	0.733038	0.812535	-7.06373
CETN3	0.063748	3.186458	0.341241	0.733285	0.812729	-6.62958
SF4	0.039495	4.17848	0.341182	0.733329	0.812729	-6.8526
CISD1	-0.05991	4.28068	-0.34103	0.73344	0.812788	-6.88721
ZCCHC3	0.060496	4.672019	0.340536	0.733815	0.813138	-6.92812
ZNF513	-0.05641	3.971731	-0.3403	0.733995	0.813209	-6.82874
CTNND1	-0.06271	9.225361	-0.34032	0.733975	0.813209	-6.99869
KATNAL2	-0.14644	0.434049	-0.34013	0.734121	0.813284	-6.15727
KLHL29	0.136002	3.452978	0.340011	0.734209	0.813318	-6.68045
TP53BP2	-0.0508	6.217018	-0.33987	0.734312	0.813367	-7.06198
SLC2A4RG	0.081329	6.151406	0.339791	0.734375	0.813372	-7.05627
CINP	-0.05583	4.07817	-0.33941	0.734661	0.813624	-6.84956
SEMA4D	-0.08997	5.426198	-0.3392	0.734821	0.813737	-7.02341
GPR183	0.140874	3.037049	0.33836	0.735452	0.814371	-6.58484
ATP6V1G1	-0.05373	6.343892	-0.33796	0.735751	0.814638	-7.06549
DECR2	0.083911	3.515642	0.337595	0.736027	0.814879	-6.70011
ZNF644	-0.04843	5.848849	-0.33751	0.736094	0.814888	-7.04887
LOC100132247	-0.08772	5.375657	-0.33731	0.736241	0.814914	-7.01928
C1S	-0.15415	7.338134	-0.33724	0.736292	0.814914	-7.05718
PIK3R2	-0.06204	7.000056	-0.33737	0.736194	0.814914	-7.06514
LOC220729	0.075221	3.391167	0.336814	0.736615	0.815142	-6.67445
OXSR1	-0.06022	6.09417	-0.33683	0.736605	0.815142	-7.05964
C3orf31	-0.04923	2.464563	-0.33657	0.736796	0.815149	-6.49585
STAT5A	0.098413	4.846143	0.3367	0.736701	0.815149	-6.94736
TOMM20	-0.05899	7.758172	-0.33661	0.736768	0.815149	-7.04942
RRP15	0.053931	4.874012	0.335967	0.737253	0.815589	-6.95603
HAUS4	0.062425	4.390504	0.335721	0.737438	0.815729	-6.88846
WDR73	0.051345	4.027557	0.335352	0.737716	0.815973	-6.82179
RSRC2	0.04047	5.649384	0.334854	0.738091	0.816323	-7.03279
SLC38A9	0.05854	3.208601	0.333945	0.738775	0.817015	-6.63656
LOC100009676	0.079088	2.069898	0.333706	0.738955	0.81715	-6.40212
SLFN12	0.123125	2.044823	0.333539	0.739082	0.817224	-6.3911
NMNAT2	0.168753	1.738495	0.333129	0.73939	0.817436	-6.32747
GRIP1	0.199769	2.341879	0.333137	0.739384	0.817436	-6.43501
UBA3	-0.04434	5.417364	-0.33178	0.740409	0.818498	-7.02188
CDK3	0.075018	2.452946	0.33137	0.740717	0.818767	-6.47323
SDK1	0.222725	4.287416	0.331299	0.74077	0.818767	-6.84638
TRIM22	-0.13293	5.456313	-0.33109	0.740926	0.818875	-7.03138
ZNF490	-0.05247	3.434985	-0.33029	0.741528	0.819476	-6.71304

TXNL4B	0.046888	4.001537	0.329965	0.741776	0.819685	-6.81912
TCF4	0.11211	5.877601	0.329628	0.74203	0.819901	-7.04543
COX6B1	-0.06658	6.990427	-0.32955	0.742091	0.819904	-7.06783
KIAA1804	0.107348	4.973715	0.329429	0.742181	0.819938	-6.96199
IKZF1	-0.1411	3.123409	-0.32935	0.742242	0.81994	-6.66156
NUDT22	-0.05696	4.027798	-0.32921	0.742345	0.819989	-6.84221
CAV2	0.134626	6.389114	0.328523	0.742865	0.820498	-7.06593
CHCHD6	-0.07925	2.297093	-0.32823	0.743083	0.820675	-6.47098
LYPD6	0.199808	2.058273	0.327768	0.743434	0.820933	-6.37894
NFATC2IP	-0.04456	5.582709	-0.3278	0.743408	0.820933	-7.03576
MPEG1	-0.13562	3.872446	-0.32725	0.743824	0.821298	-6.82933
COL9A2	0.190231	2.825125	0.327106	0.743934	0.821355	-6.52363
LIN52	0.066267	2.648363	0.326991	0.744021	0.821386	-6.51627
RPL27A	0.068813	8.088447	0.326043	0.744737	0.822111	-7.04676
ADAT2	0.072787	3.542161	0.325676	0.745014	0.822353	-6.7107
B4GALT7	0.055965	4.288346	0.324973	0.745546	0.822809	-6.87464
GLB1	-0.0766	5.841855	-0.32499	0.745536	0.822809	-7.05373
SFRS4	-0.03985	6.391606	-0.32462	0.745813	0.823039	-7.07055
CYTH2	0.053822	5.545292	0.324267	0.746079	0.823268	-7.02745
COPZ2	-0.1619	2.149211	-0.32379	0.746441	0.823537	-6.46154
LOC440944	-0.076	3.671588	-0.32384	0.7464	0.823537	-6.77173
MSTO2P	0.077034	2.176212	0.323679	0.746524	0.823563	-6.42372
ZNF8	-0.06556	3.374576	-0.32294	0.747085	0.824052	-6.70474
PDXP	-0.08761	3.43416	-0.32296	0.747067	0.824052	-6.72122
ATP6V1B2	0.051618	6.309715	0.322721	0.747248	0.824167	-7.06767
PUS7L	0.061554	3.666412	0.322522	0.747398	0.824196	-6.74396
STK17A	-0.08538	5.47333	-0.32245	0.747451	0.824196	-7.03227
LUC7L3	-0.07681	6.900361	-0.32246	0.747442	0.824196	-7.07135
ARRDC1	0.0756	5.750936	0.322241	0.747611	0.824307	-7.04147
LOC400927	0.091953	1.081652	0.322036	0.747766	0.824348	-6.23163
KIAA1274	0.112015	3.629684	0.322045	0.747759	0.824348	-6.72828
DENND2C	0.215024	3.164262	0.321953	0.747829	0.824352	-6.60442
CXorf57	0.169062	1.966577	0.321671	0.748042	0.824392	-6.37415
FAM129B	0.098617	8.603053	0.321677	0.748038	0.824392	-7.03202
MVP	0.08489	7.524305	0.321677	0.748038	0.824392	-7.06279
DNAH5	0.233764	1.041631	0.321278	0.74834	0.824525	-6.20303
ZCCHC9	-0.05236	3.85752	-0.32133	0.748299	0.824525	-6.81013
IKBKAP	0.043406	5.871546	0.321358	0.748279	0.824525	-7.051
FLT3LG	0.092426	1.973095	0.320465	0.748955	0.825115	-6.38767
SNX17	-0.04287	6.350881	-0.32041	0.748994	0.825115	-7.07121
TMC5	-0.38253	4.095205	-0.32029	0.749085	0.825151	-6.90179
ETV5	0.128835	4.686753	0.319618	0.749596	0.82563	-6.92773
ZFAND6	0.042442	5.453786	0.319562	0.749638	0.82563	-7.02216
FKBP7	-0.08741	2.951155	-0.31934	0.749803	0.825747	-6.61464
RALGPS2	0.088303	5.212122	0.319112	0.749979	0.825875	-6.99596
WDR59	-0.042	5.178969	-0.31898	0.750076	0.825917	-7.00399
PDLIM7	0.101032	6.11047	0.318877	0.750157	0.825941	-7.06087
CENPBD1	0.065433	3.58049	0.318232	0.750646	0.826377	-6.72674
NRP1	0.102148	6.080829	0.318198	0.750671	0.826377	-7.05969
PGAP1	0.103851	5.167454	0.317732	0.751024	0.8267	-6.99016
FAU	0.060879	7.097052	0.317516	0.751187	0.826815	-7.07182
COX7A2L	-0.04802	5.578142	-0.31711	0.751498	0.827092	-7.039
ESRP2	0.090592	6.402816	0.31699	0.751586	0.827124	-7.07089
ZNF673	-0.06818	2.948179	-0.31652	0.75194	0.827448	-6.60951
ENGASE	0.082268	4.294002	0.316228	0.752163	0.827609	-6.87359
SRP68	0.042802	6.375785	0.316174	0.752205	0.827609	-7.07154
CLCN7	-0.04264	6.330022	-0.3158	0.752486	0.827853	-7.07228
ZNF143	0.032602	4.154124	0.315686	0.752574	0.827885	-6.85815

COL9A3	0.247352	1.468593	0.315569	0.752663	0.827917	-6.27346
MX2	0.134587	5.186939	0.315271	0.752889	0.828101	-6.9901
ODZ4	-0.20796	3.587436	-0.31458	0.753414	0.828613	-6.79228
AP3S1	0.058954	5.309959	0.314221	0.753685	0.828846	-7.00956
PROX1	-0.22787	0.481785	-0.31394	0.753896	0.829012	-6.18252
SDSL	-0.11988	2.047422	-0.31375	0.754042	0.829108	-6.43094
CDKN2AIPNL	0.056585	3.321015	0.313624	0.754138	0.829148	-6.66975
AKR1C3	-0.23953	5.974096	-0.31336	0.754338	0.829303	-7.0684
UVRAG	-0.05548	5.090657	-0.31289	0.754692	0.829561	-6.9982
NFE2L1	-0.05889	8.27066	-0.31293	0.754667	0.829561	-7.04149
DACT2	-0.27598	0.681337	-0.3127	0.754836	0.829655	-6.22353
B9D1	0.078972	1.987351	0.31261	0.754907	0.829668	-6.39416
SNCA	-0.17496	1.014373	-0.31247	0.755015	0.829691	-6.26439
CPS1	0.345288	1.950839	0.312395	0.75507	0.829691	-6.34514
HFE	0.078742	2.782658	0.312346	0.755107	0.829691	-6.54292
NLGN2	-0.12138	4.668826	-0.31156	0.755704	0.830216	-6.95895
IMP3	0.055528	5.763408	0.311561	0.755703	0.830216	-7.04707
CTSH	0.115014	6.228583	0.311234	0.755951	0.830423	-7.06709
DDX1	-0.0429	6.678168	-0.31061	0.756425	0.830878	-7.07721
APBB1IP	0.151835	2.892156	0.310107	0.756807	0.831232	-6.55715
PCYT2	0.075883	4.954961	0.309863	0.756992	0.83127	-6.97076
NUCKS1	0.064663	8.587014	0.309827	0.757019	0.83127	-7.03529
LPCAT3	-0.07208	6.557713	-0.3099	0.756963	0.83127	-7.07728
LSM1	0.063731	4.407293	0.309501	0.757267	0.831476	-6.89944
ZC3H14	-0.03939	5.865619	-0.30891	0.757712	0.8319	-7.0585
TMEM187	-0.06926	2.827243	-0.30876	0.757832	0.831966	-6.5833
TMEM198	-0.0955	1.392624	-0.30824	0.758228	0.832282	-6.31714
ETV3	-0.06741	3.604317	-0.3081	0.758334	0.832282	-6.7625
PRPF40B	-0.073	3.817	-0.30806	0.758358	0.832282	-6.8108
SYDE1	-0.09215	3.819282	-0.3081	0.758332	0.832282	-6.81716
JOSD2	-0.06908	3.680054	-0.30796	0.758437	0.832303	-6.77676
DYNC1I1	0.200461	1.10171	0.307762	0.758588	0.832403	-6.22837
ST3GAL5	-0.09899	2.438704	-0.30741	0.758859	0.832582	-6.50954
GLI4	0.086921	3.300531	0.30739	0.75887	0.832582	-6.66059
CTSW	-0.16918	0.801504	-0.3073	0.75894	0.832594	-6.23073
LOH12CR1	0.063651	2.951367	0.306121	0.759835	0.833444	-6.58779
TAF1	-0.05181	5.798348	-0.30617	0.759796	0.833444	-7.05647
TAF1L	-0.06718	0.972254	-0.30475	0.760881	0.834299	-6.24349
MRPS14	0.044659	3.951364	0.304899	0.760764	0.834299	-6.81677
KIAA1430	-0.0568	5.646387	-0.3047	0.760913	0.834299	-7.0483
CA12	0.213394	6.496953	0.304912	0.760755	0.834299	-7.07429
CASC3	-0.06585	6.932216	-0.30486	0.760795	0.834299	-7.07655
TCTN1	0.087892	3.526178	0.304291	0.761226	0.834577	-6.71518
C21orf125	0.148694	1.177941	0.304071	0.761394	0.834696	-6.24252
PDK3	-0.08475	3.509977	-0.30377	0.761625	0.834743	-6.74658
PLXNB3	-0.17767	4.19358	-0.30371	0.761668	0.834743	-6.90306
CNOT1	-0.05322	8.485137	-0.3037	0.761676	0.834743	-7.03704
ANKRD27	0.054448	5.859256	0.30386	0.761555	0.834743	-7.05534
PRDXDD1P	-0.08122	0.509007	-0.30339	0.76191	0.834934	-6.17015
TM9SF2	-0.05972	7.455333	-0.30274	0.762409	0.835416	-7.06742
CHID1	-0.04882	5.576868	-0.30248	0.762605	0.835535	-7.04351
IARS	-0.05049	7.653473	-0.30244	0.762638	0.835535	-7.06312
EXOSC6	0.05403	4.025339	0.30194	0.763016	0.835883	-6.83255
MS4A6A	0.129782	3.476976	0.301499	0.763351	0.836185	-6.69656
DNAJC22	-0.24944	1.258087	-0.30137	0.763447	0.836225	-6.31267
EPHA10	-0.2521	0.711532	-0.3008	0.763884	0.836638	-6.22569
LY6G5B	0.067353	1.703188	0.300204	0.764338	0.836961	-6.34836
ZNF419	-0.08422	1.755793	-0.30018	0.764359	0.836961	-6.38251

CCNL2	0.061429	6.531606	0.300216	0.764328	0.836961	-7.07889
STX11	0.127319	2.778899	0.299556	0.764831	0.837412	-6.54454
VEGFC	0.136111	2.657223	0.299301	0.765025	0.837428	-6.51663
LOC148413	-0.04632	3.630065	-0.2994	0.764951	0.837428	-6.76494
STX3	-0.08249	5.476616	-0.29937	0.76497	0.837428	-7.039
GLI1	0.200807	1.073838	0.298946	0.765296	0.837611	-6.22354
PLRG1	0.042906	5.104733	0.298846	0.765372	0.837611	-6.9949
MAP7D1	0.077736	7.136298	0.298916	0.765319	0.837611	-7.07715
UTP15	-0.04722	3.964265	-0.29844	0.765683	0.837819	-6.83906
SPSB1	0.093174	5.764596	0.298504	0.765633	0.837819	-7.04935
EFS	-0.21329	3.924258	-0.29749	0.766406	0.83848	-6.86644
PLAT	0.123568	6.131805	0.29752	0.766382	0.83848	-7.06743
C3orf14	-0.17541	1.030783	-0.29735	0.766511	0.838529	-6.27424
AADAT	-0.12884	2.085931	-0.29717	0.766646	0.838568	-6.45203
FOXRED1	-0.07025	4.166199	-0.29715	0.766667	0.838568	-6.88211
CCR1	0.126067	2.209686	0.29683	0.766909	0.838739	-6.43303
VAMP3	-0.04275	6.798414	-0.29678	0.766944	0.838739	-7.08079
FAM116B	-0.06914	2.541651	-0.29562	0.767834	0.839643	-6.52835
KDM5A	0.048574	6.579268	0.295543	0.76789	0.839643	-7.08094
HELZ	-0.04692	6.478361	-0.29507	0.768254	0.839976	-7.08094
CBLL1	0.044798	5.322249	0.294944	0.768347	0.840011	-7.01774
SAP30	-0.06625	3.071309	-0.29428	0.768856	0.840436	-6.64465
C6orf47	-0.04443	4.693859	-0.29432	0.76882	0.840436	-6.95748
EEF1B2	-0.05879	6.096845	-0.29419	0.768922	0.840442	-7.07313
LMNA	-0.05904	9.128009	-0.29406	0.769019	0.840483	-7.01686
EP300	-0.04152	7.648251	-0.29362	0.769358	0.840787	-7.0661
PGBD2	0.060491	2.774496	0.293312	0.769591	0.840977	-6.55325
ZNF251	0.067587	3.599232	0.292518	0.770198	0.841574	-6.73738
NFRKB	-0.04743	5.332253	-0.29244	0.770258	0.841574	-7.02685
VASH1	0.081288	4.254283	0.292313	0.770355	0.841614	-6.8763
ZNF562	-0.05419	4.990929	-0.29191	0.770661	0.841783	-6.99392
KIF16B	0.072551	5.317017	0.291873	0.77069	0.841783	-7.01558
WBSCR16	0.041994	5.351645	0.291977	0.770611	0.841783	-7.0216
IGHMBP2	-0.0884	4.871911	-0.29155	0.770937	0.841986	-6.9841
ACP1	0.043017	5.925688	0.291178	0.771221	0.84223	-7.06302
NKX6-1	0.182616	0.17402	0.290993	0.771362	0.842319	-6.09421
INPP1	0.086973	4.81974	0.290295	0.771895	0.842703	-6.95987
KLHL24	-0.07511	6.384595	-0.29038	0.771827	0.842703	-7.08146
CUL4A	-0.05204	6.62073	-0.29035	0.771851	0.842703	-7.08324
WASH3P	0.047458	4.207118	0.290096	0.772047	0.842738	-6.87248
POSTN	0.162236	7.096067	0.290115	0.772033	0.842738	-7.0814
TBC1D3	0.06885	4.14021	0.289616	0.772414	0.843072	-6.85621
NELL2	0.283148	2.582085	0.289373	0.7726	0.843209	-6.47863
PRDM10	-0.03698	4.380875	-0.28894	0.772928	0.843369	-6.91512
GNL1	0.037594	5.341178	0.288947	0.772925	0.843369	-7.02188
TXLNA	-0.03972	6.27143	-0.28907	0.772834	0.843369	-7.07906
RILPL1	-0.07363	3.378376	-0.28859	0.773195	0.843544	-6.72114
ASF1A	-0.05726	4.492855	-0.28857	0.77321	0.843544	-6.93422
TNFRSF1A	0.051753	7.09455	0.288497	0.773269	0.843544	-7.08051
GNB5	-0.06041	4.391155	-0.2878	0.773805	0.844063	-6.92109
LGR6	-0.24895	1.937605	-0.28709	0.774346	0.84439	-6.44432
C2orf43	-0.06293	4.353106	-0.28714	0.774305	0.84439	-6.91431
MARVELD2	-0.08777	4.38413	-0.2871	0.774338	0.84439	-6.91987
CISD3	-0.07015	4.788185	-0.28714	0.774304	0.84439	-6.97307
SSR3	0.045218	7.50757	0.286795	0.77457	0.844568	-7.07299
CRCP	-0.05045	5.512584	-0.2866	0.774721	0.844666	-7.04364
TRADD	0.056199	4.734285	0.28648	0.774811	0.844699	-6.95302
RHOF	-0.15377	4.441146	-0.28626	0.774979	0.844816	-6.93788

ACP2	0.048883	5.44126	0.286079	0.775117	0.844901	-7.03057
SLC38A5	-0.17533	3.552017	-0.28594	0.775221	0.844948	-6.77446
TSSK3	-0.08764	0.666759	-0.2854	0.775638	0.845167	-6.20126
MAP1D	-0.06171	1.750229	-0.28541	0.775629	0.845167	-6.38078
RNF5P1	-0.0798	2.047721	-0.28513	0.775845	0.845167	-6.43634
RRAGD	0.137216	3.227458	0.285156	0.775823	0.845167	-6.64886
HBXIP	0.043804	5.911688	0.285246	0.775755	0.845167	-7.06403
RAI1	-0.05412	6.873793	-0.28516	0.775819	0.845167	-7.08325
MPZL2	0.113999	6.597175	0.285551	0.775522	0.845167	-7.08339
ALDH1A3	-0.1314	4.846204	-0.28483	0.776076	0.845352	-6.98855
FAM49A	-0.11077	1.767597	-0.28455	0.776291	0.84552	-6.39373
GNG4	-0.24367	1.722494	-0.28436	0.776434	0.845562	-6.4101
NDUFA9	0.049365	5.780321	0.284337	0.77645	0.845562	-7.05654
EIF3D	0.041247	7.539286	0.284134	0.776605	0.845665	-7.07301
EHD3	0.123993	4.015789	0.283925	0.776765	0.845774	-6.82861
LOC202781	-0.07844	2.78413	-0.28338	0.777181	0.84616	-6.5851
FDX1L	0.049577	2.795651	0.283279	0.777259	0.84618	-6.56335
GYS1	-0.0487	6.555521	-0.28288	0.777567	0.846448	-7.08517
THAP11	0.043128	4.992249	0.282666	0.777729	0.846559	-6.98731
ORAI3	-0.06854	4.180082	-0.2822	0.778089	0.846884	-6.89057
C9orf98	-0.132	-0.0356	-0.28201	0.778235	0.846977	-6.10359
IKBKB	0.057807	5.406512	0.281833	0.778367	0.847055	-7.02808
DACH1	-0.19379	1.568295	-0.28173	0.778443	0.847072	-6.36985
CATSPER2P1	-0.05405	0.575143	-0.28133	0.778754	0.847344	-6.18359
RARA	-0.06591	5.6316	-0.28103	0.778983	0.84745	-7.05464
PPIL2	-0.03848	5.817331	-0.28096	0.779033	0.84745	-7.06416
ACSL4	-0.0715	6.693295	-0.28106	0.778961	0.84745	-7.08581
C7orf49	-0.0481	4.73992	-0.28021	0.779606	0.848007	-6.96737
ACTR3B	0.067787	2.46943	0.280047	0.779735	0.848081	-6.49308
NOM1	0.051609	4.968942	0.27935	0.780269	0.848596	-6.98453
EFR3A	0.049183	6.982209	0.279261	0.780337	0.848604	-7.08469
C5orf62	0.082625	4.154172	0.278438	0.780968	0.849171	-6.85967
GATAD1	-0.0674	5.408373	-0.27834	0.781041	0.849171	-7.03887
GTF3A	0.06875	6.190654	0.278394	0.781001	0.849171	-7.07693
COTL1	0.090591	6.697592	0.277755	0.781491	0.849595	-7.08665
C9orf169	0.257131	1.376157	0.277185	0.781928	0.850003	-6.2701
C9orf129	-0.06787	2.091827	-0.27689	0.782154	0.850184	-6.44496
DTX1	-0.10503	2.051892	-0.27671	0.782292	0.850267	-6.44487
DPM3	-0.06833	3.550629	-0.2762	0.782682	0.850625	-6.75713
CTXN1	0.131128	1.963628	0.275833	0.782965	0.850676	-6.39631
PLA2G4F	0.224992	2.904537	0.276046	0.782802	0.850676	-6.54615
ZNF260	-0.07012	4.690581	-0.27588	0.782929	0.850676	-6.96561
RPL10	0.058195	8.349401	0.275822	0.782973	0.850676	-7.05322
ZFP62	-0.05468	4.761691	-0.27543	0.783275	0.850806	-6.9721
REPS1	0.045214	5.635299	0.275569	0.783167	0.850806	-7.0495
CLPTM1L	0.052905	6.753439	0.275437	0.783269	0.850806	-7.08748
ZNF48	-0.05515	2.935742	-0.27529	0.783378	0.850852	-6.61679
ACTN1	0.077863	8.52168	0.27507	0.78355	0.850973	-7.04818
CCR7	0.15559	1.025843	0.274568	0.783935	0.851325	-6.22845
SPTBN5	-0.11973	2.402483	-0.27447	0.784011	0.851341	-6.51478
CTBP1	0.04133	6.798327	0.273863	0.784476	0.85178	-7.08776
USP6	0.066875	0.347427	0.273307	0.784903	0.852177	-6.13453
DNASE1L1	-0.04859	4.653285	-0.27299	0.785149	0.852194	-6.95824
CCDC72	-0.04998	4.805135	-0.27297	0.785163	0.852194	-6.97746
ZNF322A	-0.06331	4.835247	-0.273	0.785139	0.852194	-6.98282
RBM3	0.050771	6.840423	0.273147	0.785026	0.852194	-7.08775
ZNF678	0.069953	1.779004	0.272881	0.78523	0.852201	-6.36887
VDAC2	-0.05675	6.795575	-0.27266	0.785398	0.852317	-7.08756

NLRC3	-0.09684	2.25555	-0.27243	0.785577	0.852379	-6.48356
ALG1	-0.05229	4.505742	-0.27248	0.785538	0.852379	-6.93874
VCPIP1	0.044218	5.58331	0.272268	0.785701	0.852447	-7.04658
NUP214	0.037696	6.826101	0.271868	0.786008	0.852714	-7.08812
TXNL4A	-0.04929	4.910765	-0.27178	0.786077	0.852722	-6.99035
RASSF4	0.096167	3.828503	0.271561	0.786244	0.852837	-6.7894
FRMD4B	-0.07148	5.370922	-0.27142	0.786354	0.85289	-7.0379
LIME1	0.107715	2.934215	0.271266	0.78647	0.852951	-6.58121
FAM173B	-0.0561	3.255874	-0.27065	0.786946	0.853401	-6.69065
CEP192	-0.05158	5.397994	-0.2703	0.787208	0.853618	-7.03892
ZFP90	-0.05749	4.483981	-0.26975	0.787637	0.854017	-6.93863
EDF1	-0.0483	6.655749	-0.2695	0.787828	0.854157	-7.08913
BLVRA	0.085229	4.556646	0.269239	0.788027	0.854307	-6.93264
NEFL	-0.32589	0.806458	-0.26816	0.78886	0.855011	-6.26809
IL17RB	0.181174	2.630916	0.268212	0.788816	0.855011	-6.50617
ZC3H4	0.029014	6.305157	0.268306	0.788744	0.855011	-7.08413
NDUFB5	-0.04763	6.075456	-0.26739	0.789445	0.85558	-7.07956
ZNF883	0.186112	0.291724	0.26708	0.789686	0.855708	-6.11366
C21orf7	0.078298	1.547888	0.267145	0.789637	0.855708	-6.3305
TMEM20	-0.10122	2.940738	-0.26672	0.789961	0.855938	-6.62855
DHX40	-0.0434	5.85905	-0.26664	0.790021	0.855938	-7.0705
LOC100129637	-0.05536	3.466193	-0.26537	0.791003	0.856863	-6.73932
AVPI1	-0.08401	4.572901	-0.26534	0.791028	0.856863	-6.9537
KIAA1522	-0.06146	8.247171	-0.26529	0.791059	0.856863	-7.05586
DHFRL1	-0.0578	3.038657	-0.26516	0.79116	0.856906	-6.64216
LRP12	0.148526	3.944954	0.264716	0.791504	0.857117	-6.81091
ZBTB45	0.048695	4.397084	0.264671	0.791539	0.857117	-6.91299
RBMS1	-0.07412	5.683899	-0.26469	0.791526	0.857117	-7.06309
ZMAT2	-0.03815	5.400944	-0.26439	0.791755	0.857218	-7.03997
CES2	0.110487	6.641029	0.264445	0.791713	0.857218	-7.08968
INPP5F	0.045672	4.677165	0.264197	0.791904	0.857246	-6.95384
C2orf69	-0.03841	4.833978	-0.26423	0.791881	0.857246	-6.98228
ZNF341	0.045334	3.006006	0.263859	0.792163	0.857461	-6.61435
KIAA0664P3	0.059931	2.07969	0.263505	0.792437	0.857491	-6.42562
HEBP1	-0.04676	4.758846	-0.26357	0.792388	0.857491	-6.97457
BCL7C	0.054756	5.040661	0.263576	0.792382	0.857491	-6.99677
SUN1	0.04038	7.336546	0.26366	0.792317	0.857491	-7.08281
PKDCC	0.217437	3.682012	0.263166	0.792697	0.857706	-6.73449
ARHGAP9	0.107133	2.271775	0.262578	0.793149	0.858063	-6.45549
ABTB2	0.103695	4.28014	0.262614	0.793122	0.858063	-6.88355
SAMD12	0.149997	4.793249	0.262169	0.793464	0.858337	-6.95637
PRR14	0.035147	5.322188	0.261699	0.793827	0.858663	-7.02772
NCF4	0.094606	1.999314	0.261391	0.794063	0.858852	-6.40959
ENC1	0.126949	5.523616	0.260978	0.794381	0.85913	-7.03796
APOL3	-0.10568	3.863771	-0.26004	0.795105	0.859846	-6.84087
SYNE2	0.062537	8.101732	0.25907	0.795851	0.860586	-7.06573
OR2A9P	0.091437	2.237388	0.258982	0.795919	0.860593	-6.45235
RASAL3	0.102277	2.498857	0.258806	0.796054	0.860673	-6.49973
SWAP70	0.044547	6.080731	0.258389	0.796376	0.860954	-7.07908
CDAN1	0.038271	4.071747	0.257558	0.797016	0.861579	-6.85609
INF2	-0.06586	7.501709	-0.25738	0.797156	0.861664	-7.07893
RNF215	0.050814	2.145045	0.257108	0.797364	0.861821	-6.44226
PIGY	0.037943	5.786641	0.256843	0.797568	0.861975	-7.06511
PCNP	-0.03396	6.529172	-0.25603	0.798198	0.862589	-7.09209
GOLGA5	-0.03308	5.827879	-0.25577	0.798396	0.86273	-7.07116
SLC35C1	-0.06657	5.801771	-0.2557	0.798452	0.86273	-7.07135
TMEM126B	-0.04821	4.275211	-0.25559	0.798532	0.862751	-6.90866
FRAS1	0.178188	3.95665	0.255249	0.798797	0.86297	-6.81116

POLR2E	0.038165	6.448979	0.255084	0.798924	0.86304	-7.09043
CCDC73	0.061941	1.579068	0.254745	0.799185	0.863082	-6.34104
NAF1	-0.04179	3.869898	-0.25487	0.79909	0.863082	-6.8312
PTPRC	0.119936	4.65733	0.254907	0.79906	0.863082	-6.94504
PAG1	0.095126	4.912533	0.254676	0.799238	0.863082	-6.97865
PTP4A1	0.058459	7.91856	0.254634	0.799271	0.863082	-7.07206
TUSC3	0.16471	3.717426	0.254419	0.799437	0.863194	-6.76114
C14orf2	0.046129	5.778618	0.254339	0.799498	0.863194	-7.06467
CACNA2D4	-0.08853	0.551181	-0.25409	0.799691	0.863335	-6.19111
SPDYE5	-0.07648	0.394678	-0.25392	0.799824	0.863345	-6.16511
RBM27	0.033395	5.649058	0.253918	0.799823	0.863345	-7.05695
CHSY1	-0.05005	5.71355	-0.25334	0.800272	0.863762	-7.06632
ZNF680	-0.06689	3.577479	-0.25312	0.800436	0.863872	-6.7701
IMPDH1	-0.06565	5.985269	-0.25278	0.800704	0.864095	-7.08055
USP11	0.050907	6.155679	0.252473	0.800938	0.864281	-7.08321
BOLA1	0.059493	2.327198	0.25228	0.801087	0.864374	-6.47492
CD40	0.109753	3.222031	0.252007	0.801298	0.864411	-6.6567
STK38L	-0.05861	6.145323	-0.252	0.801306	0.864411	-7.0862
SEC23B	-0.04472	6.30983	-0.25212	0.801211	0.864411	-7.08995
RPS6KB2	-0.05408	5.551625	-0.25176	0.801488	0.86454	-7.05606
C9orf21	-0.05884	3.060672	-0.25103	0.802052	0.865025	-6.65219
ITPRIPL2	-0.04718	6.716682	-0.25102	0.802061	0.865025	-7.09378
GPR161	0.112641	2.941576	0.250754	0.802265	0.865178	-6.59425
TBX6	0.113602	1.848969	0.250173	0.802714	0.865595	-6.38241
C5orf51	0.043463	6.055159	0.250038	0.802818	0.865641	-7.08019
ABHD10	-0.04247	4.772313	-0.24978	0.803015	0.865786	-6.97844
CD180	-0.11509	1.219854	-0.24969	0.803086	0.865796	-6.30847
IL1R2	-0.15572	3.088424	-0.24956	0.803191	0.865842	-6.67492
TANK	0.037947	5.525277	0.249207	0.80346	0.866065	-7.04849
AIMP1	-0.02877	5.549942	-0.24904	0.803592	0.866141	-7.05499
TPP2	-0.0394	5.785471	-0.24841	0.804075	0.866594	-7.0711
GPKOW	0.035453	4.036231	0.248287	0.80417	0.866631	-6.85243
EIF3J	0.03693	6.428725	0.248073	0.804336	0.866742	-7.09184
OBSCN	-0.09105	4.721773	-0.24792	0.804451	0.8668	-6.9795
BRF1	-0.03343	5.06165	-0.24749	0.804789	0.867097	-7.01131
PRR22	0.081376	0.730441	0.247331	0.804909	0.867159	-6.19685
RC3H2	-0.04949	5.33069	-0.24713	0.805068	0.867264	-7.03905
C11orf51	0.067107	4.108364	0.246944	0.805209	0.867286	-6.86217
RTN4	-0.04296	7.974951	-0.24694	0.805213	0.867286	-7.06984
PRRG1	0.09128	3.582368	0.246779	0.805336	0.867351	-6.74084
ADCK2	0.050106	4.682682	0.246308	0.8057	0.867677	-6.95829
UPF1	-0.03201	7.452889	-0.24623	0.805762	0.867677	-7.0835
TRIM45	0.067988	2.105364	0.246001	0.805937	0.867798	-6.43505
LRRC56	0.084822	1.579086	0.245484	0.806337	0.867961	-6.33757
DNAL4	0.049686	3.3487	0.245696	0.806173	0.867961	-6.69441
FZD1	-0.07814	5.4736	-0.24553	0.806301	0.867961	-7.05377
ID1	0.091352	6.467384	0.245626	0.806227	0.867961	-7.09225
MRI1	-0.06075	4.499037	-0.2451	0.806634	0.86808	-6.94684
C11orf68	-0.04189	4.932326	-0.24523	0.806529	0.86808	-6.99875
CHPF2	0.043707	6.23245	0.245114	0.806623	0.86808	-7.08773
USP7	0.030912	7.228651	0.244917	0.806775	0.868166	-7.08948
BAT4	-0.03955	3.306175	-0.2441	0.807406	0.868777	-6.70459
LOC100272228	0.075098	0.784588	0.243858	0.807594	0.868913	-6.20707
GLTSCR1	-0.03201	5.430238	-0.24366	0.807749	0.868984	-7.04717
RPSA	-0.04769	6.444234	-0.24361	0.807785	0.868984	-7.0944
DFNA5	0.152218	3.091735	0.243062	0.80821	0.869331	-6.62344
PKN2	0.037493	6.402941	0.243034	0.808232	0.869331	-7.09259
MST1R	-0.13139	5.399262	-0.24277	0.808433	0.869481	-7.05105

RFX3	-0.07555	1.629206	-0.24251	0.808637	0.869499	-6.37209
FERMT3	0.098512	3.597958	0.242594	0.808572	0.869499	-6.74318
LOXL1	0.095824	4.544985	0.242584	0.808579	0.869499	-6.93622
DDB1	-0.03512	8.048782	-0.24212	0.808938	0.869756	-7.06895
C5orf54	0.058158	1.920408	0.241926	0.809089	0.869851	-6.40502
SPRED2	0.044425	6.088551	0.241618	0.809327	0.870041	-7.08355
OVCA2	0.043777	4.301664	0.241405	0.809492	0.870084	-6.90316
SMU1	0.034387	5.626464	0.241482	0.809432	0.870084	-7.05843
RRN3	0.032672	5.837358	0.241167	0.809676	0.870215	-7.07226
TBC1D23	-0.03664	5.801635	-0.2407	0.810034	0.870533	-7.07377
SLAMF1	-0.10868	0.035335	-0.23999	0.81059	0.870857	-6.11999
MAGED4B	-0.13951	2.895718	-0.24009	0.810509	0.870857	-6.63635
LOC283070	0.108888	3.223253	0.239509	0.810959	0.870857	-6.66235
CD320	-0.0638	4.364008	-0.23952	0.810952	0.870857	-6.92808
CWC22	0.030132	5.128313	0.240078	0.810519	0.870857	-7.01431
GLRX5	-0.03434	5.182293	-0.23966	0.810846	0.870857	-7.02567
HSPA13	-0.04681	5.186386	-0.23966	0.810841	0.870857	-7.02742
WASH7P	0.040558	5.806486	0.239589	0.810897	0.870857	-7.07031
ATPIF1	-0.04556	5.977637	-0.23976	0.810769	0.870857	-7.08268
MINK1	-0.04757	7.542443	-0.23991	0.810652	0.870857	-7.08274
CCDC97	0.031707	5.504555	0.238614	0.811653	0.871535	-7.0497
KLHDC3	0.051037	6.213493	0.237948	0.812168	0.872021	-7.08867
SERF1A	0.045725	3.583813	0.237486	0.812526	0.872306	-6.75586
TXLNG	-0.048	4.053878	-0.23745	0.812558	0.872306	-6.87294
TRNAU1AP	0.038922	3.458736	0.237319	0.812655	0.872343	-6.72545
CCDC117	-0.03645	5.383672	-0.23679	0.813063	0.87258	-7.04524
IGF2R	-0.04184	7.759624	-0.23691	0.812969	0.87258	-7.07831
ETS1	-0.07538	6.236415	-0.23685	0.81302	0.87258	-7.09268
SPINK5	-0.24353	4.515292	-0.23659	0.813217	0.872678	-6.97313
LOC80054	-0.10537	1.171577	-0.23628	0.81346	0.872872	-6.29852
GCLC	0.080704	6.37259	0.235746	0.813874	0.873249	-7.09285
PLD6	-0.06186	2.358845	-0.23521	0.814289	0.873627	-6.50437
SRP19	0.033029	4.467333	0.2346	0.814763	0.874068	-6.93316
FAM178A	-0.03715	5.196306	-0.23416	0.8151	0.874314	-7.0286
PPP2CA	0.033003	7.234868	0.234143	0.815117	0.874314	-7.09197
EPS8	-0.10691	6.041466	-0.23367	0.815486	0.874643	-7.08862
SCNN1A	-0.15653	6.574959	-0.23345	0.815653	0.874754	-7.09816
MGC29506	0.186659	1.662277	0.232931	0.816056	0.875002	-6.33691
C2orf34	0.050289	2.34526	0.232939	0.81605	0.875002	-6.48332
ZNF772	-0.09077	2.823758	-0.23279	0.816166	0.875002	-6.61023
SNRNP25	-0.04854	3.683443	-0.23285	0.816119	0.875002	-6.79443
PVRL2	0.069194	7.230662	0.232749	0.816197	0.875002	-7.09294
KSR1	0.065938	5.160886	0.232574	0.816333	0.875081	-7.01683
SPTB	0.073952	3.017331	0.232215	0.816611	0.875178	-6.621
WDR5B	-0.04874	3.397875	-0.23225	0.816584	0.875178	-6.72998
JUNB	-0.06518	8.062185	-0.2323	0.816546	0.875178	-7.07013
NSF	0.039481	6.191283	0.232127	0.81668	0.875184	-7.08962
TWIST2	-0.11677	0.847218	-0.2308	0.817713	0.87609	-6.25041
ICOSLG	0.068789	3.976036	0.230867	0.817657	0.87609	-6.83697
TRPS1	0.113574	4.891989	0.230872	0.817653	0.87609	-6.9827
UBE2R2	-0.0391	6.595276	-0.23057	0.81789	0.876212	-7.09871
FBRS	-0.03435	6.839861	-0.23047	0.817967	0.876228	-7.09793
GCET2	-0.08543	1.686097	-0.23035	0.81806	0.87626	-6.38684
EXOC6	0.06146	4.132995	0.230043	0.818297	0.876379	-6.87018
PTPRG	-0.07779	4.996814	-0.23011	0.818244	0.876379	-7.01255
ARL2BP	0.042153	5.931439	0.229643	0.818607	0.876644	-7.07954
DNAJC30	0.042035	3.985612	0.229079	0.819045	0.876921	-6.84488
GEMIN4	0.043191	5.308666	0.229213	0.818941	0.876921	-7.03367

RNF13	0.037503	6.099816	0.229068	0.819054	0.876921	-7.0872
LOC728640	-0.05639	0.661698	-0.22874	0.819304	0.877091	-6.21074
IL10RA	-0.09561	3.562196	-0.2287	0.819338	0.877091	-6.78164
PLXDC2	-0.09669	4.061075	-0.22854	0.819462	0.877157	-6.88963
NDRG4	-0.1635	3.586516	-0.22843	0.819547	0.877181	-6.80369
FASN	0.051759	8.949544	0.2279	0.81996	0.877555	-7.04385
SEMA6D	-0.12727	2.434056	-0.22767	0.820139	0.87768	-6.53623
ZNF365	-0.17268	0.941846	-0.22753	0.820251	0.877732	-6.27792
QPRT	0.149757	1.789172	0.226907	0.820731	0.878178	-6.36867
MS4A7	-0.09906	3.156099	-0.22679	0.820825	0.878212	-6.68818
LOC344595	0.081312	1.606339	0.226424	0.821106	0.878445	-6.35132
ANXA5	0.042921	7.766896	0.226221	0.821263	0.878546	-7.08249
NTNG2	-0.09426	0.232697	-0.22578	0.821603	0.878842	-6.15103
TRIM25	0.045328	6.380413	0.225365	0.821928	0.879123	-7.09612
ASAP2	-0.05139	6.535514	-0.22511	0.822125	0.879266	-7.0997
PREB	0.035725	5.35814	0.224854	0.822325	0.879413	-7.03971
REG4	0.428426	1.332435	0.224672	0.822467	0.879497	-6.24216
HEPH	0.132324	4.646096	0.224495	0.822604	0.879576	-6.94892
HDDC2	-0.04608	4.973584	-0.22438	0.822693	0.879604	-7.00852
C9orf46	0.05239	3.328089	0.223947	0.82303	0.879897	-6.69525
STRN	0.046141	5.344826	0.223647	0.823263	0.88007	-7.03813
C10orf46	-0.03179	6.328069	-0.2235	0.82338	0.88007	-7.09689
MAML1	0.031451	6.476978	0.223572	0.823321	0.88007	-7.09854
ZNF443	-0.067	1.227835	-0.22294	0.82381	0.880357	-6.30684
C19orf52	0.029035	3.561022	0.222903	0.823841	0.880357	-6.75507
ZNF440	0.05102	4.305082	0.222826	0.823901	0.880357	-6.90658
PTK2B	0.057669	5.871821	0.222839	0.823891	0.880357	-7.07706
BCL9	-0.04119	5.40794	-0.22249	0.82416	0.880566	-7.05105
SLC33A1	0.039842	5.23202	0.222335	0.824283	0.88063	-7.02783
B4GALNT4	0.229016	1.2398	0.221734	0.82475	0.881062	-6.26945
GFOD2	-0.04487	4.851071	-0.22165	0.824814	0.881063	-6.99581
INO80C	-0.0545	3.438825	-0.2214	0.825013	0.881172	-6.74361
MAML2	-0.06465	4.275291	-0.22136	0.825043	0.881172	-6.92011
ARMC9	0.058432	3.152031	0.221049	0.825282	0.881294	-6.65684
LOC96610	-0.15326	8.462654	-0.22112	0.825224	0.881294	-7.05623
CD47	-0.04899	6.900925	-0.22085	0.825434	0.881388	-7.09921
ZDHHC23	0.081083	4.040043	0.220572	0.825653	0.881555	-6.84853
TMEM30B	-0.07571	6.364915	-0.22027	0.825891	0.881741	-7.099
C14orf138	0.036608	3.191596	0.21951	0.82648	0.882303	-6.66885
KLHL11	0.065224	3.291381	0.219291	0.82665	0.882398	-6.68591
THOC7	0.033871	4.968982	0.219203	0.826718	0.882398	-7.0014
PNP	0.055385	6.303534	0.219047	0.82684	0.882398	-7.09545
RAPH1	-0.04578	6.319854	-0.21908	0.826811	0.882398	-7.09795
C6orf62	-0.0372	6.650874	-0.21899	0.826885	0.882398	-7.10142
PRMT6	0.059399	4.02404	0.218891	0.826961	0.882412	-6.85283
CEP57	-0.03417	5.279943	-0.21845	0.827306	0.882712	-7.03974
MAST3	0.052013	4.682149	0.218205	0.827495	0.882779	-6.96374
LLGL2	0.101063	6.497566	0.218279	0.827437	0.882779	-7.09892
SLC25A28	-0.04226	4.75776	-0.21807	0.827596	0.88282	-6.98458
INVS	-0.03857	4.040523	-0.21786	0.827763	0.882931	-6.87364
ME1	-0.08535	5.561486	-0.21749	0.828048	0.883167	-7.06716
ZMYM3	-0.04053	5.62732	-0.2172	0.828278	0.883345	-7.06884
C18orf8	-0.04438	4.569882	-0.21704	0.828404	0.883412	-6.96066
IL10RB	-0.04011	5.188044	-0.2168	0.82859	0.883543	-7.03174
LRP5L	-0.04817	2.288375	-0.21603	0.82919	0.884115	-6.49212
GUCY1B3	0.08277	3.70581	0.215497	0.829602	0.884471	-6.78161
SLCO2B1	0.096583	4.76661	0.215435	0.82965	0.884471	-6.97094
KRT4	-0.35624	2.558341	-0.21533	0.829732	0.88449	-6.61785

RPLP1	-0.04409	9.446453	-0.21498	0.830005	0.884714	-7.0263
SEC13	-0.03631	6.079337	-0.21488	0.83008	0.884726	-7.09196
PTPN23	-0.02901	6.875507	-0.21469	0.830227	0.884816	-7.10108
ATP6V0E1	0.035346	6.911372	0.214453	0.830415	0.884948	-7.10137
FLJ39582	0.079495	0.662768	0.213948	0.830809	0.885107	-6.19597
ELOF1	0.035138	4.578122	0.213937	0.830817	0.885107	-6.95301
PEF1	0.03402	6.066802	0.213966	0.830794	0.885107	-7.08936
KIAA1267	-0.02897	6.27468	-0.21406	0.830721	0.885107	-7.09774
ARHGAP28	0.115822	2.048322	0.213783	0.830937	0.885149	-6.42476
CCDC115	-0.03235	4.627165	-0.21372	0.830984	0.885149	-6.96791
PORCN	-0.06208	3.822801	-0.21331	0.831303	0.885354	-6.83593
LOC401397	-0.05648	4.453244	-0.21339	0.831241	0.885354	-6.94737
CACNA1D	-0.17219	1.206046	-0.21286	0.831658	0.885615	-6.32022
DDIT4	0.081417	6.719181	0.212836	0.831675	0.885615	-7.10264
LYPD6B	-0.13433	2.278239	-0.21262	0.831846	0.885731	-6.49939
TDP2	0.042023	5.355354	0.212456	0.83197	0.885795	-7.04188
LOC253039	0.041605	4.393345	0.212133	0.832222	0.885996	-6.92498
ADRA2C	-0.11902	1.093351	-0.21191	0.832396	0.886113	-6.29492
PIN4	-0.04583	3.017199	-0.21174	0.832525	0.886142	-6.64844
RABGGTA	-0.04617	5.020506	-0.21171	0.83255	0.886142	-7.01647
SIGLEC1	0.120697	3.007747	0.211615	0.832626	0.886155	-6.61314
PARP15	-0.11278	0.158629	-0.2108	0.83326	0.886569	-6.14387
MRPS9	0.030484	4.580091	0.210928	0.833161	0.886569	-6.95464
NAA35	-0.03028	4.528761	-0.21079	0.833268	0.886569	-6.955
CUTA	0.039365	5.831912	0.210889	0.833192	0.886569	-7.07834
MGC16275	-0.04732	0.333818	-0.21029	0.833661	0.886758	-6.16229
MRPL32	0.031977	4.814677	0.210266	0.833677	0.886758	-6.98495
DGKQ	-0.04931	4.865273	-0.21039	0.83358	0.886758	-6.99949
UNC5B	0.074517	5.861273	0.210237	0.8337	0.886758	-7.07861
RPL7A	-0.04576	8.198106	-0.21008	0.83382	0.886819	-7.07111
HIVEP2	-0.04079	6.370392	-0.20999	0.833896	0.886831	-7.10078
SLC35B4	0.036129	4.404978	0.209873	0.833983	0.886857	-6.92909
SERINC3	-0.03167	7.081039	-0.20896	0.834691	0.887542	-7.09927
STRADB	-0.04834	2.911316	-0.20855	0.835013	0.887675	-6.62541
UIMC1	-0.02804	4.166929	-0.20854	0.835025	0.887675	-6.8988
ZFYVE27	0.044524	4.573757	0.208479	0.83507	0.887675	-6.95144
NEU1	0.064913	5.70195	0.208479	0.83507	0.887675	-7.06912
TMED2	0.024782	8.010525	0.207939	0.835491	0.888055	-7.07935
AGK	-0.04061	4.708595	-0.20755	0.835796	0.888244	-6.98084
DNAJB1	0.061431	7.938227	0.207629	0.835733	0.888244	-7.08249
GPS2	0.035405	5.766032	0.207004	0.83622	0.888627	-7.07546
RASSF5	-0.06921	5.128396	-0.20681	0.836372	0.888653	-7.0313
WAPAL	-0.02845	6.272058	-0.20689	0.83631	0.888653	-7.09916
PRRC1	-0.02655	6.311161	-0.20644	0.836661	0.888893	-7.10011
KCND1	0.068428	1.728419	0.206321	0.836753	0.888922	-6.37779
F5	0.21303	2.729859	0.206237	0.836819	0.888925	-6.5302
HLA-DOA	0.116435	2.878933	0.205857	0.837114	0.889172	-6.59037
BRWD3	0.048472	4.477899	0.205271	0.837572	0.88959	-6.93838
HHLA3	0.059603	2.241716	0.204931	0.837837	0.889714	-6.46979
GPR89A	0.032287	4.78423	0.204877	0.837879	0.889714	-6.98186
COG2	-0.03214	5.131645	-0.2049	0.837864	0.889714	-7.02792
FCHSD1	0.05699	3.650112	0.20449	0.838181	0.889802	-6.77334
TSTD1	-0.06413	3.989833	-0.20444	0.838217	0.889802	-6.87055
HSDL1	-0.03246	4.074805	-0.20461	0.838091	0.889802	-6.88285
CD151	-0.04312	7.423144	-0.20451	0.838164	0.889802	-7.0933
FAM86B1	-0.07599	1.8856	-0.20426	0.838361	0.889886	-6.4282
IRX2	-0.17147	3.995662	-0.20379	0.838731	0.890212	-6.88421
C21orf119	0.05228	1.364606	0.203301	0.839109	0.89051	-6.31712

MPG	0.037599	4.726239	0.203181	0.839203	0.89051	-6.97444
SMURF1	0.041956	6.728061	0.203181	0.839203	0.89051	-7.10473
SLA	-0.08207	3.118938	-0.20301	0.839337	0.890585	-6.68208
RPL26	-0.049	7.922741	-0.20259	0.839666	0.890866	-7.08116
TTYH2	-0.0753	2.784355	-0.20233	0.839871	0.890948	-6.6065
PPM1H	-0.12404	3.940868	-0.20238	0.839824	0.890948	-6.8651
GATSL1	0.082349	0.538487	0.201924	0.840184	0.891213	-6.17755
SLC10A6	-0.14786	0.278817	-0.20184	0.840251	0.891216	-6.17172
RNF169	0.040224	5.672437	0.201429	0.840571	0.89142	-7.0703
SLC11A2	0.042817	6.069435	0.201441	0.840561	0.89142	-7.09173
GPR34	0.072884	1.709518	0.201185	0.840761	0.891512	-6.37397
MYO5A	0.084536	5.646828	0.201154	0.840785	0.891512	-7.06575
C14orf153	-0.03133	3.904987	-0.20094	0.840953	0.891622	-6.84821
SETX	-0.03376	7.048025	-0.20072	0.841126	0.891738	-7.10147
AASDH	-0.03451	3.580047	-0.20057	0.841245	0.891796	-6.7764
PTPN3	-0.04732	5.723637	-0.20005	0.841646	0.892153	-7.07858
KLHL13	-0.11938	2.839613	-0.19862	0.842767	0.893069	-6.6303
EIF4E	-0.03124	4.134713	-0.19881	0.842613	0.893069	-6.89514
SERPINB8	0.059614	4.653093	0.198537	0.842829	0.893069	-6.96477
PRPSAP1	-0.02998	5.271217	-0.19855	0.84282	0.893069	-7.04298
SSR2	0.032345	7.36761	0.1987	0.842702	0.893069	-7.09702
RAB40C	-0.03887	5.494487	-0.19801	0.84324	0.893436	-7.06277
IMPA1	-0.03867	4.846303	-0.19776	0.843439	0.893482	-6.99888
HDAC6	-0.03378	5.411409	-0.19772	0.843471	0.893482	-7.05572
MYST3	0.03855	6.923475	0.197711	0.843475	0.893482	-7.10471
PDS5A	0.026647	7.505536	0.197587	0.843571	0.893517	-7.09427
FGR	-0.07573	2.49567	-0.19684	0.844156	0.894059	-6.54529
PRKRIP1	0.034864	4.172464	0.196608	0.844336	0.894059	-6.89066
SDCBP2	-0.11482	4.983477	-0.19672	0.844246	0.894059	-7.02054
MOBKL3	-0.02501	5.78829	-0.1966	0.844339	0.894059	-7.08216
ARAP3	0.054615	4.688659	0.196224	0.844636	0.89417	-6.96961
FAM89B	0.040422	5.558029	0.196229	0.844633	0.89417	-7.06293
SH3BP2	-0.04314	5.983849	-0.19624	0.844623	0.89417	-7.09233
PPP1R1B	0.307824	2.819856	0.19529	0.845366	0.894873	-6.53295
RNF170	-0.03852	4.490241	-0.19513	0.845492	0.894873	-6.95361
PNKD	-0.06834	5.921874	-0.19517	0.845464	0.894873	-7.09074
CEMP1	-0.0371	1.921922	-0.19481	0.845742	0.894954	-6.42942
LOC728855	-0.05293	2.580911	-0.19483	0.845729	0.894954	-6.55659
DCTN5	0.025522	6.076444	0.194786	0.845761	0.894954	-7.09397
DDHD1	-0.04639	4.758292	-0.19309	0.847084	0.896286	-6.9904
ZNF530	-0.07447	1.28869	-0.19265	0.847434	0.89652	-6.3256
BSCL2	0.047269	5.39388	0.192713	0.847382	0.89652	-7.04865
DGCR14	0.029296	4.123283	0.191799	0.848096	0.897154	-6.88391
PLOD2	-0.07178	6.088949	-0.19109	0.84865	0.897671	-7.0983
ATP2B1	0.045392	6.518584	0.190204	0.849344	0.898338	-7.10585
ANAPC5	0.023666	6.596725	0.190112	0.849417	0.898346	-7.1069
DMXL2	0.053448	5.24774	0.189977	0.849522	0.89839	-7.03449
RARRES3	0.109608	4.025955	0.188966	0.850313	0.8991	-6.84824
EGFR	0.093287	7.320811	0.188954	0.850323	0.8991	-7.10091
GPAA1	0.033054	6.812985	0.188745	0.850486	0.899205	-7.10729
FGD2	0.071227	2.178463	0.188654	0.850557	0.899212	-6.45965
TRPC4AP	-0.02526	6.749728	-0.18857	0.850627	0.899218	-7.10741
SAP30BP	0.025807	5.416701	0.18847	0.850702	0.899229	-7.0535
DISC1	0.064208	2.686788	0.188138	0.850961	0.899271	-6.56236
MINA	0.029904	5.459633	0.18809	0.850999	0.899271	-7.05704
IDH3B	0.033932	5.885631	0.188319	0.85082	0.899271	-7.08606
CCDC109A	-0.06031	6.114287	-0.18811	0.850985	0.899271	-7.09922
C2orf28	0.026667	6.436252	0.187666	0.851331	0.899554	-7.10528

PDE4B	0.075242	3.709881	0.187365	0.851567	0.899735	-6.78991
LOC100129387	-0.03776	2.66181	-0.18676	0.852044	0.900073	-6.5741
MOSPD2	0.036884	4.10481	0.186761	0.85204	0.900073	-6.87944
ATP6V1A	0.033402	6.749318	0.186709	0.85208	0.900073	-7.10794
CD163L1	-0.08939	1.183235	-0.18625	0.852438	0.900315	-6.3107
PLEKHA2	-0.03761	5.554651	-0.18633	0.852374	0.900315	-7.06958
TNFRSF19	-0.10005	3.399094	-0.18573	0.852844	0.900467	-6.75299
ZNF765	-0.03867	3.634744	-0.18569	0.852881	0.900467	-6.79161
MRPL23	0.0416	4.889538	0.185863	0.852743	0.900467	-6.99743
MAF	-0.08778	5.5813	-0.18566	0.852904	0.900467	-7.07504
REXO1	0.022186	6.076896	0.185796	0.852795	0.900467	-7.09579
SLC41A3	-0.03349	5.139328	-0.18548	0.853045	0.900548	-7.03276
PPIL4	0.027084	5.00094	0.184998	0.853421	0.900768	-7.0123
FTSJD1	0.030783	5.162303	0.184975	0.853438	0.900768	-7.02941
SPAG9	-0.03206	7.023286	-0.18496	0.853447	0.900768	-7.10493
C7orf59	0.038928	5.275837	0.184742	0.853621	0.900883	-7.03991
ZBTB26	-0.03507	2.535222	-0.1839	0.854277	0.901371	-6.54654
H2AFY2	-0.1124	3.363155	-0.18402	0.854183	0.901371	-6.75347
WARS2	-0.03005	4.075562	-0.18397	0.854229	0.901371	-6.88487
UCHL1	0.186539	3.021331	0.183724	0.854418	0.901384	-6.61695
TMEM97	0.062276	5.560484	0.183805	0.854355	0.901384	-7.06297
RAGE	0.061311	2.996136	0.183611	0.854507	0.90141	-6.62538
HRSP12	0.042008	3.799962	0.183222	0.854812	0.9016	-6.81193
BMF	-0.05881	4.172235	-0.18322	0.854816	0.9016	-6.90793
NIPBL	0.031376	7.080097	0.182631	0.855275	0.902016	-7.10534
RALBP1	0.034176	6.931036	0.182253	0.855571	0.90226	-7.10751
DOT1L	-0.03108	6.668633	-0.1821	0.855694	0.902321	-7.10882
OSBP2	0.079637	3.406268	0.181562	0.856113	0.902664	-6.71689
TCF12	-0.03347	6.802059	-0.18152	0.856148	0.902664	-7.10834
PLA2G4C	0.082163	1.575438	0.181432	0.856214	0.902666	-6.35481
SFT2D2	-0.0363	3.310016	-0.18064	0.856837	0.903253	-6.71848
LOC150776	0.033543	4.471631	0.180532	0.85692	0.903274	-6.94471
LOC221442	-0.07489	0.945147	-0.18032	0.857085	0.90331	-6.26661
NCRNA00188	0.043032	7.065591	0.180368	0.857049	0.90331	-7.10615
HDDC3	0.044004	2.996598	0.180126	0.857238	0.903404	-6.62753
C14orf21	-0.02974	3.768336	-0.17961	0.85764	0.90376	-6.82281
CX3CL1	-0.09078	4.946422	-0.17933	0.857862	0.903925	-7.02017
G6PC3	0.045524	5.612324	0.178927	0.858179	0.904191	-7.0698
SHROOM2	-0.08976	4.11915	-0.17881	0.858268	0.904216	-6.90557
FBXO32	-0.06894	4.784689	-0.17842	0.85858	0.904477	-7.00019
CPZ	-0.07831	2.618605	-0.17815	0.858791	0.904631	-6.57489
FRK	0.07097	3.358396	0.177976	0.858924	0.904703	-6.70158
MBD6	0.024904	6.69184	0.177778	0.85908	0.904799	-7.10954
FAM70B	0.062703	1.092769	0.176967	0.859716	0.905285	-6.27295
TOM1	0.038096	5.423209	0.176942	0.859735	0.905285	-7.05543
MAF1	-0.03159	6.516836	-0.17698	0.859707	0.905285	-7.10903
C11orf30	-0.02989	4.704371	-0.17646	0.860117	0.905613	-6.98483
RAB5C	0.023693	7.166929	0.17638	0.860176	0.905613	-7.10489
VAPB	-0.02453	6.096763	-0.17561	0.86078	0.90618	-7.09988
C15orf62	-0.06259	1.91626	-0.17531	0.861014	0.906358	-6.43759
NAMPT	0.060532	7.618864	0.174904	0.861334	0.906627	-7.09666
MUC13	0.331133	3.610607	0.174143	0.861931	0.907063	-6.69618
TSPAN6	0.045138	5.282367	0.174281	0.861823	0.907063	-7.04226
BRD8	-0.02594	5.262398	-0.17404	0.862009	0.907063	-7.04637
KIAA0174	-0.01862	6.243493	-0.17409	0.861974	0.907063	-7.10444
GAN	-0.05343	3.450113	-0.17381	0.862196	0.907142	-6.75726
BAT2L1	0.030931	8.478182	0.173765	0.862228	0.907142	-7.07087
TSPO	0.043622	6.462573	0.173701	0.862279	0.907142	-7.108

SNAP23	-0.02284	5.640402	-0.17352	0.862418	0.907221	-7.07698
TNFRSF1B	0.067977	5.047694	0.173213	0.862662	0.907409	-7.01559
SSFA2	-0.04188	7.912634	-0.17294	0.862879	0.907569	-7.08712
C2orf49	0.023779	3.114853	0.172751	0.863024	0.907653	-6.66191
BAI2	-0.09456	2.090558	-0.17227	0.863398	0.907978	-6.47423
THUMPD3	-0.0256	5.191959	-0.17193	0.863671	0.908196	-7.03986
ZNF133	-0.02973	3.903975	-0.17178	0.863787	0.90825	-6.85258
ZDHHC4	0.0388	4.967153	0.17158	0.863943	0.908346	-7.00958
LNP1	0.053079	1.145164	0.171302	0.864162	0.908508	-6.2866
KTN1	0.035465	8.114414	0.171144	0.864286	0.90857	-7.08352
PLCH1	-0.12504	2.122851	-0.17065	0.864671	0.908906	-6.4774
ZYX	0.039272	7.96629	0.170189	0.865036	0.909221	-7.08821
FBXO11	-0.02032	6.255788	-0.17008	0.865123	0.909245	-7.10549
TSG101	0.023883	5.848836	0.169377	0.865674	0.909755	-7.08796
DND1	0.034115	2.212674	0.169203	0.86581	0.90983	-6.47376
ZNF746	0.031987	4.821085	0.168941	0.866016	0.909978	-6.99354
METTL6	0.023437	2.892893	0.168351	0.86648	0.910397	-6.61447
MYLK4	-0.05758	0.260794	-0.16799	0.866764	0.910626	-6.16049
GNPNAT1	0.035719	6.008913	0.167907	0.866828	0.910626	-7.0955
MTMR14	0.030527	4.994129	0.167287	0.867316	0.911069	-7.01408
LDLRAP1	0.033551	5.181636	0.167028	0.867519	0.911215	-7.03425
OGG1	-0.02969	3.424233	-0.16693	0.867594	0.911225	-6.74535
MIR17HG	-0.05912	1.578145	-0.16604	0.868294	0.911892	-6.37697
CCND3	-0.04011	5.965452	-0.16591	0.868398	0.911932	-7.09695
TAGAP	-0.0731	2.02096	-0.16576	0.868516	0.911988	-6.4596
MAN2B1	-0.02927	6.770122	-0.16564	0.868606	0.912014	-7.11131
ELP3	-0.02799	4.418611	-0.16551	0.868711	0.912055	-6.94789
RIT1	-0.04064	4.849943	-0.16519	0.868962	0.91225	-7.00575
RPL32	0.037367	8.297524	0.16492	0.869176	0.912406	-7.07873
NR1H3	0.049504	3.365539	0.163796	0.870059	0.913265	-6.71442
AP4S1	0.04204	1.884495	0.163387	0.870381	0.913503	-6.41776
SMARCD2	0.02756	6.776138	0.163341	0.870417	0.913503	-7.11194
EPHB3	-0.08082	5.885079	-0.16324	0.870498	0.913519	-7.0953
SMURF2	-0.02814	4.836372	-0.16314	0.870578	0.913535	-7.00267
IER3IP1	-0.03214	5.470482	-0.16299	0.870689	0.913583	-7.06684
MRPL40	-0.03187	4.373175	-0.16284	0.870815	0.913646	-6.94152
HEY2	-0.05435	1.485201	-0.16247	0.871103	0.91374	-6.36146
FAM26E	0.06861	1.701103	0.162357	0.871191	0.91374	-6.38079
TICAM1	-0.03713	5.769936	-0.16231	0.871231	0.91374	-7.08779
RNMT	0.026798	5.901882	0.162338	0.871206	0.91374	-7.0918
PLD3	-0.0324	7.325965	-0.16256	0.871031	0.91374	-7.10325
BCAS3	-0.03159	4.870781	-0.1621	0.871394	0.913842	-7.00779
ZFP37	-0.06577	1.098296	-0.1614	0.871942	0.913868	-6.29774
FBXO48	0.030284	1.384445	0.16184	0.871598	0.913868	-6.3297
SMPD2	-0.04031	3.516296	-0.16166	0.871736	0.913868	-6.76822
MRPS23	0.02601	4.603865	0.161449	0.871905	0.913868	-6.96728
ZNF507	0.029704	5.295498	0.161491	0.871872	0.913868	-7.04687
POLD4	0.043671	5.577949	0.161838	0.871599	0.913868	-7.06986
BAT3	0.021542	8.07178	0.161702	0.871706	0.913868	-7.08599
DNAJC13	0.027314	6.542828	0.161473	0.871886	0.913868	-7.11136
KIAA1324L	-0.09803	3.108345	-0.16117	0.872126	0.913992	-6.69546
SLC27A5	0.059645	1.848457	0.160874	0.872357	0.914028	-6.40791
SMPDL3B	-0.12859	2.378318	-0.16093	0.872312	0.914028	-6.52587
ZBTB25	-0.03042	3.005451	-0.16094	0.872309	0.914028	-6.65144
NUDT11	-0.1268	0.477928	-0.16076	0.872446	0.914053	-6.20845
ZNF543	-0.0307	3.178721	-0.16033	0.872786	0.914273	-6.69277
PHIP	0.024512	7.023033	0.160333	0.872783	0.914273	-7.10996
GPR89C	-0.05006	2.746362	-0.15992	0.873109	0.91449	-6.59824

FAM127A	-0.05431	5.775362	-0.1599	0.873125	0.91449	-7.0896
CCL28	-0.10511	2.173844	-0.15933	0.873569	0.914867	-6.48826
LAMP2	0.028073	7.992654	0.159274	0.873616	0.914867	-7.08891
RASA1	-0.02665	5.395656	-0.15862	0.874132	0.91534	-7.06083
MSLN	0.192512	2.482077	0.15808	0.874555	0.915714	-6.49311
UBL7	-0.02424	5.191257	-0.15718	0.875262	0.916385	-7.04192
LOC728613	-0.06334	2.771932	-0.15688	0.875497	0.9165	-6.60615
RHOT2	-0.02221	5.663032	-0.15681	0.875554	0.9165	-7.08127
CYFIP1	0.024665	7.496666	0.156793	0.875569	0.9165	-7.10163
DAZAP2	-0.02172	7.850677	-0.15667	0.875663	0.91653	-7.09209
VWA1	-0.04487	6.70239	-0.15632	0.875937	0.916749	-7.11311
UPF2	0.024402	6.042246	0.156031	0.876168	0.916921	-7.09939
P2RX7	0.07205	0.999635	0.155907	0.876266	0.916955	-6.26392
PODXL2	-0.08878	4.298934	-0.15582	0.876338	0.916962	-6.93824
YY1AP1	-0.01767	6.56906	-0.15546	0.876619	0.917188	-7.11296
AGER	-0.04902	0.883767	-0.15512	0.876883	0.917258	-6.25901
MAP3K2	0.029913	5.556212	0.155126	0.876881	0.917258	-7.07045
NDUFAB1	-0.02684	5.621238	-0.15524	0.876794	0.917258	-7.07893
MFSD1	-0.02856	5.97554	-0.15503	0.876959	0.917269	-7.09875
TRIL	-0.06922	2.599904	-0.15476	0.877167	0.917309	-6.57195
CHKB-CPT1B	-0.03229	4.429987	-0.15475	0.877174	0.917309	-6.95097
GRB10	0.044196	5.710163	0.154727	0.877195	0.917309	-7.08065
C20orf30	0.024541	6.286869	0.154239	0.877579	0.917642	-7.1078
ISLR	0.087312	4.991671	0.153818	0.87791	0.91792	-7.01133
SLC29A3	-0.04438	3.31564	-0.15323	0.878373	0.918266	-6.72484
TMEM188	-0.02292	3.409851	-0.15329	0.878323	0.918266	-6.74385
EME2	0.039012	0.99092	0.152996	0.878558	0.918391	-6.26364
SELS	-0.02836	5.281789	-0.15264	0.878837	0.918614	-7.05141
UCKL1AS	-0.04398	1.193549	-0.15248	0.878965	0.918678	-6.31012
SCYL1	-0.01998	6.510249	-0.1523	0.879104	0.918756	-7.11291
ZG16B	-0.1631	1.116857	-0.15185	0.879458	0.919056	-6.30741
WDR81	0.027475	5.803283	0.151697	0.879581	0.919116	-7.08814
P4HA2	0.039882	5.560347	0.151484	0.879749	0.919223	-7.07058
CHMP4A	-0.02422	4.907478	-0.15102	0.880117	0.919539	-7.01269
YARS	0.024351	6.538153	0.150905	0.880205	0.919562	-7.11298
C1orf103	0.0391	4.300083	0.150488	0.880533	0.919836	-6.92117
KAZ	0.047624	4.332995	0.149694	0.881159	0.920419	-6.92828
KLHL5	0.04282	5.346785	0.149613	0.881223	0.920419	-7.05295
DCAKD	0.033575	4.659243	0.149449	0.881352	0.920485	-6.97635
ZNF600	0.049416	3.50959	0.148784	0.881876	0.920964	-6.75065
SCRN1	0.042113	6.736935	0.148553	0.882058	0.921085	-7.11434
AGT	0.133052	2.412684	0.14803	0.88247	0.92124	-6.49837
WIPF1	-0.05192	5.410706	-0.14825	0.882295	0.92124	-7.06614
PRDX4	0.031978	5.837066	0.148184	0.882349	0.92124	-7.09015
HMGCR	-0.0371	6.235398	-0.14809	0.882423	0.92124	-7.10883
LGALS3	-0.05158	7.738162	-0.14771	0.882726	0.921438	-7.09574
RPL22	0.027065	7.810478	0.147349	0.883007	0.921662	-7.09576
HSPC157	-0.03361	1.791833	-0.14713	0.883183	0.921708	-6.41388
HIRIP3	-0.02688	3.83988	-0.14713	0.88318	0.921708	-6.84324
YIPF3	-0.03033	6.800179	-0.14683	0.883417	0.921884	-7.11405
MAP3K13	0.059155	2.551058	0.146048	0.884032	0.922457	-6.53497
SLC6A15	0.182938	0.450558	0.145776	0.884247	0.922612	-6.16483
AARSD1	-0.02301	3.775409	-0.14496	0.884892	0.923177	-6.82834
BRD7	0.021944	5.531106	0.144921	0.884921	0.923177	-7.07074
CCDC130	-0.02235	4.365141	-0.14452	0.88524	0.923441	-6.94187
MPP6	-0.07331	2.293854	-0.1441	0.88557	0.923717	-6.51389
PCDHB11	-0.07585	1.423805	-0.14339	0.886132	0.924234	-6.35965
LRRC20	-0.04149	3.757045	-0.14277	0.886616	0.924669	-6.82761

KLRD1	-0.0749	0.210091	-0.14231	0.88698	0.92498	-6.15876
INTS10	-0.02297	5.500646	-0.14221	0.887059	0.924994	-7.07171
TMED10	-0.01892	8.436101	-0.14186	0.887334	0.925212	-7.07579
MIS12	-0.02008	4.270099	-0.1417	0.887463	0.925277	-6.92716
TMEM144	-0.05572	3.342202	-0.14125	0.88782	0.92558	-6.73191
PAK1	0.031005	6.415197	0.140955	0.888049	0.92575	-7.11249
MGAT4B	0.037699	7.292673	0.140813	0.888161	0.925797	-7.1084
PHACTR4	-0.02391	6.559844	-0.14043	0.88846	0.92604	-7.11515
ADAM15	-0.02846	7.43066	-0.13992	0.888863	0.926381	-7.10454
BTG1	-0.03281	7.051296	-0.13985	0.88892	0.926381	-7.11178
GCHFR	-0.04648	2.743798	-0.13958	0.889135	0.926536	-6.59944
SMCHD1	0.027113	6.582594	0.13894	0.889639	0.926992	-7.11515
DDRGK1	-0.02545	4.722967	-0.13884	0.889721	0.927008	-6.99207
PEX11B	-0.02178	4.417363	-0.13875	0.889792	0.927013	-6.95047
KLHDC4	-0.02347	3.703517	-0.13851	0.889975	0.927134	-6.8121
MLF1	-0.10361	3.067251	-0.1384	0.890068	0.927162	-6.69163
FKBP15	0.020672	5.741417	0.138303	0.890142	0.92717	-7.08656
DLG1	-0.02772	6.856616	-0.13793	0.890439	0.927411	-7.11483
AARS	0.025168	7.10164	0.137834	0.890512	0.927418	-7.11207
PARD6B	0.058295	4.084284	0.137642	0.890664	0.927507	-6.87573
TUFM	0.024023	7.113169	0.137553	0.890734	0.92751	-7.1119
PRDX3	-0.02165	6.938761	-0.13681	0.891317	0.928048	-7.11404
IGF2	0.073606	5.396471	0.136707	0.891402	0.928067	-7.05629
PTPRA	-0.01892	6.396543	-0.13651	0.891557	0.92816	-7.1136
EPS8L1	-0.08044	4.963706	-0.13621	0.891798	0.928341	-7.02527
ZNF799	-0.03737	1.485321	-0.13589	0.892051	0.928466	-6.36226
BOD1	0.022771	4.822899	0.135934	0.892012	0.928466	-6.99992
PCDHGA10	-0.08221	2.511143	-0.13463	0.89304	0.929427	-6.55888
ZNF239	-0.06971	1.735689	-0.13442	0.893208	0.929532	-6.41156
C6orf132	-0.05696	4.432904	-0.13417	0.893405	0.929668	-6.956
FLJ90757	0.031739	3.789867	0.133897	0.89362	0.929776	-6.82139
KCTD9	-0.02522	5.213827	-0.13387	0.893642	0.929776	-7.04785
LOC728758	0.027028	2.029837	0.133699	0.893777	0.929783	-6.44906
GON4L	-0.0149	6.039272	-0.13369	0.893781	0.929783	-7.10394
MVK	-0.03287	4.021112	-0.13338	0.894025	0.929967	-6.88473
STXBP3	0.018729	5.516609	0.132382	0.894817	0.930664	-7.07144
LAMB1	0.038177	7.517113	0.132368	0.894828	0.930664	-7.10497
SAP18	-0.02188	6.617418	-0.1322	0.894965	0.930736	-7.11654
PLEKHG4B	0.125671	1.146595	0.131756	0.895311	0.931028	-6.27912
KLHL23	0.055086	3.161002	0.131594	0.895439	0.931092	-6.67565
TFIP11	-0.018	5.262555	-0.13131	0.895664	0.931256	-7.05217
ZNF202	0.020069	3.646458	0.130546	0.896268	0.931691	-6.79179
C1orf63	0.030776	4.528639	0.130488	0.896313	0.931691	-6.96035
COL16A1	0.054258	5.433922	0.130538	0.896274	0.931691	-7.06174
F3	0.055663	5.49394	0.130442	0.896349	0.931691	-7.06687
RAB1A	-0.01525	7.247696	-0.13026	0.896494	0.931772	-7.10981
ZCCHC10	0.018089	3.94125	0.129781	0.896872	0.932096	-6.85822
PCDH18	-0.04975	4.010261	-0.12956	0.897045	0.932206	-6.88618
MS4A4A	0.064249	1.880667	0.129285	0.897264	0.932295	-6.41731
RPL37A	0.025627	8.915994	0.129333	0.897225	0.932295	-7.06182
COPS8	-0.01693	5.502341	-0.12906	0.897439	0.932408	-7.07333
TMEM14B	-0.02876	4.843824	-0.12831	0.898037	0.932821	-7.00867
MTSS1L	0.040425	6.079913	0.128388	0.897972	0.932821	-7.10444
EDC4	0.016345	6.473275	0.128307	0.898037	0.932821	-7.11543
CHD8	0.016976	6.837556	0.127914	0.898348	0.933074	-7.11667
DYRK3	0.037799	3.060498	0.127753	0.898474	0.933087	-6.65731
SRRM1	-0.01365	7.370446	-0.12773	0.898494	0.933087	-7.10772
CXorf40B	-0.01839	3.665332	-0.12747	0.898696	0.933228	-6.80378

NET1	0.027745	7.45228	0.127339	0.898801	0.933268	-7.1068
C9orf64	0.028133	3.935632	0.127019	0.899055	0.933404	-6.85395
CDKN2B	-0.07943	4.292638	-0.127	0.899066	0.933404	-6.94151
PICALM	-0.01921	7.781024	-0.12668	0.899326	0.933605	-7.09828
NES	-0.04276	4.750981	-0.12607	0.899803	0.93403	-6.99984
RNH1	-0.02225	6.814989	-0.12587	0.899963	0.934127	-7.11686
C12orf43	-0.01693	3.400128	-0.12565	0.900139	0.93424	-6.74389
SNAPC5	0.022325	1.833737	0.12402	0.901425	0.935288	-6.41587
DYNC2LI1	0.019585	3.668276	0.123967	0.901468	0.935288	-6.79652
FRS2	-0.03013	5.743082	-0.1241	0.90136	0.935288	-7.09144
MFGE8	0.036963	6.345609	0.12399	0.90145	0.935288	-7.11324
ATP6V0B	0.021598	6.600163	0.123948	0.901483	0.935288	-7.11731
NOL3	-0.03167	3.850761	-0.12379	0.901605	0.935345	-6.85182
C17orf76	0.067384	1.796978	0.123599	0.901758	0.935432	-6.39958
INTS12	-0.01586	3.977323	-0.12335	0.901958	0.935432	-6.87255
SYTL1	-0.05467	5.231336	-0.1235	0.901837	0.935432	-7.05257
SMARCAD1	0.018307	5.568173	0.123421	0.9019	0.935432	-7.07661
DEGS1	-0.0288	6.037327	-0.12326	0.902023	0.935432	-7.10577
DNAJC17	0.018844	2.704703	0.12305	0.902192	0.935495	-6.58075
HMG20B	0.022231	5.968048	0.123018	0.902218	0.935495	-7.10088
ZFR	0.014972	7.084926	0.122831	0.902366	0.935509	-7.11415
DBNL	0.022978	6.761574	0.122913	0.902301	0.935509	-7.11776
VPS29	0.018409	5.765454	0.122324	0.902767	0.935856	-7.09032
EIF4G3	-0.01751	6.661288	-0.1217	0.903259	0.936296	-7.11795
CCDC47	0.019784	6.956592	0.12151	0.90341	0.936383	-7.11625
ERAP1	-0.03041	6.47242	-0.12113	0.903711	0.936626	-7.11685
GPR39	0.077385	2.22193	0.120821	0.903956	0.93674	-6.46898
GPR107	0.018337	7.189783	0.12088	0.903909	0.93674	-7.11263
CD27	0.067588	1.459914	0.12072	0.904036	0.936754	-6.3436
CXorf26	0.028398	4.406569	0.120296	0.904371	0.937031	-6.94466
NCKAP1L	-0.0532	3.240672	-0.11989	0.904688	0.937291	-6.71967
SECTM1	-0.04864	4.56101	-0.11974	0.904812	0.937349	-6.97604
GGPS1	0.016836	5.072901	0.119487	0.905011	0.937486	-7.03151
AGA	0.026199	3.944021	0.11898	0.905412	0.937832	-6.85957
GSTO2	-0.06046	3.228311	-0.11845	0.905833	0.938051	-6.70917
APBA3	0.019201	4.03932	0.118311	0.905942	0.938051	-6.87879
FTH1	-0.02719	10.13623	-0.11829	0.905959	0.938051	-7.01897
TMEM168	-0.02739	5.411371	-0.11861	0.905705	0.938051	-7.06779
IDI1	-0.02456	5.813693	-0.11841	0.905864	0.938051	-7.0957
POLG	0.016876	6.100447	0.1179	0.906267	0.9383	-7.10717
SCHIP1	0.049903	3.621921	0.117706	0.90642	0.93832	-6.78821
WRN	-0.02245	4.545572	-0.11777	0.90637	0.93832	-6.97143
CIZ1	-0.01702	6.901324	-0.11754	0.90655	0.938385	-7.11701
IRX5	-0.08144	2.175309	-0.11739	0.906672	0.938423	-6.49103
FRRS1	0.043039	3.064161	0.117241	0.906788	0.938423	-6.65502
KIAA0114	0.0358	5.247619	0.117287	0.906752	0.938423	-7.04777
VPS37D	0.048638	0.271973	0.117138	0.90687	0.938438	-6.15516
KDSR	-0.02031	6.268812	-0.11696	0.907011	0.938515	-7.11346
LIPE	-0.04194	2.693064	-0.11643	0.907432	0.93888	-6.5926
COX6C	0.022413	6.628847	0.116158	0.907646	0.939032	-7.11842
LRRC23	0.02596	2.285732	0.115555	0.908123	0.939456	-6.49753
ALPK2	0.060613	0.645671	0.115239	0.908373	0.939645	-6.20934
SRCIN1	-0.05853	2.502267	-0.11508	0.908502	0.939709	-6.55615
RIC8B	-0.01903	3.962519	-0.11437	0.909059	0.940216	-6.87093
CCDC75	-0.01863	2.168861	-0.11388	0.909449	0.940349	-6.48273
COPG2	0.024644	3.199351	0.113943	0.909398	0.940349	-6.692
WFDC2	0.11245	3.404599	0.113902	0.909431	0.940349	-6.71956
TRIM38	0.023781	4.798475	0.11387	0.909457	0.940349	-6.99975

DHTKD1	-0.02519	5.390988	-0.11346	0.909784	0.940617	-7.06637
LOC642852	0.031039	3.547982	0.113232	0.909962	0.940732	-6.76844
HOXA2	-0.04515	0.487404	-0.11285	0.910267	0.940977	-6.20358
SV2A	-0.05696	2.270192	-0.11276	0.910335	0.940978	-6.51173
FBXO2	0.068804	2.659286	0.112156	0.910814	0.941309	-6.56682
KIAA0664	0.020333	7.599699	0.1121	0.910858	0.941309	-7.10527
USF2	0.017712	6.320966	0.112136	0.910829	0.941309	-7.11445
IKZF3	0.07382	1.090739	0.111985	0.910949	0.941334	-6.28087
WIZ	0.018953	6.929603	0.111333	0.911465	0.941798	-7.11776
FRMD5	-0.07333	0.256872	-0.11103	0.911702	0.941973	-6.16553
NDNL2	0.019315	3.911357	0.110662	0.911997	0.942208	-6.8549
LOC550112	0.021552	3.220485	0.110132	0.912417	0.94257	-6.69669
UXT	-0.0225	5.240636	-0.11005	0.912482	0.94257	-7.0529
KCTD11	-0.03743	4.750554	-0.10931	0.913068	0.943106	-7.00161
9-Mar	0.022784	4.195056	0.108886	0.913404	0.943382	-6.91067
TMEM140	-0.02043	4.387606	-0.10855	0.913669	0.943587	-6.94958
EPS8L2	0.034573	6.691553	0.108332	0.913842	0.943696	-7.11944
HYAL3	0.028578	1.86976	0.108224	0.913928	0.943714	-6.42135
EVI2B	0.045789	3.632404	0.107992	0.914111	0.943834	-6.7878
NCRNA00116	-0.02815	3.204985	-0.10766	0.914377	0.943969	-6.70329
PARP4	-0.02857	6.987824	-0.1077	0.914345	0.943969	-7.11682
LZIC	0.017661	4.304538	0.107491	0.914508	0.943972	-6.93074
SAT1	0.031718	8.039161	0.107482	0.914515	0.943972	-7.09452
TSLP	-0.0601	0.698338	-0.10727	0.914686	0.944009	-6.24019
NDUFAF4	-0.02319	3.976693	-0.10729	0.914671	0.944009	-6.87647
P2RY13	-0.04943	0.511814	-0.10653	0.915268	0.944539	-6.20727
ZNF169	-0.024	2.235785	-0.10623	0.915511	0.944721	-6.49566
FAM3A	-0.01851	5.061343	-0.10591	0.915761	0.944909	-7.03477
RBBP7	0.019084	6.92857	0.105686	0.915939	0.945023	-7.11839
NT5M	0.049323	0.133516	0.105348	0.916206	0.945159	-6.13688
ACSL5	-0.07773	5.316313	-0.10543	0.916144	0.945159	-7.06404
RNASEH1	0.014248	4.125834	0.104516	0.916866	0.945769	-6.89975
LOC115110	-0.06348	1.733576	-0.1039	0.917355	0.946161	-6.40931
ZNF77	0.020404	1.98424	0.103619	0.917577	0.946161	-6.44448
ST3GAL4	-0.0402	4.11722	-0.10381	0.917427	0.946161	-6.91179
FAM175B	0.01231	4.602314	0.103715	0.917501	0.946161	-6.97663
SEMA4C	0.02665	5.408775	0.103611	0.917584	0.946161	-7.0655
FCRL5	-0.08469	0.519631	-0.10323	0.917886	0.946402	-6.21058
ISM1	-0.06388	1.14647	-0.10288	0.918161	0.946587	-6.31405
THOC5	-0.01654	4.85753	-0.10266	0.918335	0.946587	-7.01252
ITPKC	-0.02968	6.168648	-0.10273	0.918283	0.946587	-7.11258
ELF1	0.019333	6.73688	0.102681	0.91832	0.946587	-7.12009
STYK1	0.03841	2.854968	0.102466	0.918491	0.946677	-6.60708
PRH1	-0.02746	0.982783	-0.10226	0.918653	0.946704	-6.27829
CAMK1D	0.049484	2.203515	0.102177	0.91872	0.946704	-6.48306
LCA5	0.028934	2.760234	0.102235	0.918674	0.946704	-6.59599
GNB2L1	-0.02066	9.667289	-0.10191	0.918935	0.946855	-7.03731
LOC440354	0.021804	3.434036	0.101762	0.919049	0.946903	-6.74582
RRAD	0.066657	2.677995	0.101473	0.919278	0.94707	-6.57822
RPRD2	0.013076	6.786353	0.101238	0.919464	0.947191	-7.12002
MDM2	0.02728	6.648684	0.101032	0.919627	0.947289	-7.12012
CLIP4	-0.05076	4.724723	-0.10073	0.919868	0.947467	-7.00185
URB1	-0.01794	6.093892	-0.1006	0.919969	0.947502	-7.10997
CANX	0.015722	9.477595	0.100118	0.920352	0.947826	-7.04511
C20orf7	0.01629	2.662483	0.100025	0.920426	0.947832	-6.57626
SAMD8	0.02399	3.352902	0.099875	0.920545	0.947885	-6.72906
MFN1	-0.01597	5.877001	-0.09971	0.920679	0.947954	-7.10067
C9orf3	-0.02664	5.13956	-0.09874	0.921446	0.948464	-7.04509

PCBD1	0.024183	5.670328	0.098874	0.921339	0.948464	-7.08619
GBP2	0.033215	5.90954	0.098742	0.921443	0.948464	-7.10034
PPP1R11	0.017793	6.4153	0.098802	0.921395	0.948464	-7.11775
ANKRD36BP1	0.025715	4.081443	0.09863	0.921532	0.948482	-6.88887
CDC73	0.012857	5.919113	0.098429	0.921692	0.948576	-7.10167
URGCP	-0.01663	5.613463	-0.0981	0.921951	0.948774	-7.08506
NPC1	-0.02387	6.517819	-0.09786	0.922144	0.948902	-7.11982
EFNB3	0.057429	0.993454	0.097771	0.922213	0.948904	-6.27055
ZFP91	-0.01232	7.012365	-0.09725	0.922627	0.94926	-7.11774
B9D2	0.019325	1.578374	0.096997	0.922827	0.949395	-6.37392
PPP1R15A	0.029139	6.437232	0.096692	0.923069	0.949575	-7.11819
MORN1	0.024234	1.293524	0.096136	0.92351	0.949959	-6.32375
SSR1	-0.0135	7.972992	-0.09594	0.923663	0.950038	-7.09646
TAX1BP3	-0.01939	6.760087	-0.09587	0.923724	0.950038	-7.12054
C21orf70	0.020622	2.35036	0.095239	0.924222	0.950341	-6.51273
NQO2	-0.0237	3.921761	-0.0953	0.92417	0.950341	-6.86452
RAB11FIP1	-0.04414	7.581061	-0.09533	0.924151	0.950341	-7.10607
RPAP1	-0.01513	5.334978	-0.09502	0.924398	0.950451	-7.0626
RALGDS	0.020672	6.942394	0.094932	0.924465	0.950451	-7.11931
KLHL6	0.042959	2.759756	0.094676	0.924668	0.950488	-6.59151
SLC39A11	0.023685	4.716565	0.094544	0.924773	0.950488	-6.98986
CDK10	0.015209	5.039592	0.094579	0.924745	0.950488	-7.03016
ZBED5	-0.01415	5.453364	-0.09471	0.924645	0.950488	-7.07297
ZNF468	-0.03729	3.927892	-0.09429	0.924973	0.950623	-6.86825
MAFG	-0.01844	5.955904	-0.09411	0.925121	0.950705	-7.10509
SETD8	0.017069	5.424816	0.093683	0.925456	0.950979	-7.06852
FSTL3	-0.04224	4.435675	-0.09343	0.92566	0.951069	-6.9627
MED21	0.019831	5.024454	0.093345	0.925724	0.951069	-7.02878
PROM2	-0.05627	6.658481	-0.09332	0.925747	0.951069	-7.12104
LOC151009	0.035003	1.433074	0.092737	0.926207	0.951318	-6.34342
KIF7	0.037748	2.49192	0.092686	0.926247	0.951318	-6.54111
CD37	0.039899	3.517032	0.092582	0.926329	0.951318	-6.76602
MAPK8	-0.0176	3.531465	-0.0928	0.926158	0.951318	-6.77809
MUS81	-0.01206	4.635279	-0.09261	0.926304	0.951318	-6.98478
FAM103A1	0.014019	3.791041	0.092214	0.926622	0.951519	-6.82988
ABCA7	0.028824	4.631881	0.092163	0.926662	0.951519	-6.97966
SFRS14	-0.01352	5.987824	-0.09208	0.926732	0.951521	-7.10641
UQCRH	0.016389	6.022393	0.09197	0.926815	0.951536	-7.10676
RBM39	-0.01331	7.465227	-0.09161	0.927104	0.951623	-7.10966
ZBED1	-0.01956	6.078321	-0.0917	0.927029	0.951623	-7.11029
TSTA3	0.02797	6.400154	0.091651	0.927068	0.951623	-7.11796
METTL2B	-0.0148	4.005897	-0.09083	0.927723	0.952189	-6.88162
LOC283922	0.01965	2.464799	0.090654	0.927859	0.952259	-6.53514
PTPRCAP	0.039718	2.218408	0.090483	0.927995	0.952329	-6.4862
COL6A1	0.037321	8.444767	0.090374	0.928082	0.952347	-7.08329
KRAS	-0.02812	6.665751	-0.09027	0.928161	0.952358	-7.1213
TRIM46	0.03459	0.758847	0.08906	0.929124	0.953277	-6.23474
SLC37A4	0.024544	4.063442	0.088736	0.929382	0.953471	-6.88379
SFRS16	-0.01107	4.783484	-0.08834	0.929696	0.953653	-7.0039
SLC2A3	-0.03581	4.78393	-0.08836	0.929676	0.953653	-7.00815
RPL9	0.024971	3.248295	0.088089	0.929895	0.953732	-6.70592
EIF2S3	0.014204	7.262239	0.088071	0.929909	0.953732	-7.11464
EML2	-0.01807	5.171774	-0.08786	0.93008	0.953767	-7.04781
UBN1	-0.01194	6.620983	-0.08791	0.930039	0.953767	-7.12139
CLIC3	0.064382	3.140837	0.087435	0.930414	0.953926	-6.67374
CCDC64B	0.046604	4.182091	0.087403	0.93044	0.953926	-6.90088
TEX261	-0.01066	7.034261	-0.08745	0.930403	0.953926	-7.11831
EVI2A	0.032797	2.113422	0.087228	0.930579	0.953999	-6.46875

CTNNBL1	0.011811	5.234065	0.086141	0.931442	0.954813	-7.05166
DNAJA4	0.027671	5.680924	0.086017	0.93154	0.954844	-7.08828
CLASP1	-0.01683	6.556246	-0.08497	0.932371	0.955626	-7.12132
TTC13	-0.01523	4.428153	-0.08467	0.932607	0.955674	-6.95652
TMEM45B	0.073852	4.700411	0.084658	0.932619	0.955674	-6.98214
C22orf29	0.01878	4.976627	0.084653	0.932623	0.955674	-7.02405
MON1A	0.016719	3.520892	0.084361	0.932855	0.955782	-6.76834
SDC4	0.024289	7.951259	0.084262	0.932934	0.955782	-7.09913
VGLL4	-0.01574	6.397248	-0.08432	0.93289	0.955782	-7.11931
SIRT7	0.018274	4.431718	0.084172	0.933005	0.955784	-6.9525
PLCG2	-0.02606	4.055122	-0.08404	0.933108	0.95582	-6.8948
C19orf12	-0.01892	4.333327	-0.08372	0.933367	0.955912	-6.94385
MTM1	0.015482	4.381081	0.083602	0.933457	0.955912	-6.94579
ACE	0.035724	5.024573	0.083584	0.933472	0.955912	-7.02702
ORMDL3	0.023462	5.273914	0.083631	0.933435	0.955912	-7.05438
NRSN2	0.027583	4.617128	0.083426	0.933597	0.955971	-6.98
ZNF212	0.015979	3.622287	0.083247	0.933739	0.956018	-6.79237
MRPS31	-0.01508	3.703	-0.08315	0.933815	0.956018	-6.8167
GGT5	-0.02999	3.960308	-0.08311	0.933849	0.956018	-6.87683
SGPP2	-0.03821	4.829676	-0.08243	0.93439	0.956502	-7.01152
TRMT5	-0.01184	4.423649	-0.08218	0.934583	0.95663	-6.95677
GJC1	0.031714	3.54767	0.081813	0.934878	0.956792	-6.77545
MRPL49	0.011822	5.933494	0.081819	0.934874	0.956792	-7.10388
CWC25	-0.01226	4.361061	-0.08165	0.935012	0.956858	-6.947
MGST2	-0.02206	5.070992	-0.0814	0.935203	0.956914	-7.03846
CDR2L	-0.02808	5.579536	-0.08146	0.935158	0.956914	-7.08454
CENPV	-0.04295	2.999236	-0.0807	0.935766	0.957352	-6.65647
ZCCHC17	0.011219	4.688561	0.080679	0.935779	0.957352	-6.99028
MED22	-0.01269	5.199089	-0.08061	0.935837	0.957352	-7.05095
FAM113A	0.016365	4.114315	0.08042	0.935985	0.957433	-6.89838
SFRS6	-0.01247	6.921108	-0.07992	0.936384	0.957772	-7.12052
DLG5	0.016967	7.041252	0.079555	0.936672	0.957996	-7.11926
RBM4B	0.012613	3.357237	0.079189	0.936962	0.958153	-6.73323
EXPH5	-0.02714	4.850549	-0.07925	0.936914	0.958153	-7.01391
CBL	-0.01394	5.957518	-0.07899	0.937121	0.958244	-7.10627
SLC4A5	-0.01742	3.32343	-0.07869	0.937362	0.958421	-6.72961
CECR1	0.034538	4.873789	0.078463	0.93754	0.958532	-7.01139
MRPL53	-0.01534	4.312095	-0.0783	0.937669	0.958594	-6.93959
UBLCP1	-0.01382	4.838006	-0.07812	0.937813	0.958672	-7.01205
LOC554202	-0.06332	0.946795	-0.07791	0.937981	0.958773	-6.27817
C22orf46	-0.01294	3.88645	-0.07713	0.938599	0.959334	-6.85733
EEF1D	0.01529	7.887721	0.076962	0.938732	0.9594	-7.10126
SCARNA2	-0.02549	0.063391	-0.07658	0.939036	0.959436	-6.13649
TBC1D10C	-0.03385	0.9655	-0.07631	0.939249	0.959436	-6.28018
PPCDC	0.014555	2.520117	0.076161	0.939368	0.959436	-6.54715
TIAM1	-0.04849	4.049098	-0.07629	0.939264	0.959436	-6.90221
ARL4A	-0.01972	4.692142	-0.07614	0.939385	0.959436	-6.99473
VANGL2	-0.05062	4.797396	-0.07647	0.939123	0.959436	-7.01272
FN1	0.041946	10.19282	0.076234	0.93931	0.959436	-7.02339
LIMS1	-0.01386	5.151152	-0.07664	0.938984	0.959436	-7.04668
UNK	0.010478	5.29394	0.076284	0.939271	0.959436	-7.05844
TMEM63A	0.029383	6.221718	0.075903	0.939573	0.959558	-7.11485
NTRK2	0.081287	4.330165	0.075105	0.940207	0.960066	-6.93156
BNIP3L	-0.01656	6.485756	-0.07515	0.94017	0.960066	-7.12138
INPP5D	-0.0342	4.384012	-0.07486	0.940401	0.960193	-6.95406
C18orf21	0.013872	2.908587	0.074506	0.940683	0.960341	-6.63102
ZNF592	0.011996	6.348583	0.07459	0.940617	0.960341	-7.11868
PLAG1	-0.03901	2.817249	-0.07434	0.940817	0.960408	-6.62293

BTBD12	-0.01113	4.413902	-0.074	0.941083	0.960609	-6.9554
DNMT3A	-0.01703	4.566696	-0.07318	0.941739	0.961208	-6.97844
POLM	-0.01325	4.701295	-0.0729	0.941956	0.96136	-6.99501
NSUN5P1	0.017838	2.791183	0.072645	0.942162	0.9615	-6.60179
NSUN3	-0.01398	2.924112	-0.07225	0.942477	0.96175	-6.63903
CDC42SE2	0.014031	5.801918	0.072148	0.942557	0.961762	-7.09748
TP53I3	-0.02395	3.782341	-0.07159	0.942999	0.962143	-6.83812
C1orf212	-0.00907	4.743823	-0.07117	0.943336	0.962346	-7.00042
DIAPH1	0.01299	7.898283	0.071226	0.94329	0.962346	-7.10133
FAM69B	0.02435	2.754265	0.071059	0.943422	0.962364	-6.59829
GALNT4	0.035018	5.588628	0.0706	0.943788	0.962666	-7.0815
MXD1	0.027629	6.765758	0.07045	0.943907	0.962718	-7.12283
ANKRD49	-0.01302	4.175875	-0.07032	0.944008	0.96275	-6.9154
DYRK4	0.016689	3.44953	0.070163	0.944135	0.96281	-6.75515
HSN2	-0.02597	0.988578	-0.06993	0.944322	0.96293	-6.28297
DBNDD2	0.020086	5.268792	0.069073	0.945001	0.963552	-7.05594
DNAJC11	0.00974	5.768492	0.068663	0.945328	0.963815	-7.09603
DPM2	0.013404	5.465445	0.068406	0.945532	0.963953	-7.07338
HLA-L	0.023471	0.248842	0.068238	0.945665	0.964018	-6.15834
MARS	-0.01065	6.534619	-0.06811	0.945764	0.964049	-7.12233
MIPEP	0.013637	4.069183	0.068005	0.94585	0.964066	-6.89117
ZC3HC1	0.01221	3.608685	0.067875	0.945953	0.964101	-6.79089
PRKRIR	-0.0136	5.876616	-0.0675	0.946249	0.964332	-7.10316
GALC	0.029306	5.171016	0.067378	0.946349	0.964364	-7.04477
INPP4A	-0.01352	5.096815	-0.06724	0.946461	0.964407	-7.04174
PLEKHG1	-0.01923	5.17807	-0.06707	0.946595	0.964474	-7.05054
COPS3	0.011531	5.636892	0.066952	0.946688	0.964498	-7.08764
UBE2F	-0.01029	4.824246	-0.06662	0.946952	0.964697	-7.01079
BCL2L14	-0.05741	1.017763	-0.06626	0.947238	0.964865	-6.28279
CDHR5	-0.08308	1.97825	-0.06606	0.947393	0.964865	-6.4536
POLR1E	0.012408	4.218303	0.066082	0.947379	0.964865	-6.92133
ZNF777	0.011505	4.796315	0.066202	0.947284	0.964865	-7.005
C10orf75	0.02126	1.978699	0.065762	0.947633	0.964969	-6.44677
ADAM23	-0.05844	2.047393	-0.06579	0.947613	0.964969	-6.47702
CLUAP1	0.01664	3.870501	0.06555	0.947803	0.965011	-6.85092
LPAR5	-0.02339	4.237863	-0.06552	0.947826	0.965011	-6.92687
COMT	-0.01242	6.291873	-0.06545	0.947883	0.965011	-7.11854
FAM83E	-0.05326	3.203447	-0.06511	0.948152	0.965215	-6.69843
ZNF572	0.028844	0.915706	0.064937	0.94829	0.965281	-6.26349
OTUD5	0.008677	5.917125	0.064855	0.948355	0.965281	-7.10448
CNIH3	0.032777	0.163629	0.064581	0.948573	0.965323	-6.14495
USE1	-0.01305	3.464949	-0.06454	0.948603	0.965323	-6.76317
ZFPM1	0.021747	4.950362	0.06459	0.948566	0.965323	-7.02076
ADC	0.023611	0.769233	0.064347	0.948759	0.965377	-6.24092
GUCY1A3	0.028537	4.827877	0.064226	0.948856	0.965377	-7.00795
NDUFS8	-0.01467	6.321339	-0.06422	0.948864	0.965377	-7.11932
YJEFN3	-0.0254	1.498187	-0.06402	0.949016	0.965462	-6.36906
MGC57346	0.012918	3.260411	0.063747	0.949237	0.965616	-6.71409
NHS	-0.02359	4.176258	-0.06298	0.949845	0.966094	-6.91926
HPS4	-0.0103	5.569597	-0.06299	0.949838	0.966094	-7.08418
CKAP4	-0.01483	7.402335	-0.06278	0.950008	0.966189	-7.11321
VPS16	-0.00874	5.058281	-0.06267	0.950093	0.966206	-7.03717
BPHL	-0.01405	3.403033	-0.06181	0.950774	0.966827	-6.74857
ZNF821	-0.01354	2.12241	-0.06172	0.950844	0.966829	-6.47923
MOSPD1	-0.01237	3.869373	-0.06145	0.951061	0.966979	-6.85682
KIAA1107	-0.01994	1.954696	-0.06127	0.95121	0.96706	-6.44998
SLC26A2	-0.0175	4.925972	-0.06113	0.951315	0.967097	-7.02411
INHBB	0.030558	3.160406	0.06092	0.951485	0.967199	-6.69299

LOC219347	-0.02999	0.730305	-0.0607	0.951656	0.967303	-6.24346
WDSUB1	-0.01122	2.878209	-0.05985	0.952333	0.96792	-6.63008
SLC16A5	0.026174	4.078408	0.058685	0.953262	0.968723	-6.88812
TAF8	-0.01102	4.64885	-0.05873	0.95323	0.968723	-6.989
PLK3	0.016351	4.460017	0.058479	0.953427	0.96882	-6.96093
SERINC5	0.017956	4.476242	0.057996	0.95381	0.969139	-6.96136
TET1	0.025557	1.991836	0.057655	0.954082	0.969345	-6.45142
LOC348926	0.01348	1.434318	0.057207	0.954438	0.969636	-6.35273
LOC283663	0.024418	0.413708	0.056137	0.95529	0.970431	-6.18393
PON3	-0.04749	0.027805	-0.05586	0.955512	0.97058	-6.13747
PRTFDC1	0.023725	3.020903	0.055777	0.955576	0.97058	-6.65214
NUFIP2	-0.00888	7.412126	-0.05561	0.955712	0.970649	-7.11354
GPATCH3	0.007397	3.056589	0.055488	0.955806	0.970653	-6.66617
AURKAIP1	-0.0105	5.704433	-0.05543	0.955856	0.970653	-7.09396
MYO6	0.015769	6.960594	0.055057	0.956149	0.97088	-7.122
RPSAP9	0.015221	0.042232	0.05465	0.956473	0.971068	-6.1296
C3orf17	-0.00856	5.5242	-0.05472	0.956415	0.971068	-7.08121
RPPH1	-0.02006	4.754011	-0.05442	0.956654	0.971182	-7.00296
FEZ1	0.023334	2.980971	0.054111	0.956901	0.971362	-6.64892
NUDC	0.008509	6.043991	0.053909	0.957062	0.971455	-7.11076
DDX26B	-0.01591	3.036236	-0.05326	0.957576	0.971886	-6.66979
APOO	0.012456	3.262721	0.0532	0.957626	0.971886	-6.7105
LRRC34	-0.01854	1.235552	-0.05296	0.957815	0.972007	-6.3242
ACCN2	-0.01989	2.562577	-0.05264	0.958075	0.972164	-6.56368
FGFR1OP2	-0.01029	4.523635	-0.05259	0.958109	0.972164	-6.97275
NBPF16	-0.02645	0.929241	-0.05244	0.958229	0.972215	-6.27357
DBP	0.01483	3.885918	0.052054	0.958538	0.972388	-6.85269
PCNXL2	0.011834	4.658725	0.052078	0.958519	0.972388	-6.98841
FAM156A	-0.01041	4.505001	-0.05099	0.959386	0.973177	-6.96992
MAP2K2	0.008302	6.634842	0.050902	0.959455	0.973177	-7.12394
SMYD2	-0.00879	4.813029	-0.05072	0.959602	0.973255	-7.00983
CD46	0.010028	8.110556	0.050394	0.959859	0.973445	-7.09622
RPL6	0.009264	7.955926	0.050129	0.96007	0.973588	-7.10092
ADORA2A	-0.01643	1.497631	-0.04976	0.960364	0.973674	-6.36994
XPNPEP3	-0.0077	4.090334	-0.04983	0.96031	0.973674	-6.90014
PITPNB	0.007947	6.488408	0.049835	0.960304	0.973674	-7.12273
ASTE1	-0.00838	3.289472	-0.04935	0.96069	0.973831	-6.72269
NLK	0.008403	4.408649	0.049303	0.960727	0.973831	-6.95285
CXCR4	0.020879	4.811623	0.04942	0.960634	0.973831	-7.0071
MFF	0.007146	5.50338	0.049176	0.960829	0.973833	-7.07876
MMADHC	0.006002	6.384069	0.049126	0.960869	0.973833	-7.12106
HCG4	0.033745	0.063236	0.04857	0.961311	0.974156	-6.1319
TLE3	0.008935	6.567979	0.04855	0.961327	0.974156	-7.12363
PRKAG1	-0.00662	5.218297	-0.04845	0.961403	0.974163	-7.05453
TMEM91	-0.01781	1.816334	-0.04827	0.961551	0.974241	-6.42692
TRMT2B	-0.00825	4.490719	-0.04796	0.961796	0.974419	-6.96767
PCYOX1L	0.010841	3.340095	0.047871	0.961868	0.974421	-6.73168
CHST7	-0.01946	2.528357	-0.04744	0.962213	0.9747	-6.55946
FAM200A	0.011817	2.995276	0.046715	0.962788	0.975008	-6.65277
RRAS2	0.015806	4.475424	0.046564	0.962908	0.975008	-6.96308
FAM55C	-0.01729	4.446086	-0.04694	0.962607	0.975008	-6.96411
LOC550643	-0.01291	4.508457	-0.04653	0.962935	0.975008	-6.97168
CARS2	0.008081	4.821757	0.046627	0.962858	0.975008	-7.00906
CC2D1A	0.007532	5.733566	0.046671	0.962822	0.975008	-7.09531
GPR35	-0.04034	2.834857	-0.04622	0.963184	0.975075	-6.61836
PTPRU	0.020893	4.98918	0.046139	0.963246	0.975075	-7.02836
VARS2	-0.00869	5.052617	-0.04609	0.963281	0.975075	-7.03732
EIF5B	0.008611	7.914966	0.046314	0.963107	0.975075	-7.10225

RICTOR	-0.01016	6.047031	-0.04593	0.963409	0.975134	-7.11194
CORIN	-0.02255	0.276458	-0.04578	0.963531	0.975187	-6.17235
ZNF90	-0.01222	3.804716	-0.04563	0.963648	0.975234	-6.84198
MAP3K9	-0.01389	3.904991	-0.04536	0.963864	0.975382	-6.86309
MOSPD3	0.010019	4.223188	0.044834	0.964285	0.975687	-6.92271
KIAA0226	-0.00771	5.529279	-0.04481	0.964304	0.975687	-7.08209
C12orf73	0.007386	2.424351	0.044553	0.964509	0.975823	-6.53222
HCCS	0.00706	4.683053	0.044188	0.964799	0.976046	-6.99231
FUK	-0.00919	4.159549	-0.04369	0.965197	0.976378	-6.9133
BTF3L4	0.006968	5.217316	0.042347	0.966265	0.977388	-7.05362
UBE2E2	0.013475	3.614588	0.041931	0.966596	0.977581	-6.79817
SF3B1	-0.00535	8.49478	-0.04196	0.96657	0.977581	-7.08331
TRAF3IP1	-0.00661	4.483757	-0.04174	0.966748	0.977664	-6.96699
INTS5	0.006666	5.326141	0.041596	0.966863	0.977709	-7.0637
ARRB2	0.007942	4.82295	0.041275	0.967118	0.977897	-7.00942
LRIG2	-0.00667	4.026035	-0.04087	0.967439	0.97815	-6.88766
GLTP	0.018674	6.24996	0.040614	0.967645	0.978288	-7.11811
GNG10	0.007563	4.326492	0.040492	0.967742	0.978315	-6.94188
TMC4	0.020756	5.044777	0.04025	0.967935	0.978382	-7.03225
RRM2B	-0.00757	5.431782	-0.04023	0.967949	0.978382	-7.07421
SPIRE2	0.021697	3.3476	0.039754	0.968329	0.978648	-6.72472
TET2	0.008838	5.630683	0.039639	0.968421	0.978648	-7.08871
ERMP1	-0.01171	6.233556	-0.03965	0.968412	0.978648	-7.11834
PATZ1	-0.00887	5.14778	-0.03878	0.969101	0.979264	-7.04788
TIMM22	-0.00642	3.863235	-0.03814	0.969612	0.979709	-6.85423
ZNF670	0.008652	2.001309	0.037965	0.969754	0.979781	-6.45401
AKAP8L	0.004838	5.0631	0.037652	0.970003	0.979963	-7.03782
CHCHD5	0.008928	3.923412	0.037373	0.970226	0.980117	-6.86252
KIAA1024	0.012931	1.233053	0.036213	0.971149	0.980979	-6.31776
ZNF414	-0.00638	3.754798	-0.03565	0.9716	0.981364	-6.82989
FBXL8	0.008319	2.942003	0.034757	0.972308	0.982008	-6.6389
SDK2	0.025715	3.066473	0.034486	0.972524	0.982155	-6.66869
CGN	-0.02392	5.365515	-0.03413	0.972811	0.982373	-7.0688
TPST1	0.011683	3.998091	0.033824	0.973052	0.982545	-6.88266
AGAP3	-0.00673	6.053841	-0.03373	0.973127	0.98255	-7.11262
CATSPER2	0.011341	1.038487	0.033289	0.973478	0.982779	-6.28572
RNF111	-0.00488	5.451551	-0.03327	0.973495	0.982779	-7.07593
C15orf61	-0.00711	2.568633	-0.03301	0.973703	0.982919	-6.56353
PARK7	-0.00491	6.503346	-0.03289	0.973795	0.98294	-7.12373
C9orf122	-0.01646	0.61516	-0.03272	0.973928	0.983004	-6.22225
NAT1	0.01279	2.274679	0.032575	0.974046	0.983052	-6.50021
HHIPL1	-0.01247	1.308778	-0.03244	0.974155	0.983091	-6.33826
POMZP3	0.008203	2.586456	0.03204	0.974473	0.98334	-6.56499
TM2D1	0.005589	3.646242	0.03186	0.974616	0.983414	-6.80319
MRPS18A	-0.00739	4.969584	-0.03128	0.975077	0.983808	-7.02854
RBBP9	0.005197	5.00409	0.031006	0.975296	0.983958	-7.03181
FAM160A1	-0.01206	3.917556	-0.03058	0.975636	0.984103	-6.86633
GTF2B	-0.00432	4.557973	-0.03063	0.975596	0.984103	-6.97761
PTPRK	0.006688	7.280597	0.030561	0.975651	0.984103	-7.11755
BRAP	-0.00413	4.717371	-0.03046	0.975733	0.984114	-6.99865
C3orf33	0.007394	1.717904	0.030226	0.975918	0.98423	-6.40439
PRKCQ	0.016117	0.722661	0.029948	0.976139	0.984382	-6.23675
RAB40B	-0.00804	4.212474	-0.02957	0.976436	0.984468	-6.92294
LOC144438	-0.00401	5.096802	-0.02961	0.976408	0.984468	-7.04258
LARP1	-0.00395	8.216444	-0.02971	0.976332	0.984468	-7.09331
LYL1	0.008779	1.271212	0.029055	0.97685	0.984815	-6.32803
GTF3C6	0.005397	4.766885	0.028835	0.977025	0.984849	-7.00369
MMP2	0.012886	7.797687	0.028846	0.977016	0.984849	-7.10619

TFF3	-0.03204	1.876677	-0.02847	0.977317	0.985073	-6.43384
PI4K2A	0.005066	5.694008	0.027998	0.977692	0.985308	-7.09378
C6orf106	-0.00383	7.297029	-0.02804	0.977661	0.985308	-7.11713
E4F1	0.003685	4.559481	0.026893	0.978572	0.986124	-6.97711
MCOLN1	0.004248	4.178986	0.026599	0.978807	0.986289	-6.91674
ZNF28	0.009448	4.077765	0.026428	0.978943	0.986355	-6.89584
ZBTB6	-0.00393	4.260195	-0.02607	0.979231	0.986558	-6.93193
RALGAPB	0.003832	6.908483	0.025998	0.979285	0.986558	-7.12371
LOC100272146	0.008259	1.475066	0.025426	0.979741	0.986751	-6.36096
UBE2MP1	0.004693	3.47291	0.025492	0.979688	0.986751	-6.76473
CD24	-0.0138	9.212167	-0.0254	0.979759	0.986751	-7.05789
FASTK	-0.00452	5.723609	-0.02554	0.979647	0.986751	-7.09604
PGAP3	0.010078	5.108772	0.024602	0.980397	0.987275	-7.04211
ARF3	-0.00322	7.720496	-0.02457	0.980421	0.987275	-7.10789
CXCR2	0.016322	0.640223	0.024173	0.980739	0.987524	-6.22533
RFTN1	0.007563	4.297508	0.024002	0.980875	0.98759	-6.93866
SEC61A1	0.003691	8.587047	0.023809	0.981029	0.987674	-7.08089
ASPHD1	0.015769	1.704664	0.023531	0.98125	0.987754	-6.39336
ARL8A	0.003671	5.608302	0.023616	0.981183	0.987754	-7.08791
TRAM1	0.003933	7.72927	0.023429	0.981332	0.987765	-7.10782
ZNF343	-0.00335	3.821386	-0.02316	0.981549	0.987913	-6.84494
PCDHB16	0.010664	2.279326	0.022822	0.981815	0.988109	-6.50757
ZNF326	-0.00346	4.95989	-0.02273	0.981888	0.988112	-7.02793
PARS2	0.003874	2.799126	0.02247	0.982096	0.988178	-6.61062
COQ5	-0.00353	3.941498	-0.02248	0.982086	0.988178	-6.86933
EML3	0.003317	5.417679	0.022138	0.98236	0.988373	-7.07289
PKDREJ	-0.00745	0.263133	-0.02204	0.982438	0.98838	-6.16744
HAX1	-0.00317	6.03693	-0.02194	0.982515	0.988386	-7.11209
C15orf29	-0.00384	4.246183	-0.02164	0.982755	0.988485	-6.93007
CCDC57	-0.00449	4.478477	-0.02165	0.982751	0.988485	-6.96596
TNFSF4	0.00831	1.245713	0.021364	0.982976	0.988606	-6.32421
WIPF2	0.004356	6.524717	0.021315	0.983016	0.988606	-7.12418
EPN2	0.004599	5.934154	0.021185	0.983119	0.988638	-7.10747
PIGM	0.004002	4.416315	0.020922	0.983329	0.988778	-6.95602
ROBO3	-0.0079	1.355702	-0.02016	0.983937	0.989078	-6.3448
RPL41	-0.00361	9.084542	-0.02037	0.98377	0.989078	-7.06305
LAMP1	0.003717	8.408614	0.020111	0.983975	0.989078	-7.08729
ZRANB2	-0.003	5.911969	-0.02028	0.983843	0.989078	-7.10659
NGRN	-0.00332	6.356443	-0.02006	0.984016	0.989078	-7.1217
PPIF	-0.00495	6.963117	-0.02001	0.984052	0.989078	-7.12306
LOC143666	0.005804	0.061235	0.019173	0.984722	0.989681	-6.13568
ZFYVE19	0.002879	4.073209	0.018967	0.984886	0.989774	-6.896
LRCH3	-0.0035	4.518261	-0.01883	0.984999	0.989817	-6.97255
CALHM2	-0.00501	3.436853	-0.01865	0.985141	0.989888	-6.76002
CLCF1	0.006356	2.983731	0.018015	0.985645	0.990252	-6.65462
MAP4K2	-0.00423	4.385736	-0.01802	0.985639	0.990252	-6.95239
FBXL2	-0.0057	1.687076	-0.01727	0.986241	0.990779	-6.40168
EGFL7	-0.00522	3.8607	-0.01707	0.986397	0.990793	-6.85371
AACS	-0.00388	5.547155	-0.01709	0.986382	0.990793	-7.08392
NMRAL1	0.003356	4.726107	0.016701	0.986692	0.991018	-6.99846
PRELID2	-0.00527	2.02108	-0.0163	0.987015	0.991234	-6.45654
HDAC7	0.002406	6.232195	0.016254	0.987048	0.991234	-7.11868
MICALL2	-0.00409	5.332153	-0.01515	0.987928	0.992046	-7.06554
CCDC88A	-0.00446	5.742951	-0.01504	0.988019	0.992066	-7.09755
SLC2A8	0.003504	3.697692	0.014869	0.988152	0.992128	-6.8143
DOM3Z	0.002669	3.619324	0.014504	0.988443	0.992349	-6.79654
ZNF174	0.001938	3.22127	0.014151	0.988724	0.99256	-6.70782
DDX59	-0.00233	3.790804	-0.01394	0.988894	0.992659	-6.83886

ZNF711	-0.00677	3.19481	-0.0137	0.989083	0.992778	-6.7009
TRIM56	-0.00273	5.405743	-0.01328	0.989415	0.992968	-7.07239
CFLAR	-0.00288	6.020141	-0.01333	0.989382	0.992968	-7.11153
TMEM185A	-0.00263	4.505627	-0.01313	0.989534	0.993016	-6.97148
TBC1D3B	-0.0037	2.761069	-0.01294	0.989688	0.993099	-6.60364
DYNC1LI1	-0.00203	5.057123	-0.01271	0.989872	0.993213	-7.0387
RPL23A	-0.00239	7.522358	-0.0126	0.989963	0.993233	-7.11275
IRF8	0.007433	3.670434	0.012162	0.990309	0.993508	-6.8069
APOL4	0.004946	2.899508	0.011882	0.990532	0.993661	-6.63537
DENND1B	-0.00286	2.980223	-0.01169	0.990684	0.993742	-6.65154
SDHC	0.001696	5.821954	0.011401	0.990915	0.993903	-7.10186
TRAPPC6A	0.003171	2.960226	0.01123	0.991051	0.993968	-6.64662
BLZF1	0.002045	5.081427	0.01102	0.991219	0.994064	-7.04072
DNAH14	-0.00747	0.798851	-0.01083	0.991368	0.994072	-6.25088
MESDC1	0.001966	5.913638	0.010871	0.991337	0.994072	-7.1066
AFG3L1	0.001819	4.060902	0.010375	0.991733	0.994366	-6.89433
ELMO1	-0.00398	4.280585	-0.01003	0.992005	0.994568	-6.93794
ATP8B4	0.003724	0.743271	0.009578	0.992368	0.99486	-6.24106
ARSE	0.009042	1.053015	0.009274	0.99261	0.994933	-6.28495
BANK1	-0.00533	1.343501	-0.00934	0.992561	0.994933	-6.34294
PRAF2	-0.00256	4.276714	-0.00922	0.992655	0.994933	-6.93647
ERP44	-0.00132	6.618291	-0.00895	0.992866	0.995073	-7.12517
TMEM92	-0.00553	2.182837	-0.00841	0.993297	0.995424	-6.47983
ANKH	0.002075	6.1508	0.008335	0.993358	0.995424	-7.11629
SLC6A9	0.002622	4.714881	0.008021	0.993608	0.995604	-6.99872
LOC729991	-0.0012	3.272484	-0.00792	0.993685	0.995609	-6.71851
MAGT1	-0.0012	6.694016	-0.00716	0.994291	0.996145	-7.12534
PPIL3	0.001199	3.586249	0.006785	0.994593	0.996376	-6.79025
CSDAP1	0.001781	2.584284	0.0059	0.995298	0.996939	-6.57
POC1B	-0.00142	4.633515	-0.00596	0.995252	0.996939	-6.98718
SCFD1	-0.00075	5.393235	-0.00528	0.995795	0.997366	-7.0712
TMEM205	0.001047	4.961136	0.005153	0.995894	0.997393	-7.0276
SOX6	-0.00246	2.543227	-0.00473	0.996233	0.997661	-6.55714
POM121	-0.00056	7.584843	-0.00373	0.997031	0.998389	-7.11144
GOLGA8A	-0.00102	5.603565	-0.00343	0.997268	0.998523	-7.08809
AHNAK2	0.002387	7.515298	0.003378	0.997308	0.998523	-7.11312
GNG5	-0.00057	6.243075	-0.00325	0.997406	0.99855	-7.11913
SOX18	-0.001	4.003458	-0.00314	0.997496	0.998568	-6.88493
ASXL2	-0.00068	5.774734	-0.00297	0.99763	0.998605	-7.09944
ANKRD52	-0.00054	6.71802	-0.00292	0.997676	0.998605	-7.12536
GGA3	-0.00039	5.681712	-0.00252	0.997995	0.998852	-7.09351
KLHL28	0.000368	4.67421	0.002171	0.99827	0.999056	-6.99284
ERCC6	-0.00036	4.169941	-0.00178	0.998579	0.999151	-6.91543
ALG2	0.000267	5.13445	0.001799	0.998566	0.999151	-7.04608
SSH3	0.000445	6.07329	0.001804	0.998562	0.999151	-7.11368
RLF	-0.00035	5.43416	-0.00163	0.998699	0.9992	-7.07502
AGTPBP1	0.000297	4.453951	0.001431	0.998859	0.999288	-6.96284
TMEM231	0.000337	2.183723	0.001021	0.999187	0.999473	-6.49167
ZNF691	-0.00016	2.783816	-0.00103	0.999178	0.999473	-6.61004
SESN3	-0.00046	3.284439	-0.00076	0.999391	0.999605	-6.72734
CCT6P1	#######	2.190213	-0.00046	0.999631	0.999774	-6.49118
SS18L1	5.61E-05	4.613859	0.000327	0.99974	0.999811	-6.98497
ZNF768	#######	5.092167	#######	0.999932	0.999932	-7.04218
