# Supplementary material for: Novel lncRNA Panel as for Prognosis in Esophageal Squamous Cell Carcinoma Based on ceRNA Network Mechanism
Source: Comput Math Methods Med. 2021 Sep 24;2021:8020879. doi: 10.1155/2021/8020879 (PMC8486540; doi:10.1155/2021/8020879)
Supplement: Supplementary Materials — Supplementary Table 1: the sample information was shown in Table S1. Supplementary Table 2: expression data of genes were gathered in Table S2. Supplementary Table 3: differently expressed lncRNAs were shown in Table S3. Supplementary Table 4: differently expressed miRNAs were shown in Table S4. Supplementary Table 5: the interaction network of between lncRNAs and miRNAs is shown in Table S5. [file 8020879.f1.zip › Table-S3.pdf]

	logFC	AveExpr	t	PValue	FDR	B
ZNF710-AS1	-2.81579	8.947842	-11.3274	1.09E-22	8.19E-19	40.71261
AC119424.1	-7.20325	-0.3813	-11.1635	3.21E-22	1.21E-18	39.35192
AC116407.1	-3.12611	5.790642	-11.094	5.07E-22	1.27E-18	39.41053
AL032819.2	-3.54633	4.725081	-10.5729	1.54E-20	2.90E-17	36.0595
AC004836.1	-5.84085	2.003717	-10.5245	2.11E-20	3.18E-17	35.60586
C9orf147	-2.48213	6.316063	-10.2023	1.71E-19	2.14E-16	33.66254
AC012409.3	-5.48454	1.381993	-10.0827	3.71E-19	3.98E-16	32.69764
AC025271.4	-5.91438	0.235762	-9.731	3.55E-18	3.33E-15	30.35751
AC005920.4	-5.40921	2.150169	-9.29285	5.73E-17	4.31E-14	27.93903
AC123912.4	-5.87568	0.567013	-9.29404	5.69E-17	4.31E-14	27.73685
AP001148.1	-2.69422	5.163215	-9.06772	2.36E-16	1.60E-13	26.61232
AC015908.3	-3.3807	5.023219	-9.05491	2.55E-16	1.60E-13	26.51768
BX284668.2	-3.48701	3.788215	-8.99666	3.67E-16	2.12E-13	26.18149
AL158847.1	-4.97797	2.506416	-8.86648	8.25E-16	4.43E-13	25.36765
AC024337.2	-3.63021	2.327624	-8.72363	2.00E-15	1.00E-12	24.37585
AL158163.2	-1.7584	6.483382	-8.68003	2.61E-15	1.20E-12	24.19438
LINC00940	-7.08558	-1.59819	-8.67315	2.72E-15	1.20E-12	23.38348
AC093583.1	-3.54573	3.358962	-8.61251	3.95E-15	1.65E-12	23.84214
AC239798.2	-4.26307	1.31613	-8.308	2.53E-14	1.00E-11	21.77447
AL157392.2	-4.57665	2.364328	-8.20101	4.82E-14	1.81E-11	21.41953
AP001528.3	-2.22856	5.625095	-8.10108	8.79E-14	3.00E-11	20.79132
AC012409.5	-3.42527	2.352883	-8.10643	8.51E-14	3.00E-11	20.7572
AC010998.3	-5.38748	3.02501	-7.99892	1.62E-13	5.28E-11	20.26313
AP001528.2	-2.11532	5.781392	-7.97377	1.88E-13	5.88E-11	20.03961
AC063919.1	-6.84004	-1.14898	-7.91409	2.68E-13	8.05E-11	19.44459
AC093797.1	-3.20573	4.556365	-7.89887	2.93E-13	8.47E-11	19.65705
AC103563.2	-6.65697	0.454781	-7.76518	6.45E-13	1.79E-10	18.89785
AC016251.1	-3.10097	2.328906	-7.46112	3.78E-12	1.02E-09	17.03256
AC108472.1	-4.16206	2.831457	-7.44489	4.16E-12	1.08E-09	17.12454
AC124312.3	-3.56594	3.560222	-7.39715	5.47E-12	1.37E-09	16.85396
AC091563.1	-2.76213	8.647079	-7.32723	8.16E-12	1.98E-09	15.95273
NGF-AS1	-6.59867	-2.57231	-7.26577	1.16E-11	2.72E-09	15.18756
LINC01996	-4.99834	1.146572	-7.2565	1.22E-11	2.78E-09	16.01335
AC244453.3	-5.54917	1.537374	-7.19164	1.76E-11	3.88E-09	15.71939
NCAM1-AS1	-6.19986	-1.44264	-7.1875	1.81E-11	3.88E-09	15.1938
LINC02489	-6.15918	1.419889	-7.17816	1.90E-11	3.97E-09	15.65559
CADM3-AS1	-3.5368	2.242452	-7.14729	2.27E-11	4.60E-09	15.45941
AL138847.2	-2.82032	3.444798	-7.12922	2.51E-11	4.96E-09	15.38737
AL158206.1	-2.13293	9.828721	-7.10563	2.87E-11	5.52E-09	14.64508
AC092834.1	-5.75476	0.833465	-7.01858	4.67E-11	8.77E-09	14.75533
AC139749.1	-3.18476	4.247475	-6.99477	5.34E-11	9.78E-09	14.62218
AP003117.1	-3.86288	2.495696	-6.91967	8.10E-11	1.45E-08	14.26262
AP000907.2	-4.87111	0.91399	-6.90346	8.87E-11	1.51E-08	14.12524
AL158163.1	-1.28508	7.052086	-6.90503	8.79E-11	1.51E-08	13.94836
AP001972.5	-2.29644	9.564367	-6.88876	9.62E-11	1.61E-08	13.46376
AC021016.2	-1.2858	7.274137	-6.87128	1.06E-10	1.73E-08	13.74576
AC005725.1	-3.8158	2.336381	-6.80993	1.49E-10	2.37E-08	13.67253
AC105074.1	-1.97377	5.300014	-6.72263	2.40E-10	3.75E-08	13.12029
AC100830.1	-1.36423	5.580657	-6.67046	3.19E-10	4.88E-08	12.86344
AC004982.1	-2.2842	5.990165	-6.65587	3.45E-10	5.18E-08	12.65846
AC103746.1	-2.55838	4.749416	-6.65176	3.53E-10	5.19E-08	12.77724
AC110609.1	-4.73222	1.014462	-6.58275	5.12E-10	7.40E-08	12.46819
AL355388.1	-4.38862	2.982993	-6.55682	5.89E-10	8.35E-08	12.3386
AC015967.1	-4.53982	1.262806	-6.54248	6.37E-10	8.68E-08	12.26076
AC053503.6	-4.05635	1.944362	-6.53501	6.63E-10	8.68E-08	12.24825
AL512328.1	-4.47128	2.792816	-6.53347	6.68E-10	8.68E-08	12.23381
SPIN4-AS1	-4.88232	0.43522	-6.54197	6.38E-10	8.68E-08	12.22986

AC073862.3	-5.36091	-0.17293	-6.53292	6.70E-10	8.68E-08	12.04932
AC011379.1	-1.87035	4.800833	-6.50595	7.74E-10	9.86E-08	12.05495
AC103563.7	-5.4748	1.443852	-6.49479	8.22E-10	1.03E-07	12.04469
AC091057.3	-2.85867	3.201258	-6.48431	8.70E-10	1.07E-07	11.99235
AC079061.1	-4.45806	1.303036	-6.46683	9.55E-10	1.16E-07	11.82578
C8orf49	-4.69666	3.182084	-6.42616	1.19E-09	1.41E-07	11.65403
AL049869.2	-5.68446	-0.25309	-6.35851	1.70E-09	1.99E-07	11.2076
AC053503.4	-5.40056	1.656928	-6.34547	1.82E-09	2.10E-07	11.28162
AC012085.2	-2.03624	3.894057	-6.32937	1.98E-09	2.26E-07	11.20239
LINC02447	-1.53479	3.892786	-6.16288	4.74E-09	5.31E-07	10.37396
AL590004.3	-3.19308	3.92622	-6.12926	5.64E-09	6.23E-07	10.15657
AP001094.2	-1.76463	4.247359	-6.11718	6.00E-09	6.54E-07	10.13588
AC113398.2	-5.06015	-0.0543	-6.07249	7.56E-09	8.12E-07	9.833174
AC091544.5	-5.01975	0.94728	-6.06505	7.86E-09	8.20E-07	9.874078
AC013726.1	-3.47315	1.51183	-6.0652	7.85E-09	8.20E-07	9.808509
AL132642.1	-3.23873	3.795823	-6.03582	9.13E-09	9.40E-07	9.675702
LINC01783	-3.50665	2.097831	-6.03275	9.27E-09	9.42E-07	9.728834
AL031429.2	-5.45481	0.398529	-6.01666	1.01E-08	1.01E-06	9.625944
AC022558.2	-3.9512	1.626843	-5.99036	1.15E-08	1.14E-06	9.509233
AL355073.1	-3.59442	2.851981	-5.95617	1.37E-08	1.34E-06	9.351778
FAM225A	4.156229	3.262988	5.907301	1.76E-08	1.69E-06	7.589251
AC007193.2	-4.30342	2.589374	-5.83374	2.55E-08	2.42E-06	8.75027
AC021766.1	-4.01535	1.363892	-5.81375	2.82E-08	2.64E-06	8.679968
C15orf54	5.343776	1.828303	5.806356	2.92E-08	2.71E-06	6.831869
AC099850.3	2.646278	11.43825	5.796643	3.07E-08	2.81E-06	8.131637
AL133355.1	-1.27252	8.061349	-5.76795	3.54E-08	3.20E-06	7.975202
AC139769.2	-3.43408	2.536717	-5.75249	3.82E-08	3.42E-06	8.395503
AC025279.1	-1.4837	3.934368	-5.7249	4.39E-08	3.88E-06	8.271298
IDH2-DT	-4.52748	2.052652	-5.70805	4.77E-08	4.17E-06	8.190754
AC246680.1	-6.17789	-1.4544	-5.69467	5.09E-08	4.40E-06	7.874605
LINC01985	-4.58747	1.082626	-5.66368	5.94E-08	5.04E-06	7.981591
AC019294.2	4.233832	2.767932	5.662537	5.97E-08	5.04E-06	6.540621
AL158212.3	-1.07743	8.30364	-5.65785	6.11E-08	5.10E-06	7.432634
AL023973.1	-3.3307	2.729176	-5.64328	6.56E-08	5.42E-06	7.881371
LINC02352	-1.25042	5.965291	-5.62617	7.14E-08	5.83E-06	7.592113
LINC02185	-2.61921	3.909642	-5.61062	7.71E-08	6.23E-06	7.661996
TUSC8	-5.76611	0.016554	-5.60692	7.85E-08	6.27E-06	7.665531
AC005180.2	-2.68176	4.946427	-5.59209	8.44E-08	6.67E-06	7.422874
AC005225.4	-1.47624	5.317754	-5.57536	9.16E-08	7.17E-06	7.407212
AP005717.1	-2.20174	3.672011	-5.55465	1.01E-07	7.76E-06	7.466065
AC023794.6	-1.35873	4.748656	-5.55268	1.02E-07	7.76E-06	7.415207
AC114810.1	-1.31783	6.194384	-5.55409	1.02E-07	7.76E-06	7.207094
AL136164.3	-1.7023	4.988421	-5.53875	1.09E-07	8.22E-06	7.277408
AP003059.1	-3.43395	3.144151	-5.5343	1.12E-07	8.32E-06	7.330864
AC073842.1	-3.3441	3.209226	-5.52494	1.17E-07	8.52E-06	7.286629
AC009078.3	-1.1354	6.272673	-5.52348	1.18E-07	8.52E-06	7.067425
AL109936.2	-1.5011	6.404837	-5.52336	1.18E-07	8.52E-06	7.007591
AC129492.3	-6.097	-0.44844	-5.52104	1.19E-07	8.53E-06	7.247011
AC012409.1	-3.84876	2.469081	-5.5164	1.22E-07	8.65E-06	7.294697
TRG-AS1	-1.67898	5.678001	-5.49306	1.37E-07	9.59E-06	6.966873
RPL34-AS1	-1.99807	3.054051	-5.47368	1.50E-07	1.04E-05	7.107763
AC007637.1	-2.61016	6.50886	-5.4516	1.67E-07	1.15E-05	6.553628
AC079210.1	-2.89087	5.636284	-5.44935	1.69E-07	1.15E-05	6.641817
AP001972.1	-2.89234	4.335416	-5.43154	1.84E-07	1.24E-05	6.76165
AL158212.1	-3.38773	2.804403	-5.42662	1.88E-07	1.26E-05	6.871341
AC244453.1	-5.57888	-0.53523	-5.40398	2.10E-07	1.39E-05	6.696008
MAGI1-AS1	-2.5643	4.46957	-5.37484	2.41E-07	1.59E-05	6.499604
AC011504.1	-4.28677	-0.47157	-5.36349	2.54E-07	1.65E-05	6.370611

SEMA3B-AS1	-2.2774	7.287554	-5.365	2.52E-07	1.65E-05	6.066192
AC079331.1	-1.73304	5.383992	-5.35367	2.66E-07	1.71E-05	6.362792
LINC00365	-2.97313	4.855835	-5.34182	2.82E-07	1.79E-05	6.286173
HCG21	-4.85469	1.114339	-5.32738	3.02E-07	1.91E-05	6.455737
AC132154.1	-5.53748	-1.5455	-5.29584	3.50E-07	2.19E-05	5.994194
AL138808.1	-4.7608	0.929234	-5.29001	3.60E-07	2.22E-05	6.290586
LINC01593	-6.00851	-0.58532	-5.29122	3.58E-07	2.22E-05	6.237038
NCOA7-AS1	-4.61418	1.509321	-5.25149	4.32E-07	2.64E-05	6.126831
AC012313.5	-1.25735	6.587916	-5.24008	4.55E-07	2.76E-05	5.701062
AC130371.2	-1.66489	7.081406	-5.23588	4.64E-07	2.79E-05	5.564357
AC008669.1	-1.25811	6.995036	-5.22379	4.91E-07	2.93E-05	5.567929
AF001548.3	-2.37488	4.438774	-5.21248	5.18E-07	3.07E-05	5.777706
AC010478.1	-3.93265	1.503576	-5.20275	5.42E-07	3.18E-05	5.908644
LINC00261	-4.76598	4.585306	-5.20125	5.46E-07	3.18E-05	5.576092
ENOX1-AS1	-2.4457	2.268886	-5.1954	5.61E-07	3.24E-05	5.848053
AC024075.1	-1.02909	9.427961	-5.17936	6.05E-07	3.47E-05	5.069109
AC091544.4	-2.52507	3.365617	-5.16985	6.32E-07	3.60E-05	5.738234
FAM13A-AS1	-1.0465	6.696415	-5.1671	6.40E-07	3.62E-05	5.381573
AC244131.2	-3.8942	1.544917	-5.16248	6.54E-07	3.67E-05	5.740321
AC005274.1	-2.92425	3.67412	-5.15993	6.62E-07	3.68E-05	5.618632
AC002116.2	1.739382	7.626682	5.157761	6.68E-07	3.69E-05	5.576026
AC129492.5	-3.25695	2.738021	-5.15596	6.74E-07	3.70E-05	5.686346
LINC02593	-1.7483	5.518044	-5.15143	6.88E-07	3.75E-05	5.40073
LINC02435	-4.03935	2.307976	-5.14854	6.98E-07	3.77E-05	5.663616
PCAT18	-5.57147	-0.80237	-5.13338	7.48E-07	4.02E-05	5.439697
ZNF30-AS1	-4.06804	1.115483	-5.12335	7.84E-07	4.18E-05	5.567776
AC018511.2	-4.19729	1.168791	-5.11998	7.96E-07	4.21E-05	5.557651
NKAIN3-IT1	-3.54735	0.891748	-5.11579	8.12E-07	4.26E-05	5.512486
AP000866.2	-1.50883	6.598141	-5.1129	8.22E-07	4.29E-05	5.096813
AL136309.2	-1.81307	3.940227	-5.09221	9.05E-07	4.69E-05	5.414819
AL008733.1	-5.57572	0.292527	-5.05145	1.09E-06	5.61E-05	5.251051
AC092667.1	-5.21058	-1.92759	-5.0374	1.16E-06	5.94E-05	4.879105
AL035701.1	-3.35874	3.474349	-5.03189	1.19E-06	6.05E-05	5.045054
AC118344.2	-0.91012	7.866186	-5.01811	1.27E-06	6.40E-05	4.57484
AC138819.1	-5.32427	-0.24213	-5.01622	1.28E-06	6.41E-05	5.050928
AL354836.1	1.39212	10.20947	5.003541	1.36E-06	6.75E-05	4.458387
AC012317.1	-4.47734	2.154639	-4.98341	1.49E-06	7.35E-05	4.936138
AC103853.2	-3.39437	2.004348	-4.97812	1.52E-06	7.47E-05	4.952005
AC018926.1	-1.07421	6.615641	-4.96888	1.59E-06	7.74E-05	4.514307
SH3BP5-AS1	-1.06709	8.288226	-4.96344	1.63E-06	7.88E-05	4.260959
ELN-AS1	-2.44923	6.112536	-4.94662	1.75E-06	8.40E-05	4.350916
AL031587.5	-1.76564	8.328168	-4.94667	1.75E-06	8.40E-05	4.107658
LINC01644	-3.86343	1.995098	-4.94437	1.77E-06	8.43E-05	4.803299
AC099521.2	-2.32376	4.942813	-4.93839	1.82E-06	8.61E-05	4.519265
AC004988.1	7.002631	3.553374	4.935108	1.85E-06	8.68E-05	3.721756
LINC01354	-2.65746	2.500522	-4.93166	1.88E-06	8.71E-05	4.759622
AC048380.2	-1.0428	5.914243	-4.93265	1.87E-06	8.71E-05	4.468527
SNHG3	1.308314	11.12816	4.915498	2.02E-06	9.30E-05	3.948535
AC244453.2	-3.26628	1.292036	-4.9063	2.10E-06	9.63E-05	4.648313
AC092718.4	1.378511	11.17417	4.905005	2.12E-06	9.63E-05	3.908937
AC025470.2	-5.36526	-1.48447	-4.90302	2.13E-06	9.66E-05	4.441949
AC135012.3	-2.47356	2.615476	-4.88548	2.31E-06	0.000104	4.560628
UGDH-AS1	-0.82407	7.194516	-4.87957	2.37E-06	0.000106	4.078375
AC026401.3	1.691952	11.33023	4.873042	2.44E-06	0.000109	3.786934
AL031846.2	-1.02446	5.368286	-4.86158	2.57E-06	0.000114	4.26974
AC010480.1	-2.60995	3.304019	-4.84109	2.81E-06	0.000124	4.316953
AL109659.2	-2.33026	3.71531	-4.83682	2.87E-06	0.000125	4.278722
AP000437.1	-0.87855	6.234202	-4.83667	2.87E-06	0.000125	4.031417

THRB-IT1	-2.22084	4.65156	-4.83367	2.91E-06	0.000126	4.118413
AL133370.1	-3.23239	5.516535	-4.8323	2.93E-06	0.000126	3.875328
AC012640.3	-4.50607	1.032435	-4.82014	3.09E-06	0.000132	4.302335
FOXP1-AS1	-1.33572	5.732277	-4.81767	3.12E-06	0.000132	3.963731
AC114546.1	-5.22104	-1.46411	-4.79611	3.43E-06	0.000145	4.097372
AC068491.3	2.412682	8.195111	4.783219	3.63E-06	0.000153	3.977285
AC024075.2	-0.8466	9.063612	-4.77735	3.73E-06	0.000156	3.382032
AC105345.1	-1.16868	4.130033	-4.76859	3.88E-06	0.000161	4.051272
AC100861.1	1.606706	8.069248	4.767022	3.90E-06	0.000161	3.794431
AC129507.2	-3.42497	3.308057	-4.76187	3.99E-06	0.000164	3.922797
Z84484.1	6.193692	6.060832	4.75817	4.06E-06	0.000166	3.435344
AL020997.2	-2.83592	4.563643	-4.75666	4.08E-06	0.000166	3.742692
AC108215.1	-2.27992	3.906561	-4.75076	4.19E-06	0.000169	3.879385
AC091544.2	-3.24993	1.855378	-4.73512	4.49E-06	0.00018	3.954797
AC106876.1	-4.98155	2.213833	-4.72906	4.61E-06	0.000184	3.871718
AC009974.1	-3.35125	2.394502	-4.72455	4.70E-06	0.000187	3.879451
AC012073.1	1.371915	8.861805	4.718978	4.82E-06	0.00019	3.428088
AC037459.2	-0.91976	5.776078	-4.69411	5.37E-06	0.000211	3.513977
AC005495.1	-2.87652	2.090592	-4.68658	5.54E-06	0.000217	3.766015
AC048380.3	-3.36351	1.623496	-4.67007	5.96E-06	0.000232	3.700497
DNAJC9-AS1	-1.12001	3.667733	-4.65247	6.43E-06	0.000249	3.62784
AL020997.3	-1.52172	6.743334	-4.64878	6.53E-06	0.000252	3.081173
AL035448.1	-1.17479	6.298826	-4.63916	6.81E-06	0.000261	3.156775
AL157996.1	-2.89025	1.58335	-4.61339	7.60E-06	0.00029	3.471744
AP003059.2	-4.30242	0.669453	-4.60577	7.86E-06	0.000298	3.44143
PSMD6-AS2	-0.88658	6.445828	-4.6011	8.02E-06	0.000303	3.014703
ALDH1L1-AS2	-3.95433	-0.09987	-4.5986	8.10E-06	0.000304	3.334447
AC005332.2	-1.66517	4.369336	-4.59691	8.16E-06	0.000305	3.271617
AP003355.2	-3.8368	-0.00878	-4.59522	8.22E-06	0.000306	3.316645
AC113189.4	4.587368	1.776268	4.585861	8.56E-06	0.000317	2.557832
AC012557.1	-0.86929	7.258775	-4.57836	8.83E-06	0.000325	2.800726
AL137793.1	-4.72388	0.306566	-4.57212	9.07E-06	0.000333	3.294549
AL160286.2	-2.91201	2.297885	-4.56463	9.37E-06	0.000342	3.277277
AC130456.2	-3.70951	2.364778	-4.56141	9.50E-06	0.000345	3.223826
CYMP-AS1	-4.46409	-0.14277	-4.55386	9.81E-06	0.000354	3.209729
AC020659.1	-1.5197	4.439921	-4.5525	9.86E-06	0.000355	3.107008
AL157834.2	-2.77152	2.777473	-4.55059	9.94E-06	0.000356	3.193369
AC108463.2	7.562406	5.536411	4.549077	1.00E-05	0.000356	2.546769
FOXD2-AS1	1.987452	9.039648	4.544378	1.02E-05	0.000362	2.779504
Z97653.2	-1.48986	5.184144	-4.54084	1.04E-05	0.000366	2.88275
CYP4A22-AS1	6.329043	6.439372	4.538832	1.05E-05	0.000367	2.710169
AC025917.1	-1.03493	7.277981	-4.529	1.09E-05	0.000381	2.574604
AC092127.1	-1.16978	4.03839	-4.5261	1.10E-05	0.000384	3.096904
AP000708.1	5.322573	1.184877	4.516435	1.15E-05	0.000398	2.290537
AC009119.3	-2.19716	4.077668	-4.50745	1.19E-05	0.000411	2.870033
PWAR5	-1.9109	5.219416	-4.50088	1.23E-05	0.000421	2.687959
AL031768.1	-2.19174	4.485951	-4.49228	1.27E-05	0.000433	2.761624
AC098869.2	-1.43839	7.126847	-4.49215	1.27E-05	0.000433	2.402773
TYMSOS	2.087604	8.542867	4.490192	1.28E-05	0.000434	2.645613
LINC01050	9.434273	2.167308	4.486836	1.30E-05	0.000439	2.208415
TMEM26-AS1	7.354857	1.239304	4.482523	1.33E-05	0.000445	2.182671
AC135178.1	-2.69108	3.072859	-4.47793	1.35E-05	0.000451	2.868419
PITPNM2-AS1	-1.17702	4.47142	-4.47196	1.39E-05	0.000457	2.813674
AC021491.2	-1.15327	5.751811	-4.47182	1.39E-05	0.000457	2.591758
AC026356.1	1.268734	8.259036	4.473406	1.38E-05	0.000457	2.507689
LINC02595	2.632799	8.690624	4.469979	1.40E-05	0.000459	2.638262
AC139887.4	-0.96378	7.605661	-4.46784	1.41E-05	0.000461	2.290844
AC069544.1	1.253102	7.32703	4.462556	1.44E-05	0.000469	2.637591

AL121584.1	-4.89264	-0.54191	-4.44991	1.52E-05	0.000492	2.798899
AC015921.1	-1.66068	4.688222	-4.44707	1.54E-05	0.000496	2.628665
AJ006995.1	-4.72524	-0.63192	-4.43683	1.61E-05	0.000515	2.754025
LINC01560	0.996704	8.231166	4.434937	1.62E-05	0.000515	2.314439
AP003486.1	-0.97244	8.700085	-4.43514	1.62E-05	0.000515	2.010188
AC138956.2	-0.95715	8.391778	-4.43212	1.64E-05	0.000519	2.042698
AC004637.1	-2.25332	4.111571	-4.41832	1.73E-05	0.000548	2.500672
AC021205.3	-1.53245	4.35103	-4.41682	1.75E-05	0.000549	2.570809
AC006942.1	-1.14234	6.288525	-4.41077	1.79E-05	0.00056	2.241466
AL731557.1	-5.19877	-1.65658	-4.40485	1.83E-05	0.000567	2.52976
SLC12A5-AS1	4.964987	4.337655	4.406008	1.83E-05	0.000567	2.170493
AL391834.2	-1.00491	8.759388	-4.40536	1.83E-05	0.000567	1.879067
AL445309.1	-1.25308	6.828774	-4.4011	1.86E-05	0.000574	2.100629
AL138930.1	-4.56567	0.519336	-4.39833	1.88E-05	0.000578	2.645606
AC093607.1	-4.79407	0.120629	-4.39564	1.91E-05	0.000582	2.621249
AGAP2-AS1	2.080937	10.55767	4.389614	1.95E-05	0.000594	1.932195
MACROD2-AS1	-4.30914	-0.43717	-4.35262	2.28E-05	0.00069	2.409881
LINC01637	1.928302	8.318692	4.348248	2.32E-05	0.000699	2.099752
AL662797.1	1.234647	7.079763	4.345826	2.34E-05	0.000704	2.202427
AC006946.3	-2.91034	3.141817	-4.3335	2.46E-05	0.000737	2.259359
GLYCTK-AS1	-1.46304	4.183062	-4.33054	2.49E-05	0.000743	2.293157
AC136469.1	-0.97807	5.694059	-4.32407	2.56E-05	0.00076	2.03664
CACNA1C-AS2	-2.30652	3.912331	-4.321	2.59E-05	0.000767	2.165068
AC012181.1	-1.17876	7.191069	-4.31555	2.65E-05	0.000781	1.721876
AC010300.1	-1.59387	5.109653	-4.31342	2.67E-05	0.000785	1.995434
AP002433.1	-2.94338	1.81581	-4.31077	2.70E-05	0.00079	2.323709
AC079331.2	-1.09662	5.977903	-4.29935	2.83E-05	0.000825	1.876517
TSC22D1-AS1	-0.86712	6.472207	-4.29726	2.86E-05	0.000829	1.800425
AC087721.1	8.374578	4.388699	4.29348	2.90E-05	0.000838	1.629009
AC004491.1	2.767856	4.366002	4.28649	2.98E-05	0.000859	1.994461
AC073476.3	-1.01412	4.583052	-4.27276	3.16E-05	0.000905	2.068195
AP000781.1	2.165695	6.815286	4.266375	3.24E-05	0.000925	2.082868
AC111182.1	-1.55789	3.436037	-4.26006	3.32E-05	0.000946	2.106994
AC136604.2	-0.68706	6.675574	-4.25442	3.40E-05	0.000964	1.628326
AC027237.4	-2.7356	2.804088	-4.24442	3.54E-05	0.000993	2.022238
AC011816.2	-2.45265	3.390944	-4.2445	3.54E-05	0.000993	1.959586
AL118508.2	5.156978	2.660821	4.245184	3.53E-05	0.000993	1.51178
AC022031.1	7.447069	-0.64945	4.236632	3.65E-05	0.00102	1.407166
AL359715.4	-1.97456	6.133047	-4.23307	3.71E-05	0.001031	1.476623
AC091825.1	-0.81686	6.21635	-4.22397	3.85E-05	0.001066	1.585928
PACRG-AS1	-4.36654	-1.35478	-4.21713	3.95E-05	0.001092	1.848699
AC084026.2	-3.58886	2.375736	-4.20968	4.07E-05	0.001117	1.897222
Z69706.1	-1.08191	6.51691	-4.21008	4.07E-05	0.001117	1.43138
AL512383.1	-3.66625	1.259974	-4.20691	4.12E-05	0.001121	1.945862
GSN-AS1	-0.91892	6.199081	-4.20731	4.11E-05	0.001121	1.497897
AC010776.2	-5.24834	-1.73631	-4.20318	4.18E-05	0.001134	1.803398
AC024075.3	-0.86532	8.708365	-4.19789	4.27E-05	0.001154	1.095267
SEMA3F-AS1	-0.67997	6.981647	-4.19579	4.31E-05	0.001157	1.354814
AC011472.4	-0.90377	6.901501	-4.1954	4.31E-05	0.001157	1.331509
LINC01625	-4.70759	-1.56011	-4.19256	4.36E-05	0.001166	1.763974
LINC01873	5.348645	4.002382	4.189328	4.42E-05	0.001177	1.432742
AL137060.1	2.605031	4.718728	4.188196	4.44E-05	0.001179	1.732967
AC013391.2	7.668755	1.928842	4.185584	4.49E-05	0.001187	1.271018
AC245100.6	6.722839	1.509369	4.18115	4.57E-05	0.001204	1.253217
AP001107.5	-1.98764	4.426909	-4.16336	4.90E-05	0.001288	1.507203
AC100823.1	-4.49629	0.55731	-4.15896	4.99E-05	0.001306	1.773379
AC022173.1	-2.5046	3.036243	-4.15708	5.03E-05	0.00131	1.671379
AC012181.2	-1.146	7.160354	-4.15648	5.04E-05	0.00131	1.12594

STK32A-AS1	-4.90924	-0.94724	-4.15367	5.10E-05	0.001316	1.705212
AC008121.2	-2.91853	2.512418	-4.15411	5.09E-05	0.001316	1.702012
AC010632.1	-3.47333	1.482784	-4.14695	5.23E-05	0.001347	1.720449
AC022893.1	-0.9126	7.054372	-4.13996	5.38E-05	0.00138	1.102618
AC104958.1	-2.89341	1.758288	-4.13576	5.47E-05	0.001398	1.683988
AC022211.3	1.398178	8.92256	4.133002	5.53E-05	0.001409	1.099009
AC004817.3	5.951601	3.229931	4.130695	5.58E-05	0.001414	1.156841
AC102945.2	-1.40777	7.59014	-4.13038	5.59E-05	0.001414	0.923542
AC010761.2	0.830064	8.817872	4.123999	5.73E-05	0.001445	1.001
AC009102.2	-3.88233	0.184303	-4.11261	6.00E-05	0.001507	1.586995
MELTF-AS1	1.876807	9.162519	4.109671	6.07E-05	0.001519	1.042585
LINC01623	5.543451	-0.61197	4.106059	6.15E-05	0.001536	1.015779
AL390208.1	-1.03941	6.802645	-4.10437	6.19E-05	0.001541	0.990462
AC005520.2	-1.14453	7.91098	-4.09657	6.39E-05	0.001584	0.794638
AC112236.2	-1.07139	5.951862	-4.08953	6.57E-05	0.001617	1.091542
EPB41L4A-DT	-1.27971	6.898056	-4.08963	6.57E-05	0.001617	0.892114
AC018926.3	-1.3991	7.077337	-4.08878	6.59E-05	0.001617	0.858195
AL138960.1	-4.3704	0.105055	-4.07556	6.94E-05	0.001687	1.475965
AC004948.1	-2.50621	3.268986	-4.0757	6.93E-05	0.001687	1.334673
AC091588.3	-2.50854	5.293709	-4.07637	6.92E-05	0.001687	0.981664
LINC02331	-4.9977	-1.10739	-4.0718	7.04E-05	0.001707	1.430093
AC002525.1	-2.13956	3.766268	-4.06943	7.11E-05	0.001717	1.295116
AC083843.4	-1.10853	6.29051	-4.06822	7.14E-05	0.00172	0.934887
AC015912.3	1.515331	9.415593	4.06538	7.22E-05	0.001733	0.781817
AL023802.1	5.25505	0.010709	4.054622	7.53E-05	0.001802	0.867067
AC020779.2	-3.78855	1.466873	-4.05363	7.56E-05	0.001803	1.393047
AC010261.1	-2.87163	2.606102	-4.05047	7.65E-05	0.00182	1.303413
AC099850.1	1.152181	7.173591	4.048264	7.72E-05	0.001824	1.072423
TMPO-AS1	1.586814	8.460835	4.048786	7.71E-05	0.001824	0.895714
AC007792.1	-2.44102	3.56242	-4.036	8.10E-05	0.001908	1.157931
AC007671.1	-3.55486	0.982378	-4.03036	8.28E-05	0.001944	1.320295
AC012409.2	-4.58401	-0.57893	-4.02588	8.42E-05	0.001972	1.2678
LINC01124	-2.3415	4.686878	-4.02177	8.56E-05	0.001997	0.91297
LINC01633	9.075744	3.937633	4.021053	8.58E-05	0.001997	0.801101
AC009630.2	5.688099	2.61129	4.009003	9.00E-05	0.002086	0.771969
AC079305.3	-4.13387	1.987514	-4.00247	9.23E-05	0.002133	1.048953
AC011511.5	3.324465	4.015485	3.990924	9.65E-05	0.002223	0.945218
AC104825.1	-1.1861	8.033107	-3.98719	9.79E-05	0.002249	0.358119
SLC12A9-AS1	1.385426	8.521312	3.985214	9.86E-05	0.002259	0.609461
AC090950.2	-1.47465	4.624759	-3.98376	9.92E-05	0.002265	0.903747
AC018445.4	-1.46247	3.933434	-3.98266	9.96E-05	0.002268	1.026429
MIR181A2HG	1.452709	7.224359	3.980622	0.0001	0.002279	0.869691
AC091059.2	-2.73305	2.750248	-3.96901	0.000105	0.002376	1.02187
AL357033.4	-1.43556	8.346929	-3.96435	0.000107	0.002412	0.212365
AC114980.1	-0.71505	5.967397	-3.96169	0.000108	0.002429	0.663616
AL121832.2	1.185692	9.333894	3.953069	0.000112	0.002503	0.349914
AC073611.2	1.000494	7.64709	3.948655	0.000114	0.002531	0.581749
CEBPB-AS1	-0.82811	6.10207	-3.94909	0.000113	0.002531	0.574901
AL359513.1	3.384857	7.63273	3.944025	0.000116	0.002569	0.966846
AC008676.1	-1.1813	4.578003	-3.93805	0.000118	0.002613	0.819729
AL162171.3	-1.23564	5.269145	-3.93874	0.000118	0.002613	0.625557
AC012531.1	7.4276	5.638803	3.936687	0.000119	0.002619	0.652123
AC093520.1	7.045308	2.740782	3.93437	0.00012	0.002634	0.539334
AC015914.1	-1.32181	6.540625	-3.93254	0.000121	0.002645	0.376242
AC096888.1	7.137599	-0.95204	3.931507	0.000121	0.002648	0.507351
LINC02060	-3.96022	-0.20958	-3.92463	0.000124	0.002703	0.931552
AC023830.3	-1.30856	4.651028	-3.92529	0.000124	0.002703	0.722504
AC103591.3	1.521076	8.6274	3.922766	0.000125	0.002714	0.380442

AC017002.3	5.792775	5.116126	3.920252	0.000127	0.002732	0.652705
FAM155A-IT1	-3.56533	0.773874	-3.9186	0.000127	0.002735	0.939231
AC034213.1	8.83054	2.743336	3.918528	0.000127	0.002735	0.493833
AC020658.4	6.876784	4.680838	3.916522	0.000128	0.002748	0.562616
AL079305.1	6.545327	0.487226	3.913938	0.00013	0.002759	0.463091
AP000808.1	-1.70331	5.739971	-3.91447	0.000129	0.002759	0.413018
AL590302.2	-4.31356	-0.51102	-3.91094	0.000131	0.002783	0.895604
AC130650.1	-1.26153	6.144332	-3.90363	0.000135	0.002853	0.324559
AP002449.1	1.34207	7.804372	3.901996	0.000136	0.002863	0.44108
AL049555.1	2.084884	11.53874	3.898438	0.000138	0.002894	-0.04404
AC068880.4	-2.3097	3.612434	-3.89602	0.000139	0.002904	0.656244
AC022144.1	1.79435	7.621504	3.896403	0.000139	0.002904	0.542544
AC009090.3	-1.19543	7.103024	-3.89451	0.00014	0.002913	0.162976
AC108449.2	-0.88428	9.444324	-3.89094	0.000141	0.002945	-0.14582
LINC01943	3.517715	6.617603	3.88147	0.000147	0.003044	0.804267
TNRC6C-AS1	1.317687	8.182394	3.880375	0.000147	0.003048	0.281764
AC104211.2	-4.64523	-1.55133	-3.8756	0.00015	0.003095	0.717985
AC007950.3	-1.63024	3.198095	-3.87324	0.000151	0.003114	0.752187
AC109454.4	-1.16425	5.834428	-3.86932	0.000154	0.003152	0.292363
AC006252.1	2.315032	5.758825	3.86831	0.000154	0.003155	0.765091
AC090952.1	-4.44652	-0.42895	-3.86441	0.000156	0.003194	0.749478
AL023803.2	3.183511	7.330257	3.863095	0.000157	0.003201	0.729444
U62317.2	1.3597	10.14687	3.854524	0.000162	0.003297	-0.10566
AC007342.6	-0.64603	5.784455	-3.85266	0.000164	0.003311	0.321105
AC104117.3	-2.09624	4.254823	-3.84873	0.000166	0.003352	0.393446
LINC02574	7.714239	1.600343	3.84585	0.000168	0.00337	0.277584
DIAPH3-AS2	7.371914	1.515551	3.846016	0.000168	0.00337	0.277279
AP001486.2	-0.82993	7.229658	-3.84365	0.000169	0.003389	0.001386
AC024619.3	-2.70589	3.063548	-3.83818	0.000173	0.00345	0.495399
AC007611.1	3.5149	5.385126	3.836099	0.000174	0.003468	0.565374
AL117382.2	-4.18283	1.639612	-3.83334	0.000176	0.003495	0.597248
AC239803.2	-3.21004	1.559911	-3.82577	0.000181	0.003576	0.606586
AC020917.4	-0.66543	7.82366	-3.82604	0.000181	0.003576	-0.12845
AC139530.2	0.842532	8.641459	3.815813	0.000188	0.003702	-0.09265
VPS9D1-AS1	1.678187	10.76669	3.814416	0.000189	0.003712	-0.29021
AL031726.1	-4.59695	-0.55126	-3.80971	0.000192	0.003768	0.565622
LOH12CR2	-0.85228	6.461707	-3.80896	0.000193	0.003769	0.011907
AF131215.4	-1.77863	4.046025	-3.80681	0.000194	0.003789	0.336212
LINC02029	5.729561	1.299685	3.80517	0.000195	0.003803	0.160816
Z94721.1	1.439146	7.89611	3.80411	0.000196	0.003808	0.108335
AC008543.4	-2.10595	3.880515	-3.79712	0.000201	0.003885	0.287939
HPN-AS1	-2.21665	4.196168	-3.79582	0.000202	0.003885	0.216159
AL132657.1	-1.37834	5.360299	-3.79464	0.000203	0.003885	0.092367
AC016738.1	-1.16298	5.727956	-3.79463	0.000203	0.003885	0.039093
AP002498.1	-1.94555	5.910017	-3.79658	0.000202	0.003885	-0.08371
AL035461.2	1.215472	8.74974	3.797836	0.000201	0.003885	-0.11316
AC010136.1	-2.93301	2.978576	-3.79231	0.000205	0.003898	0.379456
AL391244.2	0.90685	8.51224	3.792626	0.000205	0.003898	-0.14019
ZRANB2-AS1	-2.03379	3.61567	-3.78203	0.000213	0.00404	0.320209
AL139383.1	-1.71287	4.876312	-3.78109	0.000214	0.004044	0.104561
Z83844.2	2.265416	5.36338	3.775402	0.000218	0.00412	0.456534
AC112721.2	8.973448	3.120023	3.772873	0.00022	0.004148	0.085579
AC016924.1	-3.20212	2.032844	-3.7718	0.000221	0.004154	0.404961
AL138759.1	6.054767	4.424441	3.770472	0.000222	0.004164	0.174513
AC233266.2	-2.77398	3.190554	-3.76938	0.000223	0.004171	0.214574
AL670729.1	2.261242	7.515554	3.767002	0.000225	0.004187	0.219657
AC008764.2	-0.89207	9.020162	-3.767	0.000225	0.004187	-0.52775
AC007114.2	-1.07242	5.355808	-3.76552	0.000226	0.0042	0.058779

AC004947.1	-3.3644	0.24437	-3.76142	0.00023	0.004253	0.416651
AL357033.3	-1.44253	7.506955	-3.75883	0.000232	0.004284	-0.39872
AC116337.3	-4.19772	-0.00133	-3.75184	0.000238	0.004385	0.380584
AL359317.2	-1.10545	4.017533	-3.74897	0.000241	0.004421	0.276739
AC013275.1	-4.29371	0.051005	-3.74644	0.000243	0.004451	0.3717
MIR181A1HG	4.301548	1.627543	3.743498	0.000246	0.004489	0.016806
AC010226.1	-0.71021	6.775241	-3.73352	0.000255	0.004646	-0.28927
AP001630.1	-3.60895	1.165361	-3.73284	0.000255	0.004646	0.321021
DDX11-AS1	1.92235	6.331077	3.731958	0.000256	0.004649	0.263962
AC135050.6	-0.62418	10.76753	-3.73134	0.000257	0.004649	-0.8621
XXYLT1-AS2	-1.82082	5.613868	-3.72966	0.000258	0.004667	-0.23256
AP005131.4	-1.71306	4.953986	-3.72746	0.000261	0.004693	-0.10774
AL135960.1	-1.97488	1.963939	-3.72637	0.000262	0.004701	0.307657
LINC00322	6.620958	0.827765	3.725471	0.000262	0.004705	-0.06195
AC095057.3	-0.85704	7.06341	-3.72294	0.000265	0.004738	-0.397
AC015961.1	-2.2748	2.543964	-3.72055	0.000267	0.004768	0.237973
PART1	-1.62521	5.381244	-3.71794	0.00027	0.004803	-0.20063
AC122129.1	-0.83875	7.769086	-3.71695	0.000271	0.004809	-0.52096
C9orf106	-1.9579	3.297378	-3.71076	0.000277	0.004898	0.200244
AC007938.1	4.772123	1.481235	3.710596	0.000277	0.004898	-0.09088
AC025627.1	6.774218	1.775297	3.708746	0.000279	0.004898	-0.10208
KIF9-AS1	-0.65263	5.976447	-3.7087	0.000279	0.004898	-0.20894
AL356273.3	-0.95903	6.701606	-3.70896	0.000279	0.004898	-0.39402
AL590683.1	5.521883	0.53031	3.704652	0.000283	0.00496	-0.12056
AC004241.5	-0.84653	5.110401	-3.69875	0.000289	0.005056	-0.0816
AC005884.1	-2.45726	3.146495	-3.68922	0.0003	0.005223	0.028148
AC107072.2	-2.19175	4.204859	-3.68604	0.000303	0.00526	-0.15211
AC004241.3	1.181827	7.78053	3.686463	0.000303	0.00526	-0.33557
AL117332.1	1.163847	7.17588	3.685322	0.000304	0.005261	-0.23231
AC022211.2	1.42339	7.464544	3.683774	0.000306	0.005279	-0.23682
AC078925.3	-4.16019	-2.51408	-3.67819	0.000312	0.005375	0.009367
AC069029.1	-2.54662	3.735615	-3.67551	0.000315	0.005415	-0.15026
AC010997.6	-1.96979	2.987146	-3.67468	0.000316	0.005419	0.07262
AL161891.1	1.371093	6.966042	3.671289	0.00032	0.005474	-0.1598
LDLRAD4-AS1	-2.21384	2.675233	-3.66874	0.000323	0.005512	0.06025
AC022211.1	1.379091	8.724795	3.667264	0.000325	0.005529	-0.53944
AP000873.3	4.272797	4.044038	3.659651	0.000334	0.005671	-0.09164
AL353194.1	0.830308	8.745347	3.656406	0.000338	0.005725	-0.65465
AP000897.1	-4.0427	-1.42275	-3.65393	0.000341	0.005764	0.01478
AC087071.1	-2.44163	3.030314	-3.64854	0.000347	0.005864	-0.09619
AC048344.4	2.982348	6.852266	3.647144	0.000349	0.005879	0.02486
AC006378.2	-1.11766	4.656024	-3.64658	0.00035	0.005879	-0.22655
LINC01954	-4.15366	0.557534	-3.64471	0.000352	0.005906	0.048756
AC020978.4	-1.18698	7.819642	-3.64198	0.000356	0.005951	-0.81916
SCAT2	1.070877	7.922613	3.639123	0.000359	0.006	-0.53952
AC092614.1	2.166305	6.341172	3.636442	0.000363	0.006044	-0.01421
RBM5-AS1	-0.68503	6.317644	-3.63447	0.000365	0.006063	-0.54382
AC010442.1	-1.49624	11.69816	-3.63438	0.000366	0.006063	-1.35441
AL133338.1	1.081896	7.715042	3.63201	0.000369	0.006101	-0.51889
AP002884.4	-2.74643	1.405587	-3.62467	0.000379	0.006238	-0.01901
AC012676.1	1.065039	6.4863	3.624641	0.000379	0.006238	-0.2702
AC008073.2	-2.5474	1.790477	-3.62043	0.000384	0.006319	-0.03827
AL157932.1	1.191207	7.707992	3.619042	0.000386	0.006337	-0.55629
AP001831.1	-4.06664	-0.12073	-3.6179	0.000388	0.006349	-0.03454
AC090152.1	-1.35151	6.810324	-3.61717	0.000389	0.006352	-0.76456
AC113346.1	9.490673	2.399415	3.613009	0.000395	0.006434	-0.35593
CEP83-DT	2.164211	5.031223	3.607494	0.000403	0.006548	-0.08065
AC016683.1	-1.65777	4.252225	-3.6065	0.000404	0.006557	-0.33214

KMT2E-AS1	0.915344	9.796276	3.604141	0.000408	0.006599	-0.97346
AC020951.1	-1.27328	4.722792	-3.59826	0.000416	0.006711	-0.45007
AP003721.4	-1.39457	4.950277	-3.59824	0.000416	0.006711	-0.48036
AC090844.2	-1.84518	4.469654	-3.59701	0.000418	0.006726	-0.4642
AC007673.1	-2.51042	2.590813	-3.59118	0.000427	0.006853	-0.21989
AP003352.1	0.816115	9.965898	3.589731	0.000429	0.006874	-1.06501
RBM12B-AS1	0.939328	8.342044	3.583746	0.000438	0.007007	-0.82148
AL355802.2	1.421179	8.033954	3.58268	0.00044	0.007019	-0.70344
AC106795.3	-3.1481	1.875671	-3.57878	0.000446	0.007102	-0.23092
AC002401.2	-2.37426	3.72183	-3.56814	0.000463	0.007345	-0.48364
AC010210.1	-1.08956	5.036928	-3.56844	0.000463	0.007345	-0.52744
AP001453.2	2.247723	9.49337	3.567376	0.000465	0.00735	-0.89724
AC048380.1	-2.75542	2.26802	-3.55888	0.000479	0.007559	-0.29469
HID1-AS1	-1.67103	4.580129	-3.55809	0.00048	0.007564	-0.57466
LINC01981	6.049835	0.911963	3.555634	0.000484	0.007615	-0.51822
FER1L6-AS1	-4.05193	-0.37338	-3.55275	0.000489	0.007635	-0.24075
AC134026.1	-4.52367	-1.34965	-3.55319	0.000489	0.007635	-0.25473
AC009806.1	-1.87491	4.256948	-3.55253	0.00049	0.007635	-0.577
AF001548.1	-1.991	6.259778	-3.55365	0.000488	0.007635	-0.98208
AC012183.1	-2.5753	2.873529	-3.54933	0.000495	0.007686	-0.39797
AC068491.2	5.87599	4.020792	3.549915	0.000494	0.007686	-0.45599
MAGI1-IT1	-1.12727	4.645618	-3.54891	0.000496	0.007686	-0.52841
AC007224.2	-1.75193	4.280101	-3.54709	0.000499	0.007709	-0.59624
CD44-AS1	2.183826	9.20127	3.546879	0.0005	0.007709	-0.91078
ZEB1-AS1	1.281274	8.387347	3.546177	0.000501	0.007713	-0.90255
LINC00115	0.912942	7.11348	3.542361	0.000508	0.007802	-0.73515
AC007688.2	-2.48411	3.089629	-3.53466	0.000522	0.007984	-0.48234
AC022973.4	1.347629	6.814842	3.534752	0.000522	0.007984	-0.60295
AC097382.2	-1.14449	2.657788	-3.53328	0.000524	0.007991	-0.29789
AC008663.2	-1.25084	3.589135	-3.5333	0.000524	0.007991	-0.37635
HOXC-AS2	6.334638	6.365512	3.531446	0.000528	0.008006	-0.42967
AC004551.1	5.04404	4.640829	3.532053	0.000527	0.008006	-0.45121
AL596325.2	-0.92139	8.463082	-3.53103	0.000529	0.008006	-1.25051
AC084117.1	2.089519	7.938105	3.529917	0.000531	0.008021	-0.70132
AC026362.2	-0.79799	4.538359	-3.52553	0.000539	0.00813	-0.53356
AC110774.1	-2.46055	2.791797	-3.52316	0.000543	0.008181	-0.4245
AL109767.1	6.482533	2.7092	3.522258	0.000545	0.008191	-0.59324
AC124303.1	-3.66943	-0.57465	-3.52063	0.000548	0.008197	-0.35172
HEXA-AS1	-0.9273	4.3644	-3.51977	0.00055	0.008197	-0.51128
AC010331.1	1.13408	6.95164	3.520037	0.000549	0.008197	-0.71051
AC005746.1	-1.22991	5.486665	-3.52066	0.000548	0.008197	-0.8486
AL049539.1	3.29825	6.513317	3.515764	0.000558	0.008297	-0.34746
AC006026.3	-2.79642	5.384539	-3.51304	0.000563	0.008361	-1.05787
AC027228.2	2.709665	6.873521	3.510725	0.000568	0.008412	-0.42658
Z99774.1	4.984338	-1.29903	3.506742	0.000576	0.008514	-0.6533
AL359715.3	-0.79684	7.600551	-3.50542	0.000578	0.008537	-1.19204
AC017076.1	6.783203	3.19779	3.50355	0.000582	0.008576	-0.63722
PRICKLE2-AS3	-1.94149	2.316294	-3.50284	0.000584	0.008581	-0.40567
AC019069.1	1.800674	8.012744	3.500681	0.000588	0.008629	-0.90034
AL109924.4	-4.02149	0.064096	-3.49812	0.000593	0.00869	-0.4001
AC022893.2	-3.26286	0.879336	-3.49717	0.000595	0.008702	-0.40425
AC007598.3	-0.71442	5.128003	-3.49578	0.000598	0.008727	-0.73435
AC010201.2	-0.78769	5.907358	-3.49491	0.0006	0.008737	-0.94165
AC079354.2	7.856312	3.102435	3.494077	0.000602	0.008746	-0.66675
AC007849.1	-2.10882	7.585049	-3.4856	0.00062	0.008991	-1.41288
AC011468.3	-2.78996	2.181472	-3.48403	0.000623	0.009023	-0.54578
LINC02585	1.929369	7.843505	3.482852	0.000626	0.009043	-0.86603
AL590326.1	1.069717	7.611976	3.480642	0.000631	0.009096	-0.99479

HMMR-AS1	5.314595	1.737312	3.479632	0.000633	0.00911	-0.70975
AC016251.2	-3.88421	-1.29702	-3.47793	0.000637	0.009147	-0.51316
JARID2-AS1	2.836323	5.754049	3.471302	0.000652	0.009343	-0.48056
BX537318.2	2.171395	2.893594	3.469938	0.000655	0.009362	-0.59158
AC006960.3	-2.38326	3.018717	-3.46963	0.000655	0.009362	-0.68582
AP001189.1	-1.4077	4.970878	-3.46897	0.000657	0.009365	-0.93131
AC231657.1	-1.13457	6.126889	-3.4676	0.00066	0.009393	-1.11678
AL391097.1	-2.63055	2.025069	-3.46644	0.000663	0.009413	-0.54388
AC107072.1	-4.27523	0.956098	-3.46533	0.000665	0.009431	-0.52123
AC008114.1	8.101037	4.88342	3.464336	0.000668	0.009446	-0.71963
ERVE-1	-1.65905	5.571447	-3.46294	0.000671	0.009474	-1.062
AL133384.2	-2.70947	0.873169	-3.45571	0.000688	0.009697	-0.52693
AP001021.2	-3.06095	1.850475	-3.45509	0.000689	0.0097	-0.61378
AC073167.1	-2.49368	2.407686	-3.45453	0.000691	0.009701	-0.62471
AC015961.2	-2.35162	2.846826	-3.45397	0.000692	0.009701	-0.67528
AC092140.2	-0.77715	6.249552	-3.45046	0.000701	0.009802	-1.15789
AC083799.1	0.821103	10.92056	3.44801	0.000707	0.009867	-1.65692
SSBP3-AS1	-0.87071	7.03808	-3.44648	0.00071	0.009901	-1.31722
LNX1-AS2	-2.26003	3.142474	-3.44571	0.000712	0.009909	-0.73533
AC112196.1	-3.83482	0.171184	-3.44365	0.000717	0.009961	-0.56249
Z97989.1	-0.62887	6.426724	-3.43894	0.000729	0.010107	-1.19228
AC064807.2	-2.12133	4.446601	-3.43546	0.000738	0.01021	-0.94807
AL590226.1	-1.95327	3.9496	-3.43264	0.000745	0.010272	-0.89003
AC116407.2	1.210793	7.715121	3.432777	0.000745	0.010272	-1.14684
AC010551.2	-4.43855	-1.25206	-3.42091	0.000776	0.010667	-0.64468
AC068014.1	6.319746	0.651121	3.420637	0.000777	0.010667	-0.86922
LGALS8-AS1	2.025219	4.369838	3.418629	0.000782	0.010722	-0.65288
AL450992.1	2.713437	4.179703	3.417286	0.000786	0.010752	-0.68971
PYCARD-AS1	3.558049	4.108084	3.41566	0.00079	0.010793	-0.73974
AC084036.1	-0.90178	8.956345	-3.41496	0.000792	0.010799	-1.70022
Z97653.1	-1.68874	5.116271	-3.41182	0.000801	0.010897	-1.22352
AL590666.3	4.05727	0.503164	3.410516	0.000804	0.010906	-0.89326
AC079322.1	-0.65446	6.037941	-3.41074	0.000804	0.010906	-1.21974
AC091043.1	-4.41264	1.445481	-3.40933	0.000807	0.010931	-0.72472
AL590093.1	-1.59122	5.53718	-3.40622	0.000816	0.011029	-1.28146
LINC00853	3.918052	6.85171	3.399508	0.000835	0.011265	-0.69216
AC022898.2	-4.00701	-0.88051	-3.39897	0.000837	0.011266	-0.70862
GASAL1	2.341002	7.385601	3.395244	0.000847	0.01139	-0.9211
AC068491.4	1.805233	5.938585	3.394292	0.00085	0.011407	-0.77039
DCXR-DT	-1.89473	3.844994	-3.39269	0.000855	0.011449	-0.95512
AC080112.1	1.015116	9.886078	3.390987	0.00086	0.011496	-1.67704
AC009303.4	1.14156	7.295902	3.387444	0.00087	0.011615	-1.21698
AC131182.1	-2.14008	3.09874	-3.38541	0.000876	0.011676	-0.89492
AL445223.1	-1.86987	3.966119	-3.38454	0.000879	0.01169	-1.02823
LINC02038	-2.75235	4.972966	-3.38338	0.000883	0.011715	-1.35605
AL118516.1	1.006537	9.605979	3.380544	0.000891	0.011808	-1.66735
SRGAP3-AS4	-4.32027	-0.52483	-3.37875	0.000897	0.011858	-0.75414
AC144831.1	-1.2436	6.682743	-3.37829	0.000898	0.011858	-1.52954
BX470102.1	1.755019	9.220646	3.376148	0.000905	0.011924	-1.51392
NEXN-AS1	-0.75105	5.328868	-3.37359	0.000913	0.011975	-1.17085
UBE2Q1-AS1	1.107311	7.117147	3.373353	0.000913	0.011975	-1.23905
AC106820.3	0.960882	7.600003	3.374027	0.000911	0.011975	-1.35487
AC068473.4	7.186791	1.260149	3.372365	0.000916	0.011986	-0.98903
AC008105.1	1.522098	6.798296	3.37207	0.000917	0.011986	-1.07798
AL356417.2	6.511592	2.313558	3.366487	0.000935	0.012195	-0.99816
LINC01675	5.998516	1.124678	3.365913	0.000937	0.012197	-1.00617
AC079209.1	5.50675	2.29611	3.364521	0.000941	0.012234	-0.99524
LINC01711	5.865005	4.065856	3.362497	0.000948	0.012272	-0.95285

AC129507.3	-2.54279	3.830351	-3.36219	0.000949	0.012272	-1.17343
AL391834.1	-0.73219	8.591384	-3.3621	0.000949	0.012272	-1.79691
BLACE	4.51136	0.205183	3.360439	0.000954	0.012299	-1.02309
AL132639.3	1.032429	6.465742	3.360454	0.000954	0.012299	-1.11281
AL021408.1	-3.25972	1.556277	-3.35895	0.000959	0.012321	-0.85831
AC135279.3	1.183683	5.483568	3.358892	0.000959	0.012321	-0.89002
AC008434.1	2.542071	5.094901	3.349816	0.000989	0.012663	-0.84063
AC091588.1	-3.19444	3.632052	-3.34998	0.000989	0.012663	-1.17801
AL139081.1	-3.23676	2.195815	-3.34597	0.001002	0.012786	-0.95352
Z73965.1	-0.71444	5.330772	-3.34599	0.001002	0.012786	-1.2805
AL109923.1	1.793672	5.586917	3.341989	0.001016	0.012938	-0.88775
ZNF674-AS1	0.720611	8.504908	3.336302	0.001036	0.013167	-1.68588
AC067931.1	1.83037	7.185747	3.333269	0.001046	0.01328	-1.20841
AC016065.1	0.987551	8.591327	3.329521	0.00106	0.013426	-1.67635
LINC01311	1.128964	6.665867	3.328062	0.001065	0.01347	-1.26927
AC007938.2	1.713183	4.191342	3.323849	0.00108	0.013636	-0.91289
AL583856.2	-1.21466	5.63473	-3.32343	0.001082	0.013636	-1.49139
AC079922.2	0.951831	6.785191	3.318596	0.0011	0.013837	-1.34361
AC139768.2	-0.74193	7.360036	-3.31595	0.001109	0.013937	-1.75921
AC011473.3	-3.1723	3.050357	-3.31319	0.00112	0.014044	-1.17931
AC002546.1	-3.58822	0.864245	-3.31226	0.001123	0.014054	-0.96266
AL136982.3	-0.77914	6.28412	-3.31198	0.001124	0.014054	-1.58967
AL035588.1	-2.35092	2.898173	-3.31046	0.00113	0.014103	-1.14465
AC053503.2	4.104813	3.630145	3.305009	0.001151	0.01434	-1.0667
AC127521.1	-1.38216	5.959773	-3.30412	0.001154	0.014359	-1.63924
AL139230.1	-4.39529	-1.32437	-3.30164	0.001164	0.014455	-0.98576
AC036176.1	-0.77189	7.730318	-3.30021	0.00117	0.014501	-1.87232
AC096733.2	-0.901	7.401205	-3.29739	0.001181	0.014614	-1.84615
LINC02043	4.071484	4.535545	3.295002	0.00119	0.014683	-1.06178
AL031847.1	-1.14347	6.494928	-3.29503	0.00119	0.014683	-1.73484
AC139795.2	-0.88531	8.70441	-3.29437	0.001193	0.01469	-2.04026
MIR4713HG	3.263055	7.462912	3.286571	0.001224	0.015054	-1.12962
AC068189.1	2.975839	4.945216	3.283092	0.001239	0.015205	-1.04022
AC106712.1	5.336463	0.052293	3.277823	0.001261	0.015424	-1.22862
AL118505.1	1.59832	8.044431	3.278036	0.00126	0.015424	-1.64251
AC053513.1	-0.89156	6.832465	-3.27564	0.00127	0.015512	-1.81845
AC093458.1	-3.78063	0.68401	-3.27357	0.001279	0.015591	-1.07564
AC138956.1	-0.71388	7.686627	-3.27313	0.00128	0.015591	-1.93822
U62317.1	3.178565	8.815839	3.272522	0.001283	0.015598	-1.48189
LINC01281	5.398151	1.301694	3.26758	0.001304	0.015808	-1.24929
AC002398.1	0.76271	8.794508	3.267534	0.001305	0.015808	-1.93604
LINC01508	7.071435	1.11111	3.265809	0.001312	0.015873	-1.25469
AL353795.3	-0.85938	4.371881	-3.26489	0.001316	0.015875	-1.30034
AC046158.4	-1.48526	4.275149	-3.26481	0.001316	0.015875	-1.41294
AC009097.2	6.301639	5.317839	3.262436	0.001327	0.015975	-1.17717
AL035458.2	1.925444	7.110885	3.260308	0.001336	0.016063	-1.38568
Z99289.2	4.560465	2.060456	3.258905	0.001342	0.016112	-1.24626
AC007342.7	-0.57032	7.125882	-3.25812	0.001346	0.016128	-1.8848
AC005264.1	2.556698	5.203739	3.256957	0.001351	0.016158	-1.09794
AC138207.4	0.813523	8.004461	3.256135	0.001355	0.016158	-1.83269
AC074117.1	0.725947	9.406017	3.256353	0.001354	0.016158	-2.05496
OGFR-AS1	1.649941	6.552397	3.255461	0.001358	0.016168	-1.30681
AP001469.3	0.865796	7.752641	3.253598	0.001366	0.016243	-1.78681
MIR222HG	1.179478	8.626697	3.252528	0.001371	0.016275	-1.89608
AC010329.1	-2.56792	1.669857	-3.25136	0.001377	0.016312	-1.13838
AC007787.1	-2.25435	1.941089	-3.24758	0.001394	0.016492	-1.16457
AC009090.1	-0.69166	8.267679	-3.24666	0.001398	0.016516	-2.09899
LINC01385	6.016844	-1.08597	3.245634	0.001403	0.016546	-1.30968

LINC00891	-2.93949	-0.05104	-3.24465	0.001407	0.016566	-1.13496
AC010201.1	-1.19428	5.322126	-3.24433	0.001409	0.016566	-1.66499
AC011199.1	3.958998	0.467612	3.243275	0.001414	0.016598	-1.3097
LINC02416	6.050123	5.844849	3.239868	0.00143	0.016712	-1.21703
AL031667.3	1.312116	7.139771	3.240598	0.001426	0.016712	-1.62098
AC036108.3	-0.92917	6.199635	-3.2398	0.00143	0.016712	-1.83397
AC134407.2	-0.56463	6.210839	-3.23489	0.001454	0.016959	-1.78063
AP005136.2	0.971138	6.926436	3.234243	0.001457	0.016969	-1.61013
TFAP2A-AS1	3.135467	6.624366	3.233113	0.001462	0.017006	-1.18782
ZBTB20-AS3	-2.90283	2.108153	-3.22973	0.001479	0.01717	-1.26474
ZBTB20-AS5	-2.13912	2.279482	-3.22841	0.001485	0.017218	-1.24225
AC097358.2	3.684861	3.90406	3.227178	0.001491	0.017262	-1.24684
LINC02108	5.337719	-0.60129	3.22669	0.001494	0.017263	-1.35477
AC074032.1	-0.49381	7.897658	-3.22401	0.001507	0.01739	-2.08802
AL162431.2	1.198077	6.655136	3.222789	0.001513	0.017425	-1.56113
AC027117.2	-1.56566	6.421275	-3.22247	0.001514	0.017425	-2.00113
AL356489.2	-2.04908	2.841758	-3.22024	0.001526	0.017499	-1.37624
AC026356.2	0.956482	7.199441	3.220482	0.001524	0.017499	-1.73612
AP003469.2	2.776118	7.454633	3.21953	0.001529	0.017513	-1.32297
AC007405.2	6.04632	1.139218	3.211098	0.001572	0.017978	-1.38806
AC096637.2	3.954983	2.666936	3.206695	0.001595	0.018213	-1.34142
LINC02601	5.065577	2.791584	3.203729	0.001611	0.018307	-1.37107
AC008764.8	0.633722	6.153681	3.202959	0.001615	0.018307	-1.61318
AL928654.4	0.939948	8.276275	3.202811	0.001616	0.018307	-2.02023
CDC37L1-DT	-0.87378	7.263774	-3.20378	0.00161	0.018307	-2.10541
NPTN-IT1	-0.54543	8.398846	-3.20419	0.001608	0.018307	-2.23473
AC004812.2	0.852924	8.306995	3.200689	0.001627	0.018407	-2.04566
LINC02261	4.698464	-0.64133	3.1993	0.001634	0.018463	-1.42102
AL161668.4	-3.31693	0.890964	-3.19811	0.001641	0.01848	-1.28917
AL357497.1	-2.73848	1.904713	-3.19853	0.001638	0.01848	-1.33476
AL021368.1	-2.44098	2.521908	-3.19727	0.001645	0.018484	-1.42795
L3MBTL4-AS1	-1.21632	5.317565	-3.19712	0.001646	0.018484	-1.80049
PHACTR2-AS1	-1.12422	5.836196	-3.19659	0.001649	0.018488	-1.89633
AC100800.1	-2.18052	1.898742	-3.19279	0.001669	0.018677	-1.30137
AC125437.2	-0.8916	4.16468	-3.19259	0.001671	0.018677	-1.49544
HNF4A-AS1	-3.34232	2.741336	-3.19118	0.001678	0.018735	-1.5023
RNF139-AS1	0.693822	6.704756	3.187945	0.001696	0.01889	-1.78771
AC004943.2	0.907491	7.963841	3.187754	0.001697	0.01889	-2.00631
STX17-AS1	-0.9106	7.650437	-3.18577	0.001708	0.018985	-2.22397
E2F3-IT1	6.249209	2.656427	3.180543	0.001738	0.019254	-1.45196
AC009145.4	-0.87745	5.663181	-3.18082	0.001736	0.019254	-1.87856
AL117379.1	0.850259	7.842503	3.177929	0.001752	0.01939	-2.02873
AC058791.1	-0.81458	6.483383	-3.17553	0.001766	0.019514	-2.05534
AC097532.2	3.880858	2.925962	3.172686	0.001783	0.019667	-1.41776
DEPDC1-AS1	3.887399	3.872578	3.168223	0.001809	0.019777	-1.40167
AC122710.2	7.048047	2.920361	3.169532	0.001801	0.019777	-1.47996
AP000526.1	6.649522	2.522115	3.167829	0.001811	0.019777	-1.48608
LINC01419	5.956392	-0.18588	3.17042	0.001796	0.019777	-1.48915
FNDC1-IT1	5.940458	-1.16314	3.168653	0.001806	0.019777	-1.49522
AC124312.2	-2.25007	4.6901	-3.17	0.001798	0.019777	-1.91108
DLGAP1-AS2	1.564438	8.651389	3.168162	0.001809	0.019777	-2.08053
AC100791.2	6.171896	1.495344	3.166541	0.001819	0.019831	-1.49388
AC104123.1	-1.43898	4.102031	-3.16504	0.001827	0.019899	-1.63824
AC117394.2	8.471974	4.927089	3.160643	0.001854	0.020156	-1.49202
ACTA2-AS1	-1.04188	6.831789	-3.15931	0.001862	0.020214	-2.2116
LINC01612	-3.85038	1.040826	-3.15863	0.001866	0.020229	-1.41843
AC018695.7	4.478608	-0.56363	3.156323	0.00188	0.020352	-1.52362
AC022387.1	-4.27517	-1.33189	-3.1547	0.00189	0.020371	-1.38409

LINC01594	-3.73582	1.638752	-3.15483	0.001889	0.020371	-1.45946
AC015982.1	0.790797	7.849424	3.1552	0.001887	0.020371	-2.10683
AC092118.2	1.808631	6.019056	3.15305	0.0019	0.020451	-1.52786
AC026369.2	2.710032	6.865039	3.151967	0.001907	0.020494	-1.48729
AC104078.1	4.48822	0.799544	3.151451	0.00191	0.020499	-1.53085
LINC00163	-3.17756	0.319402	-3.14989	0.00192	0.02052	-1.39946
AC063977.6	5.021667	1.135829	3.150552	0.001915	0.02052	-1.53231
AC100830.2	-1.05116	7.395258	-3.14981	0.00192	0.02052	-2.31469
AC008770.3	-1.20318	7.105822	-3.14687	0.001938	0.020687	-2.29325
AC024580.1	-0.89814	7.286229	-3.1456	0.001946	0.020742	-2.28172
AC090709.1	7.105772	2.336879	3.143428	0.00196	0.020859	-1.54557
LINC00567	4.000772	0.514448	3.139645	0.001984	0.021086	-1.55879
TMEM75	2.555965	5.530159	3.139074	0.001988	0.021095	-1.42594
AC020917.3	-2.12675	2.88571	-3.13785	0.001996	0.021133	-1.56784
AL024507.2	1.120417	6.489568	3.137639	0.001997	0.021133	-1.75788
AC090515.4	-2.62308	3.3673	-3.13475	0.002016	0.021271	-1.71004
AC008731.1	-0.57268	6.938766	-3.13517	0.002013	0.021271	-2.21659
AC026401.2	3.258495	6.129917	3.134183	0.00202	0.02128	-1.43916
AP002518.1	-2.97706	-0.25833	-3.13119	0.002039	0.021411	-1.44292
AC073263.2	4.58686	-0.31521	3.131085	0.00204	0.021411	-1.58291
AL021368.2	-0.72111	5.950571	-3.13098	0.00204	0.021411	-2.07997
AC023813.3	6.027383	3.566732	3.130201	0.002046	0.021435	-1.54976
AC112721.1	6.50218	2.979827	3.12732	0.002065	0.021605	-1.57869
AC121338.1	3.547746	2.387559	3.124082	0.002086	0.021801	-1.54191
AC096656.1	6.699771	4.060391	3.120884	0.002108	0.021995	-1.57483
AC006213.3	-1.1135	4.500068	-3.11967	0.002116	0.02202	-1.82025
LINC02363	-0.99627	5.023495	-3.11974	0.002116	0.02202	-1.90833
AC134312.5	5.770729	5.291216	3.118039	0.002127	0.022105	-1.53425
AC007639.1	2.554162	6.983536	3.115377	0.002146	0.022264	-1.64295
AC012358.1	-0.78361	5.83633	-3.11444	0.002152	0.022301	-2.09563
AC009262.1	7.347357	2.490049	3.111365	0.002173	0.02249	-1.62087
AC016737.1	1.192456	6.771262	3.110951	0.002176	0.02249	-1.88342
AC079296.1	-3.66037	-0.39628	-3.1101	0.002182	0.02252	-1.49644
AC021106.3	1.240497	8.829012	3.107582	0.0022	0.022672	-2.36047
AL158196.1	1.707301	5.909624	3.105236	0.002216	0.022788	-1.61267
AC020656.2	-2.03271	4.528012	-3.10471	0.00222	0.022788	-1.99563
AC108704.2	-0.67968	6.78297	-3.10505	0.002218	0.022788	-2.29463
AC087667.1	5.351786	1.363432	3.101291	0.002245	0.022926	-1.64811
AC023043.1	1.34976	7.939642	3.101109	0.002246	0.022926	-2.21151
AC046143.1	1.024276	8.284796	3.101177	0.002245	0.022926	-2.31101
AC011444.1	-0.69442	7.009354	-3.10211	0.002239	0.022926	-2.34828
AL670729.3	5.256097	2.998801	3.099892	0.002255	0.022985	-1.62158
AC107959.2	2.722296	3.122925	3.098076	0.002268	0.023087	-1.57227
CTBP1-AS	-0.67928	7.418232	-3.09481	0.002292	0.023298	-2.42872
AC125807.2	0.976874	8.688999	3.094076	0.002297	0.023321	-2.40448
AC002066.1	5.370115	2.976578	3.093371	0.002302	0.023342	-1.64157
AP000432.2	4.755009	-2.06619	3.091021	0.00232	0.023486	-1.67982
AL359551.1	5.303649	0.416684	3.090034	0.002327	0.023529	-1.67779
MKNK1-AS1	-0.6344	5.539475	-3.08897	0.002335	0.023577	-2.05307
AC084018.2	-0.67696	5.95639	-3.08828	0.00234	0.023597	-2.17188
AC007036.2	-0.91953	7.010271	-3.0834	0.002377	0.023935	-2.42451
Z97652.1	-1.84609	4.218543	-3.0827	0.002382	0.023956	-1.99455
BX255923.2	4.000805	-0.44288	3.080777	0.002397	0.024065	-1.70109
LINC02225	4.230706	-1.07078	3.080443	0.002399	0.024065	-1.70289
AC123768.1	5.420765	2.687331	3.079317	0.002408	0.024119	-1.68103
AL353135.1	5.6086	-1.36462	3.078587	0.002413	0.024143	-1.70754
AC097382.1	-2.05751	3.25575	-3.07795	0.002418	0.024159	-1.80521
AL356740.1	-2.24946	4.25259	-3.07688	0.002426	0.02421	-2.08582

AC016590.2	-0.85476	7.377501	-3.07647	0.00243	0.02421	-2.50143
AC096734.1	5.38466	-0.36586	3.07579	0.002435	0.02423	-1.7124
AL591845.1	1.539033	8.371687	3.071637	0.002467	0.024519	-2.35268
AL117339.4	-0.89196	3.806902	-3.06933	0.002485	0.024667	-1.78313
AL355297.3	-2.56507	2.111412	-3.06796	0.002496	0.024742	-1.74914
AL121990.1	5.563828	-0.00699	3.067044	0.002503	0.024781	-1.73206
AP003071.2	-3.34587	0.51606	-3.06619	0.00251	0.024815	-1.62798
ERVH48-1	-1.1406	4.976897	-3.06328	0.002534	0.025013	-2.12853
AC108047.1	0.875657	8.240841	3.062608	0.002539	0.025033	-2.44132
AL109741.1	-1.25329	6.023516	-3.06187	0.002545	0.025059	-2.35459
AC027627.1	5.191389	3.556357	3.059258	0.002566	0.02512	-1.70462
AC117465.1	5.756881	0.08736	3.058568	0.002572	0.02512	-1.7515
AL121900.1	4.536753	0.135399	3.057805	0.002578	0.02512	-1.75332
AC021945.1	1.30086	5.861283	3.059809	0.002562	0.02512	-1.80621
AL162274.3	-1.2343	4.30056	-3.05984	0.002561	0.02512	-1.95126
AC092139.3	-0.69311	5.94151	-3.05955	0.002564	0.02512	-2.26403
AL008582.1	-1.08982	6.22388	-3.0582	0.002575	0.02512	-2.39572
AC093726.1	-0.67233	8.804726	-3.05813	0.002575	0.02512	-2.73578
AC103724.4	6.401084	1.756794	3.056045	0.002592	0.025163	-1.75302
FP671120.2	-0.90411	5.345914	-3.05625	0.002591	0.025163	-2.15619
AC074033.1	-1.83372	6.014509	-3.05607	0.002592	0.025163	-2.46252
ASH1L-AS1	0.571341	8.856812	3.055624	0.002596	0.025164	-2.59609
AC092198.1	4.344048	1.334583	3.055124	0.0026	0.025171	-1.7491
AC005324.5	1.296871	6.171925	3.053873	0.00261	0.025206	-1.89963
AC005180.1	-2.30948	3.885747	-3.05391	0.00261	0.025206	-2.07433
AC016583.1	-2.23939	1.462537	-3.05305	0.002617	0.02524	-1.6624
AC008121.3	-3.36237	0.644822	-3.04925	0.002649	0.025476	-1.67803
AF233439.1	4.95034	2.091532	3.048878	0.002652	0.025476	-1.75869
LINC02542	2.00708	8.988577	3.049337	0.002648	0.025476	-2.41379
FAM66D	-1.36569	4.823296	-3.04666	0.00267	0.025622	-2.16215
AL034347.1	-2.92866	1.816991	-3.04622	0.002674	0.025626	-1.7154
AC025176.1	6.387271	5.870277	3.045164	0.002683	0.025646	-1.71098
AL445531.1	4.736482	2.489736	3.045318	0.002682	0.025646	-1.75194
AP001363.1	-0.929	7.149289	-3.0444	0.002689	0.025675	-2.56935
AC073862.5	-3.5689	-0.98634	-3.04294	0.002702	0.02572	-1.676
AL136982.1	-1.37218	2.637911	-3.04276	0.002703	0.02572	-1.7287
AC073569.2	1.022938	9.099017	3.042631	0.002705	0.02572	-2.61037
AL139288.1	3.210071	3.478626	3.041197	0.002717	0.025772	-1.71104
AC020978.6	3.695247	1.487864	3.041292	0.002716	0.025772	-1.7644
AC093388.1	1.135162	7.289107	3.038299	0.002742	0.025976	-2.26735
LINC01655	7.760461	3.419996	3.037308	0.00275	0.025991	-1.79015
SLC25A21-AS1	-1.38242	6.512209	-3.03762	0.002748	0.025991	-2.53879
LINC01522	6.601383	0.9406	3.034163	0.002778	0.026217	-1.80586
AC079753.1	-1.79137	3.986788	-3.03375	0.002781	0.026218	-1.98206
AC073842.2	1.080948	6.761316	3.031591	0.0028	0.026364	-2.13594
LINC02154	6.707176	3.469163	3.029249	0.002821	0.026525	-1.80388
AC079790.1	-4.111	-1.63682	-3.02775	0.002834	0.026613	-1.71604
AC009121.2	4.405218	5.000507	3.026286	0.002847	0.026613	-1.73509
AL512604.3	5.071244	0.324053	3.026914	0.002842	0.026613	-1.82404
AC103858.2	5.610872	0.831374	3.026146	0.002849	0.026613	-1.8247
AC103740.1	-1.08316	3.650858	-3.02582	0.002851	0.026613	-1.88813
AC010761.4	-0.82361	8.149659	-3.02736	0.002838	0.026613	-2.74363
AC090844.3	3.704571	3.254687	3.02355	0.002872	0.02677	-1.76998
LHX1-DT	5.464499	0.746139	3.022835	0.002878	0.026797	-1.83249
AC006116.4	-3.74682	-0.2272	-3.01846	0.002918	0.027067	-1.74012
AC013643.2	-2.96509	1.109053	-3.01904	0.002913	0.027067	-1.77324
AC025048.4	1.288041	7.125012	3.018696	0.002916	0.027067	-2.24553
AC068025.1	5.821576	4.773942	3.016544	0.002936	0.027119	-1.79147

LINC01691	6.788437	2.084086	3.015372	0.002947	0.027119	-1.84564
LINC00392	8.934859	2.011251	3.014875	0.002951	0.027119	-1.84656
AC090643.1	4.10217	-1.96637	3.01681	0.002933	0.027119	-1.85081
LINC00111	-2.86558	1.997428	-3.0147	0.002953	0.027119	-1.85433
AC087741.3	0.956512	6.565645	3.015448	0.002946	0.027119	-2.199
AC010894.2	1.09982	7.429523	3.014906	0.002951	0.027119	-2.3614
AC124319.2	1.2611	7.824653	3.015881	0.002942	0.027119	-2.40812
AC092159.2	2.266373	3.209653	3.012104	0.002977	0.027308	-1.77159
AC010680.1	-3.3426	-2.11795	-3.01157	0.002982	0.027318	-1.78062
LINC01546	5.735861	1.989743	3.01081	0.002989	0.027318	-1.85544
AC108174.1	6.294012	1.605743	3.01085	0.002989	0.027318	-1.85755
AC025857.2	1.173371	10.83212	3.00966	0.003	0.027384	-2.93123
AP002884.1	-0.95429	6.887923	-3.00735	0.003021	0.027549	-2.61992
AL451074.5	4.512327	3.111203	3.004081	0.003052	0.027798	-1.83294
AL354977.2	-2.95931	1.52561	-3.00364	0.003057	0.027802	-1.84545
AL354864.1	4.935951	2.166821	2.999646	0.003095	0.028083	-1.86841
AC010203.2	-1.42006	4.979928	-2.99989	0.003093	0.028083	-2.35584
AP001469.2	1.490348	7.473254	2.994107	0.003149	0.028538	-2.36823
AC027243.2	5.14338	2.086107	2.991181	0.003178	0.028634	-1.89298
AC026250.1	1.839711	6.083804	2.991108	0.003179	0.028634	-1.95607
AC127070.2	-0.86375	4.786704	-2.9913	0.003177	0.028634	-2.20116
AL078581.1	-0.62492	6.739976	-2.99187	0.003171	0.028634	-2.59801
AC010525.1	-0.77237	7.620685	-2.99196	0.00317	0.028634	-2.75417
AC087286.4	-0.76161	6.330348	-2.98548	0.003235	0.029105	-2.56853
AC020900.1	5.146716	4.982276	2.984181	0.003248	0.029164	-1.85161
AC121761.2	-1.16251	6.708061	-2.98406	0.003249	0.029164	-2.69507
AC092135.3	-0.60424	6.761021	-2.98178	0.003272	0.029335	-2.63911
LINC01494	4.628031	2.048157	2.979702	0.003293	0.029481	-1.91185
AL121895.2	-1.74565	6.931437	-2.97942	0.003296	0.029481	-2.81213
AC005476.2	0.874866	7.013407	2.977098	0.00332	0.029659	-2.44686
AC092964.1	1.9716	5.330677	2.975683	0.003335	0.029754	-1.8752
AL355483.1	7.393871	2.766716	2.971092	0.003382	0.030145	-1.94422
AC116913.1	0.503274	7.246353	2.969491	0.003399	0.030259	-2.5821
AC113133.1	-3.91426	-2.26631	-2.96816	0.003413	0.030274	-1.87848
MAFA-AS1	7.297977	2.591704	2.967967	0.003415	0.030274	-1.95188
AC021739.2	-0.72459	6.634996	-2.96821	0.003413	0.030274	-2.66956
AL391121.1	-0.58989	10.81811	-2.96781	0.003417	0.030274	-3.27646
AL365330.1	-0.49523	8.655747	-2.96728	0.003423	0.030288	-2.95711
LINC01511	4.928476	-1.25052	2.966003	0.003436	0.030372	-1.96478
AC073934.1	5.369227	-1.31836	2.963919	0.003458	0.030532	-1.96952
AC127164.1	-0.74187	4.316739	-2.96336	0.003464	0.030549	-2.11607
AC090159.1	5.060786	0.384562	2.960949	0.00349	0.030706	-1.97373
AC115989.1	-1.35702	5.305759	-2.96112	0.003488	0.030706	-2.50567
AC009878.1	-3.57542	1.267545	-2.96016	0.003499	0.03072	-1.95975
AL133467.2	5.174006	-1.93441	2.960038	0.0035	0.03072	-1.97908
AC009118.3	-0.53728	7.748076	-2.95835	0.003518	0.030845	-2.85494
AC106900.1	8.608383	7.466435	2.957625	0.003526	0.030878	-1.92622
AL021807.1	4.406524	7.967515	2.954591	0.00356	0.031096	-1.96142
AC005840.2	1.126721	7.886965	2.954651	0.003559	0.031096	-2.63608
AC078906.1	-2.85706	2.366821	-2.95406	0.003565	0.031111	-2.04345
AC104667.1	-3.42079	-0.79484	-2.95195	0.003589	0.031277	-1.90248
AC008738.2	-2.09742	3.883506	-2.95053	0.003604	0.031378	-2.32034
AL133297.1	2.698945	5.577071	2.948069	0.003632	0.031563	-1.93217
AL365203.2	1.095187	8.609466	2.947873	0.003634	0.031563	-2.78902
AC126474.1	1.030796	6.312162	2.947008	0.003644	0.031611	-2.30025
AL023803.3	4.926657	3.292344	2.946048	0.003654	0.031639	-1.9703
AC013451.1	5.478392	0.591679	2.945053	0.003666	0.031639	-2.00898
AC011239.2	-1.53439	3.774789	-2.94534	0.003662	0.031639	-2.2357

AC013356.3	-1.14722	5.008409	-2.94485	0.003668	0.031639	-2.42975
LINC02256	-1.19227	6.359076	-2.94503	0.003666	0.031639	-2.7635
AC018653.3	0.946375	9.02924	2.943176	0.003687	0.031766	-2.89511
ATP13A4-AS1	-3.92087	-1.12886	-2.94119	0.00371	0.031876	-1.92921
AC022405.1	4.84797	4.497701	2.940768	0.003714	0.031876	-1.95771
AC106052.1	4.919572	3.763855	2.941544	0.003705	0.031876	-1.97048
FP325332.1	-0.74447	6.045939	-2.94057	0.003717	0.031876	-2.61988
AL157402.2	5.708185	1.108098	2.937424	0.003753	0.032126	-2.02498
AC022509.4	5.204391	-1.75198	2.936906	0.003759	0.032126	-2.03055
AC092803.2	1.354463	7.206392	2.937093	0.003757	0.032126	-2.449
AL118506.1	0.753576	8.821552	2.934481	0.003787	0.03233	-2.91651
AL049830.3	2.803526	4.194708	2.927566	0.003868	0.032985	-1.96599
AC145285.6	0.552978	8.189892	2.927047	0.003874	0.033	-2.86148
AC093535.2	-0.71895	6.905366	-2.9257	0.00389	0.033099	-2.83136
AC131009.3	0.782274	8.976216	2.925227	0.003896	0.033109	-2.95718
AL390037.1	6.079083	3.355565	2.922786	0.003925	0.033208	-2.0375
AC017083.1	0.923224	6.282623	2.923064	0.003922	0.033208	-2.36457
AL121655.1	-0.64407	7.121993	-2.92317	0.00392	0.033208	-2.8696
AGBL5-IT1	-0.59196	7.677286	-2.92299	0.003923	0.033208	-2.95066
AC027279.4	-1.11945	4.208055	-2.9221	0.003933	0.03324	-2.32833
AC010864.1	1.441192	5.992147	2.921078	0.003946	0.033286	-2.13954
AC093673.1	1.035534	10.41418	2.92091	0.003948	0.033286	-3.15082
NDUFB2-AS1	0.637614	6.821465	2.92007	0.003958	0.033334	-2.59451
AC026124.1	-0.94279	4.004532	-2.91839	0.003978	0.033468	-2.24146
AC092902.2	-2.58321	2.522266	-2.91638	0.004003	0.033637	-2.21045
AL035665.1	3.320792	2.79569	2.914057	0.004031	0.033823	-2.02807
HDAC11-AS1	5.312609	1.800965	2.913551	0.004037	0.033823	-2.07467
AL356805.1	-1.26267	5.790461	-2.91348	0.004038	0.033823	-2.74683
AC020910.4	0.778917	8.386383	2.912283	0.004053	0.033909	-2.90099
AC025171.1	0.948971	8.79011	2.911656	0.004061	0.033936	-2.94693
LINC01283	4.916369	-1.92546	2.9111	0.004068	0.033938	-2.08805
LINC01537	-1.15389	4.134169	-2.91054	0.004075	0.033938	-2.33587
PRR7-AS1	1.129567	7.302473	2.910801	0.004071	0.033938	-2.64514
AC147651.4	1.340394	7.190482	2.908912	0.004095	0.034032	-2.53407
AP002907.1	0.767713	7.812743	2.909215	0.004091	0.034032	-2.81861
SZT2-AS1	3.186057	3.41744	2.903897	0.004158	0.03437	-2.03691
CLEC12A-AS1	4.211498	2.45625	2.904809	0.004146	0.03437	-2.06961
AL121759.1	4.614706	2.038578	2.904474	0.004151	0.03437	-2.08343
LINC01973	3.359621	-0.17938	2.903856	0.004158	0.03437	-2.10075
AL022326.2	-2.00939	4.081469	-2.90479	0.004147	0.03437	-2.51612
AL356299.2	0.847779	6.936346	2.902277	0.004179	0.034498	-2.64161
PRC1-AS1	0.870025	6.09761	2.901441	0.004189	0.034548	-2.39502
STK24-AS1	1.177853	6.115536	2.896828	0.004248	0.034998	-2.34656
AC093484.4	0.906365	8.711727	2.896063	0.004258	0.035041	-2.98039
AC008109.1	6.114138	-1.04578	2.895584	0.004264	0.035054	-2.12109
AC009121.1	2.569191	5.813537	2.891794	0.004314	0.035409	-2.09015
AC007182.1	4.646593	2.675408	2.890462	0.004331	0.035409	-2.10492
LINC02042	6.939055	3.188775	2.890973	0.004325	0.035409	-2.12132
AC023421.1	-2.02704	2.522574	-2.89137	0.004319	0.035409	-2.19385
AC024940.6	1.069992	6.674118	2.890798	0.004327	0.035409	-2.49001
AC109322.1	0.862327	7.927882	2.889747	0.004341	0.035448	-2.87229
IBA57-DT	2.43146	5.549189	2.888473	0.004358	0.035547	-2.09599
LINC01952	5.759995	3.433686	2.885947	0.004391	0.035703	-2.11586
LINC01338	6.127366	2.305585	2.885592	0.004396	0.035703	-2.13612
LINC01524	4.095492	-0.60637	2.885754	0.004394	0.035703	-2.14231
AC005288.1	0.651449	11.50567	2.885711	0.004394	0.035703	-3.45178
AC083906.3	7.379558	3.333382	2.884956	0.004404	0.035734	-2.13444
AC098828.2	5.131526	1.229066	2.883736	0.004421	0.035814	-2.14314

AC107081.2	0.586444	7.368881	2.883182	0.004428	0.035814	-2.83181
AC007686.3	-0.49695	6.698714	-2.88315	0.004428	0.035814	-2.87693
AC021242.3	1.96307	4.343253	2.882286	0.00444	0.03583	-2.07717
AL034417.2	-1.10981	4.267922	-2.88239	0.004439	0.03583	-2.39749
AL139393.2	1.495527	7.929654	2.879365	0.004479	0.03611	-2.81714
AC007878.1	-0.69539	7.167716	-2.87622	0.004522	0.036416	-3.01561
AL139039.3	-2.55925	1.726833	-2.87265	0.004571	0.036732	-2.17349
AC090241.3	-1.10828	3.678288	-2.873	0.004567	0.036732	-2.31799
AC078850.2	-3.71546	-0.54598	-2.87214	0.004578	0.03675	-2.09981
PLCH1-AS2	5.861212	1.243866	2.871319	0.00459	0.036802	-2.17095
AC096921.2	-0.91089	6.739837	-2.8707	0.004598	0.036831	-2.98275
AC090192.2	7.639978	3.861077	2.865702	0.004668	0.037311	-2.17512
AC074351.1	5.419024	-0.15286	2.865701	0.004668	0.037311	-2.18554
POU6F2-AS1	-3.83495	0.637759	-2.86405	0.004692	0.037418	-2.16351
AC124319.4	-1.36749	4.61928	-2.86413	0.00469	0.037418	-2.61669
LINC00501	5.120029	3.271877	2.860271	0.004745	0.037766	-2.16666
AL122023.1	-0.69398	7.239922	-2.86061	0.00474	0.037766	-3.06908
AL451050.2	1.007261	6.739889	2.859695	0.004754	0.037792	-2.6484
AF015262.1	5.260553	-0.66643	2.859339	0.004759	0.037792	-2.20005
KC877982.1	6.872189	-0.15712	2.854982	0.004821	0.03825	-2.20879
AP002812.3	-0.95137	6.032041	-2.85445	0.004829	0.038271	-2.91807
AL358394.2	3.609055	0.310614	2.850555	0.004886	0.038681	-2.21581
AL109811.2	-0.62647	8.442799	-2.84909	0.004908	0.038811	-3.27144
AL161937.1	5.152539	-0.24998	2.84822	0.00492	0.038871	-2.22374
AC069360.1	4.801676	4.346108	2.845271	0.004964	0.038971	-2.18214
AC020658.3	2.998886	1.54178	2.845885	0.004955	0.038971	-2.20011
AC012377.1	3.091912	0.259353	2.845719	0.004957	0.038971	-2.22263
AC012467.1	-2.98513	2.626009	-2.84592	0.004954	0.038971	-2.43963
AC021224.1	0.891136	6.239082	2.845428	0.004962	0.038971	-2.63962
AL139423.1	-0.67428	6.380225	-2.84615	0.004951	0.038971	-2.94565
PLCXD2-AS1	5.336745	1.871391	2.842274	0.005009	0.039241	-2.2307
AL022067.1	1.981414	7.239661	2.842541	0.005005	0.039241	-2.59973
MHENCR	0.66933	9.60511	2.839934	0.005044	0.039434	-3.30464
LINC00641	-0.65792	8.736517	-2.83994	0.005044	0.039434	-3.34768
AC112496.1	1.739083	6.773963	2.836377	0.005098	0.039774	-2.54173
AC007787.2	-0.93689	6.211107	-2.83664	0.005094	0.039774	-2.99251
GAS1RR	-1.53143	3.427765	-2.83414	0.005132	0.039999	-2.46797
AP002852.1	4.024996	3.371329	2.833267	0.005146	0.040021	-2.2143
AL138724.1	0.914515	8.397456	2.833327	0.005145	0.040021	-3.10286
AL445072.1	5.560849	-1.208	2.832687	0.005155	0.040049	-2.25843
AL592301.1	1.813531	5.671694	2.832169	0.005163	0.040069	-2.33348
AC012464.3	-1.83236	1.613152	-2.83054	0.005188	0.040224	-2.2359
AC108134.2	1.647401	7.055318	2.829421	0.005205	0.040317	-2.53853
AC125611.4	2.091632	3.148862	2.826145	0.005256	0.040587	-2.21299
FO680682.1	5.981139	3.702001	2.826203	0.005256	0.040587	-2.24893
AC136603.1	-2.6626	1.838762	-2.82672	0.005247	0.040587	-2.29629
AL133325.3	-3.53434	-0.11065	-2.82446	0.005283	0.040749	-2.22374
FP671120.5	-1.08083	10.77153	-2.82372	0.005295	0.040798	-3.72278
C17orf82	2.117273	5.99207	2.822325	0.005317	0.040856	-2.35862
AC007490.1	-2.93052	3.602794	-2.82221	0.005318	0.040856	-2.64316
AC079907.1	0.971075	6.754859	2.822724	0.00531	0.040856	-2.75798
AL596247.1	1.084161	7.11369	2.821722	0.005326	0.040874	-2.83583
AC023157.3	1.083761	9.131348	2.820961	0.005338	0.040925	-3.23031
AL355482.1	-3.01169	0.177255	-2.81811	0.005384	0.041232	-2.24104
AL441992.1	0.852653	9.466226	2.814676	0.005439	0.041613	-3.32271
OCIAD1-AS1	1.299083	7.510306	2.811228	0.005495	0.042	-2.90677
GSEC	1.393462	7.001102	2.810215	0.005512	0.042083	-2.77696
AC005808.1	5.755021	0.143359	2.808965	0.005532	0.042197	-2.30793

AC092681.2	-1.83423	2.435943	-2.80817	0.005545	0.042254	-2.35377
AL031320.2	-0.64228	5.903745	-2.80769	0.005553	0.042272	-2.93079
AC004704.1	5.607193	-0.27138	2.806624	0.005571	0.042362	-2.31344
AP002478.1	6.090123	4.301269	2.804312	0.005609	0.042611	-2.28842
AL354928.1	4.843807	4.544153	2.802318	0.005643	0.04282	-2.27679
AP001962.1	5.52152	3.236829	2.799993	0.005682	0.043017	-2.30591
AC063926.3	-2.10598	1.700136	-2.79985	0.005684	0.043017	-2.31338
AC118754.2	-2.09201	3.958383	-2.79975	0.005686	0.043017	-2.80221
AP000766.1	-0.49729	7.691033	-2.79927	0.005694	0.043035	-3.27515
LINC02302	-3.45738	-0.91982	-2.79737	0.005726	0.043235	-2.27646
AC040977.1	0.728755	8.469007	2.796961	0.005733	0.043243	-3.24404
AC013565.1	-1.867	3.716576	-2.79634	0.005743	0.043279	-2.64324
AC099398.1	3.694476	-0.59415	2.795189	0.005763	0.043383	-2.33833
AC016723.1	5.131063	-0.6973	2.79486	0.005769	0.043383	-2.33909
AC004241.2	-1.27108	5.712511	-2.79402	0.005783	0.043405	-3.04119
AC016394.1	0.677925	8.446907	2.79401	0.005783	0.043405	-3.25196
AC011893.1	4.199906	-0.06786	2.791479	0.005827	0.043687	-2.3456
LINC02208	-3.27938	-1.51643	-2.79099	0.005835	0.043707	-2.29317
AC068299.1	4.428441	-0.59614	2.790011	0.005852	0.043746	-2.34936
ZNF236-DT	-0.62422	7.372959	-2.79003	0.005852	0.043746	-3.26481
AC004080.4	5.025149	1.289243	2.789109	0.005867	0.043776	-2.34806
LINC01583	4.497264	-0.35221	2.789333	0.005864	0.043776	-2.35055
AC104472.1	4.138411	2.646397	2.786099	0.00592	0.044123	-2.33002
AL355483.4	5.238638	0.350303	2.78528	0.005934	0.044186	-2.35834
AC105020.5	-0.88578	5.789832	-2.78478	0.005943	0.044207	-3.02889
AC091814.1	5.082805	-0.16375	2.7842	0.005953	0.04424	-2.36123
AC090673.1	5.85845	1.235395	2.783217	0.00597	0.044324	-2.36134
UBE2E1-AS1	4.428017	1.659869	2.782026	0.005991	0.044408	-2.355
AL357093.2	-1.27977	4.762136	-2.78191	0.005994	0.044408	-2.84169
AC090618.1	-3.78431	-1.3629	-2.78028	0.006022	0.044446	-2.31517
AC046158.2	-1.38944	5.322054	-2.78033	0.006021	0.044446	-3.03814
LINC00513	-0.8145	6.249364	-2.78091	0.006011	0.044446	-3.11995
AC127070.4	-0.77279	6.352904	-2.78125	0.006005	0.044446	-3.13253
AC005523.1	5.136126	2.962979	2.775813	0.006102	0.044945	-2.36006
AC064807.1	0.691289	8.283472	2.775726	0.006104	0.044945	-3.27472
SNHG19	0.901897	12.78239	2.775492	0.006108	0.044945	-3.89689
AC087286.2	-0.75518	6.998503	-2.7749	0.006119	0.044979	-3.26293
AC022217.2	4.981783	1.630669	2.772554	0.006161	0.045115	-2.38121
AC027279.1	-0.63849	6.118008	-2.77271	0.006158	0.045115	-3.09156
AL080317.3	-0.45544	7.211689	-2.77313	0.006151	0.045115	-3.26363
AC006329.1	1.486929	9.544195	2.772678	0.006159	0.045115	-3.36104
TGFB3-AS1	-2.29765	2.724783	-2.77095	0.00619	0.045284	-2.53325
C2-AS1	5.195433	3.551303	2.768027	0.006244	0.04563	-2.36743
AC112722.1	-0.99426	5.793938	-2.76747	0.006254	0.045661	-3.10933
AL137802.2	2.146775	5.357783	2.766305	0.006275	0.045772	-2.39805
AC084125.4	1.118456	7.160057	2.762474	0.006346	0.046245	-2.99847
RASA3-IT1	-3.76331	-0.92034	-2.75795	0.006431	0.046502	-2.37289
AC092112.1	5.938695	3.213987	2.759542	0.006401	0.046502	-2.39823
LMNB1-DT	4.552122	0.624744	2.758248	0.006425	0.046502	-2.41486
AC018616.1	5.957092	0.266018	2.758264	0.006425	0.046502	-2.41566
AC087239.1	2.475363	5.808608	2.759923	0.006394	0.046502	-2.45106
AC007327.2	-2.91868	1.250365	-2.75802	0.00643	0.046502	-2.45456
AC022400.2	0.977842	6.171701	2.7602	0.006389	0.046502	-2.79042
AC117503.2	0.667174	7.511186	2.758254	0.006425	0.046502	-3.18347
AL590708.1	2.28567	5.474894	2.756915	0.00645	0.046509	-2.41818
AC012213.3	1.410592	6.081794	2.757062	0.006448	0.046509	-2.57483
AC073896.2	-0.41698	8.250953	-2.75735	0.006442	0.046509	-3.45923
AL137246.1	-3.03611	0.531056	-2.75591	0.006469	0.046557	-2.40052

GCC2-AS1	0.891269	6.861785	2.755935	0.006469	0.046557	-2.96602
AL110115.1	1.421307	6.568033	2.753918	0.006507	0.046747	-2.80701
AC087289.3	0.577501	6.819317	2.753857	0.006508	0.046747	-3.05434
AC009652.2	4.789803	3.526356	2.751916	0.006546	0.046924	-2.39981
AC073857.1	0.639855	9.211575	2.751983	0.006544	0.046924	-3.47927
AC015853.1	0.912807	6.518145	2.750922	0.006565	0.047016	-2.8967
AL139022.2	-0.62645	6.603944	-2.74746	0.006631	0.047449	-3.25357
AC091729.3	0.882527	9.151766	2.746675	0.006647	0.047512	-3.4615
AL034374.1	5.399939	1.634882	2.745766	0.006664	0.047593	-2.43925
AP003465.1	-3.80827	-0.79312	-2.74529	0.006673	0.047614	-2.4008
AL096870.2	0.508941	6.741766	2.743788	0.006703	0.047778	-3.09059
AL355073.2	-2.0108	4.054674	-2.74317	0.006715	0.047819	-2.84246
AC092111.1	-0.95431	4.404233	-2.74279	0.006722	0.047827	-2.80999
AL162511.1	-3.73053	-1.04728	-2.74148	0.006748	0.047965	-2.4058
AL137918.1	3.058833	3.679206	2.740835	0.006761	0.04801	-2.40885
AC090409.1	3.157915	4.991552	2.738353	0.00681	0.048276	-2.41417
LINC01806	2.407757	8.416232	2.738287	0.006811	0.048276	-3.05817
AL157762.1	-1.62939	4.533896	-2.73753	0.006826	0.048338	-2.97494
AC015849.5	2.835826	4.724428	2.736731	0.006842	0.048404	-2.41878
AC007292.1	0.626028	7.708664	2.735722	0.006862	0.048501	-3.29666
AC104667.2	-1.64378	5.203954	-2.73509	0.006875	0.048545	-3.13306
AC019077.1	4.671636	3.467054	2.734579	0.006885	0.048571	-2.43757
AC022028.2	4.108876	-2.33383	2.733095	0.006915	0.048736	-2.47106
AL139424.1	4.50559	1.262633	2.732215	0.006933	0.048815	-2.46631
AL031666.1	-0.70834	6.198206	-2.73083	0.006961	0.048966	-3.20166
AC006557.1	4.025361	3.748109	2.73049	0.006968	0.048969	-2.43831
AL136162.1	1.662895	6.592826	2.728271	0.007013	0.04924	-2.79731
LINC02160	-4.02594	-0.75249	-2.7275	0.007028	0.049304	-2.44891
AC007389.3	4.035407	2.173616	2.725878	0.007062	0.049491	-2.46563
AC107952.2	-1.07954	6.417904	-2.72546	0.00707	0.049505	-3.34163
AC010913.1	1.063631	4.362162	2.724794	0.007084	0.049554	-2.49898
LINC02367	1.370814	5.401155	2.722289	0.007136	0.04987	-2.58806
AC022400.5	-0.48818	5.982938	-2.72187	0.007144	0.049884	-3.14622
AC012363.2	5.051306	0.465942	2.721509	0.007152	0.04989	-2.49261
AC073195.1	0.965672	6.853895	2.720321	0.007176	0.050016	-3.05232
LINC00601	4.804086	-1.25553	2.719629	0.007191	0.050069	-2.49816
AC135507.1	-0.60555	6.360996	-2.71609	0.007265	0.050539	-3.27307
AL160291.1	4.37344	0.617164	2.713904	0.007311	0.050813	-2.50772
AC123912.2	-3.27433	-1.74719	-2.71227	0.007346	0.050879	-2.47282
AC015818.2	5.185822	-0.59036	2.713085	0.007329	0.050879	-2.51116
ARMC2-AS1	-3.47837	0.537468	-2.71186	0.007355	0.050879	-2.57201
AC105384.1	-2.06095	2.436268	-2.71273	0.007336	0.050879	-2.62899
AC145098.1	0.94869	7.953118	2.712136	0.007349	0.050879	-3.34177
PSORS1C3	-1.89375	7.843338	-2.7115	0.007362	0.050885	-3.68305
LINC02003	3.115133	3.127684	2.708006	0.007437	0.051355	-2.48625
AC005759.1	-1.87223	2.540686	-2.70538	0.007494	0.051699	-2.64537
AC110048.2	-1.33569	2.730007	-2.7049	0.007504	0.051723	-2.5806
LINC00896	-1.04295	5.050074	-2.70349	0.007535	0.051886	-3.05197
AC024257.4	-1.60561	4.02758	-2.70257	0.007555	0.051976	-2.95074
FAM225B	2.017358	2.274473	2.70179	0.007572	0.052046	-2.4994
AC125257.1	0.736768	10.85977	2.701277	0.007583	0.052075	-3.84499
LINC02371	-3.26535	0.07153	-2.70085	0.007592	0.052091	-2.51911
AC087289.6	0.964851	6.663726	2.698387	0.007646	0.05232	-3.08976
AL596094.1	0.893179	7.14219	2.698917	0.007635	0.05232	-3.21611
AL049597.2	0.794274	7.614839	2.698692	0.00764	0.05232	-3.34118
AC008875.1	2.918438	5.168843	2.696582	0.007686	0.05249	-2.52141
AC011595.2	4.881797	-0.0727	2.695999	0.007699	0.05249	-2.54612
AC016405.3	1.314919	5.328985	2.696627	0.007685	0.05249	-2.68815

AC114947.2	0.987212	6.404977	2.696081	0.007697	0.05249	-3.03553
AC008750.7	5.14686	1.671255	2.694563	0.007731	0.052619	-2.54571
AL137804.1	5.308219	-0.38362	2.694513	0.007732	0.052619	-2.54944
AC027279.2	-1.37405	3.994431	-2.69411	0.007741	0.052632	-2.90819
SYNPR-AS1	-2.1824	5.141108	-2.69372	0.00775	0.052645	-3.30632
AC090617.2	5.197446	1.932107	2.693065	0.007764	0.052696	-2.54704
AC020763.4	0.965148	6.225211	2.691743	0.007794	0.052849	-3.00685
LINC02599	3.823102	-0.32007	2.691194	0.007806	0.052885	-2.55616
FP325330.3	4.287652	1.603356	2.690553	0.007821	0.052897	-2.54843
AC011483.1	6.610048	2.639295	2.690489	0.007822	0.052897	-2.55313
AL161644.1	-2.64018	1.224094	-2.68994	0.007835	0.052933	-2.58871
AC018809.2	-0.47602	7.632058	-2.68919	0.007852	0.053	-3.55443
AC069281.2	0.784782	8.096091	2.687545	0.007889	0.053203	-3.45619
AC004069.1	1.031687	6.329417	2.68707	0.007899	0.053228	-3.00605
AC109361.1	1.770317	3.156743	2.686377	0.007915	0.053249	-2.52839
AC027698.1	-3.61208	-0.15504	-2.68569	0.007931	0.053249	-2.54668
AL121992.1	1.16816	4.118553	2.685876	0.007927	0.053249	-2.56853
AC018629.1	3.123614	10.04884	2.686121	0.007921	0.053249	-3.4449
AC069281.1	5.119487	0.317038	2.68406	0.007968	0.05345	-2.57037
AC093642.2	-2.39641	0.540829	-2.68194	0.008017	0.053728	-2.5572
AC007406.3	2.411448	5.938007	2.681616	0.008024	0.05373	-2.6128
AL109615.2	3.715197	1.624426	2.680467	0.008051	0.05386	-2.56373
AP000695.2	1.762177	6.943673	2.679898	0.008064	0.0539	-2.97397
AC011815.3	-0.73279	7.558895	-2.67838	0.008099	0.054087	-3.60151
AL157823.2	-1.79598	3.501149	-2.67714	0.008128	0.054218	-2.88607
AC131934.1	-0.52787	7.04299	-2.67692	0.008133	0.054218	-3.49995
MCM8-AS1	3.014198	3.718822	2.676074	0.008153	0.054301	-2.55206
AC092634.3	-3.38072	-0.0089	-2.67474	0.008184	0.05446	-2.57312
AC105206.1	2.088096	3.049496	2.67392	0.008203	0.054495	-2.55615
AC138207.5	0.927775	8.349334	2.6739	0.008204	0.054495	-3.50501
AC005899.7	-1.07363	6.136177	-2.67314	0.008222	0.054566	-3.44106
AL356608.1	-1.86242	2.685493	-2.67179	0.008253	0.054729	-2.75794
Z99758.1	5.135394	0.326899	2.668445	0.008333	0.055157	-2.60244
AC092279.2	-1.06744	6.972487	-2.66862	0.008329	0.055157	-3.59367
AL133371.3	4.817201	-1.06608	2.667728	0.00835	0.055199	-2.60507
LINC01504	-1.19904	5.933734	-2.66756	0.008354	0.055199	-3.38154
AL138713.1	-2.07364	1.600796	-2.66685	0.008371	0.055263	-2.63291
AC106772.1	5.588852	3.412059	2.66619	0.008387	0.05527	-2.59122
AC011306.1	-2.04712	2.450939	-2.66649	0.008379	0.05527	-2.73737
AC134043.1	5.64307	1.293423	2.665165	0.008411	0.055335	-2.60795
LINC01179	4.567101	-1.49269	2.665457	0.008404	0.055335	-2.61005
AC015813.7	1.967879	5.609901	2.663983	0.00844	0.055473	-2.66171
AC106882.1	3.264911	3.291452	2.662844	0.008467	0.055523	-2.58391
LNCOC1	1.556597	4.808476	2.662751	0.008469	0.055523	-2.65033
CELF2-AS1	-1.39316	3.632405	-2.66313	0.00846	0.055523	-2.90654
LINC01096	5.793998	1.291114	2.660443	0.008525	0.055594	-2.61766
AC004877.1	-1.29851	2.569542	-2.66104	0.008511	0.055594	-2.70635
DAPK1-IT1	-2.42646	2.246077	-2.66023	0.00853	0.055594	-2.73674
AC092134.1	-1.1468	4.263988	-2.66017	0.008532	0.055594	-3.0484
AC009831.3	-1.89979	3.898694	-2.66054	0.008523	0.055594	-3.07369
AC007285.2	0.791527	6.433055	2.660399	0.008526	0.055594	-3.13331
AC114730.3	-0.56057	7.367133	-2.66083	0.008516	0.055594	-3.5967
AC131025.1	4.335359	4.15209	2.659719	0.008543	0.055617	-2.59082
LINC00973	5.180304	-0.04582	2.657792	0.00859	0.055875	-2.62453
AL132657.2	-2.51462	2.330486	-2.65617	0.00863	0.056038	-2.80357
AC005041.3	1.146832	7.374846	2.656172	0.00863	0.056038	-3.31108
AP000553.2	5.553246	4.159978	2.655252	0.008652	0.056135	-2.60649
FP236383.3	-1.49281	7.192538	-2.65483	0.008663	0.056155	-3.70562

AC104958.2	-1.38143	6.722395	-2.65352	0.008695	0.056316	-3.61771
AP001065.1	-1.57299	5.107441	-2.65273	0.008715	0.056394	-3.31748
LRRC2-AS1	-2.96603	0.058879	-2.65231	0.008725	0.056413	-2.61579
AC009407.1	-1.96894	4.295715	-2.65184	0.008737	0.05644	-3.16863
AC016746.1	4.548993	-1.27251	2.651364	0.008749	0.056467	-2.63861
SPATA13-AS1	-2.54912	2.281822	-2.65072	0.008765	0.056523	-2.78516
AC004257.1	5.532433	3.260972	2.649161	0.008804	0.056725	-2.62653
AC012404.2	-3.16556	0.981281	-2.64876	0.008814	0.056741	-2.68983
AC025030.2	4.674381	0.643326	2.648332	0.008824	0.056761	-2.64291
AL121672.1	-0.62923	5.257108	-2.6468	0.008863	0.05696	-3.15983
AC004835.1	3.055271	0.681173	2.646443	0.008872	0.05697	-2.6394
AC078788.1	5.472902	1.554987	2.644346	0.008925	0.057261	-2.64974
AL355607.1	6.363829	2.037039	2.643411	0.008949	0.057364	-2.65115
AP001453.4	0.855223	7.810985	2.641719	0.008992	0.057591	-3.51917
LINC02321	7.973543	5.586091	2.640267	0.009029	0.057779	-2.64502
AC004920.1	5.31885	-0.3797	2.639456	0.00905	0.057863	-2.66208
AC126773.2	2.373676	6.290956	2.638737	0.009068	0.057883	-2.78811
AC011944.2	-1.03886	5.51124	-2.63903	0.00906	0.057883	-3.35796
AC002519.1	-1.71125	1.96239	-2.63831	0.009079	0.057904	-2.72354
AL445686.2	-3.54659	-0.63736	-2.63732	0.009105	0.058017	-2.6463
Z69720.2	5.862315	3.194222	2.636926	0.009115	0.058033	-2.65569
LINC01705	9.237858	4.68865	2.635768	0.009145	0.058125	-2.66302
AC107464.2	-1.51821	4.269373	-2.63599	0.009139	0.058125	-3.13668
AP000695.1	1.610769	7.105945	2.632897	0.009219	0.05855	-3.19968
AC078850.1	-1.48499	3.5256	-2.63252	0.009229	0.058563	-2.9432
AC106738.2	-3.61308	-1.3018	-2.63185	0.009247	0.058587	-2.65094
AC021683.3	2.935729	1.112897	2.631776	0.009249	0.058587	-2.66437
AC004080.2	8.757717	4.449291	2.630057	0.009294	0.058823	-2.67464
AC108861.1	-3.60611	-0.83403	-2.62925	0.009315	0.058908	-2.67804
AP001767.3	-1.46961	5.061248	-2.6275	0.009361	0.059151	-3.39423
AL022315.1	-3.06074	1.441926	-2.62699	0.009375	0.059183	-2.76059
AC010536.1	0.89076	5.990018	2.626708	0.009382	0.059183	-3.09457
AC118755.2	5.760893	2.312097	2.625315	0.009419	0.059317	-2.68595
AC012360.3	0.62619	7.317004	2.625469	0.009415	0.059317	-3.47829
AC125494.2	-0.73197	6.42771	-2.62501	0.009427	0.059318	-3.52775
AP001094.3	-3.54504	-0.87629	-2.62465	0.009437	0.059329	-2.68114
SRGAP2-AS1	2.881816	1.643156	2.623576	0.009465	0.059459	-2.67609
AC084809.1	0.995492	7.011922	2.623283	0.009473	0.059459	-3.34816
AC137932.2	1.582695	5.117883	2.622845	0.009485	0.059483	-2.77808
LY6E-DT	2.108262	6.080115	2.621385	0.009524	0.059679	-2.83265
AC011481.4	1.178469	3.102913	2.618846	0.009593	0.060057	-2.68151
AC116348.3	-2.75789	1.158289	-2.61769	0.009624	0.060203	-2.74723
AC008079.1	-0.49636	4.628183	-2.6153	0.009689	0.060508	-3.05421
AC109992.2	0.954538	6.288045	2.615327	0.009688	0.060508	-3.19474
AL390195.2	-0.74183	6.305204	-2.61295	0.009753	0.06086	-3.55955
AC008736.3	-2.28967	2.162159	-2.61165	0.009789	0.060982	-2.87135
MAN1B1-DT	1.073919	8.407873	2.611816	0.009784	0.060982	-3.66309
AC034229.1	4.397177	-0.33098	2.610669	0.009816	0.0611	-2.72014
AC024884.2	2.678674	4.447305	2.608613	0.009873	0.061251	-2.70525
AC114546.2	-3.30894	0.400795	-2.60869	0.009871	0.061251	-2.73712
FER1L6-AS2	-2.92147	0.879209	-2.60915	0.009858	0.061251	-2.75413
AP000892.3	-0.94452	7.227181	-2.60922	0.009856	0.061251	-3.7687
AC011815.2	-0.73303	5.105063	-2.60684	0.009922	0.061506	-3.24695
AC024941.1	-3.35813	0.46318	-2.60532	0.009965	0.061718	-2.76452
LINC02323	2.784432	7.757819	2.604436	0.009989	0.061821	-3.12894
AC016705.2	-1.45583	4.058206	-2.60408	0.009999	0.061832	-3.16392
AC016877.3	4.908088	4.935165	2.601007	0.010086	0.06223	-2.71483
TRHDE-AS1	-2.71431	-0.36337	-2.60121	0.01008	0.06223	-2.7219

AC120498.4	7.911332	3.537128	2.600573	0.010098	0.06223	-2.73547
LINC01526	4.87605	0.013565	2.600426	0.010102	0.06223	-2.7404
AC008543.1	0.875881	5.582936	2.600325	0.010105	0.06223	-3.03338
C1orf147	1.090621	5.778151	2.597748	0.010179	0.062579	-3.00699
AC026740.1	1.207067	8.274366	2.597887	0.010175	0.062579	-3.63722
AL645608.7	1.401477	7.671107	2.597394	0.010189	0.06259	-3.4897
AC097372.2	3.535272	5.60664	2.596421	0.010216	0.062658	-2.75149
AC108673.2	0.943288	6.853205	2.596505	0.010214	0.062658	-3.36496
AC010247.2	1.831796	4.115081	2.595328	0.010248	0.062799	-2.75492
AC016394.2	0.618665	8.794365	2.594365	0.010275	0.062917	-3.82108
AC010359.1	1.596343	5.599728	2.593322	0.010306	0.062999	-2.93503
AC253536.3	-1.00119	7.994654	-2.59342	0.010303	0.062999	-3.91952
AC009336.1	3.872838	1.914356	2.589836	0.010406	0.063564	-2.74983
AF131215.5	-1.0204	7.762094	-2.58926	0.010423	0.063615	-3.90321
PPM1K-DT	-1.58624	2.690673	-2.5872	0.010484	0.063931	-2.92228
AC039056.2	1.164394	7.177056	2.586378	0.010508	0.064025	-3.4524
AC007038.1	-0.66756	6.815653	-2.58592	0.010521	0.064055	-3.70686
AL360093.1	3.360502	4.274313	2.585185	0.010543	0.064135	-2.74843
AC005920.1	-2.4745	2.047148	-2.58466	0.010558	0.064177	-2.90631
AL031008.1	-2.67875	1.288472	-2.58315	0.010603	0.064396	-2.85835
HOXA10-AS	7.093361	6.37579	2.582592	0.010619	0.064444	-2.75485
AP000688.2	-2.36994	1.504885	-2.58159	0.010649	0.064573	-2.85852
AP000424.2	5.411765	-0.23661	2.578525	0.01074	0.065074	-2.78418
SMC5-AS1	-0.52632	6.694227	-2.5781	0.010753	0.065099	-3.67642
LINC01775	6.531882	5.63865	2.577455	0.010772	0.065111	-2.76685
AL391069.2	1.165943	6.572725	2.577557	0.010769	0.065111	-3.2642
LINC02518	5.596405	0.762712	2.576302	0.010807	0.065215	-2.78785
AC012640.2	0.957272	7.818894	2.576327	0.010806	0.065215	-3.649
AC020661.1	1.115551	6.821166	2.575777	0.010823	0.065258	-3.40997
AC091614.1	4.302586	-0.43157	2.574814	0.010852	0.065366	-2.7917
AL592431.1	-3.54582	-0.18766	-2.57461	0.010858	0.065366	-2.80619
AC068196.1	3.907105	2.296339	2.574225	0.01087	0.065383	-2.77851
AC006116.10	-2.71941	1.647117	-2.57375	0.010884	0.065418	-2.89733
AC027088.3	4.727307	-0.7381	2.573265	0.010899	0.065454	-2.79494
AC009041.4	0.920979	6.92638	2.56903	0.011028	0.066176	-3.48489
AL512791.1	-0.6097	8.505969	-2.56807	0.011057	0.066299	-4.01428
LINC02527	5.505042	-0.06609	2.567296	0.011081	0.06639	-2.80631
TBX5-AS1	2.711098	5.370551	2.565948	0.011123	0.066586	-2.84287
AL445985.1	3.428999	2.775682	2.564749	0.01116	0.066754	-2.79389
AC021755.3	-3.48983	-0.38399	-2.56286	0.011218	0.067052	-2.81474
AC104791.1	3.229446	4.022125	2.561047	0.011275	0.067336	-2.79944
LINC01391	3.733424	0.960962	2.55983	0.011313	0.067501	-2.81683
AC034187.1	4.523651	-1.27574	2.559021	0.011338	0.067501	-2.82338
AC090970.2	1.547674	7.074162	2.559095	0.011336	0.067501	-3.41049
HIF1A-AS2	1.077054	7.346021	2.5591	0.011336	0.067501	-3.59636
AC092431.1	-2.4023	2.455973	-2.55849	0.011355	0.067524	-3.02615
AC115522.1	1.429013	7.716118	2.558326	0.01136	0.067524	-3.60474
ZMYM4-AS1	-1.59738	3.925167	-2.5564	0.011421	0.067831	-3.28161
AC110285.6	1.035834	7.405824	2.555413	0.011452	0.067963	-3.58471
PTOV1-AS2	0.469288	9.743769	2.5544	0.011485	0.0681	-4.08464
AC015726.1	-0.59254	7.55166	-2.55392	0.0115	0.068136	-3.90165
SLC25A34-AS1	4.415388	0.724081	2.550211	0.011618	0.068785	-2.83908
VAV3-AS1	-3.64241	-1.8778	-2.54882	0.011663	0.068996	-2.82513
AC004947.2	-2.3003	1.020507	-2.5471	0.011719	0.06906	-2.89014
RRS1-AS1	-1.29238	2.824948	-2.54748	0.011706	0.06906	-2.94387
AC002511.2	-2.17194	3.830128	-2.54788	0.011693	0.06906	-3.32796
RHPN1-AS1	1.201292	7.113047	2.54706	0.01172	0.06906	-3.51323
AC032044.1	-0.50446	7.513856	-2.54762	0.011702	0.06906	-3.89179

AC026782.2	6.193772	3.293664	2.546046	0.011753	0.069199	-2.84126
AC116049.2	6.849105	2.631821	2.545293	0.011777	0.069235	-2.8469
AC124283.3	0.721737	6.99443	2.545433	0.011773	0.069235	-3.59512
AL137191.1	2.293906	4.338245	2.544665	0.011798	0.069301	-2.85037
AC099568.1	4.862879	1.302543	2.543236	0.011844	0.069358	-2.85201
LINC01422	-0.96261	3.946921	-2.54325	0.011844	0.069358	-3.18972
LINC02175	-0.87828	4.747291	-2.54353	0.011835	0.069358	-3.35597
AC139887.1	-0.5998	8.180775	-2.5438	0.011826	0.069358	-4.0245
AL139023.1	6.415541	0.802954	2.542108	0.011881	0.06952	-2.85525
AC079779.2	-1.34881	3.943066	-2.54171	0.011894	0.069542	-3.24323
AC120036.4	-1.27001	4.103148	-2.54097	0.011919	0.069619	-3.26211
AC005722.3	-2.39553	4.137458	-2.54074	0.011926	0.069619	-3.46792
AL391005.1	-3.30268	-2.30004	-2.53958	0.011964	0.06968	-2.8435
AL355607.2	4.4406	1.084274	2.539864	0.011955	0.06968	-2.85828
AC016596.1	1.53668	4.246583	2.539722	0.01196	0.06968	-2.88587
AC106738.1	-2.68242	2.156625	-2.53891	0.011986	0.069755	-3.07007
AC092620.1	-1.0223	5.187421	-2.53796	0.012018	0.069884	-3.51794
HOXC-AS3	5.505137	4.268583	2.537292	0.01204	0.069938	-2.85061
AC018752.1	-1.0745	7.59514	-2.53712	0.012046	0.069938	-4
AP003119.2	2.125505	7.519093	2.536179	0.012077	0.070064	-3.42517
AC026202.2	-0.61082	6.496433	-2.53418	0.012143	0.070353	-3.77099
YTHDF3-AS1	0.69849	7.837718	2.534126	0.012145	0.070353	-3.80605
AC008938.1	2.651273	-0.62133	2.532442	0.012202	0.070625	-2.87421
AC018904.1	0.676133	8.872214	2.529813	0.01229	0.071082	-3.98703
AL136141.1	4.213057	4.256581	2.529444	0.012303	0.0711	-2.86443
AC004816.1	1.133912	9.530128	2.52831	0.012341	0.071267	-4.04753
MAPK6-DT	3.856634	4.906627	2.526848	0.012391	0.071498	-2.87253
AP000640.2	3.214277	2.87464	2.524523	0.01247	0.071676	-2.87509
AC024267.6	4.025335	1.681173	2.524945	0.012456	0.071676	-2.8829
AC119150.1	7.380687	3.672555	2.525156	0.012448	0.071676	-2.88489
AC091182.1	-0.74482	4.693267	-2.52399	0.012488	0.071676	-3.36911
AC046158.3	-1.04469	4.813043	-2.52543	0.012439	0.071676	-3.49952
AC107959.1	-0.58732	5.760818	-2.52453	0.01247	0.071676	-3.61392
AC010327.5	0.586259	7.30303	2.524248	0.012479	0.071676	-3.74277
AP002336.3	5.36249	4.177914	2.522356	0.012544	0.071888	-2.88035
AC007540.1	5.288775	0.779614	2.522558	0.012537	0.071888	-2.89353
AL359711.2	-0.75559	6.003539	-2.52123	0.012583	0.072055	-3.71544
AC111170.1	2.199355	4.206794	2.520762	0.012599	0.072093	-2.90465
SIX3-AS1	4.651196	-1.34935	2.518784	0.012668	0.072412	-2.90206
AP003390.1	3.11781	7.250679	2.518314	0.012684	0.072412	-3.21784
AL512303.1	-1.79186	3.921274	-2.51841	0.012681	0.072412	-3.38375
AC011008.2	-1.08762	4.193817	-2.5178	0.012702	0.07246	-3.38345
GHET1	1.004473	6.063119	2.514729	0.012809	0.073015	-3.32703
AP002754.1	4.12647	1.084964	2.513578	0.012849	0.07319	-2.9087
LINC00237	4.146711	-2.0219	2.512919	0.012873	0.073211	-2.91376
AC006270.1	2.820645	6.277047	2.513034	0.012869	0.073211	-3.04834
AC061992.1	3.114017	6.058948	2.511878	0.012909	0.073364	-2.96351
AC134407.1	-0.45432	7.874825	-2.51138	0.012927	0.073408	-4.03713
AC009269.2	-1.73829	3.609509	-2.51034	0.012964	0.073562	-3.30158
AC016745.2	2.334403	2.389129	2.507691	0.013058	0.073984	-2.90897
AL031283.1	1.986151	3.355504	2.507876	0.013051	0.073984	-2.91347
AL050341.2	0.635872	9.401775	2.506802	0.013089	0.074108	-4.13359
LINC01920	4.035855	-1.9047	2.505537	0.013135	0.074308	-2.92797
AL591767.1	-2.00068	3.04947	-2.50499	0.013154	0.074364	-3.22925
RASA2-IT1	1.731434	5.123282	2.502414	0.013247	0.074831	-3.04633
AC134312.1	-1.11528	3.094766	-2.50163	0.013275	0.074897	-3.11103
LINC01402	-2.58728	2.178012	-2.50154	0.013279	0.074897	-3.18461
AC126283.1	4.784328	1.373188	2.500897	0.013302	0.074972	-2.93425

AC007619.1	-0.57199	7.74498	-2.4998	0.013342	0.07514	-4.06041
AC025754.1	4.6445	2.26616	2.498583	0.013386	0.075292	-2.93368
AC099811.5	2.34595	5.130142	2.498508	0.013389	0.075292	-2.98582
AC116563.1	4.865594	-0.50449	2.496647	0.013457	0.075561	-2.94444
AC007610.4	-0.53921	4.515563	-2.49682	0.01345	0.075561	-3.31744
AL031848.1	7.306853	5.352943	2.495337	0.013505	0.075774	-2.93616
AC105101.1	-1.21553	3.840175	-2.49474	0.013527	0.07584	-3.31844
AC138150.2	1.226495	6.231323	2.494159	0.013548	0.075904	-3.35715
LINC00184	3.349053	3.783791	2.493658	0.013567	0.075951	-2.9384
AC015712.2	0.682193	6.96008	2.492488	0.01361	0.076119	-3.74379
AF131215.6	-0.94567	6.9625	-2.4923	0.013617	0.076119	-4.00642
AL513497.1	0.983157	6.194648	2.491435	0.013649	0.076241	-3.40194
AC007834.1	5.121316	1.297483	2.49102	0.013664	0.07627	-2.95399
FOXP1-IT1	-0.64933	4.508138	-2.49056	0.013681	0.076309	-3.38643
AC092919.1	5.836212	1.641533	2.489365	0.013726	0.076392	-2.95697
AC055811.4	-0.68191	8.88492	-2.48971	0.013713	0.076392	-4.26325
ILF3-DT	0.5811	10.67899	2.489337	0.013727	0.076392	-4.36861
AL450322.2	4.419602	1.871511	2.48751	0.013795	0.076658	-2.95679
AC007601.1	-0.49736	5.864898	-2.48762	0.013791	0.076658	-3.71973
LINC02555	4.039972	-0.92792	2.486122	0.013847	0.076834	-2.96489
AC092145.1	-1.39629	3.582497	-2.48617	0.013845	0.076834	-3.25515
AL133264.2	4.707191	0.20638	2.485582	0.013867	0.07689	-2.96539
AC097460.3	-1.49751	3.862562	-2.48484	0.013895	0.076988	-3.43999
AC026150.1	-1.75341	3.012746	-2.48442	0.013911	0.07702	-3.27164
AC093278.2	-0.74693	7.6133	-2.48362	0.013941	0.07713	-4.0963
PTPRD-AS1	-2.09684	2.044556	-2.48247	0.013985	0.077313	-3.16143
AL133230.1	0.745364	6.147301	2.481779	0.014011	0.077401	-3.51743
AC015727.1	-1.25794	5.672069	-2.48126	0.014031	0.077452	-3.83865
AC087289.2	0.595515	6.153449	2.479303	0.014105	0.077693	-3.54246
AF129075.1	-0.44677	6.794954	-2.47943	0.014101	0.077693	-3.93738
AC138028.4	0.590668	8.66905	2.479663	0.014092	0.077693	-4.09873
AC110813.1	-1.3591	2.64658	-2.47826	0.014145	0.077855	-3.13929
AC020928.2	2.556057	3.126513	2.476013	0.014231	0.07819	-2.9741
AL160408.3	3.018606	1.983264	2.475596	0.014248	0.07819	-2.97474
AL160274.1	-2.11044	1.856291	-2.47584	0.014238	0.07819	-3.09256
AL139089.1	0.643651	7.813809	2.475729	0.014242	0.07819	-3.94742
AC114956.2	2.336101	8.323866	2.473931	0.014312	0.078485	-3.74491
NCBP2-AS1	0.703032	6.989963	2.471359	0.014412	0.078975	-3.77156
AC046185.3	1.070559	6.781203	2.469788	0.014473	0.079252	-3.64598
AL139384.1	1.008943	6.727434	2.468987	0.014504	0.079366	-3.64206
AC099343.2	-0.53974	6.631401	-2.46808	0.01454	0.079503	-3.93981
AP000893.2	4.589068	1.172943	2.46775	0.014553	0.079516	-2.99823
AC025244.1	6.081058	1.605688	2.466629	0.014597	0.079698	-3.00074
AC019211.1	4.931386	0.322955	2.462646	0.014754	0.08042	-3.00917
AL354993.2	3.746735	7.159404	2.462475	0.014761	0.08042	-3.15982
AL592430.1	-0.70807	5.658378	-2.46256	0.014757	0.08042	-3.77352
AC004233.4	4.34797	2.936097	2.461194	0.014812	0.08064	-3.00333
LINC02450	5.472397	2.314092	2.460561	0.014837	0.08066	-3.01048
LINC01137	0.75334	9.983379	2.460648	0.014834	0.08066	-4.3094
LINC01126	0.716718	6.211456	2.460183	0.014852	0.080684	-3.60318
LINC01087	3.743558	0.258645	2.458658	0.014913	0.080889	-3.01633
AL353746.1	-2.23849	0.776686	-2.45763	0.014955	0.080889	-3.04946
AP000696.1	-2.3655	2.812332	-2.45862	0.014915	0.080889	-3.31406
AF287957.1	-1.09242	4.055944	-2.45798	0.014941	0.080889	-3.40952
AC135803.1	-1.29646	5.653847	-2.45775	0.01495	0.080889	-3.899
TNFRSF10A-AS1	0.860795	9.75815	2.458383	0.014924	0.080889	-4.27091
AC104260.2	-1.30553	2.734187	-2.45492	0.015064	0.081422	-3.18584
AC002470.1	1.190988	7.521882	2.454107	0.015097	0.081542	-3.88301

AC117490.2	0.759017	7.374127	2.453532	0.01512	0.081609	-3.89691
AL049840.5	-0.54742	8.129463	-2.45232	0.01517	0.081816	-4.22852
AC093525.9	-0.54747	5.994506	-2.45043	0.015247	0.082174	-3.84031
AC093382.1	5.031643	5.051171	2.449566	0.015282	0.082305	-3.02705
AP005264.3	4.726	-0.98907	2.448287	0.015335	0.082529	-3.03694
AC010255.1	-2.55407	1.580018	-2.4466	0.015404	0.082844	-3.17127
AL161772.1	1.382589	7.254707	2.445341	0.015456	0.083064	-3.74382
AL645608.8	3.019565	4.629225	2.444656	0.015485	0.083157	-3.05152
AC069287.3	4.434634	0.291014	2.443943	0.015514	0.083257	-3.04458
AC108681.1	4.678482	3.504576	2.443363	0.015538	0.083327	-3.03787
AC111152.2	4.023889	3.725536	2.442584	0.015571	0.083441	-3.03957
AL033381.1	-2.18049	1.413652	-2.44155	0.015614	0.083614	-3.12122
ATP6V1B1-AS1	4.386074	1.450832	2.44086	0.015643	0.083692	-3.04823
HAGLROS	2.17345	7.072474	2.440665	0.015651	0.083692	-3.52743
UPK1A-AS1	4.474216	0.063992	2.440383	0.015663	0.083695	-3.05149
AC021321.1	0.838794	5.521734	2.436517	0.015826	0.084445	-3.40807
AL133243.2	0.520112	8.936816	2.436639	0.01582	0.084445	-4.24401
AC007991.4	6.472237	3.707723	2.43557	0.015866	0.084544	-3.05537
AC020661.3	1.062716	6.718832	2.435544	0.015867	0.084544	-3.66435
RASAL2-AS1	0.710489	7.179608	2.434455	0.015913	0.084731	-3.92178
L34079.2	3.97526	2.441787	2.433548	0.015952	0.084876	-3.05766
AC109479.1	4.669434	2.482096	2.431715	0.01603	0.085187	-3.06305
AC087821.1	-1.74972	3.467194	-2.43165	0.016033	0.085187	-3.47528
AC012625.1	5.475415	1.988693	2.430032	0.016102	0.085494	-3.06955
AL445426.1	-2.71746	1.389826	-2.42975	0.016114	0.0855	-3.219
AL135924.2	6.309275	2.027313	2.427333	0.016218	0.08599	-3.07491
AL353764.1	4.695311	5.296775	2.426274	0.016264	0.086173	-3.07714
AP002812.5	-0.63153	6.919546	-2.42585	0.016283	0.086209	-4.10962
AC022413.1	0.968113	6.941842	2.422979	0.016407	0.086768	-3.81251
AC011978.2	0.54947	6.881442	2.422893	0.016411	0.086768	-3.88583
AC022929.2	3.133452	1.272229	2.422107	0.016446	0.086889	-3.08091
AC016734.2	3.158332	1.385425	2.421442	0.016475	0.08692	-3.08195
AC005498.3	-1.59895	3.121475	-2.42166	0.016465	0.08692	-3.40414
AC068580.1	0.750762	8.576875	2.4211	0.01649	0.086938	-4.19463
AP002812.2	-0.80739	6.127794	-2.41977	0.016548	0.087186	-4.00093
AC073863.1	3.918163	2.599706	2.419086	0.016578	0.087222	-3.08537
AC007009.1	-2.30882	3.797338	-2.41922	0.016572	0.087222	-3.61906
LINC01992	-3.58261	-0.41467	-2.418	0.016626	0.087351	-3.12567
AC018638.6	-0.66532	5.069978	-2.41812	0.016621	0.087351	-3.71843
SYNGAP1-AS1	2.100489	4.522938	2.415998	0.016715	0.087756	-3.13577
AL139125.1	3.059614	3.231181	2.413938	0.016807	0.088166	-3.09681
AC010255.2	4.979709	1.47735	2.413716	0.016816	0.088166	-3.10053
AC007598.1	-0.66048	5.213793	-2.41319	0.01684	0.088228	-3.75149
AC084809.2	4.154824	4.220133	2.412455	0.016873	0.088285	-3.10064
AC109347.2	-0.90526	7.21071	-2.41242	0.016874	0.088285	-4.22717
AL049552.1	-0.90813	7.150621	-2.41109	0.016934	0.088534	-4.22447
AC116025.1	-2.33212	2.283232	-2.41018	0.016975	0.088687	-3.31993
LINC02438	5.202966	0.349249	2.408901	0.017033	0.088865	-3.11044
AL121578.3	4.679112	-1.29651	2.409147	0.017021	0.088865	-3.1105
AC112484.1	0.929836	7.046481	2.408483	0.017051	0.088902	-3.89279
AC091965.5	-2.60964	2.111552	-2.40808	0.01707	0.088935	-3.35267
AC091153.4	3.345387	4.748381	2.407048	0.017116	0.089117	-3.1242
AC005291.2	4.992643	0.475848	2.405982	0.017165	0.089307	-3.11584
AP000864.1	4.452878	4.631694	2.404879	0.017215	0.089506	-3.11623
AC004231.1	5.016911	3.410642	2.403416	0.017282	0.089791	-3.1155
AL355388.2	0.677646	5.730095	2.402949	0.017303	0.08984	-3.55152
AC010531.8	4.230128	-1.3984	2.401313	0.017378	0.090167	-3.12511
AC130324.2	2.678305	5.222507	2.400437	0.017418	0.090314	-3.18719

AC005225.2	-1.75255	2.914661	-2.39987	0.017444	0.090386	-3.41992
AC004908.2	0.546033	8.285889	2.396014	0.017623	0.091249	-4.24054
AC016745.1	5.227603	3.771484	2.395641	0.01764	0.091276	-3.13046
AC015813.6	0.689167	8.724023	2.393557	0.017738	0.091716	-4.29617
AJ239322.1	-2.47763	0.121901	-2.39204	0.017809	0.09202	-3.1735
AC004080.1	4.470549	-1.4507	2.390522	0.01788	0.092273	-3.14511
LRP1-AS	2.82771	3.892996	2.390022	0.017904	0.092273	-3.15203
AC144652.1	0.973377	7.377531	2.389957	0.017907	0.092273	-4.01244
AC120049.1	-0.94296	6.100949	-2.39031	0.01789	0.092273	-4.07945
AC018638.7	-0.5965	7.173807	-2.38779	0.018009	0.092738	-4.23124
AC009054.2	0.644528	7.690275	2.387223	0.018036	0.092813	-4.13059
AC006213.7	-0.7486	4.252516	-2.38683	0.018055	0.092845	-3.58063
AC013549.3	-2.65011	1.149596	-2.3862	0.018085	0.092935	-3.25807
AC022306.3	-1.17746	5.33039	-2.38512	0.018136	0.093136	-3.95076
AC019186.1	3.347943	5.629671	2.384563	0.018163	0.093209	-3.21055
AC011731.1	4.685484	5.419697	2.383894	0.018195	0.09331	-3.16406
AP005264.7	-2.46851	0.056127	-2.38337	0.01822	0.093375	-3.18315
AC040169.1	0.85657	8.218295	2.383045	0.018236	0.093391	-4.22254
POU6F2-AS2	8.053303	3.325383	2.382155	0.018278	0.093546	-3.15871
AC024230.1	5.108042	-0.61369	2.380376	0.018364	0.093854	-3.16362
AC011498.3	3.383289	4.631468	2.380127	0.018376	0.093854	-3.17775
AC093458.2	-1.53556	4.403389	-2.38056	0.018355	0.093854	-3.79219
AC099508.2	4.331551	-1.22991	2.379468	0.018408	0.093907	-3.16545
AC104411.1	3.278684	6.660845	2.379396	0.018411	0.093907	-3.29668
AC073592.7	3.768906	3.156252	2.377945	0.018482	0.094201	-3.16522
AL138781.1	1.095065	5.967364	2.376706	0.018542	0.094444	-3.66098
AC084855.2	-1.21729	3.27067	-2.37503	0.018623	0.094795	-3.48014
AC011352.1	4.813716	0.752419	2.373454	0.018701	0.095059	-3.17592
AC092343.1	-1.8976	3.110397	-2.37363	0.018692	0.095059	-3.49976
AC089987.2	5.032514	1.185201	2.371221	0.01881	0.095553	-3.17982
TMLHE-AS1	1.931635	3.035411	2.370443	0.018849	0.095554	-3.18989
LINC01220	1.037873	5.983181	2.370529	0.018845	0.095554	-3.65232
AC012464.1	-1.06719	5.382692	-2.3705	0.018846	0.095554	-3.97877
AC131009.2	4.556481	1.985133	2.368379	0.018951	0.095981	-3.18319
AC135050.3	-0.5852	6.785897	-2.36822	0.018959	0.095981	-4.20678
AL121832.3	0.830483	6.758924	2.36707	0.019016	0.096206	-3.9183
RBAKDN	4.892166	2.503291	2.366771	0.019031	0.096217	-3.18587
AC005831.1	1.150318	9.175863	2.365609	0.019089	0.096445	-4.35526
AC027601.5	-0.55696	4.854807	-2.36534	0.019102	0.096448	-3.7221
AP001085.1	4.473655	-0.2106	2.363412	0.019199	0.09687	-3.1947
AC004449.1	4.341492	2.697982	2.361872	0.019276	0.097195	-3.19447
AL161719.1	-2.199	3.304668	-2.36129	0.019305	0.097213	-3.60187
AC013468.1	-0.6098	5.706484	-2.36152	0.019294	0.097213	-3.96712
LINC01397	4.045555	2.647453	2.3604	0.01935	0.097309	-3.19737
UMODL1-AS1	-3.06923	-2.23874	-2.3605	0.019345	0.097309	-3.20019
LINC02640	-3.1306	-0.04644	-2.35808	0.019468	0.097709	-3.26941
AC137630.1	-1.22634	5.400541	-2.35846	0.019448	0.097709	-4.06472
AL031985.3	0.706208	8.429152	2.358058	0.019469	0.097709	-4.32629
AP001046.1	-2.2952	0.851336	-2.35531	0.019609	0.098281	-3.28247
AP003110.1	-1.81635	2.735183	-2.35544	0.019602	0.098281	-3.47831
AC239868.2	3.586429	-0.7178	2.35403	0.019675	0.098281	-3.21197
LINC02287	-3.02415	-0.54418	-2.35407	0.019673	0.098281	-3.2362
AC021483.1	-2.55247	1.381869	-2.35493	0.019628	0.098281	-3.33949
AC002064.2	-0.90928	7.152773	-2.3541	0.019671	0.098281	-4.3661
ARHGAP5-AS1	0.786326	8.772652	2.354725	0.019639	0.098281	-4.38711
AC233992.3	0.820834	8.460952	2.353506	0.019701	0.09835	-4.32086
AC096564.1	-0.83119	3.787574	-2.35292	0.019731	0.098435	-3.54541
AC002310.2	1.529464	3.551315	2.352028	0.019777	0.098533	-3.25167

AC006449.5	0.973572	6.257455	2.352057	0.019776	0.098533	-3.78575
AC093083.1	5.306651	1.378291	2.351749	0.019792	0.098539	-3.2155
LINC01962	4.518112	-0.22745	2.350854	0.019838	0.09863	-3.21765
AL445363.1	3.733451	-0.58627	2.350631	0.019849	0.09863	-3.21814
AC087392.4	-0.52428	6.821571	-2.35092	0.019834	0.09863	-4.23908
AC011352.3	4.946682	0.921226	2.349363	0.019915	0.098817	-3.22001
AC007494.2	-0.6564	5.777782	-2.34946	0.01991	0.098817	-4.05694
AC024941.2	-1.34285	5.656857	-2.34914	0.019927	0.098817	-4.13605
AC061961.1	-3.71934	-0.21835	-2.34842	0.019964	0.098938	-3.28438
AC013400.1	3.881988	2.929706	2.347175	0.020029	0.099193	-3.22284
AL078644.2	0.843689	6.658026	2.345276	0.020128	0.099618	-3.95056
AC078785.2	-1.22554	3.381834	-2.34402	0.020193	0.099877	-3.53591
AC034199.1	2.9526	2.468197	2.343736	0.020209	0.099886	-3.2301
AC097468.1	-2.68883	1.084064	-2.34085	0.020361	0.100572	-3.33528
AC012640.1	3.985136	3.456322	2.340159	0.020397	0.100685	-3.23769
AL591806.1	3.058955	5.156866	2.339769	0.020418	0.100721	-3.286
AC016575.1	4.412581	3.475056	2.339297	0.020443	0.10077	-3.2381
GK-IT1	4.052264	6.939951	2.339035	0.020457	0.10077	-3.3439
AC018695.3	2.427508	5.410595	2.338823	0.020468	0.10077	-3.34752
AP005137.2	5.002374	2.96581	2.33769	0.020528	0.100911	-3.23959
AC068228.1	5.234604	2.258944	2.337527	0.020537	0.100911	-3.24068
AC022034.3	-1.63231	4.91258	-2.3379	0.020517	0.100911	-4.03468
AC138904.1	3.155978	4.227718	2.337056	0.020562	0.100968	-3.25784
PRRX2-AS1	4.794847	1.444781	2.335969	0.02062	0.101073	-3.244
AC005796.1	-3.53123	-0.67823	-2.3359	0.020624	0.101073	-3.27915
EIF3J-DT	-0.37448	8.638066	-2.33597	0.02062	0.101073	-4.54754
AL023581.2	-1.31354	4.818056	-2.3352	0.020661	0.101124	-3.97662
AC093690.1	-0.42241	6.00608	-2.33536	0.020652	0.101124	-4.08755
AL133245.1	-0.57405	6.126889	-2.33469	0.020688	0.101191	-4.15587
AC004801.6	0.553154	6.829083	2.333822	0.020735	0.101354	-4.08727
AL109954.2	4.682383	1.738497	2.33355	0.020749	0.10136	-3.24797
LINC01424	1.404585	4.919894	2.333144	0.020771	0.1014	-3.39596
AD000813.1	2.965437	3.961449	2.332483	0.020807	0.101506	-3.2679
AC123595.1	-1.26597	4.174595	-2.33224	0.02082	0.101506	-3.72728
AC099518.1	3.874608	5.62585	2.331407	0.020865	0.101659	-3.29145
AL357078.1	-1.43175	4.572646	-2.33089	0.020893	0.101728	-3.94051
AC128709.3	5.345771	1.865742	2.330504	0.020914	0.101754	-3.25393
AL117350.1	-1.03472	5.135065	-2.3303	0.020925	0.101754	-4.00163
AC092999.1	-1.22307	3.59296	-2.3298	0.020952	0.101819	-3.64118
AL138756.1	-0.62605	7.837499	-2.32931	0.020978	0.101881	-4.46789
CASK-AS1	3.931795	5.339784	2.328833	0.021004	0.101941	-3.28648
AC136618.1	3.825015	-1.96957	2.328427	0.021026	0.101982	-3.25863
LINC02139	2.835192	1.489385	2.32743	0.021081	0.102134	-3.25877
AL353803.1	-2.82602	0.586176	-2.32735	0.021085	0.102134	-3.33068
AL391152.1	3.421642	4.473794	2.326287	0.021143	0.10235	-3.2787
AL645924.1	-3.28255	-0.10569	-2.32587	0.021166	0.102394	-3.33474
AC010533.1	2.497493	3.607058	2.325501	0.021186	0.102426	-3.27981
AL162274.1	-0.87613	4.975757	-2.32475	0.021227	0.102558	-3.94635
AC010132.4	-0.81485	5.027781	-2.32289	0.021329	0.102987	-3.93782
MAGEA4-AS1	5.217305	-0.7804	2.322602	0.021345	0.102996	-3.26893
LINC01698	5.026901	0.145289	2.322319	0.021361	0.103005	-3.2693
LINC02421	-1.17598	3.515611	-2.32145	0.021408	0.103142	-3.6047
AL157392.4	-0.7689	6.947667	-2.32131	0.021416	0.103142	-4.37707
AC105389.2	-2.38036	2.164608	-2.32102	0.021433	0.103154	-3.51638
AC138028.6	0.927041	6.675352	2.320544	0.021459	0.103213	-3.99427
AC087442.1	6.26705	2.916989	2.31962	0.02151	0.103267	-3.27331
AC134312.4	-2.55733	1.101272	-2.31986	0.021497	0.103267	-3.40936
AL161669.1	-2.28944	3.681354	-2.3196	0.021511	0.103267	-3.77707

AL355304.1	3.680357	3.34582	2.317851	0.021608	0.103636	-3.28057
AC040160.1	0.792519	7.032966	2.317719	0.021615	0.103636	-4.10981
AC023509.2	-1.2151	2.658791	-2.31735	0.021636	0.103668	-3.47248
AC084262.1	-3.20619	0.252285	-2.31689	0.021662	0.103725	-3.35349
AL355596.1	3.901268	0.708046	2.316183	0.021701	0.103758	-3.27997
AL591848.3	-0.57266	5.413753	-2.31602	0.02171	0.103758	-3.99016
AC009509.4	-0.6647	7.221072	-2.31626	0.021697	0.103758	-4.40394
AC084357.2	1.064394	6.781372	2.31577	0.021724	0.10376	-3.88358
AP003351.1	4.346725	0.323639	2.314593	0.02179	0.103896	-3.28314
AL392023.2	-3.12596	-1.61952	-2.31478	0.02178	0.103896	-3.29479
AC008742.1	2.190415	4.607352	2.314519	0.021794	0.103896	-3.34369
LINC02642	4.225491	-0.53897	2.313893	0.021829	0.103932	-3.28457
AL513008.1	-1.32775	7.880568	-2.314	0.021823	0.103932	-4.60709
AC067930.5	0.554441	6.41378	2.313104	0.021874	0.104077	-4.03283
AL390066.1	0.702109	6.864303	2.312836	0.021889	0.104083	-4.09498
AC022211.4	0.872982	4.789593	2.312028	0.021934	0.104234	-3.51261
AF124730.1	5.554997	2.312581	2.311757	0.02195	0.10424	-3.28756
AC022816.1	4.192716	0.694952	2.310843	0.022001	0.104305	-3.28972
AC064805.1	2.610604	2.318848	2.310677	0.022011	0.104305	-3.29321
AC026992.2	-1.71868	1.008864	-2.3104	0.022026	0.104305	-3.34566
P3H2-AS1	-1.80684	3.786097	-2.31119	0.021982	0.104305	-3.81581
AC092535.4	0.926488	8.217802	2.310287	0.022033	0.104305	-4.35719
AC096920.1	-3.08146	-0.28552	-2.30879	0.022118	0.104642	-3.3572
AC004158.1	1.149646	3.827628	2.30854	0.022132	0.104643	-3.36651
AC012074.1	-0.67476	5.579925	-2.30804	0.02216	0.104712	-4.08296
HOMER3-AS1	3.280728	4.294656	2.306862	0.022228	0.104963	-3.31454
AC084018.1	-0.52398	9.381991	-2.3048	0.022346	0.105454	-4.74525
AL591846.2	4.795967	3.890709	2.304465	0.022365	0.105478	-3.30312
LUARIS	3.363056	3.091471	2.303566	0.022416	0.105655	-3.30697
AL121839.2	-0.9585	7.368759	-2.30292	0.022453	0.105763	-4.50853
AC013652.2	3.81132	0.043714	2.302001	0.022507	0.105881	-3.30574
AL591623.1	-0.68841	4.457259	-2.3021	0.022501	0.105881	-3.79874
AC002551.1	-0.95679	5.211078	-2.30144	0.022539	0.105966	-4.07779
AC131011.1	3.499534	4.854079	2.299674	0.022641	0.106332	-3.33747
AC073517.1	0.7301	6.232749	2.299608	0.022645	0.106332	-3.97423
AC125611.3	4.023692	-0.8722	2.298747	0.022695	0.106438	-3.31171
TBC1D8-AS1	0.759876	6.392256	2.298732	0.022696	0.106438	-3.99624
AC116021.1	4.359604	1.491518	2.29799	0.022739	0.106574	-3.31238
AP000487.1	0.948938	7.674647	2.297337	0.022777	0.106685	-4.27293
TIPARP-AS1	0.861821	6.981568	2.297021	0.022795	0.106705	-4.13389
KIAA1614-AS1	1.846709	3.99918	2.295322	0.022895	0.106984	-3.37526
AC010168.1	1.095843	8.184795	2.295273	0.022898	0.106984	-4.35104
LINC01772	-0.40077	6.934185	-2.29536	0.022893	0.106984	-4.36968
AC011466.2	4.551842	2.22488	2.294972	0.022915	0.107	-3.31781
AL356274.2	4.42909	3.109017	2.293785	0.022985	0.107258	-3.32151
AC016876.3	2.3666	5.466739	2.293117	0.023024	0.107375	-3.45598
AC124861.1	4.63313	-0.08716	2.291722	0.023106	0.107558	-3.32415
AC020922.3	2.826867	6.036188	2.291991	0.023091	0.107558	-3.47755
AL031665.2	0.605984	8.534264	2.29179	0.023102	0.107558	-4.49849
AC026782.1	4.347979	-1.38352	2.289481	0.023239	0.108108	-3.32826
AL021368.3	-3.03423	0.024897	-2.2889	0.023274	0.108202	-3.39327
AL031595.3	-0.562	4.684201	-2.2871	0.023381	0.108629	-3.82932
AC005514.1	-1.78074	4.093797	-2.28687	0.023394	0.108629	-3.92876
AC084064.1	-3.4025	-1.50745	-2.28652	0.023415	0.10866	-3.35243
AC107909.2	3.895332	2.465555	2.284885	0.023513	0.108979	-3.33752
AL391845.2	3.022372	3.713424	2.284931	0.02351	0.108979	-3.35255
AL358473.1	2.78169	0.559361	2.283537	0.023594	0.109172	-3.33832
AC007128.2	8.0789	5.282166	2.283224	0.023613	0.109172	-3.3392

AC105339.5	-0.572	6.034366	-2.28337	0.023604	0.109172	-4.2243
AC007684.1	-0.60393	6.31602	-2.28351	0.023595	0.109172	-4.31359
AL158068.2	-2.02105	2.866446	-2.28247	0.023658	0.109314	-3.63302
AC007014.2	2.587574	6.240448	2.282206	0.023674	0.109321	-3.53788
TMEM202-AS1	-0.39335	6.812707	-2.28193	0.023691	0.109331	-4.379
AC079089.1	2.770963	3.122445	2.280766	0.023761	0.109588	-3.35476
HELLPAR	0.936549	2.484083	2.280378	0.023785	0.109629	-3.372
AC007218.1	3.939291	-1.93791	2.279854	0.023817	0.109708	-3.34537
AL355076.2	-1.66903	3.806227	-2.27941	0.023843	0.109764	-3.80213
TM4SF19-AS1	2.163239	7.095523	2.278438	0.023903	0.109969	-3.88775
AC011287.2	4.853245	1.213606	2.277498	0.02396	0.110165	-3.34923
AL357500.1	-2.98574	0.779395	-2.27635	0.02403	0.110419	-3.46583
AL355483.2	4.056876	1.388029	2.275612	0.024075	0.110492	-3.35248
AL357079.1	0.553599	7.723376	2.27571	0.024069	0.110492	-4.40599
AC136475.3	2.021949	8.920335	2.274826	0.024123	0.110645	-4.35636
AC011498.7	0.504051	7.106143	2.274319	0.024154	0.110653	-4.29104
AL359643.3	0.795686	8.761654	2.274344	0.024153	0.110653	-4.5602
AC092287.1	0.826244	7.431816	2.273788	0.024187	0.110735	-4.3048
LINC01267	3.422573	0.383136	2.273046	0.024232	0.110809	-3.35709
AC008124.1	-0.3587	8.272685	-2.2732	0.024223	0.110809	-4.63409
AL162457.2	3.933965	-1.83455	2.272228	0.024283	0.110971	-3.35883
AC005332.4	0.645473	8.232648	2.271847	0.024306	0.111011	-4.49518
AC006213.2	-0.55634	4.559874	-2.27104	0.024356	0.111172	-3.8585
AL121890.4	-1.07286	4.753871	-2.26901	0.024482	0.111678	-4.02817
AC079684.1	0.706453	7.612762	2.268771	0.024497	0.111678	-4.36963
AC022874.1	4.468951	-1.11997	2.268515	0.024513	0.111682	-3.36533
AL589745.1	-2.16278	1.05856	-2.26806	0.024541	0.111743	-3.47462
AL163051.1	0.633826	6.991155	2.267576	0.024571	0.111813	-4.22639
AC100778.2	-0.88199	6.084385	-2.26685	0.024617	0.111952	-4.34281
LINC01960	2.252075	2.557151	2.266354	0.024647	0.112016	-3.37984
TGFB2-AS1	1.255518	7.414544	2.266145	0.02466	0.112016	-4.21751
AC018470.1	3.574238	-1.50708	2.26399	0.024795	0.112104	-3.37332
AC243967.2	5.552875	3.176057	2.263931	0.024799	0.112104	-3.37407
AC005954.2	3.330534	2.556676	2.264466	0.024766	0.112104	-3.37733
AC211433.2	3.56955	5.253722	2.26468	0.024752	0.112104	-3.41962
AC010173.1	1.009943	5.316626	2.264607	0.024757	0.112104	-3.67675
AC109597.2	-1.39266	3.520617	-2.26458	0.024758	0.112104	-3.77527
AC006486.2	0.708369	5.540591	2.26461	0.024757	0.112104	-3.82416
AC005753.1	-1.36464	4.71204	-2.26418	0.024784	0.112104	-4.14496
SSSCA1-AS1	0.429247	8.130695	2.263693	0.024814	0.112104	-4.5243
AL513327.2	3.093044	4.728672	2.262949	0.024861	0.112196	-3.41411
AC104971.1	1.865965	5.248234	2.262896	0.024864	0.112196	-3.56765
AL365356.4	4.458749	6.667671	2.260769	0.024998	0.112619	-3.44708
AP000695.3	1.299895	4.950985	2.260695	0.025003	0.112619	-3.59059
AC006042.1	-1.62988	7.931723	-2.26076	0.024999	0.112619	-4.7728
AC106886.2	0.942912	6.58571	2.260201	0.025034	0.112625	-4.09705
LINC00957	-0.59532	7.454273	-2.26024	0.025032	0.112625	-4.57737
AC004943.1	0.609024	5.910285	2.259819	0.025059	0.112667	-3.96124
AC138512.1	-3.30778	-0.21733	-2.2593	0.025092	0.112747	-3.44079
AC009646.2	3.927309	-1.92937	2.258923	0.025116	0.112758	-3.38223
AC135048.4	0.720828	6.247449	2.258789	0.025124	0.112758	-4.04758
LINC01305	3.675675	1.669607	2.258324	0.025154	0.112823	-3.38395
LINC02580	-1.13222	5.13746	-2.25738	0.025214	0.113026	-4.19965
AC020934.1	3.867444	-1.78589	2.256139	0.025293	0.113291	-3.3871
IQCJ-SCHIP1-AS1	4.218062	1.83086	2.255978	0.025303	0.113291	-3.38773
LINC00837	2.950789	-0.44596	2.255572	0.025329	0.11334	-3.38801
AC073592.10	4.564219	2.526713	2.255057	0.025362	0.11342	-3.39009
AC092167.1	4.613974	-0.23277	2.252649	0.025517	0.113941	-3.39316

AC020891.3	5.247408	4.051382	2.252534	0.025524	0.113941	-3.39816
FMR1-IT1	0.60218	7.291397	2.252988	0.025495	0.113941	-4.3602
AC092910.3	0.533597	7.161984	2.25208	0.025553	0.114004	-4.34229
LIF-AS1	3.579175	1.991015	2.251341	0.025601	0.114149	-3.39762
AC091931.1	4.376046	-0.92879	2.2508	0.025636	0.114237	-3.39642
AL138688.1	3.590573	0.388256	2.249757	0.025704	0.11447	-3.39819
AC067747.1	-2.09756	3.171582	-2.24915	0.025743	0.114578	-3.80073
AC093330.1	-2.33446	0.067902	-2.24823	0.025803	0.114776	-3.44896
LINC02334	-1.13318	3.055929	-2.24753	0.025848	0.114853	-3.6346
AC023043.4	0.73939	8.694171	2.247491	0.025851	0.114853	-4.61179
AL031058.1	1.273084	8.576179	2.24721	0.025869	0.114867	-4.51343
AL118556.1	1.645191	5.634942	2.246912	0.025889	0.114885	-3.72998
AC005544.1	-3.09373	-0.26096	-2.24577	0.025963	0.115148	-3.46128
AC115618.3	0.53538	9.862672	2.245195	0.026001	0.115247	-4.82
LINC02048	3.144401	0.533489	2.243252	0.026128	0.115743	-3.40979
AC090771.2	-3.13357	-1.49898	-2.24273	0.026162	0.115826	-3.43682
LINC01857	5.689	4.788079	2.242242	0.026195	0.115901	-3.41944
AL009178.2	1.683435	5.039001	2.241408	0.02625	0.116076	-3.58135
FUT8-AS1	-0.87268	6.066775	-2.24	0.026343	0.11642	-4.37967
AC092802.3	-2.868	0.850133	-2.23937	0.026384	0.116534	-3.52853
AC013549.1	-0.91197	5.516231	-2.23909	0.026403	0.116547	-4.29399
AL450263.1	-1.14347	6.790011	-2.23873	0.026427	0.116584	-4.58106
AJ003147.3	-0.54094	5.520602	-2.23782	0.026488	0.116784	-4.20892
AC093817.1	4.300255	1.735486	2.237027	0.02654	0.116948	-3.42101
LINC01254	3.474903	0.088673	2.234667	0.026698	0.117013	-3.42456
AP005432.2	3.805247	3.914592	2.235064	0.026671	0.117013	-3.43834
AC079228.1	1.109946	4.539431	2.23594	0.026613	0.117013	-3.57746
AC011997.1	2.393123	6.040114	2.235293	0.026656	0.117013	-3.63932
AC009962.1	-0.61445	4.964267	-2.23619	0.026596	0.117013	-4.04271
AC104982.1	-0.86855	4.969908	-2.23543	0.026647	0.117013	-4.13038
AC019205.1	0.932289	7.086578	2.234697	0.026696	0.117013	-4.27679
AC084125.2	0.65768	6.878906	2.234474	0.026711	0.117013	-4.28785
AC124283.2	0.767898	7.3782	2.236001	0.026609	0.117013	-4.37416
AC133552.5	-0.65545	8.148719	-2.23459	0.026703	0.117013	-4.73713
AC090578.2	4.911861	1.66874	2.233094	0.026803	0.117078	-3.42747
SKAP1-AS1	3.654328	5.102869	2.233489	0.026777	0.117078	-3.47067
AC008496.2	-2.17591	2.800916	-2.23354	0.026773	0.117078	-3.7312
AC114730.1	0.898292	5.745222	2.233234	0.026794	0.117078	-3.95176
AP005131.3	-0.99437	5.587278	-2.23341	0.026782	0.117078	-4.31548
AC010245.2	-0.48859	6.792603	-2.23159	0.026904	0.117451	-4.4962
AC016876.1	0.622433	9.425703	2.231302	0.026924	0.117468	-4.77483
AC125437.1	0.732065	6.758171	2.230955	0.026948	0.117502	-4.27858
IL10RB-DT	0.553044	6.451093	2.230353	0.026988	0.117611	-4.231
AC099560.1	4.156484	-1.59174	2.229549	0.027043	0.11778	-3.43342
ABALON	0.827874	6.880787	2.22836	0.027123	0.118063	-4.25047
AC027575.2	-0.66109	6.759733	-2.22811	0.02714	0.118068	-4.50874
AC010186.3	0.811507	7.829928	2.227872	0.027156	0.118071	-4.4865
AC027243.1	4.028825	2.792803	2.227442	0.027186	0.118129	-3.44192
AL356488.2	3.98251	1.958582	2.225618	0.02731	0.118464	-3.44192
AP006545.1	-0.81902	6.277918	-2.22607	0.027279	0.118464	-4.45256
AC007485.1	-1.19476	6.248074	-2.22566	0.027307	0.118464	-4.50768
LINC01460	2.730773	2.946091	2.225149	0.027342	0.118511	-3.45645
AC114401.1	-2.26105	0.784236	-2.22488	0.027361	0.118511	-3.52575
AC004264.2	-1.122	4.094616	-2.22477	0.027368	0.118511	-4.00287
AC115284.2	-0.66759	5.491672	-2.22433	0.027398	0.118543	-4.24933
AL590666.2	1.772966	9.827323	2.224201	0.027407	0.118543	-4.68752
AL109930.1	-1.21062	4.290462	-2.2237	0.027442	0.118624	-4.05025
AL355075.1	3.999545	1.586729	2.222707	0.02751	0.118781	-3.44631

AC138356.1	-0.6428	4.141481	-2.22275	0.027507	0.118781	-3.85842
LINC01518	5.257289	0.751376	2.221747	0.027576	0.118998	-3.44699
AC131097.3	3.268644	5.089501	2.220682	0.027649	0.119178	-3.51151
AC068831.1	0.481487	5.945131	2.220873	0.027636	0.119178	-4.0879
AC118344.1	-0.48121	6.710248	-2.21916	0.027755	0.119564	-4.51214
C7orf65	3.266972	-1.92532	2.218448	0.027804	0.119625	-3.45261
LINC01456	4.75876	-0.12868	2.217787	0.02785	0.119625	-3.45379
AC144836.1	5.009099	1.862484	2.218016	0.027834	0.119625	-3.45384
AC100791.1	4.246007	-1.32062	2.217578	0.027864	0.119625	-3.45412
AC091152.4	-0.89947	2.991271	-2.21826	0.027817	0.119625	-3.65682
AC016027.1	0.478196	6.851015	2.217978	0.027836	0.119625	-4.35175
AC008622.2	0.692114	6.274554	2.217048	0.027901	0.119715	-4.18068
LINC02320	4.975361	2.943378	2.216296	0.027953	0.11981	-3.45904
AC124016.1	0.603245	6.846887	2.216271	0.027955	0.11981	-4.33433
AC092807.2	4.836945	2.36051	2.215583	0.028003	0.119925	-3.45921
AL442071.1	-1.36621	4.00276	-2.21543	0.028014	0.119925	-4.0086
AC005358.2	-1.93877	0.747023	-2.21478	0.028059	0.120051	-3.5307
AC024581.1	5.657834	2.088739	2.213879	0.028122	0.120252	-3.4608
AC005229.4	0.585683	8.518824	2.213504	0.028149	0.120296	-4.67824
AC096887.2	0.751825	6.791139	2.212126	0.028245	0.120641	-4.30981
AC012360.2	-0.71724	6.306435	-2.21141	0.028296	0.120788	-4.45735
AL451070.1	3.963824	2.433656	2.210008	0.028395	0.121141	-3.47214
EIPR1-IT1	3.241967	4.084702	2.209756	0.028413	0.121149	-3.49729
AC017002.1	3.182332	4.6784	2.209508	0.02843	0.121154	-3.51021
AL078605.1	4.00847	-0.85994	2.208627	0.028493	0.121352	-3.46954
AC002076.1	6.22745	3.416468	2.208308	0.028515	0.121379	-3.47166
AL355483.3	3.893866	2.893709	2.204221	0.028806	0.122389	-3.48529
AC130456.1	-2.73965	1.384762	-2.20469	0.028773	0.122389	-3.62746
AL354813.1	1.321249	5.423311	2.204095	0.028815	0.122389	-3.78562
AC114956.3	0.524327	6.942613	2.204063	0.028818	0.122389	-4.39746
AC073346.2	3.377937	-1.72904	2.202022	0.028964	0.122942	-3.48084
AL136172.1	2.503737	3.385205	2.200614	0.029065	0.123302	-3.50892
AC023090.2	-0.45043	5.524121	-2.2	0.02911	0.12342	-4.25869
F10-AS1	-3.28224	-0.84543	-2.19937	0.029156	0.123545	-3.52877
AL358334.3	5.32524	4.378858	2.19874	0.029201	0.123668	-3.49691
AC125494.3	4.186036	0.908127	2.197738	0.029273	0.123835	-3.48853
CARNMT1-AS1	0.543432	6.484054	2.197904	0.029261	0.123835	-4.29972
AC005383.1	-1.18807	5.246838	-2.19697	0.02933	0.124003	-4.3252
AC022400.1	0.557717	8.112703	2.195101	0.029465	0.124507	-4.65782
AL449403.1	-2.82951	-0.37139	-2.1944	0.029517	0.124514	-3.54461
AC010976.2	-1.1852	5.962342	-2.19443	0.029514	0.124514	-4.52297
AL604028.1	-0.35922	9.126292	-2.19448	0.029511	0.124514	-4.93123
AC010761.5	1.217796	6.131509	2.193929	0.029551	0.124589	-3.94281
AC005828.2	4.007209	0.303156	2.192556	0.029652	0.124888	-3.49722
AC022306.2	-0.47655	8.164585	-2.19251	0.029655	0.124888	-4.80545
FRGCA	4.481569	-1.08691	2.190832	0.029778	0.125205	-3.5
AC005392.3	4.136747	-1.51924	2.19035	0.029814	0.125205	-3.50079
AC008474.1	3.411896	3.117282	2.190622	0.029794	0.125205	-3.51494
AC016722.2	-0.34667	6.452186	-2.19045	0.029806	0.125205	-4.50386
AC021218.1	-1.93122	5.905087	-2.19103	0.029764	0.125205	-4.601
AC005674.2	-0.57384	6.747439	-2.18922	0.029897	0.125486	-4.60183
AC132192.2	0.534727	7.542981	2.18855	0.029947	0.125553	-4.55196
AC008966.1	-0.54551	7.107592	-2.18877	0.029931	0.125553	-4.64357
AP003032.1	-2.31204	2.699456	-2.188	0.029988	0.125654	-3.85048
AC097634.1	-0.5755	4.962804	-2.18749	0.030025	0.125742	-4.17148
NAALADL2-AS2	3.791408	3.236664	2.182504	0.030397	0.127228	-3.52678
AC092692.1	-0.85571	3.370029	-2.18226	0.030416	0.127235	-3.79829
AC091946.1	4.119805	-1.42167	2.180782	0.030527	0.127556	-3.51707

AC002401.4	5.5896	4.444056	2.180794	0.030526	0.127556	-3.52713
AC106799.3	4.452045	1.284723	2.179252	0.030642	0.127896	-3.52043
AC008873.1	-3.12288	0.284911	-2.17946	0.030626	0.127896	-3.6543
AL157884.2	3.958968	0.696073	2.178148	0.030725	0.128173	-3.52203
AL121906.1	3.92077	3.408223	2.177541	0.030771	0.128294	-3.5366
LINC00880	2.776585	2.597144	2.1771	0.030805	0.128362	-3.53823
AC017067.1	-3.2798	0.257026	-2.17521	0.030948	0.128889	-3.62236
AP003355.3	5.046681	0.758666	2.174223	0.031024	0.129131	-3.52846
LINC01850	4.640586	-0.72263	2.172503	0.031155	0.129396	-3.53117
AL603839.1	4.100049	0.297245	2.17259	0.031148	0.129396	-3.5312
CD200R1L-AS1	4.479237	2.537638	2.173153	0.031105	0.129396	-3.53431
AL353622.1	-0.56605	7.277429	-2.17249	0.031156	0.129396	-4.70745
AC008280.3	-0.50329	7.548677	-2.17087	0.031281	0.129841	-4.75936
AC092653.2	-0.74189	5.556003	-2.17047	0.031311	0.129894	-4.39857
AC007952.4	3.77441	3.041092	2.168151	0.03149	0.130494	-3.55021
AL158151.4	3.65405	4.978168	2.168369	0.031473	0.130494	-3.58751
IGF2-AS	3.092044	0.753421	2.167335	0.031553	0.130683	-3.54194
TXNDC12-AS1	-1.46861	4.370004	-2.16662	0.031609	0.130842	-4.25097
AC092574.2	-0.80964	5.991528	-2.16615	0.031645	0.130887	-4.53107
LINC00852	-0.47651	7.665727	-2.16603	0.031655	0.130887	-4.7835
AC114341.1	1.821744	6.362572	2.165195	0.031719	0.131081	-3.98934
AL355336.1	-0.53457	5.260752	-2.16497	0.031737	0.131081	-4.26052
LINC02390	3.874776	0.885438	2.162808	0.031905	0.131663	-3.54846
AC138305.3	0.768151	10.86794	2.162718	0.031912	0.131663	-5.10465
AC008551.1	4.54302	0.321988	2.162367	0.03194	0.131704	-3.54845
MYOSLID	2.765847	7.257607	2.162006	0.031968	0.131749	-4.05043
LINC02598	4.313333	0.993213	2.161235	0.032029	0.131925	-3.55076
AC093227.1	-0.59355	8.51968	-2.16087	0.032057	0.13197	-4.94765
AL022322.2	-0.5009	3.84818	-2.15966	0.032152	0.132257	-3.86488
AC080080.1	0.637537	6.08892	2.159314	0.03218	0.132257	-4.22414
AC003102.1	0.571916	8.065709	2.159483	0.032166	0.132257	-4.72052
LINC01357	7.543989	5.235522	2.158701	0.032228	0.132383	-3.56049
AC231533.1	3.522829	-0.39877	2.157774	0.032301	0.132611	-3.55611
AC090517.2	0.575724	7.439217	2.15739	0.032331	0.132663	-4.60395
AC098798.1	3.537129	-1.33124	2.156562	0.032397	0.132787	-3.55798
AC104113.1	-0.46706	7.631203	-2.15659	0.032395	0.132787	-4.79939
AC015689.1	-1.58166	4.61067	-2.15588	0.032451	0.132935	-4.32892
AC026254.2	4.434517	4.198753	2.153876	0.03261	0.133516	-3.58211
AL049649.1	3.901481	-0.18567	2.153624	0.032631	0.133526	-3.56309
AC099552.4	4.046135	-1.08385	2.15335	0.032652	0.133526	-3.5634
AL683887.1	6.39001	2.241313	2.153178	0.032666	0.133526	-3.56448
AC005670.1	3.846095	2.981992	2.151945	0.032765	0.133783	-3.57786
AP000593.3	1.567011	5.924778	2.15198	0.032762	0.133783	-3.9656
CECR3	3.563075	-1.27471	2.15149	0.032801	0.13384	-3.56649
AP003031.1	3.667143	3.313343	2.151326	0.032814	0.13384	-3.58409
AC011592.1	3.169178	-1.84871	2.150282	0.032898	0.134109	-3.56844
UXT-AS1	0.552008	5.964138	2.149136	0.03299	0.134266	-4.23779
AL139161.1	-1.77643	4.230976	-2.14955	0.032957	0.134266	-4.30575
OSGEPL1-AS1	-0.41389	6.330254	-2.14919	0.032986	0.134266	-4.56604
AC006372.3	2.975773	-0.65636	2.148598	0.033033	0.134268	-3.57152
AC092724.1	2.711548	0.557333	2.148292	0.033058	0.134268	-3.57495
WWTR1-AS1	-0.76301	4.690244	-2.14796	0.033084	0.134268	-4.18508
AC105020.6	-0.42898	5.592055	-2.14798	0.033083	0.134268	-4.38887
AC020558.2	0.615783	7.05014	2.147799	0.033098	0.134268	-4.53552
AL157838.1	0.535462	7.634273	2.148907	0.033008	0.134268	-4.66484
AC022148.1	-2.39467	-0.19895	-2.14728	0.03314	0.134364	-3.63225
AL138760.1	4.499807	2.466892	2.14648	0.033204	0.134555	-3.58061
AL357874.2	-1.12568	4.404387	-2.14604	0.03324	0.134625	-4.1953

LINC01543	3.667382	-1.04224	2.145375	0.033294	0.134627	-3.57677
AC012485.1	-0.61318	4.06967	-2.1456	0.033276	0.134627	-3.99025
AC016831.5	-0.71144	5.458219	-2.14551	0.033283	0.134627	-4.41351
AC009248.2	4.450291	1.313759	2.144904	0.033332	0.134693	-3.57875
KLHL30-AS1	-2.2376	0.112406	-2.14473	0.033346	0.134693	-3.64831
AC004066.2	-3.30405	-0.03305	-2.14413	0.033395	0.134817	-3.66939
AL109976.1	1.708678	6.796841	2.143703	0.03343	0.134886	-4.19799
AC005355.1	4.495633	3.037017	2.141653	0.033597	0.135488	-3.59248
AL591468.1	4.297879	0.626866	2.13989	0.033741	0.135998	-3.58635
AC092070.4	3.657468	2.148293	2.137895	0.033906	0.136445	-3.59679
IL21R-AS1	1.998309	1.968406	2.137438	0.033943	0.136445	-3.60816
AC005954.1	3.109933	4.588878	2.138016	0.033896	0.136445	-3.64428
AC016245.1	0.786946	4.050004	2.137515	0.033937	0.136445	-3.76903
AL157394.1	0.752018	6.599789	2.137788	0.033914	0.136445	-4.39603
LINC00656	3.625555	-0.64129	2.136974	0.033981	0.136453	-3.59085
AC068338.3	-1.01094	5.67015	-2.13704	0.033976	0.136453	-4.56774
AL138995.1	-1.00034	5.255526	-2.13615	0.034049	0.136652	-4.46379
AC130469.1	4.009031	1.76568	2.13445	0.03419	0.137072	-3.59927
LINC01686	1.757089	4.98872	2.134459	0.03419	0.137072	-3.76858
AC011676.3	-2.23153	2.856617	-2.13386	0.034239	0.137122	-3.98787
Z69733.1	-1.45071	3.815575	-2.1339	0.034236	0.137122	-4.19383
AC069185.1	-0.70219	6.043783	-2.13254	0.034349	0.137414	-4.54189
AC116366.1	-0.61753	7.342908	-2.13256	0.034347	0.137414	-4.8136
Z95115.1	0.612263	7.76078	2.1305	0.03452	0.138023	-4.71423
LINC02080	-0.67297	3.650754	-2.12964	0.034592	0.138166	-3.90877
LINC00346	1.109496	7.398778	2.129683	0.034588	0.138166	-4.54824
KIAA2012-AS1	2.348842	1.038873	2.129018	0.034644	0.138299	-3.61157
MIR583HG	-1.38096	3.572129	-2.12752	0.03477	0.138727	-4.06679
AC026336.3	4.411825	2.252808	2.126152	0.034885	0.139115	-3.61394
LINC01143	4.191024	-1.26595	2.125906	0.034906	0.139123	-3.60915
AL079303.1	4.593586	3.652614	2.123681	0.035094	0.13978	-3.62717
AC016526.3	-1.5548	1.47332	-2.12352	0.035108	0.13978	-3.73851
AC103740.2	3.478481	0.864591	2.122568	0.035189	0.140011	-3.61716
AC022075.1	1.875939	7.623126	2.122401	0.035203	0.140011	-4.41444
AL136301.1	4.004248	0.37178	2.120692	0.035349	0.140508	-3.61825
AC068580.2	6.37834	3.454002	2.120497	0.035365	0.140508	-3.62133
AC008895.1	0.468274	6.827036	2.120198	0.035391	0.140535	-4.55532
AL138789.1	5.437057	6.72113	2.119649	0.035438	0.140625	-3.67427
BAIAP2-DT	0.541565	9.534522	2.119496	0.035451	0.140625	-5.03332
AC016722.1	3.759794	-0.8547	2.117262	0.035643	0.141023	-3.62352
AC022031.2	7.124368	3.427145	2.117523	0.03562	0.141023	-3.62496
LINC01968	2.197154	1.814455	2.117226	0.035646	0.141023	-3.63913
AC020891.2	5.374707	5.914375	2.117016	0.035664	0.141023	-3.66134
AC008406.3	2.286793	5.410385	2.117505	0.035622	0.141023	-3.8112
AC104083.1	0.995974	9.701079	2.117194	0.035649	0.141023	-5.00813
AC097637.2	-1.15585	2.957077	-2.11594	0.035757	0.141241	-3.9124
AC132872.1	0.475293	8.580751	2.115945	0.035756	0.141241	-4.90939
AC064801.2	-0.83524	4.852009	-2.1156	0.035786	0.141282	-4.34383
AC129510.2	3.966137	2.986765	2.112477	0.036057	0.142275	-3.64532
AP003774.3	3.605523	0.131686	2.111681	0.036126	0.142474	-3.6332
AC010969.1	3.25135	-1.23733	2.110944	0.03619	0.142563	-3.63386
AC034102.6	0.620078	6.303481	2.110836	0.036199	0.142563	-4.38986
AP000223.1	-1.04876	4.91617	-2.11077	0.036205	0.142563	-4.41222
LINC02437	-3.13395	-0.6412	-2.11018	0.036256	0.142689	-3.70636
AC125618.1	-2.94291	-0.06811	-2.10962	0.036306	0.142809	-3.7189
LINC00974	3.207758	0.155863	2.108734	0.036383	0.143038	-3.63853
LINC01671	3.711242	3.534611	2.106627	0.036568	0.14369	-3.66398
AC004884.2	1.777899	6.562911	2.106394	0.036588	0.143695	-4.12403

AL121987.2	0.515696	6.446722	2.105701	0.036649	0.143859	-4.5023
AC022898.1	-2.62234	0.253221	-2.10533	0.036682	0.143911	-3.74352
AC026310.2	3.980923	1.546021	2.104558	0.03675	0.144083	-3.64792
AC048382.1	-1.13616	4.2782	-2.1044	0.036764	0.144083	-4.22236
AL353801.3	2.915028	4.433432	2.102848	0.036901	0.144528	-3.70438
ZNF630-AS1	-2.23218	1.930238	-2.10269	0.036916	0.144528	-3.89921
MACORIS	4.355892	2.250899	2.101095	0.037057	0.144655	-3.65637
AC011270.2	-2.76454	-2.07468	-2.10168	0.037005	0.144655	-3.67037
AC120498.6	-3.29659	-1.39598	-2.10103	0.037063	0.144655	-3.69748
AC108463.3	1.966446	5.198126	2.101829	0.036992	0.144655	-3.83851
AC105219.4	1.336734	6.82803	2.10102	0.037064	0.144655	-4.39086
AL645568.1	-0.44087	6.702831	-2.1016	0.037012	0.144655	-4.73888
CU638689.5	3.45766	-1.39821	2.100556	0.037105	0.144658	-3.6509
AC012645.2	3.132998	3.038352	2.100361	0.037123	0.144658	-3.67517
AC099494.3	-1.00597	3.788842	-2.10061	0.0371	0.144658	-4.08887
AL445228.2	2.02685	4.900636	2.099426	0.037206	0.144908	-3.81335
AC104564.2	4.061713	1.519638	2.097101	0.037414	0.145571	-3.66027
ALG13-AS1	-0.53596	8.463826	-2.09709	0.037415	0.145571	-5.06105
AC005863.1	3.626239	-1.21587	2.096203	0.037494	0.14573	-3.65807
AC087273.2	3.882082	1.557771	2.096302	0.037486	0.14573	-3.66261
LINC00462	4.224776	3.072973	2.095099	0.037594	0.145823	-3.67342
LINC002481	-0.66674	5.913603	-2.09538	0.037568	0.145823	-4.61382
AC090587.1	-0.53944	7.058733	-2.09541	0.037566	0.145823	-4.83635
AC122688.3	0.494337	8.055935	2.095074	0.037596	0.145823	-4.86162
AL592435.2	3.708583	3.116232	2.093706	0.037719	0.146226	-3.68122
AC005828.5	3.998192	-0.4284	2.092657	0.037814	0.146371	-3.66405
AC084824.3	-0.42386	7.342142	-2.09266	0.037814	0.146371	-4.8816
AC090589.3	-0.3385	8.677442	-2.09265	0.037815	0.146371	-5.07468
AL138962.1	4.449186	1.673557	2.092188	0.037857	0.146456	-3.66784
AC116552.1	-0.82389	6.56277	-2.0915	0.037919	0.14662	-4.79347
AL033527.3	1.972495	4.936612	2.091248	0.037942	0.146635	-3.8154
AL606534.1	1.628023	5.949193	2.090936	0.03797	0.146669	-4.15218
AC092368.3	0.611703	8.300968	2.090405	0.038019	0.14678	-4.88829
AC244034.3	-1.13944	3.123489	-2.09012	0.038044	0.146804	-3.97061
AC091173.1	3.129519	-1.10045	2.089138	0.038134	0.147074	-3.66963
AC079385.3	3.567514	-0.04128	2.088605	0.038183	0.147186	-3.67094
AC007336.1	0.809947	5.551959	2.088303	0.03821	0.147217	-4.18613
AC024270.2	-1.44518	3.51685	-2.08766	0.038269	0.147367	-4.14576
AC121320.1	3.081529	1.313557	2.086119	0.03841	0.147811	-3.68228
AC092436.4	-1.32531	5.470619	-2.08597	0.038423	0.147811	-4.66454
AC011511.2	3.381492	1.049824	2.085522	0.038465	0.147894	-3.67986
EGOT	2.634852	4.37556	2.08495	0.038517	0.148021	-3.74943
PARAL1	3.910183	-0.16054	2.082446	0.038748	0.148801	-3.68083
AC011465.1	0.651227	8.293587	2.082319	0.03876	0.148801	-4.90328
USP12-AS2	4.428571	2.409348	2.081424	0.038843	0.148855	-3.69015
AC018878.1	3.720689	3.38811	2.080882	0.038893	0.148855	-3.70809
AC002550.1	1.887507	3.311207	2.080998	0.038882	0.148855	-3.73753
AC120114.1	0.701596	7.202647	2.081575	0.038829	0.148855	-4.67494
LINC01176	0.574567	9.145532	2.08116	0.038867	0.148855	-5.04936
LINC02604	0.457248	10.46718	2.081427	0.038842	0.148855	-5.25745
ATP2C2-AS1	0.912376	6.939456	2.08049	0.038929	0.148919	-4.58183
AL033519.3	-3.04826	-0.65873	-2.07966	0.039007	0.148966	-3.75327
AC099518.2	2.596926	5.715868	2.079503	0.039021	0.148966	-3.84733
AC040904.1	2.200041	6.648904	2.079871	0.038987	0.148966	-4.22063
POLH-AS1	0.579776	7.93948	2.08014	0.038962	0.148966	-4.85131
AC126696.1	3.84879	1.52666	2.078661	0.039099	0.149189	-3.69115
TRPM2-AS	2.748889	7.822781	2.077736	0.039186	0.149442	-4.2794
AC239799.1	3.246602	-0.33767	2.076883	0.039265	0.149518	-3.68996

AC087672.2	3.254896	3.18298	2.077301	0.039226	0.149518	-3.71376
AC004837.2	0.626624	7.072398	2.076904	0.039263	0.149518	-4.68572
AC004817.1	3.727741	0.101802	2.075969	0.039351	0.149768	-3.69156
AC139720.1	4.799863	2.738537	2.075663	0.03938	0.149802	-3.69964
AL592114.3	-2.50231	2.468924	-2.07519	0.039424	0.149894	-4.02637
AL157700.1	-0.64203	6.497437	-2.07475	0.039465	0.149975	-4.80407
AL513123.1	9.746606	5.735837	2.074314	0.039506	0.150055	-3.69726
AC112493.1	4.050961	-0.93936	2.073321	0.039599	0.150334	-3.69538
AC002540.1	3.350624	1.413974	2.071312	0.039789	0.150977	-3.70567
AC011313.1	4.1068	0.177586	2.070572	0.039859	0.151167	-3.70026
AC100782.1	4.074585	2.450981	2.070105	0.039903	0.151258	-3.71155
AP000851.1	4.101571	1.523421	2.068901	0.040017	0.151614	-3.70691
LINC01703	0.895989	7.970738	2.06832	0.040073	0.151747	-4.81854
AC083841.2	4.471633	0.803582	2.067986	0.040105	0.151791	-3.70479
SLC25A5-AS1	-0.3418	6.874769	-2.06706	0.040193	0.152048	-4.83221
AC013391.1	3.846695	0.297105	2.065965	0.040297	0.152299	-3.70793
AC027031.2	1.203129	7.069384	2.065942	0.040299	0.152299	-4.61082
AL121827.2	2.133841	2.073505	2.065544	0.040338	0.152366	-3.73186
AC048337.1	3.692043	1.313823	2.065318	0.040359	0.152371	-3.71313
AL133153.2	3.317095	5.338118	2.064765	0.040412	0.152389	-3.80367
AC092135.1	-0.6035	4.083954	-2.06479	0.040409	0.152389	-4.16132
BX322562.1	0.913312	7.703742	2.064634	0.040425	0.152389	-4.809
AC108136.1	6.001724	3.4481	2.063741	0.04051	0.152635	-3.71658
AL139351.1	4.467529	0.124074	2.063104	0.040572	0.152789	-3.71221
AC024361.2	-1.35052	3.333631	-2.06258	0.040622	0.152837	-4.1401
AC011477.2	-0.5513	8.940427	-2.06255	0.040625	0.152837	-5.20403
CASC9	3.913178	9.472412	2.062011	0.040677	0.152955	-4.5093
AC010271.2	3.806666	0.433109	2.061583	0.040718	0.153003	-3.71524
AL354793.1	3.287401	4.030303	2.061455	0.04073	0.153003	-3.75722
DLX2-DT	4.157043	0.800137	2.061209	0.040754	0.153016	-3.716
AC106881.1	-1.06468	3.065716	-2.06092	0.040781	0.153043	-4.04648
SERPINB9P1	1.376579	7.302631	2.060559	0.040817	0.153099	-4.551
LINC02004	-1.62151	4.572494	-2.05915	0.040953	0.153534	-4.54747
LINC02584	3.887743	4.270399	2.058359	0.04103	0.153744	-3.75555
AL354714.1	2.873949	1.80594	2.058126	0.041052	0.153752	-3.73391
AC073592.8	3.951375	0.370044	2.056485	0.041212	0.154043	-3.72322
LINC02631	3.452692	1.244212	2.056997	0.041162	0.154043	-3.72766
ARHGAP31-AS1	1.54557	6.333037	2.056877	0.041174	0.154043	-4.24305
AL390728.6	0.470384	10.62134	2.056508	0.04121	0.154043	-5.32795
AL031118.1	-0.78317	4.704256	-2.0557	0.041289	0.1541	-4.39548
AC020612.3	0.589865	6.656562	2.055823	0.041277	0.1541	-4.61825
AC010542.5	0.603657	8.77377	2.055974	0.041262	0.1541	-5.03856
AC113194.1	4.126982	-1.21963	2.054761	0.04138	0.154212	-3.72523
AC008498.2	-1.40081	2.053526	-2.05516	0.041341	0.154212	-3.91088
AC097359.2	-0.49061	7.190586	-2.05481	0.041376	0.154212	-4.94398
AC079145.1	-1.18506	5.216799	-2.0537	0.041484	0.15452	-4.62714
AL450384.2	-0.46039	7.63033	-2.05153	0.041697	0.155238	-5.01411
AL121987.1	-2.11665	2.080609	-2.04994	0.041854	0.155745	-3.98291
AL136131.2	4.28963	-0.21857	2.048927	0.041954	0.155886	-3.73489
AC005920.2	4.187033	6.266046	2.049219	0.041925	0.155886	-3.85164
AC046158.1	-0.96064	4.806401	-2.04907	0.04194	0.155886	-4.49836
AC009063.3	3.755861	-1.06655	2.047894	0.042056	0.156189	-3.73628
AC103702.2	3.060184	9.075748	2.047302	0.042115	0.15633	-4.62485
AC104794.3	3.965023	1.195665	2.047039	0.042141	0.156349	-3.74118
EXOC3-AS1	0.679506	8.437227	2.045638	0.042281	0.156789	-4.99992
AC073655.1	3.558252	3.61694	2.044061	0.042438	0.157271	-3.77404
TTLL11-IT1	3.011815	3.278679	2.043914	0.042452	0.157271	-3.77483
AC010595.1	5.49957	2.816476	2.043641	0.04248	0.157295	-3.74868

AC090286.1	3.288678	-1.62109	2.043162	0.042528	0.157395	-3.74367
AC024560.1	3.295284	2.798294	2.041011	0.042744	0.158115	-3.76986
AL355303.1	3.896914	1.199795	2.039842	0.042861	0.158394	-3.75267
EMC1-AS1	-0.43157	7.394835	-2.03985	0.042861	0.158394	-4.99808
AC026124.2	1.509271	6.433525	2.038211	0.043026	0.158924	-4.24891
AC009502.1	2.146693	2.281009	2.037156	0.043132	0.159084	-3.78223
AP000919.3	-1.93345	2.888497	-2.03718	0.04313	0.159084	-4.12332
AL137847.1	-1.92257	3.747393	-2.03729	0.043119	0.159084	-4.29825
AC105053.1	-2.24455	0.415882	-2.03685	0.043164	0.159121	-3.84659
C2orf27B	-2.3205	-0.18882	-2.0365	0.043199	0.159174	-3.82339
LIMD1-AS1	-1.31908	2.527551	-2.03607	0.043242	0.159254	-3.97063
AL121820.2	-1.6002	6.484992	-2.03567	0.043283	0.159327	-5.00429
AC006141.1	-0.46999	7.148229	-2.0348	0.043372	0.159576	-4.9678
AC104823.1	3.693046	0.159039	2.034005	0.043453	0.159794	-3.7592
LINC02570	4.079829	1.944934	2.032955	0.04356	0.159953	-3.76801
LINC02014	5.182316	6.710944	2.033148	0.04354	0.159953	-3.84437
AP003392.5	1.673406	6.392958	2.033082	0.043547	0.159953	-4.17996
AC089983.1	4.099662	2.846695	2.031485	0.04371	0.160045	-3.77793
AC023855.1	3.246505	3.327158	2.032015	0.043656	0.160045	-3.79548
AL161457.1	-2.9674	-1.36904	-2.0325	0.043607	0.160045	-3.81174
AC092040.1	-1.57024	0.895417	-2.03196	0.043661	0.160045	-3.85396
AC004623.1	0.481772	5.966336	2.031641	0.043694	0.160045	-4.49405
AC004865.2	0.663025	6.222815	2.03146	0.043713	0.160045	-4.54017
AL391069.3	3.782654	0.395965	2.029896	0.043873	0.160353	-3.76602
LINC01841	2.891234	2.54656	2.029953	0.043867	0.160353	-3.79006
LINC02544	4.179633	4.016023	2.029603	0.043903	0.160353	-3.79536
AL391425.1	-0.98472	3.300673	-2.03022	0.04384	0.160353	-4.11455
AC027682.1	0.653595	5.640328	2.029704	0.043893	0.160353	-4.35027
AC010999.2	2.668341	3.300887	2.028426	0.044024	0.160717	-3.80944
AL603750.1	-0.69199	8.180641	-2.02748	0.044122	0.160996	-5.17327
AC041040.1	2.279333	1.839328	2.02708	0.044163	0.161067	-3.79028
AC012150.1	3.655721	-1.28931	2.026843	0.044188	0.161079	-3.76973
AP001160.3	0.508989	8.61564	2.026614	0.044212	0.161087	-5.09046
AL603910.1	-0.81607	6.096149	-2.02558	0.044318	0.161397	-4.83957
AL353693.1	5.161667	3.594924	2.023604	0.044524	0.162068	-3.79115
LINC01415	2.007902	4.829109	2.02283	0.044604	0.162282	-3.9296
AC012442.1	0.700185	6.924414	2.022349	0.044655	0.162386	-4.69587
AC011443.1	-0.73505	6.551046	-2.02198	0.044694	0.162449	-4.9215
AP003469.1	3.674719	0.511669	2.018968	0.045009	0.16351	-3.78401
AC005695.1	-0.8175	4.090853	-2.01877	0.045029	0.16351	-4.31217
AC020907.5	1.900841	2.779415	2.01714	0.045201	0.164055	-3.83239
AC005091.1	2.272711	3.193347	2.016643	0.045253	0.164166	-3.83591
AC141586.5	-0.44675	5.128575	-2.01553	0.04537	0.164512	-4.49773
RRM1-AS1	3.686498	3.57611	2.014485	0.045481	0.164799	-3.82146
AC009093.1	1.378807	5.74609	2.014371	0.045494	0.164799	-4.17461
UBR5-AS1	0.395916	9.388998	2.013367	0.0456	0.165105	-5.2456
AC130650.2	0.510838	6.493027	2.01232	0.045711	0.165428	-4.68405
AL391261.2	-2.91497	-1.56385	-2.01156	0.045792	0.16548	-3.83481
AL356124.2	-1.99153	3.200039	-2.01178	0.045769	0.16548	-4.20453
AP001505.1	0.514642	8.857258	2.011673	0.04578	0.16548	-5.15245
AL592494.2	4.458692	3.525383	2.010964	0.045856	0.165632	-3.81671
LINC01932	6.402841	4.642374	2.010539	0.045901	0.165717	-3.80922
AL160408.5	4.491294	0.89548	2.010223	0.045935	0.165759	-3.79721
AC130456.4	-2.60075	1.867391	-2.00924	0.046041	0.166024	-4.05117
AP003086.1	-0.72893	4.809965	-2.00912	0.046053	0.166024	-4.512
AP001025.1	3.780792	2.911791	2.008659	0.046102	0.166083	-3.81974
AC067750.1	-0.40806	6.032231	-2.00856	0.046113	0.166083	-4.77848
AC040160.2	-0.39412	5.59984	-2.00828	0.046143	0.166111	-4.66519

AP000439.3	3.168787	-2.03208	2.006867	0.046295	0.166418	-3.80098
LINC02607	3.579368	-0.54884	2.007228	0.046256	0.166418	-3.801
IDH1-AS1	0.737372	6.750546	2.006989	0.046282	0.166418	-4.70091
AC092422.1	-2.53303	1.257415	-2.00616	0.046371	0.166612	-3.99054
AC025918.1	-1.23332	4.782404	-2.0059	0.046399	0.166634	-4.65101
AP006259.1	-1.42197	3.658609	-2.00522	0.046472	0.166814	-4.32678
AL117336.1	0.639431	7.226948	2.004189	0.046584	0.167136	-4.86748
AGAP11	-1.02458	3.217486	-2.00362	0.046645	0.167277	-4.18085
AC006483.2	3.637241	1.900968	2.002957	0.046717	0.167455	-3.8185
AC010327.3	3.304666	1.012431	2.001918	0.04683	0.167705	-3.81523
AF127577.3	6.235877	5.168147	2.001822	0.04684	0.167705	-3.83297
LINC00596	-2.77412	-2.34706	-2.0017	0.046854	0.167705	-3.83617
AL160408.4	3.932559	0.863636	2.000359	0.046999	0.167986	-3.81382
AC016397.2	2.510729	1.673069	2.000765	0.046955	0.167986	-3.82939
AC011611.3	3.49725	4.586282	2.00039	0.046996	0.167986	-3.8842
AC073964.1	5.590301	5.209088	1.998144	0.047241	0.16869	-3.84956
AL592071.1	3.681466	3.886253	1.998267	0.047228	0.16869	-3.85243
AL354710.2	3.512673	1.536319	1.997317	0.047332	0.168853	-3.82529
AC009902.3	1.012472	4.81268	1.997373	0.047326	0.168853	-4.15137
LINC01561	3.089361	1.932555	1.996261	0.047448	0.169186	-3.83355
AC092894.1	3.461634	0.849102	1.995323	0.047551	0.16929	-3.82386
AC087501.2	-0.61158	5.798913	-1.9956	0.04752	0.16929	-4.77027
AC008556.1	1.826657	8.300818	1.995198	0.047565	0.16929	-4.90159
AC093726.2	-0.42636	6.884778	-1.99518	0.047567	0.16929	-4.98798
LINC02234	-2.48556	0.515802	-1.99473	0.047616	0.169386	-3.9421
AL049776.1	-0.4359	6.768022	-1.99166	0.047955	0.170511	-4.97794
AL355601.1	4.425487	0.555346	1.990893	0.04804	0.170732	-3.82721
AC010486.3	4.17788	-0.55609	1.990356	0.0481	0.170783	-3.82735
SMCR2	3.616083	1.730379	1.990363	0.048099	0.170783	-3.83839
LARGE-IT1	-2.43175	1.426595	-1.98982	0.04816	0.170915	-4.02408
LINC00524	4.418323	0.730061	1.989493	0.048196	0.170962	-3.82958
FP700111.1	2.672884	-1.01375	1.988978	0.048253	0.171004	-3.82954
LINC01352	-1.47531	2.925726	-1.989	0.04825	0.171004	-4.22051
AL031705.1	1.807622	5.076747	1.988609	0.048295	0.171069	-4.03542
AC129926.1	4.207959	1.699608	1.987765	0.048389	0.171322	-3.83723
AF241728.1	2.304445	4.289228	1.985973	0.048589	0.171951	-3.92831
AL031009.1	0.460208	7.026029	1.984942	0.048705	0.172279	-4.87685
AL109804.1	2.708181	4.764483	1.982604	0.048968	0.173128	-3.94771
AC009142.1	3.57921	-0.40784	1.982291	0.049003	0.173172	-3.84007
AP006621.3	0.506217	8.313139	1.981552	0.049087	0.173303	-5.12736
AC135050.5	-0.34033	8.554561	-1.98162	0.049079	0.173303	-5.27556
AL157400.3	1.798828	3.756119	1.979764	0.049289	0.17375	-3.95291
WASIR2	2.709643	4.873051	1.97962	0.049306	0.17375	-3.96707
AC027373.1	1.301032	6.189799	1.98015	0.049246	0.17375	-4.46076
TET2-AS1	-0.84778	5.345497	-1.97986	0.049279	0.17375	-4.75932
AC010624.1	-1.56416	2.484726	-1.97938	0.049333	0.173765	-4.12212
LINC01816	0.822853	8.05158	1.978966	0.04938	0.173848	-5.02817
LINC00994	-2.45977	-0.91541	-1.97865	0.049415	0.173891	-3.8949
FOXC2-AS1	4.333735	-0.4911	1.977187	0.049582	0.174316	-3.84785
AC067956.1	3.396991	-0.62012	1.977194	0.049582	0.174316	-3.84787
LINC01910	2.636797	3.322609	1.976693	0.049639	0.174432	-3.89483
AL157400.4	2.46554	4.832249	1.976224	0.049692	0.174539	-3.97677
AC099494.1	-0.66268	4.939188	-1.97572	0.04975	0.174659	-4.5915
AC136475.5	-1.11399	3.969064	-1.97524	0.049805	0.174771	-4.39785
AC073370.1	3.883085	1.818882	1.974587	0.04988	0.174951	-3.86072
AC012317.2	3.243882	-1.43724	1.973575	0.049996	0.175113	-3.85303
AC106799.1	4.291927	0.137388	1.973632	0.049989	0.175113	-3.85372
AL031719.1	-0.66922	3.837962	-1.97391	0.049958	0.175113	-4.31026

TMCO1-AS1	1.057406	5.38333	1.973123	0.050048	0.175132	-4.26265
AL591848.4	-0.84931	4.891239	-1.9733	0.050027	0.175132	-4.6254
AC015845.1	-0.83143	6.193444	-1.9726	0.050108	0.175261	-4.96796
AL359710.1	-0.96742	3.447625	-1.97217	0.050158	0.175353	-4.26084
AC123768.4	-2.61252	0.299516	-1.97072	0.050325	0.175856	-3.9766
AL354707.2	3.744675	-0.43894	1.969637	0.05045	0.17621	-3.8596
AC012447.1	2.326172	4.900154	1.969256	0.050494	0.176282	-3.9911
CYP4F26P	6.537924	4.838427	1.968795	0.050547	0.176338	-3.87907
AC012313.8	0.822534	5.180092	1.968713	0.050557	0.176338	-4.28089
LINC01449	3.316486	-1.51386	1.968243	0.050611	0.176446	-3.86123
AC100839.2	4.158526	2.724488	1.967686	0.050676	0.17659	-3.87991
AC093159.1	3.894512	5.268955	1.966822	0.050776	0.176858	-3.94723
AL121652.1	-1.02428	5.362447	-1.96655	0.050808	0.176886	-4.8432
AL162574.2	4.315249	0.079321	1.964478	0.05105	0.177645	-3.86779
AL050344.1	3.349816	-1.41752	1.963862	0.051122	0.177731	-3.86801
AC009093.4	-1.63077	4.763099	-1.96389	0.051118	0.177731	-4.63874
LINC02195	5.011899	6.420113	1.963528	0.051161	0.177784	-3.95233
AC011944.1	2.19154	3.2307	1.962626	0.051266	0.177905	-3.93032
AC007728.3	3.638415	4.641163	1.962656	0.051263	0.177905	-3.93296
AL807757.2	0.536969	7.322081	1.962963	0.051227	0.177905	-4.96949
AC105275.2	3.478684	-1.33024	1.961999	0.05134	0.177996	-3.87091
NCKAP5-AS1	-1.73395	2.187291	-1.96202	0.051338	0.177996	-4.13857
AC121761.1	0.700829	8.260543	1.961446	0.051405	0.178139	-5.13182
DENND6A-DT	2.12848	0.922635	1.960992	0.051458	0.178207	-3.88791
AC018690.1	0.379169	8.656876	1.960875	0.051472	0.178207	-5.23849
AC008806.1	-2.01041	0.620983	-1.96058	0.051507	0.178245	-3.98565
AC008915.1	0.931117	5.01482	1.9601	0.051563	0.178277	-4.18877
AL022238.3	-0.45367	6.558402	-1.96011	0.051562	0.178277	-4.98981
AC009120.2	-0.36388	8.249168	-1.95943	0.051642	0.178468	-5.27866
AP003559.1	2.05602	5.062258	1.959177	0.051672	0.178489	-4.08136
AC233723.2	0.610114	8.028246	1.958841	0.051712	0.178543	-5.11716
ACAP2-IT1	0.614637	7.891749	1.957905	0.051823	0.178844	-5.08709
AL391097.2	-1.28312	6.22657	-1.95716	0.051911	0.178984	-5.07511
AL162231.2	-0.88209	7.25154	-1.95722	0.051904	0.178984	-5.18862
FGF10-AS1	-2.64954	-1.95576	-1.95637	0.052005	0.179144	-3.91247
CAMTA1-DT	1.023092	7.090101	1.956436	0.051997	0.179144	-4.80933
AC147067.1	1.232543	7.089496	1.955042	0.052162	0.179604	-4.66939
AC115618.1	0.469048	7.491226	1.954657	0.052208	0.179679	-5.04064
LINC01098	3.547462	-1.22582	1.954009	0.052285	0.179744	-3.88321
AC125793.1	3.092509	-2.01426	1.953695	0.052323	0.179744	-3.88336
AC092979.1	-2.15436	0.843246	-1.95387	0.052302	0.179744	-4.01111
AL359921.2	0.403953	8.868585	1.954087	0.052276	0.179744	-5.28207
MYCBP2-AS2	-1.56537	3.026478	-1.95054	0.052701	0.180877	-4.36
AL365181.3	1.68269	9.463201	1.950649	0.052687	0.180877	-5.20095
SACS-AS1	-2.40626	-0.65659	-1.94998	0.052768	0.181024	-3.95028
KCNQ1OT1	-0.61564	6.568423	-1.94944	0.052832	0.181081	-5.0421
AC092574.1	-0.80884	6.784636	-1.94964	0.052809	0.181081	-5.11432
AF186192.3	2.831005	2.198442	1.948069	0.052998	0.181419	-3.91882
AC007601.2	-0.9887	4.849354	-1.94802	0.053003	0.181419	-4.70583
AC004830.1	-0.9861	6.270388	-1.94819	0.052983	0.181419	-5.03931
SREBF2-AS1	0.368864	8.604306	1.946807	0.05315	0.181838	-5.2572
AL355385.1	2.330962	6.08603	1.946568	0.053179	0.181854	-4.17791
AP001094.1	-1.65415	3.955499	-1.94612	0.053233	0.181955	-4.57679
AC023202.1	3.827054	0.542504	1.945722	0.053281	0.181956	-3.8978
AC022497.1	-0.5845	5.010188	-1.94581	0.053271	0.181956	-4.63239
LINC01585	1.637142	3.331527	1.944519	0.053427	0.182288	-3.97865
AC104964.3	-1.18119	2.303647	-1.94458	0.05342	0.182288	-4.13039
ZNF114-AS1	4.090182	1.516277	1.943358	0.053568	0.182592	-3.90616

AC099518.6	1.140751	6.950747	1.94353	0.053547	0.182592	-4.80594
AC023906.5	0.627419	8.156215	1.943188	0.053589	0.182592	-5.15553
AC073288.2	3.097283	4.276611	1.941803	0.053758	0.183001	-3.97557
LINC01977	1.146676	5.85306	1.941955	0.053739	0.183001	-4.44229
AC083862.2	0.581134	7.278317	1.941363	0.053812	0.183101	-4.98453
AC015871.4	-0.79554	4.088038	-1.94044	0.053924	0.1834	-4.39035
AF129075.2	0.641427	5.429886	1.939726	0.054012	0.183611	-4.47145
AL049820.1	-1.31952	3.811297	-1.93954	0.054035	0.183611	-4.50134
AC138150.1	-0.66953	5.885186	-1.93827	0.054191	0.184057	-4.91729
AC022079.1	1.10334	6.353211	1.936614	0.054395	0.184666	-4.61515
AL135902.2	-2.67188	0.582663	-1.93554	0.054527	0.185015	-4.06341
AC005703.6	1.509727	5.102265	1.935381	0.054547	0.185015	-4.19799
AC007881.3	2.386492	4.230735	1.933896	0.05473	0.185555	-4.04283
LINC02242	4.09519	0.550127	1.932897	0.054854	0.185807	-3.91674
LSAMP-AS1	5.337788	5.160041	1.932965	0.054846	0.185807	-3.95715
AC118755.1	5.004831	6.121386	1.932276	0.054932	0.185974	-3.99114
AC087071.2	1.434117	6.14412	1.932102	0.054953	0.185974	-4.46111
AP005131.2	3.541225	4.170428	1.931221	0.055063	0.186262	-3.97284
AC026894.1	-0.527	5.340248	-1.92986	0.055232	0.18675	-4.75699
AL161658.1	-2.80001	-2.25718	-1.92952	0.055275	0.186811	-3.95287
AC002347.1	-1.05623	4.637278	-1.92907	0.055331	0.186918	-4.70692
AL008721.2	-0.40371	6.804307	-1.92777	0.055493	0.187381	-5.10738
AC073333.1	0.535996	7.516765	1.926702	0.055628	0.187751	-5.09076
COL4A2-AS2	2.435478	2.676384	1.926033	0.055712	0.18795	-3.96633
AC027338.2	3.655791	-1.25779	1.925348	0.055798	0.188157	-3.92677
AC078864.1	-2.90993	-0.66509	-1.92491	0.055854	0.188259	-4.02757
AC016586.1	-0.51917	7.210479	-1.92375	0.056	0.188636	-5.20487
AP001893.1	-0.54166	7.255779	-1.92362	0.056016	0.188636	-5.21196
AC026367.3	-0.53946	7.318832	-1.92336	0.05605	0.188666	-5.23153
AC122694.1	3.921625	0.026562	1.92295	0.056101	0.188754	-3.93125
HIF1A-AS1	3.049469	3.708128	1.921863	0.056239	0.189132	-3.98742
AC114939.1	-0.61746	6.064183	-1.92076	0.056379	0.189518	-4.9917
DOCK4-AS1	2.182615	1.909715	1.919713	0.056512	0.189881	-3.96643
AC105219.2	5.811182	6.73712	1.919316	0.056562	0.189966	-4.01538
AC026474.1	-1.05922	5.161684	-1.91877	0.056632	0.190113	-4.86145
AC009075.1	3.659655	3.805925	1.918519	0.056664	0.190138	-3.98013
AC023389.1	-0.47016	8.093564	-1.91829	0.056693	0.190149	-5.33663
AC009086.3	0.399944	6.962908	1.917938	0.056738	0.190216	-4.99967
AP007216.2	4.099457	0.76809	1.917446	0.056801	0.190257	-3.94069
AC005330.1	2.576273	4.734891	1.917623	0.056779	0.190257	-4.05758
AL121658.1	0.618628	6.406515	1.913945	0.05725	0.191676	-4.78815
AC004466.2	-0.90744	5.071354	-1.91279	0.057399	0.192089	-4.83348
AC006262.2	4.029242	1.245705	1.912217	0.057473	0.19225	-3.95189
AL132796.2	3.009338	-1.66707	1.911272	0.057595	0.192573	-3.94773
AL096816.1	-1.23586	3.507222	-1.91062	0.05768	0.192769	-4.40695
AC004854.2	0.497304	7.870605	1.910315	0.057719	0.192769	-5.19843
AL139246.3	-1.4215	6.40426	-1.91022	0.057731	0.192769	-5.21111
AC011337.1	1.368773	6.522269	1.909208	0.057863	0.193124	-4.71259
AC010969.2	0.392192	8.632366	1.908837	0.057911	0.193199	-5.33377
AC107993.1	0.53633	7.009298	1.906329	0.058237	0.194202	-5.01466
LINC02533	3.072236	3.252884	1.904413	0.058488	0.194878	-4.00189
AC005261.3	0.319385	9.757719	1.904383	0.058492	0.194878	-5.52103
AC022532.1	0.707726	6.326114	1.903764	0.058573	0.195062	-4.83103
AL162253.1	-1.53749	2.790926	-1.90292	0.058683	0.195256	-4.34424
AP000866.5	-1.44481	5.21705	-1.90308	0.058663	0.195256	-4.96968
AC048341.2	0.48814	8.793957	1.902458	0.058745	0.195373	-5.35733
AC110769.2	0.687269	5.446677	1.901101	0.058923	0.195881	-4.5025
AL354919.2	3.841961	4.207789	1.900088	0.059057	0.196152	-4.01592

AC080038.4	0.944367	6.238182	1.900121	0.059053	0.196152	-4.75153
AC009495.2	-1.66528	3.390244	-1.89984	0.05909	0.196176	-4.43929
LINC02444	3.391727	-1.28064	1.89947	0.059139	0.196194	-3.96556
AC095055.1	-0.41006	6.839617	-1.8994	0.059148	0.196194	-5.16904
AC090912.2	1.536655	5.105454	1.898462	0.059272	0.196519	-4.24839
AC007036.1	-0.87814	5.583461	-1.89792	0.059344	0.19667	-4.92541
AL512444.1	4.176384	2.286771	1.897623	0.059383	0.196714	-3.98394
AL589765.7	-2.44038	0.555559	-1.89705	0.059459	0.196877	-4.11725
AC012494.1	3.73704	-0.79791	1.895772	0.059629	0.197181	-3.97132
AC079921.1	0.728911	6.225461	1.895889	0.059614	0.197181	-4.74452
AC016542.2	-0.7729	7.550328	-1.89594	0.059607	0.197181	-5.33575
AC073115.2	3.467033	-1.49312	1.895273	0.059696	0.197314	-3.9717
AC073073.2	0.429882	7.672081	1.89389	0.059881	0.197837	-5.19555
AC022098.3	2.853231	4.184052	1.893103	0.059986	0.198098	-4.05949
AL138762.1	0.555812	5.477516	1.892256	0.060099	0.198385	-4.63027
AC073592.3	1.675228	3.663482	1.891521	0.060198	0.198624	-4.08601
AL157902.2	-0.53466	4.129436	-1.89114	0.060249	0.198705	-4.45466
LINC01516	2.908121	-1.02919	1.890873	0.060285	0.198736	-3.97864
AP005137.1	-2.48178	1.131284	-1.89011	0.060387	0.198898	-4.16843
AC013731.1	0.622427	6.570226	1.89026	0.060367	0.198898	-4.86339
AC009088.1	-0.84398	4.862763	-1.88973	0.060438	0.19898	-4.77766
AC069503.1	3.492895	0.806922	1.888679	0.06058	0.199099	-3.98683
AL512363.1	4.887713	7.57799	1.888986	0.060539	0.199099	-4.17418
AC011503.2	-1.10057	5.392572	-1.88921	0.060509	0.199099	-4.96602
AC084782.3	-1.08423	5.418769	-1.88885	0.060557	0.199099	-5.0064
SMILR	5.913286	3.626899	1.888144	0.060652	0.199139	-3.99529
LINC02033	2.195793	2.741371	1.887999	0.060672	0.199139	-4.03225
AC025031.3	3.151987	5.481736	1.888089	0.06066	0.199139	-4.17123
LINC00556	3.775577	0.787062	1.886612	0.06086	0.199668	-3.98849
AC007996.1	0.643698	8.787544	1.886235	0.060911	0.199748	-5.36367
AC016526.2	1.499917	6.150128	1.884418	0.061157	0.200327	-4.51349
AP000753.2	-0.82438	5.831955	-1.88415	0.061194	0.200327	-5.04585
AL353763.2	0.423513	8.76164	1.884651	0.061126	0.200327	-5.39172
AC108673.3	-0.41486	8.352432	-1.88418	0.06119	0.200327	-5.44183
AC091982.1	-0.7422	5.952461	-1.88394	0.061223	0.200334	-5.04364
AC080129.1	4.100485	-0.4218	1.883339	0.061304	0.200514	-3.98998
RUNX2-AS1	2.925416	-1.02335	1.882821	0.061375	0.200544	-3.99057
AC005332.1	0.533447	5.730845	1.882687	0.061393	0.200544	-4.69973
AL132656.2	-0.4491	6.403371	-1.88268	0.061394	0.200544	-5.11126
LINC00920	1.343074	6.707857	1.882377	0.061436	0.200594	-4.75225
SATB2-AS1	1.177004	3.399107	1.881603	0.061541	0.200852	-4.11841
LINC00167	1.89747	3.029901	1.881373	0.061573	0.200867	-4.05997
AC092701.1	2.903094	-1.1389	1.880512	0.061691	0.200949	-3.99388
AP003721.2	3.678314	1.472834	1.880678	0.061668	0.200949	-4.00439
AC005786.4	2.998616	1.540777	1.880407	0.061705	0.200949	-4.01186
AC015909.5	-0.39893	6.244379	-1.88074	0.061659	0.200949	-5.05962
AL590385.2	3.512491	-0.12737	1.880169	0.061738	0.200968	-3.99521
AC069431.1	-1.68156	1.712306	-1.87966	0.061807	0.201107	-4.19419
AC022417.1	-0.44493	5.837612	-1.87787	0.062053	0.20182	-4.96366
AC092794.1	-0.63319	7.192832	-1.87713	0.062156	0.202068	-5.30462
AC104462.2	3.826913	0.318042	1.876027	0.062308	0.202473	-4.00196
AC011825.4	3.311601	1.952226	1.874966	0.062454	0.202774	-4.02344
AC107375.1	0.341604	8.205089	1.875021	0.062447	0.202774	-5.33764
AC007952.9	3.877731	-0.55552	1.873604	0.062643	0.203187	-4.00426
AC067863.2	4.480571	1.705802	1.87337	0.062676	0.203187	-4.01093
AC100791.3	2.669884	4.859827	1.873539	0.062652	0.203187	-4.15718
AC027702.1	0.927435	6.749427	1.87327	0.06269	0.203187	-4.8652
AC000124.1	-2.66375	-1.26908	-1.87203	0.062862	0.203658	-4.05894

AC008083.3	3.492269	-1.09915	1.871257	0.062969	0.203763	-4.0074
AC005912.2	1.694723	2.086685	1.871211	0.062976	0.203763	-4.05384
AC009318.3	-0.42267	7.467011	-1.87125	0.062971	0.203763	-5.32563
AC078795.1	0.456081	6.290762	1.87089	0.063021	0.20382	-4.90363
AC017006.2	-0.77004	5.328695	-1.87037	0.063093	0.203878	-4.94168
AC032044.2	0.479438	7.850564	1.870433	0.063084	0.203878	-5.25411
CXXC4-AS1	2.601595	-0.63671	1.870073	0.063135	0.203926	-4.01045
AC096642.1	0.710146	5.151101	1.869864	0.063164	0.203932	-4.50678
AL022476.1	-0.48936	4.449513	-1.86802	0.063422	0.204677	-4.55649
LINC02637	0.823857	6.827593	1.867743	0.063461	0.204715	-4.99821
AL671710.1	0.333037	6.394149	1.867503	0.063495	0.204736	-4.96232
LINC01521	0.649048	7.198207	1.867302	0.063523	0.204739	-5.10607
AL392089.1	-2.44413	0.616791	-1.86693	0.063574	0.204817	-4.18404
AC121493.1	-1.10663	5.330555	-1.86612	0.063688	0.205009	-5.01711
SOX21-AS1	-1.4927	7.353196	-1.86631	0.063662	0.205009	-5.46107
AC006538.1	0.857873	5.630101	1.865906	0.063719	0.205019	-4.60554
AC090206.1	-2.36308	0.029027	-1.86546	0.063781	0.205022	-4.10916
AP003696.1	2.470808	4.928411	1.865318	0.063802	0.205022	-4.17289
AC011445.2	1.047962	7.868021	1.865609	0.063761	0.205022	-5.18297
AC137723.1	-2.39454	1.077008	-1.86481	0.063874	0.205091	-4.20034
AL135999.3	-2.65981	1.252566	-1.86478	0.063878	0.205091	-4.22838
AL390728.5	2.980585	1.306118	1.862782	0.06416	0.205909	-4.03551
AL365356.3	4.977061	3.311107	1.862464	0.064205	0.205966	-4.0404
AL391845.1	3.30339	2.0463	1.861759	0.064305	0.206022	-4.0439
AC044781.1	3.420714	2.258036	1.861952	0.064277	0.206022	-4.04537
AC016735.1	-1.97376	5.83916	-1.86186	0.06429	0.206022	-5.26489
AC078860.1	3.794876	1.643068	1.860543	0.064477	0.206487	-4.03472
AC087749.2	1.921565	5.819927	1.859974	0.064558	0.206658	-4.35916
AF186192.1	-2.68465	0.092017	-1.85868	0.064742	0.207158	-4.14034
AL662890.1	3.170108	3.8799	1.857074	0.064972	0.207806	-4.08991
AP003392.4	0.576099	7.80334	1.85683	0.065007	0.207829	-5.2574
AC005593.1	3.188248	1.697214	1.855494	0.065198	0.208352	-4.04985
LINC02172	2.505434	0.208713	1.854589	0.065328	0.208413	-4.04037
LINC00304	-1.17307	1.885514	-1.85489	0.065286	0.208413	-4.21416
AF230666.1	0.398428	6.022525	1.854852	0.065291	0.208413	-4.88243
DCST1-AS1	0.592596	8.244105	1.854716	0.06531	0.208413	-5.33943
AC100812.1	3.165442	3.246142	1.853639	0.065465	0.208535	-4.07901
AL162724.1	-1.12169	5.947506	-1.85378	0.065444	0.208535	-5.17749
AC004477.3	0.421939	8.364316	1.85376	0.065448	0.208535	-5.38023
AC127024.5	0.397024	9.324033	1.853552	0.065478	0.208535	-5.53698
AC100843.1	4.156654	1.246296	1.853201	0.065528	0.208607	-4.03921
AC131097.4	-1.27985	3.932798	-1.85257	0.065619	0.208776	-4.70111
AC012513.3	-0.54817	7.447088	-1.85245	0.065637	0.208776	-5.37759
MIS18A-AS1	2.03915	5.551203	1.848578	0.066197	0.210469	-4.31801
AP000785.2	4.695572	4.009727	1.846682	0.066473	0.21114	-4.08047
AC010266.2	-2.48158	-1.84831	-1.84636	0.06652	0.21114	-4.08484
AL096678.1	1.491626	5.405778	1.846712	0.066468	0.21114	-4.42766
AC010320.3	-0.44213	7.448095	-1.84637	0.066519	0.21114	-5.3743
AL590560.3	3.081355	3.45013	1.846067	0.066562	0.211184	-4.09901
AC010320.1	2.230629	3.567216	1.845693	0.066617	0.211268	-4.13505
AC008609.1	2.566955	0.218656	1.844392	0.066807	0.211693	-4.05446
LINC01344	-1.88507	1.026474	-1.84452	0.066788	0.211693	-4.19473
CLYBL-AS1	-2.51815	-0.13351	-1.84402	0.066862	0.211775	-4.1526
SMAD9-IT1	-1.6695	3.155447	-1.84185	0.06718	0.212693	-4.53508
AC005304.1	-2.89318	-1.36404	-1.84114	0.067285	0.212937	-4.11624
AC008610.1	0.605456	7.877481	1.84081	0.067333	0.213	-5.28633
AC024451.4	0.558331	6.724158	1.840412	0.067392	0.213097	-5.0355
AC023908.3	0.500691	5.661478	1.840086	0.06744	0.213159	-4.73282

AC234775.3	-0.89756	6.329889	-1.83903	0.067597	0.213564	-5.25357
PACERR	3.044491	3.796144	1.838415	0.067687	0.21362	-4.11916
AL445649.1	-2.31418	0.865179	-1.83833	0.0677	0.21362	-4.25542
HECW2-AS1	1.037105	6.02992	1.83871	0.067643	0.21362	-4.70953
AC016708.1	0.763982	6.066279	1.837978	0.067752	0.213694	-4.79602
AC130686.1	1.904086	4.092013	1.837651	0.0678	0.213757	-4.18689
AC024592.1	3.116681	3.178294	1.837272	0.067856	0.213845	-4.10491
AC009055.2	3.056754	-0.84719	1.836839	0.067921	0.213901	-4.05789
AL008635.1	-1.02974	4.830433	-1.83677	0.067931	0.213901	-4.86888
AC091181.1	1.314781	4.603847	1.835627	0.068101	0.214313	-4.27191
AL356215.1	2.199719	5.62132	1.835504	0.068119	0.214313	-4.35729
FOXN3-AS2	-2.02459	1.195999	-1.83498	0.068197	0.214469	-4.22776
AC105446.1	1.714441	8.221699	1.834343	0.068292	0.214677	-5.07214
AC009118.2	2.458614	6.115166	1.831962	0.068648	0.215705	-4.41979
AC011297.1	-2.36089	0.893493	-1.83168	0.06869	0.215749	-4.28598
AC138430.1	2.21882	-1.09308	1.831216	0.06876	0.215786	-4.06648
AL442067.1	0.558535	7.160952	1.831246	0.068755	0.215786	-5.17291
AL109947.1	-2.75973	-0.28327	-1.8306	0.068852	0.215987	-4.16956
AC023302.1	1.934051	5.357562	1.830345	0.06889	0.216015	-4.37826
AC068722.1	3.052379	-0.81888	1.827114	0.069377	0.217261	-4.07194
AC005162.2	3.917485	2.557668	1.826749	0.069432	0.217261	-4.09761
AC091057.2	-0.68967	4.359511	-1.82747	0.069323	0.217261	-4.68728
AC007598.2	-0.98436	4.524178	-1.82687	0.069414	0.217261	-4.83511
AC018628.1	0.36103	8.01622	1.826835	0.069419	0.217261	-5.38892
AC018781.1	2.955454	3.285206	1.82596	0.069551	0.217453	-4.12526
AP001350.2	0.609364	6.335956	1.826118	0.069527	0.217453	-4.96744
LINC01983	3.491133	2.312273	1.82559	0.069607	0.217537	-4.10125
AC234772.2	1.66266	5.955653	1.825161	0.069672	0.217599	-4.50185
AC009506.1	-0.38732	8.509155	-1.82508	0.069685	0.217599	-5.56833
AC004943.3	2.751777	3.344741	1.824502	0.069772	0.217752	-4.13607
AL133551.1	0.969523	4.385234	1.823991	0.06985	0.217752	-4.32385
AC046143.2	0.579534	8.167436	1.82402	0.069845	0.217752	-5.3789
PRKAG2-AS1	-0.72849	8.01919	-1.82413	0.069828	0.217752	-5.53932
AC006372.1	3.611998	-1.12589	1.822298	0.070107	0.218374	-4.07842
AC093249.2	1.347058	5.804116	1.822404	0.070091	0.218374	-4.5793
LINC01342	-1.94982	3.716864	-1.82204	0.070147	0.218407	-4.62569
AC017002.2	3.176138	0.560745	1.821449	0.070237	0.218596	-4.08624
Z94160.1	2.928105	-1.78816	1.819928	0.070469	0.219047	-4.08139
AC004053.1	3.63615	0.485469	1.820045	0.070451	0.219047	-4.08447
AC008946.1	0.639324	7.151764	1.820161	0.070434	0.219047	-5.17288
AC026523.2	1.924153	0.531468	1.81913	0.070592	0.219151	-4.10207
AC145343.1	2.16752	5.783714	1.818946	0.07062	0.219151	-4.35052
AL049569.1	1.096934	5.231225	1.819201	0.070581	0.219151	-4.49887
AC105760.2	0.514785	6.018323	1.819353	0.070557	0.219151	-4.89722
AL358472.3	0.398011	7.826824	1.81875	0.07065	0.219155	-5.36314
AC092535.1	-1.77153	1.315593	-1.81728	0.070876	0.219765	-4.26529
LINC00866	3.753459	2.412479	1.816574	0.070984	0.22001	-4.10997
AC112907.1	3.432879	0.802149	1.815788	0.071105	0.220294	-4.09461
TGFA-IT1	3.448315	-0.22607	1.814592	0.07129	0.220775	-4.09045
AC003986.2	2.509414	2.220274	1.813801	0.071412	0.221063	-4.13082
MLIP-IT1	4.263559	3.184331	1.813483	0.071461	0.221124	-4.11954
AP001793.1	0.492619	6.73205	1.813078	0.071524	0.221227	-5.11693
AC020917.2	3.045286	3.047202	1.812622	0.071595	0.221354	-4.14112
LINC01993	1.281443	3.058984	1.812142	0.071669	0.221493	-4.19543
AC011483.3	3.694546	0.469952	1.811874	0.071711	0.22153	-4.0962
AC004466.1	0.531294	5.557457	1.810444	0.071933	0.222126	-4.77869
AL121992.2	3.589043	-0.77848	1.810109	0.071985	0.222196	-4.09604
AL161733.1	3.647394	-0.58049	1.809208	0.072125	0.222537	-4.09751

AL136298.1	3.237316	-0.70803	1.80837	0.072256	0.22266	-4.09879
DSCAS	2.611763	3.89595	1.808612	0.072219	0.22266	-4.19013
SIAH2-AS1	0.555949	7.665694	1.8085	0.072236	0.22266	-5.33
AL353796.1	0.389815	7.807368	1.808195	0.072284	0.22266	-5.38025
AC113361.1	3.290237	-0.50313	1.807159	0.072446	0.222896	-4.10077
BX546450.2	3.368428	-0.3203	1.806638	0.072527	0.222896	-4.10174
AP001208.2	3.714437	-0.50904	1.806258	0.072587	0.222896	-4.10174
AC008456.1	3.564518	0.828414	1.806277	0.072584	0.222896	-4.1075
AC025164.1	0.932978	3.838138	1.806189	0.072598	0.222896	-4.30706
AC068580.3	0.465645	7.785163	1.807019	0.072468	0.222896	-5.35973
DNAJC3-DT	0.484197	8.119179	1.806624	0.07253	0.222896	-5.4223
AC073896.3	-0.43891	7.919361	-1.80735	0.072416	0.222896	-5.51508
AC055736.1	4.13136	-0.17187	1.805393	0.072723	0.223097	-4.10318
AL031666.2	-0.4252	6.077443	-1.8055	0.072706	0.223097	-5.14528
AL355075.4	2.646258	3.777123	1.804632	0.072842	0.223282	-4.18147
AL450306.1	0.465757	5.651943	1.804729	0.072827	0.223282	-4.84313
AL355353.1	0.719248	8.456902	1.804358	0.072885	0.223323	-5.44536
AP000977.1	1.85721	2.230673	1.803583	0.073007	0.223514	-4.15789
AC004846.1	1.004605	6.838883	1.803651	0.072997	0.223514	-5.04491
AC092375.2	-0.80878	5.056037	-1.80336	0.073042	0.223531	-4.99816
AC104653.2	3.344792	3.036977	1.802511	0.073176	0.223739	-4.15024
AC100793.4	-1.74772	2.82469	-1.80236	0.0732	0.223739	-4.57357
AL645933.2	-0.48402	8.267811	-1.80244	0.073188	0.223739	-5.58206
BMP7-AS1	3.560068	-0.21946	1.800686	0.073465	0.224325	-4.11021
LINC01271	1.592472	4.309932	1.800585	0.073481	0.224325	-4.28106
AC009902.2	1.356885	7.973041	1.800827	0.073443	0.224325	-5.2919
AC138474.1	-2.53519	-0.37051	-1.80039	0.073512	0.224328	-4.20439
AC082651.3	3.978028	4.836333	1.799011	0.073731	0.224904	-4.18604
AC126614.1	3.217873	1.963784	1.798105	0.073875	0.22516	-4.13731
AC008897.3	-0.93665	4.920406	-1.79813	0.073871	0.22516	-5.00582
AC005529.1	3.585649	2.555048	1.797001	0.074051	0.225605	-4.14531
LINC00525	3.147837	3.847407	1.796246	0.074171	0.225789	-4.1849
AC141586.2	-1.07516	4.337289	-1.79633	0.074158	0.225789	-4.86421
AC027018.1	-0.70316	5.584716	-1.79551	0.074288	0.226054	-5.11322
AC034243.1	-1.46225	4.486575	-1.79519	0.074339	0.226117	-4.89476
PRAL	1.289515	4.671749	1.794821	0.074399	0.226207	-4.38524
AL357315.1	3.62531	1.573688	1.794258	0.074489	0.226389	-4.13345
LINC02150	2.673259	-2.2685	1.793795	0.074563	0.226523	-4.11821
AC127502.2	0.507384	7.693716	1.793415	0.074624	0.226617	-5.36727
AL358937.1	3.270184	3.50851	1.793108	0.074673	0.226675	-4.17776
AC093424.1	-1.71078	2.949462	-1.79263	0.074749	0.226814	-4.62621
AC015712.7	1.403945	5.342878	1.792349	0.074795	0.226862	-4.5899
AC116651.1	0.854651	5.960012	1.791949	0.074859	0.226965	-4.71251
LINC01214	4.232365	1.425176	1.791049	0.075004	0.227221	-4.13027
AC121757.1	3.49956	5.757005	1.791127	0.074992	0.227221	-4.28268
AC005828.1	3.209406	3.100917	1.790643	0.07507	0.227328	-4.17032
AC026369.3	3.395423	1.94703	1.789957	0.07518	0.227571	-4.14708
AC019118.1	2.926344	3.160819	1.788773	0.075372	0.227967	-4.18056
AC012404.1	-2.78032	-0.62588	-1.7884	0.075433	0.227967	-4.22584
AL357093.1	-1.68357	2.743279	-1.78865	0.075392	0.227967	-4.50836
AL451085.2	0.460339	7.933525	1.788481	0.075419	0.227967	-5.42925
LMO7DN	2.473445	2.30619	1.787819	0.075526	0.228102	-4.17202
AC093591.2	0.932237	4.104281	1.787465	0.075583	0.228102	-4.37174
AC067930.4	1.374083	6.881158	1.787371	0.075599	0.228102	-4.9739
ABCC5-AS1	-1.03986	5.311779	-1.78751	0.075576	0.228102	-5.09531
AC068512.1	2.706725	-2.21413	1.784747	0.076025	0.229075	-4.13096
AP003550.1	-2.60096	-0.58767	-1.78438	0.076085	0.229075	-4.22227
AC132872.2	1.393791	3.183127	1.784292	0.076099	0.229075	-4.24169

AC011491.2	-1.05699	4.467305	-1.78505	0.075976	0.229075	-4.83763
AC020604.1	-0.66852	5.053913	-1.785	0.075985	0.229075	-4.97335
AC090559.2	-0.43923	7.316174	-1.78426	0.076104	0.229075	-5.4558
AC004672.2	-1.03568	4.470961	-1.78392	0.07616	0.229151	-4.90143
AC090735.1	3.737131	-1.13178	1.783592	0.076214	0.229221	-4.13331
AP003555.2	2.526314	7.852739	1.782893	0.076328	0.22938	-4.77156
DGUOK-AS1	0.638675	9.806049	1.782896	0.076327	0.22938	-5.70782
AC015660.2	4.370382	3.491987	1.782407	0.076407	0.229436	-4.17348
DBH-AS1	-0.74737	5.793335	-1.78249	0.076394	0.229436	-5.18565
AC023906.4	-0.65235	4.741383	-1.78199	0.076475	0.229547	-4.88561
AC022113.2	3.065391	-1.71145	1.781257	0.076596	0.229783	-4.13617
AC245060.6	-0.41114	5.94083	-1.78114	0.076615	0.229783	-5.17805
AC021087.4	-2.71593	-1.25919	-1.77958	0.07687	0.230457	-4.20553
CASC20	3.969554	1.773194	1.778291	0.077083	0.231003	-4.15457
AP005436.2	4.776136	3.900823	1.777567	0.077202	0.231176	-4.18028
AL117382.1	-2.01286	3.789377	-1.77769	0.077183	0.231176	-4.85066
AC007848.1	4.214779	2.86695	1.776874	0.077317	0.231426	-4.17091
AC025576.2	3.30463	3.517935	1.77633	0.077407	0.231457	-4.20295
LINC01842	4.414946	6.352454	1.7763	0.077412	0.231457	-4.28835
AL136982.6	-1.21342	3.623837	-1.77625	0.077419	0.231457	-4.75944
AL137024.1	-2.48944	0.360347	-1.77295	0.077967	0.233001	-4.29099
AC068473.1	2.605402	0.598586	1.772734	0.078003	0.233017	-4.16135
AC019155.3	4.432675	2.346981	1.771586	0.078194	0.233339	-4.16689
AC091925.1	3.607799	2.759963	1.771526	0.078204	0.233339	-4.18397
AC124067.2	4.482265	7.207039	1.771775	0.078163	0.233339	-4.38138
AC040174.1	-1.38199	3.729515	-1.77118	0.078263	0.233421	-4.72297
AC099786.1	3.291486	0.548408	1.77009	0.078444	0.233606	-4.15858
AC117382.2	1.495109	2.752149	1.770253	0.078417	0.233606	-4.23932
AC103809.1	-2.4454	0.049012	-1.76998	0.078463	0.233606	-4.2702
AC091153.3	1.116195	6.692395	1.769873	0.07848	0.233606	-5.00128
MED14OS	0.460987	7.623469	1.770152	0.078434	0.233606	-5.40565
AL451164.2	3.353754	-0.28215	1.769583	0.078529	0.233658	-4.15396
AP001269.1	-0.71981	5.505828	-1.76869	0.078679	0.234012	-5.14152
AC022784.6	3.637235	2.443116	1.767688	0.078846	0.234417	-4.18525
AC015660.1	7.506727	5.818277	1.766937	0.078972	0.23459	-4.18476
AC008700.1	-1.81162	1.930802	-1.76678	0.078998	0.23459	-4.41389
AC010422.1	-1.00235	4.562478	-1.7669	0.078979	0.23459	-4.92174
AC245128.3	3.360199	4.773232	1.766334	0.079074	0.234722	-4.26469
AL121721.1	3.449286	0.175383	1.765955	0.079138	0.234726	-4.16035
AC004847.1	0.838542	6.42913	1.765967	0.079136	0.234726	-4.99667
AL365436.2	1.54271	7.180695	1.765566	0.079203	0.234828	-5.07613
AC063948.1	2.123289	5.957391	1.765064	0.079288	0.234893	-4.47123
FRMD6-AS1	0.971579	5.858612	1.765173	0.079269	0.234893	-4.87974
AC009949.1	1.925861	1.882917	1.763746	0.07951	0.235459	-4.20536
AC243547.1	3.294177	-1.15762	1.763116	0.079617	0.235496	-4.16184
LINC01416	3.071802	-0.93859	1.763344	0.079578	0.235496	-4.16187
AC005034.5	-0.32586	9.510897	-1.7632	0.079603	0.235496	-5.81839
AC026495.1	3.104388	0.348197	1.762167	0.079778	0.235735	-4.16871
AL023803.1	5.010381	5.641989	1.762025	0.079802	0.235735	-4.24033
HYI-AS1	0.476471	6.807449	1.7619	0.079823	0.235735	-5.2384
AL049835.1	-0.69448	7.07182	-1.76207	0.079795	0.235735	-5.49235
IL21-AS1	2.52469	-0.25313	1.761195	0.079942	0.23581	-4.16944
AC087742.1	2.269731	4.156246	1.761274	0.079929	0.23581	-4.29488
AL354892.2	0.520142	9.813168	1.761488	0.079893	0.23581	-5.76115
AL353803.2	-1.89659	1.004396	-1.76023	0.080106	0.236179	-4.32044
AC026367.2	-0.83065	6.434078	-1.76009	0.080131	0.236179	-5.41347
AC019080.4	1.877834	6.934106	1.759698	0.080197	0.236282	-4.9028
AC009088.2	2.204484	3.831723	1.758366	0.080424	0.236858	-4.30726

AL354872.2	-1.01641	5.669513	-1.75651	0.080741	0.237604	-5.20348
AC069257.1	0.631441	7.755061	1.756522	0.080739	0.237604	-5.41806
AC006963.1	3.075013	0.56359	1.75542	0.080928	0.237892	-4.18224
AL008718.2	2.573691	1.470394	1.755387	0.080933	0.237892	-4.19968
AL354740.1	0.478276	3.514715	1.755683	0.080883	0.237892	-4.40122
AL359682.1	2.707231	-2.31075	1.755163	0.080972	0.237912	-4.17207
C18orf15	-1.86546	0.410679	-1.75497	0.081004	0.237914	-4.28826
SMAD1-AS2	2.408338	1.745852	1.754383	0.081106	0.238084	-4.20872
AC015802.5	1.702842	5.17781	1.754269	0.081125	0.238084	-4.52515
AL139327.2	4.108833	-0.48981	1.753931	0.081183	0.238161	-4.17508
AL049775.2	3.392968	-1.19102	1.753312	0.08129	0.238214	-4.1754
AC122688.4	2.151476	0.26032	1.753273	0.081297	0.238214	-4.19075
AL354766.2	4.665114	2.853238	1.753339	0.081285	0.238214	-4.19987
AP003390.2	3.425751	2.443646	1.752344	0.081457	0.23859	-4.20893
DDX39B-AS1	3.112445	3.32714	1.751906	0.081532	0.238625	-4.2322
AP001330.5	0.55523	8.543509	1.75203	0.081511	0.238625	-5.5861
AC027237.2	-1.19027	4.21801	-1.75164	0.081579	0.238669	-4.89201
AC040162.3	0.305004	6.759271	1.751164	0.08166	0.238814	-5.27365
LINC02288	-0.85778	3.014532	-1.75086	0.081713	0.238876	-4.4928
AL390961.3	1.828318	2.13987	1.750191	0.081828	0.238881	-4.23242
AC005332.7	-1.30549	3.99947	-1.75047	0.081781	0.238881	-4.86044
AC018529.2	0.53718	5.712822	1.750512	0.081773	0.238881	-4.91252
AC090912.1	1.131899	6.594486	1.750112	0.081842	0.238881	-4.97673
AC073046.1	0.610884	10.06415	1.749835	0.08189	0.238928	-5.80331
AL031963.3	0.527745	6.993955	1.749125	0.082013	0.239194	-5.29028
AC008063.1	3.243909	1.616	1.747236	0.082341	0.240057	-4.20466
AC091212.1	3.419392	-1.31668	1.746818	0.082414	0.240166	-4.18426
AC069148.1	2.757993	5.059084	1.746656	0.082442	0.240166	-4.34319
ZBTB46-AS1	3.396369	1.24113	1.745154	0.082704	0.240835	-4.20051
AL132801.1	2.911874	0.174179	1.74475	0.082774	0.240947	-4.19313
AC090921.1	2.90997	0.888424	1.744243	0.082863	0.241111	-4.20281
AC006213.4	-1.01694	5.449112	-1.74401	0.082903	0.241134	-5.24901
LINC01239	-1.25426	4.964258	-1.7437	0.082958	0.241201	-5.04736
AC144548.1	0.421764	6.964098	1.742989	0.083082	0.241469	-5.31933
LINC02178	5.816047	3.643325	1.741698	0.083309	0.241753	-4.21129
AL031283.2	1.889538	0.834854	1.741836	0.083285	0.241753	-4.21741
AC011773.4	1.615263	3.324941	1.742006	0.083255	0.241753	-4.30437
AC106820.2	0.887722	3.320006	1.741891	0.083275	0.241753	-4.35537
Z97200.1	1.962018	5.474768	1.741059	0.083421	0.241986	-4.52535
AL354754.1	3.284302	-1.3783	1.740377	0.083541	0.242147	-4.19305
AC102953.1	3.044177	3.080021	1.740376	0.083541	0.242147	-4.24389
AP001642.1	2.621615	-2.6618	1.739763	0.083649	0.242367	-4.19304
AC012510.1	0.369828	7.539136	1.739134	0.08376	0.242594	-5.45791
LINC02254	3.573753	2.408652	1.737781	0.083999	0.243144	-4.22895
ATP1B3-AS1	0.683274	8.346409	1.737693	0.084015	0.243144	-5.55562
AP000424.1	2.928824	3.252254	1.737129	0.084114	0.243292	-4.26666
AC025766.1	2.187553	5.222882	1.736679	0.084194	0.243292	-4.45725
AC092800.1	-1.75757	2.574655	-1.73649	0.084228	0.243292	-4.55235
AC012603.1	-0.97607	5.118684	-1.73667	0.084196	0.243292	-5.12835
ACVR2B-AS1	-0.46136	6.901757	-1.73678	0.084177	0.243292	-5.46629
AC012615.4	1.507477	5.288388	1.73629	0.084263	0.2433	-4.63467
AL359182.2	3.250229	1.927303	1.735832	0.084344	0.243441	-4.22523
AC005993.1	9.361754	5.440388	1.734959	0.084499	0.243795	-4.20975
AC022613.2	0.593036	6.737472	1.734722	0.084541	0.243823	-5.24822
AC004839.1	1.806837	4.832979	1.733974	0.084674	0.244063	-4.47413
AC026271.3	0.518648	7.457007	1.733888	0.084689	0.244063	-5.42595
AC099791.2	1.247002	5.636998	1.733149	0.084821	0.244349	-4.65323
AC131956.2	3.222964	-1.16583	1.732899	0.084866	0.244383	-4.20343

AC008894.1	-2.61194	-0.48435	-1.73222	0.084987	0.244638	-4.31024
AP002360.3	1.252072	6.769212	1.731015	0.085202	0.244998	-4.96251
AF111167.2	-0.48824	6.055136	-1.7312	0.085168	0.244998	-5.29599
AL139289.2	0.535549	8.281727	1.730654	0.085267	0.244998	-5.5671
AC009120.3	-0.37002	8.793142	-1.73067	0.085265	0.244998	-5.7767
Z99129.4	-0.38773	9.785527	-1.73061	0.085275	0.244998	-5.92271
AP000560.1	-0.38067	6.630619	-1.72966	0.085445	0.245392	-5.41702
AC105046.1	1.359832	3.58365	1.728954	0.085571	0.245662	-4.37627
AL627309.2	4.633119	4.583707	1.728567	0.085641	0.245674	-4.27421
AL160313.1	-0.83948	4.464792	-1.72859	0.085636	0.245674	-4.92197
AC015802.3	-0.63767	5.781536	-1.72798	0.085747	0.245885	-5.27448
AL512656.1	0.83187	6.430275	1.727781	0.085782	0.245891	-5.10691
AC116096.1	-2.29235	-1.72826	-1.72664	0.085988	0.246387	-4.25828
AC024084.1	3.598184	-0.10139	1.72607	0.08609	0.246492	-4.21405
AL355922.1	3.424437	1.158474	1.726113	0.086082	0.246492	-4.22576
AC084781.1	3.229099	-1.33089	1.725415	0.086208	0.246549	-4.2135
AL133330.1	1.074622	7.830382	1.725718	0.086154	0.246549	-5.40521
AL513365.2	-0.39001	7.030218	-1.72557	0.086181	0.246549	-5.51128
AC024270.4	1.234972	5.64704	1.725168	0.086253	0.246562	-4.79235
AC016205.1	1.704629	6.474204	1.725027	0.086279	0.246562	-4.82276
AC064836.3	0.486998	7.92909	1.724363	0.086398	0.24681	-5.53581
LINC01732	3.067253	-1.64486	1.722076	0.086813	0.247756	-4.2178
AC011491.3	-2.39042	-0.19607	-1.72199	0.086828	0.247756	-4.33069
DPH6-DT	-1.20347	3.26421	-1.72216	0.086797	0.247756	-4.70464
AC132872.3	0.379195	9.095289	1.721212	0.08697	0.248066	-5.73849
AC090220.1	-1.13579	2.867789	-1.72073	0.087059	0.248224	-4.59401
AC090772.2	-0.92742	4.560955	-1.72001	0.087189	0.248503	-4.99902
SLFNL1-AS1	0.408779	6.759606	1.719497	0.087282	0.248674	-5.30739
AC009951.1	-1.23404	2.172775	-1.71848	0.087469	0.248787	-4.47477
LINC01750	-0.81195	4.268485	-1.71865	0.087436	0.248787	-4.90892
SMIM15-AS1	-0.59189	5.279578	-1.71848	0.087468	0.248787	-5.1064
AC010998.2	-0.62246	5.586459	-1.7187	0.087427	0.248787	-5.25927
AC242842.1	0.685639	8.416805	1.718372	0.087488	0.248787	-5.58834
AC067863.1	2.473579	1.136763	1.717548	0.087638	0.248839	-4.24905
AC007431.2	3.135894	1.71714	1.71781	0.087591	0.248839	-4.25017
AL021937.4	2.839703	2.982308	1.717697	0.087611	0.248839	-4.27892
AC009090.5	1.013023	3.363502	1.71805	0.087547	0.248839	-4.40014
LINC02657	4.153239	6.915958	1.716644	0.087804	0.249215	-4.47561
AC254562.2	2.640782	3.83506	1.716088	0.087906	0.24941	-4.31928
AC108751.4	2.716464	3.367326	1.71569	0.087979	0.249523	-4.2989
AC007298.2	2.880066	-0.87465	1.715013	0.088103	0.24959	-4.22839
AC011290.1	3.277615	-0.01669	1.714838	0.088135	0.24959	-4.23046
RFX3-AS1	-0.54161	6.579026	-1.7149	0.088124	0.24959	-5.46327
AC092687.3	0.606922	8.015692	1.715302	0.08805	0.24959	-5.53737
AC092535.3	-2.53329	0.085792	-1.71359	0.088365	0.250145	-4.36865
AL049543.1	2.718857	-0.6742	1.712187	0.088624	0.250784	-4.23287
AC008687.2	3.865036	4.810988	1.711572	0.088738	0.250822	-4.32314
AC084876.1	1.120169	5.734912	1.71168	0.088718	0.250822	-4.77461
AC092171.4	0.497602	6.782825	1.711781	0.088699	0.250822	-5.27374
AL355001.1	-0.50808	6.211004	-1.71085	0.088871	0.251105	-5.39073
AC025154.2	-1.98495	2.587743	-1.71026	0.088981	0.251229	-4.66406
LINC00653	-0.39673	6.978522	-1.71025	0.088982	0.251229	-5.51594
AC211476.2	0.520825	6.408054	1.709693	0.089086	0.25126	-5.17044
AL731566.2	-1.08335	5.205853	-1.70965	0.089093	0.25126	-5.29169
AC008649.2	0.556398	8.452773	1.709939	0.08904	0.25126	-5.63809
AL354726.1	1.455236	5.847756	1.709088	0.089198	0.251461	-4.75799
BX088651.4	2.129727	1.939613	1.708695	0.089271	0.251478	-4.28401
AC055822.1	-0.45562	8.146859	-1.70877	0.089256	0.251478	-5.71906

AC004906.1	2.861664	2.68071	1.708131	0.089375	0.251678	-4.28673
AC109326.1	0.711046	7.312443	1.707303	0.08953	0.252018	-5.37854
AC025211.1	1.698097	5.911162	1.705295	0.089904	0.252977	-4.70528
AC096751.2	-1.91616	2.138743	-1.70476	0.090005	0.253165	-4.57021
TONSL-AS1	-0.46937	8.437244	-1.70446	0.09006	0.253228	-5.77532
AC098848.1	-1.31291	4.104475	-1.70404	0.090139	0.253354	-4.93321
LARS2-AS1	1.469931	4.583061	1.703716	0.0902	0.253429	-4.4775
AC016542.1	2.520733	5.379102	1.702152	0.090493	0.254158	-4.48933
AL390838.1	2.62933	-2.14856	1.70096	0.090717	0.254313	-4.24589
AC111149.2	3.788833	0.075357	1.701294	0.090654	0.254313	-4.24776
LINC02477	-2.61885	-1.49095	-1.70124	0.090665	0.254313	-4.31744
AC092718.1	-1.17014	3.188715	-1.70112	0.090687	0.254313	-4.70568
AL445685.1	-1.13775	3.338643	-1.70131	0.090652	0.254313	-4.73037
LINC01907	1.493834	2.55081	1.70016	0.090868	0.25464	-4.3302
AC012531.2	-2.14165	-0.50268	-1.69995	0.090907	0.254656	-4.33463
PIK3CD-AS2	0.918451	7.715432	1.699556	0.090982	0.254769	-5.44334
AC010680.5	-2.28854	0.667021	-1.69928	0.091034	0.254822	-4.43692
AC012640.5	3.505566	1.690892	1.697046	0.091456	0.255907	-4.27225
ST7-AS1	0.588961	7.325835	1.695752	0.091701	0.256498	-5.44822
AC007342.9	1.522504	8.180773	1.694946	0.091855	0.256831	-5.39192
AC005392.2	2.512472	8.680443	1.694381	0.091962	0.257035	-5.34587
AC090164.2	3.360906	0.039135	1.693903	0.092053	0.257194	-4.25839
AL512408.1	0.58685	6.361635	1.693598	0.092111	0.257261	-5.19171
LINC00677	2.811472	2.82774	1.693275	0.092173	0.257337	-4.31112
AL132989.1	-0.31782	8.927632	-1.69309	0.092209	0.257341	-5.85072
GK-AS1	1.44451	6.780528	1.692381	0.092343	0.257622	-5.10961
AC090527.3	1.151802	5.565371	1.692132	0.092391	0.257658	-4.71004
AL157904.1	2.74392	5.227028	1.691405	0.09253	0.257855	-4.47619
AC073508.3	0.521821	8.654553	1.691524	0.092507	0.257855	-5.70695
AC009955.1	3.35094	-1.19115	1.690418	0.092719	0.258286	-4.26072
MYB-AS1	3.475398	1.289244	1.689683	0.09286	0.258552	-4.27711
EXOSC10-AS1	0.66831	7.105106	1.689561	0.092883	0.258552	-5.37938
AL031710.2	0.572256	5.951172	1.689182	0.092956	0.258658	-5.03997
AC073326.1	2.542825	3.61837	1.688426	0.093101	0.25887	-4.35631
ISM1-AS1	2.419436	3.673648	1.688525	0.093082	0.25887	-4.36496
AC098828.3	3.176514	3.606931	1.687143	0.093348	0.25946	-4.33452
AC112512.1	-0.29111	6.272494	-1.68683	0.093408	0.259515	-5.38822
AC008147.1	-1.0058	6.868179	-1.68668	0.093436	0.259515	-5.63132
AP002336.1	2.669649	5.79781	1.685431	0.093678	0.26002	-4.50897
U51244.1	-1.06352	5.169216	-1.68538	0.093688	0.26002	-5.28591
AC005224.4	0.904407	6.269386	1.684725	0.093815	0.260276	-5.14611
AC023034.2	4.176579	2.022296	1.682724	0.094202	0.261255	-4.28867
AC092068.3	-1.56775	1.505329	-1.68209	0.094325	0.261499	-4.47922
AL133215.3	2.024258	4.783879	1.681724	0.094396	0.261601	-4.51321
AC005786.2	3.179533	-0.26804	1.681429	0.094454	0.261663	-4.27441
LINC01972	2.97001	-1.82431	1.680798	0.094577	0.261838	-4.273
AC118754.3	3.342203	0.587207	1.680631	0.094609	0.261838	-4.28104
AL132656.3	-0.79938	3.499269	-1.68057	0.094622	0.261838	-4.74016
AC246793.1	2.422758	-1.44374	1.680177	0.094697	0.261856	-4.27442
AC078846.1	0.364967	6.732036	1.680339	0.094666	0.261856	-5.37204
LINC02454	3.81546	3.372649	1.679394	0.09485	0.262116	-4.31942
AC008083.1	3.746591	4.891523	1.679337	0.094861	0.262116	-4.37395
AP003680.1	0.699767	3.950314	1.67896	0.094935	0.262222	-4.53967
AC012435.2	0.969573	6.633075	1.678669	0.094992	0.262283	-5.10442
AL021392.1	1.19302	6.131991	1.677538	0.095213	0.262797	-4.98692
AC020663.2	-0.9658	6.139812	-1.67682	0.095354	0.263091	-5.49351
AC079075.1	3.20203	-0.03447	1.6765	0.095416	0.263165	-4.28203
AC026368.1	-0.60907	6.85246	-1.67583	0.095548	0.263433	-5.56895

AC087741.2	1.816049	7.095039	1.674852	0.09574	0.263839	-5.11342
MIR762HG	0.349206	7.312961	1.674718	0.095766	0.263839	-5.51375
AC007406.4	2.850574	-1.76902	1.674091	0.095889	0.264029	-4.2819
LINC00239	0.965376	7.172405	1.674011	0.095905	0.264029	-5.35181
AC243571.2	1.601423	3.302919	1.673805	0.095946	0.264044	-4.40759
AL353719.1	0.744836	5.021912	1.673536	0.095999	0.264071	-4.79509
AC034198.2	-0.83181	5.268519	-1.6734	0.096026	0.264071	-5.26003
AC016396.2	2.647052	-2.24239	1.672697	0.096164	0.264161	-4.28339
AC068985.1	3.486158	-0.78799	1.672912	0.096122	0.264161	-4.28428
AC010422.2	0.54752	6.063911	1.672819	0.09614	0.264161	-5.13346
LINC01565	2.320204	-2.65318	1.67199	0.096304	0.264448	-4.28404
AC019322.4	2.107019	-1.42638	1.671439	0.096412	0.264649	-4.28626
AC012254.3	-0.50622	4.544142	-1.67126	0.096447	0.264649	-5.0062
AC148477.3	2.867724	-0.76505	1.670616	0.096575	0.264904	-4.28786
AC027288.3	1.107256	4.993056	1.670007	0.096696	0.265041	-4.69451
AC105339.3	-0.38219	6.259861	-1.67002	0.096693	0.265041	-5.41681
AL160286.3	-1.78635	1.636193	-1.66925	0.096847	0.265261	-4.48986
AL354920.1	-0.49058	7.794341	-1.66939	0.096818	0.265261	-5.7335
AC010275.1	6.215815	6.146832	1.668612	0.096973	0.265419	-4.35459
AC100803.1	2.199193	2.473975	1.668259	0.097043	0.265419	-4.35496
AC007128.1	5.844653	6.046529	1.668065	0.097081	0.265419	-4.36695
LINC00628	-1.72448	2.660927	-1.6682	0.097054	0.265419	-4.74394
AC023510.2	-1.18198	4.024823	-1.6683	0.097034	0.265419	-5.07067
TOB1-AS1	-0.37951	6.605224	-1.66754	0.097186	0.265609	-5.50331
OXCT1-AS1	-0.83114	4.043259	-1.66733	0.097227	0.265623	-4.93185
AC013391.3	2.827558	1.22064	1.66638	0.097417	0.266046	-4.31627
AC123905.1	3.301202	-0.34932	1.665264	0.09764	0.266317	-4.2953
AC245140.1	3.2442	-0.40625	1.664527	0.097787	0.266317	-4.29625
AC131532.1	6.022619	3.680311	1.664535	0.097785	0.266317	-4.31469
AC025171.3	2.356746	5.603966	1.665316	0.097629	0.266317	-4.5811
AC016542.3	-1.07336	4.601766	-1.66446	0.0978	0.266317	-5.11037
AC009318.1	-0.45158	6.394531	-1.66566	0.09756	0.266317	-5.48285
IGBP1-AS1	-0.42207	6.513966	-1.66497	0.097699	0.266317	-5.52073
RNASEH1-AS1	0.480411	9.415193	1.664853	0.097722	0.266317	-5.86411
AC010608.2	4.022757	0.912575	1.663549	0.097983	0.266327	-4.30107
GACAT2	2.554825	3.642067	1.663807	0.097931	0.266327	-4.38975
XXYLT1-AS1	3.278369	4.622576	1.663405	0.098012	0.266327	-4.40939
AC110285.3	-2.1694	1.0652	-1.66373	0.097946	0.266327	-4.47732
AC023825.2	2.343414	5.696587	1.663382	0.098016	0.266327	-4.60414
AC055811.3	-0.64945	5.18315	-1.66366	0.097961	0.266327	-5.19405
AL078645.1	3.17322	2.005209	1.662653	0.098162	0.266447	-4.33024
AC011383.1	-1.94074	1.398737	-1.66263	0.098167	0.266447	-4.55604
AC051619.4	-0.97981	4.884444	-1.66289	0.098114	0.266447	-5.23927
AL731571.1	-0.37259	7.520483	-1.66066	0.098562	0.267423	-5.69439
LINC01353	1.279991	4.33825	1.66033	0.098629	0.267509	-4.58372
AP001767.4	-0.62818	6.699611	-1.66011	0.098673	0.267531	-5.58976
AL359265.3	-0.82169	5.047366	-1.65918	0.09886	0.267942	-5.22073
AC105345.2	2.025571	3.541958	1.658938	0.09891	0.26798	-4.41144
MPRIP-AS1	0.790737	5.28792	1.658637	0.09897	0.268048	-4.80814
AL445471.2	2.358969	-2.5005	1.657977	0.099104	0.268216	-4.30252
AC138932.5	-0.4253	8.201374	-1.65808	0.099084	0.268216	-5.81291
AC112484.3	0.840341	7.638508	1.657342	0.099232	0.268467	-5.53133
HEXD-IT1	-0.52083	5.435076	-1.65683	0.099336	0.268552	-5.26156
AC026470.3	0.883861	7.008107	1.656573	0.099388	0.268552	-5.37631
AL133410.1	-1.2449	5.309401	-1.65648	0.099407	0.268552	-5.39697
AC010487.2	-1.07234	6.243457	-1.65648	0.099407	0.268552	-5.59952
AC091170.1	3.283461	0.045207	1.655781	0.099549	0.268742	-4.30931
WNT5A-AS1	1.339115	9.019457	1.655918	0.099521	0.268742	-5.72191

AC005618.1	1.018358	5.835827	1.655371	0.099632	0.26887	-4.97852
AC096947.1	-0.91312	5.24747	-1.65504	0.099698	0.268892	-5.33794
AL121753.2	-0.37686	8.343623	-1.65498	0.099712	0.268892	-5.83206
AC073641.1	2.642781	2.648221	1.654276	0.099854	0.269108	-4.36478
AC129510.1	0.440727	8.099433	1.654231	0.099863	0.269108	-5.68262
CARS-AS1	-0.56809	3.990436	-1.65339	0.100035	0.269185	-4.81734
AC103923.1	-0.74965	5.475962	-1.65364	0.099984	0.269185	-5.29291
AL138976.2	0.62513	7.358915	1.653609	0.09999	0.269185	-5.51752
UBE2R2-AS1	0.594215	7.438077	1.653407	0.100031	0.269185	-5.53573
AP002387.2	0.742091	10.21144	1.652688	0.100178	0.269471	-5.97018
AC005379.1	3.312732	-0.12004	1.652488	0.100218	0.269485	-4.31274
LINC01063	0.74547	7.047619	1.652141	0.100289	0.269579	-5.40615
LINC02026	1.238231	4.477246	1.651628	0.100394	0.269682	-4.60552
AC114956.1	1.953174	6.944907	1.651601	0.100399	0.269682	-4.94809
AC002044.2	-0.41433	6.790879	-1.65092	0.100539	0.269961	-5.58218
AC131212.4	1.371598	3.912001	1.650138	0.100699	0.270039	-4.51385
AC120057.3	0.62205	5.183654	1.650073	0.100712	0.270039	-4.90825
ERICD	0.546699	5.752544	1.650543	0.100616	0.270039	-5.08136
AC109460.1	0.307648	6.545788	1.65034	0.100657	0.270039	-5.36678
AC097381.2	2.924579	0.567642	1.649182	0.100894	0.270432	-4.32753
AC073316.2	3.282273	1.283665	1.64607	0.101534	0.27205	-4.33671
CXXC5-AS1	-2.28026	0.523295	-1.64586	0.101577	0.272067	-4.47762
AL157392.1	3.240019	1.452805	1.645454	0.101661	0.272196	-4.34127
AL606760.2	-0.32019	5.658395	-1.64506	0.101742	0.272314	-5.28541
TNK2-AS1	1.063205	5.406761	1.644795	0.101797	0.272366	-4.84718
LINC00336	2.424092	2.760646	1.644336	0.101892	0.272523	-4.38737
AL021707.6	0.384698	9.106246	1.643775	0.102008	0.272736	-5.86538
AC040174.2	-1.96007	3.072766	-1.64333	0.102101	0.272887	-4.80812
AC012363.1	5.179785	4.050793	1.642287	0.102316	0.273365	-4.36604
AC092669.1	2.970129	-1.03495	1.642016	0.102373	0.273381	-4.32472
AC112693.3	2.261853	-0.14511	1.641383	0.102504	0.273381	-4.33549
AC020934.2	2.697716	3.717837	1.641512	0.102477	0.273381	-4.42002
AC106801.1	-0.65212	4.081194	-1.64154	0.102471	0.273381	-4.90423
AL078581.3	-0.32584	6.762994	-1.64156	0.102468	0.273381	-5.58297
AL358333.2	3.028896	-0.26409	1.640716	0.102643	0.273556	-4.32843
AC004584.1	2.228937	4.300407	1.64084	0.102617	0.273556	-4.48625
AP000317.2	-1.8464	1.189464	-1.64041	0.102706	0.273629	-4.551
AC007879.4	3.430785	3.935794	1.639626	0.10287	0.273869	-4.40089
AC244517.7	2.555637	6.251442	1.639705	0.102853	0.273869	-4.71358
AL662795.2	-0.40428	9.724351	-1.63809	0.10319	0.274625	-6.07185
AC025575.1	3.214299	0.784797	1.637401	0.103334	0.274783	-4.34181
AC110491.1	-2.33178	-1.29737	-1.63711	0.103396	0.274783	-4.39937
AC005746.3	2.542958	3.563364	1.637599	0.103292	0.274783	-4.43046
AC006262.1	1.792254	4.483915	1.637148	0.103387	0.274783	-4.57799
AC108159.1	2.835377	2.595812	1.636896	0.10344	0.274802	-4.38328
AC007278.1	-1.30435	2.970276	-1.63556	0.10372	0.275449	-4.77848
AP001020.3	0.411943	6.290308	1.635272	0.10378	0.275511	-5.31741
AL136531.3	-0.78959	5.207304	-1.63496	0.103846	0.275589	-5.29468
AL352984.1	3.58837	1.614415	1.634325	0.103979	0.275666	-4.35527
AL390961.2	2.208001	5.095005	1.634154	0.104015	0.275666	-4.59616
AL161909.2	-0.77225	5.685509	-1.63453	0.103935	0.275666	-5.44645
AL035587.1	0.501555	8.628492	1.63412	0.104021	0.275666	-5.79044
LINC01186	1.601693	6.634515	1.63326	0.104202	0.276048	-5.10684
CIRBP-AS1	0.466229	8.63105	1.632583	0.104345	0.276329	-5.80364
AL022328.3	0.340171	7.976016	1.632332	0.104398	0.276371	-5.71687
AC105206.2	-0.38056	6.901391	-1.63192	0.104484	0.276502	-5.63561
AC004941.1	2.142172	-0.30248	1.631526	0.104568	0.276534	-4.34732
HMGN3-AS1	0.421562	7.671656	1.631516	0.10457	0.276534	-5.6478

AC083805.2	-1.01873	4.307792	-1.63117	0.104643	0.27663	-5.08075
HGC6.3	-2.35466	-0.64991	-1.63085	0.104711	0.27668	-4.4353
AL080317.2	-0.34914	6.814632	-1.63073	0.104735	0.27668	-5.60513
RAI1-AS1	-0.51884	5.131673	-1.63052	0.104781	0.276702	-5.22292
AC107204.1	-0.98218	2.294744	-1.63024	0.104839	0.27676	-4.59434
AC011365.1	-1.27561	1.707078	-1.62982	0.104927	0.276895	-4.53933
AC017033.1	3.052271	0.704383	1.62831	0.105248	0.277591	-4.35505
AL161773.1	2.556985	1.004207	1.628147	0.105283	0.277591	-4.36543
SCAMP1-AS1	0.379979	9.350299	1.628057	0.105302	0.277591	-5.92598
AL122010.1	-0.27729	8.931137	-1.62752	0.105417	0.277796	-5.95352
LINC02198	2.427783	2.386884	1.626739	0.105582	0.278036	-4.39779
SNHG18	-0.72588	8.966196	-1.62674	0.105581	0.278036	-6.02452
LINC02473	2.373682	1.30285	1.626247	0.105687	0.278162	-4.37821
AC135586.2	-2.32428	-0.65898	-1.62617	0.105704	0.278162	-4.45199
DIP2A-IT1	-0.35945	7.30434	-1.6251	0.10593	0.27866	-5.7183
NALCN-AS1	-1.70296	1.053383	-1.62425	0.106112	0.279041	-4.52582
AC122108.2	3.502868	1.54228	1.622837	0.106415	0.279574	-4.36898
AC120498.2	-2.31286	1.772513	-1.62278	0.106426	0.279574	-4.65317
AC010719.1	-0.62921	7.205626	-1.6229	0.1064	0.279574	-5.73387
AL158151.1	1.095635	4.248255	1.622331	0.106523	0.27973	-4.60125
LINC01833	4.058684	5.150963	1.620841	0.106842	0.280399	-4.4632
AL353763.1	-0.50386	6.538863	-1.62079	0.106853	0.280399	-5.59738
AL135937.1	2.573241	3.877883	1.620444	0.106928	0.2804	-4.45966
AC127024.4	-0.38281	8.383599	-1.62061	0.106892	0.2804	-5.89702
TTLL7-IT1	-1.81407	0.048517	-1.61999	0.107024	0.280546	-4.45764
MGAT3-AS1	-1.76898	3.618727	-1.61984	0.107058	0.280546	-4.98085
AC117422.1	3.222273	0.116162	1.619148	0.107206	0.28074	-4.35772
AL021578.1	0.654438	7.687408	1.619291	0.107176	0.28074	-5.62935
AP000525.1	4.803998	5.771033	1.61881	0.107279	0.280832	-4.45826
AC087761.1	2.868651	0.516232	1.618098	0.107433	0.281038	-4.36723
AP002336.2	0.721532	7.972896	1.618174	0.107416	0.281038	-5.67114
AL161908.1	2.998919	1.648449	1.616882	0.107695	0.281529	-4.38474
AC004381.1	-2.14541	1.231511	-1.61696	0.107678	0.281529	-4.58252
AL589765.1	3.202196	-0.12282	1.616218	0.107839	0.281806	-4.36004
LINC02600	-0.97185	4.001013	-1.61521	0.108057	0.28218	-5.04003
BX322234.1	0.56229	7.941147	1.615248	0.108049	0.28218	-5.70428
AL031600.1	0.444187	8.569501	1.614723	0.108162	0.282357	-5.82093
ASTN2-AS1	2.646711	-0.20113	1.613104	0.108514	0.283177	-4.36706
AC130456.3	-1.53159	6.345762	-1.61275	0.108591	0.283279	-5.73845
AC022973.3	0.65626	6.850548	1.611871	0.108782	0.28368	-5.44997
AC026355.2	2.769481	-1.99544	1.611671	0.108826	0.283695	-4.36261
AL360219.1	2.941306	5.084958	1.611387	0.108888	0.283758	-4.54109
AC132938.5	-0.37045	6.867265	-1.6111	0.108951	0.283824	-5.66305
AL132780.5	0.411309	6.397678	1.610513	0.109078	0.284057	-5.36036
AC037486.1	3.017976	-0.40726	1.609771	0.109241	0.284381	-4.36753
LINC01205	2.465379	-0.68421	1.608595	0.109498	0.28462	-4.37009
AC020922.4	3.170283	-0.05426	1.608486	0.109522	0.28462	-4.37046
AC093908.1	0.661094	5.345927	1.608911	0.109428	0.28462	-5.01091
PRRT3-AS1	0.556531	9.14262	1.609019	0.109405	0.28462	-5.90079
AC012306.2	0.361501	9.239568	1.608501	0.109518	0.28462	-5.94891
AC021755.2	-1.55422	2.361802	-1.60787	0.109656	0.284771	-4.6885
AC005838.2	-0.61712	7.626318	-1.60789	0.109653	0.284771	-5.82689
AC020931.1	0.422034	5.859434	1.607362	0.109768	0.284964	-5.23798
AC022154.1	0.358726	5.942637	1.606539	0.109949	0.285334	-5.25897
AC009779.5	2.86453	-1.73715	1.605677	0.110138	0.285728	-4.37044
AC008507.3	2.323009	-2.72005	1.605081	0.110269	0.285884	-4.37043
AC092117.1	0.605415	5.80942	1.605057	0.110275	0.285884	-5.14182
AC092171.5	0.449939	7.819284	1.604489	0.1104	0.28611	-5.7158

AP005131.1	-1.05606	2.716904	-1.60399	0.110511	0.286299	-4.71226
LINC01348	0.873216	6.76208	1.603721	0.110569	0.286351	-5.36197
AC009061.1	2.177257	5.290061	1.603452	0.110628	0.286406	-4.6411
AC020911.2	-2.01719	0.7516	-1.60294	0.110741	0.286598	-4.53189
AC245014.1	2.859362	1.648795	1.60266	0.110803	0.28661	-4.40878
AC099811.1	0.573956	5.411538	1.602579	0.110821	0.28661	-5.05257
AC116366.2	2.541212	3.231738	1.601922	0.110967	0.286886	-4.45446
AL590609.3	3.277711	-1.07427	1.601425	0.111077	0.287072	-4.37642
AC008750.1	2.762613	4.645637	1.601201	0.111126	0.287089	-4.52494
TBL1XR1-AS1	1.26632	5.272415	1.60105	0.11116	0.287089	-4.86393
AC087491.1	4.792024	4.890169	1.600544	0.111272	0.287281	-4.44932
AC069213.1	1.109585	4.734481	1.600224	0.111343	0.287365	-4.71958
TRPC7-AS1	3.635014	2.240319	1.599225	0.111565	0.287582	-4.41023
AC002401.1	2.628312	2.551057	1.59878	0.111664	0.287582	-4.43751
AC005821.1	1.699375	2.256587	1.598639	0.111695	0.287582	-4.45171
AC092608.1	-2.01699	2.038765	-1.59949	0.111506	0.287582	-4.69739
AC091946.2	-1.69279	2.888881	-1.59864	0.111694	0.287582	-4.85525
AL023584.2	1.438613	5.438364	1.598838	0.111651	0.287582	-4.91542
CAPN10-DT	0.332943	7.099982	1.599453	0.111514	0.287582	-5.59312
AL359851.1	2.128776	0.836589	1.597979	0.111842	0.287636	-4.4089
AC104051.2	-2.17886	-2.16705	-1.59803	0.11183	0.287636	-4.42421
AF250324.1	-2.4093	0.121374	-1.59786	0.111869	0.287636	-4.55209
PCOLCE-AS1	-0.62706	3.70912	-1.59799	0.111841	0.287636	-4.8616
AC022167.4	2.766861	1.901503	1.597274	0.111999	0.287871	-4.42084
AC010504.1	-0.4461	6.268117	-1.59701	0.112057	0.287922	-5.56279
PLA2G4E-AS1	2.813256	4.586241	1.596108	0.112259	0.288343	-4.51929
LINC02285	-1.44165	2.236032	-1.59563	0.112365	0.288516	-4.73595
AL137129.1	2.812739	-0.82068	1.594815	0.112548	0.28884	-4.38559
AC004951.1	0.277956	7.278797	1.594727	0.112568	0.28884	-5.65668
AC018645.2	0.362494	10.49489	1.594498	0.112619	0.288873	-6.15406
AC010542.4	1.362771	2.395918	1.593878	0.112758	0.28913	-4.47192
AC009955.2	3.258691	-0.16363	1.593102	0.112932	0.28928	-4.38893
AC007099.1	2.952851	0.868498	1.593199	0.11291	0.28928	-4.40433
AC099568.2	0.784863	6.865395	1.593156	0.11292	0.28928	-5.42693
AL162430.2	-0.5477	6.283146	-1.59198	0.113185	0.289829	-5.60579
AL008718.3	0.520686	7.201498	1.591494	0.113293	0.290008	-5.59167
AC090948.1	-0.41032	6.92412	-1.59125	0.113347	0.290047	-5.70638
AC109454.3	5.393338	3.959628	1.590698	0.113473	0.290269	-4.42703
AC020765.2	0.447147	7.280239	1.59012	0.113603	0.290503	-5.63282
AC012417.1	2.72274	0.0004	1.589587	0.113723	0.290625	-4.39876
AC074044.1	2.192408	4.227024	1.589566	0.113728	0.290625	-4.5435
AC011444.3	-1.90378	1.97206	-1.5886	0.113947	0.29108	-4.74075
LINC01978	-1.12754	5.361094	-1.58843	0.113983	0.29108	-5.46721
AC011298.1	2.73988	0.961428	1.586865	0.114339	0.291888	-4.41684
AC092747.1	2.806598	-1.79728	1.586561	0.114407	0.291964	-4.39448
AL139082.1	1.174118	3.15856	1.58603	0.114528	0.292173	-4.5429
AC004888.1	3.077932	-0.9404	1.584626	0.114847	0.292887	-4.39794
AC025430.1	-0.50651	6.783906	-1.5838	0.115035	0.293268	-5.70733
AC115837.2	3.001629	1.422504	1.583446	0.115116	0.293268	-4.42625
AC117503.1	2.695419	2.665459	1.583421	0.115121	0.293268	-4.46111
AL357153.1	2.163554	2.504812	1.583071	0.115201	0.293268	-4.46922
AP003117.2	-2.39093	-0.71348	-1.58318	0.115176	0.293268	-4.49277
AC005726.5	1.496881	3.512333	1.582942	0.11523	0.293268	-4.54178
AL360270.1	0.537002	6.781708	1.582672	0.115292	0.293325	-5.4956
AC005899.3	-2.2311	0.872959	-1.58159	0.115539	0.293854	-4.61014
AC068790.7	-0.53048	7.167212	-1.5802	0.115857	0.294564	-5.78225
AC074029.3	-0.83204	5.511015	-1.57988	0.115931	0.294652	-5.45565
AC022001.2	2.798728	0.343582	1.577927	0.116379	0.295016	-4.41707

AC144450.1	2.785496	0.604954	1.577723	0.116425	0.295016	-4.42059
AC010789.1	5.57943	4.093127	1.577544	0.116466	0.295016	-4.45052
AC007448.2	2.870475	3.132196	1.57841	0.116268	0.295016	-4.47886
STPG3-AS1	1.250219	4.333224	1.578355	0.11628	0.295016	-4.68516
STK4-AS1	0.596059	5.247773	1.578678	0.116206	0.295016	-4.9915
AC078909.1	-1.42762	4.05832	-1.57754	0.116467	0.295016	-5.05004
AL096828.3	1.219563	6.480443	1.578045	0.116351	0.295016	-5.22452
AC055720.2	-0.8532	7.552359	-1.57803	0.116355	0.295016	-5.89745
AC245060.2	-0.40562	9.576044	-1.57757	0.116461	0.295016	-6.14951
AP001065.2	3.331114	-0.56669	1.577017	0.116588	0.295171	-4.40797
AC009163.1	2.868414	0.687496	1.576935	0.116607	0.295171	-4.42277
LINC02561	4.801748	5.519403	1.576348	0.116742	0.295346	-4.49866
AC084757.2	1.747317	4.518759	1.576292	0.116755	0.295346	-4.64585
AP001062.2	3.587873	1.95802	1.576076	0.116804	0.295372	-4.43506
AC015813.1	-0.47081	8.727296	-1.57547	0.116943	0.295624	-6.02771
AC092171.3	0.504537	7.450433	1.575271	0.11699	0.295643	-5.68393
AC134772.1	2.528318	-1.34742	1.575094	0.117031	0.295647	-4.40957
AC022400.3	0.973398	2.453564	1.57436	0.117201	0.295855	-4.52334
AC015802.4	0.431287	5.575454	1.573995	0.117285	0.295855	-5.20404
FAM66B	-0.63861	5.028486	-1.57432	0.11721	0.295855	-5.33209
AC023830.1	-0.39534	5.576909	-1.57418	0.117241	0.295855	-5.35818
Z93930.3	-0.33458	6.553903	-1.57389	0.11731	0.295855	-5.64073
AL451042.2	0.575199	7.934911	1.572196	0.117702	0.296742	-5.77018
AC105219.1	0.359336	6.5893	1.571119	0.117952	0.297273	-5.50919
AL136040.1	0.494362	6.453982	1.57069	0.118051	0.297424	-5.44105
AP002840.2	-0.37024	7.696355	-1.57028	0.118145	0.297562	-5.86567
AL353803.4	1.518085	2.492252	1.569701	0.118281	0.297804	-4.50413
AC091729.1	1.25676	5.751132	1.569434	0.118343	0.297826	-5.02109
AC243965.2	-0.48352	4.490731	-1.56932	0.118369	0.297826	-5.11261
AC005224.1	0.418429	6.296194	1.568309	0.118606	0.298321	-5.41566
AC025171.4	0.515628	6.726314	1.567833	0.118717	0.2985	-5.52149
LINC02448	3.138562	-1.17084	1.567092	0.11889	0.298795	-4.41932
AC084125.3	2.992013	-0.63327	1.566991	0.118913	0.298795	-4.42061
AC116903.2	1.1164	5.02372	1.566382	0.119056	0.298953	-4.89802
AC073534.2	-1.41444	3.905889	-1.56654	0.11902	0.298953	-5.11055
TPRG1-AS2	3.088361	2.16114	1.565397	0.119287	0.299432	-4.46393
AL096865.1	1.838456	6.115962	1.564516	0.119493	0.299681	-4.89759
AC010680.4	-1.08206	5.357259	-1.56446	0.119505	0.299681	-5.4952
CCDC183-AS1	-0.41254	8.167313	-1.56471	0.119447	0.299681	-5.95687
AC011773.1	2.543251	5.05281	1.56424	0.119558	0.299713	-4.65663
LINC02166	0.454527	7.310219	1.56401	0.119612	0.299748	-5.66344
AL807752.5	0.493129	6.277012	1.56292	0.119868	0.30029	-5.40768
AC024267.4	0.829795	5.024481	1.562666	0.119928	0.300339	-4.8765
AL450322.1	3.327964	0.158026	1.562055	0.120072	0.3006	-4.42913
HCFC1-AS1	-1.77381	2.313083	-1.56176	0.120142	0.300603	-4.79193
AL451047.1	-1.44031	2.897492	-1.56171	0.120153	0.300603	-4.8446
AL049747.1	2.771447	-0.76808	1.561339	0.120241	0.300658	-4.42754
AC005479.1	1.779632	5.829033	1.561276	0.120255	0.300658	-4.86909
AC027281.2	1.078853	5.385602	1.560976	0.120326	0.30069	-4.96392
HDHD5-AS1	-0.53791	6.010464	-1.56088	0.120348	0.30069	-5.5461
AC012615.1	0.327806	8.307303	1.560139	0.120524	0.301029	-5.88354
LINC02037	2.501417	-1.98885	1.558709	0.120862	0.301427	-4.4289
AC012174.1	3.270568	-0.12027	1.558562	0.120897	0.301427	-4.4322
FAM242A	-2.25198	-1.80293	-1.55907	0.120776	0.301427	-4.48501
AC092068.2	-0.98584	2.700849	-1.55848	0.120917	0.301427	-4.75246
AC011444.2	-1.06355	3.833186	-1.5587	0.120865	0.301427	-5.14038
AC131953.1	-0.98566	5.053245	-1.55845	0.120924	0.301427	-5.43392
AL035425.3	-2.0597	0.356662	-1.55779	0.121079	0.301614	-4.5806

AC133644.2	1.394701	6.20482	1.557811	0.121075	0.301614	-5.09587
AC005522.1	2.124064	5.090151	1.555988	0.121508	0.302521	-4.69443
U52111.1	0.453617	6.969223	1.555921	0.121524	0.302521	-5.61263
AC018761.3	2.429388	3.906608	1.555492	0.121626	0.302674	-4.56946
AL135790.1	3.483552	0.616202	1.554971	0.12175	0.302682	-4.44149
U47924.1	2.221118	5.242718	1.555265	0.12168	0.302682	-4.70668
AC073389.1	0.461612	6.964683	1.554985	0.121747	0.302682	-5.6041
AL024508.2	0.831586	8.170808	1.554441	0.121876	0.302896	-5.7768
MRGPRF-AS1	-1.49445	1.128213	-1.55366	0.122062	0.303064	-4.62595
AC040169.3	0.464707	6.895262	1.553834	0.122021	0.303064	-5.58171
AL360091.1	0.444459	7.248749	1.55365	0.122065	0.303064	-5.68048
AL596223.2	3.864747	0.871315	1.552389	0.122366	0.303712	-4.44431
AL162727.2	-0.98718	5.462012	-1.5517	0.122532	0.304023	-5.56552
AC016877.1	2.910478	0.546397	1.551254	0.122638	0.304084	-4.45209
AL954642.1	-2.33202	-0.82851	-1.55135	0.122615	0.304084	-4.55137
AL359740.1	2.533243	3.913131	1.550552	0.122806	0.304402	-4.5601
AC005703.5	2.78567	-1.73769	1.550212	0.122887	0.304503	-4.43958
AL358334.2	2.102022	4.097834	1.549704	0.123009	0.304704	-4.59765
AC003070.1	0.558915	9.274219	1.548928	0.123196	0.305066	-6.0234
AC127024.8	0.398616	6.568226	1.5486	0.123275	0.305161	-5.5362
DNM3-IT1	2.992409	-0.37255	1.548033	0.123411	0.305297	-4.44501
AC091849.2	-1.86488	1.69836	-1.54811	0.123393	0.305297	-4.70161
EIF2AK3-DT	0.402958	6.687767	1.547747	0.12348	0.305367	-5.55749
AC006206.1	2.890624	2.5306	1.546739	0.123723	0.305767	-4.49942
AC127024.7	2.522824	5.251056	1.54678	0.123713	0.305767	-4.66725
AC144522.1	2.304161	-2.58455	1.546477	0.123786	0.305822	-4.44342
AC116914.1	2.114785	5.181583	1.545564	0.124007	0.306185	-4.71507
AC090772.4	0.52873	9.604084	1.545531	0.124014	0.306185	-6.0765
AL513329.1	1.419135	5.6565	1.545092	0.124121	0.306346	-4.9941
AC005899.5	-0.72233	5.574592	-1.54441	0.124286	0.306655	-5.55163
AC008040.1	-0.87386	1.173428	-1.54393	0.124403	0.306842	-4.58795
AC087623.3	-1.49231	2.599597	-1.54362	0.124477	0.306925	-4.82114
LINC02181	1.971957	-1.73621	1.542933	0.124644	0.30722	-4.44896
AC130352.1	-0.84436	5.104876	-1.54279	0.124679	0.30722	-5.41558
AL138921.2	-0.32897	7.359274	-1.54229	0.124799	0.307415	-5.84362
LINC00562	-0.46582	4.926918	-1.54208	0.12485	0.30744	-5.27974
AC026333.3	2.311699	0.893456	1.541601	0.124967	0.307526	-4.4792
AC131097.1	3.104901	2.669529	1.541754	0.12493	0.307526	-4.50584
AL365361.1	-0.62503	6.37737	-1.53938	0.125509	0.308758	-5.70623
AC084024.1	-0.85393	4.919885	-1.53896	0.125609	0.308905	-5.35843
AC073320.1	-0.74094	3.590278	-1.53871	0.125672	0.308958	-4.91071
AC112907.3	0.373141	6.551205	1.538265	0.12578	0.309122	-5.54742
AC026771.1	1.170175	5.498971	1.537617	0.125938	0.309309	-4.96056
AC090236.2	-0.69768	4.897318	-1.53777	0.125902	0.309309	-5.30308
KIRREL1-IT1	1.795856	6.017366	1.537188	0.126044	0.309466	-5.06538
AL021920.1	2.980373	-0.69501	1.536816	0.126135	0.309487	-4.45761
LINC01887	2.930404	-0.60907	1.536839	0.126129	0.309487	-4.4578
AL353795.2	2.710748	2.141385	1.536615	0.126184	0.309507	-4.50797
MIR200CHG	1.082778	10.19032	1.535997	0.126335	0.309777	-6.08589
AC010491.1	0.650974	5.349844	1.535775	0.12639	0.30981	-5.113
AC079777.1	1.744057	2.164978	1.535354	0.126493	0.309861	-4.53042
AC005776.2	0.300779	7.56354	1.535477	0.126463	0.309861	-5.79304
LINC01101	2.711814	-0.53511	1.534651	0.126666	0.310182	-4.46178
AC009171.2	1.544152	6.391066	1.533664	0.126908	0.310473	-5.15775
AC087645.2	0.638495	5.879067	1.533777	0.126881	0.310473	-5.27236
AC020661.2	-0.59763	6.245168	-1.53369	0.126903	0.310473	-5.69012
AP001627.1	-1.55362	1.58694	-1.53338	0.126979	0.310544	-4.69271
LINC01337	-1.33449	3.257964	-1.53259	0.127173	0.310917	-5.02277

AC025822.2	2.153497	0.178783	1.531845	0.127357	0.311266	-4.48013
AC078802.1	0.994764	5.24944	1.531323	0.127486	0.311479	-5.01877
AC104126.1	-2.23274	-0.48835	-1.52968	0.127892	0.312371	-4.5814
AC005220.1	-2.32009	-0.85836	-1.52936	0.12797	0.312459	-4.55532
AC008035.1	-0.4337	7.29074	-1.52802	0.128304	0.313173	-5.85879
AC080013.3	1.455992	6.327553	1.527671	0.12839	0.313281	-5.10462
AL137186.1	2.559191	2.532618	1.527497	0.128433	0.313285	-4.53207
AC091544.7	-2.36199	-0.00249	-1.52704	0.128546	0.313459	-4.60184
TRIM36-IT1	-1.77121	1.499547	-1.52671	0.128628	0.313556	-4.6879
AL358472.2	0.287702	7.214148	1.526042	0.128795	0.31376	-5.74481
AC008735.2	0.311325	9.494951	1.526134	0.128772	0.31376	-6.11665
AF127936.1	2.153216	4.486744	1.525491	0.128932	0.313891	-4.66487
AF127577.6	1.106208	3.772177	1.525574	0.128911	0.313891	-4.70169
AC233280.1	1.190064	5.220343	1.52437	0.129212	0.314469	-4.96117
AC006213.5	-0.85643	6.574098	-1.52333	0.129472	0.315	-5.82407
AC004009.1	3.574015	-0.38062	1.521805	0.129853	0.315605	-4.47587
AL136366.1	-1.72655	1.309582	-1.52167	0.129888	0.315605	-4.72444
AL133373.2	-0.90585	4.708203	-1.52184	0.129845	0.315605	-5.33185
AC091868.2	-1.02258	5.15056	-1.52183	0.129847	0.315605	-5.47919
FAM83C-AS1	3.068389	4.860087	1.520432	0.130197	0.316228	-4.63516
UBE2D3-AS1	-0.26058	7.890525	-1.52031	0.130229	0.316228	-5.95624
AL451064.1	1.557531	5.312342	1.51827	0.130741	0.317369	-4.85217
AC098484.1	-0.28426	8.547956	-1.51784	0.13085	0.317531	-6.07093
NRAV	0.34134	10.20539	1.517429	0.130953	0.317678	-6.23315
AC020923.1	2.216669	4.369072	1.517019	0.131056	0.3178	-4.65101
LINC01436	-1.14753	5.351052	-1.5169	0.131088	0.3178	-5.61827
AL691482.3	1.33173	8.378305	1.51646	0.131198	0.317964	-5.81521
AC022400.6	-0.26433	7.797916	-1.51581	0.131362	0.318259	-5.94686
AP000355.1	2.834426	-0.57522	1.515042	0.131556	0.318524	-4.48471
ZNF350-AS1	-0.78433	4.634591	-1.51511	0.131538	0.318524	-5.31505
AC007991.2	5.119253	3.461503	1.514312	0.131741	0.318869	-4.52261
LINC02651	2.699917	-1.65085	1.512947	0.132087	0.318988	-4.48479
MYO16-AS1	3.551642	3.532916	1.513014	0.13207	0.318988	-4.55399
AC036176.3	5.899894	6.948817	1.51341	0.13197	0.318988	-4.61721
AC084876.2	0.761706	4.933897	1.513344	0.131987	0.318988	-4.9835
AC025280.1	-1.58991	3.541231	-1.51364	0.131911	0.318988	-5.09027
AL158825.2	0.360697	7.836363	1.513213	0.13202	0.318988	-5.86501
ZBTB11-AS1	0.392396	7.972907	1.513587	0.131925	0.318988	-5.88967
AL442128.2	1.142045	4.079591	1.512282	0.132256	0.319229	-4.75446
RABGAP1L-IT1	-0.58579	6.632606	-1.51222	0.132272	0.319229	-5.79007
AL096677.1	1.499629	4.272735	1.511943	0.132342	0.319296	-4.75699
GLIS3-AS1	2.56241	3.207034	1.51171	0.132402	0.319337	-4.57548
AC005722.2	2.155494	4.079834	1.510572	0.132691	0.31983	-4.64399
LINC00960	1.359967	6.776645	1.510574	0.132691	0.31983	-5.46887
AC083900.1	1.049114	5.733333	1.509788	0.132891	0.320106	-5.05306
AC037198.2	-0.79157	6.607817	-1.50984	0.132877	0.320106	-5.86122
AC243830.2	2.245478	3.430043	1.509431	0.132982	0.32012	-4.61735
AC027682.7	-0.39132	4.628801	-1.50944	0.132979	0.32012	-5.19627
AC025183.1	4.953461	3.234221	1.50866	0.133179	0.320492	-4.52391
AC130456.7	-2.3202	0.807104	-1.50813	0.133315	0.320613	-4.67928
AC010326.3	-0.33715	10.16251	-1.50819	0.133299	0.320613	-6.32211
PAPPA-AS2	-1.90418	0.245372	-1.50715	0.133566	0.321115	-4.62249
AL161421.1	0.443831	8.275951	1.506844	0.133644	0.321199	-5.9439
AC073367.1	0.446828	6.287236	1.506548	0.13372	0.321278	-5.53386
AL354760.1	0.623066	6.302454	1.505595	0.133964	0.321735	-5.45113
AC137932.3	0.278549	6.940485	1.505305	0.134039	0.321735	-5.71868
AC011481.3	-0.45873	7.003368	-1.50536	0.134026	0.321735	-5.85205
AC084026.3	2.694276	-0.7287	1.504121	0.134343	0.322344	-4.4974

AC138028.2	0.396434	6.343184	1.503985	0.134378	0.322344	-5.55878
LINC01614	4.639472	6.46886	1.50322	0.134575	0.322714	-4.66181
BX293535.1	-0.94088	6.603205	-1.5027	0.134708	0.32293	-5.86554
AC021683.1	-0.85978	4.177903	-1.50197	0.134898	0.323252	-5.30382
CNNM3-DT	0.477633	7.436524	1.501849	0.134929	0.323252	-5.79323
AL137002.1	-1.471	4.108409	-1.50164	0.134982	0.323277	-5.30703
AC104118.1	0.44041	6.592327	1.500784	0.135204	0.323705	-5.56759
AC024060.1	-0.28957	9.520311	-1.50038	0.135308	0.323852	-6.23862
HOXC13-AS	6.016955	5.25409	1.500159	0.135365	0.323886	-4.55837
AC003681.1	-0.24163	5.489432	-1.4997	0.135484	0.324066	-5.46417
AC106786.1	2.016458	5.454528	1.499008	0.135663	0.324289	-4.88882
AL606834.2	-0.40318	7.906925	-1.49908	0.135645	0.324289	-6.01614
P4HA3-AS1	1.951137	-0.15558	1.49831	0.135845	0.324619	-4.51829
AC069222.1	1.021663	3.850677	1.498122	0.135893	0.324632	-4.74434
AC068831.6	3.175609	5.587871	1.494977	0.136712	0.326483	-4.71528
AL049647.1	-1.04279	3.722309	-1.49472	0.13678	0.326542	-5.10383
AL136979.1	-1.53843	1.769694	-1.4938	0.13702	0.326947	-4.78332
AC005899.6	-0.44948	7.95628	-1.49373	0.137037	0.326947	-6.03738
AC025031.2	1.817959	4.167667	1.492984	0.137232	0.32731	-4.71546
AL355482.2	-1.66805	2.112563	-1.49264	0.137323	0.327423	-4.8437
AL358332.1	2.699866	3.235186	1.491407	0.137645	0.328087	-4.60294
CHRM3-AS1	-1.85039	2.242433	-1.49091	0.137776	0.32819	-4.84789
LINC01023	0.504098	8.703684	1.490956	0.137764	0.32819	-6.02402
AC079866.2	1.679337	5.125007	1.490595	0.137858	0.328282	-4.83996
AC135776.1	-1.74967	-0.45013	-1.4903	0.137936	0.328363	-4.61807
AL359880.1	3.165317	4.401503	1.489709	0.138091	0.328398	-4.63439
AL512288.1	-2.246	0.139561	-1.48992	0.138037	0.328398	-4.65149
AF196972.1	0.829551	6.095645	1.489844	0.138055	0.328398	-5.38019
AC026691.1	-0.46281	5.596304	-1.48958	0.138125	0.328398	-5.54845
AL139317.1	-1.91613	2.232093	-1.4885	0.13841	0.328969	-4.88569
AC080013.5	1.837699	3.814794	1.487897	0.138568	0.329242	-4.67559
AC124276.1	2.404897	1.869118	1.487401	0.138699	0.329344	-4.56414
AC007191.1	0.343516	7.762105	1.487474	0.138679	0.329344	-5.89916
AL645608.4	2.974473	-0.45713	1.487176	0.138758	0.329381	-4.51823
SALRNA1	2.171764	2.023176	1.486472	0.138944	0.329509	-4.57578
AL158071.4	2.276737	4.27237	1.486562	0.13892	0.329509	-4.68154
AC145207.5	0.413386	9.115776	1.486511	0.138934	0.329509	-6.10685
AC007952.6	-1.47089	1.652575	-1.4862	0.139017	0.329578	-4.77941
LINC02586	1.63707	4.594095	1.485823	0.139115	0.329708	-4.81193
AC024559.1	2.623985	2.265054	1.484735	0.139403	0.330286	-4.57533
AC087749.1	-0.47911	6.528122	-1.48447	0.139474	0.330349	-5.80031
AC107953.2	2.451294	-1.33352	1.483705	0.139676	0.330724	-4.52029
AL162412.1	2.424298	-0.37487	1.483267	0.139792	0.330895	-4.52708
AC104791.2	1.425508	3.399521	1.482584	0.139974	0.331144	-4.68171
AC012442.2	-0.81213	5.482502	-1.48254	0.139986	0.331144	-5.55906
AL096828.1	2.707218	0.4851	1.482224	0.140069	0.331238	-4.5382
AL022322.1	0.701542	8.016302	1.48174	0.140198	0.331438	-5.88995
AC242988.1	2.415136	-0.17003	1.481027	0.140388	0.331782	-4.53268
LINC02562	1.399398	8.791736	1.479892	0.14069	0.332392	-5.91504
ADGRD1-AS1	-2.11881	1.424058	-1.47946	0.140805	0.332557	-4.80368
CACNA2D3-AS1	-2.09281	-1.82174	-1.479	0.140928	0.332659	-4.58261
AL360091.3	2.320485	3.72828	1.478647	0.141023	0.332659	-4.6577
AC005730.2	-1.65628	1.12208	-1.47864	0.141025	0.332659	-4.7398
AC106028.4	-0.43737	6.097892	-1.47888	0.14096	0.332659	-5.70193
AC128709.2	3.466694	2.238897	1.477804	0.141248	0.33298	-4.56687
AC004816.2	2.182346	5.780268	1.477798	0.14125	0.33298	-4.94583
AC010531.5	0.447648	6.948195	1.477137	0.141427	0.333293	-5.72473
AC106795.5	-1.5624	2.72112	-1.47679	0.14152	0.333408	-5.00145

AC016747.3	-0.28937	7.808853	-1.47611	0.141701	0.33373	-6.01701
AC244517.6	2.583402	2.45485	1.475753	0.141798	0.333853	-4.59406
AC009163.3	-0.4146	5.27101	-1.47371	0.142347	0.335041	-5.44537
AL162293.1	1.747904	1.083709	1.473217	0.142479	0.335248	-4.5782
ZSCAN16-AS1	-0.36243	8.749127	-1.47294	0.142555	0.335321	-6.17308
AL138916.1	2.886483	-0.42155	1.472608	0.142643	0.335331	-4.53566
AC134775.1	-0.73312	5.030172	-1.47259	0.142648	0.335331	-5.46044
AC008115.1	3.230188	2.61545	1.470577	0.143192	0.336082	-4.59198
AL583722.3	-1.74743	1.416294	-1.47085	0.143117	0.336082	-4.79086
AC008269.1	-1.72273	1.664575	-1.47062	0.14318	0.336082	-4.80284
AP003733.4	0.364072	7.000807	1.470814	0.143127	0.336082	-5.75954
AC011468.1	-0.40916	7.97252	-1.47115	0.143037	0.336082	-6.06678
AL135787.1	-1.01105	5.385935	-1.46991	0.143371	0.336399	-5.65929
AC107909.1	1.730699	4.329845	1.469236	0.143555	0.336618	-4.76703
AC138696.2	0.46933	8.962842	1.469331	0.143529	0.336618	-6.09962
AC020913.2	1.117312	4.414724	1.468956	0.14363	0.336691	-4.87169
AC083964.2	-0.58305	4.102645	-1.46796	0.143901	0.33722	-5.14403
NADK2-AS1	-0.43612	7.062982	-1.46678	0.144221	0.337865	-5.92348
PICART1	0.691711	5.460733	1.466375	0.144331	0.338017	-5.24039
AC016831.6	2.285971	-1.52403	1.465669	0.144523	0.338362	-4.5411
AC007448.3	0.682051	5.589338	1.46503	0.144697	0.338664	-5.30494
AL031727.2	2.944497	-1.16117	1.46338	0.145147	0.338757	-4.54389
LINC02163	7.182675	5.415774	1.463459	0.145126	0.338757	-4.5831
AC022148.2	-2.35294	-1.48024	-1.46358	0.145093	0.338757	-4.61443
AP000265.1	1.499363	2.111955	1.46377	0.145041	0.338757	-4.6286
AL162632.3	1.706782	2.843571	1.463877	0.145012	0.338757	-4.65132
AL353768.1	2.487643	3.610701	1.464461	0.144852	0.338757	-4.65228
AF230666.2	1.937216	3.203283	1.463881	0.145011	0.338757	-4.66372
ACTN1-AS1	1.434594	4.526535	1.464286	0.1449	0.338757	-4.83196
AC068987.4	0.657653	5.370036	1.463232	0.145188	0.338757	-5.20765
AC018682.1	0.400336	6.320351	1.464124	0.144944	0.338757	-5.59433
AL035045.1	2.797568	0.268001	1.461859	0.145563	0.338975	-4.55654
AC126407.1	3.488085	2.408431	1.462332	0.145434	0.338975	-4.58836
AC003005.2	2.455531	1.98383	1.461464	0.145672	0.338975	-4.60362
AL136528.1	2.413599	2.242682	1.461137	0.145761	0.338975	-4.60988
AC020558.3	1.705843	1.865769	1.461859	0.145563	0.338975	-4.6126
AC009120.4	-1.98125	0.739086	-1.46148	0.145666	0.338975	-4.72735
AL049794.1	1.895331	4.310917	1.462305	0.145442	0.338975	-4.75017
AL136379.1	-0.86695	2.669687	-1.46232	0.145438	0.338975	-4.89397
BX649632.1	-0.60083	5.037292	-1.46108	0.145778	0.338975	-5.45786
AC022784.5	0.629276	6.26403	1.461585	0.145639	0.338975	-5.48652
AC074212.1	-0.34062	6.205944	-1.4614	0.145689	0.338975	-5.73458
LINC02078	1.410136	0.81152	1.459	0.146348	0.339966	-4.59522
AL365318.1	1.735875	3.997143	1.458931	0.146367	0.339966	-4.75106
AD000864.2	0.60755	4.939878	1.459067	0.146329	0.339966	-5.08278
AL035661.1	-0.68065	10.48363	-1.45887	0.146385	0.339966	-6.47429
AL626787.1	-1.40851	3.012842	-1.45746	0.146772	0.340654	-4.99996
AP001029.1	-1.28266	5.028643	-1.4576	0.146735	0.340654	-5.62673
AP003119.1	3.187491	2.352849	1.457201	0.146843	0.340714	-4.5975
LINC02177	1.777336	-0.10873	1.456234	0.14711	0.340754	-4.57214
AP001011.1	1.979162	3.258407	1.456153	0.147132	0.340754	-4.67443
AC104662.1	-1.94858	0.951751	-1.4568	0.146954	0.340754	-4.75997
AL445673.1	-1.59658	2.970186	-1.4564	0.147064	0.340754	-5.0341
AC079336.7	-0.67263	5.048281	-1.45645	0.147051	0.340754	-5.50875
AL133346.1	-0.6857	6.661122	-1.45637	0.147073	0.340754	-5.90904
DGCR10	2.680768	3.339291	1.45551	0.14731	0.34106	-4.65141
AC018946.2	-1.0943	2.439444	-1.45457	0.147569	0.341554	-4.84667
AC104984.2	2.075111	4.48493	1.453833	0.147774	0.341924	-4.79874

LINC01166	-1.97797	-1.34221	-1.45346	0.147878	0.341953	-4.62419
AC004832.6	2.106452	2.919266	1.453552	0.147852	0.341953	-4.65428
DLG3-AS1	-1.01119	4.477129	-1.45301	0.148001	0.342133	-5.45217
AP003472.1	2.514919	0.115355	1.451858	0.148322	0.342381	-4.57066
AC023906.2	4.165989	3.779509	1.452047	0.148269	0.342381	-4.62025
LINC02145	-0.86432	3.753092	-1.45208	0.148259	0.342381	-5.16105
ITPR1-DT	-0.97463	4.354222	-1.45181	0.148334	0.342381	-5.24396
AP000350.6	-1.07408	4.89776	-1.45181	0.148336	0.342381	-5.55239
EXTL3-AS1	0.480569	5.389234	1.451294	0.148479	0.342604	-5.28313
AC012313.2	0.558484	7.242106	1.450487	0.148703	0.343017	-5.81135
AC092681.3	-1.94657	0.427178	-1.45032	0.14875	0.343019	-4.72261
AC007663.3	1.389045	5.002416	1.449888	0.14887	0.343191	-4.92163
AC026803.1	2.048348	5.937617	1.449378	0.149012	0.343413	-5.01377
LINC01250	2.18541	-0.73903	1.448508	0.149255	0.343867	-4.56598
MAST4-AS1	0.982504	6.823549	1.44828	0.149318	0.343908	-5.6482
AC008438.1	0.347455	7.55459	1.448115	0.149365	0.343909	-5.91377
AC011389.3	-2.13298	-2.23513	-1.44783	0.149444	0.343987	-4.60995
AC002091.1	1.631595	5.911587	1.447657	0.149492	0.343993	-5.11585
AL512422.1	2.560418	1.579228	1.446518	0.149811	0.344556	-4.60719
AC025423.1	0.764802	6.509615	1.446455	0.149829	0.344556	-5.57982
AC020741.1	2.054237	-0.25772	1.445958	0.149968	0.34474	-4.57727
AL035446.1	4.345653	5.410894	1.445842	0.15	0.34474	-4.68503
AC138951.2	-0.86876	4.430775	-1.44512	0.150203	0.3451	-5.34665
AC005899.1	-0.89837	4.534929	-1.44483	0.150284	0.345181	-5.43199
AC007066.2	0.278363	7.207794	1.444502	0.150376	0.345286	-5.85777
AC009486.1	-1.19486	3.705096	-1.4439	0.150547	0.345572	-5.15542
AC017002.5	2.484851	-1.03754	1.443058	0.150782	0.345696	-4.56817
AC105235.1	0.388877	6.924776	1.443326	0.150707	0.345696	-5.77842
AC013403.2	-0.2858	7.43176	-1.4435	0.150658	0.345696	-6.00024
AC004918.3	-0.34331	9.401905	-1.44305	0.150785	0.345696	-6.3138
AC131238.1	2.675316	1.852975	1.442768	0.150864	0.345771	-4.61357
LINC02126	2.495196	-0.2457	1.442228	0.151016	0.345804	-4.57556
AC100803.2	-0.60846	5.87293	-1.44236	0.150978	0.345804	-5.70473
AC005070.3	-0.28797	7.402773	-1.44254	0.150926	0.345804	-5.99206
AC008115.2	2.970351	1.957522	1.441781	0.151142	0.345906	-4.61478
AC009065.6	1.60428	4.813309	1.441742	0.151153	0.345906	-4.85047
LINC00412	-1.47587	3.033827	-1.44157	0.151202	0.345913	-5.05426
THAP7-AS1	0.567559	6.893888	1.441172	0.151313	0.346063	-5.74967
LINC01891	2.809434	0.757693	1.440795	0.15142	0.346177	-4.59004
LINC00884	1.171398	6.476308	1.440669	0.151455	0.346177	-5.31627
AC024560.3	0.419295	8.526287	1.440133	0.151607	0.346418	-6.08185
AL390726.3	2.701493	2.842863	1.439372	0.151822	0.346804	-4.65031
AL445931.1	-0.56267	5.422439	-1.43882	0.151977	0.346947	-5.58591
AC009996.1	-0.2587	7.088716	-1.43885	0.151968	0.346947	-5.93569
LYST-AS1	2.559903	1.320167	1.438119	0.152176	0.347297	-4.61057
AC019118.2	2.318677	1.034783	1.437624	0.152317	0.347406	-4.60808
AC145098.2	0.531243	5.462833	1.437669	0.152304	0.347406	-5.34002
AC073878.1	-2.2462	-0.99941	-1.43725	0.152422	0.347541	-4.66243
AC068870.1	1.207853	2.707705	1.436127	0.152742	0.348164	-4.70353
LINC02412	-1.83455	0.436788	-1.43569	0.152867	0.348344	-4.73193
KIF26B-AS1	1.017326	6.573582	1.435343	0.152965	0.348461	-5.46519
AL139354.1	-2.21013	-0.92976	-1.43485	0.153105	0.348675	-4.66744
AC009093.6	-0.95697	5.278195	-1.43424	0.153279	0.348965	-5.52158
AC093585.1	-1.61235	2.68868	-1.43405	0.153333	0.348982	-5.03994
AC133919.2	1.571407	7.111409	1.433029	0.153624	0.349539	-5.60108
AC004471.1	0.894652	6.11883	1.431999	0.153918	0.350102	-5.38198
AL590787.1	2.0944	1.19788	1.431274	0.154125	0.350467	-4.62326
AL157871.5	-0.28752	7.775643	-1.43078	0.154266	0.350681	-6.06984

AC010285.1	-1.3418	3.253587	-1.42963	0.154596	0.351326	-5.10134
AP003500.1	2.643699	2.573598	1.428901	0.154805	0.351694	-4.65335
AC124067.3	2.743999	-1.38649	1.428222	0.155001	0.351892	-4.58389
NLGN4Y-AS1	2.798716	0.212623	1.428081	0.155041	0.351892	-4.59712
AC005387.2	0.325281	6.359602	1.428318	0.154973	0.351892	-5.65858
AC006504.1	-0.50339	6.287532	-1.42795	0.15508	0.351892	-5.8342
LINC02535	2.88326	5.508211	1.427781	0.155127	0.351893	-4.82282
AC087645.3	2.629281	-0.84924	1.427537	0.155197	0.351946	-4.58618
AC023449.2	-0.54452	5.136863	-1.42673	0.155429	0.352366	-5.51124
AC010654.1	0.463121	6.75334	1.426535	0.155486	0.352388	-5.72927
AC023090.1	-1.41528	4.493579	-1.42536	0.155825	0.353051	-5.45309
AC008494.3	0.45534	6.033416	1.424682	0.156021	0.353386	-5.54302
AC012313.6	-0.39759	6.108316	-1.42395	0.156231	0.353756	-5.77516
LINC02356	2.284396	4.245428	1.423215	0.156444	0.35392	-4.77034
AC120498.9	0.65256	3.047739	1.423321	0.156414	0.35392	-4.79809
AC245100.7	1.003686	6.827996	1.423398	0.156392	0.35392	-5.64886
AC107926.1	2.795145	-0.78979	1.421684	0.156888	0.354542	-4.59266
MYHAS	2.197447	0.743271	1.421202	0.157028	0.354542	-4.62326
AC009065.1	2.187012	1.280921	1.421521	0.156935	0.354542	-4.63506
Z98257.1	2.211234	3.961817	1.421126	0.15705	0.354542	-4.7543
AL121820.1	-0.971	4.471357	-1.42134	0.156987	0.354542	-5.43718
AC083843.3	-0.37676	8.262704	-1.42186	0.156836	0.354542	-6.17501
AC018647.2	0.377212	9.857919	1.421838	0.156843	0.354542	-6.32083
AC008663.1	3.13017	-0.19836	1.420494	0.157233	0.35485	-4.59543
AC024475.1	2.373631	2.250675	1.420259	0.157302	0.354897	-4.65743
AC113382.2	-1.43505	2.103017	-1.42002	0.15737	0.354945	-4.90649
AC145676.1	-0.67013	3.790562	-1.41938	0.157559	0.355264	-5.21147
AL034405.1	2.413289	3.394789	1.418779	0.157732	0.355548	-4.69927
AP005019.1	0.942324	5.829075	1.418282	0.157877	0.35567	-5.3061
AC009318.4	-0.45916	6.693554	-1.41827	0.157881	0.35567	-5.91446
NCF4-AS1	2.367926	-0.7704	1.418099	0.157931	0.355675	-4.59853
AL353801.1	-1.7301	1.908657	-1.41728	0.15817	0.356107	-4.88416
AL392048.1	2.446103	0.82215	1.417088	0.158225	0.356118	-4.62639
AL589843.1	-1.02504	6.134228	-1.41694	0.15827	0.356118	-5.89775
AP001178.1	2.84698	2.869316	1.416179	0.158491	0.356509	-4.67168
AC015971.1	-0.37147	6.106026	-1.41545	0.158703	0.35688	-5.77461
LINC00492	2.889272	0.018937	1.415123	0.1588	0.35699	-4.60583
AC090774.2	2.606716	-1.39448	1.414182	0.159075	0.357076	-4.59985
LINC02067	-1.11971	1.700291	-1.41469	0.158925	0.357076	-4.82786
AL358075.1	-0.93263	4.185586	-1.41427	0.15905	0.357076	-5.35757
AL512413.1	0.960991	7.512159	1.414504	0.158981	0.357076	-5.81739
AC002467.1	0.383482	8.287479	1.414423	0.159005	0.357076	-6.08598
MCCC1-AS1	0.417726	7.496484	1.413137	0.159382	0.357657	-5.95147
STAM-AS1	0.423689	5.018796	1.411832	0.159765	0.358411	-5.1937
RAD21-AS1	-0.40596	4.666864	-1.41157	0.159842	0.358475	-5.35343
AL449106.1	0.699313	5.219175	1.411197	0.159952	0.358615	-5.25214
AC012555.1	-1.02376	3.606545	-1.41079	0.160073	0.358779	-5.24192
AC004707.1	-0.87283	4.877706	-1.4105	0.160157	0.358861	-5.46926
AL021328.1	-0.50626	5.763577	-1.41031	0.160214	0.358862	-5.75434
AC132872.4	0.327672	6.99723	1.410175	0.160253	0.358862	-5.85624
AC005578.1	1.935675	1.753943	1.409174	0.160549	0.359337	-4.66493
SAPCD1-AS1	-1.26997	4.093908	-1.40913	0.160561	0.359337	-5.27174
AC004264.1	1.623517	6.192118	1.408871	0.160638	0.359402	-5.29692
AC005550.2	2.839704	3.01285	1.408546	0.160734	0.35951	-4.68779
AC096741.1	-1.29451	3.567781	-1.40834	0.160794	0.359534	-5.20992
AC007773.1	0.719617	6.45839	1.408187	0.16084	0.359534	-5.61273
AP002761.1	4.074376	4.752025	1.407938	0.160914	0.359591	-4.70952
AC004596.1	0.414614	7.658173	1.407432	0.161064	0.359819	-5.98151

AL390067.1	2.170402	3.996483	1.406973	0.161199	0.35989	-4.76984
AC008080.1	-1.72001	0.663504	-1.40662	0.161303	0.35989	-4.78456
AC009120.5	-0.59759	4.249275	-1.40667	0.161288	0.35989	-5.25048
AC027682.4	-0.28659	6.07583	-1.40707	0.161172	0.35989	-5.77302
AC136475.2	0.551006	7.460379	1.406389	0.161372	0.35989	-5.93757
AC010319.4	0.279127	7.647173	1.406353	0.161383	0.35989	-5.99659
SAMSN1-AS1	2.348898	0.459399	1.405813	0.161543	0.360056	-4.6329
AC007938.3	0.709854	7.085847	1.405779	0.161553	0.360056	-5.79672
AC011603.2	0.464281	4.670749	1.405138	0.161743	0.360373	-5.11679
AC120042.2	2.439754	1.904245	1.404747	0.161859	0.360418	-4.6658
AL162741.1	0.603395	4.825553	1.404898	0.161815	0.360418	-5.16179
AC067930.3	1.353793	7.256744	1.40438	0.161968	0.360554	-5.6338
AC016866.3	3.254884	6.374375	1.404056	0.162065	0.360661	-4.93546
AC023154.1	0.896456	1.89066	1.403857	0.162124	0.360686	-4.70902
AL355312.4	0.941476	8.985926	1.403585	0.162205	0.360759	-6.13877
AC136475.1	0.484586	5.719029	1.403228	0.162311	0.360889	-5.45845
LIPC-AS1	2.02187	-0.12257	1.402644	0.162485	0.360955	-4.63006
AL008727.1	-0.54337	3.252903	-1.40268	0.162474	0.360955	-5.0339
AC005253.1	0.228562	7.594173	1.402824	0.162431	0.360955	-6.00433
LINC01067	2.488303	-0.98639	1.402446	0.162544	0.360979	-4.61408
AC246817.1	-2.012	0.769137	-1.40123	0.162908	0.361573	-4.81021
AL590133.1	1.137701	6.135059	1.40133	0.162877	0.361573	-5.39269
EPN2-AS1	0.384849	6.178963	1.400352	0.163169	0.362046	-5.63425
AC092849.2	1.923028	4.624221	1.399598	0.163394	0.362439	-4.86544
LINC02227	-1.51881	-0.28178	-1.39889	0.163607	0.362804	-4.71685
AC007610.1	2.368309	1.28699	1.398387	0.163757	0.363029	-4.65922
AP001198.1	-1.89693	-2.17173	-1.39805	0.163859	0.363148	-4.66353
AP000697.1	-1.28406	6.160372	-1.39683	0.164225	0.363852	-5.97938
AC018809.1	-0.37424	6.582251	-1.39609	0.164447	0.364166	-5.90219
AC009318.2	-0.2759	8.102264	-1.39603	0.164464	0.364166	-6.17256
AC013553.3	2.110018	3.732334	1.395857	0.164516	0.364175	-4.77416
LINC00996	0.979681	5.156784	1.395572	0.164602	0.364258	-5.0766
AC093821.1	-2.11505	-0.21141	-1.39525	0.164698	0.364362	-4.74814
AC009271.1	4.299157	4.24669	1.394528	0.164916	0.364739	-4.70058
ATXN2-AS	0.897577	5.996067	1.394319	0.164979	0.364771	-5.32838
AL049629.1	-0.89159	5.816667	-1.39384	0.165124	0.364983	-5.82966
AC098818.2	1.595925	1.786007	1.393454	0.16524	0.365132	-4.69595
AC132938.1	2.39683	2.70512	1.393126	0.165339	0.365244	-4.70889
AC131902.1	2.706636	0.522848	1.392954	0.165391	0.365251	-4.64301
AP000894.4	0.531527	7.645141	1.391306	0.165889	0.366243	-5.9766
LINC01990	-1.22831	2.170683	-1.39079	0.166044	0.366477	-4.93134
AC245041.1	6.055877	8.611905	1.390439	0.166152	0.3665	-4.92725
AC007485.2	-0.23291	9.553489	-1.39049	0.166137	0.3665	-6.38998
AC132192.1	0.646167	6.947503	1.390006	0.166283	0.366682	-5.80008
ARRDC1-AS1	0.281878	9.71031	1.389555	0.16642	0.366875	-6.34985
AC011477.3	-0.99097	7.569595	-1.38935	0.166482	0.366906	-6.1985
AC018716.2	2.683705	-0.16512	1.388187	0.166835	0.367491	-4.63565
AC016747.2	-0.71467	4.687642	-1.38815	0.166846	0.367491	-5.46932
LINC00323	-1.02187	2.770371	-1.38741	0.167071	0.36788	-5.06794
LINC02065	2.63354	1.659921	1.387132	0.167156	0.367959	-4.67559
AC068473.3	1.439595	3.420659	1.386593	0.16732	0.368212	-4.81033
LINC01807	3.797932	1.755661	1.386152	0.167454	0.368356	-4.65541
AC022762.1	2.353125	1.405746	1.386056	0.167484	0.368356	-4.67651
AL020995.1	-0.44852	4.882146	-1.38509	0.167777	0.368878	-5.45706
AC010973.1	-0.55591	5.697639	-1.3848	0.167868	0.368878	-5.76438
ZKSCAN7-AS1	-0.57513	6.319743	-1.38488	0.167841	0.368878	-5.91771
AC104596.1	-0.73129	4.478555	-1.38449	0.167963	0.368978	-5.43742
AL359091.4	0.52416	6.576823	1.383429	0.168286	0.36958	-5.73643

LINC01994	2.47547	-1.09993	1.382845	0.168465	0.369756	-4.63544
AC063965.1	-0.44751	6.745687	-1.38289	0.168452	0.369756	-5.98492
AC008278.1	2.45816	1.321765	1.382387	0.168605	0.369956	-4.67564
TCF7L1-IT1	2.74346	-0.4023	1.381921	0.168748	0.370054	-4.6393
AC117503.4	-0.24397	7.453725	-1.38207	0.168702	0.370054	-6.07809
AC078962.3	-1.47943	2.858496	-1.38079	0.169096	0.370709	-5.08508
AP000238.1	2.166003	5.049605	1.3799	0.169368	0.370971	-4.89609
AP000253.1	0.485516	5.062527	1.379753	0.169413	0.370971	-5.25526
TSPEAR-AS2	1.290912	6.651602	1.379889	0.169372	0.370971	-5.54282
AL023653.1	-0.76578	5.464982	-1.37975	0.169413	0.370971	-5.76119
AL157373.2	1.660894	4.282422	1.37943	0.169513	0.371081	-4.90806
AC022601.1	-2.18998	-1.26125	-1.37908	0.169621	0.37121	-4.72058
AC011377.1	-2.01346	0.923914	-1.37851	0.169796	0.371485	-4.85631
AL645939.4	2.785922	1.046791	1.37784	0.170003	0.371828	-4.66776
AL157871.2	0.531366	7.792726	1.377663	0.170057	0.371839	-6.02087
AC104564.4	2.791843	-0.46523	1.377112	0.170227	0.372103	-4.64388
AGAP1-IT1	-0.65958	6.453627	-1.37671	0.170352	0.372266	-5.93785
AC007687.1	2.722173	1.886947	1.37634	0.170466	0.37235	-4.69334
CRIM1-DT	0.463232	8.659255	1.376263	0.170489	0.37235	-6.19281
AC023790.2	-0.57231	4.138154	-1.37498	0.170886	0.373094	-5.24144
AC019131.2	-0.34047	7.532991	-1.37484	0.170929	0.373094	-6.12721
PPP1R26-AS1	0.474185	7.052096	1.374302	0.171096	0.37335	-5.87542
AC110792.3	-0.48021	7.366856	-1.37402	0.171183	0.373431	-6.11958
AL121603.2	0.300252	8.603669	1.373729	0.171273	0.37352	-6.21033
AC005540.1	0.913117	5.947977	1.373317	0.171401	0.373582	-5.41641
AC087392.5	-0.56729	6.401659	-1.37342	0.171369	0.373582	-5.9311
AL031600.2	2.507829	1.622088	1.371969	0.17182	0.374386	-4.69392
AL139300.2	1.375246	4.239923	1.370958	0.172134	0.374668	-4.91023
AP001363.2	1.968073	5.250565	1.371089	0.172093	0.374668	-5.04093
AC233300.1	-0.48431	6.396924	-1.37091	0.172149	0.374668	-5.93096
LINC01963	-0.39591	8.663492	-1.3712	0.172059	0.374668	-6.30897
AL359979.2	2.513869	0.240615	1.370047	0.172418	0.375145	-4.66568
AC025171.5	0.704568	6.215322	1.369381	0.172626	0.375379	-5.62069
MNX1-AS2	-1.33353	4.993235	-1.3694	0.172618	0.375379	-5.69081
LINC01730	0.875037	6.578205	1.368973	0.172753	0.375547	-5.62687
AC026979.1	1.486623	4.725858	1.368577	0.172876	0.375707	-4.96651
LINC01180	2.052585	-0.95888	1.367679	0.173157	0.376208	-4.65524
AC010280.1	3.460964	1.100037	1.36661	0.173491	0.376717	-4.66968
AC068669.1	0.626881	4.212543	1.366639	0.173482	0.376717	-5.04616
AC009133.2	2.465039	-1.17946	1.365614	0.173803	0.377263	-4.65406
AC060766.7	0.51758	7.441449	1.365487	0.173843	0.377263	-5.96091
DOCK9-AS1	-1.49891	2.295635	-1.36488	0.174035	0.377461	-5.0468
AC004477.1	0.472311	5.583467	1.364957	0.174009	0.377461	-5.43242
AC036214.2	0.354615	7.797922	1.364574	0.17413	0.377557	-6.0712
AC073111.1	2.138047	3.53952	1.364059	0.174291	0.377799	-4.79289
AC012146.3	2.500747	2.382747	1.363704	0.174403	0.377861	-4.72658
RNF217-AS1	-0.92789	4.189991	-1.36317	0.174572	0.377861	-5.42615
AL365181.2	1.450604	7.275624	1.36328	0.174536	0.377861	-5.71214
AL133243.3	0.223037	7.58133	1.36321	0.174558	0.377861	-6.0608
AC009495.3	-0.53414	7.134404	-1.36344	0.174485	0.377861	-6.08403
AC107308.1	4.194601	4.651038	1.36182	0.174996	0.378561	-4.74966
AC132938.2	1.131021	4.541684	1.361927	0.174962	0.378561	-5.01167
AL606489.1	-0.90407	8.105202	-1.36142	0.175122	0.378726	-6.30609
LINC02594	2.411519	1.08941	1.361004	0.175253	0.378812	-4.69408
AC105339.2	0.334687	5.058912	1.360973	0.175263	0.378812	-5.32754
AC034102.5	1.83225	3.515024	1.360586	0.175385	0.378966	-4.81323
AC104117.5	-1.99273	-0.40185	-1.36034	0.175462	0.379024	-4.78194
AC007785.1	6.116632	5.717132	1.360006	0.175568	0.379035	-4.73929

AL590648.3	2.14828	3.28312	1.36011	0.175535	0.379035	-4.78366
AC006538.3	-2.10918	-0.74717	-1.35977	0.175642	0.379086	-4.76384
AC022034.1	-0.65895	7.550715	-1.35798	0.176209	0.380201	-6.18166
LINC01740	2.674712	-0.7094	1.356779	0.17659	0.380395	-4.66517
AC008972.2	0.505381	6.892574	1.356892	0.176554	0.380395	-5.89474
AC091231.1	-0.50411	6.554113	-1.35674	0.176601	0.380395	-5.95897
AC108860.2	0.48869	7.731184	1.356737	0.176603	0.380395	-6.03146
SNHG22	-0.34549	7.081436	-1.35681	0.176581	0.380395	-6.07195
AL139287.1	0.276644	10.19661	1.356789	0.176586	0.380395	-6.47012
AL137058.3	1.863495	-1.7944	1.356078	0.176812	0.380737	-4.66359
AC138207.1	2.021078	3.673636	1.355777	0.176908	0.380749	-4.82142
AL034349.1	2.068243	4.115505	1.355741	0.176919	0.380749	-4.84304
AL356272.1	2.35286	-1.45314	1.354505	0.177312	0.380886	-4.66557
AC114489.1	3.384759	3.746934	1.354505	0.177312	0.380886	-4.75649
AP000442.1	-0.9984	3.682404	-1.35533	0.17705	0.380886	-5.24976
MEIS1-AS2	-0.50517	4.392718	-1.35462	0.177277	0.380886	-5.37103
AF127577.4	0.442852	7.013337	1.354425	0.177338	0.380886	-5.90098
AC034231.1	0.567192	8.181412	1.355004	0.177153	0.380886	-6.11965
AC004148.2	-0.23816	8.5198	-1.35495	0.177172	0.380886	-6.29076
LINC01564	4.683672	5.50921	1.354008	0.17747	0.38102	-4.78022
AC008687.3	3.018925	4.820063	1.35391	0.177502	0.38102	-4.85963
AC112503.2	-0.79339	5.128003	-1.35334	0.177683	0.381191	-5.68785
AC104695.4	0.736279	7.756722	1.353416	0.177659	0.381191	-6.0209
AC139099.1	1.875463	-0.59692	1.352459	0.177964	0.38125	-4.68113
AC010904.2	2.175298	1.94213	1.352603	0.177918	0.38125	-4.73275
AL138831.1	2.572115	4.340903	1.352662	0.177899	0.38125	-4.85785
AC012157.1	0.68424	5.426512	1.352773	0.177864	0.38125	-5.2755
AL137127.1	0.294114	5.795395	1.352594	0.177921	0.38125	-5.63555
MAP3K2-DT	0.562991	6.734469	1.352183	0.178052	0.381317	-5.76698
DDN-AS1	-0.29275	6.53172	-1.35204	0.178097	0.381317	-5.93637
LINC00691	1.521013	0.294243	1.350887	0.178466	0.381999	-4.70516
AC097641.1	2.312791	0.818318	1.350374	0.17863	0.382241	-4.70212
AC013264.1	2.620379	0.695186	1.350161	0.178698	0.382278	-4.69421
LINC02269	2.795935	-0.13374	1.349586	0.178883	0.38241	-4.677
AC090912.3	2.911966	5.824888	1.349781	0.17882	0.38241	-4.94382
AC013476.1	0.88499	5.472298	1.349175	0.179014	0.38241	-5.22784
AC008985.1	-0.27789	5.430155	-1.34925	0.178992	0.38241	-5.64979
AC126118.1	-0.47942	6.030596	-1.34943	0.178931	0.38241	-5.88014
AC010883.1	0.298247	7.584656	1.348745	0.179152	0.382595	-6.06811
AL021068.1	-0.41257	7.278701	-1.34834	0.179282	0.382764	-6.11466
AC254629.1	3.313026	4.038143	1.347307	0.179614	0.38282	-4.78226
AL645608.1	1.711082	4.826742	1.347481	0.179558	0.38282	-5.03817
AC007014.1	-0.78699	4.28225	-1.34776	0.179468	0.38282	-5.4276
AC106886.3	-0.85769	4.19607	-1.34781	0.179451	0.38282	-5.43401
COA6-AS1	0.492261	8.664115	1.348093	0.179361	0.38282	-6.21887
LINC01003	0.357677	9.192304	1.347361	0.179597	0.38282	-6.32607
CNOT10-AS1	2.198342	3.415454	1.346566	0.179852	0.382878	-4.80897
AC018737.2	-1.47048	0.996904	-1.34623	0.17996	0.382878	-4.85652
AC009716.2	-1.03375	1.333656	-1.34627	0.179949	0.382878	-4.86175
AC093864.1	-0.80861	3.967342	-1.34645	0.179889	0.382878	-5.36121
AC091053.2	-0.54945	5.757126	-1.34629	0.179941	0.382878	-5.78633
SH3RF3-AS1	0.527544	7.258328	1.345953	0.180049	0.382878	-5.96862
AL683813.1	-0.26193	6.85197	-1.34706	0.179693	0.382878	-6.02087
AC113143.1	0.679856	7.797893	1.346009	0.180031	0.382878	-6.04408
AC005726.2	-0.36108	5.676904	-1.34537	0.180236	0.383167	-5.69296
AC090116.1	2.754177	-0.82593	1.344827	0.180412	0.383216	-4.67719
AL158211.3	2.800291	0.77306	1.34497	0.180366	0.383216	-4.6994
AC005225.3	2.458527	2.345099	1.344873	0.180397	0.383216	-4.74619

AP001434.1	-1.43452	3.843348	-1.34415	0.180631	0.383573	-5.42912
USP27X-AS1	-0.339	6.608039	-1.34386	0.180725	0.383664	-5.99363
AL049612.1	2.392844	2.343897	1.341701	0.181422	0.385035	-4.75272
AF064860.2	2.185672	0.046875	1.341118	0.181611	0.385218	-4.69912
AC008735.4	-0.28955	7.350508	-1.34115	0.1816	0.385218	-6.12868
AL049646.2	2.459426	3.704557	1.340631	0.181768	0.385444	-4.83354
AC011389.2	-1.74243	2.260354	-1.3404	0.181844	0.385496	-5.05388
AC009041.1	2.186111	-0.71375	1.339527	0.182127	0.385768	-4.68683
AP000943.1	2.727705	2.659247	1.339531	0.182125	0.385768	-4.7544
AL449983.1	-2.14376	-0.60647	-1.33977	0.182049	0.385768	-4.79621
AL162376.1	3.322281	1.725593	1.338984	0.182303	0.385815	-4.71709
AL121970.1	2.423791	1.154339	1.339202	0.182232	0.385815	-4.71937
AL031733.2	-0.68761	3.606076	-1.33912	0.182257	0.385815	-5.19265
AC023983.2	2.384111	2.322079	1.338758	0.182377	0.385862	-4.75433
AL022328.1	-0.26368	8.546215	-1.33833	0.182514	0.386044	-6.31796
AL022329.1	2.263098	-1.3367	1.337583	0.182759	0.386344	-4.68404
AC002094.4	-0.82254	5.632604	-1.33759	0.182755	0.386344	-5.89785
AC008267.5	0.410515	8.923864	1.336746	0.183032	0.386812	-6.28574
AP003170.4	-0.4255	5.494827	-1.33611	0.183237	0.387137	-5.70917
AC011476.2	-0.65783	4.67792	-1.33537	0.18348	0.387441	-5.51644
AC008737.1	-0.60509	6.812307	-1.33536	0.183484	0.387441	-6.08962
AC005076.1	0.489084	7.069842	1.334811	0.183663	0.387709	-5.93649
AL449423.1	2.709035	1.198902	1.334188	0.183867	0.387916	-4.72218
AC009108.5	1.601712	1.962124	1.333308	0.184154	0.387916	-4.76707
AC080075.1	2.127264	2.98552	1.333088	0.184226	0.387916	-4.80076
AP001178.3	0.944462	4.061242	1.33325	0.184173	0.387916	-4.99278
AC073130.3	0.460941	4.984572	1.332934	0.184277	0.387916	-5.36506
AC078778.1	0.680347	6.022561	1.333255	0.184172	0.387916	-5.56852
AL008723.2	-0.95955	4.461053	-1.33306	0.184237	0.387916	-5.6009
LINC00638	0.465763	6.92695	1.334224	0.183855	0.387916	-5.92201
AP001062.1	-0.3188	6.759991	-1.33298	0.18426	0.387916	-6.03341
TRAPPC12-AS1	-0.32176	8.095731	-1.33366	0.184038	0.387916	-6.25525
VCAN-AS1	2.227208	1.27257	1.332206	0.184515	0.387972	-4.73482
AC007529.1	-1.65082	0.580479	-1.33235	0.184468	0.387972	-4.85278
AP001020.2	0.79491	4.869692	1.332536	0.184407	0.387972	-5.14201
LINC02560	3.590249	7.53797	1.332077	0.184558	0.387972	-5.28058
AL136295.2	0.333108	6.634053	1.332065	0.184562	0.387972	-5.84531
AC105114.1	1.938014	-2.31997	1.331495	0.184749	0.388132	-4.68854
CERNA3	2.328511	0.878352	1.331169	0.184856	0.388132	-4.72457
AC005695.3	2.735916	3.182268	1.330889	0.184948	0.388132	-4.78799
Z82217.1	-0.3715	4.030498	-1.33156	0.184727	0.388132	-5.23542
AC107214.1	0.364373	7.11238	1.331011	0.184907	0.388132	-5.97377
AC009495.1	-0.74635	6.308336	-1.33109	0.18488	0.388132	-6.0084
AC010735.1	2.125798	2.965927	1.330652	0.185025	0.388187	-4.79495
AL078621.3	-0.33921	6.569169	-1.33014	0.185194	0.388432	-6.01035
AC073896.5	-2.0281	-1.27789	-1.32987	0.185284	0.388511	-4.77025
AL031282.2	-0.26656	7.32417	-1.32955	0.185388	0.388622	-6.13334
AC243654.1	2.361967	3.889891	1.329021	0.185562	0.38866	-4.84678
AC026412.3	0.515862	6.074346	1.329052	0.185551	0.38866	-5.65697
AC008760.1	0.310148	7.860803	1.32922	0.185496	0.38866	-6.14461
AC133065.1	2.275126	2.134041	1.328633	0.18569	0.38882	-4.76275
AC093484.1	-1.82049	0.782175	-1.32637	0.186438	0.390278	-4.9062
AC079600.3	2.631487	0.53467	1.326025	0.18655	0.390404	-4.7158
LINC00626	2.625783	0.718634	1.324887	0.186927	0.391083	-4.72091
AC099487.1	3.353796	2.287149	1.324054	0.187203	0.391552	-4.74523
LINC02204	1.285927	2.12107	1.323005	0.187551	0.391843	-4.79504
LINC02280	-0.85393	2.785825	-1.3234	0.187421	0.391843	-5.06437
AC034236.2	0.319885	6.830111	1.323083	0.187524	0.391843	-5.93785

AC005261.1	0.232851	11.53311	1.323192	0.187488	0.391843	-6.71579
AC133540.1	-1.26931	2.210092	-1.32272	0.187643	0.391928	-5.01187
AC093001.1	2.713628	1.906076	1.322485	0.187723	0.391938	-4.75059
AC027271.1	-0.67993	5.373936	-1.3224	0.187753	0.391938	-5.76412
AC131235.3	1.016228	5.549957	1.322034	0.187873	0.391972	-5.27684
AC073349.1	-1.07988	3.564505	-1.32208	0.187856	0.391972	-5.42186
AC005154.4	-0.52168	6.735785	-1.32169	0.187988	0.392103	-6.0694
AC107027.3	-0.34305	7.852401	-1.32059	0.188353	0.392755	-6.24518
AL133215.1	-0.78605	6.173114	-1.31897	0.188893	0.393772	-5.99529
AL356134.1	2.705238	0.221032	1.318798	0.188951	0.393782	-4.71736
LINC00885	3.954931	5.871797	1.318251	0.189133	0.394053	-4.89229
PANDAR	1.964873	-0.43746	1.317213	0.18948	0.394667	-4.71898
PTCSC3	2.470288	-1.14559	1.316212	0.189815	0.395101	-4.7068
SLC25A30-AS1	-0.90455	4.340295	-1.31607	0.189861	0.395101	-5.37815
AC080038.2	0.962951	5.222731	1.315961	0.189899	0.395101	-5.40086
AC008870.5	-1.66392	5.074371	-1.31627	0.189796	0.395101	-5.72666
AC092611.1	0.449447	5.16719	1.315559	0.190034	0.395272	-5.41327
AC124067.4	3.224503	3.678667	1.313847	0.190608	0.396248	-4.80996
AP000648.3	0.305549	8.059263	1.313942	0.190576	0.396248	-6.19335
AL137244.1	0.486172	6.147043	1.313258	0.190806	0.396549	-5.71305
AC009292.1	2.062072	-0.81929	1.312709	0.190991	0.396659	-4.71552
AC008592.1	2.187701	-0.55259	1.312606	0.191025	0.396659	-4.71733
AC008897.2	2.250658	1.272312	1.312552	0.191044	0.396659	-4.7554
AC147067.2	-0.93159	6.350796	-1.3124	0.191095	0.396659	-6.08906
AC120349.1	-0.35673	7.351117	-1.31232	0.191123	0.396659	-6.17638
FAM66E	1.794412	-1.59226	1.311395	0.191433	0.396844	-4.71196
AC011455.1	2.075877	1.847681	1.311267	0.191476	0.396844	-4.77806
AL035425.1	-2.13344	-0.95951	-1.31157	0.191374	0.396844	-4.80358
TESC-AS1	-1.58712	1.397025	-1.31165	0.191347	0.396844	-4.92576
AC004846.2	0.38385	6.53983	1.311475	0.191406	0.396844	-5.85588
ANKRD44-IT1	-0.59168	5.497133	-1.31003	0.191892	0.397597	-5.80459
AP003774.4	1.138124	5.062816	1.309526	0.192064	0.397842	-5.20976
AC004696.2	-2.16476	-0.63238	-1.30919	0.192177	0.397967	-4.82534
AJ011932.1	2.451289	1.733174	1.308547	0.192394	0.398308	-4.76654
AL359693.1	1.923412	-2.25239	1.308388	0.192448	0.39831	-4.71288
AC007952.2	1.46972	1.987723	1.308146	0.19253	0.398369	-4.8049
AC022916.1	0.360114	6.56036	1.307844	0.192632	0.398471	-5.87861
LINC01181	2.654885	-0.63098	1.307123	0.192877	0.398757	-4.7181
AC007298.1	1.524399	3.758945	1.307192	0.192853	0.398757	-4.91483
AL022324.3	2.394405	0.217747	1.306925	0.192944	0.398785	-4.73485
LINC01971	2.136914	0.946817	1.305834	0.193314	0.39944	-4.75792
AC116351.2	1.339181	4.153775	1.305272	0.193504	0.399725	-5.0144
SMG6-IT1	2.299501	1.486284	1.304839	0.193652	0.399895	-4.76945
AC078881.1	-0.47821	5.219733	-1.30458	0.193738	0.399895	-5.70029
AC090425.2	0.295864	7.537459	1.30456	0.193746	0.399895	-6.12858
AC009948.4	-0.84941	2.124052	-1.30435	0.193818	0.399932	-4.98425
AP000320.1	2.452936	-0.96128	1.30398	0.193944	0.399979	-4.7202
AC083949.1	1.124467	4.46025	1.30397	0.193947	0.399979	-5.06048
AL731533.2	1.233451	5.105696	1.303205	0.194207	0.400406	-5.22979
AP001120.2	2.43879	0.391526	1.302592	0.194416	0.400617	-4.74299
AC079360.1	2.31336	2.599475	1.302695	0.194381	0.400617	-4.80973
AC013452.2	1.839623	2.952069	1.302015	0.194613	0.400901	-4.84383
AC127496.1	0.710232	3.659754	1.301875	0.194661	0.400901	-5.01461
AC011921.1	0.620787	5.779545	1.301114	0.194921	0.401326	-5.43315
AC090186.1	1.167198	5.558562	1.299682	0.19541	0.402224	-5.20883
AC007493.2	2.561015	-0.4218	1.298841	0.195698	0.402706	-4.72961
AL592424.1	1.03321	3.889841	1.298458	0.195829	0.402866	-5.02182
AC011483.2	2.452412	0.097768	1.297518	0.196151	0.40305	-4.74162

AC105118.1	2.618229	3.562937	1.297462	0.196171	0.40305	-4.84278
ZSWIM8-AS1	-0.47885	3.786759	-1.29741	0.196187	0.40305	-5.27833
AC006449.3	1.097519	5.46615	1.297419	0.196185	0.40305	-5.3345
AP003392.6	0.893829	5.903605	1.298028	0.195976	0.40305	-5.52101
AC007391.1	2.299773	-0.23253	1.296642	0.196452	0.403153	-4.73945
AC112504.1	-0.4588	4.125935	-1.29707	0.196304	0.403153	-5.33673
AC025165.4	-0.35983	6.659833	-1.29671	0.196429	0.403153	-6.07099
AC000123.3	-0.28437	7.760744	-1.29692	0.196358	0.403153	-6.25176
AC004817.4	1.501587	0.763663	1.294784	0.197091	0.403914	-4.7769
AC072061.1	0.295454	6.08251	1.294894	0.197053	0.403914	-5.77894
AC103810.5	-0.69122	5.284038	-1.29515	0.196966	0.403914	-5.79147
AC005005.3	0.409273	7.005029	1.294863	0.197064	0.403914	-5.99324
SEC62-AS1	-0.6279	6.778496	-1.2953	0.196913	0.403914	-6.14092
AC002398.2	-1.87712	-0.0018	-1.29392	0.197388	0.404089	-4.90138
AC007683.2	-0.96286	3.903147	-1.29409	0.197331	0.404089	-5.42989
AC027607.1	-0.4733	6.860252	-1.29416	0.197306	0.404089	-6.11043
AC079174.1	-0.47081	7.082097	-1.29391	0.197392	0.404089	-6.15737
AL139246.2	1.848575	3.79943	1.293143	0.197657	0.404301	-4.92603
AC012313.10	0.840199	5.672034	1.29344	0.197554	0.404301	-5.50179
AL163051.2	-0.23202	8.836664	-1.29329	0.197605	0.404301	-6.41501
AL138899.1	-1.26665	1.85156	-1.29261	0.197842	0.404568	-5.00038
BTBD9-AS1	-0.66737	6.481572	-1.29199	0.198054	0.404892	-6.09788
AC145146.1	2.537421	0.930335	1.291579	0.198197	0.404979	-4.76282
AC093525.4	-0.86654	4.737813	-1.29156	0.198204	0.404979	-5.69744
AC243829.2	2.412686	-0.84679	1.291387	0.198263	0.40499	-4.7343
AC080188.2	0.641537	6.685371	1.290867	0.198443	0.405247	-5.88681
AC073896.4	0.278346	10.05419	1.290688	0.198505	0.405263	-6.52947
AC091925.2	2.578475	0.382589	1.290289	0.198643	0.405435	-4.7524
AC008395.1	1.437796	5.3499	1.289874	0.198787	0.405619	-5.136
LINC01945	-1.93624	-1.28896	-1.28902	0.199084	0.406004	-4.81084
RNF157-AS1	1.115316	5.222995	1.289133	0.199044	0.406004	-5.34136
HMBOX1-IT1	-0.81026	5.40695	-1.28835	0.199317	0.406369	-5.88276
AL356414.1	2.308959	-1.20343	1.288004	0.199436	0.406439	-4.73608
C11orf72	0.753256	3.303138	1.287779	0.199514	0.406439	-4.9757
AP003071.4	-0.68759	5.452852	-1.2878	0.199507	0.406439	-5.86227
LPP-AS1	-2.00078	-1.04552	-1.28759	0.199579	0.406462	-4.82544
AC100814.1	0.338423	8.545998	1.28742	0.199639	0.406473	-6.3046
CASC18	1.496023	2.234746	1.286448	0.199977	0.407051	-4.8359
AC027807.2	-1.89026	1.074376	-1.2858	0.200203	0.407401	-4.94976
AC007406.5	-0.24433	9.431537	-1.28557	0.200283	0.407454	-6.51428
AC099794.1	2.340295	-0.0937	1.285262	0.20039	0.407561	-4.75307
AP005328.2	2.5971	-0.90882	1.284068	0.200806	0.407605	-4.7406
AC034223.2	2.992359	0.810873	1.284099	0.200795	0.407605	-4.76162
AC007879.1	2.277746	0.829299	1.284362	0.200704	0.407605	-4.77381
DDR1-DT	1.30496	0.702981	1.283955	0.200846	0.407605	-4.79342
AC087203.3	-1.69673	0.101604	-1.28434	0.200713	0.407605	-4.87729
USP12-AS1	2.474224	4.32671	1.284679	0.200593	0.407605	-4.91704
AC026333.4	0.63876	6.128249	1.284649	0.200603	0.407605	-5.71243
U73166.1	-0.44722	5.979708	-1.28464	0.200607	0.407605	-5.93304
AC078925.4	-1.69426	1.984857	-1.28357	0.200981	0.407768	-5.08928
AL158198.1	-2.02549	-0.44541	-1.2822	0.201461	0.408468	-4.85548
AL158834.2	0.89259	4.547047	1.282295	0.201426	0.408468	-5.14419
AC011374.2	-0.32831	7.837765	-1.28212	0.201489	0.408468	-6.28801
SEPSECS-AS1	-0.33164	6.53257	-1.28144	0.201726	0.408839	-6.02937
AL772363.1	1.792485	-2.38669	1.280639	0.202006	0.409297	-4.74121
AC069503.3	2.784368	-0.47303	1.280221	0.202153	0.409316	-4.74655
AC020634.1	2.14624	2.347415	1.280425	0.202081	0.409316	-4.82543
LINC02324	-1.68097	0.383958	-1.28014	0.202179	0.409316	-4.89321

AL122008.3	-1.999	-0.84554	-1.27913	0.202537	0.409774	-4.83919
AP005060.1	-1.60446	-0.38464	-1.27883	0.202642	0.409774	-4.84753
LINC00330	-1.40736	1.966968	-1.27872	0.202678	0.409774	-5.09109
AL139274.2	-0.29786	5.080376	-1.2791	0.202545	0.409774	-5.63485
AC007342.8	1.242488	8.302483	1.278961	0.202595	0.409774	-6.09204
AC079336.1	1.838266	4.785639	1.277828	0.202993	0.410301	-5.0603
AC008655.2	2.170694	4.046526	1.277492	0.203112	0.41043	-4.92057
AP003393.1	1.164993	1.044789	1.276675	0.203399	0.410642	-4.8143
IL20RB-AS1	2.983162	4.643709	1.277023	0.203277	0.410642	-4.91808
AP001525.1	-1.26495	3.428115	-1.27657	0.203435	0.410642	-5.32619
AC002553.1	-0.26209	8.925588	-1.27668	0.203399	0.410642	-6.45101
AC073957.1	2.695225	3.325844	1.276301	0.203531	0.410725	-4.85088
AC108865.1	2.616255	2.429055	1.275439	0.203835	0.411118	-4.82003
AL034428.1	1.557064	1.723833	1.275444	0.203833	0.411118	-4.82595
LINC01998	2.125358	-1.77933	1.274378	0.20421	0.411763	-4.74863
AL035420.2	2.069657	2.021308	1.273307	0.204589	0.412416	-4.82583
AL391095.3	1.103254	5.005941	1.273122	0.204654	0.412437	-5.2627
AC092919.2	0.886188	6.691771	1.272	0.205052	0.413057	-5.80028
AL391422.4	0.414619	8.462999	1.271943	0.205072	0.413057	-6.30521
AP004609.3	-0.2845	6.711329	-1.27159	0.205198	0.413202	-6.08839
LINC01754	-1.78517	0.360096	-1.27055	0.205565	0.413607	-4.92094
AD001527.1	-0.31186	8.278381	-1.27069	0.205515	0.413607	-6.37305
URB1-AS1	0.362845	9.590409	1.270791	0.205481	0.413607	-6.48345
AL049840.1	-0.25382	9.536798	-1.27021	0.205687	0.413742	-6.55193
AC009303.3	2.096922	0.271081	1.267777	0.206553	0.415261	-4.78216
AC007000.3	0.911807	5.15238	1.267809	0.206541	0.415261	-5.38204
AC024243.1	1.596853	3.036121	1.265647	0.207313	0.416456	-4.9002
AC092755.1	4.405975	6.545431	1.265645	0.207313	0.416456	-4.97991
AL033543.1	-0.3971	3.379763	-1.26593	0.207212	0.416456	-5.16501
AC005332.3	-0.28268	9.103482	-1.2654	0.2074	0.416519	-6.49839
AL157832.2	-1.65067	-1.72639	-1.26486	0.207595	0.416757	-4.81519
AL158166.1	1.220642	7.042283	1.264761	0.20763	0.416757	-5.82625
AC104316.2	2.377259	0.035198	1.263626	0.208036	0.417279	-4.77579
AC104462.1	2.373392	2.658622	1.263546	0.208065	0.417279	-4.84554
LINC01031	1.675333	2.543888	1.263442	0.208102	0.417279	-4.86945
ITCH-AS1	2.085646	3.763121	1.263415	0.208112	0.417279	-4.93126
DANT2	-0.78536	6.19521	-1.26314	0.20821	0.417365	-6.07845
AC009097.4	1.650974	3.916775	1.262559	0.208418	0.417559	-4.97624
AC100763.1	-1.28415	2.667463	-1.26259	0.208408	0.417559	-5.1662
AL355512.1	2.221803	3.376873	1.262225	0.208538	0.417577	-4.89018
AP001271.2	1.287619	4.375614	1.262249	0.20853	0.417577	-5.04357
AC109454.2	-0.28469	7.078044	-1.26193	0.208643	0.417674	-6.16685
AC011446.1	0.524994	6.837089	1.261588	0.208767	0.417812	-5.96503
UFL1-AS1	2.21791	1.588991	1.261081	0.208949	0.417994	-4.82092
AC135782.1	-1.27502	2.593619	-1.26096	0.208992	0.417994	-5.13455
AC011933.4	-0.60254	5.145855	-1.26087	0.209025	0.417994	-5.75674
AC084871.2	1.511013	4.5772	1.259978	0.209346	0.418525	-5.05657
LINC01800	1.787989	-0.31577	1.259744	0.20943	0.418582	-4.78317
AC096536.2	0.457479	7.234195	1.259386	0.209559	0.418728	-6.08687
AL356441.1	2.308123	-1.18891	1.258992	0.209701	0.418831	-4.76565
AL034550.2	1.44213	1.874043	1.258933	0.209722	0.418831	-4.84913
AC005519.1	0.264444	7.659456	1.258075	0.210032	0.419338	-6.20022
AL390774.2	1.98445	1.496598	1.257545	0.210223	0.419608	-4.82725
ZNF385D-AS1	2.255564	-1.20327	1.256781	0.210499	0.419713	-4.76808
AC097478.3	2.234997	-0.57207	1.2571	0.210384	0.419713	-4.77476
AL591368.1	-1.27339	1.836396	-1.25686	0.210469	0.419713	-5.02968
AL024508.1	-0.31201	7.245863	-1.25703	0.210409	0.419713	-6.20788
LSINCT5	-1.66572	-1.28353	-1.25508	0.211114	0.420478	-4.83773

BX649601.1	1.516384	4.21682	1.255363	0.211012	0.420478	-5.02172
AC007785.3	1.203895	4.433014	1.255422	0.21099	0.420478	-5.13364
AC012645.4	-0.36275	6.040093	-1.25524	0.211055	0.420478	-5.96018
AC087301.1	-0.4791	6.256493	-1.25495	0.211162	0.420478	-6.0456
AL357140.2	0.32781	7.398541	1.254381	0.211367	0.420775	-6.14226
ODF2-AS1	0.318979	7.233039	1.253947	0.211525	0.420976	-6.11664
AC132938.3	0.325611	8.66462	1.253318	0.211753	0.421319	-6.36423
KCNQ1-AS1	-1.81639	-0.75914	-1.25297	0.211879	0.421457	-4.86358
LINC01940	1.859786	-1.45498	1.252322	0.212115	0.421815	-4.77239
AC009570.1	0.5064	6.501274	1.252155	0.212175	0.421824	-5.91141
AC023886.1	2.171536	-0.63168	1.250996	0.212597	0.422017	-4.77983
AL139095.4	0.562662	5.874115	1.251568	0.212389	0.422017	-5.66236
AC104170.1	-0.74574	6.024661	-1.25066	0.212721	0.422017	-6.03802
AC053527.1	-0.34304	6.34971	-1.25153	0.212402	0.422017	-6.04452
AC026979.3	-0.42663	6.464476	-1.25079	0.212671	0.422017	-6.09393
AL132712.2	1.043641	8.144098	1.250653	0.212722	0.422017	-6.12302
AC002350.1	0.223589	7.619563	1.251112	0.212554	0.422017	-6.21388
AC108134.1	-0.48025	7.538079	-1.25092	0.212624	0.422017	-6.29511
AC105760.1	1.728465	-0.43542	1.250188	0.212891	0.422019	-4.79168
AL133320.1	-1.6582	-1.58772	-1.25021	0.212882	0.422019	-4.83406
AC087623.1	-0.82477	5.646342	-1.25047	0.212787	0.422019	-5.98199
AC008736.2	-0.36893	8.134282	-1.24994	0.21298	0.422084	-6.38231
AC020763.1	1.862844	3.074429	1.249343	0.213199	0.422184	-4.90498
AC061999.1	2.213471	5.990961	1.249349	0.213197	0.422184	-5.22359
AL353804.1	-0.4835	7.773341	-1.24952	0.213133	0.422184	-6.34186
LINC01215	1.437382	3.756591	1.248856	0.213377	0.422424	-5.00978
AP002768.1	-1.84702	1.545026	-1.24786	0.213739	0.422998	-5.08092
AP001610.1	0.752188	5.981464	1.247754	0.213779	0.422998	-5.69976
AC005920.3	-1.762	-1.00983	-1.24699	0.214057	0.423122	-4.85963
FAM215B	-0.44306	4.87981	-1.24697	0.214067	0.423122	-5.59809
AC007823.1	0.42961	6.332995	1.247247	0.213964	0.423122	-5.84673
AP000442.2	0.249392	8.10399	1.247202	0.213981	0.423122	-6.29515
AC115618.2	0.27952	11.18034	1.246622	0.214193	0.42326	-6.75679
AC087392.2	-0.74579	5.246982	-1.24625	0.214329	0.423417	-5.84194
AC078922.1	2.444686	1.771947	1.245153	0.214731	0.4241	-4.8358
BICRA-AS1	-0.57089	5.763334	-1.24477	0.214871	0.424153	-5.95972
AC009690.2	0.232088	6.840359	1.244873	0.214834	0.424153	-6.05092
AP001458.1	2.07102	6.576127	1.244549	0.214953	0.424203	-5.46435
AC023830.2	1.740488	-0.10074	1.243938	0.215177	0.424534	-4.80458
WASHC5-AS1	0.534034	6.761159	1.243352	0.215392	0.424844	-5.97263
AC106897.1	-0.8309	5.77785	-1.2432	0.215448	0.424844	-6.00759
AL513218.1	0.313908	7.169478	1.24247	0.215716	0.424941	-6.10676
AC244093.4	0.294672	7.417438	1.242453	0.215723	0.424941	-6.17656
AC092953.2	-0.32776	8.360804	-1.24258	0.215675	0.424941	-6.41905
AC009779.2	-0.28437	9.163349	-1.2425	0.215704	0.424941	-6.53117
TMEM92-AS1	-0.55563	6.976861	-1.24205	0.215869	0.425118	-6.21316
AP001790.1	2.042481	2.52135	1.241819	0.215956	0.425177	-4.87754
AL121906.2	-0.49449	6.65126	-1.24164	0.216021	0.425194	-6.13963
LINC00536	1.964555	0.074436	1.240982	0.216264	0.425339	-4.80746
AC008906.2	-0.81132	4.504314	-1.24107	0.216231	0.425339	-5.52208
AC091271.1	0.396483	7.852183	1.241084	0.216227	0.425339	-6.23618
AC021016.3	-0.35327	6.682835	-1.23994	0.216648	0.425982	-6.12719
AL590399.1	1.713695	-1.10695	1.239735	0.216724	0.42602	-4.79076
AC093849.1	-1.40519	0.583788	-1.23909	0.216962	0.426376	-4.93586
LINC02325	1.831043	0.039354	1.238922	0.217024	0.426387	-4.81073
AC008649.1	2.572279	2.591411	1.237321	0.217616	0.427439	-4.86465
AP003064.2	-1.9209	-0.29429	-1.23702	0.21773	0.427549	-4.91209
LINC01771	2.118541	3.173667	1.236517	0.217914	0.427688	-4.91357

AC090948.2	-0.33859	7.141502	-1.23658	0.21789	0.427688	-6.2283
AP004782.1	-1.65631	-1.85208	-1.23497	0.218488	0.42859	-4.84214
AC027279.3	-2.06863	0.593547	-1.23502	0.218469	0.42859	-4.99475
AC006435.4	2.404016	-0.92143	1.234633	0.218613	0.428612	-4.79099
LINC01238	-0.57806	4.843603	-1.23476	0.218567	0.428612	-5.69939
KBTBD11-OT1	-1.90551	0.717984	-1.23397	0.21886	0.428984	-4.99095
AC119868.2	-2.01578	-1.57912	-1.23379	0.218925	0.428999	-4.86137
AC091152.2	0.355623	4.206162	1.233555	0.219014	0.429062	-5.27278
LINC01926	2.300411	-1.04572	1.232376	0.219453	0.429698	-4.79286
AC024592.2	1.575206	6.11053	1.232377	0.219452	0.429698	-5.57951
AL121768.2	1.593999	0.351226	1.231907	0.219628	0.42991	-4.82995
ITPKB-AS1	-1.69982	0.35354	-1.23147	0.21979	0.42991	-4.95174
MED4-AS1	0.298654	6.540326	1.231622	0.219734	0.42991	-5.98005
ATP2B1-AS1	0.341332	6.779205	1.231483	0.219786	0.42991	-6.04607
HOXB-AS4	5.606401	5.975447	1.231307	0.219851	0.429918	-4.90485
AL138902.1	1.473788	4.975646	1.230947	0.219986	0.430068	-5.22344
AP001029.2	0.463382	4.404382	1.230187	0.220269	0.430399	-5.27445
AC020978.7	-0.20206	6.030252	-1.23027	0.220239	0.430399	-5.96332
AL050327.1	2.3062	-1.28405	1.229993	0.220342	0.430428	-4.79394
AL450326.1	-0.30313	7.96231	-1.22866	0.22084	0.431289	-6.36628
LINC01289	1.931927	-1.47374	1.228242	0.220997	0.431375	-4.79593
ADARB2-AS1	2.250736	0.254242	1.228083	0.221056	0.431375	-4.81889
AL355488.1	0.306628	8.865295	1.228146	0.221033	0.431375	-6.42631
AC025183.2	2.264654	-0.32176	1.227453	0.221292	0.431723	-4.80701
AC006206.2	4.202199	5.517362	1.226674	0.221584	0.432181	-4.96345
AP003071.1	-1.30529	2.396696	-1.22644	0.221674	0.432243	-5.22557
AC026304.1	0.878409	6.028738	1.225756	0.221929	0.432516	-5.69993
AL136368.1	-0.45556	6.482825	-1.2259	0.221875	0.432516	-6.13265
LINC01629	2.870591	3.707838	1.225268	0.222112	0.432761	-4.91628
AC004817.2	2.304975	-0.95949	1.224712	0.222321	0.432855	-4.80083
AL590723.1	0.336058	6.897622	1.22468	0.222333	0.432855	-6.07522
AC011472.1	-0.26569	8.380797	-1.22479	0.222291	0.432855	-6.4378
AL499616.1	-1.37257	0.175219	-1.22413	0.22254	0.433145	-4.92778
AL589739.1	2.266324	-0.14434	1.223322	0.222844	0.4334	-4.81418
AC073342.1	1.916349	1.121121	1.22317	0.222901	0.4334	-4.85039
AC006369.1	-1.65444	1.460811	-1.22349	0.222782	0.4334	-5.05652
AC103691.1	0.506363	8.811687	1.22317	0.222901	0.4334	-6.3947
LINC00970	-1.61825	0.811482	-1.22248	0.22316	0.433791	-4.98621
AC093915.1	1.948094	-1.64324	1.222295	0.223231	0.433816	-4.80095
AC122713.2	-1.24013	0.636026	-1.22133	0.223595	0.434412	-4.95295
AC022613.1	-0.85854	6.340974	-1.22059	0.223874	0.434841	-6.13391
AL591848.2	-1.12229	4.183357	-1.22034	0.223968	0.434912	-5.55434
AC004706.1	1.109245	6.349196	1.219653	0.224229	0.435158	-5.73475
AL121772.1	0.673475	7.244049	1.219547	0.224269	0.435158	-6.06378
AC232271.1	0.258017	7.410958	1.21965	0.22423	0.435158	-6.21092
LINC02292	2.003841	-1.77202	1.218892	0.224517	0.435489	-4.80384
AC099314.1	1.549656	5.016043	1.21879	0.224555	0.435489	-5.15491
AP000487.2	1.509267	5.901988	1.218591	0.224631	0.435523	-5.50183
AL159166.1	2.433178	-0.91973	1.218185	0.224784	0.435596	-4.80699
AC007599.2	1.968532	1.78255	1.218335	0.224728	0.435596	-4.87814
AL445489.1	1.568892	3.386149	1.216945	0.225255	0.436283	-4.9974
LINC01068	1.727354	4.685217	1.216945	0.225255	0.436283	-5.13241
AC134407.3	-0.21915	7.047494	-1.21624	0.225523	0.436688	-6.22287
ERI3-IT1	-0.66246	5.021766	-1.21362	0.22652	0.438507	-5.82535
AC005632.2	0.281561	6.87865	1.213257	0.226658	0.438661	-6.09107
SOS1-IT1	-0.23167	7.927544	-1.21251	0.226942	0.439098	-6.37269
AC034206.1	2.436714	3.145157	1.211726	0.227242	0.439565	-4.91889
AC243967.3	1.734612	-0.20367	1.209635	0.228042	0.440464	-4.83578

AP003498.1	2.501849	0.574182	1.209438	0.228117	0.440464	-4.83977
AL023881.1	2.270834	1.593698	1.209539	0.228078	0.440464	-4.87015
AC015849.1	1.745292	2.406308	1.209654	0.228034	0.440464	-4.91901
AC104794.4	-1.02807	3.376486	-1.2099	0.227942	0.440464	-5.34154
AL031666.3	-0.53601	5.341424	-1.20961	0.228052	0.440464	-5.82997
AL353583.1	0.358111	6.819914	1.209817	0.227972	0.440464	-6.06448
AC129502.1	1.33383	3.109653	1.208987	0.22829	0.440684	-4.98616
AP005899.1	0.358557	7.941418	1.208505	0.228474	0.440927	-6.29439
AC006205.2	2.276998	-1.25487	1.207417	0.228892	0.441071	-4.81601
AC011373.1	2.49073	-0.78788	1.206629	0.229195	0.441071	-4.81877
AC009227.1	1.835132	-1.02354	1.206968	0.229064	0.441071	-4.8213
AC099489.3	1.178291	2.321214	1.20668	0.229175	0.441071	-4.9396
AL049836.1	2.494483	4.062829	1.207126	0.229004	0.441071	-4.98156
LINC02100	3.620196	6.356421	1.207765	0.228759	0.441071	-5.07876
AL031283.3	-1.41774	2.44288	-1.20723	0.228962	0.441071	-5.1611
AC092376.2	-0.38197	4.789029	-1.20708	0.22902	0.441071	-5.68167
AC007922.2	-0.83463	4.839162	-1.20796	0.228683	0.441071	-5.77804
LINC01268	-0.58946	5.203469	-1.20667	0.229178	0.441071	-5.79035
COL4A2-AS1	-0.8338	4.872613	-1.20746	0.228874	0.441071	-5.82756
AC123912.1	-1.31963	3.579555	-1.20635	0.229304	0.441168	-5.45926
AL596244.1	-0.38328	7.638327	-1.20595	0.229454	0.441344	-6.35776
AC096992.2	0.305516	7.966654	1.205677	0.229561	0.441436	-6.3166
AC027306.1	-1.79092	-0.84923	-1.20505	0.229801	0.441784	-4.91082
AP003306.2	2.163439	-1.51447	1.203777	0.230293	0.442617	-4.8189
LINC00398	1.56614	2.684052	1.203593	0.230364	0.44264	-4.94899
AC120498.8	2.167118	0.779632	1.203324	0.230468	0.442726	-4.85697
AC092828.1	-0.30159	5.971489	-1.2029	0.23063	0.442925	-5.96502
AC087362.2	-0.74757	5.02156	-1.20268	0.230715	0.442976	-5.87855
AC005104.1	-0.2259	8.546342	-1.20252	0.230777	0.44298	-6.48455
C5orf66-AS2	-0.97479	1.673288	-1.20126	0.231265	0.443804	-5.06602
AP000255.1	-0.5531	4.746034	-1.19999	0.231756	0.44452	-5.70414
AC022762.2	-0.37762	6.838687	-1.2	0.231752	0.44452	-6.2224
AP000812.1	2.252621	-1.02387	1.199714	0.231864	0.444612	-4.82489
Z83843.1	-0.25674	8.939643	-1.19929	0.232029	0.444817	-6.55168
DNAJB5-DT	1.766089	2.850837	1.198125	0.23248	0.445548	-4.95574
AL645465.1	-1.45224	1.745612	-1.198	0.232529	0.445548	-5.08941
AC053503.5	1.193338	5.300994	1.197284	0.232806	0.445918	-5.40952
AC011511.3	0.652729	6.949456	1.197194	0.232841	0.445918	-6.02422
AL031665.1	2.212576	1.002972	1.196556	0.233089	0.445945	-4.86946
AL359697.1	0.435538	6.563727	1.196548	0.233093	0.445945	-5.98321
LINC01389	0.65172	7.594451	1.196585	0.233078	0.445945	-6.19497
AC008608.2	0.323893	7.986351	1.19661	0.233068	0.445945	-6.31967
AL358074.1	1.487354	0.84222	1.195793	0.233387	0.446184	-4.8821
AL353771.1	1.785591	2.995736	1.195782	0.233391	0.446184	-4.97049
AC009159.4	-1.4093	1.366462	-1.19562	0.233456	0.446184	-5.08162
AL603832.1	0.473537	6.234759	1.195643	0.233445	0.446184	-5.84927
AC018716.1	2.670575	-0.6852	1.194849	0.233754	0.446301	-4.82957
AL157770.1	2.140738	1.010395	1.195034	0.233682	0.446301	-4.87153
AL132765.2	2.01115	3.069064	1.19489	0.233738	0.446301	-4.95493
LPP-AS2	0.352339	8.176252	1.194905	0.233732	0.446301	-6.3581
AC005291.1	2.239452	0.146489	1.194647	0.233833	0.446338	-4.84817
AL513523.3	2.214654	1.31659	1.194142	0.23403	0.446487	-4.87797
AC104964.4	-0.58434	6.13096	-1.19416	0.234024	0.446487	-6.13647
AC068790.3	-0.3019	7.034046	-1.19316	0.234413	0.447105	-6.25596
AC005304.3	2.210192	-1.18907	1.191614	0.235017	0.447759	-4.83195
AC113410.3	2.026866	5.662482	1.191521	0.235054	0.447759	-5.25525
AC078883.3	1.754073	6.079136	1.191708	0.234981	0.447759	-5.45402
AC011445.1	1.038434	5.581649	1.191849	0.234925	0.447759	-5.52834

AC026803.2	0.334055	5.975025	1.191783	0.234951	0.447759	-5.81247
AL138963.1	0.361556	7.909701	1.191259	0.235156	0.44784	-6.30757
AC127024.2	0.348574	7.201814	1.190101	0.23561	0.44859	-6.18875
AC010186.1	2.128657	-0.39533	1.189497	0.235847	0.448927	-4.84583
AC145207.1	2.028293	0.61323	1.188898	0.236082	0.449261	-4.86937
AC011611.2	2.470407	1.786636	1.188141	0.236379	0.449611	-4.89159
AL033528.2	1.817748	1.463472	1.188125	0.236385	0.449611	-4.9003
AC073323.1	2.419948	-0.54654	1.187459	0.236647	0.449899	-4.84128
AC010320.2	-0.59315	3.516778	-1.18743	0.236657	0.449899	-5.39834
AC110801.1	-1.66683	1.828468	-1.18539	0.237461	0.451202	-5.17944
AC002044.3	-0.60923	5.461039	-1.18539	0.237462	0.451202	-5.96168
AL359852.1	1.751421	-2.49868	1.184806	0.237692	0.451509	-4.83495
AL022328.2	-0.20177	9.213628	-1.18467	0.237744	0.451509	-6.60659
PSD2-AS1	2.255181	-1.41783	1.183986	0.238016	0.451785	-4.83787
AC005281.1	-1.51604	1.634421	-1.18396	0.238028	0.451785	-5.12937
AC090515.5	-0.55152	5.635664	-1.18385	0.23807	0.451785	-5.99317
Z98884.2	0.247827	8.841679	1.183682	0.238135	0.451796	-6.48618
AC091965.4	-1.04478	4.603421	-1.18324	0.238311	0.452015	-5.65463
AL022097.1	-0.48358	5.171953	-1.1828	0.238486	0.452223	-5.79625
AC087620.1	0.323009	7.028541	1.182656	0.238541	0.452223	-6.16045
AC016747.4	0.541625	4.193778	1.18236	0.238658	0.452331	-5.28396
AC104072.1	-1.78056	0.848753	-1.18132	0.23907	0.452997	-5.05373
HHIP-AS1	-0.62191	5.882948	-1.18084	0.23926	0.453242	-6.03895
AC007679.1	-1.8355	-1.98061	-1.18043	0.239421	0.453433	-4.89822
AC007879.2	2.178623	1.303834	1.180209	0.23951	0.453488	-4.89051
AC018521.3	1.921709	0.399695	1.179997	0.239594	0.453516	-4.87512
AL078587.1	-1.0887	4.73837	-1.17987	0.239646	0.453516	-5.7368
AC116407.4	-0.21352	7.558379	-1.17949	0.239796	0.453686	-6.34964
AC104365.2	2.009847	0.494218	1.178862	0.240045	0.453814	-4.87848
AC087277.2	2.639569	2.493992	1.179024	0.23998	0.453814	-4.91853
AC100810.1	-0.2781	9.860509	-1.17896	0.240006	0.453814	-6.72301
AC022919.1	-1.79699	-0.71998	-1.17824	0.24029	0.454089	-4.94738
AC119800.1	1.91677	3.160083	1.178191	0.240311	0.454089	-4.98627
LINC01731	2.311206	0.892575	1.176937	0.24081	0.454461	-4.88154
RPS6KA2-AS1	-1.88337	-1.63	-1.1771	0.240744	0.454461	-4.91251
AC013553.4	1.754407	2.50243	1.17738	0.240634	0.454461	-4.95523
AC008073.1	-1.49544	1.346044	-1.17699	0.240787	0.454461	-5.0755
AC131009.1	0.823418	6.053498	1.177165	0.240719	0.454461	-5.75297
AL512652.2	1.177971	2.95574	1.176387	0.24103	0.454646	-5.00793
AC073592.1	0.961125	4.721104	1.176446	0.241006	0.454646	-5.28962
LINC01801	-0.65894	4.214403	-1.1757	0.241302	0.455047	-5.61156
ELDR	1.423304	1.475074	1.174746	0.241684	0.455651	-4.92274
AL135818.2	1.118652	6.079304	1.174409	0.241818	0.455676	-5.67513
AC090617.5	0.270915	8.231803	1.174425	0.241812	0.455676	-6.40502
LLPH-DT	2.387217	5.77978	1.17411	0.241937	0.455786	-5.20732
AL138688.2	-0.89301	2.809148	-1.17337	0.242233	0.456229	-5.25838
AC021231.1	-1.16199	1.901987	-1.17289	0.242425	0.456475	-5.13701
AC006237.1	-1.45151	2.200206	-1.17274	0.242485	0.456475	-5.23264
AC131157.1	2.708552	0.663614	1.172306	0.242659	0.456573	-4.87268
AL121672.2	0.491024	5.578151	1.172344	0.242644	0.456573	-5.70678
AL592528.1	1.308719	0.285828	1.171742	0.242885	0.456769	-4.89241
AC022400.9	0.191174	6.736661	1.171881	0.242829	0.456769	-6.133
AC025539.1	1.070082	3.070947	1.170613	0.243337	0.457458	-5.05464
AP003170.3	-0.85721	6.649479	-1.17052	0.243373	0.457458	-6.29446
TCF4-AS1	2.565143	0.667762	1.169712	0.243699	0.457755	-4.87788
AL512283.1	-1.58313	0.151417	-1.16962	0.243734	0.457755	-4.98951
AC119403.1	-0.46187	6.526258	-1.16952	0.243774	0.457755	-6.19778
AL928654.2	0.270168	9.610908	1.169875	0.243633	0.457755	-6.61873

AC020908.3	0.74782	6.101657	1.168693	0.244108	0.458267	-5.67845
AC008957.2	2.221797	3.536017	1.16836	0.244242	0.458404	-5.01624
AC026785.3	2.774846	-0.26048	1.167355	0.244646	0.459048	-4.85866
AC019129.2	1.791127	-0.21001	1.167094	0.244752	0.459131	-4.87685
AC012360.1	1.229749	4.506026	1.166774	0.24488	0.459143	-5.20973
AC005726.3	0.351416	8.288305	1.166854	0.244848	0.459143	-6.38952
AC037459.4	1.988848	-1.77153	1.166372	0.245043	0.45915	-4.85375
AC005277.2	-1.47276	2.618298	-1.16631	0.245066	0.45915	-5.29231
AL049780.3	-0.36473	4.42192	-1.16616	0.245129	0.45915	-5.59977
AL162311.1	-0.70494	4.696625	-1.16622	0.245104	0.45915	-5.78583
MDC1-AS1	1.887713	0.227938	1.165738	0.245298	0.459353	-4.88628
AL121656.1	2.211351	-1.54439	1.165473	0.245405	0.459439	-4.85488
AF111169.3	0.577898	5.107495	1.165188	0.24552	0.45954	-5.601
AC025754.2	1.630206	3.812691	1.16446	0.245814	0.459689	-5.09056
AL157395.1	1.28432	5.230457	1.164724	0.245707	0.459689	-5.40259
AC092756.1	0.397112	7.228877	1.164508	0.245794	0.459689	-6.20351
AP006621.5	0.369802	8.353641	1.164248	0.2459	0.459689	-6.41205
AP003392.1	-0.23514	8.401765	-1.16423	0.245906	0.459689	-6.50501
AL157834.1	-1.51286	1.856347	-1.16403	0.245988	0.459728	-5.15058
AC087500.2	-0.86323	4.747374	-1.16277	0.246495	0.460562	-5.71926
AC010768.1	1.427646	3.67414	1.162385	0.246653	0.460742	-5.09269
ITGB5-AS1	1.145709	5.226508	1.161884	0.246856	0.460909	-5.44434
AC015853.3	-0.3675	5.762471	-1.16178	0.246896	0.460909	-6.01227
SUCLA2-AS1	0.365473	7.730757	1.161711	0.246926	0.460909	-6.31796
LINC02348	2.216113	-1.17383	1.159766	0.247716	0.461924	-4.86151
AC011369.1	2.142375	-0.29465	1.159684	0.247749	0.461924	-4.87455
AL590714.1	2.126921	-0.04593	1.15935	0.247885	0.461924	-4.88102
AC080038.3	1.960196	2.603604	1.159722	0.247733	0.461924	-4.96878
AL121760.1	2.194776	3.12699	1.15921	0.247941	0.461924	-5.00128
AC103736.1	-1.39672	1.498314	-1.15916	0.247962	0.461924	-5.10639
CAHM	0.732426	5.870971	1.159691	0.247746	0.461924	-5.66912
AP005329.2	-0.33053	6.798529	-1.15958	0.24779	0.461924	-6.26225
AC138466.5	0.343458	6.237249	1.159007	0.248024	0.461925	-5.95368
AL162385.2	-1.12435	4.625355	-1.15871	0.248144	0.462034	-5.70654
AC084398.2	1.915148	1.826964	1.158442	0.248253	0.462123	-4.9367
AL138999.1	-1.51938	2.32814	-1.15792	0.248468	0.462178	-5.21841
AC005261.2	0.666812	4.807606	1.157977	0.248443	0.462178	-5.39603
AC009269.5	0.677175	5.800418	1.158079	0.248401	0.462178	-5.74766
AC087501.4	-0.2237	6.557032	-1.15773	0.248543	0.462204	-6.17538
LINC01756	1.672461	-2.46485	1.157137	0.248785	0.462311	-4.86085
AL034376.2	2.366934	-0.78252	1.157239	0.248743	0.462311	-4.86594
AC127496.2	-1.12305	3.729247	-1.15716	0.248775	0.462311	-5.55951
Z98749.1	1.839611	3.363058	1.156426	0.249075	0.462734	-5.02283
LINC01556	2.376095	3.562108	1.156155	0.249185	0.462825	-5.00594
AC110771.1	-0.47137	3.610813	-1.15594	0.249273	0.462875	-5.39831
AC010463.3	0.290084	7.359729	1.155784	0.249337	0.462878	-6.25716
AC008739.5	-1.16414	1.685946	-1.15541	0.249488	0.462918	-5.10071
AL353150.1	0.663319	6.773487	1.155278	0.249543	0.462918	-6.04021
AL139099.2	-0.28419	6.77209	-1.1555	0.249451	0.462918	-6.24469
AC120498.10	-1.45326	2.164334	-1.15379	0.250152	0.463934	-5.20396
AC010325.2	2.188439	2.92883	1.152999	0.250475	0.464302	-4.98261
AL359915.1	-1.61092	0.090614	-1.15301	0.25047	0.464302	-5.00896
AC092580.2	2.172907	-1.21986	1.152486	0.250685	0.464348	-4.86821
AC007998.5	-1.4713	2.540944	-1.15268	0.250605	0.464348	-5.32278
AC018926.2	-0.29134	6.975426	-1.15249	0.250683	0.464348	-6.28698
AP001324.3	1.39257	2.740751	1.152319	0.250753	0.46436	-5.0122
AC124276.2	2.013651	2.075039	1.152006	0.250881	0.464483	-4.95076
AC008438.2	2.029147	-1.07368	1.15124	0.251196	0.46495	-4.87169

AL356121.2	1.949813	-0.74506	1.150732	0.251404	0.465135	-4.87765
AC005790.1	-1.58151	2.040492	-1.1507	0.251419	0.465135	-5.21924
AC016396.1	2.197212	0.074415	1.149916	0.251739	0.465612	-4.89258
AC005839.1	0.181491	8.094005	1.149045	0.252097	0.466159	-6.42097
AC073569.3	0.710844	5.089696	1.148632	0.252267	0.466359	-5.43235
AC239584.1	2.16343	-1.19608	1.147806	0.252607	0.466499	-4.87263
AC104971.3	2.458662	0.038065	1.148065	0.2525	0.466499	-4.88672
AC007848.2	1.839974	0.813889	1.14724	0.25284	0.466499	-4.92068
AP006248.3	-1.91231	-1.55125	-1.14777	0.252622	0.466499	-4.94532
AC008011.2	3.105001	4.061325	1.147571	0.252704	0.466499	-5.00635
AC021491.4	-0.79477	3.280894	-1.14725	0.252838	0.466499	-5.30874
AP000345.2	-0.69628	4.435201	-1.14785	0.252587	0.466499	-5.69689
AL049840.3	-0.28795	8.983573	-1.1474	0.252773	0.466499	-6.6225
AC015795.1	1.720617	3.149221	1.146908	0.252977	0.466637	-5.01704
C10orf111	0.332059	5.352156	1.145956	0.253369	0.467247	-5.69407
AC004461.2	-0.28889	6.235498	-1.14557	0.253529	0.467391	-6.1523
AL031670.1	0.250198	8.136683	1.145465	0.253572	0.467391	-6.42831
AC012313.1	0.284889	9.449434	1.14514	0.253706	0.467524	-6.62525
HHATL-AS1	-1.65037	-1.4107	-1.14351	0.254381	0.468313	-4.94688
AC008750.2	1.664546	2.178296	1.143886	0.254225	0.468313	-4.97702
AL450344.3	-0.80321	5.057233	-1.1435	0.254384	0.468313	-5.96391
AL049780.2	0.296743	6.569971	1.143693	0.254304	0.468313	-6.10649
AC083809.1	2.685713	4.945862	1.143087	0.254556	0.468514	-5.10994
AC145207.9	0.494274	7.236657	1.142095	0.254966	0.469156	-6.1911
AC013714.1	1.804745	1.737037	1.1413	0.255296	0.469545	-4.95667
AC243964.2	-0.46848	6.374734	-1.14128	0.255303	0.469545	-6.19346
SALRNA2	-1.2695	0.658443	-1.14107	0.255392	0.469593	-5.04803
AC007216.1	-0.60391	4.674554	-1.14071	0.255542	0.46967	-5.75984
AC105935.1	-0.40543	5.574526	-1.14067	0.255559	0.46967	-5.94314
DOCK9-DT	0.51909	7.985365	1.139504	0.256042	0.470443	-6.37871
AL589765.4	0.409838	7.811583	1.139126	0.256199	0.470617	-6.35658
AC114488.3	1.327677	3.83395	1.13888	0.256301	0.47069	-5.15215
AC009127.1	1.728588	3.050625	1.138707	0.256373	0.470707	-5.02855
AL589990.1	-1.02824	3.450153	-1.13828	0.256549	0.470914	-5.41208
PIK3CD-AS1	1.196584	2.854652	1.137568	0.256848	0.471018	-5.04944
AC027228.3	1.046281	3.261055	1.137785	0.256757	0.471018	-5.08152
AC016957.2	-0.46625	7.134665	-1.13775	0.256771	0.471018	-6.35925
AP001033.2	-0.27678	8.535506	-1.13755	0.256856	0.471018	-6.56255
AC008592.5	-1.49226	1.855629	-1.13718	0.25701	0.471171	-5.15177
SLC25A25-AS1	-0.48448	9.466859	-1.13705	0.257065	0.471171	-6.72116
FP236383.2	-0.94577	7.991091	-1.13664	0.257236	0.47137	-6.55915
CACNA1G-AS1	-1.26506	0.977874	-1.13599	0.257504	0.471499	-5.07255
AP006333.2	-1.2103	2.045686	-1.13571	0.257621	0.471499	-5.21048
AC068700.1	-0.74991	2.604085	-1.1363	0.257375	0.471499	-5.27006
AC002310.1	0.292372	7.368176	1.135931	0.25753	0.471499	-6.28158
AC008894.3	0.313002	7.759142	1.13577	0.257597	0.471499	-6.36917
AC099677.4	2.309995	-0.78943	1.135555	0.257687	0.471506	-4.88628
AL731577.2	-0.28482	8.983107	-1.13532	0.257786	0.471573	-6.63582
AC010336.2	1.973639	1.706121	1.134264	0.258227	0.472263	-4.95505
AL355312.3	0.332416	9.364891	1.1341	0.258295	0.472274	-6.61355
AC138965.3	2.025135	0.859301	1.133307	0.258627	0.472754	-4.92989
AC084864.1	2.219548	2.232829	1.133146	0.258695	0.472754	-4.97
AL008628.1	-1.14141	-0.03899	-1.13302	0.258747	0.472754	-4.99184
AC087588.2	0.791346	7.331063	1.132673	0.258892	0.47287	-6.16271
FLNB-AS1	-0.34236	7.544977	-1.13257	0.258936	0.47287	-6.42048
LINC02274	1.88861	-0.36568	1.131061	0.259569	0.473891	-4.90424
AL031722.1	-1.06889	4.538678	-1.13093	0.259622	0.473891	-5.72778
AC018445.2	2.243927	1.68693	1.130482	0.259812	0.474008	-4.95069

AP005131.6	-0.44483	4.859597	-1.13055	0.259784	0.474008	-5.76475
AC012354.1	1.742239	-1.49885	1.130096	0.259974	0.474099	-4.88884
AC093525.7	-0.38738	4.25857	-1.13006	0.259988	0.474099	-5.5766
AL391832.1	2.214475	0.157329	1.129539	0.260208	0.47427	-4.91126
AC087893.1	1.001009	4.151448	1.129596	0.260184	0.47427	-5.22322
CDKN2A-DT	2.142092	-0.66394	1.129367	0.26028	0.474271	-4.89598
AC063926.1	-1.96882	-0.33569	-1.12911	0.260387	0.474271	-5.01783
AC009549.1	-0.55777	4.814639	-1.12909	0.260398	0.474271	-5.82237
AC027763.2	2.595156	5.663271	1.128678	0.26057	0.474363	-5.23974
AL138955.1	0.286973	6.07224	1.128668	0.260574	0.474363	-5.95622
AC005064.1	-1.59818	-0.05714	-1.1281	0.260813	0.474682	-5.01944
LINC01054	-1.78861	-0.6183	-1.12699	0.261283	0.475078	-5.00128
AL359091.5	1.43522	6.383286	1.127239	0.261176	0.475078	-5.59359
ARAP1-AS2	0.33975	6.329877	1.12706	0.261252	0.475078	-6.03093
LBX2-AS1	0.436099	10.0857	1.127089	0.261239	0.475078	-6.70353
AC010168.2	-0.29647	7.709622	-1.12671	0.261399	0.475173	-6.45266
LINC01120	1.766097	-1.1761	1.126182	0.261622	0.475387	-4.89557
AC244034.2	1.672713	4.53845	1.126132	0.261643	0.475387	-5.23008
AL360270.2	0.426434	5.040875	1.125746	0.261806	0.475568	-5.55601
LINC01004	0.257894	8.826402	1.125559	0.261885	0.475596	-6.55128
AL109945.1	1.863179	0.535985	1.125014	0.262116	0.4759	-4.93288
AC092127.2	-1.11555	2.803666	-1.12486	0.262181	0.475904	-5.36503
AC010280.2	-1.31161	3.005856	-1.12421	0.262453	0.476053	-5.41098
AC105429.1	0.310815	5.405626	1.124417	0.262368	0.476053	-5.71626
AC004771.5	-0.29181	7.289047	-1.12425	0.262439	0.476053	-6.37642
AC009065.5	0.707345	9.289034	1.123872	0.262598	0.476201	-6.5569
AL137802.1	2.173071	0.041492	1.122916	0.263003	0.476693	-4.91505
SFTPD-AS1	-1.14415	0.05087	-1.12267	0.263105	0.476693	-5.00644
AC007376.2	1.785187	3.236174	1.122632	0.263123	0.476693	-5.04937
AL157938.3	0.322847	4.842279	1.123039	0.262951	0.476693	-5.52542
AL133367.1	0.318379	7.164135	1.121615	0.263554	0.477359	-6.25506
AP003086.2	1.418293	5.037785	1.121296	0.26369	0.477489	-5.3192
AC090802.1	2.108945	-1.46954	1.12082	0.263892	0.47774	-4.89594
AC006058.1	-0.88213	2.750519	-1.12018	0.264162	0.478114	-5.33566
AP001775.2	1.779822	3.663004	1.119738	0.264352	0.478341	-5.10915
AC011726.3	1.919779	1.882889	1.119373	0.264507	0.47844	-4.97689
AC110597.1	-0.7895	2.493139	-1.11931	0.264534	0.47844	-5.24841
AC022445.1	1.265773	4.45925	1.118788	0.264756	0.478727	-5.31598
AC093752.3	-0.26837	8.775108	-1.11831	0.26496	0.478981	-6.61931
AC087501.1	-0.38414	6.034743	-1.11795	0.265111	0.479138	-6.13382
AC119428.2	1.804301	1.009823	1.116534	0.265716	0.479655	-4.95415
AC110769.1	1.697765	1.310777	1.11661	0.265684	0.479655	-4.96709
AL512356.3	1.935455	1.608198	1.116571	0.2657	0.479655	-4.96995
AC004990.1	2.239501	5.2293	1.116839	0.265586	0.479655	-5.25413
AL132712.1	0.600164	7.989474	1.116714	0.265639	0.479655	-6.35498
AC114730.2	1.2763	3.387949	1.116106	0.265899	0.47987	-5.12285
AC004221.1	1.749294	2.257027	1.115069	0.266341	0.480527	-5.01141
AC090772.1	-0.48668	6.232019	-1.11495	0.266391	0.480527	-6.21294
AC000068.1	-0.23482	6.146163	-1.11461	0.26654	0.48068	-6.12683
AC022558.1	0.226292	7.246994	1.114198	0.266714	0.480879	-6.288
FO393418.1	1.022365	5.670368	1.113757	0.266903	0.481104	-5.64708
TRIM52-AS1	-0.25603	9.743467	-1.11315	0.267163	0.481457	-6.76896
AL391001.1	-0.46885	6.656517	-1.11274	0.267338	0.481656	-6.30084
AC004528.1	1.842325	0.065208	1.111706	0.267781	0.48234	-4.93253
AC016910.1	-1.11547	2.278017	-1.11125	0.267978	0.482522	-5.25559
GRK5-IT1	-1.11036	3.984863	-1.11117	0.268011	0.482522	-5.62007
AC090181.2	0.678101	8.015849	1.11084	0.268153	0.482662	-6.3549
AC005625.1	1.989735	0.919196	1.109522	0.268719	0.483566	-4.95443

AC021066.1	2.010914	-0.69474	1.109117	0.268893	0.48361	-4.91563
AC019171.1	1.307785	3.222413	1.109114	0.268894	0.48361	-5.09511
AC023355.1	0.340455	5.014498	1.109016	0.268937	0.48361	-5.62079
AC027801.4	-0.78839	4.685481	-1.10865	0.269093	0.483775	-5.83509
AL022724.1	2.055441	0.675553	1.10817	0.269301	0.483878	-4.9474
AC024909.1	0.414511	5.944442	1.108071	0.269344	0.483878	-5.92212
AL109614.1	0.421435	7.552545	1.108094	0.269334	0.483878	-6.32937
AC027796.4	0.279978	8.450478	1.107816	0.269453	0.48396	-6.50825
AC009107.2	-0.24506	6.358013	-1.10751	0.269585	0.484081	-6.18684
AC006435.5	2.463629	2.209781	1.107237	0.269703	0.484133	-4.98615
ST3GAL5-AS1	1.797954	1.777133	1.107143	0.269743	0.484133	-4.98633
AC106895.2	1.786354	-2.06821	1.106851	0.269869	0.484244	-4.90707
AC009480.1	-1.15702	2.751224	-1.10663	0.269965	0.484299	-5.39001
LINC00637	0.866528	3.525038	1.106477	0.27003	0.484302	-5.16329
AL590369.1	0.295076	6.583292	1.104894	0.270714	0.485412	-6.14704
AP003385.3	1.972567	-1.70473	1.10419	0.271019	0.485445	-4.91015
AC091044.1	2.108637	-0.0535	1.104363	0.270944	0.485445	-4.93032
AC073389.2	-1.52892	0.481815	-1.104	0.271102	0.485445	-5.08663
AL358394.1	2.22125	3.978334	1.103851	0.271165	0.485445	-5.10565
AC103858.1	1.799965	3.662237	1.104045	0.271081	0.485445	-5.11639
AL031658.1	-0.55089	3.537782	-1.10427	0.270984	0.485445	-5.43926
AC135048.1	0.447638	4.732673	1.103805	0.271185	0.485445	-5.56086
AL356020.1	0.827882	3.758287	1.103119	0.271482	0.485861	-5.2302
AC005332.6	-0.20906	10.88553	-1.10262	0.271698	0.486132	-6.94252
AC055733.2	2.122049	-1.17681	1.102367	0.271808	0.486212	-4.91389
AC016949.1	0.234799	8.5663	1.102137	0.271908	0.486275	-6.53945
AL606760.3	1.082469	1.042283	1.101277	0.27228	0.486825	-4.98959
AC068860.1	0.901668	2.371255	1.101014	0.272394	0.486914	-5.06464
AL591167.1	1.973816	-1.74018	1.100709	0.272527	0.487035	-4.9132
AL355803.1	2.50695	4.535641	1.099483	0.27306	0.487409	-5.13072
AL021937.1	-0.39421	4.540256	-1.09978	0.272932	0.487409	-5.66804
AC037487.4	-0.7161	4.293479	-1.09972	0.272954	0.487409	-5.7119
AL162311.3	0.485999	6.250404	1.099528	0.27304	0.487409	-5.99895
AL512770.1	0.220488	7.028596	1.09948	0.273061	0.487409	-6.2697
AP003068.4	-0.45135	5.658179	-1.09929	0.273144	0.487442	-6.11042
KCNQ5-IT1	2.221772	-0.98282	1.098645	0.273424	0.487672	-4.91805
AC068658.1	2.178099	-0.79821	1.09884	0.273339	0.487672	-4.92079
AL356056.2	0.883351	4.869621	1.098545	0.273468	0.487672	-5.43299
AC006042.4	1.539839	3.047874	1.098201	0.273617	0.487685	-5.06872
AC093677.2	0.636178	5.009926	1.0983	0.273574	0.487685	-5.5714
AC008870.4	0.474245	5.657235	1.098081	0.27367	0.487685	-5.81111
AC079915.1	0.333881	6.169306	1.096907	0.274181	0.48848	-5.99427
AC078883.2	0.758253	6.845968	1.096731	0.274258	0.488501	-5.99408
TLR8-AS1	1.800642	-2.25612	1.096287	0.274451	0.488502	-4.91579
AC018695.2	1.886933	3.33162	1.096472	0.274371	0.488502	-5.08665
AL121782.1	-0.81525	4.630489	-1.09628	0.274453	0.488502	-5.82885
AL132639.2	-0.26323	6.893713	-1.09601	0.274571	0.488596	-6.31902
AL353898.3	-0.23194	6.759397	-1.09533	0.274871	0.488979	-6.29386
REV3L-IT1	-0.51516	6.756536	-1.09522	0.274917	0.488979	-6.34266
AL356481.2	-0.5051	5.385968	-1.09444	0.275257	0.489469	-5.98204
AC012501.1	2.113449	1.316059	1.094185	0.275369	0.489553	-4.97414
AC009163.6	-0.30286	5.55332	-1.09381	0.275535	0.489732	-5.9812
AC100778.1	-1.74733	-0.32703	-1.09358	0.275635	0.489793	-5.04785
AP000302.1	1.599825	1.23566	1.093344	0.275737	0.489859	-4.98915
AC078788.2	2.170824	1.147415	1.092725	0.276008	0.490225	-4.97209
AC023509.3	-0.2839	7.300149	-1.09241	0.276145	0.490351	-6.41834
AC007638.2	1.771274	0.198308	1.09179	0.276418	0.49066	-4.95568
AL359532.1	-0.36572	5.517016	-1.09157	0.276515	0.49066	-5.98101

AP002993.1	-0.51958	7.414327	-1.0916	0.276502	0.49066	-6.4819
AC096720.2	0.310466	6.21218	1.0911	0.276721	0.490802	-6.04443
AC243964.3	0.305225	9.639048	1.09109	0.276725	0.490802	-6.70824
SCARNA9	-0.28295	8.38242	-1.08991	0.277242	0.491602	-6.59203
AC044802.2	2.182703	0.302303	1.089763	0.277308	0.491603	-4.94979
AP002505.2	1.707989	1.686714	1.089011	0.277638	0.492073	-5.00487
AC141930.1	2.000054	0.947229	1.088648	0.277798	0.49224	-4.97273
LINC02569	-0.54796	5.31521	-1.0885	0.277863	0.49224	-5.94915
AC055713.1	0.19157	6.674213	1.088339	0.277934	0.492249	-6.16721
AP000851.2	2.174	-1.07931	1.087056	0.278499	0.492282	-4.92841
AC015909.2	1.923308	0.765799	1.087424	0.278337	0.492282	-4.97217
AP005264.1	1.938695	2.110479	1.087358	0.278366	0.492282	-5.01437
B4GALT4-AS1	1.589981	3.700206	1.087269	0.278405	0.492282	-5.16946
AF131215.2	-1.092	2.294562	-1.08793	0.278113	0.492282	-5.28708
AL163952.1	1.33026	4.875944	1.087177	0.278446	0.492282	-5.31961
AL031668.1	0.364918	6.441345	1.086957	0.278542	0.492282	-6.09293
AC104564.1	-0.30489	6.629303	-1.0878	0.278171	0.492282	-6.28818
AC015871.3	0.199245	8.353381	1.087132	0.278466	0.492282	-6.52566
AC010186.4	1.869093	-1.69565	1.085944	0.278989	0.49281	-4.92635
AC007336.2	1.908877	-1.84082	1.085362	0.279246	0.49281	-4.92635
AL807757.1	1.842413	2.357792	1.085239	0.279301	0.49281	-5.02765
AC010894.3	1.077874	3.34328	1.085486	0.279191	0.49281	-5.15248
AP001033.1	1.590925	5.704823	1.085828	0.27904	0.49281	-5.3932
Z73429.1	-1.24817	1.847545	-1.08525	0.279295	0.49281	-5.40887
AC107398.2	0.850127	5.748682	1.085851	0.27903	0.49281	-5.71972
AC090753.1	2.387675	0.024932	1.08466	0.279556	0.493118	-4.94425
AC245884.11	1.324465	1.316107	1.08425	0.279737	0.493118	-5.00733
AC022126.1	1.720754	3.599787	1.084518	0.279619	0.493118	-5.13475
AL445471.1	1.38189	4.688368	1.084364	0.279687	0.493118	-5.29686
PRR29-AS1	1.060621	1.401558	1.083847	0.279916	0.493316	-5.01585
AC010280.3	2.008933	-1.60378	1.083619	0.280016	0.493378	-4.92839
KANSL1-AS1	-0.33647	8.344679	-1.08346	0.280088	0.493389	-6.59069
AC110792.2	1.419888	1.391797	1.083226	0.28019	0.493453	-5.00843
AC092490.1	-0.72129	4.731479	-1.08299	0.280295	0.493522	-5.87339
AL445472.1	0.337366	7.585888	1.08277	0.280392	0.493577	-6.38828
AL122125.1	0.883092	6.283616	1.082353	0.280577	0.493671	-5.97824
RC3H1-IT1	-0.44238	6.188281	-1.08246	0.280531	0.493671	-6.22963
OOEP-AS1	2.040595	-1.32642	1.08211	0.280684	0.493744	-4.93092
AL357033.2	-1.66673	1.101897	-1.08196	0.280751	0.493745	-5.15534
SNAP47-AS1	1.046189	2.478234	1.080925	0.28121	0.494437	-5.07716
AL391361.3	2.383616	-0.16118	1.080025	0.281609	0.494674	-4.94528
SHANK2-AS3	1.478429	1.059061	1.079837	0.281693	0.494674	-4.99705
AC093158.1	-1.42616	-0.44694	-1.07964	0.281778	0.494674	-5.03628
AC138028.5	-1.53713	0.334705	-1.08017	0.281544	0.494674	-5.08528
AC005775.1	1.004827	4.34552	1.079582	0.281806	0.494674	-5.30576
AC025031.4	0.443486	4.970362	1.0804	0.281443	0.494674	-5.62789
AP000577.1	0.317792	6.380835	1.079907	0.281662	0.494674	-6.12631
AL844908.1	0.393333	5.97262	1.079241	0.281957	0.494825	-5.96002
AC009090.4	0.376685	4.386695	1.077849	0.282577	0.49568	-5.46235
AC006111.2	0.258883	7.945482	1.077881	0.282563	0.49568	-6.45731
AC018766.1	-0.47741	5.691587	-1.07755	0.282711	0.495685	-6.08435
ITCH-IT1	0.287932	7.635398	1.077648	0.282666	0.495685	-6.40269
AC005355.2	1.759919	3.412803	1.077164	0.282882	0.495868	-5.10015
LINC02393	-1.60153	-2.29879	-1.07617	0.283322	0.496293	-4.97919
AC025048.1	1.98539	1.064718	1.076182	0.283319	0.496293	-4.98689
AC093732.2	1.955791	2.040877	1.076315	0.28326	0.496293	-5.0221
AL592211.1	0.351741	6.841517	1.075818	0.283482	0.496456	-6.20922
AL135905.1	-0.33304	8.007961	-1.07521	0.283753	0.496816	-6.56308

AC011676.1	0.498541	6.37824	1.075007	0.283843	0.496858	-6.04053
AC083805.3	1.630732	-0.40705	1.074563	0.284042	0.496961	-4.95903
AC007681.1	-0.34967	4.688539	-1.07465	0.284003	0.496961	-5.73461
AC010997.4	-0.50329	5.746178	-1.07434	0.28414	0.496961	-6.14181
AC000068.2	-0.36368	6.758782	-1.07428	0.284167	0.496961	-6.34535
AC005740.3	-0.49673	5.371083	-1.07405	0.284271	0.497028	-6.01963
AC005736.2	2.121824	0.224317	1.073042	0.284722	0.497353	-4.96592
AP002957.1	1.706175	-0.04432	1.073396	0.284564	0.497353	-4.96643
AC009108.3	-0.66408	2.899884	-1.07318	0.284658	0.497353	-5.34595
AC011825.2	0.442908	6.296174	1.073286	0.284613	0.497353	-6.06329
AC026471.3	2.190446	3.701558	1.071655	0.285343	0.498045	-5.10095
AC096708.3	1.724682	3.38685	1.071417	0.285449	0.498045	-5.10996
AC090907.2	1.364172	3.28534	1.071799	0.285278	0.498045	-5.14141
AL356752.1	1.227588	4.554856	1.071459	0.28543	0.498045	-5.3354
AC015799.1	0.22478	7.406432	1.071644	0.285348	0.498045	-6.37337
AC147651.3	-1.89516	-1.21979	-1.07083	0.285714	0.498329	-5.02719
AP000254.1	0.208567	8.918766	1.070758	0.285745	0.498329	-6.63677
RAPGEF4-AS1	1.223435	0.124802	1.07056	0.285834	0.498368	-4.98522
AL450344.1	1.84859	0.448494	1.069782	0.286183	0.498843	-4.98044
AC073592.2	1.79296	2.702378	1.069657	0.286239	0.498843	-5.06775
AC055854.1	-1.19654	2.05426	-1.06923	0.286431	0.498972	-5.24861
AL583722.4	-1.2938	1.947576	-1.06905	0.286512	0.498972	-5.25225
HCG14	-0.94845	4.116422	-1.06918	0.286453	0.498972	-5.62209
AL390115.1	2.01773	-1.24858	1.068599	0.286714	0.499093	-4.94322
KRTAP5-AS1	-0.54419	5.501171	-1.06868	0.28668	0.499093	-6.01738
AC019226.2	1.109286	5.81414	1.067786	0.28708	0.499613	-5.62746
AC093525.3	2.025274	-0.23364	1.067415	0.287247	0.499673	-4.96081
AL353653.1	1.232572	3.851022	1.067361	0.287271	0.499673	-5.21553
AC007384.1	-0.4263	6.43238	-1.06727	0.287314	0.499673	-6.27808
IQCA1-AS1	2.0284	-1.49354	1.066315	0.287742	0.500282	-4.94364
LINC02044	1.255001	1.372506	1.066012	0.287879	0.500282	-5.02605
AC117395.1	1.230104	4.013173	1.066096	0.287841	0.500282	-5.25588
AC018521.6	-0.44691	7.916378	-1.0659	0.28793	0.500282	-6.57137
AP000892.2	-0.96544	4.184343	-1.06568	0.28803	0.500339	-5.75362
PCSK6-AS1	1.769703	1.771859	1.064403	0.288605	0.500592	-5.02613
AL513164.1	2.168534	3.598413	1.064994	0.288338	0.500592	-5.12146
AP003072.5	-1.16305	2.227495	-1.06455	0.288539	0.500592	-5.30038
LYPLAL1-DT	-1.04563	2.266403	-1.06432	0.288642	0.500592	-5.5198
AC037487.1	-0.44907	4.533783	-1.06444	0.288587	0.500592	-5.72655
AC010327.4	-0.58403	5.022896	-1.06477	0.288439	0.500592	-5.92846
LINC00294	0.253059	9.925732	1.064739	0.288453	0.500592	-6.78245
AC011939.2	-0.86706	4.656833	-1.06379	0.288883	0.500895	-5.877
AL445248.1	0.258806	7.744195	1.063393	0.289061	0.501088	-6.44314
AL118558.4	-0.29609	6.184687	-1.0629	0.289284	0.501359	-6.19461
AC136621.1	1.933491	0.193194	1.062562	0.289437	0.501508	-4.97705
INTS6L-AS1	1.811844	2.833115	1.062016	0.289684	0.501705	-5.07899
AP006545.2	-0.59849	5.824565	-1.06214	0.289626	0.501705	-6.17823
AC008957.1	-0.64232	4.017733	-1.06156	0.289889	0.501945	-5.64473
AC012254.5	1.501425	4.955995	1.060464	0.290387	0.50269	-5.38228
AC091588.2	-1.82645	-0.65413	-1.06008	0.290563	0.502764	-5.0532
AL022316.1	1.656839	5.124199	1.060196	0.290509	0.502764	-5.37923
UST-AS1	1.801539	0.575084	1.058682	0.291196	0.503706	-4.99389
AC011442.1	0.625634	6.523126	1.058581	0.291242	0.503706	-6.08535
AC011700.1	-1.26415	1.413681	-1.05839	0.291328	0.503739	-5.19327
AC010185.1	2.03238	-1.21341	1.057119	0.291907	0.504453	-4.95357
AC046136.1	1.924739	-0.52309	1.056738	0.29208	0.504453	-4.96547
AL035404.2	2.040041	-0.26307	1.056793	0.292055	0.504453	-4.97132
AP001922.3	1.515034	2.399708	1.056564	0.292159	0.504453	-5.07896

AC010320.4	0.832263	4.380723	1.056578	0.292153	0.504453	-5.37104
AL162258.2	0.289676	6.723314	1.056906	0.292003	0.504453	-6.22547
AC067852.2	0.233833	8.910101	1.056451	0.292211	0.504453	-6.64609
LINC02626	1.734065	3.223382	1.056263	0.292296	0.504485	-5.12003
AP003072.4	-1.75102	0.200814	-1.05595	0.292437	0.504613	-5.11358
AC105137.2	0.46043	5.265518	1.054798	0.292964	0.505405	-5.72047
AL603839.2	1.083542	5.911188	1.054161	0.293255	0.505674	-5.70333
AL031432.3	-0.38378	6.203545	-1.05421	0.293232	0.505674	-6.24624
AC244100.3	1.835028	-1.28118	1.053612	0.293505	0.50599	-4.95734
AC002558.2	0.249166	6.785333	1.053031	0.293771	0.506331	-6.24875
AC009093.5	2.005251	0.171572	1.052262	0.294122	0.506371	-4.98385
AC092120.1	1.705132	2.891821	1.051875	0.294299	0.506371	-5.10287
AC025166.1	2.759813	5.035803	1.052376	0.29407	0.506371	-5.19484
AC005329.3	-0.73432	2.904858	-1.05197	0.294255	0.506371	-5.3759
AC025175.1	-0.28365	6.584884	-1.05268	0.293931	0.506371	-6.31271
AL139289.1	0.232685	7.270104	1.051802	0.294333	0.506371	-6.37278
VASH1-AS1	0.192131	7.577635	1.052193	0.294154	0.506371	-6.43812
AL691432.2	-0.25115	9.025576	-1.0522	0.294149	0.506371	-6.73486
AC023824.6	-1.13341	-0.95759	-1.05078	0.294801	0.50706	-5.03069
AGBL1-AS1	-1.52351	-0.43551	-1.05044	0.294956	0.50721	-5.06612
LINC02332	-1.4237	2.024623	-1.04989	0.295209	0.507414	-5.30867
AC005962.1	-0.72243	4.514474	-1.04994	0.295186	0.507414	-5.79206
AL031186.1	0.306601	7.291863	1.049165	0.295541	0.507868	-6.36099
AL391421.1	-0.36552	4.817747	-1.04818	0.295992	0.508528	-5.83683
AC009704.2	0.467174	6.493247	1.047343	0.296378	0.508958	-6.12126
AC068473.5	0.284327	8.311601	1.047363	0.296369	0.508958	-6.5592
AC112176.1	1.712075	-1.22923	1.045308	0.297315	0.510332	-4.96587
AC090971.5	-1.04182	3.369815	-1.04542	0.297265	0.510332	-5.59057
BOK-AS1	1.95576	0.574964	1.044827	0.297536	0.510596	-5.00437
AC073569.1	0.260098	6.01184	1.044448	0.297711	0.510662	-6.05321
AC104819.3	-0.52625	6.49168	-1.04453	0.297672	0.510662	-6.34026
AC091132.3	-0.46758	3.963306	-1.04383	0.297995	0.510917	-5.58026
AL161452.1	0.427047	5.739193	1.043537	0.298131	0.510917	-5.91503
AC010997.5	-0.87353	4.827616	-1.0437	0.298056	0.510917	-5.97413
AC083880.1	0.333375	6.950247	1.043567	0.298118	0.510917	-6.27742
AC104986.2	-0.24263	8.854606	-1.0433	0.298241	0.510988	-6.71112
LINC00482	-0.60046	5.344866	-1.04309	0.298339	0.51104	-6.04688
AC020916.2	0.699836	6.407861	1.042609	0.29856	0.511301	-6.10389
TERC	1.516488	3.42994	1.041636	0.29901	0.511955	-5.18297
LINC01107	-1.33908	-0.09018	-1.04104	0.299287	0.512257	-5.08322
AC079949.2	1.036749	6.563529	1.040961	0.299322	0.512257	-5.91671
AC025188.1	1.702534	2.351616	1.040382	0.29959	0.512365	-5.0735
AC006064.1	-0.54267	4.21168	-1.04067	0.299459	0.512365	-5.75109
HM13-AS1	-0.38814	6.130105	-1.04053	0.299523	0.512365	-6.21046
AC027307.1	0.812581	6.282986	1.040162	0.299692	0.512423	-6.01433
AC092849.1	1.203679	5.331959	1.03999	0.299772	0.512442	-5.55019
AC145124.1	-0.45093	5.366277	-1.03964	0.299933	0.512602	-6.01913
AL139393.1	2.063833	0.335599	1.039432	0.30003	0.51265	-4.9989
AC136424.1	-1.4019	1.410608	-1.03929	0.300098	0.51265	-5.21935
AP001471.1	1.750084	0.114042	1.038607	0.300413	0.512722	-4.99947
AC099521.1	1.507679	0.518494	1.039007	0.300227	0.512722	-5.01556
AC110285.5	1.832722	1.865303	1.038652	0.300392	0.512722	-5.04942
AC073912.1	-0.91617	3.460097	-1.03888	0.300287	0.512722	-5.5321
AL121601.1	0.662465	6.344947	1.038104	0.300646	0.513004	-5.92005
IFT74-AS1	1.910782	-0.18832	1.036686	0.301305	0.513447	-4.9911
AC009097.1	1.798623	2.208317	1.036959	0.301178	0.513447	-5.06872
FAM53B-AS1	-0.56564	3.297973	-1.03668	0.301307	0.513447	-5.41904
AL590428.1	0.816601	4.558813	1.037179	0.301076	0.513447	-5.47112

AL161645.1	-0.97043	3.678468	-1.03666	0.301316	0.513447	-5.66837
AC233728.1	0.2745	6.712978	1.037327	0.301007	0.513447	-6.25649
AL731569.1	0.21409	6.090034	1.036165	0.301547	0.513724	-6.0747
AC015987.1	0.367551	5.492579	1.036014	0.301618	0.513728	-5.88696
AC040970.1	0.362975	7.293481	1.035832	0.301702	0.513755	-6.35212
AC005363.2	1.618489	4.461852	1.035238	0.301978	0.513992	-5.33263
AL117335.1	0.502871	5.586339	1.035272	0.301963	0.513992	-5.90053
AL591721.1	1.365216	4.040091	1.034426	0.302357	0.51452	-5.23598
Z97206.2	1.869103	-0.0412	1.033775	0.30266	0.514803	-4.9958
AC010737.1	1.974462	0.028762	1.033812	0.302643	0.514803	-4.99618
AC091182.2	4.096522	5.963908	1.033382	0.302843	0.514881	-5.19407
AC099522.2	0.350063	6.745912	1.033442	0.302815	0.514881	-6.23013
AC010435.1	-0.25863	5.802456	-1.03293	0.303053	0.515122	-6.11643
KCND3-AS1	-1.59112	-0.91924	-1.03267	0.303178	0.515203	-5.06576
LINC02344	-1.39669	2.122112	-1.03254	0.303238	0.515203	-5.31241
AC005052.1	-1.30474	-0.79711	-1.03137	0.303782	0.515894	-5.05863
AP000873.4	-0.20237	6.771149	-1.03151	0.303717	0.515894	-6.37167
AL118558.3	-0.27292	7.876035	-1.03108	0.303916	0.516005	-6.57537
AC112255.1	-1.51085	-1.29518	-1.03077	0.304064	0.51614	-5.04673
AC005779.1	-0.80316	3.890057	-1.03041	0.304232	0.516308	-5.68543
AL021707.3	0.204378	8.720194	1.030217	0.304322	0.516344	-6.64053
AC087257.1	-0.28081	5.447555	-1.03003	0.30441	0.516376	-6.00936
AC006272.1	1.400096	3.231237	1.029552	0.304633	0.516639	-5.18723
AC020910.1	-1.10881	3.01174	-1.02917	0.304812	0.516825	-5.49597
AC012414.5	-1.3401	0.855548	-1.029	0.304894	0.516847	-5.15886
AC131009.4	-0.24379	7.200094	-1.02779	0.305459	0.517688	-6.45994
LINC01527	2.387666	2.27645	1.027484	0.305603	0.517699	-5.05712
FARP1-AS1	-0.8633	4.772642	-1.02762	0.305538	0.517699	-5.94393
AC004923.4	-0.48523	6.791584	-1.02714	0.305765	0.517856	-6.43775
AL513412.1	-1.05816	2.417027	-1.02663	0.306006	0.518149	-5.3602
AL445222.1	0.449319	7.514319	1.025371	0.306596	0.518867	-6.40559
AP001527.2	0.367439	7.532078	1.02532	0.30662	0.518867	-6.42847
BACH1-IT1	0.202678	8.029223	1.025282	0.306638	0.518867	-6.53948
Z82180.1	1.625272	-2.3306	1.024576	0.30697	0.519312	-4.97643
FRY-AS1	-1.08741	2.236654	-1.02413	0.307182	0.519554	-5.30464
AP003168.2	1.523347	2.55426	1.022554	0.307923	0.520689	-5.11591
AL122017.1	-1.55831	-0.82835	-1.02233	0.30803	0.520754	-5.075
GFOD1-AS1	1.98129	1.104479	1.022025	0.308172	0.52076	-5.03684
AC073072.1	-1.06926	1.861895	-1.02216	0.308107	0.52076	-5.30766
MEIS1-AS3	1.681174	1.830442	1.020378	0.30895	0.52184	-5.07254
AC013451.2	1.677342	2.037797	1.020483	0.308901	0.52184	-5.09393
AC090425.1	0.278195	8.468006	1.020102	0.309081	0.521943	-6.60664
AP000879.2	1.528542	4.775406	1.019833	0.309208	0.521997	-5.40863
LINC00519	2.735743	6.727756	1.01974	0.309252	0.521997	-5.54796
AC005009.2	1.775664	0.170962	1.018895	0.309652	0.522555	-5.01755
PRKX-AS1	1.329592	3.743521	1.018587	0.309798	0.522568	-5.23872
AL391839.2	1.18867	4.582813	1.018585	0.309798	0.522568	-5.3502
AC092757.2	0.35417	6.630408	1.017829	0.310156	0.523054	-6.19382
AC008443.4	1.198615	5.177526	1.017508	0.310309	0.523106	-5.56914
AC025043.1	-0.37401	5.787566	-1.01747	0.310327	0.523106	-6.1892
AC093525.8	-0.20879	7.632358	-1.01711	0.310498	0.523278	-6.54435
AL353593.3	1.466729	4.819811	1.016797	0.310646	0.523312	-5.44005
AC005921.3	0.268712	6.193302	1.016772	0.310658	0.523312	-6.11303
AC087286.1	-1.1074	2.836296	-1.01594	0.311051	0.523505	-5.42238
AC064807.4	0.463083	6.503211	1.016204	0.310927	0.523505	-6.204
AC099778.1	-0.19107	6.847009	-1.01624	0.310913	0.523505	-6.39036
AC020915.2	0.250526	8.353542	1.01608	0.310986	0.523505	-6.59465
AL021878.2	-0.18731	7.67379	-1.01571	0.311164	0.523578	-6.54945

AC130448.1	1.508907	-0.46038	1.014494	0.31174	0.524228	-5.01142
AC136424.2	1.385704	3.647687	1.014574	0.311702	0.524228	-5.23919
AC015802.1	1.16857	5.42331	1.014453	0.311759	0.524228	-5.6966
LCMT1-AS2	-0.21863	4.485413	-1.01428	0.311841	0.524247	-5.71434
AC008764.10	-0.53048	4.165123	-1.01397	0.311988	0.52426	-5.64595
AC079035.1	-0.26173	5.705439	-1.01405	0.311954	0.52426	-6.11228
AFAP1-AS1	1.235838	7.558128	1.013794	0.312073	0.524285	-6.27594
AC090340.1	1.174129	0.588881	1.013203	0.312354	0.524399	-5.04805
AL691447.2	-0.48362	4.543133	-1.01308	0.312413	0.524399	-5.79514
AC003956.1	-0.39746	6.573225	-1.01307	0.31242	0.524399	-6.36064
AC002553.2	-0.21099	8.507121	-1.01315	0.312381	0.524399	-6.68332
AC002511.1	-0.86916	4.163672	-1.01079	0.313505	0.52608	-5.73749
AC083964.1	0.59183	6.870885	1.010672	0.313561	0.52608	-6.26937
AC007552.2	-0.86834	4.402751	-1.01028	0.31375	0.526279	-5.81736
AC010547.1	-0.8948	1.463654	-1.00994	0.313911	0.526431	-5.21041
AC233976.1	-1.77373	-0.85452	-1.00979	0.313985	0.526437	-5.09054
AC025741.1	-1.61948	-0.29577	-1.00955	0.314095	0.526505	-5.11203
AL022328.4	0.258748	6.852727	1.009262	0.314235	0.526622	-6.33571
AL606970.1	1.837574	0.088829	1.008887	0.314414	0.526805	-5.02242
LINC02538	1.356622	2.082829	1.008662	0.314522	0.526868	-5.10576
AC130895.1	-0.59631	4.962536	-1.00806	0.314811	0.527235	-5.98945
TTC39C-AS1	-0.38512	5.410425	-1.00782	0.314925	0.527308	-6.06084
NCKAP5-IT1	1.916018	-1.56469	1.007186	0.315228	0.527698	-4.99234
AC003965.2	1.686635	7.418671	1.006929	0.315351	0.527787	-6.04783
LINC00705	1.406009	2.292399	1.006698	0.315462	0.527855	-5.11823
AC005005.4	0.416474	2.469669	1.006434	0.315589	0.527949	-5.19547
AC084346.1	1.580397	3.41718	1.005149	0.316205	0.528863	-5.19976
AC019270.1	-1.17758	-0.22051	-1.00471	0.316415	0.528978	-5.10186
ENO1-AS1	0.870401	5.959515	1.004718	0.316412	0.528978	-5.77203
AC004490.1	0.341962	4.399471	1.003934	0.316789	0.529368	-5.56226
BX537318.1	0.268747	8.711182	1.003981	0.316766	0.529368	-6.65857
AC073655.2	0.463643	6.257372	1.002426	0.317514	0.530461	-6.08504
AC005387.1	0.612539	6.060448	1.002046	0.317698	0.53065	-6.00931
AC079584.2	1.835734	-1.68422	1.001762	0.317834	0.53076	-4.99656
DLEU2L	0.266898	5.280314	1.0013	0.318057	0.531014	-5.84144
AL590705.1	1.279816	3.748456	1.001086	0.31816	0.531068	-5.25301
LINC00391	1.58457	-2.46868	0.999985	0.318691	0.531434	-4.99594
AC023300.2	1.836148	-0.45715	0.999751	0.318804	0.531434	-5.01704
AL034399.2	1.523904	2.94816	1.000078	0.318646	0.531434	-5.15462
TRBV11-2	2.012089	3.558393	0.999833	0.318765	0.531434	-5.17328
AC012557.2	-0.6378	5.386838	-1.00005	0.318659	0.531434	-6.02205
AC008735.1	0.274946	6.424826	1.000302	0.318538	0.531434	-6.191
AL445933.2	2.037236	0.202056	0.998759	0.319283	0.531804	-5.0294
AL079307.1	1.362653	-0.19903	0.998772	0.319277	0.531804	-5.03376
AC011601.1	1.705844	4.360004	0.998778	0.319274	0.531804	-5.32799
LINC02289	-0.50294	3.526607	-0.99869	0.319314	0.531804	-5.46552
KLHL7-DT	0.621616	5.940386	0.998559	0.31938	0.531804	-5.94177
SCHLAP1	1.769332	-1.94762	0.998005	0.319648	0.531907	-4.99877
AC026726.1	-1.55623	-0.37371	-0.99799	0.319654	0.531907	-5.11242
AC020915.3	0.201321	8.985489	0.998176	0.319565	0.531907	-6.71503
AC026786.2	-1.60393	-0.39696	-0.99748	0.3199	0.532199	-5.11953
AC008267.8	0.863203	3.627256	0.99706	0.320105	0.532421	-5.30334
AL445493.3	1.931883	-1.44086	0.99654	0.320356	0.532722	-5.00181
AC006058.3	1.850594	-1.55051	0.996041	0.320598	0.53277	-5.00162
AP000962.1	-1.54212	-0.02555	-0.99604	0.320596	0.53277	-5.14575
AC078960.1	-1.43086	1.793364	-0.99622	0.320511	0.53277	-5.34939
AC090229.1	0.494518	5.567703	0.99466	0.321267	0.533658	-5.8914
AL356481.1	0.168303	7.240338	0.994645	0.321275	0.533658	-6.42715

AL162424.1	1.608037	5.270498	0.993685	0.321741	0.534315	-5.50516
PSG8-AS1	1.658944	-1.11966	0.993389	0.321885	0.534435	-5.01085
AC000061.1	2.141753	-0.80077	0.992995	0.322076	0.534634	-5.01003
AC009019.1	-0.33588	4.8947	-0.99275	0.322197	0.534717	-5.87734
AC027020.1	-0.40618	6.163282	-0.9924	0.322365	0.53486	-6.31004
AC009716.1	-0.38939	6.691727	-0.99228	0.322425	0.53486	-6.4128
AL135744.1	1.158867	3.66775	0.99167	0.32272	0.534862	-5.27429
AF235103.1	1.212273	4.308104	0.991104	0.322996	0.534862	-5.33577
AL121894.2	0.337605	7.22887	0.991201	0.322948	0.534862	-6.4018
AC011450.1	-0.33504	6.983982	-0.99198	0.322572	0.534862	-6.47578
AP001381.1	0.363933	8.091019	0.99162	0.322745	0.534862	-6.55426
AC025287.3	-0.21369	7.612084	-0.99117	0.322963	0.534862	-6.56337
TBILA	-0.35159	8.170943	-0.9913	0.322899	0.534862	-6.66021
AC067838.1	-0.24731	8.649312	-0.99147	0.322818	0.534862	-6.73286
AL365226.2	2.309916	7.149139	0.990918	0.323086	0.534894	-5.72521
AC113382.1	0.964445	2.883766	0.990567	0.323257	0.534941	-5.19168
AC138393.3	0.236679	7.171288	0.990579	0.323252	0.534941	-6.40154
LINC01563	-1.42964	0.326453	-0.99023	0.323423	0.535098	-5.15372
Z95114.2	-1.0985	0.56496	-0.98935	0.323852	0.535454	-5.16015
AC095031.1	1.486537	4.009832	0.989471	0.323792	0.535454	-5.27414
AL353804.2	-0.50287	6.056005	-0.98953	0.323763	0.535454	-6.29545
AP006748.1	2.028146	0.180772	0.988203	0.32441	0.53616	-5.03569
AC073548.1	0.181421	7.384492	0.988178	0.324422	0.53616	-6.46888
LINC02147	-1.41709	-0.1923	-0.98759	0.32471	0.536282	-5.12566
AL512506.2	1.207153	3.855048	0.987789	0.324612	0.536282	-5.30333
AL136295.6	0.185102	8.098721	0.987593	0.324708	0.536282	-6.58948
AC010619.2	-0.71449	2.901803	-0.98656	0.325215	0.536998	-5.41838
BX248123.1	1.713443	-0.88361	0.985789	0.32559	0.537499	-5.02043
AL356309.3	-1.37719	-2.5032	-0.98478	0.326083	0.537722	-5.0471
AC010247.1	1.347006	2.844213	0.985272	0.325843	0.537722	-5.17584
AC130462.1	0.969333	4.150326	0.985095	0.32593	0.537722	-5.36895
TPM1-AS	-0.40954	5.54467	-0.98496	0.325995	0.537722	-6.14033
AC012645.1	0.325002	6.681641	0.984885	0.326033	0.537722	-6.28057
AC113139.1	0.253097	8.061283	0.98461	0.326168	0.537744	-6.57692
AP001547.1	2.043993	-0.27578	0.984169	0.326384	0.537838	-5.02755
AC026469.1	0.976116	1.570004	0.983799	0.326565	0.537838	-5.11531
AC010198.1	-1.06995	1.343384	-0.98421	0.326365	0.537838	-5.2315
AC137894.1	-0.70258	4.935798	-0.98376	0.326583	0.537838	-6.0075
AC017074.1	0.537645	7.489996	0.98381	0.32656	0.537838	-6.42687
AL133260.2	1.170206	4.485195	0.983388	0.326767	0.538005	-5.46156
RB1-DT	-0.52022	4.254235	-0.98312	0.326899	0.538005	-5.76533
EIF1AX-AS1	-0.37153	6.805834	-0.98318	0.326868	0.538005	-6.45662
AC009229.2	-1.69295	-1.1236	-0.98256	0.327172	0.538336	-5.10003
LINC01607	1.405386	5.14551	0.982141	0.327379	0.538559	-5.42918
AC102945.1	0.863981	5.646469	0.98191	0.327492	0.538597	-5.89109
AC063943.1	-0.50654	5.652084	-0.9818	0.327545	0.538597	-6.16
AC020661.4	-0.41103	3.748473	-0.98084	0.328018	0.538902	-5.56938
AC104699.1	-0.50208	7.160486	-0.98104	0.327918	0.538902	-6.53927
AP000759.1	0.293755	9.834834	0.981157	0.327862	0.538902	-6.84654
AP001816.1	-0.24373	10.07835	-0.98097	0.327953	0.538902	-6.95373
AC110921.1	-1.3083	1.359047	-0.97862	0.329114	0.540476	-5.23689
AL603756.1	-0.22124	5.886982	-0.9786	0.32912	0.540476	-6.20163
AC090541.1	-1.67774	-1.05113	-0.97823	0.329301	0.540656	-5.10411
TAT-AS1	1.632855	0.146931	0.977874	0.329479	0.54083	-5.0522
AC068448.1	1.165186	-0.77118	0.97735	0.329738	0.540971	-5.04055
AL157702.2	1.406843	1.795044	0.977262	0.329781	0.540971	-5.11362
AL360270.3	0.456662	4.622464	0.977264	0.32978	0.540971	-5.60139
AC023632.2	-0.25364	5.631783	-0.97673	0.330042	0.54128	-6.10446

AC007608.1	-1.01124	1.60893	-0.97608	0.330367	0.541696	-5.2782
AC073487.1	-0.1819	7.323223	-0.97582	0.330493	0.541725	-6.53127
ACBD3-AS1	0.234241	8.424351	0.975601	0.330601	0.541725	-6.653
AC068870.2	0.297184	9.266593	0.97564	0.330582	0.541725	-6.7777
LINC02397	-0.7663	1.107558	-0.97537	0.330714	0.541792	-5.19655
LINC00427	1.641519	1.733616	0.974992	0.330903	0.541982	-5.11107
AP001160.1	0.176424	7.4216	0.9748	0.330998	0.54202	-6.47364
AC234917.1	1.795102	1.382149	0.97457	0.331112	0.542046	-5.09256
AC010240.3	1.202364	3.983241	0.974476	0.331158	0.542046	-5.34334
AL159174.1	1.597961	-1.65398	0.973829	0.331478	0.542097	-5.01994
PAN3-AS1	0.205142	6.976296	0.974224	0.331283	0.542097	-6.39901
AL391988.1	-0.31443	7.953427	-0.97403	0.33138	0.542097	-6.64848
AP001542.3	-0.32476	9.080929	-0.97388	0.331452	0.542097	-6.82646
AL391832.3	1.478145	2.255361	0.973271	0.331755	0.542432	-5.1379
AC019068.1	1.776005	-0.91358	0.972066	0.332352	0.542474	-5.02909
AL135838.1	1.587358	1.403884	0.972562	0.332106	0.542474	-5.09824
AL360014.1	-1.32579	-0.09174	-0.97205	0.332358	0.542474	-5.14118
AC022182.2	1.251901	3.763878	0.972667	0.332054	0.542474	-5.27685
LINC01433	-0.53779	5.007424	-0.97253	0.33212	0.542474	-6.00534
AC104653.1	0.387453	5.931934	0.972536	0.332119	0.542474	-6.05386
AL021707.8	0.234976	8.286516	0.972132	0.332319	0.542474	-6.63003
LINC00997	0.22671	9.533267	0.972334	0.332219	0.542474	-6.82163
AC011479.3	0.251686	6.957023	0.97189	0.332439	0.542489	-6.36357
AL353997.2	-0.82592	2.444268	-0.97161	0.332578	0.542598	-5.39252
AL139193.1	1.727198	-0.6307	0.970891	0.332935	0.543051	-5.03623
LINC00402	1.415274	-0.6863	0.970758	0.333001	0.543051	-5.04245
AC004034.1	0.485718	6.79033	0.970588	0.333086	0.543071	-6.28039
LINC01258	1.514304	-1.26687	0.970086	0.333335	0.54336	-5.0282
AC145423.3	0.277202	7.19403	0.969844	0.333456	0.543439	-6.41371
AC046168.1	1.271718	-0.17366	0.969019	0.333866	0.543853	-5.05978
AL162457.1	-1.36571	-1.98839	-0.96885	0.333949	0.543853	-5.07544
AC092139.2	0.842975	4.63813	0.968863	0.333943	0.543853	-5.55525
AP001107.6	0.455312	6.376886	0.96875	0.334	0.543853	-6.17482
AC126365.1	0.852306	3.443117	0.968223	0.334262	0.544163	-5.28835
AC009135.2	1.558072	-1.51748	0.967329	0.334707	0.544587	-5.02675
AL136038.3	0.446386	4.164186	0.967461	0.334642	0.544587	-5.49656
AC079336.2	0.41558	6.398354	0.967264	0.33474	0.544587	-6.20225
AC011773.2	-1.68636	-0.94069	-0.967	0.33487	0.54468	-5.11829
AC099811.4	1.441267	4.008308	0.9665	0.335121	0.544939	-5.30782
AL023806.1	-0.75187	2.833097	-0.96639	0.335174	0.544939	-5.48112
AC018761.1	2.163536	1.954515	0.966004	0.335368	0.545136	-5.11195
AC008781.3	0.537844	6.12262	0.96542	0.335659	0.545492	-6.07342
AC007364.1	0.661278	5.051492	0.965008	0.335865	0.545708	-5.59425
GPRACR	0.962512	1.706904	0.96486	0.335939	0.54571	-5.13802
AC005828.3	-1.44037	-0.53246	-0.96353	0.336604	0.546623	-5.13413
AP000892.1	1.162886	4.66603	0.963445	0.336646	0.546623	-5.38477
AC091180.4	1.842208	2.397383	0.962891	0.336923	0.546955	-5.139
AC007541.1	0.392293	5.785641	0.962432	0.337154	0.54721	-5.99907
AC026979.2	0.279633	8.508162	0.961992	0.337374	0.547331	-6.66308
AP002761.4	0.617791	9.265962	0.962098	0.337321	0.547331	-6.75258
AP000842.3	1.368971	1.736856	0.96178	0.33748	0.547385	-5.12587
AC141424.1	-0.91023	1.899038	-0.96135	0.337694	0.547614	-5.31283
AL356481.3	0.237347	7.305973	0.961173	0.337784	0.547642	-6.46915
AC087752.4	-0.25387	6.679415	-0.95979	0.338478	0.548411	-6.42991
AC131212.2	-0.22048	7.375089	-0.95985	0.338446	0.548411	-6.55694
AL023284.4	0.365682	9.8837	0.959939	0.338403	0.548411	-6.85828
AC026770.1	-1.40754	0.463799	-0.95935	0.338699	0.548651	-5.19526
DPYD-AS2	1.615013	-0.88368	0.957443	0.339657	0.548902	-5.04463

AC008883.2	1.82787	-0.4946	0.958196	0.339279	0.548902	-5.04795
AC100832.2	1.667982	-0.65433	0.957546	0.339606	0.548902	-5.0481
AC010468.2	1.741533	0.48372	0.958524	0.339114	0.548902	-5.07538
AL606537.1	-1.41024	-1.52719	-0.95766	0.339549	0.548902	-5.09683
AL356652.1	-0.53111	5.34432	-0.95752	0.339619	0.548902	-6.1184
AL161729.3	-0.29973	7.038257	-0.95831	0.33922	0.548902	-6.49518
AC096586.2	-0.48032	7.573095	-0.9575	0.339629	0.548902	-6.62439
AC093249.6	0.209596	8.245883	0.958494	0.339129	0.548902	-6.63848
AC006441.1	-0.29839	8.162819	-0.95843	0.339164	0.548902	-6.6907
AC025181.2	-0.21876	8.818023	-0.95778	0.33949	0.548902	-6.7897
AC010335.1	1.36849	2.450769	0.956319	0.340223	0.549698	-5.16827
LINC00052	1.530424	-2.32922	0.955647	0.340562	0.549944	-5.03103
AL161729.2	1.866756	1.185937	0.955592	0.340589	0.549944	-5.09466
AC007431.3	1.577809	1.836813	0.955449	0.340662	0.549944	-5.12878
LINC01678	-0.66145	3.764179	-0.95544	0.340668	0.549944	-5.69065
AC093865.1	1.851496	1.678655	0.955113	0.340831	0.550088	-5.11581
AC090373.1	-1.50385	-1.99093	-0.95494	0.340916	0.550107	-5.08702
AP003500.2	1.779677	1.879745	0.954744	0.341017	0.550132	-5.12569
AL022323.1	-0.34544	8.73668	-0.95462	0.341078	0.550132	-6.79728
AC074138.1	-0.40363	6.231668	-0.95437	0.341205	0.55022	-6.38127
AL450226.1	1.464623	2.334339	0.954141	0.341321	0.550289	-5.16053
AC011676.5	-0.46462	4.735186	-0.95343	0.341679	0.550408	-5.88044
AC011510.1	-0.69286	5.149886	-0.95344	0.341675	0.550408	-6.1942
AC006064.2	0.197931	7.251213	0.953414	0.341688	0.550408	-6.4556
LINC00526	-0.34816	7.459715	-0.95371	0.341539	0.550408	-6.59311
AP001596.1	-0.61601	3.385097	-0.95309	0.341853	0.550556	-5.6239
AC091179.1	2.080635	-0.375	0.952374	0.342214	0.550921	-5.05208
AC007389.5	1.624166	1.593589	0.952203	0.3423	0.550921	-5.11959
AL359220.1	0.330223	6.353297	0.952295	0.342254	0.550921	-6.23873
AC108865.2	2.013788	-0.44753	0.950919	0.34295	0.550922	-5.05194
AC245052.1	1.728002	-0.30682	0.950679	0.343071	0.550922	-5.06109
AC027808.1	1.600312	-0.40904	0.951277	0.342769	0.550922	-5.06207
AL391497.1	1.538511	1.695734	0.951852	0.342478	0.550922	-5.12614
AL049775.1	1.469602	2.306679	0.950348	0.343239	0.550922	-5.15405
AC011595.1	0.816265	4.60912	0.951667	0.342571	0.550922	-5.54814
AC073575.1	0.853644	4.718052	0.950984	0.342917	0.550922	-5.56968
AL035420.3	-1.06258	3.656872	-0.95197	0.342419	0.550922	-5.66573
AL442067.2	1.055188	5.301587	0.95045	0.343187	0.550922	-5.71205
Z97055.2	-0.61851	4.127509	-0.95032	0.343254	0.550922	-5.74535
TH2LCRR	-0.32314	5.703066	-0.95134	0.342734	0.550922	-6.15175
AC022272.1	-0.4721	5.450585	-0.95034	0.343244	0.550922	-6.18188
CR936218.1	0.249946	8.398038	0.950711	0.343055	0.550922	-6.67144
AC007040.1	-0.83241	3.412367	-0.94988	0.343475	0.551042	-5.60921
AC006273.1	0.572055	4.854661	0.949966	0.343432	0.551042	-5.66386
LINC00551	-1.37175	0.193637	-0.94973	0.343551	0.551045	-5.19672
LINC02587	1.797779	-0.30217	0.949348	0.343746	0.551093	-5.05982
AL662860.1	-1.41265	0.199159	-0.94935	0.343744	0.551093	-5.18435
AP001160.2	-0.46619	5.627431	-0.94924	0.3438	0.551093	-6.2007
AL390778.2	-1.54637	0.252298	-0.94884	0.344005	0.551303	-5.20495
LINC02404	2.00123	-0.37042	0.948205	0.344326	0.551582	-5.0552
AC139795.3	-0.37892	6.30209	-0.94834	0.344256	0.551582	-6.35956
AC092447.5	1.436145	-2.45321	0.947352	0.344759	0.551745	-5.03712
AC010834.2	1.556194	2.505567	0.947328	0.344771	0.551745	-5.17214
AC004975.2	0.939425	5.905916	0.947281	0.344795	0.551745	-5.93547
CATIP-AS2	-0.87373	4.960929	-0.94781	0.344526	0.551745	-5.96608
AP001271.1	-0.76342	6.996978	-0.94746	0.344703	0.551745	-6.57931
AL161636.1	1.635335	-1.55752	0.946063	0.345414	0.552618	-5.04166
AC006566.1	-0.94027	3.264611	-0.94546	0.345722	0.552994	-5.57595

AP005205.2	1.883571	-0.29923	0.944569	0.346174	0.553427	-5.0638
AC093904.3	1.97184	3.342763	0.94462	0.346148	0.553427	-5.20928
AC079807.1	0.191842	9.325665	0.94449	0.346214	0.553427	-6.82204
AC005481.1	0.931245	2.842785	0.944115	0.346406	0.553615	-5.23476
AL035706.1	-1.404	0.851791	-0.94396	0.346482	0.55362	-5.22354
AP001107.3	1.572141	3.512198	0.943759	0.346587	0.553626	-5.24215
AL022341.1	0.318603	6.917563	0.943668	0.346633	0.553626	-6.37724
AL590079.1	1.488425	0.722295	0.943305	0.346818	0.553803	-5.1006
AL390718.1	-1.59546	-0.96333	-0.94234	0.347309	0.554351	-5.13477
AL136980.1	0.638355	5.422134	0.942439	0.34726	0.554351	-5.75291
AL512622.1	-1.55458	-0.53198	-0.94169	0.347645	0.554534	-5.15368
AC091887.1	0.867673	5.487297	0.941541	0.347718	0.554534	-5.70766
AC008894.2	-0.21802	4.513318	-0.94182	0.347574	0.554534	-5.78037
AC008555.4	0.414224	7.92125	0.941973	0.347498	0.554534	-6.58429
AC092295.1	-1.13006	2.487953	-0.94118	0.347902	0.554709	-5.43326
AC011477.4	-1.13706	2.056498	-0.94037	0.348318	0.555254	-5.36192
LINC01679	-0.41982	6.957651	-0.94003	0.348491	0.555413	-6.5173
AL731563.2	1.317282	4.306763	0.939675	0.348672	0.555583	-5.40524
PRICKLE2-AS2	-1.54357	-0.74035	-0.93939	0.348816	0.555695	-5.14451
AC084083.1	0.271404	6.863377	0.93874	0.349151	0.55611	-6.3721
AC018529.3	0.313647	4.190959	0.938003	0.349529	0.556476	-5.57332
AP000786.1	0.524444	7.500081	0.93807	0.349494	0.556476	-6.46054
AC098679.4	1.632719	0.281472	0.936955	0.350066	0.557213	-5.09039
AL596223.1	1.595819	3.289248	0.936504	0.350297	0.557463	-5.23464
AC100839.1	1.319959	-0.22043	0.935727	0.350696	0.557979	-5.08316
AC138466.1	1.796205	-0.05515	0.934707	0.351221	0.558563	-5.07591
AL583810.1	0.317479	7.023479	0.93458	0.351286	0.558563	-6.4085
EBLN3P	-0.16747	11.37172	-0.93474	0.351204	0.558563	-7.17357
POC1B-AS1	-0.23342	6.002652	-0.93384	0.351664	0.559046	-6.26452
LINC02031	2.214062	1.58044	0.933543	0.351819	0.559174	-5.114
AC010328.1	0.908422	1.899406	0.933193	0.351999	0.559342	-5.17874
AC008883.1	1.92735	0.051618	0.932535	0.352338	0.559526	-5.07832
AC012291.1	1.622594	0.919799	0.932685	0.352261	0.559526	-5.11273
AC006511.3	-0.19877	7.099547	-0.9326	0.352305	0.559526	-6.52951
AC089998.4	1.703267	-0.8149	0.93205	0.352588	0.559804	-5.06436
AP001189.3	-0.43635	6.565765	-0.93163	0.352806	0.560032	-6.46294
AC092111.2	-1.13504	1.192335	-0.93139	0.352928	0.560107	-5.26727
ANK3-DT	-1.08008	3.489985	-0.93118	0.353036	0.560161	-5.62431
AL136439.1	1.560338	-1.13203	0.93036	0.35346	0.560714	-5.06064
AC092755.2	0.433644	6.46868	0.929919	0.353687	0.560956	-6.25992
AL356019.2	-0.20449	6.415489	-0.92961	0.353847	0.561091	-6.38665
AC051619.7	-0.8261	3.234769	-0.92927	0.354024	0.561136	-5.51383
AC027290.2	-0.23381	7.676243	-0.92933	0.353992	0.561136	-6.63733
AL139041.1	0.943116	4.985082	0.928698	0.354318	0.561483	-5.59331
AL360182.2	1.682718	0.675495	0.928506	0.354417	0.561501	-5.10486
AL162632.1	0.957239	4.573941	0.928386	0.354479	0.561501	-5.57049
AC103564.1	1.545319	0.418907	0.927606	0.354882	0.561934	-5.10397
AC006946.2	-0.72259	4.870018	-0.92742	0.354977	0.561934	-6.05111
AC048341.1	-0.18592	6.617659	-0.92751	0.354931	0.561934	-6.42364
AC026725.1	-1.25818	-0.00735	-0.92661	0.355396	0.56248	-5.17819
AC002563.1	1.213352	3.688798	0.925354	0.356049	0.563298	-5.34024
AC004691.1	0.877922	4.626144	0.925327	0.356063	0.563298	-5.58435
AC060809.1	1.766959	-0.69378	0.92501	0.356228	0.56344	-5.06968
AC010655.4	1.540326	-0.21355	0.923434	0.357045	0.563784	-5.09012
AL390755.1	1.465454	1.57339	0.923992	0.356756	0.563784	-5.14532
AC010336.5	-1.40505	-0.17179	-0.92427	0.356611	0.563784	-5.18345
AC008080.4	-0.93706	0.934337	-0.92384	0.356837	0.563784	-5.22441
AP000251.1	2.271238	4.817904	0.924051	0.356725	0.563784	-5.35163

AC107464.3	-0.91766	3.28271	-0.92403	0.356739	0.563784	-5.6289
AL645941.1	0.8864	5.343433	0.923453	0.357036	0.563784	-5.7952
AC130324.1	0.234975	6.945996	0.923721	0.356896	0.563784	-6.40892
AL136962.1	1.421684	4.130886	0.923042	0.357249	0.563987	-5.35772
AC103706.1	0.44083	6.974276	0.922407	0.357579	0.564389	-6.38302
AC008464.1	2.025203	-0.22919	0.922159	0.357709	0.564475	-5.07787
AL049870.2	1.600689	0.055228	0.921518	0.358042	0.564882	-5.09556
CLYBL-AS2	1.409998	1.389261	0.921326	0.358142	0.564921	-5.14241
AC092115.3	0.663047	6.129586	0.920375	0.358637	0.565583	-6.04085
AC124798.1	0.460446	8.466841	0.920061	0.3588	0.565722	-6.67954
AC079193.2	-1.05754	0.659407	-0.91962	0.359028	0.565844	-5.21655
AC106038.1	0.678542	2.731475	0.919751	0.358962	0.565844	-5.28255
AL513534.2	-0.31548	6.737499	-0.91936	0.359166	0.565942	-6.46683
LINC02000	1.733962	-0.40251	0.919101	0.359301	0.566036	-5.08131
AC090630.1	1.661983	-1.67926	0.918639	0.359542	0.566297	-5.0611
AC019131.1	1.650805	-0.61783	0.918006	0.359872	0.56658	-5.0782
AC022509.3	0.470151	8.039862	0.918137	0.359804	0.56658	-6.60619
AC127496.3	-1.04318	2.571439	-0.91762	0.360076	0.566782	-5.46291
AC245033.2	-1.51071	-1.16701	-0.91744	0.360167	0.566807	-5.14288
AC117500.2	0.202616	6.962821	0.917012	0.360392	0.567042	-6.4351
AC174065.1	-0.97845	-0.77049	-0.91611	0.360862	0.567161	-5.14251
AC025219.1	-1.25295	2.98771	-0.91634	0.360741	0.567161	-5.56467
AC079949.1	1.53802	6.599788	0.916	0.360921	0.567161	-5.89668
AC069200.1	0.856674	5.771321	0.916038	0.360901	0.567161	-5.90536
AC011472.5	0.248894	6.762301	0.916713	0.360548	0.567161	-6.37743
AC024575.1	-0.20812	6.631544	-0.91655	0.360633	0.567161	-6.43914
AL390195.1	-0.21715	6.105896	-0.91458	0.361666	0.568213	-6.31784
ELFN1-AS1	1.349708	7.307194	0.914388	0.361764	0.568249	-6.15213
AF001548.2	0.313207	9.63195	0.913977	0.36198	0.568468	-6.88644
AC027601.2	0.384458	5.193623	0.913131	0.362423	0.569046	-5.86358
AC021028.1	0.846074	6.042809	0.912895	0.362547	0.569105	-6.00277
AC007314.1	-0.34088	5.413854	-0.91277	0.362612	0.569105	-6.15925
AL035634.1	-0.85362	4.062732	-0.91204	0.362996	0.569588	-5.76401
AC018695.4	0.204122	7.483881	0.910885	0.363602	0.57042	-6.56405
FAM230I	-1.43018	-1.56204	-0.91019	0.363969	0.570877	-5.1328
LINC00659	-0.98894	5.606297	-0.90985	0.364144	0.571032	-6.25257
AC126178.1	1.486653	0.951615	0.909413	0.364376	0.571276	-5.13451
AC073316.3	-0.92754	3.258044	-0.90881	0.364692	0.571652	-5.5782
AC126763.1	1.407656	-0.70848	0.908381	0.364919	0.571684	-5.08898
AP006621.1	-0.28149	5.915969	-0.90834	0.36494	0.571684	-6.27624
AL591895.1	-0.27672	11.00448	-0.90857	0.364821	0.571684	-7.15957
AC087164.2	0.488515	2.604775	0.908105	0.365065	0.57176	-5.2885
AC107884.2	-1.17659	1.984343	-0.90778	0.365234	0.571906	-5.38575
AC126768.3	1.981858	0.643451	0.90734	0.365468	0.57209	-5.1135
AL365209.1	0.901786	0.081071	0.907272	0.365504	0.57209	-5.1244
LINC01901	1.924296	-0.77107	0.906717	0.365796	0.572216	-5.07857
AC092437.1	-0.84217	3.130126	-0.90675	0.365781	0.572216	-5.53952
NARF-IT1	0.251576	6.388151	0.906686	0.365813	0.572216	-6.28422
AC087164.1	-0.38203	3.235632	-0.90536	0.366514	0.572883	-5.50425
AC025575.2	2.746875	7.297504	0.905299	0.366545	0.572883	-5.63744
MORC2-AS1	0.25188	4.513806	0.905468	0.366456	0.572883	-5.66989
ASAP1-IT2	0.205722	6.547999	0.905718	0.366323	0.572883	-6.34794
AL033397.2	0.401045	7.345942	0.904991	0.366708	0.573019	-6.50119
AC083806.3	1.655525	0.187429	0.904209	0.367121	0.573068	-5.11037
AC010205.1	-1.48685	-0.67538	-0.90464	0.366895	0.573068	-5.17097
AC011092.2	1.323586	2.432808	0.904452	0.366992	0.573068	-5.21724
AC108676.1	0.991397	6.289729	0.904211	0.36712	0.573068	-5.97304
MIR210HG	0.406986	8.80532	0.904519	0.366957	0.573068	-6.7604

AC092652.2	-1.30962	-0.06148	-0.90391	0.367277	0.573193	-5.19683
LINC01080	-1.29116	-2.63082	-0.90368	0.367401	0.573268	-5.1027
AC018552.2	1.542431	0.428269	0.90333	0.367586	0.573318	-5.1194
LINC01340	-1.41461	-1.28897	-0.90335	0.367574	0.573318	-5.14642
AC105415.1	-1.26219	-0.99909	-0.90267	0.367934	0.573385	-5.1541
AC116036.2	-1.19064	2.531062	-0.90275	0.367895	0.573385	-5.45213
AC022973.2	0.382601	7.146272	0.903043	0.367738	0.573385	-6.45658
FBXL19-AS1	0.295903	7.712191	0.902798	0.367867	0.573385	-6.58331
AC092071.1	-1.42941	0.60482	-0.90214	0.368215	0.573704	-5.23789
AC090515.2	-0.22102	6.108525	-0.89967	0.369524	0.575623	-6.3263
AC053503.1	-1.4695	-1.4654	-0.8991	0.369829	0.575863	-5.14698
AC007906.1	-0.60888	4.493204	-0.89895	0.369907	0.575863	-5.94962
AC004877.2	-0.2511	6.615638	-0.899	0.369881	0.575863	-6.47924
AL354977.1	-0.91266	2.896181	-0.89869	0.370046	0.57596	-5.55703
AL110115.2	0.688582	5.912111	0.898523	0.370135	0.575978	-6.0445
LINC01315	-0.36296	7.444005	-0.8981	0.370359	0.576208	-6.66708
AC010653.2	1.284752	0.333	0.897069	0.370908	0.576823	-5.12947
AC145207.8	0.306174	7.493117	0.897164	0.370857	0.576823	-6.54962
LINC01886	-1.44811	-1.59851	-0.89651	0.371205	0.577141	-5.14566
AC116025.2	0.685133	6.300397	0.896395	0.371266	0.577141	-6.15018
AF130417.1	1.809037	0.591527	0.894724	0.372157	0.577974	-5.12697
BX005266.2	0.934724	2.932716	0.895156	0.371927	0.577974	-5.30034
ITPK1-AS1	-0.48009	2.830807	-0.89499	0.372017	0.577974	-5.45039
AC009812.3	-0.34333	4.616929	-0.89462	0.37221	0.577974	-5.87303
AP003068.2	0.321176	8.878102	0.894524	0.372264	0.577974	-6.77682
AL513165.1	-0.23609	9.906075	-0.89495	0.372036	0.577974	-7.01486
AC012254.1	1.883246	3.146125	0.891642	0.373802	0.580243	-5.23966
AC117503.3	0.19126	5.820624	0.891241	0.374017	0.580457	-6.14561
AC112236.1	1.816658	3.060973	0.890793	0.374256	0.580598	-5.22908
AL035658.1	-0.63239	4.80214	-0.89078	0.374263	0.580598	-6.05943
AC022509.2	0.580541	6.200247	0.890499	0.374414	0.580713	-6.16182
CPNE8-AS1	1.403898	5.989561	0.890118	0.374618	0.580789	-5.78424
AC244090.1	-0.21586	9.291363	-0.89022	0.374564	0.580789	-6.91436
BASP1-AS1	1.149006	0.357354	0.889901	0.374734	0.58085	-5.13906
AC007390.2	0.142588	6.909568	0.889749	0.374815	0.580856	-6.46149
SYNJ2-IT1	-0.94923	2.59974	-0.88813	0.375683	0.581805	-5.52142
AL662907.1	0.21791	6.734774	0.888147	0.375674	0.581805	-6.41874
GAS6-DT	-0.35938	6.638045	-0.88803	0.375738	0.581805	-6.50529
AC004771.1	0.202994	7.328351	0.888304	0.37559	0.581805	-6.54573
PAXIP1-AS1	0.248454	9.635154	0.887207	0.376179	0.582368	-6.91663
AC090115.1	-1.16318	1.435918	-0.88658	0.376515	0.582646	-5.32804
RN7SKP23	-0.17658	5.852561	-0.88644	0.376591	0.582646	-6.26804
AC104534.1	0.404581	8.048494	0.886549	0.376532	0.582646	-6.64081
AC145285.3	-0.20699	6.778663	-0.88607	0.376791	0.582835	-6.49359
AC107021.1	1.218429	4.11796	0.885533	0.377078	0.583159	-5.40489
AC104758.2	0.735023	5.298678	0.885077	0.377323	0.583418	-5.82693
AC022915.2	-0.94405	2.036279	-0.88478	0.377484	0.583547	-5.40344
AC104435.2	1.523627	-1.00414	0.884389	0.377693	0.58375	-5.0979
AL355102.4	1.048046	4.656874	0.883323	0.378267	0.584517	-5.63762
AC111170.3	-0.19493	7.349358	-0.88313	0.378369	0.584553	-6.61776
AC105749.1	0.285097	5.552223	0.882646	0.378632	0.58483	-6.00031
AC022400.8	0.191087	10.32269	0.882513	0.378704	0.58483	-7.02886
AC018845.3	0.27906	5.698731	0.882298	0.378819	0.584889	-6.0706
AC005089.1	0.766103	5.46079	0.881764	0.379107	0.585213	-5.86502
AC013270.1	1.606821	-0.54692	0.881077	0.379478	0.585545	-5.10953
AC015804.1	1.554448	0.30923	0.881173	0.379426	0.585545	-5.13193
Z85994.1	1.620778	-0.65917	0.880063	0.380026	0.58612	-5.10636
STARD7-AS1	0.153328	8.863997	0.880045	0.380035	0.58612	-6.81368

AL122035.1	-0.21819	8.926676	-0.87995	0.380085	0.58612	-6.87988
AC010761.7	-0.93969	3.746449	-0.87943	0.380366	0.586434	-5.73957
AC025031.5	0.723381	2.617844	0.878013	0.381134	0.587497	-5.28228
AC118658.1	1.242249	4.188949	0.877497	0.381413	0.587696	-5.41642
AL137798.1	-0.93681	3.20305	-0.87749	0.38142	0.587696	-5.6542
AC007950.2	-1.51885	-0.839	-0.87687	0.381752	0.588087	-5.18784
U73169.1	-0.33906	6.375695	-0.87659	0.381902	0.588198	-6.43512
HS1BP3-IT1	-0.37749	5.944601	-0.87609	0.382174	0.588496	-6.33921
AC109361.2	0.967142	5.604311	0.875827	0.382318	0.588598	-5.88211
AC008393.1	0.234748	7.230784	0.875509	0.38249	0.588742	-6.53393
AC008972.1	0.847039	4.76104	0.875156	0.382682	0.588917	-5.68698
AP005264.6	1.525624	-1.62313	0.874695	0.382932	0.589044	-5.09394
AL031779.1	1.484606	2.028578	0.874741	0.382907	0.589044	-5.20351
AC005865.1	0.968856	3.798445	0.87457	0.383	0.589044	-5.43483
AC126773.4	-0.38429	3.979579	-0.87399	0.383313	0.589405	-5.70872
AC004584.3	-0.38678	4.412684	-0.87362	0.383514	0.589593	-5.85222
AC005479.2	1.05684	5.283942	0.873422	0.383623	0.58964	-5.71124
AF131215.7	-0.68423	4.91444	-0.87213	0.384326	0.5906	-6.19003
AC022558.3	0.177105	6.813941	0.871724	0.384547	0.590818	-6.44632
AC011365.2	-1.1492	1.336631	-0.87074	0.385081	0.591507	-5.34187
AC090181.3	-0.38554	6.675006	-0.87061	0.385153	0.591507	-6.51966
AC025594.2	0.469575	5.17189	0.869948	0.385514	0.591941	-5.76773
AC104024.2	-0.71446	3.750622	-0.86969	0.385655	0.592036	-5.71984
AC068792.1	-0.34577	6.676801	-0.86954	0.385737	0.592041	-6.50811
AC109446.4	1.227568	-1.30997	0.869134	0.385958	0.592052	-5.1079
AC007250.1	1.68251	-0.42569	0.8692	0.385922	0.592052	-5.11678
AC034111.1	-0.94188	0.383459	-0.86909	0.385981	0.592052	-5.23321
AC068020.1	1.55086	-0.89868	0.867809	0.386681	0.592665	-5.11164
LINC01481	0.665696	4.762184	0.867894	0.386635	0.592665	-5.79207
AC025034.1	0.267633	6.697586	0.868027	0.386562	0.592665	-6.40514
AC092354.2	-0.26976	6.37735	-0.86778	0.386695	0.592665	-6.42847
AP001021.1	-1.58096	-0.87233	-0.86713	0.387051	0.593088	-5.20663
TM4SF1-AS1	0.689509	6.719758	0.866619	0.387331	0.593397	-6.2887
KDM5C-IT1	0.793695	3.667086	0.866344	0.387481	0.593506	-5.45009
AC000067.1	1.714783	-0.32455	0.864855	0.388296	0.59432	-5.12282
IGBP1-AS2	0.73487	5.409152	0.864768	0.388344	0.59432	-5.79985
AL357033.1	-0.45295	5.51211	-0.86467	0.388395	0.59432	-6.26065
AC009087.1	-0.3012	6.489897	-0.86465	0.388409	0.59432	-6.47011
AL139260.1	-0.18495	7.332596	-0.86481	0.388318	0.59432	-6.62695
AL138881.1	1.651193	0.496009	0.864073	0.388724	0.594529	-5.14752
AC093525.10	-0.22603	5.289703	-0.86397	0.388783	0.594529	-6.11756
AC107032.2	0.568446	6.269171	0.864119	0.388699	0.594529	-6.24633
AL365356.1	-0.26514	5.279784	-0.86361	0.388976	0.594583	-6.14575
AC245884.10	-0.48219	6.24264	-0.86363	0.388969	0.594583	-6.45904
AL135936.1	1.463073	1.562712	0.863241	0.38918	0.594738	-5.19327
LINC01165	-1.16131	-0.07405	-0.86314	0.389236	0.594738	-5.22153
AJ011931.1	1.229904	-1.35919	0.862714	0.389469	0.594974	-5.10954
AC036214.1	1.008335	1.80838	0.862498	0.389588	0.595034	-5.21979
LINC01293	1.380776	4.061903	0.862319	0.389686	0.595063	-5.38929
AC005480.1	-0.18367	5.106451	-0.86182	0.389957	0.595357	-6.03974
LINC00235	0.276826	6.686134	0.861582	0.39009	0.595438	-6.42494
A2ML1-AS1	1.765612	-0.02889	0.860831	0.390503	0.595858	-5.13438
AL353622.2	-0.71815	4.068097	-0.86079	0.390524	0.595858	-5.77848
AL049637.1	0.767636	5.360011	0.860588	0.390636	0.595908	-5.87854
AC087190.2	1.510436	-0.83681	0.860036	0.390939	0.59625	-5.11716
LINC01465	0.231689	6.077462	0.85976	0.391091	0.59636	-6.24384
AL162727.1	1.028922	1.605111	0.859329	0.391328	0.596601	-5.21056
AC034236.3	-1.24978	0.87377	-0.85915	0.391429	0.596607	-5.30362

AC091132.5	0.279776	6.207199	0.859033	0.391491	0.596607	-6.29806
AL139005.1	0.922527	2.955973	0.858786	0.391627	0.596693	-5.30676
AL023581.1	1.56854	-0.79308	0.858108	0.392	0.596778	-5.12092
AC078923.1	-0.72402	4.765016	-0.85852	0.391775	0.596778	-5.9975
AL162724.2	-0.28503	7.034664	-0.85825	0.39192	0.596778	-6.59205
AL359076.1	0.229951	9.577263	0.858224	0.391936	0.596778	-6.93583
AC100788.2	0.270307	5.109667	0.857897	0.392116	0.596833	-5.92423
AC104237.3	-1.4531	-1.22321	-0.85761	0.392274	0.596841	-5.18532
AC129492.2	0.63867	6.957363	0.857599	0.39228	0.596841	-6.34374
AP001267.4	-1.07761	1.810395	-0.85702	0.392599	0.597084	-5.38047
AC004921.1	1.003281	4.631949	0.857039	0.392589	0.597084	-5.56435
AC127537.1	-0.20676	5.355365	-0.85622	0.393043	0.597548	-6.14922
AC004908.3	-0.23556	7.4022	-0.85618	0.393063	0.597548	-6.65297
ARNTL2-AS1	0.662203	5.292268	0.855666	0.393346	0.597858	-5.86048
AC008378.1	-1.36822	-1.48685	-0.85544	0.39347	0.597925	-5.17437
MCF2L-AS1	-0.79694	8.146632	-0.85507	0.393672	0.598112	-6.84747
AC018904.2	1.351909	-1.63545	0.854033	0.394248	0.598724	-5.11021
CR559946.2	0.362836	4.581109	0.854021	0.394254	0.598724	-5.74795
AC016999.1	-0.47596	5.136967	-0.85391	0.394315	0.598724	-6.12852
AC012173.1	-0.41567	5.920129	-0.85314	0.39474	0.599248	-6.38156
GNA14-AS1	-0.9645	3.162596	-0.85299	0.394825	0.599257	-5.66668
AC097716.1	1.797777	1.576999	0.852243	0.395238	0.599762	-5.18846
AC104088.1	0.968324	7.028812	0.851935	0.395408	0.599899	-6.30167
AL133445.2	-0.24583	5.602338	-0.85174	0.395515	0.59994	-6.24561
BTG3-AS1	0.245991	6.488843	0.851112	0.395864	0.600348	-6.36509
LINC02069	-1.05779	-0.53539	-0.84968	0.396656	0.600943	-5.20112
AF038458.2	1.322151	2.4301	0.849705	0.396644	0.600943	-5.25029
SMYD3-IT1	1.207479	4.786452	0.850089	0.396431	0.600943	-5.5933
AC090197.1	0.307692	4.494512	0.850217	0.39636	0.600943	-5.71332
NDUFV2-AS1	-0.13229	7.941681	-0.84978	0.396604	0.600943	-6.73548
STX18-IT1	-0.892	2.524745	-0.84866	0.397224	0.601683	-5.53291
LINC02130	1.559224	-1.71415	0.84677	0.398273	0.602865	-5.11202
AC073592.5	1.399745	1.374152	0.847007	0.398142	0.602865	-5.198
AC102953.2	0.266212	7.665096	0.846675	0.398326	0.602865	-6.63204
AC023818.1	-0.13295	7.884532	-0.84682	0.398248	0.602865	-6.72748
WWC2-AS1	-1.44695	-1.01784	-0.84596	0.398722	0.603146	-5.20066
AC087276.3	0.807429	5.41601	0.846147	0.39862	0.603146	-5.83061
AL513190.1	-0.21043	6.556392	-0.84591	0.398753	0.603146	-6.49149
AC127002.1	-1.15668	1.599098	-0.84571	0.398863	0.603191	-5.36892
AC148477.4	1.466024	-1.62993	0.845075	0.399217	0.603269	-5.11532
AL138733.1	1.152718	2.005529	0.845041	0.399235	0.603269	-5.24008
ZNF197-AS1	0.670651	5.380784	0.845289	0.399097	0.603269	-5.81838
AC137630.3	-0.26069	7.023393	-0.84517	0.399166	0.603269	-6.58799
AC092135.2	-0.78141	3.220087	-0.84461	0.399474	0.603508	-5.67681
AC148477.2	-1.14299	-0.28662	-0.8443	0.399649	0.60353	-5.22288
AC083841.1	1.723783	3.988864	0.844366	0.399611	0.60353	-5.38235
CATIP-AS1	1.017877	3.73058	0.843792	0.399932	0.603836	-5.37612
HLCS-IT1	1.25925	2.501915	0.843419	0.400139	0.604028	-5.26535
AC090907.1	0.596243	5.438671	0.842312	0.400757	0.604607	-5.95224
AC008763.1	0.570933	6.208721	0.842298	0.400765	0.604607	-6.16266
AC062037.2	0.920558	7.290184	0.842528	0.400636	0.604607	-6.44728
AL049840.2	-0.20085	8.909595	-0.84215	0.40085	0.604614	-6.90399
AC093635.1	0.659144	5.607903	0.840425	0.401811	0.605821	-5.96659
USP46-AS1	-0.19819	9.082442	-0.84055	0.401742	0.605821	-6.92925
Z92544.2	-0.21143	6.762275	-0.84011	0.401986	0.605963	-6.55135
AC017048.3	1.417363	1.21345	0.839354	0.40241	0.605994	-5.19559
AC093627.7	-0.99265	1.840834	-0.83939	0.402388	0.605994	-5.38959
AL158071.5	-0.22537	5.196113	-0.83968	0.402229	0.605994	-6.07829

AC023051.1	-0.31949	5.161583	-0.83952	0.402318	0.605994	-6.13458
LINC01715	-0.25424	5.545908	-0.83973	0.402201	0.605994	-6.17033
AL359382.1	-1.2379	2.025341	-0.83804	0.403145	0.606858	-5.4805
AC068389.3	-0.28783	6.031981	-0.83814	0.403089	0.606858	-6.38715
AC087623.2	0.251572	7.318271	0.837867	0.403242	0.606882	-6.58083
AL136964.1	-1.12571	-0.3388	-0.8377	0.403336	0.606902	-5.22637
AC073115.1	1.538499	-1.09911	0.836607	0.403948	0.607204	-5.12877
AC004453.2	-0.68382	1.985637	-0.83651	0.404005	0.607204	-5.40788
AC073352.1	-0.56453	4.451083	-0.83672	0.403885	0.607204	-5.93155
AL603839.3	0.321533	7.1776	0.836843	0.403816	0.607204	-6.52583
ZNF503-AS2	-0.32505	7.613314	-0.83648	0.404022	0.607204	-6.72647
AC005785.2	-0.16733	8.001566	-0.83661	0.403949	0.607204	-6.76353
AC138123.2	1.446069	-0.81564	0.836061	0.404255	0.607433	-5.13718
AL158063.1	-0.50266	4.912661	-0.83582	0.404392	0.607518	-6.09011
CFAP58-DT	0.639526	6.093176	0.835599	0.404514	0.607579	-6.16858
AC010335.3	-1.29592	-0.56417	-0.83419	0.405303	0.608529	-5.22619
AC009560.1	-1.07156	2.317519	-0.83419	0.405308	0.608529	-5.53255
ERVK9-11	-0.35749	4.015713	-0.83397	0.405428	0.608588	-5.80821
CLSTN2-AS1	1.229807	-1.23497	0.833782	0.405534	0.608626	-5.13275
AL021707.7	0.217994	7.298281	0.833391	0.405754	0.608834	-6.57417
AC007365.1	0.827655	5.268443	0.832923	0.406017	0.609107	-5.85844
AC243960.3	-0.5739	3.841173	-0.83245	0.406282	0.609382	-5.80122
AC114812.2	1.554637	2.535136	0.83205	0.406509	0.609601	-5.25928
LINC01805	1.754445	0.717474	0.831735	0.406686	0.609745	-5.17663
OBSCN-AS1	0.36403	5.42029	0.831361	0.406896	0.609938	-5.98344
AC025871.1	1.384107	1.660882	0.831072	0.407059	0.610061	-5.21984
ANKRD33B-AS1	-1.36966	-1.2547	-0.83063	0.407309	0.610314	-5.19913
AC099804.1	-0.22958	6.078356	-0.82975	0.407804	0.610934	-6.38227
AC120057.4	1.612879	4.703046	0.829419	0.407991	0.611092	-5.45906
AC109347.1	0.188004	6.505187	0.829218	0.408104	0.61114	-6.41169
AC068870.3	-0.81581	1.943858	-0.82887	0.408299	0.61131	-5.41048
AL109917.1	0.544002	5.766057	0.82843	0.408549	0.611562	-6.06207
AC005837.1	0.462237	4.316578	0.827152	0.409271	0.612227	-5.64672
AC132219.1	0.783368	4.735841	0.827066	0.40932	0.612227	-5.72109
AC243919.1	-0.3402	5.959309	-0.82715	0.409272	0.612227	-6.37793
AL031123.2	0.323714	6.765892	0.827288	0.409194	0.612227	-6.41487
AP000317.1	1.02385	3.181018	0.826795	0.409473	0.612287	-5.37329
AL133215.2	0.255649	7.403123	0.826707	0.409523	0.612287	-6.57242
AC011374.1	2.150806	5.449575	0.826257	0.409777	0.612424	-5.6159
AC114284.1	0.830554	4.709728	0.826284	0.409762	0.612424	-5.62452
AC099066.1	1.534926	1.418862	0.825929	0.409963	0.612579	-5.20844
AP002008.1	1.375485	-1.42366	0.825676	0.410106	0.612624	-5.13209
AC092809.4	0.19752	5.624352	0.825588	0.410156	0.612624	-6.12844
Z99289.3	1.423157	-1.24865	0.824394	0.410832	0.613175	-5.13616
AL161785.2	-1.27029	-1.55416	-0.82444	0.410809	0.613175	-5.19049
AC005013.1	1.534692	1.018711	0.824131	0.410981	0.613175	-5.19849
AL139397.1	-0.68569	4.283636	-0.82407	0.411014	0.613175	-5.93986
PPP1R12A-AS1	-0.18445	5.888866	-0.82434	0.410861	0.613175	-6.303
U47924.2	0.243709	7.353385	0.824194	0.410945	0.613175	-6.59416
INKA2-AS1	-0.69848	2.125576	-0.8237	0.411223	0.613364	-5.42941
AL354694.1	1.29236	2.513934	0.823522	0.411326	0.613397	-5.28933
AL590822.1	-1.09157	2.624602	-0.82248	0.411915	0.614153	-5.53312
AL109955.1	0.266464	5.358804	0.822248	0.412049	0.61423	-6.04062
AC020658.2	0.859895	0.215134	0.822066	0.412152	0.614262	-5.19117
AC103957.2	1.408288	4.782166	0.8217	0.41236	0.61445	-5.57156
AJ239328.1	0.873203	1.814763	0.821235	0.412624	0.614722	-5.25931
AC069234.4	-0.49077	5.278226	-0.82037	0.413113	0.615193	-6.22836
AC022706.1	0.578769	6.383071	0.820449	0.41307	0.615193	-6.24825

AC022150.2	-0.32743	7.707367	-0.82025	0.413185	0.615193	-6.75702
AP000438.1	-1.32205	-1.34579	-0.81944	0.413646	0.615555	-5.20163
Z95114.4	0.700093	1.221441	0.819386	0.413675	0.615555	-5.2345
AC026355.1	1.280595	4.826528	0.819626	0.413538	0.615555	-5.62992
AC103987.2	1.506275	-1.57344	0.819108	0.413833	0.615669	-5.1326
AC025809.1	1.437359	0.681291	0.818845	0.413982	0.615769	-5.19105
BRWD1-IT1	-0.48675	4.755193	-0.81858	0.414134	0.615873	-6.0744
AC096536.1	0.703774	6.74247	0.817977	0.414476	0.61626	-6.29865
AC013640.1	1.451955	-0.72622	0.817547	0.414721	0.616378	-5.1519
AL360169.1	1.45641	3.447512	0.817406	0.414802	0.616378	-5.40542
AC027796.1	-0.38608	5.870976	-0.81761	0.414686	0.616378	-6.38146
AC022294.1	-1.28027	-1.63515	-0.81682	0.415134	0.616505	-5.19327
AC006238.1	-0.75565	3.660991	-0.817	0.415031	0.616505	-5.7113
AC018553.1	-0.81672	5.999527	-0.81692	0.415079	0.616505	-6.45766
AC010307.3	1.205913	-1.89212	0.816418	0.415365	0.616726	-5.13306
AC093510.1	-0.86013	2.598133	-0.81591	0.415653	0.617032	-5.53673
MIR31HG	-0.80725	7.20457	-0.81555	0.415858	0.617215	-6.73424
AC116158.2	1.32825	2.248769	0.815013	0.416167	0.617551	-5.27162
AL158055.1	-1.3755	-1.67393	-0.81456	0.416427	0.617815	-5.20189
AC007032.1	0.9711	4.242475	0.814361	0.416539	0.617859	-5.51736
GYG2-AS1	1.373426	-0.18357	0.811613	0.41811	0.619423	-5.1735
AC016644.1	1.259542	2.949536	0.811754	0.418029	0.619423	-5.32192
AC114811.2	0.355256	3.905797	0.811509	0.41817	0.619423	-5.60807
DNM1P35	0.29584	5.060543	0.811801	0.418003	0.619423	-5.92077
AC005911.1	0.369355	6.707072	0.811901	0.417946	0.619423	-6.41091
LINC02298	0.320751	7.883574	0.812235	0.417754	0.619423	-6.68868
AL133520.1	0.158893	8.112519	0.811709	0.418055	0.619423	-6.75533
AC104809.1	-1.12597	-1.69577	-0.81081	0.418569	0.619469	-5.1924
AL138767.3	1.42266	2.24377	0.810872	0.418534	0.619469	-5.25909
AC008592.4	-1.25674	0.562642	-0.81073	0.418614	0.619469	-5.2914
AC067930.2	-0.41926	5.048505	-0.81115	0.418375	0.619469	-6.08345
AC009065.8	0.146234	7.769317	0.811104	0.418402	0.619469	-6.69623
AC073912.2	1.442004	2.667271	0.810532	0.41873	0.619519	-5.2853
AC008494.2	-0.82856	3.969672	-0.80994	0.419069	0.619899	-5.81676
LINC01429	0.97609	2.744776	0.809695	0.419209	0.619984	-5.3266
LINC02621	1.648853	0.982809	0.809444	0.419353	0.620075	-5.20157
AP000962.2	-1.10178	1.446716	-0.8088	0.419723	0.620473	-5.39311
AC148476.1	1.288813	3.638194	0.808686	0.419788	0.620473	-5.40422
AC013356.2	-0.21434	5.626759	-0.80807	0.420143	0.620876	-6.26115
AP000919.2	-0.62728	4.619674	-0.80727	0.4206	0.62143	-5.98792
TDRKH-AS1	0.405401	5.86565	0.806408	0.421096	0.62204	-6.14136
AC000403.1	0.300227	5.522961	0.805002	0.421905	0.623112	-6.12061
AC020663.1	-1.12766	1.070042	-0.80456	0.422162	0.62337	-5.34687
AC027319.1	1.137625	3.35752	0.803467	0.422789	0.624101	-5.38436
AC009095.1	0.132809	7.332402	0.803407	0.422824	0.624101	-6.62907
AL513320.1	0.236893	6.19994	0.802737	0.42321	0.624548	-6.33202
AC097639.1	-0.37309	6.840971	-0.80249	0.423355	0.624639	-6.60213
AC073651.1	-0.9422	2.963901	-0.80181	0.423743	0.62509	-5.67687
AC006116.6	-1.23775	-1.99641	-0.80084	0.424302	0.625301	-5.1909
AC104135.1	1.764749	3.246707	0.801309	0.424034	0.625301	-5.31021
AL121574.1	1.278139	3.278695	0.80093	0.424252	0.625301	-5.35656
AC026367.1	-0.41492	4.833037	-0.80118	0.424107	0.625301	-6.08653
AC120053.1	0.180881	9.035358	0.801025	0.424197	0.625301	-6.90508
AC109927.1	1.260383	0.902771	0.800359	0.424582	0.625443	-5.21758
AL512631.1	1.217774	2.176361	0.800206	0.424671	0.625443	-5.28544
AC021422.1	-1.05956	1.61537	-0.8001	0.424732	0.625443	-5.40235
AC005757.1	-0.407	5.167426	-0.80046	0.424525	0.625443	-6.17363
LINC02360	1.438577	0.128088	0.799301	0.425194	0.625656	-5.18701

AP003469.3	1.325218	2.247958	0.799238	0.42523	0.625656	-5.27926
AP003108.5	1.1751	4.494356	0.799613	0.425013	0.625656	-5.5576
AC005534.1	0.575122	5.869537	0.799129	0.425293	0.625656	-6.0158
AC005696.1	0.281962	7.365549	0.799405	0.425133	0.625656	-6.61084
AC097059.2	1.468195	-0.79387	0.798339	0.42575	0.626206	-5.16099
AL138689.1	0.658552	6.641806	0.798148	0.42586	0.626246	-6.34864
AL162411.1	-1.22195	2.315753	-0.79799	0.425951	0.626256	-5.52488
AL035078.1	-0.73845	2.715563	-0.79748	0.426246	0.626567	-5.52184
AL357507.1	1.410505	-0.97262	0.797024	0.426511	0.626743	-5.15993
AC003035.1	-1.00412	2.284234	-0.79684	0.426615	0.626743	-5.48979
Z69666.1	-0.17648	6.997171	-0.79694	0.426562	0.626743	-6.60913
BRWD1-AS2	0.338136	6.254969	0.7967	0.426699	0.626743	-6.3638
ST20-AS1	-0.16264	7.439343	-0.79567	0.427298	0.627499	-6.69707
AC244517.1	0.649791	4.344557	0.795047	0.427658	0.627782	-5.68574
AC080037.1	0.744305	6.956343	0.795086	0.427635	0.627782	-6.37432
Z97633.1	0.180522	5.55896	0.794878	0.427755	0.627804	-6.10661
AC092168.2	-0.37567	6.990421	-0.7938	0.42838	0.628597	-6.65282
AC016252.1	-0.72703	5.368469	-0.7935	0.428558	0.628735	-6.22938
AC017104.1	0.260371	6.09115	0.793253	0.428699	0.62882	-6.31748
AC011676.2	1.064922	3.44632	0.793049	0.428818	0.628871	-5.38585
SNCA-AS1	1.130689	-2.2364	0.79255	0.429107	0.628927	-5.14621
AC011363.1	-1.10333	1.791385	-0.79259	0.429084	0.628927	-5.44202
AC024361.1	-0.2524	6.837022	-0.79266	0.429042	0.628927	-6.59556
AL136090.1	1.502056	-0.91255	0.792021	0.429415	0.628931	-5.16226
AL139339.2	1.001278	2.793167	0.792141	0.429345	0.628931	-5.35353
AP001318.1	-0.63571	3.928136	-0.79222	0.429297	0.628931	-5.87234
AC008555.7	0.247371	7.674121	0.791826	0.429528	0.628931	-6.67784
AC009237.15	-0.53443	7.440026	-0.79191	0.429481	0.628931	-6.7653
LINC02617	1.446042	-0.22874	0.791178	0.429906	0.629238	-5.18356
AC068234.2	-0.35456	5.660932	-0.79128	0.429847	0.629238	-6.32333
AC073592.6	1.414835	1.165538	0.790565	0.430262	0.629637	-5.22647
C9orf163	-0.22744	5.690087	-0.78999	0.430597	0.630006	-6.29478
AC127070.1	-0.75585	4.084575	-0.78967	0.430782	0.630154	-5.86556
JMJD1C-AS1	0.249184	6.873142	0.789326	0.430984	0.630325	-6.51068
AC009656.1	1.388354	-1.18668	0.787501	0.432048	0.631759	-5.16085
AC080078.2	1.394261	0.187005	0.78683	0.43244	0.632086	-5.19864
AC084757.3	-0.46912	3.056139	-0.78692	0.432389	0.632086	-5.5871
AC108058.1	0.342237	7.360258	0.786406	0.432687	0.632325	-6.61186
DGCR11	0.196474	7.980729	0.786158	0.432832	0.632413	-6.75016
AL391095.1	0.386433	5.854611	0.784473	0.433818	0.633606	-6.16505
AL512625.1	-0.21101	6.201637	-0.78457	0.433764	0.633606	-6.4579
AC008403.4	1.302899	2.399158	0.783358	0.43447	0.634261	-5.29576
AC098936.1	-0.82498	1.139872	-0.78327	0.434519	0.634261	-5.34161
C1orf220	0.251929	6.287874	0.783408	0.43444	0.634261	-6.35477
AL163192.1	-0.72307	3.686246	-0.78212	0.435193	0.635121	-5.76376
AC092338.2	0.471285	6.94245	0.781972	0.435282	0.635128	-6.40738
AL445437.1	-0.81873	2.487562	-0.78158	0.43551	0.635337	-5.52776
AC004233.1	1.282583	2.536354	0.781192	0.435739	0.635549	-5.30812
AL590438.1	-0.67041	3.622293	-0.7802	0.436321	0.636274	-5.69592
IRAIN	1.492036	2.815451	0.77935	0.43682	0.63667	-5.31079
AP005432.1	-0.21626	5.016569	-0.7793	0.436848	0.63667	-6.04549
AC006277.1	0.159897	6.335075	0.77958	0.436686	0.63667	-6.37823
AC115102.1	0.223088	7.036947	0.778859	0.437109	0.636928	-6.54529
AC104521.1	1.402608	2.915243	0.778543	0.437294	0.637075	-5.33339
AC008752.2	1.122991	1.84398	0.778073	0.437571	0.63723	-5.27789
LINC01144	0.254947	6.318102	0.77809	0.437561	0.63723	-6.3581
RARA-AS1	0.218784	8.277876	0.777854	0.4377	0.637295	-6.80079
AC009229.4	-1.12717	-1.63806	-0.77659	0.438443	0.638253	-5.21622

AC003101.2	0.794599	5.158576	0.776042	0.438766	0.638599	-5.80662
AL445307.1	-1.14974	0.377543	-0.77567	0.438986	0.638672	-5.31127
PITPNA-AS1	0.239762	9.222023	0.775735	0.438947	0.638672	-6.94356
AL008721.1	1.529515	-0.74791	0.774711	0.43955	0.638901	-5.17855
AC104458.1	-1.29061	-1.64358	-0.77511	0.439315	0.638901	-5.22009
AC007663.2	0.985047	1.945607	0.774535	0.439654	0.638901	-5.29382
LINC02265	-1.05196	0.765389	-0.77476	0.439519	0.638901	-5.33394
AL109806.1	0.579841	4.351554	0.774639	0.439593	0.638901	-5.62387
AC245884.1	-0.65125	3.838624	-0.77487	0.439455	0.638901	-5.84986
AC108693.2	-0.4452	6.488832	-0.77431	0.439789	0.638974	-6.56694
AC016405.2	1.299287	4.440051	0.772898	0.44062	0.640057	-5.52445
AC104134.1	0.620864	3.24713	0.772584	0.440805	0.640203	-5.4423
AL157813.1	0.683838	5.400909	0.772009	0.441145	0.640458	-5.88842
LINC01770	0.306541	7.850874	0.771998	0.441151	0.640458	-6.73293
AC138207.2	0.335329	6.445705	0.77152	0.441434	0.640744	-6.39186
Z98200.1	0.253018	6.10045	0.770166	0.442234	0.641782	-6.27901
AC135584.1	1.439326	1.984321	0.769285	0.442756	0.642415	-5.28398
AC000036.1	1.088512	-0.68023	0.768806	0.443039	0.642702	-5.19191
HSD17B3-AS1	1.183512	1.521282	0.76861	0.443155	0.642746	-5.26549
AC004522.2	-1.23793	-0.89008	-0.76809	0.443461	0.643061	-5.25012
AL606491.1	0.437463	6.3714	0.767954	0.443544	0.643061	-6.36663
AL021707.2	0.182131	7.476726	0.767502	0.443812	0.643326	-6.67728
AC093627.3	1.385587	-1.0321	0.766974	0.444125	0.643407	-5.17645
AC025262.1	1.244492	0.940957	0.767026	0.444094	0.643407	-5.24141
AC069234.5	-0.35844	6.785012	-0.76717	0.444006	0.643407	-6.63576
AL353801.2	-1.11468	-1.81222	-0.76653	0.444387	0.64353	-5.21622
AC079316.2	-0.49275	4.025329	-0.76646	0.444427	0.64353	-5.85251
AC027020.2	-0.24729	7.632152	-0.7664	0.444466	0.64353	-6.76951
LINC02422	1.234976	1.692585	0.766176	0.444598	0.643596	-5.27119
LINC02148	1.212611	-0.54487	0.765242	0.445153	0.644027	-5.19474
AL022313.2	1.279398	3.179242	0.765349	0.445089	0.644027	-5.3707
AC108516.2	1.125438	4.488946	0.765332	0.445099	0.644027	-5.57361
AC010894.4	-1.10983	1.303253	-0.76498	0.445306	0.644124	-5.39819
AP002770.1	0.970235	3.580492	0.764708	0.44547	0.644162	-5.46412
AC138932.2	-0.85177	2.179605	-0.76465	0.445503	0.644162	-5.50329
AC005746.2	1.014717	3.685032	0.764111	0.445825	0.644503	-5.4609
AL049833.3	1.028767	-1.48605	0.763538	0.446165	0.644747	-5.17593
AF127577.5	1.298502	1.131233	0.763676	0.446083	0.644747	-5.24999
FGF12-AS3	1.493571	0.237794	0.763152	0.446394	0.644954	-5.21252
AL807752.4	1.31698	1.277005	0.762583	0.446733	0.645195	-5.25512
AC135048.3	0.814347	5.068171	0.762723	0.44665	0.645195	-5.80693
AC091132.1	-0.44146	4.142309	-0.76161	0.447315	0.645416	-5.93648
AL356512.1	0.187112	6.078429	0.761762	0.447222	0.645416	-6.36312
AC009275.1	0.478606	6.659208	0.761719	0.447247	0.645416	-6.41406
AC027644.3	0.2116	7.934024	0.761881	0.447151	0.645416	-6.75606
AP001372.2	0.232206	9.130156	0.761856	0.447166	0.645416	-6.9389
AC099494.2	0.383879	6.338479	0.761448	0.447409	0.645427	-6.37053
AP001267.2	0.777444	1.933297	0.760947	0.447707	0.645609	-5.30924
AC009053.2	-0.22233	4.471361	-0.76104	0.44765	0.645609	-5.89657
DISC1-IT1	1.099778	1.874798	0.759845	0.448364	0.646158	-5.29296
AC002347.2	1.41719	3.450661	0.759443	0.448604	0.646158	-5.3944
AGBL5-AS1	0.912237	4.144754	0.759692	0.448455	0.646158	-5.54535
AC010889.1	1.159826	4.093399	0.759553	0.448538	0.646158	-5.55582
AL121992.3	0.454982	6.583678	0.759787	0.448399	0.646158	-6.39875
AC092119.2	-0.1862	6.645156	-0.75955	0.448539	0.646158	-6.55963
AC010731.2	1.666224	2.88485	0.758474	0.449182	0.646809	-5.35219
AC134349.1	0.907381	4.739971	0.758398	0.449228	0.646809	-5.62757
AC034102.3	-0.98763	2.001965	-0.75792	0.449511	0.647093	-5.53677

AC023796.2	-1.27472	-0.18789	-0.75748	0.449775	0.647349	-5.2915
AC009464.1	-0.79913	2.927896	-0.75636	0.450442	0.648185	-5.61841
BACH1-IT2	0.176774	6.002111	0.755804	0.450777	0.648543	-6.32939
AL031770.1	0.305859	4.856487	0.755551	0.450928	0.648636	-5.92206
AD000671.3	0.289048	3.777072	0.754506	0.451554	0.649334	-5.63384
EP300-AS1	-0.1688	8.050042	-0.75445	0.451587	0.649334	-6.83806
AC003965.1	0.951097	4.193653	0.753654	0.452064	0.649889	-5.5385
ACOXL-AS1	-0.36703	3.18019	-0.7535	0.452155	0.649889	-5.61336
AC011815.1	-0.19503	6.803313	-0.75337	0.452232	0.649889	-6.60315
AL357992.1	-0.87225	4.411157	-0.75258	0.452707	0.650213	-6.07238
AC073869.3	-0.41747	4.782943	-0.75256	0.452717	0.650213	-6.11842
AL080250.1	-0.24826	5.801393	-0.75266	0.452661	0.650213	-6.35256
AC078777.1	0.296509	6.393413	0.751814	0.453167	0.650735	-6.426
Z95114.3	-0.37964	4.27704	-0.75139	0.45342	0.650848	-5.91571
AC135050.4	0.534641	5.419236	0.751449	0.453386	0.650848	-5.98248
AL355490.2	0.596891	4.995428	0.750683	0.453846	0.651336	-5.8003
RORB-AS1	-1.14511	-1.55963	-0.74987	0.454336	0.651859	-5.23517
Z98885.2	-0.91916	0.752296	-0.7497	0.454434	0.651859	-5.33474
AC079174.2	-0.16484	5.856507	-0.74964	0.454471	0.651859	-6.35619
LINC02310	1.541145	-0.2903	0.749436	0.454595	0.651914	-5.20354
AL031123.3	0.319087	6.621145	0.749237	0.454715	0.651961	-6.43287
AC009955.3	1.305484	1.92553	0.74858	0.45511	0.652311	-5.29121
AC003991.2	0.393488	5.284898	0.748542	0.455133	0.652311	-6.02517
ETV5-AS1	0.979417	4.610752	0.747709	0.455634	0.652746	-5.63999
AC010655.2	0.419829	5.253442	0.747459	0.455784	0.652746	-6.09202
AL136038.5	0.21695	5.864697	0.747532	0.45574	0.652746	-6.25609
HCG15	0.284368	6.078388	0.747649	0.45567	0.652746	-6.31016
AC104365.1	0.669016	4.350001	0.74713	0.455982	0.652906	-5.67974
LINC00846	1.001657	0.427191	0.746448	0.456393	0.653369	-5.2416
AL512506.1	0.795904	5.256985	0.746099	0.456603	0.653545	-5.87216
AC018445.3	-0.84527	0.996706	-0.7459	0.456722	0.653591	-5.37393
PICSAR	1.538059	4.501684	0.744324	0.457674	0.654487	-5.54315
KRT7-AS	0.924972	6.354781	0.744286	0.457697	0.654487	-6.24257
AC004263.1	-0.20824	5.633877	-0.74439	0.457633	0.654487	-6.30169
AL049780.1	0.17037	6.746093	0.744295	0.457691	0.654487	-6.54997
AC079610.1	1.049339	-0.26266	0.743853	0.457958	0.654514	-5.22063
LINC01509	-1.21474	-0.81594	-0.74399	0.457875	0.654514	-5.26899
AL160408.1	1.083384	2.897641	0.743347	0.458263	0.654514	-5.39116
NUCB1-AS1	-0.40643	5.492569	-0.7436	0.458112	0.654514	-6.33756
AC024361.3	0.318227	6.657028	0.743567	0.45813	0.654514	-6.47747
AC078962.1	-0.33417	7.501509	-0.74338	0.458243	0.654514	-6.77298
AC080013.6	0.196926	9.382882	0.743243	0.458326	0.654514	-6.99792
AC001226.1	0.937217	3.093289	0.742946	0.458505	0.654522	-5.40851
AC093151.2	-0.59613	4.657182	-0.743	0.458475	0.654522	-6.17091
AL391863.2	1.072473	2.512077	0.741984	0.459086	0.65519	-5.34723
LINC02006	0.829652	4.617425	0.741883	0.459147	0.65519	-5.71694
AC147651.2	1.191434	2.408864	0.741737	0.459236	0.655192	-5.33467
AC008534.1	1.104112	-0.99285	0.740855	0.459769	0.655828	-5.19971
BARX1-DT	-0.9619	1.688692	-0.7403	0.460106	0.656183	-5.42512
AL355338.1	-0.24182	10.30482	-0.74005	0.460254	0.65627	-7.19174
AC008761.2	-0.87503	2.398526	-0.73986	0.460372	0.656315	-5.57282
AC092171.2	0.200761	10.16172	0.739543	0.460563	0.656462	-7.11972
AL354989.1	0.187573	6.506053	0.738962	0.460915	0.656838	-6.46218
AL031055.1	0.25822	5.797527	0.736934	0.462144	0.658465	-6.26274
AL139385.1	0.369763	6.705297	0.736689	0.462293	0.658552	-6.50904
AC002064.1	-0.97765	1.832953	-0.73607	0.46267	0.65884	-5.4552
AC005280.1	-0.99306	2.071872	-0.73607	0.462669	0.65884	-5.50218
AC005674.1	1.170411	3.303916	0.735789	0.462839	0.658956	-5.42771

AL513548.1	1.110238	-1.21803	0.735171	0.463214	0.65932	-5.19994
AC109449.1	0.293806	5.422607	0.734934	0.463358	0.65932	-6.15076
AC012170.2	0.147659	7.861632	0.735055	0.463284	0.65932	-6.77803
FLNC-AS1	1.346552	-0.6075	0.734506	0.463618	0.659531	-5.21165
AL607028.1	1.064662	4.069274	0.734242	0.463778	0.659531	-5.52848
AL513185.1	1.546622	4.78637	0.734112	0.463857	0.659531	-5.55084
AL139407.1	-0.15494	7.274315	-0.73438	0.463694	0.659531	-6.72417
AL117328.2	0.677367	1.810998	0.733789	0.464054	0.659592	-5.33302
SMC2-AS1	0.447716	2.386704	0.733609	0.464164	0.659592	-5.3921
BACH1-IT3	0.748681	4.50057	0.733658	0.464134	0.659592	-5.7871
AC116914.2	0.148607	8.349788	0.733428	0.464273	0.659623	-6.8482
AC107021.2	0.307607	5.682581	0.73323	0.464394	0.659669	-6.22609
AC107294.1	-1.07058	1.183864	-0.73253	0.464822	0.659853	-5.41931
AL162231.4	-0.43248	2.543412	-0.73262	0.464766	0.659853	-5.5409
AC110619.1	0.456433	5.33759	0.73244	0.464875	0.659853	-6.13763
AL139124.1	-0.4002	5.411076	-0.73245	0.46487	0.659853	-6.28264
AC106037.2	-0.21776	8.442962	-0.73172	0.465311	0.660348	-6.91296
AC007608.3	0.475588	2.819543	0.731559	0.465411	0.660365	-5.45745
AL121583.1	-0.22725	6.288	-0.7312	0.465632	0.660554	-6.52298
AC016590.3	0.348692	6.256393	0.730767	0.465894	0.6608	-6.35533
AL133492.1	-0.70065	1.154295	-0.73028	0.466188	0.661093	-5.36881
ZNF649-AS1	-0.82968	3.00374	-0.73004	0.466336	0.661172	-5.68382
AL359183.1	-0.21945	6.284849	-0.72976	0.466508	0.661172	-6.47793
AC016888.1	0.321128	10.8958	0.729898	0.466423	0.661172	-7.21149
AC004223.4	0.445831	6.416272	0.72893	0.467014	0.661764	-6.42773
AC104532.2	-0.24333	6.200303	-0.72767	0.467785	0.662608	-6.50362
Z82243.1	0.186228	8.907357	0.727785	0.467713	0.662608	-6.94265
CHODL-AS1	1.304068	-1.49166	0.727396	0.46795	0.662641	-5.19443
FP671120.4	-0.49349	8.655704	-0.72734	0.467985	0.662641	-7.00009
AC113615.1	-0.84796	-0.96485	-0.72672	0.468362	0.662871	-5.26078
AC007923.4	1.263049	1.068129	0.726626	0.468421	0.662871	-5.27291
AC009884.1	-0.92693	2.246449	-0.7265	0.4685	0.662871	-5.51855
AC126544.1	-0.52304	6.31614	-0.72651	0.468494	0.662871	-6.58422
RUVBL1-AS1	0.64443	4.233211	0.724971	0.469433	0.664065	-5.66048
AC007114.1	-0.21818	8.336532	-0.72422	0.469891	0.664588	-6.90138
AL592164.1	-0.929	0.267761	-0.72405	0.469995	0.66461	-5.31886
AC099667.1	0.869467	4.677833	0.723074	0.470595	0.664634	-5.67027
AL583785.1	0.925294	4.91554	0.723063	0.470601	0.664634	-5.76351
AC020763.3	0.19805	5.089295	0.723014	0.470631	0.664634	-6.02744
AC010519.1	0.393028	6.51291	0.723855	0.470116	0.664634	-6.42017
AC073575.2	0.176056	6.972606	0.723426	0.470379	0.664634	-6.59548
AL451042.1	0.290827	8.066941	0.723071	0.470597	0.664634	-6.79631
AC005046.1	0.227854	8.537093	0.723606	0.470268	0.664634	-6.88215
AC011481.2	0.676464	5.558977	0.722777	0.470777	0.664715	-6.00955
AC008759.2	0.915968	3.970377	0.722491	0.470952	0.664837	-5.58383
AC021237.1	0.340734	3.805815	0.721809	0.47137	0.665302	-5.63103
HTT-AS	-1.17485	-1.17068	-0.72098	0.47188	0.665394	-5.27172
AC091806.1	0.614742	2.964499	0.721454	0.471588	0.665394	-5.44767
BX284668.6	1.000421	3.765848	0.721014	0.471858	0.665394	-5.5223
AC020658.6	0.361539	5.235352	0.720989	0.471873	0.665394	-6.02086
AC004223.2	0.469534	5.322678	0.720836	0.471967	0.665394	-6.06481
AC007383.2	-0.12806	8.545229	-0.72107	0.471826	0.665394	-6.93356
AC027601.3	0.5741	5.713657	0.720013	0.472472	0.665982	-6.18635
AL360091.2	1.095862	2.293699	0.71969	0.472671	0.666131	-5.34621
AC007336.3	0.820254	2.495271	0.71951	0.472782	0.666131	-5.37518
Z97192.1	-0.68059	1.807143	-0.71939	0.472855	0.666131	-5.45187
AC008750.4	0.643729	5.809666	0.719263	0.472933	0.666131	-6.19504
AP005131.5	-0.57334	6.24424	-0.71865	0.473307	0.666533	-6.50093

AC107068.1	-0.14661	7.735304	-0.7182	0.473589	0.666806	-6.80901
AL024474.2	1.15923	1.583624	0.717516	0.474007	0.66727	-5.29858
AC018682.2	-0.98369	1.494261	-0.71723	0.474184	0.667393	-5.42768
ATG10-IT1	1.214325	-0.00613	0.715626	0.475171	0.668656	-5.23978
SMARCA5-AS1	0.74666	3.147834	0.71433	0.47597	0.66953	-5.45258
AC022819.1	0.184033	5.004573	0.714393	0.475931	0.66953	-6.00417
AC120042.1	1.250542	-1.35343	0.713788	0.476304	0.66965	-5.20515
AC009563.1	0.768518	3.774046	0.713759	0.476322	0.66965	-5.54262
AC090579.1	-0.19748	7.118361	-0.71389	0.476238	0.66965	-6.71553
AP000266.1	0.753855	4.391897	0.713448	0.476514	0.669785	-5.686
AC004067.1	0.169194	7.412035	0.713313	0.476597	0.669785	-6.70577
AC064852.1	0.950624	2.215492	0.712764	0.476936	0.670137	-5.3414
AL662907.2	0.141922	5.594359	0.712522	0.477085	0.670221	-6.22328
AC008870.3	0.774612	4.879771	0.712126	0.47733	0.670439	-5.76753
AC002091.2	1.034246	5.559759	0.711801	0.477531	0.670596	-5.8535
LINC01567	0.974368	-0.01084	0.711117	0.477953	0.67087	-5.24917
LRRC3-DT	-0.99993	0.657376	-0.71082	0.478138	0.67087	-5.35677
RNU6ATAC35P	-0.95981	2.24853	-0.71132	0.477826	0.67087	-5.55115
AC108449.1	-0.67582	4.165748	-0.71076	0.478173	0.67087	-5.91567
AL731563.3	-0.14001	6.236814	-0.71085	0.478119	0.67087	-6.48342
AL158839.1	0.890706	3.905439	0.710224	0.478505	0.671212	-5.53806
AC090337.1	0.89908	1.049657	0.709693	0.478834	0.671422	-5.28904
AC096540.1	0.200132	5.983564	0.709825	0.478752	0.671422	-6.36509
AC068722.2	0.730885	4.45556	0.709213	0.479131	0.671442	-5.69697
AL121790.1	-0.57274	3.870054	-0.70917	0.479156	0.671442	-5.92109
AC138207.7	0.23898	6.017531	0.709142	0.479175	0.671442	-6.33222
FLJ45513	0.231598	6.515803	0.709092	0.479206	0.671442	-6.47935
AC022382.1	-0.50586	4.81825	-0.70794	0.479919	0.672317	-6.18919
AP002892.1	1.244474	-1.07187	0.707538	0.480169	0.672415	-5.21474
AL024497.1	1.29561	-1.00833	0.707546	0.480163	0.672415	-5.21591
AL355810.1	-1.1698	-1.32781	-0.70706	0.480467	0.672708	-5.2688
EGFR-AS1	0.362029	5.790419	0.706454	0.480841	0.673105	-6.24187
AL450344.2	-0.40968	5.544088	-0.70621	0.480989	0.673188	-6.38742
AC010422.4	-0.24431	6.11804	-0.70602	0.481108	0.673228	-6.47032
SCAT8	-0.60115	4.690042	-0.70433	0.482156	0.674443	-6.12845
AL356488.3	0.182092	6.685429	0.704341	0.482152	0.674443	-6.52735
AC087521.1	-0.46443	3.376485	-0.70385	0.482458	0.67474	-5.7647
CLDN10-AS1	1.310491	0.411674	0.703374	0.482753	0.674902	-5.26094
AC116535.1	0.272929	5.321166	0.703456	0.482701	0.674902	-6.08723
AC006033.2	0.455077	4.485401	0.702883	0.483058	0.675037	-5.76962
AC079416.2	-0.29223	4.445347	-0.70278	0.483119	0.675037	-5.97755
AC008543.3	-0.41017	5.929409	-0.703	0.482983	0.675037	-6.50153
MANEA-DT	0.172072	6.259875	0.701401	0.483979	0.675999	-6.44652
AC120114.3	0.174816	8.378467	0.701387	0.483988	0.675999	-6.87863
HLA-DQB1-AS1	0.516419	7.853111	0.700759	0.484379	0.67642	-6.72116
AL121852.1	0.803983	4.215961	0.70059	0.484484	0.676441	-5.63063
LINC01269	0.743183	7.132163	0.700347	0.484636	0.676459	-6.53153
AL354696.1	-0.26805	7.656242	-0.70028	0.484677	0.676459	-6.82353
AC007496.2	-0.17782	5.98969	-0.69996	0.484879	0.676615	-6.44486
AC234781.1	0.80394	2.174359	0.699793	0.48498	0.676631	-5.36785
AL049796.1	0.215851	5.378525	0.699313	0.48528	0.676881	-6.18797
MBNL1-AS1	-0.27476	7.176025	-0.69922	0.485339	0.676881	-6.7577
LINC02446	-0.6442	4.982554	-0.69848	0.485799	0.677396	-6.19052
AC091117.2	0.803191	4.042382	0.697644	0.486321	0.677745	-5.5702
AP000692.2	0.263832	6.80916	0.697658	0.486312	0.677745	-6.58569
AL162377.1	0.173135	7.580529	0.697752	0.486254	0.677745	-6.75183
AC007163.1	0.863364	2.787082	0.697368	0.486493	0.67786	-5.41556
AC012370.1	0.515668	5.05264	0.696354	0.487126	0.678239	-5.95797

Z98885.3	0.176958	5.514688	0.696489	0.487042	0.678239	-6.224
AC008750.5	-0.19514	7.446191	-0.69649	0.487041	0.678239	-6.77683
AC010503.4	-0.23973	10.93475	-0.69672	0.486896	0.678239	-7.30905
AC105020.1	-0.1798	6.863206	-0.69597	0.487364	0.678445	-6.67391
AL391262.1	1.212635	-0.73998	0.695443	0.487695	0.678683	-5.23062
AL590666.1	-0.34602	6.792579	-0.69541	0.487716	0.678683	-6.68776
AL136531.1	0.181041	7.863214	0.695074	0.487926	0.678849	-6.79857
SEMA6A-AS2	-0.9786	1.868894	-0.69491	0.488026	0.678863	-5.49388
AC067852.3	-0.16003	7.337039	-0.69461	0.488216	0.679002	-6.76095
AC004969.1	-1.06922	0.013556	-0.69378	0.488738	0.679602	-5.33119
AC079209.2	1.218135	1.374776	0.693499	0.488911	0.679642	-5.30246
LINC01942	-1.17131	-0.21446	-0.6933	0.489038	0.679642	-5.32826
AC002550.2	0.155102	8.051183	0.693377	0.488987	0.679642	-6.83866
AC104561.1	-0.61708	4.003033	-0.69294	0.489264	0.67983	-5.87549
AC005697.2	-0.63207	1.838114	-0.69255	0.489505	0.680038	-5.45892
AL513327.3	-0.36442	5.641511	-0.69176	0.49	0.680601	-6.40946
AC069277.1	0.859403	4.131452	0.69063	0.490708	0.68121	-5.64265
AC008758.2	-0.67504	3.135641	-0.69063	0.490711	0.68121	-5.72228
AGPAT4-IT1	0.266869	6.704927	0.690854	0.490568	0.68121	-6.50777
AL357873.1	-1.13438	0.622193	-0.69023	0.490962	0.681432	-5.41296
AC072039.2	0.970343	3.863376	0.689146	0.491639	0.682088	-5.54375
AFDN-DT	0.293481	6.686226	0.689039	0.491706	0.682088	-6.54258
AC131159.2	-0.13517	6.341296	-0.68906	0.491695	0.682088	-6.54772
AC018648.1	0.369209	5.634582	0.688772	0.491874	0.682194	-6.2171
AC092376.1	-1.20117	-1.50156	-0.68746	0.492696	0.683082	-5.27497
LINC02091	-0.78517	2.2717	-0.68755	0.492638	0.683082	-5.56299
AC022431.1	-1.09187	-0.40414	-0.68672	0.493163	0.683465	-5.31535
AC136469.2	0.457869	4.305921	0.686457	0.493329	0.683465	-5.75175
AL050343.1	-0.13744	6.659505	-0.68645	0.493335	0.683465	-6.62979
AC104447.1	-0.17071	8.40613	-0.68676	0.493139	0.683465	-6.9361
AL032819.1	1.119493	2.878689	0.685673	0.493822	0.683773	-5.40541
AP001439.1	0.945726	3.750275	0.685709	0.493799	0.683773	-5.53394
AC003973.2	-0.33665	4.332348	-0.68551	0.493922	0.683773	-6.03025
AC009686.2	-0.28571	7.605051	-0.6856	0.493868	0.683773	-6.81594
AC106786.2	-0.35082	5.809129	-0.68536	0.494016	0.683777	-6.43435
Z69720.1	0.828151	3.651168	0.684858	0.494335	0.684092	-5.55107
TSPAN9-IT1	1.189455	-0.33588	0.684338	0.494662	0.684233	-5.24906
AL451069.3	0.970817	1.44775	0.683684	0.495074	0.684233	-5.32399
AC023301.1	-1.03279	0.945091	-0.68388	0.494948	0.684233	-5.40442
LIVAR	1.287303	4.796964	0.683825	0.494985	0.684233	-5.64387
AC121247.1	-0.23683	5.036366	-0.68418	0.494765	0.684233	-6.12863
AC010754.1	-0.43046	4.813747	-0.68427	0.494707	0.684233	-6.13456
UBXN10-AS1	-0.61603	4.829418	-0.68375	0.495032	0.684233	-6.31257
AL844908.2	-0.35443	5.911611	-0.68342	0.495243	0.684341	-6.46861
AL133284.1	0.868585	0.302445	0.683143	0.495415	0.684453	-5.27959
AC010343.3	-1.02847	2.601413	-0.68249	0.495825	0.684641	-5.63326
AL121989.1	0.246713	5.827214	0.682587	0.495766	0.684641	-6.27947
AP001574.1	-0.56895	5.200496	-0.6826	0.495758	0.684641	-6.35793
AP001783.1	1.358801	0.9594	0.681676	0.49634	0.684913	-5.28945
AC079340.2	-1.07183	-0.85744	-0.68187	0.496218	0.684913	-5.29527
AC096564.2	-0.6202	1.91407	-0.68159	0.496392	0.684913	-5.46375
ITIH4-AS1	-0.95357	1.533524	-0.68154	0.496429	0.684913	-5.48335
AC004540.2	0.29709	5.370261	0.681458	0.496477	0.684913	-6.10588
LINC01840	-1.0706	0.245541	-0.68123	0.496624	0.684989	-5.3596
AC092384.1	-0.58148	2.517333	-0.68101	0.496763	0.685055	-5.59297
LINC01149	0.930767	-1.42003	0.680496	0.497085	0.685374	-5.22975
AL139260.2	0.732103	0.941021	0.680351	0.497177	0.685374	-5.31072
AC012170.3	0.612704	6.179436	0.680017	0.497388	0.685539	-6.19602

AC100827.4	0.466343	3.435522	0.679283	0.497851	0.686052	-5.55932
AL139246.4	1.240098	3.816126	0.679088	0.497974	0.686096	-5.53689
AC013549.2	1.215434	0.113093	0.678899	0.498094	0.686135	-5.26667
AC020558.1	1.005315	3.856139	0.677741	0.498826	0.686766	-5.57862
AC012186.3	-0.42923	4.917617	-0.67793	0.498704	0.686766	-6.19754
AC106028.3	-0.15308	6.806917	-0.67786	0.498752	0.686766	-6.65482
AC090589.2	0.752273	5.061645	0.677086	0.499241	0.68721	-5.83966
MORF4L2-AS1	-0.17859	5.927581	-0.67667	0.499501	0.687443	-6.43559
LINC01191	0.915534	3.005465	0.676297	0.49974	0.687646	-5.43718
AC243562.3	-0.74524	2.063304	-0.67587	0.50001	0.687891	-5.49623
AC078852.2	-0.78443	2.736276	-0.67566	0.500142	0.687947	-5.66314
AC009159.2	-0.9564	1.77113	-0.67528	0.500386	0.688121	-5.49486
AC068594.1	0.292135	6.000788	0.675174	0.500452	0.688121	-6.30824
AP000356.1	-0.89817	2.314786	-0.6749	0.500622	0.68823	-5.5647
LINC02365	-0.89638	2.44525	-0.67474	0.500727	0.688248	-5.59109
AC037198.1	0.318184	6.915022	0.674318	0.500994	0.688489	-6.62725
AC109446.3	-0.54143	4.189529	-0.6739	0.50126	0.688602	-6.00493
AC021739.4	-0.29232	5.459773	-0.67391	0.501252	0.688602	-6.31884
LINC01684	0.601008	1.791056	0.673657	0.501413	0.688648	-5.36953
AC084032.1	0.941456	3.386688	0.673218	0.501692	0.688648	-5.50753
LINC01979	-0.47368	4.363244	-0.67305	0.501798	0.688648	-5.99754
AC135050.1	-0.12744	4.966624	-0.67304	0.501802	0.688648	-6.08669
AC091132.2	0.226103	6.421309	0.67298	0.501843	0.688648	-6.50267
AC006077.2	-0.14257	7.546257	-0.67322	0.501689	0.688648	-6.80443
AC092354.1	0.901002	3.592499	0.6719	0.502529	0.688953	-5.54724
AC139100.1	0.475006	5.001079	0.671763	0.502616	0.688953	-6.02826
AC132219.2	-0.17819	6.467243	-0.6718	0.50259	0.688953	-6.58506
AP4B1-AS1	-0.18347	6.747895	-0.67222	0.502327	0.688953	-6.64874
AC245884.8	-0.23168	7.299549	-0.67211	0.502394	0.688953	-6.7747
AC011451.1	0.238081	8.142524	0.672393	0.502215	0.688953	-6.85876
AL139286.2	0.328869	6.709477	0.671498	0.502784	0.689058	-6.5378
AC012676.5	-0.14453	8.147554	-0.67115	0.503005	0.689235	-6.90506
AC138028.3	1.322191	-0.71881	0.670638	0.50333	0.689555	-5.245
AC084782.1	0.849462	-0.20526	0.67021	0.503603	0.689585	-5.27232
AC020658.5	0.987582	3.258106	0.670294	0.503549	0.689585	-5.47998
AC008569.2	-0.30748	6.072292	-0.67013	0.503655	0.689585	-6.52823
AP000240.1	0.161408	8.797721	0.669882	0.503811	0.689585	-6.9685
AC024896.1	-0.1849	9.977777	-0.66994	0.503777	0.689585	-7.18444
AP003419.2	-0.19426	7.06708	-0.6695	0.504056	0.689669	-6.73459
AC020978.3	-0.12658	7.323792	-0.66953	0.504038	0.689669	-6.76679
AC104066.2	-0.89587	-0.81362	-0.66915	0.504278	0.68984	-5.30297
AL158070.2	-0.89782	-0.79702	-0.66901	0.504365	0.68984	-5.30482
AC083843.1	1.185822	0.464555	0.668399	0.504755	0.690248	-5.28448
AC104024.1	-0.6054	1.483193	-0.66636	0.506053	0.691581	-5.44545
AC006058.4	-0.77978	2.226239	-0.66629	0.506098	0.691581	-5.58266
AC005072.1	-0.17524	6.993779	-0.66638	0.50604	0.691581	-6.70475
LINC00921	-0.16236	7.129951	-0.66631	0.506087	0.691581	-6.74169
AC097652.1	1.083816	-1.42433	0.665905	0.506344	0.691789	-5.23344
LINC02253	1.679866	5.048005	0.665764	0.506434	0.691789	-5.6602
AC006947.1	0.775092	4.795123	0.665241	0.506768	0.692119	-5.90336
AC092384.3	0.869976	-0.07268	0.664329	0.507349	0.692418	-5.2765
AC026358.1	-0.89595	-0.44155	-0.66432	0.507355	0.692418	-5.31837
AP003774.1	0.656355	2.129876	0.664399	0.507305	0.692418	-5.39252
AL355001.2	0.191742	8.760897	0.664412	0.507297	0.692418	-6.97093
LINC01864	0.974663	-0.16992	0.663901	0.507623	0.692657	-5.27144
AC135782.3	-0.19234	4.021834	-0.66304	0.508169	0.693277	-5.84106
AC015883.1	0.228123	7.170599	0.662875	0.508278	0.693299	-6.67358
AC013472.3	-0.19652	5.384587	-0.66246	0.508542	0.693533	-6.29839

AC084082.1	1.066169	-1.75695	0.661532	0.509137	0.693667	-5.22943
LINC00165	1.247628	1.567065	0.66159	0.5091	0.693667	-5.32803
AL022393.1	-0.32599	4.69101	-0.66187	0.508922	0.693667	-6.11224
AC011330.2	0.202709	6.58389	0.661749	0.508998	0.693667	-6.51901
FAR1-IT1	-0.39005	6.364831	-0.66139	0.50923	0.693667	-6.52221
AC133550.2	0.142952	7.131673	0.661298	0.509286	0.693667	-6.68742
ANKRD10-IT1	0.19561	10.78441	0.661988	0.508845	0.693667	-7.26212
AL133243.1	0.759241	4.684939	0.660264	0.509948	0.694442	-5.81809
AL355102.5	0.674372	5.446431	0.659004	0.510754	0.695037	-6.09346
KLF7-IT1	-0.46826	5.233983	-0.65915	0.510662	0.695037	-6.32815
AL022311.1	-0.09024	5.9955	-0.65905	0.510723	0.695037	-6.45055
AC009309.1	-0.36401	7.134722	-0.65934	0.510539	0.695037	-6.76486
AC018442.2	0.76448	3.789595	0.658728	0.510932	0.695152	-5.5649
AC120024.1	0.658283	3.083335	0.658218	0.511259	0.695295	-5.50631
AL451062.1	0.827368	4.152617	0.657884	0.511473	0.695295	-5.61472
LINC00612	-0.46062	3.323686	-0.65809	0.511343	0.695295	-5.71024
AC092119.3	0.601625	4.56111	0.657842	0.511499	0.695295	-5.80752
AC069307.1	0.20818	7.163056	0.658295	0.511209	0.695295	-6.6747
AL359538.1	1.015275	-1.19343	0.656657	0.51226	0.695813	-5.24591
LINC02457	1.498593	0.656126	0.656526	0.512343	0.695813	-5.28685
AC006600.2	-0.88759	-0.10635	-0.65669	0.512238	0.695813	-5.3375
LMF1-AS1	0.483935	3.46022	0.657062	0.512	0.695813	-5.60042
AL354993.1	-0.26052	5.850625	-0.65653	0.512343	0.695813	-6.45696
AC099343.3	0.153284	8.490433	0.656358	0.512451	0.695834	-6.92833
LINC01555	1.006583	-0.37304	0.656141	0.51259	0.695896	-5.26797
MAGEA8-AS1	1.167919	0.465355	0.655524	0.512987	0.696309	-5.29219
LINC01792	0.985945	-0.58717	0.654942	0.51336	0.696566	-5.2647
AL591178.1	-0.90403	2.343642	-0.65494	0.513362	0.696566	-5.64642
AL365181.1	0.930027	-0.73248	0.653834	0.514072	0.696729	-5.26121
ALG9-IT1	-0.90153	0.937531	-0.65441	0.5137	0.696729	-5.4112
AC099811.6	-0.26278	4.615928	-0.65374	0.514131	0.696729	-6.0738
AC007731.3	-0.35688	5.139713	-0.65376	0.51412	0.696729	-6.21521
AL596442.2	-0.23323	5.515927	-0.65433	0.513756	0.696729	-6.35823
LINC02019	-0.16205	5.987857	-0.65426	0.513795	0.696729	-6.48449
AC090970.1	0.184036	8.121345	0.653775	0.51411	0.696729	-6.85867
AOAH-IT1	1.188868	-1.47083	0.65331	0.514409	0.696892	-5.23709
AC079834.2	0.628753	4.517073	0.653267	0.514437	0.696892	-5.83853
AC090826.1	-0.71124	0.651504	-0.65307	0.514566	0.696941	-5.38244
AL121772.3	0.240066	9.261278	0.652825	0.514721	0.697026	-7.04105
HDAC4-AS1	-0.19662	6.721705	-0.65258	0.514881	0.697117	-6.67183
AC078942.1	-1.10266	0.507048	-0.65214	0.515161	0.697344	-5.38591
AC010973.2	-0.1543	7.654604	-0.65203	0.515234	0.697344	-6.83783
AC090044.1	-0.97885	1.564113	-0.65158	0.515522	0.697607	-5.50172
AL359643.2	0.216017	4.837097	0.651343	0.515675	0.697689	-6.02169
AC244197.2	0.412774	6.544869	0.650983	0.515907	0.697877	-6.4915
AC009034.1	-0.329	4.656425	-0.6508	0.516025	0.69788	-6.11697
LIMS1-AS1	-0.15684	7.665251	-0.65069	0.516095	0.69788	-6.84615
AC124254.1	1.331894	-1.02952	0.649903	0.516602	0.698301	-5.24699
AC100847.1	-0.34448	5.532856	-0.64967	0.516754	0.698301	-6.37594
AC106028.2	-0.30856	6.561687	-0.64974	0.516709	0.698301	-6.66869
AC080013.4	0.174101	8.614093	0.649631	0.516778	0.698301	-6.95204
AL135925.1	0.143607	7.887984	0.649419	0.516915	0.69836	-6.83729
LINC00485	-0.88854	-1.63057	-0.64888	0.51726	0.69845	-5.28527
LINC02084	0.634537	4.578443	0.648897	0.517251	0.69845	-5.75599
AC011479.2	0.294453	6.557151	0.649129	0.517101	0.69845	-6.54122
AC004973.1	1.07159	-0.44775	0.648622	0.517428	0.698551	-5.26843
ESRG	1.166215	3.047537	0.647847	0.517929	0.699101	-5.43826
AC022274.1	0.821672	4.057359	0.646755	0.518633	0.699927	-5.6452

AC010809.3	-0.61814	3.341576	-0.64646	0.518826	0.700061	-5.76527
AL359922.2	0.451287	6.121473	0.64606	0.519082	0.700281	-6.39016
AC147651.1	0.331026	5.668907	0.645235	0.519615	0.70075	-6.28686
AC027228.1	-0.35656	5.5865	-0.64523	0.519617	0.70075	-6.42833
AL031651.2	0.794938	4.797967	0.644598	0.520027	0.701178	-5.81816
AC089999.2	-0.18638	6.649123	-0.64393	0.52046	0.701635	-6.65635
AC002472.1	1.138242	-1.89467	0.643237	0.520908	0.701988	-5.23548
AL162595.1	0.205472	7.243471	0.643303	0.520865	0.701988	-6.70533
AL032821.1	1.139091	0.047342	0.642924	0.52111	0.702009	-5.28652
AL133553.1	-1.02797	0.873679	-0.64293	0.521107	0.702009	-5.41648
LINC01843	-0.65526	5.560613	-0.64242	0.521436	0.702321	-6.41718
AC009133.3	-0.57174	2.863642	-0.64218	0.521592	0.702378	-5.63045
AC087392.1	0.281736	6.782678	0.642068	0.521665	0.702378	-6.57713
AC004492.1	-0.1861	8.002725	-0.64176	0.521867	0.702524	-6.91035
AC125616.1	-0.87499	2.389087	-0.64046	0.522708	0.70353	-5.53561
AC010501.1	0.594441	3.644487	0.639392	0.523399	0.704335	-5.59001
AC048382.2	-0.17917	6.719755	-0.6392	0.523522	0.704374	-6.68254
AC128709.1	1.143327	3.648905	0.639032	0.523633	0.704396	-5.51514
AC010333.1	0.873478	2.756859	0.638019	0.524291	0.705034	-5.46608
AC087392.3	0.151442	5.986769	0.638014	0.524294	0.705034	-6.36487
AC112250.2	-0.91832	1.697307	-0.63775	0.524468	0.705141	-5.49257
AL021154.1	0.547517	5.690425	0.637553	0.524593	0.705183	-6.08391
AC002056.2	-1.10518	-0.23071	-0.63715	0.524857	0.705206	-5.35023
AC024940.1	0.248889	5.361598	0.637093	0.524892	0.705206	-6.14655
AC027277.2	-0.22545	7.109112	-0.6372	0.524826	0.705206	-6.76651
AC100860.1	1.160406	-0.60128	0.636219	0.52546	0.705465	-5.26798
LINC02413	-0.87944	0.658161	-0.63627	0.525427	0.705465	-5.4012
AC245140.2	0.128532	7.346906	0.636358	0.52537	0.705465	-6.75143
AL512274.1	-0.34748	10.04101	-0.63636	0.525366	0.705465	-7.24882
AL359198.1	0.828904	4.150808	0.636039	0.525577	0.705496	-5.66579
AC015853.2	1.167188	-0.22897	0.634349	0.526677	0.705518	-5.2822
AC004012.1	1.191837	0.169181	0.634163	0.526798	0.705518	-5.29156
AC022733.2	1.202356	0.292432	0.635843	0.525705	0.705518	-5.29523
AC093010.2	-0.78333	-0.61051	-0.63468	0.526465	0.705518	-5.3253
AP001619.2	0.738958	3.500988	0.634138	0.526815	0.705518	-5.54615
AL355472.2	-0.85597	3.693092	-0.63455	0.526544	0.705518	-5.86523
AC078909.2	-0.51435	4.650247	-0.63457	0.526533	0.705518	-6.02234
GAU1	-0.49899	4.935195	-0.63491	0.526312	0.705518	-6.23391
DENND6A-AS1	-0.32445	5.510628	-0.63479	0.52639	0.705518	-6.41013
AC107214.2	-0.17856	5.884578	-0.63571	0.525792	0.705518	-6.45848
AC253536.6	-0.18856	6.554759	-0.6348	0.526386	0.705518	-6.62506
AP001273.1	0.132401	6.882964	0.634568	0.526534	0.705518	-6.65734
AP001432.1	0.165833	7.303567	0.634907	0.526314	0.705518	-6.74211
AC006480.2	-0.15506	7.022488	-0.63396	0.526927	0.705544	-6.73936
AC008440.1	-0.54221	5.073268	-0.63353	0.527211	0.705797	-6.24255
LINC01786	-0.24888	5.687051	-0.63192	0.528261	0.707077	-6.44525
AC008663.3	-1.05449	-0.58537	-0.63061	0.529111	0.707333	-5.33391
AL359881.2	-0.944	1.052196	-0.63082	0.528979	0.707333	-5.44775
ZFX-AS1	0.584145	4.916546	0.631415	0.528589	0.707333	-5.8899
AL512306.3	-0.64732	4.213547	-0.63078	0.529004	0.707333	-5.94968
AC008507.2	-0.32718	4.426388	-0.63106	0.528823	0.707333	-6.03357
AL662844.3	0.445068	5.892365	0.630614	0.529111	0.707333	-6.16376
DHRS4-AS1	-0.12952	10.69485	-0.63112	0.528784	0.707333	-7.31301
AC090001.1	-0.9903	-0.4048	-0.63044	0.529226	0.707361	-5.34211
AC093117.1	0.930449	-2.22779	0.629779	0.529656	0.707749	-5.24041
LINC02582	1.151073	2.857264	0.629705	0.529705	0.707749	-5.44593
AC096711.2	1.181229	-1.36718	0.629331	0.529949	0.707949	-5.25211
AC012100.2	-0.34928	5.394417	-0.62916	0.530058	0.70797	-6.36434

LINC01030	-1.01336	-1.14015	-0.62857	0.530445	0.70836	-5.31472
AL031728.1	0.15578	5.827092	0.627464	0.531169	0.7092	-6.33982
AL450311.1	1.172422	-1.45167	0.626606	0.531729	0.709463	-5.25186
AL355297.2	1.316237	-0.13626	0.626586	0.531743	0.709463	-5.28262
AC104779.1	1.077116	3.754256	0.626892	0.531542	0.709463	-5.58415
AC022364.1	-0.22367	8.01128	-0.62683	0.53158	0.709463	-6.9204
AC021054.1	0.186291	8.880488	0.626399	0.531865	0.709499	-7.00408
AL137026.1	0.985553	-0.74795	0.62581	0.532251	0.709888	-5.27375
INE1	0.12013	8.597254	0.625508	0.532448	0.710025	-6.97268
CTBP1-DT	0.140118	9.200796	0.625122	0.532701	0.710236	-7.06155
LINC00621	1.016371	1.551977	0.623891	0.533507	0.711059	-5.35927
LINC00443	-0.97412	1.375769	-0.62392	0.533491	0.711059	-5.49476
AL136097.2	-0.99462	0.881946	-0.62317	0.533983	0.711566	-5.43413
KIAA0087	-0.79861	-1.27745	-0.62302	0.53408	0.71157	-5.30717
AL035409.1	0.993181	3.053682	0.622346	0.53452	0.711988	-5.46928
AC016747.1	0.305972	8.14178	0.622249	0.534583	0.711988	-6.86693
AP001207.3	1.27592	5.236685	0.620518	0.535719	0.713375	-5.77454
AL365258.1	-1.01127	1.124513	-0.61968	0.536272	0.713985	-5.44469
AC084782.2	-0.62975	3.015115	-0.61834	0.537151	0.714521	-5.71071
AL118511.1	0.630981	4.34242	0.618388	0.537119	0.714521	-5.73944
AC117402.1	0.485752	5.88713	0.61839	0.537118	0.714521	-6.24862
AL732292.2	0.381995	6.103735	0.618532	0.537025	0.714521	-6.34818
AC093620.1	0.171384	7.14881	0.618591	0.536986	0.714521	-6.71753
AC084757.4	1.025413	1.189273	0.617695	0.537575	0.714579	-5.33882
LINC01562	-0.22116	3.327917	-0.61791	0.537435	0.714579	-5.69798
AC005014.2	0.296337	6.50424	0.617711	0.537564	0.714579	-6.37837
AL359921.1	0.217249	7.654073	0.617727	0.537554	0.714579	-6.8151
AC090617.1	1.080633	1.464491	0.616974	0.53805	0.715083	-5.35765
AC004672.1	0.502063	3.280803	0.616612	0.538287	0.715273	-5.57385
AC116348.1	0.608633	4.557436	0.614989	0.539356	0.716567	-5.81104
AC007494.3	-0.32948	4.828572	-0.61474	0.539523	0.716661	-6.18664
AL589986.2	1.225688	3.278984	0.613966	0.540031	0.717209	-5.51541
Z83847.1	0.492862	3.159044	0.613642	0.540244	0.717239	-5.57381
AC087276.1	-0.22593	7.945417	-0.61369	0.540215	0.717239	-6.9259
AC027348.1	0.906539	2.737266	0.6134	0.540404	0.717285	-5.45574
AL590006.1	-0.19357	6.980842	-0.6133	0.54047	0.717285	-6.74019
AC016866.1	-0.17511	6.675356	-0.61274	0.540843	0.717653	-6.6691
AC024267.5	0.563823	5.767424	0.61212	0.541248	0.718064	-6.11906
AP002439.1	-1.02061	-0.70813	-0.61153	0.54164	0.718204	-5.33997
AL162734.1	0.761775	6.156231	0.611796	0.541463	0.718204	-6.15126
AC131971.1	-0.16522	6.442101	-0.61157	0.541611	0.718204	-6.6191
AC079766.1	-0.48399	4.956218	-0.61131	0.541785	0.718269	-6.16417
AC016027.2	0.230039	4.942925	0.60938	0.543058	0.71983	-6.05568
AC116312.1	1.229654	0.333055	0.608608	0.543569	0.720253	-5.31138
AC007001.1	1.073123	0.787134	0.608672	0.543527	0.720253	-5.33157
ZNF346-IT1	-0.12537	6.943475	-0.60842	0.543696	0.720295	-6.72561
AP000943.4	-0.64779	3.863631	-0.6074	0.544368	0.72094	-6.03067
N4BP2L2-IT2	-0.12259	7.512927	-0.60739	0.544376	0.72094	-6.84194
AC090739.1	0.180278	8.525621	0.607216	0.54449	0.720965	-6.96013
AC131212.1	1.049044	-0.17079	0.606719	0.544819	0.721059	-5.29844
AC079906.1	-0.79632	1.566927	-0.60664	0.544869	0.721059	-5.49375
AC046130.2	0.711829	3.256831	0.606107	0.545225	0.721059	-5.5355
AC004925.1	-0.49285	2.514607	-0.6065	0.544967	0.721059	-5.61847
AC012568.1	0.654319	5.010429	0.606095	0.545233	0.721059	-5.85966
AC008083.2	0.514125	5.958197	0.606218	0.545152	0.721059	-6.28923
CACTIN-AS1	0.125632	6.020354	0.606247	0.545132	0.721059	-6.45502
AP001605.1	-0.81895	-2.40358	-0.60562	0.545546	0.721346	-5.28228
AC007556.1	1.199844	-0.63434	0.605244	0.545797	0.72155	-5.28027

AP003419.3	0.241849	6.042912	0.604625	0.546207	0.721966	-6.41889
AL031651.1	0.581404	2.967279	0.604343	0.546394	0.722042	-5.50252
AL023583.1	0.604605	3.690197	0.604249	0.546457	0.722042	-5.64869
AC090627.1	0.785745	1.780728	0.604027	0.546604	0.722109	-5.39695
LINC01291	0.731793	7.102885	0.602949	0.547319	0.7228	-6.518
AC020913.3	0.168592	8.299287	0.602986	0.547295	0.7228	-6.92716
PPP4R1-AS1	-0.42564	6.016499	-0.60199	0.547954	0.723511	-6.59393
AC073439.1	-0.15506	4.951024	-0.60157	0.548236	0.723629	-6.18318
AL445231.1	0.17039	6.493973	0.601622	0.5482	0.723629	-6.54857
AP006287.2	-0.58381	3.61148	-0.60117	0.5485	0.72385	-5.8069
AL078601.2	-1.03207	-0.85758	-0.6009	0.548677	0.723956	-5.34196
PINK1-AS	-0.12331	8.506049	-0.60074	0.548784	0.72397	-7.00298
Z99129.3	-0.23228	3.492982	-0.60019	0.549153	0.72433	-5.74058
AC009269.4	0.856563	2.804631	0.599773	0.549429	0.724567	-5.4623
AC005845.1	0.941965	1.06763	0.599145	0.549847	0.724991	-5.35623
AC069542.1	0.393221	4.981842	0.598947	0.549979	0.725038	-6.05466
AC091100.1	-0.50893	2.175603	-0.59878	0.550091	0.725059	-5.53819
AL583810.2	0.468608	3.619671	0.598566	0.550232	0.725118	-5.63561
ZFHX4-AS1	0.975241	0.555964	0.597305	0.551072	0.726096	-5.32978
AC010524.1	-0.71317	3.266006	-0.5966	0.551539	0.726585	-5.72673
AC093462.1	0.581339	2.495373	0.595499	0.552275	0.727427	-5.47824
AL031710.1	-0.22422	5.87726	-0.59512	0.552529	0.727634	-6.49685
AC003070.2	1.079543	0.564241	0.594833	0.552719	0.727635	-5.32763
BX539320.1	0.42263	6.328692	0.594826	0.552724	0.727635	-6.28631
AL354950.1	1.057151	-0.18243	0.593969	0.553296	0.72826	-5.30876
AC004775.1	0.935364	0.413045	0.593154	0.55384	0.728722	-5.32888
AP000350.5	0.754551	3.729647	0.593233	0.553787	0.728722	-5.64525
BNC2-AS1	1.003461	3.037177	0.592518	0.554264	0.728885	-5.48736
LINC02158	0.514612	3.975526	0.592425	0.554326	0.728885	-5.73191
AL034346.1	0.322728	5.131077	0.59248	0.55429	0.728885	-6.18726
AC024933.1	-0.28996	6.475535	-0.59239	0.554352	0.728885	-6.65648
AL357054.3	0.777479	0.222768	0.591557	0.554907	0.729415	-5.32806
AL672291.1	0.966982	1.920704	0.59149	0.554952	0.729415	-5.40497
AL139349.1	-0.16947	7.404807	-0.59135	0.555047	0.729415	-6.83201
LINC02506	1.189623	-0.24384	0.590387	0.555689	0.730131	-5.30058
AC090772.3	0.374262	6.525667	0.588299	0.557086	0.731839	-6.49333
AL445363.3	0.209205	4.65306	0.587838	0.557394	0.732116	-5.99117
AL672032.1	0.951452	-0.95532	0.587517	0.55761	0.732271	-5.28862
AC113146.1	0.885235	-0.51708	0.586866	0.558046	0.732583	-5.30463
AC015967.2	-0.97278	1.256398	-0.58673	0.55814	0.732583	-5.48126
AC006159.1	-0.6491	4.445871	-0.58687	0.558044	0.732583	-6.10872
AC006357.1	1.248984	-0.86306	0.5861	0.55856	0.733006	-5.28523
AC073389.3	0.170731	6.743349	0.585478	0.558976	0.733425	-6.6454
AC015922.3	0.267221	9.666639	0.584997	0.559299	0.73372	-7.13876
AL356055.1	0.70675	4.13182	0.584257	0.559796	0.734203	-5.77099
AC009054.1	-0.26562	5.6389	-0.58416	0.559863	0.734203	-6.40884
AL133485.1	-1.00086	-0.83148	-0.58295	0.560672	0.734781	-5.34951
AC005336.3	-0.81283	-0.35618	-0.58292	0.560695	0.734781	-5.36323
AL158151.2	1.077894	1.700268	0.583161	0.560532	0.734781	-5.37971
AC007029.1	-0.83519	1.967815	-0.58298	0.560652	0.734781	-5.54802
LINC02245	0.541136	2.098637	0.582002	0.56131	0.735331	-5.44126
AC133065.3	-0.20092	7.859002	-0.58201	0.561304	0.735331	-6.91545
AC010333.2	-0.47786	3.540939	-0.58167	0.561531	0.735364	-5.83021
LINC01589	-0.29912	7.177382	-0.58175	0.561482	0.735364	-6.82063
AC012123.1	-0.87932	-1.48842	-0.58115	0.56188	0.735692	-5.32367
AL158042.1	1.095395	-1.12015	0.580762	0.562144	0.735755	-5.28471
LINC01970	0.26865	4.504174	0.580647	0.562221	0.735755	-5.90923
AC007382.1	0.124284	8.326596	0.580813	0.56211	0.735755	-6.95351

AP002360.1	-0.16569	9.475719	-0.58036	0.562414	0.735879	-7.16418
LINC02012	0.305284	6.513144	0.579745	0.562828	0.736292	-6.5359
AC090617.9	0.633064	6.088882	0.57911	0.563256	0.736583	-6.36282
NOP53-AS1	-0.16576	6.552323	-0.57898	0.563344	0.736583	-6.66974
AC127024.6	0.13295	7.420903	0.579242	0.563167	0.736583	-6.79232
AL158166.2	0.49554	5.923594	0.578568	0.563621	0.736816	-6.26745
AL583808.1	1.497972	2.740503	0.577292	0.56448	0.737591	-5.41885
AC004160.2	-0.9069	0.636377	-0.5771	0.564606	0.737591	-5.42326
AC020611.2	0.684105	3.574698	0.577536	0.564316	0.737591	-5.63216
EHD4-AS1	-0.3929	4.69553	-0.57722	0.564529	0.737591	-6.11814
AC010273.1	-0.19518	5.652793	-0.57691	0.564738	0.737636	-6.40389
AL391987.4	-0.93025	0.527318	-0.57671	0.564869	0.737679	-5.41881
AC103982.1	1.008946	-1.50991	0.576203	0.565214	0.737891	-5.27809
AL356475.1	1.082351	0.202401	0.576182	0.565229	0.737891	-5.32428
AC093426.1	-0.81219	-1.82729	-0.57554	0.565661	0.738118	-5.31152
LINC02507	0.89739	0.491284	0.575487	0.565698	0.738118	-5.34226
AP003396.3	-0.86643	-0.27089	-0.57559	0.565626	0.738118	-5.37542
AC068790.2	-0.13558	7.062605	-0.57527	0.565844	0.738181	-6.77237
AC131902.2	0.921535	-1.1947	0.574939	0.566067	0.738216	-5.28915
AC245014.3	0.286224	5.860284	0.574966	0.566049	0.738216	-6.37862
LINC01450	0.829509	-1.87069	0.574686	0.566238	0.73831	-5.27412
AL136320.1	-0.97401	-0.44743	-0.57408	0.566646	0.73833	-5.3667
AC100835.1	-0.91303	0.215893	-0.57421	0.566561	0.73833	-5.40548
AC093525.6	0.648352	2.912598	0.574356	0.566461	0.73833	-5.51869
AL355916.2	-0.2565	5.992914	-0.57449	0.566367	0.73833	-6.58238
LINC02062	0.228693	6.062771	0.573414	0.567097	0.738789	-6.30333
AC068620.2	0.214171	6.691823	0.572903	0.567442	0.738983	-6.63278
AP003392.3	0.178439	7.191005	0.573023	0.567361	0.738983	-6.74087
AC156455.1	0.304755	5.986562	0.57262	0.567633	0.739103	-6.41993
AP005205.3	-0.96537	-0.60977	-0.57185	0.568152	0.739288	-5.36145
TRIM31-AS1	0.887689	6.689874	0.572229	0.567897	0.739288	-6.29331
ITGA6-AS1	-0.17261	6.591864	-0.57183	0.568168	0.739288	-6.67218
SPAG5-AS1	0.106306	7.453783	0.572108	0.567979	0.739288	-6.79824
AL121983.1	0.904326	2.293361	0.571514	0.568381	0.739436	-5.44178
AC016597.2	-0.9348	0.238577	-0.57108	0.568673	0.739688	-5.40066
DLGAP1-AS3	0.918946	-1.1267	0.570451	0.5691	0.740115	-5.29188
AC005034.4	-0.54186	3.471995	-0.56985	0.569508	0.740286	-5.82454
AC084859.1	0.477446	7.02235	0.569826	0.569523	0.740286	-6.58936
AC010531.6	-0.16397	7.039813	-0.56982	0.569526	0.740286	-6.77279
Z92544.1	0.744545	1.972855	0.568286	0.570565	0.741252	-5.43149
NARF-AS1	-0.17666	5.765857	-0.56837	0.570507	0.741252	-6.45296
AL021707.1	-0.13293	6.284916	-0.56833	0.570538	0.741252	-6.59997
AC044839.1	0.832704	3.445796	0.567681	0.570976	0.741563	-5.56337
AC004494.1	-0.17116	5.165386	-0.56764	0.571003	0.741563	-6.23561
AC134043.2	0.199376	5.313744	0.567123	0.571354	0.741891	-6.20226
AL033384.2	0.16302	7.637191	0.566784	0.571583	0.74206	-6.84429
AL359399.1	1.006039	1.134077	0.566535	0.571752	0.74212	-5.36267
C8orf17	-0.434	2.31373	-0.56643	0.571826	0.74212	-5.58587
AC092598.1	0.973824	-1.16759	0.565687	0.572327	0.742528	-5.29135
ERVH-1	0.950352	0.868805	0.565638	0.57236	0.742528	-5.35957
AL355490.1	0.264456	5.610859	0.565524	0.572438	0.742528	-6.24578
LINC00315	0.467949	2.407035	0.565373	0.57254	0.742533	-5.49038
AC116158.1	0.18542	5.136398	0.564665	0.573021	0.743028	-6.14495
AL645929.2	-0.56404	2.844707	-0.56365	0.573711	0.743701	-5.67359
AC098820.3	-0.559	3.065165	-0.56361	0.573738	0.743701	-5.71555
AP001107.8	0.596457	4.169912	0.563189	0.574023	0.743815	-5.7684
AC008115.3	0.135781	8.099472	0.563299	0.573948	0.743815	-6.92459
AC011005.4	0.931605	4.470431	0.562828	0.574268	0.744004	-5.74188

AL162414.1	0.981528	-1.74177	0.561721	0.575021	0.744851	-5.27974
AC246787.2	1.088858	-0.71521	0.561185	0.575385	0.744881	-5.30423
AL109811.1	0.131674	5.272013	0.561129	0.575424	0.744881	-6.18742
AL355102.1	-0.36606	5.325139	-0.56143	0.57522	0.744881	-6.44194
AC093909.6	-0.19384	8.903573	-0.5611	0.575441	0.744881	-7.09309
AC069294.1	-0.95699	-1.33973	-0.56088	0.575592	0.744948	-5.34503
WARS2-IT1	-0.2008	4.430281	-0.56007	0.576142	0.745532	-6.03546
C1QTNF1-AS1	-0.48725	4.197645	-0.55902	0.576859	0.746331	-6.04986
AC009152.1	-0.56771	3.647453	-0.55821	0.577408	0.746913	-5.93213
AC120349.3	0.381315	5.107771	0.558017	0.577542	0.746957	-6.02024
AC015813.5	0.124701	7.485187	0.557683	0.57777	0.746994	-6.81972
AL731566.1	-0.10725	7.76201	-0.55781	0.577681	0.746994	-6.90636
AC017006.1	1.003534	1.182951	0.557409	0.577956	0.747107	-5.38161
C9orf170	0.765371	-0.36666	0.557201	0.578098	0.747162	-5.32758
AC104248.1	0.823927	2.046218	0.557036	0.578211	0.747179	-5.42885
AP000721.2	-0.6322	2.023854	-0.55681	0.578367	0.747252	-5.55606
AC019130.1	0.992607	-0.06023	0.556415	0.578634	0.747258	-5.32938
AL133481.1	-0.92522	-1.61781	-0.55652	0.578565	0.747258	-5.33536
AL122058.1	-0.68794	3.405196	-0.55622	0.578769	0.747258	-5.81314
AL049870.3	-0.28212	6.362982	-0.55624	0.578753	0.747258	-6.68109
DPYD-IT1	0.965313	0.818354	0.556021	0.578903	0.747302	-5.36184
AC025580.3	-0.51429	2.392181	-0.55552	0.579244	0.747305	-5.60642
AL450226.2	-0.50102	3.906738	-0.5558	0.579053	0.747305	-5.89038
AC068620.1	0.157776	6.5252	0.555492	0.579264	0.747305	-6.59361
AC025265.1	0.209339	6.59334	0.555434	0.579303	0.747305	-6.60317
AC022098.4	0.816203	3.071811	0.55406	0.580241	0.748387	-5.5419
AC114550.1	0.934051	-1.4863	0.553607	0.58055	0.74856	-5.28992
AP000911.2	0.809265	0.8741	0.553571	0.580575	0.74856	-5.37042
GIHCG	0.231337	7.810175	0.55325	0.580795	0.748617	-6.86353
AL031714.1	0.089256	8.015598	0.553215	0.580818	0.748617	-6.92782
AC126564.1	0.793676	0.657973	0.552322	0.581429	0.749275	-5.36184
AL445183.2	0.815518	0.766525	0.551868	0.581739	0.749546	-5.36848
AC087241.3	0.851269	0.030482	0.551511	0.581983	0.749733	-5.33624
AL390760.1	1.007171	0.132301	0.551222	0.582181	0.749855	-5.33543
AC005828.4	-0.26706	5.408394	-0.55108	0.582278	0.749855	-6.4059
AC104794.2	0.160275	8.757067	0.55075	0.582504	0.750017	-7.03306
AIRN	-0.23596	2.424096	-0.54958	0.583307	0.750923	-5.57285
MACC1-AS1	0.952054	2.286607	0.549264	0.583521	0.750941	-5.45593
AC012613.2	-0.52383	1.472554	-0.54939	0.583432	0.750941	-5.49881
MAPT-IT1	-0.72208	-2.15576	-0.54837	0.584132	0.751273	-5.31315
AC007786.2	0.94976	-0.30593	0.548179	0.584264	0.751273	-5.32535
AC007786.1	-0.51463	3.307042	-0.54865	0.583942	0.751273	-5.75308
AL360181.1	1.102852	4.968453	0.548157	0.584279	0.751273	-5.81422
AC098613.1	0.503772	6.842803	0.548324	0.584164	0.751273	-6.57525
Z93241.1	0.290574	4.735442	0.547259	0.584894	0.751834	-5.98247
AL627171.1	-0.1198	6.623432	-0.54723	0.584916	0.751834	-6.69115
AL390318.1	0.9105	-2.04744	0.546606	0.585342	0.752216	-5.28291
AL139246.1	0.949238	3.713264	0.546502	0.585413	0.752216	-5.63989
ZKSCAN2-DT	0.111161	7.057568	0.546328	0.585532	0.752241	-6.74624
AL512306.2	-0.28719	5.677411	-0.54556	0.586058	0.752788	-6.48727
AC110285.2	-0.27913	6.871101	-0.54519	0.58631	0.752982	-6.76914
AC108451.2	0.987588	4.833703	0.544302	0.586923	0.753512	-5.74073
AC099689.1	-0.19525	5.495791	-0.54433	0.586907	0.753512	-6.40824
AC092171.1	-0.11915	6.371676	-0.54388	0.587211	0.753624	-6.63542
AC018521.5	-0.09969	7.209684	-0.54401	0.587127	0.753624	-6.81536
IER3-AS1	0.190911	6.871288	0.543383	0.587554	0.753936	-6.68593
SEMA5A-AS1	-0.82274	-1.96134	-0.54323	0.587656	0.753938	-5.32795
AC007370.1	1.009972	-0.96226	0.542982	0.587829	0.754014	-5.3064

WWTR1-IT1	-0.289	6.160116	-0.54286	0.587916	0.754014	-6.62059
AL359878.2	-0.57909	4.429278	-0.54256	0.588119	0.754145	-6.01738
AC023794.5	0.720266	2.473116	0.542366	0.588253	0.754188	-5.49512
AL359881.3	0.850121	-1.10978	0.542094	0.58844	0.754199	-5.30722
AC092295.2	-0.16746	7.170284	-0.54206	0.588462	0.754199	-6.82706
AC132068.1	0.609368	3.874526	0.541712	0.588702	0.754378	-5.75279
AP000808.2	0.31986	6.747758	0.541193	0.589059	0.754707	-6.62559
AP001266.2	-0.25859	6.405437	-0.54072	0.589384	0.754995	-6.69346
AC099796.1	1.083386	-0.01784	0.540545	0.589505	0.75502	-5.33583
AC127496.7	-0.14495	7.516559	-0.53993	0.589926	0.755431	-6.88574
AC087241.4	0.726078	3.87269	0.539644	0.590125	0.755557	-5.66259
AL121929.2	-0.19003	6.618455	-0.53883	0.590688	0.756149	-6.7127
AL451069.1	-0.90959	-1.41782	-0.53855	0.590875	0.756167	-5.35002
AL008726.1	0.146956	7.229033	0.538514	0.590903	0.756167	-6.78561
AP003027.1	-0.7563	-2.77923	-0.53804	0.591228	0.756186	-5.30057
AL133351.1	0.957884	0.590504	0.538092	0.591194	0.756186	-5.36063
AC124319.5	-0.14234	5.053035	-0.53799	0.591261	0.756186	-6.20982
AP006623.1	-0.20412	5.589241	-0.53791	0.591321	0.756186	-6.4339
AC009312.1	-0.92421	-1.03109	-0.53721	0.591801	0.756507	-5.3615
LINC02649	-0.16445	5.178918	-0.53672	0.592141	0.756507	-6.28393
AL031005.1	-0.31168	5.108555	-0.53667	0.592176	0.756507	-6.29369
GAS6-AS1	0.24138	6.660167	0.536925	0.591998	0.756507	-6.64356
AC141002.1	-0.29016	6.610236	-0.53705	0.591913	0.756507	-6.74123
AC091185.1	0.150727	7.374147	0.536848	0.592051	0.756507	-6.81242
AC137770.1	-0.69718	2.157959	-0.53644	0.59233	0.756575	-5.58878
AC098484.2	-0.27259	5.63163	-0.53527	0.593141	0.757353	-6.48591
TAF1A-AS1	0.155799	7.448115	0.535406	0.593045	0.757353	-6.8088
AL355432.1	0.923343	-0.53072	0.534296	0.593811	0.757822	-5.3276
LINC00028	0.920433	-0.28219	0.534004	0.594012	0.757822	-5.33279
AC109809.1	0.893292	1.091204	0.534142	0.593918	0.757822	-5.38467
AC096541.1	-0.62639	1.288429	-0.53454	0.593645	0.757822	-5.48888
AC007620.3	0.379476	5.603784	0.534116	0.593936	0.757822	-6.17004
AC010894.5	0.905862	-0.64561	0.533003	0.594704	0.758535	-5.32302
AC021549.1	0.859591	3.218127	0.532903	0.594773	0.758535	-5.59475
LINC00622	0.295023	6.112657	0.532223	0.595243	0.759005	-6.4189
BX324167.2	-0.32051	3.004467	-0.53169	0.595609	0.759343	-5.69628
AC012379.1	0.953593	-1.49842	0.53144	0.595784	0.759438	-5.30035
AP002892.2	0.918792	-0.41457	0.531192	0.595956	0.759528	-5.33035
SIDT1-AS1	0.899495	2.474554	0.530425	0.596486	0.75963	-5.47415
LEMD1-AS1	0.543002	3.33074	0.530278	0.596588	0.75963	-5.60007
U91328.3	-0.35129	4.615243	-0.53067	0.596318	0.75963	-6.20696
AC004112.1	-0.19323	5.623705	-0.53068	0.596307	0.75963	-6.43999
AC004771.3	0.152295	7.51186	0.530199	0.596643	0.75963	-6.8428
AC022146.2	-0.37672	7.432497	-0.53055	0.596397	0.75963	-6.90861
AL359510.2	-0.15662	5.111558	-0.52965	0.597024	0.759986	-6.27693
LINC02470	0.941456	-1.2195	0.528307	0.597952	0.760541	-5.30868
HAR1A	-0.36069	3.988501	-0.52855	0.597782	0.760541	-6.00681
AP000919.4	-0.23539	6.180671	-0.52816	0.598057	0.760541	-6.61193
AC068338.2	0.151136	7.307482	0.528141	0.598067	0.760541	-6.79313
AC004832.5	-0.12679	7.490381	-0.52876	0.597635	0.760541	-6.878
AL354733.3	-0.11992	9.458605	-0.5284	0.59789	0.760541	-7.19072
AL353807.2	0.65326	5.013893	0.527495	0.598515	0.760853	-6.00472
LINC01836	-0.3119	6.49247	-0.5276	0.598438	0.760853	-6.6848
AL138781.2	0.480029	2.870967	0.527222	0.598703	0.760932	-5.58264
AC034229.4	-0.29422	6.018628	-0.52711	0.59878	0.760932	-6.59288
USP30-AS1	0.254595	7.73811	0.526701	0.599065	0.761166	-6.83715
AC097504.2	0.999528	0.184835	0.526332	0.59932	0.761208	-5.35023
AL358075.2	-0.62145	4.011005	-0.52621	0.599402	0.761208	-5.94042

AC025259.3	0.264619	6.531303	0.526333	0.599319	0.761208	-6.64357
AC020908.1	0.983916	-1.0524	0.525591	0.599834	0.761628	-5.31332
AL135999.1	0.092886	7.929977	0.524712	0.600444	0.762273	-6.92709
MAST4-IT1	0.91321	-1.84421	0.524008	0.600932	0.762335	-5.29491
AP000350.7	-0.17249	3.63012	-0.52391	0.601	0.762335	-5.78869
AL096701.3	0.103371	6.293395	0.523915	0.600997	0.762335	-6.55421
AC005586.1	-0.18293	8.237885	-0.52402	0.600922	0.762335	-7.01209
AC126474.2	-0.08228	9.951037	-0.52418	0.600815	0.762335	-7.2584
AC022167.3	0.471647	4.620433	0.523588	0.601224	0.762491	-5.91119
AC000123.2	0.120706	8.44552	0.523404	0.601352	0.762524	-7.00626
AC005096.1	-0.24406	5.892105	-0.52314	0.601537	0.76263	-6.5237
AL596330.1	-0.86762	-0.43714	-0.5229	0.601703	0.762712	-5.38999
AC244090.3	0.467123	4.008232	0.522231	0.602167	0.763171	-5.85467
AC090971.2	-0.13426	6.605011	-0.52088	0.603105	0.764232	-6.70694
AC005342.1	-0.83911	-2.0109	-0.51964	0.603968	0.765195	-5.33316
AC136624.1	-0.84134	-0.98109	-0.51874	0.604591	0.765856	-5.3677
AC134682.1	0.355756	3.307031	0.518358	0.60486	0.766067	-5.66566
AL731537.1	0.963301	-1.26723	0.517954	0.605141	0.766294	-5.31044
AP003555.3	0.641817	7.665654	0.517299	0.605597	0.766743	-6.69522
AC025678.3	0.256044	3.572056	0.51712	0.605722	0.766771	-5.72062
CEBPA-DT	-0.21204	8.013175	-0.51689	0.605884	0.766848	-6.98036
LINC02664	0.575811	1.934313	0.515993	0.606507	0.767507	-5.45993
WWC3-AS1	0.84033	1.160295	0.51545	0.606886	0.767857	-5.39796
ZNF252P-AS1	-0.14502	5.443388	-0.51522	0.607048	0.767933	-6.35705
STARD4-AS1	-0.14241	6.466406	-0.51429	0.607692	0.768618	-6.65796
AP000915.1	0.929987	-1.07756	0.513979	0.607912	0.768637	-5.31773
AC027682.5	0.498565	4.500083	0.514117	0.607816	0.768637	-5.88487
AC098476.1	0.916854	3.560435	0.512441	0.608985	0.769865	-5.57718
AC244100.2	0.759138	-0.67552	0.510299	0.610481	0.771541	-5.33577
AC005037.1	0.209399	5.610891	0.510249	0.610517	0.771541	-6.28633
AC008443.3	1.024273	3.219384	0.510032	0.610668	0.771603	-5.55755
CCAT2	0.469161	2.812272	0.509207	0.611245	0.772202	-5.55681
P4HA2-AS1	-0.72352	0.784371	-0.50815	0.611983	0.773004	-5.45195
AL161669.3	0.681332	5.508637	0.507901	0.612159	0.773096	-6.03733
AL078604.2	0.258719	4.647337	0.507595	0.612373	0.773237	-5.96322
AL357078.3	0.49472	4.639801	0.507245	0.612618	0.773286	-5.88519
GTSE1-DT	0.158629	6.005696	0.507358	0.612539	0.773286	-6.45881
AL159972.1	0.894747	1.16225	0.507	0.612789	0.773372	-5.39617
AC055811.1	0.16215	5.967488	0.506296	0.613282	0.773735	-6.46818
AL160006.1	-0.11269	8.789475	-0.50639	0.613218	0.773735	-7.09889
AL161785.1	-0.22682	6.141273	-0.50556	0.613797	0.774254	-6.62029
LINC02243	-0.63394	1.519297	-0.50497	0.614214	0.774397	-5.53877
AC026150.3	-0.6742	1.784649	-0.50496	0.614219	0.774397	-5.57089
AC092117.2	-0.24391	4.523555	-0.50519	0.614061	0.774397	-6.08953
AC104996.1	0.86924	-1.35934	0.503681	0.615116	0.774487	-5.31598
AL589666.2	-0.7599	0.288727	-0.50373	0.61508	0.774487	-5.43057
LINC02636	-0.83107	0.414626	-0.5047	0.614401	0.774487	-5.43859
LINC01659	-0.75842	1.510306	-0.50402	0.614875	0.774487	-5.51894
AC011471.2	-0.69107	1.768895	-0.50417	0.614775	0.774487	-5.5657
AC010643.1	0.450445	4.211446	0.50424	0.614723	0.774487	-5.80145
AC011498.6	0.136339	6.527946	0.504532	0.614519	0.774487	-6.64065
AC079414.3	-0.13994	7.325218	-0.50376	0.61506	0.774487	-6.86114
AC013437.1	0.929743	-0.24109	0.501919	0.616352	0.775252	-5.3488
AC004808.2	0.996938	0.159241	0.500898	0.617069	0.775252	-5.36064
AC131280.1	-0.67553	-1.04321	-0.5015	0.616645	0.775252	-5.36736
AC005304.2	-0.70979	-0.98118	-0.5015	0.616648	0.775252	-5.37321
AC020928.3	-0.55067	1.334541	-0.50245	0.615978	0.775252	-5.49463
AC069234.1	0.654676	2.766624	0.501208	0.616851	0.775252	-5.56287

AC036108.2	0.450416	2.785271	0.500583	0.61729	0.775252	-5.5634
AC092162.3	-0.49684	2.511178	-0.5003	0.617492	0.775252	-5.64293
TTLL10-AS1	-0.22281	3.586581	-0.50263	0.615853	0.775252	-5.81402
AC002306.1	0.445079	4.639052	0.501645	0.616544	0.775252	-5.85798
AC091132.4	0.239813	4.122474	0.500924	0.617051	0.775252	-5.90709
AC027682.6	-0.25972	4.904526	-0.50033	0.61747	0.775252	-6.11417
AC110769.3	0.140304	5.571373	0.50062	0.617264	0.775252	-6.34541
AC012676.4	-0.18024	5.479979	-0.50043	0.617394	0.775252	-6.37035
MNX1-AS1	0.675088	6.708863	0.50017	0.61758	0.775252	-6.43326
AC104785.1	0.160563	6.054245	0.501939	0.616338	0.775252	-6.50489
AC004233.3	-0.34813	7.126524	-0.50087	0.61709	0.775252	-6.85042
ZNF32-AS2	-0.12101	7.627969	-0.50236	0.616043	0.775252	-6.91955
AC100821.2	-0.18962	6.124174	-0.49975	0.617876	0.775493	-6.60259
AC090877.2	0.393315	4.796967	0.499545	0.618019	0.775544	-6.12877
AC008543.5	-0.53517	0.537636	-0.49921	0.618252	0.775705	-5.43674
AC015909.3	0.759308	-0.99285	0.498737	0.618588	0.775743	-5.33084
AL359979.1	1.030545	-0.28981	0.498732	0.618591	0.775743	-5.34709
AC090607.1	0.39144	4.875327	0.498996	0.618406	0.775743	-6.0618
AC067817.1	0.830565	2.210802	0.498184	0.618977	0.77608	-5.46981
AL445423.1	-0.7673	1.066275	-0.49793	0.619157	0.77608	-5.47889
AL158801.2	0.209794	5.568319	0.497909	0.61917	0.77608	-6.32302
RFPL1S	0.283653	2.688166	0.497411	0.61952	0.77639	-5.56904
AC009831.4	0.679351	-1.332	0.496472	0.620181	0.776978	-5.32247
AC022107.1	-0.12866	7.113805	-0.49645	0.620197	0.776978	-6.81456
LINC02066	0.923082	-1.57396	0.495875	0.620601	0.777096	-5.31306
AC009090.2	0.866122	-1.42021	0.495968	0.620536	0.777096	-5.31731
AC107079.1	0.818105	-0.83223	0.495967	0.620537	0.777096	-5.33713
GPC6-AS1	-0.80026	-0.14621	-0.49449	0.62158	0.778192	-5.40964
AC008957.3	0.39847	4.418076	0.494095	0.621855	0.778407	-5.95522
AC004771.2	-0.2551	5.017142	-0.49301	0.622623	0.778978	-6.27417
AC005224.3	-0.17794	5.64833	-0.49328	0.622432	0.778978	-6.49652
AC023632.5	-0.1185	6.833562	-0.4931	0.622556	0.778978	-6.76267
AC021087.3	0.134508	5.323092	0.49275	0.622803	0.779074	-6.29344
AC093904.4	-0.3237	5.607085	-0.49229	0.623125	0.779347	-6.49974
AC027514.1	0.248	4.023763	0.491978	0.623348	0.779497	-5.86275
AP006216.1	0.886324	-1.2399	0.491543	0.623655	0.77975	-5.32438
RASSF1-AS1	-0.13668	5.765551	-0.49112	0.623955	0.779906	-6.45745
AC138470.1	-0.13426	6.779731	-0.49107	0.623987	0.779906	-6.76396
AP001527.1	0.707012	-0.70244	0.490622	0.624305	0.780027	-5.34398
LINC01154	-0.82006	-1.47719	-0.49052	0.624374	0.780027	-5.36278
AL133406.2	-0.13438	7.203948	-0.49049	0.624396	0.780027	-6.84809
AL117341.1	-0.836	-0.44622	-0.48981	0.62488	0.780423	-5.40073
AC127496.5	-0.29823	4.650979	-0.48946	0.625128	0.780423	-6.26929
AL139021.1	-0.22361	5.545138	-0.48956	0.625052	0.780423	-6.48487
AL035252.3	-0.18112	6.926621	-0.48962	0.625012	0.780423	-6.78908
AL022323.2	0.154703	4.93429	0.488869	0.625543	0.780812	-6.10523
AC108134.4	-0.17369	6.520015	-0.48783	0.626279	0.781601	-6.70552
AC073593.1	-0.8399	0.163721	-0.4875	0.626511	0.78176	-5.43812
AL096869.2	0.365336	3.32567	0.486473	0.627238	0.782407	-5.67405
AC005520.3	0.415574	5.585527	0.486475	0.627236	0.782407	-6.18623
AC137834.2	0.642349	2.571952	0.486042	0.627542	0.782657	-5.51936
AC135178.5	0.132062	6.97671	0.485255	0.628099	0.783092	-6.75283
AC002310.6	-0.13671	7.363718	-0.48526	0.628098	0.783092	-6.8758
AF106564.1	0.845889	-1.39965	0.484351	0.628739	0.783629	-5.32312
AC091180.5	0.60522	3.638176	0.484444	0.628673	0.783629	-5.69262
AP001970.1	0.812667	-0.34397	0.48392	0.629044	0.78388	-5.35643
AC074029.4	0.717035	-2.42996	0.482308	0.630187	0.784392	-5.30581
LINC01381	-0.79851	-1.82814	-0.48242	0.630108	0.784392	-5.35268

LINC01169	0.746467	2.682624	0.482836	0.629813	0.784392	-5.51885
AC107027.1	0.686899	2.788012	0.483067	0.629648	0.784392	-5.54356
AP000654.1	0.449631	4.066805	0.482786	0.629848	0.784392	-5.73443
AP002364.1	-0.15627	4.700212	-0.48268	0.629925	0.784392	-6.17461
LINC02041	0.717784	7.941405	0.482534	0.630027	0.784392	-6.74762
AC011466.4	0.247983	4.379816	0.481888	0.630484	0.784633	-5.9283
AC067817.2	-0.15028	7.707737	-0.48174	0.630592	0.784637	-6.94584
AC063944.3	0.860432	-0.42167	0.480951	0.631149	0.78468	-5.35397
AC091153.2	0.369689	5.468889	0.481198	0.630973	0.78468	-6.16945
AC133528.1	-0.21565	6.489128	-0.48096	0.631144	0.78468	-6.69816
AC008074.2	-0.14457	7.012	-0.48121	0.630964	0.78468	-6.82207
AL031775.1	0.096795	8.321129	0.481333	0.630878	0.78468	-7.00874
AL031587.1	0.736017	0.299731	0.480512	0.63146	0.784937	-5.38224
AC007557.3	-0.51066	0.683983	-0.48017	0.631704	0.78511	-5.45535
AC245052.4	0.07621	6.4136	0.47935	0.632285	0.785703	-6.62167
AC007608.2	-0.7093	-0.05286	-0.47846	0.632916	0.786356	-5.42147
AC093297.2	0.100066	9.141756	0.478183	0.633114	0.786472	-7.13958
AC012511.1	0.766784	2.205044	0.477782	0.633399	0.786696	-5.48317
AC078852.1	0.76596	2.228139	0.477529	0.633578	0.786789	-5.48031
AC093734.1	0.907101	0.114763	0.476525	0.634291	0.787545	-5.37296
AC106820.4	-0.22781	4.240212	-0.47586	0.634762	0.787985	-5.99677
AC087163.3	0.113517	6.424148	0.475731	0.634856	0.787985	-6.62788
AL356356.1	-0.15283	7.692637	-0.47541	0.635083	0.788137	-6.95186
C2CD4D-AS1	-0.20123	9.594372	-0.47515	0.635267	0.788234	-7.24629
AL133297.2	0.367752	5.025983	0.474732	0.635567	0.788476	-6.04999
CLIP1-AS1	0.132375	5.874332	0.47409	0.636024	0.788913	-6.44774
AL121944.1	0.122146	8.521865	0.473427	0.636496	0.789368	-7.03918
AL118522.1	-0.63007	3.448966	-0.47314	0.636702	0.789493	-5.84425
AC011899.3	-0.52621	2.818555	-0.47285	0.636908	0.789619	-5.75466
AL645940.1	0.260613	5.576238	0.472189	0.637377	0.79007	-6.30447
CU639417.5	0.30514	3.57899	0.471961	0.637539	0.790141	-5.73045
AC126696.2	-0.31315	4.53929	-0.47168	0.637741	0.79026	-6.19814
AL121950.1	-0.72306	-2.34057	-0.47116	0.638108	0.790428	-5.34114
AL109659.3	-0.63818	0.775676	-0.47105	0.638192	0.790428	-5.48131
AC104463.2	0.359867	5.77835	0.471115	0.638142	0.790428	-6.273
AC024145.1	-0.17552	6.307693	-0.47025	0.63876	0.791001	-6.67606
AC137630.2	0.366843	4.445237	0.469282	0.639449	0.791724	-5.91313
AP001267.1	0.140764	3.666883	0.468213	0.640212	0.792537	-5.785
AC020907.3	0.635068	2.90416	0.467965	0.640389	0.792626	-5.56621
AL135786.2	-0.70955	0.573183	-0.46742	0.640779	0.792978	-5.45724
AC092296.1	-0.7355	0.394511	-0.46631	0.641569	0.793826	-5.44841
NFYC-AS1	-0.10516	7.248698	-0.46604	0.641764	0.793935	-6.8619
XIAP-AS1	0.285499	6.412534	0.465761	0.641963	0.794051	-6.50116
AC007278.2	0.767436	-0.99482	0.465151	0.642399	0.794325	-5.34413
LINC02308	-0.7762	-1.02713	-0.46492	0.64256	0.794325	-5.38728
AC010609.1	-0.74567	0.708728	-0.46476	0.64268	0.794325	-5.47108
AC022098.1	0.083811	7.117368	0.464711	0.642713	0.794325	-6.80505
AC116407.3	0.086024	7.871982	0.465259	0.642322	0.794325	-6.94588
AC112187.3	0.67207	2.863884	0.464536	0.642838	0.794349	-5.58859
AC083806.2	-0.09267	5.383441	-0.46376	0.643394	0.794644	-6.3578
AL442125.1	-0.11336	6.493174	-0.46378	0.643376	0.794644	-6.7034
AL136115.2	0.212357	6.875187	0.463765	0.64339	0.794644	-6.72415
AC025871.2	-0.64726	2.602062	-0.46356	0.643535	0.794687	-5.67771
AL442663.3	0.258054	4.907098	0.463414	0.64364	0.794687	-6.02766
AC099811.3	0.318744	5.286634	0.462142	0.64455	0.79568	-6.16547
AC007220.1	0.335387	5.069263	0.46176	0.644824	0.795887	-6.02433
AC020978.1	-0.23221	5.521927	-0.46146	0.645036	0.796017	-6.44897
AC100788.1	0.424503	3.999414	0.461173	0.645244	0.796144	-5.82671

AL022329.2	-0.59408	1.787221	-0.46058	0.645672	0.796221	-5.55618
AC104564.5	-0.12187	5.451823	-0.46049	0.645731	0.796221	-6.37965
AC018946.1	0.126198	6.324025	0.460822	0.645496	0.796221	-6.56393
AC068533.3	0.098108	7.79542	0.460576	0.645671	0.796221	-6.93382
AL121787.1	-0.66137	1.072203	-0.45981	0.646223	0.796339	-5.49931
AC021739.5	0.477156	5.146969	0.459769	0.64625	0.796339	-6.12168
AC010618.3	0.109597	6.830403	0.459641	0.646342	0.796339	-6.74779
AL138831.3	-0.12434	6.734468	-0.45962	0.646356	0.796339	-6.77075
SCGB1B2P	-0.13322	7.569663	-0.45978	0.646239	0.796339	-6.92749
KCNJ2-AS1	0.209011	6.341391	0.459027	0.646781	0.796601	-6.57121
AL159169.2	-0.13128	6.946731	-0.45909	0.646734	0.796601	-6.81828
AC026801.2	0.160443	7.186975	0.458723	0.646999	0.796739	-6.80032
AC006330.1	0.103234	7.259586	0.458384	0.647242	0.796777	-6.82486
AC015849.4	-0.10412	7.173518	-0.45852	0.647141	0.796777	-6.84833
AL357552.2	-0.17348	6.744702	-0.45757	0.647824	0.797363	-6.77972
AC007370.2	-0.49781	1.054367	-0.45704	0.648206	0.797702	-5.48525
JADRR	0.683313	-1.96271	0.456565	0.648547	0.797991	-5.32256
LINC00484	-0.31831	2.596303	-0.45602	0.64894	0.798344	-5.64238
LINC02097	0.574202	1.312136	0.455804	0.649093	0.798401	-5.439
AC017100.1	-0.16919	7.36457	-0.45549	0.64932	0.798549	-6.90187
AL136084.3	-0.25667	7.40918	-0.45519	0.649531	0.798679	-6.92198
AC108718.1	0.801743	2.149316	0.454519	0.650015	0.799093	-5.48969
AC105020.2	-0.51968	2.95561	-0.45443	0.650081	0.799093	-5.71606
LAMP5-AS1	0.667967	-2.4474	0.453838	0.650505	0.799484	-5.31701
AC004771.4	0.507562	3.686579	0.453643	0.650645	0.799524	-5.71734
AL357568.1	0.587424	1.134678	0.452438	0.651511	0.800136	-5.43448
MRPL23-AS1	0.67599	1.239186	0.452676	0.65134	0.800136	-5.43795
AL139147.1	0.355945	4.664017	0.452559	0.651424	0.800136	-5.97223
AL354892.3	-0.08396	6.754787	-0.45236	0.651568	0.800136	-6.75754
AC010096.1	-0.70321	-1.36896	-0.45157	0.652138	0.800443	-5.37707
AC104984.3	-0.20437	5.957807	-0.45161	0.652104	0.800443	-6.57213
AP003068.1	-0.12632	7.011531	-0.45181	0.651964	0.800443	-6.82131
AC009166.2	0.655911	-1.6796	0.451047	0.652511	0.80077	-5.33271
AC138305.2	0.837335	2.414786	0.450164	0.653146	0.801419	-5.50377
AC093599.1	0.901601	-0.09427	0.449786	0.653418	0.801622	-5.37245
LINC00471	0.345991	5.318084	0.449491	0.653631	0.801751	-6.05717
AC092338.3	-0.11384	5.42725	-0.4486	0.654275	0.802411	-6.40788
AC087627.1	-0.59007	0.929375	-0.44838	0.654432	0.80243	-5.49272
AC022517.1	0.729698	2.749031	0.448217	0.654548	0.80243	-5.56953
AC105001.1	0.145347	6.687773	0.44813	0.654611	0.80243	-6.71063
AC074135.1	0.536588	4.012712	0.447929	0.654756	0.802476	-5.78025
AC092068.1	-0.74662	-1.67111	-0.44775	0.654885	0.802504	-5.37121
AC007546.1	0.115312	6.323717	0.447545	0.655032	0.802553	-6.60682
AC061975.1	-0.58353	-0.31787	-0.44735	0.655173	0.802595	-5.41475
AP001437.1	0.328289	5.12459	0.447081	0.655367	0.802701	-6.2251
AC132803.1	0.780691	-1.67032	0.446528	0.655765	0.802704	-5.33197
AL118508.3	-0.373	3.168366	-0.44655	0.655752	0.802704	-5.78725
C8orf37-AS1	-0.23174	4.384533	-0.44648	0.655796	0.802704	-6.11172
AC022613.3	-0.15068	5.873023	-0.44651	0.655781	0.802704	-6.56697
AC004828.1	0.792595	-1.38466	0.446211	0.655994	0.802722	-5.33824
AC090023.2	-0.53204	2.834531	-0.44617	0.656025	0.802722	-5.70173
AL731684.1	0.838804	-1.00047	0.444796	0.657014	0.803797	-5.35183
AC005614.1	0.517066	-0.75957	0.444322	0.657356	0.803797	-5.36634
AC130448.2	-0.77268	-0.4706	-0.44375	0.657767	0.803797	-5.41805
LINC01931	-0.54945	1.051209	-0.44306	0.658264	0.803797	-5.48789
AC010547.2	-0.73685	3.015549	-0.44324	0.658135	0.803797	-5.75488
AL135999.2	-0.21726	3.402331	-0.44329	0.658103	0.803797	-5.80683
AC044840.1	-0.38823	4.67889	-0.4434	0.65802	0.803797	-6.16281

AF127577.2	-0.26268	4.774123	-0.44317	0.65819	0.803797	-6.23691
AC087521.2	0.108063	5.455371	0.443041	0.65828	0.803797	-6.32638
AC009630.1	-0.21005	5.232321	-0.44406	0.657543	0.803797	-6.33867
AC103739.2	0.119288	5.781294	0.444503	0.657225	0.803797	-6.42028
AC037459.3	-0.08434	8.954293	-0.44395	0.657628	0.803797	-7.14838
AC005261.5	0.105097	9.974399	0.443022	0.658294	0.803797	-7.27933
AC022733.1	-0.74141	-0.13031	-0.4427	0.658528	0.803952	-5.43216
AC133919.1	0.746236	0.657889	0.442297	0.658817	0.804174	-5.4121
AC091932.1	-0.75917	-0.73615	-0.44065	0.660009	0.805499	-5.40513
AC018410.2	0.237498	6.418994	0.439973	0.660496	0.805962	-6.60654
AP001646.3	0.748227	0.627944	0.439523	0.660822	0.806035	-5.4107
AC095033.1	0.589079	1.154693	0.439689	0.660702	0.806035	-5.43812
AL022318.1	-0.69976	0.103766	-0.43945	0.660878	0.806035	-5.44051
AC068473.2	-0.73466	-1.57924	-0.43915	0.661089	0.806162	-5.37771
AC107982.2	0.275523	5.965629	0.438981	0.661214	0.806183	-6.32947
AC097634.2	-0.71215	-1.94386	-0.43866	0.661448	0.806207	-5.36414
AC079601.1	0.42861	1.654034	0.438772	0.661365	0.806207	-5.47922
AL138831.2	-0.34901	4.236695	-0.43827	0.66173	0.806242	-6.10695
AL163636.1	-0.42881	4.855247	-0.43817	0.661799	0.806242	-6.15011
AC084824.5	0.083495	8.191922	0.438443	0.661603	0.806242	-7.01315
AL357153.2	0.667921	1.857094	0.437431	0.662335	0.806765	-5.47584
AP003072.3	-0.72995	-1.43939	-0.43719	0.662513	0.80685	-5.37967
LINC02475	0.68993	1.579178	0.437006	0.662643	0.806877	-5.45648
LINC01447	0.580316	-0.76201	0.436233	0.663203	0.807429	-5.36643
AP004609.1	-0.54229	0.933382	-0.43576	0.663544	0.807697	-5.49548
AL606834.1	0.122381	8.292641	0.435631	0.663638	0.807697	-7.01809
TPRG1-AS1	0.373677	6.082182	0.435359	0.663835	0.807806	-6.47942
LMO7DN-IT1	0.846062	0.084965	0.434544	0.664426	0.808251	-5.38826
AC124017.1	-0.57231	-0.79953	-0.43457	0.664404	0.808251	-5.39987
LINC01641	-0.44383	0.73659	-0.43441	0.664524	0.808251	-5.47276
LINC00173	0.337997	6.164773	0.434248	0.66464	0.808262	-6.46596
LIX1-AS1	0.382069	2.677021	0.433389	0.665263	0.808888	-5.58959
AC005592.1	0.647306	-0.62397	0.432995	0.665549	0.808974	-5.36968
Z97192.2	-0.30516	3.161384	-0.43286	0.665645	0.808974	-5.77063
AP001625.2	-0.22973	7.165315	-0.43285	0.665657	0.808974	-6.88521
AC027130.1	0.399317	3.817187	0.432628	0.665815	0.809036	-5.81397
SPANXA2-OT1	0.674914	-2.37955	0.431723	0.666471	0.80964	-5.32517
AC011509.2	0.760681	-1.42758	0.431568	0.666584	0.80964	-5.34345
AC073314.1	0.382911	2.171778	0.431348	0.666743	0.80964	-5.53457
AP000346.1	-0.34103	3.964287	-0.43135	0.666739	0.80964	-5.9861
AC127496.4	0.700723	0.878671	0.430645	0.667254	0.809998	-5.42618
AC005006.1	-0.57785	3.315603	-0.4307	0.667212	0.809998	-5.82653
AC110760.2	-0.50881	0.474878	-0.42975	0.667903	0.810263	-5.46298
AL031428.1	-0.58077	1.426403	-0.43008	0.667667	0.810263	-5.53631
AL121845.4	0.186902	6.984342	0.429837	0.66784	0.810263	-6.76916
AL117209.1	-0.10412	8.008204	-0.42996	0.667748	0.810263	-7.00876
AF038458.3	-0.48624	3.31507	-0.42956	0.668044	0.810303	-5.84276
AC092140.1	0.536916	3.338054	0.428882	0.668534	0.810766	-5.65783
AC066613.2	0.636231	1.832748	0.428585	0.668749	0.810804	-5.4797
AL451085.1	0.481538	2.907755	0.428541	0.668781	0.810804	-5.61135
AC002059.1	-0.21324	5.452998	-0.42833	0.668934	0.810859	-6.4091
AC079416.3	0.608157	2.279831	0.426923	0.669957	0.811837	-5.55006
AL359881.1	0.834058	4.280934	0.427049	0.669866	0.811837	-5.75155
AC026992.1	0.710985	-0.98715	0.426269	0.670433	0.812189	-5.36089
AC004076.2	-0.09973	6.447712	-0.42623	0.670464	0.812189	-6.70451
AC015911.3	-0.28591	5.584955	-0.42595	0.670665	0.812301	-6.48696
AC005785.1	-0.06648	6.966789	-0.42575	0.670807	0.812342	-6.81875
AC243773.2	0.698755	2.708091	0.42555	0.670956	0.812391	-5.5503

LINC00324	-0.10079	7.829895	-0.42499	0.671364	0.812755	-6.98263
AL645608.6	0.733644	2.270335	0.424529	0.671699	0.812936	-5.52423
LINC01694	0.281694	4.542401	0.424486	0.67173	0.812936	-6.02556
AL133343.2	-0.74886	-1.27633	-0.42399	0.672092	0.81321	-5.38901
AL133523.1	-0.30599	5.572659	-0.42388	0.672173	0.81321	-6.53433
AC005696.3	-0.38201	5.36203	-0.42317	0.672686	0.8137	-6.34304
AC008781.2	-0.20596	3.590293	-0.42281	0.672947	0.813754	-5.85253
AC022498.2	0.10988	7.534314	0.422873	0.672904	0.813754	-6.89888
AC254562.3	-0.11318	7.297836	-0.42258	0.673117	0.813829	-6.89397
AC006435.2	-0.09194	7.866326	-0.4223	0.673322	0.813944	-6.99673
AL034546.1	0.527895	-1.2197	0.422066	0.673492	0.81402	-5.35875
AC092652.1	-0.70897	-0.8878	-0.4218	0.673687	0.814124	-5.40389
AC011840.2	-0.61795	-2.53054	-0.42146	0.673936	0.814268	-5.35018
AC011369.2	-0.66288	1.648676	-0.42119	0.674131	0.814268	-5.57369
AC106873.1	-0.59877	2.113969	-0.42127	0.674073	0.814268	-5.61147
AL137779.1	-0.149	6.197256	-0.42096	0.674299	0.814339	-6.65906
AC025165.5	-0.0832	8.171803	-0.4204	0.674709	0.814704	-7.0358
AC087354.1	0.647251	-1.33403	0.420142	0.674894	0.814797	-5.35389
AC092142.2	-0.73514	-1.58332	-0.41975	0.675181	0.815012	-5.38205
AL451064.2	-0.22822	4.503422	-0.41921	0.675575	0.815095	-6.16591
AL929236.1	0.262084	5.645276	0.419247	0.675547	0.815095	-6.34866
AC087284.1	-0.11642	7.799921	-0.4194	0.675438	0.815095	-6.9831
AC007255.1	0.482461	6.228628	0.418872	0.675821	0.81526	-6.37361
LINC00895	0.639387	-1.52152	0.418578	0.676035	0.815375	-5.34989
AC106037.1	-0.18108	7.417563	-0.41844	0.676133	0.815375	-6.92384
AC022537.1	-0.70792	-0.74956	-0.41798	0.676471	0.81539	-5.41352
AP003721.1	-0.45946	3.408677	-0.41809	0.676388	0.81539	-5.80082
AC098820.1	0.136792	6.026156	0.418008	0.676451	0.81539	-6.49102
RPS6KA2-IT1	-0.32825	4.642029	-0.41696	0.677213	0.816022	-6.17014
AC010530.1	-0.07797	6.964082	-0.41704	0.67716	0.816022	-6.81648
AL133243.4	0.106345	5.688539	0.416214	0.677761	0.816552	-6.43117
LINC00552	-0.58328	0.090955	-0.41566	0.678168	0.816888	-5.44585
AL158212.2	0.228236	5.340907	0.415534	0.678258	0.816888	-6.30931
AL158834.1	0.596109	2.25804	0.41517	0.678523	0.817077	-5.51755
AL390955.2	-0.25771	5.220143	-0.41502	0.678632	0.817077	-6.37065
AP003108.3	-0.17331	3.293199	-0.41417	0.679253	0.817563	-5.7867
AC021739.3	-0.27567	5.227914	-0.41427	0.67918	0.817563	-6.39911
EFCAB6-AS1	-0.54633	-0.08285	-0.41329	0.679899	0.81762	-5.435
AC073592.9	0.674168	1.651394	0.412997	0.680111	0.81762	-5.48171
U62317.3	-0.27917	5.202103	-0.41269	0.680338	0.81762	-6.35461
AC231981.1	0.133192	5.773312	0.413139	0.680008	0.81762	-6.42489
AL358852.1	-0.10339	6.026042	-0.41288	0.680198	0.81762	-6.61083
LINC00899	0.11719	6.476895	0.413571	0.679692	0.81762	-6.65709
AC000120.1	-0.13577	6.433542	-0.41262	0.680388	0.81762	-6.71469
AC131159.1	-0.22012	6.740697	-0.41284	0.680225	0.81762	-6.80279
AP001178.2	-0.16822	7.632331	-0.41333	0.679869	0.81762	-6.97336
AC245060.5	-0.13245	8.935495	-0.41379	0.679529	0.81762	-7.17102
AC007342.5	0.296399	8.248044	0.411802	0.680986	0.818207	-6.98807
AC069209.1	0.546747	3.001412	0.411593	0.681139	0.818259	-5.61735
AC010980.2	0.293574	5.661372	0.411296	0.681356	0.818259	-6.40323
FARSA-AS1	-0.25052	6.241909	-0.41132	0.681335	0.818259	-6.70924
AC009093.7	-0.48634	1.675174	-0.41091	0.681637	0.818412	-5.56029
AL603839.4	0.307508	4.523594	0.410824	0.681701	0.818412	-6.02485
AC009268.2	0.267249	5.531383	0.410171	0.68218	0.818856	-6.31919
AC009163.5	-0.76333	0.318901	-0.4085	0.683405	0.819725	-5.46452
STARD13-IT1	-0.66737	0.451661	-0.40868	0.683271	0.819725	-5.46963
AL022323.3	-0.12465	4.415609	-0.40887	0.683135	0.819725	-6.05472
AL731577.1	-0.10975	5.383524	-0.40814	0.683668	0.819725	-6.39518

AL662884.1	0.108094	5.822079	0.408925	0.683092	0.819725	-6.46847
AL662844.4	-0.09126	6.622216	-0.40838	0.683492	0.819725	-6.74348
AL136295.7	-0.05994	9.279863	-0.40816	0.683653	0.819725	-7.21268
AL355112.1	-0.64889	-0.91359	-0.40718	0.68437	0.820436	-5.40772
AC004825.2	0.137872	6.778344	0.406215	0.685078	0.821154	-6.76714
AL157832.1	0.685825	2.026833	0.40566	0.685486	0.821179	-5.49943
AL359258.3	0.325212	2.575869	0.40559	0.685536	0.821179	-5.58288
AC005546.1	0.15599	7.276447	0.405817	0.68537	0.821179	-6.85051
AP003469.4	-0.12191	7.989724	-0.40594	0.685282	0.821179	-7.02188
AC011462.5	-0.09706	7.830796	-0.40514	0.68587	0.821447	-6.99358
BFSP2-AS1	-0.64379	-0.47704	-0.40412	0.686616	0.822011	-5.42558
AC007570.1	0.348475	4.233192	0.403749	0.686888	0.822011	-5.94039
AP001351.1	0.247949	5.44115	0.403975	0.686721	0.822011	-6.1927
AC009137.2	0.187355	5.240254	0.40383	0.686828	0.822011	-6.22808
AC006449.2	0.101528	7.184701	0.403835	0.686824	0.822011	-6.84227
AL392183.1	-0.53192	-0.89336	-0.40277	0.687609	0.822449	-5.407
AB015752.1	0.676678	0.489699	0.402655	0.687691	0.822449	-5.42067
AP002992.1	0.613552	1.516194	0.402719	0.687644	0.822449	-5.46628
AC108727.1	0.186109	7.783802	0.402728	0.687637	0.822449	-6.93542
GOLGA8M	-0.66116	-1.87314	-0.40212	0.688085	0.822658	-5.37973
LINC01055	0.419897	2.634238	0.402184	0.688037	0.822658	-5.57501
AC111170.2	0.549712	2.174162	0.401732	0.688369	0.822862	-5.53527
AC025162.2	0.312498	4.52464	0.401588	0.688474	0.822862	-5.97623
DPP9-AS1	-0.0708	6.124801	-0.40143	0.688594	0.822874	-6.61273
AL137186.2	0.071617	6.917168	0.400897	0.688982	0.823207	-6.78947
AC124248.1	0.275847	7.482117	0.400306	0.689417	0.823595	-6.8599
AC097724.1	0.52913	4.221137	0.399978	0.689658	0.823666	-5.81155
AC016027.3	0.354473	4.938782	0.399927	0.689695	0.823666	-6.04482
AL353726.2	0.654404	-0.18121	0.399727	0.689842	0.823711	-5.3959
AC243960.7	-0.66134	-0.81289	-0.39945	0.690048	0.823824	-5.41566
AC007953.1	0.720836	-0.68076	0.398808	0.690518	0.824035	-5.37872
AC074194.1	0.597101	4.661867	0.398612	0.690662	0.824035	-5.8845
INO80-AS1	0.161003	5.373173	0.398748	0.690562	0.824035	-6.31785
BOLA3-AS1	0.163234	7.185794	0.398733	0.690573	0.824035	-6.84309
RSF1-IT2	0.251638	5.159966	0.39786	0.691216	0.824564	-6.21665
AC016168.3	0.527096	-0.79231	0.39635	0.692327	0.825657	-5.37995
AC005703.4	0.65334	0.398143	0.396167	0.692462	0.825657	-5.41871
AL606469.1	-0.60845	0.066444	-0.39631	0.692355	0.825657	-5.45196
AL355377.2	0.324271	3.071565	0.395848	0.692696	0.825711	-5.68492
AC010531.3	0.33403	5.062189	0.395807	0.692727	0.825711	-6.1072
AC018892.3	-0.33356	2.493416	-0.39544	0.692996	0.825901	-5.65209
AL365181.4	-0.61558	0.98051	-0.39522	0.693157	0.825962	-5.50402
AC245884.9	0.45486	4.346237	0.394098	0.693986	0.826687	-5.89078
AC009148.1	0.107923	4.890243	0.394212	0.693902	0.826687	-6.17567
SHANK2-AS2	0.675423	4.178216	0.393782	0.694219	0.826833	-5.73532
AC003957.1	0.3613	1.999355	0.392461	0.695193	0.827731	-5.53083
AL360268.1	0.234062	5.397114	0.392527	0.695144	0.827731	-6.32487
AC079313.1	0.307938	5.875015	0.391859	0.695636	0.827997	-6.3991
AC098851.1	-0.17266	7.021124	-0.39191	0.6956	0.827997	-6.87897
AC100786.1	0.138856	5.351367	0.391345	0.696016	0.828317	-6.29283
AC004918.1	-0.08506	9.148103	-0.39086	0.696376	0.828615	-7.1996
AC017104.3	-0.23818	5.322601	-0.3905	0.696637	0.828662	-6.43384
AC097381.3	-0.29235	5.766299	-0.39057	0.696587	0.828662	-6.55747
SPACA6P-AS	0.364556	1.922041	0.390005	0.697004	0.828968	-5.52784
AL731567.1	0.281863	6.084537	0.389435	0.697425	0.829075	-6.54547
AL137779.2	-0.09726	6.716294	-0.38944	0.697422	0.829075	-6.78021
AC253576.2	-0.21252	6.983166	-0.38973	0.697207	0.829075	-6.87009
AL353596.1	-0.52343	1.93171	-0.3892	0.6976	0.829151	-5.59397

AC022336.3	0.168724	4.366991	0.388023	0.698468	0.830052	-5.99816
AC027796.5	-0.32941	4.585851	-0.38784	0.698602	0.83008	-6.10815
AC104836.1	-0.35386	3.023961	-0.38729	0.699007	0.830431	-5.74781
AC104170.2	0.099287	6.542743	0.387139	0.699121	0.830434	-6.68177
AC078962.2	0.666809	-1.56471	0.386809	0.699365	0.830462	-5.35763
LMLN-AS1	0.612715	1.211072	0.386854	0.699332	0.830462	-5.45795
AC135983.1	0.601355	-1.57156	0.385977	0.69998	0.830502	-5.35997
KCNQ1DN	-0.60099	-2.11788	-0.3852	0.700555	0.830502	-5.37283
AC097478.2	0.718399	-0.50195	0.385986	0.699974	0.830502	-5.38791
LINC01918	0.662936	-0.24041	0.384837	0.700823	0.830502	-5.39821
AC011462.4	-0.4963	2.47133	-0.38518	0.700568	0.830502	-5.65831
AL121772.2	0.607126	3.83789	0.384822	0.700834	0.830502	-5.76977
FAM66A	-0.43123	3.052905	-0.38521	0.700545	0.830502	-5.77037
AL162413.1	1.578531	5.873466	0.38505	0.700665	0.830502	-5.88049
AC015819.2	-0.39491	3.470757	-0.38539	0.70041	0.830502	-5.90837
AC004951.4	-0.11945	5.307303	-0.38608	0.699903	0.830502	-6.41867
AL357054.4	-0.16491	5.71551	-0.3855	0.700334	0.830502	-6.50702
AC008537.3	0.119701	6.135285	0.384819	0.700836	0.830502	-6.61306
AC090948.3	0.16687	6.845361	0.385512	0.700324	0.830502	-6.76349
AP000688.4	0.680034	-1.68906	0.384486	0.701082	0.830531	-5.35556
AC104581.4	-0.06589	6.037073	-0.3846	0.701	0.830531	-6.58729
PARD3-AS1	0.35027	6.248061	0.383495	0.701816	0.831269	-6.58356
AL109910.2	0.564433	-2.68548	0.382756	0.702363	0.831655	-5.3391
AC011284.1	0.549619	3.377445	0.382759	0.702361	0.831655	-5.66181
AL353708.3	0.151025	5.918771	0.381394	0.703371	0.832586	-6.49179
AC027307.3	-0.15007	6.026692	-0.3814	0.703363	0.832586	-6.62195
LINC00565	0.227446	3.546482	0.381039	0.703634	0.832688	-5.83401
AC004908.1	-0.09873	7.992294	-0.38098	0.703679	0.832688	-7.03463
AL356750.1	-0.31678	2.961937	-0.38057	0.703983	0.832917	-5.72475
AP002884.2	-0.58746	0.41557	-0.38041	0.704098	0.832922	-5.4697
AC068790.5	-0.08721	7.526854	-0.38021	0.704249	0.832969	-6.94332
AC005021.1	-0.3079	5.754151	-0.37992	0.70446	0.833088	-6.63153
AL121890.5	0.563316	4.19431	0.379021	0.705129	0.833605	-5.80268
AC005740.4	0.252053	4.585627	0.378885	0.70523	0.833605	-5.99857
THOC7-AS1	0.300393	4.935091	0.379039	0.705116	0.833605	-6.13615
AL512631.2	0.379481	2.84849	0.37873	0.705345	0.83361	-5.63363
AC104590.1	0.324641	2.623699	0.378198	0.705739	0.833944	-5.60406
LINC00909	-0.08148	8.482493	-0.37786	0.705993	0.834114	-7.1016
STEAP3-AS1	0.155733	5.355612	0.376389	0.707081	0.835267	-6.32457
LINC01729	-0.66046	-1.30766	-0.37562	0.70765	0.835808	-5.40405
AC090061.1	0.127443	4.39019	0.375063	0.708066	0.836167	-5.99589
AL139339.1	0.450695	3.415049	0.374644	0.708376	0.836251	-5.69534
AC007292.3	-0.3827	4.058796	-0.37452	0.708471	0.836251	-6.02112
TNKS2-AS1	-0.14678	5.680628	-0.37471	0.708325	0.836251	-6.53138
AL020993.1	0.379027	4.812843	0.373405	0.709297	0.837095	-6.10082
AC034229.2	0.520021	-2.86743	0.372448	0.710007	0.837144	-5.3412
AC087045.2	0.623053	-1.88513	0.372477	0.709986	0.837144	-5.35405
AC003682.1	0.623583	1.807796	0.372867	0.709696	0.837144	-5.50362
AF064858.2	0.664204	3.234977	0.373165	0.709475	0.837144	-5.65296
CERNA1	-0.0827	6.055104	-0.3726	0.709894	0.837144	-6.61998
AL049838.1	-0.22606	6.442759	-0.3726	0.709895	0.837144	-6.84955
AC009060.1	0.513186	-1.68003	0.372031	0.710318	0.837248	-5.36254
AC133961.1	-0.48175	1.953169	-0.37212	0.710248	0.837248	-5.61012
AC090377.1	0.537897	-0.5462	0.371539	0.710683	0.837485	-5.39659
AC139149.1	-0.14496	6.233802	-0.37146	0.710742	0.837485	-6.69267
AC055855.2	0.639884	-0.29275	0.371126	0.71099	0.837646	-5.40176
OTUD6B-AS1	-0.0596	9.993907	-0.37043	0.711508	0.838125	-7.3302
AC068254.2	-0.48183	-2.25993	-0.36971	0.712043	0.838415	-5.37131

FAM87B	0.143806	4.907434	0.36973	0.712028	0.838415	-6.23178
AL049840.4	-0.07605	11.29429	-0.36965	0.712089	0.838415	-7.5233
ARHGEF38-IT1	-0.68741	1.508186	-0.36907	0.712516	0.838524	-5.56112
LINC00311	0.228439	4.015436	0.36927	0.71237	0.838524	-5.89473
AC139792.1	-0.19008	5.484267	-0.36918	0.712437	0.838524	-6.4728
Z93022.1	-0.52397	-0.44955	-0.36865	0.712828	0.83857	-5.4369
AC007998.4	0.331387	2.318509	0.368572	0.71289	0.83857	-5.57897
AL035071.1	0.079572	9.42083	0.368608	0.712863	0.83857	-7.22822
LINC01058	-0.36431	4.065094	-0.3682	0.713164	0.838761	-6.09885
AC035140.1	0.645011	-0.88806	0.368006	0.713311	0.838802	-5.38696
AL033381.2	-0.584	1.06332	-0.36725	0.713873	0.839331	-5.53597
AL158835.2	-0.27095	5.607295	-0.36708	0.714002	0.839352	-6.5246
AC006042.2	-0.35912	3.48817	-0.36616	0.714688	0.839904	-5.86821
AL022323.5	-0.14336	4.099429	-0.36615	0.714695	0.839904	-5.99384
AL033527.2	0.478543	2.952689	0.365946	0.714845	0.839949	-5.63902
AL096869.1	-0.56198	0.26417	-0.36569	0.715036	0.839992	-5.4666
AL365226.1	0.428747	4.452315	0.365596	0.715106	0.839992	-5.90915
DIO2-AS1	0.620134	-0.95605	0.364122	0.716204	0.84	-5.38322
AC079465.1	-0.39395	1.755464	-0.36464	0.715821	0.84	-5.58605
AC006111.3	0.472881	3.531913	0.364638	0.715819	0.84	-5.73015
AC007342.1	-0.1573	5.519158	-0.36468	0.715791	0.84	-6.44634
AC131212.3	-0.09956	7.171501	-0.36427	0.716092	0.84	-6.87849
AC009032.1	-0.09726	7.409478	-0.365	0.715552	0.84	-6.93392
AP001462.1	0.073915	7.698003	0.365169	0.715424	0.84	-6.95588
AC118553.1	-0.10117	7.639443	-0.36409	0.716231	0.84	-6.96504
AC005086.1	-0.09442	8.253253	-0.36482	0.715684	0.84	-7.07268
PTOV1-AS1	0.059143	9.460181	0.364487	0.715932	0.84	-7.23813
NAV2-AS3	0.626211	0.526921	0.36344	0.716713	0.840172	-5.43302
AC090587.2	-0.11995	6.911438	-0.36357	0.716616	0.840172	-6.85374
AC005034.3	-0.06175	10.39402	-0.36353	0.716646	0.840172	-7.3915
AC136604.3	0.111265	6.204932	0.363118	0.716953	0.840191	-6.55916
AC025580.1	-0.3255	6.116216	-0.36313	0.716947	0.840191	-6.69883
AL356218.1	0.681971	-1.39284	0.362684	0.717276	0.840214	-5.36979
AC008667.1	-0.52334	1.923082	-0.36234	0.717531	0.840214	-5.60852
AC124944.1	0.404701	3.277537	0.362421	0.717473	0.840214	-5.70838
Z97832.2	0.098237	5.48225	0.362634	0.717314	0.840214	-6.39331
AC010536.2	-0.18836	7.346162	-0.3629	0.717114	0.840214	-6.92662
AL356515.1	0.693621	-1.36185	0.361942	0.71783	0.840301	-5.37089
AC091534.1	0.499758	-0.93926	0.361968	0.71781	0.840301	-5.38933
AC114737.1	-0.22325	6.026273	-0.36163	0.718063	0.840444	-6.64262
LATS2-AS1	-0.34821	3.785371	-0.36127	0.718333	0.840628	-5.92631
AL450468.1	-0.63796	-0.66533	-0.3606	0.718831	0.840794	-5.42779
AC012213.4	0.798709	1.762897	0.360503	0.718904	0.840794	-5.48727
AC011933.3	0.335777	4.945453	0.360781	0.718696	0.840794	-6.09247
AL513122.2	-0.23872	4.716148	-0.36048	0.718922	0.840794	-6.21422
TMSB15B-AS1	0.293807	4.047063	0.3599	0.719354	0.840991	-5.91326
AC073573.1	-0.33805	3.833956	-0.3598	0.719426	0.840991	-5.94716
LINC00543	-0.39799	5.620605	-0.35988	0.719372	0.840991	-6.51932
AC093152.1	0.601185	-1.00025	0.358473	0.720419	0.842021	-5.3854
AC068397.1	-0.33738	4.389985	-0.35808	0.720713	0.842234	-6.08905
AC105935.2	-0.45925	3.600996	-0.35775	0.720962	0.842393	-5.84638
AC007342.4	0.24008	8.715948	0.357006	0.721515	0.842908	-7.09123
AC068594.2	0.49884	3.200285	0.356655	0.721777	0.843083	-5.65598
ITPKB-IT1	0.505161	1.10633	0.356126	0.722173	0.843152	-5.46776
AC023983.1	0.451827	4.242722	0.356297	0.722045	0.843152	-5.85551
AC104109.2	-0.08915	6.423315	-0.35614	0.722162	0.843152	-6.71299
AC022710.1	0.513139	-1.37591	0.355683	0.722504	0.843175	-5.37684
KC877392.1	-0.60781	-0.32557	-0.35527	0.722811	0.843175	-5.44438

AL121917.2	0.660059	1.329941	0.35552	0.722626	0.843175	-5.4685
LINC00574	0.197186	4.513022	0.35528	0.722805	0.843175	-6.03085
CD81-AS1	-0.1858	4.192511	-0.35569	0.722498	0.843175	-6.0955
AC002128.2	-0.15651	7.575315	-0.35509	0.72295	0.843175	-6.9663
AL162274.2	-0.08812	8.18208	-0.35505	0.722978	0.843175	-7.05673
AL079304.1	-0.54703	0.705301	-0.35483	0.723142	0.843236	-5.49373
AC093422.2	0.596097	-1.8014	0.353827	0.723892	0.843535	-5.36332
AC009145.2	0.637928	-1.56893	0.353736	0.723961	0.843535	-5.36784
AL136526.1	0.249971	3.342383	0.353959	0.723793	0.843535	-5.75372
HOXB-AS2	0.223311	6.630385	0.353868	0.723862	0.843535	-6.71034
AP000692.1	0.073596	7.964454	0.354121	0.723672	0.843535	-7.01311
AL080312.2	0.588534	-0.08012	0.352913	0.724576	0.843803	-5.42033
LINC01484	-0.34133	2.704352	-0.35313	0.724411	0.843803	-5.69984
AC090246.1	-0.22007	6.152102	-0.35297	0.72453	0.843803	-6.67527
SCAANT1	-0.22256	6.163127	-0.35269	0.724742	0.843803	-6.702
AL162171.2	-0.10172	6.305681	-0.35268	0.724752	0.843803	-6.70998
AC126773.1	-0.15394	6.475369	-0.35219	0.72512	0.8441	-6.73119
AC023310.4	-0.61548	1.092311	-0.35173	0.725463	0.844338	-5.53297
AC239800.2	-0.46552	1.307264	-0.35161	0.725549	0.844338	-5.54766
AL133342.1	0.344039	4.941561	0.351355	0.725742	0.844403	-6.09145
AC092123.1	-0.06841	6.908675	-0.35124	0.725829	0.844403	-6.82742
AC068790.9	-0.12315	3.643341	-0.3508	0.726157	0.844654	-5.88505
AC023509.1	0.260487	3.20205	0.34997	0.72678	0.845248	-5.71427
AC090617.4	0.080599	6.430744	0.349552	0.727093	0.845481	-6.68712
AC011447.6	-0.4398	1.665032	-0.349	0.72751	0.845703	-5.57143
AC025178.1	-0.08565	6.657838	-0.34909	0.727441	0.845703	-6.78564
AC025287.2	0.394933	3.851659	0.348716	0.727719	0.8458	-5.80211
LINC02656	-0.18095	4.29224	-0.34858	0.727818	0.8458	-6.11711
AP001469.1	-0.13309	6.051278	-0.34838	0.727971	0.845847	-6.65648
AC016629.2	0.492861	0.574684	0.347718	0.728468	0.846032	-5.44821
AC017101.1	0.355522	5.057546	0.348006	0.728252	0.846032	-6.07667
AC004000.1	0.160777	8.507489	0.347823	0.728389	0.846032	-7.08417
AC012613.1	0.569677	0.399966	0.347319	0.728767	0.846144	-5.43903
OSTN-AS1	-0.56242	1.019636	-0.34714	0.728903	0.846144	-5.53208
AC064801.1	-0.15827	6.532982	-0.34718	0.728869	0.846144	-6.78475
AC125603.2	0.654604	-0.77185	0.346886	0.729091	0.846232	-5.39354
AC022730.4	-0.52876	-0.20061	-0.34664	0.729279	0.84632	-5.44944
AL845552.2	0.431196	3.768248	0.346446	0.729422	0.846355	-5.77373
AC135050.7	0.080732	5.170858	0.345945	0.729798	0.84666	-6.23235
AC037487.2	0.197051	5.110687	0.345508	0.730125	0.846909	-6.23714
LINC02652	-0.63366	-0.59018	-0.34509	0.730441	0.847145	-5.43766
AL355974.2	0.614358	0.984454	0.344719	0.730717	0.847217	-5.45924
AC093567.1	0.351222	3.287687	0.344704	0.730729	0.847217	-5.74721
AC105105.1	0.478852	-1.46746	0.344338	0.731004	0.847324	-5.37766
AC110491.2	-0.55291	-1.84214	-0.34428	0.731046	0.847324	-5.3947
AL133383.1	0.549312	0.697153	0.34334	0.731753	0.848012	-5.447
AP006621.2	-0.09229	7.694582	-0.34284	0.732132	0.84832	-6.99095
AC130343.2	0.537048	3.351833	0.342067	0.732709	0.848857	-5.68598
AC124068.2	0.161449	5.011564	0.341514	0.733124	0.849208	-6.22006
AP001269.2	-0.62245	-0.97867	-0.34101	0.7335	0.849427	-5.42458
AC130651.1	0.266856	5.193448	0.340961	0.73354	0.849427	-6.18469
AL035416.1	0.413625	2.8909	0.340547	0.733851	0.849542	-5.63495
AC010834.3	0.066927	8.407629	0.340529	0.733865	0.849542	-7.08298
AC073957.3	-0.11359	8.054248	-0.34026	0.734068	0.849646	-7.05685
AC018665.1	-0.07478	9.802646	-0.3401	0.734185	0.84965	-7.31614
AC127540.1	-0.54539	-0.2448	-0.33993	0.734315	0.84967	-5.45071
AC012640.4	0.099375	4.873592	0.339735	0.734461	0.849709	-6.15532
HMGA1P4	0.114583	8.396868	0.339572	0.734583	0.849719	-7.06752

AP001056.1	0.570779	-0.22874	0.339323	0.734771	0.849805	-5.41679
AL450384.1	-0.19916	5.988544	-0.33903	0.734989	0.84985	-6.56306
AC008870.2	0.06022	7.528874	0.338971	0.735036	0.84985	-6.93778
AL358781.2	-0.20407	5.153114	-0.33865	0.735273	0.849994	-6.44651
CHL1-AS1	0.5652	-0.53703	0.338263	0.735568	0.850073	-5.40527
AC009065.3	0.335786	3.458221	0.338289	0.735548	0.850073	-5.76146
AP001453.1	0.122466	5.195425	0.338044	0.735733	0.850133	-6.28773
AL161725.2	-0.44625	3.310846	-0.33776	0.735949	0.850252	-5.81074
AC138230.1	-0.11202	6.114684	-0.33714	0.736415	0.850659	-6.61502
AC010307.2	-0.48036	2.97678	-0.33606	0.737226	0.851436	-5.78884
AL136418.1	0.213223	6.56705	0.335943	0.737314	0.851436	-6.69232
HLX-AS1	0.575858	-0.38403	0.335632	0.737548	0.851575	-5.41248
AL137003.1	0.092123	7.932385	0.335465	0.737674	0.851589	-7.00561
AC104581.3	0.346987	1.777912	0.334825	0.738156	0.851943	-5.52868
AL357874.1	-0.42323	2.119478	-0.33476	0.738207	0.851943	-5.62725
MIF-AS1	-0.12455	7.838868	-0.33433	0.738525	0.85218	-7.02861
AC007497.1	0.238102	5.353716	0.333799	0.738929	0.852432	-6.32326
AL359922.3	-0.17905	6.102284	-0.33374	0.738971	0.852432	-6.67241
AL138733.2	0.557754	-1.1299	0.332299	0.740059	0.853469	-5.38741
IL6R-AS1	0.213501	4.189951	0.332098	0.740211	0.853469	-5.8846
AC254633.1	-0.18724	7.493558	-0.33223	0.740113	0.853469	-6.9908
AC005197.1	0.51112	1.053167	0.331293	0.740818	0.853907	-5.47518
AC022167.2	-0.0611	8.094168	-0.33138	0.740752	0.853907	-7.05738
AL031673.1	0.106321	8.127969	0.330857	0.741146	0.854154	-7.03933
AL121835.2	0.497318	-1.74464	0.330413	0.741481	0.854278	-5.373
AP001107.4	-0.07405	6.978051	-0.33056	0.741371	0.854278	-6.85654
U62631.1	0.619387	-0.02304	0.330216	0.74163	0.854318	-5.42347
AC022150.4	0.168807	7.762654	0.329896	0.741871	0.854465	-6.97988
AC009084.1	0.664551	-0.71363	0.329432	0.742221	0.854738	-5.40073
MPPED2-AS1	0.596395	-1.55566	0.328755	0.742732	0.855039	-5.37613
AC097634.3	0.392955	2.446295	0.328685	0.742784	0.855039	-5.59928
NEBL-AS1	-0.25726	6.139806	-0.32858	0.742861	0.855039	-6.69991
ADNP-AS1	-0.05201	8.965405	-0.32848	0.742938	0.855039	-7.18751
AC104695.3	-0.11984	8.375422	-0.32801	0.743291	0.855314	-7.11676
ZBTB20-AS4	-0.35675	3.508495	-0.32711	0.743973	0.855968	-5.82077
AC005034.2	0.23845	4.992238	0.32683	0.744185	0.856044	-6.14965
AC103739.1	-0.08317	6.759147	-0.32672	0.744267	0.856044	-6.79939
AL359232.1	0.1903	4.294801	0.325942	0.744856	0.856358	-6.00456
AC069224.1	-0.10267	6.319737	-0.32606	0.744764	0.856358	-6.71204
FAM201A	-0.15951	7.172913	-0.32591	0.744882	0.856358	-6.91572
LINC02575	0.532763	5.797881	0.325643	0.745081	0.856456	-6.24373
AL137003.2	0.077343	7.918803	0.324852	0.745679	0.857011	-7.00955
AL031595.2	0.153589	0.85891	0.32445	0.745983	0.85723	-5.48067
AC100803.3	-0.19218	4.324874	-0.32418	0.746188	0.857335	-6.15491
AC092645.1	0.18794	5.683809	0.323102	0.747002	0.858138	-6.41891
LINC01364	0.503358	0.222655	0.322329	0.747586	0.858513	-5.43808
AL158070.1	0.506487	0.429696	0.322217	0.747671	0.858513	-5.44442
AC061975.7	-0.41153	2.666986	-0.32245	0.747498	0.858513	-5.69229
AC020910.5	-0.08287	7.695867	-0.32126	0.748397	0.859216	-6.995
LINC01838	0.482863	-2.44691	0.320301	0.74912	0.859606	-5.36052
AC108451.1	0.444015	-0.59738	0.320202	0.749195	0.859606	-5.4126
L29074.1	0.543585	1.474729	0.320402	0.749044	0.859606	-5.49375
AC093788.1	0.07971	7.556296	0.320289	0.749129	0.859606	-6.94884
AC121338.2	0.13812	6.816454	0.319609	0.749644	0.85999	-6.7599
AL355075.2	0.088391	6.950731	0.319107	0.750024	0.860231	-6.82721
AL021707.4	-0.06972	7.139909	-0.31903	0.750083	0.860231	-6.89797
AC104984.1	0.522135	0.238766	0.318708	0.750326	0.860323	-5.4406
AC006128.1	-0.07349	8.573789	-0.31862	0.750392	0.860323	-7.13826

AC016737.2	-0.51074	1.590839	-0.31753	0.751219	0.861139	-5.57709
AC005224.2	0.471862	-2.58316	0.317115	0.751533	0.861236	-5.35914
AP002761.3	0.108837	5.90262	0.317226	0.751448	0.861236	-6.55864
AL162171.4	-0.06335	5.789685	-0.31672	0.751834	0.86145	-6.55257
AC104984.4	-0.57756	0.133917	-0.31642	0.752061	0.861579	-5.48104
AC008937.1	-0.32034	2.63615	-0.31605	0.752338	0.861633	-5.70726
AC145422.1	-0.16795	5.874227	-0.31606	0.752335	0.861633	-6.60195
AC012313.4	0.090053	7.807596	0.315786	0.752539	0.861733	-6.98826
AC026765.2	0.445339	0.318106	0.315138	0.75303	0.862032	-5.44667
AP003086.3	0.549764	1.61269	0.315219	0.752969	0.862032	-5.50872
AC022467.1	0.479423	0.814875	0.314334	0.75364	0.862086	-5.47524
AC010605.1	0.503359	1.325265	0.314432	0.753566	0.862086	-5.48862
AC136475.7	-0.50208	0.941891	-0.31472	0.753343	0.862086	-5.52525
AC079848.1	-0.13586	6.081432	-0.31459	0.753448	0.862086	-6.65885
AC145423.2	0.173025	7.006542	0.314319	0.753651	0.862086	-6.81817
ELMO1-AS1	-0.52575	-1.88282	-0.3137	0.75412	0.862491	-5.39942
AC060766.4	0.10548	7.983304	0.31327	0.754447	0.862733	-7.01439
AC010632.2	0.193391	4.362566	0.312446	0.755072	0.863185	-5.965
AL136988.2	0.171123	5.98887	0.312528	0.75501	0.863185	-6.56014
AC011726.2	-0.4232	1.995668	-0.31216	0.755291	0.863305	-5.64438
AC015983.2	-0.48372	1.308203	-0.31182	0.755548	0.863336	-5.54886
AC011755.1	-0.25249	2.852338	-0.31187	0.755511	0.863336	-5.72185
AC108063.1	0.390254	3.503747	0.311525	0.75577	0.863458	-5.75331
FAM85B	-0.26179	3.792138	-0.31077	0.756343	0.863925	-5.923
AL359504.2	0.230156	5.082656	0.310684	0.756408	0.863925	-6.25041
AC032011.1	-0.29817	2.418567	-0.31022	0.756763	0.863934	-5.66701
FGF14-AS2	-0.18308	5.913988	-0.31009	0.75686	0.863934	-6.68396
AL034550.1	-0.14633	6.813718	-0.31016	0.756803	0.863934	-6.82888
AL136115.1	-0.10319	7.009769	-0.31007	0.756876	0.863934	-6.87135
AC009053.3	-0.35168	2.927031	-0.30965	0.757194	0.864165	-5.77274
AP002800.1	0.197228	6.255592	0.309415	0.757372	0.864236	-6.62392
AC018693.1	0.46416	1.503199	0.309112	0.757602	0.864341	-5.50408
AL358072.1	-0.14481	5.355475	-0.30899	0.757693	0.864341	-6.43517
SMAD1-AS1	-0.51826	-0.49511	-0.30883	0.757817	0.864351	-5.44926
TMEM72-AS1	-0.30401	1.276442	-0.30867	0.757934	0.864353	-5.53713
AC125603.4	-0.36497	1.834523	-0.30842	0.758125	0.86444	-5.60931
AC092683.2	0.460099	-1.41797	0.307852	0.758559	0.864672	-5.3895
Z95331.1	-0.06149	4.690902	-0.30794	0.758494	0.864672	-6.17675
STAU2-AS1	0.16386	4.858491	0.307479	0.758842	0.864864	-6.13513
AC099509.1	0.551956	1.546459	0.306636	0.759483	0.865463	-5.50267
AL391839.1	0.444259	3.181941	0.305717	0.760182	0.866128	-5.70585
AC073052.2	0.134755	4.520944	0.305422	0.760406	0.866252	-6.06832
BX323046.1	-0.36815	2.748381	-0.30482	0.760864	0.866515	-5.74843
AC090114.2	0.068119	8.089891	0.304815	0.760868	0.866515	-7.05192
AL355613.1	0.500535	0.848716	0.304544	0.761073	0.866618	-5.47229
AC006064.3	-0.2036	5.103682	-0.30399	0.761494	0.866966	-6.37624
FAM160A1-DT	0.137192	8.212561	0.303689	0.761724	0.867095	-7.04913
AC004832.4	-0.11414	6.728925	-0.30325	0.76206	0.867347	-6.8295
AL138820.1	-0.07499	4.665148	-0.30215	0.762895	0.867888	-6.20862
AC012464.2	-0.10287	5.159193	-0.30207	0.762953	0.867888	-6.29215
AP003096.1	-0.07914	6.496815	-0.30201	0.762997	0.867888	-6.76322
RHOA-IT1	-0.06396	7.938733	-0.30224	0.762825	0.867888	-7.03887
AL356753.1	0.418737	-0.69143	0.301404	0.763462	0.868204	-5.41472
AL035427.1	0.508727	0.416766	0.301042	0.763738	0.868204	-5.45406
AC090617.7	-0.35087	4.572552	-0.3012	0.76362	0.868204	-6.26626
AC092718.7	-0.06291	6.271373	-0.30119	0.763624	0.868204	-6.68386
SLC6A1-AS1	0.525467	-1.02756	0.29952	0.764897	0.869121	-5.40264
AC103853.1	-0.48692	-0.44652	-0.29893	0.765346	0.869121	-5.4519

AL133467.1	0.344979	3.269467	0.299228	0.765119	0.869121	-5.77899
AL357055.3	0.083317	5.983997	0.29892	0.765354	0.869121	-6.56515
AC007622.2	0.059381	6.637185	0.299226	0.765121	0.869121	-6.76549
AC092142.1	0.205039	7.122694	0.298938	0.76534	0.869121	-6.79972
AL031775.2	-0.06208	8.126394	-0.29942	0.764974	0.869121	-7.07226
AL022323.4	-0.07263	5.579288	-0.2986	0.7656	0.869269	-6.47459
AC078880.3	0.422156	0.779215	0.298338	0.765797	0.869361	-5.46996
NHS-AS1	0.54635	-0.48125	0.2976	0.766359	0.869868	-5.41943
AC107464.1	-0.42194	-1.23259	-0.29704	0.766788	0.8699	-5.42064
AP002360.2	-0.31206	3.207565	-0.29669	0.767055	0.8699	-5.8275
AC011933.2	0.084153	5.36802	0.296898	0.766895	0.8699	-6.34808
LINC02361	-0.11445	6.281291	-0.29665	0.767082	0.8699	-6.68975
AC008937.3	-0.0715	6.378871	-0.29705	0.766776	0.8699	-6.72982
AC009831.1	0.0692	6.513264	0.29688	0.766908	0.8699	-6.7313
AC080078.1	0.534459	-1.43529	0.296033	0.767554	0.870042	-5.38825
AC005410.2	-0.18998	5.343862	-0.29618	0.767439	0.870042	-6.41601
AC005391.1	0.187119	6.786184	0.29614	0.767472	0.870042	-6.77303
AC140125.2	-0.45206	-1.05599	-0.29587	0.767675	0.870047	-5.4297
OTX2-AS1	0.423643	0.130655	0.295304	0.76811	0.870408	-5.44598
AL163953.1	0.41803	3.949258	0.294954	0.768377	0.87058	-5.87954
AL133480.1	-0.28184	3.4091	-0.29431	0.768869	0.871006	-5.82673
AC112715.1	0.495573	1.796375	0.293406	0.769558	0.871129	-5.5242
AC103876.1	-0.29749	3.406579	-0.29343	0.769543	0.871129	-5.84515
AL049871.1	-0.19601	4.147454	-0.29371	0.769329	0.871129	-6.06677
AC010207.1	0.135874	4.530994	0.293807	0.769252	0.871129	-6.14816
AL359182.1	0.240797	6.202997	0.293839	0.769227	0.871129	-6.57705
AC104564.3	-0.06691	7.064194	-0.29301	0.769861	0.871342	-6.88384
LINC02671	0.454515	-1.63894	0.292244	0.770444	0.871739	-5.38587
AC099509.2	-0.56071	-1.18847	-0.29232	0.770386	0.871739	-5.42788
AC010271.1	-0.16227	5.655165	-0.29207	0.770575	0.871756	-6.55277
FOCAD-AS1	0.418453	2.687447	0.291481	0.771026	0.872135	-5.6406
AL138895.1	0.480473	4.132728	0.291096	0.77132	0.872336	-5.78232
AC007613.1	0.071875	4.657999	0.290256	0.771962	0.872704	-6.11674
WWC2-AS2	-0.10565	5.078395	-0.29021	0.771994	0.872704	-6.33951
AC012313.9	0.173044	5.634506	0.290471	0.771798	0.872704	-6.42656
AC080129.2	-0.23839	5.643988	-0.28936	0.772643	0.873307	-6.5349
CYP51A1-AS1	-0.16595	3.896087	-0.2889	0.772995	0.873573	-5.9652
ADAMTSL4-AS1	-0.08162	6.962633	-0.28753	0.774048	0.874631	-6.87402
AC243654.3	-0.15365	4.59933	-0.2862	0.775065	0.875649	-6.21397
AC008514.1	0.354773	4.064718	0.285166	0.775852	0.876406	-5.88266
TRMT2B-AS1	0.276265	5.941058	0.284875	0.776075	0.876526	-6.50905
PLS1-AS1	0.537743	1.212875	0.284638	0.776256	0.876599	-5.49346
AP000785.1	0.538714	-0.06944	0.284125	0.776649	0.876672	-5.44244
LINC02102	0.256129	1.821127	0.284281	0.776529	0.876672	-5.55742
RNF216-IT1	-0.0646	7.544677	-0.2841	0.776671	0.876672	-6.98236
AL360169.2	0.302898	3.754523	0.283629	0.777028	0.876812	-5.851
AL391335.1	0.080145	7.177735	0.283681	0.776989	0.876812	-6.88709
AC091435.2	0.500947	-1.00454	0.282944	0.777552	0.876876	-5.40627
AL731568.1	-0.46658	0.907664	-0.28308	0.777447	0.876876	-5.52369
LINC01394	-0.31933	3.285791	-0.28325	0.777317	0.876876	-5.88944
AL133371.2	0.318891	5.5642	0.283079	0.777449	0.876876	-6.31449
LINC01290	0.109687	7.255987	0.282676	0.777757	0.876976	-6.90555
SMAD5-AS1	-0.16149	3.81657	-0.28211	0.778189	0.877331	-5.97679
AC009948.2	0.133456	5.708726	0.281201	0.778887	0.877986	-6.49484
AC012366.1	0.517578	-0.90506	0.279794	0.779964	0.879068	-5.40878
AC007378.1	0.363183	2.981893	0.279231	0.780395	0.879422	-5.67727
AL512604.2	0.415362	-0.32217	0.278554	0.780914	0.879744	-5.43211
AC024901.1	0.487181	0.44033	0.278655	0.780837	0.879744	-5.45809

AL158801.3	0.326151	4.350209	0.278107	0.781257	0.879997	-6.0264
COL18A1-AS2	0.451913	1.493002	0.277438	0.781769	0.880047	-5.51719
AC069366.2	-0.39501	1.593188	-0.27749	0.781732	0.880047	-5.61316
AC098479.1	-0.41118	3.746355	-0.27747	0.781746	0.880047	-5.88645
AC091060.1	-0.07545	5.16877	-0.27764	0.781618	0.880047	-6.34196
AL596087.2	-0.47611	-1.64982	-0.27655	0.782452	0.880684	-5.41295
AL359541.1	-0.255	3.961495	-0.27578	0.78304	0.881214	-5.94438
LINC02398	-0.50778	-0.16048	-0.27543	0.783309	0.881367	-5.4687
AL136984.1	-0.12804	4.520481	-0.2753	0.783411	0.881367	-6.16191
NDST1-AS1	0.449329	2.710131	0.274809	0.783785	0.881614	-5.61584
AL589765.5	-0.38726	1.977533	-0.2747	0.783865	0.881614	-5.64741
AC005696.4	0.128558	5.603944	0.274285	0.784188	0.881844	-6.50003
AC091488.1	0.358431	3.627767	0.273755	0.784594	0.881939	-5.78949
AC092653.1	-0.06652	7.32435	-0.27372	0.784624	0.881939	-6.94698
AC005332.5	-0.0637	8.505734	-0.27374	0.784604	0.881939	-7.13743
AP003467.1	-0.17443	5.675428	-0.27341	0.784856	0.882068	-6.55793
AKT3-IT1	-0.35983	2.645467	-0.27326	0.784976	0.882071	-5.72809
AC139792.2	0.405522	1.884415	0.27277	0.78535	0.882359	-5.54817
AC074183.2	0.265912	3.440446	0.27206	0.785895	0.882839	-5.77743
AC009145.1	0.485101	-0.4766	0.271043	0.786675	0.883498	-5.42437
LINC01480	-0.16002	5.130776	-0.27099	0.786716	0.883498	-6.37309
AC010536.3	-0.38028	1.84022	-0.27024	0.787291	0.88401	-5.61118
AC097375.3	0.380566	-2.04463	0.269318	0.788	0.884147	-5.38307
SLC9A9-AS1	0.472048	-1.08195	0.269397	0.78794	0.884147	-5.40852
AL392023.1	-0.45926	-1.40664	-0.26942	0.787919	0.884147	-5.42247
LINC01936	-0.15021	3.765115	-0.26954	0.787832	0.884147	-5.9138
AC026471.6	0.110199	4.995311	0.269404	0.787934	0.884147	-6.24676
AP002358.1	0.397829	-1.60035	0.269038	0.788215	0.884256	-5.39593
AP006248.4	-0.46422	-1.28613	-0.26847	0.788652	0.88435	-5.42819
AC114291.1	-0.40263	1.135349	-0.26847	0.78865	0.88435	-5.55148
AC004241.4	-0.16101	3.876891	-0.26873	0.78845	0.88435	-5.9802
AL160163.1	0.330822	3.901909	0.26823	0.788836	0.884424	-5.8721
AC005730.3	-0.06107	7.300496	-0.26757	0.789344	0.884862	-6.92974
AC115099.1	0.386796	4.116414	0.266665	0.790039	0.884993	-5.89438
BIRC6-AS1	-0.2364	3.964705	-0.26696	0.789813	0.884993	-5.96949
AC002401.3	-0.33764	4.219449	-0.26703	0.78976	0.884993	-5.97169
AL512791.2	-0.09602	4.885584	-0.26686	0.789889	0.884993	-6.28368
AL391244.1	-0.20524	6.26777	-0.26665	0.79005	0.884993	-6.76683
AC026471.1	0.060141	9.079814	0.266238	0.790368	0.885216	-7.21489
AC010999.1	0.303768	4.483904	0.265448	0.790975	0.885764	-5.97108
AC023024.2	-0.09182	6.850364	-0.26438	0.791798	0.886554	-6.85165
PLCG1-AS1	0.062398	5.490355	0.263604	0.792394	0.887089	-6.43375
FAM41C	0.365115	-0.8813	0.263231	0.792681	0.887278	-5.4198
AC016700.3	0.460249	-0.70892	0.262143	0.793518	0.888083	-5.42215
LINC02244	0.310444	5.25359	0.261982	0.793642	0.888089	-6.17819
AL390719.2	-0.10702	10.11804	-0.26151	0.794007	0.888366	-7.37813
AC025442.1	0.248599	4.451226	0.261113	0.794311	0.888573	-5.99317
AC019193.2	0.380093	1.957623	0.259277	0.795725	0.890023	-5.54948
AC008667.3	0.45986	-0.98119	0.256976	0.797497	0.891051	-5.4129
AC020891.1	0.513646	0.307105	0.257115	0.79739	0.891051	-5.45801
AC108102.1	0.357107	2.154983	0.257217	0.797312	0.891051	-5.5948
CFAP44-AS1	0.27329	2.833699	0.257041	0.797448	0.891051	-5.68205
AC004593.1	-0.31726	3.269086	-0.2567	0.797711	0.891051	-5.88013
AF178030.1	0.316281	3.893661	0.257785	0.796874	0.891051	-6.03399
CRTC3-AS1	0.059293	5.928783	0.256777	0.797651	0.891051	-6.56087
AP001830.1	0.089421	6.89543	0.257762	0.796892	0.891051	-6.81603
AC009283.1	-0.08257	9.808377	-0.25693	0.797535	0.891051	-7.33278
MIR5689HG	-0.39956	0.564889	-0.25604	0.798219	0.891352	-5.50661

TCEAL3-AS1	-0.39684	1.537098	-0.25607	0.798194	0.891352	-5.57971
Z98884.1	0.132191	6.3904	0.255674	0.798501	0.891429	-6.65553
AC010809.1	-0.07254	7.664795	-0.25564	0.798525	0.891429	-7.01026
AL158212.4	0.16679	3.853184	0.255033	0.798995	0.891822	-5.89357
LINC02313	0.333246	4.838462	0.254879	0.799114	0.891822	-6.15278
AC104187.1	0.24477	3.233601	0.254561	0.79936	0.891963	-5.75915
AC010931.2	-0.19023	3.186311	-0.25331	0.800322	0.892783	-5.81563
AC008026.3	-0.06837	6.256364	-0.25329	0.800338	0.892783	-6.69366
AC103769.1	0.115812	7.9494	0.253146	0.800451	0.892783	-7.02264
AL359962.2	0.1249	6.618694	0.252155	0.801215	0.893503	-6.73676
AC136475.9	-0.37102	2.069706	-0.25143	0.801776	0.893996	-5.65546
AL008636.1	0.393479	-0.18319	0.251227	0.801932	0.894037	-5.44323
AP001893.3	-0.41692	-0.22926	-0.25075	0.802301	0.894316	-5.46866
LINC00857	0.158379	8.326761	0.250292	0.802654	0.894576	-7.08511
AL022069.2	-0.18989	3.10483	-0.24987	0.802981	0.894675	-5.78714
RN7SL832P	0.076016	6.485606	0.250005	0.802875	0.894675	-6.70298
AC008805.2	0.437079	-0.8763	0.249097	0.803576	0.895206	-5.42017
AL354811.1	-0.3408	2.564525	-0.24859	0.803966	0.895508	-5.69467
AC011461.1	0.09603	6.63893	0.248068	0.804371	0.895826	-6.75322
AC069547.1	0.058077	7.960474	0.247785	0.80459	0.895937	-7.04817
AC055764.2	0.111078	5.703782	0.247358	0.80492	0.896171	-6.48079
AC008147.2	-0.07009	7.16377	-0.24681	0.80534	0.896506	-6.90249
AL079343.1	-0.35846	-0.14916	-0.24508	0.80668	0.897497	-5.4721
AC015722.2	-0.42356	0.230637	-0.24504	0.806708	0.897497	-5.4927
AC015920.1	-0.21047	2.939327	-0.2451	0.806665	0.897497	-5.74738
AC002094.5	-0.08441	5.914922	-0.24549	0.806359	0.897497	-6.65751
AC097468.2	0.439054	-1.62171	0.244594	0.807056	0.897725	-5.39646
AC138811.1	-0.10334	6.176549	-0.24447	0.807152	0.897725	-6.71126
KCNMA1-AS3	-0.3109	1.893856	-0.2441	0.807435	0.897774	-5.62952
AL589765.6	0.352573	3.348441	0.244237	0.807332	0.897774	-5.72341
AC008264.2	-0.07587	3.237677	-0.24336	0.808009	0.897973	-5.80776
AC083801.2	0.274604	4.061574	0.242836	0.808415	0.897973	-5.89987
AC009754.1	-0.18584	4.177637	-0.24262	0.80858	0.897973	-6.13262
AC034102.8	-0.20561	4.237483	-0.24186	0.809168	0.897973	-6.13622
C15orf56	-0.1486	5.064696	-0.24213	0.808965	0.897973	-6.3453
AC090617.6	-0.2642	5.06497	-0.24242	0.80874	0.897973	-6.4233
AL139021.2	0.111508	5.578247	0.243295	0.80806	0.897973	-6.45468
AP001001.1	-0.05623	6.410005	-0.24249	0.80868	0.897973	-6.74896
GATA2-AS1	-0.12977	6.792843	-0.24191	0.80913	0.897973	-6.87064
ZNF426-DT	-0.08324	6.968602	-0.24208	0.808999	0.897973	-6.90015
AL442125.2	-0.07403	7.353436	-0.24371	0.807739	0.897973	-6.95259
AC068790.4	-0.12785	7.301722	-0.24224	0.808873	0.897973	-6.95623
AC007038.2	-0.05034	8.268108	-0.24259	0.808606	0.897973	-7.10607
AC006116.5	0.251602	4.106161	0.241298	0.809605	0.898325	-5.94153
AC015726.3	0.414671	0.892207	0.240852	0.80995	0.8985	-5.48716
AC007216.3	-0.0473	7.378304	-0.24079	0.810002	0.8985	-6.9556
SNHG25	0.086698	11.33238	0.2404	0.8103	0.898698	-7.54205
AC009097.3	-0.36779	-1.88784	-0.2397	0.810845	0.89917	-5.41174
FGF12-AS2	-0.36035	2.175638	-0.23882	0.811521	0.899522	-5.63398
BACH1-AS1	0.056775	5.808855	0.238967	0.81141	0.899522	-6.56155
AC139100.2	-0.0836	7.443958	-0.23909	0.81131	0.899522	-6.98367
LINC02094	-0.41591	-1.63423	-0.23652	0.813308	0.899776	-5.41949
AC011471.3	-0.41435	-1.09191	-0.23695	0.812974	0.899776	-5.43953
ERLNC1	0.377911	0.404005	0.237648	0.812431	0.899776	-5.47196
AL157931.1	0.462903	0.762573	0.236838	0.813058	0.899776	-5.48263
AC020763.5	-0.36483	1.238022	-0.23782	0.812301	0.899776	-5.55414
LINC02076	0.198475	3.774058	0.237972	0.81218	0.899776	-5.88526
AC124069.1	-0.18432	4.791983	-0.23659	0.813253	0.899776	-6.16512

AC091729.2	-0.18192	5.117186	-0.23673	0.813145	0.899776	-6.39972
TSPEAR-AS1	0.168983	5.872715	0.236872	0.813031	0.899776	-6.55014
AC103810.2	0.081203	6.002234	0.237242	0.812745	0.899776	-6.59182
AC090616.6	-0.04154	6.310323	-0.23776	0.812348	0.899776	-6.72062
AC007566.1	-0.07054	7.892994	-0.23758	0.812484	0.899776	-7.0588
HM13-IT1	0.047354	9.629778	0.236649	0.813205	0.899776	-7.30486
AC103691.2	0.350839	1.625024	0.236156	0.813586	0.89982	-5.54604
AL022069.1	0.116666	4.483677	0.236271	0.813497	0.89982	-6.02613
AL513188.1	0.313008	2.963556	0.234988	0.814491	0.900688	-5.6856
AC000123.1	0.052984	8.652147	0.234569	0.814816	0.900914	-7.15926
AL133255.1	-0.3417	2.225006	-0.23392	0.815323	0.901342	-5.66496
AC021188.1	-0.11574	5.249661	-0.23334	0.815769	0.901703	-6.37211
AC118758.3	0.076529	2.160267	0.232381	0.816512	0.901745	-5.62195
AC025165.1	0.163617	4.227216	0.232941	0.816078	0.901745	-5.98453
AC008555.2	-0.08116	4.157077	-0.23301	0.816028	0.901745	-6.07476
AC106886.4	-0.04439	5.273397	-0.23258	0.816359	0.901745	-6.39383
AC027544.2	0.062447	5.83422	0.232303	0.816573	0.901745	-6.54453
AC079336.6	-0.07396	5.696538	-0.23221	0.816648	0.901745	-6.55203
AC124045.1	-0.06035	7.73299	-0.23264	0.81631	0.901745	-7.03
AL445123.1	-0.41584	-0.0365	-0.2313	0.81735	0.901797	-5.48512
AC004702.1	0.432553	1.211477	0.231803	0.816961	0.901797	-5.5114
C1orf229	-0.09298	4.628893	-0.23127	0.817373	0.901797	-6.24518
AC011468.5	-0.05934	6.834128	-0.23153	0.81717	0.901797	-6.85365
LINC02449	-0.08668	6.861937	-0.23122	0.817415	0.901797	-6.87123
AL359962.1	-0.07822	7.835027	-0.23187	0.81691	0.901797	-7.03309
AC009320.1	-0.3713	2.499968	-0.23093	0.817637	0.901909	-5.68095
AC009686.1	-0.14263	6.201534	-0.2306	0.817891	0.902057	-6.72561
AP001350.1	0.047622	6.438806	0.230279	0.818142	0.902187	-6.73014
AC008966.2	-0.10121	7.015107	-0.23014	0.818249	0.902187	-6.89585
LINC00449	-0.09358	5.849815	-0.22971	0.818587	0.902428	-6.56485
AL031280.1	0.234528	1.861284	0.228646	0.81941	0.902954	-5.56674
AC002480.1	0.208635	3.088683	0.228471	0.819545	0.902954	-5.7373
AL691403.2	0.265081	3.489348	0.228565	0.819472	0.902954	-5.81602
AC093423.2	-0.21158	4.592339	-0.22879	0.819296	0.902954	-6.17302
CACNA1C-AS4	0.379427	-0.78279	0.228138	0.819804	0.903106	-5.42757
AL136304.1	-0.05832	7.065942	-0.22795	0.819947	0.903131	-6.90249
AL031719.2	0.056926	7.775342	0.227675	0.820163	0.903238	-7.0116
AP003969.2	-0.25688	2.22929	-0.22715	0.820574	0.903558	-5.64203
AL359317.1	-0.09957	5.10063	-0.22699	0.820696	0.903559	-6.36478
AC100797.1	0.266853	4.956369	0.226813	0.820833	0.903574	-6.22903
AL138966.2	0.158032	5.392021	0.226662	0.82095	0.903574	-6.37821
AC015660.3	0.31474	2.676545	0.226473	0.821097	0.903603	-5.65704
AC131649.1	-0.35826	-1.0844	-0.22483	0.82237	0.904156	-5.44038
AL354919.1	0.235629	1.364749	0.224891	0.822325	0.904156	-5.53172
AC131391.1	0.334808	1.698472	0.224741	0.822441	0.904156	-5.54202
AL158071.3	0.193143	5.311229	0.224769	0.82242	0.904156	-6.24345
AC245452.1	-0.03565	5.109321	-0.22537	0.82195	0.904156	-6.35046
AC092794.2	-0.12645	5.160742	-0.22537	0.821951	0.904156	-6.42964
AL358115.1	-0.04843	6.701588	-0.22503	0.822216	0.904156	-6.81797
ABHD15-AS1	0.374506	2.459502	0.224394	0.822711	0.904211	-5.62751
AL132656.4	0.071213	6.926202	0.224367	0.822732	0.904211	-6.84546
AC068987.1	-0.36538	1.377992	-0.22409	0.822945	0.904313	-5.56006
AC027449.1	-0.38205	1.543873	-0.22383	0.823151	0.904318	-5.58747
AL162390.1	-0.05287	6.613093	-0.22366	0.823278	0.904318	-6.80403
AC112484.4	0.072122	6.838186	0.223622	0.823311	0.904318	-6.84172
AC116348.2	0.316172	3.222463	0.22338	0.823499	0.904392	-5.73059
RBM15-AS1	-0.08163	5.030751	-0.22319	0.823644	0.904419	-6.28753
AL357568.2	-0.26701	1.224182	-0.22284	0.823917	0.904586	-5.55065

AP001189.6	-0.35322	-2.06231	-0.22263	0.824079	0.904632	-5.40913
AL357054.2	-0.31674	2.097206	-0.22176	0.824756	0.905244	-5.66904
AL158211.4	-0.29272	2.02015	-0.22103	0.825327	0.905652	-5.63696
AC020978.9	-0.04534	7.057029	-0.22097	0.825369	0.905652	-6.90763
PTPRJ-AS1	0.144387	6.779133	0.220675	0.825601	0.905775	-6.70475
AC007292.2	0.04775	6.655406	0.220518	0.825723	0.905776	-6.80582
AC027338.1	-0.30859	1.701372	-0.21927	0.826694	0.906709	-5.59633
AC011466.1	0.201846	4.353218	0.21909	0.826834	0.906729	-5.97745
Z99756.1	-0.29485	-0.89105	-0.21879	0.827064	0.90685	-5.44934
AL365271.1	0.395516	-0.60539	0.217414	0.828137	0.907297	-5.43696
AC008567.2	-0.33801	0.111676	-0.21725	0.828267	0.907297	-5.48825
AC092839.1	-0.33008	0.941597	-0.21736	0.828183	0.907297	-5.53668
AC093330.2	-0.18112	1.209419	-0.21751	0.828065	0.907297	-5.54826
AC009955.4	0.356756	2.164879	0.217941	0.827728	0.907297	-5.5789
AC114760.2	-0.0671	6.242116	-0.21718	0.828317	0.907297	-6.70557
AC093110.1	-0.07787	8.491661	-0.21786	0.827791	0.907297	-7.15143
AC013457.1	0.397365	-0.82009	0.216659	0.828725	0.907478	-5.42658
AL121829.2	-0.31627	2.819932	-0.21673	0.828668	0.907478	-5.76412
AL133415.1	-0.08657	7.32602	-0.21636	0.828959	0.907602	-6.96454
AL358933.1	-0.2366	0.60269	-0.2153	0.829786	0.907625	-5.51026
AL049712.1	-0.2765	1.818219	-0.21516	0.829891	0.907625	-5.61439
WWOX-AS1	0.302888	2.812036	0.215518	0.829613	0.907625	-5.6627
AL008729.2	0.270001	2.822745	0.214935	0.830067	0.907625	-5.68052
LINC02005	-0.35265	2.555029	-0.21586	0.829344	0.907625	-5.71046
AC020659.2	0.224861	3.054485	0.215145	0.829903	0.907625	-5.71983
AC011753.2	0.1512	5.089311	0.215482	0.829641	0.907625	-6.27847
AC055855.1	-0.04799	6.932741	-0.21507	0.829962	0.907625	-6.8726
AC009948.3	-0.10185	6.961017	-0.21542	0.829689	0.907625	-6.88413
AC117489.1	0.195417	3.14278	0.213591	0.831113	0.908637	-5.90224
AC007272.1	-0.07379	5.947948	-0.21317	0.83144	0.908862	-6.58503
LINC00456	0.381835	0.179327	0.21286	0.831682	0.908995	-5.46511
AL592546.1	0.156654	5.667286	0.211952	0.832389	0.909503	-6.42203
AC005841.1	-0.10217	7.08503	-0.21201	0.832347	0.909503	-6.91529
AC061975.6	-0.34646	-1.94342	-0.21107	0.833076	0.909685	-5.41278
AC068726.1	0.382644	-0.43064	0.211215	0.832964	0.909685	-5.4421
AC004994.1	-0.38742	-1.13021	-0.21049	0.833525	0.909685	-5.44344
AL513548.3	0.180944	0.921455	0.210686	0.833376	0.909685	-5.51067
AC096719.1	-0.16845	3.274482	-0.21056	0.833478	0.909685	-5.79468
AC060766.6	0.276871	4.809792	0.21123	0.832953	0.909685	-5.97885
AL662791.1	0.185033	6.340866	0.211526	0.832721	0.909685	-6.67435
AC073283.1	-0.13921	6.427182	-0.21054	0.833492	0.909685	-6.76223
AC107031.1	0.395405	-1.0582	0.209921	0.833973	0.909916	-5.42273
Z82186.1	-0.26162	-0.55984	-0.20991	0.833979	0.909916	-5.45857
Z99916.3	0.351119	-1.92058	0.207011	0.836241	0.911423	-5.39815
AP003497.2	-0.33923	-1.45741	-0.20704	0.836219	0.911423	-5.42863
AC103996.2	0.341512	0.358917	0.207043	0.836216	0.911423	-5.47491
AC010401.1	0.089838	2.887516	0.207261	0.836046	0.911423	-5.72548
AC005014.3	-0.25666	4.071537	-0.20734	0.835981	0.911423	-6.14998
LINC01852	0.061548	5.5514	0.207789	0.835634	0.911423	-6.46202
AL035681.1	-0.05497	6.893417	-0.2069	0.836331	0.911423	-6.86576
MID1IP1-AS1	-0.06092	8.209087	-0.2073	0.836016	0.911423	-7.10441
AC016292.1	-0.36093	2.364172	-0.20645	0.836677	0.911537	-5.67074
AP005131.7	-0.20766	4.263994	-0.2065	0.836639	0.911537	-6.05521
AC012150.2	0.329239	1.480141	0.206212	0.836863	0.911607	-5.53636
AL049646.1	0.199895	1.823175	0.205359	0.837529	0.912062	-5.58646
AC091167.5	0.234696	4.006375	0.205183	0.837666	0.912062	-5.93069
AC020907.4	-0.09039	7.32967	-0.20505	0.837766	0.912062	-6.95839
AC021078.1	-0.04477	9.932352	-0.20549	0.837427	0.912062	-7.36957

ITPRIP-AS1	-0.08468	6.320695	-0.20409	0.838521	0.912751	-6.77129
AC009044.1	0.253004	1.168256	0.203236	0.839186	0.913342	-5.52237
LINC00605	0.217087	4.419792	0.202891	0.839454	0.913502	-6.05578
AL645504.1	-0.06828	5.377095	-0.2022	0.839993	0.913957	-6.43919
AC036108.1	-0.19134	4.531343	-0.20174	0.840353	0.914215	-6.07406
AP001318.2	-0.04836	9.224502	-0.20101	0.840924	0.914704	-7.2661
AC137579.1	-0.32693	-1.94844	-0.20043	0.841373	0.914795	-5.41704
AC006157.1	0.385793	1.416664	0.200668	0.84119	0.914795	-5.52956
AP001922.5	0.192052	4.162218	0.20044	0.841368	0.914795	-5.98146
LINC02556	0.348729	0.359815	0.199788	0.841877	0.915078	-5.47683
AL442647.1	-0.34716	0.421126	-0.19986	0.841821	0.915078	-5.50921
AL021391.1	0.292316	2.922007	0.198918	0.842556	0.915684	-5.6812
AC092718.5	0.097223	5.588484	0.19788	0.843367	0.916433	-6.47076
LINC00608	0.237103	-1.39616	0.196772	0.844233	0.916559	-5.41778
AC084819.1	-0.37388	-0.50322	-0.19676	0.844245	0.916559	-5.46971
AL591212.1	-0.26338	1.666668	-0.19749	0.843672	0.916559	-5.61925
AC015923.1	0.211217	3.960871	0.197135	0.843949	0.916559	-5.88091
AC005225.1	-0.11969	4.585811	-0.19664	0.844337	0.916559	-6.19208
AC008764.6	-0.03634	6.449173	-0.19687	0.844156	0.916559	-6.75185
AC040934.1	0.059662	6.598231	0.196824	0.844193	0.916559	-6.77423
AC093019.2	0.310003	1.975132	0.196455	0.844481	0.916566	-5.58182
Z82188.2	0.253555	2.649896	0.196231	0.844656	0.916566	-5.66141
AC018413.1	-0.09109	6.736604	-0.19616	0.844709	0.916566	-6.83747
AC116351.1	-0.1239	3.78141	-0.19578	0.845009	0.916759	-5.94324
AC006017.1	0.081241	6.40251	0.195258	0.845416	0.917068	-6.71386
AC051619.6	0.321179	-0.98641	0.19436	0.846118	0.917167	-5.43022
AC093510.2	-0.22914	1.983566	-0.1949	0.8457	0.917167	-5.71232
AC016717.2	-0.18984	2.824843	-0.19443	0.846067	0.917167	-5.76913
AC022113.1	-0.11526	3.613105	-0.19446	0.846043	0.917167	-5.91648
AC008982.2	0.04746	8.365213	0.194665	0.84588	0.917167	-7.11747
AC105020.3	0.322073	-1.01822	0.193798	0.846558	0.91731	-5.42815
AL355472.3	-0.19698	5.38395	-0.19393	0.846456	0.91731	-6.45352
AC097534.1	-0.10139	6.085485	-0.19372	0.846616	0.91731	-6.67023
AL162151.1	0.335288	-1.74995	0.193422	0.846851	0.917433	-5.40669
AL031716.1	0.081845	6.872243	0.193233	0.846999	0.917461	-6.84898
LINC00582	-0.26387	2.631046	-0.19292	0.847245	0.917462	-5.72068
AC087481.3	-0.03081	9.423409	-0.19295	0.847217	0.917462	-7.29217
AL353597.3	-0.25818	-1.30258	-0.19262	0.847482	0.917548	-5.43515
AL118511.2	-0.36138	-0.80942	-0.19234	0.847701	0.917548	-5.45899
AL031289.1	0.252142	3.292939	0.192423	0.847633	0.917548	-5.74371
AL512598.1	-0.20918	5.908175	-0.19219	0.847813	0.917548	-6.52464
AC024651.1	-0.27994	-1.7342	-0.18905	0.850271	0.918093	-5.42306
AC015911.6	0.32664	-0.73974	0.189873	0.849628	0.918093	-5.43694
AP001257.1	0.34158	-0.21857	0.190205	0.849368	0.918093	-5.45487
AC146944.4	0.328163	-0.20172	0.190785	0.848914	0.918093	-5.45548
AL031595.1	0.177628	1.062279	0.189923	0.849589	0.918093	-5.52399
AC016229.2	-0.32035	1.292197	-0.18929	0.850085	0.918093	-5.55633
AC010273.2	0.348292	2.726466	0.189117	0.850219	0.918093	-5.63717
AP000662.1	-0.15995	3.212227	-0.18983	0.84966	0.918093	-5.8128
AC005256.1	0.335284	4.684119	0.190119	0.849435	0.918093	-6.05479
AC022079.2	0.144574	5.605953	0.189271	0.850098	0.918093	-6.35819
AC019226.1	-0.04502	6.31027	-0.1899	0.849603	0.918093	-6.73091
AC092301.1	-0.03877	7.339914	-0.18936	0.850025	0.918093	-6.95814
AP001628.1	0.072217	7.366418	0.189197	0.850156	0.918093	-6.96452
ZBTB40-IT1	-0.05265	7.302227	-0.18991	0.849601	0.918093	-6.96853
AL031717.1	0.040096	8.301207	0.189438	0.849968	0.918093	-7.11261
AC005884.2	-0.06231	8.794656	-0.18968	0.849778	0.918093	-7.20662
RSF1-IT1	0.140527	4.324591	0.18822	0.850921	0.918662	-6.05058

AL161729.1	-0.0627	5.978661	-0.18758	0.851425	0.919074	-6.65107
AC093484.2	0.242104	0.156808	0.187263	0.85167	0.919206	-5.47338
FBXW7-AS1	-0.19476	3.933047	-0.18617	0.852527	0.92	-5.99758
AL031599.1	0.298598	-0.34356	0.185498	0.853052	0.920304	-5.45273
AP002847.1	-0.14701	3.994875	-0.1855	0.853054	0.920304	-6.0081
LINC01764	-0.29528	0.34802	-0.18413	0.854126	0.921235	-5.50482
AC008443.5	-0.06066	7.478675	-0.18408	0.854162	0.921235	-6.98555
AC092747.4	0.044774	10.16249	0.18373	0.854437	0.921399	-7.39532
LINC02132	0.309806	-0.85678	0.183256	0.854808	0.921402	-5.43551
AL121748.1	-0.1392	4.483734	-0.18329	0.854779	0.921402	-6.16893
AC010680.2	-0.07191	5.870201	-0.18351	0.854609	0.921402	-6.58462
AC068305.2	0.143762	5.740523	0.182557	0.855356	0.92186	-6.49512
AC022893.3	-0.22434	3.248691	-0.18219	0.85564	0.922034	-5.83965
LINC00112	-0.31037	-1.68448	-0.18172	0.856014	0.922123	-5.42618
LINC02351	-0.34798	-1.146	-0.18156	0.856139	0.922123	-5.44364
AL358216.1	0.209247	1.368101	0.181873	0.855891	0.922123	-5.54702
AC008555.1	-0.10245	5.042446	-0.18146	0.856213	0.922123	-6.37251
AC106053.1	-0.28077	1.408115	-0.18051	0.856962	0.922769	-5.57573
AL031709.1	-0.06827	6.199839	-0.18038	0.857059	0.922769	-6.71585
LINC01123	0.066361	6.777974	0.179814	0.857505	0.923118	-6.79211
AC007347.1	-0.0709	6.769291	-0.17956	0.857702	0.923197	-6.85392
BRWD1-AS1	0.231785	2.183336	0.17737	0.859422	0.924916	-5.62418
AL021937.3	-0.31931	-0.27361	-0.17691	0.859782	0.925104	-5.47663
SPON1-AS1	-0.23401	3.895335	-0.17677	0.859894	0.925104	-5.96582
AL049795.1	0.044454	5.748047	0.176676	0.859966	0.925104	-6.51849
AL158211.1	0.159628	4.239667	0.176267	0.860287	0.925316	-6.03735
FO393415.1	0.215367	0.953245	0.175888	0.860584	0.925504	-5.51387
AL117381.1	-0.11411	6.896707	-0.17546	0.860917	0.925729	-6.88591
AC226118.1	-0.17903	4.630888	-0.17515	0.861162	0.925805	-6.1659
AP003119.3	0.070027	7.726956	0.175059	0.861234	0.925805	-7.01943
AC079921.2	-0.07778	7.226448	-0.17428	0.861848	0.926333	-6.95044
AP001160.4	0.043103	7.052428	0.174076	0.862006	0.926369	-6.87215
AC099506.1	0.20821	0.789788	0.173318	0.862601	0.926744	-5.50649
AL353608.4	0.164019	3.744972	0.173361	0.862567	0.926744	-5.88606
AL109924.2	0.265426	1.705838	0.172955	0.862886	0.926917	-5.55509
BAALC-AS2	0.273893	2.424816	0.172154	0.863514	0.927327	-5.63767
AL139384.2	-0.07901	4.82439	-0.17219	0.863487	0.927327	-6.2281
AC008635.1	0.06948	5.965939	0.171785	0.863804	0.927506	-6.61086
AC107398.3	0.13872	1.720345	0.171024	0.864401	0.928014	-5.57255
LINC01634	-0.20492	0.797837	-0.17067	0.86468	0.928138	-5.52943
LINC01106	-0.05626	7.248418	-0.17056	0.864763	0.928138	-6.93693
AC079858.1	0.318481	0.205938	0.169885	0.865296	0.928187	-5.47661
AC067931.2	0.162988	2.948923	0.169561	0.86555	0.928187	-5.73542
AP000941.1	-0.08484	5.001089	-0.1702	0.865046	0.928187	-6.23917
AC019080.5	0.055388	7.085331	0.169738	0.865411	0.928187	-6.88135
ZNF32-AS1	-0.04546	6.904943	-0.16971	0.865437	0.928187	-6.88521
AC067852.4	0.041682	7.472676	0.169588	0.865529	0.928187	-6.97519
AC244093.3	-0.12606	5.00421	-0.16883	0.866122	0.928667	-6.29471
AC084855.1	0.290887	-1.03082	0.168417	0.866449	0.928886	-5.43674
SLCO4A1-AS1	-0.11471	7.68448	-0.16793	0.866828	0.92916	-7.03796
AC020658.7	0.21541	0.26028	0.1675	0.867169	0.929393	-5.48104
AL050320.1	-0.23538	2.423267	-0.16712	0.86747	0.929518	-5.68164
LINC01871	-0.11724	8.880078	-0.16704	0.867534	0.929518	-7.21495
LINC01099	-0.25389	-2.34972	-0.16664	0.867846	0.929587	-5.40781
AC022960.1	-0.12756	5.089967	-0.16671	0.867791	0.929587	-6.35691
SHANK2-AS1	0.147947	3.993919	0.166207	0.868185	0.92978	-5.91628
AC090519.2	-0.05895	5.777123	-0.16609	0.868273	0.92978	-6.56025
AP001972.4	0.289004	-1.13962	0.164214	0.869751	0.930222	-5.42819

PTENP1-AS	0.302471	-0.29086	0.164693	0.869375	0.930222	-5.46103
LINC02300	0.312482	0.079279	0.164823	0.869273	0.930222	-5.47407
AC126768.1	0.332972	2.043506	0.165109	0.869048	0.930222	-5.59711
AP001099.1	0.218648	3.669042	0.163364	0.870419	0.930222	-5.80975
AL139241.1	0.157623	3.594014	0.163876	0.870016	0.930222	-5.89129
AC009084.2	-0.05543	5.489516	-0.16377	0.870099	0.930222	-6.49889
AC005306.1	0.041401	5.877568	0.164102	0.869839	0.930222	-6.56713
AL135791.1	-0.06462	5.871021	-0.16403	0.869898	0.930222	-6.61856
AC119396.2	0.094171	6.773468	0.165249	0.868938	0.930222	-6.82192
AC008760.2	0.114343	6.785169	0.163537	0.870283	0.930222	-6.83333
AL606760.1	-0.04776	6.822488	-0.16416	0.86979	0.930222	-6.84794
AC010260.1	-0.0481	7.00081	-0.164	0.869919	0.930222	-6.90095
AC015849.3	-0.0327	9.763025	-0.16338	0.870405	0.930222	-7.34722
AC092818.1	0.284267	0.787398	0.163029	0.870683	0.930371	-5.50396
AC008708.2	0.272367	-1.60195	0.162716	0.870929	0.930502	-5.41649
LINC00273	-0.23287	0.398189	-0.16248	0.871118	0.930572	-5.50598
AC079203.2	-0.22844	2.985776	-0.16176	0.871684	0.930776	-5.79387
AL512329.2	-0.20294	4.329635	-0.16192	0.871552	0.930776	-6.12029
AC020594.1	-0.16872	5.043694	-0.1616	0.871805	0.930776	-6.27722
GEMIN7-AS1	0.034597	7.221365	0.161771	0.871672	0.930776	-6.92715
GPC6-AS2	-0.28211	-1.47529	-0.16056	0.872626	0.931119	-5.43384
LINC02387	-0.2238	2.877915	-0.16048	0.872689	0.931119	-5.72331
AP000974.1	0.0551	4.586612	0.160663	0.872543	0.931119	-6.10807
AC005532.1	0.120026	5.396283	0.160555	0.872628	0.931119	-6.48221
AC145285.2	-0.03486	6.815559	-0.16041	0.872746	0.931119	-6.84526
LINC02526	0.291032	-1.53416	0.1598	0.873222	0.931123	-5.41655
NBAT1	0.207082	0.773561	0.159774	0.873242	0.931123	-5.5074
LINC00517	0.158646	0.80006	0.159951	0.873103	0.931123	-5.51019
AC018511.1	0.202355	3.036413	0.15977	0.873246	0.931123	-5.74326
AC090198.1	-0.02829	8.476229	-0.15936	0.873568	0.931335	-7.15624
AC078789.1	0.28059	-1.61861	0.158517	0.874231	0.931909	-5.41448
AC008050.1	-0.1949	3.040185	-0.15774	0.87484	0.931969	-5.8062
AC008467.1	-0.05407	3.759506	-0.15784	0.874766	0.931969	-5.9344
AC097381.1	-0.089	3.638173	-0.15797	0.874661	0.931969	-5.94977
AC123768.2	0.054185	5.731737	0.158182	0.874495	0.931969	-6.54864
AC008937.2	-0.04177	6.323403	-0.15748	0.875049	0.931969	-6.72672
AC106739.1	-0.05439	7.347861	-0.15734	0.875156	0.931969	-6.97607
AP005482.3	0.039337	8.412281	0.157632	0.874928	0.931969	-7.13654
AC018529.1	0.063743	6.152335	0.157053	0.875383	0.93208	-6.66973
AC008871.1	0.275997	-0.59336	0.156354	0.875934	0.932533	-5.44953
ZDHHC20-IT1	0.040015	7.167498	0.156156	0.876089	0.932567	-6.91751
AC008250.1	0.270348	-1.69554	0.154086	0.877718	0.932717	-5.41485
ZFY-AS1	-0.26897	0.14701	-0.15435	0.877508	0.932717	-5.49979
AL121821.1	-0.27159	0.257335	-0.15393	0.877844	0.932717	-5.50218
AC010463.2	0.232564	1.481719	0.15432	0.877534	0.932717	-5.54839
AC004543.1	-0.22273	2.567257	-0.15496	0.877028	0.932717	-5.70683
AC103974.1	-0.14087	4.766024	-0.15419	0.877639	0.932717	-6.19367
LNCSRLR	-0.12373	4.680581	-0.15424	0.877596	0.932717	-6.27225
AF131216.1	0.045807	4.951045	0.154017	0.877773	0.932717	-6.29202
AC008149.1	-0.07971	5.444991	-0.15471	0.877224	0.932717	-6.46829
AC093732.1	0.073665	6.627859	0.155397	0.876686	0.932717	-6.80221
AC007533.1	-0.04377	6.763046	-0.15575	0.876405	0.932717	-6.85452
U91328.1	-0.04704	7.440526	-0.15479	0.877166	0.932717	-6.98456
AL122035.2	0.030948	7.73392	0.154231	0.877604	0.932717	-7.03008
AC023510.1	-0.27342	0.184876	-0.15339	0.878267	0.932734	-5.50235
AC027801.1	-0.18471	0.938483	-0.15349	0.878189	0.932734	-5.53915
KCNIP2-AS1	-0.03865	5.609966	-0.15315	0.878457	0.932734	-6.50389
AC018607.1	0.097752	6.54723	0.152803	0.878729	0.932734	-6.73687

AC010768.2	0.04761	6.795236	0.152936	0.878624	0.932734	-6.8385
AC005856.1	-0.0674	6.975747	-0.15317	0.878442	0.932734	-6.90594
AC112491.1	0.047816	12.22319	0.152941	0.87862	0.932734	-7.68389
AC109597.1	0.186726	2.723277	0.149494	0.881335	0.935239	-5.6807
LINC01909	0.063786	6.079212	0.14949	0.881338	0.935239	-6.62109
AC008764.9	0.051638	4.462085	0.148747	0.881923	0.935728	-6.12286
AC009137.1	0.262945	1.066163	0.147569	0.882852	0.936332	-5.51902
AL022157.1	-0.07177	6.138701	-0.14778	0.882685	0.936332	-6.69486
AC015922.2	-0.08952	9.670634	-0.14755	0.882867	0.936332	-7.34405
AL596220.1	-0.0749	5.424452	-0.14712	0.883205	0.936558	-6.50852
AL161729.4	0.094438	6.061583	0.146777	0.883476	0.936714	-6.63661
AC093752.2	0.138785	4.242282	0.146225	0.883911	0.93704	-6.04974
AC092757.3	-0.05545	5.906937	-0.14607	0.884033	0.93704	-6.61142
AL590705.3	-0.07986	6.536556	-0.14549	0.884493	0.937395	-6.79077
AL022341.2	-0.07111	4.005604	-0.14475	0.885075	0.937531	-6.06193
AC017071.1	-0.04847	4.658612	-0.14469	0.885123	0.937531	-6.20527
AL356740.3	0.150858	5.582237	0.145143	0.884764	0.937531	-6.45161
AP001429.1	-0.05791	6.256457	-0.14482	0.88502	0.937531	-6.71642
AC009113.1	0.035712	7.805503	0.144533	0.885245	0.937531	-7.04023
PCAT5	-0.20646	-2.15358	-0.14432	0.885412	0.937576	-5.41435
AC112694.2	-0.03412	7.337281	-0.14367	0.885927	0.937989	-6.96898
AC079336.4	0.156231	4.132484	0.142726	0.88667	0.938644	-6.03323
AC007207.2	-0.26319	-0.19053	-0.14149	0.887642	0.939119	-5.48643
AP000753.1	0.229901	0.3352	0.141476	0.887656	0.939119	-5.48835
AC004471.2	-0.11471	3.395991	-0.14191	0.88731	0.939119	-5.88499
AL136985.2	-0.07979	5.027711	-0.14159	0.887563	0.939119	-6.3437
AL512343.2	-0.0494	6.585091	-0.14136	0.887744	0.939119	-6.81567
AL139120.1	0.039645	7.53878	0.140813	0.888179	0.939447	-6.99793
AC087362.1	-0.15252	3.614062	-0.14044	0.88847	0.939622	-5.96314
AC083843.2	0.22554	-0.12022	0.13984	0.888947	0.939861	-5.47138
AC104561.3	0.172134	3.143135	0.139846	0.888941	0.939861	-5.74536
AC107976.1	0.233586	-1.97156	0.139466	0.889242	0.940041	-5.40918
AL158207.2	-0.14312	4.326352	-0.13909	0.889536	0.94022	-6.19036
LINC00244	0.212986	1.821559	0.138771	0.88979	0.940356	-5.58153
AC092567.1	-0.24625	-0.16998	-0.13854	0.889969	0.940413	-5.48881
AC006441.4	-0.21322	-0.54786	-0.13764	0.890679	0.940629	-5.46762
AC079015.1	0.221175	1.089688	0.13752	0.890777	0.940629	-5.52383
AC012486.1	0.228764	2.891407	0.137715	0.890623	0.940629	-5.67816
LINC02202	-0.0646	4.213879	-0.13749	0.890799	0.940629	-6.02673
AC023024.1	0.044101	6.327032	0.137803	0.890554	0.940629	-6.7114
AC002044.1	0.064403	8.302601	0.137275	0.89097	0.940677	-7.11662
AL035252.4	-0.0991	6.336167	-0.137	0.891184	0.940771	-6.73974
AC026362.1	0.028696	4.269869	0.13664	0.891472	0.940942	-6.06148
AC023794.2	0.168579	3.503176	0.136435	0.891634	0.940981	-5.83317
AL132655.1	0.222997	0.390439	0.135832	0.892109	0.941351	-5.49163
AC092620.2	0.146317	1.063575	0.135533	0.892346	0.941468	-5.52712
AL596202.1	0.030835	7.254326	0.134995	0.89277	0.941732	-6.92351
AC004253.1	-0.0297	7.754772	-0.1349	0.892846	0.941732	-7.04354
AP002340.1	0.090979	6.45166	0.134238	0.893368	0.94215	-6.66084
AL157912.1	-0.20677	1.32981	-0.13385	0.893676	0.942342	-5.5694
AL359555.1	-0.2165	-1.75646	-0.13365	0.893834	0.942376	-5.42757
AC018450.1	0.192442	2.069586	0.133395	0.894034	0.942455	-5.59822
AC026462.3	0.265785	-0.07527	0.131872	0.895236	0.94301	-5.47048
AL139161.2	0.21463	1.040339	0.131616	0.895439	0.94301	-5.5226
AC099684.1	-0.17873	1.052244	-0.13239	0.894824	0.94301	-5.54685
AF131216.3	-0.2171	2.114492	-0.13163	0.895428	0.94301	-5.63416
AC127526.1	0.230582	2.537022	0.132097	0.895058	0.94301	-5.65156
AL121832.1	-0.16828	2.310959	-0.13174	0.895341	0.94301	-5.69208

AC026748.1	-0.10659	2.612476	-0.13246	0.894773	0.94301	-5.69935
LINC02453	-0.04852	5.061115	-0.13106	0.895879	0.943341	-6.30175
LINC01722	0.181426	-1.48834	0.12877	0.897687	0.944684	-5.42764
AC018521.7	-0.06038	2.704018	-0.12849	0.897909	0.944684	-5.71772
AC093904.2	0.133815	5.64224	0.128548	0.897862	0.944684	-6.4266
AC079305.1	0.046957	7.252583	0.128652	0.89778	0.944684	-6.95068
AC005840.4	0.034208	7.715589	0.128788	0.897672	0.944684	-7.01521
AC106782.2	0.034123	8.820593	0.1288	0.897663	0.944684	-7.19594
AC006333.2	-0.0279	9.250433	-0.12803	0.898273	0.944935	-7.28279
AC008759.3	-0.18175	3.75341	-0.12687	0.899189	0.945766	-5.92856
AL009181.1	-0.19519	-0.07952	-0.12522	0.900494	0.946068	-5.48417
AL096817.1	-0.09971	2.650518	-0.12515	0.900549	0.946068	-5.71206
AC068724.1	-0.12148	3.776745	-0.1256	0.900189	0.946068	-5.93333
AC018755.4	-0.21248	4.140509	-0.12593	0.899929	0.946068	-6.04762
AC100774.1	-0.07263	4.370238	-0.12563	0.900166	0.946068	-6.12087
AC005237.1	-0.12788	4.423471	-0.12572	0.900098	0.946068	-6.13744
AC092143.3	0.028517	6.54347	0.125071	0.90061	0.946068	-6.79648
AC012467.2	-0.02889	8.766302	-0.12537	0.900374	0.946068	-7.20534
BX284668.5	-0.05266	9.477731	-0.12547	0.900296	0.946068	-7.31284
LINC02206	0.203008	-2.11782	0.123909	0.901529	0.946108	-5.40716
AP001476.1	0.149231	1.00349	0.124055	0.901414	0.946108	-5.52335
AC118555.1	0.167693	2.241948	0.124335	0.901192	0.946108	-5.64257
SMCR5	0.072435	3.632652	0.124066	0.901405	0.946108	-5.86008
GLIS2-AS1	0.114335	5.990807	0.124107	0.901372	0.946108	-6.4942
GRPEL2-AS1	-0.07407	5.820105	-0.12444	0.90111	0.946108	-6.63912
AL136221.1	0.066054	6.906249	0.123913	0.901526	0.946108	-6.85549
LINC01765	0.216279	0.670091	0.123539	0.901821	0.94623	-5.50318
AC026471.4	-0.02938	9.315574	-0.12344	0.901897	0.94623	-7.29032
AC010809.2	0.032327	7.17198	0.122836	0.902377	0.946602	-6.9284
AL590729.1	0.033277	6.43846	0.122635	0.902536	0.946636	-6.74866
LINC01374	-0.21846	-1.33649	-0.12188	0.903133	0.946865	-5.44125
RERG-IT1	0.233085	0.226248	0.122139	0.902928	0.946865	-5.48387
AC008537.2	-0.03569	7.850363	-0.1219	0.903117	0.946865	-7.06334
LINC01713	-0.20863	-1.38145	-0.12161	0.903343	0.946883	-5.44026
AC008752.1	-0.14067	3.799033	-0.12112	0.903733	0.946883	-5.96228
DENND5B-AS1	-0.04942	4.807214	-0.12151	0.903427	0.946883	-6.22468
AC126768.2	0.187154	5.077331	0.121063	0.90378	0.946883	-6.2338
AC007496.1	0.027721	6.279845	0.121199	0.903672	0.946883	-6.74323
AC022296.3	0.211342	0.761135	0.119548	0.904977	0.948006	-5.50998
AC025031.1	-0.10871	3.768009	-0.11859	0.905733	0.948618	-5.92305
AC006504.8	-0.04435	7.817204	-0.11849	0.905814	0.948618	-7.05566
AC012368.2	0.104863	5.200265	0.117979	0.906219	0.948909	-6.22641
AP006621.4	0.070574	7.606487	0.11777	0.906384	0.94895	-7.00852
AL807761.4	-0.1484	-0.55807	-0.117	0.906993	0.949455	-5.47106
AC092868.2	0.04081	7.242437	0.115902	0.907862	0.950233	-6.9415
AC083805.1	0.098927	1.295523	0.115611	0.908092	0.950341	-5.55154
AC010624.2	0.201478	0.350425	0.115385	0.908271	0.950396	-5.49001
AC008686.1	-0.08738	3.188429	-0.11481	0.908724	0.950737	-5.84609
PLCH1-AS1	-0.17841	0.243624	-0.11398	0.909382	0.951036	-5.49926
Z84723.1	0.109752	3.444825	0.113801	0.909525	0.951036	-5.84413
LINC00900	-0.05079	4.485171	-0.11377	0.909549	0.951036	-6.18964
AC080112.3	-0.03171	5.525684	-0.11426	0.909165	0.951036	-6.50629
AC106782.6	-0.02543	8.013029	-0.11365	0.909643	0.951036	-7.09063
AC097478.4	0.184053	1.348621	0.113307	0.909916	0.951183	-5.54899
AC087878.1	-0.15114	2.481429	-0.11315	0.910037	0.951183	-5.68272
AC092118.1	0.069317	4.936699	0.11222	0.910777	0.951825	-6.18766
AL645608.2	0.086336	3.602451	0.111022	0.911725	0.952683	-5.91413
AC093206.1	-0.19146	-1.37449	-0.10976	0.912722	0.953592	-5.44199

AP000944.1	0.144404	3.128688	0.10929	0.913097	0.953793	-5.76159
AC243829.1	0.111252	3.459848	0.1092	0.913169	0.953793	-5.85406
AC008781.1	-0.02752	5.34056	-0.10849	0.913732	0.954249	-6.47034
AL139123.1	-0.0411	6.220393	-0.10796	0.914153	0.954557	-6.7241
AC005393.1	-0.03874	6.140505	-0.1075	0.914516	0.954802	-6.70934
CR559946.1	0.11481	3.561208	0.106866	0.915017	0.955193	-5.84786
LZTS1-AS1	0.16697	-0.09764	0.105921	0.915766	0.955444	-5.47763
AC098850.1	-0.17985	0.263998	-0.10624	0.915514	0.955444	-5.50205
AC010735.2	-0.06082	6.338149	-0.10617	0.915571	0.955444	-6.73928
AC087276.2	0.025632	6.38068	0.106043	0.915669	0.955444	-6.74337
ARHGAP26-IT1	-0.17057	2.137843	-0.10557	0.916041	0.955465	-5.65442
AP000777.2	-0.11915	3.309674	-0.10567	0.915963	0.955465	-5.85979
AC104076.1	0.195379	-0.50146	0.104626	0.916792	0.95551	-5.4598
AL359771.1	0.237615	1.441773	0.104728	0.916711	0.95551	-5.54498
AC020910.6	-0.07892	3.65361	-0.10474	0.9167	0.95551	-5.88491
AL606468.1	0.119019	3.764052	0.104755	0.916689	0.95551	-5.90407
AC079148.1	0.032229	5.395996	0.104741	0.916701	0.95551	-6.4612
AC107294.2	-0.05738	6.482526	-0.10456	0.916847	0.95551	-6.77173
AP001010.1	0.112304	3.769575	0.103774	0.917467	0.956024	-5.85204
AC007608.4	-0.06813	3.157741	-0.1028	0.91824	0.956696	-5.82135
AC026310.3	-0.10557	2.052452	-0.10247	0.918504	0.956719	-5.64339
AC069549.1	-0.04026	7.586178	-0.10245	0.918517	0.956719	-7.03838
BX255925.1	-0.04465	6.010904	-0.10141	0.919337	0.957441	-6.60856
AP002967.1	-0.15581	0.905707	-0.10108	0.919603	0.957585	-5.54483
AC131888.1	0.064733	3.649154	0.100547	0.920024	0.957891	-5.86813
AL591885.1	0.139027	-0.93155	0.099881	0.920552	0.958175	-5.44891
APOA1-AS	-0.06253	5.134703	-0.10001	0.920448	0.958175	-6.28371
AC093157.2	0.035314	6.884217	0.09893	0.921307	0.958828	-6.8627
SEC23A-AS1	-0.15619	0.527015	-0.09815	0.921928	0.959341	-5.51522
AC004217.1	0.145984	1.316539	0.097716	0.922269	0.959444	-5.54307
AC243772.2	-0.05327	4.375992	-0.09738	0.922538	0.959444	-6.13923
AC004466.3	-0.03778	6.25013	-0.09738	0.922532	0.959444	-6.7103
AC008906.1	-0.02557	7.534146	-0.09767	0.922308	0.959444	-7.00167
AL096803.3	-0.16558	-0.13176	-0.09691	0.922907	0.959696	-5.49038
AC010542.1	0.121908	4.116149	0.09522	0.924248	0.960824	-5.90633
LAMC1-AS1	-0.03859	7.171468	-0.09538	0.924123	0.960824	-6.94245
AC079610.2	0.047493	6.255859	0.094932	0.924476	0.960929	-6.74353
AC016493.1	-0.15178	-1.40548	-0.09432	0.924962	0.960934	-5.44008
AL136307.1	0.173736	-1.03767	0.09447	0.924844	0.960934	-5.44796
AMMECR1-IT1	-0.1314	1.581601	-0.09423	0.92503	0.960934	-5.60155
AC084880.3	0.160106	1.610869	0.093797	0.925377	0.960934	-5.61848
AL109935.1	-0.11998	3.297153	-0.09398	0.925233	0.960934	-5.82117
AC011773.3	-0.06892	4.335412	-0.0944	0.924896	0.960934	-6.05299
AC011120.1	-0.02289	6.447607	-0.09383	0.925353	0.960934	-6.77798
AL353593.1	0.033026	5.869978	0.092887	0.926099	0.961495	-6.61494
AL021707.5	0.049277	6.295239	0.092793	0.926173	0.961495	-6.68777
Z99943.1	0.099	3.778473	0.091708	0.927034	0.962256	-5.93208
RAET1E-AS1	0.133195	2.431623	0.091353	0.927316	0.962415	-5.69382
AL158832.1	0.158807	-0.54505	0.090852	0.927714	0.962695	-5.46188
Z82173.1	0.15054	0.914587	0.090556	0.927948	0.962805	-5.51925
FALEC	0.064879	5.604528	0.090094	0.928315	0.963053	-6.56966
AC073911.3	0.141264	-0.14344	0.088665	0.929449	0.964096	-5.4757
AP001107.1	0.014185	6.824754	0.088318	0.929725	0.964249	-6.85396
AC140912.1	0.139252	-0.70932	0.088096	0.9299	0.964298	-5.45574
AC011239.1	-0.1561	-1.19504	-0.08713	0.930668	0.964507	-5.44982
AL121890.2	0.13742	1.450604	0.087343	0.930498	0.964507	-5.5711
AL451069.2	-0.1102	2.877362	-0.08747	0.930396	0.964507	-5.93044
AL121672.3	0.077441	4.663295	0.086872	0.930872	0.964507	-6.19877

AC092384.2	0.051349	4.685026	0.086955	0.930806	0.964507	-6.2328
AC020663.3	0.016749	6.319305	0.086953	0.930808	0.964507	-6.74146
AC015917.2	0.122346	3.658205	0.086067	0.931511	0.964689	-5.81761
AL159169.3	-0.08488	3.994657	-0.08647	0.93119	0.964689	-6.05999
AC096649.1	0.064014	5.321996	0.086292	0.931332	0.964689	-6.22943
SENCR	-0.0326	6.145228	-0.086	0.931561	0.964689	-6.68658
AC009511.2	0.120381	1.797198	0.085336	0.932092	0.965105	-5.60227
AC112719.2	0.075379	2.50806	0.08434	0.932882	0.965791	-5.67858
AP001412.1	-0.02484	5.585314	-0.08392	0.933214	0.966001	-6.4908
AC084824.4	-0.01556	8.985785	-0.08375	0.933349	0.966007	-7.23983
AC093151.3	0.081546	2.608877	0.083101	0.933865	0.966276	-5.69758
LINC00943	0.075271	3.313857	0.083153	0.933824	0.966276	-5.80682
AC092839.2	0.131651	2.204651	0.082797	0.934107	0.966393	-5.62992
GRTP1-AS1	-0.03093	5.652986	-0.08262	0.934251	0.966409	-6.5389
AC097347.1	0.107666	4.625973	0.082067	0.934687	0.966726	-6.0732
AC006213.6	0.110724	4.935834	0.081158	0.935408	0.96734	-6.16566
AC068888.2	-0.01846	7.188855	-0.08091	0.935604	0.967408	-6.93353
AC027307.2	-0.02056	9.963837	-0.08071	0.935761	0.967439	-7.38982
AC090617.8	0.139629	2.66945	0.080539	0.9359	0.967449	-5.71344
AC008747.1	0.124954	1.237016	0.08012	0.936233	0.96755	-5.55031
AC026704.1	-0.02558	6.461078	-0.08009	0.936256	0.96755	-6.76206
AC104984.6	-0.03857	6.489817	-0.07991	0.936398	0.967564	-6.79109
AC006487.1	-0.10795	1.767312	-0.07952	0.936707	0.967751	-5.61698
AC004009.2	-0.15789	-0.85912	-0.07899	0.93713	0.967954	-5.45922
AC007216.4	0.025834	6.875657	0.07895	0.937162	0.967954	-6.86497
UBXN7-AS1	0.054722	6.03376	0.078723	0.937343	0.968008	-6.49026
AC103681.2	0.097544	2.663828	0.07856	0.937471	0.968008	-5.69563
AC019080.3	0.031283	7.870795	0.078244	0.937723	0.968134	-7.05733
Z97192.3	0.077487	2.159313	0.077265	0.9385	0.968804	-5.63742
LINC01700	-0.11342	-2.13999	-0.07681	0.938859	0.96904	-5.41966
AL080317.1	0.014901	9.484983	0.076504	0.939105	0.969162	-7.30646
AP001626.1	-0.10179	-0.77571	-0.0758	0.939666	0.969555	-5.46321
AF213884.3	0.034003	5.603886	0.0757	0.939744	0.969555	-6.5097
AC021860.1	-0.1343	-1.11084	-0.07527	0.940085	0.969697	-5.45354
FO393419.2	-0.01982	6.573349	-0.0752	0.94014	0.969697	-6.81239
AC080023.1	-0.03967	6.077121	-0.07435	0.940815	0.970259	-6.68435
UCKL1-AS1	0.01905	7.259655	0.074074	0.941036	0.970355	-6.94947
CARD8-AS1	0.01858	8.243646	0.073898	0.941176	0.970365	-7.11845
AC068768.1	-0.07226	4.186878	-0.07364	0.941377	0.970395	-6.03175
AL024498.1	-0.02686	5.522327	-0.07354	0.941463	0.970395	-6.4929
LINC02547	0.099494	-2.31622	0.073023	0.941871	0.970557	-5.40762
AC244093.5	0.029334	6.01949	0.073013	0.941878	0.970557	-6.62281
AC090136.3	0.077992	0.711061	0.07254	0.942255	0.970812	-5.52161
AC078820.1	0.116064	3.005289	0.072308	0.942439	0.970868	-5.72809
AP000553.3	-0.11183	1.176591	-0.07207	0.942625	0.970927	-5.55743
AC110760.1	-0.12727	-1.51175	-0.07182	0.942825	0.971	-5.43832
AC055764.1	0.042358	6.460175	0.071014	0.943467	0.971528	-6.74871
AC074011.1	-0.04683	5.118695	-0.0708	0.94364	0.971573	-6.3928
AL513477.2	-0.02261	7.215557	-0.07062	0.943778	0.971582	-6.95481
AC080188.1	-0.06512	4.774936	-0.07046	0.943909	0.971584	-6.19448
AC011447.7	0.046295	6.8922	0.068428	0.945523	0.973111	-6.85475
AC092067.1	-0.05503	4.899838	-0.06803	0.945839	0.973304	-6.28746
AC020911.1	-0.01476	6.033632	-0.06714	0.946549	0.973901	-6.61413
AC005695.2	0.058174	5.434351	0.066865	0.946765	0.97399	-6.35298
FOXN3-AS1	0.018926	8.54999	0.066617	0.946962	0.974059	-7.17113
AC100793.3	-0.06812	3.229015	-0.06627	0.947235	0.974206	-5.83918
AC013564.1	0.1114	-0.86042	0.065445	0.947894	0.974217	-5.45185
KLHL6-AS1	0.096499	2.705499	0.065876	0.947551	0.974217	-5.68867

AC074050.4	-0.05396	4.25059	-0.06555	0.947811	0.974217	-6.10535
AP003354.1	0.046843	5.253353	0.065536	0.947821	0.974217	-6.32768
ASH1L-IT1	-0.03134	5.847818	-0.06545	0.947893	0.974217	-6.58453
AC084024.3	0.088717	2.273211	0.064936	0.948299	0.974367	-5.65394
AL132989.2	0.015666	7.194187	0.065081	0.948184	0.974367	-6.93428
AC036222.2	-0.11775	-1.07667	-0.06468	0.948503	0.974443	-5.45143
RASGRF2-AS1	0.053329	3.189267	0.064052	0.949001	0.974662	-5.79848
AL139807.1	-0.03083	5.688407	-0.06395	0.94908	0.974662	-6.55665
AL132780.4	0.037681	6.823053	0.063921	0.949105	0.974662	-6.85467
AC026202.3	0.083455	1.093904	0.063137	0.949729	0.974827	-5.54183
AC007431.1	-0.0327	4.8663	-0.06317	0.949702	0.974827	-6.256
AP003108.4	-0.02491	5.030305	-0.06326	0.949635	0.974827	-6.35467
AC004232.3	-0.01451	6.929233	-0.06307	0.949785	0.974827	-6.87822
AC022165.1	0.045563	5.376148	0.062789	0.950006	0.974921	-6.42921
AL583722.1	0.055968	4.937535	0.062051	0.950593	0.97539	-6.17823
AC240565.2	-0.01826	6.297911	-0.06104	0.951393	0.976077	-6.7423
AC008033.3	0.077574	2.36985	0.060281	0.952	0.976567	-5.64552
TOLLIP-AS1	0.016279	8.085726	0.060091	0.952152	0.976589	-7.09697
AC012456.2	0.098868	0.48498	0.058219	0.95364	0.976958	-5.50832
Z95114.1	-0.02586	1.338195	-0.05929	0.952787	0.976958	-5.5656
AC097505.1	0.07352	2.787619	0.058755	0.953214	0.976958	-5.71346
NAV2-AS2	-0.06083	3.432507	-0.05828	0.953589	0.976958	-5.90617
CERS3-AS1	0.10667	3.962425	0.058372	0.953519	0.976958	-5.96292
AC007496.3	-0.0151	4.861686	-0.05817	0.953681	0.976958	-6.26772
AL359878.1	-0.01535	5.230828	-0.05916	0.95289	0.976958	-6.3664
AC100793.2	0.037797	5.355835	0.058973	0.953041	0.976958	-6.46137
AC011247.1	0.025187	6.954893	0.058725	0.953237	0.976958	-6.88663
AC104316.1	0.035024	5.15492	0.057554	0.954169	0.977324	-6.34538
AC021092.1	-0.03458	3.393348	-0.05702	0.954597	0.977629	-5.86809
AC079950.1	-0.08832	-2.16225	-0.05644	0.955058	0.977701	-5.41981
AC044849.1	0.030603	4.983391	0.056655	0.954884	0.977701	-6.28234
AC012186.2	0.02762	5.980167	0.056497	0.95501	0.977701	-6.61741
AC008115.4	0.042222	4.762396	0.056246	0.955209	0.977723	-6.12699
AL359853.2	0.051304	5.110873	0.055679	0.955661	0.978052	-6.2002
AC110285.7	-0.03064	7.506631	-0.0555	0.955804	0.978066	-6.99305
AC007663.4	-0.01407	6.593457	-0.05533	0.955935	0.978066	-6.81668
AC107294.3	-0.05191	4.963496	-0.05439	0.956688	0.978436	-6.25051
AC013489.3	0.026489	5.085867	0.054695	0.956444	0.978436	-6.34342
ADIRF-AS1	-0.02514	8.138708	-0.05447	0.956624	0.978436	-7.11431
AC068506.1	0.078975	2.127238	0.053944	0.957041	0.978665	-5.67139
AP001922.6	0.054654	4.13384	0.053249	0.957594	0.979097	-6.01578
AC012555.2	0.091955	0.021983	0.05266	0.958063	0.97931	-5.48915
AC004678.2	-0.02402	5.752488	-0.05272	0.958012	0.97931	-6.53693
AC087683.2	0.026543	6.034948	0.052171	0.958452	0.979574	-6.69195
AC092134.2	-0.07535	-2.28118	-0.05137	0.959091	0.980094	-5.41483
CAMTA1-IT1	-0.0922	-0.83902	-0.05052	0.959768	0.980401	-5.45803
AC015656.1	0.068479	1.021887	0.050498	0.959782	0.980401	-5.53991
AC012100.3	0.048142	4.051399	0.050527	0.95976	0.980401	-5.99563
AC114763.2	-0.03972	5.089553	-0.05007	0.960124	0.980616	-6.27485
AC002480.2	0.06577	2.66633	0.049541	0.960545	0.980779	-5.70172
AC016026.1	-0.01638	4.0844	-0.04964	0.960466	0.980779	-6.06075
Z95624.1	0.079594	-0.18311	0.048861	0.961086	0.980957	-5.47932
AL138921.1	-0.00895	6.055479	-0.04883	0.961111	0.980957	-6.64394
AL121761.1	-0.03463	7.928226	-0.04911	0.960884	0.980957	-7.08511
AL590822.2	-0.01908	4.055898	-0.04817	0.961633	0.981357	-6.00018
AC025262.2	-0.04004	3.479072	-0.04691	0.962636	0.982247	-5.89437
AC093503.3	-0.04321	1.659097	-0.04668	0.962825	0.982306	-5.59988
AC018755.1	0.069374	-1.4521	0.046121	0.963266	0.982623	-5.4354

AC097641.2	-0.00907	7.808677	-0.04579	0.96353	0.982758	-7.05314
AC009812.4	0.02054	6.646923	0.045547	0.963723	0.982776	-6.79617
AL138787.2	-0.0144	6.505223	-0.04544	0.963809	0.982776	-6.80095
LINC02373	0.067275	2.846615	0.045196	0.964002	0.98284	-5.73012
PSPC1-AS2	-0.0113	7.852901	-0.04491	0.964229	0.982938	-7.06121
AC097103.1	-0.08136	0.412589	-0.04443	0.964612	0.983195	-5.51124
FO704657.1	0.059208	2.743167	0.043013	0.96574	0.984064	-5.71932
AC007610.2	-0.03787	4.02406	-0.04311	0.965663	0.984064	-5.95607
AC008637.1	0.030408	6.124623	0.042865	0.965858	0.984064	-6.69021
AC114271.1	0.009776	5.952613	0.041964	0.966575	0.984661	-6.62482
NALT1	-0.01698	6.342338	-0.0415	0.966946	0.984905	-6.77317
LINC02391	0.056052	1.81773	0.041193	0.967189	0.984952	-5.60187
PRKAR2A-AS1	-0.01035	6.337503	-0.04111	0.967254	0.984952	-6.74106
AP001610.2	0.077548	0.260473	0.040578	0.967678	0.985083	-5.49953
AC092042.1	-0.05377	2.330032	-0.04029	0.967907	0.985083	-5.74918
AC068790.6	0.035492	5.46188	0.040734	0.967554	0.985083	-6.4386
AC127024.3	-0.02255	6.903138	-0.04033	0.967876	0.985083	-6.88755
AL158211.5	-0.01616	6.445509	-0.03989	0.968226	0.985275	-6.78111
AC079336.3	0.018161	6.267335	0.038005	0.969727	0.986668	-6.7377
AC134312.3	-0.0715	0.406473	-0.03768	0.969985	0.986798	-5.50621
AL360181.2	0.006764	9.194748	0.037119	0.970433	0.987119	-7.27114
AC138965.2	-0.0621	-1.6293	-0.03673	0.970745	0.987303	-5.43662
BSN-DT	0.045322	0.197189	0.036074	0.971264	0.987697	-5.49484
AC005832.1	-0.05887	0.256456	-0.03572	0.97155	0.987854	-5.50294
AC099550.1	0.05518	0.142164	0.034754	0.972315	0.987991	-5.49393
AC120349.2	-0.05715	1.852866	-0.03472	0.972342	0.987991	-5.61583
AL353708.1	0.011599	4.949019	0.034859	0.972231	0.987991	-6.32138
AC007216.2	-0.00805	7.820323	-0.03503	0.972097	0.987991	-7.0532
AC097468.3	-0.00745	9.244978	-0.03534	0.971851	0.987991	-7.2783
AL162584.1	-0.04599	1.90709	-0.0345	0.972515	0.988033	-5.60992
C10orf25	-0.01055	7.099591	-0.03398	0.97293	0.988321	-6.91718
AC131649.2	-0.01172	7.338588	-0.03342	0.973381	0.988645	-6.9812
AC133106.1	0.05701	0.463803	0.031996	0.974512	0.989125	-5.50876
AC008734.1	-0.03632	3.535972	-0.03207	0.974451	0.989125	-5.88085
AC018552.3	-0.02912	4.57344	-0.03206	0.97446	0.989125	-6.11931
AC005837.3	-0.02711	5.54068	-0.03226	0.9743	0.989125	-6.53969
AC009237.14	0.013957	9.415315	0.032118	0.974414	0.989125	-7.30279
C9orf139	-0.01192	3.791824	-0.03179	0.974672	0.989154	-5.95912
LINC01013	0.027326	2.028066	0.031542	0.974873	0.989225	-5.64483
AC069528.2	-0.00866	6.684718	-0.03057	0.975643	0.989839	-6.82797
PRKCZ-AS1	0.00836	8.581129	0.030452	0.975741	0.989839	-7.17834
AC012645.3	-0.01433	5.491213	-0.03001	0.976094	0.990062	-6.51018
LINC01388	0.053407	-0.9627	0.029778	0.976278	0.990063	-5.45507
AC009908.1	0.050867	-0.31229	0.029564	0.976448	0.990063	-5.47717
LINC02432	0.039302	2.465759	0.029512	0.97649	0.990063	-5.70052
AC128687.2	0.033139	3.522704	0.029223	0.97672	0.990163	-5.87184
AC138466.4	-0.04731	-1.76315	-0.02839	0.97738	0.990344	-5.43031
AL079342.3	-0.03119	1.254573	-0.02809	0.977621	0.990344	-5.56134
AC025809.2	0.036877	1.42607	0.02784	0.977821	0.990344	-5.56743
AC015819.1	0.017383	5.580887	0.028099	0.977615	0.990344	-6.52657
AL512652.1	0.012542	5.786724	0.027985	0.977706	0.990344	-6.60608
AL354893.2	0.013943	6.087838	0.028132	0.977588	0.990344	-6.68509
C5orf66-AS1	0.024917	7.931434	0.028337	0.977425	0.990344	-7.09268
AC079630.1	0.022006	3.943269	0.027648	0.977974	0.990365	-5.99012
AC114781.2	0.029413	3.99527	0.027248	0.978293	0.990446	-6.03881
PDCD4-AS1	0.007241	8.466542	0.027218	0.978317	0.990446	-7.16082
LINC02135	0.039224	-1.76497	0.02664	0.978777	0.990514	-5.42836
AC037450.1	0.046406	-1.21722	0.026746	0.978693	0.990514	-5.4443

AC023481.1	-0.04231	-0.4612	-0.02664	0.97878	0.990514	-5.47234
LINC02035	0.007301	9.43715	0.026427	0.978946	0.990549	-7.31134
AC087222.1	0.007774	7.311685	0.026216	0.979115	0.990586	-6.96366
AL512785.1	0.040456	3.172964	0.025721	0.979509	0.990814	-5.77543
AL035071.2	-0.00603	7.252359	-0.0256	0.979604	0.990814	-6.94886
AC016355.1	0.007308	6.777175	0.024745	0.980287	0.991371	-6.83587
AC006449.6	0.005983	7.62438	0.023716	0.981106	0.992066	-7.02557
AC012065.3	-0.01486	6.022017	-0.02345	0.981316	0.992145	-6.68003
AC022960.2	0.01574	4.174349	0.023061	0.981628	0.992327	-6.05134
AC018695.6	0.019999	6.118523	0.022197	0.982316	0.992888	-6.65364
AL132800.1	-0.00997	5.908093	-0.02154	0.982837	0.993281	-6.64708
AP000569.1	-0.02161	3.35215	-0.02032	0.983807	0.994074	-5.85348
AC138028.1	-0.00872	3.691421	-0.02023	0.983886	0.994074	-5.92742
ATP11A-AS1	0.015136	3.568163	0.019891	0.984153	0.99421	-5.90197
AC010680.3	0.021051	4.138321	0.019119	0.984768	0.994698	-6.00684
CPEB1-AS1	0.01487	1.328196	0.018906	0.984937	0.994735	-5.56504
AC004777.1	-0.03325	0.331115	-0.01824	0.985466	0.994972	-5.50636
AC009365.3	0.017896	3.08475	0.017448	0.986099	0.994972	-5.78487
AC022872.1	0.015508	3.475289	0.017451	0.986097	0.994972	-5.86703
AC104031.1	0.026466	4.509802	0.018174	0.985521	0.994972	-6.10591
LINC00528	0.009155	4.40114	0.017682	0.985913	0.994972	-6.11559
AC021087.1	-0.00957	5.387325	-0.01794	0.985709	0.994972	-6.47366
AC002128.1	0.005193	7.57213	0.01792	0.985723	0.994972	-7.01104
PCAT14	-0.02486	-0.50936	-0.01685	0.986579	0.995322	-5.47223
AC107982.3	0.007592	5.96988	0.01668	0.986711	0.995322	-6.63761
AL050343.2	-0.01538	4.838158	-0.01626	0.987045	0.995525	-6.22376
AC013565.3	-0.0254	-0.25195	-0.01545	0.987694	0.99568	-5.47889
AL162386.2	0.026617	0.834009	0.015403	0.987728	0.99568	-5.52459
AC243836.1	-0.02422	1.607174	-0.01564	0.987543	0.99568	-5.59088
AC116447.1	-0.01152	5.652155	-0.01548	0.987664	0.99568	-6.43394
LINC02266	0.023839	-0.11539	0.014586	0.988379	0.995741	-5.48378
TMEM78	0.014934	1.518706	0.014743	0.988254	0.995741	-5.58544
PHKA1-AS1	0.020217	1.864113	0.014797	0.988211	0.995741	-5.61532
AL031663.3	0.015422	1.949458	0.013621	0.989148	0.995741	-5.62038
AL845472.1	0.014212	1.959717	0.014115	0.988754	0.995741	-5.62928
AC026401.1	-0.01742	3.108342	-0.01393	0.988905	0.995741	-5.771
AC124283.5	-0.00974	3.043755	-0.01333	0.989379	0.995741	-5.77598
AC107241.1	-0.01119	4.628559	-0.01498	0.988069	0.995741	-6.14049
AL445483.1	-0.01077	4.697035	-0.0134	0.989323	0.995741	-6.21323
AP000845.1	-0.00404	4.988359	-0.01441	0.988522	0.995741	-6.32948
AP001619.1	-0.00576	5.465707	-0.01377	0.989027	0.995741	-6.48909
AC137767.1	0.00298	6.642844	0.014037	0.988816	0.995741	-6.81414
AC009159.3	0.008547	5.589273	0.012977	0.989661	0.995758	-6.53724
SND1-IT1	-0.00262	7.917242	-0.01298	0.989661	0.995758	-7.0717
LINC02182	-0.02114	-0.27937	-0.01196	0.990467	0.995959	-5.47869
AC245297.2	0.021058	0.10087	0.012356	0.990156	0.995959	-5.49411
AC022001.3	-0.01655	1.785844	-0.01173	0.990656	0.995959	-5.60488
AC141586.4	0.005112	4.231756	0.011805	0.990595	0.995959	-6.05332
U47924.3	-0.00567	5.986707	-0.01178	0.990611	0.995959	-6.60164
AC093799.1	0.003686	7.406734	0.012041	0.990406	0.995959	-6.98639
AC109309.1	0.021285	-0.54572	0.011226	0.991056	0.995961	-5.46864
FBXO36-IT1	-0.01549	2.467779	-0.01134	0.990966	0.995961	-5.70035
AC142472.1	0.002616	6.762148	0.011375	0.990937	0.995961	-6.83786
AC006557.6	-0.0103	4.96086	-0.0109	0.991314	0.996027	-6.24148
AC090510.2	0.002391	6.362332	0.01081	0.991387	0.996027	-6.75594
AL137796.1	-0.01133	3.329966	-0.00981	0.992187	0.996615	-5.84759
AC022400.10	0.008926	4.963553	0.009743	0.992237	0.996615	-6.31345
INSYN1-AS1	0.015993	-0.48027	0.009259	0.992623	0.99667	-5.47125

AC016866.2	-0.01324	1.545115	-0.00884	0.992955	0.99667	-5.57782
AL359397.1	0.005342	5.338453	0.008985	0.992841	0.99667	-6.45177
AC027514.2	0.002916	6.509026	0.008956	0.992865	0.99667	-6.81512
SNHG9	-0.0029	10.15205	-0.00915	0.992709	0.99667	-7.4221
AL162731.1	-0.01218	-2.47097	-0.00844	0.993278	0.996861	-5.41095
AL592148.3	-0.00155	7.862447	-0.00815	0.993507	0.996957	-7.07012
AC010336.1	0.003287	3.622344	0.007883	0.993719	0.997037	-5.89359
AC024267.3	-0.00166	8.491337	-0.00742	0.994087	0.997273	-7.16204
AP003071.3	-0.01046	3.363274	-0.00708	0.994361	0.997415	-5.85672
TTC3-AS1	-0.00301	5.075703	-0.00655	0.994779	0.997574	-6.33648
AC114488.1	0.006499	6.978129	0.006544	0.994786	0.997574	-6.85429
AL136146.2	-0.00909	0.672051	-0.00572	0.995444	0.997998	-5.52319
AC064836.2	0.006868	3.180601	0.00568	0.995474	0.997998	-5.8227
AC044802.1	0.001812	3.851569	0.005459	0.99565	0.998042	-5.95969
AC099684.2	0.006019	3.014245	0.004493	0.99642	0.998547	-5.767
CDC42-IT1	0.001872	6.79491	0.004598	0.996336	0.998547	-6.85451
AP003973.2	-0.00513	-1.05236	-0.0029	0.997686	0.998616	-5.45109
AL139130.1	-0.00328	0.22264	-0.00378	0.996992	0.998616	-5.50188
AC015563.1	0.004136	3.427307	0.003433	0.997265	0.998616	-5.82262
AL356740.2	0.003849	4.212691	0.003207	0.997445	0.998616	-6.03625
AC108704.1	0.002993	4.333138	0.004187	0.996664	0.998616	-6.11608
AC068025.2	0.000923	6.335373	0.003493	0.997217	0.998616	-6.72007
AC012676.3	0.00109	6.378054	0.00321	0.997442	0.998616	-6.73699
AP002490.1	0.000917	6.533581	0.003952	0.996851	0.998616	-6.80546
AC009404.1	0.000785	7.042901	0.002922	0.997672	0.998616	-6.9055
AC009970.1	-0.00344	3.818796	-0.0026	0.997926	0.998724	-5.97672
AC107871.2	0.002506	2.781382	0.001944	0.998451	0.999116	-5.71477
AC012213.2	0.001017	3.140987	0.001776	0.998585	0.999117	-5.79388
AC117383.1	-0.00179	2.510974	-0.0012	0.999043	0.999442	-5.6897
AC010457.1	0.001055	1.116197	0.000724	0.999423	0.999585	-5.56717
AC090260.1	-0.00043	3.525752	-0.00069	0.999452	0.999585	-5.86854
AC022150.3	-0.00014	0.049984	#######	0.999931	0.999931	-5.49033
