# Supplementary material for: Novel lncRNA Panel as for Prognosis in Esophageal Squamous Cell Carcinoma Based on ceRNA Network Mechanism
Source: Comput Math Methods Med. 2021 Sep 24;2021:8020879. doi: 10.1155/2021/8020879 (PMC8486540; doi:10.1155/2021/8020879)
Supplement: Supplementary Materials — Supplementary Table 1: the sample information was shown in Table S1. Supplementary Table 2: expression data of genes were gathered in Table S2. Supplementary Table 3: differently expressed lncRNAs were shown in Table S3. Supplementary Table 4: differently expressed miRNAs were shown in Table S4. Supplementary Table 5: the interaction network of between lncRNAs and miRNAs is shown in Table S5. [file 8020879.f1.zip › Table-S4.pdf]

| | logFC | logCPM | LR | PValue | FDR |
|---|---|---|---|---|---|
| hsa-mir-148a | -2.87435 | 15.01972 | 169.97 | 7.51E-39 | 2.40E-36 |
| hsa-mir-139 | -2.14686 | 6.031006 | 92.03494 | 8.52E-22 | 1.36E-19 |
| hsa-mir-204 | -4.06264 | 2.96663 | 90.07128 | 2.30E-21 | 2.44E-19 |
| hsa-mir-30c-2 | -1.49512 | 9.496275 | 84.29264 | 4.27E-20 | 3.40E-18 |
| hsa-mir-30e | -1.25292 | 12.9738 | 82.63952 | 9.85E-20 | 6.28E-18 |
| hsa-mir-28 | -1.35887 | 12.36616 | 67.0485 | 2.65E-16 | 1.41E-14 |
| hsa-mir-133a-1 | -3.13339 | 5.850166 | 65.73034 | 5.17E-16 | 2.36E-14 |
| hsa-mir-30a | -1.86452 | 13.96734 | 55.94189 | 7.46E-14 | 2.98E-12 |
| hsa-mir-664 | -1.28579 | 4.961087 | 55.49725 | 9.36E-14 | 3.32E-12 |
| hsa-mir-29c | -1.84661 | 10.92841 | 54.91749 | 1.26E-13 | 4.01E-12 |
| hsa-mir-26b | -1.00101 | 9.435123 | 49.1311 | 2.39E-12 | 6.94E-11 |
| hsa-mir-125a | -1.251 | 8.680801 | 47.93754 | 4.40E-12 | 1.17E-10 |
| hsa-mir-153-2 | -2.39081 | 4.05984 | 47.28953 | 6.12E-12 | 1.50E-10 |
| hsa-mir-145 | -2.17419 | 11.92482 | 46.5364 | 8.99E-12 | 2.05E-10 |
| hsa-mir-378c | -1.6667 | 3.570728 | 44.68261 | 2.32E-11 | 4.93E-10 |
| hsa-mir-574 | -0.95966 | 6.087378 | 42.31696 | 7.76E-11 | 1.55E-09 |
| hsa-mir-628 | -1.1949 | 2.986084 | 41.09105 | 1.45E-10 | 2.73E-09 |
| hsa-mir-101-2 | -1.19086 | 5.883343 | 35.08933 | 3.15E-09 | 5.58E-08 |
| hsa-mir-1468 | -1.92623 | 2.577733 | 33.27457 | 8.00E-09 | 1.34E-07 |
| hsa-mir-1-2 | -2.43101 | 6.478633 | 32.28518 | 1.33E-08 | 2.12E-07 |
| hsa-mir-365-2 | -1.27236 | 4.75051 | 30.59325 | 3.18E-08 | 4.83E-07 |
| hsa-mir-21 | 1.119401 | 18.3481 | 30.12802 | 4.04E-08 | 5.86E-07 |
| hsa-mir-30b | -0.97043 | 8.7941 | 28.18689 | 1.10E-07 | 1.53E-06 |
| hsa-mir-93 | 1.317575 | 13.20279 | 28.03851 | 1.19E-07 | 1.58E-06 |
| hsa-mir-365-1 | -1.22606 | 4.762678 | 27.86111 | 1.30E-07 | 1.66E-06 |
| hsa-mir-26a-2 | -0.88924 | 10.85673 | 27.60164 | 1.49E-07 | 1.83E-06 |
| hsa-mir-3648 | 5.015034 | 3.775643 | 27.21412 | 1.82E-07 | 2.15E-06 |
| hsa-mir-195 | -1.22342 | 4.953247 | 27.04181 | 1.99E-07 | 2.27E-06 |
| hsa-mir-106b | 1.160691 | 9.43227 | 24.83724 | 6.24E-07 | 6.86E-06 |
| hsa-mir-767 | 7.52815 | 6.00439 | 24.58204 | 7.12E-07 | 7.57E-06 |
| hsa-mir-143 | -1.52124 | 17.41902 | 24.30971 | 8.20E-07 | 8.44E-06 |
| hsa-mir-18a | 1.433224 | 5.288428 | 23.97912 | 9.74E-07 | 9.71E-06 |
| hsa-mir-100 | -1.52691 | 11.26983 | 22.36302 | 2.26E-06 | 2.18E-05 |
| hsa-mir-133b | -2.29169 | 2.452303 | 22.24688 | 2.40E-06 | 2.22E-05 |
| hsa-mir-224 | 3.108988 | 7.027008 | 22.21985 | 2.43E-06 | 2.22E-05 |
| hsa-mir-497 | -1.0594 | 3.904056 | 22.09857 | 2.59E-06 | 2.30E-05 |
| hsa-mir-375 | -2.55786 | 14.26816 | 21.84683 | 2.95E-06 | 2.55E-05 |
| hsa-mir-105-2 | 6.545669 | 5.153251 | 20.83254 | 5.01E-06 | 4.21E-05 |
| hsa-mir-140 | -0.82098 | 9.991457 | 20.73271 | 5.28E-06 | 4.32E-05 |
| hsa-mir-301b | 2.178781 | 1.152422 | 20.61237 | 5.62E-06 | 4.48E-05 |
| hsa-mir-23b | -0.89418 | 10.19981 | 19.96006 | 7.91E-06 | 6.15E-05 |
| hsa-mir-642a | -1.40805 | 1.416392 | 19.67407 | 9.18E-06 | 6.98E-05 |
| hsa-mir-335 | 1.594088 | 6.862292 | 18.76928 | 1.48E-05 | 0.000109 |
| hsa-mir-361 | -0.47984 | 8.718391 | 18.58684 | 1.62E-05 | 0.000115 |
| hsa-mir-937 | 2.346435 | 1.676821 | 18.60134 | 1.61E-05 | 0.000115 |
| hsa-mir-135b | 2.073406 | 5.939479 | 18.47861 | 1.72E-05 | 0.000119 |
| hsa-mir-30d | -0.92182 | 12.58777 | 18.09619 | 2.10E-05 | 0.000143 |
| hsa-mir-421 | 1.248114 | 2.48975 | 18.02772 | 2.18E-05 | 0.000145 |
| hsa-mir-877 | 1.659008 | 2.281451 | 17.97781 | 2.23E-05 | 0.000145 |
| hsa-mir-218-2 | -1.28816 | 5.027071 | 17.89221 | 2.34E-05 | 0.000149 |
| hsa-mir-24-1 | -0.7241 | 4.859689 | 17.70282 | 2.58E-05 | 0.000162 |
| hsa-mir-374b | -0.70877 | 6.007298 | 17.54441 | 2.81E-05 | 0.000172 |
| hsa-mir-1304 | 2.356043 | 1.332682 | 17.38629 | 3.05E-05 | 0.000182 |
| hsa-mir-16-2 | 0.938267 | 3.922895 | 17.37241 | 3.07E-05 | 0.000182 |
| hsa-mir-181b-1 | 1.105102 | 8.354415 | 17.10256 | 3.54E-05 | 0.000205 |
| hsa-mir-101-1 | -0.81237 | 12.50291 | 16.44103 | 5.02E-05 | 0.000262 |
| hsa-mir-129-2 | -1.89699 | 2.199726 | 16.49156 | 4.89E-05 | 0.000262 |

| | | | | | |
|---|---|---|---|---|---|
| hsa-mir-15b | 0.903174 | 8.332932 | 16.45468 | 4.98E-05 | 0.000262 |
| hsa-mir-17 | 0.87446 | 10.29596 | 16.44385 | 5.01E-05 | 0.000262 |
| hsa-mir-29b-2 | -0.8425 | 8.606744 | 16.52802 | 4.79E-05 | 0.000262 |
| hsa-mir-301a | 1.105188 | 3.795846 | 16.5758 | 4.67E-05 | 0.000262 |
| hsa-mir-181a-1 | 0.83483 | 10.43746 | 15.75772 | 7.20E-05 | 0.000366 |
| hsa-mir-615 | 2.033781 | 1.780396 | 15.75022 | 7.23E-05 | 0.000366 |
| hsa-mir-942 | 1.088743 | 3.617908 | 15.54093 | 8.07E-05 | 0.000402 |
| hsa-mir-136 | -0.8119 | 4.774481 | 15.4885 | 8.30E-05 | 0.000407 |
| hsa-mir-483 | 4.071121 | 3.679036 | 15.1363 | 0.0001 | 0.000483 |
| hsa-mir-125b-1 | -1.0705 | 8.679727 | 15.011 | 0.000107 | 0.000509 |
| hsa-mir-129-1 | -1.88567 | 2.171791 | 14.27358 | 0.000158 | 0.000733 |
| hsa-mir-452 | 1.999762 | 7.305737 | 14.26905 | 0.000158 | 0.000733 |
| hsa-mir-940 | 1.422236 | 1.461638 | 14.17162 | 0.000167 | 0.00076 |
| hsa-mir-345 | 0.946908 | 3.516088 | 14.06841 | 0.000176 | 0.000792 |
| hsa-mir-99b | -0.69274 | 13.55076 | 14.02314 | 0.000181 | 0.0008 |
| hsa-mir-337 | -0.91999 | 4.211098 | 13.89482 | 0.000193 | 0.000833 |
| hsa-mir-3662 | 3.490133 | 2.506338 | 13.9034 | 0.000192 | 0.000833 |
| hsa-mir-1180 | 1.202533 | 4.277147 | 13.82685 | 0.0002 | 0.000853 |
| hsa-mir-455 | 1.196954 | 7.736951 | 13.5517 | 0.000232 | 0.000974 |
| hsa-mir-103-2 | 0.714312 | 3.737198 | 13.49658 | 0.000239 | 0.00099 |
| hsa-mir-20a | 0.868891 | 9.392539 | 13.44063 | 0.000246 | 0.001007 |
| hsa-mir-196a-2 | 2.030831 | 3.929142 | 13.09081 | 0.000297 | 0.001198 |
| hsa-mir-3677 | 1.229533 | 2.245753 | 12.82094 | 0.000343 | 0.001367 |
| hsa-mir-146b | 0.948141 | 9.983176 | 12.58396 | 0.000389 | 0.001532 |
| hsa-mir-550a-2 | 1.373855 | 1.734517 | 12.50907 | 0.000405 | 0.001575 |
| hsa-mir-328 | -0.75475 | 3.804728 | 12.23881 | 0.000468 | 0.001799 |
| hsa-mir-196a-1 | 1.952646 | 7.514739 | 12.21457 | 0.000474 | 0.001801 |
| hsa-mir-1301 | 0.958386 | 3.281904 | 12.16055 | 0.000488 | 0.001832 |
| hsa-mir-502 | -0.73393 | 3.614464 | 11.66938 | 0.000635 | 0.002357 |
| hsa-mir-99a | -1.17414 | 8.295055 | 11.63326 | 0.000648 | 0.002375 |
| hsa-mir-550a-1 | 1.187181 | 2.109431 | 11.53627 | 0.000683 | 0.002474 |
| hsa-mir-3682 | 1.216753 | 0.843752 | 11.26426 | 0.00079 | 0.002832 |
| hsa-mir-196b | 1.73607 | 8.400215 | 11.20375 | 0.000816 | 0.002893 |
| hsa-mir-769 | 0.614238 | 4.533067 | 11.1331 | 0.000848 | 0.002973 |
| hsa-mir-1293 | 2.792077 | 2.363753 | 10.98019 | 0.000921 | 0.003189 |
| hsa-mir-491 | -0.823 | 1.051829 | 10.96274 | 0.00093 | 0.003189 |
| hsa-mir-584 | 1.390925 | 7.031106 | 10.87552 | 0.000974 | 0.003307 |
| hsa-mir-675 | 2.711128 | 5.600538 | 10.67919 | 0.001083 | 0.003638 |
| hsa-mir-495 | -0.75985 | 3.125107 | 10.65922 | 0.001095 | 0.003639 |
| hsa-mir-210 | 1.420089 | 9.252947 | 10.62609 | 0.001115 | 0.003667 |
| hsa-mir-378 | -0.77256 | 9.906692 | 10.07472 | 0.001503 | 0.004893 |
| hsa-mir-338 | -1.0828 | 10.1415 | 9.93177 | 0.001625 | 0.005235 |
| hsa-mir-27a | 0.678847 | 10.91701 | 9.886356 | 0.001665 | 0.005312 |
| hsa-mir-1269 | 3.963338 | 6.600888 | 9.842256 | 0.001705 | 0.005373 |
| hsa-mir-508 | 2.975761 | 5.076485 | 9.828691 | 0.001718 | 0.005373 |
| hsa-mir-34c | 2.124362 | 4.575087 | 9.704524 | 0.001838 | 0.005693 |
| hsa-mir-29b-1 | -0.67097 | 8.539707 | 9.683414 | 0.001859 | 0.005703 |
| hsa-mir-10a | -0.94145 | 14.6364 | 9.586268 | 0.00196 | 0.005956 |
| hsa-let-7b | -0.57099 | 13.35898 | 9.541136 | 0.002009 | 0.006046 |
| hsa-mir-625 | -0.89757 | 8.563649 | 9.464505 | 0.002095 | 0.006245 |
| hsa-mir-362 | -0.8156 | 4.337756 | 9.293367 | 0.0023 | 0.006748 |
| hsa-mir-363 | -1.11515 | 3.512502 | 9.28868 | 0.002306 | 0.006748 |
| hsa-mir-1307 | 0.695557 | 10.70529 | 9.17269 | 0.002457 | 0.007124 |
| hsa-mir-944 | 2.990999 | 7.05727 | 8.892198 | 0.002864 | 0.008231 |
| hsa-mir-1287 | -0.62505 | 4.336397 | 8.819766 | 0.00298 | 0.008487 |
| hsa-mir-183 | 1.164161 | 12.81894 | 8.705791 | 0.003172 | 0.008955 |
| hsa-mir-19a | 0.746467 | 5.875532 | 8.650538 | 0.00327 | 0.009149 |
| hsa-mir-487b | -0.74678 | 2.378522 | 8.58425 | 0.003391 | 0.009406 |

| | | | | | |
|---|---|---|---|---|---|
| hsa-mir-3065 | -1.0044 | 5.829507 | 8.550902 | 0.003454 | 0.009497 |
| hsa-mir-590 | 0.575438 | 4.368912 | 8.498943 | 0.003554 | 0.009689 |
| hsa-mir-381 | -0.68796 | 5.513902 | 8.00649 | 0.004661 | 0.0126 |
| hsa-mir-671 | 0.614641 | 3.134261 | 7.900431 | 0.004942 | 0.013249 |
| hsa-mir-205 | 2.685906 | 12.74127 | 7.72803 | 0.005437 | 0.014453 |
| hsa-mir-185 | 0.469541 | 6.546629 | 7.685443 | 0.005567 | 0.014676 |
| hsa-mir-29a | -0.55905 | 12.62755 | 7.611402 | 0.0058 | 0.015166 |
| hsa-mir-7-3 | 1.83774 | 2.01014 | 7.590664 | 0.005867 | 0.015216 |
| hsa-mir-874 | -0.57633 | 4.211429 | 7.471472 | 0.006268 | 0.016126 |
| hsa-mir-887 | -0.76322 | 1.846401 | 7.432639 | 0.006405 | 0.016346 |
| hsa-mir-19b-1 | 0.645974 | 3.56169 | 7.366694 | 0.006644 | 0.016822 |
| hsa-mir-324 | 0.52572 | 5.328255 | 7.315573 | 0.006836 | 0.016904 |
| hsa-mir-3615 | 0.684054 | 2.772225 | 7.339335 | 0.006746 | 0.016904 |
| hsa-mir-744 | 0.650282 | 4.83131 | 7.31579 | 0.006835 | 0.016904 |
| hsa-mir-25 | 0.578834 | 13.38845 | 7.266106 | 0.007027 | 0.017243 |
| hsa-mir-15a | 0.464007 | 7.8633 | 7.23186 | 0.007162 | 0.017441 |
| hsa-mir-186 | -0.35412 | 7.901088 | 7.08048 | 0.007793 | 0.018833 |
| hsa-mir-3651 | 1.488432 | 1.828204 | 7.041475 | 0.007964 | 0.019102 |
| hsa-mir-660 | -0.58341 | 6.234275 | 6.957435 | 0.008347 | 0.019871 |
| hsa-mir-331 | 0.481835 | 3.917987 | 6.710019 | 0.009587 | 0.022654 |
| hsa-mir-3613 | 0.615411 | 3.963084 | 6.619858 | 0.010085 | 0.023655 |
| hsa-mir-222 | 0.615836 | 6.766123 | 6.520026 | 0.010667 | 0.024837 |
| hsa-mir-708 | 1.228001 | 7.512739 | 6.432225 | 0.011207 | 0.025906 |
| hsa-mir-10b | -0.6122 | 14.11397 | 6.346932 | 0.011758 | 0.026602 |
| hsa-mir-200b | -0.82938 | 10.84517 | 6.359044 | 0.011678 | 0.026602 |
| hsa-mir-503 | 0.818258 | 3.314424 | 6.371972 | 0.011594 | 0.026602 |
| hsa-mir-190 | -0.78332 | 2.71981 | 6.272159 | 0.012265 | 0.027553 |
| hsa-mir-326 | -0.72612 | 1.502206 | 6.257058 | 0.01237 | 0.027594 |
| hsa-mir-34b | 1.692279 | 2.150945 | 6.048036 | 0.013922 | 0.030841 |
| hsa-mir-340 | -0.49742 | 4.625053 | 5.987446 | 0.014408 | 0.031698 |
| hsa-mir-221 | 0.558724 | 8.478022 | 5.857224 | 0.015513 | 0.033895 |
| hsa-mir-493 | 0.706562 | 3.841498 | 5.761768 | 0.016379 | 0.035543 |
| hsa-let-7c | -0.83403 | 10.61501 | 5.722416 | 0.01675 | 0.036102 |
| hsa-mir-181b-2 | 0.811766 | 3.075943 | 5.6384 | 0.017571 | 0.037619 |
| hsa-mir-582 | -0.57914 | 7.962535 | 5.578063 | 0.018187 | 0.038677 |
| hsa-mir-22 | -0.36834 | 15.9689 | 5.542721 | 0.018558 | 0.039158 |
| hsa-mir-7-2 | 1.474306 | 2.011581 | 5.53325 | 0.018658 | 0.039158 |
| hsa-mir-296 | 1.252563 | 2.028792 | 5.38262 | 0.020338 | 0.042405 |
| hsa-mir-3676 | 1.061085 | 1.550738 | 5.298539 | 0.021343 | 0.044211 |
| hsa-mir-146a | 0.890228 | 7.843515 | 5.23812 | 0.022097 | 0.045477 |
| hsa-mir-155 | 0.669474 | 8.747185 | 5.176877 | 0.022889 | 0.046806 |
| hsa-mir-323b | 1.020593 | 1.136982 | 5.118661 | 0.02367 | 0.048094 |
| hsa-mir-24-2 | 0.444484 | 11.1758 | 5.04821 | 0.024651 | 0.049771 |
| hsa-mir-188 | 0.671112 | 2.029918 | 5.032476 | 0.024876 | 0.049909 |
| hsa-mir-217 | 1.884357 | 7.220809 | 4.945976 | 0.026151 | 0.052139 |
| hsa-mir-193a | -0.4726 | 7.646882 | 4.854106 | 0.02758 | 0.054647 |
| hsa-mir-27b | -0.45545 | 11.03321 | 4.641689 | 0.031204 | 0.061445 |
| hsa-mir-374a | -0.35196 | 9.560926 | 4.596377 | 0.03204 | 0.062703 |
| hsa-mir-223 | 0.778168 | 8.88301 | 4.513121 | 0.033636 | 0.065426 |
| hsa-mir-92a-2 | 0.435757 | 13.72153 | 4.449147 | 0.034919 | 0.067509 |
| hsa-mir-16-1 | 0.316754 | 8.912111 | 4.433111 | 0.035248 | 0.067736 |
| hsa-mir-214 | 0.608281 | 4.568189 | 4.41072 | 0.035714 | 0.06822 |
| hsa-mir-3074 | 0.702801 | 2.060415 | 4.367964 | 0.036621 | 0.069536 |
| hsa-mir-142 | 0.654136 | 10.36467 | 4.339165 | 0.037245 | 0.070303 |
| hsa-mir-486 | -0.76902 | 7.27242 | 4.229272 | 0.039732 | 0.074557 |
| hsa-mir-1249 | 0.644284 | 1.28874 | 4.092837 | 0.043065 | 0.080338 |
| hsa-mir-629 | 0.467596 | 6.716113 | 4.081291 | 0.043361 | 0.080419 |
| hsa-mir-149 | 1.112519 | 6.268281 | 4.056541 | 0.044001 | 0.081134 |

| | | | | | |
|---|---|---|---|---|---|
| hsa-mir-219-1 | 0.497807 | 2.299671 | 3.943894 | 0.047042 | 0.086243 |
| hsa-mir-20b | -0.80739 | 4.576746 | 3.916061 | 0.047827 | 0.087181 |
| hsa-mir-96 | 0.669988 | 4.781227 | 3.891058 | 0.048544 | 0.087986 |
| hsa-let-7a-3 | -0.41771 | 12.9455 | 3.76479 | 0.052342 | 0.094335 |
| hsa-let-7a-2 | -0.41519 | 13.93712 | 3.681757 | 0.055011 | 0.098351 |
| hsa-mir-3647 | 0.476746 | 2.487649 | 3.676411 | 0.055188 | 0.098351 |
| hsa-mir-425 | 0.476875 | 8.385314 | 3.658315 | 0.05579 | 0.098872 |
| hsa-let-7a-1 | -0.40951 | 12.94339 | 3.588031 | 0.058197 | 0.102568 |
| hsa-mir-33b | 0.723381 | 1.21791 | 3.443356 | 0.063507 | 0.111311 |
| hsa-mir-500b | -0.47936 | 3.519374 | 3.406539 | 0.064938 | 0.113199 |
| hsa-mir-98 | 0.383935 | 5.983125 | 3.352759 | 0.067092 | 0.116318 |
| hsa-mir-199b | 0.435083 | 10.76677 | 3.297201 | 0.069398 | 0.119665 |
| hsa-mir-181a-2 | 0.412954 | 8.917549 | 3.280454 | 0.07011 | 0.120242 |
| hsa-mir-33a | -0.44729 | 4.437014 | 3.075391 | 0.079485 | 0.135593 |
| hsa-mir-1245 | 0.715036 | 1.621999 | 3.032332 | 0.081621 | 0.138495 |
| hsa-mir-3127 | 0.543237 | 2.597012 | 3.016258 | 0.082433 | 0.139134 |
| hsa-mir-1271 | 0.526493 | 1.339152 | 2.960865 | 0.085302 | 0.143218 |
| hsa-mir-92b | 0.425521 | 6.693707 | 2.95021 | 0.085866 | 0.14341 |
| hsa-mir-148b | -0.28232 | 6.795086 | 2.738474 | 0.097958 | 0.162754 |
| hsa-mir-3614 | 0.562017 | 2.494283 | 2.72564 | 0.098749 | 0.163216 |
| hsa-mir-4326 | 0.8163 | 3.441726 | 2.643909 | 0.103948 | 0.170924 |
| hsa-mir-1248 | 0.798809 | 2.342612 | 2.532435 | 0.111528 | 0.182345 |
| hsa-mir-454 | 0.308686 | 3.678287 | 2.525257 | 0.112037 | 0.182345 |
| hsa-mir-200c | -0.42317 | 13.67321 | 2.503103 | 0.113622 | 0.183987 |
| hsa-mir-212 | 0.4086 | 2.581786 | 2.476181 | 0.115583 | 0.186216 |
| hsa-mir-2355 | 0.550847 | 5.716909 | 2.430212 | 0.119017 | 0.190786 |
| hsa-mir-199a-2 | 0.380879 | 10.42618 | 2.398125 | 0.121481 | 0.193762 |
| hsa-mir-382 | 0.407328 | 4.175872 | 2.376371 | 0.123183 | 0.1955 |
| hsa-mir-496 | 0.538428 | 1.445452 | 2.340964 | 0.126012 | 0.197921 |
| hsa-mir-598 | -0.48664 | 4.848745 | 2.349414 | 0.12533 | 0.197921 |
| hsa-mir-766 | -0.3256 | 2.534532 | 2.334078 | 0.12657 | 0.197921 |
| hsa-mir-197 | 0.254564 | 8.093732 | 2.316362 | 0.128019 | 0.19921 |
| hsa-mir-103-1 | 0.207259 | 14.70111 | 2.298636 | 0.129488 | 0.200517 |
| hsa-mir-3653 | -0.42479 | 2.419597 | 2.189985 | 0.138911 | 0.21407 |
| hsa-mir-624 | 0.371002 | 1.295947 | 2.070042 | 0.150217 | 0.230381 |
| hsa-mir-106a | -0.41954 | 4.054524 | 1.984451 | 0.158922 | 0.242566 |
| hsa-mir-200a | -0.47378 | 10.83978 | 1.959364 | 0.161581 | 0.24545 |
| hsa-mir-128-1 | 0.254211 | 6.113245 | 1.936032 | 0.1641 | 0.248094 |
| hsa-let-7i | 0.25104 | 8.992773 | 1.914108 | 0.166508 | 0.249371 |
| hsa-mir-23a | 0.253058 | 11.42388 | 1.917402 | 0.166143 | 0.249371 |
| hsa-mir-320a | -0.23823 | 9.453513 | 1.852099 | 0.173539 | 0.258687 |
| hsa-mir-1296 | 0.399731 | 2.760113 | 1.808491 | 0.178689 | 0.263898 |
| hsa-mir-539 | -0.37603 | 2.311425 | 1.812453 | 0.178214 | 0.263898 |
| hsa-mir-199a-1 | 0.334119 | 9.710016 | 1.740835 | 0.187033 | 0.274947 |
| hsa-mir-3913-1 | 0.384255 | 1.707955 | 1.723801 | 0.189204 | 0.276863 |
| hsa-mir-128-2 | 0.268514 | 5.546722 | 1.698628 | 0.192468 | 0.279102 |
| hsa-mir-409 | 0.330329 | 4.507735 | 1.698502 | 0.192484 | 0.279102 |
| hsa-mir-107 | 0.248096 | 6.195389 | 1.58558 | 0.207959 | 0.300176 |
| hsa-mir-532 | -0.28396 | 9.794897 | 1.578896 | 0.20892 | 0.300205 |
| hsa-mir-151 | 0.245819 | 11.06485 | 1.533945 | 0.215521 | 0.308302 |
| hsa-mir-182 | 0.401979 | 14.19307 | 1.48376 | 0.223187 | 0.317842 |
| hsa-mir-653 | 0.572918 | 2.673129 | 1.474856 | 0.224581 | 0.318406 |
| hsa-mir-144 | -0.4577 | 7.383036 | 1.370478 | 0.24173 | 0.339704 |
| hsa-mir-32 | -0.22722 | 5.060192 | 1.370458 | 0.241733 | 0.339704 |
| hsa-mir-125b-2 | -0.47395 | 3.902172 | 1.33041 | 0.248732 | 0.348007 |
| hsa-mir-412 | 0.524022 | 2.655377 | 1.309003 | 0.252575 | 0.350659 |
| hsa-mir-511-1 | 0.382969 | 2.331164 | 1.307619 | 0.252826 | 0.350659 |
| hsa-mir-3200 | 0.494722 | 1.896433 | 1.301513 | 0.253937 | 0.350675 |

| | | | | | |
|---|---|---|---|---|---|
| hsa-mir-500a | -0.26634 | 8.18369 | 1.249075 | 0.263729 | 0.362628 |
| hsa-mir-3607 | -0.26871 | 3.892007 | 1.222075 | 0.268954 | 0.368224 |
| hsa-mir-501 | 0.285205 | 5.710485 | 1.206278 | 0.272071 | 0.3709 |
| hsa-mir-758 | 0.339679 | 3.774304 | 1.181632 | 0.277024 | 0.376045 |
| hsa-mir-651 | 0.311454 | 1.8622 | 1.135142 | 0.286681 | 0.387505 |
| hsa-mir-429 | -0.37178 | 8.032997 | 1.123943 | 0.289071 | 0.389087 |
| hsa-mir-19b-2 | 0.218413 | 7.735812 | 1.112835 | 0.291466 | 0.390663 |
| hsa-mir-134 | 0.308235 | 7.520051 | 1.076057 | 0.299581 | 0.398194 |
| hsa-mir-485 | 0.352446 | 2.191126 | 1.076191 | 0.299551 | 0.398194 |
| hsa-mir-432 | -0.29226 | 2.791299 | 1.067524 | 0.301505 | 0.399088 |
| hsa-let-7g | -0.28992 | 9.692651 | 1.058596 | 0.303536 | 0.400115 |
| hsa-mir-126 | 0.192241 | 10.92735 | 1.022193 | 0.311999 | 0.409579 |
| hsa-mir-7-1 | 0.310426 | 5.274902 | 0.978858 | 0.322481 | 0.421604 |
| hsa-mir-654 | -0.27138 | 4.015823 | 0.963713 | 0.326253 | 0.424795 |
| hsa-mir-411 | -0.26784 | 2.973451 | 0.891883 | 0.344968 | 0.447336 |
| hsa-mir-299 | -0.27568 | 2.21369 | 0.877393 | 0.348917 | 0.448808 |
| hsa-mir-484 | 0.154956 | 6.042311 | 0.878415 | 0.348636 | 0.448808 |
| hsa-mir-1274b | 0.316964 | 0.844934 | 0.831536 | 0.361829 | 0.461693 |
| hsa-mir-505 | 0.168632 | 5.202093 | 0.83629 | 0.36046 | 0.461693 |
| hsa-mir-376b | 0.300017 | 1.387663 | 0.823522 | 0.364152 | 0.462807 |
| hsa-mir-451 | -0.34563 | 9.487969 | 0.778684 | 0.377544 | 0.477922 |
| hsa-mir-543 | 0.309219 | 1.075861 | 0.762133 | 0.382662 | 0.482487 |
| hsa-mir-627 | 0.259507 | 1.148451 | 0.741191 | 0.389279 | 0.488898 |
| hsa-mir-1306 | 0.190653 | 2.495755 | 0.728214 | 0.393463 | 0.492214 |
| hsa-mir-323 | 0.347164 | 1.775521 | 0.721381 | 0.395691 | 0.493069 |
| hsa-mir-376c | -0.23562 | 2.814102 | 0.715974 | 0.397468 | 0.493355 |
| hsa-mir-379 | -0.21924 | 9.075643 | 0.687412 | 0.407046 | 0.503285 |
| hsa-mir-542 | 0.157569 | 7.487649 | 0.673776 | 0.411738 | 0.507121 |
| hsa-mir-450a-1 | -0.19258 | 2.151849 | 0.656014 | 0.417971 | 0.512818 |
| hsa-mir-511-2 | 0.278511 | 2.346899 | 0.642564 | 0.422784 | 0.516736 |
| hsa-mir-193b | 0.266226 | 6.646559 | 0.613807 | 0.433358 | 0.527638 |
| hsa-mir-132 | -0.12593 | 6.564941 | 0.59093 | 0.44206 | 0.536187 |
| hsa-mir-592 | 0.392839 | 2.643185 | 0.583865 | 0.444801 | 0.537468 |
| hsa-mir-31 | 0.39535 | 6.377505 | 0.552748 | 0.457197 | 0.548293 |
| hsa-mir-616 | 0.261322 | 1.540751 | 0.556546 | 0.455655 | 0.548293 |
| hsa-mir-192 | -0.50003 | 14.31967 | 0.539832 | 0.462502 | 0.551517 |
| hsa-mir-576 | 0.179262 | 3.793708 | 0.537806 | 0.463343 | 0.551517 |
| hsa-mir-130b | 0.164853 | 5.11391 | 0.524477 | 0.468938 | 0.556101 |
| hsa-mir-494 | 0.241378 | 1.386122 | 0.508635 | 0.47573 | 0.562066 |
| hsa-mir-589 | 0.125035 | 6.152307 | 0.488694 | 0.48451 | 0.570328 |
| hsa-mir-9-2 | 0.359218 | 9.973712 | 0.472421 | 0.491875 | 0.576869 |
| hsa-mir-9-1 | 0.352724 | 9.974775 | 0.458582 | 0.498288 | 0.582248 |
| hsa-mir-3130-1 | -0.24166 | 1.807309 | 0.443356 | 0.505507 | 0.588528 |
| hsa-mir-369 | -0.16354 | 3.470232 | 0.43496 | 0.509565 | 0.591096 |
| hsa-mir-320b-2 | 0.161734 | 2.727626 | 0.415867 | 0.519006 | 0.599866 |
| hsa-let-7d | 0.095089 | 8.871969 | 0.368546 | 0.543798 | 0.623998 |
| hsa-mir-450b | -0.14255 | 3.523652 | 0.371903 | 0.541969 | 0.623998 |
| hsa-mir-377 | 0.177437 | 2.001996 | 0.332733 | 0.564055 | 0.644923 |
| hsa-mir-34a | 0.11698 | 7.221877 | 0.32297 | 0.569828 | 0.649197 |
| hsa-mir-130a | -0.13049 | 6.224159 | 0.304243 | 0.581235 | 0.659092 |
| hsa-mir-194-2 | -0.37693 | 12.39126 | 0.301977 | 0.582646 | 0.659092 |
| hsa-mir-194-1 | -0.3773 | 12.18516 | 0.297884 | 0.585212 | 0.659656 |
| hsa-mir-655 | 0.155795 | 1.772954 | 0.264175 | 0.607266 | 0.682105 |
| hsa-mir-1247 | -0.28626 | 4.193898 | 0.260439 | 0.609818 | 0.682569 |
| hsa-mir-431 | -0.15778 | 3.26492 | 0.249849 | 0.617182 | 0.688395 |
| hsa-mir-889 | -0.13178 | 4.266447 | 0.216512 | 0.641711 | 0.71326 |
| hsa-mir-187 | 0.304213 | 4.890809 | 0.198454 | 0.655971 | 0.722513 |
| hsa-mir-339 | -0.08727 | 5.585915 | 0.197397 | 0.65683 | 0.722513 |

| | | | | | |
|---|---|---|---|---|---|
| hsa-mir-92a-1 | 0.103806 | 9.59597 | 0.20014 | 0.654607 | 0.722513 |
| hsa-let-7e | -0.13599 | 10.45781 | 0.186171 | 0.666123 | 0.730217 |
| hsa-mir-191 | 0.097771 | 8.966104 | 0.179075 | 0.67217 | 0.734322 |
| hsa-mir-150 | -0.17 | 8.835654 | 0.174226 | 0.676383 | 0.734938 |
| hsa-mir-181c | 0.080897 | 6.023416 | 0.171168 | 0.679076 | 0.734938 |
| hsa-mir-577 | -0.23075 | 4.388863 | 0.170525 | 0.679645 | 0.734938 |
| hsa-mir-652 | 0.090203 | 4.684776 | 0.161354 | 0.687913 | 0.741366 |
| hsa-mir-152 | 0.101208 | 7.655 | 0.12098 | 0.727974 | 0.781416 |
| hsa-mir-30c-1 | -0.08951 | 0.8536 | 0.119135 | 0.729975 | 0.781416 |
| hsa-mir-215 | 0.307513 | 10.57495 | 0.112144 | 0.737716 | 0.787062 |
| hsa-mir-127 | -0.07432 | 8.995259 | 0.099497 | 0.752434 | 0.800088 |
| hsa-mir-1976 | 0.053484 | 3.00367 | 0.072497 | 0.787735 | 0.834842 |
| hsa-mir-95 | 0.101194 | 3.226097 | 0.063799 | 0.800589 | 0.845655 |
| hsa-mir-203 | -0.11015 | 15.46154 | 0.045741 | 0.830647 | 0.871633 |
| hsa-mir-342 | -0.05102 | 7.06252 | 0.046211 | 0.829793 | 0.871633 |
| hsa-mir-141 | -0.04926 | 11.00725 | 0.030959 | 0.860331 | 0.899822 |
| hsa-mir-370 | -0.04163 | 2.68752 | 0.022108 | 0.881801 | 0.919263 |
| hsa-mir-3934 | 0.038551 | 2.488992 | 0.020731 | 0.885514 | 0.920127 |
| hsa-let-7f-2 | 0.035668 | 13.77072 | 0.013243 | 0.908383 | 0.928763 |
| hsa-mir-181d | 0.029434 | 4.268969 | 0.014761 | 0.9033 | 0.928763 |
| hsa-mir-2110 | 0.039894 | 1.722304 | 0.013312 | 0.908146 | 0.928763 |
| hsa-mir-330 | 0.024284 | 4.614018 | 0.01538 | 0.901302 | 0.928763 |
| hsa-mir-410 | 0.037225 | 3.475452 | 0.013388 | 0.907885 | 0.928763 |
| hsa-let-7f-1 | 0.019431 | 5.212026 | 0.005281 | 0.942071 | 0.96013 |
| hsa-mir-376a-1 | -0.01734 | 1.019369 | 0.002333 | 0.961477 | 0.976787 |
| hsa-mir-1266 | -0.01679 | 3.610097 | 0.001112 | 0.973399 | 0.980669 |
| hsa-mir-154 | 0.009668 | 2.674997 | 0.001335 | 0.970854 | 0.980669 |
| hsa-mir-424 | 0.007199 | 6.142542 | 0.00102 | 0.974521 | 0.980669 |
| hsa-mir-450a-2 | 0.005445 | 2.159061 | 0.000567 | 0.981008 | 0.984093 |
| hsa-mir-423 | -0.00188 | 7.737644 | 0.000149 | 0.990267 | 0.990267 |
