# Supplementary material for: Novel lncRNA Panel as for Prognosis in Esophageal Squamous Cell Carcinoma Based on ceRNA Network Mechanism
Source: Comput Math Methods Med. 2021 Sep 24;2021:8020879. doi: 10.1155/2021/8020879 (PMC8486540; doi:10.1155/2021/8020879)
Supplement: Supplementary Materials — Supplementary Table 1: the sample information was shown in Table S1. Supplementary Table 2: expression data of genes were gathered in Table S2. Supplementary Table 3: differently expressed lncRNAs were shown in Table S3. Supplementary Table 4: differently expressed miRNAs were shown in Table S4. Supplementary Table 5: the interaction network of between lncRNAs and miRNAs is shown in Table S5. [file 8020879.f1.zip › Table-S5.pdf]

	source	target	corr	type
1	hsa-mir-30c-2	GREM2	0.345782107	mirna_pc
2	hsa-mir-30c-2	GPER	0.393185524	mirna_pc
3	hsa-mir-30c-2	AFF3	0.348817679	mirna_pc
4	hsa-mir-30c-2	KLF15	0.447470032	mirna_pc
5	hsa-mir-30c-2	GPR133	0.440884329	mirna_pc
6	hsa-mir-30c-2	KL	0.428828446	mirna_pc
7	hsa-mir-30c-2	PTGER3	0.515474891	mirna_pc
8	hsa-mir-30c-2	SCUBE2	0.388269303	mirna_pc
9	hsa-mir-30c-2	ZNF471	0.406582651	mirna_pc
10	hsa-mir-30c-2	GFRA1	0.360153625	mirna_pc
11	hsa-mir-30c-2	GPR155	0.522009377	mirna_pc
12	hsa-mir-30c-2	SLC16A7	0.501303104	mirna_pc
13	hsa-mir-30c-2	PNPLA7	0.381760428	mirna_pc
14	hsa-mir-30c-2	STX12	0.524790843	mirna_pc
15	hsa-mir-30c-2	C3orf18	0.459782936	mirna_pc
16	hsa-mir-30c-2	GPR146	0.445300045	mirna_pc
17	hsa-mir-30c-2	RNF180	0.356489295	mirna_pc
18	hsa-mir-30c-2	MLYCD	0.417197271	mirna_pc
19	hsa-mir-30c-2	CRY2	0.419118621	mirna_pc
20	hsa-mir-30c-2	ATOH8	0.337013018	mirna_pc
21	hsa-mir-30c-2	RGMB	0.460560819	mirna_pc
22	hsa-mir-30c-2	CGNL1	0.45561563	mirna_pc
23	hsa-mir-30c-2	SLC25A4	0.47692882	mirna_pc
24	hsa-mir-30c-2	HIF3A	0.301967695	mirna_pc
25	hsa-mir-30c-2	C7orf41	0.346700842	mirna_pc
26	hsa-mir-30c-2	FAM165B	0.533741465	mirna_pc
27	hsa-mir-30c-2	DUSP19	0.587645702	mirna_pc
28	hsa-mir-30c-2	RAB11FIP2	0.628478692	mirna_pc
29	hsa-mir-30c-2	ZNF626	0.439173219	mirna_pc
30	hsa-mir-30c-2	FAM189A2	0.391131283	mirna_pc
31	hsa-mir-30c-2	KIT	0.441937935	mirna_pc
32	hsa-mir-30c-2	ALDH6A1	0.518466456	mirna_pc
33	hsa-mir-30c-2	TMEM161B	0.566518504	mirna_pc
34	hsa-mir-30c-2	KIAA0427	0.405527335	mirna_pc
35	hsa-mir-30c-2	LEPR	0.403652491	mirna_pc
36	hsa-mir-30c-2	FAM54B	0.429824067	mirna_pc
37	hsa-mir-30c-2	PRX	0.489463039	mirna_pc
38	hsa-mir-30c-2	MITF	0.373244182	mirna_pc
39	hsa-mir-30c-2	C22orf23	0.386263835	mirna_pc
40	hsa-mir-30c-2	MFSD4	0.539936411	mirna_pc
41	hsa-mir-30c-2	SIK2	0.467028408	mirna_pc
42	hsa-mir-30c-2	EFHA2	0.361455268	mirna_pc
43	hsa-mir-30c-2	ALAD	0.40977689	mirna_pc
44	hsa-mir-30c-2	GNAZ	0.388659945	mirna_pc
45	hsa-mir-30c-2	MT1G	0.424696576	mirna_pc
46	hsa-mir-30c-2	LOC400043	0.533620487	mirna_pc
47	hsa-mir-30c-2	CDH2	0.474473825	mirna_pc
48	hsa-mir-30c-2	CKB	0.512211314	mirna_pc
49	hsa-mir-30c-2	SPHAR	0.516275573	mirna_pc
50	hsa-mir-30c-2	C8orf84	0.375765342	mirna_pc
51	hsa-mir-30c-2	ACAT1	0.498210677	mirna_pc
52	hsa-mir-30c-2	DPCR1	0.341823094	mirna_pc
53	hsa-mir-30c-2	IPW	0.33706199	mirna_pc

54	hsa-mir-30c-2	ACACB	0.362959965	mirna_pc
55	hsa-mir-30c-2	LIFR	0.559755953	mirna_pc
56	hsa-mir-30c-2	KIAA1191	0.428823514	mirna_pc
57	hsa-mir-30c-2	C14orf159	0.37034641	mirna_pc
58	hsa-mir-30c-2	ROR1	0.367670273	mirna_pc
59	hsa-mir-30c-2	C5orf53	0.304816972	mirna_pc
60	hsa-mir-30c-2	ACADSB	0.485336715	mirna_pc
61	hsa-mir-30c-2	CNTD1	0.527618945	mirna_pc
62	hsa-mir-30c-2	ARHGAP24	0.49084466	mirna_pc
63	hsa-mir-30c-2	GBGT1	0.469178421	mirna_pc
64	hsa-mir-30c-2	PAIP2B	0.448626026	mirna_pc
65	hsa-mir-30c-2	SYNJ2BP	0.457325815	mirna_pc
66	hsa-mir-30c-2	RIMS3	0.356350544	mirna_pc
67	hsa-mir-30c-2	CDHR3	0.320131329	mirna_pc
68	hsa-mir-30c-2	TMEM8B	0.318316816	mirna_pc
69	hsa-mir-30c-2	GHR	0.371965241	mirna_pc
70	hsa-mir-30c-2	ZSCAN18	0.338094199	mirna_pc
71	hsa-mir-30c-2	PXMP2	0.491774832	mirna_pc
72	hsa-mir-30c-2	VSIG2	0.531523166	mirna_pc
73	hsa-mir-30c-2	SIDT2	0.414970667	mirna_pc
74	hsa-mir-30c-2	DBT	0.576309591	mirna_pc
75	hsa-mir-30c-2	GTF2IRD2P1	0.47418851	mirna_pc
76	hsa-mir-30c-2	SECISBP2L	0.422330208	mirna_pc
77	hsa-mir-30c-2	TMEM220	0.454423754	mirna_pc
78	hsa-mir-30c-2	BTD	0.324294724	mirna_pc
79	hsa-mir-30c-2	PLCL1	0.313321652	mirna_pc
80	hsa-mir-30c-2	WASF3	0.369008781	mirna_pc
81	hsa-mir-30c-2	ERO1LB	0.539005886	mirna_pc
82	hsa-mir-30c-2	PBXIP1	0.413187024	mirna_pc
83	hsa-mir-30c-2	RAB27A	0.452507458	mirna_pc
84	hsa-mir-30c-2	ITGA9	0.360457256	mirna_pc
85	hsa-mir-30c-2	GTF2IRD2	0.45595427	mirna_pc
86	hsa-mir-30c-2	PBLD	0.514036818	mirna_pc
87	hsa-mir-30c-2	PCBD2	0.39885972	mirna_pc
88	hsa-mir-30c-2	ZNF415	0.392612894	mirna_pc
89	hsa-mir-30c-2	TSPYL4	0.477956872	mirna_pc
90	hsa-mir-30c-2	PDGFD	0.531244141	mirna_pc
91	hsa-mir-30c-2	AMT	0.395248839	mirna_pc
92	hsa-mir-30c-2	SNX1	0.360222511	mirna_pc
93	hsa-mir-30c-2	APLP1	0.432359319	mirna_pc
94	hsa-mir-30c-2	ACO2	0.325395164	mirna_pc
95	hsa-mir-30c-2	PSCA	0.476314557	mirna_pc
96	hsa-mir-30c-2	KLHDC1	0.419608104	mirna_pc
97	hsa-mir-30c-2	C6orf204	0.389285322	mirna_pc
98	hsa-mir-30c-2	C4orf34	0.364379672	mirna_pc
99	hsa-mir-30c-2	BDH2	0.457642619	mirna_pc
100	hsa-mir-30c-2	FABP3	0.377265806	mirna_pc
101	hsa-mir-30c-2	PIK3C2G	0.411104562	mirna_pc
102	hsa-mir-30c-2	ZMAT1	0.400022344	mirna_pc
103	hsa-mir-30c-2	TADA2B	0.393681063	mirna_pc
104	hsa-mir-30c-2	ZNF778	0.337960383	mirna_pc
105	hsa-mir-30c-2	ANO5	0.351850034	mirna_pc
106	hsa-mir-30c-2	RNF14	0.366274877	mirna_pc
107	hsa-mir-30c-2	ME3	0.487237873	mirna_pc

108	hsa-mir-30c-2	PPP1R9A	0.33848388	mirna_pc
109	hsa-mir-30c-2	UBL3	0.468271308	mirna_pc
110	hsa-mir-30c-2	TBC1D14	0.476163537	mirna_pc
111	hsa-mir-30c-2	ZNF470	0.477876569	mirna_pc
112	hsa-mir-30c-2	METTL7A	0.516303682	mirna_pc
113	hsa-mir-30c-2	SUCLG2	0.444785181	mirna_pc
114	hsa-mir-30c-2	TAPT1	0.481786187	mirna_pc
115	hsa-mir-30c-2	MYEF2	0.430298612	mirna_pc
116	hsa-mir-30c-2	IL11RA	0.321896769	mirna_pc
117	hsa-mir-30c-2	AUH	0.404497205	mirna_pc
118	hsa-mir-30c-2	RAB4A	0.302579883	mirna_pc
119	hsa-mir-30c-2	GTF2IRD2B	0.349490026	mirna_pc
120	hsa-mir-30c-2	ZFP28	0.300635445	mirna_pc
121	hsa-mir-30c-2	FLJ33630	0.379937841	mirna_pc
122	hsa-mir-30c-2	ZNF542	0.437319016	mirna_pc
123	hsa-mir-30c-2	SYNE1	0.326476868	mirna_pc
124	hsa-mir-30c-2	ZNF844	0.415021478	mirna_pc
125	hsa-mir-30c-2	LDHD	0.362765253	mirna_pc
126	hsa-mir-30c-2	ATPAF1	0.390007772	mirna_pc
127	hsa-mir-30c-2	KCNAB1	0.418723653	mirna_pc
128	hsa-mir-30c-2	TPCN2	0.515799367	mirna_pc
129	hsa-mir-30c-2	PTPRN2	0.333323087	mirna_pc
130	hsa-mir-30c-2	TCEB3	0.422589822	mirna_pc
131	hsa-mir-30c-2	KIAA0141	0.345148238	mirna_pc
132	hsa-mir-30c-2	KIAA0494	0.369736726	mirna_pc
133	hsa-mir-30c-2	BRP44L	0.387234651	mirna_pc
134	hsa-mir-30c-2	KIAA0430	0.362456013	mirna_pc
135	hsa-mir-30c-2	BCKDHB	0.50215746	mirna_pc
136	hsa-mir-30c-2	UPRT	0.413044404	mirna_pc
137	hsa-mir-30c-2	PHF17	0.336124717	mirna_pc
138	hsa-mir-30c-2	MYST4	0.435265798	mirna_pc
139	hsa-mir-30c-2	XYLT2	0.469357869	mirna_pc
140	hsa-mir-30c-2	GIN1	0.447859085	mirna_pc
141	hsa-mir-30c-2	ZNF467	0.335048926	mirna_pc
142	hsa-mir-30c-2	MOSC2	0.462500131	mirna_pc
143	hsa-mir-30c-2	YPEL1	0.445210686	mirna_pc
144	hsa-mir-30c-2	PRKACB	0.466799019	mirna_pc
145	hsa-mir-30c-2	CYFIP2	0.505746229	mirna_pc
146	hsa-mir-30c-2	DGKD	0.50465806	mirna_pc
147	hsa-mir-30c-2	DEAF1	0.330445425	mirna_pc
148	hsa-mir-30c-2	GABRB3	0.34567543	mirna_pc
149	hsa-mir-30c-2	PECI	0.492568185	mirna_pc
150	hsa-mir-30c-2	PLA2G12A	0.405547992	mirna_pc
151	hsa-mir-30c-2	PDCD4	0.356017679	mirna_pc
152	hsa-mir-30c-2	CAPN6	0.390156777	mirna_pc
153	hsa-mir-30c-2	CCNI	0.328039386	mirna_pc
154	hsa-mir-30c-2	ZNF568	0.396387196	mirna_pc
155	hsa-mir-30c-2	USP53	0.367873334	mirna_pc
156	hsa-mir-30c-2	WIPF3	0.613175768	mirna_pc
157	hsa-mir-30c-2	CCDC149	0.447746579	mirna_pc
158	hsa-mir-30c-2	NTN4	0.417458572	mirna_pc
159	hsa-mir-30c-2	AK3	0.311516714	mirna_pc
160	hsa-mir-30c-2	RGNEF	0.372341874	mirna_pc
161	hsa-mir-30c-2	MOCS1	0.305365159	mirna_pc

162	hsa-mir-30c-2	NRTN	0.442543835	mirna_pc
163	hsa-mir-30c-2	ACADM	0.397002669	mirna_pc
164	hsa-mir-30c-2	PRDM16	0.437900687	mirna_pc
165	hsa-mir-30c-2	TSC22D3	0.304942204	mirna_pc
166	hsa-mir-30c-2	PPM1K	0.349436945	mirna_pc
167	hsa-mir-30c-2	C14orf37	0.327909058	mirna_pc
168	hsa-mir-30c-2	SLC26A9	0.447701362	mirna_pc
169	hsa-mir-30c-2	VAMP2	0.333806682	mirna_pc
170	hsa-mir-30c-2	KIAA0495	0.500667728	mirna_pc
171	hsa-mir-30c-2	FZD4	0.382992564	mirna_pc
172	hsa-mir-30c-2	GLUL	0.422641566	mirna_pc
173	hsa-mir-30c-2	HERC1	0.332432084	mirna_pc
174	hsa-mir-30c-2	ZNF737	0.379648893	mirna_pc
175	hsa-mir-30c-2	SNRPN	0.313628723	mirna_pc
176	hsa-mir-30c-2	NUCB2	0.390313667	mirna_pc
177	hsa-mir-30c-2	BBS1	0.327982732	mirna_pc
178	hsa-mir-30c-2	FBXW11	0.33326379	mirna_pc
179	hsa-mir-30c-2	ZNF229	0.363803524	mirna_pc
180	hsa-mir-30c-2	GMPR	0.465403013	mirna_pc
181	hsa-mir-30c-2	CTSF	0.391835404	mirna_pc
182	hsa-mir-30c-2	ZNF528	0.375400141	mirna_pc
183	hsa-mir-30c-2	SGSM3	0.351764543	mirna_pc
184	hsa-mir-30c-2	FBXO9	0.324512153	mirna_pc
185	hsa-mir-30c-2	PGCP	0.377956201	mirna_pc
186	hsa-mir-30c-2	GABARAPL1	0.355613685	mirna_pc
187	hsa-mir-30c-2	PARD3B	0.368599393	mirna_pc
188	hsa-mir-30c-2	EIF2C4	0.359835226	mirna_pc
189	hsa-mir-30c-2	AKD1	0.403966228	mirna_pc
190	hsa-mir-30c-2	ZBTB3	0.378807556	mirna_pc
191	hsa-mir-30c-2	NAT15	0.418231423	mirna_pc
192	hsa-mir-30c-2	LRIG1	0.492343674	mirna_pc
193	hsa-mir-30c-2	LOC646471	0.363866766	mirna_pc
194	hsa-mir-30c-2	LOC729234	0.4593945	mirna_pc
195	hsa-mir-30c-2	ZNF570	0.312762086	mirna_pc
196	hsa-mir-30c-2	SMARCD3	0.328897596	mirna_pc
197	hsa-mir-30c-2	CHRFAM7A	0.443525111	mirna_pc
198	hsa-mir-30c-2	NECAB3	0.371161916	mirna_pc
199	hsa-mir-30c-2	WDR37	0.333878251	mirna_pc
200	hsa-mir-30c-2	KCNJ11	0.477487852	mirna_pc
201	hsa-mir-30c-2	FAM174B	0.367990352	mirna_pc
202	hsa-mir-30c-2	PRKAG2	0.338332186	mirna_pc
203	hsa-mir-30c-2	FMO5	0.441299309	mirna_pc
204	hsa-mir-30c-2	DMXL1	0.318622281	mirna_pc
205	hsa-mir-30c-2	SACM1L	0.45593424	mirna_pc
206	hsa-mir-30c-2	PGRMC2	0.314050475	mirna_pc
207	hsa-mir-30c-2	MAGI1	0.403355175	mirna_pc
208	hsa-mir-30c-2	ZNF57	0.350855728	mirna_pc
209	hsa-mir-30c-2	GPT2	0.450300501	mirna_pc
210	hsa-mir-30c-2	FGD4	0.452200187	mirna_pc
211	hsa-mir-30c-2	TBC1D9	0.41718752	mirna_pc
212	hsa-mir-30c-2	MAGI3	0.339717731	mirna_pc
213	hsa-mir-30c-2	ZNF347	0.364579488	mirna_pc
214	hsa-mir-30c-2	C6orf225	0.320193809	mirna_pc
215	hsa-mir-30c-2	ZNF493	0.360920034	mirna_pc

216	hsa-mir-30c-2	TTC37	0.317318859	mirna_pc
217	hsa-mir-30c-2	ZNF85	0.498397897	mirna_pc
218	hsa-mir-30c-2	FAM13A	0.452109947	mirna_pc
219	hsa-mir-30c-2	NEK1	0.420765425	mirna_pc
220	hsa-mir-30c-2	PPAP2B	0.390697155	mirna_pc
221	hsa-mir-30c-2	NDUFA5	0.423029808	mirna_pc
222	hsa-mir-30c-2	ST5	0.313054259	mirna_pc
223	hsa-mir-30c-2	ATP6V0A2	0.436314846	mirna_pc
224	hsa-mir-30c-2	ZNF793	0.405952907	mirna_pc
225	hsa-mir-30c-2	ZNF429	0.446670189	mirna_pc
226	hsa-mir-30c-2	C18orf32	0.333608431	mirna_pc
227	hsa-mir-30c-2	SATL1	0.478756543	mirna_pc
228	hsa-mir-30c-2	CLIC6	0.556412576	mirna_pc
229	hsa-mir-30c-2	SLC4A2	0.489884004	mirna_pc
230	hsa-mir-30c-2	ZKSCAN3	0.45397748	mirna_pc
231	hsa-mir-30c-2	ECHDC2	0.435802842	mirna_pc
232	hsa-mir-30c-2	NBAS	0.360354715	mirna_pc
233	hsa-mir-30c-2	DISP1	0.305839725	mirna_pc
234	hsa-mir-30c-2	SATB1	0.320653071	mirna_pc
235	hsa-mir-30c-2	TUBE1	0.433040515	mirna_pc
236	hsa-mir-30c-2	MTMR12	0.403626067	mirna_pc
237	hsa-mir-30c-2	SYTL3	0.391486877	mirna_pc
238	hsa-mir-30c-2	PTPRM	0.3313071	mirna_pc
239	hsa-mir-30c-2	GCNT2	0.560343944	mirna_pc
240	hsa-mir-30c-2	FAM13B	0.303204394	mirna_pc
241	hsa-mir-30c-2	RNLS	0.309563944	mirna_pc
242	hsa-mir-30c-2	DNAJC27	0.395314975	mirna_pc
243	hsa-mir-30c-2	CACNB4	0.353153269	mirna_pc
244	hsa-mir-30c-2	LOC728743	0.34048126	mirna_pc
245	hsa-mir-30c-2	CCNG1	0.385487578	mirna_pc
246	hsa-mir-30c-2	NR2F2	0.338996683	mirna_pc
247	hsa-mir-30c-2	ATXN7	0.305099444	mirna_pc
248	hsa-mir-30c-2	NOSTRIN	0.334403997	mirna_pc
249	hsa-mir-30c-2	DNAJC6	0.301702623	mirna_pc
250	hsa-mir-30c-2	RELL1	0.448386895	mirna_pc
251	hsa-mir-30c-2	INPP5J	0.36318238	mirna_pc
252	hsa-mir-30c-2	NDUFS1	0.319400643	mirna_pc
253	hsa-mir-30c-2	TCEA3	0.359871035	mirna_pc
254	hsa-mir-30c-2	C17orf108	0.316792256	mirna_pc
255	hsa-mir-30c-2	COBLL1	0.381966755	mirna_pc
256	hsa-mir-30c-2	C5	0.422535906	mirna_pc
257	hsa-mir-30c-2	LRP2BP	0.373907926	mirna_pc
258	hsa-mir-30c-2	APOOL	0.341629355	mirna_pc
259	hsa-mir-30c-2	ZNF167	0.375415098	mirna_pc
260	hsa-mir-30c-2	SLC41A1	0.433226017	mirna_pc
261	hsa-mir-30c-2	ZMYND11	0.304105357	mirna_pc
262	hsa-mir-30c-2	LOC200030	0.520395268	mirna_pc
263	hsa-mir-30c-2	ZNF181	0.346056231	mirna_pc
264	hsa-mir-30c-2	SLC13A3	0.401398053	mirna_pc
265	hsa-mir-30c-2	ZNF43	0.449427125	mirna_pc
266	hsa-mir-30c-2	ZNF331	0.33683019	mirna_pc
267	hsa-mir-30c-2	TMEM167B	0.349706761	mirna_pc
268	hsa-mir-30c-2	NFIX	0.330300888	mirna_pc
269	hsa-mir-30c-2	SLC25A16	0.35711655	mirna_pc

270	hsa-mir-30c-2	RERE	0.375739435	mirna_pc
271	hsa-mir-30c-2	HDHD2	0.323195977	mirna_pc
272	hsa-mir-30c-2	ZBED3	0.419757753	mirna_pc
273	hsa-mir-30c-2	ERN 1.00	0.308254536	mirna_pc
274	hsa-mir-30c-2	TMEM108	0.302488667	mirna_pc
275	hsa-mir-30c-2	MDH1	0.323785616	mirna_pc
276	hsa-mir-30c-2	EIF4EBP3	0.308432057	mirna_pc
277	hsa-mir-30c-2	C17orf48	0.302799162	mirna_pc
278	hsa-mir-30c-2	CCPG1	0.4389123	mirna_pc
279	hsa-mir-30c-2	ZSWIM6	0.384210773	mirna_pc
280	hsa-mir-30c-2	ZC3H6	0.410009258	mirna_pc
281	hsa-mir-30c-2	ZNF569	0.341050347	mirna_pc
282	hsa-mir-30c-2	ZNF10	0.363173595	mirna_pc
283	hsa-mir-30c-2	VSIG1	0.372726108	mirna_pc
284	hsa-mir-30c-2	SLC25A34	0.302696066	mirna_pc
285	hsa-mir-30c-2	LOC202181	0.425381937	mirna_pc
286	hsa-mir-30c-2	KIAA1109	0.318323425	mirna_pc
287	hsa-mir-30c-2	MON2	0.410227956	mirna_pc
288	hsa-mir-30c-2	HOMER2	0.321846594	mirna_pc
289	hsa-mir-30c-2	N6AMT1	0.348607405	mirna_pc
290	hsa-mir-30c-2	LARGE	0.343619051	mirna_pc
291	hsa-mir-30c-2	ZNF397	0.363029337	mirna_pc
292	hsa-mir-30c-2	PTTG1IP	0.30364393	mirna_pc
293	hsa-mir-30c-2	KIAA0895	0.479008082	mirna_pc
294	hsa-mir-30c-2	XKR8	0.355458184	mirna_pc
295	hsa-mir-30c-2	RHOU	0.420388209	mirna_pc
296	hsa-mir-30c-2	MTERFD3	0.439627126	mirna_pc
297	hsa-mir-30c-2	NNT	0.374783111	mirna_pc
298	hsa-mir-30c-2	CMTM4	0.45470274	mirna_pc
299	hsa-mir-30c-2	ARRDC4	0.302394679	mirna_pc
300	hsa-mir-30c-2	ARSK	0.328478995	mirna_pc
301	hsa-mir-30c-2	CLK4	0.462069555	mirna_pc
302	hsa-mir-30c-2	ZNF439	0.349002919	mirna_pc
303	hsa-mir-30c-2	MCEE	0.393993517	mirna_pc
304	hsa-mir-30c-2	DDX17	0.370648434	mirna_pc
305	hsa-mir-30c-2	FMOD	0.452363084	mirna_pc
306	hsa-mir-30c-2	SPATA6	0.332640269	mirna_pc
307	hsa-mir-30c-2	ATP8A1	0.355476031	mirna_pc
308	hsa-mir-30c-2	POLI	0.436469766	mirna_pc
309	hsa-mir-30c-2	SLC30A9	0.422974861	mirna_pc
310	hsa-mir-30c-2	RAB33B	0.38336787	mirna_pc
311	hsa-mir-30c-2	ZNF204P	0.357762388	mirna_pc
312	hsa-mir-30c-2	C12orf72	0.351503425	mirna_pc
313	hsa-mir-30c-2	ACSL3	0.406999416	mirna_pc
314	hsa-mir-30c-2	SLC44A2	0.31389489	mirna_pc
315	hsa-mir-30c-2	FXC1	0.357671019	mirna_pc
316	hsa-mir-30c-2	ZNF345	0.310225624	mirna_pc
317	hsa-mir-30c-2	C1orf69	0.306881895	mirna_pc
318	hsa-mir-30c-2	PRKAB2	0.302539532	mirna_pc
319	hsa-mir-30c-2	KIAA1958	0.362106825	mirna_pc
320	hsa-mir-30c-2	ARL 1.00	0.385565191	mirna_pc
321	hsa-mir-30c-2	AASS	0.370862597	mirna_pc
322	hsa-mir-30c-2	TTC3	0.406567728	mirna_pc
323	hsa-mir-30c-2	LOC286367	0.415577652	mirna_pc

324	hsa-mir-30c-2	ZNF404	0.376536514	mirna_pc
325	hsa-mir-30c-2	C11orf54	0.319866043	mirna_pc
326	hsa-mir-30c-2	DCUN1D4	0.303713834	mirna_pc
327	hsa-mir-30c-2	ZNF880	0.300992854	mirna_pc
328	hsa-mir-30c-2	PAFAH2	0.34208414	mirna_pc
329	hsa-mir-30c-2	AKR7A3	0.471332864	mirna_pc
330	hsa-mir-30c-2	ZNF649	0.313409486	mirna_pc
331	hsa-mir-30c-2	ENPP5	0.454504388	mirna_pc
332	hsa-mir-30c-2	ZNF862	0.311522858	mirna_pc
333	hsa-mir-30c-2	PEX7	0.36866152	mirna_pc
334	hsa-mir-30c-2	ANKAR	0.453973609	mirna_pc
335	hsa-mir-30c-2	TMEM70	0.312021276	mirna_pc
336	hsa-mir-30c-2	TUBA8	0.316141043	mirna_pc
337	hsa-mir-30c-2	FAM13AOS	0.384865496	mirna_pc
338	hsa-mir-30c-2	ZBTB40	0.404168745	mirna_pc
339	hsa-mir-30c-2	SEMA3B	0.348339184	mirna_pc
340	hsa-mir-30c-2	HIPK2	0.316854694	mirna_pc
341	hsa-mir-30c-2	EIF2AK3	0.361238016	mirna_pc
342	hsa-mir-30c-2	AAK1	0.374814134	mirna_pc
343	hsa-mir-30c-2	RUNDC2C	0.302935108	mirna_pc
344	hsa-mir-30c-2	BTBD3	0.374616237	mirna_pc
345	hsa-mir-30c-2	CLCC1	0.519293962	mirna_pc
346	hsa-mir-30c-2	WBP1	0.435690695	mirna_pc
347	hsa-mir-30c-2	NEDD4L	0.384738965	mirna_pc
348	hsa-mir-30c-2	WDR60	0.433950705	mirna_pc
349	hsa-mir-30c-2	ZNF577	0.314380635	mirna_pc
350	hsa-mir-30c-2	SH2B1	0.335434194	mirna_pc
351	hsa-mir-30c-2	SPCS3	0.409089169	mirna_pc
352	hsa-mir-30c-2	IDH2	0.331017534	mirna_pc
353	hsa-mir-30c-2	C3orf19	0.310337285	mirna_pc
354	hsa-mir-30c-2	MEGF8	0.384637502	mirna_pc
355	hsa-mir-30c-2	FAM46C	0.352587909	mirna_pc
356	hsa-mir-30c-2	ADAMTS13	0.383544246	mirna_pc
357	hsa-mir-30c-2	TRIP11	0.313614636	mirna_pc
358	hsa-mir-30c-2	CCDC125	0.382746758	mirna_pc
359	hsa-mir-30c-2	GKAP1	0.348552751	mirna_pc
360	hsa-mir-30c-2	ZNF441	0.331621579	mirna_pc
361	hsa-mir-30c-2	KIAA0319L	0.409460294	mirna_pc
362	hsa-mir-30c-2	ANXA10	0.381738414	mirna_pc
363	hsa-mir-30c-2	RAP1GAP2	0.394276693	mirna_pc
364	hsa-mir-30c-2	SSBP3	0.418599688	mirna_pc
365	hsa-mir-30c-2	ZNF254	0.365259931	mirna_pc
366	hsa-mir-30c-2	RUFY3	0.315792043	mirna_pc
367	hsa-mir-30c-2	GOLGA4	0.356254918	mirna_pc
368	hsa-mir-30c-2	ABCD3	0.376956763	mirna_pc
369	hsa-mir-30c-2	REPS2	0.337486588	mirna_pc
370	hsa-mir-30c-2	MT1F	0.409509795	mirna_pc
371	hsa-mir-30c-2	SLC2A11	0.314142086	mirna_pc
372	hsa-mir-30c-2	SENP7	0.379083705	mirna_pc
373	hsa-mir-30c-2	PCMTD1	0.371770858	mirna_pc
374	hsa-mir-30c-2	HADH	0.306976907	mirna_pc
375	hsa-mir-30c-2	SCMH1	0.410598746	mirna_pc
376	hsa-mir-30c-2	ZNF433	0.404553558	mirna_pc
377	hsa-mir-30c-2	PLCB4	0.313440198	mirna_pc

378	hsa-mir-30c-2	ZNF175	0.470380756	mirna_pc
379	hsa-mir-30c-2	ALS2CR8	0.371071618	mirna_pc
380	hsa-mir-30c-2	PXMP4	0.300067581	mirna_pc
381	hsa-mir-30c-2	LOC100132707	0.332638755	mirna_pc
382	hsa-mir-30c-2	ING3	0.473810536	mirna_pc
383	hsa-mir-30c-2	NUDT12	0.423670902	mirna_pc
384	hsa-mir-30c-2	THUMPD1	0.343940469	mirna_pc
385	hsa-mir-30c-2	CXCL17	0.382114256	mirna_pc
386	hsa-mir-30c-2	ADAM28	0.402189778	mirna_pc
387	hsa-mir-30c-2	C5orf33	0.411264595	mirna_pc
388	hsa-mir-30c-2	C17orf107	0.375758125	mirna_pc
389	hsa-mir-30c-2	ANKRD12	0.392155553	mirna_pc
390	hsa-mir-30c-2	GGA2	0.344234126	mirna_pc
391	hsa-mir-30c-2	TMEM56	0.455946778	mirna_pc
392	hsa-mir-30c-2	TSPAN12	0.316558899	mirna_pc
393	hsa-mir-30c-2	ZNF717	0.339862888	mirna_pc
394	hsa-mir-30c-2	CD302	0.303373031	mirna_pc
395	hsa-mir-30c-2	ZRANB1	0.312734739	mirna_pc
396	hsa-mir-30c-2	ZNF652	0.378063651	mirna_pc
397	hsa-mir-30c-2	LARP1B	0.4051721	mirna_pc
398	hsa-mir-30c-2	ME2	0.342873725	mirna_pc
399	hsa-mir-30c-2	C5orf41	0.320340629	mirna_pc
400	hsa-mir-30c-2	PPP2R3A	0.311910901	mirna_pc
401	hsa-mir-30c-2	C10orf118	0.417864105	mirna_pc
402	hsa-mir-30c-2	TMEM170B	0.389604209	mirna_pc
403	hsa-mir-30c-2	ST3GAL6	0.362130259	mirna_pc
404	hsa-mir-30c-2	ELAC1	0.349323024	mirna_pc
405	hsa-mir-30c-2	ARID4A	0.387297879	mirna_pc
406	hsa-mir-30c-2	MAP9	0.434550652	mirna_pc
407	hsa-mir-30c-2	SMAD4	0.371851635	mirna_pc
408	hsa-mir-30c-2	BMPR1B	0.300331081	mirna_pc
409	hsa-mir-30c-2	IPP	0.325784663	mirna_pc
410	hsa-mir-30c-2	ZNF546	0.413939104	mirna_pc
411	hsa-mir-30c-2	STAM2	0.354211532	mirna_pc
412	hsa-mir-30c-2	C5orf32	0.403788006	mirna_pc
413	hsa-mir-30c-2	BBS4	0.337555722	mirna_pc
414	hsa-mir-30c-2	ABCA2	0.382508002	mirna_pc
415	hsa-mir-30c-2	ZNF33A	0.423629072	mirna_pc
416	hsa-mir-30c-2	CREBL2	0.371394477	mirna_pc
417	hsa-mir-30c-2	HERPUD1	0.34516648	mirna_pc
418	hsa-mir-30c-2	ZNF33B	0.443485182	mirna_pc
419	hsa-mir-30c-2	CYP20A1	0.30203446	mirna_pc
420	hsa-mir-30c-2	ANKRD43	0.372112461	mirna_pc
421	hsa-mir-30c-2	CHPT1	0.459213518	mirna_pc
422	hsa-mir-30c-2	RAP1GAP	0.425057352	mirna_pc
423	hsa-mir-30c-2	ZNF248	0.372382669	mirna_pc
424	hsa-mir-30c-2	ZNF420	0.367495484	mirna_pc
425	hsa-mir-30c-2	TUB	0.335437206	mirna_pc
426	hsa-mir-30c-2	RORC	0.310859141	mirna_pc
427	hsa-mir-30c-2	IGFBP2	0.320129769	mirna_pc
428	hsa-mir-30c-2	CABC1	0.443361602	mirna_pc
429	hsa-mir-30c-2	IKZF5	0.43258051	mirna_pc
430	hsa-mir-30c-2	PWWP2A	0.365252721	mirna_pc
431	hsa-mir-30c-2	FAR1	0.333439407	mirna_pc

432	hsa-mir-30c-2	FAM8A1	0.302927557	mirna_pc
433	hsa-mir-30c-2	EIF4EBP2	0.308666588	mirna_pc
434	hsa-mir-30c-2	ZNF571	0.337673114	mirna_pc
435	hsa-mir-30c-2	PPFIBP2	0.307820243	mirna_pc
436	hsa-mir-30c-2	MTR	0.323582	mirna_pc
437	hsa-mir-30c-2	USP16	0.332938134	mirna_pc
438	hsa-mir-30c-2	CBR4	0.343604621	mirna_pc
439	hsa-mir-30c-2	SARDH	0.336802781	mirna_pc
440	hsa-mir-30c-2	FGFR1	0.315703558	mirna_pc
441	hsa-mir-30c-2	RAPGEF2	0.350780434	mirna_pc
442	hsa-mir-30c-2	ERMAP	0.323276082	mirna_pc
443	hsa-mir-30c-2	TMBIM6	0.324764564	mirna_pc
444	hsa-mir-30c-2	CACHD1	0.427241653	mirna_pc
445	hsa-mir-30c-2	ZNF354C	0.339316039	mirna_pc
446	hsa-mir-30c-2	TATDN3	0.307405276	mirna_pc
447	hsa-mir-30c-2	GIGYF2	0.352979189	mirna_pc
448	hsa-mir-30c-2	PAPSS1	0.437216696	mirna_pc
449	hsa-mir-30c-2	LOC100130093	0.331264157	mirna_pc
450	hsa-mir-30c-2	FLJ39653	0.409781585	mirna_pc
451	hsa-mir-30c-2	TMX4	0.300948472	mirna_pc
452	hsa-mir-30c-2	PRLR	0.403982103	mirna_pc
453	hsa-mir-30c-2	APPL1	0.369396259	mirna_pc
454	hsa-mir-30c-2	RGL3	0.393309759	mirna_pc
455	hsa-mir-30c-2	ZNF280D	0.364145858	mirna_pc
456	hsa-mir-30c-2	B3GNT1	0.301114832	mirna_pc
457	hsa-mir-30c-2	MAP4K3	0.451635741	mirna_pc
458	hsa-mir-30c-2	ZNF24	0.333978758	mirna_pc
459	hsa-mir-30c-2	TTC18	0.385405997	mirna_pc
460	hsa-mir-30c-2	PER3	0.318049423	mirna_pc
461	hsa-mir-30c-2	CTBS	0.396362803	mirna_pc
462	hsa-mir-30c-2	ATRX	0.426008494	mirna_pc
463	hsa-mir-30c-2	ZFP112	0.363502785	mirna_pc
464	hsa-mir-30c-2	DET1	0.365278599	mirna_pc
465	hsa-mir-30c-2	ZNF573	0.300943685	mirna_pc
466	hsa-mir-30c-2	MCFD2	0.331055614	mirna_pc
467	hsa-mir-30c-2	ANO7	0.331835332	mirna_pc
468	hsa-mir-30c-2	ITSN2	0.38554167	mirna_pc
469	hsa-mir-30c-2	AZGP1	0.310400054	mirna_pc
470	hsa-mir-30c-2	ACVR2A	0.550879653	mirna_pc
471	hsa-mir-30c-2	FAM107B	0.358289703	mirna_pc
472	hsa-mir-30c-2	PHF10	0.362780793	mirna_pc
473	hsa-mir-30c-2	CHD2	0.326837823	mirna_pc
474	hsa-mir-30c-2	TMEM86B	0.42970138	mirna_pc
475	hsa-mir-30c-2	NAALADL2	0.315748682	mirna_pc
476	hsa-mir-30c-2	WDR19	0.430911439	mirna_pc
477	hsa-mir-30c-2	KCNQ1	0.499573514	mirna_pc
478	hsa-mir-30c-2	C4orf52	0.362004961	mirna_pc
479	hsa-mir-30c-2	ZBTB8A	0.316284996	mirna_pc
480	hsa-mir-30c-2	ZNF709	0.41161469	mirna_pc
481	hsa-mir-30c-2	TFR2	0.417858514	mirna_pc
482	hsa-mir-30c-2	SEC63	0.348188535	mirna_pc
483	hsa-mir-30c-2	PLEKHM3	0.31361627	mirna_pc
484	hsa-mir-30c-2	PHC1	0.370443644	mirna_pc
485	hsa-mir-30c-2	TMEM192	0.374307185	mirna_pc

486	hsa-mir-30c-2	MALAT1	0.462007613	mirna_pc
487	hsa-mir-30c-2	KIAA1147	0.331656027	mirna_pc
488	hsa-mir-30c-2	CCDC121	0.478077513	mirna_pc
489	hsa-mir-30c-2	SLC41A2	0.401779117	mirna_pc
490	hsa-mir-30c-2	ZNF449	0.391429331	mirna_pc
491	hsa-mir-30c-2	CTDSP2	0.335297511	mirna_pc
492	hsa-mir-30c-2	HSP90B1	0.32608454	mirna_pc
493	hsa-mir-30c-2	ZNF445	0.31935259	mirna_pc
494	hsa-mir-30c-2	C4orf41	0.322832981	mirna_pc
495	hsa-mir-30c-2	FAM168B	0.324424369	mirna_pc
496	hsa-mir-30c-2	ZNF615	0.333402969	mirna_pc
497	hsa-mir-30c-2	CDC14A	0.421066959	mirna_pc
498	hsa-mir-30c-2	C1orf26	0.425577578	mirna_pc
499	hsa-mir-30c-2	TCEANC	0.35678121	mirna_pc
500	hsa-mir-30c-2	IQGAP2	0.356945985	mirna_pc
501	hsa-mir-30c-2	GOLGA6L10	0.329905057	mirna_pc
502	hsa-mir-30c-2	BRPF3	0.331438885	mirna_pc
503	hsa-mir-30c-2	WWC2	0.349712763	mirna_pc
504	hsa-mir-30c-2	SVIP	0.39745895	mirna_pc
505	hsa-mir-30c-2	NBPF1	0.372378647	mirna_pc
506	hsa-mir-30c-2	PELI2	0.339871124	mirna_pc
507	hsa-mir-30c-2	ZNF221	0.363075772	mirna_pc
508	hsa-mir-30c-2	CPM	0.335898002	mirna_pc
509	hsa-mir-30c-2	LNX1	0.375099276	mirna_pc
510	hsa-mir-30c-2	SLC9A2	0.331638162	mirna_pc
511	hsa-mir-30c-2	SFRS18	0.460930306	mirna_pc
512	hsa-mir-30c-2	ACSS1	0.477041	mirna_pc
513	hsa-mir-30c-2	CNOT6L	0.423772481	mirna_pc
514	hsa-mir-30c-2	DPY19L3	0.445513687	mirna_pc
515	hsa-mir-30c-2	NUDT13	0.370887092	mirna_pc
516	hsa-mir-30c-2	XBP1	0.316850329	mirna_pc
517	hsa-mir-30c-2	C1orf130	0.404272359	mirna_pc
518	hsa-mir-30c-2	PIK3C2A	0.364219413	mirna_pc
519	hsa-mir-30c-2	C5orf30	0.335814613	mirna_pc
520	hsa-mir-30c-2	ZNF397OS	0.388282797	mirna_pc
521	hsa-mir-30c-2	CCDC66	0.349755033	mirna_pc
522	hsa-mir-30c-2	FBXW8	0.343301479	mirna_pc
523	hsa-mir-30c-2	ZNF14	0.415437456	mirna_pc
524	hsa-mir-30c-2	ACSF2	0.371359073	mirna_pc
525	hsa-mir-30c-2	C5orf24	0.306030138	mirna_pc
526	hsa-mir-30c-2	C2orf86	0.51841273	mirna_pc
527	hsa-mir-30c-2	B3GALNT1	0.515543643	mirna_pc
528	hsa-mir-30c-2	PPIEL	0.335820021	mirna_pc
529	hsa-mir-30c-2	FAM160B1	0.343038042	mirna_pc
530	hsa-mir-30c-2	CROCCL2	0.43424541	mirna_pc
531	hsa-mir-30c-2	FNDC3A	0.380369163	mirna_pc
532	hsa-mir-30c-2	ZNF177	0.300732784	mirna_pc
533	hsa-mir-30c-2	ZNF549	0.394072227	mirna_pc
534	hsa-mir-30c-2	SEC31B	0.32518136	mirna_pc
535	hsa-mir-30c-2	KIAA0776	0.350254351	mirna_pc
536	hsa-mir-30c-2	CSRP2BP	0.38469548	mirna_pc
537	hsa-mir-30c-2	GATA6	0.317073253	mirna_pc
538	hsa-mir-30c-2	ZNF629	0.333840898	mirna_pc
539	hsa-mir-30c-2	RBPJ	0.38212217	mirna_pc

540	hsa-mir-30c-2	KLF11	0.336785705	mirna_pc
541	hsa-mir-30c-2	ZNF704	0.300942849	mirna_pc
542	hsa-mir-30c-2	ZNF37A	0.403230703	mirna_pc
543	hsa-mir-30c-2	PDHX	0.322034529	mirna_pc
544	hsa-mir-30c-2	CDC14B	0.343061571	mirna_pc
545	hsa-mir-30c-2	ZNF552	0.418304415	mirna_pc
546	hsa-mir-30c-2	MFSD8	0.442716139	mirna_pc
547	hsa-mir-30c-2	THAP10	0.396312378	mirna_pc
548	hsa-mir-30c-2	CYP2R1	0.304474116	mirna_pc
549	hsa-mir-30c-2	BOD1L	0.448068343	mirna_pc
550	hsa-mir-30c-2	SPRN	0.309573097	mirna_pc
551	hsa-mir-30c-2	ZNF141	0.387099318	mirna_pc
552	hsa-mir-30c-2	ALDH1A1	0.50100311	mirna_pc
553	hsa-mir-30c-2	TRAPPC2	0.324914668	mirna_pc
554	hsa-mir-30c-2	HEATR5B	0.326455859	mirna_pc
555	hsa-mir-30c-2	CCDC55	0.317009865	mirna_pc
556	hsa-mir-30c-2	TRIM66	0.326957806	mirna_pc
557	hsa-mir-30c-2	NFIB	0.44030273	mirna_pc
558	hsa-mir-30c-2	FBXW7	0.330231432	mirna_pc
559	hsa-mir-30c-2	PREPL	0.327225499	mirna_pc
560	hsa-mir-30c-2	RSBN1	0.324839919	mirna_pc
561	hsa-mir-30c-2	ING5	0.371506364	mirna_pc
562	hsa-mir-30c-2	FAM73A	0.358446165	mirna_pc
563	hsa-mir-30c-2	MECOM	0.308287707	mirna_pc
564	hsa-mir-30c-2	METTL14	0.354170751	mirna_pc
565	hsa-mir-30c-2	ITFG2	0.358770265	mirna_pc
566	hsa-mir-30c-2	ZNF2	0.318301059	mirna_pc
567	hsa-mir-30c-2	AMN1	0.33923438	mirna_pc
568	hsa-mir-30c-2	DNASE1	0.341500292	mirna_pc
569	hsa-mir-30c-2	TTC17	0.331951661	mirna_pc
570	hsa-mir-30c-2	TMEM150C	0.359099401	mirna_pc
571	hsa-mir-30c-2	GDAP2	0.322382568	mirna_pc
572	hsa-mir-30c-2	HPCAL1	0.331120071	mirna_pc
573	hsa-mir-30c-2	CADM1	0.435669261	mirna_pc
574	hsa-mir-30c-2	BRWD1	0.374579625	mirna_pc
575	hsa-mir-30c-2	SERPINI1	0.366023766	mirna_pc
576	hsa-mir-30c-2	C1orf115	0.387901551	mirna_pc
577	hsa-mir-30c-2	C11orf52	0.379111371	mirna_pc
578	hsa-mir-30c-2	CPAMD8	0.431061058	mirna_pc
579	hsa-mir-30c-2	SLC35E2	0.33505903	mirna_pc
580	hsa-mir-30c-2	SIRT1	0.34489433	mirna_pc
581	hsa-mir-30c-2	SALL2	0.303534708	mirna_pc
582	hsa-mir-30c-2	GRAMD1C	0.419891503	mirna_pc
583	hsa-mir-30c-2	PCM1	0.303718782	mirna_pc
584	hsa-mir-30c-2	AGAP5	0.397549977	mirna_pc
585	hsa-mir-30c-2	MOBKL1A	0.433175273	mirna_pc
586	hsa-mir-30c-2	MAN1A2	0.320954092	mirna_pc
587	hsa-mir-30c-2	CA2	0.345445816	mirna_pc
588	hsa-mir-30c-2	ERBB2IP	0.314388929	mirna_pc
589	hsa-mir-30c-2	MTMR7	0.345304917	mirna_pc
590	hsa-mir-30c-2	ELF2	0.320645544	mirna_pc
591	hsa-mir-30c-2	FAM161A	0.473906563	mirna_pc
592	hsa-mir-30c-2	THAP2	0.329743536	mirna_pc
593	hsa-mir-30c-2	SLTM	0.418049421	mirna_pc

594	hsa-mir-30c-2	RAB17	0.460805785	mirna_pc
595	hsa-mir-30c-2	FAM193A	0.403234581	mirna_pc
596	hsa-mir-30c-2	ZNF606	0.441845508	mirna_pc
597	hsa-mir-30c-2	GPAM	0.356412036	mirna_pc
598	hsa-mir-30c-2	LOC651250	0.314420687	mirna_pc
599	hsa-mir-30c-2	OSBPL9	0.424023589	mirna_pc
600	hsa-mir-30c-2	NKTR	0.393228343	mirna_pc
601	hsa-mir-30c-2	ZNF236	0.319100562	mirna_pc
602	hsa-mir-30c-2	ZSCAN2	0.435511837	mirna_pc
603	hsa-mir-30c-2	IRAK1BP1	0.41267418	mirna_pc
604	hsa-mir-30c-2	SOS 1	0.377075573	mirna_pc
605	hsa-mir-30c-2	ZNF211	0.300963846	mirna_pc
606	hsa-mir-30c-2	LMO4	0.332884231	mirna_pc
607	hsa-mir-30c-2	CCNJL	0.382756892	mirna_pc
608	hsa-mir-30c-2	LRRN1	0.379352069	mirna_pc
609	hsa-mir-30c-2	SFRS12	0.477098734	mirna_pc
610	hsa-mir-30c-2	PPIP5K2	0.359865172	mirna_pc
611	hsa-mir-30c-2	ATXN7L3B	0.353460847	mirna_pc
612	hsa-mir-30c-2	HIST2H2BF	0.307642173	mirna_pc
613	hsa-mir-30c-2	ZNF444	0.383144904	mirna_pc
614	hsa-mir-30c-2	C6orf120	0.323840398	mirna_pc
615	hsa-mir-30c-2	TMEM181	0.302211914	mirna_pc
616	hsa-mir-30c-2	ZNF23	0.309923568	mirna_pc
617	hsa-mir-30c-2	C2orf63	0.404095481	mirna_pc
618	hsa-mir-30c-2	CDKL3	0.300086961	mirna_pc
619	hsa-mir-30c-2	ZNF225	0.361818921	mirna_pc
620	hsa-mir-30c-2	ZNF160	0.421881399	mirna_pc
621	hsa-mir-30c-2	C11orf9	0.306064094	mirna_pc
622	hsa-mir-30c-2	SLC16A10	0.415233855	mirna_pc
623	hsa-mir-30c-2	EPOR	0.352501796	mirna_pc
624	hsa-mir-30c-2	ZNF136	0.393936706	mirna_pc
625	hsa-mir-30c-2	MDM4	0.384563656	mirna_pc
626	hsa-mir-30c-2	EPB41	0.334461975	mirna_pc
627	hsa-mir-30c-2	RHOBTB1	0.309207611	mirna_pc
628	hsa-mir-30c-2	C4orf29	0.39040582	mirna_pc
629	hsa-mir-30c-2	MED13L	0.322758876	mirna_pc
630	hsa-mir-30c-2	LOC144571	0.301885132	mirna_pc
631	hsa-mir-30c-2	SH3YL1	0.370060829	mirna_pc
632	hsa-mir-30c-2	EIF5A2	0.371447381	mirna_pc
633	hsa-mir-30c-2	MAN2A2	0.313037131	mirna_pc
634	hsa-mir-30c-2	ZNF451	0.317218811	mirna_pc
635	hsa-mir-30c-2	ZNF187	0.308638844	mirna_pc
636	hsa-mir-30c-2	KLRA1	0.426830254	mirna_pc
637	hsa-mir-30c-2	ZKSCAN2	0.374442466	mirna_pc
638	hsa-mir-30c-2	ZDBF2	0.368663063	mirna_pc
639	hsa-mir-30c-2	C12orf23	0.379896353	mirna_pc
640	hsa-mir-30c-2	RCOR3	0.357192583	mirna_pc
641	hsa-mir-30c-2	ZNF684	0.618949313	mirna_pc
642	hsa-mir-30c-2	GOLGA6L9	0.315899871	mirna_pc
643	hsa-mir-30c-2	ZNF192	0.378759494	mirna_pc
644	hsa-mir-30c-2	SLC25A30	0.326269605	mirna_pc
645	hsa-mir-30c-2	NSUN7	0.320358697	mirna_pc
646	hsa-mir-30c-2	ARHGAP18	0.40954792	mirna_pc
647	hsa-mir-30c-2	TMPRSS2	0.312125157	mirna_pc

648	hsa-mir-30c-2	EPM2AIP1	0.32864323	mirna_pc
649	hsa-mir-30c-2	DNAJB9	0.30283078	mirna_pc
650	hsa-mir-30c-2	SEPSECS	0.392460304	mirna_pc
651	hsa-mir-30c-2	JMJD5	0.306296569	mirna_pc
652	hsa-mir-30c-2	FAM119B	0.30670898	mirna_pc
653	hsa-mir-30c-2	BMS1P4	0.330296879	mirna_pc
654	hsa-mir-30c-2	PTBP2	0.339037599	mirna_pc
655	hsa-mir-30c-2	PPCS	0.330532997	mirna_pc
656	hsa-mir-30c-2	ARFGEF2	0.36360846	mirna_pc
657	hsa-mir-30c-2	CENPC1	0.336171736	mirna_pc
658	hsa-mir-30c-2	SCAND2	0.339107822	mirna_pc
659	hsa-mir-30c-2	NAPEPLD	0.338334968	mirna_pc
660	hsa-mir-30c-2	USP34	0.323977555	mirna_pc
661	hsa-mir-30c-2	SNX25	0.303288926	mirna_pc
662	hsa-mir-30c-2	ZNF780B	0.362370206	mirna_pc
663	hsa-mir-30c-2	CA9	0.343513499	mirna_pc
664	hsa-mir-30c-2	ZNF630	0.319216172	mirna_pc
665	hsa-mir-30c-2	ZNF189	0.340902601	mirna_pc
666	hsa-mir-30c-2	ZNF605	0.324395404	mirna_pc
667	hsa-mir-30c-2	KDM6A	0.380120965	mirna_pc
668	hsa-mir-30c-2	SLC39A14	0.307961159	mirna_pc
669	hsa-mir-30c-2	SRBD1	0.337121767	mirna_pc
670	hsa-mir-30c-2	RASSF6	0.362135961	mirna_pc
671	hsa-mir-30c-2	RGPD3	0.341426303	mirna_pc
672	hsa-mir-30c-2	CCDC30	0.415564654	mirna_pc
673	hsa-mir-30c-2	RBM33	0.340272649	mirna_pc
674	hsa-mir-30c-2	LMAN1	0.428081113	mirna_pc
675	hsa-mir-30c-2	ZNF234	0.322798532	mirna_pc
676	hsa-mir-30c-2	ZNF230	0.305129999	mirna_pc
677	hsa-mir-30c-2	ANKRD26	0.40171558	mirna_pc
678	hsa-mir-30c-2	DPY19L4	0.43862056	mirna_pc
679	hsa-mir-30c-2	ZC3H7A	0.350175081	mirna_pc
680	hsa-mir-30c-2	RGPD4	0.302853454	mirna_pc
681	hsa-mir-30c-2	RG9MTD2	0.309603463	mirna_pc
682	hsa-mir-30c-2	GPBP1L1	0.35873685	mirna_pc
683	hsa-mir-30c-2	GOLGA2B	0.356102266	mirna_pc
684	hsa-mir-30c-2	ZNF253	0.385946266	mirna_pc
685	hsa-mir-30c-2	GABPA	0.339844061	mirna_pc
686	hsa-mir-30c-2	ZNF529	0.316571854	mirna_pc
687	hsa-mir-30c-2	ZFP161	0.302050164	mirna_pc
688	hsa-mir-30c-2	B4GALNT3	0.303392389	mirna_pc
689	hsa-mir-30c-2	RGPD6	0.399149756	mirna_pc
690	hsa-mir-30c-2	ZNF302	0.313517822	mirna_pc
691	hsa-mir-30c-2	ING4	0.319307155	mirna_pc
692	hsa-mir-30c-2	RFC1	0.445338997	mirna_pc
693	hsa-mir-30c-2	ZNF776	0.382084848	mirna_pc
694	hsa-mir-30c-2	ANKRD11	0.30377993	mirna_pc
695	hsa-mir-30c-2	ATN1	0.381467203	mirna_pc
696	hsa-mir-30c-2	CEP120	0.313283107	mirna_pc
697	hsa-mir-30c-2	ANKRD6	0.387460856	mirna_pc
698	hsa-mir-30c-2	TXNDC16	0.30069097	mirna_pc
699	hsa-mir-30c-2	ZNF566	0.354861473	mirna_pc
700	hsa-mir-30c-2	GGT7	0.392080057	mirna_pc
701	hsa-mir-30c-2	C1orf213	0.377322074	mirna_pc

702	hsa-mir-30c-2	WDR52	0.320482934	mirna_pc
703	hsa-mir-30c-2	KIAA1324	0.301606071	mirna_pc
704	hsa-mir-30c-2	WWC1	0.304513765	mirna_pc
705	hsa-mir-30c-2	ZNF417	0.317096091	mirna_pc
706	hsa-mir-30c-2	ZBTB49	0.365527939	mirna_pc
707	hsa-mir-30c-2	ARNT2	0.325446092	mirna_pc
708	hsa-mir-30c-2	TMEM98	0.330192042	mirna_pc
709	hsa-mir-30c-2	ZNF700	0.30724741	mirna_pc
710	hsa-mir-30c-2	ERP27	0.409571119	mirna_pc
711	hsa-mir-30c-2	CAPN13	0.422170891	mirna_pc
712	hsa-mir-30c-2	FUNDC1	0.338872436	mirna_pc
713	hsa-mir-30c-2	TTN	0.394178229	mirna_pc
714	hsa-mir-30c-2	ZNF84	0.439818385	mirna_pc
715	hsa-mir-30c-2	SLC25A36	0.432096381	mirna_pc
716	hsa-mir-30c-2	CRLS1	0.312649272	mirna_pc
717	hsa-mir-30c-2	RWDD3	0.408214649	mirna_pc
718	hsa-mir-30c-2	ARID4B	0.462177727	mirna_pc
719	hsa-mir-30c-2	DDX46	0.348022606	mirna_pc
720	hsa-mir-30c-2	ZDHHC17	0.435064893	mirna_pc
721	hsa-mir-30c-2	ZNF599	0.363207838	mirna_pc
722	hsa-mir-30c-2	INPPL1	0.300526191	mirna_pc
723	hsa-mir-30c-2	UBN2	0.374260075	mirna_pc
724	hsa-mir-30c-2	ZFP14	0.327908199	mirna_pc
725	hsa-mir-30c-2	HUNK	0.316551445	mirna_pc
726	hsa-mir-30c-2	CHKA	0.326902048	mirna_pc
727	hsa-mir-30c-2	SFRS13A	0.310796055	mirna_pc
728	hsa-mir-30c-2	RBM47	0.325055445	mirna_pc
729	hsa-mir-30c-2	GPR125	0.390115766	mirna_pc
730	hsa-mir-30c-2	FIZ1	0.304707162	mirna_pc
731	hsa-mir-30c-2	C1orf203	0.308810166	mirna_pc
732	hsa-mir-30c-2	PIKFYVE	0.352181574	mirna_pc
733	hsa-mir-30c-2	LOC729799	0.345191995	mirna_pc
734	hsa-mir-30c-2	FNBP1L	0.390900548	mirna_pc
735	hsa-mir-30c-2	REST	0.341998131	mirna_pc
736	hsa-mir-30c-2	ACVR2B	0.421087473	mirna_pc
737	hsa-mir-30c-2	LOC91316	0.304075322	mirna_pc
738	hsa-mir-30c-2	POGZ	0.388939623	mirna_pc
739	hsa-mir-30c-2	FAM22A	0.336974859	mirna_pc
740	hsa-mir-30c-2	UBE2K	0.323076573	mirna_pc
741	hsa-mir-30c-2	WDR35	0.366774479	mirna_pc
742	hsa-mir-30c-2	ZNF514	0.309665232	mirna_pc
743	hsa-mir-30c-2	TTC14	0.352665524	mirna_pc
744	hsa-mir-30c-2	ANKRD22	0.328231509	mirna_pc
745	hsa-mir-30c-2	ZNF773	0.328934785	mirna_pc
746	hsa-mir-30c-2	AFTPH	0.37489143	mirna_pc
747	hsa-mir-30c-2	TBC1D15	0.355204227	mirna_pc
748	hsa-mir-30c-2	C1orf27	0.307244443	mirna_pc
749	hsa-mir-30c-2	SUV420H1	0.37542259	mirna_pc
750	hsa-mir-30c-2	FLJ10213	0.356246172	mirna_pc
751	hsa-mir-30c-2	CCDC144B	0.306462539	mirna_pc
752	hsa-mir-30c-2	CCAR1	0.304673927	mirna_pc
753	hsa-mir-30c-2	NEO1	0.310272573	mirna_pc
754	hsa-mir-30c-2	EDAR	0.312384378	mirna_pc
755	hsa-mir-30c-2	ZNF304	0.303392362	mirna_pc

756	hsa-mir-30c-2	ERLEC1	0.318464238	mirna_pc
757	hsa-mir-30c-2	USP33	0.359807556	mirna_pc
758	hsa-mir-30c-2	TIGD2	0.35439587	mirna_pc
759	hsa-mir-30c-2	FOXJ3	0.4183486	mirna_pc
760	hsa-mir-30c-2	ZNF548	0.310821965	mirna_pc
761	hsa-mir-30c-2	ZNF814	0.339953305	mirna_pc
762	hsa-mir-30c-2	ZNF180	0.360926814	mirna_pc
763	hsa-mir-30c-2	ZNF780A	0.327401491	mirna_pc
764	hsa-mir-30c-2	CDK18	0.363125641	mirna_pc
765	hsa-mir-30c-2	CCNT2	0.426531954	mirna_pc
766	hsa-mir-30c-2	ZNF214	0.335079773	mirna_pc
767	hsa-mir-30c-2	ZBTB10	0.332846019	mirna_pc
768	hsa-mir-30c-2	TMEM128	0.347910655	mirna_pc
769	hsa-mir-30c-2	GOLGA8B	0.370107003	mirna_pc
770	hsa-mir-30c-2	ZNF45	0.312242087	mirna_pc
771	hsa-mir-30c-2	TTC21B	0.311263231	mirna_pc
772	hsa-mir-30c-2	PPIG	0.359121143	mirna_pc
773	hsa-mir-30c-2	FNDC3B	0.307203878	mirna_pc
774	hsa-mir-30c-2	PRPF4B	0.364004801	mirna_pc
775	hsa-mir-30c-2	LRP6	0.436496901	mirna_pc
776	hsa-mir-30c-2	EXTL2	0.322182178	mirna_pc
777	hsa-mir-30c-2	DCP2	0.430405598	mirna_pc
778	hsa-mir-30c-2	PRKD3	0.330167662	mirna_pc
779	hsa-mir-30c-2	LOC100130557	0.44562779	mirna_pc
780	hsa-mir-30c-2	TMTC4	0.311303719	mirna_pc
781	hsa-mir-30c-2	CTR9	0.330799402	mirna_pc
782	hsa-mir-30c-2	ADNP	0.309395359	mirna_pc
783	hsa-mir-30c-2	ARID1A	0.328942397	mirna_pc
784	hsa-mir-30c-2	IFT172	0.396927365	mirna_pc
785	hsa-mir-30c-2	CHD6	0.313992931	mirna_pc
786	hsa-mir-30c-2	CCDC76	0.460468898	mirna_pc
787	hsa-mir-30c-2	VASH2	0.396193623	mirna_pc
788	hsa-mir-30c-2	MCCC2	0.328968459	mirna_pc
789	hsa-mir-30c-2	ATG4C	0.327446797	mirna_pc
790	hsa-mir-30c-2	ZNF638	0.405053071	mirna_pc
791	hsa-mir-30c-2	GCA	0.362515755	mirna_pc
792	hsa-mir-30c-2	ZNF512	0.31899067	mirna_pc
793	hsa-mir-30c-2	ALMS1	0.301808933	mirna_pc
794	hsa-mir-30c-2	ZCCHC4	0.300296244	mirna_pc
795	hsa-mir-30c-2	RAD52	0.333462645	mirna_pc
796	hsa-mir-30c-2	EMID1	0.389058506	mirna_pc
797	hsa-mir-30c-2	ZMYM4	0.373280567	mirna_pc
798	hsa-mir-30c-2	TRPM7	0.308907173	mirna_pc
799	hsa-mir-30c-2	BCLAF1	0.304184232	mirna_pc
800	hsa-mir-30c-2	ZNF227	0.314062855	mirna_pc
801	hsa-mir-30c-2	MOXD1	0.326067856	mirna_pc
802	hsa-mir-30c-2	THAP9	0.474489165	mirna_pc
803	hsa-mir-30c-2	PRPF38B	0.351039646	mirna_pc
804	hsa-mir-30c-2	CEP290	0.377643238	mirna_pc
805	hsa-mir-30c-2	PHF16	0.331761239	mirna_pc
806	hsa-mir-30c-2	LOC400657	0.31483651	mirna_pc
807	hsa-mir-30c-2	ALG10B	0.475275696	mirna_pc
808	hsa-mir-30c-2	MNS1	0.322091288	mirna_pc
809	hsa-mir-30c-2	OFD1	0.397800859	mirna_pc

810	hsa-mir-30c-2	TRUB1	0.326189442	mirna_pc
811	hsa-mir-30c-2	FAM71E1	0.300343269	mirna_pc
812	hsa-mir-30c-2	PDIK1L	0.316819579	mirna_pc
813	hsa-mir-30c-2	QRICH2	0.309226129	mirna_pc
814	hsa-mir-30c-2	PHTF1	0.338630637	mirna_pc
815	hsa-mir-30c-2	DCAF16	0.356729464	mirna_pc
816	hsa-mir-30c-2	C10orf137	0.37866099	mirna_pc
817	hsa-mir-30c-2	HAUS3	0.379127953	mirna_pc
818	hsa-mir-30c-2	MANEA	0.332571372	mirna_pc
819	hsa-mir-30c-2	ZNF674	0.301908883	mirna_pc
820	hsa-mir-30c-2	ANGEL2	0.305657736	mirna_pc
821	hsa-mir-30c-2	GXYLT1	0.428185294	mirna_pc
822	hsa-mir-30c-2	N4BP2	0.339660622	mirna_pc
823	hsa-mir-30c-2	ZZZ3	0.315733166	mirna_pc
824	hsa-mir-30c-2	SFRS11	0.414220406	mirna_pc
825	hsa-mir-30c-2	SCAMP5	0.301232916	mirna_pc
826	hsa-mir-30c-2	TRO	0.331313261	mirna_pc
827	hsa-mir-30c-2	BBS10	0.30936891	mirna_pc
828	hsa-mir-30c-2	ZBTB39	0.335448541	mirna_pc
829	hsa-mir-30c-2	NCRNA00201	0.39646204	mirna_pc
830	hsa-mir-30c-2	C20orf12	0.389595996	mirna_pc
831	hsa-mir-30c-2	FANCF	0.354606238	mirna_pc
832	hsa-mir-30c-2	VPS54	0.412938868	mirna_pc
833	hsa-mir-30c-2	ZBTB24	0.346399559	mirna_pc
834	hsa-mir-30c-2	SPDYE8P	0.313642144	mirna_pc
835	hsa-mir-30c-2	NFYC	0.310687098	mirna_pc
836	hsa-mir-30c-2	C1orf9	0.361268055	mirna_pc
837	hsa-mir-30c-2	TSGA10	0.320691463	mirna_pc
838	hsa-mir-30c-2	PLGLB2	0.307623536	mirna_pc
839	hsa-mir-30c-2	SNX22	0.424559919	mirna_pc
840	hsa-mir-30c-2	FAM119A	0.301196603	mirna_pc
841	hsa-mir-30c-2	ZNF550	0.309703017	mirna_pc
842	hsa-mir-30c-2	ZNF567	0.307863796	mirna_pc
843	hsa-mir-30c-2	TIA1	0.404755024	mirna_pc
844	hsa-mir-30c-2	IL17RD	0.314923668	mirna_pc
845	hsa-mir-30c-2	ZNF664	0.341152745	mirna_pc
846	hsa-mir-30c-2	RBBP6	0.310063458	mirna_pc
847	hsa-mir-30c-2	NR1D2	0.309433786	mirna_pc
848	hsa-mir-30c-2	CSPP1	0.307605779	mirna_pc
849	hsa-mir-30c-2	SIKE1	0.316865568	mirna_pc
850	hsa-mir-30c-2	PPP4R2	0.307481613	mirna_pc
851	hsa-mir-30c-2	C2orf60	0.366324444	mirna_pc
852	hsa-mir-30c-2	GCFC1	0.348044358	mirna_pc
853	hsa-mir-30c-2	ZCCHC11	0.350230592	mirna_pc
854	hsa-mir-30c-2	ZNF644	0.344145959	mirna_pc
855	hsa-mir-30c-2	COL9A2	0.370417632	mirna_pc
856	hsa-mir-30c-2	MST02P	0.315235436	mirna_pc
857	hsa-mir-30c-2	COL9A3	0.32028086	mirna_pc
858	hsa-mir-30c-2	MARVELD2	0.319160867	mirna_pc
859	hsa-mir-30c-2	ZNF680	0.360312237	mirna_pc
860	hsa-mir-30c-2	TRIM45	0.319920675	mirna_pc
861	hsa-mir-30c-2	ZDHHC23	0.373850354	mirna_pc
862	hsa-mir-30c-2	LYPD6B	0.368651673	mirna_pc
863	hsa-mir-30c-2	PHIP	0.305698109	mirna_pc

864	hsa-mir-30c-2	SMARCAD1	0.308965244	mirna_pc
865	hsa-mir-30c-2	ZBED5	0.324985412	mirna_pc
866	hsa-mir-30c-2	SFRS6	0.383191847	mirna_pc
867	hsa-mir-30c-2	SF3B1	0.300819481	mirna_pc
868	hsa-mir-30c-2	ZNF670	0.357865205	mirna_pc
869	hsa-mir-30c-2	C3orf33	0.3027623	mirna_pc
870	hsa-mir-30c-2	GOLGA8A	0.34706593	mirna_pc
871	hsa-mir-30c-2	SS18L1	0.324449828	mirna_pc
872	hsa-mir-29c	ZBTB16	0.320016049	mirna_pc
873	hsa-mir-29c	GREM2	0.555868654	mirna_pc
874	hsa-mir-29c	GPER	0.583726277	mirna_pc
875	hsa-mir-29c	AFF3	0.460413439	mirna_pc
876	hsa-mir-29c	NCAM1	0.326145904	mirna_pc
877	hsa-mir-29c	FAM107A	0.393281777	mirna_pc
878	hsa-mir-29c	KLF15	0.492217896	mirna_pc
879	hsa-mir-29c	GPR133	0.50505869	mirna_pc
880	hsa-mir-29c	CLEC3B	0.336456607	mirna_pc
881	hsa-mir-29c	GNG7	0.471803844	mirna_pc
882	hsa-mir-29c	KL	0.548500703	mirna_pc
883	hsa-mir-29c	PTGER3	0.595210907	mirna_pc
884	hsa-mir-29c	CAB39L	0.434328934	mirna_pc
885	hsa-mir-29c	ABCA8	0.369693545	mirna_pc
886	hsa-mir-29c	SCUBE2	0.529318243	mirna_pc
887	hsa-mir-29c	GPR155	0.594478924	mirna_pc
888	hsa-mir-29c	SLC16A7	0.57564101	mirna_pc
889	hsa-mir-29c	CARNS1	0.327143846	mirna_pc
890	hsa-mir-29c	PNPLA7	0.566247255	mirna_pc
891	hsa-mir-29c	STX12	0.595417659	mirna_pc
892	hsa-mir-29c	C3orf18	0.534165323	mirna_pc
893	hsa-mir-29c	GPR146	0.607221169	mirna_pc
894	hsa-mir-29c	RNF180	0.445769553	mirna_pc
895	hsa-mir-29c	MLYCD	0.491488013	mirna_pc
896	hsa-mir-29c	CRY2	0.535768719	mirna_pc
897	hsa-mir-29c	PARK2	0.376105187	mirna_pc
898	hsa-mir-29c	ATOH8	0.345468886	mirna_pc
899	hsa-mir-29c	RGMB	0.554734411	mirna_pc
900	hsa-mir-29c	CGNL1	0.530665981	mirna_pc
901	hsa-mir-29c	SLC25A4	0.493032126	mirna_pc
902	hsa-mir-29c	C7orf41	0.390198384	mirna_pc
903	hsa-mir-29c	FAM165B	0.555906127	mirna_pc
904	hsa-mir-29c	DUSP19	0.541017171	mirna_pc
905	hsa-mir-29c	RAB11FIP2	0.584632069	mirna_pc
906	hsa-mir-29c	ZNF626	0.356311709	mirna_pc
907	hsa-mir-29c	FAM189A2	0.54581594	mirna_pc
908	hsa-mir-29c	KIT	0.554818844	mirna_pc
909	hsa-mir-29c	RGS11	0.356808542	mirna_pc
910	hsa-mir-29c	ALDH6A1	0.58045491	mirna_pc
911	hsa-mir-29c	C5orf4	0.339256143	mirna_pc
912	hsa-mir-29c	MMRN1	0.329125677	mirna_pc
913	hsa-mir-29c	GCNT4	0.489855452	mirna_pc
914	hsa-mir-29c	TMEM161B	0.569364379	mirna_pc
915	hsa-mir-29c	KIAA0427	0.406667941	mirna_pc
916	hsa-mir-29c	LEPR	0.562205198	mirna_pc
917	hsa-mir-29c	FAM54B	0.383158604	mirna_pc

918	hsa-mir-29c	PRX	0.42313842	mirna_pc
919	hsa-mir-29c	MITF	0.479065018	mirna_pc
920	hsa-mir-29c	C22orf23	0.393600441	mirna_pc
921	hsa-mir-29c	MFSD4	0.627291664	mirna_pc
922	hsa-mir-29c	SIK2	0.500117003	mirna_pc
923	hsa-mir-29c	EFHA2	0.348385172	mirna_pc
924	hsa-mir-29c	ALAD	0.567333314	mirna_pc
925	hsa-mir-29c	GNAZ	0.353565054	mirna_pc
926	hsa-mir-29c	MT1G	0.530492449	mirna_pc
927	hsa-mir-29c	LOC400043	0.612467043	mirna_pc
928	hsa-mir-29c	PDE2A	0.321995612	mirna_pc
929	hsa-mir-29c	CDH2	0.427784818	mirna_pc
930	hsa-mir-29c	CKB	0.577812031	mirna_pc
931	hsa-mir-29c	SPHAR	0.427798864	mirna_pc
932	hsa-mir-29c	RAI2	0.315837874	mirna_pc
933	hsa-mir-29c	ACAT1	0.440501606	mirna_pc
934	hsa-mir-29c	DPCR1	0.390652731	mirna_pc
935	hsa-mir-29c	ACACB	0.41731629	mirna_pc
936	hsa-mir-29c	LIFR	0.627703761	mirna_pc
937	hsa-mir-29c	KIAA1191	0.389949628	mirna_pc
938	hsa-mir-29c	C14orf159	0.537368186	mirna_pc
939	hsa-mir-29c	ROR1	0.462059777	mirna_pc
940	hsa-mir-29c	TCF21	0.350774051	mirna_pc
941	hsa-mir-29c	SORBS2	0.439213528	mirna_pc
942	hsa-mir-29c	C5orf53	0.394085982	mirna_pc
943	hsa-mir-29c	ACADSB	0.419480402	mirna_pc
944	hsa-mir-29c	SDPR	0.319733702	mirna_pc
945	hsa-mir-29c	CBX7	0.33133598	mirna_pc
946	hsa-mir-29c	FAM149A	0.449877081	mirna_pc
947	hsa-mir-29c	IL33	0.304912428	mirna_pc
948	hsa-mir-29c	CNTD1	0.556832374	mirna_pc
949	hsa-mir-29c	ARHGAP24	0.585284284	mirna_pc
950	hsa-mir-29c	CPEB3	0.465365525	mirna_pc
951	hsa-mir-29c	GBGT1	0.550910655	mirna_pc
952	hsa-mir-29c	PAIP2B	0.56984705	mirna_pc
953	hsa-mir-29c	SYNJ2BP	0.464748579	mirna_pc
954	hsa-mir-29c	GCOM1	0.385302317	mirna_pc
955	hsa-mir-29c	CDHR3	0.327626225	mirna_pc
956	hsa-mir-29c	TMEM8B	0.376642829	mirna_pc
957	hsa-mir-29c	PXMP2	0.507035608	mirna_pc
958	hsa-mir-29c	VSIG2	0.626619284	mirna_pc
959	hsa-mir-29c	SIDT2	0.582144417	mirna_pc
960	hsa-mir-29c	DBT	0.463277924	mirna_pc
961	hsa-mir-29c	GTF2IRD2P1	0.426919712	mirna_pc
962	hsa-mir-29c	SECISBP2L	0.531507408	mirna_pc
963	hsa-mir-29c	TMEM220	0.456805974	mirna_pc
964	hsa-mir-29c	BTD	0.469336048	mirna_pc
965	hsa-mir-29c	ITM2A	0.33839105	mirna_pc
966	hsa-mir-29c	ERO1LB	0.579645407	mirna_pc
967	hsa-mir-29c	PBXIP1	0.520385842	mirna_pc
968	hsa-mir-29c	NICN1	0.475744121	mirna_pc
969	hsa-mir-29c	SCNN1B	0.339146994	mirna_pc
970	hsa-mir-29c	RAB27A	0.550453966	mirna_pc
971	hsa-mir-29c	GTF2IRD2	0.446853049	mirna_pc

972	hsa-mir-29c	PBLD	0.594176113	mirna_pc
973	hsa-mir-29c	PCBD2	0.425996159	mirna_pc
974	hsa-mir-29c	ARHGEF37	0.443665784	mirna_pc
975	hsa-mir-29c	PTPN21	0.322207445	mirna_pc
976	hsa-mir-29c	TSPYL4	0.459899403	mirna_pc
977	hsa-mir-29c	PDGFD	0.631386103	mirna_pc
978	hsa-mir-29c	ETFDH	0.360104784	mirna_pc
979	hsa-mir-29c	AMT	0.418774617	mirna_pc
980	hsa-mir-29c	PCCA	0.380918372	mirna_pc
981	hsa-mir-29c	SNX1	0.328893371	mirna_pc
982	hsa-mir-29c	KANK3	0.502744268	mirna_pc
983	hsa-mir-29c	APLP1	0.534983344	mirna_pc
984	hsa-mir-29c	CIRBP	0.573225729	mirna_pc
985	hsa-mir-29c	ACO2	0.434863475	mirna_pc
986	hsa-mir-29c	PSCA	0.502517849	mirna_pc
987	hsa-mir-29c	KLHDC1	0.42407257	mirna_pc
988	hsa-mir-29c	FAM63A	0.442811438	mirna_pc
989	hsa-mir-29c	C6orf204	0.52695534	mirna_pc
990	hsa-mir-29c	TRIM3	0.323015187	mirna_pc
991	hsa-mir-29c	KAT2B	0.452792128	mirna_pc
992	hsa-mir-29c	C4orf34	0.46552224	mirna_pc
993	hsa-mir-29c	BDH2	0.412289788	mirna_pc
994	hsa-mir-29c	FABP3	0.407142347	mirna_pc
995	hsa-mir-29c	RAF1	0.484913923	mirna_pc
996	hsa-mir-29c	PIK3C2G	0.604503925	mirna_pc
997	hsa-mir-29c	TENC1	0.31560939	mirna_pc
998	hsa-mir-29c	ZMAT1	0.362217875	mirna_pc
999	hsa-mir-29c	HPGD	0.392128005	mirna_pc
1000	hsa-mir-29c	TADA2B	0.477325188	mirna_pc
1001	hsa-mir-29c	RNF14	0.321186158	mirna_pc
1002	hsa-mir-29c	ME3	0.575540436	mirna_pc
1003	hsa-mir-29c	C11orf67	0.413386764	mirna_pc
1004	hsa-mir-29c	UBL3	0.59717587	mirna_pc
1005	hsa-mir-29c	TBC1D14	0.48366521	mirna_pc
1006	hsa-mir-29c	METTL7A	0.620406949	mirna_pc
1007	hsa-mir-29c	SUCLG2	0.519485914	mirna_pc
1008	hsa-mir-29c	TAPT1	0.377247692	mirna_pc
1009	hsa-mir-29c	IL11RA	0.309038812	mirna_pc
1010	hsa-mir-29c	AUH	0.481945183	mirna_pc
1011	hsa-mir-29c	RAB4A	0.360100763	mirna_pc
1012	hsa-mir-29c	POU6F1	0.335036845	mirna_pc
1013	hsa-mir-29c	FLJ33630	0.413527121	mirna_pc
1014	hsa-mir-29c	SYNE1	0.425782605	mirna_pc
1015	hsa-mir-29c	ZNF844	0.412735414	mirna_pc
1016	hsa-mir-29c	ANKDD1A	0.40302504	mirna_pc
1017	hsa-mir-29c	FOXN3	0.403451472	mirna_pc
1018	hsa-mir-29c	LDHD	0.479350788	mirna_pc
1019	hsa-mir-29c	PINK1	0.309359372	mirna_pc
1020	hsa-mir-29c	ATPAF1	0.336607943	mirna_pc
1021	hsa-mir-29c	KCNAB1	0.432119212	mirna_pc
1022	hsa-mir-29c	TPCN2	0.395016821	mirna_pc
1023	hsa-mir-29c	PTPRN2	0.516204955	mirna_pc
1024	hsa-mir-29c	TCEB3	0.491999703	mirna_pc
1025	hsa-mir-29c	KIAA0141	0.376964288	mirna_pc

1026	hsa-mir-29c	KLF9	0.351628204	mirna_pc
1027	hsa-mir-29c	FAM114A2	0.379039244	mirna_pc
1028	hsa-mir-29c	KIAA0494	0.430701405	mirna_pc
1029	hsa-mir-29c	CRBN	0.483395574	mirna_pc
1030	hsa-mir-29c	BRP44L	0.419363414	mirna_pc
1031	hsa-mir-29c	KIAA0430	0.417790311	mirna_pc
1032	hsa-mir-29c	BCKDHB	0.438766478	mirna_pc
1033	hsa-mir-29c	LAMA2	0.335366979	mirna_pc
1034	hsa-mir-29c	UPRT	0.481051415	mirna_pc
1035	hsa-mir-29c	PHF17	0.315784776	mirna_pc
1036	hsa-mir-29c	ASAP3	0.407042771	mirna_pc
1037	hsa-mir-29c	XYLT2	0.588142546	mirna_pc
1038	hsa-mir-29c	GIN1	0.311023426	mirna_pc
1039	hsa-mir-29c	PDE1A	0.363049467	mirna_pc
1040	hsa-mir-29c	ZNF467	0.375436027	mirna_pc
1041	hsa-mir-29c	LMF1	0.50954049	mirna_pc
1042	hsa-mir-29c	NFIC	0.321645092	mirna_pc
1043	hsa-mir-29c	MOSC2	0.569824992	mirna_pc
1044	hsa-mir-29c	PRKACB	0.584934863	mirna_pc
1045	hsa-mir-29c	GPD1L	0.351015079	mirna_pc
1046	hsa-mir-29c	TOX	0.389023377	mirna_pc
1047	hsa-mir-29c	CLN8	0.388063904	mirna_pc
1048	hsa-mir-29c	CYFIP2	0.599453052	mirna_pc
1049	hsa-mir-29c	NR3C2	0.310826738	mirna_pc
1050	hsa-mir-29c	DGKD	0.513688772	mirna_pc
1051	hsa-mir-29c	DEAF1	0.44111345	mirna_pc
1052	hsa-mir-29c	FBXL5	0.386617825	mirna_pc
1053	hsa-mir-29c	STK32B	0.307735221	mirna_pc
1054	hsa-mir-29c	GABRB3	0.303630269	mirna_pc
1055	hsa-mir-29c	PECI	0.542191459	mirna_pc
1056	hsa-mir-29c	PMM1	0.329415295	mirna_pc
1057	hsa-mir-29c	PDCD4	0.573029693	mirna_pc
1058	hsa-mir-29c	CCNI	0.31481081	mirna_pc
1059	hsa-mir-29c	CALCOCO1	0.360048312	mirna_pc
1060	hsa-mir-29c	SOCS2	0.418884815	mirna_pc
1061	hsa-mir-29c	USP53	0.350509816	mirna_pc
1062	hsa-mir-29c	ACADS	0.403806415	mirna_pc
1063	hsa-mir-29c	DEPDC6	0.463447991	mirna_pc
1064	hsa-mir-29c	WIPF3	0.482410801	mirna_pc
1065	hsa-mir-29c	SCNN1G	0.341841514	mirna_pc
1066	hsa-mir-29c	CCDC149	0.513949216	mirna_pc
1067	hsa-mir-29c	NTN4	0.57383372	mirna_pc
1068	hsa-mir-29c	AK3	0.39010557	mirna_pc
1069	hsa-mir-29c	RGNEF	0.499741369	mirna_pc
1070	hsa-mir-29c	MOCS1	0.32927557	mirna_pc
1071	hsa-mir-29c	SLC25A26	0.422998931	mirna_pc
1072	hsa-mir-29c	NRTN	0.415094348	mirna_pc
1073	hsa-mir-29c	CCDC107	0.36349899	mirna_pc
1074	hsa-mir-29c	PRDM16	0.475065915	mirna_pc
1075	hsa-mir-29c	TSC22D3	0.548569639	mirna_pc
1076	hsa-mir-29c	CA5B	0.306086353	mirna_pc
1077	hsa-mir-29c	PPM1K	0.512622102	mirna_pc
1078	hsa-mir-29c	SLC26A9	0.555543572	mirna_pc
1079	hsa-mir-29c	SLC4A4	0.333840494	mirna_pc

1080	hsa-mir-29c	PXK	0.364764052	mirna_pc
1081	hsa-mir-29c	VAMP2	0.454472593	mirna_pc
1082	hsa-mir-29c	FZD4	0.495766774	mirna_pc
1083	hsa-mir-29c	GLUL	0.543944106	mirna_pc
1084	hsa-mir-29c	XPA	0.328133388	mirna_pc
1085	hsa-mir-29c	P2RX1	0.329734565	mirna_pc
1086	hsa-mir-29c	HERC1	0.396370405	mirna_pc
1087	hsa-mir-29c	ZNF737	0.316071005	mirna_pc
1088	hsa-mir-29c	SNRPN	0.412870357	mirna_pc
1089	hsa-mir-29c	NUCB2	0.465629269	mirna_pc
1090	hsa-mir-29c	FBXW11	0.371835672	mirna_pc
1091	hsa-mir-29c	GMPR	0.409584394	mirna_pc
1092	hsa-mir-29c	KLHDC2	0.356466452	mirna_pc
1093	hsa-mir-29c	CTSF	0.313831229	mirna_pc
1094	hsa-mir-29c	SGSM3	0.562657663	mirna_pc
1095	hsa-mir-29c	PGCP	0.416132477	mirna_pc
1096	hsa-mir-29c	GABARAPL1	0.399098069	mirna_pc
1097	hsa-mir-29c	PARD3B	0.376562546	mirna_pc
1098	hsa-mir-29c	AKD1	0.392759685	mirna_pc
1099	hsa-mir-29c	REC8	0.38102303	mirna_pc
1100	hsa-mir-29c	ZBTB3	0.368244829	mirna_pc
1101	hsa-mir-29c	ASB16	0.398565484	mirna_pc
1102	hsa-mir-29c	NISCH	0.303926318	mirna_pc
1103	hsa-mir-29c	NAT15	0.465398285	mirna_pc
1104	hsa-mir-29c	LRIG1	0.517785259	mirna_pc
1105	hsa-mir-29c	LOC646471	0.342951654	mirna_pc
1106	hsa-mir-29c	LOC729234	0.370133897	mirna_pc
1107	hsa-mir-29c	RAPGEF3	0.351964599	mirna_pc
1108	hsa-mir-29c	ZNF570	0.302048529	mirna_pc
1109	hsa-mir-29c	APOD	0.321086697	mirna_pc
1110	hsa-mir-29c	CHRFAM7A	0.422164585	mirna_pc
1111	hsa-mir-29c	NECAB3	0.357396581	mirna_pc
1112	hsa-mir-29c	WDR37	0.354490322	mirna_pc
1113	hsa-mir-29c	KCNJ11	0.469905127	mirna_pc
1114	hsa-mir-29c	TTC28	0.302119219	mirna_pc
1115	hsa-mir-29c	ISCU	0.456596581	mirna_pc
1116	hsa-mir-29c	FAM174B	0.51679973	mirna_pc
1117	hsa-mir-29c	FBXL17	0.36218205	mirna_pc
1118	hsa-mir-29c	PDE7B	0.352569128	mirna_pc
1119	hsa-mir-29c	STARD8	0.395459812	mirna_pc
1120	hsa-mir-29c	FMO5	0.544832444	mirna_pc
1121	hsa-mir-29c	DMXL1	0.4149992	mirna_pc
1122	hsa-mir-29c	SACM1L	0.349582722	mirna_pc
1123	hsa-mir-29c	PGRMC2	0.322745679	mirna_pc
1124	hsa-mir-29c	MAGI1	0.497658457	mirna_pc
1125	hsa-mir-29c	ZNF57	0.489546536	mirna_pc
1126	hsa-mir-29c	EPB41L4A	0.51591815	mirna_pc
1127	hsa-mir-29c	FEM1C	0.32130003	mirna_pc
1128	hsa-mir-29c	GPT2	0.567847244	mirna_pc
1129	hsa-mir-29c	FGD4	0.551781613	mirna_pc
1130	hsa-mir-29c	TBC1D9	0.486722857	mirna_pc
1131	hsa-mir-29c	SESN1	0.339348904	mirna_pc
1132	hsa-mir-29c	ACADVL	0.324976268	mirna_pc
1133	hsa-mir-29c	MAGI3	0.40407165	mirna_pc

1134	hsa-mir-29c	SLC25A42	0.315314065	mirna_pc
1135	hsa-mir-29c	PRKAB1	0.502317639	mirna_pc
1136	hsa-mir-29c	FZD8	0.334020995	mirna_pc
1137	hsa-mir-29c	KIAA0513	0.385763141	mirna_pc
1138	hsa-mir-29c	ZNF493	0.359943098	mirna_pc
1139	hsa-mir-29c	TTC37	0.344088459	mirna_pc
1140	hsa-mir-29c	ELL2	0.364311187	mirna_pc
1141	hsa-mir-29c	ZNF85	0.44834046	mirna_pc
1142	hsa-mir-29c	FAM13A	0.456114524	mirna_pc
1143	hsa-mir-29c	CMAH	0.338828929	mirna_pc
1144	hsa-mir-29c	SIK3	0.379827595	mirna_pc
1145	hsa-mir-29c	CYB5D2	0.377067913	mirna_pc
1146	hsa-mir-29c	RBM5	0.30914602	mirna_pc
1147	hsa-mir-29c	PPAP2B	0.348932115	mirna_pc
1148	hsa-mir-29c	ST5	0.308919614	mirna_pc
1149	hsa-mir-29c	SEC14L1	0.471323668	mirna_pc
1150	hsa-mir-29c	ZNF429	0.404037079	mirna_pc
1151	hsa-mir-29c	C18orf32	0.33085711	mirna_pc
1152	hsa-mir-29c	FRY	0.312339372	mirna_pc
1153	hsa-mir-29c	DMRTA1	0.492657538	mirna_pc
1154	hsa-mir-29c	CLIC6	0.609274256	mirna_pc
1155	hsa-mir-29c	SLC4A2	0.444472563	mirna_pc
1156	hsa-mir-29c	VPS39	0.316070847	mirna_pc
1157	hsa-mir-29c	ECHDC2	0.418252683	mirna_pc
1158	hsa-mir-29c	C9orf130	0.325257278	mirna_pc
1159	hsa-mir-29c	NBAS	0.304994814	mirna_pc
1160	hsa-mir-29c	SATB1	0.358479068	mirna_pc
1161	hsa-mir-29c	MTMR12	0.374249899	mirna_pc
1162	hsa-mir-29c	SYTL3	0.400068401	mirna_pc
1163	hsa-mir-29c	PTPRM	0.366190905	mirna_pc
1164	hsa-mir-29c	CCDC159	0.507818485	mirna_pc
1165	hsa-mir-29c	TM7SF2	0.462053645	mirna_pc
1166	hsa-mir-29c	GCNT2	0.416887454	mirna_pc
1167	hsa-mir-29c	PER1	0.302052055	mirna_pc
1168	hsa-mir-29c	FOXO3	0.347113551	mirna_pc
1169	hsa-mir-29c	RNLS	0.463679059	mirna_pc
1170	hsa-mir-29c	PTGDS	0.399172581	mirna_pc
1171	hsa-mir-29c	C22orf32	0.400064592	mirna_pc
1172	hsa-mir-29c	LOC728743	0.439218096	mirna_pc
1173	hsa-mir-29c	CCNG1	0.370806753	mirna_pc
1174	hsa-mir-29c	NOSTRIN	0.416752072	mirna_pc
1175	hsa-mir-29c	RELL1	0.314042441	mirna_pc
1176	hsa-mir-29c	INPP5J	0.445958466	mirna_pc
1177	hsa-mir-29c	ZFYVE28	0.369004849	mirna_pc
1178	hsa-mir-29c	TCEA3	0.479104698	mirna_pc
1179	hsa-mir-29c	ANAPC16	0.325551856	mirna_pc
1180	hsa-mir-29c	COBLL1	0.514922117	mirna_pc
1181	hsa-mir-29c	SNX18	0.332470267	mirna_pc
1182	hsa-mir-29c	ERGIC1	0.30854332	mirna_pc
1183	hsa-mir-29c	CCL21	0.314351961	mirna_pc
1184	hsa-mir-29c	SLC41A1	0.413549284	mirna_pc
1185	hsa-mir-29c	LOC200030	0.374022885	mirna_pc
1186	hsa-mir-29c	SLC13A3	0.456306341	mirna_pc
1187	hsa-mir-29c	ZNF43	0.312252474	mirna_pc

1188	hsa-mir-29c	GNAQ	0.323312248	mirna_pc
1189	hsa-mir-29c	ZBED3	0.378537306	mirna_pc
1190	hsa-mir-29c	ERN 1.00	0.457572428	mirna_pc
1191	hsa-mir-29c	MDH1	0.330019386	mirna_pc
1192	hsa-mir-29c	CPEB4	0.345636507	mirna_pc
1193	hsa-mir-29c	GIPC3	0.318331267	mirna_pc
1194	hsa-mir-29c	EIF4EBP3	0.491046944	mirna_pc
1195	hsa-mir-29c	C17orf48	0.447218581	mirna_pc
1196	hsa-mir-29c	CCPG1	0.543794875	mirna_pc
1197	hsa-mir-29c	SHE	0.307190915	mirna_pc
1198	hsa-mir-29c	VSIG1	0.412826745	mirna_pc
1199	hsa-mir-29c	LTF	0.391537092	mirna_pc
1200	hsa-mir-29c	GALNT12	0.386503603	mirna_pc
1201	hsa-mir-29c	RASGRP2	0.383871903	mirna_pc
1202	hsa-mir-29c	TMTC2	0.353150627	mirna_pc
1203	hsa-mir-29c	IL6ST	0.319517931	mirna_pc
1204	hsa-mir-29c	HOMER2	0.500060704	mirna_pc
1205	hsa-mir-29c	LARGE	0.338751223	mirna_pc
1206	hsa-mir-29c	PTTG1IP	0.326082648	mirna_pc
1207	hsa-mir-29c	KIAA0895	0.39184142	mirna_pc
1208	hsa-mir-29c	XKR8	0.423067251	mirna_pc
1209	hsa-mir-29c	RHOU	0.382276769	mirna_pc
1210	hsa-mir-29c	RNASE4	0.398623971	mirna_pc
1211	hsa-mir-29c	REEP5	0.425222101	mirna_pc
1212	hsa-mir-29c	ATP5J	0.301346649	mirna_pc
1213	hsa-mir-29c	NNT	0.329530926	mirna_pc
1214	hsa-mir-29c	CMTM4	0.358881371	mirna_pc
1215	hsa-mir-29c	ARRDC4	0.330368168	mirna_pc
1216	hsa-mir-29c	TXNIP	0.313330233	mirna_pc
1217	hsa-mir-29c	TPSB2	0.401775329	mirna_pc
1218	hsa-mir-29c	MCEE	0.458461689	mirna_pc
1219	hsa-mir-29c	DHRS7	0.327380981	mirna_pc
1220	hsa-mir-29c	FMOD	0.581879273	mirna_pc
1221	hsa-mir-29c	TEK	0.344316322	mirna_pc
1222	hsa-mir-29c	ATP8A1	0.452844256	mirna_pc
1223	hsa-mir-29c	ECHDC3	0.318492833	mirna_pc
1224	hsa-mir-29c	LDB2	0.318922153	mirna_pc
1225	hsa-mir-29c	LRRC70	0.406505901	mirna_pc
1226	hsa-mir-29c	NRIP2	0.392461175	mirna_pc
1227	hsa-mir-29c	SYBU	0.360609818	mirna_pc
1228	hsa-mir-29c	CRADD	0.344149794	mirna_pc
1229	hsa-mir-29c	KIAA1370	0.422561654	mirna_pc
1230	hsa-mir-29c	KALRN	0.309882694	mirna_pc
1231	hsa-mir-29c	HMGCL	0.336276904	mirna_pc
1232	hsa-mir-29c	C12orf72	0.310745363	mirna_pc
1233	hsa-mir-29c	ACSL3	0.412641801	mirna_pc
1234	hsa-mir-29c	SLC44A2	0.416978686	mirna_pc
1235	hsa-mir-29c	FICD	0.40528949	mirna_pc
1236	hsa-mir-29c	KIAA1958	0.453404235	mirna_pc
1237	hsa-mir-29c	TXNDC15	0.318960616	mirna_pc
1238	hsa-mir-29c	IGFBP5	0.339578503	mirna_pc
1239	hsa-mir-29c	DCAF11	0.330466512	mirna_pc
1240	hsa-mir-29c	NDRG2	0.335430369	mirna_pc
1241	hsa-mir-29c	PAFAH2	0.421258161	mirna_pc

1242	hsa-mir-29c	AKR7A3	0.519773843	mirna_pc
1243	hsa-mir-29c	ENPP5	0.374219054	mirna_pc
1244	hsa-mir-29c	FAM20A	0.305293135	mirna_pc
1245	hsa-mir-29c	CTDSP1	0.316873615	mirna_pc
1246	hsa-mir-29c	PEX7	0.374848987	mirna_pc
1247	hsa-mir-29c	ANKAR	0.327114059	mirna_pc
1248	hsa-mir-29c	TUBA8	0.39964785	mirna_pc
1249	hsa-mir-29c	PPAP2A	0.383264303	mirna_pc
1250	hsa-mir-29c	GAB2	0.357311311	mirna_pc
1251	hsa-mir-29c	PPP1R16B	0.426629208	mirna_pc
1252	hsa-mir-29c	TXNRD2	0.374280451	mirna_pc
1253	hsa-mir-29c	HIPK2	0.330323117	mirna_pc
1254	hsa-mir-29c	EIF2AK3	0.466714636	mirna_pc
1255	hsa-mir-29c	AAK1	0.355297646	mirna_pc
1256	hsa-mir-29c	TFF1	0.306258618	mirna_pc
1257	hsa-mir-29c	RUNDC2C	0.470302793	mirna_pc
1258	hsa-mir-29c	LMBRD1	0.319631586	mirna_pc
1259	hsa-mir-29c	PKIB	0.383800003	mirna_pc
1260	hsa-mir-29c	WBP1	0.324387547	mirna_pc
1261	hsa-mir-29c	SURF1	0.335073205	mirna_pc
1262	hsa-mir-29c	BCKDHA	0.31102134	mirna_pc
1263	hsa-mir-29c	NEDD4L	0.334416492	mirna_pc
1264	hsa-mir-29c	LOC643008	0.304115109	mirna_pc
1265	hsa-mir-29c	ASB8	0.318523591	mirna_pc
1266	hsa-mir-29c	CHP	0.421627899	mirna_pc
1267	hsa-mir-29c	KLHDC7A	0.345903246	mirna_pc
1268	hsa-mir-29c	SPCS3	0.367621457	mirna_pc
1269	hsa-mir-29c	IDH2	0.421737116	mirna_pc
1270	hsa-mir-29c	MXD4	0.377298104	mirna_pc
1271	hsa-mir-29c	C3orf19	0.365071565	mirna_pc
1272	hsa-mir-29c	ATG4A	0.394793633	mirna_pc
1273	hsa-mir-29c	FAM46C	0.593474835	mirna_pc
1274	hsa-mir-29c	UNC13B	0.317018531	mirna_pc
1275	hsa-mir-29c	TMEM143	0.340570051	mirna_pc
1276	hsa-mir-29c	CPA3	0.371642121	mirna_pc
1277	hsa-mir-29c	F2RL3	0.354608198	mirna_pc
1278	hsa-mir-29c	TRIP11	0.335472435	mirna_pc
1279	hsa-mir-29c	FAM3B	0.392421926	mirna_pc
1280	hsa-mir-29c	PPP3CC	0.415553459	mirna_pc
1281	hsa-mir-29c	CCDC125	0.45759085	mirna_pc
1282	hsa-mir-29c	NCOA1	0.340994945	mirna_pc
1283	hsa-mir-29c	MAN2C1	0.327287816	mirna_pc
1284	hsa-mir-29c	DNAJC4	0.428130108	mirna_pc
1285	hsa-mir-29c	KIAA0319L	0.454547007	mirna_pc
1286	hsa-mir-29c	ANXA10	0.438841652	mirna_pc
1287	hsa-mir-29c	RAP1GAP2	0.389100749	mirna_pc
1288	hsa-mir-29c	ZNF254	0.304401073	mirna_pc
1289	hsa-mir-29c	REPS2	0.332699091	mirna_pc
1290	hsa-mir-29c	MT1F	0.505901429	mirna_pc
1291	hsa-mir-29c	GIMAP6	0.426907675	mirna_pc
1292	hsa-mir-29c	TARSL2	0.300629933	mirna_pc
1293	hsa-mir-29c	PLLP	0.390502927	mirna_pc
1294	hsa-mir-29c	CXCL17	0.687071566	mirna_pc
1295	hsa-mir-29c	NFATC2	0.350853973	mirna_pc

1296	hsa-mir-29c	ADAM28	0.615978136	mirna_pc
1297	hsa-mir-29c	GIMAP5	0.418656078	mirna_pc
1298	hsa-mir-29c	C17orf107	0.312349372	mirna_pc
1299	hsa-mir-29c	ANKRD12	0.332996787	mirna_pc
1300	hsa-mir-29c	GLOD4	0.320984397	mirna_pc
1301	hsa-mir-29c	GGA2	0.341743101	mirna_pc
1302	hsa-mir-29c	TSPAN12	0.373154098	mirna_pc
1303	hsa-mir-29c	LARP1B	0.3008619	mirna_pc
1304	hsa-mir-29c	C2orf55	0.366252584	mirna_pc
1305	hsa-mir-29c	C5orf41	0.327792339	mirna_pc
1306	hsa-mir-29c	XPC	0.327433048	mirna_pc
1307	hsa-mir-29c	C10orf118	0.402714773	mirna_pc
1308	hsa-mir-29c	IFNAR1	0.320593977	mirna_pc
1309	hsa-mir-29c	SETDB2	0.344886334	mirna_pc
1310	hsa-mir-29c	ARHGEF6	0.4134299	mirna_pc
1311	hsa-mir-29c	C5orf32	0.508180925	mirna_pc
1312	hsa-mir-29c	MAN2B2	0.310160028	mirna_pc
1313	hsa-mir-29c	UQCRQ	0.384301015	mirna_pc
1314	hsa-mir-29c	SLC9A3R2	0.443518125	mirna_pc
1315	hsa-mir-29c	GALNT10	0.304642328	mirna_pc
1316	hsa-mir-29c	HSD17B4	0.322538108	mirna_pc
1317	hsa-mir-29c	ZNF33A	0.3016955	mirna_pc
1318	hsa-mir-29c	CREBL2	0.304511715	mirna_pc
1319	hsa-mir-29c	HERPUD1	0.645426465	mirna_pc
1320	hsa-mir-29c	ZNF33B	0.300877383	mirna_pc
1321	hsa-mir-29c	ZNF763	0.370826077	mirna_pc
1322	hsa-mir-29c	ZNF671	0.310668244	mirna_pc
1323	hsa-mir-29c	ANKRD43	0.3691907	mirna_pc
1324	hsa-mir-29c	MT1E	0.312905654	mirna_pc
1325	hsa-mir-29c	GPRC5C	0.381443894	mirna_pc
1326	hsa-mir-29c	CHPT1	0.344989464	mirna_pc
1327	hsa-mir-29c	SNRK	0.326266271	mirna_pc
1328	hsa-mir-29c	RAP1GAP	0.494306996	mirna_pc
1329	hsa-mir-29c	RORC	0.442275006	mirna_pc
1330	hsa-mir-29c	C9orf7	0.300747487	mirna_pc
1331	hsa-mir-29c	IGFBP2	0.351821656	mirna_pc
1332	hsa-mir-29c	PRKCB	0.391411076	mirna_pc
1333	hsa-mir-29c	TPSAB1	0.37191885	mirna_pc
1334	hsa-mir-29c	CABC1	0.439194864	mirna_pc
1335	hsa-mir-29c	GIMAP7	0.451850128	mirna_pc
1336	hsa-mir-29c	PPFIBP2	0.457066033	mirna_pc
1337	hsa-mir-29c	CST3	0.438426478	mirna_pc
1338	hsa-mir-29c	CYP2C9	0.335497316	mirna_pc
1339	hsa-mir-29c	TMBIM6	0.350235327	mirna_pc
1340	hsa-mir-29c	6-Sep	0.415652591	mirna_pc
1341	hsa-mir-29c	ZNF565	0.390697173	mirna_pc
1342	hsa-mir-29c	MAGIX	0.351118583	mirna_pc
1343	hsa-mir-29c	TMEM66	0.367236085	mirna_pc
1344	hsa-mir-29c	CTH	0.305568817	mirna_pc
1345	hsa-mir-29c	TP53INP1	0.413566327	mirna_pc
1346	hsa-mir-29c	PRLR	0.315703323	mirna_pc
1347	hsa-mir-29c	EPN1	0.376983513	mirna_pc
1348	hsa-mir-29c	RGL3	0.313516366	mirna_pc
1349	hsa-mir-29c	MAP4K3	0.335219413	mirna_pc

1350	hsa-mir-29c	TUBGCP6	0.314297303	mirna_pc
1351	hsa-mir-29c	BCAS1	0.327509277	mirna_pc
1352	hsa-mir-29c	ANO7	0.334421249	mirna_pc
1353	hsa-mir-29c	ITSN2	0.333271762	mirna_pc
1354	hsa-mir-29c	AZGP1	0.409469896	mirna_pc
1355	hsa-mir-29c	FAM107B	0.368108492	mirna_pc
1356	hsa-mir-29c	SH3BGRL2	0.327336653	mirna_pc
1357	hsa-mir-29c	HMOX2	0.33935903	mirna_pc
1358	hsa-mir-29c	HOXC5	0.326716049	mirna_pc
1359	hsa-mir-29c	RAPGEF4	0.36452317	mirna_pc
1360	hsa-mir-29c	KCNQ1	0.54980721	mirna_pc
1361	hsa-mir-29c	ARSD	0.367637078	mirna_pc
1362	hsa-mir-29c	MAP3K5	0.311239772	mirna_pc
1363	hsa-mir-29c	DCXR	0.396492322	mirna_pc
1364	hsa-mir-29c	GIMAP8	0.312060335	mirna_pc
1365	hsa-mir-29c	SLC41A2	0.439409444	mirna_pc
1366	hsa-mir-29c	TMEM59	0.343111317	mirna_pc
1367	hsa-mir-29c	CDC14A	0.360665509	mirna_pc
1368	hsa-mir-29c	C1orf26	0.366545323	mirna_pc
1369	hsa-mir-29c	TCEANC	0.311577167	mirna_pc
1370	hsa-mir-29c	PTPRB	0.345393557	mirna_pc
1371	hsa-mir-29c	IQGAP2	0.410019296	mirna_pc
1372	hsa-mir-29c	RAB11B	0.342637979	mirna_pc
1373	hsa-mir-29c	ZNF366	0.377203008	mirna_pc
1374	hsa-mir-29c	SVIP	0.311065695	mirna_pc
1375	hsa-mir-29c	ATP13A4	0.317360418	mirna_pc
1376	hsa-mir-29c	PELI2	0.336952385	mirna_pc
1377	hsa-mir-29c	CPM	0.372635529	mirna_pc
1378	hsa-mir-29c	BRP44	0.301971298	mirna_pc
1379	hsa-mir-29c	SLC9A2	0.394573007	mirna_pc
1380	hsa-mir-29c	ACSS1	0.303946041	mirna_pc
1381	hsa-mir-29c	CNOT6L	0.336702802	mirna_pc
1382	hsa-mir-29c	PLCL2	0.34689541	mirna_pc
1383	hsa-mir-29c	XBP1	0.593092592	mirna_pc
1384	hsa-mir-29c	C1orf130	0.466570732	mirna_pc
1385	hsa-mir-29c	HIP1R	0.387965982	mirna_pc
1386	hsa-mir-29c	TMEM175	0.311470596	mirna_pc
1387	hsa-mir-29c	C2orf86	0.338910226	mirna_pc
1388	hsa-mir-29c	FNDC3A	0.348693822	mirna_pc
1389	hsa-mir-29c	GATA6	0.322711493	mirna_pc
1390	hsa-mir-29c	GABARAP	0.332636558	mirna_pc
1391	hsa-mir-29c	SIL1	0.334777426	mirna_pc
1392	hsa-mir-29c	ZNF552	0.341908058	mirna_pc
1393	hsa-mir-29c	CYP2R1	0.318182495	mirna_pc
1394	hsa-mir-29c	ALDH1A1	0.475008938	mirna_pc
1395	hsa-mir-29c	FBXW7	0.315476298	mirna_pc
1396	hsa-mir-29c	TMEM219	0.361503666	mirna_pc
1397	hsa-mir-29c	RNASE1	0.328227374	mirna_pc
1398	hsa-mir-29c	RAMP3	0.360624721	mirna_pc
1399	hsa-mir-29c	MECOM	0.338295512	mirna_pc
1400	hsa-mir-29c	HPCAL1	0.343689521	mirna_pc
1401	hsa-mir-29c	IDUA	0.447447623	mirna_pc
1402	hsa-mir-29c	SERPINI1	0.463727201	mirna_pc
1403	hsa-mir-29c	CHKB	0.39779568	mirna_pc

1404	hsa-mir-29c	UBE2J1	0.33921296	mirna_pc
1405	hsa-mir-29c	C11orf52	0.493726788	mirna_pc
1406	hsa-mir-29c	RCSD1	0.356599279	mirna_pc
1407	hsa-mir-29c	MIOS	0.322166962	mirna_pc
1408	hsa-mir-29c	GRAMD1C	0.302771314	mirna_pc
1409	hsa-mir-29c	GIMAP1	0.403711954	mirna_pc
1410	hsa-mir-29c	CA2	0.317385198	mirna_pc
1411	hsa-mir-29c	CSF1R	0.346348932	mirna_pc
1412	hsa-mir-29c	CCDC56	0.442259605	mirna_pc
1413	hsa-mir-29c	RAB17	0.418954888	mirna_pc
1414	hsa-mir-29c	ZSCAN2	0.301657198	mirna_pc
1415	hsa-mir-29c	CYTIP	0.53297367	mirna_pc
1416	hsa-mir-29c	CCNJL	0.313752094	mirna_pc
1417	hsa-mir-29c	FA2H	0.342694059	mirna_pc
1418	hsa-mir-29c	C11orf9	0.362108799	mirna_pc
1419	hsa-mir-29c	OCEL1	0.314376102	mirna_pc
1420	hsa-mir-29c	SLC16A10	0.338716008	mirna_pc
1421	hsa-mir-29c	EPOR	0.308898247	mirna_pc
1422	hsa-mir-29c	RHOBTB1	0.310137148	mirna_pc
1423	hsa-mir-29c	TCN2	0.325917092	mirna_pc
1424	hsa-mir-29c	SH3YL1	0.372739888	mirna_pc
1425	hsa-mir-29c	PDE4A	0.379803459	mirna_pc
1426	hsa-mir-29c	TXNDC11	0.405497996	mirna_pc
1427	hsa-mir-29c	ANKRD44	0.334699675	mirna_pc
1428	hsa-mir-29c	OSBPL7	0.313822727	mirna_pc
1429	hsa-mir-29c	SPDEF	0.308171572	mirna_pc
1430	hsa-mir-29c	ARHGAP18	0.388851243	mirna_pc
1431	hsa-mir-29c	TMPRSS2	0.434216159	mirna_pc
1432	hsa-mir-29c	DNAJB9	0.320279688	mirna_pc
1433	hsa-mir-29c	VMAC	0.309013929	mirna_pc
1434	hsa-mir-29c	TLR9	0.353982163	mirna_pc
1435	hsa-mir-29c	CA9	0.426838435	mirna_pc
1436	hsa-mir-29c	PREX1	0.426490919	mirna_pc
1437	hsa-mir-29c	IGJ	0.502410398	mirna_pc
1438	hsa-mir-29c	CLEC14A	0.34889351	mirna_pc
1439	hsa-mir-29c	DOCK2	0.517287741	mirna_pc
1440	hsa-mir-29c	RASSF6	0.345214109	mirna_pc
1441	hsa-mir-29c	CARD8	0.348978742	mirna_pc
1442	hsa-mir-29c	SMAP2	0.433428397	mirna_pc
1443	hsa-mir-29c	C13orf15	0.30199239	mirna_pc
1444	hsa-mir-29c	B4GALNT3	0.45465329	mirna_pc
1445	hsa-mir-29c	FAM65B	0.383444879	mirna_pc
1446	hsa-mir-29c	POU2AF1	0.539874517	mirna_pc
1447	hsa-mir-29c	SYVN1	0.375127846	mirna_pc
1448	hsa-mir-29c	KIAA1324	0.364420262	mirna_pc
1449	hsa-mir-29c	P2RY8	0.457306565	mirna_pc
1450	hsa-mir-29c	ERP27	0.418361575	mirna_pc
1451	hsa-mir-29c	CAPN13	0.452540577	mirna_pc
1452	hsa-mir-29c	SH2D3C	0.305381217	mirna_pc
1453	hsa-mir-29c	SYNRG	0.354996928	mirna_pc
1454	hsa-mir-29c	PLA1A	0.33786541	mirna_pc
1455	hsa-mir-29c	EPB41L4B	0.326763335	mirna_pc
1456	hsa-mir-29c	CXCL13	0.328261035	mirna_pc
1457	hsa-mir-29c	CDC42EP1	0.336576145	mirna_pc

1458	hsa-mir-29c	AKNA	0.37714554	mirna_pc
1459	hsa-mir-29c	RBM47	0.375203317	mirna_pc
1460	hsa-mir-29c	TMEM171	0.423487781	mirna_pc
1461	hsa-mir-29c	IL16	0.326930875	mirna_pc
1462	hsa-mir-29c	CORO1A	0.30389154	mirna_pc
1463	hsa-mir-29c	PTPRJ	0.339586274	mirna_pc
1464	hsa-mir-29c	FUT8	0.312598739	mirna_pc
1465	hsa-mir-29c	ANKRD22	0.521683017	mirna_pc
1466	hsa-mir-29c	ARSA	0.361861326	mirna_pc
1467	hsa-mir-29c	AFTPH	0.396356961	mirna_pc
1468	hsa-mir-29c	GVIN1	0.359946605	mirna_pc
1469	hsa-mir-29c	FLI1	0.306041307	mirna_pc
1470	hsa-mir-29c	CPNE5	0.377588402	mirna_pc
1471	hsa-mir-29c	KCNN3	0.331809536	mirna_pc
1472	hsa-mir-29c	KLRK1	0.300802176	mirna_pc
1473	hsa-mir-29c	CD52	0.331205147	mirna_pc
1474	hsa-mir-29c	IPCEF1	0.395886156	mirna_pc
1475	hsa-mir-29c	LSP1	0.304903436	mirna_pc
1476	hsa-mir-29c	ADA	0.361450019	mirna_pc
1477	hsa-mir-29c	CD6	0.31248845	mirna_pc
1478	hsa-mir-29c	GAB3	0.31200141	mirna_pc
1479	hsa-mir-29c	CDK18	0.328974693	mirna_pc
1480	hsa-mir-29c	GIMAP4	0.355306638	mirna_pc
1481	hsa-mir-29c	RAMP2	0.305802997	mirna_pc
1482	hsa-mir-29c	ARHGAP15	0.368672198	mirna_pc
1483	hsa-mir-29c	FLJ40330	0.379080693	mirna_pc
1484	hsa-mir-29c	C1orf116	0.330260065	mirna_pc
1485	hsa-mir-29c	SASH3	0.341612772	mirna_pc
1486	hsa-mir-29c	MEI1	0.390561899	mirna_pc
1487	hsa-mir-29c	CD3D	0.303818096	mirna_pc
1488	hsa-mir-29c	CD3E	0.30930408	mirna_pc
1489	hsa-mir-29c	IRF4	0.440042646	mirna_pc
1490	hsa-mir-29c	BIN2	0.318689704	mirna_pc
1491	hsa-mir-29c	ARHGAP25	0.317707575	mirna_pc
1492	hsa-mir-29c	ITK	0.3580277	mirna_pc
1493	hsa-mir-29c	AMICA1	0.352929557	mirna_pc
1494	hsa-mir-29c	GMFG	0.318044996	mirna_pc
1495	hsa-mir-29c	SP140	0.316683778	mirna_pc
1496	hsa-mir-29c	CD79A	0.533081755	mirna_pc
1497	hsa-mir-29c	CHAC1	0.329826181	mirna_pc
1498	hsa-mir-29c	SSR4	0.457558278	mirna_pc
1499	hsa-mir-29c	CD3G	0.300491869	mirna_pc
1500	hsa-mir-29c	EPN3	0.468420741	mirna_pc
1501	hsa-mir-29c	FAIM3	0.410308276	mirna_pc
1502	hsa-mir-29c	FOXA1	0.317638905	mirna_pc
1503	hsa-mir-29c	SLAMF7	0.354806234	mirna_pc
1504	hsa-mir-29c	ADAM6	0.395678518	mirna_pc
1505	hsa-mir-29c	CSF2RB	0.321004754	mirna_pc
1506	hsa-mir-29c	MAP4K1	0.385577496	mirna_pc
1507	hsa-mir-29c	BTK	0.365106515	mirna_pc
1508	hsa-mir-29c	RHOH	0.309222898	mirna_pc
1509	hsa-mir-29c	1-Sep	0.385052672	mirna_pc
1510	hsa-mir-29c	FKBP11	0.401517313	mirna_pc
1511	hsa-mir-29c	POU2F2	0.360990485	mirna_pc

1512	hsa-mir-29c	SELL	0.338609026	mirna_pc
1513	hsa-mir-29c	ACAP1	0.346778364	mirna_pc
1514	hsa-mir-29c	DERL3	0.399053538	mirna_pc
1515	hsa-mir-29c	SLAMF6	0.387205327	mirna_pc
1516	hsa-mir-29c	NAPSB	0.351393904	mirna_pc
1517	hsa-mir-29c	PIK3CG	0.302246764	mirna_pc
1518	hsa-mir-29c	DENND1C	0.438816857	mirna_pc
1519	hsa-mir-29c	CD48	0.39254562	mirna_pc
1520	hsa-mir-29c	ITGAL	0.352089896	mirna_pc
1521	hsa-mir-29c	WDFY4	0.332387521	mirna_pc
1522	hsa-mir-29c	LAX1	0.373660251	mirna_pc
1523	hsa-mir-29c	IKZF1	0.379463964	mirna_pc
1524	hsa-mir-29c	DTX1	0.322784968	mirna_pc
1525	hsa-mir-29c	CCR7	0.36351811	mirna_pc
1526	hsa-mir-29c	NLRC3	0.329860479	mirna_pc
1527	hsa-mir-29c	ARHGAP9	0.311208379	mirna_pc
1528	hsa-mir-29c	RASAL3	0.352716463	mirna_pc
1529	hsa-mir-29c	PTPRC	0.300861797	mirna_pc
1530	hsa-mir-29c	CD180	0.38403054	mirna_pc
1531	hsa-mir-29c	SLAMF1	0.360513662	mirna_pc
1532	hsa-mir-29c	MGC29506	0.414265642	mirna_pc
1533	hsa-mir-29c	IL10RA	0.315865843	mirna_pc
1534	hsa-mir-29c	LOC96610	0.507565081	mirna_pc
1535	hsa-mir-29c	PARP15	0.307157644	mirna_pc
1536	hsa-mir-29c	FGD2	0.302788133	mirna_pc
1537	hsa-mir-29c	TAGAP	0.375859675	mirna_pc
1538	hsa-mir-29c	CD27	0.4246265	mirna_pc
1539	hsa-mir-29c	EVI2B	0.374788937	mirna_pc
1540	hsa-mir-29c	FCRL5	0.386001104	mirna_pc
1541	hsa-mir-29c	KLHL6	0.364137525	mirna_pc
1542	hsa-mir-29c	CD37	0.388117571	mirna_pc
1543	hsa-mir-29c	PTPRCAP	0.384483823	mirna_pc
1544	hsa-mir-29c	CECR1	0.329951886	mirna_pc
1545	hsa-mir-29c	TBC1D10C	0.376107976	mirna_pc
1546	hsa-mir-29c	LYL1	0.338681724	mirna_pc
1547	hsa-mir-29c	BANK1	0.325057965	mirna_pc
1548	hsa-mir-574	MT1G	0.313937156	mirna_pc
1549	hsa-mir-574	PXMP2	0.356524832	mirna_pc
1550	hsa-mir-574	CCDC149	0.31894438	mirna_pc
1551	hsa-mir-574	NRTN	0.336219322	mirna_pc
1552	hsa-mir-574	CTSF	0.312935535	mirna_pc
1553	hsa-mir-574	SGSM3	0.305395262	mirna_pc
1554	hsa-mir-574	LOC729234	0.311804245	mirna_pc
1555	hsa-mir-574	FAM64A	0.334437711	mirna_pc
1556	hsa-mir-574	XKR8	0.324522267	mirna_pc
1557	hsa-mir-574	SLC30A9	0.327974665	mirna_pc
1558	hsa-mir-574	SSBP3	0.341589065	mirna_pc
1559	hsa-mir-574	MT1F	0.387703022	mirna_pc
1560	hsa-mir-574	WDR19	0.307401836	mirna_pc
1561	hsa-mir-574	C4orf52	0.404075832	mirna_pc
1562	hsa-mir-574	QDPR	0.309458667	mirna_pc
1563	hsa-mir-574	B3GALNT1	0.31056946	mirna_pc
1564	hsa-mir-574	RBPJ	0.306622766	mirna_pc
1565	hsa-mir-574	CDRT4	0.328312071	mirna_pc

1566	hsa-mir-574	AS3MT	0.321919542	mirna_pc
1567	hsa-mir-574	LRPAP1	0.31088779	mirna_pc
1568	hsa-mir-574	MEX3A	0.306067154	mirna_pc
1569	hsa-mir-574	TBC1D19	0.310101618	mirna_pc
1570	hsa-mir-574	UNC119B	0.331735261	mirna_pc
1571	hsa-mir-574	MAD2L2	0.330601032	mirna_pc
1572	hsa-mir-574	ERI3	0.308297493	mirna_pc
1573	hsa-mir-574	C16orf58	0.320110266	mirna_pc
1574	hsa-mir-574	IGSF9	0.326930708	mirna_pc
1575	hsa-mir-574	MRFAP1	0.358044984	mirna_pc
1576	hsa-mir-574	OSTC	0.345221912	mirna_pc
1577	hsa-mir-574	DMAP1	0.326672361	mirna_pc
1578	hsa-mir-574	RFC1	0.309180564	mirna_pc
1579	hsa-mir-574	FAM200B	0.315460457	mirna_pc
1580	hsa-mir-574	RAB28	0.4626285	mirna_pc
1581	hsa-mir-574	MRFAP1L1	0.317180715	mirna_pc
1582	hsa-mir-574	ZNF599	0.310307796	mirna_pc
1583	hsa-mir-574	GPR125	0.440405941	mirna_pc
1584	hsa-mir-574	UROD	0.331074131	mirna_pc
1585	hsa-mir-574	ALX3	0.32668678	mirna_pc
1586	hsa-mir-574	AKR7A2	0.402075341	mirna_pc
1587	hsa-mir-574	PTOV1	0.323346773	mirna_pc
1588	hsa-mir-574	NUDT16L1	0.304638394	mirna_pc
1589	hsa-mir-574	DCTD	0.314632783	mirna_pc
1590	hsa-mir-574	ABHD8	0.316763467	mirna_pc
1591	hsa-mir-574	TMEM9	0.316607064	mirna_pc
1592	hsa-mir-574	AGBL5	0.354439554	mirna_pc
1593	hsa-mir-574	MED28	0.38660696	mirna_pc
1594	hsa-mir-574	LMAN2L	0.30468761	mirna_pc
1595	hsa-mir-574	TCF7L1	0.319629074	mirna_pc
1596	hsa-mir-574	IQCC	0.341452123	mirna_pc
1597	hsa-mir-574	LOC93622	0.330917224	mirna_pc
1598	hsa-mir-574	DCAF16	0.383318325	mirna_pc
1599	hsa-mir-574	PABPC4L	0.320789999	mirna_pc
1600	hsa-mir-574	TRO	0.310381322	mirna_pc
1601	hsa-mir-574	PACRGL	0.360961675	mirna_pc
1602	hsa-mir-574	ETV5	0.315941486	mirna_pc
1603	hsa-mir-574	TXNL4A	0.30822008	mirna_pc
1604	hsa-mir-574	BAI2	0.303575615	mirna_pc
1605	hsa-mir-574	UBE2MP1	0.304112479	mirna_pc
1606	hsa-mir-1-2	ZBTB16	0.679058029	mirna_pc
1607	hsa-mir-1-2	GREM2	0.538705258	mirna_pc
1608	hsa-mir-1-2	DPT	0.642118399	mirna_pc
1609	hsa-mir-1-2	AFF3	0.669750591	mirna_pc
1610	hsa-mir-1-2	NCAM1	0.577854867	mirna_pc
1611	hsa-mir-1-2	FAM107A	0.596477214	mirna_pc
1612	hsa-mir-1-2	KLF15	0.436829448	mirna_pc
1613	hsa-mir-1-2	P2RY14	0.562921082	mirna_pc
1614	hsa-mir-1-2	GPR133	0.388438907	mirna_pc
1615	hsa-mir-1-2	CLEC3B	0.63945778	mirna_pc
1616	hsa-mir-1-2	GNG7	0.718789507	mirna_pc
1617	hsa-mir-1-2	RBPMS2	0.736736244	mirna_pc
1618	hsa-mir-1-2	SLC2A4	0.824254504	mirna_pc
1619	hsa-mir-1-2	CAB39L	0.608030674	mirna_pc

1620	hsa-mir-1-2	ABCA8	0.623273515	mirna_pc
1621	hsa-mir-1-2	C7	0.689827725	mirna_pc
1622	hsa-mir-1-2	ADAMTSL1	0.635625486	mirna_pc
1623	hsa-mir-1-2	MAMDC2	0.642276085	mirna_pc
1624	hsa-mir-1-2	PLIN4	0.796182536	mirna_pc
1625	hsa-mir-1-2	ZNF471	0.544206923	mirna_pc
1626	hsa-mir-1-2	C3orf18	0.406795993	mirna_pc
1627	hsa-mir-1-2	HSPB7	0.698283583	mirna_pc
1628	hsa-mir-1-2	MYOCD	0.8783391	mirna_pc
1629	hsa-mir-1-2	LYVE1	0.538097582	mirna_pc
1630	hsa-mir-1-2	RNF180	0.599482525	mirna_pc
1631	hsa-mir-1-2	OGN	0.531304411	mirna_pc
1632	hsa-mir-1-2	LONRF2	0.575991873	mirna_pc
1633	hsa-mir-1-2	CRY2	0.475362462	mirna_pc
1634	hsa-mir-1-2	PARK2	0.591916086	mirna_pc
1635	hsa-mir-1-2	ATOH8	0.499520394	mirna_pc
1636	hsa-mir-1-2	RGMB	0.415340358	mirna_pc
1637	hsa-mir-1-2	CGNL1	0.318273271	mirna_pc
1638	hsa-mir-1-2	NEGR1	0.811930306	mirna_pc
1639	hsa-mir-1-2	SLC25A4	0.34610523	mirna_pc
1640	hsa-mir-1-2	CCL14	0.701006086	mirna_pc
1641	hsa-mir-1-2	C7orf41	0.606488608	mirna_pc
1642	hsa-mir-1-2	CHRDL1	0.793973015	mirna_pc
1643	hsa-mir-1-2	RYR2	0.786900101	mirna_pc
1644	hsa-mir-1-2	PGM5	0.865332851	mirna_pc
1645	hsa-mir-1-2	HSPB6	0.672805331	mirna_pc
1646	hsa-mir-1-2	ITGA8	0.490246535	mirna_pc
1647	hsa-mir-1-2	KIT	0.331976599	mirna_pc
1648	hsa-mir-1-2	TMOD1	0.749298736	mirna_pc
1649	hsa-mir-1-2	ADAM33	0.69856623	mirna_pc
1650	hsa-mir-1-2	C5orf4	0.482888443	mirna_pc
1651	hsa-mir-1-2	RCAN2	0.608792351	mirna_pc
1652	hsa-mir-1-2	MMRN1	0.525024754	mirna_pc
1653	hsa-mir-1-2	FAM54B	0.465886276	mirna_pc
1654	hsa-mir-1-2	PRX	0.346922055	mirna_pc
1655	hsa-mir-1-2	CFD	0.5612506	mirna_pc
1656	hsa-mir-1-2	MITF	0.497500095	mirna_pc
1657	hsa-mir-1-2	SIK2	0.32478176	mirna_pc
1658	hsa-mir-1-2	EFHA2	0.500800295	mirna_pc
1659	hsa-mir-1-2	GNAZ	0.540087791	mirna_pc
1660	hsa-mir-1-2	PDE2A	0.661371733	mirna_pc
1661	hsa-mir-1-2	CDH2	0.355305537	mirna_pc
1662	hsa-mir-1-2	RAI2	0.392597001	mirna_pc
1663	hsa-mir-1-2	C8orf84	0.493122128	mirna_pc
1664	hsa-mir-1-2	TNXB	0.58209502	mirna_pc
1665	hsa-mir-1-2	ABI3BP	0.622974718	mirna_pc
1666	hsa-mir-1-2	DCLK2	0.555549031	mirna_pc
1667	hsa-mir-1-2	IPW	0.355114088	mirna_pc
1668	hsa-mir-1-2	ACACB	0.579116427	mirna_pc
1669	hsa-mir-1-2	ADAMTS8	0.617943353	mirna_pc
1670	hsa-mir-1-2	ANK2	0.69630176	mirna_pc
1671	hsa-mir-1-2	C14orf159	0.311371182	mirna_pc
1672	hsa-mir-1-2	BVES	0.590909697	mirna_pc
1673	hsa-mir-1-2	ROR1	0.311042084	mirna_pc

1674	hsa-mir-1-2	TCF21	0.469362136	mirna_pc
1675	hsa-mir-1-2	SORBS2	0.495763924	mirna_pc
1676	hsa-mir-1-2	C5orf53	0.414658355	mirna_pc
1677	hsa-mir-1-2	LGI4	0.767976127	mirna_pc
1678	hsa-mir-1-2	C7orf58	0.711718953	mirna_pc
1679	hsa-mir-1-2	SDPR	0.646334694	mirna_pc
1680	hsa-mir-1-2	CBX7	0.727455235	mirna_pc
1681	hsa-mir-1-2	GALNTL2	0.440612221	mirna_pc
1682	hsa-mir-1-2	AOX1	0.36838864	mirna_pc
1683	hsa-mir-1-2	IL33	0.382969937	mirna_pc
1684	hsa-mir-1-2	ARHGAP24	0.302304061	mirna_pc
1685	hsa-mir-1-2	PRIMA1	0.696702683	mirna_pc
1686	hsa-mir-1-2	EPM2A	0.783425331	mirna_pc
1687	hsa-mir-1-2	NECAB1	0.680907624	mirna_pc
1688	hsa-mir-1-2	DIRAS1	0.618828195	mirna_pc
1689	hsa-mir-1-2	SYNJ2BP	0.32764386	mirna_pc
1690	hsa-mir-1-2	DAAM2	0.857004074	mirna_pc
1691	hsa-mir-1-2	CDHR3	0.53502637	mirna_pc
1692	hsa-mir-1-2	TMEM8B	0.498292878	mirna_pc
1693	hsa-mir-1-2	ZSCAN18	0.527091639	mirna_pc
1694	hsa-mir-1-2	TTLL7	0.585556651	mirna_pc
1695	hsa-mir-1-2	PTGIS	0.627594624	mirna_pc
1696	hsa-mir-1-2	ZHX3	0.656677438	mirna_pc
1697	hsa-mir-1-2	GTF2IRD2P1	0.438828274	mirna_pc
1698	hsa-mir-1-2	SECISBP2L	0.361310105	mirna_pc
1699	hsa-mir-1-2	ITIH5	0.590743714	mirna_pc
1700	hsa-mir-1-2	ADAMTSL3	0.596099834	mirna_pc
1701	hsa-mir-1-2	KCNK3	0.347381975	mirna_pc
1702	hsa-mir-1-2	GPRASP1	0.735966305	mirna_pc
1703	hsa-mir-1-2	JAM2	0.76469622	mirna_pc
1704	hsa-mir-1-2	PLCL1	0.713144774	mirna_pc
1705	hsa-mir-1-2	ITM2A	0.341235652	mirna_pc
1706	hsa-mir-1-2	PBXIP1	0.503598328	mirna_pc
1707	hsa-mir-1-2	PLN	0.824696545	mirna_pc
1708	hsa-mir-1-2	REEP1	0.660619435	mirna_pc
1709	hsa-mir-1-2	GNAO1	0.467854867	mirna_pc
1710	hsa-mir-1-2	ITGA9	0.544118448	mirna_pc
1711	hsa-mir-1-2	GTF2IRD2	0.407382967	mirna_pc
1712	hsa-mir-1-2	ADCY5	0.721789233	mirna_pc
1713	hsa-mir-1-2	CILP	0.454316121	mirna_pc
1714	hsa-mir-1-2	NPR 1.00	0.614301548	mirna_pc
1715	hsa-mir-1-2	ZBTB47	0.690928403	mirna_pc
1716	hsa-mir-1-2	PRKAA2	0.636474795	mirna_pc
1717	hsa-mir-1-2	DARC	0.684011873	mirna_pc
1718	hsa-mir-1-2	PTPN21	0.440352387	mirna_pc
1719	hsa-mir-1-2	FHL1	0.77093497	mirna_pc
1720	hsa-mir-1-2	DMD	0.712512929	mirna_pc
1721	hsa-mir-1-2	DIXDC1	0.807321212	mirna_pc
1722	hsa-mir-1-2	SNX1	0.551953804	mirna_pc
1723	hsa-mir-1-2	KANK3	0.455102536	mirna_pc
1724	hsa-mir-1-2	CIRBP	0.301243556	mirna_pc
1725	hsa-mir-1-2	PEG3	0.756366341	mirna_pc
1726	hsa-mir-1-2	C1QTNF3	0.573664795	mirna_pc
1727	hsa-mir-1-2	ITPR1	0.775744441	mirna_pc

1728	hsa-mir-1-2	KLHDC1	0.455946618	mirna_pc
1729	hsa-mir-1-2	C6orf204	0.358811388	mirna_pc
1730	hsa-mir-1-2	BCL2L2	0.494027826	mirna_pc
1731	hsa-mir-1-2	MAP6	0.681108838	mirna_pc
1732	hsa-mir-1-2	PDK4	0.519488461	mirna_pc
1733	hsa-mir-1-2	KAT2B	0.331261995	mirna_pc
1734	hsa-mir-1-2	INPP5A	0.61679049	mirna_pc
1735	hsa-mir-1-2	C16orf45	0.666199519	mirna_pc
1736	hsa-mir-1-2	MAPT	0.563063608	mirna_pc
1737	hsa-mir-1-2	MYOM1	0.751620766	mirna_pc
1738	hsa-mir-1-2	TENC1	0.693728668	mirna_pc
1739	hsa-mir-1-2	RECK	0.640992031	mirna_pc
1740	hsa-mir-1-2	PPP1R12C	0.657233774	mirna_pc
1741	hsa-mir-1-2	ABCA6	0.59895087	mirna_pc
1742	hsa-mir-1-2	ANO5	0.432020792	mirna_pc
1743	hsa-mir-1-2	KCNMB1	0.811342226	mirna_pc
1744	hsa-mir-1-2	NCRNA00087	0.318329141	mirna_pc
1745	hsa-mir-1-2	LMOD1	0.872119822	mirna_pc
1746	hsa-mir-1-2	SLIT2	0.710481491	mirna_pc
1747	hsa-mir-1-2	ZNF470	0.326888719	mirna_pc
1748	hsa-mir-1-2	IL11RA	0.457519624	mirna_pc
1749	hsa-mir-1-2	NACAD	0.425722064	mirna_pc
1750	hsa-mir-1-2	GTF2IRD2B	0.395610022	mirna_pc
1751	hsa-mir-1-2	POU6F1	0.654610065	mirna_pc
1752	hsa-mir-1-2	PRKAR2B	0.781694461	mirna_pc
1753	hsa-mir-1-2	FYCO1	0.724152124	mirna_pc
1754	hsa-mir-1-2	ZFP28	0.352847856	mirna_pc
1755	hsa-mir-1-2	ZNF542	0.329100622	mirna_pc
1756	hsa-mir-1-2	SYNE1	0.538622828	mirna_pc
1757	hsa-mir-1-2	ZNF844	0.314074264	mirna_pc
1758	hsa-mir-1-2	TMEM47	0.757443345	mirna_pc
1759	hsa-mir-1-2	HCFC2	0.475387788	mirna_pc
1760	hsa-mir-1-2	ANKDD1A	0.465784733	mirna_pc
1761	hsa-mir-1-2	FOXN3	0.679010007	mirna_pc
1762	hsa-mir-1-2	AOC3	0.849859485	mirna_pc
1763	hsa-mir-1-2	CNN1	0.877891195	mirna_pc
1764	hsa-mir-1-2	PRELP	0.720567631	mirna_pc
1765	hsa-mir-1-2	PRICKLE2	0.766643762	mirna_pc
1766	hsa-mir-1-2	KCNAB1	0.448423136	mirna_pc
1767	hsa-mir-1-2	KLF9	0.660595186	mirna_pc
1768	hsa-mir-1-2	SORBS1	0.845807051	mirna_pc
1769	hsa-mir-1-2	PDZD4	0.625089659	mirna_pc
1770	hsa-mir-1-2	KIAA0430	0.472655627	mirna_pc
1771	hsa-mir-1-2	NBEA	0.57745802	mirna_pc
1772	hsa-mir-1-2	TCEAL3	0.511641691	mirna_pc
1773	hsa-mir-1-2	LAMA2	0.463740172	mirna_pc
1774	hsa-mir-1-2	PHF17	0.464268241	mirna_pc
1775	hsa-mir-1-2	SCUBE1	0.729139	mirna_pc
1776	hsa-mir-1-2	NEXN	0.803894884	mirna_pc
1777	hsa-mir-1-2	TOR1AIP1	0.629747158	mirna_pc
1778	hsa-mir-1-2	EIF4E3	0.628065652	mirna_pc
1779	hsa-mir-1-2	SOBP	0.535045593	mirna_pc
1780	hsa-mir-1-2	MOAP1	0.409140299	mirna_pc
1781	hsa-mir-1-2	PDE1A	0.557034651	mirna_pc

1782	hsa-mir-1-2	ID4	0.369105996	mirna_pc
1783	hsa-mir-1-2	NFIC	0.613000226	mirna_pc
1784	hsa-mir-1-2	SPRYD3	0.360993696	mirna_pc
1785	hsa-mir-1-2	FAM172A	0.469189409	mirna_pc
1786	hsa-mir-1-2	GADD45B	0.691866989	mirna_pc
1787	hsa-mir-1-2	FBXL5	0.502344828	mirna_pc
1788	hsa-mir-1-2	DCLK1	0.478088547	mirna_pc
1789	hsa-mir-1-2	KCNMA1	0.727290971	mirna_pc
1790	hsa-mir-1-2	GARNL3	0.403500028	mirna_pc
1791	hsa-mir-1-2	SGSM2	0.434127787	mirna_pc
1792	hsa-mir-1-2	CSRNP1	0.545855249	mirna_pc
1793	hsa-mir-1-2	ARHGAP10	0.623122791	mirna_pc
1794	hsa-mir-1-2	FHL5	0.590907518	mirna_pc
1795	hsa-mir-1-2	CCNI	0.410184745	mirna_pc
1796	hsa-mir-1-2	CALCOCO1	0.582902179	mirna_pc
1797	hsa-mir-1-2	TSPYL2	0.442201854	mirna_pc
1798	hsa-mir-1-2	SOCS2	0.409765892	mirna_pc
1799	hsa-mir-1-2	ZNF568	0.325197797	mirna_pc
1800	hsa-mir-1-2	USP53	0.34101841	mirna_pc
1801	hsa-mir-1-2	ZFYVE20	0.521038728	mirna_pc
1802	hsa-mir-1-2	INMT	0.721022655	mirna_pc
1803	hsa-mir-1-2	CCDC149	0.332602115	mirna_pc
1804	hsa-mir-1-2	MFAP5	0.487844866	mirna_pc
1805	hsa-mir-1-2	TSC22D3	0.338243211	mirna_pc
1806	hsa-mir-1-2	CA5B	0.397574613	mirna_pc
1807	hsa-mir-1-2	PPM1K	0.347745096	mirna_pc
1808	hsa-mir-1-2	SYNPO2	0.857751721	mirna_pc
1809	hsa-mir-1-2	TGFBR3	0.719610471	mirna_pc
1810	hsa-mir-1-2	C14orf37	0.34992765	mirna_pc
1811	hsa-mir-1-2	CACNB2	0.623933524	mirna_pc
1812	hsa-mir-1-2	SRPX	0.46476345	mirna_pc
1813	hsa-mir-1-2	APBB1	0.548116811	mirna_pc
1814	hsa-mir-1-2	VAMP2	0.31582289	mirna_pc
1815	hsa-mir-1-2	FZD4	0.300373414	mirna_pc
1816	hsa-mir-1-2	CAMTA2	0.329921981	mirna_pc
1817	hsa-mir-1-2	LOC728264	0.778689668	mirna_pc
1818	hsa-mir-1-2	SMAD9	0.553731994	mirna_pc
1819	hsa-mir-1-2	P2RX1	0.395860351	mirna_pc
1820	hsa-mir-1-2	DTNA	0.740467952	mirna_pc
1821	hsa-mir-1-2	HERC1	0.462983032	mirna_pc
1822	hsa-mir-1-2	ABL1	0.648153802	mirna_pc
1823	hsa-mir-1-2	BBS1	0.464856169	mirna_pc
1824	hsa-mir-1-2	KIF1C	0.362311041	mirna_pc
1825	hsa-mir-1-2	CYP1B1	0.349089038	mirna_pc
1826	hsa-mir-1-2	SLMAP	0.730783233	mirna_pc
1827	hsa-mir-1-2	C17orf91	0.362432071	mirna_pc
1828	hsa-mir-1-2	PGCP	0.366862168	mirna_pc
1829	hsa-mir-1-2	GABARAPL1	0.432947682	mirna_pc
1830	hsa-mir-1-2	PARD3B	0.366052523	mirna_pc
1831	hsa-mir-1-2	FKBP5	0.495373969	mirna_pc
1832	hsa-mir-1-2	EIF2C4	0.485945915	mirna_pc
1833	hsa-mir-1-2	CXCL12	0.525527414	mirna_pc
1834	hsa-mir-1-2	GSN	0.669915901	mirna_pc
1835	hsa-mir-1-2	C22orf39	0.494149996	mirna_pc

1836	hsa-mir-1-2	PDZRN3	0.578617148	mirna_pc
1837	hsa-mir-1-2	NISCH	0.341293933	mirna_pc
1838	hsa-mir-1-2	ZNF25	0.632966775	mirna_pc
1839	hsa-mir-1-2	LRIG1	0.306520724	mirna_pc
1840	hsa-mir-1-2	ZNF483	0.530502744	mirna_pc
1841	hsa-mir-1-2	COL14A1	0.529059818	mirna_pc
1842	hsa-mir-1-2	ZNF570	0.410269397	mirna_pc
1843	hsa-mir-1-2	ARHGAP6	0.374261421	mirna_pc
1844	hsa-mir-1-2	SMARCD3	0.357774301	mirna_pc
1845	hsa-mir-1-2	FLNC	0.817444863	mirna_pc
1846	hsa-mir-1-2	FOXF1	0.683699613	mirna_pc
1847	hsa-mir-1-2	PRUNE2	0.778402246	mirna_pc
1848	hsa-mir-1-2	ITGA7	0.770445407	mirna_pc
1849	hsa-mir-1-2	C14orf132	0.582439029	mirna_pc
1850	hsa-mir-1-2	NACC2	0.675778097	mirna_pc
1851	hsa-mir-1-2	TTC28	0.543857686	mirna_pc
1852	hsa-mir-1-2	CORO2B	0.490886133	mirna_pc
1853	hsa-mir-1-2	PDE7B	0.600188252	mirna_pc
1854	hsa-mir-1-2	CELF2	0.73050907	mirna_pc
1855	hsa-mir-1-2	PDE4D	0.480798537	mirna_pc
1856	hsa-mir-1-2	STARD8	0.431178818	mirna_pc
1857	hsa-mir-1-2	DMXL1	0.301870101	mirna_pc
1858	hsa-mir-1-2	C10orf26	0.453255579	mirna_pc
1859	hsa-mir-1-2	MASP1	0.340087923	mirna_pc
1860	hsa-mir-1-2	TMEM25	0.302062768	mirna_pc
1861	hsa-mir-1-2	FILIP1	0.797280211	mirna_pc
1862	hsa-mir-1-2	ZCCHC24	0.750131191	mirna_pc
1863	hsa-mir-1-2	C15orf17	0.376725099	mirna_pc
1864	hsa-mir-1-2	SLIT3	0.555485748	mirna_pc
1865	hsa-mir-1-2	DDR2	0.724921865	mirna_pc
1866	hsa-mir-1-2	KIAA0232	0.401503543	mirna_pc
1867	hsa-mir-1-2	ZNF347	0.308874553	mirna_pc
1868	hsa-mir-1-2	LIMS2	0.716301453	mirna_pc
1869	hsa-mir-1-2	C6orf225	0.472854288	mirna_pc
1870	hsa-mir-1-2	ASB1	0.311245991	mirna_pc
1871	hsa-mir-1-2	ATP1B2	0.660520741	mirna_pc
1872	hsa-mir-1-2	ADAMTS1	0.71551666	mirna_pc
1873	hsa-mir-1-2	MRVI1	0.81304119	mirna_pc
1874	hsa-mir-1-2	TNFSF12	0.663742525	mirna_pc
1875	hsa-mir-1-2	CMAH	0.374525498	mirna_pc
1876	hsa-mir-1-2	ROGDI	0.312081276	mirna_pc
1877	hsa-mir-1-2	CCDC69	0.846741671	mirna_pc
1878	hsa-mir-1-2	SIK3	0.338053088	mirna_pc
1879	hsa-mir-1-2	GPM6B	0.677016544	mirna_pc
1880	hsa-mir-1-2	NEK1	0.324311928	mirna_pc
1881	hsa-mir-1-2	PPAP2B	0.428300663	mirna_pc
1882	hsa-mir-1-2	HLF	0.762984031	mirna_pc
1883	hsa-mir-1-2	CFL2	0.772456542	mirna_pc
1884	hsa-mir-1-2	LAMB2	0.598685279	mirna_pc
1885	hsa-mir-1-2	ST5	0.347412215	mirna_pc
1886	hsa-mir-1-2	S1PR1	0.6090499	mirna_pc
1887	hsa-mir-1-2	WLS	0.644946675	mirna_pc
1888	hsa-mir-1-2	WFDC1	0.515466872	mirna_pc
1889	hsa-mir-1-2	CRYZL1	0.446517443	mirna_pc

1890	hsa-mir-1-2	DACT3	0.840805302	mirna_pc
1891	hsa-mir-1-2	FGF2	0.628405099	mirna_pc
1892	hsa-mir-1-2	FILIP1L	0.69265075	mirna_pc
1893	hsa-mir-1-2	DUSP3	0.563742536	mirna_pc
1894	hsa-mir-1-2	VPS13D	0.402892504	mirna_pc
1895	hsa-mir-1-2	PPP1R12B	0.864150412	mirna_pc
1896	hsa-mir-1-2	PITPNM2	0.343583783	mirna_pc
1897	hsa-mir-1-2	PIP5K1C	0.61850728	mirna_pc
1898	hsa-mir-1-2	JPH2	0.835616666	mirna_pc
1899	hsa-mir-1-2	KIAA1737	0.371788414	mirna_pc
1900	hsa-mir-1-2	NFIA	0.554007236	mirna_pc
1901	hsa-mir-1-2	FRY	0.493285348	mirna_pc
1902	hsa-mir-1-2	OSR1	0.650273515	mirna_pc
1903	hsa-mir-1-2	FAM190B	0.541858486	mirna_pc
1904	hsa-mir-1-2	C13orf33	0.491007399	mirna_pc
1905	hsa-mir-1-2	PGR	0.71522226	mirna_pc
1906	hsa-mir-1-2	ST6GALNAC6	0.554751164	mirna_pc
1907	hsa-mir-1-2	C6orf72	0.314707549	mirna_pc
1908	hsa-mir-1-2	OMD	0.444753389	mirna_pc
1909	hsa-mir-1-2	ACOX2	0.524429595	mirna_pc
1910	hsa-mir-1-2	PHYHIP	0.437311968	mirna_pc
1911	hsa-mir-1-2	SMOC2	0.572921248	mirna_pc
1912	hsa-mir-1-2	C9orf130	0.313157357	mirna_pc
1913	hsa-mir-1-2	CACNA1H	0.788476282	mirna_pc
1914	hsa-mir-1-2	KCNE4	0.376274381	mirna_pc
1915	hsa-mir-1-2	RFX2	0.493661558	mirna_pc
1916	hsa-mir-1-2	C14orf28	0.379453656	mirna_pc
1917	hsa-mir-1-2	SSC5D	0.692423686	mirna_pc
1918	hsa-mir-1-2	PTPRM	0.499858907	mirna_pc
1919	hsa-mir-1-2	CHADL	0.314844973	mirna_pc
1920	hsa-mir-1-2	RPS6KA5	0.562752773	mirna_pc
1921	hsa-mir-1-2	CRISPLD2	0.701016833	mirna_pc
1922	hsa-mir-1-2	STARD13	0.536857975	mirna_pc
1923	hsa-mir-1-2	FAM13B	0.589073758	mirna_pc
1924	hsa-mir-1-2	PER1	0.544534366	mirna_pc
1925	hsa-mir-1-2	TLE2	0.436104182	mirna_pc
1926	hsa-mir-1-2	C20orf194	0.596679873	mirna_pc
1927	hsa-mir-1-2	DNAJC27	0.368047621	mirna_pc
1928	hsa-mir-1-2	USP47	0.324023371	mirna_pc
1929	hsa-mir-1-2	ACTG2	0.883158871	mirna_pc
1930	hsa-mir-1-2	DMPK	0.7818472	mirna_pc
1931	hsa-mir-1-2	APOLD1	0.489584635	mirna_pc
1932	hsa-mir-1-2	C9orf5	0.458804248	mirna_pc
1933	hsa-mir-1-2	ZBTB4	0.679394201	mirna_pc
1934	hsa-mir-1-2	TACR2	0.820539853	mirna_pc
1935	hsa-mir-1-2	WBSCR17	0.5788532	mirna_pc
1936	hsa-mir-1-2	LRRN4CL	0.630744396	mirna_pc
1937	hsa-mir-1-2	CLIP3	0.739836193	mirna_pc
1938	hsa-mir-1-2	ARHGEF9	0.388084573	mirna_pc
1939	hsa-mir-1-2	MAGI2	0.534755269	mirna_pc
1940	hsa-mir-1-2	SLC22A17	0.515175359	mirna_pc
1941	hsa-mir-1-2	SYNGR1	0.333107735	mirna_pc
1942	hsa-mir-1-2	LTBP4	0.570793847	mirna_pc
1943	hsa-mir-1-2	UTRN	0.45260518	mirna_pc

1944	hsa-mir-1-2	AKAP6	0.653267572	mirna_pc
1945	hsa-mir-1-2	ADAMTS15	0.444176037	mirna_pc
1946	hsa-mir-1-2	RNF150	0.56302749	mirna_pc
1947	hsa-mir-1-2	FAM13C	0.668728375	mirna_pc
1948	hsa-mir-1-2	MFAP4	0.562230979	mirna_pc
1949	hsa-mir-1-2	JAM3	0.686507618	mirna_pc
1950	hsa-mir-1-2	KANK2	0.807062849	mirna_pc
1951	hsa-mir-1-2	TOB2	0.341339348	mirna_pc
1952	hsa-mir-1-2	CSRP1	0.85480875	mirna_pc
1953	hsa-mir-1-2	GPX3	0.332802453	mirna_pc
1954	hsa-mir-1-2	AG2	0.662035974	mirna_pc
1955	hsa-mir-1-2	SEMA3G	0.497133334	mirna_pc
1956	hsa-mir-1-2	MPRIP	0.34154836	mirna_pc
1957	hsa-mir-1-2	SGEF	0.516629324	mirna_pc
1958	hsa-mir-1-2	CCL21	0.489052642	mirna_pc
1959	hsa-mir-1-2	SLC7A2	0.309074558	mirna_pc
1960	hsa-mir-1-2	PDE1B	0.511874337	mirna_pc
1961	hsa-mir-1-2	TNS1	0.834381367	mirna_pc
1962	hsa-mir-1-2	ZMYND11	0.397689935	mirna_pc
1963	hsa-mir-1-2	DYNC1LI2	0.401308781	mirna_pc
1964	hsa-mir-1-2	PTPLA	0.334106865	mirna_pc
1965	hsa-mir-1-2	PLXNA4	0.595865367	mirna_pc
1966	hsa-mir-1-2	ZNF331	0.394289309	mirna_pc
1967	hsa-mir-1-2	RSPO3	0.697692356	mirna_pc
1968	hsa-mir-1-2	NFIX	0.45080387	mirna_pc
1969	hsa-mir-1-2	SVEP1	0.496400557	mirna_pc
1970	hsa-mir-1-2	PODN	0.552107791	mirna_pc
1971	hsa-mir-1-2	PRKCE	0.402580512	mirna_pc
1972	hsa-mir-1-2	NCALD	0.500019206	mirna_pc
1973	hsa-mir-1-2	RAP1A	0.524406089	mirna_pc
1974	hsa-mir-1-2	DUSP22	0.302770942	mirna_pc
1975	hsa-mir-1-2	RERE	0.458874843	mirna_pc
1976	hsa-mir-1-2	KIAA1529	0.412948488	mirna_pc
1977	hsa-mir-1-2	C14orf139	0.356696417	mirna_pc
1978	hsa-mir-1-2	LIMCH1	0.399857017	mirna_pc
1979	hsa-mir-1-2	F8	0.653522556	mirna_pc
1980	hsa-mir-1-2	GIPC3	0.313705152	mirna_pc
1981	hsa-mir-1-2	ATL1	0.322569573	mirna_pc
1982	hsa-mir-1-2	ABCC9	0.598954488	mirna_pc
1983	hsa-mir-1-2	MYO9A	0.688830119	mirna_pc
1984	hsa-mir-1-2	NR4A3	0.575750272	mirna_pc
1985	hsa-mir-1-2	SHE	0.4866991	mirna_pc
1986	hsa-mir-1-2	GPRASP2	0.368849293	mirna_pc
1987	hsa-mir-1-2	C12orf51	0.307416802	mirna_pc
1988	hsa-mir-1-2	ZC3H6	0.368064557	mirna_pc
1989	hsa-mir-1-2	ZNF569	0.361462482	mirna_pc
1990	hsa-mir-1-2	CSDC2	0.387075135	mirna_pc
1991	hsa-mir-1-2	C4orf31	0.302410121	mirna_pc
1992	hsa-mir-1-2	ZER1	0.34507608	mirna_pc
1993	hsa-mir-1-2	RASGRP2	0.48995948	mirna_pc
1994	hsa-mir-1-2	TCEAL1	0.521256644	mirna_pc
1995	hsa-mir-1-2	IL6ST	0.565037422	mirna_pc
1996	hsa-mir-1-2	KIAA1109	0.381938865	mirna_pc
1997	hsa-mir-1-2	CAND2	0.483863056	mirna_pc

1998	hsa-mir-1-2	MXI1	0.390508758	mirna_pc
1999	hsa-mir-1-2	ADD1	0.53009929	mirna_pc
2000	hsa-mir-1-2	CYYR1	0.414777628	mirna_pc
2001	hsa-mir-1-2	IKZF4	0.568462168	mirna_pc
2002	hsa-mir-1-2	NTN1	0.746129194	mirna_pc
2003	hsa-mir-1-2	PJA2	0.603297096	mirna_pc
2004	hsa-mir-1-2	TTLL11	0.513876683	mirna_pc
2005	hsa-mir-1-2	SETBP1	0.460977861	mirna_pc
2006	hsa-mir-1-2	PARVA	0.741800575	mirna_pc
2007	hsa-mir-1-2	ZFP106	0.422028546	mirna_pc
2008	hsa-mir-1-2	MSRB3	0.838048212	mirna_pc
2009	hsa-mir-1-2	SYNM	0.8304746	mirna_pc
2010	hsa-mir-1-2	DSEL	0.312214514	mirna_pc
2011	hsa-mir-1-2	CNRIP1	0.598686234	mirna_pc
2012	hsa-mir-1-2	MAN1C1	0.582785407	mirna_pc
2013	hsa-mir-1-2	FERMT2	0.75366051	mirna_pc
2014	hsa-mir-1-2	SNED1	0.342060858	mirna_pc
2015	hsa-mir-1-2	FOXP1	0.399051304	mirna_pc
2016	hsa-mir-1-2	FAT4	0.489535391	mirna_pc
2017	hsa-mir-1-2	FOXO3B	0.343460572	mirna_pc
2018	hsa-mir-1-2	DES	0.861379272	mirna_pc
2019	hsa-mir-1-2	SASH1	0.300932375	mirna_pc
2020	hsa-mir-1-2	TXNIP	0.580959368	mirna_pc
2021	hsa-mir-1-2	DLC1	0.578176932	mirna_pc
2022	hsa-mir-1-2	TPSB2	0.34547751	mirna_pc
2023	hsa-mir-1-2	FAM129A	0.776667587	mirna_pc
2024	hsa-mir-1-2	CNST	0.427992504	mirna_pc
2025	hsa-mir-1-2	TECPR2	0.489729133	mirna_pc
2026	hsa-mir-1-2	SCN4B	0.335923901	mirna_pc
2027	hsa-mir-1-2	TEK	0.484975385	mirna_pc
2028	hsa-mir-1-2	GYPC	0.473945362	mirna_pc
2029	hsa-mir-1-2	MRGPRF	0.769265742	mirna_pc
2030	hsa-mir-1-2	CHRD	0.450767251	mirna_pc
2031	hsa-mir-1-2	LDB2	0.385599124	mirna_pc
2032	hsa-mir-1-2	LRRC70	0.381292559	mirna_pc
2033	hsa-mir-1-2	NRIP2	0.347498532	mirna_pc
2034	hsa-mir-1-2	AKAP12	0.615172048	mirna_pc
2035	hsa-mir-1-2	SMTN	0.777737281	mirna_pc
2036	hsa-mir-1-2	AKAP2	0.508220948	mirna_pc
2037	hsa-mir-1-2	HIPK3	0.584066812	mirna_pc
2038	hsa-mir-1-2	PTGS1	0.450156219	mirna_pc
2039	hsa-mir-1-2	EZH1	0.573384279	mirna_pc
2040	hsa-mir-1-2	WDTC1	0.300765129	mirna_pc
2041	hsa-mir-1-2	ZNF71	0.402349809	mirna_pc
2042	hsa-mir-1-2	SLC25A23	0.447858656	mirna_pc
2043	hsa-mir-1-2	ZNF287	0.32003693	mirna_pc
2044	hsa-mir-1-2	PPM1L	0.351900246	mirna_pc
2045	hsa-mir-1-2	TXNDC15	0.316924634	mirna_pc
2046	hsa-mir-1-2	IGFBP5	0.520450667	mirna_pc
2047	hsa-mir-1-2	PRMT10	0.399394266	mirna_pc
2048	hsa-mir-1-2	SGCD	0.616170233	mirna_pc
2049	hsa-mir-1-2	AASS	0.405317026	mirna_pc
2050	hsa-mir-1-2	VLDLR	0.346734356	mirna_pc
2051	hsa-mir-1-2	BCHE	0.484716534	mirna_pc

2052	hsa-mir-1-2	FBXL7	0.620439766	mirna_pc
2053	hsa-mir-1-2	LEPROT	0.373644405	mirna_pc
2054	hsa-mir-1-2	AFF1	0.416043764	mirna_pc
2055	hsa-mir-1-2	AFF4	0.3501101	mirna_pc
2056	hsa-mir-1-2	LOC286367	0.318805226	mirna_pc
2057	hsa-mir-1-2	IGF1	0.479923508	mirna_pc
2058	hsa-mir-1-2	NHSL2	0.680475705	mirna_pc
2059	hsa-mir-1-2	RBPMS	0.739637506	mirna_pc
2060	hsa-mir-1-2	SPARCL1	0.727807199	mirna_pc
2061	hsa-mir-1-2	DCUN1D4	0.318103651	mirna_pc
2062	hsa-mir-1-2	FBXL22	0.707740985	mirna_pc
2063	hsa-mir-1-2	CALM1	0.330294767	mirna_pc
2064	hsa-mir-1-2	SOX17	0.568762417	mirna_pc
2065	hsa-mir-1-2	TUBA8	0.32641903	mirna_pc
2066	hsa-mir-1-2	PPAP2A	0.427232507	mirna_pc
2067	hsa-mir-1-2	DSTN	0.729022132	mirna_pc
2068	hsa-mir-1-2	EMCN	0.519502916	mirna_pc
2069	hsa-mir-1-2	EML1	0.690121859	mirna_pc
2070	hsa-mir-1-2	PIK3R1	0.436090573	mirna_pc
2071	hsa-mir-1-2	C1orf183	0.543002633	mirna_pc
2072	hsa-mir-1-2	BTG2	0.454413832	mirna_pc
2073	hsa-mir-1-2	BTC	0.597508017	mirna_pc
2074	hsa-mir-1-2	NLGN3	0.458891874	mirna_pc
2075	hsa-mir-1-2	PPP3CB	0.400263007	mirna_pc
2076	hsa-mir-1-2	BOC	0.64958267	mirna_pc
2077	hsa-mir-1-2	SPOP	0.488878375	mirna_pc
2078	hsa-mir-1-2	ADARB1	0.680254979	mirna_pc
2079	hsa-mir-1-2	TLN1	0.74661996	mirna_pc
2080	hsa-mir-1-2	TSPAN2	0.741073011	mirna_pc
2081	hsa-mir-1-2	CACNA1C	0.755629634	mirna_pc
2082	hsa-mir-1-2	POPDC2	0.692957374	mirna_pc
2083	hsa-mir-1-2	PSD	0.599633623	mirna_pc
2084	hsa-mir-1-2	ARHGAP20	0.721071846	mirna_pc
2085	hsa-mir-1-2	CHRM3	0.45247435	mirna_pc
2086	hsa-mir-1-2	FNBP1	0.816979486	mirna_pc
2087	hsa-mir-1-2	MYH11	0.856554101	mirna_pc
2088	hsa-mir-1-2	DLG4	0.328843235	mirna_pc
2089	hsa-mir-1-2	C15orf52	0.469824964	mirna_pc
2090	hsa-mir-1-2	CSDE1	0.501202468	mirna_pc
2091	hsa-mir-1-2	NAP1L5	0.501852872	mirna_pc
2092	hsa-mir-1-2	HSPB8	0.692223513	mirna_pc
2093	hsa-mir-1-2	TACC1	0.667689988	mirna_pc
2094	hsa-mir-1-2	MEGF8	0.409598495	mirna_pc
2095	hsa-mir-1-2	KLHL15	0.393756766	mirna_pc
2096	hsa-mir-1-2	WDR7	0.335623566	mirna_pc
2097	hsa-mir-1-2	CPA3	0.319404759	mirna_pc
2098	hsa-mir-1-2	COX7A1	0.769818839	mirna_pc
2099	hsa-mir-1-2	ZFPM2	0.6017565	mirna_pc
2100	hsa-mir-1-2	MECP2	0.46525898	mirna_pc
2101	hsa-mir-1-2	SERINC1	0.341252759	mirna_pc
2102	hsa-mir-1-2	ZNF423	0.441003086	mirna_pc
2103	hsa-mir-1-2	SRR	0.305098066	mirna_pc
2104	hsa-mir-1-2	SNX21	0.340682946	mirna_pc
2105	hsa-mir-1-2	PREX2	0.482148991	mirna_pc

2106	hsa-mir-1-2	NCOA1	0.346061553	mirna_pc
2107	hsa-mir-1-2	PHF1	0.330458813	mirna_pc
2108	hsa-mir-1-2	PKD1	0.666650552	mirna_pc
2109	hsa-mir-1-2	LOC375190	0.32160856	mirna_pc
2110	hsa-mir-1-2	MPP2	0.432213601	mirna_pc
2111	hsa-mir-1-2	DENND2A	0.560907635	mirna_pc
2112	hsa-mir-1-2	F13A1	0.432141101	mirna_pc
2113	hsa-mir-1-2	CYBRD1	0.539562627	mirna_pc
2114	hsa-mir-1-2	POLK	0.302336575	mirna_pc
2115	hsa-mir-1-2	KCND3	0.648775962	mirna_pc
2116	hsa-mir-1-2	LRCH2	0.606888339	mirna_pc
2117	hsa-mir-1-2	FGL2	0.627164506	mirna_pc
2118	hsa-mir-1-2	CNTN4	0.392954163	mirna_pc
2119	hsa-mir-1-2	PCMTD1	0.337525231	mirna_pc
2120	hsa-mir-1-2	TSC22D1	0.308155307	mirna_pc
2121	hsa-mir-1-2	SCMH1	0.350778426	mirna_pc
2122	hsa-mir-1-2	FXYD6	0.577287693	mirna_pc
2123	hsa-mir-1-2	TRAK2	0.508227869	mirna_pc
2124	hsa-mir-1-2	GAB1	0.429997249	mirna_pc
2125	hsa-mir-1-2	ALS2CR8	0.324675708	mirna_pc
2126	hsa-mir-1-2	MAP3K3	0.556879246	mirna_pc
2127	hsa-mir-1-2	IQSEC1	0.318168394	mirna_pc
2128	hsa-mir-1-2	GIMAP6	0.326963068	mirna_pc
2129	hsa-mir-1-2	ILK	0.546359382	mirna_pc
2130	hsa-mir-1-2	TPM2	0.7858284	mirna_pc
2131	hsa-mir-1-2	MKL2	0.415436437	mirna_pc
2132	hsa-mir-1-2	LRRK2	0.538321693	mirna_pc
2133	hsa-mir-1-2	FOXP2	0.607633209	mirna_pc
2134	hsa-mir-1-2	GGTA1	0.447675465	mirna_pc
2135	hsa-mir-1-2	ASB2	0.718752152	mirna_pc
2136	hsa-mir-1-2	TNRC6C	0.386946407	mirna_pc
2137	hsa-mir-1-2	GIMAP5	0.300445485	mirna_pc
2138	hsa-mir-1-2	ZZEF1	0.303398452	mirna_pc
2139	hsa-mir-1-2	CRYBG3	0.422490514	mirna_pc
2140	hsa-mir-1-2	DIP2C	0.69884492	mirna_pc
2141	hsa-mir-1-2	SBF2	0.348372303	mirna_pc
2142	hsa-mir-1-2	KIAA0753	0.601563374	mirna_pc
2143	hsa-mir-1-2	ZNF295	0.424123524	mirna_pc
2144	hsa-mir-1-2	TAGLN	0.834197208	mirna_pc
2145	hsa-mir-1-2	BNC2	0.642704886	mirna_pc
2146	hsa-mir-1-2	TNFAIP8L3	0.443602483	mirna_pc
2147	hsa-mir-1-2	RGS2	0.640682678	mirna_pc
2148	hsa-mir-1-2	TMEM88	0.349596545	mirna_pc
2149	hsa-mir-1-2	C5orf41	0.408946997	mirna_pc
2150	hsa-mir-1-2	SH3BGRL	0.587036382	mirna_pc
2151	hsa-mir-1-2	MEF2D	0.401742428	mirna_pc
2152	hsa-mir-1-2	ITGB3	0.593421836	mirna_pc
2153	hsa-mir-1-2	PPP1R14A	0.640498797	mirna_pc
2154	hsa-mir-1-2	ZFAND5	0.49488235	mirna_pc
2155	hsa-mir-1-2	TEF	0.319994716	mirna_pc
2156	hsa-mir-1-2	REV3L	0.423282464	mirna_pc
2157	hsa-mir-1-2	BMPR1B	0.347483735	mirna_pc
2158	hsa-mir-1-2	MAPRE2	0.301894385	mirna_pc
2159	hsa-mir-1-2	GAS6	0.357272965	mirna_pc

2160	hsa-mir-1-2	IPP		0.337669457	mirna_pc
2161	hsa-mir-1-2	C3orf70		0.666510159	mirna_pc
2162	hsa-mir-1-2	ARHGEF6		0.369215227	mirna_pc
2163	hsa-mir-1-2	MYL9		0.839146734	mirna_pc
2164	hsa-mir-1-2	FAM63B		0.452461334	mirna_pc
2165	hsa-mir-1-2	DOCK3		0.661156653	mirna_pc
2166	hsa-mir-1-2		2-Mar	0.510729269	mirna_pc
2167	hsa-mir-1-2	ACTA2		0.858809912	mirna_pc
2168	hsa-mir-1-2	ARID5B		0.502666466	mirna_pc
2169	hsa-mir-1-2	PDLIM3		0.808444767	mirna_pc
2170	hsa-mir-1-2	COLEC12		0.65390418	mirna_pc
2171	hsa-mir-1-2	NFASC		0.733443678	mirna_pc
2172	hsa-mir-1-2	ZNF671		0.319221409	mirna_pc
2173	hsa-mir-1-2	MMRN2		0.52253649	mirna_pc
2174	hsa-mir-1-2	MYO1C		0.348394853	mirna_pc
2175	hsa-mir-1-2	ZNF248		0.305972844	mirna_pc
2176	hsa-mir-1-2	NR2F1		0.402000289	mirna_pc
2177	hsa-mir-1-2	ARHGEF15		0.470965772	mirna_pc
2178	hsa-mir-1-2	DNAJB5		0.457198443	mirna_pc
2179	hsa-mir-1-2	LOC401093		0.7943893	mirna_pc
2180	hsa-mir-1-2	FBLN5		0.449969956	mirna_pc
2181	hsa-mir-1-2	TPSAB1		0.348389647	mirna_pc
2182	hsa-mir-1-2	RHOB		0.451964986	mirna_pc
2183	hsa-mir-1-2	TGFBR2		0.376040339	mirna_pc
2184	hsa-mir-1-2	HDAC5		0.357101785	mirna_pc
2185	hsa-mir-1-2	CYP2U1		0.397566029	mirna_pc
2186	hsa-mir-1-2	LOC387647		0.322159656	mirna_pc
2187	hsa-mir-1-2	TPM1		0.782305037	mirna_pc
2188	hsa-mir-1-2	MBD5		0.453432671	mirna_pc
2189	hsa-mir-1-2	MYCT1		0.493468925	mirna_pc
2190	hsa-mir-1-2	MTR		0.37263043	mirna_pc
2191	hsa-mir-1-2	SH3BP5		0.492352388	mirna_pc
2192	hsa-mir-1-2	MPDZ		0.73459988	mirna_pc
2193	hsa-mir-1-2	RUNX1T1		0.365028082	mirna_pc
2194	hsa-mir-1-2	FGF7		0.455688448	mirna_pc
2195	hsa-mir-1-2	S1PR3		0.770348323	mirna_pc
2196	hsa-mir-1-2	LPP		0.788350071	mirna_pc
2197	hsa-mir-1-2	BMPR1A		0.496133153	mirna_pc
2198	hsa-mir-1-2	STON1		0.750889005	mirna_pc
2199	hsa-mir-1-2	LOC399959		0.656092923	mirna_pc
2200	hsa-mir-1-2	GAS7		0.588513174	mirna_pc
2201	hsa-mir-1-2	WDFY3		0.315701997	mirna_pc
2202	hsa-mir-1-2	SOS　2		0.318268029	mirna_pc
2203	hsa-mir-1-2	ROM1		0.464614162	mirna_pc
2204	hsa-mir-1-2	ZNF354C		0.322501285	mirna_pc
2205	hsa-mir-1-2	C21orf63		0.42068118	mirna_pc
2206	hsa-mir-1-2	ABCA10		0.320707963	mirna_pc
2207	hsa-mir-1-2	CADPS2		0.356382822	mirna_pc
2208	hsa-mir-1-2	KRBA2		0.371239635	mirna_pc
2209	hsa-mir-1-2	ROR2		0.417344737	mirna_pc
2210	hsa-mir-1-2	RUSC2		0.4595912	mirna_pc
2211	hsa-mir-1-2	PI4KA		0.323209934	mirna_pc
2212	hsa-mir-1-2	RBL2		0.300878152	mirna_pc
2213	hsa-mir-1-2	PLCD4		0.700977713	mirna_pc

2214	hsa-mir-1-2	STAT5B	0.509288789	mirna_pc
2215	hsa-mir-1-2	RADIL	0.378920144	mirna_pc
2216	hsa-mir-1-2	SPG20	0.41303505	mirna_pc
2217	hsa-mir-1-2	RGS5	0.333062778	mirna_pc
2218	hsa-mir-1-2	MYLK	0.811428535	mirna_pc
2219	hsa-mir-1-2	AVPR1A	0.616919199	mirna_pc
2220	hsa-mir-1-2	ZNF280D	0.307199928	mirna_pc
2221	hsa-mir-1-2	USP20	0.407494009	mirna_pc
2222	hsa-mir-1-2	ATP10A	0.418293626	mirna_pc
2223	hsa-mir-1-2	TIMP3	0.569796543	mirna_pc
2224	hsa-mir-1-2	PER3	0.467500922	mirna_pc
2225	hsa-mir-1-2	MID2	0.37882801	mirna_pc
2226	hsa-mir-1-2	SETD7	0.35923791	mirna_pc
2227	hsa-mir-1-2	ICA1L	0.401751835	mirna_pc
2228	hsa-mir-1-2	PAM	0.428888464	mirna_pc
2229	hsa-mir-1-2	PCDHGB7	0.342685416	mirna_pc
2230	hsa-mir-1-2	FBLN1	0.577726905	mirna_pc
2231	hsa-mir-1-2	GNG11	0.412367823	mirna_pc
2232	hsa-mir-1-2	USP12	0.450293536	mirna_pc
2233	hsa-mir-1-2	NRXN3	0.728961409	mirna_pc
2234	hsa-mir-1-2	PRMT2	0.431877227	mirna_pc
2235	hsa-mir-1-2	CCDC80	0.600175095	mirna_pc
2236	hsa-mir-1-2	DCHS1	0.459214423	mirna_pc
2237	hsa-mir-1-2	GEFT	0.719835112	mirna_pc
2238	hsa-mir-1-2	SAMD11	0.420442897	mirna_pc
2239	hsa-mir-1-2	JMY	0.447361292	mirna_pc
2240	hsa-mir-1-2	LOC284900	0.327993386	mirna_pc
2241	hsa-mir-1-2	NFATC4	0.446502849	mirna_pc
2242	hsa-mir-1-2	LOC92249	0.347953993	mirna_pc
2243	hsa-mir-1-2	REEP2	0.541444214	mirna_pc
2244	hsa-mir-1-2	ZBTB8A	0.337052282	mirna_pc
2245	hsa-mir-1-2	ADCY4	0.400471927	mirna_pc
2246	hsa-mir-1-2	PBX3	0.373176259	mirna_pc
2247	hsa-mir-1-2	DCN	0.569912623	mirna_pc
2248	hsa-mir-1-2	LONRF1	0.382916622	mirna_pc
2249	hsa-mir-1-2	ARHGEF10	0.340383657	mirna_pc
2250	hsa-mir-1-2	PHC1	0.343374336	mirna_pc
2251	hsa-mir-1-2	LTBP3	0.566875664	mirna_pc
2252	hsa-mir-1-2	PCDH7	0.518191789	mirna_pc
2253	hsa-mir-1-2	SYT15	0.415184638	mirna_pc
2254	hsa-mir-1-2	ZYG11B	0.463982298	mirna_pc
2255	hsa-mir-1-2	CTGF	0.617584834	mirna_pc
2256	hsa-mir-1-2	CC2D2A	0.57462438	mirna_pc
2257	hsa-mir-1-2	FAM114A1	0.316090283	mirna_pc
2258	hsa-mir-1-2	GIMAP8	0.374313384	mirna_pc
2259	hsa-mir-1-2	AHNAK	0.444214143	mirna_pc
2260	hsa-mir-1-2	ANXA6	0.564254063	mirna_pc
2261	hsa-mir-1-2	VAT1L	0.320284716	mirna_pc
2262	hsa-mir-1-2	LSM 11.00	0.637089524	mirna_pc
2263	hsa-mir-1-2	ENPP1	0.335915603	mirna_pc
2264	hsa-mir-1-2	LOC644538	0.559827656	mirna_pc
2265	hsa-mir-1-2	SSBP2	0.480421251	mirna_pc
2266	hsa-mir-1-2	SYNC	0.680031837	mirna_pc
2267	hsa-mir-1-2	SYDE2	0.626128082	mirna_pc

2268	hsa-mir-1-2	ULK2	0.352959068	mirna_pc
2269	hsa-mir-1-2	PDE5A	0.726209698	mirna_pc
2270	hsa-mir-1-2	PPM1A	0.316062268	mirna_pc
2271	hsa-mir-1-2	GLIPR2	0.487107362	mirna_pc
2272	hsa-mir-1-2	CPXM2	0.705825794	mirna_pc
2273	hsa-mir-1-2	DNAJC18	0.628954018	mirna_pc
2274	hsa-mir-1-2	ZFYVE21	0.330630098	mirna_pc
2275	hsa-mir-1-2	SAMD4A	0.479700887	mirna_pc
2276	hsa-mir-1-2	HSD17B6	0.330496076	mirna_pc
2277	hsa-mir-1-2	SELP	0.394025967	mirna_pc
2278	hsa-mir-1-2	PTPRB	0.309582569	mirna_pc
2279	hsa-mir-1-2	RUNDC2A	0.315084285	mirna_pc
2280	hsa-mir-1-2	PBX1	0.494638125	mirna_pc
2281	hsa-mir-1-2	ZBTB20	0.45262918	mirna_pc
2282	hsa-mir-1-2	RASL12	0.705308613	mirna_pc
2283	hsa-mir-1-2	PRKD1	0.426590565	mirna_pc
2284	hsa-mir-1-2	OPHN1	0.302704911	mirna_pc
2285	hsa-mir-1-2	PRDM5	0.30044715	mirna_pc
2286	hsa-mir-1-2	LOH3CR2A	0.326391742	mirna_pc
2287	hsa-mir-1-2	PALM2-AKAP2	0.352104797	mirna_pc
2288	hsa-mir-1-2	FAM70A	0.336444903	mirna_pc
2289	hsa-mir-1-2	SOD3	0.572566519	mirna_pc
2290	hsa-mir-1-2	SYNPO	0.493321488	mirna_pc
2291	hsa-mir-1-2	SLC25A12	0.456000381	mirna_pc
2292	hsa-mir-1-2	CXorf36	0.466305031	mirna_pc
2293	hsa-mir-1-2	FOSL2	0.390838367	mirna_pc
2294	hsa-mir-1-2	CALD1	0.80554136	mirna_pc
2295	hsa-mir-1-2	TEAD1	0.531122888	mirna_pc
2296	hsa-mir-1-2	KCNJ8	0.509185905	mirna_pc
2297	hsa-mir-1-2	C1orf21	0.488805329	mirna_pc
2298	hsa-mir-1-2	CNTNAP1	0.610518377	mirna_pc
2299	hsa-mir-1-2	RASSF2	0.413620228	mirna_pc
2300	hsa-mir-1-2	KCTD7	0.344847247	mirna_pc
2301	hsa-mir-1-2	LNPEP	0.411784053	mirna_pc
2302	hsa-mir-1-2	ZEB2	0.419335452	mirna_pc
2303	hsa-mir-1-2	LILRB5	0.353837445	mirna_pc
2304	hsa-mir-1-2	ZFYVE9	0.329218853	mirna_pc
2305	hsa-mir-1-2	RNF38	0.343722092	mirna_pc
2306	hsa-mir-1-2	SMARCA2	0.378435681	mirna_pc
2307	hsa-mir-1-2	TACC2	0.355269136	mirna_pc
2308	hsa-mir-1-2	CREB3L2	0.321354932	mirna_pc
2309	hsa-mir-1-2	SGCB	0.590863525	mirna_pc
2310	hsa-mir-1-2	ESYT2	0.319098208	mirna_pc
2311	hsa-mir-1-2	LOC283174	0.606055949	mirna_pc
2312	hsa-mir-1-2	SFMBT2	0.432889116	mirna_pc
2313	hsa-mir-1-2	ASAM	0.485387003	mirna_pc
2314	hsa-mir-1-2	GNAL	0.470463759	mirna_pc
2315	hsa-mir-1-2	PRKG1	0.679406197	mirna_pc
2316	hsa-mir-1-2	EPAS1	0.305419789	mirna_pc
2317	hsa-mir-1-2	TP53INP2	0.308376024	mirna_pc
2318	hsa-mir-1-2	CUEDC1	0.341023075	mirna_pc
2319	hsa-mir-1-2	C10orf72	0.468223111	mirna_pc
2320	hsa-mir-1-2	ANKS1A	0.563786736	mirna_pc
2321	hsa-mir-1-2	CYB5R3	0.319983283	mirna_pc

2322	hsa-mir-1-2	EBF1	0.499412042	mirna_pc
2323	hsa-mir-1-2	C9orf172	0.498817977	mirna_pc
2324	hsa-mir-1-2	KCTD2	0.309185231	mirna_pc
2325	hsa-mir-1-2	RBMS3	0.543097953	mirna_pc
2326	hsa-mir-1-2	TGFB1I1	0.672264182	mirna_pc
2327	hsa-mir-1-2	TRPC1	0.56125839	mirna_pc
2328	hsa-mir-1-2	KLF11	0.312729702	mirna_pc
2329	hsa-mir-1-2	ZNF704	0.307658894	mirna_pc
2330	hsa-mir-1-2	SUN2	0.409446278	mirna_pc
2331	hsa-mir-1-2	RHOJ	0.500334438	mirna_pc
2332	hsa-mir-1-2	VPS24	0.388958625	mirna_pc
2333	hsa-mir-1-2	EPHA3	0.430934347	mirna_pc
2334	hsa-mir-1-2	CRTAP	0.484596441	mirna_pc
2335	hsa-mir-1-2	ABLIM3	0.311370854	mirna_pc
2336	hsa-mir-1-2	SCN1B	0.310640965	mirna_pc
2337	hsa-mir-1-2	EEF2K	0.320770519	mirna_pc
2338	hsa-mir-1-2	AGL	0.392839075	mirna_pc
2339	hsa-mir-1-2	UHRF1BP1L	0.310578383	mirna_pc
2340	hsa-mir-1-2	GREB1	0.352947479	mirna_pc
2341	hsa-mir-1-2	THBS1	0.661768613	mirna_pc
2342	hsa-mir-1-2	ITPKB	0.600900361	mirna_pc
2343	hsa-mir-1-2	CD99L2	0.389211191	mirna_pc
2344	hsa-mir-1-2	IL1R1	0.389235319	mirna_pc
2345	hsa-mir-1-2	PPP1R12A	0.731356942	mirna_pc
2346	hsa-mir-1-2	PPM1M	0.324648785	mirna_pc
2347	hsa-mir-1-2	PITPNM3	0.450612256	mirna_pc
2348	hsa-mir-1-2	PALLD	0.774594394	mirna_pc
2349	hsa-mir-1-2	PKD2	0.611431906	mirna_pc
2350	hsa-mir-1-2	SMG6	0.339581767	mirna_pc
2351	hsa-mir-1-2	SLC8A1	0.643103772	mirna_pc
2352	hsa-mir-1-2	FBLN2	0.451616609	mirna_pc
2353	hsa-mir-1-2	NDN	0.339851909	mirna_pc
2354	hsa-mir-1-2	TCEAL4	0.389224816	mirna_pc
2355	hsa-mir-1-2	MAP1A	0.597206326	mirna_pc
2356	hsa-mir-1-2	MAP1B	0.556137368	mirna_pc
2357	hsa-mir-1-2	FAM184A	0.509077095	mirna_pc
2358	hsa-mir-1-2	RAB11FIP3	0.42093671	mirna_pc
2359	hsa-mir-1-2	CNTN1	0.369525126	mirna_pc
2360	hsa-mir-1-2	EID1	0.378725834	mirna_pc
2361	hsa-mir-1-2	ZNF154	0.47859031	mirna_pc
2362	hsa-mir-1-2	ATP8B2	0.479973017	mirna_pc
2363	hsa-mir-1-2	PRDM8	0.373793678	mirna_pc
2364	hsa-mir-1-2	CHURC1	0.354795765	mirna_pc
2365	hsa-mir-1-2	CTSO	0.372902674	mirna_pc
2366	hsa-mir-1-2	ZNF333	0.321099181	mirna_pc
2367	hsa-mir-1-2	PTPRS	0.311001925	mirna_pc
2368	hsa-mir-1-2	RERG	0.326019722	mirna_pc
2369	hsa-mir-1-2	MGP	0.487443542	mirna_pc
2370	hsa-mir-1-2	C9orf102	0.331737373	mirna_pc
2371	hsa-mir-1-2	NPTXR	0.302140613	mirna_pc
2372	hsa-mir-1-2	HECTD2	0.452852605	mirna_pc
2373	hsa-mir-1-2	CYTSA	0.328471171	mirna_pc
2374	hsa-mir-1-2	TTC23	0.410782786	mirna_pc
2375	hsa-mir-1-2	ZEB1	0.71002639	mirna_pc

2376	hsa-mir-1-2	ARID5A	0.61061746	mirna_pc
2377	hsa-mir-1-2	AQP1	0.651778622	mirna_pc
2378	hsa-mir-1-2	TES	0.359173859	mirna_pc
2379	hsa-mir-1-2	COL4A6	0.591802849	mirna_pc
2380	hsa-mir-1-2	CD34	0.510125695	mirna_pc
2381	hsa-mir-1-2	RORA	0.325514824	mirna_pc
2382	hsa-mir-1-2	RGMA	0.546935973	mirna_pc
2383	hsa-mir-1-2	ELN	0.396695153	mirna_pc
2384	hsa-mir-1-2	SNCG	0.38543334	mirna_pc
2385	hsa-mir-1-2	ARHGAP31	0.416693135	mirna_pc
2386	hsa-mir-1-2	FOXO1	0.429872155	mirna_pc
2387	hsa-mir-1-2	MAP4	0.378279693	mirna_pc
2388	hsa-mir-1-2	ADAMTS9	0.45331312	mirna_pc
2389	hsa-mir-1-2	OCRL	0.324630443	mirna_pc
2390	hsa-mir-1-2	TMOD2	0.40603594	mirna_pc
2391	hsa-mir-1-2	WWC3	0.362609187	mirna_pc
2392	hsa-mir-1-2	SPRY1	0.482811234	mirna_pc
2393	hsa-mir-1-2	FAM46B	0.327145424	mirna_pc
2394	hsa-mir-1-2	CBX6	0.39892107	mirna_pc
2395	hsa-mir-1-2	BACE1	0.314909877	mirna_pc
2396	hsa-mir-1-2	PCDHGA12	0.500053457	mirna_pc
2397	hsa-mir-1-2	C10orf10	0.41032021	mirna_pc
2398	hsa-mir-1-2	SPEG	0.784284799	mirna_pc
2399	hsa-mir-1-2	FOXF2	0.652143347	mirna_pc
2400	hsa-mir-1-2	FBXO30	0.575939306	mirna_pc
2401	hsa-mir-1-2	EVC	0.470342201	mirna_pc
2402	hsa-mir-1-2	CNTNAP3	0.569330074	mirna_pc
2403	hsa-mir-1-2	GPR124	0.326279659	mirna_pc
2404	hsa-mir-1-2	ATF7	0.336740494	mirna_pc
2405	hsa-mir-1-2	ST3GAL3	0.325758358	mirna_pc
2406	hsa-mir-1-2	FIBIN	0.477251303	mirna_pc
2407	hsa-mir-1-2	ZFP36	0.317076887	mirna_pc
2408	hsa-mir-1-2	C3orf64	0.541007578	mirna_pc
2409	hsa-mir-1-2	MED13L	0.321645601	mirna_pc
2410	hsa-mir-1-2	PIK3R3	0.307867575	mirna_pc
2411	hsa-mir-1-2	LOC144571	0.510584855	mirna_pc
2412	hsa-mir-1-2	H6PD	0.322324192	mirna_pc
2413	hsa-mir-1-2	CLIC4	0.625277724	mirna_pc
2414	hsa-mir-1-2	CFH	0.480532079	mirna_pc
2415	hsa-mir-1-2	MEIS1	0.549473367	mirna_pc
2416	hsa-mir-1-2	ANKRD44	0.317934257	mirna_pc
2417	hsa-mir-1-2	C18orf1	0.431430462	mirna_pc
2418	hsa-mir-1-2	EPHA7	0.343899781	mirna_pc
2419	hsa-mir-1-2	RASA4	0.367363425	mirna_pc
2420	hsa-mir-1-2	PTGFR	0.454988538	mirna_pc
2421	hsa-mir-1-2	A2M	0.557421804	mirna_pc
2422	hsa-mir-1-2	FLNA	0.771979012	mirna_pc
2423	hsa-mir-1-2	KCTD10	0.639129478	mirna_pc
2424	hsa-mir-1-2	DGKG	0.585177218	mirna_pc
2425	hsa-mir-1-2	PHLDB1	0.399500234	mirna_pc
2426	hsa-mir-1-2	RABGAP1	0.43128492	mirna_pc
2427	hsa-mir-1-2	LATS2	0.537376731	mirna_pc
2428	hsa-mir-1-2	C14orf45	0.328706995	mirna_pc
2429	hsa-mir-1-2	ACTC1	0.451946274	mirna_pc

2430	hsa-mir-1-2	EBF4	0.347921795	mirna_pc
2431	hsa-mir-1-2	SAMHD1	0.522380498	mirna_pc
2432	hsa-mir-1-2	TMTC1	0.317001563	mirna_pc
2433	hsa-mir-1-2	PCOLCE2	0.427169879	mirna_pc
2434	hsa-mir-1-2	AXL	0.347180622	mirna_pc
2435	hsa-mir-1-2	FAM168A	0.325490159	mirna_pc
2436	hsa-mir-1-2	CCDC50	0.316018288	mirna_pc
2437	hsa-mir-1-2	ANKRD13A	0.332101116	mirna_pc
2438	hsa-mir-1-2	ARHGEF17	0.574106306	mirna_pc
2439	hsa-mir-1-2	ADAMTS5	0.370118474	mirna_pc
2440	hsa-mir-1-2	DAAM1	0.362258685	mirna_pc
2441	hsa-mir-1-2	GPC6	0.406091756	mirna_pc
2442	hsa-mir-1-2	TYRP1	0.359216778	mirna_pc
2443	hsa-mir-1-2	COL4A5	0.33194511	mirna_pc
2444	hsa-mir-1-2	PKIG	0.555167872	mirna_pc
2445	hsa-mir-1-2	GEM	0.393449434	mirna_pc
2446	hsa-mir-1-2	FAM20C	0.340756111	mirna_pc
2447	hsa-mir-1-2	VWF	0.321441744	mirna_pc
2448	hsa-mir-1-2	ALDH1B1	0.569449282	mirna_pc
2449	hsa-mir-1-2	C6orf145	0.30825619	mirna_pc
2450	hsa-mir-1-2	KIAA1614	0.345523406	mirna_pc
2451	hsa-mir-1-2	C4orf3	0.300760749	mirna_pc
2452	hsa-mir-1-2	SYT11	0.356006	mirna_pc
2453	hsa-mir-1-2	MYL6	0.419722223	mirna_pc
2454	hsa-mir-1-2	PLEKHO2	0.555602005	mirna_pc
2455	hsa-mir-1-2	ERG	0.303284139	mirna_pc
2456	hsa-mir-1-2	CSPG4	0.349531727	mirna_pc
2457	hsa-mir-1-2	OLFML1	0.419376062	mirna_pc
2458	hsa-mir-1-2	OLFML3	0.353994665	mirna_pc
2459	hsa-mir-1-2	NEK9	0.440531643	mirna_pc
2460	hsa-mir-1-2	CEP68	0.359138683	mirna_pc
2461	hsa-mir-1-2	ARSB	0.517078213	mirna_pc
2462	hsa-mir-1-2	MYADM	0.322062837	mirna_pc
2463	hsa-mir-1-2	THBS3	0.518750461	mirna_pc
2464	hsa-mir-1-2	PECAM1	0.353693825	mirna_pc
2465	hsa-mir-1-2	TSPAN18	0.374259683	mirna_pc
2466	hsa-mir-1-2	WWTR1	0.604253913	mirna_pc
2467	hsa-mir-1-2	GFPT2	0.303402997	mirna_pc
2468	hsa-mir-1-2	CCDC3	0.351831756	mirna_pc
2469	hsa-mir-1-2	CCDC136	0.454709053	mirna_pc
2470	hsa-mir-1-2	DENND5A	0.50529229	mirna_pc
2471	hsa-mir-1-2	PHF21A	0.316097472	mirna_pc
2472	hsa-mir-1-2	SSPN	0.497366032	mirna_pc
2473	hsa-mir-1-2	IGSF9B	0.575593764	mirna_pc
2474	hsa-mir-1-2	GABBR1	0.387291213	mirna_pc
2475	hsa-mir-1-2	ATXN1	0.326141283	mirna_pc
2476	hsa-mir-1-2	KIF1B	0.342282517	mirna_pc
2477	hsa-mir-1-2	ECSCR	0.390795769	mirna_pc
2478	hsa-mir-1-2	JAZF1	0.461218363	mirna_pc
2479	hsa-mir-1-2	HSPG2	0.40249712	mirna_pc
2480	hsa-mir-1-2	CDK17	0.481106889	mirna_pc
2481	hsa-mir-1-2	MATN2	0.386259088	mirna_pc
2482	hsa-mir-1-2	ZFHX3	0.307605813	mirna_pc
2483	hsa-mir-1-2	LIX1L	0.43153256	mirna_pc

2484	hsa-mir-1-2	RBM9	0.48428026	mirna_pc
2485	hsa-mir-1-2	FAM124A	0.651992835	mirna_pc
2486	hsa-mir-1-2	SELM	0.500836672	mirna_pc
2487	hsa-mir-1-2	AMOTL1	0.527304919	mirna_pc
2488	hsa-mir-1-2	KIAA1377	0.343826135	mirna_pc
2489	hsa-mir-1-2	HSPA2	0.451897287	mirna_pc
2490	hsa-mir-1-2	MCAM	0.564217602	mirna_pc
2491	hsa-mir-1-2	LHFP	0.50635315	mirna_pc
2492	hsa-mir-1-2	IGSF1	0.323123593	mirna_pc
2493	hsa-mir-1-2	LOC653653	0.604277297	mirna_pc
2494	hsa-mir-1-2	SHANK3	0.368452171	mirna_pc
2495	hsa-mir-1-2	SCARA3	0.316694174	mirna_pc
2496	hsa-mir-1-2	RNF122	0.386701313	mirna_pc
2497	hsa-mir-1-2	PRDM11	0.311689341	mirna_pc
2498	hsa-mir-1-2	GPR135	0.462694829	mirna_pc
2499	hsa-mir-1-2	SVIL	0.791004158	mirna_pc
2500	hsa-mir-1-2	TOX2	0.358359075	mirna_pc
2501	hsa-mir-1-2	WHAMML1	0.48655235	mirna_pc
2502	hsa-mir-1-2	AKT3	0.483777318	mirna_pc
2503	hsa-mir-1-2	CDH5	0.350493774	mirna_pc
2504	hsa-mir-1-2	VCL	0.620737772	mirna_pc
2505	hsa-mir-1-2	ANTXR2	0.415656795	mirna_pc
2506	hsa-mir-1-2	PALMD	0.311349406	mirna_pc
2507	hsa-mir-1-2	NT5DC3	0.50497853	mirna_pc
2508	hsa-mir-1-2	TIE1	0.396373695	mirna_pc
2509	hsa-mir-1-2	ZNF34	0.315595135	mirna_pc
2510	hsa-mir-1-2	TBC1D1	0.501143937	mirna_pc
2511	hsa-mir-1-2	MEF2C	0.431355795	mirna_pc
2512	hsa-mir-1-2	PMP22	0.458259954	mirna_pc
2513	hsa-mir-1-2	ST8SIA1	0.309538451	mirna_pc
2514	hsa-mir-1-2	NUDT4	0.381163625	mirna_pc
2515	hsa-mir-1-2	ITGA1	0.437757209	mirna_pc
2516	hsa-mir-1-2	TBX5	0.398773827	mirna_pc
2517	hsa-mir-1-2	STX2	0.34458735	mirna_pc
2518	hsa-mir-1-2	CCDC46	0.445044113	mirna_pc
2519	hsa-mir-1-2	PDE3A	0.670032502	mirna_pc
2520	hsa-mir-1-2	PTRF	0.508871495	mirna_pc
2521	hsa-mir-1-2	LRRC32	0.32390858	mirna_pc
2522	hsa-mir-1-2	SORCS2	0.425729489	mirna_pc
2523	hsa-mir-1-2	PLSCR4	0.391346645	mirna_pc
2524	hsa-mir-1-2	CRIM1	0.373763762	mirna_pc
2525	hsa-mir-1-2	NPR 2.00	0.301049047	mirna_pc
2526	hsa-mir-1-2	CRIP2	0.300128848	mirna_pc
2527	hsa-mir-1-2	TSHZ3	0.453642487	mirna_pc
2528	hsa-mir-1-2	RASL11A	0.320473305	mirna_pc
2529	hsa-mir-1-2	LHX6	0.356444614	mirna_pc
2530	hsa-mir-1-2	CAMK2G	0.517350201	mirna_pc
2531	hsa-mir-1-2	CAMK1	0.329021766	mirna_pc
2532	hsa-mir-1-2	CMYA5	0.336039812	mirna_pc
2533	hsa-mir-1-2	FAM127C	0.326099546	mirna_pc
2534	hsa-mir-1-2	HSPA12B	0.40044793	mirna_pc
2535	hsa-mir-1-2	C14orf4	0.346753609	mirna_pc
2536	hsa-mir-1-2	KLF10	0.310751363	mirna_pc
2537	hsa-mir-1-2	CD93	0.324073818	mirna_pc

2538	hsa-mir-1-2	SERPINF1	0.399069785	mirna_pc
2539	hsa-mir-1-2	C3	0.342294512	mirna_pc
2540	hsa-mir-1-2	DPYSL3	0.688778488	mirna_pc
2541	hsa-mir-1-2	KIAA0355	0.332327203	mirna_pc
2542	hsa-mir-1-2	ATP2B4	0.669863353	mirna_pc
2543	hsa-mir-1-2	PROS1	0.45176612	mirna_pc
2544	hsa-mir-1-2	DDX24	0.30987146	mirna_pc
2545	hsa-mir-1-2	TIMP2	0.395257058	mirna_pc
2546	hsa-mir-1-2	FBN1	0.457770286	mirna_pc
2547	hsa-mir-1-2	ADH5	0.363645427	mirna_pc
2548	hsa-mir-1-2	SHROOM4	0.346667908	mirna_pc
2549	hsa-mir-1-2	NOD1	0.307863298	mirna_pc
2550	hsa-mir-1-2	AP1S2	0.588376568	mirna_pc
2551	hsa-mir-1-2	DOCK1	0.357474365	mirna_pc
2552	hsa-mir-1-2	KLC1	0.312740741	mirna_pc
2553	hsa-mir-1-2	CREM	0.341094005	mirna_pc
2554	hsa-mir-1-2	MXRA7	0.415048317	mirna_pc
2555	hsa-mir-1-2	IFFO1	0.361111014	mirna_pc
2556	hsa-mir-1-2	C5orf36	0.338209422	mirna_pc
2557	hsa-mir-1-2	PCNX	0.389484158	mirna_pc
2558	hsa-mir-1-2	C1RL	0.339516087	mirna_pc
2559	hsa-mir-1-2	MEOX1	0.490432126	mirna_pc
2560	hsa-mir-1-2	TTBK2	0.356416658	mirna_pc
2561	hsa-mir-1-2	ADCY9	0.484762434	mirna_pc
2562	hsa-mir-1-2	RASD2	0.510216293	mirna_pc
2563	hsa-mir-1-2	CD81	0.329018801	mirna_pc
2564	hsa-mir-1-2	VIM	0.339672157	mirna_pc
2565	hsa-mir-1-2	MN1	0.3257003	mirna_pc
2566	hsa-mir-1-2	SDC3	0.395499253	mirna_pc
2567	hsa-mir-1-2	PNMA1	0.334824852	mirna_pc
2568	hsa-mir-1-2	FAM83D	0.510379155	mirna_pc
2569	hsa-mir-1-2	ZAK	0.696383403	mirna_pc
2570	hsa-mir-1-2	IGFBP4	0.323297574	mirna_pc
2571	hsa-mir-1-2	RAB23	0.480641311	mirna_pc
2572	hsa-mir-1-2	LTBP1	0.374384203	mirna_pc
2573	hsa-mir-1-2	DOCK11	0.443010719	mirna_pc
2574	hsa-mir-1-2	MAP3K14	0.332745956	mirna_pc
2575	hsa-mir-1-2	STAB1	0.328100186	mirna_pc
2576	hsa-mir-1-2	TM6SF1	0.334058323	mirna_pc
2577	hsa-mir-1-2	EHBP1L1	0.378154771	mirna_pc
2578	hsa-mir-1-2	FLJ42709	0.402197432	mirna_pc
2579	hsa-mir-1-2	TUBA1A	0.543345051	mirna_pc
2580	hsa-mir-1-2	ELTD1	0.322997337	mirna_pc
2581	hsa-mir-1-2	SERPING1	0.390065618	mirna_pc
2582	hsa-mir-1-2	GFOD1	0.437424839	mirna_pc
2583	hsa-mir-1-2	LRP1	0.392650124	mirna_pc
2584	hsa-mir-1-2	ADAMTS4	0.356383001	mirna_pc
2585	hsa-mir-1-2	FTO	0.35054441	mirna_pc
2586	hsa-mir-1-2	ATL3	0.31930911	mirna_pc
2587	hsa-mir-1-2	MAP3K12	0.338999453	mirna_pc
2588	hsa-mir-1-2	COL15A1	0.31754362	mirna_pc
2589	hsa-mir-1-2	RHOQ	0.345917599	mirna_pc
2590	hsa-mir-1-2	ADAMTS10	0.365449536	mirna_pc
2591	hsa-mir-1-2	CRTC3	0.407266732	mirna_pc

2592	hsa-mir-1-2	SFRP2	0.398082671	mirna_pc
2593	hsa-mir-1-2	NCS1	0.512759886	mirna_pc
2594	hsa-mir-1-2	SACS	0.433965421	mirna_pc
2595	hsa-mir-1-2	SEC23A	0.384517147	mirna_pc
2596	hsa-mir-1-2	LAMA4	0.404736713	mirna_pc
2597	hsa-mir-1-2	PCDHGA6	0.316952768	mirna_pc
2598	hsa-mir-1-2	PTPLAD2	0.327349348	mirna_pc
2599	hsa-mir-1-2	CDC42EP3	0.325956659	mirna_pc
2600	hsa-mir-1-2	HSPA12A	0.416675713	mirna_pc
2601	hsa-mir-1-2	GREM1	0.452026607	mirna_pc
2602	hsa-mir-1-2	SESTD1	0.313641397	mirna_pc
2603	hsa-mir-1-2	HMCN1	0.61546874	mirna_pc
2604	hsa-mir-1-2	TEAD3	0.415759968	mirna_pc
2605	hsa-mir-1-2	ENTPD1	0.381826113	mirna_pc
2606	hsa-mir-1-2	ECM2	0.414202574	mirna_pc
2607	hsa-mir-1-2	NRP2	0.482679185	mirna_pc
2608	hsa-mir-1-2	BMPR2	0.31982592	mirna_pc
2609	hsa-mir-1-2	ANO6	0.397672765	mirna_pc
2610	hsa-mir-1-2	RAB30	0.320075909	mirna_pc
2611	hsa-mir-1-2	IGFBP7	0.324333554	mirna_pc
2612	hsa-mir-1-2	RGL1	0.382947902	mirna_pc
2613	hsa-mir-1-2	COL6A2	0.335010247	mirna_pc
2614	hsa-mir-1-2	FSTL1	0.40064072	mirna_pc
2615	hsa-mir-1-2	PLEKHO1	0.51928756	mirna_pc
2616	hsa-mir-1-2	TWSG1	0.301693479	mirna_pc
2617	hsa-mir-1-2	KLHDC5	0.366190054	mirna_pc
2618	hsa-mir-1-2	DYSF	0.383960595	mirna_pc
2619	hsa-mir-1-2	BCL6	0.363528267	mirna_pc
2620	hsa-mir-1-2	EHD2	0.358588211	mirna_pc
2621	hsa-mir-1-2	TCP11L1	0.327306605	mirna_pc
2622	hsa-mir-1-2	NFIL3	0.386886389	mirna_pc
2623	hsa-mir-1-2	TMEM43	0.365968884	mirna_pc
2624	hsa-mir-1-2	HEG1	0.300694384	mirna_pc
2625	hsa-mir-1-2	SLC4A3	0.438272026	mirna_pc
2626	hsa-mir-1-2	TSPAN4	0.302975548	mirna_pc
2627	hsa-mir-1-2	AFAP1	0.329047998	mirna_pc
2628	hsa-mir-1-2	FAM198B	0.30333088	mirna_pc
2629	hsa-mir-1-2	C4A	0.369242663	mirna_pc
2630	hsa-mir-1-2	CAP2	0.599515846	mirna_pc
2631	hsa-mir-1-2	SOCS3	0.398384352	mirna_pc
2632	hsa-mir-1-2	BICC1	0.342243471	mirna_pc
2633	hsa-mir-1-2	NDE1	0.349146158	mirna_pc
2634	hsa-mir-1-2	SH3PXD2B	0.38128591	mirna_pc
2635	hsa-mir-1-2	HOXA4	0.414366165	mirna_pc
2636	hsa-mir-1-2	C1R	0.405710481	mirna_pc
2637	hsa-mir-1-2	PRKG2	0.502063593	mirna_pc
2638	hsa-mir-1-2	MAP7D3	0.325808944	mirna_pc
2639	hsa-mir-1-2	KATNAL1	0.41026245	mirna_pc
2640	hsa-mir-1-2	STOM	0.393887528	mirna_pc
2641	hsa-mir-1-2	KCTD20	0.314861566	mirna_pc
2642	hsa-mir-1-2	PDLIM7	0.339144598	mirna_pc
2643	hsa-mir-1-2	NLGN2	0.36915598	mirna_pc
2644	hsa-mir-1-2	SYDE1	0.338673914	mirna_pc
2645	hsa-mir-1-2	NKX6-1	0.662344372	mirna_pc

2646	hsa-mir-1-2	FAM49A	0.330783029	mirna_pc
2647	hsa-mir-1-2	ACTN1	0.437973394	mirna_pc
2648	hsa-mir-1-2	ARMC9	0.460298689	mirna_pc
2649	hsa-mir-1-2	GUCY1B3	0.516710179	mirna_pc
2650	hsa-mir-1-2	FBXO32	0.588689544	mirna_pc
2651	hsa-mir-1-2	KLHL5	0.369879398	mirna_pc
2652	hsa-mir-1-2	MFGE8	0.324891823	mirna_pc
2653	hsa-mir-1-2	GBP2	0.309647261	mirna_pc
2654	hsa-mir-1-2	GUCY1A3	0.52573768	mirna_pc
2655	hsa-mir-93	MKI67	0.410994961	mirna_pc
2656	hsa-mir-93	TPX2	0.402634073	mirna_pc
2657	hsa-mir-93	KIF4B	0.403994306	mirna_pc
2658	hsa-mir-93	CENPF	0.561994517	mirna_pc
2659	hsa-mir-93	RCC2	0.442097426	mirna_pc
2660	hsa-mir-93	HOXC9	0.308360338	mirna_pc
2661	hsa-mir-93	KIF11	0.513668196	mirna_pc
2662	hsa-mir-93	ECT2	0.34730559	mirna_pc
2663	hsa-mir-93	FOXM1	0.433938631	mirna_pc
2664	hsa-mir-93	RRM2	0.388208848	mirna_pc
2665	hsa-mir-93	CDK1	0.402189799	mirna_pc
2666	hsa-mir-93	CDC25C	0.345485886	mirna_pc
2667	hsa-mir-93	MYBL2	0.322855884	mirna_pc
2668	hsa-mir-93	PLK1	0.336120715	mirna_pc
2669	hsa-mir-93	UBE2C	0.378031655	mirna_pc
2670	hsa-mir-93	BUB1	0.441478462	mirna_pc
2671	hsa-mir-93	PRC1	0.309449589	mirna_pc
2672	hsa-mir-93	NUSAP1	0.440660956	mirna_pc
2673	hsa-mir-93	MCM4	0.387885928	mirna_pc
2674	hsa-mir-93	KIFC1	0.400632547	mirna_pc
2675	hsa-mir-93	BUB1B	0.463290478	mirna_pc
2676	hsa-mir-93	HDGF	0.397890846	mirna_pc
2677	hsa-mir-93	KIF18B	0.394484213	mirna_pc
2678	hsa-mir-93	ASPM	0.493842882	mirna_pc
2679	hsa-mir-93	KIF2C	0.418724958	mirna_pc
2680	hsa-mir-93	FANCA	0.407183495	mirna_pc
2681	hsa-mir-93	NCAPG	0.470704621	mirna_pc
2682	hsa-mir-93	CLSPN	0.354703391	mirna_pc
2683	hsa-mir-93	SGOL1	0.429191773	mirna_pc
2684	hsa-mir-93	ARHGAP11A	0.37028841	mirna_pc
2685	hsa-mir-93	CCNA2	0.542367035	mirna_pc
2686	hsa-mir-93	SPC25	0.461076438	mirna_pc
2687	hsa-mir-93	NUP62	0.358719934	mirna_pc
2688	hsa-mir-93	FANCI	0.445637728	mirna_pc
2689	hsa-mir-93	NEK2	0.518349239	mirna_pc
2690	hsa-mir-93	MND1	0.566637412	mirna_pc
2691	hsa-mir-93	KIF22	0.407644542	mirna_pc
2692	hsa-mir-93	NCAPH	0.491201941	mirna_pc
2693	hsa-mir-93	GTSE1	0.424296585	mirna_pc
2694	hsa-mir-93	KIF23	0.366394046	mirna_pc
2695	hsa-mir-93	RAD54L	0.530395434	mirna_pc
2696	hsa-mir-93	CENPA	0.418846898	mirna_pc
2697	hsa-mir-93	MAD2L1	0.57910927	mirna_pc
2698	hsa-mir-93	TIMELESS	0.562256616	mirna_pc
2699	hsa-mir-93	LMNB1	0.490145061	mirna_pc

2700	hsa-mir-93	ASF1B	0.319749275	mirna_pc
2701	hsa-mir-93	CDCA8	0.403889521	mirna_pc
2702	hsa-mir-93	TROAP	0.46797362	mirna_pc
2703	hsa-mir-93	CDCA5	0.421329844	mirna_pc
2704	hsa-mir-93	TACC3	0.324158387	mirna_pc
2705	hsa-mir-93	XPO1	0.546233201	mirna_pc
2706	hsa-mir-93	CDC45	0.341135545	mirna_pc
2707	hsa-mir-93	STMN1	0.464731377	mirna_pc
2708	hsa-mir-93	EXO1	0.429790253	mirna_pc
2709	hsa-mir-93	CDCA2	0.44079886	mirna_pc
2710	hsa-mir-93	STIL	0.318877517	mirna_pc
2711	hsa-mir-93	DKC1	0.308423278	mirna_pc
2712	hsa-mir-93	UBE2T	0.317487677	mirna_pc
2713	hsa-mir-93	CKS1B	0.36008901	mirna_pc
2714	hsa-mir-93	TUBB	0.393769715	mirna_pc
2715	hsa-mir-93	MCM2	0.401463068	mirna_pc
2716	hsa-mir-93	KIF4A	0.374971432	mirna_pc
2717	hsa-mir-93	ORC1L	0.453559815	mirna_pc
2718	hsa-mir-93	KNTC1	0.488872044	mirna_pc
2719	hsa-mir-93	UHRF1	0.323503672	mirna_pc
2720	hsa-mir-93	CCNF	0.443407897	mirna_pc
2721	hsa-mir-93	CENPI	0.313260229	mirna_pc
2722	hsa-mir-93	CCNB2	0.354526588	mirna_pc
2723	hsa-mir-93	NUF2	0.528385926	mirna_pc
2724	hsa-mir-93	SFRP1	0.490130907	mirna_pc
2725	hsa-mir-93	PLK4	0.643624659	mirna_pc
2726	hsa-mir-93	FANCB	0.448170435	mirna_pc
2727	hsa-mir-93	KIF18A	0.439750982	mirna_pc
2728	hsa-mir-93	DEPDC1	0.480982292	mirna_pc
2729	hsa-mir-93	SKA3	0.337269797	mirna_pc
2730	hsa-mir-93	KIF14	0.448474841	mirna_pc
2731	hsa-mir-93	HJURP	0.428525921	mirna_pc
2732	hsa-mir-93	SKA1	0.365262453	mirna_pc
2733	hsa-mir-93	RAD51	0.383642154	mirna_pc
2734	hsa-mir-93	BLM	0.468197376	mirna_pc
2735	hsa-mir-93	CAPN6	0.30696615	mirna_pc
2736	hsa-mir-93	CKAP2L	0.343519608	mirna_pc
2737	hsa-mir-93	DNMT1	0.392813651	mirna_pc
2738	hsa-mir-93	PAICS	0.420130811	mirna_pc
2739	hsa-mir-93	CCDC150	0.459766879	mirna_pc
2740	hsa-mir-93	BIRC5	0.338240437	mirna_pc
2741	hsa-mir-93	LIG1	0.436427448	mirna_pc
2742	hsa-mir-93	OIP5	0.334679849	mirna_pc
2743	hsa-mir-93	DTL	0.606954586	mirna_pc
2744	hsa-mir-93	EME1	0.427124597	mirna_pc
2745	hsa-mir-93	RECQL4	0.365666172	mirna_pc
2746	hsa-mir-93	AURKA	0.358619844	mirna_pc
2747	hsa-mir-93	CENPK	0.401363522	mirna_pc
2748	hsa-mir-93	AURKB	0.470490111	mirna_pc
2749	hsa-mir-93	GSG2	0.419358854	mirna_pc
2750	hsa-mir-93	MCM10	0.498624093	mirna_pc
2751	hsa-mir-93	SGOL2	0.465141679	mirna_pc
2752	hsa-mir-93	CHEK2	0.392350018	mirna_pc
2753	hsa-mir-93	CENPE	0.434601651	mirna_pc

2754	hsa-mir-93	POLQ	0.433466485	mirna_pc
2755	hsa-mir-93	KIF15	0.501347468	mirna_pc
2756	hsa-mir-93	CDCA3	0.472458654	mirna_pc
2757	hsa-mir-93	C1orf112	0.593068142	mirna_pc
2758	hsa-mir-93	GINS1	0.414223861	mirna_pc
2759	hsa-mir-93	FEN1	0.32734168	mirna_pc
2760	hsa-mir-93	TRAIP	0.332208969	mirna_pc
2761	hsa-mir-93	ORC6L	0.418793306	mirna_pc
2762	hsa-mir-93	PBK	0.359360886	mirna_pc
2763	hsa-mir-93	C16orf59	0.465322894	mirna_pc
2764	hsa-mir-93	C17orf53	0.331161129	mirna_pc
2765	hsa-mir-93	FAM72B	0.328402472	mirna_pc
2766	hsa-mir-93	NEIL3	0.539851672	mirna_pc
2767	hsa-mir-93	MCM7	0.668690584	mirna_pc
2768	hsa-mir-93	ACTL6A	0.330746085	mirna_pc
2769	hsa-mir-93	POLD1	0.345779724	mirna_pc
2770	hsa-mir-93	C12orf48	0.472548958	mirna_pc
2771	hsa-mir-93	HNRNPL	0.385784911	mirna_pc
2772	hsa-mir-93	ZWILCH	0.340414305	mirna_pc
2773	hsa-mir-93	SMC4	0.431134966	mirna_pc
2774	hsa-mir-93	HMMR	0.326755455	mirna_pc
2775	hsa-mir-93	PKMYT1	0.32199815	mirna_pc
2776	hsa-mir-93	TTK	0.41972873	mirna_pc
2777	hsa-mir-93	ZWINT	0.34896858	mirna_pc
2778	hsa-mir-93	HOXC6	0.308058423	mirna_pc
2779	hsa-mir-93	BUB3	0.312237569	mirna_pc
2780	hsa-mir-93	RAD51AP1	0.367717051	mirna_pc
2781	hsa-mir-93	HELLS	0.46212272	mirna_pc
2782	hsa-mir-93	ERCC6L	0.301036961	mirna_pc
2783	hsa-mir-93	NCAPD2	0.529137191	mirna_pc
2784	hsa-mir-93	ILF2	0.322812095	mirna_pc
2785	hsa-mir-93	TCF3	0.304015957	mirna_pc
2786	hsa-mir-93	C6orf167	0.477810203	mirna_pc
2787	hsa-mir-93	BRCA1	0.408198826	mirna_pc
2788	hsa-mir-93	MCM5	0.317567473	mirna_pc
2789	hsa-mir-93	HNRNPA2B1	0.379224299	mirna_pc
2790	hsa-mir-93	TYMS	0.321497019	mirna_pc
2791	hsa-mir-93	RFWD3	0.330861765	mirna_pc
2792	hsa-mir-93	MCM6	0.550114717	mirna_pc
2793	hsa-mir-93	CDCA7	0.387510414	mirna_pc
2794	hsa-mir-93	EPR1	0.387737961	mirna_pc
2795	hsa-mir-93	KIAA1524	0.468486475	mirna_pc
2796	hsa-mir-93	BRIP1	0.478279183	mirna_pc
2797	hsa-mir-93	PIF1	0.343296434	mirna_pc
2798	hsa-mir-93	SFRS2	0.341883765	mirna_pc
2799	hsa-mir-93	UCK2	0.453148813	mirna_pc
2800	hsa-mir-93	NFKBIL2	0.425018391	mirna_pc
2801	hsa-mir-93	C15orf42	0.485043598	mirna_pc
2802	hsa-mir-93	PRIM2	0.322554869	mirna_pc
2803	hsa-mir-93	FAM60A	0.41188629	mirna_pc
2804	hsa-mir-93	FAM64A	0.359003978	mirna_pc
2805	hsa-mir-93	ATAD5	0.503766902	mirna_pc
2806	hsa-mir-93	MTBP	0.364348916	mirna_pc
2807	hsa-mir-93	FAM111B	0.338859248	mirna_pc

2808	hsa-mir-93	KIF20B	0.427543979	mirna_pc
2809	hsa-mir-93	ESCO2	0.397791827	mirna_pc
2810	hsa-mir-93	SNRPA	0.357618198	mirna_pc
2811	hsa-mir-93	FUS	0.425178501	mirna_pc
2812	hsa-mir-93	ATAD2	0.356648071	mirna_pc
2813	hsa-mir-93	DNAJC9	0.327477674	mirna_pc
2814	hsa-mir-93	U2AF2	0.372190664	mirna_pc
2815	hsa-mir-93	RFC3	0.315556301	mirna_pc
2816	hsa-mir-93	CHAF1A	0.369414925	mirna_pc
2817	hsa-mir-93	DEPDC1B	0.329966809	mirna_pc
2818	hsa-mir-93	GINS4	0.345892472	mirna_pc
2819	hsa-mir-93	ANP32E	0.367341712	mirna_pc
2820	hsa-mir-93	CENPO	0.57370471	mirna_pc
2821	hsa-mir-93	XRCC2	0.44037766	mirna_pc
2822	hsa-mir-93	BRCA2	0.323935699	mirna_pc
2823	hsa-mir-93	CENPL	0.37255429	mirna_pc
2824	hsa-mir-93	GEN1	0.456177186	mirna_pc
2825	hsa-mir-93	HMGB2	0.572816157	mirna_pc
2826	hsa-mir-93	GINS2	0.374175777	mirna_pc
2827	hsa-mir-93	DNA2	0.460990334	mirna_pc
2828	hsa-mir-93	DSN1	0.304767371	mirna_pc
2829	hsa-mir-93	SNHG1	0.335427783	mirna_pc
2830	hsa-mir-93	CSE1L	0.347085949	mirna_pc
2831	hsa-mir-93	E2F7	0.487365763	mirna_pc
2832	hsa-mir-93	PRMT1	0.361343517	mirna_pc
2833	hsa-mir-93	MLF1IP	0.445872463	mirna_pc
2834	hsa-mir-93	HEATR1	0.309076598	mirna_pc
2835	hsa-mir-93	C16orf75	0.367136365	mirna_pc
2836	hsa-mir-93	RBL1	0.361556388	mirna_pc
2837	hsa-mir-93	CAD	0.414622181	mirna_pc
2838	hsa-mir-93	CCDC99	0.310208601	mirna_pc
2839	hsa-mir-93	CDK2	0.439365952	mirna_pc
2840	hsa-mir-93	CDK4	0.332843577	mirna_pc
2841	hsa-mir-93	TMEM108	0.499798259	mirna_pc
2842	hsa-mir-93	CKAP5	0.313246364	mirna_pc
2843	hsa-mir-93	CHTF18	0.379233214	mirna_pc
2844	hsa-mir-93	HNRNPR	0.450830267	mirna_pc
2845	hsa-mir-93	DLEU2	0.32946392	mirna_pc
2846	hsa-mir-93	PRIM1	0.388739518	mirna_pc
2847	hsa-mir-93	CENPH	0.406098629	mirna_pc
2848	hsa-mir-93	CHAF1B	0.327120637	mirna_pc
2849	hsa-mir-93	DHX34	0.368122927	mirna_pc
2850	hsa-mir-93	NCAPG2	0.321276009	mirna_pc
2851	hsa-mir-93	MSH2	0.53832294	mirna_pc
2852	hsa-mir-93	DBF4	0.375138657	mirna_pc
2853	hsa-mir-93	SF3B3	0.447415332	mirna_pc
2854	hsa-mir-93	CDC7	0.477884348	mirna_pc
2855	hsa-mir-93	INTS7	0.301553018	mirna_pc
2856	hsa-mir-93	SSB	0.364464804	mirna_pc
2857	hsa-mir-93	RFC4	0.409493004	mirna_pc
2858	hsa-mir-93	FBXO5	0.541716174	mirna_pc
2859	hsa-mir-93	DCLRE1B	0.324018172	mirna_pc
2860	hsa-mir-93	FIGNL1	0.396983235	mirna_pc
2861	hsa-mir-93	E2F3	0.406515275	mirna_pc

2862	hsa-mir-93	DDX11	0.469986791	mirna_pc
2863	hsa-mir-93	CDC25A	0.484672455	mirna_pc
2864	hsa-mir-93	TOPBP1	0.350967103	mirna_pc
2865	hsa-mir-93	TMEM48	0.307547958	mirna_pc
2866	hsa-mir-93	RAD54B	0.410539217	mirna_pc
2867	hsa-mir-93	OTX1	0.313989631	mirna_pc
2868	hsa-mir-93	PRKDC	0.343906477	mirna_pc
2869	hsa-mir-93	H2AFZ	0.377710074	mirna_pc
2870	hsa-mir-93	DDX12	0.597157465	mirna_pc
2871	hsa-mir-93	SMC2	0.322748254	mirna_pc
2872	hsa-mir-93	MCM8	0.470356918	mirna_pc
2873	hsa-mir-93	CPSF3	0.3654121	mirna_pc
2874	hsa-mir-93	SNRPE	0.301728541	mirna_pc
2875	hsa-mir-93	C21orf45	0.348763133	mirna_pc
2876	hsa-mir-93	TFAP4	0.390107935	mirna_pc
2877	hsa-mir-93	PUS7	0.47669139	mirna_pc
2878	hsa-mir-93	DAZAP1	0.317757312	mirna_pc
2879	hsa-mir-93	CBX8	0.308753593	mirna_pc
2880	hsa-mir-93	MASTL	0.346259703	mirna_pc
2881	hsa-mir-93	TMEM201	0.390302054	mirna_pc
2882	hsa-mir-93	NCL	0.38119805	mirna_pc
2883	hsa-mir-93	SRRT	0.672372509	mirna_pc
2884	hsa-mir-93	CACYBP	0.370014236	mirna_pc
2885	hsa-mir-93	SASS6	0.416495685	mirna_pc
2886	hsa-mir-93	PSRC1	0.532077881	mirna_pc
2887	hsa-mir-93	C12orf32	0.352080689	mirna_pc
2888	hsa-mir-93	DBF4B	0.33950185	mirna_pc
2889	hsa-mir-93	POU2F1	0.314982163	mirna_pc
2890	hsa-mir-93	FBL	0.309111128	mirna_pc
2891	hsa-mir-93	HIST1H1E	0.354826928	mirna_pc
2892	hsa-mir-93	HNRNPM	0.401473701	mirna_pc
2893	hsa-mir-93	TMPO	0.501910926	mirna_pc
2894	hsa-mir-93	SSBP3	0.323356813	mirna_pc
2895	hsa-mir-93	C20orf72	0.350477283	mirna_pc
2896	hsa-mir-93	SMPD4	0.42614103	mirna_pc
2897	hsa-mir-93	GPR19	0.485691343	mirna_pc
2898	hsa-mir-93	GABPB1	0.514238148	mirna_pc
2899	hsa-mir-93	PPAT	0.399098906	mirna_pc
2900	hsa-mir-93	DHX9	0.500587262	mirna_pc
2901	hsa-mir-93	CCDC138	0.585670188	mirna_pc
2902	hsa-mir-93	LOC100128191	0.417233812	mirna_pc
2903	hsa-mir-93	HMGN2	0.396352229	mirna_pc
2904	hsa-mir-93	ETV4	0.373398638	mirna_pc
2905	hsa-mir-93	POLA2	0.346324049	mirna_pc
2906	hsa-mir-93	CCDC77	0.365296119	mirna_pc
2907	hsa-mir-93	PHF6	0.339925236	mirna_pc
2908	hsa-mir-93	SFRS1	0.522009623	mirna_pc
2909	hsa-mir-93	WDR43	0.315711212	mirna_pc
2910	hsa-mir-93	SKP2	0.354131236	mirna_pc
2911	hsa-mir-93	SMARCD1	0.338811485	mirna_pc
2912	hsa-mir-93	ATXN2L	0.33774495	mirna_pc
2913	hsa-mir-93	C4orf46	0.58357257	mirna_pc
2914	hsa-mir-93	C1orf107	0.316994947	mirna_pc
2915	hsa-mir-93	GPN1	0.310258284	mirna_pc

2916	hsa-mir-93	RFC5	0.423252532	mirna_pc
2917	hsa-mir-93	HSPD1	0.421377259	mirna_pc
2918	hsa-mir-93	MAZ	0.382500797	mirna_pc
2919	hsa-mir-93	SSRP1	0.341244195	mirna_pc
2920	hsa-mir-93	POLR2D	0.470389495	mirna_pc
2921	hsa-mir-93	KHDRBS1	0.477657829	mirna_pc
2922	hsa-mir-93	TUB	0.312592016	mirna_pc
2923	hsa-mir-93	SPAST	0.365579426	mirna_pc
2924	hsa-mir-93	CCDC21	0.332592279	mirna_pc
2925	hsa-mir-93	MTL 5.00	0.536537102	mirna_pc
2926	hsa-mir-93	PRPF40A	0.381512967	mirna_pc
2927	hsa-mir-93	CCDC18	0.415563746	mirna_pc
2928	hsa-mir-93	PARP1	0.422774946	mirna_pc
2929	hsa-mir-93	CCDC34	0.355590152	mirna_pc
2930	hsa-mir-93	DNAJC2	0.473335646	mirna_pc
2931	hsa-mir-93	C4orf21	0.501970621	mirna_pc
2932	hsa-mir-93	TMEM194A	0.594218022	mirna_pc
2933	hsa-mir-93	AP1S1	0.357318671	mirna_pc
2934	hsa-mir-93	SLC39A10	0.495058777	mirna_pc
2935	hsa-mir-93	SUV420H2	0.359205115	mirna_pc
2936	hsa-mir-93	ZNF695	0.523262024	mirna_pc
2937	hsa-mir-93	NEDD1	0.435654953	mirna_pc
2938	hsa-mir-93	KIF24	0.301858276	mirna_pc
2939	hsa-mir-93	HAUS6	0.421386565	mirna_pc
2940	hsa-mir-93	CENPJ	0.353242821	mirna_pc
2941	hsa-mir-93	LIN9	0.359245706	mirna_pc
2942	hsa-mir-93	CEP135	0.44102911	mirna_pc
2943	hsa-mir-93	HNRNPU	0.513720915	mirna_pc
2944	hsa-mir-93	PSMC2	0.425078009	mirna_pc
2945	hsa-mir-93	NASP	0.482079486	mirna_pc
2946	hsa-mir-93	ZNF280C	0.308878936	mirna_pc
2947	hsa-mir-93	POLR1B	0.373286017	mirna_pc
2948	hsa-mir-93	BCL2	0.450875487	mirna_pc
2949	hsa-mir-93	PTMA	0.369473855	mirna_pc
2950	hsa-mir-93	WDR76	0.36905195	mirna_pc
2951	hsa-mir-93	HMGXB4	0.309207246	mirna_pc
2952	hsa-mir-93	C11orf84	0.375122605	mirna_pc
2953	hsa-mir-93	NCBP2	0.330411077	mirna_pc
2954	hsa-mir-93	SLC12A9	0.395970396	mirna_pc
2955	hsa-mir-93	SUV39H2	0.557695916	mirna_pc
2956	hsa-mir-93	GORAB	0.440150056	mirna_pc
2957	hsa-mir-93	R3HDM1	0.404663745	mirna_pc
2958	hsa-mir-93	FANCL	0.312363194	mirna_pc
2959	hsa-mir-93	RQCD1	0.355797913	mirna_pc
2960	hsa-mir-93	HNRNPD	0.513672009	mirna_pc
2961	hsa-mir-93	TARDBP	0.36185352	mirna_pc
2962	hsa-mir-93	WDR19	0.33086237	mirna_pc
2963	hsa-mir-93	POP1	0.303846661	mirna_pc
2964	hsa-mir-93	SFRS3	0.379360193	mirna_pc
2965	hsa-mir-93	NUP160	0.361370756	mirna_pc
2966	hsa-mir-93	SHOX2	0.420916545	mirna_pc
2967	hsa-mir-93	CCT4	0.335985383	mirna_pc
2968	hsa-mir-93	RAD9A	0.329560537	mirna_pc
2969	hsa-mir-93	ELAVL1	0.343730818	mirna_pc

2970	hsa-mir-93	CCDC121	0.302827242	mirna_pc
2971	hsa-mir-93	ABCE1	0.334876363	mirna_pc
2972	hsa-mir-93	KIAA0406	0.336714535	mirna_pc
2973	hsa-mir-93	PSMC3IP	0.354193794	mirna_pc
2974	hsa-mir-93	PAK1IP1	0.302856016	mirna_pc
2975	hsa-mir-93	ATP13A3	0.335175119	mirna_pc
2976	hsa-mir-93	PMAIP1	0.374402082	mirna_pc
2977	hsa-mir-93	POP7	0.417979918	mirna_pc
2978	hsa-mir-93	DHFR	0.445109154	mirna_pc
2979	hsa-mir-93	NAA25	0.362600706	mirna_pc
2980	hsa-mir-93	ILF3	0.473525141	mirna_pc
2981	hsa-mir-93	TAF6	0.451875821	mirna_pc
2982	hsa-mir-93	C1orf96	0.419229627	mirna_pc
2983	hsa-mir-93	EHMT2	0.39973554	mirna_pc
2984	hsa-mir-93	MAGOHB	0.380017547	mirna_pc
2985	hsa-mir-93	C18orf54	0.326963004	mirna_pc
2986	hsa-mir-93	UBAP2L	0.39717074	mirna_pc
2987	hsa-mir-93	DEK	0.447847568	mirna_pc
2988	hsa-mir-93	TBC1D4	0.322664201	mirna_pc
2989	hsa-mir-93	PLOD3	0.341816954	mirna_pc
2990	hsa-mir-93	PASK	0.442595011	mirna_pc
2991	hsa-mir-93	USP42	0.300241209	mirna_pc
2992	hsa-mir-93	RPGRIP1L	0.392999586	mirna_pc
2993	hsa-mir-93	TAF4	0.302512636	mirna_pc
2994	hsa-mir-93	ZBTB33	0.308387604	mirna_pc
2995	hsa-mir-93	TGS1	0.377499632	mirna_pc
2996	hsa-mir-93	LRWD1	0.409576183	mirna_pc
2997	hsa-mir-93	HEATR2	0.434488236	mirna_pc
2998	hsa-mir-93	LSM 6.00	0.426093111	mirna_pc
2999	hsa-mir-93	PRAME	0.368794129	mirna_pc
3000	hsa-mir-93	FBXW8	0.307208941	mirna_pc
3001	hsa-mir-93	FUBP1	0.462118544	mirna_pc
3002	hsa-mir-93	IPO9	0.458468315	mirna_pc
3003	hsa-mir-93	SART3	0.435076538	mirna_pc
3004	hsa-mir-93	HNRNPK	0.325532939	mirna_pc
3005	hsa-mir-93	SR140	0.330859054	mirna_pc
3006	hsa-mir-93	TRA2B	0.492568334	mirna_pc
3007	hsa-mir-93	RSRC1	0.373344422	mirna_pc
3008	hsa-mir-93	TRAF4	0.344849445	mirna_pc
3009	hsa-mir-93	GAR1	0.426157053	mirna_pc
3010	hsa-mir-93	CEP152	0.400031075	mirna_pc
3011	hsa-mir-93	C7orf47	0.33789923	mirna_pc
3012	hsa-mir-93	C15orf23	0.379118627	mirna_pc
3013	hsa-mir-93	CDRT4	0.44978397	mirna_pc
3014	hsa-mir-93	RBM12B	0.362648365	mirna_pc
3015	hsa-mir-93	C3orf21	0.355023463	mirna_pc
3016	hsa-mir-93	LSM14B	0.323294811	mirna_pc
3017	hsa-mir-93	CPXM1	0.325910358	mirna_pc
3018	hsa-mir-93	PMS2L1	0.325615199	mirna_pc
3019	hsa-mir-93	IGF2BP2	0.330328104	mirna_pc
3020	hsa-mir-93	WHSC1	0.459105317	mirna_pc
3021	hsa-mir-93	ITGB3BP	0.435840095	mirna_pc
3022	hsa-mir-93	BCL2A1	0.32441821	mirna_pc
3023	hsa-mir-93	ENOPH1	0.317609078	mirna_pc

3024	hsa-mir-93	NFIB	0.311304548	mirna_pc
3025	hsa-mir-93	C4orf43	0.426431939	mirna_pc
3026	hsa-mir-93	ING5	0.310962182	mirna_pc
3027	hsa-mir-93	HSPA14	0.310542757	mirna_pc
3028	hsa-mir-93	CEP78	0.374335097	mirna_pc
3029	hsa-mir-93	PLEKHG4	0.321134503	mirna_pc
3030	hsa-mir-93	SFPQ	0.462330245	mirna_pc
3031	hsa-mir-93	CNOT3	0.303394107	mirna_pc
3032	hsa-mir-93	CD83	0.490993767	mirna_pc
3033	hsa-mir-93	FAM125B	0.342527859	mirna_pc
3034	hsa-mir-93	MPHOSPH9	0.368187198	mirna_pc
3035	hsa-mir-93	CADM1	0.432190125	mirna_pc
3036	hsa-mir-93	POLA1	0.391757562	mirna_pc
3037	hsa-mir-93	HDAC2	0.364533656	mirna_pc
3038	hsa-mir-93	GMNN	0.35967603	mirna_pc
3039	hsa-mir-93	C10orf119	0.383767649	mirna_pc
3040	hsa-mir-93	ANKRD32	0.387744129	mirna_pc
3041	hsa-mir-93	C10orf2	0.31874984	mirna_pc
3042	hsa-mir-93	POGK	0.33411282	mirna_pc
3043	hsa-mir-93	CPAMD8	0.399942455	mirna_pc
3044	hsa-mir-93	SALL2	0.300352925	mirna_pc
3045	hsa-mir-93	FAM86C	0.357395025	mirna_pc
3046	hsa-mir-93	DUS4L	0.366580626	mirna_pc
3047	hsa-mir-93	PTCD1	0.321504616	mirna_pc
3048	hsa-mir-93	ZNF107	0.303134413	mirna_pc
3049	hsa-mir-93	MSH6	0.513307846	mirna_pc
3050	hsa-mir-93	XRN2	0.341856799	mirna_pc
3051	hsa-mir-93	TP73	0.33515297	mirna_pc
3052	hsa-mir-93	KPNB1	0.35248888	mirna_pc
3053	hsa-mir-93	LEF1	0.470182286	mirna_pc
3054	hsa-mir-93	CASP3	0.310154564	mirna_pc
3055	hsa-mir-93	SMC3	0.462164722	mirna_pc
3056	hsa-mir-93	FLVCR1	0.346677562	mirna_pc
3057	hsa-mir-93	HNRNPA1	0.349603761	mirna_pc
3058	hsa-mir-93	KREMEN2	0.548782807	mirna_pc
3059	hsa-mir-93	TRIP6	0.395190702	mirna_pc
3060	hsa-mir-93	TMEM194B	0.306300191	mirna_pc
3061	hsa-mir-93	MEX3A	0.716093916	mirna_pc
3062	hsa-mir-93	MOGS	0.321098865	mirna_pc
3063	hsa-mir-93	CEP250	0.307861336	mirna_pc
3064	hsa-mir-93	BAT1	0.330408307	mirna_pc
3065	hsa-mir-93	COIL	0.307119539	mirna_pc
3066	hsa-mir-93	NGFR	0.313551079	mirna_pc
3067	hsa-mir-93	PDCD11	0.390775979	mirna_pc
3068	hsa-mir-93	IRAK1BP1	0.380931008	mirna_pc
3069	hsa-mir-93	TAF5	0.413072258	mirna_pc
3070	hsa-mir-93	FANCC	0.386687115	mirna_pc
3071	hsa-mir-93	LRRN1	0.324268802	mirna_pc
3072	hsa-mir-93	TIFA	0.41079053	mirna_pc
3073	hsa-mir-93	CCDC28B	0.377898696	mirna_pc
3074	hsa-mir-93	UNC119B	0.325575328	mirna_pc
3075	hsa-mir-93	SUZ12	0.337889139	mirna_pc
3076	hsa-mir-93	HNRNPA1L2	0.372501535	mirna_pc
3077	hsa-mir-93	NAA15	0.361810934	mirna_pc

3078	hsa-mir-93	PPFIA1	0.3415623	mirna_pc
3079	hsa-mir-93	BARD1	0.315216166	mirna_pc
3080	hsa-mir-93	CBX1	0.32692257	mirna_pc
3081	hsa-mir-93	EMILIN3	0.397630052	mirna_pc
3082	hsa-mir-93	ZBTB12	0.488002402	mirna_pc
3083	hsa-mir-93	TADA1	0.344401457	mirna_pc
3084	hsa-mir-93	MCTP2	0.527729946	mirna_pc
3085	hsa-mir-93	USP1	0.354631871	mirna_pc
3086	hsa-mir-93	TMEM68	0.313745765	mirna_pc
3087	hsa-mir-93	LOC642846	0.580687117	mirna_pc
3088	hsa-mir-93	TGIF2	0.574754993	mirna_pc
3089	hsa-mir-93	CHERP	0.320140811	mirna_pc
3090	hsa-mir-93	SENP1	0.40791923	mirna_pc
3091	hsa-mir-93	RCN2	0.413519287	mirna_pc
3092	hsa-mir-93	MTF2	0.41272855	mirna_pc
3093	hsa-mir-93	CCDC142	0.323111847	mirna_pc
3094	hsa-mir-93	POU6F2	0.392428812	mirna_pc
3095	hsa-mir-93	ETV6	0.354784358	mirna_pc
3096	hsa-mir-93	NOL10	0.345491415	mirna_pc
3097	hsa-mir-93	ANAPC1	0.312082391	mirna_pc
3098	hsa-mir-93	PXDN	0.3275029	mirna_pc
3099	hsa-mir-93	DDX47	0.395759923	mirna_pc
3100	hsa-mir-93	POLE	0.565233945	mirna_pc
3101	hsa-mir-93	ZKSCAN2	0.30458201	mirna_pc
3102	hsa-mir-93	CHML	0.323613652	mirna_pc
3103	hsa-mir-93	CTDSPL2	0.388248504	mirna_pc
3104	hsa-mir-93	ERF	0.305726178	mirna_pc
3105	hsa-mir-93	TAF1B	0.310796966	mirna_pc
3106	hsa-mir-93	PPP3R1	0.377358303	mirna_pc
3107	hsa-mir-93	RBM14	0.404070465	mirna_pc
3108	hsa-mir-93	EXOSC9	0.406386065	mirna_pc
3109	hsa-mir-93	UNG	0.317962243	mirna_pc
3110	hsa-mir-93	RBMX	0.501595437	mirna_pc
3111	hsa-mir-93	ORC5L	0.489942683	mirna_pc
3112	hsa-mir-93	TSEN15	0.306464724	mirna_pc
3113	hsa-mir-93	CCDC41	0.310102049	mirna_pc
3114	hsa-mir-93	NOC3L	0.360050931	mirna_pc
3115	hsa-mir-93	MDC 1.00	0.442590082	mirna_pc
3116	hsa-mir-93	XRCC5	0.305976765	mirna_pc
3117	hsa-mir-93	CTCF	0.380530875	mirna_pc
3118	hsa-mir-93	RPL39L	0.40796307	mirna_pc
3119	hsa-mir-93	ASB3	0.34396817	mirna_pc
3120	hsa-mir-93	EPHB4	0.609792406	mirna_pc
3121	hsa-mir-93	IGSF9	0.544733063	mirna_pc
3122	hsa-mir-93	LBR	0.36115082	mirna_pc
3123	hsa-mir-93	UBXN2A	0.302847058	mirna_pc
3124	hsa-mir-93	GPATCH2	0.327837749	mirna_pc
3125	hsa-mir-93	DNAJC14	0.326753878	mirna_pc
3126	hsa-mir-93	FIP1L1	0.35705211	mirna_pc
3127	hsa-mir-93	ZNF519	0.373356327	mirna_pc
3128	hsa-mir-93	GTF3C3	0.360687702	mirna_pc
3129	hsa-mir-93	CIT	0.427991669	mirna_pc
3130	hsa-mir-93	ZRANB3	0.428266247	mirna_pc
3131	hsa-mir-93	RIF1	0.422406621	mirna_pc

3132	hsa-mir-93	KDM2B	0.401520246	mirna_pc
3133	hsa-mir-93	STAG2	0.301839658	mirna_pc
3134	hsa-mir-93	NUDT21	0.321276641	mirna_pc
3135	hsa-mir-93	SCLT1	0.419199304	mirna_pc
3136	hsa-mir-93	FAM185A	0.339099391	mirna_pc
3137	hsa-mir-93	UFSP1	0.323201629	mirna_pc
3138	hsa-mir-93	SUZ12P	0.301786404	mirna_pc
3139	hsa-mir-93	FRMD4A	0.399826571	mirna_pc
3140	hsa-mir-93	MYB	0.473155176	mirna_pc
3141	hsa-mir-93	ZNF639	0.304728225	mirna_pc
3142	hsa-mir-93	ITPRIPL1	0.46582213	mirna_pc
3143	hsa-mir-93	TTLL4	0.473781352	mirna_pc
3144	hsa-mir-93	THADA	0.34995448	mirna_pc
3145	hsa-mir-93	C20orf177	0.367711388	mirna_pc
3146	hsa-mir-93	ATXN7L2	0.331739957	mirna_pc
3147	hsa-mir-93	ACBD7	0.31147219	mirna_pc
3148	hsa-mir-93	RFC1	0.340216947	mirna_pc
3149	hsa-mir-93	ATN1	0.394158478	mirna_pc
3150	hsa-mir-93	ANKRD6	0.423290994	mirna_pc
3151	hsa-mir-93	NUP133	0.373286172	mirna_pc
3152	hsa-mir-93	BCL7A	0.41656788	mirna_pc
3153	hsa-mir-93	WNT10A	0.309134693	mirna_pc
3154	hsa-mir-93	C7orf44	0.300404014	mirna_pc
3155	hsa-mir-93	TRPA1	0.49565985	mirna_pc
3156	hsa-mir-93	URB2	0.330514977	mirna_pc
3157	hsa-mir-93	PALM2	0.302819737	mirna_pc
3158	hsa-mir-93	ARNT2	0.365919823	mirna_pc
3159	hsa-mir-93	ARHGAP19	0.425999034	mirna_pc
3160	hsa-mir-93	ZNF286A	0.592590826	mirna_pc
3161	hsa-mir-93	PRPF38A	0.358183046	mirna_pc
3162	hsa-mir-93	KIAA0922	0.306493977	mirna_pc
3163	hsa-mir-93	WDR77	0.31164173	mirna_pc
3164	hsa-mir-93	SPDYE3	0.341360601	mirna_pc
3165	hsa-mir-93	MRPL21	0.50722063	mirna_pc
3166	hsa-mir-93	WBP11	0.319095499	mirna_pc
3167	hsa-mir-93	DMRTA2	0.340455312	mirna_pc
3168	hsa-mir-93	RFX7	0.314806509	mirna_pc
3169	hsa-mir-93	ILKAP	0.338436428	mirna_pc
3170	hsa-mir-93	PRR3	0.346515044	mirna_pc
3171	hsa-mir-93	SPATA5	0.336894196	mirna_pc
3172	hsa-mir-93	CNTROB	0.330052741	mirna_pc
3173	hsa-mir-93	TRRAP	0.543008159	mirna_pc
3174	hsa-mir-93	UBFD1	0.357531113	mirna_pc
3175	hsa-mir-93	ORAI2	0.435979571	mirna_pc
3176	hsa-mir-93	SEMA4F	0.406743774	mirna_pc
3177	hsa-mir-93	RBM12	0.353555329	mirna_pc
3178	hsa-mir-93	SLC25A13	0.422464595	mirna_pc
3179	hsa-mir-93	ZNF286B	0.380361196	mirna_pc
3180	hsa-mir-93	DDX20	0.46447081	mirna_pc
3181	hsa-mir-93	SAFB	0.313405976	mirna_pc
3182	hsa-mir-93	SAP130	0.3641588	mirna_pc
3183	hsa-mir-93	BAZ1B	0.365688658	mirna_pc
3184	hsa-mir-93	INPPL1	0.380808323	mirna_pc
3185	hsa-mir-93	TCERG1	0.351304213	mirna_pc

3186	hsa-mir-93	SEC61A2	0.401357623	mirna_pc
3187	hsa-mir-93	PSPC1	0.331824797	mirna_pc
3188	hsa-mir-93	ATP6V1E2	0.36293818	mirna_pc
3189	hsa-mir-93	LMLN	0.313384054	mirna_pc
3190	hsa-mir-93	MAPKAPK5	0.321735665	mirna_pc
3191	hsa-mir-93	RBMXL1	0.308282388	mirna_pc
3192	hsa-mir-93	GMCL1	0.320865223	mirna_pc
3193	hsa-mir-93	GPR3	0.453979615	mirna_pc
3194	hsa-mir-93	E2F6	0.422255873	mirna_pc
3195	hsa-mir-93	SSX2IP	0.354636207	mirna_pc
3196	hsa-mir-93	CPSF4	0.301741445	mirna_pc
3197	hsa-mir-93	HUNK	0.351237517	mirna_pc
3198	hsa-mir-93	PILRB	0.417865361	mirna_pc
3199	hsa-mir-93	SFRS13A	0.430534276	mirna_pc
3200	hsa-mir-93	SNRNP200	0.33834127	mirna_pc
3201	hsa-mir-93	LOC646851	0.329892492	mirna_pc
3202	hsa-mir-93	SCLY	0.634061947	mirna_pc
3203	hsa-mir-93	GPR125	0.423455336	mirna_pc
3204	hsa-mir-93	NUP35	0.307594417	mirna_pc
3205	hsa-mir-93	ALG10	0.373175281	mirna_pc
3206	hsa-mir-93	TFAM	0.346381169	mirna_pc
3207	hsa-mir-93	LDLRAD3	0.511498957	mirna_pc
3208	hsa-mir-93	TTC27	0.3082118	mirna_pc
3209	hsa-mir-93	COPS6	0.353333573	mirna_pc
3210	hsa-mir-93	GSTCD	0.406461143	mirna_pc
3211	hsa-mir-93	DHRS13	0.415490942	mirna_pc
3212	hsa-mir-93	ORC4L	0.370550815	mirna_pc
3213	hsa-mir-93	RTTN	0.392602172	mirna_pc
3214	hsa-mir-93	HNRNPA3	0.321565186	mirna_pc
3215	hsa-mir-93	EWSR1	0.310428274	mirna_pc
3216	hsa-mir-93	SMC1A	0.306929582	mirna_pc
3217	hsa-mir-93	DHX57	0.474909805	mirna_pc
3218	hsa-mir-93	NUMA1	0.37769429	mirna_pc
3219	hsa-mir-93	ALX3	0.517016624	mirna_pc
3220	hsa-mir-93	CHST14	0.301171413	mirna_pc
3221	hsa-mir-93	ACVR2B	0.40080901	mirna_pc
3222	hsa-mir-93	SFT2D3	0.407050572	mirna_pc
3223	hsa-mir-93	LIG3	0.326579087	mirna_pc
3224	hsa-mir-93	DPY19L2P2	0.353178518	mirna_pc
3225	hsa-mir-93	CABLES2	0.321656602	mirna_pc
3226	hsa-mir-93	WDR35	0.304687617	mirna_pc
3227	hsa-mir-93	MRPL30	0.326375158	mirna_pc
3228	hsa-mir-93	SFRS7	0.394854791	mirna_pc
3229	hsa-mir-93	ALKBH4	0.46632677	mirna_pc
3230	hsa-mir-93	MRPL42	0.369565594	mirna_pc
3231	hsa-mir-93	BEND3	0.336544236	mirna_pc
3232	hsa-mir-93	CBFA2T2	0.327888997	mirna_pc
3233	hsa-mir-93	LZTS1	0.313860189	mirna_pc
3234	hsa-mir-93	ATXN7L1	0.307068197	mirna_pc
3235	hsa-mir-93	NFXL1	0.313768112	mirna_pc
3236	hsa-mir-93	PFAS	0.391042972	mirna_pc
3237	hsa-mir-93	LRTOMT	0.386467302	mirna_pc
3238	hsa-mir-93	ARMC10	0.464774316	mirna_pc
3239	hsa-mir-93	GXYLT2	0.306860742	mirna_pc

3240	hsa-mir-93	RPL13P5	0.304341739	mirna_pc
3241	hsa-mir-93	BAT2	0.339526832	mirna_pc
3242	hsa-mir-93	ERCC3	0.326075697	mirna_pc
3243	hsa-mir-93	EDAR	0.442633885	mirna_pc
3244	hsa-mir-93	IFT81	0.429799531	mirna_pc
3245	hsa-mir-93	ORAOV1	0.311946717	mirna_pc
3246	hsa-mir-93	HNRNPH1	0.312660648	mirna_pc
3247	hsa-mir-93	ZNF184	0.374198589	mirna_pc
3248	hsa-mir-93	NUP153	0.420747421	mirna_pc
3249	hsa-mir-93	NIPSNAP1	0.303707171	mirna_pc
3250	hsa-mir-93	WEE1	0.314621822	mirna_pc
3251	hsa-mir-93	C2orf68	0.307572418	mirna_pc
3252	hsa-mir-93	ARHGEF19	0.383539266	mirna_pc
3253	hsa-mir-93	C17orf75	0.329577052	mirna_pc
3254	hsa-mir-93	THAP5	0.432021447	mirna_pc
3255	hsa-mir-93	WDR92	0.317576907	mirna_pc
3256	hsa-mir-93	CCND1	0.316729157	mirna_pc
3257	hsa-mir-93	MCM3APAS	0.411798561	mirna_pc
3258	hsa-mir-93	NFATC3	0.320011852	mirna_pc
3259	hsa-mir-93	HCFC1	0.343119878	mirna_pc
3260	hsa-mir-93	ZNF771	0.336549599	mirna_pc
3261	hsa-mir-93	GTF3C4	0.333880789	mirna_pc
3262	hsa-mir-93	CASP8AP2	0.306379139	mirna_pc
3263	hsa-mir-93	TCTN2	0.50111511	mirna_pc
3264	hsa-mir-93	BCAN	0.48020383	mirna_pc
3265	hsa-mir-93	DHX15	0.374712632	mirna_pc
3266	hsa-mir-93	ZKSCAN5	0.318143513	mirna_pc
3267	hsa-mir-93	ZNF669	0.319906341	mirna_pc
3268	hsa-mir-93	TOP3A	0.339192464	mirna_pc
3269	hsa-mir-93	LRP6	0.38427217	mirna_pc
3270	hsa-mir-93	EXTL2	0.359968917	mirna_pc
3271	hsa-mir-93	DCP2	0.385559923	mirna_pc
3272	hsa-mir-93	ZNF92	0.342692448	mirna_pc
3273	hsa-mir-93	PRKD3	0.409607275	mirna_pc
3274	hsa-mir-93	C2orf44	0.431085016	mirna_pc
3275	hsa-mir-93	SEPHS1	0.507700734	mirna_pc
3276	hsa-mir-93	SHISA2	0.48925644	mirna_pc
3277	hsa-mir-93	CBX5	0.473477857	mirna_pc
3278	hsa-mir-93	RMND1	0.319950728	mirna_pc
3279	hsa-mir-93	RYK	0.307149211	mirna_pc
3280	hsa-mir-93	ADNP	0.383480841	mirna_pc
3281	hsa-mir-93	IFT172	0.337365	mirna_pc
3282	hsa-mir-93	TTC30B	0.366503641	mirna_pc
3283	hsa-mir-93	ATAD2B	0.305233664	mirna_pc
3284	hsa-mir-93	AGBL5	0.454623277	mirna_pc
3285	hsa-mir-93	VASH2	0.563568145	mirna_pc
3286	hsa-mir-93	PHB2	0.324947573	mirna_pc
3287	hsa-mir-93	MYL6B	0.386642816	mirna_pc
3288	hsa-mir-93	ZNF498	0.51362093	mirna_pc
3289	hsa-mir-93	NRF1	0.305264167	mirna_pc
3290	hsa-mir-93	CD200	0.480993369	mirna_pc
3291	hsa-mir-93	RABL5	0.443946437	mirna_pc
3292	hsa-mir-93	MED28	0.366177413	mirna_pc
3293	hsa-mir-93	MATR3	0.331471648	mirna_pc

3294	hsa-mir-93	HNRNPUL1	0.411279868	mirna_pc
3295	hsa-mir-93	ZNF384	0.394502229	mirna_pc
3296	hsa-mir-93	GPX7	0.312432212	mirna_pc
3297	hsa-mir-93	PAN2	0.3351055	mirna_pc
3298	hsa-mir-93	NUP43	0.356250656	mirna_pc
3299	hsa-mir-93	BRD3	0.300970638	mirna_pc
3300	hsa-mir-93	RNF41	0.305872396	mirna_pc
3301	hsa-mir-93	CHST11	0.438544495	mirna_pc
3302	hsa-mir-93	BCL11A	0.507200108	mirna_pc
3303	hsa-mir-93	FBXO17	0.325465707	mirna_pc
3304	hsa-mir-93	C4orf27	0.365845601	mirna_pc
3305	hsa-mir-93	ZNF512	0.321654026	mirna_pc
3306	hsa-mir-93	PMPCB	0.442317772	mirna_pc
3307	hsa-mir-93	ZNF473	0.411516775	mirna_pc
3308	hsa-mir-93	COPS7B	0.33576008	mirna_pc
3309	hsa-mir-93	USP21	0.418357962	mirna_pc
3310	hsa-mir-93	ALMS1	0.46341515	mirna_pc
3311	hsa-mir-93	PRR12	0.303481583	mirna_pc
3312	hsa-mir-93	PMS2L3	0.313183284	mirna_pc
3313	hsa-mir-93	SNRNP48	0.350570504	mirna_pc
3314	hsa-mir-93	DCLRE1A	0.360199261	mirna_pc
3315	hsa-mir-93	ANP32A	0.387227203	mirna_pc
3316	hsa-mir-93	RAD52	0.357465056	mirna_pc
3317	hsa-mir-93	E2F5	0.317246531	mirna_pc
3318	hsa-mir-93	RNF121	0.362033299	mirna_pc
3319	hsa-mir-93	CPT1A	0.339783087	mirna_pc
3320	hsa-mir-93	EMID1	0.334720305	mirna_pc
3321	hsa-mir-93	ZMYM4	0.318820743	mirna_pc
3322	hsa-mir-93	AP4M1	0.460778849	mirna_pc
3323	hsa-mir-93	PRKAR1B	0.32871987	mirna_pc
3324	hsa-mir-93	LMAN2L	0.301735445	mirna_pc
3325	hsa-mir-93	RBM15	0.317781984	mirna_pc
3326	hsa-mir-93	TCF7L1	0.391571457	mirna_pc
3327	hsa-mir-93	LRRCC1	0.339558084	mirna_pc
3328	hsa-mir-93	FAM98B	0.378066158	mirna_pc
3329	hsa-mir-93	BCLAF1	0.344019577	mirna_pc
3330	hsa-mir-93	MOXD1	0.327850029	mirna_pc
3331	hsa-mir-93	THAP9	0.338709286	mirna_pc
3332	hsa-mir-93	SF1	0.302426981	mirna_pc
3333	hsa-mir-93	HNRNPH3	0.391327443	mirna_pc
3334	hsa-mir-93	NAP1L1	0.340364602	mirna_pc
3335	hsa-mir-93	PEX5	0.416091319	mirna_pc
3336	hsa-mir-93	RABL2A	0.423194591	mirna_pc
3337	hsa-mir-93	NSL1	0.420849659	mirna_pc
3338	hsa-mir-93	CNIH2	0.358320838	mirna_pc
3339	hsa-mir-93	TRDMT1	0.365516233	mirna_pc
3340	hsa-mir-93	IQCC	0.393858339	mirna_pc
3341	hsa-mir-93	GATAD2B	0.363607115	mirna_pc
3342	hsa-mir-93	PMS1	0.361071868	mirna_pc
3343	hsa-mir-93	ALG10B	0.371988883	mirna_pc
3344	hsa-mir-93	MNS1	0.359004051	mirna_pc
3345	hsa-mir-93	ZSCAN21	0.432468956	mirna_pc
3346	hsa-mir-93	RBM16	0.340222222	mirna_pc
3347	hsa-mir-93	TRUB1	0.327212068	mirna_pc

3348	hsa-mir-93	PEG10	0.366338521	mirna_pc
3349	hsa-mir-93	CCNJ	0.421816529	mirna_pc
3350	hsa-mir-93	MYCN	0.580102954	mirna_pc
3351	hsa-mir-93	C12orf24	0.30735644	mirna_pc
3352	hsa-mir-93	EFCAB7	0.382311396	mirna_pc
3353	hsa-mir-93	DCAF16	0.393255568	mirna_pc
3354	hsa-mir-93	RINT1	0.341258522	mirna_pc
3355	hsa-mir-93	FKBP4	0.314700607	mirna_pc
3356	hsa-mir-93	SBK1	0.473234044	mirna_pc
3357	hsa-mir-93	PPID	0.302774119	mirna_pc
3358	hsa-mir-93	PABPC4L	0.480261288	mirna_pc
3359	hsa-mir-93	C10orf137	0.344501449	mirna_pc
3360	hsa-mir-93	ADNP2	0.325682438	mirna_pc
3361	hsa-mir-93	FAM171A1	0.338508076	mirna_pc
3362	hsa-mir-93	THNSL1	0.376034144	mirna_pc
3363	hsa-mir-93	USP13	0.517711908	mirna_pc
3364	hsa-mir-93	GALNT14	0.357919478	mirna_pc
3365	hsa-mir-93	HAUS3	0.314644812	mirna_pc
3366	hsa-mir-93	RASL10B	0.355601077	mirna_pc
3367	hsa-mir-93	ZNF3	0.547317549	mirna_pc
3368	hsa-mir-93	CD69	0.45288298	mirna_pc
3369	hsa-mir-93	DNM1P35	0.307082106	mirna_pc
3370	hsa-mir-93	CP110	0.389210709	mirna_pc
3371	hsa-mir-93	ERLIN1	0.323845876	mirna_pc
3372	hsa-mir-93	C16orf46	0.418286755	mirna_pc
3373	hsa-mir-93	MSI1	0.395937837	mirna_pc
3374	hsa-mir-93	CLPB	0.342071377	mirna_pc
3375	hsa-mir-93	SMARCC1	0.303797806	mirna_pc
3376	hsa-mir-93	SERTAD4	0.361945541	mirna_pc
3377	hsa-mir-93	SCAI	0.419805965	mirna_pc
3378	hsa-mir-93	GXYLT1	0.325126239	mirna_pc
3379	hsa-mir-93	N4BP2	0.365344902	mirna_pc
3380	hsa-mir-93	ZNF740	0.344784911	mirna_pc
3381	hsa-mir-93	EP400	0.331033423	mirna_pc
3382	hsa-mir-93	TRO	0.354186181	mirna_pc
3383	hsa-mir-93	PACRGL	0.42811893	mirna_pc
3384	hsa-mir-93	SMARCA5	0.317923153	mirna_pc
3385	hsa-mir-93	PBX2	0.336732342	mirna_pc
3386	hsa-mir-93	ZNF232	0.471172051	mirna_pc
3387	hsa-mir-93	ZBTB39	0.496440164	mirna_pc
3388	hsa-mir-93	NRCAM	0.437956104	mirna_pc
3389	hsa-mir-93	ZNF827	0.314880369	mirna_pc
3390	hsa-mir-93	C20orf12	0.428880474	mirna_pc
3391	hsa-mir-93	FANCF	0.386540827	mirna_pc
3392	hsa-mir-93	VPS54	0.356282348	mirna_pc
3393	hsa-mir-93	RAC3	0.383007492	mirna_pc
3394	hsa-mir-93	ZBTB24	0.389562639	mirna_pc
3395	hsa-mir-93	RBBP4	0.398120009	mirna_pc
3396	hsa-mir-93	NCOA5	0.315239846	mirna_pc
3397	hsa-mir-93	CDKN1B	0.418301793	mirna_pc
3398	hsa-mir-93	ZNF618	0.394834897	mirna_pc
3399	hsa-mir-93	BRSK1	0.302270581	mirna_pc
3400	hsa-mir-93	C2orf3	0.396530477	mirna_pc
3401	hsa-mir-93	ZNF22	0.33890968	mirna_pc

3402	hsa-mir-93	CHD4	0.37265127	mirna_pc
3403	hsa-mir-93	POM121C	0.357503343	mirna_pc
3404	hsa-mir-93	CCDC111	0.355994466	mirna_pc
3405	hsa-mir-93	ZNF713	0.30935677	mirna_pc
3406	hsa-mir-93	GIGYF1	0.469851519	mirna_pc
3407	hsa-mir-93	TIA1	0.340241106	mirna_pc
3408	hsa-mir-93	ZNF664	0.324674222	mirna_pc
3409	hsa-mir-93	RRP7B	0.337279694	mirna_pc
3410	hsa-mir-93	RSBN1L	0.317490572	mirna_pc
3411	hsa-mir-93	LRDD	0.381768608	mirna_pc
3412	hsa-mir-93	CCDC157	0.457419139	mirna_pc
3413	hsa-mir-93	PDGFA	0.424094253	mirna_pc
3414	hsa-mir-93	SCML2	0.439064914	mirna_pc
3415	hsa-mir-93	SIKE1	0.379098552	mirna_pc
3416	hsa-mir-93	ZNF594	0.345397275	mirna_pc
3417	hsa-mir-93	MRPL19	0.34609983	mirna_pc
3418	hsa-mir-93	HSF2	0.331919102	mirna_pc
3419	hsa-mir-93	GTF2I	0.330165374	mirna_pc
3420	hsa-mir-93	MEPCE	0.480950563	mirna_pc
3421	hsa-mir-93	ZCCHC3	0.375224535	mirna_pc
3422	hsa-mir-93	TP53BP2	0.304797215	mirna_pc
3423	hsa-mir-93	ZNF644	0.30346905	mirna_pc
3424	hsa-mir-93	RRP15	0.374542493	mirna_pc
3425	hsa-mir-93	COL9A2	0.313910903	mirna_pc
3426	hsa-mir-93	MSTO2P	0.345501437	mirna_pc
3427	hsa-mir-93	ETV5	0.573403097	mirna_pc
3428	hsa-mir-93	PGAP1	0.320176826	mirna_pc
3429	hsa-mir-93	NUCKS1	0.372440188	mirna_pc
3430	hsa-mir-93	KIAA1430	0.338728825	mirna_pc
3431	hsa-mir-93	AADAT	0.440612361	mirna_pc
3432	hsa-mir-93	CBLL1	0.448576674	mirna_pc
3433	hsa-mir-93	IGHMBP2	0.584360136	mirna_pc
3434	hsa-mir-93	ACTR3B	0.319695065	mirna_pc
3435	hsa-mir-93	ABTB2	0.316637066	mirna_pc
3436	hsa-mir-93	PCNP	0.307575912	mirna_pc
3437	hsa-mir-93	NAF1	0.305022099	mirna_pc
3438	hsa-mir-93	C11orf51	0.308784308	mirna_pc
3439	hsa-mir-93	FZD1	0.422656182	mirna_pc
3440	hsa-mir-93	SPRED2	0.325914263	mirna_pc
3441	hsa-mir-93	B4GALNT4	0.379767921	mirna_pc
3442	hsa-mir-93	PRMT6	0.365976578	mirna_pc
3443	hsa-mir-93	HSDL1	0.308701392	mirna_pc
3444	hsa-mir-93	PDS5A	0.325699262	mirna_pc
3445	hsa-mir-93	PRKRIP1	0.353030159	mirna_pc
3446	hsa-mir-93	PDE4B	0.309820877	mirna_pc
3447	hsa-mir-93	H2AFY2	0.37099002	mirna_pc
3448	hsa-mir-93	FBXO48	0.301827905	mirna_pc
3449	hsa-mir-93	KIAA1324L	0.336034659	mirna_pc
3450	hsa-mir-93	PHIP	0.314172508	mirna_pc
3451	hsa-mir-93	TRIL	0.44760856	mirna_pc
3452	hsa-mir-93	PCDH18	0.367301076	mirna_pc
3453	hsa-mir-93	SMARCAD1	0.370936735	mirna_pc
3454	hsa-mir-93	DHTKD1	0.359459635	mirna_pc
3455	hsa-mir-93	SV2A	0.307032376	mirna_pc

3456	hsa-mir-93	SEMA4C	0.303518872	mirna_pc
3457	hsa-mir-93	EFNB3	0.39394379	mirna_pc
3458	hsa-mir-93	CENPV	0.336912822	mirna_pc
3459	hsa-mir-93	DNMT3A	0.554559014	mirna_pc
3460	hsa-mir-93	CKAP4	0.325791663	mirna_pc
3461	hsa-mir-93	TET1	0.433884189	mirna_pc
3462	hsa-mir-93	PRTFDC1	0.370832255	mirna_pc
3463	hsa-mir-93	FAM200A	0.515156847	mirna_pc
3464	hsa-mir-93	ZNF326	0.384597766	mirna_pc
3465	hsa-mir-93	ZNF711	0.350423081	mirna_pc
3466	hsa-mir-93	POM121	0.363862229	mirna_pc
3467	hsa-mir-3648	SEMA3E	0.471788877	mirna_pc
3468	hsa-mir-3648	ANLN	0.349893623	mirna_pc
3469	hsa-mir-3648	NCAPG	0.305663105	mirna_pc
3470	hsa-mir-3648	CLSPN	0.300716418	mirna_pc
3471	hsa-mir-3648	DLGAP5	0.30686817	mirna_pc
3472	hsa-mir-3648	EXO1	0.38658524	mirna_pc
3473	hsa-mir-3648	CKS2	0.396159206	mirna_pc
3474	hsa-mir-3648	DEPDC1	0.342033311	mirna_pc
3475	hsa-mir-3648	PTTG1	0.30206326	mirna_pc
3476	hsa-mir-3648	SKA1	0.32101457	mirna_pc
3477	hsa-mir-3648	CENPK	0.339527171	mirna_pc
3478	hsa-mir-3648	PBK	0.37375137	mirna_pc
3479	hsa-mir-3648	CDKN3	0.344531419	mirna_pc
3480	hsa-mir-3648	HMMR	0.346956217	mirna_pc
3481	hsa-mir-3648	CITED2	0.402262447	mirna_pc
3482	hsa-mir-3648	C6orf167	0.359866455	mirna_pc
3483	hsa-mir-3648	PTGES3	0.309480755	mirna_pc
3484	hsa-mir-3648	ULBP1	0.467503695	mirna_pc
3485	hsa-mir-3648	NR2F2	0.394208138	mirna_pc
3486	hsa-mir-3648	SNHG1	0.307085918	mirna_pc
3487	hsa-mir-3648	MLF1IP	0.367833564	mirna_pc
3488	hsa-mir-3648	CCDC99	0.322880516	mirna_pc
3489	hsa-mir-3648	FGF14	0.313275959	mirna_pc
3490	hsa-mir-3648	MSH2	0.323108484	mirna_pc
3491	hsa-mir-3648	CDC7	0.429708413	mirna_pc
3492	hsa-mir-3648	ARL6IP6	0.331394194	mirna_pc
3493	hsa-mir-3648	FBXO5	0.325106505	mirna_pc
3494	hsa-mir-3648	SMC2	0.568222714	mirna_pc
3495	hsa-mir-3648	ZNF367	0.526858921	mirna_pc
3496	hsa-mir-3648	NCL	0.302451167	mirna_pc
3497	hsa-mir-3648	HIST1H1E	0.334723909	mirna_pc
3498	hsa-mir-3648	C13orf37	0.340116094	mirna_pc
3499	hsa-mir-3648	THOP1	0.316351833	mirna_pc
3500	hsa-mir-3648	LOC375190	0.314853023	mirna_pc
3501	hsa-mir-3648	CRY1	0.325699312	mirna_pc
3502	hsa-mir-3648	TYW3	0.516259524	mirna_pc
3503	hsa-mir-3648	PPAT	0.325836175	mirna_pc
3504	hsa-mir-3648	ACAD10	0.381624551	mirna_pc
3505	hsa-mir-3648	TEX10	0.387647979	mirna_pc
3506	hsa-mir-3648	GAD1	0.39263429	mirna_pc
3507	hsa-mir-3648	RFC5	0.354603973	mirna_pc
3508	hsa-mir-3648	FIGN	0.489019504	mirna_pc
3509	hsa-mir-3648	HAUS6	0.321786781	mirna_pc

3510	hsa-mir-3648	RADIL	0.369676401	mirna_pc
3511	hsa-mir-3648	DENR	0.314280382	mirna_pc
3512	hsa-mir-3648	ALG6	0.310161666	mirna_pc
3513	hsa-mir-3648	PHF10	0.360850392	mirna_pc
3514	hsa-mir-3648	RIBC2	0.439438649	mirna_pc
3515	hsa-mir-3648	CAPS2	0.350543381	mirna_pc
3516	hsa-mir-3648	PBX3	0.364036784	mirna_pc
3517	hsa-mir-3648	MTHFD1	0.302982786	mirna_pc
3518	hsa-mir-3648	HABP4	0.451784199	mirna_pc
3519	hsa-mir-3648	PSMC3IP	0.344499934	mirna_pc
3520	hsa-mir-3648	C18orf54	0.300574171	mirna_pc
3521	hsa-mir-3648	CCNE2	0.37692398	mirna_pc
3522	hsa-mir-3648	CTSL2	0.371228662	mirna_pc
3523	hsa-mir-3648	CCDC104	0.305379533	mirna_pc
3524	hsa-mir-3648	HNRNPK	0.378562325	mirna_pc
3525	hsa-mir-3648	KIAA0776	0.32982999	mirna_pc
3526	hsa-mir-3648	CTNNAL1	0.450413483	mirna_pc
3527	hsa-mir-3648	TDG	0.325424449	mirna_pc
3528	hsa-mir-3648	RAD23B	0.336280166	mirna_pc
3529	hsa-mir-3648	CEP78	0.354608261	mirna_pc
3530	hsa-mir-3648	HDAC2	0.359323835	mirna_pc
3531	hsa-mir-3648	FAM184A	0.367584146	mirna_pc
3532	hsa-mir-3648	TDRD5	0.305942907	mirna_pc
3533	hsa-mir-3648	HAUS1	0.416021776	mirna_pc
3534	hsa-mir-3648	PCM1	0.311594932	mirna_pc
3535	hsa-mir-3648	DCK	0.412841304	mirna_pc
3536	hsa-mir-3648	OTUD6B	0.377353692	mirna_pc
3537	hsa-mir-3648	HNRNPA1	0.312755329	mirna_pc
3538	hsa-mir-3648	CCDC74B	0.31912676	mirna_pc
3539	hsa-mir-3648	HNRNPA1L2	0.342196216	mirna_pc
3540	hsa-mir-3648	TMX1	0.30419898	mirna_pc
3541	hsa-mir-3648	NCBP1	0.36910391	mirna_pc
3542	hsa-mir-3648	FANCM	0.300634389	mirna_pc
3543	hsa-mir-3648	USP1	0.370976276	mirna_pc
3544	hsa-mir-3648	SCG2	0.36689011	mirna_pc
3545	hsa-mir-3648	EDIL3	0.303341845	mirna_pc
3546	hsa-mir-3648	ORC3L	0.302672897	mirna_pc
3547	hsa-mir-3648	TCP1	0.330428548	mirna_pc
3548	hsa-mir-3648	SEMA3D	0.492422581	mirna_pc
3549	hsa-mir-3648	SLC25A30	0.374822323	mirna_pc
3550	hsa-mir-3648	ISCA1	0.345603422	mirna_pc
3551	hsa-mir-3648	C13orf27	0.323586745	mirna_pc
3552	hsa-mir-3648	KSR2	0.442870275	mirna_pc
3553	hsa-mir-3648	CCDC41	0.380922309	mirna_pc
3554	hsa-mir-3648	NOL8	0.303426344	mirna_pc
3555	hsa-mir-3648	GPN3	0.359835808	mirna_pc
3556	hsa-mir-3648	THSD7A	0.398507381	mirna_pc
3557	hsa-mir-3648	ISCA1P1	0.346022541	mirna_pc
3558	hsa-mir-3648	PSIP1	0.421578408	mirna_pc
3559	hsa-mir-3648	PCGF6	0.321390325	mirna_pc
3560	hsa-mir-3648	OSR2	0.300360741	mirna_pc
3561	hsa-mir-3648	C12orf76	0.361656776	mirna_pc
3562	hsa-mir-3648	C6orf170	0.339524593	mirna_pc
3563	hsa-mir-3648	KIAA0020	0.30717041	mirna_pc

3564	hsa-mir-3648	SENP6	0.305972449	mirna_pc
3565	hsa-mir-3648	METAP2	0.324625334	mirna_pc
3566	hsa-mir-3648	NUDCD2	0.360145968	mirna_pc
3567	hsa-mir-3648	PIK3C3	0.309321342	mirna_pc
3568	hsa-mir-3648	PHTF2	0.328800828	mirna_pc
3569	hsa-mir-3648	CNIH	0.31520053	mirna_pc
3570	hsa-mir-3648	PPP1CC	0.317557235	mirna_pc
3571	hsa-mir-3648	SNHG5	0.329132672	mirna_pc
3572	hsa-mir-3648	RMI1	0.418775417	mirna_pc
3573	hsa-mir-3648	TMEM67	0.340089012	mirna_pc
3574	hsa-mir-3648	DRG1	0.322395276	mirna_pc
3575	hsa-mir-3648	PSMD5	0.34970247	mirna_pc
3576	hsa-mir-3648	ACTR6	0.323797904	mirna_pc
3577	hsa-mir-3648	SFRS13A	0.33466691	mirna_pc
3578	hsa-mir-3648	KPNA5	0.306162555	mirna_pc
3579	hsa-mir-3648	MYBL1	0.455727788	mirna_pc
3580	hsa-mir-3648	PAPOLA	0.35581906	mirna_pc
3581	hsa-mir-3648	TMEM38B	0.414629505	mirna_pc
3582	hsa-mir-3648	GAS1	0.31203078	mirna_pc
3583	hsa-mir-3648	EFNA5	0.312643604	mirna_pc
3584	hsa-mir-3648	MRPL42	0.324279249	mirna_pc
3585	hsa-mir-3648	CRNKL1	0.305141688	mirna_pc
3586	hsa-mir-3648	CCDC78	0.38588161	mirna_pc
3587	hsa-mir-3648	DNM3	0.447320322	mirna_pc
3588	hsa-mir-3648	WDR78	0.405443678	mirna_pc
3589	hsa-mir-3648	CROCCL1	0.343713924	mirna_pc
3590	hsa-mir-3648	ZNF184	0.331045296	mirna_pc
3591	hsa-mir-3648	NIPSNAP1	0.301012169	mirna_pc
3592	hsa-mir-3648	C8orf37	0.468186373	mirna_pc
3593	hsa-mir-3648	PDGFC	0.43792333	mirna_pc
3594	hsa-mir-3648	SPA17	0.312899327	mirna_pc
3595	hsa-mir-3648	SFI1	0.330883847	mirna_pc
3596	hsa-mir-3648	MAP3K12	0.314995068	mirna_pc
3597	hsa-mir-3648	ZBTB10	0.341505872	mirna_pc
3598	hsa-mir-3648	TTC21B	0.338834609	mirna_pc
3599	hsa-mir-3648	ANGPT1	0.480424884	mirna_pc
3600	hsa-mir-3648	DNAJA1	0.349821962	mirna_pc
3601	hsa-mir-3648	LOC338758	0.39311322	mirna_pc
3602	hsa-mir-3648	ETV1	0.332163179	mirna_pc
3603	hsa-mir-3648	CPT1C	0.309052278	mirna_pc
3604	hsa-mir-3648	GPR137C	0.424111811	mirna_pc
3605	hsa-mir-3648	UBQLN1	0.318841906	mirna_pc
3606	hsa-mir-3648	CEP110	0.397017543	mirna_pc
3607	hsa-mir-3648	POLR3F	0.349954569	mirna_pc
3608	hsa-mir-3648	TMX3	0.32708196	mirna_pc
3609	hsa-mir-3648	GRB14	0.330197878	mirna_pc
3610	hsa-mir-3648	TIMM9	0.325235243	mirna_pc
3611	hsa-mir-3648	HTR7P1	0.347947813	mirna_pc
3612	hsa-mir-3648	TGFB2	0.360913161	mirna_pc
3613	hsa-mir-3648	BCLAF1	0.309020455	mirna_pc
3614	hsa-mir-3648	CROCC	0.357254759	mirna_pc
3615	hsa-mir-3648	B4GALT6	0.327898109	mirna_pc
3616	hsa-mir-3648	FADS1	0.316668464	mirna_pc
3617	hsa-mir-3648	C6orf182	0.321996594	mirna_pc

3618	hsa-mir-3648	CNIH2	0.313756841	mirna_pc
3619	hsa-mir-3648	EID3	0.464651858	mirna_pc
3620	hsa-mir-3648	MNS1	0.311730683	mirna_pc
3621	hsa-mir-3648	KIAA1467	0.426567869	mirna_pc
3622	hsa-mir-3648	PLAC2	0.312202994	mirna_pc
3623	hsa-mir-3648	LYPLAL1	0.302800939	mirna_pc
3624	hsa-mir-3648	C12orf24	0.536535525	mirna_pc
3625	hsa-mir-3648	GLS2	0.448812	mirna_pc
3626	hsa-mir-3648	SEMA3A	0.4990976	mirna_pc
3627	hsa-mir-3648	USP13	0.31101111	mirna_pc
3628	hsa-mir-3648	ESF1	0.346017756	mirna_pc
3629	hsa-mir-3648	IFT74	0.49390002	mirna_pc
3630	hsa-mir-3648	MDM1	0.465109042	mirna_pc
3631	hsa-mir-3648	C6orf162	0.350225877	mirna_pc
3632	hsa-mir-3648	LRRC40	0.35417441	mirna_pc
3633	hsa-mir-3648	SLITRK6	0.408784104	mirna_pc
3634	hsa-mir-3648	ERGIC2	0.404233932	mirna_pc
3635	hsa-mir-3648	HAPLN1	0.529081842	mirna_pc
3636	hsa-mir-3648	LOC221710	0.320431134	mirna_pc
3637	hsa-mir-3648	CRYZ	0.405360427	mirna_pc
3638	hsa-mir-3648	SMEK2	0.306054783	mirna_pc
3639	hsa-mir-3648	C20orf96	0.323375162	mirna_pc
3640	hsa-mir-3648	HSF2	0.341080311	mirna_pc
3641	hsa-mir-3648	RSRC2	0.30634266	mirna_pc
3642	hsa-mir-3648	IKBKAP	0.323986486	mirna_pc
3643	hsa-mir-3648	ASF1A	0.31417341	mirna_pc
3644	hsa-mir-3648	CTXN1	0.359952258	mirna_pc
3645	hsa-mir-3648	TMEM20	0.391400067	mirna_pc
3646	hsa-mir-3648	ZNF680	0.373253573	mirna_pc
3647	hsa-mir-3648	C9orf21	0.381782514	mirna_pc
3648	hsa-mir-3648	C5orf54	0.306423656	mirna_pc
3649	hsa-mir-3648	LOC283070	0.37471221	mirna_pc
3650	hsa-mir-3648	SLC11A2	0.342394294	mirna_pc
3651	hsa-mir-3648	HEY2	0.385576365	mirna_pc
3652	hsa-mir-3648	ZFP37	0.351642342	mirna_pc
3653	hsa-mir-3648	PHIP	0.317773352	mirna_pc
3654	hsa-mir-3648	NDUFAF4	0.376534358	mirna_pc
3655	hsa-mir-3648	LCA5	0.447747982	mirna_pc
3656	hsa-mir-3648	C20orf7	0.302560091	mirna_pc
3657	hsa-mir-3648	CDC73	0.308727465	mirna_pc
3658	hsa-mir-3648	SLC2A3	0.325833927	mirna_pc
3659	hsa-mir-3648	CNIH3	0.351487179	mirna_pc
3660	hsa-mir-3648	WDSUB1	0.328036271	mirna_pc
3661	hsa-mir-3648	BRAP	0.302996377	mirna_pc
3662	hsa-mir-3648	AGTPBP1	0.387696748	mirna_pc
3663	hsa-mir-18a	TPX2	0.441092708	mirna_pc
3664	hsa-mir-18a	KIF4B	0.368651013	mirna_pc
3665	hsa-mir-18a	TOP2A	0.325508349	mirna_pc
3666	hsa-mir-18a	KPNA2	0.338975802	mirna_pc
3667	hsa-mir-18a	CENPF	0.300418386	mirna_pc
3668	hsa-mir-18a	HOXC9	0.375969324	mirna_pc
3669	hsa-mir-18a	KIF11	0.354882955	mirna_pc
3670	hsa-mir-18a	ECT2	0.304920728	mirna_pc
3671	hsa-mir-18a	RRM2	0.438362433	mirna_pc

3672	hsa-mir-18a	CDK1	0.398168302	mirna_pc
3673	hsa-mir-18a	CDC25C	0.390995591	mirna_pc
3674	hsa-mir-18a	MYBL2	0.37728832	mirna_pc
3675	hsa-mir-18a	PLK1	0.326127196	mirna_pc
3676	hsa-mir-18a	UBE2C	0.517272141	mirna_pc
3677	hsa-mir-18a	BUB1	0.464997492	mirna_pc
3678	hsa-mir-18a	KIFC1	0.403977744	mirna_pc
3679	hsa-mir-18a	BUB1B	0.448691807	mirna_pc
3680	hsa-mir-18a	HDGF	0.424664415	mirna_pc
3681	hsa-mir-18a	KIF18B	0.414630599	mirna_pc
3682	hsa-mir-18a	FAM72A	0.353925158	mirna_pc
3683	hsa-mir-18a	KIF2C	0.395854915	mirna_pc
3684	hsa-mir-18a	FANCA	0.413688012	mirna_pc
3685	hsa-mir-18a	NCAPG	0.432184505	mirna_pc
3686	hsa-mir-18a	CDC20	0.332477376	mirna_pc
3687	hsa-mir-18a	CLSPN	0.401754949	mirna_pc
3688	hsa-mir-18a	SGOL1	0.485329598	mirna_pc
3689	hsa-mir-18a	ARHGAP11A	0.383135777	mirna_pc
3690	hsa-mir-18a	CCNA2	0.391791753	mirna_pc
3691	hsa-mir-18a	CDC25B	0.324763399	mirna_pc
3692	hsa-mir-18a	CBX3	0.437592508	mirna_pc
3693	hsa-mir-18a	SPC25	0.445853886	mirna_pc
3694	hsa-mir-18a	IQGAP3	0.329681889	mirna_pc
3695	hsa-mir-18a	NUP62	0.305953415	mirna_pc
3696	hsa-mir-18a	FANCI	0.332668954	mirna_pc
3697	hsa-mir-18a	FAM54A	0.370460254	mirna_pc
3698	hsa-mir-18a	NEK2	0.493287201	mirna_pc
3699	hsa-mir-18a	MND1	0.39252974	mirna_pc
3700	hsa-mir-18a	HOXC11	0.338224488	mirna_pc
3701	hsa-mir-18a	KIF22	0.374468409	mirna_pc
3702	hsa-mir-18a	NCAPH	0.413093338	mirna_pc
3703	hsa-mir-18a	GTSE1	0.359076142	mirna_pc
3704	hsa-mir-18a	KIF23	0.32812361	mirna_pc
3705	hsa-mir-18a	RAD54L	0.407763706	mirna_pc
3706	hsa-mir-18a	CENPA	0.394730108	mirna_pc
3707	hsa-mir-18a	MAD2L1	0.507553135	mirna_pc
3708	hsa-mir-18a	TIMELESS	0.394974823	mirna_pc
3709	hsa-mir-18a	LMNB1	0.363413489	mirna_pc
3710	hsa-mir-18a	DLGAP5	0.337428513	mirna_pc
3711	hsa-mir-18a	CDCA8	0.456994498	mirna_pc
3712	hsa-mir-18a	TROAP	0.405608556	mirna_pc
3713	hsa-mir-18a	CCNB1	0.330985766	mirna_pc
3714	hsa-mir-18a	CDCA5	0.351916622	mirna_pc
3715	hsa-mir-18a	TACC3	0.312583314	mirna_pc
3716	hsa-mir-18a	XPO1	0.391474073	mirna_pc
3717	hsa-mir-18a	STMN1	0.349446762	mirna_pc
3718	hsa-mir-18a	CDCA2	0.366008626	mirna_pc
3719	hsa-mir-18a	HMGA1	0.302007622	mirna_pc
3720	hsa-mir-18a	DKC1	0.367425226	mirna_pc
3721	hsa-mir-18a	UBE2T	0.333862537	mirna_pc
3722	hsa-mir-18a	CKS2	0.323728315	mirna_pc
3723	hsa-mir-18a	CKS1B	0.31661041	mirna_pc
3724	hsa-mir-18a	KIAA0101	0.330794403	mirna_pc
3725	hsa-mir-18a	KIF4A	0.401078275	mirna_pc

3726	hsa-mir-18a	ORC1L	0.356403666	mirna_pc
3727	hsa-mir-18a	SNRPB	0.326446164	mirna_pc
3728	hsa-mir-18a	KNTC1	0.421948544	mirna_pc
3729	hsa-mir-18a	CCNB2	0.327417564	mirna_pc
3730	hsa-mir-18a	NUF2	0.4995642	mirna_pc
3731	hsa-mir-18a	PLK4	0.468368981	mirna_pc
3732	hsa-mir-18a	FANCB	0.348078935	mirna_pc
3733	hsa-mir-18a	KIF18A	0.408760373	mirna_pc
3734	hsa-mir-18a	DEPDC1	0.387729287	mirna_pc
3735	hsa-mir-18a	SKA3	0.521074447	mirna_pc
3736	hsa-mir-18a	KIF14	0.311846246	mirna_pc
3737	hsa-mir-18a	HJURP	0.459035577	mirna_pc
3738	hsa-mir-18a	SKA1	0.37493929	mirna_pc
3739	hsa-mir-18a	BLM	0.435876891	mirna_pc
3740	hsa-mir-18a	NME1	0.477540862	mirna_pc
3741	hsa-mir-18a	PAICS	0.437524344	mirna_pc
3742	hsa-mir-18a	CCDC150	0.53394456	mirna_pc
3743	hsa-mir-18a	RCC1	0.430949937	mirna_pc
3744	hsa-mir-18a	OIP5	0.36085796	mirna_pc
3745	hsa-mir-18a	DTL	0.341903443	mirna_pc
3746	hsa-mir-18a	EME1	0.380886254	mirna_pc
3747	hsa-mir-18a	RECQL4	0.447733527	mirna_pc
3748	hsa-mir-18a	AURKA	0.470493332	mirna_pc
3749	hsa-mir-18a	CENPK	0.417659193	mirna_pc
3750	hsa-mir-18a	AURKB	0.406864307	mirna_pc
3751	hsa-mir-18a	MCM10	0.404373375	mirna_pc
3752	hsa-mir-18a	SGOL2	0.458415382	mirna_pc
3753	hsa-mir-18a	CHEK2	0.33489323	mirna_pc
3754	hsa-mir-18a	CENPE	0.368943583	mirna_pc
3755	hsa-mir-18a	KIF15	0.416310721	mirna_pc
3756	hsa-mir-18a	CDCA3	0.417943375	mirna_pc
3757	hsa-mir-18a	C1orf112	0.480651958	mirna_pc
3758	hsa-mir-18a	GINS1	0.403497041	mirna_pc
3759	hsa-mir-18a	FEN1	0.355823737	mirna_pc
3760	hsa-mir-18a	TRAIP	0.427953313	mirna_pc
3761	hsa-mir-18a	ORC6L	0.392720284	mirna_pc
3762	hsa-mir-18a	ERH	0.364913864	mirna_pc
3763	hsa-mir-18a	PBK	0.339280212	mirna_pc
3764	hsa-mir-18a	C16orf59	0.346740028	mirna_pc
3765	hsa-mir-18a	CDKN3	0.354024437	mirna_pc
3766	hsa-mir-18a	RAE1	0.351038112	mirna_pc
3767	hsa-mir-18a	FAM72D	0.359179289	mirna_pc
3768	hsa-mir-18a	FAM72B	0.397658088	mirna_pc
3769	hsa-mir-18a	EZH2	0.343555101	mirna_pc
3770	hsa-mir-18a	NEIL3	0.308552414	mirna_pc
3771	hsa-mir-18a	C12orf48	0.474168287	mirna_pc
3772	hsa-mir-18a	HNRNPL	0.432092523	mirna_pc
3773	hsa-mir-18a	ZWILCH	0.305493863	mirna_pc
3774	hsa-mir-18a	HMMR	0.364419639	mirna_pc
3775	hsa-mir-18a	TTK	0.404879273	mirna_pc
3776	hsa-mir-18a	ZWINT	0.445614622	mirna_pc
3777	hsa-mir-18a	TUBA1B	0.319393689	mirna_pc
3778	hsa-mir-18a	WDR67	0.325848994	mirna_pc
3779	hsa-mir-18a	HOXC6	0.339496538	mirna_pc

3780	hsa-mir-18a	BUB3	0.399836783	mirna_pc
3781	hsa-mir-18a	HELLS	0.376090971	mirna_pc
3782	hsa-mir-18a	POLE2	0.378459249	mirna_pc
3783	hsa-mir-18a	HNRNPC	0.374441998	mirna_pc
3784	hsa-mir-18a	ILF2	0.312966199	mirna_pc
3785	hsa-mir-18a	C6orf167	0.329757658	mirna_pc
3786	hsa-mir-18a	CHEK1	0.402763899	mirna_pc
3787	hsa-mir-18a	HNRNPA2B1	0.471637846	mirna_pc
3788	hsa-mir-18a	RAN	0.337900287	mirna_pc
3789	hsa-mir-18a	TYMS	0.34004037	mirna_pc
3790	hsa-mir-18a	C20orf20	0.377895202	mirna_pc
3791	hsa-mir-18a	MCM6	0.403373903	mirna_pc
3792	hsa-mir-18a	CDC6	0.31297008	mirna_pc
3793	hsa-mir-18a	CDCA7	0.368776244	mirna_pc
3794	hsa-mir-18a	EPR1	0.341279837	mirna_pc
3795	hsa-mir-18a	PIF1	0.440083582	mirna_pc
3796	hsa-mir-18a	H2AFX	0.361727858	mirna_pc
3797	hsa-mir-18a	SFRS2	0.355402943	mirna_pc
3798	hsa-mir-18a	DCAF13	0.383941343	mirna_pc
3799	hsa-mir-18a	MTHFD1L	0.309951426	mirna_pc
3800	hsa-mir-18a	PSMD14	0.314518481	mirna_pc
3801	hsa-mir-18a	UCK2	0.424581204	mirna_pc
3802	hsa-mir-18a	NFKBIL2	0.391990054	mirna_pc
3803	hsa-mir-18a	PTGES3	0.310163128	mirna_pc
3804	hsa-mir-18a	FAM60A	0.404525709	mirna_pc
3805	hsa-mir-18a	ATAD5	0.349545563	mirna_pc
3806	hsa-mir-18a	SNRPG	0.438594045	mirna_pc
3807	hsa-mir-18a	TH1L	0.313767007	mirna_pc
3808	hsa-mir-18a	KIF20B	0.382043786	mirna_pc
3809	hsa-mir-18a	SNRPA	0.41478512	mirna_pc
3810	hsa-mir-18a	FUS	0.332420024	mirna_pc
3811	hsa-mir-18a	ATAD2	0.322549712	mirna_pc
3812	hsa-mir-18a	U2AF2	0.304382918	mirna_pc
3813	hsa-mir-18a	RFC3	0.461837975	mirna_pc
3814	hsa-mir-18a	DEPDC1B	0.538250971	mirna_pc
3815	hsa-mir-18a	HOXA11	0.30294697	mirna_pc
3816	hsa-mir-18a	ANP32E	0.378732603	mirna_pc
3817	hsa-mir-18a	CENPO	0.425173354	mirna_pc
3818	hsa-mir-18a	XRCC2	0.314288316	mirna_pc
3819	hsa-mir-18a	C19orf48	0.321070615	mirna_pc
3820	hsa-mir-18a	BAX	0.309929176	mirna_pc
3821	hsa-mir-18a	CENPL	0.346895119	mirna_pc
3822	hsa-mir-18a	GEN1	0.341767531	mirna_pc
3823	hsa-mir-18a	HMGB2	0.442176162	mirna_pc
3824	hsa-mir-18a	DNA2	0.467079097	mirna_pc
3825	hsa-mir-18a	NOP56	0.458537535	mirna_pc
3826	hsa-mir-18a	SNRPD1	0.334714769	mirna_pc
3827	hsa-mir-18a	DSN1	0.393471281	mirna_pc
3828	hsa-mir-18a	SNHG1	0.38790135	mirna_pc
3829	hsa-mir-18a	CSE1L	0.344420621	mirna_pc
3830	hsa-mir-18a	DTYMK	0.466343383	mirna_pc
3831	hsa-mir-18a	MLF1IP	0.393006751	mirna_pc
3832	hsa-mir-18a	HSPE1	0.447755699	mirna_pc
3833	hsa-mir-18a	CCDC99	0.40594164	mirna_pc

3834	hsa-mir-18a	CDK2	0.400976781	mirna_pc
3835	hsa-mir-18a	CDK4	0.329454542	mirna_pc
3836	hsa-mir-18a	PFDN2	0.326939593	mirna_pc
3837	hsa-mir-18a	TPM3	0.3053522	mirna_pc
3838	hsa-mir-18a	BCL2L12	0.434462854	mirna_pc
3839	hsa-mir-18a	HNRNPR	0.316858784	mirna_pc
3840	hsa-mir-18a	DLEU2	0.401978481	mirna_pc
3841	hsa-mir-18a	PRIM1	0.40531298	mirna_pc
3842	hsa-mir-18a	KAT2A	0.308208913	mirna_pc
3843	hsa-mir-18a	PSMC4	0.403841422	mirna_pc
3844	hsa-mir-18a	GGCT	0.321548152	mirna_pc
3845	hsa-mir-18a	DDX27	0.421003808	mirna_pc
3846	hsa-mir-18a	TCOF1	0.387308229	mirna_pc
3847	hsa-mir-18a	HOXC10	0.323308407	mirna_pc
3848	hsa-mir-18a	NCAPG2	0.31147421	mirna_pc
3849	hsa-mir-18a	MSH2	0.409808619	mirna_pc
3850	hsa-mir-18a	SALL4	0.396985259	mirna_pc
3851	hsa-mir-18a	EIF3B	0.365606268	mirna_pc
3852	hsa-mir-18a	NOP58	0.424640255	mirna_pc
3853	hsa-mir-18a	DBF4	0.365230917	mirna_pc
3854	hsa-mir-18a	TOMM40	0.351403495	mirna_pc
3855	hsa-mir-18a	EIF2AK1	0.32748956	mirna_pc
3856	hsa-mir-18a	CDC7	0.388735431	mirna_pc
3857	hsa-mir-18a	SSB	0.569086939	mirna_pc
3858	hsa-mir-18a	PUS1	0.437273309	mirna_pc
3859	hsa-mir-18a	NOL11	0.377906995	mirna_pc
3860	hsa-mir-18a	SNRPC	0.441938348	mirna_pc
3861	hsa-mir-18a	FBXO5	0.476522051	mirna_pc
3862	hsa-mir-18a	WDR75	0.375724295	mirna_pc
3863	hsa-mir-18a	FIGNL1	0.322161678	mirna_pc
3864	hsa-mir-18a	MDK	0.303572796	mirna_pc
3865	hsa-mir-18a	E2F3	0.522599574	mirna_pc
3866	hsa-mir-18a	DDX11	0.320469845	mirna_pc
3867	hsa-mir-18a	SF3B14	0.341758939	mirna_pc
3868	hsa-mir-18a	RUVBL2	0.369744286	mirna_pc
3869	hsa-mir-18a	CDC25A	0.499460352	mirna_pc
3870	hsa-mir-18a	TMEM48	0.318358734	mirna_pc
3871	hsa-mir-18a	RAD54B	0.373923389	mirna_pc
3872	hsa-mir-18a	H2AFZ	0.327378637	mirna_pc
3873	hsa-mir-18a	MAGOH	0.314123771	mirna_pc
3874	hsa-mir-18a	DSCC1	0.397155578	mirna_pc
3875	hsa-mir-18a	WDR12	0.333184408	mirna_pc
3876	hsa-mir-18a	C13orf34	0.434064873	mirna_pc
3877	hsa-mir-18a	SRPK1	0.327457543	mirna_pc
3878	hsa-mir-18a	MCM8	0.459305568	mirna_pc
3879	hsa-mir-18a	CPSF3	0.418205729	mirna_pc
3880	hsa-mir-18a	SNRPE	0.430545965	mirna_pc
3881	hsa-mir-18a	C21orf45	0.441208775	mirna_pc
3882	hsa-mir-18a	ATIC	0.469729676	mirna_pc
3883	hsa-mir-18a	UTP6	0.302751099	mirna_pc
3884	hsa-mir-18a	PPM1G	0.352971962	mirna_pc
3885	hsa-mir-18a	MARCKSL1	0.337100866	mirna_pc
3886	hsa-mir-18a	NPM3	0.302833812	mirna_pc
3887	hsa-mir-18a	NUTF2	0.338129479	mirna_pc

3888	hsa-mir-18a	DAZAP1	0.37389393	mirna_pc
3889	hsa-mir-18a	CBX8	0.37282152	mirna_pc
3890	hsa-mir-18a	RPA3	0.309658215	mirna_pc
3891	hsa-mir-18a	TRMT6	0.324013078	mirna_pc
3892	hsa-mir-18a	GNPDA1	0.311385744	mirna_pc
3893	hsa-mir-18a	WDR34	0.301151326	mirna_pc
3894	hsa-mir-18a	NCL	0.447655762	mirna_pc
3895	hsa-mir-18a	NUDCD1	0.318114167	mirna_pc
3896	hsa-mir-18a	PFDN4	0.438373231	mirna_pc
3897	hsa-mir-18a	PAFAH1B3	0.319919005	mirna_pc
3898	hsa-mir-18a	SRRT	0.399421714	mirna_pc
3899	hsa-mir-18a	CACYBP	0.414086427	mirna_pc
3900	hsa-mir-18a	SASS6	0.428966321	mirna_pc
3901	hsa-mir-18a	PSRC1	0.50443289	mirna_pc
3902	hsa-mir-18a	DGUOK	0.338733124	mirna_pc
3903	hsa-mir-18a	DBF4B	0.400302086	mirna_pc
3904	hsa-mir-18a	POU2F1	0.417231392	mirna_pc
3905	hsa-mir-18a	FBL	0.422082933	mirna_pc
3906	hsa-mir-18a	C13orf37	0.372367151	mirna_pc
3907	hsa-mir-18a	HNRNPM	0.353356596	mirna_pc
3908	hsa-mir-18a	LSM 12.00	0.359766327	mirna_pc
3909	hsa-mir-18a	PNPT1	0.434329117	mirna_pc
3910	hsa-mir-18a	TMPO	0.41647127	mirna_pc
3911	hsa-mir-18a	SET	0.347589928	mirna_pc
3912	hsa-mir-18a	LOC388796	0.435979801	mirna_pc
3913	hsa-mir-18a	MRTO4	0.37535209	mirna_pc
3914	hsa-mir-18a	GABPB1	0.409719248	mirna_pc
3915	hsa-mir-18a	PPIH	0.399675318	mirna_pc
3916	hsa-mir-18a	RCN1	0.328147389	mirna_pc
3917	hsa-mir-18a	PPAT	0.340821222	mirna_pc
3918	hsa-mir-18a	DHX9	0.334691345	mirna_pc
3919	hsa-mir-18a	CCDC138	0.359992817	mirna_pc
3920	hsa-mir-18a	LOC100128191	0.37068313	mirna_pc
3921	hsa-mir-18a	HMGN2	0.39775118	mirna_pc
3922	hsa-mir-18a	ETV4	0.433500777	mirna_pc
3923	hsa-mir-18a	SMYD5	0.330838104	mirna_pc
3924	hsa-mir-18a	TSSC1	0.327640105	mirna_pc
3925	hsa-mir-18a	TLCD1	0.326536536	mirna_pc
3926	hsa-mir-18a	SNRPF	0.378046431	mirna_pc
3927	hsa-mir-18a	SFRS1	0.530298988	mirna_pc
3928	hsa-mir-18a	CASP8	0.318412171	mirna_pc
3929	hsa-mir-18a	WDR43	0.420143206	mirna_pc
3930	hsa-mir-18a	SNRPA1	0.43657584	mirna_pc
3931	hsa-mir-18a	CCDC58	0.314956009	mirna_pc
3932	hsa-mir-18a	TIPIN	0.357868559	mirna_pc
3933	hsa-mir-18a	HSPBP1	0.368116499	mirna_pc
3934	hsa-mir-18a	C4orf46	0.394345564	mirna_pc
3935	hsa-mir-18a	DNMT3B	0.312630609	mirna_pc
3936	hsa-mir-18a	DARS2	0.365947628	mirna_pc
3937	hsa-mir-18a	LYAR	0.489840457	mirna_pc
3938	hsa-mir-18a	UTP14A	0.403624582	mirna_pc
3939	hsa-mir-18a	HSPD1	0.508989085	mirna_pc
3940	hsa-mir-18a	SSRP1	0.345375211	mirna_pc
3941	hsa-mir-18a	POLR2D	0.430967717	mirna_pc

3942	hsa-mir-18a	FTSJ2	0.388861133	mirna_pc
3943	hsa-mir-18a	PTRH2	0.324116293	mirna_pc
3944	hsa-mir-18a	PA2G4	0.457039022	mirna_pc
3945	hsa-mir-18a	KHDRBS1	0.393531764	mirna_pc
3946	hsa-mir-18a	SLC5A6	0.343073479	mirna_pc
3947	hsa-mir-18a	TOMM34	0.341972077	mirna_pc
3948	hsa-mir-18a	MTL 5.00	0.387286266	mirna_pc
3949	hsa-mir-18a	PRPF40A	0.337193452	mirna_pc
3950	hsa-mir-18a	CCDC18	0.361416828	mirna_pc
3951	hsa-mir-18a	AHCY	0.348587431	mirna_pc
3952	hsa-mir-18a	CCDC34	0.462546989	mirna_pc
3953	hsa-mir-18a	DNAJC2	0.397402554	mirna_pc
3954	hsa-mir-18a	HNRNPF	0.369409978	mirna_pc
3955	hsa-mir-18a	SLC39A10	0.415058397	mirna_pc
3956	hsa-mir-18a	SUV420H2	0.360374819	mirna_pc
3957	hsa-mir-18a	ZNF695	0.526317839	mirna_pc
3958	hsa-mir-18a	PDSS1	0.444181349	mirna_pc
3959	hsa-mir-18a	HAUS6	0.340399884	mirna_pc
3960	hsa-mir-18a	PSMA1	0.304190103	mirna_pc
3961	hsa-mir-18a	CENPJ	0.317759789	mirna_pc
3962	hsa-mir-18a	GNL2	0.310570402	mirna_pc
3963	hsa-mir-18a	HNRNPU	0.355694755	mirna_pc
3964	hsa-mir-18a	NOC2L	0.302174123	mirna_pc
3965	hsa-mir-18a	NASP	0.475070065	mirna_pc
3966	hsa-mir-18a	POLR1B	0.417463104	mirna_pc
3967	hsa-mir-18a	OBFC2B	0.407287143	mirna_pc
3968	hsa-mir-18a	PTMA	0.463450424	mirna_pc
3969	hsa-mir-18a	PSMD1	0.402105468	mirna_pc
3970	hsa-mir-18a	DDX56	0.308686351	mirna_pc
3971	hsa-mir-18a	DENR	0.365674917	mirna_pc
3972	hsa-mir-18a	SCARB1	0.378485562	mirna_pc
3973	hsa-mir-18a	C11orf84	0.307574077	mirna_pc
3974	hsa-mir-18a	TBCE	0.372904548	mirna_pc
3975	hsa-mir-18a	SUV39H2	0.4738558	mirna_pc
3976	hsa-mir-18a	LSM 2.00	0.39597907	mirna_pc
3977	hsa-mir-18a	FANCL	0.355526114	mirna_pc
3978	hsa-mir-18a	HNRNPD	0.441804681	mirna_pc
3979	hsa-mir-18a	DPY30	0.359435725	mirna_pc
3980	hsa-mir-18a	MTHFD2	0.314530289	mirna_pc
3981	hsa-mir-18a	GTF2F2	0.36499423	mirna_pc
3982	hsa-mir-18a	GTPBP4	0.36765358	mirna_pc
3983	hsa-mir-18a	TARDBP	0.316150318	mirna_pc
3984	hsa-mir-18a	POP1	0.312096274	mirna_pc
3985	hsa-mir-18a	NFE2L3	0.407466029	mirna_pc
3986	hsa-mir-18a	SFRS3	0.452303482	mirna_pc
3987	hsa-mir-18a	LSM 5.00	0.466249005	mirna_pc
3988	hsa-mir-18a	CCT4	0.327043772	mirna_pc
3989	hsa-mir-18a	RAD9A	0.308150114	mirna_pc
3990	hsa-mir-18a	C7orf40	0.350623705	mirna_pc
3991	hsa-mir-18a	KIAA0406	0.355088802	mirna_pc
3992	hsa-mir-18a	DPM1	0.304942241	mirna_pc
3993	hsa-mir-18a	HSP90B1	0.41312436	mirna_pc
3994	hsa-mir-18a	NAE1	0.343164005	mirna_pc
3995	hsa-mir-18a	PSMC3IP	0.341108632	mirna_pc

3996	hsa-mir-18a	PAK1IP1	0.468038066	mirna_pc
3997	hsa-mir-18a	NUDT15	0.368731019	mirna_pc
3998	hsa-mir-18a	E2F4	0.3145801	mirna_pc
3999	hsa-mir-18a	SLMO2	0.323156698	mirna_pc
4000	hsa-mir-18a	PFDN6	0.323541945	mirna_pc
4001	hsa-mir-18a	TWISTNB	0.35758079	mirna_pc
4002	hsa-mir-18a	DHFR	0.304597165	mirna_pc
4003	hsa-mir-18a	NAA25	0.359680001	mirna_pc
4004	hsa-mir-18a	ILF3	0.353030104	mirna_pc
4005	hsa-mir-18a	NT5DC2	0.312541884	mirna_pc
4006	hsa-mir-18a	EHMT2	0.387666222	mirna_pc
4007	hsa-mir-18a	MMD	0.332725735	mirna_pc
4008	hsa-mir-18a	HNRNPAB	0.353268053	mirna_pc
4009	hsa-mir-18a	DEK	0.400426162	mirna_pc
4010	hsa-mir-18a	TBC1D4	0.316276714	mirna_pc
4011	hsa-mir-18a	SFRS9	0.336483136	mirna_pc
4012	hsa-mir-18a	TRIB3	0.345329887	mirna_pc
4013	hsa-mir-18a	PASK	0.401118121	mirna_pc
4014	hsa-mir-18a	SAAL1	0.392941421	mirna_pc
4015	hsa-mir-18a	C1orf131	0.359508352	mirna_pc
4016	hsa-mir-18a	TAF4	0.38121334	mirna_pc
4017	hsa-mir-18a	TGS1	0.306037044	mirna_pc
4018	hsa-mir-18a	SNHG3	0.316285159	mirna_pc
4019	hsa-mir-18a	HEATR2	0.533590448	mirna_pc
4020	hsa-mir-18a	LSM 6.00	0.312267308	mirna_pc
4021	hsa-mir-18a	PRAME	0.340340132	mirna_pc
4022	hsa-mir-18a	CYB5B	0.356660871	mirna_pc
4023	hsa-mir-18a	SNRPB2	0.460453953	mirna_pc
4024	hsa-mir-18a	GLO1	0.363204688	mirna_pc
4025	hsa-mir-18a	MSL3L2	0.330857703	mirna_pc
4026	hsa-mir-18a	SART3	0.304599481	mirna_pc
4027	hsa-mir-18a	TRAF4	0.34269533	mirna_pc
4028	hsa-mir-18a	GAR1	0.436230056	mirna_pc
4029	hsa-mir-18a	CEP152	0.315969295	mirna_pc
4030	hsa-mir-18a	C15orf23	0.383810293	mirna_pc
4031	hsa-mir-18a	PRMT3	0.327867934	mirna_pc
4032	hsa-mir-18a	RPL36A	0.361574248	mirna_pc
4033	hsa-mir-18a	PSMD12	0.303243628	mirna_pc
4034	hsa-mir-18a	C1orf174	0.320676984	mirna_pc
4035	hsa-mir-18a	IGF2BP2	0.315189491	mirna_pc
4036	hsa-mir-18a	WHSC1	0.311485329	mirna_pc
4037	hsa-mir-18a	RPN2	0.34340779	mirna_pc
4038	hsa-mir-18a	SAE1	0.364116274	mirna_pc
4039	hsa-mir-18a	MRPL17	0.334286471	mirna_pc
4040	hsa-mir-18a	RNF219	0.397591915	mirna_pc
4041	hsa-mir-18a	ITGB3BP	0.449243109	mirna_pc
4042	hsa-mir-18a	EIF2S2	0.411479987	mirna_pc
4043	hsa-mir-18a	SLC25A32	0.309052082	mirna_pc
4044	hsa-mir-18a	ENOPH1	0.343254846	mirna_pc
4045	hsa-mir-18a	PABPC1L	0.330833602	mirna_pc
4046	hsa-mir-18a	C17orf42	0.36223562	mirna_pc
4047	hsa-mir-18a	DNAJC7	0.331249973	mirna_pc
4048	hsa-mir-18a	TSEN54	0.347048557	mirna_pc
4049	hsa-mir-18a	ACTR5	0.364521444	mirna_pc

4050	hsa-mir-18a	HSPA14	0.300165554	mirna_pc
4051	hsa-mir-18a	DDX55	0.505104506	mirna_pc
4052	hsa-mir-18a	HPDL	0.450571315	mirna_pc
4053	hsa-mir-18a	CDK5RAP1	0.344911845	mirna_pc
4054	hsa-mir-18a	SFPQ	0.353504371	mirna_pc
4055	hsa-mir-18a	POMP	0.318354674	mirna_pc
4056	hsa-mir-18a	VPS72	0.309416549	mirna_pc
4057	hsa-mir-18a	RCOR2	0.387468266	mirna_pc
4058	hsa-mir-18a	ATAD3A	0.348843503	mirna_pc
4059	hsa-mir-18a	HDAC2	0.46738592	mirna_pc
4060	hsa-mir-18a	GMNN	0.437661788	mirna_pc
4061	hsa-mir-18a	PDCD2L	0.308775075	mirna_pc
4062	hsa-mir-18a	PA2G4P4	0.453543204	mirna_pc
4063	hsa-mir-18a	POLG2	0.329461146	mirna_pc
4064	hsa-mir-18a	GEMIN6	0.45406889	mirna_pc
4065	hsa-mir-18a	MRPL11	0.30204495	mirna_pc
4066	hsa-mir-18a	TPRKB	0.348932537	mirna_pc
4067	hsa-mir-18a	PSMD13	0.316475479	mirna_pc
4068	hsa-mir-18a	TCEB1	0.335647042	mirna_pc
4069	hsa-mir-18a	KPNB1	0.419650644	mirna_pc
4070	hsa-mir-18a	SMC3	0.363295099	mirna_pc
4071	hsa-mir-18a	FLVCR1	0.37362202	mirna_pc
4072	hsa-mir-18a	HNRNPA1	0.363398222	mirna_pc
4073	hsa-mir-18a	NPM1	0.34601644	mirna_pc
4074	hsa-mir-18a	NUDT3	0.320761586	mirna_pc
4075	hsa-mir-18a	AGMAT	0.397640174	mirna_pc
4076	hsa-mir-18a	MEX3A	0.471685746	mirna_pc
4077	hsa-mir-18a	BAT1	0.467666161	mirna_pc
4078	hsa-mir-18a	DHX37	0.300192199	mirna_pc
4079	hsa-mir-18a	COIL	0.435318634	mirna_pc
4080	hsa-mir-18a	PHF14	0.374721639	mirna_pc
4081	hsa-mir-18a	LIPG	0.332263012	mirna_pc
4082	hsa-mir-18a	MSH5	0.307476441	mirna_pc
4083	hsa-mir-18a	ERAL1	0.36989261	mirna_pc
4084	hsa-mir-18a	TAF5	0.381488664	mirna_pc
4085	hsa-mir-18a	C2orf29	0.308013928	mirna_pc
4086	hsa-mir-18a	ACAN	0.320136095	mirna_pc
4087	hsa-mir-18a	HNRNPA1L2	0.413414905	mirna_pc
4088	hsa-mir-18a	HMGB1	0.474568781	mirna_pc
4089	hsa-mir-18a	ZBTB12	0.401646506	mirna_pc
4090	hsa-mir-18a	DIAPH3	0.318498674	mirna_pc
4091	hsa-mir-18a	DUSP12	0.354400928	mirna_pc
4092	hsa-mir-18a	TGIF2	0.397667289	mirna_pc
4093	hsa-mir-18a	FARSB	0.453425449	mirna_pc
4094	hsa-mir-18a	PIK3AP1	0.333840969	mirna_pc
4095	hsa-mir-18a	SNX5	0.301130142	mirna_pc
4096	hsa-mir-18a	THUMPD2	0.362854306	mirna_pc
4097	hsa-mir-18a	FAM96A	0.340816352	mirna_pc
4098	hsa-mir-18a	SC65	0.349831776	mirna_pc
4099	hsa-mir-18a	MTF2	0.43116057	mirna_pc
4100	hsa-mir-18a	FGFR1OP	0.426675685	mirna_pc
4101	hsa-mir-18a	PHB	0.346729953	mirna_pc
4102	hsa-mir-18a	NOL10	0.450649272	mirna_pc
4103	hsa-mir-18a	POLE	0.428356759	mirna_pc

4104	hsa-mir-18a	C8orf30A	0.326922397	mirna_pc
4105	hsa-mir-18a	C1orf163	0.33781652	mirna_pc
4106	hsa-mir-18a	RRP8	0.339910438	mirna_pc
4107	hsa-mir-18a	HMBS	0.383903543	mirna_pc
4108	hsa-mir-18a	TRIAP1	0.404930633	mirna_pc
4109	hsa-mir-18a	SOX9	0.329499343	mirna_pc
4110	hsa-mir-18a	TIMM50	0.472589252	mirna_pc
4111	hsa-mir-18a	VRK1	0.379646104	mirna_pc
4112	hsa-mir-18a	RBMX2	0.335030788	mirna_pc
4113	hsa-mir-18a	C13orf27	0.458548541	mirna_pc
4114	hsa-mir-18a	DDOST	0.300731378	mirna_pc
4115	hsa-mir-18a	EXOSC9	0.365477955	mirna_pc
4116	hsa-mir-18a	TMEM209	0.306020333	mirna_pc
4117	hsa-mir-18a	MXD3	0.30173017	mirna_pc
4118	hsa-mir-18a	CCDC43	0.394765326	mirna_pc
4119	hsa-mir-18a	RBMX	0.47630788	mirna_pc
4120	hsa-mir-18a	C6orf134	0.301954546	mirna_pc
4121	hsa-mir-18a	RRS1	0.315976114	mirna_pc
4122	hsa-mir-18a	CCDC41	0.378304215	mirna_pc
4123	hsa-mir-18a	UCHL3	0.328376368	mirna_pc
4124	hsa-mir-18a	DAP3	0.405114892	mirna_pc
4125	hsa-mir-18a	CCDC90A	0.336115504	mirna_pc
4126	hsa-mir-18a	C9orf142	0.303627614	mirna_pc
4127	hsa-mir-18a	ATAD3B	0.418919894	mirna_pc
4128	hsa-mir-18a	XRCC5	0.368485103	mirna_pc
4129	hsa-mir-18a	RPF2	0.42265151	mirna_pc
4130	hsa-mir-18a	EXOSC8	0.415638851	mirna_pc
4131	hsa-mir-18a	C11orf48	0.31345319	mirna_pc
4132	hsa-mir-18a	TAF1D	0.410041875	mirna_pc
4133	hsa-mir-18a	PDIA6	0.399140085	mirna_pc
4134	hsa-mir-18a	GPN3	0.311474278	mirna_pc
4135	hsa-mir-18a	RPE	0.308287621	mirna_pc
4136	hsa-mir-18a	LBR	0.399467265	mirna_pc
4137	hsa-mir-18a	SCLT1	0.358070726	mirna_pc
4138	hsa-mir-18a	DDX10	0.361644167	mirna_pc
4139	hsa-mir-18a	LTV1	0.411894691	mirna_pc
4140	hsa-mir-18a	PCGF6	0.303289115	mirna_pc
4141	hsa-mir-18a	OSTC	0.344257274	mirna_pc
4142	hsa-mir-18a	FAM136A	0.556900247	mirna_pc
4143	hsa-mir-18a	MRPS5	0.355882367	mirna_pc
4144	hsa-mir-18a	UBE2V1	0.322043448	mirna_pc
4145	hsa-mir-18a	SMN2	0.355809439	mirna_pc
4146	hsa-mir-18a	MYB	0.359780194	mirna_pc
4147	hsa-mir-18a	ARID3A	0.320674399	mirna_pc
4148	hsa-mir-18a	MTPAP	0.323510726	mirna_pc
4149	hsa-mir-18a	ITPRIPL1	0.379273553	mirna_pc
4150	hsa-mir-18a	RBM17	0.361724615	mirna_pc
4151	hsa-mir-18a	GDPD5	0.364681083	mirna_pc
4152	hsa-mir-18a	CALM2	0.328838261	mirna_pc
4153	hsa-mir-18a	GPATCH4	0.46798915	mirna_pc
4154	hsa-mir-18a	MPHOSPH10	0.308000443	mirna_pc
4155	hsa-mir-18a	SNORA8	0.434603743	mirna_pc
4156	hsa-mir-18a	EML4	0.372092079	mirna_pc
4157	hsa-mir-18a	RPS7	0.32825702	mirna_pc

4158	hsa-mir-18a	BZW2	0.488917726	mirna_pc
4159	hsa-mir-18a	PABPN1	0.367158932	mirna_pc
4160	hsa-mir-18a	LOC401010	0.324217853	mirna_pc
4161	hsa-mir-18a	RPIA	0.489797743	mirna_pc
4162	hsa-mir-18a	ZNRD1	0.31383322	mirna_pc
4163	hsa-mir-18a	BCL7A	0.303467161	mirna_pc
4164	hsa-mir-18a	C7orf44	0.362107694	mirna_pc
4165	hsa-mir-18a	TRPA1	0.360185847	mirna_pc
4166	hsa-mir-18a	C8orf59	0.300566942	mirna_pc
4167	hsa-mir-18a	NAT9	0.324898948	mirna_pc
4168	hsa-mir-18a	RP9	0.449488682	mirna_pc
4169	hsa-mir-18a	KIAA1731	0.40194277	mirna_pc
4170	hsa-mir-18a	ZNF286A	0.315608668	mirna_pc
4171	hsa-mir-18a	SFXN4	0.390354346	mirna_pc
4172	hsa-mir-18a	PRPF38A	0.304141206	mirna_pc
4173	hsa-mir-18a	ALG8	0.351531634	mirna_pc
4174	hsa-mir-18a	WDR77	0.441857575	mirna_pc
4175	hsa-mir-18a	MRPL21	0.339248571	mirna_pc
4176	hsa-mir-18a	TCEA1	0.369284882	mirna_pc
4177	hsa-mir-18a	RBM28	0.393232059	mirna_pc
4178	hsa-mir-18a	TAF11	0.321858591	mirna_pc
4179	hsa-mir-18a	RANBP17	0.34631892	mirna_pc
4180	hsa-mir-18a	LACTB2	0.306428417	mirna_pc
4181	hsa-mir-18a	UPF3B	0.406343205	mirna_pc
4182	hsa-mir-18a	PPP1CC	0.308525395	mirna_pc
4183	hsa-mir-18a	ILKAP	0.35981802	mirna_pc
4184	hsa-mir-18a	C13orf23	0.322755601	mirna_pc
4185	hsa-mir-18a	SPATA2	0.32529922	mirna_pc
4186	hsa-mir-18a	TAF4B	0.31313051	mirna_pc
4187	hsa-mir-18a	RBM12	0.321780341	mirna_pc
4188	hsa-mir-18a	RDH14	0.301977445	mirna_pc
4189	hsa-mir-18a	SLC25A13	0.317485361	mirna_pc
4190	hsa-mir-18a	PDF	0.328211627	mirna_pc
4191	hsa-mir-18a	DDX20	0.391407348	mirna_pc
4192	hsa-mir-18a	RNF34	0.389089917	mirna_pc
4193	hsa-mir-18a	CHD7	0.419830292	mirna_pc
4194	hsa-mir-18a	TOP 1.00	0.363765413	mirna_pc
4195	hsa-mir-18a	RPGR	0.302745735	mirna_pc
4196	hsa-mir-18a	UBE2N	0.395089078	mirna_pc
4197	hsa-mir-18a	ZNF593	0.362542845	mirna_pc
4198	hsa-mir-18a	TCERG1	0.403823409	mirna_pc
4199	hsa-mir-18a	SEC61A2	0.310015876	mirna_pc
4200	hsa-mir-18a	PSPC1	0.391581473	mirna_pc
4201	hsa-mir-18a	MAPKAPK5	0.41620595	mirna_pc
4202	hsa-mir-18a	E2F6	0.465149456	mirna_pc
4203	hsa-mir-18a	ZNF833	0.377040008	mirna_pc
4204	hsa-mir-18a	NOL7	0.351703999	mirna_pc
4205	hsa-mir-18a	SLC25A15	0.420116136	mirna_pc
4206	hsa-mir-18a	B4GALT3	0.329908779	mirna_pc
4207	hsa-mir-18a	SFRS13A	0.474482942	mirna_pc
4208	hsa-mir-18a	SS18L2	0.30361034	mirna_pc
4209	hsa-mir-18a	SCLY	0.451984304	mirna_pc
4210	hsa-mir-18a	NUP35	0.390157976	mirna_pc
4211	hsa-mir-18a	TGDS	0.365235716	mirna_pc

4212	hsa-mir-18a	TFAM	0.473361937	mirna_pc
4213	hsa-mir-18a	CCDC59	0.316427083	mirna_pc
4214	hsa-mir-18a	DHRS13	0.323330431	mirna_pc
4215	hsa-mir-18a	ORC4L	0.341495286	mirna_pc
4216	hsa-mir-18a	ZC3H15	0.401850928	mirna_pc
4217	hsa-mir-18a	MTIF2	0.434770225	mirna_pc
4218	hsa-mir-18a	KRTCAP3	0.302829958	mirna_pc
4219	hsa-mir-18a	EWSR1	0.367485708	mirna_pc
4220	hsa-mir-18a	LOC222699	0.301947406	mirna_pc
4221	hsa-mir-18a	NUPL1	0.350757505	mirna_pc
4222	hsa-mir-18a	UBE2CBP	0.397235089	mirna_pc
4223	hsa-mir-18a	ACVR2B	0.334062126	mirna_pc
4224	hsa-mir-18a	WBP4	0.304466694	mirna_pc
4225	hsa-mir-18a	RP9P	0.372266244	mirna_pc
4226	hsa-mir-18a	SMUG1	0.313765864	mirna_pc
4227	hsa-mir-18a	SUGT1	0.327502233	mirna_pc
4228	hsa-mir-18a	NR2C1	0.308816351	mirna_pc
4229	hsa-mir-18a	PRELID1	0.315451284	mirna_pc
4230	hsa-mir-18a	MRPS26	0.31670217	mirna_pc
4231	hsa-mir-18a	GNL3	0.366414937	mirna_pc
4232	hsa-mir-18a	METTL5	0.340091607	mirna_pc
4233	hsa-mir-18a	DPP4	0.307154407	mirna_pc
4234	hsa-mir-18a	UPF3A	0.355237498	mirna_pc
4235	hsa-mir-18a	SFRS7	0.443441438	mirna_pc
4236	hsa-mir-18a	MRPL42	0.388283624	mirna_pc
4237	hsa-mir-18a	C9orf100	0.344185499	mirna_pc
4238	hsa-mir-18a	SLC25A19	0.401458182	mirna_pc
4239	hsa-mir-18a	BEND3	0.400552978	mirna_pc
4240	hsa-mir-18a	FGFR4	0.400401937	mirna_pc
4241	hsa-mir-18a	LOC151534	0.317687909	mirna_pc
4242	hsa-mir-18a	NFXL1	0.331687812	mirna_pc
4243	hsa-mir-18a	PFAS	0.361909608	mirna_pc
4244	hsa-mir-18a	C7orf36	0.408108311	mirna_pc
4245	hsa-mir-18a	CCAR1	0.42444992	mirna_pc
4246	hsa-mir-18a	PDCD2	0.369264238	mirna_pc
4247	hsa-mir-18a	SERBP1	0.348345838	mirna_pc
4248	hsa-mir-18a	SPA17	0.332510851	mirna_pc
4249	hsa-mir-18a	C11orf57	0.353583465	mirna_pc
4250	hsa-mir-18a	LOC147804	0.314747808	mirna_pc
4251	hsa-mir-18a	SLC35F2	0.325194044	mirna_pc
4252	hsa-mir-18a	ING1	0.318530371	mirna_pc
4253	hsa-mir-18a	TPMT	0.335698418	mirna_pc
4254	hsa-mir-18a	EEF1E1	0.431722866	mirna_pc
4255	hsa-mir-18a	ZNF697	0.330154359	mirna_pc
4256	hsa-mir-18a	MRPL44	0.364433209	mirna_pc
4257	hsa-mir-18a	PTCD3	0.344018271	mirna_pc
4258	hsa-mir-18a	SPRY4	0.375741315	mirna_pc
4259	hsa-mir-18a	IWS1	0.321897894	mirna_pc
4260	hsa-mir-18a	CASP6	0.367468353	mirna_pc
4261	hsa-mir-18a	ZNF92	0.338343859	mirna_pc
4262	hsa-mir-18a	C12orf45	0.322862892	mirna_pc
4263	hsa-mir-18a	SEPHS1	0.378281636	mirna_pc
4264	hsa-mir-18a	CTR9	0.320463591	mirna_pc
4265	hsa-mir-18a	RMND1	0.327362809	mirna_pc

4266	hsa-mir-18a	RNF216L	0.316340374	mirna_pc
4267	hsa-mir-18a	ZNF259	0.30075503	mirna_pc
4268	hsa-mir-18a	RSL1D1	0.314088774	mirna_pc
4269	hsa-mir-18a	BOLA3	0.323251258	mirna_pc
4270	hsa-mir-18a	MYL6B	0.310897866	mirna_pc
4271	hsa-mir-18a	MRP63	0.311630937	mirna_pc
4272	hsa-mir-18a	IPO5	0.441263973	mirna_pc
4273	hsa-mir-18a	C2orf47	0.380755906	mirna_pc
4274	hsa-mir-18a	C6orf211	0.352639108	mirna_pc
4275	hsa-mir-18a	GET4	0.314265014	mirna_pc
4276	hsa-mir-18a	DPAGT1	0.404837305	mirna_pc
4277	hsa-mir-18a	GPR137C	0.316909424	mirna_pc
4278	hsa-mir-18a	NARS2	0.393585293	mirna_pc
4279	hsa-mir-18a	NUP43	0.396961953	mirna_pc
4280	hsa-mir-18a	LYRM4	0.356527955	mirna_pc
4281	hsa-mir-18a	ZNF124	0.316497511	mirna_pc
4282	hsa-mir-18a	LOC388955	0.321236912	mirna_pc
4283	hsa-mir-18a	RNASEH2B	0.3281747	mirna_pc
4284	hsa-mir-18a	CHD1L	0.435095801	mirna_pc
4285	hsa-mir-18a	ZNF473	0.402336286	mirna_pc
4286	hsa-mir-18a	SNRNP48	0.363884609	mirna_pc
4287	hsa-mir-18a	DCLRE1A	0.305972152	mirna_pc
4288	hsa-mir-18a	ANP32A	0.486707313	mirna_pc
4289	hsa-mir-18a	CHCHD7	0.339704073	mirna_pc
4290	hsa-mir-18a	E2F5	0.474352033	mirna_pc
4291	hsa-mir-18a	NUFIP1	0.424560234	mirna_pc
4292	hsa-mir-18a	ZNF195	0.552394952	mirna_pc
4293	hsa-mir-18a	RBM25	0.303536185	mirna_pc
4294	hsa-mir-18a	PRKAR1B	0.301864991	mirna_pc
4295	hsa-mir-18a	LRRCC1	0.337597	mirna_pc
4296	hsa-mir-18a	ASNSD1	0.330635715	mirna_pc
4297	hsa-mir-18a	HYLS1	0.345433902	mirna_pc
4298	hsa-mir-18a	NEK3	0.334633629	mirna_pc
4299	hsa-mir-18a	HIC2	0.31779112	mirna_pc
4300	hsa-mir-18a	HNRNPH3	0.307979552	mirna_pc
4301	hsa-mir-18a	NAP1L1	0.317045515	mirna_pc
4302	hsa-mir-18a	SNF8	0.318047412	mirna_pc
4303	hsa-mir-18a	C1orf156	0.315966797	mirna_pc
4304	hsa-mir-18a	C6orf182	0.343785979	mirna_pc
4305	hsa-mir-18a	VIL1	0.369910214	mirna_pc
4306	hsa-mir-18a	GSDMB	0.308011992	mirna_pc
4307	hsa-mir-18a	TMEM170A	0.335127141	mirna_pc
4308	hsa-mir-18a	PHF16	0.329555525	mirna_pc
4309	hsa-mir-18a	BCCIP	0.33030615	mirna_pc
4310	hsa-mir-18a	IQCC	0.321023445	mirna_pc
4311	hsa-mir-18a	PMS1	0.396808882	mirna_pc
4312	hsa-mir-18a	TRMT11	0.300434468	mirna_pc
4313	hsa-mir-18a	PNN	0.329797072	mirna_pc
4314	hsa-mir-18a	MNS1	0.320798179	mirna_pc
4315	hsa-mir-18a	ZNF485	0.330788984	mirna_pc
4316	hsa-mir-18a	MORN2	0.342561428	mirna_pc
4317	hsa-mir-18a	KIN	0.315356965	mirna_pc
4318	hsa-mir-18a	UGT8	0.304632417	mirna_pc
4319	hsa-mir-18a	PEG10	0.313310665	mirna_pc

4320	hsa-mir-18a	CCNJ	0.461058816	mirna_pc
4321	hsa-mir-18a	MYCN	0.318182925	mirna_pc
4322	hsa-mir-18a	IPO7	0.365746266	mirna_pc
4323	hsa-mir-18a	FAM171A1	0.3253008	mirna_pc
4324	hsa-mir-18a	ABCC4	0.353974682	mirna_pc
4325	hsa-mir-18a	ESF1	0.345773253	mirna_pc
4326	hsa-mir-18a	CCDC84	0.317239652	mirna_pc
4327	hsa-mir-18a	SLC25A33	0.431423648	mirna_pc
4328	hsa-mir-18a	ZNF26	0.328832806	mirna_pc
4329	hsa-mir-18a	GTF2H2C	0.370809317	mirna_pc
4330	hsa-mir-18a	CTPS2	0.357173828	mirna_pc
4331	hsa-mir-18a	GXYLT1	0.349731448	mirna_pc
4332	hsa-mir-18a	HNF1A	0.309685405	mirna_pc
4333	hsa-mir-18a	BCS1L	0.347913667	mirna_pc
4334	hsa-mir-18a	C2orf56	0.348104559	mirna_pc
4335	hsa-mir-18a	DDX51	0.351770295	mirna_pc
4336	hsa-mir-18a	PBX2	0.323858255	mirna_pc
4337	hsa-mir-18a	LRRC40	0.309708267	mirna_pc
4338	hsa-mir-18a	SNCAIP	0.336659016	mirna_pc
4339	hsa-mir-18a	ZNF232	0.422255523	mirna_pc
4340	hsa-mir-18a	RNF32	0.345171506	mirna_pc
4341	hsa-mir-18a	SNRNP27	0.314026823	mirna_pc
4342	hsa-mir-18a	MRPL16	0.31171613	mirna_pc
4343	hsa-mir-18a	ENO3	0.328481616	mirna_pc
4344	hsa-mir-18a	IFT88	0.417714866	mirna_pc
4345	hsa-mir-18a	STARD3NL	0.32057269	mirna_pc
4346	hsa-mir-18a	QRSL1	0.320797707	mirna_pc
4347	hsa-mir-18a	FANCF	0.448054161	mirna_pc
4348	hsa-mir-18a	VPS54	0.317162843	mirna_pc
4349	hsa-mir-18a	HYOU1	0.328907202	mirna_pc
4350	hsa-mir-18a	XYLB	0.360287326	mirna_pc
4351	hsa-mir-18a	CIAO1	0.387395843	mirna_pc
4352	hsa-mir-18a	C2orf3	0.425003613	mirna_pc
4353	hsa-mir-18a	ZNF22	0.404950891	mirna_pc
4354	hsa-mir-18a	FAM119A	0.458030979	mirna_pc
4355	hsa-mir-18a	GOLT1A	0.402678536	mirna_pc
4356	hsa-mir-18a	PDGFA	0.304584431	mirna_pc
4357	hsa-mir-18a	CSPP1	0.305107366	mirna_pc
4358	hsa-mir-18a	C20orf96	0.352586669	mirna_pc
4359	hsa-mir-18a	SCML2	0.352777357	mirna_pc
4360	hsa-mir-18a	MRPL19	0.379442672	mirna_pc
4361	hsa-mir-18a	ZCCHC3	0.500935744	mirna_pc
4362	hsa-mir-18a	RRP15	0.389607384	mirna_pc
4363	hsa-mir-18a	ADAT2	0.390021176	mirna_pc
4364	hsa-mir-18a	LUC7L3	0.30665529	mirna_pc
4365	hsa-mir-18a	ZCCHC9	0.305998447	mirna_pc
4366	hsa-mir-18a	DDX1	0.346290254	mirna_pc
4367	hsa-mir-18a	STX3	0.334694558	mirna_pc
4368	hsa-mir-18a	AADAT	0.398799245	mirna_pc
4369	hsa-mir-18a	ACP1	0.460063923	mirna_pc
4370	hsa-mir-18a	C2orf43	0.37548386	mirna_pc
4371	hsa-mir-18a	GTF3A	0.415798928	mirna_pc
4372	hsa-mir-18a	TMEM126B	0.336606721	mirna_pc
4373	hsa-mir-18a	SPRED2	0.336547147	mirna_pc

4374	hsa-mir-18a	TXLNG	0.31260711	mirna_pc
4375	hsa-mir-18a	LOC80054	0.314377909	mirna_pc
4376	hsa-mir-18a	C2orf34	0.344642885	mirna_pc
4377	hsa-mir-18a	QPRT	0.33066784	mirna_pc
4378	hsa-mir-18a	PRMT6	0.345307566	mirna_pc
4379	hsa-mir-18a	MRPS9	0.360783129	mirna_pc
4380	hsa-mir-18a	STRADB	0.346608186	mirna_pc
4381	hsa-mir-18a	TMED2	0.306211627	mirna_pc
4382	hsa-mir-18a	PNKD	0.307628756	mirna_pc
4383	hsa-mir-18a	ZNF530	0.354636132	mirna_pc
4384	hsa-mir-18a	ZNF765	0.323124786	mirna_pc
4385	hsa-mir-18a	PPIL4	0.303925363	mirna_pc
4386	hsa-mir-18a	HRSP12	0.351026318	mirna_pc
4387	hsa-mir-18a	VAPB	0.301681318	mirna_pc
4388	hsa-mir-18a	MIR17HG	0.549408883	mirna_pc
4389	hsa-mir-18a	AARSD1	0.324297516	mirna_pc
4390	hsa-mir-18a	ZNF239	0.443102707	mirna_pc
4391	hsa-mir-18a	ZNF202	0.303552367	mirna_pc
4392	hsa-mir-18a	TMEM14B	0.361077391	mirna_pc
4393	hsa-mir-18a	ZNF77	0.351744171	mirna_pc
4394	hsa-mir-18a	ZNF468	0.394107161	mirna_pc
4395	hsa-mir-18a	LOC151009	0.339078418	mirna_pc
4396	hsa-mir-18a	SLC37A4	0.368877885	mirna_pc
4397	hsa-mir-18a	CTNNBL1	0.337928344	mirna_pc
4398	hsa-mir-18a	MRPS31	0.34839398	mirna_pc
4399	hsa-mir-18a	CENPV	0.471243952	mirna_pc
4400	hsa-mir-18a	DNMT3A	0.417045669	mirna_pc
4401	hsa-mir-18a	PRTFDC1	0.396579352	mirna_pc
4402	hsa-mir-18a	EIF5B	0.32175486	mirna_pc
4403	hsa-mir-18a	ZNF670	0.333145052	mirna_pc
4404	hsa-mir-18a	GTF3C6	0.391033993	mirna_pc
4405	hsa-mir-18a	ZNF326	0.333949488	mirna_pc
4406	hsa-mir-18a	ZRANB2	0.307235246	mirna_pc
4407	hsa-mir-18a	DOM3Z	0.307982425	mirna_pc
4408	hsa-mir-18a	ARSE	0.302889118	mirna_pc
4409	hsa-mir-301b	HOXC9	0.368835346	mirna_pc
4410	hsa-mir-301b	RRM2	0.319835372	mirna_pc
4411	hsa-mir-301b	UBE2C	0.338514385	mirna_pc
4412	hsa-mir-301b	BUB1B	0.307425768	mirna_pc
4413	hsa-mir-301b	KIF2C	0.335170449	mirna_pc
4414	hsa-mir-301b	CCNA2	0.371518591	mirna_pc
4415	hsa-mir-301b	MND1	0.41414207	mirna_pc
4416	hsa-mir-301b	KIF22	0.302814153	mirna_pc
4417	hsa-mir-301b	NCAPH	0.312587162	mirna_pc
4418	hsa-mir-301b	RAD54L	0.321280209	mirna_pc
4419	hsa-mir-301b	CENPA	0.400278118	mirna_pc
4420	hsa-mir-301b	MAD2L1	0.456971367	mirna_pc
4421	hsa-mir-301b	LMNB1	0.306553872	mirna_pc
4422	hsa-mir-301b	CDCA8	0.347493344	mirna_pc
4423	hsa-mir-301b	TROAP	0.419579798	mirna_pc
4424	hsa-mir-301b	STMN1	0.384025408	mirna_pc
4425	hsa-mir-301b	DKC1	0.341989117	mirna_pc
4426	hsa-mir-301b	CCNB2	0.336495004	mirna_pc
4427	hsa-mir-301b	NUF2	0.38108297	mirna_pc

4428	hsa-mir-301b	PLK4	0.373023565	mirna_pc
4429	hsa-mir-301b	HJURP	0.323805632	mirna_pc
4430	hsa-mir-301b	PAICS	0.319181169	mirna_pc
4431	hsa-mir-301b	BIRC5	0.418931947	mirna_pc
4432	hsa-mir-301b	RCC1	0.332430812	mirna_pc
4433	hsa-mir-301b	RECQL4	0.301004516	mirna_pc
4434	hsa-mir-301b	AURKB	0.397632031	mirna_pc
4435	hsa-mir-301b	KIF20A	0.31723587	mirna_pc
4436	hsa-mir-301b	ACTL6A	0.430060001	mirna_pc
4437	hsa-mir-301b	C12orf48	0.349838091	mirna_pc
4438	hsa-mir-301b	HNRNPL	0.312927699	mirna_pc
4439	hsa-mir-301b	SMC4	0.347865459	mirna_pc
4440	hsa-mir-301b	C11orf82	0.448089264	mirna_pc
4441	hsa-mir-301b	HNRNPC	0.308256764	mirna_pc
4442	hsa-mir-301b	ILF2	0.325268743	mirna_pc
4443	hsa-mir-301b	RNASEH2A	0.324104674	mirna_pc
4444	hsa-mir-301b	EPR1	0.359535536	mirna_pc
4445	hsa-mir-301b	SFRS2	0.322096883	mirna_pc
4446	hsa-mir-301b	UCK2	0.414375598	mirna_pc
4447	hsa-mir-301b	NFKBIL2	0.31670141	mirna_pc
4448	hsa-mir-301b	SNRPG	0.368872209	mirna_pc
4449	hsa-mir-301b	EIF4A3	0.302614575	mirna_pc
4450	hsa-mir-301b	SNRPA	0.325980308	mirna_pc
4451	hsa-mir-301b	FUS	0.315384047	mirna_pc
4452	hsa-mir-301b	GINS4	0.390159962	mirna_pc
4453	hsa-mir-301b	ANP32E	0.341210751	mirna_pc
4454	hsa-mir-301b	CENPO	0.354592344	mirna_pc
4455	hsa-mir-301b	HMGB2	0.38109693	mirna_pc
4456	hsa-mir-301b	MLF1IP	0.301781558	mirna_pc
4457	hsa-mir-301b	HIST1H2AE	0.521540675	mirna_pc
4458	hsa-mir-301b	PFDN2	0.324241665	mirna_pc
4459	hsa-mir-301b	HNRNPR	0.384532404	mirna_pc
4460	hsa-mir-301b	DLEU2	0.333721003	mirna_pc
4461	hsa-mir-301b	ACYP1	0.377614375	mirna_pc
4462	hsa-mir-301b	MSH2	0.388036177	mirna_pc
4463	hsa-mir-301b	DBF4	0.494733759	mirna_pc
4464	hsa-mir-301b	CDC7	0.40338648	mirna_pc
4465	hsa-mir-301b	YDJC	0.40058626	mirna_pc
4466	hsa-mir-301b	RFC4	0.412928745	mirna_pc
4467	hsa-mir-301b	FBXO5	0.321136857	mirna_pc
4468	hsa-mir-301b	FIGNL1	0.427723371	mirna_pc
4469	hsa-mir-301b	SF3B14	0.322029898	mirna_pc
4470	hsa-mir-301b	RAD54B	0.309992224	mirna_pc
4471	hsa-mir-301b	MAGOH	0.333561837	mirna_pc
4472	hsa-mir-301b	DDX12	0.33570956	mirna_pc
4473	hsa-mir-301b	CPSF3	0.494713385	mirna_pc
4474	hsa-mir-301b	BANF1	0.409885657	mirna_pc
4475	hsa-mir-301b	C21orf45	0.396777472	mirna_pc
4476	hsa-mir-301b	PPM1G	0.391273416	mirna_pc
4477	hsa-mir-301b	DNAJB11	0.319010348	mirna_pc
4478	hsa-mir-301b	ACAD8	0.436412653	mirna_pc
4479	hsa-mir-301b	TFAP4	0.309647719	mirna_pc
4480	hsa-mir-301b	NUDT5	0.316397224	mirna_pc
4481	hsa-mir-301b	DAZAP1	0.376306664	mirna_pc

4482	hsa-mir-301b	CBX8	0.444706319	mirna_pc
4483	hsa-mir-301b	TMEM201	0.330483865	mirna_pc
4484	hsa-mir-301b	PAFAH1B3	0.364494565	mirna_pc
4485	hsa-mir-301b	PSRC1	0.376989104	mirna_pc
4486	hsa-mir-301b	DGUOK	0.46606401	mirna_pc
4487	hsa-mir-301b	MRPL47	0.380817142	mirna_pc
4488	hsa-mir-301b	HNRNPM	0.354877125	mirna_pc
4489	hsa-mir-301b	CCT6A	0.442829775	mirna_pc
4490	hsa-mir-301b	POLR2H	0.370675386	mirna_pc
4491	hsa-mir-301b	SSBP3	0.409145402	mirna_pc
4492	hsa-mir-301b	SHFM1	0.490777071	mirna_pc
4493	hsa-mir-301b	LSG1	0.414417047	mirna_pc
4494	hsa-mir-301b	GABPB1	0.30422433	mirna_pc
4495	hsa-mir-301b	PPIH	0.373437555	mirna_pc
4496	hsa-mir-301b	LOC100128191	0.361153622	mirna_pc
4497	hsa-mir-301b	SMYD5	0.353465821	mirna_pc
4498	hsa-mir-301b	SF3A2	0.337882741	mirna_pc
4499	hsa-mir-301b	TSSC1	0.387395254	mirna_pc
4500	hsa-mir-301b	SNRPF	0.345558776	mirna_pc
4501	hsa-mir-301b	SUMO2	0.453368492	mirna_pc
4502	hsa-mir-301b	WDR43	0.32365548	mirna_pc
4503	hsa-mir-301b	SNRPA1	0.424419246	mirna_pc
4504	hsa-mir-301b	SMC6	0.362358951	mirna_pc
4505	hsa-mir-301b	UBE2I	0.30088753	mirna_pc
4506	hsa-mir-301b	TIPIN	0.368090704	mirna_pc
4507	hsa-mir-301b	ECE2	0.371263517	mirna_pc
4508	hsa-mir-301b	PA2G4	0.312217872	mirna_pc
4509	hsa-mir-301b	SNRPD3	0.460197208	mirna_pc
4510	hsa-mir-301b	KHDRBS1	0.3411315	mirna_pc
4511	hsa-mir-301b	SPAST	0.412961078	mirna_pc
4512	hsa-mir-301b	MTL 5.00	0.378158596	mirna_pc
4513	hsa-mir-301b	PRRT1	0.32791521	mirna_pc
4514	hsa-mir-301b	GNL2	0.477317884	mirna_pc
4515	hsa-mir-301b	NASP	0.368633028	mirna_pc
4516	hsa-mir-301b	PTMA	0.418036872	mirna_pc
4517	hsa-mir-301b	USP39	0.302340213	mirna_pc
4518	hsa-mir-301b	AMZ2P1	0.317373231	mirna_pc
4519	hsa-mir-301b	C11orf84	0.36367922	mirna_pc
4520	hsa-mir-301b	CLCN2	0.30633299	mirna_pc
4521	hsa-mir-301b	LRRC8D	0.351804201	mirna_pc
4522	hsa-mir-301b	NCBP2	0.386481464	mirna_pc
4523	hsa-mir-301b	FBXO22OS	0.388375401	mirna_pc
4524	hsa-mir-301b	NAALADL2	0.456879746	mirna_pc
4525	hsa-mir-301b	HNRNPD	0.43288702	mirna_pc
4526	hsa-mir-301b	DPY30	0.435523703	mirna_pc
4527	hsa-mir-301b	MTHFD2	0.312874912	mirna_pc
4528	hsa-mir-301b	CCT4	0.313774519	mirna_pc
4529	hsa-mir-301b	RAD9A	0.329871246	mirna_pc
4530	hsa-mir-301b	NCAPD3	0.503000058	mirna_pc
4531	hsa-mir-301b	TBC1D7	0.313360142	mirna_pc
4532	hsa-mir-301b	PAK1IP1	0.33579287	mirna_pc
4533	hsa-mir-301b	YWHAE	0.346566981	mirna_pc
4534	hsa-mir-301b	HSD17B12	0.339176501	mirna_pc
4535	hsa-mir-301b	C3orf57	0.504086362	mirna_pc

4536	hsa-mir-301b	RNF214	0.348512632	mirna_pc
4537	hsa-mir-301b	EHMT2	0.308249859	mirna_pc
4538	hsa-mir-301b	PPT1	0.300962212	mirna_pc
4539	hsa-mir-301b	DEK	0.312779972	mirna_pc
4540	hsa-mir-301b	PASK	0.316697452	mirna_pc
4541	hsa-mir-301b	KHSRP	0.394656779	mirna_pc
4542	hsa-mir-301b	LSM 6.00	0.301681568	mirna_pc
4543	hsa-mir-301b	PRAME	0.440065858	mirna_pc
4544	hsa-mir-301b	MRPS17	0.479918453	mirna_pc
4545	hsa-mir-301b	TNFRSF18	0.376233422	mirna_pc
4546	hsa-mir-301b	B3GALNT1	0.332343219	mirna_pc
4547	hsa-mir-301b	VPS26B	0.321577501	mirna_pc
4548	hsa-mir-301b	TRA2B	0.33996474	mirna_pc
4549	hsa-mir-301b	RSRC1	0.397858852	mirna_pc
4550	hsa-mir-301b	GAR1	0.359761671	mirna_pc
4551	hsa-mir-301b	C15orf23	0.307467202	mirna_pc
4552	hsa-mir-301b	PRMT3	0.328963921	mirna_pc
4553	hsa-mir-301b	C3orf21	0.319825725	mirna_pc
4554	hsa-mir-301b	PSMD12	0.390992366	mirna_pc
4555	hsa-mir-301b	ZNF879	0.301833565	mirna_pc
4556	hsa-mir-301b	ITGB3BP	0.315682544	mirna_pc
4557	hsa-mir-301b	ENOPH1	0.449148385	mirna_pc
4558	hsa-mir-301b	DDX55	0.303062554	mirna_pc
4559	hsa-mir-301b	PLEKHG4	0.396062923	mirna_pc
4560	hsa-mir-301b	SFPQ	0.342076832	mirna_pc
4561	hsa-mir-301b	AP3M2	0.305351474	mirna_pc
4562	hsa-mir-301b	EIF4EBP1	0.502362716	mirna_pc
4563	hsa-mir-301b	TTC17	0.389216663	mirna_pc
4564	hsa-mir-301b	CADM1	0.499739758	mirna_pc
4565	hsa-mir-301b	HDAC2	0.365345455	mirna_pc
4566	hsa-mir-301b	PDAP1	0.621376812	mirna_pc
4567	hsa-mir-301b	RNF7	0.449920851	mirna_pc
4568	hsa-mir-301b	GEMIN6	0.412428436	mirna_pc
4569	hsa-mir-301b	PTCD1	0.616115801	mirna_pc
4570	hsa-mir-301b	CRIPT	0.343961675	mirna_pc
4571	hsa-mir-301b	MSH6	0.38707863	mirna_pc
4572	hsa-mir-301b	SOX4	0.385257133	mirna_pc
4573	hsa-mir-301b	C3orf34	0.429888832	mirna_pc
4574	hsa-mir-301b	TPRKB	0.444195131	mirna_pc
4575	hsa-mir-301b	LEF1	0.358139736	mirna_pc
4576	hsa-mir-301b	POLB	0.427388456	mirna_pc
4577	hsa-mir-301b	HNRNPA1	0.308362658	mirna_pc
4578	hsa-mir-301b	PPP1R8	0.359932308	mirna_pc
4579	hsa-mir-301b	CDK2AP1	0.319956346	mirna_pc
4580	hsa-mir-301b	NUDT3	0.516767029	mirna_pc
4581	hsa-mir-301b	MEX3A	0.401726487	mirna_pc
4582	hsa-mir-301b	ATP5J2	0.597067396	mirna_pc
4583	hsa-mir-301b	IRAK1BP1	0.361146973	mirna_pc
4584	hsa-mir-301b	BUD31	0.539840214	mirna_pc
4585	hsa-mir-301b	C16orf88	0.337848634	mirna_pc
4586	hsa-mir-301b	APEX1	0.320486042	mirna_pc
4587	hsa-mir-301b	HNRNPA1L2	0.351374024	mirna_pc
4588	hsa-mir-301b	EMILIN3	0.415017174	mirna_pc
4589	hsa-mir-301b	PADI3	0.328235144	mirna_pc

4590	hsa-mir-301b	ZBTB12	0.303572904	mirna_pc
4591	hsa-mir-301b	LOC642846	0.305847943	mirna_pc
4592	hsa-mir-301b	RCN2	0.324843016	mirna_pc
4593	hsa-mir-301b	THUMPD2	0.395941612	mirna_pc
4594	hsa-mir-301b	NOL10	0.409478019	mirna_pc
4595	hsa-mir-301b	SF3A3	0.403074144	mirna_pc
4596	hsa-mir-301b	EPHA7	0.305953104	mirna_pc
4597	hsa-mir-301b	MUTYH	0.411499902	mirna_pc
4598	hsa-mir-301b	TAF1B	0.487819017	mirna_pc
4599	hsa-mir-301b	XRCC6BP1	0.392875879	mirna_pc
4600	hsa-mir-301b	RPP21	0.304936227	mirna_pc
4601	hsa-mir-301b	VRK1	0.336793985	mirna_pc
4602	hsa-mir-301b	EXOSC9	0.449577592	mirna_pc
4603	hsa-mir-301b	ALKBH2	0.418422427	mirna_pc
4604	hsa-mir-301b	ZNF642	0.347838658	mirna_pc
4605	hsa-mir-301b	RPF2	0.320789582	mirna_pc
4606	hsa-mir-301b	SCARNA7	0.309916292	mirna_pc
4607	hsa-mir-301b	NHLRC1	0.308553438	mirna_pc
4608	hsa-mir-301b	CCDC24	0.389575718	mirna_pc
4609	hsa-mir-301b	RPL39L	0.301292474	mirna_pc
4610	hsa-mir-301b	TAF1D	0.308617371	mirna_pc
4611	hsa-mir-301b	GPN3	0.321394915	mirna_pc
4612	hsa-mir-301b	ASB3	0.302018972	mirna_pc
4613	hsa-mir-301b	MEMO1	0.476856955	mirna_pc
4614	hsa-mir-301b	CCDC23	0.303708918	mirna_pc
4615	hsa-mir-301b	IGSF9	0.316201499	mirna_pc
4616	hsa-mir-301b	UBXN2A	0.35942734	mirna_pc
4617	hsa-mir-301b	FIP1L1	0.338656325	mirna_pc
4618	hsa-mir-301b	SCLT1	0.305287421	mirna_pc
4619	hsa-mir-301b	MAGEF1	0.453070532	mirna_pc
4620	hsa-mir-301b	YRDC	0.36702076	mirna_pc
4621	hsa-mir-301b	SMARCB1	0.382072178	mirna_pc
4622	hsa-mir-301b	TTC32	0.518417425	mirna_pc
4623	hsa-mir-301b	SMN2	0.3227385	mirna_pc
4624	hsa-mir-301b	U2AF1	0.371799306	mirna_pc
4625	hsa-mir-301b	ZNF639	0.339956029	mirna_pc
4626	hsa-mir-301b	ITPRIPL1	0.354028293	mirna_pc
4627	hsa-mir-301b	RBM17	0.355113545	mirna_pc
4628	hsa-mir-301b	ABT1	0.446734393	mirna_pc
4629	hsa-mir-301b	ZNF643	0.363043228	mirna_pc
4630	hsa-mir-301b	MPHOSPH10	0.394429658	mirna_pc
4631	hsa-mir-301b	AKAP9	0.583545611	mirna_pc
4632	hsa-mir-301b	RPS7	0.330223457	mirna_pc
4633	hsa-mir-301b	PABPN1	0.382083328	mirna_pc
4634	hsa-mir-301b	BCL7A	0.32501627	mirna_pc
4635	hsa-mir-301b	WNT10A	0.32157819	mirna_pc
4636	hsa-mir-301b	TIMM16	0.310190083	mirna_pc
4637	hsa-mir-301b	C7orf44	0.339285131	mirna_pc
4638	hsa-mir-301b	TRPA1	0.314159807	mirna_pc
4639	hsa-mir-301b	NUDCD2	0.328427557	mirna_pc
4640	hsa-mir-301b	NAT9	0.31959967	mirna_pc
4641	hsa-mir-301b	ZNF286A	0.307895115	mirna_pc
4642	hsa-mir-301b	WDR77	0.345165991	mirna_pc
4643	hsa-mir-301b	MRPL21	0.429388951	mirna_pc

4644	hsa-mir-301b	TCEA1	0.308565226	mirna_pc
4645	hsa-mir-301b	NLE1	0.33312186	mirna_pc
4646	hsa-mir-301b	FXR1	0.310518133	mirna_pc
4647	hsa-mir-301b	DMRTA2	0.420601179	mirna_pc
4648	hsa-mir-301b	UPF3B	0.323440163	mirna_pc
4649	hsa-mir-301b	BTG3	0.313650849	mirna_pc
4650	hsa-mir-301b	LOC645332	0.418468922	mirna_pc
4651	hsa-mir-301b	PSPH	0.357070971	mirna_pc
4652	hsa-mir-301b	PRSS21	0.310724272	mirna_pc
4653	hsa-mir-301b	MGC72080	0.567331783	mirna_pc
4654	hsa-mir-301b	TRRAP	0.436395356	mirna_pc
4655	hsa-mir-301b	TRMT61B	0.330310818	mirna_pc
4656	hsa-mir-301b	RWDD3	0.300662801	mirna_pc
4657	hsa-mir-301b	SEMA4F	0.340664909	mirna_pc
4658	hsa-mir-301b	RDH14	0.360495955	mirna_pc
4659	hsa-mir-301b	MPV17	0.359456321	mirna_pc
4660	hsa-mir-301b	DDX20	0.336879943	mirna_pc
4661	hsa-mir-301b	C1orf109	0.404659627	mirna_pc
4662	hsa-mir-301b	PCBP1	0.337014975	mirna_pc
4663	hsa-mir-301b	CHCHD2	0.438995408	mirna_pc
4664	hsa-mir-301b	PIGX	0.423651922	mirna_pc
4665	hsa-mir-301b	MTA1	0.306142502	mirna_pc
4666	hsa-mir-301b	EIF4A2	0.330031814	mirna_pc
4667	hsa-mir-301b	HTRA2	0.403357476	mirna_pc
4668	hsa-mir-301b	MTA3	0.312065739	mirna_pc
4669	hsa-mir-301b	CHAC2	0.351743698	mirna_pc
4670	hsa-mir-301b	ACN9	0.494169483	mirna_pc
4671	hsa-mir-301b	INTU	0.557898095	mirna_pc
4672	hsa-mir-301b	ATP6V1E2	0.371389976	mirna_pc
4673	hsa-mir-301b	E2F6	0.377869258	mirna_pc
4674	hsa-mir-301b	DRG1	0.310008364	mirna_pc
4675	hsa-mir-301b	NOL7	0.377976957	mirna_pc
4676	hsa-mir-301b	CPSF4	0.63470798	mirna_pc
4677	hsa-mir-301b	SFRS13A	0.353045078	mirna_pc
4678	hsa-mir-301b	GADD45GIP1	0.327364015	mirna_pc
4679	hsa-mir-301b	SCLY	0.390195877	mirna_pc
4680	hsa-mir-301b	NT5C	0.429369596	mirna_pc
4681	hsa-mir-301b	STAMBP	0.374973987	mirna_pc
4682	hsa-mir-301b	CCDC59	0.328888096	mirna_pc
4683	hsa-mir-301b	SLC4A1AP	0.435702369	mirna_pc
4684	hsa-mir-301b	TTC27	0.415592213	mirna_pc
4685	hsa-mir-301b	DHRS13	0.300400038	mirna_pc
4686	hsa-mir-301b	RPF1	0.338945403	mirna_pc
4687	hsa-mir-301b	MTIF2	0.386774374	mirna_pc
4688	hsa-mir-301b	CDK2AP2	0.321857423	mirna_pc
4689	hsa-mir-301b	KRTCAP3	0.304941068	mirna_pc
4690	hsa-mir-301b	LOC222699	0.378895833	mirna_pc
4691	hsa-mir-301b	KCNMB3	0.334083202	mirna_pc
4692	hsa-mir-301b	DHX57	0.388034278	mirna_pc
4693	hsa-mir-301b	HIST1H1C	0.455314048	mirna_pc
4694	hsa-mir-301b	ALX3	0.338224942	mirna_pc
4695	hsa-mir-301b	AAAS	0.399616091	mirna_pc
4696	hsa-mir-301b	C19orf54	0.339222241	mirna_pc
4697	hsa-mir-301b	SUPT7L	0.316349758	mirna_pc

4698	hsa-mir-301b	LIG3	0.335917781	mirna_pc
4699	hsa-mir-301b	ZNF277	0.515051816	mirna_pc
4700	hsa-mir-301b	PELP1	0.318060153	mirna_pc
4701	hsa-mir-301b	THOC1	0.330319766	mirna_pc
4702	hsa-mir-301b	C11orf45	0.457240184	mirna_pc
4703	hsa-mir-301b	SFRS7	0.401705574	mirna_pc
4704	hsa-mir-301b	IFRD1	0.514530733	mirna_pc
4705	hsa-mir-301b	INO80B	0.378638828	mirna_pc
4706	hsa-mir-301b	LOC100128292	0.316889094	mirna_pc
4707	hsa-mir-301b	CORO1B	0.415844369	mirna_pc
4708	hsa-mir-301b	ANKIB1	0.341554511	mirna_pc
4709	hsa-mir-301b	THYN1	0.557053033	mirna_pc
4710	hsa-mir-301b	PARL	0.356960537	mirna_pc
4711	hsa-mir-301b	C2orf68	0.387253575	mirna_pc
4712	hsa-mir-301b	ARPC1A	0.35938112	mirna_pc
4713	hsa-mir-301b	KCNG1	0.333756383	mirna_pc
4714	hsa-mir-301b	APLP2	0.455337868	mirna_pc
4715	hsa-mir-301b	C17orf75	0.430052544	mirna_pc
4716	hsa-mir-301b	RPL35A	0.307261403	mirna_pc
4717	hsa-mir-301b	ZBTB44	0.46426805	mirna_pc
4718	hsa-mir-301b	NUDT14	0.315277979	mirna_pc
4719	hsa-mir-301b	EXT2	0.339623786	mirna_pc
4720	hsa-mir-301b	ZNF789	0.432629433	mirna_pc
4721	hsa-mir-301b	ZNF771	0.326488255	mirna_pc
4722	hsa-mir-301b	EEF1E1	0.31002643	mirna_pc
4723	hsa-mir-301b	MRPL38	0.353314232	mirna_pc
4724	hsa-mir-301b	ATPBD4	0.372040097	mirna_pc
4725	hsa-mir-301b	GDF11	0.331400071	mirna_pc
4726	hsa-mir-301b	ZKSCAN5	0.50613084	mirna_pc
4727	hsa-mir-301b	POLR2C	0.382593155	mirna_pc
4728	hsa-mir-301b	C12orf10	0.351320458	mirna_pc
4729	hsa-mir-301b	PPP1R2	0.310545154	mirna_pc
4730	hsa-mir-301b	CLEC2B	0.397513237	mirna_pc
4731	hsa-mir-301b	C12orf45	0.386060709	mirna_pc
4732	hsa-mir-301b	C2orf44	0.302730408	mirna_pc
4733	hsa-mir-301b	RING1	0.33305914	mirna_pc
4734	hsa-mir-301b	SEPHS1	0.349789193	mirna_pc
4735	hsa-mir-301b	SUMF2	0.419433626	mirna_pc
4736	hsa-mir-301b	RPS19BP1	0.38151905	mirna_pc
4737	hsa-mir-301b	KDM4D	0.303423054	mirna_pc
4738	hsa-mir-301b	GBAS	0.480099309	mirna_pc
4739	hsa-mir-301b	C8orf40	0.437813749	mirna_pc
4740	hsa-mir-301b	AGBL5	0.312270765	mirna_pc
4741	hsa-mir-301b	AIP	0.408680481	mirna_pc
4742	hsa-mir-301b	BOLA3	0.374837048	mirna_pc
4743	hsa-mir-301b	C19orf23	0.535871957	mirna_pc
4744	hsa-mir-301b	MYL6B	0.304520043	mirna_pc
4745	hsa-mir-301b	PDCD7	0.331421654	mirna_pc
4746	hsa-mir-301b	C11orf71	0.373116245	mirna_pc
4747	hsa-mir-301b	POLR2F	0.318907044	mirna_pc
4748	hsa-mir-301b	ALKBH3	0.369145238	mirna_pc
4749	hsa-mir-301b	CD200	0.308893166	mirna_pc
4750	hsa-mir-301b	MRPS15	0.363088779	mirna_pc
4751	hsa-mir-301b	C17orf58	0.443672532	mirna_pc

4752	hsa-mir-301b	NARS2	0.476475238	mirna_pc
4753	hsa-mir-301b	LYRM4	0.403404411	mirna_pc
4754	hsa-mir-301b	FAM133B	0.575150039	mirna_pc
4755	hsa-mir-301b	BCL11A	0.327318793	mirna_pc
4756	hsa-mir-301b	BSG	0.348153031	mirna_pc
4757	hsa-mir-301b	UTP11L	0.360558011	mirna_pc
4758	hsa-mir-301b	TIMM8B	0.309064567	mirna_pc
4759	hsa-mir-301b	ALMS1	0.306435159	mirna_pc
4760	hsa-mir-301b	ANP32A	0.357711752	mirna_pc
4761	hsa-mir-301b	SPDYA	0.3295876	mirna_pc
4762	hsa-mir-301b	RBM25	0.310327333	mirna_pc
4763	hsa-mir-301b	ETAA1	0.319027858	mirna_pc
4764	hsa-mir-301b	HYLS1	0.37014883	mirna_pc
4765	hsa-mir-301b	C7orf60	0.397427081	mirna_pc
4766	hsa-mir-301b	LCMT2	0.309140226	mirna_pc
4767	hsa-mir-301b	ZNF300	0.385483002	mirna_pc
4768	hsa-mir-301b	MORN2	0.415293023	mirna_pc
4769	hsa-mir-301b	SETD6	0.368341801	mirna_pc
4770	hsa-mir-301b	RTN4RL1	0.417132768	mirna_pc
4771	hsa-mir-301b	C15orf40	0.391071161	mirna_pc
4772	hsa-mir-301b	CWC15	0.392334929	mirna_pc
4773	hsa-mir-301b	MYCN	0.310855118	mirna_pc
4774	hsa-mir-301b	MTERF	0.643230735	mirna_pc
4775	hsa-mir-301b	SBK1	0.459197237	mirna_pc
4776	hsa-mir-301b	AFMID	0.377628266	mirna_pc
4777	hsa-mir-301b	C7orf64	0.436697606	mirna_pc
4778	hsa-mir-301b	GLS2	0.319222779	mirna_pc
4779	hsa-mir-301b	SLC25A33	0.394317962	mirna_pc
4780	hsa-mir-301b	CDKAL1	0.308812264	mirna_pc
4781	hsa-mir-301b	OST4	0.325305372	mirna_pc
4782	hsa-mir-301b	TMEM18	0.36990793	mirna_pc
4783	hsa-mir-301b	ZNF394	0.432581935	mirna_pc
4784	hsa-mir-301b	PAQR6	0.357906881	mirna_pc
4785	hsa-mir-301b	DDX51	0.327209841	mirna_pc
4786	hsa-mir-301b	GTF2H2	0.373787948	mirna_pc
4787	hsa-mir-301b	ZNF232	0.355538192	mirna_pc
4788	hsa-mir-301b	ZBTB39	0.373601111	mirna_pc
4789	hsa-mir-301b	SNRNP27	0.359132077	mirna_pc
4790	hsa-mir-301b	C1orf56	0.31666526	mirna_pc
4791	hsa-mir-301b	ENO3	0.304865419	mirna_pc
4792	hsa-mir-301b	RPAIN	0.381008249	mirna_pc
4793	hsa-mir-301b	CCDC90B	0.528161084	mirna_pc
4794	hsa-mir-301b	VPS54	0.332957409	mirna_pc
4795	hsa-mir-301b	MRPL1	0.338501542	mirna_pc
4796	hsa-mir-301b	API5	0.484928319	mirna_pc
4797	hsa-mir-301b	USP28	0.384549512	mirna_pc
4798	hsa-mir-301b	C2orf3	0.502451449	mirna_pc
4799	hsa-mir-301b	KRIT1	0.496113628	mirna_pc
4800	hsa-mir-301b	DHRS2	0.434180725	mirna_pc
4801	hsa-mir-301b	MEAF6	0.426536885	mirna_pc
4802	hsa-mir-301b	FGFBP3	0.371210701	mirna_pc
4803	hsa-mir-301b	DNAJC19	0.315782987	mirna_pc
4804	hsa-mir-301b	TMEM134	0.463319753	mirna_pc
4805	hsa-mir-301b	CCDC157	0.313172359	mirna_pc

4806	hsa-mir-301b	ZNF354A	0.310340365	mirna_pc
4807	hsa-mir-301b	PEX1	0.460995712	mirna_pc
4808	hsa-mir-301b	NDUFS5	0.325415603	mirna_pc
4809	hsa-mir-301b	MRPL19	0.390604328	mirna_pc
4810	hsa-mir-301b	C11orf35	0.380636592	mirna_pc
4811	hsa-mir-301b	CETN3	0.380487409	mirna_pc
4812	hsa-mir-301b	ETV5	0.395298992	mirna_pc
4813	hsa-mir-301b	LSM 1.00	0.402878747	mirna_pc
4814	hsa-mir-301b	DYNC1I1	0.538831719	mirna_pc
4815	hsa-mir-301b	GLI4	0.379071971	mirna_pc
4816	hsa-mir-301b	AADAT	0.360212566	mirna_pc
4817	hsa-mir-301b	NFRKB	0.318620973	mirna_pc
4818	hsa-mir-301b	IGHMBP2	0.334086915	mirna_pc
4819	hsa-mir-301b	ACP1	0.354031339	mirna_pc
4820	hsa-mir-301b	FDX1L	0.316493902	mirna_pc
4821	hsa-mir-301b	GATAD1	0.363257182	mirna_pc
4822	hsa-mir-301b	ZNF322A	0.615922634	mirna_pc
4823	hsa-mir-301b	SPRED2	0.344027417	mirna_pc
4824	hsa-mir-301b	GLRX5	0.348249244	mirna_pc
4825	hsa-mir-301b	SERF1A	0.425267259	mirna_pc
4826	hsa-mir-301b	LOC728640	0.438768143	mirna_pc
4827	hsa-mir-301b	PRMT6	0.305200341	mirna_pc
4828	hsa-mir-301b	LOC401397	0.611346859	mirna_pc
4829	hsa-mir-301b	C21orf119	0.354429221	mirna_pc
4830	hsa-mir-301b	C2orf28	0.430420092	mirna_pc
4831	hsa-mir-301b	HIRIP3	0.346710052	mirna_pc
4832	hsa-mir-301b	TMEM168	0.578220826	mirna_pc
4833	hsa-mir-301b	SCHIP1	0.310571797	mirna_pc
4834	hsa-mir-301b	CCDC75	0.437834627	mirna_pc
4835	hsa-mir-301b	MRPL53	0.47012392	mirna_pc
4836	hsa-mir-301b	PPCDC	0.332842335	mirna_pc
4837	hsa-mir-301b	DNMT3A	0.436734549	mirna_pc
4838	hsa-mir-301b	ANKRD49	0.331444552	mirna_pc
4839	hsa-mir-301b	NDUFS8	0.472469382	mirna_pc
4840	hsa-mir-301b	ACCN2	0.300993988	mirna_pc
4841	hsa-mir-301b	C15orf61	0.391231478	mirna_pc
4842	hsa-mir-301b	ZNF326	0.365086391	mirna_pc
4843	hsa-mir-361	KIF4B	0.361195125	mirna_pc
4844	hsa-mir-361	HOXC9	0.359387358	mirna_pc
4845	hsa-mir-361	FANCA	0.336438963	mirna_pc
4846	hsa-mir-361	SGOL1	0.350510646	mirna_pc
4847	hsa-mir-361	MND1	0.311853034	mirna_pc
4848	hsa-mir-361	RAD54L	0.302788386	mirna_pc
4849	hsa-mir-361	MAD2L1	0.311715924	mirna_pc
4850	hsa-mir-361	TIMELESS	0.329106276	mirna_pc
4851	hsa-mir-361	XPO1	0.30417402	mirna_pc
4852	hsa-mir-361	DKC1	0.308840048	mirna_pc
4853	hsa-mir-361	KIF4A	0.361849889	mirna_pc
4854	hsa-mir-361	PLK4	0.355001617	mirna_pc
4855	hsa-mir-361	FANCB	0.327029788	mirna_pc
4856	hsa-mir-361	LIG1	0.320531077	mirna_pc
4857	hsa-mir-361	OIP5	0.305395138	mirna_pc
4858	hsa-mir-361	C1orf112	0.359998143	mirna_pc
4859	hsa-mir-361	FEN1	0.303198915	mirna_pc

4860	hsa-mir-361	HNRNPL	0.310128891	mirna_pc
4861	hsa-mir-361	ZWINT	0.359869236	mirna_pc
4862	hsa-mir-361	C15orf42	0.334031329	mirna_pc
4863	hsa-mir-361	DEPDC1B	0.322561444	mirna_pc
4864	hsa-mir-361	CENPO	0.32480399	mirna_pc
4865	hsa-mir-361	HMGB2	0.385636296	mirna_pc
4866	hsa-mir-361	CDK2	0.302841914	mirna_pc
4867	hsa-mir-361	MSH2	0.343132117	mirna_pc
4868	hsa-mir-361	SALL4	0.309642107	mirna_pc
4869	hsa-mir-361	FBXO5	0.320621943	mirna_pc
4870	hsa-mir-361	DCLRE1B	0.32936706	mirna_pc
4871	hsa-mir-361	MDK	0.346548205	mirna_pc
4872	hsa-mir-361	CDC25A	0.367860604	mirna_pc
4873	hsa-mir-361	MARCKSL1	0.318545121	mirna_pc
4874	hsa-mir-361	SRRT	0.348335752	mirna_pc
4875	hsa-mir-361	SASS6	0.307598826	mirna_pc
4876	hsa-mir-361	PSRC1	0.325955055	mirna_pc
4877	hsa-mir-361	POU2F1	0.365888504	mirna_pc
4878	hsa-mir-361	TMPO	0.308346574	mirna_pc
4879	hsa-mir-361	VBP1	0.377204004	mirna_pc
4880	hsa-mir-361	LOC100128191	0.305218331	mirna_pc
4881	hsa-mir-361	PHF6	0.327213424	mirna_pc
4882	hsa-mir-361	SFRS1	0.339499431	mirna_pc
4883	hsa-mir-361	C4orf46	0.353101236	mirna_pc
4884	hsa-mir-361	UTP14A	0.426602024	mirna_pc
4885	hsa-mir-361	POLR2D	0.343336433	mirna_pc
4886	hsa-mir-361	SLC5A6	0.302881528	mirna_pc
4887	hsa-mir-361	ZNF695	0.362423765	mirna_pc
4888	hsa-mir-361	TIMM8A	0.34789031	mirna_pc
4889	hsa-mir-361	NASP	0.35547025	mirna_pc
4890	hsa-mir-361	NONO	0.408866764	mirna_pc
4891	hsa-mir-361	ELK1	0.314255352	mirna_pc
4892	hsa-mir-361	SUV39H2	0.301149227	mirna_pc
4893	hsa-mir-361	ACVR2A	0.323289042	mirna_pc
4894	hsa-mir-361	NKRF	0.31846738	mirna_pc
4895	hsa-mir-361	MOV10	0.360177019	mirna_pc
4896	hsa-mir-361	PDRG1	0.342195164	mirna_pc
4897	hsa-mir-361	PASK	0.329564969	mirna_pc
4898	hsa-mir-361	FUBP1	0.315968613	mirna_pc
4899	hsa-mir-361	GAR1	0.385883394	mirna_pc
4900	hsa-mir-361	RPN2	0.323986421	mirna_pc
4901	hsa-mir-361	TRAPPC2	0.326559172	mirna_pc
4902	hsa-mir-361	ITGB3BP	0.327139273	mirna_pc
4903	hsa-mir-361	LAS1L	0.414400773	mirna_pc
4904	hsa-mir-361	ING5	0.33996127	mirna_pc
4905	hsa-mir-361	CADM1	0.405898554	mirna_pc
4906	hsa-mir-361	POLA1	0.360402933	mirna_pc
4907	hsa-mir-361	C10orf2	0.348135786	mirna_pc
4908	hsa-mir-361	CSTF1	0.300812006	mirna_pc
4909	hsa-mir-361	GPAM	0.36214468	mirna_pc
4910	hsa-mir-361	MEX3A	0.324256968	mirna_pc
4911	hsa-mir-361	PDCD11	0.346958184	mirna_pc
4912	hsa-mir-361	TAF5	0.339804581	mirna_pc
4913	hsa-mir-361	ZBTB12	0.302699101	mirna_pc

4914	hsa-mir-361	TGIF2	0.390378838	mirna_pc
4915	hsa-mir-361	MTF2	0.362614318	mirna_pc
4916	hsa-mir-361	CCDC142	0.313498642	mirna_pc
4917	hsa-mir-361	POLE	0.448802169	mirna_pc
4918	hsa-mir-361	MUTYH	0.317055274	mirna_pc
4919	hsa-mir-361	ABCB7	0.303859043	mirna_pc
4920	hsa-mir-361	THOC2	0.349373024	mirna_pc
4921	hsa-mir-361	DDOST	0.315723189	mirna_pc
4922	hsa-mir-361	RBMX	0.400130884	mirna_pc
4923	hsa-mir-361	ZNF200	0.331481158	mirna_pc
4924	hsa-mir-361	MSX2	0.325043221	mirna_pc
4925	hsa-mir-361	DNAJC14	0.356309821	mirna_pc
4926	hsa-mir-361	EIF1AX	0.37011097	mirna_pc
4927	hsa-mir-361	ITPRIPL1	0.387992187	mirna_pc
4928	hsa-mir-361	TTLL4	0.31526971	mirna_pc
4929	hsa-mir-361	ATXN7L2	0.305971337	mirna_pc
4930	hsa-mir-361	GGT7	0.317824239	mirna_pc
4931	hsa-mir-361	NOTUM	0.339929028	mirna_pc
4932	hsa-mir-361	MORF4L2	0.30670717	mirna_pc
4933	hsa-mir-361	NKAP	0.332262541	mirna_pc
4934	hsa-mir-361	UPF3B	0.381550881	mirna_pc
4935	hsa-mir-361	TBX3	0.344371351	mirna_pc
4936	hsa-mir-361	RBM12	0.303660396	mirna_pc
4937	hsa-mir-361	DDX20	0.403081301	mirna_pc
4938	hsa-mir-361	UBA1	0.305137462	mirna_pc
4939	hsa-mir-361	ZNF620	0.326455417	mirna_pc
4940	hsa-mir-361	RBMXL1	0.326006189	mirna_pc
4941	hsa-mir-361	HUNK	0.313384645	mirna_pc
4942	hsa-mir-361	C1orf77	0.302439637	mirna_pc
4943	hsa-mir-361	SFRS13A	0.30717435	mirna_pc
4944	hsa-mir-361	CWF19L1	0.311691923	mirna_pc
4945	hsa-mir-361	TFAM	0.304946719	mirna_pc
4946	hsa-mir-361	MED14	0.312758906	mirna_pc
4947	hsa-mir-361	FAM35B2	0.333125998	mirna_pc
4948	hsa-mir-361	EWSR1	0.366457499	mirna_pc
4949	hsa-mir-361	SMC1A	0.430266781	mirna_pc
4950	hsa-mir-361	ACVR2B	0.343409061	mirna_pc
4951	hsa-mir-361	RNF113A	0.340914065	mirna_pc
4952	hsa-mir-361	HDAC8	0.327936038	mirna_pc
4953	hsa-mir-361	SFRS7	0.339292532	mirna_pc
4954	hsa-mir-361	BEND3	0.35508685	mirna_pc
4955	hsa-mir-361	CA5BP	0.483809501	mirna_pc
4956	hsa-mir-361	CHM	0.362213497	mirna_pc
4957	hsa-mir-361	AXIN2	0.326233597	mirna_pc
4958	hsa-mir-361	FAM35B	0.329753566	mirna_pc
4959	hsa-mir-361	SYAP1	0.309592883	mirna_pc
4960	hsa-mir-361	YY2	0.306922328	mirna_pc
4961	hsa-mir-361	LOC728024	0.375542276	mirna_pc
4962	hsa-mir-361	GEMIN8	0.31461968	mirna_pc
4963	hsa-mir-361	BHLHB9	0.325136566	mirna_pc
4964	hsa-mir-361	GJB1	0.304290738	mirna_pc
4965	hsa-mir-361	C2orf44	0.316050414	mirna_pc
4966	hsa-mir-361	CTR9	0.32651733	mirna_pc
4967	hsa-mir-361	IFT172	0.308505462	mirna_pc

4968	hsa-mir-361	CCDC76	0.330972367	mirna_pc
4969	hsa-mir-361	ZNF233	0.310827537	mirna_pc
4970	hsa-mir-361	NUP43	0.308734125	mirna_pc
4971	hsa-mir-361	ZNF124	0.33340957	mirna_pc
4972	hsa-mir-361	CHD1L	0.332159128	mirna_pc
4973	hsa-mir-361	ZNF473	0.337907004	mirna_pc
4974	hsa-mir-361	ANP32A	0.341606899	mirna_pc
4975	hsa-mir-361	ZNF195	0.316378537	mirna_pc
4976	hsa-mir-361	ZMYM4	0.338830106	mirna_pc
4977	hsa-mir-361	RBM15	0.32773187	mirna_pc
4978	hsa-mir-361	HIC2	0.411235612	mirna_pc
4979	hsa-mir-361	PHF16	0.330937585	mirna_pc
4980	hsa-mir-361	FAM123B	0.30535228	mirna_pc
4981	hsa-mir-361	IQCC	0.302643733	mirna_pc
4982	hsa-mir-361	MBNL3	0.333002422	mirna_pc
4983	hsa-mir-361	AMOT	0.333177957	mirna_pc
4984	hsa-mir-361	NAIF1	0.361390097	mirna_pc
4985	hsa-mir-361	L3MBTL2	0.361800541	mirna_pc
4986	hsa-mir-361	CCNJ	0.365471119	mirna_pc
4987	hsa-mir-361	OTUD3	0.362172131	mirna_pc
4988	hsa-mir-361	DCAF16	0.358902789	mirna_pc
4989	hsa-mir-361	KDM5C	0.305776992	mirna_pc
4990	hsa-mir-361	C10orf137	0.397427583	mirna_pc
4991	hsa-mir-361	TSR2	0.303171402	mirna_pc
4992	hsa-mir-361	FAM171A1	0.347777056	mirna_pc
4993	hsa-mir-361	RASL10B	0.317225731	mirna_pc
4994	hsa-mir-361	SLC25A33	0.369498222	mirna_pc
4995	hsa-mir-361	RLIM	0.314554932	mirna_pc
4996	hsa-mir-361	CTPS2	0.365778874	mirna_pc
4997	hsa-mir-361	SPHK2	0.36815591	mirna_pc
4998	hsa-mir-361	GXYLT1	0.357759569	mirna_pc
4999	hsa-mir-361	ZBTB39	0.39396639	mirna_pc
5000	hsa-mir-361	ZNF547	0.32895466	mirna_pc
5001	hsa-mir-361	FANCF	0.365720537	mirna_pc
5002	hsa-mir-361	VPS54	0.404386302	mirna_pc
5003	hsa-mir-361	MED12	0.397117184	mirna_pc
5004	hsa-mir-361	TOE1	0.367893996	mirna_pc
5005	hsa-mir-361	ZNF551	0.317639142	mirna_pc
5006	hsa-mir-361	ZNF618	0.317527822	mirna_pc
5007	hsa-mir-361	RRP7B	0.303495191	mirna_pc
5008	hsa-mir-361	NCRNA00183	0.329259687	mirna_pc
5009	hsa-mir-361	SCML2	0.331151084	mirna_pc
5010	hsa-mir-361	SIKE1	0.350717264	mirna_pc
5011	hsa-mir-361	ASXL1	0.301414915	mirna_pc
5012	hsa-mir-361	ARMC2	0.313697931	mirna_pc
5013	hsa-mir-361	CALM3	0.317313062	mirna_pc
5014	hsa-mir-361	ZCCHC3	0.382351333	mirna_pc
5015	hsa-mir-361	RRP15	0.301269781	mirna_pc
5016	hsa-mir-361	WDR73	0.337306905	mirna_pc
5017	hsa-mir-361	COL9A2	0.327478987	mirna_pc
5018	hsa-mir-361	ETV5	0.337563032	mirna_pc
5019	hsa-mir-361	COL9A3	0.3025687	mirna_pc
5020	hsa-mir-361	PROX1	0.353344992	mirna_pc
5021	hsa-mir-361	TAF1	0.30989668	mirna_pc

5022	hsa-mir-361	TAF1L	0.30637831	mirna_pc
5023	hsa-mir-361	IGHMBP2	0.300886727	mirna_pc
5024	hsa-mir-361	NAF1	0.346314341	mirna_pc
5025	hsa-mir-361	AIMP1	0.312315168	mirna_pc
5026	hsa-mir-361	TXLNG	0.400662132	mirna_pc
5027	hsa-mir-361	PRMT6	0.329040787	mirna_pc
5028	hsa-mir-361	ZMYM3	0.387564829	mirna_pc
5029	hsa-mir-361	PIN4	0.31462568	mirna_pc
5030	hsa-mir-361	ZNF530	0.352991507	mirna_pc
5031	hsa-mir-361	ZNF239	0.309703663	mirna_pc
5032	hsa-mir-361	LOC728758	0.397903926	mirna_pc
5033	hsa-mir-361	EIF2S3	0.362124154	mirna_pc
5034	hsa-mir-361	CENPV	0.302980476	mirna_pc
5035	hsa-mir-361	SFRS6	0.335872355	mirna_pc
5036	hsa-mir-361	ZNF670	0.351103407	mirna_pc
5037	hsa-mir-361	ZNF711	0.484307756	mirna_pc
5038	hsa-mir-361	MAGT1	0.32734023	mirna_pc
5039	hsa-mir-937	CDCA8	0.300486449	mirna_pc
5040	hsa-mir-937	RCC1	0.304353543	mirna_pc
5041	hsa-mir-937	RECQL4	0.400405799	mirna_pc
5042	hsa-mir-937	ADRM1	0.304558158	mirna_pc
5043	hsa-mir-937	PIF1	0.302616471	mirna_pc
5044	hsa-mir-937	DCAF13	0.406149495	mirna_pc
5045	hsa-mir-937	NFKBIL2	0.433194968	mirna_pc
5046	hsa-mir-937	RTKN	0.335482474	mirna_pc
5047	hsa-mir-937	FAM83H	0.467845341	mirna_pc
5048	hsa-mir-937	CCT3	0.325931907	mirna_pc
5049	hsa-mir-937	GRINA	0.386281837	mirna_pc
5050	hsa-mir-937	CSE1L	0.309549516	mirna_pc
5051	hsa-mir-937	EBNA1BP2	0.30529717	mirna_pc
5052	hsa-mir-937	F12	0.303295608	mirna_pc
5053	hsa-mir-937	SF3B14	0.343167149	mirna_pc
5054	hsa-mir-937	GPR172A	0.495708721	mirna_pc
5055	hsa-mir-937	NPM3	0.321905941	mirna_pc
5056	hsa-mir-937	TMEM201	0.311169127	mirna_pc
5057	hsa-mir-937	STIP1	0.313608938	mirna_pc
5058	hsa-mir-937	TBRG4	0.337269417	mirna_pc
5059	hsa-mir-937	EIF5A	0.309858274	mirna_pc
5060	hsa-mir-937	BOP 1.00	0.38938612	mirna_pc
5061	hsa-mir-937	YWHAZ	0.300986714	mirna_pc
5062	hsa-mir-937	PA2G4	0.310832117	mirna_pc
5063	hsa-mir-937	TOMM34	0.374379485	mirna_pc
5064	hsa-mir-937	GGH	0.319366534	mirna_pc
5065	hsa-mir-937	MRPL13	0.307572393	mirna_pc
5066	hsa-mir-937	PYCRL	0.339728434	mirna_pc
5067	hsa-mir-937	GNL2	0.336570485	mirna_pc
5068	hsa-mir-937	ZC3H3	0.484546842	mirna_pc
5069	hsa-mir-937	TOP1MT	0.332176085	mirna_pc
5070	hsa-mir-937	ARHGAP39	0.466040676	mirna_pc
5071	hsa-mir-937	FBXL6	0.585894496	mirna_pc
5072	hsa-mir-937	EIF5AL1	0.310805561	mirna_pc
5073	hsa-mir-937	ADCK5	0.422430124	mirna_pc
5074	hsa-mir-937	NUPL2	0.411652128	mirna_pc
5075	hsa-mir-937	PGAM5	0.392984654	mirna_pc

5076	hsa-mir-937	PUF60	0.531465767	mirna_pc
5077	hsa-mir-937	GAR1	0.306166323	mirna_pc
5078	hsa-mir-937	PRMT3	0.304282565	mirna_pc
5079	hsa-mir-937	IGF2BP3	0.322601212	mirna_pc
5080	hsa-mir-937	RAD51L3	0.316609286	mirna_pc
5081	hsa-mir-937	TDRD5	0.344323481	mirna_pc
5082	hsa-mir-937	STK31	0.359129419	mirna_pc
5083	hsa-mir-937	PA2G4P4	0.343792444	mirna_pc
5084	hsa-mir-937	RRP12	0.314584889	mirna_pc
5085	hsa-mir-937	CHRAC1	0.358889615	mirna_pc
5086	hsa-mir-937	COMMD5	0.400469149	mirna_pc
5087	hsa-mir-937	POLR3G	0.305045522	mirna_pc
5088	hsa-mir-937	PSMG1	0.321641784	mirna_pc
5089	hsa-mir-937	C8orf73	0.451083474	mirna_pc
5090	hsa-mir-937	PDCD11	0.302561876	mirna_pc
5091	hsa-mir-937	C7orf30	0.442499239	mirna_pc
5092	hsa-mir-937	EXOSC4	0.436632781	mirna_pc
5093	hsa-mir-937	SCRIB	0.403396617	mirna_pc
5094	hsa-mir-937	TRAPPC9	0.355972749	mirna_pc
5095	hsa-mir-937	C8orf30A	0.520288544	mirna_pc
5096	hsa-mir-937	GATA3	0.325629775	mirna_pc
5097	hsa-mir-937	ZNF707	0.326966411	mirna_pc
5098	hsa-mir-937	RRP1	0.340495851	mirna_pc
5099	hsa-mir-937	SPATA5L1	0.322560841	mirna_pc
5100	hsa-mir-937	PGLYRP4	0.348411796	mirna_pc
5101	hsa-mir-937	C8orf55	0.478708173	mirna_pc
5102	hsa-mir-937	RPL30	0.308990155	mirna_pc
5103	hsa-mir-937	YRDC	0.373250617	mirna_pc
5104	hsa-mir-937	RDH16	0.336887512	mirna_pc
5105	hsa-mir-937	HMGN1	0.301635087	mirna_pc
5106	hsa-mir-937	CCT8	0.363959307	mirna_pc
5107	hsa-mir-937	TIGD5	0.39011376	mirna_pc
5108	hsa-mir-937	EIF2C2	0.393108424	mirna_pc
5109	hsa-mir-937	RPS7	0.358542672	mirna_pc
5110	hsa-mir-937	EPPK1	0.318378229	mirna_pc
5111	hsa-mir-937	C8orf51	0.32695949	mirna_pc
5112	hsa-mir-937	TFAP2E	0.313697692	mirna_pc
5113	hsa-mir-937	RPL8	0.39993859	mirna_pc
5114	hsa-mir-937	NRBP2	0.33466652	mirna_pc
5115	hsa-mir-937	FAM83A	0.344429417	mirna_pc
5116	hsa-mir-937	NLE1	0.392996339	mirna_pc
5117	hsa-mir-937	LYNX1	0.312551538	mirna_pc
5118	hsa-mir-937	ZNF7	0.347946692	mirna_pc
5119	hsa-mir-937	EIF3H	0.379432895	mirna_pc
5120	hsa-mir-937	CYP2W1	0.303868863	mirna_pc
5121	hsa-mir-937	RHPN1	0.376406228	mirna_pc
5122	hsa-mir-937	RPL5	0.334790697	mirna_pc
5123	hsa-mir-937	SLC19A1	0.323991214	mirna_pc
5124	hsa-mir-937	NAPRT1	0.378225999	mirna_pc
5125	hsa-mir-937	KIFC2	0.419440557	mirna_pc
5126	hsa-mir-937	KRT16	0.333727946	mirna_pc
5127	hsa-mir-937	SATB2	0.458819335	mirna_pc
5128	hsa-mir-937	LIG3	0.319400173	mirna_pc
5129	hsa-mir-937	ZFP41	0.358903549	mirna_pc

5130	hsa-mir-937	RPL7	0.366668573	mirna_pc
5131	hsa-mir-937	HSF1	0.472247927	mirna_pc
5132	hsa-mir-937	CPSF1	0.48104341	mirna_pc
5133	hsa-mir-937	OPLAH	0.363841751	mirna_pc
5134	hsa-mir-937	SERBP1	0.337581332	mirna_pc
5135	hsa-mir-937	SPRY3	0.357906092	mirna_pc
5136	hsa-mir-937	DGAT1	0.319060317	mirna_pc
5137	hsa-mir-937	KLHL17	0.319864414	mirna_pc
5138	hsa-mir-937	PTK2	0.300415213	mirna_pc
5139	hsa-mir-937	C10orf99	0.324649559	mirna_pc
5140	hsa-mir-937	RPL14	0.305206585	mirna_pc
5141	hsa-mir-937	ZNF16	0.350972379	mirna_pc
5142	hsa-mir-937	PTDSS2	0.3385458	mirna_pc
5143	hsa-mir-937	HEATR7A	0.325900445	mirna_pc
5144	hsa-mir-937	LRP11	0.304830042	mirna_pc
5145	hsa-mir-937	CYHR1	0.502632419	mirna_pc
5146	hsa-mir-937	UTP11L	0.316211806	mirna_pc
5147	hsa-mir-937	MFSD3	0.439475123	mirna_pc
5148	hsa-mir-937	TRA2A	0.357648952	mirna_pc
5149	hsa-mir-937	E2F5	0.348685672	mirna_pc
5150	hsa-mir-937	ZNF696	0.324863306	mirna_pc
5151	hsa-mir-937	CDC34	0.315120863	mirna_pc
5152	hsa-mir-937	MGC70857	0.30338269	mirna_pc
5153	hsa-mir-937	PSTK	0.315604343	mirna_pc
5154	hsa-mir-937	JRK	0.331025433	mirna_pc
5155	hsa-mir-937	VPS28	0.380839331	mirna_pc
5156	hsa-mir-937	PSMD6	0.310870138	mirna_pc
5157	hsa-mir-937	C21orf59	0.32089872	mirna_pc
5158	hsa-mir-937	SHARPIN	0.442805663	mirna_pc
5159	hsa-mir-937	PPP1R16A	0.408381282	mirna_pc
5160	hsa-mir-937	CEACAM19	0.318499318	mirna_pc
5161	hsa-mir-937	LRRC14	0.4153282	mirna_pc
5162	hsa-mir-937	CYCS	0.408448555	mirna_pc
5163	hsa-mir-937	CYC1	0.424039841	mirna_pc
5164	hsa-mir-937	TUBB2A	0.303563892	mirna_pc
5165	hsa-mir-937	NACA2	0.312750547	mirna_pc
5166	hsa-mir-937	EIF3E	0.411936593	mirna_pc
5167	hsa-mir-937	KIAA1274	0.326518856	mirna_pc
5168	hsa-mir-937	GPAA1	0.320053388	mirna_pc
5169	hsa-mir-937	MAF 1.00	0.316733091	mirna_pc
5170	hsa-mir-937	MYLK4	0.372338692	mirna_pc
5171	hsa-mir-937	C21orf70	0.345357645	mirna_pc
5172	hsa-mir-937	TSTA3	0.330890882	mirna_pc
5173	hsa-mir-937	EEF1D	0.337588711	mirna_pc
5174	hsa-mir-17	MKI67	0.371985585	mirna_pc
5175	hsa-mir-17	TPX2	0.411529437	mirna_pc
5176	hsa-mir-17	KIF4B	0.360280953	mirna_pc
5177	hsa-mir-17	TOP2A	0.459834234	mirna_pc
5178	hsa-mir-17	KPNA2	0.351986508	mirna_pc
5179	hsa-mir-17	CENPF	0.440610538	mirna_pc
5180	hsa-mir-17	RCC2	0.41483339	mirna_pc
5181	hsa-mir-17	HOXC9	0.335180255	mirna_pc
5182	hsa-mir-17	KIF11	0.438735698	mirna_pc
5183	hsa-mir-17	ECT2	0.358596736	mirna_pc

5184	hsa-mir-17	RRM2	0.413800126	mirna_pc
5185	hsa-mir-17	SPC24	0.306491639	mirna_pc
5186	hsa-mir-17	CDK1	0.464850542	mirna_pc
5187	hsa-mir-17	CDC25C	0.403080399	mirna_pc
5188	hsa-mir-17	MYBL2	0.376450441	mirna_pc
5189	hsa-mir-17	PLK1	0.369317759	mirna_pc
5190	hsa-mir-17	PCNA	0.34617984	mirna_pc
5191	hsa-mir-17	UBE2C	0.435440453	mirna_pc
5192	hsa-mir-17	BUB1	0.459994385	mirna_pc
5193	hsa-mir-17	PRC1	0.311225948	mirna_pc
5194	hsa-mir-17	NUSAP1	0.393624618	mirna_pc
5195	hsa-mir-17	MCM4	0.34664999	mirna_pc
5196	hsa-mir-17	KIFC1	0.434459748	mirna_pc
5197	hsa-mir-17	BUB1B	0.466508092	mirna_pc
5198	hsa-mir-17	HDGF	0.383739264	mirna_pc
5199	hsa-mir-17	KIF18B	0.401525079	mirna_pc
5200	hsa-mir-17	ASPM	0.368130534	mirna_pc
5201	hsa-mir-17	CKAP2	0.471798219	mirna_pc
5202	hsa-mir-17	FAM72A	0.397583846	mirna_pc
5203	hsa-mir-17	KIF2C	0.420928411	mirna_pc
5204	hsa-mir-17	FANCA	0.483428716	mirna_pc
5205	hsa-mir-17	NCAPG	0.502070654	mirna_pc
5206	hsa-mir-17	CDC20	0.301928328	mirna_pc
5207	hsa-mir-17	CLSPN	0.409819175	mirna_pc
5208	hsa-mir-17	SGOL1	0.486515345	mirna_pc
5209	hsa-mir-17	ARHGAP11A	0.449798265	mirna_pc
5210	hsa-mir-17	CCNA2	0.482716949	mirna_pc
5211	hsa-mir-17	CDC25B	0.307857882	mirna_pc
5212	hsa-mir-17	CBX3	0.409603471	mirna_pc
5213	hsa-mir-17	SPC25	0.502450133	mirna_pc
5214	hsa-mir-17	NUP62	0.314352532	mirna_pc
5215	hsa-mir-17	FANCI	0.416031142	mirna_pc
5216	hsa-mir-17	NEK2	0.568043024	mirna_pc
5217	hsa-mir-17	MND1	0.414521665	mirna_pc
5218	hsa-mir-17	KIF22	0.412946999	mirna_pc
5219	hsa-mir-17	NCAPH	0.472155177	mirna_pc
5220	hsa-mir-17	GTSE1	0.423883443	mirna_pc
5221	hsa-mir-17	KIF23	0.312053059	mirna_pc
5222	hsa-mir-17	RAD54L	0.461597044	mirna_pc
5223	hsa-mir-17	CENPA	0.413611492	mirna_pc
5224	hsa-mir-17	MAD2L1	0.58740245	mirna_pc
5225	hsa-mir-17	TIMELESS	0.442189446	mirna_pc
5226	hsa-mir-17	LMNB1	0.47631668	mirna_pc
5227	hsa-mir-17	ASF1B	0.346695449	mirna_pc
5228	hsa-mir-17	CDCA8	0.486729025	mirna_pc
5229	hsa-mir-17	TROAP	0.393203821	mirna_pc
5230	hsa-mir-17	CCNB1	0.316243838	mirna_pc
5231	hsa-mir-17	CDCA5	0.398452097	mirna_pc
5232	hsa-mir-17	TACC3	0.372828808	mirna_pc
5233	hsa-mir-17	XPO1	0.472048814	mirna_pc
5234	hsa-mir-17	CDC45	0.360929968	mirna_pc
5235	hsa-mir-17	STMN1	0.395644736	mirna_pc
5236	hsa-mir-17	CENPM	0.332406317	mirna_pc
5237	hsa-mir-17	EXO1	0.343699458	mirna_pc

5238	hsa-mir-17	CDCA2	0.364771395	mirna_pc
5239	hsa-mir-17	HMGA1	0.311757618	mirna_pc
5240	hsa-mir-17	DKC1	0.348488199	mirna_pc
5241	hsa-mir-17	UBE2T	0.42674514	mirna_pc
5242	hsa-mir-17	CKS2	0.376342024	mirna_pc
5243	hsa-mir-17	CKS1B	0.388949479	mirna_pc
5244	hsa-mir-17	MCM2	0.334443039	mirna_pc
5245	hsa-mir-17	KIF4A	0.36554235	mirna_pc
5246	hsa-mir-17	ORC1L	0.365420073	mirna_pc
5247	hsa-mir-17	SNRPB	0.370885333	mirna_pc
5248	hsa-mir-17	KNTC1	0.405641584	mirna_pc
5249	hsa-mir-17	UHRF1	0.347204039	mirna_pc
5250	hsa-mir-17	CCNF	0.347710389	mirna_pc
5251	hsa-mir-17	PTBP1	0.346018322	mirna_pc
5252	hsa-mir-17	CCNB2	0.35643431	mirna_pc
5253	hsa-mir-17	NUF2	0.455218841	mirna_pc
5254	hsa-mir-17	PLK4	0.500407194	mirna_pc
5255	hsa-mir-17	FANCB	0.390483388	mirna_pc
5256	hsa-mir-17	KIF18A	0.407092352	mirna_pc
5257	hsa-mir-17	DEPDC1	0.424524044	mirna_pc
5258	hsa-mir-17	SKA3	0.598904596	mirna_pc
5259	hsa-mir-17	KIF14	0.381721318	mirna_pc
5260	hsa-mir-17	HJURP	0.545347998	mirna_pc
5261	hsa-mir-17	SKA1	0.346027111	mirna_pc
5262	hsa-mir-17	RAD51	0.345519916	mirna_pc
5263	hsa-mir-17	BLM	0.486925121	mirna_pc
5264	hsa-mir-17	NME1	0.4002132	mirna_pc
5265	hsa-mir-17	DNMT1	0.363503872	mirna_pc
5266	hsa-mir-17	PAICS	0.523890539	mirna_pc
5267	hsa-mir-17	CCDC150	0.478384993	mirna_pc
5268	hsa-mir-17	BIRC5	0.338511019	mirna_pc
5269	hsa-mir-17	RCC1	0.394918678	mirna_pc
5270	hsa-mir-17	LIG1	0.338989183	mirna_pc
5271	hsa-mir-17	OIP5	0.374991962	mirna_pc
5272	hsa-mir-17	DTL	0.469807261	mirna_pc
5273	hsa-mir-17	EME1	0.48266552	mirna_pc
5274	hsa-mir-17	RECQL4	0.454991145	mirna_pc
5275	hsa-mir-17	AURKA	0.343890525	mirna_pc
5276	hsa-mir-17	CENPK	0.438030058	mirna_pc
5277	hsa-mir-17	AURKB	0.347552144	mirna_pc
5278	hsa-mir-17	GSG2	0.387957922	mirna_pc
5279	hsa-mir-17	CDT1	0.354395315	mirna_pc
5280	hsa-mir-17	CBFB	0.353422833	mirna_pc
5281	hsa-mir-17	MCM10	0.423349776	mirna_pc
5282	hsa-mir-17	SGOL2	0.438715609	mirna_pc
5283	hsa-mir-17	CHEK2	0.405762629	mirna_pc
5284	hsa-mir-17	CENPE	0.421762821	mirna_pc
5285	hsa-mir-17	POLQ	0.373155094	mirna_pc
5286	hsa-mir-17	KIF15	0.40702039	mirna_pc
5287	hsa-mir-17	CDCA3	0.38684362	mirna_pc
5288	hsa-mir-17	C1orf112	0.480185934	mirna_pc
5289	hsa-mir-17	GINS1	0.416414425	mirna_pc
5290	hsa-mir-17	FEN1	0.452440869	mirna_pc
5291	hsa-mir-17	TRAIP	0.442566569	mirna_pc

5292	hsa-mir-17	ORC6L	0.422880835	mirna_pc
5293	hsa-mir-17	ERH	0.355399074	mirna_pc
5294	hsa-mir-17	PBK	0.320500969	mirna_pc
5295	hsa-mir-17	C16orf59	0.425294241	mirna_pc
5296	hsa-mir-17	RAE1	0.363611743	mirna_pc
5297	hsa-mir-17	FAM72D	0.416269499	mirna_pc
5298	hsa-mir-17	FAM72B	0.440395694	mirna_pc
5299	hsa-mir-17	EZH2	0.398929426	mirna_pc
5300	hsa-mir-17	NEIL3	0.424846673	mirna_pc
5301	hsa-mir-17	POLD1	0.40223043	mirna_pc
5302	hsa-mir-17	C12orf48	0.449913428	mirna_pc
5303	hsa-mir-17	HNRNPL	0.425246421	mirna_pc
5304	hsa-mir-17	ZWILCH	0.341681528	mirna_pc
5305	hsa-mir-17	HMMR	0.37235801	mirna_pc
5306	hsa-mir-17	TTK	0.432447899	mirna_pc
5307	hsa-mir-17	ZWINT	0.489935435	mirna_pc
5308	hsa-mir-17	TUBA1B	0.323639004	mirna_pc
5309	hsa-mir-17	WDR67	0.315493	mirna_pc
5310	hsa-mir-17	RUVBL1	0.305733662	mirna_pc
5311	hsa-mir-17	HOXC6	0.389374589	mirna_pc
5312	hsa-mir-17	BUB3	0.47205243	mirna_pc
5313	hsa-mir-17	HELLS	0.426676259	mirna_pc
5314	hsa-mir-17	POLE2	0.33491878	mirna_pc
5315	hsa-mir-17	UBE2S	0.342029156	mirna_pc
5316	hsa-mir-17	NCAPD2	0.304091576	mirna_pc
5317	hsa-mir-17	HNRNPC	0.427762543	mirna_pc
5318	hsa-mir-17	ILF2	0.400203484	mirna_pc
5319	hsa-mir-17	TCF3	0.383898965	mirna_pc
5320	hsa-mir-17	C6orf167	0.377560457	mirna_pc
5321	hsa-mir-17	CHEK1	0.423707141	mirna_pc
5322	hsa-mir-17	BRCA1	0.38810116	mirna_pc
5323	hsa-mir-17	NDC80	0.301749127	mirna_pc
5324	hsa-mir-17	HNRNPA2B1	0.479795385	mirna_pc
5325	hsa-mir-17	RAN	0.338248382	mirna_pc
5326	hsa-mir-17	TYMS	0.374501617	mirna_pc
5327	hsa-mir-17	RFWD3	0.420436409	mirna_pc
5328	hsa-mir-17	C20orf20	0.329841933	mirna_pc
5329	hsa-mir-17	MCM6	0.526325628	mirna_pc
5330	hsa-mir-17	CDC6	0.426551317	mirna_pc
5331	hsa-mir-17	CDCA7	0.438136451	mirna_pc
5332	hsa-mir-17	EPR1	0.399557776	mirna_pc
5333	hsa-mir-17	BRIP1	0.311062002	mirna_pc
5334	hsa-mir-17	PIF1	0.40156581	mirna_pc
5335	hsa-mir-17	H2AFX	0.336951926	mirna_pc
5336	hsa-mir-17	SFRS2	0.379276751	mirna_pc
5337	hsa-mir-17	DCAF13	0.369518582	mirna_pc
5338	hsa-mir-17	MTHFD1L	0.319950107	mirna_pc
5339	hsa-mir-17	PSMD14	0.339995513	mirna_pc
5340	hsa-mir-17	UCK2	0.436970084	mirna_pc
5341	hsa-mir-17	EFTUD2	0.319311057	mirna_pc
5342	hsa-mir-17	THOC4	0.303618519	mirna_pc
5343	hsa-mir-17	NFKBIL2	0.4208907	mirna_pc
5344	hsa-mir-17	C15orf42	0.323620269	mirna_pc
5345	hsa-mir-17	PTGES3	0.347812259	mirna_pc

5346	hsa-mir-17	PRIM2	0.398858204	mirna_pc
5347	hsa-mir-17	FAM60A	0.438092512	mirna_pc
5348	hsa-mir-17	RTKN	0.302206746	mirna_pc
5349	hsa-mir-17	FAM64A	0.368390431	mirna_pc
5350	hsa-mir-17	ATAD5	0.365840669	mirna_pc
5351	hsa-mir-17	RANBP1	0.365388707	mirna_pc
5352	hsa-mir-17	E2F1	0.387202405	mirna_pc
5353	hsa-mir-17	SNRPG	0.379480969	mirna_pc
5354	hsa-mir-17	FAM111B	0.347948986	mirna_pc
5355	hsa-mir-17	KIF20B	0.37435003	mirna_pc
5356	hsa-mir-17	MCM3	0.356247163	mirna_pc
5357	hsa-mir-17	SNRPA	0.438721141	mirna_pc
5358	hsa-mir-17	FUS	0.391766243	mirna_pc
5359	hsa-mir-17	ATAD2	0.306849971	mirna_pc
5360	hsa-mir-17	C1orf135	0.312730596	mirna_pc
5361	hsa-mir-17	DNAJC9	0.358020157	mirna_pc
5362	hsa-mir-17	RFC3	0.581203272	mirna_pc
5363	hsa-mir-17	DEPDC1B	0.377837622	mirna_pc
5364	hsa-mir-17	POC1A	0.345469264	mirna_pc
5365	hsa-mir-17	ANP32E	0.320632168	mirna_pc
5366	hsa-mir-17	CENPO	0.504669561	mirna_pc
5367	hsa-mir-17	XRCC2	0.388292544	mirna_pc
5368	hsa-mir-17	C19orf48	0.40030542	mirna_pc
5369	hsa-mir-17	BRCA2	0.424554537	mirna_pc
5370	hsa-mir-17	CENPL	0.398127259	mirna_pc
5371	hsa-mir-17	GEN1	0.377980308	mirna_pc
5372	hsa-mir-17	NUDT1	0.30382759	mirna_pc
5373	hsa-mir-17	HMGB2	0.487503155	mirna_pc
5374	hsa-mir-17	CCT3	0.322791634	mirna_pc
5375	hsa-mir-17	GINS2	0.37882871	mirna_pc
5376	hsa-mir-17	DNA2	0.460498057	mirna_pc
5377	hsa-mir-17	NOP56	0.487325179	mirna_pc
5378	hsa-mir-17	SNRPD1	0.313220272	mirna_pc
5379	hsa-mir-17	DSN1	0.311000169	mirna_pc
5380	hsa-mir-17	SNHG1	0.42236183	mirna_pc
5381	hsa-mir-17	CSE1L	0.354551947	mirna_pc
5382	hsa-mir-17	DTYMK	0.497556136	mirna_pc
5383	hsa-mir-17	PRMT1	0.366229468	mirna_pc
5384	hsa-mir-17	MLF1IP	0.410295482	mirna_pc
5385	hsa-mir-17	RAVER1	0.342816149	mirna_pc
5386	hsa-mir-17	HEATR1	0.320347025	mirna_pc
5387	hsa-mir-17	HSPE1	0.35491593	mirna_pc
5388	hsa-mir-17	C16orf75	0.350006312	mirna_pc
5389	hsa-mir-17	CAD	0.37071964	mirna_pc
5390	hsa-mir-17	CCDC99	0.445257037	mirna_pc
5391	hsa-mir-17	CDK2	0.405486349	mirna_pc
5392	hsa-mir-17	CDK4	0.396273497	mirna_pc
5393	hsa-mir-17	INCENP	0.315317099	mirna_pc
5394	hsa-mir-17	HOXA10	0.366735668	mirna_pc
5395	hsa-mir-17	CKAP5	0.32873393	mirna_pc
5396	hsa-mir-17	BCL2L12	0.312610085	mirna_pc
5397	hsa-mir-17	HNRNPR	0.402006866	mirna_pc
5398	hsa-mir-17	DLEU2	0.430309001	mirna_pc
5399	hsa-mir-17	PRIM1	0.434846392	mirna_pc

5400	hsa-mir-17	KAT2A	0.326941992	mirna_pc
5401	hsa-mir-17	CENPH	0.380863878	mirna_pc
5402	hsa-mir-17	GGCT	0.31033248	mirna_pc
5403	hsa-mir-17	TCOF1	0.405343923	mirna_pc
5404	hsa-mir-17	CHAF1B	0.41230393	mirna_pc
5405	hsa-mir-17	NCAPG2	0.371367974	mirna_pc
5406	hsa-mir-17	MSH2	0.512692095	mirna_pc
5407	hsa-mir-17	PRPF19	0.364338669	mirna_pc
5408	hsa-mir-17	EIF3B	0.364308051	mirna_pc
5409	hsa-mir-17	NOP58	0.329014378	mirna_pc
5410	hsa-mir-17	TOMM40	0.346955942	mirna_pc
5411	hsa-mir-17	EIF2AK1	0.378824265	mirna_pc
5412	hsa-mir-17	SF3B3	0.445567604	mirna_pc
5413	hsa-mir-17	CDC7	0.471341084	mirna_pc
5414	hsa-mir-17	INTS7	0.320461135	mirna_pc
5415	hsa-mir-17	SSB	0.51789761	mirna_pc
5416	hsa-mir-17	PUS1	0.42793574	mirna_pc
5417	hsa-mir-17	NOL11	0.406059304	mirna_pc
5418	hsa-mir-17	SNRPC	0.386189854	mirna_pc
5419	hsa-mir-17	RFC4	0.358442735	mirna_pc
5420	hsa-mir-17	FBXO5	0.456198487	mirna_pc
5421	hsa-mir-17	WDR75	0.339889597	mirna_pc
5422	hsa-mir-17	FIGNL1	0.339682877	mirna_pc
5423	hsa-mir-17	E2F3	0.478921997	mirna_pc
5424	hsa-mir-17	DDX11	0.396173199	mirna_pc
5425	hsa-mir-17	SF3B14	0.327383752	mirna_pc
5426	hsa-mir-17	RUVBL2	0.384744411	mirna_pc
5427	hsa-mir-17	CDC25A	0.54076791	mirna_pc
5428	hsa-mir-17	TMEM48	0.345966433	mirna_pc
5429	hsa-mir-17	RAD54B	0.352878545	mirna_pc
5430	hsa-mir-17	H2AFZ	0.436422162	mirna_pc
5431	hsa-mir-17	PRR7	0.331174867	mirna_pc
5432	hsa-mir-17	DDX12	0.358586489	mirna_pc
5433	hsa-mir-17	DSCC1	0.400807487	mirna_pc
5434	hsa-mir-17	WDR12	0.332471015	mirna_pc
5435	hsa-mir-17	C13orf34	0.544383604	mirna_pc
5436	hsa-mir-17	SMC2	0.356327723	mirna_pc
5437	hsa-mir-17	SRPK1	0.348829317	mirna_pc
5438	hsa-mir-17	CLN6	0.354535738	mirna_pc
5439	hsa-mir-17	MCM8	0.473346308	mirna_pc
5440	hsa-mir-17	CPSF3	0.427490223	mirna_pc
5441	hsa-mir-17	SNRPE	0.48156577	mirna_pc
5442	hsa-mir-17	C21orf45	0.447762885	mirna_pc
5443	hsa-mir-17	ATIC	0.50063744	mirna_pc
5444	hsa-mir-17	PPM1G	0.374266957	mirna_pc
5445	hsa-mir-17	MARCKSL1	0.343914727	mirna_pc
5446	hsa-mir-17	NPM3	0.41213653	mirna_pc
5447	hsa-mir-17	TFAP4	0.373533854	mirna_pc
5448	hsa-mir-17	ZNF367	0.304796938	mirna_pc
5449	hsa-mir-17	C14orf106	0.317962553	mirna_pc
5450	hsa-mir-17	NUTF2	0.327562561	mirna_pc
5451	hsa-mir-17	DAZAP1	0.381190921	mirna_pc
5452	hsa-mir-17	CBX8	0.431489401	mirna_pc
5453	hsa-mir-17	TRMT6	0.305086577	mirna_pc

5454	hsa-mir-17	GNPDA1	0.376084862	mirna_pc
5455	hsa-mir-17	WDR34	0.38407373	mirna_pc
5456	hsa-mir-17	TMEM201	0.356819609	mirna_pc
5457	hsa-mir-17	NCL	0.490655677	mirna_pc
5458	hsa-mir-17	NUDCD1	0.304294803	mirna_pc
5459	hsa-mir-17	PFDN4	0.316581478	mirna_pc
5460	hsa-mir-17	PAFAH1B3	0.301185624	mirna_pc
5461	hsa-mir-17	SRRT	0.340996155	mirna_pc
5462	hsa-mir-17	CACYBP	0.428714207	mirna_pc
5463	hsa-mir-17	DCTPP1	0.337123001	mirna_pc
5464	hsa-mir-17	SASS6	0.35642297	mirna_pc
5465	hsa-mir-17	PSRC1	0.483581392	mirna_pc
5466	hsa-mir-17	ODF2	0.3636117	mirna_pc
5467	hsa-mir-17	DGUOK	0.30401685	mirna_pc
5468	hsa-mir-17	DBF4B	0.386892559	mirna_pc
5469	hsa-mir-17	POU2F1	0.406598415	mirna_pc
5470	hsa-mir-17	FBL	0.429240861	mirna_pc
5471	hsa-mir-17	DONSON	0.319162447	mirna_pc
5472	hsa-mir-17	C13orf37	0.526743699	mirna_pc
5473	hsa-mir-17	HNRNPM	0.344037346	mirna_pc
5474	hsa-mir-17	LSM 12.00	0.353939626	mirna_pc
5475	hsa-mir-17	PNPT1	0.459690771	mirna_pc
5476	hsa-mir-17	TMPO	0.446073792	mirna_pc
5477	hsa-mir-17	SET	0.448118798	mirna_pc
5478	hsa-mir-17	C3orf26	0.355375695	mirna_pc
5479	hsa-mir-17	MRTO4	0.358341053	mirna_pc
5480	hsa-mir-17	SMPD4	0.365764013	mirna_pc
5481	hsa-mir-17	GPR19	0.316351009	mirna_pc
5482	hsa-mir-17	PSMD3	0.342691048	mirna_pc
5483	hsa-mir-17	GABPB1	0.348614977	mirna_pc
5484	hsa-mir-17	PPIH	0.356772775	mirna_pc
5485	hsa-mir-17	RCN1	0.313330757	mirna_pc
5486	hsa-mir-17	PPAT	0.412611198	mirna_pc
5487	hsa-mir-17	DHX9	0.464313689	mirna_pc
5488	hsa-mir-17	CCDC138	0.416994087	mirna_pc
5489	hsa-mir-17	LOC100128191	0.359175351	mirna_pc
5490	hsa-mir-17	HMGN2	0.424827816	mirna_pc
5491	hsa-mir-17	ETV4	0.40266645	mirna_pc
5492	hsa-mir-17	CHRNA5	0.344188136	mirna_pc
5493	hsa-mir-17	SMYD5	0.335959328	mirna_pc
5494	hsa-mir-17	TSSC1	0.361484076	mirna_pc
5495	hsa-mir-17	POLA2	0.328101426	mirna_pc
5496	hsa-mir-17	SFRS1	0.561560232	mirna_pc
5497	hsa-mir-17	TEX10	0.367293304	mirna_pc
5498	hsa-mir-17	WDR43	0.43520647	mirna_pc
5499	hsa-mir-17	SNRPA1	0.33497434	mirna_pc
5500	hsa-mir-17	CCDC58	0.355575483	mirna_pc
5501	hsa-mir-17	SKP2	0.408033016	mirna_pc
5502	hsa-mir-17	TIPIN	0.347115755	mirna_pc
5503	hsa-mir-17	SMARCD1	0.322567119	mirna_pc
5504	hsa-mir-17	C4orf46	0.462748422	mirna_pc
5505	hsa-mir-17	DARS2	0.394727263	mirna_pc
5506	hsa-mir-17	LYAR	0.393811985	mirna_pc
5507	hsa-mir-17	UTP14A	0.346345228	mirna_pc

5508	hsa-mir-17	C1orf107	0.30301186	mirna_pc
5509	hsa-mir-17	GPN1	0.323865827	mirna_pc
5510	hsa-mir-17	RFC5	0.354498047	mirna_pc
5511	hsa-mir-17	HSPD1	0.453771974	mirna_pc
5512	hsa-mir-17	MAZ	0.348884205	mirna_pc
5513	hsa-mir-17	SSRP1	0.45100284	mirna_pc
5514	hsa-mir-17	SLBP	0.338478786	mirna_pc
5515	hsa-mir-17	POLR2D	0.492428744	mirna_pc
5516	hsa-mir-17	FTSJ2	0.416294048	mirna_pc
5517	hsa-mir-17	PA2G4	0.395656857	mirna_pc
5518	hsa-mir-17	KHDRBS1	0.463260934	mirna_pc
5519	hsa-mir-17	SLC5A6	0.311254011	mirna_pc
5520	hsa-mir-17	C20orf27	0.343425761	mirna_pc
5521	hsa-mir-17	TOMM34	0.426992396	mirna_pc
5522	hsa-mir-17	MTL 5.00	0.365204065	mirna_pc
5523	hsa-mir-17	TAF1A	0.326064166	mirna_pc
5524	hsa-mir-17	RNF220	0.376788855	mirna_pc
5525	hsa-mir-17	PRPF40A	0.408517688	mirna_pc
5526	hsa-mir-17	CCDC18	0.325388059	mirna_pc
5527	hsa-mir-17	AHCY	0.375520377	mirna_pc
5528	hsa-mir-17	PARP1	0.402214931	mirna_pc
5529	hsa-mir-17	CCDC34	0.375753002	mirna_pc
5530	hsa-mir-17	CCT7	0.403010984	mirna_pc
5531	hsa-mir-17	HNRNPF	0.337918625	mirna_pc
5532	hsa-mir-17	C4orf21	0.323217356	mirna_pc
5533	hsa-mir-17	TMEM194A	0.371712389	mirna_pc
5534	hsa-mir-17	SLC39A10	0.447348172	mirna_pc
5535	hsa-mir-17	RAD18	0.335152637	mirna_pc
5536	hsa-mir-17	NOLC1	0.328641411	mirna_pc
5537	hsa-mir-17	ZNF695	0.463551169	mirna_pc
5538	hsa-mir-17	PDSS1	0.425935703	mirna_pc
5539	hsa-mir-17	HAUS6	0.391299932	mirna_pc
5540	hsa-mir-17	CENPJ	0.476826127	mirna_pc
5541	hsa-mir-17	LIN9	0.363606449	mirna_pc
5542	hsa-mir-17	HNRNPU	0.437377651	mirna_pc
5543	hsa-mir-17	NASP	0.428539274	mirna_pc
5544	hsa-mir-17	NONO	0.35468121	mirna_pc
5545	hsa-mir-17	CCDC86	0.330544962	mirna_pc
5546	hsa-mir-17	POLR1B	0.389013982	mirna_pc
5547	hsa-mir-17	QSOX2	0.350712242	mirna_pc
5548	hsa-mir-17	OBFC2B	0.333037125	mirna_pc
5549	hsa-mir-17	PTMA	0.421191769	mirna_pc
5550	hsa-mir-17	WDR76	0.316062729	mirna_pc
5551	hsa-mir-17	PSMD1	0.345619787	mirna_pc
5552	hsa-mir-17	EXOSC2	0.398568403	mirna_pc
5553	hsa-mir-17	SCARB1	0.372053877	mirna_pc
5554	hsa-mir-17	C11orf84	0.39446924	mirna_pc
5555	hsa-mir-17	ARHGAP39	0.309382259	mirna_pc
5556	hsa-mir-17	TBCE	0.392920675	mirna_pc
5557	hsa-mir-17	CLCN2	0.348463445	mirna_pc
5558	hsa-mir-17	NCBP2	0.312719063	mirna_pc
5559	hsa-mir-17	SUV39H2	0.462452301	mirna_pc
5560	hsa-mir-17	LSM 2.00	0.331902889	mirna_pc
5561	hsa-mir-17	R3HDM1	0.329803621	mirna_pc

5562	hsa-mir-17	FANCL	0.448330268	mirna_pc
5563	hsa-mir-17	RIBC2	0.316039846	mirna_pc
5564	hsa-mir-17	RQCD1	0.337292803	mirna_pc
5565	hsa-mir-17	HNRNPD	0.484803685	mirna_pc
5566	hsa-mir-17	MTHFD2	0.416523323	mirna_pc
5567	hsa-mir-17	GTF2F2	0.45709433	mirna_pc
5568	hsa-mir-17	DDX31	0.320657428	mirna_pc
5569	hsa-mir-17	GTPBP4	0.327774476	mirna_pc
5570	hsa-mir-17	TARDBP	0.314553241	mirna_pc
5571	hsa-mir-17	POP1	0.343910684	mirna_pc
5572	hsa-mir-17	SFRS3	0.426921278	mirna_pc
5573	hsa-mir-17	NUP160	0.301287474	mirna_pc
5574	hsa-mir-17	LSM 5.00	0.44336265	mirna_pc
5575	hsa-mir-17	REXO4	0.338545659	mirna_pc
5576	hsa-mir-17	PNO1	0.371080538	mirna_pc
5577	hsa-mir-17	CCT4	0.381357607	mirna_pc
5578	hsa-mir-17	MITD1	0.329863933	mirna_pc
5579	hsa-mir-17	ELAVL1	0.421064398	mirna_pc
5580	hsa-mir-17	ABCE1	0.327422631	mirna_pc
5581	hsa-mir-17	MTHFD1	0.319332879	mirna_pc
5582	hsa-mir-17	C7orf40	0.305928211	mirna_pc
5583	hsa-mir-17	NCAPD3	0.32804079	mirna_pc
5584	hsa-mir-17	NAE1	0.386484458	mirna_pc
5585	hsa-mir-17	PSMC3IP	0.357477202	mirna_pc
5586	hsa-mir-17	PAK1IP1	0.442080393	mirna_pc
5587	hsa-mir-17	NUDT15	0.483208687	mirna_pc
5588	hsa-mir-17	SUPT16H	0.302209923	mirna_pc
5589	hsa-mir-17	E2F4	0.357499456	mirna_pc
5590	hsa-mir-17	TWISTNB	0.344859096	mirna_pc
5591	hsa-mir-17	DHFR	0.392561313	mirna_pc
5592	hsa-mir-17	NAA25	0.385412954	mirna_pc
5593	hsa-mir-17	ILF3	0.461687376	mirna_pc
5594	hsa-mir-17	NT5DC2	0.361939554	mirna_pc
5595	hsa-mir-17	EHMT2	0.415218553	mirna_pc
5596	hsa-mir-17	GAS2L3	0.311663977	mirna_pc
5597	hsa-mir-17	HNRNPAB	0.336794075	mirna_pc
5598	hsa-mir-17	KDM1B	0.332340229	mirna_pc
5599	hsa-mir-17	UBAP2L	0.302215322	mirna_pc
5600	hsa-mir-17	GART	0.338732976	mirna_pc
5601	hsa-mir-17	DEK	0.423783476	mirna_pc
5602	hsa-mir-17	TBC1D4	0.383010855	mirna_pc
5603	hsa-mir-17	SFRS9	0.333230631	mirna_pc
5604	hsa-mir-17	TRIB3	0.382656814	mirna_pc
5605	hsa-mir-17	CCNE2	0.318465815	mirna_pc
5606	hsa-mir-17	PASK	0.404063377	mirna_pc
5607	hsa-mir-17	SAAL1	0.421519127	mirna_pc
5608	hsa-mir-17	OLA1	0.332478701	mirna_pc
5609	hsa-mir-17	C1orf131	0.315931859	mirna_pc
5610	hsa-mir-17	RPGRIP1L	0.317209647	mirna_pc
5611	hsa-mir-17	TGS1	0.375717042	mirna_pc
5612	hsa-mir-17	HEATR2	0.537833736	mirna_pc
5613	hsa-mir-17	LSM 6.00	0.339876018	mirna_pc
5614	hsa-mir-17	PRAME	0.408053952	mirna_pc
5615	hsa-mir-17	CYB5B	0.381681102	mirna_pc

5616	hsa-mir-17	SNRPB2	0.38763638	mirna_pc
5617	hsa-mir-17	AZI1	0.320986193	mirna_pc
5618	hsa-mir-17	FUBP1	0.364464215	mirna_pc
5619	hsa-mir-17	C7orf27	0.310765191	mirna_pc
5620	hsa-mir-17	SUV39H1	0.343139257	mirna_pc
5621	hsa-mir-17	IPO9	0.399734766	mirna_pc
5622	hsa-mir-17	SART3	0.351664738	mirna_pc
5623	hsa-mir-17	HNRNPK	0.386770668	mirna_pc
5624	hsa-mir-17	SR140	0.304203226	mirna_pc
5625	hsa-mir-17	TRA2B	0.350937726	mirna_pc
5626	hsa-mir-17	GAR1	0.441200793	mirna_pc
5627	hsa-mir-17	CEP152	0.309352294	mirna_pc
5628	hsa-mir-17	C15orf23	0.418677974	mirna_pc
5629	hsa-mir-17	PRMT3	0.306280251	mirna_pc
5630	hsa-mir-17	SDCCAG3	0.300952201	mirna_pc
5631	hsa-mir-17	IGF2BP2	0.305907429	mirna_pc
5632	hsa-mir-17	WHSC1	0.399960028	mirna_pc
5633	hsa-mir-17	SAE1	0.367415313	mirna_pc
5634	hsa-mir-17	RNF219	0.596056108	mirna_pc
5635	hsa-mir-17	ITGB3BP	0.438655294	mirna_pc
5636	hsa-mir-17	EIF2S2	0.313780888	mirna_pc
5637	hsa-mir-17	ENOPH1	0.385727881	mirna_pc
5638	hsa-mir-17	PABPC1L	0.312037928	mirna_pc
5639	hsa-mir-17	C4orf43	0.33080824	mirna_pc
5640	hsa-mir-17	DDX55	0.391148907	mirna_pc
5641	hsa-mir-17	CEP78	0.415053832	mirna_pc
5642	hsa-mir-17	PLEKHG4	0.345788276	mirna_pc
5643	hsa-mir-17	CCDC103	0.31114224	mirna_pc
5644	hsa-mir-17	SFPQ	0.413638841	mirna_pc
5645	hsa-mir-17	POMP	0.34016808	mirna_pc
5646	hsa-mir-17	CD83	0.31482725	mirna_pc
5647	hsa-mir-17	RCOR2	0.485536391	mirna_pc
5648	hsa-mir-17	ATAD3A	0.318589661	mirna_pc
5649	hsa-mir-17	MPHOSPH9	0.303915557	mirna_pc
5650	hsa-mir-17	SHMT2	0.304313295	mirna_pc
5651	hsa-mir-17	POLA1	0.368275372	mirna_pc
5652	hsa-mir-17	HDAC2	0.473532225	mirna_pc
5653	hsa-mir-17	GMNN	0.383134766	mirna_pc
5654	hsa-mir-17	PA2G4P4	0.355747227	mirna_pc
5655	hsa-mir-17	C10orf119	0.322707993	mirna_pc
5656	hsa-mir-17	POLG2	0.309788176	mirna_pc
5657	hsa-mir-17	C10orf2	0.405344453	mirna_pc
5658	hsa-mir-17	POGK	0.314425909	mirna_pc
5659	hsa-mir-17	GEMIN6	0.362295435	mirna_pc
5660	hsa-mir-17	COX4NB	0.320541562	mirna_pc
5661	hsa-mir-17	MSH6	0.374540292	mirna_pc
5662	hsa-mir-17	XRN2	0.349153295	mirna_pc
5663	hsa-mir-17	KPNB1	0.471233495	mirna_pc
5664	hsa-mir-17	SMC3	0.367492286	mirna_pc
5665	hsa-mir-17	FLVCR1	0.386549658	mirna_pc
5666	hsa-mir-17	ACBD6	0.323908812	mirna_pc
5667	hsa-mir-17	HNRNPA1	0.410495473	mirna_pc
5668	hsa-mir-17	UBA2	0.303581663	mirna_pc
5669	hsa-mir-17	NPM1	0.345511687	mirna_pc

5670	hsa-mir-17	ZBED4	0.310328789	mirna_pc
5671	hsa-mir-17	AGMAT	0.32852341	mirna_pc
5672	hsa-mir-17	MEX3A	0.493001839	mirna_pc
5673	hsa-mir-17	BAT1	0.397201019	mirna_pc
5674	hsa-mir-17	COIL	0.41256146	mirna_pc
5675	hsa-mir-17	PHF14	0.362573234	mirna_pc
5676	hsa-mir-17	PDCD11	0.332064414	mirna_pc
5677	hsa-mir-17	TAF5	0.466482966	mirna_pc
5678	hsa-mir-17	TRIM28	0.344445495	mirna_pc
5679	hsa-mir-17	C2orf29	0.313880046	mirna_pc
5680	hsa-mir-17	WDR5	0.344114399	mirna_pc
5681	hsa-mir-17	GAS5	0.306421721	mirna_pc
5682	hsa-mir-17	SUZ12	0.346209938	mirna_pc
5683	hsa-mir-17	HNRNPA1L2	0.473255284	mirna_pc
5684	hsa-mir-17	NAA15	0.305746564	mirna_pc
5685	hsa-mir-17	CBX1	0.338890222	mirna_pc
5686	hsa-mir-17	HMGB1	0.543802722	mirna_pc
5687	hsa-mir-17	ZBTB12	0.481001172	mirna_pc
5688	hsa-mir-17	DIAPH3	0.419657121	mirna_pc
5689	hsa-mir-17	TADA1	0.321724455	mirna_pc
5690	hsa-mir-17	MCTP2	0.315422934	mirna_pc
5691	hsa-mir-17	USP1	0.311635705	mirna_pc
5692	hsa-mir-17	DUSP12	0.32054635	mirna_pc
5693	hsa-mir-17	TMEM68	0.31902011	mirna_pc
5694	hsa-mir-17	LOC642846	0.320541677	mirna_pc
5695	hsa-mir-17	TGIF2	0.424606308	mirna_pc
5696	hsa-mir-17	H3F3A	0.34550739	mirna_pc
5697	hsa-mir-17	SNAPC4	0.336094761	mirna_pc
5698	hsa-mir-17	ZC3H18	0.318237281	mirna_pc
5699	hsa-mir-17	FARSB	0.446075101	mirna_pc
5700	hsa-mir-17	PIK3AP1	0.307636545	mirna_pc
5701	hsa-mir-17	SNX5	0.34806905	mirna_pc
5702	hsa-mir-17	THUMPD2	0.311685271	mirna_pc
5703	hsa-mir-17	FAM96A	0.325251809	mirna_pc
5704	hsa-mir-17	MTF2	0.309427485	mirna_pc
5705	hsa-mir-17	TARBP2	0.301630906	mirna_pc
5706	hsa-mir-17	FGFR1OP	0.325212645	mirna_pc
5707	hsa-mir-17	PHB	0.398545584	mirna_pc
5708	hsa-mir-17	METTL13	0.314591106	mirna_pc
5709	hsa-mir-17	C12orf52	0.320184777	mirna_pc
5710	hsa-mir-17	EBPL	0.326427192	mirna_pc
5711	hsa-mir-17	NOL10	0.442405929	mirna_pc
5712	hsa-mir-17	ANAPC1	0.328547511	mirna_pc
5713	hsa-mir-17	POLE	0.437165784	mirna_pc
5714	hsa-mir-17	C1orf163	0.308018295	mirna_pc
5715	hsa-mir-17	WRNIP1	0.304052407	mirna_pc
5716	hsa-mir-17	HMBS	0.360327989	mirna_pc
5717	hsa-mir-17	TAF1B	0.301144946	mirna_pc
5718	hsa-mir-17	SOX9	0.328243794	mirna_pc
5719	hsa-mir-17	TIMM50	0.45232614	mirna_pc
5720	hsa-mir-17	ADSL	0.33160864	mirna_pc
5721	hsa-mir-17	VRK1	0.352503975	mirna_pc
5722	hsa-mir-17	C13orf27	0.530060808	mirna_pc
5723	hsa-mir-17	RBM14	0.34434079	mirna_pc

5724	hsa-mir-17	ANP32B	0.305754244	mirna_pc
5725	hsa-mir-17	EXOSC9	0.438735465	mirna_pc
5726	hsa-mir-17	UNG	0.30865742	mirna_pc
5727	hsa-mir-17	CCDC43	0.353095317	mirna_pc
5728	hsa-mir-17	RBMX	0.509445317	mirna_pc
5729	hsa-mir-17	SRP9	0.401238574	mirna_pc
5730	hsa-mir-17	NOC3L	0.310209734	mirna_pc
5731	hsa-mir-17	UCHL3	0.333728935	mirna_pc
5732	hsa-mir-17	GPR180	0.409875431	mirna_pc
5733	hsa-mir-17	DAP3	0.380510305	mirna_pc
5734	hsa-mir-17	IQCB1	0.302153402	mirna_pc
5735	hsa-mir-17	CCDC90A	0.339300284	mirna_pc
5736	hsa-mir-17	MDC 1.00	0.317524515	mirna_pc
5737	hsa-mir-17	XRCC5	0.490090775	mirna_pc
5738	hsa-mir-17	SNX25	0.302532483	mirna_pc
5739	hsa-mir-17	RPF2	0.338471052	mirna_pc
5740	hsa-mir-17	EXOSC8	0.511308549	mirna_pc
5741	hsa-mir-17	CTCF	0.396807609	mirna_pc
5742	hsa-mir-17	TAF1D	0.321611551	mirna_pc
5743	hsa-mir-17	PDIA6	0.344527135	mirna_pc
5744	hsa-mir-17	RPE	0.341563037	mirna_pc
5745	hsa-mir-17	LBR	0.404304226	mirna_pc
5746	hsa-mir-17	NAA40	0.322252222	mirna_pc
5747	hsa-mir-17	DNAJC14	0.316845043	mirna_pc
5748	hsa-mir-17	FIP1L1	0.305375183	mirna_pc
5749	hsa-mir-17	ZNF519	0.347104128	mirna_pc
5750	hsa-mir-17	GTF3C3	0.300585521	mirna_pc
5751	hsa-mir-17	ZRANB3	0.305429834	mirna_pc
5752	hsa-mir-17	NUDT21	0.328166497	mirna_pc
5753	hsa-mir-17	MAGEF1	0.300445467	mirna_pc
5754	hsa-mir-17	DDX10	0.322652677	mirna_pc
5755	hsa-mir-17	LTV1	0.301268164	mirna_pc
5756	hsa-mir-17	PCGF6	0.31309173	mirna_pc
5757	hsa-mir-17	OSTC	0.311034397	mirna_pc
5758	hsa-mir-17	FAM136A	0.509264223	mirna_pc
5759	hsa-mir-17	ZNF511	0.309575235	mirna_pc
5760	hsa-mir-17	MRPS5	0.341657249	mirna_pc
5761	hsa-mir-17	HMGN1	0.342674429	mirna_pc
5762	hsa-mir-17	MYB	0.315532648	mirna_pc
5763	hsa-mir-17	ITPRIPL1	0.38876671	mirna_pc
5764	hsa-mir-17	TTLL4	0.349327606	mirna_pc
5765	hsa-mir-17	FBXO41	0.318626291	mirna_pc
5766	hsa-mir-17	BIVM	0.336283248	mirna_pc
5767	hsa-mir-17	CALM2	0.30768804	mirna_pc
5768	hsa-mir-17	GPATCH4	0.300947748	mirna_pc
5769	hsa-mir-17	EML4	0.306073823	mirna_pc
5770	hsa-mir-17	RPS7	0.310297161	mirna_pc
5771	hsa-mir-17	BZW2	0.423284248	mirna_pc
5772	hsa-mir-17	ACBD7	0.308100838	mirna_pc
5773	hsa-mir-17	LRPPRC	0.414527443	mirna_pc
5774	hsa-mir-17	CSNK2A1	0.339674995	mirna_pc
5775	hsa-mir-17	LOC401010	0.323394825	mirna_pc
5776	hsa-mir-17	RPIA	0.474383388	mirna_pc
5777	hsa-mir-17	BCL7A	0.332598567	mirna_pc

5778	hsa-mir-17	C7orf44	0.368105025	mirna_pc
5779	hsa-mir-17	TRPA1	0.319986021	mirna_pc
5780	hsa-mir-17	URB2	0.36275405	mirna_pc
5781	hsa-mir-17	RP9	0.374902778	mirna_pc
5782	hsa-mir-17	KIAA1731	0.303012572	mirna_pc
5783	hsa-mir-17	ZNF286A	0.394240539	mirna_pc
5784	hsa-mir-17	SLC27A2	0.309782878	mirna_pc
5785	hsa-mir-17	SFXN4	0.408583145	mirna_pc
5786	hsa-mir-17	PRPF38A	0.318284606	mirna_pc
5787	hsa-mir-17	WDR77	0.368517657	mirna_pc
5788	hsa-mir-17	SURF6	0.348689735	mirna_pc
5789	hsa-mir-17	MRPL21	0.303090442	mirna_pc
5790	hsa-mir-17	TCEA1	0.335203924	mirna_pc
5791	hsa-mir-17	NLE1	0.364483741	mirna_pc
5792	hsa-mir-17	RBM28	0.354481316	mirna_pc
5793	hsa-mir-17	RANBP17	0.322835137	mirna_pc
5794	hsa-mir-17	TMEM180	0.307078715	mirna_pc
5795	hsa-mir-17	MRE11A	0.300301337	mirna_pc
5796	hsa-mir-17	WDR85	0.31838355	mirna_pc
5797	hsa-mir-17	USP37	0.300138716	mirna_pc
5798	hsa-mir-17	PPP1CC	0.311058265	mirna_pc
5799	hsa-mir-17	ILKAP	0.382456978	mirna_pc
5800	hsa-mir-17	PRR3	0.301412826	mirna_pc
5801	hsa-mir-17	TMEM106C	0.328360006	mirna_pc
5802	hsa-mir-17	DLEU1	0.434101823	mirna_pc
5803	hsa-mir-17	C13orf23	0.433119672	mirna_pc
5804	hsa-mir-17	TAF4B	0.314379574	mirna_pc
5805	hsa-mir-17	TSFM	0.30748341	mirna_pc
5806	hsa-mir-17	RBM12	0.319635778	mirna_pc
5807	hsa-mir-17	ZNF286B	0.35742871	mirna_pc
5808	hsa-mir-17	PDF	0.381217933	mirna_pc
5809	hsa-mir-17	DDX20	0.327805474	mirna_pc
5810	hsa-mir-17	RNF34	0.303088819	mirna_pc
5811	hsa-mir-17	CHD7	0.332928294	mirna_pc
5812	hsa-mir-17	TCERG1	0.391578332	mirna_pc
5813	hsa-mir-17	PSPC1	0.490756925	mirna_pc
5814	hsa-mir-17	COPZ1	0.313794639	mirna_pc
5815	hsa-mir-17	CCNB1IP1	0.383480247	mirna_pc
5816	hsa-mir-17	MAPKAPK5	0.399393685	mirna_pc
5817	hsa-mir-17	GMCL1	0.327230689	mirna_pc
5818	hsa-mir-17	E2F6	0.455095141	mirna_pc
5819	hsa-mir-17	NOL7	0.346853915	mirna_pc
5820	hsa-mir-17	SLC25A15	0.47664523	mirna_pc
5821	hsa-mir-17	B4GALT3	0.318440108	mirna_pc
5822	hsa-mir-17	SFRS13A	0.472014185	mirna_pc
5823	hsa-mir-17	SCLY	0.479954212	mirna_pc
5824	hsa-mir-17	STARD7	0.322032259	mirna_pc
5825	hsa-mir-17	GPR125	0.305652945	mirna_pc
5826	hsa-mir-17	NUP35	0.347813794	mirna_pc
5827	hsa-mir-17	SLC19A1	0.371888058	mirna_pc
5828	hsa-mir-17	TGDS	0.445591987	mirna_pc
5829	hsa-mir-17	TFAM	0.462149042	mirna_pc
5830	hsa-mir-17	LDLRAD3	0.302279365	mirna_pc
5831	hsa-mir-17	TTC27	0.321689348	mirna_pc

5832	hsa-mir-17	DHRS13	0.323441203	mirna_pc
5833	hsa-mir-17	ORC4L	0.44165052	mirna_pc
5834	hsa-mir-17	ZC3H15	0.316346342	mirna_pc
5835	hsa-mir-17	KIAA1704	0.37786584	mirna_pc
5836	hsa-mir-17	MTIF2	0.440585124	mirna_pc
5837	hsa-mir-17	HNRNPA3	0.345938798	mirna_pc
5838	hsa-mir-17	MSL1	0.363843954	mirna_pc
5839	hsa-mir-17	EWSR1	0.390344991	mirna_pc
5840	hsa-mir-17	ZNF828	0.486074412	mirna_pc
5841	hsa-mir-17	SMC1A	0.36254124	mirna_pc
5842	hsa-mir-17	DHX57	0.33486803	mirna_pc
5843	hsa-mir-17	NUPL1	0.501856449	mirna_pc
5844	hsa-mir-17	DARS	0.314923898	mirna_pc
5845	hsa-mir-17	AAAS	0.307923425	mirna_pc
5846	hsa-mir-17	UBE2CBP	0.307285796	mirna_pc
5847	hsa-mir-17	WBP4	0.333357711	mirna_pc
5848	hsa-mir-17	SFT2D3	0.33717771	mirna_pc
5849	hsa-mir-17	SUGT1	0.371007735	mirna_pc
5850	hsa-mir-17	MRPS26	0.373046915	mirna_pc
5851	hsa-mir-17	TUBGCP3	0.333037907	mirna_pc
5852	hsa-mir-17	MRPL30	0.384642052	mirna_pc
5853	hsa-mir-17	GNL3	0.361598977	mirna_pc
5854	hsa-mir-17	DHODH	0.343321429	mirna_pc
5855	hsa-mir-17	TSGA14	0.305665244	mirna_pc
5856	hsa-mir-17	UPF3A	0.425230627	mirna_pc
5857	hsa-mir-17	RPS16	0.321821189	mirna_pc
5858	hsa-mir-17	SFRS7	0.478587112	mirna_pc
5859	hsa-mir-17	MRPL42	0.35783601	mirna_pc
5860	hsa-mir-17	C9orf100	0.30866319	mirna_pc
5861	hsa-mir-17	BEND3	0.356297333	mirna_pc
5862	hsa-mir-17	CA5BP	0.315308349	mirna_pc
5863	hsa-mir-17	NFXL1	0.325058268	mirna_pc
5864	hsa-mir-17	PFAS	0.38481441	mirna_pc
5865	hsa-mir-17	SF3B2	0.307348456	mirna_pc
5866	hsa-mir-17	C10orf35	0.300774736	mirna_pc
5867	hsa-mir-17	UCHL5	0.369808356	mirna_pc
5868	hsa-mir-17	RBM10	0.31634195	mirna_pc
5869	hsa-mir-17	CDCA7L	0.427484086	mirna_pc
5870	hsa-mir-17	NUP153	0.310010633	mirna_pc
5871	hsa-mir-17	NIPSNAP1	0.325042601	mirna_pc
5872	hsa-mir-17	SERBP1	0.323958629	mirna_pc
5873	hsa-mir-17	SPA17	0.348030964	mirna_pc
5874	hsa-mir-17	WDR92	0.338146369	mirna_pc
5875	hsa-mir-17	C11orf57	0.308441585	mirna_pc
5876	hsa-mir-17	SLC35F2	0.319588083	mirna_pc
5877	hsa-mir-17	RCBTB1	0.338708156	mirna_pc
5878	hsa-mir-17	CDC16	0.413248309	mirna_pc
5879	hsa-mir-17	ING1	0.340273765	mirna_pc
5880	hsa-mir-17	EEF1E1	0.389537682	mirna_pc
5881	hsa-mir-17	CDK8	0.302245926	mirna_pc
5882	hsa-mir-17	MRPL44	0.325174558	mirna_pc
5883	hsa-mir-17	PTCD3	0.393616465	mirna_pc
5884	hsa-mir-17	ATPBD4	0.302317344	mirna_pc
5885	hsa-mir-17	BCAN	0.342096278	mirna_pc

5886	hsa-mir-17	DHX15	0.306201826	mirna_pc
5887	hsa-mir-17	IWS1	0.337811114	mirna_pc
5888	hsa-mir-17	MAP2K6	0.30724499	mirna_pc
5889	hsa-mir-17	CASP6	0.356398452	mirna_pc
5890	hsa-mir-17	RBM26	0.374152368	mirna_pc
5891	hsa-mir-17	ZNF92	0.310716496	mirna_pc
5892	hsa-mir-17	C2orf44	0.318840168	mirna_pc
5893	hsa-mir-17	TMTC4	0.400474836	mirna_pc
5894	hsa-mir-17	SEPHS1	0.424443623	mirna_pc
5895	hsa-mir-17	CBX5	0.317388718	mirna_pc
5896	hsa-mir-17	RMND1	0.41523689	mirna_pc
5897	hsa-mir-17	GEMIN5	0.304782479	mirna_pc
5898	hsa-mir-17	AZIN1	0.31744949	mirna_pc
5899	hsa-mir-17	RSL1D1	0.349738021	mirna_pc
5900	hsa-mir-17	AGBL5	0.362890508	mirna_pc
5901	hsa-mir-17	CEBPZ	0.343181967	mirna_pc
5902	hsa-mir-17	SMARCE1	0.386391651	mirna_pc
5903	hsa-mir-17	C8orf33	0.385290243	mirna_pc
5904	hsa-mir-17	MYL6B	0.376889471	mirna_pc
5905	hsa-mir-17	TASP1	0.300073942	mirna_pc
5906	hsa-mir-17	MRP63	0.38227369	mirna_pc
5907	hsa-mir-17	IPO5	0.602240574	mirna_pc
5908	hsa-mir-17	ALG11	0.349906676	mirna_pc
5909	hsa-mir-17	INTS1	0.320502386	mirna_pc
5910	hsa-mir-17	MED28	0.363054013	mirna_pc
5911	hsa-mir-17	NARS2	0.362579886	mirna_pc
5912	hsa-mir-17	NUP43	0.334120965	mirna_pc
5913	hsa-mir-17	BRD3	0.300806956	mirna_pc
5914	hsa-mir-17	LYRM4	0.368315882	mirna_pc
5915	hsa-mir-17	ZNF124	0.376233934	mirna_pc
5916	hsa-mir-17	RNASEH2B	0.488538192	mirna_pc
5917	hsa-mir-17	CHD1L	0.394667311	mirna_pc
5918	hsa-mir-17	ZNF473	0.407545068	mirna_pc
5919	hsa-mir-17	COPS7B	0.318442271	mirna_pc
5920	hsa-mir-17	USP21	0.313292811	mirna_pc
5921	hsa-mir-17	SNRNP48	0.323311155	mirna_pc
5922	hsa-mir-17	RFXAP	0.327793197	mirna_pc
5923	hsa-mir-17	DCLRE1A	0.410617283	mirna_pc
5924	hsa-mir-17	ANP32A	0.523121456	mirna_pc
5925	hsa-mir-17	DIS3	0.308327084	mirna_pc
5926	hsa-mir-17	E2F5	0.429057172	mirna_pc
5927	hsa-mir-17	STARD3	0.305530223	mirna_pc
5928	hsa-mir-17	NUFIP1	0.562182842	mirna_pc
5929	hsa-mir-17	ZNF195	0.442911	mirna_pc
5930	hsa-mir-17	ZMYM4	0.326087359	mirna_pc
5931	hsa-mir-17	PRKAR1B	0.389378017	mirna_pc
5932	hsa-mir-17	MED4	0.328424023	mirna_pc
5933	hsa-mir-17	LRRCC1	0.351007605	mirna_pc
5934	hsa-mir-17	ASNSD1	0.30567265	mirna_pc
5935	hsa-mir-17	FXN	0.320600638	mirna_pc
5936	hsa-mir-17	PPP5C	0.364140115	mirna_pc
5937	hsa-mir-17	HYLS1	0.343084648	mirna_pc
5938	hsa-mir-17	NEK3	0.364380389	mirna_pc
5939	hsa-mir-17	HNRNPH3	0.374281366	mirna_pc

5940	hsa-mir-17	NAP1L1	0.339226085	mirna_pc
5941	hsa-mir-17	POLR1D	0.307790779	mirna_pc
5942	hsa-mir-17	RRP1B	0.347430228	mirna_pc
5943	hsa-mir-17	THSD1P1	0.301573806	mirna_pc
5944	hsa-mir-17	NSL1	0.392091885	mirna_pc
5945	hsa-mir-17	VIL1	0.328497668	mirna_pc
5946	hsa-mir-17	GSDMB	0.375778458	mirna_pc
5947	hsa-mir-17	TMEM170A	0.360784417	mirna_pc
5948	hsa-mir-17	EPDR1	0.305963684	mirna_pc
5949	hsa-mir-17	KIAA1383	0.324858417	mirna_pc
5950	hsa-mir-17	BCCIP	0.320557045	mirna_pc
5951	hsa-mir-17	PTPLAD1	0.337183072	mirna_pc
5952	hsa-mir-17	IQCC	0.340035777	mirna_pc
5953	hsa-mir-17	FAM48A	0.4681308	mirna_pc
5954	hsa-mir-17	PMS1	0.389203767	mirna_pc
5955	hsa-mir-17	DNAJC15	0.323202492	mirna_pc
5956	hsa-mir-17	MNS1	0.35938333	mirna_pc
5957	hsa-mir-17	CHCHD3	0.384441056	mirna_pc
5958	hsa-mir-17	CCDC122	0.368151581	mirna_pc
5959	hsa-mir-17	TRUB1	0.312589751	mirna_pc
5960	hsa-mir-17	ZMYM2	0.379378391	mirna_pc
5961	hsa-mir-17	C2orf15	0.315303958	mirna_pc
5962	hsa-mir-17	UGT8	0.31288872	mirna_pc
5963	hsa-mir-17	CCNJ	0.508563651	mirna_pc
5964	hsa-mir-17	PIBF1	0.31482478	mirna_pc
5965	hsa-mir-17	DCAF16	0.3823489	mirna_pc
5966	hsa-mir-17	SBK1	0.404524468	mirna_pc
5967	hsa-mir-17	IPO7	0.307751293	mirna_pc
5968	hsa-mir-17	ABCC4	0.32255111	mirna_pc
5969	hsa-mir-17	KBTBD6	0.389215168	mirna_pc
5970	hsa-mir-17	USP13	0.368957695	mirna_pc
5971	hsa-mir-17	ESF1	0.318470675	mirna_pc
5972	hsa-mir-17	MOSC1	0.312582581	mirna_pc
5973	hsa-mir-17	SLC25A33	0.396722969	mirna_pc
5974	hsa-mir-17	SMARCC1	0.384413435	mirna_pc
5975	hsa-mir-17	CTPS2	0.418988129	mirna_pc
5976	hsa-mir-17	GXYLT1	0.312490831	mirna_pc
5977	hsa-mir-17	BCS1L	0.304043537	mirna_pc
5978	hsa-mir-17	PACRGL	0.355379205	mirna_pc
5979	hsa-mir-17	C2orf56	0.324526449	mirna_pc
5980	hsa-mir-17	XPO4	0.444202201	mirna_pc
5981	hsa-mir-17	TSEN2	0.310294812	mirna_pc
5982	hsa-mir-17	DDX51	0.337360471	mirna_pc
5983	hsa-mir-17	C21orf59	0.32303925	mirna_pc
5984	hsa-mir-17	ZNF232	0.382678765	mirna_pc
5985	hsa-mir-17	ZBTB39	0.348648278	mirna_pc
5986	hsa-mir-17	SNRNP27	0.304383466	mirna_pc
5987	hsa-mir-17	METTL10	0.320239675	mirna_pc
5988	hsa-mir-17	MRPL16	0.346918283	mirna_pc
5989	hsa-mir-17	IFT88	0.458823419	mirna_pc
5990	hsa-mir-17	ESD	0.388663135	mirna_pc
5991	hsa-mir-17	QRSL1	0.311473002	mirna_pc
5992	hsa-mir-17	FANCF	0.401314046	mirna_pc
5993	hsa-mir-17	MARS2	0.326036107	mirna_pc

5994	hsa-mir-17	VPS54	0.322249455	mirna_pc
5995	hsa-mir-17	ZBTB24	0.302808057	mirna_pc
5996	hsa-mir-17	RBBP4	0.351308263	mirna_pc
5997	hsa-mir-17	CIAO1	0.401487549	mirna_pc
5998	hsa-mir-17	C2orf3	0.422235479	mirna_pc
5999	hsa-mir-17	THAP4	0.312936886	mirna_pc
6000	hsa-mir-17	FAM119A	0.393801006	mirna_pc
6001	hsa-mir-17	ZNF664	0.359527707	mirna_pc
6002	hsa-mir-17	GOLT1A	0.346189443	mirna_pc
6003	hsa-mir-17	SCML2	0.362468195	mirna_pc
6004	hsa-mir-17	MRPL19	0.41120402	mirna_pc
6005	hsa-mir-17	EFHA1	0.339101343	mirna_pc
6006	hsa-mir-17	ABCB10	0.32519662	mirna_pc
6007	hsa-mir-17	KPNA3	0.338392007	mirna_pc
6008	hsa-mir-17	ZCCHC3	0.481576358	mirna_pc
6009	hsa-mir-17	RRP15	0.441084578	mirna_pc
6010	hsa-mir-17	ETV5	0.394184265	mirna_pc
6011	hsa-mir-17	COX7A2L	0.310964508	mirna_pc
6012	hsa-mir-17	CASC3	0.301580699	mirna_pc
6013	hsa-mir-17	AADAT	0.467592792	mirna_pc
6014	hsa-mir-17	ACP1	0.438785765	mirna_pc
6015	hsa-mir-17	C2orf43	0.378427918	mirna_pc
6016	hsa-mir-17	DACH1	0.352972815	mirna_pc
6017	hsa-mir-17	RARA	0.320053811	mirna_pc
6018	hsa-mir-17	ACTR3B	0.346248242	mirna_pc
6019	hsa-mir-17	GTF3A	0.463930269	mirna_pc
6020	hsa-mir-17	ZNF48	0.34010241	mirna_pc
6021	hsa-mir-17	AIMP1	0.324609813	mirna_pc
6022	hsa-mir-17	TPP2	0.420147249	mirna_pc
6023	hsa-mir-17	SPRED2	0.330745659	mirna_pc
6024	hsa-mir-17	CD320	0.321322136	mirna_pc
6025	hsa-mir-17	TXLNG	0.36263927	mirna_pc
6026	hsa-mir-17	TRIM25	0.304397163	mirna_pc
6027	hsa-mir-17	PRMT6	0.3535648	mirna_pc
6028	hsa-mir-17	MRPS9	0.352536824	mirna_pc
6029	hsa-mir-17	PPP1R1B	0.362013815	mirna_pc
6030	hsa-mir-17	ANAPC5	0.300855335	mirna_pc
6031	hsa-mir-17	NCRNA00188	0.343814158	mirna_pc
6032	hsa-mir-17	MIR17HG	0.455281076	mirna_pc
6033	hsa-mir-17	MRPS23	0.32549849	mirna_pc
6034	hsa-mir-17	AARSD1	0.392933572	mirna_pc
6035	hsa-mir-17	PRDX3	0.353508277	mirna_pc
6036	hsa-mir-17	BOD1	0.312710906	mirna_pc
6037	hsa-mir-17	ZNF239	0.330387378	mirna_pc
6038	hsa-mir-17	SAP18	0.321397711	mirna_pc
6039	hsa-mir-17	KLHL23	0.358850314	mirna_pc
6040	hsa-mir-17	TMEM14B	0.400048404	mirna_pc
6041	hsa-mir-17	SMARCAD1	0.317385872	mirna_pc
6042	hsa-mir-17	ZNF77	0.31495684	mirna_pc
6043	hsa-mir-17	C20orf7	0.304186935	mirna_pc
6044	hsa-mir-17	EIF2S3	0.344712433	mirna_pc
6045	hsa-mir-17	MRPS31	0.408630053	mirna_pc
6046	hsa-mir-17	CENPV	0.418004872	mirna_pc
6047	hsa-mir-17	DNMT3A	0.474091249	mirna_pc

6048	hsa-mir-17	MIPEP	0.420806381	mirna_pc
6049	hsa-mir-17	PRTFDC1	0.39049012	mirna_pc
6050	hsa-mir-17	CARS2	0.375547921	mirna_pc
6051	hsa-mir-17	ZNF90	0.303610087	mirna_pc
6052	hsa-mir-17	PATZ1	0.381629333	mirna_pc
6053	hsa-mir-17	ZNF670	0.314677287	mirna_pc
6054	hsa-mir-17	PARK7	0.300823503	mirna_pc
6055	hsa-mir-301a	TPX2	0.327930245	mirna_pc
6056	hsa-mir-301a	KPNA2	0.326036111	mirna_pc
6057	hsa-mir-301a	CENPF	0.316384671	mirna_pc
6058	hsa-mir-301a	RCC2	0.411125094	mirna_pc
6059	hsa-mir-301a	HOXC9	0.373597796	mirna_pc
6060	hsa-mir-301a	KIF11	0.343010715	mirna_pc
6061	hsa-mir-301a	RRM2	0.349871555	mirna_pc
6062	hsa-mir-301a	CDC25C	0.350416243	mirna_pc
6063	hsa-mir-301a	UBE2C	0.394773501	mirna_pc
6064	hsa-mir-301a	NUSAP1	0.364754295	mirna_pc
6065	hsa-mir-301a	MCM4	0.357375856	mirna_pc
6066	hsa-mir-301a	BUB1B	0.343679463	mirna_pc
6067	hsa-mir-301a	KIF18B	0.35687225	mirna_pc
6068	hsa-mir-301a	KIF2C	0.421213422	mirna_pc
6069	hsa-mir-301a	NCAPG	0.441887184	mirna_pc
6070	hsa-mir-301a	CDC20	0.324131441	mirna_pc
6071	hsa-mir-301a	CLSPN	0.381769342	mirna_pc
6072	hsa-mir-301a	CCNA2	0.410519682	mirna_pc
6073	hsa-mir-301a	FANCI	0.315180726	mirna_pc
6074	hsa-mir-301a	NEK2	0.309842922	mirna_pc
6075	hsa-mir-301a	MND1	0.467302074	mirna_pc
6076	hsa-mir-301a	KIF22	0.350424236	mirna_pc
6077	hsa-mir-301a	FAM49B	0.326631935	mirna_pc
6078	hsa-mir-301a	NCAPH	0.361747687	mirna_pc
6079	hsa-mir-301a	KIF23	0.329296703	mirna_pc
6080	hsa-mir-301a	RAD54L	0.394192874	mirna_pc
6081	hsa-mir-301a	CENPA	0.427738103	mirna_pc
6082	hsa-mir-301a	MAD2L1	0.460645884	mirna_pc
6083	hsa-mir-301a	TIMELESS	0.388596148	mirna_pc
6084	hsa-mir-301a	LMNB1	0.396993145	mirna_pc
6085	hsa-mir-301a	DLGAP5	0.305757277	mirna_pc
6086	hsa-mir-301a	TROAP	0.451063436	mirna_pc
6087	hsa-mir-301a	CCNB1	0.355140639	mirna_pc
6088	hsa-mir-301a	CDCA5	0.321211699	mirna_pc
6089	hsa-mir-301a	STMN1	0.431216902	mirna_pc
6090	hsa-mir-301a	EXO1	0.300714307	mirna_pc
6091	hsa-mir-301a	TK1	0.306798018	mirna_pc
6092	hsa-mir-301a	DKC1	0.375867243	mirna_pc
6093	hsa-mir-301a	CKS1B	0.325639689	mirna_pc
6094	hsa-mir-301a	TUBB	0.397317898	mirna_pc
6095	hsa-mir-301a	KIAA0101	0.376099086	mirna_pc
6096	hsa-mir-301a	KIF4A	0.326577735	mirna_pc
6097	hsa-mir-301a	ORC1L	0.351931213	mirna_pc
6098	hsa-mir-301a	KNTC1	0.38404283	mirna_pc
6099	hsa-mir-301a	CCNF	0.329977733	mirna_pc
6100	hsa-mir-301a	CCNB2	0.375113148	mirna_pc
6101	hsa-mir-301a	NUF2	0.520487415	mirna_pc

6102	hsa-mir-301a	PLK4	0.47443104	mirna_pc
6103	hsa-mir-301a	KIF18A	0.354966448	mirna_pc
6104	hsa-mir-301a	DEPDC1	0.34581352	mirna_pc
6105	hsa-mir-301a	PTTG1	0.310675129	mirna_pc
6106	hsa-mir-301a	HJURP	0.366191782	mirna_pc
6107	hsa-mir-301a	SKA1	0.326251769	mirna_pc
6108	hsa-mir-301a	NME1	0.31607193	mirna_pc
6109	hsa-mir-301a	DNMT1	0.326371289	mirna_pc
6110	hsa-mir-301a	PAICS	0.433343383	mirna_pc
6111	hsa-mir-301a	BIRC5	0.447794859	mirna_pc
6112	hsa-mir-301a	RCC1	0.348385946	mirna_pc
6113	hsa-mir-301a	DTL	0.368544976	mirna_pc
6114	hsa-mir-301a	EME1	0.330025766	mirna_pc
6115	hsa-mir-301a	RECQL4	0.357740135	mirna_pc
6116	hsa-mir-301a	AURKB	0.473647217	mirna_pc
6117	hsa-mir-301a	CBFB	0.312861559	mirna_pc
6118	hsa-mir-301a	MCM10	0.336093609	mirna_pc
6119	hsa-mir-301a	KIF20A	0.450727858	mirna_pc
6120	hsa-mir-301a	SGOL2	0.300280998	mirna_pc
6121	hsa-mir-301a	CENPE	0.325049266	mirna_pc
6122	hsa-mir-301a	CDCA3	0.39272272	mirna_pc
6123	hsa-mir-301a	C1orf112	0.417006044	mirna_pc
6124	hsa-mir-301a	GINS1	0.351427581	mirna_pc
6125	hsa-mir-301a	ORC6L	0.39151457	mirna_pc
6126	hsa-mir-301a	ERH	0.30589485	mirna_pc
6127	hsa-mir-301a	C16orf59	0.410236943	mirna_pc
6128	hsa-mir-301a	NEIL3	0.41361355	mirna_pc
6129	hsa-mir-301a	ACTL6A	0.473014297	mirna_pc
6130	hsa-mir-301a	ZNF229	0.378823843	mirna_pc
6131	hsa-mir-301a	C12orf48	0.364849775	mirna_pc
6132	hsa-mir-301a	HNRNPL	0.388651042	mirna_pc
6133	hsa-mir-301a	SMC4	0.418182579	mirna_pc
6134	hsa-mir-301a	PKMYT1	0.341182318	mirna_pc
6135	hsa-mir-301a	TTK	0.348892811	mirna_pc
6136	hsa-mir-301a	SF3B4	0.330846266	mirna_pc
6137	hsa-mir-301a	TUBA1B	0.342139916	mirna_pc
6138	hsa-mir-301a	HELLS	0.342418462	mirna_pc
6139	hsa-mir-301a	POLE2	0.346512859	mirna_pc
6140	hsa-mir-301a	UBE2S	0.386226271	mirna_pc
6141	hsa-mir-301a	NCAPD2	0.340063464	mirna_pc
6142	hsa-mir-301a	C11orf82	0.451919558	mirna_pc
6143	hsa-mir-301a	HNRNPC	0.41012034	mirna_pc
6144	hsa-mir-301a	ILF2	0.310540077	mirna_pc
6145	hsa-mir-301a	C6orf167	0.360127218	mirna_pc
6146	hsa-mir-301a	HNRNPA2B1	0.387224119	mirna_pc
6147	hsa-mir-301a	TYMS	0.314225311	mirna_pc
6148	hsa-mir-301a	RNASEH2A	0.366857659	mirna_pc
6149	hsa-mir-301a	EPR1	0.40084767	mirna_pc
6150	hsa-mir-301a	BRIP1	0.322152355	mirna_pc
6151	hsa-mir-301a	PIF1	0.338813102	mirna_pc
6152	hsa-mir-301a	SFRS2	0.36699176	mirna_pc
6153	hsa-mir-301a	DCAF13	0.327710328	mirna_pc
6154	hsa-mir-301a	UCK2	0.453076298	mirna_pc
6155	hsa-mir-301a	WDHD1	0.319078122	mirna_pc

6156	hsa-mir-301a	NFKBIL2	0.397121303	mirna_pc
6157	hsa-mir-301a	C15orf42	0.338369412	mirna_pc
6158	hsa-mir-301a	PRIM2	0.311200349	mirna_pc
6159	hsa-mir-301a	HMGB3	0.307696491	mirna_pc
6160	hsa-mir-301a	MTBP	0.328316034	mirna_pc
6161	hsa-mir-301a	SNRPG	0.334578645	mirna_pc
6162	hsa-mir-301a	EIF4A3	0.344381932	mirna_pc
6163	hsa-mir-301a	SNRPA	0.356058719	mirna_pc
6164	hsa-mir-301a	FUS	0.496436316	mirna_pc
6165	hsa-mir-301a	ATAD2	0.321315931	mirna_pc
6166	hsa-mir-301a	U2AF2	0.454950362	mirna_pc
6167	hsa-mir-301a	CHAF1A	0.347535714	mirna_pc
6168	hsa-mir-301a	DEPDC1B	0.317283403	mirna_pc
6169	hsa-mir-301a	GINS4	0.376977233	mirna_pc
6170	hsa-mir-301a	ANP32E	0.492075518	mirna_pc
6171	hsa-mir-301a	CENPO	0.352888916	mirna_pc
6172	hsa-mir-301a	CENPL	0.317982303	mirna_pc
6173	hsa-mir-301a	NUDT1	0.313042983	mirna_pc
6174	hsa-mir-301a	HMGB2	0.472162864	mirna_pc
6175	hsa-mir-301a	GINS2	0.353453896	mirna_pc
6176	hsa-mir-301a	DNA2	0.319331034	mirna_pc
6177	hsa-mir-301a	CDCA4	0.345217148	mirna_pc
6178	hsa-mir-301a	CSE1L	0.374917685	mirna_pc
6179	hsa-mir-301a	E2F7	0.390188841	mirna_pc
6180	hsa-mir-301a	PRMT1	0.303934105	mirna_pc
6181	hsa-mir-301a	MLF1IP	0.339673312	mirna_pc
6182	hsa-mir-301a	C16orf75	0.326631814	mirna_pc
6183	hsa-mir-301a	HIST1H2AE	0.457009226	mirna_pc
6184	hsa-mir-301a	CCDC99	0.320455567	mirna_pc
6185	hsa-mir-301a	CDK2	0.359056417	mirna_pc
6186	hsa-mir-301a	PFDN2	0.412087981	mirna_pc
6187	hsa-mir-301a	CHTF18	0.339634077	mirna_pc
6188	hsa-mir-301a	ENO1	0.374454519	mirna_pc
6189	hsa-mir-301a	HNRNPR	0.485452387	mirna_pc
6190	hsa-mir-301a	DLEU2	0.338696974	mirna_pc
6191	hsa-mir-301a	PRIM1	0.344888372	mirna_pc
6192	hsa-mir-301a	CENPH	0.360571801	mirna_pc
6193	hsa-mir-301a	PSMC4	0.322547513	mirna_pc
6194	hsa-mir-301a	TCOF1	0.334428622	mirna_pc
6195	hsa-mir-301a	CHAF1B	0.33650007	mirna_pc
6196	hsa-mir-301a	ACYP1	0.324351418	mirna_pc
6197	hsa-mir-301a	MSH2	0.441345217	mirna_pc
6198	hsa-mir-301a	DBF4	0.464190711	mirna_pc
6199	hsa-mir-301a	CDC7	0.440173893	mirna_pc
6200	hsa-mir-301a	NOL11	0.392205286	mirna_pc
6201	hsa-mir-301a	PSMB4	0.311193222	mirna_pc
6202	hsa-mir-301a	RFC4	0.450447969	mirna_pc
6203	hsa-mir-301a	FBXO5	0.430292432	mirna_pc
6204	hsa-mir-301a	FIGNL1	0.456861865	mirna_pc
6205	hsa-mir-301a	BRIX1	0.336954016	mirna_pc
6206	hsa-mir-301a	E2F3	0.350162012	mirna_pc
6207	hsa-mir-301a	SF3B14	0.306172599	mirna_pc
6208	hsa-mir-301a	RAD54B	0.390740103	mirna_pc
6209	hsa-mir-301a	H2AFZ	0.320836019	mirna_pc

6210	hsa-mir-301a	NRM	0.382782345	mirna_pc
6211	hsa-mir-301a	MAGOH	0.348131607	mirna_pc
6212	hsa-mir-301a	DDX12	0.420853627	mirna_pc
6213	hsa-mir-301a	CPSF3	0.477419658	mirna_pc
6214	hsa-mir-301a	BANF1	0.398150793	mirna_pc
6215	hsa-mir-301a	C21orf45	0.423697668	mirna_pc
6216	hsa-mir-301a	DNAJB11	0.329658274	mirna_pc
6217	hsa-mir-301a	TFAP4	0.379679962	mirna_pc
6218	hsa-mir-301a	RNPS1	0.323542658	mirna_pc
6219	hsa-mir-301a	DAZAP1	0.337252654	mirna_pc
6220	hsa-mir-301a	CBX8	0.407416087	mirna_pc
6221	hsa-mir-301a	TMEM201	0.440754002	mirna_pc
6222	hsa-mir-301a	NCL	0.318531886	mirna_pc
6223	hsa-mir-301a	PFDN4	0.41336163	mirna_pc
6224	hsa-mir-301a	PAFAH1B3	0.356288117	mirna_pc
6225	hsa-mir-301a	CACYBP	0.400384932	mirna_pc
6226	hsa-mir-301a	SASS6	0.357730129	mirna_pc
6227	hsa-mir-301a	PSRC1	0.40414866	mirna_pc
6228	hsa-mir-301a	DGUOK	0.407937227	mirna_pc
6229	hsa-mir-301a	MRPL47	0.392675892	mirna_pc
6230	hsa-mir-301a	STIP1	0.335925505	mirna_pc
6231	hsa-mir-301a	HNRNPM	0.367436986	mirna_pc
6232	hsa-mir-301a	CCT6A	0.347001463	mirna_pc
6233	hsa-mir-301a	POLR2H	0.36557062	mirna_pc
6234	hsa-mir-301a	SSBP3	0.331412544	mirna_pc
6235	hsa-mir-301a	C20orf72	0.337361594	mirna_pc
6236	hsa-mir-301a	MRTO4	0.350846155	mirna_pc
6237	hsa-mir-301a	SHFM1	0.372823336	mirna_pc
6238	hsa-mir-301a	GPR19	0.394733746	mirna_pc
6239	hsa-mir-301a	LSG1	0.341987045	mirna_pc
6240	hsa-mir-301a	GABPB1	0.452961643	mirna_pc
6241	hsa-mir-301a	PPIH	0.349585641	mirna_pc
6242	hsa-mir-301a	THOC6	0.339560877	mirna_pc
6243	hsa-mir-301a	GMPS	0.309941855	mirna_pc
6244	hsa-mir-301a	PPAT	0.363163714	mirna_pc
6245	hsa-mir-301a	DHX9	0.321660383	mirna_pc
6246	hsa-mir-301a	CCDC138	0.358669331	mirna_pc
6247	hsa-mir-301a	LOC100128191	0.454119473	mirna_pc
6248	hsa-mir-301a	HMGN2	0.339520354	mirna_pc
6249	hsa-mir-301a	SMYD5	0.335221326	mirna_pc
6250	hsa-mir-301a	SF3A2	0.316136844	mirna_pc
6251	hsa-mir-301a	TSSC1	0.303987291	mirna_pc
6252	hsa-mir-301a	POLA2	0.310019372	mirna_pc
6253	hsa-mir-301a	SNRPF	0.348746256	mirna_pc
6254	hsa-mir-301a	SUMO2	0.523885455	mirna_pc
6255	hsa-mir-301a	SFRS1	0.379058216	mirna_pc
6256	hsa-mir-301a	SNRPA1	0.416158421	mirna_pc
6257	hsa-mir-301a	SKP2	0.352082193	mirna_pc
6258	hsa-mir-301a	SMC6	0.349893721	mirna_pc
6259	hsa-mir-301a	HOXD9	0.309796832	mirna_pc
6260	hsa-mir-301a	UBE2I	0.385063079	mirna_pc
6261	hsa-mir-301a	TIPIN	0.393978555	mirna_pc
6262	hsa-mir-301a	SMARCD1	0.355820318	mirna_pc
6263	hsa-mir-301a	ATXN2L	0.335643068	mirna_pc

6264	hsa-mir-301a	HSPBP1	0.304518642	mirna_pc
6265	hsa-mir-301a	C4orf46	0.349893211	mirna_pc
6266	hsa-mir-301a	ECE2	0.367630246	mirna_pc
6267	hsa-mir-301a	RFC5	0.414668695	mirna_pc
6268	hsa-mir-301a	PA2G4	0.325232314	mirna_pc
6269	hsa-mir-301a	SNRPD3	0.333486026	mirna_pc
6270	hsa-mir-301a	KHDRBS1	0.478007658	mirna_pc
6271	hsa-mir-301a	SPAST	0.313264877	mirna_pc
6272	hsa-mir-301a	CCDC21	0.35842388	mirna_pc
6273	hsa-mir-301a	MTL 5.00	0.341086508	mirna_pc
6274	hsa-mir-301a	CCDC18	0.31317364	mirna_pc
6275	hsa-mir-301a	MTERFD1	0.311377873	mirna_pc
6276	hsa-mir-301a	C4orf21	0.319957399	mirna_pc
6277	hsa-mir-301a	TMEM194A	0.342432922	mirna_pc
6278	hsa-mir-301a	MED30	0.435554906	mirna_pc
6279	hsa-mir-301a	TMEM69	0.364855105	mirna_pc
6280	hsa-mir-301a	NEDD1	0.362007004	mirna_pc
6281	hsa-mir-301a	CEP135	0.30469206	mirna_pc
6282	hsa-mir-301a	JUB	0.304622436	mirna_pc
6283	hsa-mir-301a	GNL2	0.338444936	mirna_pc
6284	hsa-mir-301a	RAD21	0.368073049	mirna_pc
6285	hsa-mir-301a	HNRNPU	0.328998258	mirna_pc
6286	hsa-mir-301a	NOC2L	0.325471928	mirna_pc
6287	hsa-mir-301a	NASP	0.44515668	mirna_pc
6288	hsa-mir-301a	RPP40	0.341994858	mirna_pc
6289	hsa-mir-301a	ZC3H3	0.301342668	mirna_pc
6290	hsa-mir-301a	ZNF280C	0.302116807	mirna_pc
6291	hsa-mir-301a	PTMA	0.43388917	mirna_pc
6292	hsa-mir-301a	WDR76	0.324854652	mirna_pc
6293	hsa-mir-301a	DENR	0.324094381	mirna_pc
6294	hsa-mir-301a	DUSP11	0.301746602	mirna_pc
6295	hsa-mir-301a	C11orf84	0.370902794	mirna_pc
6296	hsa-mir-301a	TOP1MT	0.306050328	mirna_pc
6297	hsa-mir-301a	TBCE	0.346413023	mirna_pc
6298	hsa-mir-301a	LRRC8D	0.38120811	mirna_pc
6299	hsa-mir-301a	NCBP2	0.402520387	mirna_pc
6300	hsa-mir-301a	FBXO22OS	0.342217657	mirna_pc
6301	hsa-mir-301a	SUV39H2	0.315350798	mirna_pc
6302	hsa-mir-301a	HOXA9	0.35495471	mirna_pc
6303	hsa-mir-301a	GORAB	0.333574568	mirna_pc
6304	hsa-mir-301a	HNRNPD	0.468369608	mirna_pc
6305	hsa-mir-301a	DPY30	0.435030093	mirna_pc
6306	hsa-mir-301a	TARDBP	0.324247374	mirna_pc
6307	hsa-mir-301a	SFRS3	0.322622917	mirna_pc
6308	hsa-mir-301a	MEN1	0.340984039	mirna_pc
6309	hsa-mir-301a	CCT4	0.352045674	mirna_pc
6310	hsa-mir-301a	RAD9A	0.343588095	mirna_pc
6311	hsa-mir-301a	DUSP9	0.359570163	mirna_pc
6312	hsa-mir-301a	TFAP2A	0.324010243	mirna_pc
6313	hsa-mir-301a	NCAPD3	0.356266385	mirna_pc
6314	hsa-mir-301a	NAE1	0.358893137	mirna_pc
6315	hsa-mir-301a	PSMC3IP	0.358786194	mirna_pc
6316	hsa-mir-301a	TBC1D7	0.353415036	mirna_pc
6317	hsa-mir-301a	PAK1IP1	0.383810351	mirna_pc

6318	hsa-mir-301a	RPAP3	0.350348333	mirna_pc
6319	hsa-mir-301a	TRMT112	0.356508084	mirna_pc
6320	hsa-mir-301a	KPTN	0.394156787	mirna_pc
6321	hsa-mir-301a	PSMD2	0.323578668	mirna_pc
6322	hsa-mir-301a	ATP13A3	0.342753569	mirna_pc
6323	hsa-mir-301a	PMAIP1	0.328610673	mirna_pc
6324	hsa-mir-301a	RNF2	0.30537396	mirna_pc
6325	hsa-mir-301a	YWHAE	0.44898722	mirna_pc
6326	hsa-mir-301a	KDM1A	0.345060208	mirna_pc
6327	hsa-mir-301a	C3orf57	0.331790414	mirna_pc
6328	hsa-mir-301a	UBAP2L	0.398801695	mirna_pc
6329	hsa-mir-301a	PUF60	0.315571505	mirna_pc
6330	hsa-mir-301a	DEK	0.45623841	mirna_pc
6331	hsa-mir-301a	SKA2	0.371861146	mirna_pc
6332	hsa-mir-301a	TGS1	0.300723231	mirna_pc
6333	hsa-mir-301a	KHSRP	0.375468944	mirna_pc
6334	hsa-mir-301a	LSM 6.00	0.348672362	mirna_pc
6335	hsa-mir-301a	PRAME	0.432643429	mirna_pc
6336	hsa-mir-301a	MRPS17	0.354708971	mirna_pc
6337	hsa-mir-301a	TNFRSF18	0.331753715	mirna_pc
6338	hsa-mir-301a	FUBP1	0.344312209	mirna_pc
6339	hsa-mir-301a	TIPRL	0.313360802	mirna_pc
6340	hsa-mir-301a	TRA2B	0.423894153	mirna_pc
6341	hsa-mir-301a	RSRC1	0.424639493	mirna_pc
6342	hsa-mir-301a	GAR1	0.389876695	mirna_pc
6343	hsa-mir-301a	C15orf23	0.347230482	mirna_pc
6344	hsa-mir-301a	C3orf21	0.34266233	mirna_pc
6345	hsa-mir-301a	PSMD12	0.485228851	mirna_pc
6346	hsa-mir-301a	ZNF668	0.336216864	mirna_pc
6347	hsa-mir-301a	TDG	0.350909444	mirna_pc
6348	hsa-mir-301a	ITGB3BP	0.310763256	mirna_pc
6349	hsa-mir-301a	ENOPH1	0.444809781	mirna_pc
6350	hsa-mir-301a	C19orf47	0.374476492	mirna_pc
6351	hsa-mir-301a	C4orf43	0.378369578	mirna_pc
6352	hsa-mir-301a	XRCC3	0.314935064	mirna_pc
6353	hsa-mir-301a	YY1	0.340956819	mirna_pc
6354	hsa-mir-301a	DDX55	0.35547152	mirna_pc
6355	hsa-mir-301a	C16orf80	0.313585386	mirna_pc
6356	hsa-mir-301a	PLEKHG4	0.338837144	mirna_pc
6357	hsa-mir-301a	SFPQ	0.470379544	mirna_pc
6358	hsa-mir-301a	CNOT3	0.306566415	mirna_pc
6359	hsa-mir-301a	VPS72	0.355879957	mirna_pc
6360	hsa-mir-301a	ODF2L	0.343689232	mirna_pc
6361	hsa-mir-301a	EIF4EBP1	0.406484121	mirna_pc
6362	hsa-mir-301a	CADM1	0.483324692	mirna_pc
6363	hsa-mir-301a	HDAC2	0.347766105	mirna_pc
6364	hsa-mir-301a	GMNN	0.380677654	mirna_pc
6365	hsa-mir-301a	WDR53	0.34950464	mirna_pc
6366	hsa-mir-301a	ALOX12P2	0.353841895	mirna_pc
6367	hsa-mir-301a	ANKRD32	0.403934411	mirna_pc
6368	hsa-mir-301a	CHRAC1	0.335204148	mirna_pc
6369	hsa-mir-301a	PDAP1	0.478706982	mirna_pc
6370	hsa-mir-301a	RNF7	0.407345542	mirna_pc
6371	hsa-mir-301a	GEMIN6	0.39535225	mirna_pc

6372	hsa-mir-301a	H3F3B	0.33293694	mirna_pc
6373	hsa-mir-301a	FAM86C	0.351471698	mirna_pc
6374	hsa-mir-301a	CLSTN1	0.324230687	mirna_pc
6375	hsa-mir-301a	PTCD1	0.473208479	mirna_pc
6376	hsa-mir-301a	CRIPT	0.423072878	mirna_pc
6377	hsa-mir-301a	MSH6	0.425804132	mirna_pc
6378	hsa-mir-301a	C3orf34	0.402306653	mirna_pc
6379	hsa-mir-301a	TPRKB	0.372569297	mirna_pc
6380	hsa-mir-301a	TP73	0.377569599	mirna_pc
6381	hsa-mir-301a	KPNB1	0.30130959	mirna_pc
6382	hsa-mir-301a	LEF1	0.350546803	mirna_pc
6383	hsa-mir-301a	SMC3	0.356453169	mirna_pc
6384	hsa-mir-301a	POLB	0.342522868	mirna_pc
6385	hsa-mir-301a	HNRNPA1	0.343165044	mirna_pc
6386	hsa-mir-301a	PPP1R8	0.416850069	mirna_pc
6387	hsa-mir-301a	NPM1	0.304990498	mirna_pc
6388	hsa-mir-301a	CDK2AP1	0.373216897	mirna_pc
6389	hsa-mir-301a	KREMEN2	0.325661295	mirna_pc
6390	hsa-mir-301a	NUDT3	0.380604197	mirna_pc
6391	hsa-mir-301a	MEX3A	0.482739115	mirna_pc
6392	hsa-mir-301a	ATP5J2	0.438162201	mirna_pc
6393	hsa-mir-301a	COIL	0.398099904	mirna_pc
6394	hsa-mir-301a	BUD31	0.412052006	mirna_pc
6395	hsa-mir-301a	C16orf88	0.344249472	mirna_pc
6396	hsa-mir-301a	EXOSC4	0.310093431	mirna_pc
6397	hsa-mir-301a	B3GALT6	0.314967596	mirna_pc
6398	hsa-mir-301a	APEX1	0.308062938	mirna_pc
6399	hsa-mir-301a	MAD2L2	0.365618895	mirna_pc
6400	hsa-mir-301a	HNRNPA1L2	0.382882152	mirna_pc
6401	hsa-mir-301a	CBX1	0.342379781	mirna_pc
6402	hsa-mir-301a	EMILIN3	0.359879747	mirna_pc
6403	hsa-mir-301a	ZBTB12	0.329228336	mirna_pc
6404	hsa-mir-301a	TMEM68	0.358284995	mirna_pc
6405	hsa-mir-301a	LOC642846	0.423906656	mirna_pc
6406	hsa-mir-301a	TGIF2	0.343077529	mirna_pc
6407	hsa-mir-301a	C16orf87	0.33489034	mirna_pc
6408	hsa-mir-301a	RCN2	0.382205911	mirna_pc
6409	hsa-mir-301a	THUMPD2	0.389295904	mirna_pc
6410	hsa-mir-301a	MTF2	0.333276204	mirna_pc
6411	hsa-mir-301a	PSMD7	0.303138112	mirna_pc
6412	hsa-mir-301a	C16orf53	0.303003133	mirna_pc
6413	hsa-mir-301a	NOL10	0.397821211	mirna_pc
6414	hsa-mir-301a	DDX47	0.303934464	mirna_pc
6415	hsa-mir-301a	POLE	0.359087657	mirna_pc
6416	hsa-mir-301a	MUTYH	0.324423098	mirna_pc
6417	hsa-mir-301a	WRNIP1	0.403152055	mirna_pc
6418	hsa-mir-301a	CTDSPL2	0.305216213	mirna_pc
6419	hsa-mir-301a	TAF1B	0.398454551	mirna_pc
6420	hsa-mir-301a	XRCC6BP1	0.412909753	mirna_pc
6421	hsa-mir-301a	VRK1	0.330498968	mirna_pc
6422	hsa-mir-301a	RIOK1	0.313897375	mirna_pc
6423	hsa-mir-301a	EXOSC9	0.478572601	mirna_pc
6424	hsa-mir-301a	MPHOSPH6	0.326470998	mirna_pc
6425	hsa-mir-301a	ZNF707	0.304495691	mirna_pc

6426	hsa-mir-301a	UNG	0.323343766	mirna_pc
6427	hsa-mir-301a	CCDC43	0.359052008	mirna_pc
6428	hsa-mir-301a	NRD1	0.339525898	mirna_pc
6429	hsa-mir-301a	RBMX	0.392500687	mirna_pc
6430	hsa-mir-301a	TELO2	0.343839878	mirna_pc
6431	hsa-mir-301a	ALKBH2	0.364791637	mirna_pc
6432	hsa-mir-301a	LOC374443	0.371254178	mirna_pc
6433	hsa-mir-301a	RPF2	0.331668723	mirna_pc
6434	hsa-mir-301a	CLK2	0.326896902	mirna_pc
6435	hsa-mir-301a	NHLRC1	0.319348593	mirna_pc
6436	hsa-mir-301a	RPL39L	0.324988064	mirna_pc
6437	hsa-mir-301a	GPN3	0.383350018	mirna_pc
6438	hsa-mir-301a	ASB3	0.38785481	mirna_pc
6439	hsa-mir-301a	CHORDC1	0.306145115	mirna_pc
6440	hsa-mir-301a	MEMO1	0.464437838	mirna_pc
6441	hsa-mir-301a	CCDC23	0.3851209	mirna_pc
6442	hsa-mir-301a	IGSF9	0.422066258	mirna_pc
6443	hsa-mir-301a	UBXN2A	0.434091405	mirna_pc
6444	hsa-mir-301a	FIP1L1	0.350617788	mirna_pc
6445	hsa-mir-301a	KDM2B	0.306702554	mirna_pc
6446	hsa-mir-301a	SCLT1	0.334526442	mirna_pc
6447	hsa-mir-301a	MAGEF1	0.413249856	mirna_pc
6448	hsa-mir-301a	PSMC1	0.34701502	mirna_pc
6449	hsa-mir-301a	ENY2	0.323534199	mirna_pc
6450	hsa-mir-301a	PCGF6	0.331011312	mirna_pc
6451	hsa-mir-301a	TTC32	0.42445448	mirna_pc
6452	hsa-mir-301a	SMN2	0.431740271	mirna_pc
6453	hsa-mir-301a	U2AF1	0.300466983	mirna_pc
6454	hsa-mir-301a	FAM92A1	0.32925218	mirna_pc
6455	hsa-mir-301a	ZNF639	0.364531938	mirna_pc
6456	hsa-mir-301a	SART1	0.322115617	mirna_pc
6457	hsa-mir-301a	ITPRIPL1	0.308333969	mirna_pc
6458	hsa-mir-301a	ABT1	0.406906643	mirna_pc
6459	hsa-mir-301a	SLC16A1	0.359114937	mirna_pc
6460	hsa-mir-301a	MPHOSPH10	0.384296355	mirna_pc
6461	hsa-mir-301a	AKAP9	0.407739613	mirna_pc
6462	hsa-mir-301a	ATXN7L2	0.314916694	mirna_pc
6463	hsa-mir-301a	MKRN3	0.35256631	mirna_pc
6464	hsa-mir-301a	PABPN1	0.437046488	mirna_pc
6465	hsa-mir-301a	RFC1	0.313718746	mirna_pc
6466	hsa-mir-301a	ATN1	0.315788692	mirna_pc
6467	hsa-mir-301a	METAP2	0.393635512	mirna_pc
6468	hsa-mir-301a	BCL7A	0.390455026	mirna_pc
6469	hsa-mir-301a	CDC23	0.300800833	mirna_pc
6470	hsa-mir-301a	WNT10A	0.423830364	mirna_pc
6471	hsa-mir-301a	TIMM16	0.303697008	mirna_pc
6472	hsa-mir-301a	MRPS28	0.365368994	mirna_pc
6473	hsa-mir-301a	DVL2	0.312566097	mirna_pc
6474	hsa-mir-301a	C7orf44	0.336491802	mirna_pc
6475	hsa-mir-301a	NUDCD2	0.334607789	mirna_pc
6476	hsa-mir-301a	ZNF286A	0.3931049	mirna_pc
6477	hsa-mir-301a	MRPL21	0.433372656	mirna_pc
6478	hsa-mir-301a	TCEA1	0.383382454	mirna_pc
6479	hsa-mir-301a	FXR1	0.402016965	mirna_pc

6480	hsa-mir-301a	DMRTA2	0.381382762	mirna_pc
6481	hsa-mir-301a	PPP1R14C	0.358045955	mirna_pc
6482	hsa-mir-301a	UPF3B	0.355938111	mirna_pc
6483	hsa-mir-301a	BTG3	0.30826009	mirna_pc
6484	hsa-mir-301a	LOC645332	0.315191149	mirna_pc
6485	hsa-mir-301a	MRPS22	0.304546328	mirna_pc
6486	hsa-mir-301a	EVC2	0.341157938	mirna_pc
6487	hsa-mir-301a	MGC72080	0.410091491	mirna_pc
6488	hsa-mir-301a	CNTROB	0.337355619	mirna_pc
6489	hsa-mir-301a	TRRAP	0.306927426	mirna_pc
6490	hsa-mir-301a	SEMA4F	0.385825622	mirna_pc
6491	hsa-mir-301a	RDH14	0.354403069	mirna_pc
6492	hsa-mir-301a	MPV17	0.301544432	mirna_pc
6493	hsa-mir-301a	ZNF286B	0.329749319	mirna_pc
6494	hsa-mir-301a	DDX20	0.394783245	mirna_pc
6495	hsa-mir-301a	GBAP1	0.304994433	mirna_pc
6496	hsa-mir-301a	UBE2N	0.343553601	mirna_pc
6497	hsa-mir-301a	CHCHD2	0.324041731	mirna_pc
6498	hsa-mir-301a	TMEM67	0.302200568	mirna_pc
6499	hsa-mir-301a	PIGX	0.388269388	mirna_pc
6500	hsa-mir-301a	HTRA2	0.308696157	mirna_pc
6501	hsa-mir-301a	CHAC2	0.327980551	mirna_pc
6502	hsa-mir-301a	ACN9	0.354026662	mirna_pc
6503	hsa-mir-301a	PFN2	0.332079154	mirna_pc
6504	hsa-mir-301a	TCERG1	0.338009038	mirna_pc
6505	hsa-mir-301a	INTU	0.445322609	mirna_pc
6506	hsa-mir-301a	UBE2V2	0.336689711	mirna_pc
6507	hsa-mir-301a	ATP6V1E2	0.325913707	mirna_pc
6508	hsa-mir-301a	GPR3	0.312644242	mirna_pc
6509	hsa-mir-301a	E2F6	0.406846816	mirna_pc
6510	hsa-mir-301a	SSSCA1	0.302715312	mirna_pc
6511	hsa-mir-301a	NOL7	0.324105131	mirna_pc
6512	hsa-mir-301a	PRPSAP2	0.314108638	mirna_pc
6513	hsa-mir-301a	CPSF4	0.476926625	mirna_pc
6514	hsa-mir-301a	ACTR6	0.359194052	mirna_pc
6515	hsa-mir-301a	SFRS13A	0.393047688	mirna_pc
6516	hsa-mir-301a	GADD45GIP1	0.322310342	mirna_pc
6517	hsa-mir-301a	SCLY	0.355015534	mirna_pc
6518	hsa-mir-301a	NT5C	0.390989392	mirna_pc
6519	hsa-mir-301a	PTMS	0.328010601	mirna_pc
6520	hsa-mir-301a	CCDC59	0.318886033	mirna_pc
6521	hsa-mir-301a	SLC4A1AP	0.422729039	mirna_pc
6522	hsa-mir-301a	TTC27	0.394311206	mirna_pc
6523	hsa-mir-301a	DHRS13	0.412019468	mirna_pc
6524	hsa-mir-301a	TFAP2C	0.315974582	mirna_pc
6525	hsa-mir-301a	RTTN	0.361325739	mirna_pc
6526	hsa-mir-301a	LOC222699	0.444110397	mirna_pc
6527	hsa-mir-301a	KRR1	0.367432291	mirna_pc
6528	hsa-mir-301a	DHX57	0.38784041	mirna_pc
6529	hsa-mir-301a	HIST1H1C	0.409887494	mirna_pc
6530	hsa-mir-301a	ALX3	0.30143694	mirna_pc
6531	hsa-mir-301a	AAAS	0.368652476	mirna_pc
6532	hsa-mir-301a	CHST14	0.301873054	mirna_pc
6533	hsa-mir-301a	SUPT7L	0.310869546	mirna_pc

6534	hsa-mir-301a	C6orf147	0.304200657	mirna_pc
6535	hsa-mir-301a	ZNF277	0.342334768	mirna_pc
6536	hsa-mir-301a	PELP1	0.373732642	mirna_pc
6537	hsa-mir-301a	THOC1	0.308912448	mirna_pc
6538	hsa-mir-301a	C11orf45	0.352730595	mirna_pc
6539	hsa-mir-301a	CCDC15	0.37667386	mirna_pc
6540	hsa-mir-301a	SFRS7	0.362933862	mirna_pc
6541	hsa-mir-301a	IFRD1	0.347147228	mirna_pc
6542	hsa-mir-301a	SARS2	0.317904567	mirna_pc
6543	hsa-mir-301a	C10orf35	0.321373977	mirna_pc
6544	hsa-mir-301a	LOC100128292	0.300827156	mirna_pc
6545	hsa-mir-301a	THYN1	0.359121045	mirna_pc
6546	hsa-mir-301a	EDAR	0.320390575	mirna_pc
6547	hsa-mir-301a	AMZ2	0.329789332	mirna_pc
6548	hsa-mir-301a	EIF2B5	0.306385091	mirna_pc
6549	hsa-mir-301a	NUP153	0.313960088	mirna_pc
6550	hsa-mir-301a	PARL	0.341390097	mirna_pc
6551	hsa-mir-301a	FAM76B	0.304982662	mirna_pc
6552	hsa-mir-301a	MRPL28	0.318325273	mirna_pc
6553	hsa-mir-301a	C2orf68	0.336838013	mirna_pc
6554	hsa-mir-301a	C17orf80	0.370899175	mirna_pc
6555	hsa-mir-301a	YWHAQ	0.304167614	mirna_pc
6556	hsa-mir-301a	ARHGEF19	0.313628209	mirna_pc
6557	hsa-mir-301a	DNAJC8	0.347321764	mirna_pc
6558	hsa-mir-301a	APLP2	0.338399923	mirna_pc
6559	hsa-mir-301a	C17orf75	0.405028255	mirna_pc
6560	hsa-mir-301a	ZBTB44	0.340410305	mirna_pc
6561	hsa-mir-301a	EXT2	0.326272057	mirna_pc
6562	hsa-mir-301a	IL28RA	0.343615009	mirna_pc
6563	hsa-mir-301a	YTHDF2	0.383231964	mirna_pc
6564	hsa-mir-301a	MRPL38	0.310923305	mirna_pc
6565	hsa-mir-301a	HSPA4	0.306747236	mirna_pc
6566	hsa-mir-301a	YBX1	0.359757562	mirna_pc
6567	hsa-mir-301a	ATPBD4	0.321165965	mirna_pc
6568	hsa-mir-301a	TCTN2	0.300307926	mirna_pc
6569	hsa-mir-301a	C14orf104	0.301809089	mirna_pc
6570	hsa-mir-301a	ZKSCAN5	0.386312716	mirna_pc
6571	hsa-mir-301a	POLR2C	0.35828831	mirna_pc
6572	hsa-mir-301a	EXTL2	0.352059332	mirna_pc
6573	hsa-mir-301a	CLEC2B	0.401067184	mirna_pc
6574	hsa-mir-301a	C12orf45	0.330333133	mirna_pc
6575	hsa-mir-301a	C2orf44	0.386575587	mirna_pc
6576	hsa-mir-301a	RING1	0.360607463	mirna_pc
6577	hsa-mir-301a	SEPHS1	0.350493741	mirna_pc
6578	hsa-mir-301a	POMGNT1	0.307495953	mirna_pc
6579	hsa-mir-301a	KDM4D	0.33987972	mirna_pc
6580	hsa-mir-301a	SUB1	0.309238523	mirna_pc
6581	hsa-mir-301a	GBAS	0.377021274	mirna_pc
6582	hsa-mir-301a	AIP	0.341105906	mirna_pc
6583	hsa-mir-301a	BOLA3	0.356418557	mirna_pc
6584	hsa-mir-301a	VASH2	0.38275194	mirna_pc
6585	hsa-mir-301a	C19orf23	0.424261672	mirna_pc
6586	hsa-mir-301a	MYL6B	0.388634636	mirna_pc
6587	hsa-mir-301a	PDCD7	0.391259724	mirna_pc

6588	hsa-mir-301a	ALKBH3	0.314844014	mirna_pc
6589	hsa-mir-301a	MED28	0.333897444	mirna_pc
6590	hsa-mir-301a	MRPS15	0.357981859	mirna_pc
6591	hsa-mir-301a	C17orf58	0.415249387	mirna_pc
6592	hsa-mir-301a	NARS2	0.378135679	mirna_pc
6593	hsa-mir-301a	ZFP1	0.348588927	mirna_pc
6594	hsa-mir-301a	EIF2B3	0.325042201	mirna_pc
6595	hsa-mir-301a	LYRM4	0.420135824	mirna_pc
6596	hsa-mir-301a	FAM133B	0.417556664	mirna_pc
6597	hsa-mir-301a	CHST11	0.373968762	mirna_pc
6598	hsa-mir-301a	BCL11A	0.381085073	mirna_pc
6599	hsa-mir-301a	C4orf27	0.327836353	mirna_pc
6600	hsa-mir-301a	USP21	0.358817512	mirna_pc
6601	hsa-mir-301a	SPDYA	0.32093246	mirna_pc
6602	hsa-mir-301a	RBM25	0.3093813	mirna_pc
6603	hsa-mir-301a	CFDP1	0.310016557	mirna_pc
6604	hsa-mir-301a	LMAN2L	0.307975411	mirna_pc
6605	hsa-mir-301a	C17orf81	0.32199276	mirna_pc
6606	hsa-mir-301a	HYLS1	0.358004536	mirna_pc
6607	hsa-mir-301a	C7orf60	0.384216386	mirna_pc
6608	hsa-mir-301a	NAP1L1	0.332605457	mirna_pc
6609	hsa-mir-301a	C1orf156	0.334523816	mirna_pc
6610	hsa-mir-301a	CNIH2	0.319617139	mirna_pc
6611	hsa-mir-301a	FLJ37453	0.314173748	mirna_pc
6612	hsa-mir-301a	IQCC	0.335879076	mirna_pc
6613	hsa-mir-301a	GATAD2B	0.341007799	mirna_pc
6614	hsa-mir-301a	HNRNPA0	0.367271282	mirna_pc
6615	hsa-mir-301a	MORN2	0.393629606	mirna_pc
6616	hsa-mir-301a	SETD6	0.317537966	mirna_pc
6617	hsa-mir-301a	RTN4RL1	0.367212664	mirna_pc
6618	hsa-mir-301a	MLLT11	0.357198862	mirna_pc
6619	hsa-mir-301a	AASDHPPT	0.314848575	mirna_pc
6620	hsa-mir-301a	CWC15	0.327086958	mirna_pc
6621	hsa-mir-301a	MYCN	0.318262487	mirna_pc
6622	hsa-mir-301a	FYTTD1	0.329672137	mirna_pc
6623	hsa-mir-301a	EFCAB7	0.350561543	mirna_pc
6624	hsa-mir-301a	MTERF	0.491402503	mirna_pc
6625	hsa-mir-301a	SBK1	0.353662942	mirna_pc
6626	hsa-mir-301a	C7orf64	0.308897148	mirna_pc
6627	hsa-mir-301a	PMF1	0.343665649	mirna_pc
6628	hsa-mir-301a	USP13	0.352043018	mirna_pc
6629	hsa-mir-301a	GALNT14	0.319969413	mirna_pc
6630	hsa-mir-301a	SLC25A33	0.410172378	mirna_pc
6631	hsa-mir-301a	C1orf144	0.316105786	mirna_pc
6632	hsa-mir-301a	TMEM18	0.360495898	mirna_pc
6633	hsa-mir-301a	ZNF394	0.305261065	mirna_pc
6634	hsa-mir-301a	C1orf52	0.328898502	mirna_pc
6635	hsa-mir-301a	GTF2H2	0.333743677	mirna_pc
6636	hsa-mir-301a	ZNF232	0.318966575	mirna_pc
6637	hsa-mir-301a	SNRNP27	0.315350966	mirna_pc
6638	hsa-mir-301a	RPAIN	0.316215697	mirna_pc
6639	hsa-mir-301a	TERF1	0.313210457	mirna_pc
6640	hsa-mir-301a	CCDC90B	0.396335312	mirna_pc
6641	hsa-mir-301a	RBBP4	0.327318457	mirna_pc

6642	hsa-mir-301a	LOC221710	0.32299466	mirna_pc
6643	hsa-mir-301a	GUSBP1	0.329446432	mirna_pc
6644	hsa-mir-301a	MRPL1	0.368880269	mirna_pc
6645	hsa-mir-301a	KIAA1586	0.304321851	mirna_pc
6646	hsa-mir-301a	API5	0.350525164	mirna_pc
6647	hsa-mir-301a	USP28	0.382944935	mirna_pc
6648	hsa-mir-301a	C2orf3	0.381585288	mirna_pc
6649	hsa-mir-301a	CHD4	0.320570777	mirna_pc
6650	hsa-mir-301a	KRIT1	0.358769321	mirna_pc
6651	hsa-mir-301a	DHRS2	0.337943014	mirna_pc
6652	hsa-mir-301a	MEAF6	0.34518678	mirna_pc
6653	hsa-mir-301a	DNAJC19	0.302411228	mirna_pc
6654	hsa-mir-301a	TMEM134	0.345056589	mirna_pc
6655	hsa-mir-301a	LRDD	0.313610729	mirna_pc
6656	hsa-mir-301a	C12orf65	0.335615731	mirna_pc
6657	hsa-mir-301a	MRPL19	0.320522168	mirna_pc
6658	hsa-mir-301a	HSF2	0.370039819	mirna_pc
6659	hsa-mir-301a	ZCCHC11	0.312971639	mirna_pc
6660	hsa-mir-301a	CETN3	0.46347156	mirna_pc
6661	hsa-mir-301a	ZCCHC9	0.326875787	mirna_pc
6662	hsa-mir-301a	ETV5	0.379988644	mirna_pc
6663	hsa-mir-301a	DDX1	0.311647705	mirna_pc
6664	hsa-mir-301a	LSM 1.00	0.358934183	mirna_pc
6665	hsa-mir-301a	DYNC1I1	0.390708395	mirna_pc
6666	hsa-mir-301a	GLI4	0.338486268	mirna_pc
6667	hsa-mir-301a	AADAT	0.310935204	mirna_pc
6668	hsa-mir-301a	IGHMBP2	0.41475503	mirna_pc
6669	hsa-mir-301a	ACP1	0.303012036	mirna_pc
6670	hsa-mir-301a	FDX1L	0.351322405	mirna_pc
6671	hsa-mir-301a	ZNF322A	0.448879399	mirna_pc
6672	hsa-mir-301a	SERF1A	0.402631155	mirna_pc
6673	hsa-mir-301a	SRP19	0.332283014	mirna_pc
6674	hsa-mir-301a	PRMT6	0.305808132	mirna_pc
6675	hsa-mir-301a	LOC401397	0.460950806	mirna_pc
6676	hsa-mir-301a	GPS2	0.303606861	mirna_pc
6677	hsa-mir-301a	C21orf119	0.311109827	mirna_pc
6678	hsa-mir-301a	PDS5A	0.30229991	mirna_pc
6679	hsa-mir-301a	C2orf28	0.348654488	mirna_pc
6680	hsa-mir-301a	H2AFY2	0.411200753	mirna_pc
6681	hsa-mir-301a	TRIL	0.330745644	mirna_pc
6682	hsa-mir-301a	HIRIP3	0.399693367	mirna_pc
6683	hsa-mir-301a	SLC6A15	0.406717759	mirna_pc
6684	hsa-mir-301a	MLF 1.00	0.315460929	mirna_pc
6685	hsa-mir-301a	SRRM1	0.310999877	mirna_pc
6686	hsa-mir-301a	AGA	0.321165908	mirna_pc
6687	hsa-mir-301a	TMEM168	0.379434669	mirna_pc
6688	hsa-mir-301a	SCHIP1	0.301689479	mirna_pc
6689	hsa-mir-301a	CCDC75	0.343337607	mirna_pc
6690	hsa-mir-301a	SEMA4C	0.328351059	mirna_pc
6691	hsa-mir-301a	EFNB3	0.311995787	mirna_pc
6692	hsa-mir-301a	MRPL53	0.381476329	mirna_pc
6693	hsa-mir-301a	DNMT3A	0.353448958	mirna_pc
6694	hsa-mir-301a	COPS3	0.31218047	mirna_pc
6695	hsa-mir-301a	NDUFS8	0.3579602	mirna_pc

6696	hsa-mir-301a	ZNF326	0.382696665	mirna_pc
6697	hsa-mir-301a	NMRAL1	0.304869835	mirna_pc
6698	hsa-mir-483	MYBL2	0.426471448	mirna_pc
6699	hsa-mir-483	BUB1B	0.345732437	mirna_pc
6700	hsa-mir-483	CDCA5	0.336210708	mirna_pc
6701	hsa-mir-483	ZNF568	0.308199486	mirna_pc
6702	hsa-mir-483	RECQL4	0.340562426	mirna_pc
6703	hsa-mir-483	PSMA7	0.352356551	mirna_pc
6704	hsa-mir-483	ADRM1	0.356683751	mirna_pc
6705	hsa-mir-483	ILF2	0.329447053	mirna_pc
6706	hsa-mir-483	CDCA7	0.335395398	mirna_pc
6707	hsa-mir-483	SESN1	0.312347163	mirna_pc
6708	hsa-mir-483	ASB1	0.314773221	mirna_pc
6709	hsa-mir-483	SNTB1	0.316436241	mirna_pc
6710	hsa-mir-483	RTKN	0.336185786	mirna_pc
6711	hsa-mir-483	TH1L	0.344205416	mirna_pc
6712	hsa-mir-483	SMOC2	0.36934101	mirna_pc
6713	hsa-mir-483	CCT3	0.418262001	mirna_pc
6714	hsa-mir-483	CSE1L	0.315246695	mirna_pc
6715	hsa-mir-483	SLC7A2	0.355486832	mirna_pc
6716	hsa-mir-483	TOMM40	0.320294537	mirna_pc
6717	hsa-mir-483	CDC25A	0.404244769	mirna_pc
6718	hsa-mir-483	SRPK1	0.468960626	mirna_pc
6719	hsa-mir-483	CHRM3	0.30215241	mirna_pc
6720	hsa-mir-483	CYB561D1	0.382951686	mirna_pc
6721	hsa-mir-483	KCNIP3	0.36814677	mirna_pc
6722	hsa-mir-483	HSPD1	0.419475399	mirna_pc
6723	hsa-mir-483	SLC5A6	0.34748139	mirna_pc
6724	hsa-mir-483	TOMM34	0.425959988	mirna_pc
6725	hsa-mir-483	GGH	0.359407832	mirna_pc
6726	hsa-mir-483	ZNF695	0.371057156	mirna_pc
6727	hsa-mir-483	SCARB1	0.396777322	mirna_pc
6728	hsa-mir-483	RQCD1	0.339516803	mirna_pc
6729	hsa-mir-483	MTCH2	0.301265734	mirna_pc
6730	hsa-mir-483	NUP160	0.3258742	mirna_pc
6731	hsa-mir-483	RPS21	0.449178326	mirna_pc
6732	hsa-mir-483	SLC1A5	0.333648371	mirna_pc
6733	hsa-mir-483	ADAMTS14	0.367401411	mirna_pc
6734	hsa-mir-483	TBC1D4	0.321946884	mirna_pc
6735	hsa-mir-483	AMIGO1	0.314303119	mirna_pc
6736	hsa-mir-483	AMPD2	0.346935782	mirna_pc
6737	hsa-mir-483	LSM14B	0.329893386	mirna_pc
6738	hsa-mir-483	L1CAM	0.572429596	mirna_pc
6739	hsa-mir-483	NCRNA00176	0.632069829	mirna_pc
6740	hsa-mir-483	FAM125B	0.344602598	mirna_pc
6741	hsa-mir-483	EPHB2	0.438276768	mirna_pc
6742	hsa-mir-483	ASCL2	0.327549532	mirna_pc
6743	hsa-mir-483	HAUS2	0.340176147	mirna_pc
6744	hsa-mir-483	HDAC11	0.534928969	mirna_pc
6745	hsa-mir-483	PPAN	0.342912867	mirna_pc
6746	hsa-mir-483	PDCD11	0.324576168	mirna_pc
6747	hsa-mir-483	ZNF703	0.406953807	mirna_pc
6748	hsa-mir-483	GYLTL1B	0.407897088	mirna_pc
6749	hsa-mir-483	MAN2A2	0.314538422	mirna_pc

6750	hsa-mir-483	HDAC4	0.379504214	mirna_pc
6751	hsa-mir-483	PCBP4	0.315078554	mirna_pc
6752	hsa-mir-483	NPHP4	0.609639592	mirna_pc
6753	hsa-mir-483	ZNF350	0.422286332	mirna_pc
6754	hsa-mir-483	C2	0.315448911	mirna_pc
6755	hsa-mir-483	BAMBI	0.321704474	mirna_pc
6756	hsa-mir-483	EEF1A1P9	0.308652912	mirna_pc
6757	hsa-mir-483	TLR9	0.386523239	mirna_pc
6758	hsa-mir-483	PTGES2	0.306040788	mirna_pc
6759	hsa-mir-483	FGFRL1	0.381376329	mirna_pc
6760	hsa-mir-483	PUSL1	0.355731582	mirna_pc
6761	hsa-mir-483	NKD2	0.328206537	mirna_pc
6762	hsa-mir-483	ZSCAN5A	0.491735943	mirna_pc
6763	hsa-mir-483	KCTD17	0.467425241	mirna_pc
6764	hsa-mir-483	AGAP1	0.307102584	mirna_pc
6765	hsa-mir-483	GDPD5	0.311454443	mirna_pc
6766	hsa-mir-483	ADAMTS17	0.332726196	mirna_pc
6767	hsa-mir-483	LRPPRC	0.387725275	mirna_pc
6768	hsa-mir-483	GPC4	0.381731249	mirna_pc
6769	hsa-mir-483	ARNT2	0.379353379	mirna_pc
6770	hsa-mir-483	MME	0.416416193	mirna_pc
6771	hsa-mir-483	PTP4A3	0.340358054	mirna_pc
6772	hsa-mir-483	LCK	0.313262233	mirna_pc
6773	hsa-mir-483	USP37	0.333632225	mirna_pc
6774	hsa-mir-483	ILKAP	0.322824095	mirna_pc
6775	hsa-mir-483	PPP4R1L	0.328948534	mirna_pc
6776	hsa-mir-483	CTNNB1	0.413076769	mirna_pc
6777	hsa-mir-483	JPH1	0.343870777	mirna_pc
6778	hsa-mir-483	ZNF486	0.34969601	mirna_pc
6779	hsa-mir-483	DDX20	0.331505045	mirna_pc
6780	hsa-mir-483	FGGY	0.52007784	mirna_pc
6781	hsa-mir-483	SLC25A10	0.345875656	mirna_pc
6782	hsa-mir-483	DEPDC7	0.368027891	mirna_pc
6783	hsa-mir-483	EHD4	0.349147022	mirna_pc
6784	hsa-mir-483	C2orf89	0.401306643	mirna_pc
6785	hsa-mir-483	GGA1	0.323048519	mirna_pc
6786	hsa-mir-483	SLC29A2	0.317188844	mirna_pc
6787	hsa-mir-483	GTPBP5	0.372287482	mirna_pc
6788	hsa-mir-483	BIN1	0.393609227	mirna_pc
6789	hsa-mir-483	CABLES2	0.311685305	mirna_pc
6790	hsa-mir-483	RPS16	0.358878258	mirna_pc
6791	hsa-mir-483	SYMPK	0.471762647	mirna_pc
6792	hsa-mir-483	BEND3	0.300182394	mirna_pc
6793	hsa-mir-483	PLEKHB1	0.324061729	mirna_pc
6794	hsa-mir-483	TRAF5	0.353025073	mirna_pc
6795	hsa-mir-483	NKD1	0.395498714	mirna_pc
6796	hsa-mir-483	KLHL25	0.516074996	mirna_pc
6797	hsa-mir-483	RBM38	0.502079637	mirna_pc
6798	hsa-mir-483	IRF2BP1	0.345259412	mirna_pc
6799	hsa-mir-483	ARL 2.00	0.303521152	mirna_pc
6800	hsa-mir-483	NAALADL1	0.417448902	mirna_pc
6801	hsa-mir-483	NUP210	0.451319066	mirna_pc
6802	hsa-mir-483	ACTR3C	0.339316792	mirna_pc
6803	hsa-mir-483	FYN	0.363132575	mirna_pc

6804	hsa-mir-483	RPL14	0.315134951	mirna_pc
6805	hsa-mir-483	LMO2	0.377880124	mirna_pc
6806	hsa-mir-483	UCP2	0.323465769	mirna_pc
6807	hsa-mir-483	PIGT	0.409348939	mirna_pc
6808	hsa-mir-483	PVR	0.303387848	mirna_pc
6809	hsa-mir-483	NT5E	0.350394068	mirna_pc
6810	hsa-mir-483	MMACHC	0.328071388	mirna_pc
6811	hsa-mir-483	HES6	0.325846593	mirna_pc
6812	hsa-mir-483	LGR5	0.593037782	mirna_pc
6813	hsa-mir-483	SAMD4B	0.351140371	mirna_pc
6814	hsa-mir-483	REPIN1	0.351819417	mirna_pc
6815	hsa-mir-483	ATAD1	0.342824052	mirna_pc
6816	hsa-mir-483	VIL1	0.552392565	mirna_pc
6817	hsa-mir-483	CDC34	0.354157068	mirna_pc
6818	hsa-mir-483	LOC148189	0.36701903	mirna_pc
6819	hsa-mir-483	SMARCC1	0.319541762	mirna_pc
6820	hsa-mir-483	ACOT9	0.472404815	mirna_pc
6821	hsa-mir-483	DMWD	0.560287914	mirna_pc
6822	hsa-mir-483	RGPD5	0.368961187	mirna_pc
6823	hsa-mir-483	CST7	0.533885748	mirna_pc
6824	hsa-mir-483	ZNF142	0.386476632	mirna_pc
6825	hsa-mir-483	DUS1L	0.30030875	mirna_pc
6826	hsa-mir-483	RAC3	0.537150936	mirna_pc
6827	hsa-mir-483	RPS12	0.340233531	mirna_pc
6828	hsa-mir-483	ATG2A	0.3834696	mirna_pc
6829	hsa-mir-483	RPL4	0.3420181	mirna_pc
6830	hsa-mir-483	GOLT1A	0.394242095	mirna_pc
6831	hsa-mir-483	RPSAP58	0.308023305	mirna_pc
6832	hsa-mir-483	UBA3	0.331984124	mirna_pc
6833	hsa-mir-483	KIAA1274	0.547988799	mirna_pc
6834	hsa-mir-483	PROX1	0.502694861	mirna_pc
6835	hsa-mir-483	STX3	0.37742455	mirna_pc
6836	hsa-mir-483	GNG4	0.33858652	mirna_pc
6837	hsa-mir-483	SPTBN5	0.461008348	mirna_pc
6838	hsa-mir-483	IL17RB	0.424262702	mirna_pc
6839	hsa-mir-483	EIF3J	0.322454005	mirna_pc
6840	hsa-mir-483	RPSA	0.332484036	mirna_pc
6841	hsa-mir-483	FASN	0.398383309	mirna_pc
6842	hsa-mir-483	PRDX4	0.303143073	mirna_pc
6843	hsa-mir-483	IGF2	0.979805152	mirna_pc
6844	hsa-mir-483	NES	0.404061479	mirna_pc
6845	hsa-mir-483	DNAJA4	0.383445333	mirna_pc
6846	hsa-mir-483	DBNDD2	0.335411164	mirna_pc
6847	hsa-mir-483	INHBB	0.525578123	mirna_pc
6848	hsa-mir-483	APOO	0.385767634	mirna_pc
6849	hsa-mir-483	CD46	0.336215575	mirna_pc
6850	hsa-mir-483	ZNF90	0.435188694	mirna_pc
6851	hsa-mir-483	SLC2A8	0.331814871	mirna_pc
6852	hsa-mir-483	ANKRD52	0.315999511	mirna_pc
6853	hsa-mir-99b	HIF3A	0.358435022	mirna_pc
6854	hsa-mir-99b	ZNF415	0.409894914	mirna_pc
6855	hsa-mir-99b	KIF2C	0.360660018	mirna_pc
6856	hsa-mir-99b	NCAPG	0.363064622	mirna_pc
6857	hsa-mir-99b	CCNA2	0.329531232	mirna_pc

6858	hsa-mir-99b	NUP62	0.307499593	mirna_pc
6859	hsa-mir-99b	MND1	0.39076202	mirna_pc
6860	hsa-mir-99b	RAD54L	0.34738688	mirna_pc
6861	hsa-mir-99b	MAD2L1	0.363592378	mirna_pc
6862	hsa-mir-99b	TIMELESS	0.407075346	mirna_pc
6863	hsa-mir-99b	TROAP	0.314047821	mirna_pc
6864	hsa-mir-99b	ZNF470	0.322690084	mirna_pc
6865	hsa-mir-99b	TACC3	0.374803528	mirna_pc
6866	hsa-mir-99b	MYEF2	0.317037633	mirna_pc
6867	hsa-mir-99b	XPO1	0.334233812	mirna_pc
6868	hsa-mir-99b	STMN1	0.318506346	mirna_pc
6869	hsa-mir-99b	ZNF542	0.446344602	mirna_pc
6870	hsa-mir-99b	TUBB	0.373572594	mirna_pc
6871	hsa-mir-99b	KNTC1	0.330691822	mirna_pc
6872	hsa-mir-99b	PLK4	0.359333056	mirna_pc
6873	hsa-mir-99b	ZNF568	0.306324049	mirna_pc
6874	hsa-mir-99b	LIG1	0.302642266	mirna_pc
6875	hsa-mir-99b	C1orf112	0.348916337	mirna_pc
6876	hsa-mir-99b	APBB1	0.307284483	mirna_pc
6877	hsa-mir-99b	KIAA0495	0.320397298	mirna_pc
6878	hsa-mir-99b	RAE1	0.319076589	mirna_pc
6879	hsa-mir-99b	NEIL3	0.438727539	mirna_pc
6880	hsa-mir-99b	POLD1	0.444093073	mirna_pc
6881	hsa-mir-99b	ZNF229	0.403711064	mirna_pc
6882	hsa-mir-99b	ZNF528	0.357301247	mirna_pc
6883	hsa-mir-99b	TUBA1B	0.300521935	mirna_pc
6884	hsa-mir-99b	MCM6	0.324268573	mirna_pc
6885	hsa-mir-99b	TMEM25	0.304958691	mirna_pc
6886	hsa-mir-99b	NFKBIL2	0.360968039	mirna_pc
6887	hsa-mir-99b	FUS	0.303722663	mirna_pc
6888	hsa-mir-99b	CENPO	0.372464832	mirna_pc
6889	hsa-mir-99b	C19orf48	0.302662291	mirna_pc
6890	hsa-mir-99b	HMGB2	0.33076983	mirna_pc
6891	hsa-mir-99b	CDKN1C	0.4129528	mirna_pc
6892	hsa-mir-99b	C5	0.407255128	mirna_pc
6893	hsa-mir-99b	PRMT1	0.310840514	mirna_pc
6894	hsa-mir-99b	CAD	0.335376756	mirna_pc
6895	hsa-mir-99b	ZNF331	0.318764314	mirna_pc
6896	hsa-mir-99b	CDK2	0.412995035	mirna_pc
6897	hsa-mir-99b	CDK4	0.366654065	mirna_pc
6898	hsa-mir-99b	GPRASP2	0.333229958	mirna_pc
6899	hsa-mir-99b	PRIM1	0.379212636	mirna_pc
6900	hsa-mir-99b	CAND2	0.31340464	mirna_pc
6901	hsa-mir-99b	MSH2	0.41442068	mirna_pc
6902	hsa-mir-99b	SALL4	0.410158832	mirna_pc
6903	hsa-mir-99b	SF3B3	0.323260317	mirna_pc
6904	hsa-mir-99b	CDC7	0.309418642	mirna_pc
6905	hsa-mir-99b	ZNF439	0.317575107	mirna_pc
6906	hsa-mir-99b	ZNF853	0.404562246	mirna_pc
6907	hsa-mir-99b	FBXO5	0.319749131	mirna_pc
6908	hsa-mir-99b	MDK	0.300871188	mirna_pc
6909	hsa-mir-99b	H2AFZ	0.347630329	mirna_pc
6910	hsa-mir-99b	ZNF649	0.370111513	mirna_pc
6911	hsa-mir-99b	CCDC106	0.364771121	mirna_pc

6912	hsa-mir-99b	GNPDA1	0.343857687	mirna_pc
6913	hsa-mir-99b	TMEM201	0.344401362	mirna_pc
6914	hsa-mir-99b	PSRC1	0.355966446	mirna_pc
6915	hsa-mir-99b	MEGF8	0.403179793	mirna_pc
6916	hsa-mir-99b	ZNF329	0.38024864	mirna_pc
6917	hsa-mir-99b	MPP2	0.347079097	mirna_pc
6918	hsa-mir-99b	GPR19	0.394177929	mirna_pc
6919	hsa-mir-99b	ZNF175	0.381129669	mirna_pc
6920	hsa-mir-99b	PPAT	0.346615901	mirna_pc
6921	hsa-mir-99b	LOC100128191	0.461102113	mirna_pc
6922	hsa-mir-99b	C20orf3	0.321523409	mirna_pc
6923	hsa-mir-99b	CDH24	0.3259382	mirna_pc
6924	hsa-mir-99b	SKP2	0.306024001	mirna_pc
6925	hsa-mir-99b	SMARCD1	0.349602343	mirna_pc
6926	hsa-mir-99b	C4orf46	0.40111648	mirna_pc
6927	hsa-mir-99b	FZD2	0.433865737	mirna_pc
6928	hsa-mir-99b	TBC1D17	0.34657456	mirna_pc
6929	hsa-mir-99b	POLR2D	0.352843104	mirna_pc
6930	hsa-mir-99b	FTSJ2	0.314588013	mirna_pc
6931	hsa-mir-99b	KHDRBS1	0.30878858	mirna_pc
6932	hsa-mir-99b	TUB	0.35790254	mirna_pc
6933	hsa-mir-99b	MTL 5.00	0.389789107	mirna_pc
6934	hsa-mir-99b	SARDH	0.306792381	mirna_pc
6935	hsa-mir-99b	TMEM194A	0.396663526	mirna_pc
6936	hsa-mir-99b	SLC39A10	0.394186848	mirna_pc
6937	hsa-mir-99b	NASP	0.380383291	mirna_pc
6938	hsa-mir-99b	LOC100130093	0.382749028	mirna_pc
6939	hsa-mir-99b	SCARB1	0.36026602	mirna_pc
6940	hsa-mir-99b	LARP6	0.30672181	mirna_pc
6941	hsa-mir-99b	HIST1H2BK	0.303040731	mirna_pc
6942	hsa-mir-99b	HOXA9	0.344681795	mirna_pc
6943	hsa-mir-99b	HNRNPD	0.312387662	mirna_pc
6944	hsa-mir-99b	WDR19	0.332533917	mirna_pc
6945	hsa-mir-99b	TFR2	0.347548688	mirna_pc
6946	hsa-mir-99b	PHC1	0.368363077	mirna_pc
6947	hsa-mir-99b	LTBP3	0.303364477	mirna_pc
6948	hsa-mir-99b	APOE	0.360845414	mirna_pc
6949	hsa-mir-99b	DUSP9	0.546822569	mirna_pc
6950	hsa-mir-99b	ULK2	0.316890947	mirna_pc
6951	hsa-mir-99b	KIAA0406	0.31116967	mirna_pc
6952	hsa-mir-99b	MOV10	0.400069102	mirna_pc
6953	hsa-mir-99b	USP22	0.310425763	mirna_pc
6954	hsa-mir-99b	C4orf41	0.314475054	mirna_pc
6955	hsa-mir-99b	FAM168B	0.304687003	mirna_pc
6956	hsa-mir-99b	ZNF615	0.328759665	mirna_pc
6957	hsa-mir-99b	KPTN	0.401270563	mirna_pc
6958	hsa-mir-99b	ZNF221	0.382101146	mirna_pc
6959	hsa-mir-99b	EHMT2	0.310725051	mirna_pc
6960	hsa-mir-99b	UBAP2L	0.360175464	mirna_pc
6961	hsa-mir-99b	MEIS2	0.309687398	mirna_pc
6962	hsa-mir-99b	PRAME	0.59760461	mirna_pc
6963	hsa-mir-99b	FBXW8	0.316580337	mirna_pc
6964	hsa-mir-99b	AZI1	0.323993113	mirna_pc
6965	hsa-mir-99b	IPO9	0.3831152	mirna_pc

6966	hsa-mir-99b	SART3	0.35407017	mirna_pc
6967	hsa-mir-99b	ZNF549	0.328658279	mirna_pc
6968	hsa-mir-99b	CSRP2BP	0.337110748	mirna_pc
6969	hsa-mir-99b	RBPJ	0.312035416	mirna_pc
6970	hsa-mir-99b	ZNF704	0.304726163	mirna_pc
6971	hsa-mir-99b	GAR1	0.35335044	mirna_pc
6972	hsa-mir-99b	CPXM1	0.321403335	mirna_pc
6973	hsa-mir-99b	WHSC1	0.373764966	mirna_pc
6974	hsa-mir-99b	ZNF134	0.453765139	mirna_pc
6975	hsa-mir-99b	SAE1	0.524282978	mirna_pc
6976	hsa-mir-99b	C19orf47	0.303912248	mirna_pc
6977	hsa-mir-99b	C4orf43	0.354182353	mirna_pc
6978	hsa-mir-99b	RAD51L3	0.423345317	mirna_pc
6979	hsa-mir-99b	METTL14	0.394096131	mirna_pc
6980	hsa-mir-99b	PLEKHG4	0.322146086	mirna_pc
6981	hsa-mir-99b	CNOT3	0.310532224	mirna_pc
6982	hsa-mir-99b	CD83	0.371303412	mirna_pc
6983	hsa-mir-99b	ZNF788	0.379220207	mirna_pc
6984	hsa-mir-99b	ITIH4	0.35620396	mirna_pc
6985	hsa-mir-99b	AS3MT	0.321442497	mirna_pc
6986	hsa-mir-99b	CADM1	0.46516525	mirna_pc
6987	hsa-mir-99b	CPAMD8	0.331838485	mirna_pc
6988	hsa-mir-99b	SALL2	0.522596221	mirna_pc
6989	hsa-mir-99b	SLC39A1	0.349236382	mirna_pc
6990	hsa-mir-99b	MSH6	0.403544504	mirna_pc
6991	hsa-mir-99b	LEF1	0.333178087	mirna_pc
6992	hsa-mir-99b	FAM161A	0.328569346	mirna_pc
6993	hsa-mir-99b	FAM193A	0.345945731	mirna_pc
6994	hsa-mir-99b	KREMEN2	0.376938901	mirna_pc
6995	hsa-mir-99b	ZNF606	0.390633211	mirna_pc
6996	hsa-mir-99b	SMARCA1	0.564800671	mirna_pc
6997	hsa-mir-99b	GPAM	0.431722088	mirna_pc
6998	hsa-mir-99b	MCM3AP	0.326826946	mirna_pc
6999	hsa-mir-99b	OSBPL9	0.355610222	mirna_pc
7000	hsa-mir-99b	MEX3A	0.461104759	mirna_pc
7001	hsa-mir-99b	SDC2	0.334209033	mirna_pc
7002	hsa-mir-99b	PDCD11	0.301254362	mirna_pc
7003	hsa-mir-99b	TRIM28	0.322098829	mirna_pc
7004	hsa-mir-99b	TIFA	0.31237345	mirna_pc
7005	hsa-mir-99b	ZNF444	0.336114347	mirna_pc
7006	hsa-mir-99b	CCDC28B	0.343260018	mirna_pc
7007	hsa-mir-99b	UNC119B	0.352969841	mirna_pc
7008	hsa-mir-99b	CBX1	0.32740509	mirna_pc
7009	hsa-mir-99b	ZNF160	0.357874241	mirna_pc
7010	hsa-mir-99b	EVC	0.30527551	mirna_pc
7011	hsa-mir-99b	ZBTB12	0.365823755	mirna_pc
7012	hsa-mir-99b	TGIF2	0.404605262	mirna_pc
7013	hsa-mir-99b	SLC26A1	0.306132328	mirna_pc
7014	hsa-mir-99b	PRPF31	0.373818999	mirna_pc
7015	hsa-mir-99b	SENP1	0.318483933	mirna_pc
7016	hsa-mir-99b	RCN2	0.340718657	mirna_pc
7017	hsa-mir-99b	TARBP2	0.319619422	mirna_pc
7018	hsa-mir-99b	POU6F2	0.3298693	mirna_pc
7019	hsa-mir-99b	ZNF256	0.378406411	mirna_pc

7020	hsa-mir-99b	C11orf74	0.328554441	mirna_pc
7021	hsa-mir-99b	USP30	0.343507309	mirna_pc
7022	hsa-mir-99b	POLE	0.337198792	mirna_pc
7023	hsa-mir-99b	ZKSCAN2	0.491928778	mirna_pc
7024	hsa-mir-99b	ZDBF2	0.334857443	mirna_pc
7025	hsa-mir-99b	ERF	0.478386105	mirna_pc
7026	hsa-mir-99b	RTN4RL2	0.333586884	mirna_pc
7027	hsa-mir-99b	EXOSC9	0.317070795	mirna_pc
7028	hsa-mir-99b	UNG	0.344168023	mirna_pc
7029	hsa-mir-99b	RBMX	0.305061187	mirna_pc
7030	hsa-mir-99b	OBSL1	0.409507382	mirna_pc
7031	hsa-mir-99b	GPC3	0.512313512	mirna_pc
7032	hsa-mir-99b	QPCTL	0.324285753	mirna_pc
7033	hsa-mir-99b	KIAA1614	0.382206019	mirna_pc
7034	hsa-mir-99b	SNX25	0.326714977	mirna_pc
7035	hsa-mir-99b	MORN4	0.333740333	mirna_pc
7036	hsa-mir-99b	GPN3	0.329510564	mirna_pc
7037	hsa-mir-99b	ASB3	0.326707679	mirna_pc
7038	hsa-mir-99b	IGSF9	0.330336033	mirna_pc
7039	hsa-mir-99b	LOC440905	0.316823651	mirna_pc
7040	hsa-mir-99b	DNAJC14	0.302326633	mirna_pc
7041	hsa-mir-99b	GTF3C3	0.325634822	mirna_pc
7042	hsa-mir-99b	KDM2B	0.359769264	mirna_pc
7043	hsa-mir-99b	NAT14	0.384955427	mirna_pc
7044	hsa-mir-99b	KIAA1310	0.344992898	mirna_pc
7045	hsa-mir-99b	LYPD1	0.429125027	mirna_pc
7046	hsa-mir-99b	SOX12	0.401915035	mirna_pc
7047	hsa-mir-99b	ZNF234	0.315498284	mirna_pc
7048	hsa-mir-99b	ZNF230	0.380433925	mirna_pc
7049	hsa-mir-99b	ITPRIPL1	0.355960424	mirna_pc
7050	hsa-mir-99b	DMAP1	0.301077928	mirna_pc
7051	hsa-mir-99b	C12orf76	0.323350451	mirna_pc
7052	hsa-mir-99b	BBS5	0.303375809	mirna_pc
7053	hsa-mir-99b	C20orf177	0.375870831	mirna_pc
7054	hsa-mir-99b	ATXN7L2	0.336254544	mirna_pc
7055	hsa-mir-99b	ZNF813	0.369915204	mirna_pc
7056	hsa-mir-99b	RFC1	0.386207797	mirna_pc
7057	hsa-mir-99b	POTEF	0.340696621	mirna_pc
7058	hsa-mir-99b	ANKRD6	0.440827684	mirna_pc
7059	hsa-mir-99b	KIAA0090	0.356284426	mirna_pc
7060	hsa-mir-99b	BCL7A	0.478144529	mirna_pc
7061	hsa-mir-99b	RNFT2	0.322158792	mirna_pc
7062	hsa-mir-99b	LAMA1	0.401546222	mirna_pc
7063	hsa-mir-99b	DVL2	0.301792391	mirna_pc
7064	hsa-mir-99b	C7orf44	0.32942203	mirna_pc
7065	hsa-mir-99b	TRPA1	0.302834816	mirna_pc
7066	hsa-mir-99b	ZBTB49	0.334657891	mirna_pc
7067	hsa-mir-99b	TMEM98	0.309509579	mirna_pc
7068	hsa-mir-99b	ZNF286A	0.382783146	mirna_pc
7069	hsa-mir-99b	FAM171A2	0.369292866	mirna_pc
7070	hsa-mir-99b	KCNC3	0.357759061	mirna_pc
7071	hsa-mir-99b	KIAA0922	0.319922394	mirna_pc
7072	hsa-mir-99b	MRPL21	0.300675089	mirna_pc
7073	hsa-mir-99b	RASSF8	0.3058479	mirna_pc

7074	hsa-mir-99b	MINPP1	0.301049383	mirna_pc
7075	hsa-mir-99b	ZNF362	0.403258568	mirna_pc
7076	hsa-mir-99b	CHST10	0.34021738	mirna_pc
7077	hsa-mir-99b	TTN	0.303054668	mirna_pc
7078	hsa-mir-99b	TBX5	0.356208704	mirna_pc
7079	hsa-mir-99b	EVC2	0.41033298	mirna_pc
7080	hsa-mir-99b	LZTS2	0.329851168	mirna_pc
7081	hsa-mir-99b	PRR3	0.425754652	mirna_pc
7082	hsa-mir-99b	CIC	0.368366854	mirna_pc
7083	hsa-mir-99b	SEMA4F	0.397654342	mirna_pc
7084	hsa-mir-99b	CHD3	0.313382776	mirna_pc
7085	hsa-mir-99b	ZNF286B	0.347114019	mirna_pc
7086	hsa-mir-99b	DDX20	0.341850989	mirna_pc
7087	hsa-mir-99b	ZDHHC17	0.315701572	mirna_pc
7088	hsa-mir-99b	ZNF599	0.329804453	mirna_pc
7089	hsa-mir-99b	FKRP	0.433595949	mirna_pc
7090	hsa-mir-99b	GPR3	0.33644462	mirna_pc
7091	hsa-mir-99b	E2F6	0.454128305	mirna_pc
7092	hsa-mir-99b	ZNF833	0.33524865	mirna_pc
7093	hsa-mir-99b	ZNF222	0.460177521	mirna_pc
7094	hsa-mir-99b	HUNK	0.566296382	mirna_pc
7095	hsa-mir-99b	SCLY	0.316593752	mirna_pc
7096	hsa-mir-99b	GPR125	0.512688857	mirna_pc
7097	hsa-mir-99b	FIZ1	0.386849021	mirna_pc
7098	hsa-mir-99b	LAMA5	0.318916414	mirna_pc
7099	hsa-mir-99b	TFAM	0.329756286	mirna_pc
7100	hsa-mir-99b	ADH5	0.316383793	mirna_pc
7101	hsa-mir-99b	LDLRAD3	0.307317917	mirna_pc
7102	hsa-mir-99b	TTC27	0.306348177	mirna_pc
7103	hsa-mir-99b	HOXA5	0.326041028	mirna_pc
7104	hsa-mir-99b	DHRS13	0.430625469	mirna_pc
7105	hsa-mir-99b	SLC9A5	0.344605919	mirna_pc
7106	hsa-mir-99b	LOC222699	0.407290011	mirna_pc
7107	hsa-mir-99b	DHX57	0.391194551	mirna_pc
7108	hsa-mir-99b	ALX3	0.396310662	mirna_pc
7109	hsa-mir-99b	CHST14	0.381475589	mirna_pc
7110	hsa-mir-99b	CLSTN3	0.394063707	mirna_pc
7111	hsa-mir-99b	ACVR2B	0.44145606	mirna_pc
7112	hsa-mir-99b	NLGN4X	0.40086175	mirna_pc
7113	hsa-mir-99b	SATB2	0.344707362	mirna_pc
7114	hsa-mir-99b	LIG3	0.359901928	mirna_pc
7115	hsa-mir-99b	CABLES2	0.321698814	mirna_pc
7116	hsa-mir-99b	HOXA3	0.305533345	mirna_pc
7117	hsa-mir-99b	PELP1	0.315498125	mirna_pc
7118	hsa-mir-99b	ZNF773	0.416018271	mirna_pc
7119	hsa-mir-99b	ZNF512B	0.333524158	mirna_pc
7120	hsa-mir-99b	BCORL1	0.326392398	mirna_pc
7121	hsa-mir-99b	SERPINF2	0.470091399	mirna_pc
7122	hsa-mir-99b	CA5BP	0.343572253	mirna_pc
7123	hsa-mir-99b	MAMSTR	0.317732769	mirna_pc
7124	hsa-mir-99b	TMEM136	0.300889176	mirna_pc
7125	hsa-mir-99b	VPS33A	0.341455681	mirna_pc
7126	hsa-mir-99b	C10orf35	0.316854377	mirna_pc
7127	hsa-mir-99b	GXYLT2	0.322771942	mirna_pc

7128	hsa-mir-99b	EDAR	0.333218648	mirna_pc
7129	hsa-mir-99b	IFT81	0.345874752	mirna_pc
7130	hsa-mir-99b	ZNF304	0.347857446	mirna_pc
7131	hsa-mir-99b	LOXL3	0.335095977	mirna_pc
7132	hsa-mir-99b	PTOV1	0.335144647	mirna_pc
7133	hsa-mir-99b	C17orf80	0.305364769	mirna_pc
7134	hsa-mir-99b	ATF5	0.377231002	mirna_pc
7135	hsa-mir-99b	IRF2BP1	0.303714268	mirna_pc
7136	hsa-mir-99b	POLR3B	0.334989572	mirna_pc
7137	hsa-mir-99b	DCTD	0.370043964	mirna_pc
7138	hsa-mir-99b	ZNF180	0.374339795	mirna_pc
7139	hsa-mir-99b	ANAPC4	0.344797937	mirna_pc
7140	hsa-mir-99b	ZNF235	0.310490295	mirna_pc
7141	hsa-mir-99b	FUZ	0.439008223	mirna_pc
7142	hsa-mir-99b	ZNF771	0.34023051	mirna_pc
7143	hsa-mir-99b	ZNF45	0.465644845	mirna_pc
7144	hsa-mir-99b	PRUNE	0.310477833	mirna_pc
7145	hsa-mir-99b	NCRNA00085	0.54517107	mirna_pc
7146	hsa-mir-99b	TCTN2	0.447186081	mirna_pc
7147	hsa-mir-99b	TOP3A	0.341123959	mirna_pc
7148	hsa-mir-99b	EXTL2	0.490083795	mirna_pc
7149	hsa-mir-99b	UBE2M	0.311392539	mirna_pc
7150	hsa-mir-99b	DCP2	0.312376948	mirna_pc
7151	hsa-mir-99b	ANXA9	0.350736469	mirna_pc
7152	hsa-mir-99b	C2orf44	0.316951256	mirna_pc
7153	hsa-mir-99b	SEPHS1	0.336411723	mirna_pc
7154	hsa-mir-99b	SHISA2	0.303898286	mirna_pc
7155	hsa-mir-99b	CBX5	0.381205248	mirna_pc
7156	hsa-mir-99b	TMEM9	0.340389769	mirna_pc
7157	hsa-mir-99b	AGBL5	0.458182015	mirna_pc
7158	hsa-mir-99b	VASH2	0.385912206	mirna_pc
7159	hsa-mir-99b	ISYNA1	0.407566259	mirna_pc
7160	hsa-mir-99b	MYL6B	0.444333156	mirna_pc
7161	hsa-mir-99b	ZNF498	0.351686589	mirna_pc
7162	hsa-mir-99b	DTX3	0.496773934	mirna_pc
7163	hsa-mir-99b	ZNF517	0.318470647	mirna_pc
7164	hsa-mir-99b	CD200	0.317065729	mirna_pc
7165	hsa-mir-99b	ZNF233	0.423190817	mirna_pc
7166	hsa-mir-99b	TEAD2	0.454274832	mirna_pc
7167	hsa-mir-99b	INTS1	0.309660348	mirna_pc
7168	hsa-mir-99b	MED28	0.50514238	mirna_pc
7169	hsa-mir-99b	GPX7	0.334739941	mirna_pc
7170	hsa-mir-99b	NME4	0.357415783	mirna_pc
7171	hsa-mir-99b	CHST11	0.370967064	mirna_pc
7172	hsa-mir-99b	FBXO17	0.448553072	mirna_pc
7173	hsa-mir-99b	ZNF496	0.361571672	mirna_pc
7174	hsa-mir-99b	C4orf27	0.477235113	mirna_pc
7175	hsa-mir-99b	ZNF512	0.350591378	mirna_pc
7176	hsa-mir-99b	ZNF473	0.334325874	mirna_pc
7177	hsa-mir-99b	SIX5	0.403941102	mirna_pc
7178	hsa-mir-99b	ZNF544	0.376278016	mirna_pc
7179	hsa-mir-99b	USP21	0.331168381	mirna_pc
7180	hsa-mir-99b	PRR12	0.369443277	mirna_pc
7181	hsa-mir-99b	ZCCHC4	0.390349563	mirna_pc

7182	hsa-mir-99b	PIH1D1	0.345782762	mirna_pc
7183	hsa-mir-99b	ZMYM4	0.436945023	mirna_pc
7184	hsa-mir-99b	DPF2	0.307680301	mirna_pc
7185	hsa-mir-99b	LMAN2L	0.328561304	mirna_pc
7186	hsa-mir-99b	TCF7L1	0.396279274	mirna_pc
7187	hsa-mir-99b	PPP5C	0.495956082	mirna_pc
7188	hsa-mir-99b	ZNF227	0.445781085	mirna_pc
7189	hsa-mir-99b	HIC2	0.332894753	mirna_pc
7190	hsa-mir-99b	THAP9	0.331227724	mirna_pc
7191	hsa-mir-99b	LRFN1	0.438066025	mirna_pc
7192	hsa-mir-99b	HNRNPH3	0.327330788	mirna_pc
7193	hsa-mir-99b	NAP1L1	0.346346212	mirna_pc
7194	hsa-mir-99b	C1orf156	0.302314604	mirna_pc
7195	hsa-mir-99b	SLC26A11	0.443531204	mirna_pc
7196	hsa-mir-99b	NLRP2	0.379634981	mirna_pc
7197	hsa-mir-99b	NSL1	0.331718469	mirna_pc
7198	hsa-mir-99b	C1orf216	0.382796258	mirna_pc
7199	hsa-mir-99b	CNIH2	0.38714707	mirna_pc
7200	hsa-mir-99b	LOC400657	0.356785293	mirna_pc
7201	hsa-mir-99b	DDX19A	0.388935499	mirna_pc
7202	hsa-mir-99b	ALG10B	0.380121461	mirna_pc
7203	hsa-mir-99b	MBNL3	0.358488197	mirna_pc
7204	hsa-mir-99b	LRRC37B	0.370603635	mirna_pc
7205	hsa-mir-99b	TSPYL3	0.429143981	mirna_pc
7206	hsa-mir-99b	ZNF281	0.321386621	mirna_pc
7207	hsa-mir-99b	FAM71E1	0.408438073	mirna_pc
7208	hsa-mir-99b	PSTK	0.311207078	mirna_pc
7209	hsa-mir-99b	PEG10	0.384420705	mirna_pc
7210	hsa-mir-99b	SYCP2	0.32294964	mirna_pc
7211	hsa-mir-99b	MYCN	0.373914176	mirna_pc
7212	hsa-mir-99b	C12orf24	0.308925485	mirna_pc
7213	hsa-mir-99b	DCAF16	0.484998832	mirna_pc
7214	hsa-mir-99b	CCDC102A	0.361877124	mirna_pc
7215	hsa-mir-99b	SBK1	0.355601139	mirna_pc
7216	hsa-mir-99b	PABPC4L	0.462249087	mirna_pc
7217	hsa-mir-99b	ABCA11P	0.358233915	mirna_pc
7218	hsa-mir-99b	C21orf67	0.372895456	mirna_pc
7219	hsa-mir-99b	USP13	0.383208195	mirna_pc
7220	hsa-mir-99b	HAUS3	0.311828233	mirna_pc
7221	hsa-mir-99b	RASL10B	0.446563739	mirna_pc
7222	hsa-mir-99b	ERLIN1	0.300612142	mirna_pc
7223	hsa-mir-99b	C16orf46	0.365479379	mirna_pc
7224	hsa-mir-99b	MSI1	0.304750742	mirna_pc
7225	hsa-mir-99b	ZNF391	0.381736403	mirna_pc
7226	hsa-mir-99b	NUDT17	0.449498665	mirna_pc
7227	hsa-mir-99b	TTC39C	0.304503518	mirna_pc
7228	hsa-mir-99b	GXYLT1	0.38940822	mirna_pc
7229	hsa-mir-99b	N4BP2	0.367954356	mirna_pc
7230	hsa-mir-99b	ZNF740	0.378127807	mirna_pc
7231	hsa-mir-99b	EP400	0.302020355	mirna_pc
7232	hsa-mir-99b	TRO	0.484581375	mirna_pc
7233	hsa-mir-99b	PACRGL	0.365322783	mirna_pc
7234	hsa-mir-99b	LAMC1	0.422103125	mirna_pc
7235	hsa-mir-99b	AKT2	0.304424089	mirna_pc

7236	hsa-mir-99b	PBX2	0.531569582	mirna_pc
7237	hsa-mir-99b	NCKAP5L	0.368910733	mirna_pc
7238	hsa-mir-99b	ZNF232	0.356871525	mirna_pc
7239	hsa-mir-99b	ZBTB39	0.413994692	mirna_pc
7240	hsa-mir-99b	ENO3	0.305222165	mirna_pc
7241	hsa-mir-99b	C11orf95	0.351036507	mirna_pc
7242	hsa-mir-99b	HSD17B14	0.416058614	mirna_pc
7243	hsa-mir-99b	RBBP4	0.363330371	mirna_pc
7244	hsa-mir-99b	ODZ3	0.315317941	mirna_pc
7245	hsa-mir-99b	SPATS2	0.333381048	mirna_pc
7246	hsa-mir-99b	ZNF618	0.410585615	mirna_pc
7247	hsa-mir-99b	PLGLB2	0.367324099	mirna_pc
7248	hsa-mir-99b	BRSK1	0.497962524	mirna_pc
7249	hsa-mir-99b	PCDHB2	0.303867658	mirna_pc
7250	hsa-mir-99b	ZNF22	0.380616128	mirna_pc
7251	hsa-mir-99b	CHD4	0.333693518	mirna_pc
7252	hsa-mir-99b	ZNF550	0.390029539	mirna_pc
7253	hsa-mir-99b	C19orf63	0.321022549	mirna_pc
7254	hsa-mir-99b	CCDC111	0.351439745	mirna_pc
7255	hsa-mir-99b	LRDD	0.328757384	mirna_pc
7256	hsa-mir-99b	LOC645166	0.347830212	mirna_pc
7257	hsa-mir-99b	PDGFA	0.305560317	mirna_pc
7258	hsa-mir-99b	C17orf79	0.312290702	mirna_pc
7259	hsa-mir-99b	KDELC2	0.342220067	mirna_pc
7260	hsa-mir-99b	RAB3IL1	0.322763466	mirna_pc
7261	hsa-mir-99b	C4orf42	0.30607873	mirna_pc
7262	hsa-mir-99b	SIKE1	0.311097438	mirna_pc
7263	hsa-mir-99b	FAM171B	0.362094797	mirna_pc
7264	hsa-mir-99b	ZNF594	0.328396525	mirna_pc
7265	hsa-mir-99b	ZCCHC11	0.342112737	mirna_pc
7266	hsa-mir-99b	ASXL1	0.394011135	mirna_pc
7267	hsa-mir-99b	BCAM	0.338626318	mirna_pc
7268	hsa-mir-99b	ZCCHC3	0.369999858	mirna_pc
7269	hsa-mir-99b	KLHL29	0.420241912	mirna_pc
7270	hsa-mir-99b	ETV5	0.384987765	mirna_pc
7271	hsa-mir-99b	COL9A3	0.311023557	mirna_pc
7272	hsa-mir-99b	NLGN2	0.310714178	mirna_pc
7273	hsa-mir-99b	NUCKS1	0.306273109	mirna_pc
7274	hsa-mir-99b	KIAA1430	0.373893426	mirna_pc
7275	hsa-mir-99b	TCTN1	0.4101015	mirna_pc
7276	hsa-mir-99b	ZNF419	0.426511062	mirna_pc
7277	hsa-mir-99b	AADAT	0.415170889	mirna_pc
7278	hsa-mir-99b	IGHMBP2	0.400406415	mirna_pc
7279	hsa-mir-99b	CTXN1	0.308183931	mirna_pc
7280	hsa-mir-99b	FRAS1	0.442465872	mirna_pc
7281	hsa-mir-99b	OBSCN	0.346918864	mirna_pc
7282	hsa-mir-99b	SERF1A	0.315073046	mirna_pc
7283	hsa-mir-99b	LOC80054	0.424675767	mirna_pc
7284	hsa-mir-99b	ZNF772	0.414471133	mirna_pc
7285	hsa-mir-99b	QPRT	0.31891539	mirna_pc
7286	hsa-mir-99b	PRMT6	0.328720978	mirna_pc
7287	hsa-mir-99b	ZMYM3	0.444744118	mirna_pc
7288	hsa-mir-99b	KCND1	0.376248298	mirna_pc
7289	hsa-mir-99b	HSDL1	0.325862627	mirna_pc

7290	hsa-mir-99b	AASDH	0.300735736	mirna_pc
7291	hsa-mir-99b	ZNF530	0.481938691	mirna_pc
7292	hsa-mir-99b	H2AFY2	0.356796394	mirna_pc
7293	hsa-mir-99b	BAI2	0.362038602	mirna_pc
7294	hsa-mir-99b	TRIL	0.436139434	mirna_pc
7295	hsa-mir-99b	C1orf103	0.38563191	mirna_pc
7296	hsa-mir-99b	AGT	0.369118549	mirna_pc
7297	hsa-mir-99b	HSPC157	0.31009882	mirna_pc
7298	hsa-mir-99b	MPP6	0.388528477	mirna_pc
7299	hsa-mir-99b	BOD1	0.305574796	mirna_pc
7300	hsa-mir-99b	MTSS1L	0.309590013	mirna_pc
7301	hsa-mir-99b	SMARCAD1	0.363665997	mirna_pc
7302	hsa-mir-99b	SCHIP1	0.300460672	mirna_pc
7303	hsa-mir-99b	LOC642852	0.315328102	mirna_pc
7304	hsa-mir-99b	SV2A	0.456519942	mirna_pc
7305	hsa-mir-99b	NT5M	0.321702291	mirna_pc
7306	hsa-mir-99b	SEMA4C	0.333666774	mirna_pc
7307	hsa-mir-99b	THOC5	0.328243413	mirna_pc
7308	hsa-mir-99b	EFNB3	0.415884746	mirna_pc
7309	hsa-mir-99b	KIF7	0.366181759	mirna_pc
7310	hsa-mir-99b	NRSN2	0.407686119	mirna_pc
7311	hsa-mir-99b	DNMT3A	0.483596807	mirna_pc
7312	hsa-mir-99b	ZNF821	0.375248932	mirna_pc
7313	hsa-mir-99b	TET1	0.350838	mirna_pc
7314	hsa-mir-99b	ACCN2	0.309802749	mirna_pc
7315	hsa-mir-99b	PATZ1	0.344186471	mirna_pc
7316	hsa-mir-99b	UBE2MP1	0.32504584	mirna_pc
7317	hsa-mir-99b	TMEM231	0.324121497	mirna_pc
7318	hsa-mir-99b	ZNF691	0.358321951	mirna_pc
7319	hsa-mir-1180	TPX2	0.328317215	mirna_pc
7320	hsa-mir-1180	KPNA2	0.334113723	mirna_pc
7321	hsa-mir-1180	CENPF	0.34340257	mirna_pc
7322	hsa-mir-1180	RCC2	0.461574893	mirna_pc
7323	hsa-mir-1180	HOXC9	0.429690855	mirna_pc
7324	hsa-mir-1180	KIF11	0.359545579	mirna_pc
7325	hsa-mir-1180	ECT2	0.367153191	mirna_pc
7326	hsa-mir-1180	RRM2	0.307886759	mirna_pc
7327	hsa-mir-1180	CDC25C	0.303041738	mirna_pc
7328	hsa-mir-1180	PLK1	0.358452384	mirna_pc
7329	hsa-mir-1180	UBE2C	0.34146762	mirna_pc
7330	hsa-mir-1180	BUB1	0.351037982	mirna_pc
7331	hsa-mir-1180	NUSAP1	0.354530463	mirna_pc
7332	hsa-mir-1180	MCM4	0.341307639	mirna_pc
7333	hsa-mir-1180	KIFC1	0.327478638	mirna_pc
7334	hsa-mir-1180	BUB1B	0.407562301	mirna_pc
7335	hsa-mir-1180	KIF18B	0.350071476	mirna_pc
7336	hsa-mir-1180	FAM72A	0.302311958	mirna_pc
7337	hsa-mir-1180	KIF2C	0.413515997	mirna_pc
7338	hsa-mir-1180	NCAPG	0.329591639	mirna_pc
7339	hsa-mir-1180	CDC20	0.352067481	mirna_pc
7340	hsa-mir-1180	CCNA2	0.463181014	mirna_pc
7341	hsa-mir-1180	SPC25	0.323694818	mirna_pc
7342	hsa-mir-1180	FANCI	0.36421193	mirna_pc
7343	hsa-mir-1180	NEK2	0.355468204	mirna_pc

7344	hsa-mir-1180	MND1	0.470935223	mirna_pc
7345	hsa-mir-1180	KIF22	0.421686852	mirna_pc
7346	hsa-mir-1180	DDX39	0.330812454	mirna_pc
7347	hsa-mir-1180	NCAPH	0.420003997	mirna_pc
7348	hsa-mir-1180	GTSE1	0.340464059	mirna_pc
7349	hsa-mir-1180	RAD54L	0.409806262	mirna_pc
7350	hsa-mir-1180	CENPA	0.456565843	mirna_pc
7351	hsa-mir-1180	MAD2L1	0.554511347	mirna_pc
7352	hsa-mir-1180	TIMELESS	0.488104738	mirna_pc
7353	hsa-mir-1180	LMNB1	0.328502131	mirna_pc
7354	hsa-mir-1180	CDCA8	0.350593273	mirna_pc
7355	hsa-mir-1180	TROAP	0.451548904	mirna_pc
7356	hsa-mir-1180	CDCA5	0.302460154	mirna_pc
7357	hsa-mir-1180	XPO1	0.321386592	mirna_pc
7358	hsa-mir-1180	CDC45	0.373221395	mirna_pc
7359	hsa-mir-1180	STMN1	0.402516062	mirna_pc
7360	hsa-mir-1180	EXO1	0.333630313	mirna_pc
7361	hsa-mir-1180	CDCA2	0.337186759	mirna_pc
7362	hsa-mir-1180	DKC1	0.386169977	mirna_pc
7363	hsa-mir-1180	UBE2T	0.303647151	mirna_pc
7364	hsa-mir-1180	CKS1B	0.415385937	mirna_pc
7365	hsa-mir-1180	TUBB	0.463088751	mirna_pc
7366	hsa-mir-1180	MCM2	0.380310844	mirna_pc
7367	hsa-mir-1180	ORC1L	0.322584044	mirna_pc
7368	hsa-mir-1180	KNTC1	0.315814304	mirna_pc
7369	hsa-mir-1180	CCNF	0.332528157	mirna_pc
7370	hsa-mir-1180	RACGAP1	0.345989197	mirna_pc
7371	hsa-mir-1180	CCNB2	0.328145812	mirna_pc
7372	hsa-mir-1180	NUF2	0.390477888	mirna_pc
7373	hsa-mir-1180	PLK4	0.458307034	mirna_pc
7374	hsa-mir-1180	FANCB	0.321115218	mirna_pc
7375	hsa-mir-1180	DEPDC1	0.348686291	mirna_pc
7376	hsa-mir-1180	SKA3	0.348061525	mirna_pc
7377	hsa-mir-1180	HJURP	0.370076268	mirna_pc
7378	hsa-mir-1180	SKA1	0.304738436	mirna_pc
7379	hsa-mir-1180	RAD51	0.366508752	mirna_pc
7380	hsa-mir-1180	BLM	0.320760855	mirna_pc
7381	hsa-mir-1180	NME1	0.335676257	mirna_pc
7382	hsa-mir-1180	DNMT1	0.315695229	mirna_pc
7383	hsa-mir-1180	PAICS	0.456457013	mirna_pc
7384	hsa-mir-1180	BIRC5	0.410095028	mirna_pc
7385	hsa-mir-1180	OIP5	0.35926445	mirna_pc
7386	hsa-mir-1180	DTL	0.385796808	mirna_pc
7387	hsa-mir-1180	EME1	0.466633248	mirna_pc
7388	hsa-mir-1180	RECQL4	0.36434141	mirna_pc
7389	hsa-mir-1180	AURKB	0.467075898	mirna_pc
7390	hsa-mir-1180	GSG2	0.428166761	mirna_pc
7391	hsa-mir-1180	MCM10	0.412740694	mirna_pc
7392	hsa-mir-1180	KIF20A	0.307910051	mirna_pc
7393	hsa-mir-1180	CHEK2	0.410490792	mirna_pc
7394	hsa-mir-1180	CDCA3	0.370736612	mirna_pc
7395	hsa-mir-1180	C1orf112	0.445671693	mirna_pc
7396	hsa-mir-1180	GINS1	0.31651871	mirna_pc
7397	hsa-mir-1180	ALG3	0.411222057	mirna_pc

7398	hsa-mir-1180	FEN1	0.374511619	mirna_pc
7399	hsa-mir-1180	ORC6L	0.380415731	mirna_pc
7400	hsa-mir-1180	ERH	0.341391978	mirna_pc
7401	hsa-mir-1180	PBK	0.311106747	mirna_pc
7402	hsa-mir-1180	C16orf59	0.523669846	mirna_pc
7403	hsa-mir-1180	C17orf53	0.369198346	mirna_pc
7404	hsa-mir-1180	RAE1	0.329442469	mirna_pc
7405	hsa-mir-1180	FAM72D	0.369720141	mirna_pc
7406	hsa-mir-1180	FAM72B	0.373976114	mirna_pc
7407	hsa-mir-1180	NEIL3	0.471902814	mirna_pc
7408	hsa-mir-1180	ACTL6A	0.519829661	mirna_pc
7409	hsa-mir-1180	C12orf48	0.34841399	mirna_pc
7410	hsa-mir-1180	HNRNPL	0.40729268	mirna_pc
7411	hsa-mir-1180	SMC4	0.371842013	mirna_pc
7412	hsa-mir-1180	PKMYT1	0.325356417	mirna_pc
7413	hsa-mir-1180	TTK	0.363543206	mirna_pc
7414	hsa-mir-1180	ZWINT	0.307466922	mirna_pc
7415	hsa-mir-1180	TUBA1B	0.355121113	mirna_pc
7416	hsa-mir-1180	RUVBL1	0.401273699	mirna_pc
7417	hsa-mir-1180	UBE2S	0.402521183	mirna_pc
7418	hsa-mir-1180	C11orf82	0.46134729	mirna_pc
7419	hsa-mir-1180	HNRNPC	0.432195956	mirna_pc
7420	hsa-mir-1180	ILF2	0.492340712	mirna_pc
7421	hsa-mir-1180	C6orf167	0.31099957	mirna_pc
7422	hsa-mir-1180	TFRC	0.408605705	mirna_pc
7423	hsa-mir-1180	MCM5	0.30582204	mirna_pc
7424	hsa-mir-1180	RFWD3	0.386513304	mirna_pc
7425	hsa-mir-1180	C20orf20	0.337175442	mirna_pc
7426	hsa-mir-1180	MCM6	0.361260695	mirna_pc
7427	hsa-mir-1180	RNASEH2A	0.339353181	mirna_pc
7428	hsa-mir-1180	EPR1	0.457843199	mirna_pc
7429	hsa-mir-1180	KIAA1524	0.300067366	mirna_pc
7430	hsa-mir-1180	SFRS2	0.366267621	mirna_pc
7431	hsa-mir-1180	UCK2	0.472281533	mirna_pc
7432	hsa-mir-1180	EFTUD2	0.350714228	mirna_pc
7433	hsa-mir-1180	NFKBIL2	0.429424656	mirna_pc
7434	hsa-mir-1180	C15orf42	0.363167353	mirna_pc
7435	hsa-mir-1180	FAM60A	0.341579099	mirna_pc
7436	hsa-mir-1180	HMGB3	0.404147585	mirna_pc
7437	hsa-mir-1180	FAM64A	0.334548616	mirna_pc
7438	hsa-mir-1180	RANBP1	0.344378162	mirna_pc
7439	hsa-mir-1180	SNRPG	0.325971922	mirna_pc
7440	hsa-mir-1180	EIF4A3	0.34605746	mirna_pc
7441	hsa-mir-1180	TH1L	0.315091954	mirna_pc
7442	hsa-mir-1180	SNRPA	0.410905995	mirna_pc
7443	hsa-mir-1180	FUS	0.474140799	mirna_pc
7444	hsa-mir-1180	C1orf135	0.537640739	mirna_pc
7445	hsa-mir-1180	U2AF2	0.378044489	mirna_pc
7446	hsa-mir-1180	RFC3	0.322597078	mirna_pc
7447	hsa-mir-1180	CHAF1A	0.38530723	mirna_pc
7448	hsa-mir-1180	GINS4	0.324545734	mirna_pc
7449	hsa-mir-1180	ANP32E	0.361499307	mirna_pc
7450	hsa-mir-1180	CENPO	0.474806595	mirna_pc
7451	hsa-mir-1180	TRIP13	0.318493781	mirna_pc

7452	hsa-mir-1180	C19orf48	0.392649666	mirna_pc
7453	hsa-mir-1180	TRIM59	0.310656533	mirna_pc
7454	hsa-mir-1180	CENPL	0.317636869	mirna_pc
7455	hsa-mir-1180	ARL6IP1	0.387410186	mirna_pc
7456	hsa-mir-1180	NUDT1	0.328318225	mirna_pc
7457	hsa-mir-1180	HMGB2	0.404069393	mirna_pc
7458	hsa-mir-1180	GINS2	0.364978266	mirna_pc
7459	hsa-mir-1180	DNA2	0.31793361	mirna_pc
7460	hsa-mir-1180	NOP56	0.338847023	mirna_pc
7461	hsa-mir-1180	SNHG1	0.475061322	mirna_pc
7462	hsa-mir-1180	CSE1L	0.326824798	mirna_pc
7463	hsa-mir-1180	DTYMK	0.342191324	mirna_pc
7464	hsa-mir-1180	TMEM206	0.32825472	mirna_pc
7465	hsa-mir-1180	PRMT1	0.440690975	mirna_pc
7466	hsa-mir-1180	C16orf75	0.348391042	mirna_pc
7467	hsa-mir-1180	NOP2	0.321590059	mirna_pc
7468	hsa-mir-1180	CAD	0.405927838	mirna_pc
7469	hsa-mir-1180	ZFP64	0.504190441	mirna_pc
7470	hsa-mir-1180	CDK2	0.343043775	mirna_pc
7471	hsa-mir-1180	CDK4	0.434724624	mirna_pc
7472	hsa-mir-1180	INCENP	0.328218766	mirna_pc
7473	hsa-mir-1180	PFDN2	0.378495775	mirna_pc
7474	hsa-mir-1180	CHTF18	0.370099397	mirna_pc
7475	hsa-mir-1180	HNRNPR	0.482513094	mirna_pc
7476	hsa-mir-1180	DLEU2	0.323699031	mirna_pc
7477	hsa-mir-1180	KAT2A	0.520700701	mirna_pc
7478	hsa-mir-1180	CENPH	0.308024086	mirna_pc
7479	hsa-mir-1180	PSMC4	0.340440206	mirna_pc
7480	hsa-mir-1180	TCOF1	0.313385342	mirna_pc
7481	hsa-mir-1180	DHX34	0.340299424	mirna_pc
7482	hsa-mir-1180	MSH2	0.347043383	mirna_pc
7483	hsa-mir-1180	PRPF19	0.502445661	mirna_pc
7484	hsa-mir-1180	DBF4	0.310141689	mirna_pc
7485	hsa-mir-1180	SF3B3	0.463014043	mirna_pc
7486	hsa-mir-1180	CDC7	0.362293402	mirna_pc
7487	hsa-mir-1180	SSB	0.312588618	mirna_pc
7488	hsa-mir-1180	YDJC	0.338212461	mirna_pc
7489	hsa-mir-1180	PUS1	0.336212569	mirna_pc
7490	hsa-mir-1180	NOL11	0.368698101	mirna_pc
7491	hsa-mir-1180	SNRPC	0.358832763	mirna_pc
7492	hsa-mir-1180	TMEM41A	0.335973276	mirna_pc
7493	hsa-mir-1180	RFC4	0.468353252	mirna_pc
7494	hsa-mir-1180	FBXO5	0.330967792	mirna_pc
7495	hsa-mir-1180	FIGNL1	0.31248851	mirna_pc
7496	hsa-mir-1180	BRIX1	0.323520636	mirna_pc
7497	hsa-mir-1180	E2F3	0.370630298	mirna_pc
7498	hsa-mir-1180	CDC25A	0.373626696	mirna_pc
7499	hsa-mir-1180	TOPBP1	0.338749976	mirna_pc
7500	hsa-mir-1180	TMEM48	0.312794347	mirna_pc
7501	hsa-mir-1180	RAD54B	0.351453379	mirna_pc
7502	hsa-mir-1180	OTX1	0.402747728	mirna_pc
7503	hsa-mir-1180	H2AFZ	0.453373199	mirna_pc
7504	hsa-mir-1180	PRR7	0.366639752	mirna_pc
7505	hsa-mir-1180	CPSF3	0.374479874	mirna_pc

7506	hsa-mir-1180	BANF1	0.314738913	mirna_pc
7507	hsa-mir-1180	SNRPE	0.413853135	mirna_pc
7508	hsa-mir-1180	C21orf45	0.49171275	mirna_pc
7509	hsa-mir-1180	PPM1G	0.316801544	mirna_pc
7510	hsa-mir-1180	DNAJB11	0.346178217	mirna_pc
7511	hsa-mir-1180	SNRPD2	0.335181508	mirna_pc
7512	hsa-mir-1180	NPM3	0.346934535	mirna_pc
7513	hsa-mir-1180	TFAP4	0.444648555	mirna_pc
7514	hsa-mir-1180	RNPS1	0.412336817	mirna_pc
7515	hsa-mir-1180	DAZAP1	0.319807615	mirna_pc
7516	hsa-mir-1180	CBX8	0.451527894	mirna_pc
7517	hsa-mir-1180	TBL1XR1	0.309469188	mirna_pc
7518	hsa-mir-1180	TMEM201	0.467423778	mirna_pc
7519	hsa-mir-1180	NCL	0.305426924	mirna_pc
7520	hsa-mir-1180	PAFAH1B3	0.356845911	mirna_pc
7521	hsa-mir-1180	SRRT	0.339305556	mirna_pc
7522	hsa-mir-1180	DCTPP1	0.301373881	mirna_pc
7523	hsa-mir-1180	PSRC1	0.414900619	mirna_pc
7524	hsa-mir-1180	DGUOK	0.362912898	mirna_pc
7525	hsa-mir-1180	MRPL47	0.418095072	mirna_pc
7526	hsa-mir-1180	DBF4B	0.312986387	mirna_pc
7527	hsa-mir-1180	FBL	0.465188384	mirna_pc
7528	hsa-mir-1180	HIST1H1E	0.318020202	mirna_pc
7529	hsa-mir-1180	EXOSC5	0.350122	mirna_pc
7530	hsa-mir-1180	HNRNPM	0.38439853	mirna_pc
7531	hsa-mir-1180	PRCC	0.458199054	mirna_pc
7532	hsa-mir-1180	POLR2H	0.562728083	mirna_pc
7533	hsa-mir-1180	C3orf26	0.380580427	mirna_pc
7534	hsa-mir-1180	SMPD4	0.31132155	mirna_pc
7535	hsa-mir-1180	FLAD1	0.310738262	mirna_pc
7536	hsa-mir-1180	LSG1	0.495019033	mirna_pc
7537	hsa-mir-1180	PPIH	0.301933406	mirna_pc
7538	hsa-mir-1180	THOC6	0.365999877	mirna_pc
7539	hsa-mir-1180	GMPS	0.429949213	mirna_pc
7540	hsa-mir-1180	PPAT	0.400068186	mirna_pc
7541	hsa-mir-1180	DHX9	0.461097983	mirna_pc
7542	hsa-mir-1180	CCDC138	0.358506887	mirna_pc
7543	hsa-mir-1180	LOC100128191	0.362633283	mirna_pc
7544	hsa-mir-1180	MRPL3	0.442398159	mirna_pc
7545	hsa-mir-1180	EIF5A	0.311646867	mirna_pc
7546	hsa-mir-1180	ETV4	0.325842512	mirna_pc
7547	hsa-mir-1180	MORC2	0.362719711	mirna_pc
7548	hsa-mir-1180	CHRNA5	0.35777081	mirna_pc
7549	hsa-mir-1180	SMYD5	0.414832071	mirna_pc
7550	hsa-mir-1180	TSSC1	0.319635827	mirna_pc
7551	hsa-mir-1180	POLA2	0.309406466	mirna_pc
7552	hsa-mir-1180	SUMO2	0.350591614	mirna_pc
7553	hsa-mir-1180	SFRS1	0.427309759	mirna_pc
7554	hsa-mir-1180	CDH24	0.345549749	mirna_pc
7555	hsa-mir-1180	WDR43	0.329623814	mirna_pc
7556	hsa-mir-1180	CCDC58	0.361569176	mirna_pc
7557	hsa-mir-1180	SKP2	0.366043751	mirna_pc
7558	hsa-mir-1180	UBE2I	0.310633316	mirna_pc
7559	hsa-mir-1180	TIPIN	0.313503287	mirna_pc

7560	hsa-mir-1180	SMARCD1	0.348650549	mirna_pc
7561	hsa-mir-1180	ATXN2L	0.307520392	mirna_pc
7562	hsa-mir-1180	HSPBP1	0.309647699	mirna_pc
7563	hsa-mir-1180	C4orf46	0.414822186	mirna_pc
7564	hsa-mir-1180	FZD2	0.324619872	mirna_pc
7565	hsa-mir-1180	ECE2	0.491065051	mirna_pc
7566	hsa-mir-1180	C1orf107	0.304034064	mirna_pc
7567	hsa-mir-1180	RFC5	0.332372584	mirna_pc
7568	hsa-mir-1180	MAZ	0.453661004	mirna_pc
7569	hsa-mir-1180	SSRP1	0.352970023	mirna_pc
7570	hsa-mir-1180	POLR2D	0.404434123	mirna_pc
7571	hsa-mir-1180	FTSJ2	0.304792257	mirna_pc
7572	hsa-mir-1180	PA2G4	0.344515397	mirna_pc
7573	hsa-mir-1180	SNRPD3	0.338694738	mirna_pc
7574	hsa-mir-1180	EPT1	0.363451026	mirna_pc
7575	hsa-mir-1180	KHDRBS1	0.317809201	mirna_pc
7576	hsa-mir-1180	C20orf27	0.365332599	mirna_pc
7577	hsa-mir-1180	YEATS2	0.350844402	mirna_pc
7578	hsa-mir-1180	SPAST	0.359703498	mirna_pc
7579	hsa-mir-1180	LOC92659	0.393998458	mirna_pc
7580	hsa-mir-1180	MTL 5.00	0.440515778	mirna_pc
7581	hsa-mir-1180	RNF220	0.310037051	mirna_pc
7582	hsa-mir-1180	B3GNT5	0.340130678	mirna_pc
7583	hsa-mir-1180	PARP1	0.394564776	mirna_pc
7584	hsa-mir-1180	CCT7	0.314576237	mirna_pc
7585	hsa-mir-1180	TMEM194A	0.352363444	mirna_pc
7586	hsa-mir-1180	TDP1	0.333463952	mirna_pc
7587	hsa-mir-1180	SUV420H2	0.354047859	mirna_pc
7588	hsa-mir-1180	ZNF695	0.304518032	mirna_pc
7589	hsa-mir-1180	METTL1	0.374050227	mirna_pc
7590	hsa-mir-1180	CENPJ	0.324718372	mirna_pc
7591	hsa-mir-1180	LIN9	0.319490789	mirna_pc
7592	hsa-mir-1180	GNL2	0.316706412	mirna_pc
7593	hsa-mir-1180	WRAP53	0.374551941	mirna_pc
7594	hsa-mir-1180	SREBF1	0.397226578	mirna_pc
7595	hsa-mir-1180	KRI1	0.343459638	mirna_pc
7596	hsa-mir-1180	NOC2L	0.351669018	mirna_pc
7597	hsa-mir-1180	RPP40	0.373596589	mirna_pc
7598	hsa-mir-1180	CCDC86	0.439351494	mirna_pc
7599	hsa-mir-1180	ZNF280C	0.367463056	mirna_pc
7600	hsa-mir-1180	POLR1B	0.364149102	mirna_pc
7601	hsa-mir-1180	PTMA	0.326468909	mirna_pc
7602	hsa-mir-1180	USP39	0.405602312	mirna_pc
7603	hsa-mir-1180	HMGXB4	0.302342186	mirna_pc
7604	hsa-mir-1180	C11orf84	0.472947316	mirna_pc
7605	hsa-mir-1180	TBCE	0.437773088	mirna_pc
7606	hsa-mir-1180	CLCN2	0.469082495	mirna_pc
7607	hsa-mir-1180	NCBP2	0.469977421	mirna_pc
7608	hsa-mir-1180	SUV39H2	0.456993197	mirna_pc
7609	hsa-mir-1180	LSM 2.00	0.368316558	mirna_pc
7610	hsa-mir-1180	TMEM177	0.373501667	mirna_pc
7611	hsa-mir-1180	HNRNPD	0.420263729	mirna_pc
7612	hsa-mir-1180	DPY30	0.357183458	mirna_pc
7613	hsa-mir-1180	MTHFD2	0.420009743	mirna_pc

7614	hsa-mir-1180	DPH2	0.323528445	mirna_pc
7615	hsa-mir-1180	SFRS3	0.442784245	mirna_pc
7616	hsa-mir-1180	SHOX2	0.360875647	mirna_pc
7617	hsa-mir-1180	MEN1	0.347319079	mirna_pc
7618	hsa-mir-1180	FAM53C	0.327282041	mirna_pc
7619	hsa-mir-1180	H1FX	0.361524738	mirna_pc
7620	hsa-mir-1180	PNO1	0.308223935	mirna_pc
7621	hsa-mir-1180	CCT4	0.329956062	mirna_pc
7622	hsa-mir-1180	UMPS	0.382370427	mirna_pc
7623	hsa-mir-1180	SPNS1	0.300922034	mirna_pc
7624	hsa-mir-1180	ELAVL1	0.462921456	mirna_pc
7625	hsa-mir-1180	TSPYL5	0.310541526	mirna_pc
7626	hsa-mir-1180	ABCE1	0.351132855	mirna_pc
7627	hsa-mir-1180	PRMT5	0.319586791	mirna_pc
7628	hsa-mir-1180	MIF	0.3011867	mirna_pc
7629	hsa-mir-1180	ULK2	0.309444285	mirna_pc
7630	hsa-mir-1180	EIF4A1	0.302065975	mirna_pc
7631	hsa-mir-1180	NCAPD3	0.32757168	mirna_pc
7632	hsa-mir-1180	TRMT1	0.308875398	mirna_pc
7633	hsa-mir-1180	NAE1	0.312450685	mirna_pc
7634	hsa-mir-1180	USP22	0.416817653	mirna_pc
7635	hsa-mir-1180	PSMC3IP	0.325249019	mirna_pc
7636	hsa-mir-1180	TBC1D7	0.439641055	mirna_pc
7637	hsa-mir-1180	PAK1IP1	0.461947507	mirna_pc
7638	hsa-mir-1180	PAK2	0.327876535	mirna_pc
7639	hsa-mir-1180	TRMT112	0.312718801	mirna_pc
7640	hsa-mir-1180	PSMD2	0.381513049	mirna_pc
7641	hsa-mir-1180	ATP13A3	0.333228582	mirna_pc
7642	hsa-mir-1180	YWHAE	0.491335693	mirna_pc
7643	hsa-mir-1180	NAA25	0.37574817	mirna_pc
7644	hsa-mir-1180	KDM1A	0.300486201	mirna_pc
7645	hsa-mir-1180	PDCL3	0.352006936	mirna_pc
7646	hsa-mir-1180	ILF3	0.451390166	mirna_pc
7647	hsa-mir-1180	ADCY3	0.327567308	mirna_pc
7648	hsa-mir-1180	C1orf96	0.316907797	mirna_pc
7649	hsa-mir-1180	NT5DC2	0.369398964	mirna_pc
7650	hsa-mir-1180	EHMT2	0.503254033	mirna_pc
7651	hsa-mir-1180	C18orf54	0.307861838	mirna_pc
7652	hsa-mir-1180	UBAP2L	0.422181285	mirna_pc
7653	hsa-mir-1180	DEK	0.303851033	mirna_pc
7654	hsa-mir-1180	SFRS9	0.320999963	mirna_pc
7655	hsa-mir-1180	IPO4	0.340563525	mirna_pc
7656	hsa-mir-1180	SIX1	0.376166088	mirna_pc
7657	hsa-mir-1180	RPGRIP1L	0.352676134	mirna_pc
7658	hsa-mir-1180	MFSD2A	0.302520584	mirna_pc
7659	hsa-mir-1180	ADORA2B	0.363022014	mirna_pc
7660	hsa-mir-1180	GTF2H4	0.449437014	mirna_pc
7661	hsa-mir-1180	LSM 6.00	0.418850298	mirna_pc
7662	hsa-mir-1180	PRAME	0.481553057	mirna_pc
7663	hsa-mir-1180	THOC3	0.300340697	mirna_pc
7664	hsa-mir-1180	CBX2	0.405633178	mirna_pc
7665	hsa-mir-1180	AZI1	0.322377474	mirna_pc
7666	hsa-mir-1180	FUBP1	0.322574844	mirna_pc
7667	hsa-mir-1180	C7orf27	0.3062659	mirna_pc

7668	hsa-mir-1180	WDR74	0.392598586	mirna_pc
7669	hsa-mir-1180	ZBTB9	0.322835635	mirna_pc
7670	hsa-mir-1180	SUV39H1	0.314501786	mirna_pc
7671	hsa-mir-1180	IPO9	0.375347416	mirna_pc
7672	hsa-mir-1180	CSRP2BP	0.317011291	mirna_pc
7673	hsa-mir-1180	SR140	0.324870194	mirna_pc
7674	hsa-mir-1180	TRA2B	0.484356729	mirna_pc
7675	hsa-mir-1180	RSRC1	0.44408171	mirna_pc
7676	hsa-mir-1180	GAR1	0.463544973	mirna_pc
7677	hsa-mir-1180	C15orf23	0.435195617	mirna_pc
7678	hsa-mir-1180	CDRT4	0.409932119	mirna_pc
7679	hsa-mir-1180	RBM34	0.370806927	mirna_pc
7680	hsa-mir-1180	C3orf21	0.451576759	mirna_pc
7681	hsa-mir-1180	ZNF668	0.392064545	mirna_pc
7682	hsa-mir-1180	IGF2BP2	0.303801488	mirna_pc
7683	hsa-mir-1180	C1QBP	0.505374459	mirna_pc
7684	hsa-mir-1180	SAE1	0.300048664	mirna_pc
7685	hsa-mir-1180	ENOPH1	0.319581818	mirna_pc
7686	hsa-mir-1180	C19orf47	0.314288467	mirna_pc
7687	hsa-mir-1180	MAP6D1	0.315520382	mirna_pc
7688	hsa-mir-1180	C4orf43	0.406484065	mirna_pc
7689	hsa-mir-1180	TGIF1	0.316380356	mirna_pc
7690	hsa-mir-1180	ZNF595	0.419914332	mirna_pc
7691	hsa-mir-1180	YY1	0.309133425	mirna_pc
7692	hsa-mir-1180	C16orf80	0.361287508	mirna_pc
7693	hsa-mir-1180	MYBBP1A	0.401746947	mirna_pc
7694	hsa-mir-1180	C17orf96	0.360193952	mirna_pc
7695	hsa-mir-1180	CXCL1	0.332938187	mirna_pc
7696	hsa-mir-1180	PLEKHG4	0.45051783	mirna_pc
7697	hsa-mir-1180	CNOT2	0.366426565	mirna_pc
7698	hsa-mir-1180	TMEM223	0.347238516	mirna_pc
7699	hsa-mir-1180	SFPQ	0.434585348	mirna_pc
7700	hsa-mir-1180	CNOT3	0.31387304	mirna_pc
7701	hsa-mir-1180	GIT1	0.319947845	mirna_pc
7702	hsa-mir-1180	RCOR2	0.430938086	mirna_pc
7703	hsa-mir-1180	RHBDD3	0.302780242	mirna_pc
7704	hsa-mir-1180	MTA2	0.444773988	mirna_pc
7705	hsa-mir-1180	SHMT2	0.331212945	mirna_pc
7706	hsa-mir-1180	HDAC2	0.36201562	mirna_pc
7707	hsa-mir-1180	PDCD2L	0.3690212	mirna_pc
7708	hsa-mir-1180	PA2G4P4	0.301761237	mirna_pc
7709	hsa-mir-1180	HAP1	0.313758223	mirna_pc
7710	hsa-mir-1180	CPNE2	0.306371647	mirna_pc
7711	hsa-mir-1180	WDR53	0.303040044	mirna_pc
7712	hsa-mir-1180	ALOX12P2	0.404831077	mirna_pc
7713	hsa-mir-1180	C10orf2	0.301746952	mirna_pc
7714	hsa-mir-1180	POGK	0.328614359	mirna_pc
7715	hsa-mir-1180	RNF7	0.380495741	mirna_pc
7716	hsa-mir-1180	SALL2	0.346118914	mirna_pc
7717	hsa-mir-1180	DCK	0.350146697	mirna_pc
7718	hsa-mir-1180	RNMTL1	0.413941191	mirna_pc
7719	hsa-mir-1180	FAM86C	0.361359609	mirna_pc
7720	hsa-mir-1180	PTCD1	0.309877217	mirna_pc
7721	hsa-mir-1180	CRIPT	0.346248966	mirna_pc

7722	hsa-mir-1180	MSH6	0.486214252	mirna_pc
7723	hsa-mir-1180	SOX4	0.305714414	mirna_pc
7724	hsa-mir-1180	C3orf34	0.352861279	mirna_pc
7725	hsa-mir-1180	TAF6L	0.484632261	mirna_pc
7726	hsa-mir-1180	LEF1	0.359280625	mirna_pc
7727	hsa-mir-1180	ACBD6	0.463593427	mirna_pc
7728	hsa-mir-1180	HNRNPA1	0.408227221	mirna_pc
7729	hsa-mir-1180	UBA2	0.317037807	mirna_pc
7730	hsa-mir-1180	CDK2AP1	0.399089533	mirna_pc
7731	hsa-mir-1180	NUDT3	0.482090937	mirna_pc
7732	hsa-mir-1180	ZBED4	0.311269926	mirna_pc
7733	hsa-mir-1180	MEA1	0.310742645	mirna_pc
7734	hsa-mir-1180	MEX3A	0.541546791	mirna_pc
7735	hsa-mir-1180	MOGS	0.368379204	mirna_pc
7736	hsa-mir-1180	BAT1	0.345174765	mirna_pc
7737	hsa-mir-1180	COIL	0.348455858	mirna_pc
7738	hsa-mir-1180	VARS	0.33374945	mirna_pc
7739	hsa-mir-1180	MRPS12	0.374586388	mirna_pc
7740	hsa-mir-1180	RFC2	0.319188226	mirna_pc
7741	hsa-mir-1180	TAF5	0.337663305	mirna_pc
7742	hsa-mir-1180	TRIM28	0.403274756	mirna_pc
7743	hsa-mir-1180	GTPBP3	0.431165831	mirna_pc
7744	hsa-mir-1180	C16orf88	0.483081155	mirna_pc
7745	hsa-mir-1180	B3GALT6	0.427552488	mirna_pc
7746	hsa-mir-1180	APEX1	0.390313585	mirna_pc
7747	hsa-mir-1180	HNRNPA1L2	0.412276369	mirna_pc
7748	hsa-mir-1180	NAA15	0.340989472	mirna_pc
7749	hsa-mir-1180	CBX1	0.437930714	mirna_pc
7750	hsa-mir-1180	EMILIN3	0.355242413	mirna_pc
7751	hsa-mir-1180	YEATS4	0.335764977	mirna_pc
7752	hsa-mir-1180	HMGB1	0.312668425	mirna_pc
7753	hsa-mir-1180	RPS2	0.360993937	mirna_pc
7754	hsa-mir-1180	ZBTB12	0.570380462	mirna_pc
7755	hsa-mir-1180	BCDIN3D	0.306944366	mirna_pc
7756	hsa-mir-1180	C1orf35	0.310316485	mirna_pc
7757	hsa-mir-1180	KLHDC9	0.336249392	mirna_pc
7758	hsa-mir-1180	TGIF2	0.361405056	mirna_pc
7759	hsa-mir-1180	SENP1	0.304904405	mirna_pc
7760	hsa-mir-1180	ERI3	0.418688043	mirna_pc
7761	hsa-mir-1180	RCN2	0.452124872	mirna_pc
7762	hsa-mir-1180	THUMPD2	0.339336995	mirna_pc
7763	hsa-mir-1180	TARBP2	0.468841741	mirna_pc
7764	hsa-mir-1180	SNHG9	0.33683409	mirna_pc
7765	hsa-mir-1180	C16orf53	0.364341716	mirna_pc
7766	hsa-mir-1180	NOL10	0.444220815	mirna_pc
7767	hsa-mir-1180	ANAPC1	0.310641145	mirna_pc
7768	hsa-mir-1180	POLE	0.334243455	mirna_pc
7769	hsa-mir-1180	WRNIP1	0.418520319	mirna_pc
7770	hsa-mir-1180	C1orf159	0.306029058	mirna_pc
7771	hsa-mir-1180	CPSF6	0.324778558	mirna_pc
7772	hsa-mir-1180	ERF	0.364471845	mirna_pc
7773	hsa-mir-1180	TAF1B	0.409761066	mirna_pc
7774	hsa-mir-1180	TIMM50	0.380609204	mirna_pc
7775	hsa-mir-1180	TMEM216	0.350186637	mirna_pc

7776	hsa-mir-1180	XRCC6BP1	0.318575244	mirna_pc
7777	hsa-mir-1180	ADSL	0.350500871	mirna_pc
7778	hsa-mir-1180	RIOK1	0.321659052	mirna_pc
7779	hsa-mir-1180	RBM14	0.312745154	mirna_pc
7780	hsa-mir-1180	EXOSC9	0.449264938	mirna_pc
7781	hsa-mir-1180	FOXRED2	0.316776117	mirna_pc
7782	hsa-mir-1180	UNG	0.336830412	mirna_pc
7783	hsa-mir-1180	GRK6	0.365966903	mirna_pc
7784	hsa-mir-1180	ZNF598	0.353915908	mirna_pc
7785	hsa-mir-1180	RBMX	0.392897762	mirna_pc
7786	hsa-mir-1180	TELO2	0.328405779	mirna_pc
7787	hsa-mir-1180	TSEN15	0.32808737	mirna_pc
7788	hsa-mir-1180	CDK16	0.346225512	mirna_pc
7789	hsa-mir-1180	ALKBH2	0.373743003	mirna_pc
7790	hsa-mir-1180	USP10	0.337247352	mirna_pc
7791	hsa-mir-1180	NTHL1	0.453318188	mirna_pc
7792	hsa-mir-1180	TCTEX1D2	0.308361614	mirna_pc
7793	hsa-mir-1180	PYCR2	0.353130664	mirna_pc
7794	hsa-mir-1180	ALKBH5	0.532362031	mirna_pc
7795	hsa-mir-1180	CCDC90A	0.318496559	mirna_pc
7796	hsa-mir-1180	MDC 1.00	0.308665423	mirna_pc
7797	hsa-mir-1180	PRKCSH	0.400095835	mirna_pc
7798	hsa-mir-1180	MLF 2.00	0.308013532	mirna_pc
7799	hsa-mir-1180	SOCS7	0.308173346	mirna_pc
7800	hsa-mir-1180	CTCF	0.459360674	mirna_pc
7801	hsa-mir-1180	MED9	0.467211403	mirna_pc
7802	hsa-mir-1180	RPL39L	0.437530354	mirna_pc
7803	hsa-mir-1180	GPN3	0.331301596	mirna_pc
7804	hsa-mir-1180	ASB3	0.306020567	mirna_pc
7805	hsa-mir-1180	LOC152217	0.430739309	mirna_pc
7806	hsa-mir-1180	IGSF9	0.37389549	mirna_pc
7807	hsa-mir-1180	LOC440905	0.372960552	mirna_pc
7808	hsa-mir-1180	PRKX	0.331535355	mirna_pc
7809	hsa-mir-1180	GPATCH2	0.341850438	mirna_pc
7810	hsa-mir-1180	DNAJC14	0.377000839	mirna_pc
7811	hsa-mir-1180	FIP1L1	0.346089341	mirna_pc
7812	hsa-mir-1180	TFB2M	0.343957002	mirna_pc
7813	hsa-mir-1180	KDM2B	0.316987527	mirna_pc
7814	hsa-mir-1180	TRAP1	0.356134644	mirna_pc
7815	hsa-mir-1180	GTF3C2	0.310332557	mirna_pc
7816	hsa-mir-1180	MAGEF1	0.569583329	mirna_pc
7817	hsa-mir-1180	OSTC	0.388685391	mirna_pc
7818	hsa-mir-1180	WDR90	0.328680057	mirna_pc
7819	hsa-mir-1180	APOA1BP	0.34552663	mirna_pc
7820	hsa-mir-1180	SUPT3H	0.369332548	mirna_pc
7821	hsa-mir-1180	IMP4	0.332969025	mirna_pc
7822	hsa-mir-1180	SOX12	0.373468189	mirna_pc
7823	hsa-mir-1180	SMARCB1	0.405720094	mirna_pc
7824	hsa-mir-1180	PYGO2	0.318154088	mirna_pc
7825	hsa-mir-1180	RPA1	0.403963706	mirna_pc
7826	hsa-mir-1180	FAM92A1	0.309708692	mirna_pc
7827	hsa-mir-1180	ZNF639	0.537702986	mirna_pc
7828	hsa-mir-1180	TTC5	0.308980823	mirna_pc
7829	hsa-mir-1180	TBCCD1	0.342943721	mirna_pc

7830	hsa-mir-1180	ITPRIPL1	0.340260257	mirna_pc
7831	hsa-mir-1180	MSTO1	0.351318418	mirna_pc
7832	hsa-mir-1180	FOXK2	0.34028808	mirna_pc
7833	hsa-mir-1180	ANAPC10	0.340654943	mirna_pc
7834	hsa-mir-1180	ABT1	0.303898627	mirna_pc
7835	hsa-mir-1180	LDHB	0.314774124	mirna_pc
7836	hsa-mir-1180	UBXN7	0.394625821	mirna_pc
7837	hsa-mir-1180	FXR2	0.351441985	mirna_pc
7838	hsa-mir-1180	FAM189B	0.383484071	mirna_pc
7839	hsa-mir-1180	MKRN3	0.372754917	mirna_pc
7840	hsa-mir-1180	LRPPRC	0.310448041	mirna_pc
7841	hsa-mir-1180	LOC401010	0.366350926	mirna_pc
7842	hsa-mir-1180	DVL3	0.40312864	mirna_pc
7843	hsa-mir-1180	NUP133	0.31887483	mirna_pc
7844	hsa-mir-1180	LPHN3	0.305177991	mirna_pc
7845	hsa-mir-1180	BCL7A	0.460276445	mirna_pc
7846	hsa-mir-1180	DVL2	0.456715493	mirna_pc
7847	hsa-mir-1180	TRPA1	0.310310797	mirna_pc
7848	hsa-mir-1180	ZNF286A	0.536605727	mirna_pc
7849	hsa-mir-1180	SUMO3	0.30647773	mirna_pc
7850	hsa-mir-1180	MRPL21	0.509837406	mirna_pc
7851	hsa-mir-1180	NLE1	0.418112187	mirna_pc
7852	hsa-mir-1180	COX10	0.3636898	mirna_pc
7853	hsa-mir-1180	FXR1	0.375359834	mirna_pc
7854	hsa-mir-1180	DMRTA2	0.328634412	mirna_pc
7855	hsa-mir-1180	CHST10	0.359039012	mirna_pc
7856	hsa-mir-1180	PSPH	0.306386281	mirna_pc
7857	hsa-mir-1180	PRR3	0.414526542	mirna_pc
7858	hsa-mir-1180	MGC72080	0.315986278	mirna_pc
7859	hsa-mir-1180	MCF2L2	0.330368766	mirna_pc
7860	hsa-mir-1180	CNTROB	0.379309729	mirna_pc
7861	hsa-mir-1180	TRMT61B	0.430304183	mirna_pc
7862	hsa-mir-1180	TSFM	0.394146205	mirna_pc
7863	hsa-mir-1180	RAD1	0.302121148	mirna_pc
7864	hsa-mir-1180	UBFD1	0.387103322	mirna_pc
7865	hsa-mir-1180	SEMA4F	0.338770151	mirna_pc
7866	hsa-mir-1180	APITD1	0.304323919	mirna_pc
7867	hsa-mir-1180	GANAB	0.380442388	mirna_pc
7868	hsa-mir-1180	GRSF1	0.371220565	mirna_pc
7869	hsa-mir-1180	CHD3	0.304161415	mirna_pc
7870	hsa-mir-1180	ZNF286B	0.492153166	mirna_pc
7871	hsa-mir-1180	DDX20	0.342008508	mirna_pc
7872	hsa-mir-1180	DCUN1D1	0.449163387	mirna_pc
7873	hsa-mir-1180	C1orf109	0.342509755	mirna_pc
7874	hsa-mir-1180	ALDH3A2	0.439376612	mirna_pc
7875	hsa-mir-1180	PIGX	0.402833066	mirna_pc
7876	hsa-mir-1180	MTA1	0.349423222	mirna_pc
7877	hsa-mir-1180	SLC47A1	0.394716477	mirna_pc
7878	hsa-mir-1180	DPH1	0.382467448	mirna_pc
7879	hsa-mir-1180	CRK	0.302150518	mirna_pc
7880	hsa-mir-1180	PFN2	0.403852261	mirna_pc
7881	hsa-mir-1180	PFKM	0.316956877	mirna_pc
7882	hsa-mir-1180	PSPC1	0.356284155	mirna_pc
7883	hsa-mir-1180	INTU	0.35217077	mirna_pc

7884	hsa-mir-1180	COPZ1	0.306715626	mirna_pc
7885	hsa-mir-1180	SMAD1	0.322099057	mirna_pc
7886	hsa-mir-1180	ATP6V1E2	0.387364375	mirna_pc
7887	hsa-mir-1180	LMLN	0.346399676	mirna_pc
7888	hsa-mir-1180	CCNB1IP1	0.381213875	mirna_pc
7889	hsa-mir-1180	NHP2L1	0.353366824	mirna_pc
7890	hsa-mir-1180	MAPKAPK5	0.300980291	mirna_pc
7891	hsa-mir-1180	RBMXL1	0.309416706	mirna_pc
7892	hsa-mir-1180	SMCR8	0.326870405	mirna_pc
7893	hsa-mir-1180	GMCL1	0.310782677	mirna_pc
7894	hsa-mir-1180	GPR3	0.327844121	mirna_pc
7895	hsa-mir-1180	E2F6	0.372568017	mirna_pc
7896	hsa-mir-1180	NOL7	0.337627544	mirna_pc
7897	hsa-mir-1180	PRPSAP2	0.648026177	mirna_pc
7898	hsa-mir-1180	SENP3	0.467341185	mirna_pc
7899	hsa-mir-1180	CPSF4	0.325697064	mirna_pc
7900	hsa-mir-1180	B4GALT3	0.431546505	mirna_pc
7901	hsa-mir-1180	SNRNP200	0.309230084	mirna_pc
7902	hsa-mir-1180	SCLY	0.351345083	mirna_pc
7903	hsa-mir-1180	STARD7	0.404915447	mirna_pc
7904	hsa-mir-1180	GPR125	0.317454932	mirna_pc
7905	hsa-mir-1180	NUP35	0.313083107	mirna_pc
7906	hsa-mir-1180	SLC19A1	0.305485662	mirna_pc
7907	hsa-mir-1180	TFAM	0.307882575	mirna_pc
7908	hsa-mir-1180	TTC27	0.337914444	mirna_pc
7909	hsa-mir-1180	DHRS13	0.407291856	mirna_pc
7910	hsa-mir-1180	MCCC1	0.472297098	mirna_pc
7911	hsa-mir-1180	ZNF624	0.351341652	mirna_pc
7912	hsa-mir-1180	C18orf55	0.309369806	mirna_pc
7913	hsa-mir-1180	MRPS34	0.317493056	mirna_pc
7914	hsa-mir-1180	LOC222699	0.334533533	mirna_pc
7915	hsa-mir-1180	KCNMB3	0.362466796	mirna_pc
7916	hsa-mir-1180	DHX57	0.45319435	mirna_pc
7917	hsa-mir-1180	ALX3	0.339337063	mirna_pc
7918	hsa-mir-1180	AAAS	0.463773737	mirna_pc
7919	hsa-mir-1180	UBQLN4	0.388436458	mirna_pc
7920	hsa-mir-1180	C19orf54	0.314673893	mirna_pc
7921	hsa-mir-1180	MTFMT	0.331442328	mirna_pc
7922	hsa-mir-1180	SUPT7L	0.307665422	mirna_pc
7923	hsa-mir-1180	PEMT	0.498377226	mirna_pc
7924	hsa-mir-1180	SMUG1	0.300372762	mirna_pc
7925	hsa-mir-1180	LIG3	0.379644273	mirna_pc
7926	hsa-mir-1180	KIAA0195	0.32406182	mirna_pc
7927	hsa-mir-1180	MRPS26	0.431693981	mirna_pc
7928	hsa-mir-1180	MRPL30	0.418890878	mirna_pc
7929	hsa-mir-1180	SETD1A	0.315316332	mirna_pc
7930	hsa-mir-1180	GFM1	0.326101417	mirna_pc
7931	hsa-mir-1180	CWC27	0.347053582	mirna_pc
7932	hsa-mir-1180	PELP1	0.501469527	mirna_pc
7933	hsa-mir-1180	ACPL2	0.323795087	mirna_pc
7934	hsa-mir-1180	ZNF692	0.355234315	mirna_pc
7935	hsa-mir-1180	SFRS7	0.398010284	mirna_pc
7936	hsa-mir-1180	C9orf100	0.325441642	mirna_pc
7937	hsa-mir-1180	C18orf10	0.3405006	mirna_pc

7938	hsa-mir-1180	TRMT2A	0.375620698	mirna_pc
7939	hsa-mir-1180	PCBP2	0.323245945	mirna_pc
7940	hsa-mir-1180	UBE2O	0.326085383	mirna_pc
7941	hsa-mir-1180	NFXL1	0.322080485	mirna_pc
7942	hsa-mir-1180	TRIT1	0.317429042	mirna_pc
7943	hsa-mir-1180	PFAS	0.420182554	mirna_pc
7944	hsa-mir-1180	PET112L	0.353603649	mirna_pc
7945	hsa-mir-1180	SARS2	0.490287318	mirna_pc
7946	hsa-mir-1180	C10orf35	0.332737985	mirna_pc
7947	hsa-mir-1180	HLTF	0.340186974	mirna_pc
7948	hsa-mir-1180	BLMH	0.331947628	mirna_pc
7949	hsa-mir-1180	NUP88	0.392709066	mirna_pc
7950	hsa-mir-1180	BAT2	0.308001955	mirna_pc
7951	hsa-mir-1180	RBM19	0.330277639	mirna_pc
7952	hsa-mir-1180	ERCC3	0.308773027	mirna_pc
7953	hsa-mir-1180	DHX33	0.465061031	mirna_pc
7954	hsa-mir-1180	FADS2	0.335168568	mirna_pc
7955	hsa-mir-1180	RPUSD2	0.389126316	mirna_pc
7956	hsa-mir-1180	ABCF3	0.349706641	mirna_pc
7957	hsa-mir-1180	EIF2B5	0.469561094	mirna_pc
7958	hsa-mir-1180	NUP153	0.437006315	mirna_pc
7959	hsa-mir-1180	NIPSNAP1	0.351193439	mirna_pc
7960	hsa-mir-1180	PARL	0.43476313	mirna_pc
7961	hsa-mir-1180	PTOV1	0.349456131	mirna_pc
7962	hsa-mir-1180	C17orf80	0.343998861	mirna_pc
7963	hsa-mir-1180	ANKRD23	0.308054128	mirna_pc
7964	hsa-mir-1180	ARHGEF19	0.350777528	mirna_pc
7965	hsa-mir-1180	C17orf75	0.440832953	mirna_pc
7966	hsa-mir-1180	TMEM93	0.36638511	mirna_pc
7967	hsa-mir-1180	UST	0.331332801	mirna_pc
7968	hsa-mir-1180	RPL35A	0.378784304	mirna_pc
7969	hsa-mir-1180	FBRSL1	0.345053929	mirna_pc
7970	hsa-mir-1180	NFATC3	0.361147795	mirna_pc
7971	hsa-mir-1180	FUZ	0.309499845	mirna_pc
7972	hsa-mir-1180	ATXN10	0.404837881	mirna_pc
7973	hsa-mir-1180	NPRL3	0.365153458	mirna_pc
7974	hsa-mir-1180	ZNF771	0.504557512	mirna_pc
7975	hsa-mir-1180	EEF1E1	0.420824939	mirna_pc
7976	hsa-mir-1180	PRUNE	0.310592708	mirna_pc
7977	hsa-mir-1180	ZSWIM7	0.481676806	mirna_pc
7978	hsa-mir-1180	EIF4G1	0.321329837	mirna_pc
7979	hsa-mir-1180	TCTN2	0.323365928	mirna_pc
7980	hsa-mir-1180	C14orf104	0.36969984	mirna_pc
7981	hsa-mir-1180	POLR2C	0.312617395	mirna_pc
7982	hsa-mir-1180	C12orf10	0.356402567	mirna_pc
7983	hsa-mir-1180	TOP3A	0.519354635	mirna_pc
7984	hsa-mir-1180	UBE2M	0.305426183	mirna_pc
7985	hsa-mir-1180	C2orf44	0.407770584	mirna_pc
7986	hsa-mir-1180	RING1	0.327606497	mirna_pc
7987	hsa-mir-1180	PSMB6	0.349387101	mirna_pc
7988	hsa-mir-1180	SEPHS1	0.49904575	mirna_pc
7989	hsa-mir-1180	SHISA2	0.305636947	mirna_pc
7990	hsa-mir-1180	RMND1	0.331426887	mirna_pc
7991	hsa-mir-1180	RYK	0.375402569	mirna_pc

7992	hsa-mir-1180	GEMIN5	0.319649822	mirna_pc
7993	hsa-mir-1180	TMEM183A	0.30134592	mirna_pc
7994	hsa-mir-1180	MLST8	0.384226128	mirna_pc
7995	hsa-mir-1180	C16orf13	0.313556648	mirna_pc
7996	hsa-mir-1180	TMEM11	0.400147318	mirna_pc
7997	hsa-mir-1180	AGBL5	0.445292696	mirna_pc
7998	hsa-mir-1180	PHB2	0.349538865	mirna_pc
7999	hsa-mir-1180	ISYNA1	0.333137966	mirna_pc
8000	hsa-mir-1180	MYL6B	0.478096588	mirna_pc
8001	hsa-mir-1180	PWP2	0.367524353	mirna_pc
8002	hsa-mir-1180	PDCD7	0.357153229	mirna_pc
8003	hsa-mir-1180	CPSF7	0.385331938	mirna_pc
8004	hsa-mir-1180	SENP5	0.353753427	mirna_pc
8005	hsa-mir-1180	DTX3	0.364524998	mirna_pc
8006	hsa-mir-1180	TEAD2	0.30725096	mirna_pc
8007	hsa-mir-1180	NARS2	0.330086916	mirna_pc
8008	hsa-mir-1180	ZFP1	0.39040816	mirna_pc
8009	hsa-mir-1180	NME4	0.398613892	mirna_pc
8010	hsa-mir-1180	LYRM4	0.529809363	mirna_pc
8011	hsa-mir-1180	RNF41	0.371948705	mirna_pc
8012	hsa-mir-1180	BCL11A	0.3365972	mirna_pc
8013	hsa-mir-1180	ZNF496	0.392684006	mirna_pc
8014	hsa-mir-1180	C4orf27	0.371036031	mirna_pc
8015	hsa-mir-1180	DDX28	0.369942201	mirna_pc
8016	hsa-mir-1180	USP21	0.511722421	mirna_pc
8017	hsa-mir-1180	ALMS1	0.330997485	mirna_pc
8018	hsa-mir-1180	SLC25A11	0.39162615	mirna_pc
8019	hsa-mir-1180	ZNF74	0.395731047	mirna_pc
8020	hsa-mir-1180	METT10D	0.309613173	mirna_pc
8021	hsa-mir-1180	ANP32A	0.376730614	mirna_pc
8022	hsa-mir-1180	RNF121	0.315951973	mirna_pc
8023	hsa-mir-1180	CPT1A	0.369101976	mirna_pc
8024	hsa-mir-1180	SUPV3L1	0.371707974	mirna_pc
8025	hsa-mir-1180	GAL	0.315320785	mirna_pc
8026	hsa-mir-1180	TSNAX	0.3183682	mirna_pc
8027	hsa-mir-1180	KHDC1	0.301972204	mirna_pc
8028	hsa-mir-1180	C2orf76	0.359869015	mirna_pc
8029	hsa-mir-1180	C17orf81	0.439926254	mirna_pc
8030	hsa-mir-1180	PHF23	0.313833542	mirna_pc
8031	hsa-mir-1180	PPP5C	0.312087792	mirna_pc
8032	hsa-mir-1180	UBE2J2	0.349194802	mirna_pc
8033	hsa-mir-1180	LRFN1	0.31766129	mirna_pc
8034	hsa-mir-1180	PEX5	0.355399781	mirna_pc
8035	hsa-mir-1180	RABL2A	0.32757974	mirna_pc
8036	hsa-mir-1180	LCMT2	0.330168659	mirna_pc
8037	hsa-mir-1180	RRP1B	0.340600428	mirna_pc
8038	hsa-mir-1180	C1orf156	0.313405749	mirna_pc
8039	hsa-mir-1180	UBTF	0.366049198	mirna_pc
8040	hsa-mir-1180	ABCC1	0.30005529	mirna_pc
8041	hsa-mir-1180	NSL1	0.300970276	mirna_pc
8042	hsa-mir-1180	ZNF428	0.317933622	mirna_pc
8043	hsa-mir-1180	ELAC2	0.306148919	mirna_pc
8044	hsa-mir-1180	LOC400657	0.303857538	mirna_pc
8045	hsa-mir-1180	PTPLAD1	0.319641233	mirna_pc

8046	hsa-mir-1180	HNRNPA0	0.377415899	mirna_pc
8047	hsa-mir-1180	SNAP47	0.320744713	mirna_pc
8048	hsa-mir-1180	MORN2	0.353986872	mirna_pc
8049	hsa-mir-1180	C12orf41	0.319450198	mirna_pc
8050	hsa-mir-1180	NAIF1	0.319100965	mirna_pc
8051	hsa-mir-1180	C11orf59	0.307251674	mirna_pc
8052	hsa-mir-1180	TFDP2	0.387014361	mirna_pc
8053	hsa-mir-1180	MLLT11	0.327621849	mirna_pc
8054	hsa-mir-1180	FAM18B	0.469949593	mirna_pc
8055	hsa-mir-1180	CCNJ	0.324839388	mirna_pc
8056	hsa-mir-1180	IFT122	0.315751215	mirna_pc
8057	hsa-mir-1180	PRPF8	0.31055461	mirna_pc
8058	hsa-mir-1180	MTERF	0.302346938	mirna_pc
8059	hsa-mir-1180	SBK1	0.399803655	mirna_pc
8060	hsa-mir-1180	PABPC4L	0.356491787	mirna_pc
8061	hsa-mir-1180	HSPA9	0.340450663	mirna_pc
8062	hsa-mir-1180	KBTBD6	0.300255434	mirna_pc
8063	hsa-mir-1180	THNSL1	0.370216479	mirna_pc
8064	hsa-mir-1180	USP13	0.398083175	mirna_pc
8065	hsa-mir-1180	GALNT14	0.4710145	mirna_pc
8066	hsa-mir-1180	TSR1	0.395165155	mirna_pc
8067	hsa-mir-1180	DNM1P35	0.324129421	mirna_pc
8068	hsa-mir-1180	DRG2	0.509128494	mirna_pc
8069	hsa-mir-1180	SCO1	0.316315786	mirna_pc
8070	hsa-mir-1180	SLC25A33	0.40739122	mirna_pc
8071	hsa-mir-1180	NGFRAP1	0.325661812	mirna_pc
8072	hsa-mir-1180	MSI1	0.353644502	mirna_pc
8073	hsa-mir-1180	CLPB	0.45251316	mirna_pc
8074	hsa-mir-1180	DAK	0.353779526	mirna_pc
8075	hsa-mir-1180	ZNF740	0.349982292	mirna_pc
8076	hsa-mir-1180	CUL7	0.301521286	mirna_pc
8077	hsa-mir-1180	PACRGL	0.308571976	mirna_pc
8078	hsa-mir-1180	DDX51	0.326190121	mirna_pc
8079	hsa-mir-1180	PBX2	0.3702852	mirna_pc
8080	hsa-mir-1180	NUBP2	0.324127733	mirna_pc
8081	hsa-mir-1180	ZNF232	0.417858942	mirna_pc
8082	hsa-mir-1180	SENP2	0.373260651	mirna_pc
8083	hsa-mir-1180	ZBTB39	0.421348778	mirna_pc
8084	hsa-mir-1180	NRCAM	0.340791613	mirna_pc
8085	hsa-mir-1180	ARAP1	0.330064602	mirna_pc
8086	hsa-mir-1180	METTL12	0.387532898	mirna_pc
8087	hsa-mir-1180	ENO3	0.450105935	mirna_pc
8088	hsa-mir-1180	RPAIN	0.472015235	mirna_pc
8089	hsa-mir-1180	DTNB	0.312184343	mirna_pc
8090	hsa-mir-1180	TRIM16L	0.33324521	mirna_pc
8091	hsa-mir-1180	FAM169A	0.328973961	mirna_pc
8092	hsa-mir-1180	PIK3CA	0.331602659	mirna_pc
8093	hsa-mir-1180	PPOX	0.376751469	mirna_pc
8094	hsa-mir-1180	GTF2IP1	0.337240461	mirna_pc
8095	hsa-mir-1180	MRPL1	0.304442242	mirna_pc
8096	hsa-mir-1180	C21orf91	0.325543572	mirna_pc
8097	hsa-mir-1180	MYCL1	0.308822346	mirna_pc
8098	hsa-mir-1180	SOD1	0.322083886	mirna_pc
8099	hsa-mir-1180	SMYD4	0.329319275	mirna_pc

8100	hsa-mir-1180	CIAO1	0.309747913	mirna_pc
8101	hsa-mir-1180	C2orf3	0.321153196	mirna_pc
8102	hsa-mir-1180	THAP4	0.376936952	mirna_pc
8103	hsa-mir-1180	TUT1	0.312753281	mirna_pc
8104	hsa-mir-1180	CHD4	0.322440803	mirna_pc
8105	hsa-mir-1180	LYZ	0.348581938	mirna_pc
8106	hsa-mir-1180	GFER	0.305929377	mirna_pc
8107	hsa-mir-1180	ACOT13	0.307205696	mirna_pc
8108	hsa-mir-1180	C21orf56	0.32028196	mirna_pc
8109	hsa-mir-1180	RRP7B	0.419067817	mirna_pc
8110	hsa-mir-1180	DNAJC19	0.531360887	mirna_pc
8111	hsa-mir-1180	CCDC157	0.329824422	mirna_pc
8112	hsa-mir-1180	KILLIN	0.302473575	mirna_pc
8113	hsa-mir-1180	SCML2	0.31609001	mirna_pc
8114	hsa-mir-1180	FAM18B2	0.555217531	mirna_pc
8115	hsa-mir-1180	NMNAT3	0.304693681	mirna_pc
8116	hsa-mir-1180	MRPL19	0.35882959	mirna_pc
8117	hsa-mir-1180	HSF2	0.30708581	mirna_pc
8118	hsa-mir-1180	ZNF18	0.314682263	mirna_pc
8119	hsa-mir-1180	TOMM20	0.419130762	mirna_pc
8120	hsa-mir-1180	CHCHD6	0.369718781	mirna_pc
8121	hsa-mir-1180	ETV5	0.499746488	mirna_pc
8122	hsa-mir-1180	DACT2	0.341479393	mirna_pc
8123	hsa-mir-1180	B9D1	0.60375444	mirna_pc
8124	hsa-mir-1180	NUCKS1	0.306068204	mirna_pc
8125	hsa-mir-1180	KIAA1430	0.308711752	mirna_pc
8126	hsa-mir-1180	EXOSC6	0.320808234	mirna_pc
8127	hsa-mir-1180	PLRG1	0.321013455	mirna_pc
8128	hsa-mir-1180	AADAT	0.396341331	mirna_pc
8129	hsa-mir-1180	IGHMBP2	0.385408541	mirna_pc
8130	hsa-mir-1180	RAI1	0.410685154	mirna_pc
8131	hsa-mir-1180	FDX1L	0.321939522	mirna_pc
8132	hsa-mir-1180	THAP11	0.308300432	mirna_pc
8133	hsa-mir-1180	ACTR3B	0.333225776	mirna_pc
8134	hsa-mir-1180	ZNF48	0.481364783	mirna_pc
8135	hsa-mir-1180	PRR22	0.423614697	mirna_pc
8136	hsa-mir-1180	C11orf51	0.35035898	mirna_pc
8137	hsa-mir-1180	BAT4	0.313118804	mirna_pc
8138	hsa-mir-1180	DDB1	0.48927801	mirna_pc
8139	hsa-mir-1180	KLHDC3	0.322150692	mirna_pc
8140	hsa-mir-1180	SERF1A	0.326881878	mirna_pc
8141	hsa-mir-1180	PLD6	0.362738197	mirna_pc
8142	hsa-mir-1180	GEMIN4	0.402774706	mirna_pc
8143	hsa-mir-1180	HDDC2	0.312835179	mirna_pc
8144	hsa-mir-1180	B4GALNT4	0.433245458	mirna_pc
8145	hsa-mir-1180	PRMT6	0.343383462	mirna_pc
8146	hsa-mir-1180	LOC401397	0.330236596	mirna_pc
8147	hsa-mir-1180	HSDL1	0.354721755	mirna_pc
8148	hsa-mir-1180	C21orf119	0.332744796	mirna_pc
8149	hsa-mir-1180	H2AFY2	0.454999047	mirna_pc
8150	hsa-mir-1180	NCRNA00188	0.430083536	mirna_pc
8151	hsa-mir-1180	EPHB3	0.31523238	mirna_pc
8152	hsa-mir-1180	MRPS23	0.392982864	mirna_pc
8153	hsa-mir-1180	SLC27A5	0.330427424	mirna_pc

8154	hsa-mir-1180	AARSD1	0.307371047	mirna_pc
8155	hsa-mir-1180	TMED10	0.316493618	mirna_pc
8156	hsa-mir-1180	MIS12	0.342760711	mirna_pc
8157	hsa-mir-1180	MLF 1.00	0.337651491	mirna_pc
8158	hsa-mir-1180	BOD1	0.352128176	mirna_pc
8159	hsa-mir-1180	TMEM14B	0.305829352	mirna_pc
8160	hsa-mir-1180	SMARCAD1	0.373300052	mirna_pc
8161	hsa-mir-1180	SCHIP1	0.347960724	mirna_pc
8162	hsa-mir-1180	KIAA0114	0.326075304	mirna_pc
8163	hsa-mir-1180	VPS37D	0.427069381	mirna_pc
8164	hsa-mir-1180	SV2A	0.335245557	mirna_pc
8165	hsa-mir-1180	NT5M	0.479039605	mirna_pc
8166	hsa-mir-1180	ZNF77	0.361003589	mirna_pc
8167	hsa-mir-1180	SEMA4C	0.339868446	mirna_pc
8168	hsa-mir-1180	THOC5	0.342569073	mirna_pc
8169	hsa-mir-1180	EFNB3	0.38303316	mirna_pc
8170	hsa-mir-1180	SSR1	0.314773314	mirna_pc
8171	hsa-mir-1180	RPAP1	0.326988177	mirna_pc
8172	hsa-mir-1180	MAFG	0.303924191	mirna_pc
8173	hsa-mir-1180	CENPV	0.334781074	mirna_pc
8174	hsa-mir-1180	VANGL2	0.328123457	mirna_pc
8175	hsa-mir-1180	DNMT3A	0.447135851	mirna_pc
8176	hsa-mir-1180	FAM69B	0.350765037	mirna_pc
8177	hsa-mir-1180	DNAJC11	0.301177551	mirna_pc
8178	hsa-mir-1180	COPS3	0.531312997	mirna_pc
8179	hsa-mir-1180	ACCN2	0.353093844	mirna_pc
8180	hsa-mir-1180	VARS2	0.329232846	mirna_pc
8181	hsa-mir-1180	C12orf73	0.345366324	mirna_pc
8182	hsa-mir-1180	PATZ1	0.342029955	mirna_pc
8183	hsa-mir-1180	TIMM22	0.347923819	mirna_pc
8184	hsa-mir-1180	C3orf33	0.395822597	mirna_pc
8185	hsa-mir-1180	E4F1	0.318812636	mirna_pc
8186	hsa-mir-1180	PIGM	0.337251825	mirna_pc
8187	hsa-mir-1180	SDHC	0.318203796	mirna_pc
8188	hsa-mir-1180	TMEM231	0.319501394	mirna_pc
8189	hsa-mir-1180	ZNF768	0.333772881	mirna_pc
8190	hsa-mir-455	KPNA2	0.550210101	mirna_pc
8191	hsa-mir-455	RCC2	0.375142355	mirna_pc
8192	hsa-mir-455	KIF11	0.364125134	mirna_pc
8193	hsa-mir-455	FOXM1	0.438591087	mirna_pc
8194	hsa-mir-455	RRM2	0.473374202	mirna_pc
8195	hsa-mir-455	SPC24	0.394956668	mirna_pc
8196	hsa-mir-455	CDK1	0.384320351	mirna_pc
8197	hsa-mir-455	PLK1	0.465433568	mirna_pc
8198	hsa-mir-455	UBE2C	0.328777356	mirna_pc
8199	hsa-mir-455	PRC1	0.33756456	mirna_pc
8200	hsa-mir-455	NUSAP1	0.381590683	mirna_pc
8201	hsa-mir-455	MCM4	0.394562522	mirna_pc
8202	hsa-mir-455	BUB1B	0.380341914	mirna_pc
8203	hsa-mir-455	KIF18B	0.430090313	mirna_pc
8204	hsa-mir-455	ANLN	0.308731159	mirna_pc
8205	hsa-mir-455	KIF2C	0.342363561	mirna_pc
8206	hsa-mir-455	NCAPG	0.453307708	mirna_pc
8207	hsa-mir-455	CDC20	0.514646645	mirna_pc

8208	hsa-mir-455	CLSPN	0.313740937	mirna_pc
8209	hsa-mir-455	CCNA2	0.534379886	mirna_pc
8210	hsa-mir-455	CEP55	0.397069651	mirna_pc
8211	hsa-mir-455	SPC25	0.3347111	mirna_pc
8212	hsa-mir-455	MELK	0.329718146	mirna_pc
8213	hsa-mir-455	FANCI	0.369654287	mirna_pc
8214	hsa-mir-455	LMNB2	0.421726375	mirna_pc
8215	hsa-mir-455	NEK2	0.307022841	mirna_pc
8216	hsa-mir-455	MND1	0.464669983	mirna_pc
8217	hsa-mir-455	KIF22	0.389208523	mirna_pc
8218	hsa-mir-455	FAM49B	0.306096442	mirna_pc
8219	hsa-mir-455	NCAPH	0.428718535	mirna_pc
8220	hsa-mir-455	GTSE1	0.333594644	mirna_pc
8221	hsa-mir-455	KIF23	0.331965924	mirna_pc
8222	hsa-mir-455	RAD54L	0.353393851	mirna_pc
8223	hsa-mir-455	CENPA	0.53027456	mirna_pc
8224	hsa-mir-455	MAD2L1	0.473547887	mirna_pc
8225	hsa-mir-455	TIMELESS	0.385230213	mirna_pc
8226	hsa-mir-455	DLGAP5	0.36631062	mirna_pc
8227	hsa-mir-455	TROAP	0.338685204	mirna_pc
8228	hsa-mir-455	CCNB1	0.488341088	mirna_pc
8229	hsa-mir-455	CDCA5	0.38822414	mirna_pc
8230	hsa-mir-455	CDC45	0.398501269	mirna_pc
8231	hsa-mir-455	STMN1	0.315537508	mirna_pc
8232	hsa-mir-455	EXO1	0.307572889	mirna_pc
8233	hsa-mir-455	TK1	0.339604942	mirna_pc
8234	hsa-mir-455	CDCA2	0.312571286	mirna_pc
8235	hsa-mir-455	UBE2T	0.337277907	mirna_pc
8236	hsa-mir-455	CKS2	0.383263843	mirna_pc
8237	hsa-mir-455	CKS1B	0.475137136	mirna_pc
8238	hsa-mir-455	TUBB	0.42879522	mirna_pc
8239	hsa-mir-455	MCM2	0.412071402	mirna_pc
8240	hsa-mir-455	ORC1L	0.415587904	mirna_pc
8241	hsa-mir-455	UHRF1	0.450711325	mirna_pc
8242	hsa-mir-455	CCNF	0.393250214	mirna_pc
8243	hsa-mir-455	RACGAP1	0.341129512	mirna_pc
8244	hsa-mir-455	PTBP1	0.389797021	mirna_pc
8245	hsa-mir-455	CCNB2	0.432456851	mirna_pc
8246	hsa-mir-455	PLK4	0.445646142	mirna_pc
8247	hsa-mir-455	FANCB	0.300279403	mirna_pc
8248	hsa-mir-455	KIF18A	0.33493041	mirna_pc
8249	hsa-mir-455	PTTG1	0.330750279	mirna_pc
8250	hsa-mir-455	KIF14	0.317523613	mirna_pc
8251	hsa-mir-455	HJURP	0.350716545	mirna_pc
8252	hsa-mir-455	SKA1	0.340382059	mirna_pc
8253	hsa-mir-455	RAD51	0.40018635	mirna_pc
8254	hsa-mir-455	BLM	0.31987901	mirna_pc
8255	hsa-mir-455	SHCBP1	0.370304854	mirna_pc
8256	hsa-mir-455	CKAP2L	0.433805362	mirna_pc
8257	hsa-mir-455	DNMT1	0.354971696	mirna_pc
8258	hsa-mir-455	PAICS	0.442432871	mirna_pc
8259	hsa-mir-455	BIRC5	0.417602759	mirna_pc
8260	hsa-mir-455	RCC1	0.341170922	mirna_pc
8261	hsa-mir-455	OIP5	0.304108689	mirna_pc

8262	hsa-mir-455	DTL	0.453123098	mirna_pc
8263	hsa-mir-455	EME1	0.392231985	mirna_pc
8264	hsa-mir-455	CENPK	0.335212901	mirna_pc
8265	hsa-mir-455	AURKB	0.499135309	mirna_pc
8266	hsa-mir-455	GSG2	0.407797514	mirna_pc
8267	hsa-mir-455	CDT1	0.354298143	mirna_pc
8268	hsa-mir-455	MCM10	0.4190786	mirna_pc
8269	hsa-mir-455	KIF20A	0.352482934	mirna_pc
8270	hsa-mir-455	PSMB2	0.377710921	mirna_pc
8271	hsa-mir-455	SGOL2	0.374677878	mirna_pc
8272	hsa-mir-455	CENPE	0.301608973	mirna_pc
8273	hsa-mir-455	POLQ	0.304296999	mirna_pc
8274	hsa-mir-455	CDCA3	0.364868798	mirna_pc
8275	hsa-mir-455	GINS1	0.304516461	mirna_pc
8276	hsa-mir-455	ALG3	0.328956913	mirna_pc
8277	hsa-mir-455	FEN1	0.40904153	mirna_pc
8278	hsa-mir-455	ORC6L	0.431449244	mirna_pc
8279	hsa-mir-455	ERH	0.378546022	mirna_pc
8280	hsa-mir-455	C16orf59	0.459899478	mirna_pc
8281	hsa-mir-455	CDKN3	0.361914209	mirna_pc
8282	hsa-mir-455	C17orf53	0.32566698	mirna_pc
8283	hsa-mir-455	NEIL3	0.549820311	mirna_pc
8284	hsa-mir-455	MCM7	0.374809053	mirna_pc
8285	hsa-mir-455	ACTL6A	0.306599418	mirna_pc
8286	hsa-mir-455	POLD1	0.326134524	mirna_pc
8287	hsa-mir-455	HNRNPL	0.360380098	mirna_pc
8288	hsa-mir-455	SMC4	0.315074218	mirna_pc
8289	hsa-mir-455	PKMYT1	0.537417501	mirna_pc
8290	hsa-mir-455	TUBA1B	0.435709277	mirna_pc
8291	hsa-mir-455	RUVBL1	0.384775251	mirna_pc
8292	hsa-mir-455	POLE2	0.32423785	mirna_pc
8293	hsa-mir-455	UBE2S	0.459986824	mirna_pc
8294	hsa-mir-455	NCAPD2	0.330660609	mirna_pc
8295	hsa-mir-455	CCT5	0.3336085	mirna_pc
8296	hsa-mir-455	HNRNPC	0.40860248	mirna_pc
8297	hsa-mir-455	PPIA	0.32775453	mirna_pc
8298	hsa-mir-455	TCF3	0.432437854	mirna_pc
8299	hsa-mir-455	CHEK1	0.325441603	mirna_pc
8300	hsa-mir-455	PRR11	0.32002837	mirna_pc
8301	hsa-mir-455	PATL1	0.311212865	mirna_pc
8302	hsa-mir-455	MCM5	0.416429549	mirna_pc
8303	hsa-mir-455	RAN	0.340927331	mirna_pc
8304	hsa-mir-455	TYMS	0.317125026	mirna_pc
8305	hsa-mir-455	MCM6	0.343259236	mirna_pc
8306	hsa-mir-455	RNASEH2A	0.412914613	mirna_pc
8307	hsa-mir-455	EPR1	0.354058853	mirna_pc
8308	hsa-mir-455	BRIP1	0.313133307	mirna_pc
8309	hsa-mir-455	H2AFX	0.393496533	mirna_pc
8310	hsa-mir-455	MTHFD1L	0.33688311	mirna_pc
8311	hsa-mir-455	PSMD14	0.380636123	mirna_pc
8312	hsa-mir-455	UCK2	0.345118913	mirna_pc
8313	hsa-mir-455	WDHD1	0.406006825	mirna_pc
8314	hsa-mir-455	EFTUD2	0.374876278	mirna_pc
8315	hsa-mir-455	THOC4	0.424992906	mirna_pc

8316	hsa-mir-455	GLT25D1	0.429790178	mirna_pc
8317	hsa-mir-455	CENPN	0.310988372	mirna_pc
8318	hsa-mir-455	PRIM2	0.346733515	mirna_pc
8319	hsa-mir-455	RTKN	0.383632946	mirna_pc
8320	hsa-mir-455	FAM64A	0.382162533	mirna_pc
8321	hsa-mir-455	RANBP1	0.439690527	mirna_pc
8322	hsa-mir-455	MTBP	0.338965714	mirna_pc
8323	hsa-mir-455	SNRPG	0.31486797	mirna_pc
8324	hsa-mir-455	EIF4A3	0.339852025	mirna_pc
8325	hsa-mir-455	SNRPA	0.326236742	mirna_pc
8326	hsa-mir-455	FUS	0.419304854	mirna_pc
8327	hsa-mir-455	DNAJC9	0.505624018	mirna_pc
8328	hsa-mir-455	TUBA1C	0.311058867	mirna_pc
8329	hsa-mir-455	CHAF1A	0.397055687	mirna_pc
8330	hsa-mir-455	PSMD11	0.302875094	mirna_pc
8331	hsa-mir-455	CENPO	0.534484792	mirna_pc
8332	hsa-mir-455	SERPINH1	0.319337697	mirna_pc
8333	hsa-mir-455	NUDT1	0.556467962	mirna_pc
8334	hsa-mir-455	GINS2	0.433813949	mirna_pc
8335	hsa-mir-455	TEAD4	0.427799583	mirna_pc
8336	hsa-mir-455	CDCA4	0.573541779	mirna_pc
8337	hsa-mir-455	DTYMK	0.314978589	mirna_pc
8338	hsa-mir-455	TMEM206	0.325316731	mirna_pc
8339	hsa-mir-455	E2F7	0.402927135	mirna_pc
8340	hsa-mir-455	MYO1B	0.496675569	mirna_pc
8341	hsa-mir-455	C16orf75	0.364722865	mirna_pc
8342	hsa-mir-455	EBNA1BP2	0.355192134	mirna_pc
8343	hsa-mir-455	NOP2	0.348584647	mirna_pc
8344	hsa-mir-455	TCF19	0.31690684	mirna_pc
8345	hsa-mir-455	CAD	0.389428976	mirna_pc
8346	hsa-mir-455	CDK2	0.434605655	mirna_pc
8347	hsa-mir-455	CDK4	0.359536987	mirna_pc
8348	hsa-mir-455	C7orf11	0.364500472	mirna_pc
8349	hsa-mir-455	ENO1	0.397317559	mirna_pc
8350	hsa-mir-455	LAMC2	0.487570927	mirna_pc
8351	hsa-mir-455	CENPH	0.405688703	mirna_pc
8352	hsa-mir-455	TCOF1	0.411136932	mirna_pc
8353	hsa-mir-455	TMSB10	0.387275592	mirna_pc
8354	hsa-mir-455	ULBP2	0.346709157	mirna_pc
8355	hsa-mir-455	EIF3B	0.313643335	mirna_pc
8356	hsa-mir-455	G3BP1	0.31405884	mirna_pc
8357	hsa-mir-455	TOMM40	0.330745299	mirna_pc
8358	hsa-mir-455	F12	0.321977611	mirna_pc
8359	hsa-mir-455	NOL11	0.300478696	mirna_pc
8360	hsa-mir-455	TMEM189	0.337700668	mirna_pc
8361	hsa-mir-455	ARL6IP6	0.405806161	mirna_pc
8362	hsa-mir-455	HAUS8	0.306487133	mirna_pc
8363	hsa-mir-455	RFC4	0.454759098	mirna_pc
8364	hsa-mir-455	FBXO5	0.306201822	mirna_pc
8365	hsa-mir-455	LAMB3	0.403316765	mirna_pc
8366	hsa-mir-455	EFNB1	0.419937097	mirna_pc
8367	hsa-mir-455	ACOT7	0.326565504	mirna_pc
8368	hsa-mir-455	H2AFZ	0.507900246	mirna_pc
8369	hsa-mir-455	NRM	0.422409353	mirna_pc

8370	hsa-mir-455	MAGOH	0.329522267	mirna_pc
8371	hsa-mir-455	NUP37	0.419212813	mirna_pc
8372	hsa-mir-455	LDHA	0.395248257	mirna_pc
8373	hsa-mir-455	CPSF3	0.322700097	mirna_pc
8374	hsa-mir-455	SNRPE	0.328645516	mirna_pc
8375	hsa-mir-455	PAQR4	0.392157516	mirna_pc
8376	hsa-mir-455	PPM1G	0.357226946	mirna_pc
8377	hsa-mir-455	SNRPD2	0.307576859	mirna_pc
8378	hsa-mir-455	PLAU	0.377415996	mirna_pc
8379	hsa-mir-455	MDFI	0.325040079	mirna_pc
8380	hsa-mir-455	NPM3	0.37139801	mirna_pc
8381	hsa-mir-455	TFAP4	0.337419039	mirna_pc
8382	hsa-mir-455	PUS7	0.439338988	mirna_pc
8383	hsa-mir-455	CDH3	0.422225911	mirna_pc
8384	hsa-mir-455	NUTF2	0.36913911	mirna_pc
8385	hsa-mir-455	RNPS1	0.354579736	mirna_pc
8386	hsa-mir-455	DAZAP1	0.416222629	mirna_pc
8387	hsa-mir-455	TXNDC9	0.351914618	mirna_pc
8388	hsa-mir-455	C10orf55	0.340542104	mirna_pc
8389	hsa-mir-455	PPIL5	0.38938662	mirna_pc
8390	hsa-mir-455	TMEM201	0.336966129	mirna_pc
8391	hsa-mir-455	DCTPP1	0.326802306	mirna_pc
8392	hsa-mir-455	PSRC1	0.384626512	mirna_pc
8393	hsa-mir-455	ODF2	0.373123108	mirna_pc
8394	hsa-mir-455	DBF4B	0.322623174	mirna_pc
8395	hsa-mir-455	S100A3	0.336545972	mirna_pc
8396	hsa-mir-455	STIP1	0.375774108	mirna_pc
8397	hsa-mir-455	HIST1H1E	0.443657464	mirna_pc
8398	hsa-mir-455	PSMG3	0.333558128	mirna_pc
8399	hsa-mir-455	THOP1	0.322766691	mirna_pc
8400	hsa-mir-455	SET	0.468634854	mirna_pc
8401	hsa-mir-455	NIP7	0.320186407	mirna_pc
8402	hsa-mir-455	C3orf26	0.390870342	mirna_pc
8403	hsa-mir-455	MRTO4	0.36667135	mirna_pc
8404	hsa-mir-455	BMP1	0.300857444	mirna_pc
8405	hsa-mir-455	NUP93	0.303346587	mirna_pc
8406	hsa-mir-455	POLE3	0.496947853	mirna_pc
8407	hsa-mir-455	GABPB1	0.375551944	mirna_pc
8408	hsa-mir-455	THOC6	0.367760165	mirna_pc
8409	hsa-mir-455	GMPS	0.360765921	mirna_pc
8410	hsa-mir-455	PPAT	0.314642567	mirna_pc
8411	hsa-mir-455	EIF5A	0.386243119	mirna_pc
8412	hsa-mir-455	GNB1L	0.316142408	mirna_pc
8413	hsa-mir-455	SMYD5	0.326974781	mirna_pc
8414	hsa-mir-455	PFN1	0.448373871	mirna_pc
8415	hsa-mir-455	BOP 1.00	0.337770806	mirna_pc
8416	hsa-mir-455	POLA2	0.389784614	mirna_pc
8417	hsa-mir-455	SFN	0.35949825	mirna_pc
8418	hsa-mir-455	CCDC77	0.305160856	mirna_pc
8419	hsa-mir-455	BCL11B	0.352779704	mirna_pc
8420	hsa-mir-455	EXT1	0.317841305	mirna_pc
8421	hsa-mir-455	TEX10	0.422265999	mirna_pc
8422	hsa-mir-455	SKP2	0.37386264	mirna_pc
8423	hsa-mir-455	TRAF3	0.327975723	mirna_pc

8424	hsa-mir-455	UBE2I	0.312339421	mirna_pc
8425	hsa-mir-455	TIPIN	0.322878873	mirna_pc
8426	hsa-mir-455	CYP27B1	0.32005908	mirna_pc
8427	hsa-mir-455	POLD2	0.366920157	mirna_pc
8428	hsa-mir-455	SMARCD1	0.390589286	mirna_pc
8429	hsa-mir-455	ATXN2L	0.401833219	mirna_pc
8430	hsa-mir-455	C4orf46	0.400829772	mirna_pc
8431	hsa-mir-455	AGTRAP	0.337083815	mirna_pc
8432	hsa-mir-455	ECE2	0.400606947	mirna_pc
8433	hsa-mir-455	GPN1	0.381447363	mirna_pc
8434	hsa-mir-455	VAV2	0.339142857	mirna_pc
8435	hsa-mir-455	FANCG	0.333846286	mirna_pc
8436	hsa-mir-455	MAZ	0.432691998	mirna_pc
8437	hsa-mir-455	RANGAP1	0.335945728	mirna_pc
8438	hsa-mir-455	SSRP1	0.439119391	mirna_pc
8439	hsa-mir-455	COL7A1	0.399001251	mirna_pc
8440	hsa-mir-455	POLR2D	0.336794196	mirna_pc
8441	hsa-mir-455	FTSJ2	0.301718983	mirna_pc
8442	hsa-mir-455	PA2G4	0.326466454	mirna_pc
8443	hsa-mir-455	SNRPD3	0.353254082	mirna_pc
8444	hsa-mir-455	KHDRBS1	0.363824532	mirna_pc
8445	hsa-mir-455	YEATS2	0.388860083	mirna_pc
8446	hsa-mir-455	MTL 5.00	0.352239087	mirna_pc
8447	hsa-mir-455	FSCN1	0.523240355	mirna_pc
8448	hsa-mir-455	RAD51L1	0.352371112	mirna_pc
8449	hsa-mir-455	PRPF4	0.364108858	mirna_pc
8450	hsa-mir-455	PYCRL	0.370840706	mirna_pc
8451	hsa-mir-455	HSP90AA1	0.300786225	mirna_pc
8452	hsa-mir-455	LOC100130776	0.352861362	mirna_pc
8453	hsa-mir-455	CCT7	0.423094414	mirna_pc
8454	hsa-mir-455	SNRNP40	0.383837209	mirna_pc
8455	hsa-mir-455	ITGB4	0.352255118	mirna_pc
8456	hsa-mir-455	MED30	0.324562002	mirna_pc
8457	hsa-mir-455	GINS3	0.400131931	mirna_pc
8458	hsa-mir-455	RPLP0P2	0.322805147	mirna_pc
8459	hsa-mir-455	NEDD1	0.499537173	mirna_pc
8460	hsa-mir-455	JUB	0.388247178	mirna_pc
8461	hsa-mir-455	YWHAG	0.367475731	mirna_pc
8462	hsa-mir-455	WRAP53	0.361547113	mirna_pc
8463	hsa-mir-455	PSMC2	0.374294684	mirna_pc
8464	hsa-mir-455	PTPN12	0.306650528	mirna_pc
8465	hsa-mir-455	CCDC86	0.501957899	mirna_pc
8466	hsa-mir-455	ZP3	0.326119547	mirna_pc
8467	hsa-mir-455	WDR76	0.354270411	mirna_pc
8468	hsa-mir-455	EXOSC2	0.451059625	mirna_pc
8469	hsa-mir-455	C11orf84	0.44065864	mirna_pc
8470	hsa-mir-455	KRT17	0.38680578	mirna_pc
8471	hsa-mir-455	TOP1MT	0.34187684	mirna_pc
8472	hsa-mir-455	GAPDH	0.341333121	mirna_pc
8473	hsa-mir-455	HIST1H2BK	0.375070715	mirna_pc
8474	hsa-mir-455	NCBP2	0.302018414	mirna_pc
8475	hsa-mir-455	FBXO22OS	0.300078381	mirna_pc
8476	hsa-mir-455	SLC3A2	0.465155554	mirna_pc
8477	hsa-mir-455	HNRNPD	0.362633952	mirna_pc

8478	hsa-mir-455	DDX31	0.322535963	mirna_pc
8479	hsa-mir-455	EIF5AL1	0.407048906	mirna_pc
8480	hsa-mir-455	SHOX2	0.32424476	mirna_pc
8481	hsa-mir-455	PLOD1	0.310681089	mirna_pc
8482	hsa-mir-455	REXO4	0.467760755	mirna_pc
8483	hsa-mir-455	MEN1	0.474115038	mirna_pc
8484	hsa-mir-455	IKBIP	0.354350571	mirna_pc
8485	hsa-mir-455	C17orf49	0.354428411	mirna_pc
8486	hsa-mir-455	H1FX	0.373313791	mirna_pc
8487	hsa-mir-455	PNO1	0.320517388	mirna_pc
8488	hsa-mir-455	CCT4	0.442670819	mirna_pc
8489	hsa-mir-455	SAC3D1	0.358014836	mirna_pc
8490	hsa-mir-455	RABEPK	0.461023598	mirna_pc
8491	hsa-mir-455	ELAVL1	0.347295373	mirna_pc
8492	hsa-mir-455	WDR18	0.347012092	mirna_pc
8493	hsa-mir-455	CENPP	0.50921818	mirna_pc
8494	hsa-mir-455	MTHFD1	0.363646776	mirna_pc
8495	hsa-mir-455	MIF	0.372524095	mirna_pc
8496	hsa-mir-455	TFAP2A	0.413335154	mirna_pc
8497	hsa-mir-455	EIF4A1	0.426361665	mirna_pc
8498	hsa-mir-455	MMP12	0.423115924	mirna_pc
8499	hsa-mir-455	PSMC3IP	0.408689836	mirna_pc
8500	hsa-mir-455	PPP1R14B	0.429855525	mirna_pc
8501	hsa-mir-455	PES 1.00	0.325338774	mirna_pc
8502	hsa-mir-455	PAK2	0.344132405	mirna_pc
8503	hsa-mir-455	PSMB7	0.472527715	mirna_pc
8504	hsa-mir-455	E2F4	0.388639289	mirna_pc
8505	hsa-mir-455	GJB3	0.404530948	mirna_pc
8506	hsa-mir-455	TRMT112	0.41700596	mirna_pc
8507	hsa-mir-455	PKM2	0.355695616	mirna_pc
8508	hsa-mir-455	PLEK2	0.424978733	mirna_pc
8509	hsa-mir-455	PSMD2	0.424750054	mirna_pc
8510	hsa-mir-455	PGAM5	0.304674815	mirna_pc
8511	hsa-mir-455	PMAIP1	0.409989603	mirna_pc
8512	hsa-mir-455	YWHAE	0.420179257	mirna_pc
8513	hsa-mir-455	KDM1A	0.31814125	mirna_pc
8514	hsa-mir-455	FOXD1	0.326254741	mirna_pc
8515	hsa-mir-455	ADCY3	0.341090687	mirna_pc
8516	hsa-mir-455	SLC25A22	0.372834881	mirna_pc
8517	hsa-mir-455	GLE1	0.321836839	mirna_pc
8518	hsa-mir-455	PGF	0.381274914	mirna_pc
8519	hsa-mir-455	WDR4	0.353404665	mirna_pc
8520	hsa-mir-455	NLN	0.359951816	mirna_pc
8521	hsa-mir-455	YARS2	0.353498527	mirna_pc
8522	hsa-mir-455	ADRB2	0.306370476	mirna_pc
8523	hsa-mir-455	TM4SF19	0.329717171	mirna_pc
8524	hsa-mir-455	GLRX3	0.385636159	mirna_pc
8525	hsa-mir-455	NCLN	0.329376521	mirna_pc
8526	hsa-mir-455	C14orf80	0.420180621	mirna_pc
8527	hsa-mir-455	AHSA1	0.338991135	mirna_pc
8528	hsa-mir-455	TGFA	0.367784062	mirna_pc
8529	hsa-mir-455	SKA2	0.3099345	mirna_pc
8530	hsa-mir-455	ADORA2B	0.360062591	mirna_pc
8531	hsa-mir-455	CTSL2	0.336561349	mirna_pc

8532	hsa-mir-455	MT2A	0.315307672	mirna_pc
8533	hsa-mir-455	KHSRP	0.31910111	mirna_pc
8534	hsa-mir-455	LRWD1	0.350679018	mirna_pc
8535	hsa-mir-455	HEATR2	0.335648168	mirna_pc
8536	hsa-mir-455	SH2D5	0.50869096	mirna_pc
8537	hsa-mir-455	LSM 6.00	0.348205574	mirna_pc
8538	hsa-mir-455	PRAME	0.306257898	mirna_pc
8539	hsa-mir-455	FTSJ1	0.334663378	mirna_pc
8540	hsa-mir-455	WDR74	0.33594707	mirna_pc
8541	hsa-mir-455	SUV39H1	0.329456786	mirna_pc
8542	hsa-mir-455	POLR2G	0.313927313	mirna_pc
8543	hsa-mir-455	PSMB5	0.326720381	mirna_pc
8544	hsa-mir-455	MFAP2	0.319222615	mirna_pc
8545	hsa-mir-455	NUP188	0.418332784	mirna_pc
8546	hsa-mir-455	TRA2B	0.322591763	mirna_pc
8547	hsa-mir-455	DPF1	0.325767212	mirna_pc
8548	hsa-mir-455	C15orf23	0.437485096	mirna_pc
8549	hsa-mir-455	KDELC1	0.317521259	mirna_pc
8550	hsa-mir-455	CIRH1A	0.339686783	mirna_pc
8551	hsa-mir-455	C3orf21	0.387742971	mirna_pc
8552	hsa-mir-455	SDCCAG3	0.338056285	mirna_pc
8553	hsa-mir-455	CD276	0.507538726	mirna_pc
8554	hsa-mir-455	IGF2BP2	0.478838868	mirna_pc
8555	hsa-mir-455	CTNNAL1	0.371171729	mirna_pc
8556	hsa-mir-455	C1QBP	0.308001175	mirna_pc
8557	hsa-mir-455	RRM1	0.327408495	mirna_pc
8558	hsa-mir-455	RAD23B	0.347546678	mirna_pc
8559	hsa-mir-455	MAP4K4	0.436246165	mirna_pc
8560	hsa-mir-455	C19orf47	0.300564113	mirna_pc
8561	hsa-mir-455	C4orf43	0.434136665	mirna_pc
8562	hsa-mir-455	XRCC3	0.545329013	mirna_pc
8563	hsa-mir-455	SRD5A1	0.3158567	mirna_pc
8564	hsa-mir-455	YY1	0.31924433	mirna_pc
8565	hsa-mir-455	GJB4	0.326226231	mirna_pc
8566	hsa-mir-455	CEP78	0.307872197	mirna_pc
8567	hsa-mir-455	TUBG1	0.426899751	mirna_pc
8568	hsa-mir-455	C16orf80	0.341713397	mirna_pc
8569	hsa-mir-455	MYBBP1A	0.338540244	mirna_pc
8570	hsa-mir-455	SFPQ	0.333572576	mirna_pc
8571	hsa-mir-455	XPO6	0.354187217	mirna_pc
8572	hsa-mir-455	CD83	0.322566317	mirna_pc
8573	hsa-mir-455	SHMT2	0.312312801	mirna_pc
8574	hsa-mir-455	CISD2	0.36850917	mirna_pc
8575	hsa-mir-455	GMNN	0.337440021	mirna_pc
8576	hsa-mir-455	EMG1	0.302415782	mirna_pc
8577	hsa-mir-455	IFI27L2	0.40680444	mirna_pc
8578	hsa-mir-455	PA2G4P4	0.364406657	mirna_pc
8579	hsa-mir-455	WDR53	0.388795931	mirna_pc
8580	hsa-mir-455	ALOX12P2	0.365143376	mirna_pc
8581	hsa-mir-455	WDR54	0.351099093	mirna_pc
8582	hsa-mir-455	RPL26L1	0.359696945	mirna_pc
8583	hsa-mir-455	DUS4L	0.337887195	mirna_pc
8584	hsa-mir-455	DUT	0.38843585	mirna_pc
8585	hsa-mir-455	CLSTN1	0.333420809	mirna_pc

8586	hsa-mir-455	PCGF1	0.417392101	mirna_pc
8587	hsa-mir-455	HPRT1	0.304018249	mirna_pc
8588	hsa-mir-455	COX4NB	0.335103505	mirna_pc
8589	hsa-mir-455	MSH6	0.36204706	mirna_pc
8590	hsa-mir-455	RPS6KA4	0.359788648	mirna_pc
8591	hsa-mir-455	TYSND1	0.370963943	mirna_pc
8592	hsa-mir-455	HOXD11	0.35667227	mirna_pc
8593	hsa-mir-455	SRM	0.357409927	mirna_pc
8594	hsa-mir-455	PSMG1	0.321649775	mirna_pc
8595	hsa-mir-455	PPP1R8	0.309141145	mirna_pc
8596	hsa-mir-455	NPM1	0.319442017	mirna_pc
8597	hsa-mir-455	KREMEN2	0.394369928	mirna_pc
8598	hsa-mir-455	TRIP6	0.425447123	mirna_pc
8599	hsa-mir-455	TMEM194B	0.419800855	mirna_pc
8600	hsa-mir-455	UPP1	0.367132878	mirna_pc
8601	hsa-mir-455	RGS20	0.42836251	mirna_pc
8602	hsa-mir-455	TPI1	0.381346525	mirna_pc
8603	hsa-mir-455	MEX3A	0.315083056	mirna_pc
8604	hsa-mir-455	MOGS	0.312646859	mirna_pc
8605	hsa-mir-455	RFC2	0.405053216	mirna_pc
8606	hsa-mir-455	FANCC	0.38844226	mirna_pc
8607	hsa-mir-455	C16orf57	0.318441941	mirna_pc
8608	hsa-mir-455	WDR5	0.52933355	mirna_pc
8609	hsa-mir-455	EXOSC4	0.356887169	mirna_pc
8610	hsa-mir-455	FAM58A	0.333944545	mirna_pc
8611	hsa-mir-455	MAD2L2	0.341642316	mirna_pc
8612	hsa-mir-455	CSNK1G2	0.388288248	mirna_pc
8613	hsa-mir-455	MAN1B1	0.317255278	mirna_pc
8614	hsa-mir-455	CBX1	0.43237305	mirna_pc
8615	hsa-mir-455	COL27A1	0.649829835	mirna_pc
8616	hsa-mir-455	SNAPC1	0.360856084	mirna_pc
8617	hsa-mir-455	NOP10	0.336486028	mirna_pc
8618	hsa-mir-455	MRPL51	0.326235494	mirna_pc
8619	hsa-mir-455	FJX1	0.566730352	mirna_pc
8620	hsa-mir-455	ZDHHC12	0.354623778	mirna_pc
8621	hsa-mir-455	HIATL1	0.326356674	mirna_pc
8622	hsa-mir-455	GYLTL1B	0.310066568	mirna_pc
8623	hsa-mir-455	MED27	0.478834979	mirna_pc
8624	hsa-mir-455	C9orf30	0.533990378	mirna_pc
8625	hsa-mir-455	WDR66	0.368970186	mirna_pc
8626	hsa-mir-455	NOL10	0.310977523	mirna_pc
8627	hsa-mir-455	PXDN	0.314785182	mirna_pc
8628	hsa-mir-455	ODC1	0.456472771	mirna_pc
8629	hsa-mir-455	ITGA3	0.387378281	mirna_pc
8630	hsa-mir-455	LOC441089	0.451498964	mirna_pc
8631	hsa-mir-455	C9orf40	0.387811712	mirna_pc
8632	hsa-mir-455	HES4	0.312662956	mirna_pc
8633	hsa-mir-455	ADSL	0.361593971	mirna_pc
8634	hsa-mir-455	VRK1	0.362930878	mirna_pc
8635	hsa-mir-455	GPR176	0.311690993	mirna_pc
8636	hsa-mir-455	FPGS	0.346981884	mirna_pc
8637	hsa-mir-455	ANP32B	0.359851125	mirna_pc
8638	hsa-mir-455	EXOSC9	0.389232931	mirna_pc
8639	hsa-mir-455	KLC2	0.334067013	mirna_pc

8640	hsa-mir-455	ZNF598	0.421634214	mirna_pc
8641	hsa-mir-455	FAM89A	0.318029543	mirna_pc
8642	hsa-mir-455	TELO2	0.360299607	mirna_pc
8643	hsa-mir-455	PRPS1	0.305939256	mirna_pc
8644	hsa-mir-455	ANO1	0.300170183	mirna_pc
8645	hsa-mir-455	TCTEX1D2	0.31427504	mirna_pc
8646	hsa-mir-455	NETO2	0.302470342	mirna_pc
8647	hsa-mir-455	SPHK1	0.373009041	mirna_pc
8648	hsa-mir-455	BNIP3	0.309873392	mirna_pc
8649	hsa-mir-455	ITGA6	0.441933824	mirna_pc
8650	hsa-mir-455	RNF126	0.34283942	mirna_pc
8651	hsa-mir-455	MLF 2.00	0.31035642	mirna_pc
8652	hsa-mir-455	RAB7A	0.323503977	mirna_pc
8653	hsa-mir-455	SIGMAR1	0.330224151	mirna_pc
8654	hsa-mir-455	CSPG4	0.314090035	mirna_pc
8655	hsa-mir-455	FCF1	0.34850855	mirna_pc
8656	hsa-mir-455	GSTO1	0.347598096	mirna_pc
8657	hsa-mir-455	RPL39L	0.486283832	mirna_pc
8658	hsa-mir-455	EIF2B2	0.316171405	mirna_pc
8659	hsa-mir-455	ACD	0.370991501	mirna_pc
8660	hsa-mir-455	RAD23A	0.328014065	mirna_pc
8661	hsa-mir-455	DNLZ	0.38035124	mirna_pc
8662	hsa-mir-455	EPHB4	0.449509688	mirna_pc
8663	hsa-mir-455	LOC152217	0.35215775	mirna_pc
8664	hsa-mir-455	TANC2	0.352774064	mirna_pc
8665	hsa-mir-455	ARNTL2	0.337645582	mirna_pc
8666	hsa-mir-455	NOB1	0.435320667	mirna_pc
8667	hsa-mir-455	PLXNA1	0.342261754	mirna_pc
8668	hsa-mir-455	ADK	0.311898322	mirna_pc
8669	hsa-mir-455	RPL12	0.331738211	mirna_pc
8670	hsa-mir-455	ZMYND19	0.453073642	mirna_pc
8671	hsa-mir-455	TAF12	0.362569632	mirna_pc
8672	hsa-mir-455	ARPC5L	0.380576075	mirna_pc
8673	hsa-mir-455	TLR2	0.326371484	mirna_pc
8674	hsa-mir-455	CDC27	0.336861708	mirna_pc
8675	hsa-mir-455	SURF2	0.428920181	mirna_pc
8676	hsa-mir-455	OSTC	0.355783932	mirna_pc
8677	hsa-mir-455	METTL11A	0.476110589	mirna_pc
8678	hsa-mir-455	LEPREL1	0.463103356	mirna_pc
8679	hsa-mir-455	DUSP7	0.322036228	mirna_pc
8680	hsa-mir-455	LTBR	0.30498429	mirna_pc
8681	hsa-mir-455	PWP1	0.342645287	mirna_pc
8682	hsa-mir-455	MNAT1	0.364095543	mirna_pc
8683	hsa-mir-455	DRAP1	0.408801279	mirna_pc
8684	hsa-mir-455	SMARCB1	0.354917699	mirna_pc
8685	hsa-mir-455	B3GAT3	0.379483365	mirna_pc
8686	hsa-mir-455	MEX3D	0.395250742	mirna_pc
8687	hsa-mir-455	IL1RAP	0.374130838	mirna_pc
8688	hsa-mir-455	B4GALT2	0.4599521	mirna_pc
8689	hsa-mir-455	RPA1	0.335580309	mirna_pc
8690	hsa-mir-455	FAM92A1	0.364788175	mirna_pc
8691	hsa-mir-455	TIMM10	0.328308264	mirna_pc
8692	hsa-mir-455	TGFB1	0.346040379	mirna_pc
8693	hsa-mir-455	IRF6	0.350109515	mirna_pc

8694	hsa-mir-455	ZDHHC18	0.403037354	mirna_pc
8695	hsa-mir-455	ITPRIPL1	0.312991694	mirna_pc
8696	hsa-mir-455	FOXK2	0.412530339	mirna_pc
8697	hsa-mir-455	ANAPC10	0.309296678	mirna_pc
8698	hsa-mir-455	TIGD5	0.363369109	mirna_pc
8699	hsa-mir-455	HEPHL1	0.376743953	mirna_pc
8700	hsa-mir-455	SLC7A5	0.362272896	mirna_pc
8701	hsa-mir-455	SLC16A1	0.421805007	mirna_pc
8702	hsa-mir-455	SLC25A17	0.346837843	mirna_pc
8703	hsa-mir-455	GSDMC	0.338505537	mirna_pc
8704	hsa-mir-455	MYO19	0.352138047	mirna_pc
8705	hsa-mir-455	NMT1	0.39472245	mirna_pc
8706	hsa-mir-455	SLC2A9	0.337632496	mirna_pc
8707	hsa-mir-455	RPL35	0.369043259	mirna_pc
8708	hsa-mir-455	DVL3	0.304955049	mirna_pc
8709	hsa-mir-455	PSMC3	0.331748124	mirna_pc
8710	hsa-mir-455	BCL7A	0.304766935	mirna_pc
8711	hsa-mir-455	LGALS1	0.395088299	mirna_pc
8712	hsa-mir-455	MTX1	0.311478796	mirna_pc
8713	hsa-mir-455	DVL2	0.36681503	mirna_pc
8714	hsa-mir-455	TRPA1	0.325827127	mirna_pc
8715	hsa-mir-455	STARD5	0.382597589	mirna_pc
8716	hsa-mir-455	TTC4	0.440489739	mirna_pc
8717	hsa-mir-455	KIAA0922	0.320407721	mirna_pc
8718	hsa-mir-455	CCM2	0.338214352	mirna_pc
8719	hsa-mir-455	PANX1	0.435243479	mirna_pc
8720	hsa-mir-455	MRPL21	0.3266467	mirna_pc
8721	hsa-mir-455	UBE2Z	0.337285649	mirna_pc
8722	hsa-mir-455	GJB5	0.309117615	mirna_pc
8723	hsa-mir-455	PPP1R14C	0.406120145	mirna_pc
8724	hsa-mir-455	CYP26B1	0.404943892	mirna_pc
8725	hsa-mir-455	KCNQ5	0.391752872	mirna_pc
8726	hsa-mir-455	PSPH	0.317448175	mirna_pc
8727	hsa-mir-455	EIF2S1	0.442902426	mirna_pc
8728	hsa-mir-455	S100A2	0.410213183	mirna_pc
8729	hsa-mir-455	TTL	0.320941	mirna_pc
8730	hsa-mir-455	C9orf69	0.429750164	mirna_pc
8731	hsa-mir-455	CNTROB	0.335908426	mirna_pc
8732	hsa-mir-455	IPPK	0.384198001	mirna_pc
8733	hsa-mir-455	C5orf13	0.343923159	mirna_pc
8734	hsa-mir-455	SEMA4F	0.322596184	mirna_pc
8735	hsa-mir-455	LPAR3	0.367327594	mirna_pc
8736	hsa-mir-455	ZBED2	0.332908485	mirna_pc
8737	hsa-mir-455	PDPN	0.440853754	mirna_pc
8738	hsa-mir-455	TUSC1	0.336719855	mirna_pc
8739	hsa-mir-455	HRAS	0.352603394	mirna_pc
8740	hsa-mir-455	RMI1	0.307395798	mirna_pc
8741	hsa-mir-455	MRPS2	0.392294927	mirna_pc
8742	hsa-mir-455	MTA1	0.407863458	mirna_pc
8743	hsa-mir-455	FMNL2	0.440189676	mirna_pc
8744	hsa-mir-455	HTRA2	0.336563141	mirna_pc
8745	hsa-mir-455	RAB38	0.377821758	mirna_pc
8746	hsa-mir-455	PFN2	0.304405543	mirna_pc
8747	hsa-mir-455	ATP6V1E2	0.34347094	mirna_pc

8748	hsa-mir-455	GNAI1	0.419707319	mirna_pc
8749	hsa-mir-455	SSSCA1	0.340112355	mirna_pc
8750	hsa-mir-455	B4GALNT1	0.403026603	mirna_pc
8751	hsa-mir-455	SENP3	0.376924446	mirna_pc
8752	hsa-mir-455	PSMD5	0.321199816	mirna_pc
8753	hsa-mir-455	C1orf74	0.312718055	mirna_pc
8754	hsa-mir-455	IL12RB2	0.334243167	mirna_pc
8755	hsa-mir-455	GPC1	0.370790991	mirna_pc
8756	hsa-mir-455	C9orf114	0.39290029	mirna_pc
8757	hsa-mir-455	PTMS	0.355012999	mirna_pc
8758	hsa-mir-455	SOCS1	0.326194699	mirna_pc
8759	hsa-mir-455	STON2	0.420531361	mirna_pc
8760	hsa-mir-455	LDLRAD3	0.426011357	mirna_pc
8761	hsa-mir-455	COPS6	0.315286955	mirna_pc
8762	hsa-mir-455	MRPS34	0.331058918	mirna_pc
8763	hsa-mir-455	TRAF7	0.339701677	mirna_pc
8764	hsa-mir-455	DARS	0.419112204	mirna_pc
8765	hsa-mir-455	AAAS	0.302399593	mirna_pc
8766	hsa-mir-455	MARK3	0.377961558	mirna_pc
8767	hsa-mir-455	CHST14	0.334130893	mirna_pc
8768	hsa-mir-455	COL17A1	0.45808531	mirna_pc
8769	hsa-mir-455	INPP5E	0.384341276	mirna_pc
8770	hsa-mir-455	C9orf6	0.302962292	mirna_pc
8771	hsa-mir-455	PYGL	0.353546096	mirna_pc
8772	hsa-mir-455	KRT5	0.388748062	mirna_pc
8773	hsa-mir-455	MRPL30	0.345381655	mirna_pc
8774	hsa-mir-455	TMEFF1	0.453578429	mirna_pc
8775	hsa-mir-455	P4HA1	0.301527106	mirna_pc
8776	hsa-mir-455	FGFBP1	0.32089991	mirna_pc
8777	hsa-mir-455	IL20RB	0.393399353	mirna_pc
8778	hsa-mir-455	GOLGA7B	0.394550218	mirna_pc
8779	hsa-mir-455	ZNF385A	0.311559246	mirna_pc
8780	hsa-mir-455	ITGB1BP1	0.361688852	mirna_pc
8781	hsa-mir-455	UBE2E3	0.376196635	mirna_pc
8782	hsa-mir-455	ENDOG	0.343710143	mirna_pc
8783	hsa-mir-455	INO80B	0.333042534	mirna_pc
8784	hsa-mir-455	LY6K	0.338949215	mirna_pc
8785	hsa-mir-455	MBD3	0.379479577	mirna_pc
8786	hsa-mir-455	RXRA	0.303762886	mirna_pc
8787	hsa-mir-455	VSNL1	0.321884972	mirna_pc
8788	hsa-mir-455	HSF1	0.349162195	mirna_pc
8789	hsa-mir-455	AVEN	0.325918755	mirna_pc
8790	hsa-mir-455	PLS3	0.341996003	mirna_pc
8791	hsa-mir-455	FADS2	0.317152404	mirna_pc
8792	hsa-mir-455	CCDC85B	0.327409716	mirna_pc
8793	hsa-mir-455	HOMER3	0.372679125	mirna_pc
8794	hsa-mir-455	SERPINE1	0.365177655	mirna_pc
8795	hsa-mir-455	PHF19	0.352361708	mirna_pc
8796	hsa-mir-455	CELSR1	0.328204123	mirna_pc
8797	hsa-mir-455	MRPL28	0.351438057	mirna_pc
8798	hsa-mir-455	HOXA7	0.305455935	mirna_pc
8799	hsa-mir-455	BTBD6	0.367570025	mirna_pc
8800	hsa-mir-455	YWHAQ	0.429546255	mirna_pc
8801	hsa-mir-455	MSN	0.423878856	mirna_pc

8802	hsa-mir-455	RPL35A	0.30897535	mirna_pc
8803	hsa-mir-455	PTRH1	0.316857923	mirna_pc
8804	hsa-mir-455	C16orf74	0.373945732	mirna_pc
8805	hsa-mir-455	ZNF771	0.32845755	mirna_pc
8806	hsa-mir-455	PLEC	0.333618466	mirna_pc
8807	hsa-mir-455	COBRA1	0.330876551	mirna_pc
8808	hsa-mir-455	SERPINE2	0.359011055	mirna_pc
8809	hsa-mir-455	FN3KRP	0.304927545	mirna_pc
8810	hsa-mir-455	CNTLN	0.315399682	mirna_pc
8811	hsa-mir-455	EIF4G1	0.302712393	mirna_pc
8812	hsa-mir-455	BICD2	0.354326563	mirna_pc
8813	hsa-mir-455	PPP2R3B	0.340800882	mirna_pc
8814	hsa-mir-455	DSE	0.320434954	mirna_pc
8815	hsa-mir-455	KRT14	0.471782038	mirna_pc
8816	hsa-mir-455	C12orf45	0.347355518	mirna_pc
8817	hsa-mir-455	PSMB6	0.43132768	mirna_pc
8818	hsa-mir-455	TMEM17	0.366986072	mirna_pc
8819	hsa-mir-455	UBAC1	0.305441844	mirna_pc
8820	hsa-mir-455	MMP17	0.311811045	mirna_pc
8821	hsa-mir-455	TMEM132A	0.400776611	mirna_pc
8822	hsa-mir-455	PGAM1	0.333288567	mirna_pc
8823	hsa-mir-455	GNA12	0.416095788	mirna_pc
8824	hsa-mir-455	YKT6	0.302973404	mirna_pc
8825	hsa-mir-455	TP63	0.336817834	mirna_pc
8826	hsa-mir-455	TMEM11	0.301100703	mirna_pc
8827	hsa-mir-455	USP5	0.308317382	mirna_pc
8828	hsa-mir-455	AGBL5	0.30454802	mirna_pc
8829	hsa-mir-455	CCRN4L	0.357049586	mirna_pc
8830	hsa-mir-455	C17orf89	0.31049948	mirna_pc
8831	hsa-mir-455	PHB2	0.313357838	mirna_pc
8832	hsa-mir-455	MAPKBP1	0.342843503	mirna_pc
8833	hsa-mir-455	SNAI2	0.525807973	mirna_pc
8834	hsa-mir-455	SLC31A2	0.40950178	mirna_pc
8835	hsa-mir-455	VAC14	0.34873364	mirna_pc
8836	hsa-mir-455	SSNA1	0.309798458	mirna_pc
8837	hsa-mir-455	APLF	0.330103252	mirna_pc
8838	hsa-mir-455	PJA1	0.337023639	mirna_pc
8839	hsa-mir-455	CDK9	0.333951056	mirna_pc
8840	hsa-mir-455	BAIAP2	0.320130032	mirna_pc
8841	hsa-mir-455	SOX15	0.412910057	mirna_pc
8842	hsa-mir-455	EIF2B3	0.389687933	mirna_pc
8843	hsa-mir-455	BNC1	0.449733116	mirna_pc
8844	hsa-mir-455	NME4	0.315823852	mirna_pc
8845	hsa-mir-455	LYRM4	0.341064287	mirna_pc
8846	hsa-mir-455	NDRG1	0.330973668	mirna_pc
8847	hsa-mir-455	CHST11	0.381833638	mirna_pc
8848	hsa-mir-455	FBXO17	0.303623067	mirna_pc
8849	hsa-mir-455	DOLK	0.336629638	mirna_pc
8850	hsa-mir-455	IRX4	0.443122912	mirna_pc
8851	hsa-mir-455	SLC25A11	0.309595323	mirna_pc
8852	hsa-mir-455	C14orf149	0.367178337	mirna_pc
8853	hsa-mir-455	MARVELD1	0.323566042	mirna_pc
8854	hsa-mir-455	PIN1	0.313525173	mirna_pc
8855	hsa-mir-455	PTCD2	0.325376411	mirna_pc

8856	hsa-mir-455	C7orf10	0.413289525	mirna_pc
8857	hsa-mir-455	LMAN2L	0.402685022	mirna_pc
8858	hsa-mir-455	TCF7L1	0.331415519	mirna_pc
8859	hsa-mir-455	C17orf81	0.306454354	mirna_pc
8860	hsa-mir-455	PTHLH	0.371609987	mirna_pc
8861	hsa-mir-455	CYP27C1	0.379014262	mirna_pc
8862	hsa-mir-455	PCDHGC3	0.324523419	mirna_pc
8863	hsa-mir-455	FADS1	0.316971042	mirna_pc
8864	hsa-mir-455	PIK3CD	0.343029497	mirna_pc
8865	hsa-mir-455	CHST15	0.313577372	mirna_pc
8866	hsa-mir-455	DDX19A	0.384361009	mirna_pc
8867	hsa-mir-455	EIF4H	0.302888424	mirna_pc
8868	hsa-mir-455	MED31	0.333616722	mirna_pc
8869	hsa-mir-455	DDX50	0.310046398	mirna_pc
8870	hsa-mir-455	RTN4RL1	0.322114664	mirna_pc
8871	hsa-mir-455	PLSCR3	0.445887325	mirna_pc
8872	hsa-mir-455	MLLT11	0.416162941	mirna_pc
8873	hsa-mir-455	RAP1GDS1	0.405331878	mirna_pc
8874	hsa-mir-455	TRAPPC1	0.345333265	mirna_pc
8875	hsa-mir-455	C14orf179	0.304409395	mirna_pc
8876	hsa-mir-455	TBCA	0.333189089	mirna_pc
8877	hsa-mir-455	AP1M1	0.322104608	mirna_pc
8878	hsa-mir-455	NXN	0.425519896	mirna_pc
8879	hsa-mir-455	LRRC8A	0.412013032	mirna_pc
8880	hsa-mir-455	SRPK2	0.468143789	mirna_pc
8881	hsa-mir-455	PGAM4	0.300091613	mirna_pc
8882	hsa-mir-455	NRG1	0.457079011	mirna_pc
8883	hsa-mir-455	BPGM	0.329499588	mirna_pc
8884	hsa-mir-455	GALNT14	0.492211044	mirna_pc
8885	hsa-mir-455	XRCC1	0.393920599	mirna_pc
8886	hsa-mir-455	LRRC8C	0.317543012	mirna_pc
8887	hsa-mir-455	GALNTL4	0.475492493	mirna_pc
8888	hsa-mir-455	AFAP1L1	0.345627171	mirna_pc
8889	hsa-mir-455	FLRT2	0.411029502	mirna_pc
8890	hsa-mir-455	CXCL14	0.378370702	mirna_pc
8891	hsa-mir-455	ERLIN1	0.30575399	mirna_pc
8892	hsa-mir-455	CLPB	0.309791454	mirna_pc
8893	hsa-mir-455	ZDHHC8	0.322448222	mirna_pc
8894	hsa-mir-455	FGD1	0.31614032	mirna_pc
8895	hsa-mir-455	CCBL1	0.331141944	mirna_pc
8896	hsa-mir-455	PRNP	0.386322114	mirna_pc
8897	hsa-mir-455	PCYT1A	0.351857974	mirna_pc
8898	hsa-mir-455	SENP2	0.305531133	mirna_pc
8899	hsa-mir-455	KIRREL	0.427848223	mirna_pc
8900	hsa-mir-455	UHRF2	0.316892546	mirna_pc
8901	hsa-mir-455	BTBD2	0.383127994	mirna_pc
8902	hsa-mir-455	PACSIN3	0.304327852	mirna_pc
8903	hsa-mir-455	TMEM200B	0.376947121	mirna_pc
8904	hsa-mir-455	FAM169A	0.348488991	mirna_pc
8905	hsa-mir-455	PPP2R4	0.352998633	mirna_pc
8906	hsa-mir-455	C16orf42	0.360252451	mirna_pc
8907	hsa-mir-455	GPR68	0.376734738	mirna_pc
8908	hsa-mir-455	EIF2AK4	0.300950441	mirna_pc
8909	hsa-mir-455	ODZ3	0.496970453	mirna_pc

8910	hsa-mir-455	TMEM203	0.310655118	mirna_pc
8911	hsa-mir-455	RASL11B	0.420278855	mirna_pc
8912	hsa-mir-455	PDLIM4	0.300821186	mirna_pc
8913	hsa-mir-455	POLE4	0.334947774	mirna_pc
8914	hsa-mir-455	PCDHGC5	0.361599494	mirna_pc
8915	hsa-mir-455	DKK 3.00	0.319213342	mirna_pc
8916	hsa-mir-455	IQCG	0.327001646	mirna_pc
8917	hsa-mir-455	CCDC111	0.314566893	mirna_pc
8918	hsa-mir-455	TXNDC17	0.348241548	mirna_pc
8919	hsa-mir-455	FST	0.331212014	mirna_pc
8920	hsa-mir-455	NAP1L4	0.374161831	mirna_pc
8921	hsa-mir-455	LOC645166	0.323663713	mirna_pc
8922	hsa-mir-455	NLRP1	0.320329096	mirna_pc
8923	hsa-mir-455	ODZ2	0.423888382	mirna_pc
8924	hsa-mir-455	UBE2L3	0.377557121	mirna_pc
8925	hsa-mir-455	ARSJ	0.335226169	mirna_pc
8926	hsa-mir-455	PSMD9	0.323994531	mirna_pc
8927	hsa-mir-455	MYH10	0.427587306	mirna_pc
8928	hsa-mir-455	FAM126A	0.312134828	mirna_pc
8929	hsa-mir-455	MOBKL2A	0.433882542	mirna_pc
8930	hsa-mir-455	PC	0.366465763	mirna_pc
8931	hsa-mir-455	DULLARD	0.357132588	mirna_pc
8932	hsa-mir-455	C1orf201	0.348594399	mirna_pc
8933	hsa-mir-455	RIC8A	0.312996261	mirna_pc
8934	hsa-mir-455	CINP	0.35702803	mirna_pc
8935	hsa-mir-455	STK17A	0.348761332	mirna_pc
8936	hsa-mir-455	IKBKAP	0.355387536	mirna_pc
8937	hsa-mir-455	ETV5	0.30957457	mirna_pc
8938	hsa-mir-455	NFE2L1	0.346952908	mirna_pc
8939	hsa-mir-455	CA12	0.346260645	mirna_pc
8940	hsa-mir-455	EXOSC6	0.455387496	mirna_pc
8941	hsa-mir-455	VEGFC	0.321588678	mirna_pc
8942	hsa-mir-455	THAP11	0.343256223	mirna_pc
8943	hsa-mir-455	LRP12	0.330722791	mirna_pc
8944	hsa-mir-455	POLR2E	0.307648245	mirna_pc
8945	hsa-mir-455	FZD1	0.313891684	mirna_pc
8946	hsa-mir-455	DFNA5	0.409580362	mirna_pc
8947	hsa-mir-455	TWIST2	0.310469643	mirna_pc
8948	hsa-mir-455	GEMIN4	0.337949935	mirna_pc
8949	hsa-mir-455	ANXA5	0.305850453	mirna_pc
8950	hsa-mir-455	RPL26	0.303433626	mirna_pc
8951	hsa-mir-455	H2AFY2	0.450335528	mirna_pc
8952	hsa-mir-455	UCHL1	0.339409337	mirna_pc
8953	hsa-mir-455	MRPS23	0.361671694	mirna_pc
8954	hsa-mir-455	P4HA2	0.316970064	mirna_pc
8955	hsa-mir-455	SCHIP1	0.383440152	mirna_pc
8956	hsa-mir-455	CIZ1	0.327281064	mirna_pc
8957	hsa-mir-455	C21orf70	0.321555768	mirna_pc
8958	hsa-mir-455	GJC1	0.327065743	mirna_pc
8959	hsa-mir-455	DPM2	0.333878361	mirna_pc
8960	hsa-mir-455	ZC3HC1	0.300964764	mirna_pc
8961	hsa-mir-455	COPS3	0.339519679	mirna_pc
8962	hsa-mir-455	CKAP4	0.437602923	mirna_pc
8963	hsa-mir-455	FEZ1	0.442639657	mirna_pc

8964	hsa-mir-455	HCG4	0.334036241	mirna_pc
8965	hsa-mir-455	RRAS2	0.368844849	mirna_pc
8966	hsa-mir-455	TIMM22	0.396414812	mirna_pc
8967	hsa-mir-455	PPIF	0.418458778	mirna_pc
8968	hsa-mir-103-2	MKI67	0.322377601	mirna_pc
8969	hsa-mir-103-2	TPX2	0.40664534	mirna_pc
8970	hsa-mir-103-2	KIF4B	0.352360176	mirna_pc
8971	hsa-mir-103-2	CENPF	0.366268074	mirna_pc
8972	hsa-mir-103-2	KIF11	0.381888101	mirna_pc
8973	hsa-mir-103-2	FOXM1	0.316237493	mirna_pc
8974	hsa-mir-103-2	RRM2	0.341365175	mirna_pc
8975	hsa-mir-103-2	CDK1	0.372266431	mirna_pc
8976	hsa-mir-103-2	CDC25C	0.330094432	mirna_pc
8977	hsa-mir-103-2	MYBL2	0.313240572	mirna_pc
8978	hsa-mir-103-2	PCNA	0.380004983	mirna_pc
8979	hsa-mir-103-2	UBE2C	0.391147712	mirna_pc
8980	hsa-mir-103-2	BUB1	0.362695485	mirna_pc
8981	hsa-mir-103-2	NUSAP1	0.341381059	mirna_pc
8982	hsa-mir-103-2	MCM4	0.365195503	mirna_pc
8983	hsa-mir-103-2	BUB1B	0.370492652	mirna_pc
8984	hsa-mir-103-2	HDGF	0.387549993	mirna_pc
8985	hsa-mir-103-2	KIF18B	0.337923195	mirna_pc
8986	hsa-mir-103-2	ASPM	0.370313525	mirna_pc
8987	hsa-mir-103-2	KIF2C	0.328112785	mirna_pc
8988	hsa-mir-103-2	FANCA	0.374471843	mirna_pc
8989	hsa-mir-103-2	NCAPG	0.402843105	mirna_pc
8990	hsa-mir-103-2	CLSPN	0.346078201	mirna_pc
8991	hsa-mir-103-2	SGOL1	0.315420602	mirna_pc
8992	hsa-mir-103-2	ARHGAP11A	0.312237794	mirna_pc
8993	hsa-mir-103-2	CDC25B	0.389084101	mirna_pc
8994	hsa-mir-103-2	CBX3	0.335059942	mirna_pc
8995	hsa-mir-103-2	SPC25	0.345690297	mirna_pc
8996	hsa-mir-103-2	MELK	0.402992276	mirna_pc
8997	hsa-mir-103-2	FANCI	0.350335845	mirna_pc
8998	hsa-mir-103-2	NEK2	0.366478393	mirna_pc
8999	hsa-mir-103-2	NCAPH	0.366818442	mirna_pc
9000	hsa-mir-103-2	GTSE1	0.348092683	mirna_pc
9001	hsa-mir-103-2	KIF23	0.308855339	mirna_pc
9002	hsa-mir-103-2	TIMELESS	0.408785133	mirna_pc
9003	hsa-mir-103-2	LMNB1	0.340404829	mirna_pc
9004	hsa-mir-103-2	TROAP	0.340806148	mirna_pc
9005	hsa-mir-103-2	CCNB1	0.300597272	mirna_pc
9006	hsa-mir-103-2	CDCA5	0.313270499	mirna_pc
9007	hsa-mir-103-2	XPO1	0.313999337	mirna_pc
9008	hsa-mir-103-2	EXO1	0.320263182	mirna_pc
9009	hsa-mir-103-2	CDCA2	0.328983355	mirna_pc
9010	hsa-mir-103-2	DKC1	0.360533311	mirna_pc
9011	hsa-mir-103-2	KIF4A	0.376682768	mirna_pc
9012	hsa-mir-103-2	ORC1L	0.37775707	mirna_pc
9013	hsa-mir-103-2	SNRPB	0.409866709	mirna_pc
9014	hsa-mir-103-2	KNTC1	0.388853599	mirna_pc
9015	hsa-mir-103-2	CCNF	0.329649607	mirna_pc
9016	hsa-mir-103-2	PTBP1	0.303922555	mirna_pc
9017	hsa-mir-103-2	NUF2	0.461252585	mirna_pc

9018	hsa-mir-103-2	PLK4	0.348410716	mirna_pc
9019	hsa-mir-103-2	FANCB	0.386503307	mirna_pc
9020	hsa-mir-103-2	KIF18A	0.339485148	mirna_pc
9021	hsa-mir-103-2	DEPDC1	0.319019731	mirna_pc
9022	hsa-mir-103-2	KIF14	0.34979961	mirna_pc
9023	hsa-mir-103-2	HJURP	0.377951454	mirna_pc
9024	hsa-mir-103-2	SKA1	0.38633935	mirna_pc
9025	hsa-mir-103-2	BLM	0.380345001	mirna_pc
9026	hsa-mir-103-2	CKAP2L	0.305169979	mirna_pc
9027	hsa-mir-103-2	DNMT1	0.392786586	mirna_pc
9028	hsa-mir-103-2	PAICS	0.36264134	mirna_pc
9029	hsa-mir-103-2	CCDC150	0.329499605	mirna_pc
9030	hsa-mir-103-2	LIG1	0.323132709	mirna_pc
9031	hsa-mir-103-2	DTL	0.401408649	mirna_pc
9032	hsa-mir-103-2	RECQL4	0.417708021	mirna_pc
9033	hsa-mir-103-2	AURKA	0.370674586	mirna_pc
9034	hsa-mir-103-2	CENPK	0.406461835	mirna_pc
9035	hsa-mir-103-2	AURKB	0.344105926	mirna_pc
9036	hsa-mir-103-2	MCM10	0.38216866	mirna_pc
9037	hsa-mir-103-2	SGOL2	0.355603055	mirna_pc
9038	hsa-mir-103-2	CHEK2	0.318062301	mirna_pc
9039	hsa-mir-103-2	CENPE	0.342705351	mirna_pc
9040	hsa-mir-103-2	KIF15	0.363126665	mirna_pc
9041	hsa-mir-103-2	CDCA3	0.342150408	mirna_pc
9042	hsa-mir-103-2	C1orf112	0.466891207	mirna_pc
9043	hsa-mir-103-2	GINS1	0.503301945	mirna_pc
9044	hsa-mir-103-2	ORC6L	0.373598133	mirna_pc
9045	hsa-mir-103-2	C16orf59	0.302283476	mirna_pc
9046	hsa-mir-103-2	CDKN3	0.303216395	mirna_pc
9047	hsa-mir-103-2	RAE1	0.300275182	mirna_pc
9048	hsa-mir-103-2	EZH2	0.310415399	mirna_pc
9049	hsa-mir-103-2	POLD1	0.307535665	mirna_pc
9050	hsa-mir-103-2	C12orf48	0.416987141	mirna_pc
9051	hsa-mir-103-2	HNRNPL	0.32556688	mirna_pc
9052	hsa-mir-103-2	TTK	0.357766567	mirna_pc
9053	hsa-mir-103-2	WDR67	0.311115839	mirna_pc
9054	hsa-mir-103-2	HELLS	0.434067669	mirna_pc
9055	hsa-mir-103-2	POLE2	0.368680743	mirna_pc
9056	hsa-mir-103-2	NCAPD2	0.357125117	mirna_pc
9057	hsa-mir-103-2	C6orf167	0.379242016	mirna_pc
9058	hsa-mir-103-2	BRCA1	0.303780564	mirna_pc
9059	hsa-mir-103-2	HNRNPA2B1	0.363610958	mirna_pc
9060	hsa-mir-103-2	C20orf20	0.332647949	mirna_pc
9061	hsa-mir-103-2	MCM6	0.360695542	mirna_pc
9062	hsa-mir-103-2	BRIP1	0.317911306	mirna_pc
9063	hsa-mir-103-2	DCAF13	0.307570605	mirna_pc
9064	hsa-mir-103-2	PSMD14	0.332574554	mirna_pc
9065	hsa-mir-103-2	UCK2	0.342505922	mirna_pc
9066	hsa-mir-103-2	WDHD1	0.315481404	mirna_pc
9067	hsa-mir-103-2	NFKBIL2	0.337350177	mirna_pc
9068	hsa-mir-103-2	PRIM2	0.322029618	mirna_pc
9069	hsa-mir-103-2	ATAD5	0.395079599	mirna_pc
9070	hsa-mir-103-2	MTBP	0.395376429	mirna_pc
9071	hsa-mir-103-2	E2F1	0.318322127	mirna_pc

9072	hsa-mir-103-2	TH1L	0.342938281	mirna_pc
9073	hsa-mir-103-2	KIF20B	0.313184576	mirna_pc
9074	hsa-mir-103-2	FUS	0.327237337	mirna_pc
9075	hsa-mir-103-2	ATAD2	0.392933433	mirna_pc
9076	hsa-mir-103-2	CHAF1A	0.308609479	mirna_pc
9077	hsa-mir-103-2	DEPDC1B	0.414366593	mirna_pc
9078	hsa-mir-103-2	ANP32E	0.415420012	mirna_pc
9079	hsa-mir-103-2	CENPO	0.328146985	mirna_pc
9080	hsa-mir-103-2	CENPL	0.347876522	mirna_pc
9081	hsa-mir-103-2	GEN1	0.35499663	mirna_pc
9082	hsa-mir-103-2	DNA2	0.405954529	mirna_pc
9083	hsa-mir-103-2	NOP56	0.445749892	mirna_pc
9084	hsa-mir-103-2	DSN1	0.343651226	mirna_pc
9085	hsa-mir-103-2	CSE1L	0.372034304	mirna_pc
9086	hsa-mir-103-2	E2F7	0.332833693	mirna_pc
9087	hsa-mir-103-2	RBL1	0.324750701	mirna_pc
9088	hsa-mir-103-2	CDK2	0.384856372	mirna_pc
9089	hsa-mir-103-2	DLEU2	0.322637314	mirna_pc
9090	hsa-mir-103-2	PRIM1	0.367338097	mirna_pc
9091	hsa-mir-103-2	CENPH	0.300345731	mirna_pc
9092	hsa-mir-103-2	DDX27	0.349132403	mirna_pc
9093	hsa-mir-103-2	CHAF1B	0.352671226	mirna_pc
9094	hsa-mir-103-2	MSH2	0.391942614	mirna_pc
9095	hsa-mir-103-2	DBF4	0.332000621	mirna_pc
9096	hsa-mir-103-2	CDC7	0.340264497	mirna_pc
9097	hsa-mir-103-2	SSB	0.35434308	mirna_pc
9098	hsa-mir-103-2	PUS1	0.349771329	mirna_pc
9099	hsa-mir-103-2	FBXO5	0.373534286	mirna_pc
9100	hsa-mir-103-2	FIGNL1	0.300403368	mirna_pc
9101	hsa-mir-103-2	E2F3	0.352092234	mirna_pc
9102	hsa-mir-103-2	CDC25A	0.410423594	mirna_pc
9103	hsa-mir-103-2	RAD54B	0.377393436	mirna_pc
9104	hsa-mir-103-2	PRKDC	0.337281768	mirna_pc
9105	hsa-mir-103-2	DDX12	0.399376528	mirna_pc
9106	hsa-mir-103-2	MCM8	0.514808518	mirna_pc
9107	hsa-mir-103-2	CPSF3	0.301842142	mirna_pc
9108	hsa-mir-103-2	C21orf45	0.312061072	mirna_pc
9109	hsa-mir-103-2	PUS7	0.371706073	mirna_pc
9110	hsa-mir-103-2	DAZAP1	0.349825929	mirna_pc
9111	hsa-mir-103-2	TRMT6	0.446391671	mirna_pc
9112	hsa-mir-103-2	TMEM201	0.303181684	mirna_pc
9113	hsa-mir-103-2	NCL	0.313143511	mirna_pc
9114	hsa-mir-103-2	PFDN4	0.336160647	mirna_pc
9115	hsa-mir-103-2	SRRT	0.375959773	mirna_pc
9116	hsa-mir-103-2	CACYBP	0.376970235	mirna_pc
9117	hsa-mir-103-2	SASS6	0.339648243	mirna_pc
9118	hsa-mir-103-2	HNRNPM	0.346535005	mirna_pc
9119	hsa-mir-103-2	PNPT1	0.343638685	mirna_pc
9120	hsa-mir-103-2	TMPO	0.414808398	mirna_pc
9121	hsa-mir-103-2	VBP1	0.332268481	mirna_pc
9122	hsa-mir-103-2	C20orf72	0.36532232	mirna_pc
9123	hsa-mir-103-2	GABPB1	0.343852087	mirna_pc
9124	hsa-mir-103-2	DHX9	0.397415037	mirna_pc
9125	hsa-mir-103-2	CCDC138	0.388407823	mirna_pc

9126	hsa-mir-103-2	LOC100128191	0.346484488	mirna_pc
9127	hsa-mir-103-2	C20orf3	0.408655973	mirna_pc
9128	hsa-mir-103-2	PHF6	0.349486266	mirna_pc
9129	hsa-mir-103-2	SFRS1	0.354582634	mirna_pc
9130	hsa-mir-103-2	WDR43	0.301160087	mirna_pc
9131	hsa-mir-103-2	SKP2	0.305613932	mirna_pc
9132	hsa-mir-103-2	TIPIN	0.31086302	mirna_pc
9133	hsa-mir-103-2	UTP14A	0.311285746	mirna_pc
9134	hsa-mir-103-2	RFC5	0.350705105	mirna_pc
9135	hsa-mir-103-2	HSPD1	0.339520349	mirna_pc
9136	hsa-mir-103-2	FANCG	0.311262368	mirna_pc
9137	hsa-mir-103-2	SSRP1	0.340014383	mirna_pc
9138	hsa-mir-103-2	KHDRBS1	0.326866207	mirna_pc
9139	hsa-mir-103-2	NXT1	0.331892221	mirna_pc
9140	hsa-mir-103-2	C20orf27	0.385699844	mirna_pc
9141	hsa-mir-103-2	FKBP1A	0.310431199	mirna_pc
9142	hsa-mir-103-2	DNAJC2	0.314972214	mirna_pc
9143	hsa-mir-103-2	TMEM194A	0.303258801	mirna_pc
9144	hsa-mir-103-2	ZNF695	0.341930658	mirna_pc
9145	hsa-mir-103-2	KIF24	0.322850011	mirna_pc
9146	hsa-mir-103-2	HAUS6	0.355417112	mirna_pc
9147	hsa-mir-103-2	CEP135	0.31502865	mirna_pc
9148	hsa-mir-103-2	HNRNPU	0.39733938	mirna_pc
9149	hsa-mir-103-2	POLR1B	0.303262042	mirna_pc
9150	hsa-mir-103-2	WDR76	0.304321894	mirna_pc
9151	hsa-mir-103-2	TBCE	0.340098153	mirna_pc
9152	hsa-mir-103-2	SUV39H2	0.331300296	mirna_pc
9153	hsa-mir-103-2	POP1	0.313660821	mirna_pc
9154	hsa-mir-103-2	KIAA0406	0.302551429	mirna_pc
9155	hsa-mir-103-2	HSP90B1	0.339837726	mirna_pc
9156	hsa-mir-103-2	DHFR	0.356027622	mirna_pc
9157	hsa-mir-103-2	NAA25	0.318694096	mirna_pc
9158	hsa-mir-103-2	ILF3	0.322379618	mirna_pc
9159	hsa-mir-103-2	PUF60	0.303931729	mirna_pc
9160	hsa-mir-103-2	DEK	0.334223099	mirna_pc
9161	hsa-mir-103-2	PASK	0.322376882	mirna_pc
9162	hsa-mir-103-2	TGS1	0.308512322	mirna_pc
9163	hsa-mir-103-2	KHSRP	0.305511249	mirna_pc
9164	hsa-mir-103-2	SNRPB2	0.340207337	mirna_pc
9165	hsa-mir-103-2	ITPA	0.33742607	mirna_pc
9166	hsa-mir-103-2	CEP152	0.307649461	mirna_pc
9167	hsa-mir-103-2	BAT2L2	0.312073301	mirna_pc
9168	hsa-mir-103-2	DDX55	0.328230313	mirna_pc
9169	hsa-mir-103-2	LPGAT1	0.302112869	mirna_pc
9170	hsa-mir-103-2	FOXN2	0.300409233	mirna_pc
9171	hsa-mir-103-2	POLA1	0.345242945	mirna_pc
9172	hsa-mir-103-2	HDAC2	0.327856594	mirna_pc
9173	hsa-mir-103-2	GMNN	0.306962609	mirna_pc
9174	hsa-mir-103-2	ANKRD32	0.3405062	mirna_pc
9175	hsa-mir-103-2	CHRAC1	0.470128493	mirna_pc
9176	hsa-mir-103-2	XRN2	0.44902003	mirna_pc
9177	hsa-mir-103-2	KPNB1	0.354160996	mirna_pc
9178	hsa-mir-103-2	OTUD6B	0.303186756	mirna_pc
9179	hsa-mir-103-2	SMC3	0.405981301	mirna_pc

9180	hsa-mir-103-2	MEX3A	0.310342141	mirna_pc
9181	hsa-mir-103-2	NANP	0.422872587	mirna_pc
9182	hsa-mir-103-2	LOC642846	0.346752318	mirna_pc
9183	hsa-mir-103-2	SNX5	0.398487323	mirna_pc
9184	hsa-mir-103-2	NOL10	0.330662012	mirna_pc
9185	hsa-mir-103-2	POLE	0.40375627	mirna_pc
9186	hsa-mir-103-2	VRK1	0.348533236	mirna_pc
9187	hsa-mir-103-2	RBMX	0.356026118	mirna_pc
9188	hsa-mir-103-2	NOC3L	0.316744037	mirna_pc
9189	hsa-mir-103-2	DAP3	0.320703929	mirna_pc
9190	hsa-mir-103-2	PDIA6	0.30943948	mirna_pc
9191	hsa-mir-103-2	LTV1	0.333428881	mirna_pc
9192	hsa-mir-103-2	TTLL4	0.304007175	mirna_pc
9193	hsa-mir-103-2	EIF2C2	0.367823001	mirna_pc
9194	hsa-mir-103-2	SNORA8	0.312117122	mirna_pc
9195	hsa-mir-103-2	CSNK2A1	0.355439566	mirna_pc
9196	hsa-mir-103-2	ARHGAP19	0.312858927	mirna_pc
9197	hsa-mir-103-2	TCEA1	0.313568103	mirna_pc
9198	hsa-mir-103-2	UPF3B	0.343037978	mirna_pc
9199	hsa-mir-103-2	POLR3C	0.301501495	mirna_pc
9200	hsa-mir-103-2	TOP 1.00	0.331331427	mirna_pc
9201	hsa-mir-103-2	UBE2N	0.331963276	mirna_pc
9202	hsa-mir-103-2	TCERG1	0.304819709	mirna_pc
9203	hsa-mir-103-2	E2F6	0.314148507	mirna_pc
9204	hsa-mir-103-2	GMFB	0.300127876	mirna_pc
9205	hsa-mir-103-2	TFAM	0.31330486	mirna_pc
9206	hsa-mir-103-2	RTTN	0.33065855	mirna_pc
9207	hsa-mir-103-2	SMC1A	0.315955367	mirna_pc
9208	hsa-mir-103-2	ANKRD5	0.301658431	mirna_pc
9209	hsa-mir-103-2	MRPS26	0.368836697	mirna_pc
9210	hsa-mir-103-2	MRPL42	0.332078058	mirna_pc
9211	hsa-mir-103-2	CRNKL1	0.346049778	mirna_pc
9212	hsa-mir-103-2	SF3B2	0.333708888	mirna_pc
9213	hsa-mir-103-2	CCAR1	0.301954354	mirna_pc
9214	hsa-mir-103-2	PTK2	0.426812062	mirna_pc
9215	hsa-mir-103-2	FASTKD5	0.318036952	mirna_pc
9216	hsa-mir-103-2	TASP1	0.311707531	mirna_pc
9217	hsa-mir-103-2	NUP43	0.371341321	mirna_pc
9218	hsa-mir-103-2	POLR3F	0.417544279	mirna_pc
9219	hsa-mir-103-2	CHD1L	0.318194265	mirna_pc
9220	hsa-mir-103-2	DCLRE1A	0.365493573	mirna_pc
9221	hsa-mir-103-2	GZF1	0.30709978	mirna_pc
9222	hsa-mir-103-2	ENTPD6	0.301137823	mirna_pc
9223	hsa-mir-103-2	CCNJ	0.30584162	mirna_pc
9224	hsa-mir-103-2	ESF1	0.348130031	mirna_pc
9225	hsa-mir-103-2	SLC45A4	0.321035549	mirna_pc
9226	hsa-mir-103-2	FAM169A	0.355366652	mirna_pc
9227	hsa-mir-103-2	MKKS	0.344416833	mirna_pc
9228	hsa-mir-103-2	ZCCHC3	0.400198838	mirna_pc
9229	hsa-mir-103-2	CTNNBL1	0.300259839	mirna_pc
9230	hsa-mir-103-2	ZNF343	0.31213855	mirna_pc
9231	hsa-mir-20a	MKI67	0.346309412	mirna_pc
9232	hsa-mir-20a	TPX2	0.344858574	mirna_pc
9233	hsa-mir-20a	KIF4B	0.326472561	mirna_pc

9234	hsa-mir-20a	TOP2A	0.466533344	mirna_pc
9235	hsa-mir-20a	CENPF	0.461368538	mirna_pc
9236	hsa-mir-20a	RCC2	0.350173913	mirna_pc
9237	hsa-mir-20a	KIF11	0.392124838	mirna_pc
9238	hsa-mir-20a	ECT2	0.339484734	mirna_pc
9239	hsa-mir-20a	RRM2	0.362789285	mirna_pc
9240	hsa-mir-20a	CDK1	0.409688062	mirna_pc
9241	hsa-mir-20a	CDC25C	0.321071142	mirna_pc
9242	hsa-mir-20a	UBE2C	0.34623996	mirna_pc
9243	hsa-mir-20a	BUB1	0.426508256	mirna_pc
9244	hsa-mir-20a	NUSAP1	0.327670581	mirna_pc
9245	hsa-mir-20a	MCM4	0.307267447	mirna_pc
9246	hsa-mir-20a	KIFC1	0.355444671	mirna_pc
9247	hsa-mir-20a	BUB1B	0.41559752	mirna_pc
9248	hsa-mir-20a	HDGF	0.309098385	mirna_pc
9249	hsa-mir-20a	KIF18B	0.35123287	mirna_pc
9250	hsa-mir-20a	ASPM	0.414289216	mirna_pc
9251	hsa-mir-20a	CKAP2	0.460434701	mirna_pc
9252	hsa-mir-20a	FAM72A	0.354140987	mirna_pc
9253	hsa-mir-20a	KIF2C	0.327414903	mirna_pc
9254	hsa-mir-20a	FANCA	0.470610669	mirna_pc
9255	hsa-mir-20a	NCAPG	0.433932648	mirna_pc
9256	hsa-mir-20a	CLSPN	0.376045459	mirna_pc
9257	hsa-mir-20a	SGOL1	0.457247456	mirna_pc
9258	hsa-mir-20a	ARHGAP11A	0.45887455	mirna_pc
9259	hsa-mir-20a	CCNA2	0.39702536	mirna_pc
9260	hsa-mir-20a	CBX3	0.423556505	mirna_pc
9261	hsa-mir-20a	SPC25	0.45706232	mirna_pc
9262	hsa-mir-20a	NUP62	0.323953283	mirna_pc
9263	hsa-mir-20a	FANCI	0.378374612	mirna_pc
9264	hsa-mir-20a	NEK2	0.48549828	mirna_pc
9265	hsa-mir-20a	MND1	0.321033917	mirna_pc
9266	hsa-mir-20a	KIF22	0.30746307	mirna_pc
9267	hsa-mir-20a	NCAPH	0.412159801	mirna_pc
9268	hsa-mir-20a	GTSE1	0.411622466	mirna_pc
9269	hsa-mir-20a	KIF23	0.301176536	mirna_pc
9270	hsa-mir-20a	RAD54L	0.373573199	mirna_pc
9271	hsa-mir-20a	CENPA	0.346301659	mirna_pc
9272	hsa-mir-20a	MAD2L1	0.48160331	mirna_pc
9273	hsa-mir-20a	TIMELESS	0.383679127	mirna_pc
9274	hsa-mir-20a	LMNB1	0.421963203	mirna_pc
9275	hsa-mir-20a	ASF1B	0.303603907	mirna_pc
9276	hsa-mir-20a	CDCA8	0.38654764	mirna_pc
9277	hsa-mir-20a	TROAP	0.319472295	mirna_pc
9278	hsa-mir-20a	CDCA5	0.309339155	mirna_pc
9279	hsa-mir-20a	XPO1	0.538612482	mirna_pc
9280	hsa-mir-20a	CDC45	0.308167159	mirna_pc
9281	hsa-mir-20a	EXO1	0.319327636	mirna_pc
9282	hsa-mir-20a	CDCA2	0.363704641	mirna_pc
9283	hsa-mir-20a	DKC1	0.331600899	mirna_pc
9284	hsa-mir-20a	UBE2T	0.305030297	mirna_pc
9285	hsa-mir-20a	KIF4A	0.317628074	mirna_pc
9286	hsa-mir-20a	ORC1L	0.317869289	mirna_pc
9287	hsa-mir-20a	KNTC1	0.389479181	mirna_pc

9288	hsa-mir-20a	CCNF	0.307599196	mirna_pc
9289	hsa-mir-20a	NUF2	0.460144712	mirna_pc
9290	hsa-mir-20a	PLK4	0.472312697	mirna_pc
9291	hsa-mir-20a	FANCB	0.378104483	mirna_pc
9292	hsa-mir-20a	KIF18A	0.397034121	mirna_pc
9293	hsa-mir-20a	DEPDC1	0.40101601	mirna_pc
9294	hsa-mir-20a	SKA3	0.556112551	mirna_pc
9295	hsa-mir-20a	KIF14	0.415500515	mirna_pc
9296	hsa-mir-20a	HJURP	0.486596479	mirna_pc
9297	hsa-mir-20a	SKA1	0.324556589	mirna_pc
9298	hsa-mir-20a	BLM	0.459115563	mirna_pc
9299	hsa-mir-20a	DNMT1	0.332399538	mirna_pc
9300	hsa-mir-20a	PAICS	0.476473667	mirna_pc
9301	hsa-mir-20a	CCDC150	0.469714562	mirna_pc
9302	hsa-mir-20a	LIG1	0.328608429	mirna_pc
9303	hsa-mir-20a	DTL	0.420255566	mirna_pc
9304	hsa-mir-20a	EME1	0.439300501	mirna_pc
9305	hsa-mir-20a	RECQL4	0.360281561	mirna_pc
9306	hsa-mir-20a	AURKA	0.301754417	mirna_pc
9307	hsa-mir-20a	CENPK	0.439561281	mirna_pc
9308	hsa-mir-20a	GSG2	0.348853521	mirna_pc
9309	hsa-mir-20a	CDT1	0.300992427	mirna_pc
9310	hsa-mir-20a	CBFB	0.381079064	mirna_pc
9311	hsa-mir-20a	MCM10	0.394678354	mirna_pc
9312	hsa-mir-20a	SGOL2	0.445649374	mirna_pc
9313	hsa-mir-20a	CHEK2	0.385546943	mirna_pc
9314	hsa-mir-20a	CENPE	0.454920281	mirna_pc
9315	hsa-mir-20a	POLQ	0.405306688	mirna_pc
9316	hsa-mir-20a	KIF15	0.386507565	mirna_pc
9317	hsa-mir-20a	CDCA3	0.324089165	mirna_pc
9318	hsa-mir-20a	C1orf112	0.471864475	mirna_pc
9319	hsa-mir-20a	GINS1	0.406449342	mirna_pc
9320	hsa-mir-20a	FEN1	0.357297902	mirna_pc
9321	hsa-mir-20a	TRAIP	0.361907284	mirna_pc
9322	hsa-mir-20a	ORC6L	0.393518171	mirna_pc
9323	hsa-mir-20a	PBK	0.310675777	mirna_pc
9324	hsa-mir-20a	ARHGAP11B	0.324889199	mirna_pc
9325	hsa-mir-20a	C16orf59	0.359485994	mirna_pc
9326	hsa-mir-20a	RAE1	0.301131047	mirna_pc
9327	hsa-mir-20a	FAM72D	0.389508925	mirna_pc
9328	hsa-mir-20a	FAM72B	0.420936826	mirna_pc
9329	hsa-mir-20a	EZH2	0.385761833	mirna_pc
9330	hsa-mir-20a	NEIL3	0.378472519	mirna_pc
9331	hsa-mir-20a	POLD1	0.350128556	mirna_pc
9332	hsa-mir-20a	C12orf48	0.414336436	mirna_pc
9333	hsa-mir-20a	HNRNPL	0.339749347	mirna_pc
9334	hsa-mir-20a	ZWILCH	0.314420481	mirna_pc
9335	hsa-mir-20a	SMC4	0.317807561	mirna_pc
9336	hsa-mir-20a	HMMR	0.379580289	mirna_pc
9337	hsa-mir-20a	TTK	0.375106352	mirna_pc
9338	hsa-mir-20a	ZWINT	0.372972485	mirna_pc
9339	hsa-mir-20a	WDR67	0.310426653	mirna_pc
9340	hsa-mir-20a	HOXC6	0.354922543	mirna_pc
9341	hsa-mir-20a	BUB3	0.399047043	mirna_pc

9342	hsa-mir-20a	HELLS	0.444931256	mirna_pc
9343	hsa-mir-20a	NCAPD2	0.30623546	mirna_pc
9344	hsa-mir-20a	HNRNPC	0.34072055	mirna_pc
9345	hsa-mir-20a	TCF3	0.336480225	mirna_pc
9346	hsa-mir-20a	C6orf167	0.393401677	mirna_pc
9347	hsa-mir-20a	CHEK1	0.358504493	mirna_pc
9348	hsa-mir-20a	BRCA1	0.415540561	mirna_pc
9349	hsa-mir-20a	HNRNPA2B1	0.47747264	mirna_pc
9350	hsa-mir-20a	RFWD3	0.445424573	mirna_pc
9351	hsa-mir-20a	C20orf20	0.303135082	mirna_pc
9352	hsa-mir-20a	MCM6	0.504364635	mirna_pc
9353	hsa-mir-20a	CDC6	0.42751226	mirna_pc
9354	hsa-mir-20a	CDCA7	0.430037115	mirna_pc
9355	hsa-mir-20a	KIAA1524	0.317627482	mirna_pc
9356	hsa-mir-20a	BRIP1	0.351399248	mirna_pc
9357	hsa-mir-20a	PIF1	0.338862144	mirna_pc
9358	hsa-mir-20a	SFRS2	0.306775334	mirna_pc
9359	hsa-mir-20a	DCAF13	0.36704721	mirna_pc
9360	hsa-mir-20a	PSMD14	0.31003176	mirna_pc
9361	hsa-mir-20a	UCK2	0.325352322	mirna_pc
9362	hsa-mir-20a	NFKBIL2	0.319708293	mirna_pc
9363	hsa-mir-20a	C15orf42	0.314194524	mirna_pc
9364	hsa-mir-20a	PTGES3	0.32154419	mirna_pc
9365	hsa-mir-20a	PRIM2	0.342340468	mirna_pc
9366	hsa-mir-20a	FAM60A	0.44586468	mirna_pc
9367	hsa-mir-20a	ATAD5	0.411360519	mirna_pc
9368	hsa-mir-20a	FAM111B	0.375239289	mirna_pc
9369	hsa-mir-20a	KIF20B	0.42312424	mirna_pc
9370	hsa-mir-20a	ESCO2	0.332316968	mirna_pc
9371	hsa-mir-20a	SNRPA	0.314192551	mirna_pc
9372	hsa-mir-20a	FUS	0.32433623	mirna_pc
9373	hsa-mir-20a	ATAD2	0.335917685	mirna_pc
9374	hsa-mir-20a	RFC3	0.554610063	mirna_pc
9375	hsa-mir-20a	DEPDC1B	0.346705143	mirna_pc
9376	hsa-mir-20a	ANP32E	0.370239671	mirna_pc
9377	hsa-mir-20a	CENPO	0.411509029	mirna_pc
9378	hsa-mir-20a	XRCC2	0.45777662	mirna_pc
9379	hsa-mir-20a	C19orf48	0.329038998	mirna_pc
9380	hsa-mir-20a	BRCA2	0.469173719	mirna_pc
9381	hsa-mir-20a	CENPL	0.364102765	mirna_pc
9382	hsa-mir-20a	GEN1	0.468456621	mirna_pc
9383	hsa-mir-20a	HMGB2	0.480461134	mirna_pc
9384	hsa-mir-20a	DNA2	0.458050619	mirna_pc
9385	hsa-mir-20a	NOP56	0.437357387	mirna_pc
9386	hsa-mir-20a	SNHG1	0.386259994	mirna_pc
9387	hsa-mir-20a	CSE1L	0.320591122	mirna_pc
9388	hsa-mir-20a	DTYMK	0.397126233	mirna_pc
9389	hsa-mir-20a	PRMT1	0.333731023	mirna_pc
9390	hsa-mir-20a	MLF1IP	0.36020994	mirna_pc
9391	hsa-mir-20a	RAVER1	0.302122586	mirna_pc
9392	hsa-mir-20a	HEATR1	0.380617847	mirna_pc
9393	hsa-mir-20a	CAD	0.346560561	mirna_pc
9394	hsa-mir-20a	CCDC99	0.417215094	mirna_pc
9395	hsa-mir-20a	CDK2	0.355715973	mirna_pc

9396	hsa-mir-20a	HOXA10	0.331040044	mirna_pc
9397	hsa-mir-20a	HNRNPR	0.386896726	mirna_pc
9398	hsa-mir-20a	DLEU2	0.461295484	mirna_pc
9399	hsa-mir-20a	PRIM1	0.40354317	mirna_pc
9400	hsa-mir-20a	CENPH	0.341791046	mirna_pc
9401	hsa-mir-20a	FERMT1	0.340325157	mirna_pc
9402	hsa-mir-20a	TCOF1	0.369709964	mirna_pc
9403	hsa-mir-20a	CHAF1B	0.335657558	mirna_pc
9404	hsa-mir-20a	NCAPG2	0.392553285	mirna_pc
9405	hsa-mir-20a	MSH2	0.508892332	mirna_pc
9406	hsa-mir-20a	EIF3B	0.310075477	mirna_pc
9407	hsa-mir-20a	NOP58	0.342333102	mirna_pc
9408	hsa-mir-20a	EIF2AK1	0.326216691	mirna_pc
9409	hsa-mir-20a	SF3B3	0.393209046	mirna_pc
9410	hsa-mir-20a	CDC7	0.443523088	mirna_pc
9411	hsa-mir-20a	SSB	0.564960423	mirna_pc
9412	hsa-mir-20a	PUS1	0.386657179	mirna_pc
9413	hsa-mir-20a	NOL11	0.406334977	mirna_pc
9414	hsa-mir-20a	FBXO5	0.435731306	mirna_pc
9415	hsa-mir-20a	WDR75	0.310646261	mirna_pc
9416	hsa-mir-20a	FIGNL1	0.355873316	mirna_pc
9417	hsa-mir-20a	E2F3	0.517482187	mirna_pc
9418	hsa-mir-20a	DDX11	0.3762536	mirna_pc
9419	hsa-mir-20a	CDC25A	0.468851172	mirna_pc
9420	hsa-mir-20a	TOPBP1	0.318694627	mirna_pc
9421	hsa-mir-20a	TMEM48	0.310909804	mirna_pc
9422	hsa-mir-20a	RAD54B	0.3191362	mirna_pc
9423	hsa-mir-20a	DDX12	0.369649065	mirna_pc
9424	hsa-mir-20a	DSCC1	0.348873352	mirna_pc
9425	hsa-mir-20a	WDR12	0.315957178	mirna_pc
9426	hsa-mir-20a	C13orf34	0.564588176	mirna_pc
9427	hsa-mir-20a	SMC2	0.364592587	mirna_pc
9428	hsa-mir-20a	SRPK1	0.353203278	mirna_pc
9429	hsa-mir-20a	MCM8	0.484246452	mirna_pc
9430	hsa-mir-20a	CPSF3	0.386984276	mirna_pc
9431	hsa-mir-20a	SNRPE	0.335600722	mirna_pc
9432	hsa-mir-20a	C21orf45	0.368361328	mirna_pc
9433	hsa-mir-20a	ATIC	0.437277873	mirna_pc
9434	hsa-mir-20a	TFAP4	0.344520641	mirna_pc
9435	hsa-mir-20a	PUS7	0.30114487	mirna_pc
9436	hsa-mir-20a	C14orf106	0.362633202	mirna_pc
9437	hsa-mir-20a	CBX8	0.398764876	mirna_pc
9438	hsa-mir-20a	TRMT6	0.309375274	mirna_pc
9439	hsa-mir-20a	GNPDA1	0.323595795	mirna_pc
9440	hsa-mir-20a	NCL	0.512370945	mirna_pc
9441	hsa-mir-20a	NUDCD1	0.343249616	mirna_pc
9442	hsa-mir-20a	SRRT	0.34359164	mirna_pc
9443	hsa-mir-20a	CACYBP	0.395671961	mirna_pc
9444	hsa-mir-20a	SASS6	0.369898643	mirna_pc
9445	hsa-mir-20a	PSRC1	0.359671973	mirna_pc
9446	hsa-mir-20a	ODF2	0.327439683	mirna_pc
9447	hsa-mir-20a	DBF4B	0.3741665	mirna_pc
9448	hsa-mir-20a	POU2F1	0.331819584	mirna_pc
9449	hsa-mir-20a	FBL	0.347689575	mirna_pc

9450	hsa-mir-20a	C13orf37	0.543128356	mirna_pc
9451	hsa-mir-20a	HNRNPM	0.338390803	mirna_pc
9452	hsa-mir-20a	LSM 12.00	0.317391843	mirna_pc
9453	hsa-mir-20a	PNPT1	0.488657241	mirna_pc
9454	hsa-mir-20a	TMPO	0.422488396	mirna_pc
9455	hsa-mir-20a	SET	0.391671329	mirna_pc
9456	hsa-mir-20a	C20orf72	0.330376289	mirna_pc
9457	hsa-mir-20a	C3orf26	0.307721998	mirna_pc
9458	hsa-mir-20a	SMPD4	0.354325733	mirna_pc
9459	hsa-mir-20a	GPR19	0.312025887	mirna_pc
9460	hsa-mir-20a	PSMD3	0.310361942	mirna_pc
9461	hsa-mir-20a	GABPB1	0.356627849	mirna_pc
9462	hsa-mir-20a	PPAT	0.399955922	mirna_pc
9463	hsa-mir-20a	DHX9	0.46786131	mirna_pc
9464	hsa-mir-20a	CCDC138	0.439678268	mirna_pc
9465	hsa-mir-20a	HMGN2	0.350250452	mirna_pc
9466	hsa-mir-20a	ETV4	0.316769699	mirna_pc
9467	hsa-mir-20a	CHRNA5	0.335911571	mirna_pc
9468	hsa-mir-20a	PHF6	0.320034559	mirna_pc
9469	hsa-mir-20a	SFRS1	0.590653131	mirna_pc
9470	hsa-mir-20a	TEX10	0.336657969	mirna_pc
9471	hsa-mir-20a	WDR43	0.471194714	mirna_pc
9472	hsa-mir-20a	SKP2	0.328693186	mirna_pc
9473	hsa-mir-20a	C4orf46	0.438123912	mirna_pc
9474	hsa-mir-20a	DARS2	0.374646867	mirna_pc
9475	hsa-mir-20a	LYAR	0.350771431	mirna_pc
9476	hsa-mir-20a	UTP14A	0.322737541	mirna_pc
9477	hsa-mir-20a	C1orf107	0.310407668	mirna_pc
9478	hsa-mir-20a	RFC5	0.310882488	mirna_pc
9479	hsa-mir-20a	HSPD1	0.464968283	mirna_pc
9480	hsa-mir-20a	SSRP1	0.375233496	mirna_pc
9481	hsa-mir-20a	POLR2D	0.428040699	mirna_pc
9482	hsa-mir-20a	FTSJ2	0.367172261	mirna_pc
9483	hsa-mir-20a	PA2G4	0.303528779	mirna_pc
9484	hsa-mir-20a	EPT1	0.349099563	mirna_pc
9485	hsa-mir-20a	KHDRBS1	0.425738446	mirna_pc
9486	hsa-mir-20a	SLC5A6	0.314779693	mirna_pc
9487	hsa-mir-20a	TOMM34	0.358523671	mirna_pc
9488	hsa-mir-20a	TAF1A	0.353857625	mirna_pc
9489	hsa-mir-20a	PRPF40A	0.492515301	mirna_pc
9490	hsa-mir-20a	CCDC18	0.403722119	mirna_pc
9491	hsa-mir-20a	AHCY	0.308337781	mirna_pc
9492	hsa-mir-20a	PARP1	0.363602261	mirna_pc
9493	hsa-mir-20a	CCDC34	0.388928557	mirna_pc
9494	hsa-mir-20a	CCT7	0.301493733	mirna_pc
9495	hsa-mir-20a	HNRNPF	0.303917283	mirna_pc
9496	hsa-mir-20a	C4orf21	0.332712618	mirna_pc
9497	hsa-mir-20a	TMEM194A	0.388762541	mirna_pc
9498	hsa-mir-20a	SLC39A10	0.457745629	mirna_pc
9499	hsa-mir-20a	ZNF695	0.47877837	mirna_pc
9500	hsa-mir-20a	PDSS1	0.385204412	mirna_pc
9501	hsa-mir-20a	HAUS6	0.399753905	mirna_pc
9502	hsa-mir-20a	CENPJ	0.519687748	mirna_pc
9503	hsa-mir-20a	LIN9	0.382247195	mirna_pc

9504	hsa-mir-20a	C21orf58	0.30910045	mirna_pc
9505	hsa-mir-20a	HNRNPU	0.50656789	mirna_pc
9506	hsa-mir-20a	NASP	0.391350996	mirna_pc
9507	hsa-mir-20a	POLR1B	0.411278764	mirna_pc
9508	hsa-mir-20a	QSOX2	0.340432013	mirna_pc
9509	hsa-mir-20a	PTMA	0.366540256	mirna_pc
9510	hsa-mir-20a	PSMD1	0.325553168	mirna_pc
9511	hsa-mir-20a	EXOSC2	0.321284724	mirna_pc
9512	hsa-mir-20a	HMGXB4	0.316032657	mirna_pc
9513	hsa-mir-20a	SCARB1	0.334060403	mirna_pc
9514	hsa-mir-20a	C11orf84	0.3114457	mirna_pc
9515	hsa-mir-20a	TBCE	0.347330365	mirna_pc
9516	hsa-mir-20a	CLCN2	0.317899178	mirna_pc
9517	hsa-mir-20a	SUV39H2	0.450964502	mirna_pc
9518	hsa-mir-20a	R3HDM1	0.357793508	mirna_pc
9519	hsa-mir-20a	FANCL	0.431774365	mirna_pc
9520	hsa-mir-20a	RQCD1	0.329845847	mirna_pc
9521	hsa-mir-20a	HNRNPD	0.430061418	mirna_pc
9522	hsa-mir-20a	MTHFD2	0.399400035	mirna_pc
9523	hsa-mir-20a	GTF2F2	0.434103918	mirna_pc
9524	hsa-mir-20a	DDX31	0.307880454	mirna_pc
9525	hsa-mir-20a	GTPBP4	0.309828174	mirna_pc
9526	hsa-mir-20a	TARDBP	0.327290403	mirna_pc
9527	hsa-mir-20a	POP1	0.380461682	mirna_pc
9528	hsa-mir-20a	NFE2L3	0.311769117	mirna_pc
9529	hsa-mir-20a	SFRS3	0.383861954	mirna_pc
9530	hsa-mir-20a	NUP160	0.331964868	mirna_pc
9531	hsa-mir-20a	LSM 5.00	0.396572077	mirna_pc
9532	hsa-mir-20a	PNO1	0.370348911	mirna_pc
9533	hsa-mir-20a	CCT4	0.319647953	mirna_pc
9534	hsa-mir-20a	ELAVL1	0.35133427	mirna_pc
9535	hsa-mir-20a	ABCE1	0.346597268	mirna_pc
9536	hsa-mir-20a	KIAA0406	0.319245889	mirna_pc
9537	hsa-mir-20a	HSP90B1	0.339018023	mirna_pc
9538	hsa-mir-20a	NAE1	0.350990928	mirna_pc
9539	hsa-mir-20a	PSMC3IP	0.327652088	mirna_pc
9540	hsa-mir-20a	PAK1IP1	0.365271368	mirna_pc
9541	hsa-mir-20a	NUDT15	0.465552042	mirna_pc
9542	hsa-mir-20a	FASTKD1	0.321171115	mirna_pc
9543	hsa-mir-20a	SUPT16H	0.30886495	mirna_pc
9544	hsa-mir-20a	SLMO2	0.306363272	mirna_pc
9545	hsa-mir-20a	TWISTNB	0.369919307	mirna_pc
9546	hsa-mir-20a	ATP13A3	0.312426461	mirna_pc
9547	hsa-mir-20a	DHFR	0.415271548	mirna_pc
9548	hsa-mir-20a	NAA25	0.426696337	mirna_pc
9549	hsa-mir-20a	ILF3	0.446055179	mirna_pc
9550	hsa-mir-20a	C1orf96	0.32152203	mirna_pc
9551	hsa-mir-20a	EHMT2	0.33068405	mirna_pc
9552	hsa-mir-20a	KDM1B	0.32429092	mirna_pc
9553	hsa-mir-20a	GART	0.311220577	mirna_pc
9554	hsa-mir-20a	DEK	0.434143797	mirna_pc
9555	hsa-mir-20a	TBC1D4	0.441894087	mirna_pc
9556	hsa-mir-20a	TRIB3	0.367867521	mirna_pc
9557	hsa-mir-20a	CCNE2	0.316063807	mirna_pc

9558	hsa-mir-20a	PASK	0.422657108	mirna_pc
9559	hsa-mir-20a	USP42	0.32100959	mirna_pc
9560	hsa-mir-20a	SAAL1	0.388957386	mirna_pc
9561	hsa-mir-20a	C1orf131	0.322305157	mirna_pc
9562	hsa-mir-20a	RPGRIP1L	0.37676434	mirna_pc
9563	hsa-mir-20a	ATP11A	0.325990128	mirna_pc
9564	hsa-mir-20a	TGS1	0.402899233	mirna_pc
9565	hsa-mir-20a	HEATR2	0.475280658	mirna_pc
9566	hsa-mir-20a	PRAME	0.365880704	mirna_pc
9567	hsa-mir-20a	CYB5B	0.317367173	mirna_pc
9568	hsa-mir-20a	SNRPB2	0.353212953	mirna_pc
9569	hsa-mir-20a	FUBP1	0.380688114	mirna_pc
9570	hsa-mir-20a	IPO9	0.346844584	mirna_pc
9571	hsa-mir-20a	SART3	0.321611847	mirna_pc
9572	hsa-mir-20a	HNRNPK	0.427179507	mirna_pc
9573	hsa-mir-20a	SR140	0.377594496	mirna_pc
9574	hsa-mir-20a	TRA2B	0.335891847	mirna_pc
9575	hsa-mir-20a	GAR1	0.384175965	mirna_pc
9576	hsa-mir-20a	CEP152	0.401955276	mirna_pc
9577	hsa-mir-20a	C15orf23	0.350533432	mirna_pc
9578	hsa-mir-20a	ZC3HAV1L	0.317673842	mirna_pc
9579	hsa-mir-20a	PRMT3	0.302405952	mirna_pc
9580	hsa-mir-20a	SLMO1	0.307406192	mirna_pc
9581	hsa-mir-20a	INTS6	0.305221007	mirna_pc
9582	hsa-mir-20a	WHSC1	0.36949542	mirna_pc
9583	hsa-mir-20a	RNF219	0.651563367	mirna_pc
9584	hsa-mir-20a	ITGB3BP	0.378677903	mirna_pc
9585	hsa-mir-20a	SLC25A32	0.371598132	mirna_pc
9586	hsa-mir-20a	ENOPH1	0.366313247	mirna_pc
9587	hsa-mir-20a	PABPC1L	0.327541291	mirna_pc
9588	hsa-mir-20a	C17orf42	0.339940139	mirna_pc
9589	hsa-mir-20a	C4orf43	0.318283774	mirna_pc
9590	hsa-mir-20a	ING5	0.31629125	mirna_pc
9591	hsa-mir-20a	DDX55	0.399428618	mirna_pc
9592	hsa-mir-20a	CEP78	0.396977304	mirna_pc
9593	hsa-mir-20a	PLEKHG4	0.306875131	mirna_pc
9594	hsa-mir-20a	SFPQ	0.3919533	mirna_pc
9595	hsa-mir-20a	RCOR2	0.385235746	mirna_pc
9596	hsa-mir-20a	MPHOSPH9	0.341368744	mirna_pc
9597	hsa-mir-20a	POLA1	0.34074529	mirna_pc
9598	hsa-mir-20a	HDAC2	0.432169983	mirna_pc
9599	hsa-mir-20a	GMNN	0.31304486	mirna_pc
9600	hsa-mir-20a	POLG2	0.309322562	mirna_pc
9601	hsa-mir-20a	ANKRD32	0.314201508	mirna_pc
9602	hsa-mir-20a	C10orf2	0.407190015	mirna_pc
9603	hsa-mir-20a	POGK	0.304007643	mirna_pc
9604	hsa-mir-20a	KIAA0564	0.323947527	mirna_pc
9605	hsa-mir-20a	GEMIN6	0.306043768	mirna_pc
9606	hsa-mir-20a	MSH6	0.358199859	mirna_pc
9607	hsa-mir-20a	XRN2	0.39457673	mirna_pc
9608	hsa-mir-20a	KPNB1	0.490951189	mirna_pc
9609	hsa-mir-20a	OTUD6B	0.341088169	mirna_pc
9610	hsa-mir-20a	SMC3	0.447170743	mirna_pc
9611	hsa-mir-20a	FLVCR1	0.386309189	mirna_pc

9612	hsa-mir-20a	HNRNPA1	0.370314335	mirna_pc
9613	hsa-mir-20a	UBA2	0.348001554	mirna_pc
9614	hsa-mir-20a	ZBED4	0.341417791	mirna_pc
9615	hsa-mir-20a	MEX3A	0.475403555	mirna_pc
9616	hsa-mir-20a	BAT1	0.390668852	mirna_pc
9617	hsa-mir-20a	COIL	0.41504697	mirna_pc
9618	hsa-mir-20a	PHF14	0.370758267	mirna_pc
9619	hsa-mir-20a	PDCD11	0.319785348	mirna_pc
9620	hsa-mir-20a	TAF5	0.385006779	mirna_pc
9621	hsa-mir-20a	NANP	0.319625775	mirna_pc
9622	hsa-mir-20a	SUZ12	0.386015311	mirna_pc
9623	hsa-mir-20a	HNRNPA1L2	0.421123762	mirna_pc
9624	hsa-mir-20a	NAA15	0.323018102	mirna_pc
9625	hsa-mir-20a	BARD1	0.32715124	mirna_pc
9626	hsa-mir-20a	CBX1	0.300696274	mirna_pc
9627	hsa-mir-20a	NCBP1	0.325741727	mirna_pc
9628	hsa-mir-20a	HMGB1	0.568006091	mirna_pc
9629	hsa-mir-20a	ZBTB12	0.450307729	mirna_pc
9630	hsa-mir-20a	DIAPH3	0.407122653	mirna_pc
9631	hsa-mir-20a	TADA1	0.313849032	mirna_pc
9632	hsa-mir-20a	USP1	0.346581875	mirna_pc
9633	hsa-mir-20a	TMEM68	0.375415982	mirna_pc
9634	hsa-mir-20a	LOC642846	0.328908665	mirna_pc
9635	hsa-mir-20a	TGIF2	0.426536987	mirna_pc
9636	hsa-mir-20a	H3F3A	0.330461758	mirna_pc
9637	hsa-mir-20a	SNAPC4	0.327659299	mirna_pc
9638	hsa-mir-20a	ZC3H18	0.361258934	mirna_pc
9639	hsa-mir-20a	FARSB	0.395445021	mirna_pc
9640	hsa-mir-20a	PIK3AP1	0.3017297	mirna_pc
9641	hsa-mir-20a	SNX5	0.334922394	mirna_pc
9642	hsa-mir-20a	THUMPD2	0.345886482	mirna_pc
9643	hsa-mir-20a	MTF2	0.337674542	mirna_pc
9644	hsa-mir-20a	NOL10	0.402485893	mirna_pc
9645	hsa-mir-20a	ANAPC1	0.33692445	mirna_pc
9646	hsa-mir-20a	POLE	0.40624374	mirna_pc
9647	hsa-mir-20a	C1orf163	0.301185401	mirna_pc
9648	hsa-mir-20a	TAF1B	0.327136934	mirna_pc
9649	hsa-mir-20a	SOX9	0.362904041	mirna_pc
9650	hsa-mir-20a	PPP3R1	0.324498524	mirna_pc
9651	hsa-mir-20a	TIMM50	0.361519685	mirna_pc
9652	hsa-mir-20a	C13orf27	0.494060829	mirna_pc
9653	hsa-mir-20a	RBM14	0.316112919	mirna_pc
9654	hsa-mir-20a	EXOSC9	0.37081004	mirna_pc
9655	hsa-mir-20a	TMEM209	0.343273945	mirna_pc
9656	hsa-mir-20a	CCDC43	0.302331119	mirna_pc
9657	hsa-mir-20a	RBMX	0.496714646	mirna_pc
9658	hsa-mir-20a	SRP9	0.403343772	mirna_pc
9659	hsa-mir-20a	CCDC41	0.330488422	mirna_pc
9660	hsa-mir-20a	NOC3L	0.36681811	mirna_pc
9661	hsa-mir-20a	UCHL3	0.309544423	mirna_pc
9662	hsa-mir-20a	GPR180	0.465731597	mirna_pc
9663	hsa-mir-20a	DAP3	0.359669342	mirna_pc
9664	hsa-mir-20a	IQCB1	0.324284128	mirna_pc
9665	hsa-mir-20a	MDC 1.00	0.325631928	mirna_pc

9666	hsa-mir-20a	XRCC5	0.475895116	mirna_pc
9667	hsa-mir-20a	SNX25	0.317142252	mirna_pc
9668	hsa-mir-20a	EXOSC8	0.439723783	mirna_pc
9669	hsa-mir-20a	CTCF	0.369946439	mirna_pc
9670	hsa-mir-20a	TAF1D	0.316604372	mirna_pc
9671	hsa-mir-20a	PDIA6	0.363085608	mirna_pc
9672	hsa-mir-20a	RPE	0.382744295	mirna_pc
9673	hsa-mir-20a	LBR	0.418362045	mirna_pc
9674	hsa-mir-20a	DNAJC14	0.30519721	mirna_pc
9675	hsa-mir-20a	ZNF519	0.392502396	mirna_pc
9676	hsa-mir-20a	ORC2L	0.332095349	mirna_pc
9677	hsa-mir-20a	ZRANB3	0.352160576	mirna_pc
9678	hsa-mir-20a	RIF1	0.376534486	mirna_pc
9679	hsa-mir-20a	TFB2M	0.310493016	mirna_pc
9680	hsa-mir-20a	SRBD1	0.30888424	mirna_pc
9681	hsa-mir-20a	NUDT21	0.358235247	mirna_pc
9682	hsa-mir-20a	SCLT1	0.334397458	mirna_pc
9683	hsa-mir-20a	DDX10	0.304744709	mirna_pc
9684	hsa-mir-20a	LTV1	0.303363591	mirna_pc
9685	hsa-mir-20a	CCDC14	0.336542645	mirna_pc
9686	hsa-mir-20a	SUZ12P	0.305757067	mirna_pc
9687	hsa-mir-20a	FAM136A	0.434406854	mirna_pc
9688	hsa-mir-20a	NRAS	0.350215885	mirna_pc
9689	hsa-mir-20a	HMGN1	0.31518639	mirna_pc
9690	hsa-mir-20a	C1orf124	0.314837076	mirna_pc
9691	hsa-mir-20a	MTPAP	0.324965751	mirna_pc
9692	hsa-mir-20a	ITPRIPL1	0.349272438	mirna_pc
9693	hsa-mir-20a	TTLL4	0.37717606	mirna_pc
9694	hsa-mir-20a	FBXO41	0.33165527	mirna_pc
9695	hsa-mir-20a	BIVM	0.368843252	mirna_pc
9696	hsa-mir-20a	THADA	0.333172278	mirna_pc
9697	hsa-mir-20a	GPATCH4	0.309455476	mirna_pc
9698	hsa-mir-20a	EML4	0.34252467	mirna_pc
9699	hsa-mir-20a	BZW2	0.394567808	mirna_pc
9700	hsa-mir-20a	LRPPRC	0.422300813	mirna_pc
9701	hsa-mir-20a	CSNK2A1	0.360981682	mirna_pc
9702	hsa-mir-20a	RPIA	0.453724371	mirna_pc
9703	hsa-mir-20a	NUP133	0.323648697	mirna_pc
9704	hsa-mir-20a	C7orf44	0.382896987	mirna_pc
9705	hsa-mir-20a	URB2	0.391364566	mirna_pc
9706	hsa-mir-20a	RP9	0.323923189	mirna_pc
9707	hsa-mir-20a	KIAA1731	0.340437628	mirna_pc
9708	hsa-mir-20a	ZNF286A	0.40630124	mirna_pc
9709	hsa-mir-20a	NAA16	0.332518125	mirna_pc
9710	hsa-mir-20a	SFXN4	0.333012838	mirna_pc
9711	hsa-mir-20a	TCEA1	0.345411258	mirna_pc
9712	hsa-mir-20a	RBM28	0.396374987	mirna_pc
9713	hsa-mir-20a	RANBP17	0.341293041	mirna_pc
9714	hsa-mir-20a	MRE11A	0.341267765	mirna_pc
9715	hsa-mir-20a	WDR85	0.307239796	mirna_pc
9716	hsa-mir-20a	USP37	0.368746973	mirna_pc
9717	hsa-mir-20a	ILKAP	0.364353397	mirna_pc
9718	hsa-mir-20a	DLEU1	0.400567117	mirna_pc
9719	hsa-mir-20a	C13orf23	0.443653722	mirna_pc

9720	hsa-mir-20a	RBM12	0.413267795	mirna_pc
9721	hsa-mir-20a	ZNF286B	0.345501772	mirna_pc
9722	hsa-mir-20a	DDX20	0.305679649	mirna_pc
9723	hsa-mir-20a	CHD7	0.349404787	mirna_pc
9724	hsa-mir-20a	TOP 1.00	0.306656966	mirna_pc
9725	hsa-mir-20a	TCERG1	0.469142483	mirna_pc
9726	hsa-mir-20a	PSPC1	0.468915319	mirna_pc
9727	hsa-mir-20a	CCNB1IP1	0.333738302	mirna_pc
9728	hsa-mir-20a	TOP1P1	0.313452568	mirna_pc
9729	hsa-mir-20a	MAPKAPK5	0.340541202	mirna_pc
9730	hsa-mir-20a	GMCL1	0.361933313	mirna_pc
9731	hsa-mir-20a	E2F6	0.467874292	mirna_pc
9732	hsa-mir-20a	SLC25A15	0.440964867	mirna_pc
9733	hsa-mir-20a	SFRS13A	0.494101822	mirna_pc
9734	hsa-mir-20a	SCLY	0.423310896	mirna_pc
9735	hsa-mir-20a	FASTKD2	0.320168837	mirna_pc
9736	hsa-mir-20a	STARD7	0.314020153	mirna_pc
9737	hsa-mir-20a	NUP35	0.304978147	mirna_pc
9738	hsa-mir-20a	SLC19A1	0.308689611	mirna_pc
9739	hsa-mir-20a	TGDS	0.455976981	mirna_pc
9740	hsa-mir-20a	TFAM	0.468799904	mirna_pc
9741	hsa-mir-20a	LDLRAD3	0.3280855	mirna_pc
9742	hsa-mir-20a	ZNHIT6	0.311958672	mirna_pc
9743	hsa-mir-20a	GSTCD	0.303692829	mirna_pc
9744	hsa-mir-20a	ORC4L	0.465184713	mirna_pc
9745	hsa-mir-20a	ZC3H15	0.317577248	mirna_pc
9746	hsa-mir-20a	KIAA1704	0.426920376	mirna_pc
9747	hsa-mir-20a	MTIF2	0.460299035	mirna_pc
9748	hsa-mir-20a	HNRNPA3	0.40325923	mirna_pc
9749	hsa-mir-20a	MSL1	0.382441225	mirna_pc
9750	hsa-mir-20a	EWSR1	0.38462483	mirna_pc
9751	hsa-mir-20a	ZNF828	0.473774581	mirna_pc
9752	hsa-mir-20a	SMC1A	0.367327633	mirna_pc
9753	hsa-mir-20a	DHX57	0.351135934	mirna_pc
9754	hsa-mir-20a	NUPL1	0.595830771	mirna_pc
9755	hsa-mir-20a	DARS	0.306890284	mirna_pc
9756	hsa-mir-20a	CCDC45	0.336744317	mirna_pc
9757	hsa-mir-20a	WBP4	0.387832723	mirna_pc
9758	hsa-mir-20a	SFT2D3	0.332817048	mirna_pc
9759	hsa-mir-20a	ANKRD5	0.332744542	mirna_pc
9760	hsa-mir-20a	TUBGCP3	0.339214168	mirna_pc
9761	hsa-mir-20a	MRPL30	0.337040009	mirna_pc
9762	hsa-mir-20a	GNL3	0.300998556	mirna_pc
9763	hsa-mir-20a	TSGA14	0.334983145	mirna_pc
9764	hsa-mir-20a	TYW1	0.318257285	mirna_pc
9765	hsa-mir-20a	UPF3A	0.447533465	mirna_pc
9766	hsa-mir-20a	SFRS7	0.423029495	mirna_pc
9767	hsa-mir-20a	MRPL42	0.389470989	mirna_pc
9768	hsa-mir-20a	BEND3	0.333932229	mirna_pc
9769	hsa-mir-20a	NFXL1	0.342699543	mirna_pc
9770	hsa-mir-20a	PFAS	0.325723608	mirna_pc
9771	hsa-mir-20a	CNOT6	0.304799799	mirna_pc
9772	hsa-mir-20a	UCHL5	0.338986523	mirna_pc
9773	hsa-mir-20a	CCAR1	0.389470126	mirna_pc

9774	hsa-mir-20a	CDCA7L	0.393947648	mirna_pc
9775	hsa-mir-20a	KBTBD7	0.313782987	mirna_pc
9776	hsa-mir-20a	HNRNPH1	0.335701776	mirna_pc
9777	hsa-mir-20a	NUP153	0.312922341	mirna_pc
9778	hsa-mir-20a	NIPSNAP1	0.305863629	mirna_pc
9779	hsa-mir-20a	SERBP1	0.324709497	mirna_pc
9780	hsa-mir-20a	EPRS	0.314669477	mirna_pc
9781	hsa-mir-20a	WDR92	0.348223616	mirna_pc
9782	hsa-mir-20a	ZNF214	0.329744116	mirna_pc
9783	hsa-mir-20a	MCM3APAS	0.343752529	mirna_pc
9784	hsa-mir-20a	SLC35F2	0.328560848	mirna_pc
9785	hsa-mir-20a	RCBTB1	0.340009951	mirna_pc
9786	hsa-mir-20a	CDC16	0.40648506	mirna_pc
9787	hsa-mir-20a	ING1	0.357691464	mirna_pc
9788	hsa-mir-20a	CDK8	0.300046023	mirna_pc
9789	hsa-mir-20a	MRPL44	0.31078434	mirna_pc
9790	hsa-mir-20a	PTCD3	0.406979463	mirna_pc
9791	hsa-mir-20a	SPRY4	0.304536291	mirna_pc
9792	hsa-mir-20a	CASP8AP2	0.331857694	mirna_pc
9793	hsa-mir-20a	RNF6	0.383045874	mirna_pc
9794	hsa-mir-20a	BCAN	0.347379379	mirna_pc
9795	hsa-mir-20a	DHX15	0.346914745	mirna_pc
9796	hsa-mir-20a	ZNF669	0.315799157	mirna_pc
9797	hsa-mir-20a	PRPF4B	0.3271474	mirna_pc
9798	hsa-mir-20a	ALG5	0.302844514	mirna_pc
9799	hsa-mir-20a	IWS1	0.404514892	mirna_pc
9800	hsa-mir-20a	MAP2K6	0.342569838	mirna_pc
9801	hsa-mir-20a	CASP6	0.305327289	mirna_pc
9802	hsa-mir-20a	RBM26	0.46203967	mirna_pc
9803	hsa-mir-20a	DCP2	0.308227409	mirna_pc
9804	hsa-mir-20a	ZNF92	0.391366741	mirna_pc
9805	hsa-mir-20a	TMTC4	0.413819575	mirna_pc
9806	hsa-mir-20a	SEPHS1	0.355919941	mirna_pc
9807	hsa-mir-20a	CBX5	0.312012989	mirna_pc
9808	hsa-mir-20a	RMND1	0.360513344	mirna_pc
9809	hsa-mir-20a	ADNP	0.325923623	mirna_pc
9810	hsa-mir-20a	UGGT2	0.303425925	mirna_pc
9811	hsa-mir-20a	AZIN1	0.390685808	mirna_pc
9812	hsa-mir-20a	HNRPLL	0.322571561	mirna_pc
9813	hsa-mir-20a	RSL1D1	0.348725897	mirna_pc
9814	hsa-mir-20a	CEBPZ	0.439308318	mirna_pc
9815	hsa-mir-20a	FASTKD5	0.312245685	mirna_pc
9816	hsa-mir-20a	SMARCE1	0.39132781	mirna_pc
9817	hsa-mir-20a	C8orf33	0.359500229	mirna_pc
9818	hsa-mir-20a	TASP1	0.373924261	mirna_pc
9819	hsa-mir-20a	MRP63	0.304341747	mirna_pc
9820	hsa-mir-20a	IPO5	0.593642512	mirna_pc
9821	hsa-mir-20a	ALG11	0.377193713	mirna_pc
9822	hsa-mir-20a	MCCC2	0.327156518	mirna_pc
9823	hsa-mir-20a	NRF1	0.305878898	mirna_pc
9824	hsa-mir-20a	IDI2	0.302520025	mirna_pc
9825	hsa-mir-20a	GNPAT	0.311215041	mirna_pc
9826	hsa-mir-20a	MED28	0.307629541	mirna_pc
9827	hsa-mir-20a	MATR3	0.304670055	mirna_pc

9828	hsa-mir-20a	NARS2	0.305859713	mirna_pc
9829	hsa-mir-20a	NUP43	0.349261309	mirna_pc
9830	hsa-mir-20a	BRD3	0.304730119	mirna_pc
9831	hsa-mir-20a	MTRF1	0.329578452	mirna_pc
9832	hsa-mir-20a	CELF1	0.304571384	mirna_pc
9833	hsa-mir-20a	ZNF124	0.432382756	mirna_pc
9834	hsa-mir-20a	RNASEH2B	0.462245093	mirna_pc
9835	hsa-mir-20a	CHD1L	0.342310389	mirna_pc
9836	hsa-mir-20a	ZNF473	0.418909216	mirna_pc
9837	hsa-mir-20a	UGGT1	0.306147832	mirna_pc
9838	hsa-mir-20a	ALMS1	0.309834867	mirna_pc
9839	hsa-mir-20a	SNRNP48	0.342855301	mirna_pc
9840	hsa-mir-20a	RFXAP	0.370706808	mirna_pc
9841	hsa-mir-20a	DCLRE1A	0.402623193	mirna_pc
9842	hsa-mir-20a	ANP32A	0.451909762	mirna_pc
9843	hsa-mir-20a	DIS3	0.394768199	mirna_pc
9844	hsa-mir-20a	E2F5	0.407021121	mirna_pc
9845	hsa-mir-20a	STARD3	0.304363544	mirna_pc
9846	hsa-mir-20a	NUFIP1	0.555394403	mirna_pc
9847	hsa-mir-20a	ZNF195	0.470336618	mirna_pc
9848	hsa-mir-20a	ZMYM4	0.321938797	mirna_pc
9849	hsa-mir-20a	DCAF17	0.333689125	mirna_pc
9850	hsa-mir-20a	PRKAR1B	0.339021688	mirna_pc
9851	hsa-mir-20a	GZF1	0.312827372	mirna_pc
9852	hsa-mir-20a	BCAS2	0.341331872	mirna_pc
9853	hsa-mir-20a	MED4	0.354986552	mirna_pc
9854	hsa-mir-20a	USP45	0.314328944	mirna_pc
9855	hsa-mir-20a	LRRCC1	0.400561301	mirna_pc
9856	hsa-mir-20a	FAM98B	0.309212461	mirna_pc
9857	hsa-mir-20a	NEK3	0.377871712	mirna_pc
9858	hsa-mir-20a	FUBP3	0.315090596	mirna_pc
9859	hsa-mir-20a	HNRNPH3	0.372478695	mirna_pc
9860	hsa-mir-20a	RRP1B	0.332091627	mirna_pc
9861	hsa-mir-20a	THSD1P1	0.332265583	mirna_pc
9862	hsa-mir-20a	NSL1	0.364852368	mirna_pc
9863	hsa-mir-20a	VIL1	0.300327484	mirna_pc
9864	hsa-mir-20a	GSDMB	0.358032024	mirna_pc
9865	hsa-mir-20a	ZNF12	0.308267334	mirna_pc
9866	hsa-mir-20a	TMEM170A	0.417260756	mirna_pc
9867	hsa-mir-20a	AHCTF1	0.340608276	mirna_pc
9868	hsa-mir-20a	KIAA1383	0.320545302	mirna_pc
9869	hsa-mir-20a	FAM48A	0.528917153	mirna_pc
9870	hsa-mir-20a	PMS1	0.441278591	mirna_pc
9871	hsa-mir-20a	PNN	0.372202533	mirna_pc
9872	hsa-mir-20a	MNS1	0.394523773	mirna_pc
9873	hsa-mir-20a	CHCHD3	0.300479364	mirna_pc
9874	hsa-mir-20a	PAN3	0.365447613	mirna_pc
9875	hsa-mir-20a	CCDC122	0.433586418	mirna_pc
9876	hsa-mir-20a	TRUB1	0.405733947	mirna_pc
9877	hsa-mir-20a	ZMYM2	0.452034844	mirna_pc
9878	hsa-mir-20a	UGT8	0.307096409	mirna_pc
9879	hsa-mir-20a	CCNJ	0.519133125	mirna_pc
9880	hsa-mir-20a	WDR33	0.310924771	mirna_pc
9881	hsa-mir-20a	PIBF1	0.385701376	mirna_pc

9882	hsa-mir-20a	DCAF16	0.376166222	mirna_pc
9883	hsa-mir-20a	SBK1	0.344263418	mirna_pc
9884	hsa-mir-20a	IPO7	0.346517641	mirna_pc
9885	hsa-mir-20a	ABCC4	0.385673184	mirna_pc
9886	hsa-mir-20a	KBTBD6	0.41427795	mirna_pc
9887	hsa-mir-20a	USP13	0.380464974	mirna_pc
9888	hsa-mir-20a	ESF1	0.396375309	mirna_pc
9889	hsa-mir-20a	ZMYM5	0.340897542	mirna_pc
9890	hsa-mir-20a	SLC25A33	0.310635472	mirna_pc
9891	hsa-mir-20a	ZNF26	0.315050686	mirna_pc
9892	hsa-mir-20a	SMARCC1	0.383017907	mirna_pc
9893	hsa-mir-20a	CTPS2	0.389005981	mirna_pc
9894	hsa-mir-20a	GXYLT1	0.358755953	mirna_pc
9895	hsa-mir-20a	PACRGL	0.34738654	mirna_pc
9896	hsa-mir-20a	C2orf56	0.307804505	mirna_pc
9897	hsa-mir-20a	ZNF480	0.317167306	mirna_pc
9898	hsa-mir-20a	XPO4	0.546462783	mirna_pc
9899	hsa-mir-20a	TSEN2	0.302701065	mirna_pc
9900	hsa-mir-20a	ZNF232	0.301623101	mirna_pc
9901	hsa-mir-20a	MDN1	0.333738755	mirna_pc
9902	hsa-mir-20a	ZBTB39	0.339246633	mirna_pc
9903	hsa-mir-20a	IFT88	0.52118193	mirna_pc
9904	hsa-mir-20a	ESD	0.320322485	mirna_pc
9905	hsa-mir-20a	QRSL1	0.34232492	mirna_pc
9906	hsa-mir-20a	FANCF	0.444831327	mirna_pc
9907	hsa-mir-20a	MARS2	0.318344719	mirna_pc
9908	hsa-mir-20a	VPS54	0.333346894	mirna_pc
9909	hsa-mir-20a	ZBTB24	0.403248806	mirna_pc
9910	hsa-mir-20a	ZNF138	0.362964609	mirna_pc
9911	hsa-mir-20a	ZNF551	0.305252082	mirna_pc
9912	hsa-mir-20a	CIAO1	0.366995039	mirna_pc
9913	hsa-mir-20a	C2orf3	0.440627131	mirna_pc
9914	hsa-mir-20a	POM121C	0.310575996	mirna_pc
9915	hsa-mir-20a	FAM119A	0.417220973	mirna_pc
9916	hsa-mir-20a	TIA1	0.306382047	mirna_pc
9917	hsa-mir-20a	ZNF664	0.387052818	mirna_pc
9918	hsa-mir-20a	RRP7B	0.327260826	mirna_pc
9919	hsa-mir-20a	RBBP6	0.326960902	mirna_pc
9920	hsa-mir-20a	VPS36	0.346679621	mirna_pc
9921	hsa-mir-20a	GOLT1A	0.345268577	mirna_pc
9922	hsa-mir-20a	NCRNA00183	0.310090272	mirna_pc
9923	hsa-mir-20a	UBXN4	0.325388087	mirna_pc
9924	hsa-mir-20a	SCML2	0.353893411	mirna_pc
9925	hsa-mir-20a	SIKE1	0.313572604	mirna_pc
9926	hsa-mir-20a	MRPL19	0.481026942	mirna_pc
9927	hsa-mir-20a	GCFC1	0.339872194	mirna_pc
9928	hsa-mir-20a	EFHA1	0.339457804	mirna_pc
9929	hsa-mir-20a	ABCB10	0.354630649	mirna_pc
9930	hsa-mir-20a	KPNA3	0.372094605	mirna_pc
9931	hsa-mir-20a	ZCCHC3	0.479334153	mirna_pc
9932	hsa-mir-20a	TOMM20	0.307023839	mirna_pc
9933	hsa-mir-20a	RRP15	0.475265774	mirna_pc
9934	hsa-mir-20a	LUC7L3	0.33530099	mirna_pc
9935	hsa-mir-20a	ETV5	0.339095133	mirna_pc

9936	hsa-mir-20a	KIAA1430	0.307665436	mirna_pc
9937	hsa-mir-20a	CASC3	0.3099052	mirna_pc
9938	hsa-mir-20a	AADAT	0.42051246	mirna_pc
9939	hsa-mir-20a	CBLL1	0.310820387	mirna_pc
9940	hsa-mir-20a	ACP1	0.367097635	mirna_pc
9941	hsa-mir-20a	C2orf43	0.376572571	mirna_pc
9942	hsa-mir-20a	DACH1	0.319061295	mirna_pc
9943	hsa-mir-20a	RARA	0.322785508	mirna_pc
9944	hsa-mir-20a	ACTR3B	0.337034874	mirna_pc
9945	hsa-mir-20a	GTF3A	0.45009549	mirna_pc
9946	hsa-mir-20a	RBM27	0.303623135	mirna_pc
9947	hsa-mir-20a	ZNF680	0.33682613	mirna_pc
9948	hsa-mir-20a	TPP2	0.482263585	mirna_pc
9949	hsa-mir-20a	SPRED2	0.342719338	mirna_pc
9950	hsa-mir-20a	CWC22	0.344775992	mirna_pc
9951	hsa-mir-20a	TXLNG	0.414014797	mirna_pc
9952	hsa-mir-20a	TRIM25	0.330803434	mirna_pc
9953	hsa-mir-20a	PRMT6	0.322658184	mirna_pc
9954	hsa-mir-20a	CEP57	0.316930847	mirna_pc
9955	hsa-mir-20a	MRPS9	0.359960872	mirna_pc
9956	hsa-mir-20a	PPP1R1B	0.34774783	mirna_pc
9957	hsa-mir-20a	MIR17HG	0.510376325	mirna_pc
9958	hsa-mir-20a	PHIP	0.304875958	mirna_pc
9959	hsa-mir-20a	AARSD1	0.348555926	mirna_pc
9960	hsa-mir-20a	PRDX3	0.306113609	mirna_pc
9961	hsa-mir-20a	ZNF239	0.352374604	mirna_pc
9962	hsa-mir-20a	KLHL23	0.400071308	mirna_pc
9963	hsa-mir-20a	ZNF202	0.322827323	mirna_pc
9964	hsa-mir-20a	TMEM14B	0.310647601	mirna_pc
9965	hsa-mir-20a	SMARCAD1	0.331827173	mirna_pc
9966	hsa-mir-20a	ZNF77	0.35597679	mirna_pc
9967	hsa-mir-20a	MRPS31	0.398315197	mirna_pc
9968	hsa-mir-20a	CENPV	0.340682315	mirna_pc
9969	hsa-mir-20a	SFRS6	0.335569469	mirna_pc
9970	hsa-mir-20a	SLC4A5	0.35146049	mirna_pc
9971	hsa-mir-20a	DNMT3A	0.455061716	mirna_pc
9972	hsa-mir-20a	MIPEP	0.415013151	mirna_pc
9973	hsa-mir-20a	PRTFDC1	0.391321616	mirna_pc
9974	hsa-mir-20a	CARS2	0.308066988	mirna_pc
9975	hsa-mir-20a	PATZ1	0.377753304	mirna_pc
9976	hsa-mir-20a	ZNF670	0.381594641	mirna_pc
9977	hsa-mir-20a	ZNF326	0.342132969	mirna_pc
9978	hsa-mir-20a	ZRANB2	0.303984973	mirna_pc
9979	hsa-mir-20a	ZNF711	0.33744122	mirna_pc
9980	hsa-mir-20a	POM121	0.376476576	mirna_pc
9981	hsa-mir-20a	CCT6P1	0.343163453	mirna_pc
9982	hsa-mir-1301	TPX2	0.323428958	mirna_pc
9983	hsa-mir-1301	KPNA2	0.302063152	mirna_pc
9984	hsa-mir-1301	RCC2	0.322711171	mirna_pc
9985	hsa-mir-1301	HOXC9	0.506197284	mirna_pc
9986	hsa-mir-1301	ECT2	0.340920387	mirna_pc
9987	hsa-mir-1301	RRM2	0.390300887	mirna_pc
9988	hsa-mir-1301	HOXC8	0.301974681	mirna_pc
9989	hsa-mir-1301	CDC25C	0.319833512	mirna_pc

9990	hsa-mir-1301	UBE2C	0.408843732	mirna_pc
9991	hsa-mir-1301	NUSAP1	0.384547038	mirna_pc
9992	hsa-mir-1301	KIFC1	0.321970324	mirna_pc
9993	hsa-mir-1301	BUB1B	0.433844795	mirna_pc
9994	hsa-mir-1301	KIF18B	0.308133308	mirna_pc
9995	hsa-mir-1301	KIF2C	0.317355169	mirna_pc
9996	hsa-mir-1301	NCAPG	0.319377233	mirna_pc
9997	hsa-mir-1301	CLSPN	0.318104153	mirna_pc
9998	hsa-mir-1301	SGOL1	0.334026932	mirna_pc
9999	hsa-mir-1301	CCNA2	0.35628108	mirna_pc
10000	hsa-mir-1301	SPC25	0.335973211	mirna_pc
10001	hsa-mir-1301	FANCI	0.357009345	mirna_pc
10002	hsa-mir-1301	NEK2	0.30072393	mirna_pc
10003	hsa-mir-1301	MND1	0.369491864	mirna_pc
10004	hsa-mir-1301	KIF22	0.309506847	mirna_pc
10005	hsa-mir-1301	NCAPH	0.375148204	mirna_pc
10006	hsa-mir-1301	RAD54L	0.326380501	mirna_pc
10007	hsa-mir-1301	CENPA	0.435894593	mirna_pc
10008	hsa-mir-1301	MAD2L1	0.405230295	mirna_pc
10009	hsa-mir-1301	TIMELESS	0.420959862	mirna_pc
10010	hsa-mir-1301	LMNB1	0.342089134	mirna_pc
10011	hsa-mir-1301	CDCA8	0.33024766	mirna_pc
10012	hsa-mir-1301	TROAP	0.514341763	mirna_pc
10013	hsa-mir-1301	XPO1	0.336310159	mirna_pc
10014	hsa-mir-1301	CDC45	0.307451319	mirna_pc
10015	hsa-mir-1301	STMN1	0.392576286	mirna_pc
10016	hsa-mir-1301	CENPM	0.317618639	mirna_pc
10017	hsa-mir-1301	TK1	0.350422205	mirna_pc
10018	hsa-mir-1301	DKC1	0.347078382	mirna_pc
10019	hsa-mir-1301	TUBB	0.310904981	mirna_pc
10020	hsa-mir-1301	KIAA0101	0.338161153	mirna_pc
10021	hsa-mir-1301	KIF4A	0.331058761	mirna_pc
10022	hsa-mir-1301	ORC1L	0.320963728	mirna_pc
10023	hsa-mir-1301	KNTC1	0.386899212	mirna_pc
10024	hsa-mir-1301	CCNB2	0.352268923	mirna_pc
10025	hsa-mir-1301	NUF2	0.413641603	mirna_pc
10026	hsa-mir-1301	PLK4	0.362807321	mirna_pc
10027	hsa-mir-1301	FANCB	0.322450102	mirna_pc
10028	hsa-mir-1301	KIF18A	0.30534735	mirna_pc
10029	hsa-mir-1301	HJURP	0.381768733	mirna_pc
10030	hsa-mir-1301	RAD51	0.307016997	mirna_pc
10031	hsa-mir-1301	BLM	0.392215326	mirna_pc
10032	hsa-mir-1301	NME1	0.305866701	mirna_pc
10033	hsa-mir-1301	CCDC150	0.301832763	mirna_pc
10034	hsa-mir-1301	BIRC5	0.500968242	mirna_pc
10035	hsa-mir-1301	RCC1	0.302706814	mirna_pc
10036	hsa-mir-1301	OIP5	0.424237376	mirna_pc
10037	hsa-mir-1301	EME1	0.350603142	mirna_pc
10038	hsa-mir-1301	RECQL4	0.349645331	mirna_pc
10039	hsa-mir-1301	AURKB	0.411469491	mirna_pc
10040	hsa-mir-1301	MCM10	0.338126704	mirna_pc
10041	hsa-mir-1301	KIF20A	0.382108955	mirna_pc
10042	hsa-mir-1301	SGOL2	0.33624558	mirna_pc
10043	hsa-mir-1301	CHEK2	0.394579134	mirna_pc

10044	hsa-mir-1301	KIF15	0.356923182	mirna_pc
10045	hsa-mir-1301	CDCA3	0.390993587	mirna_pc
10046	hsa-mir-1301	C1orf112	0.395024482	mirna_pc
10047	hsa-mir-1301	TRAIP	0.345755161	mirna_pc
10048	hsa-mir-1301	ORC6L	0.311393704	mirna_pc
10049	hsa-mir-1301	C16orf59	0.426168463	mirna_pc
10050	hsa-mir-1301	RAE1	0.384968848	mirna_pc
10051	hsa-mir-1301	FAM72D	0.301312206	mirna_pc
10052	hsa-mir-1301	FAM72B	0.337070607	mirna_pc
10053	hsa-mir-1301	ACTL6A	0.480858967	mirna_pc
10054	hsa-mir-1301	POLD1	0.323983157	mirna_pc
10055	hsa-mir-1301	ZNF229	0.443579761	mirna_pc
10056	hsa-mir-1301	C12orf48	0.453700726	mirna_pc
10057	hsa-mir-1301	HNRNPL	0.48512758	mirna_pc
10058	hsa-mir-1301	SMC4	0.382247251	mirna_pc
10059	hsa-mir-1301	TTK	0.310920112	mirna_pc
10060	hsa-mir-1301	SF3B4	0.363810456	mirna_pc
10061	hsa-mir-1301	TUBA1B	0.323675269	mirna_pc
10062	hsa-mir-1301	HOXC6	0.311641546	mirna_pc
10063	hsa-mir-1301	HELLS	0.316688232	mirna_pc
10064	hsa-mir-1301	UBE2S	0.367549457	mirna_pc
10065	hsa-mir-1301	NCAPD2	0.312906	mirna_pc
10066	hsa-mir-1301	C11orf82	0.477523901	mirna_pc
10067	hsa-mir-1301	HNRNPC	0.383523917	mirna_pc
10068	hsa-mir-1301	ILF2	0.396125377	mirna_pc
10069	hsa-mir-1301	TYMS	0.314880819	mirna_pc
10070	hsa-mir-1301	C20orf20	0.303615137	mirna_pc
10071	hsa-mir-1301	RNASEH2A	0.312691809	mirna_pc
10072	hsa-mir-1301	EPR1	0.379710051	mirna_pc
10073	hsa-mir-1301	PIF1	0.341734171	mirna_pc
10074	hsa-mir-1301	H2AFX	0.302602264	mirna_pc
10075	hsa-mir-1301	SFRS2	0.388039672	mirna_pc
10076	hsa-mir-1301	UCK2	0.388687575	mirna_pc
10077	hsa-mir-1301	NFKBIL2	0.33631653	mirna_pc
10078	hsa-mir-1301	C15orf42	0.309505793	mirna_pc
10079	hsa-mir-1301	FAM60A	0.304254929	mirna_pc
10080	hsa-mir-1301	HMGB3	0.356707331	mirna_pc
10081	hsa-mir-1301	E2F1	0.330986709	mirna_pc
10082	hsa-mir-1301	SNRPG	0.402555119	mirna_pc
10083	hsa-mir-1301	EIF4A3	0.441139628	mirna_pc
10084	hsa-mir-1301	SNRPA	0.405693272	mirna_pc
10085	hsa-mir-1301	FUS	0.412657825	mirna_pc
10086	hsa-mir-1301	RFC3	0.312155136	mirna_pc
10087	hsa-mir-1301	CHAF1A	0.358295129	mirna_pc
10088	hsa-mir-1301	GINS4	0.465321432	mirna_pc
10089	hsa-mir-1301	ANP32E	0.443518902	mirna_pc
10090	hsa-mir-1301	CENPO	0.423052402	mirna_pc
10091	hsa-mir-1301	C19orf48	0.346498418	mirna_pc
10092	hsa-mir-1301	GEN1	0.354583946	mirna_pc
10093	hsa-mir-1301	NUDT1	0.312441119	mirna_pc
10094	hsa-mir-1301	HMGB2	0.343168014	mirna_pc
10095	hsa-mir-1301	GINS2	0.324804181	mirna_pc
10096	hsa-mir-1301	DNA2	0.3759197	mirna_pc
10097	hsa-mir-1301	CSE1L	0.332520312	mirna_pc

10098	hsa-mir-1301	PRMT1	0.333723503	mirna_pc
10099	hsa-mir-1301	C18orf56	0.300389278	mirna_pc
10100	hsa-mir-1301	NOP2	0.317283876	mirna_pc
10101	hsa-mir-1301	HIST1H2AE	0.508524988	mirna_pc
10102	hsa-mir-1301	CAD	0.336960685	mirna_pc
10103	hsa-mir-1301	CCDC99	0.300758414	mirna_pc
10104	hsa-mir-1301	CDK2	0.389818154	mirna_pc
10105	hsa-mir-1301	CDK4	0.426252687	mirna_pc
10106	hsa-mir-1301	PFDN2	0.380611711	mirna_pc
10107	hsa-mir-1301	BCL2L12	0.315841627	mirna_pc
10108	hsa-mir-1301	CHTF18	0.340356486	mirna_pc
10109	hsa-mir-1301	HNRNPR	0.357207836	mirna_pc
10110	hsa-mir-1301	DLEU2	0.391960833	mirna_pc
10111	hsa-mir-1301	PRIM1	0.384714706	mirna_pc
10112	hsa-mir-1301	KAT2A	0.320166651	mirna_pc
10113	hsa-mir-1301	PSMC4	0.4291599	mirna_pc
10114	hsa-mir-1301	CHAF1B	0.392018106	mirna_pc
10115	hsa-mir-1301	DHX34	0.318889094	mirna_pc
10116	hsa-mir-1301	HOXC10	0.313696725	mirna_pc
10117	hsa-mir-1301	MSH2	0.481597786	mirna_pc
10118	hsa-mir-1301	SALL4	0.365614385	mirna_pc
10119	hsa-mir-1301	NOP58	0.315337049	mirna_pc
10120	hsa-mir-1301	DBF4	0.459241988	mirna_pc
10121	hsa-mir-1301	CDC7	0.450547899	mirna_pc
10122	hsa-mir-1301	SSB	0.347495922	mirna_pc
10123	hsa-mir-1301	YDJC	0.3487213	mirna_pc
10124	hsa-mir-1301	SNRPC	0.318322441	mirna_pc
10125	hsa-mir-1301	PSMB4	0.383632554	mirna_pc
10126	hsa-mir-1301	RFC4	0.441273091	mirna_pc
10127	hsa-mir-1301	FBXO5	0.36192776	mirna_pc
10128	hsa-mir-1301	FIGNL1	0.492431349	mirna_pc
10129	hsa-mir-1301	E2F3	0.425528612	mirna_pc
10130	hsa-mir-1301	SF3B14	0.383895965	mirna_pc
10131	hsa-mir-1301	CDC25A	0.410304616	mirna_pc
10132	hsa-mir-1301	RAD54B	0.362062548	mirna_pc
10133	hsa-mir-1301	MAGOH	0.33724857	mirna_pc
10134	hsa-mir-1301	DDX12	0.392629064	mirna_pc
10135	hsa-mir-1301	NUP37	0.307972721	mirna_pc
10136	hsa-mir-1301	CPSF3	0.546329247	mirna_pc
10137	hsa-mir-1301	BANF1	0.370833883	mirna_pc
10138	hsa-mir-1301	C21orf45	0.526566532	mirna_pc
10139	hsa-mir-1301	ATIC	0.320722169	mirna_pc
10140	hsa-mir-1301	PPM1G	0.416661024	mirna_pc
10141	hsa-mir-1301	DNAJB11	0.411420048	mirna_pc
10142	hsa-mir-1301	SNRPD2	0.302155275	mirna_pc
10143	hsa-mir-1301	ACAD8	0.381129549	mirna_pc
10144	hsa-mir-1301	NUDT5	0.348327672	mirna_pc
10145	hsa-mir-1301	WBP1	0.354853939	mirna_pc
10146	hsa-mir-1301	CBX8	0.498585141	mirna_pc
10147	hsa-mir-1301	TMEM201	0.400964075	mirna_pc
10148	hsa-mir-1301	PFDN4	0.340281311	mirna_pc
10149	hsa-mir-1301	PAFAH1B3	0.473284863	mirna_pc
10150	hsa-mir-1301	CACYBP	0.316941332	mirna_pc
10151	hsa-mir-1301	SASS6	0.309676479	mirna_pc

10152	hsa-mir-1301	PSRC1	0.384842043	mirna_pc
10153	hsa-mir-1301	DGUOK	0.459984002	mirna_pc
10154	hsa-mir-1301	MRPL47	0.413280325	mirna_pc
10155	hsa-mir-1301	POU2F1	0.307477545	mirna_pc
10156	hsa-mir-1301	FBL	0.36144503	mirna_pc
10157	hsa-mir-1301	DONSON	0.394753973	mirna_pc
10158	hsa-mir-1301	HIST1H1E	0.336860808	mirna_pc
10159	hsa-mir-1301	EXOSC5	0.39362699	mirna_pc
10160	hsa-mir-1301	HNRNPM	0.413628131	mirna_pc
10161	hsa-mir-1301	PNPT1	0.35779914	mirna_pc
10162	hsa-mir-1301	TMPO	0.313031208	mirna_pc
10163	hsa-mir-1301	CCT6A	0.370992817	mirna_pc
10164	hsa-mir-1301	POLR2H	0.391396903	mirna_pc
10165	hsa-mir-1301	SHFM1	0.465107493	mirna_pc
10166	hsa-mir-1301	GPR19	0.340177101	mirna_pc
10167	hsa-mir-1301	LSG1	0.414005138	mirna_pc
10168	hsa-mir-1301	PPIH	0.337129697	mirna_pc
10169	hsa-mir-1301	DHX9	0.363103881	mirna_pc
10170	hsa-mir-1301	CCDC138	0.340290774	mirna_pc
10171	hsa-mir-1301	LOC100128191	0.496867597	mirna_pc
10172	hsa-mir-1301	SMYD5	0.459376803	mirna_pc
10173	hsa-mir-1301	SF3A2	0.351121822	mirna_pc
10174	hsa-mir-1301	TSSC1	0.387355618	mirna_pc
10175	hsa-mir-1301	TLCD1	0.325199987	mirna_pc
10176	hsa-mir-1301	SNRPF	0.397580324	mirna_pc
10177	hsa-mir-1301	SUMO2	0.456266786	mirna_pc
10178	hsa-mir-1301	SFRS1	0.326310523	mirna_pc
10179	hsa-mir-1301	WDR43	0.327712126	mirna_pc
10180	hsa-mir-1301	SNRPA1	0.477409599	mirna_pc
10181	hsa-mir-1301	SKP2	0.303778996	mirna_pc
10182	hsa-mir-1301	SMC6	0.360686046	mirna_pc
10183	hsa-mir-1301	TIPIN	0.409547188	mirna_pc
10184	hsa-mir-1301	SMARCD1	0.344770782	mirna_pc
10185	hsa-mir-1301	HSPBP1	0.383565276	mirna_pc
10186	hsa-mir-1301	DNMT3B	0.321608674	mirna_pc
10187	hsa-mir-1301	ECE2	0.392688351	mirna_pc
10188	hsa-mir-1301	GPN1	0.340675964	mirna_pc
10189	hsa-mir-1301	RFC5	0.349344415	mirna_pc
10190	hsa-mir-1301	TIGD1	0.368351976	mirna_pc
10191	hsa-mir-1301	POLR2D	0.331030663	mirna_pc
10192	hsa-mir-1301	FTSJ2	0.333575432	mirna_pc
10193	hsa-mir-1301	PA2G4	0.355736278	mirna_pc
10194	hsa-mir-1301	SNRPD3	0.42701408	mirna_pc
10195	hsa-mir-1301	KHDRBS1	0.413146241	mirna_pc
10196	hsa-mir-1301	SLC5A6	0.357388081	mirna_pc
10197	hsa-mir-1301	SPAST	0.424138318	mirna_pc
10198	hsa-mir-1301	LOC92659	0.376141546	mirna_pc
10199	hsa-mir-1301	CCDC18	0.352434534	mirna_pc
10200	hsa-mir-1301	CCT7	0.300464399	mirna_pc
10201	hsa-mir-1301	PRRT1	0.341614325	mirna_pc
10202	hsa-mir-1301	TMEM194A	0.42542431	mirna_pc
10203	hsa-mir-1301	SUV420H2	0.326322586	mirna_pc
10204	hsa-mir-1301	ZNF695	0.362447918	mirna_pc
10205	hsa-mir-1301	METTL1	0.385992894	mirna_pc

10206	hsa-mir-1301	TMEM69	0.318634176	mirna_pc
10207	hsa-mir-1301	GNL2	0.415479878	mirna_pc
10208	hsa-mir-1301	NASP	0.406107705	mirna_pc
10209	hsa-mir-1301	RPP40	0.381997975	mirna_pc
10210	hsa-mir-1301	NONO	0.322603516	mirna_pc
10211	hsa-mir-1301	CBX4	0.306519424	mirna_pc
10212	hsa-mir-1301	OBFC2B	0.348806876	mirna_pc
10213	hsa-mir-1301	PTMA	0.40416003	mirna_pc
10214	hsa-mir-1301	USP39	0.355580565	mirna_pc
10215	hsa-mir-1301	DENR	0.364449093	mirna_pc
10216	hsa-mir-1301	SCARB1	0.342892647	mirna_pc
10217	hsa-mir-1301	C11orf84	0.328939946	mirna_pc
10218	hsa-mir-1301	TBCE	0.330931895	mirna_pc
10219	hsa-mir-1301	HIST1H2BK	0.314890396	mirna_pc
10220	hsa-mir-1301	CLCN2	0.323273599	mirna_pc
10221	hsa-mir-1301	LRRC8D	0.336372761	mirna_pc
10222	hsa-mir-1301	NCBP2	0.396735833	mirna_pc
10223	hsa-mir-1301	FBXO22OS	0.344809575	mirna_pc
10224	hsa-mir-1301	SUV39H2	0.368107481	mirna_pc
10225	hsa-mir-1301	ALG6	0.305148984	mirna_pc
10226	hsa-mir-1301	FANCL	0.325659051	mirna_pc
10227	hsa-mir-1301	NAALADL2	0.421052445	mirna_pc
10228	hsa-mir-1301	HNRNPD	0.432046237	mirna_pc
10229	hsa-mir-1301	DPY30	0.450659041	mirna_pc
10230	hsa-mir-1301	MTHFD2	0.357436589	mirna_pc
10231	hsa-mir-1301	SFRS3	0.335165138	mirna_pc
10232	hsa-mir-1301	PNO1	0.38418261	mirna_pc
10233	hsa-mir-1301	CCT4	0.356412473	mirna_pc
10234	hsa-mir-1301	RAD9A	0.339715662	mirna_pc
10235	hsa-mir-1301	ELAVL1	0.343936231	mirna_pc
10236	hsa-mir-1301	CCDC121	0.458822191	mirna_pc
10237	hsa-mir-1301	NCAPD3	0.475778472	mirna_pc
10238	hsa-mir-1301	HSP90B1	0.315640554	mirna_pc
10239	hsa-mir-1301	TBC1D7	0.455575101	mirna_pc
10240	hsa-mir-1301	PAK1IP1	0.431723785	mirna_pc
10241	hsa-mir-1301	RPAP3	0.35583471	mirna_pc
10242	hsa-mir-1301	PFDN6	0.353575447	mirna_pc
10243	hsa-mir-1301	ATP13A3	0.346356477	mirna_pc
10244	hsa-mir-1301	YWHAE	0.339378108	mirna_pc
10245	hsa-mir-1301	HSD17B12	0.326779404	mirna_pc
10246	hsa-mir-1301	C3orf57	0.402869289	mirna_pc
10247	hsa-mir-1301	PDCL3	0.331319078	mirna_pc
10248	hsa-mir-1301	EHMT2	0.427383541	mirna_pc
10249	hsa-mir-1301	UBAP2L	0.31062488	mirna_pc
10250	hsa-mir-1301	DEK	0.369459856	mirna_pc
10251	hsa-mir-1301	HIST2H4A	0.334005977	mirna_pc
10252	hsa-mir-1301	PASK	0.407824011	mirna_pc
10253	hsa-mir-1301	SAAL1	0.332423227	mirna_pc
10254	hsa-mir-1301	TAF4	0.302340189	mirna_pc
10255	hsa-mir-1301	KHSRP	0.395213921	mirna_pc
10256	hsa-mir-1301	PRAME	0.550849921	mirna_pc
10257	hsa-mir-1301	MRPS17	0.414648081	mirna_pc
10258	hsa-mir-1301	MSL3L2	0.303322341	mirna_pc
10259	hsa-mir-1301	SUV39H1	0.332016358	mirna_pc

10260	hsa-mir-1301	IPO9	0.30479337	mirna_pc
10261	hsa-mir-1301	PSMD4	0.310760936	mirna_pc
10262	hsa-mir-1301	SART3	0.359741832	mirna_pc
10263	hsa-mir-1301	TRA2B	0.381471295	mirna_pc
10264	hsa-mir-1301	RSRC1	0.41670188	mirna_pc
10265	hsa-mir-1301	GAR1	0.312791947	mirna_pc
10266	hsa-mir-1301	C15orf23	0.421497094	mirna_pc
10267	hsa-mir-1301	ANAPC7	0.308059121	mirna_pc
10268	hsa-mir-1301	PRMT3	0.321840312	mirna_pc
10269	hsa-mir-1301	RBM12B	0.31148491	mirna_pc
10270	hsa-mir-1301	C3orf21	0.308820517	mirna_pc
10271	hsa-mir-1301	PSMD12	0.363992369	mirna_pc
10272	hsa-mir-1301	ZNF879	0.316864002	mirna_pc
10273	hsa-mir-1301	LSM14B	0.314323828	mirna_pc
10274	hsa-mir-1301	DIABLO	0.301191308	mirna_pc
10275	hsa-mir-1301	TDG	0.311957484	mirna_pc
10276	hsa-mir-1301	ITGB3BP	0.331340108	mirna_pc
10277	hsa-mir-1301	ENOPH1	0.42788039	mirna_pc
10278	hsa-mir-1301	PABPC1L	0.382333733	mirna_pc
10279	hsa-mir-1301	C19orf47	0.385087899	mirna_pc
10280	hsa-mir-1301	DDX55	0.390950385	mirna_pc
10281	hsa-mir-1301	C17orf96	0.409813725	mirna_pc
10282	hsa-mir-1301	PLEKHG4	0.379798322	mirna_pc
10283	hsa-mir-1301	SFPQ	0.382313433	mirna_pc
10284	hsa-mir-1301	AP3M2	0.375227644	mirna_pc
10285	hsa-mir-1301	CNOT3	0.347565506	mirna_pc
10286	hsa-mir-1301	VPS72	0.408098106	mirna_pc
10287	hsa-mir-1301	EIF4EBP1	0.4789796	mirna_pc
10288	hsa-mir-1301	TTC17	0.402602826	mirna_pc
10289	hsa-mir-1301	RHBDD3	0.344344593	mirna_pc
10290	hsa-mir-1301	CADM1	0.622638337	mirna_pc
10291	hsa-mir-1301	POLA1	0.307840741	mirna_pc
10292	hsa-mir-1301	HDAC2	0.425171827	mirna_pc
10293	hsa-mir-1301	GMNN	0.357171193	mirna_pc
10294	hsa-mir-1301	PDCD2L	0.338996782	mirna_pc
10295	hsa-mir-1301	PA2G4P4	0.346183164	mirna_pc
10296	hsa-mir-1301	WDR53	0.323674069	mirna_pc
10297	hsa-mir-1301	ALOX12P2	0.329776503	mirna_pc
10298	hsa-mir-1301	ANKRD32	0.30433234	mirna_pc
10299	hsa-mir-1301	PDAP1	0.585908013	mirna_pc
10300	hsa-mir-1301	RNF7	0.413540899	mirna_pc
10301	hsa-mir-1301	GEMIN6	0.477161339	mirna_pc
10302	hsa-mir-1301	FAM86C	0.301168227	mirna_pc
10303	hsa-mir-1301	PTCD1	0.602037354	mirna_pc
10304	hsa-mir-1301	CRIPT	0.440286931	mirna_pc
10305	hsa-mir-1301	MSH6	0.469593112	mirna_pc
10306	hsa-mir-1301	SOX4	0.379491504	mirna_pc
10307	hsa-mir-1301	C3orf34	0.431306455	mirna_pc
10308	hsa-mir-1301	TPRKB	0.47937927	mirna_pc
10309	hsa-mir-1301	LEF1	0.309059194	mirna_pc
10310	hsa-mir-1301	RNF115	0.343982066	mirna_pc
10311	hsa-mir-1301	POLB	0.46819473	mirna_pc
10312	hsa-mir-1301	ACBD6	0.336682027	mirna_pc
10313	hsa-mir-1301	HNRNPA1	0.322388061	mirna_pc

10314	hsa-mir-1301	UBA2	0.344465362	mirna_pc
10315	hsa-mir-1301	CDK2AP1	0.355528173	mirna_pc
10316	hsa-mir-1301	NUDT3	0.528606927	mirna_pc
10317	hsa-mir-1301	EIF2B4	0.327785015	mirna_pc
10318	hsa-mir-1301	MEX3A	0.419268227	mirna_pc
10319	hsa-mir-1301	MOGS	0.380896044	mirna_pc
10320	hsa-mir-1301	ATP5J2	0.560176662	mirna_pc
10321	hsa-mir-1301	BAT1	0.336682635	mirna_pc
10322	hsa-mir-1301	COIL	0.354667939	mirna_pc
10323	hsa-mir-1301	RFC2	0.30175794	mirna_pc
10324	hsa-mir-1301	BUD31	0.519107428	mirna_pc
10325	hsa-mir-1301	HIST1H4H	0.335730328	mirna_pc
10326	hsa-mir-1301	C16orf88	0.465994994	mirna_pc
10327	hsa-mir-1301	HNRNPA1L2	0.37661212	mirna_pc
10328	hsa-mir-1301	CBX1	0.308209159	mirna_pc
10329	hsa-mir-1301	EMILIN3	0.371256758	mirna_pc
10330	hsa-mir-1301	HMGB1	0.32542607	mirna_pc
10331	hsa-mir-1301	ZBTB12	0.41158497	mirna_pc
10332	hsa-mir-1301	LOC642846	0.358943922	mirna_pc
10333	hsa-mir-1301	TGIF2	0.311643175	mirna_pc
10334	hsa-mir-1301	ZNF155	0.320338764	mirna_pc
10335	hsa-mir-1301	RCN2	0.407625519	mirna_pc
10336	hsa-mir-1301	THUMPD2	0.457692573	mirna_pc
10337	hsa-mir-1301	MTF2	0.338719867	mirna_pc
10338	hsa-mir-1301	GYLTL1B	0.337096177	mirna_pc
10339	hsa-mir-1301	TARBP2	0.32727771	mirna_pc
10340	hsa-mir-1301	CCDC142	0.458682843	mirna_pc
10341	hsa-mir-1301	SNHG9	0.300173319	mirna_pc
10342	hsa-mir-1301	POU6F2	0.368193612	mirna_pc
10343	hsa-mir-1301	ZNF256	0.310576987	mirna_pc
10344	hsa-mir-1301	NOL10	0.477208836	mirna_pc
10345	hsa-mir-1301	SF3A3	0.343326606	mirna_pc
10346	hsa-mir-1301	CTU1	0.317892892	mirna_pc
10347	hsa-mir-1301	POLE	0.393596284	mirna_pc
10348	hsa-mir-1301	EPHA7	0.314091064	mirna_pc
10349	hsa-mir-1301	ZKSCAN2	0.325205065	mirna_pc
10350	hsa-mir-1301	MUTYH	0.366878477	mirna_pc
10351	hsa-mir-1301	WRNIP1	0.324448223	mirna_pc
10352	hsa-mir-1301	SNHG10	0.338882392	mirna_pc
10353	hsa-mir-1301	TAF1B	0.512984795	mirna_pc
10354	hsa-mir-1301	TIMM50	0.434119005	mirna_pc
10355	hsa-mir-1301	XRCC6BP1	0.447965206	mirna_pc
10356	hsa-mir-1301	ADSL	0.357113751	mirna_pc
10357	hsa-mir-1301	VRK1	0.309666297	mirna_pc
10358	hsa-mir-1301	DDOST	0.316182495	mirna_pc
10359	hsa-mir-1301	EXOSC9	0.387558087	mirna_pc
10360	hsa-mir-1301	FOXRED2	0.324485675	mirna_pc
10361	hsa-mir-1301	UNG	0.381649322	mirna_pc
10362	hsa-mir-1301	RBMX	0.452101391	mirna_pc
10363	hsa-mir-1301	TMCO6	0.312844931	mirna_pc
10364	hsa-mir-1301	TSEN15	0.375623071	mirna_pc
10365	hsa-mir-1301	CDK16	0.300494886	mirna_pc
10366	hsa-mir-1301	ALKBH2	0.405849575	mirna_pc
10367	hsa-mir-1301	BAMBI	0.305700159	mirna_pc

10368	hsa-mir-1301	POP5	0.383960659	mirna_pc
10369	hsa-mir-1301	CCDC90A	0.347568654	mirna_pc
10370	hsa-mir-1301	XRCC5	0.301339048	mirna_pc
10371	hsa-mir-1301	RPF2	0.328903062	mirna_pc
10372	hsa-mir-1301	CLK2	0.341997122	mirna_pc
10373	hsa-mir-1301	NHLRC1	0.427423597	mirna_pc
10374	hsa-mir-1301	EFR3B	0.301576945	mirna_pc
10375	hsa-mir-1301	CCDC24	0.397097294	mirna_pc
10376	hsa-mir-1301	PDIA6	0.37260169	mirna_pc
10377	hsa-mir-1301	GPN3	0.393395804	mirna_pc
10378	hsa-mir-1301	ASB3	0.316037087	mirna_pc
10379	hsa-mir-1301	LOC152217	0.336011517	mirna_pc
10380	hsa-mir-1301	MEMO1	0.497033177	mirna_pc
10381	hsa-mir-1301	IGSF9	0.303000995	mirna_pc
10382	hsa-mir-1301	LOC440905	0.374609682	mirna_pc
10383	hsa-mir-1301	MYLIP	0.315382534	mirna_pc
10384	hsa-mir-1301	UBXN2A	0.410199921	mirna_pc
10385	hsa-mir-1301	DNAJC14	0.394142264	mirna_pc
10386	hsa-mir-1301	KDM2B	0.365638676	mirna_pc
10387	hsa-mir-1301	GTF3C2	0.307998532	mirna_pc
10388	hsa-mir-1301	C19orf73	0.325491694	mirna_pc
10389	hsa-mir-1301	MAGEF1	0.492593157	mirna_pc
10390	hsa-mir-1301	PCGF6	0.32081739	mirna_pc
10391	hsa-mir-1301	FAM136A	0.349320045	mirna_pc
10392	hsa-mir-1301	APOA1BP	0.300101148	mirna_pc
10393	hsa-mir-1301	C7orf25	0.304620089	mirna_pc
10394	hsa-mir-1301	HMGN1	0.311941953	mirna_pc
10395	hsa-mir-1301	SMARCB1	0.313120023	mirna_pc
10396	hsa-mir-1301	TTC32	0.557793171	mirna_pc
10397	hsa-mir-1301	SMN2	0.393908481	mirna_pc
10398	hsa-mir-1301	U2AF1	0.456131049	mirna_pc
10399	hsa-mir-1301	FAM92A1	0.322888745	mirna_pc
10400	hsa-mir-1301	ZNF639	0.38182902	mirna_pc
10401	hsa-mir-1301	ITPRIPL1	0.365366006	mirna_pc
10402	hsa-mir-1301	MSTO1	0.362535365	mirna_pc
10403	hsa-mir-1301	RBM17	0.323717743	mirna_pc
10404	hsa-mir-1301	ABT1	0.460011388	mirna_pc
10405	hsa-mir-1301	THADA	0.338722221	mirna_pc
10406	hsa-mir-1301	MPHOSPH10	0.377799733	mirna_pc
10407	hsa-mir-1301	AKAP9	0.512662203	mirna_pc
10408	hsa-mir-1301	RPS7	0.325659098	mirna_pc
10409	hsa-mir-1301	ATXN7L2	0.359015068	mirna_pc
10410	hsa-mir-1301	MKRN3	0.37837525	mirna_pc
10411	hsa-mir-1301	PABPN1	0.34100291	mirna_pc
10412	hsa-mir-1301	METAP2	0.36581558	mirna_pc
10413	hsa-mir-1301	BCL7A	0.386634333	mirna_pc
10414	hsa-mir-1301	RNFT2	0.338920963	mirna_pc
10415	hsa-mir-1301	C7orf44	0.428081792	mirna_pc
10416	hsa-mir-1301	NUDCD2	0.327016723	mirna_pc
10417	hsa-mir-1301	NAT9	0.414457381	mirna_pc
10418	hsa-mir-1301	WDR77	0.373166139	mirna_pc
10419	hsa-mir-1301	MRPL21	0.350656862	mirna_pc
10420	hsa-mir-1301	NLE1	0.302607327	mirna_pc
10421	hsa-mir-1301	FXR1	0.305935997	mirna_pc

10422	hsa-mir-1301	DMRTA2	0.334389047	mirna_pc
10423	hsa-mir-1301	UPF3B	0.346010778	mirna_pc
10424	hsa-mir-1301	BTG3	0.327775943	mirna_pc
10425	hsa-mir-1301	LOC645332	0.439830767	mirna_pc
10426	hsa-mir-1301	PRR3	0.346806708	mirna_pc
10427	hsa-mir-1301	POLR3C	0.324468109	mirna_pc
10428	hsa-mir-1301	MGC72080	0.557322873	mirna_pc
10429	hsa-mir-1301	TRRAP	0.42962027	mirna_pc
10430	hsa-mir-1301	TRMT61B	0.423007001	mirna_pc
10431	hsa-mir-1301	RWDD3	0.382818188	mirna_pc
10432	hsa-mir-1301	SEMA4F	0.307286754	mirna_pc
10433	hsa-mir-1301	ZNF193	0.317863966	mirna_pc
10434	hsa-mir-1301	TBX3	0.318184453	mirna_pc
10435	hsa-mir-1301	APITD1	0.30078917	mirna_pc
10436	hsa-mir-1301	RDH14	0.36146825	mirna_pc
10437	hsa-mir-1301	SLC25A13	0.334606123	mirna_pc
10438	hsa-mir-1301	MPV17	0.369072463	mirna_pc
10439	hsa-mir-1301	DDX20	0.451322378	mirna_pc
10440	hsa-mir-1301	DCUN1D1	0.318633261	mirna_pc
10441	hsa-mir-1301	C1orf109	0.334103241	mirna_pc
10442	hsa-mir-1301	PCBP1	0.323023124	mirna_pc
10443	hsa-mir-1301	CHCHD2	0.374308775	mirna_pc
10444	hsa-mir-1301	PIGX	0.406272169	mirna_pc
10445	hsa-mir-1301	HTRA2	0.380976729	mirna_pc
10446	hsa-mir-1301	MTA3	0.400032508	mirna_pc
10447	hsa-mir-1301	CHAC2	0.346763932	mirna_pc
10448	hsa-mir-1301	ACN9	0.471673043	mirna_pc
10449	hsa-mir-1301	SEC61A2	0.347811991	mirna_pc
10450	hsa-mir-1301	PSPC1	0.354173236	mirna_pc
10451	hsa-mir-1301	INTU	0.498449602	mirna_pc
10452	hsa-mir-1301	ATP6V1E2	0.331250448	mirna_pc
10453	hsa-mir-1301	GPR3	0.334012481	mirna_pc
10454	hsa-mir-1301	E2F6	0.511675051	mirna_pc
10455	hsa-mir-1301	DRG1	0.338451384	mirna_pc
10456	hsa-mir-1301	NOL7	0.417110192	mirna_pc
10457	hsa-mir-1301	PCDHB5	0.380049347	mirna_pc
10458	hsa-mir-1301	PRPSAP2	0.33292399	mirna_pc
10459	hsa-mir-1301	CPSF4	0.607699619	mirna_pc
10460	hsa-mir-1301	HUNK	0.314954832	mirna_pc
10461	hsa-mir-1301	ACTR6	0.361209129	mirna_pc
10462	hsa-mir-1301	PCGF2	0.340103947	mirna_pc
10463	hsa-mir-1301	SFRS13A	0.345300915	mirna_pc
10464	hsa-mir-1301	COQ10A	0.305325791	mirna_pc
10465	hsa-mir-1301	SCLY	0.359793661	mirna_pc
10466	hsa-mir-1301	NT5C	0.312563105	mirna_pc
10467	hsa-mir-1301	FIZ1	0.342187154	mirna_pc
10468	hsa-mir-1301	CCDC59	0.318147841	mirna_pc
10469	hsa-mir-1301	SLC4A1AP	0.400243295	mirna_pc
10470	hsa-mir-1301	TTC27	0.442325053	mirna_pc
10471	hsa-mir-1301	DHRS13	0.380524814	mirna_pc
10472	hsa-mir-1301	MCCC1	0.32708691	mirna_pc
10473	hsa-mir-1301	MTIF2	0.382726408	mirna_pc
10474	hsa-mir-1301	EWSR1	0.302518416	mirna_pc
10475	hsa-mir-1301	LOC222699	0.576474326	mirna_pc

10476	hsa-mir-1301	KCNMB3	0.437644803	mirna_pc
10477	hsa-mir-1301	KRR1	0.305390263	mirna_pc
10478	hsa-mir-1301	DHX57	0.44859875	mirna_pc
10479	hsa-mir-1301	HIST1H1C	0.521629289	mirna_pc
10480	hsa-mir-1301	AAAS	0.46674467	mirna_pc
10481	hsa-mir-1301	SFRS8	0.314787382	mirna_pc
10482	hsa-mir-1301	CHST14	0.341284856	mirna_pc
10483	hsa-mir-1301	ACVR2B	0.406223791	mirna_pc
10484	hsa-mir-1301	C19orf54	0.39913121	mirna_pc
10485	hsa-mir-1301	DKK 1.00	0.344275292	mirna_pc
10486	hsa-mir-1301	SUPT7L	0.466225319	mirna_pc
10487	hsa-mir-1301	SMUG1	0.41667034	mirna_pc
10488	hsa-mir-1301	LIG3	0.314179187	mirna_pc
10489	hsa-mir-1301	CABLES2	0.329023646	mirna_pc
10490	hsa-mir-1301	ZNF277	0.439418127	mirna_pc
10491	hsa-mir-1301	PELP1	0.350280822	mirna_pc
10492	hsa-mir-1301	ACPL2	0.360304793	mirna_pc
10493	hsa-mir-1301	THOC1	0.35176662	mirna_pc
10494	hsa-mir-1301	C11orf45	0.396288951	mirna_pc
10495	hsa-mir-1301	CCDC15	0.305275237	mirna_pc
10496	hsa-mir-1301	SFRS7	0.458388485	mirna_pc
10497	hsa-mir-1301	GPR98	0.317432494	mirna_pc
10498	hsa-mir-1301	LOC151534	0.305390712	mirna_pc
10499	hsa-mir-1301	TRMT2A	0.325369522	mirna_pc
10500	hsa-mir-1301	IFRD1	0.401930677	mirna_pc
10501	hsa-mir-1301	PCBP2	0.344123135	mirna_pc
10502	hsa-mir-1301	UBE2O	0.314759284	mirna_pc
10503	hsa-mir-1301	PFAS	0.315733416	mirna_pc
10504	hsa-mir-1301	INO80B	0.311524042	mirna_pc
10505	hsa-mir-1301	SARS2	0.390931432	mirna_pc
10506	hsa-mir-1301	LOC100128292	0.310845417	mirna_pc
10507	hsa-mir-1301	CORO1B	0.31465318	mirna_pc
10508	hsa-mir-1301	ANKIB1	0.315438318	mirna_pc
10509	hsa-mir-1301	RPUSD2	0.325434623	mirna_pc
10510	hsa-mir-1301	THYN1	0.506130397	mirna_pc
10511	hsa-mir-1301	PARL	0.37930448	mirna_pc
10512	hsa-mir-1301	C2orf68	0.547160563	mirna_pc
10513	hsa-mir-1301	ARPC1A	0.345416918	mirna_pc
10514	hsa-mir-1301	C17orf80	0.380847062	mirna_pc
10515	hsa-mir-1301	ANKRD23	0.311542606	mirna_pc
10516	hsa-mir-1301	IRF2BP1	0.418666468	mirna_pc
10517	hsa-mir-1301	APLP2	0.379194114	mirna_pc
10518	hsa-mir-1301	C17orf75	0.459568435	mirna_pc
10519	hsa-mir-1301	ZBTB44	0.424686268	mirna_pc
10520	hsa-mir-1301	ZNF235	0.304475575	mirna_pc
10521	hsa-mir-1301	SPRYD4	0.364045637	mirna_pc
10522	hsa-mir-1301	EXT2	0.397868552	mirna_pc
10523	hsa-mir-1301	NUP210	0.312525528	mirna_pc
10524	hsa-mir-1301	ZNF789	0.439357125	mirna_pc
10525	hsa-mir-1301	ZNF771	0.335165047	mirna_pc
10526	hsa-mir-1301	EEF1E1	0.389028053	mirna_pc
10527	hsa-mir-1301	PGRMC1	0.303492919	mirna_pc
10528	hsa-mir-1301	MRPL38	0.380848215	mirna_pc
10529	hsa-mir-1301	LOC728024	0.300868323	mirna_pc

10530	hsa-mir-1301	EID2	0.374378279	mirna_pc
10531	hsa-mir-1301	ATPBD4	0.376933552	mirna_pc
10532	hsa-mir-1301	GDF11	0.391553451	mirna_pc
10533	hsa-mir-1301	C14orf104	0.307877095	mirna_pc
10534	hsa-mir-1301	ZKSCAN5	0.495513218	mirna_pc
10535	hsa-mir-1301	POLR2C	0.352038231	mirna_pc
10536	hsa-mir-1301	C12orf10	0.407655383	mirna_pc
10537	hsa-mir-1301	GNAS	0.308072347	mirna_pc
10538	hsa-mir-1301	PPP1R2	0.312805276	mirna_pc
10539	hsa-mir-1301	C12orf45	0.371201084	mirna_pc
10540	hsa-mir-1301	C2orf44	0.390061	mirna_pc
10541	hsa-mir-1301	RING1	0.391622695	mirna_pc
10542	hsa-mir-1301	SEPHS1	0.389883312	mirna_pc
10543	hsa-mir-1301	RPS19BP1	0.304156575	mirna_pc
10544	hsa-mir-1301	C22orf27	0.300807825	mirna_pc
10545	hsa-mir-1301	PNKP	0.379117688	mirna_pc
10546	hsa-mir-1301	GBAS	0.385605103	mirna_pc
10547	hsa-mir-1301	C8orf40	0.406709518	mirna_pc
10548	hsa-mir-1301	AIP	0.31303999	mirna_pc
10549	hsa-mir-1301	ERP29	0.307097584	mirna_pc
10550	hsa-mir-1301	NAB2	0.313565567	mirna_pc
10551	hsa-mir-1301	CEBPZ	0.318072045	mirna_pc
10552	hsa-mir-1301	BOLA3	0.451345639	mirna_pc
10553	hsa-mir-1301	C19orf23	0.519725172	mirna_pc
10554	hsa-mir-1301	GPS1	0.312352522	mirna_pc
10555	hsa-mir-1301	MYL6B	0.419806568	mirna_pc
10556	hsa-mir-1301	PDCD7	0.336043883	mirna_pc
10557	hsa-mir-1301	ZNRF3	0.314647794	mirna_pc
10558	hsa-mir-1301	C11orf71	0.408232845	mirna_pc
10559	hsa-mir-1301	POLR2F	0.318647145	mirna_pc
10560	hsa-mir-1301	DTX3	0.323439909	mirna_pc
10561	hsa-mir-1301	ALKBH3	0.350931409	mirna_pc
10562	hsa-mir-1301	ZNF233	0.325092218	mirna_pc
10563	hsa-mir-1301	TEAD2	0.309171011	mirna_pc
10564	hsa-mir-1301	CAMTA1	0.312657356	mirna_pc
10565	hsa-mir-1301	MRPS15	0.32953687	mirna_pc
10566	hsa-mir-1301	C17orf58	0.401858516	mirna_pc
10567	hsa-mir-1301	HNRNPUL1	0.342456087	mirna_pc
10568	hsa-mir-1301	NARS2	0.374923809	mirna_pc
10569	hsa-mir-1301	LYRM4	0.471516826	mirna_pc
10570	hsa-mir-1301	FAM133B	0.526524494	mirna_pc
10571	hsa-mir-1301	BCL11A	0.336197251	mirna_pc
10572	hsa-mir-1301	ALMS1	0.339528889	mirna_pc
10573	hsa-mir-1301	ANP32A	0.379640059	mirna_pc
10574	hsa-mir-1301	SPDYA	0.324929586	mirna_pc
10575	hsa-mir-1301	DUSP28	0.300331263	mirna_pc
10576	hsa-mir-1301	ETAA1	0.308248339	mirna_pc
10577	hsa-mir-1301	C17orf81	0.336440592	mirna_pc
10578	hsa-mir-1301	HYLS1	0.40908372	mirna_pc
10579	hsa-mir-1301	HIC2	0.34915132	mirna_pc
10580	hsa-mir-1301	LRFN1	0.423785862	mirna_pc
10581	hsa-mir-1301	C7orf60	0.35347271	mirna_pc
10582	hsa-mir-1301	NAP1L1	0.363949457	mirna_pc
10583	hsa-mir-1301	THAP7	0.313228871	mirna_pc

10584	hsa-mir-1301	LCMT2	0.383296359	mirna_pc
10585	hsa-mir-1301	SNF8	0.302067298	mirna_pc
10586	hsa-mir-1301	C1orf156	0.414391009	mirna_pc
10587	hsa-mir-1301	SLC26A11	0.422467355	mirna_pc
10588	hsa-mir-1301	ZNF428	0.330466857	mirna_pc
10589	hsa-mir-1301	RNASEH2C	0.319089591	mirna_pc
10590	hsa-mir-1301	KIAA1383	0.304791439	mirna_pc
10591	hsa-mir-1301	LOC100132287	0.315273544	mirna_pc
10592	hsa-mir-1301	HNRNPA0	0.342064525	mirna_pc
10593	hsa-mir-1301	ZNF300	0.394725453	mirna_pc
10594	hsa-mir-1301	MORN2	0.457227315	mirna_pc
10595	hsa-mir-1301	C12orf41	0.342714852	mirna_pc
10596	hsa-mir-1301	SETD6	0.360078282	mirna_pc
10597	hsa-mir-1301	C15orf40	0.400936066	mirna_pc
10598	hsa-mir-1301	SRP14	0.354416901	mirna_pc
10599	hsa-mir-1301	MESDC2	0.327194037	mirna_pc
10600	hsa-mir-1301	GOLGA7	0.317432628	mirna_pc
10601	hsa-mir-1301	MTERF	0.591782045	mirna_pc
10602	hsa-mir-1301	SBK1	0.424617195	mirna_pc
10603	hsa-mir-1301	AFMID	0.380960284	mirna_pc
10604	hsa-mir-1301	C7orf64	0.386389339	mirna_pc
10605	hsa-mir-1301	TRIM24	0.358400651	mirna_pc
10606	hsa-mir-1301	C21orf67	0.310916007	mirna_pc
10607	hsa-mir-1301	C5orf55	0.329154917	mirna_pc
10608	hsa-mir-1301	USP13	0.371238031	mirna_pc
10609	hsa-mir-1301	LIPT1	0.335111671	mirna_pc
10610	hsa-mir-1301	NDUFAF3	0.306927416	mirna_pc
10611	hsa-mir-1301	RASL10B	0.410738513	mirna_pc
10612	hsa-mir-1301	DNM1P35	0.345543816	mirna_pc
10613	hsa-mir-1301	SLC25A33	0.43357833	mirna_pc
10614	hsa-mir-1301	MSI1	0.423463708	mirna_pc
10615	hsa-mir-1301	OST4	0.327380473	mirna_pc
10616	hsa-mir-1301	GTF2H2C	0.349690406	mirna_pc
10617	hsa-mir-1301	USP36	0.30010668	mirna_pc
10618	hsa-mir-1301	GXYLT1	0.312252957	mirna_pc
10619	hsa-mir-1301	TMEM18	0.345501205	mirna_pc
10620	hsa-mir-1301	BCS1L	0.320355017	mirna_pc
10621	hsa-mir-1301	C2orf56	0.39422726	mirna_pc
10622	hsa-mir-1301	ZNF394	0.428801202	mirna_pc
10623	hsa-mir-1301	PAQR6	0.323431297	mirna_pc
10624	hsa-mir-1301	DDX51	0.405299663	mirna_pc
10625	hsa-mir-1301	WDR83	0.360719816	mirna_pc
10626	hsa-mir-1301	C6orf162	0.349001567	mirna_pc
10627	hsa-mir-1301	PBX2	0.376400765	mirna_pc
10628	hsa-mir-1301	C21orf59	0.397551355	mirna_pc
10629	hsa-mir-1301	GTF2H2	0.364315898	mirna_pc
10630	hsa-mir-1301	ZBTB39	0.447750983	mirna_pc
10631	hsa-mir-1301	NRCAM	0.334703147	mirna_pc
10632	hsa-mir-1301	SNRNP27	0.34184293	mirna_pc
10633	hsa-mir-1301	C1orf56	0.486162079	mirna_pc
10634	hsa-mir-1301	ENO3	0.439381318	mirna_pc
10635	hsa-mir-1301	RPAIN	0.362366935	mirna_pc
10636	hsa-mir-1301	FANCF	0.312273826	mirna_pc
10637	hsa-mir-1301	CCDC90B	0.462000702	mirna_pc

10638	hsa-mir-1301	VPS54	0.432157281	mirna_pc
10639	hsa-mir-1301	ASPSCR1	0.31007777	mirna_pc
10640	hsa-mir-1301	LOC221710	0.309918811	mirna_pc
10641	hsa-mir-1301	GAA	0.310940564	mirna_pc
10642	hsa-mir-1301	API5	0.448614087	mirna_pc
10643	hsa-mir-1301	CIAO1	0.361894716	mirna_pc
10644	hsa-mir-1301	USP28	0.34059331	mirna_pc
10645	hsa-mir-1301	C2orf3	0.515245339	mirna_pc
10646	hsa-mir-1301	CHD4	0.305312929	mirna_pc
10647	hsa-mir-1301	KRIT1	0.443753701	mirna_pc
10648	hsa-mir-1301	FAM119A	0.356448363	mirna_pc
10649	hsa-mir-1301	DHRS2	0.486852526	mirna_pc
10650	hsa-mir-1301	MEAF6	0.338023372	mirna_pc
10651	hsa-mir-1301	ZNF664	0.33327386	mirna_pc
10652	hsa-mir-1301	ACOT13	0.405948182	mirna_pc
10653	hsa-mir-1301	FGFBP3	0.386352393	mirna_pc
10654	hsa-mir-1301	RRP7B	0.312887821	mirna_pc
10655	hsa-mir-1301	DNAJC19	0.453746686	mirna_pc
10656	hsa-mir-1301	TMEM134	0.339612304	mirna_pc
10657	hsa-mir-1301	CCDC157	0.326642215	mirna_pc
10658	hsa-mir-1301	PEX1	0.398066476	mirna_pc
10659	hsa-mir-1301	SCML2	0.389061154	mirna_pc
10660	hsa-mir-1301	C12orf65	0.315803003	mirna_pc
10661	hsa-mir-1301	DIS3L2	0.363266976	mirna_pc
10662	hsa-mir-1301	NMNAT3	0.323492269	mirna_pc
10663	hsa-mir-1301	MRPL19	0.409092331	mirna_pc
10664	hsa-mir-1301	C11orf35	0.346926371	mirna_pc
10665	hsa-mir-1301	HSF2	0.32203668	mirna_pc
10666	hsa-mir-1301	CETN3	0.412196311	mirna_pc
10667	hsa-mir-1301	ZNF513	0.45144071	mirna_pc
10668	hsa-mir-1301	ENGASE	0.3069852	mirna_pc
10669	hsa-mir-1301	PROX1	0.318382711	mirna_pc
10670	hsa-mir-1301	B9D1	0.348896852	mirna_pc
10671	hsa-mir-1301	DDX1	0.359373333	mirna_pc
10672	hsa-mir-1301	LSM 1.00	0.436609504	mirna_pc
10673	hsa-mir-1301	PRPF40B	0.33363228	mirna_pc
10674	hsa-mir-1301	DYNC1I1	0.506149008	mirna_pc
10675	hsa-mir-1301	GLI4	0.374255675	mirna_pc
10676	hsa-mir-1301	PRDXDD1P	0.306296218	mirna_pc
10677	hsa-mir-1301	AADAT	0.368019655	mirna_pc
10678	hsa-mir-1301	ACP1	0.482882991	mirna_pc
10679	hsa-mir-1301	WASH3P	0.371501734	mirna_pc
10680	hsa-mir-1301	GATAD1	0.316100827	mirna_pc
10681	hsa-mir-1301	ZNF322A	0.578205652	mirna_pc
10682	hsa-mir-1301	CDAN1	0.321837254	mirna_pc
10683	hsa-mir-1301	PRR22	0.30186233	mirna_pc
10684	hsa-mir-1301	SERF1A	0.453872865	mirna_pc
10685	hsa-mir-1301	LOC728640	0.369962777	mirna_pc
10686	hsa-mir-1301	B4GALNT4	0.323402514	mirna_pc
10687	hsa-mir-1301	PRMT6	0.418283016	mirna_pc
10688	hsa-mir-1301	ZMYM3	0.330504815	mirna_pc
10689	hsa-mir-1301	LOC401397	0.569918496	mirna_pc
10690	hsa-mir-1301	GPS2	0.351038186	mirna_pc
10691	hsa-mir-1301	C21orf119	0.485153836	mirna_pc

10692	hsa-mir-1301	C2orf28	0.497975295	mirna_pc
10693	hsa-mir-1301	HSPC157	0.306649437	mirna_pc
10694	hsa-mir-1301	TMEM14B	0.406385897	mirna_pc
10695	hsa-mir-1301	AGA	0.311781077	mirna_pc
10696	hsa-mir-1301	TMEM168	0.5043897	mirna_pc
10697	hsa-mir-1301	CCDC75	0.306804312	mirna_pc
10698	hsa-mir-1301	SV2A	0.35242779	mirna_pc
10699	hsa-mir-1301	THOC5	0.301641767	mirna_pc
10700	hsa-mir-1301	MRPL53	0.476831418	mirna_pc
10701	hsa-mir-1301	SCARNA2	0.30429888	mirna_pc
10702	hsa-mir-1301	PPCDC	0.336398171	mirna_pc
10703	hsa-mir-1301	DNMT3A	0.485138657	mirna_pc
10704	hsa-mir-1301	NDUFS8	0.411774925	mirna_pc
10705	hsa-mir-1301	ACCN2	0.430865244	mirna_pc
10706	hsa-mir-1301	C12orf73	0.39784526	mirna_pc
10707	hsa-mir-1301	PATZ1	0.366731502	mirna_pc
10708	hsa-mir-1301	C3orf33	0.319692576	mirna_pc
10709	hsa-mir-99a	HOXC8	0.321526767	mirna_pc
10710	hsa-mir-99a	SEMA3E	0.730874593	mirna_pc
10711	hsa-mir-99a	C4orf34	0.329991064	mirna_pc
10712	hsa-mir-99a	MAPT	0.309230624	mirna_pc
10713	hsa-mir-99a	EEF1A2	0.474882023	mirna_pc
10714	hsa-mir-99a	MYEF2	0.358641252	mirna_pc
10715	hsa-mir-99a	MYST4	0.519690444	mirna_pc
10716	hsa-mir-99a	ZNF467	0.385675372	mirna_pc
10717	hsa-mir-99a	ID4	0.434604375	mirna_pc
10718	hsa-mir-99a	YPEL1	0.544036682	mirna_pc
10719	hsa-mir-99a	C22orf39	0.395035122	mirna_pc
10720	hsa-mir-99a	ZNF425	0.345819804	mirna_pc
10721	hsa-mir-99a	ZNF25	0.372491666	mirna_pc
10722	hsa-mir-99a	CITED2	0.700245964	mirna_pc
10723	hsa-mir-99a	SMARCD3	0.304054208	mirna_pc
10724	hsa-mir-99a	TBC1D9	0.330765387	mirna_pc
10725	hsa-mir-99a	ATP1B2	0.377011459	mirna_pc
10726	hsa-mir-99a	CRYZL1	0.351408841	mirna_pc
10727	hsa-mir-99a	ZNF429	0.35482366	mirna_pc
10728	hsa-mir-99a	PTGDS	0.321361623	mirna_pc
10729	hsa-mir-99a	WBSCR17	0.321754794	mirna_pc
10730	hsa-mir-99a	NR2F2	0.61754439	mirna_pc
10731	hsa-mir-99a	MAGI2	0.35405136	mirna_pc
10732	hsa-mir-99a	UTRN	0.3230453	mirna_pc
10733	hsa-mir-99a	C17orf108	0.324768524	mirna_pc
10734	hsa-mir-99a	RSPO3	0.312399046	mirna_pc
10735	hsa-mir-99a	HDHD2	0.334819544	mirna_pc
10736	hsa-mir-99a	DTWD1	0.377720501	mirna_pc
10737	hsa-mir-99a	CSDC2	0.33079765	mirna_pc
10738	hsa-mir-99a	RASGRP2	0.499028118	mirna_pc
10739	hsa-mir-99a	MXI1	0.32823222	mirna_pc
10740	hsa-mir-99a	FAT4	0.379087201	mirna_pc
10741	hsa-mir-99a	ZNF439	0.509151962	mirna_pc
10742	hsa-mir-99a	SUOX	0.314156572	mirna_pc
10743	hsa-mir-99a	POLI	0.596945734	mirna_pc
10744	hsa-mir-99a	RNF185	0.385421745	mirna_pc
10745	hsa-mir-99a	EZH1	0.320746817	mirna_pc

10746	hsa-mir-99a	TTC3	0.39853087	mirna_pc
10747	hsa-mir-99a	LOC286367	0.423515503	mirna_pc
10748	hsa-mir-99a	RAB5B	0.310184722	mirna_pc
10749	hsa-mir-99a	CCDC106	0.409379049	mirna_pc
10750	hsa-mir-99a	SLC5A12	0.408169743	mirna_pc
10751	hsa-mir-99a	DLG4	0.389737415	mirna_pc
10752	hsa-mir-99a	USP25	0.556368575	mirna_pc
10753	hsa-mir-99a	LOC375190	0.615795142	mirna_pc
10754	hsa-mir-99a	CRY1	0.307980581	mirna_pc
10755	hsa-mir-99a	MTMR3	0.360201446	mirna_pc
10756	hsa-mir-99a	HIST1H3D	0.317177784	mirna_pc
10757	hsa-mir-99a	SLC2A11	0.514827746	mirna_pc
10758	hsa-mir-99a	ZNF433	0.402555885	mirna_pc
10759	hsa-mir-99a	TYW3	0.621563665	mirna_pc
10760	hsa-mir-99a	ALS2CR8	0.337120838	mirna_pc
10761	hsa-mir-99a	MKL2	0.374897702	mirna_pc
10762	hsa-mir-99a	TNRC6C	0.331298811	mirna_pc
10763	hsa-mir-99a	PPP1R14A	0.308405471	mirna_pc
10764	hsa-mir-99a	ELAC1	0.303858818	mirna_pc
10765	hsa-mir-99a	TBC1D17	0.334557675	mirna_pc
10766	hsa-mir-99a	OXR1	0.357740745	mirna_pc
10767	hsa-mir-99a	DOCK3	0.352824042	mirna_pc
10768	hsa-mir-99a	ZNF248	0.340296208	mirna_pc
10769	hsa-mir-99a	TUB	0.402370057	mirna_pc
10770	hsa-mir-99a	FIGN	0.70593806	mirna_pc
10771	hsa-mir-99a	PRRT1	0.521398298	mirna_pc
10772	hsa-mir-99a	RADIL	0.320914612	mirna_pc
10773	hsa-mir-99a	ARSG	0.439294364	mirna_pc
10774	hsa-mir-99a	RBP7	0.306788882	mirna_pc
10775	hsa-mir-99a	SAMD11	0.52934971	mirna_pc
10776	hsa-mir-99a	PHF10	0.457187297	mirna_pc
10777	hsa-mir-99a	LOC284900	0.453404283	mirna_pc
10778	hsa-mir-99a	CAPS2	0.601171913	mirna_pc
10779	hsa-mir-99a	PHC1	0.488809416	mirna_pc
10780	hsa-mir-99a	SCD5	0.362303881	mirna_pc
10781	hsa-mir-99a	CCDC121	0.385598809	mirna_pc
10782	hsa-mir-99a	SSBP2	0.41844743	mirna_pc
10783	hsa-mir-99a	HABP4	0.648211635	mirna_pc
10784	hsa-mir-99a	TBCEL	0.367107477	mirna_pc
10785	hsa-mir-99a	C20orf160	0.561346457	mirna_pc
10786	hsa-mir-99a	CSAD	0.466876219	mirna_pc
10787	hsa-mir-99a	SFRS18	0.326882044	mirna_pc
10788	hsa-mir-99a	PRAME	0.372246626	mirna_pc
10789	hsa-mir-99a	KIAA0776	0.330572065	mirna_pc
10790	hsa-mir-99a	LRRC4B	0.403924252	mirna_pc
10791	hsa-mir-99a	CA11	0.527670717	mirna_pc
10792	hsa-mir-99a	EIF4ENIF1	0.591041753	mirna_pc
10793	hsa-mir-99a	BAIAP3	0.410048958	mirna_pc
10794	hsa-mir-99a	LOC653501	0.360541723	mirna_pc
10795	hsa-mir-99a	TUG1	0.411779026	mirna_pc
10796	hsa-mir-99a	AMN1	0.482998219	mirna_pc
10797	hsa-mir-99a	MGC21881	0.352962639	mirna_pc
10798	hsa-mir-99a	FAM184A	0.354474293	mirna_pc
10799	hsa-mir-99a	TDRD5	0.310048754	mirna_pc

10800	hsa-mir-99a	CILP2	0.468841171	mirna_pc
10801	hsa-mir-99a	ATP8B2	0.329029545	mirna_pc
10802	hsa-mir-99a	ZNF70	0.41257459	mirna_pc
10803	hsa-mir-99a	GRAMD1C	0.541355283	mirna_pc
10804	hsa-mir-99a	CTSO	0.304016386	mirna_pc
10805	hsa-mir-99a	ZNF217	0.434494113	mirna_pc
10806	hsa-mir-99a	CYTSA	0.368961407	mirna_pc
10807	hsa-mir-99a	ZNF606	0.317083151	mirna_pc
10808	hsa-mir-99a	DYRK1A	0.351233689	mirna_pc
10809	hsa-mir-99a	ZNF236	0.367617375	mirna_pc
10810	hsa-mir-99a	SOS 1	0.309799035	mirna_pc
10811	hsa-mir-99a	EFHD1	0.379702878	mirna_pc
10812	hsa-mir-99a	LPHN2	0.586420109	mirna_pc
10813	hsa-mir-99a	ZNF23	0.337558312	mirna_pc
10814	hsa-mir-99a	AMY2B	0.320798463	mirna_pc
10815	hsa-mir-99a	CDKL3	0.35488473	mirna_pc
10816	hsa-mir-99a	BARD1	0.313217538	mirna_pc
10817	hsa-mir-99a	CNTNAP3	0.413721622	mirna_pc
10818	hsa-mir-99a	ATF7	0.31521414	mirna_pc
10819	hsa-mir-99a	ZNF559	0.313251236	mirna_pc
10820	hsa-mir-99a	ZNF155	0.310946751	mirna_pc
10821	hsa-mir-99a	EDIL3	0.449687223	mirna_pc
10822	hsa-mir-99a	LOC144571	0.483304279	mirna_pc
10823	hsa-mir-99a	ZNF608	0.332136013	mirna_pc
10824	hsa-mir-99a	SEMA3D	0.735442916	mirna_pc
10825	hsa-mir-99a	HACE1	0.391188416	mirna_pc
10826	hsa-mir-99a	ZNF187	0.335046395	mirna_pc
10827	hsa-mir-99a	KLRA1	0.367621989	mirna_pc
10828	hsa-mir-99a	FLJ10038	0.364285107	mirna_pc
10829	hsa-mir-99a	ZKSCAN2	0.303916003	mirna_pc
10830	hsa-mir-99a	SLC25A30	0.437920048	mirna_pc
10831	hsa-mir-99a	ZNF658	0.438708443	mirna_pc
10832	hsa-mir-99a	RBM43	0.425653526	mirna_pc
10833	hsa-mir-99a	GATA3	0.533110665	mirna_pc
10834	hsa-mir-99a	EPM2AIP1	0.333481152	mirna_pc
10835	hsa-mir-99a	TRIM13	0.314490343	mirna_pc
10836	hsa-mir-99a	LOC374443	0.332323723	mirna_pc
10837	hsa-mir-99a	RNPC3	0.368044674	mirna_pc
10838	hsa-mir-99a	PTCH1	0.306274128	mirna_pc
10839	hsa-mir-99a	USP34	0.312085997	mirna_pc
10840	hsa-mir-99a	OXTR	0.396017639	mirna_pc
10841	hsa-mir-99a	PAQR7	0.354459972	mirna_pc
10842	hsa-mir-99a	THSD7A	0.564946403	mirna_pc
10843	hsa-mir-99a	PPM1J	0.338624377	mirna_pc
10844	hsa-mir-99a	ZNF605	0.408620815	mirna_pc
10845	hsa-mir-99a	NDST2	0.348518089	mirna_pc
10846	hsa-mir-99a	RRAGB	0.404512869	mirna_pc
10847	hsa-mir-99a	KLRAQ1	0.375708694	mirna_pc
10848	hsa-mir-99a	PHF21A	0.348752145	mirna_pc
10849	hsa-mir-99a	SSPN	0.331400051	mirna_pc
10850	hsa-mir-99a	TCEA2	0.325813048	mirna_pc
10851	hsa-mir-99a	OSR2	0.387750251	mirna_pc
10852	hsa-mir-99a	LOC338799	0.312484332	mirna_pc
10853	hsa-mir-99a	C12orf76	0.305172233	mirna_pc

10854	hsa-mir-99a	GABPA	0.413495055	mirna_pc
10855	hsa-mir-99a	ZFP161	0.387065008	mirna_pc
10856	hsa-mir-99a	ZNF503	0.402601713	mirna_pc
10857	hsa-mir-99a	ING4	0.319461744	mirna_pc
10858	hsa-mir-99a	C8orf51	0.304256472	mirna_pc
10859	hsa-mir-99a	LOC728392	0.33528485	mirna_pc
10860	hsa-mir-99a	LANCL1	0.334012584	mirna_pc
10861	hsa-mir-99a	C1orf213	0.303739564	mirna_pc
10862	hsa-mir-99a	ZNF607	0.375717885	mirna_pc
10863	hsa-mir-99a	KCNC3	0.490339396	mirna_pc
10864	hsa-mir-99a	RASSF8	0.319322686	mirna_pc
10865	hsa-mir-99a	PIK3C3	0.351299154	mirna_pc
10866	hsa-mir-99a	BTN2A2	0.308869962	mirna_pc
10867	hsa-mir-99a	ZNF362	0.374224683	mirna_pc
10868	hsa-mir-99a	CHST10	0.30239346	mirna_pc
10869	hsa-mir-99a	CLEC2D	0.373879311	mirna_pc
10870	hsa-mir-99a	ST8SIA1	0.336904579	mirna_pc
10871	hsa-mir-99a	SUSD5	0.319743375	mirna_pc
10872	hsa-mir-99a	ZNF675	0.329992444	mirna_pc
10873	hsa-mir-99a	ZNF84	0.390816827	mirna_pc
10874	hsa-mir-99a	TAF4B	0.304119979	mirna_pc
10875	hsa-mir-99a	ZNF140	0.312402374	mirna_pc
10876	hsa-mir-99a	MTA3	0.432613031	mirna_pc
10877	hsa-mir-99a	ZSCAN12	0.316977184	mirna_pc
10878	hsa-mir-99a	PUS10	0.376715007	mirna_pc
10879	hsa-mir-99a	SEC61A2	0.353648279	mirna_pc
10880	hsa-mir-99a	PSPC1	0.355784218	mirna_pc
10881	hsa-mir-99a	INTU	0.3635005	mirna_pc
10882	hsa-mir-99a	PPM1F	0.326396401	mirna_pc
10883	hsa-mir-99a	TMC8	0.373624485	mirna_pc
10884	hsa-mir-99a	NF2	0.468657797	mirna_pc
10885	hsa-mir-99a	KPNA5	0.307078014	mirna_pc
10886	hsa-mir-99a	MYBL1	0.498776812	mirna_pc
10887	hsa-mir-99a	ZNF624	0.309198507	mirna_pc
10888	hsa-mir-99a	CLSTN3	0.376485886	mirna_pc
10889	hsa-mir-99a	RIMKLB	0.352301142	mirna_pc
10890	hsa-mir-99a	GAS1	0.342063844	mirna_pc
10891	hsa-mir-99a	ZNF512B	0.30475543	mirna_pc
10892	hsa-mir-99a	EFNA5	0.552330667	mirna_pc
10893	hsa-mir-99a	C18orf10	0.376077033	mirna_pc
10894	hsa-mir-99a	OAZ2	0.301642898	mirna_pc
10895	hsa-mir-99a	C10orf41	0.414970024	mirna_pc
10896	hsa-mir-99a	MAMSTR	0.512514974	mirna_pc
10897	hsa-mir-99a	TMEM136	0.453959051	mirna_pc
10898	hsa-mir-99a	PNMAL2	0.351822545	mirna_pc
10899	hsa-mir-99a	CCDC78	0.340920916	mirna_pc
10900	hsa-mir-99a	DNM3	0.679344564	mirna_pc
10901	hsa-mir-99a	TERF2IP	0.356993696	mirna_pc
10902	hsa-mir-99a	WDR78	0.459216227	mirna_pc
10903	hsa-mir-99a	KGFLP2	0.387989076	mirna_pc
10904	hsa-mir-99a	IFT81	0.300728571	mirna_pc
10905	hsa-mir-99a	ZNF184	0.342238755	mirna_pc
10906	hsa-mir-99a	MBLAC2	0.403668271	mirna_pc
10907	hsa-mir-99a	NIPSNAP1	0.474399537	mirna_pc

10908	hsa-mir-99a	DEPDC5	0.362246755	mirna_pc
10909	hsa-mir-99a	C8orf37	0.563238248	mirna_pc
10910	hsa-mir-99a	PDGFC	0.469358544	mirna_pc
10911	hsa-mir-99a	WDR92	0.318625451	mirna_pc
10912	hsa-mir-99a	SFI1	0.55925834	mirna_pc
10913	hsa-mir-99a	MAP3K12	0.617430224	mirna_pc
10914	hsa-mir-99a	ZBTB10	0.383711229	mirna_pc
10915	hsa-mir-99a	SOCS5	0.429003743	mirna_pc
10916	hsa-mir-99a	TTC21B	0.336074167	mirna_pc
10917	hsa-mir-99a	ZNF627	0.34055822	mirna_pc
10918	hsa-mir-99a	LDB1	0.322333893	mirna_pc
10919	hsa-mir-99a	ANGPT1	0.722907975	mirna_pc
10920	hsa-mir-99a	EXTL2	0.324665791	mirna_pc
10921	hsa-mir-99a	ARID1A	0.324853886	mirna_pc
10922	hsa-mir-99a	ATAD2B	0.318525309	mirna_pc
10923	hsa-mir-99a	ZNF558	0.350705779	mirna_pc
10924	hsa-mir-99a	NAB2	0.428594393	mirna_pc
10925	hsa-mir-99a	ISYNA1	0.450684524	mirna_pc
10926	hsa-mir-99a	LOC338758	0.430098767	mirna_pc
10927	hsa-mir-99a	KIF27	0.337493342	mirna_pc
10928	hsa-mir-99a	PCMTD2	0.32827616	mirna_pc
10929	hsa-mir-99a	CPT1C	0.32224471	mirna_pc
10930	hsa-mir-99a	ZNF638	0.361834301	mirna_pc
10931	hsa-mir-99a	DGCR6L	0.316822622	mirna_pc
10932	hsa-mir-99a	ZNF20	0.38676523	mirna_pc
10933	hsa-mir-99a	PIP5K1A	0.315123117	mirna_pc
10934	hsa-mir-99a	TMX3	0.366027249	mirna_pc
10935	hsa-mir-99a	ZNF512	0.467391094	mirna_pc
10936	hsa-mir-99a	GRB14	0.437240688	mirna_pc
10937	hsa-mir-99a	ZNF74	0.412305605	mirna_pc
10938	hsa-mir-99a	HTR7P1	0.502112177	mirna_pc
10939	hsa-mir-99a	OSGEPL1	0.305424973	mirna_pc
10940	hsa-mir-99a	PEX13	0.328244658	mirna_pc
10941	hsa-mir-99a	ETAA1	0.329339169	mirna_pc
10942	hsa-mir-99a	HNRNPH3	0.314442901	mirna_pc
10943	hsa-mir-99a	B4GALT6	0.339885207	mirna_pc
10944	hsa-mir-99a	CEP290	0.310694429	mirna_pc
10945	hsa-mir-99a	SP1	0.329454667	mirna_pc
10946	hsa-mir-99a	CNIH2	0.453654997	mirna_pc
10947	hsa-mir-99a	HHAT	0.367344311	mirna_pc
10948	hsa-mir-99a	FAM22D	0.322827532	mirna_pc
10949	hsa-mir-99a	EID3	0.564002422	mirna_pc
10950	hsa-mir-99a	ALG10B	0.311551304	mirna_pc
10951	hsa-mir-99a	KIAA1467	0.325145336	mirna_pc
10952	hsa-mir-99a	PAPLN	0.312055922	mirna_pc
10953	hsa-mir-99a	ABHD12	0.301293513	mirna_pc
10954	hsa-mir-99a	GATS	0.524158411	mirna_pc
10955	hsa-mir-99a	TRUB1	0.333075214	mirna_pc
10956	hsa-mir-99a	PLAC2	0.606115286	mirna_pc
10957	hsa-mir-99a	ROBO1	0.370952139	mirna_pc
10958	hsa-mir-99a	PDIK1L	0.318488258	mirna_pc
10959	hsa-mir-99a	C2orf15	0.311191071	mirna_pc
10960	hsa-mir-99a	C12orf24	0.429888141	mirna_pc
10961	hsa-mir-99a	CCDC102A	0.31432849	mirna_pc

10962	hsa-mir-99a	ZNF436	0.338784526	mirna_pc
10963	hsa-mir-99a	NFYB	0.311000479	mirna_pc
10964	hsa-mir-99a	LCA5L	0.369326493	mirna_pc
10965	hsa-mir-99a	SEMA3A	0.588073638	mirna_pc
10966	hsa-mir-99a	THNSL1	0.303622843	mirna_pc
10967	hsa-mir-99a	USP13	0.399504443	mirna_pc
10968	hsa-mir-99a	FAM120C	0.384171889	mirna_pc
10969	hsa-mir-99a	PDGFRL	0.433184569	mirna_pc
10970	hsa-mir-99a	GPR37	0.371468937	mirna_pc
10971	hsa-mir-99a	SS18	0.355270476	mirna_pc
10972	hsa-mir-99a	ZNF391	0.383392012	mirna_pc
10973	hsa-mir-99a	IFT74	0.503258676	mirna_pc
10974	hsa-mir-99a	MAST1	0.36742823	mirna_pc
10975	hsa-mir-99a	NR5A2	0.376788036	mirna_pc
10976	hsa-mir-99a	TTC39C	0.304791697	mirna_pc
10977	hsa-mir-99a	MDM1	0.700630318	mirna_pc
10978	hsa-mir-99a	ZNF740	0.349719699	mirna_pc
10979	hsa-mir-99a	TRO	0.39578241	mirna_pc
10980	hsa-mir-99a	BBS10	0.399694953	mirna_pc
10981	hsa-mir-99a	PBX2	0.36929362	mirna_pc
10982	hsa-mir-99a	SFRS15	0.432768509	mirna_pc
10983	hsa-mir-99a	SLITRK6	0.523731259	mirna_pc
10984	hsa-mir-99a	ANKRD34A	0.396021119	mirna_pc
10985	hsa-mir-99a	HAPLN1	0.68586448	mirna_pc
10986	hsa-mir-99a	SYNGAP1	0.413614028	mirna_pc
10987	hsa-mir-99a	C8orf45	0.320381455	mirna_pc
10988	hsa-mir-99a	DBNDD1	0.341165478	mirna_pc
10989	hsa-mir-99a	PPOX	0.333782444	mirna_pc
10990	hsa-mir-99a	SAMD10	0.312936338	mirna_pc
10991	hsa-mir-99a	LOC221710	0.320987266	mirna_pc
10992	hsa-mir-99a	TSGA10	0.465113765	mirna_pc
10993	hsa-mir-99a	BRSK1	0.316191837	mirna_pc
10994	hsa-mir-99a	DGCR6	0.419187432	mirna_pc
10995	hsa-mir-99a	CRYZ	0.553733649	mirna_pc
10996	hsa-mir-99a	TIA1	0.319669846	mirna_pc
10997	hsa-mir-99a	SMEK2	0.354707493	mirna_pc
10998	hsa-mir-99a	ATL2	0.421269893	mirna_pc
10999	hsa-mir-99a	ZKSCAN4	0.330717069	mirna_pc
11000	hsa-mir-99a	IL34	0.326115305	mirna_pc
11001	hsa-mir-99a	TBX1	0.328588512	mirna_pc
11002	hsa-mir-99a	KDELC2	0.346098203	mirna_pc
11003	hsa-mir-99a	KILLIN	0.317224172	mirna_pc
11004	hsa-mir-99a	PRICKLE1	0.543791884	mirna_pc
11005	hsa-mir-99a	GCFC1	0.301002919	mirna_pc
11006	hsa-mir-99a	ZCCHC11	0.368213882	mirna_pc
11007	hsa-mir-99a	CETN3	0.306982869	mirna_pc
11008	hsa-mir-99a	ZNF644	0.328893646	mirna_pc
11009	hsa-mir-99a	PRPF40B	0.311823462	mirna_pc
11010	hsa-mir-99a	TCTN1	0.322460245	mirna_pc
11011	hsa-mir-99a	GLI1	0.349143948	mirna_pc
11012	hsa-mir-99a	CTXN1	0.548517205	mirna_pc
11013	hsa-mir-99a	RNF215	0.33499227	mirna_pc
11014	hsa-mir-99a	ZNF680	0.302306129	mirna_pc
11015	hsa-mir-99a	LOC283070	0.545093915	mirna_pc

11016	hsa-mir-99a	CCDC117	0.307731055	mirna_pc
11017	hsa-mir-99a	HSDL1	0.443302274	mirna_pc
11018	hsa-mir-99a	SLC11A2	0.575744703	mirna_pc
11019	hsa-mir-99a	LOC100129387	0.482863931	mirna_pc
11020	hsa-mir-99a	FBXO11	0.328380427	mirna_pc
11021	hsa-mir-99a	HEY2	0.406645173	mirna_pc
11022	hsa-mir-99a	ZFP37	0.311318292	mirna_pc
11023	hsa-mir-99a	HSPC157	0.316372262	mirna_pc
11024	hsa-mir-99a	AGA	0.317470814	mirna_pc
11025	hsa-mir-99a	CAMK1D	0.426139523	mirna_pc
11026	hsa-mir-99a	LCA5	0.671462648	mirna_pc
11027	hsa-mir-99a	CDC73	0.373489674	mirna_pc
11028	hsa-mir-99a	SLC2A3	0.344223163	mirna_pc
11029	hsa-mir-99a	C22orf29	0.368719186	mirna_pc
11030	hsa-mir-99a	WDSUB1	0.343606941	mirna_pc
11031	hsa-mir-99a	BTF3L4	0.386201347	mirna_pc
11032	hsa-mir-1269	GFRA1	0.641023905	mirna_pc
11033	hsa-mir-1269	CENPF	0.378070803	mirna_pc
11034	hsa-mir-1269	RCC2	0.347534113	mirna_pc
11035	hsa-mir-1269	GHR	0.369463084	mirna_pc
11036	hsa-mir-1269	ITGA9	0.496422578	mirna_pc
11037	hsa-mir-1269	KIF2C	0.303290725	mirna_pc
11038	hsa-mir-1269	NCAPG	0.367828495	mirna_pc
11039	hsa-mir-1269	CCNA2	0.422535713	mirna_pc
11040	hsa-mir-1269	NEK2	0.324333694	mirna_pc
11041	hsa-mir-1269	MND1	0.502757271	mirna_pc
11042	hsa-mir-1269	RAD54L	0.31590238	mirna_pc
11043	hsa-mir-1269	MAD2L1	0.478558439	mirna_pc
11044	hsa-mir-1269	LMNB1	0.314973886	mirna_pc
11045	hsa-mir-1269	TACC3	0.308560397	mirna_pc
11046	hsa-mir-1269	STMN1	0.392946383	mirna_pc
11047	hsa-mir-1269	TPCN2	0.343365519	mirna_pc
11048	hsa-mir-1269	CKS1B	0.318358923	mirna_pc
11049	hsa-mir-1269	TUBB	0.325963829	mirna_pc
11050	hsa-mir-1269	NUF2	0.310561011	mirna_pc
11051	hsa-mir-1269	SFRP1	0.507989192	mirna_pc
11052	hsa-mir-1269	PLK4	0.514516788	mirna_pc
11053	hsa-mir-1269	CAPN6	0.64482911	mirna_pc
11054	hsa-mir-1269	DTL	0.466742451	mirna_pc
11055	hsa-mir-1269	C1orf112	0.380634153	mirna_pc
11056	hsa-mir-1269	KIAA0495	0.320250039	mirna_pc
11057	hsa-mir-1269	C16orf59	0.329679271	mirna_pc
11058	hsa-mir-1269	NEIL3	0.415128612	mirna_pc
11059	hsa-mir-1269	ZNF528	0.338143994	mirna_pc
11060	hsa-mir-1269	NCAPD2	0.362053673	mirna_pc
11061	hsa-mir-1269	UCK2	0.340639661	mirna_pc
11062	hsa-mir-1269	FAM64A	0.3615154	mirna_pc
11063	hsa-mir-1269	FUS	0.43825738	mirna_pc
11064	hsa-mir-1269	CENPO	0.387940978	mirna_pc
11065	hsa-mir-1269	HMGB2	0.408104756	mirna_pc
11066	hsa-mir-1269	CACNB4	0.442632698	mirna_pc
11067	hsa-mir-1269	GINS2	0.310481386	mirna_pc
11068	hsa-mir-1269	RELL1	0.417532713	mirna_pc
11069	hsa-mir-1269	RNF150	0.342982844	mirna_pc

11070	hsa-mir-1269	C16orf75	0.337852807	mirna_pc
11071	hsa-mir-1269	TMEM108	0.413119155	mirna_pc
11072	hsa-mir-1269	GPRASP2	0.448617921	mirna_pc
11073	hsa-mir-1269	CENPH	0.453993932	mirna_pc
11074	hsa-mir-1269	MSH2	0.364817233	mirna_pc
11075	hsa-mir-1269	SF3B3	0.426804938	mirna_pc
11076	hsa-mir-1269	CLK4	0.317857285	mirna_pc
11077	hsa-mir-1269	RFC4	0.319867691	mirna_pc
11078	hsa-mir-1269	ZNF853	0.381423284	mirna_pc
11079	hsa-mir-1269	FBXO5	0.334400063	mirna_pc
11080	hsa-mir-1269	H2AFZ	0.373743611	mirna_pc
11081	hsa-mir-1269	DDX12	0.428938131	mirna_pc
11082	hsa-mir-1269	AASS	0.324928027	mirna_pc
11083	hsa-mir-1269	FBXL7	0.352108152	mirna_pc
11084	hsa-mir-1269	ZNF880	0.429380595	mirna_pc
11085	hsa-mir-1269	ZNF649	0.352274917	mirna_pc
11086	hsa-mir-1269	PEX19	0.349348855	mirna_pc
11087	hsa-mir-1269	PSRC1	0.39390978	mirna_pc
11088	hsa-mir-1269	NAP1L5	0.324818352	mirna_pc
11089	hsa-mir-1269	ZNF423	0.381951661	mirna_pc
11090	hsa-mir-1269	PRCC	0.32993416	mirna_pc
11091	hsa-mir-1269	SSBP3	0.498830963	mirna_pc
11092	hsa-mir-1269	C20orf72	0.381883476	mirna_pc
11093	hsa-mir-1269	GPR19	0.494683735	mirna_pc
11094	hsa-mir-1269	GABPB1	0.331081774	mirna_pc
11095	hsa-mir-1269	PPAT	0.302501568	mirna_pc
11096	hsa-mir-1269	CCDC138	0.312711222	mirna_pc
11097	hsa-mir-1269	HOXD9	0.353192916	mirna_pc
11098	hsa-mir-1269	MAP9	0.414429692	mirna_pc
11099	hsa-mir-1269	C4orf46	0.433127197	mirna_pc
11100	hsa-mir-1269	PCDHGB6	0.418792745	mirna_pc
11101	hsa-mir-1269	KHDRBS1	0.365860809	mirna_pc
11102	hsa-mir-1269	TUB	0.503144736	mirna_pc
11103	hsa-mir-1269	MTL 5.00	0.528334171	mirna_pc
11104	hsa-mir-1269	PARP1	0.374648195	mirna_pc
11105	hsa-mir-1269	TSHZ2	0.401438008	mirna_pc
11106	hsa-mir-1269	C4orf21	0.484342301	mirna_pc
11107	hsa-mir-1269	SARDH	0.31164648	mirna_pc
11108	hsa-mir-1269	TMEM194A	0.364707988	mirna_pc
11109	hsa-mir-1269	FGFR1	0.50536589	mirna_pc
11110	hsa-mir-1269	SLC39A10	0.491107328	mirna_pc
11111	hsa-mir-1269	IL4I1	0.439734859	mirna_pc
11112	hsa-mir-1269	NEDD1	0.323574837	mirna_pc
11113	hsa-mir-1269	PAPSS1	0.521745753	mirna_pc
11114	hsa-mir-1269	L3MBTL4	0.321152226	mirna_pc
11115	hsa-mir-1269	CEP135	0.389370415	mirna_pc
11116	hsa-mir-1269	ROR2	0.326550675	mirna_pc
11117	hsa-mir-1269	NASP	0.333657511	mirna_pc
11118	hsa-mir-1269	FLJ39653	0.321844353	mirna_pc
11119	hsa-mir-1269	BCL2	0.447066034	mirna_pc
11120	hsa-mir-1269	TMX4	0.398193838	mirna_pc
11121	hsa-mir-1269	SLC4A11	0.386696022	mirna_pc
11122	hsa-mir-1269	CPE	0.3238287	mirna_pc
11123	hsa-mir-1269	C11orf84	0.33834802	mirna_pc

11124	hsa-mir-1269	LARP6	0.336160859	mirna_pc
11125	hsa-mir-1269	DCHS2	0.336805838	mirna_pc
11126	hsa-mir-1269	HOXA9	0.500512964	mirna_pc
11127	hsa-mir-1269	GORAB	0.43005911	mirna_pc
11128	hsa-mir-1269	FANCL	0.34404003	mirna_pc
11129	hsa-mir-1269	HNRNPD	0.47087867	mirna_pc
11130	hsa-mir-1269	WDR19	0.596949879	mirna_pc
11131	hsa-mir-1269	SHOX2	0.505178816	mirna_pc
11132	hsa-mir-1269	PHC1	0.384839057	mirna_pc
11133	hsa-mir-1269	C4orf41	0.36545937	mirna_pc
11134	hsa-mir-1269	PDZD2	0.323868223	mirna_pc
11135	hsa-mir-1269	PBX1	0.311453986	mirna_pc
11136	hsa-mir-1269	PMAIP1	0.344824482	mirna_pc
11137	hsa-mir-1269	LNX1	0.393837715	mirna_pc
11138	hsa-mir-1269	UBAP2L	0.41797653	mirna_pc
11139	hsa-mir-1269	RPGRIP1L	0.335298214	mirna_pc
11140	hsa-mir-1269	TGFA	0.467463272	mirna_pc
11141	hsa-mir-1269	C10orf72	0.426593666	mirna_pc
11142	hsa-mir-1269	LSM 6.00	0.31214137	mirna_pc
11143	hsa-mir-1269	PARM1	0.422249114	mirna_pc
11144	hsa-mir-1269	FUBP1	0.33528581	mirna_pc
11145	hsa-mir-1269	S100B	0.563682613	mirna_pc
11146	hsa-mir-1269	B3GALNT1	0.319678777	mirna_pc
11147	hsa-mir-1269	IPO9	0.404968943	mirna_pc
11148	hsa-mir-1269	TRA2B	0.358529629	mirna_pc
11149	hsa-mir-1269	RBPJ	0.496497007	mirna_pc
11150	hsa-mir-1269	GAR1	0.302775195	mirna_pc
11151	hsa-mir-1269	CDRT4	0.438878007	mirna_pc
11152	hsa-mir-1269	C3orf21	0.390525373	mirna_pc
11153	hsa-mir-1269	MAGEE1	0.302902736	mirna_pc
11154	hsa-mir-1269	CPXM1	0.603460486	mirna_pc
11155	hsa-mir-1269	WHSC1	0.433434773	mirna_pc
11156	hsa-mir-1269	ZNF141	0.412658364	mirna_pc
11157	hsa-mir-1269	BCL2A1	0.364478378	mirna_pc
11158	hsa-mir-1269	ENOPH1	0.310791991	mirna_pc
11159	hsa-mir-1269	NFIB	0.37239865	mirna_pc
11160	hsa-mir-1269	CHI3L1	0.30372919	mirna_pc
11161	hsa-mir-1269	RAD17	0.332190978	mirna_pc
11162	hsa-mir-1269	METTL14	0.374542609	mirna_pc
11163	hsa-mir-1269	C16orf80	0.302954735	mirna_pc
11164	hsa-mir-1269	PLEKHG4	0.328600181	mirna_pc
11165	hsa-mir-1269	ITFG2	0.307744679	mirna_pc
11166	hsa-mir-1269	SFPQ	0.339663498	mirna_pc
11167	hsa-mir-1269	CD83	0.482380183	mirna_pc
11168	hsa-mir-1269	FAM125B	0.404214747	mirna_pc
11169	hsa-mir-1269	TMEM150C	0.363668701	mirna_pc
11170	hsa-mir-1269	LRPAP1	0.35198787	mirna_pc
11171	hsa-mir-1269	CADM1	0.50756855	mirna_pc
11172	hsa-mir-1269	TLL1	0.396137639	mirna_pc
11173	hsa-mir-1269	POGK	0.389914326	mirna_pc
11174	hsa-mir-1269	CPAMD8	0.593352295	mirna_pc
11175	hsa-mir-1269	IGSF10	0.375410068	mirna_pc
11176	hsa-mir-1269	SALL2	0.352511856	mirna_pc
11177	hsa-mir-1269	MOBKL1A	0.361020109	mirna_pc

11178	hsa-mir-1269	MSH6	0.362550493	mirna_pc
11179	hsa-mir-1269	HOXD11	0.317753044	mirna_pc
11180	hsa-mir-1269	TP73	0.441881616	mirna_pc
11181	hsa-mir-1269	LEF1	0.605445711	mirna_pc
11182	hsa-mir-1269	FAM161A	0.338733606	mirna_pc
11183	hsa-mir-1269	FAM193A	0.469031665	mirna_pc
11184	hsa-mir-1269	KREMEN2	0.639221044	mirna_pc
11185	hsa-mir-1269	MEX3A	0.556823004	mirna_pc
11186	hsa-mir-1269	FAM117A	0.325117941	mirna_pc
11187	hsa-mir-1269	TBC1D19	0.337470313	mirna_pc
11188	hsa-mir-1269	NGFR	0.566897612	mirna_pc
11189	hsa-mir-1269	PDCD11	0.337579309	mirna_pc
11190	hsa-mir-1269	IRAK1BP1	0.39816895	mirna_pc
11191	hsa-mir-1269	CCDC74A	0.328020431	mirna_pc
11192	hsa-mir-1269	CCDC74B	0.364973836	mirna_pc
11193	hsa-mir-1269	LRRN1	0.437423532	mirna_pc
11194	hsa-mir-1269	TIFA	0.486709092	mirna_pc
11195	hsa-mir-1269	CCDC28B	0.380116037	mirna_pc
11196	hsa-mir-1269	UNC119B	0.346466632	mirna_pc
11197	hsa-mir-1269	CDKL3	0.545586396	mirna_pc
11198	hsa-mir-1269	EMILIN3	0.425286653	mirna_pc
11199	hsa-mir-1269	MCTP2	0.607430343	mirna_pc
11200	hsa-mir-1269	ARL 3.00	0.365203327	mirna_pc
11201	hsa-mir-1269	LOC642846	0.449174165	mirna_pc
11202	hsa-mir-1269	TGIF2	0.482263978	mirna_pc
11203	hsa-mir-1269	RCN2	0.350158635	mirna_pc
11204	hsa-mir-1269	POU6F2	0.351109131	mirna_pc
11205	hsa-mir-1269	ALDH9A1	0.414995169	mirna_pc
11206	hsa-mir-1269	C9orf91	0.302375939	mirna_pc
11207	hsa-mir-1269	RCOR3	0.312506468	mirna_pc
11208	hsa-mir-1269	EBF4	0.357469416	mirna_pc
11209	hsa-mir-1269	TMTC1	0.307360161	mirna_pc
11210	hsa-mir-1269	EXOSC9	0.405516617	mirna_pc
11211	hsa-mir-1269	LYST	0.446425235	mirna_pc
11212	hsa-mir-1269	RBMX	0.306306857	mirna_pc
11213	hsa-mir-1269	NBPF3	0.311286142	mirna_pc
11214	hsa-mir-1269	GEM	0.305240316	mirna_pc
11215	hsa-mir-1269	PTBP2	0.333304733	mirna_pc
11216	hsa-mir-1269	LOC374443	0.358052899	mirna_pc
11217	hsa-mir-1269	KIAA1614	0.653918982	mirna_pc
11218	hsa-mir-1269	TWIST1	0.406101904	mirna_pc
11219	hsa-mir-1269	MORN4	0.506995823	mirna_pc
11220	hsa-mir-1269	CLK2	0.441019634	mirna_pc
11221	hsa-mir-1269	RPL39L	0.352986778	mirna_pc
11222	hsa-mir-1269	ASB3	0.483125434	mirna_pc
11223	hsa-mir-1269	IGSF9	0.59823825	mirna_pc
11224	hsa-mir-1269	FIP1L1	0.345235284	mirna_pc
11225	hsa-mir-1269	GTF3C3	0.341412727	mirna_pc
11226	hsa-mir-1269	CIT	0.340808004	mirna_pc
11227	hsa-mir-1269	SCLT1	0.371247571	mirna_pc
11228	hsa-mir-1269	MRFAP1	0.424731355	mirna_pc
11229	hsa-mir-1269	NKD2	0.358822163	mirna_pc
11230	hsa-mir-1269	FRMD4A	0.374822629	mirna_pc
11231	hsa-mir-1269	RWDD4A	0.352280395	mirna_pc

11232	hsa-mir-1269	MYB	0.323472941	mirna_pc
11233	hsa-mir-1269	ITPRIPL1	0.442321034	mirna_pc
11234	hsa-mir-1269	TTLL4	0.516254048	mirna_pc
11235	hsa-mir-1269	CYP24A1	0.591148137	mirna_pc
11236	hsa-mir-1269	FGD5	0.412345006	mirna_pc
11237	hsa-mir-1269	BBS5	0.33134494	mirna_pc
11238	hsa-mir-1269	EMILIN1	0.404678062	mirna_pc
11239	hsa-mir-1269	RFC1	0.521331331	mirna_pc
11240	hsa-mir-1269	ATN1	0.443771089	mirna_pc
11241	hsa-mir-1269	ANKRD6	0.511642026	mirna_pc
11242	hsa-mir-1269	KIAA0090	0.301867318	mirna_pc
11243	hsa-mir-1269	LPHN3	0.30870428	mirna_pc
11244	hsa-mir-1269	LOC645676	0.345827252	mirna_pc
11245	hsa-mir-1269	BCL7A	0.317361085	mirna_pc
11246	hsa-mir-1269	NID1	0.318716047	mirna_pc
11247	hsa-mir-1269	GGT7	0.350184812	mirna_pc
11248	hsa-mir-1269	WNT10A	0.447958877	mirna_pc
11249	hsa-mir-1269	LAMA1	0.328642122	mirna_pc
11250	hsa-mir-1269	FLJ10357	0.338027519	mirna_pc
11251	hsa-mir-1269	TRPA1	0.517289723	mirna_pc
11252	hsa-mir-1269	CRISPLD1	0.367006456	mirna_pc
11253	hsa-mir-1269	ZBTB49	0.489770222	mirna_pc
11254	hsa-mir-1269	PALM2	0.339909988	mirna_pc
11255	hsa-mir-1269	ARNT2	0.474677877	mirna_pc
11256	hsa-mir-1269	ARHGAP19	0.307698355	mirna_pc
11257	hsa-mir-1269	ZNF286A	0.517676492	mirna_pc
11258	hsa-mir-1269	KIAA0922	0.538262963	mirna_pc
11259	hsa-mir-1269	AHDC1	0.364682384	mirna_pc
11260	hsa-mir-1269	MRPL21	0.401016174	mirna_pc
11261	hsa-mir-1269	RASSF8	0.369355787	mirna_pc
11262	hsa-mir-1269	FBXO36	0.335231489	mirna_pc
11263	hsa-mir-1269	ZNF362	0.325631829	mirna_pc
11264	hsa-mir-1269	DMRTA2	0.395695769	mirna_pc
11265	hsa-mir-1269	ARID1B	0.333885846	mirna_pc
11266	hsa-mir-1269	CHST10	0.376463298	mirna_pc
11267	hsa-mir-1269	PPP1R14C	0.40980317	mirna_pc
11268	hsa-mir-1269	SUSD5	0.475221267	mirna_pc
11269	hsa-mir-1269	ALS2CR4	0.344222478	mirna_pc
11270	hsa-mir-1269	CUEDC2	0.331881328	mirna_pc
11271	hsa-mir-1269	TTN	0.385028984	mirna_pc
11272	hsa-mir-1269	TBX5	0.528315098	mirna_pc
11273	hsa-mir-1269	EVC2	0.57496554	mirna_pc
11274	hsa-mir-1269	PRR3	0.447512764	mirna_pc
11275	hsa-mir-1269	SPATA5	0.301834276	mirna_pc
11276	hsa-mir-1269	FAM200B	0.446377207	mirna_pc
11277	hsa-mir-1269	RAB28	0.534377032	mirna_pc
11278	hsa-mir-1269	LRRTM2	0.432808316	mirna_pc
11279	hsa-mir-1269	COPS4	0.300450587	mirna_pc
11280	hsa-mir-1269	ALDH7A1	0.304879287	mirna_pc
11281	hsa-mir-1269	ORAI2	0.309328422	mirna_pc
11282	hsa-mir-1269	SEMA4F	0.486481367	mirna_pc
11283	hsa-mir-1269	ZNF286B	0.370098901	mirna_pc
11284	hsa-mir-1269	IFT80	0.309195904	mirna_pc
11285	hsa-mir-1269	NLGN4Y	0.334763861	mirna_pc

11286	hsa-mir-1269	MRFAP1L1	0.509203544	mirna_pc
11287	hsa-mir-1269	PKIA	0.418200494	mirna_pc
11288	hsa-mir-1269	ZNF599	0.397516774	mirna_pc
11289	hsa-mir-1269	GPR3	0.474661806	mirna_pc
11290	hsa-mir-1269	E2F6	0.386437157	mirna_pc
11291	hsa-mir-1269	KIAA1755	0.434336926	mirna_pc
11292	hsa-mir-1269	HUNK	0.521328524	mirna_pc
11293	hsa-mir-1269	SCLY	0.618589025	mirna_pc
11294	hsa-mir-1269	GPR125	0.547785739	mirna_pc
11295	hsa-mir-1269	SLC44A5	0.302672759	mirna_pc
11296	hsa-mir-1269	ADH5	0.317358422	mirna_pc
11297	hsa-mir-1269	LDLRAD3	0.517822563	mirna_pc
11298	hsa-mir-1269	DHRS13	0.373953615	mirna_pc
11299	hsa-mir-1269	TFAP2C	0.497144992	mirna_pc
11300	hsa-mir-1269	DHX57	0.401004255	mirna_pc
11301	hsa-mir-1269	LPAR6	0.323249329	mirna_pc
11302	hsa-mir-1269	NUMA1	0.488078619	mirna_pc
11303	hsa-mir-1269	ALX3	0.613869613	mirna_pc
11304	hsa-mir-1269	C5orf36	0.33882086	mirna_pc
11305	hsa-mir-1269	CHST14	0.414063842	mirna_pc
11306	hsa-mir-1269	CLSTN3	0.308288629	mirna_pc
11307	hsa-mir-1269	SFT2D3	0.355140782	mirna_pc
11308	hsa-mir-1269	DPY19L2P2	0.367794364	mirna_pc
11309	hsa-mir-1269	CABLES2	0.313397939	mirna_pc
11310	hsa-mir-1269	WDR35	0.345097888	mirna_pc
11311	hsa-mir-1269	IFT140	0.33675226	mirna_pc
11312	hsa-mir-1269	TMEFF1	0.352378044	mirna_pc
11313	hsa-mir-1269	CBFA2T2	0.362917735	mirna_pc
11314	hsa-mir-1269	LZTS1	0.53964214	mirna_pc
11315	hsa-mir-1269	RBM8A	0.306983477	mirna_pc
11316	hsa-mir-1269	LRTOMT	0.326550623	mirna_pc
11317	hsa-mir-1269	GXYLT2	0.605147593	mirna_pc
11318	hsa-mir-1269	PADI2	0.503809635	mirna_pc
11319	hsa-mir-1269	BACH2	0.363659409	mirna_pc
11320	hsa-mir-1269	BAT2	0.320352815	mirna_pc
11321	hsa-mir-1269	FADS2	0.314833092	mirna_pc
11322	hsa-mir-1269	EDAR	0.747199722	mirna_pc
11323	hsa-mir-1269	IFT81	0.392479502	mirna_pc
11324	hsa-mir-1269	LOXL3	0.361717635	mirna_pc
11325	hsa-mir-1269	ZNF184	0.309672076	mirna_pc
11326	hsa-mir-1269	PITX2	0.38710607	mirna_pc
11327	hsa-mir-1269	ARHGEF19	0.440372208	mirna_pc
11328	hsa-mir-1269	DCTD	0.339247062	mirna_pc
11329	hsa-mir-1269	LRP4	0.363794653	mirna_pc
11330	hsa-mir-1269	CCND1	0.330880788	mirna_pc
11331	hsa-mir-1269	ZNF214	0.372994196	mirna_pc
11332	hsa-mir-1269	ANAPC4	0.324514122	mirna_pc
11333	hsa-mir-1269	NFATC3	0.314356681	mirna_pc
11334	hsa-mir-1269	TMEM128	0.433419306	mirna_pc
11335	hsa-mir-1269	ZNF771	0.413491766	mirna_pc
11336	hsa-mir-1269	PRUNE	0.300221261	mirna_pc
11337	hsa-mir-1269	TCTN2	0.525930828	mirna_pc
11338	hsa-mir-1269	BCAN	0.593196986	mirna_pc
11339	hsa-mir-1269	NPHP1	0.324495282	mirna_pc

11340	hsa-mir-1269	DHX15	0.402797647	mirna_pc
11341	hsa-mir-1269	BBS9	0.300735676	mirna_pc
11342	hsa-mir-1269	LRP6	0.302456641	mirna_pc
11343	hsa-mir-1269	EXTL2	0.368719097	mirna_pc
11344	hsa-mir-1269	GNAS	0.311200282	mirna_pc
11345	hsa-mir-1269	DCP2	0.349743267	mirna_pc
11346	hsa-mir-1269	PRKD3	0.346916453	mirna_pc
11347	hsa-mir-1269	C2orf44	0.340884403	mirna_pc
11348	hsa-mir-1269	ACER3	0.441828225	mirna_pc
11349	hsa-mir-1269	LOC440173	0.429628861	mirna_pc
11350	hsa-mir-1269	SHISA2	0.574808237	mirna_pc
11351	hsa-mir-1269	CBX5	0.503631653	mirna_pc
11352	hsa-mir-1269	IFT172	0.458563766	mirna_pc
11353	hsa-mir-1269	ABI2	0.351678325	mirna_pc
11354	hsa-mir-1269	TBCK	0.393454323	mirna_pc
11355	hsa-mir-1269	C2orf81	0.37025581	mirna_pc
11356	hsa-mir-1269	TMEM9	0.397899865	mirna_pc
11357	hsa-mir-1269	TMEM121	0.301934213	mirna_pc
11358	hsa-mir-1269	AGBL5	0.453354662	mirna_pc
11359	hsa-mir-1269	PUM2	0.316050596	mirna_pc
11360	hsa-mir-1269	VASH2	0.697633288	mirna_pc
11361	hsa-mir-1269	NAV2	0.414105733	mirna_pc
11362	hsa-mir-1269	MYL6B	0.412119473	mirna_pc
11363	hsa-mir-1269	SH3TC1	0.566579102	mirna_pc
11364	hsa-mir-1269	ERCC8	0.358776352	mirna_pc
11365	hsa-mir-1269	DTX3	0.320322647	mirna_pc
11366	hsa-mir-1269	CD200	0.55191451	mirna_pc
11367	hsa-mir-1269	MED28	0.563132224	mirna_pc
11368	hsa-mir-1269	MATR3	0.378696494	mirna_pc
11369	hsa-mir-1269	CPT1C	0.505100997	mirna_pc
11370	hsa-mir-1269	UNKL	0.355217744	mirna_pc
11371	hsa-mir-1269	GPX7	0.419713246	mirna_pc
11372	hsa-mir-1269	PAN2	0.324352522	mirna_pc
11373	hsa-mir-1269	ZNF518B	0.442285951	mirna_pc
11374	hsa-mir-1269	CHST11	0.700192741	mirna_pc
11375	hsa-mir-1269	BCL11A	0.499204484	mirna_pc
11376	hsa-mir-1269	FBXO17	0.415779933	mirna_pc
11377	hsa-mir-1269	C4orf27	0.371316703	mirna_pc
11378	hsa-mir-1269	SIX5	0.334344834	mirna_pc
11379	hsa-mir-1269	IRX4	0.320983169	mirna_pc
11380	hsa-mir-1269	C11orf49	0.340514369	mirna_pc
11381	hsa-mir-1269	USP21	0.515075796	mirna_pc
11382	hsa-mir-1269	L3MBTL	0.379105383	mirna_pc
11383	hsa-mir-1269	TRAF3IP3	0.440330838	mirna_pc
11384	hsa-mir-1269	ZCCHC4	0.389211923	mirna_pc
11385	hsa-mir-1269	MARVELD1	0.345872859	mirna_pc
11386	hsa-mir-1269	LOC284023	0.306081946	mirna_pc
11387	hsa-mir-1269	RNF121	0.317580292	mirna_pc
11388	hsa-mir-1269	EMID1	0.692350886	mirna_pc
11389	hsa-mir-1269	ZNF521	0.560464934	mirna_pc
11390	hsa-mir-1269	ZMYM4	0.303010269	mirna_pc
11391	hsa-mir-1269	PRKAR1B	0.358104204	mirna_pc
11392	hsa-mir-1269	LMAN2L	0.433814177	mirna_pc
11393	hsa-mir-1269	TROVE2	0.336645641	mirna_pc

11394	hsa-mir-1269	TCF7L1	0.558570683	mirna_pc
11395	hsa-mir-1269	ENTPD6	0.327484604	mirna_pc
11396	hsa-mir-1269	MOXD1	0.643251151	mirna_pc
11397	hsa-mir-1269	THAP9	0.429869339	mirna_pc
11398	hsa-mir-1269	HNRNPH3	0.390801326	mirna_pc
11399	hsa-mir-1269	COL8A2	0.352046035	mirna_pc
11400	hsa-mir-1269	RABL2A	0.343098454	mirna_pc
11401	hsa-mir-1269	APBA2	0.301808593	mirna_pc
11402	hsa-mir-1269	SH3RF3	0.627839561	mirna_pc
11403	hsa-mir-1269	NSL1	0.485897483	mirna_pc
11404	hsa-mir-1269	KIF3A	0.302324112	mirna_pc
11405	hsa-mir-1269	CNIH2	0.473506914	mirna_pc
11406	hsa-mir-1269	CHST15	0.407923432	mirna_pc
11407	hsa-mir-1269	IQCC	0.39553732	mirna_pc
11408	hsa-mir-1269	HHAT	0.334609615	mirna_pc
11409	hsa-mir-1269	GATAD2B	0.490839121	mirna_pc
11410	hsa-mir-1269	SNAP47	0.311018068	mirna_pc
11411	hsa-mir-1269	BMP7	0.430304701	mirna_pc
11412	hsa-mir-1269	LOC93622	0.368399176	mirna_pc
11413	hsa-mir-1269	MLLT11	0.40684338	mirna_pc
11414	hsa-mir-1269	TTTY15	0.376434676	mirna_pc
11415	hsa-mir-1269	QRICH2	0.444281721	mirna_pc
11416	hsa-mir-1269	ATG10	0.362757981	mirna_pc
11417	hsa-mir-1269	MYCN	0.528857897	mirna_pc
11418	hsa-mir-1269	EFCAB7	0.345718035	mirna_pc
11419	hsa-mir-1269	DCAF16	0.452307061	mirna_pc
11420	hsa-mir-1269	CCDC102A	0.400659891	mirna_pc
11421	hsa-mir-1269	SBK1	0.449142024	mirna_pc
11422	hsa-mir-1269	NINL	0.300552422	mirna_pc
11423	hsa-mir-1269	PABPC4L	0.591625766	mirna_pc
11424	hsa-mir-1269	ABCA11P	0.396290901	mirna_pc
11425	hsa-mir-1269	PMF1	0.31953072	mirna_pc
11426	hsa-mir-1269	C19orf44	0.345877537	mirna_pc
11427	hsa-mir-1269	USP13	0.384709243	mirna_pc
11428	hsa-mir-1269	GALNT14	0.351169597	mirna_pc
11429	hsa-mir-1269	VPS45	0.334077442	mirna_pc
11430	hsa-mir-1269	HAUS3	0.385914948	mirna_pc
11431	hsa-mir-1269	ZNF93	0.33442369	mirna_pc
11432	hsa-mir-1269	CD69	0.483069724	mirna_pc
11433	hsa-mir-1269	C16orf46	0.586211365	mirna_pc
11434	hsa-mir-1269	CLPB	0.332850224	mirna_pc
11435	hsa-mir-1269	TRAF1	0.49841416	mirna_pc
11436	hsa-mir-1269	ZC3H11A	0.36640661	mirna_pc
11437	hsa-mir-1269	SERTAD4	0.377301806	mirna_pc
11438	hsa-mir-1269	SCAI	0.385689978	mirna_pc
11439	hsa-mir-1269	N4BP2	0.363972185	mirna_pc
11440	hsa-mir-1269	TRO	0.577641609	mirna_pc
11441	hsa-mir-1269	PACRGL	0.529865406	mirna_pc
11442	hsa-mir-1269	C6orf48	0.327842705	mirna_pc
11443	hsa-mir-1269	LAMC1	0.42790852	mirna_pc
11444	hsa-mir-1269	PBX2	0.311487904	mirna_pc
11445	hsa-mir-1269	ZNF232	0.437142308	mirna_pc
11446	hsa-mir-1269	ZBTB39	0.301697539	mirna_pc
11447	hsa-mir-1269	NRCAM	0.392017705	mirna_pc

11448	hsa-mir-1269	LYSMD1	0.313641896	mirna_pc
11449	hsa-mir-1269	KAL1	0.314842374	mirna_pc
11450	hsa-mir-1269	RPS10P7	0.309562204	mirna_pc
11451	hsa-mir-1269	ZNF827	0.334933869	mirna_pc
11452	hsa-mir-1269	C20orf12	0.527731058	mirna_pc
11453	hsa-mir-1269	ZBTB24	0.305904626	mirna_pc
11454	hsa-mir-1269	RBBP4	0.498011959	mirna_pc
11455	hsa-mir-1269	ODZ3	0.473589821	mirna_pc
11456	hsa-mir-1269	ENTPD2	0.444815956	mirna_pc
11457	hsa-mir-1269	RGS12	0.561661809	mirna_pc
11458	hsa-mir-1269	ZNF618	0.353507161	mirna_pc
11459	hsa-mir-1269	GEMIN8P4	0.329891548	mirna_pc
11460	hsa-mir-1269	BRSK1	0.407887265	mirna_pc
11461	hsa-mir-1269	CCDC111	0.365689422	mirna_pc
11462	hsa-mir-1269	ZNF713	0.460790283	mirna_pc
11463	hsa-mir-1269	TIA1	0.339782608	mirna_pc
11464	hsa-mir-1269	FAM65C	0.328679671	mirna_pc
11465	hsa-mir-1269	NAP1L4	0.340379776	mirna_pc
11466	hsa-mir-1269	LRDD	0.444546918	mirna_pc
11467	hsa-mir-1269	CCDC157	0.430774762	mirna_pc
11468	hsa-mir-1269	PDGFA	0.425498174	mirna_pc
11469	hsa-mir-1269	MYH10	0.313102698	mirna_pc
11470	hsa-mir-1269	SIKE1	0.31486547	mirna_pc
11471	hsa-mir-1269	B4GALT5	0.310485417	mirna_pc
11472	hsa-mir-1269	SEMA4A	0.402930292	mirna_pc
11473	hsa-mir-1269	HSF2	0.376263247	mirna_pc
11474	hsa-mir-1269	COL9A2	0.481607923	mirna_pc
11475	hsa-mir-1269	MST02P	0.383036083	mirna_pc
11476	hsa-mir-1269	ETV5	0.58833725	mirna_pc
11477	hsa-mir-1269	COL9A3	0.606881941	mirna_pc
11478	hsa-mir-1269	NUCKS1	0.433885393	mirna_pc
11479	hsa-mir-1269	TCTN1	0.395587862	mirna_pc
11480	hsa-mir-1269	AADAT	0.368110813	mirna_pc
11481	hsa-mir-1269	IGHMBP2	0.678152238	mirna_pc
11482	hsa-mir-1269	LGR6	0.646391143	mirna_pc
11483	hsa-mir-1269	ZFP90	0.341854925	mirna_pc
11484	hsa-mir-1269	ABTB2	0.420306893	mirna_pc
11485	hsa-mir-1269	PIGY	0.327663319	mirna_pc
11486	hsa-mir-1269	TUSC3	0.330891457	mirna_pc
11487	hsa-mir-1269	TANK	0.379809878	mirna_pc
11488	hsa-mir-1269	FZD1	0.500094809	mirna_pc
11489	hsa-mir-1269	SERF1A	0.305253593	mirna_pc
11490	hsa-mir-1269	B4GALNT4	0.373933944	mirna_pc
11491	hsa-mir-1269	AASDH	0.301961346	mirna_pc
11492	hsa-mir-1269	PDS5A	0.329163344	mirna_pc
11493	hsa-mir-1269	PDE4B	0.370769917	mirna_pc
11494	hsa-mir-1269	H2AFY2	0.387731381	mirna_pc
11495	hsa-mir-1269	BAI2	0.455030351	mirna_pc
11496	hsa-mir-1269	ZNF133	0.309004845	mirna_pc
11497	hsa-mir-1269	HEY2	0.388363164	mirna_pc
11498	hsa-mir-1269	TRIL	0.680848807	mirna_pc
11499	hsa-mir-1269	PCDH18	0.410411612	mirna_pc
11500	hsa-mir-1269	MTSS1L	0.339810301	mirna_pc
11501	hsa-mir-1269	EDC4	0.305045655	mirna_pc

11502	hsa-mir-1269	SMARCAD1	0.303801966	mirna_pc
11503	hsa-mir-1269	SCHIP1	0.360733957	mirna_pc
11504	hsa-mir-1269	SV2A	0.496610174	mirna_pc
11505	hsa-mir-1269	NT5M	0.30294069	mirna_pc
11506	hsa-mir-1269	SEMA4C	0.326972433	mirna_pc
11507	hsa-mir-1269	RPRD2	0.320423884	mirna_pc
11508	hsa-mir-1269	EFNB3	0.520088854	mirna_pc
11509	hsa-mir-1269	ZBED5	0.344719776	mirna_pc
11510	hsa-mir-1269	KIF7	0.337855175	mirna_pc
11511	hsa-mir-1269	VGLL4	0.383457829	mirna_pc
11512	hsa-mir-1269	VANGL2	0.306346263	mirna_pc
11513	hsa-mir-1269	DNMT3A	0.460902601	mirna_pc
11514	hsa-mir-1269	ZNF821	0.380934854	mirna_pc
11515	hsa-mir-1269	LOC348926	0.304212857	mirna_pc
11516	hsa-mir-1269	CALHM2	0.324440507	mirna_pc
11517	hsa-mir-19a	CENPF	0.302898563	mirna_pc
11518	hsa-mir-19a	RRM2	0.303216723	mirna_pc
11519	hsa-mir-19a	MYBL2	0.315896504	mirna_pc
11520	hsa-mir-19a	UBE2C	0.32773778	mirna_pc
11521	hsa-mir-19a	BUB1	0.31931926	mirna_pc
11522	hsa-mir-19a	BUB1B	0.349433578	mirna_pc
11523	hsa-mir-19a	HDGF	0.308555049	mirna_pc
11524	hsa-mir-19a	FANCA	0.388669206	mirna_pc
11525	hsa-mir-19a	CLSPN	0.328603172	mirna_pc
11526	hsa-mir-19a	SGOL1	0.342578449	mirna_pc
11527	hsa-mir-19a	ARHGAP11A	0.334406867	mirna_pc
11528	hsa-mir-19a	CDC25B	0.363073186	mirna_pc
11529	hsa-mir-19a	FANCI	0.307442729	mirna_pc
11530	hsa-mir-19a	NEK2	0.345535075	mirna_pc
11531	hsa-mir-19a	HOXC11	0.314843988	mirna_pc
11532	hsa-mir-19a	GTSE1	0.343679878	mirna_pc
11533	hsa-mir-19a	RAD54L	0.305590631	mirna_pc
11534	hsa-mir-19a	MAD2L1	0.308452109	mirna_pc
11535	hsa-mir-19a	TIMELESS	0.307793485	mirna_pc
11536	hsa-mir-19a	CDCA8	0.30752225	mirna_pc
11537	hsa-mir-19a	XPO1	0.344505278	mirna_pc
11538	hsa-mir-19a	KNTC1	0.330369832	mirna_pc
11539	hsa-mir-19a	NUF2	0.401654666	mirna_pc
11540	hsa-mir-19a	PLK4	0.367404552	mirna_pc
11541	hsa-mir-19a	KIF18A	0.321833795	mirna_pc
11542	hsa-mir-19a	DEPDC1	0.315488873	mirna_pc
11543	hsa-mir-19a	SKA3	0.422290451	mirna_pc
11544	hsa-mir-19a	KIF14	0.304321706	mirna_pc
11545	hsa-mir-19a	HJURP	0.334084197	mirna_pc
11546	hsa-mir-19a	SKA1	0.303019718	mirna_pc
11547	hsa-mir-19a	BLM	0.41027619	mirna_pc
11548	hsa-mir-19a	CCDC150	0.360932857	mirna_pc
11549	hsa-mir-19a	DTL	0.324632625	mirna_pc
11550	hsa-mir-19a	CENPK	0.361050248	mirna_pc
11551	hsa-mir-19a	MCM10	0.313820875	mirna_pc
11552	hsa-mir-19a	SGOL2	0.354407448	mirna_pc
11553	hsa-mir-19a	KIF15	0.320448416	mirna_pc
11554	hsa-mir-19a	C1orf112	0.367457998	mirna_pc
11555	hsa-mir-19a	GINS1	0.318396216	mirna_pc

11556	hsa-mir-19a	TRAIP	0.316250195	mirna_pc
11557	hsa-mir-19a	C12orf48	0.356737338	mirna_pc
11558	hsa-mir-19a	HELLS	0.326298873	mirna_pc
11559	hsa-mir-19a	CDCA7	0.354934061	mirna_pc
11560	hsa-mir-19a	PIF1	0.310547952	mirna_pc
11561	hsa-mir-19a	DCAF13	0.315180147	mirna_pc
11562	hsa-mir-19a	FAM60A	0.331280049	mirna_pc
11563	hsa-mir-19a	ATAD5	0.359398978	mirna_pc
11564	hsa-mir-19a	KIF20B	0.334584883	mirna_pc
11565	hsa-mir-19a	RFC3	0.378637731	mirna_pc
11566	hsa-mir-19a	DEPDC1B	0.360758104	mirna_pc
11567	hsa-mir-19a	CENPO	0.314027025	mirna_pc
11568	hsa-mir-19a	XRCC2	0.321672439	mirna_pc
11569	hsa-mir-19a	GEN1	0.377604427	mirna_pc
11570	hsa-mir-19a	HMGB2	0.310873201	mirna_pc
11571	hsa-mir-19a	DNA2	0.378801232	mirna_pc
11572	hsa-mir-19a	NOP56	0.352688628	mirna_pc
11573	hsa-mir-19a	CDK2	0.31293739	mirna_pc
11574	hsa-mir-19a	DLEU2	0.341986436	mirna_pc
11575	hsa-mir-19a	FERMT1	0.36302597	mirna_pc
11576	hsa-mir-19a	DDX27	0.314098553	mirna_pc
11577	hsa-mir-19a	MSH2	0.313107966	mirna_pc
11578	hsa-mir-19a	CDC7	0.306679716	mirna_pc
11579	hsa-mir-19a	SSB	0.392218271	mirna_pc
11580	hsa-mir-19a	PUS1	0.365199695	mirna_pc
11581	hsa-mir-19a	FBXO5	0.340205755	mirna_pc
11582	hsa-mir-19a	E2F3	0.449010635	mirna_pc
11583	hsa-mir-19a	DDX11	0.326625967	mirna_pc
11584	hsa-mir-19a	CDC25A	0.380877015	mirna_pc
11585	hsa-mir-19a	DDX12	0.344733717	mirna_pc
11586	hsa-mir-19a	C13orf34	0.445079941	mirna_pc
11587	hsa-mir-19a	SRPK1	0.306598993	mirna_pc
11588	hsa-mir-19a	MCM8	0.4323859	mirna_pc
11589	hsa-mir-19a	PUS7	0.348538618	mirna_pc
11590	hsa-mir-19a	NCL	0.319703665	mirna_pc
11591	hsa-mir-19a	SASS6	0.341053428	mirna_pc
11592	hsa-mir-19a	PSRC1	0.316351051	mirna_pc
11593	hsa-mir-19a	DBF4B	0.321674311	mirna_pc
11594	hsa-mir-19a	C13orf37	0.3519774	mirna_pc
11595	hsa-mir-19a	PNPT1	0.381685944	mirna_pc
11596	hsa-mir-19a	TMPO	0.314989253	mirna_pc
11597	hsa-mir-19a	LOC388796	0.384873778	mirna_pc
11598	hsa-mir-19a	GABPB1	0.375396011	mirna_pc
11599	hsa-mir-19a	DHX9	0.301054	mirna_pc
11600	hsa-mir-19a	CCDC138	0.324030098	mirna_pc
11601	hsa-mir-19a	ETV4	0.324459692	mirna_pc
11602	hsa-mir-19a	SFRS1	0.39283064	mirna_pc
11603	hsa-mir-19a	WDR43	0.349697856	mirna_pc
11604	hsa-mir-19a	HSPD1	0.400902005	mirna_pc
11605	hsa-mir-19a	KHDRBS1	0.350178501	mirna_pc
11606	hsa-mir-19a	PDIA4	0.320581098	mirna_pc
11607	hsa-mir-19a	PRPF40A	0.329653828	mirna_pc
11608	hsa-mir-19a	CCDC18	0.366495413	mirna_pc
11609	hsa-mir-19a	DNAJC2	0.373946343	mirna_pc

11610	hsa-mir-19a	SLC39A10	0.338513643	mirna_pc
11611	hsa-mir-19a	ZNF695	0.408979128	mirna_pc
11612	hsa-mir-19a	HAUS6	0.355790884	mirna_pc
11613	hsa-mir-19a	CENPJ	0.327123236	mirna_pc
11614	hsa-mir-19a	HNRNPU	0.332157802	mirna_pc
11615	hsa-mir-19a	NASP	0.310827047	mirna_pc
11616	hsa-mir-19a	SCARB1	0.307734001	mirna_pc
11617	hsa-mir-19a	SUV39H2	0.354607462	mirna_pc
11618	hsa-mir-19a	GTF2F2	0.309309012	mirna_pc
11619	hsa-mir-19a	POP1	0.399711209	mirna_pc
11620	hsa-mir-19a	NFE2L3	0.309326052	mirna_pc
11621	hsa-mir-19a	KIAA0406	0.336274655	mirna_pc
11622	hsa-mir-19a	HSP90B1	0.360680482	mirna_pc
11623	hsa-mir-19a	NUDT15	0.310271755	mirna_pc
11624	hsa-mir-19a	E2F4	0.319957365	mirna_pc
11625	hsa-mir-19a	SLMO2	0.390156021	mirna_pc
11626	hsa-mir-19a	ATP13A3	0.317972585	mirna_pc
11627	hsa-mir-19a	DHFR	0.315621825	mirna_pc
11628	hsa-mir-19a	NAA25	0.343169561	mirna_pc
11629	hsa-mir-19a	DEK	0.366163503	mirna_pc
11630	hsa-mir-19a	TRIB3	0.389352668	mirna_pc
11631	hsa-mir-19a	PASK	0.372745183	mirna_pc
11632	hsa-mir-19a	ATP11A	0.306700607	mirna_pc
11633	hsa-mir-19a	HEATR2	0.366894926	mirna_pc
11634	hsa-mir-19a	RNF114	0.307261971	mirna_pc
11635	hsa-mir-19a	CEP152	0.339852505	mirna_pc
11636	hsa-mir-19a	PSMD12	0.307421606	mirna_pc
11637	hsa-mir-19a	RNF219	0.466905186	mirna_pc
11638	hsa-mir-19a	SLC25A32	0.389902563	mirna_pc
11639	hsa-mir-19a	PABPC1L	0.305829827	mirna_pc
11640	hsa-mir-19a	DDX55	0.346747193	mirna_pc
11641	hsa-mir-19a	HPDL	0.315264914	mirna_pc
11642	hsa-mir-19a	HDAC2	0.338244564	mirna_pc
11643	hsa-mir-19a	XRN2	0.307331013	mirna_pc
11644	hsa-mir-19a	KPNB1	0.352314644	mirna_pc
11645	hsa-mir-19a	CASP3	0.310498919	mirna_pc
11646	hsa-mir-19a	SMC3	0.392938331	mirna_pc
11647	hsa-mir-19a	FLVCR1	0.336997438	mirna_pc
11648	hsa-mir-19a	MEX3A	0.424665213	mirna_pc
11649	hsa-mir-19a	BAT1	0.36804254	mirna_pc
11650	hsa-mir-19a	LIPG	0.30084359	mirna_pc
11651	hsa-mir-19a	MSH5	0.324612129	mirna_pc
11652	hsa-mir-19a	ACAN	0.332577047	mirna_pc
11653	hsa-mir-19a	TMC7	0.34675388	mirna_pc
11654	hsa-mir-19a	SUZ12	0.301907701	mirna_pc
11655	hsa-mir-19a	HMGB1	0.376729787	mirna_pc
11656	hsa-mir-19a	DIAPH3	0.316051067	mirna_pc
11657	hsa-mir-19a	LOC642846	0.311311799	mirna_pc
11658	hsa-mir-19a	TGIF2	0.37098035	mirna_pc
11659	hsa-mir-19a	FARSB	0.31173629	mirna_pc
11660	hsa-mir-19a	PIK3AP1	0.316133581	mirna_pc
11661	hsa-mir-19a	SC65	0.312208177	mirna_pc
11662	hsa-mir-19a	MTF2	0.345682831	mirna_pc
11663	hsa-mir-19a	POLE	0.36844988	mirna_pc

11664	hsa-mir-19a	SOX9	0.394395055	mirna_pc
11665	hsa-mir-19a	C13orf27	0.385912987	mirna_pc
11666	hsa-mir-19a	GK3P	0.328714716	mirna_pc
11667	hsa-mir-19a	RBMX	0.313786068	mirna_pc
11668	hsa-mir-19a	NOC3L	0.302882369	mirna_pc
11669	hsa-mir-19a	GPR180	0.359811718	mirna_pc
11670	hsa-mir-19a	DAP3	0.326068642	mirna_pc
11671	hsa-mir-19a	MDC 1.00	0.319734476	mirna_pc
11672	hsa-mir-19a	EXOSC8	0.305717636	mirna_pc
11673	hsa-mir-19a	TAF1D	0.327148016	mirna_pc
11674	hsa-mir-19a	PDIA6	0.331466775	mirna_pc
11675	hsa-mir-19a	LBR	0.341064436	mirna_pc
11676	hsa-mir-19a	LTV1	0.32725926	mirna_pc
11677	hsa-mir-19a	GK	0.340022769	mirna_pc
11678	hsa-mir-19a	FAM136A	0.358225675	mirna_pc
11679	hsa-mir-19a	NRAS	0.41106544	mirna_pc
11680	hsa-mir-19a	GDPD5	0.339194527	mirna_pc
11681	hsa-mir-19a	RBCK1	0.305368504	mirna_pc
11682	hsa-mir-19a	GPATCH4	0.339438449	mirna_pc
11683	hsa-mir-19a	SNORA8	0.452657386	mirna_pc
11684	hsa-mir-19a	EML4	0.36181515	mirna_pc
11685	hsa-mir-19a	RPIA	0.382731861	mirna_pc
11686	hsa-mir-19a	KIAA1731	0.328166028	mirna_pc
11687	hsa-mir-19a	RBM28	0.374924493	mirna_pc
11688	hsa-mir-19a	C13orf23	0.342455801	mirna_pc
11689	hsa-mir-19a	SPATA2	0.354719846	mirna_pc
11690	hsa-mir-19a	RBM12	0.337487419	mirna_pc
11691	hsa-mir-19a	CHD7	0.345533427	mirna_pc
11692	hsa-mir-19a	TOP 1.00	0.409180696	mirna_pc
11693	hsa-mir-19a	TCERG1	0.364152619	mirna_pc
11694	hsa-mir-19a	TOP1P1	0.332699079	mirna_pc
11695	hsa-mir-19a	MAPKAPK5	0.31356066	mirna_pc
11696	hsa-mir-19a	E2F6	0.356555721	mirna_pc
11697	hsa-mir-19a	ZNF833	0.304812519	mirna_pc
11698	hsa-mir-19a	SLC25A15	0.30907109	mirna_pc
11699	hsa-mir-19a	SFRS13A	0.345118959	mirna_pc
11700	hsa-mir-19a	SCLY	0.328875521	mirna_pc
11701	hsa-mir-19a	TGDS	0.370997236	mirna_pc
11702	hsa-mir-19a	TFAM	0.341931347	mirna_pc
11703	hsa-mir-19a	MTIF2	0.354002749	mirna_pc
11704	hsa-mir-19a	EWSR1	0.361444495	mirna_pc
11705	hsa-mir-19a	SMC1A	0.348156314	mirna_pc
11706	hsa-mir-19a	NUPL1	0.494931671	mirna_pc
11707	hsa-mir-19a	ANKRD5	0.331283539	mirna_pc
11708	hsa-mir-19a	MRPL42	0.335056618	mirna_pc
11709	hsa-mir-19a	CCAR1	0.37696906	mirna_pc
11710	hsa-mir-19a	KIF21B	0.302054061	mirna_pc
11711	hsa-mir-19a	SPRY4	0.354916933	mirna_pc
11712	hsa-mir-19a	RNF6	0.305017929	mirna_pc
11713	hsa-mir-19a	MAP2K6	0.337349613	mirna_pc
11714	hsa-mir-19a	RBM26	0.321404642	mirna_pc
11715	hsa-mir-19a	ZNF92	0.361594562	mirna_pc
11716	hsa-mir-19a	TMTC4	0.311403882	mirna_pc
11717	hsa-mir-19a	AZIN1	0.376167819	mirna_pc

11718	hsa-mir-19a	IPO5	0.423249335	mirna_pc
11719	hsa-mir-19a	DPAGT1	0.300654062	mirna_pc
11720	hsa-mir-19a	NUP43	0.321476847	mirna_pc
11721	hsa-mir-19a	CHD1L	0.349091909	mirna_pc
11722	hsa-mir-19a	ZNF473	0.307844944	mirna_pc
11723	hsa-mir-19a	SNRNP48	0.319623646	mirna_pc
11724	hsa-mir-19a	DCLRE1A	0.335692684	mirna_pc
11725	hsa-mir-19a	ZUFSP	0.315281266	mirna_pc
11726	hsa-mir-19a	DIS3	0.306899535	mirna_pc
11727	hsa-mir-19a	E2F5	0.329039117	mirna_pc
11728	hsa-mir-19a	NUFIP1	0.397263194	mirna_pc
11729	hsa-mir-19a	ZNF195	0.444355479	mirna_pc
11730	hsa-mir-19a	BCAS2	0.372702416	mirna_pc
11731	hsa-mir-19a	USP45	0.306687782	mirna_pc
11732	hsa-mir-19a	NEK3	0.316328328	mirna_pc
11733	hsa-mir-19a	TMEM170A	0.352344738	mirna_pc
11734	hsa-mir-19a	PHF16	0.314147912	mirna_pc
11735	hsa-mir-19a	FAM48A	0.337137168	mirna_pc
11736	hsa-mir-19a	PMS1	0.321724155	mirna_pc
11737	hsa-mir-19a	PNN	0.310915731	mirna_pc
11738	hsa-mir-19a	TRUB1	0.362989775	mirna_pc
11739	hsa-mir-19a	CCNJ	0.40778969	mirna_pc
11740	hsa-mir-19a	IPO7	0.326794828	mirna_pc
11741	hsa-mir-19a	ABCC4	0.338740886	mirna_pc
11742	hsa-mir-19a	ESF1	0.337199071	mirna_pc
11743	hsa-mir-19a	DENND4B	0.307971646	mirna_pc
11744	hsa-mir-19a	GTF2H2C	0.309274087	mirna_pc
11745	hsa-mir-19a	NUS1	0.33946041	mirna_pc
11746	hsa-mir-19a	CTPS2	0.343378724	mirna_pc
11747	hsa-mir-19a	XPO4	0.443503847	mirna_pc
11748	hsa-mir-19a	SNCAIP	0.345116595	mirna_pc
11749	hsa-mir-19a	KIF12	0.31019253	mirna_pc
11750	hsa-mir-19a	RNF32	0.312663111	mirna_pc
11751	hsa-mir-19a	C20orf118	0.311914895	mirna_pc
11752	hsa-mir-19a	IFT88	0.422441301	mirna_pc
11753	hsa-mir-19a	QRSL1	0.35310281	mirna_pc
11754	hsa-mir-19a	FANCF	0.420710083	mirna_pc
11755	hsa-mir-19a	HYOU1	0.355333412	mirna_pc
11756	hsa-mir-19a	C2orf3	0.308605473	mirna_pc
11757	hsa-mir-19a	FAM119A	0.327197457	mirna_pc
11758	hsa-mir-19a	GOLT1A	0.348881874	mirna_pc
11759	hsa-mir-19a	SCML2	0.349088186	mirna_pc
11760	hsa-mir-19a	MRPL19	0.352473847	mirna_pc
11761	hsa-mir-19a	ABCB10	0.307245247	mirna_pc
11762	hsa-mir-19a	ZCCHC3	0.400113448	mirna_pc
11763	hsa-mir-19a	RRP15	0.346924546	mirna_pc
11764	hsa-mir-19a	ADAT2	0.393379041	mirna_pc
11765	hsa-mir-19a	ZNF143	0.309194407	mirna_pc
11766	hsa-mir-19a	CBLL1	0.326013431	mirna_pc
11767	hsa-mir-19a	GTF3A	0.310493229	mirna_pc
11768	hsa-mir-19a	TPP2	0.390904793	mirna_pc
11769	hsa-mir-19a	SPRED2	0.305724807	mirna_pc
11770	hsa-mir-19a	TXLNG	0.372806383	mirna_pc
11771	hsa-mir-19a	LRP5L	0.314629503	mirna_pc

11772	hsa-mir-19a	MIR17HG	0.542833749	mirna_pc
11773	hsa-mir-19a	ZNF239	0.410360668	mirna_pc
11774	hsa-mir-19a	KLHL23	0.306732984	mirna_pc
11775	hsa-mir-19a	ZNF202	0.305693049	mirna_pc
11776	hsa-mir-19a	SFRS6	0.323603282	mirna_pc
11777	hsa-mir-19a	DNMT3A	0.330886145	mirna_pc
11778	hsa-mir-19a	PRTFDC1	0.307028867	mirna_pc
11779	hsa-mir-19a	ZNF670	0.334155976	mirna_pc
11780	hsa-mir-19a	ZNF711	0.330149504	mirna_pc
11781	hsa-mir-19a	POM121	0.339147948	mirna_pc
11782	hsa-mir-590	TPX2	0.352593822	mirna_pc
11783	hsa-mir-590	RCC2	0.320469178	mirna_pc
11784	hsa-mir-590	HOXC9	0.349513627	mirna_pc
11785	hsa-mir-590	RRM2	0.387036772	mirna_pc
11786	hsa-mir-590	HOXC8	0.339871156	mirna_pc
11787	hsa-mir-590	CDK1	0.303505893	mirna_pc
11788	hsa-mir-590	MYBL2	0.353606517	mirna_pc
11789	hsa-mir-590	UBE2C	0.392142448	mirna_pc
11790	hsa-mir-590	PRC1	0.312813714	mirna_pc
11791	hsa-mir-590	NUSAP1	0.369719995	mirna_pc
11792	hsa-mir-590	KIFC1	0.328537859	mirna_pc
11793	hsa-mir-590	BUB1B	0.445642344	mirna_pc
11794	hsa-mir-590	HDGF	0.305967665	mirna_pc
11795	hsa-mir-590	NCAPG	0.315473312	mirna_pc
11796	hsa-mir-590	CDC20	0.319739278	mirna_pc
11797	hsa-mir-590	CLSPN	0.317295704	mirna_pc
11798	hsa-mir-590	CDC25B	0.327549709	mirna_pc
11799	hsa-mir-590	FANCI	0.425983584	mirna_pc
11800	hsa-mir-590	KIF23	0.397447667	mirna_pc
11801	hsa-mir-590	RAD54L	0.340364152	mirna_pc
11802	hsa-mir-590	CENPA	0.329024602	mirna_pc
11803	hsa-mir-590	TIMELESS	0.330606646	mirna_pc
11804	hsa-mir-590	CDCA8	0.344135225	mirna_pc
11805	hsa-mir-590	TROAP	0.395103975	mirna_pc
11806	hsa-mir-590	XPO1	0.310934298	mirna_pc
11807	hsa-mir-590	CDC45	0.394523334	mirna_pc
11808	hsa-mir-590	STMN1	0.324247065	mirna_pc
11809	hsa-mir-590	CENPM	0.330929856	mirna_pc
11810	hsa-mir-590	UBE2T	0.307864597	mirna_pc
11811	hsa-mir-590	CKS1B	0.310806754	mirna_pc
11812	hsa-mir-590	KIAA0101	0.317557773	mirna_pc
11813	hsa-mir-590	KNTC1	0.360030692	mirna_pc
11814	hsa-mir-590	CCNB2	0.347835938	mirna_pc
11815	hsa-mir-590	NUF2	0.3215806	mirna_pc
11816	hsa-mir-590	DEPDC1	0.343217825	mirna_pc
11817	hsa-mir-590	RAD51	0.354037019	mirna_pc
11818	hsa-mir-590	BLM	0.431812879	mirna_pc
11819	hsa-mir-590	OIP5	0.406560013	mirna_pc
11820	hsa-mir-590	RECQL4	0.387865943	mirna_pc
11821	hsa-mir-590	AURKB	0.328769084	mirna_pc
11822	hsa-mir-590	MCM10	0.31827614	mirna_pc
11823	hsa-mir-590	CHEK2	0.480421847	mirna_pc
11824	hsa-mir-590	CDCA3	0.383480993	mirna_pc
11825	hsa-mir-590	C1orf112	0.335672993	mirna_pc

11826	hsa-mir-590	ADRM1	0.342515532	mirna_pc
11827	hsa-mir-590	C12orf48	0.400310975	mirna_pc
11828	hsa-mir-590	HNRNPL	0.360271361	mirna_pc
11829	hsa-mir-590	ZWILCH	0.417575032	mirna_pc
11830	hsa-mir-590	TTK	0.328210359	mirna_pc
11831	hsa-mir-590	ZWINT	0.326050583	mirna_pc
11832	hsa-mir-590	TUBA1B	0.302362299	mirna_pc
11833	hsa-mir-590	UBE2S	0.332265853	mirna_pc
11834	hsa-mir-590	PSMA4	0.357993224	mirna_pc
11835	hsa-mir-590	C11orf82	0.304811049	mirna_pc
11836	hsa-mir-590	HNRNPC	0.33848491	mirna_pc
11837	hsa-mir-590	C6orf167	0.330147012	mirna_pc
11838	hsa-mir-590	RAN	0.369578044	mirna_pc
11839	hsa-mir-590	C20orf20	0.373374273	mirna_pc
11840	hsa-mir-590	PIF1	0.311815568	mirna_pc
11841	hsa-mir-590	DCAF13	0.319784605	mirna_pc
11842	hsa-mir-590	MTHFD1L	0.320231851	mirna_pc
11843	hsa-mir-590	UCK2	0.380623142	mirna_pc
11844	hsa-mir-590	NFKBIL2	0.35408757	mirna_pc
11845	hsa-mir-590	C15orf42	0.363691939	mirna_pc
11846	hsa-mir-590	FAM60A	0.395435094	mirna_pc
11847	hsa-mir-590	RANBP1	0.372574099	mirna_pc
11848	hsa-mir-590	MTBP	0.300507401	mirna_pc
11849	hsa-mir-590	E2F1	0.369860889	mirna_pc
11850	hsa-mir-590	SNRPA	0.362517416	mirna_pc
11851	hsa-mir-590	ATAD2	0.328056952	mirna_pc
11852	hsa-mir-590	U2AF2	0.307872153	mirna_pc
11853	hsa-mir-590	CENPO	0.324618843	mirna_pc
11854	hsa-mir-590	C19orf48	0.30180686	mirna_pc
11855	hsa-mir-590	NOP56	0.405561684	mirna_pc
11856	hsa-mir-590	CSE1L	0.40414934	mirna_pc
11857	hsa-mir-590	DTYMK	0.30441509	mirna_pc
11858	hsa-mir-590	NOP2	0.357514207	mirna_pc
11859	hsa-mir-590	CDK2	0.327947222	mirna_pc
11860	hsa-mir-590	CDK4	0.364683254	mirna_pc
11861	hsa-mir-590	PFDN2	0.356017721	mirna_pc
11862	hsa-mir-590	BCL2L12	0.315583842	mirna_pc
11863	hsa-mir-590	CHTF18	0.307631477	mirna_pc
11864	hsa-mir-590	CENPH	0.308304466	mirna_pc
11865	hsa-mir-590	MSH2	0.349637527	mirna_pc
11866	hsa-mir-590	CDC7	0.33268278	mirna_pc
11867	hsa-mir-590	SSB	0.306964738	mirna_pc
11868	hsa-mir-590	YDJC	0.357955322	mirna_pc
11869	hsa-mir-590	PUS1	0.437432065	mirna_pc
11870	hsa-mir-590	SNRPC	0.34818087	mirna_pc
11871	hsa-mir-590	FBXO5	0.305582325	mirna_pc
11872	hsa-mir-590	BRIX1	0.344895162	mirna_pc
11873	hsa-mir-590	E2F3	0.327754513	mirna_pc
11874	hsa-mir-590	SF3B14	0.315450701	mirna_pc
11875	hsa-mir-590	RUVBL2	0.343144403	mirna_pc
11876	hsa-mir-590	CDC25A	0.351815803	mirna_pc
11877	hsa-mir-590	RAD54B	0.333029041	mirna_pc
11878	hsa-mir-590	MAGOH	0.310326904	mirna_pc
11879	hsa-mir-590	DDX12	0.318964045	mirna_pc

11880	hsa-mir-590	DSCC1	0.393882035	mirna_pc
11881	hsa-mir-590	NUP37	0.374688369	mirna_pc
11882	hsa-mir-590	CLN6	0.364960046	mirna_pc
11883	hsa-mir-590	MCM8	0.342721501	mirna_pc
11884	hsa-mir-590	CPSF3	0.38350359	mirna_pc
11885	hsa-mir-590	BANF1	0.331946201	mirna_pc
11886	hsa-mir-590	C21orf45	0.332805962	mirna_pc
11887	hsa-mir-590	PPM1G	0.360274026	mirna_pc
11888	hsa-mir-590	NPM3	0.347603162	mirna_pc
11889	hsa-mir-590	NUDT5	0.344645248	mirna_pc
11890	hsa-mir-590	ZNF367	0.314804646	mirna_pc
11891	hsa-mir-590	PUS7	0.320361807	mirna_pc
11892	hsa-mir-590	DAZAP1	0.340762259	mirna_pc
11893	hsa-mir-590	CBX8	0.321370841	mirna_pc
11894	hsa-mir-590	TRMT6	0.312517236	mirna_pc
11895	hsa-mir-590	PFDN4	0.355125465	mirna_pc
11896	hsa-mir-590	PAFAH1B3	0.319018409	mirna_pc
11897	hsa-mir-590	SRRT	0.320377924	mirna_pc
11898	hsa-mir-590	SASS6	0.327980835	mirna_pc
11899	hsa-mir-590	FBL	0.339213197	mirna_pc
11900	hsa-mir-590	PNPT1	0.313474401	mirna_pc
11901	hsa-mir-590	TMPO	0.317840944	mirna_pc
11902	hsa-mir-590	MRTO4	0.355199066	mirna_pc
11903	hsa-mir-590	ADPGK	0.32034876	mirna_pc
11904	hsa-mir-590	GABPB1	0.310609933	mirna_pc
11905	hsa-mir-590	CCDC138	0.302940514	mirna_pc
11906	hsa-mir-590	LOC100128191	0.335563557	mirna_pc
11907	hsa-mir-590	EIF5A	0.312993345	mirna_pc
11908	hsa-mir-590	SF3A2	0.311351246	mirna_pc
11909	hsa-mir-590	SNRPF	0.371476015	mirna_pc
11910	hsa-mir-590	WDR43	0.361811151	mirna_pc
11911	hsa-mir-590	SNRPA1	0.464823976	mirna_pc
11912	hsa-mir-590	TIPIN	0.409194455	mirna_pc
11913	hsa-mir-590	SNX8	0.306215345	mirna_pc
11914	hsa-mir-590	GPN1	0.303101544	mirna_pc
11915	hsa-mir-590	PA2G4	0.39845404	mirna_pc
11916	hsa-mir-590	SNRPD3	0.459287052	mirna_pc
11917	hsa-mir-590	KHDRBS1	0.344068076	mirna_pc
11918	hsa-mir-590	SLC5A6	0.373558713	mirna_pc
11919	hsa-mir-590	LOC92659	0.324230186	mirna_pc
11920	hsa-mir-590	FKBP1A	0.331847022	mirna_pc
11921	hsa-mir-590	PHF5A	0.312909742	mirna_pc
11922	hsa-mir-590	CCDC18	0.340090763	mirna_pc
11923	hsa-mir-590	AHCY	0.304660336	mirna_pc
11924	hsa-mir-590	DNAJC2	0.31757796	mirna_pc
11925	hsa-mir-590	SNRNP40	0.304566185	mirna_pc
11926	hsa-mir-590	INTS8	0.326686205	mirna_pc
11927	hsa-mir-590	METTL1	0.375100514	mirna_pc
11928	hsa-mir-590	HAUS6	0.305315747	mirna_pc
11929	hsa-mir-590	NSUN5	0.339841799	mirna_pc
11930	hsa-mir-590	GNL2	0.371686262	mirna_pc
11931	hsa-mir-590	NASP	0.303273501	mirna_pc
11932	hsa-mir-590	OBFC2B	0.379554697	mirna_pc
11933	hsa-mir-590	PTMA	0.30138087	mirna_pc

11934	hsa-mir-590	DENR	0.408889997	mirna_pc
11935	hsa-mir-590	SCARB1	0.307344625	mirna_pc
11936	hsa-mir-590	TBCE	0.303944557	mirna_pc
11937	hsa-mir-590	SUV39H2	0.328659083	mirna_pc
11938	hsa-mir-590	DPY30	0.330874192	mirna_pc
11939	hsa-mir-590	MTHFD2	0.374659043	mirna_pc
11940	hsa-mir-590	CCT4	0.309033314	mirna_pc
11941	hsa-mir-590	TBC1D7	0.337860978	mirna_pc
11942	hsa-mir-590	PAK1IP1	0.461553099	mirna_pc
11943	hsa-mir-590	PES 1.00	0.341349414	mirna_pc
11944	hsa-mir-590	PGAM5	0.317905748	mirna_pc
11945	hsa-mir-590	YWHAE	0.313018664	mirna_pc
11946	hsa-mir-590	NAA25	0.381422967	mirna_pc
11947	hsa-mir-590	EHMT2	0.325925024	mirna_pc
11948	hsa-mir-590	DEK	0.327448011	mirna_pc
11949	hsa-mir-590	SFRS9	0.351266821	mirna_pc
11950	hsa-mir-590	PPIB	0.329298221	mirna_pc
11951	hsa-mir-590	PRAME	0.329699753	mirna_pc
11952	hsa-mir-590	SART3	0.309675644	mirna_pc
11953	hsa-mir-590	C15orf23	0.426105154	mirna_pc
11954	hsa-mir-590	WBSCR22	0.408871009	mirna_pc
11955	hsa-mir-590	NOC4L	0.378338006	mirna_pc
11956	hsa-mir-590	DIABLO	0.365114329	mirna_pc
11957	hsa-mir-590	TDG	0.38944465	mirna_pc
11958	hsa-mir-590	EIF2S2	0.324114185	mirna_pc
11959	hsa-mir-590	SPG21	0.347793888	mirna_pc
11960	hsa-mir-590	DDX55	0.371603248	mirna_pc
11961	hsa-mir-590	C17orf96	0.389819242	mirna_pc
11962	hsa-mir-590	CNOT3	0.314965767	mirna_pc
11963	hsa-mir-590	VPS72	0.322139079	mirna_pc
11964	hsa-mir-590	RHBDD3	0.353366663	mirna_pc
11965	hsa-mir-590	TMEM120B	0.304260946	mirna_pc
11966	hsa-mir-590	SHMT2	0.324440978	mirna_pc
11967	hsa-mir-590	HDAC2	0.442877348	mirna_pc
11968	hsa-mir-590	EMG1	0.332226766	mirna_pc
11969	hsa-mir-590	CSNK1E	0.330856755	mirna_pc
11970	hsa-mir-590	PA2G4P4	0.422139709	mirna_pc
11971	hsa-mir-590	TCEB1	0.321484444	mirna_pc
11972	hsa-mir-590	NUDT3	0.363808847	mirna_pc
11973	hsa-mir-590	BAT1	0.309592505	mirna_pc
11974	hsa-mir-590	RFC2	0.37838711	mirna_pc
11975	hsa-mir-590	TAF5	0.401087455	mirna_pc
11976	hsa-mir-590	PDIA3	0.342212527	mirna_pc
11977	hsa-mir-590	EXOSC4	0.330148488	mirna_pc
11978	hsa-mir-590	FARSB	0.34297127	mirna_pc
11979	hsa-mir-590	RCN2	0.300574521	mirna_pc
11980	hsa-mir-590	MTF2	0.328298437	mirna_pc
11981	hsa-mir-590	TARBP2	0.300658883	mirna_pc
11982	hsa-mir-590	TOMM22	0.328104357	mirna_pc
11983	hsa-mir-590	NOL10	0.345694103	mirna_pc
11984	hsa-mir-590	SEMA3D	0.300115059	mirna_pc
11985	hsa-mir-590	SF3A3	0.328859421	mirna_pc
11986	hsa-mir-590	DDX47	0.321470689	mirna_pc
11987	hsa-mir-590	POLE	0.404969861	mirna_pc

11988	hsa-mir-590	C8orf30A	0.354941349	mirna_pc
11989	hsa-mir-590	CHML	0.306852638	mirna_pc
11990	hsa-mir-590	WRNIP1	0.334890295	mirna_pc
11991	hsa-mir-590	TAF1B	0.361845913	mirna_pc
11992	hsa-mir-590	TRIAP1	0.345120877	mirna_pc
11993	hsa-mir-590	TIMM50	0.31009948	mirna_pc
11994	hsa-mir-590	ADSL	0.306538133	mirna_pc
11995	hsa-mir-590	VRK1	0.303910758	mirna_pc
11996	hsa-mir-590	CDK16	0.339322567	mirna_pc
11997	hsa-mir-590	ALKBH2	0.304876035	mirna_pc
11998	hsa-mir-590	CCDC90A	0.332076956	mirna_pc
11999	hsa-mir-590	PDIA3P	0.304970803	mirna_pc
12000	hsa-mir-590	RPF2	0.36634603	mirna_pc
12001	hsa-mir-590	GPN3	0.367779845	mirna_pc
12002	hsa-mir-590	KDM2B	0.309199585	mirna_pc
12003	hsa-mir-590	GTF3C2	0.31297031	mirna_pc
12004	hsa-mir-590	C19orf73	0.347386709	mirna_pc
12005	hsa-mir-590	C15orf63	0.320335024	mirna_pc
12006	hsa-mir-590	LTV1	0.36317519	mirna_pc
12007	hsa-mir-590	PCGF6	0.418988828	mirna_pc
12008	hsa-mir-590	FAM136A	0.364956919	mirna_pc
12009	hsa-mir-590	YRDC	0.38264342	mirna_pc
12010	hsa-mir-590	SMARCB1	0.347361299	mirna_pc
12011	hsa-mir-590	CCT8	0.413080332	mirna_pc
12012	hsa-mir-590	UFD1L	0.375530679	mirna_pc
12013	hsa-mir-590	SLC25A17	0.346832197	mirna_pc
12014	hsa-mir-590	MKRN3	0.306558458	mirna_pc
12015	hsa-mir-590	STRA13	0.416076849	mirna_pc
12016	hsa-mir-590	BCL7A	0.314846432	mirna_pc
12017	hsa-mir-590	TIMM16	0.315439367	mirna_pc
12018	hsa-mir-590	SFXN4	0.321114366	mirna_pc
12019	hsa-mir-590	PPP1CC	0.324521567	mirna_pc
12020	hsa-mir-590	C1orf109	0.33880838	mirna_pc
12021	hsa-mir-590	UBE2N	0.385649453	mirna_pc
12022	hsa-mir-590	FBXO22	0.304108812	mirna_pc
12023	hsa-mir-590	SEC61A2	0.320814834	mirna_pc
12024	hsa-mir-590	NHP2L1	0.311732269	mirna_pc
12025	hsa-mir-590	MAPKAPK5	0.403112876	mirna_pc
12026	hsa-mir-590	DRG1	0.452169757	mirna_pc
12027	hsa-mir-590	NOL7	0.343349075	mirna_pc
12028	hsa-mir-590	B4GALT3	0.354825159	mirna_pc
12029	hsa-mir-590	CCDC59	0.338914021	mirna_pc
12030	hsa-mir-590	TCTE3	0.308888263	mirna_pc
12031	hsa-mir-590	EWSR1	0.456173133	mirna_pc
12032	hsa-mir-590	KRR1	0.328236844	mirna_pc
12033	hsa-mir-590	AAAS	0.348256242	mirna_pc
12034	hsa-mir-590	SFRS8	0.318864794	mirna_pc
12035	hsa-mir-590	MTFMT	0.306313405	mirna_pc
12036	hsa-mir-590	SUPT7L	0.308193707	mirna_pc
12037	hsa-mir-590	PELP1	0.322104947	mirna_pc
12038	hsa-mir-590	ZNF692	0.33751544	mirna_pc
12039	hsa-mir-590	SFRS7	0.336413126	mirna_pc
12040	hsa-mir-590	MRPL42	0.371813859	mirna_pc
12041	hsa-mir-590	TRMT2A	0.349328304	mirna_pc

12042	hsa-mir-590	PCBP2	0.300585928	mirna_pc
12043	hsa-mir-590	COQ3	0.302800319	mirna_pc
12044	hsa-mir-590	RPUSD2	0.335331139	mirna_pc
12045	hsa-mir-590	NIPSNAP1	0.367304667	mirna_pc
12046	hsa-mir-590	SERBP1	0.301714658	mirna_pc
12047	hsa-mir-590	RDBP	0.312208016	mirna_pc
12048	hsa-mir-590	SPRYD4	0.377518457	mirna_pc
12049	hsa-mir-590	EEF1E1	0.306243524	mirna_pc
12050	hsa-mir-590	ZCCHC8	0.334455042	mirna_pc
12051	hsa-mir-590	C12orf45	0.302535908	mirna_pc
12052	hsa-mir-590	ZNF259	0.313642144	mirna_pc
12053	hsa-mir-590	LOC338758	0.346905819	mirna_pc
12054	hsa-mir-590	POLR2F	0.340158568	mirna_pc
12055	hsa-mir-590	MDH2	0.303881554	mirna_pc
12056	hsa-mir-590	LYRM4	0.323873246	mirna_pc
12057	hsa-mir-590	UTP11L	0.318832229	mirna_pc
12058	hsa-mir-590	GRB14	0.30263174	mirna_pc
12059	hsa-mir-590	ANP32A	0.372638289	mirna_pc
12060	hsa-mir-590	SUPV3L1	0.322502692	mirna_pc
12061	hsa-mir-590	SDF2L1	0.395036601	mirna_pc
12062	hsa-mir-590	LCMT2	0.336906835	mirna_pc
12063	hsa-mir-590	RSL24D1	0.325518572	mirna_pc
12064	hsa-mir-590	BUD13	0.350944884	mirna_pc
12065	hsa-mir-590	BCCIP	0.309202414	mirna_pc
12066	hsa-mir-590	TRUB1	0.324016928	mirna_pc
12067	hsa-mir-590	C12orf24	0.306747871	mirna_pc
12068	hsa-mir-590	SF3A1	0.302501083	mirna_pc
12069	hsa-mir-590	SLC25A33	0.330530092	mirna_pc
12070	hsa-mir-590	GPR37	0.311272288	mirna_pc
12071	hsa-mir-590	TIAL1	0.33609061	mirna_pc
12072	hsa-mir-590	DDX51	0.362265796	mirna_pc
12073	hsa-mir-590	MED17	0.374280185	mirna_pc
12074	hsa-mir-590	MTG1	0.346119284	mirna_pc
12075	hsa-mir-590	LOC221710	0.369673905	mirna_pc
12076	hsa-mir-590	UBE2L3	0.313518431	mirna_pc
12077	hsa-mir-590	SCML2	0.355370775	mirna_pc
12078	hsa-mir-590	DACT2	0.322481478	mirna_pc
12079	hsa-mir-590	DDX1	0.300811329	mirna_pc
12080	hsa-mir-590	C2orf43	0.309916752	mirna_pc
12081	hsa-mir-590	EIF3J	0.310489272	mirna_pc
12082	hsa-mir-590	CCDC117	0.317944798	mirna_pc
12083	hsa-mir-590	DNAJC30	0.302919493	mirna_pc
12084	hsa-mir-590	PREB	0.449351248	mirna_pc
12085	hsa-mir-590	TMED2	0.308300434	mirna_pc
12086	hsa-mir-590	TMEM14B	0.330372352	mirna_pc
12087	hsa-mir-590	C22orf29	0.311177094	mirna_pc
12088	hsa-mir-590	PPCDC	0.314300965	mirna_pc
12089	hsa-mir-590	MARS	0.309527708	mirna_pc
12090	hsa-mir-590	C12orf73	0.324738405	mirna_pc
12091	hsa-mir-590	SS18L1	0.33682863	mirna_pc
12092	hsa-mir-671	TPX2	0.369144971	mirna_pc
12093	hsa-mir-671	KIF4B	0.322336578	mirna_pc
12094	hsa-mir-671	KPNA2	0.373433718	mirna_pc
12095	hsa-mir-671	CENPF	0.330905461	mirna_pc

12096	hsa-mir-671	RCC2	0.359870122	mirna_pc
12097	hsa-mir-671	KIF11	0.372559777	mirna_pc
12098	hsa-mir-671	ECT2	0.429910431	mirna_pc
12099	hsa-mir-671	FOXM1	0.461325434	mirna_pc
12100	hsa-mir-671	RRM2	0.373921318	mirna_pc
12101	hsa-mir-671	SPC24	0.31354997	mirna_pc
12102	hsa-mir-671	CDK1	0.396013878	mirna_pc
12103	hsa-mir-671	MYBL2	0.375246914	mirna_pc
12104	hsa-mir-671	PCNA	0.314931828	mirna_pc
12105	hsa-mir-671	UBE2C	0.347760078	mirna_pc
12106	hsa-mir-671	BUB1	0.371570926	mirna_pc
12107	hsa-mir-671	PRC1	0.327106561	mirna_pc
12108	hsa-mir-671	NUSAP1	0.416988356	mirna_pc
12109	hsa-mir-671	MCM4	0.374622895	mirna_pc
12110	hsa-mir-671	KIFC1	0.313078211	mirna_pc
12111	hsa-mir-671	BUB1B	0.422827769	mirna_pc
12112	hsa-mir-671	KIF18B	0.338878665	mirna_pc
12113	hsa-mir-671	ASPM	0.33368454	mirna_pc
12114	hsa-mir-671	CKAP2	0.327819194	mirna_pc
12115	hsa-mir-671	FANCA	0.361635704	mirna_pc
12116	hsa-mir-671	NCAPG	0.368275754	mirna_pc
12117	hsa-mir-671	CDC20	0.307030327	mirna_pc
12118	hsa-mir-671	CLSPN	0.337216669	mirna_pc
12119	hsa-mir-671	SGOL1	0.316043131	mirna_pc
12120	hsa-mir-671	ARHGAP11A	0.353383336	mirna_pc
12121	hsa-mir-671	CCNA2	0.379848928	mirna_pc
12122	hsa-mir-671	CEP55	0.322701448	mirna_pc
12123	hsa-mir-671	CBX3	0.305926203	mirna_pc
12124	hsa-mir-671	SPC25	0.390512501	mirna_pc
12125	hsa-mir-671	MELK	0.378292859	mirna_pc
12126	hsa-mir-671	NUP62	0.328156643	mirna_pc
12127	hsa-mir-671	FANCI	0.420763595	mirna_pc
12128	hsa-mir-671	NEK2	0.304753307	mirna_pc
12129	hsa-mir-671	DDX39	0.339255916	mirna_pc
12130	hsa-mir-671	NCAPH	0.448542548	mirna_pc
12131	hsa-mir-671	GTSE1	0.459880302	mirna_pc
12132	hsa-mir-671	KIF23	0.416086708	mirna_pc
12133	hsa-mir-671	RAD54L	0.344820056	mirna_pc
12134	hsa-mir-671	CENPA	0.426129129	mirna_pc
12135	hsa-mir-671	MAD2L1	0.374378338	mirna_pc
12136	hsa-mir-671	TIMELESS	0.450988578	mirna_pc
12137	hsa-mir-671	ASF1B	0.414390689	mirna_pc
12138	hsa-mir-671	CDCA8	0.310658182	mirna_pc
12139	hsa-mir-671	TROAP	0.482415715	mirna_pc
12140	hsa-mir-671	CDCA5	0.375488166	mirna_pc
12141	hsa-mir-671	XPO1	0.417524485	mirna_pc
12142	hsa-mir-671	CDC45	0.541657252	mirna_pc
12143	hsa-mir-671	CENPM	0.415684907	mirna_pc
12144	hsa-mir-671	EXO1	0.353308278	mirna_pc
12145	hsa-mir-671	TK1	0.427100902	mirna_pc
12146	hsa-mir-671	CDCA2	0.332375684	mirna_pc
12147	hsa-mir-671	DKC1	0.396532491	mirna_pc
12148	hsa-mir-671	UBE2T	0.326330248	mirna_pc
12149	hsa-mir-671	CKS1B	0.359378767	mirna_pc

12150	hsa-mir-671	MCM2	0.442432305	mirna_pc
12151	hsa-mir-671	KIAA0101	0.436782833	mirna_pc
12152	hsa-mir-671	KIF4A	0.343444509	mirna_pc
12153	hsa-mir-671	ORC1L	0.420949781	mirna_pc
12154	hsa-mir-671	SNRPB	0.306638222	mirna_pc
12155	hsa-mir-671	KNTC1	0.389168388	mirna_pc
12156	hsa-mir-671	UHRF1	0.348923051	mirna_pc
12157	hsa-mir-671	CCNF	0.393450234	mirna_pc
12158	hsa-mir-671	CENPI	0.375146758	mirna_pc
12159	hsa-mir-671	RACGAP1	0.361166262	mirna_pc
12160	hsa-mir-671	CCNB2	0.385552288	mirna_pc
12161	hsa-mir-671	NUF2	0.407124249	mirna_pc
12162	hsa-mir-671	PLK4	0.367737658	mirna_pc
12163	hsa-mir-671	FANCB	0.340117181	mirna_pc
12164	hsa-mir-671	KIF18A	0.398717122	mirna_pc
12165	hsa-mir-671	DEPDC1	0.317678483	mirna_pc
12166	hsa-mir-671	SKA3	0.38928549	mirna_pc
12167	hsa-mir-671	KIF14	0.315986896	mirna_pc
12168	hsa-mir-671	HJURP	0.405375889	mirna_pc
12169	hsa-mir-671	SKA1	0.407339925	mirna_pc
12170	hsa-mir-671	RAD51	0.420560999	mirna_pc
12171	hsa-mir-671	BLM	0.408366352	mirna_pc
12172	hsa-mir-671	SHCBP1	0.313524555	mirna_pc
12173	hsa-mir-671	CKAP2L	0.306303365	mirna_pc
12174	hsa-mir-671	DNMT1	0.449483668	mirna_pc
12175	hsa-mir-671	PAICS	0.330301747	mirna_pc
12176	hsa-mir-671	BIRC5	0.462964341	mirna_pc
12177	hsa-mir-671	LIG1	0.343077144	mirna_pc
12178	hsa-mir-671	OIP5	0.396435218	mirna_pc
12179	hsa-mir-671	DTL	0.43057617	mirna_pc
12180	hsa-mir-671	EME1	0.437815898	mirna_pc
12181	hsa-mir-671	RECQL4	0.35580389	mirna_pc
12182	hsa-mir-671	AURKA	0.329866284	mirna_pc
12183	hsa-mir-671	CENPK	0.374155499	mirna_pc
12184	hsa-mir-671	AURKB	0.425005972	mirna_pc
12185	hsa-mir-671	GSG2	0.41736038	mirna_pc
12186	hsa-mir-671	CDT1	0.388719407	mirna_pc
12187	hsa-mir-671	MCM10	0.402163749	mirna_pc
12188	hsa-mir-671	KIF20A	0.414442139	mirna_pc
12189	hsa-mir-671	SGOL2	0.34745532	mirna_pc
12190	hsa-mir-671	CHEK2	0.487101147	mirna_pc
12191	hsa-mir-671	POLQ	0.439775836	mirna_pc
12192	hsa-mir-671	CDCA3	0.457797966	mirna_pc
12193	hsa-mir-671	C1orf112	0.475560872	mirna_pc
12194	hsa-mir-671	GINS1	0.340003798	mirna_pc
12195	hsa-mir-671	ALG3	0.433664187	mirna_pc
12196	hsa-mir-671	FEN1	0.328257622	mirna_pc
12197	hsa-mir-671	TRAIP	0.301546912	mirna_pc
12198	hsa-mir-671	ORC6L	0.425501288	mirna_pc
12199	hsa-mir-671	PBK	0.304774199	mirna_pc
12200	hsa-mir-671	C16orf59	0.522795136	mirna_pc
12201	hsa-mir-671	C17orf53	0.373973754	mirna_pc
12202	hsa-mir-671	RAE1	0.302505818	mirna_pc
12203	hsa-mir-671	FAM72D	0.32227459	mirna_pc

12204	hsa-mir-671	FAM72B	0.381685635	mirna_pc
12205	hsa-mir-671	EZH2	0.424207559	mirna_pc
12206	hsa-mir-671	NEIL3	0.411043879	mirna_pc
12207	hsa-mir-671	ACTL6A	0.538388388	mirna_pc
12208	hsa-mir-671	POLD1	0.425777575	mirna_pc
12209	hsa-mir-671	C12orf48	0.447879416	mirna_pc
12210	hsa-mir-671	HNRNPL	0.435681756	mirna_pc
12211	hsa-mir-671	ZWILCH	0.440820748	mirna_pc
12212	hsa-mir-671	SMC4	0.493894348	mirna_pc
12213	hsa-mir-671	HMMR	0.326531733	mirna_pc
12214	hsa-mir-671	PKMYT1	0.392734979	mirna_pc
12215	hsa-mir-671	TTK	0.386734501	mirna_pc
12216	hsa-mir-671	SF3B4	0.323611042	mirna_pc
12217	hsa-mir-671	ZWINT	0.308269929	mirna_pc
12218	hsa-mir-671	TUBA1B	0.334821115	mirna_pc
12219	hsa-mir-671	RUVBL1	0.40101332	mirna_pc
12220	hsa-mir-671	RAD51AP1	0.377500472	mirna_pc
12221	hsa-mir-671	HELLS	0.386679975	mirna_pc
12222	hsa-mir-671	ERCC6L	0.32246424	mirna_pc
12223	hsa-mir-671	POLE2	0.332141552	mirna_pc
12224	hsa-mir-671	UBE2S	0.374712111	mirna_pc
12225	hsa-mir-671	PSMA4	0.35357611	mirna_pc
12226	hsa-mir-671	NCAPD2	0.433355346	mirna_pc
12227	hsa-mir-671	C11orf82	0.368853984	mirna_pc
12228	hsa-mir-671	HNRNPC	0.389649386	mirna_pc
12229	hsa-mir-671	C6orf167	0.380503267	mirna_pc
12230	hsa-mir-671	CHEK1	0.332535821	mirna_pc
12231	hsa-mir-671	BRCA1	0.384441289	mirna_pc
12232	hsa-mir-671	NDC80	0.306252355	mirna_pc
12233	hsa-mir-671	TFRC	0.379550486	mirna_pc
12234	hsa-mir-671	MCM5	0.473069136	mirna_pc
12235	hsa-mir-671	RAN	0.394270744	mirna_pc
12236	hsa-mir-671	TYMS	0.360071989	mirna_pc
12237	hsa-mir-671	RFWD3	0.403282114	mirna_pc
12238	hsa-mir-671	C20orf20	0.332554473	mirna_pc
12239	hsa-mir-671	MCM6	0.373781198	mirna_pc
12240	hsa-mir-671	RNASEH2A	0.302301605	mirna_pc
12241	hsa-mir-671	EPR1	0.388155673	mirna_pc
12242	hsa-mir-671	KIAA1524	0.364347319	mirna_pc
12243	hsa-mir-671	BRIP1	0.373041093	mirna_pc
12244	hsa-mir-671	H2AFX	0.301697877	mirna_pc
12245	hsa-mir-671	PSMD14	0.373337069	mirna_pc
12246	hsa-mir-671	C15orf42	0.383131796	mirna_pc
12247	hsa-mir-671	CENPN	0.417849216	mirna_pc
12248	hsa-mir-671	PTGES3	0.386221602	mirna_pc
12249	hsa-mir-671	PRIM2	0.319951084	mirna_pc
12250	hsa-mir-671	FAM60A	0.338849466	mirna_pc
12251	hsa-mir-671	HMGB3	0.361669644	mirna_pc
12252	hsa-mir-671	ATAD5	0.312375601	mirna_pc
12253	hsa-mir-671	RANBP1	0.32413689	mirna_pc
12254	hsa-mir-671	MTBP	0.342829798	mirna_pc
12255	hsa-mir-671	E2F1	0.326032614	mirna_pc
12256	hsa-mir-671	EIF4A3	0.355113821	mirna_pc
12257	hsa-mir-671	KIF20B	0.304467227	mirna_pc

12258	hsa-mir-671	SNRPA	0.323470897	mirna_pc
12259	hsa-mir-671	FUS	0.371138452	mirna_pc
12260	hsa-mir-671	ATAD2	0.337222236	mirna_pc
12261	hsa-mir-671	DNAJC9	0.329737514	mirna_pc
12262	hsa-mir-671	RFC3	0.388482373	mirna_pc
12263	hsa-mir-671	CHAF1A	0.450414177	mirna_pc
12264	hsa-mir-671	GINS4	0.322959536	mirna_pc
12265	hsa-mir-671	ANP32E	0.318890721	mirna_pc
12266	hsa-mir-671	CENPO	0.41867485	mirna_pc
12267	hsa-mir-671	XRCC2	0.517285474	mirna_pc
12268	hsa-mir-671	BID	0.303553658	mirna_pc
12269	hsa-mir-671	C19orf48	0.369334603	mirna_pc
12270	hsa-mir-671	TRIM59	0.472320144	mirna_pc
12271	hsa-mir-671	CENPL	0.415062296	mirna_pc
12272	hsa-mir-671	GEN1	0.357169093	mirna_pc
12273	hsa-mir-671	NUDT1	0.314592756	mirna_pc
12274	hsa-mir-671	HMGB2	0.390261638	mirna_pc
12275	hsa-mir-671	GINS2	0.340714905	mirna_pc
12276	hsa-mir-671	DNA2	0.355762687	mirna_pc
12277	hsa-mir-671	NOP56	0.353362384	mirna_pc
12278	hsa-mir-671	CSE1L	0.384040235	mirna_pc
12279	hsa-mir-671	DTYMK	0.301156162	mirna_pc
12280	hsa-mir-671	E2F7	0.348662834	mirna_pc
12281	hsa-mir-671	PRMT1	0.397717414	mirna_pc
12282	hsa-mir-671	MLF1IP	0.358644886	mirna_pc
12283	hsa-mir-671	RAVER1	0.391743338	mirna_pc
12284	hsa-mir-671	NOP2	0.434676769	mirna_pc
12285	hsa-mir-671	TCF19	0.334882635	mirna_pc
12286	hsa-mir-671	CAD	0.324867996	mirna_pc
12287	hsa-mir-671	ZFP64	0.420642595	mirna_pc
12288	hsa-mir-671	CDK2	0.463801994	mirna_pc
12289	hsa-mir-671	CDK4	0.426730858	mirna_pc
12290	hsa-mir-671	CHTF18	0.446504995	mirna_pc
12291	hsa-mir-671	HNRNPR	0.33211207	mirna_pc
12292	hsa-mir-671	DLEU2	0.437394078	mirna_pc
12293	hsa-mir-671	PRIM1	0.350357988	mirna_pc
12294	hsa-mir-671	CENPH	0.404153882	mirna_pc
12295	hsa-mir-671	TCOF1	0.365360958	mirna_pc
12296	hsa-mir-671	CHAF1B	0.439356399	mirna_pc
12297	hsa-mir-671	NCAPG2	0.399726119	mirna_pc
12298	hsa-mir-671	MSH2	0.372834547	mirna_pc
12299	hsa-mir-671	PRPF19	0.321085472	mirna_pc
12300	hsa-mir-671	TOMM40	0.312327354	mirna_pc
12301	hsa-mir-671	CDC7	0.334887739	mirna_pc
12302	hsa-mir-671	INTS7	0.328932102	mirna_pc
12303	hsa-mir-671	SSB	0.318862896	mirna_pc
12304	hsa-mir-671	YDJC	0.32265074	mirna_pc
12305	hsa-mir-671	PUS1	0.440984165	mirna_pc
12306	hsa-mir-671	NOL11	0.372400762	mirna_pc
12307	hsa-mir-671	HAUS8	0.421982624	mirna_pc
12308	hsa-mir-671	TMEM41A	0.301142691	mirna_pc
12309	hsa-mir-671	PSMB4	0.357467074	mirna_pc
12310	hsa-mir-671	RFC4	0.511821526	mirna_pc
12311	hsa-mir-671	FBXO5	0.388086646	mirna_pc

12312	hsa-mir-671	FIGNL1	0.346961298	mirna_pc
12313	hsa-mir-671	E2F3	0.327532618	mirna_pc
12314	hsa-mir-671	CDC25A	0.364203565	mirna_pc
12315	hsa-mir-671	TOPBP1	0.399461578	mirna_pc
12316	hsa-mir-671	RAD54B	0.362707085	mirna_pc
12317	hsa-mir-671	DDX12	0.410423129	mirna_pc
12318	hsa-mir-671	DSCC1	0.328181279	mirna_pc
12319	hsa-mir-671	NUP37	0.424827782	mirna_pc
12320	hsa-mir-671	C13orf34	0.349621955	mirna_pc
12321	hsa-mir-671	MCM8	0.3275407	mirna_pc
12322	hsa-mir-671	CPSF3	0.370103629	mirna_pc
12323	hsa-mir-671	PAQR4	0.315381396	mirna_pc
12324	hsa-mir-671	C21orf45	0.415777741	mirna_pc
12325	hsa-mir-671	PPM1G	0.316681874	mirna_pc
12326	hsa-mir-671	DNAJB11	0.438240787	mirna_pc
12327	hsa-mir-671	LOC100125556	0.316207595	mirna_pc
12328	hsa-mir-671	TFAP4	0.43862183	mirna_pc
12329	hsa-mir-671	ZNF367	0.350691585	mirna_pc
12330	hsa-mir-671	PUS7	0.343838669	mirna_pc
12331	hsa-mir-671	RNPS1	0.340695755	mirna_pc
12332	hsa-mir-671	DAZAP1	0.304632511	mirna_pc
12333	hsa-mir-671	CBX8	0.343942912	mirna_pc
12334	hsa-mir-671	MASTL	0.303765887	mirna_pc
12335	hsa-mir-671	TBL1XR1	0.375085653	mirna_pc
12336	hsa-mir-671	TMEM201	0.353782379	mirna_pc
12337	hsa-mir-671	NCL	0.32140284	mirna_pc
12338	hsa-mir-671	MRPL9	0.313147377	mirna_pc
12339	hsa-mir-671	SRRT	0.32043367	mirna_pc
12340	hsa-mir-671	CACYBP	0.348608607	mirna_pc
12341	hsa-mir-671	SASS6	0.324306886	mirna_pc
12342	hsa-mir-671	ODF2	0.323230077	mirna_pc
12343	hsa-mir-671	C12orf32	0.377279689	mirna_pc
12344	hsa-mir-671	MRPL47	0.443201149	mirna_pc
12345	hsa-mir-671	DBF4B	0.319652902	mirna_pc
12346	hsa-mir-671	FBL	0.351568776	mirna_pc
12347	hsa-mir-671	DONSON	0.354827378	mirna_pc
12348	hsa-mir-671	STIP1	0.332900982	mirna_pc
12349	hsa-mir-671	HIST1H1E	0.347206039	mirna_pc
12350	hsa-mir-671	C13orf37	0.320849751	mirna_pc
12351	hsa-mir-671	HNRNPM	0.365977345	mirna_pc
12352	hsa-mir-671	LSM 12.00	0.341157151	mirna_pc
12353	hsa-mir-671	PNPT1	0.365762394	mirna_pc
12354	hsa-mir-671	TMPO	0.333267317	mirna_pc
12355	hsa-mir-671	SET	0.400313126	mirna_pc
12356	hsa-mir-671	POLR2H	0.406481113	mirna_pc
12357	hsa-mir-671	VBP1	0.361291639	mirna_pc
12358	hsa-mir-671	C3orf26	0.333455931	mirna_pc
12359	hsa-mir-671	GPR19	0.315395484	mirna_pc
12360	hsa-mir-671	LSG1	0.476094034	mirna_pc
12361	hsa-mir-671	POLE3	0.308509061	mirna_pc
12362	hsa-mir-671	GABPB1	0.313074151	mirna_pc
12363	hsa-mir-671	THOC6	0.343124544	mirna_pc
12364	hsa-mir-671	GMPS	0.472571616	mirna_pc
12365	hsa-mir-671	C3orf37	0.386874901	mirna_pc

12366	hsa-mir-671	DHX9	0.452398912	mirna_pc
12367	hsa-mir-671	CCDC138	0.38373522	mirna_pc
12368	hsa-mir-671	LOC100128191	0.391148029	mirna_pc
12369	hsa-mir-671	MRPL3	0.418778711	mirna_pc
12370	hsa-mir-671	HMGN2	0.324777415	mirna_pc
12371	hsa-mir-671	MORC2	0.387260653	mirna_pc
12372	hsa-mir-671	SOSTDC1	0.316481126	mirna_pc
12373	hsa-mir-671	SMYD5	0.383504238	mirna_pc
12374	hsa-mir-671	TSSC1	0.342150364	mirna_pc
12375	hsa-mir-671	POLA2	0.4108651	mirna_pc
12376	hsa-mir-671	CCDC77	0.391797319	mirna_pc
12377	hsa-mir-671	SFRS1	0.391631861	mirna_pc
12378	hsa-mir-671	TEX10	0.340359937	mirna_pc
12379	hsa-mir-671	WDR43	0.368635574	mirna_pc
12380	hsa-mir-671	SNRPA1	0.334919743	mirna_pc
12381	hsa-mir-671	CCDC58	0.360864949	mirna_pc
12382	hsa-mir-671	SKP2	0.326960986	mirna_pc
12383	hsa-mir-671	UBE2I	0.304464703	mirna_pc
12384	hsa-mir-671	TIPIN	0.369256196	mirna_pc
12385	hsa-mir-671	POLD2	0.311341985	mirna_pc
12386	hsa-mir-671	SMARCD1	0.429167419	mirna_pc
12387	hsa-mir-671	ATXN2L	0.301068432	mirna_pc
12388	hsa-mir-671	C4orf46	0.336983834	mirna_pc
12389	hsa-mir-671	ECE2	0.420520131	mirna_pc
12390	hsa-mir-671	GPN1	0.35034664	mirna_pc
12391	hsa-mir-671	RFC5	0.413347273	mirna_pc
12392	hsa-mir-671	HSPD1	0.320981541	mirna_pc
12393	hsa-mir-671	FANCG	0.346888229	mirna_pc
12394	hsa-mir-671	SSRP1	0.368864549	mirna_pc
12395	hsa-mir-671	POLR2D	0.320303659	mirna_pc
12396	hsa-mir-671	FTSJ2	0.350659269	mirna_pc
12397	hsa-mir-671	PA2G4	0.305667896	mirna_pc
12398	hsa-mir-671	EPT1	0.401791431	mirna_pc
12399	hsa-mir-671	KHDRBS1	0.407257849	mirna_pc
12400	hsa-mir-671	PDIA4	0.341882919	mirna_pc
12401	hsa-mir-671	YEATS2	0.398817969	mirna_pc
12402	hsa-mir-671	CCDC21	0.309576452	mirna_pc
12403	hsa-mir-671	PHF5A	0.387213277	mirna_pc
12404	hsa-mir-671	CCT7	0.362279514	mirna_pc
12405	hsa-mir-671	TMEM194A	0.432351399	mirna_pc
12406	hsa-mir-671	MED30	0.31480829	mirna_pc
12407	hsa-mir-671	METTL1	0.33675505	mirna_pc
12408	hsa-mir-671	TTC26	0.396474519	mirna_pc
12409	hsa-mir-671	NEDD1	0.338166851	mirna_pc
12410	hsa-mir-671	PSMA1	0.394412318	mirna_pc
12411	hsa-mir-671	CENPJ	0.376853982	mirna_pc
12412	hsa-mir-671	NSUN5	0.357436908	mirna_pc
12413	hsa-mir-671	LIN9	0.348669212	mirna_pc
12414	hsa-mir-671	NAT10	0.343324318	mirna_pc
12415	hsa-mir-671	YWHAG	0.386290215	mirna_pc
12416	hsa-mir-671	WRAP53	0.319505075	mirna_pc
12417	hsa-mir-671	HNRNPU	0.388677767	mirna_pc
12418	hsa-mir-671	CCDC86	0.359218621	mirna_pc
12419	hsa-mir-671	ZNF280C	0.38715877	mirna_pc

12420	hsa-mir-671	CBX4	0.309669444	mirna_pc
12421	hsa-mir-671	POLR1B	0.369638437	mirna_pc
12422	hsa-mir-671	USP39	0.359337407	mirna_pc
12423	hsa-mir-671	WDR76	0.316890921	mirna_pc
12424	hsa-mir-671	DENR	0.327434966	mirna_pc
12425	hsa-mir-671	EXOSC2	0.341024612	mirna_pc
12426	hsa-mir-671	HMGXB4	0.398552229	mirna_pc
12427	hsa-mir-671	C11orf84	0.347066739	mirna_pc
12428	hsa-mir-671	TBCE	0.306924023	mirna_pc
12429	hsa-mir-671	NCBP2	0.443328231	mirna_pc
12430	hsa-mir-671	SUV39H2	0.375452321	mirna_pc
12431	hsa-mir-671	NKRF	0.31222723	mirna_pc
12432	hsa-mir-671	ZC3H8	0.308170045	mirna_pc
12433	hsa-mir-671	HNRNPD	0.303297994	mirna_pc
12434	hsa-mir-671	MTHFD2	0.433674791	mirna_pc
12435	hsa-mir-671	REXO4	0.30705112	mirna_pc
12436	hsa-mir-671	MEN1	0.324012714	mirna_pc
12437	hsa-mir-671	H1FX	0.376425611	mirna_pc
12438	hsa-mir-671	PNO1	0.405241559	mirna_pc
12439	hsa-mir-671	CCT4	0.397314823	mirna_pc
12440	hsa-mir-671	UMPS	0.383883174	mirna_pc
12441	hsa-mir-671	CASP2	0.374585864	mirna_pc
12442	hsa-mir-671	ELAVL1	0.380667523	mirna_pc
12443	hsa-mir-671	RPN1	0.346853675	mirna_pc
12444	hsa-mir-671	NCAPD3	0.331496271	mirna_pc
12445	hsa-mir-671	HSP90B1	0.325255792	mirna_pc
12446	hsa-mir-671	PSMC3IP	0.393994267	mirna_pc
12447	hsa-mir-671	RPAP3	0.357048913	mirna_pc
12448	hsa-mir-671	NUDT15	0.302391463	mirna_pc
12449	hsa-mir-671	PES 1.00	0.345092381	mirna_pc
12450	hsa-mir-671	PAK2	0.428209321	mirna_pc
12451	hsa-mir-671	E2F4	0.344476655	mirna_pc
12452	hsa-mir-671	PSMD2	0.451620858	mirna_pc
12453	hsa-mir-671	PGAM5	0.322062485	mirna_pc
12454	hsa-mir-671	ATP13A3	0.425000077	mirna_pc
12455	hsa-mir-671	YWHAE	0.414424465	mirna_pc
12456	hsa-mir-671	DHFR	0.456575101	mirna_pc
12457	hsa-mir-671	NAA25	0.365088399	mirna_pc
12458	hsa-mir-671	PDCL3	0.30989191	mirna_pc
12459	hsa-mir-671	ILF3	0.376644559	mirna_pc
12460	hsa-mir-671	ADCY3	0.36242921	mirna_pc
12461	hsa-mir-671	C1orf96	0.33873467	mirna_pc
12462	hsa-mir-671	GLE1	0.34301689	mirna_pc
12463	hsa-mir-671	HSPBAP1	0.301658363	mirna_pc
12464	hsa-mir-671	WDR4	0.320075377	mirna_pc
12465	hsa-mir-671	MAGOHB	0.402762524	mirna_pc
12466	hsa-mir-671	UBAP2L	0.301076951	mirna_pc
12467	hsa-mir-671	NUP205	0.430839478	mirna_pc
12468	hsa-mir-671	GART	0.376048602	mirna_pc
12469	hsa-mir-671	DEK	0.350106931	mirna_pc
12470	hsa-mir-671	SFRS9	0.314034357	mirna_pc
12471	hsa-mir-671	PDCD10	0.324903944	mirna_pc
12472	hsa-mir-671	ATR	0.387241705	mirna_pc
12473	hsa-mir-671	OLA1	0.304766737	mirna_pc

12474	hsa-mir-671	VMA21	0.306754311	mirna_pc
12475	hsa-mir-671	KHSRP	0.369853993	mirna_pc
12476	hsa-mir-671	PRAME	0.304089364	mirna_pc
12477	hsa-mir-671	CBX2	0.315655806	mirna_pc
12478	hsa-mir-671	ISY1	0.39637948	mirna_pc
12479	hsa-mir-671	FUBP1	0.336254719	mirna_pc
12480	hsa-mir-671	TCF20	0.308259673	mirna_pc
12481	hsa-mir-671	SUV39H1	0.358715198	mirna_pc
12482	hsa-mir-671	SART3	0.368351412	mirna_pc
12483	hsa-mir-671	SR140	0.422155292	mirna_pc
12484	hsa-mir-671	TRA2B	0.464341506	mirna_pc
12485	hsa-mir-671	RSRC1	0.461850289	mirna_pc
12486	hsa-mir-671	CEP152	0.345069945	mirna_pc
12487	hsa-mir-671	C15orf23	0.420662859	mirna_pc
12488	hsa-mir-671	ZC3HAV1L	0.32536024	mirna_pc
12489	hsa-mir-671	PRMT3	0.337073621	mirna_pc
12490	hsa-mir-671	PSMD12	0.411650053	mirna_pc
12491	hsa-mir-671	MOBKL1B	0.35660232	mirna_pc
12492	hsa-mir-671	ATP2C1	0.330845048	mirna_pc
12493	hsa-mir-671	C1QBP	0.371893135	mirna_pc
12494	hsa-mir-671	ENOPH1	0.328565618	mirna_pc
12495	hsa-mir-671	MAP6D1	0.341070629	mirna_pc
12496	hsa-mir-671	LAS1L	0.309674735	mirna_pc
12497	hsa-mir-671	C4orf43	0.362870064	mirna_pc
12498	hsa-mir-671	XRCC3	0.310026664	mirna_pc
12499	hsa-mir-671	YY1	0.324951774	mirna_pc
12500	hsa-mir-671	C16orf80	0.31304881	mirna_pc
12501	hsa-mir-671	MYBBP1A	0.304041844	mirna_pc
12502	hsa-mir-671	C17orf96	0.310325482	mirna_pc
12503	hsa-mir-671	TUG1	0.304046552	mirna_pc
12504	hsa-mir-671	SFPQ	0.357284152	mirna_pc
12505	hsa-mir-671	CNOT3	0.385589928	mirna_pc
12506	hsa-mir-671	RHBDD3	0.413166471	mirna_pc
12507	hsa-mir-671	MPHOSPH9	0.338142457	mirna_pc
12508	hsa-mir-671	SHMT2	0.439906076	mirna_pc
12509	hsa-mir-671	POLA1	0.312155191	mirna_pc
12510	hsa-mir-671	GMNN	0.339134418	mirna_pc
12511	hsa-mir-671	EMG1	0.330751886	mirna_pc
12512	hsa-mir-671	M6PR	0.333043756	mirna_pc
12513	hsa-mir-671	HAT1	0.329293832	mirna_pc
12514	hsa-mir-671	WDR53	0.392667682	mirna_pc
12515	hsa-mir-671	ALOX12P2	0.411393126	mirna_pc
12516	hsa-mir-671	ANKRD32	0.360588327	mirna_pc
12517	hsa-mir-671	RNF7	0.365677309	mirna_pc
12518	hsa-mir-671	DUT	0.348946845	mirna_pc
12519	hsa-mir-671	AP2M1	0.36138993	mirna_pc
12520	hsa-mir-671	HPRT1	0.314549768	mirna_pc
12521	hsa-mir-671	CRIPT	0.304869305	mirna_pc
12522	hsa-mir-671	COX4NB	0.333187282	mirna_pc
12523	hsa-mir-671	MSH6	0.470755852	mirna_pc
12524	hsa-mir-671	HOXD11	0.312835223	mirna_pc
12525	hsa-mir-671	C3orf34	0.41644637	mirna_pc
12526	hsa-mir-671	KPNB1	0.333820632	mirna_pc
12527	hsa-mir-671	XRCC6	0.387285254	mirna_pc

12528	hsa-mir-671	SMC3	0.311653021	mirna_pc
12529	hsa-mir-671	ACBD6	0.319599446	mirna_pc
12530	hsa-mir-671	CDK2AP1	0.370110556	mirna_pc
12531	hsa-mir-671	TPI1	0.309157901	mirna_pc
12532	hsa-mir-671	MOGS	0.300549413	mirna_pc
12533	hsa-mir-671	COIL	0.314266314	mirna_pc
12534	hsa-mir-671	HOXD10	0.30924964	mirna_pc
12535	hsa-mir-671	RFC2	0.428542481	mirna_pc
12536	hsa-mir-671	TAF5	0.332756884	mirna_pc
12537	hsa-mir-671	TRIM28	0.321692714	mirna_pc
12538	hsa-mir-671	FANCC	0.300073884	mirna_pc
12539	hsa-mir-671	NAA15	0.303648569	mirna_pc
12540	hsa-mir-671	POLR3K	0.331469409	mirna_pc
12541	hsa-mir-671	PPHLN1	0.378956019	mirna_pc
12542	hsa-mir-671	CBX1	0.344699166	mirna_pc
12543	hsa-mir-671	SNAPC1	0.347116759	mirna_pc
12544	hsa-mir-671	HMGB1	0.329057133	mirna_pc
12545	hsa-mir-671	DIAPH3	0.321226777	mirna_pc
12546	hsa-mir-671	LOC642846	0.394511789	mirna_pc
12547	hsa-mir-671	TGIF2	0.318294124	mirna_pc
12548	hsa-mir-671	ZC3H18	0.372294745	mirna_pc
12549	hsa-mir-671	CHERP	0.337986086	mirna_pc
12550	hsa-mir-671	SENP1	0.331126853	mirna_pc
12551	hsa-mir-671	RCN2	0.418156085	mirna_pc
12552	hsa-mir-671	THUMPD2	0.345804521	mirna_pc
12553	hsa-mir-671	ORC3L	0.350617898	mirna_pc
12554	hsa-mir-671	TARBP2	0.339839787	mirna_pc
12555	hsa-mir-671	TOMM22	0.323262613	mirna_pc
12556	hsa-mir-671	NOL10	0.328262238	mirna_pc
12557	hsa-mir-671	DDX47	0.40612141	mirna_pc
12558	hsa-mir-671	POLE	0.376006451	mirna_pc
12559	hsa-mir-671	ODC1	0.30402849	mirna_pc
12560	hsa-mir-671	MBD4	0.402003503	mirna_pc
12561	hsa-mir-671	ERF	0.314620678	mirna_pc
12562	hsa-mir-671	PIAS2	0.305283252	mirna_pc
12563	hsa-mir-671	TIMM50	0.323547597	mirna_pc
12564	hsa-mir-671	XRCC6BP1	0.327664358	mirna_pc
12565	hsa-mir-671	ADSL	0.431003735	mirna_pc
12566	hsa-mir-671	EXOSC9	0.300800918	mirna_pc
12567	hsa-mir-671	TMEM209	0.37864796	mirna_pc
12568	hsa-mir-671	MPHOSPH6	0.34237533	mirna_pc
12569	hsa-mir-671	UNG	0.415110273	mirna_pc
12570	hsa-mir-671	RBMX	0.380150504	mirna_pc
12571	hsa-mir-671	TSEN15	0.405052083	mirna_pc
12572	hsa-mir-671	CDK16	0.31414537	mirna_pc
12573	hsa-mir-671	USP10	0.341999927	mirna_pc
12574	hsa-mir-671	TCTEX1D2	0.327136873	mirna_pc
12575	hsa-mir-671	PYCR2	0.318024764	mirna_pc
12576	hsa-mir-671	DAP3	0.359012748	mirna_pc
12577	hsa-mir-671	IQCB1	0.325614278	mirna_pc
12578	hsa-mir-671	LOC374443	0.326610315	mirna_pc
12579	hsa-mir-671	PRKCSH	0.327090389	mirna_pc
12580	hsa-mir-671	MLF 2.00	0.33481592	mirna_pc
12581	hsa-mir-671	RPL39L	0.39543857	mirna_pc

12582	hsa-mir-671	NAA50	0.307078222	mirna_pc
12583	hsa-mir-671	PDIA6	0.312979469	mirna_pc
12584	hsa-mir-671	GPN3	0.415148427	mirna_pc
12585	hsa-mir-671	APOBEC3F	0.306730043	mirna_pc
12586	hsa-mir-671	LOC152217	0.365912488	mirna_pc
12587	hsa-mir-671	MEMO1	0.308917836	mirna_pc
12588	hsa-mir-671	UBXN2A	0.410107046	mirna_pc
12589	hsa-mir-671	DNAJC14	0.301932228	mirna_pc
12590	hsa-mir-671	COMMD2	0.452840354	mirna_pc
12591	hsa-mir-671	CIT	0.333301918	mirna_pc
12592	hsa-mir-671	TRAP1	0.360396213	mirna_pc
12593	hsa-mir-671	NUDT21	0.300473586	mirna_pc
12594	hsa-mir-671	MAGEF1	0.441071586	mirna_pc
12595	hsa-mir-671	PCGF6	0.315131787	mirna_pc
12596	hsa-mir-671	PWP1	0.302434704	mirna_pc
12597	hsa-mir-671	SMARCB1	0.36217996	mirna_pc
12598	hsa-mir-671	DDX54	0.332131497	mirna_pc
12599	hsa-mir-671	CCT8	0.321828073	mirna_pc
12600	hsa-mir-671	U2AF1	0.315865351	mirna_pc
12601	hsa-mir-671	FAIM	0.315489142	mirna_pc
12602	hsa-mir-671	RPA1	0.382957067	mirna_pc
12603	hsa-mir-671	ZNF639	0.482487484	mirna_pc
12604	hsa-mir-671	TBCCD1	0.334365603	mirna_pc
12605	hsa-mir-671	FOXK2	0.302770607	mirna_pc
12606	hsa-mir-671	SLC25A17	0.308083853	mirna_pc
12607	hsa-mir-671	UBXN7	0.320361167	mirna_pc
12608	hsa-mir-671	LRPPRC	0.317834391	mirna_pc
12609	hsa-mir-671	ATN1	0.34410904	mirna_pc
12610	hsa-mir-671	LMBR1	0.320871941	mirna_pc
12611	hsa-mir-671	RHEB	0.396014599	mirna_pc
12612	hsa-mir-671	DVL3	0.391158761	mirna_pc
12613	hsa-mir-671	CDC23	0.323913572	mirna_pc
12614	hsa-mir-671	C16orf63	0.333929513	mirna_pc
12615	hsa-mir-671	ABCF2	0.453267822	mirna_pc
12616	hsa-mir-671	C7orf44	0.305356458	mirna_pc
12617	hsa-mir-671	SUMO3	0.321872155	mirna_pc
12618	hsa-mir-671	RBM28	0.408331614	mirna_pc
12619	hsa-mir-671	FXR1	0.406355323	mirna_pc
12620	hsa-mir-671	BTG3	0.310812506	mirna_pc
12621	hsa-mir-671	WDR85	0.313399485	mirna_pc
12622	hsa-mir-671	MRPS22	0.435243131	mirna_pc
12623	hsa-mir-671	PSPH	0.330946781	mirna_pc
12624	hsa-mir-671	ABCB6	0.303713806	mirna_pc
12625	hsa-mir-671	TTL	0.304974775	mirna_pc
12626	hsa-mir-671	C13orf23	0.300306128	mirna_pc
12627	hsa-mir-671	TRMT61B	0.393738428	mirna_pc
12628	hsa-mir-671	TSFM	0.360401333	mirna_pc
12629	hsa-mir-671	DDX20	0.305550531	mirna_pc
12630	hsa-mir-671	DCUN1D1	0.418184057	mirna_pc
12631	hsa-mir-671	IFT80	0.374792455	mirna_pc
12632	hsa-mir-671	PAXIP1	0.354607796	mirna_pc
12633	hsa-mir-671	UBE2N	0.323469549	mirna_pc
12634	hsa-mir-671	BAZ1B	0.384343565	mirna_pc
12635	hsa-mir-671	PIGX	0.453383218	mirna_pc

12636	hsa-mir-671	CHAC2	0.351720527	mirna_pc
12637	hsa-mir-671	PFN2	0.427744091	mirna_pc
12638	hsa-mir-671	ATP6V1E2	0.344113884	mirna_pc
12639	hsa-mir-671	LMLN	0.338854748	mirna_pc
12640	hsa-mir-671	E2F6	0.356441417	mirna_pc
12641	hsa-mir-671	DRG1	0.352283848	mirna_pc
12642	hsa-mir-671	PRPSAP2	0.408246616	mirna_pc
12643	hsa-mir-671	SENP3	0.356481975	mirna_pc
12644	hsa-mir-671	ACTR6	0.359506161	mirna_pc
12645	hsa-mir-671	STARD7	0.363045848	mirna_pc
12646	hsa-mir-671	CCDC59	0.307767381	mirna_pc
12647	hsa-mir-671	SLC4A1AP	0.327784025	mirna_pc
12648	hsa-mir-671	TTC27	0.3032965	mirna_pc
12649	hsa-mir-671	MCCC1	0.318068007	mirna_pc
12650	hsa-mir-671	MTIF2	0.354247951	mirna_pc
12651	hsa-mir-671	HNRNPA3	0.302576538	mirna_pc
12652	hsa-mir-671	EWSR1	0.391280952	mirna_pc
12653	hsa-mir-671	KRR1	0.313058868	mirna_pc
12654	hsa-mir-671	AAAS	0.374279354	mirna_pc
12655	hsa-mir-671	QSER1	0.302694734	mirna_pc
12656	hsa-mir-671	CHST14	0.316303277	mirna_pc
12657	hsa-mir-671	RNF168	0.300119267	mirna_pc
12658	hsa-mir-671	DHX36	0.385374407	mirna_pc
12659	hsa-mir-671	SUPT7L	0.316879332	mirna_pc
12660	hsa-mir-671	SMUG1	0.346030798	mirna_pc
12661	hsa-mir-671	MRPS26	0.311397062	mirna_pc
12662	hsa-mir-671	MRPL30	0.359467047	mirna_pc
12663	hsa-mir-671	GFM1	0.346807775	mirna_pc
12664	hsa-mir-671	PELP1	0.386875948	mirna_pc
12665	hsa-mir-671	PPP1R2P3	0.310057117	mirna_pc
12666	hsa-mir-671	C11orf45	0.32695665	mirna_pc
12667	hsa-mir-671	TSGA14	0.350054379	mirna_pc
12668	hsa-mir-671	HSCB	0.353434475	mirna_pc
12669	hsa-mir-671	SFRS7	0.305483473	mirna_pc
12670	hsa-mir-671	MRPL42	0.378926359	mirna_pc
12671	hsa-mir-671	COPB2	0.403645895	mirna_pc
12672	hsa-mir-671	CA5BP	0.327829452	mirna_pc
12673	hsa-mir-671	TRMT2A	0.350296317	mirna_pc
12674	hsa-mir-671	PCBP2	0.334418008	mirna_pc
12675	hsa-mir-671	UBE2O	0.340496216	mirna_pc
12676	hsa-mir-671	MYPOP	0.315686536	mirna_pc
12677	hsa-mir-671	C10orf35	0.350953209	mirna_pc
12678	hsa-mir-671	COQ3	0.309076654	mirna_pc
12679	hsa-mir-671	HLTF	0.367041203	mirna_pc
12680	hsa-mir-671	BLMH	0.31498455	mirna_pc
12681	hsa-mir-671	NUP88	0.308548337	mirna_pc
12682	hsa-mir-671	KPNA4	0.392507435	mirna_pc
12683	hsa-mir-671	RPUSD2	0.318728026	mirna_pc
12684	hsa-mir-671	ABCF3	0.361609846	mirna_pc
12685	hsa-mir-671	ZNF786	0.310638261	mirna_pc
12686	hsa-mir-671	EIF2B5	0.406620704	mirna_pc
12687	hsa-mir-671	NIPSNAP1	0.309462593	mirna_pc
12688	hsa-mir-671	PARL	0.413119921	mirna_pc
12689	hsa-mir-671	PCCB	0.334404186	mirna_pc

12690	hsa-mir-671	RPL35A	0.311315644	mirna_pc
12691	hsa-mir-671	AEBP2	0.327594957	mirna_pc
12692	hsa-mir-671	EIF4G1	0.37103987	mirna_pc
12693	hsa-mir-671	C12orf10	0.300952065	mirna_pc
12694	hsa-mir-671	TMEM81	0.33213819	mirna_pc
12695	hsa-mir-671	PPP1R2	0.339789964	mirna_pc
12696	hsa-mir-671	C2orf44	0.324774279	mirna_pc
12697	hsa-mir-671	LOC440173	0.318226667	mirna_pc
12698	hsa-mir-671	PIK3R4	0.316750274	mirna_pc
12699	hsa-mir-671	PSMB6	0.311039045	mirna_pc
12700	hsa-mir-671	SEPHS1	0.315230628	mirna_pc
12701	hsa-mir-671	TMEM17	0.300710873	mirna_pc
12702	hsa-mir-671	RYK	0.367623165	mirna_pc
12703	hsa-mir-671	TMEM11	0.355026941	mirna_pc
12704	hsa-mir-671	USP5	0.314100101	mirna_pc
12705	hsa-mir-671	BOLA3	0.32512244	mirna_pc
12706	hsa-mir-671	TOP3B	0.310108695	mirna_pc
12707	hsa-mir-671	LARP4	0.318083985	mirna_pc
12708	hsa-mir-671	PHB2	0.327247901	mirna_pc
12709	hsa-mir-671	PDCD7	0.337303979	mirna_pc
12710	hsa-mir-671	NRF1	0.31000761	mirna_pc
12711	hsa-mir-671	BRD4	0.330264016	mirna_pc
12712	hsa-mir-671	ZNF282	0.341122982	mirna_pc
12713	hsa-mir-671	SENP5	0.404830259	mirna_pc
12714	hsa-mir-671	HNRNPUL1	0.313590544	mirna_pc
12715	hsa-mir-671	ZNF384	0.31208578	mirna_pc
12716	hsa-mir-671	TXNRD1	0.331077151	mirna_pc
12717	hsa-mir-671	BCL11A	0.308598421	mirna_pc
12718	hsa-mir-671	LUC7L2	0.36189885	mirna_pc
12719	hsa-mir-671	FAH	0.304642636	mirna_pc
12720	hsa-mir-671	ACAD9	0.336998492	mirna_pc
12721	hsa-mir-671	FAM98B	0.344368095	mirna_pc
12722	hsa-mir-671	LRFN1	0.310490137	mirna_pc
12723	hsa-mir-671	PEX5	0.302091085	mirna_pc
12724	hsa-mir-671	SRPRB	0.357822574	mirna_pc
12725	hsa-mir-671	ARMC8	0.311124676	mirna_pc
12726	hsa-mir-671	C12orf41	0.387535432	mirna_pc
12727	hsa-mir-671	TFDP2	0.339532221	mirna_pc
12728	hsa-mir-671	FYTTD1	0.350037345	mirna_pc
12729	hsa-mir-671	C3orf58	0.301595757	mirna_pc
12730	hsa-mir-671	FKBP4	0.350367379	mirna_pc
12731	hsa-mir-671	HSPA9	0.347018978	mirna_pc
12732	hsa-mir-671	USP13	0.334914784	mirna_pc
12733	hsa-mir-671	TSR1	0.303948367	mirna_pc
12734	hsa-mir-671	CUL1	0.344166358	mirna_pc
12735	hsa-mir-671	UGT1A6	0.320603325	mirna_pc
12736	hsa-mir-671	SELT	0.32016432	mirna_pc
12737	hsa-mir-671	DDX51	0.321509601	mirna_pc
12738	hsa-mir-671	EIF2A	0.36673057	mirna_pc
12739	hsa-mir-671	SENP2	0.406712775	mirna_pc
12740	hsa-mir-671	UXS1	0.316005512	mirna_pc
12741	hsa-mir-671	TRIM16L	0.355810309	mirna_pc
12742	hsa-mir-671	FAM169A	0.359751768	mirna_pc
12743	hsa-mir-671	EIF2AK4	0.31411494	mirna_pc

12744	hsa-mir-671	GTF2IP1	0.329246958	mirna_pc
12745	hsa-mir-671	C21orf91	0.351518545	mirna_pc
12746	hsa-mir-671	SPATS2	0.320944644	mirna_pc
12747	hsa-mir-671	DNAJA3	0.315705245	mirna_pc
12748	hsa-mir-671	USP28	0.320482877	mirna_pc
12749	hsa-mir-671	POM121C	0.425788914	mirna_pc
12750	hsa-mir-671	DZIP1L	0.313589139	mirna_pc
12751	hsa-mir-671	PAX6	0.31511488	mirna_pc
12752	hsa-mir-671	MRPS33	0.302960359	mirna_pc
12753	hsa-mir-671	RRP7B	0.331952748	mirna_pc
12754	hsa-mir-671	DNAJC19	0.388072904	mirna_pc
12755	hsa-mir-671	CEP70	0.324342879	mirna_pc
12756	hsa-mir-671	OPA1	0.433411754	mirna_pc
12757	hsa-mir-671	SCML2	0.333677501	mirna_pc
12758	hsa-mir-671	STRAP	0.38056962	mirna_pc
12759	hsa-mir-671	C12orf65	0.422775659	mirna_pc
12760	hsa-mir-671	MRPL19	0.428833606	mirna_pc
12761	hsa-mir-671	GTF2I	0.336091243	mirna_pc
12762	hsa-mir-671	CNBP	0.306662741	mirna_pc
12763	hsa-mir-671	KATNAL2	0.308564054	mirna_pc
12764	hsa-mir-671	LOC220729	0.305127478	mirna_pc
12765	hsa-mir-671	B9D1	0.354376067	mirna_pc
12766	hsa-mir-671	DDX1	0.328166914	mirna_pc
12767	hsa-mir-671	WBSCR16	0.385712082	mirna_pc
12768	hsa-mir-671	ACP1	0.330715222	mirna_pc
12769	hsa-mir-671	ASF1A	0.308001212	mirna_pc
12770	hsa-mir-671	SSR3	0.336635447	mirna_pc
12771	hsa-mir-671	C7orf49	0.352430362	mirna_pc
12772	hsa-mir-671	ACTR3B	0.389417363	mirna_pc
12773	hsa-mir-671	NOM1	0.382182655	mirna_pc
12774	hsa-mir-671	NDUFB5	0.348207984	mirna_pc
12775	hsa-mir-671	TUSC3	0.340958037	mirna_pc
12776	hsa-mir-671	SLC35B4	0.33020267	mirna_pc
12777	hsa-mir-671	AGK	0.382788786	mirna_pc
12778	hsa-mir-671	ANAPC5	0.318805442	mirna_pc
12779	hsa-mir-671	MBD6	0.316219075	mirna_pc
12780	hsa-mir-671	IER3IP1	0.312479857	mirna_pc
12781	hsa-mir-671	VPS37D	0.300546469	mirna_pc
12782	hsa-mir-671	SEMA4C	0.317506726	mirna_pc
12783	hsa-mir-671	MARS	0.335412141	mirna_pc
12784	hsa-mir-671	COPS3	0.359066338	mirna_pc
12785	hsa-mir-671	ZNF777	0.330235641	mirna_pc
12786	hsa-mir-671	CKAP4	0.304688397	mirna_pc
12787	hsa-mir-671	MFF	0.302234995	mirna_pc
12788	hsa-mir-671	C12orf73	0.300136333	mirna_pc
12789	hsa-mir-671	C3orf33	0.337570068	mirna_pc
12790	hsa-mir-671	POM121	0.383822387	mirna_pc
12791	hsa-mir-19b-1	MKI67	0.306769933	mirna_pc
12792	hsa-mir-19b-1	TOP2A	0.332254448	mirna_pc
12793	hsa-mir-19b-1	CENPF	0.324318208	mirna_pc
12794	hsa-mir-19b-1	RCC2	0.329147435	mirna_pc
12795	hsa-mir-19b-1	KIF11	0.321078483	mirna_pc
12796	hsa-mir-19b-1	RRM2	0.310777047	mirna_pc
12797	hsa-mir-19b-1	CDK1	0.329771135	mirna_pc

12798	hsa-mir-19b-1	MYBL2	0.323074981	mirna_pc
12799	hsa-mir-19b-1	UBE2C	0.325478093	mirna_pc
12800	hsa-mir-19b-1	BUB1	0.353019983	mirna_pc
12801	hsa-mir-19b-1	BUB1B	0.341742376	mirna_pc
12802	hsa-mir-19b-1	HDGF	0.304028436	mirna_pc
12803	hsa-mir-19b-1	KIF18B	0.302125199	mirna_pc
12804	hsa-mir-19b-1	ASPM	0.309712109	mirna_pc
12805	hsa-mir-19b-1	CKAP2	0.342702195	mirna_pc
12806	hsa-mir-19b-1	KIF2C	0.303723253	mirna_pc
12807	hsa-mir-19b-1	FANCA	0.498739659	mirna_pc
12808	hsa-mir-19b-1	NCAPG	0.327748713	mirna_pc
12809	hsa-mir-19b-1	SGOL1	0.379652722	mirna_pc
12810	hsa-mir-19b-1	ARHGAP11A	0.389961613	mirna_pc
12811	hsa-mir-19b-1	CCNA2	0.300650591	mirna_pc
12812	hsa-mir-19b-1	CDC25B	0.348651725	mirna_pc
12813	hsa-mir-19b-1	CBX3	0.313596718	mirna_pc
12814	hsa-mir-19b-1	SPC25	0.400130784	mirna_pc
12815	hsa-mir-19b-1	NUP62	0.311289686	mirna_pc
12816	hsa-mir-19b-1	FANCI	0.309228858	mirna_pc
12817	hsa-mir-19b-1	NEK2	0.378680023	mirna_pc
12818	hsa-mir-19b-1	HOXC11	0.302171772	mirna_pc
12819	hsa-mir-19b-1	NCAPH	0.373111569	mirna_pc
12820	hsa-mir-19b-1	GTSE1	0.346370383	mirna_pc
12821	hsa-mir-19b-1	RAD54L	0.324430312	mirna_pc
12822	hsa-mir-19b-1	MAD2L1	0.3889445	mirna_pc
12823	hsa-mir-19b-1	TIMELESS	0.31765924	mirna_pc
12824	hsa-mir-19b-1	LMNB1	0.330720975	mirna_pc
12825	hsa-mir-19b-1	ASF1B	0.301551996	mirna_pc
12826	hsa-mir-19b-1	CDCA8	0.34833993	mirna_pc
12827	hsa-mir-19b-1	CDCA5	0.303665033	mirna_pc
12828	hsa-mir-19b-1	XPO1	0.39022915	mirna_pc
12829	hsa-mir-19b-1	CDCA2	0.33839972	mirna_pc
12830	hsa-mir-19b-1	KNTC1	0.303746235	mirna_pc
12831	hsa-mir-19b-1	NUF2	0.302289928	mirna_pc
12832	hsa-mir-19b-1	PLK4	0.366816487	mirna_pc
12833	hsa-mir-19b-1	FANCB	0.360730061	mirna_pc
12834	hsa-mir-19b-1	DEPDC1	0.303544799	mirna_pc
12835	hsa-mir-19b-1	SKA3	0.482254729	mirna_pc
12836	hsa-mir-19b-1	KIF14	0.311386314	mirna_pc
12837	hsa-mir-19b-1	HJURP	0.443461107	mirna_pc
12838	hsa-mir-19b-1	BLM	0.389534795	mirna_pc
12839	hsa-mir-19b-1	DNMT1	0.340529204	mirna_pc
12840	hsa-mir-19b-1	PAICS	0.41355457	mirna_pc
12841	hsa-mir-19b-1	CCDC150	0.376900716	mirna_pc
12842	hsa-mir-19b-1	LIG1	0.334294236	mirna_pc
12843	hsa-mir-19b-1	DTL	0.336569796	mirna_pc
12844	hsa-mir-19b-1	EME1	0.409013038	mirna_pc
12845	hsa-mir-19b-1	RECQL4	0.300336181	mirna_pc
12846	hsa-mir-19b-1	CENPK	0.342580631	mirna_pc
12847	hsa-mir-19b-1	CDT1	0.333366382	mirna_pc
12848	hsa-mir-19b-1	CBFB	0.356240644	mirna_pc
12849	hsa-mir-19b-1	MCM10	0.33031527	mirna_pc
12850	hsa-mir-19b-1	SGOL2	0.315586371	mirna_pc
12851	hsa-mir-19b-1	CENPE	0.345959886	mirna_pc

12852	hsa-mir-19b-1	POLQ	0.331211624	mirna_pc
12853	hsa-mir-19b-1	KIF15	0.3402889	mirna_pc
12854	hsa-mir-19b-1	C1orf112	0.394329625	mirna_pc
12855	hsa-mir-19b-1	GINS1	0.333823438	mirna_pc
12856	hsa-mir-19b-1	FEN1	0.33360618	mirna_pc
12857	hsa-mir-19b-1	TRAIP	0.355058825	mirna_pc
12858	hsa-mir-19b-1	ORC6L	0.345930774	mirna_pc
12859	hsa-mir-19b-1	ARHGAP11B	0.321110127	mirna_pc
12860	hsa-mir-19b-1	C16orf59	0.330320885	mirna_pc
12861	hsa-mir-19b-1	RAE1	0.321448685	mirna_pc
12862	hsa-mir-19b-1	FAM72D	0.305540124	mirna_pc
12863	hsa-mir-19b-1	FAM72B	0.324603542	mirna_pc
12864	hsa-mir-19b-1	EZH2	0.374293782	mirna_pc
12865	hsa-mir-19b-1	NEIL3	0.307799525	mirna_pc
12866	hsa-mir-19b-1	POLD1	0.389740697	mirna_pc
12867	hsa-mir-19b-1	C12orf48	0.334458431	mirna_pc
12868	hsa-mir-19b-1	ZWILCH	0.300322286	mirna_pc
12869	hsa-mir-19b-1	ZWINT	0.316079726	mirna_pc
12870	hsa-mir-19b-1	BUB3	0.309546976	mirna_pc
12871	hsa-mir-19b-1	HELLS	0.350405839	mirna_pc
12872	hsa-mir-19b-1	TCF3	0.416771629	mirna_pc
12873	hsa-mir-19b-1	CHEK1	0.317056467	mirna_pc
12874	hsa-mir-19b-1	BRCA1	0.345201684	mirna_pc
12875	hsa-mir-19b-1	PATL1	0.300395982	mirna_pc
12876	hsa-mir-19b-1	HNRNPA2B1	0.385280938	mirna_pc
12877	hsa-mir-19b-1	RFWD3	0.422330959	mirna_pc
12878	hsa-mir-19b-1	C20orf20	0.302807442	mirna_pc
12879	hsa-mir-19b-1	MCM6	0.458978739	mirna_pc
12880	hsa-mir-19b-1	CDCA7	0.425919657	mirna_pc
12881	hsa-mir-19b-1	MTHFD1L	0.331051417	mirna_pc
12882	hsa-mir-19b-1	FAM60A	0.356521277	mirna_pc
12883	hsa-mir-19b-1	ATAD5	0.339453082	mirna_pc
12884	hsa-mir-19b-1	FAM111B	0.303486289	mirna_pc
12885	hsa-mir-19b-1	KIF20B	0.33298218	mirna_pc
12886	hsa-mir-19b-1	ESCO2	0.312448115	mirna_pc
12887	hsa-mir-19b-1	SNRPA	0.319002515	mirna_pc
12888	hsa-mir-19b-1	RFC3	0.464107407	mirna_pc
12889	hsa-mir-19b-1	CENPO	0.355056654	mirna_pc
12890	hsa-mir-19b-1	XRCC2	0.398353008	mirna_pc
12891	hsa-mir-19b-1	C19orf48	0.329416712	mirna_pc
12892	hsa-mir-19b-1	BRCA2	0.398598105	mirna_pc
12893	hsa-mir-19b-1	GEN1	0.431039109	mirna_pc
12894	hsa-mir-19b-1	HMGB2	0.391936722	mirna_pc
12895	hsa-mir-19b-1	DNA2	0.420701737	mirna_pc
12896	hsa-mir-19b-1	NOP56	0.438959676	mirna_pc
12897	hsa-mir-19b-1	DSN1	0.302352493	mirna_pc
12898	hsa-mir-19b-1	SNHG1	0.379307433	mirna_pc
12899	hsa-mir-19b-1	DTYMK	0.352710523	mirna_pc
12900	hsa-mir-19b-1	PRMT1	0.3549483	mirna_pc
12901	hsa-mir-19b-1	RAVER1	0.343279047	mirna_pc
12902	hsa-mir-19b-1	HEATR1	0.327818578	mirna_pc
12903	hsa-mir-19b-1	RBL1	0.323646024	mirna_pc
12904	hsa-mir-19b-1	CAD	0.304700399	mirna_pc
12905	hsa-mir-19b-1	DLEU2	0.428892017	mirna_pc

12906	hsa-mir-19b-1	PRIM1	0.322930077	mirna_pc
12907	hsa-mir-19b-1	FERMT1	0.453625965	mirna_pc
12908	hsa-mir-19b-1	TCOF1	0.309024883	mirna_pc
12909	hsa-mir-19b-1	CHAF1B	0.300890724	mirna_pc
12910	hsa-mir-19b-1	NCAPG2	0.348718631	mirna_pc
12911	hsa-mir-19b-1	MSH2	0.342721589	mirna_pc
12912	hsa-mir-19b-1	SF3B3	0.379743314	mirna_pc
12913	hsa-mir-19b-1	CDC7	0.31073392	mirna_pc
12914	hsa-mir-19b-1	SSB	0.442199385	mirna_pc
12915	hsa-mir-19b-1	PUS1	0.343352832	mirna_pc
12916	hsa-mir-19b-1	NOL11	0.33306253	mirna_pc
12917	hsa-mir-19b-1	FBXO5	0.334568636	mirna_pc
12918	hsa-mir-19b-1	E2F3	0.386241782	mirna_pc
12919	hsa-mir-19b-1	DDX11	0.333026978	mirna_pc
12920	hsa-mir-19b-1	CDC25A	0.456216794	mirna_pc
12921	hsa-mir-19b-1	TMEM48	0.316985624	mirna_pc
12922	hsa-mir-19b-1	DSCC1	0.302583034	mirna_pc
12923	hsa-mir-19b-1	C13orf34	0.494177711	mirna_pc
12924	hsa-mir-19b-1	SRPK1	0.330921057	mirna_pc
12925	hsa-mir-19b-1	MCM8	0.444864008	mirna_pc
12926	hsa-mir-19b-1	ATIC	0.417205306	mirna_pc
12927	hsa-mir-19b-1	TFAP4	0.347613144	mirna_pc
12928	hsa-mir-19b-1	DAZAP1	0.320361625	mirna_pc
12929	hsa-mir-19b-1	CBX8	0.309483015	mirna_pc
12930	hsa-mir-19b-1	NCL	0.472876144	mirna_pc
12931	hsa-mir-19b-1	SRRT	0.304258932	mirna_pc
12932	hsa-mir-19b-1	FBL	0.307137188	mirna_pc
12933	hsa-mir-19b-1	C13orf37	0.422114778	mirna_pc
12934	hsa-mir-19b-1	HNRNPM	0.324559495	mirna_pc
12935	hsa-mir-19b-1	LSM 12.00	0.301974325	mirna_pc
12936	hsa-mir-19b-1	PNPT1	0.419012646	mirna_pc
12937	hsa-mir-19b-1	TMPO	0.370159823	mirna_pc
12938	hsa-mir-19b-1	LOC388796	0.332287212	mirna_pc
12939	hsa-mir-19b-1	SMPD4	0.3436935	mirna_pc
12940	hsa-mir-19b-1	PPAT	0.323498116	mirna_pc
12941	hsa-mir-19b-1	DHX9	0.400737014	mirna_pc
12942	hsa-mir-19b-1	CCDC138	0.36093725	mirna_pc
12943	hsa-mir-19b-1	CHRNA5	0.323713168	mirna_pc
12944	hsa-mir-19b-1	SFRS1	0.48788369	mirna_pc
12945	hsa-mir-19b-1	WDR43	0.395177566	mirna_pc
12946	hsa-mir-19b-1	C4orf46	0.383245685	mirna_pc
12947	hsa-mir-19b-1	DARS2	0.337001865	mirna_pc
12948	hsa-mir-19b-1	UTP14A	0.351224334	mirna_pc
12949	hsa-mir-19b-1	C1orf107	0.313770556	mirna_pc
12950	hsa-mir-19b-1	HSPD1	0.421577875	mirna_pc
12951	hsa-mir-19b-1	SSRP1	0.333487682	mirna_pc
12952	hsa-mir-19b-1	POLR2D	0.369710316	mirna_pc
12953	hsa-mir-19b-1	FTSJ2	0.326964442	mirna_pc
12954	hsa-mir-19b-1	KHDRBS1	0.344087711	mirna_pc
12955	hsa-mir-19b-1	SLC5A6	0.387408128	mirna_pc
12956	hsa-mir-19b-1	PDIA4	0.346985409	mirna_pc
12957	hsa-mir-19b-1	TOMM34	0.399150854	mirna_pc
12958	hsa-mir-19b-1	TAF1A	0.307283817	mirna_pc
12959	hsa-mir-19b-1	PRPF40A	0.448401234	mirna_pc

12960	hsa-mir-19b-1	AHCY	0.326823194	mirna_pc
12961	hsa-mir-19b-1	SLC39A10	0.357612034	mirna_pc
12962	hsa-mir-19b-1	NOLC1	0.307796048	mirna_pc
12963	hsa-mir-19b-1	ZNF695	0.405610654	mirna_pc
12964	hsa-mir-19b-1	PDSS1	0.353008534	mirna_pc
12965	hsa-mir-19b-1	HAUS6	0.350075355	mirna_pc
12966	hsa-mir-19b-1	CENPJ	0.388747552	mirna_pc
12967	hsa-mir-19b-1	HNRNPU	0.39833706	mirna_pc
12968	hsa-mir-19b-1	NASP	0.304431965	mirna_pc
12969	hsa-mir-19b-1	POLR1B	0.379604119	mirna_pc
12970	hsa-mir-19b-1	PTMA	0.317107444	mirna_pc
12971	hsa-mir-19b-1	SCARB1	0.30077597	mirna_pc
12972	hsa-mir-19b-1	CLCN2	0.336460889	mirna_pc
12973	hsa-mir-19b-1	SUV39H2	0.3708281	mirna_pc
12974	hsa-mir-19b-1	R3HDM1	0.333264458	mirna_pc
12975	hsa-mir-19b-1	FANCL	0.32911482	mirna_pc
12976	hsa-mir-19b-1	RQCD1	0.330738018	mirna_pc
12977	hsa-mir-19b-1	HNRNPD	0.318807372	mirna_pc
12978	hsa-mir-19b-1	MTHFD2	0.353975635	mirna_pc
12979	hsa-mir-19b-1	GTF2F2	0.333285765	mirna_pc
12980	hsa-mir-19b-1	POP1	0.376144478	mirna_pc
12981	hsa-mir-19b-1	PNO1	0.309221256	mirna_pc
12982	hsa-mir-19b-1	CASP2	0.303532423	mirna_pc
12983	hsa-mir-19b-1	ELAVL1	0.329508487	mirna_pc
12984	hsa-mir-19b-1	KIAA0406	0.34373882	mirna_pc
12985	hsa-mir-19b-1	HSP90B1	0.309275071	mirna_pc
12986	hsa-mir-19b-1	NUDT15	0.399279521	mirna_pc
12987	hsa-mir-19b-1	FASTKD1	0.390175913	mirna_pc
12988	hsa-mir-19b-1	E2F4	0.314259877	mirna_pc
12989	hsa-mir-19b-1	SLMO2	0.382246799	mirna_pc
12990	hsa-mir-19b-1	TWISTNB	0.311980598	mirna_pc
12991	hsa-mir-19b-1	DHFR	0.308749779	mirna_pc
12992	hsa-mir-19b-1	NAA25	0.419691954	mirna_pc
12993	hsa-mir-19b-1	ILF3	0.416386477	mirna_pc
12994	hsa-mir-19b-1	TBC1D4	0.31559534	mirna_pc
12995	hsa-mir-19b-1	TRIB3	0.356927908	mirna_pc
12996	hsa-mir-19b-1	PASK	0.349166013	mirna_pc
12997	hsa-mir-19b-1	OLA1	0.307642748	mirna_pc
12998	hsa-mir-19b-1	RPGRIP1L	0.32786125	mirna_pc
12999	hsa-mir-19b-1	ATP11A	0.306222088	mirna_pc
13000	hsa-mir-19b-1	TGS1	0.340051854	mirna_pc
13001	hsa-mir-19b-1	HEATR2	0.401486387	mirna_pc
13002	hsa-mir-19b-1	CYB5B	0.345674328	mirna_pc
13003	hsa-mir-19b-1	TRAF4	0.308192277	mirna_pc
13004	hsa-mir-19b-1	GAR1	0.319370921	mirna_pc
13005	hsa-mir-19b-1	CEP152	0.331150779	mirna_pc
13006	hsa-mir-19b-1	ZC3HAV1L	0.301235181	mirna_pc
13007	hsa-mir-19b-1	RPN2	0.301348863	mirna_pc
13008	hsa-mir-19b-1	TNFRSF10B	0.361562215	mirna_pc
13009	hsa-mir-19b-1	RNF219	0.546231693	mirna_pc
13010	hsa-mir-19b-1	ITGB3BP	0.30389126	mirna_pc
13011	hsa-mir-19b-1	SLC25A32	0.402981268	mirna_pc
13012	hsa-mir-19b-1	CEP78	0.322130704	mirna_pc
13013	hsa-mir-19b-1	SFPQ	0.324260839	mirna_pc

13014	hsa-mir-19b-1	RCOR2	0.364156973	mirna_pc
13015	hsa-mir-19b-1	POLA1	0.336782553	mirna_pc
13016	hsa-mir-19b-1	HDAC2	0.362622526	mirna_pc
13017	hsa-mir-19b-1	C10orf119	0.307878601	mirna_pc
13018	hsa-mir-19b-1	C10orf2	0.401854113	mirna_pc
13019	hsa-mir-19b-1	CSTF1	0.304108684	mirna_pc
13020	hsa-mir-19b-1	XRN2	0.320108518	mirna_pc
13021	hsa-mir-19b-1	KPNB1	0.421598261	mirna_pc
13022	hsa-mir-19b-1	SMC3	0.338264769	mirna_pc
13023	hsa-mir-19b-1	FLVCR1	0.336874907	mirna_pc
13024	hsa-mir-19b-1	HNRNPA1	0.331470866	mirna_pc
13025	hsa-mir-19b-1	AGMAT	0.337416647	mirna_pc
13026	hsa-mir-19b-1	MEX3A	0.357895674	mirna_pc
13027	hsa-mir-19b-1	TMC7	0.368066988	mirna_pc
13028	hsa-mir-19b-1	NANP	0.335306304	mirna_pc
13029	hsa-mir-19b-1	SUZ12	0.362530374	mirna_pc
13030	hsa-mir-19b-1	HNRNPA1L2	0.339083142	mirna_pc
13031	hsa-mir-19b-1	HMGB1	0.418151589	mirna_pc
13032	hsa-mir-19b-1	ZBTB12	0.300336103	mirna_pc
13033	hsa-mir-19b-1	DIAPH3	0.33696105	mirna_pc
13034	hsa-mir-19b-1	TGIF2	0.401493276	mirna_pc
13035	hsa-mir-19b-1	ZC3H18	0.363934204	mirna_pc
13036	hsa-mir-19b-1	FARSB	0.390381467	mirna_pc
13037	hsa-mir-19b-1	PIK3AP1	0.301803117	mirna_pc
13038	hsa-mir-19b-1	SNX5	0.326138299	mirna_pc
13039	hsa-mir-19b-1	NOL10	0.34635156	mirna_pc
13040	hsa-mir-19b-1	ANAPC1	0.333006121	mirna_pc
13041	hsa-mir-19b-1	POLE	0.370367715	mirna_pc
13042	hsa-mir-19b-1	SOX9	0.509577077	mirna_pc
13043	hsa-mir-19b-1	TIMM50	0.307978926	mirna_pc
13044	hsa-mir-19b-1	C13orf27	0.351732979	mirna_pc
13045	hsa-mir-19b-1	RBM14	0.307115017	mirna_pc
13046	hsa-mir-19b-1	RBMX	0.417731142	mirna_pc
13047	hsa-mir-19b-1	NOC3L	0.300214718	mirna_pc
13048	hsa-mir-19b-1	GPR180	0.414870219	mirna_pc
13049	hsa-mir-19b-1	DAP3	0.324848567	mirna_pc
13050	hsa-mir-19b-1	XRCC5	0.423025196	mirna_pc
13051	hsa-mir-19b-1	EXOSC8	0.333418967	mirna_pc
13052	hsa-mir-19b-1	CTCF	0.334302576	mirna_pc
13053	hsa-mir-19b-1	PDIA6	0.353973208	mirna_pc
13054	hsa-mir-19b-1	GRWD1	0.307899458	mirna_pc
13055	hsa-mir-19b-1	RPE	0.309165024	mirna_pc
13056	hsa-mir-19b-1	LBR	0.335959096	mirna_pc
13057	hsa-mir-19b-1	ORC2L	0.337328291	mirna_pc
13058	hsa-mir-19b-1	ZRANB3	0.302403537	mirna_pc
13059	hsa-mir-19b-1	RIF1	0.340040888	mirna_pc
13060	hsa-mir-19b-1	NUDT21	0.352177726	mirna_pc
13061	hsa-mir-19b-1	EFCAB4B	0.337874177	mirna_pc
13062	hsa-mir-19b-1	FAM136A	0.358141245	mirna_pc
13063	hsa-mir-19b-1	NRAS	0.479286436	mirna_pc
13064	hsa-mir-19b-1	TTLL4	0.30468462	mirna_pc
13065	hsa-mir-19b-1	FBXO41	0.368949732	mirna_pc
13066	hsa-mir-19b-1	RBCK1	0.333789224	mirna_pc
13067	hsa-mir-19b-1	EML4	0.301121312	mirna_pc

13068	hsa-mir-19b-1	BZW2	0.35353457	mirna_pc
13069	hsa-mir-19b-1	LRPPRC	0.369031078	mirna_pc
13070	hsa-mir-19b-1	CSNK2A1	0.358814557	mirna_pc
13071	hsa-mir-19b-1	RPIA	0.358587008	mirna_pc
13072	hsa-mir-19b-1	MOCS3	0.311046095	mirna_pc
13073	hsa-mir-19b-1	URB2	0.401000048	mirna_pc
13074	hsa-mir-19b-1	SFXN4	0.38028194	mirna_pc
13075	hsa-mir-19b-1	RBM28	0.403679316	mirna_pc
13076	hsa-mir-19b-1	MRE11A	0.316085551	mirna_pc
13077	hsa-mir-19b-1	PABPC3	0.340854397	mirna_pc
13078	hsa-mir-19b-1	ILKAP	0.373305212	mirna_pc
13079	hsa-mir-19b-1	PLB1	0.330276753	mirna_pc
13080	hsa-mir-19b-1	FOXK1	0.305717415	mirna_pc
13081	hsa-mir-19b-1	C13orf23	0.406520062	mirna_pc
13082	hsa-mir-19b-1	SPATA2	0.339508855	mirna_pc
13083	hsa-mir-19b-1	RBM12	0.423366992	mirna_pc
13084	hsa-mir-19b-1	PDF	0.320014942	mirna_pc
13085	hsa-mir-19b-1	TOP 1.00	0.33399302	mirna_pc
13086	hsa-mir-19b-1	TCERG1	0.356332651	mirna_pc
13087	hsa-mir-19b-1	PSPC1	0.42228509	mirna_pc
13088	hsa-mir-19b-1	TOP1P1	0.379415374	mirna_pc
13089	hsa-mir-19b-1	MAPKAPK5	0.37623201	mirna_pc
13090	hsa-mir-19b-1	E2F6	0.351089315	mirna_pc
13091	hsa-mir-19b-1	SLC25A15	0.353178954	mirna_pc
13092	hsa-mir-19b-1	SFRS13A	0.356637503	mirna_pc
13093	hsa-mir-19b-1	SCLY	0.352433213	mirna_pc
13094	hsa-mir-19b-1	FASTKD2	0.323537322	mirna_pc
13095	hsa-mir-19b-1	SLC19A1	0.313677673	mirna_pc
13096	hsa-mir-19b-1	TGDS	0.439108137	mirna_pc
13097	hsa-mir-19b-1	TFAM	0.355511787	mirna_pc
13098	hsa-mir-19b-1	ZDHHC7	0.309322199	mirna_pc
13099	hsa-mir-19b-1	ORC4L	0.379636559	mirna_pc
13100	hsa-mir-19b-1	ZC3H15	0.319193155	mirna_pc
13101	hsa-mir-19b-1	KIAA1704	0.39298024	mirna_pc
13102	hsa-mir-19b-1	RPRD1B	0.326009341	mirna_pc
13103	hsa-mir-19b-1	MTIF2	0.38921761	mirna_pc
13104	hsa-mir-19b-1	HNRNPA3	0.31424431	mirna_pc
13105	hsa-mir-19b-1	ZNF828	0.413005084	mirna_pc
13106	hsa-mir-19b-1	SMC1A	0.36693194	mirna_pc
13107	hsa-mir-19b-1	NUPL1	0.548422175	mirna_pc
13108	hsa-mir-19b-1	CCDC45	0.304073569	mirna_pc
13109	hsa-mir-19b-1	ANKRD5	0.300556766	mirna_pc
13110	hsa-mir-19b-1	GNL3	0.302319524	mirna_pc
13111	hsa-mir-19b-1	DHODH	0.324140964	mirna_pc
13112	hsa-mir-19b-1	UPF3A	0.336070342	mirna_pc
13113	hsa-mir-19b-1	SLC7A5P2	0.301062684	mirna_pc
13114	hsa-mir-19b-1	SFRS7	0.366570496	mirna_pc
13115	hsa-mir-19b-1	C9orf100	0.303572774	mirna_pc
13116	hsa-mir-19b-1	BEND3	0.323585854	mirna_pc
13117	hsa-mir-19b-1	NFXL1	0.312677853	mirna_pc
13118	hsa-mir-19b-1	SF3B2	0.304713641	mirna_pc
13119	hsa-mir-19b-1	UQCC	0.316599501	mirna_pc
13120	hsa-mir-19b-1	ANKS6	0.308864739	mirna_pc
13121	hsa-mir-19b-1	HNRNPH1	0.301835345	mirna_pc

13122	hsa-mir-19b-1	ATP7B	0.317812903	mirna_pc
13123	hsa-mir-19b-1	EPRS	0.305273269	mirna_pc
13124	hsa-mir-19b-1	SLC35F2	0.325619747	mirna_pc
13125	hsa-mir-19b-1	CDC16	0.337050511	mirna_pc
13126	hsa-mir-19b-1	MRPL44	0.303171484	mirna_pc
13127	hsa-mir-19b-1	PTCD3	0.347345604	mirna_pc
13128	hsa-mir-19b-1	RNF6	0.334711954	mirna_pc
13129	hsa-mir-19b-1	DHX15	0.30928217	mirna_pc
13130	hsa-mir-19b-1	IWS1	0.343496246	mirna_pc
13131	hsa-mir-19b-1	MAP2K6	0.323597082	mirna_pc
13132	hsa-mir-19b-1	CASP6	0.302392132	mirna_pc
13133	hsa-mir-19b-1	RBM26	0.332996532	mirna_pc
13134	hsa-mir-19b-1	ZNF92	0.32209414	mirna_pc
13135	hsa-mir-19b-1	TMTC4	0.343473707	mirna_pc
13136	hsa-mir-19b-1	RMND1	0.351837261	mirna_pc
13137	hsa-mir-19b-1	ADNP	0.335795705	mirna_pc
13138	hsa-mir-19b-1	AZIN1	0.419513591	mirna_pc
13139	hsa-mir-19b-1	RSL1D1	0.309518124	mirna_pc
13140	hsa-mir-19b-1	CEBPZ	0.338424904	mirna_pc
13141	hsa-mir-19b-1	FASTKD5	0.354956277	mirna_pc
13142	hsa-mir-19b-1	IPO5	0.546150849	mirna_pc
13143	hsa-mir-19b-1	ALG11	0.336595705	mirna_pc
13144	hsa-mir-19b-1	MCCC2	0.325964057	mirna_pc
13145	hsa-mir-19b-1	NRF1	0.304911112	mirna_pc
13146	hsa-mir-19b-1	IDI2	0.301271845	mirna_pc
13147	hsa-mir-19b-1	NUP43	0.347387179	mirna_pc
13148	hsa-mir-19b-1	ZNF124	0.408639109	mirna_pc
13149	hsa-mir-19b-1	RNASEH2B	0.366048816	mirna_pc
13150	hsa-mir-19b-1	ZNF473	0.423029645	mirna_pc
13151	hsa-mir-19b-1	COPS7B	0.303542345	mirna_pc
13152	hsa-mir-19b-1	UGGT1	0.336713643	mirna_pc
13153	hsa-mir-19b-1	DCLRE1A	0.374323278	mirna_pc
13154	hsa-mir-19b-1	ZNF749	0.304858301	mirna_pc
13155	hsa-mir-19b-1	ANP32A	0.369089703	mirna_pc
13156	hsa-mir-19b-1	DIS3	0.366549806	mirna_pc
13157	hsa-mir-19b-1	E2F5	0.348791249	mirna_pc
13158	hsa-mir-19b-1	NUFIP1	0.455332809	mirna_pc
13159	hsa-mir-19b-1	ZNF195	0.348496705	mirna_pc
13160	hsa-mir-19b-1	DCAF17	0.374752018	mirna_pc
13161	hsa-mir-19b-1	PRKAR1B	0.347933967	mirna_pc
13162	hsa-mir-19b-1	BCAS2	0.456184293	mirna_pc
13163	hsa-mir-19b-1	HEATR3	0.310129335	mirna_pc
13164	hsa-mir-19b-1	USP45	0.301588776	mirna_pc
13165	hsa-mir-19b-1	LRRCC1	0.305437242	mirna_pc
13166	hsa-mir-19b-1	PHOSPHO2	0.300598241	mirna_pc
13167	hsa-mir-19b-1	ASNSD1	0.309931655	mirna_pc
13168	hsa-mir-19b-1	PPP5C	0.31034875	mirna_pc
13169	hsa-mir-19b-1	NEK3	0.309047746	mirna_pc
13170	hsa-mir-19b-1	SF1	0.309484876	mirna_pc
13171	hsa-mir-19b-1	RRP1B	0.331306863	mirna_pc
13172	hsa-mir-19b-1	VIL1	0.310765629	mirna_pc
13173	hsa-mir-19b-1	TMEM170A	0.472029709	mirna_pc
13174	hsa-mir-19b-1	FAM48A	0.401133372	mirna_pc
13175	hsa-mir-19b-1	PMS1	0.378950411	mirna_pc

13176	hsa-mir-19b-1	CHCHD3	0.307104076	mirna_pc
13177	hsa-mir-19b-1	CCDC122	0.313331629	mirna_pc
13178	hsa-mir-19b-1	TRUB1	0.353731332	mirna_pc
13179	hsa-mir-19b-1	ZMYM2	0.359533107	mirna_pc
13180	hsa-mir-19b-1	UGT8	0.317837984	mirna_pc
13181	hsa-mir-19b-1	CCNJ	0.476609696	mirna_pc
13182	hsa-mir-19b-1	ABCC4	0.350374833	mirna_pc
13183	hsa-mir-19b-1	ESF1	0.304203691	mirna_pc
13184	hsa-mir-19b-1	SMARCC1	0.367138842	mirna_pc
13185	hsa-mir-19b-1	CTPS2	0.385581647	mirna_pc
13186	hsa-mir-19b-1	XPO4	0.541693014	mirna_pc
13187	hsa-mir-19b-1	TSEN2	0.310158648	mirna_pc
13188	hsa-mir-19b-1	MDN1	0.309142462	mirna_pc
13189	hsa-mir-19b-1	ZBTB39	0.309400721	mirna_pc
13190	hsa-mir-19b-1	IFT88	0.411311376	mirna_pc
13191	hsa-mir-19b-1	TP53RK	0.354145217	mirna_pc
13192	hsa-mir-19b-1	QRSL1	0.400301445	mirna_pc
13193	hsa-mir-19b-1	FANCF	0.380863697	mirna_pc
13194	hsa-mir-19b-1	MARS2	0.385196709	mirna_pc
13195	hsa-mir-19b-1	ZBTB24	0.362404344	mirna_pc
13196	hsa-mir-19b-1	ZNF551	0.340132831	mirna_pc
13197	hsa-mir-19b-1	CIAO1	0.336760745	mirna_pc
13198	hsa-mir-19b-1	C2orf3	0.33903232	mirna_pc
13199	hsa-mir-19b-1	FAM119A	0.333963518	mirna_pc
13200	hsa-mir-19b-1	RRP7B	0.306030438	mirna_pc
13201	hsa-mir-19b-1	VPS36	0.317754391	mirna_pc
13202	hsa-mir-19b-1	GOLT1A	0.335018068	mirna_pc
13203	hsa-mir-19b-1	CAPN10	0.315799791	mirna_pc
13204	hsa-mir-19b-1	GALNT5	0.310966203	mirna_pc
13205	hsa-mir-19b-1	STK35	0.3062779	mirna_pc
13206	hsa-mir-19b-1	SCML2	0.31476852	mirna_pc
13207	hsa-mir-19b-1	MRPL19	0.374835771	mirna_pc
13208	hsa-mir-19b-1	ABCB10	0.331281045	mirna_pc
13209	hsa-mir-19b-1	CLCN4	0.389585843	mirna_pc
13210	hsa-mir-19b-1	ZCCHC3	0.418790965	mirna_pc
13211	hsa-mir-19b-1	RRP15	0.395125553	mirna_pc
13212	hsa-mir-19b-1	IARS	0.30514013	mirna_pc
13213	hsa-mir-19b-1	AADAT	0.346816687	mirna_pc
13214	hsa-mir-19b-1	DACH1	0.311048298	mirna_pc
13215	hsa-mir-19b-1	NOM1	0.335499839	mirna_pc
13216	hsa-mir-19b-1	GTF3A	0.342449253	mirna_pc
13217	hsa-mir-19b-1	ZNF48	0.346339149	mirna_pc
13218	hsa-mir-19b-1	LOC100129637	0.343531277	mirna_pc
13219	hsa-mir-19b-1	AIMP1	0.33175056	mirna_pc
13220	hsa-mir-19b-1	TPP2	0.421946374	mirna_pc
13221	hsa-mir-19b-1	TXLNG	0.437081293	mirna_pc
13222	hsa-mir-19b-1	TRIM25	0.413858064	mirna_pc
13223	hsa-mir-19b-1	MIR17HG	0.583844301	mirna_pc
13224	hsa-mir-19b-1	AARS	0.35463616	mirna_pc
13225	hsa-mir-19b-1	PRDX3	0.311544753	mirna_pc
13226	hsa-mir-19b-1	ZNF239	0.33927797	mirna_pc
13227	hsa-mir-19b-1	KLHL23	0.430913127	mirna_pc
13228	hsa-mir-19b-1	ZNF202	0.30730944	mirna_pc
13229	hsa-mir-19b-1	CANX	0.326243135	mirna_pc

13230	hsa-mir-19b-1	CENPV	0.31133671	mirna_pc
13231	hsa-mir-19b-1	SFRS6	0.327594453	mirna_pc
13232	hsa-mir-19b-1	SLC4A5	0.336902046	mirna_pc
13233	hsa-mir-19b-1	DNMT3A	0.340217416	mirna_pc
13234	hsa-mir-19b-1	MIPEP	0.378202179	mirna_pc
13235	hsa-mir-19b-1	ZNF670	0.308165795	mirna_pc
13236	hsa-mir-19b-1	ZNF343	0.301486212	mirna_pc
13237	hsa-mir-19b-1	ZNF711	0.325937589	mirna_pc
13238	hsa-mir-19b-1	POM121	0.347524521	mirna_pc
13239	hsa-mir-324	TPX2	0.358250098	mirna_pc
13240	hsa-mir-324	KIF4B	0.314174082	mirna_pc
13241	hsa-mir-324	KPNA2	0.30084	mirna_pc
13242	hsa-mir-324	CENPF	0.341614682	mirna_pc
13243	hsa-mir-324	RCC2	0.406927274	mirna_pc
13244	hsa-mir-324	HOXC9	0.393371443	mirna_pc
13245	hsa-mir-324	KIF11	0.362716469	mirna_pc
13246	hsa-mir-324	RRM2	0.336329087	mirna_pc
13247	hsa-mir-324	CDK1	0.314421588	mirna_pc
13248	hsa-mir-324	CDC25C	0.345308575	mirna_pc
13249	hsa-mir-324	UBE2C	0.403218952	mirna_pc
13250	hsa-mir-324	NUSAP1	0.41110009	mirna_pc
13251	hsa-mir-324	MCM4	0.447405372	mirna_pc
13252	hsa-mir-324	BUB1B	0.408321046	mirna_pc
13253	hsa-mir-324	KIF18B	0.318398346	mirna_pc
13254	hsa-mir-324	ASPM	0.300685146	mirna_pc
13255	hsa-mir-324	KIF2C	0.427379757	mirna_pc
13256	hsa-mir-324	FANCA	0.336142238	mirna_pc
13257	hsa-mir-324	NCAPG	0.482223698	mirna_pc
13258	hsa-mir-324	CDC20	0.326836507	mirna_pc
13259	hsa-mir-324	CLSPN	0.365220208	mirna_pc
13260	hsa-mir-324	CCNA2	0.491496401	mirna_pc
13261	hsa-mir-324	SPC25	0.314301599	mirna_pc
13262	hsa-mir-324	FANCI	0.35827464	mirna_pc
13263	hsa-mir-324	NEK2	0.363907602	mirna_pc
13264	hsa-mir-324	MND1	0.451048794	mirna_pc
13265	hsa-mir-324	KIF22	0.347536488	mirna_pc
13266	hsa-mir-324	NCAPH	0.438163344	mirna_pc
13267	hsa-mir-324	RAD54L	0.409092676	mirna_pc
13268	hsa-mir-324	CENPA	0.413966785	mirna_pc
13269	hsa-mir-324	MAD2L1	0.504295989	mirna_pc
13270	hsa-mir-324	TIMELESS	0.490614807	mirna_pc
13271	hsa-mir-324	LMNB1	0.451821466	mirna_pc
13272	hsa-mir-324	DLGAP5	0.31232143	mirna_pc
13273	hsa-mir-324	CDCA8	0.321100826	mirna_pc
13274	hsa-mir-324	TROAP	0.485168018	mirna_pc
13275	hsa-mir-324	CDCA5	0.392880619	mirna_pc
13276	hsa-mir-324	XPO1	0.347725856	mirna_pc
13277	hsa-mir-324	CDC45	0.367989146	mirna_pc
13278	hsa-mir-324	STMN1	0.458070395	mirna_pc
13279	hsa-mir-324	EXO1	0.311536486	mirna_pc
13280	hsa-mir-324	DKC1	0.383438654	mirna_pc
13281	hsa-mir-324	UBE2T	0.322016351	mirna_pc
13282	hsa-mir-324	CKS1B	0.418625394	mirna_pc
13283	hsa-mir-324	TUBB	0.399921085	mirna_pc

13284	hsa-mir-324	MCM2	0.411348995	mirna_pc
13285	hsa-mir-324	KIF4A	0.332859297	mirna_pc
13286	hsa-mir-324	ORC1L	0.392169495	mirna_pc
13287	hsa-mir-324	SNRPB	0.324974287	mirna_pc
13288	hsa-mir-324	KNTC1	0.387520019	mirna_pc
13289	hsa-mir-324	UHRF1	0.335700091	mirna_pc
13290	hsa-mir-324	RACGAP1	0.313897594	mirna_pc
13291	hsa-mir-324	CCNB2	0.358411693	mirna_pc
13292	hsa-mir-324	NUF2	0.433794534	mirna_pc
13293	hsa-mir-324	PLK4	0.46539046	mirna_pc
13294	hsa-mir-324	FANCB	0.300208267	mirna_pc
13295	hsa-mir-324	KIF18A	0.316664435	mirna_pc
13296	hsa-mir-324	DEPDC1	0.368677333	mirna_pc
13297	hsa-mir-324	HJURP	0.377728837	mirna_pc
13298	hsa-mir-324	SKA1	0.371878783	mirna_pc
13299	hsa-mir-324	BLM	0.32098806	mirna_pc
13300	hsa-mir-324	ESPL1	0.30279245	mirna_pc
13301	hsa-mir-324	DNMT1	0.319881136	mirna_pc
13302	hsa-mir-324	PAICS	0.480749331	mirna_pc
13303	hsa-mir-324	BIRC5	0.438869777	mirna_pc
13304	hsa-mir-324	RCC1	0.326511722	mirna_pc
13305	hsa-mir-324	LIG1	0.316828867	mirna_pc
13306	hsa-mir-324	OIP5	0.301046348	mirna_pc
13307	hsa-mir-324	DTL	0.43160607	mirna_pc
13308	hsa-mir-324	EME1	0.365385681	mirna_pc
13309	hsa-mir-324	RECQL4	0.463007789	mirna_pc
13310	hsa-mir-324	AURKB	0.549302393	mirna_pc
13311	hsa-mir-324	GSG2	0.395202669	mirna_pc
13312	hsa-mir-324	CDT1	0.303150211	mirna_pc
13313	hsa-mir-324	MCM10	0.391001561	mirna_pc
13314	hsa-mir-324	KIF20A	0.405543034	mirna_pc
13315	hsa-mir-324	CHEK2	0.460112118	mirna_pc
13316	hsa-mir-324	CENPE	0.357335068	mirna_pc
13317	hsa-mir-324	KIF15	0.308347836	mirna_pc
13318	hsa-mir-324	CDCA3	0.350633991	mirna_pc
13319	hsa-mir-324	C1orf112	0.404863054	mirna_pc
13320	hsa-mir-324	GINS1	0.425073101	mirna_pc
13321	hsa-mir-324	FEN1	0.324237773	mirna_pc
13322	hsa-mir-324	ORC6L	0.408983281	mirna_pc
13323	hsa-mir-324	ERH	0.376056107	mirna_pc
13324	hsa-mir-324	C16orf59	0.436039736	mirna_pc
13325	hsa-mir-324	C17orf53	0.319115069	mirna_pc
13326	hsa-mir-324	RAE1	0.380394051	mirna_pc
13327	hsa-mir-324	NEIL3	0.489889291	mirna_pc
13328	hsa-mir-324	ACTL6A	0.464662501	mirna_pc
13329	hsa-mir-324	POLD1	0.420859552	mirna_pc
13330	hsa-mir-324	ZNF229	0.311311907	mirna_pc
13331	hsa-mir-324	C12orf48	0.358556682	mirna_pc
13332	hsa-mir-324	HNRNPL	0.441641268	mirna_pc
13333	hsa-mir-324	SMC4	0.331365637	mirna_pc
13334	hsa-mir-324	TTK	0.379722174	mirna_pc
13335	hsa-mir-324	TUBA1B	0.40508067	mirna_pc
13336	hsa-mir-324	RUVBL1	0.315802859	mirna_pc
13337	hsa-mir-324	HELLS	0.330024747	mirna_pc

13338	hsa-mir-324	FAHD2B	0.32198001	mirna_pc
13339	hsa-mir-324	POLE2	0.336884384	mirna_pc
13340	hsa-mir-324	UBE2S	0.388570077	mirna_pc
13341	hsa-mir-324	NCAPD2	0.352995222	mirna_pc
13342	hsa-mir-324	C11orf82	0.35866305	mirna_pc
13343	hsa-mir-324	HNRNPC	0.466177404	mirna_pc
13344	hsa-mir-324	ILF2	0.411572876	mirna_pc
13345	hsa-mir-324	C6orf167	0.325613869	mirna_pc
13346	hsa-mir-324	CHEK1	0.368193035	mirna_pc
13347	hsa-mir-324	MCM5	0.336291634	mirna_pc
13348	hsa-mir-324	HNRNPA2B1	0.310786008	mirna_pc
13349	hsa-mir-324	RAN	0.33783311	mirna_pc
13350	hsa-mir-324	TYMS	0.397516554	mirna_pc
13351	hsa-mir-324	RFWD3	0.324970932	mirna_pc
13352	hsa-mir-324	MCM6	0.364621421	mirna_pc
13353	hsa-mir-324	RNASEH2A	0.33990425	mirna_pc
13354	hsa-mir-324	EPR1	0.388250522	mirna_pc
13355	hsa-mir-324	BRIP1	0.329919683	mirna_pc
13356	hsa-mir-324	SFRS2	0.33354931	mirna_pc
13357	hsa-mir-324	DCAF13	0.334841887	mirna_pc
13358	hsa-mir-324	UCK2	0.486046437	mirna_pc
13359	hsa-mir-324	WDHD1	0.355595382	mirna_pc
13360	hsa-mir-324	THOC4	0.305351266	mirna_pc
13361	hsa-mir-324	NFKBIL2	0.455913612	mirna_pc
13362	hsa-mir-324	C15orf42	0.365076863	mirna_pc
13363	hsa-mir-324	PTGES3	0.321489766	mirna_pc
13364	hsa-mir-324	PRIM2	0.416367764	mirna_pc
13365	hsa-mir-324	FAM60A	0.308210231	mirna_pc
13366	hsa-mir-324	HMGB3	0.395825815	mirna_pc
13367	hsa-mir-324	FAM64A	0.507607556	mirna_pc
13368	hsa-mir-324	RANBP1	0.372076431	mirna_pc
13369	hsa-mir-324	MTBP	0.340490479	mirna_pc
13370	hsa-mir-324	E2F1	0.305959879	mirna_pc
13371	hsa-mir-324	EIF4A3	0.380760375	mirna_pc
13372	hsa-mir-324	SNRPA	0.427244599	mirna_pc
13373	hsa-mir-324	FUS	0.45043752	mirna_pc
13374	hsa-mir-324	DNAJC9	0.348986824	mirna_pc
13375	hsa-mir-324	U2AF2	0.382120223	mirna_pc
13376	hsa-mir-324	CHAF1A	0.339162136	mirna_pc
13377	hsa-mir-324	GINS4	0.335222976	mirna_pc
13378	hsa-mir-324	ANP32E	0.408956681	mirna_pc
13379	hsa-mir-324	PSMD11	0.330503271	mirna_pc
13380	hsa-mir-324	CENPO	0.421404368	mirna_pc
13381	hsa-mir-324	C19orf48	0.373881263	mirna_pc
13382	hsa-mir-324	CENPL	0.322144693	mirna_pc
13383	hsa-mir-324	NUDT1	0.300914865	mirna_pc
13384	hsa-mir-324	HMGB2	0.459377944	mirna_pc
13385	hsa-mir-324	GINS2	0.403176646	mirna_pc
13386	hsa-mir-324	DNA2	0.304138898	mirna_pc
13387	hsa-mir-324	NOP56	0.309346494	mirna_pc
13388	hsa-mir-324	CDCA4	0.33016045	mirna_pc
13389	hsa-mir-324	CSE1L	0.396632655	mirna_pc
13390	hsa-mir-324	TMEM206	0.314374558	mirna_pc
13391	hsa-mir-324	E2F7	0.355028473	mirna_pc

13392	hsa-mir-324	PRMT1	0.374598223	mirna_pc
13393	hsa-mir-324	MLF1IP	0.349318932	mirna_pc
13394	hsa-mir-324	C16orf75	0.330543366	mirna_pc
13395	hsa-mir-324	NOP2	0.337679261	mirna_pc
13396	hsa-mir-324	HIST1H2AE	0.303427458	mirna_pc
13397	hsa-mir-324	CAD	0.449350398	mirna_pc
13398	hsa-mir-324	ZFP64	0.399203812	mirna_pc
13399	hsa-mir-324	CCDC99	0.323180196	mirna_pc
13400	hsa-mir-324	CDK2	0.466930938	mirna_pc
13401	hsa-mir-324	CDK4	0.44945246	mirna_pc
13402	hsa-mir-324	PFDN2	0.35557995	mirna_pc
13403	hsa-mir-324	CHTF18	0.328599076	mirna_pc
13404	hsa-mir-324	HNRNPR	0.488203047	mirna_pc
13405	hsa-mir-324	PRIM1	0.426310303	mirna_pc
13406	hsa-mir-324	KAT2A	0.332690298	mirna_pc
13407	hsa-mir-324	CENPH	0.356641337	mirna_pc
13408	hsa-mir-324	TCOF1	0.351523274	mirna_pc
13409	hsa-mir-324	CHAF1B	0.43582826	mirna_pc
13410	hsa-mir-324	ACYP1	0.312336395	mirna_pc
13411	hsa-mir-324	MSH2	0.52899194	mirna_pc
13412	hsa-mir-324	DBF4	0.357422991	mirna_pc
13413	hsa-mir-324	TOMM40	0.324281656	mirna_pc
13414	hsa-mir-324	SF3B3	0.428244427	mirna_pc
13415	hsa-mir-324	CDC7	0.468845447	mirna_pc
13416	hsa-mir-324	YDJC	0.361897892	mirna_pc
13417	hsa-mir-324	NOL11	0.37456987	mirna_pc
13418	hsa-mir-324	SNRPC	0.311475316	mirna_pc
13419	hsa-mir-324	RFC4	0.490988591	mirna_pc
13420	hsa-mir-324	FBXO5	0.40211425	mirna_pc
13421	hsa-mir-324	DCLRE1B	0.300543542	mirna_pc
13422	hsa-mir-324	FIGNL1	0.356979672	mirna_pc
13423	hsa-mir-324	BRIX1	0.397792352	mirna_pc
13424	hsa-mir-324	E2F3	0.34964547	mirna_pc
13425	hsa-mir-324	SF3B14	0.346349039	mirna_pc
13426	hsa-mir-324	RUVBL2	0.307292986	mirna_pc
13427	hsa-mir-324	CDC25A	0.361915304	mirna_pc
13428	hsa-mir-324	RAD54B	0.375073834	mirna_pc
13429	hsa-mir-324	OTX1	0.346563824	mirna_pc
13430	hsa-mir-324	PRKDC	0.353621354	mirna_pc
13431	hsa-mir-324	H2AFZ	0.451172256	mirna_pc
13432	hsa-mir-324	NRM	0.305038055	mirna_pc
13433	hsa-mir-324	MAGOH	0.314297881	mirna_pc
13434	hsa-mir-324	DDX12	0.390313269	mirna_pc
13435	hsa-mir-324	NUP37	0.320727521	mirna_pc
13436	hsa-mir-324	CPSF3	0.445210803	mirna_pc
13437	hsa-mir-324	BANF1	0.417730684	mirna_pc
13438	hsa-mir-324	SNRPE	0.395219626	mirna_pc
13439	hsa-mir-324	C21orf45	0.501442553	mirna_pc
13440	hsa-mir-324	PPM1G	0.329243827	mirna_pc
13441	hsa-mir-324	SNRPD2	0.33334836	mirna_pc
13442	hsa-mir-324	NPM3	0.344994942	mirna_pc
13443	hsa-mir-324	TFAP4	0.419255157	mirna_pc
13444	hsa-mir-324	RNPS1	0.342185251	mirna_pc
13445	hsa-mir-324	DAZAP1	0.397481712	mirna_pc

13446	hsa-mir-324	CBX8	0.429622157	mirna_pc
13447	hsa-mir-324	TRMT6	0.325702342	mirna_pc
13448	hsa-mir-324	TMEM201	0.479970627	mirna_pc
13449	hsa-mir-324	NCL	0.34404036	mirna_pc
13450	hsa-mir-324	PAFAH1B3	0.420823861	mirna_pc
13451	hsa-mir-324	CACYBP	0.341751119	mirna_pc
13452	hsa-mir-324	SASS6	0.302514003	mirna_pc
13453	hsa-mir-324	PSRC1	0.395343493	mirna_pc
13454	hsa-mir-324	DGUOK	0.36237172	mirna_pc
13455	hsa-mir-324	MRPL47	0.392227483	mirna_pc
13456	hsa-mir-324	FBL	0.353004884	mirna_pc
13457	hsa-mir-324	DONSON	0.362526744	mirna_pc
13458	hsa-mir-324	STIP1	0.312870614	mirna_pc
13459	hsa-mir-324	HIST1H1E	0.323480225	mirna_pc
13460	hsa-mir-324	PTK7	0.306309516	mirna_pc
13461	hsa-mir-324	EXOSC5	0.370465648	mirna_pc
13462	hsa-mir-324	HNRNPM	0.369542118	mirna_pc
13463	hsa-mir-324	TMPO	0.323287533	mirna_pc
13464	hsa-mir-324	CCT6A	0.329942982	mirna_pc
13465	hsa-mir-324	CDC123	0.304885351	mirna_pc
13466	hsa-mir-324	SET	0.354029358	mirna_pc
13467	hsa-mir-324	POLR2H	0.353735337	mirna_pc
13468	hsa-mir-324	C20orf72	0.304779119	mirna_pc
13469	hsa-mir-324	C3orf26	0.3543403	mirna_pc
13470	hsa-mir-324	MRTO4	0.347425979	mirna_pc
13471	hsa-mir-324	GPR19	0.330482597	mirna_pc
13472	hsa-mir-324	NUP93	0.318659733	mirna_pc
13473	hsa-mir-324	LSG1	0.391822142	mirna_pc
13474	hsa-mir-324	PPIH	0.334014543	mirna_pc
13475	hsa-mir-324	THOC6	0.332866494	mirna_pc
13476	hsa-mir-324	GMPS	0.368266478	mirna_pc
13477	hsa-mir-324	LAPTM4B	0.301547971	mirna_pc
13478	hsa-mir-324	PPAT	0.44400955	mirna_pc
13479	hsa-mir-324	DHX9	0.378078497	mirna_pc
13480	hsa-mir-324	CCDC138	0.415050286	mirna_pc
13481	hsa-mir-324	LOC100128191	0.48100997	mirna_pc
13482	hsa-mir-324	MRPL3	0.338363191	mirna_pc
13483	hsa-mir-324	EIF5A	0.408849397	mirna_pc
13484	hsa-mir-324	HMGN2	0.305066402	mirna_pc
13485	hsa-mir-324	MORC2	0.305549921	mirna_pc
13486	hsa-mir-324	SMYD5	0.382543859	mirna_pc
13487	hsa-mir-324	TSSC1	0.333581431	mirna_pc
13488	hsa-mir-324	C20orf3	0.386603349	mirna_pc
13489	hsa-mir-324	PFN1	0.312156788	mirna_pc
13490	hsa-mir-324	POLA2	0.361972603	mirna_pc
13491	hsa-mir-324	CCDC77	0.324152104	mirna_pc
13492	hsa-mir-324	PHF6	0.307461822	mirna_pc
13493	hsa-mir-324	SUMO2	0.370467263	mirna_pc
13494	hsa-mir-324	SFRS1	0.331354184	mirna_pc
13495	hsa-mir-324	CDH24	0.412732128	mirna_pc
13496	hsa-mir-324	TEX10	0.349782957	mirna_pc
13497	hsa-mir-324	WDR43	0.376826532	mirna_pc
13498	hsa-mir-324	SNRPA1	0.322086364	mirna_pc
13499	hsa-mir-324	SKP2	0.495859019	mirna_pc

13500	hsa-mir-324	TIPIN	0.414184545	mirna_pc
13501	hsa-mir-324	SMARCD1	0.469287998	mirna_pc
13502	hsa-mir-324	ATXN2L	0.304224258	mirna_pc
13503	hsa-mir-324	HSPBP1	0.314237218	mirna_pc
13504	hsa-mir-324	C4orf46	0.41019975	mirna_pc
13505	hsa-mir-324	ECE2	0.385942233	mirna_pc
13506	hsa-mir-324	GPN1	0.3903683	mirna_pc
13507	hsa-mir-324	RFC5	0.4197726	mirna_pc
13508	hsa-mir-324	MAZ	0.322176213	mirna_pc
13509	hsa-mir-324	SSRP1	0.379906329	mirna_pc
13510	hsa-mir-324	TIGD1	0.314982913	mirna_pc
13511	hsa-mir-324	SLBP	0.30418825	mirna_pc
13512	hsa-mir-324	PA2G4	0.369662421	mirna_pc
13513	hsa-mir-324	SNRPD3	0.413445671	mirna_pc
13514	hsa-mir-324	KHDRBS1	0.453874816	mirna_pc
13515	hsa-mir-324	C20orf27	0.395654458	mirna_pc
13516	hsa-mir-324	YEATS2	0.339667299	mirna_pc
13517	hsa-mir-324	SPAST	0.346764784	mirna_pc
13518	hsa-mir-324	LOC92659	0.329707062	mirna_pc
13519	hsa-mir-324	CCDC21	0.38203783	mirna_pc
13520	hsa-mir-324	MTL 5.00	0.329732876	mirna_pc
13521	hsa-mir-324	PHF5A	0.311878037	mirna_pc
13522	hsa-mir-324	PARP1	0.370848135	mirna_pc
13523	hsa-mir-324	CCT7	0.357491283	mirna_pc
13524	hsa-mir-324	MTERFD1	0.359934754	mirna_pc
13525	hsa-mir-324	C4orf21	0.310245544	mirna_pc
13526	hsa-mir-324	TMEM194A	0.382550526	mirna_pc
13527	hsa-mir-324	TDP1	0.332069048	mirna_pc
13528	hsa-mir-324	MED30	0.369460733	mirna_pc
13529	hsa-mir-324	METTL1	0.305134442	mirna_pc
13530	hsa-mir-324	TMEM69	0.344981855	mirna_pc
13531	hsa-mir-324	GINS3	0.323808544	mirna_pc
13532	hsa-mir-324	NEDD1	0.346670214	mirna_pc
13533	hsa-mir-324	JUB	0.307511457	mirna_pc
13534	hsa-mir-324	GNL2	0.3337458	mirna_pc
13535	hsa-mir-324	WRAP53	0.512367422	mirna_pc
13536	hsa-mir-324	RAD21	0.329419917	mirna_pc
13537	hsa-mir-324	HNRNPU	0.309191538	mirna_pc
13538	hsa-mir-324	NASP	0.408449032	mirna_pc
13539	hsa-mir-324	RPP40	0.379166061	mirna_pc
13540	hsa-mir-324	CCDC86	0.342205286	mirna_pc
13541	hsa-mir-324	ZNF280C	0.33370712	mirna_pc
13542	hsa-mir-324	PTMA	0.33971561	mirna_pc
13543	hsa-mir-324	USP39	0.326336312	mirna_pc
13544	hsa-mir-324	WDR76	0.39467876	mirna_pc
13545	hsa-mir-324	EXOSC2	0.355975459	mirna_pc
13546	hsa-mir-324	C11orf84	0.454115381	mirna_pc
13547	hsa-mir-324	TOP1MT	0.374590799	mirna_pc
13548	hsa-mir-324	ARHGAP39	0.301982998	mirna_pc
13549	hsa-mir-324	TBCE	0.425854274	mirna_pc
13550	hsa-mir-324	LRRC8D	0.370983117	mirna_pc
13551	hsa-mir-324	NCBP2	0.396749551	mirna_pc
13552	hsa-mir-324	SUV39H2	0.373250271	mirna_pc
13553	hsa-mir-324	ZC3H8	0.327451674	mirna_pc

13554	hsa-mir-324	FANCL	0.300520588	mirna_pc
13555	hsa-mir-324	HNRNPD	0.492918313	mirna_pc
13556	hsa-mir-324	DPY30	0.397355648	mirna_pc
13557	hsa-mir-324	MTHFD2	0.353031215	mirna_pc
13558	hsa-mir-324	EIF5AL1	0.388312158	mirna_pc
13559	hsa-mir-324	SFRS3	0.303115426	mirna_pc
13560	hsa-mir-324	MEN1	0.40502915	mirna_pc
13561	hsa-mir-324	C17orf49	0.380329874	mirna_pc
13562	hsa-mir-324	H1FX	0.326855782	mirna_pc
13563	hsa-mir-324	PNO1	0.313759011	mirna_pc
13564	hsa-mir-324	CCT4	0.41295932	mirna_pc
13565	hsa-mir-324	RAD9A	0.311210765	mirna_pc
13566	hsa-mir-324	ELAVL1	0.40160835	mirna_pc
13567	hsa-mir-324	ABCE1	0.319598577	mirna_pc
13568	hsa-mir-324	DUSP9	0.445599003	mirna_pc
13569	hsa-mir-324	MTHFD1	0.30420546	mirna_pc
13570	hsa-mir-324	PRMT5	0.349413104	mirna_pc
13571	hsa-mir-324	MIF	0.303334983	mirna_pc
13572	hsa-mir-324	EIF4A1	0.44833676	mirna_pc
13573	hsa-mir-324	NCAPD3	0.381177454	mirna_pc
13574	hsa-mir-324	NAE1	0.36611437	mirna_pc
13575	hsa-mir-324	USP22	0.319894646	mirna_pc
13576	hsa-mir-324	PSMC3IP	0.371392351	mirna_pc
13577	hsa-mir-324	TBC1D7	0.366811863	mirna_pc
13578	hsa-mir-324	PAK1IP1	0.497038503	mirna_pc
13579	hsa-mir-324	RPAP3	0.355930488	mirna_pc
13580	hsa-mir-324	SUPT16H	0.373967724	mirna_pc
13581	hsa-mir-324	TRMT112	0.36083915	mirna_pc
13582	hsa-mir-324	KPTN	0.311782848	mirna_pc
13583	hsa-mir-324	PSMD2	0.369066932	mirna_pc
13584	hsa-mir-324	PGAM5	0.306652456	mirna_pc
13585	hsa-mir-324	PMAIP1	0.307825112	mirna_pc
13586	hsa-mir-324	YWHAE	0.564236593	mirna_pc
13587	hsa-mir-324	KDM1A	0.349343511	mirna_pc
13588	hsa-mir-324	PDCL3	0.302543984	mirna_pc
13589	hsa-mir-324	ILF3	0.361505382	mirna_pc
13590	hsa-mir-324	NT5DC2	0.322534441	mirna_pc
13591	hsa-mir-324	EHMT2	0.364125313	mirna_pc
13592	hsa-mir-324	NUP155	0.303004992	mirna_pc
13593	hsa-mir-324	UBAP2L	0.425596596	mirna_pc
13594	hsa-mir-324	PUF60	0.364085519	mirna_pc
13595	hsa-mir-324	DEK	0.404956163	mirna_pc
13596	hsa-mir-324	SFRS9	0.328604528	mirna_pc
13597	hsa-mir-324	IPO4	0.322231774	mirna_pc
13598	hsa-mir-324	SIX1	0.369190318	mirna_pc
13599	hsa-mir-324	SAAL1	0.308274305	mirna_pc
13600	hsa-mir-324	ADORA2B	0.328128697	mirna_pc
13601	hsa-mir-324	GTF2H4	0.320306518	mirna_pc
13602	hsa-mir-324	TGS1	0.30981222	mirna_pc
13603	hsa-mir-324	KHSRP	0.335194253	mirna_pc
13604	hsa-mir-324	LSM 6.00	0.375532507	mirna_pc
13605	hsa-mir-324	PRAME	0.55501345	mirna_pc
13606	hsa-mir-324	MRPS17	0.351202606	mirna_pc
13607	hsa-mir-324	CBX2	0.364149052	mirna_pc

13608	hsa-mir-324	AZI1	0.379923509	mirna_pc
13609	hsa-mir-324	TNFRSF18	0.365810481	mirna_pc
13610	hsa-mir-324	FUBP1	0.343797884	mirna_pc
13611	hsa-mir-324	WDR74	0.309434983	mirna_pc
13612	hsa-mir-324	IPO9	0.375836775	mirna_pc
13613	hsa-mir-324	NUP188	0.300079176	mirna_pc
13614	hsa-mir-324	SART3	0.32842675	mirna_pc
13615	hsa-mir-324	TRA2B	0.417412764	mirna_pc
13616	hsa-mir-324	RSRC1	0.387562128	mirna_pc
13617	hsa-mir-324	GAR1	0.467575047	mirna_pc
13618	hsa-mir-324	C15orf23	0.421018499	mirna_pc
13619	hsa-mir-324	CIRH1A	0.310076015	mirna_pc
13620	hsa-mir-324	C3orf21	0.363429028	mirna_pc
13621	hsa-mir-324	SLMO1	0.32988392	mirna_pc
13622	hsa-mir-324	PSMD12	0.306852182	mirna_pc
13623	hsa-mir-324	ZNF668	0.32098992	mirna_pc
13624	hsa-mir-324	DIABLO	0.341044634	mirna_pc
13625	hsa-mir-324	C1QBP	0.501917279	mirna_pc
13626	hsa-mir-324	SAE1	0.389886516	mirna_pc
13627	hsa-mir-324	ITGB3BP	0.322962901	mirna_pc
13628	hsa-mir-324	ENOPH1	0.38993245	mirna_pc
13629	hsa-mir-324	C19orf47	0.391521713	mirna_pc
13630	hsa-mir-324	C4orf43	0.436116375	mirna_pc
13631	hsa-mir-324	XRCC3	0.305838857	mirna_pc
13632	hsa-mir-324	YY1	0.304644381	mirna_pc
13633	hsa-mir-324	DDX55	0.315045815	mirna_pc
13634	hsa-mir-324	C16orf80	0.377652879	mirna_pc
13635	hsa-mir-324	MYBBP1A	0.401357853	mirna_pc
13636	hsa-mir-324	PLEKHG4	0.392475296	mirna_pc
13637	hsa-mir-324	SFPQ	0.438439658	mirna_pc
13638	hsa-mir-324	VPS72	0.322818572	mirna_pc
13639	hsa-mir-324	EIF4EBP1	0.347005575	mirna_pc
13640	hsa-mir-324	CADM1	0.415813902	mirna_pc
13641	hsa-mir-324	SHMT2	0.315879493	mirna_pc
13642	hsa-mir-324	POLA1	0.324967131	mirna_pc
13643	hsa-mir-324	HDAC2	0.36778382	mirna_pc
13644	hsa-mir-324	GMNN	0.364141213	mirna_pc
13645	hsa-mir-324	EMG1	0.30783578	mirna_pc
13646	hsa-mir-324	PA2G4P4	0.325885796	mirna_pc
13647	hsa-mir-324	CPNE2	0.363698499	mirna_pc
13648	hsa-mir-324	ALOX12P2	0.383911603	mirna_pc
13649	hsa-mir-324	ANKRD32	0.32276418	mirna_pc
13650	hsa-mir-324	CHRAC1	0.312625551	mirna_pc
13651	hsa-mir-324	PDAP1	0.33261437	mirna_pc
13652	hsa-mir-324	RNF7	0.390686743	mirna_pc
13653	hsa-mir-324	SALL2	0.352694652	mirna_pc
13654	hsa-mir-324	DCK	0.310820693	mirna_pc
13655	hsa-mir-324	GEMIN6	0.313896137	mirna_pc
13656	hsa-mir-324	RNMTL1	0.329791622	mirna_pc
13657	hsa-mir-324	FAM86C	0.318155552	mirna_pc
13658	hsa-mir-324	ANKRD39	0.347423263	mirna_pc
13659	hsa-mir-324	PTCD1	0.362440634	mirna_pc
13660	hsa-mir-324	CRIPT	0.415992226	mirna_pc
13661	hsa-mir-324	MSH6	0.527628749	mirna_pc

13662	hsa-mir-324	SOX4	0.322402479	mirna_pc
13663	hsa-mir-324	XRN2	0.311071046	mirna_pc
13664	hsa-mir-324	C3orf34	0.370796736	mirna_pc
13665	hsa-mir-324	TPRKB	0.319848537	mirna_pc
13666	hsa-mir-324	TP73	0.338854364	mirna_pc
13667	hsa-mir-324	C17orf100	0.30235379	mirna_pc
13668	hsa-mir-324	KPNB1	0.349182415	mirna_pc
13669	hsa-mir-324	XRCC6	0.311851044	mirna_pc
13670	hsa-mir-324	LEF1	0.362673075	mirna_pc
13671	hsa-mir-324	SMC3	0.305177563	mirna_pc
13672	hsa-mir-324	POLB	0.318059194	mirna_pc
13673	hsa-mir-324	ACBD6	0.365301696	mirna_pc
13674	hsa-mir-324	HNRNPA1	0.371246055	mirna_pc
13675	hsa-mir-324	PPP1R8	0.395139192	mirna_pc
13676	hsa-mir-324	CDK2AP1	0.370599223	mirna_pc
13677	hsa-mir-324	ANKRD13B	0.311054695	mirna_pc
13678	hsa-mir-324	NUDT3	0.386094271	mirna_pc
13679	hsa-mir-324	SMARCA1	0.310379647	mirna_pc
13680	hsa-mir-324	MEX3A	0.49349182	mirna_pc
13681	hsa-mir-324	COIL	0.30777397	mirna_pc
13682	hsa-mir-324	RFC2	0.38255546	mirna_pc
13683	hsa-mir-324	TAF5	0.351415093	mirna_pc
13684	hsa-mir-324	TRIM28	0.372022341	mirna_pc
13685	hsa-mir-324	EXOSC4	0.337259389	mirna_pc
13686	hsa-mir-324	SCRIB	0.308439706	mirna_pc
13687	hsa-mir-324	APEX1	0.380334781	mirna_pc
13688	hsa-mir-324	MAD2L2	0.327617559	mirna_pc
13689	hsa-mir-324	HNRNPA1L2	0.406772999	mirna_pc
13690	hsa-mir-324	NAA15	0.320388219	mirna_pc
13691	hsa-mir-324	CBX1	0.48513721	mirna_pc
13692	hsa-mir-324	EMILIN3	0.414194799	mirna_pc
13693	hsa-mir-324	ZBTB12	0.39333699	mirna_pc
13694	hsa-mir-324	TADA1	0.312024625	mirna_pc
13695	hsa-mir-324	DUSP12	0.331220522	mirna_pc
13696	hsa-mir-324	LOC642846	0.379006028	mirna_pc
13697	hsa-mir-324	TGIF2	0.355389136	mirna_pc
13698	hsa-mir-324	C16orf87	0.307940186	mirna_pc
13699	hsa-mir-324	SENP1	0.370840829	mirna_pc
13700	hsa-mir-324	ERI3	0.400641672	mirna_pc
13701	hsa-mir-324	RCN2	0.412659454	mirna_pc
13702	hsa-mir-324	THUMPD2	0.348680633	mirna_pc
13703	hsa-mir-324	TARBP2	0.39468917	mirna_pc
13704	hsa-mir-324	ZNF256	0.366378173	mirna_pc
13705	hsa-mir-324	NOL10	0.414472931	mirna_pc
13706	hsa-mir-324	DDX47	0.347180992	mirna_pc
13707	hsa-mir-324	POLE	0.378720877	mirna_pc
13708	hsa-mir-324	C8orf30A	0.326417042	mirna_pc
13709	hsa-mir-324	WRNIP1	0.415443508	mirna_pc
13710	hsa-mir-324	ERF	0.324474779	mirna_pc
13711	hsa-mir-324	TAF1B	0.358160662	mirna_pc
13712	hsa-mir-324	OGFOD1	0.31014665	mirna_pc
13713	hsa-mir-324	TIMM50	0.333913575	mirna_pc
13714	hsa-mir-324	XRCC6BP1	0.375284575	mirna_pc
13715	hsa-mir-324	ADSL	0.366902019	mirna_pc

13716	hsa-mir-324	VRK1	0.32508223	mirna_pc
13717	hsa-mir-324	RIOK1	0.38917811	mirna_pc
13718	hsa-mir-324	EXOSC9	0.544755346	mirna_pc
13719	hsa-mir-324	FOXRED2	0.367413871	mirna_pc
13720	hsa-mir-324	UNG	0.466960446	mirna_pc
13721	hsa-mir-324	RBMX	0.385565871	mirna_pc
13722	hsa-mir-324	CDK16	0.332442764	mirna_pc
13723	hsa-mir-324	ALKBH2	0.407954258	mirna_pc
13724	hsa-mir-324	USP10	0.312685491	mirna_pc
13725	hsa-mir-324	GPC3	0.351004879	mirna_pc
13726	hsa-mir-324	MLF 2.00	0.339364316	mirna_pc
13727	hsa-mir-324	XRCC5	0.313481496	mirna_pc
13728	hsa-mir-324	RPF2	0.317849822	mirna_pc
13729	hsa-mir-324	CTCF	0.336595077	mirna_pc
13730	hsa-mir-324	RPL39L	0.335709098	mirna_pc
13731	hsa-mir-324	ACD	0.300074108	mirna_pc
13732	hsa-mir-324	GPN3	0.412098821	mirna_pc
13733	hsa-mir-324	ASB3	0.363139037	mirna_pc
13734	hsa-mir-324	LOC152217	0.368064658	mirna_pc
13735	hsa-mir-324	MEMO1	0.344255178	mirna_pc
13736	hsa-mir-324	IGSF9	0.404644808	mirna_pc
13737	hsa-mir-324	UBXN2A	0.349636045	mirna_pc
13738	hsa-mir-324	DNAJC14	0.319994296	mirna_pc
13739	hsa-mir-324	FIP1L1	0.413819753	mirna_pc
13740	hsa-mir-324	KDM2B	0.357223943	mirna_pc
13741	hsa-mir-324	TRAP1	0.328882223	mirna_pc
13742	hsa-mir-324	NUDT21	0.332738318	mirna_pc
13743	hsa-mir-324	GTF3C2	0.310066288	mirna_pc
13744	hsa-mir-324	MAGEF1	0.464886507	mirna_pc
13745	hsa-mir-324	PCGF6	0.341266847	mirna_pc
13746	hsa-mir-324	SUPT3H	0.351949586	mirna_pc
13747	hsa-mir-324	SOX12	0.343229468	mirna_pc
13748	hsa-mir-324	MNAT1	0.311211145	mirna_pc
13749	hsa-mir-324	SMARCB1	0.427498464	mirna_pc
13750	hsa-mir-324	PYGO2	0.321515876	mirna_pc
13751	hsa-mir-324	CCT8	0.320847258	mirna_pc
13752	hsa-mir-324	TTC32	0.316755031	mirna_pc
13753	hsa-mir-324	SMN2	0.305635268	mirna_pc
13754	hsa-mir-324	U2AF1	0.339572608	mirna_pc
13755	hsa-mir-324	B4GALT2	0.315346361	mirna_pc
13756	hsa-mir-324	RPA1	0.502073109	mirna_pc
13757	hsa-mir-324	FAM92A1	0.379766876	mirna_pc
13758	hsa-mir-324	ZNF639	0.454642516	mirna_pc
13759	hsa-mir-324	TTC5	0.315935509	mirna_pc
13760	hsa-mir-324	SART1	0.304120993	mirna_pc
13761	hsa-mir-324	ITPRIPL1	0.356549896	mirna_pc
13762	hsa-mir-324	FOXK2	0.36547743	mirna_pc
13763	hsa-mir-324	ANAPC10	0.322225404	mirna_pc
13764	hsa-mir-324	ABT1	0.306616302	mirna_pc
13765	hsa-mir-324	TIGD5	0.336075585	mirna_pc
13766	hsa-mir-324	METAP1	0.339765016	mirna_pc
13767	hsa-mir-324	C20orf177	0.343412197	mirna_pc
13768	hsa-mir-324	SLC25A17	0.329658452	mirna_pc
13769	hsa-mir-324	UBXN7	0.329808672	mirna_pc

13770	hsa-mir-324	FXR2	0.447703227	mirna_pc
13771	hsa-mir-324	ATXN7L2	0.332200591	mirna_pc
13772	hsa-mir-324	FAM189B	0.357763241	mirna_pc
13773	hsa-mir-324	MKRN3	0.384323125	mirna_pc
13774	hsa-mir-324	PABPN1	0.320494525	mirna_pc
13775	hsa-mir-324	METAP2	0.378938069	mirna_pc
13776	hsa-mir-324	CLNS1A	0.359657195	mirna_pc
13777	hsa-mir-324	NEURL4	0.330198492	mirna_pc
13778	hsa-mir-324	DVL3	0.333838539	mirna_pc
13779	hsa-mir-324	BCL7A	0.475799243	mirna_pc
13780	hsa-mir-324	LAMA1	0.338655733	mirna_pc
13781	hsa-mir-324	MRPS28	0.367464297	mirna_pc
13782	hsa-mir-324	DVL2	0.578704847	mirna_pc
13783	hsa-mir-324	C7orf44	0.324122865	mirna_pc
13784	hsa-mir-324	NUDCD2	0.303830222	mirna_pc
13785	hsa-mir-324	GCAT	0.334318724	mirna_pc
13786	hsa-mir-324	WDR70	0.342214332	mirna_pc
13787	hsa-mir-324	ARHGAP19	0.363728661	mirna_pc
13788	hsa-mir-324	ZNF286A	0.468983134	mirna_pc
13789	hsa-mir-324	FAM171A2	0.380981646	mirna_pc
13790	hsa-mir-324	PARP2	0.311954032	mirna_pc
13791	hsa-mir-324	MRPL21	0.410844035	mirna_pc
13792	hsa-mir-324	TCEA1	0.348277534	mirna_pc
13793	hsa-mir-324	NLE1	0.414264759	mirna_pc
13794	hsa-mir-324	FXR1	0.392060279	mirna_pc
13795	hsa-mir-324	DMRTA2	0.355929102	mirna_pc
13796	hsa-mir-324	CHST10	0.309379566	mirna_pc
13797	hsa-mir-324	MRPS22	0.307940489	mirna_pc
13798	hsa-mir-324	ALS2CR4	0.304640863	mirna_pc
13799	hsa-mir-324	PSPH	0.446489047	mirna_pc
13800	hsa-mir-324	EVC2	0.302192331	mirna_pc
13801	hsa-mir-324	PRR3	0.397172479	mirna_pc
13802	hsa-mir-324	MGC72080	0.314652678	mirna_pc
13803	hsa-mir-324	CYB5D1	0.304735881	mirna_pc
13804	hsa-mir-324	CNTROB	0.526871733	mirna_pc
13805	hsa-mir-324	TRMT61B	0.388467747	mirna_pc
13806	hsa-mir-324	TSFM	0.314462906	mirna_pc
13807	hsa-mir-324	RAD1	0.337653221	mirna_pc
13808	hsa-mir-324	SEMA4F	0.412904611	mirna_pc
13809	hsa-mir-324	RNASEN	0.334662293	mirna_pc
13810	hsa-mir-324	MPV17	0.301513997	mirna_pc
13811	hsa-mir-324	ZNF286B	0.43900241	mirna_pc
13812	hsa-mir-324	DDX20	0.389443028	mirna_pc
13813	hsa-mir-324	C1orf109	0.338105609	mirna_pc
13814	hsa-mir-324	CHCHD2	0.31439795	mirna_pc
13815	hsa-mir-324	PIGX	0.365774139	mirna_pc
13816	hsa-mir-324	MTA1	0.386298515	mirna_pc
13817	hsa-mir-324	HTRA2	0.353351279	mirna_pc
13818	hsa-mir-324	MTA3	0.303641241	mirna_pc
13819	hsa-mir-324	DPH1	0.3597389	mirna_pc
13820	hsa-mir-324	PFN2	0.370902126	mirna_pc
13821	hsa-mir-324	INTU	0.388734305	mirna_pc
13822	hsa-mir-324	ATP6V1E2	0.373566382	mirna_pc
13823	hsa-mir-324	CCNB1IP1	0.433600031	mirna_pc

13824	hsa-mir-324	NHP2L1	0.305435899	mirna_pc
13825	hsa-mir-324	GPR3	0.360125009	mirna_pc
13826	hsa-mir-324	E2F6	0.377657724	mirna_pc
13827	hsa-mir-324	DRG1	0.351703805	mirna_pc
13828	hsa-mir-324	SSSCA1	0.302714627	mirna_pc
13829	hsa-mir-324	NOL7	0.382556163	mirna_pc
13830	hsa-mir-324	PRPSAP2	0.43758955	mirna_pc
13831	hsa-mir-324	SENP3	0.537867713	mirna_pc
13832	hsa-mir-324	CPSF4	0.34523197	mirna_pc
13833	hsa-mir-324	HUNK	0.321518056	mirna_pc
13834	hsa-mir-324	ACTR6	0.328767173	mirna_pc
13835	hsa-mir-324	SCLY	0.306084991	mirna_pc
13836	hsa-mir-324	RNF167	0.300475356	mirna_pc
13837	hsa-mir-324	NT5C	0.302833459	mirna_pc
13838	hsa-mir-324	STARD7	0.308667103	mirna_pc
13839	hsa-mir-324	GPR125	0.328933849	mirna_pc
13840	hsa-mir-324	PTMS	0.340241575	mirna_pc
13841	hsa-mir-324	SLC4A1AP	0.362473765	mirna_pc
13842	hsa-mir-324	TTC27	0.516861751	mirna_pc
13843	hsa-mir-324	DHRS13	0.428516592	mirna_pc
13844	hsa-mir-324	MCCC1	0.323165586	mirna_pc
13845	hsa-mir-324	ELFN1	0.331557239	mirna_pc
13846	hsa-mir-324	RPF1	0.301511929	mirna_pc
13847	hsa-mir-324	RTTN	0.316854962	mirna_pc
13848	hsa-mir-324	FIBP	0.334641401	mirna_pc
13849	hsa-mir-324	EWSR1	0.309138761	mirna_pc
13850	hsa-mir-324	LOC222699	0.446193446	mirna_pc
13851	hsa-mir-324	KRR1	0.342342079	mirna_pc
13852	hsa-mir-324	DHX57	0.469940703	mirna_pc
13853	hsa-mir-324	ALX3	0.31932339	mirna_pc
13854	hsa-mir-324	AAAS	0.476620358	mirna_pc
13855	hsa-mir-324	CHST14	0.384961672	mirna_pc
13856	hsa-mir-324	TMEM107	0.318497297	mirna_pc
13857	hsa-mir-324	SUPT7L	0.316898653	mirna_pc
13858	hsa-mir-324	PEMT	0.402687246	mirna_pc
13859	hsa-mir-324	LIG3	0.379372734	mirna_pc
13860	hsa-mir-324	MRPS26	0.391101575	mirna_pc
13861	hsa-mir-324	MRPL30	0.435859159	mirna_pc
13862	hsa-mir-324	PELP1	0.523327113	mirna_pc
13863	hsa-mir-324	METT11D1	0.301165369	mirna_pc
13864	hsa-mir-324	ACPL2	0.304456884	mirna_pc
13865	hsa-mir-324	DHODH	0.312456391	mirna_pc
13866	hsa-mir-324	C11orf45	0.342356042	mirna_pc
13867	hsa-mir-324	CCDC15	0.359086601	mirna_pc
13868	hsa-mir-324	SFRS7	0.383952236	mirna_pc
13869	hsa-mir-324	C18orf10	0.37725655	mirna_pc
13870	hsa-mir-324	CA5BP	0.380902975	mirna_pc
13871	hsa-mir-324	TRMT2A	0.343630474	mirna_pc
13872	hsa-mir-324	PCBP2	0.316899178	mirna_pc
13873	hsa-mir-324	PFAS	0.473471746	mirna_pc
13874	hsa-mir-324	PET112L	0.391321069	mirna_pc
13875	hsa-mir-324	SF3B2	0.348533853	mirna_pc
13876	hsa-mir-324	SARS2	0.337385817	mirna_pc
13877	hsa-mir-324	C10orf35	0.33694666	mirna_pc

13878	hsa-mir-324	NUP88	0.421307671	mirna_pc
13879	hsa-mir-324	DHX33	0.432582129	mirna_pc
13880	hsa-mir-324	RPUSD2	0.311095598	mirna_pc
13881	hsa-mir-324	THYN1	0.323340736	mirna_pc
13882	hsa-mir-324	EIF2B5	0.31132134	mirna_pc
13883	hsa-mir-324	NUP153	0.405764811	mirna_pc
13884	hsa-mir-324	NIPSNAP1	0.367077465	mirna_pc
13885	hsa-mir-324	SERBP1	0.320830802	mirna_pc
13886	hsa-mir-324	PARL	0.356822082	mirna_pc
13887	hsa-mir-324	PTOV1	0.36760828	mirna_pc
13888	hsa-mir-324	HOXA7	0.303387661	mirna_pc
13889	hsa-mir-324	C2orf68	0.334898633	mirna_pc
13890	hsa-mir-324	ATF5	0.359033093	mirna_pc
13891	hsa-mir-324	ANKRD23	0.315545854	mirna_pc
13892	hsa-mir-324	ARHGEF19	0.34904782	mirna_pc
13893	hsa-mir-324	C17orf75	0.430912843	mirna_pc
13894	hsa-mir-324	TMEM93	0.358753395	mirna_pc
13895	hsa-mir-324	RPL35A	0.324682981	mirna_pc
13896	hsa-mir-324	FBRSL1	0.345372069	mirna_pc
13897	hsa-mir-324	ATXN10	0.324235958	mirna_pc
13898	hsa-mir-324	ZNF771	0.372408282	mirna_pc
13899	hsa-mir-324	EEF1E1	0.355142565	mirna_pc
13900	hsa-mir-324	YBX1	0.325068532	mirna_pc
13901	hsa-mir-324	TCTN2	0.346940412	mirna_pc
13902	hsa-mir-324	C14orf104	0.330900502	mirna_pc
13903	hsa-mir-324	POLR2C	0.34768604	mirna_pc
13904	hsa-mir-324	PPP2R3B	0.303954072	mirna_pc
13905	hsa-mir-324	C12orf10	0.399353939	mirna_pc
13906	hsa-mir-324	TOP3A	0.456121392	mirna_pc
13907	hsa-mir-324	EXTL2	0.360040781	mirna_pc
13908	hsa-mir-324	UBE2M	0.307898152	mirna_pc
13909	hsa-mir-324	C2orf44	0.407431714	mirna_pc
13910	hsa-mir-324	PSMB6	0.413574384	mirna_pc
13911	hsa-mir-324	SEPHS1	0.433527561	mirna_pc
13912	hsa-mir-324	RMND1	0.314375268	mirna_pc
13913	hsa-mir-324	RYK	0.311113258	mirna_pc
13914	hsa-mir-324	KDM4D	0.355770061	mirna_pc
13915	hsa-mir-324	TMEM11	0.363390034	mirna_pc
13916	hsa-mir-324	GBAS	0.34538072	mirna_pc
13917	hsa-mir-324	AGBL5	0.444131239	mirna_pc
13918	hsa-mir-324	AIP	0.314334203	mirna_pc
13919	hsa-mir-324	NAB2	0.312249066	mirna_pc
13920	hsa-mir-324	VASH2	0.321157153	mirna_pc
13921	hsa-mir-324	C19orf23	0.329198123	mirna_pc
13922	hsa-mir-324	PHB2	0.354014402	mirna_pc
13923	hsa-mir-324	C8orf33	0.362020078	mirna_pc
13924	hsa-mir-324	ISYNA1	0.325560559	mirna_pc
13925	hsa-mir-324	GPS1	0.312037183	mirna_pc
13926	hsa-mir-324	MYL6B	0.502711928	mirna_pc
13927	hsa-mir-324	PDCD7	0.363391666	mirna_pc
13928	hsa-mir-324	POLR2F	0.316248043	mirna_pc
13929	hsa-mir-324	DTX3	0.360108349	mirna_pc
13930	hsa-mir-324	MED28	0.400233905	mirna_pc
13931	hsa-mir-324	MRPS15	0.333773782	mirna_pc

13932	hsa-mir-324	NARS2	0.399434354	mirna_pc
13933	hsa-mir-324	ZFP1	0.307581092	mirna_pc
13934	hsa-mir-324	NME4	0.332086916	mirna_pc
13935	hsa-mir-324	LYRM4	0.51222241	mirna_pc
13936	hsa-mir-324	BCL11A	0.391183628	mirna_pc
13937	hsa-mir-324	ZNF496	0.338409581	mirna_pc
13938	hsa-mir-324	C4orf27	0.438968727	mirna_pc
13939	hsa-mir-324	USP21	0.401810082	mirna_pc
13940	hsa-mir-324	ALMS1	0.331538778	mirna_pc
13941	hsa-mir-324	SLC25A11	0.430578871	mirna_pc
13942	hsa-mir-324	ZNF74	0.365311686	mirna_pc
13943	hsa-mir-324	METT10D	0.39985031	mirna_pc
13944	hsa-mir-324	ANP32A	0.356200479	mirna_pc
13945	hsa-mir-324	LOC284023	0.349539515	mirna_pc
13946	hsa-mir-324	SUPV3L1	0.346381005	mirna_pc
13947	hsa-mir-324	DPF2	0.382000819	mirna_pc
13948	hsa-mir-324	LMAN2L	0.370733364	mirna_pc
13949	hsa-mir-324	TCF7L1	0.312231404	mirna_pc
13950	hsa-mir-324	C17orf81	0.505067939	mirna_pc
13951	hsa-mir-324	PHF23	0.352352988	mirna_pc
13952	hsa-mir-324	PPP5C	0.398606946	mirna_pc
13953	hsa-mir-324	HYLS1	0.319985369	mirna_pc
13954	hsa-mir-324	HNRNPH3	0.329775146	mirna_pc
13955	hsa-mir-324	NAP1L1	0.374455266	mirna_pc
13956	hsa-mir-324	PEX5	0.324408328	mirna_pc
13957	hsa-mir-324	RRP1B	0.344952086	mirna_pc
13958	hsa-mir-324	UBTF	0.303854641	mirna_pc
13959	hsa-mir-324	NSL1	0.323351305	mirna_pc
13960	hsa-mir-324	ZNF428	0.305976723	mirna_pc
13961	hsa-mir-324	CNIH2	0.394193161	mirna_pc
13962	hsa-mir-324	ELAC2	0.300956449	mirna_pc
13963	hsa-mir-324	LOC400657	0.326316362	mirna_pc
13964	hsa-mir-324	IQCC	0.337744257	mirna_pc
13965	hsa-mir-324	MGC70857	0.303440512	mirna_pc
13966	hsa-mir-324	DDX19A	0.323235163	mirna_pc
13967	hsa-mir-324	LSMD1	0.303853628	mirna_pc
13968	hsa-mir-324	HNRNPA0	0.367873689	mirna_pc
13969	hsa-mir-324	MORN2	0.343290886	mirna_pc
13970	hsa-mir-324	BMP7	0.31089492	mirna_pc
13971	hsa-mir-324	DERL2	0.332323131	mirna_pc
13972	hsa-mir-324	C12orf41	0.350967118	mirna_pc
13973	hsa-mir-324	SETD6	0.316275092	mirna_pc
13974	hsa-mir-324	RTN4RL1	0.352530267	mirna_pc
13975	hsa-mir-324	TFDP2	0.335855917	mirna_pc
13976	hsa-mir-324	MLLT11	0.384946912	mirna_pc
13977	hsa-mir-324	TSPYL3	0.310724288	mirna_pc
13978	hsa-mir-324	AASDHPPT	0.312924204	mirna_pc
13979	hsa-mir-324	TRAPPC1	0.363532663	mirna_pc
13980	hsa-mir-324	C12orf24	0.322987099	mirna_pc
13981	hsa-mir-324	PRPF8	0.324706608	mirna_pc
13982	hsa-mir-324	EFCAB7	0.332415488	mirna_pc
13983	hsa-mir-324	DCAF16	0.378139072	mirna_pc
13984	hsa-mir-324	MTERF	0.345518513	mirna_pc
13985	hsa-mir-324	SBK1	0.40926803	mirna_pc

13986	hsa-mir-324	PABPC4L	0.311268151	mirna_pc
13987	hsa-mir-324	C21orf67	0.338595594	mirna_pc
13988	hsa-mir-324	THNSL1	0.31603332	mirna_pc
13989	hsa-mir-324	USP13	0.418983137	mirna_pc
13990	hsa-mir-324	GALNT14	0.450480766	mirna_pc
13991	hsa-mir-324	TSR1	0.439854694	mirna_pc
13992	hsa-mir-324	DRG2	0.375417286	mirna_pc
13993	hsa-mir-324	SCO1	0.334042471	mirna_pc
13994	hsa-mir-324	SLC25A33	0.386326322	mirna_pc
13995	hsa-mir-324	RANGRF	0.401482645	mirna_pc
13996	hsa-mir-324	HDGFRP3	0.301560644	mirna_pc
13997	hsa-mir-324	CLPB	0.315192427	mirna_pc
13998	hsa-mir-324	FGD1	0.312645836	mirna_pc
13999	hsa-mir-324	ZNF740	0.331538903	mirna_pc
14000	hsa-mir-324	TRO	0.379716576	mirna_pc
14001	hsa-mir-324	DDX51	0.350964031	mirna_pc
14002	hsa-mir-324	PBX2	0.33660295	mirna_pc
14003	hsa-mir-324	ZNF232	0.402163604	mirna_pc
14004	hsa-mir-324	ZBTB39	0.381443287	mirna_pc
14005	hsa-mir-324	ENO3	0.343760131	mirna_pc
14006	hsa-mir-324	RPAIN	0.493561788	mirna_pc
14007	hsa-mir-324	DTNB	0.340004538	mirna_pc
14008	hsa-mir-324	RAC3	0.343749014	mirna_pc
14009	hsa-mir-324	RBBP4	0.387337128	mirna_pc
14010	hsa-mir-324	LOC221710	0.359490476	mirna_pc
14011	hsa-mir-324	MRPL1	0.374044259	mirna_pc
14012	hsa-mir-324	C21orf91	0.311161571	mirna_pc
14013	hsa-mir-324	SOD1	0.367443009	mirna_pc
14014	hsa-mir-324	SPATS2	0.345410757	mirna_pc
14015	hsa-mir-324	SMYD4	0.372535839	mirna_pc
14016	hsa-mir-324	USP28	0.42069908	mirna_pc
14017	hsa-mir-324	C2orf3	0.396646041	mirna_pc
14018	hsa-mir-324	DGCR6	0.313615077	mirna_pc
14019	hsa-mir-324	C18orf22	0.30744612	mirna_pc
14020	hsa-mir-324	MEAF6	0.300851838	mirna_pc
14021	hsa-mir-324	RRP7B	0.317682837	mirna_pc
14022	hsa-mir-324	DNAJC19	0.339612174	mirna_pc
14023	hsa-mir-324	LRDD	0.30023836	mirna_pc
14024	hsa-mir-324	CCDC157	0.307361596	mirna_pc
14025	hsa-mir-324	MYH10	0.329772449	mirna_pc
14026	hsa-mir-324	SCML2	0.301905912	mirna_pc
14027	hsa-mir-324	C12orf65	0.303138627	mirna_pc
14028	hsa-mir-324	ZNF594	0.317966557	mirna_pc
14029	hsa-mir-324	MRPL19	0.329095098	mirna_pc
14030	hsa-mir-324	HSF2	0.364611285	mirna_pc
14031	hsa-mir-324	ZCCHC11	0.332105397	mirna_pc
14032	hsa-mir-324	BCAM	0.316026434	mirna_pc
14033	hsa-mir-324	DULLARD	0.391409512	mirna_pc
14034	hsa-mir-324	LLGL1	0.315788635	mirna_pc
14035	hsa-mir-324	CETN3	0.37828366	mirna_pc
14036	hsa-mir-324	ETV5	0.397398741	mirna_pc
14037	hsa-mir-324	B9D1	0.388701348	mirna_pc
14038	hsa-mir-324	DYNC1I1	0.309801977	mirna_pc
14039	hsa-mir-324	KIAA1430	0.321789319	mirna_pc

14040	hsa-mir-324	EXOSC6	0.338399891	mirna_pc
14041	hsa-mir-324	PLRG1	0.32498722	mirna_pc
14042	hsa-mir-324	AADAT	0.332380171	mirna_pc
14043	hsa-mir-324	IGHMBP2	0.357698311	mirna_pc
14044	hsa-mir-324	FDX1L	0.36806038	mirna_pc
14045	hsa-mir-324	ACTR3B	0.353211758	mirna_pc
14046	hsa-mir-324	ZNF322A	0.316844016	mirna_pc
14047	hsa-mir-324	CDAN1	0.311012652	mirna_pc
14048	hsa-mir-324	C11orf51	0.331433607	mirna_pc
14049	hsa-mir-324	MAGED4B	0.358271049	mirna_pc
14050	hsa-mir-324	SERF1A	0.392560897	mirna_pc
14051	hsa-mir-324	PLD6	0.30441229	mirna_pc
14052	hsa-mir-324	GEMIN4	0.354452077	mirna_pc
14053	hsa-mir-324	B4GALNT4	0.377087605	mirna_pc
14054	hsa-mir-324	LOC401397	0.31730453	mirna_pc
14055	hsa-mir-324	GPS2	0.36179715	mirna_pc
14056	hsa-mir-324	HSDL1	0.351867671	mirna_pc
14057	hsa-mir-324	C21orf119	0.354783711	mirna_pc
14058	hsa-mir-324	KLHL13	0.323953314	mirna_pc
14059	hsa-mir-324	H2AFY2	0.457086677	mirna_pc
14060	hsa-mir-324	MIS12	0.375033249	mirna_pc
14061	hsa-mir-324	MLF 1.00	0.364928372	mirna_pc
14062	hsa-mir-324	BOD1	0.326632013	mirna_pc
14063	hsa-mir-324	TMEM14B	0.324265968	mirna_pc
14064	hsa-mir-324	SMARCAD1	0.338331692	mirna_pc
14065	hsa-mir-324	SCHIP1	0.323520412	mirna_pc
14066	hsa-mir-324	VPS37D	0.371396253	mirna_pc
14067	hsa-mir-324	SV2A	0.344075773	mirna_pc
14068	hsa-mir-324	NT5M	0.381889188	mirna_pc
14069	hsa-mir-324	SEMA4C	0.426473285	mirna_pc
14070	hsa-mir-324	THOC5	0.377005366	mirna_pc
14071	hsa-mir-324	EFNB3	0.45093282	mirna_pc
14072	hsa-mir-324	C21orf70	0.360645813	mirna_pc
14073	hsa-mir-324	NRSN2	0.316147894	mirna_pc
14074	hsa-mir-324	VANGL2	0.307416252	mirna_pc
14075	hsa-mir-324	DNMT3A	0.416902	mirna_pc
14076	hsa-mir-324	COPS3	0.456813634	mirna_pc
14077	hsa-mir-324	BPHL	0.326111848	mirna_pc
14078	hsa-mir-324	ACCN2	0.330886664	mirna_pc
14079	hsa-mir-324	C12orf73	0.326323619	mirna_pc
14080	hsa-mir-324	PATZ1	0.344624688	mirna_pc
14081	hsa-mir-324	TIMM22	0.393706434	mirna_pc
14082	hsa-mir-324	ZNF326	0.324443416	mirna_pc
14083	hsa-mir-3615	KPNA2	0.354638577	mirna_pc
14084	hsa-mir-3615	RCC2	0.324791367	mirna_pc
14085	hsa-mir-3615	HOXC9	0.311050488	mirna_pc
14086	hsa-mir-3615	RRM2	0.410607809	mirna_pc
14087	hsa-mir-3615	BUB1B	0.307366119	mirna_pc
14088	hsa-mir-3615	CCNA2	0.345017271	mirna_pc
14089	hsa-mir-3615	FANCI	0.30446714	mirna_pc
14090	hsa-mir-3615	DDX39	0.381170551	mirna_pc
14091	hsa-mir-3615	NCAPH	0.32224781	mirna_pc
14092	hsa-mir-3615	CENPA	0.342792724	mirna_pc
14093	hsa-mir-3615	MAD2L1	0.362072905	mirna_pc

14094	hsa-mir-3615	TIMELESS	0.306435627	mirna_pc
14095	hsa-mir-3615	LMNB1	0.329652006	mirna_pc
14096	hsa-mir-3615	TROAP	0.383805249	mirna_pc
14097	hsa-mir-3615	CDC45	0.33999149	mirna_pc
14098	hsa-mir-3615	TK1	0.361412847	mirna_pc
14099	hsa-mir-3615	DKC1	0.342813133	mirna_pc
14100	hsa-mir-3615	TUBB	0.324344181	mirna_pc
14101	hsa-mir-3615	KIAA0101	0.306110637	mirna_pc
14102	hsa-mir-3615	CCNF	0.312246799	mirna_pc
14103	hsa-mir-3615	PTBP1	0.319808364	mirna_pc
14104	hsa-mir-3615	PLK4	0.331689828	mirna_pc
14105	hsa-mir-3615	SKA3	0.338328707	mirna_pc
14106	hsa-mir-3615	HJURP	0.320430698	mirna_pc
14107	hsa-mir-3615	BLM	0.318171015	mirna_pc
14108	hsa-mir-3615	NME1	0.385720978	mirna_pc
14109	hsa-mir-3615	PAICS	0.330235323	mirna_pc
14110	hsa-mir-3615	BIRC5	0.489779666	mirna_pc
14111	hsa-mir-3615	EME1	0.366235487	mirna_pc
14112	hsa-mir-3615	AURKB	0.314066423	mirna_pc
14113	hsa-mir-3615	CDT1	0.31447888	mirna_pc
14114	hsa-mir-3615	KIF20A	0.300030865	mirna_pc
14115	hsa-mir-3615	CHEK2	0.315287708	mirna_pc
14116	hsa-mir-3615	CDCA3	0.332695527	mirna_pc
14117	hsa-mir-3615	C1orf112	0.304413421	mirna_pc
14118	hsa-mir-3615	C16orf59	0.368270059	mirna_pc
14119	hsa-mir-3615	NEIL3	0.308207123	mirna_pc
14120	hsa-mir-3615	ACTL6A	0.388226296	mirna_pc
14121	hsa-mir-3615	POLD1	0.307389076	mirna_pc
14122	hsa-mir-3615	C12orf48	0.316086501	mirna_pc
14123	hsa-mir-3615	HNRNPL	0.38961297	mirna_pc
14124	hsa-mir-3615	UBE2S	0.330953242	mirna_pc
14125	hsa-mir-3615	C11orf82	0.363970087	mirna_pc
14126	hsa-mir-3615	HNRNPC	0.414644184	mirna_pc
14127	hsa-mir-3615	RAN	0.31748316	mirna_pc
14128	hsa-mir-3615	RFWD3	0.330332258	mirna_pc
14129	hsa-mir-3615	RNASEH2A	0.341902385	mirna_pc
14130	hsa-mir-3615	EPR1	0.325675923	mirna_pc
14131	hsa-mir-3615	SFRS2	0.448290385	mirna_pc
14132	hsa-mir-3615	UCK2	0.378486455	mirna_pc
14133	hsa-mir-3615	NFKBIL2	0.317433642	mirna_pc
14134	hsa-mir-3615	C15orf42	0.300513347	mirna_pc
14135	hsa-mir-3615	RANBP1	0.301453457	mirna_pc
14136	hsa-mir-3615	SNRPG	0.356160532	mirna_pc
14137	hsa-mir-3615	EIF4A3	0.403078427	mirna_pc
14138	hsa-mir-3615	SNRPA	0.432422048	mirna_pc
14139	hsa-mir-3615	FUS	0.347899867	mirna_pc
14140	hsa-mir-3615	RFC3	0.339905673	mirna_pc
14141	hsa-mir-3615	CHAF1A	0.37879538	mirna_pc
14142	hsa-mir-3615	GINS4	0.417539162	mirna_pc
14143	hsa-mir-3615	ANP32E	0.316747996	mirna_pc
14144	hsa-mir-3615	CENPO	0.338260624	mirna_pc
14145	hsa-mir-3615	C19orf48	0.322939383	mirna_pc
14146	hsa-mir-3615	GEN1	0.347521093	mirna_pc
14147	hsa-mir-3615	HMGB2	0.348180487	mirna_pc

14148	hsa-mir-3615	GINS2	0.333810229	mirna_pc
14149	hsa-mir-3615	NOP56	0.316400134	mirna_pc
14150	hsa-mir-3615	DTYMK	0.306748934	mirna_pc
14151	hsa-mir-3615	PRMT1	0.419100463	mirna_pc
14152	hsa-mir-3615	C18orf56	0.318202398	mirna_pc
14153	hsa-mir-3615	NOP2	0.366411787	mirna_pc
14154	hsa-mir-3615	HIST1H2AE	0.422158295	mirna_pc
14155	hsa-mir-3615	CDK4	0.341078191	mirna_pc
14156	hsa-mir-3615	PFDN2	0.361070735	mirna_pc
14157	hsa-mir-3615	CHTF18	0.370505832	mirna_pc
14158	hsa-mir-3615	DLEU2	0.371941311	mirna_pc
14159	hsa-mir-3615	KAT2A	0.409710832	mirna_pc
14160	hsa-mir-3615	CHAF1B	0.310517344	mirna_pc
14161	hsa-mir-3615	DHX34	0.301631486	mirna_pc
14162	hsa-mir-3615	MSH2	0.330753284	mirna_pc
14163	hsa-mir-3615	DBF4	0.403326623	mirna_pc
14164	hsa-mir-3615	SF3B3	0.362792196	mirna_pc
14165	hsa-mir-3615	CDC7	0.345918467	mirna_pc
14166	hsa-mir-3615	YDJC	0.400920893	mirna_pc
14167	hsa-mir-3615	PUS1	0.34493609	mirna_pc
14168	hsa-mir-3615	NOL11	0.389011154	mirna_pc
14169	hsa-mir-3615	RFC4	0.342525129	mirna_pc
14170	hsa-mir-3615	FIGNL1	0.404557829	mirna_pc
14171	hsa-mir-3615	E2F3	0.362068126	mirna_pc
14172	hsa-mir-3615	SF3B14	0.334415804	mirna_pc
14173	hsa-mir-3615	CDC25A	0.3327673	mirna_pc
14174	hsa-mir-3615	H2AFZ	0.364201317	mirna_pc
14175	hsa-mir-3615	DDX12	0.310870851	mirna_pc
14176	hsa-mir-3615	CPSF3	0.493601302	mirna_pc
14177	hsa-mir-3615	C21orf45	0.436565166	mirna_pc
14178	hsa-mir-3615	PPM1G	0.465545657	mirna_pc
14179	hsa-mir-3615	TFAP4	0.349333947	mirna_pc
14180	hsa-mir-3615	NUDT5	0.364844735	mirna_pc
14181	hsa-mir-3615	RNPS1	0.301301139	mirna_pc
14182	hsa-mir-3615	DAZAP1	0.370111621	mirna_pc
14183	hsa-mir-3615	CBX8	0.445375618	mirna_pc
14184	hsa-mir-3615	NCL	0.327853218	mirna_pc
14185	hsa-mir-3615	PAFAH1B3	0.444053222	mirna_pc
14186	hsa-mir-3615	DGUOK	0.414967495	mirna_pc
14187	hsa-mir-3615	MRPL47	0.312992238	mirna_pc
14188	hsa-mir-3615	FBL	0.356032152	mirna_pc
14189	hsa-mir-3615	THOP1	0.316588742	mirna_pc
14190	hsa-mir-3615	EXOSC5	0.403645765	mirna_pc
14191	hsa-mir-3615	HNRNPM	0.394579139	mirna_pc
14192	hsa-mir-3615	LSM 12.00	0.351139564	mirna_pc
14193	hsa-mir-3615	PNPT1	0.327304873	mirna_pc
14194	hsa-mir-3615	CCT6A	0.335677948	mirna_pc
14195	hsa-mir-3615	SHFM1	0.394237743	mirna_pc
14196	hsa-mir-3615	LSG1	0.339650516	mirna_pc
14197	hsa-mir-3615	PPIH	0.322199479	mirna_pc
14198	hsa-mir-3615	DHX9	0.30788099	mirna_pc
14199	hsa-mir-3615	LOC100128191	0.348952572	mirna_pc
14200	hsa-mir-3615	SMYD5	0.406008333	mirna_pc
14201	hsa-mir-3615	SF3A2	0.312763989	mirna_pc

14202	hsa-mir-3615	TSSC1	0.430472741	mirna_pc
14203	hsa-mir-3615	TLCD1	0.303976616	mirna_pc
14204	hsa-mir-3615	SNRPF	0.349794148	mirna_pc
14205	hsa-mir-3615	SUMO2	0.457925045	mirna_pc
14206	hsa-mir-3615	SFRS1	0.415119448	mirna_pc
14207	hsa-mir-3615	WDR43	0.360659054	mirna_pc
14208	hsa-mir-3615	SNRPA1	0.355194244	mirna_pc
14209	hsa-mir-3615	SMC6	0.318934848	mirna_pc
14210	hsa-mir-3615	UBE2I	0.329530708	mirna_pc
14211	hsa-mir-3615	TIPIN	0.405427086	mirna_pc
14212	hsa-mir-3615	SMARCD1	0.347649744	mirna_pc
14213	hsa-mir-3615	HSPBP1	0.355863742	mirna_pc
14214	hsa-mir-3615	TIGD1	0.318415334	mirna_pc
14215	hsa-mir-3615	PA2G4	0.416817114	mirna_pc
14216	hsa-mir-3615	SNRPD3	0.414649403	mirna_pc
14217	hsa-mir-3615	KHDRBS1	0.408320394	mirna_pc
14218	hsa-mir-3615	LOC92659	0.305813639	mirna_pc
14219	hsa-mir-3615	CCT7	0.327787866	mirna_pc
14220	hsa-mir-3615	METTL1	0.332841826	mirna_pc
14221	hsa-mir-3615	TMEM69	0.313529595	mirna_pc
14222	hsa-mir-3615	GNL2	0.38894835	mirna_pc
14223	hsa-mir-3615	KRI1	0.309375739	mirna_pc
14224	hsa-mir-3615	RPP40	0.411041726	mirna_pc
14225	hsa-mir-3615	OBFC2B	0.308202984	mirna_pc
14226	hsa-mir-3615	PTMA	0.434197607	mirna_pc
14227	hsa-mir-3615	USP39	0.307417985	mirna_pc
14228	hsa-mir-3615	LRRC8D	0.321273672	mirna_pc
14229	hsa-mir-3615	FBXO22OS	0.302178646	mirna_pc
14230	hsa-mir-3615	SUV39H2	0.334141274	mirna_pc
14231	hsa-mir-3615	NAALADL2	0.351633295	mirna_pc
14232	hsa-mir-3615	HNRNPD	0.429717041	mirna_pc
14233	hsa-mir-3615	DPY30	0.346927962	mirna_pc
14234	hsa-mir-3615	MTHFD2	0.383768492	mirna_pc
14235	hsa-mir-3615	SFRS3	0.313506231	mirna_pc
14236	hsa-mir-3615	PNO1	0.366281414	mirna_pc
14237	hsa-mir-3615	CCT4	0.305271474	mirna_pc
14238	hsa-mir-3615	ELAVL1	0.385382558	mirna_pc
14239	hsa-mir-3615	NCAPD3	0.425233126	mirna_pc
14240	hsa-mir-3615	TBC1D7	0.435350969	mirna_pc
14241	hsa-mir-3615	PAK1IP1	0.423076092	mirna_pc
14242	hsa-mir-3615	E2F4	0.318234542	mirna_pc
14243	hsa-mir-3615	PFDN6	0.321260951	mirna_pc
14244	hsa-mir-3615	PGAM5	0.329135214	mirna_pc
14245	hsa-mir-3615	YWHAE	0.328679299	mirna_pc
14246	hsa-mir-3615	C3orf57	0.333291214	mirna_pc
14247	hsa-mir-3615	PDCL3	0.335186845	mirna_pc
14248	hsa-mir-3615	ILF3	0.336372539	mirna_pc
14249	hsa-mir-3615	EHMT2	0.352702892	mirna_pc
14250	hsa-mir-3615	HNRNPAB	0.324560167	mirna_pc
14251	hsa-mir-3615	UBAP2L	0.316228811	mirna_pc
14252	hsa-mir-3615	SFRS9	0.307385236	mirna_pc
14253	hsa-mir-3615	PASK	0.325500557	mirna_pc
14254	hsa-mir-3615	SAAL1	0.314778101	mirna_pc
14255	hsa-mir-3615	KHSRP	0.442267578	mirna_pc

14256	hsa-mir-3615	LSM 6.00	0.334887457	mirna_pc
14257	hsa-mir-3615	PRAME	0.351047021	mirna_pc
14258	hsa-mir-3615	MRPS17	0.371675693	mirna_pc
14259	hsa-mir-3615	CBX2	0.357136582	mirna_pc
14260	hsa-mir-3615	SUV39H1	0.30092784	mirna_pc
14261	hsa-mir-3615	TRA2B	0.315804075	mirna_pc
14262	hsa-mir-3615	GAR1	0.417911727	mirna_pc
14263	hsa-mir-3615	ANAPC7	0.352738237	mirna_pc
14264	hsa-mir-3615	CIRH1A	0.308425184	mirna_pc
14265	hsa-mir-3615	PRMT3	0.34462696	mirna_pc
14266	hsa-mir-3615	PSMD12	0.405498216	mirna_pc
14267	hsa-mir-3615	C1QBP	0.316448947	mirna_pc
14268	hsa-mir-3615	ENOPH1	0.428036124	mirna_pc
14269	hsa-mir-3615	C19orf47	0.364369604	mirna_pc
14270	hsa-mir-3615	LAS1L	0.334174394	mirna_pc
14271	hsa-mir-3615	C4orf43	0.326596706	mirna_pc
14272	hsa-mir-3615	TUBG1	0.346886298	mirna_pc
14273	hsa-mir-3615	C17orf96	0.340135706	mirna_pc
14274	hsa-mir-3615	PLEKHG4	0.360951866	mirna_pc
14275	hsa-mir-3615	SFPQ	0.361720191	mirna_pc
14276	hsa-mir-3615	AP3M2	0.310210237	mirna_pc
14277	hsa-mir-3615	CNOT3	0.307047479	mirna_pc
14278	hsa-mir-3615	EIF4EBP1	0.461379768	mirna_pc
14279	hsa-mir-3615	CADM1	0.32783012	mirna_pc
14280	hsa-mir-3615	HDAC2	0.322457394	mirna_pc
14281	hsa-mir-3615	PA2G4P4	0.374839265	mirna_pc
14282	hsa-mir-3615	WDR53	0.306455556	mirna_pc
14283	hsa-mir-3615	ALOX12P2	0.304689566	mirna_pc
14284	hsa-mir-3615	PDAP1	0.477203266	mirna_pc
14285	hsa-mir-3615	RNF7	0.416670088	mirna_pc
14286	hsa-mir-3615	GEMIN6	0.436824084	mirna_pc
14287	hsa-mir-3615	H3F3B	0.348359262	mirna_pc
14288	hsa-mir-3615	PTCD1	0.493931786	mirna_pc
14289	hsa-mir-3615	COX4NB	0.33360395	mirna_pc
14290	hsa-mir-3615	MSH6	0.379388322	mirna_pc
14291	hsa-mir-3615	SOX4	0.346873759	mirna_pc
14292	hsa-mir-3615	C3orf34	0.357147846	mirna_pc
14293	hsa-mir-3615	TPRKB	0.42213886	mirna_pc
14294	hsa-mir-3615	POLB	0.419501963	mirna_pc
14295	hsa-mir-3615	ACBD6	0.354753159	mirna_pc
14296	hsa-mir-3615	HNRNPA1	0.309592636	mirna_pc
14297	hsa-mir-3615	NPM1	0.378018333	mirna_pc
14298	hsa-mir-3615	CDK2AP1	0.374446907	mirna_pc
14299	hsa-mir-3615	NUDT3	0.44583347	mirna_pc
14300	hsa-mir-3615	MEX3A	0.364440113	mirna_pc
14301	hsa-mir-3615	MOGS	0.32319266	mirna_pc
14302	hsa-mir-3615	ATP5J2	0.460321853	mirna_pc
14303	hsa-mir-3615	COIL	0.38304723	mirna_pc
14304	hsa-mir-3615	PPAN	0.319159849	mirna_pc
14305	hsa-mir-3615	BUD31	0.410616566	mirna_pc
14306	hsa-mir-3615	FAM117B	0.325249129	mirna_pc
14307	hsa-mir-3615	KARS	0.325072524	mirna_pc
14308	hsa-mir-3615	C16orf88	0.43623784	mirna_pc
14309	hsa-mir-3615	HNRNPA1L2	0.331861844	mirna_pc

14310	hsa-mir-3615	NAA15	0.337266501	mirna_pc
14311	hsa-mir-3615	EMILIN3	0.335563382	mirna_pc
14312	hsa-mir-3615	ZC3H18	0.350786903	mirna_pc
14313	hsa-mir-3615	CHERP	0.313252474	mirna_pc
14314	hsa-mir-3615	FARSB	0.309790751	mirna_pc
14315	hsa-mir-3615	RCN2	0.321486025	mirna_pc
14316	hsa-mir-3615	THUMPD2	0.381242917	mirna_pc
14317	hsa-mir-3615	NOL10	0.514631428	mirna_pc
14318	hsa-mir-3615	SF3A3	0.308315171	mirna_pc
14319	hsa-mir-3615	CTU1	0.344881712	mirna_pc
14320	hsa-mir-3615	POLE	0.339846766	mirna_pc
14321	hsa-mir-3615	WRNIP1	0.316128491	mirna_pc
14322	hsa-mir-3615	TAF1B	0.508181846	mirna_pc
14323	hsa-mir-3615	TIMM50	0.365359449	mirna_pc
14324	hsa-mir-3615	SNORD1C	0.341119083	mirna_pc
14325	hsa-mir-3615	XRCC6BP1	0.318872403	mirna_pc
14326	hsa-mir-3615	ADSL	0.321532575	mirna_pc
14327	hsa-mir-3615	EXOSC9	0.419412959	mirna_pc
14328	hsa-mir-3615	MXD3	0.307889718	mirna_pc
14329	hsa-mir-3615	UNG	0.342418439	mirna_pc
14330	hsa-mir-3615	RBMX	0.339630273	mirna_pc
14331	hsa-mir-3615	ALKBH2	0.411686897	mirna_pc
14332	hsa-mir-3615	RRP1	0.332193113	mirna_pc
14333	hsa-mir-3615	CCDC90A	0.377101313	mirna_pc
14334	hsa-mir-3615	MLF 2.00	0.329429615	mirna_pc
14335	hsa-mir-3615	XRCC5	0.338740618	mirna_pc
14336	hsa-mir-3615	NHLRC1	0.329430258	mirna_pc
14337	hsa-mir-3615	CCDC24	0.329108719	mirna_pc
14338	hsa-mir-3615	PDIA6	0.300955469	mirna_pc
14339	hsa-mir-3615	LOC152217	0.305836431	mirna_pc
14340	hsa-mir-3615	MEMO1	0.44880656	mirna_pc
14341	hsa-mir-3615	TRAP1	0.365092739	mirna_pc
14342	hsa-mir-3615	GTF3C2	0.347012619	mirna_pc
14343	hsa-mir-3615	MAGEF1	0.340377931	mirna_pc
14344	hsa-mir-3615	ICT1	0.396529506	mirna_pc
14345	hsa-mir-3615	OSTC	0.32640099	mirna_pc
14346	hsa-mir-3615	FAM136A	0.305554592	mirna_pc
14347	hsa-mir-3615	SMARCB1	0.31270272	mirna_pc
14348	hsa-mir-3615	CCT8	0.328309578	mirna_pc
14349	hsa-mir-3615	TTC32	0.401260788	mirna_pc
14350	hsa-mir-3615	SMN2	0.377690387	mirna_pc
14351	hsa-mir-3615	U2AF1	0.42535245	mirna_pc
14352	hsa-mir-3615	ZNF639	0.340723603	mirna_pc
14353	hsa-mir-3615	RPL38	0.326933322	mirna_pc
14354	hsa-mir-3615	MSTO1	0.327817464	mirna_pc
14355	hsa-mir-3615	ABT1	0.449075832	mirna_pc
14356	hsa-mir-3615	METAP1	0.3082616	mirna_pc
14357	hsa-mir-3615	NOP16	0.330992117	mirna_pc
14358	hsa-mir-3615	MPHOSPH10	0.369918927	mirna_pc
14359	hsa-mir-3615	AKAP9	0.398699101	mirna_pc
14360	hsa-mir-3615	RPS7	0.352458593	mirna_pc
14361	hsa-mir-3615	MKRN3	0.34728214	mirna_pc
14362	hsa-mir-3615	METAP2	0.322588348	mirna_pc
14363	hsa-mir-3615	TIMM16	0.332768921	mirna_pc

14364	hsa-mir-3615	NUDCD2	0.329703335	mirna_pc
14365	hsa-mir-3615	NAT9	0.484086865	mirna_pc
14366	hsa-mir-3615	WDR77	0.360526316	mirna_pc
14367	hsa-mir-3615	C17orf95	0.304745558	mirna_pc
14368	hsa-mir-3615	NLE1	0.398611376	mirna_pc
14369	hsa-mir-3615	DMRTA2	0.304096098	mirna_pc
14370	hsa-mir-3615	BTG3	0.304255244	mirna_pc
14371	hsa-mir-3615	LOC645332	0.38933301	mirna_pc
14372	hsa-mir-3615	MRPS22	0.341685962	mirna_pc
14373	hsa-mir-3615	PSPH	0.327455534	mirna_pc
14374	hsa-mir-3615	MGC72080	0.417135619	mirna_pc
14375	hsa-mir-3615	C13orf23	0.302891701	mirna_pc
14376	hsa-mir-3615	TRRAP	0.310632008	mirna_pc
14377	hsa-mir-3615	TRMT61B	0.41397649	mirna_pc
14378	hsa-mir-3615	MPV17	0.334064729	mirna_pc
14379	hsa-mir-3615	KRT15	0.390133379	mirna_pc
14380	hsa-mir-3615	DCUN1D1	0.308296579	mirna_pc
14381	hsa-mir-3615	C1orf109	0.341573729	mirna_pc
14382	hsa-mir-3615	PCBP1	0.344022397	mirna_pc
14383	hsa-mir-3615	UBE2N	0.308151603	mirna_pc
14384	hsa-mir-3615	CHCHD2	0.309285663	mirna_pc
14385	hsa-mir-3615	PIGX	0.331784891	mirna_pc
14386	hsa-mir-3615	HTRA2	0.334546324	mirna_pc
14387	hsa-mir-3615	MTA3	0.358373008	mirna_pc
14388	hsa-mir-3615	CHAC2	0.325965316	mirna_pc
14389	hsa-mir-3615	ACN9	0.428482864	mirna_pc
14390	hsa-mir-3615	PSPC1	0.33102611	mirna_pc
14391	hsa-mir-3615	INTU	0.40680831	mirna_pc
14392	hsa-mir-3615	NHP2L1	0.320984072	mirna_pc
14393	hsa-mir-3615	MAPKAPK5	0.310005679	mirna_pc
14394	hsa-mir-3615	E2F6	0.378318231	mirna_pc
14395	hsa-mir-3615	DRG1	0.315226258	mirna_pc
14396	hsa-mir-3615	NOL7	0.436185282	mirna_pc
14397	hsa-mir-3615	PRPSAP2	0.303973676	mirna_pc
14398	hsa-mir-3615	CPSF4	0.498336172	mirna_pc
14399	hsa-mir-3615	GADD45GIP1	0.314740057	mirna_pc
14400	hsa-mir-3615	NT5C	0.377288283	mirna_pc
14401	hsa-mir-3615	CTU2	0.352497236	mirna_pc
14402	hsa-mir-3615	SLC4A1AP	0.36936926	mirna_pc
14403	hsa-mir-3615	TTC27	0.402591475	mirna_pc
14404	hsa-mir-3615	DHRS13	0.382658949	mirna_pc
14405	hsa-mir-3615	MCCC1	0.315774167	mirna_pc
14406	hsa-mir-3615	MTIF2	0.364141072	mirna_pc
14407	hsa-mir-3615	KRTCAP3	0.305554123	mirna_pc
14408	hsa-mir-3615	LOC222699	0.411577761	mirna_pc
14409	hsa-mir-3615	DHX57	0.346973793	mirna_pc
14410	hsa-mir-3615	HIST1H1C	0.411680606	mirna_pc
14411	hsa-mir-3615	AAAS	0.358596945	mirna_pc
14412	hsa-mir-3615	FDXR	0.330795102	mirna_pc
14413	hsa-mir-3615	C2orf79	0.301405935	mirna_pc
14414	hsa-mir-3615	SUPT7L	0.330574361	mirna_pc
14415	hsa-mir-3615	PEMT	0.339357935	mirna_pc
14416	hsa-mir-3615	LIG3	0.349913593	mirna_pc
14417	hsa-mir-3615	MRPL30	0.318718443	mirna_pc

14418	hsa-mir-3615	ZNF277	0.392008038	mirna_pc
14419	hsa-mir-3615	PELP1	0.300084181	mirna_pc
14420	hsa-mir-3615	DHODH	0.33502163	mirna_pc
14421	hsa-mir-3615	C11orf45	0.380800542	mirna_pc
14422	hsa-mir-3615	SFRS7	0.441400429	mirna_pc
14423	hsa-mir-3615	GPR98	0.306736738	mirna_pc
14424	hsa-mir-3615	TRMT2A	0.370930795	mirna_pc
14425	hsa-mir-3615	IFRD1	0.347084181	mirna_pc
14426	hsa-mir-3615	UBE2O	0.36435976	mirna_pc
14427	hsa-mir-3615	INO80B	0.314796041	mirna_pc
14428	hsa-mir-3615	SARS2	0.312863313	mirna_pc
14429	hsa-mir-3615	RBM10	0.306649951	mirna_pc
14430	hsa-mir-3615	PDCD2	0.323096682	mirna_pc
14431	hsa-mir-3615	CORO1B	0.301418691	mirna_pc
14432	hsa-mir-3615	RPUSD2	0.362919291	mirna_pc
14433	hsa-mir-3615	THYN1	0.43170155	mirna_pc
14434	hsa-mir-3615	AMZ2	0.346986497	mirna_pc
14435	hsa-mir-3615	PARL	0.300467086	mirna_pc
14436	hsa-mir-3615	C2orf68	0.423236876	mirna_pc
14437	hsa-mir-3615	ARPC1A	0.303447103	mirna_pc
14438	hsa-mir-3615	C17orf80	0.336146524	mirna_pc
14439	hsa-mir-3615	ANKRD23	0.302025802	mirna_pc
14440	hsa-mir-3615	C17orf75	0.423232862	mirna_pc
14441	hsa-mir-3615	ZBTB44	0.317717427	mirna_pc
14442	hsa-mir-3615	FBF1	0.333259352	mirna_pc
14443	hsa-mir-3615	SPRYD4	0.36435199	mirna_pc
14444	hsa-mir-3615	ZNF789	0.313281825	mirna_pc
14445	hsa-mir-3615	EEF1E1	0.341017354	mirna_pc
14446	hsa-mir-3615	YTHDF2	0.30767442	mirna_pc
14447	hsa-mir-3615	MRPL38	0.453943674	mirna_pc
14448	hsa-mir-3615	ATPBD4	0.344428188	mirna_pc
14449	hsa-mir-3615	ZKSCAN5	0.375587621	mirna_pc
14450	hsa-mir-3615	C12orf10	0.371542204	mirna_pc
14451	hsa-mir-3615	C12orf45	0.323970505	mirna_pc
14452	hsa-mir-3615	C2orf44	0.375371092	mirna_pc
14453	hsa-mir-3615	RING1	0.339926691	mirna_pc
14454	hsa-mir-3615	SEPHS1	0.308876376	mirna_pc
14455	hsa-mir-3615	PNKP	0.304482027	mirna_pc
14456	hsa-mir-3615	C8orf40	0.308161917	mirna_pc
14457	hsa-mir-3615	BOLA3	0.384365353	mirna_pc
14458	hsa-mir-3615	C19orf23	0.40861986	mirna_pc
14459	hsa-mir-3615	MYL6B	0.386940221	mirna_pc
14460	hsa-mir-3615	PWP2	0.318478498	mirna_pc
14461	hsa-mir-3615	PDCD7	0.35245345	mirna_pc
14462	hsa-mir-3615	C11orf71	0.318126724	mirna_pc
14463	hsa-mir-3615	PSMG4	0.306929051	mirna_pc
14464	hsa-mir-3615	FAM104A	0.309306365	mirna_pc
14465	hsa-mir-3615	MRPS15	0.331722009	mirna_pc
14466	hsa-mir-3615	C17orf58	0.321930363	mirna_pc
14467	hsa-mir-3615	NARS2	0.31624058	mirna_pc
14468	hsa-mir-3615	LYRM4	0.399717694	mirna_pc
14469	hsa-mir-3615	FAM133B	0.423813882	mirna_pc
14470	hsa-mir-3615	BCL11A	0.422704703	mirna_pc
14471	hsa-mir-3615	UTP11L	0.311513718	mirna_pc

14472	hsa-mir-3615	TIMM8B	0.316165423	mirna_pc
14473	hsa-mir-3615	ANP32A	0.329478627	mirna_pc
14474	hsa-mir-3615	NUFIP1	0.374988739	mirna_pc
14475	hsa-mir-3615	CFDP1	0.302267208	mirna_pc
14476	hsa-mir-3615	HYLS1	0.313942354	mirna_pc
14477	hsa-mir-3615	LCMT2	0.314672447	mirna_pc
14478	hsa-mir-3615	SNF8	0.307827823	mirna_pc
14479	hsa-mir-3615	HNRNPA0	0.353725036	mirna_pc
14480	hsa-mir-3615	ZNF300	0.331942158	mirna_pc
14481	hsa-mir-3615	MORN2	0.366393004	mirna_pc
14482	hsa-mir-3615	MTERF	0.484865215	mirna_pc
14483	hsa-mir-3615	AFMID	0.300953786	mirna_pc
14484	hsa-mir-3615	ATP5H	0.356114768	mirna_pc
14485	hsa-mir-3615	C7orf64	0.317364896	mirna_pc
14486	hsa-mir-3615	SLC25A33	0.321819665	mirna_pc
14487	hsa-mir-3615	OST4	0.306299676	mirna_pc
14488	hsa-mir-3615	BCS1L	0.345031112	mirna_pc
14489	hsa-mir-3615	ZNF394	0.329263523	mirna_pc
14490	hsa-mir-3615	DDX51	0.393922021	mirna_pc
14491	hsa-mir-3615	WDR83	0.317303098	mirna_pc
14492	hsa-mir-3615	UTP3	0.303632567	mirna_pc
14493	hsa-mir-3615	GTF2H2	0.425324585	mirna_pc
14494	hsa-mir-3615	ZBTB39	0.310766641	mirna_pc
14495	hsa-mir-3615	RPAIN	0.326477916	mirna_pc
14496	hsa-mir-3615	CCDC90B	0.36990397	mirna_pc
14497	hsa-mir-3615	API5	0.363635585	mirna_pc
14498	hsa-mir-3615	CIAO1	0.307936905	mirna_pc
14499	hsa-mir-3615	C2orf3	0.339560954	mirna_pc
14500	hsa-mir-3615	THAP4	0.338905132	mirna_pc
14501	hsa-mir-3615	KRIT1	0.355778883	mirna_pc
14502	hsa-mir-3615	DHRS2	0.414472994	mirna_pc
14503	hsa-mir-3615	ACOT13	0.347327306	mirna_pc
14504	hsa-mir-3615	FGFBP3	0.333494324	mirna_pc
14505	hsa-mir-3615	RRP7B	0.329234627	mirna_pc
14506	hsa-mir-3615	DNAJC19	0.399591328	mirna_pc
14507	hsa-mir-3615	TMEM134	0.341317376	mirna_pc
14508	hsa-mir-3615	PEX1	0.335433636	mirna_pc
14509	hsa-mir-3615	DIS3L2	0.438410017	mirna_pc
14510	hsa-mir-3615	MRPL19	0.336665769	mirna_pc
14511	hsa-mir-3615	CETN3	0.374216132	mirna_pc
14512	hsa-mir-3615	CDK3	0.304627819	mirna_pc
14513	hsa-mir-3615	B9D1	0.390572222	mirna_pc
14514	hsa-mir-3615	LSM 1.00	0.403022365	mirna_pc
14515	hsa-mir-3615	DYNC1I1	0.3986548	mirna_pc
14516	hsa-mir-3615	UTP15	0.329972813	mirna_pc
14517	hsa-mir-3615	AADAT	0.328766753	mirna_pc
14518	hsa-mir-3615	ACP1	0.330876699	mirna_pc
14519	hsa-mir-3615	ZNF322A	0.462345475	mirna_pc
14520	hsa-mir-3615	SERF1A	0.398907775	mirna_pc
14521	hsa-mir-3615	PRMT6	0.302991268	mirna_pc
14522	hsa-mir-3615	LOC401397	0.504761385	mirna_pc
14523	hsa-mir-3615	C21orf119	0.39764801	mirna_pc
14524	hsa-mir-3615	PRPSAP1	0.314586588	mirna_pc
14525	hsa-mir-3615	C2orf28	0.350079097	mirna_pc

14526	hsa-mir-3615	MRPL40	0.31909761	mirna_pc
14527	hsa-mir-3615	AARSD1	0.389580079	mirna_pc
14528	hsa-mir-3615	TMEM14B	0.321539229	mirna_pc
14529	hsa-mir-3615	AGA	0.306117641	mirna_pc
14530	hsa-mir-3615	TMEM168	0.42577852	mirna_pc
14531	hsa-mir-3615	MRPL53	0.428678979	mirna_pc
14532	hsa-mir-3615	DNMT3A	0.310566629	mirna_pc
14533	hsa-mir-3615	NDUFS8	0.374884383	mirna_pc
14534	hsa-mir-3615	C12orf73	0.330350936	mirna_pc
14535	hsa-mir-3615	NMRAL1	0.312662631	mirna_pc
14536	hsa-mir-25	MKI67	0.315825822	mirna_pc
14537	hsa-mir-25	TPX2	0.340559721	mirna_pc
14538	hsa-mir-25	CENPF	0.462508824	mirna_pc
14539	hsa-mir-25	RCC2	0.400135264	mirna_pc
14540	hsa-mir-25	HOXC9	0.365366052	mirna_pc
14541	hsa-mir-25	KIF11	0.419919488	mirna_pc
14542	hsa-mir-25	FOXM1	0.336634755	mirna_pc
14543	hsa-mir-25	CDC25C	0.310417907	mirna_pc
14544	hsa-mir-25	MYBL2	0.353825009	mirna_pc
14545	hsa-mir-25	UBE2C	0.339970268	mirna_pc
14546	hsa-mir-25	BUB1	0.325420811	mirna_pc
14547	hsa-mir-25	NUSAP1	0.360772014	mirna_pc
14548	hsa-mir-25	KIFC1	0.361385317	mirna_pc
14549	hsa-mir-25	BUB1B	0.361342198	mirna_pc
14550	hsa-mir-25	HDGF	0.388133295	mirna_pc
14551	hsa-mir-25	KIF18B	0.35009805	mirna_pc
14552	hsa-mir-25	ASPM	0.370761589	mirna_pc
14553	hsa-mir-25	KIF2C	0.359258469	mirna_pc
14554	hsa-mir-25	FANCA	0.325406691	mirna_pc
14555	hsa-mir-25	NCAPG	0.353344695	mirna_pc
14556	hsa-mir-25	SGOL1	0.331762214	mirna_pc
14557	hsa-mir-25	CCNA2	0.478974818	mirna_pc
14558	hsa-mir-25	SPC25	0.376271338	mirna_pc
14559	hsa-mir-25	FANCI	0.400120826	mirna_pc
14560	hsa-mir-25	NEK2	0.445221692	mirna_pc
14561	hsa-mir-25	MND1	0.541344525	mirna_pc
14562	hsa-mir-25	KIF22	0.332428737	mirna_pc
14563	hsa-mir-25	NCAPH	0.383419368	mirna_pc
14564	hsa-mir-25	GTSE1	0.361851558	mirna_pc
14565	hsa-mir-25	KIF23	0.308042914	mirna_pc
14566	hsa-mir-25	RAD54L	0.515780931	mirna_pc
14567	hsa-mir-25	CENPA	0.381108938	mirna_pc
14568	hsa-mir-25	MAD2L1	0.528730117	mirna_pc
14569	hsa-mir-25	TIMELESS	0.534836302	mirna_pc
14570	hsa-mir-25	LMNB1	0.377650083	mirna_pc
14571	hsa-mir-25	CDCA8	0.36633785	mirna_pc
14572	hsa-mir-25	TROAP	0.436042623	mirna_pc
14573	hsa-mir-25	CDCA5	0.364168946	mirna_pc
14574	hsa-mir-25	TACC3	0.312144167	mirna_pc
14575	hsa-mir-25	XPO1	0.405239539	mirna_pc
14576	hsa-mir-25	CDC45	0.315002585	mirna_pc
14577	hsa-mir-25	STMN1	0.477442162	mirna_pc
14578	hsa-mir-25	EXO1	0.35764948	mirna_pc
14579	hsa-mir-25	CDCA2	0.302861222	mirna_pc

14580	hsa-mir-25	UBE2T	0.309750253	mirna_pc
14581	hsa-mir-25	CKS1B	0.369596962	mirna_pc
14582	hsa-mir-25	TUBB	0.401704103	mirna_pc
14583	hsa-mir-25	MCM2	0.411452632	mirna_pc
14584	hsa-mir-25	ORC1L	0.378664677	mirna_pc
14585	hsa-mir-25	KNTC1	0.432913211	mirna_pc
14586	hsa-mir-25	CCNF	0.33563408	mirna_pc
14587	hsa-mir-25	CCNB2	0.330101375	mirna_pc
14588	hsa-mir-25	NUF2	0.383366914	mirna_pc
14589	hsa-mir-25	SFRP1	0.57079772	mirna_pc
14590	hsa-mir-25	PLK4	0.549613887	mirna_pc
14591	hsa-mir-25	FANCB	0.370728338	mirna_pc
14592	hsa-mir-25	KIF18A	0.395432176	mirna_pc
14593	hsa-mir-25	DEPDC1	0.4084805	mirna_pc
14594	hsa-mir-25	KIF14	0.333208244	mirna_pc
14595	hsa-mir-25	HJURP	0.328890871	mirna_pc
14596	hsa-mir-25	RAD51	0.341556645	mirna_pc
14597	hsa-mir-25	BLM	0.421971606	mirna_pc
14598	hsa-mir-25	DNMT1	0.319014985	mirna_pc
14599	hsa-mir-25	PAICS	0.301510442	mirna_pc
14600	hsa-mir-25	CCDC150	0.365855689	mirna_pc
14601	hsa-mir-25	BIRC5	0.320804989	mirna_pc
14602	hsa-mir-25	LIG1	0.366996122	mirna_pc
14603	hsa-mir-25	DTL	0.556017145	mirna_pc
14604	hsa-mir-25	EME1	0.366353476	mirna_pc
14605	hsa-mir-25	CENPK	0.301398239	mirna_pc
14606	hsa-mir-25	AURKB	0.426299059	mirna_pc
14607	hsa-mir-25	GSG2	0.354583823	mirna_pc
14608	hsa-mir-25	MCM10	0.3862809	mirna_pc
14609	hsa-mir-25	SGOL2	0.360880012	mirna_pc
14610	hsa-mir-25	POLQ	0.377239388	mirna_pc
14611	hsa-mir-25	KIF15	0.418704431	mirna_pc
14612	hsa-mir-25	CDCA3	0.41756799	mirna_pc
14613	hsa-mir-25	C1orf112	0.453436828	mirna_pc
14614	hsa-mir-25	GINS1	0.30504724	mirna_pc
14615	hsa-mir-25	FEN1	0.308951251	mirna_pc
14616	hsa-mir-25	TRAIP	0.318135459	mirna_pc
14617	hsa-mir-25	ORC6L	0.329258789	mirna_pc
14618	hsa-mir-25	C16orf59	0.432450795	mirna_pc
14619	hsa-mir-25	C17orf53	0.325680459	mirna_pc
14620	hsa-mir-25	NEIL3	0.475373296	mirna_pc
14621	hsa-mir-25	MCM7	0.710196168	mirna_pc
14622	hsa-mir-25	C12orf48	0.374515211	mirna_pc
14623	hsa-mir-25	HNRNPL	0.332135544	mirna_pc
14624	hsa-mir-25	SMC4	0.339301954	mirna_pc
14625	hsa-mir-25	ZWINT	0.341810236	mirna_pc
14626	hsa-mir-25	TUBA1B	0.324139507	mirna_pc
14627	hsa-mir-25	HELLS	0.341379788	mirna_pc
14628	hsa-mir-25	NCAPD2	0.409087396	mirna_pc
14629	hsa-mir-25	ILF2	0.323416672	mirna_pc
14630	hsa-mir-25	TCF3	0.303089593	mirna_pc
14631	hsa-mir-25	C6orf167	0.352717534	mirna_pc
14632	hsa-mir-25	BRCA1	0.348998376	mirna_pc
14633	hsa-mir-25	MCM5	0.328249881	mirna_pc

14634	hsa-mir-25	MCM6	0.437814313	mirna_pc
14635	hsa-mir-25	CDCA7	0.362829986	mirna_pc
14636	hsa-mir-25	EPR1	0.397078652	mirna_pc
14637	hsa-mir-25	KIAA1524	0.358081739	mirna_pc
14638	hsa-mir-25	BRIP1	0.380005467	mirna_pc
14639	hsa-mir-25	PIF1	0.328977372	mirna_pc
14640	hsa-mir-25	UCK2	0.389924059	mirna_pc
14641	hsa-mir-25	THOC4	0.303045705	mirna_pc
14642	hsa-mir-25	NFKBIL2	0.338289124	mirna_pc
14643	hsa-mir-25	C15orf42	0.449246815	mirna_pc
14644	hsa-mir-25	FAM60A	0.315974742	mirna_pc
14645	hsa-mir-25	FAM64A	0.359053833	mirna_pc
14646	hsa-mir-25	ATAD5	0.432473763	mirna_pc
14647	hsa-mir-25	E2F1	0.342272482	mirna_pc
14648	hsa-mir-25	SNRPA	0.345746468	mirna_pc
14649	hsa-mir-25	FUS	0.321526537	mirna_pc
14650	hsa-mir-25	U2AF2	0.316057809	mirna_pc
14651	hsa-mir-25	CHAF1A	0.330749847	mirna_pc
14652	hsa-mir-25	CENPO	0.568907455	mirna_pc
14653	hsa-mir-25	XRCC2	0.323373051	mirna_pc
14654	hsa-mir-25	GEN1	0.351712982	mirna_pc
14655	hsa-mir-25	NUDT1	0.304369397	mirna_pc
14656	hsa-mir-25	HMGB2	0.482098337	mirna_pc
14657	hsa-mir-25	GINS2	0.372184327	mirna_pc
14658	hsa-mir-25	DNA2	0.337695888	mirna_pc
14659	hsa-mir-25	E2F7	0.439616705	mirna_pc
14660	hsa-mir-25	MLF1IP	0.390534753	mirna_pc
14661	hsa-mir-25	C16orf75	0.368325327	mirna_pc
14662	hsa-mir-25	CAD	0.396240897	mirna_pc
14663	hsa-mir-25	CDK2	0.397674081	mirna_pc
14664	hsa-mir-25	CDK4	0.3849468	mirna_pc
14665	hsa-mir-25	TMEM108	0.606549098	mirna_pc
14666	hsa-mir-25	CHTF18	0.323070366	mirna_pc
14667	hsa-mir-25	HNRNPR	0.30460043	mirna_pc
14668	hsa-mir-25	PRIM1	0.315270818	mirna_pc
14669	hsa-mir-25	CENPH	0.35028527	mirna_pc
14670	hsa-mir-25	DHX34	0.309890045	mirna_pc
14671	hsa-mir-25	MSH2	0.399501529	mirna_pc
14672	hsa-mir-25	SF3B3	0.435348244	mirna_pc
14673	hsa-mir-25	CDC7	0.389080764	mirna_pc
14674	hsa-mir-25	RFC4	0.402179886	mirna_pc
14675	hsa-mir-25	FBXO5	0.431375394	mirna_pc
14676	hsa-mir-25	MDK	0.327975808	mirna_pc
14677	hsa-mir-25	DDX11	0.410957412	mirna_pc
14678	hsa-mir-25	CDC25A	0.371297252	mirna_pc
14679	hsa-mir-25	TOPBP1	0.349316153	mirna_pc
14680	hsa-mir-25	OTX1	0.300141125	mirna_pc
14681	hsa-mir-25	H2AFZ	0.388676079	mirna_pc
14682	hsa-mir-25	DDX12	0.511761347	mirna_pc
14683	hsa-mir-25	MCM8	0.432266974	mirna_pc
14684	hsa-mir-25	SNRPE	0.303825477	mirna_pc
14685	hsa-mir-25	MARCKSL1	0.363983398	mirna_pc
14686	hsa-mir-25	TFAP4	0.342027979	mirna_pc
14687	hsa-mir-25	PUS7	0.348225104	mirna_pc

14688	hsa-mir-25	CBX8	0.310273739	mirna_pc
14689	hsa-mir-25	TMEM201	0.358186922	mirna_pc
14690	hsa-mir-25	SRRT	0.624063312	mirna_pc
14691	hsa-mir-25	PSRC1	0.536472435	mirna_pc
14692	hsa-mir-25	DBF4B	0.320534765	mirna_pc
14693	hsa-mir-25	POU2F1	0.324418263	mirna_pc
14694	hsa-mir-25	HNRNPM	0.340503901	mirna_pc
14695	hsa-mir-25	TMPO	0.406629875	mirna_pc
14696	hsa-mir-25	SSBP3	0.315453952	mirna_pc
14697	hsa-mir-25	SMPD4	0.348931647	mirna_pc
14698	hsa-mir-25	GPR19	0.386366701	mirna_pc
14699	hsa-mir-25	GABPB1	0.44330802	mirna_pc
14700	hsa-mir-25	DHX9	0.379223856	mirna_pc
14701	hsa-mir-25	CCDC138	0.407079814	mirna_pc
14702	hsa-mir-25	LOC100128191	0.392422828	mirna_pc
14703	hsa-mir-25	HMGN2	0.37398434	mirna_pc
14704	hsa-mir-25	ETV4	0.416840385	mirna_pc
14705	hsa-mir-25	POLA2	0.315760621	mirna_pc
14706	hsa-mir-25	SFRS1	0.356302886	mirna_pc
14707	hsa-mir-25	SKP2	0.357352111	mirna_pc
14708	hsa-mir-25	SMARCD1	0.334886869	mirna_pc
14709	hsa-mir-25	ATXN2L	0.316215449	mirna_pc
14710	hsa-mir-25	C4orf46	0.526286032	mirna_pc
14711	hsa-mir-25	FZD2	0.319384403	mirna_pc
14712	hsa-mir-25	RFC5	0.331591421	mirna_pc
14713	hsa-mir-25	MAZ	0.353309052	mirna_pc
14714	hsa-mir-25	SSRP1	0.316733012	mirna_pc
14715	hsa-mir-25	POLR2D	0.436911312	mirna_pc
14716	hsa-mir-25	KHDRBS1	0.389666417	mirna_pc
14717	hsa-mir-25	TUB	0.309551707	mirna_pc
14718	hsa-mir-25	SPAST	0.353877644	mirna_pc
14719	hsa-mir-25	MTL 5.00	0.573273806	mirna_pc
14720	hsa-mir-25	PARP1	0.340600836	mirna_pc
14721	hsa-mir-25	DNAJC2	0.371246183	mirna_pc
14722	hsa-mir-25	C4orf21	0.458403164	mirna_pc
14723	hsa-mir-25	TMEM194A	0.523406975	mirna_pc
14724	hsa-mir-25	AP1S1	0.387178685	mirna_pc
14725	hsa-mir-25	SLC39A10	0.441646134	mirna_pc
14726	hsa-mir-25	SUV420H2	0.339865797	mirna_pc
14727	hsa-mir-25	ZNF695	0.374220079	mirna_pc
14728	hsa-mir-25	NEDD1	0.356094148	mirna_pc
14729	hsa-mir-25	HAUS6	0.321769363	mirna_pc
14730	hsa-mir-25	CEP135	0.357929252	mirna_pc
14731	hsa-mir-25	HNRNPU	0.335835982	mirna_pc
14732	hsa-mir-25	PSMC2	0.397897121	mirna_pc
14733	hsa-mir-25	NASP	0.41718667	mirna_pc
14734	hsa-mir-25	BCL2	0.518806863	mirna_pc
14735	hsa-mir-25	PTMA	0.327700567	mirna_pc
14736	hsa-mir-25	ELK1	0.308210993	mirna_pc
14737	hsa-mir-25	C11orf84	0.353108391	mirna_pc
14738	hsa-mir-25	SLC12A9	0.401612975	mirna_pc
14739	hsa-mir-25	DCHS2	0.317794128	mirna_pc
14740	hsa-mir-25	SUV39H2	0.426206256	mirna_pc
14741	hsa-mir-25	GORAB	0.383192292	mirna_pc

14742	hsa-mir-25	HNRNPD	0.417943958	mirna_pc
14743	hsa-mir-25	SHOX2	0.433746215	mirna_pc
14744	hsa-mir-25	H1FX	0.317280759	mirna_pc
14745	hsa-mir-25	RAD9A	0.33811719	mirna_pc
14746	hsa-mir-25	ELAVL1	0.324729472	mirna_pc
14747	hsa-mir-25	KIAA0406	0.324946599	mirna_pc
14748	hsa-mir-25	PSMC3IP	0.330993304	mirna_pc
14749	hsa-mir-25	PMAIP1	0.353871397	mirna_pc
14750	hsa-mir-25	POP7	0.47236363	mirna_pc
14751	hsa-mir-25	DHFR	0.343840152	mirna_pc
14752	hsa-mir-25	ILF3	0.373493348	mirna_pc
14753	hsa-mir-25	TAF6	0.531100012	mirna_pc
14754	hsa-mir-25	C1orf96	0.329432655	mirna_pc
14755	hsa-mir-25	EHMT2	0.432358975	mirna_pc
14756	hsa-mir-25	UBAP2L	0.344747836	mirna_pc
14757	hsa-mir-25	DEK	0.3466388	mirna_pc
14758	hsa-mir-25	PLOD3	0.376793168	mirna_pc
14759	hsa-mir-25	PASK	0.407445877	mirna_pc
14760	hsa-mir-25	RPGRIP1L	0.310277244	mirna_pc
14761	hsa-mir-25	LRWD1	0.465442906	mirna_pc
14762	hsa-mir-25	HEATR2	0.401298723	mirna_pc
14763	hsa-mir-25	LSM 6.00	0.39986637	mirna_pc
14764	hsa-mir-25	PRAME	0.365500924	mirna_pc
14765	hsa-mir-25	FBXW8	0.307659162	mirna_pc
14766	hsa-mir-25	FUBP1	0.338555363	mirna_pc
14767	hsa-mir-25	SUV39H1	0.377777314	mirna_pc
14768	hsa-mir-25	IPO9	0.405487566	mirna_pc
14769	hsa-mir-25	SART3	0.409044469	mirna_pc
14770	hsa-mir-25	TRA2B	0.457249237	mirna_pc
14771	hsa-mir-25	RSRC1	0.334705183	mirna_pc
14772	hsa-mir-25	TRAF4	0.348374998	mirna_pc
14773	hsa-mir-25	GAR1	0.353621997	mirna_pc
14774	hsa-mir-25	C7orf47	0.442171537	mirna_pc
14775	hsa-mir-25	CDRT4	0.492780193	mirna_pc
14776	hsa-mir-25	C3orf21	0.363467503	mirna_pc
14777	hsa-mir-25	CPXM1	0.325971252	mirna_pc
14778	hsa-mir-25	PMS2L1	0.418668045	mirna_pc
14779	hsa-mir-25	IGF2BP2	0.346513907	mirna_pc
14780	hsa-mir-25	WHSC1	0.39851748	mirna_pc
14781	hsa-mir-25	ITGB3BP	0.371276712	mirna_pc
14782	hsa-mir-25	BCL2A1	0.335304837	mirna_pc
14783	hsa-mir-25	C4orf43	0.310238871	mirna_pc
14784	hsa-mir-25	SFPQ	0.369374637	mirna_pc
14785	hsa-mir-25	CD83	0.505727888	mirna_pc
14786	hsa-mir-25	FAM125B	0.444414089	mirna_pc
14787	hsa-mir-25	AS3MT	0.31461206	mirna_pc
14788	hsa-mir-25	CADM1	0.405839939	mirna_pc
14789	hsa-mir-25	POLA1	0.354109159	mirna_pc
14790	hsa-mir-25	C10orf119	0.30113019	mirna_pc
14791	hsa-mir-25	POGK	0.31047839	mirna_pc
14792	hsa-mir-25	CPAMD8	0.431979291	mirna_pc
14793	hsa-mir-25	SALL2	0.347257602	mirna_pc
14794	hsa-mir-25	FAM86C	0.389490578	mirna_pc
14795	hsa-mir-25	MSH6	0.457513507	mirna_pc

14796	hsa-mir-25	LEF1	0.507793855	mirna_pc
14797	hsa-mir-25	SMC3	0.309671802	mirna_pc
14798	hsa-mir-25	FLVCR1	0.316184432	mirna_pc
14799	hsa-mir-25	KREMEN2	0.501481929	mirna_pc
14800	hsa-mir-25	TRIP6	0.384608242	mirna_pc
14801	hsa-mir-25	MEX3A	0.694160664	mirna_pc
14802	hsa-mir-25	MOGS	0.306576293	mirna_pc
14803	hsa-mir-25	IRAK1BP1	0.328592611	mirna_pc
14804	hsa-mir-25	TAF5	0.376979592	mirna_pc
14805	hsa-mir-25	FANCC	0.384490631	mirna_pc
14806	hsa-mir-25	TIFA	0.439911728	mirna_pc
14807	hsa-mir-25	CCDC28B	0.361325033	mirna_pc
14808	hsa-mir-25	UNC119B	0.378270901	mirna_pc
14809	hsa-mir-25	HNRNPA1L2	0.330271843	mirna_pc
14810	hsa-mir-25	PPFIA1	0.347960114	mirna_pc
14811	hsa-mir-25	EMILIN3	0.345224011	mirna_pc
14812	hsa-mir-25	ZBTB12	0.435785713	mirna_pc
14813	hsa-mir-25	MCTP2	0.521988543	mirna_pc
14814	hsa-mir-25	LOC642846	0.478275341	mirna_pc
14815	hsa-mir-25	TGIF2	0.539740016	mirna_pc
14816	hsa-mir-25	SENP1	0.371457752	mirna_pc
14817	hsa-mir-25	RCN2	0.397736374	mirna_pc
14818	hsa-mir-25	MTF2	0.366680316	mirna_pc
14819	hsa-mir-25	ETV6	0.304797255	mirna_pc
14820	hsa-mir-25	PXDN	0.345928255	mirna_pc
14821	hsa-mir-25	POLE	0.540514825	mirna_pc
14822	hsa-mir-25	MUTYH	0.321080373	mirna_pc
14823	hsa-mir-25	RBM14	0.356164137	mirna_pc
14824	hsa-mir-25	EXOSC9	0.349073895	mirna_pc
14825	hsa-mir-25	RBMX	0.416248899	mirna_pc
14826	hsa-mir-25	ORC5L	0.524718952	mirna_pc
14827	hsa-mir-25	MDC 1.00	0.419230583	mirna_pc
14828	hsa-mir-25	CTCF	0.384646218	mirna_pc
14829	hsa-mir-25	RPL39L	0.419651624	mirna_pc
14830	hsa-mir-25	EPHB4	0.639469899	mirna_pc
14831	hsa-mir-25	IGSF9	0.521314735	mirna_pc
14832	hsa-mir-25	DNAJC14	0.325278542	mirna_pc
14833	hsa-mir-25	FIP1L1	0.3248551	mirna_pc
14834	hsa-mir-25	ZNF519	0.352666088	mirna_pc
14835	hsa-mir-25	CIT	0.377249606	mirna_pc
14836	hsa-mir-25	KDM2B	0.391885158	mirna_pc
14837	hsa-mir-25	UFSP1	0.366900766	mirna_pc
14838	hsa-mir-25	VWA2	0.338796703	mirna_pc
14839	hsa-mir-25	NKD2	0.329160088	mirna_pc
14840	hsa-mir-25	FRMD4A	0.470322162	mirna_pc
14841	hsa-mir-25	MYB	0.501964781	mirna_pc
14842	hsa-mir-25	ITPRIPL1	0.462222269	mirna_pc
14843	hsa-mir-25	TTLL4	0.362678356	mirna_pc
14844	hsa-mir-25	C20orf177	0.338616113	mirna_pc
14845	hsa-mir-25	EMILIN1	0.305261728	mirna_pc
14846	hsa-mir-25	ATXN7L2	0.357600599	mirna_pc
14847	hsa-mir-25	ACBD7	0.319692398	mirna_pc
14848	hsa-mir-25	POTEF	0.320367321	mirna_pc
14849	hsa-mir-25	ATN1	0.318087816	mirna_pc

14850	hsa-mir-25	ANKRD6	0.450589222	mirna_pc
14851	hsa-mir-25	BCL7A	0.414259454	mirna_pc
14852	hsa-mir-25	TRPA1	0.54221849	mirna_pc
14853	hsa-mir-25	PALM2	0.303925767	mirna_pc
14854	hsa-mir-25	ARNT2	0.387233319	mirna_pc
14855	hsa-mir-25	ARHGAP19	0.3863653	mirna_pc
14856	hsa-mir-25	ZNF286A	0.535098184	mirna_pc
14857	hsa-mir-25	SPDYE3	0.365956239	mirna_pc
14858	hsa-mir-25	MRPL21	0.538803449	mirna_pc
14859	hsa-mir-25	DMRTA2	0.349029199	mirna_pc
14860	hsa-mir-25	ZNF84	0.302347304	mirna_pc
14861	hsa-mir-25	PRR3	0.327447511	mirna_pc
14862	hsa-mir-25	TRIM4	0.330305711	mirna_pc
14863	hsa-mir-25	CNTROB	0.333527897	mirna_pc
14864	hsa-mir-25	TRRAP	0.526401508	mirna_pc
14865	hsa-mir-25	ORAI2	0.451819894	mirna_pc
14866	hsa-mir-25	MMP25	0.30380801	mirna_pc
14867	hsa-mir-25	SEMA4F	0.408632585	mirna_pc
14868	hsa-mir-25	SLC25A13	0.35388318	mirna_pc
14869	hsa-mir-25	ZNF286B	0.41852184	mirna_pc
14870	hsa-mir-25	DDX20	0.351140344	mirna_pc
14871	hsa-mir-25	INPPL1	0.491957681	mirna_pc
14872	hsa-mir-25	SEC61A2	0.343081073	mirna_pc
14873	hsa-mir-25	GPR3	0.421731811	mirna_pc
14874	hsa-mir-25	E2F6	0.362249541	mirna_pc
14875	hsa-mir-25	HUNK	0.3137111	mirna_pc
14876	hsa-mir-25	PILRB	0.475080541	mirna_pc
14877	hsa-mir-25	SFRS13A	0.32421942	mirna_pc
14878	hsa-mir-25	GNB2	0.32641279	mirna_pc
14879	hsa-mir-25	SCLY	0.590512561	mirna_pc
14880	hsa-mir-25	GPR125	0.416345398	mirna_pc
14881	hsa-mir-25	FIZ1	0.322412697	mirna_pc
14882	hsa-mir-25	LDLRAD3	0.441266102	mirna_pc
14883	hsa-mir-25	COPS6	0.424444716	mirna_pc
14884	hsa-mir-25	GSTCD	0.341598685	mirna_pc
14885	hsa-mir-25	DHRS13	0.406550435	mirna_pc
14886	hsa-mir-25	RTTN	0.316588775	mirna_pc
14887	hsa-mir-25	SMC1A	0.332654753	mirna_pc
14888	hsa-mir-25	DHX57	0.403967695	mirna_pc
14889	hsa-mir-25	TSC22D4	0.327889595	mirna_pc
14890	hsa-mir-25	NUMA1	0.459920935	mirna_pc
14891	hsa-mir-25	ALX3	0.575121891	mirna_pc
14892	hsa-mir-25	ACVR2B	0.412199394	mirna_pc
14893	hsa-mir-25	SFT2D3	0.360552863	mirna_pc
14894	hsa-mir-25	LIG3	0.30669093	mirna_pc
14895	hsa-mir-25	DPY19L2P2	0.329684708	mirna_pc
14896	hsa-mir-25	POLR2J	0.420968577	mirna_pc
14897	hsa-mir-25	ZKSCAN1	0.304188112	mirna_pc
14898	hsa-mir-25	CNPY4	0.304222272	mirna_pc
14899	hsa-mir-25	SFRS7	0.347327926	mirna_pc
14900	hsa-mir-25	ALKBH4	0.472995252	mirna_pc
14901	hsa-mir-25	PFAS	0.341595093	mirna_pc
14902	hsa-mir-25	LRTOMT	0.482608095	mirna_pc
14903	hsa-mir-25	ARMC10	0.406584793	mirna_pc

14904	hsa-mir-25	RBM10		0.319877552	mirna_pc
14905	hsa-mir-25	BAT2		0.346673377	mirna_pc
14906	hsa-mir-25	EDAR		0.390216251	mirna_pc
14907	hsa-mir-25	IFT81		0.332615364	mirna_pc
14908	hsa-mir-25	ORAOV1		0.390599757	mirna_pc
14909	hsa-mir-25	ZNF184		0.345891624	mirna_pc
14910	hsa-mir-25	NUP153		0.341619332	mirna_pc
14911	hsa-mir-25	WEE1		0.30119151	mirna_pc
14912	hsa-mir-25	ARHGEF19		0.360228061	mirna_pc
14913	hsa-mir-25	C17orf75		0.333049356	mirna_pc
14914	hsa-mir-25	THAP5		0.391627922	mirna_pc
14915	hsa-mir-25	CCND1		0.381338078	mirna_pc
14916	hsa-mir-25	NFATC3		0.327295322	mirna_pc
14917	hsa-mir-25	HCFC1		0.337546377	mirna_pc
14918	hsa-mir-25	ZNF771		0.394918362	mirna_pc
14919	hsa-mir-25		3-Sep	0.305666721	mirna_pc
14920	hsa-mir-25	TCTN2		0.532606321	mirna_pc
14921	hsa-mir-25	BCAN		0.41791726	mirna_pc
14922	hsa-mir-25	ZNF669		0.308263235	mirna_pc
14923	hsa-mir-25	LRP6		0.360418462	mirna_pc
14924	hsa-mir-25	EXTL2		0.300796785	mirna_pc
14925	hsa-mir-25	DCP2		0.332474121	mirna_pc
14926	hsa-mir-25	PRKD3		0.420918261	mirna_pc
14927	hsa-mir-25	C2orf44		0.419506702	mirna_pc
14928	hsa-mir-25	SEPHS1		0.473092197	mirna_pc
14929	hsa-mir-25	SHISA2		0.519208668	mirna_pc
14930	hsa-mir-25	CBX5		0.454164937	mirna_pc
14931	hsa-mir-25	RYK		0.320990197	mirna_pc
14932	hsa-mir-25	ADNP		0.327364069	mirna_pc
14933	hsa-mir-25	IFT172		0.328619231	mirna_pc
14934	hsa-mir-25	TTC30B		0.381513694	mirna_pc
14935	hsa-mir-25	MSL2		0.335109832	mirna_pc
14936	hsa-mir-25	AGBL5		0.538417759	mirna_pc
14937	hsa-mir-25	VASH2		0.493443449	mirna_pc
14938	hsa-mir-25	NAV2		0.339338628	mirna_pc
14939	hsa-mir-25	MYL6B		0.432604011	mirna_pc
14940	hsa-mir-25	IRF2BP2		0.31745048	mirna_pc
14941	hsa-mir-25	ZNF498		0.535987785	mirna_pc
14942	hsa-mir-25	CD200		0.508100146	mirna_pc
14943	hsa-mir-25	RABL5		0.521203736	mirna_pc
14944	hsa-mir-25	HNRNPUL1		0.31797849	mirna_pc
14945	hsa-mir-25	CHST11		0.399584792	mirna_pc
14946	hsa-mir-25	BCL11A		0.470441524	mirna_pc
14947	hsa-mir-25	C4orf27		0.314476836	mirna_pc
14948	hsa-mir-25	ZNF512		0.346650177	mirna_pc
14949	hsa-mir-25	PMPCB		0.379730839	mirna_pc
14950	hsa-mir-25	ZNF473		0.366191993	mirna_pc
14951	hsa-mir-25	COPS7B		0.323598133	mirna_pc
14952	hsa-mir-25	USP21		0.440088636	mirna_pc
14953	hsa-mir-25	ALMS1		0.417343502	mirna_pc
14954	hsa-mir-25	C7orf43		0.340357906	mirna_pc
14955	hsa-mir-25	TRAF3IP3		0.33850928	mirna_pc
14956	hsa-mir-25	DCLRE1A		0.318070187	mirna_pc
14957	hsa-mir-25	ANP32A		0.398036654	mirna_pc

14958	hsa-mir-25	RNF121	0.423603938	mirna_pc
14959	hsa-mir-25	CPT1A	0.361766569	mirna_pc
14960	hsa-mir-25	ZMYM4	0.338983029	mirna_pc
14961	hsa-mir-25	AP4M1	0.551919837	mirna_pc
14962	hsa-mir-25	LMAN2L	0.309328725	mirna_pc
14963	hsa-mir-25	TCF7L1	0.401807498	mirna_pc
14964	hsa-mir-25	C17orf81	0.318938955	mirna_pc
14965	hsa-mir-25	THAP9	0.305695142	mirna_pc
14966	hsa-mir-25	HNRNPH3	0.328136302	mirna_pc
14967	hsa-mir-25	NAP1L1	0.310979015	mirna_pc
14968	hsa-mir-25	RABL2A	0.358493517	mirna_pc
14969	hsa-mir-25	NSL1	0.354324397	mirna_pc
14970	hsa-mir-25	CNIH2	0.326195271	mirna_pc
14971	hsa-mir-25	LRCH4	0.328149112	mirna_pc
14972	hsa-mir-25	TRDMT1	0.329189069	mirna_pc
14973	hsa-mir-25	ZNHIT1	0.347128098	mirna_pc
14974	hsa-mir-25	IQCC	0.355750789	mirna_pc
14975	hsa-mir-25	GATAD2B	0.308528593	mirna_pc
14976	hsa-mir-25	ALG10B	0.326975852	mirna_pc
14977	hsa-mir-25	ZSCAN21	0.50121224	mirna_pc
14978	hsa-mir-25	C11orf59	0.409540355	mirna_pc
14979	hsa-mir-25	GATS	0.349061247	mirna_pc
14980	hsa-mir-25	PKP4	0.301813522	mirna_pc
14981	hsa-mir-25	PEG10	0.398253326	mirna_pc
14982	hsa-mir-25	CCNJ	0.340274261	mirna_pc
14983	hsa-mir-25	MYCN	0.612656308	mirna_pc
14984	hsa-mir-25	IFT122	0.319197425	mirna_pc
14985	hsa-mir-25	EFCAB7	0.307616692	mirna_pc
14986	hsa-mir-25	DCAF16	0.392205119	mirna_pc
14987	hsa-mir-25	SBK1	0.494572815	mirna_pc
14988	hsa-mir-25	PABPC4L	0.546856583	mirna_pc
14989	hsa-mir-25	FAM171A1	0.317819938	mirna_pc
14990	hsa-mir-25	THNSL1	0.306847981	mirna_pc
14991	hsa-mir-25	USP13	0.450338357	mirna_pc
14992	hsa-mir-25	RASL10B	0.301290313	mirna_pc
14993	hsa-mir-25	ZNF3	0.631126229	mirna_pc
14994	hsa-mir-25	CD69	0.541903123	mirna_pc
14995	hsa-mir-25	DNM1P35	0.34694073	mirna_pc
14996	hsa-mir-25	MLL5	0.302148063	mirna_pc
14997	hsa-mir-25	ERLIN1	0.328133557	mirna_pc
14998	hsa-mir-25	C16orf46	0.368117544	mirna_pc
14999	hsa-mir-25	MSI1	0.440271871	mirna_pc
15000	hsa-mir-25	CLPB	0.376790962	mirna_pc
15001	hsa-mir-25	SERTAD4	0.355175165	mirna_pc
15002	hsa-mir-25	SCAI	0.359349514	mirna_pc
15003	hsa-mir-25	LMTK2	0.301029689	mirna_pc
15004	hsa-mir-25	GXYLT1	0.308446419	mirna_pc
15005	hsa-mir-25	N4BP2	0.301287292	mirna_pc
15006	hsa-mir-25	ZNF740	0.372652726	mirna_pc
15007	hsa-mir-25	EP400	0.325714566	mirna_pc
15008	hsa-mir-25	TRO	0.356585047	mirna_pc
15009	hsa-mir-25	PACRGL	0.342266326	mirna_pc
15010	hsa-mir-25	PBX2	0.306298485	mirna_pc
15011	hsa-mir-25	ZNF232	0.505363843	mirna_pc

15012	hsa-mir-25	ZBTB39	0.549707051	mirna_pc
15013	hsa-mir-25	NRCAM	0.354734841	mirna_pc
15014	hsa-mir-25	C20orf12	0.336836177	mirna_pc
15015	hsa-mir-25	FANCF	0.349272115	mirna_pc
15016	hsa-mir-25	VPS54	0.349740329	mirna_pc
15017	hsa-mir-25	RBBP4	0.37069106	mirna_pc
15018	hsa-mir-25	NCOA5	0.32852008	mirna_pc
15019	hsa-mir-25	CDKN1B	0.324029534	mirna_pc
15020	hsa-mir-25	ZNF618	0.427055785	mirna_pc
15021	hsa-mir-25	BRSK1	0.31363491	mirna_pc
15022	hsa-mir-25	CCDC111	0.320396813	mirna_pc
15023	hsa-mir-25	ZNF713	0.307149936	mirna_pc
15024	hsa-mir-25	GIGYF1	0.545547805	mirna_pc
15025	hsa-mir-25	RRP7B	0.302279735	mirna_pc
15026	hsa-mir-25	LRDD	0.383769287	mirna_pc
15027	hsa-mir-25	CCDC157	0.480862747	mirna_pc
15028	hsa-mir-25	PDGFA	0.425234342	mirna_pc
15029	hsa-mir-25	SCML2	0.452028253	mirna_pc
15030	hsa-mir-25	ZNF594	0.309039041	mirna_pc
15031	hsa-mir-25	MEPCE	0.56115149	mirna_pc
15032	hsa-mir-25	ZCCHC3	0.355275309	mirna_pc
15033	hsa-mir-25	RRP15	0.317373312	mirna_pc
15034	hsa-mir-25	COL9A2	0.316957919	mirna_pc
15035	hsa-mir-25	MSTO2P	0.308389415	mirna_pc
15036	hsa-mir-25	ETV5	0.624353029	mirna_pc
15037	hsa-mir-25	DACT2	0.352238377	mirna_pc
15038	hsa-mir-25	NUCKS1	0.318358711	mirna_pc
15039	hsa-mir-25	AADAT	0.435638797	mirna_pc
15040	hsa-mir-25	CBLL1	0.373568991	mirna_pc
15041	hsa-mir-25	IGHMBP2	0.590503712	mirna_pc
15042	hsa-mir-25	ABTB2	0.338665943	mirna_pc
15043	hsa-mir-25	C11orf51	0.431757413	mirna_pc
15044	hsa-mir-25	DNAL4	0.303489238	mirna_pc
15045	hsa-mir-25	FZD1	0.430781644	mirna_pc
15046	hsa-mir-25	B4GALNT4	0.398106212	mirna_pc
15047	hsa-mir-25	PRMT6	0.343841635	mirna_pc
15048	hsa-mir-25	HSDL1	0.32905798	mirna_pc
15049	hsa-mir-25	PRKRIP1	0.424545413	mirna_pc
15050	hsa-mir-25	PDE4B	0.364096173	mirna_pc
15051	hsa-mir-25	C7orf59	0.371770432	mirna_pc
15052	hsa-mir-25	H2AFY2	0.345367002	mirna_pc
15053	hsa-mir-25	MBD6	0.36268246	mirna_pc
15054	hsa-mir-25	TRIL	0.425326869	mirna_pc
15055	hsa-mir-25	KLHL23	0.305692171	mirna_pc
15056	hsa-mir-25	PCDH18	0.392787514	mirna_pc
15057	hsa-mir-25	SMARCAD1	0.374953676	mirna_pc
15058	hsa-mir-25	SCHIP1	0.321976219	mirna_pc
15059	hsa-mir-25	VPS37D	0.301883606	mirna_pc
15060	hsa-mir-25	SV2A	0.33158148	mirna_pc
15061	hsa-mir-25	EFNB3	0.368958163	mirna_pc
15062	hsa-mir-25	CENPV	0.326979469	mirna_pc
15063	hsa-mir-25	DNMT3A	0.584275342	mirna_pc
15064	hsa-mir-25	CKAP4	0.309004006	mirna_pc
15065	hsa-mir-25	TET1	0.442258767	mirna_pc

15066	hsa-mir-25	PRTFDC1	0.301848794	mirna_pc
15067	hsa-mir-25	FAM200A	0.438979652	mirna_pc
15068	hsa-mir-25	MOSPD3	0.349654739	mirna_pc
15069	hsa-mir-25	ZNF711	0.320865777	mirna_pc
15070	hsa-mir-25	TRIM56	0.315547583	mirna_pc
15071	hsa-mir-25	POM121	0.300808424	mirna_pc
15072	hsa-mir-15a	MKI67	0.31545403	mirna_pc
15073	hsa-mir-15a	BUB1	0.313411748	mirna_pc
15074	hsa-mir-15a	KIFC1	0.309663904	mirna_pc
15075	hsa-mir-15a	CKAP2	0.442786403	mirna_pc
15076	hsa-mir-15a	FANCA	0.395732876	mirna_pc
15077	hsa-mir-15a	SGOL1	0.378452785	mirna_pc
15078	hsa-mir-15a	ARHGAP11A	0.337241451	mirna_pc
15079	hsa-mir-15a	CCNA2	0.303186357	mirna_pc
15080	hsa-mir-15a	CBX3	0.38134324	mirna_pc
15081	hsa-mir-15a	SPC25	0.367491691	mirna_pc
15082	hsa-mir-15a	NUP62	0.331533483	mirna_pc
15083	hsa-mir-15a	NEK2	0.300438683	mirna_pc
15084	hsa-mir-15a	GTSE1	0.369812155	mirna_pc
15085	hsa-mir-15a	MAD2L1	0.320417551	mirna_pc
15086	hsa-mir-15a	LMNB1	0.35242167	mirna_pc
15087	hsa-mir-15a	ASF1B	0.313471756	mirna_pc
15088	hsa-mir-15a	XPO1	0.315929827	mirna_pc
15089	hsa-mir-15a	CCNF	0.301135932	mirna_pc
15090	hsa-mir-15a	NUF2	0.345153344	mirna_pc
15091	hsa-mir-15a	PLK4	0.315771572	mirna_pc
15092	hsa-mir-15a	SKA3	0.445679486	mirna_pc
15093	hsa-mir-15a	HJURP	0.350707428	mirna_pc
15094	hsa-mir-15a	BLM	0.320271772	mirna_pc
15095	hsa-mir-15a	CCDC150	0.331564388	mirna_pc
15096	hsa-mir-15a	LIG1	0.305901666	mirna_pc
15097	hsa-mir-15a	EME1	0.315420876	mirna_pc
15098	hsa-mir-15a	CENPK	0.386512901	mirna_pc
15099	hsa-mir-15a	SGOL2	0.347222801	mirna_pc
15100	hsa-mir-15a	CENPE	0.345022122	mirna_pc
15101	hsa-mir-15a	C1orf112	0.342775207	mirna_pc
15102	hsa-mir-15a	GINS1	0.401108957	mirna_pc
15103	hsa-mir-15a	TRAIP	0.315429818	mirna_pc
15104	hsa-mir-15a	EZH2	0.32584098	mirna_pc
15105	hsa-mir-15a	HELLS	0.336536885	mirna_pc
15106	hsa-mir-15a	BRCA1	0.33816716	mirna_pc
15107	hsa-mir-15a	HNRNPA2B1	0.39438443	mirna_pc
15108	hsa-mir-15a	MCM6	0.363016286	mirna_pc
15109	hsa-mir-15a	PIF1	0.361540994	mirna_pc
15110	hsa-mir-15a	SFRS2	0.335566455	mirna_pc
15111	hsa-mir-15a	ATAD5	0.331422114	mirna_pc
15112	hsa-mir-15a	FAM111B	0.314197025	mirna_pc
15113	hsa-mir-15a	ATAD2	0.367260322	mirna_pc
15114	hsa-mir-15a	RFC3	0.459495689	mirna_pc
15115	hsa-mir-15a	DEPDC1B	0.309172662	mirna_pc
15116	hsa-mir-15a	HOXA11	0.325232119	mirna_pc
15117	hsa-mir-15a	ANP32E	0.322582727	mirna_pc
15118	hsa-mir-15a	BRCA2	0.455786548	mirna_pc
15119	hsa-mir-15a	GEN1	0.459947349	mirna_pc

15120	hsa-mir-15a	HMGB2	0.382293347	mirna_pc
15121	hsa-mir-15a	DNA2	0.322217787	mirna_pc
15122	hsa-mir-15a	NOP56	0.34394453	mirna_pc
15123	hsa-mir-15a	TCF19	0.3219807	mirna_pc
15124	hsa-mir-15a	HOXA10	0.3703557	mirna_pc
15125	hsa-mir-15a	DLEU2	0.486294935	mirna_pc
15126	hsa-mir-15a	SSB	0.362466707	mirna_pc
15127	hsa-mir-15a	PUS1	0.310456919	mirna_pc
15128	hsa-mir-15a	FBXO5	0.313059614	mirna_pc
15129	hsa-mir-15a	FIGNL1	0.343205582	mirna_pc
15130	hsa-mir-15a	E2F3	0.439949388	mirna_pc
15131	hsa-mir-15a	DSCC1	0.314453456	mirna_pc
15132	hsa-mir-15a	C13orf34	0.509214723	mirna_pc
15133	hsa-mir-15a	CLN6	0.31001133	mirna_pc
15134	hsa-mir-15a	MCM8	0.376664908	mirna_pc
15135	hsa-mir-15a	DNAJB11	0.308639049	mirna_pc
15136	hsa-mir-15a	C14orf106	0.347883757	mirna_pc
15137	hsa-mir-15a	CBX8	0.330778625	mirna_pc
15138	hsa-mir-15a	TRMT6	0.313503801	mirna_pc
15139	hsa-mir-15a	SRRT	0.359125656	mirna_pc
15140	hsa-mir-15a	DBF4B	0.308853373	mirna_pc
15141	hsa-mir-15a	MARVELD3	0.306462293	mirna_pc
15142	hsa-mir-15a	C13orf37	0.422326492	mirna_pc
15143	hsa-mir-15a	HNRNPM	0.331982082	mirna_pc
15144	hsa-mir-15a	LSM 12.00	0.301250896	mirna_pc
15145	hsa-mir-15a	HMGN2	0.301925593	mirna_pc
15146	hsa-mir-15a	TRPM2	0.306587837	mirna_pc
15147	hsa-mir-15a	SFRS1	0.474668376	mirna_pc
15148	hsa-mir-15a	C4orf46	0.326472524	mirna_pc
15149	hsa-mir-15a	FTSJ2	0.353245348	mirna_pc
15150	hsa-mir-15a	PDSS1	0.305793146	mirna_pc
15151	hsa-mir-15a	CENPJ	0.409144183	mirna_pc
15152	hsa-mir-15a	C21orf58	0.305156164	mirna_pc
15153	hsa-mir-15a	PTMA	0.320686004	mirna_pc
15154	hsa-mir-15a	SUV39H2	0.300149305	mirna_pc
15155	hsa-mir-15a	FANCL	0.310245634	mirna_pc
15156	hsa-mir-15a	HNRNPD	0.323907151	mirna_pc
15157	hsa-mir-15a	GTF2F2	0.35933927	mirna_pc
15158	hsa-mir-15a	POP1	0.348676712	mirna_pc
15159	hsa-mir-15a	HSP90B1	0.324513823	mirna_pc
15160	hsa-mir-15a	NUDT15	0.384300496	mirna_pc
15161	hsa-mir-15a	FASTKD1	0.350715724	mirna_pc
15162	hsa-mir-15a	ATP13A3	0.317265119	mirna_pc
15163	hsa-mir-15a	DHFR	0.359246016	mirna_pc
15164	hsa-mir-15a	DEK	0.309768779	mirna_pc
15165	hsa-mir-15a	TRIB3	0.347531532	mirna_pc
15166	hsa-mir-15a	CCNE2	0.311451887	mirna_pc
15167	hsa-mir-15a	PASK	0.30772653	mirna_pc
15168	hsa-mir-15a	C1orf131	0.353114929	mirna_pc
15169	hsa-mir-15a	HEATR2	0.306865446	mirna_pc
15170	hsa-mir-15a	SNRPB2	0.347516506	mirna_pc
15171	hsa-mir-15a	HNRNPK	0.303052406	mirna_pc
15172	hsa-mir-15a	SR140	0.320686884	mirna_pc
15173	hsa-mir-15a	GAR1	0.334013541	mirna_pc

15174	hsa-mir-15a	CEP152	0.331278358	mirna_pc
15175	hsa-mir-15a	RNF219	0.535490929	mirna_pc
15176	hsa-mir-15a	ENOPH1	0.320071067	mirna_pc
15177	hsa-mir-15a	C17orf42	0.392429497	mirna_pc
15178	hsa-mir-15a	POMP	0.309938196	mirna_pc
15179	hsa-mir-15a	KIAA0564	0.30308752	mirna_pc
15180	hsa-mir-15a	XRN2	0.362895679	mirna_pc
15181	hsa-mir-15a	CASP3	0.360540807	mirna_pc
15182	hsa-mir-15a	SMC3	0.311950857	mirna_pc
15183	hsa-mir-15a	BAT1	0.353255542	mirna_pc
15184	hsa-mir-15a	COIL	0.311191926	mirna_pc
15185	hsa-mir-15a	NANP	0.349303001	mirna_pc
15186	hsa-mir-15a	SUZ12	0.315020783	mirna_pc
15187	hsa-mir-15a	HMGB1	0.471443794	mirna_pc
15188	hsa-mir-15a	ZC3H18	0.375526443	mirna_pc
15189	hsa-mir-15a	PIK3AP1	0.363027562	mirna_pc
15190	hsa-mir-15a	SNX5	0.35434589	mirna_pc
15191	hsa-mir-15a	THUMPD2	0.306301509	mirna_pc
15192	hsa-mir-15a	FAM96A	0.307952671	mirna_pc
15193	hsa-mir-15a	SOX9	0.353243721	mirna_pc
15194	hsa-mir-15a	SNORD1C	0.340791485	mirna_pc
15195	hsa-mir-15a	C13orf27	0.34524696	mirna_pc
15196	hsa-mir-15a	EXOSC9	0.300703163	mirna_pc
15197	hsa-mir-15a	GK3P	0.303452453	mirna_pc
15198	hsa-mir-15a	RBMX	0.341073372	mirna_pc
15199	hsa-mir-15a	GPR180	0.356846258	mirna_pc
15200	hsa-mir-15a	EXOSC8	0.310036364	mirna_pc
15201	hsa-mir-15a	PDIA6	0.306758067	mirna_pc
15202	hsa-mir-15a	ZNF519	0.335840033	mirna_pc
15203	hsa-mir-15a	SCLT1	0.312009492	mirna_pc
15204	hsa-mir-15a	WDR90	0.305171263	mirna_pc
15205	hsa-mir-15a	CCDC14	0.344411346	mirna_pc
15206	hsa-mir-15a	SUZ12P	0.312960914	mirna_pc
15207	hsa-mir-15a	FAM136A	0.343252099	mirna_pc
15208	hsa-mir-15a	COG3	0.300524834	mirna_pc
15209	hsa-mir-15a	RBCK1	0.337803618	mirna_pc
15210	hsa-mir-15a	EML4	0.320621622	mirna_pc
15211	hsa-mir-15a	BZW2	0.365138676	mirna_pc
15212	hsa-mir-15a	CSNK2A1	0.343451623	mirna_pc
15213	hsa-mir-15a	RPIA	0.40249618	mirna_pc
15214	hsa-mir-15a	C7orf44	0.323700335	mirna_pc
15215	hsa-mir-15a	NAT9	0.338756176	mirna_pc
15216	hsa-mir-15a	NAA16	0.388607093	mirna_pc
15217	hsa-mir-15a	SLC27A2	0.341431238	mirna_pc
15218	hsa-mir-15a	UTP14C	0.363241851	mirna_pc
15219	hsa-mir-15a	DLEU1	0.36924777	mirna_pc
15220	hsa-mir-15a	C13orf23	0.376796845	mirna_pc
15221	hsa-mir-15a	TCERG1	0.352747288	mirna_pc
15222	hsa-mir-15a	PSPC1	0.32752557	mirna_pc
15223	hsa-mir-15a	TOP1P1	0.315625141	mirna_pc
15224	hsa-mir-15a	E2F6	0.340209556	mirna_pc
15225	hsa-mir-15a	SLC25A15	0.433706817	mirna_pc
15226	hsa-mir-15a	SFRS13A	0.34398126	mirna_pc
15227	hsa-mir-15a	TGDS	0.392593305	mirna_pc

15228	hsa-mir-15a	KIAA1704	0.358036537	mirna_pc
15229	hsa-mir-15a	ZNF828	0.335660273	mirna_pc
15230	hsa-mir-15a	SMC1A	0.331921836	mirna_pc
15231	hsa-mir-15a	NUPL1	0.437741384	mirna_pc
15232	hsa-mir-15a	WBP4	0.351486562	mirna_pc
15233	hsa-mir-15a	SUGT1	0.319196224	mirna_pc
15234	hsa-mir-15a	ANKRD5	0.383984297	mirna_pc
15235	hsa-mir-15a	DPP4	0.359892009	mirna_pc
15236	hsa-mir-15a	UPF3A	0.322105515	mirna_pc
15237	hsa-mir-15a	SFRS7	0.327194173	mirna_pc
15238	hsa-mir-15a	CCAR1	0.313916083	mirna_pc
15239	hsa-mir-15a	RBM10	0.347820929	mirna_pc
15240	hsa-mir-15a	UFM1	0.374407507	mirna_pc
15241	hsa-mir-15a	KBTBD7	0.367471441	mirna_pc
15242	hsa-mir-15a	ATP7B	0.307558933	mirna_pc
15243	hsa-mir-15a	RENBP	0.310250904	mirna_pc
15244	hsa-mir-15a	SYAP1	0.300368691	mirna_pc
15245	hsa-mir-15a	RCBTB1	0.346348114	mirna_pc
15246	hsa-mir-15a	SPRY4	0.309668786	mirna_pc
15247	hsa-mir-15a	RNF6	0.383676277	mirna_pc
15248	hsa-mir-15a	ALG5	0.372730806	mirna_pc
15249	hsa-mir-15a	IWS1	0.319433902	mirna_pc
15250	hsa-mir-15a	MAP2K6	0.333755815	mirna_pc
15251	hsa-mir-15a	CASP6	0.320195919	mirna_pc
15252	hsa-mir-15a	RBM26	0.315368605	mirna_pc
15253	hsa-mir-15a	ZNF92	0.335558982	mirna_pc
15254	hsa-mir-15a	FASTKD5	0.41185733	mirna_pc
15255	hsa-mir-15a	TASP1	0.326778581	mirna_pc
15256	hsa-mir-15a	IPO5	0.32727349	mirna_pc
15257	hsa-mir-15a	ALG11	0.377624005	mirna_pc
15258	hsa-mir-15a	MCCC2	0.305754223	mirna_pc
15259	hsa-mir-15a	MTRF1	0.373047348	mirna_pc
15260	hsa-mir-15a	ZNF124	0.40388247	mirna_pc
15261	hsa-mir-15a	RNASEH2B	0.482156646	mirna_pc
15262	hsa-mir-15a	RFXAP	0.359852897	mirna_pc
15263	hsa-mir-15a	ANP32A	0.327323251	mirna_pc
15264	hsa-mir-15a	DIS3	0.338928774	mirna_pc
15265	hsa-mir-15a	STARD3	0.345470352	mirna_pc
15266	hsa-mir-15a	NUFIP1	0.409671801	mirna_pc
15267	hsa-mir-15a	ZNF195	0.3963912	mirna_pc
15268	hsa-mir-15a	GZF1	0.349519884	mirna_pc
15269	hsa-mir-15a	MED4	0.364462988	mirna_pc
15270	hsa-mir-15a	NEK3	0.451254588	mirna_pc
15271	hsa-mir-15a	THSD1P1	0.430530854	mirna_pc
15272	hsa-mir-15a	TMEM170A	0.316696071	mirna_pc
15273	hsa-mir-15a	FAM48A	0.442794419	mirna_pc
15274	hsa-mir-15a	DNAJC15	0.30294623	mirna_pc
15275	hsa-mir-15a	PNN	0.303664579	mirna_pc
15276	hsa-mir-15a	PAN3	0.3204284	mirna_pc
15277	hsa-mir-15a	ZMYM2	0.302724738	mirna_pc
15278	hsa-mir-15a	CCNJ	0.300792664	mirna_pc
15279	hsa-mir-15a	PIBF1	0.311845929	mirna_pc
15280	hsa-mir-15a	KBTBD6	0.338231263	mirna_pc
15281	hsa-mir-15a	ESF1	0.301320325	mirna_pc

15282	hsa-mir-15a	SUGT1L1	0.357819805	mirna_pc
15283	hsa-mir-15a	CTPS2	0.344365818	mirna_pc
15284	hsa-mir-15a	XPO4	0.356052588	mirna_pc
15285	hsa-mir-15a	PDX1	0.329939054	mirna_pc
15286	hsa-mir-15a	SNCAIP	0.304716408	mirna_pc
15287	hsa-mir-15a	IFT88	0.354496626	mirna_pc
15288	hsa-mir-15a	RPS10P7	0.30638741	mirna_pc
15289	hsa-mir-15a	FANCF	0.349334399	mirna_pc
15290	hsa-mir-15a	PDS5B	0.300549271	mirna_pc
15291	hsa-mir-15a	CIAO1	0.30987621	mirna_pc
15292	hsa-mir-15a	VPS36	0.403901658	mirna_pc
15293	hsa-mir-15a	NCRNA00183	0.303518759	mirna_pc
15294	hsa-mir-15a	KPNA3	0.405158081	mirna_pc
15295	hsa-mir-15a	ZCCHC3	0.422798414	mirna_pc
15296	hsa-mir-15a	KATNAL2	0.346845295	mirna_pc
15297	hsa-mir-15a	GTF3A	0.31525767	mirna_pc
15298	hsa-mir-15a	TPP2	0.325410484	mirna_pc
15299	hsa-mir-15a	ZNF530	0.303182875	mirna_pc
15300	hsa-mir-15a	MIR17HG	0.437195993	mirna_pc
15301	hsa-mir-15a	AARSD1	0.324879239	mirna_pc
15302	hsa-mir-15a	ELF1	0.386869381	mirna_pc
15303	hsa-mir-15a	MRPS31	0.392976708	mirna_pc
15304	hsa-mir-15a	DNMT3A	0.306607248	mirna_pc
15305	hsa-mir-15a	ZNF670	0.305890016	mirna_pc
15306	hsa-mir-3651	CDK1	0.398643161	mirna_pc
15307	hsa-mir-3651	ANLN	0.387850099	mirna_pc
15308	hsa-mir-3651	NCAPG	0.315107496	mirna_pc
15309	hsa-mir-3651	CLSPN	0.317284629	mirna_pc
15310	hsa-mir-3651	MELK	0.31703632	mirna_pc
15311	hsa-mir-3651	DLGAP5	0.33531159	mirna_pc
15312	hsa-mir-3651	FANCB	0.330274586	mirna_pc
15313	hsa-mir-3651	CKAP2L	0.317195957	mirna_pc
15314	hsa-mir-3651	CENPK	0.426969366	mirna_pc
15315	hsa-mir-3651	CENPE	0.317012881	mirna_pc
15316	hsa-mir-3651	CDKN3	0.301565446	mirna_pc
15317	hsa-mir-3651	HMMR	0.361624649	mirna_pc
15318	hsa-mir-3651	HELLS	0.31418878	mirna_pc
15319	hsa-mir-3651	NCAPD2	0.301906794	mirna_pc
15320	hsa-mir-3651	C6orf167	0.311349283	mirna_pc
15321	hsa-mir-3651	PRR11	0.303157527	mirna_pc
15322	hsa-mir-3651	KIAA1524	0.320184803	mirna_pc
15323	hsa-mir-3651	BRIP1	0.388120702	mirna_pc
15324	hsa-mir-3651	WDHD1	0.305442797	mirna_pc
15325	hsa-mir-3651	ATAD2	0.333619467	mirna_pc
15326	hsa-mir-3651	DNAJC9	0.340136236	mirna_pc
15327	hsa-mir-3651	TEAD4	0.362506041	mirna_pc
15328	hsa-mir-3651	DNA2	0.301609365	mirna_pc
15329	hsa-mir-3651	CDCA4	0.300134565	mirna_pc
15330	hsa-mir-3651	E2F7	0.340103499	mirna_pc
15331	hsa-mir-3651	NOP2	0.318473397	mirna_pc
15332	hsa-mir-3651	CDC7	0.328209157	mirna_pc
15333	hsa-mir-3651	FBXO5	0.300830036	mirna_pc
15334	hsa-mir-3651	SMC2	0.48113875	mirna_pc
15335	hsa-mir-3651	C17orf51	0.424174198	mirna_pc

15336	hsa-mir-3651	ZNF367	0.378345832	mirna_pc
15337	hsa-mir-3651	PUS7	0.368356956	mirna_pc
15338	hsa-mir-3651	EIF2AK2	0.305221536	mirna_pc
15339	hsa-mir-3651	NUDCD1	0.374576471	mirna_pc
15340	hsa-mir-3651	PPAT	0.301429664	mirna_pc
15341	hsa-mir-3651	TEX10	0.378168249	mirna_pc
15342	hsa-mir-3651	MICB	0.305227602	mirna_pc
15343	hsa-mir-3651	VAV2	0.410340373	mirna_pc
15344	hsa-mir-3651	FIGN	0.304551186	mirna_pc
15345	hsa-mir-3651	HSP90AA1	0.322311475	mirna_pc
15346	hsa-mir-3651	CCDC18	0.337816008	mirna_pc
15347	hsa-mir-3651	ROD1	0.300226661	mirna_pc
15348	hsa-mir-3651	HAUS6	0.34621789	mirna_pc
15349	hsa-mir-3651	PTPN12	0.368567994	mirna_pc
15350	hsa-mir-3651	PBX3	0.330887255	mirna_pc
15351	hsa-mir-3651	CCL4L2	0.304822831	mirna_pc
15352	hsa-mir-3651	MMP12	0.475368922	mirna_pc
15353	hsa-mir-3651	PLEK2	0.534848391	mirna_pc
15354	hsa-mir-3651	CCNE2	0.323381086	mirna_pc
15355	hsa-mir-3651	CTSL2	0.40070675	mirna_pc
15356	hsa-mir-3651	C14orf145	0.345312032	mirna_pc
15357	hsa-mir-3651	STX17	0.308241926	mirna_pc
15358	hsa-mir-3651	DDX21	0.308867967	mirna_pc
15359	hsa-mir-3651	NUP188	0.303597688	mirna_pc
15360	hsa-mir-3651	HNRNPK	0.369590209	mirna_pc
15361	hsa-mir-3651	CTNNAL1	0.65172003	mirna_pc
15362	hsa-mir-3651	RAD23B	0.30453571	mirna_pc
15363	hsa-mir-3651	CEP78	0.417422113	mirna_pc
15364	hsa-mir-3651	RUFY2	0.300983384	mirna_pc
15365	hsa-mir-3651	M6PR	0.315077491	mirna_pc
15366	hsa-mir-3651	KPNB1	0.335450736	mirna_pc
15367	hsa-mir-3651	OTUD6B	0.458027127	mirna_pc
15368	hsa-mir-3651	RECQL	0.394513638	mirna_pc
15369	hsa-mir-3651	TMX1	0.312675534	mirna_pc
15370	hsa-mir-3651	NCBP1	0.397263115	mirna_pc
15371	hsa-mir-3651	FANCM	0.338619419	mirna_pc
15372	hsa-mir-3651	USP1	0.324369907	mirna_pc
15373	hsa-mir-3651	HIATL1	0.350866285	mirna_pc
15374	hsa-mir-3651	C9orf30	0.319912647	mirna_pc
15375	hsa-mir-3651	SEMA3D	0.30185939	mirna_pc
15376	hsa-mir-3651	DDX47	0.317845139	mirna_pc
15377	hsa-mir-3651	C9orf40	0.376941765	mirna_pc
15378	hsa-mir-3651	PPP3R1	0.340405138	mirna_pc
15379	hsa-mir-3651	ERCC4	0.30959723	mirna_pc
15380	hsa-mir-3651	CAB39	0.381781072	mirna_pc
15381	hsa-mir-3651	CCDC41	0.349204498	mirna_pc
15382	hsa-mir-3651	LOC647121	0.320650889	mirna_pc
15383	hsa-mir-3651	NOL8	0.333352024	mirna_pc
15384	hsa-mir-3651	CSPG4	0.36214812	mirna_pc
15385	hsa-mir-3651	PIGH	0.301460625	mirna_pc
15386	hsa-mir-3651	CHORDC1	0.380032492	mirna_pc
15387	hsa-mir-3651	RPE	0.308028103	mirna_pc
15388	hsa-mir-3651	ARNTL2	0.393978506	mirna_pc
15389	hsa-mir-3651	ISCA1P1	0.323197278	mirna_pc

15390	hsa-mir-3651	GOLT1B	0.400238828	mirna_pc
15391	hsa-mir-3651	KIAA0020	0.306123909	mirna_pc
15392	hsa-mir-3651	HEPHL1	0.416067621	mirna_pc
15393	hsa-mir-3651	FER	0.361964143	mirna_pc
15394	hsa-mir-3651	PHTF2	0.392048239	mirna_pc
15395	hsa-mir-3651	NBN	0.31024395	mirna_pc
15396	hsa-mir-3651	CYP26B1	0.375763976	mirna_pc
15397	hsa-mir-3651	GLMN	0.302799474	mirna_pc
15398	hsa-mir-3651	RMI1	0.43977288	mirna_pc
15399	hsa-mir-3651	TMEM67	0.367103614	mirna_pc
15400	hsa-mir-3651	BAZ1B	0.357534981	mirna_pc
15401	hsa-mir-3651	FMNL2	0.307397474	mirna_pc
15402	hsa-mir-3651	GNAI1	0.326801713	mirna_pc
15403	hsa-mir-3651	PSMD5	0.437749645	mirna_pc
15404	hsa-mir-3651	IL12RB2	0.352973303	mirna_pc
15405	hsa-mir-3651	SPTLC1	0.374218428	mirna_pc
15406	hsa-mir-3651	MYBL1	0.316395969	mirna_pc
15407	hsa-mir-3651	COL17A1	0.331922244	mirna_pc
15408	hsa-mir-3651	COL13A1	0.303696327	mirna_pc
15409	hsa-mir-3651	PAPOLA	0.337453714	mirna_pc
15410	hsa-mir-3651	TMEM38B	0.331232194	mirna_pc
15411	hsa-mir-3651	PTPDC1	0.319072142	mirna_pc
15412	hsa-mir-3651	KIAA1033	0.394808331	mirna_pc
15413	hsa-mir-3651	WDR78	0.323315971	mirna_pc
15414	hsa-mir-3651	C8orf37	0.340198011	mirna_pc
15415	hsa-mir-3651	PDGFC	0.352491015	mirna_pc
15416	hsa-mir-3651	CREB5	0.344920055	mirna_pc
15417	hsa-mir-3651	THAP5	0.347451982	mirna_pc
15418	hsa-mir-3651	GTF3C4	0.370242414	mirna_pc
15419	hsa-mir-3651	SERPINE2	0.389821912	mirna_pc
15420	hsa-mir-3651	SPIN1	0.356273714	mirna_pc
15421	hsa-mir-3651	SPOPL	0.323994351	mirna_pc
15422	hsa-mir-3651	DNAJA1	0.375484398	mirna_pc
15423	hsa-mir-3651	SLC31A2	0.51665634	mirna_pc
15424	hsa-mir-3651	KIF27	0.317148684	mirna_pc
15425	hsa-mir-3651	PPP2R5E	0.332274677	mirna_pc
15426	hsa-mir-3651	PDCD1LG2	0.334785188	mirna_pc
15427	hsa-mir-3651	UBQLN1	0.438046453	mirna_pc
15428	hsa-mir-3651	LPXN	0.33499919	mirna_pc
15429	hsa-mir-3651	CEP110	0.46908113	mirna_pc
15430	hsa-mir-3651	HERC4	0.441811688	mirna_pc
15431	hsa-mir-3651	BCLAF1	0.315610567	mirna_pc
15432	hsa-mir-3651	FUBP3	0.304992363	mirna_pc
15433	hsa-mir-3651	RTN4RL1	0.32400448	mirna_pc
15434	hsa-mir-3651	IL7R	0.431686169	mirna_pc
15435	hsa-mir-3651	NRG1	0.47108503	mirna_pc
15436	hsa-mir-3651	SEMA3A	0.426673131	mirna_pc
15437	hsa-mir-3651	GAPVD1	0.30313023	mirna_pc
15438	hsa-mir-3651	FLRT2	0.328802329	mirna_pc
15439	hsa-mir-3651	IFT74	0.323661323	mirna_pc
15440	hsa-mir-3651	UHRF2	0.329274311	mirna_pc
15441	hsa-mir-3651	ERGIC2	0.386594275	mirna_pc
15442	hsa-mir-3651	HAPLN1	0.348364005	mirna_pc
15443	hsa-mir-3651	SGPP1	0.359617982	mirna_pc

15444	hsa-mir-3651	HIATL2	0.316441704	mirna_pc
15445	hsa-mir-3651	DNAJC25	0.309136	mirna_pc
15446	hsa-mir-3651	IQCG	0.352825121	mirna_pc
15447	hsa-mir-3651	MFAP3L	0.35395069	mirna_pc
15448	hsa-mir-3651	RAB14	0.350109079	mirna_pc
15449	hsa-mir-3651	CREB1	0.300977544	mirna_pc
15450	hsa-mir-3651	IKBKAP	0.49429714	mirna_pc
15451	hsa-mir-3651	C9orf21	0.383398307	mirna_pc
15452	hsa-mir-3651	RC3H2	0.429151763	mirna_pc
15453	hsa-mir-3651	C10orf46	0.310715825	mirna_pc
15454	hsa-mir-3651	FKBP15	0.312957033	mirna_pc
15455	hsa-mir-3651	F3	0.397962001	mirna_pc
15456	hsa-mir-3651	GJC1	0.303098209	mirna_pc
15457	hsa-mir-3651	AGTPBP1	0.411566316	mirna_pc
15458	hsa-let-7c	HOXC8	0.390585933	mirna_pc
15459	hsa-let-7c	SEMA3E	0.862683824	mirna_pc
15460	hsa-let-7c	C4orf34	0.309684505	mirna_pc
15461	hsa-let-7c	EEF1A2	0.533364117	mirna_pc
15462	hsa-let-7c	MYEF2	0.329871559	mirna_pc
15463	hsa-let-7c	MYST4	0.507878522	mirna_pc
15464	hsa-let-7c	ZNF467	0.394503392	mirna_pc
15465	hsa-let-7c	ID4	0.422696398	mirna_pc
15466	hsa-let-7c	YPEL1	0.547173827	mirna_pc
15467	hsa-let-7c	CHEK2	0.336022376	mirna_pc
15468	hsa-let-7c	C22orf39	0.35190729	mirna_pc
15469	hsa-let-7c	ZNF425	0.310093791	mirna_pc
15470	hsa-let-7c	ZNF25	0.331043852	mirna_pc
15471	hsa-let-7c	CITED2	0.799532695	mirna_pc
15472	hsa-let-7c	FAM82B	0.312042799	mirna_pc
15473	hsa-let-7c	TBC1D9	0.311115941	mirna_pc
15474	hsa-let-7c	ATP1B2	0.308022329	mirna_pc
15475	hsa-let-7c	CRYZL1	0.306462581	mirna_pc
15476	hsa-let-7c	NR2F2	0.717109702	mirna_pc
15477	hsa-let-7c	HDHD2	0.355622922	mirna_pc
15478	hsa-let-7c	DTWD1	0.373989968	mirna_pc
15479	hsa-let-7c	RASGRP2	0.407355207	mirna_pc
15480	hsa-let-7c	MTERFD3	0.320476728	mirna_pc
15481	hsa-let-7c	MSH2	0.364513499	mirna_pc
15482	hsa-let-7c	FAT4	0.369915487	mirna_pc
15483	hsa-let-7c	ZNF439	0.482883306	mirna_pc
15484	hsa-let-7c	SUOX	0.303516586	mirna_pc
15485	hsa-let-7c	POLI	0.611880151	mirna_pc
15486	hsa-let-7c	RNF185	0.34939741	mirna_pc
15487	hsa-let-7c	TTC3	0.366815062	mirna_pc
15488	hsa-let-7c	LOC286367	0.330710066	mirna_pc
15489	hsa-let-7c	ZNF367	0.337115209	mirna_pc
15490	hsa-let-7c	CCDC106	0.389118921	mirna_pc
15491	hsa-let-7c	SLC5A12	0.431136906	mirna_pc
15492	hsa-let-7c	DLG4	0.391324503	mirna_pc
15493	hsa-let-7c	USP25	0.597664793	mirna_pc
15494	hsa-let-7c	LOC375190	0.673619923	mirna_pc
15495	hsa-let-7c	CRY1	0.393277011	mirna_pc
15496	hsa-let-7c	MTMR3	0.327899926	mirna_pc
15497	hsa-let-7c	HIST1H3D	0.400160104	mirna_pc

15498	hsa-let-7c	SLC2A11	0.568126802	mirna_pc
15499	hsa-let-7c	ZNF433	0.402962342	mirna_pc
15500	hsa-let-7c	TYW3	0.716218103	mirna_pc
15501	hsa-let-7c	TDRD3	0.326749654	mirna_pc
15502	hsa-let-7c	MKL2	0.34572796	mirna_pc
15503	hsa-let-7c	GAD1	0.365594179	mirna_pc
15504	hsa-let-7c	OXR1	0.417217119	mirna_pc
15505	hsa-let-7c	BBS4	0.308382929	mirna_pc
15506	hsa-let-7c	ZNF248	0.321761499	mirna_pc
15507	hsa-let-7c	TUB	0.372249018	mirna_pc
15508	hsa-let-7c	FIGN	0.846863723	mirna_pc
15509	hsa-let-7c	PRRT1	0.498806205	mirna_pc
15510	hsa-let-7c	ARSG	0.370961898	mirna_pc
15511	hsa-let-7c	SAMD11	0.56461491	mirna_pc
15512	hsa-let-7c	PHF10	0.474566106	mirna_pc
15513	hsa-let-7c	LOC284900	0.3974711	mirna_pc
15514	hsa-let-7c	RGL2	0.303615169	mirna_pc
15515	hsa-let-7c	CAPS2	0.702692127	mirna_pc
15516	hsa-let-7c	PHC1	0.421077525	mirna_pc
15517	hsa-let-7c	SCD5	0.344744562	mirna_pc
15518	hsa-let-7c	CCDC121	0.403462194	mirna_pc
15519	hsa-let-7c	SSBP2	0.326319443	mirna_pc
15520	hsa-let-7c	TFAP2A	0.303213411	mirna_pc
15521	hsa-let-7c	HABP4	0.774902461	mirna_pc
15522	hsa-let-7c	TBCEL	0.375111215	mirna_pc
15523	hsa-let-7c	C20orf160	0.53888005	mirna_pc
15524	hsa-let-7c	CSAD	0.431403017	mirna_pc
15525	hsa-let-7c	RNF2	0.302419938	mirna_pc
15526	hsa-let-7c	SFRS18	0.335143086	mirna_pc
15527	hsa-let-7c	CTSL2	0.40616331	mirna_pc
15528	hsa-let-7c	PRAME	0.410467903	mirna_pc
15529	hsa-let-7c	KIAA0776	0.427811337	mirna_pc
15530	hsa-let-7c	CA11	0.520553223	mirna_pc
15531	hsa-let-7c	TDG	0.317377785	mirna_pc
15532	hsa-let-7c	HEATR5B	0.303091541	mirna_pc
15533	hsa-let-7c	EIF4ENIF1	0.608061044	mirna_pc
15534	hsa-let-7c	BAIAP3	0.490952104	mirna_pc
15535	hsa-let-7c	LOC653501	0.336529725	mirna_pc
15536	hsa-let-7c	TUG1	0.454550337	mirna_pc
15537	hsa-let-7c	KIAA0182	0.307098522	mirna_pc
15538	hsa-let-7c	AMN1	0.532775056	mirna_pc
15539	hsa-let-7c	MGC21881	0.308394316	mirna_pc
15540	hsa-let-7c	FAM184A	0.376081205	mirna_pc
15541	hsa-let-7c	TDRD5	0.400956113	mirna_pc
15542	hsa-let-7c	CILP2	0.528728256	mirna_pc
15543	hsa-let-7c	ATP8B2	0.308242267	mirna_pc
15544	hsa-let-7c	ZNF70	0.399182622	mirna_pc
15545	hsa-let-7c	GRAMD1C	0.577949277	mirna_pc
15546	hsa-let-7c	ZNF217	0.531645351	mirna_pc
15547	hsa-let-7c	CYTSA	0.386288073	mirna_pc
15548	hsa-let-7c	FAM161A	0.326527723	mirna_pc
15549	hsa-let-7c	DYRK1A	0.34966192	mirna_pc
15550	hsa-let-7c	ZNF236	0.355341968	mirna_pc
15551	hsa-let-7c	EFHD1	0.425019787	mirna_pc

15552	hsa-let-7c	LPHN2	0.663846018	mirna_pc
15553	hsa-let-7c	HIST3H2A	0.351306561	mirna_pc
15554	hsa-let-7c	C6orf120	0.339474951	mirna_pc
15555	hsa-let-7c	ZNF23	0.313166915	mirna_pc
15556	hsa-let-7c	AMY2B	0.318672551	mirna_pc
15557	hsa-let-7c	CDKL3	0.332654237	mirna_pc
15558	hsa-let-7c	BARD1	0.412805402	mirna_pc
15559	hsa-let-7c	CNTNAP3	0.458280532	mirna_pc
15560	hsa-let-7c	RPL23AP53	0.338338105	mirna_pc
15561	hsa-let-7c	C16orf87	0.367223261	mirna_pc
15562	hsa-let-7c	EDIL3	0.515962403	mirna_pc
15563	hsa-let-7c	LOC144571	0.406241968	mirna_pc
15564	hsa-let-7c	ZNF608	0.415088782	mirna_pc
15565	hsa-let-7c	METTL13	0.36945727	mirna_pc
15566	hsa-let-7c	SEMA3D	0.876879951	mirna_pc
15567	hsa-let-7c	HACE1	0.41567808	mirna_pc
15568	hsa-let-7c	ZNF451	0.303464062	mirna_pc
15569	hsa-let-7c	KLRA1	0.344220985	mirna_pc
15570	hsa-let-7c	SLC25A30	0.526844625	mirna_pc
15571	hsa-let-7c	ZNF658	0.421342087	mirna_pc
15572	hsa-let-7c	ERCC4	0.303794909	mirna_pc
15573	hsa-let-7c	RBM43	0.408095863	mirna_pc
15574	hsa-let-7c	GATA3	0.586508381	mirna_pc
15575	hsa-let-7c	NBPF3	0.306487248	mirna_pc
15576	hsa-let-7c	LOC162632	0.30112691	mirna_pc
15577	hsa-let-7c	RNPC3	0.395063156	mirna_pc
15578	hsa-let-7c	OXTR	0.486007535	mirna_pc
15579	hsa-let-7c	PAQR7	0.367797624	mirna_pc
15580	hsa-let-7c	PEBP1	0.341473969	mirna_pc
15581	hsa-let-7c	THSD7A	0.636507814	mirna_pc
15582	hsa-let-7c	PPM1J	0.434199239	mirna_pc
15583	hsa-let-7c	ZNF605	0.460544437	mirna_pc
15584	hsa-let-7c	NDST2	0.346061894	mirna_pc
15585	hsa-let-7c	RRAGB	0.370625029	mirna_pc
15586	hsa-let-7c	KLRAQ1	0.401236773	mirna_pc
15587	hsa-let-7c	PCGF6	0.324202558	mirna_pc
15588	hsa-let-7c	OSR2	0.467282014	mirna_pc
15589	hsa-let-7c	C12orf76	0.377745324	mirna_pc
15590	hsa-let-7c	C6orf170	0.397726037	mirna_pc
15591	hsa-let-7c	GABPA	0.405218072	mirna_pc
15592	hsa-let-7c	ZFP161	0.324685014	mirna_pc
15593	hsa-let-7c	MEX3B	0.321085256	mirna_pc
15594	hsa-let-7c	SENP6	0.302646853	mirna_pc
15595	hsa-let-7c	ZNF503	0.440772129	mirna_pc
15596	hsa-let-7c	C8orf51	0.383558441	mirna_pc
15597	hsa-let-7c	LOC728392	0.312677007	mirna_pc
15598	hsa-let-7c	LANCL1	0.32417976	mirna_pc
15599	hsa-let-7c	AP3M1	0.336393852	mirna_pc
15600	hsa-let-7c	ZNF607	0.364013418	mirna_pc
15601	hsa-let-7c	FMR1	0.326948644	mirna_pc
15602	hsa-let-7c	RGS16	0.305519241	mirna_pc
15603	hsa-let-7c	KCNC3	0.569529993	mirna_pc
15604	hsa-let-7c	ZFYVE16	0.325985248	mirna_pc
15605	hsa-let-7c	PRRT2	0.322236749	mirna_pc

15606	hsa-let-7c	RASSF8	0.354567537	mirna_pc
15607	hsa-let-7c	PIK3C3	0.402034175	mirna_pc
15608	hsa-let-7c	ZNF362	0.354112112	mirna_pc
15609	hsa-let-7c	CHST10	0.303881342	mirna_pc
15610	hsa-let-7c	NBN	0.308777229	mirna_pc
15611	hsa-let-7c	ST8SIA1	0.341006144	mirna_pc
15612	hsa-let-7c	ZNF84	0.398536298	mirna_pc
15613	hsa-let-7c	TAF4B	0.393256395	mirna_pc
15614	hsa-let-7c	ZNF140	0.327821788	mirna_pc
15615	hsa-let-7c	TMEM67	0.314152779	mirna_pc
15616	hsa-let-7c	MTA3	0.441018063	mirna_pc
15617	hsa-let-7c	ZSCAN12	0.301236125	mirna_pc
15618	hsa-let-7c	PUS10	0.369480002	mirna_pc
15619	hsa-let-7c	SCAND3	0.308378871	mirna_pc
15620	hsa-let-7c	SEC61A2	0.453730707	mirna_pc
15621	hsa-let-7c	PSPC1	0.42980328	mirna_pc
15622	hsa-let-7c	INTU	0.322499298	mirna_pc
15623	hsa-let-7c	DRG1	0.33298845	mirna_pc
15624	hsa-let-7c	SSX2IP	0.326675925	mirna_pc
15625	hsa-let-7c	TMC8	0.300232439	mirna_pc
15626	hsa-let-7c	NF2	0.529003857	mirna_pc
15627	hsa-let-7c	KPNA5	0.351579248	mirna_pc
15628	hsa-let-7c	MYBL1	0.605499741	mirna_pc
15629	hsa-let-7c	ZNF624	0.318857161	mirna_pc
15630	hsa-let-7c	TCTE3	0.320575993	mirna_pc
15631	hsa-let-7c	WBP4	0.348149453	mirna_pc
15632	hsa-let-7c	CHRNB1	0.316663586	mirna_pc
15633	hsa-let-7c	CYR61	0.30292716	mirna_pc
15634	hsa-let-7c	RIMKLB	0.355070759	mirna_pc
15635	hsa-let-7c	GAS1	0.423810867	mirna_pc
15636	hsa-let-7c	EFNA5	0.626222067	mirna_pc
15637	hsa-let-7c	HCG18	0.357725152	mirna_pc
15638	hsa-let-7c	C18orf10	0.388322333	mirna_pc
15639	hsa-let-7c	C10orf41	0.381486917	mirna_pc
15640	hsa-let-7c	SUDS3	0.377644028	mirna_pc
15641	hsa-let-7c	MAMSTR	0.562068515	mirna_pc
15642	hsa-let-7c	TMEM136	0.491312538	mirna_pc
15643	hsa-let-7c	CCDC78	0.429619856	mirna_pc
15644	hsa-let-7c	DNM3	0.814523028	mirna_pc
15645	hsa-let-7c	RPL7	0.366181242	mirna_pc
15646	hsa-let-7c	SEMA3C	0.353000395	mirna_pc
15647	hsa-let-7c	PEX2	0.370908253	mirna_pc
15648	hsa-let-7c	WDR78	0.568926899	mirna_pc
15649	hsa-let-7c	KGFLP2	0.408658571	mirna_pc
15650	hsa-let-7c	IFT81	0.353633	mirna_pc
15651	hsa-let-7c	ZNF184	0.396445485	mirna_pc
15652	hsa-let-7c	MBLAC2	0.402035342	mirna_pc
15653	hsa-let-7c	NIPSNAP1	0.535319903	mirna_pc
15654	hsa-let-7c	DEPDC5	0.37823237	mirna_pc
15655	hsa-let-7c	C8orf37	0.693788665	mirna_pc
15656	hsa-let-7c	PDGFC	0.549770342	mirna_pc
15657	hsa-let-7c	SPA17	0.34953477	mirna_pc
15658	hsa-let-7c	WDR92	0.364761676	mirna_pc
15659	hsa-let-7c	SFI1	0.603289655	mirna_pc

15660	hsa-let-7c	MAP3K12	0.637404249	mirna_pc
15661	hsa-let-7c	ZBTB10	0.433574755	mirna_pc
15662	hsa-let-7c	SOCS5	0.425040971	mirna_pc
15663	hsa-let-7c	TTC21B	0.392566487	mirna_pc
15664	hsa-let-7c	ZNF627	0.335272522	mirna_pc
15665	hsa-let-7c	ANGPT1	0.859005583	mirna_pc
15666	hsa-let-7c	EXTL2	0.387476453	mirna_pc
15667	hsa-let-7c	ARID1A	0.355317308	mirna_pc
15668	hsa-let-7c	ATAD2B	0.350132319	mirna_pc
15669	hsa-let-7c	ZNF558	0.349277096	mirna_pc
15670	hsa-let-7c	NAB2	0.406713859	mirna_pc
15671	hsa-let-7c	FDXACB1	0.358850544	mirna_pc
15672	hsa-let-7c	ISYNA1	0.475448605	mirna_pc
15673	hsa-let-7c	LOC338758	0.503458397	mirna_pc
15674	hsa-let-7c	KIF27	0.377950987	mirna_pc
15675	hsa-let-7c	7-Mar	0.300528051	mirna_pc
15676	hsa-let-7c	PCMTD2	0.341395175	mirna_pc
15677	hsa-let-7c	PIM1	0.32558941	mirna_pc
15678	hsa-let-7c	CPT1C	0.324752543	mirna_pc
15679	hsa-let-7c	ZNF638	0.373808498	mirna_pc
15680	hsa-let-7c	ZNF20	0.357494852	mirna_pc
15681	hsa-let-7c	PIP5K1A	0.39446933	mirna_pc
15682	hsa-let-7c	TMX3	0.422571064	mirna_pc
15683	hsa-let-7c	ZNF512	0.40183685	mirna_pc
15684	hsa-let-7c	GRB14	0.550049282	mirna_pc
15685	hsa-let-7c	USP21	0.319987238	mirna_pc
15686	hsa-let-7c	ZNF74	0.408025279	mirna_pc
15687	hsa-let-7c	HTR7P1	0.578826455	mirna_pc
15688	hsa-let-7c	OSGEPL1	0.350930477	mirna_pc
15689	hsa-let-7c	PEX13	0.365910324	mirna_pc
15690	hsa-let-7c	DCAF17	0.313385317	mirna_pc
15691	hsa-let-7c	ETAA1	0.348893352	mirna_pc
15692	hsa-let-7c	HNRNPH3	0.364187702	mirna_pc
15693	hsa-let-7c	HMGN3	0.322934744	mirna_pc
15694	hsa-let-7c	STAU2	0.344426168	mirna_pc
15695	hsa-let-7c	B4GALT6	0.425602383	mirna_pc
15696	hsa-let-7c	CEP290	0.342848236	mirna_pc
15697	hsa-let-7c	SP1	0.355757397	mirna_pc
15698	hsa-let-7c	KIF3A	0.312092687	mirna_pc
15699	hsa-let-7c	CNIH2	0.501810457	mirna_pc
15700	hsa-let-7c	HHAT	0.364184013	mirna_pc
15701	hsa-let-7c	PMS1	0.330798492	mirna_pc
15702	hsa-let-7c	EID3	0.668332909	mirna_pc
15703	hsa-let-7c	ALG10B	0.316859463	mirna_pc
15704	hsa-let-7c	KIAA1467	0.365392072	mirna_pc
15705	hsa-let-7c	ABHD12	0.378692168	mirna_pc
15706	hsa-let-7c	PAN3	0.313413611	mirna_pc
15707	hsa-let-7c	UBE2W	0.355077719	mirna_pc
15708	hsa-let-7c	GATS	0.550209565	mirna_pc
15709	hsa-let-7c	TRUB1	0.43237721	mirna_pc
15710	hsa-let-7c	PLAC2	0.686484885	mirna_pc
15711	hsa-let-7c	ROBO1	0.355493883	mirna_pc
15712	hsa-let-7c	PDIK1L	0.340520699	mirna_pc
15713	hsa-let-7c	C2orf15	0.406149547	mirna_pc

15714	hsa-let-7c	C12orf24	0.545686044	mirna_pc
15715	hsa-let-7c	TAF3	0.328000976	mirna_pc
15716	hsa-let-7c	NFYB	0.318279741	mirna_pc
15717	hsa-let-7c	LCA5L	0.403888529	mirna_pc
15718	hsa-let-7c	SEMA3A	0.72221461	mirna_pc
15719	hsa-let-7c	THNSL1	0.355607807	mirna_pc
15720	hsa-let-7c	USP13	0.434092505	mirna_pc
15721	hsa-let-7c	FAM120C	0.389440657	mirna_pc
15722	hsa-let-7c	ESF1	0.32902746	mirna_pc
15723	hsa-let-7c	PDGFRL	0.447718892	mirna_pc
15724	hsa-let-7c	GPR37	0.430882105	mirna_pc
15725	hsa-let-7c	MIB1	0.333497976	mirna_pc
15726	hsa-let-7c	SS18	0.41530792	mirna_pc
15727	hsa-let-7c	ZNF391	0.434344738	mirna_pc
15728	hsa-let-7c	ZBTB41	0.34132565	mirna_pc
15729	hsa-let-7c	IFT74	0.620583519	mirna_pc
15730	hsa-let-7c	MAST1	0.372774865	mirna_pc
15731	hsa-let-7c	NR5A2	0.471593508	mirna_pc
15732	hsa-let-7c	TTC39C	0.337377298	mirna_pc
15733	hsa-let-7c	CDK19	0.325508733	mirna_pc
15734	hsa-let-7c	MDM1	0.804498051	mirna_pc
15735	hsa-let-7c	C10orf88	0.332658798	mirna_pc
15736	hsa-let-7c	ZNF740	0.324034746	mirna_pc
15737	hsa-let-7c	TRO	0.366891739	mirna_pc
15738	hsa-let-7c	BBS10	0.455032063	mirna_pc
15739	hsa-let-7c	PBX2	0.399771697	mirna_pc
15740	hsa-let-7c	SFRS15	0.441502599	mirna_pc
15741	hsa-let-7c	MLLT10	0.306179394	mirna_pc
15742	hsa-let-7c	SLITRK6	0.649747615	mirna_pc
15743	hsa-let-7c	PPTC7	0.304492345	mirna_pc
15744	hsa-let-7c	ANKRD34A	0.449812668	mirna_pc
15745	hsa-let-7c	HAPLN1	0.82199648	mirna_pc
15746	hsa-let-7c	C6orf70	0.342621478	mirna_pc
15747	hsa-let-7c	RTF1	0.330868663	mirna_pc
15748	hsa-let-7c	SYNGAP1	0.398745469	mirna_pc
15749	hsa-let-7c	TBC1D22B	0.316356798	mirna_pc
15750	hsa-let-7c	C8orf45	0.408151224	mirna_pc
15751	hsa-let-7c	DBNDD1	0.392846824	mirna_pc
15752	hsa-let-7c	PPOX	0.352582901	mirna_pc
15753	hsa-let-7c	NEBL	0.31827415	mirna_pc
15754	hsa-let-7c	SAMD10	0.301923943	mirna_pc
15755	hsa-let-7c	LOC221710	0.424178012	mirna_pc
15756	hsa-let-7c	DKFZP686I15217	0.302740713	mirna_pc
15757	hsa-let-7c	TSGA10	0.534451711	mirna_pc
15758	hsa-let-7c	BRSK1	0.316815198	mirna_pc
15759	hsa-let-7c	DGCR6	0.431884684	mirna_pc
15760	hsa-let-7c	CRYZ	0.695961267	mirna_pc
15761	hsa-let-7c	ZNF664	0.353081528	mirna_pc
15762	hsa-let-7c	SMEK2	0.418979764	mirna_pc
15763	hsa-let-7c	ATL2	0.464802872	mirna_pc
15764	hsa-let-7c	RBBP6	0.312004014	mirna_pc
15765	hsa-let-7c	CES8	0.320300259	mirna_pc
15766	hsa-let-7c	TBX1	0.390894791	mirna_pc
15767	hsa-let-7c	KDELC2	0.420710134	mirna_pc

15768	hsa-let-7c	KILLIN	0.344804107	mirna_pc
15769	hsa-let-7c	PRICKLE1	0.621027813	mirna_pc
15770	hsa-let-7c	GCFC1	0.301196223	mirna_pc
15771	hsa-let-7c	UBE3A	0.371888173	mirna_pc
15772	hsa-let-7c	ZCCHC11	0.364491079	mirna_pc
15773	hsa-let-7c	CETN3	0.303482559	mirna_pc
15774	hsa-let-7c	ZNF644	0.37379765	mirna_pc
15775	hsa-let-7c	PRPF40B	0.305219411	mirna_pc
15776	hsa-let-7c	TCTN1	0.334296208	mirna_pc
15777	hsa-let-7c	CTXN1	0.641308883	mirna_pc
15778	hsa-let-7c	RNF215	0.338902593	mirna_pc
15779	hsa-let-7c	ZNF680	0.335015462	mirna_pc
15780	hsa-let-7c	LOC283070	0.595663745	mirna_pc
15781	hsa-let-7c	CCDC117	0.380776849	mirna_pc
15782	hsa-let-7c	C10orf46	0.345612349	mirna_pc
15783	hsa-let-7c	HSDL1	0.450701162	mirna_pc
15784	hsa-let-7c	SLC11A2	0.67783522	mirna_pc
15785	hsa-let-7c	LOC100129387	0.49600704	mirna_pc
15786	hsa-let-7c	FBXO11	0.349462317	mirna_pc
15787	hsa-let-7c	HEY2	0.457708181	mirna_pc
15788	hsa-let-7c	ZFP37	0.352288191	mirna_pc
15789	hsa-let-7c	HSPC157	0.334735627	mirna_pc
15790	hsa-let-7c	THOC5	0.313588622	mirna_pc
15791	hsa-let-7c	CAMK1D	0.445829061	mirna_pc
15792	hsa-let-7c	LCA5	0.784510203	mirna_pc
15793	hsa-let-7c	CDC73	0.468453022	mirna_pc
15794	hsa-let-7c	SLC2A3	0.440504618	mirna_pc
15795	hsa-let-7c	C22orf29	0.392075489	mirna_pc
15796	hsa-let-7c	KIAA1107	0.359785891	mirna_pc
15797	hsa-let-7c	WDSUB1	0.426277811	mirna_pc
15798	hsa-let-7c	BTF3L4	0.414199783	mirna_pc
15799	hsa-let-7c	DDX59	0.324809765	mirna_pc
15800	hsa-let-7c	SS18L1	0.37164014	mirna_pc
15801	hsa-mir-181b-2	TTYH3	0.318456938	mirna_pc
15802	hsa-mir-181b-2	GABPB1	0.337704292	mirna_pc
15803	hsa-mir-181b-2	SLC39A10	0.385898501	mirna_pc
15804	hsa-mir-181b-2	HSP90B1	0.306407993	mirna_pc
15805	hsa-mir-181b-2	CMTM1	0.416953604	mirna_pc
15806	hsa-mir-181b-2	RNF219	0.309971208	mirna_pc
15807	hsa-mir-181b-2	SMC3	0.315520789	mirna_pc
15808	hsa-mir-181b-2	SLC1A1	0.334612255	mirna_pc
15809	hsa-mir-181b-2	SC65	0.331155264	mirna_pc
15810	hsa-mir-181b-2	SOX9	0.310565641	mirna_pc
15811	hsa-mir-181b-2	PPP3R1	0.30680951	mirna_pc
15812	hsa-mir-181b-2	CXorf38	0.361606733	mirna_pc
15813	hsa-mir-181b-2	ZBTB34	0.326929424	mirna_pc
15814	hsa-mir-181b-2	MED14	0.307150774	mirna_pc
15815	hsa-mir-181b-2	SMC1A	0.402495409	mirna_pc
15816	hsa-mir-181b-2	SH3KBP1	0.332571564	mirna_pc
15817	hsa-mir-181b-2	ARHGAP12	0.351966237	mirna_pc
15818	hsa-mir-181b-2	SYAP1	0.325656373	mirna_pc
15819	hsa-mir-181b-2	DHX15	0.373721392	mirna_pc
15820	hsa-mir-181b-2	MAP2K6	0.301495414	mirna_pc
15821	hsa-mir-181b-2	PRKD3	0.318192845	mirna_pc

15822	hsa-mir-181b-2	KIF5B	0.315218977	mirna_pc
15823	hsa-mir-181b-2	HS3ST1	0.351943625	mirna_pc
15824	hsa-mir-181b-2	TRUB1	0.306768127	mirna_pc
15825	hsa-mir-181b-2	KDM5C	0.341551095	mirna_pc
15826	hsa-mir-181b-2	TCF7L2	0.318879037	mirna_pc
15827	hsa-mir-181b-2	SNRNP27	0.418988001	mirna_pc
15828	hsa-mir-181b-2	HMG20A	0.300899024	mirna_pc
15829	hsa-mir-181b-2	TANK	0.307859625	mirna_pc
15830	hsa-mir-24-2	KPNA2	0.53315743	mirna_pc
15831	hsa-mir-24-2	RCC2	0.366387708	mirna_pc
15832	hsa-mir-24-2	RRM2	0.366481982	mirna_pc
15833	hsa-mir-24-2	PLK1	0.350572617	mirna_pc
15834	hsa-mir-24-2	MCM4	0.325305229	mirna_pc
15835	hsa-mir-24-2	NCAPG	0.331185009	mirna_pc
15836	hsa-mir-24-2	CDC20	0.528972199	mirna_pc
15837	hsa-mir-24-2	CCNA2	0.348745494	mirna_pc
15838	hsa-mir-24-2	CEP55	0.329107258	mirna_pc
15839	hsa-mir-24-2	LMNB2	0.391144519	mirna_pc
15840	hsa-mir-24-2	MND1	0.322619662	mirna_pc
15841	hsa-mir-24-2	KIF22	0.352257998	mirna_pc
15842	hsa-mir-24-2	DDX39	0.488086486	mirna_pc
15843	hsa-mir-24-2	FAM49B	0.344630748	mirna_pc
15844	hsa-mir-24-2	NCAPH	0.30675351	mirna_pc
15845	hsa-mir-24-2	RAD54L	0.307760375	mirna_pc
15846	hsa-mir-24-2	CENPA	0.46974741	mirna_pc
15847	hsa-mir-24-2	TROAP	0.328911305	mirna_pc
15848	hsa-mir-24-2	CCNB1	0.477622785	mirna_pc
15849	hsa-mir-24-2	CDC45	0.359090335	mirna_pc
15850	hsa-mir-24-2	STMN1	0.331164691	mirna_pc
15851	hsa-mir-24-2	CENPM	0.340764337	mirna_pc
15852	hsa-mir-24-2	TK1	0.437965463	mirna_pc
15853	hsa-mir-24-2	UBE2T	0.337215021	mirna_pc
15854	hsa-mir-24-2	CKS2	0.313098469	mirna_pc
15855	hsa-mir-24-2	CKS1B	0.477029848	mirna_pc
15856	hsa-mir-24-2	TUBB	0.359722595	mirna_pc
15857	hsa-mir-24-2	MCM2	0.30364663	mirna_pc
15858	hsa-mir-24-2	ORC1L	0.316278114	mirna_pc
15859	hsa-mir-24-2	UHRF1	0.346588553	mirna_pc
15860	hsa-mir-24-2	HN1	0.325548838	mirna_pc
15861	hsa-mir-24-2	RACGAP1	0.361598072	mirna_pc
15862	hsa-mir-24-2	PTBP1	0.46726939	mirna_pc
15863	hsa-mir-24-2	CCNB2	0.390611836	mirna_pc
15864	hsa-mir-24-2	PTTG1	0.554054168	mirna_pc
15865	hsa-mir-24-2	RAD51	0.347961295	mirna_pc
15866	hsa-mir-24-2	NME1	0.377345104	mirna_pc
15867	hsa-mir-24-2	PAICS	0.354478093	mirna_pc
15868	hsa-mir-24-2	BIRC5	0.49062882	mirna_pc
15869	hsa-mir-24-2	RCC1	0.414504695	mirna_pc
15870	hsa-mir-24-2	AURKB	0.402135554	mirna_pc
15871	hsa-mir-24-2	CDT1	0.404867276	mirna_pc
15872	hsa-mir-24-2	KIF20A	0.405327188	mirna_pc
15873	hsa-mir-24-2	PSMB2	0.499141573	mirna_pc
15874	hsa-mir-24-2	CDCA3	0.346083993	mirna_pc
15875	hsa-mir-24-2	ALG3	0.448760411	mirna_pc

15876	hsa-mir-24-2	TMEM191A	0.342322163	mirna_pc
15877	hsa-mir-24-2	ORC6L	0.335904746	mirna_pc
15878	hsa-mir-24-2	ERH	0.387009727	mirna_pc
15879	hsa-mir-24-2	PSMA7	0.303770906	mirna_pc
15880	hsa-mir-24-2	C16orf59	0.388474201	mirna_pc
15881	hsa-mir-24-2	CDKN3	0.376837375	mirna_pc
15882	hsa-mir-24-2	ADRM1	0.335075137	mirna_pc
15883	hsa-mir-24-2	ACTL6A	0.374577719	mirna_pc
15884	hsa-mir-24-2	HNRNPL	0.411546847	mirna_pc
15885	hsa-mir-24-2	PKMYT1	0.483544637	mirna_pc
15886	hsa-mir-24-2	SF3B4	0.330568174	mirna_pc
15887	hsa-mir-24-2	TUBA1B	0.474328764	mirna_pc
15888	hsa-mir-24-2	UBE2S	0.521922159	mirna_pc
15889	hsa-mir-24-2	PSMA4	0.430998634	mirna_pc
15890	hsa-mir-24-2	CCT5	0.396529	mirna_pc
15891	hsa-mir-24-2	C11orf82	0.37295801	mirna_pc
15892	hsa-mir-24-2	HNRNPC	0.495687374	mirna_pc
15893	hsa-mir-24-2	PPIA	0.618111343	mirna_pc
15894	hsa-mir-24-2	ILF2	0.398414927	mirna_pc
15895	hsa-mir-24-2	TCF3	0.320452815	mirna_pc
15896	hsa-mir-24-2	MCM5	0.338781781	mirna_pc
15897	hsa-mir-24-2	RAN	0.470755962	mirna_pc
15898	hsa-mir-24-2	TYMS	0.356273528	mirna_pc
15899	hsa-mir-24-2	LAD1	0.446398995	mirna_pc
15900	hsa-mir-24-2	RNASEH2A	0.519357297	mirna_pc
15901	hsa-mir-24-2	EPR1	0.375305603	mirna_pc
15902	hsa-mir-24-2	H2AFX	0.38524109	mirna_pc
15903	hsa-mir-24-2	SFRS2	0.354154796	mirna_pc
15904	hsa-mir-24-2	SH3BP1	0.495820669	mirna_pc
15905	hsa-mir-24-2	MTHFD1L	0.312061804	mirna_pc
15906	hsa-mir-24-2	PSMD14	0.345123351	mirna_pc
15907	hsa-mir-24-2	UCK2	0.343456653	mirna_pc
15908	hsa-mir-24-2	THOC4	0.427833173	mirna_pc
15909	hsa-mir-24-2	GLT25D1	0.353290673	mirna_pc
15910	hsa-mir-24-2	CENPN	0.321290804	mirna_pc
15911	hsa-mir-24-2	HMGB3	0.330505162	mirna_pc
15912	hsa-mir-24-2	RTKN	0.320506075	mirna_pc
15913	hsa-mir-24-2	RANBP1	0.473134372	mirna_pc
15914	hsa-mir-24-2	FAM83H	0.446945978	mirna_pc
15915	hsa-mir-24-2	SNRPG	0.456330195	mirna_pc
15916	hsa-mir-24-2	EIF4A3	0.512370296	mirna_pc
15917	hsa-mir-24-2	SNRPA	0.431873208	mirna_pc
15918	hsa-mir-24-2	FUS	0.435335356	mirna_pc
15919	hsa-mir-24-2	CENPW	0.491355649	mirna_pc
15920	hsa-mir-24-2	DNAJC9	0.403479905	mirna_pc
15921	hsa-mir-24-2	TUBA1C	0.553503377	mirna_pc
15922	hsa-mir-24-2	CHAF1A	0.387235049	mirna_pc
15923	hsa-mir-24-2	PSMD11	0.394140684	mirna_pc
15924	hsa-mir-24-2	NUDT1	0.515435462	mirna_pc
15925	hsa-mir-24-2	CCT3	0.320979443	mirna_pc
15926	hsa-mir-24-2	GINS2	0.425749341	mirna_pc
15927	hsa-mir-24-2	PSMA3	0.343147566	mirna_pc
15928	hsa-mir-24-2	TEAD4	0.340906007	mirna_pc
15929	hsa-mir-24-2	SNRPD1	0.332722824	mirna_pc

15930	hsa-mir-24-2	S100A11	0.532306383	mirna_pc
15931	hsa-mir-24-2	CDCA4	0.536708919	mirna_pc
15932	hsa-mir-24-2	ATP1B3	0.410015106	mirna_pc
15933	hsa-mir-24-2	MYO1B	0.323833085	mirna_pc
15934	hsa-mir-24-2	C16orf75	0.333635845	mirna_pc
15935	hsa-mir-24-2	EBNA1BP2	0.487371051	mirna_pc
15936	hsa-mir-24-2	C18orf56	0.401205976	mirna_pc
15937	hsa-mir-24-2	CFL1	0.446758837	mirna_pc
15938	hsa-mir-24-2	NOP2	0.32463835	mirna_pc
15939	hsa-mir-24-2	HIST1H2AE	0.348562103	mirna_pc
15940	hsa-mir-24-2	STX6	0.355551996	mirna_pc
15941	hsa-mir-24-2	CDK4	0.320961686	mirna_pc
15942	hsa-mir-24-2	FAM110A	0.376846061	mirna_pc
15943	hsa-mir-24-2	TACSTD2	0.332864868	mirna_pc
15944	hsa-mir-24-2	TYMP	0.317501247	mirna_pc
15945	hsa-mir-24-2	PFDN2	0.484194669	mirna_pc
15946	hsa-mir-24-2	C7orf11	0.42865845	mirna_pc
15947	hsa-mir-24-2	CHTF18	0.320219828	mirna_pc
15948	hsa-mir-24-2	ENO1	0.530894745	mirna_pc
15949	hsa-mir-24-2	LAMC2	0.31893702	mirna_pc
15950	hsa-mir-24-2	YIF1B	0.32313174	mirna_pc
15951	hsa-mir-24-2	CENPH	0.412434706	mirna_pc
15952	hsa-mir-24-2	TCOF1	0.325349464	mirna_pc
15953	hsa-mir-24-2	ACYP1	0.343750276	mirna_pc
15954	hsa-mir-24-2	TMSB10	0.502774394	mirna_pc
15955	hsa-mir-24-2	ULBP2	0.475817344	mirna_pc
15956	hsa-mir-24-2	G3BP1	0.370818428	mirna_pc
15957	hsa-mir-24-2	TOMM40	0.338560239	mirna_pc
15958	hsa-mir-24-2	F12	0.475169417	mirna_pc
15959	hsa-mir-24-2	GAMT	0.326634076	mirna_pc
15960	hsa-mir-24-2	YDJC	0.403594989	mirna_pc
15961	hsa-mir-24-2	TMEM189	0.51186384	mirna_pc
15962	hsa-mir-24-2	ARL6IP6	0.322205477	mirna_pc
15963	hsa-mir-24-2	PKP3	0.338504725	mirna_pc
15964	hsa-mir-24-2	HAUS8	0.374118165	mirna_pc
15965	hsa-mir-24-2	TMEM41A	0.316447519	mirna_pc
15966	hsa-mir-24-2	PSMB4	0.308744263	mirna_pc
15967	hsa-mir-24-2	RFC4	0.417603114	mirna_pc
15968	hsa-mir-24-2	BRIX1	0.332015639	mirna_pc
15969	hsa-mir-24-2	SF3B14	0.435994689	mirna_pc
15970	hsa-mir-24-2	EFNB1	0.392231625	mirna_pc
15971	hsa-mir-24-2	MKI67IP	0.303232843	mirna_pc
15972	hsa-mir-24-2	CAPZA1	0.31796797	mirna_pc
15973	hsa-mir-24-2	ACOT7	0.375022242	mirna_pc
15974	hsa-mir-24-2	ANXA2	0.353091765	mirna_pc
15975	hsa-mir-24-2	H2AFZ	0.431522675	mirna_pc
15976	hsa-mir-24-2	NRM	0.381531227	mirna_pc
15977	hsa-mir-24-2	MAGOH	0.397323746	mirna_pc
15978	hsa-mir-24-2	NUP37	0.448912615	mirna_pc
15979	hsa-mir-24-2	LDHA	0.515020342	mirna_pc
15980	hsa-mir-24-2	CPSF3	0.316692908	mirna_pc
15981	hsa-mir-24-2	BANF1	0.491633477	mirna_pc
15982	hsa-mir-24-2	DCAF15	0.457725631	mirna_pc
15983	hsa-mir-24-2	SNRPE	0.356067099	mirna_pc

15984	hsa-mir-24-2	SLC2A1	0.379022084	mirna_pc
15985	hsa-mir-24-2	PYCARD	0.437801545	mirna_pc
15986	hsa-mir-24-2	PPM1G	0.464536596	mirna_pc
15987	hsa-mir-24-2	SDC1	0.536129789	mirna_pc
15988	hsa-mir-24-2	SNRPD2	0.412725937	mirna_pc
15989	hsa-mir-24-2	PLAU	0.31351646	mirna_pc
15990	hsa-mir-24-2	MDFI	0.410002493	mirna_pc
15991	hsa-mir-24-2	NPM3	0.434365308	mirna_pc
15992	hsa-mir-24-2	SIP1	0.306247789	mirna_pc
15993	hsa-mir-24-2	NUDT5	0.405177684	mirna_pc
15994	hsa-mir-24-2	APEX2	0.388394209	mirna_pc
15995	hsa-mir-24-2	CDH3	0.393011838	mirna_pc
15996	hsa-mir-24-2	NUTF2	0.375404962	mirna_pc
15997	hsa-mir-24-2	RNPS1	0.458247633	mirna_pc
15998	hsa-mir-24-2	DAZAP1	0.476018699	mirna_pc
15999	hsa-mir-24-2	BOLA2	0.415589867	mirna_pc
16000	hsa-mir-24-2	PSMA6	0.327529653	mirna_pc
16001	hsa-mir-24-2	TXNDC9	0.331068835	mirna_pc
16002	hsa-mir-24-2	C10orf55	0.458745781	mirna_pc
16003	hsa-mir-24-2	TMEM201	0.347378763	mirna_pc
16004	hsa-mir-24-2	MRPL9	0.332044298	mirna_pc
16005	hsa-mir-24-2	DCTPP1	0.352021027	mirna_pc
16006	hsa-mir-24-2	DGUOK	0.437228587	mirna_pc
16007	hsa-mir-24-2	MRPL47	0.438799531	mirna_pc
16008	hsa-mir-24-2	DPP3	0.360583821	mirna_pc
16009	hsa-mir-24-2	S100A3	0.37107244	mirna_pc
16010	hsa-mir-24-2	STIP1	0.392621723	mirna_pc
16011	hsa-mir-24-2	HIST1H1E	0.352308147	mirna_pc
16012	hsa-mir-24-2	PSMG3	0.435188867	mirna_pc
16013	hsa-mir-24-2	THOP1	0.530150408	mirna_pc
16014	hsa-mir-24-2	EXOSC5	0.328412874	mirna_pc
16015	hsa-mir-24-2	SCNM1	0.404347708	mirna_pc
16016	hsa-mir-24-2	ANXA2P2	0.35110629	mirna_pc
16017	hsa-mir-24-2	BCAP31	0.355715642	mirna_pc
16018	hsa-mir-24-2	RELL2	0.324645791	mirna_pc
16019	hsa-mir-24-2	PRCC	0.325233289	mirna_pc
16020	hsa-mir-24-2	SET	0.31937607	mirna_pc
16021	hsa-mir-24-2	POLR2H	0.305073888	mirna_pc
16022	hsa-mir-24-2	NIP7	0.300769321	mirna_pc
16023	hsa-mir-24-2	SERPINB5	0.358655634	mirna_pc
16024	hsa-mir-24-2	NR2C2AP	0.418006301	mirna_pc
16025	hsa-mir-24-2	C3orf26	0.321588644	mirna_pc
16026	hsa-mir-24-2	MRTO4	0.455202797	mirna_pc
16027	hsa-mir-24-2	LSM 7.00	0.437897842	mirna_pc
16028	hsa-mir-24-2	SCD	0.351906384	mirna_pc
16029	hsa-mir-24-2	FLAD1	0.411049152	mirna_pc
16030	hsa-mir-24-2	NUP93	0.327231273	mirna_pc
16031	hsa-mir-24-2	POLE3	0.352938813	mirna_pc
16032	hsa-mir-24-2	PPIH	0.393893948	mirna_pc
16033	hsa-mir-24-2	THOC6	0.427001603	mirna_pc
16034	hsa-mir-24-2	GMPS	0.41201475	mirna_pc
16035	hsa-mir-24-2	C3orf37	0.349114318	mirna_pc
16036	hsa-mir-24-2	MRPL3	0.321528599	mirna_pc
16037	hsa-mir-24-2	EIF5A	0.613988886	mirna_pc

16038	hsa-mir-24-2	GNB1L	0.385853636	mirna_pc
16039	hsa-mir-24-2	PPP4C	0.41114869	mirna_pc
16040	hsa-mir-24-2	SMYD5	0.320881229	mirna_pc
16041	hsa-mir-24-2	SF3A2	0.509876866	mirna_pc
16042	hsa-mir-24-2	TSSC1	0.396870541	mirna_pc
16043	hsa-mir-24-2	REEP4	0.557257354	mirna_pc
16044	hsa-mir-24-2	PFN1	0.478153082	mirna_pc
16045	hsa-mir-24-2	BOP 1.00	0.392746832	mirna_pc
16046	hsa-mir-24-2	POLA2	0.301808007	mirna_pc
16047	hsa-mir-24-2	SFN	0.55014909	mirna_pc
16048	hsa-mir-24-2	SNRPF	0.476219636	mirna_pc
16049	hsa-mir-24-2	SUMO2	0.505735336	mirna_pc
16050	hsa-mir-24-2	BCL11B	0.30108768	mirna_pc
16051	hsa-mir-24-2	CTSC	0.348469842	mirna_pc
16052	hsa-mir-24-2	CDH24	0.347953314	mirna_pc
16053	hsa-mir-24-2	SNRPA1	0.39569713	mirna_pc
16054	hsa-mir-24-2	SKP2	0.317736178	mirna_pc
16055	hsa-mir-24-2	NME2	0.408179569	mirna_pc
16056	hsa-mir-24-2	UBE2I	0.50555965	mirna_pc
16057	hsa-mir-24-2	GSTP1	0.449441798	mirna_pc
16058	hsa-mir-24-2	YWHAZ	0.335402175	mirna_pc
16059	hsa-mir-24-2	TIPIN	0.428430831	mirna_pc
16060	hsa-mir-24-2	KCTD5	0.342563606	mirna_pc
16061	hsa-mir-24-2	CYP27B1	0.323832485	mirna_pc
16062	hsa-mir-24-2	NDUFS4	0.332065552	mirna_pc
16063	hsa-mir-24-2	POLD2	0.460415544	mirna_pc
16064	hsa-mir-24-2	ATXN2L	0.322186843	mirna_pc
16065	hsa-mir-24-2	AGTRAP	0.35859297	mirna_pc
16066	hsa-mir-24-2	H2AFY	0.313131326	mirna_pc
16067	hsa-mir-24-2	ECE2	0.5340312	mirna_pc
16068	hsa-mir-24-2	GPN1	0.360641887	mirna_pc
16069	hsa-mir-24-2	RFC5	0.329171538	mirna_pc
16070	hsa-mir-24-2	PPP1CA	0.480941786	mirna_pc
16071	hsa-mir-24-2	MAZ	0.374570216	mirna_pc
16072	hsa-mir-24-2	RANGAP1	0.430954099	mirna_pc
16073	hsa-mir-24-2	SSRP1	0.380055578	mirna_pc
16074	hsa-mir-24-2	SLBP	0.305213285	mirna_pc
16075	hsa-mir-24-2	COL7A1	0.322474117	mirna_pc
16076	hsa-mir-24-2	PA2G4	0.43780239	mirna_pc
16077	hsa-mir-24-2	SNRPD3	0.534283976	mirna_pc
16078	hsa-mir-24-2	CAPG	0.304305558	mirna_pc
16079	hsa-mir-24-2	KHDRBS1	0.327979705	mirna_pc
16080	hsa-mir-24-2	C20orf27	0.329787577	mirna_pc
16081	hsa-mir-24-2	LOC92659	0.373795686	mirna_pc
16082	hsa-mir-24-2	FSCN1	0.526558439	mirna_pc
16083	hsa-mir-24-2	GGH	0.381274938	mirna_pc
16084	hsa-mir-24-2	TMEM138	0.30184157	mirna_pc
16085	hsa-mir-24-2	PYCRL	0.405276468	mirna_pc
16086	hsa-mir-24-2	B3GNT5	0.334437706	mirna_pc
16087	hsa-mir-24-2	LOC100130776	0.496178337	mirna_pc
16088	hsa-mir-24-2	ALG1L	0.328365978	mirna_pc
16089	hsa-mir-24-2	CCT7	0.482007779	mirna_pc
16090	hsa-mir-24-2	UBE2A	0.306054597	mirna_pc
16091	hsa-mir-24-2	MTERFD1	0.326745898	mirna_pc

16092	hsa-mir-24-2	HOXC13	0.350176381	mirna_pc
16093	hsa-mir-24-2	SNRNP40	0.405872037	mirna_pc
16094	hsa-mir-24-2	ITGB4	0.36928426	mirna_pc
16095	hsa-mir-24-2	HES1	0.348139766	mirna_pc
16096	hsa-mir-24-2	MED30	0.368282492	mirna_pc
16097	hsa-mir-24-2	METTL1	0.336976936	mirna_pc
16098	hsa-mir-24-2	FAM174A	0.30564971	mirna_pc
16099	hsa-mir-24-2	TMEM69	0.532883475	mirna_pc
16100	hsa-mir-24-2	GINS3	0.389626121	mirna_pc
16101	hsa-mir-24-2	AP2S1	0.462611996	mirna_pc
16102	hsa-mir-24-2	DCTN2	0.315514871	mirna_pc
16103	hsa-mir-24-2	NSUN5	0.310674769	mirna_pc
16104	hsa-mir-24-2	JUB	0.327412288	mirna_pc
16105	hsa-mir-24-2	PVRL1	0.496156563	mirna_pc
16106	hsa-mir-24-2	BRMS1	0.313214335	mirna_pc
16107	hsa-mir-24-2	CCDC137	0.410813544	mirna_pc
16108	hsa-mir-24-2	SREBF1	0.365995429	mirna_pc
16109	hsa-mir-24-2	KRI1	0.372807865	mirna_pc
16110	hsa-mir-24-2	NOC2L	0.330421102	mirna_pc
16111	hsa-mir-24-2	RPP40	0.408751133	mirna_pc
16112	hsa-mir-24-2	LAGE3	0.304581928	mirna_pc
16113	hsa-mir-24-2	CCDC86	0.52220704	mirna_pc
16114	hsa-mir-24-2	ZC3H3	0.402291829	mirna_pc
16115	hsa-mir-24-2	USP39	0.348396768	mirna_pc
16116	hsa-mir-24-2	LSM 4.00	0.455903647	mirna_pc
16117	hsa-mir-24-2	PSMB1	0.425772065	mirna_pc
16118	hsa-mir-24-2	DENR	0.32196153	mirna_pc
16119	hsa-mir-24-2	EXOSC2	0.310957363	mirna_pc
16120	hsa-mir-24-2	TRIM29	0.407603652	mirna_pc
16121	hsa-mir-24-2	DUSP11	0.440132764	mirna_pc
16122	hsa-mir-24-2	C11orf84	0.310769625	mirna_pc
16123	hsa-mir-24-2	KRT17	0.472689477	mirna_pc
16124	hsa-mir-24-2	TOP1MT	0.409517522	mirna_pc
16125	hsa-mir-24-2	TBCE	0.319142944	mirna_pc
16126	hsa-mir-24-2	GAPDH	0.509936986	mirna_pc
16127	hsa-mir-24-2	LRRC8D	0.336523024	mirna_pc
16128	hsa-mir-24-2	KLF16	0.337107184	mirna_pc
16129	hsa-mir-24-2	FBXO22OS	0.468048703	mirna_pc
16130	hsa-mir-24-2	CARM1	0.356929688	mirna_pc
16131	hsa-mir-24-2	SLC3A2	0.548911668	mirna_pc
16132	hsa-mir-24-2	DDX49	0.44306408	mirna_pc
16133	hsa-mir-24-2	DPY30	0.436563017	mirna_pc
16134	hsa-mir-24-2	RRP9	0.306796677	mirna_pc
16135	hsa-mir-24-2	TUBB2C	0.37044148	mirna_pc
16136	hsa-mir-24-2	MTCH2	0.353401043	mirna_pc
16137	hsa-mir-24-2	EIF5AL1	0.570299368	mirna_pc
16138	hsa-mir-24-2	S100A10	0.350184732	mirna_pc
16139	hsa-mir-24-2	CCDC109B	0.347770675	mirna_pc
16140	hsa-mir-24-2	MEN1	0.439319219	mirna_pc
16141	hsa-mir-24-2	C17orf49	0.446887704	mirna_pc
16142	hsa-mir-24-2	PLEKHG5	0.349740704	mirna_pc
16143	hsa-mir-24-2	H1FX	0.323314913	mirna_pc
16144	hsa-mir-24-2	CCT4	0.46073088	mirna_pc
16145	hsa-mir-24-2	SAC3D1	0.534484765	mirna_pc

16146	hsa-mir-24-2	RABEPK	0.417306626	mirna_pc
16147	hsa-mir-24-2	PRICKLE3	0.528399526	mirna_pc
16148	hsa-mir-24-2	ELAVL1	0.368617748	mirna_pc
16149	hsa-mir-24-2	MRPL4	0.496725838	mirna_pc
16150	hsa-mir-24-2	WDR18	0.501688734	mirna_pc
16151	hsa-mir-24-2	CENPP	0.336815707	mirna_pc
16152	hsa-mir-24-2	MIF	0.593049926	mirna_pc
16153	hsa-mir-24-2	RAC1	0.337694231	mirna_pc
16154	hsa-mir-24-2	ARPC3	0.403842861	mirna_pc
16155	hsa-mir-24-2	TFAP2A	0.44125663	mirna_pc
16156	hsa-mir-24-2	EIF4A1	0.547805457	mirna_pc
16157	hsa-mir-24-2	TRMT1	0.389051314	mirna_pc
16158	hsa-mir-24-2	NAE1	0.323952715	mirna_pc
16159	hsa-mir-24-2	PPP1R13L	0.327433407	mirna_pc
16160	hsa-mir-24-2	PSMC3IP	0.3086439	mirna_pc
16161	hsa-mir-24-2	TBC1D7	0.416422216	mirna_pc
16162	hsa-mir-24-2	TNFAIP8L1	0.337798773	mirna_pc
16163	hsa-mir-24-2	PPP1R14B	0.575424206	mirna_pc
16164	hsa-mir-24-2	IFI27	0.301642785	mirna_pc
16165	hsa-mir-24-2	C10orf116	0.329655168	mirna_pc
16166	hsa-mir-24-2	PES 1.00	0.330423162	mirna_pc
16167	hsa-mir-24-2	PSMB7	0.447390528	mirna_pc
16168	hsa-mir-24-2	E2F4	0.329049289	mirna_pc
16169	hsa-mir-24-2	GJB3	0.554711438	mirna_pc
16170	hsa-mir-24-2	C12orf56	0.302278645	mirna_pc
16171	hsa-mir-24-2	TRMT112	0.574301097	mirna_pc
16172	hsa-mir-24-2	PKM2	0.508026077	mirna_pc
16173	hsa-mir-24-2	TMEM102	0.308557247	mirna_pc
16174	hsa-mir-24-2	STAP2	0.399044991	mirna_pc
16175	hsa-mir-24-2	PSMD2	0.442428688	mirna_pc
16176	hsa-mir-24-2	PGAM5	0.373358341	mirna_pc
16177	hsa-mir-24-2	PMAIP1	0.307930593	mirna_pc
16178	hsa-mir-24-2	YWHAE	0.473154202	mirna_pc
16179	hsa-mir-24-2	KDM1A	0.314586657	mirna_pc
16180	hsa-mir-24-2	FOXD1	0.326670921	mirna_pc
16181	hsa-mir-24-2	NAA10	0.389893779	mirna_pc
16182	hsa-mir-24-2	TBCB	0.311697045	mirna_pc
16183	hsa-mir-24-2	PGF	0.419935072	mirna_pc
16184	hsa-mir-24-2	FBXL19	0.325504643	mirna_pc
16185	hsa-mir-24-2	YARS2	0.325809277	mirna_pc
16186	hsa-mir-24-2	PUF60	0.413141417	mirna_pc
16187	hsa-mir-24-2	ADRB2	0.350323266	mirna_pc
16188	hsa-mir-24-2	MAPK6	0.426018711	mirna_pc
16189	hsa-mir-24-2	TM4SF19	0.37793213	mirna_pc
16190	hsa-mir-24-2	SFRS9	0.458013993	mirna_pc
16191	hsa-mir-24-2	GLRX3	0.474286101	mirna_pc
16192	hsa-mir-24-2	NCLN	0.384912303	mirna_pc
16193	hsa-mir-24-2	NMB	0.377159584	mirna_pc
16194	hsa-mir-24-2	OLA1	0.337068014	mirna_pc
16195	hsa-mir-24-2	C7orf70	0.37785021	mirna_pc
16196	hsa-mir-24-2	C14orf80	0.378897312	mirna_pc
16197	hsa-mir-24-2	AHSA1	0.326418317	mirna_pc
16198	hsa-mir-24-2	ADORA2B	0.378568408	mirna_pc
16199	hsa-mir-24-2	MAD1L1	0.324961473	mirna_pc

16200	hsa-mir-24-2	KHSRP	0.396715181	mirna_pc
16201	hsa-mir-24-2	SH2D5	0.47291315	mirna_pc
16202	hsa-mir-24-2	FTSJ1	0.356120883	mirna_pc
16203	hsa-mir-24-2	THOC3	0.402820009	mirna_pc
16204	hsa-mir-24-2	TKT	0.384288862	mirna_pc
16205	hsa-mir-24-2	ARMC6	0.328447853	mirna_pc
16206	hsa-mir-24-2	MRPS17	0.343158789	mirna_pc
16207	hsa-mir-24-2	TNFRSF18	0.409905867	mirna_pc
16208	hsa-mir-24-2	PERP	0.531080675	mirna_pc
16209	hsa-mir-24-2	C7orf27	0.321679186	mirna_pc
16210	hsa-mir-24-2	WDR74	0.43141355	mirna_pc
16211	hsa-mir-24-2	AKIRIN2	0.347275411	mirna_pc
16212	hsa-mir-24-2	HIST2H2AA3	0.371405858	mirna_pc
16213	hsa-mir-24-2	PRDX1	0.428446627	mirna_pc
16214	hsa-mir-24-2	MREG	0.335829328	mirna_pc
16215	hsa-mir-24-2	POLR2G	0.423150393	mirna_pc
16216	hsa-mir-24-2	PSMB5	0.421698173	mirna_pc
16217	hsa-mir-24-2	PLP2	0.592813558	mirna_pc
16218	hsa-mir-24-2	TPST2	0.306112397	mirna_pc
16219	hsa-mir-24-2	PSMD4	0.40781852	mirna_pc
16220	hsa-mir-24-2	NUP188	0.360384969	mirna_pc
16221	hsa-mir-24-2	FARSA	0.429458798	mirna_pc
16222	hsa-mir-24-2	RUSC1	0.367947439	mirna_pc
16223	hsa-mir-24-2	C15orf23	0.30426262	mirna_pc
16224	hsa-mir-24-2	NIT2	0.318244733	mirna_pc
16225	hsa-mir-24-2	GJB2	0.315965493	mirna_pc
16226	hsa-mir-24-2	PLEKHN1	0.311951923	mirna_pc
16227	hsa-mir-24-2	ANAPC7	0.381008073	mirna_pc
16228	hsa-mir-24-2	CIRH1A	0.384886007	mirna_pc
16229	hsa-mir-24-2	PRMT3	0.309806531	mirna_pc
16230	hsa-mir-24-2	C3orf21	0.356078556	mirna_pc
16231	hsa-mir-24-2	PSMD12	0.350203814	mirna_pc
16232	hsa-mir-24-2	S100A16	0.317405191	mirna_pc
16233	hsa-mir-24-2	C22orf9	0.35832841	mirna_pc
16234	hsa-mir-24-2	ZNF668	0.461351258	mirna_pc
16235	hsa-mir-24-2	DIABLO	0.343790254	mirna_pc
16236	hsa-mir-24-2	OVOL1	0.390038856	mirna_pc
16237	hsa-mir-24-2	CD276	0.321909854	mirna_pc
16238	hsa-mir-24-2	LRRC42	0.406839676	mirna_pc
16239	hsa-mir-24-2	C1QBP	0.419876664	mirna_pc
16240	hsa-mir-24-2	MRPL17	0.328094349	mirna_pc
16241	hsa-mir-24-2	RAD23B	0.360235767	mirna_pc
16242	hsa-mir-24-2	GPI	0.35743006	mirna_pc
16243	hsa-mir-24-2	C19orf47	0.345461371	mirna_pc
16244	hsa-mir-24-2	NSDHL	0.31670785	mirna_pc
16245	hsa-mir-24-2	XRCC3	0.345224902	mirna_pc
16246	hsa-mir-24-2	PDCD6	0.342677486	mirna_pc
16247	hsa-mir-24-2	GJB4	0.468514184	mirna_pc
16248	hsa-mir-24-2	TUBG1	0.478606162	mirna_pc
16249	hsa-mir-24-2	C16orf80	0.377376728	mirna_pc
16250	hsa-mir-24-2	PRRG4	0.364850862	mirna_pc
16251	hsa-mir-24-2	PVRL4	0.357578527	mirna_pc
16252	hsa-mir-24-2	ORMDL2	0.489518578	mirna_pc
16253	hsa-mir-24-2	VPS72	0.303116685	mirna_pc

16254	hsa-mir-24-2	EIF4EBP1	0.490852035	mirna_pc
16255	hsa-mir-24-2	NACC1	0.387140428	mirna_pc
16256	hsa-mir-24-2	ATAD3A	0.349706778	mirna_pc
16257	hsa-mir-24-2	TXN	0.361064437	mirna_pc
16258	hsa-mir-24-2	SHMT2	0.307428761	mirna_pc
16259	hsa-mir-24-2	C1orf172	0.365813929	mirna_pc
16260	hsa-mir-24-2	MRPL12	0.452090094	mirna_pc
16261	hsa-mir-24-2	RAP2B	0.385343749	mirna_pc
16262	hsa-mir-24-2	EMG1	0.409216087	mirna_pc
16263	hsa-mir-24-2	CSNK1E	0.380463935	mirna_pc
16264	hsa-mir-24-2	IFI27L2	0.446106044	mirna_pc
16265	hsa-mir-24-2	HAUS1	0.317453243	mirna_pc
16266	hsa-mir-24-2	PA2G4P4	0.470730488	mirna_pc
16267	hsa-mir-24-2	WDR53	0.416676414	mirna_pc
16268	hsa-mir-24-2	ALOX12P2	0.359275502	mirna_pc
16269	hsa-mir-24-2	RNF7	0.466912879	mirna_pc
16270	hsa-mir-24-2	CNIH4	0.391654876	mirna_pc
16271	hsa-mir-24-2	WDR54	0.348005473	mirna_pc
16272	hsa-mir-24-2	SLC10A3	0.398303936	mirna_pc
16273	hsa-mir-24-2	LOC728554	0.324458397	mirna_pc
16274	hsa-mir-24-2	AK2	0.319890713	mirna_pc
16275	hsa-mir-24-2	TBC1D10B	0.337569539	mirna_pc
16276	hsa-mir-24-2	RPL26L1	0.464552387	mirna_pc
16277	hsa-mir-24-2	H3F3B	0.403421957	mirna_pc
16278	hsa-mir-24-2	MRPL11	0.334440463	mirna_pc
16279	hsa-mir-24-2	TSPAN14	0.326732623	mirna_pc
16280	hsa-mir-24-2	DUT	0.370543694	mirna_pc
16281	hsa-mir-24-2	ANKRD39	0.329986842	mirna_pc
16282	hsa-mir-24-2	AP2M1	0.42635682	mirna_pc
16283	hsa-mir-24-2	HN1L	0.36836171	mirna_pc
16284	hsa-mir-24-2	CLSTN1	0.367673982	mirna_pc
16285	hsa-mir-24-2	PCGF1	0.535112865	mirna_pc
16286	hsa-mir-24-2	GJA3	0.304738888	mirna_pc
16287	hsa-mir-24-2	SERINC2	0.307247723	mirna_pc
16288	hsa-mir-24-2	COX4NB	0.383026878	mirna_pc
16289	hsa-mir-24-2	STUB1	0.391314063	mirna_pc
16290	hsa-mir-24-2	RPS6KA4	0.370967544	mirna_pc
16291	hsa-mir-24-2	POLR3G	0.337744437	mirna_pc
16292	hsa-mir-24-2	TYSND1	0.462839836	mirna_pc
16293	hsa-mir-24-2	HOXD11	0.409865404	mirna_pc
16294	hsa-mir-24-2	C3orf34	0.382000458	mirna_pc
16295	hsa-mir-24-2	TPRKB	0.363156186	mirna_pc
16296	hsa-mir-24-2	SRM	0.470847264	mirna_pc
16297	hsa-mir-24-2	TCEB1	0.409501729	mirna_pc
16298	hsa-mir-24-2	XRCC6	0.383605614	mirna_pc
16299	hsa-mir-24-2	TAF6L	0.348938228	mirna_pc
16300	hsa-mir-24-2	MRPL18	0.394238116	mirna_pc
16301	hsa-mir-24-2	BCKDK	0.301769609	mirna_pc
16302	hsa-mir-24-2	FANCE	0.443926185	mirna_pc
16303	hsa-mir-24-2	PSMG1	0.44342192	mirna_pc
16304	hsa-mir-24-2	PPP1R8	0.422310937	mirna_pc
16305	hsa-mir-24-2	NPM1	0.482981152	mirna_pc
16306	hsa-mir-24-2	CDK2AP1	0.450921836	mirna_pc
16307	hsa-mir-24-2	ANKRD13B	0.307886898	mirna_pc

16308	hsa-mir-24-2	C19orf22	0.409681371	mirna_pc
16309	hsa-mir-24-2	UPP1	0.390781918	mirna_pc
16310	hsa-mir-24-2	RGS20	0.473988309	mirna_pc
16311	hsa-mir-24-2	COQ2	0.333505217	mirna_pc
16312	hsa-mir-24-2	TPI1	0.58716384	mirna_pc
16313	hsa-mir-24-2	ATP5J2	0.311051554	mirna_pc
16314	hsa-mir-24-2	MED19	0.416033519	mirna_pc
16315	hsa-mir-24-2	PPAN	0.411545264	mirna_pc
16316	hsa-mir-24-2	FXYD3	0.344034025	mirna_pc
16317	hsa-mir-24-2	PSMA2	0.323075723	mirna_pc
16318	hsa-mir-24-2	OTUB1	0.465469709	mirna_pc
16319	hsa-mir-24-2	RFC2	0.43430719	mirna_pc
16320	hsa-mir-24-2	WDR5	0.331671444	mirna_pc
16321	hsa-mir-24-2	GTPBP3	0.307608143	mirna_pc
16322	hsa-mir-24-2	KARS	0.323000065	mirna_pc
16323	hsa-mir-24-2	EXOSC4	0.560067574	mirna_pc
16324	hsa-mir-24-2	IL1A	0.335296754	mirna_pc
16325	hsa-mir-24-2	FAM58A	0.402426537	mirna_pc
16326	hsa-mir-24-2	C19orf24	0.508790854	mirna_pc
16327	hsa-mir-24-2	SLC25A39	0.404122985	mirna_pc
16328	hsa-mir-24-2	SCRIB	0.345303468	mirna_pc
16329	hsa-mir-24-2	CHST1	0.312660376	mirna_pc
16330	hsa-mir-24-2	ULBP3	0.326500624	mirna_pc
16331	hsa-mir-24-2	TSEN34	0.300237367	mirna_pc
16332	hsa-mir-24-2	MAD2L2	0.399500876	mirna_pc
16333	hsa-mir-24-2	CSNK1G2	0.365533357	mirna_pc
16334	hsa-mir-24-2	CD9	0.320201512	mirna_pc
16335	hsa-mir-24-2	POLR3K	0.343510628	mirna_pc
16336	hsa-mir-24-2	TMEM39B	0.36257584	mirna_pc
16337	hsa-mir-24-2	SNAPC1	0.301336531	mirna_pc
16338	hsa-mir-24-2	NOP10	0.529310667	mirna_pc
16339	hsa-mir-24-2	RHOV	0.332571505	mirna_pc
16340	hsa-mir-24-2	TMEM161A	0.309022915	mirna_pc
16341	hsa-mir-24-2	RPL28	0.303279234	mirna_pc
16342	hsa-mir-24-2	RPS2	0.439109012	mirna_pc
16343	hsa-mir-24-2	MRPL51	0.480005989	mirna_pc
16344	hsa-mir-24-2	C1orf35	0.378390269	mirna_pc
16345	hsa-mir-24-2	METRN	0.3020087	mirna_pc
16346	hsa-mir-24-2	CYB5R2	0.306324518	mirna_pc
16347	hsa-mir-24-2	FJX1	0.371763268	mirna_pc
16348	hsa-mir-24-2	CDKN2D	0.325406862	mirna_pc
16349	hsa-mir-24-2	SAMD1	0.344457225	mirna_pc
16350	hsa-mir-24-2	ZDHHC12	0.411329227	mirna_pc
16351	hsa-mir-24-2	TPRXL	0.408616588	mirna_pc
16352	hsa-mir-24-2	ERI3	0.394064724	mirna_pc
16353	hsa-mir-24-2	PSMD7	0.422771355	mirna_pc
16354	hsa-mir-24-2	GYLTL1B	0.321676928	mirna_pc
16355	hsa-mir-24-2	MED27	0.459167283	mirna_pc
16356	hsa-mir-24-2	TOMM22	0.460836749	mirna_pc
16357	hsa-mir-24-2	EBPL	0.308984839	mirna_pc
16358	hsa-mir-24-2	VTI1B	0.314422671	mirna_pc
16359	hsa-mir-24-2	TCP1	0.384666442	mirna_pc
16360	hsa-mir-24-2	C9orf30	0.303815205	mirna_pc
16361	hsa-mir-24-2	WDR66	0.504967723	mirna_pc

16362	hsa-mir-24-2	TAGLN2	0.426591547	mirna_pc
16363	hsa-mir-24-2	C8orf30A	0.354038655	mirna_pc
16364	hsa-mir-24-2	ANKRD9	0.394639014	mirna_pc
16365	hsa-mir-24-2	ODC1	0.303204454	mirna_pc
16366	hsa-mir-24-2	FZD6	0.303989226	mirna_pc
16367	hsa-mir-24-2	WRNIP1	0.314180402	mirna_pc
16368	hsa-mir-24-2	TECR	0.443538852	mirna_pc
16369	hsa-mir-24-2	BAD	0.387122783	mirna_pc
16370	hsa-mir-24-2	PDZD11	0.309440296	mirna_pc
16371	hsa-mir-24-2	PPME1	0.318587588	mirna_pc
16372	hsa-mir-24-2	LOC441089	0.420695615	mirna_pc
16373	hsa-mir-24-2	SEMA4B	0.356124129	mirna_pc
16374	hsa-mir-24-2	TUBA4A	0.368731602	mirna_pc
16375	hsa-mir-24-2	HES4	0.437594397	mirna_pc
16376	hsa-mir-24-2	DUSP14	0.353062263	mirna_pc
16377	hsa-mir-24-2	ANXA2P1	0.370213716	mirna_pc
16378	hsa-mir-24-2	VPS4A	0.364569673	mirna_pc
16379	hsa-mir-24-2	DSP	0.324494454	mirna_pc
16380	hsa-mir-24-2	XRCC6BP1	0.363281475	mirna_pc
16381	hsa-mir-24-2	RPP21	0.3811563	mirna_pc
16382	hsa-mir-24-2	IFI16	0.369748003	mirna_pc
16383	hsa-mir-24-2	RIOK1	0.30815398	mirna_pc
16384	hsa-mir-24-2	C3orf1	0.347391891	mirna_pc
16385	hsa-mir-24-2	NXPH4	0.449245603	mirna_pc
16386	hsa-mir-24-2	MXD3	0.301852573	mirna_pc
16387	hsa-mir-24-2	MPHOSPH6	0.403724406	mirna_pc
16388	hsa-mir-24-2	TMPRSS13	0.317651269	mirna_pc
16389	hsa-mir-24-2	KLC2	0.404916881	mirna_pc
16390	hsa-mir-24-2	NRD1	0.348589376	mirna_pc
16391	hsa-mir-24-2	ZNF598	0.507822547	mirna_pc
16392	hsa-mir-24-2	FAM89A	0.382076407	mirna_pc
16393	hsa-mir-24-2	FGFR3	0.340545013	mirna_pc
16394	hsa-mir-24-2	C19orf53	0.50676673	mirna_pc
16395	hsa-mir-24-2	TELO2	0.435227661	mirna_pc
16396	hsa-mir-24-2	CDK16	0.357591588	mirna_pc
16397	hsa-mir-24-2	ALKBH2	0.375823691	mirna_pc
16398	hsa-mir-24-2	RRP1	0.365988905	mirna_pc
16399	hsa-mir-24-2	POP5	0.356420982	mirna_pc
16400	hsa-mir-24-2	TCTEX1D2	0.378703669	mirna_pc
16401	hsa-mir-24-2	NETO2	0.309752883	mirna_pc
16402	hsa-mir-24-2	SPHK1	0.385992316	mirna_pc
16403	hsa-mir-24-2	BNIP3	0.429512363	mirna_pc
16404	hsa-mir-24-2	RNF126	0.60706554	mirna_pc
16405	hsa-mir-24-2	MLF 2.00	0.491530281	mirna_pc
16406	hsa-mir-24-2	FAM108A1	0.406568458	mirna_pc
16407	hsa-mir-24-2	RAB7A	0.366380087	mirna_pc
16408	hsa-mir-24-2	MRPL36	0.378862806	mirna_pc
16409	hsa-mir-24-2	SIGMAR1	0.305992826	mirna_pc
16410	hsa-mir-24-2	RHOG	0.339522144	mirna_pc
16411	hsa-mir-24-2	CSNK2B	0.360443958	mirna_pc
16412	hsa-mir-24-2	RPF2	0.362367894	mirna_pc
16413	hsa-mir-24-2	C1orf170	0.407525684	mirna_pc
16414	hsa-mir-24-2	ARL6IP4	0.320011333	mirna_pc
16415	hsa-mir-24-2	FBXW9	0.302336072	mirna_pc

16416	hsa-mir-24-2	GSTO1	0.402503549	mirna_pc
16417	hsa-mir-24-2	DDB2	0.380093393	mirna_pc
16418	hsa-mir-24-2	RPL39L	0.362253233	mirna_pc
16419	hsa-mir-24-2	KATNB1	0.321471512	mirna_pc
16420	hsa-mir-24-2	EIF2B2	0.313384222	mirna_pc
16421	hsa-mir-24-2	ACD	0.415159272	mirna_pc
16422	hsa-mir-24-2	C14orf33	0.319438864	mirna_pc
16423	hsa-mir-24-2	GPN3	0.355448847	mirna_pc
16424	hsa-mir-24-2	RAD23A	0.446055834	mirna_pc
16425	hsa-mir-24-2	DNLZ	0.363276833	mirna_pc
16426	hsa-mir-24-2	RPUSD1	0.499378829	mirna_pc
16427	hsa-mir-24-2	LOC152217	0.424062847	mirna_pc
16428	hsa-mir-24-2	USP14	0.323125418	mirna_pc
16429	hsa-mir-24-2	MRPL37	0.422491221	mirna_pc
16430	hsa-mir-24-2	PLA2G4E	0.347150902	mirna_pc
16431	hsa-mir-24-2	GPR109B	0.376963619	mirna_pc
16432	hsa-mir-24-2	MEMO1	0.532393642	mirna_pc
16433	hsa-mir-24-2	PPM1J	0.406623516	mirna_pc
16434	hsa-mir-24-2	CCDC23	0.370035363	mirna_pc
16435	hsa-mir-24-2	FKBP8	0.402469916	mirna_pc
16436	hsa-mir-24-2	NDUFS6	0.32346223	mirna_pc
16437	hsa-mir-24-2	NOB1	0.412525329	mirna_pc
16438	hsa-mir-24-2	EIF3I	0.553543104	mirna_pc
16439	hsa-mir-24-2	UBXN2A	0.337311353	mirna_pc
16440	hsa-mir-24-2	ADK	0.486228965	mirna_pc
16441	hsa-mir-24-2	RPL12	0.445139473	mirna_pc
16442	hsa-mir-24-2	C9orf89	0.306282672	mirna_pc
16443	hsa-mir-24-2	WDR47	0.302540481	mirna_pc
16444	hsa-mir-24-2	ZMYND19	0.391505289	mirna_pc
16445	hsa-mir-24-2	NDUFA8	0.319861929	mirna_pc
16446	hsa-mir-24-2	NAA20	0.370089523	mirna_pc
16447	hsa-mir-24-2	GAS2L1	0.369351137	mirna_pc
16448	hsa-mir-24-2	TRAP1	0.440185446	mirna_pc
16449	hsa-mir-24-2	TAF12	0.441173353	mirna_pc
16450	hsa-mir-24-2	ARPC5L	0.378877363	mirna_pc
16451	hsa-mir-24-2	C19orf73	0.334441965	mirna_pc
16452	hsa-mir-24-2	FAM96B	0.342297466	mirna_pc
16453	hsa-mir-24-2	DDX41	0.372889894	mirna_pc
16454	hsa-mir-24-2	HOXD8	0.352663423	mirna_pc
16455	hsa-mir-24-2	ICT1	0.404417584	mirna_pc
16456	hsa-mir-24-2	C15orf63	0.432990625	mirna_pc
16457	hsa-mir-24-2	STX8	0.327414195	mirna_pc
16458	hsa-mir-24-2	LTB4R	0.387954385	mirna_pc
16459	hsa-mir-24-2	SURF2	0.539812	mirna_pc
16460	hsa-mir-24-2	METTL11A	0.359763029	mirna_pc
16461	hsa-mir-24-2	TRPT1	0.301832009	mirna_pc
16462	hsa-mir-24-2	PUSL1	0.350696162	mirna_pc
16463	hsa-mir-24-2	APOA1BP	0.43145661	mirna_pc
16464	hsa-mir-24-2	RFXANK	0.409121265	mirna_pc
16465	hsa-mir-24-2	HES2	0.344286163	mirna_pc
16466	hsa-mir-24-2	MED10	0.40718522	mirna_pc
16467	hsa-mir-24-2	DUSP7	0.460760898	mirna_pc
16468	hsa-mir-24-2	GNA15	0.46299125	mirna_pc
16469	hsa-mir-24-2	LTBR	0.367977498	mirna_pc

16470	hsa-mir-24-2	PWP1	0.405899123	mirna_pc
16471	hsa-mir-24-2	S1PR5	0.331773758	mirna_pc
16472	hsa-mir-24-2	MNAT1	0.363958092	mirna_pc
16473	hsa-mir-24-2	RPL37	0.372585909	mirna_pc
16474	hsa-mir-24-2	DRAP1	0.557583355	mirna_pc
16475	hsa-mir-24-2	SMARCB1	0.38243175	mirna_pc
16476	hsa-mir-24-2	CHIC2	0.305356637	mirna_pc
16477	hsa-mir-24-2	DDX54	0.339803167	mirna_pc
16478	hsa-mir-24-2	TBC1D2	0.424731482	mirna_pc
16479	hsa-mir-24-2	CCT8	0.354378164	mirna_pc
16480	hsa-mir-24-2	B3GAT3	0.400274514	mirna_pc
16481	hsa-mir-24-2	MEX3D	0.477315576	mirna_pc
16482	hsa-mir-24-2	B4GALT2	0.528104518	mirna_pc
16483	hsa-mir-24-2	UFD1L	0.391332585	mirna_pc
16484	hsa-mir-24-2	RPA1	0.334893958	mirna_pc
16485	hsa-mir-24-2	FAM92A1	0.375989996	mirna_pc
16486	hsa-mir-24-2	CNN2	0.432259246	mirna_pc
16487	hsa-mir-24-2	SART1	0.316986928	mirna_pc
16488	hsa-mir-24-2	TIMM10	0.429997752	mirna_pc
16489	hsa-mir-24-2	TGFB1	0.407181274	mirna_pc
16490	hsa-mir-24-2	IRF6	0.420292085	mirna_pc
16491	hsa-mir-24-2	ZDHHC18	0.461926834	mirna_pc
16492	hsa-mir-24-2	FZR1	0.309233306	mirna_pc
16493	hsa-mir-24-2	RPL38	0.517711961	mirna_pc
16494	hsa-mir-24-2	FOXK2	0.332178235	mirna_pc
16495	hsa-mir-24-2	ANAPC10	0.308154692	mirna_pc
16496	hsa-mir-24-2	HSD17B10	0.382313084	mirna_pc
16497	hsa-mir-24-2	ABT1	0.343615481	mirna_pc
16498	hsa-mir-24-2	FAM86A	0.407292501	mirna_pc
16499	hsa-mir-24-2	TIGD5	0.479384334	mirna_pc
16500	hsa-mir-24-2	HEPHL1	0.336478561	mirna_pc
16501	hsa-mir-24-2	NUDT8	0.413515697	mirna_pc
16502	hsa-mir-24-2	NOP16	0.339607255	mirna_pc
16503	hsa-mir-24-2	IL1F9	0.350538701	mirna_pc
16504	hsa-mir-24-2	SLC7A5	0.400600244	mirna_pc
16505	hsa-mir-24-2	SLC16A1	0.399817766	mirna_pc
16506	hsa-mir-24-2	ACTR1A	0.309343957	mirna_pc
16507	hsa-mir-24-2	SLC25A17	0.42111407	mirna_pc
16508	hsa-mir-24-2	IKBKG	0.301479492	mirna_pc
16509	hsa-mir-24-2	CDK7	0.33582197	mirna_pc
16510	hsa-mir-24-2	C19orf6	0.405511025	mirna_pc
16511	hsa-mir-24-2	NDUFS7	0.41734151	mirna_pc
16512	hsa-mir-24-2	KLC3	0.406490929	mirna_pc
16513	hsa-mir-24-2	RPS7	0.486208966	mirna_pc
16514	hsa-mir-24-2	THSD1	0.304197929	mirna_pc
16515	hsa-mir-24-2	SH3BGRL3	0.427493725	mirna_pc
16516	hsa-mir-24-2	LOC100133161	0.35084046	mirna_pc
16517	hsa-mir-24-2	STYXL1	0.338967369	mirna_pc
16518	hsa-mir-24-2	BLOC1S2	0.393839357	mirna_pc
16519	hsa-mir-24-2	GSDMC	0.353574415	mirna_pc
16520	hsa-mir-24-2	EPPK1	0.324667533	mirna_pc
16521	hsa-mir-24-2	C11orf17	0.309503761	mirna_pc
16522	hsa-mir-24-2	MKRN3	0.382203167	mirna_pc
16523	hsa-mir-24-2	LOC401010	0.302033331	mirna_pc

16524	hsa-mir-24-2	METAP2	0.422433387	mirna_pc
16525	hsa-mir-24-2	ATP5D	0.439204678	mirna_pc
16526	hsa-mir-24-2	CLNS1A	0.388357027	mirna_pc
16527	hsa-mir-24-2	SLC2A9	0.378932528	mirna_pc
16528	hsa-mir-24-2	C6orf1	0.460016418	mirna_pc
16529	hsa-mir-24-2	STRA13	0.392737549	mirna_pc
16530	hsa-mir-24-2	RPL35	0.491157927	mirna_pc
16531	hsa-mir-24-2	RRP7A	0.359818097	mirna_pc
16532	hsa-mir-24-2	HGS	0.443267763	mirna_pc
16533	hsa-mir-24-2	TMEM154	0.395519391	mirna_pc
16534	hsa-mir-24-2	NHP2	0.416450718	mirna_pc
16535	hsa-mir-24-2	PSMC3	0.478490098	mirna_pc
16536	hsa-mir-24-2	FER	0.302336507	mirna_pc
16537	hsa-mir-24-2	TIMM16	0.487169205	mirna_pc
16538	hsa-mir-24-2	MTX1	0.455862956	mirna_pc
16539	hsa-mir-24-2	MIER2	0.402165964	mirna_pc
16540	hsa-mir-24-2	MRPS28	0.3785583	mirna_pc
16541	hsa-mir-24-2	DVL2	0.354535062	mirna_pc
16542	hsa-mir-24-2	STARD5	0.432027257	mirna_pc
16543	hsa-mir-24-2	GPR109A	0.393018253	mirna_pc
16544	hsa-mir-24-2	TTC4	0.462005137	mirna_pc
16545	hsa-mir-24-2	LOC100289341	0.304602283	mirna_pc
16546	hsa-mir-24-2	NUDCD2	0.430694397	mirna_pc
16547	hsa-mir-24-2	RPL8	0.425740302	mirna_pc
16548	hsa-mir-24-2	CCM2	0.332364344	mirna_pc
16549	hsa-mir-24-2	NRBP2	0.322951304	mirna_pc
16550	hsa-mir-24-2	C3orf67	0.366385261	mirna_pc
16551	hsa-mir-24-2	SUMO3	0.308605939	mirna_pc
16552	hsa-mir-24-2	PANX1	0.413549543	mirna_pc
16553	hsa-mir-24-2	FAM83A	0.353610115	mirna_pc
16554	hsa-mir-24-2	SKP1	0.327669863	mirna_pc
16555	hsa-mir-24-2	GJB5	0.528796475	mirna_pc
16556	hsa-mir-24-2	HK2	0.304751888	mirna_pc
16557	hsa-mir-24-2	FOSL1	0.302103459	mirna_pc
16558	hsa-mir-24-2	DGKA	0.399932055	mirna_pc
16559	hsa-mir-24-2	ACTB	0.310223304	mirna_pc
16560	hsa-mir-24-2	CMIP	0.334615995	mirna_pc
16561	hsa-mir-24-2	PPP1R14C	0.416973364	mirna_pc
16562	hsa-mir-24-2	LTB4R2	0.390811272	mirna_pc
16563	hsa-mir-24-2	ANXA8L2	0.380664206	mirna_pc
16564	hsa-mir-24-2	MRPS22	0.424205992	mirna_pc
16565	hsa-mir-24-2	S100A13	0.308288162	mirna_pc
16566	hsa-mir-24-2	IFFO2	0.35611013	mirna_pc
16567	hsa-mir-24-2	CDK5R1	0.357773007	mirna_pc
16568	hsa-mir-24-2	EIF2S1	0.306921064	mirna_pc
16569	hsa-mir-24-2	S100A2	0.597232095	mirna_pc
16570	hsa-mir-24-2	NDUFB10	0.397102508	mirna_pc
16571	hsa-mir-24-2	MGC72080	0.323617747	mirna_pc
16572	hsa-mir-24-2	DUS3L	0.40064526	mirna_pc
16573	hsa-mir-24-2	C9orf69	0.357356234	mirna_pc
16574	hsa-mir-24-2	MRPS21	0.30799095	mirna_pc
16575	hsa-mir-24-2	RIN1	0.326021665	mirna_pc
16576	hsa-mir-24-2	IPPK	0.397958403	mirna_pc
16577	hsa-mir-24-2	ADAMTSL5	0.375807619	mirna_pc

16578	hsa-mir-24-2	LYPD3	0.31168493	mirna_pc
16579	hsa-mir-24-2	ANAPC11	0.445377722	mirna_pc
16580	hsa-mir-24-2	TARS	0.349994346	mirna_pc
16581	hsa-mir-24-2	C11orf83	0.329615378	mirna_pc
16582	hsa-mir-24-2	MPV17	0.509281304	mirna_pc
16583	hsa-mir-24-2	LPAR3	0.385362476	mirna_pc
16584	hsa-mir-24-2	TUSC1	0.426902663	mirna_pc
16585	hsa-mir-24-2	CARHSP1	0.434924869	mirna_pc
16586	hsa-mir-24-2	PAK6	0.305243421	mirna_pc
16587	hsa-mir-24-2	GTF2A2	0.349151251	mirna_pc
16588	hsa-mir-24-2	NDUFA6	0.353754364	mirna_pc
16589	hsa-mir-24-2	NDUFB7	0.451950385	mirna_pc
16590	hsa-mir-24-2	PCBP1	0.336069023	mirna_pc
16591	hsa-mir-24-2	HRAS	0.598390342	mirna_pc
16592	hsa-mir-24-2	NRBP1	0.437826236	mirna_pc
16593	hsa-mir-24-2	UBE2N	0.339009313	mirna_pc
16594	hsa-mir-24-2	CHCHD2	0.337344332	mirna_pc
16595	hsa-mir-24-2	TPBG	0.317364051	mirna_pc
16596	hsa-mir-24-2	MRPS2	0.314359105	mirna_pc
16597	hsa-mir-24-2	NDUFAF2	0.328504831	mirna_pc
16598	hsa-mir-24-2	PIGX	0.357074345	mirna_pc
16599	hsa-mir-24-2	HTRA2	0.450436496	mirna_pc
16600	hsa-mir-24-2	RAB38	0.516853502	mirna_pc
16601	hsa-mir-24-2	KEAP1	0.307541909	mirna_pc
16602	hsa-mir-24-2	DOHH	0.375201452	mirna_pc
16603	hsa-mir-24-2	IPO13	0.325513699	mirna_pc
16604	hsa-mir-24-2	PDLIM1	0.361289121	mirna_pc
16605	hsa-mir-24-2	GRHL3	0.310773158	mirna_pc
16606	hsa-mir-24-2	SLC48A1	0.3451129	mirna_pc
16607	hsa-mir-24-2	PFN2	0.352850581	mirna_pc
16608	hsa-mir-24-2	C14orf156	0.427178845	mirna_pc
16609	hsa-mir-24-2	FAM127C	0.3022566	mirna_pc
16610	hsa-mir-24-2	C10orf58	0.471909828	mirna_pc
16611	hsa-mir-24-2	UBE2V2	0.382683688	mirna_pc
16612	hsa-mir-24-2	MTX2	0.36056393	mirna_pc
16613	hsa-mir-24-2	JMJD8	0.391592321	mirna_pc
16614	hsa-mir-24-2	NHP2L1	0.425899101	mirna_pc
16615	hsa-mir-24-2	DCTN1	0.318622776	mirna_pc
16616	hsa-mir-24-2	ATP6V1F	0.476414348	mirna_pc
16617	hsa-mir-24-2	SLC25A10	0.317277292	mirna_pc
16618	hsa-mir-24-2	CIAPIN1	0.345397847	mirna_pc
16619	hsa-mir-24-2	MRPL24	0.362693959	mirna_pc
16620	hsa-mir-24-2	DRG1	0.479854999	mirna_pc
16621	hsa-mir-24-2	GNAI1	0.372348009	mirna_pc
16622	hsa-mir-24-2	SLC39A3	0.430133142	mirna_pc
16623	hsa-mir-24-2	SSSCA1	0.515674921	mirna_pc
16624	hsa-mir-24-2	NOL7	0.351253212	mirna_pc
16625	hsa-mir-24-2	B4GALNT1	0.334569564	mirna_pc
16626	hsa-mir-24-2	HSPB1	0.552372573	mirna_pc
16627	hsa-mir-24-2	C1orf74	0.378903808	mirna_pc
16628	hsa-mir-24-2	MAP2K1	0.34635773	mirna_pc
16629	hsa-mir-24-2	ASNA1	0.429978837	mirna_pc
16630	hsa-mir-24-2	GPC1	0.403414052	mirna_pc
16631	hsa-mir-24-2	EXOSC1	0.376810336	mirna_pc

16632	hsa-mir-24-2	GADD45GIP1	0.571082265	mirna_pc
16633	hsa-mir-24-2	DEPDC7	0.330235616	mirna_pc
16634	hsa-mir-24-2	PKP1	0.404584412	mirna_pc
16635	hsa-mir-24-2	C9orf114	0.328794809	mirna_pc
16636	hsa-mir-24-2	ACAP3	0.307163435	mirna_pc
16637	hsa-mir-24-2	C16orf91	0.391149127	mirna_pc
16638	hsa-mir-24-2	NT5C	0.439779947	mirna_pc
16639	hsa-mir-24-2	PTMS	0.476077194	mirna_pc
16640	hsa-mir-24-2	STON2	0.390033614	mirna_pc
16641	hsa-mir-24-2	STAMBP	0.332744034	mirna_pc
16642	hsa-mir-24-2	SH3GL1	0.390483551	mirna_pc
16643	hsa-mir-24-2	DSC2	0.3074081	mirna_pc
16644	hsa-mir-24-2	CAPN1	0.349152119	mirna_pc
16645	hsa-mir-24-2	HR	0.36109712	mirna_pc
16646	hsa-mir-24-2	APOBEC3A	0.305832538	mirna_pc
16647	hsa-mir-24-2	SLC4A1AP	0.36182202	mirna_pc
16648	hsa-mir-24-2	TTC27	0.319772409	mirna_pc
16649	hsa-mir-24-2	FLJ45445	0.372614973	mirna_pc
16650	hsa-mir-24-2	ATP5L	0.311427108	mirna_pc
16651	hsa-mir-24-2	C1orf151	0.31523952	mirna_pc
16652	hsa-mir-24-2	C11orf73	0.358262811	mirna_pc
16653	hsa-mir-24-2	CCDC124	0.542895994	mirna_pc
16654	hsa-mir-24-2	RPF1	0.341846623	mirna_pc
16655	hsa-mir-24-2	NEDD8	0.384170762	mirna_pc
16656	hsa-mir-24-2	RNF187	0.461415133	mirna_pc
16657	hsa-mir-24-2	FAM83C	0.339546226	mirna_pc
16658	hsa-mir-24-2	C16orf61	0.314702009	mirna_pc
16659	hsa-mir-24-2	FIBP	0.406096004	mirna_pc
16660	hsa-mir-24-2	MRPS34	0.53279182	mirna_pc
16661	hsa-mir-24-2	TXN2	0.351517208	mirna_pc
16662	hsa-mir-24-2	DUS2L	0.415881198	mirna_pc
16663	hsa-mir-24-2	KIFC2	0.356045089	mirna_pc
16664	hsa-mir-24-2	LRRC28	0.311930457	mirna_pc
16665	hsa-mir-24-2	EIF3M	0.318824958	mirna_pc
16666	hsa-mir-24-2	TRAF7	0.507572007	mirna_pc
16667	hsa-mir-24-2	DARS	0.301461607	mirna_pc
16668	hsa-mir-24-2	AAAS	0.414780801	mirna_pc
16669	hsa-mir-24-2	KRT16	0.320850168	mirna_pc
16670	hsa-mir-24-2	MARK3	0.304121193	mirna_pc
16671	hsa-mir-24-2	PRDX2	0.351013306	mirna_pc
16672	hsa-mir-24-2	COX5B	0.345198392	mirna_pc
16673	hsa-mir-24-2	C3orf54	0.490955684	mirna_pc
16674	hsa-mir-24-2	KATNA1	0.348487245	mirna_pc
16675	hsa-mir-24-2	TNFAIP8	0.32539521	mirna_pc
16676	hsa-mir-24-2	TMEM107	0.339272192	mirna_pc
16677	hsa-mir-24-2	C17orf90	0.324299413	mirna_pc
16678	hsa-mir-24-2	C1orf57	0.377377826	mirna_pc
16679	hsa-mir-24-2	COL17A1	0.393535359	mirna_pc
16680	hsa-mir-24-2	FDXR	0.524006081	mirna_pc
16681	hsa-mir-24-2	C2orf79	0.426298062	mirna_pc
16682	hsa-mir-24-2	MTFMT	0.314162022	mirna_pc
16683	hsa-mir-24-2	DSG3	0.393998784	mirna_pc
16684	hsa-mir-24-2	TPD52L1	0.327708303	mirna_pc
16685	hsa-mir-24-2	PEMT	0.349425378	mirna_pc

16686	hsa-mir-24-2	PYGL	0.43702114	mirna_pc
16687	hsa-mir-24-2	DHRS4	0.390117198	mirna_pc
16688	hsa-mir-24-2	KRT5	0.614613087	mirna_pc
16689	hsa-mir-24-2	LASS4	0.404704298	mirna_pc
16690	hsa-mir-24-2	FGF11	0.358439547	mirna_pc
16691	hsa-mir-24-2	DERA	0.337897093	mirna_pc
16692	hsa-mir-24-2	ARHGDIA	0.437277231	mirna_pc
16693	hsa-mir-24-2	PRELID1	0.331924977	mirna_pc
16694	hsa-mir-24-2	MRPL30	0.306010209	mirna_pc
16695	hsa-mir-24-2	TALDO1	0.358133507	mirna_pc
16696	hsa-mir-24-2	SNAPIN	0.46159319	mirna_pc
16697	hsa-mir-24-2	METTL5	0.360787304	mirna_pc
16698	hsa-mir-24-2	KRT6A	0.439334108	mirna_pc
16699	hsa-mir-24-2	DYNLL1	0.528342123	mirna_pc
16700	hsa-mir-24-2	PGP	0.395853765	mirna_pc
16701	hsa-mir-24-2	KRT6B	0.336862008	mirna_pc
16702	hsa-mir-24-2	FGFBP1	0.547509169	mirna_pc
16703	hsa-mir-24-2	NOL12	0.313819875	mirna_pc
16704	hsa-mir-24-2	C11orf45	0.334679743	mirna_pc
16705	hsa-mir-24-2	TTLL12	0.363746812	mirna_pc
16706	hsa-mir-24-2	IL20RB	0.451555745	mirna_pc
16707	hsa-mir-24-2	GOLGA7B	0.323191319	mirna_pc
16708	hsa-mir-24-2	GPR87	0.410726576	mirna_pc
16709	hsa-mir-24-2	ZNF385A	0.505563161	mirna_pc
16710	hsa-mir-24-2	ITGB1BP1	0.535158892	mirna_pc
16711	hsa-mir-24-2	RPS16	0.30454385	mirna_pc
16712	hsa-mir-24-2	POLR3H	0.369816651	mirna_pc
16713	hsa-mir-24-2	OSTF1	0.352765472	mirna_pc
16714	hsa-mir-24-2	COMMD8	0.310358666	mirna_pc
16715	hsa-mir-24-2	C18orf10	0.367544532	mirna_pc
16716	hsa-mir-24-2	GPR98	0.3224016	mirna_pc
16717	hsa-mir-24-2	PEX10	0.386659149	mirna_pc
16718	hsa-mir-24-2	NDUFB1	0.302905332	mirna_pc
16719	hsa-mir-24-2	C9orf119	0.385969219	mirna_pc
16720	hsa-mir-24-2	EMD	0.396533038	mirna_pc
16721	hsa-mir-24-2	MRPL52	0.390215807	mirna_pc
16722	hsa-mir-24-2	RPS24	0.359941689	mirna_pc
16723	hsa-mir-24-2	HSD17B1	0.458983263	mirna_pc
16724	hsa-mir-24-2	DSC3	0.416451692	mirna_pc
16725	hsa-mir-24-2	RNF40	0.305965386	mirna_pc
16726	hsa-mir-24-2	CCDC53	0.410715352	mirna_pc
16727	hsa-mir-24-2	RAET1G	0.356369815	mirna_pc
16728	hsa-mir-24-2	CAPZB	0.351559623	mirna_pc
16729	hsa-mir-24-2	STRADA	0.311514268	mirna_pc
16730	hsa-mir-24-2	SARNP	0.41806101	mirna_pc
16731	hsa-mir-24-2	PGLS	0.427015939	mirna_pc
16732	hsa-mir-24-2	ENDOG	0.405814891	mirna_pc
16733	hsa-mir-24-2	SIAH2	0.313738284	mirna_pc
16734	hsa-mir-24-2	INO80B	0.504942349	mirna_pc
16735	hsa-mir-24-2	LY6K	0.378995039	mirna_pc
16736	hsa-mir-24-2	MBD3	0.397771144	mirna_pc
16737	hsa-mir-24-2	WASF2	0.430876053	mirna_pc
16738	hsa-mir-24-2	COQ3	0.386120822	mirna_pc
16739	hsa-mir-24-2	RPL7	0.326264123	mirna_pc

16740	hsa-mir-24-2	NOD2	0.308931024	mirna_pc
16741	hsa-mir-24-2	RPL17	0.428404438	mirna_pc
16742	hsa-mir-24-2	ANXA8	0.351454234	mirna_pc
16743	hsa-mir-24-2	ARTN	0.389329222	mirna_pc
16744	hsa-mir-24-2	STK11	0.352918846	mirna_pc
16745	hsa-mir-24-2	RXRA	0.408455198	mirna_pc
16746	hsa-mir-24-2	VSNL1	0.511469202	mirna_pc
16747	hsa-mir-24-2	HSF1	0.488324931	mirna_pc
16748	hsa-mir-24-2	ARHGAP23	0.318234649	mirna_pc
16749	hsa-mir-24-2	FAM83F	0.418331077	mirna_pc
16750	hsa-mir-24-2	DCI	0.333055343	mirna_pc
16751	hsa-mir-24-2	JARID2	0.310299791	mirna_pc
16752	hsa-mir-24-2	AVEN	0.350936761	mirna_pc
16753	hsa-mir-24-2	PLS3	0.423359066	mirna_pc
16754	hsa-mir-24-2	IL1F5	0.35903823	mirna_pc
16755	hsa-mir-24-2	CCDC85B	0.47895279	mirna_pc
16756	hsa-mir-24-2	FABP5	0.425791118	mirna_pc
16757	hsa-mir-24-2	HOMER3	0.480241422	mirna_pc
16758	hsa-mir-24-2	ABCF3	0.407093629	mirna_pc
16759	hsa-mir-24-2	TIMM13	0.45640152	mirna_pc
16760	hsa-mir-24-2	ADAT3	0.347832547	mirna_pc
16761	hsa-mir-24-2	ATP6V1E1	0.30555553	mirna_pc
16762	hsa-mir-24-2	AMZ2	0.411799888	mirna_pc
16763	hsa-mir-24-2	BTBD11	0.38578618	mirna_pc
16764	hsa-mir-24-2	SHC1	0.346597231	mirna_pc
16765	hsa-mir-24-2	C17orf61	0.322758574	mirna_pc
16766	hsa-mir-24-2	CTNNBIP1	0.335766989	mirna_pc
16767	hsa-mir-24-2	CELSR1	0.318234797	mirna_pc
16768	hsa-mir-24-2	PARL	0.37884039	mirna_pc
16769	hsa-mir-24-2	PITX2	0.310070644	mirna_pc
16770	hsa-mir-24-2	ALDOA	0.542756805	mirna_pc
16771	hsa-mir-24-2	MRPL28	0.571892313	mirna_pc
16772	hsa-mir-24-2	HOXA7	0.414923144	mirna_pc
16773	hsa-mir-24-2	NDUFC1	0.32071791	mirna_pc
16774	hsa-mir-24-2	FAM43A	0.356710731	mirna_pc
16775	hsa-mir-24-2	BTBD6	0.346324181	mirna_pc
16776	hsa-mir-24-2	RDBP	0.326141694	mirna_pc
16777	hsa-mir-24-2	MECR	0.341062701	mirna_pc
16778	hsa-mir-24-2	YWHAQ	0.484199574	mirna_pc
16779	hsa-mir-24-2	ARPC2	0.403524285	mirna_pc
16780	hsa-mir-24-2	UBTD1	0.305278956	mirna_pc
16781	hsa-mir-24-2	PIR	0.39113916	mirna_pc
16782	hsa-mir-24-2	RGS14	0.345259982	mirna_pc
16783	hsa-mir-24-2	MSN	0.301209156	mirna_pc
16784	hsa-mir-24-2	DNAJC8	0.384469198	mirna_pc
16785	hsa-mir-24-2	NDUFA4L2	0.401642326	mirna_pc
16786	hsa-mir-24-2	HAS3	0.321296605	mirna_pc
16787	hsa-mir-24-2	TMEM93	0.426089987	mirna_pc
16788	hsa-mir-24-2	KCTD1	0.331030908	mirna_pc
16789	hsa-mir-24-2	C19orf29	0.326057014	mirna_pc
16790	hsa-mir-24-2	ZFPL1	0.343061284	mirna_pc
16791	hsa-mir-24-2	RPL35A	0.45868628	mirna_pc
16792	hsa-mir-24-2	NME1-NME2	0.367415263	mirna_pc
16793	hsa-mir-24-2	LGALS7	0.363785143	mirna_pc

16794	hsa-mir-24-2	KLHL17	0.329468255	mirna_pc
16795	hsa-mir-24-2	LOC642587	0.458048038	mirna_pc
16796	hsa-mir-24-2	ARL 2.00	0.419729222	mirna_pc
16797	hsa-mir-24-2	TMEM79	0.308899984	mirna_pc
16798	hsa-mir-24-2	NUDT14	0.341289613	mirna_pc
16799	hsa-mir-24-2	SPPL3	0.365434064	mirna_pc
16800	hsa-mir-24-2	COMMD1	0.356685007	mirna_pc
16801	hsa-mir-24-2	WDR41	0.304302046	mirna_pc
16802	hsa-mir-24-2	TINF2	0.320762538	mirna_pc
16803	hsa-mir-24-2	PTRH1	0.315899963	mirna_pc
16804	hsa-mir-24-2	RPS15A	0.386506167	mirna_pc
16805	hsa-mir-24-2	PGAP2	0.376992059	mirna_pc
16806	hsa-mir-24-2	UQCR10	0.314233491	mirna_pc
16807	hsa-mir-24-2	RPS23	0.32157009	mirna_pc
16808	hsa-mir-24-2	INO80E	0.351068447	mirna_pc
16809	hsa-mir-24-2	IAH1	0.371548193	mirna_pc
16810	hsa-mir-24-2	NPRL3	0.395498795	mirna_pc
16811	hsa-mir-24-2	COX8A	0.359506157	mirna_pc
16812	hsa-mir-24-2	C16orf74	0.629715699	mirna_pc
16813	hsa-mir-24-2	GPR153	0.31913816	mirna_pc
16814	hsa-mir-24-2	ZNF771	0.302000225	mirna_pc
16815	hsa-mir-24-2	RPS29	0.330517237	mirna_pc
16816	hsa-mir-24-2	FAM125A	0.380976414	mirna_pc
16817	hsa-mir-24-2	C7orf68	0.311062457	mirna_pc
16818	hsa-mir-24-2	ADPRHL2	0.30723996	mirna_pc
16819	hsa-mir-24-2	MRPL38	0.318090406	mirna_pc
16820	hsa-mir-24-2	TCEB2	0.447601646	mirna_pc
16821	hsa-mir-24-2	FN3KRP	0.300360764	mirna_pc
16822	hsa-mir-24-2	MRPL55	0.36344938	mirna_pc
16823	hsa-mir-24-2	SEPX1	0.326134526	mirna_pc
16824	hsa-mir-24-2	HSPA4	0.39025272	mirna_pc
16825	hsa-mir-24-2	YBX1	0.365349586	mirna_pc
16826	hsa-mir-24-2	BICD2	0.422371151	mirna_pc
16827	hsa-mir-24-2	GJB6	0.349453945	mirna_pc
16828	hsa-mir-24-2	ZBTB8OS	0.414960249	mirna_pc
16829	hsa-mir-24-2	A4GALT	0.30982471	mirna_pc
16830	hsa-mir-24-2	IMPA2	0.351135223	mirna_pc
16831	hsa-mir-24-2	SLC6A8	0.362434343	mirna_pc
16832	hsa-mir-24-2	RNF181	0.429047423	mirna_pc
16833	hsa-mir-24-2	PPP2R3B	0.300148698	mirna_pc
16834	hsa-mir-24-2	C12orf10	0.345222547	mirna_pc
16835	hsa-mir-24-2	GIPC1	0.361398626	mirna_pc
16836	hsa-mir-24-2	TRIM7	0.485094141	mirna_pc
16837	hsa-mir-24-2	KRT14	0.500203844	mirna_pc
16838	hsa-mir-24-2	C12orf45	0.403964593	mirna_pc
16839	hsa-mir-24-2	C19orf43	0.442512453	mirna_pc
16840	hsa-mir-24-2	P2RY1	0.332531382	mirna_pc
16841	hsa-mir-24-2	PSMB6	0.527122887	mirna_pc
16842	hsa-mir-24-2	C19orf20	0.344756146	mirna_pc
16843	hsa-mir-24-2	RBX1	0.493865805	mirna_pc
16844	hsa-mir-24-2	UBAC1	0.45285572	mirna_pc
16845	hsa-mir-24-2	GPX4	0.404442707	mirna_pc
16846	hsa-mir-24-2	CSDA	0.45702888	mirna_pc
16847	hsa-mir-24-2	CDC42SE1	0.398065382	mirna_pc

16848	hsa-mir-24-2	RPS19BP1	0.316086719	mirna_pc
16849	hsa-mir-24-2	MMP17	0.384662696	mirna_pc
16850	hsa-mir-24-2	TMEM132A	0.325339057	mirna_pc
16851	hsa-mir-24-2	SRRM3	0.457665815	mirna_pc
16852	hsa-mir-24-2	TAF10	0.317442085	mirna_pc
16853	hsa-mir-24-2	C11orf10	0.302500988	mirna_pc
16854	hsa-mir-24-2	CORO1C	0.392704864	mirna_pc
16855	hsa-mir-24-2	PGAM1	0.487290086	mirna_pc
16856	hsa-mir-24-2	HSPB11	0.311148018	mirna_pc
16857	hsa-mir-24-2	SLC27A4	0.339526461	mirna_pc
16858	hsa-mir-24-2	YKT6	0.325487924	mirna_pc
16859	hsa-mir-24-2	TP63	0.447966888	mirna_pc
16860	hsa-mir-24-2	HSBP1	0.357636718	mirna_pc
16861	hsa-mir-24-2	POLRMT	0.400105986	mirna_pc
16862	hsa-mir-24-2	ATP10D	0.333314972	mirna_pc
16863	hsa-mir-24-2	MLST8	0.498030985	mirna_pc
16864	hsa-mir-24-2	C16orf13	0.453201205	mirna_pc
16865	hsa-mir-24-2	TMEM11	0.354796269	mirna_pc
16866	hsa-mir-24-2	RPL39	0.311446529	mirna_pc
16867	hsa-mir-24-2	SUB1	0.33956928	mirna_pc
16868	hsa-mir-24-2	USP5	0.328824344	mirna_pc
16869	hsa-mir-24-2	BOLA3	0.409744263	mirna_pc
16870	hsa-mir-24-2	C19orf23	0.363760417	mirna_pc
16871	hsa-mir-24-2	TSKU	0.30926097	mirna_pc
16872	hsa-mir-24-2	C17orf89	0.499568681	mirna_pc
16873	hsa-mir-24-2	PHB2	0.381927497	mirna_pc
16874	hsa-mir-24-2	GPS1	0.310406264	mirna_pc
16875	hsa-mir-24-2	RPS17	0.437757525	mirna_pc
16876	hsa-mir-24-2	MAPKBP1	0.350143495	mirna_pc
16877	hsa-mir-24-2	TRAPPC3	0.466035724	mirna_pc
16878	hsa-mir-24-2	SNAI2	0.425444726	mirna_pc
16879	hsa-mir-24-2	FAM83G	0.398307537	mirna_pc
16880	hsa-mir-24-2	KIAA1609	0.396060464	mirna_pc
16881	hsa-mir-24-2	NUDT18	0.303804834	mirna_pc
16882	hsa-mir-24-2	POLR2F	0.478805153	mirna_pc
16883	hsa-mir-24-2	MRPS24	0.444366576	mirna_pc
16884	hsa-mir-24-2	ARHGEF4	0.315949528	mirna_pc
16885	hsa-mir-24-2	ALKBH3	0.368385541	mirna_pc
16886	hsa-mir-24-2	MDH2	0.338901241	mirna_pc
16887	hsa-mir-24-2	FAM127B	0.50928027	mirna_pc
16888	hsa-mir-24-2	UQCRHL	0.4038366	mirna_pc
16889	hsa-mir-24-2	SSNA1	0.390947823	mirna_pc
16890	hsa-mir-24-2	MRPS15	0.58978385	mirna_pc
16891	hsa-mir-24-2	TRAPPC5	0.531663594	mirna_pc
16892	hsa-mir-24-2	APRT	0.461212852	mirna_pc
16893	hsa-mir-24-2	RPL11	0.34590213	mirna_pc
16894	hsa-mir-24-2	GDI1	0.313845358	mirna_pc
16895	hsa-mir-24-2	RPS20	0.345720622	mirna_pc
16896	hsa-mir-24-2	SPRR1B	0.399990073	mirna_pc
16897	hsa-mir-24-2	BAIAP2	0.468681963	mirna_pc
16898	hsa-mir-24-2	MRPS18C	0.366779281	mirna_pc
16899	hsa-mir-24-2	SOX15	0.601425439	mirna_pc
16900	hsa-mir-24-2	CSNK1A1	0.353112116	mirna_pc
16901	hsa-mir-24-2	EIF2B3	0.436468985	mirna_pc

16902	hsa-mir-24-2	BNC1	0.478662535	mirna_pc
16903	hsa-mir-24-2	DGCR6L	0.424809264	mirna_pc
16904	hsa-mir-24-2	LYRM4	0.397774845	mirna_pc
16905	hsa-mir-24-2	PLD2	0.375637427	mirna_pc
16906	hsa-mir-24-2	NDRG1	0.418881964	mirna_pc
16907	hsa-mir-24-2	C4orf10	0.308913606	mirna_pc
16908	hsa-mir-24-2	MRPS6	0.356839395	mirna_pc
16909	hsa-mir-24-2	NDUFA11	0.486949456	mirna_pc
16910	hsa-mir-24-2	CYHR1	0.319267874	mirna_pc
16911	hsa-mir-24-2	LASS3	0.375072437	mirna_pc
16912	hsa-mir-24-2	SPRR2D	0.344056106	mirna_pc
16913	hsa-mir-24-2	TMEM160	0.321855317	mirna_pc
16914	hsa-mir-24-2	TIMM8B	0.448240025	mirna_pc
16915	hsa-mir-24-2	MRPL41	0.366069714	mirna_pc
16916	hsa-mir-24-2	IRX4	0.356286995	mirna_pc
16917	hsa-mir-24-2	ATG4D	0.487876805	mirna_pc
16918	hsa-mir-24-2	MFSD3	0.368373319	mirna_pc
16919	hsa-mir-24-2	SLC25A11	0.374229693	mirna_pc
16920	hsa-mir-24-2	DPCD	0.305183419	mirna_pc
16921	hsa-mir-24-2	PLEKHM2	0.363142393	mirna_pc
16922	hsa-mir-24-2	NDUFB3	0.349365246	mirna_pc
16923	hsa-mir-24-2	RPS15	0.4695579	mirna_pc
16924	hsa-mir-24-2	ADM	0.414093537	mirna_pc
16925	hsa-mir-24-2	PIN1	0.482597369	mirna_pc
16926	hsa-mir-24-2	NDUFB4	0.4218859	mirna_pc
16927	hsa-mir-24-2	RPL18A	0.428179376	mirna_pc
16928	hsa-mir-24-2	MAP3K6	0.399039377	mirna_pc
16929	hsa-mir-24-2	ARF5	0.400907582	mirna_pc
16930	hsa-mir-24-2	RHOD	0.485496179	mirna_pc
16931	hsa-mir-24-2	C7orf10	0.326626559	mirna_pc
16932	hsa-mir-24-2	ATP5G2	0.331623369	mirna_pc
16933	hsa-mir-24-2	C19orf70	0.43671403	mirna_pc
16934	hsa-mir-24-2	FAM104B	0.301087363	mirna_pc
16935	hsa-mir-24-2	LSM 10.00	0.436089894	mirna_pc
16936	hsa-mir-24-2	EIF3G	0.377749049	mirna_pc
16937	hsa-mir-24-2	TRAPPC2L	0.313263291	mirna_pc
16938	hsa-mir-24-2	PSMC5	0.415127575	mirna_pc
16939	hsa-mir-24-2	C2orf76	0.329668239	mirna_pc
16940	hsa-mir-24-2	RPL27	0.371385223	mirna_pc
16941	hsa-mir-24-2	UBA52	0.492589089	mirna_pc
16942	hsa-mir-24-2	MARK4	0.350537946	mirna_pc
16943	hsa-mir-24-2	PTHLH	0.386018339	mirna_pc
16944	hsa-mir-24-2	MBD2	0.446714733	mirna_pc
16945	hsa-mir-24-2	PIAS4	0.392838655	mirna_pc
16946	hsa-mir-24-2	CLCA2	0.370579556	mirna_pc
16947	hsa-mir-24-2	CALML3	0.418014162	mirna_pc
16948	hsa-mir-24-2	UNC45A	0.316367743	mirna_pc
16949	hsa-mir-24-2	C14orf166	0.466445301	mirna_pc
16950	hsa-mir-24-2	HS6ST2	0.349036117	mirna_pc
16951	hsa-mir-24-2	PHLDB3	0.337032073	mirna_pc
16952	hsa-mir-24-2	PTPN13	0.366785655	mirna_pc
16953	hsa-mir-24-2	PEX16	0.324687848	mirna_pc
16954	hsa-mir-24-2	NDUFC2	0.335024984	mirna_pc
16955	hsa-mir-24-2	HS3ST3A1	0.367454462	mirna_pc

16956	hsa-mir-24-2	RNASEH2C	0.318100362	mirna_pc
16957	hsa-mir-24-2	C19orf56	0.340090087	mirna_pc
16958	hsa-mir-24-2	FEM1A	0.31127248	mirna_pc
16959	hsa-mir-24-2	UBL5	0.483879537	mirna_pc
16960	hsa-mir-24-2	CDC34	0.36901986	mirna_pc
16961	hsa-mir-24-2	STX10	0.380351066	mirna_pc
16962	hsa-mir-24-2	ATRIP	0.332041275	mirna_pc
16963	hsa-mir-24-2	MGC70857	0.380038234	mirna_pc
16964	hsa-mir-24-2	EIF4H	0.317015884	mirna_pc
16965	hsa-mir-24-2	KCMF1	0.465149609	mirna_pc
16966	hsa-mir-24-2	MED31	0.497370058	mirna_pc
16967	hsa-mir-24-2	LSMD1	0.442903949	mirna_pc
16968	hsa-mir-24-2	RPS10	0.336451501	mirna_pc
16969	hsa-mir-24-2	GM2A	0.407194406	mirna_pc
16970	hsa-mir-24-2	FLYWCH2	0.413036336	mirna_pc
16971	hsa-mir-24-2	PLSCR3	0.509045851	mirna_pc
16972	hsa-mir-24-2	RAP1GDS1	0.335138731	mirna_pc
16973	hsa-mir-24-2	TRAPPC1	0.458162709	mirna_pc
16974	hsa-mir-24-2	SRP14	0.340350799	mirna_pc
16975	hsa-mir-24-2	TSSC4	0.308785517	mirna_pc
16976	hsa-mir-24-2	NDUFB9	0.313603601	mirna_pc
16977	hsa-mir-24-2	PARD6G	0.424368489	mirna_pc
16978	hsa-mir-24-2	TBCA	0.474836132	mirna_pc
16979	hsa-mir-24-2	CWC15	0.323063418	mirna_pc
16980	hsa-mir-24-2	FLYWCH1	0.433757898	mirna_pc
16981	hsa-mir-24-2	NXN	0.386327381	mirna_pc
16982	hsa-mir-24-2	LRRC8A	0.445511975	mirna_pc
16983	hsa-mir-24-2	ELOVL1	0.332644774	mirna_pc
16984	hsa-mir-24-2	ST6GALNAC2	0.355342585	mirna_pc
16985	hsa-mir-24-2	FKBP4	0.3119989	mirna_pc
16986	hsa-mir-24-2	MYEOV2	0.411909225	mirna_pc
16987	hsa-mir-24-2	UBE2G1	0.3012833	mirna_pc
16988	hsa-mir-24-2	PGAM4	0.444753915	mirna_pc
16989	hsa-mir-24-2	FAM110C	0.359183557	mirna_pc
16990	hsa-mir-24-2	NARFL	0.314909159	mirna_pc
16991	hsa-mir-24-2	ATP5H	0.53821633	mirna_pc
16992	hsa-mir-24-2	NRG1	0.362472564	mirna_pc
16993	hsa-mir-24-2	BPGM	0.325462589	mirna_pc
16994	hsa-mir-24-2	PMF1	0.320952642	mirna_pc
16995	hsa-mir-24-2	TMEM159	0.324618677	mirna_pc
16996	hsa-mir-24-2	C5orf55	0.464755452	mirna_pc
16997	hsa-mir-24-2	GALNT14	0.310695494	mirna_pc
16998	hsa-mir-24-2	NTAN1	0.401303731	mirna_pc
16999	hsa-mir-24-2	FAM128B	0.353187928	mirna_pc
17000	hsa-mir-24-2	HINT1	0.486095828	mirna_pc
17001	hsa-mir-24-2	GALNTL4	0.349235738	mirna_pc
17002	hsa-mir-24-2	AFAP1L1	0.347447157	mirna_pc
17003	hsa-mir-24-2	NDUFA12	0.521219951	mirna_pc
17004	hsa-mir-24-2	PPP2R2C	0.414709181	mirna_pc
17005	hsa-mir-24-2	DRG2	0.339446406	mirna_pc
17006	hsa-mir-24-2	PTS	0.391933419	mirna_pc
17007	hsa-mir-24-2	C19orf50	0.425185752	mirna_pc
17008	hsa-mir-24-2	LOC100134229	0.399474211	mirna_pc
17009	hsa-mir-24-2	CXCL14	0.378530131	mirna_pc

17010	hsa-mir-24-2	PL-5283	0.382804456	mirna_pc
17011	hsa-mir-24-2	HCFC1R1	0.446248893	mirna_pc
17012	hsa-mir-24-2	ZDHHC8	0.315091525	mirna_pc
17013	hsa-mir-24-2	RPS27A	0.356824804	mirna_pc
17014	hsa-mir-24-2	OST4	0.500767553	mirna_pc
17015	hsa-mir-24-2	PPPDE2	0.386865243	mirna_pc
17016	hsa-mir-24-2	C1orf50	0.343493469	mirna_pc
17017	hsa-mir-24-2	RPLP0	0.421979437	mirna_pc
17018	hsa-mir-24-2	VPS28	0.392969674	mirna_pc
17019	hsa-mir-24-2	EXTL3	0.3265632	mirna_pc
17020	hsa-mir-24-2	MRPL20	0.330124825	mirna_pc
17021	hsa-mir-24-2	CCBL1	0.316326501	mirna_pc
17022	hsa-mir-24-2	EPS15L1	0.378439001	mirna_pc
17023	hsa-mir-24-2	TMEM18	0.300776344	mirna_pc
17024	hsa-mir-24-2	NIPAL4	0.357877987	mirna_pc
17025	hsa-mir-24-2	SEMA3F	0.317586057	mirna_pc
17026	hsa-mir-24-2	RPL31	0.302591577	mirna_pc
17027	hsa-mir-24-2	COX6A1	0.330103235	mirna_pc
17028	hsa-mir-24-2	C19orf26	0.343961933	mirna_pc
17029	hsa-mir-24-2	XAB2	0.312289828	mirna_pc
17030	hsa-mir-24-2	PRNP	0.31388155	mirna_pc
17031	hsa-mir-24-2	PCYT1A	0.37644698	mirna_pc
17032	hsa-mir-24-2	NUBP2	0.455925103	mirna_pc
17033	hsa-mir-24-2	SHARPIN	0.428808719	mirna_pc
17034	hsa-mir-24-2	BTF3	0.38073061	mirna_pc
17035	hsa-mir-24-2	CEACAM19	0.307394681	mirna_pc
17036	hsa-mir-24-2	UBE2D2	0.439577411	mirna_pc
17037	hsa-mir-24-2	SGTA	0.391126689	mirna_pc
17038	hsa-mir-24-2	TRAF3IP2	0.365380463	mirna_pc
17039	hsa-mir-24-2	BTBD2	0.430325705	mirna_pc
17040	hsa-mir-24-2	BDKRB1	0.34165261	mirna_pc
17041	hsa-mir-24-2	POLR2L	0.546047468	mirna_pc
17042	hsa-mir-24-2	RPS4X	0.308773782	mirna_pc
17043	hsa-mir-24-2	SYPL1	0.315948451	mirna_pc
17044	hsa-mir-24-2	MAFB	0.313405285	mirna_pc
17045	hsa-mir-24-2	PACSIN3	0.479947457	mirna_pc
17046	hsa-mir-24-2	VKORC1	0.337943257	mirna_pc
17047	hsa-mir-24-2	RPS8	0.352042671	mirna_pc
17048	hsa-mir-24-2	PPP2R4	0.402102311	mirna_pc
17049	hsa-mir-24-2	C8orf58	0.339367053	mirna_pc
17050	hsa-mir-24-2	C16orf42	0.528809374	mirna_pc
17051	hsa-mir-24-2	SF3B5	0.411121477	mirna_pc
17052	hsa-mir-24-2	RAB1B	0.473175946	mirna_pc
17053	hsa-mir-24-2	LTA4H	0.315527814	mirna_pc
17054	hsa-mir-24-2	ACTG1	0.336652829	mirna_pc
17055	hsa-mir-24-2	HSPA4L	0.320676499	mirna_pc
17056	hsa-mir-24-2	SNAPC2	0.361651809	mirna_pc
17057	hsa-mir-24-2	GOT2	0.349276008	mirna_pc
17058	hsa-mir-24-2	MIB2	0.42664773	mirna_pc
17059	hsa-mir-24-2	TMEM85	0.315735346	mirna_pc
17060	hsa-mir-24-2	C1orf128	0.305634882	mirna_pc
17061	hsa-mir-24-2	CCDC127	0.367853999	mirna_pc
17062	hsa-mir-24-2	CHMP6	0.3335797	mirna_pc
17063	hsa-mir-24-2	ATP6V0C	0.459765417	mirna_pc

17064	hsa-mir-24-2	NACA	0.415506132	mirna_pc
17065	hsa-mir-24-2	PHLDA3	0.414399801	mirna_pc
17066	hsa-mir-24-2	ALDOC	0.302366128	mirna_pc
17067	hsa-mir-24-2	FAM131C	0.323061549	mirna_pc
17068	hsa-mir-24-2	C19orf25	0.302990645	mirna_pc
17069	hsa-mir-24-2	CYC1	0.371457612	mirna_pc
17070	hsa-mir-24-2	RASL11B	0.30658808	mirna_pc
17071	hsa-mir-24-2	SNX3	0.332425383	mirna_pc
17072	hsa-mir-24-2	C17orf106	0.438337747	mirna_pc
17073	hsa-mir-24-2	DNAJA3	0.374390748	mirna_pc
17074	hsa-mir-24-2	RPS12	0.363945002	mirna_pc
17075	hsa-mir-24-2	PPARD	0.32509268	mirna_pc
17076	hsa-mir-24-2	POLE4	0.446525456	mirna_pc
17077	hsa-mir-24-2	JMJD7-PLA2G4B	0.321016167	mirna_pc
17078	hsa-mir-24-2	NDE1	0.316818873	mirna_pc
17079	hsa-mir-24-2	PTPMT1	0.320972377	mirna_pc
17080	hsa-mir-24-2	NACA2	0.397472304	mirna_pc
17081	hsa-mir-24-2	DGCR6	0.351866769	mirna_pc
17082	hsa-mir-24-2	BDH1	0.328600274	mirna_pc
17083	hsa-mir-24-2	DHRS4L2	0.410879076	mirna_pc
17084	hsa-mir-24-2	RPL24	0.418499215	mirna_pc
17085	hsa-mir-24-2	MIF4GD	0.317352861	mirna_pc
17086	hsa-mir-24-2	RPL36	0.384603531	mirna_pc
17087	hsa-mir-24-2	RPL4	0.377759265	mirna_pc
17088	hsa-mir-24-2	TXNDC17	0.531895409	mirna_pc
17089	hsa-mir-24-2	FST	0.397483868	mirna_pc
17090	hsa-mir-24-2	C21orf56	0.307664407	mirna_pc
17091	hsa-mir-24-2	MMAB	0.326111538	mirna_pc
17092	hsa-mir-24-2	DNAJC19	0.334350252	mirna_pc
17093	hsa-mir-24-2	DHPS	0.302377352	mirna_pc
17094	hsa-mir-24-2	TMEM134	0.480116122	mirna_pc
17095	hsa-mir-24-2	TBX1	0.328806117	mirna_pc
17096	hsa-mir-24-2	ARL4D	0.39708694	mirna_pc
17097	hsa-mir-24-2	PFDN1	0.42288654	mirna_pc
17098	hsa-mir-24-2	TBL3	0.444713663	mirna_pc
17099	hsa-mir-24-2	MRPS7	0.381387796	mirna_pc
17100	hsa-mir-24-2	RPL13	0.324840652	mirna_pc
17101	hsa-mir-24-2	ODZ2	0.343910014	mirna_pc
17102	hsa-mir-24-2	C1orf122	0.336147724	mirna_pc
17103	hsa-mir-24-2	ZNF205	0.362659299	mirna_pc
17104	hsa-mir-24-2	UBE2L3	0.406956804	mirna_pc
17105	hsa-mir-24-2	RAB24	0.311007643	mirna_pc
17106	hsa-mir-24-2	MPV17L2	0.303321216	mirna_pc
17107	hsa-mir-24-2	PSMD9	0.559566528	mirna_pc
17108	hsa-mir-24-2	FAM126A	0.302634089	mirna_pc
17109	hsa-mir-24-2	OAZ1	0.498655392	mirna_pc
17110	hsa-mir-24-2	TIMM44	0.446214344	mirna_pc
17111	hsa-mir-24-2	MOBKL2A	0.450753193	mirna_pc
17112	hsa-mir-24-2	COMTD1	0.354735619	mirna_pc
17113	hsa-mir-24-2	NDUFA7	0.392729338	mirna_pc
17114	hsa-mir-24-2	FRMD6	0.305482621	mirna_pc
17115	hsa-mir-24-2	COPS7A	0.388573144	mirna_pc
17116	hsa-mir-24-2	NDUFS5	0.300774813	mirna_pc
17117	hsa-mir-24-2	ERCC1	0.437058794	mirna_pc

17118	hsa-mir-24-2	LOC407835	0.403239407	mirna_pc
17119	hsa-mir-24-2	CLPP	0.403633201	mirna_pc
17120	hsa-mir-24-2	HDAC3	0.374780308	mirna_pc
17121	hsa-mir-24-2	COPE	0.425826942	mirna_pc
17122	hsa-mir-24-2	PC	0.434959466	mirna_pc
17123	hsa-mir-24-2	DULLARD	0.380475424	mirna_pc
17124	hsa-mir-24-2	CCDC94	0.392128317	mirna_pc
17125	hsa-mir-24-2	RPL10A	0.372696963	mirna_pc
17126	hsa-mir-24-2	CNBP	0.333362931	mirna_pc
17127	hsa-mir-24-2	TUBB6	0.417696888	mirna_pc
17128	hsa-mir-24-2	C1orf201	0.469495398	mirna_pc
17129	hsa-mir-24-2	MRPL22	0.324270771	mirna_pc
17130	hsa-mir-24-2	RIC8A	0.381973947	mirna_pc
17131	hsa-mir-24-2	CETN3	0.416546036	mirna_pc
17132	hsa-mir-24-2	CINP	0.447491976	mirna_pc
17133	hsa-mir-24-2	NUDT22	0.384324888	mirna_pc
17134	hsa-mir-24-2	COPZ2	0.324660298	mirna_pc
17135	hsa-mir-24-2	STK17A	0.304806348	mirna_pc
17136	hsa-mir-24-2	SNX17	0.40611828	mirna_pc
17137	hsa-mir-24-2	FAU	0.460838288	mirna_pc
17138	hsa-mir-24-2	SRP68	0.404111986	mirna_pc
17139	hsa-mir-24-2	AP3S1	0.42602705	mirna_pc
17140	hsa-mir-24-2	CDKN2AIPNL	0.320519404	mirna_pc
17141	hsa-mir-24-2	IMP3	0.423798088	mirna_pc
17142	hsa-mir-24-2	GLI4	0.389227605	mirna_pc
17143	hsa-mir-24-2	LOH12CR1	0.395734192	mirna_pc
17144	hsa-mir-24-2	CA12	0.435181419	mirna_pc
17145	hsa-mir-24-2	EXOSC6	0.389331319	mirna_pc
17146	hsa-mir-24-2	PLRG1	0.30972856	mirna_pc
17147	hsa-mir-24-2	MAP7D1	0.415027159	mirna_pc
17148	hsa-mir-24-2	EFS	0.357253915	mirna_pc
17149	hsa-mir-24-2	LMNA	0.300997606	mirna_pc
17150	hsa-mir-24-2	RILPL1	0.345561347	mirna_pc
17151	hsa-mir-24-2	MPZL2	0.345766697	mirna_pc
17152	hsa-mir-24-2	EIF3D	0.353502246	mirna_pc
17153	hsa-mir-24-2	FDX1L	0.381896502	mirna_pc
17154	hsa-mir-24-2	GYS1	0.306517019	mirna_pc
17155	hsa-mir-24-2	THAP11	0.302346804	mirna_pc
17156	hsa-mir-24-2	RPL10	0.351136764	mirna_pc
17157	hsa-mir-24-2	REPS1	0.309865418	mirna_pc
17158	hsa-mir-24-2	VDAC2	0.542894605	mirna_pc
17159	hsa-mir-24-2	BLVRA	0.329180596	mirna_pc
17160	hsa-mir-24-2	NEFL	0.340582176	mirna_pc
17161	hsa-mir-24-2	NDUFB5	0.356888532	mirna_pc
17162	hsa-mir-24-2	ZMAT2	0.306747343	mirna_pc
17163	hsa-mir-24-2	BCL7C	0.487637979	mirna_pc
17164	hsa-mir-24-2	PIGY	0.324872958	mirna_pc
17165	hsa-mir-24-2	POLR2E	0.603757916	mirna_pc
17166	hsa-mir-24-2	C14orf2	0.401128291	mirna_pc
17167	hsa-mir-24-2	IMPDH1	0.358167573	mirna_pc
17168	hsa-mir-24-2	RPS6KB2	0.389788448	mirna_pc
17169	hsa-mir-24-2	RTN4	0.329132395	mirna_pc
17170	hsa-mir-24-2	GLRX5	0.322582951	mirna_pc
17171	hsa-mir-24-2	MINK1	0.306910835	mirna_pc

17172	hsa-mir-24-2	TRNAU1AP	0.35572127	mirna_pc
17173	hsa-mir-24-2	PPP2CA	0.386043106	mirna_pc
17174	hsa-mir-24-2	SNRNP25	0.438069032	mirna_pc
17175	hsa-mir-24-2	ARL2BP	0.329603226	mirna_pc
17176	hsa-mir-24-2	HDDC2	0.419074813	mirna_pc
17177	hsa-mir-24-2	INO80C	0.379190017	mirna_pc
17178	hsa-mir-24-2	SLC25A28	0.355980337	mirna_pc
17179	hsa-mir-24-2	RPLP1	0.385241145	mirna_pc
17180	hsa-mir-24-2	ATP6V0E1	0.337854844	mirna_pc
17181	hsa-mir-24-2	ELOF1	0.470176254	mirna_pc
17182	hsa-mir-24-2	PEF1	0.411166896	mirna_pc
17183	hsa-mir-24-2	RABGGTA	0.340623275	mirna_pc
17184	hsa-mir-24-2	RPL7A	0.344566967	mirna_pc
17185	hsa-mir-24-2	GPS2	0.36618473	mirna_pc
17186	hsa-mir-24-2	HHLA3	0.320965039	mirna_pc
17187	hsa-mir-24-2	C21orf119	0.329893003	mirna_pc
17188	hsa-mir-24-2	MPG	0.375925579	mirna_pc
17189	hsa-mir-24-2	RPL26	0.394314691	mirna_pc
17190	hsa-mir-24-2	SLC10A6	0.316661003	mirna_pc
17191	hsa-mir-24-2	RAB40C	0.322304009	mirna_pc
17192	hsa-mir-24-2	FAM89B	0.422098373	mirna_pc
17193	hsa-mir-24-2	DGCR14	0.310559753	mirna_pc
17194	hsa-mir-24-2	MRPL23	0.311635621	mirna_pc
17195	hsa-mir-24-2	H2AFY2	0.346795694	mirna_pc
17196	hsa-mir-24-2	MAF 1.00	0.385995931	mirna_pc
17197	hsa-mir-24-2	TSPO	0.334199993	mirna_pc
17198	hsa-mir-24-2	MRPL40	0.438537862	mirna_pc
17199	hsa-mir-24-2	FAM127A	0.366763867	mirna_pc
17200	hsa-mir-24-2	UBL7	0.339477411	mirna_pc
17201	hsa-mir-24-2	RHOT2	0.319343727	mirna_pc
17202	hsa-mir-24-2	SCYL1	0.301786581	mirna_pc
17203	hsa-mir-24-2	BRD7	0.375562755	mirna_pc
17204	hsa-mir-24-2	MVK	0.362037326	mirna_pc
17205	hsa-mir-24-2	RNH1	0.400239497	mirna_pc
17206	hsa-mir-24-2	NOL3	0.337614839	mirna_pc
17207	hsa-mir-24-2	SYTL1	0.397897556	mirna_pc
17208	hsa-mir-24-2	HMG20B	0.36011955	mirna_pc
17209	hsa-mir-24-2	VPS29	0.363696087	mirna_pc
17210	hsa-mir-24-2	KCTD11	0.354123613	mirna_pc
17211	hsa-mir-24-2	HYAL3	0.348338233	mirna_pc
17212	hsa-mir-24-2	GNB2L1	0.330360403	mirna_pc
17213	hsa-mir-24-2	C9orf3	0.383169721	mirna_pc
17214	hsa-mir-24-2	TAX1BP3	0.335907065	mirna_pc
17215	hsa-mir-24-2	C21orf70	0.363648638	mirna_pc
17216	hsa-mir-24-2	NQO2	0.322540083	mirna_pc
17217	hsa-mir-24-2	SETD8	0.363966311	mirna_pc
17218	hsa-mir-24-2	UQCRH	0.3521279	mirna_pc
17219	hsa-mir-24-2	MGST2	0.326813403	mirna_pc
17220	hsa-mir-24-2	ZCCHC17	0.321340114	mirna_pc
17221	hsa-mir-24-2	MRPL53	0.497163574	mirna_pc
17222	hsa-mir-24-2	LOC554202	0.349021326	mirna_pc
17223	hsa-mir-24-2	EEF1D	0.439399844	mirna_pc
17224	hsa-mir-24-2	C1orf212	0.409479407	mirna_pc
17225	hsa-mir-24-2	COPS3	0.417374117	mirna_pc

17226	hsa-mir-24-2	UBE2F	0.342633489	mirna_pc
17227	hsa-mir-24-2	COMT	0.312075496	mirna_pc
17228	hsa-mir-24-2	USE1	0.421236005	mirna_pc
17229	hsa-mir-24-2	NDUFS8	0.428829461	mirna_pc
17230	hsa-mir-24-2	AURKAIP1	0.339880886	mirna_pc
17231	hsa-mir-24-2	FEZ1	0.332806927	mirna_pc
17232	hsa-mir-24-2	NUDC	0.303487106	mirna_pc
17233	hsa-mir-24-2	MAP2K2	0.452401223	mirna_pc
17234	hsa-mir-24-2	RPL6	0.321336098	mirna_pc
17235	hsa-mir-24-2	RRAS2	0.446689425	mirna_pc
17236	hsa-mir-24-2	TIMM22	0.457281752	mirna_pc
17237	hsa-mir-24-2	CHCHD5	0.34432194	mirna_pc
17238	hsa-mir-24-2	ZNF414	0.394571612	mirna_pc
17239	hsa-mir-24-2	C15orf61	0.323758867	mirna_pc
17240	hsa-mir-24-2	PI4K2A	0.334218452	mirna_pc
17241	hsa-mir-24-2	E4F1	0.408243907	mirna_pc
17242	hsa-mir-24-2	HAX1	0.35224177	mirna_pc
17243	hsa-mir-24-2	EPN2	0.31786513	mirna_pc
17244	hsa-mir-24-2	PPIF	0.452210798	mirna_pc
17245	hsa-mir-24-2	LOC143666	0.419857927	mirna_pc
17246	hsa-mir-24-2	NMRAL1	0.483430114	mirna_pc
17247	hsa-mir-24-2	CSDAP1	0.420089764	mirna_pc
17248	hsa-mir-24-2	AHNAK2	0.319566342	mirna_pc
17249	hsa-mir-188	HOXC9	0.381088185	mirna_pc
17250	hsa-mir-188	CDC25C	0.349094577	mirna_pc
17251	hsa-mir-188	SGOL1	0.322079299	mirna_pc
17252	hsa-mir-188	IQGAP3	0.31543623	mirna_pc
17253	hsa-mir-188	HOXC11	0.348207471	mirna_pc
17254	hsa-mir-188	DDX39	0.322509809	mirna_pc
17255	hsa-mir-188	FANCB	0.311698275	mirna_pc
17256	hsa-mir-188	AURKA	0.35625081	mirna_pc
17257	hsa-mir-188	C12orf48	0.3263782	mirna_pc
17258	hsa-mir-188	ZWINT	0.36504959	mirna_pc
17259	hsa-mir-188	PIF1	0.344071674	mirna_pc
17260	hsa-mir-188	E2F1	0.302836937	mirna_pc
17261	hsa-mir-188	DEPDC1B	0.314372911	mirna_pc
17262	hsa-mir-188	CLDN7	0.31107048	mirna_pc
17263	hsa-mir-188	PSMC4	0.339356455	mirna_pc
17264	hsa-mir-188	HOXC10	0.362709675	mirna_pc
17265	hsa-mir-188	TOMM40	0.309719344	mirna_pc
17266	hsa-mir-188	CDC25A	0.32788802	mirna_pc
17267	hsa-mir-188	CLN6	0.306263829	mirna_pc
17268	hsa-mir-188	XK	0.348036727	mirna_pc
17269	hsa-mir-188	MARCKSL1	0.360363806	mirna_pc
17270	hsa-mir-188	ACAD8	0.313013518	mirna_pc
17271	hsa-mir-188	CBX8	0.302278376	mirna_pc
17272	hsa-mir-188	PAFAH1B3	0.364799439	mirna_pc
17273	hsa-mir-188	ST14	0.34934805	mirna_pc
17274	hsa-mir-188	MARVELD3	0.307705251	mirna_pc
17275	hsa-mir-188	PPAP2C	0.320560297	mirna_pc
17276	hsa-mir-188	ETV4	0.306144038	mirna_pc
17277	hsa-mir-188	CHRNA5	0.343824525	mirna_pc
17278	hsa-mir-188	TLCD1	0.317664836	mirna_pc
17279	hsa-mir-188	C2orf88	0.336948276	mirna_pc

17280	hsa-mir-188	HSPBP1	0.332634926	mirna_pc
17281	hsa-mir-188	SNX8	0.3425361	mirna_pc
17282	hsa-mir-188	SLC5A6	0.310271546	mirna_pc
17283	hsa-mir-188	TUSC2	0.329238435	mirna_pc
17284	hsa-mir-188	AHCY	0.339861109	mirna_pc
17285	hsa-mir-188	PDSS1	0.324740738	mirna_pc
17286	hsa-mir-188	PDHA1	0.35250422	mirna_pc
17287	hsa-mir-188	OBFC2B	0.316836347	mirna_pc
17288	hsa-mir-188	PTMA	0.349209056	mirna_pc
17289	hsa-mir-188	LSM 4.00	0.381278979	mirna_pc
17290	hsa-mir-188	ELK1	0.31215222	mirna_pc
17291	hsa-mir-188	RRP9	0.330154987	mirna_pc
17292	hsa-mir-188	SVIP	0.336693381	mirna_pc
17293	hsa-mir-188	EPCAM	0.403898997	mirna_pc
17294	hsa-mir-188	CTSA	0.312312597	mirna_pc
17295	hsa-mir-188	PDRG1	0.317480987	mirna_pc
17296	hsa-mir-188	ANG	0.321188381	mirna_pc
17297	hsa-mir-188	SLC35A2	0.521727989	mirna_pc
17298	hsa-mir-188	SAE1	0.334587947	mirna_pc
17299	hsa-mir-188	MRPL17	0.376409458	mirna_pc
17300	hsa-mir-188	EIF2S2	0.304927467	mirna_pc
17301	hsa-mir-188	TSEN54	0.402394214	mirna_pc
17302	hsa-mir-188	HPDL	0.356172011	mirna_pc
17303	hsa-mir-188	ABHD11	0.302069393	mirna_pc
17304	hsa-mir-188	ARFIP2	0.303307439	mirna_pc
17305	hsa-mir-188	ATAD3A	0.33135736	mirna_pc
17306	hsa-mir-188	SMS	0.33302682	mirna_pc
17307	hsa-mir-188	CDX2	0.327375746	mirna_pc
17308	hsa-mir-188	ASCL2	0.30898323	mirna_pc
17309	hsa-mir-188	BRI3BP	0.393884102	mirna_pc
17310	hsa-mir-188	UBE4A	0.313410096	mirna_pc
17311	hsa-mir-188	UCA1	0.353838238	mirna_pc
17312	hsa-mir-188	CAPS	0.381117703	mirna_pc
17313	hsa-mir-188	AP1M2	0.349464486	mirna_pc
17314	hsa-mir-188	AGMAT	0.462194049	mirna_pc
17315	hsa-mir-188	ERAL1	0.304023095	mirna_pc
17316	hsa-mir-188	C2orf29	0.308769182	mirna_pc
17317	hsa-mir-188	C19orf24	0.300362144	mirna_pc
17318	hsa-mir-188	ZNF444	0.333903333	mirna_pc
17319	hsa-mir-188	POLR3K	0.336816507	mirna_pc
17320	hsa-mir-188	FAM96A	0.321018986	mirna_pc
17321	hsa-mir-188	FGFR1OP	0.31470985	mirna_pc
17322	hsa-mir-188	EBP	0.427048202	mirna_pc
17323	hsa-mir-188	PHB	0.319025312	mirna_pc
17324	hsa-mir-188	MID1IP1	0.335807976	mirna_pc
17325	hsa-mir-188	TIMM50	0.309032078	mirna_pc
17326	hsa-mir-188	IL22RA1	0.395812365	mirna_pc
17327	hsa-mir-188	DDOST	0.340315488	mirna_pc
17328	hsa-mir-188	MXD3	0.304113704	mirna_pc
17329	hsa-mir-188	GK3P	0.312942606	mirna_pc
17330	hsa-mir-188	CDK16	0.307275965	mirna_pc
17331	hsa-mir-188	ATAD3B	0.321449418	mirna_pc
17332	hsa-mir-188	C11orf48	0.432627484	mirna_pc
17333	hsa-mir-188	C8orf55	0.325058627	mirna_pc

17334	hsa-mir-188	NFKBIB	0.359190128	mirna_pc
17335	hsa-mir-188	FAM136A	0.403629178	mirna_pc
17336	hsa-mir-188	BAIAP2L2	0.325577045	mirna_pc
17337	hsa-mir-188	CHCHD8	0.332099888	mirna_pc
17338	hsa-mir-188	GIPC2	0.32627063	mirna_pc
17339	hsa-mir-188	GNL3L	0.301473134	mirna_pc
17340	hsa-mir-188	KRT18	0.416392006	mirna_pc
17341	hsa-mir-188	STRA13	0.422652141	mirna_pc
17342	hsa-mir-188	CXorf38	0.311993054	mirna_pc
17343	hsa-mir-188	TMEM126A	0.368712821	mirna_pc
17344	hsa-mir-188	TMEM98	0.329496467	mirna_pc
17345	hsa-mir-188	SLC27A2	0.363928084	mirna_pc
17346	hsa-mir-188	ALG8	0.367512369	mirna_pc
17347	hsa-mir-188	FUNDC1	0.320521581	mirna_pc
17348	hsa-mir-188	TMEM180	0.325009155	mirna_pc
17349	hsa-mir-188	TMEM106C	0.311004932	mirna_pc
17350	hsa-mir-188	CRLS1	0.3356797	mirna_pc
17351	hsa-mir-188	C11orf83	0.379735194	mirna_pc
17352	hsa-mir-188	UBA1	0.349034636	mirna_pc
17353	hsa-mir-188	GTF2A2	0.303179193	mirna_pc
17354	hsa-mir-188	RNF34	0.366434219	mirna_pc
17355	hsa-mir-188	C14orf176	0.363817863	mirna_pc
17356	hsa-mir-188	RPGR	0.37523677	mirna_pc
17357	hsa-mir-188	ZNF593	0.363682688	mirna_pc
17358	hsa-mir-188	CYP2W1	0.349153901	mirna_pc
17359	hsa-mir-188	WDR45	0.345001981	mirna_pc
17360	hsa-mir-188	REEP6	0.338112046	mirna_pc
17361	hsa-mir-188	DDAH1	0.320744318	mirna_pc
17362	hsa-mir-188	LYPLA2	0.35505424	mirna_pc
17363	hsa-mir-188	NOSIP	0.30347703	mirna_pc
17364	hsa-mir-188	ATP5L	0.341315482	mirna_pc
17365	hsa-mir-188	NAPRT1	0.354883398	mirna_pc
17366	hsa-mir-188	KRTCAP3	0.316939933	mirna_pc
17367	hsa-mir-188	COMMD7	0.323201959	mirna_pc
17368	hsa-mir-188	SORD	0.375152642	mirna_pc
17369	hsa-mir-188	ICA1	0.317803265	mirna_pc
17370	hsa-mir-188	SSBP4	0.319579342	mirna_pc
17371	hsa-mir-188	PRELID1	0.310544089	mirna_pc
17372	hsa-mir-188	CLDN3	0.382800619	mirna_pc
17373	hsa-mir-188	METTL7B	0.42351228	mirna_pc
17374	hsa-mir-188	NSMCE4A	0.311868599	mirna_pc
17375	hsa-mir-188	ZNF30	0.346482921	mirna_pc
17376	hsa-mir-188	C19orf10	0.315169135	mirna_pc
17377	hsa-mir-188	SLC25A19	0.380591547	mirna_pc
17378	hsa-mir-188	BEND3	0.304708898	mirna_pc
17379	hsa-mir-188	SH3KBP1	0.310326855	mirna_pc
17380	hsa-mir-188	CA5BP	0.354418636	mirna_pc
17381	hsa-mir-188	FZD5	0.315288512	mirna_pc
17382	hsa-mir-188	FGFR4	0.41486214	mirna_pc
17383	hsa-mir-188	LOC151534	0.324579887	mirna_pc
17384	hsa-mir-188	ASS1	0.348936339	mirna_pc
17385	hsa-mir-188	C7orf36	0.355810162	mirna_pc
17386	hsa-mir-188	SARS2	0.319225244	mirna_pc
17387	hsa-mir-188	PQBP1	0.418018379	mirna_pc

17388	hsa-mir-188	NDUFA3	0.368525758	mirna_pc
17389	hsa-mir-188	C6orf130	0.300382637	mirna_pc
17390	hsa-mir-188	ZSCAN16	0.327816221	mirna_pc
17391	hsa-mir-188	RPS6KA3	0.3016873	mirna_pc
17392	hsa-mir-188	DGAT1	0.347993361	mirna_pc
17393	hsa-mir-188	SYAP1	0.373494338	mirna_pc
17394	hsa-mir-188	ACAA2	0.308237828	mirna_pc
17395	hsa-mir-188	CHDH	0.300880599	mirna_pc
17396	hsa-mir-188	TMEM54	0.326197093	mirna_pc
17397	hsa-mir-188	GJB1	0.438775873	mirna_pc
17398	hsa-mir-188	ERGIC3	0.334766421	mirna_pc
17399	hsa-mir-188	MSL3	0.323990174	mirna_pc
17400	hsa-mir-188	TIMM17B	0.421222358	mirna_pc
17401	hsa-mir-188	C20orf54	0.307784966	mirna_pc
17402	hsa-mir-188	CDH17	0.343643316	mirna_pc
17403	hsa-mir-188	HDHD3	0.302782306	mirna_pc
17404	hsa-mir-188	C11orf10	0.313163706	mirna_pc
17405	hsa-mir-188	ZNF259	0.474007451	mirna_pc
17406	hsa-mir-188	SSU72	0.323222433	mirna_pc
17407	hsa-mir-188	ALG14	0.306179202	mirna_pc
17408	hsa-mir-188	GLYCTK	0.319730953	mirna_pc
17409	hsa-mir-188	DDAH2	0.345085471	mirna_pc
17410	hsa-mir-188	SIGIRR	0.306561442	mirna_pc
17411	hsa-mir-188	CCDC22	0.313883653	mirna_pc
17412	hsa-mir-188	GET4	0.325416393	mirna_pc
17413	hsa-mir-188	DPAGT1	0.336205708	mirna_pc
17414	hsa-mir-188	PDDC1	0.324036279	mirna_pc
17415	hsa-mir-188	NARS2	0.387951196	mirna_pc
17416	hsa-mir-188	DGAT2	0.384178684	mirna_pc
17417	hsa-mir-188	TMEM62	0.314243196	mirna_pc
17418	hsa-mir-188	BSG	0.301303474	mirna_pc
17419	hsa-mir-188	FUCA2	0.337905607	mirna_pc
17420	hsa-mir-188	EPS8L3	0.313196693	mirna_pc
17421	hsa-mir-188	C11orf75	0.370726201	mirna_pc
17422	hsa-mir-188	TP53I11	0.310714912	mirna_pc
17423	hsa-mir-188	NDUFS3	0.323024664	mirna_pc
17424	hsa-mir-188	ZNF195	0.30764987	mirna_pc
17425	hsa-mir-188	ZW10	0.307604985	mirna_pc
17426	hsa-mir-188	LGALS4	0.336614733	mirna_pc
17427	hsa-mir-188	SCML1	0.306283821	mirna_pc
17428	hsa-mir-188	MANF	0.331412751	mirna_pc
17429	hsa-mir-188	HYLS1	0.321266952	mirna_pc
17430	hsa-mir-188	SDF2L1	0.32497482	mirna_pc
17431	hsa-mir-188	STT3A	0.315838819	mirna_pc
17432	hsa-mir-188	C1orf93	0.383777437	mirna_pc
17433	hsa-mir-188	VIL1	0.30590928	mirna_pc
17434	hsa-mir-188	SPCS2	0.356373475	mirna_pc
17435	hsa-mir-188	NDUFC2	0.45805036	mirna_pc
17436	hsa-mir-188	GLT25D2	0.3021217	mirna_pc
17437	hsa-mir-188	PHF16	0.429889678	mirna_pc
17438	hsa-mir-188	POF1B	0.320704132	mirna_pc
17439	hsa-mir-188	BUD13	0.424403946	mirna_pc
17440	hsa-mir-188	GMDS	0.304006668	mirna_pc
17441	hsa-mir-188	CMC1	0.304822521	mirna_pc

17442	hsa-mir-188	FCGRT	0.322683406	mirna_pc
17443	hsa-mir-188	MORC4	0.326893941	mirna_pc
17444	hsa-mir-188	FOXA2	0.400479793	mirna_pc
17445	hsa-mir-188	SERPINB6	0.383140272	mirna_pc
17446	hsa-mir-188	OTUD3	0.351325032	mirna_pc
17447	hsa-mir-188	GDF15	0.464405883	mirna_pc
17448	hsa-mir-188	GMPPA	0.33959412	mirna_pc
17449	hsa-mir-188	CAMK2N1	0.372565676	mirna_pc
17450	hsa-mir-188	TMEM53	0.349327731	mirna_pc
17451	hsa-mir-188	USH1C	0.40504251	mirna_pc
17452	hsa-mir-188	RNF157	0.32223323	mirna_pc
17453	hsa-mir-188	CALML4	0.315248424	mirna_pc
17454	hsa-mir-188	CCDC84	0.330391417	mirna_pc
17455	hsa-mir-188	SLC25A33	0.449447274	mirna_pc
17456	hsa-mir-188	CRB3	0.35576762	mirna_pc
17457	hsa-mir-188	C2orf72	0.410022223	mirna_pc
17458	hsa-mir-188	CTPS2	0.371424286	mirna_pc
17459	hsa-mir-188	KIF9	0.369482406	mirna_pc
17460	hsa-mir-188	MRPL20	0.354671611	mirna_pc
17461	hsa-mir-188	AGR2	0.318906723	mirna_pc
17462	hsa-mir-188	NDUFB11	0.42904494	mirna_pc
17463	hsa-mir-188	HNF1A	0.364646494	mirna_pc
17464	hsa-mir-188	KRT8	0.394236722	mirna_pc
17465	hsa-mir-188	PDX1	0.360615967	mirna_pc
17466	hsa-mir-188	OCLN	0.354419924	mirna_pc
17467	hsa-mir-188	C20orf118	0.310428437	mirna_pc
17468	hsa-mir-188	CYB561D2	0.325631052	mirna_pc
17469	hsa-mir-188	CYCS	0.322085242	mirna_pc
17470	hsa-mir-188	ZNF823	0.30966455	mirna_pc
17471	hsa-mir-188	PRR13	0.338314149	mirna_pc
17472	hsa-mir-188	ID2	0.338174805	mirna_pc
17473	hsa-mir-188	SLC35D2	0.330221101	mirna_pc
17474	hsa-mir-188	CLCN5	0.492627057	mirna_pc
17475	hsa-mir-188	XYLB	0.304761599	mirna_pc
17476	hsa-mir-188	TOE1	0.33773172	mirna_pc
17477	hsa-mir-188	SLC40A1	0.327073494	mirna_pc
17478	hsa-mir-188	SLCO4A1	0.317273023	mirna_pc
17479	hsa-mir-188	ERBB3	0.387790528	mirna_pc
17480	hsa-mir-188	SEMA4G	0.325667008	mirna_pc
17481	hsa-mir-188	LOC653566	0.302179444	mirna_pc
17482	hsa-mir-188	CCNI2	0.355170963	mirna_pc
17483	hsa-mir-188	DKFZp686024166	0.320058593	mirna_pc
17484	hsa-mir-188	GLB1L2	0.362010608	mirna_pc
17485	hsa-mir-188	ADAP1	0.335082256	mirna_pc
17486	hsa-mir-188	ZNF787	0.365096839	mirna_pc
17487	hsa-mir-188	TMEM51	0.327286039	mirna_pc
17488	hsa-mir-188	CALM3	0.381697775	mirna_pc
17489	hsa-mir-188	STX3	0.322712713	mirna_pc
17490	hsa-mir-188	CCDC72	0.339921702	mirna_pc
17491	hsa-mir-188	RBM3	0.323733253	mirna_pc
17492	hsa-mir-188	IL17RB	0.34453368	mirna_pc
17493	hsa-mir-188	PKDCC	0.308805156	mirna_pc
17494	hsa-mir-188	TMEM126B	0.33531964	mirna_pc
17495	hsa-mir-188	GPKOW	0.470650703	mirna_pc

17496	hsa-mir-188	CD320	0.316260097	mirna_pc
17497	hsa-mir-188	C2orf34	0.337392105	mirna_pc
17498	hsa-mir-188	PVRL2	0.394170513	mirna_pc
17499	hsa-mir-188	PREB	0.40321278	mirna_pc
17500	hsa-mir-188	ZNF443	0.431372267	mirna_pc
17501	hsa-mir-188	LLGL2	0.354835602	mirna_pc
17502	hsa-mir-188	PIN4	0.315635146	mirna_pc
17503	hsa-mir-188	SSR2	0.32419671	mirna_pc
17504	hsa-mir-188	HDAC6	0.307074753	mirna_pc
17505	hsa-mir-188	PNKD	0.383301583	mirna_pc
17506	hsa-mir-188	SMPDL3B	0.306645841	mirna_pc
17507	hsa-mir-188	PRDX4	0.359664402	mirna_pc
17508	hsa-mir-188	GCHFR	0.373932339	mirna_pc
17509	hsa-mir-188	ZNF799	0.376449968	mirna_pc
17510	hsa-mir-188	UXT	0.404719629	mirna_pc
17511	hsa-mir-188	RBBP7	0.340591785	mirna_pc
17512	hsa-mir-188	ACSL5	0.331770855	mirna_pc
17513	hsa-mir-188	PCBD1	0.375880122	mirna_pc
17514	hsa-mir-188	ZNF468	0.42420449	mirna_pc
17515	hsa-mir-188	TSTA3	0.328195717	mirna_pc
17516	hsa-mir-188	SLC37A4	0.357757219	mirna_pc
17517	hsa-mir-188	EIF2S3	0.437812599	mirna_pc
17518	hsa-mir-188	TMEM45B	0.335010845	mirna_pc
17519	hsa-mir-188	CENPV	0.432003977	mirna_pc
17520	hsa-mir-188	ANKRD49	0.301169793	mirna_pc
17521	hsa-mir-188	AURKAIP1	0.329822473	mirna_pc
17522	hsa-mir-188	FAM156A	0.342592221	mirna_pc
17523	hsa-mir-188	HCCS	0.37448235	mirna_pc
17524	hsa-mir-188	PARK7	0.333872168	mirna_pc
17525	hsa-mir-188	PRELID2	0.322066011	mirna_pc
17526	hsa-mir-188	ARSE	0.44664183	mirna_pc
17527	hsa-mir-92a-2	MKI67	0.309865455	mirna_pc
17528	hsa-mir-92a-2	TPX2	0.364258635	mirna_pc
17529	hsa-mir-92a-2	KIF4B	0.305498323	mirna_pc
17530	hsa-mir-92a-2	TOP2A	0.41503287	mirna_pc
17531	hsa-mir-92a-2	KPNA2	0.357544758	mirna_pc
17532	hsa-mir-92a-2	CENPF	0.436294397	mirna_pc
17533	hsa-mir-92a-2	RCC2	0.408187647	mirna_pc
17534	hsa-mir-92a-2	HOXC9	0.332768298	mirna_pc
17535	hsa-mir-92a-2	KIF11	0.394664509	mirna_pc
17536	hsa-mir-92a-2	FOXM1	0.30288228	mirna_pc
17537	hsa-mir-92a-2	RRM2	0.379202613	mirna_pc
17538	hsa-mir-92a-2	SPC24	0.348922625	mirna_pc
17539	hsa-mir-92a-2	CDK1	0.377716595	mirna_pc
17540	hsa-mir-92a-2	CDC25C	0.317407521	mirna_pc
17541	hsa-mir-92a-2	MYBL2	0.386156524	mirna_pc
17542	hsa-mir-92a-2	PLK1	0.34305804	mirna_pc
17543	hsa-mir-92a-2	PCNA	0.324925613	mirna_pc
17544	hsa-mir-92a-2	UBE2C	0.355055516	mirna_pc
17545	hsa-mir-92a-2	BUB1	0.406004795	mirna_pc
17546	hsa-mir-92a-2	NUSAP1	0.353424334	mirna_pc
17547	hsa-mir-92a-2	MCM4	0.378717353	mirna_pc
17548	hsa-mir-92a-2	KIFC1	0.364642628	mirna_pc
17549	hsa-mir-92a-2	BUB1B	0.423976366	mirna_pc

17550	hsa-mir-92a-2	HDGF	0.35881285	mirna_pc
17551	hsa-mir-92a-2	KIF18B	0.432791347	mirna_pc
17552	hsa-mir-92a-2	ASPM	0.349917911	mirna_pc
17553	hsa-mir-92a-2	CKAP2	0.434894833	mirna_pc
17554	hsa-mir-92a-2	FAM72A	0.311185522	mirna_pc
17555	hsa-mir-92a-2	KIF2C	0.341734673	mirna_pc
17556	hsa-mir-92a-2	FANCA	0.468477066	mirna_pc
17557	hsa-mir-92a-2	NCAPG	0.421385229	mirna_pc
17558	hsa-mir-92a-2	CLSPN	0.32951136	mirna_pc
17559	hsa-mir-92a-2	SGOL1	0.372858816	mirna_pc
17560	hsa-mir-92a-2	ARHGAP11A	0.380396674	mirna_pc
17561	hsa-mir-92a-2	CCNA2	0.464224107	mirna_pc
17562	hsa-mir-92a-2	CDC25B	0.321688705	mirna_pc
17563	hsa-mir-92a-2	CBX3	0.340356709	mirna_pc
17564	hsa-mir-92a-2	SPC25	0.437265341	mirna_pc
17565	hsa-mir-92a-2	NUP62	0.338281947	mirna_pc
17566	hsa-mir-92a-2	FANCI	0.414440831	mirna_pc
17567	hsa-mir-92a-2	LMNB2	0.300510204	mirna_pc
17568	hsa-mir-92a-2	NEK2	0.479412807	mirna_pc
17569	hsa-mir-92a-2	MND1	0.403874731	mirna_pc
17570	hsa-mir-92a-2	KIF22	0.374962108	mirna_pc
17571	hsa-mir-92a-2	DDX39	0.312911881	mirna_pc
17572	hsa-mir-92a-2	NCAPH	0.48069301	mirna_pc
17573	hsa-mir-92a-2	GTSE1	0.423850598	mirna_pc
17574	hsa-mir-92a-2	RAD54L	0.437296883	mirna_pc
17575	hsa-mir-92a-2	CENPA	0.392020695	mirna_pc
17576	hsa-mir-92a-2	MAD2L1	0.536622325	mirna_pc
17577	hsa-mir-92a-2	TIMELESS	0.453214196	mirna_pc
17578	hsa-mir-92a-2	LMNB1	0.428791349	mirna_pc
17579	hsa-mir-92a-2	ASF1B	0.34246607	mirna_pc
17580	hsa-mir-92a-2	CDCA8	0.416284061	mirna_pc
17581	hsa-mir-92a-2	TROAP	0.36409296	mirna_pc
17582	hsa-mir-92a-2	CDCA5	0.386036853	mirna_pc
17583	hsa-mir-92a-2	TACC3	0.315685506	mirna_pc
17584	hsa-mir-92a-2	XPO1	0.461718109	mirna_pc
17585	hsa-mir-92a-2	CDC45	0.403681102	mirna_pc
17586	hsa-mir-92a-2	STMN1	0.353645046	mirna_pc
17587	hsa-mir-92a-2	CENPM	0.342811114	mirna_pc
17588	hsa-mir-92a-2	EXO1	0.333620727	mirna_pc
17589	hsa-mir-92a-2	CDCA2	0.31052379	mirna_pc
17590	hsa-mir-92a-2	HMGA1	0.304623982	mirna_pc
17591	hsa-mir-92a-2	DKC1	0.344850926	mirna_pc
17592	hsa-mir-92a-2	UBE2T	0.385162912	mirna_pc
17593	hsa-mir-92a-2	CKS1B	0.380181222	mirna_pc
17594	hsa-mir-92a-2	TUBB	0.310471834	mirna_pc
17595	hsa-mir-92a-2	MCM2	0.414130805	mirna_pc
17596	hsa-mir-92a-2	KIF4A	0.306450246	mirna_pc
17597	hsa-mir-92a-2	ORC1L	0.348500378	mirna_pc
17598	hsa-mir-92a-2	SNRPB	0.374928667	mirna_pc
17599	hsa-mir-92a-2	KNTC1	0.399353609	mirna_pc
17600	hsa-mir-92a-2	UHRF1	0.377490138	mirna_pc
17601	hsa-mir-92a-2	CCNF	0.33418244	mirna_pc
17602	hsa-mir-92a-2	PTBP1	0.351606431	mirna_pc
17603	hsa-mir-92a-2	CCNB2	0.318598568	mirna_pc

17604	hsa-mir-92a-2	NUF2	0.403228637	mirna_pc
17605	hsa-mir-92a-2	PLK4	0.480495804	mirna_pc
17606	hsa-mir-92a-2	FANCB	0.386428235	mirna_pc
17607	hsa-mir-92a-2	KIF18A	0.345071674	mirna_pc
17608	hsa-mir-92a-2	DEPDC1	0.331104709	mirna_pc
17609	hsa-mir-92a-2	SKA3	0.523068211	mirna_pc
17610	hsa-mir-92a-2	KIF14	0.358185966	mirna_pc
17611	hsa-mir-92a-2	HJURP	0.494362833	mirna_pc
17612	hsa-mir-92a-2	SKA1	0.31486092	mirna_pc
17613	hsa-mir-92a-2	RAD51	0.337795084	mirna_pc
17614	hsa-mir-92a-2	BLM	0.466714151	mirna_pc
17615	hsa-mir-92a-2	NME1	0.40086011	mirna_pc
17616	hsa-mir-92a-2	DNMT1	0.402647887	mirna_pc
17617	hsa-mir-92a-2	PAICS	0.524178253	mirna_pc
17618	hsa-mir-92a-2	CCDC150	0.399851801	mirna_pc
17619	hsa-mir-92a-2	BIRC5	0.339745502	mirna_pc
17620	hsa-mir-92a-2	RCC1	0.348962791	mirna_pc
17621	hsa-mir-92a-2	LIG1	0.382787097	mirna_pc
17622	hsa-mir-92a-2	OIP5	0.313305194	mirna_pc
17623	hsa-mir-92a-2	DTL	0.488375407	mirna_pc
17624	hsa-mir-92a-2	EME1	0.493392349	mirna_pc
17625	hsa-mir-92a-2	RECQL4	0.402860355	mirna_pc
17626	hsa-mir-92a-2	CENPK	0.358951281	mirna_pc
17627	hsa-mir-92a-2	AURKB	0.338293291	mirna_pc
17628	hsa-mir-92a-2	GSG2	0.355585633	mirna_pc
17629	hsa-mir-92a-2	CDT1	0.388695075	mirna_pc
17630	hsa-mir-92a-2	CBFB	0.321952385	mirna_pc
17631	hsa-mir-92a-2	MCM10	0.40432271	mirna_pc
17632	hsa-mir-92a-2	SGOL2	0.378290589	mirna_pc
17633	hsa-mir-92a-2	CHEK2	0.412688738	mirna_pc
17634	hsa-mir-92a-2	CENPE	0.36129643	mirna_pc
17635	hsa-mir-92a-2	POLQ	0.422254673	mirna_pc
17636	hsa-mir-92a-2	KIF15	0.355451128	mirna_pc
17637	hsa-mir-92a-2	CDCA3	0.369670433	mirna_pc
17638	hsa-mir-92a-2	C1orf112	0.428543912	mirna_pc
17639	hsa-mir-92a-2	GINS1	0.436743208	mirna_pc
17640	hsa-mir-92a-2	FEN1	0.409411503	mirna_pc
17641	hsa-mir-92a-2	TRAIP	0.407172	mirna_pc
17642	hsa-mir-92a-2	ORC6L	0.429357236	mirna_pc
17643	hsa-mir-92a-2	ERH	0.305113683	mirna_pc
17644	hsa-mir-92a-2	C16orf59	0.448610011	mirna_pc
17645	hsa-mir-92a-2	C17orf53	0.317121822	mirna_pc
17646	hsa-mir-92a-2	RAE1	0.342927157	mirna_pc
17647	hsa-mir-92a-2	FAM72D	0.327085546	mirna_pc
17648	hsa-mir-92a-2	FAM72B	0.354178199	mirna_pc
17649	hsa-mir-92a-2	EZH2	0.455413359	mirna_pc
17650	hsa-mir-92a-2	NEIL3	0.445484312	mirna_pc
17651	hsa-mir-92a-2	MCM7	0.303165107	mirna_pc
17652	hsa-mir-92a-2	POLD1	0.481294088	mirna_pc
17653	hsa-mir-92a-2	C12orf48	0.404539244	mirna_pc
17654	hsa-mir-92a-2	HNRNPL	0.404240518	mirna_pc
17655	hsa-mir-92a-2	TTK	0.316647435	mirna_pc
17656	hsa-mir-92a-2	ZWINT	0.403773858	mirna_pc
17657	hsa-mir-92a-2	TUBA1B	0.328119956	mirna_pc

17658	hsa-mir-92a-2	RUVBL1	0.372878084	mirna_pc
17659	hsa-mir-92a-2	HOXC6	0.332763829	mirna_pc
17660	hsa-mir-92a-2	BUB3	0.35948853	mirna_pc
17661	hsa-mir-92a-2	HELLS	0.410610722	mirna_pc
17662	hsa-mir-92a-2	POLE2	0.324288744	mirna_pc
17663	hsa-mir-92a-2	UBE2S	0.329042243	mirna_pc
17664	hsa-mir-92a-2	NCAPD2	0.320163536	mirna_pc
17665	hsa-mir-92a-2	HNRNPC	0.409870125	mirna_pc
17666	hsa-mir-92a-2	ILF2	0.373314403	mirna_pc
17667	hsa-mir-92a-2	TCF3	0.454813134	mirna_pc
17668	hsa-mir-92a-2	C6orf167	0.343309413	mirna_pc
17669	hsa-mir-92a-2	CHEK1	0.388631006	mirna_pc
17670	hsa-mir-92a-2	BRCA1	0.419034815	mirna_pc
17671	hsa-mir-92a-2	TFRC	0.31897493	mirna_pc
17672	hsa-mir-92a-2	MCM5	0.326007382	mirna_pc
17673	hsa-mir-92a-2	HNRNPA2B1	0.409861296	mirna_pc
17674	hsa-mir-92a-2	RAN	0.314086274	mirna_pc
17675	hsa-mir-92a-2	TYMS	0.344491741	mirna_pc
17676	hsa-mir-92a-2	RFWD3	0.479232798	mirna_pc
17677	hsa-mir-92a-2	MCM6	0.517713516	mirna_pc
17678	hsa-mir-92a-2	CDC6	0.417542593	mirna_pc
17679	hsa-mir-92a-2	CDCA7	0.458709177	mirna_pc
17680	hsa-mir-92a-2	EPR1	0.382346572	mirna_pc
17681	hsa-mir-92a-2	BRIP1	0.359986934	mirna_pc
17682	hsa-mir-92a-2	PIF1	0.328496877	mirna_pc
17683	hsa-mir-92a-2	H2AFX	0.327999438	mirna_pc
17684	hsa-mir-92a-2	SFRS2	0.366702317	mirna_pc
17685	hsa-mir-92a-2	DCAF13	0.32494455	mirna_pc
17686	hsa-mir-92a-2	MTHFD1L	0.319205556	mirna_pc
17687	hsa-mir-92a-2	UCK2	0.422475117	mirna_pc
17688	hsa-mir-92a-2	EFTUD2	0.379497683	mirna_pc
17689	hsa-mir-92a-2	THOC4	0.329471118	mirna_pc
17690	hsa-mir-92a-2	NFKBIL2	0.375947235	mirna_pc
17691	hsa-mir-92a-2	C15orf42	0.36865966	mirna_pc
17692	hsa-mir-92a-2	PTGES3	0.300173814	mirna_pc
17693	hsa-mir-92a-2	PRIM2	0.357024954	mirna_pc
17694	hsa-mir-92a-2	FAM60A	0.411756831	mirna_pc
17695	hsa-mir-92a-2	FAM64A	0.356565197	mirna_pc
17696	hsa-mir-92a-2	ATAD5	0.383410034	mirna_pc
17697	hsa-mir-92a-2	RANBP1	0.391488868	mirna_pc
17698	hsa-mir-92a-2	E2F1	0.375460042	mirna_pc
17699	hsa-mir-92a-2	SNRPG	0.327021396	mirna_pc
17700	hsa-mir-92a-2	TH1L	0.332315059	mirna_pc
17701	hsa-mir-92a-2	MCM3	0.322602587	mirna_pc
17702	hsa-mir-92a-2	SNRPA	0.431732163	mirna_pc
17703	hsa-mir-92a-2	FUS	0.381687776	mirna_pc
17704	hsa-mir-92a-2	DNAJC9	0.324833999	mirna_pc
17705	hsa-mir-92a-2	U2AF2	0.308028711	mirna_pc
17706	hsa-mir-92a-2	RFC3	0.522559088	mirna_pc
17707	hsa-mir-92a-2	CHAF1A	0.321368974	mirna_pc
17708	hsa-mir-92a-2	DEPDC1B	0.32737648	mirna_pc
17709	hsa-mir-92a-2	CENPO	0.517455375	mirna_pc
17710	hsa-mir-92a-2	XRCC2	0.458985157	mirna_pc
17711	hsa-mir-92a-2	C19orf48	0.443899989	mirna_pc

17712	hsa-mir-92a-2	BRCA2	0.389453874	mirna_pc
17713	hsa-mir-92a-2	CENPL	0.336803318	mirna_pc
17714	hsa-mir-92a-2	GEN1	0.418489468	mirna_pc
17715	hsa-mir-92a-2	NUDT1	0.332102818	mirna_pc
17716	hsa-mir-92a-2	HMGB2	0.50401403	mirna_pc
17717	hsa-mir-92a-2	GINS2	0.423291952	mirna_pc
17718	hsa-mir-92a-2	DNA2	0.425703169	mirna_pc
17719	hsa-mir-92a-2	NOP56	0.49002303	mirna_pc
17720	hsa-mir-92a-2	SNHG1	0.358950008	mirna_pc
17721	hsa-mir-92a-2	CSE1L	0.325373936	mirna_pc
17722	hsa-mir-92a-2	DTYMK	0.459309351	mirna_pc
17723	hsa-mir-92a-2	TMEM206	0.331187977	mirna_pc
17724	hsa-mir-92a-2	PRMT1	0.427533177	mirna_pc
17725	hsa-mir-92a-2	MLF1IP	0.390387562	mirna_pc
17726	hsa-mir-92a-2	RAVER1	0.392876965	mirna_pc
17727	hsa-mir-92a-2	HEATR1	0.321702715	mirna_pc
17728	hsa-mir-92a-2	HSPE1	0.305122976	mirna_pc
17729	hsa-mir-92a-2	C16orf75	0.356519935	mirna_pc
17730	hsa-mir-92a-2	NOP2	0.302245894	mirna_pc
17731	hsa-mir-92a-2	CAD	0.453887562	mirna_pc
17732	hsa-mir-92a-2	ZFP64	0.381577552	mirna_pc
17733	hsa-mir-92a-2	CCDC99	0.378799885	mirna_pc
17734	hsa-mir-92a-2	CDK2	0.396475404	mirna_pc
17735	hsa-mir-92a-2	CDK4	0.432409073	mirna_pc
17736	hsa-mir-92a-2	CKAP5	0.323366204	mirna_pc
17737	hsa-mir-92a-2	CHTF18	0.344447743	mirna_pc
17738	hsa-mir-92a-2	HNRNPR	0.350881706	mirna_pc
17739	hsa-mir-92a-2	DLEU2	0.390093559	mirna_pc
17740	hsa-mir-92a-2	PRIM1	0.391220466	mirna_pc
17741	hsa-mir-92a-2	KAT2A	0.372186429	mirna_pc
17742	hsa-mir-92a-2	CENPH	0.384950495	mirna_pc
17743	hsa-mir-92a-2	FERMT1	0.340641563	mirna_pc
17744	hsa-mir-92a-2	TCOF1	0.368560348	mirna_pc
17745	hsa-mir-92a-2	CHAF1B	0.392952722	mirna_pc
17746	hsa-mir-92a-2	DHX34	0.346560136	mirna_pc
17747	hsa-mir-92a-2	NCAPG2	0.363124717	mirna_pc
17748	hsa-mir-92a-2	MSH2	0.454099192	mirna_pc
17749	hsa-mir-92a-2	PRPF19	0.341259432	mirna_pc
17750	hsa-mir-92a-2	EIF3B	0.311018274	mirna_pc
17751	hsa-mir-92a-2	TOMM40	0.300307574	mirna_pc
17752	hsa-mir-92a-2	EIF2AK1	0.310363588	mirna_pc
17753	hsa-mir-92a-2	SF3B3	0.479530667	mirna_pc
17754	hsa-mir-92a-2	CDC7	0.416431561	mirna_pc
17755	hsa-mir-92a-2	INTS7	0.339075742	mirna_pc
17756	hsa-mir-92a-2	SSB	0.428447468	mirna_pc
17757	hsa-mir-92a-2	PUS1	0.422661344	mirna_pc
17758	hsa-mir-92a-2	NOL11	0.421723706	mirna_pc
17759	hsa-mir-92a-2	RFC4	0.383576685	mirna_pc
17760	hsa-mir-92a-2	FBXO5	0.384246138	mirna_pc
17761	hsa-mir-92a-2	FIGNL1	0.327248035	mirna_pc
17762	hsa-mir-92a-2	E2F3	0.431646861	mirna_pc
17763	hsa-mir-92a-2	DDX11	0.389295309	mirna_pc
17764	hsa-mir-92a-2	RUVBL2	0.374057664	mirna_pc
17765	hsa-mir-92a-2	CDC25A	0.462654679	mirna_pc

17766	hsa-mir-92a-2	TOPBP1	0.362649475	mirna_pc
17767	hsa-mir-92a-2	RAD54B	0.34329275	mirna_pc
17768	hsa-mir-92a-2	OTX1	0.388128583	mirna_pc
17769	hsa-mir-92a-2	PRKDC	0.31751187	mirna_pc
17770	hsa-mir-92a-2	H2AFZ	0.444941236	mirna_pc
17771	hsa-mir-92a-2	PRR7	0.35120855	mirna_pc
17772	hsa-mir-92a-2	DDX12	0.364578606	mirna_pc
17773	hsa-mir-92a-2	DSCC1	0.367904865	mirna_pc
17774	hsa-mir-92a-2	C13orf34	0.49827038	mirna_pc
17775	hsa-mir-92a-2	SMC2	0.302230264	mirna_pc
17776	hsa-mir-92a-2	CLN6	0.353528091	mirna_pc
17777	hsa-mir-92a-2	MCM8	0.474040678	mirna_pc
17778	hsa-mir-92a-2	CPSF3	0.409080075	mirna_pc
17779	hsa-mir-92a-2	SNRPE	0.45670747	mirna_pc
17780	hsa-mir-92a-2	C21orf45	0.349744145	mirna_pc
17781	hsa-mir-92a-2	ATIC	0.421125195	mirna_pc
17782	hsa-mir-92a-2	PPM1G	0.373804315	mirna_pc
17783	hsa-mir-92a-2	NPM3	0.397583907	mirna_pc
17784	hsa-mir-92a-2	TFAP4	0.47354942	mirna_pc
17785	hsa-mir-92a-2	PUS7	0.314334114	mirna_pc
17786	hsa-mir-92a-2	NUTF2	0.303963086	mirna_pc
17787	hsa-mir-92a-2	RNPS1	0.307448304	mirna_pc
17788	hsa-mir-92a-2	DAZAP1	0.402898438	mirna_pc
17789	hsa-mir-92a-2	CBX8	0.442621087	mirna_pc
17790	hsa-mir-92a-2	TRMT6	0.320978179	mirna_pc
17791	hsa-mir-92a-2	GNPDA1	0.342387306	mirna_pc
17792	hsa-mir-92a-2	WDR34	0.304426353	mirna_pc
17793	hsa-mir-92a-2	TMEM201	0.320602019	mirna_pc
17794	hsa-mir-92a-2	NCL	0.455007637	mirna_pc
17795	hsa-mir-92a-2	PAFAH1B3	0.303519745	mirna_pc
17796	hsa-mir-92a-2	SRRT	0.339475676	mirna_pc
17797	hsa-mir-92a-2	CACYBP	0.367941223	mirna_pc
17798	hsa-mir-92a-2	DCTPP1	0.338819812	mirna_pc
17799	hsa-mir-92a-2	PSRC1	0.424746914	mirna_pc
17800	hsa-mir-92a-2	ODF2	0.309177131	mirna_pc
17801	hsa-mir-92a-2	DBF4B	0.436438069	mirna_pc
17802	hsa-mir-92a-2	POU2F1	0.376163058	mirna_pc
17803	hsa-mir-92a-2	FBL	0.427832662	mirna_pc
17804	hsa-mir-92a-2	C13orf37	0.448404003	mirna_pc
17805	hsa-mir-92a-2	EXOSC5	0.30708531	mirna_pc
17806	hsa-mir-92a-2	HNRNPM	0.368076633	mirna_pc
17807	hsa-mir-92a-2	LSM 12.00	0.346068738	mirna_pc
17808	hsa-mir-92a-2	PNPT1	0.440664099	mirna_pc
17809	hsa-mir-92a-2	TMPO	0.401023775	mirna_pc
17810	hsa-mir-92a-2	SET	0.417105085	mirna_pc
17811	hsa-mir-92a-2	NIP7	0.30711522	mirna_pc
17812	hsa-mir-92a-2	C20orf72	0.304083152	mirna_pc
17813	hsa-mir-92a-2	C3orf26	0.406684553	mirna_pc
17814	hsa-mir-92a-2	MRTO4	0.31568813	mirna_pc
17815	hsa-mir-92a-2	SMPD4	0.417798177	mirna_pc
17816	hsa-mir-92a-2	LSG1	0.335964187	mirna_pc
17817	hsa-mir-92a-2	PSMD3	0.335739358	mirna_pc
17818	hsa-mir-92a-2	GABPB1	0.349976071	mirna_pc
17819	hsa-mir-92a-2	PPIH	0.314406589	mirna_pc

17820	hsa-mir-92a-2	PPAT	0.385408892	mirna_pc
17821	hsa-mir-92a-2	DHX9	0.47291932	mirna_pc
17822	hsa-mir-92a-2	CCDC138	0.389951691	mirna_pc
17823	hsa-mir-92a-2	LOC100128191	0.327202625	mirna_pc
17824	hsa-mir-92a-2	MRPL3	0.328485286	mirna_pc
17825	hsa-mir-92a-2	HMGN2	0.345378778	mirna_pc
17826	hsa-mir-92a-2	ETV4	0.382940653	mirna_pc
17827	hsa-mir-92a-2	SMYD5	0.400294794	mirna_pc
17828	hsa-mir-92a-2	TSSC1	0.338803834	mirna_pc
17829	hsa-mir-92a-2	POLA2	0.354514222	mirna_pc
17830	hsa-mir-92a-2	SFRS1	0.546058842	mirna_pc
17831	hsa-mir-92a-2	TEX10	0.362019546	mirna_pc
17832	hsa-mir-92a-2	WDR43	0.428818029	mirna_pc
17833	hsa-mir-92a-2	CCDC58	0.364791896	mirna_pc
17834	hsa-mir-92a-2	SKP2	0.429631373	mirna_pc
17835	hsa-mir-92a-2	TIPIN	0.307135076	mirna_pc
17836	hsa-mir-92a-2	SMARCD1	0.410548176	mirna_pc
17837	hsa-mir-92a-2	ATXN2L	0.324879442	mirna_pc
17838	hsa-mir-92a-2	C4orf46	0.480937288	mirna_pc
17839	hsa-mir-92a-2	DARS2	0.316709021	mirna_pc
17840	hsa-mir-92a-2	UTP14A	0.314555148	mirna_pc
17841	hsa-mir-92a-2	C1orf107	0.339920576	mirna_pc
17842	hsa-mir-92a-2	GPN1	0.350397203	mirna_pc
17843	hsa-mir-92a-2	RFC5	0.333329942	mirna_pc
17844	hsa-mir-92a-2	HSPD1	0.367827189	mirna_pc
17845	hsa-mir-92a-2	MAZ	0.378567093	mirna_pc
17846	hsa-mir-92a-2	SSRP1	0.459103055	mirna_pc
17847	hsa-mir-92a-2	POLR2D	0.513915819	mirna_pc
17848	hsa-mir-92a-2	FTSJ2	0.378807142	mirna_pc
17849	hsa-mir-92a-2	PA2G4	0.349914816	mirna_pc
17850	hsa-mir-92a-2	EPT1	0.339148524	mirna_pc
17851	hsa-mir-92a-2	KHDRBS1	0.411160113	mirna_pc
17852	hsa-mir-92a-2	SLC5A6	0.342688342	mirna_pc
17853	hsa-mir-92a-2	C20orf27	0.407485672	mirna_pc
17854	hsa-mir-92a-2	TOMM34	0.354718999	mirna_pc
17855	hsa-mir-92a-2	MTL 5.00	0.358361073	mirna_pc
17856	hsa-mir-92a-2	TAF1A	0.306774637	mirna_pc
17857	hsa-mir-92a-2	RNF220	0.335168357	mirna_pc
17858	hsa-mir-92a-2	PRPF40A	0.406813362	mirna_pc
17859	hsa-mir-92a-2	PHF5A	0.334099817	mirna_pc
17860	hsa-mir-92a-2	AHCY	0.368475429	mirna_pc
17861	hsa-mir-92a-2	PARP1	0.401702485	mirna_pc
17862	hsa-mir-92a-2	CCT7	0.402803695	mirna_pc
17863	hsa-mir-92a-2	C4orf21	0.341137218	mirna_pc
17864	hsa-mir-92a-2	TMEM194A	0.359554629	mirna_pc
17865	hsa-mir-92a-2	SLC39A10	0.403434311	mirna_pc
17866	hsa-mir-92a-2	TDP1	0.303647136	mirna_pc
17867	hsa-mir-92a-2	NOLC1	0.316621844	mirna_pc
17868	hsa-mir-92a-2	ZNF695	0.376525507	mirna_pc
17869	hsa-mir-92a-2	PDSS1	0.346499231	mirna_pc
17870	hsa-mir-92a-2	HAUS6	0.357699188	mirna_pc
17871	hsa-mir-92a-2	CENPJ	0.49352621	mirna_pc
17872	hsa-mir-92a-2	LIN9	0.30268928	mirna_pc
17873	hsa-mir-92a-2	HNRNPU	0.437356966	mirna_pc

17874	hsa-mir-92a-2	NASP	0.388464941	mirna_pc
17875	hsa-mir-92a-2	NONO	0.345825261	mirna_pc
17876	hsa-mir-92a-2	CCDC86	0.390126111	mirna_pc
17877	hsa-mir-92a-2	ZNF280C	0.321407108	mirna_pc
17878	hsa-mir-92a-2	POLR1B	0.388819579	mirna_pc
17879	hsa-mir-92a-2	QSOX2	0.306453046	mirna_pc
17880	hsa-mir-92a-2	PTMA	0.374076302	mirna_pc
17881	hsa-mir-92a-2	USP39	0.32480025	mirna_pc
17882	hsa-mir-92a-2	WDR76	0.301237112	mirna_pc
17883	hsa-mir-92a-2	EXOSC2	0.391947535	mirna_pc
17884	hsa-mir-92a-2	HMGXB4	0.325183705	mirna_pc
17885	hsa-mir-92a-2	SCARB1	0.324112252	mirna_pc
17886	hsa-mir-92a-2	C11orf84	0.435602714	mirna_pc
17887	hsa-mir-92a-2	SMG7	0.313195234	mirna_pc
17888	hsa-mir-92a-2	TBCE	0.378463129	mirna_pc
17889	hsa-mir-92a-2	NCBP2	0.342241821	mirna_pc
17890	hsa-mir-92a-2	SUV39H2	0.409140206	mirna_pc
17891	hsa-mir-92a-2	ZC3H8	0.333568168	mirna_pc
17892	hsa-mir-92a-2	R3HDM1	0.390172262	mirna_pc
17893	hsa-mir-92a-2	FANCL	0.404424441	mirna_pc
17894	hsa-mir-92a-2	RQCD1	0.323232806	mirna_pc
17895	hsa-mir-92a-2	HNRNPD	0.459196007	mirna_pc
17896	hsa-mir-92a-2	MTHFD2	0.439041152	mirna_pc
17897	hsa-mir-92a-2	GTF2F2	0.422667857	mirna_pc
17898	hsa-mir-92a-2	DDX31	0.334554222	mirna_pc
17899	hsa-mir-92a-2	DPH2	0.300407401	mirna_pc
17900	hsa-mir-92a-2	POP1	0.382559863	mirna_pc
17901	hsa-mir-92a-2	SFRS3	0.349126475	mirna_pc
17902	hsa-mir-92a-2	LSM 5.00	0.375161277	mirna_pc
17903	hsa-mir-92a-2	SHOX2	0.302391544	mirna_pc
17904	hsa-mir-92a-2	REXO4	0.331674717	mirna_pc
17905	hsa-mir-92a-2	H1FX	0.306821387	mirna_pc
17906	hsa-mir-92a-2	PNO1	0.35936719	mirna_pc
17907	hsa-mir-92a-2	CCT4	0.344034189	mirna_pc
17908	hsa-mir-92a-2	UMPS	0.371255964	mirna_pc
17909	hsa-mir-92a-2	CASP2	0.350807238	mirna_pc
17910	hsa-mir-92a-2	ELAVL1	0.457201841	mirna_pc
17911	hsa-mir-92a-2	ABCE1	0.314075548	mirna_pc
17912	hsa-mir-92a-2	MTHFD1	0.305177331	mirna_pc
17913	hsa-mir-92a-2	KIAA0406	0.301223479	mirna_pc
17914	hsa-mir-92a-2	NAE1	0.359977309	mirna_pc
17915	hsa-mir-92a-2	USP22	0.319616621	mirna_pc
17916	hsa-mir-92a-2	PSMC3IP	0.397914868	mirna_pc
17917	hsa-mir-92a-2	PAK1IP1	0.372935202	mirna_pc
17918	hsa-mir-92a-2	NUDT15	0.454101841	mirna_pc
17919	hsa-mir-92a-2	PES 1.00	0.318795624	mirna_pc
17920	hsa-mir-92a-2	E2F4	0.389027692	mirna_pc
17921	hsa-mir-92a-2	TWISTNB	0.30821125	mirna_pc
17922	hsa-mir-92a-2	PGAM5	0.30222318	mirna_pc
17923	hsa-mir-92a-2	DHFR	0.344852312	mirna_pc
17924	hsa-mir-92a-2	NAA25	0.361372584	mirna_pc
17925	hsa-mir-92a-2	ILF3	0.483638715	mirna_pc
17926	hsa-mir-92a-2	NT5DC2	0.353845652	mirna_pc
17927	hsa-mir-92a-2	EHMT2	0.395612784	mirna_pc

17928	hsa-mir-92a-2	HNRNPAB	0.329127664	mirna_pc
17929	hsa-mir-92a-2	UBAP2L	0.336238788	mirna_pc
17930	hsa-mir-92a-2	NUP205	0.306460167	mirna_pc
17931	hsa-mir-92a-2	DEK	0.36810141	mirna_pc
17932	hsa-mir-92a-2	TBC1D4	0.324992815	mirna_pc
17933	hsa-mir-92a-2	SFRS9	0.318253331	mirna_pc
17934	hsa-mir-92a-2	TRIB3	0.39055099	mirna_pc
17935	hsa-mir-92a-2	PASK	0.383644588	mirna_pc
17936	hsa-mir-92a-2	SAAL1	0.373426943	mirna_pc
17937	hsa-mir-92a-2	OLA1	0.318218007	mirna_pc
17938	hsa-mir-92a-2	RPGRIP1L	0.317536945	mirna_pc
17939	hsa-mir-92a-2	TGS1	0.408036009	mirna_pc
17940	hsa-mir-92a-2	HEATR2	0.48636982	mirna_pc
17941	hsa-mir-92a-2	LSM 6.00	0.357158516	mirna_pc
17942	hsa-mir-92a-2	PRAME	0.396894456	mirna_pc
17943	hsa-mir-92a-2	CYB5B	0.32587631	mirna_pc
17944	hsa-mir-92a-2	SNRPB2	0.30663625	mirna_pc
17945	hsa-mir-92a-2	FUBP1	0.311346564	mirna_pc
17946	hsa-mir-92a-2	SUV39H1	0.368881379	mirna_pc
17947	hsa-mir-92a-2	IPO9	0.397267372	mirna_pc
17948	hsa-mir-92a-2	SART3	0.34704507	mirna_pc
17949	hsa-mir-92a-2	HNRNPK	0.323980418	mirna_pc
17950	hsa-mir-92a-2	SR140	0.359449065	mirna_pc
17951	hsa-mir-92a-2	TRA2B	0.383117258	mirna_pc
17952	hsa-mir-92a-2	RSRC1	0.301431387	mirna_pc
17953	hsa-mir-92a-2	GAR1	0.461455436	mirna_pc
17954	hsa-mir-92a-2	CEP152	0.313248429	mirna_pc
17955	hsa-mir-92a-2	C15orf23	0.364612671	mirna_pc
17956	hsa-mir-92a-2	ZC3HAV1L	0.402599399	mirna_pc
17957	hsa-mir-92a-2	CIRH1A	0.334894175	mirna_pc
17958	hsa-mir-92a-2	SLMO1	0.354830187	mirna_pc
17959	hsa-mir-92a-2	IGF2BP2	0.332709401	mirna_pc
17960	hsa-mir-92a-2	WHSC1	0.349900719	mirna_pc
17961	hsa-mir-92a-2	SAE1	0.337865851	mirna_pc
17962	hsa-mir-92a-2	RNF219	0.595683966	mirna_pc
17963	hsa-mir-92a-2	ITGB3BP	0.329144675	mirna_pc
17964	hsa-mir-92a-2	ENOPH1	0.358706187	mirna_pc
17965	hsa-mir-92a-2	C4orf43	0.380523999	mirna_pc
17966	hsa-mir-92a-2	DDX55	0.323276035	mirna_pc
17967	hsa-mir-92a-2	CEP78	0.370812861	mirna_pc
17968	hsa-mir-92a-2	PLEKHG4	0.321619423	mirna_pc
17969	hsa-mir-92a-2	SFPQ	0.422711313	mirna_pc
17970	hsa-mir-92a-2	CD83	0.325752761	mirna_pc
17971	hsa-mir-92a-2	GIT1	0.374095996	mirna_pc
17972	hsa-mir-92a-2	RCOR2	0.453345849	mirna_pc
17973	hsa-mir-92a-2	SHMT2	0.318456497	mirna_pc
17974	hsa-mir-92a-2	POLA1	0.370416925	mirna_pc
17975	hsa-mir-92a-2	HDAC2	0.379181838	mirna_pc
17976	hsa-mir-92a-2	GMNN	0.320050469	mirna_pc
17977	hsa-mir-92a-2	PA2G4P4	0.313976134	mirna_pc
17978	hsa-mir-92a-2	C10orf119	0.336352249	mirna_pc
17979	hsa-mir-92a-2	POLG2	0.308648123	mirna_pc
17980	hsa-mir-92a-2	C10orf2	0.415531469	mirna_pc
17981	hsa-mir-92a-2	POGK	0.350273387	mirna_pc

17982	hsa-mir-92a-2	GEMIN6	0.315355926	mirna_pc
17983	hsa-mir-92a-2	MRPL11	0.303223814	mirna_pc
17984	hsa-mir-92a-2	COX4NB	0.335996986	mirna_pc
17985	hsa-mir-92a-2	MSH6	0.440006392	mirna_pc
17986	hsa-mir-92a-2	XRN2	0.353093497	mirna_pc
17987	hsa-mir-92a-2	KPNB1	0.463163454	mirna_pc
17988	hsa-mir-92a-2	SMC3	0.335611411	mirna_pc
17989	hsa-mir-92a-2	FLVCR1	0.356890953	mirna_pc
17990	hsa-mir-92a-2	ACBD6	0.368501913	mirna_pc
17991	hsa-mir-92a-2	HNRNPA1	0.368560248	mirna_pc
17992	hsa-mir-92a-2	NPM1	0.321410991	mirna_pc
17993	hsa-mir-92a-2	ANKRD13B	0.316035441	mirna_pc
17994	hsa-mir-92a-2	ZBED4	0.401815872	mirna_pc
17995	hsa-mir-92a-2	MEX3A	0.520208808	mirna_pc
17996	hsa-mir-92a-2	CEP250	0.306931008	mirna_pc
17997	hsa-mir-92a-2	COIL	0.412038468	mirna_pc
17998	hsa-mir-92a-2	ERAL1	0.311881163	mirna_pc
17999	hsa-mir-92a-2	TAF5	0.437653576	mirna_pc
18000	hsa-mir-92a-2	TRIM28	0.436192672	mirna_pc
18001	hsa-mir-92a-2	FANCC	0.312122058	mirna_pc
18002	hsa-mir-92a-2	WDR5	0.355510796	mirna_pc
18003	hsa-mir-92a-2	APEX1	0.306793463	mirna_pc
18004	hsa-mir-92a-2	NANP	0.300464713	mirna_pc
18005	hsa-mir-92a-2	GAS5	0.317161929	mirna_pc
18006	hsa-mir-92a-2	SUZ12	0.30711224	mirna_pc
18007	hsa-mir-92a-2	HNRNPA1L2	0.426756341	mirna_pc
18008	hsa-mir-92a-2	NAA15	0.32123976	mirna_pc
18009	hsa-mir-92a-2	CBX1	0.405506093	mirna_pc
18010	hsa-mir-92a-2	HMGB1	0.474205042	mirna_pc
18011	hsa-mir-92a-2	ZBTB12	0.451740921	mirna_pc
18012	hsa-mir-92a-2	DIAPH3	0.383023169	mirna_pc
18013	hsa-mir-92a-2	C1orf35	0.309576753	mirna_pc
18014	hsa-mir-92a-2	TADA1	0.334352666	mirna_pc
18015	hsa-mir-92a-2	LOC642846	0.328328265	mirna_pc
18016	hsa-mir-92a-2	TGIF2	0.456786551	mirna_pc
18017	hsa-mir-92a-2	ZC3H18	0.34839824	mirna_pc
18018	hsa-mir-92a-2	CHERP	0.395172778	mirna_pc
18019	hsa-mir-92a-2	FARSB	0.384456802	mirna_pc
18020	hsa-mir-92a-2	SNX5	0.326825831	mirna_pc
18021	hsa-mir-92a-2	THUMPD2	0.308581088	mirna_pc
18022	hsa-mir-92a-2	CIB2	0.328725459	mirna_pc
18023	hsa-mir-92a-2	TARBP2	0.343158779	mirna_pc
18024	hsa-mir-92a-2	PHB	0.382333659	mirna_pc
18025	hsa-mir-92a-2	EBPL	0.307107131	mirna_pc
18026	hsa-mir-92a-2	NOL10	0.408920834	mirna_pc
18027	hsa-mir-92a-2	ANAPC1	0.365060709	mirna_pc
18028	hsa-mir-92a-2	POLE	0.446926014	mirna_pc
18029	hsa-mir-92a-2	HMBS	0.334272047	mirna_pc
18030	hsa-mir-92a-2	SOX9	0.350597335	mirna_pc
18031	hsa-mir-92a-2	TIMM50	0.41684135	mirna_pc
18032	hsa-mir-92a-2	ADSL	0.384478115	mirna_pc
18033	hsa-mir-92a-2	VRK1	0.303890369	mirna_pc
18034	hsa-mir-92a-2	C13orf27	0.431516007	mirna_pc
18035	hsa-mir-92a-2	RBM14	0.415915842	mirna_pc

18036	hsa-mir-92a-2	EXOSC9	0.420995903	mirna_pc
18037	hsa-mir-92a-2	UNG	0.332787722	mirna_pc
18038	hsa-mir-92a-2	CCDC43	0.363094211	mirna_pc
18039	hsa-mir-92a-2	RBMX	0.472412858	mirna_pc
18040	hsa-mir-92a-2	SRP9	0.334387404	mirna_pc
18041	hsa-mir-92a-2	TSEN15	0.311472558	mirna_pc
18042	hsa-mir-92a-2	USP10	0.348193913	mirna_pc
18043	hsa-mir-92a-2	GPR180	0.33032834	mirna_pc
18044	hsa-mir-92a-2	DAP3	0.310666954	mirna_pc
18045	hsa-mir-92a-2	IQCB1	0.390671484	mirna_pc
18046	hsa-mir-92a-2	XRCC5	0.451366269	mirna_pc
18047	hsa-mir-92a-2	EXOSC8	0.442870873	mirna_pc
18048	hsa-mir-92a-2	CTCF	0.444114612	mirna_pc
18049	hsa-mir-92a-2	PDIA6	0.304035219	mirna_pc
18050	hsa-mir-92a-2	LBR	0.354969287	mirna_pc
18051	hsa-mir-92a-2	NOB1	0.336157347	mirna_pc
18052	hsa-mir-92a-2	NAA40	0.304103189	mirna_pc
18053	hsa-mir-92a-2	DNAJC14	0.31774202	mirna_pc
18054	hsa-mir-92a-2	FIP1L1	0.320000747	mirna_pc
18055	hsa-mir-92a-2	ZNF519	0.412039325	mirna_pc
18056	hsa-mir-92a-2	GTF3C3	0.322434982	mirna_pc
18057	hsa-mir-92a-2	CIT	0.305637024	mirna_pc
18058	hsa-mir-92a-2	ZRANB3	0.308005805	mirna_pc
18059	hsa-mir-92a-2	TRAP1	0.322055377	mirna_pc
18060	hsa-mir-92a-2	NUDT21	0.391732147	mirna_pc
18061	hsa-mir-92a-2	GTF3C2	0.336709186	mirna_pc
18062	hsa-mir-92a-2	MAGEF1	0.30626273	mirna_pc
18063	hsa-mir-92a-2	PSAT1	0.331545685	mirna_pc
18064	hsa-mir-92a-2	POLR1A	0.313673845	mirna_pc
18065	hsa-mir-92a-2	FAM136A	0.417342279	mirna_pc
18066	hsa-mir-92a-2	NKD2	0.301633419	mirna_pc
18067	hsa-mir-92a-2	SUPT3H	0.35193279	mirna_pc
18068	hsa-mir-92a-2	SOX12	0.338935624	mirna_pc
18069	hsa-mir-92a-2	SMARCB1	0.316732484	mirna_pc
18070	hsa-mir-92a-2	TMEM151B	0.304727625	mirna_pc
18071	hsa-mir-92a-2	ITPRIPL1	0.377452033	mirna_pc
18072	hsa-mir-92a-2	FBXO41	0.418076608	mirna_pc
18073	hsa-mir-92a-2	BZW2	0.360093706	mirna_pc
18074	hsa-mir-92a-2	LRPPRC	0.382131444	mirna_pc
18075	hsa-mir-92a-2	CSNK2A1	0.376106096	mirna_pc
18076	hsa-mir-92a-2	RPIA	0.414627073	mirna_pc
18077	hsa-mir-92a-2	NUP133	0.303604083	mirna_pc
18078	hsa-mir-92a-2	BCL7A	0.359383965	mirna_pc
18079	hsa-mir-92a-2	ABCF2	0.305251352	mirna_pc
18080	hsa-mir-92a-2	C7orf44	0.385880754	mirna_pc
18081	hsa-mir-92a-2	URB2	0.385991431	mirna_pc
18082	hsa-mir-92a-2	RP9	0.337195992	mirna_pc
18083	hsa-mir-92a-2	ZNF286A	0.429836796	mirna_pc
18084	hsa-mir-92a-2	SFXN4	0.335000816	mirna_pc
18085	hsa-mir-92a-2	WDR77	0.302847457	mirna_pc
18086	hsa-mir-92a-2	SURF6	0.327416697	mirna_pc
18087	hsa-mir-92a-2	MRPL21	0.303245312	mirna_pc
18088	hsa-mir-92a-2	TCEA1	0.312254603	mirna_pc
18089	hsa-mir-92a-2	NLE1	0.381071885	mirna_pc

18090	hsa-mir-92a-2	RBM28	0.386980128	mirna_pc
18091	hsa-mir-92a-2	RFX7	0.304705972	mirna_pc
18092	hsa-mir-92a-2	WDR85	0.30157392	mirna_pc
18093	hsa-mir-92a-2	PSPH	0.331813463	mirna_pc
18094	hsa-mir-92a-2	ILKAP	0.326596177	mirna_pc
18095	hsa-mir-92a-2	FOXK1	0.363448422	mirna_pc
18096	hsa-mir-92a-2	DLEU1	0.388829041	mirna_pc
18097	hsa-mir-92a-2	C13orf23	0.46170124	mirna_pc
18098	hsa-mir-92a-2	TRMT61B	0.31913753	mirna_pc
18099	hsa-mir-92a-2	SEMA4F	0.324334312	mirna_pc
18100	hsa-mir-92a-2	RBM12	0.334889217	mirna_pc
18101	hsa-mir-92a-2	ZNF286B	0.437230303	mirna_pc
18102	hsa-mir-92a-2	PDF	0.385836323	mirna_pc
18103	hsa-mir-92a-2	C1orf109	0.300933299	mirna_pc
18104	hsa-mir-92a-2	MTA1	0.397623392	mirna_pc
18105	hsa-mir-92a-2	TCERG1	0.329079393	mirna_pc
18106	hsa-mir-92a-2	PSPC1	0.432728682	mirna_pc
18107	hsa-mir-92a-2	ATP6V1E2	0.311493511	mirna_pc
18108	hsa-mir-92a-2	CCNB1IP1	0.43024795	mirna_pc
18109	hsa-mir-92a-2	MAPKAPK5	0.336909224	mirna_pc
18110	hsa-mir-92a-2	E2F6	0.450204448	mirna_pc
18111	hsa-mir-92a-2	SLC25A15	0.435298395	mirna_pc
18112	hsa-mir-92a-2	SFRS13A	0.380414182	mirna_pc
18113	hsa-mir-92a-2	SCLY	0.44639478	mirna_pc
18114	hsa-mir-92a-2	STARD7	0.40480042	mirna_pc
18115	hsa-mir-92a-2	GPR125	0.321378411	mirna_pc
18116	hsa-mir-92a-2	NUP35	0.322857313	mirna_pc
18117	hsa-mir-92a-2	FIZ1	0.327110149	mirna_pc
18118	hsa-mir-92a-2	SLC19A1	0.338522402	mirna_pc
18119	hsa-mir-92a-2	TGDS	0.351840568	mirna_pc
18120	hsa-mir-92a-2	TFAM	0.360174346	mirna_pc
18121	hsa-mir-92a-2	LDLRAD3	0.335144082	mirna_pc
18122	hsa-mir-92a-2	TTC27	0.336677275	mirna_pc
18123	hsa-mir-92a-2	DHRS13	0.384024636	mirna_pc
18124	hsa-mir-92a-2	ORC4L	0.40922614	mirna_pc
18125	hsa-mir-92a-2	KIAA1704	0.371153168	mirna_pc
18126	hsa-mir-92a-2	MTIF2	0.375454404	mirna_pc
18127	hsa-mir-92a-2	HNRNPA3	0.385947194	mirna_pc
18128	hsa-mir-92a-2	KRTCAP3	0.310270734	mirna_pc
18129	hsa-mir-92a-2	MSL1	0.352775431	mirna_pc
18130	hsa-mir-92a-2	EWSR1	0.397874949	mirna_pc
18131	hsa-mir-92a-2	ZNF828	0.459250427	mirna_pc
18132	hsa-mir-92a-2	SMC1A	0.372231745	mirna_pc
18133	hsa-mir-92a-2	DHX57	0.370875493	mirna_pc
18134	hsa-mir-92a-2	ALX3	0.335347145	mirna_pc
18135	hsa-mir-92a-2	NUPL1	0.449912626	mirna_pc
18136	hsa-mir-92a-2	DARS	0.316830553	mirna_pc
18137	hsa-mir-92a-2	AAAS	0.302207182	mirna_pc
18138	hsa-mir-92a-2	SFT2D3	0.319541122	mirna_pc
18139	hsa-mir-92a-2	LIG3	0.317411265	mirna_pc
18140	hsa-mir-92a-2	ANKRD5	0.300509532	mirna_pc
18141	hsa-mir-92a-2	MRPS26	0.398234243	mirna_pc
18142	hsa-mir-92a-2	TUBGCP3	0.353922212	mirna_pc
18143	hsa-mir-92a-2	MRPL30	0.421989035	mirna_pc

18144	hsa-mir-92a-2	DHODH	0.377538932	mirna_pc
18145	hsa-mir-92a-2	TSGA14	0.312718032	mirna_pc
18146	hsa-mir-92a-2	UPF3A	0.396739627	mirna_pc
18147	hsa-mir-92a-2	SFRS7	0.466604984	mirna_pc
18148	hsa-mir-92a-2	MRPL42	0.300598653	mirna_pc
18149	hsa-mir-92a-2	C18orf10	0.318626728	mirna_pc
18150	hsa-mir-92a-2	CA5BP	0.32314637	mirna_pc
18151	hsa-mir-92a-2	TRMT2A	0.303694889	mirna_pc
18152	hsa-mir-92a-2	TRIT1	0.316399637	mirna_pc
18153	hsa-mir-92a-2	PFAS	0.347308695	mirna_pc
18154	hsa-mir-92a-2	SF3B2	0.344283974	mirna_pc
18155	hsa-mir-92a-2	RBM10	0.389425721	mirna_pc
18156	hsa-mir-92a-2	CDCA7L	0.390116847	mirna_pc
18157	hsa-mir-92a-2	ERCC3	0.325236	mirna_pc
18158	hsa-mir-92a-2	HNRNPH1	0.338571717	mirna_pc
18159	hsa-mir-92a-2	HIRA	0.312763527	mirna_pc
18160	hsa-mir-92a-2	NUP153	0.307219445	mirna_pc
18161	hsa-mir-92a-2	NIPSNAP1	0.340267095	mirna_pc
18162	hsa-mir-92a-2	ARHGEF19	0.310899865	mirna_pc
18163	hsa-mir-92a-2	MRPL10	0.327640456	mirna_pc
18164	hsa-mir-92a-2	C17orf75	0.30130993	mirna_pc
18165	hsa-mir-92a-2	WDR92	0.344879556	mirna_pc
18166	hsa-mir-92a-2	RCBTB1	0.324550743	mirna_pc
18167	hsa-mir-92a-2	CDC16	0.362264073	mirna_pc
18168	hsa-mir-92a-2	HCFC1	0.324447448	mirna_pc
18169	hsa-mir-92a-2	PTCD3	0.343569263	mirna_pc
18170	hsa-mir-92a-2	ZNF669	0.303096275	mirna_pc
18171	hsa-mir-92a-2	TOP3A	0.303654429	mirna_pc
18172	hsa-mir-92a-2	CASP6	0.356347779	mirna_pc
18173	hsa-mir-92a-2	RBM26	0.357282452	mirna_pc
18174	hsa-mir-92a-2	C2orf44	0.382857802	mirna_pc
18175	hsa-mir-92a-2	TMTC4	0.373902942	mirna_pc
18176	hsa-mir-92a-2	SEPHS1	0.422546779	mirna_pc
18177	hsa-mir-92a-2	CBX5	0.345864941	mirna_pc
18178	hsa-mir-92a-2	RMND1	0.33016888	mirna_pc
18179	hsa-mir-92a-2	RYK	0.308711278	mirna_pc
18180	hsa-mir-92a-2	GEMIN5	0.301548591	mirna_pc
18181	hsa-mir-92a-2	AZIN1	0.300656054	mirna_pc
18182	hsa-mir-92a-2	RSL1D1	0.329736395	mirna_pc
18183	hsa-mir-92a-2	AGBL5	0.464655435	mirna_pc
18184	hsa-mir-92a-2	CEBPZ	0.327315092	mirna_pc
18185	hsa-mir-92a-2	SMARCE1	0.364982681	mirna_pc
18186	hsa-mir-92a-2	PHB2	0.321034085	mirna_pc
18187	hsa-mir-92a-2	C8orf33	0.403066119	mirna_pc
18188	hsa-mir-92a-2	MYL6B	0.402900567	mirna_pc
18189	hsa-mir-92a-2	MRP63	0.334502245	mirna_pc
18190	hsa-mir-92a-2	IPO5	0.554488392	mirna_pc
18191	hsa-mir-92a-2	ALG11	0.324778006	mirna_pc
18192	hsa-mir-92a-2	NRF1	0.356851634	mirna_pc
18193	hsa-mir-92a-2	INTS1	0.31455745	mirna_pc
18194	hsa-mir-92a-2	MED28	0.320690601	mirna_pc
18195	hsa-mir-92a-2	BRD3	0.307742167	mirna_pc
18196	hsa-mir-92a-2	LYRM4	0.353819821	mirna_pc
18197	hsa-mir-92a-2	ZNF124	0.42023162	mirna_pc

18198	hsa-mir-92a-2	BCL11A	0.372325194	mirna_pc
18199	hsa-mir-92a-2	ZNF496	0.384059445	mirna_pc
18200	hsa-mir-92a-2	ZNF512	0.302385455	mirna_pc
18201	hsa-mir-92a-2	RNASEH2B	0.424016066	mirna_pc
18202	hsa-mir-92a-2	CHD1L	0.323540652	mirna_pc
18203	hsa-mir-92a-2	ZNF473	0.428203679	mirna_pc
18204	hsa-mir-92a-2	COPS7B	0.341657969	mirna_pc
18205	hsa-mir-92a-2	USP21	0.314870114	mirna_pc
18206	hsa-mir-92a-2	ALMS1	0.306888345	mirna_pc
18207	hsa-mir-92a-2	RFXAP	0.310562216	mirna_pc
18208	hsa-mir-92a-2	DCLRE1A	0.389974644	mirna_pc
18209	hsa-mir-92a-2	ZNF749	0.302411774	mirna_pc
18210	hsa-mir-92a-2	ZNF74	0.319207935	mirna_pc
18211	hsa-mir-92a-2	ANP32A	0.503983256	mirna_pc
18212	hsa-mir-92a-2	DIS3	0.305432504	mirna_pc
18213	hsa-mir-92a-2	E2F5	0.33159737	mirna_pc
18214	hsa-mir-92a-2	NUFIP1	0.556513267	mirna_pc
18215	hsa-mir-92a-2	ZNF195	0.36741074	mirna_pc
18216	hsa-mir-92a-2	ZMYM4	0.326769799	mirna_pc
18217	hsa-mir-92a-2	PRKAR1B	0.396037445	mirna_pc
18218	hsa-mir-92a-2	MED4	0.313678959	mirna_pc
18219	hsa-mir-92a-2	LRRCC1	0.322707043	mirna_pc
18220	hsa-mir-92a-2	PPP5C	0.371323014	mirna_pc
18221	hsa-mir-92a-2	SF1	0.346046832	mirna_pc
18222	hsa-mir-92a-2	HNRNPH3	0.360712025	mirna_pc
18223	hsa-mir-92a-2	NAP1L1	0.306516532	mirna_pc
18224	hsa-mir-92a-2	PEX5	0.308176045	mirna_pc
18225	hsa-mir-92a-2	POLR1D	0.333291083	mirna_pc
18226	hsa-mir-92a-2	RRP1B	0.311404152	mirna_pc
18227	hsa-mir-92a-2	UBTF	0.314099905	mirna_pc
18228	hsa-mir-92a-2	NSL1	0.37690126	mirna_pc
18229	hsa-mir-92a-2	GSDMB	0.365881973	mirna_pc
18230	hsa-mir-92a-2	TMEM170A	0.315769423	mirna_pc
18231	hsa-mir-92a-2	PHF16	0.302635433	mirna_pc
18232	hsa-mir-92a-2	EPDR1	0.303311712	mirna_pc
18233	hsa-mir-92a-2	KIAA1383	0.311151477	mirna_pc
18234	hsa-mir-92a-2	LOC400657	0.327366265	mirna_pc
18235	hsa-mir-92a-2	PTPLAD1	0.308179352	mirna_pc
18236	hsa-mir-92a-2	FAM48A	0.431273608	mirna_pc
18237	hsa-mir-92a-2	PMS1	0.348244289	mirna_pc
18238	hsa-mir-92a-2	DDX19A	0.330305042	mirna_pc
18239	hsa-mir-92a-2	CHCHD3	0.369292671	mirna_pc
18240	hsa-mir-92a-2	CCDC122	0.33418445	mirna_pc
18241	hsa-mir-92a-2	TFDP2	0.323263833	mirna_pc
18242	hsa-mir-92a-2	ZMYM2	0.359035936	mirna_pc
18243	hsa-mir-92a-2	CCNJ	0.446365584	mirna_pc
18244	hsa-mir-92a-2	DCAF16	0.392139917	mirna_pc
18245	hsa-mir-92a-2	SBK1	0.426470448	mirna_pc
18246	hsa-mir-92a-2	PABPC4L	0.301879079	mirna_pc
18247	hsa-mir-92a-2	RPS5	0.320153324	mirna_pc
18248	hsa-mir-92a-2	ABCC4	0.325370665	mirna_pc
18249	hsa-mir-92a-2	KBTBD6	0.423469812	mirna_pc
18250	hsa-mir-92a-2	USP13	0.359813215	mirna_pc
18251	hsa-mir-92a-2	RASL10B	0.311284324	mirna_pc

18252	hsa-mir-92a-2	CLPB	0.301987415	mirna_pc
18253	hsa-mir-92a-2	SMARCC1	0.397179103	mirna_pc
18254	hsa-mir-92a-2	CTPS2	0.378355353	mirna_pc
18255	hsa-mir-92a-2	PACRGL	0.323309443	mirna_pc
18256	hsa-mir-92a-2	XPO4	0.414750292	mirna_pc
18257	hsa-mir-92a-2	DDX51	0.353590807	mirna_pc
18258	hsa-mir-92a-2	ZNF232	0.346720666	mirna_pc
18259	hsa-mir-92a-2	ZBTB39	0.426645041	mirna_pc
18260	hsa-mir-92a-2	CECR5	0.313665417	mirna_pc
18261	hsa-mir-92a-2	IFT88	0.39824679	mirna_pc
18262	hsa-mir-92a-2	ESD	0.336247413	mirna_pc
18263	hsa-mir-92a-2	FANCF	0.373608323	mirna_pc
18264	hsa-mir-92a-2	FAM169A	0.305511757	mirna_pc
18265	hsa-mir-92a-2	MARS2	0.302470671	mirna_pc
18266	hsa-mir-92a-2	VPS54	0.313163778	mirna_pc
18267	hsa-mir-92a-2	ZNF551	0.328133985	mirna_pc
18268	hsa-mir-92a-2	MYCL1	0.311400393	mirna_pc
18269	hsa-mir-92a-2	UBAC2	0.305838041	mirna_pc
18270	hsa-mir-92a-2	CIAO1	0.37714908	mirna_pc
18271	hsa-mir-92a-2	C2orf3	0.370937464	mirna_pc
18272	hsa-mir-92a-2	THAP4	0.308096751	mirna_pc
18273	hsa-mir-92a-2	POM121C	0.327438946	mirna_pc
18274	hsa-mir-92a-2	ZNF664	0.30603492	mirna_pc
18275	hsa-mir-92a-2	RRP7B	0.364503594	mirna_pc
18276	hsa-mir-92a-2	STK35	0.3188771	mirna_pc
18277	hsa-mir-92a-2	SCML2	0.396468814	mirna_pc
18278	hsa-mir-92a-2	MRPL19	0.398625434	mirna_pc
18279	hsa-mir-92a-2	ABCB10	0.302150372	mirna_pc
18280	hsa-mir-92a-2	ZCCHC3	0.463553063	mirna_pc
18281	hsa-mir-92a-2	RRP15	0.437107801	mirna_pc
18282	hsa-mir-92a-2	ETV5	0.423824292	mirna_pc
18283	hsa-mir-92a-2	DACT2	0.317906635	mirna_pc
18284	hsa-mir-92a-2	KIAA1430	0.326477833	mirna_pc
18285	hsa-mir-92a-2	AADAT	0.459962352	mirna_pc
18286	hsa-mir-92a-2	CBLL1	0.330106226	mirna_pc
18287	hsa-mir-92a-2	ACP1	0.352503328	mirna_pc
18288	hsa-mir-92a-2	C2orf43	0.33237271	mirna_pc
18289	hsa-mir-92a-2	DACH1	0.309823205	mirna_pc
18290	hsa-mir-92a-2	ACTR3B	0.401461048	mirna_pc
18291	hsa-mir-92a-2	NOM1	0.307195521	mirna_pc
18292	hsa-mir-92a-2	GTF3A	0.428193319	mirna_pc
18293	hsa-mir-92a-2	ZNF48	0.343768411	mirna_pc
18294	hsa-mir-92a-2	AIMP1	0.304749176	mirna_pc
18295	hsa-mir-92a-2	TPP2	0.355337107	mirna_pc
18296	hsa-mir-92a-2	TXLNG	0.355899523	mirna_pc
18297	hsa-mir-92a-2	TRIM25	0.335544289	mirna_pc
18298	hsa-mir-92a-2	B4GALNT4	0.315417857	mirna_pc
18299	hsa-mir-92a-2	HSDL1	0.341962556	mirna_pc
18300	hsa-mir-92a-2	PPP1R1B	0.325643365	mirna_pc
18301	hsa-mir-92a-2	ZNF530	0.303211354	mirna_pc
18302	hsa-mir-92a-2	NCRNA00188	0.351577118	mirna_pc
18303	hsa-mir-92a-2	MIR17HG	0.46890158	mirna_pc
18304	hsa-mir-92a-2	SMARCD2	0.303159831	mirna_pc
18305	hsa-mir-92a-2	MRPS23	0.316194777	mirna_pc

18306	hsa-mir-92a-2	SLC27A5	0.347534598	mirna_pc
18307	hsa-mir-92a-2	AARSD1	0.408244188	mirna_pc
18308	hsa-mir-92a-2	AARS	0.354615945	mirna_pc
18309	hsa-mir-92a-2	BOD1	0.382586332	mirna_pc
18310	hsa-mir-92a-2	KLHL23	0.353587123	mirna_pc
18311	hsa-mir-92a-2	SMARCAD1	0.393457418	mirna_pc
18312	hsa-mir-92a-2	VPS37D	0.300635485	mirna_pc
18313	hsa-mir-92a-2	MRPS31	0.361715938	mirna_pc
18314	hsa-mir-92a-2	CENPV	0.358713161	mirna_pc
18315	hsa-mir-92a-2	SLC4A5	0.312103142	mirna_pc
18316	hsa-mir-92a-2	DNMT3A	0.525916747	mirna_pc
18317	hsa-mir-92a-2	MIPEP	0.336566433	mirna_pc
18318	hsa-mir-92a-2	MGC57346	0.337142005	mirna_pc
18319	hsa-mir-92a-2	TET1	0.301325584	mirna_pc
18320	hsa-mir-92a-2	PRTFDC1	0.358470225	mirna_pc
18321	hsa-mir-92a-2	CARS2	0.35377413	mirna_pc
18322	hsa-mir-92a-2	PATZ1	0.432519768	mirna_pc
18323	hsa-mir-92a-2	ZNF670	0.337625115	mirna_pc
18324	hsa-mir-92a-2	ZNF343	0.340415293	mirna_pc
18325	hsa-mir-92a-2	ZNF711	0.300202698	mirna_pc
18326	hsa-mir-92a-2	POM121	0.363895071	mirna_pc
18327	hsa-mir-16-1	TPX2	0.390152391	mirna_pc
18328	hsa-mir-16-1	KPNA2	0.374340726	mirna_pc
18329	hsa-mir-16-1	RCC2	0.401490954	mirna_pc
18330	hsa-mir-16-1	HOXC9	0.475754313	mirna_pc
18331	hsa-mir-16-1	KIF11	0.397241647	mirna_pc
18332	hsa-mir-16-1	ECT2	0.366936892	mirna_pc
18333	hsa-mir-16-1	FOXM1	0.3064214	mirna_pc
18334	hsa-mir-16-1	RRM2	0.408771037	mirna_pc
18335	hsa-mir-16-1	SPC24	0.329146133	mirna_pc
18336	hsa-mir-16-1	CDK1	0.325789285	mirna_pc
18337	hsa-mir-16-1	CDC25C	0.38062879	mirna_pc
18338	hsa-mir-16-1	MYBL2	0.329846801	mirna_pc
18339	hsa-mir-16-1	PLK1	0.412335645	mirna_pc
18340	hsa-mir-16-1	PCNA	0.393004374	mirna_pc
18341	hsa-mir-16-1	UBE2C	0.415961871	mirna_pc
18342	hsa-mir-16-1	NUSAP1	0.389399898	mirna_pc
18343	hsa-mir-16-1	KIFC1	0.388586982	mirna_pc
18344	hsa-mir-16-1	BUB1B	0.344797696	mirna_pc
18345	hsa-mir-16-1	HDGF	0.392095111	mirna_pc
18346	hsa-mir-16-1	KIF18B	0.371748498	mirna_pc
18347	hsa-mir-16-1	KIF2C	0.382657053	mirna_pc
18348	hsa-mir-16-1	FANCA	0.350749627	mirna_pc
18349	hsa-mir-16-1	NCAPG	0.363922608	mirna_pc
18350	hsa-mir-16-1	CDC20	0.353098984	mirna_pc
18351	hsa-mir-16-1	SGOL1	0.317817191	mirna_pc
18352	hsa-mir-16-1	CCNA2	0.490578011	mirna_pc
18353	hsa-mir-16-1	SPC25	0.408754018	mirna_pc
18354	hsa-mir-16-1	FANCI	0.35777371	mirna_pc
18355	hsa-mir-16-1	NEK2	0.384616846	mirna_pc
18356	hsa-mir-16-1	MND1	0.512311068	mirna_pc
18357	hsa-mir-16-1	KIF22	0.427302572	mirna_pc
18358	hsa-mir-16-1	DDX39	0.406365125	mirna_pc
18359	hsa-mir-16-1	NCAPH	0.391765488	mirna_pc

18360	hsa-mir-16-1	GTSE1	0.333327562	mirna_pc
18361	hsa-mir-16-1	RAD54L	0.420519085	mirna_pc
18362	hsa-mir-16-1	CENPA	0.497457875	mirna_pc
18363	hsa-mir-16-1	MAD2L1	0.545528451	mirna_pc
18364	hsa-mir-16-1	TIMELESS	0.440424711	mirna_pc
18365	hsa-mir-16-1	LMNB1	0.395468916	mirna_pc
18366	hsa-mir-16-1	ASF1B	0.408027933	mirna_pc
18367	hsa-mir-16-1	CDCA8	0.378505818	mirna_pc
18368	hsa-mir-16-1	TROAP	0.454345705	mirna_pc
18369	hsa-mir-16-1	CCNB1	0.372692726	mirna_pc
18370	hsa-mir-16-1	CDCA5	0.312398942	mirna_pc
18371	hsa-mir-16-1	TACC3	0.346347082	mirna_pc
18372	hsa-mir-16-1	CDC45	0.373460246	mirna_pc
18373	hsa-mir-16-1	STMN1	0.503758111	mirna_pc
18374	hsa-mir-16-1	CENPM	0.447968499	mirna_pc
18375	hsa-mir-16-1	EXO1	0.314009577	mirna_pc
18376	hsa-mir-16-1	TK1	0.428419109	mirna_pc
18377	hsa-mir-16-1	UBE2T	0.391696343	mirna_pc
18378	hsa-mir-16-1	CKS2	0.342250162	mirna_pc
18379	hsa-mir-16-1	CKS1B	0.454080434	mirna_pc
18380	hsa-mir-16-1	TUBB	0.378786959	mirna_pc
18381	hsa-mir-16-1	MCM2	0.456062863	mirna_pc
18382	hsa-mir-16-1	KIAA0101	0.35018084	mirna_pc
18383	hsa-mir-16-1	ORC1L	0.323623914	mirna_pc
18384	hsa-mir-16-1	SNRPB	0.396317594	mirna_pc
18385	hsa-mir-16-1	KNTC1	0.326859048	mirna_pc
18386	hsa-mir-16-1	UHRF1	0.360986104	mirna_pc
18387	hsa-mir-16-1	CCNF	0.354266482	mirna_pc
18388	hsa-mir-16-1	RACGAP1	0.310503508	mirna_pc
18389	hsa-mir-16-1	PTBP1	0.346464504	mirna_pc
18390	hsa-mir-16-1	CCNB2	0.467229084	mirna_pc
18391	hsa-mir-16-1	NUF2	0.312590458	mirna_pc
18392	hsa-mir-16-1	PLK4	0.4184388	mirna_pc
18393	hsa-mir-16-1	KIF18A	0.341686839	mirna_pc
18394	hsa-mir-16-1	DEPDC1	0.317101489	mirna_pc
18395	hsa-mir-16-1	PTTG1	0.368951872	mirna_pc
18396	hsa-mir-16-1	SKA3	0.354332253	mirna_pc
18397	hsa-mir-16-1	HJURP	0.363971101	mirna_pc
18398	hsa-mir-16-1	RAD51	0.400756247	mirna_pc
18399	hsa-mir-16-1	BLM	0.346678569	mirna_pc
18400	hsa-mir-16-1	NME1	0.332149443	mirna_pc
18401	hsa-mir-16-1	PAICS	0.365219595	mirna_pc
18402	hsa-mir-16-1	BIRC5	0.497616924	mirna_pc
18403	hsa-mir-16-1	OIP5	0.389802595	mirna_pc
18404	hsa-mir-16-1	DTL	0.421575792	mirna_pc
18405	hsa-mir-16-1	EME1	0.412623018	mirna_pc
18406	hsa-mir-16-1	CENPK	0.345448237	mirna_pc
18407	hsa-mir-16-1	AURKB	0.442058426	mirna_pc
18408	hsa-mir-16-1	GSG2	0.35268619	mirna_pc
18409	hsa-mir-16-1	CDT1	0.432507037	mirna_pc
18410	hsa-mir-16-1	MCM10	0.328177703	mirna_pc
18411	hsa-mir-16-1	CDCA3	0.459459169	mirna_pc
18412	hsa-mir-16-1	C1orf112	0.360273124	mirna_pc
18413	hsa-mir-16-1	GINS1	0.311786541	mirna_pc

18414	hsa-mir-16-1	ALG3	0.410123301	mirna_pc
18415	hsa-mir-16-1	FEN1	0.455378755	mirna_pc
18416	hsa-mir-16-1	TRAIP	0.395786173	mirna_pc
18417	hsa-mir-16-1	ORC6L	0.382572182	mirna_pc
18418	hsa-mir-16-1	C16orf59	0.517757154	mirna_pc
18419	hsa-mir-16-1	C17orf53	0.324051721	mirna_pc
18420	hsa-mir-16-1	FAM72B	0.322362665	mirna_pc
18421	hsa-mir-16-1	NEIL3	0.384875109	mirna_pc
18422	hsa-mir-16-1	MCM7	0.367778229	mirna_pc
18423	hsa-mir-16-1	ACTL6A	0.417028408	mirna_pc
18424	hsa-mir-16-1	POLD1	0.3285222	mirna_pc
18425	hsa-mir-16-1	C12orf48	0.382275858	mirna_pc
18426	hsa-mir-16-1	HNRNPL	0.448515404	mirna_pc
18427	hsa-mir-16-1	SMC4	0.397569536	mirna_pc
18428	hsa-mir-16-1	PKMYT1	0.41393169	mirna_pc
18429	hsa-mir-16-1	SF3B4	0.313552387	mirna_pc
18430	hsa-mir-16-1	ZWINT	0.426371398	mirna_pc
18431	hsa-mir-16-1	TUBA1B	0.422537274	mirna_pc
18432	hsa-mir-16-1	RUVBL1	0.373170523	mirna_pc
18433	hsa-mir-16-1	BUB3	0.325978062	mirna_pc
18434	hsa-mir-16-1	UBE2S	0.456088983	mirna_pc
18435	hsa-mir-16-1	PSMA4	0.301522474	mirna_pc
18436	hsa-mir-16-1	NCAPD2	0.307324239	mirna_pc
18437	hsa-mir-16-1	HNRNPC	0.400817766	mirna_pc
18438	hsa-mir-16-1	PPIA	0.311632588	mirna_pc
18439	hsa-mir-16-1	ILF2	0.418025609	mirna_pc
18440	hsa-mir-16-1	TCF3	0.304329532	mirna_pc
18441	hsa-mir-16-1	TFRC	0.324512476	mirna_pc
18442	hsa-mir-16-1	MCM5	0.392219181	mirna_pc
18443	hsa-mir-16-1	RAN	0.353759676	mirna_pc
18444	hsa-mir-16-1	TYMS	0.385854795	mirna_pc
18445	hsa-mir-16-1	RFWD3	0.348984155	mirna_pc
18446	hsa-mir-16-1	C20orf20	0.331726762	mirna_pc
18447	hsa-mir-16-1	MCM6	0.377123592	mirna_pc
18448	hsa-mir-16-1	RNASEH2A	0.403170172	mirna_pc
18449	hsa-mir-16-1	EPR1	0.506258957	mirna_pc
18450	hsa-mir-16-1	PIF1	0.407068081	mirna_pc
18451	hsa-mir-16-1	H2AFX	0.480734262	mirna_pc
18452	hsa-mir-16-1	SFRS2	0.487743911	mirna_pc
18453	hsa-mir-16-1	UCK2	0.417151265	mirna_pc
18454	hsa-mir-16-1	EFTUD2	0.304029068	mirna_pc
18455	hsa-mir-16-1	THOC4	0.445625832	mirna_pc
18456	hsa-mir-16-1	NFKBIL2	0.356538887	mirna_pc
18457	hsa-mir-16-1	C15orf42	0.312892718	mirna_pc
18458	hsa-mir-16-1	CENPN	0.31554697	mirna_pc
18459	hsa-mir-16-1	PRIM2	0.30088759	mirna_pc
18460	hsa-mir-16-1	FAM60A	0.300293609	mirna_pc
18461	hsa-mir-16-1	HMGB3	0.317412784	mirna_pc
18462	hsa-mir-16-1	FAM64A	0.410356126	mirna_pc
18463	hsa-mir-16-1	RANBP1	0.388889972	mirna_pc
18464	hsa-mir-16-1	E2F1	0.471819127	mirna_pc
18465	hsa-mir-16-1	E2F2	0.314252071	mirna_pc
18466	hsa-mir-16-1	SNRPG	0.39068897	mirna_pc
18467	hsa-mir-16-1	MCM3	0.341463413	mirna_pc

18468	hsa-mir-16-1	SNRPA	0.510207936	mirna_pc
18469	hsa-mir-16-1	FUS	0.423370891	mirna_pc
18470	hsa-mir-16-1	DNAJC9	0.360688055	mirna_pc
18471	hsa-mir-16-1	U2AF2	0.339310415	mirna_pc
18472	hsa-mir-16-1	RFC3	0.336520816	mirna_pc
18473	hsa-mir-16-1	CHAF1A	0.413152209	mirna_pc
18474	hsa-mir-16-1	POC1A	0.358885538	mirna_pc
18475	hsa-mir-16-1	CENPO	0.538103676	mirna_pc
18476	hsa-mir-16-1	C19orf48	0.41714286	mirna_pc
18477	hsa-mir-16-1	NUDT1	0.496592245	mirna_pc
18478	hsa-mir-16-1	HMGB2	0.414124705	mirna_pc
18479	hsa-mir-16-1	GINS2	0.49963142	mirna_pc
18480	hsa-mir-16-1	NOP56	0.367642307	mirna_pc
18481	hsa-mir-16-1	SNRPD1	0.347079985	mirna_pc
18482	hsa-mir-16-1	SNHG1	0.305046684	mirna_pc
18483	hsa-mir-16-1	DTYMK	0.396228013	mirna_pc
18484	hsa-mir-16-1	PRMT1	0.363982282	mirna_pc
18485	hsa-mir-16-1	MLF1IP	0.374391891	mirna_pc
18486	hsa-mir-16-1	C16orf75	0.48223364	mirna_pc
18487	hsa-mir-16-1	TCF19	0.336743447	mirna_pc
18488	hsa-mir-16-1	CAD	0.347560379	mirna_pc
18489	hsa-mir-16-1	ZFP64	0.361294599	mirna_pc
18490	hsa-mir-16-1	CDK2	0.396599102	mirna_pc
18491	hsa-mir-16-1	CDK4	0.459497674	mirna_pc
18492	hsa-mir-16-1	CHTF18	0.363896241	mirna_pc
18493	hsa-mir-16-1	DLEU2	0.373998909	mirna_pc
18494	hsa-mir-16-1	KAT2A	0.343897485	mirna_pc
18495	hsa-mir-16-1	CENPH	0.412866735	mirna_pc
18496	hsa-mir-16-1	RALY	0.343063308	mirna_pc
18497	hsa-mir-16-1	MSH2	0.302292592	mirna_pc
18498	hsa-mir-16-1	PRPF19	0.325477592	mirna_pc
18499	hsa-mir-16-1	SF3B3	0.453031232	mirna_pc
18500	hsa-mir-16-1	CDC7	0.310836866	mirna_pc
18501	hsa-mir-16-1	YDJC	0.3169221	mirna_pc
18502	hsa-mir-16-1	PUS1	0.361859554	mirna_pc
18503	hsa-mir-16-1	SNRPC	0.377859105	mirna_pc
18504	hsa-mir-16-1	HAUS8	0.310265671	mirna_pc
18505	hsa-mir-16-1	PSMB4	0.326500922	mirna_pc
18506	hsa-mir-16-1	RFC4	0.517418472	mirna_pc
18507	hsa-mir-16-1	FBXO5	0.353588709	mirna_pc
18508	hsa-mir-16-1	FIGNL1	0.312995793	mirna_pc
18509	hsa-mir-16-1	MDK	0.316504134	mirna_pc
18510	hsa-mir-16-1	CDC25A	0.338060113	mirna_pc
18511	hsa-mir-16-1	TOPBP1	0.324286335	mirna_pc
18512	hsa-mir-16-1	H2AFZ	0.539895826	mirna_pc
18513	hsa-mir-16-1	NRM	0.312834703	mirna_pc
18514	hsa-mir-16-1	PRR7	0.309799689	mirna_pc
18515	hsa-mir-16-1	MAGOH	0.33900742	mirna_pc
18516	hsa-mir-16-1	DDX12	0.316599355	mirna_pc
18517	hsa-mir-16-1	C13orf34	0.300081364	mirna_pc
18518	hsa-mir-16-1	CLN6	0.314526467	mirna_pc
18519	hsa-mir-16-1	MCM8	0.385400303	mirna_pc
18520	hsa-mir-16-1	CPSF3	0.329962934	mirna_pc
18521	hsa-mir-16-1	BANF1	0.333902423	mirna_pc

18522	hsa-mir-16-1	SNRPE	0.393053393	mirna_pc
18523	hsa-mir-16-1	C21orf45	0.369527985	mirna_pc
18524	hsa-mir-16-1	PPM1G	0.449105542	mirna_pc
18525	hsa-mir-16-1	DNAJB11	0.438084599	mirna_pc
18526	hsa-mir-16-1	MARCKSL1	0.388720531	mirna_pc
18527	hsa-mir-16-1	SNRPD2	0.324960357	mirna_pc
18528	hsa-mir-16-1	NPM3	0.304849732	mirna_pc
18529	hsa-mir-16-1	TFAP4	0.450729382	mirna_pc
18530	hsa-mir-16-1	NUTF2	0.349438049	mirna_pc
18531	hsa-mir-16-1	RNPS1	0.40805919	mirna_pc
18532	hsa-mir-16-1	DAZAP1	0.394835166	mirna_pc
18533	hsa-mir-16-1	CBX8	0.402841322	mirna_pc
18534	hsa-mir-16-1	RPA3	0.307409597	mirna_pc
18535	hsa-mir-16-1	WDR34	0.324543556	mirna_pc
18536	hsa-mir-16-1	TMEM201	0.368023226	mirna_pc
18537	hsa-mir-16-1	MRPL9	0.35729896	mirna_pc
18538	hsa-mir-16-1	PAFAH1B3	0.404139469	mirna_pc
18539	hsa-mir-16-1	SRRT	0.364426957	mirna_pc
18540	hsa-mir-16-1	DCTPP1	0.467646774	mirna_pc
18541	hsa-mir-16-1	PSRC1	0.435389491	mirna_pc
18542	hsa-mir-16-1	DGUOK	0.401838801	mirna_pc
18543	hsa-mir-16-1	MRPL47	0.388568714	mirna_pc
18544	hsa-mir-16-1	DBF4B	0.327428574	mirna_pc
18545	hsa-mir-16-1	POU2F1	0.335899845	mirna_pc
18546	hsa-mir-16-1	FBL	0.367684357	mirna_pc
18547	hsa-mir-16-1	HIST1H1E	0.392975772	mirna_pc
18548	hsa-mir-16-1	PSMG3	0.310077211	mirna_pc
18549	hsa-mir-16-1	THOP1	0.324559654	mirna_pc
18550	hsa-mir-16-1	EXOSC5	0.347799303	mirna_pc
18551	hsa-mir-16-1	HNRNPM	0.416518679	mirna_pc
18552	hsa-mir-16-1	TMPO	0.335142903	mirna_pc
18553	hsa-mir-16-1	PRCC	0.395415377	mirna_pc
18554	hsa-mir-16-1	POLR2H	0.433263818	mirna_pc
18555	hsa-mir-16-1	VBP1	0.300351521	mirna_pc
18556	hsa-mir-16-1	C3orf26	0.386974309	mirna_pc
18557	hsa-mir-16-1	GPR19	0.327905104	mirna_pc
18558	hsa-mir-16-1	LSM 7.00	0.308360128	mirna_pc
18559	hsa-mir-16-1	LSG1	0.386414432	mirna_pc
18560	hsa-mir-16-1	GABPB1	0.348781125	mirna_pc
18561	hsa-mir-16-1	PPIH	0.419668319	mirna_pc
18562	hsa-mir-16-1	THOC6	0.329698783	mirna_pc
18563	hsa-mir-16-1	GMPS	0.346699589	mirna_pc
18564	hsa-mir-16-1	RCN1	0.34025083	mirna_pc
18565	hsa-mir-16-1	LOC100128191	0.430320907	mirna_pc
18566	hsa-mir-16-1	MRPL3	0.374620942	mirna_pc
18567	hsa-mir-16-1	HMGN2	0.354593095	mirna_pc
18568	hsa-mir-16-1	ETV4	0.355743695	mirna_pc
18569	hsa-mir-16-1	SMYD5	0.394133516	mirna_pc
18570	hsa-mir-16-1	SF3A2	0.309883027	mirna_pc
18571	hsa-mir-16-1	TSSC1	0.354949967	mirna_pc
18572	hsa-mir-16-1	POLA2	0.332608492	mirna_pc
18573	hsa-mir-16-1	SNRPF	0.369859841	mirna_pc
18574	hsa-mir-16-1	SUMO2	0.351133604	mirna_pc
18575	hsa-mir-16-1	SFRS1	0.401856353	mirna_pc

18576	hsa-mir-16-1	CCDC58	0.369143653	mirna_pc
18577	hsa-mir-16-1	SKP2	0.343923565	mirna_pc
18578	hsa-mir-16-1	UBE2I	0.386669501	mirna_pc
18579	hsa-mir-16-1	TIPIN	0.325510447	mirna_pc
18580	hsa-mir-16-1	POLD2	0.354948893	mirna_pc
18581	hsa-mir-16-1	SMARCD1	0.340600566	mirna_pc
18582	hsa-mir-16-1	ATXN2L	0.312531841	mirna_pc
18583	hsa-mir-16-1	HSPBP1	0.335490654	mirna_pc
18584	hsa-mir-16-1	C4orf46	0.419416984	mirna_pc
18585	hsa-mir-16-1	ECE2	0.406933332	mirna_pc
18586	hsa-mir-16-1	GPN1	0.366255367	mirna_pc
18587	hsa-mir-16-1	RFC5	0.353644097	mirna_pc
18588	hsa-mir-16-1	MAZ	0.380525831	mirna_pc
18589	hsa-mir-16-1	SSRP1	0.422399017	mirna_pc
18590	hsa-mir-16-1	POLR2D	0.40871224	mirna_pc
18591	hsa-mir-16-1	FTSJ2	0.396353555	mirna_pc
18592	hsa-mir-16-1	PA2G4	0.348575626	mirna_pc
18593	hsa-mir-16-1	SNRPD3	0.357080518	mirna_pc
18594	hsa-mir-16-1	KHDRBS1	0.411303039	mirna_pc
18595	hsa-mir-16-1	NXT1	0.356957031	mirna_pc
18596	hsa-mir-16-1	SLC5A6	0.361936208	mirna_pc
18597	hsa-mir-16-1	C20orf27	0.36706445	mirna_pc
18598	hsa-mir-16-1	LOC92659	0.339985494	mirna_pc
18599	hsa-mir-16-1	MTL 5.00	0.440120996	mirna_pc
18600	hsa-mir-16-1	AHCY	0.303462693	mirna_pc
18601	hsa-mir-16-1	CCT7	0.336499403	mirna_pc
18602	hsa-mir-16-1	C4orf21	0.328073691	mirna_pc
18603	hsa-mir-16-1	SNRNP40	0.329550386	mirna_pc
18604	hsa-mir-16-1	METTL1	0.346945789	mirna_pc
18605	hsa-mir-16-1	GINS3	0.322128772	mirna_pc
18606	hsa-mir-16-1	C6orf129	0.403748103	mirna_pc
18607	hsa-mir-16-1	TIMM8A	0.307160652	mirna_pc
18608	hsa-mir-16-1	WRAP53	0.351108396	mirna_pc
18609	hsa-mir-16-1	NASP	0.313953555	mirna_pc
18610	hsa-mir-16-1	LAGE3	0.307868407	mirna_pc
18611	hsa-mir-16-1	NONO	0.367898129	mirna_pc
18612	hsa-mir-16-1	CCDC86	0.416765612	mirna_pc
18613	hsa-mir-16-1	OBFC2B	0.403989732	mirna_pc
18614	hsa-mir-16-1	PTMA	0.448842283	mirna_pc
18615	hsa-mir-16-1	USP39	0.379409291	mirna_pc
18616	hsa-mir-16-1	LSM 4.00	0.360839297	mirna_pc
18617	hsa-mir-16-1	C11orf84	0.391980586	mirna_pc
18618	hsa-mir-16-1	HIST1H2BK	0.316126525	mirna_pc
18619	hsa-mir-16-1	NCBP2	0.362276755	mirna_pc
18620	hsa-mir-16-1	SUV39H2	0.349057217	mirna_pc
18621	hsa-mir-16-1	LSM 2.00	0.401991798	mirna_pc
18622	hsa-mir-16-1	RIBC2	0.314559927	mirna_pc
18623	hsa-mir-16-1	HNRNPD	0.42523245	mirna_pc
18624	hsa-mir-16-1	DPY30	0.399699518	mirna_pc
18625	hsa-mir-16-1	MTHFD2	0.380971226	mirna_pc
18626	hsa-mir-16-1	POC5	0.306061096	mirna_pc
18627	hsa-mir-16-1	SFRS3	0.3971023	mirna_pc
18628	hsa-mir-16-1	SCAMP3	0.323737752	mirna_pc
18629	hsa-mir-16-1	C17orf49	0.357428261	mirna_pc

18630	hsa-mir-16-1	H1FX	0.46771519	mirna_pc
18631	hsa-mir-16-1	CCT4	0.361586058	mirna_pc
18632	hsa-mir-16-1	UMPS	0.370138164	mirna_pc
18633	hsa-mir-16-1	SPNS1	0.317583475	mirna_pc
18634	hsa-mir-16-1	ELAVL1	0.43208365	mirna_pc
18635	hsa-mir-16-1	RPN1	0.330772828	mirna_pc
18636	hsa-mir-16-1	WDR18	0.312036411	mirna_pc
18637	hsa-mir-16-1	HSP90B1	0.300498569	mirna_pc
18638	hsa-mir-16-1	PSMC3IP	0.318952654	mirna_pc
18639	hsa-mir-16-1	TBC1D7	0.348443019	mirna_pc
18640	hsa-mir-16-1	E2F4	0.35767979	mirna_pc
18641	hsa-mir-16-1	PSMD2	0.319035775	mirna_pc
18642	hsa-mir-16-1	PMAIP1	0.320875205	mirna_pc
18643	hsa-mir-16-1	YWHAE	0.338001579	mirna_pc
18644	hsa-mir-16-1	DHFR	0.324155392	mirna_pc
18645	hsa-mir-16-1	ILF3	0.341430458	mirna_pc
18646	hsa-mir-16-1	NT5DC2	0.317703946	mirna_pc
18647	hsa-mir-16-1	EHMT2	0.387909023	mirna_pc
18648	hsa-mir-16-1	HNRNPAB	0.320312197	mirna_pc
18649	hsa-mir-16-1	UBAP2L	0.317932757	mirna_pc
18650	hsa-mir-16-1	SFRS9	0.32853519	mirna_pc
18651	hsa-mir-16-1	COMMD4	0.396793209	mirna_pc
18652	hsa-mir-16-1	FAM100B	0.310969109	mirna_pc
18653	hsa-mir-16-1	PPIB	0.304809553	mirna_pc
18654	hsa-mir-16-1	HEATR2	0.371882204	mirna_pc
18655	hsa-mir-16-1	LSM 6.00	0.48420333	mirna_pc
18656	hsa-mir-16-1	PRAME	0.379226151	mirna_pc
18657	hsa-mir-16-1	AZI1	0.303149712	mirna_pc
18658	hsa-mir-16-1	C7orf27	0.308856535	mirna_pc
18659	hsa-mir-16-1	WDR74	0.358546285	mirna_pc
18660	hsa-mir-16-1	SUV39H1	0.449505889	mirna_pc
18661	hsa-mir-16-1	POLR2G	0.300232389	mirna_pc
18662	hsa-mir-16-1	TRA2B	0.493675583	mirna_pc
18663	hsa-mir-16-1	RSRC1	0.374710954	mirna_pc
18664	hsa-mir-16-1	GAR1	0.370761829	mirna_pc
18665	hsa-mir-16-1	C15orf23	0.349340743	mirna_pc
18666	hsa-mir-16-1	CDRT4	0.323875227	mirna_pc
18667	hsa-mir-16-1	C3orf21	0.374026391	mirna_pc
18668	hsa-mir-16-1	NOC4L	0.382079513	mirna_pc
18669	hsa-mir-16-1	DIABLO	0.352924297	mirna_pc
18670	hsa-mir-16-1	IGF2BP2	0.362011531	mirna_pc
18671	hsa-mir-16-1	LIMD2	0.303692212	mirna_pc
18672	hsa-mir-16-1	ITGB3BP	0.339019866	mirna_pc
18673	hsa-mir-16-1	ENOPH1	0.328255898	mirna_pc
18674	hsa-mir-16-1	LAS1L	0.326723285	mirna_pc
18675	hsa-mir-16-1	C4orf43	0.317395612	mirna_pc
18676	hsa-mir-16-1	TUBG1	0.30145782	mirna_pc
18677	hsa-mir-16-1	C16orf80	0.381043562	mirna_pc
18678	hsa-mir-16-1	PLEKHG4	0.315311029	mirna_pc
18679	hsa-mir-16-1	SFPQ	0.382052656	mirna_pc
18680	hsa-mir-16-1	VPS72	0.339670413	mirna_pc
18681	hsa-mir-16-1	CD83	0.376007229	mirna_pc
18682	hsa-mir-16-1	RCOR2	0.331992755	mirna_pc
18683	hsa-mir-16-1	SHMT2	0.321029743	mirna_pc

18684	hsa-mir-16-1	HDAC2	0.325891801	mirna_pc
18685	hsa-mir-16-1	CISD2	0.321248749	mirna_pc
18686	hsa-mir-16-1	MRPL12	0.323856939	mirna_pc
18687	hsa-mir-16-1	GMNN	0.330492139	mirna_pc
18688	hsa-mir-16-1	EMG1	0.313654911	mirna_pc
18689	hsa-mir-16-1	PA2G4P4	0.343424345	mirna_pc
18690	hsa-mir-16-1	C10orf119	0.300696899	mirna_pc
18691	hsa-mir-16-1	WDR53	0.30320021	mirna_pc
18692	hsa-mir-16-1	RNF7	0.349653693	mirna_pc
18693	hsa-mir-16-1	GEMIN6	0.305324889	mirna_pc
18694	hsa-mir-16-1	MRPL11	0.379503186	mirna_pc
18695	hsa-mir-16-1	CRIPT	0.312387975	mirna_pc
18696	hsa-mir-16-1	COX4NB	0.35029904	mirna_pc
18697	hsa-mir-16-1	MSH6	0.402149253	mirna_pc
18698	hsa-mir-16-1	TAF6L	0.331297495	mirna_pc
18699	hsa-mir-16-1	LEF1	0.323740406	mirna_pc
18700	hsa-mir-16-1	ACBD6	0.320975386	mirna_pc
18701	hsa-mir-16-1	HNRNPA1	0.374320797	mirna_pc
18702	hsa-mir-16-1	NPM1	0.315390921	mirna_pc
18703	hsa-mir-16-1	NUDT3	0.314025033	mirna_pc
18704	hsa-mir-16-1	MEA1	0.318008947	mirna_pc
18705	hsa-mir-16-1	MEX3A	0.437910165	mirna_pc
18706	hsa-mir-16-1	MOGS	0.311673467	mirna_pc
18707	hsa-mir-16-1	COIL	0.307153417	mirna_pc
18708	hsa-mir-16-1	ERAL1	0.358225134	mirna_pc
18709	hsa-mir-16-1	C12orf47	0.303377633	mirna_pc
18710	hsa-mir-16-1	TAF5	0.443649672	mirna_pc
18711	hsa-mir-16-1	TRIM28	0.373768689	mirna_pc
18712	hsa-mir-16-1	TIFA	0.343004105	mirna_pc
18713	hsa-mir-16-1	MAD2L2	0.301447032	mirna_pc
18714	hsa-mir-16-1	HNRNPA1L2	0.408119681	mirna_pc
18715	hsa-mir-16-1	POLR3K	0.352762055	mirna_pc
18716	hsa-mir-16-1	CBX1	0.311841677	mirna_pc
18717	hsa-mir-16-1	TMEM39B	0.328659742	mirna_pc
18718	hsa-mir-16-1	ZBTB12	0.327224733	mirna_pc
18719	hsa-mir-16-1	MRPL51	0.327177992	mirna_pc
18720	hsa-mir-16-1	MCTP2	0.308507603	mirna_pc
18721	hsa-mir-16-1	TGIF2	0.34184882	mirna_pc
18722	hsa-mir-16-1	ERI3	0.381939635	mirna_pc
18723	hsa-mir-16-1	RCN2	0.324023051	mirna_pc
18724	hsa-mir-16-1	CIB2	0.311771415	mirna_pc
18725	hsa-mir-16-1	FAM96A	0.303737365	mirna_pc
18726	hsa-mir-16-1	TARBP2	0.368488384	mirna_pc
18727	hsa-mir-16-1	TOMM22	0.315235745	mirna_pc
18728	hsa-mir-16-1	PHB	0.359114412	mirna_pc
18729	hsa-mir-16-1	C12orf52	0.332782222	mirna_pc
18730	hsa-mir-16-1	NOL10	0.329607626	mirna_pc
18731	hsa-mir-16-1	POLE	0.409627947	mirna_pc
18732	hsa-mir-16-1	MUTYH	0.308795913	mirna_pc
18733	hsa-mir-16-1	HMBS	0.323519776	mirna_pc
18734	hsa-mir-16-1	TIMM50	0.301551206	mirna_pc
18735	hsa-mir-16-1	TMEM216	0.362939635	mirna_pc
18736	hsa-mir-16-1	RBMX2	0.301984149	mirna_pc
18737	hsa-mir-16-1	RBM14	0.330294274	mirna_pc

18738	hsa-mir-16-1	EXOSC9	0.415547899	mirna_pc
18739	hsa-mir-16-1	MXD3	0.311562615	mirna_pc
18740	hsa-mir-16-1	UNG	0.312420761	mirna_pc
18741	hsa-mir-16-1	GRK6	0.342772885	mirna_pc
18742	hsa-mir-16-1	RBMX	0.450111033	mirna_pc
18743	hsa-mir-16-1	TSEN15	0.307454473	mirna_pc
18744	hsa-mir-16-1	CDK16	0.44303118	mirna_pc
18745	hsa-mir-16-1	ALKBH2	0.377158346	mirna_pc
18746	hsa-mir-16-1	USP10	0.319646534	mirna_pc
18747	hsa-mir-16-1	NTHL1	0.366740758	mirna_pc
18748	hsa-mir-16-1	PRKCSH	0.338258305	mirna_pc
18749	hsa-mir-16-1	MLF 2.00	0.306784149	mirna_pc
18750	hsa-mir-16-1	CTCF	0.431118995	mirna_pc
18751	hsa-mir-16-1	C11orf48	0.310884989	mirna_pc
18752	hsa-mir-16-1	RPL39L	0.447703573	mirna_pc
18753	hsa-mir-16-1	KATNB1	0.309945751	mirna_pc
18754	hsa-mir-16-1	ACD	0.398048021	mirna_pc
18755	hsa-mir-16-1	LOC152217	0.452503031	mirna_pc
18756	hsa-mir-16-1	MRPL37	0.309064924	mirna_pc
18757	hsa-mir-16-1	CCDC23	0.338698903	mirna_pc
18758	hsa-mir-16-1	IGSF9	0.346100612	mirna_pc
18759	hsa-mir-16-1	NOB1	0.336390206	mirna_pc
18760	hsa-mir-16-1	DNAJC14	0.345256547	mirna_pc
18761	hsa-mir-16-1	FIP1L1	0.343008563	mirna_pc
18762	hsa-mir-16-1	KDM2B	0.300796353	mirna_pc
18763	hsa-mir-16-1	TRAP1	0.354706878	mirna_pc
18764	hsa-mir-16-1	GTF3C2	0.314143942	mirna_pc
18765	hsa-mir-16-1	MAGEF1	0.423912584	mirna_pc
18766	hsa-mir-16-1	ICT1	0.330298727	mirna_pc
18767	hsa-mir-16-1	OSTC	0.335302368	mirna_pc
18768	hsa-mir-16-1	FAM136A	0.354681248	mirna_pc
18769	hsa-mir-16-1	ZNF511	0.327797501	mirna_pc
18770	hsa-mir-16-1	SUPT3H	0.30820984	mirna_pc
18771	hsa-mir-16-1	SOX12	0.340691341	mirna_pc
18772	hsa-mir-16-1	SMARCB1	0.333259122	mirna_pc
18773	hsa-mir-16-1	B3GAT3	0.362413582	mirna_pc
18774	hsa-mir-16-1	SMN2	0.302076542	mirna_pc
18775	hsa-mir-16-1	ZNF639	0.391309691	mirna_pc
18776	hsa-mir-16-1	TIMM10	0.326259599	mirna_pc
18777	hsa-mir-16-1	ITPRIPL1	0.362374794	mirna_pc
18778	hsa-mir-16-1	HSD17B10	0.352056271	mirna_pc
18779	hsa-mir-16-1	ABT1	0.304294489	mirna_pc
18780	hsa-mir-16-1	RPS7	0.306775249	mirna_pc
18781	hsa-mir-16-1	CSNK2A1	0.331596514	mirna_pc
18782	hsa-mir-16-1	STRA13	0.509939618	mirna_pc
18783	hsa-mir-16-1	DVL3	0.303819987	mirna_pc
18784	hsa-mir-16-1	BCL7A	0.360394741	mirna_pc
18785	hsa-mir-16-1	TIMM16	0.328428441	mirna_pc
18786	hsa-mir-16-1	MTX1	0.316039449	mirna_pc
18787	hsa-mir-16-1	TRPA1	0.385884852	mirna_pc
18788	hsa-mir-16-1	SFXN4	0.324765192	mirna_pc
18789	hsa-mir-16-1	MRPL21	0.449998523	mirna_pc
18790	hsa-mir-16-1	NLE1	0.337037639	mirna_pc
18791	hsa-mir-16-1	TMEM180	0.319910023	mirna_pc

18792	hsa-mir-16-1	CUEDC2	0.344705994	mirna_pc
18793	hsa-mir-16-1	PSPH	0.32079861	mirna_pc
18794	hsa-mir-16-1	TMEM106C	0.301159463	mirna_pc
18795	hsa-mir-16-1	TRMT61B	0.300791572	mirna_pc
18796	hsa-mir-16-1	TSFM	0.316069364	mirna_pc
18797	hsa-mir-16-1	GANAB	0.357136689	mirna_pc
18798	hsa-mir-16-1	ANAPC11	0.33301515	mirna_pc
18799	hsa-mir-16-1	ZNF286B	0.365646951	mirna_pc
18800	hsa-mir-16-1	PDF	0.376823253	mirna_pc
18801	hsa-mir-16-1	EEFSEC	0.318390878	mirna_pc
18802	hsa-mir-16-1	CHCHD2	0.311140308	mirna_pc
18803	hsa-mir-16-1	PIGX	0.319037436	mirna_pc
18804	hsa-mir-16-1	PFN2	0.318430494	mirna_pc
18805	hsa-mir-16-1	COPZ1	0.354480866	mirna_pc
18806	hsa-mir-16-1	GPR3	0.326593402	mirna_pc
18807	hsa-mir-16-1	E2F6	0.373322492	mirna_pc
18808	hsa-mir-16-1	PRPSAP2	0.325523829	mirna_pc
18809	hsa-mir-16-1	B4GALT3	0.330613356	mirna_pc
18810	hsa-mir-16-1	CWF19L1	0.308079268	mirna_pc
18811	hsa-mir-16-1	EXOSC1	0.360310179	mirna_pc
18812	hsa-mir-16-1	SCLY	0.413287227	mirna_pc
18813	hsa-mir-16-1	NUP35	0.337821268	mirna_pc
18814	hsa-mir-16-1	CTU2	0.336135947	mirna_pc
18815	hsa-mir-16-1	DHRS13	0.404387084	mirna_pc
18816	hsa-mir-16-1	KRTCAP3	0.33310841	mirna_pc
18817	hsa-mir-16-1	MRPS34	0.300228496	mirna_pc
18818	hsa-mir-16-1	SMC1A	0.30332278	mirna_pc
18819	hsa-mir-16-1	ALX3	0.334754418	mirna_pc
18820	hsa-mir-16-1	AAAS	0.413289364	mirna_pc
18821	hsa-mir-16-1	C2orf79	0.394642698	mirna_pc
18822	hsa-mir-16-1	SUPT7L	0.318988773	mirna_pc
18823	hsa-mir-16-1	SMUG1	0.33644319	mirna_pc
18824	hsa-mir-16-1	MRPS26	0.409990633	mirna_pc
18825	hsa-mir-16-1	MRPL30	0.310725554	mirna_pc
18826	hsa-mir-16-1	PELP1	0.313104539	mirna_pc
18827	hsa-mir-16-1	NSMCE4A	0.309108298	mirna_pc
18828	hsa-mir-16-1	PGP	0.305698838	mirna_pc
18829	hsa-mir-16-1	PYCR1	0.340418999	mirna_pc
18830	hsa-mir-16-1	DHODH	0.335682812	mirna_pc
18831	hsa-mir-16-1	SFRS7	0.46371371	mirna_pc
18832	hsa-mir-16-1	C18orf10	0.316942636	mirna_pc
18833	hsa-mir-16-1	EMD	0.300674426	mirna_pc
18834	hsa-mir-16-1	CA5BP	0.336842488	mirna_pc
18835	hsa-mir-16-1	PCBP2	0.314153606	mirna_pc
18836	hsa-mir-16-1	UBE2O	0.30936176	mirna_pc
18837	hsa-mir-16-1	C10orf35	0.398696405	mirna_pc
18838	hsa-mir-16-1	LRRC45	0.372234031	mirna_pc
18839	hsa-mir-16-1	RBM10	0.456481774	mirna_pc
18840	hsa-mir-16-1	RPUSD2	0.322211751	mirna_pc
18841	hsa-mir-16-1	EIF2B5	0.320997891	mirna_pc
18842	hsa-mir-16-1	PARL	0.417443245	mirna_pc
18843	hsa-mir-16-1	ARHGEF19	0.300766753	mirna_pc
18844	hsa-mir-16-1	C17orf75	0.387923245	mirna_pc
18845	hsa-mir-16-1	RPL35A	0.385328006	mirna_pc

18846	hsa-mir-16-1	CCND1		0.351704833	mirna_pc
18847	hsa-mir-16-1	NFATC3		0.345557068	mirna_pc
18848	hsa-mir-16-1	ZNF771		0.440130416	mirna_pc
18849	hsa-mir-16-1		3-Sep	0.325879165	mirna_pc
18850	hsa-mir-16-1	MRPL38		0.386954232	mirna_pc
18851	hsa-mir-16-1	FN3KRP		0.303141542	mirna_pc
18852	hsa-mir-16-1	TCTN2		0.346541741	mirna_pc
18853	hsa-mir-16-1	POLR2C		0.353601135	mirna_pc
18854	hsa-mir-16-1	PPP2R3B		0.31057432	mirna_pc
18855	hsa-mir-16-1	C12orf10		0.358279073	mirna_pc
18856	hsa-mir-16-1	UBE2M		0.355968608	mirna_pc
18857	hsa-mir-16-1	C12orf45		0.325061069	mirna_pc
18858	hsa-mir-16-1	C2orf44		0.392812147	mirna_pc
18859	hsa-mir-16-1	TIMM17B		0.388889972	mirna_pc
18860	hsa-mir-16-1	SEPHS1		0.388751706	mirna_pc
18861	hsa-mir-16-1	C11orf10		0.30172119	mirna_pc
18862	hsa-mir-16-1	ZNF259		0.373139474	mirna_pc
18863	hsa-mir-16-1	MLST8		0.316648295	mirna_pc
18864	hsa-mir-16-1	C16orf13		0.300904571	mirna_pc
18865	hsa-mir-16-1	AGBL5		0.438654081	mirna_pc
18866	hsa-mir-16-1	C17orf89		0.303019554	mirna_pc
18867	hsa-mir-16-1	PHB2		0.356902207	mirna_pc
18868	hsa-mir-16-1	GPS1		0.334932091	mirna_pc
18869	hsa-mir-16-1	MYL6B		0.441182653	mirna_pc
18870	hsa-mir-16-1	C7orf50		0.32086692	mirna_pc
18871	hsa-mir-16-1	SENP5		0.323250697	mirna_pc
18872	hsa-mir-16-1	VAC14		0.318628543	mirna_pc
18873	hsa-mir-16-1	CD200		0.31023761	mirna_pc
18874	hsa-mir-16-1	C20orf196		0.355858889	mirna_pc
18875	hsa-mir-16-1	NME4		0.316739946	mirna_pc
18876	hsa-mir-16-1	LYRM4		0.347683155	mirna_pc
18877	hsa-mir-16-1	BCL11A		0.305415219	mirna_pc
18878	hsa-mir-16-1	DDX28		0.320789079	mirna_pc
18879	hsa-mir-16-1	COPS7B		0.334065281	mirna_pc
18880	hsa-mir-16-1	USP21		0.380641476	mirna_pc
18881	hsa-mir-16-1	DPCD		0.315021369	mirna_pc
18882	hsa-mir-16-1	ANP32A		0.464029253	mirna_pc
18883	hsa-mir-16-1	NUFIP1		0.330963659	mirna_pc
18884	hsa-mir-16-1	ATP5G2		0.328255926	mirna_pc
18885	hsa-mir-16-1	LMAN2L		0.32279729	mirna_pc
18886	hsa-mir-16-1	FAM104B		0.313272489	mirna_pc
18887	hsa-mir-16-1	C17orf81		0.38983934	mirna_pc
18888	hsa-mir-16-1	SPCS2		0.321471788	mirna_pc
18889	hsa-mir-16-1	BUD13		0.464188675	mirna_pc
18890	hsa-mir-16-1	MORN2		0.376183108	mirna_pc
18891	hsa-mir-16-1	NAIF1		0.305591806	mirna_pc
18892	hsa-mir-16-1	TFDP2		0.302092151	mirna_pc
18893	hsa-mir-16-1	SBK1		0.387772095	mirna_pc
18894	hsa-mir-16-1	PABPC4L		0.315281386	mirna_pc
18895	hsa-mir-16-1	PMF1		0.416443354	mirna_pc
18896	hsa-mir-16-1	CD69		0.354855781	mirna_pc
18897	hsa-mir-16-1	C12orf44		0.306013771	mirna_pc
18898	hsa-mir-16-1	SLC25A33		0.335294726	mirna_pc
18899	hsa-mir-16-1	MSI1		0.320664722	mirna_pc

18900	hsa-mir-16-1	CLPB	0.319787563	mirna_pc
18901	hsa-mir-16-1	RPS27A	0.308028352	mirna_pc
18902	hsa-mir-16-1	OST4	0.325110622	mirna_pc
18903	hsa-mir-16-1	DAK	0.32291786	mirna_pc
18904	hsa-mir-16-1	NDUFB11	0.362348984	mirna_pc
18905	hsa-mir-16-1	TMEM18	0.315638541	mirna_pc
18906	hsa-mir-16-1	ZNF232	0.400955285	mirna_pc
18907	hsa-mir-16-1	SENP2	0.365583777	mirna_pc
18908	hsa-mir-16-1	ZBTB39	0.328505448	mirna_pc
18909	hsa-mir-16-1	ASPSCR1	0.312515505	mirna_pc
18910	hsa-mir-16-1	RBBP4	0.31135391	mirna_pc
18911	hsa-mir-16-1	PTPMT1	0.313067309	mirna_pc
18912	hsa-mir-16-1	BDH1	0.316064072	mirna_pc
18913	hsa-mir-16-1	DNAJC19	0.379378236	mirna_pc
18914	hsa-mir-16-1	LRDD	0.325633658	mirna_pc
18915	hsa-mir-16-1	MCRS1	0.325274989	mirna_pc
18916	hsa-mir-16-1	SCML2	0.371707507	mirna_pc
18917	hsa-mir-16-1	NMNAT3	0.325601545	mirna_pc
18918	hsa-mir-16-1	ZCCHC3	0.388370382	mirna_pc
18919	hsa-mir-16-1	CHCHD6	0.347989904	mirna_pc
18920	hsa-mir-16-1	SNX17	0.319278172	mirna_pc
18921	hsa-mir-16-1	ETV5	0.449613055	mirna_pc
18922	hsa-mir-16-1	COX7A2L	0.347560579	mirna_pc
18923	hsa-mir-16-1	DACT2	0.433206691	mirna_pc
18924	hsa-mir-16-1	EXOSC6	0.347359341	mirna_pc
18925	hsa-mir-16-1	AADAT	0.325063899	mirna_pc
18926	hsa-mir-16-1	IGHMBP2	0.343986504	mirna_pc
18927	hsa-mir-16-1	ACP1	0.323552987	mirna_pc
18928	hsa-mir-16-1	THAP11	0.351365868	mirna_pc
18929	hsa-mir-16-1	TXNL4A	0.308002203	mirna_pc
18930	hsa-mir-16-1	SERF1A	0.382981749	mirna_pc
18931	hsa-mir-16-1	SNRNP25	0.35026447	mirna_pc
18932	hsa-mir-16-1	PREB	0.47646038	mirna_pc
18933	hsa-mir-16-1	B4GALNT4	0.330559815	mirna_pc
18934	hsa-mir-16-1	SSR2	0.33649032	mirna_pc
18935	hsa-mir-16-1	SLC41A3	0.300725494	mirna_pc
18936	hsa-mir-16-1	H2AFY2	0.331512	mirna_pc
18937	hsa-mir-16-1	MRPS23	0.302213915	mirna_pc
18938	hsa-mir-16-1	BOD1	0.328211697	mirna_pc
18939	hsa-mir-16-1	UXT	0.346295394	mirna_pc
18940	hsa-mir-16-1	RBBP7	0.306850415	mirna_pc
18941	hsa-mir-16-1	TEX261	0.324536598	mirna_pc
18942	hsa-mir-16-1	DNMT3A	0.420293191	mirna_pc
18943	hsa-mir-16-1	ZNF821	0.322012609	mirna_pc
18944	hsa-mir-16-1	C12orf73	0.337797721	mirna_pc
18945	hsa-mir-16-1	C15orf61	0.320994067	mirna_pc
18946	hsa-mir-16-1	UBE2MP1	0.303223765	mirna_pc
18947	hsa-mir-16-1	HAX1	0.346847641	mirna_pc
18948	hsa-mir-3074	TPX2	0.301019446	mirna_pc
18949	hsa-mir-3074	SEMA3E	0.436716488	mirna_pc
18950	hsa-mir-3074	NUSAP1	0.323311211	mirna_pc
18951	hsa-mir-3074	BUB1B	0.316337141	mirna_pc
18952	hsa-mir-3074	NCAPG	0.322449435	mirna_pc
18953	hsa-mir-3074	NEK2	0.335835825	mirna_pc

18954	hsa-mir-3074	TIMELESS	0.309315154	mirna_pc
18955	hsa-mir-3074	XPO1	0.310604223	mirna_pc
18956	hsa-mir-3074	KNTC1	0.348807689	mirna_pc
18957	hsa-mir-3074	YPEL1	0.347801709	mirna_pc
18958	hsa-mir-3074	DEPDC1	0.328340317	mirna_pc
18959	hsa-mir-3074	HJURP	0.302107634	mirna_pc
18960	hsa-mir-3074	BLM	0.302693289	mirna_pc
18961	hsa-mir-3074	SGOL2	0.304755099	mirna_pc
18962	hsa-mir-3074	CHEK2	0.351921163	mirna_pc
18963	hsa-mir-3074	C12orf48	0.363571854	mirna_pc
18964	hsa-mir-3074	WDR67	0.360268853	mirna_pc
18965	hsa-mir-3074	CITED2	0.380587331	mirna_pc
18966	hsa-mir-3074	PIF1	0.33369023	mirna_pc
18967	hsa-mir-3074	E2F1	0.301607467	mirna_pc
18968	hsa-mir-3074	ATAD2	0.34025867	mirna_pc
18969	hsa-mir-3074	ANP32E	0.348507324	mirna_pc
18970	hsa-mir-3074	NR2F2	0.37546968	mirna_pc
18971	hsa-mir-3074	DNAJC6	0.34114475	mirna_pc
18972	hsa-mir-3074	HDHD2	0.32476576	mirna_pc
18973	hsa-mir-3074	MSH2	0.432643693	mirna_pc
18974	hsa-mir-3074	CDC7	0.444964319	mirna_pc
18975	hsa-mir-3074	E2F3	0.326415014	mirna_pc
18976	hsa-mir-3074	SMC2	0.388312283	mirna_pc
18977	hsa-mir-3074	MCM8	0.339589441	mirna_pc
18978	hsa-mir-3074	ZNF367	0.402626717	mirna_pc
18979	hsa-mir-3074	ODF2	0.34357641	mirna_pc
18980	hsa-mir-3074	TMPO	0.39217162	mirna_pc
18981	hsa-mir-3074	SET	0.313543836	mirna_pc
18982	hsa-mir-3074	CRY1	0.328889583	mirna_pc
18983	hsa-mir-3074	SLC2A11	0.309752807	mirna_pc
18984	hsa-mir-3074	TYW3	0.349132833	mirna_pc
18985	hsa-mir-3074	LOC100128191	0.336995303	mirna_pc
18986	hsa-mir-3074	C20orf3	0.329276168	mirna_pc
18987	hsa-mir-3074	TEX10	0.326071906	mirna_pc
18988	hsa-mir-3074	GAD1	0.424483325	mirna_pc
18989	hsa-mir-3074	TMEM170B	0.301213344	mirna_pc
18990	hsa-mir-3074	TOMM34	0.300833308	mirna_pc
18991	hsa-mir-3074	FIGN	0.43906745	mirna_pc
18992	hsa-mir-3074	CCDC18	0.300810395	mirna_pc
18993	hsa-mir-3074	WDR76	0.306722312	mirna_pc
18994	hsa-mir-3074	SCARB1	0.312024614	mirna_pc
18995	hsa-mir-3074	PHF10	0.318681744	mirna_pc
18996	hsa-mir-3074	CAPS2	0.32556262	mirna_pc
18997	hsa-mir-3074	HABP4	0.439494498	mirna_pc
18998	hsa-mir-3074	HSP90B1	0.359912529	mirna_pc
18999	hsa-mir-3074	EHMT2	0.343395066	mirna_pc
19000	hsa-mir-3074	KDM1B	0.343001303	mirna_pc
19001	hsa-mir-3074	DEK	0.338218213	mirna_pc
19002	hsa-mir-3074	PRAME	0.361752145	mirna_pc
19003	hsa-mir-3074	CEP78	0.302812814	mirna_pc
19004	hsa-mir-3074	TMEM120B	0.31884181	mirna_pc
19005	hsa-mir-3074	HDAC2	0.32883817	mirna_pc
19006	hsa-mir-3074	TDRD5	0.339820178	mirna_pc
19007	hsa-mir-3074	CILP2	0.341434813	mirna_pc

19008	hsa-mir-3074	PA2G4P4	0.30847288	mirna_pc
19009	hsa-mir-3074	ZNF217	0.328289731	mirna_pc
19010	hsa-mir-3074	XRN2	0.39946794	mirna_pc
19011	hsa-mir-3074	MEX3A	0.304495763	mirna_pc
19012	hsa-mir-3074	BAT1	0.357653362	mirna_pc
19013	hsa-mir-3074	TAF5	0.315895803	mirna_pc
19014	hsa-mir-3074	FANCC	0.319697435	mirna_pc
19015	hsa-mir-3074	ACAN	0.420461104	mirna_pc
19016	hsa-mir-3074	C6orf120	0.300912298	mirna_pc
19017	hsa-mir-3074	SUZ12	0.331386218	mirna_pc
19018	hsa-mir-3074	BARD1	0.322645703	mirna_pc
19019	hsa-mir-3074	USP1	0.323460279	mirna_pc
19020	hsa-mir-3074	MTF2	0.338370604	mirna_pc
19021	hsa-mir-3074	METTL13	0.380658167	mirna_pc
19022	hsa-mir-3074	SEMA3D	0.456943746	mirna_pc
19023	hsa-mir-3074	POLE	0.328161852	mirna_pc
19024	hsa-mir-3074	CTDSPL2	0.301752374	mirna_pc
19025	hsa-mir-3074	C13orf27	0.316977266	mirna_pc
19026	hsa-mir-3074	CCDC41	0.319166021	mirna_pc
19027	hsa-mir-3074	OXTR	0.326649018	mirna_pc
19028	hsa-mir-3074	THSD7A	0.301617285	mirna_pc
19029	hsa-mir-3074	ZNF605	0.333880368	mirna_pc
19030	hsa-mir-3074	FAM136A	0.300802512	mirna_pc
19031	hsa-mir-3074	CABYR	0.303945348	mirna_pc
19032	hsa-mir-3074	C12orf76	0.311185265	mirna_pc
19033	hsa-mir-3074	GDPD5	0.386699737	mirna_pc
19034	hsa-mir-3074	C8orf51	0.30077628	mirna_pc
19035	hsa-mir-3074	ARHGAP19	0.311725529	mirna_pc
19036	hsa-mir-3074	KIAA1731	0.397888863	mirna_pc
19037	hsa-mir-3074	KCNC3	0.307272288	mirna_pc
19038	hsa-mir-3074	CHST10	0.303814486	mirna_pc
19039	hsa-mir-3074	MRE11A	0.36642372	mirna_pc
19040	hsa-mir-3074	ZNF84	0.43464758	mirna_pc
19041	hsa-mir-3074	TAF4B	0.315583467	mirna_pc
19042	hsa-mir-3074	ZNF140	0.321148589	mirna_pc
19043	hsa-mir-3074	WASF1	0.309960685	mirna_pc
19044	hsa-mir-3074	SEC61A2	0.444998741	mirna_pc
19045	hsa-mir-3074	PSPC1	0.37740294	mirna_pc
19046	hsa-mir-3074	SSX2IP	0.302712724	mirna_pc
19047	hsa-mir-3074	B4GALT3	0.356102518	mirna_pc
19048	hsa-mir-3074	NF2	0.33334549	mirna_pc
19049	hsa-mir-3074	MYBL1	0.321055897	mirna_pc
19050	hsa-mir-3074	SLC9A5	0.361413914	mirna_pc
19051	hsa-mir-3074	EWSR1	0.34333896	mirna_pc
19052	hsa-mir-3074	DHX57	0.321300304	mirna_pc
19053	hsa-mir-3074	KIAA1598	0.302364186	mirna_pc
19054	hsa-mir-3074	PAPOLA	0.356816897	mirna_pc
19055	hsa-mir-3074	TMEM38B	0.341826799	mirna_pc
19056	hsa-mir-3074	MRPL42	0.314138676	mirna_pc
19057	hsa-mir-3074	CRNKL1	0.332021153	mirna_pc
19058	hsa-mir-3074	TMEM136	0.329885088	mirna_pc
19059	hsa-mir-3074	DNM3	0.429155446	mirna_pc
19060	hsa-mir-3074	ZNF184	0.349231855	mirna_pc
19061	hsa-mir-3074	NIPSNAP1	0.348751139	mirna_pc

19062	hsa-mir-3074	C8orf37	0.379103311	mirna_pc
19063	hsa-mir-3074	SFI1	0.367020733	mirna_pc
19064	hsa-mir-3074	ZBTB10	0.358633984	mirna_pc
19065	hsa-mir-3074	TTC21B	0.34705605	mirna_pc
19066	hsa-mir-3074	SPRY4	0.306151267	mirna_pc
19067	hsa-mir-3074	ANGPT1	0.438618591	mirna_pc
19068	hsa-mir-3074	EXTL2	0.334150123	mirna_pc
19069	hsa-mir-3074	TMTC4	0.360535145	mirna_pc
19070	hsa-mir-3074	LOC338758	0.386599121	mirna_pc
19071	hsa-mir-3074	PCMTD2	0.330814327	mirna_pc
19072	hsa-mir-3074	ANKRD36B	0.319000608	mirna_pc
19073	hsa-mir-3074	GPR137C	0.440390992	mirna_pc
19074	hsa-mir-3074	PIP5K1A	0.326815264	mirna_pc
19075	hsa-mir-3074	GRB14	0.37598471	mirna_pc
19076	hsa-mir-3074	DCLRE1A	0.315848722	mirna_pc
19077	hsa-mir-3074	ANP32A	0.349414043	mirna_pc
19078	hsa-mir-3074	HTR7P1	0.334908975	mirna_pc
19079	hsa-mir-3074	E2F5	0.30086049	mirna_pc
19080	hsa-mir-3074	B4GALT6	0.384190787	mirna_pc
19081	hsa-mir-3074	PHF16	0.325953868	mirna_pc
19082	hsa-mir-3074	CNIH2	0.337425651	mirna_pc
19083	hsa-mir-3074	BUD13	0.33455597	mirna_pc
19084	hsa-mir-3074	EID3	0.452923616	mirna_pc
19085	hsa-mir-3074	KIAA1467	0.334335647	mirna_pc
19086	hsa-mir-3074	ABHD12	0.349065739	mirna_pc
19087	hsa-mir-3074	GATS	0.356611687	mirna_pc
19088	hsa-mir-3074	TRUB1	0.311704678	mirna_pc
19089	hsa-mir-3074	C2orf15	0.366834604	mirna_pc
19090	hsa-mir-3074	C12orf24	0.408660167	mirna_pc
19091	hsa-mir-3074	DCAF16	0.335936284	mirna_pc
19092	hsa-mir-3074	SBK1	0.364868721	mirna_pc
19093	hsa-mir-3074	SEMA3A	0.36986418	mirna_pc
19094	hsa-mir-3074	USP13	0.433097382	mirna_pc
19095	hsa-mir-3074	ESF1	0.325118163	mirna_pc
19096	hsa-mir-3074	HS2ST1	0.404171795	mirna_pc
19097	hsa-mir-3074	GPR37	0.349193385	mirna_pc
19098	hsa-mir-3074	ZNF26	0.369301102	mirna_pc
19099	hsa-mir-3074	NR5A2	0.3987307	mirna_pc
19100	hsa-mir-3074	MDM1	0.426937981	mirna_pc
19101	hsa-mir-3074	C10orf88	0.331028755	mirna_pc
19102	hsa-mir-3074	MCOLN3	0.332849297	mirna_pc
19103	hsa-mir-3074	PBX2	0.30156268	mirna_pc
19104	hsa-mir-3074	SLITRK6	0.346380491	mirna_pc
19105	hsa-mir-3074	ENO3	0.309011731	mirna_pc
19106	hsa-mir-3074	HAPLN1	0.45232455	mirna_pc
19107	hsa-mir-3074	MED17	0.384862981	mirna_pc
19108	hsa-mir-3074	HYOU1	0.345571127	mirna_pc
19109	hsa-mir-3074	RBBP4	0.305389704	mirna_pc
19110	hsa-mir-3074	LOC221710	0.431071429	mirna_pc
19111	hsa-mir-3074	CRYZ	0.378347183	mirna_pc
19112	hsa-mir-3074	ZNF664	0.383823411	mirna_pc
19113	hsa-mir-3074	SMEK2	0.313903207	mirna_pc
19114	hsa-mir-3074	C20orf96	0.479990308	mirna_pc
19115	hsa-mir-3074	SCML2	0.394749844	mirna_pc

19116	hsa-mir-3074	FAM171B	0.307625861	mirna_pc
19117	hsa-mir-3074	ZCCHC11	0.305763636	mirna_pc
19118	hsa-mir-3074	ZCCHC3	0.39207187	mirna_pc
19119	hsa-mir-3074	WDR73	0.335254577	mirna_pc
19120	hsa-mir-3074	C2orf43	0.317474476	mirna_pc
19121	hsa-mir-3074	CTXN1	0.367721918	mirna_pc
19122	hsa-mir-3074	CEP192	0.30929117	mirna_pc
19123	hsa-mir-3074	CCDC117	0.339405672	mirna_pc
19124	hsa-mir-3074	PRMT6	0.312258801	mirna_pc
19125	hsa-mir-3074	SLC11A2	0.358539899	mirna_pc
19126	hsa-mir-3074	BMF	0.314255508	mirna_pc
19127	hsa-mir-3074	BAI2	0.312100157	mirna_pc
19128	hsa-mir-3074	ZFP37	0.326509416	mirna_pc
19129	hsa-mir-3074	MPP6	0.303742493	mirna_pc
19130	hsa-mir-3074	LCA5	0.387441102	mirna_pc
19131	hsa-mir-3074	DNMT3A	0.351459226	mirna_pc
19132	hsa-mir-3074	WDSUB1	0.353705926	mirna_pc
19133	hsa-mir-3074	TET1	0.313855362	mirna_pc
19134	hsa-mir-3074	PATZ1	0.337150707	mirna_pc
19135	hsa-mir-3074	ZNF711	0.303064668	mirna_pc
19136	hsa-mir-3074	SS18L1	0.342207427	mirna_pc
19137	hsa-mir-3647	HOXC8	0.426021255	mirna_pc
19138	hsa-mir-3647	FANCA	0.302752548	mirna_pc
19139	hsa-mir-3647	XPO1	0.353520417	mirna_pc
19140	hsa-mir-3647	KIAA0101	0.346465503	mirna_pc
19141	hsa-mir-3647	KNTC1	0.36896664	mirna_pc
19142	hsa-mir-3647	UHRF1	0.342166508	mirna_pc
19143	hsa-mir-3647	PLK4	0.344794812	mirna_pc
19144	hsa-mir-3647	DNMT1	0.332575096	mirna_pc
19145	hsa-mir-3647	CENPK	0.337237859	mirna_pc
19146	hsa-mir-3647	CDT1	0.300024261	mirna_pc
19147	hsa-mir-3647	CBFB	0.389231598	mirna_pc
19148	hsa-mir-3647	ORC6L	0.371107233	mirna_pc
19149	hsa-mir-3647	C16orf59	0.301617223	mirna_pc
19150	hsa-mir-3647	ACTL6A	0.307018974	mirna_pc
19151	hsa-mir-3647	POLD1	0.305237097	mirna_pc
19152	hsa-mir-3647	SMC4	0.409794015	mirna_pc
19153	hsa-mir-3647	HELLS	0.350886057	mirna_pc
19154	hsa-mir-3647	HNRNPC	0.305708899	mirna_pc
19155	hsa-mir-3647	RFWD3	0.343876333	mirna_pc
19156	hsa-mir-3647	BRIP1	0.307247454	mirna_pc
19157	hsa-mir-3647	C15orf42	0.307585786	mirna_pc
19158	hsa-mir-3647	FAM60A	0.334781878	mirna_pc
19159	hsa-mir-3647	CHAF1A	0.362336315	mirna_pc
19160	hsa-mir-3647	ULBP1	0.321943122	mirna_pc
19161	hsa-mir-3647	ANP32E	0.362145817	mirna_pc
19162	hsa-mir-3647	TRIM59	0.324798655	mirna_pc
19163	hsa-mir-3647	GEN1	0.372811303	mirna_pc
19164	hsa-mir-3647	HMGB2	0.385698256	mirna_pc
19165	hsa-mir-3647	E2F7	0.316686217	mirna_pc
19166	hsa-mir-3647	MLF1IP	0.328180479	mirna_pc
19167	hsa-mir-3647	HIST1H2AE	0.376081166	mirna_pc
19168	hsa-mir-3647	CCDC99	0.307802283	mirna_pc
19169	hsa-mir-3647	CHTF18	0.306531198	mirna_pc

19170	hsa-mir-3647	HNRNPR	0.313530223	mirna_pc
19171	hsa-mir-3647	DLEU2	0.430764319	mirna_pc
19172	hsa-mir-3647	MSH2	0.349847581	mirna_pc
19173	hsa-mir-3647	SF3B3	0.312943708	mirna_pc
19174	hsa-mir-3647	CDC7	0.35070123	mirna_pc
19175	hsa-mir-3647	ARL6IP6	0.336768906	mirna_pc
19176	hsa-mir-3647	FBXO5	0.350789539	mirna_pc
19177	hsa-mir-3647	DDX12	0.317169334	mirna_pc
19178	hsa-mir-3647	CLDN1	0.357520172	mirna_pc
19179	hsa-mir-3647	CPSF3	0.370391797	mirna_pc
19180	hsa-mir-3647	TFAP4	0.365912561	mirna_pc
19181	hsa-mir-3647	DAZAP1	0.312634851	mirna_pc
19182	hsa-mir-3647	EIF2AK2	0.318853313	mirna_pc
19183	hsa-mir-3647	SLC5A12	0.324831626	mirna_pc
19184	hsa-mir-3647	NCL	0.360794584	mirna_pc
19185	hsa-mir-3647	SASS6	0.352881535	mirna_pc
19186	hsa-mir-3647	HNRNPM	0.375407988	mirna_pc
19187	hsa-mir-3647	PNPT1	0.344457756	mirna_pc
19188	hsa-mir-3647	NIP7	0.342248158	mirna_pc
19189	hsa-mir-3647	C3orf26	0.329085698	mirna_pc
19190	hsa-mir-3647	HIST1H3D	0.302223372	mirna_pc
19191	hsa-mir-3647	NUP93	0.340238882	mirna_pc
19192	hsa-mir-3647	GABPB1	0.362895614	mirna_pc
19193	hsa-mir-3647	WDR43	0.386462464	mirna_pc
19194	hsa-mir-3647	SMC6	0.447175503	mirna_pc
19195	hsa-mir-3647	HOXD9	0.328644518	mirna_pc
19196	hsa-mir-3647	SMARCD1	0.304547497	mirna_pc
19197	hsa-mir-3647	ATXN2L	0.326897353	mirna_pc
19198	hsa-mir-3647	C4orf46	0.307025668	mirna_pc
19199	hsa-mir-3647	GPN1	0.300533199	mirna_pc
19200	hsa-mir-3647	KHDRBS1	0.365381332	mirna_pc
19201	hsa-mir-3647	PRPF40A	0.335916928	mirna_pc
19202	hsa-mir-3647	CCDC18	0.365464612	mirna_pc
19203	hsa-mir-3647	C4orf21	0.334672972	mirna_pc
19204	hsa-mir-3647	SMNDC1	0.350153791	mirna_pc
19205	hsa-mir-3647	NEDD1	0.312498239	mirna_pc
19206	hsa-mir-3647	ZNF267	0.311317787	mirna_pc
19207	hsa-mir-3647	NASP	0.342142914	mirna_pc
19208	hsa-mir-3647	PTMA	0.370730211	mirna_pc
19209	hsa-mir-3647	SP3	0.31810508	mirna_pc
19210	hsa-mir-3647	DENR	0.35310465	mirna_pc
19211	hsa-mir-3647	FANCL	0.314312156	mirna_pc
19212	hsa-mir-3647	CCT4	0.302970489	mirna_pc
19213	hsa-mir-3647	ABCE1	0.326431312	mirna_pc
19214	hsa-mir-3647	ACTR2	0.33025723	mirna_pc
19215	hsa-mir-3647	PTPN2	0.329074395	mirna_pc
19216	hsa-mir-3647	RPAP3	0.34771205	mirna_pc
19217	hsa-mir-3647	CSAD	0.321036461	mirna_pc
19218	hsa-mir-3647	PMAIP1	0.34047195	mirna_pc
19219	hsa-mir-3647	NAA25	0.318229609	mirna_pc
19220	hsa-mir-3647	C18orf54	0.305117326	mirna_pc
19221	hsa-mir-3647	KHSRP	0.313703752	mirna_pc
19222	hsa-mir-3647	LSM 6.00	0.308006344	mirna_pc
19223	hsa-mir-3647	SART3	0.338936271	mirna_pc

19224	hsa-mir-3647	HNRNPK	0.308625227	mirna_pc
19225	hsa-mir-3647	SR140	0.306775239	mirna_pc
19226	hsa-mir-3647	TRA2B	0.303431878	mirna_pc
19227	hsa-mir-3647	RSRC1	0.301266236	mirna_pc
19228	hsa-mir-3647	CEP152	0.313975875	mirna_pc
19229	hsa-mir-3647	SLMO1	0.344393688	mirna_pc
19230	hsa-mir-3647	TDG	0.307595018	mirna_pc
19231	hsa-mir-3647	C4orf43	0.350015901	mirna_pc
19232	hsa-mir-3647	DDX55	0.349023581	mirna_pc
19233	hsa-mir-3647	ODF2L	0.314218328	mirna_pc
19234	hsa-mir-3647	RBBP8	0.302635221	mirna_pc
19235	hsa-mir-3647	HDAC2	0.371250003	mirna_pc
19236	hsa-mir-3647	ANKRD32	0.356577307	mirna_pc
19237	hsa-mir-3647	CRIPT	0.306440794	mirna_pc
19238	hsa-mir-3647	MSH6	0.334271074	mirna_pc
19239	hsa-mir-3647	XRN2	0.329559423	mirna_pc
19240	hsa-mir-3647	TP73	0.322645003	mirna_pc
19241	hsa-mir-3647	OTUD6B	0.32045118	mirna_pc
19242	hsa-mir-3647	SMC3	0.42973017	mirna_pc
19243	hsa-mir-3647	TAF5	0.330287751	mirna_pc
19244	hsa-mir-3647	PPHLN1	0.314223781	mirna_pc
19245	hsa-mir-3647	PAPOLG	0.477977579	mirna_pc
19246	hsa-mir-3647	USP1	0.309460068	mirna_pc
19247	hsa-mir-3647	SAMD1	0.352260744	mirna_pc
19248	hsa-mir-3647	C16orf87	0.415117651	mirna_pc
19249	hsa-mir-3647	THUMPD2	0.31267157	mirna_pc
19250	hsa-mir-3647	MTF2	0.324487054	mirna_pc
19251	hsa-mir-3647	PSMD7	0.312846719	mirna_pc
19252	hsa-mir-3647	DDX47	0.318830539	mirna_pc
19253	hsa-mir-3647	CTDSPL2	0.301707668	mirna_pc
19254	hsa-mir-3647	TAF1B	0.329272749	mirna_pc
19255	hsa-mir-3647	OGFOD1	0.362569219	mirna_pc
19256	hsa-mir-3647	PPP3R1	0.455444411	mirna_pc
19257	hsa-mir-3647	EXOSC9	0.321301972	mirna_pc
19258	hsa-mir-3647	NETO2	0.393029357	mirna_pc
19259	hsa-mir-3647	LOC374443	0.323868387	mirna_pc
19260	hsa-mir-3647	GPN3	0.321106656	mirna_pc
19261	hsa-mir-3647	ASB3	0.309856492	mirna_pc
19262	hsa-mir-3647	ZNF200	0.3408254	mirna_pc
19263	hsa-mir-3647	C5orf15	0.325575231	mirna_pc
19264	hsa-mir-3647	UBXN2A	0.381200575	mirna_pc
19265	hsa-mir-3647	COMMD2	0.312830882	mirna_pc
19266	hsa-mir-3647	NUDT21	0.417799531	mirna_pc
19267	hsa-mir-3647	SCLT1	0.329798271	mirna_pc
19268	hsa-mir-3647	PCGF6	0.376937379	mirna_pc
19269	hsa-mir-3647	S1PR5	0.385844217	mirna_pc
19270	hsa-mir-3647	TTC32	0.324466014	mirna_pc
19271	hsa-mir-3647	SMN2	0.404001421	mirna_pc
19272	hsa-mir-3647	ZNF639	0.315443723	mirna_pc
19273	hsa-mir-3647	SART1	0.306682027	mirna_pc
19274	hsa-mir-3647	C12orf76	0.373115535	mirna_pc
19275	hsa-mir-3647	MEX3B	0.308668703	mirna_pc
19276	hsa-mir-3647	MPHOSPH10	0.397481597	mirna_pc
19277	hsa-mir-3647	ANKRD11	0.367888108	mirna_pc

19278	hsa-mir-3647	IL27RA	0.426177699	mirna_pc
19279	hsa-mir-3647	WNT10A	0.318211101	mirna_pc
19280	hsa-mir-3647	TCEA1	0.366916313	mirna_pc
19281	hsa-mir-3647	FXR1	0.368496472	mirna_pc
19282	hsa-mir-3647	CLEC2D	0.333767612	mirna_pc
19283	hsa-mir-3647	UPF3B	0.303375767	mirna_pc
19284	hsa-mir-3647	TAF4B	0.309437882	mirna_pc
19285	hsa-mir-3647	RWDD3	0.302208606	mirna_pc
19286	hsa-mir-3647	IFT80	0.304857224	mirna_pc
19287	hsa-mir-3647	DDX46	0.31001572	mirna_pc
19288	hsa-mir-3647	PUS10	0.399925417	mirna_pc
19289	hsa-mir-3647	TCERG1	0.361309106	mirna_pc
19290	hsa-mir-3647	INTU	0.327839236	mirna_pc
19291	hsa-mir-3647	E2F6	0.33374903	mirna_pc
19292	hsa-mir-3647	SFRS13A	0.339965838	mirna_pc
19293	hsa-mir-3647	CCDC59	0.354164581	mirna_pc
19294	hsa-mir-3647	SLC4A1AP	0.443569839	mirna_pc
19295	hsa-mir-3647	TTC27	0.370289942	mirna_pc
19296	hsa-mir-3647	MTIF2	0.304914356	mirna_pc
19297	hsa-mir-3647	HNRNPA3	0.355301674	mirna_pc
19298	hsa-mir-3647	EWSR1	0.339275296	mirna_pc
19299	hsa-mir-3647	KRR1	0.368409775	mirna_pc
19300	hsa-mir-3647	DHX57	0.355891911	mirna_pc
19301	hsa-mir-3647	TNFAIP8	0.317192615	mirna_pc
19302	hsa-mir-3647	CCDC45	0.304107009	mirna_pc
19303	hsa-mir-3647	SUPT7L	0.32797014	mirna_pc
19304	hsa-mir-3647	RIMKLB	0.301744687	mirna_pc
19305	hsa-mir-3647	SFRS7	0.32583094	mirna_pc
19306	hsa-mir-3647	MRPL42	0.3495846	mirna_pc
19307	hsa-mir-3647	SF3B2	0.367226669	mirna_pc
19308	hsa-mir-3647	KGFLP2	0.355529787	mirna_pc
19309	hsa-mir-3647	AEBP2	0.393947656	mirna_pc
19310	hsa-mir-3647	SLC35F2	0.305365987	mirna_pc
19311	hsa-mir-3647	IL15	0.331837329	mirna_pc
19312	hsa-mir-3647	DNM1L	0.318143523	mirna_pc
19313	hsa-mir-3647	ZCCHC8	0.358718003	mirna_pc
19314	hsa-mir-3647	CLEC2B	0.339148839	mirna_pc
19315	hsa-mir-3647	DCP2	0.305222738	mirna_pc
19316	hsa-mir-3647	PRKD3	0.352455601	mirna_pc
19317	hsa-mir-3647	SRFBP1	0.325795002	mirna_pc
19318	hsa-mir-3647	CEBPZ	0.317723276	mirna_pc
19319	hsa-mir-3647	CCDC76	0.304055154	mirna_pc
19320	hsa-mir-3647	MATR3	0.319027904	mirna_pc
19321	hsa-mir-3647	ZFP1	0.342625759	mirna_pc
19322	hsa-mir-3647	ARMC1	0.312061035	mirna_pc
19323	hsa-mir-3647	BCL11A	0.374673272	mirna_pc
19324	hsa-mir-3647	POLR3F	0.308748881	mirna_pc
19325	hsa-mir-3647	PAPD5	0.318254554	mirna_pc
19326	hsa-mir-3647	PEX13	0.407120691	mirna_pc
19327	hsa-mir-3647	DCAF17	0.3657913	mirna_pc
19328	hsa-mir-3647	ETAA1	0.339341315	mirna_pc
19329	hsa-mir-3647	HNRNPH3	0.315220851	mirna_pc
19330	hsa-mir-3647	C7orf60	0.30266107	mirna_pc
19331	hsa-mir-3647	NAP1L1	0.308020499	mirna_pc

19332	hsa-mir-3647	UBE2W	0.312358281	mirna_pc
19333	hsa-mir-3647	RTN4RL1	0.391444233	mirna_pc
19334	hsa-mir-3647	TRUB1	0.411289438	mirna_pc
19335	hsa-mir-3647	SLC38A2	0.329712101	mirna_pc
19336	hsa-mir-3647	C12orf24	0.312718678	mirna_pc
19337	hsa-mir-3647	CCDC102A	0.316264848	mirna_pc
19338	hsa-mir-3647	MTERF	0.305781556	mirna_pc
19339	hsa-mir-3647	SF3A1	0.301390255	mirna_pc
19340	hsa-mir-3647	USP13	0.323913541	mirna_pc
19341	hsa-mir-3647	IRX3	0.317047261	mirna_pc
19342	hsa-mir-3647	ESF1	0.325290896	mirna_pc
19343	hsa-mir-3647	GOPC	0.32021317	mirna_pc
19344	hsa-mir-3647	SLITRK6	0.330699848	mirna_pc
19345	hsa-mir-3647	SFRS2IP	0.314172931	mirna_pc
19346	hsa-mir-3647	PPTC7	0.326083999	mirna_pc
19347	hsa-mir-3647	KIAA1586	0.304487367	mirna_pc
19348	hsa-mir-3647	C2orf3	0.420788009	mirna_pc
19349	hsa-mir-3647	TIA1	0.3698788	mirna_pc
19350	hsa-mir-3647	ATL2	0.345793949	mirna_pc
19351	hsa-mir-3647	PDE7A	0.379196292	mirna_pc
19352	hsa-mir-3647	CREB1	0.311140072	mirna_pc
19353	hsa-mir-3647	C12orf65	0.320048605	mirna_pc
19354	hsa-mir-3647	MRPL19	0.360098118	mirna_pc
19355	hsa-mir-3647	ZCCHC11	0.324542897	mirna_pc
19356	hsa-mir-3647	CETN3	0.388908166	mirna_pc
19357	hsa-mir-3647	RSRC2	0.366444654	mirna_pc
19358	hsa-mir-3647	KIAA1430	0.306234696	mirna_pc
19359	hsa-mir-3647	KDM5A	0.313221317	mirna_pc
19360	hsa-mir-3647	CWC22	0.300095364	mirna_pc
19361	hsa-mir-3647	DDIT4	0.37984178	mirna_pc
19362	hsa-mir-3647	HSDL1	0.341087777	mirna_pc
19363	hsa-mir-3647	FBXO11	0.421674803	mirna_pc
19364	hsa-mir-3647	TMEM188	0.336044814	mirna_pc
19365	hsa-mir-3647	ZCCHC10	0.316194846	mirna_pc
19366	hsa-mir-3647	DNMT3A	0.304705553	mirna_pc
19367	hsa-mir-3647	TET1	0.301422437	mirna_pc
19368	hsa-mir-3647	CCDC88A	0.366865202	mirna_pc
19369	hsa-mir-425	MKI67	0.302175508	mirna_pc
19370	hsa-mir-425	TPX2	0.324738329	mirna_pc
19371	hsa-mir-425	KIF4B	0.318643172	mirna_pc
19372	hsa-mir-425	HOXC9	0.464425286	mirna_pc
19373	hsa-mir-425	RRM2	0.309475131	mirna_pc
19374	hsa-mir-425	CDC25C	0.364116001	mirna_pc
19375	hsa-mir-425	UBE2C	0.350913248	mirna_pc
19376	hsa-mir-425	KIFC1	0.390809025	mirna_pc
19377	hsa-mir-425	BUB1B	0.354114064	mirna_pc
19378	hsa-mir-425	FAM72A	0.34018474	mirna_pc
19379	hsa-mir-425	CLSPN	0.307406375	mirna_pc
19380	hsa-mir-425	SGOL1	0.481136786	mirna_pc
19381	hsa-mir-425	ARHGAP11A	0.368440839	mirna_pc
19382	hsa-mir-425	NEK2	0.336984607	mirna_pc
19383	hsa-mir-425	HOXC11	0.350524953	mirna_pc
19384	hsa-mir-425	CDCA8	0.4079201	mirna_pc
19385	hsa-mir-425	STIL	0.351023528	mirna_pc

19386	hsa-mir-425	KIF4A	0.369142976	mirna_pc
19387	hsa-mir-425	DEPDC1	0.321596892	mirna_pc
19388	hsa-mir-425	SKA3	0.327486599	mirna_pc
19389	hsa-mir-425	BLM	0.403944139	mirna_pc
19390	hsa-mir-425	CCDC150	0.303418404	mirna_pc
19391	hsa-mir-425	OIP5	0.408815817	mirna_pc
19392	hsa-mir-425	AURKA	0.363942981	mirna_pc
19393	hsa-mir-425	CENPK	0.320028792	mirna_pc
19394	hsa-mir-425	SGOL2	0.340772057	mirna_pc
19395	hsa-mir-425	HBB	0.413885586	mirna_pc
19396	hsa-mir-425	KIF15	0.44910389	mirna_pc
19397	hsa-mir-425	FEN1	0.327908395	mirna_pc
19398	hsa-mir-425	TRAIP	0.461128045	mirna_pc
19399	hsa-mir-425	FAM72D	0.375244242	mirna_pc
19400	hsa-mir-425	FAM72B	0.335185497	mirna_pc
19401	hsa-mir-425	C12orf48	0.386985599	mirna_pc
19402	hsa-mir-425	HMMR	0.328046465	mirna_pc
19403	hsa-mir-425	ZWINT	0.40175676	mirna_pc
19404	hsa-mir-425	BUB3	0.327825455	mirna_pc
19405	hsa-mir-425	PDHB	0.345276352	mirna_pc
19406	hsa-mir-425	CDCA7	0.311862443	mirna_pc
19407	hsa-mir-425	PIF1	0.370427445	mirna_pc
19408	hsa-mir-425	DNAH3	0.355479039	mirna_pc
19409	hsa-mir-425	E2F1	0.315809867	mirna_pc
19410	hsa-mir-425	E2F2	0.301467135	mirna_pc
19411	hsa-mir-425	DEPDC1B	0.352042791	mirna_pc
19412	hsa-mir-425	POC1A	0.391921343	mirna_pc
19413	hsa-mir-425	DNA2	0.365673141	mirna_pc
19414	hsa-mir-425	NEURL3	0.369441377	mirna_pc
19415	hsa-mir-425	SNHG1	0.321309776	mirna_pc
19416	hsa-mir-425	CCDC99	0.308174904	mirna_pc
19417	hsa-mir-425	HOXA10	0.330683203	mirna_pc
19418	hsa-mir-425	DLEU2	0.320042471	mirna_pc
19419	hsa-mir-425	PRIM1	0.341478009	mirna_pc
19420	hsa-mir-425	DDX27	0.363805164	mirna_pc
19421	hsa-mir-425	HOXC10	0.335687632	mirna_pc
19422	hsa-mir-425	MSH2	0.316234807	mirna_pc
19423	hsa-mir-425	SALL4	0.349757645	mirna_pc
19424	hsa-mir-425	NOP58	0.304797342	mirna_pc
19425	hsa-mir-425	SSB	0.347555432	mirna_pc
19426	hsa-mir-425	SNRPC	0.35033412	mirna_pc
19427	hsa-mir-425	E2F3	0.423934026	mirna_pc
19428	hsa-mir-425	MEG3	0.312984807	mirna_pc
19429	hsa-mir-425	CDC25A	0.447595237	mirna_pc
19430	hsa-mir-425	TMEM48	0.334779679	mirna_pc
19431	hsa-mir-425	SRPK1	0.332780062	mirna_pc
19432	hsa-mir-425	MCM8	0.345264715	mirna_pc
19433	hsa-mir-425	ATIC	0.301923143	mirna_pc
19434	hsa-mir-425	MARCKSL1	0.331939544	mirna_pc
19435	hsa-mir-425	CBX8	0.352670189	mirna_pc
19436	hsa-mir-425	SASS6	0.335661871	mirna_pc
19437	hsa-mir-425	TMPO	0.352804351	mirna_pc
19438	hsa-mir-425	LOC388796	0.336062421	mirna_pc
19439	hsa-mir-425	RPP14	0.39480322	mirna_pc

19440	hsa-mir-425	CHRNA5	0.391856217	mirna_pc
19441	hsa-mir-425	SFRS1	0.329208344	mirna_pc
19442	hsa-mir-425	CASP8	0.321819651	mirna_pc
19443	hsa-mir-425	GAD1	0.329480624	mirna_pc
19444	hsa-mir-425	DNMT3B	0.369274871	mirna_pc
19445	hsa-mir-425	DARS2	0.30979098	mirna_pc
19446	hsa-mir-425	HSPD1	0.348592485	mirna_pc
19447	hsa-mir-425	PA2G4	0.347416952	mirna_pc
19448	hsa-mir-425	SLC5A6	0.398242488	mirna_pc
19449	hsa-mir-425	TOMM34	0.347441109	mirna_pc
19450	hsa-mir-425	MAPRE1	0.324538533	mirna_pc
19451	hsa-mir-425	CCDC18	0.315521481	mirna_pc
19452	hsa-mir-425	AHCY	0.348225779	mirna_pc
19453	hsa-mir-425	CCNE1	0.323263667	mirna_pc
19454	hsa-mir-425	ZNF695	0.424644937	mirna_pc
19455	hsa-mir-425	PDSS1	0.31458792	mirna_pc
19456	hsa-mir-425	HAUS6	0.315391831	mirna_pc
19457	hsa-mir-425	OBFC2B	0.45700272	mirna_pc
19458	hsa-mir-425	PTMA	0.301172162	mirna_pc
19459	hsa-mir-425	SCARB1	0.460208839	mirna_pc
19460	hsa-mir-425	SUV39H2	0.328242127	mirna_pc
19461	hsa-mir-425	LSM 2.00	0.329164732	mirna_pc
19462	hsa-mir-425	ALG6	0.336263852	mirna_pc
19463	hsa-mir-425	RIBC2	0.315791207	mirna_pc
19464	hsa-mir-425	RRP9	0.322513377	mirna_pc
19465	hsa-mir-425	TFR2	0.319726629	mirna_pc
19466	hsa-mir-425	SFRS3	0.361007579	mirna_pc
19467	hsa-mir-425	C6orf223	0.303906442	mirna_pc
19468	hsa-mir-425	HSP90B1	0.487113926	mirna_pc
19469	hsa-mir-425	RBM6	0.34665387	mirna_pc
19470	hsa-mir-425	PAK1IP1	0.355130836	mirna_pc
19471	hsa-mir-425	EDEM2	0.385535043	mirna_pc
19472	hsa-mir-425	C1orf59	0.318790683	mirna_pc
19473	hsa-mir-425	EPCAM	0.356325454	mirna_pc
19474	hsa-mir-425	EHMT2	0.426329308	mirna_pc
19475	hsa-mir-425	GAS2L3	0.320713062	mirna_pc
19476	hsa-mir-425	PDRG1	0.376030744	mirna_pc
19477	hsa-mir-425	PASK	0.323099298	mirna_pc
19478	hsa-mir-425	WDR46	0.330518424	mirna_pc
19479	hsa-mir-425	TAF4	0.380757436	mirna_pc
19480	hsa-mir-425	SNRPB2	0.318403849	mirna_pc
19481	hsa-mir-425	GLO1	0.312875145	mirna_pc
19482	hsa-mir-425	ZBTB9	0.368370861	mirna_pc
19483	hsa-mir-425	CSRP2BP	0.353097613	mirna_pc
19484	hsa-mir-425	RPN2	0.403944533	mirna_pc
19485	hsa-mir-425	KCTD6	0.46994914	mirna_pc
19486	hsa-mir-425	ITGB3BP	0.319411339	mirna_pc
19487	hsa-mir-425	EIF2S2	0.321360689	mirna_pc
19488	hsa-mir-425	PABPC1L	0.352723954	mirna_pc
19489	hsa-mir-425	C17orf42	0.381249015	mirna_pc
19490	hsa-mir-425	DNAJC7	0.320419708	mirna_pc
19491	hsa-mir-425	TSEN54	0.362688436	mirna_pc
19492	hsa-mir-425	ACTR5	0.325438343	mirna_pc
19493	hsa-mir-425	ZNF595	0.343974262	mirna_pc

19494	hsa-mir-425	DDX55	0.340523652	mirna_pc
19495	hsa-mir-425	HPDL	0.320624368	mirna_pc
19496	hsa-mir-425	CDK5RAP1	0.3483867	mirna_pc
19497	hsa-mir-425	RCOR2	0.322027284	mirna_pc
19498	hsa-mir-425	ATAD3A	0.357389233	mirna_pc
19499	hsa-mir-425	POLA1	0.30423333	mirna_pc
19500	hsa-mir-425	HDAC2	0.395968806	mirna_pc
19501	hsa-mir-425	GMNN	0.327266812	mirna_pc
19502	hsa-mir-425	PA2G4P4	0.40751613	mirna_pc
19503	hsa-mir-425	ASCL2	0.348161445	mirna_pc
19504	hsa-mir-425	BRI3BP	0.30182051	mirna_pc
19505	hsa-mir-425	FLVCR1	0.32959496	mirna_pc
19506	hsa-mir-425	NUDT3	0.307857375	mirna_pc
19507	hsa-mir-425	AGMAT	0.365430012	mirna_pc
19508	hsa-mir-425	BAT1	0.460841641	mirna_pc
19509	hsa-mir-425	VARS	0.35353818	mirna_pc
19510	hsa-mir-425	ZSCAN2	0.326914625	mirna_pc
19511	hsa-mir-425	MSH5	0.381555002	mirna_pc
19512	hsa-mir-425	PDIA3	0.341501038	mirna_pc
19513	hsa-mir-425	C2orf29	0.30721791	mirna_pc
19514	hsa-mir-425	ACAN	0.518416359	mirna_pc
19515	hsa-mir-425	B3GALT6	0.323846253	mirna_pc
19516	hsa-mir-425	SUZ12	0.355033412	mirna_pc
19517	hsa-mir-425	ZBTB12	0.387594551	mirna_pc
19518	hsa-mir-425	PBRM1	0.336116382	mirna_pc
19519	hsa-mir-425	PIK3AP1	0.407831616	mirna_pc
19520	hsa-mir-425	CIB2	0.364403722	mirna_pc
19521	hsa-mir-425	FAM96A	0.384477358	mirna_pc
19522	hsa-mir-425	SC65	0.387098171	mirna_pc
19523	hsa-mir-425	FGFR1OP	0.346940937	mirna_pc
19524	hsa-mir-425	METTL13	0.317519811	mirna_pc
19525	hsa-mir-425	POLE	0.303609425	mirna_pc
19526	hsa-mir-425	C1orf163	0.325482996	mirna_pc
19527	hsa-mir-425	TRIAP1	0.335564727	mirna_pc
19528	hsa-mir-425	SOX9	0.301982659	mirna_pc
19529	hsa-mir-425	C13orf27	0.332393669	mirna_pc
19530	hsa-mir-425	DDOST	0.327818474	mirna_pc
19531	hsa-mir-425	GK3P	0.319553303	mirna_pc
19532	hsa-mir-425	RBMX	0.314750818	mirna_pc
19533	hsa-mir-425	C9orf142	0.319950685	mirna_pc
19534	hsa-mir-425	PDIA3P	0.311827157	mirna_pc
19535	hsa-mir-425	MDC 1.00	0.325067495	mirna_pc
19536	hsa-mir-425	ATAD3B	0.427365629	mirna_pc
19537	hsa-mir-425	C11orf48	0.352768137	mirna_pc
19538	hsa-mir-425	PDIA6	0.33031557	mirna_pc
19539	hsa-mir-425	LINGO1	0.346913605	mirna_pc
19540	hsa-mir-425	DNAJC14	0.405963469	mirna_pc
19541	hsa-mir-425	DTNBP1	0.333437726	mirna_pc
19542	hsa-mir-425	PUSL1	0.315309432	mirna_pc
19543	hsa-mir-425	GK	0.337876558	mirna_pc
19544	hsa-mir-425	ABCF1	0.307319165	mirna_pc
19545	hsa-mir-425	CNPY2	0.324403349	mirna_pc
19546	hsa-mir-425	MPV17L	0.325730552	mirna_pc
19547	hsa-mir-425	FAM136A	0.390583107	mirna_pc

19548	hsa-mir-425	GDPD5	0.561342053	mirna_pc
19549	hsa-mir-425	SNORA8	0.338419646	mirna_pc
19550	hsa-mir-425	EML4	0.382724879	mirna_pc
19551	hsa-mir-425	LOC401010	0.302410301	mirna_pc
19552	hsa-mir-425	STRA13	0.341708852	mirna_pc
19553	hsa-mir-425	RPIA	0.305185657	mirna_pc
19554	hsa-mir-425	NOTUM	0.344357135	mirna_pc
19555	hsa-mir-425	NAT9	0.382671337	mirna_pc
19556	hsa-mir-425	KIAA1731	0.439197858	mirna_pc
19557	hsa-mir-425	SLC27A2	0.318433744	mirna_pc
19558	hsa-mir-425	PNRC2	0.303511254	mirna_pc
19559	hsa-mir-425	SPCS1	0.386780291	mirna_pc
19560	hsa-mir-425	SFXN4	0.335981065	mirna_pc
19561	hsa-mir-425	TAF11	0.320025562	mirna_pc
19562	hsa-mir-425	MINPP1	0.31759806	mirna_pc
19563	hsa-mir-425	RANBP17	0.390178073	mirna_pc
19564	hsa-mir-425	TMEM180	0.303723219	mirna_pc
19565	hsa-mir-425	ACOT4	0.326982705	mirna_pc
19566	hsa-mir-425	DAGLA	0.315061031	mirna_pc
19567	hsa-mir-425	CRLS1	0.361570862	mirna_pc
19568	hsa-mir-425	RWDD3	0.306961001	mirna_pc
19569	hsa-mir-425	ZNF620	0.311266853	mirna_pc
19570	hsa-mir-425	RNF34	0.340460035	mirna_pc
19571	hsa-mir-425	CHD7	0.345160504	mirna_pc
19572	hsa-mir-425	TOP 1.00	0.330460655	mirna_pc
19573	hsa-mir-425	FKBP10	0.322188985	mirna_pc
19574	hsa-mir-425	SEC61A2	0.373266319	mirna_pc
19575	hsa-mir-425	ZNF35	0.369025272	mirna_pc
19576	hsa-mir-425	MAPKAPK5	0.383773181	mirna_pc
19577	hsa-mir-425	GMCL1	0.344455686	mirna_pc
19578	hsa-mir-425	ZNF833	0.35503808	mirna_pc
19579	hsa-mir-425	SLC25A15	0.302254035	mirna_pc
19580	hsa-mir-425	B4GALT3	0.330435532	mirna_pc
19581	hsa-mir-425	SFRS13A	0.325481901	mirna_pc
19582	hsa-mir-425	TFAM	0.405312913	mirna_pc
19583	hsa-mir-425	NME6	0.314019275	mirna_pc
19584	hsa-mir-425	EAF1	0.320194074	mirna_pc
19585	hsa-mir-425	MORF4L1	0.301595003	mirna_pc
19586	hsa-mir-425	ACVR2B	0.318969248	mirna_pc
19587	hsa-mir-425	DCP1A	0.525550684	mirna_pc
19588	hsa-mir-425	GNL3	0.515725031	mirna_pc
19589	hsa-mir-425	METTL7B	0.343255568	mirna_pc
19590	hsa-mir-425	HCG18	0.331533582	mirna_pc
19591	hsa-mir-425	SLC25A19	0.358442463	mirna_pc
19592	hsa-mir-425	BEND3	0.387454856	mirna_pc
19593	hsa-mir-425	CRNKL1	0.332042527	mirna_pc
19594	hsa-mir-425	FZD5	0.323014442	mirna_pc
19595	hsa-mir-425	FGFR4	0.40313676	mirna_pc
19596	hsa-mir-425	CCAR1	0.35473688	mirna_pc
19597	hsa-mir-425	C3orf63	0.3122266	mirna_pc
19598	hsa-mir-425	ZNF184	0.306096803	mirna_pc
19599	hsa-mir-425	ATXN2	0.305944047	mirna_pc
19600	hsa-mir-425	SFMBT1	0.502760999	mirna_pc
19601	hsa-mir-425	GGT1	0.381089608	mirna_pc

19602	hsa-mir-425	ZSCAN16	0.320960077	mirna_pc
19603	hsa-mir-425	SEC11A	0.342219559	mirna_pc
19604	hsa-mir-425	ZNF780A	0.387592551	mirna_pc
19605	hsa-mir-425	CHDH	0.439067664	mirna_pc
19606	hsa-mir-425	EEF1E1	0.350651488	mirna_pc
19607	hsa-mir-425	ZNF697	0.377913158	mirna_pc
19608	hsa-mir-425	MRPL44	0.321737186	mirna_pc
19609	hsa-mir-425	SPRY4	0.422256762	mirna_pc
19610	hsa-mir-425	GJB1	0.401601654	mirna_pc
19611	hsa-mir-425	ZNF92	0.385631169	mirna_pc
19612	hsa-mir-425	TMTC4	0.30969529	mirna_pc
19613	hsa-mir-425	ACY1	0.302036652	mirna_pc
19614	hsa-mir-425	ZNF259	0.315563098	mirna_pc
19615	hsa-mir-425	RPS6KL1	0.345424309	mirna_pc
19616	hsa-mir-425	GLYCTK	0.350798588	mirna_pc
19617	hsa-mir-425	DDAH2	0.350495414	mirna_pc
19618	hsa-mir-425	NEK4	0.529212684	mirna_pc
19619	hsa-mir-425	CNNM3	0.347282515	mirna_pc
19620	hsa-mir-425	PCMTD2	0.329849622	mirna_pc
19621	hsa-mir-425	DPAGT1	0.427332436	mirna_pc
19622	hsa-mir-425	YBX2	0.371780728	mirna_pc
19623	hsa-mir-425	GPR137C	0.351696276	mirna_pc
19624	hsa-mir-425	NUP43	0.32967557	mirna_pc
19625	hsa-mir-425	RNF41	0.375386297	mirna_pc
19626	hsa-mir-425	ZNF124	0.388859324	mirna_pc
19627	hsa-mir-425	DGAT2	0.318489466	mirna_pc
19628	hsa-mir-425	TMEM62	0.348751745	mirna_pc
19629	hsa-mir-425	GRB14	0.336713788	mirna_pc
19630	hsa-mir-425	CHD1L	0.342978285	mirna_pc
19631	hsa-mir-425	ZNF473	0.309534875	mirna_pc
19632	hsa-mir-425	SNRNP48	0.335205994	mirna_pc
19633	hsa-mir-425	RFT1	0.415567454	mirna_pc
19634	hsa-mir-425	DCLRE1A	0.347398522	mirna_pc
19635	hsa-mir-425	RNF5	0.351302575	mirna_pc
19636	hsa-mir-425	ANP32A	0.354867233	mirna_pc
19637	hsa-mir-425	E2F5	0.38445207	mirna_pc
19638	hsa-mir-425	TMED5	0.345037884	mirna_pc
19639	hsa-mir-425	SEPHS2	0.324639672	mirna_pc
19640	hsa-mir-425	ZNF195	0.444441752	mirna_pc
19641	hsa-mir-425	MANF	0.461140853	mirna_pc
19642	hsa-mir-425	UBE2J2	0.30339705	mirna_pc
19643	hsa-mir-425	GLT8D1	0.437079558	mirna_pc
19644	hsa-mir-425	KIF3B	0.304759121	mirna_pc
19645	hsa-mir-425	SERPINA1	0.346707087	mirna_pc
19646	hsa-mir-425	VIL1	0.357412746	mirna_pc
19647	hsa-mir-425	SPCS2	0.347402098	mirna_pc
19648	hsa-mir-425	ASRGL1	0.367244306	mirna_pc
19649	hsa-mir-425	VPRBP	0.311148095	mirna_pc
19650	hsa-mir-425	PHF16	0.417955524	mirna_pc
19651	hsa-mir-425	BUD13	0.314264599	mirna_pc
19652	hsa-mir-425	MANEAL	0.307424335	mirna_pc
19653	hsa-mir-425	APAF1	0.303214611	mirna_pc
19654	hsa-mir-425	ZNF485	0.322053265	mirna_pc
19655	hsa-mir-425	KIN	0.324752826	mirna_pc

19656	hsa-mir-425	CMC1	0.320685382	mirna_pc
19657	hsa-mir-425	TRUB1	0.300680648	mirna_pc
19658	hsa-mir-425	C2orf15	0.384419908	mirna_pc
19659	hsa-mir-425	SLFN13	0.342846216	mirna_pc
19660	hsa-mir-425	CCNJ	0.437997144	mirna_pc
19661	hsa-mir-425	FOXA2	0.396560818	mirna_pc
19662	hsa-mir-425	AEN	0.300922536	mirna_pc
19663	hsa-mir-425	GDF15	0.379076492	mirna_pc
19664	hsa-mir-425	TMEM52	0.457449814	mirna_pc
19665	hsa-mir-425	FAM171A1	0.315258331	mirna_pc
19666	hsa-mir-425	ESF1	0.324368343	mirna_pc
19667	hsa-mir-425	HS2ST1	0.469027349	mirna_pc
19668	hsa-mir-425	SLC25A33	0.421262523	mirna_pc
19669	hsa-mir-425	GTF2H2C	0.368721892	mirna_pc
19670	hsa-mir-425	SMARCC1	0.341431351	mirna_pc
19671	hsa-mir-425	C2orf72	0.364432734	mirna_pc
19672	hsa-mir-425	NR5A2	0.369074442	mirna_pc
19673	hsa-mir-425	CTPS2	0.382476156	mirna_pc
19674	hsa-mir-425	KIF9	0.392119638	mirna_pc
19675	hsa-mir-425	GXYLT1	0.359337992	mirna_pc
19676	hsa-mir-425	RND1	0.302132423	mirna_pc
19677	hsa-mir-425	C10orf88	0.393590216	mirna_pc
19678	hsa-mir-425	MRPL20	0.303158859	mirna_pc
19679	hsa-mir-425	HNF1A	0.347042705	mirna_pc
19680	hsa-mir-425	C20orf46	0.383664732	mirna_pc
19681	hsa-mir-425	SNCAIP	0.386164654	mirna_pc
19682	hsa-mir-425	KIF12	0.528637634	mirna_pc
19683	hsa-mir-425	RNF32	0.423196632	mirna_pc
19684	hsa-mir-425	BMI1	0.30761929	mirna_pc
19685	hsa-mir-425	CYB561D2	0.325044665	mirna_pc
19686	hsa-mir-425	METTL12	0.308435229	mirna_pc
19687	hsa-mir-425	ENO3	0.384793646	mirna_pc
19688	hsa-mir-425	MED17	0.413843686	mirna_pc
19689	hsa-mir-425	VPS54	0.339033035	mirna_pc
19690	hsa-mir-425	ID2	0.424446556	mirna_pc
19691	hsa-mir-425	HYOU1	0.422785059	mirna_pc
19692	hsa-mir-425	C3orf75	0.399481266	mirna_pc
19693	hsa-mir-425	XYLB	0.36469966	mirna_pc
19694	hsa-mir-425	LOC221710	0.342398326	mirna_pc
19695	hsa-mir-425	ERBB3	0.3767217	mirna_pc
19696	hsa-mir-425	PLIN2	0.400569764	mirna_pc
19697	hsa-mir-425	LOC100130581	0.329977915	mirna_pc
19698	hsa-mir-425	LOC653566	0.313474755	mirna_pc
19699	hsa-mir-425	SYS1	0.330232411	mirna_pc
19700	hsa-mir-425	CCNI2	0.394424184	mirna_pc
19701	hsa-mir-425	CRYZ	0.300301678	mirna_pc
19702	hsa-mir-425	DHRS2	0.347521308	mirna_pc
19703	hsa-mir-425	ZNF664	0.382480747	mirna_pc
19704	hsa-mir-425	SLC39A7	0.339983105	mirna_pc
19705	hsa-mir-425	GOLT1A	0.316440792	mirna_pc
19706	hsa-mir-425	C20orf96	0.421379361	mirna_pc
19707	hsa-mir-425	PPP4R2	0.439330117	mirna_pc
19708	hsa-mir-425	ZCCHC3	0.379985403	mirna_pc
19709	hsa-mir-425	WDR73	0.505014249	mirna_pc

19710	hsa-mir-425	UBA3	0.317023961	mirna_pc
19711	hsa-mir-425	PROX1	0.379864461	mirna_pc
19712	hsa-mir-425	STX3	0.31079022	mirna_pc
19713	hsa-mir-425	C2orf43	0.336891269	mirna_pc
19714	hsa-mir-425	RNF5P1	0.345482484	mirna_pc
19715	hsa-mir-425	IL17RB	0.452979281	mirna_pc
19716	hsa-mir-425	ZNF341	0.315416713	mirna_pc
19717	hsa-mir-425	SEC23B	0.327379938	mirna_pc
19718	hsa-mir-425	CD320	0.324799291	mirna_pc
19719	hsa-mir-425	QPRT	0.322162636	mirna_pc
19720	hsa-mir-425	PREB	0.311778682	mirna_pc
19721	hsa-mir-425	ZNF443	0.377234356	mirna_pc
19722	hsa-mir-425	PRMT6	0.34312912	mirna_pc
19723	hsa-mir-425	CACNA1D	0.387168718	mirna_pc
19724	hsa-mir-425	NEU1	0.363228157	mirna_pc
19725	hsa-mir-425	TMED2	0.315288267	mirna_pc
19726	hsa-mir-425	SLC11A2	0.301377675	mirna_pc
19727	hsa-mir-425	PNKD	0.390211855	mirna_pc
19728	hsa-mir-425	BMF	0.320737176	mirna_pc
19729	hsa-mir-425	BAT3	0.309056355	mirna_pc
19730	hsa-mir-425	PRDX3	0.315594652	mirna_pc
19731	hsa-mir-425	ZNF799	0.408072205	mirna_pc
19732	hsa-mir-425	ZNF239	0.40350361	mirna_pc
19733	hsa-mir-425	TMEM14B	0.36714348	mirna_pc
19734	hsa-mir-425	NES	0.365545047	mirna_pc
19735	hsa-mir-425	SRCIN1	0.361480075	mirna_pc
19736	hsa-mir-425	ZNF77	0.4692047	mirna_pc
19737	hsa-mir-425	ZNF468	0.396721012	mirna_pc
19738	hsa-mir-425	SLC37A4	0.364620064	mirna_pc
19739	hsa-mir-425	EIF2S3	0.306316173	mirna_pc
19740	hsa-mir-425	CENPV	0.409422082	mirna_pc
19741	hsa-mir-425	WDSUB1	0.320803321	mirna_pc
19742	hsa-mir-425	NLK	0.309767925	mirna_pc
19743	hsa-mir-425	ZNF670	0.335890542	mirna_pc
19744	hsa-mir-425	ZNF28	0.308215982	mirna_pc
19745	hsa-mir-425	COQ5	0.33177327	mirna_pc
19746	hsa-mir-425	PIGM	0.361131058	mirna_pc
19747	hsa-mir-425	SLC2A8	0.382661199	mirna_pc
19748	hsa-mir-425	DOM3Z	0.376176554	mirna_pc
19749	hsa-mir-425	ARSE	0.340605955	mirna_pc
19750	hsa-mir-425	SS18L1	0.36662161	mirna_pc
19751	hsa-mir-92b	TPX2	0.344108214	mirna_pc
19752	hsa-mir-92b	CENPF	0.318232699	mirna_pc
19753	hsa-mir-92b	RCC2	0.3033912	mirna_pc
19754	hsa-mir-92b	HOXC9	0.395330212	mirna_pc
19755	hsa-mir-92b	KIF11	0.352124393	mirna_pc
19756	hsa-mir-92b	ECT2	0.309724323	mirna_pc
19757	hsa-mir-92b	CDK1	0.333294514	mirna_pc
19758	hsa-mir-92b	CDC25C	0.308666546	mirna_pc
19759	hsa-mir-92b	SEMA3E	0.431326878	mirna_pc
19760	hsa-mir-92b	PLK1	0.356830225	mirna_pc
19761	hsa-mir-92b	UBE2C	0.373963358	mirna_pc
19762	hsa-mir-92b	BUB1	0.320324397	mirna_pc
19763	hsa-mir-92b	PRC1	0.385891243	mirna_pc

19764	hsa-mir-92b	NUSAP1	0.380710752	mirna_pc
19765	hsa-mir-92b	KIFC1	0.383529508	mirna_pc
19766	hsa-mir-92b	BUB1B	0.370863076	mirna_pc
19767	hsa-mir-92b	HDGF	0.428137512	mirna_pc
19768	hsa-mir-92b	FAM72A	0.388148453	mirna_pc
19769	hsa-mir-92b	KIF2C	0.32379199	mirna_pc
19770	hsa-mir-92b	FANCA	0.319603443	mirna_pc
19771	hsa-mir-92b	NCAPG	0.411729585	mirna_pc
19772	hsa-mir-92b	CCNA2	0.354739975	mirna_pc
19773	hsa-mir-92b	CEP55	0.306622753	mirna_pc
19774	hsa-mir-92b	SPC25	0.405303996	mirna_pc
19775	hsa-mir-92b	IQGAP3	0.309982144	mirna_pc
19776	hsa-mir-92b	FANCI	0.316571673	mirna_pc
19777	hsa-mir-92b	NEK2	0.51399111	mirna_pc
19778	hsa-mir-92b	MND1	0.381679248	mirna_pc
19779	hsa-mir-92b	KIF22	0.424690579	mirna_pc
19780	hsa-mir-92b	NCAPH	0.344563509	mirna_pc
19781	hsa-mir-92b	RAD54L	0.383170523	mirna_pc
19782	hsa-mir-92b	CENPA	0.326860632	mirna_pc
19783	hsa-mir-92b	MAD2L1	0.46158417	mirna_pc
19784	hsa-mir-92b	TIMELESS	0.343610529	mirna_pc
19785	hsa-mir-92b	CDCA8	0.330598359	mirna_pc
19786	hsa-mir-92b	TROAP	0.314702323	mirna_pc
19787	hsa-mir-92b	CDCA5	0.353829506	mirna_pc
19788	hsa-mir-92b	XPO1	0.326053671	mirna_pc
19789	hsa-mir-92b	CDC45	0.300624557	mirna_pc
19790	hsa-mir-92b	STMN1	0.426653204	mirna_pc
19791	hsa-mir-92b	EXO1	0.346031632	mirna_pc
19792	hsa-mir-92b	UBE2T	0.504623037	mirna_pc
19793	hsa-mir-92b	CKS2	0.381863795	mirna_pc
19794	hsa-mir-92b	CKS1B	0.486857411	mirna_pc
19795	hsa-mir-92b	TUBB	0.330784394	mirna_pc
19796	hsa-mir-92b	NUF2	0.317781142	mirna_pc
19797	hsa-mir-92b	PLK4	0.323928252	mirna_pc
19798	hsa-mir-92b	DEPDC1	0.343712701	mirna_pc
19799	hsa-mir-92b	HJURP	0.320570812	mirna_pc
19800	hsa-mir-92b	RAD51	0.328311282	mirna_pc
19801	hsa-mir-92b	BLM	0.336416051	mirna_pc
19802	hsa-mir-92b	PAICS	0.320275887	mirna_pc
19803	hsa-mir-92b	BIRC5	0.312659544	mirna_pc
19804	hsa-mir-92b	OIP5	0.387167792	mirna_pc
19805	hsa-mir-92b	DTL	0.399943738	mirna_pc
19806	hsa-mir-92b	RECQL4	0.346257321	mirna_pc
19807	hsa-mir-92b	CBFB	0.306054435	mirna_pc
19808	hsa-mir-92b	CHEK2	0.385853278	mirna_pc
19809	hsa-mir-92b	C1orf112	0.367673701	mirna_pc
19810	hsa-mir-92b	FEN1	0.408668597	mirna_pc
19811	hsa-mir-92b	C16orf59	0.414772574	mirna_pc
19812	hsa-mir-92b	FAM72D	0.440290507	mirna_pc
19813	hsa-mir-92b	FAM72B	0.367541455	mirna_pc
19814	hsa-mir-92b	ZWINT	0.430960225	mirna_pc
19815	hsa-mir-92b	TUBA1B	0.321274358	mirna_pc
19816	hsa-mir-92b	WDR67	0.397134111	mirna_pc
19817	hsa-mir-92b	HOXC6	0.310615269	mirna_pc

19818	hsa-mir-92b	BUB3	0.438225558	mirna_pc
19819	hsa-mir-92b	CITED2	0.356195061	mirna_pc
19820	hsa-mir-92b	UBE2S	0.386538848	mirna_pc
19821	hsa-mir-92b	HNRNPC	0.332460138	mirna_pc
19822	hsa-mir-92b	ILF2	0.388697512	mirna_pc
19823	hsa-mir-92b	CHEK1	0.305869285	mirna_pc
19824	hsa-mir-92b	TYMS	0.358139896	mirna_pc
19825	hsa-mir-92b	MCM6	0.333048493	mirna_pc
19826	hsa-mir-92b	EPR1	0.325780413	mirna_pc
19827	hsa-mir-92b	H2AFX	0.31978975	mirna_pc
19828	hsa-mir-92b	UCK2	0.485481353	mirna_pc
19829	hsa-mir-92b	NFKBIL2	0.335609331	mirna_pc
19830	hsa-mir-92b	PRIM2	0.354649318	mirna_pc
19831	hsa-mir-92b	FAM60A	0.328369371	mirna_pc
19832	hsa-mir-92b	FAM64A	0.411066141	mirna_pc
19833	hsa-mir-92b	RANBP1	0.309229584	mirna_pc
19834	hsa-mir-92b	E2F1	0.374919992	mirna_pc
19835	hsa-mir-92b	MCM3	0.324415029	mirna_pc
19836	hsa-mir-92b	SNRPA	0.309298875	mirna_pc
19837	hsa-mir-92b	FUS	0.379961892	mirna_pc
19838	hsa-mir-92b	POC1A	0.337214784	mirna_pc
19839	hsa-mir-92b	ANP32E	0.35372783	mirna_pc
19840	hsa-mir-92b	CENPO	0.420484942	mirna_pc
19841	hsa-mir-92b	CENPL	0.342372958	mirna_pc
19842	hsa-mir-92b	CCT3	0.328255867	mirna_pc
19843	hsa-mir-92b	GINS2	0.331788255	mirna_pc
19844	hsa-mir-92b	NR2F2	0.320109835	mirna_pc
19845	hsa-mir-92b	SNRPD1	0.374246906	mirna_pc
19846	hsa-mir-92b	DSN1	0.341092828	mirna_pc
19847	hsa-mir-92b	SNHG1	0.318322841	mirna_pc
19848	hsa-mir-92b	TMEM206	0.320547012	mirna_pc
19849	hsa-mir-92b	C16orf75	0.354495216	mirna_pc
19850	hsa-mir-92b	TCF19	0.300717111	mirna_pc
19851	hsa-mir-92b	CAD	0.337887705	mirna_pc
19852	hsa-mir-92b	CDK2	0.372037549	mirna_pc
19853	hsa-mir-92b	CDK4	0.331271829	mirna_pc
19854	hsa-mir-92b	HOXA10	0.346370972	mirna_pc
19855	hsa-mir-92b	PFDN2	0.313146534	mirna_pc
19856	hsa-mir-92b	CKAP5	0.325078382	mirna_pc
19857	hsa-mir-92b	HNRNPR	0.384509962	mirna_pc
19858	hsa-mir-92b	CENPH	0.319368466	mirna_pc
19859	hsa-mir-92b	MSH2	0.401036619	mirna_pc
19860	hsa-mir-92b	PRPF19	0.313293993	mirna_pc
19861	hsa-mir-92b	EIF2AK1	0.311507601	mirna_pc
19862	hsa-mir-92b	SF3B3	0.424553736	mirna_pc
19863	hsa-mir-92b	CDC7	0.329377562	mirna_pc
19864	hsa-mir-92b	SSB	0.346928008	mirna_pc
19865	hsa-mir-92b	SNRPC	0.390763066	mirna_pc
19866	hsa-mir-92b	TXNDC12	0.345900176	mirna_pc
19867	hsa-mir-92b	PSMB4	0.308333767	mirna_pc
19868	hsa-mir-92b	RFC4	0.309369471	mirna_pc
19869	hsa-mir-92b	WDR75	0.372517474	mirna_pc
19870	hsa-mir-92b	MDK	0.333341958	mirna_pc
19871	hsa-mir-92b	BRIX1	0.372718701	mirna_pc

19872	hsa-mir-92b	H2AFZ	0.359485585	mirna_pc
19873	hsa-mir-92b	DSCC1	0.336455995	mirna_pc
19874	hsa-mir-92b	SMC2	0.334022351	mirna_pc
19875	hsa-mir-92b	SNRPE	0.473835747	mirna_pc
19876	hsa-mir-92b	C21orf45	0.312792174	mirna_pc
19877	hsa-mir-92b	ATIC	0.3143128	mirna_pc
19878	hsa-mir-92b	C5orf34	0.352435164	mirna_pc
19879	hsa-mir-92b	MARCKSL1	0.411561001	mirna_pc
19880	hsa-mir-92b	NPM3	0.38268146	mirna_pc
19881	hsa-mir-92b	ZNF367	0.34517517	mirna_pc
19882	hsa-mir-92b	NUTF2	0.35774574	mirna_pc
19883	hsa-mir-92b	RPA3	0.325619428	mirna_pc
19884	hsa-mir-92b	WDR34	0.444746368	mirna_pc
19885	hsa-mir-92b	MRPL9	0.382076515	mirna_pc
19886	hsa-mir-92b	PFDN4	0.307786896	mirna_pc
19887	hsa-mir-92b	DCTPP1	0.358352446	mirna_pc
19888	hsa-mir-92b	PSRC1	0.377337447	mirna_pc
19889	hsa-mir-92b	ODF2	0.386264258	mirna_pc
19890	hsa-mir-92b	HIST1H1E	0.314548565	mirna_pc
19891	hsa-mir-92b	PRCC	0.342146309	mirna_pc
19892	hsa-mir-92b	SET	0.331019331	mirna_pc
19893	hsa-mir-92b	SLC2A11	0.309545699	mirna_pc
19894	hsa-mir-92b	TYW3	0.419825486	mirna_pc
19895	hsa-mir-92b	LAPTM4B	0.355542376	mirna_pc
19896	hsa-mir-92b	PPAT	0.343146354	mirna_pc
19897	hsa-mir-92b	DHX9	0.373075534	mirna_pc
19898	hsa-mir-92b	CCDC138	0.325609781	mirna_pc
19899	hsa-mir-92b	LOC100128191	0.300584881	mirna_pc
19900	hsa-mir-92b	SFRS1	0.309194846	mirna_pc
19901	hsa-mir-92b	TEX10	0.315600672	mirna_pc
19902	hsa-mir-92b	SKP2	0.338647884	mirna_pc
19903	hsa-mir-92b	GAD1	0.336946008	mirna_pc
19904	hsa-mir-92b	C4orf46	0.33626448	mirna_pc
19905	hsa-mir-92b	MAZ	0.37998844	mirna_pc
19906	hsa-mir-92b	SSRP1	0.345710621	mirna_pc
19907	hsa-mir-92b	POLR2D	0.337364764	mirna_pc
19908	hsa-mir-92b	KHDRBS1	0.337743627	mirna_pc
19909	hsa-mir-92b	TUB	0.306138297	mirna_pc
19910	hsa-mir-92b	LOC92659	0.342475215	mirna_pc
19911	hsa-mir-92b	TOMM34	0.406580819	mirna_pc
19912	hsa-mir-92b	MTL 5.00	0.417853565	mirna_pc
19913	hsa-mir-92b	MAPRE1	0.315495785	mirna_pc
19914	hsa-mir-92b	C16orf93	0.35048613	mirna_pc
19915	hsa-mir-92b	FIGN	0.45079348	mirna_pc
19916	hsa-mir-92b	PARP1	0.378554419	mirna_pc
19917	hsa-mir-92b	SNRNP40	0.324218658	mirna_pc
19918	hsa-mir-92b	SLC39A10	0.449102754	mirna_pc
19919	hsa-mir-92b	SUV420H2	0.310206207	mirna_pc
19920	hsa-mir-92b	NOLC1	0.329521984	mirna_pc
19921	hsa-mir-92b	METTL1	0.305097413	mirna_pc
19922	hsa-mir-92b	LIN9	0.355803086	mirna_pc
19923	hsa-mir-92b	QSOX2	0.304712489	mirna_pc
19924	hsa-mir-92b	EXOSC2	0.335848645	mirna_pc
19925	hsa-mir-92b	SCARB1	0.30981264	mirna_pc

19926	hsa-mir-92b	C11orf84	0.389623828	mirna_pc
19927	hsa-mir-92b	TBCE	0.377524832	mirna_pc
19928	hsa-mir-92b	SUV39H2	0.312034372	mirna_pc
19929	hsa-mir-92b	HOXA9	0.34248595	mirna_pc
19930	hsa-mir-92b	LSM 2.00	0.390441982	mirna_pc
19931	hsa-mir-92b	GORAB	0.333451894	mirna_pc
19932	hsa-mir-92b	DPY30	0.341528867	mirna_pc
19933	hsa-mir-92b	SFRS3	0.335342724	mirna_pc
19934	hsa-mir-92b	SHOX2	0.376126913	mirna_pc
19935	hsa-mir-92b	SCAMP3	0.423738353	mirna_pc
19936	hsa-mir-92b	CCT4	0.31941724	mirna_pc
19937	hsa-mir-92b	ELAVL1	0.394684514	mirna_pc
19938	hsa-mir-92b	HABP4	0.413383489	mirna_pc
19939	hsa-mir-92b	NAE1	0.371433985	mirna_pc
19940	hsa-mir-92b	PAK1IP1	0.335570373	mirna_pc
19941	hsa-mir-92b	TWISTNB	0.344513011	mirna_pc
19942	hsa-mir-92b	ILF3	0.303280218	mirna_pc
19943	hsa-mir-92b	EHMT2	0.393628388	mirna_pc
19944	hsa-mir-92b	C1orf85	0.30174558	mirna_pc
19945	hsa-mir-92b	UBAP2L	0.360762673	mirna_pc
19946	hsa-mir-92b	TOR3A	0.313889044	mirna_pc
19947	hsa-mir-92b	RPGRIP1L	0.377063264	mirna_pc
19948	hsa-mir-92b	GTF2H4	0.411751254	mirna_pc
19949	hsa-mir-92b	HEATR2	0.410375676	mirna_pc
19950	hsa-mir-92b	LSM 6.00	0.303634309	mirna_pc
19951	hsa-mir-92b	PRAME	0.354516473	mirna_pc
19952	hsa-mir-92b	AZI1	0.314281406	mirna_pc
19953	hsa-mir-92b	C7orf27	0.338453651	mirna_pc
19954	hsa-mir-92b	WRB	0.310756697	mirna_pc
19955	hsa-mir-92b	IPO9	0.454205548	mirna_pc
19956	hsa-mir-92b	C15orf23	0.355670818	mirna_pc
19957	hsa-mir-92b	KDELC1	0.329306672	mirna_pc
19958	hsa-mir-92b	SLMO1	0.319675524	mirna_pc
19959	hsa-mir-92b	PMS2L1	0.300774913	mirna_pc
19960	hsa-mir-92b	ITGB3BP	0.379744635	mirna_pc
19961	hsa-mir-92b	SPG21	0.403853685	mirna_pc
19962	hsa-mir-92b	C16orf80	0.340119491	mirna_pc
19963	hsa-mir-92b	TMEM223	0.324185219	mirna_pc
19964	hsa-mir-92b	VPS72	0.383430996	mirna_pc
19965	hsa-mir-92b	RCOR2	0.443113178	mirna_pc
19966	hsa-mir-92b	FAM125B	0.306534627	mirna_pc
19967	hsa-mir-92b	AS3MT	0.369445203	mirna_pc
19968	hsa-mir-92b	HDAC2	0.36094552	mirna_pc
19969	hsa-mir-92b	CILP2	0.305626729	mirna_pc
19970	hsa-mir-92b	HAUS1	0.322961681	mirna_pc
19971	hsa-mir-92b	RRP12	0.351322882	mirna_pc
19972	hsa-mir-92b	POGK	0.33212609	mirna_pc
19973	hsa-mir-92b	SLC39A1	0.350936673	mirna_pc
19974	hsa-mir-92b	UBE2Q1	0.314364483	mirna_pc
19975	hsa-mir-92b	KREMEN2	0.300400104	mirna_pc
19976	hsa-mir-92b	NUDT3	0.343143099	mirna_pc
19977	hsa-mir-92b	MEX3A	0.332909161	mirna_pc
19978	hsa-mir-92b	BAT1	0.303423983	mirna_pc
19979	hsa-mir-92b	CCDC74A	0.479863089	mirna_pc

19980	hsa-mir-92b	TAF5	0.395923354	mirna_pc
19981	hsa-mir-92b	PDIA3	0.314234581	mirna_pc
19982	hsa-mir-92b	CCDC74B	0.463628986	mirna_pc
19983	hsa-mir-92b	RFX5	0.338230376	mirna_pc
19984	hsa-mir-92b	HIST3H2A	0.379662716	mirna_pc
19985	hsa-mir-92b	TSEN34	0.32237396	mirna_pc
19986	hsa-mir-92b	MAD2L2	0.347087376	mirna_pc
19987	hsa-mir-92b	HNRNPA1L2	0.324078405	mirna_pc
19988	hsa-mir-92b	RCCD1	0.400330756	mirna_pc
19989	hsa-mir-92b	ZBTB12	0.31976984	mirna_pc
19990	hsa-mir-92b	TADA1	0.326641798	mirna_pc
19991	hsa-mir-92b	DUSP12	0.49583785	mirna_pc
19992	hsa-mir-92b	ARL 3.00	0.376937585	mirna_pc
19993	hsa-mir-92b	H3F3A	0.33506738	mirna_pc
19994	hsa-mir-92b	PPIL6	0.363826955	mirna_pc
19995	hsa-mir-92b	METTL13	0.479635904	mirna_pc
19996	hsa-mir-92b	SEMA3D	0.430631516	mirna_pc
19997	hsa-mir-92b	C13orf27	0.331812557	mirna_pc
19998	hsa-mir-92b	RBMX	0.316702268	mirna_pc
19999	hsa-mir-92b	SRP9	0.336438113	mirna_pc
20000	hsa-mir-92b	DAP3	0.357389852	mirna_pc
20001	hsa-mir-92b	QPCTL	0.334128407	mirna_pc
20002	hsa-mir-92b	PDIA3P	0.32163743	mirna_pc
20003	hsa-mir-92b	MDC 1.00	0.325285491	mirna_pc
20004	hsa-mir-92b	OXTR	0.312235141	mirna_pc
20005	hsa-mir-92b	CLK2	0.348372394	mirna_pc
20006	hsa-mir-92b	ACD	0.367407201	mirna_pc
20007	hsa-mir-92b	GPN3	0.323195176	mirna_pc
20008	hsa-mir-92b	MRPL37	0.308838088	mirna_pc
20009	hsa-mir-92b	LOC440905	0.30146346	mirna_pc
20010	hsa-mir-92b	NAA40	0.307202512	mirna_pc
20011	hsa-mir-92b	GPATCH2	0.348449754	mirna_pc
20012	hsa-mir-92b	NAT14	0.301544451	mirna_pc
20013	hsa-mir-92b	PCGF6	0.384515735	mirna_pc
20014	hsa-mir-92b	WDR90	0.300340987	mirna_pc
20015	hsa-mir-92b	CNPY2	0.345108832	mirna_pc
20016	hsa-mir-92b	APOA1BP	0.314005286	mirna_pc
20017	hsa-mir-92b	CASC2	0.30319838	mirna_pc
20018	hsa-mir-92b	PYGO2	0.352851622	mirna_pc
20019	hsa-mir-92b	B3GAT3	0.340636168	mirna_pc
20020	hsa-mir-92b	FAM92A1	0.316157285	mirna_pc
20021	hsa-mir-92b	C12orf76	0.312827612	mirna_pc
20022	hsa-mir-92b	FAM189B	0.370051384	mirna_pc
20023	hsa-mir-92b	LRPPRC	0.313914279	mirna_pc
20024	hsa-mir-92b	POTEF	0.333489292	mirna_pc
20025	hsa-mir-92b	NUP133	0.332112665	mirna_pc
20026	hsa-mir-92b	LOC645676	0.327850287	mirna_pc
20027	hsa-mir-92b	BCL7A	0.329295338	mirna_pc
20028	hsa-mir-92b	WNT10A	0.30091019	mirna_pc
20029	hsa-mir-92b	AP3M1	0.310009192	mirna_pc
20030	hsa-mir-92b	MTX1	0.339019676	mirna_pc
20031	hsa-mir-92b	TRPA1	0.364317688	mirna_pc
20032	hsa-mir-92b	WDR70	0.332066134	mirna_pc
20033	hsa-mir-92b	RGS16	0.361803699	mirna_pc

20034	hsa-mir-92b	KCNC3	0.302259119	mirna_pc
20035	hsa-mir-92b	SETDB1	0.328066182	mirna_pc
20036	hsa-mir-92b	CHST10	0.374548125	mirna_pc
20037	hsa-mir-92b	ARF1	0.320315098	mirna_pc
20038	hsa-mir-92b	CUEDC2	0.328742815	mirna_pc
20039	hsa-mir-92b	PRR3	0.388643219	mirna_pc
20040	hsa-mir-92b	TMEM106C	0.313304545	mirna_pc
20041	hsa-mir-92b	RAB28	0.380848122	mirna_pc
20042	hsa-mir-92b	TAF4B	0.371389597	mirna_pc
20043	hsa-mir-92b	CDK20	0.309180892	mirna_pc
20044	hsa-mir-92b	RAD1	0.350254481	mirna_pc
20045	hsa-mir-92b	UBFD1	0.304541701	mirna_pc
20046	hsa-mir-92b	GANAB	0.360088843	mirna_pc
20047	hsa-mir-92b	GBAP1	0.303707272	mirna_pc
20048	hsa-mir-92b	TMEM67	0.331068113	mirna_pc
20049	hsa-mir-92b	SEC61A2	0.413795499	mirna_pc
20050	hsa-mir-92b	PSPC1	0.345122414	mirna_pc
20051	hsa-mir-92b	COPZ1	0.359355792	mirna_pc
20052	hsa-mir-92b	GPR3	0.462984725	mirna_pc
20053	hsa-mir-92b	B4GALT3	0.548660184	mirna_pc
20054	hsa-mir-92b	UROS	0.304229011	mirna_pc
20055	hsa-mir-92b	COQ10A	0.339098053	mirna_pc
20056	hsa-mir-92b	SCLY	0.404678646	mirna_pc
20057	hsa-mir-92b	GPR125	0.321655119	mirna_pc
20058	hsa-mir-92b	CTU2	0.313008766	mirna_pc
20059	hsa-mir-92b	NF2	0.303676596	mirna_pc
20060	hsa-mir-92b	GSTCD	0.316876989	mirna_pc
20061	hsa-mir-92b	MYBL1	0.336287874	mirna_pc
20062	hsa-mir-92b	DHX57	0.333366067	mirna_pc
20063	hsa-mir-92b	UBQLN4	0.380109414	mirna_pc
20064	hsa-mir-92b	CHST14	0.303650782	mirna_pc
20065	hsa-mir-92b	TMEM107	0.330720528	mirna_pc
20066	hsa-mir-92b	SFT2D3	0.406461712	mirna_pc
20067	hsa-mir-92b	TMEFF1	0.407827406	mirna_pc
20068	hsa-mir-92b	AKR7A2	0.316182865	mirna_pc
20069	hsa-mir-92b	SFRS7	0.348165953	mirna_pc
20070	hsa-mir-92b	C18orf10	0.326680451	mirna_pc
20071	hsa-mir-92b	PFAS	0.431697642	mirna_pc
20072	hsa-mir-92b	PAIP1	0.374619198	mirna_pc
20073	hsa-mir-92b	C10orf35	0.319083995	mirna_pc
20074	hsa-mir-92b	DNM3	0.422838993	mirna_pc
20075	hsa-mir-92b	LRRC45	0.30768265	mirna_pc
20076	hsa-mir-92b	CDCA7L	0.346922271	mirna_pc
20077	hsa-mir-92b	WDR78	0.304788261	mirna_pc
20078	hsa-mir-92b	IFT81	0.424024733	mirna_pc
20079	hsa-mir-92b	ZNF184	0.315647649	mirna_pc
20080	hsa-mir-92b	NIPSNAP1	0.426960614	mirna_pc
20081	hsa-mir-92b	C8orf37	0.415481746	mirna_pc
20082	hsa-mir-92b	C2orf77	0.336056748	mirna_pc
20083	hsa-mir-92b	SPA17	0.420084759	mirna_pc
20084	hsa-mir-92b	LRP4	0.321293936	mirna_pc
20085	hsa-mir-92b	SFI1	0.302561423	mirna_pc
20086	hsa-mir-92b	ZBTB10	0.340487158	mirna_pc
20087	hsa-mir-92b	TIMM17A	0.361498681	mirna_pc

20088	hsa-mir-92b	ZNF771	0.398099843	mirna_pc
20089	hsa-mir-92b	EEF1E1	0.33454105	mirna_pc
20090	hsa-mir-92b	PRUNE	0.333762648	mirna_pc
20091	hsa-mir-92b	TCTN2	0.436483495	mirna_pc
20092	hsa-mir-92b	NPHP1	0.351883929	mirna_pc
20093	hsa-mir-92b	ANGPT1	0.424882239	mirna_pc
20094	hsa-mir-92b	EXTL2	0.365115922	mirna_pc
20095	hsa-mir-92b	LOC440173	0.322611793	mirna_pc
20096	hsa-mir-92b	SEPHS1	0.381812886	mirna_pc
20097	hsa-mir-92b	SHISA2	0.321013566	mirna_pc
20098	hsa-mir-92b	TMEM183A	0.389575202	mirna_pc
20099	hsa-mir-92b	TMEM9	0.462441802	mirna_pc
20100	hsa-mir-92b	AGBL5	0.316644861	mirna_pc
20101	hsa-mir-92b	ISYNA1	0.310214866	mirna_pc
20102	hsa-mir-92b	MYL6B	0.305001283	mirna_pc
20103	hsa-mir-92b	LOC338758	0.367972242	mirna_pc
20104	hsa-mir-92b	PIPSL	0.412090442	mirna_pc
20105	hsa-mir-92b	RNF138	0.315022549	mirna_pc
20106	hsa-mir-92b	CD200	0.308963644	mirna_pc
20107	hsa-mir-92b	GPR137C	0.32347273	mirna_pc
20108	hsa-mir-92b	BRD3	0.357221744	mirna_pc
20109	hsa-mir-92b	TMEM14C	0.383379012	mirna_pc
20110	hsa-mir-92b	NME4	0.327341029	mirna_pc
20111	hsa-mir-92b	LYRM4	0.370696796	mirna_pc
20112	hsa-mir-92b	PIP5K1A	0.435113278	mirna_pc
20113	hsa-mir-92b	C9orf116	0.373662753	mirna_pc
20114	hsa-mir-92b	GRB14	0.390271205	mirna_pc
20115	hsa-mir-92b	CCDC113	0.400779893	mirna_pc
20116	hsa-mir-92b	CHD1L	0.384947293	mirna_pc
20117	hsa-mir-92b	C11orf49	0.357147035	mirna_pc
20118	hsa-mir-92b	USP21	0.521184642	mirna_pc
20119	hsa-mir-92b	DPCD	0.446587407	mirna_pc
20120	hsa-mir-92b	KLHL12	0.349713099	mirna_pc
20121	hsa-mir-92b	RNF5	0.33738051	mirna_pc
20122	hsa-mir-92b	ANP32A	0.401762246	mirna_pc
20123	hsa-mir-92b	OSGEPL1	0.355838649	mirna_pc
20124	hsa-mir-92b	E2F5	0.326714043	mirna_pc
20125	hsa-mir-92b	SUPV3L1	0.338077859	mirna_pc
20126	hsa-mir-92b	LMAN2L	0.305382028	mirna_pc
20127	hsa-mir-92b	FAM104B	0.301992154	mirna_pc
20128	hsa-mir-92b	C17orf81	0.367483236	mirna_pc
20129	hsa-mir-92b	HNRNPH3	0.36000539	mirna_pc
20130	hsa-mir-92b	B4GALT6	0.314974267	mirna_pc
20131	hsa-mir-92b	NSL1	0.349058641	mirna_pc
20132	hsa-mir-92b	CNIH2	0.533771883	mirna_pc
20133	hsa-mir-92b	BCCIP	0.326824694	mirna_pc
20134	hsa-mir-92b	IQCC	0.309981737	mirna_pc
20135	hsa-mir-92b	DDX19A	0.349463263	mirna_pc
20136	hsa-mir-92b	EID3	0.401610768	mirna_pc
20137	hsa-mir-92b	MORN2	0.367325571	mirna_pc
20138	hsa-mir-92b	GATS	0.300492562	mirna_pc
20139	hsa-mir-92b	MLLT11	0.400144445	mirna_pc
20140	hsa-mir-92b	TRUB1	0.312953633	mirna_pc
20141	hsa-mir-92b	PSTK	0.314711497	mirna_pc

20142	hsa-mir-92b	C2orf15	0.343613326	mirna_pc
20143	hsa-mir-92b	C12orf24	0.509551618	mirna_pc
20144	hsa-mir-92b	SBK1	0.366566485	mirna_pc
20145	hsa-mir-92b	NFYB	0.322096186	mirna_pc
20146	hsa-mir-92b	NCRNA00094	0.40453076	mirna_pc
20147	hsa-mir-92b	SEMA3A	0.347297019	mirna_pc
20148	hsa-mir-92b	THNSL1	0.357231562	mirna_pc
20149	hsa-mir-92b	USP13	0.423398738	mirna_pc
20150	hsa-mir-92b	UFC1	0.30437452	mirna_pc
20151	hsa-mir-92b	CP110	0.306388734	mirna_pc
20152	hsa-mir-92b	NGFRAP1	0.301032575	mirna_pc
20153	hsa-mir-92b	MSI1	0.305117293	mirna_pc
20154	hsa-mir-92b	IFT74	0.373852463	mirna_pc
20155	hsa-mir-92b	NR5A2	0.333428242	mirna_pc
20156	hsa-mir-92b	C16orf48	0.416544253	mirna_pc
20157	hsa-mir-92b	FGD1	0.331454573	mirna_pc
20158	hsa-mir-92b	MDM1	0.394746522	mirna_pc
20159	hsa-mir-92b	C10orf88	0.359393411	mirna_pc
20160	hsa-mir-92b	TRO	0.371310003	mirna_pc
20161	hsa-mir-92b	PACRGL	0.362064694	mirna_pc
20162	hsa-mir-92b	PBX2	0.34604053	mirna_pc
20163	hsa-mir-92b	RAB36	0.380814535	mirna_pc
20164	hsa-mir-92b	SLITRK6	0.383750429	mirna_pc
20165	hsa-mir-92b	ZNF232	0.32943229	mirna_pc
20166	hsa-mir-92b	LYSMD1	0.3258525	mirna_pc
20167	hsa-mir-92b	METTL10	0.382581217	mirna_pc
20168	hsa-mir-92b	HAPLN1	0.402513857	mirna_pc
20169	hsa-mir-92b	RAC3	0.359097554	mirna_pc
20170	hsa-mir-92b	C1orf43	0.30162074	mirna_pc
20171	hsa-mir-92b	PPOX	0.414639506	mirna_pc
20172	hsa-mir-92b	RBBP4	0.42373122	mirna_pc
20173	hsa-mir-92b	LOC221710	0.34257399	mirna_pc
20174	hsa-mir-92b	DKFZP686I15217	0.325021148	mirna_pc
20175	hsa-mir-92b	BRSK1	0.357092321	mirna_pc
20176	hsa-mir-92b	IQCE	0.325475949	mirna_pc
20177	hsa-mir-92b	CRYZ	0.469123733	mirna_pc
20178	hsa-mir-92b	SPIN2B	0.316716543	mirna_pc
20179	hsa-mir-92b	LRDD	0.302523643	mirna_pc
20180	hsa-mir-92b	PDGFA	0.360696746	mirna_pc
20181	hsa-mir-92b	KDELC2	0.302286662	mirna_pc
20182	hsa-mir-92b	C20orf96	0.309247624	mirna_pc
20183	hsa-mir-92b	TCTN3	0.409151945	mirna_pc
20184	hsa-mir-92b	HSF2	0.324688097	mirna_pc
20185	hsa-mir-92b	RRP15	0.356947111	mirna_pc
20186	hsa-mir-92b	ETV5	0.35715857	mirna_pc
20187	hsa-mir-92b	NUCKS1	0.329286612	mirna_pc
20188	hsa-mir-92b	TCTN1	0.418325811	mirna_pc
20189	hsa-mir-92b	CHID1	0.312860439	mirna_pc
20190	hsa-mir-92b	EXOSC6	0.317986067	mirna_pc
20191	hsa-mir-92b	CTXN1	0.570255805	mirna_pc
20192	hsa-mir-92b	B4GALNT4	0.363865737	mirna_pc
20193	hsa-mir-92b	PRMT6	0.311353824	mirna_pc
20194	hsa-mir-92b	BAI2	0.42269189	mirna_pc
20195	hsa-mir-92b	LNP1	0.342718822	mirna_pc

20196	hsa-mir-92b	PRDX3	0.368368609	mirna_pc
20197	hsa-mir-92b	TMEM14B	0.41828582	mirna_pc
20198	hsa-mir-92b	DYNC2LI1	0.340531574	mirna_pc
20199	hsa-mir-92b	RBBP7	0.304228861	mirna_pc
20200	hsa-mir-92b	NT5M	0.352887841	mirna_pc
20201	hsa-mir-92b	LCA5	0.408902278	mirna_pc
20202	hsa-mir-92b	CDC73	0.328224084	mirna_pc
20203	hsa-mir-92b	DNMT3A	0.301277865	mirna_pc
20204	hsa-mir-92b	WDSUB1	0.314314764	mirna_pc
20205	hsa-mir-92b	TET1	0.317872372	mirna_pc
20206	hsa-mir-92b	APOO	0.301869268	mirna_pc
20207	hsa-mir-92b	C12orf73	0.322034177	mirna_pc
20208	hsa-mir-92b	DDX59	0.308587952	mirna_pc
20209	hsa-mir-92b	PRAF2	0.306043307	mirna_pc
20210	hsa-mir-92b	TMEM231	0.608532109	mirna_pc
20211	hsa-mir-4326	TPX2	0.317409637	mirna_pc
20212	hsa-mir-4326	KIF4B	0.300766708	mirna_pc
20213	hsa-mir-4326	UBE2C	0.353775733	mirna_pc
20214	hsa-mir-4326	BUB1	0.329686606	mirna_pc
20215	hsa-mir-4326	ASPM	0.325156574	mirna_pc
20216	hsa-mir-4326	FAM72A	0.375315869	mirna_pc
20217	hsa-mir-4326	FANCA	0.340445579	mirna_pc
20218	hsa-mir-4326	NCAPG	0.317342095	mirna_pc
20219	hsa-mir-4326	CLSPN	0.324870773	mirna_pc
20220	hsa-mir-4326	SGOL1	0.445137403	mirna_pc
20221	hsa-mir-4326	ARHGAP11A	0.315902972	mirna_pc
20222	hsa-mir-4326	CBX3	0.328720185	mirna_pc
20223	hsa-mir-4326	IQGAP3	0.308609832	mirna_pc
20224	hsa-mir-4326	NEK2	0.330911479	mirna_pc
20225	hsa-mir-4326	KIF23	0.303481537	mirna_pc
20226	hsa-mir-4326	TROAP	0.345805877	mirna_pc
20227	hsa-mir-4326	XPO1	0.375629225	mirna_pc
20228	hsa-mir-4326	CDCA2	0.317334765	mirna_pc
20229	hsa-mir-4326	DKC1	0.341051229	mirna_pc
20230	hsa-mir-4326	KIF4A	0.354871621	mirna_pc
20231	hsa-mir-4326	CENPI	0.302593028	mirna_pc
20232	hsa-mir-4326	NUF2	0.356819562	mirna_pc
20233	hsa-mir-4326	FANCB	0.324417662	mirna_pc
20234	hsa-mir-4326	HJURP	0.300649176	mirna_pc
20235	hsa-mir-4326	OIP5	0.352869695	mirna_pc
20236	hsa-mir-4326	RECQL4	0.33036031	mirna_pc
20237	hsa-mir-4326	AURKA	0.501408223	mirna_pc
20238	hsa-mir-4326	SGOL2	0.301406541	mirna_pc
20239	hsa-mir-4326	CHEK2	0.329638082	mirna_pc
20240	hsa-mir-4326	KIF15	0.377764239	mirna_pc
20241	hsa-mir-4326	C1orf112	0.382582809	mirna_pc
20242	hsa-mir-4326	FAM72D	0.382819389	mirna_pc
20243	hsa-mir-4326	FAM72B	0.380938521	mirna_pc
20244	hsa-mir-4326	C12orf48	0.345814912	mirna_pc
20245	hsa-mir-4326	TTK	0.313727319	mirna_pc
20246	hsa-mir-4326	C6orf167	0.308257514	mirna_pc
20247	hsa-mir-4326	HNRNPA2B1	0.303744426	mirna_pc
20248	hsa-mir-4326	C20orf20	0.327567552	mirna_pc
20249	hsa-mir-4326	KIAA1524	0.306160075	mirna_pc

20250	hsa-mir-4326	KIF20B	0.339368976	mirna_pc
20251	hsa-mir-4326	ATAD2	0.304086637	mirna_pc
20252	hsa-mir-4326	C1orf135	0.369142023	mirna_pc
20253	hsa-mir-4326	ANP32E	0.38973346	mirna_pc
20254	hsa-mir-4326	CENPL	0.37976649	mirna_pc
20255	hsa-mir-4326	DNA2	0.315058415	mirna_pc
20256	hsa-mir-4326	DSN1	0.30642539	mirna_pc
20257	hsa-mir-4326	CSE1L	0.322141234	mirna_pc
20258	hsa-mir-4326	TPM3	0.30161436	mirna_pc
20259	hsa-mir-4326	HNRNPR	0.324840862	mirna_pc
20260	hsa-mir-4326	MSH2	0.348730628	mirna_pc
20261	hsa-mir-4326	DBF4	0.310996191	mirna_pc
20262	hsa-mir-4326	EIF2AK1	0.326116582	mirna_pc
20263	hsa-mir-4326	SSB	0.375554434	mirna_pc
20264	hsa-mir-4326	FBXO5	0.322352937	mirna_pc
20265	hsa-mir-4326	E2F3	0.320061307	mirna_pc
20266	hsa-mir-4326	CDC25A	0.303311298	mirna_pc
20267	hsa-mir-4326	RAD54B	0.373288943	mirna_pc
20268	hsa-mir-4326	C21orf45	0.305799205	mirna_pc
20269	hsa-mir-4326	C5orf34	0.381337	mirna_pc
20270	hsa-mir-4326	NCL	0.310258972	mirna_pc
20271	hsa-mir-4326	SRRT	0.310194955	mirna_pc
20272	hsa-mir-4326	SASS6	0.382914948	mirna_pc
20273	hsa-mir-4326	PNPT1	0.324104245	mirna_pc
20274	hsa-mir-4326	CASP8	0.327941094	mirna_pc
20275	hsa-mir-4326	CCDC18	0.4034447	mirna_pc
20276	hsa-mir-4326	DNAJC2	0.347378915	mirna_pc
20277	hsa-mir-4326	INTS8	0.366076722	mirna_pc
20278	hsa-mir-4326	SUV420H2	0.327993596	mirna_pc
20279	hsa-mir-4326	ZNF695	0.334532356	mirna_pc
20280	hsa-mir-4326	LIN9	0.327822043	mirna_pc
20281	hsa-mir-4326	POLR1B	0.340929409	mirna_pc
20282	hsa-mir-4326	ACTR2	0.384826641	mirna_pc
20283	hsa-mir-4326	HSP90B1	0.406746559	mirna_pc
20284	hsa-mir-4326	YTHDF1	0.344965077	mirna_pc
20285	hsa-mir-4326	ATP13A3	0.334396434	mirna_pc
20286	hsa-mir-4326	NAA25	0.317745203	mirna_pc
20287	hsa-mir-4326	MAGOHB	0.385978863	mirna_pc
20288	hsa-mir-4326	DEK	0.32486707	mirna_pc
20289	hsa-mir-4326	PLOD3	0.30652341	mirna_pc
20290	hsa-mir-4326	TAF4	0.434044598	mirna_pc
20291	hsa-mir-4326	AMPD2	0.30116432	mirna_pc
20292	hsa-mir-4326	RPN2	0.355763925	mirna_pc
20293	hsa-mir-4326	BAT2L2	0.338610279	mirna_pc
20294	hsa-mir-4326	TDG	0.352661782	mirna_pc
20295	hsa-mir-4326	HSPA14	0.322711038	mirna_pc
20296	hsa-mir-4326	UCKL1	0.336185328	mirna_pc
20297	hsa-mir-4326	DDX55	0.310076946	mirna_pc
20298	hsa-mir-4326	CDK5RAP1	0.334763988	mirna_pc
20299	hsa-mir-4326	CNOT3	0.300815803	mirna_pc
20300	hsa-mir-4326	SMC3	0.340897843	mirna_pc
20301	hsa-mir-4326	BAT1	0.320685355	mirna_pc
20302	hsa-mir-4326	USP1	0.325387479	mirna_pc
20303	hsa-mir-4326	TMEM68	0.318759133	mirna_pc

20304	hsa-mir-4326	FARSB	0.314688062	mirna_pc
20305	hsa-mir-4326	MTF2	0.330526486	mirna_pc
20306	hsa-mir-4326	FGFR1OP	0.349365743	mirna_pc
20307	hsa-mir-4326	METTL13	0.31647456	mirna_pc
20308	hsa-mir-4326	DDOST	0.369504336	mirna_pc
20309	hsa-mir-4326	ATAD3B	0.348112972	mirna_pc
20310	hsa-mir-4326	ZNF137	0.311990382	mirna_pc
20311	hsa-mir-4326	PDIA6	0.314822518	mirna_pc
20312	hsa-mir-4326	LOC440905	0.321739599	mirna_pc
20313	hsa-mir-4326	LTV1	0.331543486	mirna_pc
20314	hsa-mir-4326	MTPAP	0.301670368	mirna_pc
20315	hsa-mir-4326	ZNF701	0.315528389	mirna_pc
20316	hsa-mir-4326	ARFGAP1	0.390940678	mirna_pc
20317	hsa-mir-4326	ISPD	0.326534949	mirna_pc
20318	hsa-mir-4326	KCNC3	0.357490191	mirna_pc
20319	hsa-mir-4326	MORF4L2	0.320571556	mirna_pc
20320	hsa-mir-4326	KCNQ1OT1	0.411726555	mirna_pc
20321	hsa-mir-4326	NKAP	0.301488005	mirna_pc
20322	hsa-mir-4326	UPF3B	0.349788925	mirna_pc
20323	hsa-mir-4326	MGC72080	0.306232152	mirna_pc
20324	hsa-mir-4326	TBX3	0.304511111	mirna_pc
20325	hsa-mir-4326	SLC25A13	0.382748128	mirna_pc
20326	hsa-mir-4326	TOP 1.00	0.306535899	mirna_pc
20327	hsa-mir-4326	SEC61A2	0.303040161	mirna_pc
20328	hsa-mir-4326	SSX2IP	0.32322523	mirna_pc
20329	hsa-mir-4326	KDELR2	0.442931805	mirna_pc
20330	hsa-mir-4326	SFRS13A	0.32837721	mirna_pc
20331	hsa-mir-4326	C5orf28	0.406691312	mirna_pc
20332	hsa-mir-4326	SS18L2	0.390155645	mirna_pc
20333	hsa-mir-4326	MYBL1	0.303655179	mirna_pc
20334	hsa-mir-4326	MTIF2	0.326028387	mirna_pc
20335	hsa-mir-4326	FAM35B2	0.354851767	mirna_pc
20336	hsa-mir-4326	TCTE3	0.363293246	mirna_pc
20337	hsa-mir-4326	DPH3B	0.302524482	mirna_pc
20338	hsa-mir-4326	THOC1	0.337137432	mirna_pc
20339	hsa-mir-4326	MRPL42	0.312270036	mirna_pc
20340	hsa-mir-4326	C7orf36	0.32077335	mirna_pc
20341	hsa-mir-4326	AXIN2	0.310388391	mirna_pc
20342	hsa-mir-4326	CCAR1	0.379218398	mirna_pc
20343	hsa-mir-4326	FAM122C	0.317256117	mirna_pc
20344	hsa-mir-4326	FAM35B	0.30108747	mirna_pc
20345	hsa-mir-4326	EEF1E1	0.324485071	mirna_pc
20346	hsa-mir-4326	PRPF4B	0.302222291	mirna_pc
20347	hsa-mir-4326	ZNF525	0.306246609	mirna_pc
20348	hsa-mir-4326	YIPF4	0.321295281	mirna_pc
20349	hsa-mir-4326	TSPAN13	0.351135074	mirna_pc
20350	hsa-mir-4326	C6orf211	0.506265491	mirna_pc
20351	hsa-mir-4326	NUP43	0.35545682	mirna_pc
20352	hsa-mir-4326	COPA	0.3665361	mirna_pc
20353	hsa-mir-4326	SNRNP48	0.301867419	mirna_pc
20354	hsa-mir-4326	FAM98B	0.355114123	mirna_pc
20355	hsa-mir-4326	LRFN1	0.316124653	mirna_pc
20356	hsa-mir-4326	PRPF38B	0.307699068	mirna_pc
20357	hsa-mir-4326	C6orf182	0.336896141	mirna_pc

20358	hsa-mir-4326	TRUB1	0.321807758	mirna_pc
20359	hsa-mir-4326	RGPD1	0.32242172	mirna_pc
20360	hsa-mir-4326	LCA5L	0.329051166	mirna_pc
20361	hsa-mir-4326	SEMA3A	0.314901306	mirna_pc
20362	hsa-mir-4326	COPB1	0.364949919	mirna_pc
20363	hsa-mir-4326	RTN4IP1	0.314791424	mirna_pc
20364	hsa-mir-4326	HS2ST1	0.332954762	mirna_pc
20365	hsa-mir-4326	C10orf88	0.339234931	mirna_pc
20366	hsa-mir-4326	LOC727896	0.320716767	mirna_pc
20367	hsa-mir-4326	BMI1	0.423684829	mirna_pc
20368	hsa-mir-4326	FANCF	0.316095977	mirna_pc
20369	hsa-mir-4326	XYLB	0.328698086	mirna_pc
20370	hsa-mir-4326	RBBP6	0.30268956	mirna_pc
20371	hsa-mir-4326	MRPL19	0.332110568	mirna_pc
20372	hsa-mir-4326	PGM3	0.305934118	mirna_pc
20373	hsa-mir-4326	GLB1	0.305801972	mirna_pc
20374	hsa-mir-4326	DDX1	0.373965278	mirna_pc
20375	hsa-mir-4326	C21orf125	0.388957515	mirna_pc
20376	hsa-mir-4326	C6orf62	0.304741606	mirna_pc
20377	hsa-mir-4326	TMED2	0.319308981	mirna_pc
20378	hsa-mir-4326	GPR89A	0.311796129	mirna_pc
20379	hsa-mir-4326	ZNF765	0.360584937	mirna_pc
20380	hsa-mir-4326	PPIL4	0.319578952	mirna_pc
20381	hsa-mir-4326	HRSP12	0.325853865	mirna_pc
20382	hsa-mir-4326	PHIP	0.302423893	mirna_pc
20383	hsa-mir-4326	MAPK8	0.347046126	mirna_pc
20384	hsa-mir-4326	SLC2A3	0.301120388	mirna_pc
20385	hsa-mir-4326	SS18L1	0.325796674	mirna_pc
20386	hsa-mir-1248	HOXC8	0.358142103	mirna_pc
20387	hsa-mir-1248	SEMA3E	0.622382141	mirna_pc
20388	hsa-mir-1248	EEF1A2	0.376980785	mirna_pc
20389	hsa-mir-1248	XPO1	0.302387472	mirna_pc
20390	hsa-mir-1248	MYST4	0.346742882	mirna_pc
20391	hsa-mir-1248	YPEL1	0.333696245	mirna_pc
20392	hsa-mir-1248	CITED2	0.549212755	mirna_pc
20393	hsa-mir-1248	NR2F2	0.522829613	mirna_pc
20394	hsa-mir-1248	HIST1H2AE	0.303878947	mirna_pc
20395	hsa-mir-1248	ZNF10	0.305814469	mirna_pc
20396	hsa-mir-1248	MSH2	0.331782365	mirna_pc
20397	hsa-mir-1248	CDC7	0.390104322	mirna_pc
20398	hsa-mir-1248	POLI	0.411580942	mirna_pc
20399	hsa-mir-1248	SMC2	0.492101563	mirna_pc
20400	hsa-mir-1248	ZNF367	0.470858203	mirna_pc
20401	hsa-mir-1248	USP25	0.391179045	mirna_pc
20402	hsa-mir-1248	LOC375190	0.427038882	mirna_pc
20403	hsa-mir-1248	CRY1	0.40217198	mirna_pc
20404	hsa-mir-1248	HIST1H3D	0.433083597	mirna_pc
20405	hsa-mir-1248	SLC2A11	0.402850114	mirna_pc
20406	hsa-mir-1248	ZNF433	0.377995546	mirna_pc
20407	hsa-mir-1248	TYW3	0.551582911	mirna_pc
20408	hsa-mir-1248	ACAD10	0.372529313	mirna_pc
20409	hsa-mir-1248	TEX10	0.349205084	mirna_pc
20410	hsa-mir-1248	GAD1	0.373263632	mirna_pc
20411	hsa-mir-1248	OXR1	0.320385273	mirna_pc

20412	hsa-mir-1248	FIGN	0.628583479	mirna_pc
20413	hsa-mir-1248	CCDC18	0.319975707	mirna_pc
20414	hsa-mir-1248	PRRT1	0.32463768	mirna_pc
20415	hsa-mir-1248	SAMD11	0.355594403	mirna_pc
20416	hsa-mir-1248	PHF10	0.37972789	mirna_pc
20417	hsa-mir-1248	CAPS2	0.506075062	mirna_pc
20418	hsa-mir-1248	PBX3	0.371977746	mirna_pc
20419	hsa-mir-1248	CCDC121	0.336698233	mirna_pc
20420	hsa-mir-1248	HABP4	0.589684503	mirna_pc
20421	hsa-mir-1248	TBCEL	0.306744416	mirna_pc
20422	hsa-mir-1248	C20orf160	0.32270474	mirna_pc
20423	hsa-mir-1248	RNF2	0.307522213	mirna_pc
20424	hsa-mir-1248	SFRS18	0.316309898	mirna_pc
20425	hsa-mir-1248	CCNE2	0.338567199	mirna_pc
20426	hsa-mir-1248	CTSL2	0.513757968	mirna_pc
20427	hsa-mir-1248	PTGER4	0.337466255	mirna_pc
20428	hsa-mir-1248	PRAME	0.313886763	mirna_pc
20429	hsa-mir-1248	HNRNPK	0.331273219	mirna_pc
20430	hsa-mir-1248	KIAA0776	0.383146177	mirna_pc
20431	hsa-mir-1248	CA11	0.320821406	mirna_pc
20432	hsa-mir-1248	TTC33	0.394553419	mirna_pc
20433	hsa-mir-1248	CTNNAL1	0.338648611	mirna_pc
20434	hsa-mir-1248	TDG	0.450523473	mirna_pc
20435	hsa-mir-1248	EIF4ENIF1	0.33430356	mirna_pc
20436	hsa-mir-1248	BAIAP3	0.315665331	mirna_pc
20437	hsa-mir-1248	RAD23B	0.351690976	mirna_pc
20438	hsa-mir-1248	TUG1	0.373749288	mirna_pc
20439	hsa-mir-1248	ODF2L	0.335262305	mirna_pc
20440	hsa-mir-1248	AMN1	0.43093494	mirna_pc
20441	hsa-mir-1248	FAM184A	0.39614544	mirna_pc
20442	hsa-mir-1248	TDRD5	0.358785632	mirna_pc
20443	hsa-mir-1248	CILP2	0.379444646	mirna_pc
20444	hsa-mir-1248	GRAMD1C	0.413564372	mirna_pc
20445	hsa-mir-1248	PCM1	0.427732443	mirna_pc
20446	hsa-mir-1248	ZNF217	0.43563385	mirna_pc
20447	hsa-mir-1248	OTUD6B	0.422607203	mirna_pc
20448	hsa-mir-1248	LPHN2	0.382256839	mirna_pc
20449	hsa-mir-1248	HIST1H4H	0.307863387	mirna_pc
20450	hsa-mir-1248	BARD1	0.390135592	mirna_pc
20451	hsa-mir-1248	ZNF131	0.397287211	mirna_pc
20452	hsa-mir-1248	CNTNAP3	0.340827661	mirna_pc
20453	hsa-mir-1248	USP1	0.484326036	mirna_pc
20454	hsa-mir-1248	C16orf87	0.306752598	mirna_pc
20455	hsa-mir-1248	SCG2	0.314900046	mirna_pc
20456	hsa-mir-1248	EDIL3	0.435640599	mirna_pc
20457	hsa-mir-1248	ZNF608	0.334502807	mirna_pc
20458	hsa-mir-1248	SEMA3D	0.646550864	mirna_pc
20459	hsa-mir-1248	HACE1	0.357646112	mirna_pc
20460	hsa-mir-1248	ZNF451	0.315570692	mirna_pc
20461	hsa-mir-1248	CTDSPL2	0.345134888	mirna_pc
20462	hsa-mir-1248	HSPH1	0.362672622	mirna_pc
20463	hsa-mir-1248	SLC25A30	0.501564763	mirna_pc
20464	hsa-mir-1248	PPP3R1	0.348579415	mirna_pc
20465	hsa-mir-1248	ZNF658	0.311513687	mirna_pc

20466	hsa-mir-1248	ERCC4	0.344781289	mirna_pc
20467	hsa-mir-1248	GATA3	0.345831024	mirna_pc
20468	hsa-mir-1248	KSR2	0.3626368	mirna_pc
20469	hsa-mir-1248	CCDC41	0.368692524	mirna_pc
20470	hsa-mir-1248	LOC162632	0.372138309	mirna_pc
20471	hsa-mir-1248	NOL8	0.301268722	mirna_pc
20472	hsa-mir-1248	OXTR	0.375690863	mirna_pc
20473	hsa-mir-1248	CHORDC1	0.352644226	mirna_pc
20474	hsa-mir-1248	THSD7A	0.502553084	mirna_pc
20475	hsa-mir-1248	PPM1J	0.321239313	mirna_pc
20476	hsa-mir-1248	RPE	0.312714161	mirna_pc
20477	hsa-mir-1248	ZNF605	0.390910579	mirna_pc
20478	hsa-mir-1248	PSIP1	0.317913061	mirna_pc
20479	hsa-mir-1248	PCGF6	0.316745741	mirna_pc
20480	hsa-mir-1248	ZBTB43	0.301839252	mirna_pc
20481	hsa-mir-1248	TTC32	0.307459566	mirna_pc
20482	hsa-mir-1248	DPY19L4	0.312115196	mirna_pc
20483	hsa-mir-1248	OSR2	0.390378074	mirna_pc
20484	hsa-mir-1248	C12orf76	0.368740109	mirna_pc
20485	hsa-mir-1248	C6orf170	0.427154133	mirna_pc
20486	hsa-mir-1248	SENP6	0.373852839	mirna_pc
20487	hsa-mir-1248	HIST1H2BC	0.488294691	mirna_pc
20488	hsa-mir-1248	VPS37A	0.345568418	mirna_pc
20489	hsa-mir-1248	CRISPLD1	0.321691205	mirna_pc
20490	hsa-mir-1248	RGS16	0.357924281	mirna_pc
20491	hsa-mir-1248	KCNC3	0.408763801	mirna_pc
20492	hsa-mir-1248	ZFYVE16	0.384226203	mirna_pc
20493	hsa-mir-1248	RASSF8	0.308981097	mirna_pc
20494	hsa-mir-1248	PIK3C3	0.341667606	mirna_pc
20495	hsa-mir-1248	PHTF2	0.352093888	mirna_pc
20496	hsa-mir-1248	NBN	0.308052262	mirna_pc
20497	hsa-mir-1248	ZNF84	0.374834646	mirna_pc
20498	hsa-mir-1248	TAF4B	0.323271224	mirna_pc
20499	hsa-mir-1248	RMI1	0.36370385	mirna_pc
20500	hsa-mir-1248	ZDHHC17	0.333113804	mirna_pc
20501	hsa-mir-1248	TMEM67	0.393062478	mirna_pc
20502	hsa-mir-1248	ZBTB34	0.326855065	mirna_pc
20503	hsa-mir-1248	ZNF782	0.382526355	mirna_pc
20504	hsa-mir-1248	SEC61A2	0.352406487	mirna_pc
20505	hsa-mir-1248	HIST2H2BE	0.370118032	mirna_pc
20506	hsa-mir-1248	SFRS13A	0.30993823	mirna_pc
20507	hsa-mir-1248	C5orf28	0.332928998	mirna_pc
20508	hsa-mir-1248	NF2	0.358152321	mirna_pc
20509	hsa-mir-1248	MYSM1	0.309230939	mirna_pc
20510	hsa-mir-1248	ASB7	0.36514074	mirna_pc
20511	hsa-mir-1248	ATP6V1C1	0.314353534	mirna_pc
20512	hsa-mir-1248	MYBL1	0.52926215	mirna_pc
20513	hsa-mir-1248	PAPOLA	0.326134409	mirna_pc
20514	hsa-mir-1248	GAS1	0.447239392	mirna_pc
20515	hsa-mir-1248	PTPDC1	0.344579575	mirna_pc
20516	hsa-mir-1248	EFNA5	0.47746475	mirna_pc
20517	hsa-mir-1248	MRPL42	0.324089482	mirna_pc
20518	hsa-mir-1248	SUDS3	0.304048965	mirna_pc
20519	hsa-mir-1248	TMEM136	0.325410112	mirna_pc

20520	hsa-mir-1248	PAIP1	0.346196609	mirna_pc
20521	hsa-mir-1248	CCDC78	0.420450108	mirna_pc
20522	hsa-mir-1248	DNM3	0.587350438	mirna_pc
20523	hsa-mir-1248	KIAA1033	0.344498811	mirna_pc
20524	hsa-mir-1248	SEMA3C	0.342745617	mirna_pc
20525	hsa-mir-1248	WDR78	0.462616283	mirna_pc
20526	hsa-mir-1248	LOC153684	0.309234851	mirna_pc
20527	hsa-mir-1248	IFT81	0.303376719	mirna_pc
20528	hsa-mir-1248	ZNF184	0.425051871	mirna_pc
20529	hsa-mir-1248	MBLAC2	0.300833361	mirna_pc
20530	hsa-mir-1248	ATXN2	0.310469477	mirna_pc
20531	hsa-mir-1248	NIPSNAP1	0.355851478	mirna_pc
20532	hsa-mir-1248	FAM76B	0.302577891	mirna_pc
20533	hsa-mir-1248	C8orf37	0.565315979	mirna_pc
20534	hsa-mir-1248	PDGFC	0.583536542	mirna_pc
20535	hsa-mir-1248	CNOT7	0.344918401	mirna_pc
20536	hsa-mir-1248	SFI1	0.412398277	mirna_pc
20537	hsa-mir-1248	MAP3K12	0.413850298	mirna_pc
20538	hsa-mir-1248	MAP2	0.314592914	mirna_pc
20539	hsa-mir-1248	ZBTB10	0.475579755	mirna_pc
20540	hsa-mir-1248	TTC21B	0.360038238	mirna_pc
20541	hsa-mir-1248	SPIN1	0.370000483	mirna_pc
20542	hsa-mir-1248	CASP8AP2	0.326452293	mirna_pc
20543	hsa-mir-1248	ANGPT1	0.621601563	mirna_pc
20544	hsa-mir-1248	PRPF4B	0.309806762	mirna_pc
20545	hsa-mir-1248	EXTL2	0.316975099	mirna_pc
20546	hsa-mir-1248	ZNF525	0.343439702	mirna_pc
20547	hsa-mir-1248	TASP1	0.305945655	mirna_pc
20548	hsa-mir-1248	LOC338758	0.427733068	mirna_pc
20549	hsa-mir-1248	KIF27	0.377285674	mirna_pc
20550	hsa-mir-1248	ATG4C	0.306434447	mirna_pc
20551	hsa-mir-1248	ZNF638	0.312689524	mirna_pc
20552	hsa-mir-1248	GPR137C	0.365505807	mirna_pc
20553	hsa-mir-1248	CEP110	0.3677926	mirna_pc
20554	hsa-mir-1248	PIP5K1A	0.350392094	mirna_pc
20555	hsa-mir-1248	TMX3	0.371367965	mirna_pc
20556	hsa-mir-1248	RB1CC1	0.306131692	mirna_pc
20557	hsa-mir-1248	GRB14	0.430038506	mirna_pc
20558	hsa-mir-1248	HTR7P1	0.420296519	mirna_pc
20559	hsa-mir-1248	EMB	0.323668829	mirna_pc
20560	hsa-mir-1248	KIAA0528	0.367463349	mirna_pc
20561	hsa-mir-1248	ETAA1	0.325009643	mirna_pc
20562	hsa-mir-1248	HERC4	0.35439513	mirna_pc
20563	hsa-mir-1248	BCLAF1	0.343281536	mirna_pc
20564	hsa-mir-1248	C14orf135	0.321065366	mirna_pc
20565	hsa-mir-1248	B4GALT6	0.391119813	mirna_pc
20566	hsa-mir-1248	TMEM167A	0.302347222	mirna_pc
20567	hsa-mir-1248	CEP290	0.389489472	mirna_pc
20568	hsa-mir-1248	CNIH2	0.367478859	mirna_pc
20569	hsa-mir-1248	EID3	0.569501212	mirna_pc
20570	hsa-mir-1248	KIAA1467	0.410869185	mirna_pc
20571	hsa-mir-1248	UBE2W	0.455230605	mirna_pc
20572	hsa-mir-1248	ETNK1	0.308121768	mirna_pc
20573	hsa-mir-1248	GATS	0.393424495	mirna_pc

20574	hsa-mir-1248	TRUB1	0.368975259	mirna_pc
20575	hsa-mir-1248	HIST1H3E	0.329114833	mirna_pc
20576	hsa-mir-1248	PLAC2	0.461213787	mirna_pc
20577	hsa-mir-1248	SNX16	0.344602898	mirna_pc
20578	hsa-mir-1248	HIST4H4	0.380034978	mirna_pc
20579	hsa-mir-1248	C12orf24	0.476467062	mirna_pc
20580	hsa-mir-1248	ZNF484	0.303570892	mirna_pc
20581	hsa-mir-1248	GLS2	0.311384912	mirna_pc
20582	hsa-mir-1248	SEMA3A	0.623850039	mirna_pc
20583	hsa-mir-1248	USP13	0.341790894	mirna_pc
20584	hsa-mir-1248	ESF1	0.386084215	mirna_pc
20585	hsa-mir-1248	MIB1	0.309972304	mirna_pc
20586	hsa-mir-1248	ZNF26	0.342688836	mirna_pc
20587	hsa-mir-1248	SS18	0.314980068	mirna_pc
20588	hsa-mir-1248	ZNF391	0.375639031	mirna_pc
20589	hsa-mir-1248	ZBTB41	0.378353574	mirna_pc
20590	hsa-mir-1248	IFT74	0.49139768	mirna_pc
20591	hsa-mir-1248	NR5A2	0.351604026	mirna_pc
20592	hsa-mir-1248	ZZZ3	0.316470703	mirna_pc
20593	hsa-mir-1248	MDM1	0.598562784	mirna_pc
20594	hsa-mir-1248	C10orf88	0.334229319	mirna_pc
20595	hsa-mir-1248	MCOLN3	0.329229744	mirna_pc
20596	hsa-mir-1248	C1orf52	0.317469727	mirna_pc
20597	hsa-mir-1248	BBS10	0.378767818	mirna_pc
20598	hsa-mir-1248	SLITRK6	0.663389046	mirna_pc
20599	hsa-mir-1248	ERGIC2	0.486901937	mirna_pc
20600	hsa-mir-1248	PPTC7	0.321968134	mirna_pc
20601	hsa-mir-1248	ANKRD34A	0.343660063	mirna_pc
20602	hsa-mir-1248	HAPLN1	0.68909811	mirna_pc
20603	hsa-mir-1248	C8orf45	0.318597983	mirna_pc
20604	hsa-mir-1248	HOOK1	0.344312562	mirna_pc
20605	hsa-mir-1248	HIATL2	0.338863651	mirna_pc
20606	hsa-mir-1248	LOC221710	0.409418305	mirna_pc
20607	hsa-mir-1248	KIAA1586	0.307903695	mirna_pc
20608	hsa-mir-1248	TSGA10	0.386008981	mirna_pc
20609	hsa-mir-1248	CRYZ	0.514877757	mirna_pc
20610	hsa-mir-1248	ZNF664	0.359813238	mirna_pc
20611	hsa-mir-1248	TMEM106B	0.330668331	mirna_pc
20612	hsa-mir-1248	SMEK2	0.421940188	mirna_pc
20613	hsa-mir-1248	TBX1	0.308593206	mirna_pc
20614	hsa-mir-1248	KDELC2	0.343825088	mirna_pc
20615	hsa-mir-1248	C20orf96	0.326176722	mirna_pc
20616	hsa-mir-1248	SLC4A7	0.320729569	mirna_pc
20617	hsa-mir-1248	PRICKLE1	0.36688737	mirna_pc
20618	hsa-mir-1248	GRINL1A	0.316524449	mirna_pc
20619	hsa-mir-1248	PHF3	0.307181268	mirna_pc
20620	hsa-mir-1248	ZNF644	0.329428104	mirna_pc
20621	hsa-mir-1248	RSRC2	0.386739365	mirna_pc
20622	hsa-mir-1248	KLHL24	0.31035026	mirna_pc
20623	hsa-mir-1248	CTXN1	0.42983885	mirna_pc
20624	hsa-mir-1248	ZNF883	0.312806997	mirna_pc
20625	hsa-mir-1248	RBM27	0.30311948	mirna_pc
20626	hsa-mir-1248	ZNF680	0.415180755	mirna_pc
20627	hsa-mir-1248	C9orf21	0.38156336	mirna_pc

20628	hsa-mir-1248	LOC283070	0.43064282	mirna_pc
20629	hsa-mir-1248	CCDC117	0.416275593	mirna_pc
20630	hsa-mir-1248	FAM178A	0.330444833	mirna_pc
20631	hsa-mir-1248	C10orf46	0.315900075	mirna_pc
20632	hsa-mir-1248	SLC11A2	0.457233046	mirna_pc
20633	hsa-mir-1248	BAI2	0.304877067	mirna_pc
20634	hsa-mir-1248	HEY2	0.386222895	mirna_pc
20635	hsa-mir-1248	ZFP37	0.454455398	mirna_pc
20636	hsa-mir-1248	PHIP	0.372771188	mirna_pc
20637	hsa-mir-1248	CAMK1D	0.302511512	mirna_pc
20638	hsa-mir-1248	LCA5	0.5974896	mirna_pc
20639	hsa-mir-1248	CDC73	0.382496413	mirna_pc
20640	hsa-mir-1248	SLC2A3	0.452706954	mirna_pc
20641	hsa-mir-1248	KIAA1107	0.339349172	mirna_pc
20642	hsa-mir-1248	BRAP	0.310625439	mirna_pc
20643	hsa-mir-1248	ZNF326	0.33347824	mirna_pc
20644	hsa-mir-1248	ZRANB2	0.323901713	mirna_pc
20645	hsa-mir-1248	CCDC88A	0.306206516	mirna_pc
20646	hsa-mir-1248	AGTPBP1	0.31159869	mirna_pc
20647	hsa-mir-1248	SS18L1	0.326076581	mirna_pc
20648	hsa-mir-454	TPX2	0.308626152	mirna_pc
20649	hsa-mir-454	ECT2	0.322030679	mirna_pc
20650	hsa-mir-454	RRM2	0.384539766	mirna_pc
20651	hsa-mir-454	CDK1	0.335066614	mirna_pc
20652	hsa-mir-454	CDC25C	0.371228536	mirna_pc
20653	hsa-mir-454	UBE2C	0.348239744	mirna_pc
20654	hsa-mir-454	NUSAP1	0.313226502	mirna_pc
20655	hsa-mir-454	KIF18B	0.356834113	mirna_pc
20656	hsa-mir-454	ASPM	0.310613617	mirna_pc
20657	hsa-mir-454	FANCA	0.339049644	mirna_pc
20658	hsa-mir-454	NCAPG	0.37263321	mirna_pc
20659	hsa-mir-454	CLSPN	0.411422429	mirna_pc
20660	hsa-mir-454	SGOL1	0.350101491	mirna_pc
20661	hsa-mir-454	ARHGAP11A	0.312532069	mirna_pc
20662	hsa-mir-454	CBX3	0.306254976	mirna_pc
20663	hsa-mir-454	SPC25	0.310991563	mirna_pc
20664	hsa-mir-454	FANCI	0.335690154	mirna_pc
20665	hsa-mir-454	NCAPH	0.301570164	mirna_pc
20666	hsa-mir-454	GTSE1	0.394868688	mirna_pc
20667	hsa-mir-454	KIF23	0.325707516	mirna_pc
20668	hsa-mir-454	CENPA	0.348482323	mirna_pc
20669	hsa-mir-454	MAD2L1	0.310115886	mirna_pc
20670	hsa-mir-454	TIMELESS	0.356464355	mirna_pc
20671	hsa-mir-454	LMNB1	0.365614745	mirna_pc
20672	hsa-mir-454	ASF1B	0.345691645	mirna_pc
20673	hsa-mir-454	CDCA8	0.303690463	mirna_pc
20674	hsa-mir-454	TROAP	0.366475916	mirna_pc
20675	hsa-mir-454	XPO1	0.347696328	mirna_pc
20676	hsa-mir-454	TK1	0.32498295	mirna_pc
20677	hsa-mir-454	CDCA2	0.322235267	mirna_pc
20678	hsa-mir-454	DKC1	0.321315296	mirna_pc
20679	hsa-mir-454	KIAA0101	0.437979575	mirna_pc
20680	hsa-mir-454	KIF4A	0.351433725	mirna_pc
20681	hsa-mir-454	ORC1L	0.348002529	mirna_pc

20682	hsa-mir-454	KNTC1	0.359296597	mirna_pc
20683	hsa-mir-454	CCNF	0.318328569	mirna_pc
20684	hsa-mir-454	CCNB2	0.300330784	mirna_pc
20685	hsa-mir-454	NUF2	0.479544769	mirna_pc
20686	hsa-mir-454	PLK4	0.39230365	mirna_pc
20687	hsa-mir-454	KIF18A	0.370526451	mirna_pc
20688	hsa-mir-454	SKA3	0.300109115	mirna_pc
20689	hsa-mir-454	HJURP	0.373312987	mirna_pc
20690	hsa-mir-454	SKA1	0.376092224	mirna_pc
20691	hsa-mir-454	BLM	0.337204233	mirna_pc
20692	hsa-mir-454	DNMT1	0.361702119	mirna_pc
20693	hsa-mir-454	PAICS	0.305079473	mirna_pc
20694	hsa-mir-454	CCDC150	0.309221285	mirna_pc
20695	hsa-mir-454	BIRC5	0.356832035	mirna_pc
20696	hsa-mir-454	DTL	0.350981756	mirna_pc
20697	hsa-mir-454	EME1	0.357965333	mirna_pc
20698	hsa-mir-454	CENPK	0.483625185	mirna_pc
20699	hsa-mir-454	AURKB	0.356439058	mirna_pc
20700	hsa-mir-454	MCM10	0.350671349	mirna_pc
20701	hsa-mir-454	KIF20A	0.366501502	mirna_pc
20702	hsa-mir-454	SGOL2	0.342205016	mirna_pc
20703	hsa-mir-454	CENPE	0.322135933	mirna_pc
20704	hsa-mir-454	KIF15	0.39314364	mirna_pc
20705	hsa-mir-454	CDCA3	0.366100465	mirna_pc
20706	hsa-mir-454	C1orf112	0.397107326	mirna_pc
20707	hsa-mir-454	GINS1	0.382776253	mirna_pc
20708	hsa-mir-454	TRAIP	0.348733696	mirna_pc
20709	hsa-mir-454	ORC6L	0.343634864	mirna_pc
20710	hsa-mir-454	C16orf59	0.338653675	mirna_pc
20711	hsa-mir-454	FAM72B	0.336900894	mirna_pc
20712	hsa-mir-454	NEIL3	0.324501371	mirna_pc
20713	hsa-mir-454	ACTL6A	0.336090783	mirna_pc
20714	hsa-mir-454	ZNF229	0.307217501	mirna_pc
20715	hsa-mir-454	C12orf48	0.43938469	mirna_pc
20716	hsa-mir-454	HNRNPL	0.349421693	mirna_pc
20717	hsa-mir-454	SMC4	0.418807108	mirna_pc
20718	hsa-mir-454	HMMR	0.341762647	mirna_pc
20719	hsa-mir-454	TTK	0.307778917	mirna_pc
20720	hsa-mir-454	SF3B4	0.309858559	mirna_pc
20721	hsa-mir-454	HELLS	0.422301221	mirna_pc
20722	hsa-mir-454	ERCC6L	0.301045767	mirna_pc
20723	hsa-mir-454	POLE2	0.320832083	mirna_pc
20724	hsa-mir-454	NCAPD2	0.320175846	mirna_pc
20725	hsa-mir-454	C11orf82	0.315735181	mirna_pc
20726	hsa-mir-454	C6orf167	0.335491546	mirna_pc
20727	hsa-mir-454	BRCA1	0.324881587	mirna_pc
20728	hsa-mir-454	HNRNPA2B1	0.349509912	mirna_pc
20729	hsa-mir-454	MCM6	0.302052577	mirna_pc
20730	hsa-mir-454	EPR1	0.304651187	mirna_pc
20731	hsa-mir-454	BRIP1	0.405999497	mirna_pc
20732	hsa-mir-454	PIF1	0.32898168	mirna_pc
20733	hsa-mir-454	SFRS2	0.300698179	mirna_pc
20734	hsa-mir-454	C15orf42	0.318122946	mirna_pc
20735	hsa-mir-454	FAM60A	0.317333019	mirna_pc

20736	hsa-mir-454	ATAD5		0.408405317	mirna_pc
20737	hsa-mir-454	KIF20B		0.311123024	mirna_pc
20738	hsa-mir-454	ESCO2		0.319761296	mirna_pc
20739	hsa-mir-454	ATAD2		0.346785337	mirna_pc
20740	hsa-mir-454	CHAF1A		0.336060839	mirna_pc
20741	hsa-mir-454	DEPDC1B		0.381960575	mirna_pc
20742	hsa-mir-454	GINS4		0.31600921	mirna_pc
20743	hsa-mir-454	ANP32E		0.478324633	mirna_pc
20744	hsa-mir-454	CENPO		0.321875409	mirna_pc
20745	hsa-mir-454	XRCC2		0.350794482	mirna_pc
20746	hsa-mir-454	TRIM59		0.311746093	mirna_pc
20747	hsa-mir-454	CENPL		0.327246936	mirna_pc
20748	hsa-mir-454	GEN1		0.374383731	mirna_pc
20749	hsa-mir-454	HMGB2		0.44491488	mirna_pc
20750	hsa-mir-454	DNA2		0.412061666	mirna_pc
20751	hsa-mir-454	DTYMK		0.314825921	mirna_pc
20752	hsa-mir-454	E2F7		0.35120139	mirna_pc
20753	hsa-mir-454	RBL1		0.307673889	mirna_pc
20754	hsa-mir-454	CCDC99		0.348226643	mirna_pc
20755	hsa-mir-454	CDK2		0.371372836	mirna_pc
20756	hsa-mir-454	HNRNPR		0.306033776	mirna_pc
20757	hsa-mir-454	DLEU2		0.374635391	mirna_pc
20758	hsa-mir-454	PRIM1		0.375921514	mirna_pc
20759	hsa-mir-454	PSMC4		0.34550002	mirna_pc
20760	hsa-mir-454	CHAF1B		0.300373653	mirna_pc
20761	hsa-mir-454	MSH2		0.394217946	mirna_pc
20762	hsa-mir-454	DBF4		0.424824294	mirna_pc
20763	hsa-mir-454	CDC7		0.369496689	mirna_pc
20764	hsa-mir-454	SSB		0.358551287	mirna_pc
20765	hsa-mir-454	NOL11		0.367627209	mirna_pc
20766	hsa-mir-454	PSMB4		0.312429649	mirna_pc
20767	hsa-mir-454	RFC4		0.336435565	mirna_pc
20768	hsa-mir-454	FBXO5		0.438215889	mirna_pc
20769	hsa-mir-454	FIGNL1		0.454802298	mirna_pc
20770	hsa-mir-454	E2F3		0.382255576	mirna_pc
20771	hsa-mir-454	CDC25A		0.304509268	mirna_pc
20772	hsa-mir-454	RAD54B		0.326833381	mirna_pc
20773	hsa-mir-454	DDX12		0.390025121	mirna_pc
20774	hsa-mir-454	C13orf34		0.334669236	mirna_pc
20775	hsa-mir-454	MCM8		0.328136637	mirna_pc
20776	hsa-mir-454	CPSF3		0.358039134	mirna_pc
20777	hsa-mir-454	UTP6		0.315559978	mirna_pc
20778	hsa-mir-454	DNAJB11		0.388696856	mirna_pc
20779	hsa-mir-454	PUS7		0.312775406	mirna_pc
20780	hsa-mir-454	CBX8		0.332783745	mirna_pc
20781	hsa-mir-454	TMEM201		0.314527826	mirna_pc
20782	hsa-mir-454	NCL		0.307334321	mirna_pc
20783	hsa-mir-454	SRRT		0.353340386	mirna_pc
20784	hsa-mir-454	CACYBP		0.368486231	mirna_pc
20785	hsa-mir-454	SASS6		0.448518161	mirna_pc
20786	hsa-mir-454	PSRC1		0.307854964	mirna_pc
20787	hsa-mir-454	DBF4B		0.39367141	mirna_pc
20788	hsa-mir-454	HNRNPM		0.353528827	mirna_pc
20789	hsa-mir-454	LSM	12.00	0.32224719	mirna_pc

20790	hsa-mir-454	PNPT1	0.306320384	mirna_pc
20791	hsa-mir-454	TMPO	0.407289653	mirna_pc
20792	hsa-mir-454	POLR2H	0.306540926	mirna_pc
20793	hsa-mir-454	GPR19	0.378133608	mirna_pc
20794	hsa-mir-454	LSG1	0.302867672	mirna_pc
20795	hsa-mir-454	GABPB1	0.406138958	mirna_pc
20796	hsa-mir-454	CCDC138	0.347702023	mirna_pc
20797	hsa-mir-454	LOC100128191	0.362941953	mirna_pc
20798	hsa-mir-454	HMGN2	0.365683501	mirna_pc
20799	hsa-mir-454	SUMO2	0.327177944	mirna_pc
20800	hsa-mir-454	SFRS1	0.423207561	mirna_pc
20801	hsa-mir-454	TIPIN	0.33243421	mirna_pc
20802	hsa-mir-454	C4orf46	0.324958847	mirna_pc
20803	hsa-mir-454	RFC5	0.330324991	mirna_pc
20804	hsa-mir-454	HSPD1	0.332930246	mirna_pc
20805	hsa-mir-454	FTSJ2	0.322340859	mirna_pc
20806	hsa-mir-454	KHDRBS1	0.407621997	mirna_pc
20807	hsa-mir-454	HSP90AA1	0.303673252	mirna_pc
20808	hsa-mir-454	CCDC18	0.422323872	mirna_pc
20809	hsa-mir-454	DNAJC2	0.341841455	mirna_pc
20810	hsa-mir-454	C4orf21	0.306204257	mirna_pc
20811	hsa-mir-454	TMEM194A	0.365572794	mirna_pc
20812	hsa-mir-454	TTC26	0.310062601	mirna_pc
20813	hsa-mir-454	HAUS6	0.315218746	mirna_pc
20814	hsa-mir-454	HNRNPU	0.413986773	mirna_pc
20815	hsa-mir-454	NASP	0.371418055	mirna_pc
20816	hsa-mir-454	PTMA	0.41058076	mirna_pc
20817	hsa-mir-454	NCBP2	0.31781885	mirna_pc
20818	hsa-mir-454	SUV39H2	0.347825889	mirna_pc
20819	hsa-mir-454	FANCL	0.327462563	mirna_pc
20820	hsa-mir-454	HNRNPD	0.302336051	mirna_pc
20821	hsa-mir-454	POP1	0.318869925	mirna_pc
20822	hsa-mir-454	POC5	0.358910307	mirna_pc
20823	hsa-mir-454	HSP90B1	0.417481602	mirna_pc
20824	hsa-mir-454	PSMC3IP	0.344441452	mirna_pc
20825	hsa-mir-454	ATP13A3	0.45353126	mirna_pc
20826	hsa-mir-454	DHFR	0.432726952	mirna_pc
20827	hsa-mir-454	DEK	0.395709523	mirna_pc
20828	hsa-mir-454	PASK	0.3561148	mirna_pc
20829	hsa-mir-454	SKA2	0.363535528	mirna_pc
20830	hsa-mir-454	TGS1	0.328419259	mirna_pc
20831	hsa-mir-454	KHSRP	0.309215529	mirna_pc
20832	hsa-mir-454	C14orf145	0.300034285	mirna_pc
20833	hsa-mir-454	FUBP1	0.353087398	mirna_pc
20834	hsa-mir-454	SART3	0.332455827	mirna_pc
20835	hsa-mir-454	SR140	0.304800708	mirna_pc
20836	hsa-mir-454	TRA2B	0.335977617	mirna_pc
20837	hsa-mir-454	RSRC1	0.360526094	mirna_pc
20838	hsa-mir-454	CEP152	0.376425315	mirna_pc
20839	hsa-mir-454	RBM12B	0.303417112	mirna_pc
20840	hsa-mir-454	PSMD12	0.391514552	mirna_pc
20841	hsa-mir-454	C1orf174	0.310580214	mirna_pc
20842	hsa-mir-454	ENOPH1	0.315464106	mirna_pc
20843	hsa-mir-454	C17orf42	0.425427324	mirna_pc

20844	hsa-mir-454	DDX55	0.368343697	mirna_pc
20845	hsa-mir-454	SFPQ	0.348063702	mirna_pc
20846	hsa-mir-454	MPHOSPH9	0.302178324	mirna_pc
20847	hsa-mir-454	CADM1	0.378853753	mirna_pc
20848	hsa-mir-454	GMNN	0.336863457	mirna_pc
20849	hsa-mir-454	POLG2	0.433989321	mirna_pc
20850	hsa-mir-454	ANKRD32	0.505657164	mirna_pc
20851	hsa-mir-454	PDAP1	0.321900192	mirna_pc
20852	hsa-mir-454	GEMIN6	0.385936794	mirna_pc
20853	hsa-mir-454	PTCD1	0.351822467	mirna_pc
20854	hsa-mir-454	CRIPT	0.301893198	mirna_pc
20855	hsa-mir-454	KPNB1	0.315197727	mirna_pc
20856	hsa-mir-454	SMC3	0.403016364	mirna_pc
20857	hsa-mir-454	FLVCR1	0.304849855	mirna_pc
20858	hsa-mir-454	MEX3A	0.348108702	mirna_pc
20859	hsa-mir-454	COIL	0.400703343	mirna_pc
20860	hsa-mir-454	SFRS12	0.363422907	mirna_pc
20861	hsa-mir-454	SUZ12	0.358193575	mirna_pc
20862	hsa-mir-454	TMEM68	0.339711376	mirna_pc
20863	hsa-mir-454	LOC642846	0.373983933	mirna_pc
20864	hsa-mir-454	TGIF2	0.323194684	mirna_pc
20865	hsa-mir-454	ZC3H18	0.304516279	mirna_pc
20866	hsa-mir-454	THUMPD2	0.366344252	mirna_pc
20867	hsa-mir-454	FAM96A	0.309166578	mirna_pc
20868	hsa-mir-454	MTF2	0.389273168	mirna_pc
20869	hsa-mir-454	POLE	0.353521327	mirna_pc
20870	hsa-mir-454	C13orf27	0.305247685	mirna_pc
20871	hsa-mir-454	EXOSC9	0.320974933	mirna_pc
20872	hsa-mir-454	TMEM209	0.32802877	mirna_pc
20873	hsa-mir-454	RBMX	0.388441438	mirna_pc
20874	hsa-mir-454	NOC3L	0.319378729	mirna_pc
20875	hsa-mir-454	PDIA6	0.331358172	mirna_pc
20876	hsa-mir-454	ZNF200	0.379504377	mirna_pc
20877	hsa-mir-454	UBXN2A	0.303065765	mirna_pc
20878	hsa-mir-454	DNAJC14	0.343091783	mirna_pc
20879	hsa-mir-454	CIT	0.309197966	mirna_pc
20880	hsa-mir-454	SCLT1	0.339293023	mirna_pc
20881	hsa-mir-454	SMN2	0.38768884	mirna_pc
20882	hsa-mir-454	GPATCH4	0.334951308	mirna_pc
20883	hsa-mir-454	MPHOSPH10	0.361043214	mirna_pc
20884	hsa-mir-454	ATXN7L2	0.309222343	mirna_pc
20885	hsa-mir-454	C17orf67	0.326604291	mirna_pc
20886	hsa-mir-454	C7orf44	0.339262663	mirna_pc
20887	hsa-mir-454	NAT9	0.339285757	mirna_pc
20888	hsa-mir-454	UPF3B	0.354222494	mirna_pc
20889	hsa-mir-454	RDH14	0.344831038	mirna_pc
20890	hsa-mir-454	DDX20	0.35855012	mirna_pc
20891	hsa-mir-454	IFT80	0.342206258	mirna_pc
20892	hsa-mir-454	RNF34	0.318890453	mirna_pc
20893	hsa-mir-454	DDX46	0.413405236	mirna_pc
20894	hsa-mir-454	UBE2N	0.333345396	mirna_pc
20895	hsa-mir-454	RNFT1	0.310828726	mirna_pc
20896	hsa-mir-454	CHAC2	0.32671371	mirna_pc
20897	hsa-mir-454	TCERG1	0.353860146	mirna_pc

20898	hsa-mir-454	E2F6	0.422542869	mirna_pc
20899	hsa-mir-454	ZNF833	0.330862119	mirna_pc
20900	hsa-mir-454	FTSJ3	0.314698119	mirna_pc
20901	hsa-mir-454	CPSF4	0.350130295	mirna_pc
20902	hsa-mir-454	SFRS13A	0.408203173	mirna_pc
20903	hsa-mir-454	NUP35	0.311493975	mirna_pc
20904	hsa-mir-454	TFAM	0.344740881	mirna_pc
20905	hsa-mir-454	MTIF2	0.334727432	mirna_pc
20906	hsa-mir-454	EWSR1	0.308536191	mirna_pc
20907	hsa-mir-454	LOC222699	0.322709704	mirna_pc
20908	hsa-mir-454	HIST1H1C	0.329727867	mirna_pc
20909	hsa-mir-454	CCDC45	0.406695151	mirna_pc
20910	hsa-mir-454	SFRS7	0.366145082	mirna_pc
20911	hsa-mir-454	MRPL42	0.406236698	mirna_pc
20912	hsa-mir-454	CCAR1	0.333729257	mirna_pc
20913	hsa-mir-454	C2orf68	0.335479367	mirna_pc
20914	hsa-mir-454	C17orf80	0.363336286	mirna_pc
20915	hsa-mir-454	ZKSCAN5	0.323033586	mirna_pc
20916	hsa-mir-454	DCP2	0.324756833	mirna_pc
20917	hsa-mir-454	ZNF92	0.320007515	mirna_pc
20918	hsa-mir-454	C2orf44	0.387299639	mirna_pc
20919	hsa-mir-454	CCDC76	0.391270998	mirna_pc
20920	hsa-mir-454	C17orf58	0.365525795	mirna_pc
20921	hsa-mir-454	MATR3	0.322933346	mirna_pc
20922	hsa-mir-454	ZFP1	0.306287868	mirna_pc
20923	hsa-mir-454	NUP43	0.343977614	mirna_pc
20924	hsa-mir-454	ZNF473	0.33386526	mirna_pc
20925	hsa-mir-454	DCLRE1A	0.323230933	mirna_pc
20926	hsa-mir-454	RBM25	0.37860765	mirna_pc
20927	hsa-mir-454	PRPF38B	0.351078477	mirna_pc
20928	hsa-mir-454	TRDMT1	0.310666346	mirna_pc
20929	hsa-mir-454	PNN	0.345299245	mirna_pc
20930	hsa-mir-454	MNS1	0.363785098	mirna_pc
20931	hsa-mir-454	MTERF	0.3395526	mirna_pc
20932	hsa-mir-454	SLC25A33	0.322837137	mirna_pc
20933	hsa-mir-454	GTF2H2C	0.403544057	mirna_pc
20934	hsa-mir-454	SNCAIP	0.30194983	mirna_pc
20935	hsa-mir-454	GTF2H2	0.307871332	mirna_pc
20936	hsa-mir-454	FANCF	0.356366623	mirna_pc
20937	hsa-mir-454	FAM169A	0.322765799	mirna_pc
20938	hsa-mir-454	FAM119A	0.304452104	mirna_pc
20939	hsa-mir-454	C12orf65	0.384949094	mirna_pc
20940	hsa-mir-454	MRPL19	0.347918086	mirna_pc
20941	hsa-mir-454	SPRED1	0.325414104	mirna_pc
20942	hsa-mir-454	CETN3	0.304161065	mirna_pc
20943	hsa-mir-454	ZCCHC3	0.310599563	mirna_pc
20944	hsa-mir-454	ZCCHC9	0.350110856	mirna_pc
20945	hsa-mir-454	AADAT	0.306565931	mirna_pc
20946	hsa-mir-454	ACP1	0.325535269	mirna_pc
20947	hsa-mir-454	PRMT6	0.335619185	mirna_pc
20948	hsa-mir-454	SLC35B4	0.343355089	mirna_pc
20949	hsa-mir-454	ZNF239	0.317838363	mirna_pc
20950	hsa-mir-454	CCDC75	0.313969667	mirna_pc
20951	hsa-mir-454	PRTFDC1	0.308874333	mirna_pc

20952	hsa-mir-454	ZNF670	0.321751442	mirna_pc
20953	hsa-mir-454	C3orf33	0.356174083	mirna_pc
20954	hsa-mir-454	ZNF326	0.337657901	mirna_pc
20955	hsa-mir-454	CCT6P1	0.301170562	mirna_pc
20956	hsa-mir-766	RRM2	0.344234722	mirna_pc
20957	hsa-mir-766	TIMELESS	0.354884962	mirna_pc
20958	hsa-mir-766	CDCA8	0.304803607	mirna_pc
20959	hsa-mir-766	ORC1L	0.316344907	mirna_pc
20960	hsa-mir-766	KNTC1	0.317812118	mirna_pc
20961	hsa-mir-766	DNMT1	0.309790622	mirna_pc
20962	hsa-mir-766	TRAIP	0.307709209	mirna_pc
20963	hsa-mir-766	POLD1	0.325382042	mirna_pc
20964	hsa-mir-766	ZNF229	0.362186237	mirna_pc
20965	hsa-mir-766	TUBA1B	0.309549088	mirna_pc
20966	hsa-mir-766	CHEK1	0.325870991	mirna_pc
20967	hsa-mir-766	RAN	0.317900254	mirna_pc
20968	hsa-mir-766	EFTUD2	0.307277415	mirna_pc
20969	hsa-mir-766	DEPDC1B	0.361268095	mirna_pc
20970	hsa-mir-766	CDKN1C	0.325499012	mirna_pc
20971	hsa-mir-766	NEURL3	0.343399726	mirna_pc
20972	hsa-mir-766	C5	0.400054352	mirna_pc
20973	hsa-mir-766	CDK2	0.408360196	mirna_pc
20974	hsa-mir-766	CDK4	0.333239282	mirna_pc
20975	hsa-mir-766	PRIM1	0.39147673	mirna_pc
20976	hsa-mir-766	SALL4	0.383143384	mirna_pc
20977	hsa-mir-766	E2F3	0.343549571	mirna_pc
20978	hsa-mir-766	CDC25A	0.367700341	mirna_pc
20979	hsa-mir-766	WNT3	0.306916614	mirna_pc
20980	hsa-mir-766	CPSF3	0.341421543	mirna_pc
20981	hsa-mir-766	IFI30	0.332727329	mirna_pc
20982	hsa-mir-766	DONSON	0.315029703	mirna_pc
20983	hsa-mir-766	LOC100128191	0.41103542	mirna_pc
20984	hsa-mir-766	INPP5B	0.367881796	mirna_pc
20985	hsa-mir-766	DNMT3B	0.313107224	mirna_pc
20986	hsa-mir-766	FZD2	0.373950774	mirna_pc
20987	hsa-mir-766	CTPS	0.394310778	mirna_pc
20988	hsa-mir-766	PA2G4	0.341600769	mirna_pc
20989	hsa-mir-766	AHCY	0.308264884	mirna_pc
20990	hsa-mir-766	6-Sep	0.388868551	mirna_pc
20991	hsa-mir-766	GNL2	0.393249635	mirna_pc
20992	hsa-mir-766	NASP	0.304374063	mirna_pc
20993	hsa-mir-766	CBX4	0.340783513	mirna_pc
20994	hsa-mir-766	OBFC2B	0.324510715	mirna_pc
20995	hsa-mir-766	SCARB1	0.382231257	mirna_pc
20996	hsa-mir-766	TFR2	0.40392687	mirna_pc
20997	hsa-mir-766	APOE	0.353709887	mirna_pc
20998	hsa-mir-766	DUSP9	0.379222444	mirna_pc
20999	hsa-mir-766	MOV10	0.408645422	mirna_pc
21000	hsa-mir-766	PAK1IP1	0.300387833	mirna_pc
21001	hsa-mir-766	PPT1	0.375232546	mirna_pc
21002	hsa-mir-766	PPP2R1B	0.362270733	mirna_pc
21003	hsa-mir-766	PRAME	0.379327922	mirna_pc
21004	hsa-mir-766	NOC4L	0.323765883	mirna_pc
21005	hsa-mir-766	SAE1	0.388980875	mirna_pc

21006	hsa-mir-766	THTPA	0.324966705	mirna_pc
21007	hsa-mir-766	ITIH4	0.360267452	mirna_pc
21008	hsa-mir-766	ZC3H10	0.349081921	mirna_pc
21009	hsa-mir-766	CADM1	0.359597343	mirna_pc
21010	hsa-mir-766	POLA1	0.307274097	mirna_pc
21011	hsa-mir-766	GMNN	0.325685596	mirna_pc
21012	hsa-mir-766	PA2G4P4	0.337242851	mirna_pc
21013	hsa-mir-766	ANKRD32	0.300255117	mirna_pc
21014	hsa-mir-766	SMARCA1	0.369156768	mirna_pc
21015	hsa-mir-766	GPAM	0.351493162	mirna_pc
21016	hsa-mir-766	ACAN	0.32702089	mirna_pc
21017	hsa-mir-766	AKIRIN1	0.376833924	mirna_pc
21018	hsa-mir-766	PIK3AP1	0.376087739	mirna_pc
21019	hsa-mir-766	CIB2	0.310417862	mirna_pc
21020	hsa-mir-766	TARBP2	0.353968198	mirna_pc
21021	hsa-mir-766	SF3A3	0.46074104	mirna_pc
21022	hsa-mir-766	POLE	0.395138096	mirna_pc
21023	hsa-mir-766	CHML	0.352162541	mirna_pc
21024	hsa-mir-766	TRIAP1	0.388783986	mirna_pc
21025	hsa-mir-766	RTN4RL2	0.318780621	mirna_pc
21026	hsa-mir-766	ADSL	0.311102759	mirna_pc
21027	hsa-mir-766	UNG	0.314511437	mirna_pc
21028	hsa-mir-766	GPC3	0.433557352	mirna_pc
21029	hsa-mir-766	POP5	0.309037479	mirna_pc
21030	hsa-mir-766	ZNF642	0.302925457	mirna_pc
21031	hsa-mir-766	GPN3	0.300642226	mirna_pc
21032	hsa-mir-766	DNAJC14	0.330073639	mirna_pc
21033	hsa-mir-766	KDM2B	0.349478034	mirna_pc
21034	hsa-mir-766	YRDC	0.326825481	mirna_pc
21035	hsa-mir-766	LYPD1	0.381596005	mirna_pc
21036	hsa-mir-766	ITPRIPL1	0.35333882	mirna_pc
21037	hsa-mir-766	GDPD5	0.317298505	mirna_pc
21038	hsa-mir-766	ZNF643	0.38879674	mirna_pc
21039	hsa-mir-766	BCL7A	0.338785602	mirna_pc
21040	hsa-mir-766	PLAGL2	0.361742348	mirna_pc
21041	hsa-mir-766	FRAT1	0.303167305	mirna_pc
21042	hsa-mir-766	GATC	0.382358269	mirna_pc
21043	hsa-mir-766	DDX20	0.308565873	mirna_pc
21044	hsa-mir-766	RNF34	0.361047336	mirna_pc
21045	hsa-mir-766	C1orf109	0.38850838	mirna_pc
21046	hsa-mir-766	PABPC4	0.365455908	mirna_pc
21047	hsa-mir-766	ZNF833	0.337321377	mirna_pc
21048	hsa-mir-766	LOC222699	0.445157422	mirna_pc
21049	hsa-mir-766	AAAS	0.342816738	mirna_pc
21050	hsa-mir-766	ACVR2B	0.318031654	mirna_pc
21051	hsa-mir-766	DKK 1.00	0.362343535	mirna_pc
21052	hsa-mir-766	BCORL1	0.30662009	mirna_pc
21053	hsa-mir-766	SERPINF2	0.457760988	mirna_pc
21054	hsa-mir-766	CA5BP	0.38330222	mirna_pc
21055	hsa-mir-766	VPS33A	0.470875849	mirna_pc
21056	hsa-mir-766	KCNG1	0.404926157	mirna_pc
21057	hsa-mir-766	GGT1	0.335121339	mirna_pc
21058	hsa-mir-766	POLR3B	0.341457149	mirna_pc
21059	hsa-mir-766	RNF26	0.366960492	mirna_pc

21060	hsa-mir-766	MYCBP	0.334658989	mirna_pc
21061	hsa-mir-766	GDF11	0.410352089	mirna_pc
21062	hsa-mir-766	ANXA9	0.369212856	mirna_pc
21063	hsa-mir-766	GLYCTK	0.304755449	mirna_pc
21064	hsa-mir-766	MYL6B	0.317597474	mirna_pc
21065	hsa-mir-766	ARID3B	0.416330681	mirna_pc
21066	hsa-mir-766	RNF41	0.414567921	mirna_pc
21067	hsa-mir-766	UTP11L	0.394941978	mirna_pc
21068	hsa-mir-766	ZW10	0.33701912	mirna_pc
21069	hsa-mir-766	HIC2	0.456534627	mirna_pc
21070	hsa-mir-766	LRFN1	0.30795655	mirna_pc
21071	hsa-mir-766	NLRP2	0.384858521	mirna_pc
21072	hsa-mir-766	PHF16	0.344859579	mirna_pc
21073	hsa-mir-766	ESYT1	0.344685109	mirna_pc
21074	hsa-mir-766	MBNL3	0.376347355	mirna_pc
21075	hsa-mir-766	TSPYL3	0.316266249	mirna_pc
21076	hsa-mir-766	SF3A1	0.306971807	mirna_pc
21077	hsa-mir-766	TRIM24	0.419440827	mirna_pc
21078	hsa-mir-766	ABCC4	0.310499063	mirna_pc
21079	hsa-mir-766	C21orf67	0.33569961	mirna_pc
21080	hsa-mir-766	PPIE	0.375342296	mirna_pc
21081	hsa-mir-766	RASL10B	0.33912536	mirna_pc
21082	hsa-mir-766	CCDC134	0.38199971	mirna_pc
21083	hsa-mir-766	NUDT17	0.310383479	mirna_pc
21084	hsa-mir-766	C2orf72	0.32514197	mirna_pc
21085	hsa-mir-766	EP400	0.345393437	mirna_pc
21086	hsa-mir-766	FHL3	0.34094432	mirna_pc
21087	hsa-mir-766	PBX2	0.353472767	mirna_pc
21088	hsa-mir-766	SNCAIP	0.315444536	mirna_pc
21089	hsa-mir-766	RRAGC	0.36490729	mirna_pc
21090	hsa-mir-766	ZBTB39	0.356507865	mirna_pc
21091	hsa-mir-766	HYOU1	0.383385009	mirna_pc
21092	hsa-mir-766	HSD17B14	0.360023427	mirna_pc
21093	hsa-mir-766	NFYC	0.322704231	mirna_pc
21094	hsa-mir-766	PLIN2	0.388189456	mirna_pc
21095	hsa-mir-766	DHRS2	0.393767688	mirna_pc
21096	hsa-mir-766	GCN1L1	0.360430178	mirna_pc
21097	hsa-mir-766	LOC645166	0.330979918	mirna_pc
21098	hsa-mir-766	TAF9	0.334242364	mirna_pc
21099	hsa-mir-766	NDUFS5	0.310659482	mirna_pc
21100	hsa-mir-766	ASXL1	0.310455676	mirna_pc
21101	hsa-mir-766	SNIP1	0.318732998	mirna_pc
21102	hsa-mir-766	UTP15	0.324105608	mirna_pc
21103	hsa-mir-766	NFRKB	0.300072859	mirna_pc
21104	hsa-mir-766	ACP2	0.406006295	mirna_pc
21105	hsa-mir-766	FRAS1	0.431872407	mirna_pc
21106	hsa-mir-766	CD40	0.307926765	mirna_pc
21107	hsa-mir-766	LOC80054	0.32449444	mirna_pc
21108	hsa-mir-766	SEMA6D	0.324271695	mirna_pc
21109	hsa-mir-766	ZMYM3	0.326327412	mirna_pc
21110	hsa-mir-766	ZNF530	0.300587628	mirna_pc
21111	hsa-mir-766	AGT	0.324660698	mirna_pc
21112	hsa-mir-766	C12orf43	0.320125225	mirna_pc
21113	hsa-mir-766	SLC37A4	0.403630118	mirna_pc

21114	hsa-mir-766	MARS	0.356788419	mirna_pc
21115	hsa-mir-197	TPX2	0.45116458	mirna_pc
21116	hsa-mir-197	KIF4B	0.388143022	mirna_pc
21117	hsa-mir-197	CENPF	0.308566977	mirna_pc
21118	hsa-mir-197	RCC2	0.415791953	mirna_pc
21119	hsa-mir-197	HOXC9	0.402233771	mirna_pc
21120	hsa-mir-197	KIF11	0.359601974	mirna_pc
21121	hsa-mir-197	RRM2	0.381806498	mirna_pc
21122	hsa-mir-197	CDK1	0.325191649	mirna_pc
21123	hsa-mir-197	CDC25C	0.340057403	mirna_pc
21124	hsa-mir-197	MYBL2	0.55060664	mirna_pc
21125	hsa-mir-197	PCNA	0.307113202	mirna_pc
21126	hsa-mir-197	UBE2C	0.479833341	mirna_pc
21127	hsa-mir-197	BUB1	0.313404955	mirna_pc
21128	hsa-mir-197	PRC1	0.412589976	mirna_pc
21129	hsa-mir-197	NUSAP1	0.438158615	mirna_pc
21130	hsa-mir-197	MCM4	0.301473598	mirna_pc
21131	hsa-mir-197	KIFC1	0.426670928	mirna_pc
21132	hsa-mir-197	BUB1B	0.505302379	mirna_pc
21133	hsa-mir-197	HDGF	0.3633938	mirna_pc
21134	hsa-mir-197	KIF18B	0.30298011	mirna_pc
21135	hsa-mir-197	FAM72A	0.32459701	mirna_pc
21136	hsa-mir-197	KIF2C	0.446234241	mirna_pc
21137	hsa-mir-197	FANCA	0.354790899	mirna_pc
21138	hsa-mir-197	NCAPG	0.448428342	mirna_pc
21139	hsa-mir-197	CDC20	0.431406455	mirna_pc
21140	hsa-mir-197	CLSPN	0.352002067	mirna_pc
21141	hsa-mir-197	SGOL1	0.385197332	mirna_pc
21142	hsa-mir-197	CCNA2	0.358964353	mirna_pc
21143	hsa-mir-197	CDC25B	0.331327932	mirna_pc
21144	hsa-mir-197	SPC25	0.344846598	mirna_pc
21145	hsa-mir-197	FANCI	0.456762946	mirna_pc
21146	hsa-mir-197	NEK2	0.413870424	mirna_pc
21147	hsa-mir-197	MND1	0.398694429	mirna_pc
21148	hsa-mir-197	NCAPH	0.399674208	mirna_pc
21149	hsa-mir-197	GTSE1	0.367161336	mirna_pc
21150	hsa-mir-197	KIF23	0.348156083	mirna_pc
21151	hsa-mir-197	RAD54L	0.551565679	mirna_pc
21152	hsa-mir-197	CENPA	0.346606501	mirna_pc
21153	hsa-mir-197	MAD2L1	0.419117715	mirna_pc
21154	hsa-mir-197	TIMELESS	0.500801616	mirna_pc
21155	hsa-mir-197	CDCA8	0.456193731	mirna_pc
21156	hsa-mir-197	TROAP	0.408256363	mirna_pc
21157	hsa-mir-197	CCNB1	0.315026714	mirna_pc
21158	hsa-mir-197	CDCA5	0.46961763	mirna_pc
21159	hsa-mir-197	CDC45	0.387182137	mirna_pc
21160	hsa-mir-197	STMN1	0.524420748	mirna_pc
21161	hsa-mir-197	CENPM	0.383797202	mirna_pc
21162	hsa-mir-197	EXO1	0.307370101	mirna_pc
21163	hsa-mir-197	UBE2T	0.436754191	mirna_pc
21164	hsa-mir-197	CKS2	0.323980242	mirna_pc
21165	hsa-mir-197	CKS1B	0.537756858	mirna_pc
21166	hsa-mir-197	TUBB	0.420912438	mirna_pc
21167	hsa-mir-197	MCM2	0.383590954	mirna_pc

21168	hsa-mir-197	KIF4A	0.420754906	mirna_pc
21169	hsa-mir-197	ORC1L	0.421914758	mirna_pc
21170	hsa-mir-197	KNTC1	0.404544398	mirna_pc
21171	hsa-mir-197	RACGAP1	0.362972407	mirna_pc
21172	hsa-mir-197	PTBP1	0.32485231	mirna_pc
21173	hsa-mir-197	CCNB2	0.342009711	mirna_pc
21174	hsa-mir-197	NUF2	0.304208281	mirna_pc
21175	hsa-mir-197	PLK4	0.367899825	mirna_pc
21176	hsa-mir-197	FANCB	0.32183862	mirna_pc
21177	hsa-mir-197	KIF18A	0.325069145	mirna_pc
21178	hsa-mir-197	DEPDC1	0.430448587	mirna_pc
21179	hsa-mir-197	HJURP	0.366917113	mirna_pc
21180	hsa-mir-197	SKA1	0.35494749	mirna_pc
21181	hsa-mir-197	RAD51	0.457533281	mirna_pc
21182	hsa-mir-197	BLM	0.442919613	mirna_pc
21183	hsa-mir-197	DNMT1	0.398555461	mirna_pc
21184	hsa-mir-197	BIRC5	0.369637557	mirna_pc
21185	hsa-mir-197	RCC1	0.402588165	mirna_pc
21186	hsa-mir-197	OIP5	0.495297454	mirna_pc
21187	hsa-mir-197	DTL	0.389762337	mirna_pc
21188	hsa-mir-197	RECQL4	0.475140722	mirna_pc
21189	hsa-mir-197	AURKA	0.374005898	mirna_pc
21190	hsa-mir-197	AURKB	0.408090658	mirna_pc
21191	hsa-mir-197	GSG2	0.329547542	mirna_pc
21192	hsa-mir-197	CDT1	0.306534437	mirna_pc
21193	hsa-mir-197	MCM10	0.408920788	mirna_pc
21194	hsa-mir-197	SGOL2	0.358032012	mirna_pc
21195	hsa-mir-197	CHEK2	0.416305039	mirna_pc
21196	hsa-mir-197	KIF15	0.403097232	mirna_pc
21197	hsa-mir-197	CDCA3	0.379233207	mirna_pc
21198	hsa-mir-197	C1orf112	0.403862363	mirna_pc
21199	hsa-mir-197	FEN1	0.394042048	mirna_pc
21200	hsa-mir-197	TRAIP	0.418477229	mirna_pc
21201	hsa-mir-197	PSMA7	0.499750135	mirna_pc
21202	hsa-mir-197	CASC5	0.302984182	mirna_pc
21203	hsa-mir-197	C16orf59	0.407781878	mirna_pc
21204	hsa-mir-197	RAE1	0.465255924	mirna_pc
21205	hsa-mir-197	ADRM1	0.498505358	mirna_pc
21206	hsa-mir-197	FAM72B	0.318261113	mirna_pc
21207	hsa-mir-197	NEIL3	0.307256728	mirna_pc
21208	hsa-mir-197	MCM7	0.337377193	mirna_pc
21209	hsa-mir-197	POLD1	0.409512587	mirna_pc
21210	hsa-mir-197	C12orf48	0.399486921	mirna_pc
21211	hsa-mir-197	HNRNPL	0.421871332	mirna_pc
21212	hsa-mir-197	ZWILCH	0.361432657	mirna_pc
21213	hsa-mir-197	TTK	0.372244701	mirna_pc
21214	hsa-mir-197	ZWINT	0.436718448	mirna_pc
21215	hsa-mir-197	TUBA1B	0.463282594	mirna_pc
21216	hsa-mir-197	UBE2S	0.415355212	mirna_pc
21217	hsa-mir-197	NCAPD2	0.315241812	mirna_pc
21218	hsa-mir-197	HNRNPC	0.33145281	mirna_pc
21219	hsa-mir-197	ILF2	0.55023233	mirna_pc
21220	hsa-mir-197	C6orf167	0.325609632	mirna_pc
21221	hsa-mir-197	MCM5	0.35081756	mirna_pc

21222	hsa-mir-197	RAN	0.418587794	mirna_pc
21223	hsa-mir-197	TYMS	0.397869467	mirna_pc
21224	hsa-mir-197	MCM6	0.363611611	mirna_pc
21225	hsa-mir-197	CDCA7	0.307915111	mirna_pc
21226	hsa-mir-197	EPR1	0.330475713	mirna_pc
21227	hsa-mir-197	H2AFX	0.306786288	mirna_pc
21228	hsa-mir-197	UCK2	0.475329618	mirna_pc
21229	hsa-mir-197	NFKBIL2	0.395136438	mirna_pc
21230	hsa-mir-197	C15orf42	0.40369764	mirna_pc
21231	hsa-mir-197	PTGES3	0.373446355	mirna_pc
21232	hsa-mir-197	PRIM2	0.429793733	mirna_pc
21233	hsa-mir-197	HMGB3	0.319255688	mirna_pc
21234	hsa-mir-197	RTKN	0.334004135	mirna_pc
21235	hsa-mir-197	FAM64A	0.402757083	mirna_pc
21236	hsa-mir-197	RANBP1	0.440529183	mirna_pc
21237	hsa-mir-197	E2F1	0.479322876	mirna_pc
21238	hsa-mir-197	E2F2	0.307331574	mirna_pc
21239	hsa-mir-197	SNRPG	0.305145731	mirna_pc
21240	hsa-mir-197	TH1L	0.464851044	mirna_pc
21241	hsa-mir-197	MCM3	0.383389337	mirna_pc
21242	hsa-mir-197	SNRPA	0.411169788	mirna_pc
21243	hsa-mir-197	DNAJC9	0.328355296	mirna_pc
21244	hsa-mir-197	DEPDC1B	0.313274049	mirna_pc
21245	hsa-mir-197	POC1A	0.417081304	mirna_pc
21246	hsa-mir-197	CENPO	0.45285638	mirna_pc
21247	hsa-mir-197	NUDT1	0.300449884	mirna_pc
21248	hsa-mir-197	CCT3	0.426554473	mirna_pc
21249	hsa-mir-197	GINS2	0.319492389	mirna_pc
21250	hsa-mir-197	DNA2	0.342526924	mirna_pc
21251	hsa-mir-197	NOP56	0.315948818	mirna_pc
21252	hsa-mir-197	CSE1L	0.497196258	mirna_pc
21253	hsa-mir-197	DTYMK	0.368109973	mirna_pc
21254	hsa-mir-197	MLF1IP	0.342175331	mirna_pc
21255	hsa-mir-197	HSPE1	0.331623675	mirna_pc
21256	hsa-mir-197	EBNA1BP2	0.314547852	mirna_pc
21257	hsa-mir-197	CFL1	0.357232364	mirna_pc
21258	hsa-mir-197	TCF19	0.312292174	mirna_pc
21259	hsa-mir-197	CAD	0.376336948	mirna_pc
21260	hsa-mir-197	CDK2	0.456905497	mirna_pc
21261	hsa-mir-197	CDK4	0.508155515	mirna_pc
21262	hsa-mir-197	CKAP5	0.380431064	mirna_pc
21263	hsa-mir-197	BCL2L12	0.300291014	mirna_pc
21264	hsa-mir-197	HNRNPR	0.312391686	mirna_pc
21265	hsa-mir-197	PRIM1	0.394079491	mirna_pc
21266	hsa-mir-197	CENPH	0.380130992	mirna_pc
21267	hsa-mir-197	CHAF1B	0.503638143	mirna_pc
21268	hsa-mir-197	RALY	0.332515069	mirna_pc
21269	hsa-mir-197	MSH2	0.368250004	mirna_pc
21270	hsa-mir-197	SF3B3	0.347433938	mirna_pc
21271	hsa-mir-197	CDC7	0.382704351	mirna_pc
21272	hsa-mir-197	PUS1	0.343091748	mirna_pc
21273	hsa-mir-197	SNRPC	0.461256222	mirna_pc
21274	hsa-mir-197	PSMB4	0.306650962	mirna_pc
21275	hsa-mir-197	RFC4	0.369498445	mirna_pc

21276	hsa-mir-197	FBXO5	0.391853861	mirna_pc
21277	hsa-mir-197	DCLRE1B	0.431951709	mirna_pc
21278	hsa-mir-197	MDK	0.321580296	mirna_pc
21279	hsa-mir-197	E2F3	0.325490962	mirna_pc
21280	hsa-mir-197	RUVBL2	0.43090059	mirna_pc
21281	hsa-mir-197	CDC25A	0.493193505	mirna_pc
21282	hsa-mir-197	TMEM48	0.325677285	mirna_pc
21283	hsa-mir-197	RAD54B	0.359747121	mirna_pc
21284	hsa-mir-197	H2AFZ	0.367097611	mirna_pc
21285	hsa-mir-197	MAGOH	0.392315471	mirna_pc
21286	hsa-mir-197	DDX12	0.300444772	mirna_pc
21287	hsa-mir-197	DSCC1	0.315135019	mirna_pc
21288	hsa-mir-197	NUP37	0.35479221	mirna_pc
21289	hsa-mir-197	CLN6	0.32843583	mirna_pc
21290	hsa-mir-197	MCM8	0.363812637	mirna_pc
21291	hsa-mir-197	CPSF3	0.328351115	mirna_pc
21292	hsa-mir-197	BANF1	0.380898245	mirna_pc
21293	hsa-mir-197	SNRPE	0.456522765	mirna_pc
21294	hsa-mir-197	C21orf45	0.376731986	mirna_pc
21295	hsa-mir-197	ATIC	0.317702832	mirna_pc
21296	hsa-mir-197	PPM1G	0.340879226	mirna_pc
21297	hsa-mir-197	MARCKSL1	0.333797566	mirna_pc
21298	hsa-mir-197	NPM3	0.322651549	mirna_pc
21299	hsa-mir-197	NUDT5	0.332984187	mirna_pc
21300	hsa-mir-197	DAZAP1	0.32659798	mirna_pc
21301	hsa-mir-197	CBX8	0.313386204	mirna_pc
21302	hsa-mir-197	WDR34	0.301927956	mirna_pc
21303	hsa-mir-197	TMEM201	0.444043391	mirna_pc
21304	hsa-mir-197	MRPL9	0.305751417	mirna_pc
21305	hsa-mir-197	PFDN4	0.36030371	mirna_pc
21306	hsa-mir-197	PAFAH1B3	0.325641884	mirna_pc
21307	hsa-mir-197	SRRT	0.301916936	mirna_pc
21308	hsa-mir-197	CACYBP	0.301029697	mirna_pc
21309	hsa-mir-197	SASS6	0.357310387	mirna_pc
21310	hsa-mir-197	PSRC1	0.596412337	mirna_pc
21311	hsa-mir-197	POU2F1	0.384050638	mirna_pc
21312	hsa-mir-197	STIP1	0.373157882	mirna_pc
21313	hsa-mir-197	CEP72	0.34306697	mirna_pc
21314	hsa-mir-197	TMPO	0.393648593	mirna_pc
21315	hsa-mir-197	VBP1	0.343124858	mirna_pc
21316	hsa-mir-197	MRTO4	0.433177587	mirna_pc
21317	hsa-mir-197	PPIH	0.368142437	mirna_pc
21318	hsa-mir-197	RCN1	0.325890251	mirna_pc
21319	hsa-mir-197	CCDC138	0.311482979	mirna_pc
21320	hsa-mir-197	LOC100128191	0.443154174	mirna_pc
21321	hsa-mir-197	HMGN2	0.376447264	mirna_pc
21322	hsa-mir-197	POLA2	0.36384628	mirna_pc
21323	hsa-mir-197	SNRPF	0.316142991	mirna_pc
21324	hsa-mir-197	SNRPA1	0.416349643	mirna_pc
21325	hsa-mir-197	SKP2	0.436007568	mirna_pc
21326	hsa-mir-197	TIPIN	0.366607207	mirna_pc
21327	hsa-mir-197	HSPBP1	0.304819367	mirna_pc
21328	hsa-mir-197	C4orf46	0.325929595	mirna_pc
21329	hsa-mir-197	DNMT3B	0.316984882	mirna_pc

21330	hsa-mir-197	UTP14A	0.326195157	mirna_pc
21331	hsa-mir-197	CTPS	0.306184439	mirna_pc
21332	hsa-mir-197	GPN1	0.308193004	mirna_pc
21333	hsa-mir-197	RFC5	0.360705941	mirna_pc
21334	hsa-mir-197	MAZ	0.319966148	mirna_pc
21335	hsa-mir-197	SSRP1	0.409780578	mirna_pc
21336	hsa-mir-197	POLR2D	0.360787738	mirna_pc
21337	hsa-mir-197	PA2G4	0.442956989	mirna_pc
21338	hsa-mir-197	SNRPD3	0.366453196	mirna_pc
21339	hsa-mir-197	KHDRBS1	0.375782304	mirna_pc
21340	hsa-mir-197	SLC5A6	0.418596801	mirna_pc
21341	hsa-mir-197	C20orf27	0.36792161	mirna_pc
21342	hsa-mir-197	TOMM34	0.487351365	mirna_pc
21343	hsa-mir-197	CCDC21	0.371542354	mirna_pc
21344	hsa-mir-197	MTL 5.00	0.328388262	mirna_pc
21345	hsa-mir-197	GGH	0.481663937	mirna_pc
21346	hsa-mir-197	RNF220	0.349068871	mirna_pc
21347	hsa-mir-197	AHCY	0.318439211	mirna_pc
21348	hsa-mir-197	PARP1	0.310857035	mirna_pc
21349	hsa-mir-197	CCT7	0.335858837	mirna_pc
21350	hsa-mir-197	TMEM194A	0.391821596	mirna_pc
21351	hsa-mir-197	SNRNP40	0.490640419	mirna_pc
21352	hsa-mir-197	SUV420H2	0.329373352	mirna_pc
21353	hsa-mir-197	ZNF695	0.389546455	mirna_pc
21354	hsa-mir-197	TMEM69	0.323872138	mirna_pc
21355	hsa-mir-197	TIMM8A	0.363125445	mirna_pc
21356	hsa-mir-197	GNL2	0.335485338	mirna_pc
21357	hsa-mir-197	WRAP53	0.316774758	mirna_pc
21358	hsa-mir-197	NOC2L	0.300720411	mirna_pc
21359	hsa-mir-197	NASP	0.421686173	mirna_pc
21360	hsa-mir-197	LAGE3	0.324648058	mirna_pc
21361	hsa-mir-197	NONO	0.513813436	mirna_pc
21362	hsa-mir-197	CCDC86	0.32571941	mirna_pc
21363	hsa-mir-197	OBFC2B	0.499413057	mirna_pc
21364	hsa-mir-197	WDR76	0.383365367	mirna_pc
21365	hsa-mir-197	LSM 4.00	0.308141399	mirna_pc
21366	hsa-mir-197	DENR	0.346422849	mirna_pc
21367	hsa-mir-197	SCARB1	0.528402992	mirna_pc
21368	hsa-mir-197	ELK1	0.339597378	mirna_pc
21369	hsa-mir-197	C11orf84	0.362484581	mirna_pc
21370	hsa-mir-197	TBCE	0.3223955	mirna_pc
21371	hsa-mir-197	SUV39H2	0.378848628	mirna_pc
21372	hsa-mir-197	LSM 2.00	0.383401436	mirna_pc
21373	hsa-mir-197	RIBC2	0.324816622	mirna_pc
21374	hsa-mir-197	DPY30	0.3021789	mirna_pc
21375	hsa-mir-197	RRP9	0.333442416	mirna_pc
21376	hsa-mir-197	DPH2	0.422862013	mirna_pc
21377	hsa-mir-197	GTPBP4	0.311696989	mirna_pc
21378	hsa-mir-197	MTCH2	0.392429704	mirna_pc
21379	hsa-mir-197	SCAMP3	0.339065074	mirna_pc
21380	hsa-mir-197	MEN1	0.369553332	mirna_pc
21381	hsa-mir-197	CCT4	0.358035709	mirna_pc
21382	hsa-mir-197	SAC3D1	0.406305697	mirna_pc
21383	hsa-mir-197	ELAVL1	0.346503122	mirna_pc

21384	hsa-mir-197	MRPL4	0.326332853	mirna_pc
21385	hsa-mir-197	MOV10	0.308151333	mirna_pc
21386	hsa-mir-197	RPS21	0.528980333	mirna_pc
21387	hsa-mir-197	TBC1D7	0.327507397	mirna_pc
21388	hsa-mir-197	PAK1IP1	0.44943517	mirna_pc
21389	hsa-mir-197	SLC1A5	0.359750549	mirna_pc
21390	hsa-mir-197	PES 1.00	0.307785185	mirna_pc
21391	hsa-mir-197	PGAM5	0.404005281	mirna_pc
21392	hsa-mir-197	KDM1A	0.388603115	mirna_pc
21393	hsa-mir-197	ILF3	0.312427617	mirna_pc
21394	hsa-mir-197	NT5DC2	0.344320304	mirna_pc
21395	hsa-mir-197	EHMT2	0.451066074	mirna_pc
21396	hsa-mir-197	DEK	0.318922163	mirna_pc
21397	hsa-mir-197	SFRS9	0.380556586	mirna_pc
21398	hsa-mir-197	PDRG1	0.345199296	mirna_pc
21399	hsa-mir-197	SAAL1	0.310627741	mirna_pc
21400	hsa-mir-197	TAF4	0.358575601	mirna_pc
21401	hsa-mir-197	HEATR2	0.362226185	mirna_pc
21402	hsa-mir-197	PRAME	0.376421481	mirna_pc
21403	hsa-mir-197	SUV39H1	0.3630449	mirna_pc
21404	hsa-mir-197	IPO9	0.403633352	mirna_pc
21405	hsa-mir-197	SART3	0.345693521	mirna_pc
21406	hsa-mir-197	C15orf23	0.426794932	mirna_pc
21407	hsa-mir-197	AMPD2	0.383141065	mirna_pc
21408	hsa-mir-197	LSM14B	0.460075503	mirna_pc
21409	hsa-mir-197	NOC4L	0.331586215	mirna_pc
21410	hsa-mir-197	DIABLO	0.360427845	mirna_pc
21411	hsa-mir-197	LRRC42	0.390610282	mirna_pc
21412	hsa-mir-197	SAE1	0.374044302	mirna_pc
21413	hsa-mir-197	ITGB3BP	0.36498951	mirna_pc
21414	hsa-mir-197	PABPC1L	0.336038573	mirna_pc
21415	hsa-mir-197	LAS1L	0.32131383	mirna_pc
21416	hsa-mir-197	PDCD6	0.324859304	mirna_pc
21417	hsa-mir-197	NCRNA00176	0.361709785	mirna_pc
21418	hsa-mir-197	SFPQ	0.320025285	mirna_pc
21419	hsa-mir-197	VPS72	0.320148687	mirna_pc
21420	hsa-mir-197	FAM125B	0.458006903	mirna_pc
21421	hsa-mir-197	EPHB2	0.355949371	mirna_pc
21422	hsa-mir-197	AS3MT	0.319594656	mirna_pc
21423	hsa-mir-197	ATAD3A	0.431386732	mirna_pc
21424	hsa-mir-197	SMS	0.349701959	mirna_pc
21425	hsa-mir-197	CADM1	0.391868905	mirna_pc
21426	hsa-mir-197	SHMT2	0.359859473	mirna_pc
21427	hsa-mir-197	POLA1	0.441473728	mirna_pc
21428	hsa-mir-197	HDAC2	0.447010001	mirna_pc
21429	hsa-mir-197	MRPL12	0.336544136	mirna_pc
21430	hsa-mir-197	GMNN	0.389137808	mirna_pc
21431	hsa-mir-197	CSNK1E	0.382579942	mirna_pc
21432	hsa-mir-197	PA2G4P4	0.472269164	mirna_pc
21433	hsa-mir-197	RRP12	0.301336369	mirna_pc
21434	hsa-mir-197	ASCL2	0.385287506	mirna_pc
21435	hsa-mir-197	SLC39A1	0.363477003	mirna_pc
21436	hsa-mir-197	BRI3BP	0.304040895	mirna_pc
21437	hsa-mir-197	CSTF1	0.335200924	mirna_pc

21438	hsa-mir-197	DUT	0.355776239	mirna_pc
21439	hsa-mir-197	MSH6	0.36705088	mirna_pc
21440	hsa-mir-197	SRM	0.31997496	mirna_pc
21441	hsa-mir-197	XRCC6	0.336851132	mirna_pc
21442	hsa-mir-197	PSMG1	0.307363391	mirna_pc
21443	hsa-mir-197	PPP1R8	0.321225754	mirna_pc
21444	hsa-mir-197	NUDT3	0.346939394	mirna_pc
21445	hsa-mir-197	MEX3A	0.409974023	mirna_pc
21446	hsa-mir-197	DHX37	0.337964071	mirna_pc
21447	hsa-mir-197	PPAN	0.380670016	mirna_pc
21448	hsa-mir-197	PDCD11	0.329728817	mirna_pc
21449	hsa-mir-197	RFC2	0.351370522	mirna_pc
21450	hsa-mir-197	TAF5	0.370960875	mirna_pc
21451	hsa-mir-197	PDIA3	0.306548605	mirna_pc
21452	hsa-mir-197	TRIM28	0.330963737	mirna_pc
21453	hsa-mir-197	CCDC28B	0.320783117	mirna_pc
21454	hsa-mir-197	TSEN34	0.332832873	mirna_pc
21455	hsa-mir-197	UNC119B	0.370611292	mirna_pc
21456	hsa-mir-197	MAD2L2	0.366882272	mirna_pc
21457	hsa-mir-197	HNRNPA1L2	0.307274297	mirna_pc
21458	hsa-mir-197	SARS	0.316551719	mirna_pc
21459	hsa-mir-197	SUMO1P3	0.336436389	mirna_pc
21460	hsa-mir-197	TMEM39B	0.328280984	mirna_pc
21461	hsa-mir-197	PRPF31	0.33986561	mirna_pc
21462	hsa-mir-197	FARSB	0.301025027	mirna_pc
21463	hsa-mir-197	ERI3	0.384911777	mirna_pc
21464	hsa-mir-197	RCN2	0.364889047	mirna_pc
21465	hsa-mir-197	CIB2	0.414193071	mirna_pc
21466	hsa-mir-197	MTF2	0.328795606	mirna_pc
21467	hsa-mir-197	TARBP2	0.423425833	mirna_pc
21468	hsa-mir-197	TOMM22	0.334348491	mirna_pc
21469	hsa-mir-197	EBP	0.30818068	mirna_pc
21470	hsa-mir-197	C12orf52	0.413974267	mirna_pc
21471	hsa-mir-197	TCP1	0.32999419	mirna_pc
21472	hsa-mir-197	SF3A3	0.374354287	mirna_pc
21473	hsa-mir-197	POLE	0.525355847	mirna_pc
21474	hsa-mir-197	C8orf30A	0.322116516	mirna_pc
21475	hsa-mir-197	MUTYH	0.378701042	mirna_pc
21476	hsa-mir-197	CHML	0.31009358	mirna_pc
21477	hsa-mir-197	WRNIP1	0.370258809	mirna_pc
21478	hsa-mir-197	TRIAP1	0.341101288	mirna_pc
21479	hsa-mir-197	ADSL	0.398141012	mirna_pc
21480	hsa-mir-197	NPHP4	0.495819325	mirna_pc
21481	hsa-mir-197	ZNF350	0.323186256	mirna_pc
21482	hsa-mir-197	UNG	0.362530647	mirna_pc
21483	hsa-mir-197	RBMX	0.335778693	mirna_pc
21484	hsa-mir-197	CDK16	0.418327494	mirna_pc
21485	hsa-mir-197	ALKBH2	0.336802375	mirna_pc
21486	hsa-mir-197	BAMBI	0.34455325	mirna_pc
21487	hsa-mir-197	CCDC90A	0.394074058	mirna_pc
21488	hsa-mir-197	MDC 1.00	0.331692185	mirna_pc
21489	hsa-mir-197	CSNK2B	0.323232804	mirna_pc
21490	hsa-mir-197	PDIA6	0.305423743	mirna_pc
21491	hsa-mir-197	GPN3	0.367132857	mirna_pc

21492	hsa-mir-197	MRPL37	0.433864395	mirna_pc
21493	hsa-mir-197	EIF3I	0.383249907	mirna_pc
21494	hsa-mir-197	DNAJC14	0.361684171	mirna_pc
21495	hsa-mir-197	KDM2B	0.33885751	mirna_pc
21496	hsa-mir-197	PCGF6	0.318162279	mirna_pc
21497	hsa-mir-197	PUSL1	0.34952231	mirna_pc
21498	hsa-mir-197	CNPY2	0.31814369	mirna_pc
21499	hsa-mir-197	FAM136A	0.345028219	mirna_pc
21500	hsa-mir-197	NKD2	0.396181692	mirna_pc
21501	hsa-mir-197	APOA1BP	0.324111082	mirna_pc
21502	hsa-mir-197	ZSCAN5A	0.418715386	mirna_pc
21503	hsa-mir-197	YRDC	0.359969977	mirna_pc
21504	hsa-mir-197	SMARCB1	0.336566902	mirna_pc
21505	hsa-mir-197	DDX54	0.308845337	mirna_pc
21506	hsa-mir-197	PYGO2	0.317936745	mirna_pc
21507	hsa-mir-197	CCT8	0.31338446	mirna_pc
21508	hsa-mir-197	FAM40A	0.311981955	mirna_pc
21509	hsa-mir-197	KCTD17	0.400970439	mirna_pc
21510	hsa-mir-197	ITPRIPL1	0.426698373	mirna_pc
21511	hsa-mir-197	GDPD5	0.357713052	mirna_pc
21512	hsa-mir-197	RPS7	0.3121169	mirna_pc
21513	hsa-mir-197	ATXN7L2	0.409391973	mirna_pc
21514	hsa-mir-197	LOC401010	0.370748814	mirna_pc
21515	hsa-mir-197	STRA13	0.405099805	mirna_pc
21516	hsa-mir-197	KIAA0090	0.327106971	mirna_pc
21517	hsa-mir-197	BCL7A	0.420528985	mirna_pc
21518	hsa-mir-197	ST13	0.321810695	mirna_pc
21519	hsa-mir-197	LAMA1	0.310714046	mirna_pc
21520	hsa-mir-197	NOTUM	0.385983636	mirna_pc
21521	hsa-mir-197	ARNT2	0.443588146	mirna_pc
21522	hsa-mir-197	PRPF38A	0.30435814	mirna_pc
21523	hsa-mir-197	WDR77	0.449626194	mirna_pc
21524	hsa-mir-197	MRPL21	0.330710834	mirna_pc
21525	hsa-mir-197	ILKAP	0.31725521	mirna_pc
21526	hsa-mir-197	PRR3	0.31286704	mirna_pc
21527	hsa-mir-197	MGC72080	0.305584027	mirna_pc
21528	hsa-mir-197	CTNNB1	0.323696917	mirna_pc
21529	hsa-mir-197	SEMA4F	0.317448905	mirna_pc
21530	hsa-mir-197	GANAB	0.383797811	mirna_pc
21531	hsa-mir-197	DDX20	0.455463585	mirna_pc
21532	hsa-mir-197	RNF34	0.315624975	mirna_pc
21533	hsa-mir-197	C1orf109	0.327487884	mirna_pc
21534	hsa-mir-197	FGGY	0.348596742	mirna_pc
21535	hsa-mir-197	UBE2N	0.375123342	mirna_pc
21536	hsa-mir-197	SEC61A2	0.361104738	mirna_pc
21537	hsa-mir-197	C20orf43	0.312142232	mirna_pc
21538	hsa-mir-197	COPZ1	0.379210545	mirna_pc
21539	hsa-mir-197	SLC25A10	0.302854619	mirna_pc
21540	hsa-mir-197	MAPKAPK5	0.447789408	mirna_pc
21541	hsa-mir-197	GPR3	0.339396604	mirna_pc
21542	hsa-mir-197	DRG1	0.37817847	mirna_pc
21543	hsa-mir-197	NOL7	0.375756073	mirna_pc
21544	hsa-mir-197	HUNK	0.329669837	mirna_pc
21545	hsa-mir-197	B4GALT3	0.32184721	mirna_pc

21546	hsa-mir-197	DEPDC7	0.390984563	mirna_pc
21547	hsa-mir-197	UBL4A	0.329726582	mirna_pc
21548	hsa-mir-197	SCLY	0.344591579	mirna_pc
21549	hsa-mir-197	RPL5	0.337114827	mirna_pc
21550	hsa-mir-197	FIZ1	0.381474114	mirna_pc
21551	hsa-mir-197	GGA1	0.339139294	mirna_pc
21552	hsa-mir-197	DHRS13	0.317544336	mirna_pc
21553	hsa-mir-197	RPF1	0.394012005	mirna_pc
21554	hsa-mir-197	EWSR1	0.382873485	mirna_pc
21555	hsa-mir-197	KRR1	0.313832013	mirna_pc
21556	hsa-mir-197	ALX3	0.371822368	mirna_pc
21557	hsa-mir-197	GTPBP5	0.380559838	mirna_pc
21558	hsa-mir-197	AAAS	0.447431595	mirna_pc
21559	hsa-mir-197	UBQLN4	0.318228915	mirna_pc
21560	hsa-mir-197	CHST14	0.331912652	mirna_pc
21561	hsa-mir-197	ACVR2B	0.429548838	mirna_pc
21562	hsa-mir-197	CABLES2	0.408898761	mirna_pc
21563	hsa-mir-197	MRPS26	0.313135029	mirna_pc
21564	hsa-mir-197	GNL3	0.337752838	mirna_pc
21565	hsa-mir-197	NOL12	0.300403688	mirna_pc
21566	hsa-mir-197	BCORL1	0.308531807	mirna_pc
21567	hsa-mir-197	RPS16	0.319768032	mirna_pc
21568	hsa-mir-197	AKR7A2	0.342297028	mirna_pc
21569	hsa-mir-197	BEND3	0.39267282	mirna_pc
21570	hsa-mir-197	C18orf10	0.314135673	mirna_pc
21571	hsa-mir-197	EMD	0.328630911	mirna_pc
21572	hsa-mir-197	CA5BP	0.36894583	mirna_pc
21573	hsa-mir-197	PCBP2	0.362823887	mirna_pc
21574	hsa-mir-197	NKD1	0.470488612	mirna_pc
21575	hsa-mir-197	MRPS16	0.317067792	mirna_pc
21576	hsa-mir-197	PFAS	0.389702575	mirna_pc
21577	hsa-mir-197	SF3B2	0.306574111	mirna_pc
21578	hsa-mir-197	COQ3	0.306346866	mirna_pc
21579	hsa-mir-197	RPL7	0.341978867	mirna_pc
21580	hsa-mir-197	RBM38	0.393448121	mirna_pc
21581	hsa-mir-197	SNUPN	0.340402378	mirna_pc
21582	hsa-mir-197	RPUSD2	0.332702404	mirna_pc
21583	hsa-mir-197	NUP153	0.359690663	mirna_pc
21584	hsa-mir-197	NIPSNAP1	0.315287652	mirna_pc
21585	hsa-mir-197	SERBP1	0.32398994	mirna_pc
21586	hsa-mir-197	RDBP	0.341536381	mirna_pc
21587	hsa-mir-197	FAM10A4	0.351515574	mirna_pc
21588	hsa-mir-197	ARL 2.00	0.344643788	mirna_pc
21589	hsa-mir-197	EEF1E1	0.346251809	mirna_pc
21590	hsa-mir-197	YBX1	0.348442243	mirna_pc
21591	hsa-mir-197	TCTN2	0.314102167	mirna_pc
21592	hsa-mir-197	RPL14	0.357229586	mirna_pc
21593	hsa-mir-197	UBE2M	0.300396799	mirna_pc
21594	hsa-mir-197	TIMM17B	0.32895764	mirna_pc
21595	hsa-mir-197	SEPHS1	0.434011696	mirna_pc
21596	hsa-mir-197	POMGNT1	0.340823985	mirna_pc
21597	hsa-mir-197	DPH5	0.304605788	mirna_pc
21598	hsa-mir-197	TMEM9	0.337209283	mirna_pc
21599	hsa-mir-197	SUB1	0.309691745	mirna_pc

21600	hsa-mir-197	AGBL5	0.327358197	mirna_pc
21601	hsa-mir-197	ERP29	0.304659321	mirna_pc
21602	hsa-mir-197	MYL6B	0.44226322	mirna_pc
21603	hsa-mir-197	RPS17	0.303724352	mirna_pc
21604	hsa-mir-197	PIGT	0.407441309	mirna_pc
21605	hsa-mir-197	POLR2F	0.416836707	mirna_pc
21606	hsa-mir-197	PIPSL	0.311441926	mirna_pc
21607	hsa-mir-197	ATP5E	0.33261051	mirna_pc
21608	hsa-mir-197	UQCRHL	0.312669305	mirna_pc
21609	hsa-mir-197	CAMTA1	0.338829929	mirna_pc
21610	hsa-mir-197	EIF2B3	0.300156066	mirna_pc
21611	hsa-mir-197	TMEM14C	0.32262726	mirna_pc
21612	hsa-mir-197	NME4	0.332464839	mirna_pc
21613	hsa-mir-197	LYRM4	0.378240647	mirna_pc
21614	hsa-mir-197	MMACHC	0.439429184	mirna_pc
21615	hsa-mir-197	RNF41	0.348321442	mirna_pc
21616	hsa-mir-197	USP21	0.321513653	mirna_pc
21617	hsa-mir-197	ANP32A	0.39684601	mirna_pc
21618	hsa-mir-197	ZNF775	0.364805251	mirna_pc
21619	hsa-mir-197	SUPV3L1	0.34638765	mirna_pc
21620	hsa-mir-197	DPF2	0.342686586	mirna_pc
21621	hsa-mir-197	RTCD1	0.304135484	mirna_pc
21622	hsa-mir-197	C17orf81	0.314233907	mirna_pc
21623	hsa-mir-197	HIC2	0.306442634	mirna_pc
21624	hsa-mir-197	UBE2J2	0.300117038	mirna_pc
21625	hsa-mir-197	NAP1L1	0.394284909	mirna_pc
21626	hsa-mir-197	SMG5	0.370028453	mirna_pc
21627	hsa-mir-197	GLT25D2	0.352305993	mirna_pc
21628	hsa-mir-197	PHF16	0.32042541	mirna_pc
21629	hsa-mir-197	CDKN2A	0.309308719	mirna_pc
21630	hsa-mir-197	BUD13	0.324396075	mirna_pc
21631	hsa-mir-197	CDC34	0.33812451	mirna_pc
21632	hsa-mir-197	MANEAL	0.300426773	mirna_pc
21633	hsa-mir-197	RPS10	0.346904488	mirna_pc
21634	hsa-mir-197	MYCN	0.359947409	mirna_pc
21635	hsa-mir-197	SBK1	0.340395702	mirna_pc
21636	hsa-mir-197	SF3A1	0.35138627	mirna_pc
21637	hsa-mir-197	RPS5	0.386340985	mirna_pc
21638	hsa-mir-197	USP13	0.330858152	mirna_pc
21639	hsa-mir-197	CCDC134	0.320754054	mirna_pc
21640	hsa-mir-197	HS2ST1	0.340048337	mirna_pc
21641	hsa-mir-197	SLC25A33	0.361582597	mirna_pc
21642	hsa-mir-197	RPLP2	0.321998257	mirna_pc
21643	hsa-mir-197	GPR37	0.336858047	mirna_pc
21644	hsa-mir-197	RANGRF	0.31892473	mirna_pc
21645	hsa-mir-197	MSI1	0.446004288	mirna_pc
21646	hsa-mir-197	CLPB	0.324269193	mirna_pc
21647	hsa-mir-197	SMARCC1	0.414517442	mirna_pc
21648	hsa-mir-197	CTPS2	0.34300462	mirna_pc
21649	hsa-mir-197	FGD1	0.312953028	mirna_pc
21650	hsa-mir-197	RPLP0	0.311873318	mirna_pc
21651	hsa-mir-197	MRPL20	0.338742782	mirna_pc
21652	hsa-mir-197	ZNF740	0.304219535	mirna_pc
21653	hsa-mir-197	DDX51	0.349778554	mirna_pc

21654	hsa-mir-197	CST7	0.364081115	mirna_pc
21655	hsa-mir-197	ZNF232	0.314941329	mirna_pc
21656	hsa-mir-197	ZBTB39	0.38915342	mirna_pc
21657	hsa-mir-197	SGTA	0.302090788	mirna_pc
21658	hsa-mir-197	POLR2L	0.309550703	mirna_pc
21659	hsa-mir-197	MED17	0.306033152	mirna_pc
21660	hsa-mir-197	C3orf75	0.320415927	mirna_pc
21661	hsa-mir-197	ASPSCR1	0.322641922	mirna_pc
21662	hsa-mir-197	TOE1	0.360749958	mirna_pc
21663	hsa-mir-197	RBBP4	0.350250238	mirna_pc
21664	hsa-mir-197	LOC221710	0.312152944	mirna_pc
21665	hsa-mir-197	NACA	0.302284146	mirna_pc
21666	hsa-mir-197	RPS12	0.348431123	mirna_pc
21667	hsa-mir-197	PTPMT1	0.338771254	mirna_pc
21668	hsa-mir-197	NACA2	0.334796572	mirna_pc
21669	hsa-mir-197	FAF1	0.317858713	mirna_pc
21670	hsa-mir-197	LONP1	0.321387977	mirna_pc
21671	hsa-mir-197	LRDD	0.327159788	mirna_pc
21672	hsa-mir-197	SCML2	0.404862694	mirna_pc
21673	hsa-mir-197	RPSAP58	0.378065421	mirna_pc
21674	hsa-mir-197	ANKRD54	0.303549525	mirna_pc
21675	hsa-mir-197	PDXP	0.509095813	mirna_pc
21676	hsa-mir-197	KIAA1274	0.342781229	mirna_pc
21677	hsa-mir-197	ETV5	0.349913078	mirna_pc
21678	hsa-mir-197	COX7A2L	0.300109946	mirna_pc
21679	hsa-mir-197	PROX1	0.481367618	mirna_pc
21680	hsa-mir-197	DACT2	0.336989648	mirna_pc
21681	hsa-mir-197	CHID1	0.396073621	mirna_pc
21682	hsa-mir-197	IGHMBP2	0.301059755	mirna_pc
21683	hsa-mir-197	RNF5P1	0.364928299	mirna_pc
21684	hsa-mir-197	HBXIP	0.39267579	mirna_pc
21685	hsa-mir-197	GNG4	0.377996582	mirna_pc
21686	hsa-mir-197	IL17RB	0.30423996	mirna_pc
21687	hsa-mir-197	CDAN1	0.345150182	mirna_pc
21688	hsa-mir-197	RPSA	0.393047474	mirna_pc
21689	hsa-mir-197	CD320	0.307233541	mirna_pc
21690	hsa-mir-197	SERF1A	0.344081169	mirna_pc
21691	hsa-mir-197	PREB	0.4450282	mirna_pc
21692	hsa-mir-197	PRMT6	0.354249294	mirna_pc
21693	hsa-mir-197	ZMYM3	0.301475769	mirna_pc
21694	hsa-mir-197	ANAPC5	0.313392743	mirna_pc
21695	hsa-mir-197	VAPB	0.428445583	mirna_pc
21696	hsa-mir-197	UBL7	0.305263805	mirna_pc
21697	hsa-mir-197	YARS	0.32028194	mirna_pc
21698	hsa-mir-197	PRDX4	0.394627693	mirna_pc
21699	hsa-mir-197	IGF2	0.305701811	mirna_pc
21700	hsa-mir-197	TMEM14B	0.37632223	mirna_pc
21701	hsa-mir-197	RBBP7	0.30691779	mirna_pc
21702	hsa-mir-197	THOC5	0.312836571	mirna_pc
21703	hsa-mir-197	PCBD1	0.321592053	mirna_pc
21704	hsa-mir-197	ZBED1	0.434602624	mirna_pc
21705	hsa-mir-197	EIF2S3	0.399459277	mirna_pc
21706	hsa-mir-197	CENPV	0.326333288	mirna_pc
21707	hsa-mir-197	DNMT3A	0.351415764	mirna_pc

21708	hsa-mir-197	MARS	0.349318105	mirna_pc
21709	hsa-mir-197	C10orf75	0.333290676	mirna_pc
21710	hsa-mir-197	CKAP4	0.329603015	mirna_pc
21711	hsa-mir-197	INHBB	0.415286564	mirna_pc
21712	hsa-mir-197	TET1	0.357649508	mirna_pc
21713	hsa-mir-197	APOO	0.533714154	mirna_pc
21714	hsa-mir-197	C12orf73	0.371459859	mirna_pc
21715	hsa-mir-197	DOM3Z	0.313239333	mirna_pc
21716	hsa-mir-197	ANKRD52	0.324963499	mirna_pc
21717	hsa-mir-103-1	TPX2	0.409967104	mirna_pc
21718	hsa-mir-103-1	KIF4B	0.334286704	mirna_pc
21719	hsa-mir-103-1	CENPF	0.35984651	mirna_pc
21720	hsa-mir-103-1	RCC2	0.338094148	mirna_pc
21721	hsa-mir-103-1	KIF11	0.374836837	mirna_pc
21722	hsa-mir-103-1	CDK1	0.346683744	mirna_pc
21723	hsa-mir-103-1	CDC25C	0.488271576	mirna_pc
21724	hsa-mir-103-1	PCNA	0.464921069	mirna_pc
21725	hsa-mir-103-1	UBE2C	0.393776968	mirna_pc
21726	hsa-mir-103-1	BUB1	0.33367835	mirna_pc
21727	hsa-mir-103-1	NUSAP1	0.401129703	mirna_pc
21728	hsa-mir-103-1	MCM4	0.308361334	mirna_pc
21729	hsa-mir-103-1	KIFC1	0.361253632	mirna_pc
21730	hsa-mir-103-1	BUB1B	0.318479876	mirna_pc
21731	hsa-mir-103-1	HDGF	0.351041416	mirna_pc
21732	hsa-mir-103-1	KIF18B	0.315505941	mirna_pc
21733	hsa-mir-103-1	CKAP2	0.310872187	mirna_pc
21734	hsa-mir-103-1	KIF2C	0.406749762	mirna_pc
21735	hsa-mir-103-1	FANCA	0.327735367	mirna_pc
21736	hsa-mir-103-1	NCAPG	0.459910844	mirna_pc
21737	hsa-mir-103-1	CLSPN	0.328272104	mirna_pc
21738	hsa-mir-103-1	SGOL1	0.40247244	mirna_pc
21739	hsa-mir-103-1	CCNA2	0.406431766	mirna_pc
21740	hsa-mir-103-1	CBX3	0.335235659	mirna_pc
21741	hsa-mir-103-1	SPC25	0.386111939	mirna_pc
21742	hsa-mir-103-1	FANCI	0.304444169	mirna_pc
21743	hsa-mir-103-1	NEK2	0.42995095	mirna_pc
21744	hsa-mir-103-1	MND1	0.379509461	mirna_pc
21745	hsa-mir-103-1	KIF22	0.346471587	mirna_pc
21746	hsa-mir-103-1	DDX39	0.35975839	mirna_pc
21747	hsa-mir-103-1	NCAPH	0.360196446	mirna_pc
21748	hsa-mir-103-1	GTSE1	0.326658962	mirna_pc
21749	hsa-mir-103-1	RAD54L	0.338118507	mirna_pc
21750	hsa-mir-103-1	CENPA	0.321510299	mirna_pc
21751	hsa-mir-103-1	MAD2L1	0.471765388	mirna_pc
21752	hsa-mir-103-1	TIMELESS	0.4042282	mirna_pc
21753	hsa-mir-103-1	LMNB1	0.518959741	mirna_pc
21754	hsa-mir-103-1	CDCA8	0.383729165	mirna_pc
21755	hsa-mir-103-1	TROAP	0.434023444	mirna_pc
21756	hsa-mir-103-1	TACC3	0.330154121	mirna_pc
21757	hsa-mir-103-1	XPO1	0.328827954	mirna_pc
21758	hsa-mir-103-1	STMN1	0.425743852	mirna_pc
21759	hsa-mir-103-1	CENPM	0.319358969	mirna_pc
21760	hsa-mir-103-1	EXO1	0.30959964	mirna_pc
21761	hsa-mir-103-1	UBE2T	0.333447834	mirna_pc

21762	hsa-mir-103-1	CKS1B	0.31165771	mirna_pc
21763	hsa-mir-103-1	KIF4A	0.343623977	mirna_pc
21764	hsa-mir-103-1	ORC1L	0.306855802	mirna_pc
21765	hsa-mir-103-1	SNRPB	0.481168586	mirna_pc
21766	hsa-mir-103-1	KNTC1	0.311196468	mirna_pc
21767	hsa-mir-103-1	CCNF	0.325662091	mirna_pc
21768	hsa-mir-103-1	PTBP1	0.315608707	mirna_pc
21769	hsa-mir-103-1	CCNB2	0.339078786	mirna_pc
21770	hsa-mir-103-1	NUF2	0.434654755	mirna_pc
21771	hsa-mir-103-1	PLK4	0.386890162	mirna_pc
21772	hsa-mir-103-1	DEPDC1	0.35218856	mirna_pc
21773	hsa-mir-103-1	PTTG1	0.311892286	mirna_pc
21774	hsa-mir-103-1	SKA3	0.300973592	mirna_pc
21775	hsa-mir-103-1	HJURP	0.469969354	mirna_pc
21776	hsa-mir-103-1	SKA1	0.386344797	mirna_pc
21777	hsa-mir-103-1	PAICS	0.395761814	mirna_pc
21778	hsa-mir-103-1	CCDC150	0.404623451	mirna_pc
21779	hsa-mir-103-1	RCC1	0.316386279	mirna_pc
21780	hsa-mir-103-1	LIG1	0.376158428	mirna_pc
21781	hsa-mir-103-1	DTL	0.340803969	mirna_pc
21782	hsa-mir-103-1	EME1	0.317555884	mirna_pc
21783	hsa-mir-103-1	RECQL4	0.430770154	mirna_pc
21784	hsa-mir-103-1	AURKA	0.336353334	mirna_pc
21785	hsa-mir-103-1	CENPK	0.322998747	mirna_pc
21786	hsa-mir-103-1	AURKB	0.315374661	mirna_pc
21787	hsa-mir-103-1	CENPE	0.361811023	mirna_pc
21788	hsa-mir-103-1	KIF15	0.383253554	mirna_pc
21789	hsa-mir-103-1	CDCA3	0.343917616	mirna_pc
21790	hsa-mir-103-1	C1orf112	0.431049732	mirna_pc
21791	hsa-mir-103-1	GINS1	0.410913808	mirna_pc
21792	hsa-mir-103-1	FEN1	0.305809092	mirna_pc
21793	hsa-mir-103-1	TRAIP	0.365164627	mirna_pc
21794	hsa-mir-103-1	ORC6L	0.300688972	mirna_pc
21795	hsa-mir-103-1	C16orf59	0.317953102	mirna_pc
21796	hsa-mir-103-1	RAE1	0.398440957	mirna_pc
21797	hsa-mir-103-1	FAM72B	0.301007647	mirna_pc
21798	hsa-mir-103-1	EZH2	0.341006079	mirna_pc
21799	hsa-mir-103-1	NEIL3	0.336116133	mirna_pc
21800	hsa-mir-103-1	ACTL6A	0.301329992	mirna_pc
21801	hsa-mir-103-1	POLD1	0.338030836	mirna_pc
21802	hsa-mir-103-1	C12orf48	0.372266464	mirna_pc
21803	hsa-mir-103-1	HNRNPL	0.420365288	mirna_pc
21804	hsa-mir-103-1	TTK	0.391463639	mirna_pc
21805	hsa-mir-103-1	ZWINT	0.36112999	mirna_pc
21806	hsa-mir-103-1	TUBA1B	0.302835009	mirna_pc
21807	hsa-mir-103-1	HOXC6	0.353231326	mirna_pc
21808	hsa-mir-103-1	BUB3	0.341450693	mirna_pc
21809	hsa-mir-103-1	HELLS	0.30734111	mirna_pc
21810	hsa-mir-103-1	UBE2S	0.330032718	mirna_pc
21811	hsa-mir-103-1	NCAPD2	0.339986234	mirna_pc
21812	hsa-mir-103-1	HNRNPC	0.389179759	mirna_pc
21813	hsa-mir-103-1	ILF2	0.415021572	mirna_pc
21814	hsa-mir-103-1	C6orf167	0.32636992	mirna_pc
21815	hsa-mir-103-1	HNRNPA2B1	0.436575016	mirna_pc

21816	hsa-mir-103-1	TYMS	0.349094231	mirna_pc
21817	hsa-mir-103-1	MCM6	0.389335191	mirna_pc
21818	hsa-mir-103-1	EPR1	0.391192677	mirna_pc
21819	hsa-mir-103-1	PIF1	0.432812201	mirna_pc
21820	hsa-mir-103-1	SFRS2	0.394589354	mirna_pc
21821	hsa-mir-103-1	DCAF13	0.306904199	mirna_pc
21822	hsa-mir-103-1	UCK2	0.427241391	mirna_pc
21823	hsa-mir-103-1	NFKBIL2	0.456193179	mirna_pc
21824	hsa-mir-103-1	PTGES3	0.311433647	mirna_pc
21825	hsa-mir-103-1	FAM64A	0.367508337	mirna_pc
21826	hsa-mir-103-1	E2F1	0.328312237	mirna_pc
21827	hsa-mir-103-1	E2F2	0.336603824	mirna_pc
21828	hsa-mir-103-1	TH1L	0.341199752	mirna_pc
21829	hsa-mir-103-1	SNRPA	0.410359478	mirna_pc
21830	hsa-mir-103-1	FUS	0.439149112	mirna_pc
21831	hsa-mir-103-1	ATAD2	0.322375853	mirna_pc
21832	hsa-mir-103-1	U2AF2	0.355358363	mirna_pc
21833	hsa-mir-103-1	RFC3	0.352468772	mirna_pc
21834	hsa-mir-103-1	DEPDC1B	0.304736346	mirna_pc
21835	hsa-mir-103-1	ANP32E	0.357575298	mirna_pc
21836	hsa-mir-103-1	CENPO	0.349776581	mirna_pc
21837	hsa-mir-103-1	C19orf48	0.365328007	mirna_pc
21838	hsa-mir-103-1	CENPL	0.339832322	mirna_pc
21839	hsa-mir-103-1	HMGB2	0.427922522	mirna_pc
21840	hsa-mir-103-1	DNA2	0.353631108	mirna_pc
21841	hsa-mir-103-1	NOP56	0.464529285	mirna_pc
21842	hsa-mir-103-1	SNRPD1	0.329668582	mirna_pc
21843	hsa-mir-103-1	DSN1	0.342957656	mirna_pc
21844	hsa-mir-103-1	SNHG1	0.368840111	mirna_pc
21845	hsa-mir-103-1	CSE1L	0.320230907	mirna_pc
21846	hsa-mir-103-1	DTYMK	0.380990037	mirna_pc
21847	hsa-mir-103-1	PIGU	0.347174724	mirna_pc
21848	hsa-mir-103-1	PRMT1	0.341158957	mirna_pc
21849	hsa-mir-103-1	MLF1IP	0.361992076	mirna_pc
21850	hsa-mir-103-1	C16orf75	0.308903034	mirna_pc
21851	hsa-mir-103-1	PVT1	0.383346726	mirna_pc
21852	hsa-mir-103-1	CCDC99	0.317178881	mirna_pc
21853	hsa-mir-103-1	CDK2	0.321882913	mirna_pc
21854	hsa-mir-103-1	CDK4	0.330396311	mirna_pc
21855	hsa-mir-103-1	HOXA10	0.379502913	mirna_pc
21856	hsa-mir-103-1	HNRNPR	0.389417856	mirna_pc
21857	hsa-mir-103-1	DLEU2	0.313088141	mirna_pc
21858	hsa-mir-103-1	PRIM1	0.413548307	mirna_pc
21859	hsa-mir-103-1	KAT2A	0.340365972	mirna_pc
21860	hsa-mir-103-1	CENPH	0.357731207	mirna_pc
21861	hsa-mir-103-1	CHAF1B	0.404816741	mirna_pc
21862	hsa-mir-103-1	DHX34	0.338334068	mirna_pc
21863	hsa-mir-103-1	MSH2	0.467745408	mirna_pc
21864	hsa-mir-103-1	SF3B3	0.313934124	mirna_pc
21865	hsa-mir-103-1	CDC7	0.473616625	mirna_pc
21866	hsa-mir-103-1	SSB	0.318680434	mirna_pc
21867	hsa-mir-103-1	RFC4	0.337235967	mirna_pc
21868	hsa-mir-103-1	FBXO5	0.456001171	mirna_pc
21869	hsa-mir-103-1	FIGNL1	0.363335112	mirna_pc

21870	hsa-mir-103-1	E2F3	0.324032717	mirna_pc
21871	hsa-mir-103-1	RUVBL2	0.330146095	mirna_pc
21872	hsa-mir-103-1	CDC25A	0.410702073	mirna_pc
21873	hsa-mir-103-1	RAD54B	0.319390787	mirna_pc
21874	hsa-mir-103-1	H2AFZ	0.404525618	mirna_pc
21875	hsa-mir-103-1	DDX12	0.390971888	mirna_pc
21876	hsa-mir-103-1	SMC2	0.328708104	mirna_pc
21877	hsa-mir-103-1	MCM8	0.424615938	mirna_pc
21878	hsa-mir-103-1	SNRPE	0.386707115	mirna_pc
21879	hsa-mir-103-1	C21orf45	0.498000545	mirna_pc
21880	hsa-mir-103-1	ATIC	0.320820849	mirna_pc
21881	hsa-mir-103-1	MARCKSL1	0.327733091	mirna_pc
21882	hsa-mir-103-1	ZNF207	0.339026599	mirna_pc
21883	hsa-mir-103-1	DAZAP1	0.300270655	mirna_pc
21884	hsa-mir-103-1	CBX8	0.441992053	mirna_pc
21885	hsa-mir-103-1	TRMT6	0.441580067	mirna_pc
21886	hsa-mir-103-1	GNPDA1	0.366345076	mirna_pc
21887	hsa-mir-103-1	TMEM201	0.37706524	mirna_pc
21888	hsa-mir-103-1	NCL	0.342326276	mirna_pc
21889	hsa-mir-103-1	PAFAH1B3	0.365908772	mirna_pc
21890	hsa-mir-103-1	SRRT	0.34757297	mirna_pc
21891	hsa-mir-103-1	CACYBP	0.410063037	mirna_pc
21892	hsa-mir-103-1	PSRC1	0.403298159	mirna_pc
21893	hsa-mir-103-1	POU2F1	0.331867178	mirna_pc
21894	hsa-mir-103-1	FBL	0.320308975	mirna_pc
21895	hsa-mir-103-1	HIST1H1E	0.364276804	mirna_pc
21896	hsa-mir-103-1	C13orf37	0.318992885	mirna_pc
21897	hsa-mir-103-1	EXOSC5	0.315651111	mirna_pc
21898	hsa-mir-103-1	HNRNPM	0.360827449	mirna_pc
21899	hsa-mir-103-1	TMPO	0.44442444	mirna_pc
21900	hsa-mir-103-1	PRCC	0.337915983	mirna_pc
21901	hsa-mir-103-1	POLR2H	0.317702379	mirna_pc
21902	hsa-mir-103-1	C20orf72	0.378674523	mirna_pc
21903	hsa-mir-103-1	GPR19	0.443196242	mirna_pc
21904	hsa-mir-103-1	PPAT	0.362964107	mirna_pc
21905	hsa-mir-103-1	DHX9	0.354830221	mirna_pc
21906	hsa-mir-103-1	CCDC138	0.322117152	mirna_pc
21907	hsa-mir-103-1	LOC100128191	0.416529092	mirna_pc
21908	hsa-mir-103-1	HMGN2	0.364901431	mirna_pc
21909	hsa-mir-103-1	ETV4	0.301445567	mirna_pc
21910	hsa-mir-103-1	CHRNA5	0.309743683	mirna_pc
21911	hsa-mir-103-1	C20orf3	0.419228183	mirna_pc
21912	hsa-mir-103-1	SFRS1	0.378786117	mirna_pc
21913	hsa-mir-103-1	CDH24	0.314641651	mirna_pc
21914	hsa-mir-103-1	SKP2	0.362446892	mirna_pc
21915	hsa-mir-103-1	HSPBP1	0.346599528	mirna_pc
21916	hsa-mir-103-1	C4orf46	0.333615891	mirna_pc
21917	hsa-mir-103-1	RFC5	0.366066445	mirna_pc
21918	hsa-mir-103-1	SSRP1	0.304853663	mirna_pc
21919	hsa-mir-103-1	TIGD1	0.322939648	mirna_pc
21920	hsa-mir-103-1	SLBP	0.324416906	mirna_pc
21921	hsa-mir-103-1	FTSJ2	0.317840848	mirna_pc
21922	hsa-mir-103-1	KHDRBS1	0.456934544	mirna_pc
21923	hsa-mir-103-1	NXT1	0.370906292	mirna_pc

21924	hsa-mir-103-1	C20orf27	0.41971151	mirna_pc
21925	hsa-mir-103-1	TOMM34	0.324177469	mirna_pc
21926	hsa-mir-103-1	AHCY	0.309107269	mirna_pc
21927	hsa-mir-103-1	PARP1	0.354600345	mirna_pc
21928	hsa-mir-103-1	C4orf21	0.350230942	mirna_pc
21929	hsa-mir-103-1	TMEM194A	0.334096282	mirna_pc
21930	hsa-mir-103-1	ZNF695	0.309925248	mirna_pc
21931	hsa-mir-103-1	HNRNPU	0.33442914	mirna_pc
21932	hsa-mir-103-1	NASP	0.306623153	mirna_pc
21933	hsa-mir-103-1	POLR1B	0.30493358	mirna_pc
21934	hsa-mir-103-1	OBFC2B	0.306973851	mirna_pc
21935	hsa-mir-103-1	PTMA	0.404033083	mirna_pc
21936	hsa-mir-103-1	WDR76	0.350813829	mirna_pc
21937	hsa-mir-103-1	ARHGAP39	0.338357612	mirna_pc
21938	hsa-mir-103-1	TBCE	0.351718427	mirna_pc
21939	hsa-mir-103-1	CLCN2	0.309088414	mirna_pc
21940	hsa-mir-103-1	SUV39H2	0.371142768	mirna_pc
21941	hsa-mir-103-1	HOXA9	0.342841164	mirna_pc
21942	hsa-mir-103-1	FANCL	0.410695922	mirna_pc
21943	hsa-mir-103-1	RIBC2	0.34393748	mirna_pc
21944	hsa-mir-103-1	HNRNPD	0.443808163	mirna_pc
21945	hsa-mir-103-1	DPY30	0.324839339	mirna_pc
21946	hsa-mir-103-1	TARDBP	0.327488439	mirna_pc
21947	hsa-mir-103-1	SFRS3	0.386232638	mirna_pc
21948	hsa-mir-103-1	CCT4	0.326696327	mirna_pc
21949	hsa-mir-103-1	ELAVL1	0.331738155	mirna_pc
21950	hsa-mir-103-1	TBC1D7	0.306180717	mirna_pc
21951	hsa-mir-103-1	NUPL2	0.406489661	mirna_pc
21952	hsa-mir-103-1	PAK1IP1	0.375819091	mirna_pc
21953	hsa-mir-103-1	ILF3	0.377215597	mirna_pc
21954	hsa-mir-103-1	NT5DC2	0.331514132	mirna_pc
21955	hsa-mir-103-1	EHMT2	0.347226021	mirna_pc
21956	hsa-mir-103-1	HNRNPAB	0.322007611	mirna_pc
21957	hsa-mir-103-1	UBAP2L	0.321991877	mirna_pc
21958	hsa-mir-103-1	PUF60	0.344931428	mirna_pc
21959	hsa-mir-103-1	DEK	0.324565853	mirna_pc
21960	hsa-mir-103-1	SFRS9	0.302326866	mirna_pc
21961	hsa-mir-103-1	TRIB3	0.310614022	mirna_pc
21962	hsa-mir-103-1	CCNE2	0.371930983	mirna_pc
21963	hsa-mir-103-1	PDRG1	0.316117995	mirna_pc
21964	hsa-mir-103-1	PASK	0.384700453	mirna_pc
21965	hsa-mir-103-1	SAAL1	0.363372998	mirna_pc
21966	hsa-mir-103-1	LSM 6.00	0.332545834	mirna_pc
21967	hsa-mir-103-1	PRAME	0.38507244	mirna_pc
21968	hsa-mir-103-1	SNRPB2	0.38753588	mirna_pc
21969	hsa-mir-103-1	AZI1	0.329497615	mirna_pc
21970	hsa-mir-103-1	ITPA	0.338257473	mirna_pc
21971	hsa-mir-103-1	FUBP1	0.303192254	mirna_pc
21972	hsa-mir-103-1	IPO9	0.301771642	mirna_pc
21973	hsa-mir-103-1	HNRNPK	0.318419772	mirna_pc
21974	hsa-mir-103-1	CSRP2BP	0.362033135	mirna_pc
21975	hsa-mir-103-1	TRA2B	0.337809147	mirna_pc
21976	hsa-mir-103-1	CA8	0.323397393	mirna_pc
21977	hsa-mir-103-1	GAR1	0.428446784	mirna_pc

21978	hsa-mir-103-1	LSM14B	0.347659845	mirna_pc
21979	hsa-mir-103-1	WHSC1	0.302577248	mirna_pc
21980	hsa-mir-103-1	SAE1	0.344202839	mirna_pc
21981	hsa-mir-103-1	ITGB3BP	0.314927816	mirna_pc
21982	hsa-mir-103-1	ENOPH1	0.35371587	mirna_pc
21983	hsa-mir-103-1	C17orf42	0.322640856	mirna_pc
21984	hsa-mir-103-1	TSEN54	0.306279236	mirna_pc
21985	hsa-mir-103-1	C4orf43	0.3043751	mirna_pc
21986	hsa-mir-103-1	DDX55	0.337176631	mirna_pc
21987	hsa-mir-103-1	CEP78	0.319531082	mirna_pc
21988	hsa-mir-103-1	RAD51L3	0.372984564	mirna_pc
21989	hsa-mir-103-1	PLEKHG4	0.366490361	mirna_pc
21990	hsa-mir-103-1	SFPQ	0.401534335	mirna_pc
21991	hsa-mir-103-1	RCOR2	0.301289744	mirna_pc
21992	hsa-mir-103-1	CADM1	0.314493176	mirna_pc
21993	hsa-mir-103-1	HDAC2	0.431704607	mirna_pc
21994	hsa-mir-103-1	TDRD5	0.308787258	mirna_pc
21995	hsa-mir-103-1	GMNN	0.341764183	mirna_pc
21996	hsa-mir-103-1	HAUS1	0.304064141	mirna_pc
21997	hsa-mir-103-1	POGK	0.302027991	mirna_pc
21998	hsa-mir-103-1	DCK	0.360630657	mirna_pc
21999	hsa-mir-103-1	COMMD5	0.305813048	mirna_pc
22000	hsa-mir-103-1	CRIPT	0.309485858	mirna_pc
22001	hsa-mir-103-1	MSH6	0.329264806	mirna_pc
22002	hsa-mir-103-1	XRN2	0.407324241	mirna_pc
22003	hsa-mir-103-1	SMC3	0.310874565	mirna_pc
22004	hsa-mir-103-1	HNRNPA1	0.359010937	mirna_pc
22005	hsa-mir-103-1	MEX3A	0.39799573	mirna_pc
22006	hsa-mir-103-1	BAT1	0.365516775	mirna_pc
22007	hsa-mir-103-1	COIL	0.313513956	mirna_pc
22008	hsa-mir-103-1	C12orf47	0.330365367	mirna_pc
22009	hsa-mir-103-1	IRAK1BP1	0.331432425	mirna_pc
22010	hsa-mir-103-1	TAF5	0.366033356	mirna_pc
22011	hsa-mir-103-1	C7orf30	0.308621966	mirna_pc
22012	hsa-mir-103-1	NANP	0.31004161	mirna_pc
22013	hsa-mir-103-1	CCDC28B	0.363093579	mirna_pc
22014	hsa-mir-103-1	SUZ12	0.332232231	mirna_pc
22015	hsa-mir-103-1	HNRNPA1L2	0.382275787	mirna_pc
22016	hsa-mir-103-1	HMGB1	0.358428695	mirna_pc
22017	hsa-mir-103-1	ZBTB12	0.402688575	mirna_pc
22018	hsa-mir-103-1	TADA1	0.378723996	mirna_pc
22019	hsa-mir-103-1	DUSP12	0.305991943	mirna_pc
22020	hsa-mir-103-1	TMEM68	0.312119167	mirna_pc
22021	hsa-mir-103-1	LOC642846	0.371341088	mirna_pc
22022	hsa-mir-103-1	TGIF2	0.374518943	mirna_pc
22023	hsa-mir-103-1	H3F3A	0.327071558	mirna_pc
22024	hsa-mir-103-1	CHERP	0.312753564	mirna_pc
22025	hsa-mir-103-1	SNX5	0.481699058	mirna_pc
22026	hsa-mir-103-1	RCN2	0.319858669	mirna_pc
22027	hsa-mir-103-1	TARBP2	0.323520378	mirna_pc
22028	hsa-mir-103-1	FGFR1OP	0.349206819	mirna_pc
22029	hsa-mir-103-1	C12orf52	0.329096148	mirna_pc
22030	hsa-mir-103-1	NOL10	0.308428046	mirna_pc
22031	hsa-mir-103-1	POLE	0.355728221	mirna_pc

22032	hsa-mir-103-1	WRNIP1	0.317636522	mirna_pc
22033	hsa-mir-103-1	EXOSC9	0.429875253	mirna_pc
22034	hsa-mir-103-1	KSR2	0.354349115	mirna_pc
22035	hsa-mir-103-1	UNG	0.322686491	mirna_pc
22036	hsa-mir-103-1	RBMX	0.437464248	mirna_pc
22037	hsa-mir-103-1	ALKBH2	0.392927007	mirna_pc
22038	hsa-mir-103-1	NTHL1	0.315843145	mirna_pc
22039	hsa-mir-103-1	PRKCSH	0.309678981	mirna_pc
22040	hsa-mir-103-1	XRCC5	0.340291669	mirna_pc
22041	hsa-mir-103-1	ASB3	0.370085496	mirna_pc
22042	hsa-mir-103-1	IGSF9	0.329575725	mirna_pc
22043	hsa-mir-103-1	FIP1L1	0.359229549	mirna_pc
22044	hsa-mir-103-1	C8orf55	0.318207211	mirna_pc
22045	hsa-mir-103-1	CIT	0.305756226	mirna_pc
22046	hsa-mir-103-1	SCLT1	0.318526891	mirna_pc
22047	hsa-mir-103-1	MAGEF1	0.335257705	mirna_pc
22048	hsa-mir-103-1	AP1AR	0.300509712	mirna_pc
22049	hsa-mir-103-1	PCDHB8	0.307323629	mirna_pc
22050	hsa-mir-103-1	FAM136A	0.343176172	mirna_pc
22051	hsa-mir-103-1	SOX12	0.391278614	mirna_pc
22052	hsa-mir-103-1	UBXN11	0.326889854	mirna_pc
22053	hsa-mir-103-1	TTLL4	0.339577446	mirna_pc
22054	hsa-mir-103-1	ATXN7L2	0.346315332	mirna_pc
22055	hsa-mir-103-1	ZNF813	0.313592959	mirna_pc
22056	hsa-mir-103-1	CSNK2A1	0.370690549	mirna_pc
22057	hsa-mir-103-1	C7orf44	0.33105684	mirna_pc
22058	hsa-mir-103-1	ZNF286A	0.353989662	mirna_pc
22059	hsa-mir-103-1	SLC27A2	0.367332779	mirna_pc
22060	hsa-mir-103-1	PRPF38A	0.351710357	mirna_pc
22061	hsa-mir-103-1	WDR77	0.319463873	mirna_pc
22062	hsa-mir-103-1	NLE1	0.412705817	mirna_pc
22063	hsa-mir-103-1	ILKAP	0.34225333	mirna_pc
22064	hsa-mir-103-1	PRR3	0.320852232	mirna_pc
22065	hsa-mir-103-1	POLR3C	0.311780405	mirna_pc
22066	hsa-mir-103-1	TMEM106C	0.354176666	mirna_pc
22067	hsa-mir-103-1	PANK2	0.3723153	mirna_pc
22068	hsa-mir-103-1	APITD1	0.314808367	mirna_pc
22069	hsa-mir-103-1	ZNF286B	0.33725028	mirna_pc
22070	hsa-mir-103-1	DDX20	0.32708574	mirna_pc
22071	hsa-mir-103-1	TCERG1	0.325074084	mirna_pc
22072	hsa-mir-103-1	SEC61A2	0.305629245	mirna_pc
22073	hsa-mir-103-1	PSPC1	0.427038264	mirna_pc
22074	hsa-mir-103-1	GDAP1	0.351568085	mirna_pc
22075	hsa-mir-103-1	CCNB1IP1	0.396964164	mirna_pc
22076	hsa-mir-103-1	MAPKAPK5	0.310388803	mirna_pc
22077	hsa-mir-103-1	GMCL1	0.327542714	mirna_pc
22078	hsa-mir-103-1	GPR3	0.310591863	mirna_pc
22079	hsa-mir-103-1	E2F6	0.363249267	mirna_pc
22080	hsa-mir-103-1	RHPN1	0.336674238	mirna_pc
22081	hsa-mir-103-1	B4GALT3	0.34012298	mirna_pc
22082	hsa-mir-103-1	SFRS13A	0.403925463	mirna_pc
22083	hsa-mir-103-1	SCLY	0.370446661	mirna_pc
22084	hsa-mir-103-1	SLC19A1	0.306106419	mirna_pc
22085	hsa-mir-103-1	TFAM	0.32518996	mirna_pc

22086	hsa-mir-103-1	DHRS13	0.445003317	mirna_pc
22087	hsa-mir-103-1	MCCC1	0.308159191	mirna_pc
22088	hsa-mir-103-1	ZNF613	0.313998718	mirna_pc
22089	hsa-mir-103-1	AAAS	0.309889282	mirna_pc
22090	hsa-mir-103-1	SATB2	0.332169209	mirna_pc
22091	hsa-mir-103-1	LIG3	0.366096349	mirna_pc
22092	hsa-mir-103-1	CABLES2	0.315747168	mirna_pc
22093	hsa-mir-103-1	MRPS26	0.477137021	mirna_pc
22094	hsa-mir-103-1	C8orf38	0.300358856	mirna_pc
22095	hsa-mir-103-1	ZNF692	0.349026699	mirna_pc
22096	hsa-mir-103-1	SFRS7	0.360248974	mirna_pc
22097	hsa-mir-103-1	CRNKL1	0.457930434	mirna_pc
22098	hsa-mir-103-1	CA5BP	0.313160674	mirna_pc
22099	hsa-mir-103-1	NFXL1	0.360486992	mirna_pc
22100	hsa-mir-103-1	CCDC78	0.326072959	mirna_pc
22101	hsa-mir-103-1	SARS2	0.31706474	mirna_pc
22102	hsa-mir-103-1	CDCA7L	0.318103041	mirna_pc
22103	hsa-mir-103-1	ZNF614	0.323429785	mirna_pc
22104	hsa-mir-103-1	HNRNPH1	0.349706316	mirna_pc
22105	hsa-mir-103-1	ZNF184	0.318730058	mirna_pc
22106	hsa-mir-103-1	RPA2	0.327532962	mirna_pc
22107	hsa-mir-103-1	ATF5	0.351929047	mirna_pc
22108	hsa-mir-103-1	C17orf75	0.423502217	mirna_pc
22109	hsa-mir-103-1	LOC147804	0.341332847	mirna_pc
22110	hsa-mir-103-1	YTHDF2	0.300767339	mirna_pc
22111	hsa-mir-103-1	BCAN	0.3189345	mirna_pc
22112	hsa-mir-103-1	C12orf10	0.323933042	mirna_pc
22113	hsa-mir-103-1	SEPHS1	0.357531208	mirna_pc
22114	hsa-mir-103-1	RMND1	0.352738409	mirna_pc
22115	hsa-mir-103-1	FASTKD5	0.358010837	mirna_pc
22116	hsa-mir-103-1	VASH2	0.314253101	mirna_pc
22117	hsa-mir-103-1	PHB2	0.310852386	mirna_pc
22118	hsa-mir-103-1	MYL6B	0.366770092	mirna_pc
22119	hsa-mir-103-1	TASP1	0.321567798	mirna_pc
22120	hsa-mir-103-1	ALG11	0.334993043	mirna_pc
22121	hsa-mir-103-1	RNF138	0.329239124	mirna_pc
22122	hsa-mir-103-1	ETV1	0.307794947	mirna_pc
22123	hsa-mir-103-1	MED28	0.409805296	mirna_pc
22124	hsa-mir-103-1	GPR137C	0.3915322	mirna_pc
22125	hsa-mir-103-1	NUP43	0.363749258	mirna_pc
22126	hsa-mir-103-1	POLR3F	0.377308683	mirna_pc
22127	hsa-mir-103-1	ZNF473	0.312480081	mirna_pc
22128	hsa-mir-103-1	COPS7B	0.316904393	mirna_pc
22129	hsa-mir-103-1	USP21	0.363386421	mirna_pc
22130	hsa-mir-103-1	DCLRE1A	0.390453656	mirna_pc
22131	hsa-mir-103-1	ANP32A	0.403189127	mirna_pc
22132	hsa-mir-103-1	PIH1D1	0.311614025	mirna_pc
22133	hsa-mir-103-1	GZF1	0.347952308	mirna_pc
22134	hsa-mir-103-1	ZNF696	0.31017727	mirna_pc
22135	hsa-mir-103-1	C2orf16	0.324996952	mirna_pc
22136	hsa-mir-103-1	HTATSF1	0.343007485	mirna_pc
22137	hsa-mir-103-1	ENTPD6	0.35352475	mirna_pc
22138	hsa-mir-103-1	PPP5C	0.404738649	mirna_pc
22139	hsa-mir-103-1	NAP1L1	0.308011904	mirna_pc

22140	hsa-mir-103-1	PEX5	0.333928226	mirna_pc
22141	hsa-mir-103-1	C1orf156	0.332788708	mirna_pc
22142	hsa-mir-103-1	NSL1	0.366915487	mirna_pc
22143	hsa-mir-103-1	IQCC	0.389505981	mirna_pc
22144	hsa-mir-103-1	LRRC37B	0.346993925	mirna_pc
22145	hsa-mir-103-1	HNRNPA0	0.312175502	mirna_pc
22146	hsa-mir-103-1	MNS1	0.33887251	mirna_pc
22147	hsa-mir-103-1	KIAA1467	0.35439211	mirna_pc
22148	hsa-mir-103-1	C14orf93	0.307334008	mirna_pc
22149	hsa-mir-103-1	DCAF16	0.378492808	mirna_pc
22150	hsa-mir-103-1	SBK1	0.370172877	mirna_pc
22151	hsa-mir-103-1	ABCA11P	0.351033047	mirna_pc
22152	hsa-mir-103-1	GLS2	0.325825242	mirna_pc
22153	hsa-mir-103-1	USP13	0.335374509	mirna_pc
22154	hsa-mir-103-1	ESF1	0.331585894	mirna_pc
22155	hsa-mir-103-1	SLC25A33	0.447155331	mirna_pc
22156	hsa-mir-103-1	BNIP1	0.312419028	mirna_pc
22157	hsa-mir-103-1	PACRGL	0.322650667	mirna_pc
22158	hsa-mir-103-1	ZNF232	0.395673765	mirna_pc
22159	hsa-mir-103-1	ENO3	0.410359208	mirna_pc
22160	hsa-mir-103-1	CYCS	0.314882629	mirna_pc
22161	hsa-mir-103-1	ALDH5A1	0.340020149	mirna_pc
22162	hsa-mir-103-1	C20orf12	0.315927849	mirna_pc
22163	hsa-mir-103-1	ZBTB24	0.323976745	mirna_pc
22164	hsa-mir-103-1	RBBP4	0.451769074	mirna_pc
22165	hsa-mir-103-1	ZNF761	0.37025418	mirna_pc
22166	hsa-mir-103-1	CYC1	0.309486451	mirna_pc
22167	hsa-mir-103-1	FAM105A	0.300803873	mirna_pc
22168	hsa-mir-103-1	MKKS	0.305675014	mirna_pc
22169	hsa-mir-103-1	ZNF664	0.306498781	mirna_pc
22170	hsa-mir-103-1	LRDD	0.323231398	mirna_pc
22171	hsa-mir-103-1	C20orf96	0.417821602	mirna_pc
22172	hsa-mir-103-1	STK35	0.304616507	mirna_pc
22173	hsa-mir-103-1	ZCCHC3	0.513182843	mirna_pc
22174	hsa-mir-103-1	ETV5	0.326661856	mirna_pc
22175	hsa-mir-103-1	AADAT	0.392129513	mirna_pc
22176	hsa-mir-103-1	ACP1	0.30923657	mirna_pc
22177	hsa-mir-103-1	DACH1	0.329134927	mirna_pc
22178	hsa-mir-103-1	ZNF883	0.324970204	mirna_pc
22179	hsa-mir-103-1	RFX3	0.362708481	mirna_pc
22180	hsa-mir-103-1	CD320	0.306976263	mirna_pc
22181	hsa-mir-103-1	SERF1A	0.334149365	mirna_pc
22182	hsa-mir-103-1	PRMT6	0.32953764	mirna_pc
22183	hsa-mir-103-1	IDH3B	0.441439161	mirna_pc
22184	hsa-mir-103-1	BAI2	0.338925222	mirna_pc
22185	hsa-mir-103-1	C20orf30	0.33088935	mirna_pc
22186	hsa-mir-103-1	MPP6	0.396922839	mirna_pc
22187	hsa-mir-103-1	DDRGK1	0.335775367	mirna_pc
22188	hsa-mir-103-1	C20orf7	0.465224519	mirna_pc
22189	hsa-mir-103-1	FAM113A	0.303096341	mirna_pc
22190	hsa-mir-103-1	VPS16	0.369086659	mirna_pc
22191	hsa-mir-103-1	C12orf73	0.304278322	mirna_pc
22192	hsa-mir-103-1	RBBP9	0.305066269	mirna_pc
22193	hsa-mir-103-1	ZNF326	0.306153754	mirna_pc

22194	hsa-mir-103-1	DOM3Z	0.313054921	mirna_pc
22195	hsa-mir-106a	HOXC9	0.309575377	mirna_pc
22196	hsa-mir-106a	ACADSB	0.430468606	mirna_pc
22197	hsa-mir-106a	ZNF415	0.360615288	mirna_pc
22198	hsa-mir-106a	FABP3	0.348196253	mirna_pc
22199	hsa-mir-106a	TIMELESS	0.312436244	mirna_pc
22200	hsa-mir-106a	ZNF542	0.31396554	mirna_pc
22201	hsa-mir-106a	SKA1	0.376459684	mirna_pc
22202	hsa-mir-106a	ZNF229	0.400799145	mirna_pc
22203	hsa-mir-106a	C12orf48	0.303609573	mirna_pc
22204	hsa-mir-106a	HOXC6	0.300215313	mirna_pc
22205	hsa-mir-106a	CDKN1C	0.303712038	mirna_pc
22206	hsa-mir-106a	DNAJC6	0.430515606	mirna_pc
22207	hsa-mir-106a	C5	0.557374958	mirna_pc
22208	hsa-mir-106a	CDK2	0.315310552	mirna_pc
22209	hsa-mir-106a	ZBED3	0.333958189	mirna_pc
22210	hsa-mir-106a	PRIM1	0.485832516	mirna_pc
22211	hsa-mir-106a	SALL4	0.357480679	mirna_pc
22212	hsa-mir-106a	MCM8	0.341185067	mirna_pc
22213	hsa-mir-106a	TRMT6	0.314270006	mirna_pc
22214	hsa-mir-106a	PAFAH1B3	0.31604259	mirna_pc
22215	hsa-mir-106a	TMPO	0.36595241	mirna_pc
22216	hsa-mir-106a	GPR19	0.314525903	mirna_pc
22217	hsa-mir-106a	LOC100128191	0.480386875	mirna_pc
22218	hsa-mir-106a	PHF6	0.343076361	mirna_pc
22219	hsa-mir-106a	TMEM170B	0.310939536	mirna_pc
22220	hsa-mir-106a	FZD2	0.303586113	mirna_pc
22221	hsa-mir-106a	BSN	0.304596744	mirna_pc
22222	hsa-mir-106a	SLC5A6	0.310590522	mirna_pc
22223	hsa-mir-106a	TMEM194A	0.314325708	mirna_pc
22224	hsa-mir-106a	C10orf4	0.31047275	mirna_pc
22225	hsa-mir-106a	CBX4	0.367130853	mirna_pc
22226	hsa-mir-106a	OBFC2B	0.300060047	mirna_pc
22227	hsa-mir-106a	SCARB1	0.31082826	mirna_pc
22228	hsa-mir-106a	TMEM86B	0.398492799	mirna_pc
22229	hsa-mir-106a	TFR2	0.554397257	mirna_pc
22230	hsa-mir-106a	SAT2	0.337192241	mirna_pc
22231	hsa-mir-106a	APOE	0.35261171	mirna_pc
22232	hsa-mir-106a	DUSP9	0.554314938	mirna_pc
22233	hsa-mir-106a	TRIB3	0.366656266	mirna_pc
22234	hsa-mir-106a	PPP2R1B	0.403303811	mirna_pc
22235	hsa-mir-106a	PRAME	0.395650365	mirna_pc
22236	hsa-mir-106a	CSRP2BP	0.374771884	mirna_pc
22237	hsa-mir-106a	SAE1	0.375028602	mirna_pc
22238	hsa-mir-106a	C17orf42	0.308785367	mirna_pc
22239	hsa-mir-106a	DDX55	0.313043462	mirna_pc
22240	hsa-mir-106a	RAD51L3	0.396588472	mirna_pc
22241	hsa-mir-106a	ITIH4	0.493778096	mirna_pc
22242	hsa-mir-106a	CADM1	0.306172125	mirna_pc
22243	hsa-mir-106a	GMNN	0.410141427	mirna_pc
22244	hsa-mir-106a	KPNB1	0.323380057	mirna_pc
22245	hsa-mir-106a	CAPS	0.328444529	mirna_pc
22246	hsa-mir-106a	SMARCA1	0.527542557	mirna_pc
22247	hsa-mir-106a	GPAM	0.368183681	mirna_pc

22248	hsa-mir-106a	LOC651250	0.300544693	mirna_pc
22249	hsa-mir-106a	COIL	0.335752418	mirna_pc
22250	hsa-mir-106a	TRIM28	0.300169584	mirna_pc
22251	hsa-mir-106a	ZNF444	0.390838665	mirna_pc
22252	hsa-mir-106a	ZBTB12	0.347628538	mirna_pc
22253	hsa-mir-106a	SLC26A1	0.323038169	mirna_pc
22254	hsa-mir-106a	PIK3AP1	0.402521704	mirna_pc
22255	hsa-mir-106a	USP30	0.325042702	mirna_pc
22256	hsa-mir-106a	POLE	0.309710176	mirna_pc
22257	hsa-mir-106a	ZKSCAN2	0.381858361	mirna_pc
22258	hsa-mir-106a	GAL3ST1	0.387873694	mirna_pc
22259	hsa-mir-106a	RTN4RL2	0.384877339	mirna_pc
22260	hsa-mir-106a	FOXRED2	0.432145212	mirna_pc
22261	hsa-mir-106a	RBMX	0.322665282	mirna_pc
22262	hsa-mir-106a	GPC3	0.692735554	mirna_pc
22263	hsa-mir-106a	STXBP4	0.359144563	mirna_pc
22264	hsa-mir-106a	DNAJC14	0.319370729	mirna_pc
22265	hsa-mir-106a	LYPD1	0.498793274	mirna_pc
22266	hsa-mir-106a	SOX12	0.3617954	mirna_pc
22267	hsa-mir-106a	ZNF230	0.313059162	mirna_pc
22268	hsa-mir-106a	ARID3A	0.340178419	mirna_pc
22269	hsa-mir-106a	APOM	0.375723574	mirna_pc
22270	hsa-mir-106a	PGAM2	0.354320648	mirna_pc
22271	hsa-mir-106a	BCL7A	0.324013751	mirna_pc
22272	hsa-mir-106a	TMEM98	0.359659227	mirna_pc
22273	hsa-mir-106a	SLC27A2	0.315373438	mirna_pc
22274	hsa-mir-106a	FRAT1	0.418958559	mirna_pc
22275	hsa-mir-106a	HNF1B	0.347245279	mirna_pc
22276	hsa-mir-106a	SLC46A1	0.305121255	mirna_pc
22277	hsa-mir-106a	FMO1	0.310390741	mirna_pc
22278	hsa-mir-106a	GYG2	0.312701017	mirna_pc
22279	hsa-mir-106a	ZNF286B	0.318461533	mirna_pc
22280	hsa-mir-106a	RNF34	0.33417112	mirna_pc
22281	hsa-mir-106a	GMCL1	0.40603276	mirna_pc
22282	hsa-mir-106a	E2F6	0.31540976	mirna_pc
22283	hsa-mir-106a	ZNF833	0.427280792	mirna_pc
22284	hsa-mir-106a	ZNF222	0.354986333	mirna_pc
22285	hsa-mir-106a	HUNK	0.338627989	mirna_pc
22286	hsa-mir-106a	FIZ1	0.309905044	mirna_pc
22287	hsa-mir-106a	TFAM	0.317209849	mirna_pc
22288	hsa-mir-106a	FAM122B	0.328266902	mirna_pc
22289	hsa-mir-106a	LOC222699	0.421381907	mirna_pc
22290	hsa-mir-106a	NLGN4X	0.303016926	mirna_pc
22291	hsa-mir-106a	HHEX	0.373172545	mirna_pc
22292	hsa-mir-106a	SMUG1	0.358582265	mirna_pc
22293	hsa-mir-106a	C6orf147	0.352854678	mirna_pc
22294	hsa-mir-106a	METTL7B	0.328382374	mirna_pc
22295	hsa-mir-106a	PHKA2	0.306208947	mirna_pc
22296	hsa-mir-106a	SERPINF2	0.578252113	mirna_pc
22297	hsa-mir-106a	CA5BP	0.354875004	mirna_pc
22298	hsa-mir-106a	C19orf57	0.377985245	mirna_pc
22299	hsa-mir-106a	VPS33A	0.317790507	mirna_pc
22300	hsa-mir-106a	ZNF184	0.300943554	mirna_pc
22301	hsa-mir-106a	LIMS3-LOC440895	0.360942723	mirna_pc

22302	hsa-mir-106a	ATF5	0.358489781	mirna_pc
22303	hsa-mir-106a	AKAP7	0.378932177	mirna_pc
22304	hsa-mir-106a	IRF2BP1	0.327718765	mirna_pc
22305	hsa-mir-106a	GGT1	0.37106832	mirna_pc
22306	hsa-mir-106a	POLR3B	0.314602192	mirna_pc
22307	hsa-mir-106a	C17orf75	0.306054946	mirna_pc
22308	hsa-mir-106a	ZNF180	0.3429807	mirna_pc
22309	hsa-mir-106a	CEBPA	0.423424505	mirna_pc
22310	hsa-mir-106a	LOC147804	0.3357606	mirna_pc
22311	hsa-mir-106a	FUZ	0.317857622	mirna_pc
22312	hsa-mir-106a	SLC43A3	0.364866218	mirna_pc
22313	hsa-mir-106a	ZNF45	0.444031577	mirna_pc
22314	hsa-mir-106a	ANXA9	0.50305542	mirna_pc
22315	hsa-mir-106a	MAK16	0.300337961	mirna_pc
22316	hsa-mir-106a	ZNF498	0.381113613	mirna_pc
22317	hsa-mir-106a	MCCC2	0.300100469	mirna_pc
22318	hsa-mir-106a	ZNF233	0.372130318	mirna_pc
22319	hsa-mir-106a	GPR137C	0.367745348	mirna_pc
22320	hsa-mir-106a	NUP43	0.303068122	mirna_pc
22321	hsa-mir-106a	SMAD5	0.301790082	mirna_pc
22322	hsa-mir-106a	DDIT3	0.325605329	mirna_pc
22323	hsa-mir-106a	ZNF124	0.304795826	mirna_pc
22324	hsa-mir-106a	ZNF611	0.343736977	mirna_pc
22325	hsa-mir-106a	ZNF473	0.422230518	mirna_pc
22326	hsa-mir-106a	ZNF544	0.340719904	mirna_pc
22327	hsa-mir-106a	DCLRE1A	0.37245785	mirna_pc
22328	hsa-mir-106a	SEPHS2	0.359190137	mirna_pc
22329	hsa-mir-106a	PPP5C	0.369745308	mirna_pc
22330	hsa-mir-106a	ZNF227	0.355848367	mirna_pc
22331	hsa-mir-106a	PROC	0.390919471	mirna_pc
22332	hsa-mir-106a	SLC26A11	0.323405676	mirna_pc
22333	hsa-mir-106a	NLRP2	0.36105103	mirna_pc
22334	hsa-mir-106a	SERPINA1	0.311731567	mirna_pc
22335	hsa-mir-106a	PHF16	0.34481482	mirna_pc
22336	hsa-mir-106a	BUD13	0.30531342	mirna_pc
22337	hsa-mir-106a	LOC400657	0.317517107	mirna_pc
22338	hsa-mir-106a	MANEAL	0.332707753	mirna_pc
22339	hsa-mir-106a	MBNL3	0.374118032	mirna_pc
22340	hsa-mir-106a	LRRC37B	0.317439019	mirna_pc
22341	hsa-mir-106a	FOXA2	0.330889716	mirna_pc
22342	hsa-mir-106a	C10orf137	0.324028366	mirna_pc
22343	hsa-mir-106a	TRIM24	0.328399174	mirna_pc
22344	hsa-mir-106a	ABCC4	0.306612579	mirna_pc
22345	hsa-mir-106a	C7orf13	0.348845981	mirna_pc
22346	hsa-mir-106a	ACE2	0.39875248	mirna_pc
22347	hsa-mir-106a	GTF2H2C	0.37984814	mirna_pc
22348	hsa-mir-106a	NUDT17	0.445969261	mirna_pc
22349	hsa-mir-106a	C2orf72	0.436830738	mirna_pc
22350	hsa-mir-106a	GXYLT1	0.374612585	mirna_pc
22351	hsa-mir-106a	ZBTB39	0.324036015	mirna_pc
22352	hsa-mir-106a	ENO3	0.366690419	mirna_pc
22353	hsa-mir-106a	ALDH5A1	0.316442663	mirna_pc
22354	hsa-mir-106a	FAM169A	0.343506747	mirna_pc
22355	hsa-mir-106a	ID2	0.357130753	mirna_pc

22356	hsa-mir-106a	HSD17B14		0.365177232	mirna_pc
22357	hsa-mir-106a	PLGLB2		0.462121094	mirna_pc
22358	hsa-mir-106a	LASS2		0.300248876	mirna_pc
22359	hsa-mir-106a	ZNF550		0.31774187	mirna_pc
22360	hsa-mir-106a	ZNF664		0.363579859	mirna_pc
22361	hsa-mir-106a	PECR		0.466371821	mirna_pc
22362	hsa-mir-106a	LRDD		0.314724605	mirna_pc
22363	hsa-mir-106a	CSPP1		0.339523238	mirna_pc
22364	hsa-mir-106a	SCML2		0.305336564	mirna_pc
22365	hsa-mir-106a	ZCCHC3		0.522792871	mirna_pc
22366	hsa-mir-106a	KLHL29		0.415187541	mirna_pc
22367	hsa-mir-106a	PROX1		0.336209491	mirna_pc
22368	hsa-mir-106a	DACT2		0.326031784	mirna_pc
22369	hsa-mir-106a	AADAT		0.338618207	mirna_pc
22370	hsa-mir-106a	FRAS1		0.466833745	mirna_pc
22371	hsa-mir-106a	LOC80054		0.469000295	mirna_pc
22372	hsa-mir-106a	ZNF772		0.358916221	mirna_pc
22373	hsa-mir-106a	PNKD		0.354343766	mirna_pc
22374	hsa-mir-106a	ZNF530		0.343531195	mirna_pc
22375	hsa-mir-106a	TMEM97		0.349551927	mirna_pc
22376	hsa-mir-106a	BMF		0.340768689	mirna_pc
22377	hsa-mir-106a	MBD6		0.306436147	mirna_pc
22378	hsa-mir-106a	ELP3		0.326012235	mirna_pc
22379	hsa-mir-106a	C20orf30		0.362111945	mirna_pc
22380	hsa-mir-106a	AGT		0.457423969	mirna_pc
22381	hsa-mir-106a	MPP6		0.405324701	mirna_pc
22382	hsa-mir-106a		9-Mar	0.307016017	mirna_pc
22383	hsa-mir-106a	ZNF468		0.330892054	mirna_pc
22384	hsa-mir-106a	SLC37A4		0.379842928	mirna_pc
22385	hsa-mir-106a	NRSN2		0.321613026	mirna_pc
22386	hsa-mir-106a	MGC57346		0.307951361	mirna_pc
22387	hsa-mir-106a	ZNF343		0.380877264	mirna_pc
22388	hsa-mir-409	BUB1B		0.311981392	mirna_pc
22389	hsa-mir-409	KIF2C		0.363192056	mirna_pc
22390	hsa-mir-409	CDC20		0.343894404	mirna_pc
22391	hsa-mir-409	CLSPN		0.322476205	mirna_pc
22392	hsa-mir-409	DLGAP5		0.306124846	mirna_pc
22393	hsa-mir-409	PLK4		0.339789521	mirna_pc
22394	hsa-mir-409	BIRC5		0.300331387	mirna_pc
22395	hsa-mir-409	AURKB		0.303288235	mirna_pc
22396	hsa-mir-409	HBB		0.443007412	mirna_pc
22397	hsa-mir-409	SERPINH1		0.376679813	mirna_pc
22398	hsa-mir-409	ZNF167		0.302997019	mirna_pc
22399	hsa-mir-409	CDK2		0.304924101	mirna_pc
22400	hsa-mir-409	MEG3		0.793371657	mirna_pc
22401	hsa-mir-409	BMP1		0.384128698	mirna_pc
22402	hsa-mir-409	C17orf107		0.309466639	mirna_pc
22403	hsa-mir-409	TRAF3		0.342508838	mirna_pc
22404	hsa-mir-409	DNMT3B		0.41388305	mirna_pc
22405	hsa-mir-409	PA2G4		0.316121809	mirna_pc
22406	hsa-mir-409	CCNE1		0.306381364	mirna_pc
22407	hsa-mir-409	NEDD1		0.317943874	mirna_pc
22408	hsa-mir-409	LOXL2		0.337255609	mirna_pc
22409	hsa-mir-409	LEPRE1		0.337652367	mirna_pc

22410	hsa-mir-409	IKBIP	0.333660138	mirna_pc
22411	hsa-mir-409	EHMT2	0.309622562	mirna_pc
22412	hsa-mir-409	FHOD1	0.371971314	mirna_pc
22413	hsa-mir-409	C14orf145	0.314834056	mirna_pc
22414	hsa-mir-409	MFAP2	0.316270494	mirna_pc
22415	hsa-mir-409	CD276	0.314339277	mirna_pc
22416	hsa-mir-409	BCL2A1	0.307559818	mirna_pc
22417	hsa-mir-409	ZNF788	0.302759007	mirna_pc
22418	hsa-mir-409	SLC2A6	0.309397348	mirna_pc
22419	hsa-mir-409	COL4A1	0.309758897	mirna_pc
22420	hsa-mir-409	PA2G4P4	0.311745901	mirna_pc
22421	hsa-mir-409	NKIRAS2	0.309835508	mirna_pc
22422	hsa-mir-409	MEX3A	0.361747692	mirna_pc
22423	hsa-mir-409	ACAN	0.561949754	mirna_pc
22424	hsa-mir-409	COL27A1	0.302769982	mirna_pc
22425	hsa-mir-409	C16orf70	0.357717194	mirna_pc
22426	hsa-mir-409	FCGR2A	0.324058642	mirna_pc
22427	hsa-mir-409	CHN1	0.323120426	mirna_pc
22428	hsa-mir-409	SC65	0.337139153	mirna_pc
22429	hsa-mir-409	PXDN	0.35472467	mirna_pc
22430	hsa-mir-409	SPON2	0.317650495	mirna_pc
22431	hsa-mir-409	ENPEP	0.325568702	mirna_pc
22432	hsa-mir-409	NID2	0.316307325	mirna_pc
22433	hsa-mir-409	GDPD5	0.349619595	mirna_pc
22434	hsa-mir-409	SNORA8	0.359894925	mirna_pc
22435	hsa-mir-409	LOC401010	0.318832153	mirna_pc
22436	hsa-mir-409	CHSY3	0.442171485	mirna_pc
22437	hsa-mir-409	ADAMTS7	0.316207074	mirna_pc
22438	hsa-mir-409	NID1	0.366790003	mirna_pc
22439	hsa-mir-409	NAT9	0.31719251	mirna_pc
22440	hsa-mir-409	KIAA1731	0.326231233	mirna_pc
22441	hsa-mir-409	PANX1	0.374826006	mirna_pc
22442	hsa-mir-409	C5orf13	0.313606429	mirna_pc
22443	hsa-mir-409	FKBP10	0.335571417	mirna_pc
22444	hsa-mir-409	PCDHB5	0.365313319	mirna_pc
22445	hsa-mir-409	LDLRAD3	0.316229434	mirna_pc
22446	hsa-mir-409	PRR16	0.326113894	mirna_pc
22447	hsa-mir-409	RDBP	0.317386982	mirna_pc
22448	hsa-mir-409	LY86	0.401483342	mirna_pc
22449	hsa-mir-409	EDNRA	0.330596625	mirna_pc
22450	hsa-mir-409	ZNF697	0.34972078	mirna_pc
22451	hsa-mir-409	ZFHX4	0.32291503	mirna_pc
22452	hsa-mir-409	GPX7	0.354675886	mirna_pc
22453	hsa-mir-409	EIF2B3	0.323680165	mirna_pc
22454	hsa-mir-409	RNF41	0.342749124	mirna_pc
22455	hsa-mir-409	GGCX	0.312397275	mirna_pc
22456	hsa-mir-409	EGFLAM	0.435294255	mirna_pc
22457	hsa-mir-409	GLT8D1	0.330852132	mirna_pc
22458	hsa-mir-409	NLRP3	0.310375535	mirna_pc
22459	hsa-mir-409	ESYT1	0.30731714	mirna_pc
22460	hsa-mir-409	C9orf84	0.40572509	mirna_pc
22461	hsa-mir-409	BCAT1	0.3799275	mirna_pc
22462	hsa-mir-409	N4BP2	0.324609623	mirna_pc
22463	hsa-mir-409	PBX2	0.320505577	mirna_pc

22464	hsa-mir-409	SNCAIP	0.519821841	mirna_pc
22465	hsa-mir-409	KIF12	0.441264896	mirna_pc
22466	hsa-mir-409	C4A	0.332237286	mirna_pc
22467	hsa-mir-409	MED17	0.346364482	mirna_pc
22468	hsa-mir-409	HYOU1	0.372587562	mirna_pc
22469	hsa-mir-409	DHRS2	0.625334452	mirna_pc
22470	hsa-mir-409	CCDC102B	0.394103684	mirna_pc
22471	hsa-mir-409	AGPAT4	0.323413946	mirna_pc
22472	hsa-mir-409	LOC220594	0.329895649	mirna_pc
22473	hsa-mir-409	LOC645166	0.539368813	mirna_pc
22474	hsa-mir-409	NT5C3L	0.3202735	mirna_pc
22475	hsa-mir-409	WDR73	0.425783364	mirna_pc
22476	hsa-mir-409	H2AFY2	0.305152264	mirna_pc
22477	hsa-mir-409	NES	0.325095248	mirna_pc
22478	hsa-mir-409	GJC1	0.338763244	mirna_pc
22479	hsa-mir-32	AUH	0.309074234	mirna_pc
22480	hsa-mir-32	CKS2	0.347256925	mirna_pc
22481	hsa-mir-32	PEX7	0.300257158	mirna_pc
22482	hsa-mir-32	GKAP1	0.316659896	mirna_pc
22483	hsa-mir-32	CABC1	0.306141776	mirna_pc
22484	hsa-mir-32	C6orf129	0.308231313	mirna_pc
22485	hsa-mir-32	DCXR	0.334147122	mirna_pc
22486	hsa-mir-32	SECISBP2	0.32593526	mirna_pc
22487	hsa-mir-32	COMMD4	0.368727486	mirna_pc
22488	hsa-mir-32	APIP	0.318855663	mirna_pc
22489	hsa-mir-32	EIF2S2	0.340469494	mirna_pc
22490	hsa-mir-32	ARFIP2	0.303569263	mirna_pc
22491	hsa-mir-32	HDAC2	0.343910337	mirna_pc
22492	hsa-mir-32	MTIF3	0.317694663	mirna_pc
22493	hsa-mir-32	LCN2	0.300086812	mirna_pc
22494	hsa-mir-32	CCDC56	0.313197098	mirna_pc
22495	hsa-mir-32	COX7A2	0.350517855	mirna_pc
22496	hsa-mir-32	C9orf16	0.322060422	mirna_pc
22497	hsa-mir-32	FN3K	0.304388334	mirna_pc
22498	hsa-mir-32	FAM96A	0.350596727	mirna_pc
22499	hsa-mir-32	TOR2A	0.324506544	mirna_pc
22500	hsa-mir-32	HACL1	0.333601317	mirna_pc
22501	hsa-mir-32	ANP32B	0.312468579	mirna_pc
22502	hsa-mir-32	C11orf48	0.308579346	mirna_pc
22503	hsa-mir-32	PTGES2	0.305128287	mirna_pc
22504	hsa-mir-32	PLEKHJ1	0.350658567	mirna_pc
22505	hsa-mir-32	C15orf63	0.320980635	mirna_pc
22506	hsa-mir-32	BLOC1S1	0.344229049	mirna_pc
22507	hsa-mir-32	FAM136A	0.346374418	mirna_pc
22508	hsa-mir-32	MRPL48	0.354475033	mirna_pc
22509	hsa-mir-32	STRA13	0.346731176	mirna_pc
22510	hsa-mir-32	SPCS1	0.336746026	mirna_pc
22511	hsa-mir-32	SFXN4	0.302470308	mirna_pc
22512	hsa-mir-32	EPB41L4B	0.30958011	mirna_pc
22513	hsa-mir-32	COPZ1	0.35642478	mirna_pc
22514	hsa-mir-32	CHKA	0.316188269	mirna_pc
22515	hsa-mir-32	GNL3	0.373834823	mirna_pc
22516	hsa-mir-32	NSMCE4A	0.344709801	mirna_pc
22517	hsa-mir-32	RPS3	0.31411312	mirna_pc

22518	hsa-mir-32	COX5A	0.401847184	mirna_pc
22519	hsa-mir-32	PQBP1	0.301715124	mirna_pc
22520	hsa-mir-32	NDUFA3	0.32328807	mirna_pc
22521	hsa-mir-32	SERF2	0.303669884	mirna_pc
22522	hsa-mir-32	MRPS11	0.306125327	mirna_pc
22523	hsa-mir-32	MRPL50	0.348717478	mirna_pc
22524	hsa-mir-32	MRPL46	0.303777482	mirna_pc
22525	hsa-mir-32	TIMM17B	0.319848862	mirna_pc
22526	hsa-mir-32	HDHD3	0.30085712	mirna_pc
22527	hsa-mir-32	C11orf10	0.334085741	mirna_pc
22528	hsa-mir-32	ERP29	0.305088935	mirna_pc
22529	hsa-mir-32	RPS17	0.306044305	mirna_pc
22530	hsa-mir-32	RNF5	0.346522729	mirna_pc
22531	hsa-mir-32	ANP32A	0.346702402	mirna_pc
22532	hsa-mir-32	C11orf75	0.305936654	mirna_pc
22533	hsa-mir-32	ATP5G2	0.381877118	mirna_pc
22534	hsa-mir-32	FXN	0.325114703	mirna_pc
22535	hsa-mir-32	RSL24D1	0.318546958	mirna_pc
22536	hsa-mir-32	URM1	0.340631651	mirna_pc
22537	hsa-mir-32	HINT2	0.319309416	mirna_pc
22538	hsa-mir-32	SRP14	0.301240482	mirna_pc
22539	hsa-mir-32	LSM 3.00	0.328899808	mirna_pc
22540	hsa-mir-32	RPLP2	0.31279885	mirna_pc
22541	hsa-mir-32	NDUFB11	0.33079131	mirna_pc
22542	hsa-mir-32	LOC440957	0.304244635	mirna_pc
22543	hsa-mir-32	ASPSCR1	0.329305273	mirna_pc
22544	hsa-mir-32	UBB	0.307249012	mirna_pc
22545	hsa-mir-32	RMRP	0.316840753	mirna_pc
22546	hsa-mir-32	CCDC72	0.31447535	mirna_pc
22547	hsa-mir-32	TMEM14B	0.327493463	mirna_pc
22548	hsa-mir-32	UXT	0.404621444	mirna_pc
22549	hsa-mir-32	NDUFAF4	0.337254262	mirna_pc
22550	hsa-mir-32	PPCDC	0.364684989	mirna_pc
22551	hsa-mir-32	DPM2	0.30875769	mirna_pc
22552	hsa-mir-32	RPPH1	0.315023548	mirna_pc
22553	hsa-mir-32	APOO	0.308700009	mirna_pc
22554	hsa-mir-32	DOM3Z	0.331910299	mirna_pc
22555	hsa-mir-125b-2	HOXC8	0.442726051	mirna_pc
22556	hsa-mir-125b-2	SEMA3E	0.777788346	mirna_pc
22557	hsa-mir-125b-2	EEF1A2	0.495754684	mirna_pc
22558	hsa-mir-125b-2	MYST4	0.450360252	mirna_pc
22559	hsa-mir-125b-2	ZNF467	0.350631466	mirna_pc
22560	hsa-mir-125b-2	ID4	0.400784091	mirna_pc
22561	hsa-mir-125b-2	YPEL1	0.470782413	mirna_pc
22562	hsa-mir-125b-2	CHEK2	0.348826889	mirna_pc
22563	hsa-mir-125b-2	C22orf39	0.330660135	mirna_pc
22564	hsa-mir-125b-2	RAD51AP1	0.306750786	mirna_pc
22565	hsa-mir-125b-2	CITED2	0.707066489	mirna_pc
22566	hsa-mir-125b-2	NR2F2	0.6198416	mirna_pc
22567	hsa-mir-125b-2	MAGI2	0.308385672	mirna_pc
22568	hsa-mir-125b-2	STX6	0.306295443	mirna_pc
22569	hsa-mir-125b-2	HDHD2	0.346078223	mirna_pc
22570	hsa-mir-125b-2	DTWD1	0.406305325	mirna_pc
22571	hsa-mir-125b-2	RASGRP2	0.301790912	mirna_pc

22572	hsa-mir-125b-2	MSH2	0.389245776	mirna_pc
22573	hsa-mir-125b-2	ZNF439	0.403332241	mirna_pc
22574	hsa-mir-125b-2	POLI	0.588916803	mirna_pc
22575	hsa-mir-125b-2	RNF185	0.300914055	mirna_pc
22576	hsa-mir-125b-2	TTC3	0.34745779	mirna_pc
22577	hsa-mir-125b-2	ZNF367	0.356492839	mirna_pc
22578	hsa-mir-125b-2	CCDC106	0.389911021	mirna_pc
22579	hsa-mir-125b-2	SLC5A12	0.542115831	mirna_pc
22580	hsa-mir-125b-2	DLG4	0.309478505	mirna_pc
22581	hsa-mir-125b-2	USP25	0.527303663	mirna_pc
22582	hsa-mir-125b-2	LOC375190	0.603317497	mirna_pc
22583	hsa-mir-125b-2	CRY1	0.306492795	mirna_pc
22584	hsa-mir-125b-2	HIST1H3D	0.372933687	mirna_pc
22585	hsa-mir-125b-2	SLC2A11	0.495121143	mirna_pc
22586	hsa-mir-125b-2	ZNF433	0.322523618	mirna_pc
22587	hsa-mir-125b-2	TYW3	0.632837358	mirna_pc
22588	hsa-mir-125b-2	GAD1	0.317983467	mirna_pc
22589	hsa-mir-125b-2	OXR1	0.350561064	mirna_pc
22590	hsa-mir-125b-2	KHDRBS1	0.316566941	mirna_pc
22591	hsa-mir-125b-2	TUB	0.352137596	mirna_pc
22592	hsa-mir-125b-2	PRR19	0.341801361	mirna_pc
22593	hsa-mir-125b-2	FIGN	0.762160724	mirna_pc
22594	hsa-mir-125b-2	PRRT1	0.427255573	mirna_pc
22595	hsa-mir-125b-2	SAMD11	0.470013562	mirna_pc
22596	hsa-mir-125b-2	PHF10	0.446519348	mirna_pc
22597	hsa-mir-125b-2	LOC284900	0.38724923	mirna_pc
22598	hsa-mir-125b-2	CAPS2	0.627337411	mirna_pc
22599	hsa-mir-125b-2	PHC1	0.386057495	mirna_pc
22600	hsa-mir-125b-2	SCD5	0.370688334	mirna_pc
22601	hsa-mir-125b-2	ELAVL1	0.308436569	mirna_pc
22602	hsa-mir-125b-2	CCDC121	0.363023888	mirna_pc
22603	hsa-mir-125b-2	SSBP2	0.375038453	mirna_pc
22604	hsa-mir-125b-2	TFAP2A	0.341775252	mirna_pc
22605	hsa-mir-125b-2	HABP4	0.67837349	mirna_pc
22606	hsa-mir-125b-2	TBCEL	0.323379048	mirna_pc
22607	hsa-mir-125b-2	C20orf160	0.459848392	mirna_pc
22608	hsa-mir-125b-2	CSAD	0.555017941	mirna_pc
22609	hsa-mir-125b-2	RNF2	0.311718895	mirna_pc
22610	hsa-mir-125b-2	CTSL2	0.367016895	mirna_pc
22611	hsa-mir-125b-2	PRAME	0.404350147	mirna_pc
22612	hsa-mir-125b-2	HNRNPK	0.302974439	mirna_pc
22613	hsa-mir-125b-2	KIAA0776	0.34824002	mirna_pc
22614	hsa-mir-125b-2	CA11	0.48354752	mirna_pc
22615	hsa-mir-125b-2	TDG	0.328800743	mirna_pc
22616	hsa-mir-125b-2	EIF4ENIF1	0.553086457	mirna_pc
22617	hsa-mir-125b-2	BAIAP3	0.455376394	mirna_pc
22618	hsa-mir-125b-2	LOC653501	0.316142788	mirna_pc
22619	hsa-mir-125b-2	TUG1	0.430998997	mirna_pc
22620	hsa-mir-125b-2	ITFG2	0.304741713	mirna_pc
22621	hsa-mir-125b-2	AMN1	0.500799174	mirna_pc
22622	hsa-mir-125b-2	HDAC2	0.31249068	mirna_pc
22623	hsa-mir-125b-2	FAM184A	0.311999023	mirna_pc
22624	hsa-mir-125b-2	TDRD5	0.391841256	mirna_pc
22625	hsa-mir-125b-2	CILP2	0.467684147	mirna_pc

22626	hsa-mir-125b-2	ZNF70	0.325260707	mirna_pc
22627	hsa-mir-125b-2	GRAMD1C	0.501570589	mirna_pc
22628	hsa-mir-125b-2	ZNF217	0.457150707	mirna_pc
22629	hsa-mir-125b-2	SOX4	0.321969369	mirna_pc
22630	hsa-mir-125b-2	CYTSA	0.321651824	mirna_pc
22631	hsa-mir-125b-2	FAM161A	0.308317639	mirna_pc
22632	hsa-mir-125b-2	DYRK1A	0.337212396	mirna_pc
22633	hsa-mir-125b-2	ZNF236	0.352857781	mirna_pc
22634	hsa-mir-125b-2	EFHD1	0.377825714	mirna_pc
22635	hsa-mir-125b-2	LPHN2	0.584309922	mirna_pc
22636	hsa-mir-125b-2	HIST3H2A	0.333798321	mirna_pc
22637	hsa-mir-125b-2	C6orf120	0.31177165	mirna_pc
22638	hsa-mir-125b-2	ZNF23	0.302815786	mirna_pc
22639	hsa-mir-125b-2	BARD1	0.375179292	mirna_pc
22640	hsa-mir-125b-2	PAPOLG	0.324280749	mirna_pc
22641	hsa-mir-125b-2	CNTNAP3	0.392253553	mirna_pc
22642	hsa-mir-125b-2	RPL23AP53	0.320792711	mirna_pc
22643	hsa-mir-125b-2	SAMD1	0.308507668	mirna_pc
22644	hsa-mir-125b-2	C16orf87	0.381111629	mirna_pc
22645	hsa-mir-125b-2	EDIL3	0.455626396	mirna_pc
22646	hsa-mir-125b-2	LOC144571	0.333671954	mirna_pc
22647	hsa-mir-125b-2	ZNF608	0.321277851	mirna_pc
22648	hsa-mir-125b-2	METTL13	0.324404551	mirna_pc
22649	hsa-mir-125b-2	SEMA3D	0.805861673	mirna_pc
22650	hsa-mir-125b-2	HACE1	0.348935726	mirna_pc
22651	hsa-mir-125b-2	KLRA1	0.344725144	mirna_pc
22652	hsa-mir-125b-2	DDX47	0.322141067	mirna_pc
22653	hsa-mir-125b-2	SLC25A30	0.461138479	mirna_pc
22654	hsa-mir-125b-2	VRK2	0.34644445	mirna_pc
22655	hsa-mir-125b-2	ZNF658	0.404215803	mirna_pc
22656	hsa-mir-125b-2	RBM43	0.371362335	mirna_pc
22657	hsa-mir-125b-2	GATA3	0.568065826	mirna_pc
22658	hsa-mir-125b-2	NETO2	0.381417407	mirna_pc
22659	hsa-mir-125b-2	LOC374443	0.310547598	mirna_pc
22660	hsa-mir-125b-2	RNPC3	0.333739861	mirna_pc
22661	hsa-mir-125b-2	OXTR	0.426833347	mirna_pc
22662	hsa-mir-125b-2	PAQR7	0.310731737	mirna_pc
22663	hsa-mir-125b-2	PEBP1	0.32600734	mirna_pc
22664	hsa-mir-125b-2	THSD7A	0.538085827	mirna_pc
22665	hsa-mir-125b-2	PPM1J	0.444111415	mirna_pc
22666	hsa-mir-125b-2	LOC440905	0.301417615	mirna_pc
22667	hsa-mir-125b-2	ZNF605	0.438988587	mirna_pc
22668	hsa-mir-125b-2	RRAGB	0.373302603	mirna_pc
22669	hsa-mir-125b-2	KLRAQ1	0.376841736	mirna_pc
22670	hsa-mir-125b-2	PCGF6	0.324098062	mirna_pc
22671	hsa-mir-125b-2	TCEA2	0.314986868	mirna_pc
22672	hsa-mir-125b-2	S1PR5	0.344796224	mirna_pc
22673	hsa-mir-125b-2	OSR2	0.426381826	mirna_pc
22674	hsa-mir-125b-2	C12orf76	0.373944401	mirna_pc
22675	hsa-mir-125b-2	C6orf170	0.392262565	mirna_pc
22676	hsa-mir-125b-2	GABPA	0.350734057	mirna_pc
22677	hsa-mir-125b-2	ZFP161	0.323053167	mirna_pc
22678	hsa-mir-125b-2	MEX3B	0.375679682	mirna_pc
22679	hsa-mir-125b-2	ZNF503	0.45027566	mirna_pc

22680	hsa-mir-125b-2	C8orf51	0.337720431	mirna_pc
22681	hsa-mir-125b-2	LOC728392	0.319981907	mirna_pc
22682	hsa-mir-125b-2	LANCL1	0.327215387	mirna_pc
22683	hsa-mir-125b-2	IL27RA	0.378990068	mirna_pc
22684	hsa-mir-125b-2	FMO4	0.368159598	mirna_pc
22685	hsa-mir-125b-2	ZNF607	0.328328225	mirna_pc
22686	hsa-mir-125b-2	KCNC3	0.51908932	mirna_pc
22687	hsa-mir-125b-2	PIK3C3	0.374459739	mirna_pc
22688	hsa-mir-125b-2	ZNF362	0.338302367	mirna_pc
22689	hsa-mir-125b-2	ZNF84	0.382216071	mirna_pc
22690	hsa-mir-125b-2	GAS8	0.332084747	mirna_pc
22691	hsa-mir-125b-2	TAF4B	0.380874153	mirna_pc
22692	hsa-mir-125b-2	ZNF140	0.346216662	mirna_pc
22693	hsa-mir-125b-2	GALNT11	0.328822725	mirna_pc
22694	hsa-mir-125b-2	MTA3	0.441563923	mirna_pc
22695	hsa-mir-125b-2	PUS10	0.400431494	mirna_pc
22696	hsa-mir-125b-2	SCAND3	0.309039999	mirna_pc
22697	hsa-mir-125b-2	SEC61A2	0.431962514	mirna_pc
22698	hsa-mir-125b-2	PSPC1	0.443236345	mirna_pc
22699	hsa-mir-125b-2	OLFM2	0.373385633	mirna_pc
22700	hsa-mir-125b-2	DRG1	0.340910363	mirna_pc
22701	hsa-mir-125b-2	SSX2IP	0.354059219	mirna_pc
22702	hsa-mir-125b-2	TMC8	0.313807884	mirna_pc
22703	hsa-mir-125b-2	NF2	0.478836487	mirna_pc
22704	hsa-mir-125b-2	TTC27	0.330679656	mirna_pc
22705	hsa-mir-125b-2	KPNA5	0.300321271	mirna_pc
22706	hsa-mir-125b-2	MYBL1	0.545406829	mirna_pc
22707	hsa-mir-125b-2	RNF187	0.315462203	mirna_pc
22708	hsa-mir-125b-2	TCTE3	0.311751258	mirna_pc
22709	hsa-mir-125b-2	CLSTN3	0.30600575	mirna_pc
22710	hsa-mir-125b-2	WBP4	0.32359392	mirna_pc
22711	hsa-mir-125b-2	CHRNB1	0.301655667	mirna_pc
22712	hsa-mir-125b-2	RIMKLB	0.378116528	mirna_pc
22713	hsa-mir-125b-2	GAS1	0.368268406	mirna_pc
22714	hsa-mir-125b-2	ZNF692	0.315836219	mirna_pc
22715	hsa-mir-125b-2	EFNA5	0.547911741	mirna_pc
22716	hsa-mir-125b-2	HCG18	0.307317838	mirna_pc
22717	hsa-mir-125b-2	C18orf10	0.44465097	mirna_pc
22718	hsa-mir-125b-2	C10orf41	0.424722606	mirna_pc
22719	hsa-mir-125b-2	SUDS3	0.377391949	mirna_pc
22720	hsa-mir-125b-2	MAMSTR	0.564832531	mirna_pc
22721	hsa-mir-125b-2	TMEM136	0.431918779	mirna_pc
22722	hsa-mir-125b-2	PNMAL2	0.432453864	mirna_pc
22723	hsa-mir-125b-2	CCDC78	0.41342354	mirna_pc
22724	hsa-mir-125b-2	DNM3	0.733066571	mirna_pc
22725	hsa-mir-125b-2	RPL7	0.326028358	mirna_pc
22726	hsa-mir-125b-2	SEMA3C	0.329871499	mirna_pc
22727	hsa-mir-125b-2	PEX2	0.35755102	mirna_pc
22728	hsa-mir-125b-2	WDR78	0.511103338	mirna_pc
22729	hsa-mir-125b-2	KGFLP2	0.388562416	mirna_pc
22730	hsa-mir-125b-2	IFT81	0.354739948	mirna_pc
22731	hsa-mir-125b-2	ZNF786	0.300591196	mirna_pc
22732	hsa-mir-125b-2	ZNF184	0.364494586	mirna_pc
22733	hsa-mir-125b-2	MBLAC2	0.390252532	mirna_pc

22734	hsa-mir-125b-2	NIPSNAP1	0.521806353	mirna_pc
22735	hsa-mir-125b-2	DEPDC5	0.344162968	mirna_pc
22736	hsa-mir-125b-2	C8orf37	0.654038133	mirna_pc
22737	hsa-mir-125b-2	PDGFC	0.493619458	mirna_pc
22738	hsa-mir-125b-2	SPA17	0.322588634	mirna_pc
22739	hsa-mir-125b-2	WDR92	0.377911564	mirna_pc
22740	hsa-mir-125b-2	PCSK4	0.380564587	mirna_pc
22741	hsa-mir-125b-2	SFI1	0.581921228	mirna_pc
22742	hsa-mir-125b-2	AEBP2	0.370083175	mirna_pc
22743	hsa-mir-125b-2	MAP3K12	0.539404496	mirna_pc
22744	hsa-mir-125b-2	ZBTB10	0.372271951	mirna_pc
22745	hsa-mir-125b-2	SOCS5	0.372504973	mirna_pc
22746	hsa-mir-125b-2	YTHDF2	0.311371197	mirna_pc
22747	hsa-mir-125b-2	TTC21B	0.353890412	mirna_pc
22748	hsa-mir-125b-2	ZNF627	0.331052775	mirna_pc
22749	hsa-mir-125b-2	LDB1	0.32584038	mirna_pc
22750	hsa-mir-125b-2	ANGPT1	0.788439678	mirna_pc
22751	hsa-mir-125b-2	EXTL2	0.345722579	mirna_pc
22752	hsa-mir-125b-2	LOC440173	0.30525984	mirna_pc
22753	hsa-mir-125b-2	ARID1A	0.331005049	mirna_pc
22754	hsa-mir-125b-2	ATAD2B	0.351633809	mirna_pc
22755	hsa-mir-125b-2	ZNF558	0.397334896	mirna_pc
22756	hsa-mir-125b-2	NAB2	0.45643018	mirna_pc
22757	hsa-mir-125b-2	EMP2	0.348082439	mirna_pc
22758	hsa-mir-125b-2	FDXACB1	0.344757624	mirna_pc
22759	hsa-mir-125b-2	ISYNA1	0.532169886	mirna_pc
22760	hsa-mir-125b-2	LOC338758	0.470652375	mirna_pc
22761	hsa-mir-125b-2	SCPEP1	0.316036462	mirna_pc
22762	hsa-mir-125b-2	KIF27	0.342186862	mirna_pc
22763	hsa-mir-125b-2	TEAD2	0.300181079	mirna_pc
22764	hsa-mir-125b-2	PCMTD2	0.322867769	mirna_pc
22765	hsa-mir-125b-2	ZNF638	0.342925692	mirna_pc
22766	hsa-mir-125b-2	ZNF384	0.310799302	mirna_pc
22767	hsa-mir-125b-2	SMO	0.319434134	mirna_pc
22768	hsa-mir-125b-2	ZNF20	0.332110772	mirna_pc
22769	hsa-mir-125b-2	FBXO17	0.343595988	mirna_pc
22770	hsa-mir-125b-2	LUC7L2	0.319237821	mirna_pc
22771	hsa-mir-125b-2	PIP5K1A	0.366916302	mirna_pc
22772	hsa-mir-125b-2	TMX3	0.395591364	mirna_pc
22773	hsa-mir-125b-2	ZNF512	0.425609171	mirna_pc
22774	hsa-mir-125b-2	GRB14	0.494840816	mirna_pc
22775	hsa-mir-125b-2	USP21	0.307252244	mirna_pc
22776	hsa-mir-125b-2	ZNF74	0.427104331	mirna_pc
22777	hsa-mir-125b-2	HTR7P1	0.534201477	mirna_pc
22778	hsa-mir-125b-2	OSGEPL1	0.362429944	mirna_pc
22779	hsa-mir-125b-2	PEX13	0.403886255	mirna_pc
22780	hsa-mir-125b-2	DCAF17	0.379697009	mirna_pc
22781	hsa-mir-125b-2	ETAA1	0.355991874	mirna_pc
22782	hsa-mir-125b-2	HNRNPH3	0.409490001	mirna_pc
22783	hsa-mir-125b-2	HMGN3	0.356977065	mirna_pc
22784	hsa-mir-125b-2	B4GALT6	0.392700721	mirna_pc
22785	hsa-mir-125b-2	UBTF	0.300296677	mirna_pc
22786	hsa-mir-125b-2	CEP290	0.343021446	mirna_pc
22787	hsa-mir-125b-2	CNIH2	0.479191783	mirna_pc

22788	hsa-mir-125b-2	HHAT	0.39221862	mirna_pc
22789	hsa-mir-125b-2	PMS1	0.326226383	mirna_pc
22790	hsa-mir-125b-2	EID3	0.617720035	mirna_pc
22791	hsa-mir-125b-2	KIAA1467	0.365991129	mirna_pc
22792	hsa-mir-125b-2	PAPLN	0.351109265	mirna_pc
22793	hsa-mir-125b-2	ABHD12	0.320277066	mirna_pc
22794	hsa-mir-125b-2	UBE2W	0.316073206	mirna_pc
22795	hsa-mir-125b-2	GATS	0.504424889	mirna_pc
22796	hsa-mir-125b-2	TRUB1	0.42703578	mirna_pc
22797	hsa-mir-125b-2	PLAC2	0.679954629	mirna_pc
22798	hsa-mir-125b-2	ROBO1	0.434872859	mirna_pc
22799	hsa-mir-125b-2	C2orf15	0.350773025	mirna_pc
22800	hsa-mir-125b-2	C12orf24	0.499255788	mirna_pc
22801	hsa-mir-125b-2	CCDC102A	0.323471506	mirna_pc
22802	hsa-mir-125b-2	TULP3	0.361197514	mirna_pc
22803	hsa-mir-125b-2	NFYB	0.30464284	mirna_pc
22804	hsa-mir-125b-2	LCA5L	0.372654582	mirna_pc
22805	hsa-mir-125b-2	SEMA3A	0.645274701	mirna_pc
22806	hsa-mir-125b-2	THNSL1	0.338523848	mirna_pc
22807	hsa-mir-125b-2	USP13	0.426316963	mirna_pc
22808	hsa-mir-125b-2	FAM120C	0.383810084	mirna_pc
22809	hsa-mir-125b-2	ESF1	0.311191546	mirna_pc
22810	hsa-mir-125b-2	PDGFRL	0.50960884	mirna_pc
22811	hsa-mir-125b-2	GPR37	0.379152588	mirna_pc
22812	hsa-mir-125b-2	MIB1	0.304695683	mirna_pc
22813	hsa-mir-125b-2	SS18	0.389070082	mirna_pc
22814	hsa-mir-125b-2	ZNF391	0.395575574	mirna_pc
22815	hsa-mir-125b-2	IFT74	0.568751369	mirna_pc
22816	hsa-mir-125b-2	MAST1	0.423821763	mirna_pc
22817	hsa-mir-125b-2	NR5A2	0.415817594	mirna_pc
22818	hsa-mir-125b-2	TTC39C	0.334245203	mirna_pc
22819	hsa-mir-125b-2	MYNN	0.303604623	mirna_pc
22820	hsa-mir-125b-2	MDM1	0.737946556	mirna_pc
22821	hsa-mir-125b-2	ZNF740	0.312553154	mirna_pc
22822	hsa-mir-125b-2	TNPO2	0.303453679	mirna_pc
22823	hsa-mir-125b-2	TRO	0.336190393	mirna_pc
22824	hsa-mir-125b-2	BBS10	0.421206942	mirna_pc
22825	hsa-mir-125b-2	PBX2	0.390571215	mirna_pc
22826	hsa-mir-125b-2	SFRS15	0.456143428	mirna_pc
22827	hsa-mir-125b-2	SLITRK6	0.593485432	mirna_pc
22828	hsa-mir-125b-2	UXS1	0.336378161	mirna_pc
22829	hsa-mir-125b-2	ANKRD34A	0.387882206	mirna_pc
22830	hsa-mir-125b-2	HAPLN1	0.74237811	mirna_pc
22831	hsa-mir-125b-2	C6orf70	0.335412441	mirna_pc
22832	hsa-mir-125b-2	RTF1	0.340524902	mirna_pc
22833	hsa-mir-125b-2	SYNGAP1	0.353957384	mirna_pc
22834	hsa-mir-125b-2	C8orf45	0.378957448	mirna_pc
22835	hsa-mir-125b-2	DBNDD1	0.395128027	mirna_pc
22836	hsa-mir-125b-2	PPOX	0.327226848	mirna_pc
22837	hsa-mir-125b-2	GTF2IP1	0.316025111	mirna_pc
22838	hsa-mir-125b-2	LOC221710	0.397272291	mirna_pc
22839	hsa-mir-125b-2	TSGA10	0.474691791	mirna_pc
22840	hsa-mir-125b-2	BRSK1	0.30285509	mirna_pc
22841	hsa-mir-125b-2	DGCR6	0.439893543	mirna_pc

22842	hsa-mir-125b-2	CRYZ	0.623560729	mirna_pc
22843	hsa-mir-125b-2	TIA1	0.340589348	mirna_pc
22844	hsa-mir-125b-2	ZNF664	0.323306223	mirna_pc
22845	hsa-mir-125b-2	SMEK2	0.372127341	mirna_pc
22846	hsa-mir-125b-2	ATL2	0.524177398	mirna_pc
22847	hsa-mir-125b-2	TBX1	0.402657369	mirna_pc
22848	hsa-mir-125b-2	KDELC2	0.357769932	mirna_pc
22849	hsa-mir-125b-2	KILLIN	0.323373674	mirna_pc
22850	hsa-mir-125b-2	PRICKLE1	0.635540983	mirna_pc
22851	hsa-mir-125b-2	GCFC1	0.310793626	mirna_pc
22852	hsa-mir-125b-2	UBE3A	0.328658974	mirna_pc
22853	hsa-mir-125b-2	ZCCHC11	0.357922337	mirna_pc
22854	hsa-mir-125b-2	CETN3	0.319166907	mirna_pc
22855	hsa-mir-125b-2	ZNF644	0.316318864	mirna_pc
22856	hsa-mir-125b-2	PIK3R2	0.300340592	mirna_pc
22857	hsa-mir-125b-2	PRPF40B	0.341022248	mirna_pc
22858	hsa-mir-125b-2	TCTN1	0.321762503	mirna_pc
22859	hsa-mir-125b-2	CTXN1	0.6208915	mirna_pc
22860	hsa-mir-125b-2	RNF215	0.346764406	mirna_pc
22861	hsa-mir-125b-2	ZNF680	0.310045586	mirna_pc
22862	hsa-mir-125b-2	LOC283070	0.5476287	mirna_pc
22863	hsa-mir-125b-2	CCDC117	0.335604761	mirna_pc
22864	hsa-mir-125b-2	C10orf46	0.304697007	mirna_pc
22865	hsa-mir-125b-2	HSDL1	0.474628877	mirna_pc
22866	hsa-mir-125b-2	SLC11A2	0.611060393	mirna_pc
22867	hsa-mir-125b-2	LOC100129387	0.493505427	mirna_pc
22868	hsa-mir-125b-2	FBXO11	0.365993867	mirna_pc
22869	hsa-mir-125b-2	HEY2	0.398673628	mirna_pc
22870	hsa-mir-125b-2	ZFP37	0.328157452	mirna_pc
22871	hsa-mir-125b-2	HSPC157	0.30916847	mirna_pc
22872	hsa-mir-125b-2	IRX5	0.301031447	mirna_pc
22873	hsa-mir-125b-2	THOC5	0.307247678	mirna_pc
22874	hsa-mir-125b-2	CAMK1D	0.401495473	mirna_pc
22875	hsa-mir-125b-2	LCA5	0.689330411	mirna_pc
22876	hsa-mir-125b-2	CDC73	0.419795012	mirna_pc
22877	hsa-mir-125b-2	SLC2A3	0.357671199	mirna_pc
22878	hsa-mir-125b-2	C22orf29	0.390624797	mirna_pc
22879	hsa-mir-125b-2	WDSUB1	0.40307348	mirna_pc
22880	hsa-mir-125b-2	PITPNB	0.301151335	mirna_pc
22881	hsa-mir-125b-2	BTF3L4	0.401838442	mirna_pc
22882	hsa-mir-125b-2	DDX59	0.314808422	mirna_pc
22883	hsa-mir-125b-2	AFG3L1	0.309196624	mirna_pc
22884	hsa-mir-125b-2	SOX6	0.309811429	mirna_pc
22885	hsa-mir-125b-2	SS18L1	0.33131726	mirna_pc
22886	hsa-mir-19b-2	CENPF	0.334148081	mirna_pc
22887	hsa-mir-19b-2	ASPM	0.302714831	mirna_pc
22888	hsa-mir-19b-2	FANCA	0.348598705	mirna_pc
22889	hsa-mir-19b-2	NCAPG	0.316532104	mirna_pc
22890	hsa-mir-19b-2	CDC25B	0.358872737	mirna_pc
22891	hsa-mir-19b-2	CBX3	0.331208065	mirna_pc
22892	hsa-mir-19b-2	NEK2	0.351386613	mirna_pc
22893	hsa-mir-19b-2	MAD2L1	0.325925405	mirna_pc
22894	hsa-mir-19b-2	XPO1	0.333923789	mirna_pc
22895	hsa-mir-19b-2	KNTC1	0.324549464	mirna_pc

22896	hsa-mir-19b-2	NUF2	0.388969397	mirna_pc
22897	hsa-mir-19b-2	PLK4	0.353732149	mirna_pc
22898	hsa-mir-19b-2	KIF18A	0.314773677	mirna_pc
22899	hsa-mir-19b-2	DEPDC1	0.317460744	mirna_pc
22900	hsa-mir-19b-2	SKA3	0.405199918	mirna_pc
22901	hsa-mir-19b-2	KIF14	0.316448376	mirna_pc
22902	hsa-mir-19b-2	HJURP	0.346573777	mirna_pc
22903	hsa-mir-19b-2	BLM	0.340282137	mirna_pc
22904	hsa-mir-19b-2	PAICS	0.313988674	mirna_pc
22905	hsa-mir-19b-2	CCDC150	0.36262277	mirna_pc
22906	hsa-mir-19b-2	DTL	0.365507321	mirna_pc
22907	hsa-mir-19b-2	CENPK	0.36130243	mirna_pc
22908	hsa-mir-19b-2	SGOL2	0.32761588	mirna_pc
22909	hsa-mir-19b-2	C1orf112	0.37224214	mirna_pc
22910	hsa-mir-19b-2	GINS1	0.314475013	mirna_pc
22911	hsa-mir-19b-2	C12orf48	0.319144409	mirna_pc
22912	hsa-mir-19b-2	HELLS	0.323024941	mirna_pc
22913	hsa-mir-19b-2	HNRNPA2B1	0.313011924	mirna_pc
22914	hsa-mir-19b-2	MCM6	0.326372117	mirna_pc
22915	hsa-mir-19b-2	CDCA7	0.30469779	mirna_pc
22916	hsa-mir-19b-2	FAM60A	0.343157962	mirna_pc
22917	hsa-mir-19b-2	ATAD5	0.320282586	mirna_pc
22918	hsa-mir-19b-2	RFC3	0.37737204	mirna_pc
22919	hsa-mir-19b-2	DEPDC1B	0.312934661	mirna_pc
22920	hsa-mir-19b-2	GEN1	0.332169193	mirna_pc
22921	hsa-mir-19b-2	HMGB2	0.329998549	mirna_pc
22922	hsa-mir-19b-2	DNA2	0.314222101	mirna_pc
22923	hsa-mir-19b-2	NOP56	0.362392264	mirna_pc
22924	hsa-mir-19b-2	DLEU2	0.340922794	mirna_pc
22925	hsa-mir-19b-2	FERMT1	0.35078527	mirna_pc
22926	hsa-mir-19b-2	MSH2	0.324818429	mirna_pc
22927	hsa-mir-19b-2	CDC7	0.315728728	mirna_pc
22928	hsa-mir-19b-2	SSB	0.370464976	mirna_pc
22929	hsa-mir-19b-2	FBXO5	0.337570593	mirna_pc
22930	hsa-mir-19b-2	E2F3	0.376423986	mirna_pc
22931	hsa-mir-19b-2	DDX11	0.311232327	mirna_pc
22932	hsa-mir-19b-2	CDC25A	0.333252823	mirna_pc
22933	hsa-mir-19b-2	DDX12	0.333353497	mirna_pc
22934	hsa-mir-19b-2	C13orf34	0.455824146	mirna_pc
22935	hsa-mir-19b-2	MCM8	0.445892434	mirna_pc
22936	hsa-mir-19b-2	PUS7	0.326170971	mirna_pc
22937	hsa-mir-19b-2	NCL	0.320943439	mirna_pc
22938	hsa-mir-19b-2	PSRC1	0.306791143	mirna_pc
22939	hsa-mir-19b-2	C13orf37	0.42403202	mirna_pc
22940	hsa-mir-19b-2	PNPT1	0.333981652	mirna_pc
22941	hsa-mir-19b-2	TMPO	0.311853997	mirna_pc
22942	hsa-mir-19b-2	SET	0.300798155	mirna_pc
22943	hsa-mir-19b-2	LOC388796	0.326901812	mirna_pc
22944	hsa-mir-19b-2	GABPB1	0.355370145	mirna_pc
22945	hsa-mir-19b-2	CCDC138	0.309535253	mirna_pc
22946	hsa-mir-19b-2	HMGN2	0.309745512	mirna_pc
22947	hsa-mir-19b-2	ETV4	0.32926348	mirna_pc
22948	hsa-mir-19b-2	SFRS1	0.359196015	mirna_pc
22949	hsa-mir-19b-2	WDR43	0.303955455	mirna_pc

22950	hsa-mir-19b-2	C4orf46	0.332793599	mirna_pc
22951	hsa-mir-19b-2	HSPD1	0.345755574	mirna_pc
22952	hsa-mir-19b-2	SSRP1	0.319560705	mirna_pc
22953	hsa-mir-19b-2	KHDRBS1	0.366910112	mirna_pc
22954	hsa-mir-19b-2	PRPF40A	0.363015123	mirna_pc
22955	hsa-mir-19b-2	CCDC18	0.31136646	mirna_pc
22956	hsa-mir-19b-2	DNAJC2	0.344498447	mirna_pc
22957	hsa-mir-19b-2	SLC39A10	0.372866206	mirna_pc
22958	hsa-mir-19b-2	ZNF695	0.33745839	mirna_pc
22959	hsa-mir-19b-2	HAUS6	0.340739587	mirna_pc
22960	hsa-mir-19b-2	CENPJ	0.368478553	mirna_pc
22961	hsa-mir-19b-2	HNRNPU	0.32423682	mirna_pc
22962	hsa-mir-19b-2	NASP	0.320692192	mirna_pc
22963	hsa-mir-19b-2	PTMA	0.324973584	mirna_pc
22964	hsa-mir-19b-2	SUV39H2	0.30252791	mirna_pc
22965	hsa-mir-19b-2	GTF2F2	0.341955177	mirna_pc
22966	hsa-mir-19b-2	POP1	0.357810989	mirna_pc
22967	hsa-mir-19b-2	HSP90B1	0.336183469	mirna_pc
22968	hsa-mir-19b-2	NUDT15	0.333773598	mirna_pc
22969	hsa-mir-19b-2	SLMO2	0.357624226	mirna_pc
22970	hsa-mir-19b-2	DHFR	0.320328063	mirna_pc
22971	hsa-mir-19b-2	NAA25	0.306868722	mirna_pc
22972	hsa-mir-19b-2	DEK	0.366285663	mirna_pc
22973	hsa-mir-19b-2	TRIB3	0.352301007	mirna_pc
22974	hsa-mir-19b-2	PASK	0.348115199	mirna_pc
22975	hsa-mir-19b-2	HEATR2	0.34610901	mirna_pc
22976	hsa-mir-19b-2	CYB5B	0.336603866	mirna_pc
22977	hsa-mir-19b-2	CEP152	0.302951255	mirna_pc
22978	hsa-mir-19b-2	CDRT4	0.32079313	mirna_pc
22979	hsa-mir-19b-2	SLMO1	0.304030551	mirna_pc
22980	hsa-mir-19b-2	RNF219	0.495605951	mirna_pc
22981	hsa-mir-19b-2	SLC25A32	0.356562109	mirna_pc
22982	hsa-mir-19b-2	DDX55	0.313871384	mirna_pc
22983	hsa-mir-19b-2	HDAC2	0.36134548	mirna_pc
22984	hsa-mir-19b-2	XRN2	0.34356846	mirna_pc
22985	hsa-mir-19b-2	KPNB1	0.369592842	mirna_pc
22986	hsa-mir-19b-2	SMC3	0.398568258	mirna_pc
22987	hsa-mir-19b-2	MEX3A	0.476921296	mirna_pc
22988	hsa-mir-19b-2	BAT1	0.306956335	mirna_pc
22989	hsa-mir-19b-2	HMGB1	0.389268196	mirna_pc
22990	hsa-mir-19b-2	DIAPH3	0.344750995	mirna_pc
22991	hsa-mir-19b-2	MCTP2	0.307023864	mirna_pc
22992	hsa-mir-19b-2	LOC642846	0.305702856	mirna_pc
22993	hsa-mir-19b-2	TGIF2	0.389051244	mirna_pc
22994	hsa-mir-19b-2	MTF2	0.321970751	mirna_pc
22995	hsa-mir-19b-2	POLE	0.333639075	mirna_pc
22996	hsa-mir-19b-2	SOX9	0.352154672	mirna_pc
22997	hsa-mir-19b-2	C13orf27	0.424810227	mirna_pc
22998	hsa-mir-19b-2	RBMX	0.346978577	mirna_pc
22999	hsa-mir-19b-2	GPR180	0.37963882	mirna_pc
23000	hsa-mir-19b-2	DAP3	0.31318424	mirna_pc
23001	hsa-mir-19b-2	MDC 1.00	0.310656512	mirna_pc
23002	hsa-mir-19b-2	XRCC5	0.32685411	mirna_pc
23003	hsa-mir-19b-2	EXOSC8	0.339613873	mirna_pc

23004	hsa-mir-19b-2	TAF1D	0.336456026	mirna_pc
23005	hsa-mir-19b-2	FAM136A	0.343432999	mirna_pc
23006	hsa-mir-19b-2	NRAS	0.382031839	mirna_pc
23007	hsa-mir-19b-2	ITPRIPL1	0.300196225	mirna_pc
23008	hsa-mir-19b-2	SNORA8	0.38516619	mirna_pc
23009	hsa-mir-19b-2	CSNK2A1	0.309639781	mirna_pc
23010	hsa-mir-19b-2	RPIA	0.313368016	mirna_pc
23011	hsa-mir-19b-2	ZNF286A	0.317975314	mirna_pc
23012	hsa-mir-19b-2	RBM28	0.312056321	mirna_pc
23013	hsa-mir-19b-2	ANKRD46	0.304007497	mirna_pc
23014	hsa-mir-19b-2	C13orf23	0.35937115	mirna_pc
23015	hsa-mir-19b-2	TOP 1.00	0.360863751	mirna_pc
23016	hsa-mir-19b-2	TCERG1	0.348048689	mirna_pc
23017	hsa-mir-19b-2	PSPC1	0.301521045	mirna_pc
23018	hsa-mir-19b-2	TOP1P1	0.307560878	mirna_pc
23019	hsa-mir-19b-2	E2F6	0.36724661	mirna_pc
23020	hsa-mir-19b-2	SFRS13A	0.371861014	mirna_pc
23021	hsa-mir-19b-2	SCLY	0.367525418	mirna_pc
23022	hsa-mir-19b-2	TGDS	0.392169176	mirna_pc
23023	hsa-mir-19b-2	KIAA1704	0.353634456	mirna_pc
23024	hsa-mir-19b-2	MTIF2	0.340637603	mirna_pc
23025	hsa-mir-19b-2	EWSR1	0.340507103	mirna_pc
23026	hsa-mir-19b-2	ZNF828	0.312233177	mirna_pc
23027	hsa-mir-19b-2	SMC1A	0.312403597	mirna_pc
23028	hsa-mir-19b-2	NUPL1	0.491953185	mirna_pc
23029	hsa-mir-19b-2	ANKRD5	0.303004001	mirna_pc
23030	hsa-mir-19b-2	UPF3A	0.363463339	mirna_pc
23031	hsa-mir-19b-2	MRPL42	0.341778859	mirna_pc
23032	hsa-mir-19b-2	SF3B2	0.317191965	mirna_pc
23033	hsa-mir-19b-2	CCAR1	0.333177179	mirna_pc
23034	hsa-mir-19b-2	UFM1	0.330395493	mirna_pc
23035	hsa-mir-19b-2	CDC16	0.343004336	mirna_pc
23036	hsa-mir-19b-2	ING1	0.312269413	mirna_pc
23037	hsa-mir-19b-2	RNF6	0.310653291	mirna_pc
23038	hsa-mir-19b-2	DHX15	0.334006536	mirna_pc
23039	hsa-mir-19b-2	MAP2K6	0.353556999	mirna_pc
23040	hsa-mir-19b-2	RBM26	0.385399738	mirna_pc
23041	hsa-mir-19b-2	ZNF92	0.331616149	mirna_pc
23042	hsa-mir-19b-2	TMTC4	0.361355942	mirna_pc
23043	hsa-mir-19b-2	AZIN1	0.372207017	mirna_pc
23044	hsa-mir-19b-2	IPO5	0.459706036	mirna_pc
23045	hsa-mir-19b-2	NUP43	0.303826629	mirna_pc
23046	hsa-mir-19b-2	CHD1L	0.302643228	mirna_pc
23047	hsa-mir-19b-2	SNRNP48	0.329259946	mirna_pc
23048	hsa-mir-19b-2	RFXAP	0.301591242	mirna_pc
23049	hsa-mir-19b-2	DCLRE1A	0.35313181	mirna_pc
23050	hsa-mir-19b-2	ANP32A	0.334853885	mirna_pc
23051	hsa-mir-19b-2	DIS3	0.368503555	mirna_pc
23052	hsa-mir-19b-2	E2F5	0.302495575	mirna_pc
23053	hsa-mir-19b-2	NUFIP1	0.40880479	mirna_pc
23054	hsa-mir-19b-2	ZNF195	0.401728527	mirna_pc
23055	hsa-mir-19b-2	BCAS2	0.347703902	mirna_pc
23056	hsa-mir-19b-2	FAM48A	0.356387618	mirna_pc
23057	hsa-mir-19b-2	PMS1	0.322431119	mirna_pc

23058	hsa-mir-19b-2	PNN	0.317548698	mirna_pc
23059	hsa-mir-19b-2	PAN3	0.31721485	mirna_pc
23060	hsa-mir-19b-2	CCDC122	0.353466139	mirna_pc
23061	hsa-mir-19b-2	TRUB1	0.338189658	mirna_pc
23062	hsa-mir-19b-2	ZMYM2	0.328986361	mirna_pc
23063	hsa-mir-19b-2	CCNJ	0.350222335	mirna_pc
23064	hsa-mir-19b-2	ABCC4	0.320891251	mirna_pc
23065	hsa-mir-19b-2	KBTBD6	0.3064278	mirna_pc
23066	hsa-mir-19b-2	ESF1	0.363466746	mirna_pc
23067	hsa-mir-19b-2	CTPS2	0.328743369	mirna_pc
23068	hsa-mir-19b-2	XPO4	0.414289254	mirna_pc
23069	hsa-mir-19b-2	SNCAIP	0.304611638	mirna_pc
23070	hsa-mir-19b-2	IFT88	0.420465576	mirna_pc
23071	hsa-mir-19b-2	QRSL1	0.333759969	mirna_pc
23072	hsa-mir-19b-2	FANCF	0.390307857	mirna_pc
23073	hsa-mir-19b-2	C2orf3	0.314539702	mirna_pc
23074	hsa-mir-19b-2	FAM119A	0.304922257	mirna_pc
23075	hsa-mir-19b-2	SCML2	0.360636171	mirna_pc
23076	hsa-mir-19b-2	MRPL19	0.322112983	mirna_pc
23077	hsa-mir-19b-2	ZCCHC3	0.410861099	mirna_pc
23078	hsa-mir-19b-2	RRP15	0.40010738	mirna_pc
23079	hsa-mir-19b-2	ADAT2	0.324681485	mirna_pc
23080	hsa-mir-19b-2	ETV5	0.324996787	mirna_pc
23081	hsa-mir-19b-2	CBLL1	0.327539165	mirna_pc
23082	hsa-mir-19b-2	GTF3A	0.349167037	mirna_pc
23083	hsa-mir-19b-2	TPP2	0.415063418	mirna_pc
23084	hsa-mir-19b-2	CEP57	0.312591174	mirna_pc
23085	hsa-mir-19b-2	MIR17HG	0.502529453	mirna_pc
23086	hsa-mir-19b-2	ZNF239	0.333368472	mirna_pc
23087	hsa-mir-19b-2	SFRS6	0.334880258	mirna_pc
23088	hsa-mir-19b-2	SLC4A5	0.31594374	mirna_pc
23089	hsa-mir-19b-2	DNMT3A	0.354217176	mirna_pc
23090	hsa-mir-19b-2	PRTFDC1	0.301772433	mirna_pc
23091	hsa-mir-19b-2	ZNF711	0.357528517	mirna_pc
23092	hsa-mir-19b-2	POM121	0.300586183	mirna_pc
23093	hsa-mir-134	CDC20	0.349411089	mirna_pc
23094	hsa-mir-134	PLK4	0.31175138	mirna_pc
23095	hsa-mir-134	BIRC5	0.306311485	mirna_pc
23096	hsa-mir-134	C6orf167	0.314330094	mirna_pc
23097	hsa-mir-134	PRR11	0.324120848	mirna_pc
23098	hsa-mir-134	EPR1	0.304814521	mirna_pc
23099	hsa-mir-134	CENPO	0.343792588	mirna_pc
23100	hsa-mir-134	CDCA4	0.339723407	mirna_pc
23101	hsa-mir-134	MEG3	0.636552934	mirna_pc
23102	hsa-mir-134	PPP3CC	0.305076846	mirna_pc
23103	hsa-mir-134	BMP1	0.494881994	mirna_pc
23104	hsa-mir-134	EIF5A	0.312788161	mirna_pc
23105	hsa-mir-134	FSCN1	0.319867259	mirna_pc
23106	hsa-mir-134	ISG15	0.347886557	mirna_pc
23107	hsa-mir-134	NEDD1	0.312979923	mirna_pc
23108	hsa-mir-134	ROR2	0.300628077	mirna_pc
23109	hsa-mir-134	LEPRE1	0.315301289	mirna_pc
23110	hsa-mir-134	EIF5AL1	0.302447858	mirna_pc
23111	hsa-mir-134	GJB3	0.303432928	mirna_pc

23112	hsa-mir-134	PMAIP1	0.321917855	mirna_pc
23113	hsa-mir-134	MT2A	0.347225383	mirna_pc
23114	hsa-mir-134	SH2D5	0.328299628	mirna_pc
23115	hsa-mir-134	MFAP2	0.387816198	mirna_pc
23116	hsa-mir-134	CD276	0.357302985	mirna_pc
23117	hsa-mir-134	IGF2BP2	0.367517988	mirna_pc
23118	hsa-mir-134	MAP4K4	0.300886423	mirna_pc
23119	hsa-mir-134	PCDHB4	0.304596043	mirna_pc
23120	hsa-mir-134	GBP1	0.323341254	mirna_pc
23121	hsa-mir-134	GJB4	0.336706269	mirna_pc
23122	hsa-mir-134	AK3L1	0.31149819	mirna_pc
23123	hsa-mir-134	TNFAIP3	0.324143954	mirna_pc
23124	hsa-mir-134	IFIT3	0.352800647	mirna_pc
23125	hsa-mir-134	FJX1	0.317739203	mirna_pc
23126	hsa-mir-134	PXDN	0.319699369	mirna_pc
23127	hsa-mir-134	ITGA3	0.301197774	mirna_pc
23128	hsa-mir-134	PLEKHG2	0.304819476	mirna_pc
23129	hsa-mir-134	GPR176	0.3352555	mirna_pc
23130	hsa-mir-134	NOV	0.310581266	mirna_pc
23131	hsa-mir-134	ITGA6	0.387156147	mirna_pc
23132	hsa-mir-134	WARS	0.300742869	mirna_pc
23133	hsa-mir-134	RPL39L	0.305257502	mirna_pc
23134	hsa-mir-134	LEPREL1	0.327709965	mirna_pc
23135	hsa-mir-134	DRAP1	0.327497703	mirna_pc
23136	hsa-mir-134	CHSY3	0.453291325	mirna_pc
23137	hsa-mir-134	FER	0.384008534	mirna_pc
23138	hsa-mir-134	KIAA0922	0.319813146	mirna_pc
23139	hsa-mir-134	PANX1	0.417692	mirna_pc
23140	hsa-mir-134	KCNQ5	0.322801902	mirna_pc
23141	hsa-mir-134	EVC2	0.329192286	mirna_pc
23142	hsa-mir-134	ADAMTSL5	0.390460164	mirna_pc
23143	hsa-mir-134	C5orf13	0.325953555	mirna_pc
23144	hsa-mir-134	TUSC1	0.301719058	mirna_pc
23145	hsa-mir-134	KIAA1949	0.305902037	mirna_pc
23146	hsa-mir-134	IL12RB2	0.379026531	mirna_pc
23147	hsa-mir-134	MOBKL2B	0.314999762	mirna_pc
23148	hsa-mir-134	COL17A1	0.353969148	mirna_pc
23149	hsa-mir-134	RPTOR	0.345571723	mirna_pc
23150	hsa-mir-134	PLS3	0.303482614	mirna_pc
23151	hsa-mir-134	MSN	0.302075474	mirna_pc
23152	hsa-mir-134	LY86	0.711232893	mirna_pc
23153	hsa-mir-134	ZFHX4	0.482324711	mirna_pc
23154	hsa-mir-134	ELK3	0.319481988	mirna_pc
23155	hsa-mir-134	SNAI2	0.413456051	mirna_pc
23156	hsa-mir-134	IRX4	0.357764538	mirna_pc
23157	hsa-mir-134	MARVELD1	0.33090302	mirna_pc
23158	hsa-mir-134	ADM	0.316819626	mirna_pc
23159	hsa-mir-134	PHF23	0.307210003	mirna_pc
23160	hsa-mir-134	EGFLAM	0.332067898	mirna_pc
23161	hsa-mir-134	ESYT1	0.331384277	mirna_pc
23162	hsa-mir-134	FKBP14	0.325805712	mirna_pc
23163	hsa-mir-134	TULP4	0.322279321	mirna_pc
23164	hsa-mir-134	CD38	0.316370942	mirna_pc
23165	hsa-mir-134	GALNT14	0.349491665	mirna_pc

23166	hsa-mir-134	AFAP1L1	0.372497365	mirna_pc
23167	hsa-mir-134	BCAT1	0.390575405	mirna_pc
23168	hsa-mir-134	EXTL3	0.382438184	mirna_pc
23169	hsa-mir-134	KIRREL	0.364414911	mirna_pc
23170	hsa-mir-134	PXN	0.341757195	mirna_pc
23171	hsa-mir-134	ODZ3	0.449508662	mirna_pc
23172	hsa-mir-134	DHRS2	0.494351528	mirna_pc
23173	hsa-mir-134	AGPAT4	0.528827737	mirna_pc
23174	hsa-mir-134	LOC645166	0.525183499	mirna_pc
23175	hsa-mir-134	ODZ2	0.311123968	mirna_pc
23176	hsa-mir-134	MTAP	0.515926574	mirna_pc
23177	hsa-mir-134	MOBKL2A	0.34560815	mirna_pc
23178	hsa-mir-134	STK17A	0.324286525	mirna_pc
23179	hsa-mir-134	VEGFC	0.308564881	mirna_pc
23180	hsa-mir-134	H2AFY2	0.336731029	mirna_pc
23181	hsa-mir-134	PCDHB11	0.397228003	mirna_pc
23182	hsa-mir-134	FSTL3	0.422076335	mirna_pc
23183	hsa-mir-134	TIMM22	0.308794126	mirna_pc
23184	hsa-mir-484	TPX2	0.313328114	mirna_pc
23185	hsa-mir-484	KPNA2	0.386861607	mirna_pc
23186	hsa-mir-484	HOXC9	0.383185877	mirna_pc
23187	hsa-mir-484	RRM2	0.447581937	mirna_pc
23188	hsa-mir-484	CDC25C	0.314404696	mirna_pc
23189	hsa-mir-484	PLK1	0.352873707	mirna_pc
23190	hsa-mir-484	UBE2C	0.404313726	mirna_pc
23191	hsa-mir-484	BUB1	0.305631611	mirna_pc
23192	hsa-mir-484	BUB1B	0.331979961	mirna_pc
23193	hsa-mir-484	KIF18B	0.360637447	mirna_pc
23194	hsa-mir-484	KIF2C	0.316314131	mirna_pc
23195	hsa-mir-484	FANCA	0.366673548	mirna_pc
23196	hsa-mir-484	CDC20	0.341653941	mirna_pc
23197	hsa-mir-484	CLSPN	0.336192323	mirna_pc
23198	hsa-mir-484	CCNA2	0.341566726	mirna_pc
23199	hsa-mir-484	SPC25	0.316964827	mirna_pc
23200	hsa-mir-484	MND1	0.369351275	mirna_pc
23201	hsa-mir-484	KIF22	0.443541014	mirna_pc
23202	hsa-mir-484	DDX39	0.453173923	mirna_pc
23203	hsa-mir-484	NCAPH	0.345036388	mirna_pc
23204	hsa-mir-484	RAD54L	0.315496976	mirna_pc
23205	hsa-mir-484	CENPA	0.404422683	mirna_pc
23206	hsa-mir-484	MAD2L1	0.388218566	mirna_pc
23207	hsa-mir-484	TIMELESS	0.355914136	mirna_pc
23208	hsa-mir-484	LMNB1	0.344860991	mirna_pc
23209	hsa-mir-484	ASF1B	0.346588382	mirna_pc
23210	hsa-mir-484	TROAP	0.432318982	mirna_pc
23211	hsa-mir-484	CCNB1	0.348671393	mirna_pc
23212	hsa-mir-484	STMN1	0.343396357	mirna_pc
23213	hsa-mir-484	CENPM	0.313544584	mirna_pc
23214	hsa-mir-484	TK1	0.36816195	mirna_pc
23215	hsa-mir-484	CDCA2	0.361179521	mirna_pc
23216	hsa-mir-484	DKC1	0.405410284	mirna_pc
23217	hsa-mir-484	TUBB	0.366477269	mirna_pc
23218	hsa-mir-484	KIAA0101	0.459304153	mirna_pc
23219	hsa-mir-484	ORC1L	0.303002328	mirna_pc

23220	hsa-mir-484	KNTC1	0.344318109	mirna_pc
23221	hsa-mir-484	CCNF	0.346249675	mirna_pc
23222	hsa-mir-484	CCNB2	0.326848786	mirna_pc
23223	hsa-mir-484	NUF2	0.360819942	mirna_pc
23224	hsa-mir-484	PLK4	0.394919664	mirna_pc
23225	hsa-mir-484	FANCB	0.337099004	mirna_pc
23226	hsa-mir-484	PTTG1	0.326497339	mirna_pc
23227	hsa-mir-484	SKA3	0.301957021	mirna_pc
23228	hsa-mir-484	HJURP	0.317106771	mirna_pc
23229	hsa-mir-484	BLM	0.303596849	mirna_pc
23230	hsa-mir-484	NME1	0.461217886	mirna_pc
23231	hsa-mir-484	DNMT1	0.355170652	mirna_pc
23232	hsa-mir-484	PAICS	0.354136961	mirna_pc
23233	hsa-mir-484	BIRC5	0.455349142	mirna_pc
23234	hsa-mir-484	RCC1	0.341348757	mirna_pc
23235	hsa-mir-484	OIP5	0.335372808	mirna_pc
23236	hsa-mir-484	EME1	0.364110238	mirna_pc
23237	hsa-mir-484	RECQL4	0.35645676	mirna_pc
23238	hsa-mir-484	CENPK	0.332455558	mirna_pc
23239	hsa-mir-484	AURKB	0.406418027	mirna_pc
23240	hsa-mir-484	CDT1	0.372194527	mirna_pc
23241	hsa-mir-484	MCM10	0.314764069	mirna_pc
23242	hsa-mir-484	KIF20A	0.365550968	mirna_pc
23243	hsa-mir-484	CDCA3	0.421390775	mirna_pc
23244	hsa-mir-484	C1orf112	0.393800314	mirna_pc
23245	hsa-mir-484	ORC6L	0.4212089	mirna_pc
23246	hsa-mir-484	ERH	0.350883271	mirna_pc
23247	hsa-mir-484	C16orf59	0.480941039	mirna_pc
23248	hsa-mir-484	CDKN3	0.312783576	mirna_pc
23249	hsa-mir-484	ADRM1	0.310017809	mirna_pc
23250	hsa-mir-484	NEIL3	0.334096287	mirna_pc
23251	hsa-mir-484	ACTL6A	0.446868893	mirna_pc
23252	hsa-mir-484	POLD1	0.360221521	mirna_pc
23253	hsa-mir-484	C12orf48	0.344800023	mirna_pc
23254	hsa-mir-484	HNRNPL	0.425263322	mirna_pc
23255	hsa-mir-484	SMC4	0.364594892	mirna_pc
23256	hsa-mir-484	PKMYT1	0.37212842	mirna_pc
23257	hsa-mir-484	TTK	0.312440233	mirna_pc
23258	hsa-mir-484	SF3B4	0.3121044	mirna_pc
23259	hsa-mir-484	TUBA1B	0.342331516	mirna_pc
23260	hsa-mir-484	HELLS	0.317783266	mirna_pc
23261	hsa-mir-484	POLE2	0.337156181	mirna_pc
23262	hsa-mir-484	UBE2S	0.381134791	mirna_pc
23263	hsa-mir-484	C11orf82	0.442300691	mirna_pc
23264	hsa-mir-484	HNRNPC	0.432567328	mirna_pc
23265	hsa-mir-484	PPIA	0.320435684	mirna_pc
23266	hsa-mir-484	C6orf167	0.309170591	mirna_pc
23267	hsa-mir-484	CHEK1	0.336414216	mirna_pc
23268	hsa-mir-484	HNRNPA2B1	0.336974866	mirna_pc
23269	hsa-mir-484	RAN	0.328609309	mirna_pc
23270	hsa-mir-484	TYMS	0.375936546	mirna_pc
23271	hsa-mir-484	C20orf20	0.376502865	mirna_pc
23272	hsa-mir-484	MCM6	0.305347669	mirna_pc
23273	hsa-mir-484	RNASEH2A	0.420083184	mirna_pc

23274	hsa-mir-484	PIF1	0.350048639	mirna_pc
23275	hsa-mir-484	H2AFX	0.318255915	mirna_pc
23276	hsa-mir-484	SFRS2	0.472859338	mirna_pc
23277	hsa-mir-484	UCK2	0.341124924	mirna_pc
23278	hsa-mir-484	NFKBIL2	0.389988753	mirna_pc
23279	hsa-mir-484	C15orf42	0.329507336	mirna_pc
23280	hsa-mir-484	CENPN	0.307958033	mirna_pc
23281	hsa-mir-484	SNRPG	0.438899178	mirna_pc
23282	hsa-mir-484	EIF4A3	0.414396528	mirna_pc
23283	hsa-mir-484	SNRPA	0.509353378	mirna_pc
23284	hsa-mir-484	FUS	0.453615059	mirna_pc
23285	hsa-mir-484	C1orf135	0.313160243	mirna_pc
23286	hsa-mir-484	U2AF2	0.344295549	mirna_pc
23287	hsa-mir-484	CHAF1A	0.435829383	mirna_pc
23288	hsa-mir-484	GINS4	0.487228191	mirna_pc
23289	hsa-mir-484	ANP32E	0.346471867	mirna_pc
23290	hsa-mir-484	CENPO	0.31461926	mirna_pc
23291	hsa-mir-484	C19orf48	0.317649056	mirna_pc
23292	hsa-mir-484	GEN1	0.406184708	mirna_pc
23293	hsa-mir-484	NUDT1	0.345401366	mirna_pc
23294	hsa-mir-484	HMGB2	0.386459589	mirna_pc
23295	hsa-mir-484	GINS2	0.351017566	mirna_pc
23296	hsa-mir-484	DNA2	0.324215443	mirna_pc
23297	hsa-mir-484	NOP56	0.341479835	mirna_pc
23298	hsa-mir-484	SNHG1	0.344703035	mirna_pc
23299	hsa-mir-484	DTYMK	0.325957373	mirna_pc
23300	hsa-mir-484	PRMT1	0.448612158	mirna_pc
23301	hsa-mir-484	RAVER1	0.322313608	mirna_pc
23302	hsa-mir-484	HSPE1	0.306016421	mirna_pc
23303	hsa-mir-484	C16orf75	0.358747791	mirna_pc
23304	hsa-mir-484	C18orf56	0.358886563	mirna_pc
23305	hsa-mir-484	NOP2	0.384711258	mirna_pc
23306	hsa-mir-484	HIST1H2AE	0.438103646	mirna_pc
23307	hsa-mir-484	CDK2	0.318311093	mirna_pc
23308	hsa-mir-484	CDK4	0.317981837	mirna_pc
23309	hsa-mir-484	PFDN2	0.422752515	mirna_pc
23310	hsa-mir-484	BCL2L12	0.383886133	mirna_pc
23311	hsa-mir-484	CHTF18	0.477206077	mirna_pc
23312	hsa-mir-484	HNRNPR	0.369215147	mirna_pc
23313	hsa-mir-484	DLEU2	0.459355208	mirna_pc
23314	hsa-mir-484	PRIM1	0.329012871	mirna_pc
23315	hsa-mir-484	KAT2A	0.436556329	mirna_pc
23316	hsa-mir-484	PSMC4	0.361954297	mirna_pc
23317	hsa-mir-484	TCOF1	0.391458909	mirna_pc
23318	hsa-mir-484	CHAF1B	0.310825109	mirna_pc
23319	hsa-mir-484	ACYP1	0.314605897	mirna_pc
23320	hsa-mir-484	DHX34	0.321380304	mirna_pc
23321	hsa-mir-484	HOXC10	0.31275387	mirna_pc
23322	hsa-mir-484	G3BP1	0.309165691	mirna_pc
23323	hsa-mir-484	NOP58	0.395886666	mirna_pc
23324	hsa-mir-484	DBF4	0.474700095	mirna_pc
23325	hsa-mir-484	TOMM40	0.30658742	mirna_pc
23326	hsa-mir-484	SF3B3	0.301421087	mirna_pc
23327	hsa-mir-484	CDC7	0.366583075	mirna_pc

23328	hsa-mir-484	NUP85	0.320258062	mirna_pc
23329	hsa-mir-484	YDJC	0.3196895	mirna_pc
23330	hsa-mir-484	PUS1	0.340156937	mirna_pc
23331	hsa-mir-484	NOL11	0.33518572	mirna_pc
23332	hsa-mir-484	SNRPC	0.345830528	mirna_pc
23333	hsa-mir-484	ARL6IP6	0.306919246	mirna_pc
23334	hsa-mir-484	HAUS8	0.343039728	mirna_pc
23335	hsa-mir-484	RFC4	0.348357257	mirna_pc
23336	hsa-mir-484	FBXO5	0.378696974	mirna_pc
23337	hsa-mir-484	FIGNL1	0.409077395	mirna_pc
23338	hsa-mir-484	E2F3	0.32193125	mirna_pc
23339	hsa-mir-484	SF3B14	0.309988887	mirna_pc
23340	hsa-mir-484	RUVBL2	0.309084622	mirna_pc
23341	hsa-mir-484	CDC25A	0.32748441	mirna_pc
23342	hsa-mir-484	RAD54B	0.348746612	mirna_pc
23343	hsa-mir-484	H2AFZ	0.327695727	mirna_pc
23344	hsa-mir-484	MAGOH	0.3928097	mirna_pc
23345	hsa-mir-484	DDX12	0.347290413	mirna_pc
23346	hsa-mir-484	NUP37	0.345644289	mirna_pc
23347	hsa-mir-484	CPSF3	0.436293831	mirna_pc
23348	hsa-mir-484	BANF1	0.410826596	mirna_pc
23349	hsa-mir-484	C21orf45	0.509057448	mirna_pc
23350	hsa-mir-484	PPM1G	0.384865665	mirna_pc
23351	hsa-mir-484	DNAJB11	0.383707217	mirna_pc
23352	hsa-mir-484	SNRPD2	0.353915525	mirna_pc
23353	hsa-mir-484	TFAP4	0.407992251	mirna_pc
23354	hsa-mir-484	NUDT5	0.367296526	mirna_pc
23355	hsa-mir-484	RNPS1	0.421767567	mirna_pc
23356	hsa-mir-484	DAZAP1	0.378536878	mirna_pc
23357	hsa-mir-484	BOLA2	0.340505506	mirna_pc
23358	hsa-mir-484	CBX8	0.359572103	mirna_pc
23359	hsa-mir-484	NCL	0.33993271	mirna_pc
23360	hsa-mir-484	CALR	0.325155838	mirna_pc
23361	hsa-mir-484	PFDN4	0.322962541	mirna_pc
23362	hsa-mir-484	PAFAH1B3	0.450779474	mirna_pc
23363	hsa-mir-484	SRRT	0.437072484	mirna_pc
23364	hsa-mir-484	CACYBP	0.344493222	mirna_pc
23365	hsa-mir-484	SASS6	0.303169776	mirna_pc
23366	hsa-mir-484	DGUOK	0.462327144	mirna_pc
23367	hsa-mir-484	MRPL47	0.391382208	mirna_pc
23368	hsa-mir-484	DBF4B	0.302806212	mirna_pc
23369	hsa-mir-484	FBL	0.399006699	mirna_pc
23370	hsa-mir-484	EXOSC5	0.381894595	mirna_pc
23371	hsa-mir-484	HNRNPM	0.509391557	mirna_pc
23372	hsa-mir-484	TMPO	0.302500192	mirna_pc
23373	hsa-mir-484	CCT6A	0.37010262	mirna_pc
23374	hsa-mir-484	POLR2H	0.356826867	mirna_pc
23375	hsa-mir-484	LOC388796	0.327240718	mirna_pc
23376	hsa-mir-484	VBP1	0.325167731	mirna_pc
23377	hsa-mir-484	C3orf26	0.327712633	mirna_pc
23378	hsa-mir-484	MRTO4	0.366020043	mirna_pc
23379	hsa-mir-484	SHFM1	0.465845242	mirna_pc
23380	hsa-mir-484	GPR19	0.324711679	mirna_pc
23381	hsa-mir-484	LSM 7.00	0.327841579	mirna_pc

23382	hsa-mir-484	NUP93	0.306675838	mirna_pc
23383	hsa-mir-484	PPIH	0.415079344	mirna_pc
23384	hsa-mir-484	THOC6	0.414677841	mirna_pc
23385	hsa-mir-484	CCDC138	0.323960517	mirna_pc
23386	hsa-mir-484	LOC100128191	0.393084996	mirna_pc
23387	hsa-mir-484	SMYD5	0.431164618	mirna_pc
23388	hsa-mir-484	SF3A2	0.396397995	mirna_pc
23389	hsa-mir-484	TSSC1	0.363547855	mirna_pc
23390	hsa-mir-484	SNRPF	0.451920696	mirna_pc
23391	hsa-mir-484	SUMO2	0.471631648	mirna_pc
23392	hsa-mir-484	SFRS1	0.472030738	mirna_pc
23393	hsa-mir-484	WDR43	0.342910758	mirna_pc
23394	hsa-mir-484	SNRPA1	0.425463488	mirna_pc
23395	hsa-mir-484	SMC6	0.415912267	mirna_pc
23396	hsa-mir-484	UBE2I	0.4148299	mirna_pc
23397	hsa-mir-484	TIPIN	0.427174855	mirna_pc
23398	hsa-mir-484	ATXN2L	0.334893454	mirna_pc
23399	hsa-mir-484	HSPBP1	0.424834082	mirna_pc
23400	hsa-mir-484	UTP14A	0.308315926	mirna_pc
23401	hsa-mir-484	ECE2	0.329717806	mirna_pc
23402	hsa-mir-484	MAZ	0.399982294	mirna_pc
23403	hsa-mir-484	POLR2D	0.314126652	mirna_pc
23404	hsa-mir-484	PA2G4	0.385051303	mirna_pc
23405	hsa-mir-484	SNRPD3	0.399731175	mirna_pc
23406	hsa-mir-484	KHDRBS1	0.38605164	mirna_pc
23407	hsa-mir-484	SLC5A6	0.317536794	mirna_pc
23408	hsa-mir-484	B3GNT5	0.350479417	mirna_pc
23409	hsa-mir-484	CCDC18	0.338484236	mirna_pc
23410	hsa-mir-484	DNAJC2	0.336208955	mirna_pc
23411	hsa-mir-484	ZNF695	0.305217815	mirna_pc
23412	hsa-mir-484	METTL1	0.398660077	mirna_pc
23413	hsa-mir-484	NSUN5	0.369647153	mirna_pc
23414	hsa-mir-484	GNL2	0.329037791	mirna_pc
23415	hsa-mir-484	KRI1	0.420225833	mirna_pc
23416	hsa-mir-484	NOC2L	0.327303027	mirna_pc
23417	hsa-mir-484	NASP	0.380139747	mirna_pc
23418	hsa-mir-484	RPP40	0.381872916	mirna_pc
23419	hsa-mir-484	LAGE3	0.33037289	mirna_pc
23420	hsa-mir-484	POLR1B	0.314106225	mirna_pc
23421	hsa-mir-484	OBFC2B	0.350635442	mirna_pc
23422	hsa-mir-484	PTMA	0.493080958	mirna_pc
23423	hsa-mir-484	LSM 4.00	0.317504245	mirna_pc
23424	hsa-mir-484	PSMB1	0.307468945	mirna_pc
23425	hsa-mir-484	TBCE	0.302468807	mirna_pc
23426	hsa-mir-484	FBXO22OS	0.331622861	mirna_pc
23427	hsa-mir-484	SUV39H2	0.349085643	mirna_pc
23428	hsa-mir-484	NKRF	0.300162504	mirna_pc
23429	hsa-mir-484	NAALADL2	0.31410659	mirna_pc
23430	hsa-mir-484	TMEM177	0.30642022	mirna_pc
23431	hsa-mir-484	HNRNPD	0.424982445	mirna_pc
23432	hsa-mir-484	DPY30	0.416029795	mirna_pc
23433	hsa-mir-484	MTHFD2	0.310760896	mirna_pc
23434	hsa-mir-484	SFRS3	0.448159406	mirna_pc
23435	hsa-mir-484	CCT4	0.332883169	mirna_pc

23436	hsa-mir-484	RAD9A	0.336931221	mirna_pc
23437	hsa-mir-484	ELAVL1	0.374652235	mirna_pc
23438	hsa-mir-484	ABCE1	0.351036476	mirna_pc
23439	hsa-mir-484	NCAPD3	0.424975533	mirna_pc
23440	hsa-mir-484	HSP90B1	0.358954951	mirna_pc
23441	hsa-mir-484	TRMT1	0.358522403	mirna_pc
23442	hsa-mir-484	TBC1D7	0.380336937	mirna_pc
23443	hsa-mir-484	PAK1IP1	0.377046513	mirna_pc
23444	hsa-mir-484	PFDN6	0.351970921	mirna_pc
23445	hsa-mir-484	ATP13A3	0.316600924	mirna_pc
23446	hsa-mir-484	YWHAE	0.335722375	mirna_pc
23447	hsa-mir-484	C3orf57	0.316155254	mirna_pc
23448	hsa-mir-484	ILF3	0.371687587	mirna_pc
23449	hsa-mir-484	HNRNPAB	0.343415457	mirna_pc
23450	hsa-mir-484	MAGOHB	0.383971094	mirna_pc
23451	hsa-mir-484	PASK	0.310334448	mirna_pc
23452	hsa-mir-484	IL8	0.346251871	mirna_pc
23453	hsa-mir-484	KHSRP	0.394730968	mirna_pc
23454	hsa-mir-484	LSM 6.00	0.464277672	mirna_pc
23455	hsa-mir-484	MRPS17	0.390867541	mirna_pc
23456	hsa-mir-484	GLO1	0.305228357	mirna_pc
23457	hsa-mir-484	WDR74	0.332864165	mirna_pc
23458	hsa-mir-484	TRA2B	0.370962727	mirna_pc
23459	hsa-mir-484	GAR1	0.381670898	mirna_pc
23460	hsa-mir-484	C15orf23	0.338321958	mirna_pc
23461	hsa-mir-484	WBSCR22	0.321759143	mirna_pc
23462	hsa-mir-484	RPL36A	0.354103307	mirna_pc
23463	hsa-mir-484	PSMD12	0.38551333	mirna_pc
23464	hsa-mir-484	NOC4L	0.310365468	mirna_pc
23465	hsa-mir-484	ZNF668	0.378940294	mirna_pc
23466	hsa-mir-484	MRPL17	0.311296694	mirna_pc
23467	hsa-mir-484	ITGB3BP	0.352462681	mirna_pc
23468	hsa-mir-484	ENOPH1	0.370329569	mirna_pc
23469	hsa-mir-484	C19orf47	0.385117589	mirna_pc
23470	hsa-mir-484	TSEN54	0.321708622	mirna_pc
23471	hsa-mir-484	C4orf43	0.338094944	mirna_pc
23472	hsa-mir-484	TGIF1	0.305076681	mirna_pc
23473	hsa-mir-484	DDX55	0.336094979	mirna_pc
23474	hsa-mir-484	CXCL1	0.382062028	mirna_pc
23475	hsa-mir-484	PLEKHG4	0.326606477	mirna_pc
23476	hsa-mir-484	TMEM223	0.305534619	mirna_pc
23477	hsa-mir-484	SFPQ	0.378798753	mirna_pc
23478	hsa-mir-484	AP3M2	0.306698067	mirna_pc
23479	hsa-mir-484	CNOT3	0.334739143	mirna_pc
23480	hsa-mir-484	VPS72	0.313032442	mirna_pc
23481	hsa-mir-484	EIF4EBP1	0.435042062	mirna_pc
23482	hsa-mir-484	HDAC2	0.37319699	mirna_pc
23483	hsa-mir-484	GMNN	0.322098477	mirna_pc
23484	hsa-mir-484	EMG1	0.313931984	mirna_pc
23485	hsa-mir-484	PA2G4P4	0.338898093	mirna_pc
23486	hsa-mir-484	WDR53	0.319880513	mirna_pc
23487	hsa-mir-484	PDAP1	0.530234361	mirna_pc
23488	hsa-mir-484	RNF7	0.432739492	mirna_pc
23489	hsa-mir-484	GEMIN6	0.472917554	mirna_pc

23490	hsa-mir-484	RPL26L1	0.311048633	mirna_pc
23491	hsa-mir-484	H3F3B	0.301130941	mirna_pc
23492	hsa-mir-484	MRPL11	0.31894802	mirna_pc
23493	hsa-mir-484	FAM86C	0.307282413	mirna_pc
23494	hsa-mir-484	PTCD1	0.497343572	mirna_pc
23495	hsa-mir-484	PCGF1	0.320812475	mirna_pc
23496	hsa-mir-484	HPRT1	0.341538826	mirna_pc
23497	hsa-mir-484	CRIPT	0.315001332	mirna_pc
23498	hsa-mir-484	MSH6	0.304790742	mirna_pc
23499	hsa-mir-484	SOX4	0.303390529	mirna_pc
23500	hsa-mir-484	C3orf34	0.320357378	mirna_pc
23501	hsa-mir-484	TPRKB	0.440203188	mirna_pc
23502	hsa-mir-484	IRF3	0.308282389	mirna_pc
23503	hsa-mir-484	MRPL18	0.418818384	mirna_pc
23504	hsa-mir-484	POLB	0.434610154	mirna_pc
23505	hsa-mir-484	HNRNPA1	0.38957495	mirna_pc
23506	hsa-mir-484	NPM1	0.378803847	mirna_pc
23507	hsa-mir-484	CDK2AP1	0.32991219	mirna_pc
23508	hsa-mir-484	NUDT3	0.371411251	mirna_pc
23509	hsa-mir-484	MOGS	0.34662573	mirna_pc
23510	hsa-mir-484	ATP5J2	0.512921865	mirna_pc
23511	hsa-mir-484	TNFAIP3	0.330112797	mirna_pc
23512	hsa-mir-484	COIL	0.347944991	mirna_pc
23513	hsa-mir-484	PPAN	0.364993538	mirna_pc
23514	hsa-mir-484	RFC2	0.337845928	mirna_pc
23515	hsa-mir-484	BUD31	0.490212829	mirna_pc
23516	hsa-mir-484	KARS	0.302798732	mirna_pc
23517	hsa-mir-484	C16orf88	0.591689069	mirna_pc
23518	hsa-mir-484	FAM58A	0.300120704	mirna_pc
23519	hsa-mir-484	HNRNPA1L2	0.389909795	mirna_pc
23520	hsa-mir-484	NAA15	0.326528536	mirna_pc
23521	hsa-mir-484	POLR3K	0.36953262	mirna_pc
23522	hsa-mir-484	LOC642846	0.317870457	mirna_pc
23523	hsa-mir-484	PRPF31	0.317269018	mirna_pc
23524	hsa-mir-484	FARSB	0.327717216	mirna_pc
23525	hsa-mir-484	THUMPD2	0.381060294	mirna_pc
23526	hsa-mir-484	TARBP2	0.307288011	mirna_pc
23527	hsa-mir-484	FGFR1OP	0.301740367	mirna_pc
23528	hsa-mir-484	CCDC142	0.347055609	mirna_pc
23529	hsa-mir-484	SNHG9	0.394597158	mirna_pc
23530	hsa-mir-484	TCP1	0.344177868	mirna_pc
23531	hsa-mir-484	C16orf53	0.448160401	mirna_pc
23532	hsa-mir-484	NOL10	0.47149982	mirna_pc
23533	hsa-mir-484	CTU1	0.362937506	mirna_pc
23534	hsa-mir-484	POLE	0.381238942	mirna_pc
23535	hsa-mir-484	MUTYH	0.339054967	mirna_pc
23536	hsa-mir-484	AUP1	0.340919814	mirna_pc
23537	hsa-mir-484	WRNIP1	0.346936296	mirna_pc
23538	hsa-mir-484	TAF1B	0.380549749	mirna_pc
23539	hsa-mir-484	TIMM50	0.402240581	mirna_pc
23540	hsa-mir-484	SNORD1C	0.361940647	mirna_pc
23541	hsa-mir-484	XRCC6BP1	0.327874506	mirna_pc
23542	hsa-mir-484	RPP21	0.325962531	mirna_pc
23543	hsa-mir-484	VRK1	0.355459731	mirna_pc

23544	hsa-mir-484	RBMX2	0.328205712	mirna_pc
23545	hsa-mir-484	DDOST	0.304927458	mirna_pc
23546	hsa-mir-484	EXOSC9	0.412445926	mirna_pc
23547	hsa-mir-484	TMEM60	0.311390227	mirna_pc
23548	hsa-mir-484	MXD3	0.318572557	mirna_pc
23549	hsa-mir-484	MPHOSPH6	0.305205597	mirna_pc
23550	hsa-mir-484	ZNF598	0.335804097	mirna_pc
23551	hsa-mir-484	RBMX	0.447105048	mirna_pc
23552	hsa-mir-484	C19orf53	0.330761431	mirna_pc
23553	hsa-mir-484	TELO2	0.393190314	mirna_pc
23554	hsa-mir-484	TMCO6	0.360502164	mirna_pc
23555	hsa-mir-484	ALKBH2	0.384843313	mirna_pc
23556	hsa-mir-484	NTHL1	0.355600189	mirna_pc
23557	hsa-mir-484	POP5	0.358126446	mirna_pc
23558	hsa-mir-484	NETO2	0.301671916	mirna_pc
23559	hsa-mir-484	CCDC90A	0.301118398	mirna_pc
23560	hsa-mir-484	PRKCSH	0.379001953	mirna_pc
23561	hsa-mir-484	MLF 2.00	0.315691528	mirna_pc
23562	hsa-mir-484	ATAD3B	0.30769711	mirna_pc
23563	hsa-mir-484	RPF2	0.352012436	mirna_pc
23564	hsa-mir-484	NHLRC1	0.331927352	mirna_pc
23565	hsa-mir-484	CCDC24	0.310154115	mirna_pc
23566	hsa-mir-484	PDIA6	0.390444221	mirna_pc
23567	hsa-mir-484	GPN3	0.322298163	mirna_pc
23568	hsa-mir-484	MEMO1	0.47172994	mirna_pc
23569	hsa-mir-484	LOC440905	0.380762764	mirna_pc
23570	hsa-mir-484	TRAP1	0.375154626	mirna_pc
23571	hsa-mir-484	SCLT1	0.341847409	mirna_pc
23572	hsa-mir-484	LTV1	0.359815336	mirna_pc
23573	hsa-mir-484	OSTC	0.41291979	mirna_pc
23574	hsa-mir-484	CCT8	0.34561056	mirna_pc
23575	hsa-mir-484	TTC32	0.403992948	mirna_pc
23576	hsa-mir-484	SMN2	0.40495404	mirna_pc
23577	hsa-mir-484	U2AF1	0.44804921	mirna_pc
23578	hsa-mir-484	FAM92A1	0.333315519	mirna_pc
23579	hsa-mir-484	RPL38	0.306400368	mirna_pc
23580	hsa-mir-484	MSTO1	0.329729159	mirna_pc
23581	hsa-mir-484	RBM17	0.324061895	mirna_pc
23582	hsa-mir-484	ANAPC10	0.354693685	mirna_pc
23583	hsa-mir-484	ABT1	0.424141574	mirna_pc
23584	hsa-mir-484	FAM86A	0.412313535	mirna_pc
23585	hsa-mir-484	MPHOSPH10	0.378217474	mirna_pc
23586	hsa-mir-484	AKAP9	0.443542905	mirna_pc
23587	hsa-mir-484	RPS7	0.317128563	mirna_pc
23588	hsa-mir-484	MKRN3	0.318560862	mirna_pc
23589	hsa-mir-484	PABPN1	0.39010198	mirna_pc
23590	hsa-mir-484	C14orf169	0.331929573	mirna_pc
23591	hsa-mir-484	METAP2	0.33758152	mirna_pc
23592	hsa-mir-484	TIMM16	0.432520852	mirna_pc
23593	hsa-mir-484	MRPS28	0.304704345	mirna_pc
23594	hsa-mir-484	NUDCD2	0.34071586	mirna_pc
23595	hsa-mir-484	NAT9	0.42043242	mirna_pc
23596	hsa-mir-484	MRPL21	0.373886063	mirna_pc
23597	hsa-mir-484	ANKS3	0.328826094	mirna_pc

23598	hsa-mir-484	BTG3	0.346319616	mirna_pc
23599	hsa-mir-484	LOC645332	0.387133079	mirna_pc
23600	hsa-mir-484	PRSS21	0.348796604	mirna_pc
23601	hsa-mir-484	MGC72080	0.514082222	mirna_pc
23602	hsa-mir-484	MCF2L2	0.332558001	mirna_pc
23603	hsa-mir-484	TRRAP	0.373147318	mirna_pc
23604	hsa-mir-484	TRMT61B	0.325748213	mirna_pc
23605	hsa-mir-484	TSFM	0.31221571	mirna_pc
23606	hsa-mir-484	QTRT1	0.339715707	mirna_pc
23607	hsa-mir-484	SLC25A13	0.437572214	mirna_pc
23608	hsa-mir-484	DDX20	0.355660758	mirna_pc
23609	hsa-mir-484	KRT15	0.349685762	mirna_pc
23610	hsa-mir-484	DCUN1D1	0.301535931	mirna_pc
23611	hsa-mir-484	LUC7L	0.31203074	mirna_pc
23612	hsa-mir-484	UBE2N	0.338818408	mirna_pc
23613	hsa-mir-484	CHCHD2	0.328920557	mirna_pc
23614	hsa-mir-484	BAZ1B	0.306396316	mirna_pc
23615	hsa-mir-484	ZNF593	0.301705948	mirna_pc
23616	hsa-mir-484	HTRA2	0.309059365	mirna_pc
23617	hsa-mir-484	CHAC2	0.319532476	mirna_pc
23618	hsa-mir-484	ACN9	0.457386606	mirna_pc
23619	hsa-mir-484	TCERG1	0.346639956	mirna_pc
23620	hsa-mir-484	C14orf156	0.334636303	mirna_pc
23621	hsa-mir-484	INTU	0.380028988	mirna_pc
23622	hsa-mir-484	NHP2L1	0.347288825	mirna_pc
23623	hsa-mir-484	MAPKAPK5	0.307417316	mirna_pc
23624	hsa-mir-484	E2F6	0.327479263	mirna_pc
23625	hsa-mir-484	SSSCA1	0.319994376	mirna_pc
23626	hsa-mir-484	NOL7	0.372483623	mirna_pc
23627	hsa-mir-484	PRPSAP2	0.356009978	mirna_pc
23628	hsa-mir-484	CPSF4	0.526072126	mirna_pc
23629	hsa-mir-484	SFRS13A	0.365622574	mirna_pc
23630	hsa-mir-484	GADD45GIP1	0.359919285	mirna_pc
23631	hsa-mir-484	C16orf91	0.302004118	mirna_pc
23632	hsa-mir-484	NT5C	0.374276413	mirna_pc
23633	hsa-mir-484	PTMS	0.326809054	mirna_pc
23634	hsa-mir-484	CTU2	0.350800842	mirna_pc
23635	hsa-mir-484	CCDC59	0.355101029	mirna_pc
23636	hsa-mir-484	SLC4A1AP	0.338822952	mirna_pc
23637	hsa-mir-484	TTC27	0.389124898	mirna_pc
23638	hsa-mir-484	DHRS13	0.321162761	mirna_pc
23639	hsa-mir-484	MCCC1	0.338368543	mirna_pc
23640	hsa-mir-484	MTIF2	0.369238674	mirna_pc
23641	hsa-mir-484	CDK2AP2	0.347809988	mirna_pc
23642	hsa-mir-484	KRTCAP3	0.320511493	mirna_pc
23643	hsa-mir-484	MRPS34	0.384752054	mirna_pc
23644	hsa-mir-484	LOC222699	0.395146081	mirna_pc
23645	hsa-mir-484	KCNMB3	0.449820916	mirna_pc
23646	hsa-mir-484	HIST1H1C	0.426288873	mirna_pc
23647	hsa-mir-484	AAAS	0.427547933	mirna_pc
23648	hsa-mir-484	C11orf24	0.386991468	mirna_pc
23649	hsa-mir-484	C2orf79	0.323860737	mirna_pc
23650	hsa-mir-484	SUPT7L	0.341525178	mirna_pc
23651	hsa-mir-484	PEMT	0.340094668	mirna_pc

23652	hsa-mir-484	SETD1A	0.301020223	mirna_pc
23653	hsa-mir-484	METTL5	0.36011348	mirna_pc
23654	hsa-mir-484	ZNF277	0.372698139	mirna_pc
23655	hsa-mir-484	PELP1	0.340059019	mirna_pc
23656	hsa-mir-484	PGP	0.3929092	mirna_pc
23657	hsa-mir-484	THOC1	0.365221037	mirna_pc
23658	hsa-mir-484	C11orf45	0.333383439	mirna_pc
23659	hsa-mir-484	CCDC15	0.332990487	mirna_pc
23660	hsa-mir-484	GCDH	0.347251827	mirna_pc
23661	hsa-mir-484	SFRS7	0.50280845	mirna_pc
23662	hsa-mir-484	SLC25A19	0.304015129	mirna_pc
23663	hsa-mir-484	MYL12B	0.345637514	mirna_pc
23664	hsa-mir-484	IFRD1	0.352121128	mirna_pc
23665	hsa-mir-484	PHKG2	0.319013644	mirna_pc
23666	hsa-mir-484	SARS2	0.38057809	mirna_pc
23667	hsa-mir-484	COQ3	0.331989609	mirna_pc
23668	hsa-mir-484	PDCD2	0.375432209	mirna_pc
23669	hsa-mir-484	CORO1B	0.309851259	mirna_pc
23670	hsa-mir-484	ANKIB1	0.350126628	mirna_pc
23671	hsa-mir-484	RPUSD2	0.317152541	mirna_pc
23672	hsa-mir-484	THYN1	0.423920287	mirna_pc
23673	hsa-mir-484	MRPL28	0.356492279	mirna_pc
23674	hsa-mir-484	C2orf68	0.314271663	mirna_pc
23675	hsa-mir-484	NDUFC1	0.322593585	mirna_pc
23676	hsa-mir-484	ARPC1A	0.409260343	mirna_pc
23677	hsa-mir-484	FLJ35776	0.311137355	mirna_pc
23678	hsa-mir-484	C17orf75	0.363812897	mirna_pc
23679	hsa-mir-484	TMEM93	0.310194052	mirna_pc
23680	hsa-mir-484	INO80E	0.360638314	mirna_pc
23681	hsa-mir-484	NPRL3	0.358331096	mirna_pc
23682	hsa-mir-484	MYST1	0.307395732	mirna_pc
23683	hsa-mir-484	ZNF789	0.387315312	mirna_pc
23684	hsa-mir-484	ZNF771	0.310448086	mirna_pc
23685	hsa-mir-484	EEF1E1	0.42034915	mirna_pc
23686	hsa-mir-484	PGRMC1	0.329736456	mirna_pc
23687	hsa-mir-484	MRPL38	0.396696044	mirna_pc
23688	hsa-mir-484	YBX1	0.320333662	mirna_pc
23689	hsa-mir-484	ATPBD4	0.35106239	mirna_pc
23690	hsa-mir-484	ZKSCAN5	0.440586521	mirna_pc
23691	hsa-mir-484	POLR2C	0.350935137	mirna_pc
23692	hsa-mir-484	C12orf10	0.37700259	mirna_pc
23693	hsa-mir-484	FAM100A	0.331214441	mirna_pc
23694	hsa-mir-484	CLEC2B	0.321239798	mirna_pc
23695	hsa-mir-484	C12orf45	0.401382564	mirna_pc
23696	hsa-mir-484	LOC147727	0.316232081	mirna_pc
23697	hsa-mir-484	RING1	0.342004112	mirna_pc
23698	hsa-mir-484	SUMF2	0.306177914	mirna_pc
23699	hsa-mir-484	C22orf27	0.306775148	mirna_pc
23700	hsa-mir-484	PNKP	0.329947973	mirna_pc
23701	hsa-mir-484	C16orf13	0.407975972	mirna_pc
23702	hsa-mir-484	RSL1D1	0.315132242	mirna_pc
23703	hsa-mir-484	GBAS	0.301879236	mirna_pc
23704	hsa-mir-484	AIP	0.304838633	mirna_pc
23705	hsa-mir-484	BOLA3	0.489982392	mirna_pc

23706	hsa-mir-484	C19orf23	0.364026073	mirna_pc
23707	hsa-mir-484	MYL6B	0.376738874	mirna_pc
23708	hsa-mir-484	POLR2F	0.317211802	mirna_pc
23709	hsa-mir-484	MYL12A	0.308978993	mirna_pc
23710	hsa-mir-484	CAMTA1	0.318423928	mirna_pc
23711	hsa-mir-484	MRPS15	0.357739638	mirna_pc
23712	hsa-mir-484	C17orf58	0.327121771	mirna_pc
23713	hsa-mir-484	BET1	0.38801493	mirna_pc
23714	hsa-mir-484	NARS2	0.382806626	mirna_pc
23715	hsa-mir-484	LYRM4	0.413832935	mirna_pc
23716	hsa-mir-484	FAM133B	0.483110691	mirna_pc
23717	hsa-mir-484	BCL11A	0.317888677	mirna_pc
23718	hsa-mir-484	ANP32A	0.311305765	mirna_pc
23719	hsa-mir-484	RBM25	0.306165463	mirna_pc
23720	hsa-mir-484	CFDP1	0.341481103	mirna_pc
23721	hsa-mir-484	C17orf81	0.331924201	mirna_pc
23722	hsa-mir-484	HYLS1	0.356227191	mirna_pc
23723	hsa-mir-484	WTAP	0.319958547	mirna_pc
23724	hsa-mir-484	SNF8	0.390623962	mirna_pc
23725	hsa-mir-484	C1orf156	0.336565094	mirna_pc
23726	hsa-mir-484	LOC100132287	0.311720907	mirna_pc
23727	hsa-mir-484	HNRNPA0	0.31244309	mirna_pc
23728	hsa-mir-484	ZNF300	0.321695906	mirna_pc
23729	hsa-mir-484	MORN2	0.393017104	mirna_pc
23730	hsa-mir-484	DERL2	0.302779021	mirna_pc
23731	hsa-mir-484	C6orf125	0.332068809	mirna_pc
23732	hsa-mir-484	MESDC2	0.322804076	mirna_pc
23733	hsa-mir-484	MTERF	0.515980831	mirna_pc
23734	hsa-mir-484	ATP5H	0.311613018	mirna_pc
23735	hsa-mir-484	C7orf64	0.38781223	mirna_pc
23736	hsa-mir-484	NDUFA12	0.303726502	mirna_pc
23737	hsa-mir-484	DRG2	0.310168752	mirna_pc
23738	hsa-mir-484	SLC25A33	0.365785605	mirna_pc
23739	hsa-mir-484	CXCL2	0.328137122	mirna_pc
23740	hsa-mir-484	OST4	0.340074027	mirna_pc
23741	hsa-mir-484	BCS1L	0.376987517	mirna_pc
23742	hsa-mir-484	ZNF394	0.411564805	mirna_pc
23743	hsa-mir-484	DDX51	0.348445788	mirna_pc
23744	hsa-mir-484	WDR83	0.382781315	mirna_pc
23745	hsa-mir-484	CCDC101	0.364590142	mirna_pc
23746	hsa-mir-484	NUBP2	0.332280743	mirna_pc
23747	hsa-mir-484	ENO3	0.349741824	mirna_pc
23748	hsa-mir-484	CCDC90B	0.374125354	mirna_pc
23749	hsa-mir-484	CCL2	0.329188377	mirna_pc
23750	hsa-mir-484	MRPL1	0.335829228	mirna_pc
23751	hsa-mir-484	SOD1	0.300171519	mirna_pc
23752	hsa-mir-484	API5	0.308932096	mirna_pc
23753	hsa-mir-484	C2orf3	0.355006355	mirna_pc
23754	hsa-mir-484	CHD4	0.304272028	mirna_pc
23755	hsa-mir-484	KRIT1	0.419138504	mirna_pc
23756	hsa-mir-484	DHRS2	0.413360255	mirna_pc
23757	hsa-mir-484	FGFBP3	0.306749131	mirna_pc
23758	hsa-mir-484	DNAJC19	0.472836213	mirna_pc
23759	hsa-mir-484	TMEM134	0.332343573	mirna_pc

23760	hsa-mir-484	MRPS7	0.313874384	mirna_pc
23761	hsa-mir-484	SAPS3	0.335705098	mirna_pc
23762	hsa-mir-484	PEX1	0.392415395	mirna_pc
23763	hsa-mir-484	OAZ1	0.345760228	mirna_pc
23764	hsa-mir-484	TIMM44	0.307724941	mirna_pc
23765	hsa-mir-484	NDUFA7	0.302486738	mirna_pc
23766	hsa-mir-484	DIS3L2	0.335789745	mirna_pc
23767	hsa-mir-484	FAM18B2	0.311620247	mirna_pc
23768	hsa-mir-484	MRPL19	0.318328298	mirna_pc
23769	hsa-mir-484	NME3	0.31461824	mirna_pc
23770	hsa-mir-484	CETN3	0.356907011	mirna_pc
23771	hsa-mir-484	ZNF513	0.302389148	mirna_pc
23772	hsa-mir-484	CDK3	0.325706266	mirna_pc
23773	hsa-mir-484	CDKN2AIPNL	0.337878088	mirna_pc
23774	hsa-mir-484	B9D1	0.413005035	mirna_pc
23775	hsa-mir-484	LSM 1.00	0.396694702	mirna_pc
23776	hsa-mir-484	DYNC1I1	0.389773098	mirna_pc
23777	hsa-mir-484	ACP1	0.324613224	mirna_pc
23778	hsa-mir-484	WASH3P	0.319540351	mirna_pc
23779	hsa-mir-484	FDX1L	0.335195608	mirna_pc
23780	hsa-mir-484	GATAD1	0.339758367	mirna_pc
23781	hsa-mir-484	ZNF322A	0.430028662	mirna_pc
23782	hsa-mir-484	PRR22	0.411750775	mirna_pc
23783	hsa-mir-484	SRP19	0.317841174	mirna_pc
23784	hsa-mir-484	SNRNP25	0.361559733	mirna_pc
23785	hsa-mir-484	LOC728640	0.394610594	mirna_pc
23786	hsa-mir-484	LOC401397	0.51465138	mirna_pc
23787	hsa-mir-484	GPS2	0.350048661	mirna_pc
23788	hsa-mir-484	C21orf119	0.376338302	mirna_pc
23789	hsa-mir-484	C2orf28	0.399173385	mirna_pc
23790	hsa-mir-484	MRPL40	0.304430073	mirna_pc
23791	hsa-mir-484	RHOT2	0.395685864	mirna_pc
23792	hsa-mir-484	HIRIP3	0.369693934	mirna_pc
23793	hsa-mir-484	AARSD1	0.312603916	mirna_pc
23794	hsa-mir-484	TMEM168	0.431258106	mirna_pc
23795	hsa-mir-484	MRPL53	0.471286578	mirna_pc
23796	hsa-mir-484	COPS3	0.349036981	mirna_pc
23797	hsa-mir-484	NDUFS8	0.437265127	mirna_pc
23798	hsa-mir-484	NUDC	0.30605749	mirna_pc
23799	hsa-mir-484	C12orf73	0.300965162	mirna_pc
23800	hsa-mir-484	NMRAL1	0.396898068	mirna_pc
23801	hsa-mir-1274b	RCC2	0.301832479	mirna_pc
23802	hsa-mir-1274b	CDCA3	0.325234337	mirna_pc
23803	hsa-mir-1274b	EZH2	0.307228245	mirna_pc
23804	hsa-mir-1274b	TUBA1B	0.30847178	mirna_pc
23805	hsa-mir-1274b	EFTUD2	0.307615914	mirna_pc
23806	hsa-mir-1274b	SNRPA	0.327521343	mirna_pc
23807	hsa-mir-1274b	DEPDC1B	0.300934308	mirna_pc
23808	hsa-mir-1274b	C19orf48	0.359088823	mirna_pc
23809	hsa-mir-1274b	BCL2L12	0.310586102	mirna_pc
23810	hsa-mir-1274b	PSRC1	0.301828354	mirna_pc
23811	hsa-mir-1274b	UCA1	0.323181219	mirna_pc
23812	hsa-mir-1274b	TRIM28	0.317426898	mirna_pc
23813	hsa-mir-1274b	PAX9	0.308391576	mirna_pc

23814	hsa-mir-1274b	DNAJC5	0.337623994	mirna_pc
23815	hsa-mir-1274b	ASS1	0.31127575	mirna_pc
23816	hsa-mir-1274b	RBM10	0.30799093	mirna_pc
23817	hsa-mir-1274b	ACPP	0.302727987	mirna_pc
23818	hsa-mir-1274b	PPP2R1A	0.33937173	mirna_pc
23819	hsa-mir-1274b	DACT2	0.301182501	mirna_pc
23820	hsa-mir-1274b	PREB	0.351500387	mirna_pc
23821	hsa-mir-1274b	LRRC20	0.315332278	mirna_pc
23822	hsa-mir-1274b	UBE2MP1	0.302059374	mirna_pc
23823	hsa-mir-1306	TPX2	0.350585263	mirna_pc
23824	hsa-mir-1306	KIF4B	0.32686545	mirna_pc
23825	hsa-mir-1306	KPNA2	0.412257195	mirna_pc
23826	hsa-mir-1306	RCC2	0.401701152	mirna_pc
23827	hsa-mir-1306	HOXC9	0.479861175	mirna_pc
23828	hsa-mir-1306	KIF11	0.338048245	mirna_pc
23829	hsa-mir-1306	ECT2	0.31950985	mirna_pc
23830	hsa-mir-1306	RRM2	0.422577015	mirna_pc
23831	hsa-mir-1306	CDK1	0.335166195	mirna_pc
23832	hsa-mir-1306	CDC25C	0.345920492	mirna_pc
23833	hsa-mir-1306	PLK1	0.360712239	mirna_pc
23834	hsa-mir-1306	UBE2C	0.365641094	mirna_pc
23835	hsa-mir-1306	BUB1	0.323074029	mirna_pc
23836	hsa-mir-1306	PRC1	0.403806853	mirna_pc
23837	hsa-mir-1306	NUSAP1	0.474288882	mirna_pc
23838	hsa-mir-1306	KIFC1	0.407006024	mirna_pc
23839	hsa-mir-1306	BUB1B	0.503141171	mirna_pc
23840	hsa-mir-1306	KIF18B	0.398411863	mirna_pc
23841	hsa-mir-1306	CKAP2	0.348616358	mirna_pc
23842	hsa-mir-1306	KIF2C	0.430254666	mirna_pc
23843	hsa-mir-1306	FANCA	0.300210086	mirna_pc
23844	hsa-mir-1306	NCAPG	0.371126276	mirna_pc
23845	hsa-mir-1306	CDC20	0.466647786	mirna_pc
23846	hsa-mir-1306	CLSPN	0.33897371	mirna_pc
23847	hsa-mir-1306	ARHGAP11A	0.349949704	mirna_pc
23848	hsa-mir-1306	CCNA2	0.433859133	mirna_pc
23849	hsa-mir-1306	SPC25	0.305035796	mirna_pc
23850	hsa-mir-1306	FANCI	0.438060982	mirna_pc
23851	hsa-mir-1306	NEK2	0.380420133	mirna_pc
23852	hsa-mir-1306	MND1	0.381325969	mirna_pc
23853	hsa-mir-1306	HOXC11	0.303369245	mirna_pc
23854	hsa-mir-1306	KIF22	0.360850442	mirna_pc
23855	hsa-mir-1306	NCAPH	0.419239525	mirna_pc
23856	hsa-mir-1306	GTSE1	0.365621133	mirna_pc
23857	hsa-mir-1306	KIF23	0.379576874	mirna_pc
23858	hsa-mir-1306	RAD54L	0.436057582	mirna_pc
23859	hsa-mir-1306	CENPA	0.490328239	mirna_pc
23860	hsa-mir-1306	MAD2L1	0.470109321	mirna_pc
23861	hsa-mir-1306	TIMELESS	0.383911762	mirna_pc
23862	hsa-mir-1306	LMNB1	0.331517457	mirna_pc
23863	hsa-mir-1306	CDCA8	0.478199163	mirna_pc
23864	hsa-mir-1306	TROAP	0.45290036	mirna_pc
23865	hsa-mir-1306	CCNB1	0.318887779	mirna_pc
23866	hsa-mir-1306	CDCA5	0.317917183	mirna_pc
23867	hsa-mir-1306	TACC3	0.334475076	mirna_pc

23868	hsa-mir-1306	CDC45	0.398668681	mirna_pc
23869	hsa-mir-1306	STMN1	0.380271561	mirna_pc
23870	hsa-mir-1306	CENPM	0.364505292	mirna_pc
23871	hsa-mir-1306	TK1	0.315536371	mirna_pc
23872	hsa-mir-1306	UBE2T	0.378356967	mirna_pc
23873	hsa-mir-1306	CKS1B	0.375001301	mirna_pc
23874	hsa-mir-1306	TUBB	0.348671455	mirna_pc
23875	hsa-mir-1306	KIF4A	0.383877232	mirna_pc
23876	hsa-mir-1306	ORC1L	0.335909136	mirna_pc
23877	hsa-mir-1306	UHRF1	0.313089376	mirna_pc
23878	hsa-mir-1306	CCNF	0.31494384	mirna_pc
23879	hsa-mir-1306	RACGAP1	0.376018199	mirna_pc
23880	hsa-mir-1306	PTBP1	0.334072251	mirna_pc
23881	hsa-mir-1306	CCNB2	0.467182994	mirna_pc
23882	hsa-mir-1306	PLK4	0.386701461	mirna_pc
23883	hsa-mir-1306	DEPDC1	0.38732352	mirna_pc
23884	hsa-mir-1306	HJURP	0.392113391	mirna_pc
23885	hsa-mir-1306	SKA1	0.333642573	mirna_pc
23886	hsa-mir-1306	RAD51	0.369533241	mirna_pc
23887	hsa-mir-1306	BLM	0.404870363	mirna_pc
23888	hsa-mir-1306	NME1	0.343179539	mirna_pc
23889	hsa-mir-1306	PAICS	0.354773997	mirna_pc
23890	hsa-mir-1306	BIRC5	0.546259373	mirna_pc
23891	hsa-mir-1306	RCC1	0.411387381	mirna_pc
23892	hsa-mir-1306	OIP5	0.507727611	mirna_pc
23893	hsa-mir-1306	EME1	0.334806619	mirna_pc
23894	hsa-mir-1306	RECQL4	0.344916333	mirna_pc
23895	hsa-mir-1306	AURKB	0.400628922	mirna_pc
23896	hsa-mir-1306	GSG2	0.304374776	mirna_pc
23897	hsa-mir-1306	CDT1	0.305073956	mirna_pc
23898	hsa-mir-1306	MCM10	0.304554484	mirna_pc
23899	hsa-mir-1306	KIF20A	0.334840913	mirna_pc
23900	hsa-mir-1306	SGOL2	0.312943273	mirna_pc
23901	hsa-mir-1306	CHEK2	0.46407858	mirna_pc
23902	hsa-mir-1306	KIF15	0.348658903	mirna_pc
23903	hsa-mir-1306	CDCA3	0.359585363	mirna_pc
23904	hsa-mir-1306	FEN1	0.348206875	mirna_pc
23905	hsa-mir-1306	TRAIP	0.392842454	mirna_pc
23906	hsa-mir-1306	ORC6L	0.333073623	mirna_pc
23907	hsa-mir-1306	CASC5	0.334656031	mirna_pc
23908	hsa-mir-1306	C16orf59	0.372817145	mirna_pc
23909	hsa-mir-1306	FAM72D	0.322499209	mirna_pc
23910	hsa-mir-1306	FAM72B	0.31318798	mirna_pc
23911	hsa-mir-1306	NEIL3	0.335622721	mirna_pc
23912	hsa-mir-1306	ACTL6A	0.305867592	mirna_pc
23913	hsa-mir-1306	C12orf48	0.425097679	mirna_pc
23914	hsa-mir-1306	HNRNPL	0.41528858	mirna_pc
23915	hsa-mir-1306	ZWILCH	0.36399494	mirna_pc
23916	hsa-mir-1306	TTK	0.306877244	mirna_pc
23917	hsa-mir-1306	ZWINT	0.45529	mirna_pc
23918	hsa-mir-1306	TUBA1B	0.444360679	mirna_pc
23919	hsa-mir-1306	UBE2S	0.445530413	mirna_pc
23920	hsa-mir-1306	PSMA4	0.338303724	mirna_pc
23921	hsa-mir-1306	C11orf82	0.388735912	mirna_pc

23922	hsa-mir-1306	HNRNPC	0.381702178	mirna_pc
23923	hsa-mir-1306	ILF2	0.352591082	mirna_pc
23924	hsa-mir-1306	TCF3	0.349264911	mirna_pc
23925	hsa-mir-1306	CHEK1	0.337605258	mirna_pc
23926	hsa-mir-1306	MCM5	0.316984646	mirna_pc
23927	hsa-mir-1306	RAN	0.342106951	mirna_pc
23928	hsa-mir-1306	TYMS	0.345152964	mirna_pc
23929	hsa-mir-1306	RNASEH2A	0.326067425	mirna_pc
23930	hsa-mir-1306	EPR1	0.45931217	mirna_pc
23931	hsa-mir-1306	PIF1	0.388068659	mirna_pc
23932	hsa-mir-1306	H2AFX	0.365297183	mirna_pc
23933	hsa-mir-1306	SFRS2	0.348886312	mirna_pc
23934	hsa-mir-1306	UCK2	0.409334305	mirna_pc
23935	hsa-mir-1306	EFTUD2	0.321011013	mirna_pc
23936	hsa-mir-1306	NFKBIL2	0.391587592	mirna_pc
23937	hsa-mir-1306	C15orf42	0.404769484	mirna_pc
23938	hsa-mir-1306	PRIM2	0.331770453	mirna_pc
23939	hsa-mir-1306	HMGB3	0.301017617	mirna_pc
23940	hsa-mir-1306	FAM64A	0.31379413	mirna_pc
23941	hsa-mir-1306	RANBP1	0.524454518	mirna_pc
23942	hsa-mir-1306	E2F1	0.359525575	mirna_pc
23943	hsa-mir-1306	SNRPG	0.358287287	mirna_pc
23944	hsa-mir-1306	EIF4A3	0.33269878	mirna_pc
23945	hsa-mir-1306	MCM3	0.306934454	mirna_pc
23946	hsa-mir-1306	SNRPA	0.429067266	mirna_pc
23947	hsa-mir-1306	DNAJC9	0.307494732	mirna_pc
23948	hsa-mir-1306	U2AF2	0.339204971	mirna_pc
23949	hsa-mir-1306	POC1A	0.346669336	mirna_pc
23950	hsa-mir-1306	CENPO	0.49745963	mirna_pc
23951	hsa-mir-1306	NUDT1	0.33860678	mirna_pc
23952	hsa-mir-1306	GINS2	0.308222322	mirna_pc
23953	hsa-mir-1306	DNA2	0.317689383	mirna_pc
23954	hsa-mir-1306	SNRPD1	0.332047036	mirna_pc
23955	hsa-mir-1306	DTYMK	0.329694345	mirna_pc
23956	hsa-mir-1306	HSPE1	0.301571971	mirna_pc
23957	hsa-mir-1306	C16orf75	0.305548933	mirna_pc
23958	hsa-mir-1306	EBNA1BP2	0.314538793	mirna_pc
23959	hsa-mir-1306	HIST1H2AE	0.33646744	mirna_pc
23960	hsa-mir-1306	CAD	0.370526377	mirna_pc
23961	hsa-mir-1306	CCDC99	0.322441831	mirna_pc
23962	hsa-mir-1306	CDK2	0.401181025	mirna_pc
23963	hsa-mir-1306	CDK4	0.380163536	mirna_pc
23964	hsa-mir-1306	INCENP	0.32532637	mirna_pc
23965	hsa-mir-1306	CKAP5	0.347243738	mirna_pc
23966	hsa-mir-1306	BCL2L12	0.308387041	mirna_pc
23967	hsa-mir-1306	HNRNPR	0.373737842	mirna_pc
23968	hsa-mir-1306	KAT2A	0.342758092	mirna_pc
23969	hsa-mir-1306	CHAF1B	0.32862643	mirna_pc
23970	hsa-mir-1306	HOXC10	0.374724864	mirna_pc
23971	hsa-mir-1306	MSH2	0.397566801	mirna_pc
23972	hsa-mir-1306	TOMM40	0.359699284	mirna_pc
23973	hsa-mir-1306	SF3B3	0.346012403	mirna_pc
23974	hsa-mir-1306	CDC7	0.406410629	mirna_pc
23975	hsa-mir-1306	YDJC	0.458341007	mirna_pc

23976	hsa-mir-1306	PUS1	0.33663817	mirna_pc
23977	hsa-mir-1306	SNRPC	0.364837503	mirna_pc
23978	hsa-mir-1306	PSMB4	0.328423056	mirna_pc
23979	hsa-mir-1306	RFC4	0.381033784	mirna_pc
23980	hsa-mir-1306	DCLRE1B	0.342449536	mirna_pc
23981	hsa-mir-1306	CDC25A	0.393068688	mirna_pc
23982	hsa-mir-1306	TMEM48	0.342671786	mirna_pc
23983	hsa-mir-1306	RAD54B	0.35954717	mirna_pc
23984	hsa-mir-1306	H2AFZ	0.389055997	mirna_pc
23985	hsa-mir-1306	MAGOH	0.398483917	mirna_pc
23986	hsa-mir-1306	NUP37	0.361479897	mirna_pc
23987	hsa-mir-1306	CLN6	0.325578261	mirna_pc
23988	hsa-mir-1306	CPSF3	0.426650912	mirna_pc
23989	hsa-mir-1306	BANF1	0.34304612	mirna_pc
23990	hsa-mir-1306	SNRPE	0.326204303	mirna_pc
23991	hsa-mir-1306	C21orf45	0.447929336	mirna_pc
23992	hsa-mir-1306	ATIC	0.315167025	mirna_pc
23993	hsa-mir-1306	PPM1G	0.443851159	mirna_pc
23994	hsa-mir-1306	MARCKSL1	0.327665411	mirna_pc
23995	hsa-mir-1306	NPM3	0.365698476	mirna_pc
23996	hsa-mir-1306	NUDT5	0.358760345	mirna_pc
23997	hsa-mir-1306	NUTF2	0.301585119	mirna_pc
23998	hsa-mir-1306	DAZAP1	0.357672375	mirna_pc
23999	hsa-mir-1306	CBX8	0.385795385	mirna_pc
24000	hsa-mir-1306	TMEM201	0.438862407	mirna_pc
24001	hsa-mir-1306	TFDP1	0.335442749	mirna_pc
24002	hsa-mir-1306	PAFAH1B3	0.35699483	mirna_pc
24003	hsa-mir-1306	PSRC1	0.437779508	mirna_pc
24004	hsa-mir-1306	DGUOK	0.315118397	mirna_pc
24005	hsa-mir-1306	DBF4B	0.313133294	mirna_pc
24006	hsa-mir-1306	FBL	0.324764901	mirna_pc
24007	hsa-mir-1306	DONSON	0.347959299	mirna_pc
24008	hsa-mir-1306	EXOSC5	0.330512791	mirna_pc
24009	hsa-mir-1306	TMPO	0.333187739	mirna_pc
24010	hsa-mir-1306	POLR2H	0.320937967	mirna_pc
24011	hsa-mir-1306	MRTO4	0.361593478	mirna_pc
24012	hsa-mir-1306	PPIH	0.405227699	mirna_pc
24013	hsa-mir-1306	DHX9	0.301309739	mirna_pc
24014	hsa-mir-1306	LOC100128191	0.427436958	mirna_pc
24015	hsa-mir-1306	GNB1L	0.366465276	mirna_pc
24016	hsa-mir-1306	SMYD5	0.352920653	mirna_pc
24017	hsa-mir-1306	TSSC1	0.336551874	mirna_pc
24018	hsa-mir-1306	SNRPF	0.333196214	mirna_pc
24019	hsa-mir-1306	SUMO2	0.367945148	mirna_pc
24020	hsa-mir-1306	WDR43	0.303717819	mirna_pc
24021	hsa-mir-1306	SNRPA1	0.405353875	mirna_pc
24022	hsa-mir-1306	SKP2	0.404900505	mirna_pc
24023	hsa-mir-1306	TIPIN	0.475929399	mirna_pc
24024	hsa-mir-1306	HSPBP1	0.356984738	mirna_pc
24025	hsa-mir-1306	ECE2	0.369332057	mirna_pc
24026	hsa-mir-1306	GPN1	0.342552908	mirna_pc
24027	hsa-mir-1306	RFC5	0.335958922	mirna_pc
24028	hsa-mir-1306	MAZ	0.415281708	mirna_pc
24029	hsa-mir-1306	SSRP1	0.311690888	mirna_pc

24030	hsa-mir-1306	POLR2D	0.315065852	mirna_pc
24031	hsa-mir-1306	PA2G4	0.394516582	mirna_pc
24032	hsa-mir-1306	SNRPD3	0.5507795	mirna_pc
24033	hsa-mir-1306	KHDRBS1	0.426409471	mirna_pc
24034	hsa-mir-1306	SLC5A6	0.321897564	mirna_pc
24035	hsa-mir-1306	LOC92659	0.301217492	mirna_pc
24036	hsa-mir-1306	RTN4R	0.304023805	mirna_pc
24037	hsa-mir-1306	CCT7	0.303968201	mirna_pc
24038	hsa-mir-1306	TMEM194A	0.311973748	mirna_pc
24039	hsa-mir-1306	SNRNP40	0.402598024	mirna_pc
24040	hsa-mir-1306	TMEM69	0.30170997	mirna_pc
24041	hsa-mir-1306	GNL2	0.366819922	mirna_pc
24042	hsa-mir-1306	NASP	0.304695931	mirna_pc
24043	hsa-mir-1306	RPP40	0.310297837	mirna_pc
24044	hsa-mir-1306	NONO	0.329084271	mirna_pc
24045	hsa-mir-1306	CCDC86	0.363455567	mirna_pc
24046	hsa-mir-1306	OBFC2B	0.343725975	mirna_pc
24047	hsa-mir-1306	PTMA	0.344064309	mirna_pc
24048	hsa-mir-1306	USP39	0.338065429	mirna_pc
24049	hsa-mir-1306	WDR76	0.378536896	mirna_pc
24050	hsa-mir-1306	LSM 4.00	0.336146573	mirna_pc
24051	hsa-mir-1306	C11orf84	0.303285249	mirna_pc
24052	hsa-mir-1306	TBCE	0.308757054	mirna_pc
24053	hsa-mir-1306	HIST1H2BK	0.323820558	mirna_pc
24054	hsa-mir-1306	FBXO22OS	0.347838514	mirna_pc
24055	hsa-mir-1306	SUV39H2	0.300755128	mirna_pc
24056	hsa-mir-1306	LSM 2.00	0.327996282	mirna_pc
24057	hsa-mir-1306	ALG6	0.340556648	mirna_pc
24058	hsa-mir-1306	HNRNPD	0.343392673	mirna_pc
24059	hsa-mir-1306	DPY30	0.317702873	mirna_pc
24060	hsa-mir-1306	MTHFD2	0.332903286	mirna_pc
24061	hsa-mir-1306	DPH2	0.407251235	mirna_pc
24062	hsa-mir-1306	MEN1	0.341280523	mirna_pc
24063	hsa-mir-1306	PNO1	0.33026299	mirna_pc
24064	hsa-mir-1306	CCT4	0.311787259	mirna_pc
24065	hsa-mir-1306	ELAVL1	0.379137096	mirna_pc
24066	hsa-mir-1306	MIF	0.313509414	mirna_pc
24067	hsa-mir-1306	NCAPD3	0.3975045	mirna_pc
24068	hsa-mir-1306	NAE1	0.326089385	mirna_pc
24069	hsa-mir-1306	PSMC3IP	0.30288494	mirna_pc
24070	hsa-mir-1306	TBC1D7	0.430278601	mirna_pc
24071	hsa-mir-1306	PAK1IP1	0.460094525	mirna_pc
24072	hsa-mir-1306	PES 1.00	0.322342374	mirna_pc
24073	hsa-mir-1306	E2F4	0.336714685	mirna_pc
24074	hsa-mir-1306	C18orf45	0.313043479	mirna_pc
24075	hsa-mir-1306	PGAM5	0.347512639	mirna_pc
24076	hsa-mir-1306	NAA25	0.302149634	mirna_pc
24077	hsa-mir-1306	KDM1A	0.367733873	mirna_pc
24078	hsa-mir-1306	ILF3	0.323170495	mirna_pc
24079	hsa-mir-1306	EHMT2	0.410867451	mirna_pc
24080	hsa-mir-1306	SFRS9	0.363253314	mirna_pc
24081	hsa-mir-1306	COMMD4	0.328433177	mirna_pc
24082	hsa-mir-1306	C14orf80	0.344755082	mirna_pc
24083	hsa-mir-1306	GTF2H4	0.304172054	mirna_pc

24084	hsa-mir-1306	KHSRP	0.399769388	mirna_pc
24085	hsa-mir-1306	HEATR2	0.316108098	mirna_pc
24086	hsa-mir-1306	LSM 6.00	0.31036539	mirna_pc
24087	hsa-mir-1306	PRAME	0.478005403	mirna_pc
24088	hsa-mir-1306	SUV39H1	0.379519941	mirna_pc
24089	hsa-mir-1306	IPO9	0.31704705	mirna_pc
24090	hsa-mir-1306	PSMD4	0.312885606	mirna_pc
24091	hsa-mir-1306	SART3	0.329155865	mirna_pc
24092	hsa-mir-1306	GAR1	0.319353547	mirna_pc
24093	hsa-mir-1306	C15orf23	0.486535324	mirna_pc
24094	hsa-mir-1306	NOC4L	0.302373378	mirna_pc
24095	hsa-mir-1306	DIABLO	0.306487974	mirna_pc
24096	hsa-mir-1306	ITGB3BP	0.337013852	mirna_pc
24097	hsa-mir-1306	ENOPH1	0.30962068	mirna_pc
24098	hsa-mir-1306	SPG21	0.300153426	mirna_pc
24099	hsa-mir-1306	XRCC3	0.31951802	mirna_pc
24100	hsa-mir-1306	TUBG1	0.334316772	mirna_pc
24101	hsa-mir-1306	C17orf96	0.321480238	mirna_pc
24102	hsa-mir-1306	PLEKHG4	0.333396839	mirna_pc
24103	hsa-mir-1306	SFPQ	0.37625051	mirna_pc
24104	hsa-mir-1306	VPS72	0.378158721	mirna_pc
24105	hsa-mir-1306	RCOR2	0.401846812	mirna_pc
24106	hsa-mir-1306	EIF4EBP1	0.317932426	mirna_pc
24107	hsa-mir-1306	RHBDD3	0.356385776	mirna_pc
24108	hsa-mir-1306	ATAD3A	0.360737159	mirna_pc
24109	hsa-mir-1306	GMNN	0.314489144	mirna_pc
24110	hsa-mir-1306	PA2G4P4	0.421965568	mirna_pc
24111	hsa-mir-1306	PDAP1	0.305051287	mirna_pc
24112	hsa-mir-1306	BRI3BP	0.322830241	mirna_pc
24113	hsa-mir-1306	GEMIN6	0.3632898	mirna_pc
24114	hsa-mir-1306	DUT	0.308045201	mirna_pc
24115	hsa-mir-1306	PDCD5	0.362561299	mirna_pc
24116	hsa-mir-1306	PTCD1	0.340693103	mirna_pc
24117	hsa-mir-1306	MSH6	0.368017669	mirna_pc
24118	hsa-mir-1306	C3orf34	0.303462397	mirna_pc
24119	hsa-mir-1306	SRM	0.302399072	mirna_pc
24120	hsa-mir-1306	XRCC6	0.316679179	mirna_pc
24121	hsa-mir-1306	PSMG1	0.339472122	mirna_pc
24122	hsa-mir-1306	PPP1R8	0.359941123	mirna_pc
24123	hsa-mir-1306	NPM1	0.311779325	mirna_pc
24124	hsa-mir-1306	CDK2AP1	0.313811439	mirna_pc
24125	hsa-mir-1306	NUDT3	0.470245652	mirna_pc
24126	hsa-mir-1306	MEX3A	0.340656411	mirna_pc
24127	hsa-mir-1306	ATP5J2	0.304147606	mirna_pc
24128	hsa-mir-1306	ERAL1	0.34768648	mirna_pc
24129	hsa-mir-1306	TAF5	0.398293638	mirna_pc
24130	hsa-mir-1306	APEX1	0.339018417	mirna_pc
24131	hsa-mir-1306	MAD2L2	0.309305222	mirna_pc
24132	hsa-mir-1306	HNRNPA1L2	0.311994005	mirna_pc
24133	hsa-mir-1306	RCCD1	0.331904714	mirna_pc
24134	hsa-mir-1306	C15orf44	0.352274506	mirna_pc
24135	hsa-mir-1306	FARSB	0.328123228	mirna_pc
24136	hsa-mir-1306	ERI3	0.365253237	mirna_pc
24137	hsa-mir-1306	RCN2	0.340722081	mirna_pc

24138	hsa-mir-1306	CIB2	0.307713894	mirna_pc
24139	hsa-mir-1306	TARBP2	0.328269276	mirna_pc
24140	hsa-mir-1306	TOMM22	0.327582682	mirna_pc
24141	hsa-mir-1306	PHB	0.324007352	mirna_pc
24142	hsa-mir-1306	C12orf52	0.311259582	mirna_pc
24143	hsa-mir-1306	NOL10	0.402915118	mirna_pc
24144	hsa-mir-1306	SF3A3	0.34676152	mirna_pc
24145	hsa-mir-1306	POLE	0.393910303	mirna_pc
24146	hsa-mir-1306	C8orf30A	0.348519936	mirna_pc
24147	hsa-mir-1306	MUTYH	0.431308399	mirna_pc
24148	hsa-mir-1306	WRNIP1	0.354609935	mirna_pc
24149	hsa-mir-1306	TAF1B	0.358271696	mirna_pc
24150	hsa-mir-1306	TIMM50	0.367724285	mirna_pc
24151	hsa-mir-1306	ADSL	0.398436653	mirna_pc
24152	hsa-mir-1306	VRK1	0.323221279	mirna_pc
24153	hsa-mir-1306	EXOSC9	0.344767544	mirna_pc
24154	hsa-mir-1306	UNG	0.365409481	mirna_pc
24155	hsa-mir-1306	GRK6	0.345967404	mirna_pc
24156	hsa-mir-1306	CDK16	0.370121746	mirna_pc
24157	hsa-mir-1306	ALKBH2	0.408697396	mirna_pc
24158	hsa-mir-1306	POP5	0.336612122	mirna_pc
24159	hsa-mir-1306	CCDC90A	0.324787073	mirna_pc
24160	hsa-mir-1306	SOCS7	0.314857503	mirna_pc
24161	hsa-mir-1306	NHLRC1	0.366073644	mirna_pc
24162	hsa-mir-1306	CCDC24	0.327596143	mirna_pc
24163	hsa-mir-1306	GPN3	0.355425023	mirna_pc
24164	hsa-mir-1306	LOC152217	0.306977154	mirna_pc
24165	hsa-mir-1306	MRPL37	0.340511665	mirna_pc
24166	hsa-mir-1306	MEMO1	0.30962483	mirna_pc
24167	hsa-mir-1306	CCDC23	0.346569467	mirna_pc
24168	hsa-mir-1306	EIF3I	0.392124282	mirna_pc
24169	hsa-mir-1306	LINGO1	0.353070592	mirna_pc
24170	hsa-mir-1306	DNAJC14	0.353237442	mirna_pc
24171	hsa-mir-1306	KDM2B	0.301130805	mirna_pc
24172	hsa-mir-1306	GTF3C2	0.322874075	mirna_pc
24173	hsa-mir-1306	MAGEF1	0.367678703	mirna_pc
24174	hsa-mir-1306	C15orf63	0.303335141	mirna_pc
24175	hsa-mir-1306	PCGF6	0.330448285	mirna_pc
24176	hsa-mir-1306	YRDC	0.306819155	mirna_pc
24177	hsa-mir-1306	SMARCB1	0.443077936	mirna_pc
24178	hsa-mir-1306	CCT8	0.333911628	mirna_pc
24179	hsa-mir-1306	TTC32	0.304940307	mirna_pc
24180	hsa-mir-1306	U2AF1	0.341985202	mirna_pc
24181	hsa-mir-1306	B4GALT2	0.300322704	mirna_pc
24182	hsa-mir-1306	UFD1L	0.308247077	mirna_pc
24183	hsa-mir-1306	ZNF639	0.316657102	mirna_pc
24184	hsa-mir-1306	ITPRIPL1	0.316969088	mirna_pc
24185	hsa-mir-1306	ABT1	0.337778067	mirna_pc
24186	hsa-mir-1306	SLC25A17	0.315598795	mirna_pc
24187	hsa-mir-1306	RPS7	0.317946133	mirna_pc
24188	hsa-mir-1306	MKRN3	0.356209543	mirna_pc
24189	hsa-mir-1306	LOC401010	0.330850971	mirna_pc
24190	hsa-mir-1306	STRA13	0.345727659	mirna_pc
24191	hsa-mir-1306	BCL7A	0.3811872	mirna_pc

24192	hsa-mir-1306	MTX1	0.303390459	mirna_pc
24193	hsa-mir-1306	NUDCD2	0.311065777	mirna_pc
24194	hsa-mir-1306	ARHGAP19	0.323304084	mirna_pc
24195	hsa-mir-1306	WDR77	0.35673019	mirna_pc
24196	hsa-mir-1306	MRPL21	0.307390002	mirna_pc
24197	hsa-mir-1306	NLE1	0.341296312	mirna_pc
24198	hsa-mir-1306	PPP1CC	0.30594034	mirna_pc
24199	hsa-mir-1306	TMEM106C	0.317189734	mirna_pc
24200	hsa-mir-1306	TAF4B	0.339595297	mirna_pc
24201	hsa-mir-1306	TRMT61B	0.313486545	mirna_pc
24202	hsa-mir-1306	MPV17	0.315346261	mirna_pc
24203	hsa-mir-1306	DDX20	0.358427183	mirna_pc
24204	hsa-mir-1306	GTF2A2	0.385105173	mirna_pc
24205	hsa-mir-1306	C1orf109	0.358567646	mirna_pc
24206	hsa-mir-1306	MTA1	0.340400514	mirna_pc
24207	hsa-mir-1306	HTRA2	0.303458877	mirna_pc
24208	hsa-mir-1306	FBXO22	0.343440869	mirna_pc
24209	hsa-mir-1306	MTA3	0.388250816	mirna_pc
24210	hsa-mir-1306	GRTP1	0.306359506	mirna_pc
24211	hsa-mir-1306	INTU	0.314551477	mirna_pc
24212	hsa-mir-1306	MAPKAPK5	0.356889226	mirna_pc
24213	hsa-mir-1306	DRG1	0.432721267	mirna_pc
24214	hsa-mir-1306	NOL7	0.411726278	mirna_pc
24215	hsa-mir-1306	CPSF4	0.326228261	mirna_pc
24216	hsa-mir-1306	NT5C	0.312731705	mirna_pc
24217	hsa-mir-1306	ALG10	0.306677597	mirna_pc
24218	hsa-mir-1306	NF2	0.322777087	mirna_pc
24219	hsa-mir-1306	TTC27	0.378658334	mirna_pc
24220	hsa-mir-1306	DHRS13	0.369069696	mirna_pc
24221	hsa-mir-1306	RPF1	0.364333844	mirna_pc
24222	hsa-mir-1306	EWSR1	0.349588425	mirna_pc
24223	hsa-mir-1306	LOC222699	0.326113862	mirna_pc
24224	hsa-mir-1306	DHX57	0.328192842	mirna_pc
24225	hsa-mir-1306	AAAS	0.437069378	mirna_pc
24226	hsa-mir-1306	CHST14	0.300171347	mirna_pc
24227	hsa-mir-1306	MTFMT	0.386044326	mirna_pc
24228	hsa-mir-1306	SUPT7L	0.302411743	mirna_pc
24229	hsa-mir-1306	MRPL30	0.30952098	mirna_pc
24230	hsa-mir-1306	DHODH	0.309962465	mirna_pc
24231	hsa-mir-1306	AKR7A2	0.395797073	mirna_pc
24232	hsa-mir-1306	SFRS7	0.426872285	mirna_pc
24233	hsa-mir-1306	PEX10	0.311737109	mirna_pc
24234	hsa-mir-1306	TRMT2A	0.50267627	mirna_pc
24235	hsa-mir-1306	PFAS	0.368537681	mirna_pc
24236	hsa-mir-1306	SARS2	0.303854168	mirna_pc
24237	hsa-mir-1306	LRRC45	0.303480086	mirna_pc
24238	hsa-mir-1306	SNUPN	0.346725165	mirna_pc
24239	hsa-mir-1306	RBM10	0.302120664	mirna_pc
24240	hsa-mir-1306	RPUSD2	0.36805155	mirna_pc
24241	hsa-mir-1306	THYN1	0.341554406	mirna_pc
24242	hsa-mir-1306	HIRA	0.330159596	mirna_pc
24243	hsa-mir-1306	NUP153	0.348356528	mirna_pc
24244	hsa-mir-1306	NIPSNAP1	0.386549435	mirna_pc
24245	hsa-mir-1306	SPA17	0.338485062	mirna_pc

24246	hsa-mir-1306	C17orf75	0.335028195	mirna_pc
24247	hsa-mir-1306	SEC11A	0.309806314	mirna_pc
24248	hsa-mir-1306	PGAP2	0.311537455	mirna_pc
24249	hsa-mir-1306	ZNF771	0.335309661	mirna_pc
24250	hsa-mir-1306	EEF1E1	0.374364779	mirna_pc
24251	hsa-mir-1306	YBX1	0.314329405	mirna_pc
24252	hsa-mir-1306	ATPBD4	0.348511701	mirna_pc
24253	hsa-mir-1306	C14orf104	0.334453988	mirna_pc
24254	hsa-mir-1306	MTP 18.00	0.322958748	mirna_pc
24255	hsa-mir-1306	POLR2C	0.30451354	mirna_pc
24256	hsa-mir-1306	C12orf10	0.306875739	mirna_pc
24257	hsa-mir-1306	C12orf45	0.318178175	mirna_pc
24258	hsa-mir-1306	C2orf44	0.392530324	mirna_pc
24259	hsa-mir-1306	SEPHS1	0.367495299	mirna_pc
24260	hsa-mir-1306	POMGNT1	0.321835343	mirna_pc
24261	hsa-mir-1306	RPS19BP1	0.34669394	mirna_pc
24262	hsa-mir-1306	AGBL5	0.350940033	mirna_pc
24263	hsa-mir-1306	BOLA3	0.32941493	mirna_pc
24264	hsa-mir-1306	C19orf23	0.306727823	mirna_pc
24265	hsa-mir-1306	GPS1	0.306038556	mirna_pc
24266	hsa-mir-1306	MYL6B	0.361264821	mirna_pc
24267	hsa-mir-1306	PDCD7	0.341460533	mirna_pc
24268	hsa-mir-1306	POLR2F	0.338777989	mirna_pc
24269	hsa-mir-1306	PIPSL	0.312950399	mirna_pc
24270	hsa-mir-1306	MRPS15	0.350162336	mirna_pc
24271	hsa-mir-1306	NARS2	0.321006832	mirna_pc
24272	hsa-mir-1306	EIF2B3	0.351198123	mirna_pc
24273	hsa-mir-1306	TMEM14C	0.308385175	mirna_pc
24274	hsa-mir-1306	TUBGCP4	0.366961268	mirna_pc
24275	hsa-mir-1306	DGCR6L	0.354108456	mirna_pc
24276	hsa-mir-1306	LYRM4	0.391136948	mirna_pc
24277	hsa-mir-1306	MMACHC	0.382535996	mirna_pc
24278	hsa-mir-1306	UTP11L	0.301129505	mirna_pc
24279	hsa-mir-1306	USP21	0.314492293	mirna_pc
24280	hsa-mir-1306	RFT1	0.301058742	mirna_pc
24281	hsa-mir-1306	ZNF74	0.467064743	mirna_pc
24282	hsa-mir-1306	ANP32A	0.389791517	mirna_pc
24283	hsa-mir-1306	SUPV3L1	0.342811553	mirna_pc
24284	hsa-mir-1306	HYLS1	0.317716355	mirna_pc
24285	hsa-mir-1306	HIC2	0.33142556	mirna_pc
24286	hsa-mir-1306	SDF2L1	0.336381826	mirna_pc
24287	hsa-mir-1306	THAP7	0.3865015	mirna_pc
24288	hsa-mir-1306	LCMT2	0.404601467	mirna_pc
24289	hsa-mir-1306	RRP1B	0.325584035	mirna_pc
24290	hsa-mir-1306	IQCC	0.31062855	mirna_pc
24291	hsa-mir-1306	PCID2	0.302667831	mirna_pc
24292	hsa-mir-1306	MORN2	0.302025032	mirna_pc
24293	hsa-mir-1306	C15orf40	0.339996278	mirna_pc
24294	hsa-mir-1306	MESDC2	0.315303202	mirna_pc
24295	hsa-mir-1306	SBK1	0.364108962	mirna_pc
24296	hsa-mir-1306	AFMID	0.326979237	mirna_pc
24297	hsa-mir-1306	SF3A1	0.330243688	mirna_pc
24298	hsa-mir-1306	USP13	0.314808133	mirna_pc
24299	hsa-mir-1306	SLC25A33	0.422436699	mirna_pc

24300	hsa-mir-1306	MSI1	0.361265731	mirna_pc
24301	hsa-mir-1306	APCDD1	0.312314526	mirna_pc
24302	hsa-mir-1306	BCS1L	0.356067568	mirna_pc
24303	hsa-mir-1306	DDX51	0.355012164	mirna_pc
24304	hsa-mir-1306	PBX2	0.310281472	mirna_pc
24305	hsa-mir-1306	ZBTB39	0.352523152	mirna_pc
24306	hsa-mir-1306	EDC3	0.321400337	mirna_pc
24307	hsa-mir-1306	PACSIN3	0.332045416	mirna_pc
24308	hsa-mir-1306	MED17	0.323485559	mirna_pc
24309	hsa-mir-1306	VPS54	0.320883676	mirna_pc
24310	hsa-mir-1306	EIF2AK4	0.31096083	mirna_pc
24311	hsa-mir-1306	RBBP4	0.319210842	mirna_pc
24312	hsa-mir-1306	LOC221710	0.322232305	mirna_pc
24313	hsa-mir-1306	API5	0.313491303	mirna_pc
24314	hsa-mir-1306	C2orf3	0.366612091	mirna_pc
24315	hsa-mir-1306	PTPMT1	0.303600166	mirna_pc
24316	hsa-mir-1306	DHRS2	0.345669251	mirna_pc
24317	hsa-mir-1306	MEAF6	0.326190427	mirna_pc
24318	hsa-mir-1306	RRP7B	0.310789276	mirna_pc
24319	hsa-mir-1306	TBX1	0.392014073	mirna_pc
24320	hsa-mir-1306	SRRD	0.35518987	mirna_pc
24321	hsa-mir-1306	UBE2L3	0.416421284	mirna_pc
24322	hsa-mir-1306	MFAP1	0.302432367	mirna_pc
24323	hsa-mir-1306	COX7A2L	0.320165665	mirna_pc
24324	hsa-mir-1306	EXOSC6	0.330998738	mirna_pc
24325	hsa-mir-1306	AADAT	0.30173736	mirna_pc
24326	hsa-mir-1306	ACP1	0.373900056	mirna_pc
24327	hsa-mir-1306	ZNF322A	0.303710312	mirna_pc
24328	hsa-mir-1306	CDAN1	0.389508343	mirna_pc
24329	hsa-mir-1306	SERF1A	0.371519248	mirna_pc
24330	hsa-mir-1306	CCDC117	0.332144118	mirna_pc
24331	hsa-mir-1306	PREB	0.371268515	mirna_pc
24332	hsa-mir-1306	PRMT6	0.368951099	mirna_pc
24333	hsa-mir-1306	LOC401397	0.309566398	mirna_pc
24334	hsa-mir-1306	DGCR14	0.32313463	mirna_pc
24335	hsa-mir-1306	C2orf28	0.305809933	mirna_pc
24336	hsa-mir-1306	H2AFY2	0.312139568	mirna_pc
24337	hsa-mir-1306	MRPL40	0.375157751	mirna_pc
24338	hsa-mir-1306	UBL7	0.355963477	mirna_pc
24339	hsa-mir-1306	TMEM14B	0.380546866	mirna_pc
24340	hsa-mir-1306	THOC5	0.342457435	mirna_pc
24341	hsa-mir-1306	C21orf70	0.325144429	mirna_pc
24342	hsa-mir-1306	C22orf29	0.333757451	mirna_pc
24343	hsa-mir-1306	MRPL53	0.329032933	mirna_pc
24344	hsa-mir-1306	ZNF592	0.310589619	mirna_pc
24345	hsa-mir-1306	DNMT3A	0.354780761	mirna_pc
24346	hsa-mir-1306	TET1	0.314692271	mirna_pc
24347	hsa-mir-1306	C12orf73	0.315797072	mirna_pc
24348	hsa-mir-1306	PATZ1	0.306286787	mirna_pc
24349	hsa-mir-1306	C15orf61	0.332029132	mirna_pc
24350	hsa-mir-1306	SLC2A8	0.320411983	mirna_pc
24351	hsa-mir-1306	ZNF691	0.304759181	mirna_pc
24352	hsa-mir-323	IPW	0.370598814	mirna_pc
24353	hsa-mir-323	KIF2C	0.326126498	mirna_pc

24354	hsa-mir-323	PLK4	0.327944568	mirna_pc
24355	hsa-mir-323	HBB	0.55595234	mirna_pc
24356	hsa-mir-323	HBA1	0.379938594	mirna_pc
24357	hsa-mir-323	CENPO	0.339415976	mirna_pc
24358	hsa-mir-323	CDKN1C	0.309563051	mirna_pc
24359	hsa-mir-323	ZNF167	0.315340148	mirna_pc
24360	hsa-mir-323	MEG3	0.78568005	mirna_pc
24361	hsa-mir-323	HBA2	0.352285967	mirna_pc
24362	hsa-mir-323	PSRC1	0.327913843	mirna_pc
24363	hsa-mir-323	C17orf107	0.368579724	mirna_pc
24364	hsa-mir-323	TRAF3	0.321809756	mirna_pc
24365	hsa-mir-323	DNMT3B	0.397678611	mirna_pc
24366	hsa-mir-323	PA2G4	0.311854035	mirna_pc
24367	hsa-mir-323	MTL 5.00	0.332152197	mirna_pc
24368	hsa-mir-323	CCNE1	0.3376807	mirna_pc
24369	hsa-mir-323	RGL2	0.310924727	mirna_pc
24370	hsa-mir-323	TMEM150A	0.306988468	mirna_pc
24371	hsa-mir-323	EHMT2	0.389815732	mirna_pc
24372	hsa-mir-323	FHOD1	0.346269942	mirna_pc
24373	hsa-mir-323	CDRT4	0.36885825	mirna_pc
24374	hsa-mir-323	DNAJC7	0.302984603	mirna_pc
24375	hsa-mir-323	ZNF595	0.320478083	mirna_pc
24376	hsa-mir-323	SLC2A6	0.331244479	mirna_pc
24377	hsa-mir-323	PA2G4P4	0.345738455	mirna_pc
24378	hsa-mir-323	NKIRAS2	0.33611914	mirna_pc
24379	hsa-mir-323	MEX3A	0.447838225	mirna_pc
24380	hsa-mir-323	VARS	0.311610562	mirna_pc
24381	hsa-mir-323	ACAN	0.648387217	mirna_pc
24382	hsa-mir-323	C16orf70	0.33730649	mirna_pc
24383	hsa-mir-323	SC65	0.423045526	mirna_pc
24384	hsa-mir-323	PXDN	0.318545583	mirna_pc
24385	hsa-mir-323	ATAD3B	0.300797243	mirna_pc
24386	hsa-mir-323	SCAND2	0.327112518	mirna_pc
24387	hsa-mir-323	ENPEP	0.300709507	mirna_pc
24388	hsa-mir-323	ITPRIPL1	0.301459194	mirna_pc
24389	hsa-mir-323	GDPD5	0.402603788	mirna_pc
24390	hsa-mir-323	SNORA8	0.363301632	mirna_pc
24391	hsa-mir-323	NID1	0.400579386	mirna_pc
24392	hsa-mir-323	TRPA1	0.352043884	mirna_pc
24393	hsa-mir-323	NAT9	0.338895979	mirna_pc
24394	hsa-mir-323	KIAA1731	0.323412519	mirna_pc
24395	hsa-mir-323	FKBP10	0.41855555	mirna_pc
24396	hsa-mir-323	PCDHB5	0.312014685	mirna_pc
24397	hsa-mir-323	RDBP	0.36175945	mirna_pc
24398	hsa-mir-323	SFMBT1	0.302393205	mirna_pc
24399	hsa-mir-323	LY86	0.32288597	mirna_pc
24400	hsa-mir-323	ZNF697	0.35493423	mirna_pc
24401	hsa-mir-323	CD200	0.306489971	mirna_pc
24402	hsa-mir-323	GPX7	0.342815336	mirna_pc
24403	hsa-mir-323	RNF41	0.434474925	mirna_pc
24404	hsa-mir-323	RFT1	0.340828479	mirna_pc
24405	hsa-mir-323	EGFLAM	0.377170875	mirna_pc
24406	hsa-mir-323	GLT8D1	0.373767022	mirna_pc
24407	hsa-mir-323	C9orf84	0.378173499	mirna_pc

24408	hsa-mir-323	GALNT14	0.340273034	mirna_pc
24409	hsa-mir-323	CD69	0.336174156	mirna_pc
24410	hsa-mir-323	BCAT1	0.343280036	mirna_pc
24411	hsa-mir-323	N4BP2	0.310778015	mirna_pc
24412	hsa-mir-323	PBX2	0.356653669	mirna_pc
24413	hsa-mir-323	SNCAIP	0.576635837	mirna_pc
24414	hsa-mir-323	ZNF232	0.324928553	mirna_pc
24415	hsa-mir-323	KIF12	0.565467081	mirna_pc
24416	hsa-mir-323	RNF32	0.311943392	mirna_pc
24417	hsa-mir-323	C4A	0.399747727	mirna_pc
24418	hsa-mir-323	MED17	0.341887119	mirna_pc
24419	hsa-mir-323	HYOU1	0.470485542	mirna_pc
24420	hsa-mir-323	DHRS2	0.620588547	mirna_pc
24421	hsa-mir-323	CCDC102B	0.338857127	mirna_pc
24422	hsa-mir-323	LOC220594	0.330472769	mirna_pc
24423	hsa-mir-323	SMARCC2	0.321900689	mirna_pc
24424	hsa-mir-323	LOC645166	0.514931385	mirna_pc
24425	hsa-mir-323	NT5C3L	0.408583913	mirna_pc
24426	hsa-mir-323	WDR73	0.531274594	mirna_pc
24427	hsa-mir-323	PCDH18	0.34220886	mirna_pc
24428	hsa-mir-323	NES	0.331632656	mirna_pc
24429	hsa-mir-323	GJC1	0.320816165	mirna_pc
24430	hsa-mir-323	DNMT3A	0.351160572	mirna_pc
24431	hsa-mir-323	FAM69B	0.332349522	mirna_pc
24432	hsa-mir-323	SLC2A8	0.355370637	mirna_pc
24433	hsa-mir-511-2	HBB	0.409969405	mirna_pc
24434	hsa-mir-511-2	LOC541471	0.357309159	mirna_pc
24435	hsa-mir-511-2	GLT25D1	0.384204776	mirna_pc
24436	hsa-mir-511-2	HBA1	0.394026803	mirna_pc
24437	hsa-mir-511-2	FAM64A	0.316715357	mirna_pc
24438	hsa-mir-511-2	NCRNA00152	0.305536923	mirna_pc
24439	hsa-mir-511-2	SERPINH1	0.305906274	mirna_pc
24440	hsa-mir-511-2	MYO1B	0.396381941	mirna_pc
24441	hsa-mir-511-2	SIGLEC7	0.425324382	mirna_pc
24442	hsa-mir-511-2	ENO1	0.302972401	mirna_pc
24443	hsa-mir-511-2	LAMC2	0.43731863	mirna_pc
24444	hsa-mir-511-2	TMSB10	0.422240837	mirna_pc
24445	hsa-mir-511-2	CD80	0.481333411	mirna_pc
24446	hsa-mir-511-2	LDHA	0.355610674	mirna_pc
24447	hsa-mir-511-2	HBA2	0.33869217	mirna_pc
24448	hsa-mir-511-2	RELT	0.425370169	mirna_pc
24449	hsa-mir-511-2	PLAU	0.374678518	mirna_pc
24450	hsa-mir-511-2	IFI30	0.316759235	mirna_pc
24451	hsa-mir-511-2	CLEC5A	0.42698678	mirna_pc
24452	hsa-mir-511-2	SNX10	0.321178558	mirna_pc
24453	hsa-mir-511-2	S100A3	0.421193944	mirna_pc
24454	hsa-mir-511-2	CD300LF	0.409364176	mirna_pc
24455	hsa-mir-511-2	PLA2G7	0.37336392	mirna_pc
24456	hsa-mir-511-2	PGK 1.00	0.310119039	mirna_pc
24457	hsa-mir-511-2	IFITM3	0.358181257	mirna_pc
24458	hsa-mir-511-2	CCL3	0.556267822	mirna_pc
24459	hsa-mir-511-2	EXT1	0.31474842	mirna_pc
24460	hsa-mir-511-2	AGTRAP	0.319894876	mirna_pc
24461	hsa-mir-511-2	COL7A1	0.329949762	mirna_pc

24462	hsa-mir-511-2	CAPG	0.302927081	mirna_pc
24463	hsa-mir-511-2	FSCN1	0.327325147	mirna_pc
24464	hsa-mir-511-2	RGS19	0.383268113	mirna_pc
24465	hsa-mir-511-2	AP2S1	0.333501059	mirna_pc
24466	hsa-mir-511-2	NSUN5	0.302698101	mirna_pc
24467	hsa-mir-511-2	UBE2L6	0.372705044	mirna_pc
24468	hsa-mir-511-2	CCDC109B	0.302886991	mirna_pc
24469	hsa-mir-511-2	PLOD1	0.315362587	mirna_pc
24470	hsa-mir-511-2	C17orf49	0.302692978	mirna_pc
24471	hsa-mir-511-2	CCL4L2	0.335903225	mirna_pc
24472	hsa-mir-511-2	FCGR1A	0.343341478	mirna_pc
24473	hsa-mir-511-2	MMP12	0.581051464	mirna_pc
24474	hsa-mir-511-2	OSCAR	0.578583875	mirna_pc
24475	hsa-mir-511-2	FOXD1	0.301026463	mirna_pc
24476	hsa-mir-511-2	SLC25A22	0.329331094	mirna_pc
24477	hsa-mir-511-2	WDR4	0.302734827	mirna_pc
24478	hsa-mir-511-2	RAC2	0.321750247	mirna_pc
24479	hsa-mir-511-2	TPD52L2	0.338504478	mirna_pc
24480	hsa-mir-511-2	LILRB5	0.316231182	mirna_pc
24481	hsa-mir-511-2	MT2A	0.328676216	mirna_pc
24482	hsa-mir-511-2	LRWD1	0.365376364	mirna_pc
24483	hsa-mir-511-2	FTSJ1	0.311958776	mirna_pc
24484	hsa-mir-511-2	KDELC1	0.377696683	mirna_pc
24485	hsa-mir-511-2	FCGR3A	0.42375667	mirna_pc
24486	hsa-mir-511-2	LILRB4	0.333170425	mirna_pc
24487	hsa-mir-511-2	BCL2A1	0.317472045	mirna_pc
24488	hsa-mir-511-2	NCF2	0.323651673	mirna_pc
24489	hsa-mir-511-2	TGFBI	0.345704381	mirna_pc
24490	hsa-mir-511-2	SLC2A6	0.341984268	mirna_pc
24491	hsa-mir-511-2	SEMA5B	0.430792331	mirna_pc
24492	hsa-mir-511-2	IGSF6	0.384409057	mirna_pc
24493	hsa-mir-511-2	CD86	0.376165872	mirna_pc
24494	hsa-mir-511-2	ITGAX	0.361041723	mirna_pc
24495	hsa-mir-511-2	TPI1	0.301560333	mirna_pc
24496	hsa-mir-511-2	FCER1G	0.459590705	mirna_pc
24497	hsa-mir-511-2	HAVCR2	0.374850323	mirna_pc
24498	hsa-mir-511-2	ACAN	0.317603146	mirna_pc
24499	hsa-mir-511-2	LDOC1	0.314176814	mirna_pc
24500	hsa-mir-511-2	MAN1B1	0.308580834	mirna_pc
24501	hsa-mir-511-2	COL27A1	0.328729692	mirna_pc
24502	hsa-mir-511-2	MNDA	0.436222192	mirna_pc
24503	hsa-mir-511-2	CD300A	0.431579457	mirna_pc
24504	hsa-mir-511-2	FCGR2A	0.604140404	mirna_pc
24505	hsa-mir-511-2	FAM26F	0.310489299	mirna_pc
24506	hsa-mir-511-2	C9orf30	0.319382216	mirna_pc
24507	hsa-mir-511-2	CLEC4A	0.479011618	mirna_pc
24508	hsa-mir-511-2	IL2RA	0.407920822	mirna_pc
24509	hsa-mir-511-2	GPSM1	0.373731625	mirna_pc
24510	hsa-mir-511-2	SLAMF8	0.403648401	mirna_pc
24511	hsa-mir-511-2	HK3	0.611825386	mirna_pc
24512	hsa-mir-511-2	ITGA3	0.360866129	mirna_pc
24513	hsa-mir-511-2	HES4	0.307633039	mirna_pc
24514	hsa-mir-511-2	LAPTM5	0.305428512	mirna_pc
24515	hsa-mir-511-2	C13orf27	0.303151644	mirna_pc

24516	hsa-mir-511-2	RAB7A	0.319483337	mirna_pc
24517	hsa-mir-511-2	MSR1	0.308134345	mirna_pc
24518	hsa-mir-511-2	RHOG	0.38341813	mirna_pc
24519	hsa-mir-511-2	CCL4	0.456715028	mirna_pc
24520	hsa-mir-511-2	CD14	0.522777702	mirna_pc
24521	hsa-mir-511-2	PLA2G15	0.32335523	mirna_pc
24522	hsa-mir-511-2	TLR2	0.323876436	mirna_pc
24523	hsa-mir-511-2	PILRA	0.407086682	mirna_pc
24524	hsa-mir-511-2	DRAP1	0.40282866	mirna_pc
24525	hsa-mir-511-2	B3GAT3	0.378184703	mirna_pc
24526	hsa-mir-511-2	C1QC	0.417510333	mirna_pc
24527	hsa-mir-511-2	TYROBP	0.368854327	mirna_pc
24528	hsa-mir-511-2	FAM65A	0.304472992	mirna_pc
24529	hsa-mir-511-2	SLC11A1	0.369355672	mirna_pc
24530	hsa-mir-511-2	C1QB	0.414340473	mirna_pc
24531	hsa-mir-511-2	LILRB2	0.424590059	mirna_pc
24532	hsa-mir-511-2	PSMC3	0.323849051	mirna_pc
24533	hsa-mir-511-2	LGALS1	0.390137895	mirna_pc
24534	hsa-mir-511-2	FOLR2	0.416180606	mirna_pc
24535	hsa-mir-511-2	ITGB2	0.387257026	mirna_pc
24536	hsa-mir-511-2	LST1	0.358868415	mirna_pc
24537	hsa-mir-511-2	AQP9	0.368607392	mirna_pc
24538	hsa-mir-511-2	CCM2	0.302652625	mirna_pc
24539	hsa-mir-511-2	PANX1	0.406957043	mirna_pc
24540	hsa-mir-511-2	PRSS23	0.326086574	mirna_pc
24541	hsa-mir-511-2	ACTB	0.33220284	mirna_pc
24542	hsa-mir-511-2	CYP26B1	0.332924192	mirna_pc
24543	hsa-mir-511-2	CCL18	0.425598198	mirna_pc
24544	hsa-mir-511-2	SH2B2	0.302178536	mirna_pc
24545	hsa-mir-511-2	FPR3	0.37968391	mirna_pc
24546	hsa-mir-511-2	MMP25	0.322059198	mirna_pc
24547	hsa-mir-511-2	SAMSN1	0.319637924	mirna_pc
24548	hsa-mir-511-2	SPI1	0.42122702	mirna_pc
24549	hsa-mir-511-2	PDPN	0.33415366	mirna_pc
24550	hsa-mir-511-2	CD4	0.329591839	mirna_pc
24551	hsa-mir-511-2	NECAP2	0.3517066	mirna_pc
24552	hsa-mir-511-2	LCP2	0.334479018	mirna_pc
24553	hsa-mir-511-2	AIF1	0.448702691	mirna_pc
24554	hsa-mir-511-2	KIAA1949	0.30318954	mirna_pc
24555	hsa-mir-511-2	LAIR1	0.360604974	mirna_pc
24556	hsa-mir-511-2	KMO	0.3081093	mirna_pc
24557	hsa-mir-511-2	MYO1G	0.375014742	mirna_pc
24558	hsa-mir-511-2	TMSL3	0.311135976	mirna_pc
24559	hsa-mir-511-2	CTSL1	0.367620113	mirna_pc
24560	hsa-mir-511-2	UROD	0.358675222	mirna_pc
24561	hsa-mir-511-2	TNFAIP8L2	0.356143743	mirna_pc
24562	hsa-mir-511-2	EMILIN2	0.412487914	mirna_pc
24563	hsa-mir-511-2	SIRPB1	0.34337278	mirna_pc
24564	hsa-mir-511-2	TMEM107	0.303611493	mirna_pc
24565	hsa-mir-511-2	C5AR1	0.314987018	mirna_pc
24566	hsa-mir-511-2	KIAA1644	0.323926362	mirna_pc
24567	hsa-mir-511-2	TLR8	0.340168064	mirna_pc
24568	hsa-mir-511-2	ITGA5	0.344166587	mirna_pc
24569	hsa-mir-511-2	LILRB1	0.373534146	mirna_pc

24570	hsa-mir-511-2	VIM	0.388191133	mirna_pc
24571	hsa-mir-511-2	TMEFF1	0.364643318	mirna_pc
24572	hsa-mir-511-2	GOLGA7B	0.305069905	mirna_pc
24573	hsa-mir-511-2	C1QA	0.446385492	mirna_pc
24574	hsa-mir-511-2	LRRC3	0.320964121	mirna_pc
24575	hsa-mir-511-2	RASGRP4	0.348391534	mirna_pc
24576	hsa-mir-511-2	SLC15A3	0.304327791	mirna_pc
24577	hsa-mir-511-2	MRC1	0.675876926	mirna_pc
24578	hsa-mir-511-2	C1QL1	0.370430907	mirna_pc
24579	hsa-mir-511-2	CCDC85B	0.335292339	mirna_pc
24580	hsa-mir-511-2	SERPINE1	0.524783663	mirna_pc
24581	hsa-mir-511-2	SIRPA	0.304036473	mirna_pc
24582	hsa-mir-511-2	CYBB	0.372395552	mirna_pc
24583	hsa-mir-511-2	MSN	0.365388676	mirna_pc
24584	hsa-mir-511-2	LILRA6	0.422677413	mirna_pc
24585	hsa-mir-511-2	PTRH1	0.356120286	mirna_pc
24586	hsa-mir-511-2	FCGR2B	0.379095134	mirna_pc
24587	hsa-mir-511-2	ADORA3	0.366631522	mirna_pc
24588	hsa-mir-511-2	SLC39A13	0.316497975	mirna_pc
24589	hsa-mir-511-2	CPA4	0.435616086	mirna_pc
24590	hsa-mir-511-2	GNA12	0.366122697	mirna_pc
24591	hsa-mir-511-2	ITGAM	0.333472529	mirna_pc
24592	hsa-mir-511-2	COL4A2	0.300744632	mirna_pc
24593	hsa-mir-511-2	LRRC25	0.388293881	mirna_pc
24594	hsa-mir-511-2	FADS3	0.390680664	mirna_pc
24595	hsa-mir-511-2	SNAI2	0.301534397	mirna_pc
24596	hsa-mir-511-2	SLC31A2	0.329693873	mirna_pc
24597	hsa-mir-511-2	GDI1	0.328380237	mirna_pc
24598	hsa-mir-511-2	CYTH4	0.403374713	mirna_pc
24599	hsa-mir-511-2	EIF2B3	0.359646251	mirna_pc
24600	hsa-mir-511-2	MRPS6	0.367306761	mirna_pc
24601	hsa-mir-511-2	ARHGAP22	0.302812682	mirna_pc
24602	hsa-mir-511-2	LILRB3	0.36372581	mirna_pc
24603	hsa-mir-511-2	NLRP3	0.317250946	mirna_pc
24604	hsa-mir-511-2	PIK3CD	0.388862054	mirna_pc
24605	hsa-mir-511-2	CAV1	0.323896257	mirna_pc
24606	hsa-mir-511-2	ATOX1	0.375433008	mirna_pc
24607	hsa-mir-511-2	C3AR1	0.416100143	mirna_pc
24608	hsa-mir-511-2	CD209	0.49874141	mirna_pc
24609	hsa-mir-511-2	CD163	0.39648075	mirna_pc
24610	hsa-mir-511-2	LRRC33	0.314325456	mirna_pc
24611	hsa-mir-511-2	RAP1GDS1	0.367159733	mirna_pc
24612	hsa-mir-511-2	CD53	0.310981224	mirna_pc
24613	hsa-mir-511-2	TSSC4	0.327675579	mirna_pc
24614	hsa-mir-511-2	DOK2	0.428867862	mirna_pc
24615	hsa-mir-511-2	SRPK2	0.320346305	mirna_pc
24616	hsa-mir-511-2	NRG1	0.31090985	mirna_pc
24617	hsa-mir-511-2	VSIG4	0.379815591	mirna_pc
24618	hsa-mir-511-2	AFAP1L1	0.355122129	mirna_pc
24619	hsa-mir-511-2	SRGN	0.45670061	mirna_pc
24620	hsa-mir-511-2	HGF	0.369047532	mirna_pc
24621	hsa-mir-511-2	STK10	0.306054722	mirna_pc
24622	hsa-mir-511-2	VKORC1	0.307035769	mirna_pc
24623	hsa-mir-511-2	SGTB	0.325619294	mirna_pc

24624	hsa-mir-511-2	HYOU1	0.323181178	mirna_pc
24625	hsa-mir-511-2	GPR68	0.360840959	mirna_pc
24626	hsa-mir-511-2	EMP3	0.355160616	mirna_pc
24627	hsa-mir-511-2	LPL	0.311086439	mirna_pc
24628	hsa-mir-511-2	P2RX5	0.302071106	mirna_pc
24629	hsa-mir-511-2	PEA15	0.349108159	mirna_pc
24630	hsa-mir-511-2	DKK 3.00	0.399081016	mirna_pc
24631	hsa-mir-511-2	C1orf162	0.370261178	mirna_pc
24632	hsa-mir-511-2	KIFC3	0.307378892	mirna_pc
24633	hsa-mir-511-2	FST	0.339995825	mirna_pc
24634	hsa-mir-511-2	HAS2	0.413913796	mirna_pc
24635	hsa-mir-511-2	LOC645166	0.366874817	mirna_pc
24636	hsa-mir-511-2	ODZ2	0.304406334	mirna_pc
24637	hsa-mir-511-2	FAM195B	0.307697586	mirna_pc
24638	hsa-mir-511-2	C9orf37	0.328649678	mirna_pc
24639	hsa-mir-511-2	MOBKL2A	0.311691002	mirna_pc
24640	hsa-mir-511-2	OLFML2A	0.332028076	mirna_pc
24641	hsa-mir-511-2	CMKLR1	0.33924185	mirna_pc
24642	hsa-mir-511-2	RIC8A	0.338535997	mirna_pc
24643	hsa-mir-511-2	MS4A6A	0.310042196	mirna_pc
24644	hsa-mir-511-2	VEGFC	0.415334476	mirna_pc
24645	hsa-mir-511-2	CCR1	0.33667113	mirna_pc
24646	hsa-mir-511-2	ACP2	0.312475384	mirna_pc
24647	hsa-mir-511-2	C21orf7	0.477874387	mirna_pc
24648	hsa-mir-511-2	DFNA5	0.413687249	mirna_pc
24649	hsa-mir-511-2	MS4A7	0.336678297	mirna_pc
24650	hsa-mir-511-2	SIGLEC1	0.355838767	mirna_pc
24651	hsa-mir-511-2	HHLA3	0.342450235	mirna_pc
24652	hsa-mir-511-2	FGR	0.314913502	mirna_pc
24653	hsa-mir-511-2	FAM70B	0.307814731	mirna_pc
24654	hsa-mir-511-2	P4HA2	0.308720664	mirna_pc
24655	hsa-mir-511-2	MS4A4A	0.418691541	mirna_pc
24656	hsa-mir-511-2	DPM2	0.313329156	mirna_pc
24657	hsa-mir-511-2	ZC3HC1	0.342677157	mirna_pc
24658	hsa-mir-511-2	FEZ1	0.35078897	mirna_pc
24659	hsa-mir-132	TDO2	0.355593029	mirna_pc
24660	hsa-mir-132	BUB3	0.323333855	mirna_pc
24661	hsa-mir-132	IL11	0.382755105	mirna_pc
24662	hsa-mir-132	SH2D2A	0.323339454	mirna_pc
24663	hsa-mir-132	PLAU	0.304264466	mirna_pc
24664	hsa-mir-132	IFI30	0.313500079	mirna_pc
24665	hsa-mir-132	CD300LF	0.330256855	mirna_pc
24666	hsa-mir-132	PLA2G7	0.321145315	mirna_pc
24667	hsa-mir-132	RCN1	0.314906296	mirna_pc
24668	hsa-mir-132	CCL3	0.324632821	mirna_pc
24669	hsa-mir-132	IL24	0.308753057	mirna_pc
24670	hsa-mir-132	IKBIP	0.326049244	mirna_pc
24671	hsa-mir-132	MMP1	0.318423642	mirna_pc
24672	hsa-mir-132	TIMP1	0.331019896	mirna_pc
24673	hsa-mir-132	BCL2A1	0.357051621	mirna_pc
24674	hsa-mir-132	CXCL6	0.352573508	mirna_pc
24675	hsa-mir-132	CD300A	0.412715528	mirna_pc
24676	hsa-mir-132	FCGR2A	0.316345799	mirna_pc
24677	hsa-mir-132	ASAP1	0.373804053	mirna_pc

24678	hsa-mir-132	CLEC4A	0.355479834	mirna_pc
24679	hsa-mir-132	C9orf110	0.360990749	mirna_pc
24680	hsa-mir-132	HK3	0.31573056	mirna_pc
24681	hsa-mir-132	LAPTM5	0.347550522	mirna_pc
24682	hsa-mir-132	HMGA2	0.305217664	mirna_pc
24683	hsa-mir-132	C5orf15	0.307366721	mirna_pc
24684	hsa-mir-132	GK	0.310082884	mirna_pc
24685	hsa-mir-132	LAMC3	0.36091897	mirna_pc
24686	hsa-mir-132	LILRB2	0.328234911	mirna_pc
24687	hsa-mir-132	LST1	0.302520267	mirna_pc
24688	hsa-mir-132	ORAI2	0.313072837	mirna_pc
24689	hsa-mir-132	MMP25	0.374939933	mirna_pc
24690	hsa-mir-132	SAMSN1	0.376217404	mirna_pc
24691	hsa-mir-132	LCP2	0.303631481	mirna_pc
24692	hsa-mir-132	AIF1	0.335727086	mirna_pc
24693	hsa-mir-132	LAT2	0.355432685	mirna_pc
24694	hsa-mir-132	COQ10A	0.340356659	mirna_pc
24695	hsa-mir-132	DOK3	0.322245908	mirna_pc
24696	hsa-mir-132	TFAM	0.318108127	mirna_pc
24697	hsa-mir-132	NRGN	0.307804556	mirna_pc
24698	hsa-mir-132	PTGIR	0.303493779	mirna_pc
24699	hsa-mir-132	PRR16	0.321304343	mirna_pc
24700	hsa-mir-132	LILRB1	0.343121964	mirna_pc
24701	hsa-mir-132	C8orf37	0.307638285	mirna_pc
24702	hsa-mir-132	EDNRA	0.304468432	mirna_pc
24703	hsa-mir-132	SLC35F2	0.300898262	mirna_pc
24704	hsa-mir-132	ZBTB10	0.320903245	mirna_pc
24705	hsa-mir-132	SLC43A3	0.303661434	mirna_pc
24706	hsa-mir-132	NPW	0.309808288	mirna_pc
24707	hsa-mir-132	SIPA1	0.304009706	mirna_pc
24708	hsa-mir-132	GRB14	0.429322977	mirna_pc
24709	hsa-mir-132	TRA2A	0.301602419	mirna_pc
24710	hsa-mir-132	LILRB3	0.310452033	mirna_pc
24711	hsa-mir-132	SERPINA1	0.337834586	mirna_pc
24712	hsa-mir-132	NLRP3	0.349598439	mirna_pc
24713	hsa-mir-132	TMEM22	0.30656851	mirna_pc
24714	hsa-mir-132	BCCIP	0.324742871	mirna_pc
24715	hsa-mir-132	HLA-DRB5	0.344114323	mirna_pc
24716	hsa-mir-132	PSTK	0.30210361	mirna_pc
24717	hsa-mir-132	GPR97	0.32599299	mirna_pc
24718	hsa-mir-132	FMNL1	0.314970197	mirna_pc
24719	hsa-mir-132	SRGN	0.397908072	mirna_pc
24720	hsa-mir-132	PGBD3	0.300140726	mirna_pc
24721	hsa-mir-132	C10orf88	0.386683438	mirna_pc
24722	hsa-mir-132	GTF2H2B	0.319570862	mirna_pc
24723	hsa-mir-132	STARD3NL	0.324240853	mirna_pc
24724	hsa-mir-132	HAPLN1	0.301307658	mirna_pc
24725	hsa-mir-132	POU2F2	0.305179261	mirna_pc
24726	hsa-mir-132	HSD17B14	0.30738148	mirna_pc
24727	hsa-mir-132	CCDC102B	0.319235776	mirna_pc
24728	hsa-mir-132	KDELC2	0.318230202	mirna_pc
24729	hsa-mir-132	FAM101B	0.321579498	mirna_pc
24730	hsa-mir-132	C21orf7	0.350397357	mirna_pc
24731	hsa-mir-132	LAMB1	0.323148071	mirna_pc

24732	hsa-mir-132	SLC2A3	0.351606578	mirna_pc
24733	hsa-mir-616	KIFC1	0.327348097	mirna_pc
24734	hsa-mir-616	CENPM	0.306390382	mirna_pc
24735	hsa-mir-616	BLM	0.333453359	mirna_pc
24736	hsa-mir-616	OIP5	0.302706467	mirna_pc
24737	hsa-mir-616	RAE1	0.301700641	mirna_pc
24738	hsa-mir-616	ZWILCH	0.367927518	mirna_pc
24739	hsa-mir-616	CDCA7	0.302591779	mirna_pc
24740	hsa-mir-616	SNTB1	0.447169737	mirna_pc
24741	hsa-mir-616	MTBP	0.353534212	mirna_pc
24742	hsa-mir-616	E2F1	0.347392531	mirna_pc
24743	hsa-mir-616	KLF2	0.407286732	mirna_pc
24744	hsa-mir-616	RFC3	0.398033714	mirna_pc
24745	hsa-mir-616	C5	0.335880487	mirna_pc
24746	hsa-mir-616	PSMB3	0.699559862	mirna_pc
24747	hsa-mir-616	TCF19	0.328256285	mirna_pc
24748	hsa-mir-616	CDK2	0.336438511	mirna_pc
24749	hsa-mir-616	CDK4	0.398982828	mirna_pc
24750	hsa-mir-616	PRIM1	0.313296794	mirna_pc
24751	hsa-mir-616	DSCC1	0.530857205	mirna_pc
24752	hsa-mir-616	CLN6	0.434455824	mirna_pc
24753	hsa-mir-616	ATIC	0.381790857	mirna_pc
24754	hsa-mir-616	GSTP1	0.485197354	mirna_pc
24755	hsa-mir-616	SNX8	0.357875135	mirna_pc
24756	hsa-mir-616	FAM122A	0.464419913	mirna_pc
24757	hsa-mir-616	MRPL13	0.339252468	mirna_pc
24758	hsa-mir-616	TMEM194A	0.358446556	mirna_pc
24759	hsa-mir-616	NOLC1	0.303603765	mirna_pc
24760	hsa-mir-616	METTL1	0.356059328	mirna_pc
24761	hsa-mir-616	HIF1AN	0.306190815	mirna_pc
24762	hsa-mir-616	SCARB1	0.338761159	mirna_pc
24763	hsa-mir-616	ANKRD13D	0.485201087	mirna_pc
24764	hsa-mir-616	ANO7	0.304866076	mirna_pc
24765	hsa-mir-616	TFR2	0.30307206	mirna_pc
24766	hsa-mir-616	CARD9	0.648014345	mirna_pc
24767	hsa-mir-616	YTHDF1	0.31092477	mirna_pc
24768	hsa-mir-616	RAB8A	0.656251792	mirna_pc
24769	hsa-mir-616	TRIB3	0.468406734	mirna_pc
24770	hsa-mir-616	IPO4	0.309145319	mirna_pc
24771	hsa-mir-616	GSTT1	0.317258649	mirna_pc
24772	hsa-mir-616	GRB7	0.475012026	mirna_pc
24773	hsa-mir-616	SPG21	0.362421595	mirna_pc
24774	hsa-mir-616	NMU	0.369517036	mirna_pc
24775	hsa-mir-616	MLLT6	0.334403813	mirna_pc
24776	hsa-mir-616	C17orf96	0.425870166	mirna_pc
24777	hsa-mir-616	SHMT2	0.432178819	mirna_pc
24778	hsa-mir-616	GPAM	0.330841856	mirna_pc
24779	hsa-mir-616	VARS	0.426650543	mirna_pc
24780	hsa-mir-616	C2orf29	0.320510552	mirna_pc
24781	hsa-mir-616	ADRBK1	0.748870489	mirna_pc
24782	hsa-mir-616	NCRNA00081	0.314733424	mirna_pc
24783	hsa-mir-616	C15orf44	0.307495916	mirna_pc
24784	hsa-mir-616	STK16	0.342110302	mirna_pc
24785	hsa-mir-616	FAM96A	0.313454705	mirna_pc

24786	hsa-mir-616	CDK12	0.603382488	mirna_pc
24787	hsa-mir-616	NAGLU	0.335732072	mirna_pc
24788	hsa-mir-616	SPDEF	0.330617696	mirna_pc
24789	hsa-mir-616	GAL3ST1	0.353178391	mirna_pc
24790	hsa-mir-616	FAM188B	0.303372884	mirna_pc
24791	hsa-mir-616	GPC3	0.351863967	mirna_pc
24792	hsa-mir-616	C22orf13	0.351105175	mirna_pc
24793	hsa-mir-616	C19orf73	0.327825644	mirna_pc
24794	hsa-mir-616	EFCAB4B	0.458735252	mirna_pc
24795	hsa-mir-616	TTLL4	0.305398815	mirna_pc
24796	hsa-mir-616	NUDT8	0.430899564	mirna_pc
24797	hsa-mir-616	SFXN4	0.332097077	mirna_pc
24798	hsa-mir-616	HNF1B	0.30226661	mirna_pc
24799	hsa-mir-616	NDUFV1	0.729684515	mirna_pc
24800	hsa-mir-616	ILVBL	0.470794041	mirna_pc
24801	hsa-mir-616	PCGF2	0.323692425	mirna_pc
24802	hsa-mir-616	CWF19L1	0.345453788	mirna_pc
24803	hsa-mir-616	LOC222699	0.311159929	mirna_pc
24804	hsa-mir-616	IDH1	0.387536896	mirna_pc
24805	hsa-mir-616	HHEX	0.469581208	mirna_pc
24806	hsa-mir-616	SMUG1	0.341687219	mirna_pc
24807	hsa-mir-616	PPP1R10	0.34565485	mirna_pc
24808	hsa-mir-616	S100P	0.353588668	mirna_pc
24809	hsa-mir-616	PYCR1	0.344034477	mirna_pc
24810	hsa-mir-616	SERPINF2	0.346467108	mirna_pc
24811	hsa-mir-616	CREB3L4	0.504515307	mirna_pc
24812	hsa-mir-616	FZD5	0.381711354	mirna_pc
24813	hsa-mir-616	LOC151534	0.40836627	mirna_pc
24814	hsa-mir-616	SLC35A1	0.362348379	mirna_pc
24815	hsa-mir-616	TJP2	0.317064278	mirna_pc
24816	hsa-mir-616	TPRN	0.3646486	mirna_pc
24817	hsa-mir-616	RPL23	0.669031268	mirna_pc
24818	hsa-mir-616	GGT1	0.460941535	mirna_pc
24819	hsa-mir-616	SPRYD4	0.4325888	mirna_pc
24820	hsa-mir-616	COASY	0.338955233	mirna_pc
24821	hsa-mir-616	ANXA9	0.441704497	mirna_pc
24822	hsa-mir-616	TRMT12	0.551984339	mirna_pc
24823	hsa-mir-616	MYEOV	0.457788115	mirna_pc
24824	hsa-mir-616	S100A4	0.3901834	mirna_pc
24825	hsa-mir-616	STAT6	0.368121206	mirna_pc
24826	hsa-mir-616	C17orf37	0.386667609	mirna_pc
24827	hsa-mir-616	BRD4	0.301330234	mirna_pc
24828	hsa-mir-616	DDIT3	0.305424385	mirna_pc
24829	hsa-mir-616	FXN	0.409242227	mirna_pc
24830	hsa-mir-616	AKAP8	0.346669152	mirna_pc
24831	hsa-mir-616	NQO1	0.341149451	mirna_pc
24832	hsa-mir-616	SFXN2	0.344462856	mirna_pc
24833	hsa-mir-616	SUPT4H1	0.334234943	mirna_pc
24834	hsa-mir-616	CABIN1	0.316279558	mirna_pc
24835	hsa-mir-616	ACY3	0.621956373	mirna_pc
24836	hsa-mir-616	AP1M1	0.447997632	mirna_pc
24837	hsa-mir-616	ERBB2	0.552663202	mirna_pc
24838	hsa-mir-616	FBXL14	0.365923501	mirna_pc
24839	hsa-mir-616	MNX1	0.318080129	mirna_pc

24840	hsa-mir-616	C2orf72	0.457285933	mirna_pc
24841	hsa-mir-616	LFNG	0.358267588	mirna_pc
24842	hsa-mir-616	MTG1	0.371444676	mirna_pc
24843	hsa-mir-616	PIP4K2B	0.810163108	mirna_pc
24844	hsa-mir-616	ZDHHC6	0.327156866	mirna_pc
24845	hsa-mir-616	TMEM2	0.414221018	mirna_pc
24846	hsa-mir-616	SH3BP4	0.378255264	mirna_pc
24847	hsa-mir-616	FAM32A	0.458766616	mirna_pc
24848	hsa-mir-616	DECR2	0.349136164	mirna_pc
24849	hsa-mir-616	PCYT2	0.380172034	mirna_pc
24850	hsa-mir-616	IARS	0.318070523	mirna_pc
24851	hsa-mir-616	C2orf43	0.3059468	mirna_pc
24852	hsa-mir-616	CISD3	0.465576876	mirna_pc
24853	hsa-mir-616	LOC80054	0.313059487	mirna_pc
24854	hsa-mir-616	PNKD	0.511797294	mirna_pc
24855	hsa-mir-616	HDDC3	0.331651474	mirna_pc
24856	hsa-mir-616	MBD6	0.350916	mirna_pc
24857	hsa-mir-616	POLD4	0.701372359	mirna_pc
24858	hsa-mir-616	AARS	0.319929583	mirna_pc
24859	hsa-mir-616	PRDX3	0.332988598	mirna_pc
24860	hsa-mir-616	SNAPC5	0.438072465	mirna_pc
24861	hsa-mir-616	WIZ	0.464041367	mirna_pc
24862	hsa-mir-616	CWC25	0.333025952	mirna_pc
24863	hsa-mir-616	MARS	0.539806044	mirna_pc
24864	hsa-mir-616	PGAP3	0.696147877	mirna_pc
24865	hsa-mir-616	SSH3	0.652391186	mirna_pc
24866	hsa-mir-576	CENPF	0.326282445	mirna_pc
24867	hsa-mir-576	RCC2	0.409778538	mirna_pc
24868	hsa-mir-576	CCNA2	0.415560965	mirna_pc
24869	hsa-mir-576	FANCI	0.333128591	mirna_pc
24870	hsa-mir-576	MND1	0.329015185	mirna_pc
24871	hsa-mir-576	RAD54L	0.31920588	mirna_pc
24872	hsa-mir-576	MAD2L1	0.374564335	mirna_pc
24873	hsa-mir-576	TIMELESS	0.313525934	mirna_pc
24874	hsa-mir-576	TROAP	0.32212957	mirna_pc
24875	hsa-mir-576	XPO1	0.365597812	mirna_pc
24876	hsa-mir-576	MCM2	0.31871014	mirna_pc
24877	hsa-mir-576	CCNF	0.340715727	mirna_pc
24878	hsa-mir-576	PLK4	0.406462741	mirna_pc
24879	hsa-mir-576	BLM	0.333185076	mirna_pc
24880	hsa-mir-576	DTL	0.346735786	mirna_pc
24881	hsa-mir-576	POLQ	0.363827169	mirna_pc
24882	hsa-mir-576	C1orf112	0.301067916	mirna_pc
24883	hsa-mir-576	NEIL3	0.380979328	mirna_pc
24884	hsa-mir-576	NCAPD2	0.317216054	mirna_pc
24885	hsa-mir-576	UCK2	0.368117378	mirna_pc
24886	hsa-mir-576	C15orf42	0.330467485	mirna_pc
24887	hsa-mir-576	CENPO	0.414945507	mirna_pc
24888	hsa-mir-576	XRCC2	0.308338403	mirna_pc
24889	hsa-mir-576	HMGB2	0.349586641	mirna_pc
24890	hsa-mir-576	CAD	0.349553345	mirna_pc
24891	hsa-mir-576	ZFP64	0.306781959	mirna_pc
24892	hsa-mir-576	CDK4	0.301333416	mirna_pc
24893	hsa-mir-576	TMEM108	0.324336253	mirna_pc

24894	hsa-mir-576	CHTF18	0.327467412	mirna_pc
24895	hsa-mir-576	MSH2	0.34730302	mirna_pc
24896	hsa-mir-576	SF3B3	0.33959862	mirna_pc
24897	hsa-mir-576	TOPBP1	0.325987856	mirna_pc
24898	hsa-mir-576	OTX1	0.307577902	mirna_pc
24899	hsa-mir-576	DDX12	0.413449719	mirna_pc
24900	hsa-mir-576	SRRT	0.301477264	mirna_pc
24901	hsa-mir-576	PSRC1	0.326506541	mirna_pc
24902	hsa-mir-576	PTK7	0.374476583	mirna_pc
24903	hsa-mir-576	GPR19	0.326102715	mirna_pc
24904	hsa-mir-576	GABPB1	0.366648075	mirna_pc
24905	hsa-mir-576	PPAT	0.311714894	mirna_pc
24906	hsa-mir-576	MORC2	0.353310703	mirna_pc
24907	hsa-mir-576	SFRS1	0.307664421	mirna_pc
24908	hsa-mir-576	ATXN2L	0.316554154	mirna_pc
24909	hsa-mir-576	C4orf46	0.352322472	mirna_pc
24910	hsa-mir-576	POLR2D	0.336308591	mirna_pc
24911	hsa-mir-576	TUB	0.356417983	mirna_pc
24912	hsa-mir-576	YEATS2	0.33376048	mirna_pc
24913	hsa-mir-576	PARP1	0.300897126	mirna_pc
24914	hsa-mir-576	C4orf21	0.396562395	mirna_pc
24915	hsa-mir-576	TMEM194A	0.442744293	mirna_pc
24916	hsa-mir-576	CEP135	0.310895108	mirna_pc
24917	hsa-mir-576	ELK1	0.304028031	mirna_pc
24918	hsa-mir-576	XPO5	0.311164125	mirna_pc
24919	hsa-mir-576	ANKRD13D	0.316373535	mirna_pc
24920	hsa-mir-576	TET3	0.300404732	mirna_pc
24921	hsa-mir-576	HNRNPD	0.31567943	mirna_pc
24922	hsa-mir-576	C4orf41	0.331581977	mirna_pc
24923	hsa-mir-576	QTRTD1	0.306766034	mirna_pc
24924	hsa-mir-576	EHMT2	0.327046085	mirna_pc
24925	hsa-mir-576	UBAP2L	0.37844754	mirna_pc
24926	hsa-mir-576	PASK	0.359118153	mirna_pc
24927	hsa-mir-576	RPGRIP1L	0.30710645	mirna_pc
24928	hsa-mir-576	MFSD2A	0.463318838	mirna_pc
24929	hsa-mir-576	LSM 6.00	0.318687661	mirna_pc
24930	hsa-mir-576	PRAME	0.399664312	mirna_pc
24931	hsa-mir-576	FUBP1	0.37281537	mirna_pc
24932	hsa-mir-576	SART3	0.333905015	mirna_pc
24933	hsa-mir-576	SR140	0.356175827	mirna_pc
24934	hsa-mir-576	TRA2B	0.34713291	mirna_pc
24935	hsa-mir-576	GAR1	0.417282821	mirna_pc
24936	hsa-mir-576	ENOPH1	0.314477438	mirna_pc
24937	hsa-mir-576	ING5	0.31269741	mirna_pc
24938	hsa-mir-576	SFPQ	0.34922571	mirna_pc
24939	hsa-mir-576	CNOT3	0.344373515	mirna_pc
24940	hsa-mir-576	CADM1	0.36903374	mirna_pc
24941	hsa-mir-576	C10orf2	0.306489994	mirna_pc
24942	hsa-mir-576	POGK	0.359129624	mirna_pc
24943	hsa-mir-576	CPAMD8	0.365712342	mirna_pc
24944	hsa-mir-576	FAM86C	0.344903374	mirna_pc
24945	hsa-mir-576	ZNF107	0.334027374	mirna_pc
24946	hsa-mir-576	MSH6	0.374231357	mirna_pc
24947	hsa-mir-576	TP73	0.400150427	mirna_pc

24948	hsa-mir-576	LEF1	0.319437106	mirna_pc
24949	hsa-mir-576	KREMEN2	0.351142291	mirna_pc
24950	hsa-mir-576	ZBED4	0.326575991	mirna_pc
24951	hsa-mir-576	MEX3A	0.422906705	mirna_pc
24952	hsa-mir-576	ZNF236	0.362181832	mirna_pc
24953	hsa-mir-576	NGFR	0.300965444	mirna_pc
24954	hsa-mir-576	PDCD11	0.390397026	mirna_pc
24955	hsa-mir-576	TRIM28	0.40639392	mirna_pc
24956	hsa-mir-576	ADRBK1	0.396132846	mirna_pc
24957	hsa-mir-576	NAA15	0.365393674	mirna_pc
24958	hsa-mir-576	ZBTB12	0.347612091	mirna_pc
24959	hsa-mir-576	LOC642846	0.418695249	mirna_pc
24960	hsa-mir-576	TGIF2	0.376719449	mirna_pc
24961	hsa-mir-576	ZC3H18	0.310189483	mirna_pc
24962	hsa-mir-576	C15orf44	0.304001883	mirna_pc
24963	hsa-mir-576	RCN2	0.368517993	mirna_pc
24964	hsa-mir-576	POLE	0.42206082	mirna_pc
24965	hsa-mir-576	RBM14	0.306437538	mirna_pc
24966	hsa-mir-576	GMEB2	0.340007227	mirna_pc
24967	hsa-mir-576	CDK16	0.314481119	mirna_pc
24968	hsa-mir-576	IGSF9	0.511634134	mirna_pc
24969	hsa-mir-576	PLXNA1	0.320488321	mirna_pc
24970	hsa-mir-576	FIP1L1	0.312900592	mirna_pc
24971	hsa-mir-576	GTF3C2	0.316705428	mirna_pc
24972	hsa-mir-576	WDR90	0.325378901	mirna_pc
24973	hsa-mir-576	TMEM151B	0.455189511	mirna_pc
24974	hsa-mir-576	ZC3H7B	0.33194877	mirna_pc
24975	hsa-mir-576	RG9MTD2	0.322414823	mirna_pc
24976	hsa-mir-576	MTMR15	0.315586327	mirna_pc
24977	hsa-mir-576	TTLL4	0.455592427	mirna_pc
24978	hsa-mir-576	FOXK2	0.341613075	mirna_pc
24979	hsa-mir-576	C20orf177	0.305674823	mirna_pc
24980	hsa-mir-576	UBXN7	0.304526671	mirna_pc
24981	hsa-mir-576	ATN1	0.359544476	mirna_pc
24982	hsa-mir-576	DVL3	0.35940992	mirna_pc
24983	hsa-mir-576	PPP2R5D	0.307308484	mirna_pc
24984	hsa-mir-576	ARNT2	0.382363926	mirna_pc
24985	hsa-mir-576	ZNF286A	0.337650121	mirna_pc
24986	hsa-mir-576	PRRT2	0.307078888	mirna_pc
24987	hsa-mir-576	MLL2	0.309140694	mirna_pc
24988	hsa-mir-576	RFX7	0.305248809	mirna_pc
24989	hsa-mir-576	NDUFV1	0.306621457	mirna_pc
24990	hsa-mir-576	ZNF497	0.321766502	mirna_pc
24991	hsa-mir-576	ZNF84	0.316905421	mirna_pc
24992	hsa-mir-576	LRRTM2	0.318213543	mirna_pc
24993	hsa-mir-576	ZNF286B	0.303808268	mirna_pc
24994	hsa-mir-576	INPPL1	0.353825535	mirna_pc
24995	hsa-mir-576	HUNK	0.320485636	mirna_pc
24996	hsa-mir-576	CRAMP1L	0.353079119	mirna_pc
24997	hsa-mir-576	LOC646851	0.300492373	mirna_pc
24998	hsa-mir-576	SCLY	0.364523807	mirna_pc
24999	hsa-mir-576	CAP1	0.43437505	mirna_pc
25000	hsa-mir-576	GSTCD	0.390796497	mirna_pc
25001	hsa-mir-576	ELFN1	0.306668934	mirna_pc

25002	hsa-mir-576	HNRNPA3	0.306398284	mirna_pc
25003	hsa-mir-576	EWSR1	0.34855134	mirna_pc
25004	hsa-mir-576	DHX57	0.32400373	mirna_pc
25005	hsa-mir-576	NUMA1	0.352510527	mirna_pc
25006	hsa-mir-576	ALX3	0.431274229	mirna_pc
25007	hsa-mir-576	SUPT7L	0.312766952	mirna_pc
25008	hsa-mir-576	DGCR8	0.318208468	mirna_pc
25009	hsa-mir-576	ZNF512B	0.300056027	mirna_pc
25010	hsa-mir-576	SFRS7	0.300277932	mirna_pc
25011	hsa-mir-576	TRIT1	0.382651386	mirna_pc
25012	hsa-mir-576	RBM10	0.332113854	mirna_pc
25013	hsa-mir-576	BAT2	0.32632857	mirna_pc
25014	hsa-mir-576	EDAR	0.389354952	mirna_pc
25015	hsa-mir-576	ZNF304	0.317875695	mirna_pc
25016	hsa-mir-576	NIPSNAP1	0.312776753	mirna_pc
25017	hsa-mir-576	WEE1	0.300765711	mirna_pc
25018	hsa-mir-576	KDM4A	0.341665728	mirna_pc
25019	hsa-mir-576	MGC2752	0.355774794	mirna_pc
25020	hsa-mir-576	TIGD2	0.338813398	mirna_pc
25021	hsa-mir-576	ATF5	0.348472448	mirna_pc
25022	hsa-mir-576	DCTD	0.313461065	mirna_pc
25023	hsa-mir-576	MCM3APAS	0.348183744	mirna_pc
25024	hsa-mir-576	HCFC1	0.308850914	mirna_pc
25025	hsa-mir-576	EIF4G1	0.307896833	mirna_pc
25026	hsa-mir-576	BCAN	0.498126025	mirna_pc
25027	hsa-mir-576	PPFIA4	0.302552527	mirna_pc
25028	hsa-mir-576	LRP6	0.316017483	mirna_pc
25029	hsa-mir-576	DCP2	0.302923828	mirna_pc
25030	hsa-mir-576	C2orf44	0.337243443	mirna_pc
25031	hsa-mir-576	SEPHS1	0.320480149	mirna_pc
25032	hsa-mir-576	CBX5	0.359250989	mirna_pc
25033	hsa-mir-576	RYK	0.373082108	mirna_pc
25034	hsa-mir-576	ADNP	0.318738711	mirna_pc
25035	hsa-mir-576	IFT172	0.302674654	mirna_pc
25036	hsa-mir-576	TTC30B	0.331229598	mirna_pc
25037	hsa-mir-576	MSL2	0.347532195	mirna_pc
25038	hsa-mir-576	AGBL5	0.379593035	mirna_pc
25039	hsa-mir-576	VASH2	0.432938931	mirna_pc
25040	hsa-mir-576	PDCD7	0.345876933	mirna_pc
25041	hsa-mir-576	SENP5	0.372380335	mirna_pc
25042	hsa-mir-576	ZNF384	0.32219648	mirna_pc
25043	hsa-mir-576	AARS2	0.310308061	mirna_pc
25044	hsa-mir-576	PAN2	0.301721867	mirna_pc
25045	hsa-mir-576	ZNF518B	0.308629473	mirna_pc
25046	hsa-mir-576	CHST11	0.318045316	mirna_pc
25047	hsa-mir-576	BCL11A	0.485225994	mirna_pc
25048	hsa-mir-576	USP21	0.313891978	mirna_pc
25049	hsa-mir-576	ALMS1	0.370494448	mirna_pc
25050	hsa-mir-576	PRR12	0.360904221	mirna_pc
25051	hsa-mir-576	ZNF74	0.309247544	mirna_pc
25052	hsa-mir-576	RAD52	0.340164147	mirna_pc
25053	hsa-mir-576	SRF	0.317745464	mirna_pc
25054	hsa-mir-576	EMID1	0.371943499	mirna_pc
25055	hsa-mir-576	ZMYM4	0.369497634	mirna_pc

25056	hsa-mir-576	MOXD1	0.31122219	mirna_pc
25057	hsa-mir-576	THAP9	0.304503966	mirna_pc
25058	hsa-mir-576	CNIH2	0.306705999	mirna_pc
25059	hsa-mir-576	LOC400657	0.39750956	mirna_pc
25060	hsa-mir-576	POLH	0.310123715	mirna_pc
25061	hsa-mir-576	TJAP1	0.301033109	mirna_pc
25062	hsa-mir-576	TFDP2	0.356487227	mirna_pc
25063	hsa-mir-576	QRICH2	0.375453495	mirna_pc
25064	hsa-mir-576	MYCN	0.313236781	mirna_pc
25065	hsa-mir-576	WDR33	0.316079039	mirna_pc
25066	hsa-mir-576	DCAF16	0.333737075	mirna_pc
25067	hsa-mir-576	SBK1	0.307632472	mirna_pc
25068	hsa-mir-576	PABPC4L	0.357809117	mirna_pc
25069	hsa-mir-576	USP13	0.355869162	mirna_pc
25070	hsa-mir-576	DENND4B	0.313778081	mirna_pc
25071	hsa-mir-576	C16orf46	0.352720539	mirna_pc
25072	hsa-mir-576	CLPB	0.35181208	mirna_pc
25073	hsa-mir-576	ZNF740	0.305707389	mirna_pc
25074	hsa-mir-576	EP400	0.334811327	mirna_pc
25075	hsa-mir-576	CUL7	0.357408201	mirna_pc
25076	hsa-mir-576	TRO	0.369225845	mirna_pc
25077	hsa-mir-576	DDX51	0.332298871	mirna_pc
25078	hsa-mir-576	NSD1	0.330747575	mirna_pc
25079	hsa-mir-576	ZBTB39	0.393197922	mirna_pc
25080	hsa-mir-576	EDC3	0.326712676	mirna_pc
25081	hsa-mir-576	RPS10P7	0.315254556	mirna_pc
25082	hsa-mir-576	ZNF827	0.380833369	mirna_pc
25083	hsa-mir-576	C20orf12	0.393413037	mirna_pc
25084	hsa-mir-576	VPS54	0.376236207	mirna_pc
25085	hsa-mir-576	PIP4K2B	0.444846546	mirna_pc
25086	hsa-mir-576	ENTPD2	0.31994883	mirna_pc
25087	hsa-mir-576	MYCL1	0.377947175	mirna_pc
25088	hsa-mir-576	ZNF618	0.348572632	mirna_pc
25089	hsa-mir-576	POM121C	0.337152472	mirna_pc
25090	hsa-mir-576	C22orf30	0.357438121	mirna_pc
25091	hsa-mir-576	CCDC111	0.323462034	mirna_pc
25092	hsa-mir-576	CCDC157	0.372763961	mirna_pc
25093	hsa-mir-576	STK35	0.352530848	mirna_pc
25094	hsa-mir-576	SCML2	0.368058558	mirna_pc
25095	hsa-mir-576	SH3BP5L	0.312402644	mirna_pc
25096	hsa-mir-576	DIS3L2	0.301396753	mirna_pc
25097	hsa-mir-576	COL9A2	0.335467877	mirna_pc
25098	hsa-mir-576	ZNF8	0.310640185	mirna_pc
25099	hsa-mir-576	ETV5	0.365396829	mirna_pc
25100	hsa-mir-576	COL9A3	0.342562145	mirna_pc
25101	hsa-mir-576	KIAA1430	0.317102746	mirna_pc
25102	hsa-mir-576	IGHMBP2	0.355746281	mirna_pc
25103	hsa-mir-576	LGR6	0.320444454	mirna_pc
25104	hsa-mir-576	ZBTB45	0.334613046	mirna_pc
25105	hsa-mir-576	DNAL4	0.326658502	mirna_pc
25106	hsa-mir-576	KLHDC3	0.327009698	mirna_pc
25107	hsa-mir-576	MAML1	0.301853746	mirna_pc
25108	hsa-mir-576	B4GALNT4	0.409319794	mirna_pc
25109	hsa-mir-576	HSDL1	0.323147918	mirna_pc

25110	hsa-mir-576	MBD6	0.323824489	mirna_pc
25111	hsa-mir-576	ZNF543	0.305495239	mirna_pc
25112	hsa-mir-576	SMARCAD1	0.404477637	mirna_pc
25113	hsa-mir-576	C22orf29	0.410673528	mirna_pc
25114	hsa-mir-576	DNMT3A	0.435925242	mirna_pc
25115	hsa-mir-576	POM121	0.35741528	mirna_pc
25116	hsa-mir-130b	TPX2	0.339732301	mirna_pc
25117	hsa-mir-130b	CENPF	0.33822267	mirna_pc
25118	hsa-mir-130b	RCC2	0.394527718	mirna_pc
25119	hsa-mir-130b	HOXC9	0.461154646	mirna_pc
25120	hsa-mir-130b	KIF11	0.363604859	mirna_pc
25121	hsa-mir-130b	ECT2	0.308747853	mirna_pc
25122	hsa-mir-130b	RRM2	0.426670386	mirna_pc
25123	hsa-mir-130b	SPC24	0.306380128	mirna_pc
25124	hsa-mir-130b	CDK1	0.314652805	mirna_pc
25125	hsa-mir-130b	CDC25C	0.307410097	mirna_pc
25126	hsa-mir-130b	MYBL2	0.300710286	mirna_pc
25127	hsa-mir-130b	PLK1	0.35541486	mirna_pc
25128	hsa-mir-130b	UBE2C	0.33642474	mirna_pc
25129	hsa-mir-130b	BUB1	0.330819086	mirna_pc
25130	hsa-mir-130b	PRC1	0.326235855	mirna_pc
25131	hsa-mir-130b	NUSAP1	0.39489544	mirna_pc
25132	hsa-mir-130b	KIFC1	0.384254057	mirna_pc
25133	hsa-mir-130b	BUB1B	0.404008945	mirna_pc
25134	hsa-mir-130b	KIF18B	0.374046398	mirna_pc
25135	hsa-mir-130b	KIF2C	0.383509534	mirna_pc
25136	hsa-mir-130b	FANCA	0.308163815	mirna_pc
25137	hsa-mir-130b	NCAPG	0.333056105	mirna_pc
25138	hsa-mir-130b	CCNA2	0.487321394	mirna_pc
25139	hsa-mir-130b	SPC25	0.404421007	mirna_pc
25140	hsa-mir-130b	FANCI	0.436126558	mirna_pc
25141	hsa-mir-130b	NEK2	0.422640659	mirna_pc
25142	hsa-mir-130b	MND1	0.530291169	mirna_pc
25143	hsa-mir-130b	KIF22	0.422993037	mirna_pc
25144	hsa-mir-130b	NCAPH	0.444199247	mirna_pc
25145	hsa-mir-130b	GTSE1	0.398766091	mirna_pc
25146	hsa-mir-130b	RAD54L	0.506255901	mirna_pc
25147	hsa-mir-130b	CENPA	0.465509882	mirna_pc
25148	hsa-mir-130b	MAD2L1	0.553432535	mirna_pc
25149	hsa-mir-130b	TIMELESS	0.467432995	mirna_pc
25150	hsa-mir-130b	LMNB1	0.361000728	mirna_pc
25151	hsa-mir-130b	CDCA8	0.418099444	mirna_pc
25152	hsa-mir-130b	TROAP	0.434851322	mirna_pc
25153	hsa-mir-130b	CDCA5	0.33428224	mirna_pc
25154	hsa-mir-130b	TACC3	0.31484534	mirna_pc
25155	hsa-mir-130b	XPO1	0.305677646	mirna_pc
25156	hsa-mir-130b	CDC45	0.391714219	mirna_pc
25157	hsa-mir-130b	STMN1	0.512022053	mirna_pc
25158	hsa-mir-130b	CENPM	0.386962983	mirna_pc
25159	hsa-mir-130b	EXO1	0.358165117	mirna_pc
25160	hsa-mir-130b	UBE2T	0.360243326	mirna_pc
25161	hsa-mir-130b	CKS1B	0.391953035	mirna_pc
25162	hsa-mir-130b	TUBB	0.375710232	mirna_pc
25163	hsa-mir-130b	MCM2	0.383772346	mirna_pc

25164	hsa-mir-130b	KIF4A	0.338367995	mirna_pc
25165	hsa-mir-130b	ORC1L	0.346600978	mirna_pc
25166	hsa-mir-130b	KNTC1	0.391895834	mirna_pc
25167	hsa-mir-130b	UHRF1	0.329718272	mirna_pc
25168	hsa-mir-130b	CCNF	0.306213491	mirna_pc
25169	hsa-mir-130b	RACGAP1	0.313041097	mirna_pc
25170	hsa-mir-130b	PTBP1	0.322129453	mirna_pc
25171	hsa-mir-130b	CCNB2	0.40637793	mirna_pc
25172	hsa-mir-130b	NUF2	0.349493178	mirna_pc
25173	hsa-mir-130b	SFRP1	0.31460628	mirna_pc
25174	hsa-mir-130b	PLK4	0.486171396	mirna_pc
25175	hsa-mir-130b	KIF18A	0.331226369	mirna_pc
25176	hsa-mir-130b	DEPDC1	0.368395832	mirna_pc
25177	hsa-mir-130b	HJURP	0.358390101	mirna_pc
25178	hsa-mir-130b	RAD51	0.346866214	mirna_pc
25179	hsa-mir-130b	BLM	0.43686743	mirna_pc
25180	hsa-mir-130b	PAICS	0.385373496	mirna_pc
25181	hsa-mir-130b	BIRC5	0.398744574	mirna_pc
25182	hsa-mir-130b	RCC1	0.309092617	mirna_pc
25183	hsa-mir-130b	OIP5	0.356739329	mirna_pc
25184	hsa-mir-130b	DTL	0.429270651	mirna_pc
25185	hsa-mir-130b	EME1	0.343788784	mirna_pc
25186	hsa-mir-130b	AURKB	0.445634686	mirna_pc
25187	hsa-mir-130b	GSG2	0.316137821	mirna_pc
25188	hsa-mir-130b	CDT1	0.327834992	mirna_pc
25189	hsa-mir-130b	MCM10	0.362172112	mirna_pc
25190	hsa-mir-130b	SGOL2	0.325441534	mirna_pc
25191	hsa-mir-130b	CHEK2	0.388446371	mirna_pc
25192	hsa-mir-130b	KIF15	0.332645976	mirna_pc
25193	hsa-mir-130b	CDCA3	0.423403679	mirna_pc
25194	hsa-mir-130b	C1orf112	0.342793188	mirna_pc
25195	hsa-mir-130b	GINS1	0.319556035	mirna_pc
25196	hsa-mir-130b	TRAIP	0.303209776	mirna_pc
25197	hsa-mir-130b	ORC6L	0.343449744	mirna_pc
25198	hsa-mir-130b	C16orf59	0.415769491	mirna_pc
25199	hsa-mir-130b	NEIL3	0.389731883	mirna_pc
25200	hsa-mir-130b	MCM7	0.343039102	mirna_pc
25201	hsa-mir-130b	ACTL6A	0.337652212	mirna_pc
25202	hsa-mir-130b	C12orf48	0.480370596	mirna_pc
25203	hsa-mir-130b	HNRNPL	0.371684127	mirna_pc
25204	hsa-mir-130b	SMC4	0.307236672	mirna_pc
25205	hsa-mir-130b	PKMYT1	0.303921856	mirna_pc
25206	hsa-mir-130b	ZWINT	0.43602304	mirna_pc
25207	hsa-mir-130b	TUBA1B	0.402136856	mirna_pc
25208	hsa-mir-130b	HELLS	0.303932001	mirna_pc
25209	hsa-mir-130b	UBE2S	0.326362683	mirna_pc
25210	hsa-mir-130b	NCAPD2	0.340742301	mirna_pc
25211	hsa-mir-130b	HNRNPC	0.321644018	mirna_pc
25212	hsa-mir-130b	ILF2	0.413239146	mirna_pc
25213	hsa-mir-130b	TCF3	0.358345287	mirna_pc
25214	hsa-mir-130b	MCM5	0.339359995	mirna_pc
25215	hsa-mir-130b	TYMS	0.317012247	mirna_pc
25216	hsa-mir-130b	RFWD3	0.302534134	mirna_pc
25217	hsa-mir-130b	MCM6	0.361737975	mirna_pc

25218	hsa-mir-130b	RNASEH2A	0.320644422	mirna_pc
25219	hsa-mir-130b	EPR1	0.454283077	mirna_pc
25220	hsa-mir-130b	H2AFX	0.363978146	mirna_pc
25221	hsa-mir-130b	SFRS2	0.322885769	mirna_pc
25222	hsa-mir-130b	UCK2	0.46385603	mirna_pc
25223	hsa-mir-130b	THOC4	0.363748413	mirna_pc
25224	hsa-mir-130b	NFKBIL2	0.319220077	mirna_pc
25225	hsa-mir-130b	C15orf42	0.397555987	mirna_pc
25226	hsa-mir-130b	FAM60A	0.33049733	mirna_pc
25227	hsa-mir-130b	FAM64A	0.372877087	mirna_pc
25228	hsa-mir-130b	RANBP1	0.449456684	mirna_pc
25229	hsa-mir-130b	E2F1	0.373999211	mirna_pc
25230	hsa-mir-130b	SNRPG	0.320296901	mirna_pc
25231	hsa-mir-130b	MCM3	0.307701309	mirna_pc
25232	hsa-mir-130b	SNRPA	0.401102023	mirna_pc
25233	hsa-mir-130b	FUS	0.301380759	mirna_pc
25234	hsa-mir-130b	U2AF2	0.339041925	mirna_pc
25235	hsa-mir-130b	CHAF1A	0.320143994	mirna_pc
25236	hsa-mir-130b	CENPO	0.577599263	mirna_pc
25237	hsa-mir-130b	C19orf48	0.300185411	mirna_pc
25238	hsa-mir-130b	NUDT1	0.40588839	mirna_pc
25239	hsa-mir-130b	HMGB2	0.410132074	mirna_pc
25240	hsa-mir-130b	GINS2	0.398383224	mirna_pc
25241	hsa-mir-130b	DNA2	0.323527753	mirna_pc
25242	hsa-mir-130b	SNRPD1	0.307669364	mirna_pc
25243	hsa-mir-130b	DTYMK	0.397464967	mirna_pc
25244	hsa-mir-130b	E2F7	0.335242644	mirna_pc
25245	hsa-mir-130b	MLF1IP	0.363520236	mirna_pc
25246	hsa-mir-130b	HSPE1	0.301274619	mirna_pc
25247	hsa-mir-130b	C16orf75	0.434635492	mirna_pc
25248	hsa-mir-130b	CAD	0.432523288	mirna_pc
25249	hsa-mir-130b	CCDC99	0.310323429	mirna_pc
25250	hsa-mir-130b	CDK2	0.358987871	mirna_pc
25251	hsa-mir-130b	CDK4	0.40290826	mirna_pc
25252	hsa-mir-130b	TMEM108	0.360019515	mirna_pc
25253	hsa-mir-130b	HNRNPR	0.343155259	mirna_pc
25254	hsa-mir-130b	CENPH	0.335816965	mirna_pc
25255	hsa-mir-130b	MSH2	0.421842241	mirna_pc
25256	hsa-mir-130b	SF3B3	0.415554027	mirna_pc
25257	hsa-mir-130b	CDC7	0.37742739	mirna_pc
25258	hsa-mir-130b	YDJC	0.35858523	mirna_pc
25259	hsa-mir-130b	RFC4	0.455152255	mirna_pc
25260	hsa-mir-130b	FBXO5	0.393360033	mirna_pc
25261	hsa-mir-130b	FIGNL1	0.320894347	mirna_pc
25262	hsa-mir-130b	DDX11	0.365387551	mirna_pc
25263	hsa-mir-130b	CDC25A	0.353604289	mirna_pc
25264	hsa-mir-130b	RAD54B	0.320898618	mirna_pc
25265	hsa-mir-130b	OTX1	0.365198263	mirna_pc
25266	hsa-mir-130b	H2AFZ	0.46755455	mirna_pc
25267	hsa-mir-130b	DDX12	0.345430169	mirna_pc
25268	hsa-mir-130b	MCM8	0.369337873	mirna_pc
25269	hsa-mir-130b	CPSF3	0.418009785	mirna_pc
25270	hsa-mir-130b	SNRPE	0.379006597	mirna_pc
25271	hsa-mir-130b	PPM1G	0.415500683	mirna_pc

25272	hsa-mir-130b	MARCKSL1	0.315345311	mirna_pc
25273	hsa-mir-130b	TFAP4	0.365983293	mirna_pc
25274	hsa-mir-130b	DAZAP1	0.402714962	mirna_pc
25275	hsa-mir-130b	CBX8	0.362261876	mirna_pc
25276	hsa-mir-130b	TMEM201	0.337445588	mirna_pc
25277	hsa-mir-130b	PAFAH1B3	0.308600126	mirna_pc
25278	hsa-mir-130b	DCTPP1	0.438897443	mirna_pc
25279	hsa-mir-130b	PSRC1	0.53149512	mirna_pc
25280	hsa-mir-130b	DGUOK	0.31533492	mirna_pc
25281	hsa-mir-130b	FBL	0.348297344	mirna_pc
25282	hsa-mir-130b	HIST1H1E	0.318169467	mirna_pc
25283	hsa-mir-130b	HNRNPM	0.332972272	mirna_pc
25284	hsa-mir-130b	TMPO	0.440505063	mirna_pc
25285	hsa-mir-130b	POLR2H	0.319009346	mirna_pc
25286	hsa-mir-130b	SSBP3	0.368085348	mirna_pc
25287	hsa-mir-130b	LSG1	0.354546311	mirna_pc
25288	hsa-mir-130b	GABPB1	0.372769915	mirna_pc
25289	hsa-mir-130b	PPIH	0.35855049	mirna_pc
25290	hsa-mir-130b	PPAT	0.342601351	mirna_pc
25291	hsa-mir-130b	DHX9	0.337479857	mirna_pc
25292	hsa-mir-130b	CCDC138	0.315127929	mirna_pc
25293	hsa-mir-130b	LOC100128191	0.443592086	mirna_pc
25294	hsa-mir-130b	ETV4	0.423609972	mirna_pc
25295	hsa-mir-130b	SMYD5	0.33738372	mirna_pc
25296	hsa-mir-130b	POLA2	0.341431682	mirna_pc
25297	hsa-mir-130b	SFRS1	0.355961858	mirna_pc
25298	hsa-mir-130b	SNRPA1	0.314701419	mirna_pc
25299	hsa-mir-130b	SKP2	0.348311805	mirna_pc
25300	hsa-mir-130b	TIPIN	0.317153253	mirna_pc
25301	hsa-mir-130b	SMARCD1	0.312744294	mirna_pc
25302	hsa-mir-130b	ATXN2L	0.354940085	mirna_pc
25303	hsa-mir-130b	C4orf46	0.442119463	mirna_pc
25304	hsa-mir-130b	ECE2	0.339678729	mirna_pc
25305	hsa-mir-130b	GPN1	0.341775411	mirna_pc
25306	hsa-mir-130b	RFC5	0.382603194	mirna_pc
25307	hsa-mir-130b	MAZ	0.431759423	mirna_pc
25308	hsa-mir-130b	SSRP1	0.349989476	mirna_pc
25309	hsa-mir-130b	POLR2D	0.408104211	mirna_pc
25310	hsa-mir-130b	FTSJ2	0.302410878	mirna_pc
25311	hsa-mir-130b	SNRPD3	0.411379157	mirna_pc
25312	hsa-mir-130b	KHDRBS1	0.388267816	mirna_pc
25313	hsa-mir-130b	SLC5A6	0.371594155	mirna_pc
25314	hsa-mir-130b	SPAST	0.425092003	mirna_pc
25315	hsa-mir-130b	LOC92659	0.333084981	mirna_pc
25316	hsa-mir-130b	MTL 5.00	0.528990144	mirna_pc
25317	hsa-mir-130b	PARP1	0.344838403	mirna_pc
25318	hsa-mir-130b	CCT7	0.304025573	mirna_pc
25319	hsa-mir-130b	C4orf21	0.303763391	mirna_pc
25320	hsa-mir-130b	TMEM194A	0.360567271	mirna_pc
25321	hsa-mir-130b	SNRNP40	0.310663257	mirna_pc
25322	hsa-mir-130b	SLC39A10	0.324812389	mirna_pc
25323	hsa-mir-130b	NEDD1	0.337427093	mirna_pc
25324	hsa-mir-130b	WRAP53	0.306132464	mirna_pc
25325	hsa-mir-130b	NASP	0.359308107	mirna_pc

25326	hsa-mir-130b	NONO	0.38399858	mirna_pc
25327	hsa-mir-130b	CCDC86	0.349517853	mirna_pc
25328	hsa-mir-130b	BCL2	0.380484336	mirna_pc
25329	hsa-mir-130b	OBFC2B	0.349266946	mirna_pc
25330	hsa-mir-130b	PTMA	0.352169922	mirna_pc
25331	hsa-mir-130b	USP39	0.434905608	mirna_pc
25332	hsa-mir-130b	WDR76	0.314691219	mirna_pc
25333	hsa-mir-130b	LSM 4.00	0.30779779	mirna_pc
25334	hsa-mir-130b	C11orf84	0.422606534	mirna_pc
25335	hsa-mir-130b	NCBP2	0.313029041	mirna_pc
25336	hsa-mir-130b	SUV39H2	0.367732899	mirna_pc
25337	hsa-mir-130b	LSM 2.00	0.391302275	mirna_pc
25338	hsa-mir-130b	R3HDM1	0.305155267	mirna_pc
25339	hsa-mir-130b	RIBC2	0.310211767	mirna_pc
25340	hsa-mir-130b	HNRNPD	0.419681773	mirna_pc
25341	hsa-mir-130b	DPY30	0.334061226	mirna_pc
25342	hsa-mir-130b	MTHFD2	0.324227588	mirna_pc
25343	hsa-mir-130b	DPH2	0.30751278	mirna_pc
25344	hsa-mir-130b	SFRS3	0.307259259	mirna_pc
25345	hsa-mir-130b	SHOX2	0.379074229	mirna_pc
25346	hsa-mir-130b	H1FX	0.339607166	mirna_pc
25347	hsa-mir-130b	CCT4	0.33270616	mirna_pc
25348	hsa-mir-130b	ELAVL1	0.407718831	mirna_pc
25349	hsa-mir-130b	USP22	0.323200224	mirna_pc
25350	hsa-mir-130b	PSMC3IP	0.342121773	mirna_pc
25351	hsa-mir-130b	PAK1IP1	0.301924701	mirna_pc
25352	hsa-mir-130b	PES 1.00	0.30363201	mirna_pc
25353	hsa-mir-130b	PMAIP1	0.340239103	mirna_pc
25354	hsa-mir-130b	NT5DC2	0.341530871	mirna_pc
25355	hsa-mir-130b	EHMT2	0.404974993	mirna_pc
25356	hsa-mir-130b	DEK	0.304349391	mirna_pc
25357	hsa-mir-130b	SFRS9	0.338937663	mirna_pc
25358	hsa-mir-130b	PASK	0.316209118	mirna_pc
25359	hsa-mir-130b	HEATR2	0.412619448	mirna_pc
25360	hsa-mir-130b	LSM 6.00	0.37931098	mirna_pc
25361	hsa-mir-130b	PRAME	0.457871331	mirna_pc
25362	hsa-mir-130b	SUV39H1	0.386114051	mirna_pc
25363	hsa-mir-130b	IPO9	0.31235999	mirna_pc
25364	hsa-mir-130b	SART3	0.353200119	mirna_pc
25365	hsa-mir-130b	TRA2B	0.418409833	mirna_pc
25366	hsa-mir-130b	RSRC1	0.343534591	mirna_pc
25367	hsa-mir-130b	GAR1	0.360188013	mirna_pc
25368	hsa-mir-130b	C15orf23	0.372111058	mirna_pc
25369	hsa-mir-130b	CDRT4	0.382728216	mirna_pc
25370	hsa-mir-130b	C3orf21	0.363829197	mirna_pc
25371	hsa-mir-130b	DIABLO	0.303065759	mirna_pc
25372	hsa-mir-130b	IGF2BP2	0.347887269	mirna_pc
25373	hsa-mir-130b	WHSC1	0.322952167	mirna_pc
25374	hsa-mir-130b	ITGB3BP	0.374867936	mirna_pc
25375	hsa-mir-130b	ENOPH1	0.315719553	mirna_pc
25376	hsa-mir-130b	PLEKHG4	0.343875674	mirna_pc
25377	hsa-mir-130b	SFPQ	0.406610318	mirna_pc
25378	hsa-mir-130b	CD83	0.344662608	mirna_pc
25379	hsa-mir-130b	RCOR2	0.469920216	mirna_pc

25380	hsa-mir-130b	FAM125B	0.354019668	mirna_pc
25381	hsa-mir-130b	AS3MT	0.355302627	mirna_pc
25382	hsa-mir-130b	CADM1	0.390014385	mirna_pc
25383	hsa-mir-130b	POLA1	0.305034452	mirna_pc
25384	hsa-mir-130b	HDAC2	0.336529	mirna_pc
25385	hsa-mir-130b	GMNN	0.305059239	mirna_pc
25386	hsa-mir-130b	POGK	0.307297022	mirna_pc
25387	hsa-mir-130b	SALL2	0.318813004	mirna_pc
25388	hsa-mir-130b	GEMIN6	0.332807461	mirna_pc
25389	hsa-mir-130b	MSH6	0.465311972	mirna_pc
25390	hsa-mir-130b	SOX4	0.33296662	mirna_pc
25391	hsa-mir-130b	LEF1	0.42720653	mirna_pc
25392	hsa-mir-130b	FAM161A	0.318881289	mirna_pc
25393	hsa-mir-130b	HNRNPA1	0.30033862	mirna_pc
25394	hsa-mir-130b	KREMEN2	0.386155321	mirna_pc
25395	hsa-mir-130b	NUDT3	0.377241	mirna_pc
25396	hsa-mir-130b	MEX3A	0.559193449	mirna_pc
25397	hsa-mir-130b	MOGS	0.325581921	mirna_pc
25398	hsa-mir-130b	ERAL1	0.314631594	mirna_pc
25399	hsa-mir-130b	IRAK1BP1	0.350144065	mirna_pc
25400	hsa-mir-130b	TAF5	0.434061499	mirna_pc
25401	hsa-mir-130b	TRIM28	0.302930722	mirna_pc
25402	hsa-mir-130b	TIFA	0.374839659	mirna_pc
25403	hsa-mir-130b	ATXN7L3B	0.319188602	mirna_pc
25404	hsa-mir-130b	APEX1	0.344038987	mirna_pc
25405	hsa-mir-130b	UNC119B	0.402792079	mirna_pc
25406	hsa-mir-130b	HNRNPA1L2	0.363371548	mirna_pc
25407	hsa-mir-130b	CBX1	0.354145181	mirna_pc
25408	hsa-mir-130b	EMILIN3	0.392597575	mirna_pc
25409	hsa-mir-130b	ZBTB12	0.378957172	mirna_pc
25410	hsa-mir-130b	MCTP2	0.33767204	mirna_pc
25411	hsa-mir-130b	TGIF2	0.435395176	mirna_pc
25412	hsa-mir-130b	ERI3	0.327756391	mirna_pc
25413	hsa-mir-130b	RCN2	0.394994438	mirna_pc
25414	hsa-mir-130b	CIB2	0.368556729	mirna_pc
25415	hsa-mir-130b	TARBP2	0.314360321	mirna_pc
25416	hsa-mir-130b	NOL10	0.343467388	mirna_pc
25417	hsa-mir-130b	SF3A3	0.339641993	mirna_pc
25418	hsa-mir-130b	POLE	0.446400567	mirna_pc
25419	hsa-mir-130b	ZKSCAN2	0.333130799	mirna_pc
25420	hsa-mir-130b	MUTYH	0.352977496	mirna_pc
25421	hsa-mir-130b	ADSL	0.355491557	mirna_pc
25422	hsa-mir-130b	RBM14	0.318930355	mirna_pc
25423	hsa-mir-130b	EXOSC9	0.350251269	mirna_pc
25424	hsa-mir-130b	FOXRED2	0.340859913	mirna_pc
25425	hsa-mir-130b	UNG	0.34917657	mirna_pc
25426	hsa-mir-130b	RBMX	0.399742504	mirna_pc
25427	hsa-mir-130b	TSEN15	0.318767576	mirna_pc
25428	hsa-mir-130b	CDK16	0.36568868	mirna_pc
25429	hsa-mir-130b	ALKBH2	0.380630201	mirna_pc
25430	hsa-mir-130b	SOCS7	0.300301017	mirna_pc
25431	hsa-mir-130b	CTCF	0.434394638	mirna_pc
25432	hsa-mir-130b	RPL39L	0.418750348	mirna_pc
25433	hsa-mir-130b	PDIA6	0.304635843	mirna_pc

25434	hsa-mir-130b	GPN3	0.348500206	mirna_pc
25435	hsa-mir-130b	EPHB4	0.356664749	mirna_pc
25436	hsa-mir-130b	MRPL37	0.315510775	mirna_pc
25437	hsa-mir-130b	CCDC23	0.31938738	mirna_pc
25438	hsa-mir-130b	IGSF9	0.401184091	mirna_pc
25439	hsa-mir-130b	FIP1L1	0.330473766	mirna_pc
25440	hsa-mir-130b	CIT	0.343741534	mirna_pc
25441	hsa-mir-130b	KDM2B	0.395958298	mirna_pc
25442	hsa-mir-130b	GTF3C2	0.37503772	mirna_pc
25443	hsa-mir-130b	MAGEF1	0.420751351	mirna_pc
25444	hsa-mir-130b	VWA2	0.324834158	mirna_pc
25445	hsa-mir-130b	FAM136A	0.369734985	mirna_pc
25446	hsa-mir-130b	NKD2	0.306435783	mirna_pc
25447	hsa-mir-130b	SUPT3H	0.334342605	mirna_pc
25448	hsa-mir-130b	SMARCB1	0.487929886	mirna_pc
25449	hsa-mir-130b	MYB	0.382336287	mirna_pc
25450	hsa-mir-130b	ZNF639	0.375631512	mirna_pc
25451	hsa-mir-130b	ITPRIPL1	0.478360553	mirna_pc
25452	hsa-mir-130b	UBXN7	0.317949878	mirna_pc
25453	hsa-mir-130b	ANKRD6	0.343838862	mirna_pc
25454	hsa-mir-130b	STRA13	0.464051654	mirna_pc
25455	hsa-mir-130b	DVL3	0.325019849	mirna_pc
25456	hsa-mir-130b	BCL7A	0.44384331	mirna_pc
25457	hsa-mir-130b	DVL2	0.307018564	mirna_pc
25458	hsa-mir-130b	TRPA1	0.458738528	mirna_pc
25459	hsa-mir-130b	ARHGAP19	0.354030227	mirna_pc
25460	hsa-mir-130b	ZNF286A	0.421909622	mirna_pc
25461	hsa-mir-130b	WDR77	0.370373954	mirna_pc
25462	hsa-mir-130b	MRPL21	0.455287325	mirna_pc
25463	hsa-mir-130b	NLE1	0.307435559	mirna_pc
25464	hsa-mir-130b	DMRTA2	0.363385774	mirna_pc
25465	hsa-mir-130b	CHST10	0.324488816	mirna_pc
25466	hsa-mir-130b	CUEDC2	0.315405544	mirna_pc
25467	hsa-mir-130b	PSPH	0.322622556	mirna_pc
25468	hsa-mir-130b	PPP1CC	0.405383267	mirna_pc
25469	hsa-mir-130b	TMEM106C	0.332614532	mirna_pc
25470	hsa-mir-130b	TAF4B	0.355944637	mirna_pc
25471	hsa-mir-130b	TRMT61B	0.301453945	mirna_pc
25472	hsa-mir-130b	SEMA4F	0.407262493	mirna_pc
25473	hsa-mir-130b	ZNF286B	0.418725933	mirna_pc
25474	hsa-mir-130b	PDF	0.320937899	mirna_pc
25475	hsa-mir-130b	C1orf109	0.369981897	mirna_pc
25476	hsa-mir-130b	PIGX	0.301156411	mirna_pc
25477	hsa-mir-130b	MTA1	0.353263356	mirna_pc
25478	hsa-mir-130b	ATP6V1E2	0.344141299	mirna_pc
25479	hsa-mir-130b	MAPKAPK5	0.329663069	mirna_pc
25480	hsa-mir-130b	GPR3	0.388662712	mirna_pc
25481	hsa-mir-130b	E2F6	0.388891562	mirna_pc
25482	hsa-mir-130b	DRG1	0.334312987	mirna_pc
25483	hsa-mir-130b	B4GALT3	0.303240445	mirna_pc
25484	hsa-mir-130b	SFRS13A	0.312751853	mirna_pc
25485	hsa-mir-130b	SCLY	0.498963459	mirna_pc
25486	hsa-mir-130b	GPR125	0.352647532	mirna_pc
25487	hsa-mir-130b	NUP35	0.346962606	mirna_pc

25488	hsa-mir-130b	ALG10	0.383088164	mirna_pc
25489	hsa-mir-130b	SLC4A1AP	0.317706682	mirna_pc
25490	hsa-mir-130b	TTC27	0.344998187	mirna_pc
25491	hsa-mir-130b	DHRS13	0.376459925	mirna_pc
25492	hsa-mir-130b	KRTCAP3	0.345221883	mirna_pc
25493	hsa-mir-130b	EWSR1	0.318232955	mirna_pc
25494	hsa-mir-130b	DHX57	0.415173449	mirna_pc
25495	hsa-mir-130b	ALX3	0.494397531	mirna_pc
25496	hsa-mir-130b	AAAS	0.393232526	mirna_pc
25497	hsa-mir-130b	CHST14	0.300534433	mirna_pc
25498	hsa-mir-130b	ACVR2B	0.358624667	mirna_pc
25499	hsa-mir-130b	C2orf79	0.304970321	mirna_pc
25500	hsa-mir-130b	LIG3	0.301131284	mirna_pc
25501	hsa-mir-130b	MRPS26	0.306866285	mirna_pc
25502	hsa-mir-130b	MRPL30	0.344269951	mirna_pc
25503	hsa-mir-130b	PYCR1	0.363450456	mirna_pc
25504	hsa-mir-130b	SFRS7	0.437610118	mirna_pc
25505	hsa-mir-130b	C18orf10	0.333861902	mirna_pc
25506	hsa-mir-130b	TRMT2A	0.346714392	mirna_pc
25507	hsa-mir-130b	PCBP2	0.346277889	mirna_pc
25508	hsa-mir-130b	PFAS	0.321123999	mirna_pc
25509	hsa-mir-130b	C10orf35	0.340493815	mirna_pc
25510	hsa-mir-130b	LRRC45	0.315300028	mirna_pc
25511	hsa-mir-130b	BLMH	0.307887933	mirna_pc
25512	hsa-mir-130b	RBM10	0.308660211	mirna_pc
25513	hsa-mir-130b	ORAOV1	0.308116411	mirna_pc
25514	hsa-mir-130b	EIF2B5	0.303501254	mirna_pc
25515	hsa-mir-130b	NIPSNAP1	0.385458541	mirna_pc
25516	hsa-mir-130b	C2orf68	0.300853182	mirna_pc
25517	hsa-mir-130b	ARHGEF19	0.363360164	mirna_pc
25518	hsa-mir-130b	C17orf75	0.371213491	mirna_pc
25519	hsa-mir-130b	CCND1	0.366094943	mirna_pc
25520	hsa-mir-130b	MYST1	0.335276786	mirna_pc
25521	hsa-mir-130b	ZNF771	0.454096422	mirna_pc
25522	hsa-mir-130b	3-Sep	0.303953973	mirna_pc
25523	hsa-mir-130b	TCTN2	0.41825305	mirna_pc
25524	hsa-mir-130b	MTP 18.00	0.328712186	mirna_pc
25525	hsa-mir-130b	POLR2C	0.338352046	mirna_pc
25526	hsa-mir-130b	C12orf45	0.319527616	mirna_pc
25527	hsa-mir-130b	C2orf44	0.408326269	mirna_pc
25528	hsa-mir-130b	SEPHS1	0.475382979	mirna_pc
25529	hsa-mir-130b	SHISA2	0.402978229	mirna_pc
25530	hsa-mir-130b	CBX5	0.342055479	mirna_pc
25531	hsa-mir-130b	RPS19BP1	0.375854442	mirna_pc
25532	hsa-mir-130b	AGBL5	0.529915323	mirna_pc
25533	hsa-mir-130b	VASH2	0.361658771	mirna_pc
25534	hsa-mir-130b	MYL6B	0.390110919	mirna_pc
25535	hsa-mir-130b	CD200	0.40013384	mirna_pc
25536	hsa-mir-130b	GPX7	0.301593566	mirna_pc
25537	hsa-mir-130b	NME4	0.304607336	mirna_pc
25538	hsa-mir-130b	LYRM4	0.344857758	mirna_pc
25539	hsa-mir-130b	BCL11A	0.336390599	mirna_pc
25540	hsa-mir-130b	USP21	0.42740595	mirna_pc
25541	hsa-mir-130b	ALMS1	0.307081245	mirna_pc

25542	hsa-mir-130b	DCLRE1A	0.324157568	mirna_pc
25543	hsa-mir-130b	ZNF74	0.408188526	mirna_pc
25544	hsa-mir-130b	ANP32A	0.507327729	mirna_pc
25545	hsa-mir-130b	CPT1A	0.365446922	mirna_pc
25546	hsa-mir-130b	ZMYM4	0.343808677	mirna_pc
25547	hsa-mir-130b	TCF7L1	0.334056461	mirna_pc
25548	hsa-mir-130b	C17orf81	0.315914393	mirna_pc
25549	hsa-mir-130b	HYLS1	0.301523996	mirna_pc
25550	hsa-mir-130b	HIC2	0.313196144	mirna_pc
25551	hsa-mir-130b	SDF2L1	0.328902947	mirna_pc
25552	hsa-mir-130b	NAP1L1	0.338505217	mirna_pc
25553	hsa-mir-130b	CNIH2	0.327740831	mirna_pc
25554	hsa-mir-130b	BUD13	0.396969804	mirna_pc
25555	hsa-mir-130b	PTPLAD1	0.31943048	mirna_pc
25556	hsa-mir-130b	MORN2	0.345105357	mirna_pc
25557	hsa-mir-130b	CCNJ	0.318431291	mirna_pc
25558	hsa-mir-130b	MYCN	0.43973196	mirna_pc
25559	hsa-mir-130b	C12orf24	0.305083013	mirna_pc
25560	hsa-mir-130b	SBK1	0.618148652	mirna_pc
25561	hsa-mir-130b	PABPC4L	0.411847533	mirna_pc
25562	hsa-mir-130b	SF3A1	0.335098474	mirna_pc
25563	hsa-mir-130b	PMF1	0.350307734	mirna_pc
25564	hsa-mir-130b	FAM171A1	0.313824254	mirna_pc
25565	hsa-mir-130b	USP13	0.437951912	mirna_pc
25566	hsa-mir-130b	GALNT14	0.317175689	mirna_pc
25567	hsa-mir-130b	CD69	0.395053279	mirna_pc
25568	hsa-mir-130b	SLC25A33	0.326548839	mirna_pc
25569	hsa-mir-130b	MSI1	0.473653974	mirna_pc
25570	hsa-mir-130b	CLPB	0.336650593	mirna_pc
25571	hsa-mir-130b	TMEM18	0.31951512	mirna_pc
25572	hsa-mir-130b	TRO	0.324839837	mirna_pc
25573	hsa-mir-130b	DDX51	0.312595126	mirna_pc
25574	hsa-mir-130b	ZNF232	0.484188862	mirna_pc
25575	hsa-mir-130b	SENP2	0.324962516	mirna_pc
25576	hsa-mir-130b	ZBTB39	0.477116954	mirna_pc
25577	hsa-mir-130b	SNRNP27	0.302567481	mirna_pc
25578	hsa-mir-130b	CECR5	0.305616159	mirna_pc
25579	hsa-mir-130b	DTNB	0.308678191	mirna_pc
25580	hsa-mir-130b	FAM169A	0.327403312	mirna_pc
25581	hsa-mir-130b	VPS54	0.462745951	mirna_pc
25582	hsa-mir-130b	RAC3	0.300955455	mirna_pc
25583	hsa-mir-130b	RBBP4	0.371984181	mirna_pc
25584	hsa-mir-130b	RASL11B	0.303614915	mirna_pc
25585	hsa-mir-130b	C2orf3	0.372733585	mirna_pc
25586	hsa-mir-130b	ZNF22	0.302871874	mirna_pc
25587	hsa-mir-130b	CCDC157	0.335458614	mirna_pc
25588	hsa-mir-130b	PDGFA	0.342496452	mirna_pc
25589	hsa-mir-130b	SRRD	0.328150455	mirna_pc
25590	hsa-mir-130b	UBE2L3	0.303885258	mirna_pc
25591	hsa-mir-130b	SCML2	0.47995366	mirna_pc
25592	hsa-mir-130b	HSF2	0.324127158	mirna_pc
25593	hsa-mir-130b	ZCCHC3	0.328995582	mirna_pc
25594	hsa-mir-130b	ETV5	0.537101868	mirna_pc
25595	hsa-mir-130b	PGAP1	0.304670063	mirna_pc

25596	hsa-mir-130b	DACT2	0.541278585	mirna_pc
25597	hsa-mir-130b	EXOSC6	0.301187287	mirna_pc
25598	hsa-mir-130b	AADAT	0.42907272	mirna_pc
25599	hsa-mir-130b	IGHMBP2	0.448871065	mirna_pc
25600	hsa-mir-130b	ACP1	0.353196736	mirna_pc
25601	hsa-mir-130b	C2orf43	0.320471592	mirna_pc
25602	hsa-mir-130b	ACTR3B	0.307225876	mirna_pc
25603	hsa-mir-130b	ZNF48	0.373953656	mirna_pc
25604	hsa-mir-130b	TXNL4A	0.330783952	mirna_pc
25605	hsa-mir-130b	DNAL4	0.329767595	mirna_pc
25606	hsa-mir-130b	SPRED2	0.381819433	mirna_pc
25607	hsa-mir-130b	SERF1A	0.355869177	mirna_pc
25608	hsa-mir-130b	CCDC117	0.341140236	mirna_pc
25609	hsa-mir-130b	PREB	0.466702915	mirna_pc
25610	hsa-mir-130b	B4GALNT4	0.415239335	mirna_pc
25611	hsa-mir-130b	PRMT6	0.394609886	mirna_pc
25612	hsa-mir-130b	C2orf28	0.306242995	mirna_pc
25613	hsa-mir-130b	H2AFY2	0.345334767	mirna_pc
25614	hsa-mir-130b	HIRIP3	0.327687385	mirna_pc
25615	hsa-mir-130b	BOD1	0.354982295	mirna_pc
25616	hsa-mir-130b	DYNC2LI1	0.305418786	mirna_pc
25617	hsa-mir-130b	SCHIP1	0.329883187	mirna_pc
25618	hsa-mir-130b	VPS37D	0.310896229	mirna_pc
25619	hsa-mir-130b	SV2A	0.31497941	mirna_pc
25620	hsa-mir-130b	NT5M	0.329107325	mirna_pc
25621	hsa-mir-130b	THOC5	0.360332005	mirna_pc
25622	hsa-mir-130b	EFNB3	0.324737324	mirna_pc
25623	hsa-mir-130b	TEX261	0.308890872	mirna_pc
25624	hsa-mir-130b	CENPV	0.361065223	mirna_pc
25625	hsa-mir-130b	DNMT3A	0.57933621	mirna_pc
25626	hsa-mir-130b	ZNF821	0.307115452	mirna_pc
25627	hsa-mir-130b	TET1	0.398239354	mirna_pc
25628	hsa-mir-130b	C12orf73	0.343811588	mirna_pc
25629	hsa-mir-130b	PATZ1	0.402320089	mirna_pc
25630	hsa-mir-130b	C15orf61	0.340338889	mirna_pc
25631	hsa-mir-130b	ZNF711	0.33075093	mirna_pc
25632	hsa-mir-320b-2	HOXC9	0.37420085	mirna_pc
25633	hsa-mir-320b-2	MYBL2	0.389649347	mirna_pc
25634	hsa-mir-320b-2	PCNA	0.331816652	mirna_pc
25635	hsa-mir-320b-2	UBE2C	0.422204853	mirna_pc
25636	hsa-mir-320b-2	HDGF	0.354505179	mirna_pc
25637	hsa-mir-320b-2	FANCA	0.360623547	mirna_pc
25638	hsa-mir-320b-2	NCAPG	0.301225982	mirna_pc
25639	hsa-mir-320b-2	SPC25	0.356109286	mirna_pc
25640	hsa-mir-320b-2	MND1	0.368501247	mirna_pc
25641	hsa-mir-320b-2	DDX39	0.302940583	mirna_pc
25642	hsa-mir-320b-2	RAD54L	0.374042742	mirna_pc
25643	hsa-mir-320b-2	MAD2L1	0.327152426	mirna_pc
25644	hsa-mir-320b-2	TIMELESS	0.311664293	mirna_pc
25645	hsa-mir-320b-2	ASF1B	0.311080409	mirna_pc
25646	hsa-mir-320b-2	TROAP	0.346754115	mirna_pc
25647	hsa-mir-320b-2	CDCA5	0.325394076	mirna_pc
25648	hsa-mir-320b-2	STMN1	0.403249639	mirna_pc
25649	hsa-mir-320b-2	CENPM	0.408387835	mirna_pc

25650	hsa-mir-320b-2	EXO1	0.318308439	mirna_pc
25651	hsa-mir-320b-2	CDCA2	0.333423441	mirna_pc
25652	hsa-mir-320b-2	DKC1	0.338843303	mirna_pc
25653	hsa-mir-320b-2	UBE2T	0.374144439	mirna_pc
25654	hsa-mir-320b-2	CKS2	0.309497502	mirna_pc
25655	hsa-mir-320b-2	CKS1B	0.393534654	mirna_pc
25656	hsa-mir-320b-2	SNRPB	0.307045631	mirna_pc
25657	hsa-mir-320b-2	PTBP1	0.318746532	mirna_pc
25658	hsa-mir-320b-2	RCC1	0.336635851	mirna_pc
25659	hsa-mir-320b-2	DTL	0.3068445	mirna_pc
25660	hsa-mir-320b-2	EME1	0.300328114	mirna_pc
25661	hsa-mir-320b-2	RECQL4	0.32357846	mirna_pc
25662	hsa-mir-320b-2	AURKB	0.330083056	mirna_pc
25663	hsa-mir-320b-2	CDT1	0.354468463	mirna_pc
25664	hsa-mir-320b-2	CDCA3	0.342194493	mirna_pc
25665	hsa-mir-320b-2	C1orf112	0.336666035	mirna_pc
25666	hsa-mir-320b-2	FEN1	0.332114828	mirna_pc
25667	hsa-mir-320b-2	PSMA7	0.366061401	mirna_pc
25668	hsa-mir-320b-2	PBK	0.344529589	mirna_pc
25669	hsa-mir-320b-2	C16orf59	0.343894865	mirna_pc
25670	hsa-mir-320b-2	RAE1	0.335247725	mirna_pc
25671	hsa-mir-320b-2	ADRM1	0.337014466	mirna_pc
25672	hsa-mir-320b-2	C12orf48	0.393038894	mirna_pc
25673	hsa-mir-320b-2	HNRNPL	0.331717811	mirna_pc
25674	hsa-mir-320b-2	ZWINT	0.392384643	mirna_pc
25675	hsa-mir-320b-2	TUBA1B	0.380715775	mirna_pc
25676	hsa-mir-320b-2	UBE2S	0.360181721	mirna_pc
25677	hsa-mir-320b-2	ILF2	0.329288406	mirna_pc
25678	hsa-mir-320b-2	TCF3	0.316093075	mirna_pc
25679	hsa-mir-320b-2	TYMS	0.302827015	mirna_pc
25680	hsa-mir-320b-2	C20orf20	0.342484678	mirna_pc
25681	hsa-mir-320b-2	RNASEH2A	0.378158477	mirna_pc
25682	hsa-mir-320b-2	EPR1	0.351935428	mirna_pc
25683	hsa-mir-320b-2	H2AFX	0.411915273	mirna_pc
25684	hsa-mir-320b-2	SFRS2	0.305826488	mirna_pc
25685	hsa-mir-320b-2	UCK2	0.358044798	mirna_pc
25686	hsa-mir-320b-2	THOC4	0.437461719	mirna_pc
25687	hsa-mir-320b-2	RANBP1	0.32418162	mirna_pc
25688	hsa-mir-320b-2	E2F1	0.400498505	mirna_pc
25689	hsa-mir-320b-2	TH1L	0.38208294	mirna_pc
25690	hsa-mir-320b-2	SNRPA	0.410804991	mirna_pc
25691	hsa-mir-320b-2	FUS	0.350950308	mirna_pc
25692	hsa-mir-320b-2	U2AF2	0.32739522	mirna_pc
25693	hsa-mir-320b-2	CENPO	0.31745322	mirna_pc
25694	hsa-mir-320b-2	C19orf48	0.339546079	mirna_pc
25695	hsa-mir-320b-2	BAX	0.301690011	mirna_pc
25696	hsa-mir-320b-2	NUDT1	0.38508083	mirna_pc
25697	hsa-mir-320b-2	GINS2	0.342261011	mirna_pc
25698	hsa-mir-320b-2	NOP56	0.338724471	mirna_pc
25699	hsa-mir-320b-2	DSN1	0.30606021	mirna_pc
25700	hsa-mir-320b-2	SNHG1	0.302179674	mirna_pc
25701	hsa-mir-320b-2	CSE1L	0.34247756	mirna_pc
25702	hsa-mir-320b-2	DTYMK	0.326426552	mirna_pc
25703	hsa-mir-320b-2	PIGU	0.366997875	mirna_pc

25704	hsa-mir-320b-2	HSPE1	0.348278092	mirna_pc
25705	hsa-mir-320b-2	C16orf75	0.326244844	mirna_pc
25706	hsa-mir-320b-2	CDK4	0.320221769	mirna_pc
25707	hsa-mir-320b-2	BCL2L12	0.319994616	mirna_pc
25708	hsa-mir-320b-2	CENPH	0.368681876	mirna_pc
25709	hsa-mir-320b-2	DDX27	0.320399538	mirna_pc
25710	hsa-mir-320b-2	RALY	0.456040764	mirna_pc
25711	hsa-mir-320b-2	MSH2	0.303641335	mirna_pc
25712	hsa-mir-320b-2	TOMM40	0.305965265	mirna_pc
25713	hsa-mir-320b-2	RFC4	0.311376823	mirna_pc
25714	hsa-mir-320b-2	CDC25A	0.352461768	mirna_pc
25715	hsa-mir-320b-2	H2AFZ	0.319433089	mirna_pc
25716	hsa-mir-320b-2	MAGOH	0.315031827	mirna_pc
25717	hsa-mir-320b-2	NUP37	0.305040429	mirna_pc
25718	hsa-mir-320b-2	MCM8	0.33515586	mirna_pc
25719	hsa-mir-320b-2	BANF1	0.308536464	mirna_pc
25720	hsa-mir-320b-2	SNRPE	0.413445394	mirna_pc
25721	hsa-mir-320b-2	MARCKSL1	0.365399179	mirna_pc
25722	hsa-mir-320b-2	SNRPD2	0.362979945	mirna_pc
25723	hsa-mir-320b-2	DAZAP1	0.335993472	mirna_pc
25724	hsa-mir-320b-2	RPA3	0.320266443	mirna_pc
25725	hsa-mir-320b-2	TMEM201	0.371469311	mirna_pc
25726	hsa-mir-320b-2	MRPL9	0.333377235	mirna_pc
25727	hsa-mir-320b-2	PFDN4	0.376361632	mirna_pc
25728	hsa-mir-320b-2	PAFAH1B3	0.35105092	mirna_pc
25729	hsa-mir-320b-2	SRRT	0.349978775	mirna_pc
25730	hsa-mir-320b-2	CACYBP	0.325165375	mirna_pc
25731	hsa-mir-320b-2	DCTPP1	0.345614915	mirna_pc
25732	hsa-mir-320b-2	PSRC1	0.335462328	mirna_pc
25733	hsa-mir-320b-2	DGUOK	0.309457773	mirna_pc
25734	hsa-mir-320b-2	POU2F1	0.306017054	mirna_pc
25735	hsa-mir-320b-2	FBL	0.32559726	mirna_pc
25736	hsa-mir-320b-2	STIP1	0.327196551	mirna_pc
25737	hsa-mir-320b-2	SCNM1	0.336561082	mirna_pc
25738	hsa-mir-320b-2	TMPO	0.323893862	mirna_pc
25739	hsa-mir-320b-2	NR2C2AP	0.334549744	mirna_pc
25740	hsa-mir-320b-2	MRTO4	0.315862483	mirna_pc
25741	hsa-mir-320b-2	LSM 7.00	0.331502114	mirna_pc
25742	hsa-mir-320b-2	PPIH	0.332479981	mirna_pc
25743	hsa-mir-320b-2	LOC100128191	0.322362182	mirna_pc
25744	hsa-mir-320b-2	HMGN2	0.333925644	mirna_pc
25745	hsa-mir-320b-2	ETV4	0.330421668	mirna_pc
25746	hsa-mir-320b-2	POLA2	0.303124038	mirna_pc
25747	hsa-mir-320b-2	SNRPF	0.34593563	mirna_pc
25748	hsa-mir-320b-2	HSPBP1	0.343025707	mirna_pc
25749	hsa-mir-320b-2	SSRP1	0.354996949	mirna_pc
25750	hsa-mir-320b-2	POLR2D	0.329722105	mirna_pc
25751	hsa-mir-320b-2	KHDRBS1	0.332933337	mirna_pc
25752	hsa-mir-320b-2	SLC5A6	0.382424102	mirna_pc
25753	hsa-mir-320b-2	C20orf27	0.333682497	mirna_pc
25754	hsa-mir-320b-2	LOC92659	0.416214879	mirna_pc
25755	hsa-mir-320b-2	AHCY	0.359442036	mirna_pc
25756	hsa-mir-320b-2	SNRNP40	0.35204058	mirna_pc
25757	hsa-mir-320b-2	NONO	0.449287186	mirna_pc

25758	hsa-mir-320b-2	OBFC2B	0.39134088	mirna_pc
25759	hsa-mir-320b-2	PTMA	0.334715186	mirna_pc
25760	hsa-mir-320b-2	LSM 4.00	0.402954575	mirna_pc
25761	hsa-mir-320b-2	C11orf84	0.339654936	mirna_pc
25762	hsa-mir-320b-2	DPY30	0.330988988	mirna_pc
25763	hsa-mir-320b-2	SCAMP3	0.327861075	mirna_pc
25764	hsa-mir-320b-2	SAC3D1	0.307460245	mirna_pc
25765	hsa-mir-320b-2	RPS21	0.339759279	mirna_pc
25766	hsa-mir-320b-2	MAGOHB	0.311899356	mirna_pc
25767	hsa-mir-320b-2	COMMD4	0.326903197	mirna_pc
25768	hsa-mir-320b-2	PDRG1	0.341390032	mirna_pc
25769	hsa-mir-320b-2	C20orf199	0.369434737	mirna_pc
25770	hsa-mir-320b-2	HEATR2	0.304380565	mirna_pc
25771	hsa-mir-320b-2	AZI1	0.303645073	mirna_pc
25772	hsa-mir-320b-2	WDR74	0.323655958	mirna_pc
25773	hsa-mir-320b-2	POLR2G	0.337725751	mirna_pc
25774	hsa-mir-320b-2	C1orf174	0.31264776	mirna_pc
25775	hsa-mir-320b-2	MRPL17	0.356033053	mirna_pc
25776	hsa-mir-320b-2	EIF2S2	0.300156707	mirna_pc
25777	hsa-mir-320b-2	LAS1L	0.360382998	mirna_pc
25778	hsa-mir-320b-2	SFPQ	0.321103468	mirna_pc
25779	hsa-mir-320b-2	VPS72	0.335882297	mirna_pc
25780	hsa-mir-320b-2	CADM1	0.332607241	mirna_pc
25781	hsa-mir-320b-2	MRPL12	0.38021203	mirna_pc
25782	hsa-mir-320b-2	EMG1	0.32462699	mirna_pc
25783	hsa-mir-320b-2	ASCL2	0.301965741	mirna_pc
25784	hsa-mir-320b-2	CSTF1	0.307183521	mirna_pc
25785	hsa-mir-320b-2	MRPL11	0.360863226	mirna_pc
25786	hsa-mir-320b-2	HNRNPA1	0.361569308	mirna_pc
25787	hsa-mir-320b-2	PPP1R8	0.349165792	mirna_pc
25788	hsa-mir-320b-2	MEX3A	0.331180058	mirna_pc
25789	hsa-mir-320b-2	C19orf24	0.322179671	mirna_pc
25790	hsa-mir-320b-2	HNRNPA1L2	0.38855103	mirna_pc
25791	hsa-mir-320b-2	MACROD1	0.316042977	mirna_pc
25792	hsa-mir-320b-2	POLR3K	0.333590931	mirna_pc
25793	hsa-mir-320b-2	HAUS7	0.306016597	mirna_pc
25794	hsa-mir-320b-2	MRPL51	0.30197891	mirna_pc
25795	hsa-mir-320b-2	C1orf35	0.33541021	mirna_pc
25796	hsa-mir-320b-2	TGIF2	0.321068226	mirna_pc
25797	hsa-mir-320b-2	EBP	0.326109647	mirna_pc
25798	hsa-mir-320b-2	SLC35C2	0.324351652	mirna_pc
25799	hsa-mir-320b-2	POLE	0.33291251	mirna_pc
25800	hsa-mir-320b-2	MUTYH	0.385715375	mirna_pc
25801	hsa-mir-320b-2	C11orf31	0.338755853	mirna_pc
25802	hsa-mir-320b-2	TMEM216	0.321976714	mirna_pc
25803	hsa-mir-320b-2	RBMX2	0.320259076	mirna_pc
25804	hsa-mir-320b-2	RBMX	0.424613912	mirna_pc
25805	hsa-mir-320b-2	ALKBH2	0.384448569	mirna_pc
25806	hsa-mir-320b-2	BAMBI	0.306613842	mirna_pc
25807	hsa-mir-320b-2	C19orf61	0.318733882	mirna_pc
25808	hsa-mir-320b-2	C11orf48	0.374478605	mirna_pc
25809	hsa-mir-320b-2	ACD	0.340666455	mirna_pc
25810	hsa-mir-320b-2	MRPL37	0.347206588	mirna_pc
25811	hsa-mir-320b-2	CCDC23	0.303319933	mirna_pc

25812	hsa-mir-320b-2	EIF3I	0.320416038	mirna_pc
25813	hsa-mir-320b-2	MSX2	0.300094401	mirna_pc
25814	hsa-mir-320b-2	APOA1BP	0.379642869	mirna_pc
25815	hsa-mir-320b-2	ZNF511	0.311625067	mirna_pc
25816	hsa-mir-320b-2	B3GAT3	0.400023195	mirna_pc
25817	hsa-mir-320b-2	TIMM10	0.349525448	mirna_pc
25818	hsa-mir-320b-2	ITPRIPL1	0.319937851	mirna_pc
25819	hsa-mir-320b-2	STRA13	0.600098278	mirna_pc
25820	hsa-mir-320b-2	TIMM16	0.316496637	mirna_pc
25821	hsa-mir-320b-2	NOTUM	0.437848264	mirna_pc
25822	hsa-mir-320b-2	SNHG6	0.354231503	mirna_pc
25823	hsa-mir-320b-2	TMEM180	0.306816174	mirna_pc
25824	hsa-mir-320b-2	CUEDC2	0.312277872	mirna_pc
25825	hsa-mir-320b-2	TBX3	0.353017822	mirna_pc
25826	hsa-mir-320b-2	GANAB	0.350007243	mirna_pc
25827	hsa-mir-320b-2	ANAPC11	0.341984425	mirna_pc
25828	hsa-mir-320b-2	C11orf83	0.38827479	mirna_pc
25829	hsa-mir-320b-2	ZNF593	0.363511567	mirna_pc
25830	hsa-mir-320b-2	DYNLRB1	0.349600126	mirna_pc
25831	hsa-mir-320b-2	COPZ1	0.325791915	mirna_pc
25832	hsa-mir-320b-2	GPR3	0.406654942	mirna_pc
25833	hsa-mir-320b-2	EXOSC1	0.341382797	mirna_pc
25834	hsa-mir-320b-2	SCLY	0.344393727	mirna_pc
25835	hsa-mir-320b-2	COMMD7	0.34599567	mirna_pc
25836	hsa-mir-320b-2	AAAS	0.389112533	mirna_pc
25837	hsa-mir-320b-2	DKK 1.00	0.33994252	mirna_pc
25838	hsa-mir-320b-2	C2orf79	0.317743923	mirna_pc
25839	hsa-mir-320b-2	MRPS26	0.345920921	mirna_pc
25840	hsa-mir-320b-2	PYCR1	0.32441855	mirna_pc
25841	hsa-mir-320b-2	SFRS7	0.306387873	mirna_pc
25842	hsa-mir-320b-2	MANBAL	0.318145255	mirna_pc
25843	hsa-mir-320b-2	PEX10	0.32217579	mirna_pc
25844	hsa-mir-320b-2	EMD	0.307886519	mirna_pc
25845	hsa-mir-320b-2	RPS3	0.360133822	mirna_pc
25846	hsa-mir-320b-2	NKD1	0.388536023	mirna_pc
25847	hsa-mir-320b-2	C10orf35	0.332016613	mirna_pc
25848	hsa-mir-320b-2	LRRC45	0.338957378	mirna_pc
25849	hsa-mir-320b-2	AXIN2	0.341216812	mirna_pc
25850	hsa-mir-320b-2	SCAND1	0.33607472	mirna_pc
25851	hsa-mir-320b-2	CNOT7	0.398869593	mirna_pc
25852	hsa-mir-320b-2	PGAP2	0.317121657	mirna_pc
25853	hsa-mir-320b-2	LOC728024	0.304330003	mirna_pc
25854	hsa-mir-320b-2	FN3KRP	0.342624826	mirna_pc
25855	hsa-mir-320b-2	MRPL55	0.302148947	mirna_pc
25856	hsa-mir-320b-2	MTP 18.00	0.302926975	mirna_pc
25857	hsa-mir-320b-2	ERGIC3	0.396124586	mirna_pc
25858	hsa-mir-320b-2	TIMM17B	0.38910271	mirna_pc
25859	hsa-mir-320b-2	SEPHS1	0.303993326	mirna_pc
25860	hsa-mir-320b-2	TAF10	0.30737696	mirna_pc
25861	hsa-mir-320b-2	C11orf10	0.327448209	mirna_pc
25862	hsa-mir-320b-2	ZNF259	0.432523819	mirna_pc
25863	hsa-mir-320b-2	C12orf57	0.317661672	mirna_pc
25864	hsa-mir-320b-2	MLST8	0.318261338	mirna_pc
25865	hsa-mir-320b-2	C16orf13	0.306753124	mirna_pc

25866	hsa-mir-320b-2	RPL39	0.314685568	mirna_pc
25867	hsa-mir-320b-2	C17orf89	0.318978546	mirna_pc
25868	hsa-mir-320b-2	MYL6B	0.303875556	mirna_pc
25869	hsa-mir-320b-2	UQCRHL	0.335877755	mirna_pc
25870	hsa-mir-320b-2	CAMTA1	0.366410342	mirna_pc
25871	hsa-mir-320b-2	RPS20	0.316614217	mirna_pc
25872	hsa-mir-320b-2	C20orf196	0.336406405	mirna_pc
25873	hsa-mir-320b-2	TMEM160	0.310248861	mirna_pc
25874	hsa-mir-320b-2	BIN3	0.36652681	mirna_pc
25875	hsa-mir-320b-2	ATP5G2	0.331591508	mirna_pc
25876	hsa-mir-320b-2	C17orf81	0.332302353	mirna_pc
25877	hsa-mir-320b-2	RPS27	0.342847684	mirna_pc
25878	hsa-mir-320b-2	NAP1L1	0.366659278	mirna_pc
25879	hsa-mir-320b-2	SMG5	0.302577201	mirna_pc
25880	hsa-mir-320b-2	SPCS2	0.396803862	mirna_pc
25881	hsa-mir-320b-2	NDUFC2	0.348176562	mirna_pc
25882	hsa-mir-320b-2	GLT25D2	0.306927656	mirna_pc
25883	hsa-mir-320b-2	BUD13	0.476897718	mirna_pc
25884	hsa-mir-320b-2	LSM 3.00	0.312107568	mirna_pc
25885	hsa-mir-320b-2	MYCN	0.336936766	mirna_pc
25886	hsa-mir-320b-2	PMF1	0.385818672	mirna_pc
25887	hsa-mir-320b-2	RPLP2	0.321086412	mirna_pc
25888	hsa-mir-320b-2	MSI1	0.312702795	mirna_pc
25889	hsa-mir-320b-2	NDUFB11	0.355595529	mirna_pc
25890	hsa-mir-320b-2	ZNF232	0.311236261	mirna_pc
25891	hsa-mir-320b-2	ASPSCR1	0.475740012	mirna_pc
25892	hsa-mir-320b-2	POLR3D	0.326342331	mirna_pc
25893	hsa-mir-320b-2	RBBP4	0.335189747	mirna_pc
25894	hsa-mir-320b-2	XPO7	0.300382629	mirna_pc
25895	hsa-mir-320b-2	PTPMT1	0.300290481	mirna_pc
25896	hsa-mir-320b-2	LOC653566	0.367056141	mirna_pc
25897	hsa-mir-320b-2	KIAA1967	0.417067401	mirna_pc
25898	hsa-mir-320b-2	MCAT	0.300334324	mirna_pc
25899	hsa-mir-320b-2	SCML2	0.32326019	mirna_pc
25900	hsa-mir-320b-2	PDXP	0.314453107	mirna_pc
25901	hsa-mir-320b-2	ETV5	0.330505202	mirna_pc
25902	hsa-mir-320b-2	COL9A3	0.312091401	mirna_pc
25903	hsa-mir-320b-2	DACT2	0.34051274	mirna_pc
25904	hsa-mir-320b-2	SNRNP25	0.36490063	mirna_pc
25905	hsa-mir-320b-2	PREB	0.452038603	mirna_pc
25906	hsa-mir-320b-2	PIN4	0.317258547	mirna_pc
25907	hsa-mir-320b-2	SSR2	0.366451901	mirna_pc
25908	hsa-mir-320b-2	MRPL23	0.323153128	mirna_pc
25909	hsa-mir-320b-2	VAPB	0.310616685	mirna_pc
25910	hsa-mir-320b-2	UXT	0.398326025	mirna_pc
25911	hsa-mir-320b-2	PCBD1	0.31830087	mirna_pc
25912	hsa-mir-320b-2	UQCRH	0.304535149	mirna_pc
25913	hsa-mir-320b-2	CTNNBL1	0.309939368	mirna_pc
25914	hsa-mir-320b-2	NUDC	0.349150252	mirna_pc
25915	hsa-mir-320b-2	PARK7	0.349531575	mirna_pc
25916	hsa-mir-320b-2	ZNF711	0.311843641	mirna_pc
25917	hsa-let-7d	TPX2	0.313168125	mirna_pc
25918	hsa-let-7d	KPNA2	0.311207162	mirna_pc
25919	hsa-let-7d	CENPF	0.326700699	mirna_pc

25920	hsa-let-7d	RCC2	0.331328872	mirna_pc
25921	hsa-let-7d	KIF11	0.374541543	mirna_pc
25922	hsa-let-7d	ECT2	0.436081766	mirna_pc
25923	hsa-let-7d	FOXM1	0.312213545	mirna_pc
25924	hsa-let-7d	SPC24	0.316832284	mirna_pc
25925	hsa-let-7d	CDK1	0.415281217	mirna_pc
25926	hsa-let-7d	CDC25C	0.353457213	mirna_pc
25927	hsa-let-7d	MYBL2	0.367980274	mirna_pc
25928	hsa-let-7d	PLK1	0.309236737	mirna_pc
25929	hsa-let-7d	PCNA	0.328183285	mirna_pc
25930	hsa-let-7d	UBE2C	0.429329379	mirna_pc
25931	hsa-let-7d	PRC1	0.36758301	mirna_pc
25932	hsa-let-7d	NUSAP1	0.530592367	mirna_pc
25933	hsa-let-7d	MCM4	0.368715679	mirna_pc
25934	hsa-let-7d	KIFC1	0.374087582	mirna_pc
25935	hsa-let-7d	BUB1B	0.453096677	mirna_pc
25936	hsa-let-7d	HDGF	0.362124588	mirna_pc
25937	hsa-let-7d	CKAP2	0.303340734	mirna_pc
25938	hsa-let-7d	FAM72A	0.328199262	mirna_pc
25939	hsa-let-7d	KIF2C	0.321405685	mirna_pc
25940	hsa-let-7d	NCAPG	0.401213874	mirna_pc
25941	hsa-let-7d	CDC20	0.385773543	mirna_pc
25942	hsa-let-7d	CCNA2	0.381711706	mirna_pc
25943	hsa-let-7d	CEP55	0.390888379	mirna_pc
25944	hsa-let-7d	CDC25B	0.327079072	mirna_pc
25945	hsa-let-7d	SPC25	0.381089412	mirna_pc
25946	hsa-let-7d	FANCI	0.370589258	mirna_pc
25947	hsa-let-7d	LMNB2	0.33631058	mirna_pc
25948	hsa-let-7d	NEK2	0.411182366	mirna_pc
25949	hsa-let-7d	MND1	0.315519991	mirna_pc
25950	hsa-let-7d	KIF22	0.32706576	mirna_pc
25951	hsa-let-7d	DDX39	0.349116828	mirna_pc
25952	hsa-let-7d	NCAPH	0.366662201	mirna_pc
25953	hsa-let-7d	KIF23	0.33061207	mirna_pc
25954	hsa-let-7d	RAD54L	0.323299653	mirna_pc
25955	hsa-let-7d	CENPA	0.412446422	mirna_pc
25956	hsa-let-7d	MAD2L1	0.362397676	mirna_pc
25957	hsa-let-7d	LMNB1	0.341175323	mirna_pc
25958	hsa-let-7d	ASF1B	0.382274245	mirna_pc
25959	hsa-let-7d	CDCA8	0.358487627	mirna_pc
25960	hsa-let-7d	TROAP	0.359905953	mirna_pc
25961	hsa-let-7d	CDCA5	0.435294302	mirna_pc
25962	hsa-let-7d	CDC45	0.429613449	mirna_pc
25963	hsa-let-7d	STMN1	0.365266787	mirna_pc
25964	hsa-let-7d	CENPM	0.391654121	mirna_pc
25965	hsa-let-7d	TK1	0.334916401	mirna_pc
25966	hsa-let-7d	DKC1	0.35479769	mirna_pc
25967	hsa-let-7d	UBE2T	0.502809032	mirna_pc
25968	hsa-let-7d	CKS2	0.465929089	mirna_pc
25969	hsa-let-7d	CKS1B	0.491311254	mirna_pc
25970	hsa-let-7d	MCM2	0.371783437	mirna_pc
25971	hsa-let-7d	KIF4A	0.302988984	mirna_pc
25972	hsa-let-7d	ORC1L	0.326611263	mirna_pc
25973	hsa-let-7d	SNRPB	0.345189933	mirna_pc

25974	hsa-let-7d	RACGAP1	0.353246453	mirna_pc
25975	hsa-let-7d	PTBP1	0.315329564	mirna_pc
25976	hsa-let-7d	CCNB2	0.455674108	mirna_pc
25977	hsa-let-7d	NUF2	0.333432297	mirna_pc
25978	hsa-let-7d	DEPDC1	0.314084669	mirna_pc
25979	hsa-let-7d	PTTG1	0.323742384	mirna_pc
25980	hsa-let-7d	SKA3	0.354646354	mirna_pc
25981	hsa-let-7d	HJURP	0.438247475	mirna_pc
25982	hsa-let-7d	RAD51	0.441535536	mirna_pc
25983	hsa-let-7d	BLM	0.363265073	mirna_pc
25984	hsa-let-7d	PAICS	0.367873703	mirna_pc
25985	hsa-let-7d	BIRC5	0.396824512	mirna_pc
25986	hsa-let-7d	RCC1	0.398823901	mirna_pc
25987	hsa-let-7d	OIP5	0.469065809	mirna_pc
25988	hsa-let-7d	DTL	0.364569791	mirna_pc
25989	hsa-let-7d	RECQL4	0.487192467	mirna_pc
25990	hsa-let-7d	AURKB	0.432188154	mirna_pc
25991	hsa-let-7d	GSG2	0.362846603	mirna_pc
25992	hsa-let-7d	KIF20A	0.305371774	mirna_pc
25993	hsa-let-7d	CHEK2	0.503446321	mirna_pc
25994	hsa-let-7d	CDCA3	0.370871588	mirna_pc
25995	hsa-let-7d	ALG3	0.479056474	mirna_pc
25996	hsa-let-7d	FEN1	0.336287428	mirna_pc
25997	hsa-let-7d	TRAIP	0.317857642	mirna_pc
25998	hsa-let-7d	ORC6L	0.324787013	mirna_pc
25999	hsa-let-7d	ERH	0.304522457	mirna_pc
26000	hsa-let-7d	PSMA7	0.344777499	mirna_pc
26001	hsa-let-7d	C16orf59	0.349922299	mirna_pc
26002	hsa-let-7d	CDKN3	0.347126005	mirna_pc
26003	hsa-let-7d	FAM72D	0.324648183	mirna_pc
26004	hsa-let-7d	FAM72B	0.332886801	mirna_pc
26005	hsa-let-7d	NEIL3	0.324560336	mirna_pc
26006	hsa-let-7d	ACTL6A	0.402270335	mirna_pc
26007	hsa-let-7d	C12orf48	0.366057453	mirna_pc
26008	hsa-let-7d	HNRNPL	0.394874104	mirna_pc
26009	hsa-let-7d	ZWILCH	0.426043039	mirna_pc
26010	hsa-let-7d	PKMYT1	0.312481262	mirna_pc
26011	hsa-let-7d	TTK	0.403985796	mirna_pc
26012	hsa-let-7d	ZWINT	0.416402266	mirna_pc
26013	hsa-let-7d	TUBA1B	0.311621644	mirna_pc
26014	hsa-let-7d	WDR67	0.338446038	mirna_pc
26015	hsa-let-7d	RUVBL1	0.331013209	mirna_pc
26016	hsa-let-7d	BUB3	0.300633273	mirna_pc
26017	hsa-let-7d	UBE2S	0.433632508	mirna_pc
26018	hsa-let-7d	PSMA4	0.323214851	mirna_pc
26019	hsa-let-7d	NCAPD2	0.347384274	mirna_pc
26020	hsa-let-7d	HNRNPC	0.375141854	mirna_pc
26021	hsa-let-7d	ILF2	0.399582321	mirna_pc
26022	hsa-let-7d	TFRC	0.344153626	mirna_pc
26023	hsa-let-7d	TYMS	0.317296684	mirna_pc
26024	hsa-let-7d	RNASEH2A	0.345744713	mirna_pc
26025	hsa-let-7d	EPR1	0.416958698	mirna_pc
26026	hsa-let-7d	H2AFX	0.325999834	mirna_pc
26027	hsa-let-7d	DCAF13	0.33959804	mirna_pc

26028	hsa-let-7d	UCK2	0.443394973	mirna_pc
26029	hsa-let-7d	THOC4	0.351023461	mirna_pc
26030	hsa-let-7d	NFKBIL2	0.343926297	mirna_pc
26031	hsa-let-7d	PTGES3	0.361677224	mirna_pc
26032	hsa-let-7d	PRIM2	0.371198055	mirna_pc
26033	hsa-let-7d	FAM60A	0.314301061	mirna_pc
26034	hsa-let-7d	HMGB3	0.368920754	mirna_pc
26035	hsa-let-7d	FANCD2	0.301978027	mirna_pc
26036	hsa-let-7d	FAM64A	0.427923894	mirna_pc
26037	hsa-let-7d	RANBP1	0.402439611	mirna_pc
26038	hsa-let-7d	MTBP	0.367457277	mirna_pc
26039	hsa-let-7d	E2F1	0.40581033	mirna_pc
26040	hsa-let-7d	EIF4A3	0.315771358	mirna_pc
26041	hsa-let-7d	TH1L	0.348323085	mirna_pc
26042	hsa-let-7d	MCM3	0.302477457	mirna_pc
26043	hsa-let-7d	SNRPA	0.383704616	mirna_pc
26044	hsa-let-7d	CENPW	0.33087172	mirna_pc
26045	hsa-let-7d	DNAJC9	0.427447732	mirna_pc
26046	hsa-let-7d	POC1A	0.368979399	mirna_pc
26047	hsa-let-7d	CENPO	0.371789528	mirna_pc
26048	hsa-let-7d	CENPL	0.323470217	mirna_pc
26049	hsa-let-7d	NUDT1	0.335991138	mirna_pc
26050	hsa-let-7d	CCT3	0.378662243	mirna_pc
26051	hsa-let-7d	GINS2	0.343171158	mirna_pc
26052	hsa-let-7d	C9orf140	0.41196592	mirna_pc
26053	hsa-let-7d	TEAD4	0.317017106	mirna_pc
26054	hsa-let-7d	NOP56	0.327550313	mirna_pc
26055	hsa-let-7d	GRINA	0.318431421	mirna_pc
26056	hsa-let-7d	CDCA4	0.316979	mirna_pc
26057	hsa-let-7d	CSE1L	0.397853782	mirna_pc
26058	hsa-let-7d	DTYMK	0.405518928	mirna_pc
26059	hsa-let-7d	TMEM206	0.312717771	mirna_pc
26060	hsa-let-7d	HSPE1	0.328668334	mirna_pc
26061	hsa-let-7d	EBNA1BP2	0.322648766	mirna_pc
26062	hsa-let-7d	CFL1	0.32844252	mirna_pc
26063	hsa-let-7d	NOP2	0.335906877	mirna_pc
26064	hsa-let-7d	CAD	0.307521497	mirna_pc
26065	hsa-let-7d	ZFP64	0.374267543	mirna_pc
26066	hsa-let-7d	CDK4	0.358283596	mirna_pc
26067	hsa-let-7d	HNRNPR	0.336596252	mirna_pc
26068	hsa-let-7d	CENPH	0.339462822	mirna_pc
26069	hsa-let-7d	CHAF1B	0.354967682	mirna_pc
26070	hsa-let-7d	ACYP1	0.349290097	mirna_pc
26071	hsa-let-7d	MSH2	0.386904351	mirna_pc
26072	hsa-let-7d	TOMM40	0.316987913	mirna_pc
26073	hsa-let-7d	CDC7	0.347257635	mirna_pc
26074	hsa-let-7d	YDJC	0.422651408	mirna_pc
26075	hsa-let-7d	HAUS8	0.343436093	mirna_pc
26076	hsa-let-7d	RFC4	0.475885379	mirna_pc
26077	hsa-let-7d	BRIX1	0.325539958	mirna_pc
26078	hsa-let-7d	DDX11	0.309029972	mirna_pc
26079	hsa-let-7d	SF3B14	0.308398831	mirna_pc
26080	hsa-let-7d	CDC25A	0.33103076	mirna_pc
26081	hsa-let-7d	RAD54B	0.342599654	mirna_pc

26082	hsa-let-7d	H2AFZ	0.347844761	mirna_pc
26083	hsa-let-7d	PRR7	0.304637778	mirna_pc
26084	hsa-let-7d	MAGOH	0.324287113	mirna_pc
26085	hsa-let-7d	DDX12	0.312414597	mirna_pc
26086	hsa-let-7d	DSCC1	0.379732629	mirna_pc
26087	hsa-let-7d	NUP37	0.365318071	mirna_pc
26088	hsa-let-7d	SMC2	0.402901667	mirna_pc
26089	hsa-let-7d	IRAK1	0.320384258	mirna_pc
26090	hsa-let-7d	CLN6	0.417269878	mirna_pc
26091	hsa-let-7d	BANF1	0.39927058	mirna_pc
26092	hsa-let-7d	SNRPE	0.38250457	mirna_pc
26093	hsa-let-7d	C21orf45	0.316375221	mirna_pc
26094	hsa-let-7d	ATIC	0.33691389	mirna_pc
26095	hsa-let-7d	PPM1G	0.330424777	mirna_pc
26096	hsa-let-7d	NPM3	0.415176229	mirna_pc
26097	hsa-let-7d	TFAP4	0.341257776	mirna_pc
26098	hsa-let-7d	NUDT5	0.3630218	mirna_pc
26099	hsa-let-7d	ZNF367	0.519668599	mirna_pc
26100	hsa-let-7d	DAZAP1	0.401812487	mirna_pc
26101	hsa-let-7d	CBX8	0.361392423	mirna_pc
26102	hsa-let-7d	PPIL5	0.320734308	mirna_pc
26103	hsa-let-7d	WDR34	0.439627957	mirna_pc
26104	hsa-let-7d	TMEM201	0.426818474	mirna_pc
26105	hsa-let-7d	NCL	0.332234487	mirna_pc
26106	hsa-let-7d	MRPL9	0.37554211	mirna_pc
26107	hsa-let-7d	CACYBP	0.310002737	mirna_pc
26108	hsa-let-7d	DCTPP1	0.334644279	mirna_pc
26109	hsa-let-7d	PSRC1	0.327619127	mirna_pc
26110	hsa-let-7d	ODF2	0.436045451	mirna_pc
26111	hsa-let-7d	MRPL47	0.364762956	mirna_pc
26112	hsa-let-7d	STIP1	0.321992765	mirna_pc
26113	hsa-let-7d	HIST1H1E	0.306611189	mirna_pc
26114	hsa-let-7d	THOP1	0.384091543	mirna_pc
26115	hsa-let-7d	EXOSC5	0.323710478	mirna_pc
26116	hsa-let-7d	SCNM1	0.30022491	mirna_pc
26117	hsa-let-7d	CDC123	0.319680212	mirna_pc
26118	hsa-let-7d	SET	0.524263512	mirna_pc
26119	hsa-let-7d	POLR2H	0.431097379	mirna_pc
26120	hsa-let-7d	NR2C2AP	0.339813513	mirna_pc
26121	hsa-let-7d	C3orf26	0.346482263	mirna_pc
26122	hsa-let-7d	LSG1	0.330954348	mirna_pc
26123	hsa-let-7d	POLE3	0.492302262	mirna_pc
26124	hsa-let-7d	PPIH	0.310119964	mirna_pc
26125	hsa-let-7d	GMPS	0.359875475	mirna_pc
26126	hsa-let-7d	PPAT	0.356901183	mirna_pc
26127	hsa-let-7d	DHX9	0.318736533	mirna_pc
26128	hsa-let-7d	CCDC138	0.325304403	mirna_pc
26129	hsa-let-7d	MRPL3	0.327550439	mirna_pc
26130	hsa-let-7d	TSSC1	0.314943959	mirna_pc
26131	hsa-let-7d	C20orf3	0.302271311	mirna_pc
26132	hsa-let-7d	BOP 1.00	0.31491299	mirna_pc
26133	hsa-let-7d	POLA2	0.371840084	mirna_pc
26134	hsa-let-7d	CCDC77	0.313774856	mirna_pc
26135	hsa-let-7d	TEX10	0.471503815	mirna_pc

26136	hsa-let-7d	SNRPA1	0.360451994	mirna_pc
26137	hsa-let-7d	CCDC58	0.324936841	mirna_pc
26138	hsa-let-7d	SKP2	0.390737769	mirna_pc
26139	hsa-let-7d	TIPIN	0.409444229	mirna_pc
26140	hsa-let-7d	ECE2	0.452286871	mirna_pc
26141	hsa-let-7d	PTDSS1	0.351097343	mirna_pc
26142	hsa-let-7d	RFC5	0.352394455	mirna_pc
26143	hsa-let-7d	FANCG	0.321033642	mirna_pc
26144	hsa-let-7d	MAZ	0.311213358	mirna_pc
26145	hsa-let-7d	SSRP1	0.334419511	mirna_pc
26146	hsa-let-7d	SNRPD3	0.369386375	mirna_pc
26147	hsa-let-7d	GLA	0.331656545	mirna_pc
26148	hsa-let-7d	KHDRBS1	0.370121369	mirna_pc
26149	hsa-let-7d	C20orf27	0.412763357	mirna_pc
26150	hsa-let-7d	LOC92659	0.360925358	mirna_pc
26151	hsa-let-7d	TOMM34	0.423232839	mirna_pc
26152	hsa-let-7d	CCDC21	0.387894252	mirna_pc
26153	hsa-let-7d	RNF220	0.341779705	mirna_pc
26154	hsa-let-7d	PRPF4	0.319167016	mirna_pc
26155	hsa-let-7d	PYCRL	0.352727759	mirna_pc
26156	hsa-let-7d	PARP1	0.31357951	mirna_pc
26157	hsa-let-7d	CCT7	0.368384389	mirna_pc
26158	hsa-let-7d	MTERFD1	0.338189971	mirna_pc
26159	hsa-let-7d	SNRNP40	0.41584797	mirna_pc
26160	hsa-let-7d	MED30	0.387606181	mirna_pc
26161	hsa-let-7d	NOLC1	0.334872251	mirna_pc
26162	hsa-let-7d	TMEM69	0.317510942	mirna_pc
26163	hsa-let-7d	PSMA1	0.311192343	mirna_pc
26164	hsa-let-7d	LIN9	0.302047743	mirna_pc
26165	hsa-let-7d	WRAP53	0.350694587	mirna_pc
26166	hsa-let-7d	NONO	0.345629993	mirna_pc
26167	hsa-let-7d	CCDC86	0.372060125	mirna_pc
26168	hsa-let-7d	QSOX2	0.390717839	mirna_pc
26169	hsa-let-7d	PTMA	0.304085567	mirna_pc
26170	hsa-let-7d	USP39	0.354189245	mirna_pc
26171	hsa-let-7d	WDR76	0.348326916	mirna_pc
26172	hsa-let-7d	LSM 4.00	0.406713032	mirna_pc
26173	hsa-let-7d	EXOSC2	0.548677616	mirna_pc
26174	hsa-let-7d	C11orf84	0.375057597	mirna_pc
26175	hsa-let-7d	GSS	0.315513473	mirna_pc
26176	hsa-let-7d	TOP1MT	0.309659795	mirna_pc
26177	hsa-let-7d	TBCE	0.336546117	mirna_pc
26178	hsa-let-7d	CLCN2	0.310000524	mirna_pc
26179	hsa-let-7d	NCBP2	0.347981875	mirna_pc
26180	hsa-let-7d	KLF16	0.311757277	mirna_pc
26181	hsa-let-7d	RQCD1	0.306133042	mirna_pc
26182	hsa-let-7d	DPY30	0.315653625	mirna_pc
26183	hsa-let-7d	MTHFD2	0.336752495	mirna_pc
26184	hsa-let-7d	RRP9	0.301780145	mirna_pc
26185	hsa-let-7d	DDX31	0.374786528	mirna_pc
26186	hsa-let-7d	DPH2	0.349232766	mirna_pc
26187	hsa-let-7d	MTCH2	0.304638427	mirna_pc
26188	hsa-let-7d	REXO4	0.416968669	mirna_pc
26189	hsa-let-7d	MEN1	0.396291781	mirna_pc

26190	hsa-let-7d	C17orf49	0.305670002	mirna_pc
26191	hsa-let-7d	PNO1	0.356498656	mirna_pc
26192	hsa-let-7d	CCT4	0.370020262	mirna_pc
26193	hsa-let-7d	SAC3D1	0.408077636	mirna_pc
26194	hsa-let-7d	RABEPK	0.437071928	mirna_pc
26195	hsa-let-7d	ELAVL1	0.381145248	mirna_pc
26196	hsa-let-7d	MRPL4	0.359255014	mirna_pc
26197	hsa-let-7d	CENPP	0.302922942	mirna_pc
26198	hsa-let-7d	MTHFD1	0.306756951	mirna_pc
26199	hsa-let-7d	NCAPD3	0.351600322	mirna_pc
26200	hsa-let-7d	NAE1	0.339568662	mirna_pc
26201	hsa-let-7d	GTF3C5	0.37342075	mirna_pc
26202	hsa-let-7d	PAK1IP1	0.341408656	mirna_pc
26203	hsa-let-7d	SLC1A5	0.360085946	mirna_pc
26204	hsa-let-7d	PSMB7	0.395544551	mirna_pc
26205	hsa-let-7d	E2F4	0.301929843	mirna_pc
26206	hsa-let-7d	PSMD2	0.348290443	mirna_pc
26207	hsa-let-7d	YWHAE	0.373455621	mirna_pc
26208	hsa-let-7d	DHFR	0.304192155	mirna_pc
26209	hsa-let-7d	GLE1	0.450455664	mirna_pc
26210	hsa-let-7d	WBSCR27	0.399765138	mirna_pc
26211	hsa-let-7d	YARS2	0.320551631	mirna_pc
26212	hsa-let-7d	PUF60	0.341595425	mirna_pc
26213	hsa-let-7d	NUP205	0.319643726	mirna_pc
26214	hsa-let-7d	DEK	0.339121959	mirna_pc
26215	hsa-let-7d	SIX1	0.358697493	mirna_pc
26216	hsa-let-7d	COMMD4	0.315726441	mirna_pc
26217	hsa-let-7d	C14orf80	0.386542153	mirna_pc
26218	hsa-let-7d	TKT	0.337873772	mirna_pc
26219	hsa-let-7d	C7orf27	0.321746672	mirna_pc
26220	hsa-let-7d	WDR74	0.302293087	mirna_pc
26221	hsa-let-7d	PRDX1	0.300941068	mirna_pc
26222	hsa-let-7d	SUV39H1	0.403369359	mirna_pc
26223	hsa-let-7d	NUP188	0.407879297	mirna_pc
26224	hsa-let-7d	RSRC1	0.317795957	mirna_pc
26225	hsa-let-7d	C15orf23	0.45278165	mirna_pc
26226	hsa-let-7d	SDCCAG3	0.352035839	mirna_pc
26227	hsa-let-7d	C1QBP	0.39604415	mirna_pc
26228	hsa-let-7d	MRPL17	0.33661752	mirna_pc
26229	hsa-let-7d	RAD23B	0.407654672	mirna_pc
26230	hsa-let-7d	SPG21	0.410548652	mirna_pc
26231	hsa-let-7d	CEP78	0.407293167	mirna_pc
26232	hsa-let-7d	UCK1	0.333923452	mirna_pc
26233	hsa-let-7d	VPS72	0.35170008	mirna_pc
26234	hsa-let-7d	GIT1	0.32460088	mirna_pc
26235	hsa-let-7d	SHMT2	0.362852299	mirna_pc
26236	hsa-let-7d	HDAC2	0.322587016	mirna_pc
26237	hsa-let-7d	MRPL12	0.346286897	mirna_pc
26238	hsa-let-7d	HAUS2	0.316784826	mirna_pc
26239	hsa-let-7d	MRPL11	0.315298842	mirna_pc
26240	hsa-let-7d	AP2M1	0.324034055	mirna_pc
26241	hsa-let-7d	C3orf34	0.307667863	mirna_pc
26242	hsa-let-7d	PSMG1	0.345629744	mirna_pc
26243	hsa-let-7d	PPP1R8	0.360461804	mirna_pc

26244	hsa-let-7d	ANKRD13B	0.347798284	mirna_pc
26245	hsa-let-7d	NUDT3	0.3581128	mirna_pc
26246	hsa-let-7d	DOLPP1	0.425139035	mirna_pc
26247	hsa-let-7d	RFC2	0.337903551	mirna_pc
26248	hsa-let-7d	TAF5	0.356656892	mirna_pc
26249	hsa-let-7d	FANCC	0.378719376	mirna_pc
26250	hsa-let-7d	WDR5	0.505825308	mirna_pc
26251	hsa-let-7d	HNRNPA1L2	0.330924136	mirna_pc
26252	hsa-let-7d	MACROD1	0.359074201	mirna_pc
26253	hsa-let-7d	MAN1B1	0.325761806	mirna_pc
26254	hsa-let-7d	TMEM39B	0.302387142	mirna_pc
26255	hsa-let-7d	RCCD1	0.390541474	mirna_pc
26256	hsa-let-7d	TMEM161A	0.312642401	mirna_pc
26257	hsa-let-7d	C9orf16	0.405345115	mirna_pc
26258	hsa-let-7d	C1orf35	0.325158331	mirna_pc
26259	hsa-let-7d	TMEM68	0.314137777	mirna_pc
26260	hsa-let-7d	ZNF703	0.340816953	mirna_pc
26261	hsa-let-7d	ZDHHC12	0.364940015	mirna_pc
26262	hsa-let-7d	SNAPC4	0.322966438	mirna_pc
26263	hsa-let-7d	C15orf44	0.359860684	mirna_pc
26264	hsa-let-7d	TMEM44	0.386315571	mirna_pc
26265	hsa-let-7d	FARSB	0.317145455	mirna_pc
26266	hsa-let-7d	ERI3	0.370006517	mirna_pc
26267	hsa-let-7d	MED27	0.409153059	mirna_pc
26268	hsa-let-7d	TOMM22	0.305271585	mirna_pc
26269	hsa-let-7d	PHB	0.336103902	mirna_pc
26270	hsa-let-7d	C12orf52	0.309142501	mirna_pc
26271	hsa-let-7d	C8orf30A	0.354728965	mirna_pc
26272	hsa-let-7d	MUTYH	0.420862146	mirna_pc
26273	hsa-let-7d	WRNIP1	0.349880487	mirna_pc
26274	hsa-let-7d	LOC441089	0.368307015	mirna_pc
26275	hsa-let-7d	MRRF	0.339152502	mirna_pc
26276	hsa-let-7d	ADSL	0.308538835	mirna_pc
26277	hsa-let-7d	VRK1	0.320569192	mirna_pc
26278	hsa-let-7d	FPGS	0.423063212	mirna_pc
26279	hsa-let-7d	ANP32B	0.424704258	mirna_pc
26280	hsa-let-7d	UNG	0.359698142	mirna_pc
26281	hsa-let-7d	GRK6	0.330233909	mirna_pc
26282	hsa-let-7d	CDK16	0.367407663	mirna_pc
26283	hsa-let-7d	ALKBH2	0.319797274	mirna_pc
26284	hsa-let-7d	TOR1A	0.334660444	mirna_pc
26285	hsa-let-7d	PYCR2	0.340362428	mirna_pc
26286	hsa-let-7d	DAP3	0.302912435	mirna_pc
26287	hsa-let-7d	CCDC90A	0.302515229	mirna_pc
26288	hsa-let-7d	SIGMAR1	0.362163025	mirna_pc
26289	hsa-let-7d	RPL39L	0.321325607	mirna_pc
26290	hsa-let-7d	ACD	0.321286443	mirna_pc
26291	hsa-let-7d	GPN3	0.326162677	mirna_pc
26292	hsa-let-7d	RPUSD1	0.313366449	mirna_pc
26293	hsa-let-7d	LOC152217	0.392506784	mirna_pc
26294	hsa-let-7d	PTGES2	0.460676388	mirna_pc
26295	hsa-let-7d	MRPL37	0.341593491	mirna_pc
26296	hsa-let-7d	EIF3I	0.326614184	mirna_pc
26297	hsa-let-7d	RPL12	0.391487562	mirna_pc

26298	hsa-let-7d	C9orf89	0.329964639	mirna_pc
26299	hsa-let-7d	ZMYND19	0.445509572	mirna_pc
26300	hsa-let-7d	NDUFA8	0.368154021	mirna_pc
26301	hsa-let-7d	TRAP1	0.325518043	mirna_pc
26302	hsa-let-7d	MAGEF1	0.348139516	mirna_pc
26303	hsa-let-7d	PSAT1	0.331540798	mirna_pc
26304	hsa-let-7d	C15orf63	0.348767706	mirna_pc
26305	hsa-let-7d	RPL30	0.322320329	mirna_pc
26306	hsa-let-7d	PCGF6	0.343580997	mirna_pc
26307	hsa-let-7d	SURF2	0.388187641	mirna_pc
26308	hsa-let-7d	METTL11A	0.479033928	mirna_pc
26309	hsa-let-7d	APOA1BP	0.343670199	mirna_pc
26310	hsa-let-7d	ZNF511	0.368567432	mirna_pc
26311	hsa-let-7d	MRPS5	0.322402505	mirna_pc
26312	hsa-let-7d	SMARCB1	0.346473588	mirna_pc
26313	hsa-let-7d	CCT8	0.323941457	mirna_pc
26314	hsa-let-7d	RPA1	0.364193987	mirna_pc
26315	hsa-let-7d	ZNF639	0.357636293	mirna_pc
26316	hsa-let-7d	TIGD5	0.30399929	mirna_pc
26317	hsa-let-7d	SLC25A17	0.302809453	mirna_pc
26318	hsa-let-7d	RPS7	0.30422684	mirna_pc
26319	hsa-let-7d	FAM189B	0.319880412	mirna_pc
26320	hsa-let-7d	LRPPRC	0.336481972	mirna_pc
26321	hsa-let-7d	CLNS1A	0.30352913	mirna_pc
26322	hsa-let-7d	STRA13	0.325121537	mirna_pc
26323	hsa-let-7d	RPL35	0.370371209	mirna_pc
26324	hsa-let-7d	RRP7A	0.32041325	mirna_pc
26325	hsa-let-7d	TIMM16	0.329993345	mirna_pc
26326	hsa-let-7d	DVL2	0.300214567	mirna_pc
26327	hsa-let-7d	FRAT2	0.3090401	mirna_pc
26328	hsa-let-7d	LOC100289341	0.328400726	mirna_pc
26329	hsa-let-7d	SNHG6	0.347667185	mirna_pc
26330	hsa-let-7d	GCAT	0.306435186	mirna_pc
26331	hsa-let-7d	TATDN1	0.344259241	mirna_pc
26332	hsa-let-7d	ARHGAP19	0.300614677	mirna_pc
26333	hsa-let-7d	RPL8	0.343407114	mirna_pc
26334	hsa-let-7d	ADO	0.322546567	mirna_pc
26335	hsa-let-7d	SURF6	0.464201409	mirna_pc
26336	hsa-let-7d	NLE1	0.337534017	mirna_pc
26337	hsa-let-7d	FXR1	0.342286438	mirna_pc
26338	hsa-let-7d	WDR85	0.309191943	mirna_pc
26339	hsa-let-7d	TRMT61B	0.301290149	mirna_pc
26340	hsa-let-7d	GANAB	0.301775911	mirna_pc
26341	hsa-let-7d	MPV17	0.390704632	mirna_pc
26342	hsa-let-7d	RMI1	0.346069236	mirna_pc
26343	hsa-let-7d	MRPS2	0.49690658	mirna_pc
26344	hsa-let-7d	PIGX	0.333388606	mirna_pc
26345	hsa-let-7d	HTRA2	0.301305486	mirna_pc
26346	hsa-let-7d	PFN2	0.327541704	mirna_pc
26347	hsa-let-7d	COPZ1	0.318871165	mirna_pc
26348	hsa-let-7d	MRPL24	0.330190437	mirna_pc
26349	hsa-let-7d	DRG1	0.392835868	mirna_pc
26350	hsa-let-7d	NOL7	0.348734858	mirna_pc
26351	hsa-let-7d	SENP3	0.33945024	mirna_pc

26352	hsa-let-7d	PSMD5	0.313965202	mirna_pc
26353	hsa-let-7d	B4GALT3	0.307645193	mirna_pc
26354	hsa-let-7d	DEPDC7	0.302303221	mirna_pc
26355	hsa-let-7d	C9orf114	0.480919126	mirna_pc
26356	hsa-let-7d	SLC19A1	0.336838546	mirna_pc
26357	hsa-let-7d	TTC27	0.304407258	mirna_pc
26358	hsa-let-7d	PDCL	0.372583407	mirna_pc
26359	hsa-let-7d	FIBP	0.346010372	mirna_pc
26360	hsa-let-7d	AAAS	0.341145768	mirna_pc
26361	hsa-let-7d	INPP5E	0.318813978	mirna_pc
26362	hsa-let-7d	C9orf6	0.367166947	mirna_pc
26363	hsa-let-7d	MTFMT	0.400288579	mirna_pc
26364	hsa-let-7d	MRPS26	0.329579355	mirna_pc
26365	hsa-let-7d	C9orf78	0.338326613	mirna_pc
26366	hsa-let-7d	MRPL30	0.319863099	mirna_pc
26367	hsa-let-7d	TALDO1	0.328986379	mirna_pc
26368	hsa-let-7d	PYCR1	0.414625653	mirna_pc
26369	hsa-let-7d	DHODH	0.31081607	mirna_pc
26370	hsa-let-7d	PKN3	0.331225589	mirna_pc
26371	hsa-let-7d	AKR7A2	0.336245757	mirna_pc
26372	hsa-let-7d	TRUB2	0.306504014	mirna_pc
26373	hsa-let-7d	C18orf10	0.330249479	mirna_pc
26374	hsa-let-7d	PEX10	0.304706136	mirna_pc
26375	hsa-let-7d	C9orf119	0.353440177	mirna_pc
26376	hsa-let-7d	MRPS16	0.336557285	mirna_pc
26377	hsa-let-7d	PFAS	0.332322338	mirna_pc
26378	hsa-let-7d	ENDOG	0.38307533	mirna_pc
26379	hsa-let-7d	C10orf35	0.303472085	mirna_pc
26380	hsa-let-7d	RPL7	0.333547578	mirna_pc
26381	hsa-let-7d	SNUPN	0.325144244	mirna_pc
26382	hsa-let-7d	UCHL5	0.301666904	mirna_pc
26383	hsa-let-7d	HSF1	0.326796844	mirna_pc
26384	hsa-let-7d	RPUSD2	0.339735458	mirna_pc
26385	hsa-let-7d	ABCF3	0.319782889	mirna_pc
26386	hsa-let-7d	PHF19	0.303840999	mirna_pc
26387	hsa-let-7d	NIPSNAP1	0.383310087	mirna_pc
26388	hsa-let-7d	PARL	0.344748902	mirna_pc
26389	hsa-let-7d	PIR	0.353275401	mirna_pc
26390	hsa-let-7d	PCCB	0.309448724	mirna_pc
26391	hsa-let-7d	CHCHD1	0.309141193	mirna_pc
26392	hsa-let-7d	PPP6C	0.305068167	mirna_pc
26393	hsa-let-7d	SPA17	0.304860323	mirna_pc
26394	hsa-let-7d	RPL35A	0.385368775	mirna_pc
26395	hsa-let-7d	ARL　2.00	0.363225456	mirna_pc
26396	hsa-let-7d	C9orf86	0.328474081	mirna_pc
26397	hsa-let-7d	FBRSL1	0.317456049	mirna_pc
26398	hsa-let-7d	PTRH1	0.40150583	mirna_pc
26399	hsa-let-7d	COBRA1	0.422340861	mirna_pc
26400	hsa-let-7d	MRPS11	0.312340736	mirna_pc
26401	hsa-let-7d	MRPL50	0.384893282	mirna_pc
26402	hsa-let-7d	YBX1	0.369576042	mirna_pc
26403	hsa-let-7d	ZBTB8OS	0.307227731	mirna_pc
26404	hsa-let-7d	MTP　18.00	0.306265686	mirna_pc
26405	hsa-let-7d	C12orf10	0.300523005	mirna_pc

26406	hsa-let-7d	SEPHS1	0.345591139	mirna_pc
26407	hsa-let-7d	GPX4	0.304651622	mirna_pc
26408	hsa-let-7d	TMEM183A	0.369434994	mirna_pc
26409	hsa-let-7d	PHB2	0.345398768	mirna_pc
26410	hsa-let-7d	C8orf33	0.397282443	mirna_pc
26411	hsa-let-7d	RPS17	0.303576229	mirna_pc
26412	hsa-let-7d	POLR2F	0.362354861	mirna_pc
26413	hsa-let-7d	PSMG4	0.33434267	mirna_pc
26414	hsa-let-7d	MDH2	0.331377372	mirna_pc
26415	hsa-let-7d	SSNA1	0.377797714	mirna_pc
26416	hsa-let-7d	MRPS15	0.304857106	mirna_pc
26417	hsa-let-7d	NARS2	0.322977797	mirna_pc
26418	hsa-let-7d	TMEM14C	0.310743763	mirna_pc
26419	hsa-let-7d	TXNRD1	0.30621475	mirna_pc
26420	hsa-let-7d	UBQLN1	0.326321632	mirna_pc
26421	hsa-let-7d	AIFM2	0.302909088	mirna_pc
26422	hsa-let-7d	DOLK	0.316898925	mirna_pc
26423	hsa-let-7d	USP21	0.362326372	mirna_pc
26424	hsa-let-7d	SLC25A11	0.302734574	mirna_pc
26425	hsa-let-7d	KLHL12	0.307419265	mirna_pc
26426	hsa-let-7d	ANP32A	0.333749665	mirna_pc
26427	hsa-let-7d	C9orf80	0.345534617	mirna_pc
26428	hsa-let-7d	SUPV3L1	0.417938798	mirna_pc
26429	hsa-let-7d	NELF	0.35316891	mirna_pc
26430	hsa-let-7d	C17orf81	0.348212548	mirna_pc
26431	hsa-let-7d	FXN	0.322952133	mirna_pc
26432	hsa-let-7d	HYLS1	0.316304434	mirna_pc
26433	hsa-let-7d	URM1	0.370613764	mirna_pc
26434	hsa-let-7d	MGC70857	0.315847293	mirna_pc
26435	hsa-let-7d	CHCHD3	0.35443418	mirna_pc
26436	hsa-let-7d	TFDP2	0.31684236	mirna_pc
26437	hsa-let-7d	C15orf40	0.325957352	mirna_pc
26438	hsa-let-7d	PMPCA	0.308119402	mirna_pc
26439	hsa-let-7d	C12orf24	0.320728027	mirna_pc
26440	hsa-let-7d	FKBP4	0.31142665	mirna_pc
26441	hsa-let-7d	RPS5	0.311531032	mirna_pc
26442	hsa-let-7d	THNSL1	0.303252191	mirna_pc
26443	hsa-let-7d	BCS1L	0.32981264	mirna_pc
26444	hsa-let-7d	PPP2R4	0.411821766	mirna_pc
26445	hsa-let-7d	C1orf43	0.344451649	mirna_pc
26446	hsa-let-7d	TMEM203	0.33075373	mirna_pc
26447	hsa-let-7d	CYC1	0.399515705	mirna_pc
26448	hsa-let-7d	SOD1	0.383979312	mirna_pc
26449	hsa-let-7d	THAP4	0.310150539	mirna_pc
26450	hsa-let-7d	PTPMT1	0.306895044	mirna_pc
26451	hsa-let-7d	C9orf37	0.300195923	mirna_pc
26452	hsa-let-7d	TCTN3	0.314194837	mirna_pc
26453	hsa-let-7d	RMRP	0.30927029	mirna_pc
26454	hsa-let-7d	SIX2	0.31181593	mirna_pc
26455	hsa-let-7d	PDXP	0.364783868	mirna_pc
26456	hsa-let-7d	COX7A2L	0.31036857	mirna_pc
26457	hsa-let-7d	CHID1	0.356390526	mirna_pc
26458	hsa-let-7d	IARS	0.379162438	mirna_pc
26459	hsa-let-7d	ACP1	0.335500873	mirna_pc

26460	hsa-let-7d	DNAL4	0.310387385	mirna_pc
26461	hsa-let-7d	CD320	0.326078603	mirna_pc
26462	hsa-let-7d	SNRNP25	0.341869158	mirna_pc
26463	hsa-let-7d	NAA35	0.314081732	mirna_pc
26464	hsa-let-7d	RPL7A	0.355993231	mirna_pc
26465	hsa-let-7d	GPAA1	0.30048748	mirna_pc
26466	hsa-let-7d	NCRNA00188	0.333478372	mirna_pc
26467	hsa-let-7d	MAF 1.00	0.345722346	mirna_pc
26468	hsa-let-7d	BAT2L1	0.353466507	mirna_pc
26469	hsa-let-7d	UBL7	0.307770969	mirna_pc
26470	hsa-let-7d	TMEM14B	0.342035027	mirna_pc
26471	hsa-let-7d	SNAPC5	0.3126906	mirna_pc
26472	hsa-let-7d	CIZ1	0.432314067	mirna_pc
26473	hsa-let-7d	NDNL2	0.304091414	mirna_pc
26474	hsa-let-7d	NQO2	0.319427707	mirna_pc
26475	hsa-let-7d	MAFG	0.360127536	mirna_pc
26476	hsa-let-7d	MED22	0.356684629	mirna_pc
26477	hsa-let-7d	SCARNA2	0.301970073	mirna_pc
26478	hsa-let-7d	DPM2	0.41454802	mirna_pc
26479	hsa-let-7d	C10orf75	0.355787782	mirna_pc
26480	hsa-let-7d	APOO	0.349693645	mirna_pc
26481	hsa-let-7d	C15orf61	0.325553828	mirna_pc
26482	hsa-mir-450b	MAD2L1	0.310835063	mirna_pc
26483	hsa-mir-450b	NUF2	0.339586481	mirna_pc
26484	hsa-mir-450b	BIRC5	0.34443125	mirna_pc
26485	hsa-mir-450b	C1orf112	0.307667492	mirna_pc
26486	hsa-mir-450b	ACTL6A	0.30005602	mirna_pc
26487	hsa-mir-450b	CENPO	0.332858884	mirna_pc
26488	hsa-mir-450b	CENPL	0.361962565	mirna_pc
26489	hsa-mir-450b	MSH2	0.327538026	mirna_pc
26490	hsa-mir-450b	DBF4	0.314815737	mirna_pc
26491	hsa-mir-450b	PSMB4	0.315703176	mirna_pc
26492	hsa-mir-450b	RFC4	0.319726365	mirna_pc
26493	hsa-mir-450b	DNAJB11	0.308889536	mirna_pc
26494	hsa-mir-450b	SSBP3	0.307248253	mirna_pc
26495	hsa-mir-450b	LOC100128191	0.332537544	mirna_pc
26496	hsa-mir-450b	MTL 5.00	0.335027395	mirna_pc
26497	hsa-mir-450b	PSMC2	0.314067146	mirna_pc
26498	hsa-mir-450b	GORAB	0.335904817	mirna_pc
26499	hsa-mir-450b	SHOX2	0.322468635	mirna_pc
26500	hsa-mir-450b	DUSP9	0.307373047	mirna_pc
26501	hsa-mir-450b	PSMD2	0.306638342	mirna_pc
26502	hsa-mir-450b	PRAME	0.393953603	mirna_pc
26503	hsa-mir-450b	TRA2B	0.301077685	mirna_pc
26504	hsa-mir-450b	RSRC1	0.327824319	mirna_pc
26505	hsa-mir-450b	C3orf21	0.364854773	mirna_pc
26506	hsa-mir-450b	CPXM1	0.305719369	mirna_pc
26507	hsa-mir-450b	PLEKHG4	0.329994559	mirna_pc
26508	hsa-mir-450b	TTC17	0.33155	mirna_pc
26509	hsa-mir-450b	CADM1	0.388647402	mirna_pc
26510	hsa-mir-450b	MSH6	0.304077414	mirna_pc
26511	hsa-mir-450b	C3orf34	0.333108016	mirna_pc
26512	hsa-mir-450b	LEF1	0.338716278	mirna_pc
26513	hsa-mir-450b	KREMEN2	0.327131907	mirna_pc

26514	hsa-mir-450b	MEX3A	0.344523934	mirna_pc
26515	hsa-mir-450b	MOGS	0.335372072	mirna_pc
26516	hsa-mir-450b	IRAK1BP1	0.327022224	mirna_pc
26517	hsa-mir-450b	NOL10	0.317313259	mirna_pc
26518	hsa-mir-450b	ZKSCAN2	0.307413903	mirna_pc
26519	hsa-mir-450b	RPL39L	0.332339724	mirna_pc
26520	hsa-mir-450b	MEMO1	0.32542131	mirna_pc
26521	hsa-mir-450b	IGSF9	0.362866378	mirna_pc
26522	hsa-mir-450b	UBXN2A	0.300534427	mirna_pc
26523	hsa-mir-450b	MAGEF1	0.356795235	mirna_pc
26524	hsa-mir-450b	ZNF639	0.302552119	mirna_pc
26525	hsa-mir-450b	DVL3	0.322096038	mirna_pc
26526	hsa-mir-450b	TRPA1	0.345279958	mirna_pc
26527	hsa-mir-450b	RDH14	0.301364542	mirna_pc
26528	hsa-mir-450b	PIGX	0.322688095	mirna_pc
26529	hsa-mir-450b	ATP6V1E2	0.356234896	mirna_pc
26530	hsa-mir-450b	E2F6	0.330441058	mirna_pc
26531	hsa-mir-450b	HUNK	0.33382807	mirna_pc
26532	hsa-mir-450b	SCLY	0.345806309	mirna_pc
26533	hsa-mir-450b	SLC4A1AP	0.321441745	mirna_pc
26534	hsa-mir-450b	DHX57	0.301972	mirna_pc
26535	hsa-mir-450b	ALX3	0.364343119	mirna_pc
26536	hsa-mir-450b	EXT2	0.323951893	mirna_pc
26537	hsa-mir-450b	EXTL2	0.333802436	mirna_pc
26538	hsa-mir-450b	SEPHS1	0.300386295	mirna_pc
26539	hsa-mir-450b	SHISA2	0.305867671	mirna_pc
26540	hsa-mir-450b	MLLT11	0.314546809	mirna_pc
26541	hsa-mir-450b	MYCN	0.341986015	mirna_pc
26542	hsa-mir-450b	PABPC4L	0.352101507	mirna_pc
26543	hsa-mir-450b	MSI1	0.324681085	mirna_pc
26544	hsa-mir-450b	TRO	0.300672368	mirna_pc
26545	hsa-mir-450b	C2orf3	0.315342607	mirna_pc
26546	hsa-mir-450b	LRDD	0.331401893	mirna_pc
26547	hsa-mir-450b	ETV5	0.301049856	mirna_pc
26548	hsa-mir-450b	DDX1	0.322663971	mirna_pc
26549	hsa-mir-450b	IGHMBP2	0.304294535	mirna_pc
26550	hsa-mir-450b	ACP1	0.310228246	mirna_pc
26551	hsa-mir-450b	FZD1	0.315397778	mirna_pc
26552	hsa-mir-450b	C2orf28	0.306784686	mirna_pc
26553	hsa-mir-450b	H2AFY2	0.344463061	mirna_pc
26554	hsa-mir-450b	FBXO48	0.315187517	mirna_pc
26555	hsa-mir-450b	TRIL	0.304548252	mirna_pc
26556	hsa-mir-450b	DNMT3A	0.366133622	mirna_pc
26557	hsa-mir-92a-1	TOP2A	0.441652356	mirna_pc
26558	hsa-mir-92a-1	CENPF	0.302197949	mirna_pc
26559	hsa-mir-92a-1	CKAP2	0.319499816	mirna_pc
26560	hsa-mir-92a-1	FANCA	0.387544074	mirna_pc
26561	hsa-mir-92a-1	SGOL1	0.34452498	mirna_pc
26562	hsa-mir-92a-1	ARHGAP11A	0.319585362	mirna_pc
26563	hsa-mir-92a-1	NEK2	0.321270347	mirna_pc
26564	hsa-mir-92a-1	GTSE1	0.308550235	mirna_pc
26565	hsa-mir-92a-1	MAD2L1	0.314942729	mirna_pc
26566	hsa-mir-92a-1	TIMELESS	0.305856102	mirna_pc
26567	hsa-mir-92a-1	XPO1	0.362161826	mirna_pc

26568	hsa-mir-92a-1	PLK4	0.314956992	mirna_pc
26569	hsa-mir-92a-1	FANCB	0.31351961	mirna_pc
26570	hsa-mir-92a-1	SKA3	0.388320596	mirna_pc
26571	hsa-mir-92a-1	HJURP	0.343117636	mirna_pc
26572	hsa-mir-92a-1	BLM	0.366649615	mirna_pc
26573	hsa-mir-92a-1	PAICS	0.337652388	mirna_pc
26574	hsa-mir-92a-1	CCDC150	0.33543615	mirna_pc
26575	hsa-mir-92a-1	EME1	0.312398343	mirna_pc
26576	hsa-mir-92a-1	CENPK	0.315991484	mirna_pc
26577	hsa-mir-92a-1	CENPE	0.302445527	mirna_pc
26578	hsa-mir-92a-1	C1orf112	0.303065075	mirna_pc
26579	hsa-mir-92a-1	GINS1	0.343917991	mirna_pc
26580	hsa-mir-92a-1	TRAIP	0.30023605	mirna_pc
26581	hsa-mir-92a-1	EZH2	0.320034666	mirna_pc
26582	hsa-mir-92a-1	POLD1	0.305997781	mirna_pc
26583	hsa-mir-92a-1	C12orf48	0.318174703	mirna_pc
26584	hsa-mir-92a-1	HELLS	0.311034017	mirna_pc
26585	hsa-mir-92a-1	TCF3	0.304484019	mirna_pc
26586	hsa-mir-92a-1	C6orf167	0.312823177	mirna_pc
26587	hsa-mir-92a-1	BRCA1	0.371063521	mirna_pc
26588	hsa-mir-92a-1	HNRNPA2B1	0.308223028	mirna_pc
26589	hsa-mir-92a-1	MCM6	0.346185526	mirna_pc
26590	hsa-mir-92a-1	CDC6	0.394507226	mirna_pc
26591	hsa-mir-92a-1	CDCA7	0.370673318	mirna_pc
26592	hsa-mir-92a-1	BRIP1	0.319364284	mirna_pc
26593	hsa-mir-92a-1	DCAF13	0.317183141	mirna_pc
26594	hsa-mir-92a-1	ATAD5	0.354571721	mirna_pc
26595	hsa-mir-92a-1	RFC3	0.420259774	mirna_pc
26596	hsa-mir-92a-1	CENPO	0.319703137	mirna_pc
26597	hsa-mir-92a-1	XRCC2	0.319825784	mirna_pc
26598	hsa-mir-92a-1	BRCA2	0.364308729	mirna_pc
26599	hsa-mir-92a-1	GEN1	0.427200712	mirna_pc
26600	hsa-mir-92a-1	HMGB2	0.344352609	mirna_pc
26601	hsa-mir-92a-1	DNA2	0.353794255	mirna_pc
26602	hsa-mir-92a-1	NOP56	0.329288186	mirna_pc
26603	hsa-mir-92a-1	DLEU2	0.341016791	mirna_pc
26604	hsa-mir-92a-1	PRIM1	0.361887426	mirna_pc
26605	hsa-mir-92a-1	FERMT1	0.364913965	mirna_pc
26606	hsa-mir-92a-1	MSH2	0.360610609	mirna_pc
26607	hsa-mir-92a-1	CDC7	0.348783187	mirna_pc
26608	hsa-mir-92a-1	SSB	0.37669	mirna_pc
26609	hsa-mir-92a-1	PUS1	0.302042113	mirna_pc
26610	hsa-mir-92a-1	NOL11	0.330122812	mirna_pc
26611	hsa-mir-92a-1	FBXO5	0.328602776	mirna_pc
26612	hsa-mir-92a-1	E2F3	0.445003062	mirna_pc
26613	hsa-mir-92a-1	CDC25A	0.341446124	mirna_pc
26614	hsa-mir-92a-1	DDX12	0.306438914	mirna_pc
26615	hsa-mir-92a-1	C13orf34	0.426120061	mirna_pc
26616	hsa-mir-92a-1	MCM8	0.394038977	mirna_pc
26617	hsa-mir-92a-1	ATIC	0.375007029	mirna_pc
26618	hsa-mir-92a-1	CBX8	0.353337006	mirna_pc
26619	hsa-mir-92a-1	GNPDA1	0.312161308	mirna_pc
26620	hsa-mir-92a-1	NCL	0.362912451	mirna_pc
26621	hsa-mir-92a-1	C13orf37	0.340810332	mirna_pc

26622	hsa-mir-92a-1	PNPT1	0.386448708	mirna_pc
26623	hsa-mir-92a-1	TMPO	0.350621577	mirna_pc
26624	hsa-mir-92a-1	DHX9	0.314004767	mirna_pc
26625	hsa-mir-92a-1	SFRS1	0.451640979	mirna_pc
26626	hsa-mir-92a-1	WDR43	0.337326696	mirna_pc
26627	hsa-mir-92a-1	C4orf46	0.327152678	mirna_pc
26628	hsa-mir-92a-1	DARS2	0.321427037	mirna_pc
26629	hsa-mir-92a-1	HSPD1	0.307683054	mirna_pc
26630	hsa-mir-92a-1	POLR2D	0.320135871	mirna_pc
26631	hsa-mir-92a-1	KHDRBS1	0.313398722	mirna_pc
26632	hsa-mir-92a-1	SLC5A6	0.310447211	mirna_pc
26633	hsa-mir-92a-1	PRPF40A	0.358226437	mirna_pc
26634	hsa-mir-92a-1	CCDC18	0.300189204	mirna_pc
26635	hsa-mir-92a-1	TMEM194A	0.306086383	mirna_pc
26636	hsa-mir-92a-1	SLC39A10	0.335019273	mirna_pc
26637	hsa-mir-92a-1	ZNF695	0.340853659	mirna_pc
26638	hsa-mir-92a-1	PDSS1	0.340833025	mirna_pc
26639	hsa-mir-92a-1	HAUS6	0.342242888	mirna_pc
26640	hsa-mir-92a-1	CENPJ	0.391567281	mirna_pc
26641	hsa-mir-92a-1	HNRNPU	0.332721779	mirna_pc
26642	hsa-mir-92a-1	POLR1B	0.306265432	mirna_pc
26643	hsa-mir-92a-1	QSOX2	0.303733066	mirna_pc
26644	hsa-mir-92a-1	SCARB1	0.329995348	mirna_pc
26645	hsa-mir-92a-1	SUV39H2	0.302214495	mirna_pc
26646	hsa-mir-92a-1	FANCL	0.358719087	mirna_pc
26647	hsa-mir-92a-1	HNRNPD	0.32272358	mirna_pc
26648	hsa-mir-92a-1	MTHFD2	0.319859625	mirna_pc
26649	hsa-mir-92a-1	DDX31	0.304135209	mirna_pc
26650	hsa-mir-92a-1	POP1	0.347520548	mirna_pc
26651	hsa-mir-92a-1	NUDT15	0.333936109	mirna_pc
26652	hsa-mir-92a-1	SLMO2	0.319023137	mirna_pc
26653	hsa-mir-92a-1	NAA25	0.307577528	mirna_pc
26654	hsa-mir-92a-1	ILF3	0.323322159	mirna_pc
26655	hsa-mir-92a-1	DEK	0.316342577	mirna_pc
26656	hsa-mir-92a-1	TBC1D4	0.332841349	mirna_pc
26657	hsa-mir-92a-1	TRIB3	0.416354063	mirna_pc
26658	hsa-mir-92a-1	PASK	0.360404783	mirna_pc
26659	hsa-mir-92a-1	TGS1	0.323061836	mirna_pc
26660	hsa-mir-92a-1	HEATR2	0.328639963	mirna_pc
26661	hsa-mir-92a-1	PRAME	0.322077091	mirna_pc
26662	hsa-mir-92a-1	CMTM1	0.316083121	mirna_pc
26663	hsa-mir-92a-1	CSRP2BP	0.31084869	mirna_pc
26664	hsa-mir-92a-1	CEP152	0.323991443	mirna_pc
26665	hsa-mir-92a-1	RNF219	0.567646589	mirna_pc
26666	hsa-mir-92a-1	SLC25A32	0.350503962	mirna_pc
26667	hsa-mir-92a-1	ENOPH1	0.30725816	mirna_pc
26668	hsa-mir-92a-1	PABPC1L	0.333381064	mirna_pc
26669	hsa-mir-92a-1	C17orf42	0.34918958	mirna_pc
26670	hsa-mir-92a-1	DDX55	0.327983621	mirna_pc
26671	hsa-mir-92a-1	CEP78	0.372406551	mirna_pc
26672	hsa-mir-92a-1	POLA1	0.338785616	mirna_pc
26673	hsa-mir-92a-1	HDAC2	0.322634673	mirna_pc
26674	hsa-mir-92a-1	POLG2	0.338245086	mirna_pc
26675	hsa-mir-92a-1	C10orf2	0.420198062	mirna_pc

26676	hsa-mir-92a-1	XRN2	0.378688092	mirna_pc
26677	hsa-mir-92a-1	KPNB1	0.399514509	mirna_pc
26678	hsa-mir-92a-1	SMC3	0.379015988	mirna_pc
26679	hsa-mir-92a-1	FLVCR1	0.329835731	mirna_pc
26680	hsa-mir-92a-1	ZBED4	0.317309285	mirna_pc
26681	hsa-mir-92a-1	AGMAT	0.343089586	mirna_pc
26682	hsa-mir-92a-1	MEX3A	0.392944408	mirna_pc
26683	hsa-mir-92a-1	BAT1	0.338816494	mirna_pc
26684	hsa-mir-92a-1	COIL	0.347053853	mirna_pc
26685	hsa-mir-92a-1	MSH5	0.34202461	mirna_pc
26686	hsa-mir-92a-1	MRM1	0.314368027	mirna_pc
26687	hsa-mir-92a-1	TMC7	0.311549497	mirna_pc
26688	hsa-mir-92a-1	NANP	0.309300783	mirna_pc
26689	hsa-mir-92a-1	SUZ12	0.383871893	mirna_pc
26690	hsa-mir-92a-1	HMGB1	0.412854285	mirna_pc
26691	hsa-mir-92a-1	ZBTB12	0.432586082	mirna_pc
26692	hsa-mir-92a-1	TMEM68	0.318282997	mirna_pc
26693	hsa-mir-92a-1	TGIF2	0.372918253	mirna_pc
26694	hsa-mir-92a-1	FARSB	0.340267665	mirna_pc
26695	hsa-mir-92a-1	PIK3AP1	0.377742276	mirna_pc
26696	hsa-mir-92a-1	CIB2	0.302422309	mirna_pc
26697	hsa-mir-92a-1	FAM96A	0.300235691	mirna_pc
26698	hsa-mir-92a-1	SC65	0.304393668	mirna_pc
26699	hsa-mir-92a-1	POLE	0.351673429	mirna_pc
26700	hsa-mir-92a-1	SOX9	0.458031108	mirna_pc
26701	hsa-mir-92a-1	NSUN7	0.301176824	mirna_pc
26702	hsa-mir-92a-1	C13orf27	0.351479427	mirna_pc
26703	hsa-mir-92a-1	RBMX	0.374519839	mirna_pc
26704	hsa-mir-92a-1	GPR180	0.358318017	mirna_pc
26705	hsa-mir-92a-1	XRCC5	0.399955749	mirna_pc
26706	hsa-mir-92a-1	EXOSC8	0.330432723	mirna_pc
26707	hsa-mir-92a-1	LBR	0.307680034	mirna_pc
26708	hsa-mir-92a-1	DNAJC14	0.332918526	mirna_pc
26709	hsa-mir-92a-1	ZNF519	0.351093825	mirna_pc
26710	hsa-mir-92a-1	ORC2L	0.305168811	mirna_pc
26711	hsa-mir-92a-1	EFCAB4B	0.30908208	mirna_pc
26712	hsa-mir-92a-1	FAM136A	0.366665855	mirna_pc
26713	hsa-mir-92a-1	NRAS	0.436599765	mirna_pc
26714	hsa-mir-92a-1	C12orf49	0.310693189	mirna_pc
26715	hsa-mir-92a-1	COG3	0.31965195	mirna_pc
26716	hsa-mir-92a-1	EIF1AX	0.301308906	mirna_pc
26717	hsa-mir-92a-1	FBXO41	0.357188828	mirna_pc
26718	hsa-mir-92a-1	BIVM	0.378706827	mirna_pc
26719	hsa-mir-92a-1	RBCK1	0.329189003	mirna_pc
26720	hsa-mir-92a-1	EML4	0.38390274	mirna_pc
26721	hsa-mir-92a-1	LRPPRC	0.30220877	mirna_pc
26722	hsa-mir-92a-1	CSNK2A1	0.35359933	mirna_pc
26723	hsa-mir-92a-1	RPIA	0.39942769	mirna_pc
26724	hsa-mir-92a-1	CXorf38	0.300276809	mirna_pc
26725	hsa-mir-92a-1	C7orf44	0.34609095	mirna_pc
26726	hsa-mir-92a-1	URB2	0.343443808	mirna_pc
26727	hsa-mir-92a-1	ZNF286A	0.316948277	mirna_pc
26728	hsa-mir-92a-1	NAA16	0.329495465	mirna_pc
26729	hsa-mir-92a-1	SLC27A2	0.322325991	mirna_pc

26730	hsa-mir-92a-1	SFXN4	0.337591752	mirna_pc
26731	hsa-mir-92a-1	SURF6	0.325809229	mirna_pc
26732	hsa-mir-92a-1	RBM28	0.320414015	mirna_pc
26733	hsa-mir-92a-1	RANBP17	0.301346338	mirna_pc
26734	hsa-mir-92a-1	MRE11A	0.330438376	mirna_pc
26735	hsa-mir-92a-1	ANKRD46	0.302814752	mirna_pc
26736	hsa-mir-92a-1	LTK	0.308007792	mirna_pc
26737	hsa-mir-92a-1	UTP14C	0.322717893	mirna_pc
26738	hsa-mir-92a-1	GOSR1	0.30873343	mirna_pc
26739	hsa-mir-92a-1	USP37	0.306573761	mirna_pc
26740	hsa-mir-92a-1	PLB1	0.317282983	mirna_pc
26741	hsa-mir-92a-1	ZNF84	0.301540662	mirna_pc
26742	hsa-mir-92a-1	C13orf23	0.426466263	mirna_pc
26743	hsa-mir-92a-1	RBM12	0.319879168	mirna_pc
26744	hsa-mir-92a-1	TCERG1	0.31843825	mirna_pc
26745	hsa-mir-92a-1	PSPC1	0.367256752	mirna_pc
26746	hsa-mir-92a-1	GMCL1	0.37603279	mirna_pc
26747	hsa-mir-92a-1	E2F6	0.387143517	mirna_pc
26748	hsa-mir-92a-1	ZNF833	0.324109129	mirna_pc
26749	hsa-mir-92a-1	SLC25A15	0.398031412	mirna_pc
26750	hsa-mir-92a-1	SFRS13A	0.365649466	mirna_pc
26751	hsa-mir-92a-1	SCLY	0.332261087	mirna_pc
26752	hsa-mir-92a-1	TGDS	0.399742432	mirna_pc
26753	hsa-mir-92a-1	TFAM	0.347264036	mirna_pc
26754	hsa-mir-92a-1	ORC4L	0.347315508	mirna_pc
26755	hsa-mir-92a-1	KIAA1704	0.414901207	mirna_pc
26756	hsa-mir-92a-1	MTIF2	0.383956025	mirna_pc
26757	hsa-mir-92a-1	MSL1	0.413339403	mirna_pc
26758	hsa-mir-92a-1	ZNF828	0.390369019	mirna_pc
26759	hsa-mir-92a-1	SMC1A	0.39641898	mirna_pc
26760	hsa-mir-92a-1	NUPL1	0.48319038	mirna_pc
26761	hsa-mir-92a-1	CCDC45	0.368186573	mirna_pc
26762	hsa-mir-92a-1	WBP4	0.328415111	mirna_pc
26763	hsa-mir-92a-1	ANKRD5	0.37543006	mirna_pc
26764	hsa-mir-92a-1	ZNF514	0.319020306	mirna_pc
26765	hsa-mir-92a-1	PHKA2	0.330685955	mirna_pc
26766	hsa-mir-92a-1	UPF3A	0.376760801	mirna_pc
26767	hsa-mir-92a-1	SFRS7	0.320987219	mirna_pc
26768	hsa-mir-92a-1	MRPL42	0.30369838	mirna_pc
26769	hsa-mir-92a-1	CA5BP	0.321445234	mirna_pc
26770	hsa-mir-92a-1	SPRY2	0.332422305	mirna_pc
26771	hsa-mir-92a-1	RBM10	0.302343529	mirna_pc
26772	hsa-mir-92a-1	UFM1	0.311953097	mirna_pc
26773	hsa-mir-92a-1	KBTBD7	0.342771725	mirna_pc
26774	hsa-mir-92a-1	ANKS6	0.312523215	mirna_pc
26775	hsa-mir-92a-1	ATP7B	0.364136687	mirna_pc
26776	hsa-mir-92a-1	ZNF214	0.307601576	mirna_pc
26777	hsa-mir-92a-1	C11orf57	0.317438532	mirna_pc
26778	hsa-mir-92a-1	SYAP1	0.318602112	mirna_pc
26779	hsa-mir-92a-1	RCBTB1	0.331809556	mirna_pc
26780	hsa-mir-92a-1	CDC16	0.35729097	mirna_pc
26781	hsa-mir-92a-1	PTCD3	0.336034711	mirna_pc
26782	hsa-mir-92a-1	SPRY4	0.322581334	mirna_pc
26783	hsa-mir-92a-1	RNF6	0.343556482	mirna_pc

26784	hsa-mir-92a-1	BCAN	0.309199418	mirna_pc
26785	hsa-mir-92a-1	ALG5	0.34519964	mirna_pc
26786	hsa-mir-92a-1	MAP2K6	0.425191406	mirna_pc
26787	hsa-mir-92a-1	CASP6	0.316617779	mirna_pc
26788	hsa-mir-92a-1	RBM26	0.362526587	mirna_pc
26789	hsa-mir-92a-1	ZNF92	0.359789076	mirna_pc
26790	hsa-mir-92a-1	TMTC4	0.39209201	mirna_pc
26791	hsa-mir-92a-1	LOC151162	0.338513364	mirna_pc
26792	hsa-mir-92a-1	RMND1	0.321950439	mirna_pc
26793	hsa-mir-92a-1	AZIN1	0.34953705	mirna_pc
26794	hsa-mir-92a-1	GLYCTK	0.314557273	mirna_pc
26795	hsa-mir-92a-1	CEBPZ	0.347296459	mirna_pc
26796	hsa-mir-92a-1	FASTKD5	0.300764356	mirna_pc
26797	hsa-mir-92a-1	SMARCE1	0.406528804	mirna_pc
26798	hsa-mir-92a-1	C8orf33	0.30490789	mirna_pc
26799	hsa-mir-92a-1	TASP1	0.343763528	mirna_pc
26800	hsa-mir-92a-1	IPO5	0.468456499	mirna_pc
26801	hsa-mir-92a-1	ALG11	0.421005665	mirna_pc
26802	hsa-mir-92a-1	MLXIPL	0.301090555	mirna_pc
26803	hsa-mir-92a-1	NUP43	0.316764162	mirna_pc
26804	hsa-mir-92a-1	MTRF1	0.346932241	mirna_pc
26805	hsa-mir-92a-1	ZNF124	0.483806559	mirna_pc
26806	hsa-mir-92a-1	RNASEH2B	0.406178026	mirna_pc
26807	hsa-mir-92a-1	CHD1L	0.329268024	mirna_pc
26808	hsa-mir-92a-1	ZNF473	0.438305213	mirna_pc
26809	hsa-mir-92a-1	RFXAP	0.381109554	mirna_pc
26810	hsa-mir-92a-1	DCLRE1A	0.418827967	mirna_pc
26811	hsa-mir-92a-1	ANP32A	0.403630791	mirna_pc
26812	hsa-mir-92a-1	E2F5	0.363204601	mirna_pc
26813	hsa-mir-92a-1	NUFIP1	0.481229412	mirna_pc
26814	hsa-mir-92a-1	ZNF195	0.466262237	mirna_pc
26815	hsa-mir-92a-1	BCAS2	0.402273638	mirna_pc
26816	hsa-mir-92a-1	USP45	0.34600684	mirna_pc
26817	hsa-mir-92a-1	LRRCC1	0.360150375	mirna_pc
26818	hsa-mir-92a-1	NEK3	0.392868335	mirna_pc
26819	hsa-mir-92a-1	THSD1P1	0.337668029	mirna_pc
26820	hsa-mir-92a-1	TMEM170A	0.373782147	mirna_pc
26821	hsa-mir-92a-1	PHF16	0.333954191	mirna_pc
26822	hsa-mir-92a-1	FAM48A	0.48624691	mirna_pc
26823	hsa-mir-92a-1	PMS1	0.346200529	mirna_pc
26824	hsa-mir-92a-1	PNN	0.309817701	mirna_pc
26825	hsa-mir-92a-1	MNS1	0.328451959	mirna_pc
26826	hsa-mir-92a-1	ZNF485	0.339142034	mirna_pc
26827	hsa-mir-92a-1	PAN3	0.334989072	mirna_pc
26828	hsa-mir-92a-1	CCDC122	0.41424479	mirna_pc
26829	hsa-mir-92a-1	OFD1	0.31262892	mirna_pc
26830	hsa-mir-92a-1	TRUB1	0.345359325	mirna_pc
26831	hsa-mir-92a-1	ZMYM2	0.377273927	mirna_pc
26832	hsa-mir-92a-1	C2orf15	0.311117194	mirna_pc
26833	hsa-mir-92a-1	CCNJ	0.482044277	mirna_pc
26834	hsa-mir-92a-1	PM20D2	0.321300146	mirna_pc
26835	hsa-mir-92a-1	DCAF16	0.348220563	mirna_pc
26836	hsa-mir-92a-1	SBK1	0.329403513	mirna_pc
26837	hsa-mir-92a-1	ABCC4	0.383159177	mirna_pc

26838	hsa-mir-92a-1	KBTBD6	0.333494739	mirna_pc
26839	hsa-mir-92a-1	ESF1	0.353815678	mirna_pc
26840	hsa-mir-92a-1	MLEC	0.302336493	mirna_pc
26841	hsa-mir-92a-1	RNF157	0.300217604	mirna_pc
26842	hsa-mir-92a-1	GTF2H2C	0.326827538	mirna_pc
26843	hsa-mir-92a-1	SMARCC1	0.332982419	mirna_pc
26844	hsa-mir-92a-1	SUGT1L1	0.305663244	mirna_pc
26845	hsa-mir-92a-1	CTPS2	0.440969097	mirna_pc
26846	hsa-mir-92a-1	GXYLT1	0.320413684	mirna_pc
26847	hsa-mir-92a-1	XPO4	0.482783159	mirna_pc
26848	hsa-mir-92a-1	MDN1	0.300439733	mirna_pc
26849	hsa-mir-92a-1	KIF12	0.313717279	mirna_pc
26850	hsa-mir-92a-1	ZBTB39	0.352055884	mirna_pc
26851	hsa-mir-92a-1	IFT88	0.440969325	mirna_pc
26852	hsa-mir-92a-1	QRSL1	0.376417958	mirna_pc
26853	hsa-mir-92a-1	FANCF	0.451312785	mirna_pc
26854	hsa-mir-92a-1	MARS2	0.334898772	mirna_pc
26855	hsa-mir-92a-1	VPS54	0.329486157	mirna_pc
26856	hsa-mir-92a-1	ZBTB24	0.352370762	mirna_pc
26857	hsa-mir-92a-1	ZNF138	0.341016343	mirna_pc
26858	hsa-mir-92a-1	ZNF551	0.31553289	mirna_pc
26859	hsa-mir-92a-1	CIAO1	0.361689581	mirna_pc
26860	hsa-mir-92a-1	C2orf3	0.371792262	mirna_pc
26861	hsa-mir-92a-1	ZSCAN29	0.3337651	mirna_pc
26862	hsa-mir-92a-1	ZNF664	0.390743167	mirna_pc
26863	hsa-mir-92a-1	VPS36	0.358513258	mirna_pc
26864	hsa-mir-92a-1	GOLT1A	0.392381845	mirna_pc
26865	hsa-mir-92a-1	NCRNA00183	0.31913596	mirna_pc
26866	hsa-mir-92a-1	STK35	0.30081461	mirna_pc
26867	hsa-mir-92a-1	PPP4R2	0.30410883	mirna_pc
26868	hsa-mir-92a-1	MRPL19	0.375892872	mirna_pc
26869	hsa-mir-92a-1	ZCCHC3	0.47316127	mirna_pc
26870	hsa-mir-92a-1	RRP15	0.351256063	mirna_pc
26871	hsa-mir-92a-1	CASC3	0.373329652	mirna_pc
26872	hsa-mir-92a-1	IARS	0.304411202	mirna_pc
26873	hsa-mir-92a-1	AADAT	0.377512837	mirna_pc
26874	hsa-mir-92a-1	CBLL1	0.355776025	mirna_pc
26875	hsa-mir-92a-1	ACP1	0.31116609	mirna_pc
26876	hsa-mir-92a-1	C2orf43	0.373996788	mirna_pc
26877	hsa-mir-92a-1	DACH1	0.340898354	mirna_pc
26878	hsa-mir-92a-1	RARA	0.327618065	mirna_pc
26879	hsa-mir-92a-1	GTF3A	0.378408636	mirna_pc
26880	hsa-mir-92a-1	LOC100129637	0.308177831	mirna_pc
26881	hsa-mir-92a-1	TPP2	0.411592127	mirna_pc
26882	hsa-mir-92a-1	SPRED2	0.312116158	mirna_pc
26883	hsa-mir-92a-1	TXLNG	0.470641885	mirna_pc
26884	hsa-mir-92a-1	TRIM25	0.376056146	mirna_pc
26885	hsa-mir-92a-1	PRMT6	0.321110375	mirna_pc
26886	hsa-mir-92a-1	CEP57	0.304814325	mirna_pc
26887	hsa-mir-92a-1	NAA35	0.300131643	mirna_pc
26888	hsa-mir-92a-1	PPP1R1B	0.320389673	mirna_pc
26889	hsa-mir-92a-1	ZNF530	0.310531501	mirna_pc
26890	hsa-mir-92a-1	RAGE	0.305076934	mirna_pc
26891	hsa-mir-92a-1	MIR17HG	0.548467486	mirna_pc

26892	hsa-mir-92a-1	C20orf30	0.313452686	mirna_pc
26893	hsa-mir-92a-1	AARSD1	0.366954537	mirna_pc
26894	hsa-mir-92a-1	ZNF239	0.403972196	mirna_pc
26895	hsa-mir-92a-1	KLHL23	0.417975485	mirna_pc
26896	hsa-mir-92a-1	ZNF202	0.364983993	mirna_pc
26897	hsa-mir-92a-1	ZNF77	0.325785703	mirna_pc
26898	hsa-mir-92a-1	MRPS31	0.35882991	mirna_pc
26899	hsa-mir-92a-1	CENPV	0.319553477	mirna_pc
26900	hsa-mir-92a-1	SFRS6	0.324665723	mirna_pc
26901	hsa-mir-92a-1	SLC4A5	0.331971492	mirna_pc
26902	hsa-mir-92a-1	DNMT3A	0.418887045	mirna_pc
26903	hsa-mir-92a-1	MIPEP	0.329999937	mirna_pc
26904	hsa-mir-92a-1	PRTFDC1	0.356029013	mirna_pc
26905	hsa-mir-92a-1	PATZ1	0.385195237	mirna_pc
26906	hsa-mir-92a-1	ZNF670	0.39126945	mirna_pc
26907	hsa-mir-92a-1	ZNF343	0.357897649	mirna_pc
26908	hsa-mir-92a-1	WIPF2	0.370296036	mirna_pc
26909	hsa-mir-92a-1	ZNF711	0.405912038	mirna_pc
26910	hsa-mir-92a-1	ARSE	0.315599999	mirna_pc
26911	hsa-mir-92a-1	POM121	0.327277287	mirna_pc
26912	hsa-mir-191	MKI67	0.312623036	mirna_pc
26913	hsa-mir-191	TPX2	0.329048952	mirna_pc
26914	hsa-mir-191	KIF4B	0.345375902	mirna_pc
26915	hsa-mir-191	HOXC9	0.430451683	mirna_pc
26916	hsa-mir-191	CDC25C	0.400285646	mirna_pc
26917	hsa-mir-191	UBE2C	0.319307761	mirna_pc
26918	hsa-mir-191	KIFC1	0.379199915	mirna_pc
26919	hsa-mir-191	BUB1B	0.332575386	mirna_pc
26920	hsa-mir-191	FAM72A	0.381863205	mirna_pc
26921	hsa-mir-191	SGOL1	0.542424885	mirna_pc
26922	hsa-mir-191	ARHGAP11A	0.373051588	mirna_pc
26923	hsa-mir-191	IQGAP3	0.361821553	mirna_pc
26924	hsa-mir-191	NEK2	0.382908192	mirna_pc
26925	hsa-mir-191	HOXC11	0.384538194	mirna_pc
26926	hsa-mir-191	LMNB1	0.30577566	mirna_pc
26927	hsa-mir-191	CDCA8	0.437498718	mirna_pc
26928	hsa-mir-191	STIL	0.403074636	mirna_pc
26929	hsa-mir-191	KIF4A	0.352051625	mirna_pc
26930	hsa-mir-191	HJURP	0.322754695	mirna_pc
26931	hsa-mir-191	BLM	0.314052438	mirna_pc
26932	hsa-mir-191	CCDC150	0.313148104	mirna_pc
26933	hsa-mir-191	RCC1	0.32271287	mirna_pc
26934	hsa-mir-191	OIP5	0.466366724	mirna_pc
26935	hsa-mir-191	RECQL4	0.310450166	mirna_pc
26936	hsa-mir-191	AURKA	0.46441225	mirna_pc
26937	hsa-mir-191	CHEK2	0.319172775	mirna_pc
26938	hsa-mir-191	KIF15	0.486002053	mirna_pc
26939	hsa-mir-191	FEN1	0.352045532	mirna_pc
26940	hsa-mir-191	TRAIP	0.512865994	mirna_pc
26941	hsa-mir-191	ARHGAP11B	0.314922224	mirna_pc
26942	hsa-mir-191	CASC5	0.315944589	mirna_pc
26943	hsa-mir-191	RAE1	0.353406144	mirna_pc
26944	hsa-mir-191	FAM72D	0.43505107	mirna_pc
26945	hsa-mir-191	FAM72B	0.350858268	mirna_pc

26946	hsa-mir-191	C12orf48	0.351556436	mirna_pc
26947	hsa-mir-191	TTK	0.304547443	mirna_pc
26948	hsa-mir-191	ZWINT	0.50358736	mirna_pc
26949	hsa-mir-191	BUB3	0.378132973	mirna_pc
26950	hsa-mir-191	PDHB	0.356675627	mirna_pc
26951	hsa-mir-191	PIF1	0.349778942	mirna_pc
26952	hsa-mir-191	DNAH3	0.327933083	mirna_pc
26953	hsa-mir-191	E2F1	0.349721765	mirna_pc
26954	hsa-mir-191	C1orf135	0.360664555	mirna_pc
26955	hsa-mir-191	HOXA11	0.364358151	mirna_pc
26956	hsa-mir-191	POC1A	0.515675659	mirna_pc
26957	hsa-mir-191	ARL6IP1	0.314613612	mirna_pc
26958	hsa-mir-191	DNA2	0.387253481	mirna_pc
26959	hsa-mir-191	NEURL3	0.302362856	mirna_pc
26960	hsa-mir-191	DNAJC6	0.303403988	mirna_pc
26961	hsa-mir-191	DSN1	0.311581111	mirna_pc
26962	hsa-mir-191	DTYMK	0.317690082	mirna_pc
26963	hsa-mir-191	PIGU	0.300659004	mirna_pc
26964	hsa-mir-191	C5	0.328431511	mirna_pc
26965	hsa-mir-191	CLDN7	0.328415658	mirna_pc
26966	hsa-mir-191	ZBED3	0.305377949	mirna_pc
26967	hsa-mir-191	HOXA10	0.439314035	mirna_pc
26968	hsa-mir-191	PRIM1	0.385030182	mirna_pc
26969	hsa-mir-191	PSMC4	0.312463494	mirna_pc
26970	hsa-mir-191	DDX27	0.403802931	mirna_pc
26971	hsa-mir-191	RHOU	0.316169488	mirna_pc
26972	hsa-mir-191	HOXC10	0.403716053	mirna_pc
26973	hsa-mir-191	MSH2	0.36328565	mirna_pc
26974	hsa-mir-191	SALL4	0.416296402	mirna_pc
26975	hsa-mir-191	EIF2AK1	0.308228846	mirna_pc
26976	hsa-mir-191	SSB	0.336984244	mirna_pc
26977	hsa-mir-191	SNRPC	0.361493512	mirna_pc
26978	hsa-mir-191	SUOX	0.341489895	mirna_pc
26979	hsa-mir-191	MDK	0.347755241	mirna_pc
26980	hsa-mir-191	E2F3	0.393974143	mirna_pc
26981	hsa-mir-191	CDC25A	0.510086437	mirna_pc
26982	hsa-mir-191	TMEM48	0.425518718	mirna_pc
26983	hsa-mir-191	MGC12982	0.303665662	mirna_pc
26984	hsa-mir-191	FXC1	0.357519155	mirna_pc
26985	hsa-mir-191	SRPK1	0.41193511	mirna_pc
26986	hsa-mir-191	CLN6	0.31980894	mirna_pc
26987	hsa-mir-191	C21orf45	0.343312615	mirna_pc
26988	hsa-mir-191	ATIC	0.458527935	mirna_pc
26989	hsa-mir-191	ISOC1	0.343390422	mirna_pc
26990	hsa-mir-191	XK	0.321983653	mirna_pc
26991	hsa-mir-191	UTP6	0.306703054	mirna_pc
26992	hsa-mir-191	MARCKSL1	0.425717487	mirna_pc
26993	hsa-mir-191	HOXA13	0.355875257	mirna_pc
26994	hsa-mir-191	CBX8	0.383908404	mirna_pc
26995	hsa-mir-191	GNPDA1	0.341421756	mirna_pc
26996	hsa-mir-191	ARIH2	0.452958259	mirna_pc
26997	hsa-mir-191	PAFAH1B3	0.329988449	mirna_pc
26998	hsa-mir-191	SASS6	0.312900056	mirna_pc
26999	hsa-mir-191	PSRC1	0.302774973	mirna_pc

27000	hsa-mir-191	POU2F1	0.358912376	mirna_pc
27001	hsa-mir-191	MARVELD3	0.381854923	mirna_pc
27002	hsa-mir-191	TMPO	0.406685004	mirna_pc
27003	hsa-mir-191	RPP14	0.425145248	mirna_pc
27004	hsa-mir-191	LOC100128191	0.344673076	mirna_pc
27005	hsa-mir-191	CHRNA5	0.425572203	mirna_pc
27006	hsa-mir-191	TMEM56	0.31143315	mirna_pc
27007	hsa-mir-191	SLC25A20	0.40970504	mirna_pc
27008	hsa-mir-191	PHF6	0.330023793	mirna_pc
27009	hsa-mir-191	ZNF717	0.32160959	mirna_pc
27010	hsa-mir-191	TRPM2	0.393680761	mirna_pc
27011	hsa-mir-191	SFRS1	0.303649539	mirna_pc
27012	hsa-mir-191	CASP8	0.415714809	mirna_pc
27013	hsa-mir-191	WDR48	0.322835388	mirna_pc
27014	hsa-mir-191	GAD1	0.307971222	mirna_pc
27015	hsa-mir-191	DARS2	0.41559049	mirna_pc
27016	hsa-mir-191	UTP14A	0.32695642	mirna_pc
27017	hsa-mir-191	SNX8	0.333585051	mirna_pc
27018	hsa-mir-191	ZNF33A	0.32411542	mirna_pc
27019	hsa-mir-191	IP6K1	0.330269075	mirna_pc
27020	hsa-mir-191	ANKRD43	0.345259584	mirna_pc
27021	hsa-mir-191	CHPT1	0.314502389	mirna_pc
27022	hsa-mir-191	SLC5A6	0.447478203	mirna_pc
27023	hsa-mir-191	TOMM34	0.48978923	mirna_pc
27024	hsa-mir-191	KIAA1143	0.397267261	mirna_pc
27025	hsa-mir-191	TUSC2	0.466028427	mirna_pc
27026	hsa-mir-191	AHCY	0.392627477	mirna_pc
27027	hsa-mir-191	EIF4EBP2	0.368663617	mirna_pc
27028	hsa-mir-191	HNRNPF	0.369360958	mirna_pc
27029	hsa-mir-191	PHYH	0.338140432	mirna_pc
27030	hsa-mir-191	INTS8	0.300333684	mirna_pc
27031	hsa-mir-191	RAD18	0.400600146	mirna_pc
27032	hsa-mir-191	CCNE1	0.306043412	mirna_pc
27033	hsa-mir-191	NR6A1	0.322786874	mirna_pc
27034	hsa-mir-191	SUV420H2	0.303617294	mirna_pc
27035	hsa-mir-191	ZNF695	0.46360912	mirna_pc
27036	hsa-mir-191	ADCY6	0.436617431	mirna_pc
27037	hsa-mir-191	PDSS1	0.452289414	mirna_pc
27038	hsa-mir-191	PDHA1	0.301352598	mirna_pc
27039	hsa-mir-191	C3orf23	0.351074134	mirna_pc
27040	hsa-mir-191	NOC2L	0.319833235	mirna_pc
27041	hsa-mir-191	NONO	0.313965974	mirna_pc
27042	hsa-mir-191	RNF123	0.408065339	mirna_pc
27043	hsa-mir-191	POLR1B	0.313086799	mirna_pc
27044	hsa-mir-191	OBFC2B	0.434377235	mirna_pc
27045	hsa-mir-191	APPL1	0.421691142	mirna_pc
27046	hsa-mir-191	SCARB1	0.611291247	mirna_pc
27047	hsa-mir-191	ELK1	0.420789993	mirna_pc
27048	hsa-mir-191	ARHGAP39	0.359910518	mirna_pc
27049	hsa-mir-191	USP19	0.43753623	mirna_pc
27050	hsa-mir-191	ACTR8	0.344197938	mirna_pc
27051	hsa-mir-191	CLCN2	0.501299563	mirna_pc
27052	hsa-mir-191	GORASP1	0.35587558	mirna_pc
27053	hsa-mir-191	SUV39H2	0.342364084	mirna_pc

27054	hsa-mir-191	C9orf152	0.361067249	mirna_pc
27055	hsa-mir-191	LSM 2.00	0.332287188	mirna_pc
27056	hsa-mir-191	FBXL6	0.31563952	mirna_pc
27057	hsa-mir-191	FOXD2	0.307880849	mirna_pc
27058	hsa-mir-191	SH3BGRL2	0.301756833	mirna_pc
27059	hsa-mir-191	TMEM86B	0.371382626	mirna_pc
27060	hsa-mir-191	RRP9	0.393839322	mirna_pc
27061	hsa-mir-191	AATK	0.308676524	mirna_pc
27062	hsa-mir-191	GTPBP4	0.329777795	mirna_pc
27063	hsa-mir-191	NFE2L3	0.338675711	mirna_pc
27064	hsa-mir-191	TFR2	0.340429908	mirna_pc
27065	hsa-mir-191	RDH5	0.321285534	mirna_pc
27066	hsa-mir-191	CCDC121	0.307005133	mirna_pc
27067	hsa-mir-191	HABP4	0.320751973	mirna_pc
27068	hsa-mir-191	C6orf223	0.358805523	mirna_pc
27069	hsa-mir-191	HSP90B1	0.394606967	mirna_pc
27070	hsa-mir-191	ZNF445	0.300569926	mirna_pc
27071	hsa-mir-191	RBM6	0.398905589	mirna_pc
27072	hsa-mir-191	PAK1IP1	0.337607972	mirna_pc
27073	hsa-mir-191	PGPEP1	0.372846048	mirna_pc
27074	hsa-mir-191	BRPF3	0.393942024	mirna_pc
27075	hsa-mir-191	EDEM2	0.402754779	mirna_pc
27076	hsa-mir-191	SLMO2	0.320175482	mirna_pc
27077	hsa-mir-191	C1orf59	0.462238466	mirna_pc
27078	hsa-mir-191	C3orf39	0.373636047	mirna_pc
27079	hsa-mir-191	SVIP	0.326676201	mirna_pc
27080	hsa-mir-191	EPCAM	0.497484008	mirna_pc
27081	hsa-mir-191	ARHGAP8	0.307970053	mirna_pc
27082	hsa-mir-191	CTSA	0.35124292	mirna_pc
27083	hsa-mir-191	IVD	0.392840301	mirna_pc
27084	hsa-mir-191	EHMT2	0.431211795	mirna_pc
27085	hsa-mir-191	GAS2L3	0.403960556	mirna_pc
27086	hsa-mir-191	BEND7	0.355792787	mirna_pc
27087	hsa-mir-191	TRIB3	0.300678547	mirna_pc
27088	hsa-mir-191	LOC113230	0.329960252	mirna_pc
27089	hsa-mir-191	PDRG1	0.504175214	mirna_pc
27090	hsa-mir-191	PASK	0.365269985	mirna_pc
27091	hsa-mir-191	ATP11A	0.354889824	mirna_pc
27092	hsa-mir-191	WDR46	0.334685141	mirna_pc
27093	hsa-mir-191	TAF4	0.446906944	mirna_pc
27094	hsa-mir-191	ZC3H12B	0.31986768	mirna_pc
27095	hsa-mir-191	PPP2R1B	0.364559665	mirna_pc
27096	hsa-mir-191	MEIS2	0.315457288	mirna_pc
27097	hsa-mir-191	RNF114	0.39903878	mirna_pc
27098	hsa-mir-191	ANG	0.362112013	mirna_pc
27099	hsa-mir-191	PRAME	0.324057459	mirna_pc
27100	hsa-mir-191	ZBTB9	0.394030911	mirna_pc
27101	hsa-mir-191	RPP25	0.317471051	mirna_pc
27102	hsa-mir-191	KIAA0776	0.306934703	mirna_pc
27103	hsa-mir-191	CSRP2BP	0.441050269	mirna_pc
27104	hsa-mir-191	POFUT1	0.31911034	mirna_pc
27105	hsa-mir-191	TRAF4	0.304948167	mirna_pc
27106	hsa-mir-191	NGLY1	0.340632133	mirna_pc
27107	hsa-mir-191	MRPS25	0.416185624	mirna_pc

27108	hsa-mir-191	SLC35A2	0.485866736	mirna_pc
27109	hsa-mir-191	LSM14B	0.314854944	mirna_pc
27110	hsa-mir-191	P4HTM	0.38577811	mirna_pc
27111	hsa-mir-191	C1orf174	0.386367135	mirna_pc
27112	hsa-mir-191	RPN2	0.421775342	mirna_pc
27113	hsa-mir-191	KCTD6	0.505845772	mirna_pc
27114	hsa-mir-191	SAE1	0.331644951	mirna_pc
27115	hsa-mir-191	ZNF621	0.307122465	mirna_pc
27116	hsa-mir-191	PABPC1L	0.424082214	mirna_pc
27117	hsa-mir-191	C17orf42	0.324164185	mirna_pc
27118	hsa-mir-191	TSEN54	0.384906826	mirna_pc
27119	hsa-mir-191	ACTR5	0.328269909	mirna_pc
27120	hsa-mir-191	SLC43A1	0.412228316	mirna_pc
27121	hsa-mir-191	HSPA14	0.346796273	mirna_pc
27122	hsa-mir-191	ZNF595	0.393973862	mirna_pc
27123	hsa-mir-191	AMIGO3	0.436040492	mirna_pc
27124	hsa-mir-191	DDX55	0.342942077	mirna_pc
27125	hsa-mir-191	HPDL	0.42955154	mirna_pc
27126	hsa-mir-191	FCHO1	0.326269903	mirna_pc
27127	hsa-mir-191	CDK5RAP1	0.377644337	mirna_pc
27128	hsa-mir-191	CCDC103	0.308184426	mirna_pc
27129	hsa-mir-191	PDE12	0.417963553	mirna_pc
27130	hsa-mir-191	RCOR2	0.424104899	mirna_pc
27131	hsa-mir-191	ITIH4	0.352652297	mirna_pc
27132	hsa-mir-191	EPHB2	0.418824651	mirna_pc
27133	hsa-mir-191	ATAD3A	0.382549628	mirna_pc
27134	hsa-mir-191	TM9SF3	0.347019647	mirna_pc
27135	hsa-mir-191	POLA1	0.326541202	mirna_pc
27136	hsa-mir-191	HDAC2	0.362060728	mirna_pc
27137	hsa-mir-191	SLC25A38	0.38457389	mirna_pc
27138	hsa-mir-191	PDCD2L	0.377992631	mirna_pc
27139	hsa-mir-191	C1orf115	0.407977653	mirna_pc
27140	hsa-mir-191	PA2G4P4	0.316900407	mirna_pc
27141	hsa-mir-191	CDX2	0.303662741	mirna_pc
27142	hsa-mir-191	ASCL2	0.412707584	mirna_pc
27143	hsa-mir-191	C10orf2	0.411450495	mirna_pc
27144	hsa-mir-191	RWDD2A	0.349524922	mirna_pc
27145	hsa-mir-191	ZBTB42	0.310985728	mirna_pc
27146	hsa-mir-191	SLC35E2	0.356410886	mirna_pc
27147	hsa-mir-191	SLC39A1	0.350806771	mirna_pc
27148	hsa-mir-191	BRI3BP	0.466684505	mirna_pc
27149	hsa-mir-191	CSTF1	0.38186936	mirna_pc
27150	hsa-mir-191	UBE4A	0.368318514	mirna_pc
27151	hsa-mir-191	FLVCR1	0.379685902	mirna_pc
27152	hsa-mir-191	AGPAT1	0.320997486	mirna_pc
27153	hsa-mir-191	CAPS	0.338963674	mirna_pc
27154	hsa-mir-191	RAB17	0.339942475	mirna_pc
27155	hsa-mir-191	AP1M2	0.388043221	mirna_pc
27156	hsa-mir-191	GPAM	0.48092616	mirna_pc
27157	hsa-mir-191	AGMAT	0.574326248	mirna_pc
27158	hsa-mir-191	TTC9C	0.378278639	mirna_pc
27159	hsa-mir-191	BAT1	0.434388396	mirna_pc
27160	hsa-mir-191	VARS	0.400332344	mirna_pc
27161	hsa-mir-191	MRPS12	0.312875618	mirna_pc

27162	hsa-mir-191	ZSCAN2	0.361170947	mirna_pc
27163	hsa-mir-191	MSH5	0.424069494	mirna_pc
27164	hsa-mir-191	NOXO1	0.369536236	mirna_pc
27165	hsa-mir-191	C12orf47	0.351321706	mirna_pc
27166	hsa-mir-191	PDIA3	0.385970075	mirna_pc
27167	hsa-mir-191	C2orf29	0.444309786	mirna_pc
27168	hsa-mir-191	PLEKHA6	0.318396313	mirna_pc
27169	hsa-mir-191	MRM1	0.361389547	mirna_pc
27170	hsa-mir-191	ACAN	0.325869957	mirna_pc
27171	hsa-mir-191	RFX5	0.342282237	mirna_pc
27172	hsa-mir-191	EPB41L5	0.318955236	mirna_pc
27173	hsa-mir-191	B3GALT6	0.394476874	mirna_pc
27174	hsa-mir-191	IFRD2	0.417424069	mirna_pc
27175	hsa-mir-191	ZNF444	0.433913316	mirna_pc
27176	hsa-mir-191	SUZ12	0.437519046	mirna_pc
27177	hsa-mir-191	ABCC6	0.383831669	mirna_pc
27178	hsa-mir-191	CCL20	0.303417316	mirna_pc
27179	hsa-mir-191	FN3K	0.365648211	mirna_pc
27180	hsa-mir-191	ZBTB12	0.477367729	mirna_pc
27181	hsa-mir-191	SLC12A2	0.381965861	mirna_pc
27182	hsa-mir-191	BCDIN3D	0.310191567	mirna_pc
27183	hsa-mir-191	KLHDC9	0.319313671	mirna_pc
27184	hsa-mir-191	ABHD14B	0.342376566	mirna_pc
27185	hsa-mir-191	KAZALD1	0.361268437	mirna_pc
27186	hsa-mir-191	EPB41	0.321547554	mirna_pc
27187	hsa-mir-191	SLC26A1	0.400492746	mirna_pc
27188	hsa-mir-191	SLC7A7	0.34819788	mirna_pc
27189	hsa-mir-191	DOK7	0.303529841	mirna_pc
27190	hsa-mir-191	FARSB	0.318131904	mirna_pc
27191	hsa-mir-191	ANKMY2	0.322333151	mirna_pc
27192	hsa-mir-191	PBRM1	0.363796881	mirna_pc
27193	hsa-mir-191	PIK3AP1	0.494557547	mirna_pc
27194	hsa-mir-191	CIB2	0.37462053	mirna_pc
27195	hsa-mir-191	FAM96A	0.418162979	mirna_pc
27196	hsa-mir-191	ZNF608	0.40043816	mirna_pc
27197	hsa-mir-191	TOR2A	0.310169224	mirna_pc
27198	hsa-mir-191	TARBP2	0.365881032	mirna_pc
27199	hsa-mir-191	TRIM15	0.315216101	mirna_pc
27200	hsa-mir-191	FGFR1OP	0.482622301	mirna_pc
27201	hsa-mir-191	METTL13	0.498263708	mirna_pc
27202	hsa-mir-191	C12orf52	0.346094841	mirna_pc
27203	hsa-mir-191	HACL1	0.371368163	mirna_pc
27204	hsa-mir-191	BPNT1	0.33193159	mirna_pc
27205	hsa-mir-191	MAN2A2	0.407128371	mirna_pc
27206	hsa-mir-191	POLE	0.337737653	mirna_pc
27207	hsa-mir-191	C1orf163	0.400642662	mirna_pc
27208	hsa-mir-191	WDR6	0.483558753	mirna_pc
27209	hsa-mir-191	MKRN2	0.317860968	mirna_pc
27210	hsa-mir-191	C1orf159	0.346245844	mirna_pc
27211	hsa-mir-191	TRIAP1	0.341009529	mirna_pc
27212	hsa-mir-191	SOX9	0.312960625	mirna_pc
27213	hsa-mir-191	TMEM216	0.308670698	mirna_pc
27214	hsa-mir-191	TMF1	0.308136385	mirna_pc
27215	hsa-mir-191	GAL3ST1	0.478192145	mirna_pc

27216	hsa-mir-191	NPRL2	0.473682094	mirna_pc
27217	hsa-mir-191	DDOST	0.488709188	mirna_pc
27218	hsa-mir-191	FAM188B	0.374563629	mirna_pc
27219	hsa-mir-191	GK3P	0.330405438	mirna_pc
27220	hsa-mir-191	RBMX	0.31431301	mirna_pc
27221	hsa-mir-191	C6orf134	0.342237426	mirna_pc
27222	hsa-mir-191	SEC23IP	0.346508342	mirna_pc
27223	hsa-mir-191	SEPSECS	0.327668884	mirna_pc
27224	hsa-mir-191	GPC3	0.319855212	mirna_pc
27225	hsa-mir-191	PIP5K1B	0.306550389	mirna_pc
27226	hsa-mir-191	QPCTL	0.3092117	mirna_pc
27227	hsa-mir-191	C9orf142	0.319462456	mirna_pc
27228	hsa-mir-191	PDIA3P	0.384716767	mirna_pc
27229	hsa-mir-191	ARFGEF2	0.338660819	mirna_pc
27230	hsa-mir-191	ATAD3B	0.422813067	mirna_pc
27231	hsa-mir-191	XRCC5	0.313448618	mirna_pc
27232	hsa-mir-191	ABCD1	0.320339623	mirna_pc
27233	hsa-mir-191	MYO5C	0.321648092	mirna_pc
27234	hsa-mir-191	APEH	0.395447883	mirna_pc
27235	hsa-mir-191	ZNF780B	0.343392482	mirna_pc
27236	hsa-mir-191	C19orf61	0.358088602	mirna_pc
27237	hsa-mir-191	STT3B	0.341359232	mirna_pc
27238	hsa-mir-191	C11orf48	0.445762269	mirna_pc
27239	hsa-mir-191	ZNF137	0.303661827	mirna_pc
27240	hsa-mir-191	PDIA6	0.300145161	mirna_pc
27241	hsa-mir-191	SGK2	0.347837295	mirna_pc
27242	hsa-mir-191	PLEKHH1	0.33153603	mirna_pc
27243	hsa-mir-191	C22orf13	0.305776246	mirna_pc
27244	hsa-mir-191	AIFM1	0.361564794	mirna_pc
27245	hsa-mir-191	SCAP	0.31843653	mirna_pc
27246	hsa-mir-191	LIMD1	0.458070083	mirna_pc
27247	hsa-mir-191	PTGES2	0.368339029	mirna_pc
27248	hsa-mir-191	LBR	0.322530418	mirna_pc
27249	hsa-mir-191	LOC440905	0.309716872	mirna_pc
27250	hsa-mir-191	PDSS2	0.327017506	mirna_pc
27251	hsa-mir-191	PDXDC1	0.357195915	mirna_pc
27252	hsa-mir-191	NAA40	0.399621006	mirna_pc
27253	hsa-mir-191	LINGO1	0.401535514	mirna_pc
27254	hsa-mir-191	MSX2	0.309240458	mirna_pc
27255	hsa-mir-191	DNAJC14	0.463840231	mirna_pc
27256	hsa-mir-191	C17orf28	0.306240632	mirna_pc
27257	hsa-mir-191	FGFRL1	0.450195255	mirna_pc
27258	hsa-mir-191	PEX11A	0.374117349	mirna_pc
27259	hsa-mir-191	CELSR3	0.328969621	mirna_pc
27260	hsa-mir-191	DTNBP1	0.395214827	mirna_pc
27261	hsa-mir-191	PUSL1	0.349009907	mirna_pc
27262	hsa-mir-191	ABCF1	0.334248858	mirna_pc
27263	hsa-mir-191	CNPY2	0.363588024	mirna_pc
27264	hsa-mir-191	MPV17L	0.34086977	mirna_pc
27265	hsa-mir-191	FAM136A	0.481531756	mirna_pc
27266	hsa-mir-191	TCTA	0.360094863	mirna_pc
27267	hsa-mir-191	BAIAP2L2	0.376863558	mirna_pc
27268	hsa-mir-191	KDELR1	0.328968966	mirna_pc
27269	hsa-mir-191	C12orf49	0.324591406	mirna_pc

27270	hsa-mir-191	TMEM115	0.342408009	mirna_pc
27271	hsa-mir-191	HMGN1	0.304674392	mirna_pc
27272	hsa-mir-191	OXNAD1	0.421849472	mirna_pc
27273	hsa-mir-191	PRKCD	0.370568505	mirna_pc
27274	hsa-mir-191	PDE8A	0.303221585	mirna_pc
27275	hsa-mir-191	AGAP1	0.350379442	mirna_pc
27276	hsa-mir-191	ARID3A	0.363030414	mirna_pc
27277	hsa-mir-191	MTPAP	0.320217975	mirna_pc
27278	hsa-mir-191	EIF1AX	0.311432725	mirna_pc
27279	hsa-mir-191	DLG3	0.303000053	mirna_pc
27280	hsa-mir-191	EFCAB4A	0.310393799	mirna_pc
27281	hsa-mir-191	GDPD5	0.452990345	mirna_pc
27282	hsa-mir-191	GIPC2	0.373586678	mirna_pc
27283	hsa-mir-191	GNL3L	0.306190971	mirna_pc
27284	hsa-mir-191	EML4	0.449531338	mirna_pc
27285	hsa-mir-191	BZW2	0.321842914	mirna_pc
27286	hsa-mir-191	QARS	0.45479504	mirna_pc
27287	hsa-mir-191	ACBD7	0.348683082	mirna_pc
27288	hsa-mir-191	LRPPRC	0.324318539	mirna_pc
27289	hsa-mir-191	C8orf51	0.321503978	mirna_pc
27290	hsa-mir-191	LOC401010	0.391855523	mirna_pc
27291	hsa-mir-191	KRT18	0.393959852	mirna_pc
27292	hsa-mir-191	KBTBD11	0.338847014	mirna_pc
27293	hsa-mir-191	STRA13	0.304049567	mirna_pc
27294	hsa-mir-191	RPIA	0.364696073	mirna_pc
27295	hsa-mir-191	CXorf38	0.340667125	mirna_pc
27296	hsa-mir-191	ZNF860	0.348584061	mirna_pc
27297	hsa-mir-191	RNFT2	0.39485797	mirna_pc
27298	hsa-mir-191	NOTUM	0.329045521	mirna_pc
27299	hsa-mir-191	FUCA1	0.346879933	mirna_pc
27300	hsa-mir-191	CA13	0.325056223	mirna_pc
27301	hsa-mir-191	AP3M1	0.315814158	mirna_pc
27302	hsa-mir-191	MST1P9	0.303480395	mirna_pc
27303	hsa-mir-191	CYB5RL	0.328803659	mirna_pc
27304	hsa-mir-191	ZNF607	0.313163424	mirna_pc
27305	hsa-mir-191	FRAT2	0.399291189	mirna_pc
27306	hsa-mir-191	PRR15L	0.308016081	mirna_pc
27307	hsa-mir-191	C2CD4D	0.300666255	mirna_pc
27308	hsa-mir-191	NAT9	0.319952115	mirna_pc
27309	hsa-mir-191	TMEM98	0.36564423	mirna_pc
27310	hsa-mir-191	KIAA1731	0.357250061	mirna_pc
27311	hsa-mir-191	SLC27A2	0.449535545	mirna_pc
27312	hsa-mir-191	PNRC2	0.423014718	mirna_pc
27313	hsa-mir-191	SPCS1	0.462652013	mirna_pc
27314	hsa-mir-191	SFXN4	0.431060107	mirna_pc
27315	hsa-mir-191	ZNF619	0.354449583	mirna_pc
27316	hsa-mir-191	NDUFS2	0.414096586	mirna_pc
27317	hsa-mir-191	SLC35B3	0.363863427	mirna_pc
27318	hsa-mir-191	OXSM	0.313340142	mirna_pc
27319	hsa-mir-191	FRAT1	0.449697276	mirna_pc
27320	hsa-mir-191	HNF1B	0.464258128	mirna_pc
27321	hsa-mir-191	MINPP1	0.387901415	mirna_pc
27322	hsa-mir-191	GATM	0.373964065	mirna_pc
27323	hsa-mir-191	RANBP17	0.385037711	mirna_pc

27324	hsa-mir-191	TMEM180	0.453023215	mirna_pc
27325	hsa-mir-191	ProSAPiP1	0.343081855	mirna_pc
27326	hsa-mir-191	LACTB2	0.328339917	mirna_pc
27327	hsa-mir-191	MRE11A	0.315979249	mirna_pc
27328	hsa-mir-191	QRICH1	0.374826525	mirna_pc
27329	hsa-mir-191	LTK	0.334725724	mirna_pc
27330	hsa-mir-191	LRRC27	0.343231977	mirna_pc
27331	hsa-mir-191	GOSR1	0.361461156	mirna_pc
27332	hsa-mir-191	ACOT4	0.363410927	mirna_pc
27333	hsa-mir-191	USP37	0.315464706	mirna_pc
27334	hsa-mir-191	ZNF84	0.307725207	mirna_pc
27335	hsa-mir-191	TMEM179B	0.325240758	mirna_pc
27336	hsa-mir-191	TMEM106C	0.375920343	mirna_pc
27337	hsa-mir-191	CTNNB1	0.300370553	mirna_pc
27338	hsa-mir-191	DAGLA	0.494027505	mirna_pc
27339	hsa-mir-191	SPATA2	0.41596126	mirna_pc
27340	hsa-mir-191	CRLS1	0.429592862	mirna_pc
27341	hsa-mir-191	DHX30	0.513803285	mirna_pc
27342	hsa-mir-191	GYG2	0.417639334	mirna_pc
27343	hsa-mir-191	GANAB	0.303419149	mirna_pc
27344	hsa-mir-191	AVL9	0.359683932	mirna_pc
27345	hsa-mir-191	ZNF620	0.519611272	mirna_pc
27346	hsa-mir-191	RNF34	0.443569042	mirna_pc
27347	hsa-mir-191	CHD7	0.395407984	mirna_pc
27348	hsa-mir-191	PANK1	0.464038569	mirna_pc
27349	hsa-mir-191	ALAS1	0.39580749	mirna_pc
27350	hsa-mir-191	CD164	0.336146765	mirna_pc
27351	hsa-mir-191	TOX3	0.315483469	mirna_pc
27352	hsa-mir-191	SUCLG1	0.3266441	mirna_pc
27353	hsa-mir-191	AGPHD1	0.37527056	mirna_pc
27354	hsa-mir-191	SEC61A2	0.443600081	mirna_pc
27355	hsa-mir-191	DYX1C1	0.33619925	mirna_pc
27356	hsa-mir-191	PSPC1	0.382919433	mirna_pc
27357	hsa-mir-191	COPZ1	0.343514018	mirna_pc
27358	hsa-mir-191	REEP6	0.30964002	mirna_pc
27359	hsa-mir-191	ZNF35	0.489717357	mirna_pc
27360	hsa-mir-191	MAPKAPK5	0.476212435	mirna_pc
27361	hsa-mir-191	GMCL1	0.539250066	mirna_pc
27362	hsa-mir-191	DDAH1	0.438285411	mirna_pc
27363	hsa-mir-191	KDELR2	0.300833992	mirna_pc
27364	hsa-mir-191	PCSK6	0.307198824	mirna_pc
27365	hsa-mir-191	ZNF100	0.317936787	mirna_pc
27366	hsa-mir-191	ZNF833	0.446108363	mirna_pc
27367	hsa-mir-191	RHPN1	0.307922165	mirna_pc
27368	hsa-mir-191	SLC25A15	0.365197727	mirna_pc
27369	hsa-mir-191	C1orf77	0.314603282	mirna_pc
27370	hsa-mir-191	CCHCR1	0.316807489	mirna_pc
27371	hsa-mir-191	CHKA	0.373592046	mirna_pc
27372	hsa-mir-191	RASSF1	0.406805698	mirna_pc
27373	hsa-mir-191	B4GALT3	0.392609351	mirna_pc
27374	hsa-mir-191	NADK	0.356264755	mirna_pc
27375	hsa-mir-191	SFRS13A	0.314994522	mirna_pc
27376	hsa-mir-191	DENND5B	0.304598092	mirna_pc
27377	hsa-mir-191	SS18L2	0.300217969	mirna_pc

27378	hsa-mir-191	TGDS	0.313414072	mirna_pc
27379	hsa-mir-191	HGD	0.312192305	mirna_pc
27380	hsa-mir-191	TFAM	0.50643584	mirna_pc
27381	hsa-mir-191	ANXA4	0.379412384	mirna_pc
27382	hsa-mir-191	MACC1	0.315665501	mirna_pc
27383	hsa-mir-191	TTC38	0.362956402	mirna_pc
27384	hsa-mir-191	ZNF321	0.341744222	mirna_pc
27385	hsa-mir-191	NME6	0.415884398	mirna_pc
27386	hsa-mir-191	ORC4L	0.308469914	mirna_pc
27387	hsa-mir-191	EAF1	0.419542237	mirna_pc
27388	hsa-mir-191	MORF4L1	0.304968302	mirna_pc
27389	hsa-mir-191	SLC29A2	0.378754285	mirna_pc
27390	hsa-mir-191	CHCHD4	0.317397738	mirna_pc
27391	hsa-mir-191	LOC222699	0.352293261	mirna_pc
27392	hsa-mir-191	COX19	0.414038501	mirna_pc
27393	hsa-mir-191	IDH1	0.356088608	mirna_pc
27394	hsa-mir-191	SMC1A	0.317418478	mirna_pc
27395	hsa-mir-191	FNBP1L	0.349862744	mirna_pc
27396	hsa-mir-191	SLC44A3	0.327273671	mirna_pc
27397	hsa-mir-191	ZDHHC9	0.308516719	mirna_pc
27398	hsa-mir-191	SORD	0.305476706	mirna_pc
27399	hsa-mir-191	GTPBP5	0.463853696	mirna_pc
27400	hsa-mir-191	SHROOM1	0.366056833	mirna_pc
27401	hsa-mir-191	UBE2CBP	0.377099453	mirna_pc
27402	hsa-mir-191	C4orf19	0.31645079	mirna_pc
27403	hsa-mir-191	MGAT4A	0.367823437	mirna_pc
27404	hsa-mir-191	ACVR2B	0.422774361	mirna_pc
27405	hsa-mir-191	NEK8	0.300339899	mirna_pc
27406	hsa-mir-191	SATB2	0.338639288	mirna_pc
27407	hsa-mir-191	ICA1	0.409350284	mirna_pc
27408	hsa-mir-191	SSBP4	0.332645211	mirna_pc
27409	hsa-mir-191	POGZ	0.312066202	mirna_pc
27410	hsa-mir-191	ZNF702P	0.343639289	mirna_pc
27411	hsa-mir-191	BIN1	0.313649151	mirna_pc
27412	hsa-mir-191	SMUG1	0.353508892	mirna_pc
27413	hsa-mir-191	DCP1A	0.539331973	mirna_pc
27414	hsa-mir-191	CLDN3	0.419433769	mirna_pc
27415	hsa-mir-191	GNL3	0.61441602	mirna_pc
27416	hsa-mir-191	METTL7B	0.526984136	mirna_pc
27417	hsa-mir-191	PHKA2	0.320927076	mirna_pc
27418	hsa-mir-191	ZNF518A	0.365957883	mirna_pc
27419	hsa-mir-191	PLEKHA8	0.303586238	mirna_pc
27420	hsa-mir-191	NSMCE4A	0.380488059	mirna_pc
27421	hsa-mir-191	PYCR1	0.311559491	mirna_pc
27422	hsa-mir-191	PKN3	0.319426823	mirna_pc
27423	hsa-mir-191	BCORL1	0.358860016	mirna_pc
27424	hsa-mir-191	AKR7A2	0.30510943	mirna_pc
27425	hsa-mir-191	C9orf100	0.384738436	mirna_pc
27426	hsa-mir-191	SERPINF2	0.353630582	mirna_pc
27427	hsa-mir-191	HCG18	0.377944089	mirna_pc
27428	hsa-mir-191	SLC25A19	0.361456421	mirna_pc
27429	hsa-mir-191	BEND3	0.553405688	mirna_pc
27430	hsa-mir-191	HIBADH	0.321087682	mirna_pc
27431	hsa-mir-191	ARHGEF16	0.316097345	mirna_pc

27432	hsa-mir-191	CA5BP	0.355236647	mirna_pc
27433	hsa-mir-191	RAVER2	0.359737279	mirna_pc
27434	hsa-mir-191	FZD5	0.548624606	mirna_pc
27435	hsa-mir-191	AAMP	0.315856423	mirna_pc
27436	hsa-mir-191	FLJ10213	0.308635692	mirna_pc
27437	hsa-mir-191	FGFR4	0.518209969	mirna_pc
27438	hsa-mir-191	LOC151534	0.379123732	mirna_pc
27439	hsa-mir-191	AQP11	0.358708551	mirna_pc
27440	hsa-mir-191	NFXL1	0.364891991	mirna_pc
27441	hsa-mir-191	CNNM4	0.363321835	mirna_pc
27442	hsa-mir-191	KCTD14	0.377378391	mirna_pc
27443	hsa-mir-191	VPS33A	0.360499935	mirna_pc
27444	hsa-mir-191	CCDC78	0.34076443	mirna_pc
27445	hsa-mir-191	SARS2	0.373734641	mirna_pc
27446	hsa-mir-191	RAB20	0.337712741	mirna_pc
27447	hsa-mir-191	LRRC45	0.35239572	mirna_pc
27448	hsa-mir-191	RBM38	0.323278484	mirna_pc
27449	hsa-mir-191	GHDC	0.347334434	mirna_pc
27450	hsa-mir-191	CXXC5	0.382411892	mirna_pc
27451	hsa-mir-191	CCAR1	0.308427917	mirna_pc
27452	hsa-mir-191	VHL	0.314819834	mirna_pc
27453	hsa-mir-191	SLC35A1	0.351235176	mirna_pc
27454	hsa-mir-191	ITPKA	0.418679021	mirna_pc
27455	hsa-mir-191	C3orf63	0.348146014	mirna_pc
27456	hsa-mir-191	SLC5A1	0.306183878	mirna_pc
27457	hsa-mir-191	ZNF184	0.30038372	mirna_pc
27458	hsa-mir-191	ATXN2	0.377629729	mirna_pc
27459	hsa-mir-191	TPRN	0.310261926	mirna_pc
27460	hsa-mir-191	LIMS3-LOC440895	0.300343844	mirna_pc
27461	hsa-mir-191	ATP7B	0.492082984	mirna_pc
27462	hsa-mir-191	CNOT10	0.431258817	mirna_pc
27463	hsa-mir-191	ECHS1	0.342717924	mirna_pc
27464	hsa-mir-191	ERN 2.00	0.305049096	mirna_pc
27465	hsa-mir-191	SFMBT1	0.586984312	mirna_pc
27466	hsa-mir-191	AKAP7	0.313660752	mirna_pc
27467	hsa-mir-191	FITM2	0.315138702	mirna_pc
27468	hsa-mir-191	ATP9A	0.302122281	mirna_pc
27469	hsa-mir-191	CHMP4C	0.309523017	mirna_pc
27470	hsa-mir-191	GGT1	0.483951881	mirna_pc
27471	hsa-mir-191	C10orf81	0.311029585	mirna_pc
27472	hsa-mir-191	C6orf130	0.417024708	mirna_pc
27473	hsa-mir-191	ZSCAN16	0.43880233	mirna_pc
27474	hsa-mir-191	SLC17A5	0.425202016	mirna_pc
27475	hsa-mir-191	RPS6KA3	0.379676864	mirna_pc
27476	hsa-mir-191	ZNF814	0.301044377	mirna_pc
27477	hsa-mir-191	DGAT1	0.33461038	mirna_pc
27478	hsa-mir-191	TDRKH	0.398772023	mirna_pc
27479	hsa-mir-191	ZNF780A	0.505584003	mirna_pc
27480	hsa-mir-191	FAM109A	0.381535595	mirna_pc
27481	hsa-mir-191	ZNF214	0.300297473	mirna_pc
27482	hsa-mir-191	NEU3	0.360346318	mirna_pc
27483	hsa-mir-191	LOC147804	0.359830953	mirna_pc
27484	hsa-mir-191	SYAP1	0.341637565	mirna_pc
27485	hsa-mir-191	DAG1	0.450939316	mirna_pc

27486	hsa-mir-191	ACAA2	0.332473725	mirna_pc
27487	hsa-mir-191	CHDH	0.641526092	mirna_pc
27488	hsa-mir-191	SLC43A3	0.38219171	mirna_pc
27489	hsa-mir-191	ZNF816A	0.391369546	mirna_pc
27490	hsa-mir-191	C2orf24	0.318980509	mirna_pc
27491	hsa-mir-191	KIF21B	0.370176715	mirna_pc
27492	hsa-mir-191	EEF1E1	0.359296554	mirna_pc
27493	hsa-mir-191	ZNF697	0.320700768	mirna_pc
27494	hsa-mir-191	MRPL44	0.410926716	mirna_pc
27495	hsa-mir-191	PTCD3	0.316398392	mirna_pc
27496	hsa-mir-191	MST1P2	0.353075256	mirna_pc
27497	hsa-mir-191	SPRY4	0.363767452	mirna_pc
27498	hsa-mir-191	PPARG	0.31448167	mirna_pc
27499	hsa-mir-191	GJB1	0.531117666	mirna_pc
27500	hsa-mir-191	ERGIC3	0.336101797	mirna_pc
27501	hsa-mir-191	CASP6	0.416290026	mirna_pc
27502	hsa-mir-191	ZNF92	0.304337666	mirna_pc
27503	hsa-mir-191	ANXA9	0.411478829	mirna_pc
27504	hsa-mir-191	TMTC4	0.343619425	mirna_pc
27505	hsa-mir-191	MMP15	0.320919997	mirna_pc
27506	hsa-mir-191	CYP2J2	0.333387732	mirna_pc
27507	hsa-mir-191	CTR9	0.308411409	mirna_pc
27508	hsa-mir-191	CDH17	0.302781958	mirna_pc
27509	hsa-mir-191	ACY1	0.456574984	mirna_pc
27510	hsa-mir-191	DNAJC1	0.35638197	mirna_pc
27511	hsa-mir-191	SDHB	0.321792728	mirna_pc
27512	hsa-mir-191	C17orf86	0.30278184	mirna_pc
27513	hsa-mir-191	ZNF259	0.340631623	mirna_pc
27514	hsa-mir-191	SSU72	0.303405055	mirna_pc
27515	hsa-mir-191	LZTFL1	0.340390317	mirna_pc
27516	hsa-mir-191	GPR114	0.330594416	mirna_pc
27517	hsa-mir-191	ALG14	0.337358212	mirna_pc
27518	hsa-mir-191	RPS6KL1	0.362841701	mirna_pc
27519	hsa-mir-191	GLYCTK	0.578631297	mirna_pc
27520	hsa-mir-191	ARF4	0.320823021	mirna_pc
27521	hsa-mir-191	ERP29	0.334836319	mirna_pc
27522	hsa-mir-191	DDAH2	0.487525522	mirna_pc
27523	hsa-mir-191	GALE	0.302412384	mirna_pc
27524	hsa-mir-191	FDXACB1	0.306918201	mirna_pc
27525	hsa-mir-191	NEK4	0.626892614	mirna_pc
27526	hsa-mir-191	TJP3	0.312071085	mirna_pc
27527	hsa-mir-191	PIGT	0.404290955	mirna_pc
27528	hsa-mir-191	CNNM3	0.534188351	mirna_pc
27529	hsa-mir-191	SIGIRR	0.329052563	mirna_pc
27530	hsa-mir-191	ALG11	0.375089918	mirna_pc
27531	hsa-mir-191	MCCC2	0.332614783	mirna_pc
27532	hsa-mir-191	TSPAN13	0.322287445	mirna_pc
27533	hsa-mir-191	MLXIPL	0.336483522	mirna_pc
27534	hsa-mir-191	HKDC1	0.359903048	mirna_pc
27535	hsa-mir-191	TMED4	0.4113986	mirna_pc
27536	hsa-mir-191	ZSWIM5	0.326407443	mirna_pc
27537	hsa-mir-191	ZNF233	0.451889426	mirna_pc
27538	hsa-mir-191	PCMTD2	0.378404033	mirna_pc
27539	hsa-mir-191	GET4	0.3636618	mirna_pc

27540	hsa-mir-191	HAAO	0.358620658	mirna_pc
27541	hsa-mir-191	PVR	0.313988804	mirna_pc
27542	hsa-mir-191	CCRL2	0.315045944	mirna_pc
27543	hsa-mir-191	DPAGT1	0.425407941	mirna_pc
27544	hsa-mir-191	YBX2	0.44615098	mirna_pc
27545	hsa-mir-191	PDDC1	0.310872374	mirna_pc
27546	hsa-mir-191	ASL	0.389617033	mirna_pc
27547	hsa-mir-191	GPR137C	0.338296911	mirna_pc
27548	hsa-mir-191	GCA	0.352503012	mirna_pc
27549	hsa-mir-191	NUP43	0.422996892	mirna_pc
27550	hsa-mir-191	MMACHC	0.4076744	mirna_pc
27551	hsa-mir-191	RTN3	0.348216459	mirna_pc
27552	hsa-mir-191	RNF41	0.396231853	mirna_pc
27553	hsa-mir-191	ZNF124	0.504287148	mirna_pc
27554	hsa-mir-191	DGAT2	0.417702844	mirna_pc
27555	hsa-mir-191	TMEM62	0.533797385	mirna_pc
27556	hsa-mir-191	EIF2C1	0.322593579	mirna_pc
27557	hsa-mir-191	ATE1	0.318648304	mirna_pc
27558	hsa-mir-191	ZNF611	0.362382935	mirna_pc
27559	hsa-mir-191	GUSB	0.364558032	mirna_pc
27560	hsa-mir-191	AIFM2	0.301237905	mirna_pc
27561	hsa-mir-191	TGOLN2	0.308876095	mirna_pc
27562	hsa-mir-191	LARS2	0.364802007	mirna_pc
27563	hsa-mir-191	GRB14	0.414346719	mirna_pc
27564	hsa-mir-191	NFYA	0.300641462	mirna_pc
27565	hsa-mir-191	CHD1L	0.439272421	mirna_pc
27566	hsa-mir-191	ZNF473	0.438487309	mirna_pc
27567	hsa-mir-191	FUCA2	0.343171863	mirna_pc
27568	hsa-mir-191	KIAA1211	0.307240185	mirna_pc
27569	hsa-mir-191	EPS8L3	0.407635285	mirna_pc
27570	hsa-mir-191	CEACAM5	0.300779894	mirna_pc
27571	hsa-mir-191	RFT1	0.459082936	mirna_pc
27572	hsa-mir-191	DCLRE1A	0.469342108	mirna_pc
27573	hsa-mir-191	RNF5	0.457356731	mirna_pc
27574	hsa-mir-191	ANP32A	0.423910372	mirna_pc
27575	hsa-mir-191	C11orf75	0.368167495	mirna_pc
27576	hsa-mir-191	ATP10B	0.306795938	mirna_pc
27577	hsa-mir-191	E2F5	0.543144629	mirna_pc
27578	hsa-mir-191	TMED5	0.302413888	mirna_pc
27579	hsa-mir-191	PIH1D1	0.316072435	mirna_pc
27580	hsa-mir-191	BACE2	0.303103808	mirna_pc
27581	hsa-mir-191	SEPHS2	0.481054095	mirna_pc
27582	hsa-mir-191	TNFRSF10D	0.309798154	mirna_pc
27583	hsa-mir-191	ZNF195	0.389204043	mirna_pc
27584	hsa-mir-191	REPIN1	0.315999188	mirna_pc
27585	hsa-mir-191	LGALS4	0.393661934	mirna_pc
27586	hsa-mir-191	P2RX4	0.351925562	mirna_pc
27587	hsa-mir-191	MANF	0.350047242	mirna_pc
27588	hsa-mir-191	ZNF320	0.361941246	mirna_pc
27589	hsa-mir-191	ACP6	0.353189863	mirna_pc
27590	hsa-mir-191	HYLS1	0.336110772	mirna_pc
27591	hsa-mir-191	HSD17B11	0.342217035	mirna_pc
27592	hsa-mir-191	HIC2	0.316401583	mirna_pc
27593	hsa-mir-191	UBE2J2	0.364346842	mirna_pc

27594	hsa-mir-191	OXER1	0.333777472	mirna_pc
27595	hsa-mir-191	C1orf93	0.34634139	mirna_pc
27596	hsa-mir-191	SMG5	0.341540707	mirna_pc
27597	hsa-mir-191	B4GALT6	0.363352462	mirna_pc
27598	hsa-mir-191	GLT8D1	0.409496268	mirna_pc
27599	hsa-mir-191	KIF3B	0.427512755	mirna_pc
27600	hsa-mir-191	NLRP2	0.325093249	mirna_pc
27601	hsa-mir-191	SERPINA1	0.325837201	mirna_pc
27602	hsa-mir-191	SFXN2	0.401165595	mirna_pc
27603	hsa-mir-191	VIL1	0.531481546	mirna_pc
27604	hsa-mir-191	ASRGL1	0.434080782	mirna_pc
27605	hsa-mir-191	USP3	0.305556323	mirna_pc
27606	hsa-mir-191	VPRBP	0.417537449	mirna_pc
27607	hsa-mir-191	TMEM170A	0.390495667	mirna_pc
27608	hsa-mir-191	GLT25D2	0.318830215	mirna_pc
27609	hsa-mir-191	PHF16	0.501211812	mirna_pc
27610	hsa-mir-191	BAT5	0.402143086	mirna_pc
27611	hsa-mir-191	ELL3	0.395715276	mirna_pc
27612	hsa-mir-191	BUD13	0.315721147	mirna_pc
27613	hsa-mir-191	PTPLAD1	0.419399237	mirna_pc
27614	hsa-mir-191	TMEM125	0.342158487	mirna_pc
27615	hsa-mir-191	MANEAL	0.436512149	mirna_pc
27616	hsa-mir-191	ZNF587	0.323258428	mirna_pc
27617	hsa-mir-191	APAF1	0.383794738	mirna_pc
27618	hsa-mir-191	GMDS	0.305214603	mirna_pc
27619	hsa-mir-191	MBNL3	0.342589385	mirna_pc
27620	hsa-mir-191	AMOT	0.326518394	mirna_pc
27621	hsa-mir-191	MNS1	0.302578962	mirna_pc
27622	hsa-mir-191	ZNF485	0.454374394	mirna_pc
27623	hsa-mir-191	KIN	0.346204874	mirna_pc
27624	hsa-mir-191	CMC1	0.428559295	mirna_pc
27625	hsa-mir-191	ABHD12	0.369070329	mirna_pc
27626	hsa-mir-191	NAIF1	0.309308073	mirna_pc
27627	hsa-mir-191	FCGRT	0.312971134	mirna_pc
27628	hsa-mir-191	RINL	0.378400716	mirna_pc
27629	hsa-mir-191	TRUB1	0.308263178	mirna_pc
27630	hsa-mir-191	C2orf15	0.556855879	mirna_pc
27631	hsa-mir-191	SLFN13	0.339654706	mirna_pc
27632	hsa-mir-191	LYRM2	0.341166869	mirna_pc
27633	hsa-mir-191	CCNJ	0.536333647	mirna_pc
27634	hsa-mir-191	FOXA2	0.484756552	mirna_pc
27635	hsa-mir-191	GALM	0.422966968	mirna_pc
27636	hsa-mir-191	C6orf64	0.34358988	mirna_pc
27637	hsa-mir-191	DNAJC16	0.378965704	mirna_pc
27638	hsa-mir-191	OTUD3	0.363256812	mirna_pc
27639	hsa-mir-191	LOC646762	0.421623287	mirna_pc
27640	hsa-mir-191	GDF15	0.440105255	mirna_pc
27641	hsa-mir-191	AFMID	0.30886144	mirna_pc
27642	hsa-mir-191	GMPPA	0.301671334	mirna_pc
27643	hsa-mir-191	DTX4	0.459293672	mirna_pc
27644	hsa-mir-191	HINFP	0.301065355	mirna_pc
27645	hsa-mir-191	C10orf137	0.397890858	mirna_pc
27646	hsa-mir-191	RPUSD3	0.308222502	mirna_pc
27647	hsa-mir-191	CAMK2N1	0.339415812	mirna_pc

27648	hsa-mir-191	LCA5L	0.302985873	mirna_pc
27649	hsa-mir-191	TMEM52	0.396054268	mirna_pc
27650	hsa-mir-191	TMEM53	0.434421291	mirna_pc
27651	hsa-mir-191	SDF4	0.338373016	mirna_pc
27652	hsa-mir-191	USH1C	0.439211186	mirna_pc
27653	hsa-mir-191	TSPAN15	0.306931401	mirna_pc
27654	hsa-mir-191	TRIM24	0.329948757	mirna_pc
27655	hsa-mir-191	IMPDH2	0.383813719	mirna_pc
27656	hsa-mir-191	FAM171A1	0.43909068	mirna_pc
27657	hsa-mir-191	CFTR	0.321861991	mirna_pc
27658	hsa-mir-191	RRBP1	0.308752469	mirna_pc
27659	hsa-mir-191	JRK	0.303593718	mirna_pc
27660	hsa-mir-191	MLEC	0.458094889	mirna_pc
27661	hsa-mir-191	ACE2	0.317952609	mirna_pc
27662	hsa-mir-191	UFC1	0.418286576	mirna_pc
27663	hsa-mir-191	CCDC134	0.35796666	mirna_pc
27664	hsa-mir-191	MOSC1	0.350065376	mirna_pc
27665	hsa-mir-191	RNF157	0.458214046	mirna_pc
27666	hsa-mir-191	RTN4IP1	0.312571497	mirna_pc
27667	hsa-mir-191	CALML4	0.325249828	mirna_pc
27668	hsa-mir-191	RAB11FIP4	0.423871491	mirna_pc
27669	hsa-mir-191	HS2ST1	0.448407422	mirna_pc
27670	hsa-mir-191	SLC25A33	0.549761156	mirna_pc
27671	hsa-mir-191	C20orf134	0.341223131	mirna_pc
27672	hsa-mir-191	CMTM8	0.480774639	mirna_pc
27673	hsa-mir-191	PLS1	0.311985604	mirna_pc
27674	hsa-mir-191	CPD	0.322795652	mirna_pc
27675	hsa-mir-191	MNX1	0.393753282	mirna_pc
27676	hsa-mir-191	ACBD5	0.410150943	mirna_pc
27677	hsa-mir-191	RAB15	0.309979783	mirna_pc
27678	hsa-mir-191	GTF2H2C	0.403584415	mirna_pc
27679	hsa-mir-191	SMARCC1	0.467629491	mirna_pc
27680	hsa-mir-191	DAK	0.302134361	mirna_pc
27681	hsa-mir-191	SUSD1	0.337387139	mirna_pc
27682	hsa-mir-191	C2orf72	0.551628481	mirna_pc
27683	hsa-mir-191	NR5A2	0.550904324	mirna_pc
27684	hsa-mir-191	C6orf192	0.348168054	mirna_pc
27685	hsa-mir-191	CTPS2	0.501540683	mirna_pc
27686	hsa-mir-191	SPHK2	0.348569147	mirna_pc
27687	hsa-mir-191	KIF9	0.551445631	mirna_pc
27688	hsa-mir-191	GXYLT1	0.455196299	mirna_pc
27689	hsa-mir-191	C10orf88	0.423737564	mirna_pc
27690	hsa-mir-191	MRPL20	0.343928596	mirna_pc
27691	hsa-mir-191	HNF1A	0.551092408	mirna_pc
27692	hsa-mir-191	TSEN2	0.429797459	mirna_pc
27693	hsa-mir-191	C20orf46	0.426916123	mirna_pc
27694	hsa-mir-191	KRT8	0.394064085	mirna_pc
27695	hsa-mir-191	PDX1	0.367969821	mirna_pc
27696	hsa-mir-191	C21orf59	0.339853953	mirna_pc
27697	hsa-mir-191	OCLN	0.41598333	mirna_pc
27698	hsa-mir-191	PPP1R16A	0.366668919	mirna_pc
27699	hsa-mir-191	GTF2H2	0.321052473	mirna_pc
27700	hsa-mir-191	ZNF232	0.32615567	mirna_pc
27701	hsa-mir-191	MDN1	0.346773947	mirna_pc

27702	hsa-mir-191	KIF12	0.412957469	mirna_pc
27703	hsa-mir-191	RNF32	0.311506361	mirna_pc
27704	hsa-mir-191	ZNF792	0.338218693	mirna_pc
27705	hsa-mir-191	BMI1	0.382600228	mirna_pc
27706	hsa-mir-191	C20orf118	0.304963361	mirna_pc
27707	hsa-mir-191	GFPT1	0.390628715	mirna_pc
27708	hsa-mir-191	METTL10	0.305242077	mirna_pc
27709	hsa-mir-191	CYB561D2	0.500882627	mirna_pc
27710	hsa-mir-191	MRPL16	0.309552868	mirna_pc
27711	hsa-mir-191	METTL12	0.410114937	mirna_pc
27712	hsa-mir-191	ENO3	0.385584183	mirna_pc
27713	hsa-mir-191	TMPRSS3	0.31016395	mirna_pc
27714	hsa-mir-191	HAPLN1	0.33014353	mirna_pc
27715	hsa-mir-191	NHEJ1	0.355076837	mirna_pc
27716	hsa-mir-191	ALDH5A1	0.344276443	mirna_pc
27717	hsa-mir-191	QRSL1	0.322919637	mirna_pc
27718	hsa-mir-191	FANCF	0.334286408	mirna_pc
27719	hsa-mir-191	MED17	0.315050383	mirna_pc
27720	hsa-mir-191	PRR13	0.367282896	mirna_pc
27721	hsa-mir-191	VPS54	0.35751021	mirna_pc
27722	hsa-mir-191	SMAD6	0.368482768	mirna_pc
27723	hsa-mir-191	ID2	0.444323003	mirna_pc
27724	hsa-mir-191	ZNF738	0.321910061	mirna_pc
27725	hsa-mir-191	C3orf75	0.49505162	mirna_pc
27726	hsa-mir-191	ZSCAN12P1	0.362799857	mirna_pc
27727	hsa-mir-191	SLC35D2	0.429398778	mirna_pc
27728	hsa-mir-191	CANT1	0.344125048	mirna_pc
27729	hsa-mir-191	CLCN5	0.396383201	mirna_pc
27730	hsa-mir-191	XYLB	0.559447741	mirna_pc
27731	hsa-mir-191	MTG1	0.37530256	mirna_pc
27732	hsa-mir-191	PPOX	0.30131228	mirna_pc
27733	hsa-mir-191	MED12	0.3280811	mirna_pc
27734	hsa-mir-191	TOE1	0.390877955	mirna_pc
27735	hsa-mir-191	HSD17B14	0.347596477	mirna_pc
27736	hsa-mir-191	SCCPDH	0.351245524	mirna_pc
27737	hsa-mir-191	ANUBL1	0.324360835	mirna_pc
27738	hsa-mir-191	B3GNT3	0.335770549	mirna_pc
27739	hsa-mir-191	NCOA5	0.317350725	mirna_pc
27740	hsa-mir-191	DFFB	0.351068274	mirna_pc
27741	hsa-mir-191	DIDO1	0.30337537	mirna_pc
27742	hsa-mir-191	TSGA10	0.330839811	mirna_pc
27743	hsa-mir-191	SPR	0.366253515	mirna_pc
27744	hsa-mir-191	CIAO1	0.421127081	mirna_pc
27745	hsa-mir-191	PLGLB2	0.352171507	mirna_pc
27746	hsa-mir-191	ULK4	0.315462422	mirna_pc
27747	hsa-mir-191	ERBB3	0.563985941	mirna_pc
27748	hsa-mir-191	ST6GAL1	0.377516966	mirna_pc
27749	hsa-mir-191	TUT1	0.356642557	mirna_pc
27750	hsa-mir-191	PLIN2	0.410414273	mirna_pc
27751	hsa-mir-191	JAGN1	0.323028441	mirna_pc
27752	hsa-mir-191	LOC100130581	0.328646521	mirna_pc
27753	hsa-mir-191	TNS3	0.416666465	mirna_pc
27754	hsa-mir-191	LASS2	0.413388639	mirna_pc
27755	hsa-mir-191	SEMA4G	0.540457229	mirna_pc

27756	hsa-mir-191	HMG20A	0.320779445	mirna_pc
27757	hsa-mir-191	FAM119A	0.322294363	mirna_pc
27758	hsa-mir-191	SYS1	0.387207094	mirna_pc
27759	hsa-mir-191	TRIM27	0.349986924	mirna_pc
27760	hsa-mir-191	CCNI2	0.515545664	mirna_pc
27761	hsa-mir-191	PDZD8	0.341491222	mirna_pc
27762	hsa-mir-191	SLC30A1	0.332121924	mirna_pc
27763	hsa-mir-191	IQCE	0.386291418	mirna_pc
27764	hsa-mir-191	ALDH18A1	0.396493898	mirna_pc
27765	hsa-mir-191	CRYZ	0.394318775	mirna_pc
27766	hsa-mir-191	NCKIPSD	0.400936848	mirna_pc
27767	hsa-mir-191	CTBP2	0.346224599	mirna_pc
27768	hsa-mir-191	HNF4A	0.409673869	mirna_pc
27769	hsa-mir-191	ZNF664	0.531240321	mirna_pc
27770	hsa-mir-191	SLC39A7	0.435029554	mirna_pc
27771	hsa-mir-191	PECR	0.415913645	mirna_pc
27772	hsa-mir-191	TMEM2	0.300773011	mirna_pc
27773	hsa-mir-191	ZNF774	0.414581504	mirna_pc
27774	hsa-mir-191	GLB1L2	0.343848721	mirna_pc
27775	hsa-mir-191	PTP4A2	0.310081324	mirna_pc
27776	hsa-mir-191	C6orf136	0.3787069	mirna_pc
27777	hsa-mir-191	CD97	0.326618943	mirna_pc
27778	hsa-mir-191	GOLT1A	0.471765837	mirna_pc
27779	hsa-mir-191	GCN1L1	0.315354094	mirna_pc
27780	hsa-mir-191	MST1	0.400295799	mirna_pc
27781	hsa-mir-191	CAPN10	0.356812979	mirna_pc
27782	hsa-mir-191	PSEN2	0.339241526	mirna_pc
27783	hsa-mir-191	THEM4	0.35415297	mirna_pc
27784	hsa-mir-191	ADAP1	0.366005031	mirna_pc
27785	hsa-mir-191	CSPP1	0.346669681	mirna_pc
27786	hsa-mir-191	C20orf96	0.492198795	mirna_pc
27787	hsa-mir-191	CBARA1	0.340299193	mirna_pc
27788	hsa-mir-191	NCRNA00095	0.357928275	mirna_pc
27789	hsa-mir-191	C1orf210	0.370560912	mirna_pc
27790	hsa-mir-191	PAPSS2	0.401546117	mirna_pc
27791	hsa-mir-191	OSBPL10	0.32283808	mirna_pc
27792	hsa-mir-191	PPP4R2	0.554333238	mirna_pc
27793	hsa-mir-191	VSIG10	0.407725665	mirna_pc
27794	hsa-mir-191	STAMBPL1	0.331565235	mirna_pc
27795	hsa-mir-191	C20orf112	0.346150378	mirna_pc
27796	hsa-mir-191	IP6K2	0.308663149	mirna_pc
27797	hsa-mir-191	KCNK5	0.304970196	mirna_pc
27798	hsa-mir-191	SHQ1	0.42792079	mirna_pc
27799	hsa-mir-191	ASXL1	0.358659746	mirna_pc
27800	hsa-mir-191	TMEM51	0.402990037	mirna_pc
27801	hsa-mir-191	SLC37A1	0.342309978	mirna_pc
27802	hsa-mir-191	SLC26A6	0.371067698	mirna_pc
27803	hsa-mir-191	HNF4G	0.364893661	mirna_pc
27804	hsa-mir-191	CALM3	0.429583423	mirna_pc
27805	hsa-mir-191	LOC81691	0.366402724	mirna_pc
27806	hsa-mir-191	ZCCHC3	0.422913142	mirna_pc
27807	hsa-mir-191	WDR73	0.412637448	mirna_pc
27808	hsa-mir-191	UBA3	0.35532691	mirna_pc
27809	hsa-mir-191	LYPD6	0.36332147	mirna_pc

27810	hsa-mir-191	GLB1	0.487262184	mirna_pc
27811	hsa-mir-191	LOC440944	0.338567405	mirna_pc
27812	hsa-mir-191	COX7A2L	0.307455954	mirna_pc
27813	hsa-mir-191	PROX1	0.46370661	mirna_pc
27814	hsa-mir-191	PCYT2	0.43006474	mirna_pc
27815	hsa-mir-191	DNAJC22	0.393033085	mirna_pc
27816	hsa-mir-191	EPHA10	0.370230314	mirna_pc
27817	hsa-mir-191	LY6G5B	0.306795947	mirna_pc
27818	hsa-mir-191	STX3	0.441787405	mirna_pc
27819	hsa-mir-191	ACP1	0.325769344	mirna_pc
27820	hsa-mir-191	C2orf43	0.346466601	mirna_pc
27821	hsa-mir-191	MARVELD2	0.327638084	mirna_pc
27822	hsa-mir-191	RNF5P1	0.444368872	mirna_pc
27823	hsa-mir-191	ZNF48	0.307262072	mirna_pc
27824	hsa-mir-191	CCDC72	0.343950396	mirna_pc
27825	hsa-mir-191	ALG1	0.309865626	mirna_pc
27826	hsa-mir-191	IL17RB	0.526922232	mirna_pc
27827	hsa-mir-191	ZNF341	0.337531278	mirna_pc
27828	hsa-mir-191	PKDCC	0.341133627	mirna_pc
27829	hsa-mir-191	FRAS1	0.350802067	mirna_pc
27830	hsa-mir-191	SEC23B	0.372161748	mirna_pc
27831	hsa-mir-191	GPKOW	0.327270186	mirna_pc
27832	hsa-mir-191	BAT4	0.311899834	mirna_pc
27833	hsa-mir-191	DDB1	0.333397755	mirna_pc
27834	hsa-mir-191	CD320	0.370744416	mirna_pc
27835	hsa-mir-191	TXLNG	0.375035305	mirna_pc
27836	hsa-mir-191	LOC80054	0.45154721	mirna_pc
27837	hsa-mir-191	C2orf34	0.332001905	mirna_pc
27838	hsa-mir-191	PVRL2	0.389812965	mirna_pc
27839	hsa-mir-191	EXOC6	0.423662635	mirna_pc
27840	hsa-mir-191	QPRT	0.3911707	mirna_pc
27841	hsa-mir-191	PREB	0.320058119	mirna_pc
27842	hsa-mir-191	ZNF443	0.401349893	mirna_pc
27843	hsa-mir-191	ZDHHC23	0.304100743	mirna_pc
27844	hsa-mir-191	PRMT6	0.429130603	mirna_pc
27845	hsa-mir-191	MAST3	0.310781032	mirna_pc
27846	hsa-mir-191	LLGL2	0.401985266	mirna_pc
27847	hsa-mir-191	CACNA1D	0.459373579	mirna_pc
27848	hsa-mir-191	NEU1	0.380474589	mirna_pc
27849	hsa-mir-191	TMED2	0.343148375	mirna_pc
27850	hsa-mir-191	COG2	0.311482754	mirna_pc
27851	hsa-mir-191	PPM1H	0.357834563	mirna_pc
27852	hsa-mir-191	SLC11A2	0.399168253	mirna_pc
27853	hsa-mir-191	SSR2	0.321303688	mirna_pc
27854	hsa-mir-191	HDAC6	0.402915874	mirna_pc
27855	hsa-mir-191	PNKD	0.597014897	mirna_pc
27856	hsa-mir-191	ZNF530	0.319580808	mirna_pc
27857	hsa-mir-191	DMXL2	0.350784825	mirna_pc
27858	hsa-mir-191	ZNF765	0.408336141	mirna_pc
27859	hsa-mir-191	TMEM97	0.375369261	mirna_pc
27860	hsa-mir-191	BMF	0.343341235	mirna_pc
27861	hsa-mir-191	VAPB	0.300530792	mirna_pc
27862	hsa-mir-191	MUC13	0.317504338	mirna_pc
27863	hsa-mir-191	PLCH1	0.390140203	mirna_pc

27864	hsa-mir-191	METTL6	0.312464458	mirna_pc
27865	hsa-mir-191	OGG1	0.332124866	mirna_pc
27866	hsa-mir-191	MIR17HG	0.31207277	mirna_pc
27867	hsa-mir-191	BAT3	0.342470123	mirna_pc
27868	hsa-mir-191	SMPDL3B	0.375739857	mirna_pc
27869	hsa-mir-191	C20orf30	0.367337528	mirna_pc
27870	hsa-mir-191	SLC29A3	0.375193741	mirna_pc
27871	hsa-mir-191	ZNF600	0.327494909	mirna_pc
27872	hsa-mir-191	AGT	0.360441317	mirna_pc
27873	hsa-mir-191	PRDX4	0.352433064	mirna_pc
27874	hsa-mir-191	GCHFR	0.359575751	mirna_pc
27875	hsa-mir-191	DDRGK1	0.310994695	mirna_pc
27876	hsa-mir-191	PARD6B	0.307615653	mirna_pc
27877	hsa-mir-191	PRDX3	0.46935106	mirna_pc
27878	hsa-mir-191	ZNF799	0.399466767	mirna_pc
27879	hsa-mir-191	ZNF239	0.524863518	mirna_pc
27880	hsa-mir-191	KLHL23	0.323826684	mirna_pc
27881	hsa-mir-191	TMEM14B	0.389380668	mirna_pc
27882	hsa-mir-191	C9orf64	0.304861014	mirna_pc
27883	hsa-mir-191	C17orf76	0.345263837	mirna_pc
27884	hsa-mir-191	GSTO2	0.365847612	mirna_pc
27885	hsa-mir-191	SRCIN1	0.476242943	mirna_pc
27886	hsa-mir-191	RBBP7	0.300865255	mirna_pc
27887	hsa-mir-191	ACSL5	0.362178635	mirna_pc
27888	hsa-mir-191	ZNF77	0.403530272	mirna_pc
27889	hsa-mir-191	PCBD1	0.43671486	mirna_pc
27890	hsa-mir-191	ZNF468	0.537475981	mirna_pc
27891	hsa-mir-191	LOC151009	0.372538057	mirna_pc
27892	hsa-mir-191	TSTA3	0.339320127	mirna_pc
27893	hsa-mir-191	SLC37A4	0.470408941	mirna_pc
27894	hsa-mir-191	EIF2S3	0.443190808	mirna_pc
27895	hsa-mir-191	TTC13	0.322921816	mirna_pc
27896	hsa-mir-191	MON1A	0.369102904	mirna_pc
27897	hsa-mir-191	CENPV	0.528967427	mirna_pc
27898	hsa-mir-191	SFRS6	0.331856341	mirna_pc
27899	hsa-mir-191	PPCDC	0.307368798	mirna_pc
27900	hsa-mir-191	TMEM63A	0.429831989	mirna_pc
27901	hsa-mir-191	ZNF592	0.31946443	mirna_pc
27902	hsa-mir-191	DNAJC11	0.336226486	mirna_pc
27903	hsa-mir-191	BCL2L14	0.320664234	mirna_pc
27904	hsa-mir-191	KIAA1107	0.339347915	mirna_pc
27905	hsa-mir-191	WDSUB1	0.378931155	mirna_pc
27906	hsa-mir-191	MYO6	0.332966796	mirna_pc
27907	hsa-mir-191	RPSAP9	0.363125978	mirna_pc
27908	hsa-mir-191	ACCN2	0.309594561	mirna_pc
27909	hsa-mir-191	NLK	0.341582872	mirna_pc
27910	hsa-mir-191	GPR35	0.399033021	mirna_pc
27911	hsa-mir-191	VARS2	0.319525214	mirna_pc
27912	hsa-mir-191	TMC4	0.319045228	mirna_pc
27913	hsa-mir-191	PATZ1	0.355073978	mirna_pc
27914	hsa-mir-191	ZNF670	0.364332275	mirna_pc
27915	hsa-mir-191	CGN	0.442597014	mirna_pc
27916	hsa-mir-191	PARK7	0.306744994	mirna_pc
27917	hsa-mir-191	C3orf33	0.314322879	mirna_pc

27918	hsa-mir-191	ZNF28	0.488410241	mirna_pc
27919	hsa-mir-191	PARS2	0.302116788	mirna_pc
27920	hsa-mir-191	COQ5	0.300456646	mirna_pc
27921	hsa-mir-191	PIGM	0.483569201	mirna_pc
27922	hsa-mir-191	PRELID2	0.321775019	mirna_pc
27923	hsa-mir-191	SLC2A8	0.344981684	mirna_pc
27924	hsa-mir-191	DOM3Z	0.445038082	mirna_pc
27925	hsa-mir-191	ARSE	0.482730406	mirna_pc
27926	hsa-mir-191	SS18L1	0.382730564	mirna_pc
27927	hsa-mir-191	ZNF768	0.302667496	mirna_pc
27928	hsa-mir-330	ANP32E	0.331323908	mirna_pc
27929	hsa-mir-330	TPM3	0.414662931	mirna_pc
27930	hsa-mir-330	BCL2L12	0.363206472	mirna_pc
27931	hsa-mir-330	PSMC4	0.392727015	mirna_pc
27932	hsa-mir-330	HOXC10	0.364145192	mirna_pc
27933	hsa-mir-330	CPSF3	0.313275394	mirna_pc
27934	hsa-mir-330	TLCD1	0.300260537	mirna_pc
27935	hsa-mir-330	MTHFR	0.324791795	mirna_pc
27936	hsa-mir-330	SNRPA1	0.378510592	mirna_pc
27937	hsa-mir-330	HSPBP1	0.334769346	mirna_pc
27938	hsa-mir-330	LYAR	0.370503783	mirna_pc
27939	hsa-mir-330	GNL2	0.317371486	mirna_pc
27940	hsa-mir-330	PTMA	0.306501065	mirna_pc
27941	hsa-mir-330	DPY30	0.30259759	mirna_pc
27942	hsa-mir-330	TARDBP	0.352428053	mirna_pc
27943	hsa-mir-330	SP140L	0.311659002	mirna_pc
27944	hsa-mir-330	EPCAM	0.301603517	mirna_pc
27945	hsa-mir-330	MIIP	0.419286056	mirna_pc
27946	hsa-mir-330	SLC39A4	0.351957633	mirna_pc
27947	hsa-mir-330	GLO1	0.305128017	mirna_pc
27948	hsa-mir-330	DDX55	0.324321175	mirna_pc
27949	hsa-mir-330	HPDL	0.3495942	mirna_pc
27950	hsa-mir-330	S100A6	0.312639123	mirna_pc
27951	hsa-mir-330	GEMIN6	0.317088381	mirna_pc
27952	hsa-mir-330	IRF3	0.315069844	mirna_pc
27953	hsa-mir-330	FARSB	0.322091016	mirna_pc
27954	hsa-mir-330	TRIM15	0.322261653	mirna_pc
27955	hsa-mir-330	MFN2	0.309583516	mirna_pc
27956	hsa-mir-330	ATAD3B	0.354295472	mirna_pc
27957	hsa-mir-330	RPF2	0.318108327	mirna_pc
27958	hsa-mir-330	NFKBIB	0.394460715	mirna_pc
27959	hsa-mir-330	PUSL1	0.397424121	mirna_pc
27960	hsa-mir-330	FAM136A	0.307475277	mirna_pc
27961	hsa-mir-330	BAIAP2L2	0.306064594	mirna_pc
27962	hsa-mir-330	TTC32	0.318690169	mirna_pc
27963	hsa-mir-330	U2AF1	0.326965136	mirna_pc
27964	hsa-mir-330	RAG1AP1	0.314827457	mirna_pc
27965	hsa-mir-330	TMCO1	0.316267379	mirna_pc
27966	hsa-mir-330	GPATCH4	0.310449667	mirna_pc
27967	hsa-mir-330	SH3BGRL3	0.305683139	mirna_pc
27968	hsa-mir-330	KRT18	0.301665822	mirna_pc
27969	hsa-mir-330	ZNRD1	0.320199642	mirna_pc
27970	hsa-mir-330	FBXO6	0.445251596	mirna_pc
27971	hsa-mir-330	EXOSC10	0.418286248	mirna_pc

27972	hsa-mir-330	UPF3B	0.352683611	mirna_pc
27973	hsa-mir-330	DECR1	0.320494235	mirna_pc
27974	hsa-mir-330	ZNF165	0.410590469	mirna_pc
27975	hsa-mir-330	APITD1	0.392109104	mirna_pc
27976	hsa-mir-330	RNF34	0.339645644	mirna_pc
27977	hsa-mir-330	GLMN	0.330860302	mirna_pc
27978	hsa-mir-330	ZNF593	0.318969282	mirna_pc
27979	hsa-mir-330	REEP6	0.325617551	mirna_pc
27980	hsa-mir-330	GEMIN7	0.333887661	mirna_pc
27981	hsa-mir-330	NAPRT1	0.406100758	mirna_pc
27982	hsa-mir-330	HIST1H1C	0.310661481	mirna_pc
27983	hsa-mir-330	UBE2CBP	0.319021378	mirna_pc
27984	hsa-mir-330	MTMR11	0.329643864	mirna_pc
27985	hsa-mir-330	CNO	0.332750988	mirna_pc
27986	hsa-mir-330	C7orf36	0.376206407	mirna_pc
27987	hsa-mir-330	KIAA2013	0.367407954	mirna_pc
27988	hsa-mir-330	SARS2	0.313707236	mirna_pc
27989	hsa-mir-330	DGAT1	0.337573316	mirna_pc
27990	hsa-mir-330	RER1	0.375217154	mirna_pc
27991	hsa-mir-330	SSU72	0.333941244	mirna_pc
27992	hsa-mir-330	PQLC2	0.303027411	mirna_pc
27993	hsa-mir-330	C6orf211	0.329009192	mirna_pc
27994	hsa-mir-330	C17orf58	0.332339453	mirna_pc
27995	hsa-mir-330	BSG	0.343358865	mirna_pc
27996	hsa-mir-330	STX18	0.331281281	mirna_pc
27997	hsa-mir-330	ZUFSP	0.309228704	mirna_pc
27998	hsa-mir-330	SPDYA	0.328183785	mirna_pc
27999	hsa-mir-330	RBM25	0.3635043	mirna_pc
28000	hsa-mir-330	RTCD1	0.328252909	mirna_pc
28001	hsa-mir-330	LGALS4	0.318927713	mirna_pc
28002	hsa-mir-330	C1orf93	0.311043521	mirna_pc
28003	hsa-mir-330	SNRNP35	0.324624882	mirna_pc
28004	hsa-mir-330	MTERF	0.314616393	mirna_pc
28005	hsa-mir-330	SDF4	0.306507923	mirna_pc
28006	hsa-mir-330	RAB13	0.30090261	mirna_pc
28007	hsa-mir-330	SLC25A33	0.379353959	mirna_pc
28008	hsa-mir-330	UBIAD1	0.369154067	mirna_pc
28009	hsa-mir-330	PEX14	0.399632529	mirna_pc
28010	hsa-mir-330	KRT8	0.305624764	mirna_pc
28011	hsa-mir-330	GTF2H2	0.301340384	mirna_pc
28012	hsa-mir-330	CPSF3L	0.304630351	mirna_pc
28013	hsa-mir-330	CLCN5	0.314898617	mirna_pc
28014	hsa-mir-330	C2orf3	0.300401428	mirna_pc
28015	hsa-mir-330	CDK11B	0.369577675	mirna_pc
28016	hsa-mir-330	CDK11A	0.402515669	mirna_pc
28017	hsa-mir-330	ZNF787	0.340059974	mirna_pc
28018	hsa-mir-330	ZNF513	0.386622817	mirna_pc
28019	hsa-mir-330	ZCCHC9	0.315350919	mirna_pc
28020	hsa-mir-330	DDX1	0.330362113	mirna_pc
28021	hsa-mir-330	ACP1	0.350392443	mirna_pc
28022	hsa-mir-330	C2orf34	0.363262184	mirna_pc
28023	hsa-mir-330	TNFRSF1B	0.312152957	mirna_pc
28024	hsa-mir-330	CXorf26	0.305039547	mirna_pc
28025	hsa-mir-330	ZNF468	0.350739534	mirna_pc

28026	hsa-mir-330	TSTA3	0.386741107	mirna_pc
28027	hsa-mir-330	AURKAIP1	0.327488892	mirna_pc
28028	hsa-mir-330	GTF2B	0.350161555	mirna_pc
28029	hsa-mir-330	GTF3C6	0.312819659	mirna_pc
28030	hsa-mir-424	TPX2	0.304467532	mirna_pc
28031	hsa-mir-424	RCC2	0.325495761	mirna_pc
28032	hsa-mir-424	UBE2C	0.365750197	mirna_pc
28033	hsa-mir-424	KIF2C	0.307662262	mirna_pc
28034	hsa-mir-424	CDC20	0.329450567	mirna_pc
28035	hsa-mir-424	CCNA2	0.337937124	mirna_pc
28036	hsa-mir-424	MND1	0.375385491	mirna_pc
28037	hsa-mir-424	RAD54L	0.321373853	mirna_pc
28038	hsa-mir-424	CENPA	0.332784928	mirna_pc
28039	hsa-mir-424	MAD2L1	0.351199941	mirna_pc
28040	hsa-mir-424	TIMELESS	0.316290081	mirna_pc
28041	hsa-mir-424	CCNB1	0.325125071	mirna_pc
28042	hsa-mir-424	STMN1	0.343137048	mirna_pc
28043	hsa-mir-424	CKS2	0.300589206	mirna_pc
28044	hsa-mir-424	CKS1B	0.407867927	mirna_pc
28045	hsa-mir-424	TUBB	0.316266044	mirna_pc
28046	hsa-mir-424	RAD51	0.333565119	mirna_pc
28047	hsa-mir-424	BIRC5	0.313826519	mirna_pc
28048	hsa-mir-424	EME1	0.32733604	mirna_pc
28049	hsa-mir-424	AURKB	0.356349211	mirna_pc
28050	hsa-mir-424	CHEK2	0.306677585	mirna_pc
28051	hsa-mir-424	C1orf112	0.344998744	mirna_pc
28052	hsa-mir-424	ORC6L	0.372287554	mirna_pc
28053	hsa-mir-424	C16orf59	0.360685644	mirna_pc
28054	hsa-mir-424	TTK	0.310008512	mirna_pc
28055	hsa-mir-424	PSMA4	0.32940518	mirna_pc
28056	hsa-mir-424	CCT5	0.401025128	mirna_pc
28057	hsa-mir-424	HNRNPC	0.335863975	mirna_pc
28058	hsa-mir-424	ILF2	0.351114266	mirna_pc
28059	hsa-mir-424	HNRNPA2B1	0.327901895	mirna_pc
28060	hsa-mir-424	RAN	0.317126976	mirna_pc
28061	hsa-mir-424	TYMS	0.31204159	mirna_pc
28062	hsa-mir-424	RNASEH2A	0.342867073	mirna_pc
28063	hsa-mir-424	EPR1	0.30386493	mirna_pc
28064	hsa-mir-424	H2AFX	0.300101947	mirna_pc
28065	hsa-mir-424	CENPN	0.312022461	mirna_pc
28066	hsa-mir-424	RANBP1	0.324470799	mirna_pc
28067	hsa-mir-424	FUS	0.379144931	mirna_pc
28068	hsa-mir-424	CENPO	0.316032807	mirna_pc
28069	hsa-mir-424	SERPINH1	0.373079445	mirna_pc
28070	hsa-mir-424	NUDT1	0.393163847	mirna_pc
28071	hsa-mir-424	CDKN1C	0.305206068	mirna_pc
28072	hsa-mir-424	GINS2	0.327075802	mirna_pc
28073	hsa-mir-424	C7orf11	0.376705519	mirna_pc
28074	hsa-mir-424	CENPH	0.331508625	mirna_pc
28075	hsa-mir-424	TCOF1	0.321911245	mirna_pc
28076	hsa-mir-424	SNRPC	0.306729123	mirna_pc
28077	hsa-mir-424	RFC4	0.394388048	mirna_pc
28078	hsa-mir-424	WDR75	0.366855247	mirna_pc
28079	hsa-mir-424	BRIX1	0.371765633	mirna_pc

28080	hsa-mir-424	CDC25A	0.319138567	mirna_pc
28081	hsa-mir-424	H2AFZ	0.304832915	mirna_pc
28082	hsa-mir-424	BANF1	0.32017549	mirna_pc
28083	hsa-mir-424	C21orf45	0.311410343	mirna_pc
28084	hsa-mir-424	DNAJB11	0.306099332	mirna_pc
28085	hsa-mir-424	NUTF2	0.379802761	mirna_pc
28086	hsa-mir-424	DAZAP1	0.337828372	mirna_pc
28087	hsa-mir-424	MRPL9	0.365170617	mirna_pc
28088	hsa-mir-424	PFDN4	0.351732558	mirna_pc
28089	hsa-mir-424	PSRC1	0.30365609	mirna_pc
28090	hsa-mir-424	MRPL47	0.356109654	mirna_pc
28091	hsa-mir-424	STIP1	0.333777395	mirna_pc
28092	hsa-mir-424	PSMG3	0.30377032	mirna_pc
28093	hsa-mir-424	HNRNPM	0.324438986	mirna_pc
28094	hsa-mir-424	C3orf26	0.341882982	mirna_pc
28095	hsa-mir-424	MRTO4	0.369231631	mirna_pc
28096	hsa-mir-424	PPIH	0.350329753	mirna_pc
28097	hsa-mir-424	CCDC58	0.328728676	mirna_pc
28098	hsa-mir-424	SKP2	0.301128835	mirna_pc
28099	hsa-mir-424	POLD2	0.318622937	mirna_pc
28100	hsa-mir-424	LYAR	0.316436251	mirna_pc
28101	hsa-mir-424	ECE2	0.313176604	mirna_pc
28102	hsa-mir-424	GPN1	0.331834222	mirna_pc
28103	hsa-mir-424	SSRP1	0.3630648	mirna_pc
28104	hsa-mir-424	PA2G4	0.364636306	mirna_pc
28105	hsa-mir-424	HOXA1	0.321854813	mirna_pc
28106	hsa-mir-424	MTL 5.00	0.330266416	mirna_pc
28107	hsa-mir-424	FSCN1	0.300646438	mirna_pc
28108	hsa-mir-424	PHF5A	0.319884499	mirna_pc
28109	hsa-mir-424	SNRNP40	0.305768703	mirna_pc
28110	hsa-mir-424	NOC2L	0.306717778	mirna_pc
28111	hsa-mir-424	NASP	0.311150037	mirna_pc
28112	hsa-mir-424	CCDC86	0.37636139	mirna_pc
28113	hsa-mir-424	C11orf84	0.398531867	mirna_pc
28114	hsa-mir-424	TBCE	0.315240917	mirna_pc
28115	hsa-mir-424	NCBP2	0.302698701	mirna_pc
28116	hsa-mir-424	SLC3A2	0.327597279	mirna_pc
28117	hsa-mir-424	DPY30	0.379280858	mirna_pc
28118	hsa-mir-424	LSM 5.00	0.306673827	mirna_pc
28119	hsa-mir-424	MEN1	0.3109981	mirna_pc
28120	hsa-mir-424	CCT4	0.39680652	mirna_pc
28121	hsa-mir-424	SAC3D1	0.305198469	mirna_pc
28122	hsa-mir-424	NAE1	0.325106606	mirna_pc
28123	hsa-mir-424	E2F4	0.320261655	mirna_pc
28124	hsa-mir-424	TRMT112	0.323803439	mirna_pc
28125	hsa-mir-424	PSMD2	0.300952549	mirna_pc
28126	hsa-mir-424	PMAIP1	0.342851984	mirna_pc
28127	hsa-mir-424	YWHAE	0.388003768	mirna_pc
28128	hsa-mir-424	C1QTNF6	0.304804471	mirna_pc
28129	hsa-mir-424	PSMD10	0.346594225	mirna_pc
28130	hsa-mir-424	LSM 6.00	0.382112194	mirna_pc
28131	hsa-mir-424	WDR74	0.344779337	mirna_pc
28132	hsa-mir-424	POLR2G	0.332810851	mirna_pc
28133	hsa-mir-424	MFAP2	0.33946653	mirna_pc

28134	hsa-mir-424	TRA2B	0.3423457	mirna_pc
28135	hsa-mir-424	C15orf23	0.364733033	mirna_pc
28136	hsa-mir-424	C3orf21	0.37130674	mirna_pc
28137	hsa-mir-424	DIABLO	0.306914995	mirna_pc
28138	hsa-mir-424	IGF2BP2	0.33027108	mirna_pc
28139	hsa-mir-424	EIF2S2	0.373416463	mirna_pc
28140	hsa-mir-424	C16orf80	0.324581056	mirna_pc
28141	hsa-mir-424	GMNN	0.33048362	mirna_pc
28142	hsa-mir-424	PA2G4P4	0.326054769	mirna_pc
28143	hsa-mir-424	MRPL11	0.334194396	mirna_pc
28144	hsa-mir-424	CRIPT	0.356378394	mirna_pc
28145	hsa-mir-424	HOXD11	0.325466574	mirna_pc
28146	hsa-mir-424	MED19	0.349415605	mirna_pc
28147	hsa-mir-424	PSMA2	0.357576842	mirna_pc
28148	hsa-mir-424	TAF5	0.368083753	mirna_pc
28149	hsa-mir-424	KARS	0.317357141	mirna_pc
28150	hsa-mir-424	FAM58A	0.374020217	mirna_pc
28151	hsa-mir-424	MAD2L2	0.393816174	mirna_pc
28152	hsa-mir-424	FJX1	0.304081804	mirna_pc
28153	hsa-mir-424	DUSP12	0.325340384	mirna_pc
28154	hsa-mir-424	C16orf87	0.320213382	mirna_pc
28155	hsa-mir-424	NOL10	0.317010275	mirna_pc
28156	hsa-mir-424	TRIAP1	0.331231487	mirna_pc
28157	hsa-mir-424	VRK1	0.314259013	mirna_pc
28158	hsa-mir-424	ANP32B	0.300849642	mirna_pc
28159	hsa-mir-424	PRPS1	0.300113548	mirna_pc
28160	hsa-mir-424	MRPL36	0.375051774	mirna_pc
28161	hsa-mir-424	TWIST1	0.324837097	mirna_pc
28162	hsa-mir-424	RPL39L	0.403111664	mirna_pc
28163	hsa-mir-424	ACD	0.378137986	mirna_pc
28164	hsa-mir-424	GPN3	0.32314801	mirna_pc
28165	hsa-mir-424	LOC152217	0.323864687	mirna_pc
28166	hsa-mir-424	NDUFS6	0.37718854	mirna_pc
28167	hsa-mir-424	MAGEF1	0.339029457	mirna_pc
28168	hsa-mir-424	PCGF6	0.304356034	mirna_pc
28169	hsa-mir-424	OSTC	0.361121108	mirna_pc
28170	hsa-mir-424	MRPS5	0.314078978	mirna_pc
28171	hsa-mir-424	RPL37	0.327651629	mirna_pc
28172	hsa-mir-424	FAM92A1	0.331438567	mirna_pc
28173	hsa-mir-424	TIMM10	0.359130212	mirna_pc
28174	hsa-mir-424	ITPRIPL1	0.311554986	mirna_pc
28175	hsa-mir-424	MRPS30	0.354712426	mirna_pc
28176	hsa-mir-424	ANAPC10	0.310111232	mirna_pc
28177	hsa-mir-424	LDHB	0.316597463	mirna_pc
28178	hsa-mir-424	SLC25A17	0.333327579	mirna_pc
28179	hsa-mir-424	PSMC3	0.37857773	mirna_pc
28180	hsa-mir-424	TRPA1	0.310248957	mirna_pc
28181	hsa-mir-424	RP9	0.385006527	mirna_pc
28182	hsa-mir-424	WDR70	0.334253517	mirna_pc
28183	hsa-mir-424	MRPL21	0.330765652	mirna_pc
28184	hsa-mir-424	FXR1	0.326841586	mirna_pc
28185	hsa-mir-424	RAD1	0.353456431	mirna_pc
28186	hsa-mir-424	C5orf13	0.301244724	mirna_pc
28187	hsa-mir-424	TARS	0.304617295	mirna_pc

28188	hsa-mir-424	GPR3	0.347091025	mirna_pc
28189	hsa-mir-424	ZCRB1	0.327139271	mirna_pc
28190	hsa-mir-424	SLC4A1AP	0.328926467	mirna_pc
28191	hsa-mir-424	TTC27	0.319608178	mirna_pc
28192	hsa-mir-424	C18orf55	0.351012501	mirna_pc
28193	hsa-mir-424	DUS2L	0.301467176	mirna_pc
28194	hsa-mir-424	COMMD9	0.300179863	mirna_pc
28195	hsa-mir-424	AAAS	0.324147795	mirna_pc
28196	hsa-mir-424	PELP1	0.344612664	mirna_pc
28197	hsa-mir-424	THOC1	0.302103142	mirna_pc
28198	hsa-mir-424	PAIP1	0.317480171	mirna_pc
28199	hsa-mir-424	COQ3	0.326175932	mirna_pc
28200	hsa-mir-424	PARL	0.324441704	mirna_pc
28201	hsa-mir-424	TMED1	0.313523028	mirna_pc
28202	hsa-mir-424	RPL35A	0.301335629	mirna_pc
28203	hsa-mir-424	NFATC3	0.324375727	mirna_pc
28204	hsa-mir-424	C12orf45	0.305493795	mirna_pc
28205	hsa-mir-424	BRD9	0.365490933	mirna_pc
28206	hsa-mir-424	PSMB6	0.340866405	mirna_pc
28207	hsa-mir-424	BASP1	0.337979301	mirna_pc
28208	hsa-mir-424	FRG1	0.36563719	mirna_pc
28209	hsa-mir-424	SUB1	0.381648395	mirna_pc
28210	hsa-mir-424	MRPS24	0.328805804	mirna_pc
28211	hsa-mir-424	PQLC2	0.317788416	mirna_pc
28212	hsa-mir-424	GPX7	0.417129926	mirna_pc
28213	hsa-mir-424	C4orf27	0.306434026	mirna_pc
28214	hsa-mir-424	DDX28	0.364836694	mirna_pc
28215	hsa-mir-424	OSGEPL1	0.315355733	mirna_pc
28216	hsa-mir-424	LMAN2L	0.304468416	mirna_pc
28217	hsa-mir-424	C17orf81	0.34976135	mirna_pc
28218	hsa-mir-424	C1D	0.328200775	mirna_pc
28219	hsa-mir-424	DDX19A	0.329414788	mirna_pc
28220	hsa-mir-424	MORN2	0.300020764	mirna_pc
28221	hsa-mir-424	DERL2	0.307105357	mirna_pc
28222	hsa-mir-424	MLLT11	0.350368655	mirna_pc
28223	hsa-mir-424	PMF1	0.329066773	mirna_pc
28224	hsa-mir-424	GALNT14	0.329885471	mirna_pc
28225	hsa-mir-424	NGFRAP1	0.325617637	mirna_pc
28226	hsa-mir-424	RANGRF	0.302156882	mirna_pc
28227	hsa-mir-424	WBP5	0.319787126	mirna_pc
28228	hsa-mir-424	RPP30	0.313629231	mirna_pc
28229	hsa-mir-424	ZNF622	0.323809398	mirna_pc
28230	hsa-mir-424	RASL11B	0.313904442	mirna_pc
28231	hsa-mir-424	PDGFA	0.31090004	mirna_pc
28232	hsa-mir-424	DULLARD	0.307686116	mirna_pc
28233	hsa-mir-424	EXOSC6	0.30780173	mirna_pc
28234	hsa-mir-424	FDX1L	0.344647159	mirna_pc
28235	hsa-mir-424	THAP11	0.355717669	mirna_pc
28236	hsa-mir-424	CLPTM1L	0.320691371	mirna_pc
28237	hsa-mir-424	NDUFB5	0.305302733	mirna_pc
28238	hsa-mir-424	H2AFY2	0.375171761	mirna_pc
28239	hsa-mir-424	C12orf43	0.304594128	mirna_pc
28240	hsa-mir-424	ALPK2	0.316918494	mirna_pc
28241	hsa-mir-424	ZCCHC17	0.315304361	mirna_pc

28242	hsa-mir-424	C18orf21	0.325173873	mirna_pc
28243	hsa-mir-424	CKAP4	0.316998789	mirna_pc
28244	hsa-mir-424	TET1	0.304973629	mirna_pc
28245	hsa-mir-424	SMYD2	0.318126196	mirna_pc
28246	hsa-mir-424	TIMM22	0.357081397	mirna_pc
28247	hsa-mir-424	MAP4K2	0.344259796	mirna_pc
28248	hsa-mir-30c-2	ZNF710-AS1	0.499660645	mirna_lnc
28249	hsa-mir-30c-2	AC116407.1	0.459251789	mirna_lnc
28250	hsa-mir-30c-2	AC004836.1	0.470363361	mirna_lnc
28251	hsa-mir-30c-2	C9orf147	0.408513497	mirna_lnc
28252	hsa-mir-30c-2	AC012409.3	0.519749423	mirna_lnc
28253	hsa-mir-30c-2	AC025271.4	0.592205729	mirna_lnc
28254	hsa-mir-30c-2	AC123912.4	0.44184452	mirna_lnc
28255	hsa-mir-30c-2	AP001148.1	0.540593893	mirna_lnc
28256	hsa-mir-30c-2	AC015908.3	0.454098591	mirna_lnc
28257	hsa-mir-30c-2	BX284668.2	0.361464522	mirna_lnc
28258	hsa-mir-30c-2	AL158847.1	0.548144048	mirna_lnc
28259	hsa-mir-30c-2	AC024337.2	0.362163107	mirna_lnc
28260	hsa-mir-30c-2	AL158163.2	0.561439758	mirna_lnc
28261	hsa-mir-30c-2	LINC00940	0.50986163	mirna_lnc
28262	hsa-mir-30c-2	AC093583.1	0.462396486	mirna_lnc
28263	hsa-mir-30c-2	AC239798.2	0.508381927	mirna_lnc
28264	hsa-mir-30c-2	AL157392.2	0.431185073	mirna_lnc
28265	hsa-mir-30c-2	AP001528.3	0.35909057	mirna_lnc
28266	hsa-mir-30c-2	AC012409.5	0.364911701	mirna_lnc
28267	hsa-mir-30c-2	AP001528.2	0.416870344	mirna_lnc
28268	hsa-mir-30c-2	AC063919.1	0.42039342	mirna_lnc
28269	hsa-mir-30c-2	AC093797.1	0.319092832	mirna_lnc
28270	hsa-mir-30c-2	AC016251.1	0.413523654	mirna_lnc
28271	hsa-mir-30c-2	AC108472.1	0.321475423	mirna_lnc
28272	hsa-mir-30c-2	AC124312.3	0.372497999	mirna_lnc
28273	hsa-mir-30c-2	AC091563.1	0.342587585	mirna_lnc
28274	hsa-mir-30c-2	NGF-AS1	0.452808751	mirna_lnc
28275	hsa-mir-30c-2	AC244453.3	0.505444338	mirna_lnc
28276	hsa-mir-30c-2	LINC02489	0.360018423	mirna_lnc
28277	hsa-mir-30c-2	AL138847.2	0.560629183	mirna_lnc
28278	hsa-mir-30c-2	AL158206.1	0.354267016	mirna_lnc
28279	hsa-mir-30c-2	AP003117.1	0.496809102	mirna_lnc
28280	hsa-mir-30c-2	AL158163.1	0.558244541	mirna_lnc
28281	hsa-mir-30c-2	AP001972.5	0.358004738	mirna_lnc
28282	hsa-mir-30c-2	AC021016.2	0.486078264	mirna_lnc
28283	hsa-mir-30c-2	AC105074.1	0.349636934	mirna_lnc
28284	hsa-mir-30c-2	AC004982.1	0.497042699	mirna_lnc
28285	hsa-mir-30c-2	AL512328.1	0.374594656	mirna_lnc
28286	hsa-mir-30c-2	SPIN4-AS1	0.323107603	mirna_lnc
28287	hsa-mir-30c-2	AC073862.3	0.447422555	mirna_lnc
28288	hsa-mir-30c-2	AC091057.3	0.400838972	mirna_lnc
28289	hsa-mir-30c-2	AC079061.1	0.418767743	mirna_lnc
28290	hsa-mir-30c-2	LINC02447	0.382355591	mirna_lnc
28291	hsa-mir-30c-2	AP001094.2	0.467979683	mirna_lnc
28292	hsa-mir-30c-2	AC091544.5	0.32057192	mirna_lnc
28293	hsa-mir-30c-2	AC013726.1	0.478266242	mirna_lnc
28294	hsa-mir-30c-2	LINC01783	0.378962336	mirna_lnc
28295	hsa-mir-30c-2	AL031429.2	0.52328246	mirna_lnc

28296	hsa-mir-30c-2	AC022558.2	0.307674673	mirna_lnc
28297	hsa-mir-30c-2	AL355073.1	0.360846142	mirna_lnc
28298	hsa-mir-30c-2	AC007193.2	0.323443058	mirna_lnc
28299	hsa-mir-30c-2	AC021766.1	0.336155981	mirna_lnc
28300	hsa-mir-30c-2	IDH2-DT	0.480918509	mirna_lnc
28301	hsa-mir-30c-2	AC246680.1	0.482836695	mirna_lnc
28302	hsa-mir-30c-2	LINC01985	0.310318603	mirna_lnc
28303	hsa-mir-30c-2	AL158212.3	0.302117784	mirna_lnc
28304	hsa-mir-30c-2	AL023973.1	0.316854215	mirna_lnc
28305	hsa-mir-30c-2	AC005225.4	0.352959404	mirna_lnc
28306	hsa-mir-30c-2	AC023794.6	0.385161155	mirna_lnc
28307	hsa-mir-30c-2	AC114810.1	0.437939176	mirna_lnc
28308	hsa-mir-30c-2	AL136164.3	0.497829019	mirna_lnc
28309	hsa-mir-30c-2	AC009078.3	0.321418467	mirna_lnc
28310	hsa-mir-30c-2	AL109936.2	0.428274672	mirna_lnc
28311	hsa-mir-30c-2	AC129492.3	0.493929041	mirna_lnc
28312	hsa-mir-30c-2	AC012409.1	0.492295439	mirna_lnc
28313	hsa-mir-30c-2	TRG-AS1	0.437371308	mirna_lnc
28314	hsa-mir-30c-2	RPL34-AS1	0.443874842	mirna_lnc
28315	hsa-mir-30c-2	AC007637.1	0.378078189	mirna_lnc
28316	hsa-mir-30c-2	AP001972.1	0.344227976	mirna_lnc
28317	hsa-mir-30c-2	AC244453.1	0.45312088	mirna_lnc
28318	hsa-mir-30c-2	MAGI1-AS1	0.378543174	mirna_lnc
28319	hsa-mir-30c-2	SEMA3B-AS1	0.530534095	mirna_lnc
28320	hsa-mir-30c-2	LINC00365	0.503763238	mirna_lnc
28321	hsa-mir-30c-2	HCG21	0.426364015	mirna_lnc
28322	hsa-mir-30c-2	AC132154.1	0.49546877	mirna_lnc
28323	hsa-mir-30c-2	AL138808.1	0.35403387	mirna_lnc
28324	hsa-mir-30c-2	NCOA7-AS1	0.519801329	mirna_lnc
28325	hsa-mir-30c-2	AC008669.1	0.42995727	mirna_lnc
28326	hsa-mir-30c-2	AC024075.1	0.352395405	mirna_lnc
28327	hsa-mir-30c-2	FAM13A-AS1	0.37245695	mirna_lnc
28328	hsa-mir-30c-2	AC244131.2	0.325276282	mirna_lnc
28329	hsa-mir-30c-2	AC129492.5	0.368197196	mirna_lnc
28330	hsa-mir-30c-2	LINC02435	0.397570716	mirna_lnc
28331	hsa-mir-30c-2	PCAT18	0.519928031	mirna_lnc
28332	hsa-mir-30c-2	AL136309.2	0.344837981	mirna_lnc
28333	hsa-mir-30c-2	AL035701.1	0.354444371	mirna_lnc
28334	hsa-mir-30c-2	AC118344.2	0.38936191	mirna_lnc
28335	hsa-mir-30c-2	AC138819.1	0.382129966	mirna_lnc
28336	hsa-mir-30c-2	AC018926.1	0.483976923	mirna_lnc
28337	hsa-mir-30c-2	SH3BP5-AS1	0.435081004	mirna_lnc
28338	hsa-mir-30c-2	AL031587.5	0.372130491	mirna_lnc
28339	hsa-mir-30c-2	AC244453.2	0.493268243	mirna_lnc
28340	hsa-mir-30c-2	AC025470.2	0.339473512	mirna_lnc
28341	hsa-mir-30c-2	UGDH-AS1	0.432562654	mirna_lnc
28342	hsa-mir-30c-2	AL109659.2	0.425746154	mirna_lnc
28343	hsa-mir-30c-2	FOXP1-AS1	0.404883048	mirna_lnc
28344	hsa-mir-30c-2	AC024075.2	0.435891449	mirna_lnc
28345	hsa-mir-30c-2	AC037459.2	0.384904485	mirna_lnc
28346	hsa-mir-30c-2	AC005495.1	0.328942872	mirna_lnc
28347	hsa-mir-30c-2	DNAJC9-AS1	0.431894191	mirna_lnc
28348	hsa-mir-30c-2	AL020997.3	0.523024075	mirna_lnc
28349	hsa-mir-30c-2	AL035448.1	0.473967126	mirna_lnc

28350	hsa-mir-30c-2	PSMD6-AS2	0.407805133	mirna_lnc
28351	hsa-mir-30c-2	AP003355.2	0.309315274	mirna_lnc
28352	hsa-mir-30c-2	AC012557.1	0.437108357	mirna_lnc
28353	hsa-mir-30c-2	AL137793.1	0.351734121	mirna_lnc
28354	hsa-mir-30c-2	AL160286.2	0.428701671	mirna_lnc
28355	hsa-mir-30c-2	AC025917.1	0.361009085	mirna_lnc
28356	hsa-mir-30c-2	AC009119.3	0.362065202	mirna_lnc
28357	hsa-mir-30c-2	PWAR5	0.306767356	mirna_lnc
28358	hsa-mir-30c-2	AC098869.2	0.324938069	mirna_lnc
28359	hsa-mir-30c-2	AC021491.2	0.340745111	mirna_lnc
28360	hsa-mir-30c-2	AC139887.4	0.443551531	mirna_lnc
28361	hsa-mir-30c-2	AP003486.1	0.319062729	mirna_lnc
28362	hsa-mir-30c-2	AC138956.2	0.421305346	mirna_lnc
28363	hsa-mir-30c-2	AC021205.3	0.408367526	mirna_lnc
28364	hsa-mir-30c-2	AC006942.1	0.401424002	mirna_lnc
28365	hsa-mir-30c-2	AL731557.1	0.369773391	mirna_lnc
28366	hsa-mir-30c-2	AL391834.2	0.308384138	mirna_lnc
28367	hsa-mir-30c-2	AL138930.1	0.463783983	mirna_lnc
28368	hsa-mir-30c-2	AC093607.1	0.333112981	mirna_lnc
28369	hsa-mir-30c-2	AC006946.3	0.385840017	mirna_lnc
28370	hsa-mir-30c-2	CACNA1C-AS2	0.316524543	mirna_lnc
28371	hsa-mir-30c-2	AC012181.1	0.386218202	mirna_lnc
28372	hsa-mir-30c-2	AC073476.3	0.387319758	mirna_lnc
28373	hsa-mir-30c-2	AC111182.1	0.488017102	mirna_lnc
28374	hsa-mir-30c-2	AC011816.2	0.320178142	mirna_lnc
28375	hsa-mir-30c-2	PACRG-AS1	0.334742864	mirna_lnc
28376	hsa-mir-30c-2	Z69706.1	0.368233686	mirna_lnc
28377	hsa-mir-30c-2	AC010776.2	0.392840887	mirna_lnc
28378	hsa-mir-30c-2	AC024075.3	0.319369021	mirna_lnc
28379	hsa-mir-30c-2	AC100823.1	0.510955004	mirna_lnc
28380	hsa-mir-30c-2	AC012181.2	0.31736611	mirna_lnc
28381	hsa-mir-30c-2	AC008121.2	0.363988203	mirna_lnc
28382	hsa-mir-30c-2	AC010632.1	0.313157447	mirna_lnc
28383	hsa-mir-30c-2	AC022893.1	0.455594407	mirna_lnc
28384	hsa-mir-30c-2	AC112236.2	0.314477642	mirna_lnc
28385	hsa-mir-30c-2	AC018926.3	0.511438508	mirna_lnc
28386	hsa-mir-30c-2	AC083843.4	0.322834146	mirna_lnc
28387	hsa-mir-30c-2	AC012409.2	0.35929337	mirna_lnc
28388	hsa-mir-30c-2	AC079305.3	0.507247342	mirna_lnc
28389	hsa-mir-30c-2	AC018445.4	0.303882722	mirna_lnc
28390	hsa-mir-30c-2	CEBPB-AS1	0.389598315	mirna_lnc
28391	hsa-mir-30c-2	AL162171.3	0.368115455	mirna_lnc
28392	hsa-mir-30c-2	LINC02060	0.303465217	mirna_lnc
28393	hsa-mir-30c-2	AC023830.3	0.331241083	mirna_lnc
28394	hsa-mir-30c-2	AC130650.1	0.338864339	mirna_lnc
28395	hsa-mir-30c-2	AC068880.4	0.300562837	mirna_lnc
28396	hsa-mir-30c-2	AC009090.3	0.387204425	mirna_lnc
28397	hsa-mir-30c-2	AC108449.2	0.303432184	mirna_lnc
28398	hsa-mir-30c-2	AC007950.3	0.324210025	mirna_lnc
28399	hsa-mir-30c-2	AC109454.4	0.411761878	mirna_lnc
28400	hsa-mir-30c-2	AC007342.6	0.330224943	mirna_lnc
28401	hsa-mir-30c-2	AC104117.3	0.320634999	mirna_lnc
28402	hsa-mir-30c-2	AL031726.1	0.351674528	mirna_lnc
28403	hsa-mir-30c-2	LOH12CR2	0.437115832	mirna_lnc

28404	hsa-mir-30c-2	AC010136.1	0.309926071	mirna_lnc
28405	hsa-mir-30c-2	ZRANB2-AS1	0.498012905	mirna_lnc
28406	hsa-mir-30c-2	AC016924.1	0.365327787	mirna_lnc
28407	hsa-mir-30c-2	AC008764.2	0.30026458	mirna_lnc
28408	hsa-mir-30c-2	AC010226.1	0.34542538	mirna_lnc
28409	hsa-mir-30c-2	AC135050.6	0.344683227	mirna_lnc
28410	hsa-mir-30c-2	AC095057.3	0.508670017	mirna_lnc
28411	hsa-mir-30c-2	AC015961.1	0.350532859	mirna_lnc
28412	hsa-mir-30c-2	C9orf106	0.325646656	mirna_lnc
28413	hsa-mir-30c-2	KIF9-AS1	0.303151855	mirna_lnc
28414	hsa-mir-30c-2	AL356273.3	0.414776988	mirna_lnc
28415	hsa-mir-30c-2	AC107072.2	0.3001112	mirna_lnc
28416	hsa-mir-30c-2	AC010997.6	0.322081989	mirna_lnc
28417	hsa-mir-30c-2	LINC01954	0.38817808	mirna_lnc
28418	hsa-mir-30c-2	AC020951.1	0.319229834	mirna_lnc
28419	hsa-mir-30c-2	AP003721.4	0.399592712	mirna_lnc
28420	hsa-mir-30c-2	AC010210.1	0.321639043	mirna_lnc
28421	hsa-mir-30c-2	AC008663.2	0.331976707	mirna_lnc
28422	hsa-mir-30c-2	HEXA-AS1	0.304276195	mirna_lnc
28423	hsa-mir-30c-2	AC005746.1	0.324726868	mirna_lnc
28424	hsa-mir-30c-2	AL359715.3	0.34402204	mirna_lnc
28425	hsa-mir-30c-2	AC010201.2	0.487771359	mirna_lnc
28426	hsa-mir-30c-2	AC016251.2	0.319802957	mirna_lnc
28427	hsa-mir-30c-2	AC006960.3	0.310361467	mirna_lnc
28428	hsa-mir-30c-2	ERVE-1	0.388177032	mirna_lnc
28429	hsa-mir-30c-2	AC073167.1	0.346109646	mirna_lnc
28430	hsa-mir-30c-2	AC015961.2	0.367077532	mirna_lnc
28431	hsa-mir-30c-2	SSBP3-AS1	0.428039598	mirna_lnc
28432	hsa-mir-30c-2	Z97989.1	0.383662364	mirna_lnc
28433	hsa-mir-30c-2	AC064807.2	0.474477444	mirna_lnc
28434	hsa-mir-30c-2	AC084036.1	0.522367842	mirna_lnc
28435	hsa-mir-30c-2	AC079322.1	0.454148284	mirna_lnc
28436	hsa-mir-30c-2	AC022898.2	0.398818577	mirna_lnc
28437	hsa-mir-30c-2	DCXR-DT	0.305574899	mirna_lnc
28438	hsa-mir-30c-2	AL445223.1	0.337236131	mirna_lnc
28439	hsa-mir-30c-2	AL391834.1	0.301504741	mirna_lnc
28440	hsa-mir-30c-2	AC091588.1	0.350059679	mirna_lnc
28441	hsa-mir-30c-2	Z73965.1	0.402451459	mirna_lnc
28442	hsa-mir-30c-2	AC139768.2	0.324497584	mirna_lnc
28443	hsa-mir-30c-2	AL136982.3	0.411197473	mirna_lnc
28444	hsa-mir-30c-2	AC036176.1	0.349416831	mirna_lnc
28445	hsa-mir-30c-2	AC139795.2	0.34819624	mirna_lnc
28446	hsa-mir-30c-2	AC053513.1	0.370064762	mirna_lnc
28447	hsa-mir-30c-2	AC138956.1	0.347462249	mirna_lnc
28448	hsa-mir-30c-2	AC007342.7	0.375310912	mirna_lnc
28449	hsa-mir-30c-2	AC009090.1	0.358550613	mirna_lnc
28450	hsa-mir-30c-2	AC010201.1	0.423744896	mirna_lnc
28451	hsa-mir-30c-2	AC074032.1	0.363708602	mirna_lnc
28452	hsa-mir-30c-2	AC027117.2	0.370685589	mirna_lnc
28453	hsa-mir-30c-2	L3MBTL4-AS1	0.401462754	mirna_lnc
28454	hsa-mir-30c-2	AC125437.2	0.404093029	mirna_lnc
28455	hsa-mir-30c-2	AC124312.2	0.424696868	mirna_lnc
28456	hsa-mir-30c-2	AC100830.2	0.368801478	mirna_lnc
28457	hsa-mir-30c-2	AC024580.1	0.406425207	mirna_lnc

28458	hsa-mir-30c-2	AC008731.1	0.315754231	mirna_lnc
28459	hsa-mir-30c-2	AL021368.2	0.350299802	mirna_lnc
28460	hsa-mir-30c-2	AC012358.1	0.430346536	mirna_lnc
28461	hsa-mir-30c-2	AC108704.2	0.392743632	mirna_lnc
28462	hsa-mir-30c-2	AC084018.2	0.325475964	mirna_lnc
28463	hsa-mir-30c-2	Z97652.1	0.395350519	mirna_lnc
28464	hsa-mir-30c-2	AL356740.1	0.315948687	mirna_lnc
28465	hsa-mir-30c-2	AC016590.2	0.302325438	mirna_lnc
28466	hsa-mir-30c-2	AL117339.4	0.355136594	mirna_lnc
28467	hsa-mir-30c-2	AL008582.1	0.328169219	mirna_lnc
28468	hsa-mir-30c-2	AC093726.1	0.518972683	mirna_lnc
28469	hsa-mir-30c-2	AC008121.3	0.336025421	mirna_lnc
28470	hsa-mir-30c-2	AL034347.1	0.427652978	mirna_lnc
28471	hsa-mir-30c-2	AC006116.4	0.377969757	mirna_lnc
28472	hsa-mir-30c-2	AC010525.1	0.316941592	mirna_lnc
28473	hsa-mir-30c-2	AC121761.2	0.328237332	mirna_lnc
28474	hsa-mir-30c-2	AL121895.2	0.405477052	mirna_lnc
28475	hsa-mir-30c-2	AL365330.1	0.382559278	mirna_lnc
28476	hsa-mir-30c-2	AC127164.1	0.350713046	mirna_lnc
28477	hsa-mir-30c-2	AC009118.3	0.443341149	mirna_lnc
28478	hsa-mir-30c-2	AC078906.1	0.519749418	mirna_lnc
28479	hsa-mir-30c-2	AC013356.3	0.305153396	mirna_lnc
28480	hsa-mir-30c-2	AL121655.1	0.374623935	mirna_lnc
28481	hsa-mir-30c-2	AC007878.1	0.39349629	mirna_lnc
28482	hsa-mir-30c-2	POU6F2-AS1	0.47636783	mirna_lnc
28483	hsa-mir-30c-2	AP002812.3	0.342426823	mirna_lnc
28484	hsa-mir-30c-2	AC007787.2	0.338371533	mirna_lnc
28485	hsa-mir-30c-2	OCIAD1-AS1	0.338692201	mirna_lnc
28486	hsa-mir-30c-2	AP000766.1	0.36276542	mirna_lnc
28487	hsa-mir-30c-2	AC004241.2	0.310729447	mirna_lnc
28488	hsa-mir-30c-2	AC027279.1	0.346956314	mirna_lnc
28489	hsa-mir-30c-2	AL080317.3	0.326973025	mirna_lnc
28490	hsa-mir-30c-2	AC112722.1	0.358164372	mirna_lnc
28491	hsa-mir-30c-2	AL355073.2	0.363021922	mirna_lnc
28492	hsa-mir-30c-2	AL162511.1	0.444043878	mirna_lnc
28493	hsa-mir-30c-2	AL157762.1	0.303735232	mirna_lnc
28494	hsa-mir-30c-2	AC107952.2	0.399823382	mirna_lnc
28495	hsa-mir-30c-2	LINC02367	0.330205893	mirna_lnc
28496	hsa-mir-30c-2	AC027698.1	0.321738451	mirna_lnc
28497	hsa-mir-30c-2	AC011815.3	0.350102375	mirna_lnc
28498	hsa-mir-30c-2	AL157823.2	0.417473111	mirna_lnc
28499	hsa-mir-30c-2	AC092279.2	0.301278666	mirna_lnc
28500	hsa-mir-30c-2	AC114730.3	0.335240646	mirna_lnc
28501	hsa-mir-30c-2	AC012404.2	0.312500876	mirna_lnc
28502	hsa-mir-30c-2	AC011944.2	0.342062695	mirna_lnc
28503	hsa-mir-30c-2	AL445686.2	0.31177873	mirna_lnc
28504	hsa-mir-30c-2	AC108861.1	0.345544014	mirna_lnc
28505	hsa-mir-30c-2	AC012360.3	0.357530078	mirna_lnc
28506	hsa-mir-30c-2	AC125494.2	0.371504054	mirna_lnc
28507	hsa-mir-30c-2	AP001094.3	0.507940859	mirna_lnc
28508	hsa-mir-30c-2	AC008079.1	0.344546166	mirna_lnc
28509	hsa-mir-30c-2	AC011815.2	0.316112006	mirna_lnc
28510	hsa-mir-30c-2	AC024941.1	0.400228874	mirna_lnc
28511	hsa-mir-30c-2	AC016705.2	0.363267838	mirna_lnc

28512	hsa-mir-30c-2	AC007038.1	0.361154695	mirna_lnc
28513	hsa-mir-30c-2	AC006116.10	0.55542668	mirna_lnc
28514	hsa-mir-30c-2	AC015726.1	0.388683872	mirna_lnc
28515	hsa-mir-30c-2	RRS1-AS1	0.396493905	mirna_lnc
28516	hsa-mir-30c-2	AC139887.1	0.42902802	mirna_lnc
28517	hsa-mir-30c-2	AC092620.1	0.315525038	mirna_lnc
28518	hsa-mir-30c-2	AC007619.1	0.423251141	mirna_lnc
28519	hsa-mir-30c-2	FOXP1-IT1	0.327639253	mirna_lnc
28520	hsa-mir-30c-2	AC097460.3	0.330864939	mirna_lnc
28521	hsa-mir-30c-2	AF129075.1	0.357212432	mirna_lnc
28522	hsa-mir-30c-2	AL353746.1	0.417039857	mirna_lnc
28523	hsa-mir-30c-2	AL133243.2	0.354433571	mirna_lnc
28524	hsa-mir-30c-2	AP002812.2	0.327991001	mirna_lnc
28525	hsa-mir-30c-2	AC018638.6	0.354768673	mirna_lnc
28526	hsa-mir-30c-2	AC109347.2	0.403649906	mirna_lnc
28527	hsa-mir-30c-2	AC091965.5	0.349030925	mirna_lnc
28528	hsa-mir-30c-2	AC018638.7	0.444366452	mirna_lnc
28529	hsa-mir-30c-2	AC092343.1	0.304111367	mirna_lnc
28530	hsa-mir-30c-2	AC013468.1	0.453161325	mirna_lnc
28531	hsa-mir-30c-2	UMODL1-AS1	0.335124844	mirna_lnc
28532	hsa-mir-30c-2	LINC02640	0.433962104	mirna_lnc
28533	hsa-mir-30c-2	AC021483.1	0.319580729	mirna_lnc
28534	hsa-mir-30c-2	EIF3J-DT	0.319875485	mirna_lnc
28535	hsa-mir-30c-2	AC093690.1	0.3537342	mirna_lnc
28536	hsa-mir-30c-2	AL117350.1	0.379662039	mirna_lnc
28537	hsa-mir-30c-2	AL157392.4	0.338013533	mirna_lnc
28538	hsa-mir-30c-2	AC023509.2	0.353157814	mirna_lnc
28539	hsa-mir-30c-2	AC084262.1	0.451632441	mirna_lnc
28540	hsa-mir-30c-2	AC026992.2	0.30789251	mirna_lnc
28541	hsa-mir-30c-2	AC012074.1	0.338240732	mirna_lnc
28542	hsa-mir-30c-2	LINC01772	0.350273371	mirna_lnc
28543	hsa-mir-30c-2	AC105339.5	0.367143436	mirna_lnc
28544	hsa-mir-30c-2	AC007684.1	0.38750238	mirna_lnc
28545	hsa-mir-30c-2	AC138512.1	0.395574851	mirna_lnc
28546	hsa-mir-30c-2	AC093330.1	0.399676595	mirna_lnc
28547	hsa-mir-30c-2	AC010186.3	0.33123149	mirna_lnc
28548	hsa-mir-30c-2	AC118344.1	0.315397396	mirna_lnc
28549	hsa-mir-30c-2	AL604028.1	0.313821368	mirna_lnc
28550	hsa-mir-30c-2	AC022306.2	0.365126719	mirna_lnc
28551	hsa-mir-30c-2	AC016722.2	0.442137537	mirna_lnc
28552	hsa-mir-30c-2	AC005674.2	0.311288946	mirna_lnc
28553	hsa-mir-30c-2	AC097634.1	0.373610001	mirna_lnc
28554	hsa-mir-30c-2	AC092692.1	0.340644833	mirna_lnc
28555	hsa-mir-30c-2	AC093227.1	0.353879582	mirna_lnc
28556	hsa-mir-30c-2	AC104113.1	0.300852622	mirna_lnc
28557	hsa-mir-30c-2	AL357874.2	0.341043837	mirna_lnc
28558	hsa-mir-30c-2	AC022898.1	0.334402672	mirna_lnc
28559	hsa-mir-30c-2	AL645568.1	0.323010427	mirna_lnc
28560	hsa-mir-30c-2	AC084824.3	0.387802927	mirna_lnc
28561	hsa-mir-30c-2	AC020612.3	0.309489747	mirna_lnc
28562	hsa-mir-30c-2	AC079145.1	0.410057384	mirna_lnc
28563	hsa-mir-30c-2	LIMD1-AS1	0.319241286	mirna_lnc
28564	hsa-mir-30c-2	AL356124.2	0.45084582	mirna_lnc
28565	hsa-mir-30c-2	AC093726.2	0.429074399	mirna_lnc

28566	hsa-mir-30c-2	LINC02234	0.440509909	mirna_lnc
28567	hsa-mir-30c-2	TET2-AS1	0.313427908	mirna_lnc
28568	hsa-mir-30c-2	TMCO1-AS1	0.342377285	mirna_lnc
28569	hsa-mir-30c-2	AL359710.1	0.365662881	mirna_lnc
28570	hsa-mir-30c-2	AL121652.1	0.352573266	mirna_lnc
28571	hsa-mir-30c-2	AP001094.1	0.364768845	mirna_lnc
28572	hsa-mir-30c-2	AC022497.1	0.446930639	mirna_lnc
28573	hsa-mir-30c-2	AL008721.2	0.335225725	mirna_lnc
28574	hsa-mir-30c-2	AC114939.1	0.391909886	mirna_lnc
28575	hsa-mir-30c-2	AC084782.3	0.355542258	mirna_lnc
28576	hsa-mir-30c-2	AL132656.2	0.34770947	mirna_lnc
28577	hsa-mir-30c-2	AC000124.1	0.319504062	mirna_lnc
28578	hsa-mir-30c-2	AC009318.3	0.403050358	mirna_lnc
28579	hsa-mir-30c-2	AC090206.1	0.38637196	mirna_lnc
28580	hsa-mir-30c-2	AC234775.3	0.376374555	mirna_lnc
28581	hsa-mir-30c-2	FOXN3-AS2	0.38231909	mirna_lnc
28582	hsa-mir-30c-2	AL109947.1	0.312122158	mirna_lnc
28583	hsa-mir-30c-2	AC073896.3	0.46798138	mirna_lnc
28584	hsa-mir-30c-2	AC005034.5	0.3397978	mirna_lnc
28585	hsa-mir-30c-2	AL354872.2	0.346323298	mirna_lnc
28586	hsa-mir-30c-2	AL513365.2	0.356742124	mirna_lnc
28587	hsa-mir-30c-2	DPH6-DT	0.316121471	mirna_lnc
28588	hsa-mir-30c-2	AC090772.2	0.359721516	mirna_lnc
28589	hsa-mir-30c-2	SLFNL1-AS1	0.300749283	mirna_lnc
28590	hsa-mir-30c-2	LINC00653	0.346504706	mirna_lnc
28591	hsa-mir-30c-2	AC055822.1	0.350411144	mirna_lnc
28592	hsa-mir-30c-2	AC092718.1	0.343040101	mirna_lnc
28593	hsa-mir-30c-2	AL132989.1	0.331130674	mirna_lnc
28594	hsa-mir-30c-2	AC012435.2	0.306542388	mirna_lnc
28595	hsa-mir-30c-2	AC105339.3	0.338197783	mirna_lnc
28596	hsa-mir-30c-2	AC103923.1	0.307549895	mirna_lnc
28597	hsa-mir-30c-2	AL606760.2	0.401982269	mirna_lnc
28598	hsa-mir-30c-2	HGC6.3	0.352420623	mirna_lnc
28599	hsa-mir-30c-2	AC135586.2	0.450161231	mirna_lnc
28600	hsa-mir-30c-2	AC127024.4	0.316506956	mirna_lnc
28601	hsa-mir-30c-2	AC005838.2	0.452762019	mirna_lnc
28602	hsa-mir-30c-2	AC025430.1	0.31460995	mirna_lnc
28603	hsa-mir-30c-2	AC074029.3	0.368208673	mirna_lnc
28604	hsa-mir-30c-2	Z93930.3	0.307738543	mirna_lnc
28605	hsa-mir-30c-2	AC073534.2	0.435022033	mirna_lnc
28606	hsa-mir-30c-2	AC006206.1	0.303349768	mirna_lnc
28607	hsa-mir-30c-2	AC006213.5	0.381232845	mirna_lnc
28608	hsa-mir-30c-2	UBE2D3-AS1	0.345772287	mirna_lnc
28609	hsa-mir-30c-2	AC022400.6	0.378169568	mirna_lnc
28610	hsa-mir-30c-2	AC027682.7	0.344727572	mirna_lnc
28611	hsa-mir-30c-2	AC010326.3	0.312690122	mirna_lnc
28612	hsa-mir-30c-2	CHRM3-AS1	0.303968923	mirna_lnc
28613	hsa-mir-30c-2	AC135776.1	0.318766915	mirna_lnc
28614	hsa-mir-30c-2	AC016747.3	0.404004694	mirna_lnc
28615	hsa-mir-30c-2	NADK2-AS1	0.391707826	mirna_lnc
28616	hsa-mir-30c-2	AC126407.1	0.321565377	mirna_lnc
28617	hsa-mir-30c-2	ITPR1-DT	0.318704809	mirna_lnc
28618	hsa-mir-30c-2	LINC00412	0.313840788	mirna_lnc
28619	hsa-mir-30c-2	LINC01891	0.304723172	mirna_lnc

28620	hsa-mir-30c-2	AC010285.1	0.35165893	mirna_lnc
28621	hsa-mir-30c-2	AC018809.1	0.352028265	mirna_lnc
28622	hsa-mir-30c-2	AC007485.2	0.32338153	mirna_lnc
28623	hsa-mir-30c-2	AC011477.3	0.348381191	mirna_lnc
28624	hsa-mir-30c-2	AC104596.1	0.396672563	mirna_lnc
28625	hsa-mir-30c-2	AC063965.1	0.328753613	mirna_lnc
28626	hsa-mir-30c-2	AC117503.4	0.338498328	mirna_lnc
28627	hsa-mir-30c-2	AC110792.3	0.313409013	mirna_lnc
28628	hsa-mir-30c-2	AC004148.2	0.342651751	mirna_lnc
28629	hsa-mir-30c-2	DDN-AS1	0.317445856	mirna_lnc
28630	hsa-mir-30c-2	AP001178.3	0.328879093	mirna_lnc
28631	hsa-mir-30c-2	AL078621.3	0.303309891	mirna_lnc
28632	hsa-mir-30c-2	AC027271.1	0.33947882	mirna_lnc
28633	hsa-mir-30c-2	AC090425.2	0.349047611	mirna_lnc
28634	hsa-mir-30c-2	AC090186.1	0.322852357	mirna_lnc
28635	hsa-mir-30c-2	AC027607.1	0.328772521	mirna_lnc
28636	hsa-mir-30c-2	AL163051.2	0.322486534	mirna_lnc
28637	hsa-mir-30c-2	AC007406.5	0.31075039	mirna_lnc
28638	hsa-mir-30c-2	U73166.1	0.32826627	mirna_lnc
28639	hsa-mir-30c-2	AC005332.3	0.368745048	mirna_lnc
28640	hsa-mir-30c-2	AC053527.1	0.332911468	mirna_lnc
28641	hsa-mir-30c-2	AC087623.1	0.301295362	mirna_lnc
28642	hsa-mir-30c-2	AC092953.2	0.345045786	mirna_lnc
28643	hsa-mir-30c-2	AC009779.2	0.343010993	mirna_lnc
28644	hsa-mir-30c-2	AL121906.2	0.304728966	mirna_lnc
28645	hsa-mir-30c-2	AL136368.1	0.333540852	mirna_lnc
28646	hsa-mir-30c-2	SOS1-IT1	0.474129817	mirna_lnc
28647	hsa-mir-30c-2	AC005104.1	0.31344546	mirna_lnc
28648	hsa-mir-30c-2	AL603832.1	0.322594431	mirna_lnc
28649	hsa-mir-30c-2	AP003170.3	0.48989566	mirna_lnc
28650	hsa-mir-30c-2	AC087500.2	0.33813226	mirna_lnc
28651	hsa-mir-30c-2	AC087501.4	0.3186921	mirna_lnc
28652	hsa-mir-30c-2	AC008739.5	0.352201824	mirna_lnc
28653	hsa-mir-30c-2	AC021491.4	0.456957833	mirna_lnc
28654	hsa-mir-30c-2	AL589990.1	0.304094586	mirna_lnc
28655	hsa-mir-30c-2	AC093525.7	0.306346479	mirna_lnc
28656	hsa-mir-30c-2	AC010168.2	0.353459149	mirna_lnc
28657	hsa-mir-30c-2	AC004771.5	0.308838329	mirna_lnc
28658	hsa-mir-30c-2	AC093752.3	0.32298936	mirna_lnc
28659	hsa-mir-30c-2	AC090772.1	0.354879017	mirna_lnc
28660	hsa-mir-30c-2	AC000068.1	0.419804236	mirna_lnc
28661	hsa-mir-30c-2	AC016910.1	0.315662378	mirna_lnc
28662	hsa-mir-30c-2	AC023355.1	0.319225283	mirna_lnc
28663	hsa-mir-30c-2	AC009107.2	0.351797977	mirna_lnc
28664	hsa-mir-30c-2	AC055713.1	0.339868041	mirna_lnc
28665	hsa-mir-30c-2	AC015871.3	0.33238907	mirna_lnc
28666	hsa-mir-30c-2	AL135905.1	0.312033761	mirna_lnc
28667	hsa-mir-30c-2	AC000068.2	0.378416192	mirna_lnc
28668	hsa-mir-30c-2	AC009108.3	0.319115645	mirna_lnc
28669	hsa-mir-30c-2	AP000254.1	0.324263152	mirna_lnc
28670	hsa-mir-30c-2	AL031186.1	0.479969935	mirna_lnc
28671	hsa-mir-30c-2	AC002553.2	0.340327587	mirna_lnc
28672	hsa-mir-30c-2	AC010547.1	0.32631019	mirna_lnc
28673	hsa-mir-30c-2	AC130895.1	0.300285146	mirna_lnc

28674	hsa-mir-30c-2	AC012557.2	0.471208298	mirna_lnc
28675	hsa-mir-30c-2	AC011450.1	0.325860886	mirna_lnc
28676	hsa-mir-30c-2	AC113139.1	0.30370776	mirna_lnc
28677	hsa-mir-30c-2	AC046168.1	0.31899524	mirna_lnc
28678	hsa-mir-30c-2	AC007541.1	0.34569307	mirna_lnc
28679	hsa-mir-30c-2	AC022272.1	0.388009542	mirna_lnc
28680	hsa-mir-30c-2	CATIP-AS2	0.306770771	mirna_lnc
28681	hsa-mir-30c-2	AP001271.1	0.322195634	mirna_lnc
28682	hsa-mir-30c-2	AC092111.2	0.321440159	mirna_lnc
28683	hsa-mir-30c-2	ANK3-DT	0.33977702	mirna_lnc
28684	hsa-mir-30c-2	AC006946.2	0.433559939	mirna_lnc
28685	hsa-mir-30c-2	AC048341.1	0.365020977	mirna_lnc
28686	hsa-mir-30c-2	AC107464.3	0.362265092	mirna_lnc
28687	hsa-mir-30c-2	AL513534.2	0.332596849	mirna_lnc
28688	hsa-mir-30c-2	AC025219.1	0.341402523	mirna_lnc
28689	hsa-mir-30c-2	AL390195.1	0.322018185	mirna_lnc
28690	hsa-mir-30c-2	AC009812.3	0.335351114	mirna_lnc
28691	hsa-mir-30c-2	AC244090.1	0.329731944	mirna_lnc
28692	hsa-mir-30c-2	AC007390.2	0.310516925	mirna_lnc
28693	hsa-mir-30c-2	AC005865.1	0.356252693	mirna_lnc
28694	hsa-mir-30c-2	AP001021.1	0.316334237	mirna_lnc
28695	hsa-mir-30c-2	AC004908.3	0.334848074	mirna_lnc
28696	hsa-mir-30c-2	CATIP-AS1	0.301795286	mirna_lnc
28697	hsa-mir-30c-2	AC073869.3	0.34056242	mirna_lnc
28698	hsa-mir-30c-2	AL355490.2	0.335677257	mirna_lnc
28699	hsa-mir-30c-2	AC079174.2	0.339264378	mirna_lnc
28700	hsa-mir-30c-2	ETV5-AS1	0.445489388	mirna_lnc
28701	hsa-mir-30c-2	AC107068.1	0.488139301	mirna_lnc
28702	hsa-mir-30c-2	AL731563.3	0.336077894	mirna_lnc
28703	hsa-mir-30c-2	AC072039.2	0.306688253	mirna_lnc
28704	hsa-mir-30c-2	AL050343.1	0.350251112	mirna_lnc
28705	hsa-mir-30c-2	AC096564.2	0.315015603	mirna_lnc
28706	hsa-mir-30c-2	FAR1-IT1	0.455355122	mirna_lnc
28707	hsa-mir-30c-2	AC010973.2	0.303556945	mirna_lnc
28708	hsa-mir-30c-2	AC125616.1	0.344723912	mirna_lnc
28709	hsa-mir-30c-2	AC078909.2	0.322833961	mirna_lnc
28710	hsa-mir-30c-2	AC008440.1	0.334752049	mirna_lnc
28711	hsa-mir-30c-2	AC084782.2	0.364653668	mirna_lnc
28712	hsa-mir-30c-2	AL118511.1	0.318621508	mirna_lnc
28713	hsa-mir-30c-2	AC005014.2	0.332666757	mirna_lnc
28714	hsa-mir-30c-2	AC131971.1	0.324217345	mirna_lnc
28715	hsa-mir-30c-2	AC079766.1	0.353596041	mirna_lnc
28716	hsa-mir-30c-2	AC046130.2	0.302069487	mirna_lnc
28717	hsa-mir-30c-2	AC009269.4	0.356543449	mirna_lnc
28718	hsa-mir-30c-2	AC003070.2	0.305749364	mirna_lnc
28719	hsa-mir-30c-2	LINC02062	0.32013276	mirna_lnc
28720	hsa-mir-30c-2	AC005034.4	0.426716105	mirna_lnc
28721	hsa-mir-30c-2	AL731566.1	0.301302909	mirna_lnc
28722	hsa-mir-30c-2	AC104794.2	0.311611733	mirna_lnc
28723	hsa-mir-30c-2	AP006623.1	0.315153384	mirna_lnc
28724	hsa-mir-30c-2	AC098484.2	0.347850421	mirna_lnc
28725	hsa-mir-30c-2	AL359510.2	0.335066434	mirna_lnc
28726	hsa-mir-30c-2	STARD4-AS1	0.358368859	mirna_lnc
28727	hsa-mir-30c-2	AC022107.1	0.304896706	mirna_lnc

28728	hsa-mir-30c-2	AL031587.1	0.322682503	mirna_lnc
28729	hsa-mir-30c-2	NFYC-AS1	0.455186952	mirna_lnc
28730	hsa-mir-30c-2	LINC02097	0.41002338	mirna_lnc
28731	hsa-mir-30c-2	AC017100.1	0.300625286	mirna_lnc
28732	hsa-mir-30c-2	AC004076.2	0.350503033	mirna_lnc
28733	hsa-mir-30c-2	AC098820.1	0.301316194	mirna_lnc
28734	hsa-mir-30c-2	AP001178.2	0.320950091	mirna_lnc
28735	hsa-mir-30c-2	AL662884.1	0.313353056	mirna_lnc
28736	hsa-mir-30c-2	AC011462.5	0.314361683	mirna_lnc
28737	hsa-mir-30c-2	AC108727.1	0.307401314	mirna_lnc
28738	hsa-mir-30c-2	AC018892.3	0.344534812	mirna_lnc
28739	hsa-mir-30c-2	AC004918.1	0.307849175	mirna_lnc
28740	hsa-mir-30c-2	AC104581.4	0.35354372	mirna_lnc
28741	hsa-mir-30c-2	LINC00909	0.305735227	mirna_lnc
28742	hsa-mir-30c-2	AL139339.1	0.34693995	mirna_lnc
28743	hsa-mir-30c-2	AL049838.1	0.51146581	mirna_lnc
28744	hsa-mir-30c-2	PTOV1-AS1	0.395529215	mirna_lnc
28745	hsa-mir-30c-2	AC005034.3	0.302158824	mirna_lnc
28746	hsa-mir-30c-2	AC068397.1	0.305908482	mirna_lnc
28747	hsa-mir-30c-2	AL121917.2	0.300871645	mirna_lnc
28748	hsa-mir-30c-2	AC025178.1	0.306494458	mirna_lnc
28749	hsa-mir-30c-2	AC127540.1	0.33334218	mirna_lnc
28750	hsa-mir-30c-2	AL450384.1	0.302687253	mirna_lnc
28751	hsa-mir-30c-2	AC008870.2	0.341528696	mirna_lnc
28752	hsa-mir-30c-2	AL161725.2	0.300181877	mirna_lnc
28753	hsa-mir-30c-2	ADNP-AS1	0.400742652	mirna_lnc
28754	hsa-mir-30c-2	ZBTB20-AS4	0.327847632	mirna_lnc
28755	hsa-mir-30c-2	AC005034.2	0.311968097	mirna_lnc
28756	hsa-mir-30c-2	AC103739.1	0.30206946	mirna_lnc
28757	hsa-mir-30c-2	AL137003.2	0.321975618	mirna_lnc
28758	hsa-mir-30c-2	AC108451.1	0.31770116	mirna_lnc
28759	hsa-mir-30c-2	AC093788.1	0.406614946	mirna_lnc
28760	hsa-mir-30c-2	AC145422.1	0.353817133	mirna_lnc
28761	hsa-mir-30c-2	AC108063.1	0.403808732	mirna_lnc
28762	hsa-mir-30c-2	AC032011.1	0.347722213	mirna_lnc
28763	hsa-mir-30c-2	AC107464.1	0.384511581	mirna_lnc
28764	hsa-mir-30c-2	AC139792.2	0.312562345	mirna_lnc
28765	hsa-mir-30c-2	AC005730.3	0.305289182	mirna_lnc
28766	hsa-mir-30c-2	AC010999.1	0.421514086	mirna_lnc
28767	hsa-mir-30c-2	AC009754.1	0.325455859	mirna_lnc
28768	hsa-mir-30c-2	AC007038.2	0.346881442	mirna_lnc
28769	hsa-mir-30c-2	AC124069.1	0.301613232	mirna_lnc
28770	hsa-mir-30c-2	AL358115.1	0.331801082	mirna_lnc
28771	hsa-mir-30c-2	AC055855.1	0.326884617	mirna_lnc
28772	hsa-mir-30c-2	AC060766.6	0.356171554	mirna_lnc
28773	hsa-mir-30c-2	AL049646.1	0.319280177	mirna_lnc
28774	hsa-mir-30c-2	AC092718.5	0.328375244	mirna_lnc
28775	hsa-mir-30c-2	AC006017.1	0.33316404	mirna_lnc
28776	hsa-mir-30c-2	AC097534.1	0.348045303	mirna_lnc
28777	hsa-mir-30c-2	AC022893.3	0.375030245	mirna_lnc
28778	hsa-mir-30c-2	AL512343.2	0.30881927	mirna_lnc
28779	hsa-mir-30c-2	AL596202.1	0.326952935	mirna_lnc
28780	hsa-mir-30c-2	LAMC1-AS1	0.341480912	mirna_lnc
28781	hsa-mir-30c-2	AC011239.1	0.304107524	mirna_lnc

28782	hsa-mir-30c-2	AC084824.4	0.452685572	mirna_lnc
28783	hsa-mir-30c-2	AC092839.2	0.348025019	mirna_lnc
28784	hsa-mir-30c-2	AC006213.6	0.358572237	mirna_lnc
28785	hsa-mir-30c-2	AC006487.1	0.324221663	mirna_lnc
28786	hsa-mir-30c-2	AL513477.2	0.349097146	mirna_lnc
28787	hsa-mir-30c-2	AL359878.1	0.302203968	mirna_lnc
28788	hsa-mir-30c-2	AC114763.2	0.305937615	mirna_lnc
28789	hsa-mir-30c-2	AC009812.4	0.337343179	mirna_lnc
28790	hsa-mir-30c-2	AC022001.3	0.319278722	mirna_lnc
28791	hsa-mir-30c-2	AC108704.1	0.345282029	mirna_lnc
28792	hsa-mir-29c	ZNF710-AS1	0.568114117	mirna_lnc
28793	hsa-mir-29c	AC116407.1	0.453308071	mirna_lnc
28794	hsa-mir-29c	AC004836.1	0.482435212	mirna_lnc
28795	hsa-mir-29c	C9orf147	0.544335308	mirna_lnc
28796	hsa-mir-29c	AC012409.3	0.595992684	mirna_lnc
28797	hsa-mir-29c	AC025271.4	0.494801743	mirna_lnc
28798	hsa-mir-29c	AC005920.4	0.53889732	mirna_lnc
28799	hsa-mir-29c	AC123912.4	0.461216018	mirna_lnc
28800	hsa-mir-29c	AP001148.1	0.588554534	mirna_lnc
28801	hsa-mir-29c	AC015908.3	0.602138573	mirna_lnc
28802	hsa-mir-29c	BX284668.2	0.443610431	mirna_lnc
28803	hsa-mir-29c	AL158847.1	0.536886748	mirna_lnc
28804	hsa-mir-29c	AC024337.2	0.506023492	mirna_lnc
28805	hsa-mir-29c	AL158163.2	0.609551548	mirna_lnc
28806	hsa-mir-29c	LINC00940	0.60609712	mirna_lnc
28807	hsa-mir-29c	AC093583.1	0.467096725	mirna_lnc
28808	hsa-mir-29c	AC239798.2	0.520767313	mirna_lnc
28809	hsa-mir-29c	AL157392.2	0.37172945	mirna_lnc
28810	hsa-mir-29c	AP001528.3	0.496768754	mirna_lnc
28811	hsa-mir-29c	AC012409.5	0.468816831	mirna_lnc
28812	hsa-mir-29c	AP001528.2	0.564990981	mirna_lnc
28813	hsa-mir-29c	AC063919.1	0.363010698	mirna_lnc
28814	hsa-mir-29c	AC093797.1	0.468485216	mirna_lnc
28815	hsa-mir-29c	AC016251.1	0.511583654	mirna_lnc
28816	hsa-mir-29c	AC108472.1	0.474033436	mirna_lnc
28817	hsa-mir-29c	AC124312.3	0.348614413	mirna_lnc
28818	hsa-mir-29c	AC091563.1	0.478173738	mirna_lnc
28819	hsa-mir-29c	NGF-AS1	0.498009011	mirna_lnc
28820	hsa-mir-29c	AC244453.3	0.576176456	mirna_lnc
28821	hsa-mir-29c	LINC02489	0.403238118	mirna_lnc
28822	hsa-mir-29c	CADM3-AS1	0.322262902	mirna_lnc
28823	hsa-mir-29c	AL138847.2	0.514675493	mirna_lnc
28824	hsa-mir-29c	AL158206.1	0.413798618	mirna_lnc
28825	hsa-mir-29c	AC139749.1	0.345479523	mirna_lnc
28826	hsa-mir-29c	AP003117.1	0.386550674	mirna_lnc
28827	hsa-mir-29c	AL158163.1	0.530193662	mirna_lnc
28828	hsa-mir-29c	AP001972.5	0.328515798	mirna_lnc
28829	hsa-mir-29c	AC021016.2	0.54700606	mirna_lnc
28830	hsa-mir-29c	AC005725.1	0.320317748	mirna_lnc
28831	hsa-mir-29c	AC105074.1	0.306239386	mirna_lnc
28832	hsa-mir-29c	AC004982.1	0.553697626	mirna_lnc
28833	hsa-mir-29c	AL512328.1	0.468570904	mirna_lnc
28834	hsa-mir-29c	AC073862.3	0.414496345	mirna_lnc
28835	hsa-mir-29c	AC091057.3	0.471632676	mirna_lnc

28836	hsa-mir-29c	AC079061.1	0.466131599	mirna_lnc
28837	hsa-mir-29c	LINC02447	0.386477545	mirna_lnc
28838	hsa-mir-29c	AP001094.2	0.392211014	mirna_lnc
28839	hsa-mir-29c	AC091544.5	0.438335916	mirna_lnc
28840	hsa-mir-29c	AC013726.1	0.426300428	mirna_lnc
28841	hsa-mir-29c	AL031429.2	0.594244154	mirna_lnc
28842	hsa-mir-29c	AC022558.2	0.303801652	mirna_lnc
28843	hsa-mir-29c	AL355073.1	0.461743041	mirna_lnc
28844	hsa-mir-29c	AC021766.1	0.405962989	mirna_lnc
28845	hsa-mir-29c	AL133355.1	0.439220328	mirna_lnc
28846	hsa-mir-29c	AC025279.1	0.491652676	mirna_lnc
28847	hsa-mir-29c	IDH2-DT	0.477564708	mirna_lnc
28848	hsa-mir-29c	AC246680.1	0.425029671	mirna_lnc
28849	hsa-mir-29c	LINC01985	0.393786464	mirna_lnc
28850	hsa-mir-29c	LINC02352	0.415906448	mirna_lnc
28851	hsa-mir-29c	AP005717.1	0.414854672	mirna_lnc
28852	hsa-mir-29c	AC023794.6	0.443250957	mirna_lnc
28853	hsa-mir-29c	AC114810.1	0.336146523	mirna_lnc
28854	hsa-mir-29c	AL136164.3	0.310258573	mirna_lnc
28855	hsa-mir-29c	AL109936.2	0.428693632	mirna_lnc
28856	hsa-mir-29c	AC129492.3	0.537912365	mirna_lnc
28857	hsa-mir-29c	AC012409.1	0.581756925	mirna_lnc
28858	hsa-mir-29c	TRG-AS1	0.667347627	mirna_lnc
28859	hsa-mir-29c	RPL34-AS1	0.352824702	mirna_lnc
28860	hsa-mir-29c	AC007637.1	0.474374908	mirna_lnc
28861	hsa-mir-29c	AL158212.1	0.360152365	mirna_lnc
28862	hsa-mir-29c	AC244453.1	0.535920208	mirna_lnc
28863	hsa-mir-29c	MAGI1-AS1	0.403826357	mirna_lnc
28864	hsa-mir-29c	SEMA3B-AS1	0.577187437	mirna_lnc
28865	hsa-mir-29c	LINC00365	0.509611619	mirna_lnc
28866	hsa-mir-29c	HCG21	0.446544324	mirna_lnc
28867	hsa-mir-29c	AC132154.1	0.559693242	mirna_lnc
28868	hsa-mir-29c	NCOA7-AS1	0.616902029	mirna_lnc
28869	hsa-mir-29c	AC008669.1	0.309746765	mirna_lnc
28870	hsa-mir-29c	LINC00261	0.380341046	mirna_lnc
28871	hsa-mir-29c	ENOX1-AS1	0.307755773	mirna_lnc
28872	hsa-mir-29c	AC024075.1	0.33293815	mirna_lnc
28873	hsa-mir-29c	LINC02435	0.339681478	mirna_lnc
28874	hsa-mir-29c	PCAT18	0.587942786	mirna_lnc
28875	hsa-mir-29c	AP000866.2	0.337280889	mirna_lnc
28876	hsa-mir-29c	AL035701.1	0.365552308	mirna_lnc
28877	hsa-mir-29c	AC118344.2	0.315062035	mirna_lnc
28878	hsa-mir-29c	AC138819.1	0.423564733	mirna_lnc
28879	hsa-mir-29c	AC018926.1	0.455557364	mirna_lnc
28880	hsa-mir-29c	SH3BP5-AS1	0.327246666	mirna_lnc
28881	hsa-mir-29c	ELN-AS1	0.317041357	mirna_lnc
28882	hsa-mir-29c	AL031587.5	0.387775925	mirna_lnc
28883	hsa-mir-29c	AC244453.2	0.499652751	mirna_lnc
28884	hsa-mir-29c	AL031846.2	0.336355389	mirna_lnc
28885	hsa-mir-29c	FOXP1-AS1	0.371949604	mirna_lnc
28886	hsa-mir-29c	AC024075.2	0.300974814	mirna_lnc
28887	hsa-mir-29c	AC105345.1	0.315479509	mirna_lnc
28888	hsa-mir-29c	AC037459.2	0.574148959	mirna_lnc
28889	hsa-mir-29c	AC005495.1	0.323775999	mirna_lnc

28890	hsa-mir-29c	AL020997.3	0.449359204	mirna_lnc
28891	hsa-mir-29c	AP003355.2	0.48926645	mirna_lnc
28892	hsa-mir-29c	AC012557.1	0.341628082	mirna_lnc
28893	hsa-mir-29c	AC025917.1	0.435838256	mirna_lnc
28894	hsa-mir-29c	AC009119.3	0.416925089	mirna_lnc
28895	hsa-mir-29c	AC098869.2	0.371900059	mirna_lnc
28896	hsa-mir-29c	AC021491.2	0.449912818	mirna_lnc
28897	hsa-mir-29c	AC139887.4	0.300332868	mirna_lnc
28898	hsa-mir-29c	AP003486.1	0.383665962	mirna_lnc
28899	hsa-mir-29c	AC021205.3	0.300874904	mirna_lnc
28900	hsa-mir-29c	AC006942.1	0.345186482	mirna_lnc
28901	hsa-mir-29c	AL731557.1	0.50751235	mirna_lnc
28902	hsa-mir-29c	AL391834.2	0.314526581	mirna_lnc
28903	hsa-mir-29c	AL138930.1	0.553751529	mirna_lnc
28904	hsa-mir-29c	AC093607.1	0.304499172	mirna_lnc
28905	hsa-mir-29c	AC012181.1	0.495071699	mirna_lnc
28906	hsa-mir-29c	AC111182.1	0.473829596	mirna_lnc
28907	hsa-mir-29c	AL512383.1	0.316060869	mirna_lnc
28908	hsa-mir-29c	AC010776.2	0.393387071	mirna_lnc
28909	hsa-mir-29c	AC024075.3	0.347687728	mirna_lnc
28910	hsa-mir-29c	AC022173.1	0.323169557	mirna_lnc
28911	hsa-mir-29c	AC012181.2	0.528520719	mirna_lnc
28912	hsa-mir-29c	AL390208.1	0.312997892	mirna_lnc
28913	hsa-mir-29c	AC112236.2	0.420975172	mirna_lnc
28914	hsa-mir-29c	EPB41L4A-DT	0.332586679	mirna_lnc
28915	hsa-mir-29c	AC018926.3	0.57949964	mirna_lnc
28916	hsa-mir-29c	AC012409.2	0.319904926	mirna_lnc
28917	hsa-mir-29c	AC079305.3	0.603613669	mirna_lnc
28918	hsa-mir-29c	AC104825.1	0.323920173	mirna_lnc
28919	hsa-mir-29c	AL357033.4	0.435544494	mirna_lnc
28920	hsa-mir-29c	AC114980.1	0.398393928	mirna_lnc
28921	hsa-mir-29c	AC008676.1	0.428154678	mirna_lnc
28922	hsa-mir-29c	AC009090.3	0.30359963	mirna_lnc
28923	hsa-mir-29c	AL031726.1	0.322329011	mirna_lnc
28924	hsa-mir-29c	ZRANB2-AS1	0.397903993	mirna_lnc
28925	hsa-mir-29c	AL357033.3	0.341728021	mirna_lnc
28926	hsa-mir-29c	AC116337.3	0.322636326	mirna_lnc
28927	hsa-mir-29c	AC010226.1	0.45375372	mirna_lnc
28928	hsa-mir-29c	AC135050.6	0.308316719	mirna_lnc
28929	hsa-mir-29c	AL135960.1	0.337379464	mirna_lnc
28930	hsa-mir-29c	C9orf106	0.428362456	mirna_lnc
28931	hsa-mir-29c	AL356273.3	0.310851429	mirna_lnc
28932	hsa-mir-29c	LINC01954	0.361113741	mirna_lnc
28933	hsa-mir-29c	AC020978.4	0.318098006	mirna_lnc
28934	hsa-mir-29c	AC090152.1	0.404709108	mirna_lnc
28935	hsa-mir-29c	AP003721.4	0.557493381	mirna_lnc
28936	hsa-mir-29c	AC009806.1	0.342324081	mirna_lnc
28937	hsa-mir-29c	AL596325.2	0.374299452	mirna_lnc
28938	hsa-mir-29c	AC007598.3	0.374862849	mirna_lnc
28939	hsa-mir-29c	AC006960.3	0.300654152	mirna_lnc
28940	hsa-mir-29c	AC231657.1	0.306070063	mirna_lnc
28941	hsa-mir-29c	ERVE-1	0.51111164	mirna_lnc
28942	hsa-mir-29c	AC064807.2	0.489889453	mirna_lnc
28943	hsa-mir-29c	AL590226.1	0.368135425	mirna_lnc

28944	hsa-mir-29c	AC084036.1	0.389415524	mirna_lnc
28945	hsa-mir-29c	AC022898.2	0.394907624	mirna_lnc
28946	hsa-mir-29c	AC091588.1	0.361286782	mirna_lnc
28947	hsa-mir-29c	AC139768.2	0.322467162	mirna_lnc
28948	hsa-mir-29c	AC127521.1	0.331521706	mirna_lnc
28949	hsa-mir-29c	AC036176.1	0.323140176	mirna_lnc
28950	hsa-mir-29c	AC096733.2	0.382954573	mirna_lnc
28951	hsa-mir-29c	AL031847.1	0.319183489	mirna_lnc
28952	hsa-mir-29c	AC009090.1	0.379993167	mirna_lnc
28953	hsa-mir-29c	AC074032.1	0.362763507	mirna_lnc
28954	hsa-mir-29c	AC124312.2	0.445476448	mirna_lnc
28955	hsa-mir-29c	AC008770.3	0.308982251	mirna_lnc
28956	hsa-mir-29c	AC008731.1	0.324807506	mirna_lnc
28957	hsa-mir-29c	LINC02363	0.311962985	mirna_lnc
28958	hsa-mir-29c	AC007036.2	0.313872394	mirna_lnc
28959	hsa-mir-29c	AL034347.1	0.4226719	mirna_lnc
28960	hsa-mir-29c	SLC25A21-AS1	0.341985873	mirna_lnc
28961	hsa-mir-29c	AC010525.1	0.496558121	mirna_lnc
28962	hsa-mir-29c	AL121895.2	0.384230407	mirna_lnc
28963	hsa-mir-29c	AL391121.1	0.361107825	mirna_lnc
28964	hsa-mir-29c	AC078906.1	0.426750179	mirna_lnc
28965	hsa-mir-29c	LINC02256	0.31505507	mirna_lnc
28966	hsa-mir-29c	AGBL5-IT1	0.316277069	mirna_lnc
28967	hsa-mir-29c	POU6F2-AS1	0.40024568	mirna_lnc
28968	hsa-mir-29c	AL122023.1	0.320807388	mirna_lnc
28969	hsa-mir-29c	FP671120.5	0.3400247	mirna_lnc
28970	hsa-mir-29c	AC007490.1	0.348249298	mirna_lnc
28971	hsa-mir-29c	AC087286.2	0.320753969	mirna_lnc
28972	hsa-mir-29c	AL355073.2	0.452649483	mirna_lnc
28973	hsa-mir-29c	AL162511.1	0.488520634	mirna_lnc
28974	hsa-mir-29c	AC107952.2	0.317301226	mirna_lnc
28975	hsa-mir-29c	AC135507.1	0.377834704	mirna_lnc
28976	hsa-mir-29c	LINC01504	0.452073105	mirna_lnc
28977	hsa-mir-29c	AC009831.3	0.333044702	mirna_lnc
28978	hsa-mir-29c	AC108861.1	0.527204201	mirna_lnc
28979	hsa-mir-29c	AP001094.3	0.452984387	mirna_lnc
28980	hsa-mir-29c	AL512791.1	0.309181347	mirna_lnc
28981	hsa-mir-29c	VAV3-AS1	0.430103532	mirna_lnc
28982	hsa-mir-29c	LINC01422	0.308300356	mirna_lnc
28983	hsa-mir-29c	AC092620.1	0.377628436	mirna_lnc
28984	hsa-mir-29c	AC026202.2	0.382231156	mirna_lnc
28985	hsa-mir-29c	AC093278.2	0.398442157	mirna_lnc
28986	hsa-mir-29c	AC099343.2	0.319530879	mirna_lnc
28987	hsa-mir-29c	UMODL1-AS1	0.303316735	mirna_lnc
28988	hsa-mir-29c	LINC02640	0.41214808	mirna_lnc
28989	hsa-mir-29c	AC005796.1	0.305772081	mirna_lnc
28990	hsa-mir-29c	AC084262.1	0.461997859	mirna_lnc
28991	hsa-mir-29c	AL392023.2	0.31765885	mirna_lnc
28992	hsa-mir-29c	AL513008.1	0.34860527	mirna_lnc
28993	hsa-mir-29c	AL355076.2	0.331775396	mirna_lnc
28994	hsa-mir-29c	AC138512.1	0.452184445	mirna_lnc
28995	hsa-mir-29c	LINC01857	0.355815483	mirna_lnc
28996	hsa-mir-29c	FUT8-AS1	0.343297228	mirna_lnc
28997	hsa-mir-29c	AL138995.1	0.307018907	mirna_lnc

28998	hsa-mir-29c	AC097637.2	0.326961991	mirna_lnc
28999	hsa-mir-29c	LIMD1-AS1	0.440221258	mirna_lnc
29000	hsa-mir-29c	AL356124.2	0.332820284	mirna_lnc
29001	hsa-mir-29c	AC040160.2	0.319638883	mirna_lnc
29002	hsa-mir-29c	AP001094.1	0.464112141	mirna_lnc
29003	hsa-mir-29c	AC026474.1	0.302773851	mirna_lnc
29004	hsa-mir-29c	AL096816.1	0.303325811	mirna_lnc
29005	hsa-mir-29c	AC000124.1	0.434232761	mirna_lnc
29006	hsa-mir-29c	AC234775.3	0.302124822	mirna_lnc
29007	hsa-mir-29c	AL049835.1	0.310502685	mirna_lnc
29008	hsa-mir-29c	AC026367.2	0.319174576	mirna_lnc
29009	hsa-mir-29c	AL354872.2	0.374210711	mirna_lnc
29010	hsa-mir-29c	AC011491.3	0.321820889	mirna_lnc
29011	hsa-mir-29c	AC010998.2	0.335476777	mirna_lnc
29012	hsa-mir-29c	AC092068.3	0.384105637	mirna_lnc
29013	hsa-mir-29c	AC135586.2	0.406184559	mirna_lnc
29014	hsa-mir-29c	AC120498.2	0.503211585	mirna_lnc
29015	hsa-mir-29c	AC005838.2	0.307460055	mirna_lnc
29016	hsa-mir-29c	AC092068.2	0.381177155	mirna_lnc
29017	hsa-mir-29c	AL365361.1	0.454291665	mirna_lnc
29018	hsa-mir-29c	AC008035.1	0.302201897	mirna_lnc
29019	hsa-mir-29c	RABGAP1L-IT1	0.315492319	mirna_lnc
29020	hsa-mir-29c	AC135776.1	0.339259906	mirna_lnc
29021	hsa-mir-29c	AC009093.6	0.320483161	mirna_lnc
29022	hsa-mir-29c	AL023653.1	0.34563903	mirna_lnc
29023	hsa-mir-29c	AC009495.3	0.310578044	mirna_lnc
29024	hsa-mir-29c	AL031733.2	0.374100417	mirna_lnc
29025	hsa-mir-29c	ANKRD44-IT1	0.479778291	mirna_lnc
29026	hsa-mir-29c	HMBOX1-IT1	0.301179997	mirna_lnc
29027	hsa-mir-29c	TMEM92-AS1	0.312957139	mirna_lnc
29028	hsa-mir-29c	AL136368.1	0.303619197	mirna_lnc
29029	hsa-mir-29c	AC006369.1	0.338236947	mirna_lnc
29030	hsa-mir-29c	AC021491.4	0.408570029	mirna_lnc
29031	hsa-mir-29c	AC009108.3	0.33073276	mirna_lnc
29032	hsa-mir-29c	AC136424.2	0.311421876	mirna_lnc
29033	hsa-mir-29c	AC012557.2	0.311409823	mirna_lnc
29034	hsa-mir-29c	AC104699.1	0.430767738	mirna_lnc
29035	hsa-mir-29c	AC006441.1	0.312599783	mirna_lnc
29036	hsa-mir-29c	AL662860.1	0.344181831	mirna_lnc
29037	hsa-mir-29c	AC008080.4	0.404057554	mirna_lnc
29038	hsa-mir-29c	AC092652.2	0.436042516	mirna_lnc
29039	hsa-mir-29c	AC244090.1	0.313911308	mirna_lnc
29040	hsa-mir-29c	CATIP-AS1	0.318771364	mirna_lnc
29041	hsa-mir-29c	AC115102.1	0.344004028	mirna_lnc
29042	hsa-mir-29c	LINC02422	0.390045646	mirna_lnc
29043	hsa-mir-29c	AC078962.1	0.320307483	mirna_lnc
29044	hsa-mir-29c	AC008569.2	0.342329487	mirna_lnc
29045	hsa-mir-29c	AC104024.1	0.333703054	mirna_lnc
29046	hsa-mir-29c	FAR1-IT1	0.415523724	mirna_lnc
29047	hsa-mir-29c	AC093010.2	0.423100825	mirna_lnc
29048	hsa-mir-29c	LINC02245	0.439985322	mirna_lnc
29049	hsa-mir-29c	AL136320.1	0.336851312	mirna_lnc
29050	hsa-mir-29c	AC246787.2	0.475591623	mirna_lnc
29051	hsa-mir-29c	LINC01154	0.340236125	mirna_lnc

29052	hsa-mir-29c	AC010609.1	0.31202765	mirna_lnc
29053	hsa-mir-29c	AL137779.1	0.306615752	mirna_lnc
29054	hsa-mir-29c	BFSP2-AS1	0.353496043	mirna_lnc
29055	hsa-mir-29c	LINC01055	0.403194457	mirna_lnc
29056	hsa-mir-29c	AC003957.1	0.31186243	mirna_lnc
29057	hsa-mir-29c	AL049838.1	0.520077604	mirna_lnc
29058	hsa-mir-29c	AC007342.1	0.311872006	mirna_lnc
29059	hsa-mir-29c	ZBTB20-AS4	0.32420598	mirna_lnc
29060	hsa-mir-29c	AL133467.1	0.381954624	mirna_lnc
29061	hsa-mir-29c	AC021188.1	0.301031631	mirna_lnc
29062	hsa-mir-29c	AL359962.1	0.346337768	mirna_lnc
29063	hsa-mir-29c	AC008982.2	0.337070457	mirna_lnc
29064	hsa-mir-29c	LINC00582	0.336270275	mirna_lnc
29065	hsa-mir-29c	AC119396.2	0.377301965	mirna_lnc
29066	hsa-mir-29c	AC026748.1	0.359746586	mirna_lnc
29067	hsa-mir-29c	AC092118.1	0.307542061	mirna_lnc
29068	hsa-mir-29c	AC012645.3	0.373856113	mirna_lnc
29069	hsa-mir-29c	LINC00528	0.326689166	mirna_lnc
29070	hsa-mir-574	LINC01096	0.367411609	mirna_lnc
29071	hsa-mir-574	AC109347.2	0.367602572	mirna_lnc
29072	hsa-mir-574	AC024230.1	0.351659339	mirna_lnc
29073	hsa-mir-574	EMC1-AS1	0.307457123	mirna_lnc
29074	hsa-mir-574	LINC02026	0.320304715	mirna_lnc
29075	hsa-mir-574	SEPSECS-AS1	0.308697476	mirna_lnc
29076	hsa-mir-574	AC091965.4	0.320107104	mirna_lnc
29077	hsa-mir-574	GIHCG	0.300690608	mirna_lnc
29078	hsa-mir-574	AC007786.2	0.401015375	mirna_lnc
29079	hsa-mir-574	AC026801.2	0.362466303	mirna_lnc
29080	hsa-mir-574	LINC00449	0.319674887	mirna_lnc
29081	hsa-mir-1-2	AC119424.1	0.436504043	mirna_lnc
29082	hsa-mir-1-2	AC004836.1	0.385531817	mirna_lnc
29083	hsa-mir-1-2	AC024337.2	0.462448994	mirna_lnc
29084	hsa-mir-1-2	AP001528.3	0.574590046	mirna_lnc
29085	hsa-mir-1-2	AP001528.2	0.461994384	mirna_lnc
29086	hsa-mir-1-2	AC124312.3	0.520383309	mirna_lnc
29087	hsa-mir-1-2	NCAM1-AS1	0.337598429	mirna_lnc
29088	hsa-mir-1-2	CADM3-AS1	0.611763452	mirna_lnc
29089	hsa-mir-1-2	AP003117.1	0.301262618	mirna_lnc
29090	hsa-mir-1-2	AC100830.1	0.527593482	mirna_lnc
29091	hsa-mir-1-2	AC053503.6	0.836501205	mirna_lnc
29092	hsa-mir-1-2	AC073862.3	0.336982384	mirna_lnc
29093	hsa-mir-1-2	AC053503.4	0.833326853	mirna_lnc
29094	hsa-mir-1-2	AC012085.2	0.356856566	mirna_lnc
29095	hsa-mir-1-2	AP001094.2	0.311991388	mirna_lnc
29096	hsa-mir-1-2	AL132642.1	0.647144872	mirna_lnc
29097	hsa-mir-1-2	AC007193.2	0.379762027	mirna_lnc
29098	hsa-mir-1-2	LINC01985	0.370298466	mirna_lnc
29099	hsa-mir-1-2	AL158212.3	0.412368151	mirna_lnc
29100	hsa-mir-1-2	AL023973.1	0.33425092	mirna_lnc
29101	hsa-mir-1-2	LINC02185	0.354447649	mirna_lnc
29102	hsa-mir-1-2	AC005180.2	0.730103337	mirna_lnc
29103	hsa-mir-1-2	AC005225.4	0.487469239	mirna_lnc
29104	hsa-mir-1-2	AC007637.1	0.304988326	mirna_lnc
29105	hsa-mir-1-2	AC011504.1	0.476527477	mirna_lnc

29106	hsa-mir-1-2	AC008669.1	0.44846082	mirna_lnc
29107	hsa-mir-1-2	AF001548.3	0.678244426	mirna_lnc
29108	hsa-mir-1-2	AC024075.1	0.3425266	mirna_lnc
29109	hsa-mir-1-2	LINC02593	0.338391617	mirna_lnc
29110	hsa-mir-1-2	NKAIN3-IT1	0.646694684	mirna_lnc
29111	hsa-mir-1-2	ELN-AS1	0.308340819	mirna_lnc
29112	hsa-mir-1-2	LINC01354	0.45047327	mirna_lnc
29113	hsa-mir-1-2	AC135012.3	0.731566444	mirna_lnc
29114	hsa-mir-1-2	AL157996.1	0.564984154	mirna_lnc
29115	hsa-mir-1-2	PWAR5	0.578924419	mirna_lnc
29116	hsa-mir-1-2	AC135178.1	0.66054629	mirna_lnc
29117	hsa-mir-1-2	AP003486.1	0.381444741	mirna_lnc
29118	hsa-mir-1-2	AC004637.1	0.615918635	mirna_lnc
29119	hsa-mir-1-2	AC091825.1	0.35534055	mirna_lnc
29120	hsa-mir-1-2	GSN-AS1	0.416413351	mirna_lnc
29121	hsa-mir-1-2	AC024075.3	0.309563563	mirna_lnc
29122	hsa-mir-1-2	AC011472.4	0.507799775	mirna_lnc
29123	hsa-mir-1-2	AP001107.5	0.397533237	mirna_lnc
29124	hsa-mir-1-2	AC022893.1	0.381298545	mirna_lnc
29125	hsa-mir-1-2	AC102945.2	0.605191876	mirna_lnc
29126	hsa-mir-1-2	AC009102.2	0.489160198	mirna_lnc
29127	hsa-mir-1-2	AC007671.1	0.579160973	mirna_lnc
29128	hsa-mir-1-2	AC104825.1	0.304005388	mirna_lnc
29129	hsa-mir-1-2	AC130650.1	0.395599373	mirna_lnc
29130	hsa-mir-1-2	AC104117.3	0.395722851	mirna_lnc
29131	hsa-mir-1-2	AP001486.2	0.402353012	mirna_lnc
29132	hsa-mir-1-2	AC008764.2	0.354791205	mirna_lnc
29133	hsa-mir-1-2	AL135960.1	0.371274257	mirna_lnc
29134	hsa-mir-1-2	AC009806.1	0.47107983	mirna_lnc
29135	hsa-mir-1-2	AF001548.1	0.821052374	mirna_lnc
29136	hsa-mir-1-2	PRICKLE2-AS3	0.33757468	mirna_lnc
29137	hsa-mir-1-2	AP001189.1	0.395996254	mirna_lnc
29138	hsa-mir-1-2	AL590226.1	0.369154215	mirna_lnc
29139	hsa-mir-1-2	AC010551.2	0.461374819	mirna_lnc
29140	hsa-mir-1-2	AC139768.2	0.375239138	mirna_lnc
29141	hsa-mir-1-2	AC002546.1	0.486529874	mirna_lnc
29142	hsa-mir-1-2	AC007342.7	0.373311605	mirna_lnc
29143	hsa-mir-1-2	LINC00891	0.31132461	mirna_lnc
29144	hsa-mir-1-2	AC036108.3	0.821639422	mirna_lnc
29145	hsa-mir-1-2	AL356489.2	0.562549248	mirna_lnc
29146	hsa-mir-1-2	AC124312.2	0.466402778	mirna_lnc
29147	hsa-mir-1-2	ACTA2-AS1	0.836431064	mirna_lnc
29148	hsa-mir-1-2	LINC00163	0.499520757	mirna_lnc
29149	hsa-mir-1-2	AL021368.2	0.408924218	mirna_lnc
29150	hsa-mir-1-2	AC011444.1	0.412305206	mirna_lnc
29151	hsa-mir-1-2	AL109741.1	0.517007771	mirna_lnc
29152	hsa-mir-1-2	AC005180.1	0.739212007	mirna_lnc
29153	hsa-mir-1-2	AC073862.5	0.559078223	mirna_lnc
29154	hsa-mir-1-2	AC103740.1	0.504421695	mirna_lnc
29155	hsa-mir-1-2	AC013643.2	0.30269979	mirna_lnc
29156	hsa-mir-1-2	AC010680.1	0.321985576	mirna_lnc
29157	hsa-mir-1-2	AC011239.2	0.746778477	mirna_lnc
29158	hsa-mir-1-2	LINC02256	0.560498115	mirna_lnc
29159	hsa-mir-1-2	AC093535.2	0.466757141	mirna_lnc

29160	hsa-mir-1-2	AC026124.1	0.476385651	mirna_lnc
29161	hsa-mir-1-2	LINC00641	0.471108353	mirna_lnc
29162	hsa-mir-1-2	GAS1RR	0.326030073	mirna_lnc
29163	hsa-mir-1-2	AC087286.2	0.417861427	mirna_lnc
29164	hsa-mir-1-2	AL139022.2	0.416302374	mirna_lnc
29165	hsa-mir-1-2	AC092111.1	0.313544447	mirna_lnc
29166	hsa-mir-1-2	AC015813.7	0.365549497	mirna_lnc
29167	hsa-mir-1-2	AP000892.3	0.746780863	mirna_lnc
29168	hsa-mir-1-2	TBX5-AS1	0.39830915	mirna_lnc
29169	hsa-mir-1-2	LINC01422	0.331024227	mirna_lnc
29170	hsa-mir-1-2	AC018752.1	0.480002181	mirna_lnc
29171	hsa-mir-1-2	AC107959.1	0.666506908	mirna_lnc
29172	hsa-mir-1-2	AC134312.1	0.457315082	mirna_lnc
29173	hsa-mir-1-2	AC104260.2	0.416028628	mirna_lnc
29174	hsa-mir-1-2	AL445426.1	0.616469738	mirna_lnc
29175	hsa-mir-1-2	AC120049.1	0.386320482	mirna_lnc
29176	hsa-mir-1-2	AC022034.3	0.639989247	mirna_lnc
29177	hsa-mir-1-2	AC092999.1	0.402785242	mirna_lnc
29178	hsa-mir-1-2	AC005358.2	0.599780128	mirna_lnc
29179	hsa-mir-1-2	KLHL30-AS1	0.42803501	mirna_lnc
29180	hsa-mir-1-2	AC104083.1	0.527554586	mirna_lnc
29181	hsa-mir-1-2	AC022898.1	0.330889943	mirna_lnc
29182	hsa-mir-1-2	AL031118.1	0.457496991	mirna_lnc
29183	hsa-mir-1-2	AL161457.1	0.675586327	mirna_lnc
29184	hsa-mir-1-2	AC092040.1	0.412945979	mirna_lnc
29185	hsa-mir-1-2	AL603750.1	0.463360434	mirna_lnc
29186	hsa-mir-1-2	AC067750.1	0.59141865	mirna_lnc
29187	hsa-mir-1-2	AC092422.1	0.330030874	mirna_lnc
29188	hsa-mir-1-2	LINC01352	0.603416017	mirna_lnc
29189	hsa-mir-1-2	AL359710.1	0.310624307	mirna_lnc
29190	hsa-mir-1-2	SACS-AS1	0.416325142	mirna_lnc
29191	hsa-mir-1-2	AC131097.4	0.324783693	mirna_lnc
29192	hsa-mir-1-2	AC234775.3	0.319678118	mirna_lnc
29193	hsa-mir-1-2	AF111167.2	0.354506945	mirna_lnc
29194	hsa-mir-1-2	Z99129.4	0.348615681	mirna_lnc
29195	hsa-mir-1-2	AP000317.2	0.305747579	mirna_lnc
29196	hsa-mir-1-2	AC110491.1	0.472287073	mirna_lnc
29197	hsa-mir-1-2	AC120498.2	0.326943342	mirna_lnc
29198	hsa-mir-1-2	LINC02600	0.326920473	mirna_lnc
29199	hsa-mir-1-2	AC011444.3	0.403601877	mirna_lnc
29200	hsa-mir-1-2	AL954642.1	0.419449378	mirna_lnc
29201	hsa-mir-1-2	AC098484.1	0.339429451	mirna_lnc
29202	hsa-mir-1-2	AC025280.1	0.607193751	mirna_lnc
29203	hsa-mir-1-2	AC037198.2	0.616718129	mirna_lnc
29204	hsa-mir-1-2	AC021683.1	0.752720735	mirna_lnc
29205	hsa-mir-1-2	AL133346.1	0.551899619	mirna_lnc
29206	hsa-mir-1-2	AC013403.2	0.354976601	mirna_lnc
29207	hsa-mir-1-2	AC100803.2	0.504810396	mirna_lnc
29208	hsa-mir-1-2	AC145676.1	0.342799221	mirna_lnc
29209	hsa-mir-1-2	AC246817.1	0.314442581	mirna_lnc
29210	hsa-mir-1-2	AC013553.3	0.343323327	mirna_lnc
29211	hsa-mir-1-2	LINC01990	0.371213217	mirna_lnc
29212	hsa-mir-1-2	AL139300.2	0.493918062	mirna_lnc
29213	hsa-mir-1-2	AC022034.1	0.484016754	mirna_lnc

29214	hsa-mir-1-2	LINC02269	0.473516045	mirna_lnc
29215	hsa-mir-1-2	FAM66E	0.384298136	mirna_lnc
29216	hsa-mir-1-2	AC002398.2	0.696362782	mirna_lnc
29217	hsa-mir-1-2	AP003071.4	0.505533283	mirna_lnc
29218	hsa-mir-1-2	AC027807.2	0.471640333	mirna_lnc
29219	hsa-mir-1-2	AC020634.1	0.390462653	mirna_lnc
29220	hsa-mir-1-2	AC011472.1	0.424355623	mirna_lnc
29221	hsa-mir-1-2	AC092376.2	0.306092713	mirna_lnc
29222	hsa-mir-1-2	COL4A2-AS1	0.382952045	mirna_lnc
29223	hsa-mir-1-2	AL596244.1	0.309301128	mirna_lnc
29224	hsa-mir-1-2	AC027306.1	0.582614575	mirna_lnc
29225	hsa-mir-1-2	AC053503.5	0.401078748	mirna_lnc
29226	hsa-mir-1-2	AL157770.1	0.360185845	mirna_lnc
29227	hsa-mir-1-2	AP001324.3	0.323384773	mirna_lnc
29228	hsa-mir-1-2	AC068700.1	0.38503189	mirna_lnc
29229	hsa-mir-1-2	AC009549.1	0.548647573	mirna_lnc
29230	hsa-mir-1-2	AC018521.6	0.47898176	mirna_lnc
29231	hsa-mir-1-2	LYPLAL1-DT	0.485724756	mirna_lnc
29232	hsa-mir-1-2	MEIS1-AS3	0.439411189	mirna_lnc
29233	hsa-mir-1-2	AL691447.2	0.461463055	mirna_lnc
29234	hsa-mir-1-2	AC011510.1	0.385220305	mirna_lnc
29235	hsa-mir-1-2	AL662860.1	0.514303096	mirna_lnc
29236	hsa-mir-1-2	AP001189.3	0.40868552	mirna_lnc
29237	hsa-mir-1-2	AC092111.2	0.362668247	mirna_lnc
29238	hsa-mir-1-2	AF001548.2	0.405527137	mirna_lnc
29239	hsa-mir-1-2	AC053503.1	0.47407906	mirna_lnc
29240	hsa-mir-1-2	AL122035.1	0.494640919	mirna_lnc
29241	hsa-mir-1-2	AC011365.2	0.386434005	mirna_lnc
29242	hsa-mir-1-2	AC025809.1	0.301776069	mirna_lnc
29243	hsa-mir-1-2	AC068234.2	0.461121184	mirna_lnc
29244	hsa-mir-1-2	BARX1-DT	0.326414775	mirna_lnc
29245	hsa-mir-1-2	SMC2-AS1	0.336263391	mirna_lnc
29246	hsa-mir-1-2	AL583785.1	0.504815056	mirna_lnc
29247	hsa-mir-1-2	AC007336.3	0.373444145	mirna_lnc
29248	hsa-mir-1-2	AC087521.1	0.557363611	mirna_lnc
29249	hsa-mir-1-2	MBNL1-AS1	0.796884628	mirna_lnc
29250	hsa-mir-1-2	AC092376.1	0.319340953	mirna_lnc
29251	hsa-mir-1-2	AC106786.2	0.328038796	mirna_lnc
29252	hsa-mir-1-2	AC121247.1	0.345090519	mirna_lnc
29253	hsa-mir-1-2	UBXN10-AS1	0.591276691	mirna_lnc
29254	hsa-mir-1-2	AC037198.1	0.528411604	mirna_lnc
29255	hsa-mir-1-2	AC090044.1	0.54925574	mirna_lnc
29256	hsa-mir-1-2	AL137026.1	0.403142986	mirna_lnc
29257	hsa-mir-1-2	BNC2-AS1	0.330199698	mirna_lnc
29258	hsa-mir-1-2	AL034346.1	0.416717536	mirna_lnc
29259	hsa-mir-1-2	LINC01589	0.377819036	mirna_lnc
29260	hsa-mir-1-2	AP000721.2	0.390871726	mirna_lnc
29261	hsa-mir-1-2	AL133481.1	0.515195919	mirna_lnc
29262	hsa-mir-1-2	AP000915.1	0.484946103	mirna_lnc
29263	hsa-mir-1-2	AC036108.2	0.778218421	mirna_lnc
29264	hsa-mir-1-2	AC090877.2	0.329511729	mirna_lnc
29265	hsa-mir-1-2	AL445423.1	0.527761953	mirna_lnc
29266	hsa-mir-1-2	LINC01154	0.369438931	mirna_lnc
29267	hsa-mir-1-2	AL136084.3	0.442247196	mirna_lnc

29268	hsa-mir-1-2	AP004609.1	0.353400314	mirna_lnc
29269	hsa-mir-1-2	AC004825.2	0.347785221	mirna_lnc
29270	hsa-mir-1-2	AL357054.4	0.592181773	mirna_lnc
29271	hsa-mir-1-2	HLX-AS1	0.65035763	mirna_lnc
29272	hsa-mir-1-2	AC254633.1	0.428140333	mirna_lnc
29273	hsa-mir-1-2	AC022467.1	0.312574578	mirna_lnc
29274	hsa-mir-1-2	FGF14-AS2	0.371350795	mirna_lnc
29275	hsa-mir-1-2	AP003096.1	0.388946702	mirna_lnc
29276	hsa-mir-1-2	AL391335.1	0.448728283	mirna_lnc
29277	hsa-mir-1-2	LINC01394	0.619743686	mirna_lnc
29278	hsa-mir-1-2	AL079343.1	0.353086178	mirna_lnc
29279	hsa-mir-1-2	KCNMA1-AS3	0.440109709	mirna_lnc
29280	hsa-mir-1-2	AC025165.1	0.530413819	mirna_lnc
29281	hsa-mir-1-2	AC009320.1	0.35254404	mirna_lnc
29282	hsa-mir-1-2	Z99756.1	0.338721438	mirna_lnc
29283	hsa-mir-1-2	AL133415.1	0.515626325	mirna_lnc
29284	hsa-mir-1-2	AC117489.1	0.528721898	mirna_lnc
29285	hsa-mir-1-2	AC093510.2	0.324881046	mirna_lnc
29286	hsa-mir-1-2	AP000662.1	0.313700834	mirna_lnc
29287	hsa-mir-1-2	AP000941.1	0.375363291	mirna_lnc
29288	hsa-mir-1-2	AC106739.1	0.375627109	mirna_lnc
29289	hsa-mir-1-2	AL122035.2	0.406350168	mirna_lnc
29290	hsa-mir-1-2	LINC02202	0.454175189	mirna_lnc
29291	hsa-mir-1-2	AC100793.3	0.3766043	mirna_lnc
29292	hsa-mir-1-2	RASGRF2-AS1	0.300488783	mirna_lnc
29293	hsa-mir-1-2	AC068506.1	0.473078114	mirna_lnc
29294	hsa-mir-1-2	NALT1	0.529870154	mirna_lnc
29295	hsa-mir-1-2	AC134312.3	0.365623286	mirna_lnc
29296	hsa-mir-1-2	LINC01013	0.305191788	mirna_lnc
29297	hsa-mir-1-2	AC079630.1	0.38164573	mirna_lnc
29298	hsa-mir-1-2	AL162386.2	0.400421132	mirna_lnc
29299	hsa-mir-1-2	AP003071.3	0.583392393	mirna_lnc
29300	hsa-mir-1-2	AP003973.2	0.307824891	mirna_lnc
29301	hsa-mir-93	AC092667.1	0.355051107	mirna_lnc
29302	hsa-mir-93	AC025470.2	0.303843605	mirna_lnc
29303	hsa-mir-93	AC012073.1	0.514709769	mirna_lnc
29304	hsa-mir-93	AC026356.1	0.318375249	mirna_lnc
29305	hsa-mir-93	TMPO-AS1	0.387443986	mirna_lnc
29306	hsa-mir-93	SLC12A9-AS1	0.386072893	mirna_lnc
29307	hsa-mir-93	AP000808.1	0.496573511	mirna_lnc
29308	hsa-mir-93	DDX11-AS1	0.340094697	mirna_lnc
29309	hsa-mir-93	AL161891.1	0.333642826	mirna_lnc
29310	hsa-mir-93	AC092614.1	0.422063249	mirna_lnc
29311	hsa-mir-93	CEP83-DT	0.391395141	mirna_lnc
29312	hsa-mir-93	HOXC-AS2	0.337896145	mirna_lnc
29313	hsa-mir-93	AL049539.1	0.310581947	mirna_lnc
29314	hsa-mir-93	AC074117.1	0.311800876	mirna_lnc
29315	hsa-mir-93	AP001469.3	0.42593357	mirna_lnc
29316	hsa-mir-93	AC011199.1	0.344016831	mirna_lnc
29317	hsa-mir-93	AC004943.2	0.311784945	mirna_lnc
29318	hsa-mir-93	AP003071.2	0.487546753	mirna_lnc
29319	hsa-mir-93	AC021945.1	0.302381356	mirna_lnc
29320	hsa-mir-93	AC025176.1	0.332110975	mirna_lnc
29321	hsa-mir-93	AC073842.2	0.415876266	mirna_lnc

29322	hsa-mir-93	LINC01494	0.413757777	mirna_lnc
29323	hsa-mir-93	AC090159.1	0.341097223	mirna_lnc
29324	hsa-mir-93	AC092803.2	0.350430123	mirna_lnc
29325	hsa-mir-93	AC147651.4	0.388738721	mirna_lnc
29326	hsa-mir-93	CLEC12A-AS1	0.307687734	mirna_lnc
29327	hsa-mir-93	AL121759.1	0.33192292	mirna_lnc
29328	hsa-mir-93	AC009121.1	0.426339119	mirna_lnc
29329	hsa-mir-93	AC024940.6	0.331522074	mirna_lnc
29330	hsa-mir-93	AC006329.1	0.338148756	mirna_lnc
29331	hsa-mir-93	AC073857.1	0.30523559	mirna_lnc
29332	hsa-mir-93	AC015849.5	0.342222612	mirna_lnc
29333	hsa-mir-93	LINC02367	0.557279676	mirna_lnc
29334	hsa-mir-93	LINC01096	0.494074647	mirna_lnc
29335	hsa-mir-93	AC012360.3	0.502491995	mirna_lnc
29336	hsa-mir-93	AC016705.2	0.341492099	mirna_lnc
29337	hsa-mir-93	AL391069.2	0.334810038	mirna_lnc
29338	hsa-mir-93	AC116049.2	0.498165103	mirna_lnc
29339	hsa-mir-93	AC024267.6	0.330660081	mirna_lnc
29340	hsa-mir-93	AP002336.3	0.310347526	mirna_lnc
29341	hsa-mir-93	AL513497.1	0.301056082	mirna_lnc
29342	hsa-mir-93	AL133243.2	0.347669494	mirna_lnc
29343	hsa-mir-93	AC109347.2	0.346306309	mirna_lnc
29344	hsa-mir-93	AC024230.1	0.54314308	mirna_lnc
29345	hsa-mir-93	LINC01397	0.457045524	mirna_lnc
29346	hsa-mir-93	AC138904.1	0.371379299	mirna_lnc
29347	hsa-mir-93	AC073517.1	0.316993832	mirna_lnc
29348	hsa-mir-93	AP000487.1	0.539126842	mirna_lnc
29349	hsa-mir-93	AC104971.1	0.315761511	mirna_lnc
29350	hsa-mir-93	AC004943.1	0.381450646	mirna_lnc
29351	hsa-mir-93	AC010186.3	0.348812893	mirna_lnc
29352	hsa-mir-93	AC002076.1	0.474214757	mirna_lnc
29353	hsa-mir-93	LINC01686	0.362163773	mirna_lnc
29354	hsa-mir-93	LINC01143	0.389518693	mirna_lnc
29355	hsa-mir-93	AC018878.1	0.351729177	mirna_lnc
29356	hsa-mir-93	AC100782.1	0.392111765	mirna_lnc
29357	hsa-mir-93	AL354714.1	0.365257688	mirna_lnc
29358	hsa-mir-93	AC104823.1	0.303714903	mirna_lnc
29359	hsa-mir-93	SREBF2-AS1	0.347883892	mirna_lnc
29360	hsa-mir-93	AC078864.1	0.480973777	mirna_lnc
29361	hsa-mir-93	AC073073.2	0.369469782	mirna_lnc
29362	hsa-mir-93	AC044781.1	0.422497644	mirna_lnc
29363	hsa-mir-93	AC100812.1	0.309108889	mirna_lnc
29364	hsa-mir-93	AC011297.1	0.43697187	mirna_lnc
29365	hsa-mir-93	AC004943.3	0.3236431	mirna_lnc
29366	hsa-mir-93	AC003986.2	0.364963966	mirna_lnc
29367	hsa-mir-93	AL354892.2	0.359391331	mirna_lnc
29368	hsa-mir-93	AC106820.2	0.40425172	mirna_lnc
29369	hsa-mir-93	AC026271.3	0.477282418	mirna_lnc
29370	hsa-mir-93	AC016205.1	0.47223765	mirna_lnc
29371	hsa-mir-93	AC090527.3	0.341706898	mirna_lnc
29372	hsa-mir-93	MYB-AS1	0.487687416	mirna_lnc
29373	hsa-mir-93	AP002336.1	0.319129892	mirna_lnc
29374	hsa-mir-93	AC023034.2	0.336236062	mirna_lnc
29375	hsa-mir-93	AC123905.1	0.329045482	mirna_lnc

29376	hsa-mir-93	RNASEH1-AS1	0.34165189	mirna_lnc
29377	hsa-mir-93	AL157392.1	0.310193744	mirna_lnc
29378	hsa-mir-93	AC092669.1	0.400764107	mirna_lnc
29379	hsa-mir-93	HGC6.3	0.470873697	mirna_lnc
29380	hsa-mir-93	AP002336.2	0.551300531	mirna_lnc
29381	hsa-mir-93	AC099568.2	0.340318515	mirna_lnc
29382	hsa-mir-93	AC011773.1	0.453660334	mirna_lnc
29383	hsa-mir-93	AC006206.1	0.349219862	mirna_lnc
29384	hsa-mir-93	AC069222.1	0.336852776	mirna_lnc
29385	hsa-mir-93	LINC02562	0.358105869	mirna_lnc
29386	hsa-mir-93	AC126407.1	0.309870536	mirna_lnc
29387	hsa-mir-93	AC008115.2	0.344435826	mirna_lnc
29388	hsa-mir-93	AC009271.1	0.310194229	mirna_lnc
29389	hsa-mir-93	AC098818.2	0.358441963	mirna_lnc
29390	hsa-mir-93	AC068473.3	0.360675719	mirna_lnc
29391	hsa-mir-93	AC104596.1	0.387386914	mirna_lnc
29392	hsa-mir-93	TCF7L1-IT1	0.313031094	mirna_lnc
29393	hsa-mir-93	AC107214.1	0.374462718	mirna_lnc
29394	hsa-mir-93	LINC00885	0.377330159	mirna_lnc
29395	hsa-mir-93	AL022324.3	0.327394436	mirna_lnc
29396	hsa-mir-93	AC007683.2	0.349142716	mirna_lnc
29397	hsa-mir-93	AL157832.2	0.461074546	mirna_lnc
29398	hsa-mir-93	AC006206.2	0.342765241	mirna_lnc
29399	hsa-mir-93	AP003071.1	0.432476943	mirna_lnc
29400	hsa-mir-93	AP000487.2	0.316078651	mirna_lnc
29401	hsa-mir-93	AL603832.1	0.331309103	mirna_lnc
29402	hsa-mir-93	AC007848.2	0.331715059	mirna_lnc
29403	hsa-mir-93	AL591167.1	0.407339338	mirna_lnc
29404	hsa-mir-93	AL031186.1	0.404347755	mirna_lnc
29405	hsa-mir-93	LINC01258	0.355149012	mirna_lnc
29406	hsa-mir-93	AC046168.1	0.369868626	mirna_lnc
29407	hsa-mir-93	AP000892.1	0.302368913	mirna_lnc
29408	hsa-mir-93	AC007541.1	0.311455992	mirna_lnc
29409	hsa-mir-93	AC006946.2	0.322808241	mirna_lnc
29410	hsa-mir-93	AC025219.1	0.342885252	mirna_lnc
29411	hsa-mir-93	AC005865.1	0.338776975	mirna_lnc
29412	hsa-mir-93	AC007250.1	0.36418487	mirna_lnc
29413	hsa-mir-93	AC090197.1	0.301590322	mirna_lnc
29414	hsa-mir-93	AL138767.3	0.474391084	mirna_lnc
29415	hsa-mir-93	AC098936.1	0.482259117	mirna_lnc
29416	hsa-mir-93	ETV5-AS1	0.530550461	mirna_lnc
29417	hsa-mir-93	AC107068.1	0.331961576	mirna_lnc
29418	hsa-mir-93	SEMA6A-AS2	0.335689482	mirna_lnc
29419	hsa-mir-93	AC004969.1	0.32773811	mirna_lnc
29420	hsa-mir-93	AP001783.1	0.429216393	mirna_lnc
29421	hsa-mir-93	LINC02457	0.432021178	mirna_lnc
29422	hsa-mir-93	AC084782.2	0.323365026	mirna_lnc
29423	hsa-mir-93	AL118511.1	0.47318506	mirna_lnc
29424	hsa-mir-93	AC027348.1	0.375341402	mirna_lnc
29425	hsa-mir-93	AC008115.3	0.326036962	mirna_lnc
29426	hsa-mir-93	GIHCG	0.308712413	mirna_lnc
29427	hsa-mir-93	AL359881.3	0.330905405	mirna_lnc
29428	hsa-mir-93	AP000808.2	0.473775372	mirna_lnc
29429	hsa-mir-93	LINC02470	0.455133242	mirna_lnc

29430	hsa-mir-93	AC008443.3	0.326556423	mirna_lnc
29431	hsa-mir-93	AC106820.4	0.571492236	mirna_lnc
29432	hsa-mir-93	LINC02097	0.3266773	mirna_lnc
29433	hsa-mir-93	LINC00173	0.381303213	mirna_lnc
29434	hsa-mir-93	AC131159.1	0.337911895	mirna_lnc
29435	hsa-mir-93	AL662884.1	0.300927622	mirna_lnc
29436	hsa-mir-93	AC007497.1	0.363429682	mirna_lnc
29437	hsa-mir-93	ADNP-AS1	0.301385465	mirna_lnc
29438	hsa-mir-93	AC108451.1	0.322452064	mirna_lnc
29439	hsa-mir-93	AC093788.1	0.30159129	mirna_lnc
29440	hsa-mir-93	AC125603.4	0.385115312	mirna_lnc
29441	hsa-mir-93	LINC01936	0.359251252	mirna_lnc
29442	hsa-mir-93	AL358115.1	0.387709763	mirna_lnc
29443	hsa-mir-93	AL049646.1	0.360411608	mirna_lnc
29444	hsa-mir-93	AC092718.5	0.323322749	mirna_lnc
29445	hsa-mir-93	AC097534.1	0.394460403	mirna_lnc
29446	hsa-mir-93	AC016229.2	0.353743506	mirna_lnc
29447	hsa-mir-93	AL596202.1	0.433841308	mirna_lnc
29448	hsa-mir-93	AC006487.1	0.323924517	mirna_lnc
29449	hsa-mir-93	AF213884.3	0.370329727	mirna_lnc
29450	hsa-mir-93	AC008033.3	0.450475227	mirna_lnc
29451	hsa-mir-93	AC108704.1	0.353511167	mirna_lnc
29452	hsa-mir-93	AP002490.1	0.318199705	mirna_lnc
29453	hsa-mir-93	AC009970.1	0.337365376	mirna_lnc
29454	hsa-mir-3648	AC103746.1	0.460500226	mirna_lnc
29455	hsa-mir-3648	AC019294.2	0.39505932	mirna_lnc
29456	hsa-mir-3648	AC129492.5	0.310924857	mirna_lnc
29457	hsa-mir-3648	LINC02593	0.36413245	mirna_lnc
29458	hsa-mir-3648	LINC01625	0.449704415	mirna_lnc
29459	hsa-mir-3648	SCAT2	0.391290866	mirna_lnc
29460	hsa-mir-3648	LINC02043	0.360183766	mirna_lnc
29461	hsa-mir-3648	DEPDC1-AS1	0.401647911	mirna_lnc
29462	hsa-mir-3648	AC022387.1	0.302304617	mirna_lnc
29463	hsa-mir-3648	AL139288.1	0.349710996	mirna_lnc
29464	hsa-mir-3648	AL354977.2	0.347214874	mirna_lnc
29465	hsa-mir-3648	AC104667.1	0.328143129	mirna_lnc
29466	hsa-mir-3648	AC021224.1	0.301482837	mirna_lnc
29467	hsa-mir-3648	AL137802.2	0.395114566	mirna_lnc
29468	hsa-mir-3648	AC093642.2	0.396391301	mirna_lnc
29469	hsa-mir-3648	AL157823.2	0.307314091	mirna_lnc
29470	hsa-mir-3648	AP000553.2	0.401532171	mirna_lnc
29471	hsa-mir-3648	AC110813.1	0.361075336	mirna_lnc
29472	hsa-mir-3648	LINC01087	0.352232769	mirna_lnc
29473	hsa-mir-3648	AC135803.1	0.455706125	mirna_lnc
29474	hsa-mir-3648	AC138904.1	0.393976738	mirna_lnc
29475	hsa-mir-3648	LINC01267	0.310229247	mirna_lnc
29476	hsa-mir-3648	TGFB2-AS1	0.325376097	mirna_lnc
29477	hsa-mir-3648	LINC02334	0.358958522	mirna_lnc
29478	hsa-mir-3648	AC090578.2	0.377709753	mirna_lnc
29479	hsa-mir-3648	AL078605.1	0.398053433	mirna_lnc
29480	hsa-mir-3648	AC012150.1	0.36848688	mirna_lnc
29481	hsa-mir-3648	AC130650.2	0.306277944	mirna_lnc
29482	hsa-mir-3648	AL592494.2	0.354515568	mirna_lnc
29483	hsa-mir-3648	AC007996.1	0.345904237	mirna_lnc

29484	hsa-mir-3648	AP003696.1	0.308866551	mirna_lnc
29485	hsa-mir-3648	AC016708.1	0.471018259	mirna_lnc
29486	hsa-mir-3648	AC099786.1	0.449835713	mirna_lnc
29487	hsa-mir-3648	AC109326.1	0.322471767	mirna_lnc
29488	hsa-mir-3648	AF250324.1	0.420737899	mirna_lnc
29489	hsa-mir-3648	AL135790.1	0.325162319	mirna_lnc
29490	hsa-mir-3648	DNM3-IT1	0.452660462	mirna_lnc
29491	hsa-mir-3648	AL451064.1	0.436988883	mirna_lnc
29492	hsa-mir-3648	AP000355.1	0.305124667	mirna_lnc
29493	hsa-mir-3648	AL645608.4	0.435461511	mirna_lnc
29494	hsa-mir-3648	AC008115.1	0.309072048	mirna_lnc
29495	hsa-mir-3648	AL136528.1	0.367653493	mirna_lnc
29496	hsa-mir-3648	AC007663.3	0.305728703	mirna_lnc
29497	hsa-mir-3648	LINC02594	0.356149099	mirna_lnc
29498	hsa-mir-3648	AL162376.1	0.394696715	mirna_lnc
29499	hsa-mir-3648	AC010735.1	0.429243581	mirna_lnc
29500	hsa-mir-3648	AC079600.3	0.300100345	mirna_lnc
29501	hsa-mir-3648	AP000320.1	0.373366324	mirna_lnc
29502	hsa-mir-3648	UFL1-AS1	0.304474321	mirna_lnc
29503	hsa-mir-3648	AC008649.1	0.323229762	mirna_lnc
29504	hsa-mir-3648	LINC01238	0.367808775	mirna_lnc
29505	hsa-mir-3648	ATP2B1-AS1	0.406229744	mirna_lnc
29506	hsa-mir-3648	AP000255.1	0.42385379	mirna_lnc
29507	hsa-mir-3648	AC104072.1	0.513382835	mirna_lnc
29508	hsa-mir-3648	CAHM	0.321026347	mirna_lnc
29509	hsa-mir-3648	AC010336.2	0.30973097	mirna_lnc
29510	hsa-mir-3648	AC092447.5	0.312483619	mirna_lnc
29511	hsa-mir-3648	AC010336.5	0.381208413	mirna_lnc
29512	hsa-mir-3648	LINC01901	0.321143991	mirna_lnc
29513	hsa-mir-3648	SMC2-AS1	0.431074888	mirna_lnc
29514	hsa-mir-3648	AL355810.1	0.347467681	mirna_lnc
29515	hsa-mir-3648	MAGEA8-AS1	0.319285953	mirna_lnc
29516	hsa-mir-3648	AOAH-IT1	0.31324789	mirna_lnc
29517	hsa-mir-3648	AC004925.1	0.368949759	mirna_lnc
29518	hsa-mir-3648	AP000350.5	0.541045027	mirna_lnc
29519	hsa-mir-3648	AC073389.3	0.311734977	mirna_lnc
29520	hsa-mir-3648	MAPT-IT1	0.370950607	mirna_lnc
29521	hsa-mir-3648	U91328.3	0.308038701	mirna_lnc
29522	hsa-mir-3648	AP000350.7	0.357782043	mirna_lnc
29523	hsa-mir-3648	AL596330.1	0.401801069	mirna_lnc
29524	hsa-mir-3648	LINC02636	0.402737366	mirna_lnc
29525	hsa-mir-3648	AL138831.2	0.328221001	mirna_lnc
29526	hsa-mir-3648	AL645608.6	0.332099611	mirna_lnc
29527	hsa-mir-3648	AL451064.2	0.405162231	mirna_lnc
29528	hsa-mir-3648	AL353726.2	0.332083119	mirna_lnc
29529	hsa-mir-3648	SPACA6P-AS	0.34368913	mirna_lnc
29530	hsa-mir-3648	AL109910.2	0.379790531	mirna_lnc
29531	hsa-mir-3648	L29074.1	0.344185898	mirna_lnc
29532	hsa-mir-3648	AL359541.1	0.345631504	mirna_lnc
29533	hsa-mir-3648	AL157931.1	0.497259371	mirna_lnc
29534	hsa-mir-3648	AL354919.1	0.498375289	mirna_lnc
29535	hsa-mir-3648	AL161729.4	0.432287854	mirna_lnc
29536	hsa-mir-3648	AL645608.2	0.370837532	mirna_lnc
29537	hsa-mir-3648	AC010735.2	0.403961949	mirna_lnc

29538	hsa-mir-3648	AL024498.1	0.363769012	mirna_lnc
29539	hsa-mir-3648	AC100793.2	0.341369004	mirna_lnc
29540	hsa-mir-18a	AC012073.1	0.373334959	mirna_lnc
29541	hsa-mir-18a	AC245100.6	0.313323883	mirna_lnc
29542	hsa-mir-18a	AC010761.2	0.352072438	mirna_lnc
29543	hsa-mir-18a	TMPO-AS1	0.363671777	mirna_lnc
29544	hsa-mir-18a	AP000808.1	0.311886026	mirna_lnc
29545	hsa-mir-18a	AL035461.2	0.3702715	mirna_lnc
29546	hsa-mir-18a	AL138759.1	0.370441176	mirna_lnc
29547	hsa-mir-18a	AC004241.3	0.302821482	mirna_lnc
29548	hsa-mir-18a	AL161891.1	0.41922603	mirna_lnc
29549	hsa-mir-18a	AP000873.3	0.329560785	mirna_lnc
29550	hsa-mir-18a	SCAT2	0.388937687	mirna_lnc
29551	hsa-mir-18a	AC092614.1	0.360730049	mirna_lnc
29552	hsa-mir-18a	AP003352.1	0.335596899	mirna_lnc
29553	hsa-mir-18a	AL049539.1	0.36224343	mirna_lnc
29554	hsa-mir-18a	AC074117.1	0.352438683	mirna_lnc
29555	hsa-mir-18a	AC021945.1	0.422905801	mirna_lnc
29556	hsa-mir-18a	AC025176.1	0.315152704	mirna_lnc
29557	hsa-mir-18a	AC090844.3	0.397363742	mirna_lnc
29558	hsa-mir-18a	AC008738.2	0.314393577	mirna_lnc
29559	hsa-mir-18a	AC131009.3	0.444641303	mirna_lnc
29560	hsa-mir-18a	AC017083.1	0.312085241	mirna_lnc
29561	hsa-mir-18a	AC147651.4	0.315317863	mirna_lnc
29562	hsa-mir-18a	AL356299.2	0.331494644	mirna_lnc
29563	hsa-mir-18a	AC093484.4	0.387971048	mirna_lnc
29564	hsa-mir-18a	AC024940.6	0.423462446	mirna_lnc
29565	hsa-mir-18a	LINC01952	0.345604295	mirna_lnc
29566	hsa-mir-18a	AC107081.2	0.383758581	mirna_lnc
29567	hsa-mir-18a	AL451050.2	0.315580811	mirna_lnc
29568	hsa-mir-18a	AC079907.1	0.396233345	mirna_lnc
29569	hsa-mir-18a	AC125257.1	0.319644398	mirna_lnc
29570	hsa-mir-18a	AC004835.1	0.321982495	mirna_lnc
29571	hsa-mir-18a	AC010536.1	0.310325432	mirna_lnc
29572	hsa-mir-18a	AC012360.3	0.314047872	mirna_lnc
29573	hsa-mir-18a	AC008543.1	0.408569549	mirna_lnc
29574	hsa-mir-18a	AC016394.2	0.3724236	mirna_lnc
29575	hsa-mir-18a	HOXC-AS3	0.338388587	mirna_lnc
29576	hsa-mir-18a	AC130324.2	0.311597883	mirna_lnc
29577	hsa-mir-18a	AJ239322.1	0.32071231	mirna_lnc
29578	hsa-mir-18a	AC004080.1	0.332986847	mirna_lnc
29579	hsa-mir-18a	AL121832.3	0.344663034	mirna_lnc
29580	hsa-mir-18a	GK-IT1	0.309447517	mirna_lnc
29581	hsa-mir-18a	LINC01424	0.367630036	mirna_lnc
29582	hsa-mir-18a	AC026992.2	0.31115063	mirna_lnc
29583	hsa-mir-18a	AL358473.1	0.407938411	mirna_lnc
29584	hsa-mir-18a	AC079684.1	0.308465768	mirna_lnc
29585	hsa-mir-18a	AL163051.1	0.358498758	mirna_lnc
29586	hsa-mir-18a	AC104971.1	0.324575854	mirna_lnc
29587	hsa-mir-18a	AC004943.1	0.33487424	mirna_lnc
29588	hsa-mir-18a	AC132192.2	0.304680239	mirna_lnc
29589	hsa-mir-18a	LINC01357	0.453821679	mirna_lnc
29590	hsa-mir-18a	LINC01686	0.325559644	mirna_lnc
29591	hsa-mir-18a	AC018878.1	0.357094797	mirna_lnc

29592	hsa-mir-18a	AC002550.1	0.373013758	mirna_lnc
29593	hsa-mir-18a	AC099518.2	0.329412223	mirna_lnc
29594	hsa-mir-18a	TRPM2-AS	0.329805709	mirna_lnc
29595	hsa-mir-18a	AC012150.1	0.31314936	mirna_lnc
29596	hsa-mir-18a	AP003086.1	0.301047543	mirna_lnc
29597	hsa-mir-18a	AF186192.3	0.300719517	mirna_lnc
29598	hsa-mir-18a	SREBF2-AS1	0.324766082	mirna_lnc
29599	hsa-mir-18a	AP003696.1	0.302518138	mirna_lnc
29600	hsa-mir-18a	AC004943.3	0.431304247	mirna_lnc
29601	hsa-mir-18a	AL117382.1	0.332309489	mirna_lnc
29602	hsa-mir-18a	AL121721.1	0.307747271	mirna_lnc
29603	hsa-mir-18a	AL354892.2	0.316320388	mirna_lnc
29604	hsa-mir-18a	AL390961.3	0.340570066	mirna_lnc
29605	hsa-mir-18a	AP002360.3	0.317769373	mirna_lnc
29606	hsa-mir-18a	AC016205.1	0.338168909	mirna_lnc
29607	hsa-mir-18a	PIK3CD-AS2	0.309394998	mirna_lnc
29608	hsa-mir-18a	AC090527.3	0.308404435	mirna_lnc
29609	hsa-mir-18a	MYB-AS1	0.369530081	mirna_lnc
29610	hsa-mir-18a	RNASEH1-AS1	0.514030909	mirna_lnc
29611	hsa-mir-18a	MPRIP-AS1	0.328016631	mirna_lnc
29612	hsa-mir-18a	AC005379.1	0.405690159	mirna_lnc
29613	hsa-mir-18a	AL390961.2	0.374873244	mirna_lnc
29614	hsa-mir-18a	AL139082.1	0.301252676	mirna_lnc
29615	hsa-mir-18a	AC084757.2	0.373331753	mirna_lnc
29616	hsa-mir-18a	AC005224.1	0.35344941	mirna_lnc
29617	hsa-mir-18a	AC011773.1	0.399842815	mirna_lnc
29618	hsa-mir-18a	AL024508.2	0.393658856	mirna_lnc
29619	hsa-mir-18a	AL359880.1	0.310750949	mirna_lnc
29620	hsa-mir-18a	AC008115.1	0.304035105	mirna_lnc
29621	hsa-mir-18a	AC083843.3	0.306896022	mirna_lnc
29622	hsa-mir-18a	AC007938.3	0.368680129	mirna_lnc
29623	hsa-mir-18a	AC098818.2	0.345869986	mirna_lnc
29624	hsa-mir-18a	AC113143.1	0.372924705	mirna_lnc
29625	hsa-mir-18a	LINC02204	0.319781179	mirna_lnc
29626	hsa-mir-18a	AC103810.5	0.302978314	mirna_lnc
29627	hsa-mir-18a	ODF2-AS1	0.309031413	mirna_lnc
29628	hsa-mir-18a	AL603832.1	0.361205769	mirna_lnc
29629	hsa-mir-18a	AL512652.2	0.328745113	mirna_lnc
29630	hsa-mir-18a	AC019129.2	0.3027722	mirna_lnc
29631	hsa-mir-18a	AP006621.5	0.397909917	mirna_lnc
29632	hsa-mir-18a	LBX2-AS1	0.318744864	mirna_lnc
29633	hsa-mir-18a	AP003086.2	0.435582051	mirna_lnc
29634	hsa-mir-18a	AP001775.2	0.407531974	mirna_lnc
29635	hsa-mir-18a	AC079915.1	0.309237702	mirna_lnc
29636	hsa-mir-18a	AC007638.2	0.321496193	mirna_lnc
29637	hsa-mir-18a	AC006111.2	0.386442095	mirna_lnc
29638	hsa-mir-18a	AC090907.2	0.370278034	mirna_lnc
29639	hsa-mir-18a	AC011700.1	0.339331087	mirna_lnc
29640	hsa-mir-18a	AC009704.2	0.312650578	mirna_lnc
29641	hsa-mir-18a	AP001160.1	0.347081148	mirna_lnc
29642	hsa-mir-18a	AC124798.1	0.417065286	mirna_lnc
29643	hsa-mir-18a	AC116025.2	0.358557348	mirna_lnc
29644	hsa-mir-18a	AC005089.1	0.332824645	mirna_lnc
29645	hsa-mir-18a	MCF2L-AS1	0.309776719	mirna_lnc

29646	hsa-mir-18a	AL138767.3	0.32173338	mirna_lnc
29647	hsa-mir-18a	AC098936.1	0.34169758	mirna_lnc
29648	hsa-mir-18a	AL607028.1	0.362127037	mirna_lnc
29649	hsa-mir-18a	AC005046.1	0.407313654	mirna_lnc
29650	hsa-mir-18a	BX284668.6	0.3021141	mirna_lnc
29651	hsa-mir-18a	AL356488.3	0.306560055	mirna_lnc
29652	hsa-mir-18a	LINC02365	0.436462372	mirna_lnc
29653	hsa-mir-18a	LINC01979	0.311092203	mirna_lnc
29654	hsa-mir-18a	AL118511.1	0.44170077	mirna_lnc
29655	hsa-mir-18a	AC093620.1	0.336186123	mirna_lnc
29656	hsa-mir-18a	AC007001.1	0.341321381	mirna_lnc
29657	hsa-mir-18a	AC090627.1	0.318769859	mirna_lnc
29658	hsa-mir-18a	AP003392.3	0.431391854	mirna_lnc
29659	hsa-mir-18a	AL133351.1	0.377252143	mirna_lnc
29660	hsa-mir-18a	LINC02470	0.328359662	mirna_lnc
29661	hsa-mir-18a	CEBPA-DT	0.305279751	mirna_lnc
29662	hsa-mir-18a	AC106820.4	0.331348922	mirna_lnc
29663	hsa-mir-18a	AC133961.1	0.320062673	mirna_lnc
29664	hsa-mir-18a	LINC00311	0.35659535	mirna_lnc
29665	hsa-mir-18a	LINC00543	0.342144208	mirna_lnc
29666	hsa-mir-18a	LINC02575	0.324660716	mirna_lnc
29667	hsa-mir-18a	AC005391.1	0.383390976	mirna_lnc
29668	hsa-mir-18a	AC080129.2	0.315010081	mirna_lnc
29669	hsa-mir-18a	AC004593.1	0.322595134	mirna_lnc
29670	hsa-mir-18a	AC007038.2	0.327487682	mirna_lnc
29671	hsa-mir-18a	LINC01106	0.34492531	mirna_lnc
29672	hsa-mir-18a	AP001972.4	0.333102378	mirna_lnc
29673	hsa-mir-18a	AL596202.1	0.30427723	mirna_lnc
29674	hsa-mir-18a	AC026462.3	0.307529733	mirna_lnc
29675	hsa-mir-18a	AP001476.1	0.318264478	mirna_lnc
29676	hsa-mir-18a	AL136221.1	0.355218364	mirna_lnc
29677	hsa-mir-18a	AL080317.1	0.363966109	mirna_lnc
29678	hsa-mir-18a	AC008033.3	0.30013874	mirna_lnc
29679	hsa-mir-18a	AC021092.1	0.304960282	mirna_lnc
29680	hsa-mir-18a	PSPC1-AS2	0.307968233	mirna_lnc
29681	hsa-mir-18a	AC099684.2	0.344589127	mirna_lnc
29682	hsa-mir-18a	AL356740.2	0.317073889	mirna_lnc
29683	hsa-mir-301b	AP000708.1	0.395777177	mirna_lnc
29684	hsa-mir-301b	LINC02595	0.440118752	mirna_lnc
29685	hsa-mir-301b	AL662797.1	0.331669883	mirna_lnc
29686	hsa-mir-301b	LINC01873	0.487143599	mirna_lnc
29687	hsa-mir-301b	MELTF-AS1	0.34937902	mirna_lnc
29688	hsa-mir-301b	TMPO-AS1	0.355779816	mirna_lnc
29689	hsa-mir-301b	AC103591.3	0.477323045	mirna_lnc
29690	hsa-mir-301b	AC004241.3	0.565735132	mirna_lnc
29691	hsa-mir-301b	CEP83-DT	0.307993543	mirna_lnc
29692	hsa-mir-301b	AL590326.1	0.319510372	mirna_lnc
29693	hsa-mir-301b	JARID2-AS1	0.571015483	mirna_lnc
29694	hsa-mir-301b	LINC00853	0.369904292	mirna_lnc
29695	hsa-mir-301b	AC074117.1	0.332707982	mirna_lnc
29696	hsa-mir-301b	AL031667.3	0.576675543	mirna_lnc
29697	hsa-mir-301b	TFAP2A-AS1	0.482626226	mirna_lnc
29698	hsa-mir-301b	AC100791.2	0.407995054	mirna_lnc
29699	hsa-mir-301b	LINC02542	0.443353494	mirna_lnc

29700	hsa-mir-301b	AC092159.2	0.344324881	mirna_lnc
29701	hsa-mir-301b	AL023803.3	0.446231165	mirna_lnc
29702	hsa-mir-301b	AC017083.1	0.321438668	mirna_lnc
29703	hsa-mir-301b	AL121655.1	0.463929616	mirna_lnc
29704	hsa-mir-301b	AC109322.1	0.41798747	mirna_lnc
29705	hsa-mir-301b	LINC01952	0.325853236	mirna_lnc
29706	hsa-mir-301b	AC074351.1	0.449480116	mirna_lnc
29707	hsa-mir-301b	KC877982.1	0.377814461	mirna_lnc
29708	hsa-mir-301b	AL445072.1	0.379912407	mirna_lnc
29709	hsa-mir-301b	AL592301.1	0.318414603	mirna_lnc
29710	hsa-mir-301b	AC023157.3	0.360263766	mirna_lnc
29711	hsa-mir-301b	AL137802.2	0.376005086	mirna_lnc
29712	hsa-mir-301b	AC087289.3	0.378046224	mirna_lnc
29713	hsa-mir-301b	AL136162.1	0.568326336	mirna_lnc
29714	hsa-mir-301b	AC073195.1	0.500473225	mirna_lnc
29715	hsa-mir-301b	AC015818.2	0.411933813	mirna_lnc
29716	hsa-mir-301b	AL596094.1	0.509813579	mirna_lnc
29717	hsa-mir-301b	AC004069.1	0.579539654	mirna_lnc
29718	hsa-mir-301b	LINC01096	0.348692597	mirna_lnc
29719	hsa-mir-301b	AC131025.1	0.365212908	mirna_lnc
29720	hsa-mir-301b	AC004920.1	0.636369611	mirna_lnc
29721	hsa-mir-301b	AC021683.3	0.652854809	mirna_lnc
29722	hsa-mir-301b	AC109992.2	0.302752707	mirna_lnc
29723	hsa-mir-301b	AC016877.3	0.360710106	mirna_lnc
29724	hsa-mir-301b	AL391069.2	0.393861102	mirna_lnc
29725	hsa-mir-301b	AC012640.2	0.477641613	mirna_lnc
29726	hsa-mir-301b	AC090970.2	0.42921189	mirna_lnc
29727	hsa-mir-301b	AC026782.2	0.436012313	mirna_lnc
29728	hsa-mir-301b	AC116049.2	0.357196326	mirna_lnc
29729	hsa-mir-301b	AP003119.2	0.4888287	mirna_lnc
29730	hsa-mir-301b	AL513497.1	0.351840568	mirna_lnc
29731	hsa-mir-301b	AC025244.1	0.337481817	mirna_lnc
29732	hsa-mir-301b	ATP6V1B1-AS1	0.322524585	mirna_lnc
29733	hsa-mir-301b	AL353764.1	0.487189523	mirna_lnc
29734	hsa-mir-301b	AC024230.1	0.318197761	mirna_lnc
29735	hsa-mir-301b	AL031985.3	0.350563405	mirna_lnc
29736	hsa-mir-301b	AC013400.1	0.517828424	mirna_lnc
29737	hsa-mir-301b	AC138904.1	0.321747645	mirna_lnc
29738	hsa-mir-301b	LINC01424	0.498866308	mirna_lnc
29739	hsa-mir-301b	TBC1D8-AS1	0.565615043	mirna_lnc
29740	hsa-mir-301b	AP000487.1	0.301721614	mirna_lnc
29741	hsa-mir-301b	KIAA1614-AS1	0.462139793	mirna_lnc
29742	hsa-mir-301b	AC016876.3	0.628483198	mirna_lnc
29743	hsa-mir-301b	AL031665.2	0.395957071	mirna_lnc
29744	hsa-mir-301b	AL163051.1	0.3711727	mirna_lnc
29745	hsa-mir-301b	AL513327.2	0.635896286	mirna_lnc
29746	hsa-mir-301b	IQCJ-SCHIP1-AS1	0.393991029	mirna_lnc
29747	hsa-mir-301b	AC084125.2	0.35454715	mirna_lnc
29748	hsa-mir-301b	AC138356.1	0.630651627	mirna_lnc
29749	hsa-mir-301b	LINC01456	0.347124786	mirna_lnc
29750	hsa-mir-301b	AC092807.2	0.420600614	mirna_lnc
29751	hsa-mir-301b	LINC01850	0.405569242	mirna_lnc
29752	hsa-mir-301b	LINC01357	0.345609437	mirna_lnc
29753	hsa-mir-301b	AL683887.1	0.362959732	mirna_lnc

29754	hsa-mir-301b	AP000593.3	0.380891749	mirna_lnc
29755	hsa-mir-301b	WWTR1-AS1	0.349245361	mirna_lnc
29756	hsa-mir-301b	LINC01686	0.486487408	mirna_lnc
29757	hsa-mir-301b	MIR583HG	0.390789047	mirna_lnc
29758	hsa-mir-301b	LINC01143	0.325153144	mirna_lnc
29759	hsa-mir-301b	AL033527.3	0.316947836	mirna_lnc
29760	hsa-mir-301b	AC002550.1	0.31536885	mirna_lnc
29761	hsa-mir-301b	AC010595.1	0.337516887	mirna_lnc
29762	hsa-mir-301b	LINC02607	0.663954973	mirna_lnc
29763	hsa-mir-301b	WASIR2	0.309354001	mirna_lnc
29764	hsa-mir-301b	LINC01816	0.695496157	mirna_lnc
29765	hsa-mir-301b	AL121652.1	0.345893	mirna_lnc
29766	hsa-mir-301b	SREBF2-AS1	0.415691461	mirna_lnc
29767	hsa-mir-301b	AC099518.6	0.626388805	mirna_lnc
29768	hsa-mir-301b	AC007881.3	0.531103879	mirna_lnc
29769	hsa-mir-301b	AL121658.1	0.42355364	mirna_lnc
29770	hsa-mir-301b	AC010969.2	0.406327416	mirna_lnc
29771	hsa-mir-301b	AC022098.3	0.448686103	mirna_lnc
29772	hsa-mir-301b	AC005332.1	0.327617318	mirna_lnc
29773	hsa-mir-301b	LINC00167	0.634864412	mirna_lnc
29774	hsa-mir-301b	AC011825.4	0.304618905	mirna_lnc
29775	hsa-mir-301b	AC011297.1	0.307780768	mirna_lnc
29776	hsa-mir-301b	AC023302.1	0.331757869	mirna_lnc
29777	hsa-mir-301b	AC004943.3	0.353126155	mirna_lnc
29778	hsa-mir-301b	AC046143.2	0.486652368	mirna_lnc
29779	hsa-mir-301b	AC145343.1	0.570508245	mirna_lnc
29780	hsa-mir-301b	AC112907.1	0.311748349	mirna_lnc
29781	hsa-mir-301b	AL121992.2	0.351194775	mirna_lnc
29782	hsa-mir-301b	AC093424.1	0.586718357	mirna_lnc
29783	hsa-mir-301b	AC091925.1	0.341318281	mirna_lnc
29784	hsa-mir-301b	AC117382.2	0.559850084	mirna_lnc
29785	hsa-mir-301b	AC091153.3	0.401730145	mirna_lnc
29786	hsa-mir-301b	AC063948.1	0.463082827	mirna_lnc
29787	hsa-mir-301b	AC087742.1	0.420518091	mirna_lnc
29788	hsa-mir-301b	AL354892.2	0.422421043	mirna_lnc
29789	hsa-mir-301b	AC015802.5	0.376285339	mirna_lnc
29790	hsa-mir-301b	AL139327.2	0.500750453	mirna_lnc
29791	hsa-mir-301b	DDX39B-AS1	0.422480571	mirna_lnc
29792	hsa-mir-301b	AC069148.1	0.406309683	mirna_lnc
29793	hsa-mir-301b	AC106820.2	0.316416868	mirna_lnc
29794	hsa-mir-301b	AC005993.1	0.335319563	mirna_lnc
29795	hsa-mir-301b	AC026271.3	0.329517675	mirna_lnc
29796	hsa-mir-301b	AC016205.1	0.306452368	mirna_lnc
29797	hsa-mir-301b	AC064836.3	0.429204222	mirna_lnc
29798	hsa-mir-301b	BX088651.4	0.38845682	mirna_lnc
29799	hsa-mir-301b	AC025211.1	0.353987609	mirna_lnc
29800	hsa-mir-301b	PIK3CD-AS2	0.357109312	mirna_lnc
29801	hsa-mir-301b	AC012640.5	0.321425399	mirna_lnc
29802	hsa-mir-301b	AL512408.1	0.532263158	mirna_lnc
29803	hsa-mir-301b	AC090527.3	0.329673985	mirna_lnc
29804	hsa-mir-301b	MYB-AS1	0.324151989	mirna_lnc
29805	hsa-mir-301b	AC087741.2	0.339626518	mirna_lnc
29806	hsa-mir-301b	AC010422.2	0.380665641	mirna_lnc
29807	hsa-mir-301b	RNASEH1-AS1	0.411014444	mirna_lnc

29808	hsa-mir-301b	AP002387.2	0.322031701	mirna_lnc
29809	hsa-mir-301b	AC005379.1	0.302732016	mirna_lnc
29810	hsa-mir-301b	LINC01063	0.627032282	mirna_lnc
29811	hsa-mir-301b	AC092669.1	0.545809649	mirna_lnc
29812	hsa-mir-301b	CIRBP-AS1	0.513983625	mirna_lnc
29813	hsa-mir-301b	AP002336.2	0.347322382	mirna_lnc
29814	hsa-mir-301b	AC069213.1	0.336081572	mirna_lnc
29815	hsa-mir-301b	AC091946.2	0.459116082	mirna_lnc
29816	hsa-mir-301b	AL096828.3	0.312866456	mirna_lnc
29817	hsa-mir-301b	AC084757.2	0.39256634	mirna_lnc
29818	hsa-mir-301b	AC040169.3	0.365023573	mirna_lnc
29819	hsa-mir-301b	EIF2AK3-DT	0.32134655	mirna_lnc
29820	hsa-mir-301b	AL021920.1	0.321027889	mirna_lnc
29821	hsa-mir-301b	AC009171.2	0.481615489	mirna_lnc
29822	hsa-mir-301b	CNNM3-DT	0.394695703	mirna_lnc
29823	hsa-mir-301b	AC068831.6	0.440841128	mirna_lnc
29824	hsa-mir-301b	AL355482.2	0.424487441	mirna_lnc
29825	hsa-mir-301b	AC080013.5	0.372470276	mirna_lnc
29826	hsa-mir-301b	AL645608.4	0.394139853	mirna_lnc
29827	hsa-mir-301b	LINC02562	0.411511616	mirna_lnc
29828	hsa-mir-301b	AC138696.2	0.513897344	mirna_lnc
29829	hsa-mir-301b	MAST4-AS1	0.43487012	mirna_lnc
29830	hsa-mir-301b	AC093585.1	0.318553547	mirna_lnc
29831	hsa-mir-301b	AC090774.2	0.310593198	mirna_lnc
29832	hsa-mir-301b	AC007773.1	0.300721084	mirna_lnc
29833	hsa-mir-301b	AC098818.2	0.329201611	mirna_lnc
29834	hsa-mir-301b	AC022762.1	0.365933883	mirna_lnc
29835	hsa-mir-301b	AC090912.3	0.301763257	mirna_lnc
29836	hsa-mir-301b	AC008267.5	0.409575049	mirna_lnc
29837	hsa-mir-301b	AC005695.3	0.348517778	mirna_lnc
29838	hsa-mir-301b	AC107214.1	0.397058132	mirna_lnc
29839	hsa-mir-301b	LINC00885	0.434293452	mirna_lnc
29840	hsa-mir-301b	AL022324.3	0.316127772	mirna_lnc
29841	hsa-mir-301b	AC005005.3	0.474184662	mirna_lnc
29842	hsa-mir-301b	AC073896.4	0.44938395	mirna_lnc
29843	hsa-mir-301b	DDR1-DT	0.368072078	mirna_lnc
29844	hsa-mir-301b	AC108865.1	0.420612257	mirna_lnc
29845	hsa-mir-301b	URB1-AS1	0.370112828	mirna_lnc
29846	hsa-mir-301b	AC024243.1	0.420706771	mirna_lnc
29847	hsa-mir-301b	AC011446.1	0.418553166	mirna_lnc
29848	hsa-mir-301b	AL132712.2	0.376281172	mirna_lnc
29849	hsa-mir-301b	AC008736.2	0.512707461	mirna_lnc
29850	hsa-mir-301b	AC091271.1	0.374198038	mirna_lnc
29851	hsa-mir-301b	AL590399.1	0.442018522	mirna_lnc
29852	hsa-mir-301b	AC026304.1	0.408273773	mirna_lnc
29853	hsa-mir-301b	AC096992.2	0.458267668	mirna_lnc
29854	hsa-mir-301b	LPP-AS2	0.303969078	mirna_lnc
29855	hsa-mir-301b	AC005304.3	0.382601986	mirna_lnc
29856	hsa-mir-301b	AC010186.1	0.58480573	mirna_lnc
29857	hsa-mir-301b	ELDR	0.621615672	mirna_lnc
29858	hsa-mir-301b	AC090617.5	0.335812868	mirna_lnc
29859	hsa-mir-301b	AP003170.3	0.340859809	mirna_lnc
29860	hsa-mir-301b	AC019129.2	0.414538185	mirna_lnc
29861	hsa-mir-301b	AC005790.1	0.5212235	mirna_lnc

29862	hsa-mir-301b	AC010336.2	0.324855829	mirna_lnc
29863	hsa-mir-301b	AP003086.2	0.359562292	mirna_lnc
29864	hsa-mir-301b	AP001775.2	0.467484594	mirna_lnc
29865	hsa-mir-301b	AL132712.1	0.430314234	mirna_lnc
29866	hsa-mir-301b	AC023355.1	0.391415712	mirna_lnc
29867	hsa-mir-301b	AL606760.3	0.31130631	mirna_lnc
29868	hsa-mir-301b	AC093677.2	0.543343106	mirna_lnc
29869	hsa-mir-301b	Z73429.1	0.326971384	mirna_lnc
29870	hsa-mir-301b	IQCA1-AS1	0.437422682	mirna_lnc
29871	hsa-mir-301b	INTS6L-AS1	0.425980751	mirna_lnc
29872	hsa-mir-301b	AC083880.1	0.451177097	mirna_lnc
29873	hsa-mir-301b	AL121601.1	0.337811558	mirna_lnc
29874	hsa-mir-301b	AP001547.1	0.37296472	mirna_lnc
29875	hsa-mir-301b	LINC01607	0.53434304	mirna_lnc
29876	hsa-mir-301b	AL161729.2	0.365246712	mirna_lnc
29877	hsa-mir-301b	AC007389.5	0.566942562	mirna_lnc
29878	hsa-mir-301b	AC108865.2	0.620322771	mirna_lnc
29879	hsa-mir-301b	AC010834.2	0.512504057	mirna_lnc
29880	hsa-mir-301b	AL022341.1	0.379118423	mirna_lnc
29881	hsa-mir-301b	AP000251.1	0.597983442	mirna_lnc
29882	hsa-mir-301b	AL390195.1	0.386104823	mirna_lnc
29883	hsa-mir-301b	AC025575.2	0.395574365	mirna_lnc
29884	hsa-mir-301b	BX005266.2	0.363755632	mirna_lnc
29885	hsa-mir-301b	CPNE8-AS1	0.395379426	mirna_lnc
29886	hsa-mir-301b	AC118658.1	0.37459303	mirna_lnc
29887	hsa-mir-301b	AL138881.1	0.596262144	mirna_lnc
29888	hsa-mir-301b	AC087276.3	0.392807468	mirna_lnc
29889	hsa-mir-301b	AC090907.1	0.553802904	mirna_lnc
29890	hsa-mir-301b	AC093635.1	0.442518393	mirna_lnc
29891	hsa-mir-301b	LINC01715	0.55873348	mirna_lnc
29892	hsa-mir-301b	AL603839.3	0.36889469	mirna_lnc
29893	hsa-mir-301b	AC009560.1	0.413959893	mirna_lnc
29894	hsa-mir-301b	AL590822.1	0.513641808	mirna_lnc
29895	hsa-mir-301b	BRWD1-AS2	0.327431598	mirna_lnc
29896	hsa-mir-301b	LINC01144	0.305163859	mirna_lnc
29897	hsa-mir-301b	AC016405.2	0.333322465	mirna_lnc
29898	hsa-mir-301b	AL157813.1	0.324878867	mirna_lnc
29899	hsa-mir-301b	AC009275.1	0.312180922	mirna_lnc
29900	hsa-mir-301b	AC027644.3	0.440164349	mirna_lnc
29901	hsa-mir-301b	AGBL5-AS1	0.36404054	mirna_lnc
29902	hsa-mir-301b	AC079174.2	0.300595902	mirna_lnc
29903	hsa-mir-301b	AC004263.1	0.350366902	mirna_lnc
29904	hsa-mir-301b	AC007114.1	0.410562273	mirna_lnc
29905	hsa-mir-301b	AC018682.2	0.306614905	mirna_lnc
29906	hsa-mir-301b	AC096540.1	0.340503822	mirna_lnc
29907	hsa-mir-301b	SCAT8	0.627967043	mirna_lnc
29908	hsa-mir-301b	AL356488.3	0.482279203	mirna_lnc
29909	hsa-mir-301b	AP000692.2	0.349230821	mirna_lnc
29910	hsa-mir-301b	AC004969.1	0.362694204	mirna_lnc
29911	hsa-mir-301b	AC069277.1	0.316815658	mirna_lnc
29912	hsa-mir-301b	AL032819.1	0.563392918	mirna_lnc
29913	hsa-mir-301b	AL844908.2	0.452261889	mirna_lnc
29914	hsa-mir-301b	AC092119.3	0.391003384	mirna_lnc
29915	hsa-mir-301b	AC069307.1	0.467718406	mirna_lnc

29916	hsa-mir-301b	AC099343.3	0.315934275	mirna_lnc
29917	hsa-mir-301b	AC079834.2	0.388603103	mirna_lnc
29918	hsa-mir-301b	HDAC4-AS1	0.443605689	mirna_lnc
29919	hsa-mir-301b	ESRG	0.367485554	mirna_lnc
29920	hsa-mir-301b	AL021154.1	0.543220649	mirna_lnc
29921	hsa-mir-301b	AL359198.1	0.376474205	mirna_lnc
29922	hsa-mir-301b	AC004012.1	0.643824539	mirna_lnc
29923	hsa-mir-301b	AL118511.1	0.304719865	mirna_lnc
29924	hsa-mir-301b	AC087276.1	0.422144899	mirna_lnc
29925	hsa-mir-301b	AC090739.1	0.338899959	mirna_lnc
29926	hsa-mir-301b	AL583808.1	0.504991754	mirna_lnc
29927	hsa-mir-301b	AC068620.2	0.301444775	mirna_lnc
29928	hsa-mir-301b	AP003392.3	0.393537707	mirna_lnc
29929	hsa-mir-301b	AC005034.4	0.3117946	mirna_lnc
29930	hsa-mir-301b	Z93241.1	0.455492387	mirna_lnc
29931	hsa-mir-301b	AL627171.1	0.342424014	mirna_lnc
29932	hsa-mir-301b	AL359878.2	0.463678302	mirna_lnc
29933	hsa-mir-301b	LINC02470	0.373726317	mirna_lnc
29934	hsa-mir-301b	AC068338.2	0.31481741	mirna_lnc
29935	hsa-mir-301b	AL731537.1	0.565652501	mirna_lnc
29936	hsa-mir-301b	GTSE1-DT	0.353548553	mirna_lnc
29937	hsa-mir-301b	AC135178.5	0.504295749	mirna_lnc
29938	hsa-mir-301b	KCNJ2-AS1	0.497086078	mirna_lnc
29939	hsa-mir-301b	C8orf37-AS1	0.33978942	mirna_lnc
29940	hsa-mir-301b	AC002059.1	0.599961671	mirna_lnc
29941	hsa-mir-301b	AL645608.6	0.429681143	mirna_lnc
29942	hsa-mir-301b	AC092652.1	0.353935219	mirna_lnc
29943	hsa-mir-301b	AC011840.2	0.313936697	mirna_lnc
29944	hsa-mir-301b	AC000120.1	0.431911821	mirna_lnc
29945	hsa-mir-301b	AC010980.2	0.432816478	mirna_lnc
29946	hsa-mir-301b	AL603839.4	0.314795851	mirna_lnc
29947	hsa-mir-301b	AC025162.2	0.316709793	mirna_lnc
29948	hsa-mir-301b	PARD3-AS1	0.351167312	mirna_lnc
29949	hsa-mir-301b	AC136604.3	0.413375746	mirna_lnc
29950	hsa-mir-301b	AC005197.1	0.400834628	mirna_lnc
29951	hsa-mir-301b	AC145423.2	0.328352782	mirna_lnc
29952	hsa-mir-301b	AC092142.1	0.497976297	mirna_lnc
29953	hsa-mir-301b	CYP51A1-AS1	0.580691734	mirna_lnc
29954	hsa-mir-301b	AC005696.4	0.455097728	mirna_lnc
29955	hsa-mir-301b	AL512791.2	0.425974561	mirna_lnc
29956	hsa-mir-301b	AP001893.3	0.34925452	mirna_lnc
29957	hsa-mir-301b	RN7SL832P	0.486314223	mirna_lnc
29958	hsa-mir-301b	AC008555.2	0.321728649	mirna_lnc
29959	hsa-mir-301b	AL138966.2	0.346919307	mirna_lnc
29960	hsa-mir-301b	AL358933.1	0.353702645	mirna_lnc
29961	hsa-mir-301b	AP001922.5	0.3081601	mirna_lnc
29962	hsa-mir-301b	AC084819.1	0.384510626	mirna_lnc
29963	hsa-mir-301b	AL512598.1	0.335640846	mirna_lnc
29964	hsa-mir-301b	LINC00112	0.371511143	mirna_lnc
29965	hsa-mir-301b	AP001972.4	0.394757026	mirna_lnc
29966	hsa-mir-301b	AL590705.3	0.650698978	mirna_lnc
29967	hsa-mir-301b	AC099684.1	0.327088974	mirna_lnc
29968	hsa-mir-301b	AC106782.2	0.368516892	mirna_lnc
29969	hsa-mir-301b	LINC00900	0.333735592	mirna_lnc

29970	hsa-mir-301b	AL645608.2	0.320107243	mirna_lnc
29971	hsa-mir-301b	AC015917.2	0.364806607	mirna_lnc
29972	hsa-mir-301b	FOXN3-AS1	0.360765214	mirna_lnc
29973	hsa-mir-301b	TOLLIP-AS1	0.385893564	mirna_lnc
29974	hsa-mir-301b	AC021092.1	0.320142435	mirna_lnc
29975	hsa-mir-301b	AC012676.3	0.393552699	mirna_lnc
29976	hsa-mir-361	AC012073.1	0.372787753	mirna_lnc
29977	hsa-mir-361	AC020658.4	0.313806872	mirna_lnc
29978	hsa-mir-361	HOXC-AS2	0.342587763	mirna_lnc
29979	hsa-mir-361	AP003071.2	0.36457816	mirna_lnc
29980	hsa-mir-361	AC009121.2	0.36709978	mirna_lnc
29981	hsa-mir-361	PRC1-AS1	0.33457637	mirna_lnc
29982	hsa-mir-361	AC008750.7	0.355527154	mirna_lnc
29983	hsa-mir-361	AC024884.2	0.316640608	mirna_lnc
29984	hsa-mir-361	AC016596.1	0.303032547	mirna_lnc
29985	hsa-mir-361	AC130324.2	0.311217298	mirna_lnc
29986	hsa-mir-361	AC024230.1	0.30429622	mirna_lnc
29987	hsa-mir-361	LINC01397	0.305003193	mirna_lnc
29988	hsa-mir-361	AC021483.1	0.319395276	mirna_lnc
29989	hsa-mir-361	AC026992.2	0.349465	mirna_lnc
29990	hsa-mir-361	AC018878.1	0.305126559	mirna_lnc
29991	hsa-mir-361	AC020612.3	0.321140965	mirna_lnc
29992	hsa-mir-361	WASIR2	0.301627566	mirna_lnc
29993	hsa-mir-361	SREBF2-AS1	0.359903126	mirna_lnc
29994	hsa-mir-361	AC004943.3	0.330829211	mirna_lnc
29995	hsa-mir-361	AC073896.3	0.361382836	mirna_lnc
29996	hsa-mir-361	AC126614.1	0.32417401	mirna_lnc
29997	hsa-mir-361	AC090527.3	0.304553037	mirna_lnc
29998	hsa-mir-361	AC012435.2	0.369923202	mirna_lnc
29999	hsa-mir-361	AL359851.1	0.31661217	mirna_lnc
30000	hsa-mir-361	AC099568.2	0.305362222	mirna_lnc
30001	hsa-mir-361	EXTL3-AS1	0.31195923	mirna_lnc
30002	hsa-mir-361	AL512413.1	0.324475042	mirna_lnc
30003	hsa-mir-361	AL590133.1	0.341125238	mirna_lnc
30004	hsa-mir-361	AC105339.2	0.343096261	mirna_lnc
30005	hsa-mir-361	AC107214.1	0.313504447	mirna_lnc
30006	hsa-mir-361	AP005328.2	0.327504238	mirna_lnc
30007	hsa-mir-361	AC026333.4	0.3209645	mirna_lnc
30008	hsa-mir-361	AP003071.1	0.3091502	mirna_lnc
30009	hsa-mir-361	AL603832.1	0.352331397	mirna_lnc
30010	hsa-mir-361	AP006621.5	0.310274025	mirna_lnc
30011	hsa-mir-361	AL109945.1	0.32069128	mirna_lnc
30012	hsa-mir-361	AC007638.2	0.326600377	mirna_lnc
30013	hsa-mir-361	AC055713.1	0.331573532	mirna_lnc
30014	hsa-mir-361	AC011700.1	0.379062851	mirna_lnc
30015	hsa-mir-361	AL031186.1	0.300968378	mirna_lnc
30016	hsa-mir-361	AL731569.1	0.309830415	mirna_lnc
30017	hsa-mir-361	AL606537.1	0.362000657	mirna_lnc
30018	hsa-mir-361	AC073575.1	0.312794681	mirna_lnc
30019	hsa-mir-361	AC022272.1	0.324088493	mirna_lnc
30020	hsa-mir-361	AC025219.1	0.344212939	mirna_lnc
30021	hsa-mir-361	AC093510.1	0.315408596	mirna_lnc
30022	hsa-mir-361	AL138767.3	0.407875509	mirna_lnc
30023	hsa-mir-361	AC020663.1	0.332150217	mirna_lnc

30024	hsa-mir-361	ETV5-AS1	0.327580461	mirna_lnc
30025	hsa-mir-361	AL607028.1	0.309950009	mirna_lnc
30026	hsa-mir-361	AC011481.2	0.304723074	mirna_lnc
30027	hsa-mir-361	AC107068.1	0.305416414	mirna_lnc
30028	hsa-mir-361	AC012370.1	0.301713967	mirna_lnc
30029	hsa-mir-361	AL158070.2	0.317151645	mirna_lnc
30030	hsa-mir-361	LINC02413	0.322053604	mirna_lnc
30031	hsa-mir-361	AC078909.2	0.309141065	mirna_lnc
30032	hsa-mir-361	AL118511.1	0.343994757	mirna_lnc
30033	hsa-mir-361	AL359881.3	0.33173991	mirna_lnc
30034	hsa-mir-361	AP000808.2	0.313166673	mirna_lnc
30035	hsa-mir-361	AC008443.3	0.361594452	mirna_lnc
30036	hsa-mir-361	AC007608.2	0.390770304	mirna_lnc
30037	hsa-mir-361	LIX1-AS1	0.31643153	mirna_lnc
30038	hsa-mir-361	AP002992.1	0.315124049	mirna_lnc
30039	hsa-mir-361	AC017104.3	0.308653858	mirna_lnc
30040	hsa-mir-361	AC145422.1	0.363447609	mirna_lnc
30041	hsa-mir-361	AC026765.2	0.309021549	mirna_lnc
30042	hsa-mir-361	AL358115.1	0.306862562	mirna_lnc
30043	hsa-mir-361	AC068987.1	0.32810677	mirna_lnc
30044	hsa-mir-361	AL021391.1	0.361101005	mirna_lnc
30045	hsa-mir-361	AL596202.1	0.355004871	mirna_lnc
30046	hsa-mir-361	AL590729.1	0.329792161	mirna_lnc
30047	hsa-mir-361	AC007608.4	0.371680222	mirna_lnc
30048	hsa-mir-361	AC004232.3	0.309767621	mirna_lnc
30049	hsa-mir-361	AC008033.3	0.357212351	mirna_lnc
30050	hsa-mir-361	AC021092.1	0.311617024	mirna_lnc
30051	hsa-mir-361	AC114763.2	0.309239139	mirna_lnc
30052	hsa-mir-361	AC114781.2	0.364474993	mirna_lnc
30053	hsa-mir-361	AC108704.1	0.343908788	mirna_lnc
30054	hsa-mir-361	AC090260.1	0.32198839	mirna_lnc
30055	hsa-mir-937	BLACE	0.375929913	mirna_lnc
30056	hsa-mir-937	AC084125.4	0.303035964	mirna_lnc
30057	hsa-mir-937	AP001453.4	0.366467838	mirna_lnc
30058	hsa-mir-937	AC084809.1	0.310166241	mirna_lnc
30059	hsa-mir-937	TRHDE-AS1	0.336134877	mirna_lnc
30060	hsa-mir-937	HOXA10-AS	0.366709398	mirna_lnc
30061	hsa-mir-937	RHPN1-AS1	0.318667456	mirna_lnc
30062	hsa-mir-937	AC233992.3	0.499007827	mirna_lnc
30063	hsa-mir-937	AC068228.1	0.303998647	mirna_lnc
30064	hsa-mir-937	AC067930.5	0.482266697	mirna_lnc
30065	hsa-mir-937	AC084125.2	0.311725023	mirna_lnc
30066	hsa-mir-937	AC006372.3	0.405108883	mirna_lnc
30067	hsa-mir-937	AC105219.4	0.418567963	mirna_lnc
30068	hsa-mir-937	AC113194.1	0.329874776	mirna_lnc
30069	hsa-mir-937	AP003559.1	0.308374235	mirna_lnc
30070	hsa-mir-937	AC006262.2	0.385291978	mirna_lnc
30071	hsa-mir-937	SATB2-AS1	0.537053162	mirna_lnc
30072	hsa-mir-937	AC006372.1	0.400944603	mirna_lnc
30073	hsa-mir-937	AC067930.4	0.352877326	mirna_lnc
30074	hsa-mir-937	AC025211.1	0.31012275	mirna_lnc
30075	hsa-mir-937	AC090164.2	0.364777777	mirna_lnc
30076	hsa-mir-937	AC100803.1	0.339413473	mirna_lnc
30077	hsa-mir-937	AL138916.1	0.33766841	mirna_lnc

30078	hsa-mir-937	AC008080.1	0.316114675	mirna_lnc
30079	hsa-mir-937	AP003774.4	0.310078881	mirna_lnc
30080	hsa-mir-937	AL353150.1	0.408332848	mirna_lnc
30081	hsa-mir-937	KLHL7-DT	0.318193117	mirna_lnc
30082	hsa-mir-937	AC137894.1	0.318863823	mirna_lnc
30083	hsa-mir-937	AC083841.1	0.301418973	mirna_lnc
30084	hsa-mir-937	AP000317.1	0.311064001	mirna_lnc
30085	hsa-mir-937	AC104076.1	0.396037115	mirna_lnc
30086	hsa-mir-17	AC012073.1	0.379970606	mirna_lnc
30087	hsa-mir-17	AL137060.1	0.316352758	mirna_lnc
30088	hsa-mir-17	AC010761.2	0.300391904	mirna_lnc
30089	hsa-mir-17	TMPO-AS1	0.337188971	mirna_lnc
30090	hsa-mir-17	AL035461.2	0.339044942	mirna_lnc
30091	hsa-mir-17	AL138759.1	0.353282922	mirna_lnc
30092	hsa-mir-17	DDX11-AS1	0.37886469	mirna_lnc
30093	hsa-mir-17	AL161891.1	0.545233488	mirna_lnc
30094	hsa-mir-17	AC092614.1	0.356504143	mirna_lnc
30095	hsa-mir-17	HOXC-AS2	0.350675061	mirna_lnc
30096	hsa-mir-17	AC019069.1	0.320104881	mirna_lnc
30097	hsa-mir-17	AC021945.1	0.356402352	mirna_lnc
30098	hsa-mir-17	AC025176.1	0.331588136	mirna_lnc
30099	hsa-mir-17	AC090844.3	0.34677705	mirna_lnc
30100	hsa-mir-17	AC090159.1	0.356174568	mirna_lnc
30101	hsa-mir-17	STK24-AS1	0.314889533	mirna_lnc
30102	hsa-mir-17	AC024940.6	0.351027602	mirna_lnc
30103	hsa-mir-17	AC015849.5	0.310823532	mirna_lnc
30104	hsa-mir-17	LINC01096	0.317116442	mirna_lnc
30105	hsa-mir-17	AC012360.3	0.305074873	mirna_lnc
30106	hsa-mir-17	AL139089.1	0.364021491	mirna_lnc
30107	hsa-mir-17	AL161772.1	0.361878004	mirna_lnc
30108	hsa-mir-17	AC004080.1	0.351028816	mirna_lnc
30109	hsa-mir-17	AC024230.1	0.301258374	mirna_lnc
30110	hsa-mir-17	AC138904.1	0.410922319	mirna_lnc
30111	hsa-mir-17	AL591846.2	0.323898258	mirna_lnc
30112	hsa-mir-17	AC004943.1	0.321983153	mirna_lnc
30113	hsa-mir-17	LINC01460	0.377696594	mirna_lnc
30114	hsa-mir-17	LINC01357	0.364266822	mirna_lnc
30115	hsa-mir-17	LINC01686	0.314996251	mirna_lnc
30116	hsa-mir-17	TRPM2-AS	0.349949394	mirna_lnc
30117	hsa-mir-17	AC004943.3	0.319789107	mirna_lnc
30118	hsa-mir-17	DNAJC3-DT	0.341545844	mirna_lnc
30119	hsa-mir-17	AL354892.2	0.322214612	mirna_lnc
30120	hsa-mir-17	AC026271.3	0.347701127	mirna_lnc
30121	hsa-mir-17	AC016205.1	0.384031228	mirna_lnc
30122	hsa-mir-17	PIK3CD-AS2	0.372597703	mirna_lnc
30123	hsa-mir-17	MYB-AS1	0.319660566	mirna_lnc
30124	hsa-mir-17	AC007128.1	0.37355215	mirna_lnc
30125	hsa-mir-17	RNASEH1-AS1	0.457455156	mirna_lnc
30126	hsa-mir-17	AC087491.1	0.347603955	mirna_lnc
30127	hsa-mir-17	AC005224.1	0.340933991	mirna_lnc
30128	hsa-mir-17	AC011773.1	0.341573951	mirna_lnc
30129	hsa-mir-17	AL442128.2	0.34361946	mirna_lnc
30130	hsa-mir-17	AC145207.5	0.316338973	mirna_lnc
30131	hsa-mir-17	AC007938.3	0.380995273	mirna_lnc

30132	hsa-mir-17	AC098818.2	0.318219126	mirna_lnc
30133	hsa-mir-17	MAP3K2-DT	0.301093163	mirna_lnc
30134	hsa-mir-17	AL137244.1	0.300033359	mirna_lnc
30135	hsa-mir-17	AL512652.2	0.368638375	mirna_lnc
30136	hsa-mir-17	AC112255.1	0.301367512	mirna_lnc
30137	hsa-mir-17	MCF2L-AS1	0.448432366	mirna_lnc
30138	hsa-mir-17	AL138767.3	0.325419011	mirna_lnc
30139	hsa-mir-17	AC098936.1	0.321230235	mirna_lnc
30140	hsa-mir-17	AC107068.1	0.309825834	mirna_lnc
30141	hsa-mir-17	AL118511.1	0.37424431	mirna_lnc
30142	hsa-mir-17	AC106820.4	0.391929815	mirna_lnc
30143	hsa-mir-17	LINC02575	0.304754488	mirna_lnc
30144	hsa-mir-17	LINC00449	0.327639237	mirna_lnc
30145	hsa-mir-17	LINC00456	0.311997277	mirna_lnc
30146	hsa-mir-17	AL161729.4	0.315347779	mirna_lnc
30147	hsa-mir-17	PSPC1-AS2	0.311167214	mirna_lnc
30148	hsa-mir-301a	AP000708.1	0.350663048	mirna_lnc
30149	hsa-mir-301a	LINC02595	0.324500965	mirna_lnc
30150	hsa-mir-301a	AL662797.1	0.341752532	mirna_lnc
30151	hsa-mir-301a	LINC01873	0.399862409	mirna_lnc
30152	hsa-mir-301a	TMPO-AS1	0.460273537	mirna_lnc
30153	hsa-mir-301a	AL359513.1	0.346461399	mirna_lnc
30154	hsa-mir-301a	AC103591.3	0.433790644	mirna_lnc
30155	hsa-mir-301a	AC004241.3	0.504796802	mirna_lnc
30156	hsa-mir-301a	AL117332.1	0.384379345	mirna_lnc
30157	hsa-mir-301a	CEP83-DT	0.380554034	mirna_lnc
30158	hsa-mir-301a	JARID2-AS1	0.46333297	mirna_lnc
30159	hsa-mir-301a	AC106820.3	0.312783342	mirna_lnc
30160	hsa-mir-301a	AC074117.1	0.353944179	mirna_lnc
30161	hsa-mir-301a	AL031667.3	0.490896267	mirna_lnc
30162	hsa-mir-301a	TFAP2A-AS1	0.468474457	mirna_lnc
30163	hsa-mir-301a	AC100791.2	0.346216295	mirna_lnc
30164	hsa-mir-301a	LINC02542	0.370770229	mirna_lnc
30165	hsa-mir-301a	AC092159.2	0.336664853	mirna_lnc
30166	hsa-mir-301a	LINC01494	0.420760976	mirna_lnc
30167	hsa-mir-301a	AL023803.3	0.420445739	mirna_lnc
30168	hsa-mir-301a	AC017083.1	0.338719032	mirna_lnc
30169	hsa-mir-301a	AL121655.1	0.463310563	mirna_lnc
30170	hsa-mir-301a	AC009121.1	0.347283501	mirna_lnc
30171	hsa-mir-301a	AC109322.1	0.385351432	mirna_lnc
30172	hsa-mir-301a	LINC01952	0.301822862	mirna_lnc
30173	hsa-mir-301a	AC074351.1	0.390695383	mirna_lnc
30174	hsa-mir-301a	AL445072.1	0.352886348	mirna_lnc
30175	hsa-mir-301a	AC108134.2	0.301033218	mirna_lnc
30176	hsa-mir-301a	AC079907.1	0.318537096	mirna_lnc
30177	hsa-mir-301a	AC023157.3	0.308170444	mirna_lnc
30178	hsa-mir-301a	AC064807.1	0.358455402	mirna_lnc
30179	hsa-mir-301a	AL137802.2	0.361826215	mirna_lnc
30180	hsa-mir-301a	AC087289.3	0.359449238	mirna_lnc
30181	hsa-mir-301a	AL136162.1	0.519538025	mirna_lnc
30182	hsa-mir-301a	LINC02367	0.346702017	mirna_lnc
30183	hsa-mir-301a	AC073195.1	0.411919339	mirna_lnc
30184	hsa-mir-301a	AC015818.2	0.37001179	mirna_lnc
30185	hsa-mir-301a	AL596094.1	0.400953115	mirna_lnc

30186	hsa-mir-301a	AC004069.1	0.491689318	mirna_lnc
30187	hsa-mir-301a	LINC01096	0.371432872	mirna_lnc
30188	hsa-mir-301a	AC131025.1	0.312323508	mirna_lnc
30189	hsa-mir-301a	AC078788.1	0.378433512	mirna_lnc
30190	hsa-mir-301a	AC004920.1	0.47971955	mirna_lnc
30191	hsa-mir-301a	AC021683.3	0.504963004	mirna_lnc
30192	hsa-mir-301a	AC010359.1	0.325838452	mirna_lnc
30193	hsa-mir-301a	AL391069.2	0.374275896	mirna_lnc
30194	hsa-mir-301a	AC012640.2	0.462045238	mirna_lnc
30195	hsa-mir-301a	AC090970.2	0.37463806	mirna_lnc
30196	hsa-mir-301a	AC026782.2	0.41738232	mirna_lnc
30197	hsa-mir-301a	AC116049.2	0.42186134	mirna_lnc
30198	hsa-mir-301a	AP003119.2	0.419386201	mirna_lnc
30199	hsa-mir-301a	YTHDF3-AS1	0.310367315	mirna_lnc
30200	hsa-mir-301a	AL513497.1	0.315552182	mirna_lnc
30201	hsa-mir-301a	AC025244.1	0.323702623	mirna_lnc
30202	hsa-mir-301a	AC012625.1	0.318094043	mirna_lnc
30203	hsa-mir-301a	AL353764.1	0.419171106	mirna_lnc
30204	hsa-mir-301a	AC024230.1	0.398000883	mirna_lnc
30205	hsa-mir-301a	AC013400.1	0.410935332	mirna_lnc
30206	hsa-mir-301a	LINC01424	0.451543635	mirna_lnc
30207	hsa-mir-301a	AC022211.4	0.330210248	mirna_lnc
30208	hsa-mir-301a	HOMER3-AS1	0.320776474	mirna_lnc
30209	hsa-mir-301a	TBC1D8-AS1	0.486136878	mirna_lnc
30210	hsa-mir-301a	AP000487.1	0.390280695	mirna_lnc
30211	hsa-mir-301a	KIAA1614-AS1	0.423039351	mirna_lnc
30212	hsa-mir-301a	AC016876.3	0.473210612	mirna_lnc
30213	hsa-mir-301a	AL163051.1	0.374940802	mirna_lnc
30214	hsa-mir-301a	AL513327.2	0.491741483	mirna_lnc
30215	hsa-mir-301a	AC104971.1	0.315036865	mirna_lnc
30216	hsa-mir-301a	IQCJ-SCHIP1-AS1	0.322462735	mirna_lnc
30217	hsa-mir-301a	AC084125.2	0.334272136	mirna_lnc
30218	hsa-mir-301a	AC138356.1	0.444729081	mirna_lnc
30219	hsa-mir-301a	AC092807.2	0.425355026	mirna_lnc
30220	hsa-mir-301a	AC002076.1	0.319991655	mirna_lnc
30221	hsa-mir-301a	LINC01850	0.406496882	mirna_lnc
30222	hsa-mir-301a	AL683887.1	0.327769994	mirna_lnc
30223	hsa-mir-301a	AP000593.3	0.359809671	mirna_lnc
30224	hsa-mir-301a	WWTR1-AS1	0.329203246	mirna_lnc
30225	hsa-mir-301a	LINC01686	0.387686466	mirna_lnc
30226	hsa-mir-301a	MIR583HG	0.357214605	mirna_lnc
30227	hsa-mir-301a	AC239799.1	0.369002716	mirna_lnc
30228	hsa-mir-301a	AC100782.1	0.335686788	mirna_lnc
30229	hsa-mir-301a	CASC9	0.350467221	mirna_lnc
30230	hsa-mir-301a	AC010595.1	0.398718969	mirna_lnc
30231	hsa-mir-301a	AL391069.3	0.334325671	mirna_lnc
30232	hsa-mir-301a	LINC02607	0.516258521	mirna_lnc
30233	hsa-mir-301a	WASIR2	0.321163107	mirna_lnc
30234	hsa-mir-301a	LINC01816	0.525390787	mirna_lnc
30235	hsa-mir-301a	SREBF2-AS1	0.361069405	mirna_lnc
30236	hsa-mir-301a	AC099518.6	0.476841335	mirna_lnc
30237	hsa-mir-301a	AC007881.3	0.424564098	mirna_lnc
30238	hsa-mir-301a	DOCK4-AS1	0.30663688	mirna_lnc
30239	hsa-mir-301a	AL121658.1	0.383018061	mirna_lnc

30240	hsa-mir-301a	AC010969.2	0.364127364	mirna_lnc
30241	hsa-mir-301a	AC022098.3	0.411671989	mirna_lnc
30242	hsa-mir-301a	AC005332.1	0.304036727	mirna_lnc
30243	hsa-mir-301a	LINC00167	0.47264394	mirna_lnc
30244	hsa-mir-301a	AC100812.1	0.393698912	mirna_lnc
30245	hsa-mir-301a	AC004943.3	0.380372831	mirna_lnc
30246	hsa-mir-301a	AC046143.2	0.360416678	mirna_lnc
30247	hsa-mir-301a	AC145343.1	0.456137971	mirna_lnc
30248	hsa-mir-301a	AL121992.2	0.328001677	mirna_lnc
30249	hsa-mir-301a	AC093424.1	0.421325431	mirna_lnc
30250	hsa-mir-301a	AC091925.1	0.372175587	mirna_lnc
30251	hsa-mir-301a	AC117382.2	0.472922331	mirna_lnc
30252	hsa-mir-301a	AC091153.3	0.416358583	mirna_lnc
30253	hsa-mir-301a	AC063948.1	0.336127901	mirna_lnc
30254	hsa-mir-301a	AC087742.1	0.414438369	mirna_lnc
30255	hsa-mir-301a	AL354892.2	0.38938372	mirna_lnc
30256	hsa-mir-301a	AC015802.5	0.352761087	mirna_lnc
30257	hsa-mir-301a	AL139327.2	0.373926471	mirna_lnc
30258	hsa-mir-301a	DDX39B-AS1	0.373492362	mirna_lnc
30259	hsa-mir-301a	AL031963.3	0.349906695	mirna_lnc
30260	hsa-mir-301a	AC106820.2	0.318565834	mirna_lnc
30261	hsa-mir-301a	AC026271.3	0.349862747	mirna_lnc
30262	hsa-mir-301a	AC016205.1	0.394505984	mirna_lnc
30263	hsa-mir-301a	AC064836.3	0.371043399	mirna_lnc
30264	hsa-mir-301a	AC011290.1	0.323842251	mirna_lnc
30265	hsa-mir-301a	BX088651.4	0.308797397	mirna_lnc
30266	hsa-mir-301a	AC109326.1	0.316257307	mirna_lnc
30267	hsa-mir-301a	PIK3CD-AS2	0.301006466	mirna_lnc
30268	hsa-mir-301a	AC012640.5	0.330872943	mirna_lnc
30269	hsa-mir-301a	AL512408.1	0.565998981	mirna_lnc
30270	hsa-mir-301a	AC090527.3	0.329662325	mirna_lnc
30271	hsa-mir-301a	AC087741.2	0.405170501	mirna_lnc
30272	hsa-mir-301a	AL353719.1	0.319251637	mirna_lnc
30273	hsa-mir-301a	AC010422.2	0.323996089	mirna_lnc
30274	hsa-mir-301a	AC010608.2	0.330630667	mirna_lnc
30275	hsa-mir-301a	AP002387.2	0.306603291	mirna_lnc
30276	hsa-mir-301a	LINC01063	0.500254439	mirna_lnc
30277	hsa-mir-301a	AC092669.1	0.457567759	mirna_lnc
30278	hsa-mir-301a	CIRBP-AS1	0.400452705	mirna_lnc
30279	hsa-mir-301a	HGC6.3	0.306615679	mirna_lnc
30280	hsa-mir-301a	AP002336.2	0.410981715	mirna_lnc
30281	hsa-mir-301a	AC091946.2	0.384639389	mirna_lnc
30282	hsa-mir-301a	AC115837.2	0.315322913	mirna_lnc
30283	hsa-mir-301a	AC084757.2	0.329588807	mirna_lnc
30284	hsa-mir-301a	AC092171.3	0.310704905	mirna_lnc
30285	hsa-mir-301a	AC011773.1	0.323189528	mirna_lnc
30286	hsa-mir-301a	AC040169.3	0.37895214	mirna_lnc
30287	hsa-mir-301a	AC009171.2	0.395802574	mirna_lnc
30288	hsa-mir-301a	CNNM3-DT	0.352055907	mirna_lnc
30289	hsa-mir-301a	HOXC13-AS	0.313324243	mirna_lnc
30290	hsa-mir-301a	AC068831.6	0.405806428	mirna_lnc
30291	hsa-mir-301a	AL355482.2	0.340229866	mirna_lnc
30292	hsa-mir-301a	AC080013.5	0.360157891	mirna_lnc
30293	hsa-mir-301a	AL645608.4	0.316192234	mirna_lnc

30294	hsa-mir-301a	LINC02562	0.360611438	mirna_lnc
30295	hsa-mir-301a	AC138696.2	0.478936347	mirna_lnc
30296	hsa-mir-301a	AC007448.3	0.312825173	mirna_lnc
30297	hsa-mir-301a	MAST4-AS1	0.320382253	mirna_lnc
30298	hsa-mir-301a	AC090774.2	0.311922253	mirna_lnc
30299	hsa-mir-301a	AC098818.2	0.313091077	mirna_lnc
30300	hsa-mir-301a	LINC01807	0.363028817	mirna_lnc
30301	hsa-mir-301a	AC022762.1	0.301004566	mirna_lnc
30302	hsa-mir-301a	TCF7L1-IT1	0.316492325	mirna_lnc
30303	hsa-mir-301a	AC008267.5	0.355804011	mirna_lnc
30304	hsa-mir-301a	AC107214.1	0.429031059	mirna_lnc
30305	hsa-mir-301a	AC034236.2	0.353927558	mirna_lnc
30306	hsa-mir-301a	LINC00885	0.383188865	mirna_lnc
30307	hsa-mir-301a	LINC01971	0.331729691	mirna_lnc
30308	hsa-mir-301a	AC073896.4	0.408617816	mirna_lnc
30309	hsa-mir-301a	AP005328.2	0.3107882	mirna_lnc
30310	hsa-mir-301a	DDR1-DT	0.366779158	mirna_lnc
30311	hsa-mir-301a	AC108865.1	0.356466082	mirna_lnc
30312	hsa-mir-301a	URB1-AS1	0.325219618	mirna_lnc
30313	hsa-mir-301a	AC024243.1	0.331944934	mirna_lnc
30314	hsa-mir-301a	AC011446.1	0.438501554	mirna_lnc
30315	hsa-mir-301a	AC008736.2	0.38977524	mirna_lnc
30316	hsa-mir-301a	AC091271.1	0.384442378	mirna_lnc
30317	hsa-mir-301a	AL590399.1	0.319441707	mirna_lnc
30318	hsa-mir-301a	AC026304.1	0.382002224	mirna_lnc
30319	hsa-mir-301a	AC096992.2	0.402408837	mirna_lnc
30320	hsa-mir-301a	LPP-AS2	0.340573513	mirna_lnc
30321	hsa-mir-301a	AC010186.1	0.42678237	mirna_lnc
30322	hsa-mir-301a	ELDR	0.543657081	mirna_lnc
30323	hsa-mir-301a	AC090617.5	0.318878009	mirna_lnc
30324	hsa-mir-301a	AC019129.2	0.371054568	mirna_lnc
30325	hsa-mir-301a	AC005790.1	0.422529939	mirna_lnc
30326	hsa-mir-301a	AL359091.5	0.376560056	mirna_lnc
30327	hsa-mir-301a	AP003086.2	0.304655638	mirna_lnc
30328	hsa-mir-301a	AP001775.2	0.320584241	mirna_lnc
30329	hsa-mir-301a	AL132712.1	0.320723867	mirna_lnc
30330	hsa-mir-301a	AC023355.1	0.393220244	mirna_lnc
30331	hsa-mir-301a	AL606760.3	0.411148255	mirna_lnc
30332	hsa-mir-301a	AC093677.2	0.472346765	mirna_lnc
30333	hsa-mir-301a	AC096708.3	0.332676935	mirna_lnc
30334	hsa-mir-301a	AC090907.2	0.348746211	mirna_lnc
30335	hsa-mir-301a	IQCA1-AS1	0.380968711	mirna_lnc
30336	hsa-mir-301a	AC117395.1	0.3096726	mirna_lnc
30337	hsa-mir-301a	INTS6L-AS1	0.366756137	mirna_lnc
30338	hsa-mir-301a	AL031432.3	0.319610289	mirna_lnc
30339	hsa-mir-301a	AL031186.1	0.365590376	mirna_lnc
30340	hsa-mir-301a	AC083880.1	0.372507337	mirna_lnc
30341	hsa-mir-301a	AL121601.1	0.362743296	mirna_lnc
30342	hsa-mir-301a	AC099522.2	0.306774397	mirna_lnc
30343	hsa-mir-301a	AL034399.2	0.302665947	mirna_lnc
30344	hsa-mir-301a	AP001547.1	0.427867328	mirna_lnc
30345	hsa-mir-301a	LINC01607	0.40211093	mirna_lnc
30346	hsa-mir-301a	LINC01258	0.329001443	mirna_lnc
30347	hsa-mir-301a	AC145423.3	0.315947195	mirna_lnc

30348	hsa-mir-301a	AC046168.1	0.312265059	mirna_lnc
30349	hsa-mir-301a	AL136038.3	0.314788772	mirna_lnc
30350	hsa-mir-301a	AP000892.1	0.333735853	mirna_lnc
30351	hsa-mir-301a	AC007541.1	0.321966368	mirna_lnc
30352	hsa-mir-301a	AC007389.5	0.437885774	mirna_lnc
30353	hsa-mir-301a	AC108865.2	0.466688443	mirna_lnc
30354	hsa-mir-301a	AC010834.2	0.429063671	mirna_lnc
30355	hsa-mir-301a	AL022341.1	0.364679511	mirna_lnc
30356	hsa-mir-301a	AP000251.1	0.526578272	mirna_lnc
30357	hsa-mir-301a	AC025575.2	0.358670619	mirna_lnc
30358	hsa-mir-301a	BX005266.2	0.310107861	mirna_lnc
30359	hsa-mir-301a	CPNE8-AS1	0.332593848	mirna_lnc
30360	hsa-mir-301a	AL355102.4	0.301152315	mirna_lnc
30361	hsa-mir-301a	AC118658.1	0.310311344	mirna_lnc
30362	hsa-mir-301a	AC005865.1	0.316669835	mirna_lnc
30363	hsa-mir-301a	AL138881.1	0.445249144	mirna_lnc
30364	hsa-mir-301a	AC090907.1	0.461058072	mirna_lnc
30365	hsa-mir-301a	AC093635.1	0.361296328	mirna_lnc
30366	hsa-mir-301a	LINC01715	0.442802638	mirna_lnc
30367	hsa-mir-301a	AC009560.1	0.309586343	mirna_lnc
30368	hsa-mir-301a	AL590822.1	0.400120824	mirna_lnc
30369	hsa-mir-301a	AC018553.1	0.321822026	mirna_lnc
30370	hsa-mir-301a	AC027644.3	0.390297543	mirna_lnc
30371	hsa-mir-301a	AGBL5-AS1	0.309081395	mirna_lnc
30372	hsa-mir-301a	AC079174.2	0.303503756	mirna_lnc
30373	hsa-mir-301a	ETV5-AS1	0.309130165	mirna_lnc
30374	hsa-mir-301a	SCAT8	0.470131205	mirna_lnc
30375	hsa-mir-301a	AL356488.3	0.489886796	mirna_lnc
30376	hsa-mir-301a	MANEA-DT	0.339855614	mirna_lnc
30377	hsa-mir-301a	SEMA6A-AS2	0.367049514	mirna_lnc
30378	hsa-mir-301a	AC004969.1	0.345629178	mirna_lnc
30379	hsa-mir-301a	AC069277.1	0.365692016	mirna_lnc
30380	hsa-mir-301a	AL032819.1	0.479067727	mirna_lnc
30381	hsa-mir-301a	AL844908.2	0.408140214	mirna_lnc
30382	hsa-mir-301a	AP001783.1	0.331100656	mirna_lnc
30383	hsa-mir-301a	AC092119.3	0.414358555	mirna_lnc
30384	hsa-mir-301a	AC069307.1	0.394396779	mirna_lnc
30385	hsa-mir-301a	AC099343.3	0.314671427	mirna_lnc
30386	hsa-mir-301a	AC079834.2	0.324636397	mirna_lnc
30387	hsa-mir-301a	AL021154.1	0.390987399	mirna_lnc
30388	hsa-mir-301a	AC004012.1	0.476057221	mirna_lnc
30389	hsa-mir-301a	AC087276.1	0.313971675	mirna_lnc
30390	hsa-mir-301a	AL583808.1	0.450605677	mirna_lnc
30391	hsa-mir-301a	AC068620.2	0.35918319	mirna_lnc
30392	hsa-mir-301a	AP003392.3	0.379817981	mirna_lnc
30393	hsa-mir-301a	AP005205.3	0.313632546	mirna_lnc
30394	hsa-mir-301a	AC005034.4	0.377868703	mirna_lnc
30395	hsa-mir-301a	Z93241.1	0.354469284	mirna_lnc
30396	hsa-mir-301a	AL359878.2	0.361845838	mirna_lnc
30397	hsa-mir-301a	AP000808.2	0.339956299	mirna_lnc
30398	hsa-mir-301a	AC068338.2	0.300543845	mirna_lnc
30399	hsa-mir-301a	AL731537.1	0.415050054	mirna_lnc
30400	hsa-mir-301a	AC135178.5	0.37366557	mirna_lnc
30401	hsa-mir-301a	AC106820.4	0.320624152	mirna_lnc

30402	hsa-mir-301a	KCNJ2-AS1	0.336428689	mirna_lnc
30403	hsa-mir-301a	TPRG1-AS1	0.343404735	mirna_lnc
30404	hsa-mir-301a	AC002059.1	0.443738043	mirna_lnc
30405	hsa-mir-301a	AL645608.6	0.323064912	mirna_lnc
30406	hsa-mir-301a	AC000120.1	0.327659807	mirna_lnc
30407	hsa-mir-301a	AC010980.2	0.3883337	mirna_lnc
30408	hsa-mir-301a	AC025162.2	0.311575682	mirna_lnc
30409	hsa-mir-301a	AC136604.3	0.321934144	mirna_lnc
30410	hsa-mir-301a	AC130343.2	0.311181762	mirna_lnc
30411	hsa-mir-301a	AC005197.1	0.37520585	mirna_lnc
30412	hsa-mir-301a	AC145422.1	0.312755651	mirna_lnc
30413	hsa-mir-301a	AC145423.2	0.370471634	mirna_lnc
30414	hsa-mir-301a	AC092142.1	0.344605811	mirna_lnc
30415	hsa-mir-301a	CYP51A1-AS1	0.456092101	mirna_lnc
30416	hsa-mir-301a	AL512791.2	0.351906225	mirna_lnc
30417	hsa-mir-301a	AP001893.3	0.307764894	mirna_lnc
30418	hsa-mir-301a	RN7SL832P	0.420865266	mirna_lnc
30419	hsa-mir-301a	LINC02076	0.36526921	mirna_lnc
30420	hsa-mir-301a	AL358933.1	0.380778197	mirna_lnc
30421	hsa-mir-301a	AP001972.4	0.35053792	mirna_lnc
30422	hsa-mir-301a	AL590705.3	0.471960553	mirna_lnc
30423	hsa-mir-301a	AC002044.1	0.409112921	mirna_lnc
30424	hsa-mir-301a	LINC00900	0.326047489	mirna_lnc
30425	hsa-mir-301a	AC015917.2	0.317172284	mirna_lnc
30426	hsa-mir-301a	FOXN3-AS1	0.308338101	mirna_lnc
30427	hsa-mir-301a	TOLLIP-AS1	0.318587563	mirna_lnc
30428	hsa-mir-301a	AC021092.1	0.329755172	mirna_lnc
30429	hsa-mir-301a	AC012676.3	0.368947921	mirna_lnc
30430	hsa-mir-483	AC020659.1	0.459961171	mirna_lnc
30431	hsa-mir-483	LINC00322	0.341934855	mirna_lnc
30432	hsa-mir-483	AC093458.1	0.487700242	mirna_lnc
30433	hsa-mir-483	AC090709.1	0.552937331	mirna_lnc
30434	hsa-mir-483	AC021739.2	0.433324449	mirna_lnc
30435	hsa-mir-483	AC013565.1	0.356560644	mirna_lnc
30436	hsa-mir-483	AL590708.1	0.308718085	mirna_lnc
30437	hsa-mir-483	AC012363.2	0.337672168	mirna_lnc
30438	hsa-mir-483	AP000696.1	0.505617429	mirna_lnc
30439	hsa-mir-483	AC093458.2	0.479921925	mirna_lnc
30440	hsa-mir-483	AC131011.1	0.452785769	mirna_lnc
30441	hsa-mir-483	AL162457.2	0.605951723	mirna_lnc
30442	hsa-mir-483	AC009646.2	0.636406347	mirna_lnc
30443	hsa-mir-483	AL590666.2	0.383834011	mirna_lnc
30444	hsa-mir-483	IGF2-AS	0.636938483	mirna_lnc
30445	hsa-mir-483	AC099552.4	0.350864449	mirna_lnc
30446	hsa-mir-483	AC073592.8	0.421768481	mirna_lnc
30447	hsa-mir-483	AC011944.1	0.393362647	mirna_lnc
30448	hsa-mir-483	AC078860.1	0.39465204	mirna_lnc
30449	hsa-mir-483	AC010266.2	0.369848953	mirna_lnc
30450	hsa-mir-483	AC015660.2	0.410759371	mirna_lnc
30451	hsa-mir-483	AC007099.1	0.68730129	mirna_lnc
30452	hsa-mir-483	AC011444.3	0.360479851	mirna_lnc
30453	hsa-mir-483	AC011298.1	0.319960412	mirna_lnc
30454	hsa-mir-483	LINC01101	0.417302954	mirna_lnc
30455	hsa-mir-483	CHRM3-AS1	0.329619318	mirna_lnc

30456	hsa-mir-483	AC090116.1	0.479943848	mirna_lnc
30457	hsa-mir-483	AL031665.1	0.341567838	mirna_lnc
30458	hsa-mir-483	AC012360.1	0.358724628	mirna_lnc
30459	hsa-mir-483	AP006248.3	0.325336375	mirna_lnc
30460	hsa-mir-483	AC243964.3	0.420275385	mirna_lnc
30461	hsa-mir-483	AC015909.2	0.71336776	mirna_lnc
30462	hsa-mir-483	AC025166.1	0.640940933	mirna_lnc
30463	hsa-mir-483	AL606537.1	0.49683701	mirna_lnc
30464	hsa-mir-483	AC010328.1	0.638662554	mirna_lnc
30465	hsa-mir-483	AL590438.1	0.558390519	mirna_lnc
30466	hsa-mir-483	Z98885.2	0.447636531	mirna_lnc
30467	hsa-mir-483	AC078942.1	0.417841794	mirna_lnc
30468	hsa-mir-483	AC100835.1	0.32641661	mirna_lnc
30469	hsa-mir-483	AC096541.1	0.378562979	mirna_lnc
30470	hsa-mir-483	LINC00622	0.394064365	mirna_lnc
30471	hsa-mir-483	AC022146.2	0.423291424	mirna_lnc
30472	hsa-mir-483	AC005586.1	0.45387349	mirna_lnc
30473	hsa-mir-483	TTLL10-AS1	0.429203174	mirna_lnc
30474	hsa-mir-483	AC021739.5	0.525358735	mirna_lnc
30475	hsa-mir-483	AC021739.3	0.625138062	mirna_lnc
30476	hsa-mir-483	AC016168.3	0.357296007	mirna_lnc
30477	hsa-mir-483	AL360268.1	0.411048772	mirna_lnc
30478	hsa-mir-483	AC100803.3	0.421795698	mirna_lnc
30479	hsa-mir-483	AL163953.1	0.495832254	mirna_lnc
30480	hsa-mir-483	AP006248.4	0.376082458	mirna_lnc
30481	hsa-mir-483	LINC02244	0.301864414	mirna_lnc
30482	hsa-mir-483	AC092301.1	0.420468537	mirna_lnc
30483	hsa-mir-483	AL121832.1	0.737364285	mirna_lnc
30484	hsa-mir-483	AC012467.2	0.446780504	mirna_lnc
30485	hsa-mir-483	AC103681.2	0.424210146	mirna_lnc
30486	hsa-mir-483	AC078820.1	0.311741559	mirna_lnc
30487	hsa-mir-483	AC011447.7	0.479489144	mirna_lnc
30488	hsa-mir-483	CAMTA1-IT1	0.459053411	mirna_lnc
30489	hsa-mir-483	AC109309.1	0.636128206	mirna_lnc
30490	hsa-mir-99b	LINC01783	0.329818675	mirna_lnc
30491	hsa-mir-99b	AC007193.2	0.305817216	mirna_lnc
30492	hsa-mir-99b	ZNF30-AS1	0.300692363	mirna_lnc
30493	hsa-mir-99b	AC092667.1	0.348256843	mirna_lnc
30494	hsa-mir-99b	AL160286.2	0.425864925	mirna_lnc
30495	hsa-mir-99b	TMPO-AS1	0.452578837	mirna_lnc
30496	hsa-mir-99b	AP000808.1	0.328123459	mirna_lnc
30497	hsa-mir-99b	AL670729.1	0.302198382	mirna_lnc
30498	hsa-mir-99b	AL117332.1	0.426367419	mirna_lnc
30499	hsa-mir-99b	AC092614.1	0.323199397	mirna_lnc
30500	hsa-mir-99b	CEP83-DT	0.334350295	mirna_lnc
30501	hsa-mir-99b	HOXC-AS2	0.318251133	mirna_lnc
30502	hsa-mir-99b	AC027117.2	0.32948466	mirna_lnc
30503	hsa-mir-99b	AC097532.2	0.32403361	mirna_lnc
30504	hsa-mir-99b	LINC01419	0.503908947	mirna_lnc
30505	hsa-mir-99b	AC063977.6	0.421749176	mirna_lnc
30506	hsa-mir-99b	LINC00567	0.338888704	mirna_lnc
30507	hsa-mir-99b	AC096656.1	0.302669811	mirna_lnc
30508	hsa-mir-99b	AC006213.3	0.349249178	mirna_lnc
30509	hsa-mir-99b	AP003071.2	0.339491245	mirna_lnc

30510	hsa-mir-99b	AF233439.1	0.438275514	mirna_lnc
30511	hsa-mir-99b	LINC01494	0.408615234	mirna_lnc
30512	hsa-mir-99b	AC092803.2	0.349133782	mirna_lnc
30513	hsa-mir-99b	OCIAD1-AS1	0.303231651	mirna_lnc
30514	hsa-mir-99b	LINC02302	0.41362966	mirna_lnc
30515	hsa-mir-99b	AC016723.1	0.455254199	mirna_lnc
30516	hsa-mir-99b	UBE2E1-AS1	0.365922096	mirna_lnc
30517	hsa-mir-99b	AC092111.1	0.316927323	mirna_lnc
30518	hsa-mir-99b	AL139424.1	0.377709379	mirna_lnc
30519	hsa-mir-99b	AL133371.3	0.330843702	mirna_lnc
30520	hsa-mir-99b	LINC01096	0.447943296	mirna_lnc
30521	hsa-mir-99b	AC011481.4	0.436030356	mirna_lnc
30522	hsa-mir-99b	AC008543.1	0.368857186	mirna_lnc
30523	hsa-mir-99b	HOXA10-AS	0.387732186	mirna_lnc
30524	hsa-mir-99b	AL391069.2	0.324230167	mirna_lnc
30525	hsa-mir-99b	AC006116.10	0.351626497	mirna_lnc
30526	hsa-mir-99b	AC116049.2	0.47519883	mirna_lnc
30527	hsa-mir-99b	AL139023.1	0.322420709	mirna_lnc
30528	hsa-mir-99b	AP000640.2	0.312556463	mirna_lnc
30529	hsa-mir-99b	AC020928.2	0.333674203	mirna_lnc
30530	hsa-mir-99b	AL354993.2	0.385594245	mirna_lnc
30531	hsa-mir-99b	ATP6V1B1-AS1	0.467363245	mirna_lnc
30532	hsa-mir-99b	AJ239322.1	0.491373147	mirna_lnc
30533	hsa-mir-99b	AC024230.1	0.4757663	mirna_lnc
30534	hsa-mir-99b	LINC01397	0.301017421	mirna_lnc
30535	hsa-mir-99b	AL391152.1	0.418457829	mirna_lnc
30536	hsa-mir-99b	MAGEA4-AS1	0.307394119	mirna_lnc
30537	hsa-mir-99b	AC026992.2	0.450345669	mirna_lnc
30538	hsa-mir-99b	AC012074.1	0.320577271	mirna_lnc
30539	hsa-mir-99b	AL358473.1	0.364386845	mirna_lnc
30540	hsa-mir-99b	AC006486.2	0.31802173	mirna_lnc
30541	hsa-mir-99b	AC002076.1	0.441828659	mirna_lnc
30542	hsa-mir-99b	Z95115.1	0.371182385	mirna_lnc
30543	hsa-mir-99b	LINC01143	0.320479517	mirna_lnc
30544	hsa-mir-99b	AC022031.2	0.322974074	mirna_lnc
30545	hsa-mir-99b	LINC01671	0.396697889	mirna_lnc
30546	hsa-mir-99b	AC087273.2	0.45540743	mirna_lnc
30547	hsa-mir-99b	AC100782.1	0.441701289	mirna_lnc
30548	hsa-mir-99b	ARHGAP31-AS1	0.360626324	mirna_lnc
30549	hsa-mir-99b	AC079145.1	0.362312596	mirna_lnc
30550	hsa-mir-99b	AC010595.1	0.302674884	mirna_lnc
30551	hsa-mir-99b	TMCO1-AS1	0.40429733	mirna_lnc
30552	hsa-mir-99b	SATB2-AS1	0.406764592	mirna_lnc
30553	hsa-mir-99b	LINC01521	0.311116362	mirna_lnc
30554	hsa-mir-99b	AC100812.1	0.317528239	mirna_lnc
30555	hsa-mir-99b	AC011297.1	0.307344056	mirna_lnc
30556	hsa-mir-99b	AC126614.1	0.396114567	mirna_lnc
30557	hsa-mir-99b	AL121721.1	0.56577912	mirna_lnc
30558	hsa-mir-99b	AL354892.2	0.346408718	mirna_lnc
30559	hsa-mir-99b	AC011773.4	0.303985174	mirna_lnc
30560	hsa-mir-99b	AC106820.2	0.322462771	mirna_lnc
30561	hsa-mir-99b	AC026271.3	0.400802914	mirna_lnc
30562	hsa-mir-99b	AC016205.1	0.375455592	mirna_lnc
30563	hsa-mir-99b	AC009955.1	0.303753274	mirna_lnc

30564	hsa-mir-99b	AC246793.1	0.408727449	mirna_lnc
30565	hsa-mir-99b	HGC6.3	0.393208626	mirna_lnc
30566	hsa-mir-99b	AC107204.1	0.318594891	mirna_lnc
30567	hsa-mir-99b	AP000525.1	0.377515864	mirna_lnc
30568	hsa-mir-99b	PCOLCE-AS1	0.35434253	mirna_lnc
30569	hsa-mir-99b	AP001065.2	0.374844949	mirna_lnc
30570	hsa-mir-99b	AC092171.3	0.443464307	mirna_lnc
30571	hsa-mir-99b	FAM66B	0.326165311	mirna_lnc
30572	hsa-mir-99b	AC006213.5	0.363269484	mirna_lnc
30573	hsa-mir-99b	AC084876.2	0.30126226	mirna_lnc
30574	hsa-mir-99b	AC083900.1	0.424019	mirna_lnc
30575	hsa-mir-99b	AC068987.4	0.491885867	mirna_lnc
30576	hsa-mir-99b	AC126407.1	0.300771202	mirna_lnc
30577	hsa-mir-99b	LINC02177	0.390159833	mirna_lnc
30578	hsa-mir-99b	AL445673.1	0.383836713	mirna_lnc
30579	hsa-mir-99b	AC012313.2	0.301254516	mirna_lnc
30580	hsa-mir-99b	AC009271.1	0.30596134	mirna_lnc
30581	hsa-mir-99b	AC098818.2	0.314775949	mirna_lnc
30582	hsa-mir-99b	AL137058.3	0.52224965	mirna_lnc
30583	hsa-mir-99b	USP27X-AS1	0.301461734	mirna_lnc
30584	hsa-mir-99b	AC107214.1	0.31580353	mirna_lnc
30585	hsa-mir-99b	FAM66E	0.319019745	mirna_lnc
30586	hsa-mir-99b	AP005328.2	0.518257328	mirna_lnc
30587	hsa-mir-99b	ZNF385D-AS1	0.515716106	mirna_lnc
30588	hsa-mir-99b	AC053527.1	0.327319669	mirna_lnc
30589	hsa-mir-99b	AP003064.2	0.404107271	mirna_lnc
30590	hsa-mir-99b	AC093915.1	0.327533517	mirna_lnc
30591	hsa-mir-99b	AL603832.1	0.301706401	mirna_lnc
30592	hsa-mir-99b	AL132765.2	0.301943465	mirna_lnc
30593	hsa-mir-99b	AC011445.1	0.390744618	mirna_lnc
30594	hsa-mir-99b	LINC01756	0.322951515	mirna_lnc
30595	hsa-mir-99b	AC106895.2	0.350439175	mirna_lnc
30596	hsa-mir-99b	AC073389.2	0.38107383	mirna_lnc
30597	hsa-mir-99b	AL591167.1	0.411711059	mirna_lnc
30598	hsa-mir-99b	AC007638.2	0.377201375	mirna_lnc
30599	hsa-mir-99b	AC090907.2	0.301035061	mirna_lnc
30600	hsa-mir-99b	AC011700.1	0.323382408	mirna_lnc
30601	hsa-mir-99b	AL031186.1	0.311723208	mirna_lnc
30602	hsa-mir-99b	AC005052.1	0.326011185	mirna_lnc
30603	hsa-mir-99b	AL353593.3	0.34207015	mirna_lnc
30604	hsa-mir-99b	AC025741.1	0.301937373	mirna_lnc
30605	hsa-mir-99b	AL590705.1	0.340138774	mirna_lnc
30606	hsa-mir-99b	AC011450.1	0.562612531	mirna_lnc
30607	hsa-mir-99b	AC012645.1	0.422866242	mirna_lnc
30608	hsa-mir-99b	AC145423.3	0.348418619	mirna_lnc
30609	hsa-mir-99b	AC007541.1	0.499909058	mirna_lnc
30610	hsa-mir-99b	AC116025.2	0.388194823	mirna_lnc
30611	hsa-mir-99b	AC034236.3	0.304845983	mirna_lnc
30612	hsa-mir-99b	WWC2-AS1	0.322545506	mirna_lnc
30613	hsa-mir-99b	ZNF197-AS1	0.300781585	mirna_lnc
30614	hsa-mir-99b	AC243960.3	0.323210118	mirna_lnc
30615	hsa-mir-99b	AP000317.1	0.305846369	mirna_lnc
30616	hsa-mir-99b	AL158055.1	0.327328208	mirna_lnc
30617	hsa-mir-99b	AL138767.3	0.370074529	mirna_lnc

30618	hsa-mir-99b	AC098936.1	0.320586293	mirna_lnc
30619	hsa-mir-99b	AC138207.2	0.342349295	mirna_lnc
30620	hsa-mir-99b	AC000036.1	0.435928316	mirna_lnc
30621	hsa-mir-99b	LINC02148	0.383392799	mirna_lnc
30622	hsa-mir-99b	HCG15	0.495668886	mirna_lnc
30623	hsa-mir-99b	AL162231.4	0.313965304	mirna_lnc
30624	hsa-mir-99b	ZNF649-AS1	0.349283703	mirna_lnc
30625	hsa-mir-99b	SEMA6A-AS2	0.452411924	mirna_lnc
30626	hsa-mir-99b	AL513327.3	0.335385849	mirna_lnc
30627	hsa-mir-99b	LINC02365	0.38732148	mirna_lnc
30628	hsa-mir-99b	AP003774.1	0.30702892	mirna_lnc
30629	hsa-mir-99b	MAGEA8-AS1	0.31724016	mirna_lnc
30630	hsa-mir-99b	LINC00485	0.307850496	mirna_lnc
30631	hsa-mir-99b	AC010501.1	0.449482671	mirna_lnc
30632	hsa-mir-99b	AC002056.2	0.300520603	mirna_lnc
30633	hsa-mir-99b	AL118511.1	0.407314194	mirna_lnc
30634	hsa-mir-99b	AC117402.1	0.511430079	mirna_lnc
30635	hsa-mir-99b	AC027348.1	0.517242843	mirna_lnc
30636	hsa-mir-99b	ZFHX4-AS1	0.436761652	mirna_lnc
30637	hsa-mir-99b	AC010524.1	0.313267787	mirna_lnc
30638	hsa-mir-99b	AC003070.2	0.445316737	mirna_lnc
30639	hsa-mir-99b	LINC02506	0.344337305	mirna_lnc
30640	hsa-mir-99b	AC005336.3	0.502345192	mirna_lnc
30641	hsa-mir-99b	GIHCG	0.376291926	mirna_lnc
30642	hsa-mir-99b	AC097504.2	0.322065954	mirna_lnc
30643	hsa-mir-99b	CEBPA-DT	0.417870862	mirna_lnc
30644	hsa-mir-99b	AC010643.1	0.380287705	mirna_lnc
30645	hsa-mir-99b	AC022107.1	0.475552275	mirna_lnc
30646	hsa-mir-99b	AC106820.4	0.414323605	mirna_lnc
30647	hsa-mir-99b	AC116407.3	0.321256942	mirna_lnc
30648	hsa-mir-99b	AC026801.2	0.309109594	mirna_lnc
30649	hsa-mir-99b	AC017100.1	0.308340432	mirna_lnc
30650	hsa-mir-99b	LINC00471	0.322464646	mirna_lnc
30651	hsa-mir-99b	AC074135.1	0.379907092	mirna_lnc
30652	hsa-mir-99b	LINC01447	0.32041884	mirna_lnc
30653	hsa-mir-99b	LIX1-AS1	0.356237743	mirna_lnc
30654	hsa-mir-99b	SPANXA2-OT1	0.568641929	mirna_lnc
30655	hsa-mir-99b	BOLA3-AS1	0.347776924	mirna_lnc
30656	hsa-mir-99b	AC018892.3	0.302045372	mirna_lnc
30657	hsa-mir-99b	SPACA6P-AS	0.476238678	mirna_lnc
30658	hsa-mir-99b	LINC01918	0.373840116	mirna_lnc
30659	hsa-mir-99b	FAM66A	0.314273478	mirna_lnc
30660	hsa-mir-99b	LINC00909	0.349353579	mirna_lnc
30661	hsa-mir-99b	PTOV1-AS1	0.357421995	mirna_lnc
30662	hsa-mir-99b	LINC02575	0.322409053	mirna_lnc
30663	hsa-mir-99b	AC145422.1	0.47517193	mirna_lnc
30664	hsa-mir-99b	AC145423.2	0.324410164	mirna_lnc
30665	hsa-mir-99b	AC032011.1	0.339020861	mirna_lnc
30666	hsa-mir-99b	AC107464.1	0.319564522	mirna_lnc
30667	hsa-mir-99b	WWC2-AS2	0.350634524	mirna_lnc
30668	hsa-mir-99b	AL512604.2	0.31042252	mirna_lnc
30669	hsa-mir-99b	AC019193.2	0.310187178	mirna_lnc
30670	hsa-mir-99b	AL354811.1	0.324323838	mirna_lnc
30671	hsa-mir-99b	AC068987.1	0.40770287	mirna_lnc

30672	hsa-mir-99b	AC061975.6	0.506837892	mirna_lnc
30673	hsa-mir-99b	AL662791.1	0.488289553	mirna_lnc
30674	hsa-mir-99b	LINC00605	0.340413497	mirna_lnc
30675	hsa-mir-99b	AC016229.2	0.397377561	mirna_lnc
30676	hsa-mir-99b	AP000662.1	0.473992784	mirna_lnc
30677	hsa-mir-99b	LINC01099	0.357320776	mirna_lnc
30678	hsa-mir-99b	GPC6-AS2	0.376332243	mirna_lnc
30679	hsa-mir-99b	AL132655.1	0.370826476	mirna_lnc
30680	hsa-mir-99b	AP001476.1	0.378622189	mirna_lnc
30681	hsa-mir-99b	AC010624.2	0.314776718	mirna_lnc
30682	hsa-mir-99b	AC006213.6	0.338568702	mirna_lnc
30683	hsa-mir-99b	AP001626.1	0.378902339	mirna_lnc
30684	hsa-mir-99b	AF213884.3	0.362360581	mirna_lnc
30685	hsa-mir-99b	AC021092.1	0.447783137	mirna_lnc
30686	hsa-mir-99b	AC127024.3	0.321100996	mirna_lnc
30687	hsa-mir-99b	BSN-DT	0.301204055	mirna_lnc
30688	hsa-mir-99b	AC243836.1	0.329965032	mirna_lnc
30689	hsa-mir-99b	AC099684.2	0.436982775	mirna_lnc
30690	hsa-mir-1180	AC012073.1	0.343522476	mirna_lnc
30691	hsa-mir-1180	AL662797.1	0.372724358	mirna_lnc
30692	hsa-mir-1180	TMPO-AS1	0.347572678	mirna_lnc
30693	hsa-mir-1180	AP000808.1	0.307809961	mirna_lnc
30694	hsa-mir-1180	AL391244.2	0.319931983	mirna_lnc
30695	hsa-mir-1180	AC025627.1	0.595058943	mirna_lnc
30696	hsa-mir-1180	AL590683.1	0.306632364	mirna_lnc
30697	hsa-mir-1180	AC004241.3	0.315088063	mirna_lnc
30698	hsa-mir-1180	AL117332.1	0.429463874	mirna_lnc
30699	hsa-mir-1180	AC022211.2	0.320575767	mirna_lnc
30700	hsa-mir-1180	SCAT2	0.303213381	mirna_lnc
30701	hsa-mir-1180	AC092614.1	0.310570256	mirna_lnc
30702	hsa-mir-1180	HOXC-AS2	0.328172092	mirna_lnc
30703	hsa-mir-1180	AL049539.1	0.305494125	mirna_lnc
30704	hsa-mir-1180	JARID2-AS1	0.304638697	mirna_lnc
30705	hsa-mir-1180	AC016065.1	0.31470807	mirna_lnc
30706	hsa-mir-1180	AC074117.1	0.32709681	mirna_lnc
30707	hsa-mir-1180	AP001469.3	0.335908115	mirna_lnc
30708	hsa-mir-1180	AL031667.3	0.370367047	mirna_lnc
30709	hsa-mir-1180	TFAP2A-AS1	0.30505184	mirna_lnc
30710	hsa-mir-1180	AP000526.1	0.31711635	mirna_lnc
30711	hsa-mir-1180	DLGAP1-AS2	0.330466497	mirna_lnc
30712	hsa-mir-1180	AC063977.6	0.389341011	mirna_lnc
30713	hsa-mir-1180	AC005324.5	0.464615264	mirna_lnc
30714	hsa-mir-1180	AL023803.3	0.335684295	mirna_lnc
30715	hsa-mir-1180	AC093484.4	0.300242026	mirna_lnc
30716	hsa-mir-1180	AC109322.1	0.304529835	mirna_lnc
30717	hsa-mir-1180	PLCH1-AS2	0.402518707	mirna_lnc
30718	hsa-mir-1180	AC074351.1	0.377550093	mirna_lnc
30719	hsa-mir-1180	AL138724.1	0.300709113	mirna_lnc
30720	hsa-mir-1180	AL445072.1	0.393903507	mirna_lnc
30721	hsa-mir-1180	AP002478.1	0.336365327	mirna_lnc
30722	hsa-mir-1180	AC016723.1	0.355709908	mirna_lnc
30723	hsa-mir-1180	AC104472.1	0.336281496	mirna_lnc
30724	hsa-mir-1180	SNHG19	0.317805066	mirna_lnc
30725	hsa-mir-1180	AL137802.2	0.315954308	mirna_lnc

30726	hsa-mir-1180	AC091729.3	0.324654075	mirna_lnc
30727	hsa-mir-1180	LINC01806	0.31797025	mirna_lnc
30728	hsa-mir-1180	AL136162.1	0.383146984	mirna_lnc
30729	hsa-mir-1180	LINC02367	0.33171183	mirna_lnc
30730	hsa-mir-1180	AC073195.1	0.384221246	mirna_lnc
30731	hsa-mir-1180	AC015818.2	0.357205907	mirna_lnc
30732	hsa-mir-1180	LINC01096	0.311750995	mirna_lnc
30733	hsa-mir-1180	AC021683.3	0.302769504	mirna_lnc
30734	hsa-mir-1180	AL391069.2	0.324295127	mirna_lnc
30735	hsa-mir-1180	AC012640.2	0.33335112	mirna_lnc
30736	hsa-mir-1180	AC116049.2	0.357060851	mirna_lnc
30737	hsa-mir-1180	AL139023.1	0.309896468	mirna_lnc
30738	hsa-mir-1180	AL513497.1	0.344881823	mirna_lnc
30739	hsa-mir-1180	AL161772.1	0.365927293	mirna_lnc
30740	hsa-mir-1180	AC024230.1	0.375043908	mirna_lnc
30741	hsa-mir-1180	LINC01397	0.335360018	mirna_lnc
30742	hsa-mir-1180	LINC01424	0.367873298	mirna_lnc
30743	hsa-mir-1180	AL591846.2	0.300139729	mirna_lnc
30744	hsa-mir-1180	TBC1D8-AS1	0.33631007	mirna_lnc
30745	hsa-mir-1180	AP000487.1	0.33947619	mirna_lnc
30746	hsa-mir-1180	KIAA1614-AS1	0.370326555	mirna_lnc
30747	hsa-mir-1180	AC016876.3	0.388275611	mirna_lnc
30748	hsa-mir-1180	AL391845.2	0.339151566	mirna_lnc
30749	hsa-mir-1180	AL359643.3	0.376881874	mirna_lnc
30750	hsa-mir-1180	AL163051.1	0.307660792	mirna_lnc
30751	hsa-mir-1180	AC104971.1	0.313987525	mirna_lnc
30752	hsa-mir-1180	LINC01456	0.36849319	mirna_lnc
30753	hsa-mir-1180	AC002076.1	0.355573891	mirna_lnc
30754	hsa-mir-1180	LINC01850	0.415493626	mirna_lnc
30755	hsa-mir-1180	AC007952.4	0.431125128	mirna_lnc
30756	hsa-mir-1180	AP000593.3	0.300607043	mirna_lnc
30757	hsa-mir-1180	WWTR1-AS1	0.338655679	mirna_lnc
30758	hsa-mir-1180	AC020558.2	0.362823972	mirna_lnc
30759	hsa-mir-1180	LINC01686	0.335212843	mirna_lnc
30760	hsa-mir-1180	LINC01143	0.311699943	mirna_lnc
30761	hsa-mir-1180	AC022031.2	0.324718036	mirna_lnc
30762	hsa-mir-1180	LINC01968	0.320947697	mirna_lnc
30763	hsa-mir-1180	AC125618.1	0.342543093	mirna_lnc
30764	hsa-mir-1180	AC002550.1	0.344677627	mirna_lnc
30765	hsa-mir-1180	AC010271.2	0.304146139	mirna_lnc
30766	hsa-mir-1180	AL354714.1	0.339945468	mirna_lnc
30767	hsa-mir-1180	AC010595.1	0.302325469	mirna_lnc
30768	hsa-mir-1180	AL391069.3	0.322581031	mirna_lnc
30769	hsa-mir-1180	AC006483.2	0.399788444	mirna_lnc
30770	hsa-mir-1180	AL592071.1	0.357304326	mirna_lnc
30771	hsa-mir-1180	SMCR2	0.300135859	mirna_lnc
30772	hsa-mir-1180	LINC01816	0.327443097	mirna_lnc
30773	hsa-mir-1180	AC007881.3	0.308221363	mirna_lnc
30774	hsa-mir-1180	AC010969.2	0.355805519	mirna_lnc
30775	hsa-mir-1180	LINC01521	0.338409445	mirna_lnc
30776	hsa-mir-1180	AC100812.1	0.331173448	mirna_lnc
30777	hsa-mir-1180	AC011297.1	0.300817221	mirna_lnc
30778	hsa-mir-1180	AC004943.3	0.326671054	mirna_lnc
30779	hsa-mir-1180	AC046143.2	0.301138817	mirna_lnc

30780	hsa-mir-1180	AC112907.1	0.331283016	mirna_lnc
30781	hsa-mir-1180	AC126614.1	0.319684935	mirna_lnc
30782	hsa-mir-1180	AL451085.2	0.32000942	mirna_lnc
30783	hsa-mir-1180	AC117382.2	0.31358125	mirna_lnc
30784	hsa-mir-1180	AC091153.3	0.409794588	mirna_lnc
30785	hsa-mir-1180	AC063948.1	0.311883697	mirna_lnc
30786	hsa-mir-1180	AC087742.1	0.360048676	mirna_lnc
30787	hsa-mir-1180	AL354892.2	0.476707242	mirna_lnc
30788	hsa-mir-1180	AC122688.4	0.343246744	mirna_lnc
30789	hsa-mir-1180	DDX39B-AS1	0.311678563	mirna_lnc
30790	hsa-mir-1180	AC106820.2	0.367005712	mirna_lnc
30791	hsa-mir-1180	AC026271.3	0.454229097	mirna_lnc
30792	hsa-mir-1180	AC012640.5	0.331015086	mirna_lnc
30793	hsa-mir-1180	AL512408.1	0.325766492	mirna_lnc
30794	hsa-mir-1180	AC090527.3	0.318284052	mirna_lnc
30795	hsa-mir-1180	MIR762HG	0.366772276	mirna_lnc
30796	hsa-mir-1180	AC010422.2	0.31897172	mirna_lnc
30797	hsa-mir-1180	AC131532.1	0.431297833	mirna_lnc
30798	hsa-mir-1180	RNASEH1-AS1	0.308623946	mirna_lnc
30799	hsa-mir-1180	LINC01063	0.364398277	mirna_lnc
30800	hsa-mir-1180	AC092669.1	0.388344634	mirna_lnc
30801	hsa-mir-1180	AP002336.2	0.341680658	mirna_lnc
30802	hsa-mir-1180	TBL1XR1-AS1	0.360168399	mirna_lnc
30803	hsa-mir-1180	AC010542.4	0.331519985	mirna_lnc
30804	hsa-mir-1180	AC092171.3	0.300330977	mirna_lnc
30805	hsa-mir-1180	AC078802.1	0.350485273	mirna_lnc
30806	hsa-mir-1180	AC080013.5	0.30246624	mirna_lnc
30807	hsa-mir-1180	LINC02562	0.321338327	mirna_lnc
30808	hsa-mir-1180	ADGRD1-AS1	0.335407334	mirna_lnc
30809	hsa-mir-1180	AC005730.2	0.318001455	mirna_lnc
30810	hsa-mir-1180	AC020741.1	0.301290859	mirna_lnc
30811	hsa-mir-1180	AC024560.3	0.385912457	mirna_lnc
30812	hsa-mir-1180	AC107926.1	0.426028381	mirna_lnc
30813	hsa-mir-1180	MCCC1-AS1	0.378621663	mirna_lnc
30814	hsa-mir-1180	AC009271.1	0.398685003	mirna_lnc
30815	hsa-mir-1180	AC098818.2	0.380948703	mirna_lnc
30816	hsa-mir-1180	AC022762.1	0.315076656	mirna_lnc
30817	hsa-mir-1180	LINC01994	0.309893551	mirna_lnc
30818	hsa-mir-1180	AC012146.3	0.308197727	mirna_lnc
30819	hsa-mir-1180	AC008267.5	0.318336166	mirna_lnc
30820	hsa-mir-1180	AC005695.3	0.363066533	mirna_lnc
30821	hsa-mir-1180	AC107214.1	0.351578251	mirna_lnc
30822	hsa-mir-1180	AC131235.3	0.339774042	mirna_lnc
30823	hsa-mir-1180	LINC00885	0.40479193	mirna_lnc
30824	hsa-mir-1180	AL022324.3	0.306724817	mirna_lnc
30825	hsa-mir-1180	AP005328.2	0.313772351	mirna_lnc
30826	hsa-mir-1180	DDR1-DT	0.3505879	mirna_lnc
30827	hsa-mir-1180	AC078925.4	0.364549952	mirna_lnc
30828	hsa-mir-1180	URB1-AS1	0.328707704	mirna_lnc
30829	hsa-mir-1180	AC091271.1	0.332097318	mirna_lnc
30830	hsa-mir-1180	AP004782.1	0.349576862	mirna_lnc
30831	hsa-mir-1180	AC026304.1	0.31129252	mirna_lnc
30832	hsa-mir-1180	HHIP-AS1	0.309287238	mirna_lnc
30833	hsa-mir-1180	ELDR	0.320594514	mirna_lnc

30834	hsa-mir-1180	AC090617.5	0.313044972	mirna_lnc
30835	hsa-mir-1180	AC019129.2	0.346843601	mirna_lnc
30836	hsa-mir-1180	AC005790.1	0.325479733	mirna_lnc
30837	hsa-mir-1180	AC002310.1	0.332860691	mirna_lnc
30838	hsa-mir-1180	AC005064.1	0.32980775	mirna_lnc
30839	hsa-mir-1180	AL606760.3	0.332798763	mirna_lnc
30840	hsa-mir-1180	AL591167.1	0.313771235	mirna_lnc
30841	hsa-mir-1180	AC093677.2	0.316062931	mirna_lnc
30842	hsa-mir-1180	AC023509.3	0.309059221	mirna_lnc
30843	hsa-mir-1180	AL592211.1	0.349814621	mirna_lnc
30844	hsa-mir-1180	INTS6L-AS1	0.306951386	mirna_lnc
30845	hsa-mir-1180	TERC	0.422022996	mirna_lnc
30846	hsa-mir-1180	AP001160.1	0.32667089	mirna_lnc
30847	hsa-mir-1180	AP001596.1	0.327028813	mirna_lnc
30848	hsa-mir-1180	AC007389.5	0.308540249	mirna_lnc
30849	hsa-mir-1180	AP000251.1	0.414850755	mirna_lnc
30850	hsa-mir-1180	AC025219.1	0.318568242	mirna_lnc
30851	hsa-mir-1180	AF130417.1	0.319653246	mirna_lnc
30852	hsa-mir-1180	OBSCN-AS1	0.319164164	mirna_lnc
30853	hsa-mir-1180	AC007032.1	0.301027807	mirna_lnc
30854	hsa-mir-1180	AC098936.1	0.344067749	mirna_lnc
30855	hsa-mir-1180	PITPNA-AS1	0.373100063	mirna_lnc
30856	hsa-mir-1180	AC138207.2	0.325363914	mirna_lnc
30857	hsa-mir-1180	ETV5-AS1	0.329741002	mirna_lnc
30858	hsa-mir-1180	LINC02006	0.318784031	mirna_lnc
30859	hsa-mir-1180	AC107294.1	0.378354178	mirna_lnc
30860	hsa-mir-1180	AL024497.1	0.343296247	mirna_lnc
30861	hsa-mir-1180	SCAT8	0.327313464	mirna_lnc
30862	hsa-mir-1180	AC002550.2	0.321267657	mirna_lnc
30863	hsa-mir-1180	AL032819.1	0.398074707	mirna_lnc
30864	hsa-mir-1180	AC124254.1	0.306108931	mirna_lnc
30865	hsa-mir-1180	AC010501.1	0.331017434	mirna_lnc
30866	hsa-mir-1180	AC087392.3	0.329213328	mirna_lnc
30867	hsa-mir-1180	LINC01030	0.314292894	mirna_lnc
30868	hsa-mir-1180	AL118511.1	0.357260688	mirna_lnc
30869	hsa-mir-1180	LINC02506	0.456470958	mirna_lnc
30870	hsa-mir-1180	AL583808.1	0.378791209	mirna_lnc
30871	hsa-mir-1180	GIHCG	0.441928913	mirna_lnc
30872	hsa-mir-1180	Z93241.1	0.375559434	mirna_lnc
30873	hsa-mir-1180	AC110285.2	0.348736008	mirna_lnc
30874	hsa-mir-1180	LINC02470	0.347018135	mirna_lnc
30875	hsa-mir-1180	AC008443.3	0.317712134	mirna_lnc
30876	hsa-mir-1180	GTSE1-DT	0.33964159	mirna_lnc
30877	hsa-mir-1180	AC135178.5	0.355299456	mirna_lnc
30878	hsa-mir-1180	AC106820.4	0.31929686	mirna_lnc
30879	hsa-mir-1180	AC010096.1	0.414557079	mirna_lnc
30880	hsa-mir-1180	AC093599.1	0.31453089	mirna_lnc
30881	hsa-mir-1180	AL645608.6	0.316037504	mirna_lnc
30882	hsa-mir-1180	AC011840.2	0.387372151	mirna_lnc
30883	hsa-mir-1180	AC010980.2	0.327263903	mirna_lnc
30884	hsa-mir-1180	AC025162.2	0.332391264	mirna_lnc
30885	hsa-mir-1180	LINC01918	0.315027612	mirna_lnc
30886	hsa-mir-1180	PARD3-AS1	0.315113201	mirna_lnc
30887	hsa-mir-1180	AC090061.1	0.318791754	mirna_lnc

30888	hsa-mir-1180	FAM87B	0.338973351	mirna_lnc
30889	hsa-mir-1180	AL033527.2	0.310465319	mirna_lnc
30890	hsa-mir-1180	AC025287.2	0.339210487	mirna_lnc
30891	hsa-mir-1180	AC007497.1	0.445992687	mirna_lnc
30892	hsa-mir-1180	AC005197.1	0.365685897	mirna_lnc
30893	hsa-mir-1180	AL359504.2	0.369090003	mirna_lnc
30894	hsa-mir-1180	RN7SL832P	0.385198602	mirna_lnc
30895	hsa-mir-1180	LINC02094	0.329505116	mirna_lnc
30896	hsa-mir-1180	AC016229.2	0.30600284	mirna_lnc
30897	hsa-mir-1180	AP001972.4	0.398145495	mirna_lnc
30898	hsa-mir-1180	AL132655.1	0.395555793	mirna_lnc
30899	hsa-mir-1180	AC018450.1	0.3023279	mirna_lnc
30900	hsa-mir-1180	AC010624.2	0.306256192	mirna_lnc
30901	hsa-mir-1180	AL645608.2	0.318484012	mirna_lnc
30902	hsa-mir-1180	AC107294.2	0.400017707	mirna_lnc
30903	hsa-mir-1180	AC015917.2	0.304170161	mirna_lnc
30904	hsa-mir-1180	UBXN7-AS1	0.30600777	mirna_lnc
30905	hsa-mir-1180	LINC01700	0.327439265	mirna_lnc
30906	hsa-mir-1180	AF213884.3	0.333738647	mirna_lnc
30907	hsa-mir-1180	AC021092.1	0.338714461	mirna_lnc
30908	hsa-mir-1180	AC107294.3	0.384120175	mirna_lnc
30909	hsa-mir-1180	LINC02373	0.339896684	mirna_lnc
30910	hsa-mir-1180	AC114271.1	0.305068615	mirna_lnc
30911	hsa-mir-1180	CPEB1-AS1	0.334050129	mirna_lnc
30912	hsa-mir-1180	AC107982.3	0.405560745	mirna_lnc
30913	hsa-mir-1180	SNHG9	0.335632911	mirna_lnc
30914	hsa-mir-1180	AC012676.3	0.318880717	mirna_lnc
30915	hsa-mir-455	AC026401.3	0.411964196	mirna_lnc
30916	hsa-mir-455	AC012073.1	0.327110114	mirna_lnc
30917	hsa-mir-455	AGAP2-AS1	0.373366492	mirna_lnc
30918	hsa-mir-455	AC034213.1	0.357178638	mirna_lnc
30919	hsa-mir-455	VPS9D1-AS1	0.401421362	mirna_lnc
30920	hsa-mir-455	AC092614.1	0.402651067	mirna_lnc
30921	hsa-mir-455	AP001453.2	0.31449636	mirna_lnc
30922	hsa-mir-455	HOXC-AS2	0.372281207	mirna_lnc
30923	hsa-mir-455	AC019069.1	0.326088781	mirna_lnc
30924	hsa-mir-455	AC004943.2	0.362154671	mirna_lnc
30925	hsa-mir-455	AC117394.2	0.318888153	mirna_lnc
30926	hsa-mir-455	AC093388.1	0.305162543	mirna_lnc
30927	hsa-mir-455	AC147651.4	0.341617908	mirna_lnc
30928	hsa-mir-455	CLEC12A-AS1	0.480669783	mirna_lnc
30929	hsa-mir-455	AC069360.1	0.318200794	mirna_lnc
30930	hsa-mir-455	AL138724.1	0.493614323	mirna_lnc
30931	hsa-mir-455	AL441992.1	0.402621396	mirna_lnc
30932	hsa-mir-455	AC006329.1	0.343710094	mirna_lnc
30933	hsa-mir-455	LINC01096	0.300518101	mirna_lnc
30934	hsa-mir-455	AC126773.2	0.35073418	mirna_lnc
30935	hsa-mir-455	MAPK6-DT	0.310286074	mirna_lnc
30936	hsa-mir-455	AL450322.2	0.348989468	mirna_lnc
30937	hsa-mir-455	AC114956.2	0.399898763	mirna_lnc
30938	hsa-mir-455	ATP6V1B1-AS1	0.306116999	mirna_lnc
30939	hsa-mir-455	AC024230.1	0.352499818	mirna_lnc
30940	hsa-mir-455	AL078644.2	0.3646993	mirna_lnc
30941	hsa-mir-455	P3H2-AS1	0.420743609	mirna_lnc

30942	hsa-mir-455	AC010168.1	0.380181377	mirna_lnc
30943	hsa-mir-455	TM4SF19-AS1	0.318712828	mirna_lnc
30944	hsa-mir-455	AC104971.1	0.332510275	mirna_lnc
30945	hsa-mir-455	AC019205.1	0.443411332	mirna_lnc
30946	hsa-mir-455	AC002401.4	0.338419471	mirna_lnc
30947	hsa-mir-455	MYOSLID	0.388553501	mirna_lnc
30948	hsa-mir-455	AC092070.4	0.309427859	mirna_lnc
30949	hsa-mir-455	AL138789.1	0.307027979	mirna_lnc
30950	hsa-mir-455	AC022031.2	0.310849065	mirna_lnc
30951	hsa-mir-455	AL136131.2	0.300111036	mirna_lnc
30952	hsa-mir-455	TTLL11-IT1	0.502684043	mirna_lnc
30953	hsa-mir-455	AC010595.1	0.401506416	mirna_lnc
30954	hsa-mir-455	AC069503.1	0.363748192	mirna_lnc
30955	hsa-mir-455	AC100812.1	0.352070868	mirna_lnc
30956	hsa-mir-455	AC011297.1	0.355640418	mirna_lnc
30957	hsa-mir-455	AC055736.1	0.465226905	mirna_lnc
30958	hsa-mir-455	AC004846.1	0.393400506	mirna_lnc
30959	hsa-mir-455	AC016205.1	0.360946105	mirna_lnc
30960	hsa-mir-455	LINC02657	0.31869071	mirna_lnc
30961	hsa-mir-455	AC091170.1	0.448377529	mirna_lnc
30962	hsa-mir-455	AC114956.1	0.306488046	mirna_lnc
30963	hsa-mir-455	BX322234.1	0.360712857	mirna_lnc
30964	hsa-mir-455	AL358334.2	0.428486965	mirna_lnc
30965	hsa-mir-455	AC025183.1	0.370062582	mirna_lnc
30966	hsa-mir-455	NLGN4Y-AS1	0.318677121	mirna_lnc
30967	hsa-mir-455	AC009271.1	0.34501491	mirna_lnc
30968	hsa-mir-455	AC034223.2	0.32325536	mirna_lnc
30969	hsa-mir-455	AC025183.2	0.439391077	mirna_lnc
30970	hsa-mir-455	AC034206.1	0.345037492	mirna_lnc
30971	hsa-mir-455	AL135818.2	0.397320338	mirna_lnc
30972	hsa-mir-455	AL359091.5	0.366312056	mirna_lnc
30973	hsa-mir-455	AC091182.2	0.39532722	mirna_lnc
30974	hsa-mir-455	AL034399.2	0.477286552	mirna_lnc
30975	hsa-mir-455	AC126768.3	0.30987157	mirna_lnc
30976	hsa-mir-455	MIR31HG	0.305389882	mirna_lnc
30977	hsa-mir-455	AC010343.3	0.34177314	mirna_lnc
30978	hsa-mir-455	AC084032.1	0.30373329	mirna_lnc
30979	hsa-mir-455	AL390760.1	0.320003011	mirna_lnc
30980	hsa-mir-455	LINC02470	0.323317912	mirna_lnc
30981	hsa-mir-455	AL162413.1	0.39041148	mirna_lnc
30982	hsa-mir-455	AC126768.1	0.303777328	mirna_lnc
30983	hsa-mir-455	AC126768.2	0.375029613	mirna_lnc
30984	hsa-mir-455	AL359771.1	0.331608696	mirna_lnc
30985	hsa-mir-455	AF213884.3	0.301143859	mirna_lnc
30986	hsa-mir-455	AC104031.1	0.309919209	mirna_lnc
30987	hsa-mir-455	AL445483.1	0.34756867	mirna_lnc
30988	hsa-mir-103-2	AC012073.1	0.312284488	mirna_lnc
30989	hsa-mir-103-2	TMPO-AS1	0.323878108	mirna_lnc
30990	hsa-mir-103-2	AL035461.2	0.389676304	mirna_lnc
30991	hsa-mir-103-2	AC022211.1	0.300816661	mirna_lnc
30992	hsa-mir-103-2	AP001469.3	0.316639576	mirna_lnc
30993	hsa-mir-103-2	AC131009.3	0.312622359	mirna_lnc
30994	hsa-mir-103-2	LINC02367	0.318181656	mirna_lnc
30995	hsa-mir-103-2	AC012360.3	0.303850668	mirna_lnc

30996	hsa-mir-103-2	GK-IT1	0.315463254	mirna_lnc
30997	hsa-mir-103-2	AC005379.1	0.313943842	mirna_lnc
30998	hsa-mir-103-2	AC011773.1	0.383887085	mirna_lnc
30999	hsa-mir-103-2	AC105235.1	0.32466907	mirna_lnc
31000	hsa-mir-103-2	AC083843.3	0.327035183	mirna_lnc
31001	hsa-mir-103-2	AL603832.1	0.329158376	mirna_lnc
31002	hsa-mir-103-2	AC011676.5	0.336494266	mirna_lnc
31003	hsa-mir-103-2	AC011676.2	0.308400827	mirna_lnc
31004	hsa-mir-103-2	AC003991.2	0.331620981	mirna_lnc
31005	hsa-mir-103-2	AC099684.2	0.329022995	mirna_lnc
31006	hsa-mir-20a	AC012073.1	0.395585371	mirna_lnc
31007	hsa-mir-20a	AC010761.2	0.341264811	mirna_lnc
31008	hsa-mir-20a	AC012531.1	0.331474917	mirna_lnc
31009	hsa-mir-20a	AL035461.2	0.311192732	mirna_lnc
31010	hsa-mir-20a	AL138759.1	0.353816378	mirna_lnc
31011	hsa-mir-20a	DDX11-AS1	0.336025254	mirna_lnc
31012	hsa-mir-20a	AL161891.1	0.572857029	mirna_lnc
31013	hsa-mir-20a	AC092614.1	0.312829969	mirna_lnc
31014	hsa-mir-20a	AL157932.1	0.362835353	mirna_lnc
31015	hsa-mir-20a	HOXC-AS2	0.325749499	mirna_lnc
31016	hsa-mir-20a	AC019069.1	0.31385504	mirna_lnc
31017	hsa-mir-20a	AP001469.3	0.334565579	mirna_lnc
31018	hsa-mir-20a	AC004943.2	0.309997233	mirna_lnc
31019	hsa-mir-20a	AL356740.1	0.331284628	mirna_lnc
31020	hsa-mir-20a	AC027627.1	0.306452808	mirna_lnc
31021	hsa-mir-20a	AC021945.1	0.390961096	mirna_lnc
31022	hsa-mir-20a	AC025176.1	0.371075456	mirna_lnc
31023	hsa-mir-20a	AC090844.3	0.374400778	mirna_lnc
31024	hsa-mir-20a	AC090159.1	0.382346957	mirna_lnc
31025	hsa-mir-20a	AC024940.6	0.368427728	mirna_lnc
31026	hsa-mir-20a	GCC2-AS1	0.308860263	mirna_lnc
31027	hsa-mir-20a	AC015849.5	0.318875316	mirna_lnc
31028	hsa-mir-20a	AC125257.1	0.308729677	mirna_lnc
31029	hsa-mir-20a	AC012360.3	0.388147562	mirna_lnc
31030	hsa-mir-20a	AL139089.1	0.349442823	mirna_lnc
31031	hsa-mir-20a	AL161772.1	0.345868621	mirna_lnc
31032	hsa-mir-20a	AL355388.2	0.340243154	mirna_lnc
31033	hsa-mir-20a	AC004080.1	0.325616257	mirna_lnc
31034	hsa-mir-20a	AC005831.1	0.319127465	mirna_lnc
31035	hsa-mir-20a	AC138904.1	0.36310567	mirna_lnc
31036	hsa-mir-20a	AC004943.1	0.398416733	mirna_lnc
31037	hsa-mir-20a	LINC01460	0.432960693	mirna_lnc
31038	hsa-mir-20a	AC018878.1	0.33306298	mirna_lnc
31039	hsa-mir-20a	TRPM2-AS	0.339318677	mirna_lnc
31040	hsa-mir-20a	UBR5-AS1	0.312806558	mirna_lnc
31041	hsa-mir-20a	SREBF2-AS1	0.303378665	mirna_lnc
31042	hsa-mir-20a	AC004943.3	0.320686425	mirna_lnc
31043	hsa-mir-20a	DNAJC3-DT	0.391358788	mirna_lnc
31044	hsa-mir-20a	AC026271.3	0.347425738	mirna_lnc
31045	hsa-mir-20a	AP002360.3	0.319210446	mirna_lnc
31046	hsa-mir-20a	AC016205.1	0.325802729	mirna_lnc
31047	hsa-mir-20a	PIK3CD-AS2	0.325657643	mirna_lnc
31048	hsa-mir-20a	AC007128.1	0.375324433	mirna_lnc
31049	hsa-mir-20a	RNASEH1-AS1	0.381287434	mirna_lnc

31050	hsa-mir-20a	AC087491.1	0.337609032	mirna_lnc
31051	hsa-mir-20a	AL139082.1	0.320377453	mirna_lnc
31052	hsa-mir-20a	AC005224.1	0.387388829	mirna_lnc
31053	hsa-mir-20a	AC011773.1	0.362184059	mirna_lnc
31054	hsa-mir-20a	AL442128.2	0.380194322	mirna_lnc
31055	hsa-mir-20a	AC145207.5	0.316997732	mirna_lnc
31056	hsa-mir-20a	STAM-AS1	0.328188727	mirna_lnc
31057	hsa-mir-20a	AC007938.3	0.378589183	mirna_lnc
31058	hsa-mir-20a	AL137244.1	0.361777006	mirna_lnc
31059	hsa-mir-20a	AL022324.3	0.30115339	mirna_lnc
31060	hsa-mir-20a	LINC01238	0.306808483	mirna_lnc
31061	hsa-mir-20a	AL512652.2	0.378333631	mirna_lnc
31062	hsa-mir-20a	AP003086.2	0.303892508	mirna_lnc
31063	hsa-mir-20a	AL161452.1	0.31005708	mirna_lnc
31064	hsa-mir-20a	AC112255.1	0.35633925	mirna_lnc
31065	hsa-mir-20a	MCF2L-AS1	0.422028001	mirna_lnc
31066	hsa-mir-20a	AL138767.3	0.322829702	mirna_lnc
31067	hsa-mir-20a	ETV5-AS1	0.302187382	mirna_lnc
31068	hsa-mir-20a	ZNF649-AS1	0.305914924	mirna_lnc
31069	hsa-mir-20a	AC107068.1	0.371690866	mirna_lnc
31070	hsa-mir-20a	AL136531.1	0.310831909	mirna_lnc
31071	hsa-mir-20a	LINC01555	0.30280289	mirna_lnc
31072	hsa-mir-20a	AL118511.1	0.376227702	mirna_lnc
31073	hsa-mir-20a	AC007001.1	0.325515341	mirna_lnc
31074	hsa-mir-20a	AC106820.4	0.358527632	mirna_lnc
31075	hsa-mir-20a	LINC02097	0.305597096	mirna_lnc
31076	hsa-mir-20a	AC005034.3	0.331464047	mirna_lnc
31077	hsa-mir-20a	AC007497.1	0.311194377	mirna_lnc
31078	hsa-mir-20a	LINC00449	0.301404365	mirna_lnc
31079	hsa-mir-20a	LINC00456	0.336686143	mirna_lnc
31080	hsa-mir-20a	AL049646.1	0.356848425	mirna_lnc
31081	hsa-mir-20a	AL161729.4	0.303564231	mirna_lnc
31082	hsa-mir-20a	AL596202.1	0.323898298	mirna_lnc
31083	hsa-mir-20a	AC080112.3	0.31543646	mirna_lnc
31084	hsa-mir-20a	AL080317.1	0.31294643	mirna_lnc
31085	hsa-mir-20a	PSPC1-AS2	0.375253225	mirna_lnc
31086	hsa-mir-1301	AC012073.1	0.459566587	mirna_lnc
31087	hsa-mir-1301	AP000708.1	0.361107332	mirna_lnc
31088	hsa-mir-1301	TYMSOS	0.310632983	mirna_lnc
31089	hsa-mir-1301	LINC02595	0.380391893	mirna_lnc
31090	hsa-mir-1301	AL662797.1	0.363136415	mirna_lnc
31091	hsa-mir-1301	LINC01873	0.43313153	mirna_lnc
31092	hsa-mir-1301	AL137060.1	0.328351216	mirna_lnc
31093	hsa-mir-1301	MELTF-AS1	0.37146492	mirna_lnc
31094	hsa-mir-1301	TMPO-AS1	0.500372283	mirna_lnc
31095	hsa-mir-1301	AL121832.2	0.423227017	mirna_lnc
31096	hsa-mir-1301	AC103591.3	0.433108327	mirna_lnc
31097	hsa-mir-1301	AC006252.1	0.382787739	mirna_lnc
31098	hsa-mir-1301	AC004241.3	0.505563651	mirna_lnc
31099	hsa-mir-1301	AL117332.1	0.44289815	mirna_lnc
31100	hsa-mir-1301	AC022211.2	0.304114649	mirna_lnc
31101	hsa-mir-1301	SCAT2	0.323628127	mirna_lnc
31102	hsa-mir-1301	AL133338.1	0.30241428	mirna_lnc
31103	hsa-mir-1301	CEP83-DT	0.410308019	mirna_lnc

31104	hsa-mir-1301	HOXC-AS2	0.348275697	mirna_lnc
31105	hsa-mir-1301	AL049539.1	0.315605136	mirna_lnc
31106	hsa-mir-1301	AL590326.1	0.343065385	mirna_lnc
31107	hsa-mir-1301	JARID2-AS1	0.562572749	mirna_lnc
31108	hsa-mir-1301	LINC00853	0.324806166	mirna_lnc
31109	hsa-mir-1301	AC074117.1	0.548577028	mirna_lnc
31110	hsa-mir-1301	AP001469.3	0.319906599	mirna_lnc
31111	hsa-mir-1301	AL031667.3	0.564339854	mirna_lnc
31112	hsa-mir-1301	TFAP2A-AS1	0.447835153	mirna_lnc
31113	hsa-mir-1301	LINC02601	0.337321356	mirna_lnc
31114	hsa-mir-1301	AC004812.2	0.330959984	mirna_lnc
31115	hsa-mir-1301	AC100791.2	0.370811615	mirna_lnc
31116	hsa-mir-1301	AC015982.1	0.421808282	mirna_lnc
31117	hsa-mir-1301	AL355297.3	0.358411663	mirna_lnc
31118	hsa-mir-1301	LINC02542	0.374221734	mirna_lnc
31119	hsa-mir-1301	AC009121.2	0.301539935	mirna_lnc
31120	hsa-mir-1301	AC010894.2	0.380900245	mirna_lnc
31121	hsa-mir-1301	AC092159.2	0.301189594	mirna_lnc
31122	hsa-mir-1301	AC005840.2	0.382748185	mirna_lnc
31123	hsa-mir-1301	AC008738.2	0.302946987	mirna_lnc
31124	hsa-mir-1301	AL023803.3	0.44053599	mirna_lnc
31125	hsa-mir-1301	AC131009.3	0.306066904	mirna_lnc
31126	hsa-mir-1301	AC017083.1	0.461465563	mirna_lnc
31127	hsa-mir-1301	AL121655.1	0.443852193	mirna_lnc
31128	hsa-mir-1301	AC109322.1	0.409215754	mirna_lnc
31129	hsa-mir-1301	LINC01952	0.413915342	mirna_lnc
31130	hsa-mir-1301	AC107081.2	0.319210853	mirna_lnc
31131	hsa-mir-1301	AC074351.1	0.360628867	mirna_lnc
31132	hsa-mir-1301	KC877982.1	0.328343657	mirna_lnc
31133	hsa-mir-1301	AL445072.1	0.410638079	mirna_lnc
31134	hsa-mir-1301	AL592301.1	0.310587885	mirna_lnc
31135	hsa-mir-1301	AC079907.1	0.325405594	mirna_lnc
31136	hsa-mir-1301	AC023157.3	0.341055798	mirna_lnc
31137	hsa-mir-1301	AL137802.2	0.465841712	mirna_lnc
31138	hsa-mir-1301	AC087289.3	0.400697321	mirna_lnc
31139	hsa-mir-1301	AC091729.3	0.354202277	mirna_lnc
31140	hsa-mir-1301	AC007292.1	0.306519705	mirna_lnc
31141	hsa-mir-1301	AL136162.1	0.614057577	mirna_lnc
31142	hsa-mir-1301	AC010913.1	0.301117173	mirna_lnc
31143	hsa-mir-1301	LINC02367	0.347764704	mirna_lnc
31144	hsa-mir-1301	AC073195.1	0.522698062	mirna_lnc
31145	hsa-mir-1301	AL160291.1	0.384453302	mirna_lnc
31146	hsa-mir-1301	AC015818.2	0.413602568	mirna_lnc
31147	hsa-mir-1301	AL596094.1	0.523911913	mirna_lnc
31148	hsa-mir-1301	AC004069.1	0.581297842	mirna_lnc
31149	hsa-mir-1301	AC131025.1	0.336538412	mirna_lnc
31150	hsa-mir-1301	AC004920.1	0.558273455	mirna_lnc
31151	hsa-mir-1301	AC021683.3	0.604135721	mirna_lnc
31152	hsa-mir-1301	AC012360.3	0.423282953	mirna_lnc
31153	hsa-mir-1301	AC010359.1	0.398522	mirna_lnc
31154	hsa-mir-1301	AL391069.2	0.422884805	mirna_lnc
31155	hsa-mir-1301	AC012640.2	0.4832523	mirna_lnc
31156	hsa-mir-1301	AC090970.2	0.451434865	mirna_lnc
31157	hsa-mir-1301	AC110285.6	0.364190205	mirna_lnc

31158	hsa-mir-1301	AC026782.2	0.450163709	mirna_lnc
31159	hsa-mir-1301	AC116049.2	0.346419316	mirna_lnc
31160	hsa-mir-1301	AL139023.1	0.374919506	mirna_lnc
31161	hsa-mir-1301	HOXC-AS3	0.33907093	mirna_lnc
31162	hsa-mir-1301	AP003119.2	0.448816075	mirna_lnc
31163	hsa-mir-1301	YTHDF3-AS1	0.325991889	mirna_lnc
31164	hsa-mir-1301	AL513497.1	0.336367348	mirna_lnc
31165	hsa-mir-1301	ILF3-DT	0.330903581	mirna_lnc
31166	hsa-mir-1301	AC025244.1	0.339379523	mirna_lnc
31167	hsa-mir-1301	AL354993.2	0.347739372	mirna_lnc
31168	hsa-mir-1301	AC002470.1	0.309176533	mirna_lnc
31169	hsa-mir-1301	HAGLROS	0.31612363	mirna_lnc
31170	hsa-mir-1301	AL133243.2	0.323766898	mirna_lnc
31171	hsa-mir-1301	AC012625.1	0.36828011	mirna_lnc
31172	hsa-mir-1301	AL353764.1	0.527976377	mirna_lnc
31173	hsa-mir-1301	AC022413.1	0.360303636	mirna_lnc
31174	hsa-mir-1301	SYNGAP1-AS1	0.383846681	mirna_lnc
31175	hsa-mir-1301	AC024230.1	0.338677589	mirna_lnc
31176	hsa-mir-1301	AL031985.3	0.304744957	mirna_lnc
31177	hsa-mir-1301	AC006449.5	0.312264201	mirna_lnc
31178	hsa-mir-1301	AC013400.1	0.564914797	mirna_lnc
31179	hsa-mir-1301	AC138904.1	0.30832254	mirna_lnc
31180	hsa-mir-1301	LINC01424	0.635564433	mirna_lnc
31181	hsa-mir-1301	AC099518.1	0.347311178	mirna_lnc
31182	hsa-mir-1301	AL391152.1	0.353701673	mirna_lnc
31183	hsa-mir-1301	AL355304.1	0.314120516	mirna_lnc
31184	hsa-mir-1301	TBC1D8-AS1	0.596824566	mirna_lnc
31185	hsa-mir-1301	TIPARP-AS1	0.404627072	mirna_lnc
31186	hsa-mir-1301	KIAA1614-AS1	0.52197512	mirna_lnc
31187	hsa-mir-1301	AC016876.3	0.597947324	mirna_lnc
31188	hsa-mir-1301	AL031665.2	0.427977206	mirna_lnc
31189	hsa-mir-1301	AC079684.1	0.316089128	mirna_lnc
31190	hsa-mir-1301	AL163051.1	0.464821965	mirna_lnc
31191	hsa-mir-1301	AL513327.2	0.569890053	mirna_lnc
31192	hsa-mir-1301	AC104971.1	0.372202415	mirna_lnc
31193	hsa-mir-1301	IQCJ-SCHIP1-AS1	0.391414734	mirna_lnc
31194	hsa-mir-1301	AC138356.1	0.566134822	mirna_lnc
31195	hsa-mir-1301	LINC01456	0.433302937	mirna_lnc
31196	hsa-mir-1301	AC124016.1	0.335023559	mirna_lnc
31197	hsa-mir-1301	AC092807.2	0.420676056	mirna_lnc
31198	hsa-mir-1301	AC096887.2	0.399205498	mirna_lnc
31199	hsa-mir-1301	AC002076.1	0.322974522	mirna_lnc
31200	hsa-mir-1301	AC132192.2	0.305173182	mirna_lnc
31201	hsa-mir-1301	LINC01850	0.426341829	mirna_lnc
31202	hsa-mir-1301	AC007952.4	0.38195254	mirna_lnc
31203	hsa-mir-1301	AC090517.2	0.403029368	mirna_lnc
31204	hsa-mir-1301	AL683887.1	0.340325703	mirna_lnc
31205	hsa-mir-1301	AC005670.1	0.310798687	mirna_lnc
31206	hsa-mir-1301	AP000593.3	0.414569028	mirna_lnc
31207	hsa-mir-1301	WWTR1-AS1	0.366279152	mirna_lnc
31208	hsa-mir-1301	LINC01686	0.460142411	mirna_lnc
31209	hsa-mir-1301	MIR583HG	0.302277139	mirna_lnc
31210	hsa-mir-1301	LINC01143	0.307573713	mirna_lnc
31211	hsa-mir-1301	AL445228.2	0.35476954	mirna_lnc

31212	hsa-mir-1301	AL033527.3	0.316315393	mirna_lnc
31213	hsa-mir-1301	AC002550.1	0.38094855	mirna_lnc
31214	hsa-mir-1301	AC239799.1	0.334938579	mirna_lnc
31215	hsa-mir-1301	EXOC3-AS1	0.328396056	mirna_lnc
31216	hsa-mir-1301	AC010595.1	0.376590443	mirna_lnc
31217	hsa-mir-1301	AL391069.3	0.321143986	mirna_lnc
31218	hsa-mir-1301	LINC02607	0.594770421	mirna_lnc
31219	hsa-mir-1301	AL592071.1	0.356845	mirna_lnc
31220	hsa-mir-1301	WASIR2	0.395639287	mirna_lnc
31221	hsa-mir-1301	LINC01816	0.653832088	mirna_lnc
31222	hsa-mir-1301	TMCO1-AS1	0.417803942	mirna_lnc
31223	hsa-mir-1301	AL121652.1	0.336901734	mirna_lnc
31224	hsa-mir-1301	SREBF2-AS1	0.399628478	mirna_lnc
31225	hsa-mir-1301	AC099518.6	0.582812119	mirna_lnc
31226	hsa-mir-1301	AC007881.3	0.56187322	mirna_lnc
31227	hsa-mir-1301	AL121658.1	0.502491873	mirna_lnc
31228	hsa-mir-1301	AC010969.2	0.482377038	mirna_lnc
31229	hsa-mir-1301	AC022098.3	0.399818603	mirna_lnc
31230	hsa-mir-1301	AC005332.1	0.357723262	mirna_lnc
31231	hsa-mir-1301	LINC00167	0.581080828	mirna_lnc
31232	hsa-mir-1301	AC011825.4	0.344545201	mirna_lnc
31233	hsa-mir-1301	AC027702.1	0.30225868	mirna_lnc
31234	hsa-mir-1301	LINC02637	0.320478947	mirna_lnc
31235	hsa-mir-1301	LINC01521	0.389096414	mirna_lnc
31236	hsa-mir-1301	AC023302.1	0.335225898	mirna_lnc
31237	hsa-mir-1301	AC004943.3	0.437763369	mirna_lnc
31238	hsa-mir-1301	AC046143.2	0.452960006	mirna_lnc
31239	hsa-mir-1301	AC145343.1	0.527863648	mirna_lnc
31240	hsa-mir-1301	AL358472.3	0.320543349	mirna_lnc
31241	hsa-mir-1301	AC112907.1	0.336694741	mirna_lnc
31242	hsa-mir-1301	AL121992.2	0.317428981	mirna_lnc
31243	hsa-mir-1301	AC093424.1	0.576766837	mirna_lnc
31244	hsa-mir-1301	AC091925.1	0.479036206	mirna_lnc
31245	hsa-mir-1301	AC117382.2	0.557715157	mirna_lnc
31246	hsa-mir-1301	AC091153.3	0.53551419	mirna_lnc
31247	hsa-mir-1301	AC063948.1	0.506143435	mirna_lnc
31248	hsa-mir-1301	AL023803.1	0.305865847	mirna_lnc
31249	hsa-mir-1301	AC087742.1	0.518446207	mirna_lnc
31250	hsa-mir-1301	AL354892.2	0.482319347	mirna_lnc
31251	hsa-mir-1301	AC015802.5	0.417009836	mirna_lnc
31252	hsa-mir-1301	AL139327.2	0.462702633	mirna_lnc
31253	hsa-mir-1301	DDX39B-AS1	0.371545786	mirna_lnc
31254	hsa-mir-1301	AL031963.3	0.348125984	mirna_lnc
31255	hsa-mir-1301	AC069148.1	0.365175392	mirna_lnc
31256	hsa-mir-1301	AC106820.2	0.303403493	mirna_lnc
31257	hsa-mir-1301	AC012603.1	0.31081794	mirna_lnc
31258	hsa-mir-1301	AC016205.1	0.303535237	mirna_lnc
31259	hsa-mir-1301	AC064836.3	0.526087328	mirna_lnc
31260	hsa-mir-1301	AC011290.1	0.302317338	mirna_lnc
31261	hsa-mir-1301	BX088651.4	0.420129901	mirna_lnc
31262	hsa-mir-1301	AC109326.1	0.315493629	mirna_lnc
31263	hsa-mir-1301	PIK3CD-AS2	0.328715641	mirna_lnc
31264	hsa-mir-1301	AC012640.5	0.432197009	mirna_lnc
31265	hsa-mir-1301	AL512408.1	0.530806301	mirna_lnc

31266	hsa-mir-1301	AC090527.3	0.510099536	mirna_lnc
31267	hsa-mir-1301	AC087741.2	0.358862452	mirna_lnc
31268	hsa-mir-1301	AC010422.2	0.335449468	mirna_lnc
31269	hsa-mir-1301	AC010275.1	0.311449558	mirna_lnc
31270	hsa-mir-1301	AC100803.1	0.35475002	mirna_lnc
31271	hsa-mir-1301	RNASEH1-AS1	0.466987848	mirna_lnc
31272	hsa-mir-1301	AC005379.1	0.338716227	mirna_lnc
31273	hsa-mir-1301	LINC01063	0.629769002	mirna_lnc
31274	hsa-mir-1301	AC131212.4	0.307359202	mirna_lnc
31275	hsa-mir-1301	AC092669.1	0.454415343	mirna_lnc
31276	hsa-mir-1301	CIRBP-AS1	0.492605339	mirna_lnc
31277	hsa-mir-1301	AC092171.5	0.372637404	mirna_lnc
31278	hsa-mir-1301	AC069213.1	0.40753878	mirna_lnc
31279	hsa-mir-1301	AC091946.2	0.334681776	mirna_lnc
31280	hsa-mir-1301	AC099568.2	0.403378543	mirna_lnc
31281	hsa-mir-1301	AC020765.2	0.34217476	mirna_lnc
31282	hsa-mir-1301	STPG3-AS1	0.329033984	mirna_lnc
31283	hsa-mir-1301	AL096828.3	0.411404594	mirna_lnc
31284	hsa-mir-1301	AC084757.2	0.453381017	mirna_lnc
31285	hsa-mir-1301	AC092171.3	0.380773189	mirna_lnc
31286	hsa-mir-1301	AC015802.4	0.355219114	mirna_lnc
31287	hsa-mir-1301	AL136040.1	0.348574472	mirna_lnc
31288	hsa-mir-1301	EIF2AK3-DT	0.491979269	mirna_lnc
31289	hsa-mir-1301	AC009171.2	0.460877828	mirna_lnc
31290	hsa-mir-1301	AC243830.2	0.415529399	mirna_lnc
31291	hsa-mir-1301	CNNM3-DT	0.532076336	mirna_lnc
31292	hsa-mir-1301	AC068831.6	0.382413395	mirna_lnc
31293	hsa-mir-1301	AL355482.2	0.410614371	mirna_lnc
31294	hsa-mir-1301	AC026691.1	0.303828597	mirna_lnc
31295	hsa-mir-1301	AC080013.5	0.405379236	mirna_lnc
31296	hsa-mir-1301	AL645608.4	0.439131248	mirna_lnc
31297	hsa-mir-1301	LINC02562	0.306256668	mirna_lnc
31298	hsa-mir-1301	AC138696.2	0.510098358	mirna_lnc
31299	hsa-mir-1301	LINC02163	0.340481844	mirna_lnc
31300	hsa-mir-1301	AC068987.4	0.379433032	mirna_lnc
31301	hsa-mir-1301	DGCR10	0.317937194	mirna_lnc
31302	hsa-mir-1301	AC007663.3	0.317612738	mirna_lnc
31303	hsa-mir-1301	MAST4-AS1	0.475776024	mirna_lnc
31304	hsa-mir-1301	LINC00884	0.356558409	mirna_lnc
31305	hsa-mir-1301	AC010654.1	0.396268165	mirna_lnc
31306	hsa-mir-1301	AC090774.2	0.428111044	mirna_lnc
31307	hsa-mir-1301	MCCC1-AS1	0.306761533	mirna_lnc
31308	hsa-mir-1301	STAM-AS1	0.304238884	mirna_lnc
31309	hsa-mir-1301	AC007773.1	0.472552256	mirna_lnc
31310	hsa-mir-1301	ATXN2-AS	0.357854073	mirna_lnc
31311	hsa-mir-1301	AC132938.1	0.322543408	mirna_lnc
31312	hsa-mir-1301	AC022762.1	0.393620961	mirna_lnc
31313	hsa-mir-1301	AL133243.3	0.317397817	mirna_lnc
31314	hsa-mir-1301	AC010883.1	0.322925011	mirna_lnc
31315	hsa-mir-1301	AC008267.5	0.434927015	mirna_lnc
31316	hsa-mir-1301	AC005695.3	0.385982489	mirna_lnc
31317	hsa-mir-1301	AC107214.1	0.439771186	mirna_lnc
31318	hsa-mir-1301	AC034236.2	0.447639792	mirna_lnc
31319	hsa-mir-1301	AC131235.3	0.386169993	mirna_lnc

31320	hsa-mir-1301	LINC00885	0.35078269	mirna_lnc
31321	hsa-mir-1301	AL022324.3	0.323279504	mirna_lnc
31322	hsa-mir-1301	LINC01971	0.322593431	mirna_lnc
31323	hsa-mir-1301	AC072061.1	0.329790852	mirna_lnc
31324	hsa-mir-1301	AC005005.3	0.522871634	mirna_lnc
31325	hsa-mir-1301	AC073896.4	0.438978142	mirna_lnc
31326	hsa-mir-1301	C11orf72	0.349348945	mirna_lnc
31327	hsa-mir-1301	AP005328.2	0.362323239	mirna_lnc
31328	hsa-mir-1301	DDR1-DT	0.322193073	mirna_lnc
31329	hsa-mir-1301	AL139274.2	0.302532986	mirna_lnc
31330	hsa-mir-1301	AC108865.1	0.353829091	mirna_lnc
31331	hsa-mir-1301	URB1-AS1	0.488699632	mirna_lnc
31332	hsa-mir-1301	AC024243.1	0.440393574	mirna_lnc
31333	hsa-mir-1301	AC011446.1	0.395207761	mirna_lnc
31334	hsa-mir-1301	AL132712.2	0.400988192	mirna_lnc
31335	hsa-mir-1301	AC008736.2	0.496468566	mirna_lnc
31336	hsa-mir-1301	AL121906.2	0.307351346	mirna_lnc
31337	hsa-mir-1301	AC091271.1	0.530904625	mirna_lnc
31338	hsa-mir-1301	AL590399.1	0.412078423	mirna_lnc
31339	hsa-mir-1301	AC026304.1	0.485904998	mirna_lnc
31340	hsa-mir-1301	AC096992.2	0.470145886	mirna_lnc
31341	hsa-mir-1301	AL603832.1	0.310251514	mirna_lnc
31342	hsa-mir-1301	LPP-AS2	0.3911491	mirna_lnc
31343	hsa-mir-1301	AC005304.3	0.359504994	mirna_lnc
31344	hsa-mir-1301	AC011445.1	0.415250347	mirna_lnc
31345	hsa-mir-1301	AC026803.2	0.346615151	mirna_lnc
31346	hsa-mir-1301	AC010186.1	0.538143535	mirna_lnc
31347	hsa-mir-1301	ELDR	0.525315003	mirna_lnc
31348	hsa-mir-1301	AC090617.5	0.401197378	mirna_lnc
31349	hsa-mir-1301	AP003170.3	0.34456852	mirna_lnc
31350	hsa-mir-1301	AC019129.2	0.477017681	mirna_lnc
31351	hsa-mir-1301	AC005790.1	0.4593586	mirna_lnc
31352	hsa-mir-1301	AC010336.2	0.333563391	mirna_lnc
31353	hsa-mir-1301	LBX2-AS1	0.302905396	mirna_lnc
31354	hsa-mir-1301	AP003086.2	0.334064065	mirna_lnc
31355	hsa-mir-1301	AP001775.2	0.45949933	mirna_lnc
31356	hsa-mir-1301	AL132712.1	0.43948366	mirna_lnc
31357	hsa-mir-1301	AC023355.1	0.501627885	mirna_lnc
31358	hsa-mir-1301	AL606760.3	0.31840405	mirna_lnc
31359	hsa-mir-1301	AC093677.2	0.544488025	mirna_lnc
31360	hsa-mir-1301	AC023509.3	0.360167101	mirna_lnc
31361	hsa-mir-1301	Z73429.1	0.301279941	mirna_lnc
31362	hsa-mir-1301	AC018766.1	0.301347841	mirna_lnc
31363	hsa-mir-1301	AL135905.1	0.366813474	mirna_lnc
31364	hsa-mir-1301	AC083805.3	0.309057579	mirna_lnc
31365	hsa-mir-1301	AC090907.2	0.35771549	mirna_lnc
31366	hsa-mir-1301	IQCA1-AS1	0.461446521	mirna_lnc
31367	hsa-mir-1301	INTS6L-AS1	0.392187338	mirna_lnc
31368	hsa-mir-1301	AP006545.2	0.348924828	mirna_lnc
31369	hsa-mir-1301	AC011700.1	0.318792335	mirna_lnc
31370	hsa-mir-1301	AC025166.1	0.332747604	mirna_lnc
31371	hsa-mir-1301	AL031186.1	0.328047604	mirna_lnc
31372	hsa-mir-1301	AC083880.1	0.498626659	mirna_lnc
31373	hsa-mir-1301	TERC	0.387729146	mirna_lnc

31374	hsa-mir-1301	AL121601.1	0.410651502	mirna_lnc
31375	hsa-mir-1301	AC099522.2	0.333218907	mirna_lnc
31376	hsa-mir-1301	AL606970.1	0.307688965	mirna_lnc
31377	hsa-mir-1301	AP001547.1	0.33229946	mirna_lnc
31378	hsa-mir-1301	RB1-DT	0.310362229	mirna_lnc
31379	hsa-mir-1301	LINC01607	0.505756221	mirna_lnc
31380	hsa-mir-1301	AC145423.3	0.315415806	mirna_lnc
31381	hsa-mir-1301	AL136038.3	0.324374403	mirna_lnc
31382	hsa-mir-1301	AC007541.1	0.38125212	mirna_lnc
31383	hsa-mir-1301	AL606537.1	0.329742654	mirna_lnc
31384	hsa-mir-1301	AL161729.2	0.325212028	mirna_lnc
31385	hsa-mir-1301	AC007389.5	0.495433616	mirna_lnc
31386	hsa-mir-1301	AC108865.2	0.55521312	mirna_lnc
31387	hsa-mir-1301	AC092447.5	0.367441224	mirna_lnc
31388	hsa-mir-1301	AC010834.2	0.491347824	mirna_lnc
31389	hsa-mir-1301	AL022341.1	0.362866979	mirna_lnc
31390	hsa-mir-1301	AC008883.1	0.328354957	mirna_lnc
31391	hsa-mir-1301	AP000251.1	0.51568501	mirna_lnc
31392	hsa-mir-1301	AL390195.1	0.328125328	mirna_lnc
31393	hsa-mir-1301	AC025575.2	0.307727507	mirna_lnc
31394	hsa-mir-1301	BX005266.2	0.366518951	mirna_lnc
31395	hsa-mir-1301	CPNE8-AS1	0.444166485	mirna_lnc
31396	hsa-mir-1301	AC118658.1	0.35514092	mirna_lnc
31397	hsa-mir-1301	AL138881.1	0.530698936	mirna_lnc
31398	hsa-mir-1301	AC036214.1	0.347504195	mirna_lnc
31399	hsa-mir-1301	LINC01465	0.313035605	mirna_lnc
31400	hsa-mir-1301	AC087276.3	0.343107669	mirna_lnc
31401	hsa-mir-1301	AC090907.1	0.536537617	mirna_lnc
31402	hsa-mir-1301	AC093635.1	0.469560859	mirna_lnc
31403	hsa-mir-1301	LINC01715	0.517084121	mirna_lnc
31404	hsa-mir-1301	AC009560.1	0.404689043	mirna_lnc
31405	hsa-mir-1301	AL590822.1	0.442754759	mirna_lnc
31406	hsa-mir-1301	AC093510.1	0.373706242	mirna_lnc
31407	hsa-mir-1301	BRWD1-AS2	0.430730563	mirna_lnc
31408	hsa-mir-1301	LINC01144	0.359983267	mirna_lnc
31409	hsa-mir-1301	PITPNA-AS1	0.300857892	mirna_lnc
31410	hsa-mir-1301	AL157813.1	0.326295231	mirna_lnc
31411	hsa-mir-1301	AC027644.3	0.508401807	mirna_lnc
31412	hsa-mir-1301	AGBL5-AS1	0.324326634	mirna_lnc
31413	hsa-mir-1301	AC079174.2	0.392325078	mirna_lnc
31414	hsa-mir-1301	AC004263.1	0.330784915	mirna_lnc
31415	hsa-mir-1301	AL513548.1	0.332753447	mirna_lnc
31416	hsa-mir-1301	AC007114.1	0.357321468	mirna_lnc
31417	hsa-mir-1301	AC096540.1	0.399595143	mirna_lnc
31418	hsa-mir-1301	SCAT8	0.604308127	mirna_lnc
31419	hsa-mir-1301	AL356488.3	0.58106132	mirna_lnc
31420	hsa-mir-1301	MANEA-DT	0.309758824	mirna_lnc
31421	hsa-mir-1301	AC004969.1	0.3407095	mirna_lnc
31422	hsa-mir-1301	AC069277.1	0.438182815	mirna_lnc
31423	hsa-mir-1301	AL032819.1	0.55039757	mirna_lnc
31424	hsa-mir-1301	AL844908.2	0.514298236	mirna_lnc
31425	hsa-mir-1301	AL355001.2	0.360984367	mirna_lnc
31426	hsa-mir-1301	AC092119.3	0.442418146	mirna_lnc
31427	hsa-mir-1301	AC069307.1	0.384894318	mirna_lnc

31428	hsa-mir-1301	AC099343.3	0.413409188	mirna_lnc
31429	hsa-mir-1301	AC079834.2	0.356120674	mirna_lnc
31430	hsa-mir-1301	HDAC4-AS1	0.418125095	mirna_lnc
31431	hsa-mir-1301	AL021154.1	0.534450758	mirna_lnc
31432	hsa-mir-1301	AL359198.1	0.388530529	mirna_lnc
31433	hsa-mir-1301	AC004012.1	0.599993378	mirna_lnc
31434	hsa-mir-1301	AL118511.1	0.385456902	mirna_lnc
31435	hsa-mir-1301	Z83847.1	0.308622369	mirna_lnc
31436	hsa-mir-1301	AC087276.1	0.345306727	mirna_lnc
31437	hsa-mir-1301	AC090739.1	0.3371639	mirna_lnc
31438	hsa-mir-1301	AL583808.1	0.467390161	mirna_lnc
31439	hsa-mir-1301	AC245014.3	0.374990669	mirna_lnc
31440	hsa-mir-1301	AC068620.2	0.354527978	mirna_lnc
31441	hsa-mir-1301	AP003392.3	0.459465342	mirna_lnc
31442	hsa-mir-1301	AP005205.3	0.374463153	mirna_lnc
31443	hsa-mir-1301	AC005034.4	0.398864446	mirna_lnc
31444	hsa-mir-1301	AC025580.3	0.3704576	mirna_lnc
31445	hsa-mir-1301	AC068620.1	0.321650013	mirna_lnc
31446	hsa-mir-1301	AC025265.1	0.31298009	mirna_lnc
31447	hsa-mir-1301	GIHCG	0.345986695	mirna_lnc
31448	hsa-mir-1301	Z93241.1	0.390537354	mirna_lnc
31449	hsa-mir-1301	AL627171.1	0.303435742	mirna_lnc
31450	hsa-mir-1301	AC110285.2	0.422588162	mirna_lnc
31451	hsa-mir-1301	AL359878.2	0.479128769	mirna_lnc
31452	hsa-mir-1301	LINC02470	0.306716193	mirna_lnc
31453	hsa-mir-1301	AC068338.2	0.372076513	mirna_lnc
31454	hsa-mir-1301	AC134682.1	0.300808641	mirna_lnc
31455	hsa-mir-1301	AL731537.1	0.529085197	mirna_lnc
31456	hsa-mir-1301	GTSE1-DT	0.395396235	mirna_lnc
31457	hsa-mir-1301	AC010643.1	0.325946256	mirna_lnc
31458	hsa-mir-1301	AC135178.5	0.442999481	mirna_lnc
31459	hsa-mir-1301	KCNJ2-AS1	0.51796463	mirna_lnc
31460	hsa-mir-1301	AC074135.1	0.304880972	mirna_lnc
31461	hsa-mir-1301	C8orf37-AS1	0.35176823	mirna_lnc
31462	hsa-mir-1301	AC002059.1	0.606558902	mirna_lnc
31463	hsa-mir-1301	AL645608.6	0.458327173	mirna_lnc
31464	hsa-mir-1301	AC092652.1	0.338771247	mirna_lnc
31465	hsa-mir-1301	AC231981.1	0.323904564	mirna_lnc
31466	hsa-mir-1301	AC000120.1	0.375831324	mirna_lnc
31467	hsa-mir-1301	AC010980.2	0.440720986	mirna_lnc
31468	hsa-mir-1301	AC025162.2	0.404569021	mirna_lnc
31469	hsa-mir-1301	PARD3-AS1	0.328965437	mirna_lnc
31470	hsa-mir-1301	AC090061.1	0.324275141	mirna_lnc
31471	hsa-mir-1301	AC136604.3	0.391718255	mirna_lnc
31472	hsa-mir-1301	AC007497.1	0.315901612	mirna_lnc
31473	hsa-mir-1301	AC005197.1	0.397680162	mirna_lnc
31474	hsa-mir-1301	AC069224.1	0.344533161	mirna_lnc
31475	hsa-mir-1301	AC145422.1	0.383119637	mirna_lnc
31476	hsa-mir-1301	AC145423.2	0.451564497	mirna_lnc
31477	hsa-mir-1301	AC092142.1	0.426614001	mirna_lnc
31478	hsa-mir-1301	AL163953.1	0.312176128	mirna_lnc
31479	hsa-mir-1301	AC012313.9	0.315654074	mirna_lnc
31480	hsa-mir-1301	CYP51A1-AS1	0.568148563	mirna_lnc
31481	hsa-mir-1301	AC005696.4	0.483625623	mirna_lnc

31482	hsa-mir-1301	AL512791.2	0.439699004	mirna_lnc
31483	hsa-mir-1301	AP001893.3	0.334489755	mirna_lnc
31484	hsa-mir-1301	RN7SL832P	0.593965944	mirna_lnc
31485	hsa-mir-1301	LINC02094	0.33035957	mirna_lnc
31486	hsa-mir-1301	LINC02076	0.315616653	mirna_lnc
31487	hsa-mir-1301	AC008555.2	0.417147742	mirna_lnc
31488	hsa-mir-1301	AL138966.2	0.397306255	mirna_lnc
31489	hsa-mir-1301	AL358933.1	0.367503928	mirna_lnc
31490	hsa-mir-1301	AP001922.5	0.350529667	mirna_lnc
31491	hsa-mir-1301	AC084819.1	0.316377153	mirna_lnc
31492	hsa-mir-1301	AL512598.1	0.338908217	mirna_lnc
31493	hsa-mir-1301	LINC00112	0.324621938	mirna_lnc
31494	hsa-mir-1301	AP001972.4	0.475079305	mirna_lnc
31495	hsa-mir-1301	AL590705.3	0.594078956	mirna_lnc
31496	hsa-mir-1301	AL132655.1	0.316136081	mirna_lnc
31497	hsa-mir-1301	AL121832.1	0.311466835	mirna_lnc
31498	hsa-mir-1301	AC106782.2	0.353309102	mirna_lnc
31499	hsa-mir-1301	GLIS2-AS1	0.319833396	mirna_lnc
31500	hsa-mir-1301	AC008752.1	0.304617847	mirna_lnc
31501	hsa-mir-1301	LINC00900	0.350596435	mirna_lnc
31502	hsa-mir-1301	AL645608.2	0.397414705	mirna_lnc
31503	hsa-mir-1301	SEC23A-AS1	0.324696301	mirna_lnc
31504	hsa-mir-1301	AC079610.2	0.311654545	mirna_lnc
31505	hsa-mir-1301	AC015917.2	0.433357937	mirna_lnc
31506	hsa-mir-1301	AP001412.1	0.342198298	mirna_lnc
31507	hsa-mir-1301	TOLLIP-AS1	0.448028845	mirna_lnc
31508	hsa-mir-1301	AC011247.1	0.314334402	mirna_lnc
31509	hsa-mir-1301	AC021092.1	0.434685232	mirna_lnc
31510	hsa-mir-1301	AC006449.6	0.313756289	mirna_lnc
31511	hsa-mir-1301	AC137767.1	0.353229964	mirna_lnc
31512	hsa-mir-1301	SNHG9	0.326532048	mirna_lnc
31513	hsa-mir-1301	AC099684.2	0.383706341	mirna_lnc
31514	hsa-mir-1301	AC108704.1	0.339655083	mirna_lnc
31515	hsa-mir-1301	AC012676.3	0.458665945	mirna_lnc
31516	hsa-mir-99a	AC103746.1	0.692162609	mirna_lnc
31517	hsa-mir-99a	AL023973.1	0.309177766	mirna_lnc
31518	hsa-mir-99a	AC129492.5	0.44659389	mirna_lnc
31519	hsa-mir-99a	LINC02593	0.697106431	mirna_lnc
31520	hsa-mir-99a	AL160286.2	0.315990548	mirna_lnc
31521	hsa-mir-99a	AJ006995.1	0.444767947	mirna_lnc
31522	hsa-mir-99a	LINC01560	0.313452947	mirna_lnc
31523	hsa-mir-99a	AC006942.1	0.315480979	mirna_lnc
31524	hsa-mir-99a	PACRG-AS1	0.473632019	mirna_lnc
31525	hsa-mir-99a	LINC01625	0.718596977	mirna_lnc
31526	hsa-mir-99a	AC008121.2	0.301728438	mirna_lnc
31527	hsa-mir-99a	AC012409.2	0.531643358	mirna_lnc
31528	hsa-mir-99a	TNRC6C-AS1	0.362281426	mirna_lnc
31529	hsa-mir-99a	LOH12CR2	0.448083905	mirna_lnc
31530	hsa-mir-99a	AC016251.2	0.374366737	mirna_lnc
31531	hsa-mir-99a	AC008105.1	0.392625914	mirna_lnc
31532	hsa-mir-99a	LINC01311	0.316623666	mirna_lnc
31533	hsa-mir-99a	AC139768.2	0.312506573	mirna_lnc
31534	hsa-mir-99a	AC096733.2	0.330296453	mirna_lnc
31535	hsa-mir-99a	TFAP2A-AS1	0.38577133	mirna_lnc

31536	hsa-mir-99a	AC022387.1	0.480564146	mirna_lnc
31537	hsa-mir-99a	AC063977.6	0.345768047	mirna_lnc
31538	hsa-mir-99a	AC002066.1	0.404293457	mirna_lnc
31539	hsa-mir-99a	FAM66D	0.414317564	mirna_lnc
31540	hsa-mir-99a	AL139288.1	0.52481017	mirna_lnc
31541	hsa-mir-99a	MAFA-AS1	0.377517234	mirna_lnc
31542	hsa-mir-99a	AC104667.1	0.507724229	mirna_lnc
31543	hsa-mir-99a	AC098828.2	0.321937437	mirna_lnc
31544	hsa-mir-99a	AL137802.2	0.483695842	mirna_lnc
31545	hsa-mir-99a	AC004069.1	0.329880273	mirna_lnc
31546	hsa-mir-99a	AC093642.2	0.672838411	mirna_lnc
31547	hsa-mir-99a	AL157823.2	0.48138709	mirna_lnc
31548	hsa-mir-99a	AP000553.2	0.659000323	mirna_lnc
31549	hsa-mir-99a	LINC02175	0.566580261	mirna_lnc
31550	hsa-mir-99a	AC110813.1	0.668885062	mirna_lnc
31551	hsa-mir-99a	LINC01126	0.414598251	mirna_lnc
31552	hsa-mir-99a	LINC01087	0.502603647	mirna_lnc
31553	hsa-mir-99a	AC135803.1	0.69986902	mirna_lnc
31554	hsa-mir-99a	AL133243.2	0.304594321	mirna_lnc
31555	hsa-mir-99a	SYNGAP1-AS1	0.334487454	mirna_lnc
31556	hsa-mir-99a	MAGEA4-AS1	0.379264565	mirna_lnc
31557	hsa-mir-99a	AC105339.5	0.501472493	mirna_lnc
31558	hsa-mir-99a	AL359643.3	0.311066433	mirna_lnc
31559	hsa-mir-99a	AC005332.4	0.337331203	mirna_lnc
31560	hsa-mir-99a	AC090578.2	0.596495345	mirna_lnc
31561	hsa-mir-99a	AL078605.1	0.516338756	mirna_lnc
31562	hsa-mir-99a	AC026254.2	0.307961565	mirna_lnc
31563	hsa-mir-99a	AL138760.1	0.384388122	mirna_lnc
31564	hsa-mir-99a	LINC01686	0.326253143	mirna_lnc
31565	hsa-mir-99a	Z95115.1	0.51250187	mirna_lnc
31566	hsa-mir-99a	LINC01143	0.497534523	mirna_lnc
31567	hsa-mir-99a	AC079145.1	0.374976441	mirna_lnc
31568	hsa-mir-99a	AC005695.1	0.334125853	mirna_lnc
31569	hsa-mir-99a	AC020907.5	0.504142339	mirna_lnc
31570	hsa-mir-99a	UBR5-AS1	0.315226503	mirna_lnc
31571	hsa-mir-99a	AC130650.2	0.403371803	mirna_lnc
31572	hsa-mir-99a	AL008721.2	0.374474726	mirna_lnc
31573	hsa-mir-99a	AC110769.2	0.420310565	mirna_lnc
31574	hsa-mir-99a	AL138762.1	0.384659519	mirna_lnc
31575	hsa-mir-99a	AC007996.1	0.479833689	mirna_lnc
31576	hsa-mir-99a	AC096642.1	0.35958628	mirna_lnc
31577	hsa-mir-99a	LINC01521	0.350733491	mirna_lnc
31578	hsa-mir-99a	AC016708.1	0.731630456	mirna_lnc
31579	hsa-mir-99a	AC091057.2	0.347534201	mirna_lnc
31580	hsa-mir-99a	AC009506.1	0.513801214	mirna_lnc
31581	hsa-mir-99a	AC007848.1	0.388071159	mirna_lnc
31582	hsa-mir-99a	AC099786.1	0.704286347	mirna_lnc
31583	hsa-mir-99a	AC063948.1	0.347892556	mirna_lnc
31584	hsa-mir-99a	AC087742.1	0.374866848	mirna_lnc
31585	hsa-mir-99a	AL354892.2	0.356071421	mirna_lnc
31586	hsa-mir-99a	AP001330.5	0.34738324	mirna_lnc
31587	hsa-mir-99a	AC011773.4	0.402336977	mirna_lnc
31588	hsa-mir-99a	AC015802.3	0.33977926	mirna_lnc
31589	hsa-mir-99a	AL513365.2	0.334648563	mirna_lnc

31590	hsa-mir-99a	AC064836.3	0.33132735	mirna_lnc
31591	hsa-mir-99a	BX088651.4	0.3253941	mirna_lnc
31592	hsa-mir-99a	AL512408.1	0.330926843	mirna_lnc
31593	hsa-mir-99a	AC023034.2	0.4321962	mirna_lnc
31594	hsa-mir-99a	AC092068.3	0.331954142	mirna_lnc
31595	hsa-mir-99a	AC246793.1	0.425613269	mirna_lnc
31596	hsa-mir-99a	AC105339.3	0.320998598	mirna_lnc
31597	hsa-mir-99a	AC131532.1	0.381097505	mirna_lnc
31598	hsa-mir-99a	ERICD	0.507611394	mirna_lnc
31599	hsa-mir-99a	AL662795.2	0.357465056	mirna_lnc
31600	hsa-mir-99a	AC006262.1	0.305574101	mirna_lnc
31601	hsa-mir-99a	LINC02600	0.343329517	mirna_lnc
31602	hsa-mir-99a	AC092608.1	0.390311942	mirna_lnc
31603	hsa-mir-99a	PCOLCE-AS1	0.347235008	mirna_lnc
31604	hsa-mir-99a	Z93930.3	0.423427874	mirna_lnc
31605	hsa-mir-99a	AL136040.1	0.395731496	mirna_lnc
31606	hsa-mir-99a	AC092068.2	0.324356361	mirna_lnc
31607	hsa-mir-99a	AC073389.1	0.437627831	mirna_lnc
31608	hsa-mir-99a	DNM3-IT1	0.652818669	mirna_lnc
31609	hsa-mir-99a	AC006206.1	0.377952039	mirna_lnc
31610	hsa-mir-99a	AC090772.4	0.416197221	mirna_lnc
31611	hsa-mir-99a	UBE2D3-AS1	0.315678693	mirna_lnc
31612	hsa-mir-99a	AL451064.1	0.694480047	mirna_lnc
31613	hsa-mir-99a	AC084876.2	0.347216715	mirna_lnc
31614	hsa-mir-99a	AL442128.2	0.300924743	mirna_lnc
31615	hsa-mir-99a	AC026691.1	0.339296509	mirna_lnc
31616	hsa-mir-99a	AL645608.4	0.636174918	mirna_lnc
31617	hsa-mir-99a	AC005730.2	0.317878232	mirna_lnc
31618	hsa-mir-99a	AC008115.1	0.313501359	mirna_lnc
31619	hsa-mir-99a	AC126407.1	0.318167029	mirna_lnc
31620	hsa-mir-99a	AP001029.1	0.363892269	mirna_lnc
31621	hsa-mir-99a	AC007663.3	0.614822325	mirna_lnc
31622	hsa-mir-99a	AL590787.1	0.373361647	mirna_lnc
31623	hsa-mir-99a	AC009120.5	0.377039437	mirna_lnc
31624	hsa-mir-99a	AC093821.1	0.38446226	mirna_lnc
31625	hsa-mir-99a	AP000253.1	0.392345494	mirna_lnc
31626	hsa-mir-99a	AC034102.5	0.314665981	mirna_lnc
31627	hsa-mir-99a	AL138831.1	0.48183104	mirna_lnc
31628	hsa-mir-99a	AC010883.1	0.331606102	mirna_lnc
31629	hsa-mir-99a	AP001178.3	0.31890768	mirna_lnc
31630	hsa-mir-99a	AC010735.1	0.551023055	mirna_lnc
31631	hsa-mir-99a	AC079600.3	0.494417362	mirna_lnc
31632	hsa-mir-99a	FAM66E	0.316432109	mirna_lnc
31633	hsa-mir-99a	AC022916.1	0.331832932	mirna_lnc
31634	hsa-mir-99a	AL022324.3	0.452809332	mirna_lnc
31635	hsa-mir-99a	AC105118.1	0.421291703	mirna_lnc
31636	hsa-mir-99a	AC007391.1	0.329233474	mirna_lnc
31637	hsa-mir-99a	AC005005.3	0.345236149	mirna_lnc
31638	hsa-mir-99a	AC100814.1	0.302245848	mirna_lnc
31639	hsa-mir-99a	UFL1-AS1	0.313265476	mirna_lnc
31640	hsa-mir-99a	LINC01800	0.313014729	mirna_lnc
31641	hsa-mir-99a	AC008736.2	0.346061255	mirna_lnc
31642	hsa-mir-99a	LINC01771	0.324701885	mirna_lnc
31643	hsa-mir-99a	LINC01238	0.573221928	mirna_lnc

31644	hsa-mir-99a	ATP2B1-AS1	0.43730258	mirna_lnc
31645	hsa-mir-99a	AP001029.2	0.368234325	mirna_lnc
31646	hsa-mir-99a	AP000255.1	0.748088807	mirna_lnc
31647	hsa-mir-99a	AL132765.2	0.415494877	mirna_lnc
31648	hsa-mir-99a	AC104072.1	0.516434385	mirna_lnc
31649	hsa-mir-99a	HHIP-AS1	0.307537891	mirna_lnc
31650	hsa-mir-99a	CAHM	0.375777376	mirna_lnc
31651	hsa-mir-99a	AP005329.2	0.420967312	mirna_lnc
31652	hsa-mir-99a	AC010336.2	0.329526843	mirna_lnc
31653	hsa-mir-99a	AC090772.1	0.310928598	mirna_lnc
31654	hsa-mir-99a	AC073389.2	0.4504773	mirna_lnc
31655	hsa-mir-99a	AL031658.1	0.624747364	mirna_lnc
31656	hsa-mir-99a	AC010997.4	0.599095717	mirna_lnc
31657	hsa-mir-99a	HCG14	0.363165023	mirna_lnc
31658	hsa-mir-99a	AC068473.5	0.388454194	mirna_lnc
31659	hsa-mir-99a	AC010997.5	0.3429416	mirna_lnc
31660	hsa-mir-99a	HM13-AS1	0.324740525	mirna_lnc
31661	hsa-mir-99a	AC092849.1	0.319592512	mirna_lnc
31662	hsa-mir-99a	AC233976.1	0.338930882	mirna_lnc
31663	hsa-mir-99a	TTC39C-AS1	0.655353896	mirna_lnc
31664	hsa-mir-99a	AP000962.1	0.442844703	mirna_lnc
31665	hsa-mir-99a	RB1-DT	0.413162773	mirna_lnc
31666	hsa-mir-99a	AC023632.2	0.345143898	mirna_lnc
31667	hsa-mir-99a	PAN3-AS1	0.437726889	mirna_lnc
31668	hsa-mir-99a	AL161729.3	0.302391247	mirna_lnc
31669	hsa-mir-99a	AC092447.5	0.548311765	mirna_lnc
31670	hsa-mir-99a	AC006511.3	0.377202445	mirna_lnc
31671	hsa-mir-99a	AC092111.2	0.372624729	mirna_lnc
31672	hsa-mir-99a	AC010336.5	0.622561381	mirna_lnc
31673	hsa-mir-99a	AC107464.3	0.677999755	mirna_lnc
31674	hsa-mir-99a	AL513534.2	0.377613605	mirna_lnc
31675	hsa-mir-99a	AC025219.1	0.329238859	mirna_lnc
31676	hsa-mir-99a	LINC01901	0.589534899	mirna_lnc
31677	hsa-mir-99a	AC022400.8	0.376953778	mirna_lnc
31678	hsa-mir-99a	AC107032.2	0.377498783	mirna_lnc
31679	hsa-mir-99a	LINC01465	0.422736418	mirna_lnc
31680	hsa-mir-99a	ZNF503-AS2	0.404148883	mirna_lnc
31681	hsa-mir-99a	AL158063.1	0.381349085	mirna_lnc
31682	hsa-mir-99a	ERVK9-11	0.321897512	mirna_lnc
31683	hsa-mir-99a	OBSCN-AS1	0.301172179	mirna_lnc
31684	hsa-mir-99a	AC120057.4	0.351642967	mirna_lnc
31685	hsa-mir-99a	AC109347.1	0.349750869	mirna_lnc
31686	hsa-mir-99a	AL158055.1	0.365774346	mirna_lnc
31687	hsa-mir-99a	AP000962.2	0.49064529	mirna_lnc
31688	hsa-mir-99a	AL512625.1	0.409062443	mirna_lnc
31689	hsa-mir-99a	LINC01144	0.365411738	mirna_lnc
31690	hsa-mir-99a	AL008721.1	0.327788308	mirna_lnc
31691	hsa-mir-99a	AF127577.5	0.48179283	mirna_lnc
31692	hsa-mir-99a	FGF12-AS3	0.404743996	mirna_lnc
31693	hsa-mir-99a	AC092119.2	0.342088379	mirna_lnc
31694	hsa-mir-99a	AL355490.2	0.355111516	mirna_lnc
31695	hsa-mir-99a	AC079610.1	0.344616357	mirna_lnc
31696	hsa-mir-99a	SMC2-AS1	0.538482288	mirna_lnc
31697	hsa-mir-99a	AC110619.1	0.362649196	mirna_lnc

31698	hsa-mir-99a	AC107068.1	0.423251063	mirna_lnc
31699	hsa-mir-99a	AC090579.1	0.342129539	mirna_lnc
31700	hsa-mir-99a	MANEA-DT	0.363309994	mirna_lnc
31701	hsa-mir-99a	AL049796.1	0.383077464	mirna_lnc
31702	hsa-mir-99a	AL136531.1	0.398143049	mirna_lnc
31703	hsa-mir-99a	AC133550.2	0.334457397	mirna_lnc
31704	hsa-mir-99a	AL451062.1	0.357813153	mirna_lnc
31705	hsa-mir-99a	AC092119.3	0.32491087	mirna_lnc
31706	hsa-mir-99a	MAGEA8-AS1	0.590543336	mirna_lnc
31707	hsa-mir-99a	AC116348.1	0.37998909	mirna_lnc
31708	hsa-mir-99a	Z83847.1	0.314663581	mirna_lnc
31709	hsa-mir-99a	AC004925.1	0.539215224	mirna_lnc
31710	hsa-mir-99a	AP001605.1	0.332578145	mirna_lnc
31711	hsa-mir-99a	Z99129.3	0.426155669	mirna_lnc
31712	hsa-mir-99a	AC093462.1	0.315614183	mirna_lnc
31713	hsa-mir-99a	AP000350.5	0.555153584	mirna_lnc
31714	hsa-mir-99a	AC073389.3	0.617447291	mirna_lnc
31715	hsa-mir-99a	AC005034.4	0.334912604	mirna_lnc
31716	hsa-mir-99a	AC011005.4	0.315892785	mirna_lnc
31717	hsa-mir-99a	AC104794.2	0.400703329	mirna_lnc
31718	hsa-mir-99a	MAPT-IT1	0.570669365	mirna_lnc
31719	hsa-mir-99a	AL627171.1	0.354434431	mirna_lnc
31720	hsa-mir-99a	AL451069.1	0.377404678	mirna_lnc
31721	hsa-mir-99a	AC009312.1	0.424574985	mirna_lnc
31722	hsa-mir-99a	AC098484.2	0.311669753	mirna_lnc
31723	hsa-mir-99a	AL359510.2	0.322169734	mirna_lnc
31724	hsa-mir-99a	AP000350.7	0.534290513	mirna_lnc
31725	hsa-mir-99a	AL096701.3	0.357640988	mirna_lnc
31726	hsa-mir-99a	AC079414.3	0.401870127	mirna_lnc
31727	hsa-mir-99a	AC108134.4	0.354917586	mirna_lnc
31728	hsa-mir-99a	AC020978.1	0.354401218	mirna_lnc
31729	hsa-mir-99a	AL138831.3	0.488952353	mirna_lnc
31730	hsa-mir-99a	AC017100.1	0.356162295	mirna_lnc
31731	hsa-mir-99a	AC079601.1	0.336409479	mirna_lnc
31732	hsa-mir-99a	AL138831.2	0.512046985	mirna_lnc
31733	hsa-mir-99a	LINC01447	0.348009764	mirna_lnc
31734	hsa-mir-99a	LINC00173	0.35450231	mirna_lnc
31735	hsa-mir-99a	AC005006.1	0.338026027	mirna_lnc
31736	hsa-mir-99a	AL645608.6	0.610258176	mirna_lnc
31737	hsa-mir-99a	AL451064.2	0.696089878	mirna_lnc
31738	hsa-mir-99a	AL353726.2	0.474989724	mirna_lnc
31739	hsa-mir-99a	AC009148.1	0.340227365	mirna_lnc
31740	hsa-mir-99a	SPACA6P-AS	0.579156466	mirna_lnc
31741	hsa-mir-99a	AC090061.1	0.523777981	mirna_lnc
31742	hsa-mir-99a	AC055855.2	0.31499441	mirna_lnc
31743	hsa-mir-99a	AC104109.2	0.396388431	mirna_lnc
31744	hsa-mir-99a	LINC02652	0.353943612	mirna_lnc
31745	hsa-mir-99a	AC007497.1	0.383461963	mirna_lnc
31746	hsa-mir-99a	AL137003.2	0.421734158	mirna_lnc
31747	hsa-mir-99a	L29074.1	0.552815307	mirna_lnc
31748	hsa-mir-99a	FAM160A1-DT	0.345620998	mirna_lnc
31749	hsa-mir-99a	AC107464.1	0.334862688	mirna_lnc
31750	hsa-mir-99a	AC140125.2	0.325061965	mirna_lnc
31751	hsa-mir-99a	AL359541.1	0.519690774	mirna_lnc

31752	hsa-mir-99a	RN7SL832P	0.330573184	mirna_lnc
31753	hsa-mir-99a	AL354811.1	0.305501838	mirna_lnc
31754	hsa-mir-99a	AL157931.1	0.723076761	mirna_lnc
31755	hsa-mir-99a	AL133255.1	0.39069519	mirna_lnc
31756	hsa-mir-99a	AL354919.1	0.724407797	mirna_lnc
31757	hsa-mir-99a	AC116348.2	0.32846176	mirna_lnc
31758	hsa-mir-99a	AL662791.1	0.427025766	mirna_lnc
31759	hsa-mir-99a	AC103996.2	0.326787152	mirna_lnc
31760	hsa-mir-99a	AC093510.2	0.325994064	mirna_lnc
31761	hsa-mir-99a	AP000662.1	0.341098935	mirna_lnc
31762	hsa-mir-99a	AC008555.1	0.376902238	mirna_lnc
31763	hsa-mir-99a	AL158211.1	0.363383403	mirna_lnc
31764	hsa-mir-99a	AC009084.2	0.329755004	mirna_lnc
31765	hsa-mir-99a	AL135791.1	0.520219432	mirna_lnc
31766	hsa-mir-99a	AL161729.4	0.365922864	mirna_lnc
31767	hsa-mir-99a	AL022341.2	0.399855355	mirna_lnc
31768	hsa-mir-99a	AC009113.1	0.381135627	mirna_lnc
31769	hsa-mir-99a	AC083843.2	0.378782135	mirna_lnc
31770	hsa-mir-99a	AC026471.4	0.333114584	mirna_lnc
31771	hsa-mir-99a	AL645608.2	0.614905274	mirna_lnc
31772	hsa-mir-99a	AC010735.2	0.463471268	mirna_lnc
31773	hsa-mir-99a	AC079610.2	0.415823487	mirna_lnc
31774	hsa-mir-99a	AP001412.1	0.354133183	mirna_lnc
31775	hsa-mir-99a	AL024498.1	0.552149913	mirna_lnc
31776	hsa-mir-99a	AC100793.3	0.353695978	mirna_lnc
31777	hsa-mir-99a	AC012456.2	0.350429675	mirna_lnc
31778	hsa-mir-99a	AC100793.2	0.33718448	mirna_lnc
31779	hsa-mir-99a	AC007663.4	0.495810395	mirna_lnc
31780	hsa-mir-99a	AC087683.2	0.307797128	mirna_lnc
31781	hsa-mir-99a	AL138921.1	0.326456332	mirna_lnc
31782	hsa-mir-99a	C9orf139	0.305574346	mirna_lnc
31783	hsa-mir-99a	AC079630.1	0.35024052	mirna_lnc
31784	hsa-mir-99a	AP000845.1	0.314521062	mirna_lnc
31785	hsa-mir-99a	AC137767.1	0.43134222	mirna_lnc
31786	hsa-mir-1269	AC092667.1	0.567462701	mirna_lnc
31787	hsa-mir-1269	AC025470.2	0.722885027	mirna_lnc
31788	hsa-mir-1269	UGDH-AS1	0.301473588	mirna_lnc
31789	hsa-mir-1269	AC009974.1	0.30976676	mirna_lnc
31790	hsa-mir-1269	PACRG-AS1	0.342859589	mirna_lnc
31791	hsa-mir-1269	AC100823.1	0.396776271	mirna_lnc
31792	hsa-mir-1269	AP000808.1	0.459818998	mirna_lnc
31793	hsa-mir-1269	LOH12CR2	0.366599687	mirna_lnc
31794	hsa-mir-1269	DDX11-AS1	0.366507441	mirna_lnc
31795	hsa-mir-1269	AC095057.3	0.369937092	mirna_lnc
31796	hsa-mir-1269	AC092614.1	0.331013588	mirna_lnc
31797	hsa-mir-1269	AC106795.3	0.32377137	mirna_lnc
31798	hsa-mir-1269	AC019069.1	0.320437908	mirna_lnc
31799	hsa-mir-1269	SSBP3-AS1	0.306820666	mirna_lnc
31800	hsa-mir-1269	Z97989.1	0.485476395	mirna_lnc
31801	hsa-mir-1269	AC096637.2	0.355947966	mirna_lnc
31802	hsa-mir-1269	AL356740.1	0.332317116	mirna_lnc
31803	hsa-mir-1269	AP003071.2	0.44558517	mirna_lnc
31804	hsa-mir-1269	AC025176.1	0.377829818	mirna_lnc
31805	hsa-mir-1269	LINC01494	0.633736855	mirna_lnc

31806	hsa-mir-1269	AC092803.2	0.352789859	mirna_lnc
31807	hsa-mir-1269	AC147651.4	0.420207152	mirna_lnc
31808	hsa-mir-1269	OCIAD1-AS1	0.39717818	mirna_lnc
31809	hsa-mir-1269	LINC02367	0.356049181	mirna_lnc
31810	hsa-mir-1269	AC020763.4	0.402404682	mirna_lnc
31811	hsa-mir-1269	LINC01096	0.80792814	mirna_lnc
31812	hsa-mir-1269	AC012360.3	0.371589118	mirna_lnc
31813	hsa-mir-1269	AC125494.2	0.338727589	mirna_lnc
31814	hsa-mir-1269	AC016705.2	0.507360338	mirna_lnc
31815	hsa-mir-1269	HOXA10-AS	0.424105971	mirna_lnc
31816	hsa-mir-1269	AL391069.2	0.41646817	mirna_lnc
31817	hsa-mir-1269	AC116049.2	0.761652502	mirna_lnc
31818	hsa-mir-1269	AC139887.1	0.3101481	mirna_lnc
31819	hsa-mir-1269	AC079779.2	0.337331395	mirna_lnc
31820	hsa-mir-1269	AC024267.6	0.33058937	mirna_lnc
31821	hsa-mir-1269	LINC01126	0.31833278	mirna_lnc
31822	hsa-mir-1269	AL353746.1	0.46186294	mirna_lnc
31823	hsa-mir-1269	AF287957.1	0.49213017	mirna_lnc
31824	hsa-mir-1269	AC022929.2	0.318966244	mirna_lnc
31825	hsa-mir-1269	AC109347.2	0.404404991	mirna_lnc
31826	hsa-mir-1269	LINC02438	0.421847759	mirna_lnc
31827	hsa-mir-1269	AC024230.1	0.664774584	mirna_lnc
31828	hsa-mir-1269	LINC01397	0.409371468	mirna_lnc
31829	hsa-mir-1269	AC138904.1	0.425784873	mirna_lnc
31830	hsa-mir-1269	AL355304.1	0.384444037	mirna_lnc
31831	hsa-mir-1269	AC040160.1	0.405499772	mirna_lnc
31832	hsa-mir-1269	AL355596.1	0.34277605	mirna_lnc
31833	hsa-mir-1269	AC012074.1	0.558636563	mirna_lnc
31834	hsa-mir-1269	AP000487.1	0.522213172	mirna_lnc
31835	hsa-mir-1269	AC004943.1	0.33213207	mirna_lnc
31836	hsa-mir-1269	AC091931.1	0.344455033	mirna_lnc
31837	hsa-mir-1269	AP005432.2	0.683355335	mirna_lnc
31838	hsa-mir-1269	AC011997.1	0.319268032	mirna_lnc
31839	hsa-mir-1269	AC010186.3	0.464553512	mirna_lnc
31840	hsa-mir-1269	AC124016.1	0.320012119	mirna_lnc
31841	hsa-mir-1269	AC002076.1	0.416879246	mirna_lnc
31842	hsa-mir-1269	AC097634.1	0.328699527	mirna_lnc
31843	hsa-mir-1269	AL109976.1	0.313368638	mirna_lnc
31844	hsa-mir-1269	LINC01686	0.352079952	mirna_lnc
31845	hsa-mir-1269	LINC01143	0.374295972	mirna_lnc
31846	hsa-mir-1269	AC125618.1	0.317068675	mirna_lnc
31847	hsa-mir-1269	AC120114.1	0.393709824	mirna_lnc
31848	hsa-mir-1269	AC100782.1	0.504889911	mirna_lnc
31849	hsa-mir-1269	AC079145.1	0.367798905	mirna_lnc
31850	hsa-mir-1269	EMC1-AS1	0.361520857	mirna_lnc
31851	hsa-mir-1269	AC104823.1	0.324017773	mirna_lnc
31852	hsa-mir-1269	AL031705.1	0.303464981	mirna_lnc
31853	hsa-mir-1269	AC078864.1	0.378616309	mirna_lnc
31854	hsa-mir-1269	AC044781.1	0.394375371	mirna_lnc
31855	hsa-mir-1269	AC011297.1	0.50665206	mirna_lnc
31856	hsa-mir-1269	AC092535.1	0.405159588	mirna_lnc
31857	hsa-mir-1269	AC003986.2	0.456348768	mirna_lnc
31858	hsa-mir-1269	BMP7-AS1	0.334750393	mirna_lnc
31859	hsa-mir-1269	AL354892.2	0.314661454	mirna_lnc

31860	hsa-mir-1269	AC009088.2	0.325993253	mirna_lnc
31861	hsa-mir-1269	AC106820.2	0.348863584	mirna_lnc
31862	hsa-mir-1269	AC026271.3	0.452631594	mirna_lnc
31863	hsa-mir-1269	AC105046.1	0.333899535	mirna_lnc
31864	hsa-mir-1269	AC016205.1	0.487544909	mirna_lnc
31865	hsa-mir-1269	DPH6-DT	0.304554611	mirna_lnc
31866	hsa-mir-1269	AC092535.3	0.398793839	mirna_lnc
31867	hsa-mir-1269	LINC00653	0.308453008	mirna_lnc
31868	hsa-mir-1269	MYB-AS1	0.360586748	mirna_lnc
31869	hsa-mir-1269	AC092669.1	0.450748766	mirna_lnc
31870	hsa-mir-1269	AC110491.1	0.435214877	mirna_lnc
31871	hsa-mir-1269	HGC6.3	0.814427523	mirna_lnc
31872	hsa-mir-1269	AP002336.2	0.514687102	mirna_lnc
31873	hsa-mir-1269	AC099568.2	0.303985954	mirna_lnc
31874	hsa-mir-1269	FAM66B	0.355726099	mirna_lnc
31875	hsa-mir-1269	AC011773.1	0.341632517	mirna_lnc
31876	hsa-mir-1269	LINC02037	0.357293793	mirna_lnc
31877	hsa-mir-1269	AC087623.3	0.381346597	mirna_lnc
31878	hsa-mir-1269	AL136366.1	0.395365871	mirna_lnc
31879	hsa-mir-1269	AL442128.2	0.310690391	mirna_lnc
31880	hsa-mir-1269	HOXC13-AS	0.338274829	mirna_lnc
31881	hsa-mir-1269	LINC02562	0.518058063	mirna_lnc
31882	hsa-mir-1269	AC011389.3	0.465929881	mirna_lnc
31883	hsa-mir-1269	LINC01891	0.493713324	mirna_lnc
31884	hsa-mir-1269	NLGN4Y-AS1	0.372932605	mirna_lnc
31885	hsa-mir-1269	AC098818.2	0.325042895	mirna_lnc
31886	hsa-mir-1269	AC068473.3	0.426611844	mirna_lnc
31887	hsa-mir-1269	AC104596.1	0.491018195	mirna_lnc
31888	hsa-mir-1269	TCF7L1-IT1	0.573212945	mirna_lnc
31889	hsa-mir-1269	AC007687.1	0.40891817	mirna_lnc
31890	hsa-mir-1269	AC060766.7	0.314263136	mirna_lnc
31891	hsa-mir-1269	SH3RF3-AS1	0.561215712	mirna_lnc
31892	hsa-mir-1269	AC105114.1	0.357417387	mirna_lnc
31893	hsa-mir-1269	AC107214.1	0.394757462	mirna_lnc
31894	hsa-mir-1269	LINC00885	0.36369643	mirna_lnc
31895	hsa-mir-1269	AL157832.2	0.470616216	mirna_lnc
31896	hsa-mir-1269	AL591368.1	0.513082838	mirna_lnc
31897	hsa-mir-1269	AC053527.1	0.449746004	mirna_lnc
31898	hsa-mir-1269	AC087623.1	0.451086988	mirna_lnc
31899	hsa-mir-1269	AC025183.2	0.387220885	mirna_lnc
31900	hsa-mir-1269	AC006206.2	0.37038386	mirna_lnc
31901	hsa-mir-1269	AC093915.1	0.332475996	mirna_lnc
31902	hsa-mir-1269	AC009227.1	0.388530597	mirna_lnc
31903	hsa-mir-1269	AL121760.1	0.308462069	mirna_lnc
31904	hsa-mir-1269	AC007848.2	0.312769893	mirna_lnc
31905	hsa-mir-1269	AC015795.1	0.314382843	mirna_lnc
31906	hsa-mir-1269	AC068700.1	0.413555051	mirna_lnc
31907	hsa-mir-1269	AC073389.2	0.411801138	mirna_lnc
31908	hsa-mir-1269	AL591167.1	0.335510711	mirna_lnc
31909	hsa-mir-1269	AL445471.1	0.351887349	mirna_lnc
31910	hsa-mir-1269	AC083805.3	0.346905161	mirna_lnc
31911	hsa-mir-1269	AC055854.1	0.395297351	mirna_lnc
31912	hsa-mir-1269	AL031432.3	0.330998742	mirna_lnc
31913	hsa-mir-1269	AL031186.1	0.60091511	mirna_lnc

31914	hsa-mir-1269	AC112176.1	0.412480819	mirna_lnc
31915	hsa-mir-1269	AC010198.1	0.322804973	mirna_lnc
31916	hsa-mir-1269	LINC01258	0.641943873	mirna_lnc
31917	hsa-mir-1269	AC007541.1	0.374129339	mirna_lnc
31918	hsa-mir-1269	AC139795.3	0.304160065	mirna_lnc
31919	hsa-mir-1269	AC092111.2	0.416791975	mirna_lnc
31920	hsa-mir-1269	AC006946.2	0.429874121	mirna_lnc
31921	hsa-mir-1269	AC022509.2	0.528755679	mirna_lnc
31922	hsa-mir-1269	AC005865.1	0.55942483	mirna_lnc
31923	hsa-mir-1269	AC011365.2	0.355538421	mirna_lnc
31924	hsa-mir-1269	AC007250.1	0.398054304	mirna_lnc
31925	hsa-mir-1269	AC034236.3	0.355199777	mirna_lnc
31926	hsa-mir-1269	AC090197.1	0.409290053	mirna_lnc
31927	hsa-mir-1269	AP000317.1	0.306827297	mirna_lnc
31928	hsa-mir-1269	U47924.2	0.313905574	mirna_lnc
31929	hsa-mir-1269	INKA2-AS1	0.300729824	mirna_lnc
31930	hsa-mir-1269	AL139339.2	0.480217575	mirna_lnc
31931	hsa-mir-1269	AC098936.1	0.479442909	mirna_lnc
31932	hsa-mir-1269	IRAIN	0.329415891	mirna_lnc
31933	hsa-mir-1269	AC138207.2	0.321696186	mirna_lnc
31934	hsa-mir-1269	AC079316.2	0.445543885	mirna_lnc
31935	hsa-mir-1269	AL807752.4	0.379232676	mirna_lnc
31936	hsa-mir-1269	AC073869.3	0.395694795	mirna_lnc
31937	hsa-mir-1269	ETV5-AS1	0.631682484	mirna_lnc
31938	hsa-mir-1269	AC079610.1	0.314735496	mirna_lnc
31939	hsa-mir-1269	AC109449.1	0.37643069	mirna_lnc
31940	hsa-mir-1269	AL162231.4	0.375380486	mirna_lnc
31941	hsa-mir-1269	ZNF649-AS1	0.339470601	mirna_lnc
31942	hsa-mir-1269	AC107068.1	0.309013905	mirna_lnc
31943	hsa-mir-1269	SEMA6A-AS2	0.37034422	mirna_lnc
31944	hsa-mir-1269	AC004969.1	0.421426308	mirna_lnc
31945	hsa-mir-1269	AP001783.1	0.430403628	mirna_lnc
31946	hsa-mir-1269	LINC02457	0.429661049	mirna_lnc
31947	hsa-mir-1269	AL354993.1	0.372132348	mirna_lnc
31948	hsa-mir-1269	AL031651.2	0.543827385	mirna_lnc
31949	hsa-mir-1269	AL118511.1	0.42944598	mirna_lnc
31950	hsa-mir-1269	AC027348.1	0.571295745	mirna_lnc
31951	hsa-mir-1269	AC046130.2	0.562512563	mirna_lnc
31952	hsa-mir-1269	AL354950.1	0.398566517	mirna_lnc
31953	hsa-mir-1269	BNC2-AS1	0.448193011	mirna_lnc
31954	hsa-mir-1269	AC068620.2	0.351775215	mirna_lnc
31955	hsa-mir-1269	AC005034.4	0.463886791	mirna_lnc
31956	hsa-mir-1269	GIHCG	0.419764943	mirna_lnc
31957	hsa-mir-1269	AL390760.1	0.370403753	mirna_lnc
31958	hsa-mir-1269	AC007370.1	0.31275525	mirna_lnc
31959	hsa-mir-1269	AL359881.3	0.327816388	mirna_lnc
31960	hsa-mir-1269	AP000808.2	0.500683324	mirna_lnc
31961	hsa-mir-1269	LINC00028	0.486092101	mirna_lnc
31962	hsa-mir-1269	AL359510.2	0.437325813	mirna_lnc
31963	hsa-mir-1269	LINC02470	0.461116869	mirna_lnc
31964	hsa-mir-1269	AC020928.3	0.35859479	mirna_lnc
31965	hsa-mir-1269	AC022107.1	0.408206492	mirna_lnc
31966	hsa-mir-1269	AL031587.1	0.570456088	mirna_lnc
31967	hsa-mir-1269	AC106820.4	0.53076812	mirna_lnc

31968	hsa-mir-1269	AC026801.2	0.458724419	mirna_lnc
31969	hsa-mir-1269	LINC02097	0.307310378	mirna_lnc
31970	hsa-mir-1269	LINC00471	0.389520408	mirna_lnc
31971	hsa-mir-1269	TPRG1-AS1	0.319615964	mirna_lnc
31972	hsa-mir-1269	LINC00173	0.395173429	mirna_lnc
31973	hsa-mir-1269	AL929236.1	0.357011026	mirna_lnc
31974	hsa-mir-1269	LINC00552	0.364090892	mirna_lnc
31975	hsa-mir-1269	AC010980.2	0.341570294	mirna_lnc
31976	hsa-mir-1269	AC018892.3	0.313209697	mirna_lnc
31977	hsa-mir-1269	LINC01918	0.442332986	mirna_lnc
31978	hsa-mir-1269	AC104581.4	0.307364886	mirna_lnc
31979	hsa-mir-1269	AL139339.1	0.554595416	mirna_lnc
31980	hsa-mir-1269	AL121917.2	0.448305261	mirna_lnc
31981	hsa-mir-1269	CD81-AS1	0.340012331	mirna_lnc
31982	hsa-mir-1269	AC108451.1	0.38371676	mirna_lnc
31983	hsa-mir-1269	AC015983.2	0.398741796	mirna_lnc
31984	hsa-mir-1269	AC032011.1	0.419846734	mirna_lnc
31985	hsa-mir-1269	AC125603.4	0.349964551	mirna_lnc
31986	hsa-mir-1269	AC107464.1	0.306048732	mirna_lnc
31987	hsa-mir-1269	AP002360.2	0.302211269	mirna_lnc
31988	hsa-mir-1269	LINC01936	0.360300259	mirna_lnc
31989	hsa-mir-1269	AC097468.2	0.359536226	mirna_lnc
31990	hsa-mir-1269	AL358115.1	0.330241448	mirna_lnc
31991	hsa-mir-1269	AL049712.1	0.381627419	mirna_lnc
31992	hsa-mir-1269	AC060766.6	0.426446956	mirna_lnc
31993	hsa-mir-1269	AC092718.5	0.415574404	mirna_lnc
31994	hsa-mir-1269	AC146944.4	0.30644829	mirna_lnc
31995	hsa-mir-1269	AC010680.2	0.334576843	mirna_lnc
31996	hsa-mir-1269	AC126768.1	0.330448208	mirna_lnc
31997	hsa-mir-1269	AC008537.2	0.336309162	mirna_lnc
31998	hsa-mir-1269	AL807761.4	0.301859565	mirna_lnc
31999	hsa-mir-1269	LINC00900	0.361736184	mirna_lnc
32000	hsa-mir-1269	LZTS1-AS1	0.409035558	mirna_lnc
32001	hsa-mir-1269	LAMC1-AS1	0.347040844	mirna_lnc
32002	hsa-mir-1269	AC011239.1	0.671406639	mirna_lnc
32003	hsa-mir-1269	AC006487.1	0.570973955	mirna_lnc
32004	hsa-mir-1269	AF213884.3	0.396524088	mirna_lnc
32005	hsa-mir-1269	AC008033.3	0.383073923	mirna_lnc
32006	hsa-mir-1269	AC097505.1	0.339295814	mirna_lnc
32007	hsa-mir-1269	NAV2-AS2	0.469275858	mirna_lnc
32008	hsa-mir-1269	CAMTA1-IT1	0.331070595	mirna_lnc
32009	hsa-mir-1269	AC010680.3	0.436643172	mirna_lnc
32010	hsa-mir-1269	AC022001.3	0.407671966	mirna_lnc
32011	hsa-mir-1269	AP002490.1	0.356198344	mirna_lnc
32012	hsa-mir-19a	AC012073.1	0.330077097	mirna_lnc
32013	hsa-mir-19a	AL161891.1	0.385458371	mirna_lnc
32014	hsa-mir-19a	AC124303.1	0.309635209	mirna_lnc
32015	hsa-mir-19a	AC021945.1	0.321525139	mirna_lnc
32016	hsa-mir-19a	AC090844.3	0.31129821	mirna_lnc
32017	hsa-mir-19a	AC131009.3	0.374449842	mirna_lnc
32018	hsa-mir-19a	AC024940.6	0.349728892	mirna_lnc
32019	hsa-mir-19a	LINC02003	0.341210359	mirna_lnc
32020	hsa-mir-19a	AC125257.1	0.37053495	mirna_lnc
32021	hsa-mir-19a	AC004835.1	0.322145961	mirna_lnc

32022	hsa-mir-19a	AC005831.1	0.337334945	mirna_lnc
32023	hsa-mir-19a	AL358473.1	0.323190213	mirna_lnc
32024	hsa-mir-19a	AC004943.1	0.303147982	mirna_lnc
32025	hsa-mir-19a	LINC01357	0.300243944	mirna_lnc
32026	hsa-mir-19a	AC099518.2	0.38350147	mirna_lnc
32027	hsa-mir-19a	AL390728.6	0.347264494	mirna_lnc
32028	hsa-mir-19a	UBR5-AS1	0.323404531	mirna_lnc
32029	hsa-mir-19a	SREBF2-AS1	0.312792013	mirna_lnc
32030	hsa-mir-19a	AC004943.3	0.314875492	mirna_lnc
32031	hsa-mir-19a	RNASEH1-AS1	0.326942016	mirna_lnc
32032	hsa-mir-19a	AC005379.1	0.315804911	mirna_lnc
32033	hsa-mir-19a	AC011773.1	0.323107564	mirna_lnc
32034	hsa-mir-19a	AC107909.1	0.318660748	mirna_lnc
32035	hsa-mir-19a	AC132192.1	0.332056484	mirna_lnc
32036	hsa-mir-19a	AL022324.3	0.358014139	mirna_lnc
32037	hsa-mir-19a	AL603832.1	0.373790226	mirna_lnc
32038	hsa-mir-19a	AP006621.5	0.320333669	mirna_lnc
32039	hsa-mir-19a	AP003086.2	0.327433893	mirna_lnc
32040	hsa-mir-19a	AC090907.2	0.325674977	mirna_lnc
32041	hsa-mir-19a	LINC00482	0.313902768	mirna_lnc
32042	hsa-mir-19a	AC112255.1	0.318924245	mirna_lnc
32043	hsa-mir-19a	LINC01433	0.335448113	mirna_lnc
32044	hsa-mir-19a	AC116025.2	0.303265439	mirna_lnc
32045	hsa-mir-19a	AC005089.1	0.304793929	mirna_lnc
32046	hsa-mir-19a	MCF2L-AS1	0.312143926	mirna_lnc
32047	hsa-mir-19a	AL138767.3	0.326724068	mirna_lnc
32048	hsa-mir-19a	AL008721.1	0.322174752	mirna_lnc
32049	hsa-mir-19a	AL049833.3	0.304591577	mirna_lnc
32050	hsa-mir-19a	AC005046.1	0.373788697	mirna_lnc
32051	hsa-mir-19a	LINC02365	0.38543452	mirna_lnc
32052	hsa-mir-19a	AL118511.1	0.347446874	mirna_lnc
32053	hsa-mir-19a	AP003392.3	0.419854628	mirna_lnc
32054	hsa-mir-19a	AC133961.1	0.359358636	mirna_lnc
32055	hsa-mir-19a	AC005391.1	0.306343048	mirna_lnc
32056	hsa-mir-19a	AC004593.1	0.300995067	mirna_lnc
32057	hsa-mir-19a	AC007038.2	0.30964826	mirna_lnc
32058	hsa-mir-19a	LINC00456	0.345335196	mirna_lnc
32059	hsa-mir-19a	AL049646.1	0.335656659	mirna_lnc
32060	hsa-mir-19a	PSPC1-AS2	0.301015358	mirna_lnc
32061	hsa-mir-19a	AC114488.1	0.317619867	mirna_lnc
32062	hsa-mir-19a	AL356740.2	0.310927386	mirna_lnc
32063	hsa-mir-590	AC019294.2	0.347500739	mirna_lnc
32064	hsa-mir-590	SNHG3	0.314058392	mirna_lnc
32065	hsa-mir-590	AC069544.1	0.335225872	mirna_lnc
32066	hsa-mir-590	TMPO-AS1	0.328918371	mirna_lnc
32067	hsa-mir-590	AL121832.2	0.357470299	mirna_lnc
32068	hsa-mir-590	AC020658.4	0.376427548	mirna_lnc
32069	hsa-mir-590	AL035461.2	0.314248525	mirna_lnc
32070	hsa-mir-590	AC048344.4	0.324699322	mirna_lnc
32071	hsa-mir-590	SCAT2	0.341417972	mirna_lnc
32072	hsa-mir-590	CEP83-DT	0.337806577	mirna_lnc
32073	hsa-mir-590	AL049539.1	0.319134121	mirna_lnc
32074	hsa-mir-590	AL590326.1	0.347411295	mirna_lnc
32075	hsa-mir-590	LINC01311	0.413954109	mirna_lnc

32076	hsa-mir-590	AC074117.1	0.400737573	mirna_lnc
32077	hsa-mir-590	AP001469.3	0.301087273	mirna_lnc
32078	hsa-mir-590	TFAP2A-AS1	0.357587336	mirna_lnc
32079	hsa-mir-590	AC022387.1	0.384365171	mirna_lnc
32080	hsa-mir-590	AC027243.2	0.304010722	mirna_lnc
32081	hsa-mir-590	AL023803.3	0.306567352	mirna_lnc
32082	hsa-mir-590	AC131009.3	0.454530842	mirna_lnc
32083	hsa-mir-590	AC024940.6	0.328953821	mirna_lnc
32084	hsa-mir-590	AC109322.1	0.31781986	mirna_lnc
32085	hsa-mir-590	AC098828.2	0.342382728	mirna_lnc
32086	hsa-mir-590	AC012377.1	0.342775959	mirna_lnc
32087	hsa-mir-590	AC079907.1	0.340908492	mirna_lnc
32088	hsa-mir-590	AL137802.2	0.344982209	mirna_lnc
32089	hsa-mir-590	LINC02367	0.313705012	mirna_lnc
32090	hsa-mir-590	AC073195.1	0.382977491	mirna_lnc
32091	hsa-mir-590	AP000553.2	0.304094009	mirna_lnc
32092	hsa-mir-590	AC016394.2	0.323382362	mirna_lnc
32093	hsa-mir-590	AL031985.3	0.355218683	mirna_lnc
32094	hsa-mir-590	LINC01424	0.348459841	mirna_lnc
32095	hsa-mir-590	AL163051.1	0.326990513	mirna_lnc
32096	hsa-mir-590	AC090578.2	0.313032987	mirna_lnc
32097	hsa-mir-590	AC090517.2	0.316727148	mirna_lnc
32098	hsa-mir-590	Z95115.1	0.347740866	mirna_lnc
32099	hsa-mir-590	LINC01143	0.339263468	mirna_lnc
32100	hsa-mir-590	AC004884.2	0.338116555	mirna_lnc
32101	hsa-mir-590	AP001025.1	0.345657553	mirna_lnc
32102	hsa-mir-590	AL121658.1	0.302955226	mirna_lnc
32103	hsa-mir-590	AC091153.3	0.321577176	mirna_lnc
32104	hsa-mir-590	AC063948.1	0.358994712	mirna_lnc
32105	hsa-mir-590	AL354892.2	0.36845054	mirna_lnc
32106	hsa-mir-590	AC090527.3	0.399560158	mirna_lnc
32107	hsa-mir-590	AC005379.1	0.305803978	mirna_lnc
32108	hsa-mir-590	AC131212.4	0.334527668	mirna_lnc
32109	hsa-mir-590	Z93930.3	0.301335333	mirna_lnc
32110	hsa-mir-590	AC011773.1	0.329083709	mirna_lnc
32111	hsa-mir-590	AL645608.4	0.33472733	mirna_lnc
32112	hsa-mir-590	AC008115.1	0.352398681	mirna_lnc
32113	hsa-mir-590	AC007663.3	0.330619115	mirna_lnc
32114	hsa-mir-590	AC004471.1	0.345508149	mirna_lnc
32115	hsa-mir-590	AC136475.1	0.300673692	mirna_lnc
32116	hsa-mir-590	AC113143.1	0.303275591	mirna_lnc
32117	hsa-mir-590	AC107214.1	0.339550326	mirna_lnc
32118	hsa-mir-590	AL022324.3	0.391489988	mirna_lnc
32119	hsa-mir-590	AC005005.3	0.368010622	mirna_lnc
32120	hsa-mir-590	ATP2B1-AS1	0.346109621	mirna_lnc
32121	hsa-mir-590	AP000255.1	0.312653751	mirna_lnc
32122	hsa-mir-590	AL132765.2	0.306439154	mirna_lnc
32123	hsa-mir-590	AC019129.2	0.313428854	mirna_lnc
32124	hsa-mir-590	AC092756.1	0.31110929	mirna_lnc
32125	hsa-mir-590	AC090907.2	0.305130592	mirna_lnc
32126	hsa-mir-590	AC092447.5	0.342911029	mirna_lnc
32127	hsa-mir-590	LINC01901	0.345240345	mirna_lnc
32128	hsa-mir-590	AL008721.1	0.305008746	mirna_lnc
32129	hsa-mir-590	AL121989.1	0.335366917	mirna_lnc

32130	hsa-mir-590	AC090970.1	0.313903343	mirna_lnc
32131	hsa-mir-590	AC004925.1	0.358686081	mirna_lnc
32132	hsa-mir-590	AP000350.5	0.303918704	mirna_lnc
32133	hsa-mir-590	AC073389.3	0.32394466	mirna_lnc
32134	hsa-mir-590	AP003392.3	0.369979911	mirna_lnc
32135	hsa-mir-590	DLGAP1-AS3	0.30507128	mirna_lnc
32136	hsa-mir-590	MAPT-IT1	0.331004172	mirna_lnc
32137	hsa-mir-590	AC005006.1	0.348592824	mirna_lnc
32138	hsa-mir-590	AL353726.2	0.340824853	mirna_lnc
32139	hsa-mir-590	AC007497.1	0.349377087	mirna_lnc
32140	hsa-mir-590	AC145423.2	0.457237476	mirna_lnc
32141	hsa-mir-590	RN7SL832P	0.307440948	mirna_lnc
32142	hsa-mir-590	AL157931.1	0.317405851	mirna_lnc
32143	hsa-mir-590	AL354919.1	0.327924024	mirna_lnc
32144	hsa-mir-590	AP001972.4	0.302295391	mirna_lnc
32145	hsa-mir-590	AC004232.3	0.307902298	mirna_lnc
32146	hsa-mir-590	AC137767.1	0.443163409	mirna_lnc
32147	hsa-mir-671	AC092718.4	0.374770098	mirna_lnc
32148	hsa-mir-671	AC012073.1	0.347539147	mirna_lnc
32149	hsa-mir-671	TMPO-AS1	0.38050176	mirna_lnc
32150	hsa-mir-671	AC020658.4	0.345226558	mirna_lnc
32151	hsa-mir-671	AL117332.1	0.338174025	mirna_lnc
32152	hsa-mir-671	AC083799.1	0.312519073	mirna_lnc
32153	hsa-mir-671	AC106820.3	0.318531288	mirna_lnc
32154	hsa-mir-671	AP001469.3	0.383811977	mirna_lnc
32155	hsa-mir-671	AC004943.2	0.320045681	mirna_lnc
32156	hsa-mir-671	AC096656.1	0.309043958	mirna_lnc
32157	hsa-mir-671	AC005840.2	0.409121339	mirna_lnc
32158	hsa-mir-671	AC074351.1	0.391379821	mirna_lnc
32159	hsa-mir-671	AC073857.1	0.331800655	mirna_lnc
32160	hsa-mir-671	LINC01806	0.358104682	mirna_lnc
32161	hsa-mir-671	AC007389.3	0.357864181	mirna_lnc
32162	hsa-mir-671	LINC02367	0.304465309	mirna_lnc
32163	hsa-mir-671	AL137804.1	0.35935523	mirna_lnc
32164	hsa-mir-671	AC069281.1	0.323166685	mirna_lnc
32165	hsa-mir-671	AC012360.3	0.307538334	mirna_lnc
32166	hsa-mir-671	AC027088.3	0.341367335	mirna_lnc
32167	hsa-mir-671	GHET1	0.38711014	mirna_lnc
32168	hsa-mir-671	AL161772.1	0.340560121	mirna_lnc
32169	hsa-mir-671	LINC01850	0.388929966	mirna_lnc
32170	hsa-mir-671	WWTR1-AS1	0.302931062	mirna_lnc
32171	hsa-mir-671	LINC01968	0.327184785	mirna_lnc
32172	hsa-mir-671	AC125618.1	0.301904122	mirna_lnc
32173	hsa-mir-671	AC004884.2	0.316748672	mirna_lnc
32174	hsa-mir-671	AL391069.3	0.30243082	mirna_lnc
32175	hsa-mir-671	AC012442.1	0.322574814	mirna_lnc
32176	hsa-mir-671	SREBF2-AS1	0.316330847	mirna_lnc
32177	hsa-mir-671	AC110769.2	0.303745302	mirna_lnc
32178	hsa-mir-671	AC100812.1	0.37802084	mirna_lnc
32179	hsa-mir-671	AC046143.2	0.347222957	mirna_lnc
32180	hsa-mir-671	AC112907.1	0.30526112	mirna_lnc
32181	hsa-mir-671	AC010275.1	0.329044853	mirna_lnc
32182	hsa-mir-671	LINC01063	0.321587359	mirna_lnc
32183	hsa-mir-671	LINC02198	0.30547473	mirna_lnc

32184	hsa-mir-671	AC069213.1	0.301774017	mirna_lnc
32185	hsa-mir-671	AC009271.1	0.313747039	mirna_lnc
32186	hsa-mir-671	AC107214.1	0.328197311	mirna_lnc
32187	hsa-mir-671	AC131235.3	0.364244228	mirna_lnc
32188	hsa-mir-671	AP005328.2	0.320651936	mirna_lnc
32189	hsa-mir-671	AL132712.2	0.331678836	mirna_lnc
32190	hsa-mir-671	AC007823.1	0.352496785	mirna_lnc
32191	hsa-mir-671	AC096992.2	0.351115571	mirna_lnc
32192	hsa-mir-671	LPP-AS2	0.332093075	mirna_lnc
32193	hsa-mir-671	AC026803.2	0.304717238	mirna_lnc
32194	hsa-mir-671	ELDR	0.339127168	mirna_lnc
32195	hsa-mir-671	AC026785.3	0.310847952	mirna_lnc
32196	hsa-mir-671	AC019129.2	0.334185547	mirna_lnc
32197	hsa-mir-671	AL132712.1	0.311613521	mirna_lnc
32198	hsa-mir-671	AC008883.2	0.311403937	mirna_lnc
32199	hsa-mir-671	CPNE8-AS1	0.333885436	mirna_lnc
32200	hsa-mir-671	AC036214.1	0.381021791	mirna_lnc
32201	hsa-mir-671	LINC02006	0.311658107	mirna_lnc
32202	hsa-mir-671	AL024497.1	0.385167653	mirna_lnc
32203	hsa-mir-671	AC010501.1	0.309494654	mirna_lnc
32204	hsa-mir-671	AC126768.2	0.308924361	mirna_lnc
32205	hsa-mir-19b-1	AC012073.1	0.317605524	mirna_lnc
32206	hsa-mir-19b-1	AC010761.2	0.381994737	mirna_lnc
32207	hsa-mir-19b-1	AC012531.1	0.324698482	mirna_lnc
32208	hsa-mir-19b-1	AL161891.1	0.499081438	mirna_lnc
32209	hsa-mir-19b-1	AC027228.2	0.306356349	mirna_lnc
32210	hsa-mir-19b-1	AC027627.1	0.316072607	mirna_lnc
32211	hsa-mir-19b-1	AC021945.1	0.361619921	mirna_lnc
32212	hsa-mir-19b-1	AC090844.3	0.30407234	mirna_lnc
32213	hsa-mir-19b-1	GCC2-AS1	0.305305771	mirna_lnc
32214	hsa-mir-19b-1	AL355388.2	0.306758093	mirna_lnc
32215	hsa-mir-19b-1	AC005831.1	0.375263207	mirna_lnc
32216	hsa-mir-19b-1	LINC01460	0.397275832	mirna_lnc
32217	hsa-mir-19b-1	AC099518.2	0.391438603	mirna_lnc
32218	hsa-mir-19b-1	AC007128.1	0.324304611	mirna_lnc
32219	hsa-mir-19b-1	RNASEH1-AS1	0.302104726	mirna_lnc
32220	hsa-mir-19b-1	AC087491.1	0.323701105	mirna_lnc
32221	hsa-mir-19b-1	AP001065.2	0.326983223	mirna_lnc
32222	hsa-mir-19b-1	AC145207.5	0.34530379	mirna_lnc
32223	hsa-mir-19b-1	LINC02065	0.327225926	mirna_lnc
32224	hsa-mir-19b-1	AL137244.1	0.34309709	mirna_lnc
32225	hsa-mir-19b-1	AL391095.3	0.356338258	mirna_lnc
32226	hsa-mir-19b-1	AL512652.2	0.301171996	mirna_lnc
32227	hsa-mir-19b-1	LINC01433	0.302403812	mirna_lnc
32228	hsa-mir-19b-1	LINC00659	0.345097381	mirna_lnc
32229	hsa-mir-19b-1	MCF2L-AS1	0.368434161	mirna_lnc
32230	hsa-mir-19b-1	ZNF649-AS1	0.313821362	mirna_lnc
32231	hsa-mir-19b-1	LINC01555	0.321942457	mirna_lnc
32232	hsa-mir-19b-1	LINC02097	0.326784817	mirna_lnc
32233	hsa-mir-19b-1	AC017104.3	0.301034058	mirna_lnc
32234	hsa-mir-19b-1	AC097478.2	0.30461613	mirna_lnc
32235	hsa-mir-19b-1	LINC00456	0.406473394	mirna_lnc
32236	hsa-mir-19b-1	AL049646.1	0.357766673	mirna_lnc
32237	hsa-mir-19b-1	SLCO4A1-AS1	0.351914098	mirna_lnc

32238	hsa-mir-19b-1	LINC02547	0.307678138	mirna_lnc
32239	hsa-mir-19b-1	AC114488.1	0.316277775	mirna_lnc
32240	hsa-mir-19b-1	AL136146.2	0.369952107	mirna_lnc
32241	hsa-mir-324	AC012073.1	0.303023256	mirna_lnc
32242	hsa-mir-324	LINC01873	0.308213651	mirna_lnc
32243	hsa-mir-324	MELTF-AS1	0.315124792	mirna_lnc
32244	hsa-mir-324	TMPO-AS1	0.467851355	mirna_lnc
32245	hsa-mir-324	AL121832.2	0.332247853	mirna_lnc
32246	hsa-mir-324	DDX11-AS1	0.320858957	mirna_lnc
32247	hsa-mir-324	AC004241.3	0.39798382	mirna_lnc
32248	hsa-mir-324	AL117332.1	0.518639196	mirna_lnc
32249	hsa-mir-324	AC022211.2	0.3151941	mirna_lnc
32250	hsa-mir-324	CEP83-DT	0.323811709	mirna_lnc
32251	hsa-mir-324	HOXC-AS2	0.348372674	mirna_lnc
32252	hsa-mir-324	JARID2-AS1	0.328666407	mirna_lnc
32253	hsa-mir-324	AC074117.1	0.377157307	mirna_lnc
32254	hsa-mir-324	AL031667.3	0.378065706	mirna_lnc
32255	hsa-mir-324	TFAP2A-AS1	0.396184934	mirna_lnc
32256	hsa-mir-324	AC100791.2	0.318098486	mirna_lnc
32257	hsa-mir-324	AC063977.6	0.330631454	mirna_lnc
32258	hsa-mir-324	AC092159.2	0.30617217	mirna_lnc
32259	hsa-mir-324	AL023803.3	0.36843748	mirna_lnc
32260	hsa-mir-324	AC109322.1	0.323236643	mirna_lnc
32261	hsa-mir-324	AC074351.1	0.424947783	mirna_lnc
32262	hsa-mir-324	AL138724.1	0.339744964	mirna_lnc
32263	hsa-mir-324	AL445072.1	0.395289024	mirna_lnc
32264	hsa-mir-324	OCIAD1-AS1	0.302377181	mirna_lnc
32265	hsa-mir-324	AC016723.1	0.403168514	mirna_lnc
32266	hsa-mir-324	AC064807.1	0.307988816	mirna_lnc
32267	hsa-mir-324	AL137802.2	0.348806043	mirna_lnc
32268	hsa-mir-324	AC091729.3	0.324149194	mirna_lnc
32269	hsa-mir-324	AL139424.1	0.302716656	mirna_lnc
32270	hsa-mir-324	AL136162.1	0.401120665	mirna_lnc
32271	hsa-mir-324	AC007389.3	0.36910296	mirna_lnc
32272	hsa-mir-324	LINC02367	0.312092676	mirna_lnc
32273	hsa-mir-324	AC073195.1	0.375310384	mirna_lnc
32274	hsa-mir-324	AC015818.2	0.342174938	mirna_lnc
32275	hsa-mir-324	AC004069.1	0.378778494	mirna_lnc
32276	hsa-mir-324	LNCOC1	0.303198671	mirna_lnc
32277	hsa-mir-324	LINC01096	0.358005246	mirna_lnc
32278	hsa-mir-324	AC004920.1	0.350018643	mirna_lnc
32279	hsa-mir-324	AC021683.3	0.370064117	mirna_lnc
32280	hsa-mir-324	AC011481.4	0.302010961	mirna_lnc
32281	hsa-mir-324	AL391069.2	0.318688634	mirna_lnc
32282	hsa-mir-324	AC012640.2	0.397252625	mirna_lnc
32283	hsa-mir-324	AC026782.2	0.310498978	mirna_lnc
32284	hsa-mir-324	AC116049.2	0.416455676	mirna_lnc
32285	hsa-mir-324	AL139023.1	0.361589348	mirna_lnc
32286	hsa-mir-324	AP003119.2	0.331155842	mirna_lnc
32287	hsa-mir-324	AL513497.1	0.31844307	mirna_lnc
32288	hsa-mir-324	ATP6V1B1-AS1	0.358980679	mirna_lnc
32289	hsa-mir-324	AJ239322.1	0.350905916	mirna_lnc
32290	hsa-mir-324	AC024230.1	0.382979548	mirna_lnc
32291	hsa-mir-324	LINC01424	0.415952674	mirna_lnc

32292	hsa-mir-324	AL391152.1	0.313121714	mirna_lnc
32293	hsa-mir-324	TBC1D8-AS1	0.439919827	mirna_lnc
32294	hsa-mir-324	AP000487.1	0.304092631	mirna_lnc
32295	hsa-mir-324	KIAA1614-AS1	0.334207881	mirna_lnc
32296	hsa-mir-324	AC016876.3	0.440056966	mirna_lnc
32297	hsa-mir-324	AL359643.3	0.354916293	mirna_lnc
32298	hsa-mir-324	AL513327.2	0.389824596	mirna_lnc
32299	hsa-mir-324	LINC01456	0.332102556	mirna_lnc
32300	hsa-mir-324	AC092807.2	0.36063062	mirna_lnc
32301	hsa-mir-324	AC002076.1	0.350512239	mirna_lnc
32302	hsa-mir-324	LINC01850	0.386996896	mirna_lnc
32303	hsa-mir-324	LINC01686	0.366701274	mirna_lnc
32304	hsa-mir-324	Z95115.1	0.346921617	mirna_lnc
32305	hsa-mir-324	LINC01143	0.327694348	mirna_lnc
32306	hsa-mir-324	AC022031.2	0.305825253	mirna_lnc
32307	hsa-mir-324	AC100782.1	0.334010953	mirna_lnc
32308	hsa-mir-324	AC010595.1	0.316876624	mirna_lnc
32309	hsa-mir-324	AL391069.3	0.32190616	mirna_lnc
32310	hsa-mir-324	LINC02607	0.395286648	mirna_lnc
32311	hsa-mir-324	LINC01816	0.403546733	mirna_lnc
32312	hsa-mir-324	TMCO1-AS1	0.323932668	mirna_lnc
32313	hsa-mir-324	AC099518.6	0.342107526	mirna_lnc
32314	hsa-mir-324	AC007881.3	0.343150968	mirna_lnc
32315	hsa-mir-324	AL121658.1	0.308547509	mirna_lnc
32316	hsa-mir-324	LINC00167	0.328598107	mirna_lnc
32317	hsa-mir-324	LINC01521	0.398749765	mirna_lnc
32318	hsa-mir-324	AC100812.1	0.406167666	mirna_lnc
32319	hsa-mir-324	AC004943.3	0.309556636	mirna_lnc
32320	hsa-mir-324	AC145343.1	0.318098621	mirna_lnc
32321	hsa-mir-324	AC126614.1	0.353415801	mirna_lnc
32322	hsa-mir-324	AC093424.1	0.331797675	mirna_lnc
32323	hsa-mir-324	AL451085.2	0.360081246	mirna_lnc
32324	hsa-mir-324	AC091925.1	0.344054484	mirna_lnc
32325	hsa-mir-324	AC117382.2	0.349168458	mirna_lnc
32326	hsa-mir-324	AC091153.3	0.46276069	mirna_lnc
32327	hsa-mir-324	AL121721.1	0.311239866	mirna_lnc
32328	hsa-mir-324	AC087742.1	0.422657031	mirna_lnc
32329	hsa-mir-324	AL354892.2	0.46973536	mirna_lnc
32330	hsa-mir-324	AL139327.2	0.325113785	mirna_lnc
32331	hsa-mir-324	AL031963.3	0.318049761	mirna_lnc
32332	hsa-mir-324	AC106820.2	0.315255756	mirna_lnc
32333	hsa-mir-324	AC005993.1	0.307654788	mirna_lnc
32334	hsa-mir-324	AC026271.3	0.41770241	mirna_lnc
32335	hsa-mir-324	AC016205.1	0.405476092	mirna_lnc
32336	hsa-mir-324	AC064836.3	0.326559427	mirna_lnc
32337	hsa-mir-324	BX088651.4	0.321841414	mirna_lnc
32338	hsa-mir-324	PIK3CD-AS2	0.353419466	mirna_lnc
32339	hsa-mir-324	AC012640.5	0.365485451	mirna_lnc
32340	hsa-mir-324	AL512408.1	0.439759059	mirna_lnc
32341	hsa-mir-324	AC090527.3	0.305424994	mirna_lnc
32342	hsa-mir-324	AL353719.1	0.300378725	mirna_lnc
32343	hsa-mir-324	AC010422.2	0.304348825	mirna_lnc
32344	hsa-mir-324	AC010275.1	0.332524834	mirna_lnc
32345	hsa-mir-324	AC010608.2	0.323279839	mirna_lnc

32346	hsa-mir-324	AP002387.2	0.336926413	mirna_lnc
32347	hsa-mir-324	AC005379.1	0.304010568	mirna_lnc
32348	hsa-mir-324	LINC01063	0.387928244	mirna_lnc
32349	hsa-mir-324	AC092669.1	0.439095634	mirna_lnc
32350	hsa-mir-324	CIRBP-AS1	0.316083551	mirna_lnc
32351	hsa-mir-324	AP002336.2	0.314898107	mirna_lnc
32352	hsa-mir-324	AC091946.2	0.350317117	mirna_lnc
32353	hsa-mir-324	AC092171.3	0.33647397	mirna_lnc
32354	hsa-mir-324	CNNM3-DT	0.384709847	mirna_lnc
32355	hsa-mir-324	AC068831.6	0.311901258	mirna_lnc
32356	hsa-mir-324	AL645608.4	0.342175979	mirna_lnc
32357	hsa-mir-324	LINC02562	0.31419159	mirna_lnc
32358	hsa-mir-324	AC138696.2	0.343509059	mirna_lnc
32359	hsa-mir-324	AC009271.1	0.39912928	mirna_lnc
32360	hsa-mir-324	AC098818.2	0.338321071	mirna_lnc
32361	hsa-mir-324	AC022762.1	0.304146238	mirna_lnc
32362	hsa-mir-324	AC012146.3	0.300953703	mirna_lnc
32363	hsa-mir-324	AC008267.5	0.349765816	mirna_lnc
32364	hsa-mir-324	AC005695.3	0.305647192	mirna_lnc
32365	hsa-mir-324	AC107214.1	0.364179855	mirna_lnc
32366	hsa-mir-324	LINC00885	0.433343707	mirna_lnc
32367	hsa-mir-324	AP005328.2	0.360276649	mirna_lnc
32368	hsa-mir-324	URB1-AS1	0.358582275	mirna_lnc
32369	hsa-mir-324	AC091271.1	0.357520789	mirna_lnc
32370	hsa-mir-324	AL590399.1	0.352848859	mirna_lnc
32371	hsa-mir-324	AC096992.2	0.305717232	mirna_lnc
32372	hsa-mir-324	AC010186.1	0.302549895	mirna_lnc
32373	hsa-mir-324	HHIP-AS1	0.338968199	mirna_lnc
32374	hsa-mir-324	ELDR	0.461540241	mirna_lnc
32375	hsa-mir-324	AC090617.5	0.365106332	mirna_lnc
32376	hsa-mir-324	AC005790.1	0.407516077	mirna_lnc
32377	hsa-mir-324	AC010336.2	0.357696478	mirna_lnc
32378	hsa-mir-324	AL359091.5	0.347849455	mirna_lnc
32379	hsa-mir-324	AC023355.1	0.326293368	mirna_lnc
32380	hsa-mir-324	AL606760.3	0.352850239	mirna_lnc
32381	hsa-mir-324	AL591167.1	0.305820492	mirna_lnc
32382	hsa-mir-324	AC093677.2	0.365096032	mirna_lnc
32383	hsa-mir-324	IQCA1-AS1	0.32360054	mirna_lnc
32384	hsa-mir-324	AC117395.1	0.354110422	mirna_lnc
32385	hsa-mir-324	INTS6L-AS1	0.310201472	mirna_lnc
32386	hsa-mir-324	AC083880.1	0.326415092	mirna_lnc
32387	hsa-mir-324	TERC	0.358990658	mirna_lnc
32388	hsa-mir-324	AL121601.1	0.300306087	mirna_lnc
32389	hsa-mir-324	AC012645.1	0.319925562	mirna_lnc
32390	hsa-mir-324	LINC01607	0.307700327	mirna_lnc
32391	hsa-mir-324	AP000892.1	0.319132806	mirna_lnc
32392	hsa-mir-324	AC007541.1	0.328372463	mirna_lnc
32393	hsa-mir-324	AC007389.5	0.359291496	mirna_lnc
32394	hsa-mir-324	AC108865.2	0.32134663	mirna_lnc
32395	hsa-mir-324	AC010834.2	0.316035478	mirna_lnc
32396	hsa-mir-324	AP005205.2	0.318845474	mirna_lnc
32397	hsa-mir-324	AL022341.1	0.327893099	mirna_lnc
32398	hsa-mir-324	AP000251.1	0.534964537	mirna_lnc
32399	hsa-mir-324	AC025219.1	0.356916392	mirna_lnc

32400	hsa-mir-324	LINC01901	0.383551033	mirna_lnc
32401	hsa-mir-324	AC025575.2	0.300710741	mirna_lnc
32402	hsa-mir-324	BX005266.2	0.327638121	mirna_lnc
32403	hsa-mir-324	AC090907.1	0.313853253	mirna_lnc
32404	hsa-mir-324	LINC01715	0.328912623	mirna_lnc
32405	hsa-mir-324	OBSCN-AS1	0.324578869	mirna_lnc
32406	hsa-mir-324	PITPNA-AS1	0.429180018	mirna_lnc
32407	hsa-mir-324	SCAT8	0.346739284	mirna_lnc
32408	hsa-mir-324	AL356488.3	0.332806048	mirna_lnc
32409	hsa-mir-324	SEMA6A-AS2	0.327002558	mirna_lnc
32410	hsa-mir-324	AL032819.1	0.355051467	mirna_lnc
32411	hsa-mir-324	AL844908.2	0.385253125	mirna_lnc
32412	hsa-mir-324	AC092119.3	0.308603195	mirna_lnc
32413	hsa-mir-324	AC069307.1	0.319343403	mirna_lnc
32414	hsa-mir-324	AC010501.1	0.306955011	mirna_lnc
32415	hsa-mir-324	AC004012.1	0.323532892	mirna_lnc
32416	hsa-mir-324	AC117402.1	0.388394882	mirna_lnc
32417	hsa-mir-324	ZFHX4-AS1	0.32355535	mirna_lnc
32418	hsa-mir-324	LINC02506	0.308464515	mirna_lnc
32419	hsa-mir-324	AC005336.3	0.336544532	mirna_lnc
32420	hsa-mir-324	AL583808.1	0.429184496	mirna_lnc
32421	hsa-mir-324	AP005205.3	0.31742147	mirna_lnc
32422	hsa-mir-324	AC005034.4	0.318767214	mirna_lnc
32423	hsa-mir-324	GIHCG	0.403793844	mirna_lnc
32424	hsa-mir-324	Z93241.1	0.308802086	mirna_lnc
32425	hsa-mir-324	AP000808.2	0.302144835	mirna_lnc
32426	hsa-mir-324	AL731537.1	0.335932813	mirna_lnc
32427	hsa-mir-324	GTSE1-DT	0.309882953	mirna_lnc
32428	hsa-mir-324	AC079414.3	0.309221502	mirna_lnc
32429	hsa-mir-324	AC135178.5	0.44878909	mirna_lnc
32430	hsa-mir-324	AC107027.1	0.300577507	mirna_lnc
32431	hsa-mir-324	AC106820.4	0.347136105	mirna_lnc
32432	hsa-mir-324	AL645608.6	0.362070044	mirna_lnc
32433	hsa-mir-324	AC231981.1	0.323167392	mirna_lnc
32434	hsa-mir-324	AC010980.2	0.303105861	mirna_lnc
32435	hsa-mir-324	AC025162.2	0.307007413	mirna_lnc
32436	hsa-mir-324	LINC01918	0.336369467	mirna_lnc
32437	hsa-mir-324	PARD3-AS1	0.309450797	mirna_lnc
32438	hsa-mir-324	LINC00909	0.316516908	mirna_lnc
32439	hsa-mir-324	AC007497.1	0.328055839	mirna_lnc
32440	hsa-mir-324	AC005197.1	0.343570056	mirna_lnc
32441	hsa-mir-324	AC145423.2	0.360042161	mirna_lnc
32442	hsa-mir-324	AL359504.2	0.384117121	mirna_lnc
32443	hsa-mir-324	AC012313.9	0.302216412	mirna_lnc
32444	hsa-mir-324	CYP51A1-AS1	0.330622882	mirna_lnc
32445	hsa-mir-324	RN7SL832P	0.385437698	mirna_lnc
32446	hsa-mir-324	AL358933.1	0.325553257	mirna_lnc
32447	hsa-mir-324	AL662791.1	0.326892469	mirna_lnc
32448	hsa-mir-324	AP001972.4	0.333116381	mirna_lnc
32449	hsa-mir-324	AL590705.3	0.315459517	mirna_lnc
32450	hsa-mir-324	AL132655.1	0.404659018	mirna_lnc
32451	hsa-mir-324	AL645608.2	0.343373592	mirna_lnc
32452	hsa-mir-324	AC015917.2	0.321808343	mirna_lnc
32453	hsa-mir-324	AC099684.2	0.301969257	mirna_lnc

32454	hsa-mir-324	AC108704.1	0.346535253	mirna_lnc
32455	hsa-mir-3615	TYMSOS	0.321100313	mirna_lnc
32456	hsa-mir-3615	LINC02595	0.326667424	mirna_lnc
32457	hsa-mir-3615	LINC01873	0.399669777	mirna_lnc
32458	hsa-mir-3615	TMPO-AS1	0.344278788	mirna_lnc
32459	hsa-mir-3615	AL121832.2	0.364422283	mirna_lnc
32460	hsa-mir-3615	AC103591.3	0.350030963	mirna_lnc
32461	hsa-mir-3615	AC004241.3	0.429852998	mirna_lnc
32462	hsa-mir-3615	AL117332.1	0.387403168	mirna_lnc
32463	hsa-mir-3615	AC022211.2	0.309242361	mirna_lnc
32464	hsa-mir-3615	CEP83-DT	0.309927968	mirna_lnc
32465	hsa-mir-3615	AC019069.1	0.368618588	mirna_lnc
32466	hsa-mir-3615	AL590326.1	0.320722878	mirna_lnc
32467	hsa-mir-3615	JARID2-AS1	0.50136574	mirna_lnc
32468	hsa-mir-3615	AC016065.1	0.302689041	mirna_lnc
32469	hsa-mir-3615	AC074117.1	0.429168448	mirna_lnc
32470	hsa-mir-3615	AL031667.3	0.44982063	mirna_lnc
32471	hsa-mir-3615	TFAP2A-AS1	0.448257908	mirna_lnc
32472	hsa-mir-3615	AC097358.2	0.318802349	mirna_lnc
32473	hsa-mir-3615	AC004812.2	0.330576324	mirna_lnc
32474	hsa-mir-3615	AC100791.2	0.357706107	mirna_lnc
32475	hsa-mir-3615	AC015982.1	0.316512815	mirna_lnc
32476	hsa-mir-3615	LINC02542	0.33604022	mirna_lnc
32477	hsa-mir-3615	AC092159.2	0.301276939	mirna_lnc
32478	hsa-mir-3615	AC005840.2	0.326393699	mirna_lnc
32479	hsa-mir-3615	AL023803.3	0.396722565	mirna_lnc
32480	hsa-mir-3615	AC017083.1	0.411283696	mirna_lnc
32481	hsa-mir-3615	AL121655.1	0.318170732	mirna_lnc
32482	hsa-mir-3615	AC109322.1	0.40523982	mirna_lnc
32483	hsa-mir-3615	AC074351.1	0.357431583	mirna_lnc
32484	hsa-mir-3615	KC877982.1	0.326733161	mirna_lnc
32485	hsa-mir-3615	AL445072.1	0.398070603	mirna_lnc
32486	hsa-mir-3615	AL592301.1	0.336138524	mirna_lnc
32487	hsa-mir-3615	AL137802.2	0.353007074	mirna_lnc
32488	hsa-mir-3615	AC087289.3	0.349789688	mirna_lnc
32489	hsa-mir-3615	LINC01806	0.3840394	mirna_lnc
32490	hsa-mir-3615	AL136162.1	0.531531711	mirna_lnc
32491	hsa-mir-3615	AC073195.1	0.477946404	mirna_lnc
32492	hsa-mir-3615	AC015818.2	0.33176352	mirna_lnc
32493	hsa-mir-3615	AL596094.1	0.438276137	mirna_lnc
32494	hsa-mir-3615	AC004069.1	0.453889233	mirna_lnc
32495	hsa-mir-3615	AC131025.1	0.306519697	mirna_lnc
32496	hsa-mir-3615	AC004920.1	0.470115361	mirna_lnc
32497	hsa-mir-3615	AC021683.3	0.51136684	mirna_lnc
32498	hsa-mir-3615	AC012640.2	0.39827773	mirna_lnc
32499	hsa-mir-3615	AC090970.2	0.42240636	mirna_lnc
32500	hsa-mir-3615	AC026782.2	0.3620771	mirna_lnc
32501	hsa-mir-3615	AP003119.2	0.362858664	mirna_lnc
32502	hsa-mir-3615	ILF3-DT	0.323460295	mirna_lnc
32503	hsa-mir-3615	AC002470.1	0.317899368	mirna_lnc
32504	hsa-mir-3615	AC012625.1	0.311185849	mirna_lnc
32505	hsa-mir-3615	AL353764.1	0.377024128	mirna_lnc
32506	hsa-mir-3615	AC040169.1	0.339895946	mirna_lnc
32507	hsa-mir-3615	AC013400.1	0.440425124	mirna_lnc

32508	hsa-mir-3615	LINC01424	0.479120202	mirna_lnc
32509	hsa-mir-3615	TBC1D8-AS1	0.461928486	mirna_lnc
32510	hsa-mir-3615	TIPARP-AS1	0.322470746	mirna_lnc
32511	hsa-mir-3615	KIAA1614-AS1	0.439871672	mirna_lnc
32512	hsa-mir-3615	AC016876.3	0.480711163	mirna_lnc
32513	hsa-mir-3615	AL031665.2	0.36051966	mirna_lnc
32514	hsa-mir-3615	AL359643.3	0.344016197	mirna_lnc
32515	hsa-mir-3615	AL163051.1	0.386770663	mirna_lnc
32516	hsa-mir-3615	AL513327.2	0.422082642	mirna_lnc
32517	hsa-mir-3615	AC138356.1	0.455570817	mirna_lnc
32518	hsa-mir-3615	LINC01456	0.318097927	mirna_lnc
32519	hsa-mir-3615	LINC01850	0.404924378	mirna_lnc
32520	hsa-mir-3615	AC007952.4	0.368508989	mirna_lnc
32521	hsa-mir-3615	AL683887.1	0.342220519	mirna_lnc
32522	hsa-mir-3615	AC005670.1	0.430187029	mirna_lnc
32523	hsa-mir-3615	AL445228.2	0.301569752	mirna_lnc
32524	hsa-mir-3615	AC239799.1	0.340537567	mirna_lnc
32525	hsa-mir-3615	AC010595.1	0.387940907	mirna_lnc
32526	hsa-mir-3615	AL391069.3	0.353002989	mirna_lnc
32527	hsa-mir-3615	LINC02607	0.495507305	mirna_lnc
32528	hsa-mir-3615	LINC01816	0.514296039	mirna_lnc
32529	hsa-mir-3615	TMCO1-AS1	0.308169934	mirna_lnc
32530	hsa-mir-3615	AC099518.6	0.492950995	mirna_lnc
32531	hsa-mir-3615	AC007881.3	0.481062454	mirna_lnc
32532	hsa-mir-3615	AL121658.1	0.458461319	mirna_lnc
32533	hsa-mir-3615	AC010969.2	0.498573865	mirna_lnc
32534	hsa-mir-3615	AC022098.3	0.304641071	mirna_lnc
32535	hsa-mir-3615	LINC00167	0.478240769	mirna_lnc
32536	hsa-mir-3615	AC046143.2	0.35684731	mirna_lnc
32537	hsa-mir-3615	AC145343.1	0.405726021	mirna_lnc
32538	hsa-mir-3615	AC093424.1	0.411247637	mirna_lnc
32539	hsa-mir-3615	DGUOK-AS1	0.321226339	mirna_lnc
32540	hsa-mir-3615	AC091925.1	0.361489133	mirna_lnc
32541	hsa-mir-3615	AC117382.2	0.458463615	mirna_lnc
32542	hsa-mir-3615	AC091153.3	0.449530919	mirna_lnc
32543	hsa-mir-3615	AC063948.1	0.439768298	mirna_lnc
32544	hsa-mir-3615	AL023803.1	0.330760933	mirna_lnc
32545	hsa-mir-3615	AC087742.1	0.406771513	mirna_lnc
32546	hsa-mir-3615	AL354892.2	0.4250946	mirna_lnc
32547	hsa-mir-3615	AC015802.5	0.325687705	mirna_lnc
32548	hsa-mir-3615	AL139327.2	0.404829226	mirna_lnc
32549	hsa-mir-3615	DDX39B-AS1	0.362514615	mirna_lnc
32550	hsa-mir-3615	AL031963.3	0.328000928	mirna_lnc
32551	hsa-mir-3615	AC069148.1	0.315819951	mirna_lnc
32552	hsa-mir-3615	AC064836.3	0.43841203	mirna_lnc
32553	hsa-mir-3615	AC012640.5	0.316577526	mirna_lnc
32554	hsa-mir-3615	AL512408.1	0.337435308	mirna_lnc
32555	hsa-mir-3615	AC090527.3	0.391223435	mirna_lnc
32556	hsa-mir-3615	AC010422.2	0.375080034	mirna_lnc
32557	hsa-mir-3615	RNASEH1-AS1	0.371804008	mirna_lnc
32558	hsa-mir-3615	LINC01063	0.475889484	mirna_lnc
32559	hsa-mir-3615	AC092669.1	0.426785247	mirna_lnc
32560	hsa-mir-3615	CIRBP-AS1	0.388121684	mirna_lnc
32561	hsa-mir-3615	AC099568.2	0.331316211	mirna_lnc

32562	hsa-mir-3615	AC020765.2	0.360537615	mirna_lnc
32563	hsa-mir-3615	AC084757.2	0.386912552	mirna_lnc
32564	hsa-mir-3615	AC040169.3	0.303778903	mirna_lnc
32565	hsa-mir-3615	EIF2AK3-DT	0.331748918	mirna_lnc
32566	hsa-mir-3615	AC009171.2	0.398228024	mirna_lnc
32567	hsa-mir-3615	AC243830.2	0.333243293	mirna_lnc
32568	hsa-mir-3615	CNNM3-DT	0.341828743	mirna_lnc
32569	hsa-mir-3615	AC068831.6	0.321974563	mirna_lnc
32570	hsa-mir-3615	AL355482.2	0.322661146	mirna_lnc
32571	hsa-mir-3615	AC080013.5	0.35896011	mirna_lnc
32572	hsa-mir-3615	AL645608.4	0.342475308	mirna_lnc
32573	hsa-mir-3615	AC138696.2	0.367249951	mirna_lnc
32574	hsa-mir-3615	MAST4-AS1	0.396846067	mirna_lnc
32575	hsa-mir-3615	AC010654.1	0.316027281	mirna_lnc
32576	hsa-mir-3615	AC090774.2	0.327900773	mirna_lnc
32577	hsa-mir-3615	AC007773.1	0.301781997	mirna_lnc
32578	hsa-mir-3615	AP000894.4	0.311311661	mirna_lnc
32579	hsa-mir-3615	AC022762.1	0.353302468	mirna_lnc
32580	hsa-mir-3615	AC008267.5	0.366173916	mirna_lnc
32581	hsa-mir-3615	AC005695.3	0.34610943	mirna_lnc
32582	hsa-mir-3615	AC107214.1	0.364016228	mirna_lnc
32583	hsa-mir-3615	AC034236.2	0.35014766	mirna_lnc
32584	hsa-mir-3615	AC131235.3	0.305471627	mirna_lnc
32585	hsa-mir-3615	AC005005.3	0.374498698	mirna_lnc
32586	hsa-mir-3615	AC073896.4	0.359788948	mirna_lnc
32587	hsa-mir-3615	C11orf72	0.342175606	mirna_lnc
32588	hsa-mir-3615	AC108865.1	0.381719461	mirna_lnc
32589	hsa-mir-3615	URB1-AS1	0.407284949	mirna_lnc
32590	hsa-mir-3615	AC024243.1	0.312164369	mirna_lnc
32591	hsa-mir-3615	AC011446.1	0.355410644	mirna_lnc
32592	hsa-mir-3615	AL132712.2	0.31670115	mirna_lnc
32593	hsa-mir-3615	AC008736.2	0.367952494	mirna_lnc
32594	hsa-mir-3615	AC091271.1	0.394790111	mirna_lnc
32595	hsa-mir-3615	AL590399.1	0.365925787	mirna_lnc
32596	hsa-mir-3615	AC026304.1	0.442359164	mirna_lnc
32597	hsa-mir-3615	AC096992.2	0.363550441	mirna_lnc
32598	hsa-mir-3615	LPP-AS2	0.316563869	mirna_lnc
32599	hsa-mir-3615	AC005304.3	0.343670417	mirna_lnc
32600	hsa-mir-3615	AC010186.1	0.461046745	mirna_lnc
32601	hsa-mir-3615	ELDR	0.440214704	mirna_lnc
32602	hsa-mir-3615	AC019129.2	0.41533648	mirna_lnc
32603	hsa-mir-3615	AC005790.1	0.386409303	mirna_lnc
32604	hsa-mir-3615	AP001775.2	0.368109123	mirna_lnc
32605	hsa-mir-3615	AL132712.1	0.330405695	mirna_lnc
32606	hsa-mir-3615	AC023355.1	0.341123472	mirna_lnc
32607	hsa-mir-3615	AC093677.2	0.473916409	mirna_lnc
32608	hsa-mir-3615	IQCA1-AS1	0.322888358	mirna_lnc
32609	hsa-mir-3615	INTS6L-AS1	0.332977632	mirna_lnc
32610	hsa-mir-3615	AC083880.1	0.4333188	mirna_lnc
32611	hsa-mir-3615	TERC	0.302861424	mirna_lnc
32612	hsa-mir-3615	AC099522.2	0.351362165	mirna_lnc
32613	hsa-mir-3615	LINC01607	0.366328301	mirna_lnc
32614	hsa-mir-3615	AC008883.2	0.305464714	mirna_lnc
32615	hsa-mir-3615	AC007389.5	0.440323681	mirna_lnc

32616	hsa-mir-3615	AC108865.2	0.494049611	mirna_lnc
32617	hsa-mir-3615	AC010834.2	0.378255747	mirna_lnc
32618	hsa-mir-3615	AL022341.1	0.342050123	mirna_lnc
32619	hsa-mir-3615	AP000251.1	0.484958586	mirna_lnc
32620	hsa-mir-3615	AC025575.2	0.346193873	mirna_lnc
32621	hsa-mir-3615	CPNE8-AS1	0.342809102	mirna_lnc
32622	hsa-mir-3615	AL138881.1	0.437311205	mirna_lnc
32623	hsa-mir-3615	AC090907.1	0.403220609	mirna_lnc
32624	hsa-mir-3615	AC093635.1	0.49601056	mirna_lnc
32625	hsa-mir-3615	LINC01715	0.429600902	mirna_lnc
32626	hsa-mir-3615	AC009560.1	0.357640612	mirna_lnc
32627	hsa-mir-3615	AL590822.1	0.32793631	mirna_lnc
32628	hsa-mir-3615	AC093510.1	0.311125986	mirna_lnc
32629	hsa-mir-3615	BRWD1-AS2	0.316964058	mirna_lnc
32630	hsa-mir-3615	AL157813.1	0.425291654	mirna_lnc
32631	hsa-mir-3615	AC027644.3	0.442738428	mirna_lnc
32632	hsa-mir-3615	AGBL5-AS1	0.319058864	mirna_lnc
32633	hsa-mir-3615	AC007114.1	0.326948716	mirna_lnc
32634	hsa-mir-3615	SCAT8	0.501569696	mirna_lnc
32635	hsa-mir-3615	AL356488.3	0.444192371	mirna_lnc
32636	hsa-mir-3615	AP000692.2	0.303673902	mirna_lnc
32637	hsa-mir-3615	AC069277.1	0.367674723	mirna_lnc
32638	hsa-mir-3615	AL032819.1	0.490665172	mirna_lnc
32639	hsa-mir-3615	AL844908.2	0.398981857	mirna_lnc
32640	hsa-mir-3615	AC092119.3	0.435384209	mirna_lnc
32641	hsa-mir-3615	AC069307.1	0.371995093	mirna_lnc
32642	hsa-mir-3615	AC099343.3	0.353882162	mirna_lnc
32643	hsa-mir-3615	HDAC4-AS1	0.401226314	mirna_lnc
32644	hsa-mir-3615	AL021154.1	0.40557412	mirna_lnc
32645	hsa-mir-3615	AL359198.1	0.339399174	mirna_lnc
32646	hsa-mir-3615	AC004012.1	0.483540719	mirna_lnc
32647	hsa-mir-3615	AL583808.1	0.437854422	mirna_lnc
32648	hsa-mir-3615	AP003392.3	0.400900847	mirna_lnc
32649	hsa-mir-3615	Z93241.1	0.333099492	mirna_lnc
32650	hsa-mir-3615	AL359878.2	0.404184134	mirna_lnc
32651	hsa-mir-3615	AC068338.2	0.343592431	mirna_lnc
32652	hsa-mir-3615	AL731537.1	0.387281858	mirna_lnc
32653	hsa-mir-3615	GTSE1-DT	0.315427622	mirna_lnc
32654	hsa-mir-3615	AC079414.3	0.310009826	mirna_lnc
32655	hsa-mir-3615	AC135178.5	0.320720976	mirna_lnc
32656	hsa-mir-3615	KCNJ2-AS1	0.473958898	mirna_lnc
32657	hsa-mir-3615	AC002059.1	0.469105599	mirna_lnc
32658	hsa-mir-3615	AL645608.6	0.347111585	mirna_lnc
32659	hsa-mir-3615	AC000120.1	0.301840902	mirna_lnc
32660	hsa-mir-3615	AC010980.2	0.363721863	mirna_lnc
32661	hsa-mir-3615	PARD3-AS1	0.329643576	mirna_lnc
32662	hsa-mir-3615	AC136604.3	0.327177587	mirna_lnc
32663	hsa-mir-3615	AC130343.2	0.313009673	mirna_lnc
32664	hsa-mir-3615	AC145423.2	0.347934441	mirna_lnc
32665	hsa-mir-3615	AC092142.1	0.408187211	mirna_lnc
32666	hsa-mir-3615	AC012313.9	0.319050611	mirna_lnc
32667	hsa-mir-3615	CYP51A1-AS1	0.431837163	mirna_lnc
32668	hsa-mir-3615	AC005696.4	0.32907295	mirna_lnc
32669	hsa-mir-3615	AL512791.2	0.30284802	mirna_lnc

32670	hsa-mir-3615	RN7SL832P	0.506226572	mirna_lnc
32671	hsa-mir-3615	LINC02094	0.304893744	mirna_lnc
32672	hsa-mir-3615	AL138966.2	0.396126283	mirna_lnc
32673	hsa-mir-3615	AL358933.1	0.355663713	mirna_lnc
32674	hsa-mir-3615	LINC00112	0.325110083	mirna_lnc
32675	hsa-mir-3615	AP001972.4	0.380843784	mirna_lnc
32676	hsa-mir-3615	AL590705.3	0.481447366	mirna_lnc
32677	hsa-mir-3615	AL132655.1	0.330175485	mirna_lnc
32678	hsa-mir-3615	AC079610.2	0.378050453	mirna_lnc
32679	hsa-mir-3615	AC015917.2	0.32350222	mirna_lnc
32680	hsa-mir-3615	AP001412.1	0.303335823	mirna_lnc
32681	hsa-mir-3615	TOLLIP-AS1	0.327277845	mirna_lnc
32682	hsa-mir-3615	AL353708.1	0.327408235	mirna_lnc
32683	hsa-mir-3615	AC012676.3	0.412145902	mirna_lnc
32684	hsa-mir-25	AC092667.1	0.409000066	mirna_lnc
32685	hsa-mir-25	AC012073.1	0.495624778	mirna_lnc
32686	hsa-mir-25	TMPO-AS1	0.360675358	mirna_lnc
32687	hsa-mir-25	SLC12A9-AS1	0.435212142	mirna_lnc
32688	hsa-mir-25	AP000808.1	0.540909526	mirna_lnc
32689	hsa-mir-25	AC092614.1	0.422680937	mirna_lnc
32690	hsa-mir-25	CEP83-DT	0.343431378	mirna_lnc
32691	hsa-mir-25	HOXC-AS2	0.352490229	mirna_lnc
32692	hsa-mir-25	AP001469.3	0.338847515	mirna_lnc
32693	hsa-mir-25	AC011199.1	0.34447831	mirna_lnc
32694	hsa-mir-25	AP003071.2	0.490828973	mirna_lnc
32695	hsa-mir-25	AC073842.2	0.421240342	mirna_lnc
32696	hsa-mir-25	AC090159.1	0.312989377	mirna_lnc
32697	hsa-mir-25	AC092803.2	0.321044744	mirna_lnc
32698	hsa-mir-25	AC147651.4	0.451869127	mirna_lnc
32699	hsa-mir-25	AL121759.1	0.311665383	mirna_lnc
32700	hsa-mir-25	AC009121.1	0.318525676	mirna_lnc
32701	hsa-mir-25	LINC02042	0.317442108	mirna_lnc
32702	hsa-mir-25	AC073857.1	0.302864231	mirna_lnc
32703	hsa-mir-25	AC015849.5	0.300548621	mirna_lnc
32704	hsa-mir-25	LINC02367	0.54802207	mirna_lnc
32705	hsa-mir-25	LINC01096	0.487504373	mirna_lnc
32706	hsa-mir-25	AC012360.3	0.389843849	mirna_lnc
32707	hsa-mir-25	AC125494.2	0.300072594	mirna_lnc
32708	hsa-mir-25	AC024884.2	0.318821231	mirna_lnc
32709	hsa-mir-25	AC016705.2	0.343832159	mirna_lnc
32710	hsa-mir-25	AL391069.2	0.379072988	mirna_lnc
32711	hsa-mir-25	AC116049.2	0.471318199	mirna_lnc
32712	hsa-mir-25	AC024267.6	0.3043409	mirna_lnc
32713	hsa-mir-25	AL133243.2	0.331622893	mirna_lnc
32714	hsa-mir-25	AC109347.2	0.401519473	mirna_lnc
32715	hsa-mir-25	AC130324.2	0.305916793	mirna_lnc
32716	hsa-mir-25	AC024230.1	0.525568891	mirna_lnc
32717	hsa-mir-25	LINC01397	0.440967938	mirna_lnc
32718	hsa-mir-25	AC138904.1	0.346918867	mirna_lnc
32719	hsa-mir-25	AC073517.1	0.365698967	mirna_lnc
32720	hsa-mir-25	AP000487.1	0.569489866	mirna_lnc
32721	hsa-mir-25	AC104971.1	0.350258085	mirna_lnc
32722	hsa-mir-25	AC002076.1	0.410761998	mirna_lnc
32723	hsa-mir-25	LINC01686	0.324206498	mirna_lnc

32724	hsa-mir-25	LINC01143	0.401836012	mirna_lnc
32725	hsa-mir-25	AC100782.1	0.358246911	mirna_lnc
32726	hsa-mir-25	AL354714.1	0.32569328	mirna_lnc
32727	hsa-mir-25	AC104823.1	0.414724049	mirna_lnc
32728	hsa-mir-25	SREBF2-AS1	0.35737024	mirna_lnc
32729	hsa-mir-25	AC078864.1	0.468738923	mirna_lnc
32730	hsa-mir-25	AC122694.1	0.370193112	mirna_lnc
32731	hsa-mir-25	AC073073.2	0.308231802	mirna_lnc
32732	hsa-mir-25	AC044781.1	0.502737636	mirna_lnc
32733	hsa-mir-25	AC011297.1	0.479304085	mirna_lnc
32734	hsa-mir-25	AC004943.3	0.323520934	mirna_lnc
32735	hsa-mir-25	AC003986.2	0.412586684	mirna_lnc
32736	hsa-mir-25	AL354892.2	0.389413137	mirna_lnc
32737	hsa-mir-25	AC106820.2	0.399018183	mirna_lnc
32738	hsa-mir-25	AC026271.3	0.513622772	mirna_lnc
32739	hsa-mir-25	AC016205.1	0.422755751	mirna_lnc
32740	hsa-mir-25	AC090527.3	0.304830592	mirna_lnc
32741	hsa-mir-25	MYB-AS1	0.5406744	mirna_lnc
32742	hsa-mir-25	AC123905.1	0.349280669	mirna_lnc
32743	hsa-mir-25	RNASEH1-AS1	0.337017528	mirna_lnc
32744	hsa-mir-25	AP002387.2	0.300993096	mirna_lnc
32745	hsa-mir-25	AC131212.4	0.309687984	mirna_lnc
32746	hsa-mir-25	AL157392.1	0.397060397	mirna_lnc
32747	hsa-mir-25	AC092669.1	0.429324877	mirna_lnc
32748	hsa-mir-25	HGC6.3	0.409499072	mirna_lnc
32749	hsa-mir-25	AP002336.2	0.545109092	mirna_lnc
32750	hsa-mir-25	AC005838.2	0.322025223	mirna_lnc
32751	hsa-mir-25	PCOLCE-AS1	0.382259151	mirna_lnc
32752	hsa-mir-25	AC099568.2	0.309848498	mirna_lnc
32753	hsa-mir-25	AC011773.1	0.384615486	mirna_lnc
32754	hsa-mir-25	AC069222.1	0.372526948	mirna_lnc
32755	hsa-mir-25	LINC02562	0.363366601	mirna_lnc
32756	hsa-mir-25	AC126407.1	0.345482002	mirna_lnc
32757	hsa-mir-25	AC024560.3	0.329062101	mirna_lnc
32758	hsa-mir-25	NLGN4Y-AS1	0.311586421	mirna_lnc
32759	hsa-mir-25	AC009271.1	0.322090889	mirna_lnc
32760	hsa-mir-25	AC098818.2	0.361458183	mirna_lnc
32761	hsa-mir-25	AC068473.3	0.334372342	mirna_lnc
32762	hsa-mir-25	AC104596.1	0.360859554	mirna_lnc
32763	hsa-mir-25	AC107214.1	0.336535836	mirna_lnc
32764	hsa-mir-25	LINC00885	0.430433248	mirna_lnc
32765	hsa-mir-25	AL022324.3	0.322443414	mirna_lnc
32766	hsa-mir-25	AL157832.2	0.429773105	mirna_lnc
32767	hsa-mir-25	AP003071.1	0.496876394	mirna_lnc
32768	hsa-mir-25	AP000487.2	0.317803791	mirna_lnc
32769	hsa-mir-25	AL603832.1	0.308762221	mirna_lnc
32770	hsa-mir-25	HHIP-AS1	0.316257338	mirna_lnc
32771	hsa-mir-25	AL591167.1	0.406650299	mirna_lnc
32772	hsa-mir-25	AC083805.3	0.348161009	mirna_lnc
32773	hsa-mir-25	AC007384.1	0.307414645	mirna_lnc
32774	hsa-mir-25	AL031186.1	0.316480064	mirna_lnc
32775	hsa-mir-25	AC011450.1	0.306790842	mirna_lnc
32776	hsa-mir-25	LINC01258	0.303306804	mirna_lnc
32777	hsa-mir-25	AC007541.1	0.302288646	mirna_lnc

32778	hsa-mir-25	AC006946.2	0.381676546	mirna_lnc
32779	hsa-mir-25	AC025219.1	0.328214976	mirna_lnc
32780	hsa-mir-25	LINC01315	0.302754055	mirna_lnc
32781	hsa-mir-25	AC007250.1	0.369883621	mirna_lnc
32782	hsa-mir-25	AL138767.3	0.563955795	mirna_lnc
32783	hsa-mir-25	AC098936.1	0.528586477	mirna_lnc
32784	hsa-mir-25	AC138207.2	0.32206091	mirna_lnc
32785	hsa-mir-25	ETV5-AS1	0.554539317	mirna_lnc
32786	hsa-mir-25	SEMA6A-AS2	0.305104814	mirna_lnc
32787	hsa-mir-25	AP001783.1	0.405671695	mirna_lnc
32788	hsa-mir-25	LINC02457	0.427300017	mirna_lnc
32789	hsa-mir-25	AL118511.1	0.472987798	mirna_lnc
32790	hsa-mir-25	AC027348.1	0.435902832	mirna_lnc
32791	hsa-mir-25	GIHCG	0.370929252	mirna_lnc
32792	hsa-mir-25	AL359881.3	0.377896388	mirna_lnc
32793	hsa-mir-25	AP000808.2	0.524249765	mirna_lnc
32794	hsa-mir-25	LINC02470	0.584549071	mirna_lnc
32795	hsa-mir-25	AC008443.3	0.342998943	mirna_lnc
32796	hsa-mir-25	AC106820.4	0.565839282	mirna_lnc
32797	hsa-mir-25	LINC00173	0.366385886	mirna_lnc
32798	hsa-mir-25	AC131159.1	0.330108963	mirna_lnc
32799	hsa-mir-25	LINC01918	0.322375418	mirna_lnc
32800	hsa-mir-25	AC007497.1	0.375498919	mirna_lnc
32801	hsa-mir-25	AC093788.1	0.316577964	mirna_lnc
32802	hsa-mir-25	AC145422.1	0.316008394	mirna_lnc
32803	hsa-mir-25	AC125603.4	0.425709424	mirna_lnc
32804	hsa-mir-25	LINC01936	0.434532191	mirna_lnc
32805	hsa-mir-25	AL358115.1	0.316452111	mirna_lnc
32806	hsa-mir-25	AC092718.5	0.309655962	mirna_lnc
32807	hsa-mir-25	AC097534.1	0.417318545	mirna_lnc
32808	hsa-mir-25	AC016229.2	0.337477394	mirna_lnc
32809	hsa-mir-25	AL596202.1	0.344892047	mirna_lnc
32810	hsa-mir-25	UBXN7-AS1	0.310729971	mirna_lnc
32811	hsa-mir-25	AF213884.3	0.475200779	mirna_lnc
32812	hsa-mir-25	AC008033.3	0.545615276	mirna_lnc
32813	hsa-mir-25	CAMTA1-IT1	0.400747553	mirna_lnc
32814	hsa-mir-25	AC108704.1	0.368786093	mirna_lnc
32815	hsa-mir-25	AP002490.1	0.373210627	mirna_lnc
32816	hsa-mir-25	AC009970.1	0.315721256	mirna_lnc
32817	hsa-mir-15a	AL137060.1	0.388071046	mirna_lnc
32818	hsa-mir-15a	AC245100.6	0.368104804	mirna_lnc
32819	hsa-mir-15a	AC010761.2	0.33734722	mirna_lnc
32820	hsa-mir-15a	AC009630.2	0.308046335	mirna_lnc
32821	hsa-mir-15a	Z94721.1	0.31945712	mirna_lnc
32822	hsa-mir-15a	AL035461.2	0.330680167	mirna_lnc
32823	hsa-mir-15a	AL161891.1	0.432710641	mirna_lnc
32824	hsa-mir-15a	AL353194.1	0.313215197	mirna_lnc
32825	hsa-mir-15a	AL157932.1	0.452531814	mirna_lnc
32826	hsa-mir-15a	AC002401.2	0.310511327	mirna_lnc
32827	hsa-mir-15a	AL049539.1	0.34086879	mirna_lnc
32828	hsa-mir-15a	LINC02585	0.301802713	mirna_lnc
32829	hsa-mir-15a	AC106820.3	0.309132105	mirna_lnc
32830	hsa-mir-15a	AC079209.1	0.325713583	mirna_lnc
32831	hsa-mir-15a	AC007639.1	0.365379559	mirna_lnc

32832	hsa-mir-15a	AC123768.1	0.315859593	mirna_lnc
32833	hsa-mir-15a	AC021945.1	0.357688773	mirna_lnc
32834	hsa-mir-15a	AC025176.1	0.342318503	mirna_lnc
32835	hsa-mir-15a	AC026250.1	0.301692449	mirna_lnc
32836	hsa-mir-15a	AC009121.1	0.323643402	mirna_lnc
32837	hsa-mir-15a	LINC00513	0.331177674	mirna_lnc
32838	hsa-mir-15a	AC009652.2	0.330447714	mirna_lnc
32839	hsa-mir-15a	AC006557.1	0.310533272	mirna_lnc
32840	hsa-mir-15a	LINC02003	0.375664678	mirna_lnc
32841	hsa-mir-15a	MCM8-AS1	0.331038725	mirna_lnc
32842	hsa-mir-15a	AC109992.2	0.339987123	mirna_lnc
32843	hsa-mir-15a	AL139089.1	0.345661439	mirna_lnc
32844	hsa-mir-15a	AL355388.2	0.332509939	mirna_lnc
32845	hsa-mir-15a	AC004080.1	0.316429279	mirna_lnc
32846	hsa-mir-15a	AL121832.3	0.310888585	mirna_lnc
32847	hsa-mir-15a	AC005831.1	0.344785425	mirna_lnc
32848	hsa-mir-15a	AL358473.1	0.309481409	mirna_lnc
32849	hsa-mir-15a	AC004943.1	0.309193568	mirna_lnc
32850	hsa-mir-15a	LINC01460	0.330610808	mirna_lnc
32851	hsa-mir-15a	TRPM2-AS	0.338122154	mirna_lnc
32852	hsa-mir-15a	AL390728.6	0.357549607	mirna_lnc
32853	hsa-mir-15a	AL109804.1	0.416783934	mirna_lnc
32854	hsa-mir-15a	LINC01977	0.305304846	mirna_lnc
32855	hsa-mir-15a	AC100791.3	0.318688894	mirna_lnc
32856	hsa-mir-15a	AC127024.5	0.336928094	mirna_lnc
32857	hsa-mir-15a	AC004943.3	0.305851232	mirna_lnc
32858	hsa-mir-15a	DNAJC3-DT	0.375290535	mirna_lnc
32859	hsa-mir-15a	AC211476.2	0.305804878	mirna_lnc
32860	hsa-mir-15a	AC007128.1	0.337314529	mirna_lnc
32861	hsa-mir-15a	AL139082.1	0.321317622	mirna_lnc
32862	hsa-mir-15a	AP001065.2	0.322695362	mirna_lnc
32863	hsa-mir-15a	ZNF350-AS1	0.302689059	mirna_lnc
32864	hsa-mir-15a	AC083900.1	0.309400183	mirna_lnc
32865	hsa-mir-15a	AC007485.2	0.307808738	mirna_lnc
32866	hsa-mir-15a	LINC02204	0.336824752	mirna_lnc
32867	hsa-mir-15a	AC124067.4	0.354448797	mirna_lnc
32868	hsa-mir-15a	AL137244.1	0.380148554	mirna_lnc
32869	hsa-mir-15a	AC020763.1	0.300262632	mirna_lnc
32870	hsa-mir-15a	AC244093.4	0.309508034	mirna_lnc
32871	hsa-mir-15a	AL603832.1	0.335044101	mirna_lnc
32872	hsa-mir-15a	AC127024.2	0.307858576	mirna_lnc
32873	hsa-mir-15a	AL512652.2	0.326757159	mirna_lnc
32874	hsa-mir-15a	LBX2-AS1	0.300908532	mirna_lnc
32875	hsa-mir-15a	AC006111.2	0.323403479	mirna_lnc
32876	hsa-mir-15a	AC009704.2	0.317763199	mirna_lnc
32877	hsa-mir-15a	AC064807.4	0.326125124	mirna_lnc
32878	hsa-mir-15a	AC025741.1	0.328333764	mirna_lnc
32879	hsa-mir-15a	AL133260.2	0.334947682	mirna_lnc
32880	hsa-mir-15a	AL583810.1	0.300898693	mirna_lnc
32881	hsa-mir-15a	AC130324.1	0.301351254	mirna_lnc
32882	hsa-mir-15a	AC019131.1	0.315183969	mirna_lnc
32883	hsa-mir-15a	AC116025.2	0.371490385	mirna_lnc
32884	hsa-mir-15a	AC093627.7	0.329744112	mirna_lnc
32885	hsa-mir-15a	AC080037.1	0.313799848	mirna_lnc

32886	hsa-mir-15a	AC147651.2	0.330354639	mirna_lnc
32887	hsa-mir-15a	AC091806.1	0.362300612	mirna_lnc
32888	hsa-mir-15a	AC018648.1	0.317651867	mirna_lnc
32889	hsa-mir-15a	LINC02365	0.300272548	mirna_lnc
32890	hsa-mir-15a	LMF1-AS1	0.356219244	mirna_lnc
32891	hsa-mir-15a	AL035252.3	0.360660796	mirna_lnc
32892	hsa-mir-15a	AC017104.3	0.310258873	mirna_lnc
32893	hsa-mir-15a	LINC00543	0.309861741	mirna_lnc
32894	hsa-mir-15a	AL080312.2	0.320432444	mirna_lnc
32895	hsa-mir-15a	AC004593.1	0.333596283	mirna_lnc
32896	hsa-mir-15a	PSPC1-AS2	0.337547822	mirna_lnc
32897	hsa-mir-15a	AL512652.1	0.32551418	mirna_lnc
32898	hsa-mir-3651	AC099850.3	0.307773743	mirna_lnc
32899	hsa-mir-3651	AC004988.1	0.33273273	mirna_lnc
32900	hsa-mir-3651	FER1L6-AS2	0.531400481	mirna_lnc
32901	hsa-mir-3651	AL353746.1	0.361100479	mirna_lnc
32902	hsa-mir-3651	AC090578.2	0.315121245	mirna_lnc
32903	hsa-mir-3651	AC011313.1	0.302178969	mirna_lnc
32904	hsa-mir-3651	TTLL11-IT1	0.450923706	mirna_lnc
32905	hsa-mir-3651	LINC01750	0.406389968	mirna_lnc
32906	hsa-mir-3651	AC109326.1	0.379208794	mirna_lnc
32907	hsa-mir-3651	LINC02594	0.677743803	mirna_lnc
32908	hsa-mir-3651	AC010735.1	0.322796307	mirna_lnc
32909	hsa-mir-3651	AC034223.2	0.350908049	mirna_lnc
32910	hsa-mir-3651	AC010343.3	0.416868391	mirna_lnc
32911	hsa-mir-3651	AC100786.1	0.425380507	mirna_lnc
32912	hsa-mir-3651	AC010735.2	0.31028188	mirna_lnc
32913	hsa-mir-3651	SENCR	0.350643784	mirna_lnc
32914	hsa-let-7c	AC103746.1	0.826361517	mirna_lnc
32915	hsa-let-7c	AC019294.2	0.346206561	mirna_lnc
32916	hsa-let-7c	AC129492.5	0.493717429	mirna_lnc
32917	hsa-let-7c	LINC02593	0.77267814	mirna_lnc
32918	hsa-let-7c	AL160286.2	0.328833371	mirna_lnc
32919	hsa-let-7c	AJ006995.1	0.435549415	mirna_lnc
32920	hsa-let-7c	LINC01560	0.359135032	mirna_lnc
32921	hsa-let-7c	PACRG-AS1	0.481069777	mirna_lnc
32922	hsa-let-7c	LINC01625	0.84359444	mirna_lnc
32923	hsa-let-7c	AC008121.2	0.332527431	mirna_lnc
32924	hsa-let-7c	AC012409.2	0.584896443	mirna_lnc
32925	hsa-let-7c	AC093520.1	0.304049583	mirna_lnc
32926	hsa-let-7c	TNRC6C-AS1	0.369245529	mirna_lnc
32927	hsa-let-7c	LOH12CR2	0.434322297	mirna_lnc
32928	hsa-let-7c	AC016251.2	0.400155967	mirna_lnc
32929	hsa-let-7c	AC008105.1	0.366757813	mirna_lnc
32930	hsa-let-7c	LINC01311	0.35127704	mirna_lnc
32931	hsa-let-7c	TFAP2A-AS1	0.43980011	mirna_lnc
32932	hsa-let-7c	AC022387.1	0.584287027	mirna_lnc
32933	hsa-let-7c	AC016737.1	0.310831337	mirna_lnc
32934	hsa-let-7c	AC002066.1	0.5098745	mirna_lnc
32935	hsa-let-7c	FAM66D	0.369433912	mirna_lnc
32936	hsa-let-7c	AL139288.1	0.557945649	mirna_lnc
32937	hsa-let-7c	AL078581.1	0.320733619	mirna_lnc
32938	hsa-let-7c	MAFA-AS1	0.452827267	mirna_lnc
32939	hsa-let-7c	AC104667.1	0.585243673	mirna_lnc

32940	hsa-let-7c	AC098828.2	0.393442176	mirna_lnc
32941	hsa-let-7c	AC012377.1	0.31649538	mirna_lnc
32942	hsa-let-7c	AP002852.1	0.352101651	mirna_lnc
32943	hsa-let-7c	AL137802.2	0.544709101	mirna_lnc
32944	hsa-let-7c	AC004069.1	0.303897767	mirna_lnc
32945	hsa-let-7c	AC093642.2	0.708310964	mirna_lnc
32946	hsa-let-7c	AL157823.2	0.517246917	mirna_lnc
32947	hsa-let-7c	AP000553.2	0.760677411	mirna_lnc
32948	hsa-let-7c	RHPN1-AS1	0.372772046	mirna_lnc
32949	hsa-let-7c	LINC02175	0.558756769	mirna_lnc
32950	hsa-let-7c	SIX3-AS1	0.347628681	mirna_lnc
32951	hsa-let-7c	AC110813.1	0.733230514	mirna_lnc
32952	hsa-let-7c	LINC01126	0.40174432	mirna_lnc
32953	hsa-let-7c	LINC01087	0.625366464	mirna_lnc
32954	hsa-let-7c	AC135803.1	0.81059329	mirna_lnc
32955	hsa-let-7c	SYNGAP1-AS1	0.323241723	mirna_lnc
32956	hsa-let-7c	AL391152.1	0.358796549	mirna_lnc
32957	hsa-let-7c	MAGEA4-AS1	0.443622228	mirna_lnc
32958	hsa-let-7c	AC105339.5	0.531442807	mirna_lnc
32959	hsa-let-7c	AC090578.2	0.704799448	mirna_lnc
32960	hsa-let-7c	AC092807.2	0.325279213	mirna_lnc
32961	hsa-let-7c	EIPR1-IT1	0.307292188	mirna_lnc
32962	hsa-let-7c	AL078605.1	0.598939333	mirna_lnc
32963	hsa-let-7c	AC026254.2	0.309436181	mirna_lnc
32964	hsa-let-7c	AL157838.1	0.351079871	mirna_lnc
32965	hsa-let-7c	AL138760.1	0.432159728	mirna_lnc
32966	hsa-let-7c	LINC01686	0.350228069	mirna_lnc
32967	hsa-let-7c	Z95115.1	0.560027851	mirna_lnc
32968	hsa-let-7c	LINC01143	0.571876399	mirna_lnc
32969	hsa-let-7c	AC004884.2	0.323793607	mirna_lnc
32970	hsa-let-7c	AL606534.1	0.302412568	mirna_lnc
32971	hsa-let-7c	AC024361.2	0.302956014	mirna_lnc
32972	hsa-let-7c	AC010271.2	0.324632371	mirna_lnc
32973	hsa-let-7c	AC079145.1	0.400816403	mirna_lnc
32974	hsa-let-7c	AC020907.5	0.58600746	mirna_lnc
32975	hsa-let-7c	UBR5-AS1	0.385655777	mirna_lnc
32976	hsa-let-7c	AC130650.2	0.494562245	mirna_lnc
32977	hsa-let-7c	AC129926.1	0.315618014	mirna_lnc
32978	hsa-let-7c	AL157400.4	0.318365425	mirna_lnc
32979	hsa-let-7c	AL008721.2	0.355450414	mirna_lnc
32980	hsa-let-7c	AC110769.2	0.405953631	mirna_lnc
32981	hsa-let-7c	AL138762.1	0.399148787	mirna_lnc
32982	hsa-let-7c	AC007996.1	0.579249486	mirna_lnc
32983	hsa-let-7c	AC096642.1	0.353801532	mirna_lnc
32984	hsa-let-7c	LINC01521	0.352904455	mirna_lnc
32985	hsa-let-7c	AC005593.1	0.30002385	mirna_lnc
32986	hsa-let-7c	AC016708.1	0.866057105	mirna_lnc
32987	hsa-let-7c	AC091057.2	0.373272696	mirna_lnc
32988	hsa-let-7c	AC009506.1	0.541913645	mirna_lnc
32989	hsa-let-7c	AC007848.1	0.371530378	mirna_lnc
32990	hsa-let-7c	AC099786.1	0.840450047	mirna_lnc
32991	hsa-let-7c	AC063948.1	0.371230285	mirna_lnc
32992	hsa-let-7c	AC087742.1	0.344693295	mirna_lnc
32993	hsa-let-7c	AL354892.2	0.363126083	mirna_lnc

32994	hsa-let-7c	AP001330.5	0.324936326	mirna_lnc
32995	hsa-let-7c	AC011773.4	0.43881915	mirna_lnc
32996	hsa-let-7c	AL513365.2	0.346022407	mirna_lnc
32997	hsa-let-7c	AC016205.1	0.328999287	mirna_lnc
32998	hsa-let-7c	AC064836.3	0.301126358	mirna_lnc
32999	hsa-let-7c	BX088651.4	0.314523176	mirna_lnc
33000	hsa-let-7c	AL512408.1	0.349845174	mirna_lnc
33001	hsa-let-7c	AC023034.2	0.502551515	mirna_lnc
33002	hsa-let-7c	AC246793.1	0.44772809	mirna_lnc
33003	hsa-let-7c	AC131532.1	0.41843272	mirna_lnc
33004	hsa-let-7c	ERICD	0.566376522	mirna_lnc
33005	hsa-let-7c	AL662795.2	0.43521214	mirna_lnc
33006	hsa-let-7c	AC006262.1	0.398298936	mirna_lnc
33007	hsa-let-7c	LINC02600	0.322286044	mirna_lnc
33008	hsa-let-7c	AC092608.1	0.407292711	mirna_lnc
33009	hsa-let-7c	PCOLCE-AS1	0.344003676	mirna_lnc
33010	hsa-let-7c	Z93930.3	0.41900056	mirna_lnc
33011	hsa-let-7c	AL136040.1	0.420025584	mirna_lnc
33012	hsa-let-7c	AC073389.1	0.492837328	mirna_lnc
33013	hsa-let-7c	DNM3-IT1	0.776614719	mirna_lnc
33014	hsa-let-7c	AC006206.1	0.359417506	mirna_lnc
33015	hsa-let-7c	AC090772.4	0.458026256	mirna_lnc
33016	hsa-let-7c	AL451064.1	0.80289186	mirna_lnc
33017	hsa-let-7c	AC084876.2	0.396873399	mirna_lnc
33018	hsa-let-7c	AL645608.4	0.698773499	mirna_lnc
33019	hsa-let-7c	AC005730.2	0.32463595	mirna_lnc
33020	hsa-let-7c	AC008115.1	0.366053677	mirna_lnc
33021	hsa-let-7c	AC126407.1	0.310864327	mirna_lnc
33022	hsa-let-7c	AP001029.1	0.433670819	mirna_lnc
33023	hsa-let-7c	AC007663.3	0.662377476	mirna_lnc
33024	hsa-let-7c	AL590787.1	0.37882591	mirna_lnc
33025	hsa-let-7c	AC007773.1	0.329596771	mirna_lnc
33026	hsa-let-7c	AC009120.5	0.330763227	mirna_lnc
33027	hsa-let-7c	AC093821.1	0.38534196	mirna_lnc
33028	hsa-let-7c	AP000253.1	0.388355967	mirna_lnc
33029	hsa-let-7c	AC034102.5	0.30527344	mirna_lnc
33030	hsa-let-7c	AL138831.1	0.549730768	mirna_lnc
33031	hsa-let-7c	MAP3K2-DT	0.313212374	mirna_lnc
33032	hsa-let-7c	AP001178.3	0.336334758	mirna_lnc
33033	hsa-let-7c	AC010735.1	0.675807436	mirna_lnc
33034	hsa-let-7c	AC079600.3	0.549276146	mirna_lnc
33035	hsa-let-7c	AL022324.3	0.52957481	mirna_lnc
33036	hsa-let-7c	AP000320.1	0.326053564	mirna_lnc
33037	hsa-let-7c	AC105118.1	0.525667728	mirna_lnc
33038	hsa-let-7c	AC007391.1	0.401864975	mirna_lnc
33039	hsa-let-7c	AC005005.3	0.376220097	mirna_lnc
33040	hsa-let-7c	AC100814.1	0.324833694	mirna_lnc
33041	hsa-let-7c	AL158834.2	0.320769429	mirna_lnc
33042	hsa-let-7c	UFL1-AS1	0.313359899	mirna_lnc
33043	hsa-let-7c	BX649601.1	0.324257384	mirna_lnc
33044	hsa-let-7c	AC008736.2	0.36075861	mirna_lnc
33045	hsa-let-7c	LINC01771	0.373451496	mirna_lnc
33046	hsa-let-7c	LINC01238	0.550235142	mirna_lnc
33047	hsa-let-7c	ATP2B1-AS1	0.512411623	mirna_lnc

33048	hsa-let-7c	AP001029.2	0.413480183	mirna_lnc
33049	hsa-let-7c	AP000255.1	0.838274726	mirna_lnc
33050	hsa-let-7c	AL132765.2	0.457644341	mirna_lnc
33051	hsa-let-7c	AC104072.1	0.624647194	mirna_lnc
33052	hsa-let-7c	CAHM	0.376087799	mirna_lnc
33053	hsa-let-7c	AP005329.2	0.467360266	mirna_lnc
33054	hsa-let-7c	AC010336.2	0.402395497	mirna_lnc
33055	hsa-let-7c	AC073389.2	0.458315712	mirna_lnc
33056	hsa-let-7c	AL031658.1	0.622713764	mirna_lnc
33057	hsa-let-7c	AC025031.4	0.300068875	mirna_lnc
33058	hsa-let-7c	AC010997.4	0.598986739	mirna_lnc
33059	hsa-let-7c	HCG14	0.359143636	mirna_lnc
33060	hsa-let-7c	AC068473.5	0.35422701	mirna_lnc
33061	hsa-let-7c	AC010997.5	0.314120333	mirna_lnc
33062	hsa-let-7c	HM13-AS1	0.393819851	mirna_lnc
33063	hsa-let-7c	AC092849.1	0.353814723	mirna_lnc
33064	hsa-let-7c	TTC39C-AS1	0.674195284	mirna_lnc
33065	hsa-let-7c	KLHL7-DT	0.327842904	mirna_lnc
33066	hsa-let-7c	AP000962.1	0.398265286	mirna_lnc
33067	hsa-let-7c	RB1-DT	0.386599445	mirna_lnc
33068	hsa-let-7c	AC023632.2	0.347879116	mirna_lnc
33069	hsa-let-7c	PAN3-AS1	0.44488617	mirna_lnc
33070	hsa-let-7c	AL049775.1	0.303849573	mirna_lnc
33071	hsa-let-7c	AC092447.5	0.607311096	mirna_lnc
33072	hsa-let-7c	AC006511.3	0.417198346	mirna_lnc
33073	hsa-let-7c	AC010336.5	0.71663558	mirna_lnc
33074	hsa-let-7c	AP000251.1	0.307887165	mirna_lnc
33075	hsa-let-7c	AC107464.3	0.65985235	mirna_lnc
33076	hsa-let-7c	AL136962.1	0.307161286	mirna_lnc
33077	hsa-let-7c	AC106038.1	0.300563151	mirna_lnc
33078	hsa-let-7c	AL513534.2	0.363160027	mirna_lnc
33079	hsa-let-7c	AC025219.1	0.312337148	mirna_lnc
33080	hsa-let-7c	LINC01901	0.674774463	mirna_lnc
33081	hsa-let-7c	AC022400.8	0.385900288	mirna_lnc
33082	hsa-let-7c	AC107032.2	0.392786737	mirna_lnc
33083	hsa-let-7c	LINC01465	0.430541836	mirna_lnc
33084	hsa-let-7c	AC008763.1	0.31754652	mirna_lnc
33085	hsa-let-7c	ZNF503-AS2	0.376435198	mirna_lnc
33086	hsa-let-7c	AL158063.1	0.389015337	mirna_lnc
33087	hsa-let-7c	ERVK9-11	0.365310198	mirna_lnc
33088	hsa-let-7c	AC120057.4	0.37584782	mirna_lnc
33089	hsa-let-7c	AL139397.1	0.3412834	mirna_lnc
33090	hsa-let-7c	AP000962.2	0.476397636	mirna_lnc
33091	hsa-let-7c	AL121574.1	0.35389179	mirna_lnc
33092	hsa-let-7c	AC026367.1	0.300281065	mirna_lnc
33093	hsa-let-7c	AL512625.1	0.374349976	mirna_lnc
33094	hsa-let-7c	LINC01144	0.408527188	mirna_lnc
33095	hsa-let-7c	AL008721.1	0.383713945	mirna_lnc
33096	hsa-let-7c	AF127577.5	0.561285199	mirna_lnc
33097	hsa-let-7c	FGF12-AS3	0.486287887	mirna_lnc
33098	hsa-let-7c	AC092119.2	0.357697558	mirna_lnc
33099	hsa-let-7c	AL355490.2	0.414057333	mirna_lnc
33100	hsa-let-7c	HCG15	0.324865667	mirna_lnc
33101	hsa-let-7c	AC079610.1	0.340958458	mirna_lnc

33102	hsa-let-7c	SMC2-AS1	0.618905414	mirna_lnc
33103	hsa-let-7c	AC110619.1	0.386664274	mirna_lnc
33104	hsa-let-7c	AC107068.1	0.444102141	mirna_lnc
33105	hsa-let-7c	AC090579.1	0.345266666	mirna_lnc
33106	hsa-let-7c	MANEA-DT	0.36538995	mirna_lnc
33107	hsa-let-7c	AL049796.1	0.381923669	mirna_lnc
33108	hsa-let-7c	AL136531.1	0.380045242	mirna_lnc
33109	hsa-let-7c	AC133550.2	0.302236023	mirna_lnc
33110	hsa-let-7c	AL451062.1	0.333924867	mirna_lnc
33111	hsa-let-7c	AC092119.3	0.311644087	mirna_lnc
33112	hsa-let-7c	MAGEA8-AS1	0.694700501	mirna_lnc
33113	hsa-let-7c	AC116348.1	0.354564201	mirna_lnc
33114	hsa-let-7c	AC004925.1	0.617347162	mirna_lnc
33115	hsa-let-7c	AP001605.1	0.326852041	mirna_lnc
33116	hsa-let-7c	Z99129.3	0.492947764	mirna_lnc
33117	hsa-let-7c	AC093462.1	0.335666989	mirna_lnc
33118	hsa-let-7c	AP000350.5	0.674980435	mirna_lnc
33119	hsa-let-7c	AC073389.3	0.684212843	mirna_lnc
33120	hsa-let-7c	LINC01970	0.346491999	mirna_lnc
33121	hsa-let-7c	AC104794.2	0.398020748	mirna_lnc
33122	hsa-let-7c	MAPT-IT1	0.660125688	mirna_lnc
33123	hsa-let-7c	AL627171.1	0.306769723	mirna_lnc
33124	hsa-let-7c	AP001266.2	0.350521133	mirna_lnc
33125	hsa-let-7c	AL451069.1	0.434531034	mirna_lnc
33126	hsa-let-7c	AC009312.1	0.4315409	mirna_lnc
33127	hsa-let-7c	AC098484.2	0.316390846	mirna_lnc
33128	hsa-let-7c	AC021549.1	0.302953409	mirna_lnc
33129	hsa-let-7c	U91328.3	0.30735869	mirna_lnc
33130	hsa-let-7c	AL359510.2	0.303871102	mirna_lnc
33131	hsa-let-7c	AP000350.7	0.618158931	mirna_lnc
33132	hsa-let-7c	AL096701.3	0.344432455	mirna_lnc
33133	hsa-let-7c	AC079414.3	0.392964882	mirna_lnc
33134	hsa-let-7c	AC108134.4	0.312244485	mirna_lnc
33135	hsa-let-7c	AP002364.1	0.3390901	mirna_lnc
33136	hsa-let-7c	AL645940.1	0.30892641	mirna_lnc
33137	hsa-let-7c	AC020978.1	0.333617663	mirna_lnc
33138	hsa-let-7c	AL138831.3	0.50993211	mirna_lnc
33139	hsa-let-7c	AC017100.1	0.435793634	mirna_lnc
33140	hsa-let-7c	LINC00471	0.307865003	mirna_lnc
33141	hsa-let-7c	C8orf37-AS1	0.310668101	mirna_lnc
33142	hsa-let-7c	AC079601.1	0.33034465	mirna_lnc
33143	hsa-let-7c	AL138831.2	0.55031339	mirna_lnc
33144	hsa-let-7c	LINC01447	0.397342247	mirna_lnc
33145	hsa-let-7c	LINC00173	0.347001046	mirna_lnc
33146	hsa-let-7c	AC005006.1	0.416504969	mirna_lnc
33147	hsa-let-7c	AL645608.6	0.664193158	mirna_lnc
33148	hsa-let-7c	AL451064.2	0.792135022	mirna_lnc
33149	hsa-let-7c	AC231981.1	0.306592548	mirna_lnc
33150	hsa-let-7c	AC245060.5	0.318677926	mirna_lnc
33151	hsa-let-7c	AL353726.2	0.586127009	mirna_lnc
33152	hsa-let-7c	AC009148.1	0.336779925	mirna_lnc
33153	hsa-let-7c	SPACA6P-AS	0.674186016	mirna_lnc
33154	hsa-let-7c	AC104590.1	0.326050206	mirna_lnc
33155	hsa-let-7c	AC090061.1	0.523815442	mirna_lnc

33156	hsa-let-7c	AC055855.2	0.335356594	mirna_lnc
33157	hsa-let-7c	AC104109.2	0.405874482	mirna_lnc
33158	hsa-let-7c	LINC02652	0.437762285	mirna_lnc
33159	hsa-let-7c	AC007497.1	0.413239935	mirna_lnc
33160	hsa-let-7c	AL137003.2	0.480543545	mirna_lnc
33161	hsa-let-7c	L29074.1	0.661658913	mirna_lnc
33162	hsa-let-7c	AC145423.2	0.307630649	mirna_lnc
33163	hsa-let-7c	FAM160A1-DT	0.410540539	mirna_lnc
33164	hsa-let-7c	AC140125.2	0.347001547	mirna_lnc
33165	hsa-let-7c	WWC2-AS2	0.327493608	mirna_lnc
33166	hsa-let-7c	AL359541.1	0.545599336	mirna_lnc
33167	hsa-let-7c	AL512791.2	0.319214222	mirna_lnc
33168	hsa-let-7c	CRTC3-AS1	0.30372426	mirna_lnc
33169	hsa-let-7c	RN7SL832P	0.341278282	mirna_lnc
33170	hsa-let-7c	AL354811.1	0.303184405	mirna_lnc
33171	hsa-let-7c	AL157931.1	0.869914373	mirna_lnc
33172	hsa-let-7c	AL133255.1	0.427737543	mirna_lnc
33173	hsa-let-7c	AL354919.1	0.871787711	mirna_lnc
33174	hsa-let-7c	AC068987.1	0.322787778	mirna_lnc
33175	hsa-let-7c	AC116348.2	0.359464031	mirna_lnc
33176	hsa-let-7c	AL662791.1	0.50873382	mirna_lnc
33177	hsa-let-7c	AC103996.2	0.344368027	mirna_lnc
33178	hsa-let-7c	AC008555.1	0.395822917	mirna_lnc
33179	hsa-let-7c	AL158211.1	0.382081665	mirna_lnc
33180	hsa-let-7c	AC009084.2	0.386656731	mirna_lnc
33181	hsa-let-7c	AL135791.1	0.55947071	mirna_lnc
33182	hsa-let-7c	AL161729.4	0.365763182	mirna_lnc
33183	hsa-let-7c	AL022341.2	0.433525417	mirna_lnc
33184	hsa-let-7c	AC009113.1	0.366481886	mirna_lnc
33185	hsa-let-7c	AC083843.2	0.417322844	mirna_lnc
33186	hsa-let-7c	AL645608.2	0.686044235	mirna_lnc
33187	hsa-let-7c	AC010735.2	0.587654218	mirna_lnc
33188	hsa-let-7c	AC079610.2	0.328499192	mirna_lnc
33189	hsa-let-7c	AP001412.1	0.32997245	mirna_lnc
33190	hsa-let-7c	AL024498.1	0.616138642	mirna_lnc
33191	hsa-let-7c	AC100793.3	0.371165102	mirna_lnc
33192	hsa-let-7c	AC012456.2	0.369637451	mirna_lnc
33193	hsa-let-7c	AC100793.2	0.347652705	mirna_lnc
33194	hsa-let-7c	AC007663.4	0.544273674	mirna_lnc
33195	hsa-let-7c	AC087683.2	0.354293354	mirna_lnc
33196	hsa-let-7c	AL138921.1	0.373468537	mirna_lnc
33197	hsa-let-7c	AP000845.1	0.304269071	mirna_lnc
33198	hsa-let-7c	AC137767.1	0.464844217	mirna_lnc
33199	hsa-mir-181b-2	AL390728.6	0.31460436	mirna_lnc
33200	hsa-mir-181b-2	AC048341.2	0.301821628	mirna_lnc
33201	hsa-mir-181b-2	AC084782.3	0.307000022	mirna_lnc
33202	hsa-mir-181b-2	LINC00920	0.436262756	mirna_lnc
33203	hsa-mir-181b-2	AC004943.3	0.303877591	mirna_lnc
33204	hsa-mir-181b-2	AL355312.4	0.33033359	mirna_lnc
33205	hsa-mir-181b-2	AC073316.3	0.322825356	mirna_lnc
33206	hsa-mir-181b-2	AC009269.4	0.312573989	mirna_lnc
33207	hsa-mir-181b-2	AC097504.2	0.346205642	mirna_lnc
33208	hsa-mir-181b-2	AC005034.3	0.407045715	mirna_lnc
33209	hsa-mir-24-2	SNHG3	0.329701161	mirna_lnc

33210	hsa-mir-24-2	AC092718.4	0.362729197	mirna_lnc
33211	hsa-mir-24-2	AC026401.3	0.43147167	mirna_lnc
33212	hsa-mir-24-2	AC113189.4	0.35015786	mirna_lnc
33213	hsa-mir-24-2	TYMSOS	0.418727472	mirna_lnc
33214	hsa-mir-24-2	AGAP2-AS1	0.520303719	mirna_lnc
33215	hsa-mir-24-2	AC099850.1	0.306788348	mirna_lnc
33216	hsa-mir-24-2	AL359513.1	0.305898893	mirna_lnc
33217	hsa-mir-24-2	AC034213.1	0.309975743	mirna_lnc
33218	hsa-mir-24-2	AL049555.1	0.312188809	mirna_lnc
33219	hsa-mir-24-2	VPS9D1-AS1	0.435598355	mirna_lnc
33220	hsa-mir-24-2	AC004241.3	0.311492725	mirna_lnc
33221	hsa-mir-24-2	AP001453.2	0.52368825	mirna_lnc
33222	hsa-mir-24-2	JARID2-AS1	0.363074728	mirna_lnc
33223	hsa-mir-24-2	PYCARD-AS1	0.301540927	mirna_lnc
33224	hsa-mir-24-2	AC016065.1	0.38144718	mirna_lnc
33225	hsa-mir-24-2	LINC02043	0.38959435	mirna_lnc
33226	hsa-mir-24-2	MIR4713HG	0.441987243	mirna_lnc
33227	hsa-mir-24-2	U62317.1	0.348246902	mirna_lnc
33228	hsa-mir-24-2	AL031667.3	0.305482745	mirna_lnc
33229	hsa-mir-24-2	TFAP2A-AS1	0.411351347	mirna_lnc
33230	hsa-mir-24-2	AC004812.2	0.328000366	mirna_lnc
33231	hsa-mir-24-2	DEPDC1-AS1	0.365153932	mirna_lnc
33232	hsa-mir-24-2	AC100791.2	0.315871373	mirna_lnc
33233	hsa-mir-24-2	AC117394.2	0.316740848	mirna_lnc
33234	hsa-mir-24-2	AC010894.2	0.312982974	mirna_lnc
33235	hsa-mir-24-2	AL023803.3	0.300730802	mirna_lnc
33236	hsa-mir-24-2	CLEC12A-AS1	0.354025123	mirna_lnc
33237	hsa-mir-24-2	AC109322.1	0.329371015	mirna_lnc
33238	hsa-mir-24-2	AL138724.1	0.394576004	mirna_lnc
33239	hsa-mir-24-2	AC108134.2	0.318039075	mirna_lnc
33240	hsa-mir-24-2	AL441992.1	0.37728571	mirna_lnc
33241	hsa-mir-24-2	AC016394.1	0.329188785	mirna_lnc
33242	hsa-mir-24-2	SNHG19	0.349603453	mirna_lnc
33243	hsa-mir-24-2	AC090409.1	0.40394232	mirna_lnc
33244	hsa-mir-24-2	AL136162.1	0.370919973	mirna_lnc
33245	hsa-mir-24-2	AC015818.2	0.347917395	mirna_lnc
33246	hsa-mir-24-2	AC078788.1	0.325307939	mirna_lnc
33247	hsa-mir-24-2	AP001453.4	0.55591745	mirna_lnc
33248	hsa-mir-24-2	AC126773.2	0.386546689	mirna_lnc
33249	hsa-mir-24-2	MAN1B1-DT	0.331429011	mirna_lnc
33250	hsa-mir-24-2	AC012640.2	0.405732574	mirna_lnc
33251	hsa-mir-24-2	AC090970.2	0.45263992	mirna_lnc
33252	hsa-mir-24-2	AC026782.2	0.368332661	mirna_lnc
33253	hsa-mir-24-2	AP003119.2	0.410000206	mirna_lnc
33254	hsa-mir-24-2	MAPK6-DT	0.333635068	mirna_lnc
33255	hsa-mir-24-2	AC007540.1	0.315353141	mirna_lnc
33256	hsa-mir-24-2	AP003390.1	0.410134048	mirna_lnc
33257	hsa-mir-24-2	AC114956.2	0.46110969	mirna_lnc
33258	hsa-mir-24-2	AC025244.1	0.378209942	mirna_lnc
33259	hsa-mir-24-2	AC002470.1	0.399117103	mirna_lnc
33260	hsa-mir-24-2	HAGLROS	0.338974469	mirna_lnc
33261	hsa-mir-24-2	AL353764.1	0.319875746	mirna_lnc
33262	hsa-mir-24-2	AC040169.1	0.396574362	mirna_lnc
33263	hsa-mir-24-2	AL078644.2	0.363137708	mirna_lnc

33264	hsa-mir-24-2	HOMER3-AS1	0.362870626	mirna_lnc
33265	hsa-mir-24-2	AC010168.1	0.396074229	mirna_lnc
33266	hsa-mir-24-2	AC016876.3	0.356525145	mirna_lnc
33267	hsa-mir-24-2	AL031665.2	0.322346369	mirna_lnc
33268	hsa-mir-24-2	TM4SF19-AS1	0.383644515	mirna_lnc
33269	hsa-mir-24-2	AL359643.3	0.371835629	mirna_lnc
33270	hsa-mir-24-2	AC020891.3	0.304803647	mirna_lnc
33271	hsa-mir-24-2	AL031058.1	0.392053778	mirna_lnc
33272	hsa-mir-24-2	AC019205.1	0.347530456	mirna_lnc
33273	hsa-mir-24-2	AC016876.1	0.313304065	mirna_lnc
33274	hsa-mir-24-2	AC024581.1	0.428987903	mirna_lnc
33275	hsa-mir-24-2	AC002401.4	0.336698723	mirna_lnc
33276	hsa-mir-24-2	AC092070.4	0.388431126	mirna_lnc
33277	hsa-mir-24-2	LINC00656	0.314459583	mirna_lnc
33278	hsa-mir-24-2	AC022075.1	0.322945889	mirna_lnc
33279	hsa-mir-24-2	AL138789.1	0.36425549	mirna_lnc
33280	hsa-mir-24-2	AC020891.2	0.302886848	mirna_lnc
33281	hsa-mir-24-2	AC239799.1	0.31704877	mirna_lnc
33282	hsa-mir-24-2	DLX2-DT	0.33140938	mirna_lnc
33283	hsa-mir-24-2	AC113194.1	0.317146864	mirna_lnc
33284	hsa-mir-24-2	AL136131.2	0.304522566	mirna_lnc
33285	hsa-mir-24-2	EXOC3-AS1	0.49062977	mirna_lnc
33286	hsa-mir-24-2	TTLL11-IT1	0.309083135	mirna_lnc
33287	hsa-mir-24-2	AC010595.1	0.401093503	mirna_lnc
33288	hsa-mir-24-2	AP001160.3	0.379757792	mirna_lnc
33289	hsa-mir-24-2	LINC01932	0.316476988	mirna_lnc
33290	hsa-mir-24-2	AC010327.3	0.322850304	mirna_lnc
33291	hsa-mir-24-2	AC008556.1	0.49066558	mirna_lnc
33292	hsa-mir-24-2	SMCR2	0.308352552	mirna_lnc
33293	hsa-mir-24-2	LINC01816	0.367827366	mirna_lnc
33294	hsa-mir-24-2	AC099518.6	0.313222438	mirna_lnc
33295	hsa-mir-24-2	AC007881.3	0.340754882	mirna_lnc
33296	hsa-mir-24-2	LINC02533	0.377785457	mirna_lnc
33297	hsa-mir-24-2	AC069503.1	0.325479093	mirna_lnc
33298	hsa-mir-24-2	AC011445.2	0.312540672	mirna_lnc
33299	hsa-mir-24-2	DCST1-AS1	0.323181715	mirna_lnc
33300	hsa-mir-24-2	AC009118.2	0.375864962	mirna_lnc
33301	hsa-mir-24-2	AC046143.2	0.329593293	mirna_lnc
33302	hsa-mir-24-2	AL121992.2	0.326076959	mirna_lnc
33303	hsa-mir-24-2	AC055736.1	0.355108565	mirna_lnc
33304	hsa-mir-24-2	AC004846.1	0.301723429	mirna_lnc
33305	hsa-mir-24-2	AC009902.2	0.335741417	mirna_lnc
33306	hsa-mir-24-2	AC082651.3	0.487683431	mirna_lnc
33307	hsa-mir-24-2	AL451085.2	0.350707954	mirna_lnc
33308	hsa-mir-24-2	DGUOK-AS1	0.521423095	mirna_lnc
33309	hsa-mir-24-2	AP005436.2	0.363764196	mirna_lnc
33310	hsa-mir-24-2	AC026495.1	0.309584758	mirna_lnc
33311	hsa-mir-24-2	AL139327.2	0.359238651	mirna_lnc
33312	hsa-mir-24-2	LINC02178	0.337999014	mirna_lnc
33313	hsa-mir-24-2	AL157904.1	0.363849903	mirna_lnc
33314	hsa-mir-24-2	AC087741.2	0.376137038	mirna_lnc
33315	hsa-mir-24-2	AL353719.1	0.425886379	mirna_lnc
33316	hsa-mir-24-2	AC010275.1	0.388115236	mirna_lnc
33317	hsa-mir-24-2	AC091170.1	0.381310199	mirna_lnc

33318	hsa-mir-24-2	LINC01063	0.38268713	mirna_lnc
33319	hsa-mir-24-2	AC114956.1	0.304537065	mirna_lnc
33320	hsa-mir-24-2	CIRBP-AS1	0.37829328	mirna_lnc
33321	hsa-mir-24-2	BX322234.1	0.337517672	mirna_lnc
33322	hsa-mir-24-2	AC109454.3	0.305654529	mirna_lnc
33323	hsa-mir-24-2	AC040169.3	0.319780659	mirna_lnc
33324	hsa-mir-24-2	AL358334.2	0.520456348	mirna_lnc
33325	hsa-mir-24-2	AC009171.2	0.43005741	mirna_lnc
33326	hsa-mir-24-2	AC025183.1	0.509746359	mirna_lnc
33327	hsa-mir-24-2	AC068831.6	0.552837152	mirna_lnc
33328	hsa-mir-24-2	LINC01023	0.328862775	mirna_lnc
33329	hsa-mir-24-2	AC138696.2	0.411550595	mirna_lnc
33330	hsa-mir-24-2	PICART1	0.302554226	mirna_lnc
33331	hsa-mir-24-2	AC023906.2	0.452272351	mirna_lnc
33332	hsa-mir-24-2	MAST4-AS1	0.415152725	mirna_lnc
33333	hsa-mir-24-2	AC004471.1	0.330444393	mirna_lnc
33334	hsa-mir-24-2	AC018716.2	0.305457764	mirna_lnc
33335	hsa-mir-24-2	LINC01807	0.382257705	mirna_lnc
33336	hsa-mir-24-2	AC114489.1	0.341403359	mirna_lnc
33337	hsa-mir-24-2	LINC01564	0.317621534	mirna_lnc
33338	hsa-mir-24-2	COA6-AS1	0.368073535	mirna_lnc
33339	hsa-mir-24-2	AC008267.5	0.308893012	mirna_lnc
33340	hsa-mir-24-2	AC099487.1	0.337924713	mirna_lnc
33341	hsa-mir-24-2	LINC00885	0.342365342	mirna_lnc
33342	hsa-mir-24-2	LINC01971	0.319846538	mirna_lnc
33343	hsa-mir-24-2	IL20RB-AS1	0.338145058	mirna_lnc
33344	hsa-mir-24-2	AC108865.1	0.424842079	mirna_lnc
33345	hsa-mir-24-2	LINC01998	0.304661252	mirna_lnc
33346	hsa-mir-24-2	URB1-AS1	0.352640278	mirna_lnc
33347	hsa-mir-24-2	AC096536.2	0.356555261	mirna_lnc
33348	hsa-mir-24-2	AC132938.3	0.329835539	mirna_lnc
33349	hsa-mir-24-2	AP001790.1	0.320467707	mirna_lnc
33350	hsa-mir-24-2	AL138902.1	0.310027424	mirna_lnc
33351	hsa-mir-24-2	AC026304.1	0.397118543	mirna_lnc
33352	hsa-mir-24-2	AC034206.1	0.338672297	mirna_lnc
33353	hsa-mir-24-2	AC131009.1	0.387789802	mirna_lnc
33354	hsa-mir-24-2	ELDR	0.368205202	mirna_lnc
33355	hsa-mir-24-2	AC131157.1	0.407203837	mirna_lnc
33356	hsa-mir-24-2	DOCK9-DT	0.400818518	mirna_lnc
33357	hsa-mir-24-2	AL355312.3	0.403514779	mirna_lnc
33358	hsa-mir-24-2	AL359091.5	0.368015651	mirna_lnc
33359	hsa-mir-24-2	AC093677.2	0.317317087	mirna_lnc
33360	hsa-mir-24-2	AC018695.2	0.34184813	mirna_lnc
33361	hsa-mir-24-2	AL122125.1	0.312507413	mirna_lnc
33362	hsa-mir-24-2	AP002957.1	0.346521942	mirna_lnc
33363	hsa-mir-24-2	AC083880.1	0.343875043	mirna_lnc
33364	hsa-mir-24-2	AC091182.2	0.333886225	mirna_lnc
33365	hsa-mir-24-2	LINC01527	0.312552309	mirna_lnc
33366	hsa-mir-24-2	LINC00519	0.315921013	mirna_lnc
33367	hsa-mir-24-2	AC008443.4	0.317365592	mirna_lnc
33368	hsa-mir-24-2	AL034399.2	0.453960302	mirna_lnc
33369	hsa-mir-24-2	AP001547.1	0.304745556	mirna_lnc
33370	hsa-mir-24-2	AC068870.2	0.307624091	mirna_lnc
33371	hsa-mir-24-2	AC108865.2	0.333587521	mirna_lnc

33372	hsa-mir-24-2	AC010834.2	0.322175396	mirna_lnc
33373	hsa-mir-24-2	AL022341.1	0.439824195	mirna_lnc
33374	hsa-mir-24-2	AP000251.1	0.406784743	mirna_lnc
33375	hsa-mir-24-2	AC092115.3	0.385231804	mirna_lnc
33376	hsa-mir-24-2	AC025575.2	0.440471141	mirna_lnc
33377	hsa-mir-24-2	MIR210HG	0.395855752	mirna_lnc
33378	hsa-mir-24-2	AC112236.1	0.367361661	mirna_lnc
33379	hsa-mir-24-2	AC093635.1	0.366286296	mirna_lnc
33380	hsa-mir-24-2	LINC01715	0.330842313	mirna_lnc
33381	hsa-mir-24-2	ZNF503-AS2	0.309716248	mirna_lnc
33382	hsa-mir-24-2	CFAP58-DT	0.32666135	mirna_lnc
33383	hsa-mir-24-2	AC011374.1	0.307931183	mirna_lnc
33384	hsa-mir-24-2	MIR31HG	0.354732765	mirna_lnc
33385	hsa-mir-24-2	PITPNA-AS1	0.346610366	mirna_lnc
33386	hsa-mir-24-2	AC027644.3	0.333027428	mirna_lnc
33387	hsa-mir-24-2	AC016888.1	0.305689264	mirna_lnc
33388	hsa-mir-24-2	AC138207.7	0.34060401	mirna_lnc
33389	hsa-mir-24-2	AP000692.2	0.365552625	mirna_lnc
33390	hsa-mir-24-2	AC084032.1	0.311471386	mirna_lnc
33391	hsa-mir-24-2	AC139100.1	0.423987956	mirna_lnc
33392	hsa-mir-24-2	AC069307.1	0.301397639	mirna_lnc
33393	hsa-mir-24-2	AC079834.2	0.383488055	mirna_lnc
33394	hsa-mir-24-2	AL583808.1	0.422462226	mirna_lnc
33395	hsa-mir-24-2	AC068338.2	0.309355558	mirna_lnc
33396	hsa-mir-24-2	AL358852.1	0.300278533	mirna_lnc
33397	hsa-mir-24-2	AC079465.1	0.398873669	mirna_lnc
33398	hsa-mir-24-2	AC130343.2	0.311323678	mirna_lnc
33399	hsa-mir-24-2	AC124068.2	0.338606617	mirna_lnc
33400	hsa-mir-24-2	AC145423.2	0.364056543	mirna_lnc
33401	hsa-mir-24-2	AP002800.1	0.37883759	mirna_lnc
33402	hsa-mir-24-2	AL391244.1	0.312105304	mirna_lnc
33403	hsa-mir-24-2	AC004471.2	0.332338658	mirna_lnc
33404	hsa-mir-24-2	AC126768.2	0.327541549	mirna_lnc
33405	hsa-mir-24-2	AL121672.3	0.423016476	mirna_lnc
33406	hsa-mir-24-2	FOXN3-AS1	0.423113828	mirna_lnc
33407	hsa-mir-24-2	ADIRF-AS1	0.473410105	mirna_lnc
33408	hsa-mir-24-2	AC018695.6	0.448044962	mirna_lnc
33409	hsa-mir-24-2	AC104031.1	0.442416404	mirna_lnc
33410	hsa-mir-24-2	AL445483.1	0.372691326	mirna_lnc
33411	hsa-mir-188	SNHG3	0.307127598	mirna_lnc
33412	hsa-mir-188	AC106876.1	0.303900696	mirna_lnc
33413	hsa-mir-188	AL035461.2	0.30035312	mirna_lnc
33414	hsa-mir-188	AC027228.2	0.34620461	mirna_lnc
33415	hsa-mir-188	LINC02585	0.339780716	mirna_lnc
33416	hsa-mir-188	HNF4A-AS1	0.340405465	mirna_lnc
33417	hsa-mir-188	AC131009.3	0.315029663	mirna_lnc
33418	hsa-mir-188	AL451050.2	0.328817512	mirna_lnc
33419	hsa-mir-188	AC104472.1	0.302744647	mirna_lnc
33420	hsa-mir-188	AC009407.1	0.310748923	mirna_lnc
33421	hsa-mir-188	AC004080.2	0.32407923	mirna_lnc
33422	hsa-mir-188	HOXC-AS3	0.351927323	mirna_lnc
33423	hsa-mir-188	AC007009.1	0.348823083	mirna_lnc
33424	hsa-mir-188	AL121578.3	0.308811315	mirna_lnc
33425	hsa-mir-188	AC067930.5	0.320171245	mirna_lnc

33426	hsa-mir-188	LINC01357	0.332602758	mirna_lnc
33427	hsa-mir-188	TRPM2-AS	0.324231234	mirna_lnc
33428	hsa-mir-188	AP000866.5	0.325332436	mirna_lnc
33429	hsa-mir-188	AC023302.1	0.305872546	mirna_lnc
33430	hsa-mir-188	AL117382.1	0.397674798	mirna_lnc
33431	hsa-mir-188	DDX39B-AS1	0.301651165	mirna_lnc
33432	hsa-mir-188	AP003680.1	0.318190996	mirna_lnc
33433	hsa-mir-188	LINC00628	0.38108572	mirna_lnc
33434	hsa-mir-188	AL390961.2	0.323234258	mirna_lnc
33435	hsa-mir-188	SNHG18	0.336038691	mirna_lnc
33436	hsa-mir-188	AP003733.4	0.338867761	mirna_lnc
33437	hsa-mir-188	AC113143.1	0.303018127	mirna_lnc
33438	hsa-mir-188	KBTBD11-OT1	0.308877215	mirna_lnc
33439	hsa-mir-188	AL591848.2	0.34731798	mirna_lnc
33440	hsa-mir-188	AC006205.2	0.35102652	mirna_lnc
33441	hsa-mir-188	AC022762.2	0.302138549	mirna_lnc
33442	hsa-mir-188	LINC01389	0.330665297	mirna_lnc
33443	hsa-mir-188	AC092756.1	0.347453472	mirna_lnc
33444	hsa-mir-188	AP006621.5	0.30065056	mirna_lnc
33445	hsa-mir-188	LBX2-AS1	0.330816893	mirna_lnc
33446	hsa-mir-188	AC079915.1	0.32091897	mirna_lnc
33447	hsa-mir-188	AC124798.1	0.460012194	mirna_lnc
33448	hsa-mir-188	AC116025.2	0.313619649	mirna_lnc
33449	hsa-mir-188	AL137798.1	0.333708013	mirna_lnc
33450	hsa-mir-188	AL121992.3	0.313172768	mirna_lnc
33451	hsa-mir-188	AC091117.2	0.316602763	mirna_lnc
33452	hsa-mir-188	AC010503.4	0.344678182	mirna_lnc
33453	hsa-mir-188	AL121989.1	0.344899221	mirna_lnc
33454	hsa-mir-188	LINC01979	0.317756788	mirna_lnc
33455	hsa-mir-188	AL591178.1	0.339516035	mirna_lnc
33456	hsa-mir-188	LINC00543	0.393973052	mirna_lnc
33457	hsa-mir-188	AC005696.4	0.305648425	mirna_lnc
33458	hsa-mir-188	AC016292.1	0.338080136	mirna_lnc
33459	hsa-mir-92a-2	AC012073.1	0.399695032	mirna_lnc
33460	hsa-mir-92a-2	AC010761.2	0.306976143	mirna_lnc
33461	hsa-mir-92a-2	AC020658.4	0.304830372	mirna_lnc
33462	hsa-mir-92a-2	AL035461.2	0.305244598	mirna_lnc
33463	hsa-mir-92a-2	AL138759.1	0.340220892	mirna_lnc
33464	hsa-mir-92a-2	DDX11-AS1	0.330825392	mirna_lnc
33465	hsa-mir-92a-2	AL117332.1	0.303391524	mirna_lnc
33466	hsa-mir-92a-2	AL161891.1	0.510551617	mirna_lnc
33467	hsa-mir-92a-2	AC092614.1	0.360375691	mirna_lnc
33468	hsa-mir-92a-2	AL157932.1	0.336439901	mirna_lnc
33469	hsa-mir-92a-2	HOXC-AS2	0.376420612	mirna_lnc
33470	hsa-mir-92a-2	AC019069.1	0.342077615	mirna_lnc
33471	hsa-mir-92a-2	AC074117.1	0.311919321	mirna_lnc
33472	hsa-mir-92a-2	AP001469.3	0.325490665	mirna_lnc
33473	hsa-mir-92a-2	AC004943.2	0.315354662	mirna_lnc
33474	hsa-mir-92a-2	AC027627.1	0.329828359	mirna_lnc
33475	hsa-mir-92a-2	AC021945.1	0.365288129	mirna_lnc
33476	hsa-mir-92a-2	AC090844.3	0.322275937	mirna_lnc
33477	hsa-mir-92a-2	AC090159.1	0.323173072	mirna_lnc
33478	hsa-mir-92a-2	AC009121.1	0.306192154	mirna_lnc
33479	hsa-mir-92a-2	AC024940.6	0.341558282	mirna_lnc

33480	hsa-mir-92a-2	AC016723.1	0.30404878	mirna_lnc
33481	hsa-mir-92a-2	GCC2-AS1	0.335765857	mirna_lnc
33482	hsa-mir-92a-2	AC015849.5	0.302205584	mirna_lnc
33483	hsa-mir-92a-2	LINC01096	0.316363486	mirna_lnc
33484	hsa-mir-92a-2	AC024267.6	0.331618898	mirna_lnc
33485	hsa-mir-92a-2	AL139089.1	0.301521917	mirna_lnc
33486	hsa-mir-92a-2	AL161772.1	0.370642885	mirna_lnc
33487	hsa-mir-92a-2	AC004080.1	0.308033309	mirna_lnc
33488	hsa-mir-92a-2	AC024230.1	0.313681977	mirna_lnc
33489	hsa-mir-92a-2	AC138904.1	0.354338762	mirna_lnc
33490	hsa-mir-92a-2	LINC01460	0.434608797	mirna_lnc
33491	hsa-mir-92a-2	AC018878.1	0.307703246	mirna_lnc
33492	hsa-mir-92a-2	TRPM2-AS	0.303701478	mirna_lnc
33493	hsa-mir-92a-2	SREBF2-AS1	0.320942601	mirna_lnc
33494	hsa-mir-92a-2	DNAJC3-DT	0.308657394	mirna_lnc
33495	hsa-mir-92a-2	AC106820.2	0.337306755	mirna_lnc
33496	hsa-mir-92a-2	AC026271.3	0.427291901	mirna_lnc
33497	hsa-mir-92a-2	AC016205.1	0.359459277	mirna_lnc
33498	hsa-mir-92a-2	PIK3CD-AS2	0.370028268	mirna_lnc
33499	hsa-mir-92a-2	MYB-AS1	0.321776513	mirna_lnc
33500	hsa-mir-92a-2	AP003680.1	0.310603116	mirna_lnc
33501	hsa-mir-92a-2	AC007128.1	0.337436938	mirna_lnc
33502	hsa-mir-92a-2	RNASEH1-AS1	0.410219131	mirna_lnc
33503	hsa-mir-92a-2	AC092669.1	0.305509012	mirna_lnc
33504	hsa-mir-92a-2	AC005224.1	0.303555092	mirna_lnc
33505	hsa-mir-92a-2	AC011773.1	0.331819916	mirna_lnc
33506	hsa-mir-92a-2	AL442128.2	0.356701772	mirna_lnc
33507	hsa-mir-92a-2	AC145207.5	0.345911124	mirna_lnc
33508	hsa-mir-92a-2	AC007938.3	0.386952765	mirna_lnc
33509	hsa-mir-92a-2	AC009271.1	0.307694392	mirna_lnc
33510	hsa-mir-92a-2	AC098818.2	0.309517674	mirna_lnc
33511	hsa-mir-92a-2	AL137244.1	0.34867177	mirna_lnc
33512	hsa-mir-92a-2	HHIP-AS1	0.333209885	mirna_lnc
33513	hsa-mir-92a-2	AL512652.2	0.358294502	mirna_lnc
33514	hsa-mir-92a-2	AL138688.2	0.309759958	mirna_lnc
33515	hsa-mir-92a-2	AC117395.1	0.318570665	mirna_lnc
33516	hsa-mir-92a-2	AL161452.1	0.310400611	mirna_lnc
33517	hsa-mir-92a-2	MCF2L-AS1	0.415993495	mirna_lnc
33518	hsa-mir-92a-2	AL138767.3	0.326763291	mirna_lnc
33519	hsa-mir-92a-2	AC098936.1	0.335452664	mirna_lnc
33520	hsa-mir-92a-2	AC138207.2	0.329767667	mirna_lnc
33521	hsa-mir-92a-2	AC147651.2	0.314395342	mirna_lnc
33522	hsa-mir-92a-2	ZNF649-AS1	0.31727396	mirna_lnc
33523	hsa-mir-92a-2	AL118511.1	0.322888039	mirna_lnc
33524	hsa-mir-92a-2	LINC02470	0.312374492	mirna_lnc
33525	hsa-mir-92a-2	AC106820.4	0.392376484	mirna_lnc
33526	hsa-mir-92a-2	AC004076.2	0.302570034	mirna_lnc
33527	hsa-mir-92a-2	AC017104.3	0.301952515	mirna_lnc
33528	hsa-mir-92a-2	LINC00909	0.325162479	mirna_lnc
33529	hsa-mir-92a-2	AC007497.1	0.307363927	mirna_lnc
33530	hsa-mir-92a-2	AL161729.4	0.326983421	mirna_lnc
33531	hsa-mir-92a-2	AC012486.1	0.322516877	mirna_lnc
33532	hsa-mir-92a-2	AL132655.1	0.304119524	mirna_lnc
33533	hsa-mir-92a-2	AL136146.2	0.305987054	mirna_lnc

33534	hsa-mir-16-1	AC026401.3	0.319701916	mirna_lnc
33535	hsa-mir-16-1	AC012073.1	0.363388579	mirna_lnc
33536	hsa-mir-16-1	MELTF-AS1	0.351292499	mirna_lnc
33537	hsa-mir-16-1	TMPO-AS1	0.418484294	mirna_lnc
33538	hsa-mir-16-1	AC020658.4	0.429836356	mirna_lnc
33539	hsa-mir-16-1	AP000808.1	0.346794928	mirna_lnc
33540	hsa-mir-16-1	AL035461.2	0.381258629	mirna_lnc
33541	hsa-mir-16-1	DDX11-AS1	0.316438593	mirna_lnc
33542	hsa-mir-16-1	AL117332.1	0.363645462	mirna_lnc
33543	hsa-mir-16-1	AC092614.1	0.370144293	mirna_lnc
33544	hsa-mir-16-1	HOXC-AS2	0.357032925	mirna_lnc
33545	hsa-mir-16-1	AC004943.2	0.347612068	mirna_lnc
33546	hsa-mir-16-1	AC009121.2	0.318141801	mirna_lnc
33547	hsa-mir-16-1	AL023803.3	0.311246036	mirna_lnc
33548	hsa-mir-16-1	AC147651.4	0.362699311	mirna_lnc
33549	hsa-mir-16-1	LINC01952	0.304464382	mirna_lnc
33550	hsa-mir-16-1	AC074351.1	0.310075599	mirna_lnc
33551	hsa-mir-16-1	LINC02367	0.310132386	mirna_lnc
33552	hsa-mir-16-1	AC008750.7	0.319632392	mirna_lnc
33553	hsa-mir-16-1	AL391069.2	0.376374374	mirna_lnc
33554	hsa-mir-16-1	AC116049.2	0.335022744	mirna_lnc
33555	hsa-mir-16-1	AL513497.1	0.325192317	mirna_lnc
33556	hsa-mir-16-1	AC024230.1	0.362876934	mirna_lnc
33557	hsa-mir-16-1	LINC01397	0.322240355	mirna_lnc
33558	hsa-mir-16-1	AP000487.1	0.424899394	mirna_lnc
33559	hsa-mir-16-1	AL031665.2	0.304505576	mirna_lnc
33560	hsa-mir-16-1	AC104971.1	0.400144315	mirna_lnc
33561	hsa-mir-16-1	AC010999.2	0.326944022	mirna_lnc
33562	hsa-mir-16-1	AP001025.1	0.423634678	mirna_lnc
33563	hsa-mir-16-1	SREBF2-AS1	0.301787245	mirna_lnc
33564	hsa-mir-16-1	AC100812.1	0.302036143	mirna_lnc
33565	hsa-mir-16-1	AC011297.1	0.356612808	mirna_lnc
33566	hsa-mir-16-1	AC004943.3	0.352969168	mirna_lnc
33567	hsa-mir-16-1	AC091153.3	0.311910341	mirna_lnc
33568	hsa-mir-16-1	AL354892.2	0.412221342	mirna_lnc
33569	hsa-mir-16-1	AC106820.2	0.371614403	mirna_lnc
33570	hsa-mir-16-1	AC026271.3	0.314422768	mirna_lnc
33571	hsa-mir-16-1	AC016205.1	0.359841104	mirna_lnc
33572	hsa-mir-16-1	RNASEH1-AS1	0.329848166	mirna_lnc
33573	hsa-mir-16-1	AC131212.4	0.398576913	mirna_lnc
33574	hsa-mir-16-1	AC092669.1	0.312653054	mirna_lnc
33575	hsa-mir-16-1	AP002336.2	0.374416912	mirna_lnc
33576	hsa-mir-16-1	AC145207.5	0.311523709	mirna_lnc
33577	hsa-mir-16-1	LINC02562	0.303184173	mirna_lnc
33578	hsa-mir-16-1	AC008115.1	0.34973389	mirna_lnc
33579	hsa-mir-16-1	AC024560.3	0.329338731	mirna_lnc
33580	hsa-mir-16-1	AC136475.1	0.318480739	mirna_lnc
33581	hsa-mir-16-1	AC009271.1	0.317019155	mirna_lnc
33582	hsa-mir-16-1	AC098818.2	0.368808022	mirna_lnc
33583	hsa-mir-16-1	AC107214.1	0.378165098	mirna_lnc
33584	hsa-mir-16-1	LINC00885	0.316484537	mirna_lnc
33585	hsa-mir-16-1	AC079174.1	0.341370736	mirna_lnc
33586	hsa-mir-16-1	DDR1-DT	0.3009276	mirna_lnc
33587	hsa-mir-16-1	AC079915.1	0.361523554	mirna_lnc

33588	hsa-mir-16-1	AC007250.1	0.325467004	mirna_lnc
33589	hsa-mir-16-1	AC098936.1	0.427744828	mirna_lnc
33590	hsa-mir-16-1	AC138207.2	0.32663037	mirna_lnc
33591	hsa-mir-16-1	AC080013.6	0.300556284	mirna_lnc
33592	hsa-mir-16-1	AL121989.1	0.318308609	mirna_lnc
33593	hsa-mir-16-1	AL359198.1	0.318719175	mirna_lnc
33594	hsa-mir-16-1	AL118511.1	0.307465425	mirna_lnc
33595	hsa-mir-16-1	DLGAP1-AS3	0.383039842	mirna_lnc
33596	hsa-mir-16-1	GIHCG	0.382554992	mirna_lnc
33597	hsa-mir-16-1	Z93241.1	0.35861656	mirna_lnc
33598	hsa-mir-16-1	LINC02470	0.56138929	mirna_lnc
33599	hsa-mir-16-1	AC135178.5	0.335208166	mirna_lnc
33600	hsa-mir-16-1	AC106820.4	0.350601825	mirna_lnc
33601	hsa-mir-16-1	AC107982.2	0.322960672	mirna_lnc
33602	hsa-mir-16-1	AC017104.3	0.305037052	mirna_lnc
33603	hsa-mir-16-1	AC007497.1	0.346062641	mirna_lnc
33604	hsa-mir-16-1	AP001972.4	0.300122636	mirna_lnc
33605	hsa-mir-16-1	AF213884.3	0.373116968	mirna_lnc
33606	hsa-mir-16-1	CAMTA1-IT1	0.339199885	mirna_lnc
33607	hsa-mir-3074	AC103746.1	0.434385112	mirna_lnc
33608	hsa-mir-3074	LINC01560	0.328254602	mirna_lnc
33609	hsa-mir-3074	LINC01625	0.398149625	mirna_lnc
33610	hsa-mir-3074	AC245100.6	0.310679391	mirna_lnc
33611	hsa-mir-3074	TMPO-AS1	0.329970078	mirna_lnc
33612	hsa-mir-3074	AL353194.1	0.335136844	mirna_lnc
33613	hsa-mir-3074	CEP83-DT	0.350121597	mirna_lnc
33614	hsa-mir-3074	AC124303.1	0.367085581	mirna_lnc
33615	hsa-mir-3074	LINC02585	0.323387013	mirna_lnc
33616	hsa-mir-3074	AL035458.2	0.373481923	mirna_lnc
33617	hsa-mir-3074	AC074117.1	0.320415845	mirna_lnc
33618	hsa-mir-3074	AC007405.2	0.364601977	mirna_lnc
33619	hsa-mir-3074	AC022387.1	0.366864053	mirna_lnc
33620	hsa-mir-3074	AL139288.1	0.328985287	mirna_lnc
33621	hsa-mir-3074	AC104667.1	0.4160988	mirna_lnc
33622	hsa-mir-3074	AL137802.2	0.333302043	mirna_lnc
33623	hsa-mir-3074	AC093642.2	0.437137809	mirna_lnc
33624	hsa-mir-3074	AL157823.2	0.317606878	mirna_lnc
33625	hsa-mir-3074	AP000553.2	0.480817128	mirna_lnc
33626	hsa-mir-3074	AC004835.1	0.33017976	mirna_lnc
33627	hsa-mir-3074	AC110813.1	0.310453482	mirna_lnc
33628	hsa-mir-3074	LINC01126	0.314436495	mirna_lnc
33629	hsa-mir-3074	LINC01087	0.514575472	mirna_lnc
33630	hsa-mir-3074	AC135803.1	0.377787834	mirna_lnc
33631	hsa-mir-3074	AL121832.3	0.342244845	mirna_lnc
33632	hsa-mir-3074	AL031985.3	0.327794145	mirna_lnc
33633	hsa-mir-3074	AL391152.1	0.342818835	mirna_lnc
33634	hsa-mir-3074	MAGEA4-AS1	0.333135605	mirna_lnc
33635	hsa-mir-3074	AC090578.2	0.346468162	mirna_lnc
33636	hsa-mir-3074	AC096887.2	0.31709287	mirna_lnc
33637	hsa-mir-3074	AL078605.1	0.330234713	mirna_lnc
33638	hsa-mir-3074	Z95115.1	0.396827749	mirna_lnc
33639	hsa-mir-3074	LINC01143	0.365964694	mirna_lnc
33640	hsa-mir-3074	AC034102.6	0.363756493	mirna_lnc
33641	hsa-mir-3074	AC024361.2	0.306235267	mirna_lnc

33642	hsa-mir-3074	ARHGAP31-AS1	0.342509277	mirna_lnc
33643	hsa-mir-3074	AL592494.2	0.333561338	mirna_lnc
33644	hsa-mir-3074	AC007996.1	0.395789526	mirna_lnc
33645	hsa-mir-3074	LINC01521	0.303669246	mirna_lnc
33646	hsa-mir-3074	AC016708.1	0.430430516	mirna_lnc
33647	hsa-mir-3074	DNAJC3-DT	0.306395635	mirna_lnc
33648	hsa-mir-3074	AC099786.1	0.48984575	mirna_lnc
33649	hsa-mir-3074	AL354892.2	0.370307654	mirna_lnc
33650	hsa-mir-3074	AC011773.4	0.32447394	mirna_lnc
33651	hsa-mir-3074	AP002360.3	0.35446549	mirna_lnc
33652	hsa-mir-3074	AC016205.1	0.318147101	mirna_lnc
33653	hsa-mir-3074	AC005379.1	0.334460035	mirna_lnc
33654	hsa-mir-3074	ERICD	0.359109154	mirna_lnc
33655	hsa-mir-3074	DNM3-IT1	0.427094437	mirna_lnc
33656	hsa-mir-3074	AL451064.1	0.4255557	mirna_lnc
33657	hsa-mir-3074	AL096677.1	0.325267519	mirna_lnc
33658	hsa-mir-3074	CNNM3-DT	0.300426195	mirna_lnc
33659	hsa-mir-3074	AL645608.4	0.380260164	mirna_lnc
33660	hsa-mir-3074	AL135787.1	0.311795832	mirna_lnc
33661	hsa-mir-3074	AC126407.1	0.408076394	mirna_lnc
33662	hsa-mir-3074	AC007663.3	0.345911856	mirna_lnc
33663	hsa-mir-3074	AC002091.1	0.307233739	mirna_lnc
33664	hsa-mir-3074	AC136475.1	0.355018408	mirna_lnc
33665	hsa-mir-3074	AL138831.1	0.31658509	mirna_lnc
33666	hsa-mir-3074	MAP3K2-DT	0.323924227	mirna_lnc
33667	hsa-mir-3074	AC023983.2	0.322630156	mirna_lnc
33668	hsa-mir-3074	AC010735.1	0.385378044	mirna_lnc
33669	hsa-mir-3074	AL022324.3	0.445218142	mirna_lnc
33670	hsa-mir-3074	AC007391.1	0.33669258	mirna_lnc
33671	hsa-mir-3074	BX649601.1	0.311095269	mirna_lnc
33672	hsa-mir-3074	LINC01238	0.375460485	mirna_lnc
33673	hsa-mir-3074	AC091152.2	0.411010273	mirna_lnc
33674	hsa-mir-3074	ATP2B1-AS1	0.392128968	mirna_lnc
33675	hsa-mir-3074	AL355488.1	0.314546703	mirna_lnc
33676	hsa-mir-3074	AP000255.1	0.394478133	mirna_lnc
33677	hsa-mir-3074	AL132765.2	0.306028784	mirna_lnc
33678	hsa-mir-3074	AC127024.2	0.375826296	mirna_lnc
33679	hsa-mir-3074	AC104072.1	0.43586173	mirna_lnc
33680	hsa-mir-3074	AC119403.1	0.310322252	mirna_lnc
33681	hsa-mir-3074	AP006621.5	0.344984211	mirna_lnc
33682	hsa-mir-3074	AC010336.2	0.336768927	mirna_lnc
33683	hsa-mir-3074	AL391001.1	0.317402665	mirna_lnc
33684	hsa-mir-3074	AL031658.1	0.33531592	mirna_lnc
33685	hsa-mir-3074	AC023824.6	0.306550368	mirna_lnc
33686	hsa-mir-3074	HM13-AS1	0.325056446	mirna_lnc
33687	hsa-mir-3074	TTC39C-AS1	0.323456794	mirna_lnc
33688	hsa-mir-3074	KLHL7-DT	0.347922244	mirna_lnc
33689	hsa-mir-3074	AL390778.2	0.331024957	mirna_lnc
33690	hsa-mir-3074	AC092447.5	0.485120155	mirna_lnc
33691	hsa-mir-3074	AC010336.5	0.370552484	mirna_lnc
33692	hsa-mir-3074	AC107464.3	0.449365235	mirna_lnc
33693	hsa-mir-3074	AL513534.2	0.333255026	mirna_lnc
33694	hsa-mir-3074	LINC01901	0.332204203	mirna_lnc
33695	hsa-mir-3074	AL008721.1	0.312594371	mirna_lnc

33696	hsa-mir-3074	FGF12-AS3	0.400374411	mirna_lnc
33697	hsa-mir-3074	SMC2-AS1	0.342374731	mirna_lnc
33698	hsa-mir-3074	AL162231.4	0.316092469	mirna_lnc
33699	hsa-mir-3074	AC099667.1	0.304012382	mirna_lnc
33700	hsa-mir-3074	AL136531.1	0.30336035	mirna_lnc
33701	hsa-mir-3074	MAGEA8-AS1	0.309646579	mirna_lnc
33702	hsa-mir-3074	AL118511.1	0.330308519	mirna_lnc
33703	hsa-mir-3074	AC004925.1	0.387291459	mirna_lnc
33704	hsa-mir-3074	AP000350.5	0.364028551	mirna_lnc
33705	hsa-mir-3074	AC090772.3	0.463497089	mirna_lnc
33706	hsa-mir-3074	AC073389.3	0.381268651	mirna_lnc
33707	hsa-mir-3074	MAPT-IT1	0.361235169	mirna_lnc
33708	hsa-mir-3074	AP000350.7	0.338606681	mirna_lnc
33709	hsa-mir-3074	AL138831.3	0.31117548	mirna_lnc
33710	hsa-mir-3074	AC017100.1	0.390982182	mirna_lnc
33711	hsa-mir-3074	AC005006.1	0.537030676	mirna_lnc
33712	hsa-mir-3074	AL645608.6	0.319423828	mirna_lnc
33713	hsa-mir-3074	AL451064.2	0.413672544	mirna_lnc
33714	hsa-mir-3074	AL353726.2	0.395807539	mirna_lnc
33715	hsa-mir-3074	SPACA6P-AS	0.364804264	mirna_lnc
33716	hsa-mir-3074	AC133961.1	0.389535244	mirna_lnc
33717	hsa-mir-3074	AL161725.2	0.302186019	mirna_lnc
33718	hsa-mir-3074	AL137003.2	0.368050676	mirna_lnc
33719	hsa-mir-3074	L29074.1	0.407706392	mirna_lnc
33720	hsa-mir-3074	AC005391.1	0.405239399	mirna_lnc
33721	hsa-mir-3074	CRTC3-AS1	0.329122638	mirna_lnc
33722	hsa-mir-3074	FGF12-AS2	0.351309426	mirna_lnc
33723	hsa-mir-3074	AL157931.1	0.473170831	mirna_lnc
33724	hsa-mir-3074	AC118758.3	0.318872757	mirna_lnc
33725	hsa-mir-3074	AL354919.1	0.456206895	mirna_lnc
33726	hsa-mir-3074	AL161729.4	0.363900704	mirna_lnc
33727	hsa-mir-3074	AP001476.1	0.309657961	mirna_lnc
33728	hsa-mir-3074	AL645608.2	0.368990046	mirna_lnc
33729	hsa-mir-3074	AC010735.2	0.399275058	mirna_lnc
33730	hsa-mir-3074	AC092839.2	0.314018188	mirna_lnc
33731	hsa-mir-3074	AP001626.1	0.338010169	mirna_lnc
33732	hsa-mir-3074	AL024498.1	0.360183927	mirna_lnc
33733	hsa-mir-3074	AP001922.6	0.315629239	mirna_lnc
33734	hsa-mir-3074	AC127024.3	0.357889838	mirna_lnc
33735	hsa-mir-3074	AC137767.1	0.34338916	mirna_lnc
33736	hsa-mir-3647	AP000708.1	0.310140255	mirna_lnc
33737	hsa-mir-3647	AC026356.1	0.393217654	mirna_lnc
33738	hsa-mir-3647	AC022031.1	0.302993574	mirna_lnc
33739	hsa-mir-3647	LINC01873	0.317800583	mirna_lnc
33740	hsa-mir-3647	AC020658.4	0.319819855	mirna_lnc
33741	hsa-mir-3647	AC004241.3	0.307014288	mirna_lnc
33742	hsa-mir-3647	AC048344.4	0.308892704	mirna_lnc
33743	hsa-mir-3647	CEP83-DT	0.311480975	mirna_lnc
33744	hsa-mir-3647	AC012183.1	0.344475524	mirna_lnc
33745	hsa-mir-3647	HOXC-AS2	0.316215676	mirna_lnc
33746	hsa-mir-3647	AP001021.2	0.361750451	mirna_lnc
33747	hsa-mir-3647	AL031667.3	0.301010289	mirna_lnc
33748	hsa-mir-3647	L3MBTL4-AS1	0.326198187	mirna_lnc
33749	hsa-mir-3647	AC004943.2	0.307277213	mirna_lnc

33750	hsa-mir-3647	AP000526.1	0.300716886	mirna_lnc
33751	hsa-mir-3647	AC073263.2	0.308193041	mirna_lnc
33752	hsa-mir-3647	AC002066.1	0.345185965	mirna_lnc
33753	hsa-mir-3647	AC027627.1	0.324235397	mirna_lnc
33754	hsa-mir-3647	LINC02542	0.302512558	mirna_lnc
33755	hsa-mir-3647	LINC01691	0.303813566	mirna_lnc
33756	hsa-mir-3647	AC092159.2	0.34098418	mirna_lnc
33757	hsa-mir-3647	AC092964.1	0.395814803	mirna_lnc
33758	hsa-mir-3647	AL390037.1	0.351379045	mirna_lnc
33759	hsa-mir-3647	AC024940.6	0.30125166	mirna_lnc
33760	hsa-mir-3647	AC090673.1	0.300890734	mirna_lnc
33761	hsa-mir-3647	AC027279.1	0.308027614	mirna_lnc
33762	hsa-mir-3647	AC004069.1	0.308735337	mirna_lnc
33763	hsa-mir-3647	AC131025.1	0.302784029	mirna_lnc
33764	hsa-mir-3647	AC004920.1	0.308577902	mirna_lnc
33765	hsa-mir-3647	AC021683.3	0.336808534	mirna_lnc
33766	hsa-mir-3647	AC026782.2	0.348490515	mirna_lnc
33767	hsa-mir-3647	AP000640.2	0.307536957	mirna_lnc
33768	hsa-mir-3647	LINC02450	0.378447121	mirna_lnc
33769	hsa-mir-3647	AC004158.1	0.328754378	mirna_lnc
33770	hsa-mir-3647	AC026782.1	0.311370957	mirna_lnc
33771	hsa-mir-3647	HELLPAR	0.36492153	mirna_lnc
33772	hsa-mir-3647	AC079684.1	0.349884986	mirna_lnc
33773	hsa-mir-3647	AC090578.2	0.362446905	mirna_lnc
33774	hsa-mir-3647	AC092807.2	0.317224914	mirna_lnc
33775	hsa-mir-3647	LINC01850	0.409858061	mirna_lnc
33776	hsa-mir-3647	LINC01143	0.312506246	mirna_lnc
33777	hsa-mir-3647	AC013391.1	0.322548048	mirna_lnc
33778	hsa-mir-3647	AC009502.1	0.325922584	mirna_lnc
33779	hsa-mir-3647	UBR5-AS1	0.32411702	mirna_lnc
33780	hsa-mir-3647	AC130650.2	0.312555457	mirna_lnc
33781	hsa-mir-3647	LINC02607	0.337049246	mirna_lnc
33782	hsa-mir-3647	AL157400.4	0.319268948	mirna_lnc
33783	hsa-mir-3647	AL121652.1	0.315055537	mirna_lnc
33784	hsa-mir-3647	NCKAP5-AS1	0.323631374	mirna_lnc
33785	hsa-mir-3647	SREBF2-AS1	0.333259546	mirna_lnc
33786	hsa-mir-3647	AC118755.1	0.318103481	mirna_lnc
33787	hsa-mir-3647	AC010969.2	0.366680695	mirna_lnc
33788	hsa-mir-3647	AC110769.2	0.30819357	mirna_lnc
33789	hsa-mir-3647	CXXC4-AS1	0.349193032	mirna_lnc
33790	hsa-mir-3647	AC005593.1	0.347600322	mirna_lnc
33791	hsa-mir-3647	AC016708.1	0.316871636	mirna_lnc
33792	hsa-mir-3647	AC004943.3	0.367333099	mirna_lnc
33793	hsa-mir-3647	AC004053.1	0.301334547	mirna_lnc
33794	hsa-mir-3647	AC093424.1	0.305944391	mirna_lnc
33795	hsa-mir-3647	AC093591.2	0.305501922	mirna_lnc
33796	hsa-mir-3647	AC019155.3	0.386948514	mirna_lnc
33797	hsa-mir-3647	AC117382.2	0.355207199	mirna_lnc
33798	hsa-mir-3647	AC063948.1	0.340086729	mirna_lnc
33799	hsa-mir-3647	LINC01416	0.341930949	mirna_lnc
33800	hsa-mir-3647	AC087742.1	0.347312679	mirna_lnc
33801	hsa-mir-3647	AP001642.1	0.315143849	mirna_lnc
33802	hsa-mir-3647	AC012640.5	0.356716299	mirna_lnc
33803	hsa-mir-3647	AC005379.1	0.319622972	mirna_lnc

33804	hsa-mir-3647	AC092669.1	0.339976994	mirna_lnc
33805	hsa-mir-3647	AP002336.2	0.308059293	mirna_lnc
33806	hsa-mir-3647	AC091946.2	0.338460379	mirna_lnc
33807	hsa-mir-3647	DNM3-IT1	0.33657052	mirna_lnc
33808	hsa-mir-3647	AC006206.1	0.324781535	mirna_lnc
33809	hsa-mir-3647	AC090772.4	0.34915446	mirna_lnc
33810	hsa-mir-3647	KIRREL1-IT1	0.305428294	mirna_lnc
33811	hsa-mir-3647	AL451064.1	0.3176191	mirna_lnc
33812	hsa-mir-3647	AL645608.4	0.312468765	mirna_lnc
33813	hsa-mir-3647	AC008115.1	0.378895478	mirna_lnc
33814	hsa-mir-3647	AC138951.2	0.322987981	mirna_lnc
33815	hsa-mir-3647	AC008115.2	0.359829991	mirna_lnc
33816	hsa-mir-3647	AC009120.5	0.326517322	mirna_lnc
33817	hsa-mir-3647	AC022762.1	0.33176117	mirna_lnc
33818	hsa-mir-3647	AL031600.2	0.31741934	mirna_lnc
33819	hsa-mir-3647	AC107308.1	0.360549588	mirna_lnc
33820	hsa-mir-3647	AP001178.3	0.354039425	mirna_lnc
33821	hsa-mir-3647	AC107214.1	0.334958802	mirna_lnc
33822	hsa-mir-3647	AC079600.3	0.325732126	mirna_lnc
33823	hsa-mir-3647	AL022324.3	0.320319112	mirna_lnc
33824	hsa-mir-3647	AC073896.4	0.376768652	mirna_lnc
33825	hsa-mir-3647	AP005060.1	0.341707939	mirna_lnc
33826	hsa-mir-3647	AC011446.1	0.369555029	mirna_lnc
33827	hsa-mir-3647	AP001029.2	0.320155145	mirna_lnc
33828	hsa-mir-3647	AC096992.2	0.372278916	mirna_lnc
33829	hsa-mir-3647	AC006237.1	0.318897494	mirna_lnc
33830	hsa-mir-3647	AC087501.4	0.308341474	mirna_lnc
33831	hsa-mir-3647	AC068860.1	0.303420035	mirna_lnc
33832	hsa-mir-3647	AL122125.1	0.313066775	mirna_lnc
33833	hsa-mir-3647	AC064807.4	0.3402361	mirna_lnc
33834	hsa-mir-3647	AP000962.1	0.362252459	mirna_lnc
33835	hsa-mir-3647	AC007389.5	0.37004713	mirna_lnc
33836	hsa-mir-3647	AC010834.2	0.313625118	mirna_lnc
33837	hsa-mir-3647	AC060809.1	0.311123069	mirna_lnc
33838	hsa-mir-3647	AL513534.2	0.314242694	mirna_lnc
33839	hsa-mir-3647	AC019131.1	0.318458569	mirna_lnc
33840	hsa-mir-3647	AC025219.1	0.341713769	mirna_lnc
33841	hsa-mir-3647	AL138881.1	0.305975737	mirna_lnc
33842	hsa-mir-3647	AC090907.1	0.311382806	mirna_lnc
33843	hsa-mir-3647	AP000962.2	0.367721858	mirna_lnc
33844	hsa-mir-3647	AL008721.1	0.301060145	mirna_lnc
33845	hsa-mir-3647	AC018682.2	0.311943321	mirna_lnc
33846	hsa-mir-3647	AL032819.1	0.346116048	mirna_lnc
33847	hsa-mir-3647	MAGEA8-AS1	0.342473005	mirna_lnc
33848	hsa-mir-3647	LINC00485	0.376882251	mirna_lnc
33849	hsa-mir-3647	AC087392.1	0.329702881	mirna_lnc
33850	hsa-mir-3647	AC004012.1	0.316988473	mirna_lnc
33851	hsa-mir-3647	Z99129.3	0.311192533	mirna_lnc
33852	hsa-mir-3647	AP000350.5	0.307229946	mirna_lnc
33853	hsa-mir-3647	AL583808.1	0.311866738	mirna_lnc
33854	hsa-mir-3647	AP005205.3	0.3003414	mirna_lnc
33855	hsa-mir-3647	AL627171.1	0.337958692	mirna_lnc
[truncated: 173,068 more chars]
