# Supplementary figures and images for: Novel lncRNA Panel as for Prognosis in Esophageal Squamous Cell Carcinoma Based on ceRNA Network Mechanism
Source: Comput Math Methods Med. 2021 Sep 24;2021:8020879. doi: 10.1155/2021/8020879 (PMC8486540; doi:10.1155/2021/8020879)

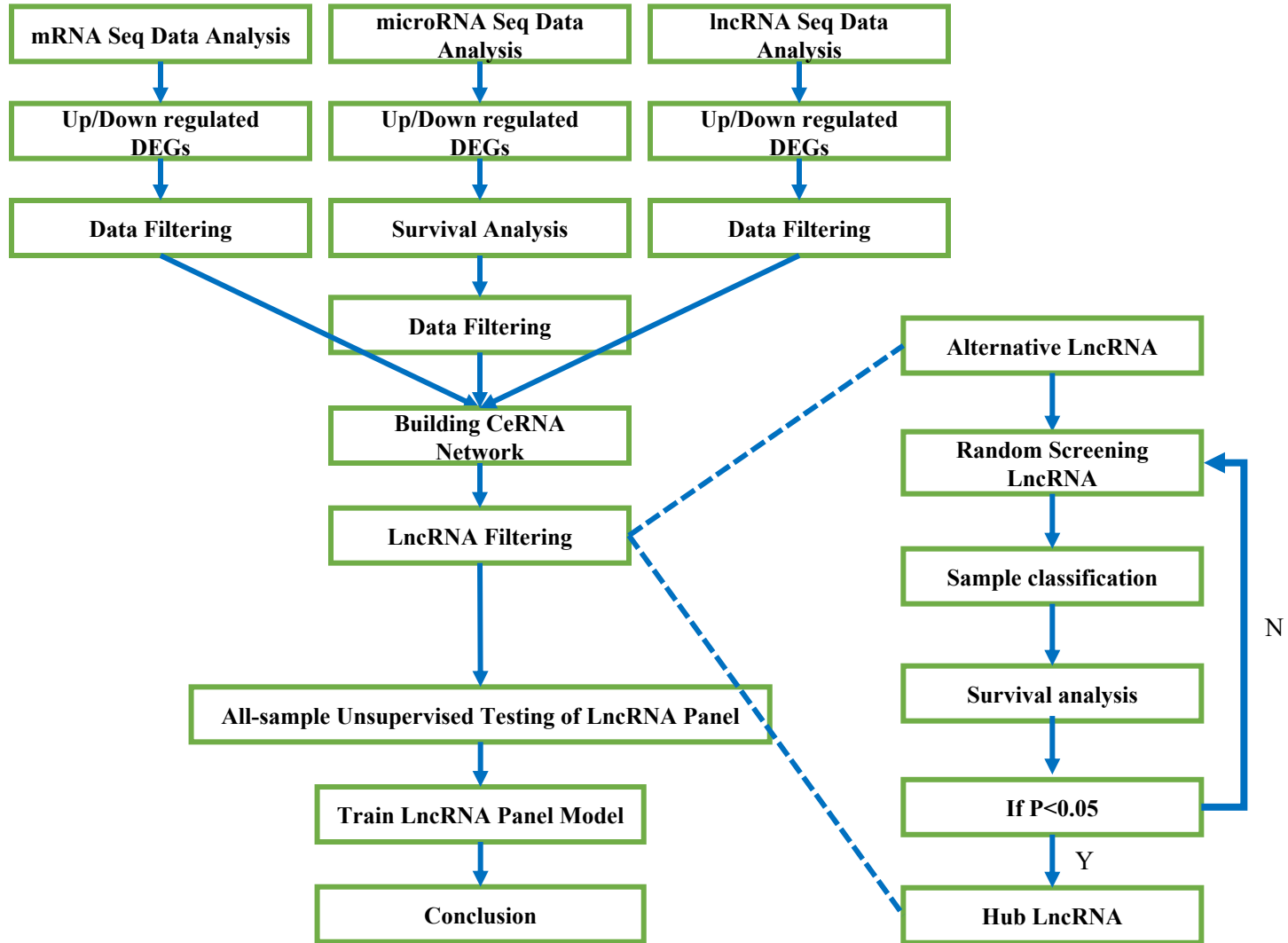

Supplement: Supplementary Materials — Supplementary Table 1: the sample information was shown in Table S1. Supplementary Table 2: expression data of genes were gathered in Table S2. Supplementary Table 3: differently expressed lncRNAs were shown in Table S3. Supplementary Table 4: differently expressed miRNAs were shown in Table S4. Supplementary Table 5: the interaction network of between lncRNAs and miRNAs is shown in Table S5. [file 8020879.f1.zip › Figure S1.pdf]
